# Fibroblast polarization over the myocardial infarction time continuum shifts roles from inflammation to angiogenesis

**DOI:** 10.1007/s00395-019-0715-4

**Published:** 2019-01-11

**Authors:** Alan J. Mouton, Yonggang Ma, Osvaldo J. Rivera Gonzalez, Michael J. Daseke, Elizabeth R. Flynn, Tom C. Freeman, Michael R. Garrett, Kristine Y. DeLeon-Pennell, Merry L. Lindsey

**Affiliations:** 1 Department of Physiology and Biophysics, Mississippi Center for Heart Research, University of Mississippi Medical Center, 2500 North State St, Jackson, MS 39216-4505 USA; 2 The Roslin Institute, University of Edinburgh, Easter Bush, Midlothian, EH25 9RG UK; 3 Department of Pharmacology and Toxicology, University of Mississippi Medical Center, Jackson, MS 39216 USA; 4 Research Service, G.V. (Sonny) Montgomery Veterans Affairs Medical Center, Jackson, MS 39216 USA

**Keywords:** Myocardial infarction, Cardiac remodeling, Fibroblast, Transcriptome, Angiogenesis, Extracellular matrix

## Abstract

Cardiac fibroblasts are the major producers of extracellular matrix (ECM) to form infarct scar. We hypothesized that fibroblasts undergo a spectrum of phenotype states over the course of myocardial infarction (MI) from early onset to scar formation. Fibroblasts were isolated from the infarct region of C57BL/6J male mice (3–6 months old, *n* = 60) at days 0 (no MI control) and 1, 3, or 7 after MI. Whole transcriptome analysis was performed by RNA-sequencing. Of the genes sequenced, 3371 were differentially expressed after MI. Enrichment analysis revealed that MI day 1 fibroblasts displayed pro-inflammatory, leukocyte-recruiting, pro-survival, and anti-migratory phenotype through *Tnfrsf9* and CD137 signaling. MI day 3 fibroblasts had a proliferative, pro-fibrotic, and pro-angiogenic profile with elevated* Il4ra* signaling. MI day 7 fibroblasts showed an anti-angiogenic homeostatic-like myofibroblast profile and with a step-wise increase in *Acta2* expression. MI day 7 fibroblasts relied on *Pik3r3* signaling to mediate *Tgfb1* effects and *Fgfr2* to regulate *PI3K* signaling. In vitro, the day 3 MI fibroblast secretome stimulated angiogenesis, while day 7 MI fibroblast secretome repressed angiogenesis through Thbs1 signaling. Our results reveal novel mechanisms for fibroblasts in expressing pro-inflammatory molecules and regulating angiogenesis following MI.

**Electronic supplementary material:**

The online version of this article (10.1007/s00395-019-0715-4) contains supplementary material, which is available to authorized users.

## Introduction

Myocardial infarction (MI) occurs when coronary blood flow to the left ventricle (LV) is blocked for a sufficient duration to result in tissue necrosis. The cardiac wound healing response involves robust inflammation, extracellular matrix (ECM) remodeling, revascularization, and scar formation [23, 41, 53]. While the cardiac fibroblast serves a baseline homeostasis role or transforms to an activated myofibroblast in response to injury, intermediary phenotypes exist, and fibroblasts display a range of phenotypes in response to individual in vitro stimuli [1, 61]. For example, cardiac fibroblasts stimulated with lipopolysaccharide show increased pro-inflammatory cytokine production and decreased collagen synthesis [37, 59]. Fibroblasts stimulated with transforming growth factor (TGF)β1 increase collagen synthesis and differentiate to a myofibroblast phenotype [62, 65].

Angiogenesis is the formation of new microvessels that sprout from pre-existing endothelial cells to re-establish blood flow to the infarcted myocardium, making it a promising therapeutic strategy for MI [7]. While endothelial cells can self-activate to promote proliferation after injury, other cell types such as macrophages mediate endothelial cell activation and the formation of new capillaries [7]. Anti-inflammatory macrophages secrete factors that promote proliferation of both fibroblasts (TGFβ1) and endothelial cells (vascular endothelial growth factor, VEGF) to form granulation tissue [3]. The role of cardiac fibroblasts in regulating angiogenesis in the heart has not been thoroughly investigated.

The goals of this study were to combine transcriptomics and cell physiology to provide a map of fibroblast phenotypes in response to MI and to determine the mechanisms of the infarct fibroblast in mediating inflammation and angiogenesis. We analyzed transcriptomic changes at MI days 1, 3, and 7 which represented the early inflammatory, proliferative, and maturation phases of cardiac wound healing. We hypothesized that fibroblasts undergo a spectrum of phenotype states over the course of MI from early onset to scar formation. This cell phenotype range begins with resident cells maintaining homeostasis and ends with a new type of resident cell that maintains the MI reset in homeostasis. To our knowledge, this is the first study to detail the full transcriptome changes that occur in cardiac fibroblasts to mediate MI inflammation and angiogenesis.

## Materials and methods (detailed methods in supplemental materials)

### Animal use

All procedures involving animals were approved by the Institutional Animal Care and Use Committee at the University of Mississippi Medical Center. A total of 69 C57BL/6 J adult (3–6 months old) male mice were included, 60 for transcriptome analysis, and 9 for multiplex immunofluorescence. Groups were randomly assigned prior to the surgeries by one investigator (KYDP) and another investigator (YM) performed the majority of surgeries. To reduce animal use as recommended by the National Centre for the Replacement, Refinement, and Reduction of Animals in Research [46], the fibroblasts used in this study were obtained from a subset of the same mice used for macrophage isolation [36].

### Coronary artery ligation

To produce permanent MI, mice underwent coronary artery ligation surgery as described previously and according to the Guidelines for Experimental Models of Ischemia and Infarction [10, 21, 24, 31, 36]. Mice were anesthetized with 2% isoflurane, intubated, and ventilated. The left coronary artery was ligated with 8-0 suture, and MI was confirmed by LV blanching and ST-segment elevation on the EKG. Buprenorphine (0.5 mg/kg body weight) was administered to the mice immediately before surgery. Mice not receiving surgery were used as no MI controls (day 0), as sham-operated mice do not differ in physiology or inflammatory gene expression from non-surgical controls [18].

### Echocardiography and necropsy

LV physiology was determined by transthoracic echocardiography (Vevo 2100, VisualSonics; Toronto, CA) as described before and according to the Guidelines for Measuring Cardiac Physiology in Mice [10, 21, 27, 31, 36]. Mice were anesthetized under 1–2% isoflurane, and both long- and short-axis images were obtained. Measurements were taken on the terminal day and were averaged from three cardiac cycles for each mouse. Following imaging, the hearts were removed and the left ventricle (LV) divided into remote and infarct (which included border zone) regions. Each region was separately weighed for infarct area estimation.

### Isolation of LV infarct fibroblasts

LV fibroblasts were isolated from the infarct by immunomagnetic separation as described previously [10, 21, 36]. Excised LV tissue was rinsed and immediately minced and digested by collagenase II (Worthington; Lakewood, NJ) and DNase solution in Hanks-buffered saline solution. After digestion, a single-cell suspension was generated and filtered through a 30 µm pre-separation column. Cell suspensions were incubated at 4 °C for 10 min with an anti-Ly6G-biotin antibody followed by anti-biotin microbeads contained in the anti-Ly-6G microbead kit (#130-092-332; Miltenyi Biotech; Bergisch Gladbach, Germany) to remove neutrophils. Cells were incubated with anti-CD11b-microbeads (Miltenyi #130-049-601) for 15 min to remove macrophages. Cells conjugated to the antibody microbeads were separated by magnetic columns (Miltenyi #130-042-201), and the effluent (Ly6G- and Cd11b- cells) was plated in T25 flasks in Dulbecco’s Modified Eagle Medium (DMEM) supplemented with 10% fetal bovine serum (FBS) and 1× antibiotic–antimycotic solution (Gibco #15240-062). Fibroblasts at passage 2 were used for RNA-Sequencing (RNA-seq), proliferation assays, and secretome collection; fibroblasts at passage 3 were used for the migration assay. For cell and secretome collection, 4 × 10^4^ cells were seeded into 6-well plates and grown to 80% confluence in DMEM supplemented with 10% fetal bovine serum (FBS). Cells were incubated in DMEM with 0.1% FBS for 16 h, after which the media was replaced with fresh media and collected after 24 h. The cell fraction was collected for RNA-seq, and the secretome was collected for mass spectrometry and immunoblotting examination and endothelial cell stimulation experiments.

### RNA-seq

Fibroblasts were pooled to obtain three biological replicates for each day. Sample sizes were: day 0-*n* = 10 mice/pool; day 1-*n* = 3–6 mice/pool, day 3-*n* = 3 mice/pool, and day 7-*n* = 2–3 mice/pool. Whole transcriptome analysis was performed as described previously [21, 31, 36]. RNA was extracted using the Pure Link RNA Mini Kit (Ambion; Foster City, CA, USA) according to the manufacturer’s instructions and assessed for quality control parameters of minimum concentration and size range. The cDNA libraries were developed using the TruSeq Total Stranded RNA with RiboZero Kit (Ambion), Set-A, quantified with the Qubit System (Invitrogen; Carlsbad, CA, USA), and assessed for quality and size with the Experion DNA 1 K Chip (Bio-Rad; Hercules, CA). All samples passed quality control standards of minimum concentration and RNA Quality Indicator > 9, with discrete 18S and 28S bands. The library of *n* = 12 pooled samples was sequenced using the NextSeq 500 High Output Kit (300 cycles, paired end 100 bp) on the Illumina NextSeq 500 platform (Illumina; San Diego, CA, USA). Sequenced reads and Fastq sequence files were used to align reads to the reference genome USCS-GRCm38/mm10 in the RNA-Seq Alignment Application with STAR aligner using Illumina Cloud Computing Platform. Fragments Per Kilobase of transcript per Million mapped reads (FPKM) values of reference genes and transcripts were generated using Cufflinks 2.

### Bioinformatic analyses

Analyses tools available in the online resource Metaboanalyst 3.0 (http://www.metaboanalyst.ca/) and GraphPad Prism were used for graphical and statistical analyses. FPKM values were uploaded into Metaboanalyst, and one-way ANOVA with Tukey’s post hoc test was performed to determine differentially expressed genes (defined as *p* < 0.05). Important feature analysis was used to identify the top five ranked genes at each MI day. Partial least-squares determinant analysis (PLS-DA) and heat mapping were used to evaluate changes in signaling genes. To assess differential expression at individual MI days, volcano plot analysis was performed comparing the MI day to day 0, with a cut-off fold-change threshold of > 1.2 and *p* < 0.05. A gene correlation network was constructed using the software Graphia Pro (Kajeka; Edinburgh, UK) and a Pearson correlation threshold of *r* > 0.95. The resultant graph was subjected to Markov clustering analysis to identify co-expression modules. Enrichment analysis for differentially expressed genes from each cluster was performed using Enrichr (http://amp.pharm.mssm.edu/Enrichr/) for both Panther Pathways and Gene Ontology (GO) Biological Processes. For MI signaling profiles, we performed partial least-squares discriminant analysis (PLS-DA) and heat mapping for receptors and intracellular signaling genes.

### RT-PCR validation

Quantitative RT-PCR was used as a secondary validation of gene expression in the same samples used for RNA-Seq. Extracted RNA from cardiac fibroblasts was used to synthesize cDNA with the High Capacity RNA-to-cDNA kit (Applied Biosystems 4387406), and RT-PCR was performed using the Taqman Gene Expression Assay (Applied Biosystems). *Acta2, Col3a1,* and *Postn* were chosen as known fibroblast markers, while *Cx3cl1* and *Mmp14* were chosen to represent genes differentially expressed at different time points. Gene expression was calculated as 2^−Δ*C*t^ to the housekeeping gene Hprt1 [26]. RT-PCR experiments were performed according to the Minimum Information for Publication of Quantitative Real-Time PCR Experiments (MIQE) with the exception that *Hprt1* was used as the housekeeping gene [19].

### Fibroblast cell physiology

Fibroblast proliferation was assessed by bromodeoxyuridine (BrdU) enzyme-linked immunosorbent assay (Roche Diagnostics #11647229001; Mannheim, Germany) as described previously using the same pooled samples for RNA-Seq at passage 2 [10, 21]. Cells were seeded into 96-well plates and 24 h later serum starved for 16–18 h. The plate was then incubated for 24 h in DMEM and antibiotics with either 0.1% or 10% FBS. Cells were incubated with BrdU (10 µM), fixed, and incubated with an anti-BrdU antibody. Absorbance at 370 nm and 492 nm was determined using a microplate reader, with measurements taken at 30 min and subtracting the limit value of 492 nm from 370 nm. Fibroblast migration was determined by electrical-cell impedance sensing (ECIS; Applied Biophysics) cells as described previously [10, 21]. Cells (4 × 10^4^) were plated in 96-well plates with gold-film surface electrodes and cultured for 48 h. Cell monolayers were wounded, and rate of migration was assessed as change in impedance over time.

### Multiplex immunofluorescence

LV mid-sections were fixed in 10% zinc-buffered formalin, paraffin-embedded, and sectioned at 5 µm. Slides were deparaffinized and rehydrated. Immunohistochemical staining was performed using the Opal 7-color Automation Kit (Perkin Elmer #NEL80100KT; Boston, MA, USA). Sections were stained with the following primary antibodies conjugated to Opal fluorophores: PDGFRα (1:100, R&D Systems; #AF1062; Minneapolis, MN) conjugated to Opal 520, and CX3CL1 (1:500; R&D #MAB571), CCL5/RANTES (R&D #AF478), VEGF (Abcam #ab51745), or thrombospondin 1 (Thbs1; 1:100; R&D Systems #AF3074) conjugated to Opal 650. Nuclei were counterstained with DAPI. Opal 520 and 650 fluorophores were visualized with FITC and Cy5 channels, respectively. Images were obtained using the Mantra System (Perkin Elmer) and quantified using inForm software (Perkin Elmer).

### Mass spectrometry

Fibroblast secretomes (500 µl) from day 0 or MI day 7 were cleaned using Sep-Pak Vac C18 cartridge (Waters, Milford, MA), and then reduced, alkylated, and trypsin-digested into peptides. The peptides were cleaned again using another Sep-Pak Vac C18 cartridge (Waters, Milford, MA) and analyzed label-free by liquid chromatography–tandem mass spectrometry using a Q Exactive (ThermoFisher, Waltham, MA, USA). A 15 cm × 75 μm C18 column (5 μm particles with 100 Å pore size) was used and the nano-UPLC ran at 300 nL/min with a 150-min linear acetonitrile gradient (from 5 to 35% B over 150 min; *A* = 0.2% formic acid in water; *B* = 0.2% formic acid in 90% acetonitrile). Tandem mass spectrometry (MS/MS) was set with an exclusion of 25 s, and the samples were run with high-energy collisional dissociation fragmentation at normalized collision energy of 30 and an isolation width of 2 m/z. The resolution setting was 70,000 for target values of the MS at 1e6 ions and in MS2 at resolution setting of 17,500 for 1x10^5^ ions. The identified peptides were quantified using spectral counting and total spectral counts of each sample were used for normalization, and a sample report was generated in Scaffold version 4.8.7 (Proteome Software, Portland, OR). Data (quantitative values of normalized total spectra) were analyzed by *t* test. The mass spectrometry proteomics data have been deposited to the ProteomeXchange Consortium via the PRIDE partner repository with the dataset identifier PXD011778 [64].

### Immunoblotting

Immunoblotting was performed according to the Guidelines for authors and reviewers on antibody use in physiology studies [5]. Secretome (20 µl) was separated by 4–12% Criterion^™^ XT Bis–Tris gels (Bio-Rad) and transferred to nitrocellulose membranes (Bio-Rad). Total protein was stained with MemCode™ Reversible Protein Stain Kit (Thermo Scientific #24580; Waltham, MA). Membranes were blocked for 1 h in 5% milk at room temperature and incubated with a primary antibody against Thbs1 (R&D Systems; #AF3074; 1:500) at 4 °C overnight. Recombinant Thbs1 (R&D Systems #7589-TH; 10 ng) was used as a positive control. Membranes were washed and incubated with secondary antibody (Vector #PI-1000; Malvern, PA, USA) at room temperature for 2 h. Images were detected using ECL Prime Western Blotting Detection Substrate (Amersham; Little Chalfont, UK). Protein expression was analyzed by densitometry using IQ-TL image analysis software (GE Healthcare; Waukesha, WI) and normalized to the total protein.

### Angiogenesis assay

Mouse microvascular endothelial cells (Cell Biologics #C57-6024; Chicago, IL, USA; 2.5 × 10^4^ cells/well, *n* = 2 technical replicates per condition) were seeded in 48-well plates coated with basement membrane extract (Matrigel^®^ BD #354230; Franklin Lakes, NJ, USA). Cells were stimulated with either basal endothelial cell medium (CellBiologics #M1168), basal medium supplemented with 10% cardiac fibroblast secretome from the same samples used for RNA-Seq (*n* = 3 biological replicates), or endothelial growth medium (basal medium supplemented with VEGF, endothelial cell growth supplement, heparin, epidermal growth factor, hydrocortisone, l-glutamine, antibiotics, and FBS). Thbs1 was inhibited with a blocking antibody (10 µg/ml; Novus Biologicals #100-2059) as described previously [32]. As negative controls, endothelial cells were stimulated with the Thbs1 blocking antibody alone, an IgG1 isotype control (R&D #MAB002) alone, or basal medium diluted 1:10 with DMEM + 0.1% FBS. Images were acquired at 10X magnification following 22 h of stimulation. Angiogenesis variables were quantified using the ImageJ Angiogenesis Analyzer feature (http://image.bio.methods.free.fr/ImageJ/?Angiogenesis-Analyzer-for-ImageJ&artpage=2-6).

### Statistics

Statistical analysis was performed according to the guidelines, Statistical Considerations in Reporting Cardiovascular Research [25]. All experiments were performed and analyzed in a blinded design. Data are presented as mean ± SEM unless otherwise noted. Survival rate was analyzed by Kaplan–Meier survival analysis. For physiology assessment, multiple group comparisons were made using one-way ANOVA followed by Newman–Keuls post hoc test. Statistics and bioinformatics for the RNA-seq are described above. RNA-seq comparisons to quantitative RT-PCR were made by Pearson’s linear regression analysis. Cell physiology comparisons were analyzed by two-tailed unpaired *t* test or one-sample *t* test. A value of *p* < 0.05 was considered statistically significant.

## Results

### Fibroblasts continually polarize over the MI spectrum

#### Proof of successful MI

Survival for the MI day 7 cohort was 52% (13/25), with 58% of mortality (7/12) due to cardiac rupture assessed at autopsy **(**Online Resource 1a). Infarct area (percent of LVI mass to total LV mass) did not differ between days 1, 3, and 7 (Online Resource 1b), indicating uniform infarct sizes across times and in line with the immediate myocyte necrosis that occurs after MI. As expected, MI led to infarct LV wall thinning, dilation, and decreased fractional shortening (Online Resource 1c–f). This is similar to what we and others have previously reported for mouse MI [9, 10, 21, 31, 66, 73].

#### MI differential gene expression and enrichment

**Fig. 1:**
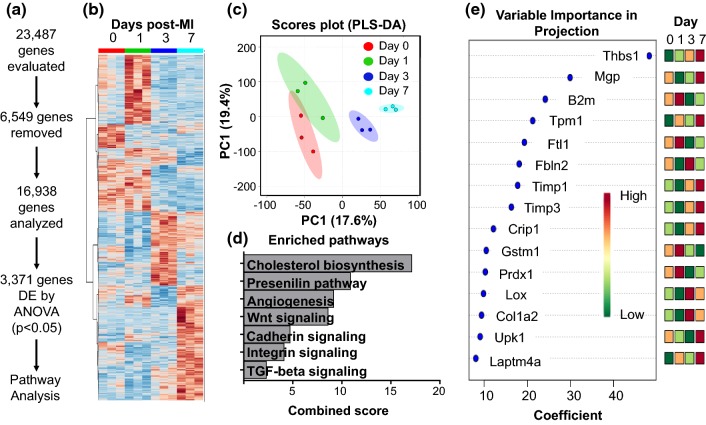
**a** Workflow for analyzing differential gene expression. Of 23,487 genes sequenced, 175 were duplicates and 6,374 did not meet quality control criteria and were removed, leaving 16,938 for analysis. Of the remaining genes, 3,371 were differentially expressed by one-way ANOVA with Tukey’s post test (*p* < 0.05). **b** Heat map displaying differentially expressed genes across the MI time course. **c** Partial least-squares discriminant analysis (PLS-DA) plot. MI day 1 showed overlap with day 0, while days 3 and 7 were unique. **d** Enrichment analysis (Panther) for significant pathways across the MI time course. **e** Top 15 genes ranked by feature analysis and relative expression across each day (red—high; green—low)

Of 23,487 genes sequenced, 175 were duplicates and 6374 were removed either because all FPKM values were 0 or there were less than 3 replicates with values greater than 0 for any one group, leaving 16,938 genes for analysis (Fig. 1a; Online Table 1a). The FPKM values for all genes are displayed in Online Table 1. Of these, 3371 were differentially expressed by one-way ANOVA with Tukey’s post hoc test (Fig. 1a, b). Cell-specific markers for fibroblasts were highly expressed, while markers for other cell types were nearly undetectable (Online Resource 2a). *Acta2* expression positively correlated with time after MI (*r* = 0.70, *p* = 0.01; Online Resource 2b). By mass spectrometry analysis of the day 0 and MI day 7 fibroblast secretomes, collagen I alpha 1 and 2 chains, secreted protein acidic and rich in cysteine (SPARC), and lysyl oxidase were significantly increased in the day 7 MI fibroblast secretome, while periostin showed no change (Online Resource 2b). Periostin gene expression was significantly increased in the LV infarct at days 1, 3, 5, and 7 (Online Resource 2c). Our results indicate the fibroblast activation marker collagens and collagen cross-linking enzymes are regulated at the protein level in MI cardiac fibroblasts.

For RT-PCR confirmation, four of the five genes (all except MMP-14) showed strong correlations between RNA-seq and RT-PCR (Online Resource 3). PLS-DA (Fig. 1c) indicated that each MI day was a separate cluster, with MI day 7 being the most separated from the day 0 no MI control. Significantly enriched pathways by Panther (all *p* < 0.05) are displayed in Fig. 1d. The top 15 genes ranked by important gene feature analysis are displayed in Fig. 1e, with the top-ranked gene being *Thbs1*.

**Fig. 2:**
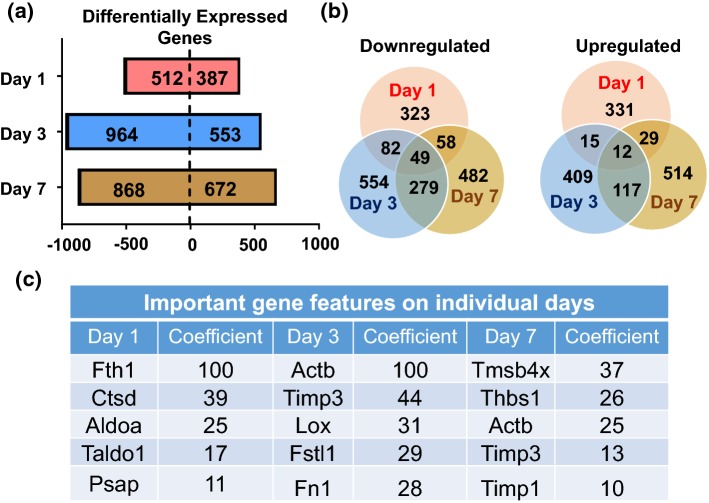
MI yields distinct time-dependent gene expression profiles in cardiac fibroblasts. **a** Differentially expressed genes (upregulated or downregulated) at each MI day. **b** Venn diagrams indicating expression overlap among MI days. **c** Important feature analysis for the top five upregulated genes at each MI day

By Venn diagram analysis of fold changes compared to day 0 with a fold-change cut-off of 1.2 and *p* < 0.05, (Fig. 2a), 387 genes were upregulated and 512 were downregulated at MI day 1, 553 were upregulated and 964 downregulated at MI day 3, and 672 upregulated and 868 downregulated at MI day 7. Very few of the top-ranked genes overlapped among days, with the majority being unique to only one time point (Fig. 2b). The top 5 genes at each MI day ranked by variable importance in projection coefficients are displayed in Fig. 2c.

**Fig. 3:**
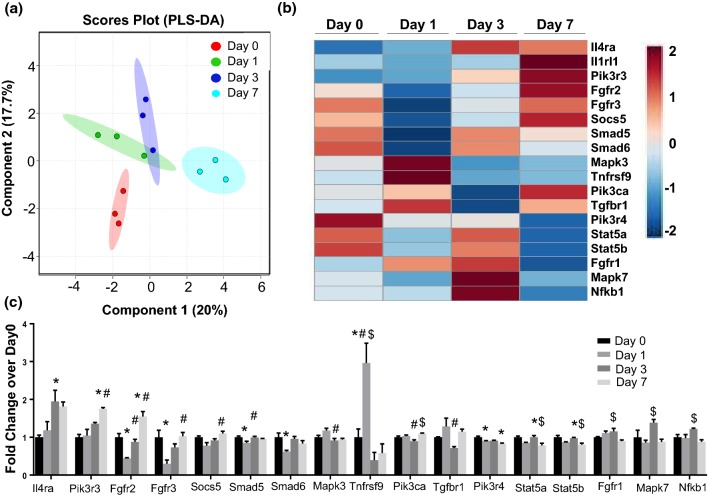
MI cardiac fibroblast signaling profiles. **a** Partial least-squares discriminant analysis (PLS-DA) for the most upregulated changes in signaling genes. **b** Heat map of differentially expressed signaling genes. **c** Gene expression (fold change over day 0) for differentially expressed signaling genes. *n* = 3 per group. *p < 0.05 versus day 0, ^#^*p* < 0.05 versus MI day 1, and ^$^*p* < 0.05 versus MI day 3

PLS-DA analysis and heat mapping for intracellular signaling genes (including *FGF, IL*-*1, IL*-*4, MAPK, NFκB, PI3K, Smad, Socs, STAT, TGFβ1,* and *TNF*) revealed that all MI days displayed unique signaling profiles compared to day 0, with MI days 1 and 3 showing the greatest overlap (Fig. 3a). By examining differentially expressed signaling genes in the heat map (Fig. 3b, c), day 0 fibroblasts use *Pik3r4* and *Stat5* signaling, while MI day 1 fibroblasts use *Tnfrsf9* (CD137) signaling to induce inflammation [20], MI day 3 fibroblasts use interleukin-4 receptor alpha (*Il4ra*) signaling to stimulate anti-inflammatory and pro-fibrotic wound healing [44], and MI day 7 fibroblasts use *Pik3r3* signaling to mediate TGFβ1 effects [65] and *Fgfr2* to regulate *PI3K* signaling [42].

#### Cell-surface marker analysis

Using Enrichr, upregulated genes that encode for cell-surface proteins were compared across MI days to determine genes that distinguish fibroblasts and MI day. A total of 28 genes were selectively upregulated at MI day 1, 32 at MI day 3, and 31 at MI day 7. A total of 314 genes were all elevated with MI and did not change across MI time, and these can be considered potential pan MI fibroblast cell-surface markers. The top 25 ranked genes for each time are listed in Online Table 2. Recent mapping of the human cardiac proteome across cell types revealed a number of novel cardiac fibroblast cell-surface markers, including *Acvr1*, *Met*, *Npr3,* and *Ror1* [11]. Our results indicate that *Met* and *Npr3* were not affected by MI, while *Acvr1* and *Ror1* were both upregulated at MI day 7. These results provide a framework for identifying novel cardiac fibroblast cell-surface markers in the healthy and injured myocardium.

#### Cluster analysis of differentially expressed genes

**Fig. 4:**
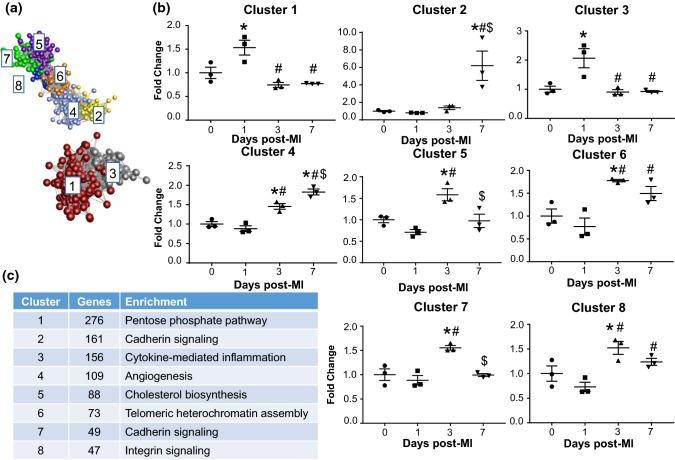
Distinct gene expression patterns by clustering. **a** Markov clustering analysis revealed eight distinct clusters of MI gene expression patterns. **b** Average fold change of genes in each cluster. **c** Number of genes and major enriched pathway for each gene cluster. *n* = 3 per group. *p < 0.05 versus day 0, ^#^*p* < 0.05 versus MI day 1, and ^$^*p* < 0.05 versus MI day 3

We used Markov clustering of the gene correlation network to assess gene expression patterns over the MI time course. Differentially expressed genes fell into one of eight unique pattern clusters (Fig. 4a, b). Cluster 1 (276 genes) and 3 (156 genes) both represented genes upregulated only at day 1. The top enriched processes (Fig. 4) for these two clusters were pentose phosphate pathway and cytokine/chemokine-mediated inflammation, corroborating the results from Fig. 2a. Cluster 5 (88 genes) and 7 (49 genes) were increased at day 3 only, and were enriched for cholesterol biosynthesis (cluster 5) and cadherin signaling (cluster 7). Cluster 4 genes (109 genes) were upregulated at day 3 and further increased at day 7, and were enriched for angiogenesis. Cluster 2 genes (161 genes) were upregulated at day 7, and were enriched for cadherin signaling.

#### Day 0 fibroblasts were homeostatic

Cardiac fibroblasts in the uninjured heart were not quiescent. Rather, they served as sentinel cells to maintain the homeostasis of the myocardium by producing replacement ECM at low concentrations necessary for normal turnover [6]. We developed a list of pan fibroblast genes, which included genes that did not change across any of the times and ranked by FPKM value; the top 500 are shown in Online Table 3. Entering the top 500 ranked genes into the Mouse Gene Atlas (http://www.mouseatlas.org/) revealed close alignment with mouse embryonic fibroblasts and osteoblasts (Online Table 4). The major enriched pathways represent pathways that are highly active in fibroblasts, regardless of injury state, including integrin signaling and glycolysis. To characterize the day 0 phenotype, we analyzed genes that were high only at day 0 (genes significantly decreased at all MI days, *n *= 63 genes). By Panther analysis, day 0 genes were enriched for 5-hydroxytryptamine degradation, mannose metabolism, and de novo pyrimidine ribonucleotide biosynthesis **(**Online Table 5).

#### MI day 1 fibroblasts demonstrated a pro-inflammatory and leukocyte-recruiting, pro-survival, and anti-migratory signature (Fig. 5, Online Table 6)

**Fig. 5:**
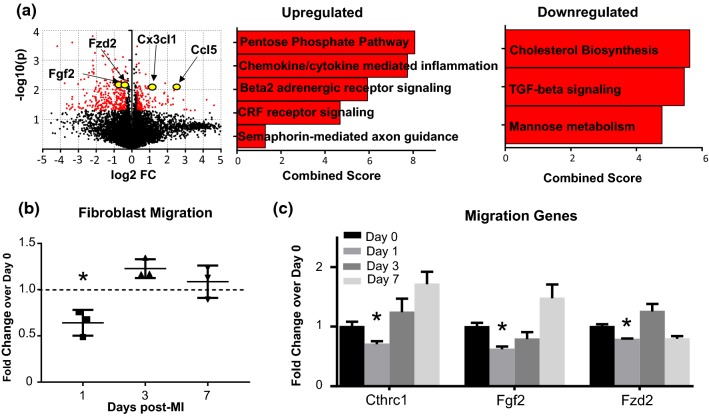
MI day 1 fibroblasts display a pro-inflammatory phenotype profile. **a** Volcano plot and enriched processes for upregulated and downregulated genes. **b** Fibroblast migration was significantly decreased at MI day 1 by one-sample *t* test. **c** Migration-related genes were significantly decreased at MI day 1 by unpaired *t* test. *n* = 3 per group. *p < 0.05 versus day 0

Upregulated and downregulated genes are displayed by volcano plot (Fig. 5a). By Panther pathway analysis, chemokine- and cytokine-mediated inflammation was a major upregulated pathway. Interestingly, while *Tgfb1* was increased at MI day 1 (Online Resource 4a), TGFβ signaling was downregulated (Fig. 5a). By GO pathway evaluation, regulation of T helper 17 cell lineage commitment (i.e. *Il23a*), positive regulation of memory T cell differentiation, negative regulation of interleukin-10 production, and positive regulation of macrophage chemotaxis (i.e., *Ccl5*, *Csf1,* and *Cx3cl1*) were upregulated biological processes (Online Table 6), indicating that MI day 1 fibroblasts provide leukocyte-recruiting signals to bring cells into the infarct region [68]. Out of ten apoptosis genes evaluated, only one, the anti-apoptotic *Bcl2*, was elevated at MI day 1 (Online Resource 4b), indicating pro-survival signaling. Fibroblast migration rates were significantly decreased at MI day 1 (Fig. 5b), and expression of migration-related genes *Cthrc1*, *Fgf2*, and *Fzd2* was significantly decreased (Fig. 5c).

#### MI day 3 fibroblasts demonstrated a proliferative, pro-fibrotic, and pro-angiogenic signature

**Fig. 6:**
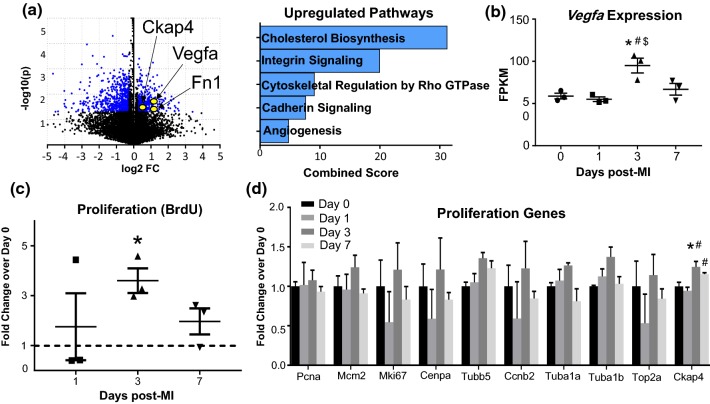
MI day 3 fibroblasts had a proliferative and pro-angiogenic phenotype profile. **a** Volcano plot and major upregulated pathways. **b** *Vegfa* expression was increased only at MI day 3. **c** Fibroblast proliferation by BrdU assay was increased by *t* test. **d** Of ten proliferation genes evaluated, Ckap4 increased at MI day 3. *n* = 3 per group. *p < 0.05 versus day 0, ^#^*p* < 0.05 versus MI day 1, and ^$^*p* < 0.05 versus MI day 3

Upregulated and downregulated genes are plotted by volcano plot (Fig. 6a). By Panther pathway analysis (Fig. 6b), integrin signaling (i.e. *Fn1* and *Itga5*), cadherin signaling (*Cdh2*), and angiogenesis (i.e., *Vegfa*) were major upregulated pathways (Online Table 5). By GO analysis, ECM organization (i.e., *Fn1* and *Lox*) and cell migration involved in sprouting angiogenesis and blood vessel endothelial cell migration (i.e. *Slit2, Ephb4,* and *Vegfa*) were major upregulated biological processes (Online Table 6). *Vegfa* was significantly increased only at MI day 3. MI Day 3 fibroblasts were more proliferative than day 0 (Fig. 6c). The increase in fibroblast proliferation after MI is similar to what we and others have shown [13, 54]. Of the ten proliferation markers examined (Fig. 6d), the recently identified maker of fibroblast proliferation *Ckap4* was elevated at MI day 3 [14]. In addition, expression of *Casp3* was significantly reduced at MI day 3 (Online Resource 4b), indicating continuation of the anti-apoptotic and pro-survival signature. We observed robust changes in genes encoding for cholesterol biosynthesis (decreased at MI day 1 and increased at MI day 3). The increase in genes involved in cholesterol metabolism coincide with increased cell proliferation at MI day 3, which requires cholesterol biosynthesis to support new cell membrane formation, proper function of new cell membrane proteins, and lipid rafts/caveolae to mediate signaling pathways involved in cell proliferation [2, 51].

#### MI day 7 fibroblasts demonstrated a homeostatic-like, anti-angiogenic signature

**Fig. 7:**
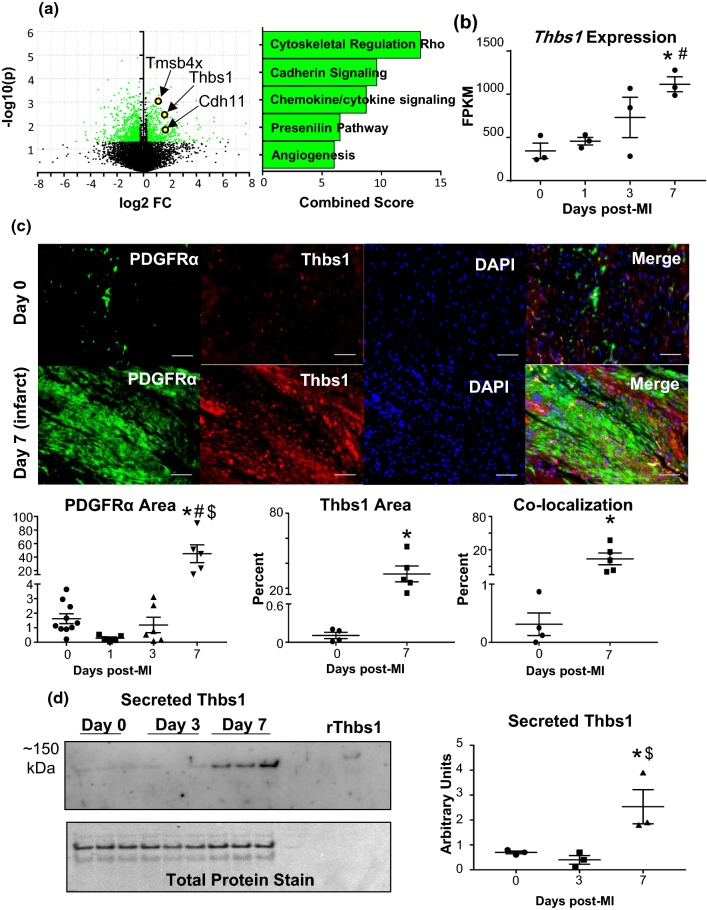
MI day 7 fibroblasts showed an anti-angiogenic myofibroblast phenotype profile. **a** Volcano plot and major upregulated processes. **b** *Thbs1* expression was significantly increased; *n* = 3 per group. **c** LV infarct region from MI day 7 stained for cardiac fibroblast marker PDGFRα (green), Thbs1 (red), and DAPI (blue). Thbs1 was significantly increased within regions staining positive for PDGFRα; *n* = 4 day 0, *n* = 5 MI day 7. **d** Immunoblot analysis of Thbs1 in cardiac fibroblast secretome. MI significantly increased cardiac fibroblast secretion of Thbs1 at MI day 7; *n* = 3 per group; densitometry normalized to total membrane stain. *p < 0.05 versus day 0; ^$^*p* < 0.05 versus MI day 3

Upregulated and downregulated genes are plotted by volcano plot (Fig. 7a). By Panther pathway analysis (Online Table 5), cadherin signaling (i.e., *Cdh11*) and angiogenesis (i.e., *Pdgfc,* and *Thbs1*) were major upregulated pathways. By GO analysis (Online Table 6), positive regulation of bone mineralization (i.e., *Grem1* and *Tgfb3*), wound healing, and regulation of endothelial cell chemotaxis (*Sema5a*, *Tmsb4x*, and *Thbs1*) were major upregulated processes. At MI day 7, the majority of fibroblasts are transdifferentiated into the myofibroblast phenotype [1]. Several myofibroblast-related genes were elevated only at MI day 7 (Online Resource 4a), including *Cdh2* (N-cadherin) [48, 58], *Cdh11* (cadherin-11) [53], *Itga11* (integrin alpha-11) [1, 57], *Timp1* and *Timp3* [56], and *Tmsb4x* (thymosin beta-4) [4]. We also found that *Wt1* and *Twist1*, markers of epicardial cell origin [52], were increased at day 7 (Online Resource 4c). *Thbs1* was the highest ranked feature for differentially expressed genes by one-way ANOVA, being 3.2-fold increased at MI day 7 over day 0. By MI day 7, the fibroblast signature was moving towards a homeostatic-like phenotype, returning towards the new baseline established by the beginning formation of the infarct scar.

As Thbs1 is a potent inhibitor of angiogenesis, we investigated cardiac fibroblast-derived Thbs1 as a potential factor regulating MI angiogenesis. Platelet-derived growth factor (PDGF) receptor alpha (PDGFRα)-positive cardiac fibroblasts demonstrated robust staining in the infarcted myocardium at MI day 7 relative to day 0 (Fig. 7c) consistent with other reports [15, 34, 35, 45]. Thbs1 immunostaining in cardiac fibroblasts (PDGFRα + cells) was significantly increased in day 7 infarcts (Fig. 7c). By immunoblotting, Thbs1 significantly increased in the secretome from isolated day 7 fibroblasts, indicating that MI day 7 fibroblasts actively secrete Thbs1 (Fig. 7d). CX3CL1 and CCL5 at MI day 1 and VEGF at MI day 3 did not localize to fibroblasts within the infarct, confirming that these proteins were secreted and not intracellular (Online Resource 6).

#### MI fibroblast dampening of angiogenesis is Thsb1-dependent

**Fig. 8:**
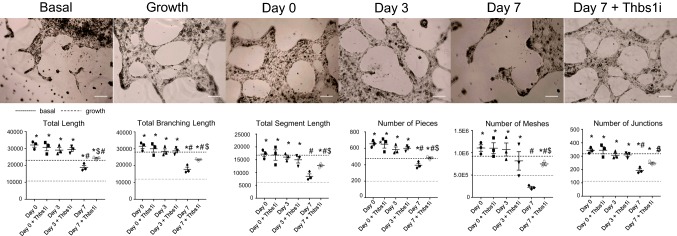
Effects of MI fibroblast secretome on in vitro endothelial cell tube formation. Day 0 and MI day 3 cardiac fibroblast secretome increased, while MI day 7 secretome decreased angiogenic variables relative to the effects of the day 0 secretome. MI day 7 secretome effects were inhibited by a Thbs1 blocking antibody. Scale bar = 200 µm. *n* = 3 per group. *p < 0.05 versus basal, ^#^*p* < 0.05 versus day 0, ^$^*p* < 0.05 versus MI day 7

As angiogenic factors were differentially expressed after MI (Online Table 6), we investigated the effects of the MI fibroblast secretome on endothelial cell tube formation in vitro. The day 0 and MI day 3 secretomes stimulated angiogenesis, as assessed by increased total length, total branching length, total segment length, number of pieces, number of meshes, and number of junctions relative to basal media (Fig. 8). MI day 7 secretome decreased angiogenic variables relative to day 0, indicating that MI day 7 fibroblasts shut off angiogenesis. To determine whether Thbs1 explained the day 7 secretome effects on endothelial cells, Thbs1 was inhibited with a blocking antibody. Inhibition of Thbs1 reduced the effect of the day 7 secretome on angiogenesis variables, indicating that the inhibitory effect of the day 7 secretome on angiogenesis was Thbs1-dependent. Thbs1 inhibition did not alter the effects of the day 0 or MI day 3 secretome, and the Thbs1 blocking antibody alone control, IgG1 isotype antibody control, or the basal media diluted 1:10 with DMEM and 0.1% FBS media only control did not show any differences.

## Discussion

**Fig. 9:**
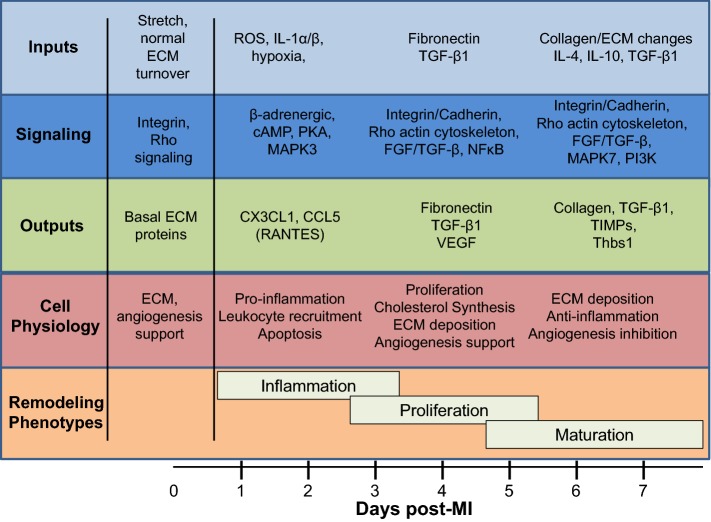
Map of fibroblast phenotypic changes over the MI time course. Fibroblast profiles (inputs, outputs, signaling pathways, and cell physiology) over the major MI remodeling phases (inflammation, proliferation, and maturation)

Cardiac fibroblasts have been implicated in MI remodeling and scar formation [15, 16, 33]. The goals of this study were to map the phenotypes of cardiac fibroblasts over the MI wound healing period and to explore new roles for fibroblasts in regulating inflammation and angiogenesis. The key findings were as follows: 1) day 0 no MI cardiac fibroblasts served as sentinel cells to actively maintain myocardial homeostasis; 2) cardiac fibroblasts underwent distinct transcriptomic changes at MI days 1, 3, and 7, with day 1 fibroblasts showing a pro-inflammatory signature, day 3 fibroblasts showing a proliferative, reparative, and pro-angiogenic profile, and day 7 fibroblasts showing homeostatic-like features and negatively regulating angiogenesis; and 3) the negative regulation of angiogenesis by day 7 fibroblasts occurred through Thbs1 signaling. Our results provide new insights into fibroblast phenotypes across the MI spectrum, with an emphasis on the roles of fibroblasts in regulating inflammation and angiogenesis (Fig. 9).

Identifying pan cell-surface markers for all cardiac fibroblasts has been a considerable challenge [6, 13, 14, 16, 17, 28]. Furthermore, a map of cell-surface markers across the MI injury state is lacking, as fibroblasts may increase or decrease marker expression in response to the changing microenvironment. Recent studies have identified novel markers in human cardiac fibroblasts from non-injured hearts, including the tyrosine kinase *Ror1* and the activin receptor *Acvr1* [11]. Our data validate these findings, as fibroblasts expressed both genes at every time point examined. The selective upregulation at MI day 7 indicates that these two genes may be particularly useful in identifying fibroblasts at MI day 7, when peak myofibroblast activation occurs [13]. Our findings provide a framework for identifying both novel constitutive and inducible markers.

In the absence of injury, the cardiac fibroblast is the major cell type producing and maintaining the ECM. Cardiac fibroblasts also support electro-chemical coupling, myocyte hypertrophy, and maintenance of the vasculature [17, 40]. Integrin signaling was the major enriched process for the pan fibroblast phenotype and was also enriched at MI days 3 and 7, indicating that MI fibroblasts selectively express integrins to adapt to the changing ECM environment. Integrin switching includes *Itga5*, the major fibronectin-binding integrin, at MI day 3, and *Itga11*, the major collagen-binding integrin, at MI day 7 [1]. Inflammation mediated by chemokine/cytokine signaling was enriched in the basal phenotype and at MI days 1 and 7, indicating that fibroblasts cross talk with resident immune cells regardless of injury state [29]. The day 0 secretome stimulated tube formation in vitro, indicating that fibroblasts in the uninjured heart play an active role in promoting a healthy vasculature.

At MI day 1, fibroblasts displayed an inflammatory signature, consistent with other reports that fibroblasts initiate and contribute to inflammation following injury [22, 38]. CCL5 and CX3CL1 are pro-inflammatory chemoattractants for monocytes and neutrophils [68]. Cardiac fibroblasts produce CX3CL1 in response to interferon γ stimulus [43]. Expression of *Il23a*, a pro-inflammatory cytokine that activates T helper 17 cells, was also increased at MI day 1 [67]. Colony stimulating facor-1 is critical for macrophage recruitment after MI [69]. TGFβ1 is an important anti-inflammatory cytokine that promotes fibroblast activation to myofibroblasts. TGFβ signaling was a major downregulated process, indicating that MI day 1 fibroblasts are unresponsive to increased TGFβ1 expression. Our results indicate that, in addition to other cell sources, fibroblasts are a source of pro-inflammatory chemokines and cytokines to recruit inflammatory cells to the infarct region. *Bcl2* was highly increased at MI day 1, indicating that fibroblasts respond to ischemia by promoting cell survival signaling [13].

At MI day 3, fibroblasts showed a proliferative, pro-fibrotic, and pro-angiogenic profile. Cholesterol biosynthesis, a major upregulated process at day 3, is important in supporting cardiac fibroblast proliferation. Treatment of mice with simvastatin, a potent inhibitor of cholesterol biosynthesis, decreases LV collagen deposition and fibroblast activity after MI [55], and in vitro, simvastatin induces cardiac fibroblast apoptosis, with a stronger effect on quiescent fibroblasts than activated myofibroblasts [8]. In line with these results, cholesterol biosynthesis was elevated at MI day 3, at which point fibroblasts were highly proliferative [13]. Thus, cholesterol biosynthesis may be required for MI fibroblast proliferation during the early granulation phase prior to scar formation and maturation.

MI day 3 fibroblasts showed a pro-angiogenic profile. Cardiac fibroblasts secrete several factors that regulate angiogenesis, including VEGF [40, 60, 71, 72]. The positive regulation of angiogenesis at MI day 3 coincides with the peak of vessel formation and endothelial cell proliferation in the mouse, indicating that fibroblasts are a significant in vivo contributor to endothelial cell activation [63].

At MI day 7, fibroblasts displayed a homeostatic-like, anti-angiogenic signature. The epicardial cell markers* Wt1* and* Twist1* were significantly increased, consistent with other reports, indicating that MI day 7 fibroblasts are derived from epicardial progenitors that underwent epithelial-to-mesenchymal transition [13, 47, 52]. In contrast to MI day 3, day 7 fibroblasts showed an anti-angiogenic profile. Fibroblast-derived factors can inhibit angiogenesis [30]. Our results suggest that day 7 fibroblasts may inhibit angiogenesis by altering the balance between pro-angiogenic (*Vegfa*) and anti-angiogenic (*Cxcl14*, *Sema3a, Lypd1*, and *Thbs1*) soluble mediators. Thbs1 has been heavily implicated as a potent inhibitor of angiogenesis, mainly through pro-apoptotic and anti-VEGF actions on cardiac microvascular endothelial cells [71]. Thbs1 expression is induced in the infarcted heart by macrophages, platelets, and fibroblasts, peaking 24 h and remaining elevated as long as 28 days after MI in the rat heart [12, 50, 70]. Mice lacking *Thbs1* show a prolonged inflammatory response and expansion of granulation tissue following MI [12]. Our results indicate that cardiac fibroblasts secrete Thbs1 at day 7 but not day 3, and that blocking Thbs1 reverses the anti-angiogenic properties of the MI day 7 secretome, indicating that fibroblast-derived Thbs1 tempers excessive angiogenesis.

Fibroblasts from day 0 healthy hearts potently stimulated angiogenesis, indicating that rather than being quiescent, fibroblasts in the healthy heart actively support angiogenesis and vessel homeostasis. Our results indicate a dichotomous role in fibroblast regulation of angiogenesis following MI injury, as day 0 and day 3 fibroblasts promoted angiogenesis, while day 7 fibroblasts show decreased angiogenic potential. Angiogenesis is typically considered beneficial for MI remodeling and correlates with better outcomes [31], thus targeting cardiac fibroblasts could be a potential therapeutic strategy to improve MI angiogenesis. MI day 7 fibroblasts may temper excessive and uncontrolled angiogenesis that could otherwise perturb proper scar formation [49]. Anti-inflammatory and pro-reparative resident macrophage and circulating monocyte populations promote angiogenesis in the MI heart. It is possible that macrophages are the predominant cell types in the MI heart that promote angiogenesis, while fibroblasts dial up or down as needed [39]. In addition to inhibiting angiogenesis, Thbs1 promotes the resolution of inflammation, suggesting that anti-inflammatory signals such as TGFβ1 may induce Thbs1 expression and promote anti-angiogenic actions during scar maturation [12].

Our results reveal that some concepts of classical fibroblast activation are in play after MI, while others have been preconceived ideas. Of note, we did not observe the expected increase in the myofibroblast marker *Postn* in MI fibroblasts. Periostin gene or protein expression did not change after MI in isolated fibroblasts, while gene expression did increase in the LV infarct at days 1, 3, 5, and 7. A recent report has shown increased *Postn* in the Tcf21+ subpopulation of cardiac cells examined [13], indicating that a subset of fibroblasts (Tcf21+ cells) and non-fibroblast cells (e.g., smooth muscle cells and macrophages) are the source of periostin. We recently reported on the MI macrophage polarization time course [36]. Tcf21 was expressed in no MI resident cardiac macrophages at 25–50-fold higher levels than MI days 1, 3, or 7 macrophages and lower expression than fibroblasts (all *p* < 0.01), revealing fluidity in cell marker expression across cell types (fibroblasts and macrophages) and across MI times. We evaluated the total fibroblast pool (all fibroblasts adhering to culture dishes), indicating that periostin is a selective marker for a subset of cells. Despite culturing, our cells retained the features of myofibroblast activation including increased collagen, SPARC, and lysyl oxidase secretion. Our results reveal that, rather than a binary on or off pattern,* Acta2* gene expression actually shows a continual increase over time (linear regression correlation coefficient *r* = 0.70, *p* = 0.01), indicating a persistent shift in time to the myofibroblast phenotype. Based on our evaluation, fibroblasts retained the in vivo MI phenotype in vitro through passage 3.

In conclusion, cardiac fibroblasts show distinct transcriptomic profiles at different time points over the first week of MI wound healing. Our findings provide novel insights into the potential mechanisms and pathways that regulate fibroblast physiology during the wound healing phases of cardiac remodeling. Overall, our work suggests that attention to temporal profiles should be given when considering interventions that target fibroblasts.

## Electronic supplementary material

Below is the link to the electronic supplementary material.

Supplementary material 1 (PDF 10492 kb)

Supplementary material 2 (PDF 13 kb)

Supplementary material 3 (PDF 30 kb)

Supplementary material 4 (PDF 21 kb)

Supplementary material 5 (PDF 457 kb)
