# Supplementary material for: Fibroblast polarization over the myocardial infarction time continuum shifts roles from inflammation to angiogenesis
Source: Basic Res Cardiol. 2019 Jan 11;114(2):6. doi: 10.1007/s00395-019-0715-4 (PMC6329742; doi:10.1007/s00395-019-0715-4)
Supplement: Supplementary file 1 — Supplementary material 1 (PDF 10492 kb) [file 395_2019_715_MOESM1_ESM.pdf]

| Online Table 1. FPKM values for each post-MI fibroblast sample. | | | | | | | | | | | | |
|---|---|---|---|---|---|---|---|---|---|---|---|---|
| | 1 | 2 | 3 | 4 | 5 | 6 | 7 | 8 | 9 | 10 | 11 | 12 |
| gene | A D0 | A D0 | A D0 | B D1 | B D1 | B D1 | C D3 | C D3 | C D3 | D D7 | D D7 | D D7 |
| 42430 | 0.1 | 0.0 | 0.1 | 1.0 | 1.2 | 0.0 | 0.1 | 0.1 | 0.0 | 0.1 | 0.1 | 0.1 |
| 42431 | 44.6 | 35.3 | 27.7 | 43.1 | 31.3 | 43.0 | 30.4 | 26.4 | 28.8 | 33.0 | 34.1 | 32.3 |
| 42432 | 10.1 | 10.6 | 11.1 | 7.9 | 13.1 | 7.2 | 13.2 | 11.6 | 12.0 | 8.7 | 8.4 | 10.8 |
| 42434 | 32.3 | 26.1 | 27.6 | 26.7 | 24.7 | 25.4 | 24.5 | 25.8 | 27.5 | 29.8 | 26.6 | 25.1 |
| 42435 | 6.4 | 7.4 | 6.7 | 6.5 | 7.0 | 6.3 | 6.5 | 6.6 | 6.8 | 7.7 | 6.8 | 7.0 |
| 42436 | 17.7 | 17.3 | 18.9 | 16.3 | 17.1 | 16.9 | 16.4 | 17.8 | 17.6 | 18.0 | 18.9 | 18.9 |
| 42437 | 25.4 | 20.6 | 18.3 | 23.6 | 20.6 | 23.7 | 19.1 | 19.4 | 21.8 | 20.6 | 21.4 | 22.6 |
| 42438 | 1.7 | 1.6 | 1.4 | 1.7 | 1.2 | 2.9 | 0.8 | 0.8 | 0.8 | 1.4 | 0.9 | 0.9 |
| 42440 | 0.0 | 0.0 | 0.0 | 0.0 | 0.1 | 0.0 | 0.1 | 0.0 | 0.0 | 0.1 | 0.0 | 0.0 |
| 42614 | 0.4 | 0.2 | 0.3 | 0.3 | 0.3 | 0.7 | 0.2 | 0.6 | 0.3 | 0.1 | 0.3 | 0.3 |
| 42615 | 126.1 | 124.0 | 117.0 | 141.7 | 119.5 | 131.4 | 118.9 | 127.3 | 124.0 | 114.3 | 139.3 | 130.3 |
| 42616 | 0.4 | 0.2 | 0.1 | 0.4 | 0.1 | 0.6 | 0.1 | 0.1 | 0.1 | 0.1 | 0.1 | 0.1 |
| 42617 | 1.0 | 2.6 | 2.6 | 1.3 | 1.3 | 0.5 | 0.5 | 1.0 | 1.0 | 1.2 | 0.8 | 1.1 |
| 42618 | 18.9 | 22.9 | 22.6 | 14.7 | 16.8 | 20.1 | 27.5 | 32.8 | 27.6 | 13.5 | 24.2 | 19.4 |
| 42619 | 1.3 | 1.7 | 2.3 | 1.1 | 1.4 | 1.5 | 1.6 | 2.6 | 1.7 | 1.5 | 1.8 | 1.3 |
| 42620 | 125.0 | 127.3 | 151.2 | 152.4 | 122.0 | 118.7 | 140.0 | 142.7 | 139.0 | 123.3 | 157.3 | 173.2 |
| 42621 | 57.7 | 64.9 | 66.8 | 63.6 | 57.2 | 62.1 | 77.6 | 89.8 | 79.9 | 45.5 | 65.8 | 66.6 |
| 42622 | 22.5 | 20.1 | 15.1 | 27.8 | 24.6 | 23.5 | 27.8 | 17.6 | 23.7 | 28.8 | 26.7 | 25.9 |
| 42623 | 19.9 | 21.2 | 20.9 | 15.9 | 17.7 | 17.0 | 19.0 | 18.0 | 16.8 | 15.6 | 19.1 | 19.5 |
| 42624 | 72.1 | 91.6 | 107.3 | 84.9 | 82.3 | 79.1 | 116.4 | 114.9 | 110.0 | 70.2 | 108.7 | 108.4 |
| 42627 | 0.1 | 0.1 | 0.2 | 0.1 | 0.0 | 0.0 | 0.0 | 0.0 | 0.0 | 0.0 | 0.0 | 0.0 |
| 42628 | 244.6 | 180.8 | 151.8 | 253.7 | 190.6 | 261.4 | 185.2 | 190.1 | 186.8 | 218.8 | 232.0 | 221.6 |
| 0610005C13Rik | 0.1 | 0.0 | 0.0 | 0.0 | 0.1 | 0.2 | 0.1 | 0.1 | 0.2 | 0.0 | 0.1 | 0.2 |
| 0610007N19Rik | 21.5 | 15.3 | 16.6 | 17.8 | 13.2 | 23.4 | 17.0 | 20.3 | 16.2 | 22.3 | 19.0 | 19.6 |
| 0610007P14Rik | 13.4 | 16.7 | 21.6 | 9.0 | 13.4 | 13.7 | 25.1 | 29.3 | 20.2 | 11.5 | 21.8 | 13.9 |
| 0610009B22Rik | 26.7 | 25.4 | 24.2 | 21.3 | 20.3 | 26.9 | 19.0 | 21.8 | 25.0 | 26.6 | 22.4 | 24.5 |
| 0610009D07Rik | 35.5 | 32.7 | 31.4 | 39.3 | 32.4 | 37.2 | 30.1 | 33.5 | 29.9 | 32.9 | 34.7 | 35.9 |
| 0610009L18Rik | 1.8 | 1.6 | 2.0 | 1.8 | 2.3 | 1.8 | 1.6 | 1.7 | 2.0 | 3.1 | 2.0 | 1.8 |
| 0610009O20Rik | 8.6 | 7.5 | 7.2 | 9.4 | 7.1 | 7.6 | 10.6 | 7.8 | 8.1 | 7.8 | 8.8 | 7.2 |
| 0610010B08Rik | 4.2 | 3.9 | 3.1 | 3.3 | 3.3 | 4.9 | 3.0 | 3.2 | 3.5 | 4.8 | 3.7 | 4.6 |
| 0610010F05Rik | 10.9 | 8.7 | 7.6 | 12.4 | 8.9 | 10.1 | 8.3 | 6.5 | 8.1 | 8.5 | 10.8 | 9.3 |
| 0610010K14Rik | 9.3 | 10.0 | 9.8 | 11.2 | 9.0 | 12.9 | 12.0 | 14.8 | 12.3 | 9.2 | 9.1 | 10.9 |
| 0610011F06Rik | 11.6 | 12.9 | 9.6 | 9.6 | 9.2 | 19.9 | 9.2 | 8.0 | 8.6 | 13.3 | 10.0 | 9.3 |
| 0610012G03Rik | 11.0 | 8.5 | 7.0 | 9.7 | 10.0 | 11.8 | 8.2 | 9.1 | 9.5 | 12.2 | 10.3 | 9.9 |
| 0610012H03Rik | 0.0 | 0.0 | 0.1 | 0.0 | 0.0 | 0.0 | 0.0 | 0.0 | 0.0 | 0.0 | 0.0 | 0.0 |
| 0610030E20Rik | 1.9 | 2.6 | 2.6 | 2.1 | 2.1 | 2.2 | 2.0 | 2.4 | 2.3 | 1.8 | 2.0 | 2.2 |
| 0610031J06Rik | 73.2 | 61.9 | 56.2 | 92.4 | 81.4 | 86.1 | 58.4 | 55.4 | 53.1 | 69.0 | 59.3 | 63.2 |
| 0610037L13Rik | 13.3 | 12.8 | 11.1 | 15.4 | 11.9 | 12.9 | 12.3 | 13.6 | 13.0 | 12.1 | 14.9 | 14.3 |
| 0610038B21Rik | 1.3 | 1.2 | 0.8 | 1.3 | 1.0 | 1.2 | 1.1 | 1.0 | 0.7 | 1.0 | 1.2 | 0.5 |
| 0610038L08Rik | 0.0 | 0.2 | 0.0 | 0.0 | 0.0 | 0.4 | 0.1 | 0.1 | 0.0 | 0.0 | 0.0 | 0.0 |
| 0610039K10Rik | 1.9 | 1.7 | 1.2 | 2.1 | 1.8 | 1.3 | 1.9 | 1.8 | 1.6 | 1.9 | 1.2 | 1.4 |
| 0610040B10Rik | 2.3 | 1.8 | 2.4 | 1.5 | 1.7 | 2.3 | 1.0 | 1.1 | 2.8 | 1.9 | 2.1 | 2.1 |
| 0610040F04Rik | 0.0 | 0.0 | 0.0 | 0.0 | 0.1 | 0.0 | 0.0 | 0.1 | 0.0 | 0.1 | 0.0 | 0.0 |
| 0610043K17Rik | 0.1 | 0.1 | 0.5 | 0.4 | 0.1 | 0.3 | 0.1 | 0.0 | 0.0 | 0.2 | 0.1 | 0.4 |
| 1100001G20Rik | 0.0 | 0.1 | 0.6 | 0.0 | 0.7 | 0.0 | 0.4 | 0.6 | 0.1 | 0.3 | 0.2 | 0.1 |
| 1110001A16Rik | 9.1 | 7.0 | 8.9 | 10.7 | 7.5 | 11.8 | 8.0 | 7.8 | 6.7 | 8.4 | 9.1 | 9.5 |
| 1110001J03Rik | 5.2 | 3.6 | 4.0 | 3.3 | 3.3 | 3.7 | 2.7 | 5.8 | 4.4 | 7.2 | 3.4 | 4.4 |
| 1110002L01Rik | 8.5 | 7.3 | 5.9 | 7.8 | 7.0 | 7.3 | 6.5 | 6.1 | 5.5 | 7.2 | 8.0 | 7.1 |
| 1110004E09Rik | 11.3 | 8.4 | 7.7 | 9.8 | 7.3 | 9.4 | 7.5 | 6.3 | 6.9 | 6.9 | 7.5 | 8.7 |
| 1110004F10Rik | 42.8 | 41.9 | 47.4 | 49.5 | 40.2 | 45.1 | 48.3 | 44.1 | 44.6 | 46.0 | 48.7 | 47.9 |
| 1110006O24Rik | 0.4 | 0.4 | 0.3 | 0.4 | 0.8 | 0.5 | 0.3 | 0.3 | 0.4 | 0.5 | 0.5 | 0.3 |
| 1110007C09Rik | 74.9 | 58.3 | 49.1 | 74.4 | 63.7 | 57.3 | 73.1 | 66.4 | 52.2 | 61.1 | 86.2 | 61.0 |
| 1110008F13Rik | 36.3 | 26.8 | 26.7 | 32.9 | 31.6 | 23.7 | 33.4 | 31.3 | 30.7 | 39.2 | 32.3 | 33.0 |
| 1110008J03Rik | 2.6 | 2.5 | 2.7 | 2.2 | 2.4 | 3.2 | 2.4 | 3.2 | 2.2 | 2.6 | 3.3 | 2.2 |

| | | | | | | | | | | | | |
|---|---|---|---|---|---|---|---|---|---|---|---|---|
| 1110008L16Rik | 4.5 | 4.3 | 3.9 | 4.4 | 3.3 | 3.6 | 3.0 | 3.7 | 3.2 | 3.7 | 3.6 | 3.6 |
| 1110008P14Rik | 18.6 | 13.1 | 15.9 | 21.6 | 15.0 | 17.3 | 11.2 | 15.2 | 10.0 | 13.7 | 12.4 | 9.7 |
| 1110012L19Rik | 27.5 | 25.2 | 23.6 | 23.8 | 18.8 | 26.0 | 21.1 | 21.7 | 17.8 | 26.0 | 24.3 | 25.3 |
| 1110017D15Rik | 0.2 | 0.1 | 0.0 | 0.0 | 0.1 | 0.1 | 0.1 | 0.1 | 0.2 | 0.1 | 0.6 | 0.1 |
| 1110018G07Rik | 10.1 | 9.5 | 9.0 | 11.0 | 10.3 | 10.3 | 9.8 | 10.5 | 10.7 | 10.9 | 9.7 | 10.1 |
| 1110019D14Rik | 6.4 | 5.4 | 5.0 | 6.2 | 5.2 | 5.4 | 4.4 | 4.3 | 4.3 | 5.1 | 5.5 | 5.8 |
| 1110020A21Rik | 0.4 | 0.0 | 0.2 | 0.3 | 0.5 | 0.2 | 0.2 | 0.2 | 0.1 | 0.2 | 0.3 | 0.3 |
| 1110032A03Rik | 16.2 | 11.9 | 13.0 | 21.3 | 10.7 | 20.4 | 12.2 | 9.1 | 9.1 | 9.9 | 14.1 | 13.8 |
| 1110034G24Rik | 2.4 | 2.0 | 1.5 | 1.8 | 1.8 | 2.2 | 1.5 | 1.6 | 1.6 | 1.9 | 2.4 | 1.6 |
| 1110035M17Rik | 0.4 | 0.5 | 0.3 | 0.7 | 0.4 | 1.4 | 0.2 | 0.0 | 0.2 | 0.4 | 0.3 | 0.7 |
| 1110036E04Rik | 0.0 | 0.2 | 0.0 | 0.0 | 0.0 | 0.0 | 0.0 | 0.0 | 0.0 | 0.0 | 0.1 | 0.0 |
| 1110037F02Rik | 20.4 | 20.0 | 18.6 | 19.6 | 18.4 | 19.8 | 17.6 | 16.7 | 16.4 | 17.7 | 19.1 | 19.3 |
| 1110038B12Rik | 81.8 | 50.0 | 43.8 | 70.0 | 52.6 | 82.2 | 63.3 | 59.0 | 54.2 | 67.2 | 63.5 | 54.5 |
| 1110038F14Rik | 10.7 | 12.2 | 13.4 | 13.5 | 10.3 | 12.4 | 10.7 | 14.5 | 12.7 | 13.2 | 13.1 | 11.0 |
| 1110046J04Rik | 12.6 | 10.8 | 7.9 | 10.4 | 10.2 | 18.2 | 8.1 | 9.2 | 8.9 | 6.9 | 8.4 | 8.7 |
| 1110051M20Rik | 10.6 | 9.3 | 10.9 | 11.0 | 8.8 | 11.7 | 9.5 | 12.5 | 9.7 | 9.2 | 10.3 | 10.1 |
| 1110054M08Rik | 1.9 | 2.4 | 2.6 | 1.7 | 2.6 | 2.4 | 1.6 | 2.4 | 1.2 | 2.5 | 1.2 | 1.8 |
| 1110057K04Rik | 12.1 | 11.8 | 10.6 | 13.9 | 11.6 | 10.9 | 11.6 | 12.4 | 10.9 | 11.4 | 12.0 | 12.1 |
| 1110058L19Rik | 34.9 | 34.9 | 22.2 | 24.4 | 27.4 | 31.6 | 21.0 | 26.1 | 26.1 | 30.2 | 31.6 | 25.4 |
| 1110059E24Rik | 10.5 | 10.5 | 11.5 | 11.2 | 10.3 | 11.1 | 10.0 | 10.9 | 10.9 | 10.5 | 11.2 | 11.6 |
| 1110059G10Rik | 3.0 | 2.8 | 3.2 | 2.6 | 2.8 | 3.3 | 2.9 | 3.4 | 2.9 | 2.9 | 3.3 | 3.1 |
| 1110059M19Rik | 0.3 | 0.0 | 0.0 | 0.2 | 0.0 | 0.0 | 0.1 | 0.0 | 0.0 | 0.2 | 0.2 | 0.0 |
| 1110065P20Rik | 21.1 | 17.4 | 15.7 | 15.9 | 17.6 | 18.0 | 19.7 | 21.5 | 25.0 | 22.6 | 19.6 | 18.0 |
| 1190002F15Rik | 3.3 | 4.0 | 6.2 | 0.6 | 5.9 | 2.0 | 7.8 | 1.6 | 4.1 | 2.8 | 4.5 | 3.5 |
| 1190002N15Rik | 28.7 | 27.2 | 24.8 | 18.7 | 18.1 | 19.6 | 27.3 | 25.0 | 31.4 | 34.2 | 41.5 | 29.8 |
| 1190003J15Rik | 0.2 | 0.1 | 0.1 | 0.3 | 0.1 | 0.4 | 0.1 | 0.1 | 0.0 | 0.1 | 0.0 | 0.1 |
| 1190005I06Rik | 1.0 | 0.9 | 0.9 | 0.3 | 0.7 | 1.1 | 0.5 | 0.9 | 0.8 | 1.3 | 0.4 | 1.0 |
| 1190007I07Rik | 3.6 | 3.6 | 3.1 | 6.7 | 3.0 | 4.3 | 2.4 | 2.2 | 2.3 | 4.2 | 4.6 | 2.7 |
| 1200011I18Rik | 9.1 | 9.8 | 10.7 | 10.6 | 9.1 | 10.2 | 8.9 | 9.8 | 8.4 | 9.5 | 11.4 | 11.3 |
| 1200014J11Rik | 5.8 | 5.9 | 6.6 | 5.1 | 4.9 | 5.1 | 6.8 | 6.7 | 6.1 | 5.4 | 7.1 | 6.3 |
| 1300002E11Rik | 5.2 | 3.5 | 5.0 | 6.8 | 4.2 | 5.8 | 4.6 | 5.6 | 4.8 | 3.3 | 5.6 | 5.4 |
| 1300002K09Rik | 0.1 | 0.1 | 0.1 | 0.2 | 0.2 | 0.0 | 0.1 | 0.1 | 0.0 | 0.1 | 0.0 | 0.0 |
| 1300017J02Rik | 0.0 | 0.1 | 0.0 | 0.1 | 0.1 | 0.0 | 0.0 | 0.1 | 0.0 | 0.0 | 0.1 | 0.1 |
| 1300018J18Rik | 7.3 | 6.1 | 6.1 | 8.4 | 6.6 | 7.8 | 8.1 | 6.2 | 6.5 | 7.5 | 7.4 | 6.8 |
| 1500002O10Rik | 0.2 | 0.0 | 0.1 | 0.1 | 0.0 | 0.1 | 0.0 | 0.0 | 0.0 | 0.1 | 0.0 | 0.1 |
| 1500004A13Rik | 1.4 | 1.6 | 1.5 | 1.4 | 1.6 | 1.6 | 1.6 | 1.6 | 1.0 | 1.2 | 1.5 | 1.7 |
| 1500009L16Rik | 5.2 | 7.6 | 9.3 | 8.1 | 13.8 | 10.0 | 6.7 | 3.5 | 3.6 | 3.0 | 2.9 | 3.4 |
| 1500011B03Rik | 4.6 | 5.2 | 4.8 | 6.5 | 5.5 | 6.1 | 4.9 | 4.8 | 4.8 | 4.9 | 4.6 | 3.8 |
| 1500011K16Rik | 20.1 | 15.3 | 13.2 | 21.1 | 19.0 | 22.6 | 14.7 | 17.5 | 15.3 | 13.6 | 16.0 | 14.4 |
| 1500012F01Rik | 26.8 | 15.4 | 17.8 | 18.2 | 18.4 | 24.9 | 19.8 | 23.8 | 23.6 | 33.3 | 30.8 | 32.2 |
| 1500015A07Rik | 0.7 | 0.4 | 0.6 | 1.1 | 0.9 | 0.8 | 0.7 | 0.7 | 0.6 | 0.3 | 0.6 | 0.9 |
| 1500015L24Rik | 0.0 | 0.0 | 0.0 | 0.0 | 0.0 | 0.1 | 0.0 | 0.0 | 0.0 | 0.0 | 0.0 | 0.0 |
| 1500015O10Rik | 7.1 | 8.5 | 9.6 | 5.9 | 6.6 | 5.9 | 10.6 | 7.7 | 11.9 | 12.2 | 7.9 | 8.4 |
| 1500017E21Rik | 0.2 | 0.3 | 0.4 | 0.6 | 0.6 | 0.1 | 0.5 | 0.2 | 0.2 | 0.2 | 0.5 | 0.3 |
| 1500032L24Rik | 77.3 | 60.4 | 61.8 | 73.9 | 75.7 | 71.6 | 76.8 | 73.1 | 73.1 | 87.9 | 84.7 | 94.0 |
| 1600002H07Rik | 5.1 | 5.7 | 5.1 | 9.3 | 5.7 | 9.5 | 4.3 | 3.3 | 3.0 | 3.7 | 3.6 | 2.8 |
| 1600002K03Rik | 21.1 | 14.6 | 11.1 | 19.5 | 15.7 | 16.5 | 16.7 | 13.2 | 16.5 | 18.3 | 17.3 | 17.6 |
| 1600010M07Rik | 0.1 | 0.0 | 0.2 | 0.8 | 0.7 | 0.0 | 0.0 | 0.0 | 0.0 | 0.2 | 0.0 | 0.1 |
| 1600012H06Rik | 12.6 | 13.0 | 15.0 | 14.0 | 14.3 | 15.8 | 15.5 | 17.0 | 18.3 | 19.4 | 20.1 | 21.5 |
| 1600014C10Rik | 12.6 | 11.1 | 11.0 | 12.9 | 12.3 | 10.9 | 12.6 | 11.1 | 11.9 | 12.5 | 13.6 | 13.2 |
| 1600014C23Rik | 0.0 | 0.0 | 0.1 | 0.3 | 0.2 | 0.0 | 0.0 | 0.1 | 0.0 | 0.0 | 0.3 | 0.1 |
| 1600016N20Rik | 0.0 | 0.3 | 0.1 | 0.0 | 0.0 | 0.0 | 0.1 | 0.1 | 0.0 | 0.1 | 0.1 | 0.0 |
| 1600020E01Rik | 5.6 | 4.4 | 3.5 | 2.8 | 3.9 | 4.5 | 2.2 | 3.8 | 3.1 | 4.8 | 3.5 | 4.0 |
| 1600023N17Rik | 0.1 | 0.1 | 0.1 | 0.2 | 0.2 | 0.1 | 0.2 | 0.3 | 0.1 | 0.0 | 0.1 | 0.0 |
| 1600029D21Rik | 0.2 | 0.0 | 0.0 | 0.1 | 0.0 | 0.0 | 0.2 | 0.0 | 0.0 | 0.1 | 0.1 | 0.0 |
| 1700001C19Rik | 0.0 | 0.0 | 0.0 | 0.0 | 0.1 | 0.1 | 0.0 | 0.0 | 0.0 | 0.0 | 0.0 | 0.0 |
| 1700001G11Rik | 0.6 | 0.9 | 0.9 | 0.4 | 0.8 | 0.9 | 0.5 | 0.9 | 0.7 | 0.9 | 0.2 | 0.6 |

| | | | | | | | | | | | | |
|---|---|---|---|---|---|---|---|---|---|---|---|---|
| 1700001G17Rik | 0.1 | 0.1 | 0.3 | 0.1 | 0.3 | 0.1 | 0.3 | 0.4 | 0.1 | 0.2 | 0.2 | 0.1 |
| 1700001J03Rik | 0.0 | 0.1 | 0.0 | 0.0 | 0.1 | 0.1 | 0.2 | 0.1 | 0.1 | 0.0 | 0.0 | 0.0 |
| 1700001J11Rik | 0.1 | 0.1 | 0.1 | 0.0 | 0.1 | 0.0 | 0.0 | 0.1 | 0.0 | 0.1 | 0.0 | 0.0 |
| 1700001K19Rik | 0.2 | 0.1 | 0.1 | 0.3 | 0.2 | 0.3 | 0.2 | 0.0 | 0.1 | 0.1 | 0.1 | 0.1 |
| 1700001K23Rik | 0.1 | 0.2 | 0.1 | 0.0 | 0.0 | 0.0 | 0.2 | 0.1 | 0.2 | 0.0 | 0.0 | 0.2 |
| 1700001L05Rik | 0.4 | 0.7 | 0.4 | 0.3 | 0.6 | 0.4 | 0.4 | 0.3 | 0.3 | 0.5 | 0.4 | 0.3 |
| 1700001L19Rik | 0.0 | 0.0 | 0.0 | 0.0 | 0.1 | 0.0 | 0.1 | 0.0 | 0.0 | 0.0 | 0.0 | 0.0 |
| 1700001O22Rik | 0.1 | 0.2 | 0.2 | 0.1 | 0.2 | 0.2 | 0.2 | 0.2 | 0.2 | 0.1 | 0.2 | 0.0 |
| 1700001P01Rik | 0.0 | 0.0 | 0.0 | 0.0 | 0.0 | 0.1 | 0.0 | 0.1 | 0.1 | 0.1 | 0.1 | 0.0 |
| 1700003E16Rik | 0.6 | 0.9 | 0.8 | 0.4 | 0.8 | 0.8 | 0.7 | 0.2 | 0.4 | 0.2 | 0.3 | 0.7 |
| 1700003F12Rik | 0.8 | 0.6 | 0.2 | 0.6 | 0.5 | 0.7 | 0.2 | 0.1 | 0.2 | 0.3 | 0.6 | 0.2 |
| 1700003M02Rik | 0.2 | 0.2 | 0.0 | 0.3 | 0.2 | 0.1 | 0.1 | 0.0 | 0.1 | 0.2 | 0.1 | 0.2 |
| 1700003M07Rik | 1.0 | 0.9 | 0.6 | 0.8 | 0.9 | 0.6 | 0.9 | 0.5 | 0.5 | 1.0 | 1.0 | 0.7 |
| 1700007B14Rik | 0.0 | 0.0 | 0.1 | 0.0 | 0.0 | 0.0 | 0.0 | 0.0 | 0.1 | 0.0 | 0.0 | 0.0 |
| 1700007G11Rik | 0.2 | 0.2 | 0.1 | 0.1 | 0.1 | 0.1 | 0.0 | 0.1 | 0.1 | 0.4 | 0.0 | 0.1 |
| 1700007J10Rik | 0.8 | 1.0 | 0.7 | 0.7 | 1.1 | 0.8 | 1.2 | 1.3 | 1.0 | 1.1 | 1.0 | 1.0 |
| 1700007K13Rik | 6.9 | 6.9 | 7.4 | 4.1 | 7.7 | 5.9 | 3.6 | 3.2 | 3.2 | 6.1 | 5.8 | 6.0 |
| 1700007L15Rik | 2.4 | 1.7 | 2.1 | 1.0 | 1.1 | 1.8 | 1.1 | 1.5 | 1.6 | 1.1 | 2.1 | 0.5 |
| 1700007P06Rik | 0.1 | 0.0 | 0.1 | 0.0 | 0.1 | 0.1 | 0.2 | 0.0 | 0.0 | 0.0 | 0.1 | 0.0 |
| 1700008F21Rik | 0.0 | 0.0 | 0.0 | 0.0 | 0.0 | 0.0 | 0.0 | 0.0 | 0.0 | 0.1 | 0.1 | 0.0 |
| 1700008I05Rik | 0.1 | 0.0 | 0.0 | 0.1 | 0.2 | 0.2 | 0.0 | 0.0 | 0.0 | 0.0 | 0.0 | 0.0 |
| 1700008J07Rik | 0.9 | 0.9 | 0.6 | 0.6 | 1.1 | 1.1 | 1.2 | 0.6 | 0.7 | 1.2 | 1.2 | 0.9 |
| 1700008O03Rik | 0.0 | 0.0 | 0.0 | 0.0 | 0.0 | 0.4 | 0.1 | 0.1 | 0.0 | 0.0 | 0.1 | 0.2 |
| 1700009N14Rik | 0.2 | 0.2 | 0.0 | 0.0 | 0.0 | 0.0 | 0.1 | 0.1 | 0.1 | 0.1 | 0.2 | 0.1 |
| 1700009P17Rik | 1.8 | 2.1 | 1.7 | 1.5 | 1.8 | 1.8 | 1.6 | 1.3 | 1.1 | 1.4 | 1.6 | 1.9 |
| 1700010I14Rik | 2.3 | 1.7 | 1.6 | 2.5 | 1.7 | 2.1 | 1.4 | 1.1 | 1.1 | 1.9 | 1.6 | 1.5 |
| 1700011J10Rik | 1.8 | 1.6 | 1.6 | 1.4 | 1.2 | 1.9 | 1.4 | 2.3 | 1.6 | 1.9 | 1.1 | 1.6 |
| 1700012B15Rik | 2.7 | 3.1 | 2.0 | 3.3 | 3.4 | 3.8 | 3.0 | 2.2 | 2.4 | 3.1 | 3.0 | 2.9 |
| 1700012D01Rik | 1.0 | 1.1 | 2.1 | 0.0 | 1.3 | 1.2 | 1.5 | 1.4 | 1.5 | 1.1 | 0.8 | 0.9 |
| 1700012D14Rik | 0.2 | 0.2 | 0.3 | 0.6 | 0.3 | 0.2 | 0.3 | 0.1 | 0.1 | 0.2 | 0.2 | 0.2 |
| 1700013G23Rik | 1.8 | 0.6 | 1.1 | 1.0 | 1.4 | 1.0 | 1.5 | 0.7 | 0.8 | 0.5 | 0.6 | 1.3 |
| 1700015F17Rik | 0.2 | 0.1 | 0.0 | 0.0 | 0.0 | 0.1 | 0.0 | 0.0 | 0.2 | 0.0 | 0.2 | 0.0 |
| 1700016K19Rik | 0.0 | 0.0 | 0.1 | 0.0 | 0.0 | 0.1 | 0.0 | 0.0 | 0.0 | 0.0 | 0.0 | 0.0 |
| 1700016L04Rik | 0.0 | 0.0 | 0.0 | 0.0 | 0.1 | 0.0 | 0.0 | 0.0 | 0.0 | 0.0 | 0.1 | 0.0 |
| 1700017B05Rik | 1.8 | 2.1 | 1.9 | 2.3 | 3.7 | 1.6 | 2.9 | 3.4 | 3.0 | 3.0 | 2.9 | 2.6 |
| 1700018L02Rik | 3.3 | 3.4 | 2.6 | 2.1 | 2.3 | 2.5 | 4.0 | 4.4 | 2.9 | 3.3 | 2.7 | 4.0 |
| 1700019B21Rik | 0.3 | 0.3 | 0.1 | 0.2 | 0.2 | 0.2 | 0.0 | 0.0 | 0.0 | 0.3 | 0.1 | 0.2 |
| 1700019G17Rik | 0.7 | 0.6 | 0.4 | 0.3 | 0.3 | 0.2 | 0.2 | 0.3 | 0.4 | 0.5 | 0.3 | 0.2 |
| 1700019L03Rik | 1.3 | 0.5 | 0.3 | 1.4 | 0.8 | 0.7 | 0.9 | 0.4 | 0.6 | 0.8 | 0.5 | 0.6 |
| 1700020D05Rik | 0.2 | 0.8 | 0.3 | 0.5 | 0.4 | 0.4 | 0.3 | 0.4 | 0.3 | 0.3 | 0.6 | 0.3 |
| 1700020I14Rik | 39.6 | 41.4 | 39.4 | 52.5 | 41.2 | 46.4 | 38.3 | 31.2 | 38.1 | 36.3 | 42.9 | 40.5 |
| 1700020L24Rik | 0.8 | 0.7 | 0.8 | 0.9 | 0.7 | 1.3 | 0.8 | 0.8 | 0.9 | 0.5 | 0.6 | 0.6 |
| 1700021F05Rik | 20.8 | 16.7 | 20.5 | 28.1 | 16.1 | 25.1 | 23.5 | 18.3 | 15.9 | 17.7 | 21.4 | 25.8 |
| 1700021K19Rik | 8.4 | 9.0 | 7.9 | 9.9 | 8.3 | 10.1 | 8.2 | 8.7 | 9.0 | 7.5 | 8.2 | 7.5 |
| 1700022I11Rik | 0.1 | 0.2 | 0.1 | 0.0 | 0.1 | 0.1 | 0.1 | 0.1 | 0.0 | 0.2 | 0.1 | 0.1 |
| 1700023A16Rik | 0.1 | 0.1 | 0.0 | 0.1 | 0.0 | 0.0 | 0.1 | 0.0 | 0.0 | 0.0 | 0.1 | 0.1 |
| 1700023F06Rik | 0.2 | 0.2 | 0.0 | 0.1 | 0.1 | 0.4 | 0.1 | 0.0 | 0.0 | 0.1 | 0.2 | 0.1 |
| 1700024P16Rik | 0.2 | 0.3 | 0.0 | 0.6 | 0.3 | 0.4 | 0.3 | 0.1 | 0.1 | 0.2 | 0.3 | 0.3 |
| 1700025E21Rik | 0.6 | 0.2 | 0.1 | 0.4 | 0.1 | 0.3 | 0.3 | 0.0 | 0.1 | 0.2 | 0.1 | 0.3 |
| 1700025G04Rik | 2.4 | 2.1 | 2.7 | 1.9 | 2.2 | 2.7 | 2.1 | 2.9 | 2.0 | 2.6 | 2.9 | 2.5 |
| 1700025N23Rik | 0.0 | 0.0 | 0.1 | 0.3 | 0.0 | 0.1 | 0.0 | 0.0 | 0.0 | 0.0 | 0.0 | 0.1 |
| 1700026D08Rik | 0.1 | 0.1 | 0.1 | 0.2 | 0.0 | 0.0 | 0.2 | 0.0 | 0.0 | 0.0 | 0.0 | 0.0 |
| 1700026L06Rik | 0.4 | 0.4 | 0.2 | 0.4 | 0.5 | 0.3 | 0.2 | 0.3 | 0.4 | 0.4 | 0.4 | 0.1 |
| 1700028D13Rik | 0.1 | 0.1 | 0.1 | 0.0 | 0.1 | 0.2 | 0.1 | 0.1 | 0.0 | 0.1 | 0.3 | 0.0 |
| 1700028E10Rik | 0.0 | 0.1 | 0.0 | 0.0 | 0.0 | 0.0 | 0.1 | 0.0 | 0.0 | 0.0 | 0.0 | 0.0 |
| 1700028I16Rik | 0.1 | 0.1 | 0.1 | 0.0 | 0.2 | 0.1 | 0.0 | 0.1 | 0.1 | 0.3 | 0.2 | 0.0 |
| 1700028J19Rik | 0.1 | 0.3 | 0.2 | 0.1 | 0.2 | 0.2 | 0.3 | 0.2 | 0.2 | 0.1 | 0.1 | 0.1 |

| | | | | | | | | | | | | |
|---|---|---|---|---|---|---|---|---|---|---|---|---|
| 1700028K03Rik | 0.1 | 0.0 | 0.1 | 0.1 | 0.1 | 0.2 | 0.1 | 0.1 | 0.1 | 0.2 | 0.1 | 0.1 |
| 1700028P14Rik | 0.1 | 0.0 | 0.0 | 0.1 | 0.0 | 0.0 | 0.1 | 0.0 | 0.0 | 0.0 | 0.0 | 0.0 |
| 1700029H14Rik | 0.0 | 0.0 | 0.1 | 0.0 | 0.1 | 0.0 | 0.1 | 0.1 | 0.0 | 0.0 | 0.0 | 0.1 |
| 1700029I15Rik | 0.0 | 0.2 | 0.1 | 0.7 | 0.0 | 0.0 | 0.0 | 0.0 | 0.0 | 0.0 | 1.0 | 0.2 |
| 1700029J07Rik | 1.4 | 1.0 | 1.2 | 1.2 | 0.7 | 1.9 | 0.5 | 0.9 | 0.7 | 0.8 | 0.9 | 0.8 |
| 1700030C10Rik | 1.3 | 0.8 | 0.7 | 1.1 | 0.5 | 1.3 | 0.8 | 0.5 | 0.6 | 0.8 | 0.5 | 0.5 |
| 1700030J22Rik | 0.3 | 0.3 | 0.2 | 0.3 | 0.3 | 0.4 | 0.3 | 0.3 | 0.2 | 0.3 | 0.2 | 0.2 |
| 1700030K09Rik | 2.9 | 2.5 | 2.5 | 3.1 | 2.5 | 2.8 | 2.5 | 2.5 | 2.4 | 3.2 | 2.3 | 2.5 |
| 1700030L20Rik | 0.9 | 1.1 | 0.6 | 0.5 | 1.2 | 0.6 | 0.9 | 0.7 | 0.5 | 1.2 | 1.0 | 0.4 |
| 1700034E13Rik | 0.3 | 0.0 | 0.2 | 0.3 | 0.0 | 0.0 | 0.0 | 0.0 | 0.3 | 0.1 | 0.7 | 0.3 |
| 1700034F02Rik | 0.2 | 0.2 | 0.2 | 0.1 | 0.2 | 0.2 | 0.2 | 0.2 | 0.1 | 0.4 | 0.2 | 0.3 |
| 1700034H15Rik | 0.0 | 0.1 | 0.0 | 0.0 | 0.0 | 0.0 | 0.0 | 0.0 | 0.0 | 0.0 | 0.0 | 0.0 |
| 1700034I23Rik | 0.0 | 0.1 | 0.0 | 0.0 | 0.0 | 0.0 | 0.0 | 0.1 | 0.1 | 0.0 | 0.0 | 0.0 |
| 1700034J05Rik | 0.4 | 1.0 | 0.6 | 0.4 | 0.4 | 0.6 | 0.5 | 0.7 | 0.4 | 0.6 | 0.6 | 0.6 |
| 1700034P13Rik | 2.9 | 3.9 | 3.9 | 3.8 | 4.1 | 3.1 | 4.0 | 3.7 | 3.9 | 4.0 | 3.9 | 3.8 |
| 1700037C18Rik | 0.6 | 0.5 | 0.7 | 1.0 | 0.4 | 1.2 | 0.6 | 0.6 | 0.9 | 0.6 | 0.4 | 0.3 |
| 1700037H04Rik | 15.4 | 16.2 | 11.1 | 20.1 | 15.2 | 21.3 | 21.0 | 13.0 | 23.6 | 16.9 | 17.6 | 16.1 |
| 1700040L02Rik | 1.1 | 0.9 | 0.4 | 0.6 | 0.5 | 0.5 | 0.5 | 0.3 | 0.3 | 0.5 | 0.8 | 0.4 |
| 1700042O10Rik | 0.1 | 0.1 | 0.1 | 0.2 | 0.2 | 0.0 | 0.0 | 0.0 | 0.0 | 0.0 | 0.0 | 0.1 |
| 1700047G03Rik | 0.2 | 0.1 | 0.0 | 0.0 | 0.0 | 0.0 | 0.0 | 0.1 | 0.0 | 0.0 | 0.2 | 0.1 |
| 1700047M11Rik | 0.1 | 0.2 | 0.4 | 0.0 | 0.1 | 0.1 | 0.1 | 0.1 | 0.1 | 0.0 | 0.0 | 0.0 |
| 1700048M11Rik | 0.2 | 0.5 | 0.1 | 0.6 | 0.5 | 0.3 | 0.3 | 0.3 | 0.4 | 0.1 | 0.0 | 0.1 |
| 1700048O20Rik | 0.6 | 0.5 | 0.2 | 0.2 | 0.3 | 0.4 | 0.3 | 0.2 | 0.5 | 0.3 | 0.2 | 0.3 |
| 1700049G17Rik | 1.8 | 2.0 | 2.1 | 1.4 | 1.7 | 2.0 | 1.5 | 1.8 | 1.7 | 1.9 | 1.6 | 2.2 |
| 1700052K11Rik | 2.4 | 2.7 | 2.9 | 2.2 | 2.3 | 2.0 | 2.4 | 3.3 | 3.1 | 3.0 | 3.2 | 3.5 |
| 1700052N19Rik | 6.1 | 8.6 | 9.7 | 5.4 | 6.9 | 4.8 | 6.6 | 6.7 | 7.0 | 6.8 | 6.5 | 7.6 |
| 1700055N04Rik | 0.0 | 0.0 | 0.0 | 0.0 | 0.1 | 0.1 | 0.0 | 0.0 | 0.0 | 0.1 | 0.0 | 0.0 |
| 1700056E22Rik | 0.4 | 0.3 | 0.2 | 0.5 | 0.4 | 0.1 | 0.1 | 0.4 | 0.4 | 0.5 | 0.4 | 0.1 |
| 1700057G04Rik | 0.0 | 0.2 | 0.0 | 0.1 | 0.0 | 0.1 | 0.0 | 0.0 | 0.0 | 0.0 | 0.0 | 0.0 |
| 1700058G18Rik | 0.0 | 0.0 | 0.0 | 0.0 | 0.0 | 0.1 | 0.1 | 0.0 | 0.0 | 0.0 | 0.0 | 0.1 |
| 1700061G19Rik | 0.3 | 0.2 | 0.1 | 0.2 | 0.1 | 0.3 | 0.2 | 0.2 | 0.1 | 0.0 | 0.3 | 0.1 |
| 1700061I17Rik | 0.2 | 0.2 | 0.5 | 0.0 | 0.2 | 0.0 | 0.3 | 0.1 | 0.1 | 0.2 | 0.0 | 0.1 |
| 1700063D05Rik | 0.1 | 0.2 | 0.1 | 0.1 | 0.0 | 0.2 | 0.0 | 0.0 | 0.1 | 0.3 | 0.1 | 0.2 |
| 1700066B19Rik | 0.2 | 0.2 | 0.1 | 0.1 | 0.1 | 0.1 | 0.1 | 0.2 | 0.1 | 0.0 | 0.0 | 0.1 |
| 1700066J24Rik | 2.4 | 2.3 | 2.0 | 3.5 | 2.3 | 3.3 | 3.1 | 2.0 | 2.2 | 3.3 | 2.3 | 2.4 |
| 1700066M21Rik | 2.7 | 3.3 | 4.4 | 3.4 | 2.9 | 3.3 | 2.9 | 2.9 | 3.7 | 3.8 | 4.0 | 3.6 |
| 1700067K01Rik | 0.3 | 0.0 | 0.2 | 0.3 | 0.1 | 0.0 | 0.1 | 0.3 | 0.1 | 0.1 | 0.1 | 0.2 |
| 1700071K01Rik | 0.0 | 0.0 | 0.0 | 0.1 | 0.1 | 0.0 | 0.0 | 0.0 | 0.1 | 0.1 | 0.0 | 0.0 |
| 1700071M16Rik | 0.1 | 0.0 | 0.0 | 0.8 | 0.6 | 0.0 | 0.0 | 0.1 | 0.0 | 0.1 | 0.1 | 0.0 |
| 1700073E17Rik | 0.0 | 0.0 | 0.0 | 0.0 | 0.0 | 0.0 | 0.0 | 0.0 | 0.0 | 0.0 | 0.0 | 0.0 |
| 1700084C01Rik | 0.0 | 0.1 | 0.0 | 0.1 | 0.0 | 0.0 | 0.0 | 0.0 | 0.0 | 0.0 | 0.0 | 0.0 |
| 1700084E18Rik | 0.1 | 0.4 | 0.6 | 0.0 | 0.7 | 0.0 | 0.2 | 0.5 | 0.4 | 0.1 | 0.2 | 0.5 |
| 1700084J12Rik | 0.1 | 0.1 | 0.1 | 0.4 | 0.0 | 0.0 | 0.1 | 0.0 | 0.0 | 0.1 | 0.2 | 0.0 |
| 1700086O06Rik | 0.8 | 0.1 | 0.4 | 1.1 | 0.8 | 0.3 | 0.4 | 0.5 | 0.3 | 0.6 | 0.4 | 0.4 |
| 1700088E04Rik | 4.6 | 4.9 | 3.1 | 4.0 | 3.6 | 4.8 | 3.3 | 3.8 | 3.8 | 4.5 | 5.4 | 3.5 |
| 1700093K21Rik | 0.0 | 0.1 | 0.0 | 0.2 | 0.0 | 0.0 | 0.0 | 0.0 | 0.0 | 0.0 | 0.0 | 0.0 |
| 1700094D03Rik | 1.0 | 0.7 | 0.7 | 1.1 | 0.8 | 1.2 | 0.6 | 0.5 | 0.5 | 0.7 | 0.7 | 0.5 |
| 1700094J05Rik | 0.1 | 0.1 | 0.5 | 0.2 | 0.4 | 0.4 | 0.3 | 0.5 | 0.2 | 0.4 | 0.3 | 0.1 |
| 1700096K18Rik | 0.4 | 0.4 | 0.4 | 0.8 | 0.4 | 0.8 | 0.5 | 0.6 | 0.3 | 0.4 | 0.7 | 0.8 |
| 1700101E01Rik | 0.0 | 0.4 | 0.1 | 0.0 | 0.0 | 0.2 | 0.0 | 0.0 | 0.1 | 0.0 | 0.0 | 0.0 |
| 1700101I11Rik | 0.0 | 0.2 | 0.4 | 0.1 | 0.2 | 0.2 | 0.0 | 0.1 | 0.2 | 0.5 | 0.0 | 0.2 |
| 1700102P08Rik | 0.0 | 0.3 | 0.1 | 0.0 | 0.1 | 0.0 | 0.1 | 0.2 | 0.2 | 0.1 | 0.2 | 0.1 |
| 1700105P06Rik | 0.4 | 0.4 | 0.1 | 0.1 | 0.2 | 0.3 | 0.0 | 0.2 | 0.2 | 0.1 | 0.1 | 0.1 |
| 1700108F19Rik | 0.0 | 0.0 | 0.0 | 0.1 | 0.1 | 0.0 | 0.0 | 0.0 | 0.0 | 0.1 | 0.0 | 0.1 |
| 1700109F18Rik | 0.0 | 0.0 | 0.0 | 0.0 | 0.0 | 0.0 | 0.0 | 0.1 | 0.0 | 0.0 | 0.0 | 0.1 |
| 1700109H08Rik | 0.3 | 0.1 | 0.2 | 0.3 | 0.3 | 0.2 | 0.6 | 0.4 | 0.2 | 0.4 | 0.2 | 0.3 |
| 1700110C19Rik | 0.0 | 0.0 | 0.1 | 0.1 | 0.0 | 0.0 | 0.0 | 0.0 | 0.0 | 0.0 | 0.0 | 0.1 |

| | | | | | | | | | | | | |
|---|---|---|---|---|---|---|---|---|---|---|---|---|
| 1700111N16Rik | 0.0 | 0.0 | 0.0 | 0.0 | 0.0 | 0.0 | 0.0 | 0.0 | 0.0 | 0.0 | 0.0 | 0.0 |
| 1700112E06Rik | 4.0 | 3.2 | 3.7 | 4.9 | 3.9 | 6.3 | 3.5 | 2.9 | 2.0 | 3.4 | 1.8 | 3.3 |
| 1700113A16Rik | 1.0 | 0.9 | 0.8 | 1.5 | 0.8 | 1.1 | 0.7 | 0.7 | 1.1 | 0.7 | 0.8 | 0.8 |
| 1700120C14Rik | 0.4 | 0.2 | 0.1 | 1.6 | 0.3 | 0.3 | 0.0 | 0.4 | 0.1 | 0.0 | 0.2 | 0.8 |
| 1700120K04Rik | 0.2 | 0.4 | 0.0 | 0.4 | 0.1 | 0.0 | 0.0 | 0.0 | 0.2 | 0.3 | 0.6 | 0.4 |
| 1700123I01Rik | 0.0 | 0.0 | 0.0 | 0.0 | 0.0 | 0.0 | 0.0 | 0.0 | 0.0 | 0.0 | 0.0 | 0.1 |
| 1700123L14Rik | 0.1 | 0.2 | 0.1 | 0.2 | 0.1 | 0.1 | 0.1 | 0.1 | 0.2 | 0.0 | 0.1 | 0.1 |
| 1700123M08Rik | 0.5 | 0.3 | 0.2 | 0.2 | 0.2 | 0.5 | 0.2 | 0.5 | 0.5 | 0.1 | 0.4 | 0.5 |
| 1700123O20Rik | 11.8 | 10.2 | 10.1 | 11.3 | 11.5 | 12.2 | 11.7 | 11.8 | 12.1 | 12.2 | 13.6 | 11.3 |
| 1700125G22Rik | 0.1 | 0.2 | 0.0 | 0.0 | 0.1 | 0.0 | 0.0 | 0.1 | 0.0 | 0.0 | 0.0 | 0.1 |
| 1700125H03Rik | 0.0 | 0.2 | 0.3 | 0.0 | 0.5 | 0.4 | 0.8 | 0.1 | 0.3 | 0.1 | 0.0 | 0.2 |
| 1700125H20Rik | 0.0 | 0.2 | 0.2 | 0.0 | 0.0 | 0.2 | 0.0 | 0.0 | 0.0 | 0.0 | 0.0 | 0.1 |
| 1700128F08Rik | 0.0 | 0.0 | 0.0 | 0.2 | 0.0 | 0.0 | 0.0 | 0.0 | 0.0 | 0.0 | 0.0 | 0.0 |
| 1810008I18Rik | 0.5 | 0.6 | 0.1 | 0.1 | 0.1 | 0.2 | 0.2 | 0.3 | 0.1 | 0.0 | 0.6 | 0.1 |
| 1810009A15Rik | 62.9 | 61.1 | 59.7 | 73.2 | 47.2 | 62.8 | 55.1 | 60.8 | 42.3 | 56.7 | 63.8 | 61.1 |
| 1810010D01Rik | 0.3 | 0.1 | 0.0 | 0.0 | 0.1 | 0.0 | 0.0 | 0.0 | 0.1 | 0.6 | 0.0 | 0.0 |
| 1810010H24Rik | 0.2 | 0.3 | 0.6 | 0.1 | 0.5 | 0.6 | 0.1 | 0.2 | 0.5 | 0.3 | 0.2 | 0.3 |
| 1810011H11Rik | 0.5 | 0.0 | 0.0 | 2.5 | 5.4 | 0.0 | 0.8 | 0.1 | 0.1 | 0.5 | 0.0 | 0.3 |
| 1810011O10Rik | 0.3 | 0.4 | 0.9 | 0.1 | 0.4 | 0.1 | 0.6 | 1.5 | 0.9 | 3.0 | 1.6 | 1.3 |
| 1810012K16Rik | 0.1 | 0.3 | 0.4 | 0.0 | 0.4 | 0.6 | 0.2 | 0.1 | 0.3 | 0.1 | 0.0 | 0.3 |
| 1810013D10Rik | 16.1 | 13.2 | 9.6 | 11.1 | 11.3 | 12.2 | 12.9 | 13.2 | 12.0 | 14.3 | 13.0 | 12.9 |
| 1810013L24Rik | 16.8 | 20.2 | 18.6 | 19.0 | 17.7 | 19.3 | 17.2 | 17.7 | 20.4 | 19.6 | 20.2 | 19.4 |
| 1810014B01Rik | 0.6 | 0.7 | 0.6 | 1.2 | 0.4 | 0.9 | 0.4 | 0.6 | 0.6 | 0.7 | 0.6 | 0.7 |
| 1810019D21Rik | 0.8 | 0.8 | 1.2 | 0.6 | 0.7 | 0.5 | 1.4 | 0.7 | 1.1 | 1.6 | 1.6 | 1.3 |
| 1810019J16Rik | 0.0 | 0.0 | 0.1 | 0.0 | 0.0 | 0.0 | 0.1 | 0.0 | 0.0 | 0.0 | 0.0 | 0.0 |
| 1810020O05Rik | 0.1 | 0.1 | 0.0 | 0.1 | 0.0 | 0.2 | 0.2 | 0.0 | 0.0 | 0.0 | 0.0 | 0.0 |
| 1810021B22Rik | 1.3 | 1.1 | 1.5 | 2.3 | 1.4 | 1.8 | 2.4 | 2.0 | 1.3 | 1.8 | 1.2 | 2.0 |
| 1810022K09Rik | 117.2 | 123.9 | 111.2 | 141.4 | 104.4 | 130.1 | 97.7 | 114.4 | 103.8 | 123.8 | 139.4 | 130.4 |
| 1810024B03Rik | 0.1 | 0.1 | 0.1 | 0.2 | 0.1 | 0.2 | 0.2 | 0.1 | 0.1 | 0.1 | 0.1 | 0.1 |
| 1810026B05Rik | 0.9 | 1.0 | 1.3 | 0.9 | 1.1 | 1.2 | 0.9 | 1.3 | 1.4 | 1.7 | 1.3 | 1.4 |
| 1810026J23Rik | 9.4 | 9.3 | 8.6 | 9.4 | 9.3 | 8.3 | 9.0 | 8.3 | 9.9 | 10.3 | 8.8 | 9.6 |
| 1810030O07Rik | 14.4 | 13.3 | 12.6 | 14.7 | 11.4 | 13.4 | 11.7 | 13.3 | 11.3 | 12.2 | 12.6 | 12.9 |
| 1810032O08Rik | 2.8 | 2.5 | 2.2 | 2.3 | 3.5 | 2.6 | 3.4 | 2.6 | 3.3 | 2.3 | 2.1 | 2.0 |
| 1810033B17Rik | 0.1 | 0.1 | 0.1 | 0.0 | 0.1 | 0.1 | 0.1 | 0.0 | 0.0 | 0.1 | 0.1 | 0.0 |
| 1810034E14Rik | 1.8 | 1.8 | 1.1 | 1.4 | 1.5 | 2.2 | 1.4 | 1.5 | 1.0 | 0.6 | 1.5 | 1.6 |
| 1810037I17Rik | 62.6 | 61.9 | 54.6 | 67.2 | 63.9 | 62.6 | 61.4 | 53.5 | 69.4 | 80.0 | 76.2 | 80.4 |
| 1810041L15Rik | 0.0 | 0.0 | 0.0 | 0.0 | 0.0 | 0.0 | 0.0 | 0.0 | 0.0 | 0.0 | 0.0 | 0.0 |
| 1810043G02Rik | 5.7 | 6.2 | 6.7 | 7.0 | 6.9 | 8.1 | 6.8 | 6.3 | 5.7 | 7.2 | 5.9 | 6.2 |
| 1810043H04Rik | 21.8 | 21.6 | 14.7 | 21.1 | 17.6 | 21.2 | 17.1 | 19.0 | 20.8 | 19.8 | 17.9 | 17.4 |
| 1810044D09Rik | 2.5 | 2.1 | 1.3 | 1.6 | 2.1 | 2.8 | 1.2 | 0.6 | 1.5 | 3.2 | 1.6 | 1.8 |
| 1810055G02Rik | 19.4 | 18.2 | 20.2 | 23.2 | 22.1 | 19.2 | 29.3 | 21.5 | 20.0 | 18.2 | 23.4 | 20.3 |
| 1810058I24Rik | 33.2 | 27.9 | 25.9 | 32.9 | 28.7 | 33.6 | 25.7 | 32.0 | 30.3 | 35.8 | 29.0 | 29.5 |
| 1810062G17Rik | 1.7 | 2.0 | 1.4 | 1.9 | 1.2 | 1.5 | 1.2 | 1.2 | 1.0 | 0.7 | 1.2 | 1.2 |
| 1810062O18Rik | 0.6 | 0.4 | 0.5 | 0.8 | 0.7 | 0.3 | 0.6 | 0.5 | 0.6 | 0.9 | 1.1 | 0.8 |
| 1810064F22Rik | 0.0 | 0.0 | 0.0 | 0.0 | 0.0 | 0.0 | 0.0 | 0.0 | 0.0 | 0.0 | 0.0 | 0.0 |
| 1810065E05Rik | 0.3 | 0.2 | 0.2 | 0.1 | 0.1 | 0.0 | 0.1 | 0.1 | 0.0 | 0.2 | 0.2 | 0.2 |
| 2010001E11Rik | 0.2 | 0.1 | 0.1 | 0.2 | 0.1 | 0.0 | 0.1 | 0.1 | 0.1 | 0.1 | 0.0 | 0.0 |
| 2010001M06Rik | 6.7 | 8.0 | 10.5 | 5.8 | 4.6 | 14.3 | 3.0 | 3.1 | 2.9 | 7.3 | 6.0 | 8.3 |
| 2010002M12Rik | 0.6 | 0.5 | 0.4 | 0.8 | 0.4 | 0.6 | 0.3 | 0.2 | 0.2 | 0.2 | 0.2 | 0.4 |
| 2010003K11Rik | 0.0 | 0.0 | 0.1 | 0.0 | 0.1 | 0.0 | 0.1 | 0.0 | 0.0 | 0.0 | 0.1 | 0.0 |
| 2010003O02Rik | 24.9 | 18.9 | 14.9 | 19.7 | 20.9 | 27.3 | 13.7 | 19.6 | 21.0 | 23.5 | 19.2 | 19.6 |
| 2010009K17Rik | 0.1 | 0.0 | 0.0 | 0.0 | 0.3 | 0.1 | 0.0 | 0.0 | 0.0 | 0.1 | 0.1 | 0.0 |
| 2010010A06Rik | 0.0 | 0.0 | 0.1 | 0.1 | 0.1 | 0.0 | 0.0 | 0.1 | 0.1 | 0.0 | 0.0 | 0.3 |
| 2010012O05Rik | 2.6 | 2.4 | 2.0 | 2.7 | 2.3 | 2.4 | 3.1 | 3.2 | 2.3 | 2.6 | 2.8 | 3.3 |
| 2010015L04Rik | 1.2 | 1.1 | 1.2 | 1.3 | 1.1 | 1.1 | 1.4 | 1.1 | 0.5 | 1.1 | 1.3 | 0.9 |
| 2010016I18Rik | 0.1 | 0.0 | 0.0 | 0.0 | 0.0 | 0.1 | 0.0 | 0.0 | 0.0 | 0.0 | 0.0 | 0.0 |
| 2010107E04Rik | 162.1 | 126.3 | 100.0 | 122.8 | 138.8 | 147.6 | 101.6 | 117.4 | 128.4 | 143.0 | 121.9 | 121.2 |

| | | | | | | | | | | | | |
|---|---|---|---|---|---|---|---|---|---|---|---|---|
| 2010107G12Rik | 0.0 | 0.1 | 0.0 | 0.0 | 0.0 | 0.0 | 0.0 | 0.1 | 0.0 | 0.1 | 0.0 | 0.0 |
| 2010107G23Rik | 2.6 | 1.2 | 0.9 | 1.7 | 1.0 | 2.9 | 0.8 | 0.6 | 0.7 | 1.6 | 1.1 | 0.9 |
| 2010109A12Rik | 0.1 | 1.1 | 0.3 | 0.5 | 0.4 | 0.4 | 0.9 | 0.4 | 0.3 | 0.3 | 0.4 | 0.5 |
| 2010111I01Rik | 16.0 | 12.7 | 9.1 | 15.7 | 12.5 | 13.0 | 9.2 | 8.4 | 7.7 | 11.3 | 10.5 | 9.2 |
| 2010204K13Rik | 1.2 | 0.4 | 0.7 | 0.4 | 0.1 | 1.9 | 0.6 | 0.8 | 0.5 | 0.6 | 0.4 | 1.0 |
| 2010300C02Rik | 0.0 | 0.0 | 0.0 | 0.0 | 0.0 | 0.1 | 0.0 | 0.0 | 0.0 | 0.1 | 0.0 | 0.0 |
| 2010310C07Rik | 0.0 | 0.0 | 0.0 | 0.0 | 0.0 | 0.0 | 0.1 | 0.0 | 0.0 | 0.3 | 0.1 | 0.1 |
| 2010315B03Rik | 2.8 | 3.4 | 4.6 | 3.7 | 3.3 | 4.0 | 2.9 | 3.2 | 3.5 | 3.7 | 3.7 | 4.1 |
| 2010320M18Rik | 7.0 | 5.4 | 8.4 | 6.2 | 6.9 | 8.0 | 6.7 | 7.8 | 8.7 | 11.8 | 8.2 | 9.4 |
| 2200002D01Rik | 16.7 | 8.6 | 5.3 | 18.1 | 8.4 | 17.9 | 12.2 | 12.3 | 10.5 | 9.5 | 13.6 | 9.0 |
| 2210010C04Rik | 0.0 | 0.1 | 0.0 | 0.0 | 0.0 | 0.0 | 0.0 | 0.1 | 0.0 | 0.0 | 0.0 | 0.1 |
| 2210013O21Rik | 37.7 | 30.5 | 27.4 | 32.4 | 25.7 | 39.1 | 22.1 | 32.5 | 29.7 | 28.7 | 31.0 | 26.3 |
| 2210015D19Rik | 2.9 | 1.8 | 1.7 | 2.6 | 1.9 | 2.8 | 2.5 | 2.3 | 2.2 | 2.5 | 2.2 | 2.2 |
| 2210016F16Rik | 15.7 | 14.4 | 14.3 | 15.2 | 14.0 | 13.9 | 15.0 | 14.3 | 14.6 | 15.3 | 15.2 | 16.1 |
| 2210016L21Rik | 18.4 | 14.4 | 14.6 | 19.0 | 14.1 | 18.2 | 13.7 | 15.4 | 13.2 | 13.8 | 14.9 | 14.5 |
| 2210018M11Rik | 3.8 | 4.3 | 5.6 | 4.0 | 4.4 | 3.6 | 6.0 | 5.4 | 4.7 | 3.9 | 5.5 | 5.3 |
| 2210039B01Rik | 0.5 | 0.5 | 0.8 | 0.8 | 0.5 | 0.9 | 0.5 | 1.0 | 0.4 | 0.4 | 0.4 | 0.6 |
| 2210404J11Rik | 3.8 | 3.1 | 2.9 | 3.3 | 3.5 | 3.1 | 2.4 | 2.7 | 3.0 | 2.7 | 2.7 | 2.8 |
| 2210404O07Rik | 0.2 | 0.6 | 0.4 | 0.3 | 0.3 | 0.0 | 0.1 | 0.6 | 0.2 | 0.6 | 0.5 | 0.1 |
| 2210404O09Rik | 1.9 | 2.6 | 2.5 | 1.5 | 2.2 | 2.3 | 1.9 | 2.2 | 2.1 | 2.3 | 2.4 | 2.7 |
| 2210408F21Rik | 11.8 | 13.8 | 9.9 | 11.5 | 10.0 | 11.3 | 7.6 | 9.6 | 10.1 | 12.4 | 9.8 | 11.4 |
| 2210408I21Rik | 1.1 | 1.4 | 1.4 | 1.7 | 1.5 | 1.8 | 1.7 | 1.5 | 1.5 | 2.1 | 1.7 | 1.8 |
| 2210416O15Rik | 0.3 | 0.1 | 0.1 | 0.3 | 0.1 | 0.4 | 0.2 | 0.3 | 0.3 | 0.4 | 0.1 | 0.3 |
| 2210417K05Rik | 0.5 | 0.1 | 0.6 | 0.4 | 0.2 | 0.8 | 0.6 | 0.4 | 0.3 | 0.5 | 0.7 | 0.4 |
| 2300009A05Rik | 5.5 | 4.3 | 4.3 | 5.8 | 4.6 | 6.0 | 4.1 | 5.5 | 4.1 | 7.8 | 3.7 | 4.8 |
| 2310001H17Rik | 0.9 | 1.0 | 0.8 | 1.1 | 1.5 | 0.5 | 1.4 | 0.9 | 0.9 | 1.4 | 1.3 | 1.0 |
| 2310001K24Rik | 0.0 | 0.2 | 0.4 | 0.2 | 0.0 | 0.1 | 0.0 | 0.0 | 0.2 | 0.2 | 0.0 | 0.0 |
| 2310002F09Rik | 0.1 | 0.0 | 0.0 | 0.0 | 0.0 | 0.0 | 0.0 | 0.0 | 0.0 | 0.0 | 0.0 | 0.0 |
| 2310003H01Rik | 1.3 | 1.4 | 1.6 | 1.0 | 1.3 | 2.0 | 1.4 | 1.6 | 1.6 | 1.3 | 1.3 | 1.0 |
| 2310005E17Rik | 0.0 | 0.0 | 0.0 | 0.0 | 0.0 | 0.0 | 0.0 | 0.0 | 0.0 | 0.0 | 0.1 | 0.0 |
| 2310005G13Rik | 0.1 | 0.1 | 0.1 | 0.1 | 0.0 | 0.0 | 0.1 | 0.0 | 0.0 | 0.1 | 0.0 | 0.0 |
| 2310007B03Rik | 0.1 | 0.0 | 0.0 | 0.0 | 0.0 | 0.1 | 0.0 | 0.0 | 0.0 | 0.0 | 0.0 | 0.0 |
| 2310008H04Rik | 11.0 | 10.7 | 13.4 | 7.8 | 10.7 | 9.0 | 10.4 | 13.3 | 10.1 | 8.3 | 11.4 | 10.8 |
| 2310008H09Rik | 12.0 | 11.5 | 10.7 | 11.7 | 8.9 | 10.1 | 10.6 | 9.7 | 8.5 | 9.5 | 11.5 | 9.8 |
| 2310008N11Rik | 0.4 | 0.2 | 0.1 | 0.2 | 0.1 | 0.0 | 0.0 | 0.3 | 0.2 | 0.2 | 0.3 | 0.2 |
| 2310009A05Rik | 11.3 | 14.7 | 20.9 | 20.0 | 15.7 | 19.2 | 18.2 | 17.5 | 15.8 | 16.2 | 18.5 | 16.6 |
| 2310009B15Rik | 10.3 | 7.5 | 8.6 | 8.9 | 8.3 | 11.7 | 10.0 | 11.5 | 10.4 | 6.4 | 8.6 | 8.9 |
| 2310010J17Rik | 2.7 | 1.9 | 1.7 | 3.1 | 3.9 | 3.2 | 2.7 | 2.9 | 2.2 | 3.2 | 3.8 | 6.1 |
| 2310010M20Rik | 0.0 | 0.1 | 0.2 | 0.2 | 0.0 | 0.0 | 0.2 | 0.1 | 0.0 | 0.1 | 0.0 | 0.0 |
| 2310011J03Rik | 16.2 | 15.0 | 12.8 | 17.5 | 15.4 | 16.6 | 15.4 | 14.7 | 15.7 | 16.5 | 12.8 | 15.6 |
| 2310014F07Rik | 0.2 | 0.2 | 0.1 | 0.0 | 0.0 | 0.2 | 0.0 | 0.0 | 0.0 | 0.0 | 0.0 | 0.1 |
| 2310014L17Rik | 0.4 | 0.4 | 0.4 | 0.3 | 0.5 | 0.3 | 0.4 | 0.2 | 0.7 | 0.7 | 0.3 | 0.4 |
| 2310015A10Rik | 1.1 | 0.7 | 0.7 | 0.8 | 0.7 | 0.8 | 0.6 | 0.7 | 0.5 | 0.6 | 0.7 | 0.8 |
| 2310015D24Rik | 0.0 | 0.0 | 0.0 | 0.0 | 0.0 | 0.0 | 0.0 | 0.0 | 0.0 | 0.1 | 0.0 | 0.0 |
| 2310022A10Rik | 1.3 | 2.0 | 1.4 | 2.0 | 2.1 | 1.4 | 1.4 | 1.4 | 1.5 | 1.6 | 1.4 | 1.2 |
| 2310022B05Rik | 23.9 | 33.9 | 27.3 | 24.9 | 21.6 | 23.6 | 31.1 | 27.7 | 43.5 | 22.4 | 26.5 | 30.3 |
| 2310030G06Rik | 1.1 | 0.8 | 0.9 | 1.5 | 0.4 | 0.5 | 0.5 | 1.0 | 0.8 | 3.9 | 2.3 | 1.5 |
| 2310033P09Rik | 5.3 | 6.1 | 8.6 | 5.4 | 5.7 | 5.7 | 7.0 | 7.6 | 6.1 | 6.7 | 7.1 | 7.8 |
| 2310034G01Rik | 0.3 | 0.2 | 0.5 | 0.6 | 0.4 | 0.2 | 0.5 | 0.3 | 0.4 | 0.5 | 0.5 | 0.5 |
| 2310035C23Rik | 9.0 | 8.8 | 7.5 | 7.8 | 8.4 | 6.9 | 8.8 | 7.8 | 8.9 | 8.9 | 9.8 | 9.5 |
| 2310036O22Rik | 69.1 | 57.6 | 57.8 | 61.6 | 59.3 | 49.5 | 77.5 | 74.5 | 69.1 | 74.3 | 73.1 | 65.8 |
| 2310039H08Rik | 8.5 | 7.6 | 6.2 | 7.3 | 7.7 | 9.0 | 9.4 | 9.4 | 7.7 | 9.5 | 10.4 | 8.0 |
| 2310039L15Rik | 1.0 | 1.4 | 1.4 | 1.0 | 1.2 | 1.1 | 1.5 | 0.6 | 0.8 | 0.9 | 0.7 | 0.6 |
| 2310040G24Rik | 0.5 | 0.6 | 0.4 | 1.2 | 0.8 | 0.7 | 0.7 | 0.5 | 0.5 | 0.3 | 0.6 | 0.9 |
| 2310042D19Rik | 0.3 | 0.3 | 0.3 | 0.3 | 0.4 | 0.4 | 0.2 | 0.1 | 0.2 | 0.2 | 0.2 | 0.2 |
| 2310044G17Rik | 3.8 | 4.3 | 3.3 | 5.6 | 4.7 | 4.5 | 3.7 | 3.9 | 3.9 | 4.4 | 3.9 | 3.6 |
| 2310045N01Rik | 41.6 | 36.8 | 35.5 | 40.9 | 29.3 | 39.4 | 36.6 | 43.9 | 30.6 | 40.3 | 42.7 | 36.5 |

| | | | | | | | | | | | | |
|---|---|---|---|---|---|---|---|---|---|---|---|---|
| 2310047M10Rik | 6.5 | 7.0 | 6.8 | 6.4 | 6.7 | 6.5 | 8.7 | 7.7 | 7.9 | 7.7 | 7.6 | 6.8 |
| 2310057M21Rik | 2.8 | 3.7 | 2.9 | 3.4 | 3.6 | 3.1 | 3.1 | 2.6 | 3.2 | 3.3 | 3.8 | 3.3 |
| 2310061I04Rik | 2.4 | 2.8 | 2.9 | 2.9 | 2.5 | 3.1 | 3.4 | 3.2 | 2.5 | 2.2 | 2.8 | 2.6 |
| 2310061J03Rik | 1.7 | 2.5 | 1.3 | 1.6 | 1.1 | 1.8 | 2.2 | 2.2 | 2.8 | 3.0 | 2.2 | 2.2 |
| 2310067B10Rik | 1.6 | 2.4 | 2.8 | 1.9 | 2.2 | 2.0 | 2.1 | 2.9 | 2.7 | 2.3 | 1.9 | 1.8 |
| 2310068J16Rik | 0.1 | 0.1 | 0.3 | 0.2 | 0.2 | 0.2 | 0.1 | 0.2 | 0.3 | 0.3 | 0.2 | 0.2 |
| 2310069B03Rik | 0.0 | 0.0 | 0.0 | 0.0 | 0.0 | 0.2 | 0.0 | 0.0 | 0.0 | 0.0 | 0.0 | 0.0 |
| 2410002F23Rik | 8.1 | 6.1 | 6.0 | 9.5 | 8.9 | 7.3 | 9.4 | 7.6 | 7.7 | 7.0 | 8.2 | 7.5 |
| 2410003L11Rik | 0.1 | 0.0 | 0.1 | 0.5 | 0.1 | 0.0 | 0.1 | 0.0 | 0.1 | 0.2 | 0.2 | 0.0 |
| 2410004B18Rik | 10.6 | 11.0 | 10.4 | 11.9 | 9.6 | 8.6 | 13.3 | 11.5 | 11.8 | 13.8 | 12.4 | 12.7 |
| 2410004I01Rik | 0.1 | 0.2 | 0.0 | 0.0 | 0.0 | 0.2 | 0.0 | 0.0 | 0.0 | 0.0 | 0.0 | 0.0 |
| 2410004N09Rik | 16.4 | 9.7 | 9.7 | 11.0 | 13.0 | 22.3 | 11.9 | 10.0 | 10.6 | 14.1 | 12.7 | 11.8 |
| 2410004P03Rik | 0.1 | 0.4 | 0.7 | 0.4 | 0.4 | 0.3 | 0.2 | 0.1 | 0.2 | 0.1 | 0.1 | 0.2 |
| 2410006H16Rik | 105.3 | 82.6 | 84.9 | 75.3 | 79.1 | 143.7 | 83.3 | 116.8 | 109.7 | 106.8 | 99.6 | 89.3 |
| 2410015M20Rik | 70.2 | 63.4 | 51.7 | 57.7 | 58.8 | 64.6 | 53.9 | 57.1 | 57.0 | 68.3 | 63.8 | 56.3 |
| 2410016O06Rik | 1.9 | 2.2 | 1.4 | 1.3 | 2.0 | 1.7 | 2.0 | 1.8 | 2.4 | 3.0 | 2.3 | 2.0 |
| 2410018L13Rik | 1.9 | 1.1 | 0.8 | 1.6 | 0.9 | 1.8 | 1.4 | 1.1 | 1.5 | 1.6 | 1.2 | 1.2 |
| 2410018M08Rik | 5.7 | 5.0 | 3.8 | 5.8 | 5.0 | 5.0 | 4.4 | 4.6 | 5.0 | 5.7 | 4.6 | 5.1 |
| 2410021H03Rik | 0.0 | 0.2 | 0.0 | 0.0 | 0.1 | 0.1 | 0.1 | 0.1 | 0.0 | 0.0 | 0.1 | 0.0 |
| 2410057H14Rik | 0.1 | 0.0 | 0.0 | 0.1 | 0.1 | 0.0 | 0.1 | 0.0 | 0.2 | 0.3 | 0.0 | 0.2 |
| 2410066E13Rik | 13.4 | 11.7 | 9.8 | 15.4 | 12.1 | 14.3 | 9.0 | 7.9 | 8.2 | 7.7 | 9.4 | 9.5 |
| 2410076I21Rik | 0.2 | 0.2 | 0.1 | 0.1 | 0.2 | 0.2 | 0.0 | 0.0 | 0.0 | 0.2 | 0.2 | 0.2 |
| 2410089E03Rik | 2.9 | 4.0 | 4.2 | 2.8 | 3.2 | 3.6 | 3.3 | 3.8 | 3.5 | 2.9 | 3.2 | 3.0 |
| 2410127L17Rik | 10.0 | 11.0 | 12.1 | 10.3 | 11.0 | 13.2 | 9.9 | 11.8 | 10.2 | 11.6 | 14.2 | 13.4 |
| 2410131K14Rik | 5.7 | 4.1 | 3.3 | 5.9 | 5.0 | 5.7 | 4.8 | 4.0 | 4.5 | 4.9 | 4.4 | 3.9 |
| 2500004C02Rik | 0.9 | 0.8 | 1.0 | 0.7 | 0.7 | 0.7 | 0.6 | 0.7 | 1.0 | 1.3 | 1.0 | 1.0 |
| 2510002D24Rik | 2.2 | 2.6 | 3.2 | 2.5 | 2.9 | 3.1 | 2.4 | 2.8 | 2.6 | 2.0 | 2.6 | 3.3 |
| 2510003E04Rik | 22.7 | 24.5 | 22.2 | 24.7 | 20.3 | 25.8 | 23.8 | 21.4 | 21.5 | 22.4 | 23.4 | 23.2 |
| 2510009E07Rik | 3.0 | 4.2 | 5.2 | 4.5 | 4.9 | 4.2 | 3.8 | 5.5 | 4.5 | 4.1 | 4.5 | 5.2 |
| 2510039O18Rik | 18.1 | 16.0 | 13.6 | 18.1 | 16.8 | 16.8 | 18.5 | 15.4 | 17.7 | 17.8 | 15.8 | 15.0 |
| 2510049J12Rik | 0.0 | 0.0 | 0.0 | 0.0 | 0.0 | 0.0 | 0.0 | 0.0 | 0.0 | 0.0 | 0.0 | 0.0 |
| 2600006L11Rik | 1.1 | 1.1 | 0.9 | 2.2 | 0.8 | 1.2 | 0.4 | 0.6 | 0.8 | 2.0 | 0.6 | 0.7 |
| 2610001J05Rik | 9.8 | 10.0 | 9.3 | 10.6 | 13.5 | 10.6 | 10.9 | 10.4 | 13.9 | 13.1 | 12.0 | 11.6 |
| 2610002J02Rik | 18.1 | 18.6 | 14.8 | 19.9 | 15.8 | 19.8 | 15.9 | 16.4 | 16.6 | 14.1 | 13.5 | 14.1 |
| 2610002M06Rik | 2.6 | 2.9 | 3.4 | 2.5 | 2.6 | 3.2 | 3.0 | 3.3 | 2.9 | 3.2 | 3.0 | 3.6 |
| 2610005L07Rik | 14.0 | 15.2 | 16.1 | 16.3 | 13.3 | 15.9 | 16.0 | 14.8 | 15.1 | 12.2 | 14.8 | 14.5 |
| 2610008E11Rik | 24.9 | 24.3 | 18.4 | 29.5 | 18.4 | 29.4 | 18.8 | 13.7 | 21.4 | 17.5 | 23.5 | 21.7 |
| 2610015P09Rik | 3.0 | 2.5 | 3.1 | 3.2 | 2.0 | 3.0 | 2.0 | 1.9 | 1.9 | 2.4 | 2.6 | 2.5 |
| 2610018G03Rik | 0.5 | 0.3 | 0.5 | 0.1 | 0.3 | 0.1 | 0.1 | 0.4 | 1.2 | 11.9 | 5.7 | 3.0 |
| 2610019E17Rik | 6.5 | 6.0 | 3.9 | 6.0 | 7.3 | 4.2 | 5.6 | 11.6 | 3.9 | 6.0 | 10.2 | 7.3 |
| 2610019F03Rik | 13.4 | 10.8 | 10.3 | 13.3 | 8.3 | 9.5 | 8.9 | 8.0 | 10.2 | 16.2 | 11.8 | 12.8 |
| 2610020C07Rik | 0.3 | 0.3 | 0.6 | 0.2 | 0.2 | 0.4 | 0.3 | 0.4 | 0.2 | 0.2 | 0.5 | 0.3 |
| 2610020H08Rik | 2.1 | 2.2 | 1.9 | 0.9 | 1.7 | 1.0 | 2.3 | 1.6 | 2.3 | 2.0 | 1.6 | 1.9 |
| 2610027K06Rik | 0.3 | 0.3 | 0.3 | 0.6 | 0.3 | 0.4 | 0.7 | 0.4 | 0.4 | 0.3 | 0.2 | 0.2 |
| 2610034B18Rik | 7.3 | 7.3 | 9.5 | 8.7 | 7.8 | 8.8 | 10.9 | 7.9 | 8.8 | 6.6 | 9.1 | 9.3 |
| 2610035D17Rik | 1.6 | 2.0 | 1.9 | 2.8 | 2.2 | 2.7 | 2.0 | 2.4 | 2.3 | 2.0 | 2.1 | 1.6 |
| 2610037D02Rik | 0.0 | 0.0 | 0.0 | 0.0 | 0.0 | 0.0 | 0.0 | 0.0 | 0.0 | 0.0 | 0.0 | 0.0 |
| 2610044O15Rik8 | 3.9 | 3.3 | 3.8 | 3.1 | 4.4 | 2.9 | 3.8 | 3.7 | 3.7 | 3.2 | 4.5 | 3.6 |
| 2610203C20Rik | 6.1 | 8.6 | 8.4 | 6.7 | 7.0 | 8.4 | 7.5 | 5.7 | 5.9 | 7.7 | 8.0 | 7.0 |
| 2610203C22Rik | 0.3 | 0.3 | 0.1 | 0.9 | 1.0 | 0.5 | 0.5 | 0.2 | 0.3 | 0.4 | 0.2 | 0.4 |
| 2610204G22Rik | 0.9 | 1.5 | 0.9 | 1.1 | 0.5 | 1.2 | 0.7 | 0.9 | 1.1 | 0.8 | 0.2 | 0.8 |
| 2610301B20Rik | 9.9 | 8.5 | 8.9 | 10.2 | 7.5 | 10.3 | 6.8 | 8.0 | 6.8 | 7.8 | 8.5 | 8.2 |
| 2610301G19Rik | 6.4 | 5.7 | 6.1 | 5.6 | 6.0 | 6.1 | 6.1 | 6.7 | 5.8 | 5.4 | 5.1 | 4.6 |
| 2610305D13Rik | 1.7 | 2.3 | 2.9 | 2.9 | 2.4 | 3.4 | 2.8 | 2.5 | 1.9 | 1.9 | 3.1 | 2.9 |
| 2610306M01Rik | 2.7 | 2.9 | 2.0 | 1.7 | 2.2 | 2.6 | 2.1 | 1.7 | 1.9 | 2.5 | 2.8 | 2.8 |
| 2610307P16Rik | 0.2 | 0.1 | 0.1 | 0.1 | 0.1 | 0.1 | 0.0 | 0.1 | 0.1 | 0.2 | 0.1 | 0.2 |
| 2610318N02Rik | 0.0 | 0.0 | 0.0 | 0.1 | 0.3 | 0.0 | 0.1 | 0.0 | 0.0 | 0.0 | 0.0 | 0.0 |

| | | | | | | | | | | | | |
|---|---|---|---|---|---|---|---|---|---|---|---|---|
| 2610507B11Rik | 26.2 | 22.6 | 22.5 | 35.6 | 33.5 | 25.5 | 23.8 | 22.5 | 20.0 | 26.8 | 24.4 | 25.1 |
| 2610507I01Rik | 2.2 | 2.5 | 2.6 | 3.1 | 2.2 | 3.4 | 1.8 | 2.8 | 1.8 | 1.8 | 2.1 | 1.9 |
| 2610524H06Rik | 4.1 | 4.2 | 3.2 | 4.2 | 4.6 | 4.6 | 4.3 | 4.9 | 4.2 | 3.7 | 3.3 | 3.1 |
| 2610528A11Rik | 0.1 | 0.1 | 0.0 | 0.5 | 0.1 | 0.1 | 0.1 | 0.0 | 0.0 | 0.0 | 0.1 | 0.0 |
| 2700029M09Rik | 10.0 | 10.0 | 11.0 | 8.7 | 12.0 | 11.2 | 11.6 | 10.4 | 10.0 | 8.8 | 10.2 | 10.8 |
| 2700038G22Rik | 0.1 | 0.1 | 0.2 | 0.1 | 0.3 | 0.1 | 0.3 | 0.1 | 0.1 | 0.1 | 0.2 | 0.2 |
| 2700046G09Rik | 1.2 | 1.1 | 1.3 | 1.4 | 1.4 | 1.2 | 1.2 | 1.4 | 0.9 | 1.1 | 1.7 | 1.2 |
| 2700049A03Rik | 2.4 | 3.2 | 3.5 | 2.1 | 2.6 | 2.3 | 2.6 | 2.9 | 2.5 | 2.3 | 2.5 | 2.7 |
| 2700050L05Rik | 3.8 | 4.1 | 3.7 | 2.6 | 3.6 | 3.4 | 3.4 | 3.2 | 3.6 | 4.5 | 4.0 | 3.7 |
| 2700054A10Rik | 0.2 | 0.3 | 0.2 | 0.1 | 0.2 | 0.2 | 0.1 | 0.2 | 0.1 | 0.2 | 0.2 | 0.2 |
| 2700060E02Rik | 103.3 | 79.7 | 88.5 | 89.5 | 79.5 | 94.1 | 81.9 | 84.5 | 74.0 | 82.8 | 90.5 | 93.6 |
| 2700062C07Rik | 7.9 | 8.2 | 7.8 | 9.7 | 6.2 | 8.6 | 7.7 | 10.8 | 9.4 | 8.8 | 10.1 | 7.3 |
| 2700069I18Rik | 0.0 | 0.1 | 0.0 | 0.0 | 0.0 | 0.0 | 0.0 | 0.0 | 0.0 | 0.1 | 0.1 | 0.0 |
| 2700070H01Rik | 0.6 | 0.2 | 0.4 | 0.3 | 0.3 | 0.5 | 0.2 | 0.0 | 0.1 | 0.1 | 0.1 | 0.2 |
| 2700081L22Rik | 0.1 | 0.2 | 0.7 | 0.2 | 0.0 | 0.4 | 0.0 | 0.2 | 0.1 | 0.1 | 0.5 | 0.1 |
| 2700081O15Rik | 2.2 | 2.2 | 2.6 | 1.4 | 1.6 | 2.5 | 2.7 | 4.7 | 5.1 | 3.9 | 3.4 | 2.9 |
| 2700089E24Rik | 33.0 | 29.6 | 34.3 | 40.5 | 29.7 | 40.5 | 35.6 | 36.8 | 31.8 | 34.6 | 39.7 | 39.1 |
| 2700094K13Rik | 4.7 | 5.8 | 6.0 | 3.3 | 6.3 | 6.9 | 6.2 | 4.9 | 6.3 | 7.1 | 5.5 | 5.9 |
| 2700097O09Rik | 4.7 | 4.4 | 2.8 | 4.1 | 4.9 | 4.0 | 3.7 | 4.3 | 4.6 | 5.0 | 4.4 | 3.4 |
| 2700099C18Rik | 0.7 | 1.9 | 2.1 | 0.7 | 1.7 | 0.2 | 1.7 | 0.4 | 1.7 | 0.6 | 0.8 | 0.6 |
| 2810001G20Rik | 1.3 | 1.0 | 0.9 | 0.8 | 1.2 | 1.8 | 1.0 | 1.3 | 1.5 | 1.6 | 1.2 | 1.1 |
| 2810002D19Rik | 1.9 | 1.6 | 2.6 | 2.1 | 2.5 | 3.0 | 2.3 | 3.1 | 2.0 | 2.7 | 3.3 | 2.8 |
| 2810004N23Rik | 22.1 | 18.4 | 16.9 | 24.0 | 17.4 | 20.6 | 17.7 | 18.2 | 15.2 | 19.1 | 21.3 | 20.7 |
| 2810006K23Rik | 4.7 | 5.4 | 6.1 | 5.1 | 5.2 | 5.2 | 4.6 | 5.8 | 4.1 | 4.8 | 6.2 | 5.7 |
| 2810008D09Rik | 4.6 | 4.2 | 3.7 | 2.3 | 4.7 | 5.3 | 4.0 | 2.3 | 4.7 | 4.6 | 2.1 | 2.8 |
| 2810013P06Rik | 2.5 | 2.3 | 2.0 | 3.0 | 2.6 | 3.7 | 2.1 | 2.6 | 2.9 | 4.3 | 2.4 | 2.8 |
| 2810021J22Rik | 1.6 | 1.7 | 1.6 | 1.8 | 1.3 | 1.5 | 1.4 | 1.1 | 1.3 | 1.3 | 1.7 | 1.4 |
| 2810025M15Rik | 6.0 | 4.8 | 4.8 | 6.5 | 5.4 | 6.6 | 5.8 | 5.7 | 5.8 | 6.8 | 5.4 | 5.8 |
| 2810029C07Rik | 0.5 | 0.7 | 0.4 | 0.3 | 0.5 | 0.6 | 0.4 | 0.2 | 0.3 | 0.3 | 0.4 | 0.6 |
| 2810032G03Rik | 2.0 | 2.3 | 1.9 | 1.6 | 1.0 | 1.9 | 0.9 | 0.9 | 0.6 | 0.9 | 0.9 | 1.2 |
| 2810047C21Rik1 | 4.6 | 3.4 | 3.4 | 4.8 | 4.1 | 4.3 | 2.9 | 3.1 | 4.3 | 1.9 | 2.6 | 2.8 |
| 2810055G20Rik | 1.8 | 1.4 | 1.7 | 1.9 | 1.0 | 1.3 | 1.3 | 1.3 | 1.0 | 1.5 | 1.8 | 1.8 |
| 2810403A07Rik | 12.9 | 14.0 | 13.6 | 14.1 | 13.9 | 14.0 | 14.0 | 12.4 | 13.6 | 12.4 | 12.9 | 13.0 |
| 2810403D21Rik | 0.2 | 0.1 | 0.4 | 0.0 | 0.1 | 0.0 | 0.0 | 0.1 | 0.1 | 0.1 | 0.3 | 0.0 |
| 2810405F15Rik | 0.0 | 0.1 | 0.1 | 0.0 | 0.0 | 0.0 | 0.0 | 0.0 | 0.0 | 0.4 | 0.0 | 0.1 |
| 2810407C02Rik | 28.8 | 24.8 | 27.3 | 34.8 | 27.6 | 36.3 | 25.8 | 26.8 | 25.0 | 27.8 | 36.6 | 35.5 |
| 2810408A11Rik | 0.9 | 0.6 | 0.7 | 0.7 | 0.5 | 0.6 | 0.2 | 0.6 | 0.5 | 0.5 | 0.6 | 0.9 |
| 2810408I11Rik | 2.0 | 2.7 | 2.4 | 1.2 | 1.8 | 0.6 | 2.6 | 2.7 | 2.5 | 2.2 | 3.3 | 2.2 |
| 2810408M09Rik | 4.1 | 3.8 | 3.5 | 3.7 | 4.3 | 4.0 | 3.8 | 3.6 | 3.8 | 4.1 | 4.1 | 3.9 |
| 2810410L24Rik | 0.5 | 0.5 | 0.4 | 0.3 | 0.6 | 0.4 | 0.2 | 0.5 | 0.8 | 0.3 | 0.5 | 0.4 |
| 2810417H13Rik | 2.6 | 4.8 | 9.8 | 0.8 | 7.9 | 1.1 | 8.3 | 4.5 | 6.5 | 4.0 | 6.6 | 4.4 |
| 2810428I15Rik | 34.2 | 28.7 | 26.4 | 31.2 | 33.9 | 28.9 | 27.4 | 40.9 | 35.7 | 29.9 | 31.1 | 31.0 |
| 2810429I04Rik | 0.0 | 0.1 | 0.1 | 0.0 | 0.0 | 0.0 | 0.2 | 0.2 | 0.1 | 0.0 | 0.0 | 0.0 |
| 2810433D01Rik | 2.7 | 2.5 | 3.1 | 2.5 | 2.4 | 1.8 | 1.9 | 2.9 | 2.6 | 3.0 | 1.8 | 3.0 |
| 2810442I21Rik | 0.0 | 0.0 | 0.1 | 0.0 | 0.0 | 0.0 | 0.1 | 0.0 | 0.0 | 0.0 | 0.1 | 0.0 |
| 2810442N19Rik | 0.2 | 0.0 | 0.3 | 0.1 | 0.1 | 0.1 | 0.3 | 0.6 | 0.3 | 0.1 | 0.1 | 0.2 |
| 2810454H06Rik | 0.3 | 0.6 | 0.5 | 0.3 | 0.5 | 0.4 | 0.5 | 0.8 | 0.5 | 0.4 | 0.5 | 0.5 |
| 2810459M11Rik | 0.1 | 0.0 | 0.0 | 0.0 | 0.0 | 0.0 | 0.0 | 0.0 | 0.0 | 0.0 | 0.0 | 0.0 |
| 2810468N07Rik | 1.0 | 1.0 | 0.5 | 0.9 | 0.9 | 1.4 | 0.8 | 0.7 | 0.7 | 0.5 | 0.7 | 0.5 |
| 2810474O19Rik | 6.0 | 6.2 | 6.9 | 6.4 | 7.8 | 5.7 | 5.9 | 5.6 | 7.4 | 9.0 | 8.3 | 10.4 |
| 2900002K06Rik | 0.8 | 0.4 | 0.9 | 0.5 | 0.6 | 0.6 | 0.6 | 0.7 | 1.0 | 0.2 | 0.6 | 1.1 |
| 2900005J15Rik | 0.4 | 0.5 | 0.3 | 0.5 | 0.2 | 0.6 | 0.4 | 0.3 | 0.5 | 0.2 | 0.2 | 0.2 |
| 2900008C10Rik | 0.1 | 0.0 | 0.1 | 0.0 | 0.1 | 0.0 | 0.1 | 0.0 | 0.1 | 0.1 | 0.0 | 0.0 |
| 2900009J06Rik | 0.8 | 0.6 | 0.5 | 0.6 | 0.8 | 0.9 | 1.0 | 0.6 | 0.3 | 1.1 | 0.3 | 0.5 |
| 2900011O08Rik | 0.1 | 0.0 | 0.0 | 0.0 | 0.0 | 0.0 | 0.0 | 0.0 | 0.0 | 0.0 | 0.0 | 0.0 |
| 2900026A02Rik | 4.2 | 3.4 | 2.9 | 3.7 | 4.2 | 4.0 | 7.6 | 5.2 | 5.7 | 4.1 | 6.5 | 4.6 |
| 2900041M22Rik | 1.0 | 0.9 | 0.7 | 0.4 | 0.6 | 1.0 | 0.5 | 0.5 | 0.4 | 0.3 | 0.4 | 0.5 |

| | | | | | | | | | | | | |
|---|---|---|---|---|---|---|---|---|---|---|---|---|
| 2900056M20Rik | 0.3 | 0.3 | 0.6 | 0.6 | 0.5 | 0.6 | 0.5 | 0.5 | 0.3 | 0.5 | 0.6 | 0.6 |
| 2900057B20Rik | 0.2 | 0.4 | 0.1 | 0.2 | 0.2 | 0.4 | 0.2 | 0.1 | 0.1 | 0.2 | 0.2 | 0.1 |
| 2900060B14Rik | 3.1 | 2.6 | 4.7 | 4.2 | 5.6 | 2.8 | 3.8 | 3.3 | 5.3 | 3.8 | 2.4 | 4.7 |
| 2900076A07Rik | 1.4 | 0.9 | 1.3 | 2.7 | 1.8 | 2.4 | 1.1 | 0.6 | 1.2 | 0.8 | 1.5 | 0.8 |
| 2900092C05Rik | 0.1 | 0.0 | 0.0 | 0.1 | 0.0 | 0.0 | 0.1 | 0.0 | 0.2 | 0.1 | 0.0 | 0.0 |
| 2900097C17Rik | 52.7 | 55.0 | 55.2 | 59.8 | 56.6 | 57.1 | 50.8 | 58.3 | 57.3 | 63.8 | 59.5 | 65.3 |
| 3000002C10Rik | 1.5 | 1.5 | 1.4 | 1.9 | 1.0 | 1.7 | 1.7 | 1.9 | 1.0 | 1.2 | 1.2 | 1.2 |
| 3010001F23Rik | 0.1 | 0.2 | 0.8 | 0.1 | 0.2 | 0.5 | 0.8 | 0.5 | 0.2 | 0.1 | 0.2 | 0.1 |
| 3010026O09Rik | 2.6 | 2.5 | 3.4 | 2.6 | 2.2 | 3.6 | 2.9 | 2.4 | 2.6 | 2.0 | 2.3 | 2.0 |
| 3100003L05Rik | 0.0 | 0.0 | 0.0 | 0.0 | 0.0 | 0.0 | 0.0 | 0.0 | 0.1 | 0.0 | 0.0 | 0.0 |
| 3110001D03Rik | 25.2 | 21.6 | 20.7 | 21.2 | 23.0 | 22.3 | 25.1 | 22.6 | 27.0 | 25.1 | 25.1 | 25.6 |
| 3110001I22Rik | 1.4 | 0.9 | 1.0 | 0.7 | 1.2 | 0.8 | 0.6 | 0.8 | 0.9 | 1.2 | 1.3 | 1.2 |
| 3110002H16Rik | 15.3 | 13.7 | 11.7 | 16.6 | 17.5 | 13.8 | 14.7 | 13.5 | 13.8 | 13.4 | 14.8 | 14.1 |
| 3110007F17Rik | 0.8 | 1.3 | 1.2 | 0.4 | 0.8 | 1.5 | 1.1 | 1.4 | 1.0 | 0.8 | 0.8 | 1.3 |
| 3110009E18Rik | 1.9 | 2.1 | 2.6 | 1.3 | 2.2 | 3.1 | 1.3 | 1.7 | 2.0 | 2.8 | 3.0 | 2.7 |
| 3110021A11Rik | 0.3 | 0.2 | 0.3 | 0.1 | 0.1 | 0.1 | 0.3 | 0.3 | 0.3 | 0.2 | 0.4 | 0.1 |
| 3110021N24Rik | 2.9 | 2.9 | 2.3 | 3.1 | 2.0 | 3.4 | 2.8 | 3.4 | 2.0 | 2.4 | 2.7 | 3.2 |
| 3110035E14Rik | 0.0 | 0.0 | 0.0 | 0.0 | 0.1 | 0.0 | 0.0 | 0.0 | 0.0 | 0.4 | 0.2 | 0.1 |
| 3110039I08Rik | 0.7 | 0.3 | 0.6 | 0.3 | 0.2 | 0.3 | 0.4 | 0.2 | 0.2 | 0.4 | 0.2 | 0.2 |
| 3110040N11Rik | 15.5 | 15.4 | 20.2 | 18.9 | 13.5 | 19.9 | 17.6 | 16.7 | 12.7 | 14.7 | 18.6 | 17.5 |
| 3110043O21Rik | 6.2 | 6.5 | 5.7 | 6.3 | 6.9 | 5.5 | 4.7 | 4.4 | 6.1 | 6.7 | 5.2 | 5.9 |
| 3110045C21Rik | 1.7 | 2.7 | 2.7 | 1.4 | 1.7 | 1.2 | 2.1 | 1.8 | 2.2 | 1.5 | 2.5 | 1.0 |
| 3110047P20Rik | 0.0 | 0.0 | 0.0 | 0.0 | 0.0 | 0.0 | 0.0 | 0.0 | 0.0 | 0.0 | 0.0 | 0.0 |
| 3110052M02Rik | 1.3 | 1.8 | 1.5 | 1.4 | 1.1 | 2.1 | 1.4 | 1.3 | 1.4 | 1.4 | 1.5 | 1.3 |
| 3110056K07Rik | 2.0 | 2.3 | 1.9 | 2.9 | 2.1 | 2.8 | 3.0 | 2.3 | 1.9 | 2.8 | 1.7 | 2.2 |
| 3110057O12Rik | 1.1 | 1.3 | 1.5 | 1.1 | 0.8 | 1.2 | 0.9 | 0.9 | 1.0 | 0.9 | 1.0 | 1.4 |
| 3110062M04Rik | 9.4 | 7.4 | 6.9 | 11.5 | 9.2 | 11.9 | 7.6 | 5.8 | 6.5 | 7.5 | 7.3 | 8.9 |
| 3110070M22Rik | 1.0 | 0.9 | 0.9 | 0.8 | 0.7 | 0.9 | 0.9 | 0.8 | 0.9 | 0.8 | 0.4 | 1.4 |
| 3110082I17Rik | 3.3 | 4.2 | 2.7 | 4.9 | 3.5 | 4.9 | 3.8 | 3.8 | 2.4 | 2.8 | 4.0 | 3.5 |
| 3110082J24Rik | 0.9 | 0.7 | 0.6 | 0.2 | 1.0 | 0.4 | 0.7 | 1.0 | 1.0 | 0.4 | 0.5 | 0.7 |
| 3200001D21Rik | 0.1 | 0.0 | 0.1 | 0.0 | 0.1 | 0.0 | 0.0 | 0.1 | 0.0 | 0.1 | 0.1 | 0.0 |
| 3230401D17Rik | 18.0 | 17.4 | 16.9 | 20.8 | 18.5 | 16.6 | 17.8 | 15.6 | 17.5 | 18.8 | 19.5 | 17.8 |
| 3300002I08Rik | 2.8 | 3.0 | 2.4 | 1.3 | 2.1 | 1.7 | 1.5 | 1.6 | 1.9 | 3.1 | 1.6 | 1.7 |
| 3300005D01Rik | 33.3 | 24.3 | 20.9 | 42.1 | 27.7 | 44.5 | 33.9 | 23.4 | 19.9 | 17.5 | 22.7 | 25.1 |
| 3425401B19Rik | 0.2 | 0.1 | 0.1 | 0.1 | 0.1 | 0.2 | 0.0 | 0.1 | 0.0 | 0.1 | 0.0 | 0.1 |
| 3632451O06Rik | 3.6 | 4.0 | 5.5 | 2.7 | 1.7 | 2.5 | 4.1 | 2.9 | 2.1 | 2.8 | 3.3 | 4.1 |
| 3632454L22Rik | 0.0 | 0.0 | 0.0 | 0.1 | 0.0 | 0.0 | 0.0 | 0.0 | 0.1 | 0.0 | 0.0 | 0.0 |
| 3830403N18Rik | 2.6 | 2.1 | 2.4 | 2.7 | 2.7 | 2.7 | 2.5 | 2.5 | 2.2 | 2.2 | 4.7 | 2.3 |
| 3830406C13Rik | 10.8 | 10.9 | 11.9 | 12.4 | 12.0 | 11.2 | 11.5 | 11.6 | 10.3 | 13.4 | 13.9 | 14.0 |
| 3830408C21Rik | 0.4 | 0.4 | 0.7 | 0.3 | 0.4 | 0.3 | 0.3 | 0.4 | 0.4 | 0.3 | 0.3 | 0.3 |
| 3930402G23Rik | 0.1 | 0.1 | 0.1 | 0.1 | 0.1 | 0.0 | 0.1 | 0.1 | 0.1 | 0.1 | 0.1 | 0.1 |
| 4430402I18Rik | 0.4 | 0.5 | 0.5 | 0.4 | 0.3 | 0.9 | 0.3 | 0.7 | 0.4 | 0.4 | 0.3 | 0.5 |
| 4632415K11Rik | 4.5 | 4.0 | 3.7 | 4.1 | 3.4 | 5.0 | 3.4 | 3.6 | 3.2 | 4.3 | 4.0 | 3.5 |
| 4632415L05Rik | 1.8 | 1.4 | 1.8 | 2.3 | 1.8 | 2.3 | 2.2 | 1.9 | 1.6 | 1.8 | 1.9 | 2.1 |
| 4632427E13Rik | 4.5 | 4.9 | 4.3 | 5.2 | 4.4 | 4.1 | 4.3 | 5.2 | 6.0 | 4.4 | 3.5 | 5.1 |
| 4632428C04Rik | 0.2 | 0.1 | 0.2 | 0.2 | 0.1 | 0.2 | 0.1 | 0.1 | 0.1 | 0.1 | 0.1 | 0.3 |
| 4632428N05Rik | 7.3 | 11.6 | 13.8 | 9.4 | 13.6 | 6.5 | 8.9 | 9.0 | 10.1 | 11.3 | 8.4 | 12.2 |
| 4632434I11Rik | 6.9 | 9.1 | 10.0 | 4.1 | 9.9 | 3.9 | 8.5 | 4.8 | 8.1 | 8.1 | 8.1 | 7.2 |
| 4732415M23Rik | 0.6 | 1.0 | 0.7 | 0.9 | 0.4 | 2.0 | 0.4 | 0.6 | 0.6 | 0.6 | 1.2 | 1.5 |
| 4732416N19Rik | 0.1 | 0.0 | 0.0 | 0.4 | 0.1 | 0.1 | 0.0 | 0.1 | 0.1 | 0.1 | 0.3 | 0.0 |
| 4732471J01Rik | 0.2 | 0.2 | 0.3 | 0.2 | 0.2 | 0.1 | 0.3 | 0.2 | 0.1 | 0.5 | 0.1 | 0.1 |
| 4732491K20Rik | 0.7 | 0.4 | 0.4 | 0.9 | 0.4 | 0.7 | 0.6 | 0.4 | 0.3 | 0.5 | 0.6 | 0.4 |
| 4831440E17Rik | 0.1 | 0.2 | 0.3 | 0.2 | 0.2 | 0.2 | 0.2 | 0.1 | 0.2 | 0.1 | 0.0 | 0.1 |
| 4833403I15Rik | 0.1 | 0.1 | 0.2 | 0.0 | 0.1 | 0.0 | 0.0 | 0.1 | 0.1 | 0.4 | 0.4 | 0.0 |
| 4833412C05Rik | 3.3 | 1.8 | 1.1 | 3.2 | 2.4 | 4.3 | 1.6 | 0.7 | 1.2 | 0.9 | 3.7 | 1.0 |
| 4833417C18Rik | 0.2 | 0.3 | 0.3 | 0.3 | 0.2 | 0.0 | 0.2 | 0.2 | 0.1 | 0.0 | 0.1 | 0.3 |
| 4833418N02Rik | 1.1 | 1.8 | 1.3 | 1.4 | 1.6 | 1.0 | 1.3 | 1.1 | 1.5 | 1.5 | 1.2 | 1.6 |

| | | | | | | | | | | | | |
|---|---|---|---|---|---|---|---|---|---|---|---|---|
| 4833419F23Rik | 0.9 | 0.8 | 1.3 | 2.0 | 1.0 | 1.2 | 1.4 | 1.5 | 1.1 | 0.8 | 1.4 | 1.2 |
| 4833420G17Rik | 8.0 | 9.7 | 9.1 | 8.8 | 8.0 | 8.7 | 7.1 | 7.9 | 7.8 | 8.5 | 8.3 | 8.0 |
| 4833422C13Rik | 0.9 | 0.7 | 1.3 | 1.0 | 1.4 | 0.5 | 1.0 | 1.0 | 1.2 | 1.1 | 0.8 | 1.0 |
| 4833427F10Rik | 0.0 | 0.0 | 0.0 | 0.0 | 0.0 | 0.0 | 0.0 | 0.0 | 0.0 | 0.1 | 0.1 | 0.0 |
| 4833427G06Rik | 3.2 | 3.2 | 2.6 | 1.2 | 2.1 | 2.6 | 1.0 | 1.1 | 1.5 | 1.8 | 0.9 | 2.2 |
| 4833439L19Rik | 53.0 | 48.4 | 39.6 | 59.6 | 44.7 | 56.7 | 42.7 | 37.7 | 39.9 | 38.6 | 43.2 | 39.7 |
| 4921507L20Rik | 0.1 | 0.1 | 0.1 | 0.0 | 0.1 | 0.0 | 0.2 | 0.1 | 0.0 | 0.2 | 0.1 | 0.0 |
| 4921507P07Rik | 0.0 | 0.1 | 0.1 | 0.1 | 0.0 | 0.1 | 0.0 | 0.1 | 0.0 | 0.1 | 0.1 | 0.1 |
| 4921508A21Rik | 0.3 | 0.5 | 0.1 | 0.4 | 0.4 | 0.3 | 0.2 | 0.4 | 0.4 | 0.4 | 0.1 | 0.1 |
| 4921511C10Rik | 0.1 | 0.1 | 0.1 | 0.0 | 0.1 | 0.1 | 0.0 | 0.2 | 0.0 | 0.1 | 0.1 | 0.0 |
| 4921511I17Rik | 0.0 | 0.1 | 0.0 | 0.1 | 0.0 | 0.1 | 0.0 | 0.1 | 0.1 | 0.2 | 0.0 | 0.0 |
| 4921513I03Rik | 0.0 | 0.1 | 0.0 | 0.0 | 0.0 | 0.1 | 0.0 | 0.1 | 0.1 | 0.1 | 0.2 | 0.1 |
| 4921524J17Rik | 15.6 | 13.5 | 19.2 | 13.7 | 14.1 | 12.1 | 14.1 | 11.9 | 13.0 | 16.4 | 17.9 | 20.3 |
| 4921525O09Rik | 0.0 | 0.0 | 0.0 | 0.0 | 0.0 | 0.0 | 0.0 | 0.0 | 0.1 | 0.0 | 0.0 | 0.0 |
| 4921530D09Rik | 0.0 | 0.1 | 0.0 | 0.0 | 0.1 | 0.1 | 0.0 | 0.0 | 0.0 | 0.0 | 0.0 | 0.0 |
| 4921530L18Rik | 0.5 | 0.5 | 0.2 | 0.9 | 0.6 | 0.7 | 0.8 | 0.7 | 0.8 | 0.5 | 0.7 | 0.5 |
| 4921531C22Rik | 1.5 | 1.5 | 1.3 | 1.5 | 1.3 | 1.3 | 1.1 | 1.8 | 1.1 | 1.2 | 1.7 | 1.7 |
| 4921536K21Rik | 0.4 | 0.3 | 0.3 | 0.5 | 0.3 | 0.3 | 0.3 | 0.1 | 0.1 | 0.1 | 0.4 | 0.1 |
| 4921539E11Rik | 0.0 | 0.0 | 0.0 | 0.0 | 0.0 | 0.0 | 0.0 | 0.0 | 0.0 | 0.0 | 0.0 | 0.0 |
| 4922501C03Rik | 5.0 | 5.2 | 6.5 | 6.4 | 5.3 | 5.2 | 5.1 | 5.1 | 3.9 | 3.5 | 5.8 | 5.3 |
| 4922501L14Rik | 0.0 | 0.0 | 0.0 | 0.0 | 0.0 | 0.0 | 0.0 | 0.0 | 0.0 | 0.0 | 0.0 | 0.1 |
| 4930402H24Rik | 17.7 | 14.1 | 12.8 | 19.4 | 18.8 | 15.0 | 16.7 | 13.3 | 15.4 | 14.4 | 16.8 | 14.2 |
| 4930404H11Rik | 0.1 | 0.1 | 0.0 | 0.0 | 0.0 | 0.0 | 0.1 | 0.0 | 0.0 | 0.1 | 0.0 | 0.0 |
| 4930404I05Rik | 0.1 | 0.0 | 0.1 | 0.5 | 0.1 | 0.0 | 0.1 | 0.1 | 0.1 | 0.1 | 0.1 | 0.4 |
| 4930404N11Rik | 0.8 | 1.5 | 1.6 | 1.1 | 1.9 | 0.8 | 1.4 | 0.9 | 1.3 | 1.2 | 0.9 | 1.3 |
| 4930405A21Rik | 0.4 | 0.3 | 0.3 | 0.2 | 0.3 | 0.1 | 0.4 | 0.1 | 0.2 | 0.2 | 0.1 | 0.3 |
| 4930405P13Rik | 0.1 | 0.0 | 0.0 | 0.2 | 0.1 | 0.1 | 0.0 | 0.0 | 0.0 | 0.1 | 0.0 | 0.0 |
| 4930412C18Rik | 0.4 | 0.6 | 0.6 | 0.5 | 0.3 | 0.5 | 0.4 | 0.5 | 0.5 | 0.4 | 0.5 | 0.4 |
| 4930413G21Rik | 1.5 | 1.5 | 1.5 | 1.0 | 1.5 | 1.2 | 1.6 | 1.9 | 2.1 | 2.5 | 1.1 | 1.6 |
| 4930414L22Rik | 3.0 | 3.1 | 2.4 | 3.3 | 3.2 | 3.1 | 3.3 | 3.0 | 3.5 | 4.1 | 2.5 | 2.7 |
| 4930414N06Rik | 0.7 | 0.7 | 0.9 | 0.0 | 1.3 | 0.7 | 0.6 | 0.7 | 0.8 | 0.6 | 0.2 | 0.4 |
| 4930415O20Rik | 0.1 | 0.1 | 0.1 | 0.1 | 0.1 | 0.0 | 0.0 | 0.2 | 0.0 | 0.2 | 0.0 | 0.0 |
| 4930422G04Rik | 1.0 | 1.4 | 2.1 | 0.7 | 1.8 | 0.9 | 1.9 | 1.0 | 1.4 | 0.8 | 1.7 | 1.3 |
| 4930426L09Rik | 0.2 | 0.4 | 0.3 | 0.4 | 0.3 | 0.4 | 0.6 | 0.2 | 0.8 | 0.6 | 0.5 | 0.7 |
| 4930427A07Rik | 0.8 | 1.5 | 1.7 | 0.4 | 1.9 | 0.3 | 2.5 | 1.0 | 1.7 | 1.0 | 1.4 | 0.7 |
| 4930429B21Rik | 1.2 | 0.7 | 0.8 | 1.5 | 1.4 | 1.5 | 1.7 | 1.2 | 1.4 | 1.8 | 2.3 | 1.5 |
| 4930429F24Rik | 0.2 | 0.1 | 0.1 | 0.1 | 0.1 | 0.0 | 0.1 | 0.2 | 0.1 | 0.0 | 0.1 | 0.1 |
| 4930430F08Rik | 4.2 | 4.3 | 5.0 | 5.0 | 3.8 | 5.7 | 3.6 | 3.1 | 3.2 | 4.1 | 4.4 | 4.5 |
| 4930431F12Rik | 0.1 | 0.1 | 0.1 | 0.0 | 0.1 | 0.1 | 0.1 | 0.1 | 0.1 | 0.1 | 0.1 | 0.0 |
| 4930431P03Rik | 0.0 | 0.0 | 0.0 | 0.0 | 0.0 | 0.0 | 0.0 | 0.0 | 0.0 | 0.0 | 0.0 | 0.0 |
| 4930432K21Rik | 0.3 | 0.4 | 0.4 | 0.3 | 0.2 | 0.3 | 0.2 | 0.2 | 0.1 | 0.1 | 0.3 | 0.2 |
| 4930441O14Rik | 0.2 | 0.2 | 0.3 | 0.1 | 0.2 | 0.2 | 0.2 | 0.2 | 0.2 | 0.2 | 0.2 | 0.1 |
| 4930443O20Rik | 0.2 | 0.3 | 0.2 | 0.0 | 0.1 | 0.3 | 0.4 | 0.5 | 0.3 | 0.3 | 0.1 | 0.3 |
| 4930444A02Rik | 7.4 | 6.5 | 5.0 | 8.2 | 6.0 | 8.1 | 5.9 | 6.4 | 5.6 | 6.2 | 6.8 | 6.4 |
| 4930444G20Rik | 0.0 | 0.0 | 0.0 | 0.2 | 0.0 | 0.0 | 0.0 | 0.0 | 0.0 | 0.0 | 0.0 | 0.0 |
| 4930447C04Rik | 0.1 | 0.1 | 0.1 | 0.3 | 0.0 | 0.3 | 0.1 | 0.0 | 0.0 | 0.0 | 0.2 | 0.2 |
| 4930447K03Rik | 0.1 | 0.1 | 0.3 | 0.0 | 0.2 | 0.2 | 0.2 | 0.2 | 0.1 | 0.0 | 0.1 | 0.0 |
| 4930447N08Rik | 0.1 | 0.1 | 0.0 | 0.0 | 0.0 | 0.0 | 0.0 | 0.0 | 0.0 | 0.0 | 0.1 | 0.0 |
| 4930451G09Rik | 0.3 | 0.2 | 0.2 | 0.1 | 0.2 | 0.4 | 0.2 | 0.2 | 0.1 | 0.2 | 0.1 | 0.3 |
| 4930452B06Rik | 0.7 | 0.5 | 0.7 | 0.3 | 0.7 | 1.0 | 0.7 | 0.3 | 0.4 | 0.3 | 0.5 | 0.4 |
| 4930453N24Rik | 26.2 | 22.1 | 24.8 | 26.0 | 21.5 | 22.9 | 22.8 | 22.0 | 20.4 | 23.0 | 27.2 | 24.6 |
| 4930455C13Rik | 6.5 | 5.7 | 4.2 | 7.0 | 4.7 | 8.7 | 7.2 | 5.1 | 4.7 | 5.2 | 7.0 | 5.3 |
| 4930455F23Rik | 10.4 | 6.9 | 6.2 | 10.2 | 5.7 | 10.9 | 6.1 | 6.2 | 4.9 | 5.8 | 7.4 | 6.9 |
| 4930465K10Rik | 0.0 | 0.0 | 0.0 | 0.1 | 0.0 | 0.0 | 0.1 | 0.1 | 0.0 | 0.2 | 0.1 | 0.1 |
| 4930467E23Rik | 0.2 | 0.2 | 0.2 | 0.0 | 0.1 | 0.1 | 0.2 | 0.0 | 0.0 | 0.1 | 0.0 | 0.2 |
| 4930470H14Rik | 6.9 | 9.7 | 8.5 | 7.3 | 7.9 | 7.1 | 9.1 | 6.1 | 5.1 | 7.5 | 6.6 | 7.5 |
| 4930471I20Rik | 0.0 | 0.0 | 0.1 | 0.1 | 0.2 | 0.1 | 0.0 | 0.3 | 0.1 | 0.0 | 0.0 | 0.0 |

| | | | | | | | | | | | | |
|---|---|---|---|---|---|---|---|---|---|---|---|---|
| 4930471M09Rik | 0.0 | 0.0 | 0.0 | 0.0 | 0.0 | 0.1 | 0.0 | 0.0 | 0.0 | 0.0 | 0.0 | 0.1 |
| 4930473A02Rik | 0.4 | 0.3 | 0.4 | 0.4 | 0.3 | 0.2 | 1.0 | 0.5 | 0.4 | 0.4 | 0.5 | 0.7 |
| 4930478L05Rik | 0.1 | 0.0 | 0.1 | 0.0 | 0.0 | 0.0 | 0.0 | 0.1 | 0.0 | 0.2 | 0.0 | 0.0 |
| 4930479D17Rik | 0.1 | 0.0 | 0.0 | 0.2 | 0.1 | 0.1 | 0.0 | 0.0 | 0.0 | 0.1 | 0.0 | 0.1 |
| 4930480G23Rik | 0.1 | 0.1 | 0.0 | 0.0 | 0.0 | 0.1 | 0.1 | 0.0 | 0.1 | 0.1 | 0.1 | 0.1 |
| 4930481A15Rik | 2.0 | 1.4 | 1.2 | 1.7 | 2.0 | 1.7 | 2.2 | 2.8 | 2.0 | 1.2 | 1.6 | 1.6 |
| 4930483J18Rik | 0.1 | 0.1 | 0.0 | 0.1 | 0.0 | 0.0 | 0.0 | 0.0 | 0.0 | 0.0 | 0.0 | 0.1 |
| 4930483K19Rik | 0.5 | 0.5 | 0.5 | 0.2 | 0.4 | 0.2 | 0.5 | 0.4 | 0.4 | 0.1 | 0.4 | 0.1 |
| 4930486L24Rik | 4.6 | 0.8 | 0.2 | 4.4 | 1.0 | 3.9 | 0.5 | 0.3 | 0.1 | 0.3 | 0.5 | 0.5 |
| 4930487H11Rik | 0.0 | 0.1 | 0.0 | 0.5 | 0.2 | 0.2 | 0.1 | 0.1 | 0.0 | 0.0 | 0.1 | 0.1 |
| 4930488L21Rik | 0.1 | 0.1 | 0.0 | 0.0 | 0.0 | 0.2 | 0.1 | 0.0 | 0.1 | 0.1 | 0.0 | 0.1 |
| 4930500J02Rik | 0.8 | 0.3 | 0.3 | 0.0 | 0.3 | 0.1 | 0.2 | 0.2 | 0.1 | 0.3 | 0.3 | 0.2 |
| 4930502A04Rik | 0.0 | 0.1 | 0.0 | 0.0 | 0.0 | 0.0 | 0.0 | 0.0 | 0.0 | 0.0 | 0.0 | 0.0 |
| 4930502E09Rik | 0.1 | 0.1 | 0.1 | 0.0 | 0.3 | 0.3 | 0.3 | 0.1 | 0.1 | 0.2 | 0.2 | 0.1 |
| 4930502E18Rik | 0.2 | 0.0 | 0.0 | 0.1 | 0.0 | 0.1 | 0.1 | 0.1 | 0.0 | 0.0 | 0.0 | 0.0 |
| 4930503B20Rik | 0.0 | 0.0 | 0.0 | 0.0 | 0.0 | 0.0 | 0.1 | 0.0 | 0.0 | 0.0 | 0.1 | 0.2 |
| 4930503L19Rik | 5.8 | 6.9 | 7.1 | 6.9 | 9.0 | 5.6 | 7.0 | 6.3 | 7.5 | 5.6 | 6.7 | 6.9 |
| 4930505A04Rik | 0.9 | 0.5 | 0.7 | 0.6 | 0.7 | 0.9 | 0.9 | 0.6 | 0.2 | 0.4 | 0.5 | 0.3 |
| 4930506C21Rik | 0.8 | 0.7 | 0.7 | 1.1 | 0.5 | 1.0 | 0.2 | 0.1 | 0.3 | 0.7 | 0.2 | 0.4 |
| 4930506M07Rik | 2.4 | 1.0 | 1.4 | 4.6 | 5.0 | 1.9 | 1.3 | 0.7 | 0.4 | 1.2 | 1.1 | 1.2 |
| 4930507D05Rik | 0.7 | 0.3 | 0.4 | 0.3 | 0.6 | 0.4 | 0.6 | 0.5 | 0.3 | 0.7 | 0.6 | 0.3 |
| 4930509E16Rik | 0.7 | 0.7 | 0.3 | 0.8 | 0.5 | 0.6 | 0.2 | 0.1 | 0.1 | 0.1 | 0.4 | 0.1 |
| 4930511M06Rik | 0.6 | 0.7 | 0.6 | 0.4 | 0.6 | 0.5 | 0.6 | 0.4 | 0.4 | 0.3 | 0.5 | 0.4 |
| 4930511M11Rik | 0.0 | 0.1 | 0.0 | 0.0 | 0.0 | 0.0 | 0.1 | 0.0 | 0.0 | 0.0 | 0.1 | 0.1 |
| 4930512B01Rik | 0.2 | 0.1 | 0.1 | 0.2 | 0.2 | 0.3 | 0.4 | 0.3 | 0.4 | 0.2 | 0.2 | 0.3 |
| 4930513N10Rik | 0.4 | 0.6 | 0.5 | 0.4 | 0.6 | 0.5 | 0.4 | 0.4 | 0.4 | 0.6 | 0.3 | 0.3 |
| 4930515G01Rik | 0.7 | 0.9 | 0.9 | 0.4 | 0.6 | 0.9 | 0.9 | 0.8 | 0.7 | 1.1 | 0.9 | 1.3 |
| 4930519F09Rik | 0.3 | 0.4 | 0.6 | 0.4 | 0.3 | 0.6 | 0.5 | 0.5 | 0.3 | 0.3 | 0.2 | 0.5 |
| 4930519G04Rik | 0.0 | 0.0 | 0.0 | 0.0 | 0.0 | 0.0 | 0.4 | 0.0 | 0.0 | 0.0 | 0.0 | 0.0 |
| 4930520O04Rik | 0.2 | 0.2 | 0.2 | 0.8 | 0.2 | 0.3 | 0.2 | 0.3 | 0.1 | 0.4 | 0.1 | 0.2 |
| 4930521E06Rik | 0.1 | 0.1 | 0.1 | 0.1 | 0.0 | 0.0 | 0.0 | 0.0 | 0.0 | 0.0 | 0.1 | 0.0 |
| 4930523C07Rik | 2.8 | 2.8 | 3.3 | 3.2 | 2.4 | 2.8 | 2.5 | 3.5 | 3.2 | 1.7 | 2.2 | 2.6 |
| 4930524L23Rik | 0.1 | 0.1 | 0.1 | 0.0 | 0.1 | 0.2 | 0.1 | 0.0 | 0.0 | 0.1 | 0.1 | 0.0 |
| 4930525G20Rik | 0.2 | 0.2 | 0.2 | 0.1 | 0.1 | 0.1 | 0.1 | 0.3 | 0.3 | 0.2 | 0.1 | 0.2 |
| 4930526I15Rik | 2.4 | 2.3 | 2.1 | 3.5 | 2.4 | 2.8 | 2.6 | 2.7 | 2.2 | 2.7 | 2.2 | 1.6 |
| 4930528A17Rik | 0.1 | 0.3 | 0.4 | 0.1 | 0.3 | 0.2 | 0.3 | 0.2 | 0.3 | 0.2 | 0.3 | 0.3 |
| 4930528D03Rik | 0.0 | 0.3 | 0.1 | 0.0 | 0.1 | 0.0 | 0.1 | 0.1 | 0.1 | 0.0 | 0.2 | 0.0 |
| 4930529C04Rik | 0.0 | 0.0 | 0.0 | 0.1 | 0.0 | 0.0 | 0.1 | 0.1 | 0.0 | 0.1 | 0.1 | 0.1 |
| 4930533B01Rik | 0.0 | 0.1 | 0.1 | 0.1 | 0.0 | 0.0 | 0.1 | 0.1 | 0.0 | 0.0 | 0.1 | 0.0 |
| 4930538K18Rik | 0.0 | 0.0 | 0.1 | 0.3 | 0.0 | 0.0 | 0.0 | 0.0 | 0.0 | 0.0 | 0.0 | 0.1 |
| 4930539E08Rik | 0.1 | 0.1 | 0.0 | 0.1 | 0.0 | 0.0 | 0.1 | 0.0 | 0.0 | 0.0 | 0.0 | 0.0 |
| 4930539J05Rik | 1.9 | 1.4 | 1.5 | 3.2 | 2.0 | 1.2 | 2.3 | 2.0 | 3.0 | 2.5 | 2.8 | 2.7 |
| 4930549G23Rik | 0.6 | 0.7 | 0.3 | 0.5 | 0.7 | 0.4 | 0.7 | 0.2 | 0.6 | 0.4 | 0.1 | 0.2 |
| 4930550C14Rik | 1.9 | 2.1 | 1.4 | 1.9 | 1.6 | 2.2 | 0.9 | 0.8 | 0.7 | 1.4 | 0.9 | 1.2 |
| 4930552P12Rik | 0.1 | 0.0 | 0.0 | 0.0 | 0.0 | 0.0 | 0.0 | 0.0 | 0.1 | 0.0 | 0.0 | 0.0 |
| 4930555G01Rik | 0.1 | 0.3 | 0.1 | 0.1 | 0.1 | 0.1 | 0.1 | 0.1 | 0.1 | 0.0 | 0.1 | 0.1 |
| 4930556M19Rik | 2.3 | 1.7 | 1.6 | 2.4 | 2.0 | 2.2 | 1.4 | 1.0 | 1.6 | 1.5 | 1.4 | 2.1 |
| 4930558J18Rik | 0.1 | 0.2 | 0.3 | 0.1 | 0.4 | 0.1 | 0.3 | 0.1 | 0.2 | 0.1 | 0.4 | 0.2 |
| 4930562C15Rik | 0.0 | 0.0 | 0.0 | 0.0 | 0.0 | 0.0 | 0.0 | 0.0 | 0.0 | 0.0 | 0.0 | 0.0 |
| 4930562F07Rik | 1.7 | 2.0 | 2.7 | 2.2 | 1.5 | 1.0 | 0.8 | 1.3 | 1.6 | 0.8 | 1.9 | 2.2 |
| 4930563D23Rik | 0.0 | 0.0 | 0.0 | 0.0 | 0.0 | 0.0 | 0.0 | 0.0 | 0.0 | 0.0 | 0.0 | 0.0 |
| 4930563E18Rik | 0.0 | 0.2 | 0.0 | 0.0 | 0.1 | 0.1 | 0.1 | 0.1 | 0.0 | 0.0 | 0.0 | 0.1 |
| 4930563E22Rik | 0.2 | 0.3 | 0.3 | 0.3 | 0.3 | 0.5 | 0.1 | 0.3 | 0.3 | 0.2 | 0.1 | 0.2 |
| 4930564C03Rik | 0.0 | 0.0 | 0.0 | 0.0 | 0.0 | 0.0 | 0.0 | 0.0 | 0.0 | 0.0 | 0.0 | 0.0 |
| 4930565N06Rik | 0.7 | 0.9 | 1.0 | 0.5 | 0.7 | 0.8 | 0.5 | 0.7 | 0.7 | 0.6 | 0.5 | 0.9 |
| 4930568K20Rik | 0.1 | 0.0 | 0.0 | 0.0 | 0.1 | 0.0 | 0.0 | 0.0 | 0.0 | 0.0 | 0.1 | 0.0 |
| 4930570G19Rik | 0.3 | 0.2 | 0.2 | 0.0 | 0.1 | 0.0 | 0.1 | 0.3 | 0.1 | 0.0 | 0.1 | 0.0 |

| | | | | | | | | | | | | |
|---|---|---|---|---|---|---|---|---|---|---|---|---|
| 4930577N17Rik | 0.6 | 0.7 | 0.7 | 1.2 | 1.0 | 0.8 | 0.9 | 1.1 | 0.8 | 1.0 | 0.8 | 0.8 |
| 4930578M01Rik | 0.3 | 0.6 | 0.2 | 0.4 | 0.3 | 0.8 | 0.6 | 0.3 | 0.3 | 0.1 | 0.3 | 0.3 |
| 4930579G18Rik | 0.4 | 0.3 | 0.2 | 0.3 | 0.5 | 0.7 | 0.3 | 0.5 | 0.2 | 0.6 | 0.4 | 0.3 |
| 4930579G24Rik | 1.7 | 2.2 | 3.3 | 1.3 | 3.7 | 2.1 | 4.3 | 2.4 | 3.8 | 2.1 | 2.8 | 2.0 |
| 4930579K19Rik | 0.5 | 0.2 | 0.4 | 0.4 | 0.6 | 0.5 | 0.4 | 0.4 | 0.6 | 0.3 | 0.4 | 0.4 |
| 4930581F22Rik | 0.8 | 0.9 | 0.8 | 0.6 | 0.8 | 0.9 | 0.5 | 1.0 | 0.7 | 0.7 | 0.8 | 0.6 |
| 4930590J08Rik | 0.0 | 0.0 | 0.0 | 0.0 | 0.0 | 0.0 | 0.0 | 0.0 | 0.0 | 0.0 | 0.0 | 0.0 |
| 4930592A05Rik | 5.2 | 5.9 | 3.7 | 3.2 | 3.8 | 5.5 | 3.2 | 1.9 | 3.2 | 4.3 | 4.1 | 2.9 |
| 4930592I03Rik | 0.1 | 0.1 | 0.2 | 0.1 | 0.3 | 0.2 | 0.2 | 0.2 | 0.1 | 0.3 | 0.1 | 0.5 |
| 4930594C11Rik | 0.1 | 0.1 | 0.1 | 0.0 | 0.2 | 0.1 | 0.1 | 0.0 | 0.0 | 0.0 | 0.1 | 0.0 |
| 4930599N23Rik | 0.6 | 0.2 | 0.3 | 0.1 | 0.4 | 0.3 | 0.3 | 0.7 | 0.2 | 0.5 | 0.3 | 0.3 |
| 4931402G19Rik | 0.1 | 0.2 | 0.1 | 0.0 | 0.2 | 0.1 | 0.1 | 0.2 | 0.1 | 0.1 | 0.1 | 0.1 |
| 4931403E22Rik | 0.3 | 0.0 | 0.1 | 0.0 | 0.2 | 0.0 | 0.0 | 0.0 | 0.1 | 0.0 | 0.1 | 0.1 |
| 4931403G20Rik | 0.2 | 0.4 | 0.3 | 0.2 | 0.3 | 0.2 | 0.4 | 0.1 | 0.4 | 0.3 | 0.3 | 0.2 |
| 4931406C07Rik | 4.0 | 4.5 | 4.0 | 3.3 | 4.3 | 4.6 | 3.8 | 4.2 | 3.9 | 4.4 | 4.3 | 5.2 |
| 4931406H21Rik | 0.2 | 0.2 | 0.2 | 0.2 | 0.3 | 0.1 | 0.3 | 0.3 | 0.2 | 0.2 | 0.2 | 0.2 |
| 4931406P16Rik | 15.6 | 20.1 | 20.3 | 14.6 | 17.8 | 13.7 | 21.7 | 30.5 | 29.8 | 17.9 | 21.8 | 22.9 |
| 4931408D14Rik | 0.3 | 0.2 | 0.3 | 0.1 | 0.3 | 0.3 | 0.1 | 0.3 | 0.2 | 0.2 | 0.3 | 0.2 |
| 4931414P19Rik | 0.8 | 1.3 | 1.5 | 0.9 | 1.1 | 1.2 | 0.8 | 1.3 | 1.3 | 1.5 | 1.2 | 1.1 |
| 4931417G12Rik | 0.1 | 0.1 | 0.0 | 0.0 | 0.2 | 0.0 | 0.0 | 0.1 | 0.1 | 0.0 | 0.1 | 0.1 |
| 4931428F04Rik | 1.4 | 1.3 | 1.6 | 2.0 | 1.6 | 1.8 | 1.7 | 1.7 | 1.7 | 1.1 | 1.9 | 1.9 |
| 4931430N09Rik | 0.0 | 0.0 | 0.0 | 0.0 | 0.1 | 0.0 | 0.0 | 0.0 | 0.0 | 0.0 | 0.0 | 0.0 |
| 4931431C16Rik | 0.2 | 0.2 | 0.3 | 0.2 | 0.3 | 0.3 | 0.2 | 0.1 | 0.1 | 0.2 | 0.2 | 0.1 |
| 4931440F15Rik | 0.1 | 0.1 | 0.1 | 0.2 | 0.1 | 0.1 | 0.1 | 0.1 | 0.1 | 0.1 | 0.1 | 0.1 |
| 4931440L10Rik | 0.0 | 0.0 | 0.0 | 0.0 | 0.0 | 0.0 | 0.1 | 0.0 | 0.0 | 0.1 | 0.0 | 0.0 |
| 4931440P22Rik | 0.2 | 0.2 | 0.1 | 0.2 | 0.1 | 0.4 | 0.1 | 0.2 | 0.2 | 0.2 | 0.1 | 0.1 |
| 4932413F04Rik | 0.0 | 0.0 | 0.0 | 0.0 | 0.0 | 0.0 | 0.0 | 0.0 | 0.0 | 0.1 | 0.0 | 0.1 |
| 4932415G12Rik | 2.3 | 2.5 | 2.5 | 2.3 | 2.5 | 2.9 | 1.8 | 1.9 | 2.3 | 1.7 | 2.0 | 2.0 |
| 4932416H05Rik | 0.7 | 0.7 | 0.8 | 0.6 | 0.8 | 0.7 | 0.6 | 1.1 | 0.6 | 0.7 | 0.7 | 0.9 |
| 4932418E24Rik | 0.0 | 0.1 | 0.1 | 0.0 | 0.1 | 0.0 | 0.1 | 0.0 | 0.0 | 0.0 | 0.0 | 0.0 |
| 4932435O22Rik | 0.0 | 0.0 | 0.0 | 0.1 | 0.0 | 0.0 | 0.0 | 0.0 | 0.0 | 0.0 | 0.0 | 0.0 |
| 4932438A13Rik | 6.7 | 7.3 | 6.4 | 7.1 | 6.3 | 6.6 | 5.5 | 5.8 | 5.1 | 5.0 | 5.6 | 5.4 |
| 4932438H23Rik | 0.0 | 0.1 | 0.0 | 0.0 | 0.1 | 0.1 | 0.0 | 0.0 | 0.0 | 0.1 | 0.0 | 0.0 |
| 4932441J04Rik | 0.0 | 0.1 | 0.2 | 0.0 | 0.0 | 0.2 | 0.0 | 0.2 | 0.1 | 0.0 | 0.0 | 0.2 |
| 4933400A11Rik | 0.4 | 0.7 | 0.6 | 0.2 | 0.2 | 0.2 | 0.4 | 0.2 | 0.4 | 0.2 | 0.0 | 0.1 |
| 4933400F21Rik | 0.1 | 0.0 | 0.1 | 0.0 | 0.0 | 0.0 | 0.0 | 0.1 | 0.0 | 0.1 | 0.0 | 0.1 |
| 4933402D24Rik | 0.0 | 0.0 | 0.1 | 0.1 | 0.0 | 0.0 | 0.0 | 0.0 | 0.0 | 0.0 | 0.0 | 0.0 |
| 4933402N22Rik | 0.1 | 0.1 | 0.0 | 0.0 | 0.1 | 0.0 | 0.1 | 0.3 | 0.0 | 0.0 | 0.0 | 0.0 |
| 4933403G14Rik | 1.6 | 1.4 | 1.8 | 1.8 | 1.3 | 1.4 | 1.0 | 1.7 | 1.4 | 1.5 | 1.6 | 1.8 |
| 4933404K08Rik | 0.0 | 0.0 | 0.1 | 0.0 | 0.0 | 0.1 | 0.0 | 0.0 | 0.0 | 0.0 | 0.0 | 0.0 |
| 4933404O12Rik | 0.3 | 0.5 | 0.7 | 0.4 | 0.5 | 0.5 | 0.6 | 0.5 | 0.8 | 0.7 | 0.4 | 0.5 |
| 4933405D12Rik | 0.1 | 0.5 | 0.5 | 0.3 | 0.2 | 0.1 | 0.0 | 0.2 | 0.3 | 0.1 | 0.1 | 0.3 |
| 4933405L10Rik | 0.0 | 0.0 | 0.1 | 0.0 | 0.1 | 0.0 | 0.0 | 0.1 | 0.0 | 0.1 | 0.0 | 0.0 |
| 4933406C10Rik | 0.1 | 0.2 | 0.2 | 0.1 | 0.1 | 0.2 | 0.0 | 0.0 | 0.0 | 0.1 | 0.1 | 0.2 |
| 4933406I18Rik | 0.1 | 0.1 | 0.1 | 0.0 | 0.1 | 0.1 | 0.1 | 0.1 | 0.0 | 0.0 | 0.1 | 0.1 |
| 4933406M09Rik | 0.0 | 0.0 | 0.0 | 0.0 | 0.0 | 0.0 | 0.0 | 0.0 | 0.0 | 0.0 | 0.0 | 0.1 |
| 4933407E24Rik | 0.0 | 0.1 | 0.0 | 0.0 | 0.1 | 0.0 | 0.0 | 0.0 | 0.0 | 0.0 | 0.0 | 0.0 |
| 4933407K13Rik | 0.6 | 0.7 | 0.7 | 0.8 | 0.7 | 0.4 | 0.6 | 0.8 | 0.7 | 0.6 | 0.6 | 0.6 |
| 4933408B17Rik | 0.1 | 0.1 | 0.2 | 0.0 | 0.2 | 0.1 | 0.1 | 0.0 | 0.1 | 0.1 | 0.1 | 0.1 |
| 4933408J17Rik | 0.4 | 0.4 | 0.3 | 0.3 | 0.4 | 0.4 | 0.4 | 0.4 | 0.6 | 0.4 | 0.2 | 0.4 |
| 4933408N05Rik | 0.0 | 0.0 | 0.0 | 0.0 | 0.1 | 0.0 | 0.0 | 0.0 | 0.0 | 0.1 | 0.0 | 0.0 |
| 4933409K07Rik | 4.8 | 4.8 | 4.0 | 4.6 | 4.5 | 4.1 | 3.9 | 4.1 | 3.4 | 3.1 | 3.9 | 3.6 |
| 4933411E08Rik | 0.0 | 0.1 | 0.1 | 0.1 | 0.0 | 0.1 | 0.1 | 0.1 | 0.0 | 0.0 | 0.2 | 0.1 |
| 4933411G11Rik | 0.0 | 0.0 | 0.0 | 0.0 | 0.0 | 0.0 | 0.1 | 0.1 | 0.0 | 0.0 | 0.0 | 0.0 |
| 4933411K16Rik | 0.2 | 0.1 | 0.3 | 0.2 | 0.2 | 0.3 | 0.3 | 0.2 | 0.2 | 0.1 | 0.2 | 0.2 |
| 4933411K20Rik | 5.3 | 6.3 | 7.2 | 5.9 | 4.8 | 5.9 | 5.1 | 5.7 | 4.9 | 5.6 | 5.8 | 5.9 |
| 4933412E12Rik | 8.2 | 6.9 | 8.2 | 9.1 | 6.8 | 7.5 | 6.7 | 6.0 | 5.9 | 4.6 | 5.7 | 5.4 |

| | | | | | | | | | | | | |
|---|---|---|---|---|---|---|---|---|---|---|---|---|
| 4933412O06Rik | 0.2 | 0.0 | 0.0 | 0.0 | 0.1 | 0.1 | 0.1 | 0.1 | 0.0 | 0.0 | 0.0 | 0.0 |
| 4933413J09Rik | 0.0 | 0.0 | 0.0 | 0.0 | 0.1 | 0.0 | 0.1 | 0.0 | 0.0 | 0.0 | 0.0 | 0.0 |
| 4933416M07Rik | 0.3 | 0.3 | 0.3 | 0.4 | 0.5 | 0.4 | 0.1 | 0.2 | 0.2 | 0.7 | 0.2 | 0.4 |
| 4933417D19Rik | 0.3 | 0.1 | 0.1 | 0.5 | 0.3 | 0.6 | 0.3 | 0.1 | 0.2 | 0.2 | 0.2 | 0.5 |
| 4933417E11Rik | 0.0 | 0.0 | 0.0 | 0.1 | 0.0 | 0.0 | 0.0 | 0.0 | 0.1 | 0.0 | 0.0 | 0.0 |
| 4933417G07Rik | 0.0 | 0.1 | 0.0 | 0.1 | 0.1 | 0.0 | 0.1 | 0.1 | 0.0 | 0.1 | 0.0 | 0.0 |
| 4933417O13Rik | 0.0 | 0.1 | 0.0 | 0.1 | 0.0 | 0.0 | 0.1 | 0.1 | 0.0 | 0.1 | 0.0 | 0.0 |
| 4933421O10Rik | 1.3 | 1.6 | 1.5 | 2.0 | 1.5 | 1.5 | 1.5 | 1.7 | 1.1 | 1.3 | 1.5 | 1.6 |
| 4933422A05Rik | 0.0 | 0.0 | 0.0 | 0.0 | 0.0 | 0.0 | 0.0 | 0.0 | 0.0 | 0.0 | 0.0 | 0.0 |
| 4933424G05Rik | 0.0 | 0.1 | 0.0 | 0.0 | 0.0 | 0.0 | 0.0 | 0.0 | 0.0 | 0.0 | 0.0 | 0.0 |
| 4933424G06Rik | 0.1 | 0.0 | 0.1 | 0.2 | 0.1 | 0.1 | 0.1 | 0.2 | 0.1 | 0.0 | 0.1 | 0.0 |
| 4933425H06Rik | 0.3 | 0.2 | 0.1 | 0.0 | 0.1 | 0.1 | 0.1 | 0.1 | 0.0 | 0.0 | 0.1 | 0.2 |
| 4933426D04Rik | 0.1 | 0.0 | 0.0 | 0.1 | 0.0 | 0.0 | 0.0 | 0.0 | 0.0 | 0.2 | 0.0 | 0.0 |
| 4933426M11Rik | 42.3 | 56.4 | 63.3 | 39.3 | 57.7 | 37.3 | 68.7 | 55.9 | 66.8 | 47.0 | 47.3 | 58.1 |
| 4933427D06Rik | 0.0 | 0.0 | 0.0 | 0.0 | 0.0 | 0.0 | 0.0 | 0.0 | 0.0 | 0.0 | 0.0 | 0.0 |
| 4933427D14Rik | 2.4 | 2.2 | 2.3 | 2.9 | 2.1 | 2.5 | 2.0 | 2.2 | 2.1 | 2.3 | 2.6 | 2.2 |
| 4933427E11Rik | 0.3 | 0.1 | 0.2 | 0.0 | 0.1 | 0.1 | 0.1 | 0.2 | 0.2 | 0.2 | 0.2 | 0.1 |
| 4933427G17Rik | 0.6 | 0.7 | 0.6 | 0.6 | 0.6 | 0.9 | 0.5 | 0.1 | 0.2 | 0.4 | 0.5 | 0.5 |
| 4933430I17Rik | 0.1 | 0.2 | 0.3 | 0.1 | 0.2 | 0.1 | 0.1 | 0.2 | 0.1 | 0.0 | 0.2 | 0.2 |
| 4933431E20Rik | 2.6 | 3.1 | 3.2 | 2.4 | 2.3 | 3.0 | 2.2 | 3.0 | 2.0 | 1.9 | 2.3 | 1.7 |
| 4933431G14Rik | 0.0 | 0.0 | 0.1 | 0.1 | 0.1 | 0.1 | 0.0 | 0.0 | 0.1 | 0.0 | 0.0 | 0.2 |
| 4933432I09Rik | 0.0 | 0.0 | 0.0 | 0.2 | 0.0 | 0.0 | 0.0 | 0.0 | 0.0 | 0.1 | 0.0 | 0.0 |
| 4933433G15Rik | 0.2 | 0.2 | 0.3 | 0.1 | 0.5 | 0.2 | 0.3 | 0.3 | 0.4 | 0.2 | 0.4 | 0.2 |
| 4933433G19Rik | 0.2 | 0.2 | 0.1 | 0.2 | 0.3 | 0.0 | 0.1 | 0.3 | 0.1 | 0.4 | 0.0 | 0.1 |
| 4933433H22Rik | 0.1 | 0.1 | 0.1 | 0.1 | 0.1 | 0.1 | 0.1 | 0.0 | 0.0 | 0.0 | 0.1 | 0.0 |
| 4933434E20Rik | 36.5 | 30.2 | 23.9 | 35.4 | 28.9 | 37.2 | 27.8 | 23.1 | 25.2 | 31.0 | 25.6 | 28.8 |
| 4933436C20Rik | 0.5 | 0.5 | 0.3 | 0.8 | 0.6 | 0.2 | 0.6 | 0.4 | 0.5 | 0.4 | 0.4 | 0.7 |
| 4933439C10Rik | 2.1 | 2.0 | 2.8 | 1.7 | 2.2 | 1.9 | 2.1 | 1.9 | 3.2 | 1.9 | 1.7 | 2.0 |
| 4933439K11Rik | 0.1 | 0.2 | 0.1 | 0.2 | 0.4 | 0.0 | 0.2 | 0.1 | 0.0 | 0.2 | 0.4 | 0.0 |
| 4933440M02Rik | 0.0 | 0.0 | 0.0 | 0.0 | 0.0 | 0.0 | 0.0 | 0.0 | 0.0 | 0.0 | 0.0 | 0.0 |
| 5031410I06Rik | 0.3 | 0.4 | 0.4 | 0.2 | 0.2 | 0.3 | 0.1 | 0.1 | 0.1 | 0.4 | 0.2 | 0.2 |
| 5031414D18Rik | 0.1 | 0.1 | 0.0 | 0.4 | 0.9 | 0.2 | 0.2 | 0.1 | 0.1 | 0.3 | 0.2 | 0.1 |
| 5031425E22Rik | 2.6 | 3.5 | 2.0 | 1.9 | 3.3 | 2.6 | 3.6 | 2.9 | 3.0 | 3.5 | 2.9 | 3.0 |
| 5031426D15Rik | 0.2 | 0.5 | 0.4 | 0.1 | 0.2 | 0.3 | 0.4 | 0.2 | 0.3 | 0.2 | 0.2 | 0.1 |
| 5031434C07Rik | 0.1 | 0.2 | 0.3 | 0.1 | 0.1 | 0.1 | 0.2 | 0.2 | 0.2 | 0.1 | 0.2 | 0.1 |
| 5031434O11Rik | 0.2 | 0.1 | 0.1 | 0.1 | 0.0 | 0.7 | 0.0 | 0.2 | 0.1 | 0.1 | 0.3 | 0.2 |
| 5031439G07Rik | 10.2 | 8.0 | 7.8 | 11.1 | 13.8 | 8.6 | 9.6 | 7.6 | 8.2 | 8.4 | 8.5 | 7.8 |
| 5033406O09Rik | 2.8 | 2.7 | 1.7 | 5.0 | 3.2 | 5.6 | 2.3 | 1.1 | 2.1 | 1.6 | 1.9 | 1.6 |
| 5033411D12Rik | 1.7 | 1.3 | 1.0 | 0.7 | 0.8 | 1.4 | 0.6 | 0.7 | 0.7 | 1.3 | 0.9 | 0.9 |
| 5330411J11Rik | 0.0 | 0.0 | 0.0 | 0.0 | 0.0 | 0.0 | 0.0 | 0.0 | 0.0 | 0.1 | 0.0 | 0.0 |
| 5330417C22Rik | 0.0 | 0.0 | 0.0 | 0.0 | 0.0 | 0.0 | 0.0 | 0.0 | 0.0 | 0.0 | 0.0 | 0.0 |
| 5330426P16Rik | 4.4 | 4.5 | 4.7 | 6.3 | 5.2 | 5.3 | 6.3 | 3.9 | 4.7 | 4.1 | 4.1 | 4.4 |
| 5430402O13Rik | 0.3 | 0.4 | 0.5 | 0.6 | 0.2 | 0.5 | 0.4 | 0.1 | 0.3 | 0.5 | 0.3 | 0.2 |
| 5430403N17Rik | 0.2 | 0.1 | 0.0 | 0.1 | 0.1 | 0.0 | 0.1 | 0.2 | 0.1 | 0.1 | 0.1 | 0.0 |
| 5430405H02Rik | 1.6 | 2.3 | 1.6 | 3.2 | 1.7 | 2.0 | 1.8 | 1.4 | 1.8 | 1.9 | 1.9 | 1.6 |
| 5430416N02Rik | 4.8 | 4.4 | 4.6 | 4.9 | 4.4 | 4.8 | 4.8 | 4.1 | 4.4 | 4.3 | 4.0 | 3.1 |
| 5430416O09Rik | 1.1 | 0.8 | 0.6 | 1.3 | 0.8 | 1.7 | 0.4 | 0.3 | 0.3 | 0.4 | 0.4 | 0.3 |
| 5430417L22Rik | 16.6 | 26.0 | 28.0 | 15.6 | 18.2 | 19.4 | 20.1 | 26.1 | 26.5 | 16.8 | 18.8 | 23.0 |
| 5430419D17Rik | 0.0 | 0.0 | 0.0 | 0.0 | 0.0 | 0.0 | 0.0 | 0.0 | 0.0 | 0.0 | 0.0 | 0.0 |
| 5430421F17Rik | 0.0 | 0.1 | 0.0 | 0.1 | 0.1 | 0.0 | 0.0 | 0.1 | 0.0 | 0.0 | 0.1 | 0.0 |
| 5430427O19Rik | 0.2 | 0.3 | 0.2 | 0.8 | 0.9 | 0.3 | 0.1 | 0.2 | 0.1 | 0.1 | 0.2 | 0.2 |
| 5430435G22Rik | 14.6 | 10.4 | 10.1 | 19.3 | 17.6 | 15.0 | 12.1 | 9.9 | 8.1 | 10.0 | 12.1 | 11.0 |
| 5430437J10Rik | 0.0 | 0.1 | 0.0 | 0.2 | 1.1 | 0.0 | 0.0 | 0.0 | 0.0 | 0.1 | 0.1 | 0.0 |
| 5530601H04Rik | 0.7 | 0.6 | 0.7 | 0.7 | 0.5 | 0.8 | 0.7 | 0.6 | 0.7 | 0.6 | 0.5 | 0.6 |
| 5730405O15Rik | 0.7 | 0.9 | 0.8 | 0.5 | 0.6 | 1.3 | 0.8 | 0.7 | 0.5 | 0.7 | 0.6 | 0.4 |
| 5730408K05Rik | 12.0 | 15.9 | 16.5 | 6.5 | 14.3 | 10.6 | 8.7 | 15.1 | 23.1 | 14.9 | 9.7 | 14.0 |
| 5730409E04Rik | 20.6 | 16.5 | 15.1 | 24.8 | 17.3 | 23.6 | 18.2 | 14.5 | 13.9 | 14.9 | 16.0 | 15.1 |

| | | | | | | | | | | | | |
|---|---|---|---|---|---|---|---|---|---|---|---|---|
| 5730416F02Rik | 0.1 | 0.0 | 0.0 | 0.1 | 0.1 | 0.0 | 0.1 | 0.1 | 0.1 | 0.0 | 0.0 | 0.0 |
| 5730422E09Rik | 0.0 | 0.1 | 0.0 | 0.0 | 0.1 | 0.0 | 0.0 | 0.0 | 0.1 | 0.1 | 0.0 | 0.1 |
| 5730455P16Rik | 7.3 | 7.3 | 8.2 | 6.8 | 5.4 | 8.8 | 5.8 | 7.0 | 6.0 | 6.5 | 7.0 | 7.5 |
| 5730480H06Rik | 0.2 | 0.2 | 0.2 | 0.1 | 0.0 | 0.3 | 0.2 | 0.1 | 0.1 | 0.2 | 0.2 | 0.2 |
| 5730507C01Rik | 2.6 | 2.9 | 3.0 | 2.7 | 2.9 | 2.6 | 3.1 | 3.1 | 2.9 | 3.6 | 3.5 | 3.0 |
| 5730508B09Rik | 1.2 | 1.0 | 1.5 | 0.7 | 1.4 | 0.7 | 0.8 | 0.6 | 1.0 | 0.7 | 1.2 | 1.5 |
| 5730559C18Rik | 0.2 | 0.1 | 0.1 | 0.1 | 0.5 | 0.2 | 0.5 | 0.4 | 0.5 | 0.4 | 0.3 | 0.1 |
| 5730577I03Rik | 0.7 | 1.1 | 0.8 | 0.5 | 0.5 | 0.8 | 0.5 | 0.6 | 0.5 | 0.9 | 0.9 | 0.6 |
| 5830403L16Rik | 0.0 | 0.0 | 0.0 | 0.1 | 0.0 | 0.0 | 0.1 | 0.0 | 0.1 | 0.0 | 0.0 | 0.0 |
| 5830415F09Rik | 1.6 | 1.1 | 1.6 | 1.7 | 1.2 | 1.4 | 1.5 | 1.4 | 1.7 | 1.5 | 1.9 | 1.3 |
| 5830416P10Rik | 0.0 | 0.0 | 0.0 | 0.0 | 0.0 | 0.0 | 0.0 | 0.0 | 0.0 | 0.2 | 0.0 | 0.0 |
| 5830417I10Rik | 3.9 | 4.7 | 4.7 | 4.0 | 4.2 | 4.4 | 4.1 | 4.9 | 4.2 | 4.3 | 4.3 | 4.3 |
| 5830418K08Rik | 2.4 | 3.3 | 3.4 | 2.4 | 2.7 | 2.4 | 3.5 | 2.9 | 2.7 | 2.5 | 2.9 | 2.9 |
| 5830418P13Rik | 0.1 | 0.0 | 0.0 | 0.0 | 0.1 | 0.1 | 0.1 | 0.0 | 0.0 | 0.2 | 0.1 | 0.0 |
| 5830432E09Rik | 0.3 | 0.1 | 0.2 | 0.9 | 0.7 | 0.4 | 0.5 | 0.2 | 0.2 | 0.3 | 0.1 | 0.1 |
| 5830454E08Rik | 0.4 | 0.2 | 0.3 | 0.8 | 0.6 | 0.6 | 0.3 | 0.4 | 0.3 | 0.2 | 0.3 | 0.2 |
| 5830473C10Rik | 0.0 | 0.0 | 0.0 | 0.0 | 0.0 | 0.0 | 0.0 | 0.0 | 0.0 | 0.0 | 0.0 | 0.0 |
| 5930403L14Rik | 0.0 | 0.0 | 0.0 | 0.0 | 0.0 | 0.0 | 0.0 | 0.0 | 0.0 | 0.0 | 0.0 | 0.0 |
| 6030407O03Rik | 0.1 | 0.0 | 0.0 | 0.0 | 0.0 | 0.0 | 0.0 | 0.0 | 0.0 | 0.0 | 0.0 | 0.1 |
| 6030408B16Rik | 0.2 | 0.3 | 0.7 | 0.1 | 0.4 | 0.1 | 0.3 | 0.6 | 0.3 | 0.1 | 0.1 | 0.2 |
| 6030419C18Rik | 13.4 | 9.8 | 9.0 | 16.4 | 11.3 | 16.4 | 13.2 | 8.0 | 10.7 | 14.3 | 11.6 | 11.0 |
| 6030458C11Rik | 3.7 | 3.4 | 4.5 | 4.3 | 4.3 | 3.8 | 4.2 | 4.5 | 3.9 | 3.8 | 4.3 | 3.6 |
| 6030466F02Rik | 0.0 | 0.0 | 0.0 | 0.0 | 0.0 | 0.0 | 0.0 | 0.0 | 0.0 | 0.1 | 0.0 | 0.1 |
| 6030468B19Rik | 0.0 | 0.0 | 0.1 | 0.0 | 0.0 | 0.0 | 0.0 | 0.0 | 0.1 | 0.0 | 0.1 | 0.0 |
| 6030498E09Rik | 0.0 | 0.0 | 0.0 | 0.0 | 0.0 | 0.0 | 0.0 | 0.0 | 0.0 | 0.0 | 0.0 | 0.0 |
| 6230400D17Rik | 0.2 | 0.3 | 0.3 | 0.3 | 0.4 | 0.3 | 0.6 | 0.5 | 0.6 | 0.7 | 0.4 | 0.6 |
| 6330403A02Rik | 0.0 | 0.0 | 0.0 | 0.0 | 0.0 | 0.0 | 0.0 | 0.0 | 0.1 | 0.0 | 0.0 | 0.0 |
| 6330403K07Rik | 0.2 | 0.2 | 0.2 | 0.1 | 0.1 | 0.3 | 0.0 | 0.0 | 0.1 | 0.2 | 0.0 | 0.2 |
| 6330403M23Rik | 1.6 | 2.0 | 2.3 | 2.4 | 1.3 | 3.1 | 1.3 | 1.4 | 1.7 | 2.2 | 2.5 | 1.8 |
| 6330407A03Rik | 0.0 | 0.0 | 0.0 | 0.0 | 0.1 | 0.1 | 0.1 | 0.0 | 0.0 | 0.0 | 0.0 | 0.0 |
| 6330408A02Rik | 3.2 | 3.0 | 2.9 | 4.0 | 2.4 | 4.4 | 2.7 | 2.5 | 1.8 | 2.3 | 2.2 | 2.8 |
| 6330409D20Rik | 0.1 | 0.0 | 0.0 | 0.1 | 0.1 | 0.0 | 0.1 | 0.0 | 0.1 | 0.0 | 0.0 | 0.0 |
| 6330416G13Rik | 1.9 | 2.0 | 1.8 | 2.1 | 1.9 | 1.9 | 2.2 | 2.1 | 2.3 | 1.4 | 2.0 | 1.7 |
| 6330418K02Rik | 1.3 | 0.7 | 1.5 | 1.2 | 1.4 | 1.2 | 1.4 | 1.1 | 1.3 | 1.3 | 0.7 | 1.4 |
| 6330549D23Rik | 0.2 | 0.2 | 0.4 | 0.3 | 0.2 | 0.2 | 0.3 | 0.1 | 0.2 | 0.2 | 0.1 | 0.3 |
| 6430411K18Rik | 1.5 | 0.8 | 0.5 | 0.7 | 0.9 | 1.3 | 1.2 | 1.5 | 1.7 | 1.9 | 1.3 | 1.7 |
| 6430531B16Rik | 0.3 | 0.7 | 0.2 | 0.1 | 0.3 | 0.5 | 0.1 | 0.3 | 0.2 | 0.1 | 0.1 | 0.1 |
| 6430548M08Rik | 1.6 | 1.7 | 1.5 | 2.3 | 2.7 | 1.5 | 1.4 | 1.6 | 1.5 | 1.9 | 1.7 | 1.7 |
| 6430550D23Rik | 0.2 | 0.5 | 0.3 | 0.2 | 0.2 | 0.2 | 0.2 | 0.3 | 0.1 | 0.2 | 0.2 | 0.1 |
| 6430562O15Rik | 0.8 | 0.9 | 0.8 | 1.0 | 0.6 | 1.0 | 0.4 | 0.9 | 1.0 | 0.8 | 0.7 | 0.7 |
| 6430571L13Rik | 0.2 | 0.2 | 0.1 | 0.1 | 0.1 | 0.4 | 0.0 | 0.1 | 0.0 | 0.0 | 0.1 | 0.1 |
| 6430573F11Rik | 0.1 | 0.1 | 0.0 | 0.0 | 0.0 | 0.1 | 0.0 | 0.0 | 0.0 | 0.0 | 0.0 | 0.0 |
| 6430584L05Rik | 0.7 | 0.7 | 0.5 | 0.4 | 0.5 | 0.3 | 0.4 | 0.5 | 0.2 | 0.4 | 0.5 | 0.4 |
| 6430706D22Rik | 7.3 | 9.9 | 12.1 | 5.9 | 12.7 | 5.5 | 14.0 | 9.7 | 14.5 | 7.2 | 9.2 | 7.9 |
| 6530402F18Rik | 1.4 | 1.1 | 0.9 | 1.5 | 1.2 | 1.3 | 1.4 | 1.2 | 1.3 | 1.2 | 1.0 | 0.9 |
| 6720401G13Rik | 2.4 | 2.4 | 2.0 | 2.2 | 2.0 | 3.0 | 2.3 | 2.3 | 2.7 | 2.1 | 2.0 | 2.1 |
| 6720456H20Rik | 7.3 | 7.6 | 7.5 | 6.5 | 7.0 | 6.5 | 7.3 | 8.7 | 7.8 | 7.8 | 7.6 | 8.0 |
| 6720468P15Rik | 0.0 | 0.1 | 0.0 | 0.2 | 0.0 | 0.0 | 0.1 | 0.0 | 0.0 | 0.1 | 0.1 | 0.0 |
| 6720483E21Rik | 0.1 | 0.0 | 0.0 | 0.0 | 0.1 | 0.2 | 0.0 | 0.0 | 0.1 | 0.0 | 0.0 | 0.1 |
| 6720489N17Rik | 0.9 | 0.9 | 1.4 | 0.7 | 1.2 | 1.4 | 1.0 | 1.1 | 1.0 | 0.9 | 1.2 | 1.0 |
| 6820408C15Rik | 0.1 | 0.0 | 0.0 | 0.1 | 0.0 | 0.0 | 0.0 | 0.0 | 0.1 | 0.2 | 0.1 | 0.1 |
| 6820431F20Rik | 24.8 | 29.8 | 35.3 | 33.8 | 29.1 | 33.7 | 32.6 | 33.1 | 32.0 | 26.2 | 33.8 | 29.9 |
| 7420700N18Rik | 0.1 | 0.0 | 0.0 | 0.0 | 0.0 | 0.0 | 0.0 | 0.0 | 0.0 | 0.0 | 0.0 | 0.0 |
| 7530420F21Rik | 0.1 | 0.2 | 0.3 | 0.2 | 0.0 | 0.0 | 0.1 | 0.3 | 0.2 | 0.0 | 0.3 | 0.2 |
| 8030423F21Rik | 0.0 | 0.0 | 0.0 | 0.0 | 0.0 | 0.0 | 0.0 | 0.0 | 0.0 | 0.0 | 0.0 | 0.0 |
| 8030442B05Rik | 0.1 | 0.1 | 0.1 | 0.1 | 0.1 | 0.0 | 0.2 | 0.2 | 0.2 | 0.4 | 0.3 | 0.2 |
| 8030462N17Rik | 5.9 | 6.3 | 6.1 | 6.2 | 5.1 | 6.0 | 6.4 | 5.2 | 5.8 | 5.1 | 6.2 | 6.2 |

| | | | | | | | | | | | | |
|---|---|---|---|---|---|---|---|---|---|---|---|---|
| 8430403D17Rik | 0.0 | 0.1 | 0.1 | 0.0 | 0.0 | 0.0 | 0.0 | 0.0 | 0.0 | 0.0 | 0.0 | 0.1 |
| 8430408G22Rik | 0.1 | 0.0 | 0.1 | 0.2 | 0.2 | 0.0 | 0.2 | 0.2 | 0.2 | 0.3 | 0.0 | 0.0 |
| 8430410A17Rik | 10.0 | 8.1 | 5.7 | 7.6 | 6.3 | 8.0 | 7.1 | 6.6 | 7.4 | 6.7 | 6.6 | 6.9 |
| 8430419L09Rik | 2.3 | 3.0 | 3.0 | 2.1 | 3.0 | 2.6 | 2.4 | 3.0 | 2.6 | 2.3 | 2.5 | 2.0 |
| 8430423G03Rik | 1.0 | 0.8 | 0.1 | 1.0 | 0.6 | 1.0 | 0.6 | 0.4 | 0.4 | 0.5 | 0.9 | 0.6 |
| 8430427H17Rik | 0.5 | 0.8 | 0.7 | 0.5 | 0.6 | 0.4 | 0.5 | 0.5 | 0.4 | 0.5 | 0.5 | 0.5 |
| 8430429K09Rik | 1.8 | 1.4 | 1.7 | 2.7 | 1.9 | 1.8 | 1.5 | 2.5 | 1.7 | 2.2 | 2.2 | 1.8 |
| 8430431K14Rik | 0.1 | 0.5 | 0.3 | 0.1 | 0.2 | 0.1 | 0.4 | 0.1 | 0.2 | 0.2 | 0.1 | 0.1 |
| 8430436N08Rik | 0.0 | 0.0 | 0.0 | 0.0 | 0.0 | 0.0 | 0.0 | 0.0 | 0.0 | 0.0 | 0.1 | 0.0 |
| 9030025P20Rik | 2.3 | 2.3 | 2.2 | 2.1 | 2.0 | 2.6 | 1.9 | 2.2 | 2.1 | 1.9 | 1.9 | 2.3 |
| 9030612E09Rik | 0.1 | 0.1 | 0.1 | 0.0 | 0.1 | 0.1 | 0.1 | 0.2 | 0.1 | 0.1 | 0.1 | 0.0 |
| 9030617O03Rik | 15.9 | 16.2 | 15.3 | 17.7 | 16.6 | 19.1 | 14.7 | 10.9 | 13.3 | 18.7 | 17.0 | 14.7 |
| 9030624G23Rik | 3.1 | 3.1 | 2.1 | 2.4 | 2.5 | 3.5 | 2.1 | 2.6 | 2.4 | 2.4 | 2.3 | 2.5 |
| 9030624J02Rik | 32.6 | 28.2 | 24.9 | 32.1 | 27.8 | 34.7 | 24.9 | 27.7 | 23.5 | 26.3 | 26.1 | 25.2 |
| 9130008F23Rik | 2.8 | 2.2 | 2.3 | 1.7 | 2.0 | 2.4 | 2.3 | 1.3 | 2.3 | 2.3 | 1.9 | 2.3 |
| 9130011E15Rik | 4.3 | 3.4 | 3.0 | 4.8 | 3.3 | 3.6 | 3.8 | 3.9 | 3.7 | 3.4 | 4.0 | 3.8 |
| 9130015A21Rik | 0.2 | 0.2 | 0.1 | 0.2 | 0.1 | 0.2 | 0.1 | 0.1 | 0.0 | 0.2 | 0.1 | 0.1 |
| 9130017N09Rik | 0.1 | 0.1 | 0.1 | 0.1 | 0.0 | 0.2 | 0.1 | 0.1 | 0.0 | 0.0 | 0.1 | 0.1 |
| 9130019O22Rik | 1.3 | 1.3 | 1.2 | 1.3 | 1.5 | 1.1 | 1.5 | 1.5 | 1.5 | 1.2 | 1.4 | 1.5 |
| 9130019P16Rik | 0.0 | 0.0 | 0.0 | 0.0 | 0.0 | 0.0 | 0.0 | 0.0 | 0.0 | 0.0 | 0.0 | 0.0 |
| 9130023H24Rik | 2.7 | 2.6 | 2.7 | 2.5 | 2.7 | 2.9 | 2.7 | 3.6 | 3.1 | 2.5 | 2.7 | 2.8 |
| 9130024F11Rik | 0.0 | 0.0 | 0.1 | 0.0 | 0.1 | 0.1 | 0.0 | 0.1 | 0.1 | 0.1 | 0.1 | 0.0 |
| 9130206I24Rik | 0.4 | 0.4 | 0.5 | 0.3 | 0.5 | 0.4 | 0.7 | 0.7 | 0.6 | 0.7 | 0.5 | 0.5 |
| 9130221F21Rik | 0.0 | 0.0 | 0.0 | 0.0 | 0.0 | 0.0 | 0.0 | 0.0 | 0.0 | 0.0 | 0.0 | 0.0 |
| 9130221H12Rik | 5.7 | 6.1 | 7.5 | 6.7 | 6.3 | 6.1 | 6.8 | 6.3 | 5.3 | 6.3 | 6.4 | 7.1 |
| 9130401M01Rik | 26.3 | 21.2 | 20.1 | 24.0 | 19.7 | 23.4 | 20.8 | 21.6 | 18.3 | 20.1 | 19.4 | 22.4 |
| 9130409I23Rik | 0.1 | 0.0 | 0.0 | 0.1 | 0.0 | 0.1 | 0.1 | 0.1 | 0.0 | 0.0 | 0.0 | 0.0 |
| 9230110C19Rik | 8.4 | 7.4 | 5.4 | 5.4 | 6.4 | 6.6 | 3.4 | 5.3 | 4.2 | 6.5 | 5.9 | 6.2 |
| 9230112J17Rik | 0.0 | 0.0 | 0.1 | 0.0 | 0.0 | 0.0 | 0.0 | 0.0 | 0.0 | 0.0 | 0.0 | 0.1 |
| 9230114K14Rik | 7.7 | 6.6 | 6.6 | 7.4 | 6.1 | 6.2 | 7.4 | 5.5 | 6.3 | 7.3 | 7.1 | 8.2 |
| 9230115E21Rik | 0.1 | 0.1 | 0.2 | 0.0 | 0.1 | 0.0 | 0.3 | 0.1 | 0.2 | 0.1 | 0.1 | 0.1 |
| 9230116N13Rik | 0.1 | 0.1 | 0.2 | 0.1 | 0.1 | 0.1 | 0.0 | 0.1 | 0.1 | 0.1 | 0.2 | 0.3 |
| 9330020H09Rik | 0.5 | 0.6 | 0.5 | 0.6 | 0.5 | 0.5 | 0.7 | 0.3 | 0.4 | 0.5 | 0.5 | 0.4 |
| 9330111N05Rik | 0.0 | 0.0 | 0.0 | 0.0 | 0.0 | 0.0 | 0.0 | 0.0 | 0.0 | 0.0 | 0.0 | 0.0 |
| 9330117O12Rik | 0.0 | 0.0 | 0.1 | 0.0 | 0.0 | 0.0 | 0.0 | 0.0 | 0.0 | 0.0 | 0.0 | 0.0 |
| 9330133O14Rik | 0.6 | 0.5 | 0.4 | 0.4 | 0.4 | 0.7 | 0.5 | 0.6 | 0.4 | 0.5 | 0.6 | 0.5 |
| 9330151L19Rik | 1.2 | 1.2 | 1.2 | 0.8 | 1.2 | 1.2 | 1.2 | 1.0 | 1.0 | 1.2 | 1.1 | 1.3 |
| 9330159F19Rik | 0.1 | 0.0 | 0.0 | 0.0 | 0.0 | 0.0 | 0.0 | 0.0 | 0.0 | 0.0 | 0.0 | 0.0 |
| 9330159M07Rik | 1.3 | 1.7 | 1.3 | 0.9 | 1.2 | 1.6 | 1.1 | 1.6 | 1.4 | 1.2 | 1.3 | 1.3 |
| 9330175E14Rik | 0.2 | 0.2 | 0.1 | 0.1 | 0.2 | 0.1 | 0.1 | 0.1 | 0.0 | 0.0 | 0.1 | 0.0 |
| 9330175M20Rik | 0.0 | 0.0 | 0.0 | 0.1 | 0.0 | 0.0 | 0.0 | 0.0 | 0.0 | 0.0 | 0.0 | 0.0 |
| 9330179D12Rik | 0.4 | 0.4 | 0.2 | 0.2 | 0.2 | 0.5 | 0.3 | 0.4 | 0.2 | 0.4 | 0.3 | 0.3 |
| 9330182L06Rik | 0.2 | 0.1 | 0.0 | 0.2 | 0.1 | 0.2 | 0.1 | 0.1 | 0.1 | 2.3 | 1.0 | 0.3 |
| 9430008C03Rik | 1.8 | 1.9 | 1.7 | 1.8 | 1.6 | 2.0 | 1.7 | 1.3 | 1.7 | 1.8 | 2.3 | 1.5 |
| 9430015G10Rik | 2.1 | 2.3 | 2.7 | 1.8 | 2.4 | 1.7 | 3.1 | 2.1 | 2.6 | 2.2 | 2.4 | 1.9 |
| 9430016H08Rik | 7.7 | 7.2 | 5.9 | 7.1 | 6.3 | 5.9 | 6.5 | 6.8 | 6.4 | 7.1 | 7.4 | 7.8 |
| 9430018G01Rik | 0.1 | 0.1 | 0.1 | 0.0 | 0.1 | 0.1 | 0.0 | 0.1 | 0.1 | 0.2 | 0.2 | 0.0 |
| 9430020K01Rik | 29.5 | 23.4 | 18.2 | 40.4 | 19.7 | 37.4 | 20.2 | 15.9 | 14.9 | 14.4 | 19.2 | 17.5 |
| 9430023L20Rik | 15.5 | 15.3 | 11.9 | 15.5 | 14.9 | 15.2 | 13.9 | 13.8 | 13.4 | 15.9 | 12.5 | 10.8 |
| 9430037G07Rik | 1.9 | 2.1 | 1.6 | 1.8 | 1.7 | 2.0 | 1.4 | 1.8 | 1.9 | 1.5 | 1.8 | 1.8 |
| 9430038I01Rik | 1.5 | 1.1 | 1.4 | 1.7 | 1.6 | 1.1 | 1.7 | 2.5 | 1.7 | 1.6 | 1.8 | 2.0 |
| 9430041J12Rik | 0.0 | 0.0 | 0.0 | 0.0 | 0.0 | 0.0 | 0.0 | 0.0 | 0.0 | 0.0 | 0.0 | 0.0 |
| 9430060I03Rik | 0.1 | 0.1 | 0.2 | 0.1 | 0.3 | 0.3 | 0.1 | 0.3 | 0.3 | 0.1 | 0.3 | 0.3 |
| 9430076C15Rik | 0.1 | 0.0 | 0.0 | 0.2 | 0.1 | 0.0 | 0.0 | 0.0 | 0.0 | 0.1 | 0.0 | 0.0 |
| 9430083A17Rik | 0.1 | 0.1 | 0.1 | 0.1 | 0.1 | 0.1 | 0.1 | 0.1 | 0.1 | 0.1 | 0.1 | 0.1 |
| 9430091E24Rik | 0.2 | 0.4 | 0.3 | 0.0 | 0.3 | 0.2 | 0.2 | 0.5 | 0.3 | 0.3 | 0.3 | 0.3 |
| 9530026F06Rik | 0.0 | 0.0 | 0.0 | 0.0 | 0.1 | 0.0 | 0.0 | 0.1 | 0.0 | 0.1 | 0.0 | 0.0 |

| | | | | | | | | | | | | |
|---|---|---|---|---|---|---|---|---|---|---|---|---|
| 9530026P05Rik | 0.4 | 0.7 | 0.3 | 0.3 | 0.3 | 0.1 | 0.3 | 0.2 | 0.3 | 0.9 | 0.4 | 0.6 |
| 9530027J09Rik | 2.0 | 1.4 | 1.6 | 1.5 | 1.6 | 1.6 | 1.4 | 1.6 | 1.0 | 1.0 | 1.8 | 1.5 |
| 9530051G07Rik | 0.3 | 0.2 | 0.2 | 0.1 | 0.2 | 0.3 | 0.0 | 0.2 | 0.1 | 0.2 | 0.1 | 0.2 |
| 9530052E02Rik | 0.0 | 0.1 | 0.0 | 0.1 | 0.1 | 0.1 | 0.0 | 0.2 | 0.0 | 0.1 | 0.0 | 0.0 |
| 9530053A07Rik | 0.3 | 0.3 | 0.3 | 0.4 | 0.4 | 0.4 | 0.2 | 0.2 | 0.1 | 0.2 | 0.2 | 0.2 |
| 9530059O14Rik | 0.0 | 0.0 | 0.0 | 0.0 | 0.0 | 0.0 | 0.0 | 0.0 | 0.0 | 0.0 | 0.0 | 0.0 |
| 9530068E07Rik | 126.9 | 111.6 | 108.9 | 142.7 | 130.6 | 136.0 | 116.0 | 104.6 | 110.4 | 120.1 | 117.3 | 113.8 |
| 9530077C05Rik | 0.7 | 0.3 | 0.5 | 0.3 | 0.3 | 0.6 | 0.4 | 0.3 | 0.3 | 0.4 | 0.7 | 0.4 |
| 9530082P21Rik | 4.2 | 4.8 | 4.4 | 5.4 | 4.3 | 5.1 | 4.4 | 4.5 | 4.4 | 4.1 | 5.1 | 3.8 |
| 9530091C08Rik | 0.4 | 0.7 | 0.7 | 0.4 | 0.5 | 0.5 | 0.9 | 0.4 | 0.3 | 0.4 | 0.4 | 0.3 |
| 9630028B13Rik | 0.1 | 0.1 | 0.0 | 0.0 | 0.1 | 0.0 | 0.0 | 0.0 | 0.1 | 0.0 | 0.1 | 0.0 |
| 9630028H03Rik | 0.0 | 0.0 | 0.0 | 0.0 | 0.0 | 0.1 | 0.0 | 0.0 | 0.0 | 0.0 | 0.0 | 0.0 |
| 9630033F20Rik | 3.4 | 3.5 | 3.4 | 4.1 | 3.1 | 3.7 | 2.8 | 3.1 | 2.7 | 2.8 | 2.7 | 3.1 |
| 9830001H06Rik | 2.4 | 2.7 | 2.6 | 2.4 | 2.9 | 2.4 | 3.2 | 2.3 | 2.6 | 2.4 | 2.2 | 2.1 |
| 9830132P13Rik | 0.0 | 0.0 | 0.0 | 0.0 | 0.0 | 0.0 | 0.0 | 0.0 | 0.0 | 0.0 | 0.0 | 0.0 |
| 9830147E19Rik | 1.4 | 1.6 | 2.0 | 1.2 | 1.1 | 1.5 | 1.3 | 1.3 | 1.3 | 1.7 | 1.5 | 1.8 |
| 9930012K11Rik | 4.0 | 7.4 | 5.7 | 2.9 | 3.5 | 4.0 | 5.3 | 8.8 | 7.0 | 10.7 | 7.5 | 7.1 |
| 9930013L23Rik | 583.9 | 669.6 | 851.5 | 418.3 | 535.7 | 464.0 | 689.2 | 878.4 | 834.1 | 461.6 | 629.6 | 690.1 |
| 9930014A18Rik | 1.1 | 0.7 | 0.5 | 1.1 | 0.8 | 1.3 | 0.8 | 0.5 | 0.4 | 1.0 | 0.8 | 0.9 |
| 9930021J03Rik | 3.5 | 4.7 | 4.5 | 3.8 | 3.7 | 3.6 | 3.7 | 3.6 | 3.6 | 3.8 | 4.2 | 4.0 |
| 9930104L06Rik | 3.4 | 2.5 | 2.6 | 3.6 | 2.9 | 3.1 | 3.2 | 2.6 | 2.6 | 3.0 | 3.1 | 2.6 |
| 9930111J21Rik1 | 63.6 | 45.5 | 28.7 | 64.5 | 39.0 | 55.5 | 42.9 | 18.2 | 25.1 | 26.2 | 39.1 | 34.1 |
| 9930111J21Rik2 | 34.6 | 26.2 | 14.8 | 32.1 | 22.3 | 30.7 | 21.3 | 9.1 | 14.0 | 15.0 | 20.8 | 16.9 |
| A030009H04Rik | 0.5 | 0.5 | 0.6 | 0.8 | 0.4 | 0.9 | 0.3 | 0.5 | 0.3 | 0.8 | 0.4 | 0.7 |
| A130010J15Rik | 1.2 | 1.5 | 1.5 | 1.0 | 1.0 | 1.0 | 0.9 | 1.6 | 1.6 | 1.5 | 1.1 | 1.2 |
| A130049A11Rik | 0.9 | 1.1 | 1.7 | 1.1 | 1.0 | 1.4 | 1.3 | 1.1 | 1.2 | 1.3 | 1.8 | 1.1 |
| A130077B15Rik | 5.7 | 9.5 | 7.2 | 4.7 | 6.7 | 5.5 | 7.1 | 5.3 | 5.3 | 7.3 | 5.2 | 5.3 |
| A230020J21Rik | 0.6 | 0.6 | 0.6 | 1.0 | 0.9 | 0.3 | 0.9 | 0.6 | 0.8 | 1.0 | 0.6 | 0.5 |
| A230028O05Rik | 0.0 | 0.0 | 0.0 | 0.2 | 0.2 | 0.0 | 0.0 | 0.0 | 0.0 | 0.0 | 0.0 | 0.0 |
| A230046K03Rik | 21.5 | 21.3 | 21.7 | 23.4 | 22.0 | 24.2 | 19.0 | 18.7 | 20.4 | 19.7 | 23.8 | 23.0 |
| A230050P20Rik | 0.9 | 1.2 | 1.9 | 1.2 | 1.2 | 1.6 | 1.2 | 1.6 | 1.2 | 1.2 | 0.9 | 1.1 |
| A230056J06Rik | 0.0 | 0.0 | 0.0 | 0.0 | 0.0 | 0.0 | 0.0 | 0.0 | 0.0 | 0.0 | 0.0 | 0.0 |
| A230056P14Rik | 1.2 | 1.2 | 1.2 | 1.7 | 1.0 | 1.5 | 0.9 | 1.0 | 0.9 | 1.4 | 1.8 | 1.1 |
| A230057D06Rik | 0.2 | 0.2 | 0.2 | 0.1 | 0.1 | 0.3 | 0.0 | 0.2 | 0.1 | 0.3 | 0.1 | 0.1 |
| A230065H16Rik | 0.1 | 0.1 | 0.0 | 0.1 | 0.0 | 0.2 | 0.1 | 0.0 | 0.0 | 0.3 | 0.0 | 0.2 |
| A230070E04Rik | 0.2 | 0.0 | 0.0 | 0.1 | 0.1 | 0.1 | 0.1 | 0.0 | 0.1 | 0.2 | 0.0 | 0.0 |
| A230072C01Rik | 1.2 | 1.8 | 2.1 | 1.7 | 1.8 | 1.9 | 1.7 | 2.0 | 1.4 | 1.7 | 1.7 | 1.8 |
| A230072E10Rik | 0.1 | 0.0 | 0.0 | 0.0 | 0.1 | 0.1 | 0.1 | 0.1 | 0.0 | 0.1 | 0.1 | 0.1 |
| A230073K19Rik | 0.4 | 0.5 | 0.6 | 0.7 | 0.5 | 0.4 | 0.5 | 0.4 | 0.5 | 0.7 | 0.6 | 0.7 |
| A230108P19Rik | 0.0 | 0.0 | 0.0 | 0.0 | 0.0 | 0.0 | 0.0 | 0.0 | 0.0 | 0.0 | 0.0 | 0.0 |
| A2m | 0.1 | 0.0 | 0.1 | 0.0 | 0.0 | 0.0 | 0.0 | 0.1 | 0.0 | 0.0 | 0.0 | 0.0 |
| A330009N23Rik | 0.6 | 0.5 | 0.5 | 0.5 | 0.3 | 0.5 | 0.8 | 0.6 | 0.5 | 0.3 | 0.6 | 0.2 |
| A330021E22Rik | 0.8 | 1.0 | 0.8 | 0.5 | 0.8 | 1.1 | 0.8 | 0.8 | 0.9 | 1.3 | 1.1 | 1.0 |
| A330023F24Rik | 1.0 | 0.9 | 0.8 | 1.1 | 1.1 | 1.0 | 0.7 | 0.7 | 0.6 | 0.6 | 0.7 | 0.8 |
| A330032B11Rik | 0.0 | 0.1 | 0.1 | 0.0 | 0.0 | 0.1 | 0.1 | 0.1 | 0.1 | 0.1 | 0.2 | 0.0 |
| A330035P11Rik | 1.1 | 1.2 | 1.3 | 1.9 | 0.9 | 1.1 | 0.8 | 1.4 | 0.9 | 1.1 | 1.5 | 1.1 |
| A330040F15Rik | 0.1 | 0.3 | 0.2 | 0.0 | 0.1 | 0.1 | 0.1 | 0.0 | 0.0 | 0.0 | 0.2 | 0.0 |
| A330041J22Rik | 0.1 | 0.0 | 0.0 | 0.2 | 0.1 | 0.1 | 0.1 | 0.0 | 0.2 | 0.1 | 0.2 | 0.0 |
| A330048O09Rik | 0.0 | 0.0 | 0.0 | 0.0 | 0.0 | 0.0 | 0.0 | 0.0 | 0.0 | 0.1 | 0.1 | 0.1 |
| A330049N07Rik | 0.0 | 0.0 | 0.0 | 0.0 | 0.0 | 0.0 | 0.0 | 0.0 | 0.0 | 0.0 | 0.0 | 0.0 |
| A330050B17Rik | 0.2 | 0.2 | 0.1 | 0.3 | 0.2 | 0.3 | 0.2 | 0.1 | 0.1 | 0.2 | 0.2 | 0.2 |
| A330069E16Rik | 0.4 | 0.3 | 0.6 | 0.1 | 0.6 | 0.5 | 0.8 | 0.7 | 0.4 | 0.5 | 0.4 | 0.7 |
| A330076H08Rik | 0.0 | 0.1 | 0.2 | 0.0 | 0.0 | 0.1 | 0.0 | 0.2 | 0.1 | 0.3 | 0.3 | 0.1 |
| A330102I10Rik | 0.1 | 0.1 | 0.1 | 0.0 | 0.1 | 0.2 | 0.1 | 0.0 | 0.1 | 0.0 | 0.0 | 0.1 |
| A3galt2 | 0.1 | 0.1 | 0.0 | 0.2 | 0.0 | 0.1 | 0.0 | 0.0 | 0.0 | 0.1 | 0.1 | 0.0 |
| A430005L14Rik | 8.9 | 9.0 | 7.8 | 9.4 | 9.8 | 8.2 | 10.5 | 9.9 | 9.1 | 9.8 | 11.0 | 8.8 |
| A430033K04Rik | 1.8 | 2.2 | 2.4 | 1.6 | 1.6 | 2.2 | 1.8 | 2.4 | 1.8 | 2.1 | 1.9 | 1.9 |

| | | | | | | | | | | | | |
|---|---|---|---|---|---|---|---|---|---|---|---|---|
| A430035B10Rik | 0.0 | 0.1 | 0.1 | 0.1 | 0.1 | 0.1 | 0.1 | 0.1 | 0.1 | 0.1 | 0.1 | 0.1 |
| A430071A18Rik | 0.0 | 0.1 | 0.1 | 0.2 | 0.1 | 0.0 | 0.1 | 0.1 | 0.1 | 0.0 | 0.1 | 0.1 |
| A430078G23Rik | 1.3 | 1.2 | 0.9 | 1.0 | 1.3 | 1.4 | 1.3 | 1.0 | 1.0 | 1.4 | 1.0 | 1.2 |
| A430088P11Rik | 0.0 | 0.0 | 0.0 | 0.1 | 0.1 | 0.1 | 0.0 | 0.0 | 0.0 | 0.0 | 0.0 | 0.0 |
| A430090L17Rik | 0.0 | 0.0 | 0.0 | 0.0 | 0.0 | 0.0 | 0.0 | 0.0 | 0.0 | 0.0 | 0.0 | 0.0 |
| A430092G05Rik | 0.0 | 0.0 | 0.0 | 0.0 | 0.0 | 0.0 | 0.0 | 0.0 | 0.0 | 0.0 | 0.0 | 0.0 |
| A430105I19Rik | 19.5 | 15.8 | 12.6 | 21.6 | 17.5 | 17.8 | 26.2 | 17.6 | 23.2 | 27.2 | 30.7 | 24.0 |
| A430107P09Rik | 0.0 | 0.1 | 0.0 | 0.0 | 0.0 | 0.0 | 0.0 | 0.0 | 0.0 | 0.0 | 0.1 | 0.1 |
| A4galt | 5.8 | 4.7 | 8.0 | 9.6 | 11.8 | 6.2 | 4.9 | 7.6 | 3.3 | 4.2 | 2.6 | 4.7 |
| A4gnt | 0.0 | 0.0 | 0.0 | 0.1 | 0.0 | 0.0 | 0.0 | 0.0 | 0.0 | 0.1 | 0.1 | 0.0 |
| A530013C23Rik | 0.7 | 0.7 | 0.6 | 0.5 | 0.8 | 0.9 | 0.5 | 0.6 | 0.6 | 0.7 | 0.6 | 0.5 |
| A530016L24Rik | 0.1 | 0.1 | 0.0 | 0.1 | 0.1 | 0.0 | 0.0 | 0.1 | 0.1 | 0.0 | 0.0 | 0.0 |
| A530032D15Rik | 0.4 | 0.6 | 0.6 | 0.6 | 0.6 | 0.4 | 0.3 | 0.3 | 0.3 | 0.4 | 0.3 | 0.4 |
| A530046M15Rik | 0.4 | 0.3 | 0.1 | 0.4 | 0.1 | 0.1 | 0.2 | 0.2 | 0.1 | 0.1 | 0.1 | 0.1 |
| A530054K11Rik | 2.7 | 2.7 | 2.9 | 2.9 | 3.0 | 3.0 | 2.3 | 2.8 | 2.3 | 2.8 | 2.9 | 3.2 |
| A530064D06Rik | 0.3 | 0.0 | 0.0 | 0.7 | 0.9 | 0.0 | 0.0 | 0.1 | 0.0 | 0.0 | 0.1 | 0.1 |
| A530072M11Rik | 0.9 | 0.9 | 0.7 | 0.6 | 0.4 | 0.8 | 0.9 | 0.4 | 0.7 | 0.6 | 0.8 | 0.6 |
| A530088E08Rik | 0.0 | 0.0 | 0.0 | 0.1 | 0.6 | 0.0 | 0.1 | 0.0 | 0.0 | 0.0 | 0.0 | 0.1 |
| A630001G21Rik | 0.7 | 0.7 | 0.3 | 1.5 | 2.2 | 0.6 | 0.7 | 0.4 | 0.4 | 0.6 | 0.7 | 0.5 |
| A630007B06Rik | 15.0 | 15.7 | 17.8 | 16.8 | 14.8 | 17.1 | 15.2 | 16.6 | 13.8 | 15.0 | 18.8 | 19.8 |
| A630019I02Rik | 0.1 | 0.1 | 0.0 | 0.0 | 0.0 | 0.0 | 0.1 | 0.1 | 0.1 | 0.0 | 0.1 | 0.1 |
| A630020A06 | 0.0 | 0.0 | 0.0 | 0.0 | 0.0 | 0.0 | 0.0 | 0.0 | 0.0 | 0.0 | 0.0 | 0.0 |
| A630023A22Rik | 0.2 | 0.2 | 0.1 | 0.1 | 0.3 | 0.1 | 0.3 | 0.2 | 0.1 | 0.4 | 0.1 | 0.1 |
| A630033H20Rik | 0.1 | 0.0 | 0.0 | 0.5 | 0.8 | 0.0 | 0.4 | 0.1 | 0.2 | 0.1 | 0.1 | 0.1 |
| A630066F11Rik | 0.8 | 1.7 | 1.0 | 1.2 | 0.9 | 1.2 | 0.9 | 1.5 | 1.1 | 0.7 | 1.2 | 1.2 |
| A630072M18Rik | 0.8 | 0.8 | 0.8 | 0.6 | 0.9 | 0.6 | 0.7 | 1.0 | 0.9 | 1.0 | 0.5 | 0.8 |
| A630075F10Rik | 0.0 | 0.0 | 0.0 | 0.0 | 0.0 | 0.0 | 0.0 | 0.0 | 0.0 | 0.1 | 0.0 | 0.1 |
| A630077J23Rik | 0.8 | 0.4 | 0.6 | 1.2 | 0.7 | 1.2 | 0.3 | 0.2 | 0.0 | 0.4 | 0.2 | 0.3 |
| A630089N07Rik | 3.0 | 3.6 | 4.5 | 2.7 | 3.0 | 3.3 | 4.3 | 3.1 | 2.1 | 3.6 | 2.7 | 3.4 |
| A730008H23Rik | 3.9 | 6.1 | 8.5 | 3.5 | 6.0 | 3.1 | 7.2 | 4.7 | 5.2 | 3.8 | 4.9 | 4.0 |
| A730017L22Rik | 0.7 | 1.1 | 0.8 | 1.3 | 1.1 | 0.6 | 0.8 | 0.4 | 0.7 | 0.8 | 0.6 | 0.7 |
| A730020M07Rik | 0.9 | 1.0 | 0.9 | 1.9 | 1.2 | 1.2 | 0.7 | 0.4 | 0.5 | 0.7 | 1.7 | 0.9 |
| A730056A06Rik | 0.1 | 0.1 | 0.1 | 0.1 | 0.0 | 0.1 | 0.0 | 0.2 | 0.0 | 0.1 | 0.0 | 0.0 |
| A730085A09Rik | 0.2 | 0.0 | 0.0 | 0.1 | 0.1 | 0.0 | 0.0 | 0.0 | 0.0 | 0.2 | 0.1 | 0.1 |
| A730090H04Rik | 0.1 | 0.0 | 0.0 | 0.1 | 0.0 | 0.1 | 0.0 | 0.0 | 0.0 | 0.2 | 0.0 | 0.1 |
| A730090N16Rik | 0.3 | 0.6 | 0.2 | 0.4 | 0.2 | 0.5 | 0.2 | 0.3 | 0.2 | 0.3 | 0.2 | 0.2 |
| A730098P11Rik | 1.4 | 1.7 | 1.9 | 2.3 | 2.1 | 1.7 | 1.9 | 2.1 | 1.9 | 2.2 | 2.0 | 2.2 |
| A830009L08Rik | 0.0 | 0.1 | 0.1 | 0.0 | 0.1 | 0.1 | 0.1 | 0.1 | 0.1 | 0.1 | 0.0 | 0.0 |
| A830010M20Rik | 1.5 | 1.5 | 1.7 | 1.3 | 1.3 | 1.8 | 1.5 | 1.3 | 1.2 | 1.3 | 1.7 | 1.5 |
| A830018L16Rik | 0.1 | 0.0 | 0.0 | 0.1 | 0.0 | 0.0 | 0.0 | 0.0 | 0.0 | 0.0 | 0.0 | 0.0 |
| A830052D11Rik | 0.3 | 0.4 | 0.4 | 0.2 | 0.2 | 0.2 | 0.2 | 0.5 | 0.2 | 0.3 | 0.3 | 0.0 |
| A830080D01Rik | 2.2 | 3.2 | 3.8 | 2.9 | 2.8 | 3.6 | 3.1 | 3.0 | 3.1 | 4.0 | 3.8 | 3.7 |
| A830082K12Rik | 0.1 | 0.1 | 0.4 | 0.3 | 0.2 | 0.5 | 0.2 | 0.6 | 0.4 | 0.4 | 0.6 | 0.4 |
| A830082N09Rik | 0.1 | 0.1 | 0.2 | 0.2 | 0.1 | 0.2 | 0.1 | 0.1 | 0.1 | 0.2 | 0.2 | 0.2 |
| A930001C03Rik | 0.7 | 0.7 | 0.5 | 0.7 | 0.6 | 0.4 | 0.4 | 0.5 | 0.2 | 0.3 | 0.5 | 0.2 |
| A930003A15Rik | 0.1 | 0.2 | 0.2 | 0.2 | 0.3 | 0.0 | 0.3 | 0.1 | 0.3 | 0.3 | 0.6 | 0.3 |
| A930003O13Rik | 0.0 | 0.0 | 0.0 | 0.0 | 0.0 | 0.0 | 0.0 | 0.0 | 0.0 | 0.0 | 0.0 | 0.0 |
| A930004D18Rik | 1.9 | 1.9 | 1.7 | 1.4 | 1.6 | 2.4 | 1.9 | 2.6 | 1.9 | 1.7 | 2.3 | 1.9 |
| A930005H10Rik | 2.7 | 2.2 | 2.8 | 3.4 | 1.7 | 2.3 | 1.8 | 2.7 | 1.0 | 1.2 | 2.3 | 2.0 |
| A930006K02Rik | 0.1 | 0.1 | 0.5 | 0.2 | 0.1 | 0.0 | 0.1 | 0.1 | 0.1 | 0.1 | 0.1 | 0.0 |
| A930007I19Rik | 0.0 | 0.1 | 0.0 | 0.0 | 0.1 | 0.1 | 0.0 | 0.1 | 0.0 | 0.1 | 0.1 | 0.0 |
| A930011G23Rik | 0.0 | 0.0 | 0.0 | 0.0 | 0.0 | 0.0 | 0.0 | 0.0 | 0.0 | 0.0 | 0.0 | 0.0 |
| A930012L18Rik | 1.2 | 1.0 | 0.6 | 0.6 | 0.8 | 1.2 | 0.7 | 0.7 | 0.5 | 0.7 | 0.5 | 0.4 |
| A930013F10Rik | 0.5 | 0.6 | 0.7 | 0.4 | 0.3 | 0.5 | 0.4 | 0.5 | 0.5 | 0.4 | 0.2 | 0.4 |
| A930015D03Rik | 0.5 | 0.3 | 0.4 | 0.2 | 0.5 | 0.3 | 0.6 | 0.1 | 0.3 | 0.7 | 0.0 | 0.5 |
| A930016O22Rik | 0.0 | 0.0 | 0.0 | 0.0 | 0.0 | 0.0 | 0.0 | 0.0 | 0.0 | 0.0 | 0.1 | 0.0 |
| A930017M01Rik | 0.0 | 0.0 | 0.0 | 0.0 | 0.0 | 0.0 | 0.0 | 0.0 | 0.0 | 0.0 | 0.0 | 0.0 |

| | | | | | | | | | | | | |
|---|---|---|---|---|---|---|---|---|---|---|---|---|
| A930018P22Rik | 0.0 | 0.2 | 0.0 | 0.1 | 0.1 | 0.2 | 0.2 | 0.1 | 0.0 | 0.1 | 0.3 | 0.2 |
| A930019D19Rik | 0.1 | 0.0 | 0.0 | 0.0 | 0.1 | 0.0 | 0.1 | 0.0 | 0.0 | 0.1 | 0.0 | 0.0 |
| A930024E05Rik | 0.5 | 0.4 | 0.7 | 0.5 | 0.3 | 0.4 | 0.2 | 0.1 | 0.3 | 0.2 | 0.4 | 0.6 |
| A930041C12Rik | 0.1 | 0.2 | 0.0 | 0.2 | 0.0 | 0.2 | 0.0 | 0.0 | 0.0 | 0.0 | 0.0 | 0.2 |
| AA388235 | 0.7 | 0.6 | 0.6 | 0.7 | 0.4 | 0.7 | 0.5 | 0.7 | 0.3 | 0.6 | 0.6 | 0.7 |
| AA414768 | 9.7 | 14.6 | 10.8 | 10.5 | 10.7 | 9.3 | 13.9 | 9.6 | 17.7 | 14.7 | 15.1 | 17.9 |
| AA415398 | 1.2 | 1.1 | 1.1 | 1.5 | 0.8 | 1.2 | 1.2 | 1.1 | 0.7 | 1.0 | 1.1 | 1.0 |
| AA465934 | 0.0 | 0.0 | 1.1 | 0.4 | 0.3 | 0.2 | 0.3 | 0.6 | 0.4 | 0.5 | 0.2 | 0.6 |
| AA467197 | 2.4 | 0.3 | 0.9 | 6.7 | 14.0 | 1.6 | 2.3 | 1.0 | 0.8 | 1.1 | 0.7 | 1.3 |
| AA474331 | 2.7 | 2.2 | 1.8 | 1.2 | 1.0 | 1.8 | 1.6 | 1.1 | 2.1 | 1.5 | 1.6 | 1.3 |
| AA536875 | 0.0 | 0.0 | 0.0 | 0.0 | 0.0 | 0.0 | 0.0 | 0.0 | 0.0 | 0.0 | 0.0 | 0.0 |
| AA543186 | 0.2 | 0.7 | 0.5 | 0.5 | 0.5 | 0.7 | 0.5 | 0.6 | 0.5 | 0.6 | 0.5 | 0.7 |
| AA792892 | 0.0 | 0.0 | 0.0 | 0.0 | 0.0 | 0.0 | 0.0 | 0.0 | 0.0 | 0.1 | 0.0 | 0.0 |
| AA986860 | 0.9 | 0.6 | 0.8 | 0.8 | 0.4 | 0.7 | 0.6 | 0.6 | 0.3 | 0.5 | 0.7 | 0.6 |
| AA987161 | 2.6 | 3.2 | 3.4 | 3.3 | 3.6 | 3.3 | 2.5 | 3.1 | 2.9 | 3.5 | 3.5 | 3.9 |
| Aaas | 18.3 | 18.9 | 19.2 | 18.7 | 16.2 | 19.5 | 19.7 | 14.7 | 15.0 | 18.8 | 17.7 | 17.0 |
| Aacs | 21.2 | 20.0 | 18.3 | 13.0 | 19.2 | 12.4 | 33.2 | 32.2 | 29.0 | 16.3 | 26.1 | 16.3 |
| Aadac | 0.0 | 0.0 | 0.0 | 0.0 | 0.0 | 0.0 | 0.0 | 0.0 | 0.0 | 0.0 | 0.0 | 0.0 |
| Aadat | 0.0 | 0.0 | 0.0 | 0.1 | 0.0 | 0.0 | 0.0 | 0.0 | 0.0 | 0.0 | 0.0 | 0.0 |
| Aaed1 | 34.6 | 28.2 | 24.8 | 25.7 | 29.4 | 31.6 | 24.4 | 23.5 | 23.2 | 29.3 | 28.6 | 30.9 |
| Aagab | 16.6 | 13.8 | 12.5 | 18.1 | 13.9 | 16.2 | 12.5 | 11.9 | 12.0 | 14.1 | 14.9 | 14.1 |
| Aak1 | 2.2 | 2.6 | 2.6 | 1.9 | 2.2 | 2.1 | 2.4 | 2.1 | 2.0 | 2.1 | 2.1 | 1.8 |
| Aamdc | 16.1 | 16.3 | 14.3 | 17.3 | 14.1 | 20.1 | 14.7 | 16.6 | 15.2 | 18.3 | 18.3 | 18.6 |
| Aamp | 67.9 | 69.2 | 66.6 | 69.8 | 62.3 | 65.1 | 71.3 | 70.6 | 73.0 | 72.6 | 67.6 | 73.8 |
| Aanat | 0.0 | 0.0 | 0.0 | 0.0 | 0.0 | 0.0 | 0.1 | 0.0 | 0.1 | 0.1 | 0.0 | 0.1 |
| Aar2 | 6.5 | 7.0 | 5.3 | 7.9 | 6.5 | 7.2 | 6.3 | 5.9 | 5.9 | 6.8 | 5.7 | 6.2 |
| Aard | 2.2 | 0.8 | 0.5 | 2.4 | 0.5 | 2.0 | 0.3 | 0.2 | 0.2 | 0.1 | 0.6 | 0.2 |
| Aars | 53.7 | 39.5 | 34.8 | 52.3 | 40.6 | 52.6 | 49.8 | 66.7 | 51.6 | 41.1 | 54.9 | 44.5 |
| Aars2 | 2.7 | 3.0 | 2.9 | 2.5 | 3.0 | 3.5 | 3.2 | 3.1 | 3.2 | 2.9 | 2.9 | 2.7 |
| Aarsd1 | 11.4 | 9.4 | 10.9 | 12.5 | 8.9 | 11.6 | 10.2 | 10.8 | 10.7 | 9.3 | 9.5 | 10.6 |
| Aasdh | 2.2 | 2.7 | 3.1 | 2.5 | 2.0 | 3.0 | 1.5 | 2.0 | 2.0 | 2.3 | 1.9 | 1.9 |
| Aasdhppt | 8.3 | 7.8 | 7.6 | 7.9 | 7.3 | 8.7 | 6.5 | 6.8 | 7.0 | 9.7 | 8.4 | 8.3 |
| Aass | 2.9 | 2.5 | 2.6 | 1.2 | 1.1 | 2.0 | 1.2 | 1.1 | 0.9 | 2.3 | 2.0 | 1.3 |
| Aatf | 16.1 | 15.3 | 14.2 | 17.4 | 12.3 | 14.1 | 16.4 | 14.0 | 12.5 | 16.9 | 18.0 | 16.9 |
| Aatk | 0.3 | 0.1 | 0.1 | 0.9 | 0.7 | 0.4 | 0.1 | 0.1 | 0.1 | 0.1 | 0.0 | 0.1 |
| AB099516 | 0.0 | 0.0 | 0.0 | 0.0 | 0.0 | 0.0 | 0.0 | 0.0 | 0.0 | 0.0 | 0.1 | 0.0 |
| AB124611 | 0.3 | 0.1 | 0.2 | 2.7 | 6.0 | 0.2 | 0.4 | 0.1 | 0.0 | 0.4 | 0.1 | 0.5 |
| Abat | 14.2 | 14.4 | 16.7 | 11.8 | 12.3 | 12.3 | 11.5 | 12.2 | 10.9 | 25.2 | 17.6 | 15.5 |
| Abca1 | 13.0 | 17.4 | 18.4 | 12.9 | 11.8 | 20.6 | 7.4 | 8.1 | 11.1 | 17.3 | 8.8 | 10.8 |
| Abca12 | 0.0 | 0.0 | 0.0 | 0.0 | 0.0 | 0.0 | 0.0 | 0.0 | 0.0 | 0.0 | 0.0 | 0.0 |
| Abca13 | 0.0 | 0.0 | 0.0 | 0.0 | 0.0 | 0.0 | 0.0 | 0.0 | 0.0 | 0.0 | 0.0 | 0.0 |
| Abca17 | 0.1 | 0.1 | 0.1 | 0.1 | 0.1 | 0.1 | 0.1 | 0.0 | 0.0 | 0.1 | 0.1 | 0.1 |
| Abca2 | 6.0 | 4.6 | 4.1 | 5.4 | 4.5 | 6.5 | 3.8 | 4.1 | 3.6 | 3.9 | 3.1 | 3.0 |
| Abca3 | 6.1 | 5.3 | 5.1 | 6.0 | 5.4 | 6.1 | 3.7 | 3.7 | 4.1 | 4.8 | 4.1 | 3.8 |
| Abca4 | 0.4 | 0.2 | 0.2 | 0.2 | 0.1 | 0.1 | 0.1 | 0.2 | 0.2 | 0.2 | 0.2 | 0.1 |
| Abca5 | 1.8 | 2.3 | 2.6 | 1.8 | 2.2 | 1.8 | 2.1 | 2.1 | 2.4 | 1.6 | 2.1 | 2.5 |
| Abca6 | 1.2 | 1.7 | 2.3 | 0.7 | 0.9 | 0.6 | 0.8 | 1.2 | 1.0 | 1.5 | 1.7 | 1.1 |
| Abca7 | 2.3 | 2.8 | 2.7 | 2.0 | 3.0 | 2.5 | 1.9 | 3.0 | 2.5 | 1.4 | 1.3 | 1.7 |
| Abca8a | 8.5 | 18.6 | 50.2 | 2.5 | 10.3 | 2.5 | 8.8 | 16.3 | 13.7 | 18.1 | 8.2 | 20.2 |
| Abca8b | 5.4 | 7.6 | 11.0 | 3.9 | 4.7 | 4.5 | 3.9 | 7.0 | 5.5 | 5.5 | 5.0 | 7.5 |
| Abca9 | 1.9 | 3.4 | 6.9 | 1.0 | 1.9 | 0.8 | 1.9 | 4.9 | 3.4 | 3.2 | 2.5 | 3.6 |
| Abcb10 | 6.0 | 6.1 | 5.1 | 5.2 | 6.2 | 6.4 | 8.0 | 6.6 | 8.0 | 5.5 | 7.1 | 5.5 |
| Abcb11 | 0.0 | 0.0 | 0.0 | 0.0 | 0.0 | 0.0 | 0.0 | 0.0 | 0.0 | 0.0 | 0.0 | 0.0 |
| Abcb1a | 1.8 | 0.7 | 0.6 | 1.0 | 1.0 | 0.6 | 0.9 | 0.6 | 0.6 | 0.5 | 0.6 | 0.4 |
| Abcb1b | 11.0 | 6.0 | 5.7 | 9.1 | 10.8 | 6.5 | 8.8 | 4.6 | 5.2 | 5.8 | 7.0 | 5.5 |
| Abcb4 | 0.1 | 0.1 | 0.0 | 0.7 | 1.9 | 0.0 | 0.1 | 0.0 | 0.0 | 0.2 | 0.0 | 0.2 |
| Abcb6 | 6.9 | 8.4 | 6.1 | 8.0 | 7.2 | 7.7 | 6.6 | 7.1 | 8.1 | 5.0 | 6.3 | 4.8 |

| | | | | | | | | | | | | |
|---|---|---|---|---|---|---|---|---|---|---|---|---|
| Abcb7 | 3.6 | 3.5 | 3.6 | 4.3 | 4.4 | 3.2 | 2.7 | 3.4 | 3.2 | 3.8 | 4.7 | 4.2 |
| Abcb8 | 4.8 | 3.8 | 3.3 | 4.2 | 3.9 | 4.4 | 3.9 | 4.7 | 4.8 | 4.9 | 4.2 | 3.9 |
| Abcb9 | 0.4 | 0.5 | 0.3 | 0.5 | 0.4 | 0.6 | 0.3 | 0.2 | 0.4 | 0.5 | 0.6 | 0.4 |
| Abcc1 | 20.0 | 17.2 | 14.8 | 29.8 | 31.3 | 29.0 | 18.3 | 18.6 | 14.2 | 18.9 | 17.2 | 13.9 |
| Abcc10 | 0.5 | 0.8 | 0.5 | 0.6 | 0.5 | 0.6 | 0.6 | 0.6 | 0.7 | 0.6 | 0.5 | 0.3 |
| Abcc12 | 0.1 | 0.0 | 0.0 | 0.0 | 0.0 | 0.0 | 0.0 | 0.0 | 0.0 | 0.1 | 0.0 | 0.0 |
| Abcc2 | 0.0 | 0.0 | 0.0 | 0.1 | 0.0 | 0.0 | 0.0 | 0.0 | 0.0 | 0.0 | 0.0 | 0.0 |
| Abcc3 | 1.8 | 1.6 | 1.3 | 4.5 | 7.2 | 2.1 | 2.1 | 1.3 | 1.3 | 1.1 | 1.0 | 1.0 |
| Abcc4 | 5.2 | 4.8 | 4.7 | 6.8 | 7.2 | 8.3 | 4.4 | 5.7 | 3.2 | 3.7 | 4.0 | 3.2 |
| Abcc5 | 16.8 | 15.9 | 16.4 | 12.1 | 14.4 | 14.2 | 15.1 | 19.7 | 17.7 | 17.3 | 15.4 | 14.1 |
| Abcc6 | 0.0 | 0.0 | 0.0 | 0.0 | 0.0 | 0.0 | 0.0 | 0.0 | 0.0 | 0.0 | 0.0 | 0.0 |
| Abcc9 | 0.0 | 0.1 | 0.1 | 0.0 | 0.0 | 0.0 | 0.0 | 0.1 | 0.1 | 0.0 | 0.1 | 0.0 |
| Abcd1 | 3.2 | 3.9 | 3.8 | 5.1 | 4.5 | 4.2 | 4.5 | 4.9 | 4.5 | 3.8 | 4.2 | 3.1 |
| Abcd2 | 0.2 | 0.3 | 0.3 | 0.1 | 0.2 | 0.1 | 0.1 | 0.3 | 0.2 | 0.2 | 0.2 | 0.3 |
| Abcd3 | 24.2 | 26.6 | 28.0 | 23.2 | 23.3 | 24.3 | 25.1 | 29.2 | 26.9 | 26.8 | 30.0 | 30.6 |
| Abcd4 | 4.9 | 5.6 | 6.0 | 5.2 | 5.0 | 8.6 | 5.1 | 5.9 | 5.7 | 5.0 | 5.3 | 4.1 |
| Abce1 | 56.5 | 52.0 | 49.7 | 57.2 | 53.9 | 53.5 | 48.1 | 49.1 | 49.3 | 53.2 | 57.3 | 54.0 |
| Abcf1 | 38.3 | 34.1 | 36.9 | 46.3 | 35.6 | 40.0 | 40.6 | 37.0 | 28.9 | 31.4 | 37.5 | 37.8 |
| Abcf2 | 41.3 | 40.5 | 38.4 | 41.5 | 39.9 | 37.9 | 44.9 | 41.3 | 35.8 | 39.3 | 44.5 | 39.1 |
| Abcf3 | 14.6 | 14.6 | 14.2 | 16.5 | 13.3 | 15.4 | 14.1 | 13.5 | 12.8 | 14.5 | 13.5 | 15.1 |
| Abcg1 | 1.0 | 0.4 | 0.5 | 3.8 | 4.1 | 1.8 | 0.8 | 0.3 | 0.4 | 0.7 | 0.1 | 0.4 |
| Abcg2 | 10.2 | 9.3 | 7.7 | 8.1 | 9.1 | 7.6 | 6.7 | 8.2 | 5.7 | 6.2 | 6.9 | 4.1 |
| Abcg3 | 0.0 | 0.0 | 0.0 | 0.2 | 0.2 | 0.0 | 0.0 | 0.0 | 0.0 | 0.0 | 0.0 | 0.0 |
| Abcg4 | 0.1 | 0.1 | 0.1 | 0.0 | 0.0 | 0.1 | 0.1 | 0.1 | 0.1 | 0.0 | 0.0 | 0.0 |
| Abcg8 | 0.0 | 0.0 | 0.0 | 0.1 | 0.0 | 0.0 | 0.0 | 0.0 | 0.0 | 0.0 | 0.0 | 0.0 |
| Abhd1 | 0.4 | 0.6 | 0.3 | 0.4 | 0.2 | 0.5 | 0.4 | 0.7 | 0.3 | 0.2 | 0.3 | 0.3 |
| Abhd10 | 2.3 | 2.4 | 3.0 | 2.5 | 2.5 | 3.2 | 1.6 | 3.3 | 2.3 | 2.5 | 2.0 | 2.8 |
| Abhd11 | 14.2 | 13.9 | 12.1 | 9.1 | 12.8 | 12.4 | 11.3 | 12.1 | 12.3 | 10.0 | 9.4 | 10.7 |
| Abhd12 | 30.6 | 20.5 | 20.0 | 44.0 | 51.8 | 27.6 | 25.3 | 20.0 | 21.2 | 27.7 | 22.5 | 24.3 |
| Abhd13 | 7.0 | 7.4 | 7.1 | 6.8 | 6.9 | 6.4 | 6.3 | 6.2 | 6.9 | 8.0 | 7.2 | 7.3 |
| Abhd14a | 15.1 | 12.4 | 11.7 | 14.6 | 12.1 | 15.7 | 10.8 | 10.8 | 11.4 | 13.0 | 11.3 | 9.9 |
| Abhd14b | 7.7 | 7.4 | 7.3 | 8.8 | 7.2 | 5.9 | 7.4 | 8.3 | 8.5 | 5.9 | 6.5 | 6.0 |
| Abhd15 | 3.2 | 3.4 | 2.0 | 5.2 | 5.6 | 4.0 | 7.1 | 3.5 | 3.8 | 4.0 | 4.1 | 4.3 |
| Abhd16a | 12.8 | 12.0 | 13.5 | 16.7 | 15.9 | 15.3 | 17.7 | 14.4 | 15.7 | 15.0 | 13.6 | 14.9 |
| Abhd16b | 0.3 | 0.3 | 0.2 | 0.1 | 0.2 | 0.3 | 0.2 | 0.2 | 0.1 | 0.3 | 0.1 | 0.3 |
| Abhd2 | 21.7 | 22.5 | 18.8 | 28.3 | 23.2 | 23.5 | 25.7 | 23.7 | 26.8 | 22.2 | 21.1 | 24.5 |
| Abhd3 | 3.4 | 4.0 | 4.2 | 3.6 | 2.1 | 4.1 | 2.1 | 2.8 | 2.3 | 2.0 | 2.2 | 3.0 |
| Abhd4 | 74.2 | 63.8 | 54.5 | 78.7 | 61.5 | 87.7 | 49.5 | 46.8 | 43.3 | 64.4 | 55.4 | 52.8 |
| Abhd5 | 18.3 | 14.9 | 13.6 | 23.0 | 17.3 | 21.7 | 13.4 | 12.8 | 12.9 | 14.2 | 14.7 | 14.6 |
| Abhd6 | 1.7 | 1.7 | 1.8 | 2.2 | 1.2 | 1.8 | 1.5 | 1.3 | 1.2 | 1.6 | 1.9 | 1.6 |
| Abhd8 | 28.9 | 29.4 | 24.5 | 22.3 | 23.6 | 33.3 | 26.8 | 23.9 | 26.1 | 27.1 | 24.1 | 21.0 |
| Abi1 | 21.2 | 22.8 | 21.0 | 27.2 | 26.8 | 23.7 | 24.5 | 22.0 | 22.2 | 21.9 | 24.7 | 24.4 |
| Abi2 | 6.4 | 8.6 | 8.6 | 5.1 | 6.1 | 7.2 | 6.5 | 7.8 | 8.3 | 7.6 | 8.0 | 8.2 |
| Abi3 | 0.4 | 0.4 | 0.3 | 1.2 | 1.3 | 0.6 | 0.3 | 0.3 | 0.5 | 0.4 | 0.3 | 0.4 |
| Abi3bp | 0.2 | 0.3 | 2.4 | 0.3 | 0.4 | 0.1 | 0.1 | 0.8 | 0.2 | 0.6 | 0.9 | 0.6 |
| Abl1 | 11.6 | 13.1 | 13.2 | 9.7 | 12.1 | 10.9 | 13.5 | 13.2 | 13.5 | 12.2 | 11.7 | 10.2 |
| Abl2 | 5.2 | 5.3 | 4.4 | 4.8 | 5.1 | 4.6 | 4.8 | 4.9 | 4.7 | 6.1 | 5.7 | 5.0 |
| Ablim1 | 14.2 | 11.7 | 12.9 | 11.1 | 10.5 | 8.3 | 12.1 | 20.9 | 9.7 | 11.8 | 13.0 | 10.7 |
| Ablim2 | 0.2 | 0.2 | 0.1 | 0.1 | 0.1 | 0.2 | 0.0 | 0.1 | 0.1 | 0.1 | 0.1 | 0.1 |
| Ablim3 | 2.5 | 1.7 | 2.0 | 1.1 | 0.6 | 1.3 | 1.2 | 3.2 | 1.1 | 1.8 | 1.6 | 1.5 |
| Abp1 | 0.0 | 0.0 | 0.1 | 0.0 | 0.1 | 0.0 | 0.0 | 0.1 | 0.0 | 0.0 | 0.0 | 0.0 |
| Abr | 6.9 | 5.6 | 5.3 | 13.3 | 16.7 | 7.3 | 8.7 | 6.6 | 6.1 | 6.8 | 6.5 | 7.0 |
| Abra | 0.1 | 0.0 | 0.1 | 0.2 | 0.0 | 0.1 | 0.0 | 0.1 | 0.0 | 0.1 | 0.1 | 0.0 |
| Abracl | 31.3 | 27.0 | 25.6 | 33.4 | 37.1 | 27.6 | 38.2 | 25.3 | 32.1 | 49.1 | 57.5 | 46.4 |
| Abt1 | 2.0 | 2.1 | 1.6 | 2.3 | 1.7 | 2.3 | 2.2 | 1.8 | 2.0 | 2.3 | 2.5 | 2.4 |
| Abtb1 | 5.3 | 5.0 | 4.6 | 5.0 | 5.6 | 7.1 | 4.5 | 5.8 | 5.0 | 5.0 | 4.7 | 4.4 |
| Abtb2 | 1.4 | 1.2 | 0.7 | 1.5 | 1.5 | 1.5 | 1.5 | 1.2 | 1.6 | 1.3 | 1.1 | 1.1 |

| | | | | | | | | | | | | |
|---|---|---|---|---|---|---|---|---|---|---|---|---|
| Acaa1a | 29.7 | 26.7 | 21.8 | 23.5 | 26.6 | 24.9 | 22.4 | 23.7 | 25.6 | 29.8 | 22.5 | 23.2 |
| Acaa1b | 8.9 | 8.4 | 6.7 | 6.0 | 7.6 | 7.2 | 5.9 | 5.7 | 7.6 | 9.0 | 6.2 | 5.8 |
| Acaa2 | 11.4 | 9.8 | 6.3 | 10.1 | 8.0 | 11.2 | 7.6 | 10.5 | 9.1 | 19.8 | 12.4 | 11.3 |
| Acaca | 10.0 | 9.7 | 8.6 | 7.5 | 7.4 | 7.5 | 10.3 | 11.4 | 9.2 | 6.1 | 10.2 | 7.5 |
| Acacb | 0.5 | 0.8 | 0.9 | 0.5 | 0.5 | 0.8 | 0.5 | 0.7 | 0.6 | 0.4 | 0.4 | 0.5 |
| Acad10 | 1.5 | 1.2 | 1.1 | 1.5 | 1.4 | 1.6 | 1.0 | 1.3 | 1.0 | 1.2 | 0.7 | 1.0 |
| Acad11 | 22.4 | 20.8 | 20.2 | 19.3 | 19.2 | 22.1 | 14.5 | 15.5 | 14.8 | 17.1 | 16.8 | 17.1 |
| Acad12 | 2.7 | 2.5 | 2.5 | 2.6 | 2.5 | 2.6 | 2.0 | 2.2 | 2.4 | 2.2 | 2.5 | 2.7 |
| Acad8 | 11.3 | 11.4 | 10.2 | 10.9 | 9.9 | 11.5 | 9.2 | 8.2 | 9.8 | 10.2 | 10.4 | 9.8 |
| Acad9 | 8.9 | 7.3 | 6.5 | 6.6 | 7.8 | 6.9 | 7.5 | 6.8 | 6.2 | 7.0 | 7.7 | 7.4 |
| Acadl | 16.0 | 14.1 | 14.2 | 14.2 | 14.0 | 17.0 | 14.1 | 16.9 | 15.0 | 14.9 | 15.7 | 18.6 |
| Acadm | 22.6 | 20.4 | 17.7 | 24.5 | 18.6 | 24.7 | 16.2 | 20.6 | 20.4 | 20.7 | 20.3 | 21.5 |
| Acads | 9.3 | 8.5 | 9.3 | 9.7 | 10.4 | 11.1 | 10.1 | 11.3 | 10.9 | 11.4 | 9.2 | 8.0 |
| Acadsb | 46.8 | 46.4 | 55.6 | 45.4 | 38.7 | 43.5 | 42.8 | 47.9 | 42.6 | 45.4 | 51.6 | 56.9 |
| Acadvl | 65.3 | 58.8 | 52.4 | 57.5 | 55.2 | 57.2 | 48.5 | 45.7 | 48.7 | 56.2 | 49.7 | 57.0 |
| Acan | 0.1 | 0.1 | 0.1 | 0.0 | 0.0 | 0.0 | 0.1 | 0.3 | 0.3 | 0.1 | 0.1 | 0.2 |
| Acap1 | 0.0 | 0.0 | 0.0 | 0.5 | 1.3 | 0.0 | 0.2 | 0.0 | 0.0 | 0.1 | 0.0 | 0.0 |
| Acap2 | 15.1 | 18.9 | 20.7 | 13.8 | 15.5 | 14.8 | 15.8 | 16.8 | 16.2 | 16.2 | 15.8 | 17.0 |
| Acap3 | 4.4 | 5.0 | 4.6 | 5.6 | 5.0 | 4.5 | 5.3 | 5.4 | 5.8 | 5.1 | 4.2 | 4.3 |
| Acat1 | 18.8 | 15.7 | 17.2 | 14.8 | 14.3 | 14.9 | 14.1 | 14.7 | 12.9 | 17.1 | 16.8 | 18.0 |
| Acat2 | 12.0 | 17.1 | 14.4 | 5.2 | 8.6 | 8.5 | 18.2 | 20.4 | 16.4 | 7.0 | 17.1 | 6.5 |
| Acat3 | 25.7 | 22.9 | 18.6 | 21.5 | 20.5 | 19.1 | 20.4 | 19.9 | 19.8 | 21.3 | 24.5 | 21.0 |
| Acbd3 | 26.7 | 26.9 | 28.8 | 30.7 | 27.4 | 28.8 | 30.2 | 25.6 | 25.7 | 25.5 | 31.6 | 33.0 |
| Acbd4 | 6.0 | 6.8 | 6.2 | 6.8 | 4.7 | 6.9 | 5.7 | 5.3 | 6.4 | 5.8 | 5.3 | 5.6 |
| Acbd5 | 21.5 | 18.9 | 18.8 | 28.0 | 19.0 | 26.5 | 21.1 | 15.0 | 17.8 | 19.2 | 23.1 | 26.0 |
| Acbd6 | 19.6 | 15.7 | 15.0 | 15.4 | 13.6 | 14.9 | 14.3 | 16.5 | 15.9 | 15.4 | 13.5 | 15.1 |
| Acbd7 | 0.1 | 0.0 | 0.1 | 0.3 | 0.0 | 0.2 | 0.0 | 0.0 | 0.0 | 0.0 | 0.0 | 0.1 |
| Accs | 1.5 | 1.8 | 1.4 | 1.5 | 1.2 | 2.1 | 1.5 | 2.3 | 1.7 | 1.3 | 1.5 | 1.8 |
| Acd | 5.6 | 5.4 | 4.3 | 5.8 | 5.3 | 5.7 | 5.7 | 5.8 | 4.8 | 4.6 | 4.7 | 3.4 |
| Ace | 0.6 | 0.6 | 1.2 | 0.5 | 0.7 | 1.0 | 0.5 | 1.3 | 0.4 | 0.5 | 0.5 | 0.3 |
| Ace2 | 0.1 | 0.0 | 0.0 | 0.1 | 0.1 | 0.0 | 0.0 | 0.0 | 0.0 | 0.2 | 0.1 | 0.1 |
| Acer2 | 0.3 | 0.3 | 0.2 | 0.4 | 0.3 | 0.4 | 0.1 | 0.1 | 0.1 | 0.2 | 0.2 | 0.1 |
| Acer3 | 5.1 | 3.8 | 4.7 | 4.5 | 4.7 | 4.0 | 4.4 | 4.7 | 4.2 | 4.6 | 5.8 | 5.0 |
| Ache | 0.1 | 0.1 | 0.1 | 0.1 | 0.1 | 0.2 | 0.0 | 0.1 | 0.1 | 0.0 | 0.2 | 0.0 |
| Acin1 | 16.9 | 17.0 | 20.2 | 21.4 | 18.2 | 16.3 | 24.0 | 22.8 | 19.3 | 19.6 | 21.6 | 21.5 |
| Acly | 47.9 | 51.8 | 46.9 | 36.5 | 42.1 | 41.5 | 59.7 | 73.8 | 55.0 | 41.6 | 64.2 | 40.3 |
| Acmsd | 0.0 | 0.2 | 0.0 | 0.1 | 0.1 | 0.0 | 0.0 | 0.0 | 0.0 | 0.0 | 0.1 | 0.0 |
| Acn9 | 6.7 | 4.1 | 5.1 | 6.2 | 4.0 | 7.8 | 5.8 | 3.7 | 5.3 | 7.4 | 4.0 | 4.2 |
| Acnat1 | 0.0 | 0.0 | 0.0 | 0.0 | 0.0 | 0.0 | 0.0 | 0.0 | 0.0 | 0.0 | 0.0 | 0.0 |
| Aco1 | 13.1 | 14.3 | 13.1 | 11.8 | 13.2 | 11.3 | 14.0 | 16.6 | 15.6 | 13.9 | 14.3 | 13.4 |
| Aco2 | 67.7 | 61.0 | 54.3 | 77.6 | 61.7 | 66.8 | 56.8 | 60.7 | 49.7 | 61.2 | 62.4 | 59.2 |
| Acot1 | 0.6 | 0.3 | 1.0 | 0.3 | 0.5 | 0.7 | 0.7 | 2.3 | 1.3 | 1.2 | 1.2 | 0.7 |
| Acot10 | 0.1 | 0.1 | 0.0 | 0.0 | 0.0 | 0.1 | 0.0 | 0.0 | 0.0 | 0.0 | 0.0 | 0.0 |
| Acot11 | 7.8 | 5.8 | 6.0 | 9.0 | 6.5 | 8.2 | 7.0 | 5.6 | 6.4 | 5.7 | 7.0 | 8.7 |
| Acot12 | 0.1 | 0.0 | 0.0 | 0.0 | 0.0 | 0.0 | 0.0 | 0.0 | 0.0 | 0.0 | 0.0 | 0.0 |
| Acot13 | 80.8 | 58.4 | 46.1 | 75.4 | 47.5 | 82.6 | 41.2 | 44.1 | 34.9 | 52.8 | 56.2 | 70.5 |
| Acot2 | 5.3 | 2.2 | 1.6 | 4.4 | 1.8 | 4.5 | 3.1 | 5.0 | 2.6 | 2.9 | 3.8 | 2.5 |
| Acot3 | 0.0 | 0.0 | 0.0 | 0.0 | 0.0 | 0.0 | 0.0 | 0.0 | 0.0 | 0.0 | 0.0 | 0.0 |
| Acot4 | 0.3 | 0.2 | 0.1 | 0.2 | 0.2 | 0.2 | 0.2 | 0.1 | 0.1 | 0.4 | 0.3 | 0.1 |
| Acot5 | 0.0 | 0.0 | 0.0 | 0.0 | 0.0 | 0.1 | 0.1 | 0.0 | 0.0 | 0.0 | 0.0 | 0.0 |
| Acot6 | 1.6 | 1.4 | 1.0 | 1.1 | 1.1 | 1.7 | 1.1 | 1.3 | 1.2 | 1.4 | 0.9 | 1.1 |
| Acot7 | 51.4 | 38.8 | 29.9 | 70.7 | 59.3 | 56.5 | 64.4 | 51.1 | 46.1 | 49.4 | 56.2 | 39.9 |
| Acot8 | 5.9 | 5.4 | 4.9 | 6.5 | 5.2 | 5.7 | 5.9 | 4.4 | 5.6 | 6.1 | 5.7 | 4.6 |
| Acot9 | 40.8 | 32.3 | 25.9 | 32.8 | 32.1 | 33.7 | 32.1 | 29.8 | 34.4 | 32.4 | 39.0 | 34.1 |
| Acox1 | 41.6 | 34.8 | 30.8 | 44.6 | 35.5 | 43.1 | 32.6 | 29.0 | 29.1 | 40.7 | 36.9 | 42.1 |
| Acox2 | 0.2 | 0.1 | 0.1 | 0.2 | 0.2 | 0.0 | 0.2 | 0.1 | 0.2 | 0.1 | 0.2 | 0.1 |
| Acox3 | 7.6 | 6.2 | 5.9 | 9.7 | 8.7 | 7.3 | 6.0 | 5.4 | 4.9 | 5.5 | 5.0 | 5.6 |

| | | | | | | | | | | | | |
|---|---|---|---|---|---|---|---|---|---|---|---|---|
| Acoxl | 0.1 | 0.0 | 0.0 | 0.1 | 0.0 | 0.0 | 0.0 | 0.0 | 0.0 | 0.0 | 0.0 | 0.1 |
| Acp1 | 21.0 | 18.8 | 17.9 | 22.1 | 17.4 | 20.5 | 18.6 | 18.0 | 16.1 | 18.9 | 20.5 | 20.1 |
| Acp2 | 11.3 | 10.2 | 10.0 | 17.0 | 12.2 | 14.1 | 11.8 | 12.1 | 11.2 | 10.2 | 11.7 | 11.9 |
| Acp5 | 0.8 | 0.7 | 0.8 | 0.5 | 1.2 | 0.3 | 0.7 | 0.8 | 0.7 | 1.4 | 1.0 | 1.0 |
| Acp6 | 7.2 | 6.0 | 4.8 | 6.2 | 4.8 | 5.7 | 5.0 | 5.8 | 4.8 | 5.5 | 5.0 | 5.1 |
| Acpl2 | 4.7 | 6.3 | 8.1 | 4.6 | 5.9 | 7.8 | 11.2 | 9.5 | 11.8 | 8.9 | 8.6 | 12.8 |
| Acpp | 0.2 | 0.1 | 0.1 | 0.2 | 0.0 | 0.0 | 0.0 | 0.1 | 0.1 | 0.1 | 0.0 | 0.0 |
| Acrbp | 0.4 | 1.2 | 0.6 | 0.5 | 0.8 | 0.3 | 0.6 | 0.3 | 0.8 | 0.4 | 0.5 | 0.6 |
| Acsbg1 | 0.2 | 0.1 | 0.0 | 0.3 | 0.6 | 0.4 | 0.1 | 0.0 | 0.1 | 0.1 | 0.2 | 0.2 |
| Acsf2 | 1.8 | 2.4 | 2.1 | 1.7 | 1.2 | 1.8 | 1.1 | 1.5 | 1.8 | 1.8 | 1.1 | 1.4 |
| Acsf3 | 2.3 | 2.8 | 2.4 | 2.8 | 2.3 | 2.7 | 2.1 | 2.4 | 2.6 | 2.9 | 2.3 | 2.2 |
| Acsl1 | 6.6 | 5.3 | 6.6 | 7.2 | 6.3 | 5.5 | 6.3 | 5.9 | 4.7 | 4.9 | 5.4 | 5.4 |
| Acsl3 | 6.7 | 11.9 | 12.7 | 4.8 | 6.3 | 6.8 | 13.0 | 15.2 | 11.5 | 4.4 | 11.1 | 7.0 |
| Acsl4 | 11.6 | 11.8 | 12.9 | 14.1 | 16.4 | 8.6 | 15.7 | 11.2 | 12.6 | 14.0 | 17.0 | 14.0 |
| Acsl5 | 31.1 | 26.3 | 27.2 | 36.9 | 36.4 | 28.4 | 33.6 | 25.3 | 30.7 | 26.8 | 31.4 | 30.3 |
| Acsl6 | 0.0 | 0.0 | 0.1 | 0.0 | 0.0 | 0.0 | 0.0 | 0.1 | 0.1 | 0.0 | 0.0 | 0.1 |
| Acsm3 | 0.0 | 0.0 | 0.0 | 0.0 | 0.1 | 0.0 | 0.1 | 0.0 | 0.1 | 0.0 | 0.0 | 0.0 |
| Acsm4 | 0.0 | 0.0 | 0.0 | 0.0 | 0.0 | 0.0 | 0.0 | 0.0 | 0.0 | 0.0 | 0.1 | 0.0 |
| Acss1 | 0.1 | 0.1 | 0.0 | 0.6 | 1.4 | 0.0 | 0.3 | 0.0 | 0.0 | 0.1 | 0.0 | 0.1 |
| Acss2 | 6.4 | 10.8 | 9.9 | 3.5 | 5.8 | 6.1 | 7.5 | 14.1 | 7.9 | 3.2 | 7.7 | 3.4 |
| Acss3 | 0.1 | 0.1 | 0.1 | 0.1 | 0.1 | 0.0 | 0.0 | 0.1 | 0.0 | 0.0 | 0.0 | 0.0 |
| Acta1 | 1.3 | 0.7 | 0.6 | 2.0 | 1.4 | 0.8 | 1.5 | 0.6 | 1.2 | 1.8 | 2.0 | 1.0 |
| Acta2 | 401.3 | 299.2 | 266.7 | 430.6 | 444.2 | 259.6 | 674.9 | 330.0 | 548.1 | 497.1 | 865.8 | 554.4 |
| Actb | 1815.6 | 1530.6 | 1385.1 | 2181.2 | 2798.9 | 1302.3 | 2815.4 | 1902.9 | 2479.7 | 2234.8 | 2644.7 | 2112.5 |
| Actc1 | 0.0 | 0.0 | 0.0 | 0.0 | 0.0 | 0.0 | 0.1 | 0.0 | 0.0 | 0.0 | 0.0 | 0.0 |
| Actg1 | 886.8 | 700.5 | 577.6 | 898.4 | 1044.2 | 678.7 | 1076.0 | 737.4 | 1027.1 | 1013.3 | 1137.0 | 905.1 |
| Actg2 | 2.7 | 1.7 | 0.7 | 3.1 | 4.6 | 2.0 | 4.8 | 1.8 | 5.7 | 15.1 | 25.1 | 9.0 |
| Actl10 | 0.1 | 0.1 | 0.0 | 0.0 | 0.0 | 0.1 | 0.1 | 0.1 | 0.1 | 0.1 | 0.0 | 0.1 |
| Actl11 | 0.0 | 0.0 | 0.0 | 0.0 | 0.0 | 0.0 | 0.0 | 0.0 | 0.0 | 0.0 | 0.0 | 0.0 |
| Actl6a | 15.4 | 17.2 | 16.0 | 14.8 | 16.3 | 13.8 | 17.0 | 16.6 | 15.8 | 15.7 | 15.7 | 15.4 |
| Actl7a | 0.2 | 0.1 | 0.0 | 0.0 | 0.0 | 0.1 | 0.0 | 0.1 | 0.0 | 0.1 | 0.1 | 0.0 |
| Actl7b | 0.0 | 0.1 | 0.1 | 0.1 | 0.0 | 0.0 | 0.0 | 0.0 | 0.0 | 0.0 | 0.0 | 0.0 |
| Actl9 | 0.0 | 0.0 | 0.1 | 0.0 | 0.0 | 0.1 | 0.0 | 0.1 | 0.0 | 0.0 | 0.0 | 0.0 |
| Actn1 | 183.0 | 154.3 | 136.9 | 182.7 | 156.9 | 148.3 | 218.2 | 176.6 | 170.4 | 178.0 | 223.4 | 181.2 |
| Actn2 | 0.0 | 0.0 | 0.0 | 0.0 | 0.0 | 0.0 | 0.0 | 0.0 | 0.0 | 0.0 | 0.0 | 0.0 |
| Actn3 | 0.7 | 1.4 | 1.3 | 0.4 | 0.7 | 0.5 | 0.8 | 0.7 | 0.7 | 1.9 | 1.7 | 1.3 |
| Actn4 | 28.9 | 37.6 | 42.1 | 33.4 | 51.0 | 28.9 | 59.5 | 54.6 | 52.8 | 55.0 | 50.7 | 54.5 |
| Actr10 | 20.1 | 19.7 | 15.2 | 18.3 | 18.1 | 19.3 | 16.4 | 16.5 | 17.6 | 19.4 | 18.9 | 17.6 |
| Actr1a | 53.9 | 51.5 | 47.4 | 57.7 | 52.4 | 54.8 | 59.6 | 55.5 | 55.1 | 53.5 | 56.4 | 52.3 |
| Actr1b | 52.8 | 43.3 | 34.8 | 62.2 | 43.5 | 55.1 | 46.7 | 35.9 | 40.7 | 43.5 | 44.6 | 39.4 |
| Actr2 | 58.6 | 50.7 | 42.0 | 70.1 | 76.7 | 61.7 | 49.9 | 43.0 | 52.1 | 59.0 | 54.6 | 58.4 |
| Actr3 | 68.6 | 62.0 | 57.6 | 85.3 | 100.0 | 61.8 | 85.3 | 61.5 | 76.1 | 74.8 | 93.7 | 78.4 |
| Actr3b | 0.6 | 0.7 | 0.7 | 0.7 | 0.8 | 0.6 | 0.5 | 0.6 | 0.5 | 0.6 | 0.4 | 0.7 |
| Actr5 | 2.0 | 2.0 | 1.8 | 1.5 | 2.1 | 2.1 | 2.1 | 2.2 | 2.1 | 1.8 | 1.8 | 1.2 |
| Actr6 | 5.9 | 6.4 | 5.8 | 5.9 | 5.2 | 6.1 | 3.7 | 4.2 | 5.1 | 6.6 | 5.8 | 6.5 |
| Actr8 | 11.2 | 10.2 | 10.1 | 12.1 | 10.4 | 11.5 | 11.3 | 10.7 | 11.3 | 11.0 | 12.3 | 11.2 |
| Acvr1 | 4.8 | 6.1 | 6.7 | 4.9 | 5.9 | 6.2 | 7.6 | 6.6 | 7.8 | 8.1 | 8.9 | 7.9 |
| Acvr1b | 4.7 | 8.8 | 14.1 | 4.2 | 9.1 | 3.5 | 8.3 | 11.4 | 8.0 | 5.5 | 5.3 | 7.5 |
| Acvr1c | 0.0 | 0.0 | 0.0 | 0.0 | 0.0 | 0.0 | 0.0 | 0.0 | 0.0 | 0.0 | 0.0 | 0.0 |
| Acvr2a | 3.3 | 3.6 | 4.2 | 4.1 | 3.7 | 3.4 | 4.1 | 6.0 | 5.2 | 4.9 | 5.9 | 4.8 |
| Acvr2b | 0.3 | 0.3 | 0.4 | 0.2 | 0.2 | 0.3 | 0.2 | 0.2 | 0.3 | 0.4 | 0.0 | 0.2 |
| Acvrl1 | 53.5 | 61.2 | 73.7 | 55.6 | 68.3 | 50.5 | 80.3 | 78.8 | 65.3 | 36.4 | 43.6 | 50.1 |
| Acy1 | 7.8 | 8.4 | 6.3 | 7.3 | 6.4 | 9.0 | 6.9 | 6.5 | 8.2 | 7.4 | 7.1 | 7.0 |
| Acy3 | 7.6 | 8.2 | 7.8 | 9.2 | 5.6 | 8.3 | 5.7 | 5.0 | 5.6 | 4.9 | 6.7 | 5.1 |
| Acyp1 | 12.9 | 14.5 | 18.0 | 12.0 | 11.3 | 16.3 | 12.9 | 15.4 | 14.8 | 17.6 | 14.6 | 18.9 |
| Acyp2 | 21.9 | 18.3 | 17.5 | 32.4 | 21.0 | 24.2 | 18.5 | 19.8 | 19.8 | 13.7 | 25.8 | 24.6 |
| Ada | 17.2 | 16.5 | 23.7 | 26.8 | 20.2 | 16.0 | 19.8 | 31.1 | 21.1 | 12.3 | 13.9 | 18.7 |

| | | | | | | | | | | | | |
|---|---|---|---|---|---|---|---|---|---|---|---|---|
| Adal | 2.9 | 2.9 | 3.1 | 3.3 | 3.3 | 3.5 | 2.4 | 3.4 | 3.2 | 3.3 | 3.2 | 3.2 |
| Adam10 | 19.0 | 23.3 | 28.5 | 19.8 | 27.4 | 20.6 | 28.3 | 31.9 | 28.8 | 29.8 | 33.0 | 33.3 |
| Adam11 | 0.1 | 0.1 | 0.2 | 0.1 | 0.1 | 0.1 | 0.1 | 0.3 | 0.2 | 0.3 | 0.1 | 0.1 |
| Adam12 | 1.1 | 2.7 | 9.7 | 0.7 | 4.7 | 0.5 | 6.7 | 18.5 | 11.8 | 8.5 | 7.0 | 9.7 |
| Adam15 | 47.7 | 44.4 | 38.2 | 55.7 | 61.7 | 48.6 | 56.2 | 51.9 | 52.7 | 47.1 | 44.2 | 40.4 |
| Adam17 | 16.3 | 18.0 | 21.8 | 21.3 | 28.3 | 17.9 | 17.6 | 19.7 | 18.2 | 16.0 | 14.8 | 18.9 |
| Adam18 | 0.0 | 0.1 | 0.0 | 0.0 | 0.1 | 0.0 | 0.1 | 0.0 | 0.1 | 0.0 | 0.0 | 0.0 |
| Adam19 | 12.7 | 16.7 | 15.6 | 14.0 | 20.9 | 15.3 | 26.9 | 18.1 | 20.8 | 13.3 | 14.3 | 13.3 |
| Adam1a | 0.3 | 0.2 | 0.2 | 0.3 | 0.4 | 0.2 | 0.4 | 0.3 | 0.4 | 0.4 | 0.4 | 0.2 |
| Adam1b | 0.0 | 0.0 | 0.0 | 0.0 | 0.0 | 0.1 | 0.1 | 0.0 | 0.0 | 0.0 | 0.1 | 0.0 |
| Adam21 | 0.0 | 0.1 | 0.0 | 0.0 | 0.0 | 0.0 | 0.0 | 0.1 | 0.0 | 0.1 | 0.0 | 0.0 |
| Adam22 | 0.2 | 0.1 | 0.2 | 0.2 | 0.1 | 0.2 | 0.2 | 0.2 | 0.3 | 0.5 | 0.2 | 0.4 |
| Adam23 | 7.2 | 10.7 | 10.9 | 13.5 | 9.8 | 14.1 | 11.8 | 9.1 | 8.2 | 20.0 | 22.1 | 12.0 |
| Adam28 | 0.0 | 0.0 | 0.0 | 0.0 | 0.0 | 0.0 | 0.0 | 0.0 | 0.0 | 0.1 | 0.0 | 0.0 |
| Adam3 | 0.2 | 0.0 | 0.0 | 0.1 | 0.0 | 0.1 | 0.0 | 0.0 | 0.0 | 0.0 | 0.0 | 0.0 |
| Adam32 | 0.1 | 0.1 | 0.2 | 0.2 | 0.1 | 0.3 | 0.1 | 0.1 | 0.2 | 0.2 | 0.1 | 0.1 |
| Adam33 | 0.3 | 0.1 | 0.1 | 0.1 | 0.0 | 0.1 | 0.0 | 0.0 | 0.1 | 0.5 | 0.2 | 0.0 |
| Adam39 | 0.0 | 0.0 | 0.0 | 0.0 | 0.0 | 0.0 | 0.0 | 0.0 | 0.0 | 0.0 | 0.0 | 0.0 |
| Adam4 | 0.1 | 0.1 | 0.1 | 0.1 | 0.2 | 0.1 | 0.1 | 0.2 | 0.1 | 0.1 | 0.1 | 0.2 |
| Adam5 | 0.8 | 0.8 | 0.8 | 0.3 | 0.8 | 0.6 | 0.8 | 0.6 | 0.6 | 0.3 | 0.6 | 0.5 |
| Adam8 | 6.1 | 0.3 | 0.6 | 49.1 | 79.4 | 0.4 | 6.5 | 1.3 | 1.2 | 4.6 | 1.4 | 2.9 |
| Adam9 | 152.6 | 148.4 | 138.1 | 160.4 | 172.4 | 136.7 | 179.3 | 136.3 | 149.1 | 169.8 | 182.0 | 170.1 |
| Adamts1 | 109.1 | 174.2 | 168.0 | 151.7 | 137.6 | 107.3 | 138.0 | 146.9 | 192.2 | 71.0 | 99.4 | 82.2 |
| Adamts10 | 12.8 | 15.9 | 19.6 | 9.3 | 14.1 | 10.9 | 14.3 | 17.5 | 22.6 | 11.7 | 12.0 | 12.8 |
| Adamts12 | 5.2 | 5.0 | 5.0 | 5.0 | 8.0 | 3.7 | 10.5 | 11.2 | 14.5 | 9.6 | 8.6 | 8.5 |
| Adamts13 | 0.0 | 0.0 | 0.0 | 0.0 | 0.0 | 0.0 | 0.0 | 0.0 | 0.0 | 0.0 | 0.0 | 0.0 |
| Adamts14 | 0.3 | 0.3 | 0.2 | 0.2 | 0.4 | 0.2 | 0.6 | 0.3 | 0.7 | 0.8 | 0.4 | 0.5 |
| Adamts15 | 0.0 | 0.1 | 0.2 | 0.0 | 0.0 | 0.0 | 0.0 | 0.4 | 0.3 | 0.4 | 0.4 | 0.5 |
| Adamts16 | 0.0 | 0.0 | 0.1 | 0.0 | 0.0 | 0.0 | 0.0 | 0.0 | 0.1 | 0.0 | 0.1 | 0.0 |
| Adamts17 | 0.0 | 0.0 | 0.0 | 0.0 | 0.0 | 0.0 | 0.0 | 0.0 | 0.0 | 0.0 | 0.0 | 0.0 |
| Adamts19 | 0.0 | 0.0 | 0.0 | 0.0 | 0.0 | 0.0 | 0.0 | 0.0 | 0.0 | 0.0 | 0.0 | 0.0 |
| Adamts2 | 38.5 | 64.7 | 96.8 | 31.1 | 65.3 | 29.1 | 79.3 | 109.4 | 94.6 | 57.4 | 51.2 | 70.6 |
| Adamts20 | 0.2 | 0.1 | 0.2 | 0.2 | 0.3 | 0.1 | 0.2 | 0.5 | 0.4 | 0.2 | 0.2 | 0.3 |
| Adamts3 | 0.0 | 0.1 | 0.4 | 0.0 | 0.1 | 0.0 | 0.1 | 0.2 | 0.1 | 0.3 | 0.1 | 0.1 |
| Adamts4 | 0.9 | 1.0 | 1.3 | 0.3 | 2.2 | 0.9 | 1.4 | 1.9 | 2.5 | 3.3 | 1.7 | 1.7 |
| Adamts5 | 117.9 | 149.2 | 161.2 | 141.1 | 116.2 | 128.0 | 140.6 | 141.0 | 140.4 | 79.8 | 112.8 | 101.0 |
| Adamts6 | 1.2 | 1.1 | 1.0 | 0.4 | 1.0 | 0.9 | 1.4 | 1.8 | 1.3 | 1.0 | 1.6 | 1.1 |
| Adamts7 | 1.4 | 1.7 | 2.1 | 1.1 | 2.6 | 1.2 | 1.7 | 2.0 | 1.5 | 1.6 | 0.9 | 1.0 |
| Adamts8 | 0.3 | 0.1 | 0.1 | 0.5 | 0.4 | 0.3 | 0.2 | 0.1 | 0.1 | 0.1 | 0.0 | 0.0 |
| Adamts9 | 11.5 | 16.4 | 14.0 | 12.9 | 17.8 | 8.9 | 15.7 | 8.0 | 11.1 | 17.9 | 13.8 | 9.7 |
| Adamtsl1 | 0.5 | 0.4 | 0.6 | 0.3 | 0.3 | 0.2 | 0.4 | 0.3 | 0.9 | 1.8 | 1.2 | 0.7 |
| Adamtsl2 | 16.9 | 37.2 | 102.2 | 11.0 | 20.8 | 9.5 | 17.7 | 74.1 | 24.0 | 5.8 | 8.3 | 20.0 |
| Adamtsl3 | 28.1 | 33.6 | 60.7 | 15.4 | 36.1 | 12.8 | 68.0 | 77.0 | 76.9 | 43.9 | 60.6 | 73.1 |
| Adamtsl4 | 3.2 | 5.5 | 5.7 | 2.5 | 3.8 | 3.7 | 7.5 | 8.8 | 6.4 | 4.3 | 4.2 | 3.8 |
| Adamtsl5 | 20.4 | 15.2 | 10.3 | 21.3 | 15.2 | 26.1 | 16.4 | 9.6 | 11.8 | 9.6 | 13.4 | 11.4 |
| Adap1 | 1.0 | 0.9 | 0.6 | 2.1 | 3.7 | 0.3 | 0.9 | 1.1 | 0.5 | 0.5 | 1.1 | 0.4 |
| Adap2 | 1.3 | 1.5 | 0.6 | 4.8 | 4.4 | 1.1 | 1.2 | 0.4 | 1.0 | 0.5 | 0.6 | 0.7 |
| Adar | 9.4 | 7.3 | 7.0 | 9.6 | 8.3 | 7.8 | 8.0 | 6.3 | 6.9 | 6.1 | 6.5 | 8.5 |
| Adarb1 | 1.6 | 1.8 | 3.2 | 1.6 | 2.3 | 1.4 | 2.3 | 3.5 | 3.1 | 2.6 | 2.1 | 3.1 |
| Adat1 | 1.0 | 1.1 | 1.2 | 1.1 | 1.1 | 1.2 | 1.1 | 0.9 | 0.8 | 0.8 | 0.9 | 0.8 |
| Adat2 | 2.2 | 2.2 | 2.0 | 2.3 | 2.3 | 2.4 | 2.2 | 2.2 | 2.1 | 1.9 | 2.8 | 2.5 |
| Adat3 | 0.8 | 1.0 | 0.9 | 0.7 | 0.9 | 0.5 | 1.3 | 1.1 | 1.1 | 0.8 | 0.9 | 0.6 |
| Adc | 10.5 | 11.0 | 14.3 | 8.9 | 10.6 | 9.3 | 12.0 | 15.9 | 17.2 | 12.6 | 10.3 | 13.4 |
| Adck1 | 5.4 | 5.5 | 4.5 | 4.4 | 4.8 | 5.2 | 4.9 | 4.8 | 5.2 | 5.3 | 4.5 | 4.7 |
| Adck2 | 2.2 | 2.0 | 2.0 | 1.7 | 1.7 | 2.2 | 2.1 | 2.3 | 2.3 | 2.4 | 1.8 | 1.8 |
| Adck3 | 1.8 | 1.7 | 1.7 | 1.7 | 1.6 | 1.8 | 1.2 | 2.0 | 1.7 | 2.0 | 1.8 | 2.1 |
| Adck4 | 6.2 | 6.5 | 7.7 | 5.5 | 7.3 | 5.0 | 6.3 | 8.7 | 6.8 | 7.3 | 5.4 | 7.7 |

| | | | | | | | | | | | | |
|---|---|---|---|---|---|---|---|---|---|---|---|---|
| Adck5 | 2.7 | 3.0 | 2.8 | 3.5 | 3.0 | 3.9 | 3.4 | 3.0 | 3.9 | 3.3 | 2.5 | 2.7 |
| Adcy1 | 2.2 | 2.1 | 1.5 | 1.2 | 1.2 | 0.8 | 2.3 | 1.5 | 2.8 | 2.2 | 2.4 | 1.6 |
| Adcy10 | 0.0 | 0.0 | 0.0 | 0.0 | 0.0 | 0.0 | 0.0 | 0.0 | 0.0 | 0.0 | 0.0 | 0.0 |
| Adcy2 | 1.2 | 0.9 | 1.4 | 2.3 | 2.2 | 0.6 | 1.0 | 0.9 | 0.8 | 0.6 | 0.6 | 0.7 |
| Adcy3 | 4.3 | 4.7 | 5.1 | 5.3 | 4.6 | 5.9 | 6.5 | 6.3 | 5.9 | 6.7 | 5.2 | 6.6 |
| Adcy4 | 0.6 | 0.6 | 0.6 | 0.8 | 0.5 | 0.5 | 0.6 | 0.8 | 0.6 | 0.6 | 0.4 | 0.6 |
| Adcy5 | 0.3 | 0.5 | 0.8 | 0.2 | 0.4 | 0.2 | 0.4 | 0.7 | 0.3 | 1.1 | 0.7 | 0.5 |
| Adcy6 | 8.5 | 9.6 | 10.0 | 8.2 | 7.9 | 8.7 | 10.5 | 8.0 | 10.5 | 9.9 | 9.4 | 8.8 |
| Adcy7 | 12.3 | 8.7 | 6.6 | 13.8 | 14.5 | 12.7 | 12.4 | 7.0 | 9.1 | 8.2 | 9.8 | 8.1 |
| Adcy8 | 0.1 | 0.1 | 0.1 | 0.1 | 0.1 | 0.3 | 0.1 | 0.0 | 0.0 | 0.1 | 0.1 | 0.0 |
| Adcy9 | 2.5 | 2.4 | 3.0 | 1.8 | 2.3 | 2.4 | 2.3 | 1.9 | 2.4 | 1.9 | 2.1 | 2.0 |
| Adcyap1r1 | 11.2 | 16.7 | 7.8 | 9.7 | 7.1 | 13.4 | 6.2 | 3.3 | 2.7 | 3.8 | 5.3 | 2.5 |
| Add1 | 70.9 | 89.7 | 108.8 | 67.4 | 81.4 | 68.0 | 98.0 | 117.9 | 99.5 | 82.0 | 93.2 | 99.7 |
| Add2 | 0.1 | 0.1 | 0.1 | 0.1 | 0.1 | 0.1 | 0.2 | 0.0 | 0.0 | 0.1 | 0.0 | 0.0 |
| Add3 | 36.6 | 34.9 | 35.9 | 32.7 | 25.0 | 28.4 | 35.6 | 41.9 | 36.1 | 30.4 | 43.4 | 36.1 |
| Adgb | 0.2 | 0.1 | 0.1 | 0.2 | 0.2 | 0.3 | 0.1 | 0.1 | 0.0 | 0.1 | 0.2 | 0.1 |
| Adh1 | 0.8 | 0.7 | 1.6 | 0.6 | 0.2 | 0.4 | 0.1 | 0.5 | 0.1 | 0.3 | 0.7 | 0.2 |
| Adh5 | 71.3 | 60.7 | 51.0 | 66.7 | 55.1 | 68.2 | 47.8 | 47.6 | 42.6 | 60.4 | 56.3 | 53.2 |
| Adh7 | 5.1 | 2.6 | 2.3 | 5.0 | 6.3 | 5.3 | 0.4 | 2.3 | 0.2 | 0.7 | 0.8 | 0.7 |
| Adhfe1 | 1.3 | 1.0 | 1.6 | 0.6 | 1.0 | 1.2 | 0.6 | 1.2 | 0.7 | 0.8 | 0.6 | 1.0 |
| Adi1 | 6.4 | 6.9 | 6.7 | 7.3 | 7.2 | 8.9 | 6.3 | 6.7 | 5.6 | 6.8 | 7.0 | 7.4 |
| Adipor1 | 134.7 | 98.6 | 79.8 | 140.1 | 104.0 | 130.1 | 106.5 | 90.9 | 97.0 | 107.1 | 113.5 | 101.8 |
| Adipor2 | 19.3 | 20.4 | 19.1 | 17.0 | 18.5 | 17.2 | 20.9 | 22.6 | 20.4 | 19.4 | 24.2 | 18.8 |
| Adk | 68.4 | 54.1 | 48.0 | 45.7 | 43.1 | 51.9 | 45.9 | 46.9 | 43.5 | 48.7 | 53.8 | 40.3 |
| Adm | 270.0 | 423.3 | 472.8 | 320.8 | 304.4 | 337.7 | 307.2 | 173.5 | 253.0 | 141.2 | 144.5 | 191.0 |
| Adm2 | 0.9 | 0.5 | 0.6 | 1.0 | 0.1 | 0.9 | 0.4 | 0.3 | 0.5 | 0.5 | 0.3 | 0.4 |
| Adnp | 5.7 | 7.0 | 7.1 | 6.4 | 6.6 | 6.3 | 6.6 | 7.3 | 6.9 | 7.2 | 7.4 | 6.9 |
| Adnp2 | 4.5 | 4.7 | 4.8 | 4.2 | 5.2 | 4.7 | 5.1 | 4.8 | 5.7 | 5.4 | 5.4 | 5.1 |
| Ado | 10.2 | 10.8 | 11.3 | 9.5 | 11.9 | 10.6 | 13.3 | 11.5 | 13.1 | 14.7 | 12.8 | 12.4 |
| Adora1 | 0.0 | 0.0 | 0.0 | 0.0 | 0.0 | 0.0 | 0.0 | 0.1 | 0.0 | 0.0 | 0.0 | 0.0 |
| Adora2a | 0.3 | 0.9 | 1.6 | 0.7 | 0.7 | 0.4 | 1.1 | 0.8 | 2.3 | 0.4 | 0.4 | 1.1 |
| Adora2b | 2.7 | 7.9 | 7.0 | 1.5 | 5.5 | 1.8 | 4.7 | 3.9 | 6.1 | 3.7 | 2.6 | 5.3 |
| Adora3 | 0.4 | 0.4 | 0.5 | 0.5 | 0.8 | 0.2 | 0.5 | 0.5 | 0.5 | 1.0 | 0.7 | 0.9 |
| Adpgk | 17.1 | 15.0 | 13.5 | 15.3 | 14.1 | 15.3 | 15.2 | 15.1 | 15.9 | 14.1 | 14.6 | 12.7 |
| Adprh | 30.4 | 27.5 | 23.6 | 29.8 | 29.0 | 30.7 | 30.6 | 25.1 | 30.0 | 29.8 | 27.2 | 28.0 |
| Adprhl2 | 9.4 | 7.8 | 6.6 | 6.3 | 7.9 | 8.2 | 6.7 | 7.3 | 8.7 | 9.6 | 7.2 | 7.1 |
| Adprm | 8.6 | 7.8 | 7.0 | 9.1 | 7.6 | 9.3 | 6.9 | 6.9 | 7.1 | 8.3 | 7.7 | 7.5 |
| Adra1a | 0.0 | 0.0 | 0.0 | 0.0 | 0.0 | 0.0 | 0.0 | 0.0 | 0.0 | 0.0 | 0.0 | 0.0 |
| Adra1b | 2.0 | 1.4 | 0.4 | 2.6 | 0.4 | 2.0 | 0.7 | 0.3 | 0.6 | 0.5 | 0.6 | 0.7 |
| Adra1d | 0.9 | 0.7 | 0.4 | 0.4 | 0.3 | 0.5 | 0.6 | 0.2 | 0.8 | 1.0 | 0.8 | 0.7 |
| Adra2a | 0.1 | 0.0 | 0.0 | 0.0 | 0.0 | 0.0 | 0.0 | 0.0 | 0.0 | 0.2 | 0.1 | 0.1 |
| Adra2b | 0.0 | 0.0 | 0.0 | 0.0 | 0.0 | 0.0 | 0.0 | 0.0 | 0.0 | 0.0 | 0.0 | 0.0 |
| Adrb1 | 0.0 | 0.0 | 0.0 | 0.1 | 0.2 | 0.0 | 0.1 | 0.0 | 0.0 | 0.0 | 0.0 | 0.0 |
| Adrb2 | 5.1 | 3.7 | 3.0 | 6.6 | 5.7 | 7.1 | 4.0 | 2.6 | 2.5 | 2.8 | 2.9 | 2.5 |
| Adrb3 | 0.4 | 0.4 | 0.3 | 0.3 | 0.3 | 0.2 | 0.5 | 0.2 | 0.6 | 0.2 | 0.4 | 0.1 |
| Adrbk1 | 14.7 | 14.5 | 13.1 | 16.0 | 20.3 | 13.6 | 16.0 | 13.5 | 13.7 | 12.1 | 13.7 | 11.9 |
| Adrbk2 | 0.0 | 0.1 | 0.0 | 0.1 | 0.3 | 0.0 | 0.0 | 0.0 | 0.0 | 0.0 | 0.0 | 0.0 |
| Adrm1 | 43.2 | 43.2 | 39.0 | 51.4 | 42.5 | 42.3 | 51.7 | 44.4 | 46.7 | 43.1 | 43.6 | 38.2 |
| Adsl | 7.5 | 7.5 | 6.5 | 7.1 | 6.7 | 6.9 | 8.3 | 7.6 | 7.2 | 6.8 | 7.5 | 6.8 |
| Adss | 26.7 | 23.6 | 22.7 | 27.9 | 24.9 | 26.3 | 24.0 | 20.8 | 23.1 | 29.1 | 29.5 | 28.5 |
| Adssl1 | 1.8 | 0.9 | 0.5 | 3.0 | 3.4 | 3.9 | 0.9 | 0.6 | 0.5 | 1.5 | 1.1 | 1.9 |
| Adtrp | 0.3 | 0.1 | 0.1 | 0.8 | 0.3 | 0.2 | 0.1 | 0.0 | 0.1 | 0.0 | 0.0 | 0.0 |
| Aebp1 | 56.1 | 68.4 | 102.8 | 39.6 | 69.8 | 60.3 | 92.5 | 93.3 | 91.0 | 117.6 | 88.1 | 118.4 |
| Aebp2 | 21.9 | 20.3 | 21.9 | 22.5 | 23.2 | 19.7 | 27.3 | 20.5 | 22.8 | 24.2 | 22.3 | 23.3 |
| Aen | 43.9 | 36.0 | 31.3 | 44.6 | 39.5 | 40.3 | 42.2 | 29.5 | 35.8 | 40.1 | 41.4 | 39.4 |
| Aes | 206.0 | 165.6 | 134.2 | 237.9 | 148.4 | 236.8 | 149.0 | 148.2 | 133.9 | 132.7 | 147.4 | 129.3 |
| AF251705 | 1.4 | 0.0 | 0.0 | 12.7 | 19.3 | 0.0 | 1.9 | 0.3 | 0.4 | 1.2 | 0.6 | 1.3 |

| | | | | | | | | | | | | |
|---|---|---|---|---|---|---|---|---|---|---|---|---|
| AF357425 | 111.2 | 143.2 | 150.1 | 236.2 | 228.1 | 0.0 | 181.2 | 326.1 | 434.8 | 651.5 | 0.0 | 0.0 |
| AF366264 | 0.0 | 0.0 | 0.0 | 0.0 | 0.0 | 0.1 | 0.0 | 0.0 | 0.0 | 0.0 | 0.0 | 0.0 |
| Afap1 | 14.7 | 15.3 | 13.5 | 17.3 | 14.1 | 16.7 | 19.4 | 14.2 | 16.2 | 15.1 | 17.7 | 14.2 |
| Afap1l1 | 0.8 | 0.4 | 0.5 | 0.3 | 0.2 | 0.2 | 0.4 | 0.8 | 0.6 | 0.1 | 0.2 | 0.4 |
| Afap1l2 | 6.8 | 4.1 | 3.7 | 5.7 | 5.0 | 3.7 | 6.2 | 3.7 | 6.1 | 6.1 | 7.5 | 6.1 |
| Aff1 | 7.6 | 10.2 | 10.2 | 7.6 | 10.7 | 6.7 | 11.4 | 9.1 | 9.9 | 8.4 | 9.0 | 9.3 |
| Aff2 | 2.0 | 1.9 | 2.2 | 1.5 | 1.1 | 1.4 | 1.1 | 1.5 | 0.9 | 1.4 | 1.1 | 1.8 |
| Aff3 | 6.4 | 5.0 | 4.7 | 5.5 | 3.9 | 5.6 | 3.6 | 3.9 | 3.3 | 2.7 | 3.0 | 3.7 |
| Aff4 | 28.1 | 29.8 | 28.6 | 25.7 | 23.4 | 28.5 | 25.0 | 25.7 | 26.0 | 25.7 | 28.3 | 29.2 |
| Afg3l1 | 8.1 | 8.5 | 8.8 | 10.4 | 10.0 | 8.4 | 8.9 | 9.3 | 9.1 | 8.6 | 8.5 | 8.8 |
| Afg3l2 | 13.3 | 13.0 | 11.1 | 15.6 | 14.8 | 15.3 | 14.7 | 11.5 | 11.5 | 13.6 | 13.5 | 13.0 |
| Afmid | 1.1 | 1.1 | 1.4 | 1.1 | 0.8 | 0.9 | 1.0 | 1.0 | 0.8 | 1.1 | 1.1 | 0.9 |
| Afp | 0.3 | 0.1 | 0.0 | 0.6 | 1.2 | 0.1 | 0.1 | 0.1 | 0.0 | 0.1 | 0.1 | 0.2 |
| Aftph | 13.9 | 13.1 | 10.6 | 14.5 | 12.1 | 14.7 | 10.1 | 9.8 | 10.6 | 12.0 | 12.4 | 12.0 |
| Aga | 116.8 | 94.6 | 89.6 | 108.1 | 84.9 | 117.5 | 69.7 | 62.3 | 63.7 | 89.9 | 84.5 | 90.0 |
| Agap1 | 3.2 | 2.5 | 2.2 | 3.3 | 2.7 | 2.6 | 2.2 | 2.0 | 2.1 | 4.0 | 3.3 | 2.3 |
| Agap2 | 0.0 | 0.0 | 0.0 | 0.2 | 0.4 | 0.0 | 0.1 | 0.0 | 0.0 | 0.1 | 0.0 | 0.0 |
| Agap3 | 10.5 | 8.4 | 8.8 | 11.0 | 10.5 | 12.5 | 10.2 | 8.4 | 9.5 | 9.1 | 8.4 | 9.1 |
| Agbl1 | 0.0 | 0.0 | 0.0 | 0.0 | 0.0 | 0.0 | 0.0 | 0.0 | 0.0 | 0.0 | 0.0 | 0.0 |
| Agbl2 | 1.0 | 1.1 | 1.2 | 0.9 | 0.8 | 1.0 | 1.0 | 0.9 | 1.1 | 0.9 | 1.0 | 1.3 |
| Agbl3 | 0.8 | 0.8 | 0.8 | 0.7 | 0.7 | 0.5 | 0.6 | 0.8 | 0.7 | 0.9 | 0.7 | 0.8 |
| Agbl4 | 0.0 | 0.0 | 0.0 | 0.0 | 0.0 | 0.0 | 0.0 | 0.0 | 0.0 | 0.0 | 0.0 | 0.0 |
| Agbl5 | 0.7 | 0.7 | 1.0 | 0.8 | 0.9 | 1.0 | 0.9 | 0.9 | 0.9 | 1.0 | 0.9 | 1.1 |
| Ager | 1.0 | 0.6 | 0.7 | 1.6 | 1.5 | 1.5 | 0.8 | 0.4 | 0.4 | 0.1 | 0.6 | 0.4 |
| Agfg1 | 13.5 | 14.8 | 13.5 | 12.5 | 14.5 | 12.7 | 13.5 | 14.1 | 16.1 | 15.9 | 15.7 | 15.6 |
| Agfg2 | 5.3 | 7.9 | 8.1 | 4.4 | 5.5 | 5.8 | 7.6 | 9.8 | 8.9 | 5.8 | 7.6 | 5.9 |
| Aggf1 | 17.5 | 16.8 | 17.8 | 17.8 | 16.8 | 17.0 | 17.0 | 18.7 | 16.1 | 18.4 | 16.0 | 20.2 |
| Agk | 7.1 | 8.5 | 7.9 | 7.4 | 6.8 | 7.4 | 8.9 | 8.2 | 7.5 | 6.1 | 7.0 | 7.4 |
| Agl | 8.5 | 7.0 | 5.7 | 9.7 | 7.3 | 9.1 | 7.1 | 5.8 | 6.4 | 7.4 | 7.5 | 7.3 |
| Agmo | 0.8 | 0.7 | 0.9 | 1.2 | 0.5 | 1.1 | 0.4 | 0.6 | 0.2 | 0.2 | 0.2 | 0.1 |
| Agpat1 | 34.8 | 34.1 | 27.4 | 37.4 | 35.0 | 38.1 | 33.2 | 30.1 | 31.6 | 31.2 | 34.8 | 28.5 |
| Agpat2 | 10.3 | 10.7 | 10.4 | 12.6 | 10.5 | 11.3 | 10.0 | 10.9 | 10.3 | 9.3 | 7.6 | 8.4 |
| Agpat3 | 29.8 | 26.7 | 26.5 | 31.5 | 26.0 | 32.8 | 31.6 | 28.6 | 28.2 | 27.0 | 30.5 | 30.1 |
| Agpat4 | 17.1 | 13.2 | 12.0 | 13.5 | 12.9 | 14.9 | 11.5 | 10.6 | 9.9 | 13.4 | 11.6 | 13.5 |
| Agpat5 | 7.3 | 6.2 | 5.2 | 5.5 | 6.2 | 6.1 | 5.4 | 4.8 | 5.5 | 6.4 | 6.4 | 6.1 |
| Agpat6 | 22.6 | 20.7 | 20.2 | 23.0 | 21.4 | 21.0 | 23.8 | 23.8 | 22.6 | 21.3 | 23.3 | 22.4 |
| Agpat9 | 0.5 | 0.3 | 0.3 | 0.7 | 0.4 | 0.7 | 0.5 | 0.5 | 0.7 | 0.5 | 0.6 | 0.5 |
| Agphd1 | 1.7 | 2.4 | 2.5 | 2.0 | 1.9 | 2.1 | 2.1 | 2.2 | 1.9 | 2.1 | 2.5 | 2.3 |
| Agps | 10.5 | 10.9 | 9.9 | 9.4 | 11.5 | 8.7 | 10.6 | 11.6 | 11.6 | 10.3 | 13.0 | 11.5 |
| Agrn | 4.2 | 4.3 | 5.5 | 3.6 | 4.2 | 3.5 | 3.6 | 4.7 | 4.4 | 5.3 | 3.1 | 3.9 |
| Agrp | 0.1 | 0.1 | 0.0 | 0.0 | 0.0 | 0.2 | 0.2 | 0.1 | 0.0 | 0.0 | 0.0 | 0.1 |
| Agt | 0.2 | 0.5 | 0.6 | 0.5 | 1.0 | 0.2 | 0.4 | 0.8 | 0.7 | 0.4 | 0.6 | 0.4 |
| Agtpbp1 | 12.0 | 12.0 | 12.5 | 12.6 | 11.3 | 10.4 | 11.3 | 9.8 | 9.9 | 10.2 | 11.1 | 11.9 |
| Agtr1a | 0.2 | 0.2 | 0.3 | 0.7 | 0.2 | 0.1 | 0.1 | 0.3 | 0.6 | 1.6 | 1.1 | 0.4 |
| Agtr1b | 0.0 | 0.0 | 0.0 | 0.0 | 0.0 | 0.0 | 0.0 | 0.0 | 0.0 | 0.0 | 0.0 | 0.0 |
| Agtr2 | 0.0 | 0.0 | 0.0 | 0.0 | 0.0 | 0.0 | 0.0 | 0.0 | 0.0 | 0.0 | 0.1 | 0.0 |
| Agtrap | 9.3 | 7.7 | 8.4 | 9.6 | 8.2 | 7.9 | 9.3 | 8.6 | 9.0 | 8.5 | 8.5 | 9.9 |
| Agxt | 0.0 | 0.1 | 0.0 | 0.0 | 0.0 | 0.0 | 0.0 | 0.0 | 0.2 | 0.1 | 0.0 | 0.1 |
| Agxt2l2 | 4.0 | 3.8 | 3.7 | 3.6 | 3.2 | 4.0 | 3.6 | 3.6 | 3.4 | 3.1 | 4.1 | 3.4 |
| Ahctf1 | 10.4 | 10.8 | 11.0 | 10.3 | 10.6 | 9.9 | 11.3 | 11.9 | 12.1 | 11.0 | 12.4 | 11.2 |
| Ahcy | 18.1 | 16.5 | 18.0 | 17.3 | 18.4 | 18.8 | 19.3 | 15.6 | 15.9 | 21.2 | 19.7 | 17.2 |
| Ahcyl1 | 56.8 | 62.9 | 58.4 | 60.2 | 63.7 | 64.3 | 57.9 | 51.4 | 53.4 | 59.7 | 62.7 | 56.5 |
| Ahcyl2 | 6.9 | 5.4 | 4.1 | 7.9 | 6.0 | 5.8 | 4.1 | 4.0 | 4.2 | 6.0 | 5.0 | 5.0 |
| Ahdc1 | 1.7 | 2.8 | 2.6 | 1.5 | 2.1 | 2.0 | 2.7 | 2.7 | 2.8 | 2.6 | 2.0 | 1.9 |
| Ahi1 | 5.5 | 5.0 | 5.1 | 4.8 | 4.2 | 6.0 | 4.0 | 4.2 | 3.0 | 4.3 | 4.9 | 4.3 |
| Ahnak | 463.3 | 476.3 | 416.9 | 384.4 | 345.7 | 473.9 | 419.3 | 482.0 | 413.0 | 384.5 | 501.6 | 463.1 |
| Ahr | 2.7 | 2.3 | 3.4 | 1.1 | 2.2 | 1.2 | 2.2 | 3.0 | 4.5 | 10.9 | 5.6 | 8.2 |

| | | | | | | | | | | | | |
|---|---|---|---|---|---|---|---|---|---|---|---|---|
| Ahrr | 0.3 | 0.2 | 0.1 | 0.5 | 0.1 | 0.3 | 0.3 | 0.3 | 0.2 | 0.3 | 0.2 | 0.2 |
| Ahsa1 | 91.4 | 97.9 | 88.4 | 113.6 | 90.3 | 85.7 | 86.7 | 76.6 | 78.6 | 88.6 | 101.6 | 94.9 |
| Ahsa2 | 22.7 | 24.9 | 21.0 | 19.5 | 19.6 | 20.4 | 18.7 | 17.3 | 20.4 | 23.6 | 24.8 | 21.6 |
| Ahsg | 0.0 | 0.1 | 0.1 | 0.0 | 0.0 | 0.0 | 0.0 | 0.0 | 0.0 | 0.2 | 0.2 | 0.0 |
| AI115009 | 0.2 | 0.0 | 0.1 | 0.1 | 0.1 | 0.3 | 0.3 | 0.1 | 0.1 | 0.2 | 0.3 | 0.3 |
| AI118078 | 0.0 | 0.0 | 0.0 | 0.0 | 0.0 | 0.1 | 0.0 | 0.0 | 0.0 | 0.0 | 0.0 | 0.0 |
| AI182371 | 0.2 | 0.1 | 0.3 | 0.0 | 0.1 | 0.2 | 0.1 | 0.2 | 0.4 | 0.0 | 0.0 | 0.1 |
| AI197445 | 0.1 | 0.1 | 0.1 | 0.0 | 0.1 | 0.0 | 0.1 | 0.1 | 0.0 | 0.0 | 0.0 | 0.0 |
| AI314180 | 21.6 | 22.8 | 20.1 | 21.1 | 20.3 | 21.7 | 19.5 | 21.5 | 20.5 | 20.1 | 21.0 | 21.5 |
| AI314831 | 0.0 | 0.0 | 0.0 | 0.0 | 0.0 | 0.0 | 0.0 | 0.0 | 0.1 | 0.3 | 0.1 | 0.1 |
| AI316807 | 14.8 | 12.7 | 11.1 | 19.3 | 11.3 | 16.2 | 11.9 | 12.7 | 11.4 | 11.1 | 13.5 | 13.2 |
| AI317395 | 0.6 | 0.4 | 0.4 | 0.5 | 0.3 | 0.7 | 0.2 | 0.5 | 0.2 | 0.2 | 0.3 | 0.3 |
| AI413582 | 20.6 | 17.0 | 11.6 | 26.6 | 25.1 | 24.9 | 16.4 | 12.4 | 10.9 | 19.0 | 12.8 | 13.8 |
| AI414108 | 1.5 | 1.2 | 0.7 | 1.1 | 0.9 | 1.0 | 0.7 | 0.2 | 0.4 | 0.9 | 1.0 | 0.8 |
| AI427809 | 1.0 | 0.6 | 0.6 | 1.7 | 1.3 | 1.2 | 0.6 | 0.4 | 0.6 | 0.7 | 0.6 | 0.7 |
| AI429214 | 1.9 | 1.6 | 2.1 | 1.6 | 1.1 | 1.9 | 1.1 | 1.9 | 1.9 | 1.4 | 1.7 | 2.0 |
| AI450353 | 0.1 | 0.3 | 0.7 | 0.2 | 0.4 | 0.3 | 0.5 | 0.3 | 0.4 | 0.3 | 0.1 | 0.3 |
| AI462493 | 17.7 | 15.7 | 17.0 | 17.9 | 15.8 | 19.1 | 15.7 | 15.7 | 19.6 | 23.7 | 18.0 | 21.7 |
| AI463170 | 0.1 | 0.0 | 0.0 | 0.0 | 0.1 | 0.0 | 0.4 | 0.3 | 0.2 | 0.5 | 0.0 | 0.2 |
| AI464131 | 3.2 | 3.6 | 3.8 | 2.4 | 2.6 | 3.2 | 3.5 | 5.2 | 4.2 | 3.2 | 3.6 | 3.1 |
| AI467606 | 0.2 | 0.0 | 0.0 | 1.6 | 2.2 | 0.0 | 0.2 | 0.0 | 0.0 | 0.1 | 0.0 | 0.2 |
| AI480653 | 12.4 | 9.4 | 7.7 | 11.1 | 11.0 | 13.3 | 11.3 | 8.7 | 9.9 | 9.8 | 9.1 | 9.4 |
| AI504432 | 0.0 | 0.0 | 0.0 | 0.2 | 0.3 | 0.0 | 0.0 | 0.0 | 0.0 | 0.0 | 0.0 | 0.0 |
| AI506816 | 0.5 | 2.4 | 0.8 | 4.9 | 0.7 | 0.7 | 10.3 | 8.7 | 1.2 | 5.7 | 9.5 | 0.4 |
| AI507597 | 0.0 | 0.1 | 0.0 | 0.1 | 0.2 | 0.0 | 0.0 | 0.0 | 0.0 | 0.1 | 0.0 | 0.0 |
| AI597468 | 14.3 | 17.5 | 17.4 | 15.1 | 16.4 | 16.9 | 16.8 | 18.7 | 20.2 | 18.9 | 22.2 | 21.0 |
| AI597479 | 14.7 | 14.4 | 12.7 | 17.3 | 12.7 | 18.2 | 11.6 | 9.9 | 11.0 | 14.0 | 14.0 | 14.4 |
| AI607873 | 0.7 | 0.4 | 0.7 | 2.9 | 2.1 | 0.5 | 0.5 | 0.4 | 0.2 | 0.3 | 0.4 | 0.6 |
| AI661453 | 0.0 | 0.1 | 0.1 | 0.1 | 0.1 | 0.0 | 0.0 | 0.0 | 0.0 | 0.1 | 0.0 | 0.0 |
| AI662270 | 0.6 | 0.1 | 0.1 | 2.8 | 5.5 | 0.0 | 0.8 | 0.1 | 0.2 | 0.6 | 0.5 | 0.6 |
| AI837181 | 9.9 | 8.5 | 8.9 | 9.7 | 9.6 | 9.2 | 9.0 | 11.4 | 9.8 | 11.3 | 10.9 | 9.0 |
| AI846148 | 6.6 | 6.6 | 4.9 | 3.9 | 5.0 | 5.1 | 6.8 | 5.4 | 6.0 | 6.1 | 6.6 | 5.7 |
| AI848285 | 0.1 | 0.0 | 0.0 | 0.0 | 0.0 | 0.0 | 0.1 | 0.0 | 0.1 | 0.0 | 0.0 | 0.0 |
| AI854703 | 0.0 | 0.1 | 0.2 | 0.1 | 0.1 | 0.1 | 0.0 | 0.0 | 0.1 | 0.0 | 0.1 | 0.1 |
| AI987944 | 8.9 | 9.1 | 11.1 | 9.0 | 8.5 | 9.7 | 8.3 | 8.8 | 8.2 | 8.3 | 11.1 | 11.0 |
| Aida | 33.6 | 32.2 | 29.8 | 50.9 | 34.3 | 40.8 | 37.4 | 28.2 | 29.7 | 25.9 | 35.1 | 33.6 |
| Aif1 | 1.3 | 0.1 | 0.0 | 8.3 | 11.1 | 0.2 | 0.9 | 0.0 | 0.6 | 0.6 | 0.7 | 1.3 |
| Aif1l | 1.8 | 0.7 | 0.5 | 1.1 | 0.6 | 1.2 | 0.9 | 1.1 | 1.3 | 4.2 | 3.2 | 1.8 |
| Aifm1 | 10.7 | 11.7 | 13.8 | 9.5 | 10.0 | 10.1 | 10.9 | 12.5 | 10.0 | 12.4 | 12.0 | 12.7 |
| Aifm2 | 8.1 | 6.7 | 5.9 | 8.3 | 6.2 | 8.0 | 7.1 | 6.0 | 7.7 | 6.9 | 7.9 | 6.9 |
| Aifm3 | 0.0 | 0.0 | 0.1 | 0.0 | 0.0 | 0.1 | 0.0 | 0.0 | 0.0 | 0.0 | 0.0 | 0.0 |
| Aig1 | 5.8 | 4.2 | 3.5 | 4.0 | 4.4 | 6.3 | 4.9 | 5.2 | 5.4 | 7.6 | 7.6 | 4.1 |
| Aim1 | 0.2 | 0.1 | 0.2 | 0.5 | 0.8 | 0.1 | 0.2 | 0.2 | 0.2 | 0.3 | 0.2 | 0.2 |
| Aim1l | 0.0 | 0.0 | 0.0 | 0.0 | 0.0 | 0.0 | 0.0 | 0.0 | 0.0 | 0.0 | 0.0 | 0.0 |
| Aim2 | 4.2 | 3.4 | 3.6 | 5.9 | 6.0 | 3.9 | 6.0 | 2.9 | 3.1 | 1.4 | 2.6 | 2.9 |
| Aimp1 | 57.7 | 47.3 | 39.6 | 48.5 | 43.5 | 57.3 | 42.4 | 42.1 | 41.3 | 51.1 | 49.0 | 48.0 |
| Aimp2 | 17.1 | 15.8 | 13.5 | 19.2 | 15.6 | 15.1 | 19.6 | 13.9 | 17.0 | 16.5 | 16.9 | 14.1 |
| Aip | 18.2 | 17.8 | 19.6 | 25.0 | 20.6 | 21.9 | 19.5 | 20.5 | 18.1 | 19.4 | 18.4 | 19.4 |
| Aipl1 | 0.1 | 0.2 | 0.3 | 0.0 | 0.3 | 0.2 | 0.2 | 0.1 | 0.1 | 0.1 | 0.0 | 0.1 |
| Airn | 1.8 | 2.3 | 2.1 | 0.9 | 1.4 | 0.9 | 3.0 | 1.5 | 1.8 | 3.1 | 0.9 | 2.5 |
| Ajuba | 10.9 | 8.1 | 5.4 | 10.0 | 6.9 | 8.6 | 8.0 | 7.5 | 10.1 | 12.9 | 11.2 | 8.0 |
| AK010878 | 2.3 | 2.1 | 2.6 | 2.6 | 2.9 | 2.3 | 2.1 | 2.5 | 2.0 | 3.7 | 2.0 | 2.2 |
| Ak1 | 126.1 | 133.0 | 136.3 | 127.8 | 139.3 | 127.1 | 175.5 | 156.7 | 165.9 | 148.7 | 162.6 | 144.8 |
| AK129341 | 1.2 | 0.7 | 0.5 | 2.1 | 0.8 | 1.4 | 0.5 | 0.4 | 0.4 | 0.7 | 0.9 | 0.6 |
| Ak2 | 16.9 | 17.7 | 17.3 | 18.3 | 19.5 | 16.0 | 21.0 | 21.2 | 18.8 | 18.6 | 22.2 | 19.8 |
| Ak3 | 44.3 | 50.8 | 48.7 | 40.9 | 37.7 | 43.9 | 37.1 | 46.0 | 40.8 | 38.4 | 46.4 | 42.7 |
| Ak4 | 8.2 | 8.9 | 5.4 | 6.8 | 8.0 | 10.3 | 7.1 | 3.5 | 5.4 | 6.5 | 5.7 | 5.7 |

| | | | | | | | | | | | | |
|---|---|---|---|---|---|---|---|---|---|---|---|---|
| Ak5 | 4.4 | 7.0 | 7.7 | 5.3 | 6.0 | 7.3 | 7.9 | 5.7 | 7.1 | 10.9 | 10.7 | 9.5 |
| Ak7 | 0.1 | 0.0 | 0.1 | 0.0 | 0.0 | 0.1 | 0.1 | 0.0 | 0.0 | 0.0 | 0.0 | 0.0 |
| Ak8 | 0.5 | 0.2 | 0.5 | 0.3 | 0.3 | 0.6 | 0.1 | 0.2 | 0.3 | 0.1 | 0.3 | 0.1 |
| Akap1 | 9.4 | 9.4 | 8.4 | 10.2 | 8.9 | 10.9 | 9.2 | 7.2 | 8.0 | 9.4 | 9.3 | 8.7 |
| Akap10 | 7.8 | 8.6 | 7.7 | 9.0 | 9.4 | 7.7 | 7.3 | 7.5 | 8.1 | 8.0 | 7.7 | 7.4 |
| Akap11 | 10.5 | 10.8 | 10.1 | 10.2 | 9.9 | 11.1 | 9.5 | 8.8 | 9.5 | 11.1 | 11.2 | 11.3 |
| Akap12 | 113.0 | 113.8 | 112.5 | 138.4 | 136.2 | 139.7 | 179.0 | 112.3 | 120.9 | 119.7 | 161.9 | 122.4 |
| Akap13 | 11.3 | 12.7 | 13.0 | 12.0 | 12.3 | 9.5 | 14.2 | 12.2 | 12.1 | 9.8 | 11.0 | 12.5 |
| Akap14 | 0.0 | 0.0 | 0.0 | 0.0 | 0.0 | 0.0 | 0.0 | 0.0 | 0.0 | 0.0 | 0.0 | 0.0 |
| Akap17b | 2.9 | 3.7 | 3.5 | 3.4 | 2.5 | 2.9 | 2.4 | 2.3 | 2.7 | 2.4 | 3.1 | 3.2 |
| Akap2 | 121.9 | 125.8 | 110.9 | 113.0 | 98.8 | 89.6 | 97.5 | 79.2 | 90.5 | 91.1 | 112.2 | 123.9 |
| Akap3 | 0.1 | 0.0 | 0.0 | 0.2 | 0.2 | 0.0 | 0.1 | 0.0 | 0.0 | 0.0 | 0.0 | 0.1 |
| Akap5 | 2.2 | 0.6 | 0.5 | 1.5 | 0.7 | 1.3 | 1.0 | 0.4 | 1.0 | 11.4 | 5.1 | 4.0 |
| Akap6 | 1.4 | 1.1 | 1.3 | 1.9 | 1.2 | 1.0 | 0.8 | 1.2 | 0.8 | 0.5 | 0.6 | 0.7 |
| Akap7 | 5.2 | 5.0 | 5.4 | 4.9 | 4.0 | 6.3 | 5.7 | 4.4 | 4.1 | 4.2 | 4.9 | 5.9 |
| Akap8 | 8.4 | 8.2 | 10.1 | 9.7 | 9.9 | 7.9 | 11.3 | 9.0 | 9.3 | 9.3 | 9.6 | 10.8 |
| Akap8l | 18.4 | 17.0 | 17.0 | 19.1 | 13.6 | 18.4 | 15.4 | 13.8 | 14.2 | 18.9 | 15.9 | 16.4 |
| Akap9 | 12.5 | 11.8 | 11.1 | 13.7 | 9.8 | 13.1 | 10.9 | 10.3 | 9.1 | 9.9 | 12.3 | 12.4 |
| Akip1 | 21.8 | 22.3 | 18.5 | 22.7 | 17.5 | 17.9 | 15.9 | 17.5 | 17.4 | 14.6 | 17.9 | 17.4 |
| Akirin1 | 25.6 | 24.2 | 20.5 | 34.4 | 26.8 | 28.3 | 24.0 | 20.0 | 21.5 | 22.5 | 26.6 | 25.3 |
| Akirin2 | 30.9 | 30.2 | 28.1 | 29.5 | 28.9 | 28.7 | 28.2 | 26.0 | 30.8 | 29.6 | 29.5 | 29.3 |
| Akna | 0.9 | 0.7 | 0.7 | 1.7 | 2.8 | 0.9 | 1.3 | 1.3 | 1.0 | 0.5 | 0.7 | 0.6 |
| Aknad1 | 0.0 | 0.0 | 0.0 | 0.1 | 0.0 | 0.0 | 0.0 | 0.0 | 0.0 | 0.0 | 0.0 | 0.0 |
| Akr1a1 | 283.1 | 223.6 | 186.9 | 377.1 | 380.4 | 285.8 | 228.6 | 233.9 | 223.8 | 253.9 | 243.9 | 236.1 |
| Akr1b10 | 19.6 | 17.7 | 15.5 | 23.3 | 21.8 | 22.7 | 15.5 | 12.9 | 13.1 | 21.6 | 17.7 | 16.8 |
| Akr1b3 | 7.8 | 5.6 | 7.1 | 6.0 | 8.3 | 9.3 | 5.2 | 10.7 | 14.0 | 6.8 | 3.4 | 6.4 |
| Akr1b7 | 0.1 | 0.1 | 0.0 | 0.1 | 0.0 | 0.1 | 0.0 | 0.0 | 0.0 | 0.1 | 0.1 | 0.1 |
| Akr1b8 | 51.6 | 39.6 | 36.3 | 68.1 | 63.8 | 55.7 | 54.6 | 52.6 | 44.4 | 38.7 | 44.8 | 40.4 |
| Akr1c12 | 2.0 | 1.7 | 1.4 | 3.7 | 3.4 | 1.8 | 1.3 | 0.9 | 0.4 | 0.7 | 0.7 | 0.8 |
| Akr1c13 | 14.0 | 10.6 | 7.7 | 18.0 | 14.9 | 10.7 | 5.0 | 4.8 | 2.7 | 3.7 | 4.6 | 5.1 |
| Akr1c14 | 14.6 | 14.0 | 14.4 | 11.3 | 7.6 | 12.5 | 3.7 | 8.4 | 4.4 | 7.5 | 8.1 | 9.4 |
| Akr1c18 | 0.2 | 0.0 | 0.0 | 0.3 | 0.0 | 0.0 | 0.0 | 0.0 | 0.0 | 0.2 | 0.1 | 0.0 |
| Akr1c19 | 0.3 | 0.4 | 0.6 | 0.0 | 0.1 | 0.1 | 0.1 | 0.0 | 0.1 | 0.1 | 0.1 | 0.1 |
| Akr1cl | 0.1 | 0.0 | 0.0 | 0.0 | 0.1 | 0.2 | 0.1 | 0.1 | 0.0 | 0.0 | 0.0 | 0.0 |
| Akr1e1 | 19.8 | 19.3 | 20.3 | 18.7 | 15.0 | 18.8 | 17.8 | 18.0 | 16.2 | 18.2 | 17.0 | 21.2 |
| Akr7a5 | 14.8 | 13.9 | 10.4 | 11.5 | 9.9 | 14.1 | 11.5 | 11.9 | 11.0 | 15.1 | 12.5 | 11.5 |
| Akt1 | 77.2 | 68.8 | 62.6 | 84.3 | 75.1 | 74.6 | 93.1 | 74.8 | 75.7 | 75.3 | 86.7 | 76.3 |
| Akt1s1 | 42.0 | 30.6 | 29.5 | 46.8 | 31.1 | 44.8 | 34.7 | 34.1 | 29.3 | 39.2 | 34.6 | 32.2 |
| Akt2 | 36.3 | 33.8 | 34.1 | 36.8 | 28.8 | 35.9 | 30.9 | 31.8 | 30.8 | 34.3 | 33.2 | 32.6 |
| Akt3 | 13.4 | 18.4 | 16.1 | 15.6 | 10.7 | 19.9 | 11.5 | 11.9 | 11.4 | 10.6 | 12.5 | 12.9 |
| Aktip | 43.5 | 35.3 | 31.5 | 55.6 | 28.7 | 58.5 | 31.9 | 24.3 | 26.4 | 27.0 | 35.0 | 31.9 |
| Alad | 2.9 | 3.1 | 2.7 | 2.8 | 2.8 | 3.4 | 2.6 | 2.5 | 2.3 | 4.8 | 3.3 | 2.3 |
| Alas1 | 48.8 | 53.3 | 45.1 | 70.3 | 51.5 | 53.1 | 25.9 | 33.4 | 21.7 | 24.4 | 25.9 | 25.2 |
| Alas2 | 0.1 | 0.1 | 0.1 | 0.0 | 0.1 | 0.2 | 0.1 | 0.0 | 0.1 | 0.7 | 0.4 | 0.1 |
| Alcam | 3.2 | 1.5 | 1.0 | 11.3 | 14.3 | 2.3 | 3.5 | 1.8 | 2.4 | 3.4 | 3.8 | 3.0 |
| Aldh16a1 | 4.5 | 5.2 | 4.4 | 4.3 | 5.8 | 3.9 | 5.2 | 5.2 | 5.1 | 4.9 | 3.9 | 4.5 |
| Aldh18a1 | 31.7 | 23.0 | 19.7 | 25.7 | 22.4 | 25.0 | 25.8 | 34.6 | 28.5 | 22.4 | 29.8 | 20.9 |
| Aldh1a1 | 1447.1 | 1418.0 | 1053.5 | 1493.7 | 1015.5 | 1495.7 | 648.3 | 532.5 | 505.1 | 846.5 | 812.4 | 736.2 |
| Aldh1a2 | 14.4 | 33.2 | 65.5 | 13.9 | 41.4 | 8.5 | 28.0 | 34.7 | 25.2 | 46.6 | 20.9 | 38.4 |
| Aldh1a3 | 17.6 | 47.5 | 73.3 | 11.0 | 28.0 | 21.2 | 30.7 | 145.4 | 30.5 | 90.8 | 117.7 | 86.1 |
| Aldh1a7 | 18.4 | 18.0 | 14.0 | 17.2 | 13.8 | 19.4 | 9.5 | 8.6 | 8.3 | 10.4 | 9.2 | 10.4 |
| Aldh1b1 | 2.1 | 1.4 | 1.1 | 1.8 | 1.3 | 2.2 | 1.5 | 0.7 | 1.0 | 1.5 | 1.2 | 1.5 |
| Aldh1l1 | 5.2 | 8.0 | 11.6 | 2.5 | 5.0 | 3.0 | 6.2 | 8.7 | 6.5 | 3.9 | 5.8 | 6.3 |
| Aldh1l2 | 9.3 | 5.6 | 6.2 | 8.6 | 3.8 | 10.3 | 6.6 | 18.5 | 8.9 | 4.7 | 10.5 | 6.1 |
| Aldh2 | 136.5 | 116.1 | 97.2 | 145.2 | 124.0 | 149.0 | 87.3 | 90.1 | 82.7 | 102.6 | 86.4 | 89.3 |
| Aldh3a1 | 10.3 | 7.2 | 10.1 | 3.0 | 2.1 | 3.9 | 0.7 | 4.7 | 0.5 | 0.9 | 0.7 | 1.8 |
| Aldh3a2 | 33.4 | 28.5 | 24.3 | 30.2 | 26.1 | 31.0 | 23.9 | 24.9 | 25.7 | 26.0 | 25.5 | 25.9 |

| | | | | | | | | | | | | |
|---|---|---|---|---|---|---|---|---|---|---|---|---|
| Aldh3b1 | 9.1 | 6.8 | 7.2 | 11.9 | 11.1 | 8.9 | 4.9 | 6.6 | 5.5 | 5.9 | 5.5 | 4.9 |
| Aldh3b2 | 0.0 | 0.0 | 0.1 | 0.0 | 0.0 | 0.0 | 0.0 | 0.0 | 0.0 | 0.0 | 0.0 | 0.0 |
| Aldh4a1 | 10.0 | 10.3 | 10.0 | 6.8 | 10.2 | 10.9 | 9.4 | 8.4 | 9.3 | 11.1 | 9.6 | 8.9 |
| Aldh5a1 | 1.5 | 1.6 | 1.5 | 1.0 | 1.0 | 1.7 | 0.9 | 1.5 | 1.2 | 1.1 | 1.1 | 1.3 |
| Aldh6a1 | 17.7 | 21.3 | 24.0 | 16.2 | 14.9 | 19.3 | 13.4 | 18.1 | 16.0 | 23.1 | 20.9 | 21.2 |
| Aldh7a1 | 28.5 | 25.0 | 29.8 | 24.0 | 21.5 | 26.1 | 24.0 | 24.3 | 22.4 | 33.3 | 26.8 | 26.6 |
| Aldh8a1 | 0.0 | 0.0 | 0.0 | 0.0 | 0.0 | 0.0 | 0.0 | 0.0 | 0.0 | 0.0 | 0.0 | 0.0 |
| Aldh9a1 | 5.1 | 5.0 | 5.3 | 6.0 | 6.7 | 6.3 | 4.9 | 5.8 | 4.9 | 5.5 | 5.0 | 5.0 |
| Aldoa | 543.3 | 457.2 | 371.2 | 734.0 | 585.8 | 680.1 | 482.5 | 443.9 | 404.1 | 453.0 | 451.5 | 451.9 |
| Aldoart1 | 0.2 | 0.2 | 0.1 | 0.3 | 0.2 | 0.2 | 0.2 | 0.1 | 0.1 | 0.1 | 0.2 | 0.1 |
| Aldoart2 | 0.1 | 0.0 | 0.0 | 0.1 | 0.0 | 0.0 | 0.0 | 0.0 | 0.0 | 0.0 | 0.0 | 0.0 |
| Aldob | 0.0 | 0.0 | 0.0 | 0.1 | 0.0 | 0.0 | 0.0 | 0.0 | 0.0 | 0.0 | 0.0 | 0.0 |
| Aldoc | 0.9 | 0.3 | 0.1 | 1.0 | 2.9 | 0.2 | 0.5 | 0.7 | 0.2 | 0.9 | 0.7 | 0.3 |
| Alg1 | 12.7 | 12.3 | 10.3 | 12.2 | 11.6 | 11.5 | 9.5 | 12.1 | 12.3 | 12.6 | 11.0 | 11.0 |
| Alg10b | 11.3 | 12.1 | 12.7 | 12.0 | 12.9 | 13.4 | 12.0 | 12.7 | 13.0 | 12.8 | 14.1 | 13.2 |
| Alg11 | 7.2 | 8.1 | 7.1 | 7.9 | 7.8 | 7.6 | 8.4 | 7.9 | 8.5 | 8.1 | 8.2 | 8.2 |
| Alg12 | 4.4 | 3.6 | 3.7 | 4.8 | 3.6 | 3.5 | 3.4 | 4.3 | 3.9 | 3.4 | 4.1 | 3.3 |
| Alg13 | 1.4 | 2.1 | 1.6 | 1.3 | 2.5 | 1.7 | 1.8 | 1.5 | 1.5 | 1.4 | 1.7 | 1.2 |
| Alg14 | 32.7 | 24.7 | 25.9 | 37.2 | 26.1 | 32.6 | 34.1 | 26.7 | 30.2 | 22.7 | 31.3 | 28.1 |
| Alg2 | 6.0 | 6.6 | 6.1 | 6.1 | 6.7 | 6.3 | 5.8 | 6.7 | 6.5 | 7.2 | 6.5 | 6.6 |
| Alg3 | 5.2 | 6.2 | 6.1 | 8.7 | 6.6 | 7.1 | 8.0 | 7.9 | 5.2 | 4.6 | 6.8 | 4.8 |
| Alg5 | 22.2 | 16.4 | 18.4 | 23.6 | 19.6 | 21.0 | 25.0 | 21.9 | 20.8 | 23.4 | 25.0 | 22.7 |
| Alg6 | 1.1 | 1.2 | 1.8 | 1.1 | 1.1 | 1.1 | 1.0 | 1.4 | 1.3 | 0.8 | 1.0 | 1.2 |
| Alg8 | 3.1 | 3.2 | 3.1 | 4.1 | 4.1 | 2.5 | 3.9 | 3.2 | 3.3 | 3.2 | 3.7 | 2.8 |
| Alg9 | 7.1 | 8.5 | 7.3 | 7.6 | 7.8 | 7.4 | 9.4 | 9.1 | 9.6 | 6.9 | 9.1 | 7.5 |
| Alk | 0.0 | 0.0 | 0.0 | 0.0 | 0.0 | 0.0 | 0.0 | 0.0 | 0.0 | 0.0 | 0.0 | 0.0 |
| Alkbh1 | 4.3 | 4.1 | 4.1 | 5.0 | 5.0 | 3.9 | 5.0 | 4.0 | 4.3 | 4.4 | 5.2 | 4.5 |
| Alkbh2 | 5.0 | 3.5 | 4.1 | 5.4 | 4.9 | 4.2 | 4.5 | 4.4 | 4.9 | 5.7 | 5.4 | 4.8 |
| Alkbh3 | 16.1 | 15.1 | 15.8 | 20.5 | 13.6 | 15.9 | 15.3 | 16.4 | 12.8 | 14.0 | 16.9 | 16.4 |
| Alkbh4 | 1.8 | 1.7 | 1.5 | 1.8 | 1.8 | 1.9 | 2.1 | 1.5 | 2.1 | 2.2 | 1.3 | 1.5 |
| Alkbh5 | 12.3 | 12.6 | 11.6 | 11.4 | 13.2 | 13.0 | 12.4 | 11.7 | 13.6 | 13.5 | 11.7 | 12.3 |
| Alkbh6 | 10.8 | 10.3 | 6.9 | 13.1 | 7.5 | 10.9 | 7.0 | 8.2 | 8.0 | 8.9 | 6.7 | 9.2 |
| Alkbh7 | 6.9 | 7.4 | 4.6 | 5.1 | 5.4 | 4.6 | 4.6 | 5.5 | 7.8 | 6.2 | 5.8 | 6.7 |
| Alkbh8 | 4.4 | 3.3 | 4.0 | 3.7 | 3.1 | 4.3 | 2.8 | 3.2 | 3.4 | 4.4 | 3.2 | 4.1 |
| Alms1 | 1.4 | 1.8 | 2.1 | 1.0 | 1.2 | 1.5 | 1.1 | 1.8 | 1.4 | 1.9 | 1.7 | 1.6 |
| Alms1-ps2 | 0.4 | 0.7 | 0.7 | 0.3 | 0.5 | 0.6 | 0.4 | 0.7 | 0.5 | 0.7 | 0.6 | 0.6 |
| Alox12 | 0.0 | 0.1 | 0.0 | 0.0 | 0.1 | 0.1 | 0.1 | 0.1 | 0.0 | 0.0 | 0.0 | 0.0 |
| Alox5 | 1.5 | 0.8 | 0.6 | 2.2 | 3.7 | 0.7 | 0.7 | 0.1 | 0.1 | 0.2 | 0.4 | 0.3 |
| Alox5ap | 5.1 | 0.2 | 0.3 | 34.3 | 66.1 | 0.1 | 5.0 | 1.5 | 1.4 | 3.9 | 1.0 | 1.9 |
| Alox8 | 0.0 | 0.0 | 0.0 | 0.0 | 0.0 | 0.1 | 0.0 | 0.1 | 0.0 | 0.1 | 0.0 | 0.0 |
| Aloxe3 | 0.0 | 0.0 | 0.0 | 0.1 | 0.0 | 0.0 | 0.0 | 0.0 | 0.0 | 0.1 | 0.0 | 0.0 |
| Alpk1 | 4.7 | 7.0 | 9.7 | 3.3 | 6.3 | 3.7 | 6.6 | 5.9 | 6.1 | 4.7 | 5.5 | 6.4 |
| Alpk2 | 0.0 | 0.0 | 0.0 | 0.0 | 0.0 | 0.0 | 0.0 | 0.0 | 0.0 | 0.0 | 0.0 | 0.0 |
| Alpk3 | 0.1 | 0.2 | 0.2 | 0.1 | 0.1 | 0.0 | 0.1 | 0.2 | 0.1 | 0.1 | 0.1 | 0.1 |
| Alpl | 1.6 | 3.2 | 3.8 | 0.9 | 1.3 | 0.2 | 0.7 | 1.9 | 0.6 | 0.8 | 1.0 | 1.8 |
| Als2 | 5.3 | 5.4 | 4.6 | 4.7 | 4.9 | 4.9 | 5.0 | 5.2 | 4.8 | 4.0 | 5.0 | 4.5 |
| Als2cl | 1.3 | 0.9 | 0.8 | 1.1 | 1.0 | 0.8 | 0.9 | 0.9 | 0.7 | 0.4 | 0.4 | 0.5 |
| Als2cr12 | 0.2 | 0.1 | 0.1 | 0.1 | 0.2 | 0.1 | 0.3 | 0.0 | 0.1 | 0.2 | 0.2 | 0.1 |
| Alx1 | 0.0 | 0.0 | 0.0 | 0.0 | 0.0 | 0.0 | 0.0 | 0.0 | 0.0 | 0.1 | 0.4 | 0.0 |
| Alx3 | 0.0 | 0.0 | 0.4 | 0.0 | 0.0 | 0.0 | 0.0 | 0.0 | 0.0 | 0.1 | 0.1 | 0.0 |
| Alx4 | 0.3 | 0.4 | 0.4 | 0.2 | 0.2 | 0.3 | 0.2 | 0.1 | 0.2 | 0.2 | 0.2 | 0.2 |
| Alyref | 30.6 | 26.8 | 30.9 | 30.9 | 33.2 | 28.7 | 39.0 | 33.4 | 33.2 | 32.0 | 33.8 | 33.2 |
| Alyref2 | 0.9 | 0.8 | 1.4 | 1.3 | 1.4 | 0.7 | 1.4 | 1.5 | 1.3 | 1.8 | 2.2 | 1.6 |
| Amacr | 4.5 | 4.4 | 3.4 | 3.3 | 3.9 | 4.0 | 3.9 | 4.6 | 4.8 | 4.2 | 4.3 | 4.4 |
| Ambp | 0.1 | 0.0 | 0.0 | 0.2 | 0.6 | 0.0 | 0.1 | 0.0 | 0.1 | 0.2 | 0.1 | 0.0 |
| Ambra1 | 4.4 | 4.9 | 4.8 | 4.1 | 4.7 | 4.7 | 5.3 | 5.4 | 4.9 | 4.6 | 4.4 | 4.6 |
| Amd1 | 0.0 | 0.0 | 0.0 | 5.9 | 3.4 | 0.0 | 0.0 | 0.0 | 0.0 | 0.0 | 0.0 | 0.0 |

| | | | | | | | | | | | | |
|---|---|---|---|---|---|---|---|---|---|---|---|---|
| Amd2 | 20.4 | 19.1 | 18.1 | 14.1 | 19.3 | 21.0 | 18.2 | 20.4 | 19.0 | 21.2 | 20.9 | 17.5 |
| Amdhd1 | 0.1 | 0.0 | 0.0 | 0.0 | 0.0 | 0.0 | 0.0 | 0.0 | 0.0 | 0.0 | 0.0 | 0.0 |
| Amdhd2 | 10.2 | 9.4 | 8.9 | 9.5 | 10.0 | 11.9 | 7.8 | 10.0 | 8.7 | 8.0 | 6.9 | 8.7 |
| Amer1 | 2.0 | 2.5 | 2.6 | 1.5 | 1.4 | 1.8 | 1.9 | 2.7 | 2.4 | 2.4 | 2.6 | 2.4 |
| Amfr | 70.4 | 62.1 | 48.7 | 67.8 | 58.5 | 72.2 | 65.2 | 55.6 | 65.9 | 64.8 | 64.8 | 61.9 |
| Amh | 0.1 | 0.1 | 0.0 | 0.0 | 0.1 | 0.2 | 0.1 | 0.0 | 0.1 | 0.0 | 0.0 | 0.1 |
| Amhr2 | 0.1 | 0.0 | 0.0 | 0.0 | 0.0 | 0.2 | 0.0 | 0.0 | 0.1 | 0.2 | 0.0 | 0.1 |
| Amica1 | 0.2 | 0.1 | 0.1 | 0.2 | 0.3 | 0.3 | 0.0 | 0.0 | 0.0 | 0.1 | 0.0 | 0.0 |
| Amigo1 | 1.4 | 1.6 | 1.6 | 1.8 | 1.3 | 2.5 | 1.8 | 1.1 | 1.6 | 1.1 | 1.3 | 1.4 |
| Amigo2 | 0.5 | 0.4 | 0.2 | 0.7 | 0.4 | 0.8 | 0.4 | 0.5 | 0.4 | 1.3 | 0.5 | 0.5 |
| Amigo3 | 0.8 | 0.9 | 0.8 | 0.7 | 1.0 | 0.6 | 0.9 | 1.0 | 1.0 | 1.0 | 0.7 | 0.8 |
| Ammecr1 | 3.1 | 2.9 | 3.0 | 3.1 | 3.9 | 2.3 | 4.2 | 4.0 | 3.9 | 5.2 | 5.3 | 5.3 |
| Ammecr1l | 6.2 | 6.4 | 6.0 | 6.3 | 6.8 | 5.9 | 6.4 | 6.4 | 6.5 | 7.0 | 6.4 | 6.3 |
| Amn | 0.0 | 0.0 | 0.1 | 0.0 | 0.1 | 0.1 | 0.0 | 0.0 | 0.1 | 0.1 | 0.0 | 0.0 |
| Amn1 | 8.5 | 8.2 | 6.6 | 7.3 | 6.9 | 7.0 | 6.9 | 6.1 | 9.7 | 9.4 | 8.6 | 9.5 |
| Amot | 3.4 | 2.6 | 1.8 | 4.2 | 2.4 | 4.0 | 1.9 | 1.4 | 1.2 | 2.5 | 2.5 | 1.5 |
| Amotl1 | 31.0 | 24.7 | 19.6 | 36.9 | 23.1 | 34.9 | 23.4 | 16.6 | 19.3 | 22.9 | 24.5 | 22.1 |
| Amotl2 | 65.8 | 87.2 | 95.8 | 69.7 | 67.8 | 79.3 | 98.7 | 104.4 | 103.8 | 106.0 | 86.8 | 89.7 |
| Ampd1 | 0.0 | 0.0 | 0.0 | 0.1 | 0.0 | 0.0 | 0.0 | 0.0 | 0.0 | 0.0 | 0.1 | 0.0 |
| Ampd2 | 9.1 | 9.8 | 9.2 | 7.7 | 10.6 | 9.8 | 9.3 | 8.6 | 9.2 | 9.0 | 8.6 | 8.7 |
| Ampd3 | 8.9 | 8.3 | 5.3 | 18.1 | 12.8 | 17.9 | 6.2 | 4.2 | 3.4 | 3.4 | 3.3 | 3.0 |
| Amph | 0.1 | 0.0 | 0.0 | 0.0 | 0.0 | 0.0 | 0.0 | 0.0 | 0.0 | 0.0 | 0.0 | 0.0 |
| Amt | 0.9 | 0.8 | 0.9 | 0.8 | 0.6 | 1.1 | 0.6 | 1.3 | 0.9 | 0.4 | 0.5 | 0.4 |
| Amy1 | 0.8 | 0.3 | 0.5 | 0.4 | 0.2 | 0.3 | 0.4 | 0.4 | 0.1 | 0.1 | 0.3 | 0.2 |
| Amy2a2 | 0.0 | 0.0 | 0.0 | 0.0 | 0.0 | 0.0 | 0.0 | 0.0 | 0.0 | 0.0 | 0.0 | 0.0 |
| Amy2a4 | 0.0 | 0.0 | 0.0 | 0.0 | 0.0 | 0.0 | 0.0 | 0.0 | 0.0 | 0.0 | 0.0 | 0.0 |
| Amz1 | 0.6 | 0.3 | 0.1 | 1.8 | 1.9 | 0.9 | 0.4 | 0.1 | 0.2 | 0.3 | 0.4 | 0.2 |
| Amz2 | 12.7 | 12.6 | 10.8 | 11.4 | 9.9 | 14.5 | 9.9 | 12.2 | 11.4 | 10.3 | 11.4 | 11.0 |
| Anapc1 | 20.2 | 20.0 | 18.4 | 18.3 | 18.2 | 19.9 | 18.0 | 18.7 | 17.5 | 21.7 | 20.8 | 17.9 |
| Anapc10 | 6.3 | 6.1 | 6.9 | 6.6 | 5.4 | 5.5 | 6.4 | 5.8 | 5.0 | 5.5 | 6.7 | 7.3 |
| Anapc11 | 6.1 | 6.5 | 4.5 | 6.7 | 6.7 | 7.7 | 5.4 | 5.6 | 6.4 | 7.0 | 5.7 | 6.3 |
| Anapc13 | 63.2 | 65.8 | 69.0 | 71.4 | 54.8 | 72.8 | 64.5 | 69.7 | 61.6 | 72.2 | 85.7 | 87.1 |
| Anapc15 | 20.3 | 19.8 | 15.9 | 28.0 | 19.0 | 20.4 | 20.9 | 16.6 | 16.4 | 19.3 | 21.7 | 20.9 |
| Anapc16 | 31.1 | 20.7 | 18.8 | 31.9 | 23.5 | 28.8 | 25.2 | 23.4 | 24.8 | 25.5 | 25.8 | 24.6 |
| Anapc2 | 19.5 | 21.3 | 21.2 | 18.5 | 22.9 | 20.0 | 20.3 | 21.8 | 18.6 | 19.6 | 19.1 | 20.2 |
| Anapc4 | 36.2 | 31.9 | 30.6 | 32.6 | 31.1 | 33.9 | 31.1 | 32.2 | 31.8 | 32.8 | 33.9 | 34.4 |
| Anapc5 | 75.7 | 68.6 | 66.5 | 69.8 | 61.7 | 70.2 | 68.5 | 73.8 | 72.5 | 54.9 | 70.4 | 62.8 |
| Anapc7 | 11.4 | 11.3 | 11.8 | 10.9 | 11.3 | 11.2 | 11.0 | 11.2 | 10.9 | 10.3 | 10.1 | 11.4 |
| Ang | 9.9 | 10.2 | 11.9 | 10.1 | 11.5 | 8.1 | 12.4 | 14.2 | 11.0 | 8.8 | 12.3 | 8.8 |
| Ang2 | 0.0 | 0.3 | 0.0 | 0.0 | 0.0 | 0.0 | 0.2 | 0.4 | 0.2 | 0.2 | 0.2 | 0.3 |
| Angel1 | 0.6 | 0.9 | 0.8 | 0.5 | 0.6 | 0.6 | 0.7 | 0.8 | 0.6 | 0.6 | 0.5 | 0.8 |
| Angel2 | 11.5 | 11.9 | 10.9 | 8.9 | 9.5 | 9.6 | 10.4 | 11.6 | 10.7 | 10.9 | 11.9 | 10.6 |
| Angpt1 | 0.4 | 0.8 | 1.4 | 0.4 | 0.7 | 0.3 | 1.2 | 1.5 | 1.3 | 1.8 | 0.8 | 1.9 |
| Angpt2 | 5.9 | 2.0 | 3.0 | 1.8 | 0.7 | 1.0 | 2.9 | 4.6 | 4.1 | 1.7 | 1.2 | 2.9 |
| Angpt4 | 10.0 | 9.2 | 8.6 | 14.6 | 11.2 | 17.3 | 7.0 | 5.7 | 7.0 | 7.3 | 4.9 | 9.2 |
| Angptl1 | 1.6 | 1.7 | 1.8 | 2.8 | 1.4 | 0.8 | 2.3 | 1.9 | 1.6 | 2.8 | 5.9 | 1.2 |
| Angptl2 | 25.1 | 21.3 | 22.6 | 29.6 | 38.8 | 38.0 | 27.4 | 29.0 | 17.7 | 20.8 | 21.8 | 19.2 |
| Angptl3 | 0.1 | 0.0 | 0.1 | 0.2 | 0.0 | 0.1 | 0.0 | 0.0 | 0.0 | 0.0 | 0.0 | 0.0 |
| Angptl4 | 16.2 | 5.6 | 8.8 | 18.6 | 27.4 | 5.7 | 13.6 | 30.3 | 11.0 | 40.2 | 10.6 | 22.8 |
| Angptl6 | 2.1 | 0.9 | 0.7 | 2.1 | 0.6 | 2.5 | 0.7 | 1.2 | 1.0 | 0.8 | 1.4 | 0.9 |
| Angptl7 | 9.4 | 4.5 | 12.7 | 1.7 | 0.9 | 0.7 | 2.0 | 6.5 | 5.0 | 84.8 | 24.8 | 18.7 |
| Ank | 14.3 | 12.4 | 10.4 | 15.4 | 12.9 | 17.5 | 13.2 | 9.8 | 11.0 | 12.3 | 12.8 | 11.1 |
| Ank1 | 0.5 | 0.3 | 0.3 | 0.6 | 0.3 | 0.7 | 0.3 | 0.2 | 0.2 | 0.4 | 0.3 | 0.3 |
| Ank2 | 11.4 | 9.4 | 10.4 | 10.3 | 7.5 | 10.1 | 8.1 | 9.6 | 6.4 | 4.6 | 7.2 | 5.5 |
| Ank3 | 0.6 | 0.4 | 0.4 | 0.5 | 0.4 | 0.3 | 0.4 | 0.3 | 0.3 | 0.2 | 0.3 | 0.3 |
| Ankar | 0.0 | 0.0 | 0.0 | 0.0 | 0.0 | 0.0 | 0.0 | 0.0 | 0.0 | 0.0 | 0.0 | 0.0 |
| Ankdd1b | 0.3 | 0.3 | 0.3 | 0.5 | 0.3 | 0.3 | 0.3 | 0.4 | 0.3 | 0.4 | 0.5 | 0.5 |

| | | | | | | | | | | | | |
|---|---|---|---|---|---|---|---|---|---|---|---|---|
| Ankef1 | 0.3 | 0.2 | 0.2 | 0.2 | 0.1 | 0.2 | 0.2 | 0.1 | 0.1 | 0.1 | 0.1 | 0.2 |
| Ankfy1 | 16.9 | 16.1 | 14.9 | 17.5 | 17.2 | 16.2 | 13.9 | 13.4 | 13.7 | 13.5 | 14.7 | 14.7 |
| Ankhd1 | 6.0 | 5.9 | 6.5 | 6.8 | 6.2 | 6.1 | 6.4 | 6.9 | 5.4 | 5.1 | 6.7 | 6.8 |
| Ankib1 | 31.2 | 34.3 | 29.9 | 31.2 | 25.9 | 33.2 | 29.5 | 25.9 | 34.8 | 26.8 | 33.1 | 36.5 |
| Ankk1 | 0.0 | 0.0 | 0.0 | 0.0 | 0.0 | 0.0 | 0.0 | 0.0 | 0.0 | 0.0 | 0.0 | 0.0 |
| Ankle1 | 0.4 | 1.0 | 1.5 | 0.1 | 1.3 | 0.2 | 1.9 | 0.6 | 1.6 | 0.5 | 0.7 | 0.4 |
| Ankle2 | 10.2 | 10.8 | 10.8 | 10.0 | 9.6 | 11.4 | 10.9 | 10.0 | 9.9 | 10.3 | 11.1 | 11.0 |
| Ankmy1 | 0.1 | 0.1 | 0.0 | 0.0 | 0.1 | 0.1 | 0.0 | 0.0 | 0.1 | 0.0 | 0.0 | 0.0 |
| Ankmy2 | 9.9 | 9.3 | 9.4 | 7.7 | 6.5 | 8.7 | 8.6 | 9.7 | 8.4 | 8.5 | 9.3 | 9.7 |
| Ankra2 | 10.5 | 11.5 | 9.3 | 12.8 | 9.2 | 11.2 | 11.0 | 9.4 | 9.3 | 8.5 | 10.1 | 9.9 |
| Ankrd1 | 517.7 | 346.6 | 184.2 | 1121.4 | 436.2 | 684.2 | 466.8 | 100.5 | 195.5 | 313.9 | 381.7 | 266.6 |
| Ankrd10 | 3.6 | 4.1 | 3.9 | 4.1 | 5.1 | 4.3 | 4.4 | 5.1 | 5.8 | 6.3 | 6.1 | 4.4 |
| Ankrd11 | 17.3 | 20.4 | 22.3 | 20.6 | 18.3 | 16.9 | 26.5 | 20.6 | 19.8 | 16.6 | 22.8 | 22.0 |
| Ankrd12 | 7.4 | 8.4 | 9.5 | 10.2 | 7.7 | 9.2 | 9.0 | 7.3 | 6.7 | 8.5 | 10.3 | 11.2 |
| Ankrd13a | 29.4 | 26.7 | 25.4 | 33.4 | 31.8 | 29.5 | 32.2 | 23.8 | 28.4 | 25.7 | 29.7 | 29.9 |
| Ankrd13b | 1.6 | 1.9 | 1.9 | 2.3 | 1.9 | 2.1 | 2.1 | 1.7 | 1.9 | 1.5 | 1.6 | 1.4 |
| Ankrd13c | 20.8 | 19.2 | 17.6 | 21.5 | 18.5 | 19.7 | 18.1 | 16.4 | 15.9 | 18.6 | 20.8 | 19.0 |
| Ankrd13d | 2.4 | 2.1 | 1.8 | 1.7 | 1.9 | 3.0 | 1.6 | 0.7 | 1.2 | 1.8 | 1.4 | 1.6 |
| Ankrd16 | 2.4 | 2.5 | 2.1 | 3.1 | 2.7 | 2.4 | 2.7 | 2.5 | 2.7 | 2.7 | 2.1 | 2.1 |
| Ankrd17 | 13.6 | 14.4 | 15.8 | 14.2 | 14.2 | 13.2 | 16.2 | 15.9 | 12.9 | 13.0 | 15.3 | 15.0 |
| Ankrd2 | 0.0 | 0.0 | 0.1 | 0.1 | 0.0 | 0.0 | 0.0 | 0.0 | 0.0 | 0.0 | 0.1 | 0.0 |
| Ankrd23 | 0.8 | 1.1 | 0.9 | 0.8 | 0.7 | 1.0 | 0.9 | 1.1 | 1.0 | 0.8 | 0.9 | 0.7 |
| Ankrd24 | 0.9 | 1.2 | 1.0 | 1.1 | 0.8 | 1.4 | 0.9 | 1.0 | 0.8 | 0.6 | 0.8 | 0.9 |
| Ankrd26 | 3.1 | 3.6 | 3.8 | 3.1 | 3.1 | 2.9 | 3.5 | 2.7 | 2.7 | 3.3 | 4.0 | 3.6 |
| Ankrd27 | 18.2 | 16.9 | 14.9 | 19.1 | 14.5 | 17.8 | 16.4 | 15.2 | 15.8 | 12.5 | 17.9 | 14.9 |
| Ankrd28 | 12.1 | 10.6 | 10.0 | 13.1 | 12.2 | 14.5 | 7.9 | 6.6 | 6.7 | 9.2 | 7.5 | 8.8 |
| Ankrd29 | 0.9 | 1.1 | 1.1 | 0.9 | 0.9 | 1.2 | 1.0 | 0.8 | 0.7 | 0.9 | 1.3 | 0.9 |
| Ankrd32 | 2.5 | 3.5 | 3.5 | 2.4 | 3.0 | 2.6 | 2.7 | 2.6 | 3.0 | 3.3 | 3.5 | 3.8 |
| Ankrd33 | 0.0 | 0.1 | 0.1 | 0.0 | 0.0 | 0.0 | 0.1 | 0.0 | 0.0 | 0.0 | 0.0 | 0.0 |
| Ankrd33b | 0.0 | 0.1 | 0.0 | 0.1 | 0.3 | 0.0 | 0.1 | 0.1 | 0.1 | 0.1 | 0.1 | 0.1 |
| Ankrd34a | 0.2 | 0.4 | 0.4 | 0.3 | 0.3 | 0.3 | 0.3 | 0.3 | 0.2 | 0.6 | 0.3 | 0.4 |
| Ankrd34c | 0.1 | 0.1 | 0.0 | 0.0 | 0.0 | 0.0 | 0.0 | 0.0 | 0.0 | 0.0 | 0.0 | 0.0 |
| Ankrd35 | 0.3 | 0.4 | 0.6 | 0.1 | 0.1 | 0.2 | 0.2 | 0.4 | 0.2 | 0.3 | 0.2 | 0.4 |
| Ankrd37 | 0.6 | 0.6 | 0.5 | 1.0 | 0.9 | 1.3 | 0.8 | 0.9 | 1.2 | 1.0 | 1.6 | 1.3 |
| Ankrd39 | 3.3 | 3.2 | 2.8 | 2.3 | 2.7 | 3.1 | 3.4 | 2.4 | 3.5 | 2.8 | 2.3 | 2.7 |
| Ankrd40 | 30.3 | 31.4 | 35.9 | 47.2 | 30.6 | 34.1 | 36.3 | 31.1 | 28.9 | 25.6 | 30.5 | 34.8 |
| Ankrd42 | 5.1 | 5.0 | 4.7 | 5.6 | 3.8 | 6.5 | 4.9 | 3.8 | 4.2 | 4.3 | 5.3 | 5.5 |
| Ankrd44 | 2.9 | 4.0 | 4.8 | 2.5 | 4.3 | 2.7 | 3.1 | 3.2 | 3.6 | 3.9 | 4.5 | 3.8 |
| Ankrd46 | 21.6 | 19.5 | 17.9 | 18.7 | 15.5 | 20.6 | 17.2 | 17.5 | 17.3 | 18.5 | 18.9 | 19.0 |
| Ankrd49 | 6.1 | 5.6 | 5.9 | 6.6 | 6.3 | 5.3 | 6.3 | 6.7 | 5.9 | 5.9 | 7.2 | 6.4 |
| Ankrd50 | 8.9 | 9.7 | 8.9 | 7.0 | 10.3 | 7.0 | 10.3 | 9.4 | 10.3 | 11.3 | 9.6 | 9.5 |
| Ankrd52 | 4.8 | 4.8 | 4.4 | 4.8 | 5.3 | 4.6 | 5.4 | 5.2 | 4.9 | 4.4 | 4.7 | 4.2 |
| Ankrd53 | 0.0 | 0.0 | 0.0 | 0.0 | 0.1 | 0.0 | 0.0 | 0.0 | 0.0 | 0.1 | 0.0 | 0.0 |
| Ankrd54 | 7.5 | 9.2 | 8.8 | 6.9 | 7.6 | 7.0 | 8.0 | 8.4 | 8.1 | 7.3 | 7.7 | 8.1 |
| Ankrd55 | 2.9 | 4.8 | 8.6 | 2.0 | 3.1 | 2.4 | 2.5 | 5.6 | 4.5 | 0.9 | 1.6 | 3.7 |
| Ankrd6 | 0.7 | 1.0 | 0.8 | 0.7 | 1.1 | 1.4 | 0.7 | 0.8 | 0.6 | 0.4 | 0.4 | 0.6 |
| Ankrd61 | 0.0 | 0.1 | 0.2 | 0.0 | 0.1 | 0.3 | 0.1 | 0.1 | 0.1 | 0.1 | 0.3 | 0.1 |
| Ankrd9 | 1.5 | 1.5 | 1.2 | 1.8 | 1.3 | 1.4 | 1.4 | 1.7 | 1.3 | 0.9 | 1.3 | 0.8 |
| Anks1 | 3.8 | 4.4 | 4.2 | 3.4 | 3.6 | 3.4 | 3.8 | 4.0 | 4.2 | 4.1 | 3.6 | 3.4 |
| Anks1b | 1.3 | 1.3 | 1.2 | 0.6 | 1.0 | 0.7 | 1.0 | 0.4 | 0.6 | 0.5 | 0.6 | 0.5 |
| Anks3 | 11.1 | 9.9 | 9.1 | 12.2 | 9.7 | 12.0 | 11.6 | 11.0 | 12.1 | 10.4 | 11.3 | 11.7 |
| Anks6 | 0.9 | 1.3 | 1.3 | 1.3 | 1.3 | 1.3 | 1.6 | 1.4 | 1.3 | 1.8 | 1.2 | 1.1 |
| Ankzf1 | 5.9 | 8.2 | 7.1 | 5.5 | 6.4 | 7.1 | 6.3 | 6.8 | 6.9 | 5.4 | 5.9 | 6.1 |
| Anln | 4.9 | 7.9 | 11.3 | 1.7 | 10.4 | 1.6 | 14.0 | 4.7 | 10.2 | 6.4 | 11.0 | 7.0 |
| Ano1 | 1.5 | 0.4 | 0.4 | 0.2 | 0.1 | 0.1 | 0.1 | 0.5 | 0.1 | 0.1 | 0.1 | 0.4 |
| Ano10 | 34.9 | 29.9 | 25.1 | 33.8 | 28.8 | 33.0 | 32.7 | 27.1 | 29.0 | 32.1 | 34.0 | 31.2 |
| Ano2 | 0.0 | 0.0 | 0.0 | 0.1 | 0.1 | 0.0 | 0.0 | 0.0 | 0.0 | 0.0 | 0.0 | 0.0 |

| | | | | | | | | | | | | |
|---|---|---|---|---|---|---|---|---|---|---|---|---|
| Ano3 | 9.2 | 7.4 | 6.9 | 6.4 | 7.2 | 8.8 | 6.5 | 4.6 | 5.3 | 7.1 | 8.4 | 7.2 |
| Ano4 | 0.0 | 0.1 | 0.1 | 0.0 | 0.1 | 0.1 | 0.0 | 0.1 | 0.0 | 0.0 | 0.1 | 0.1 |
| Ano5 | 0.0 | 0.0 | 0.0 | 0.0 | 0.0 | 0.0 | 0.0 | 0.0 | 0.0 | 0.0 | 0.0 | 0.0 |
| Ano6 | 24.6 | 24.1 | 24.4 | 23.8 | 25.1 | 21.9 | 29.1 | 24.7 | 25.6 | 30.0 | 30.8 | 28.5 |
| Ano7 | 0.1 | 0.0 | 0.1 | 0.1 | 0.1 | 0.1 | 0.1 | 0.1 | 0.1 | 0.1 | 0.2 | 0.0 |
| Ano8 | 4.4 | 3.6 | 3.7 | 4.0 | 3.7 | 3.9 | 5.0 | 4.4 | 4.9 | 3.9 | 3.4 | 3.8 |
| Anp32a | 20.8 | 19.4 | 29.4 | 26.5 | 22.6 | 26.1 | 25.6 | 30.0 | 20.9 | 29.4 | 31.1 | 34.6 |
| Anp32b | 42.0 | 38.4 | 56.3 | 53.5 | 41.7 | 45.5 | 48.6 | 48.1 | 29.5 | 32.5 | 41.3 | 46.2 |
| Anp32e | 7.8 | 7.4 | 9.9 | 11.4 | 8.6 | 9.5 | 10.1 | 8.0 | 7.2 | 9.1 | 11.0 | 9.8 |
| Anpep | 0.7 | 1.0 | 1.6 | 5.0 | 4.1 | 1.1 | 0.6 | 1.3 | 0.7 | 0.7 | 0.5 | 0.7 |
| Antxr1 | 12.7 | 16.1 | 22.1 | 13.5 | 14.7 | 14.2 | 16.3 | 22.8 | 21.7 | 21.2 | 17.7 | 22.5 |
| Antxr2 | 13.3 | 13.3 | 13.2 | 16.1 | 15.9 | 14.3 | 13.0 | 17.1 | 15.1 | 15.0 | 14.3 | 13.6 |
| Anxa1 | 1974.6 | 1389.0 | 806.6 | 1704.8 | 1127.8 | 1410.6 | 1164.1 | 524.6 | 999.2 | 1147.7 | 1342.4 | 1245.7 |
| Anxa11 | 7.2 | 8.0 | 8.7 | 8.7 | 8.4 | 7.4 | 7.4 | 7.1 | 7.9 | 7.5 | 7.3 | 7.2 |
| Anxa2 | 658.1 | 505.7 | 438.0 | 640.3 | 568.8 | 526.2 | 651.6 | 641.0 | 540.8 | 640.3 | 680.3 | 517.8 |
| Anxa3 | 337.6 | 258.0 | 178.4 | 302.8 | 205.2 | 316.4 | 199.4 | 151.9 | 200.6 | 218.1 | 240.3 | 194.7 |
| Anxa4 | 37.1 | 36.3 | 40.4 | 52.2 | 49.1 | 40.9 | 30.8 | 47.0 | 33.0 | 32.6 | 37.2 | 41.4 |
| Anxa5 | 374.2 | 210.2 | 159.5 | 373.3 | 252.7 | 345.1 | 192.2 | 175.3 | 164.2 | 217.0 | 257.9 | 268.9 |
| Anxa6 | 94.4 | 96.0 | 90.9 | 103.9 | 97.4 | 92.6 | 105.4 | 87.5 | 108.7 | 96.1 | 110.3 | 101.0 |
| Anxa7 | 59.3 | 54.9 | 60.3 | 69.5 | 57.5 | 58.7 | 64.9 | 64.4 | 56.3 | 54.2 | 60.7 | 63.3 |
| Anxa8 | 27.6 | 15.5 | 15.5 | 10.4 | 11.7 | 6.7 | 11.5 | 19.2 | 8.0 | 10.1 | 14.5 | 13.9 |
| Anxa9 | 0.6 | 0.6 | 0.5 | 0.9 | 0.6 | 1.0 | 0.4 | 0.9 | 0.5 | 0.8 | 0.4 | 0.5 |
| Aoah | 0.4 | 0.0 | 0.0 | 2.3 | 4.1 | 0.0 | 0.6 | 0.0 | 0.1 | 0.3 | 0.1 | 0.2 |
| Aoc2 | 0.3 | 0.4 | 0.2 | 0.5 | 0.4 | 0.5 | 0.3 | 0.3 | 0.2 | 0.3 | 0.2 | 0.3 |
| Aoc3 | 0.1 | 0.4 | 0.8 | 0.1 | 0.4 | 0.0 | 0.4 | 0.9 | 1.1 | 0.9 | 0.9 | 0.5 |
| Aox1 | 20.3 | 18.9 | 10.3 | 29.8 | 19.1 | 29.4 | 5.6 | 5.1 | 3.9 | 11.5 | 7.4 | 6.1 |
| Aox3 | 0.0 | 0.1 | 0.0 | 0.0 | 0.0 | 0.0 | 0.0 | 0.1 | 0.0 | 0.2 | 0.3 | 0.1 |
| Aox3l1 | 0.0 | 0.0 | 0.0 | 0.0 | 0.0 | 0.0 | 0.0 | 0.0 | 0.0 | 0.0 | 0.0 | 0.0 |
| Aox4 | 0.0 | 0.0 | 0.0 | 0.0 | 0.0 | 0.0 | 0.0 | 0.0 | 0.0 | 0.0 | 0.0 | 0.0 |
| Ap1ar | 15.0 | 15.5 | 16.7 | 16.4 | 14.2 | 15.5 | 15.6 | 14.8 | 14.1 | 15.3 | 16.5 | 16.1 |
| Ap1b1 | 17.5 | 15.4 | 13.3 | 17.9 | 18.7 | 16.4 | 14.6 | 15.6 | 13.9 | 15.5 | 14.8 | 14.7 |
| Ap1g1 | 12.9 | 14.0 | 12.5 | 14.4 | 13.5 | 13.8 | 12.3 | 12.2 | 12.0 | 13.2 | 13.3 | 12.3 |
| Ap1g2 | 2.4 | 3.5 | 2.8 | 3.1 | 3.0 | 2.6 | 2.2 | 2.6 | 2.7 | 1.8 | 2.7 | 2.2 |
| Ap1m1 | 10.6 | 9.7 | 8.8 | 10.3 | 11.5 | 8.6 | 11.1 | 11.2 | 11.4 | 10.9 | 9.8 | 10.4 |
| Ap1m2 | 0.0 | 0.0 | 0.1 | 0.0 | 0.0 | 0.0 | 0.0 | 0.0 | 0.0 | 0.0 | 0.0 | 0.0 |
| Ap1s1 | 38.4 | 40.4 | 31.8 | 43.8 | 45.9 | 37.3 | 41.5 | 42.6 | 42.4 | 42.3 | 40.9 | 33.1 |
| Ap1s2 | 2.7 | 2.9 | 3.6 | 6.4 | 8.4 | 3.3 | 3.6 | 3.2 | 4.0 | 3.4 | 4.0 | 3.5 |
| Ap1s3 | 1.2 | 1.4 | 1.3 | 1.2 | 1.4 | 1.1 | 1.1 | 1.0 | 1.1 | 0.9 | 0.9 | 0.9 |
| Ap2a1 | 20.8 | 21.5 | 19.7 | 21.4 | 20.4 | 23.6 | 22.8 | 22.3 | 21.5 | 19.4 | 20.5 | 17.7 |
| Ap2a2 | 75.4 | 75.4 | 57.7 | 59.9 | 56.7 | 56.6 | 78.8 | 75.9 | 97.8 | 71.8 | 85.3 | 81.0 |
| Ap2b1 | 40.9 | 39.0 | 38.4 | 39.7 | 40.7 | 37.8 | 37.4 | 40.4 | 36.8 | 34.7 | 38.6 | 38.2 |
| Ap2m1 | 98.7 | 89.1 | 88.4 | 104.3 | 87.9 | 95.8 | 95.3 | 101.8 | 95.7 | 85.2 | 95.4 | 94.6 |
| Ap2s1 | 84.2 | 70.9 | 67.4 | 80.9 | 88.5 | 73.1 | 73.8 | 84.4 | 79.5 | 94.5 | 74.4 | 79.2 |
| Ap3b1 | 28.1 | 25.3 | 27.2 | 30.9 | 26.4 | 29.8 | 25.6 | 25.9 | 22.8 | 24.7 | 30.7 | 31.2 |
| Ap3b2 | 0.1 | 0.0 | 0.0 | 0.0 | 0.0 | 0.0 | 0.0 | 0.0 | 0.0 | 0.0 | 0.1 | 0.0 |
| Ap3d1 | 39.0 | 36.7 | 34.1 | 45.7 | 36.9 | 37.6 | 44.4 | 38.9 | 36.2 | 35.4 | 41.4 | 41.5 |
| Ap3m1 | 20.1 | 17.7 | 16.8 | 18.9 | 18.1 | 18.8 | 16.8 | 20.6 | 17.2 | 18.0 | 21.1 | 19.2 |
| Ap3m2 | 9.7 | 7.7 | 7.4 | 11.5 | 7.9 | 12.3 | 8.4 | 6.2 | 6.2 | 6.4 | 7.5 | 8.5 |
| Ap3s1 | 56.0 | 58.1 | 72.5 | 72.4 | 54.9 | 71.9 | 63.8 | 52.9 | 62.7 | 61.5 | 75.3 | 80.6 |
| Ap3s2 | 4.4 | 3.7 | 3.2 | 4.3 | 3.6 | 3.6 | 2.9 | 3.8 | 3.3 | 3.9 | 3.7 | 3.1 |
| Ap4b1 | 11.3 | 11.0 | 9.0 | 8.9 | 9.3 | 9.8 | 9.8 | 10.1 | 11.1 | 12.0 | 10.7 | 10.8 |
| Ap4e1 | 2.8 | 3.8 | 3.5 | 2.8 | 3.4 | 2.8 | 2.9 | 3.1 | 3.8 | 3.3 | 3.4 | 3.2 |
| Ap4m1 | 5.1 | 4.9 | 5.3 | 3.2 | 4.8 | 4.0 | 4.6 | 4.8 | 4.9 | 4.3 | 5.2 | 4.9 |
| Ap4s1 | 6.7 | 6.4 | 5.5 | 6.5 | 7.4 | 6.8 | 5.0 | 5.9 | 7.7 | 6.7 | 6.7 | 6.5 |
| Ap5b1 | 1.4 | 1.3 | 1.1 | 1.8 | 2.9 | 1.2 | 1.1 | 1.5 | 1.3 | 1.4 | 1.1 | 1.3 |
| Ap5m1 | 3.8 | 3.8 | 4.4 | 3.9 | 4.3 | 3.7 | 3.3 | 3.7 | 4.3 | 4.1 | 3.8 | 4.4 |
| Ap5s1 | 3.0 | 4.4 | 4.3 | 3.8 | 3.5 | 4.4 | 3.1 | 3.6 | 2.9 | 2.7 | 2.5 | 2.9 |

| | | | | | | | | | | | | |
|---|---|---|---|---|---|---|---|---|---|---|---|---|
| Ap5z1 | 3.9 | 3.4 | 3.0 | 4.3 | 3.9 | 3.8 | 3.2 | 3.4 | 3.5 | 3.9 | 2.9 | 3.2 |
| Apaf1 | 9.4 | 10.4 | 9.1 | 8.0 | 10.0 | 7.4 | 8.2 | 9.0 | 10.4 | 9.2 | 9.1 | 8.5 |
| Apba1 | 1.9 | 3.1 | 4.4 | 1.9 | 2.8 | 1.9 | 3.9 | 3.9 | 3.1 | 2.0 | 2.5 | 3.0 |
| Apba2 | 0.0 | 0.1 | 0.1 | 0.0 | 0.1 | 0.0 | 0.1 | 0.0 | 0.1 | 0.7 | 0.4 | 0.4 |
| Apba3 | 19.7 | 21.7 | 17.0 | 16.8 | 17.5 | 19.1 | 16.6 | 17.3 | 20.1 | 20.1 | 17.3 | 16.9 |
| Apbb1 | 12.5 | 12.4 | 12.0 | 11.4 | 7.9 | 14.3 | 10.1 | 9.4 | 10.0 | 8.6 | 9.5 | 10.3 |
| Apbb1ip | 2.3 | 3.7 | 4.5 | 3.7 | 4.1 | 2.6 | 3.2 | 2.5 | 1.8 | 2.4 | 1.7 | 2.2 |
| Apbb2 | 14.9 | 17.6 | 17.4 | 19.8 | 19.2 | 19.4 | 22.0 | 16.8 | 18.3 | 19.1 | 20.5 | 19.6 |
| Apbb3 | 2.0 | 2.2 | 2.5 | 2.0 | 2.0 | 1.7 | 2.1 | 2.8 | 2.9 | 2.2 | 2.0 | 2.0 |
| Apc | 12.4 | 11.6 | 9.5 | 15.3 | 10.4 | 12.7 | 11.0 | 8.5 | 8.8 | 8.7 | 11.2 | 9.9 |
| Apc2 | 1.2 | 1.5 | 1.2 | 1.4 | 1.2 | 1.6 | 1.0 | 0.7 | 0.9 | 0.9 | 0.8 | 0.6 |
| Apcdd1 | 2.6 | 2.8 | 6.4 | 1.7 | 2.4 | 1.2 | 2.4 | 4.1 | 2.3 | 2.3 | 1.7 | 2.7 |
| Apeh | 14.1 | 16.4 | 13.8 | 12.8 | 15.2 | 15.2 | 16.0 | 16.7 | 16.6 | 17.5 | 16.1 | 16.1 |
| Apex1 | 14.6 | 11.2 | 13.3 | 17.7 | 11.2 | 15.3 | 15.8 | 14.1 | 10.4 | 13.6 | 16.4 | 14.9 |
| Apex2 | 1.6 | 1.4 | 1.8 | 1.5 | 1.8 | 1.7 | 2.1 | 1.9 | 1.4 | 1.9 | 1.7 | 1.4 |
| Aph1a | 20.0 | 18.2 | 16.5 | 16.8 | 20.2 | 16.4 | 19.7 | 20.7 | 22.0 | 20.5 | 18.2 | 18.2 |
| Aph1b | 4.9 | 5.9 | 5.5 | 4.4 | 4.9 | 4.9 | 5.6 | 6.3 | 7.9 | 5.4 | 6.7 | 6.1 |
| Aph1c | 0.4 | 0.8 | 0.6 | 1.1 | 0.8 | 0.4 | 0.5 | 0.5 | 0.7 | 0.6 | 0.4 | 0.5 |
| Api5 | 21.8 | 23.4 | 22.6 | 22.3 | 23.8 | 22.3 | 24.1 | 23.9 | 23.2 | 24.6 | 24.7 | 24.3 |
| Apip | 18.3 | 18.0 | 19.7 | 18.6 | 16.0 | 16.4 | 16.5 | 19.2 | 17.9 | 18.4 | 19.5 | 20.2 |
| Apitd1 | 0.7 | 1.6 | 1.6 | 1.6 | 1.5 | 0.8 | 2.2 | 1.3 | 1.7 | 0.8 | 0.9 | 0.6 |
| Aplf | 2.5 | 3.0 | 3.5 | 1.6 | 2.0 | 2.1 | 2.7 | 2.6 | 2.0 | 2.2 | 2.4 | 2.4 |
| Apln | 7.7 | 2.4 | 5.8 | 1.6 | 1.1 | 0.7 | 5.5 | 11.6 | 6.9 | 1.0 | 1.0 | 1.0 |
| Aplnr | 0.0 | 0.0 | 0.0 | 0.0 | 0.0 | 0.0 | 0.0 | 0.0 | 0.0 | 0.0 | 0.0 | 0.0 |
| Aplp1 | 43.9 | 39.3 | 25.0 | 33.7 | 25.4 | 44.6 | 39.7 | 20.3 | 26.2 | 44.6 | 36.7 | 34.8 |
| Aplp2 | 124.7 | 110.5 | 134.5 | 125.7 | 131.4 | 126.9 | 124.3 | 114.1 | 109.1 | 107.4 | 111.1 | 115.8 |
| Apmap | 13.7 | 12.7 | 10.7 | 14.8 | 12.1 | 13.5 | 9.3 | 8.7 | 10.7 | 11.2 | 10.3 | 11.0 |
| Apoa1bp | 61.5 | 60.3 | 57.3 | 58.7 | 47.1 | 66.7 | 53.6 | 57.7 | 51.8 | 46.9 | 55.8 | 51.6 |
| Apoa2 | 0.6 | 0.6 | 0.5 | 2.1 | 1.3 | 1.4 | 1.2 | 0.7 | 0.8 | 0.2 | 0.7 | 0.9 |
| Apoa5 | 0.0 | 0.0 | 0.0 | 0.0 | 0.0 | 0.0 | 0.0 | 0.0 | 0.0 | 0.0 | 0.0 | 0.0 |
| Apob | 0.0 | 0.0 | 0.0 | 0.0 | 0.0 | 0.0 | 0.0 | 0.0 | 0.0 | 0.0 | 0.0 | 0.0 |
| Apobec1 | 4.8 | 3.3 | 4.9 | 8.7 | 11.0 | 2.8 | 4.0 | 3.6 | 2.7 | 2.9 | 2.4 | 4.3 |
| Apobec2 | 0.1 | 0.1 | 0.0 | 0.1 | 0.1 | 0.0 | 0.0 | 0.0 | 0.1 | 0.2 | 0.1 | 0.0 |
| Apobec3 | 2.3 | 4.1 | 4.8 | 2.6 | 4.4 | 2.4 | 4.1 | 4.5 | 3.4 | 2.6 | 2.2 | 3.3 |
| Apobr | 7.6 | 3.7 | 3.8 | 10.3 | 9.4 | 5.6 | 8.6 | 11.0 | 5.3 | 2.8 | 6.2 | 5.3 |
| Apoc1 | 0.2 | 0.4 | 0.3 | 0.0 | 0.0 | 0.0 | 0.0 | 0.1 | 0.1 | 0.5 | 0.0 | 0.3 |
| Apoc2 | 0.2 | 0.0 | 0.0 | 1.0 | 1.4 | 0.0 | 0.0 | 0.1 | 0.0 | 0.1 | 0.0 | 0.0 |
| Apod | 3.3 | 3.2 | 6.6 | 4.5 | 4.1 | 2.0 | 2.4 | 3.2 | 1.2 | 1.1 | 1.5 | 1.8 |
| Apoe | 42.8 | 55.5 | 187.1 | 53.0 | 31.3 | 11.8 | 12.3 | 44.3 | 20.8 | 31.5 | 18.5 | 35.2 |
| Apof | 0.0 | 0.2 | 0.1 | 0.0 | 0.0 | 0.1 | 0.1 | 0.0 | 0.0 | 0.0 | 0.0 | 0.1 |
| Apoh | 0.8 | 0.7 | 0.4 | 0.6 | 0.6 | 0.8 | 0.1 | 0.3 | 0.4 | 0.3 | 0.4 | 0.6 |
| Apol10b | 0.4 | 0.3 | 0.7 | 0.9 | 0.7 | 0.4 | 0.4 | 0.3 | 0.3 | 0.3 | 0.4 | 0.1 |
| Apol11b | 0.0 | 0.0 | 0.0 | 0.0 | 0.0 | 0.0 | 0.0 | 0.0 | 0.0 | 0.0 | 0.0 | 0.0 |
| Apol6 | 2.7 | 4.5 | 5.8 | 1.1 | 2.8 | 0.8 | 2.7 | 5.8 | 3.1 | 2.3 | 4.0 | 3.6 |
| Apol7a | 0.3 | 0.3 | 0.3 | 0.4 | 0.3 | 0.4 | 0.5 | 0.3 | 0.3 | 0.4 | 0.4 | 0.3 |
| Apol7b | 0.0 | 0.0 | 0.0 | 0.0 | 0.0 | 0.0 | 0.0 | 0.0 | 0.0 | 0.0 | 0.0 | 0.0 |
| Apol7c | 0.0 | 0.0 | 0.0 | 0.0 | 0.0 | 0.0 | 0.0 | 0.0 | 0.0 | 0.0 | 0.0 | 0.0 |
| Apol7d | 0.2 | 0.1 | 0.3 | 0.3 | 0.1 | 0.2 | 0.2 | 0.1 | 0.1 | 0.1 | 0.1 | 0.3 |
| Apol7e | 0.0 | 0.0 | 0.0 | 0.0 | 0.0 | 0.0 | 0.0 | 0.0 | 0.0 | 0.0 | 0.0 | 0.0 |
| Apol8 | 0.1 | 0.0 | 0.0 | 0.2 | 0.0 | 0.1 | 0.0 | 0.0 | 0.0 | 0.1 | 0.0 | 0.1 |
| Apol9a | 4.5 | 1.8 | 1.9 | 6.4 | 2.8 | 2.7 | 4.3 | 0.8 | 2.1 | 2.2 | 1.7 | 4.0 |
| Apol9b | 3.7 | 1.5 | 1.5 | 5.5 | 2.4 | 2.9 | 3.0 | 0.7 | 1.5 | 1.9 | 1.7 | 3.7 |
| Apold1 | 0.2 | 0.2 | 0.1 | 0.1 | 0.2 | 0.1 | 0.1 | 0.1 | 0.2 | 0.1 | 0.1 | 0.1 |
| Apom | 0.3 | 0.1 | 0.0 | 0.1 | 0.0 | 0.1 | 0.1 | 0.1 | 0.0 | 0.1 | 0.0 | 0.0 |
| Apoo | 9.0 | 14.4 | 14.6 | 14.7 | 15.7 | 15.6 | 11.5 | 10.7 | 13.0 | 17.8 | 15.4 | 17.1 |
| Apool | 11.2 | 10.0 | 9.7 | 9.2 | 10.2 | 9.3 | 8.0 | 10.5 | 10.0 | 9.8 | 10.9 | 9.1 |
| Apoo-ps | 11.6 | 2.8 | 0.0 | 1.2 | 0.0 | 0.2 | 0.0 | 0.0 | 2.2 | 0.0 | 0.0 | 0.2 |

| | | | | | | | | | | | | |
|---|---|---|---|---|---|---|---|---|---|---|---|---|
| Apopt1 | 15.0 | 12.6 | 14.9 | 19.0 | 11.0 | 15.2 | 13.2 | 15.9 | 12.8 | 12.6 | 14.6 | 15.5 |
| App | 312.6 | 263.5 | 284.8 | 259.4 | 292.1 | 259.1 | 272.2 | 284.1 | 253.9 | 289.6 | 266.7 | 270.7 |
| Appbp2 | 14.5 | 16.0 | 13.9 | 13.3 | 14.9 | 12.3 | 14.5 | 15.6 | 16.2 | 16.9 | 15.7 | 14.3 |
| Appl1 | 6.9 | 7.5 | 7.6 | 7.7 | 7.3 | 8.0 | 7.1 | 6.6 | 6.7 | 6.9 | 7.8 | 8.5 |
| Appl2 | 9.5 | 9.0 | 10.8 | 9.7 | 10.2 | 10.9 | 7.5 | 9.0 | 8.0 | 7.9 | 7.6 | 9.6 |
| Aprt | 46.2 | 39.4 | 36.2 | 48.6 | 48.5 | 46.3 | 38.1 | 39.7 | 38.6 | 55.6 | 44.9 | 39.3 |
| Aptx | 4.3 | 3.5 | 3.5 | 4.4 | 3.2 | 4.0 | 3.6 | 3.1 | 3.2 | 3.1 | 3.5 | 3.6 |
| Aqp1 | 1.3 | 0.1 | 0.2 | 0.7 | 0.5 | 0.3 | 0.8 | 0.4 | 0.3 | 0.9 | 0.8 | 0.5 |
| Aqp11 | 0.2 | 0.2 | 0.1 | 0.5 | 0.3 | 0.4 | 0.2 | 0.2 | 0.1 | 0.3 | 0.3 | 0.1 |
| Aqp2 | 0.3 | 0.1 | 0.4 | 0.0 | 0.0 | 0.0 | 0.1 | 0.5 | 0.4 | 0.4 | 0.2 | 0.5 |
| Aqp3 | 0.1 | 0.0 | 0.0 | 0.0 | 0.1 | 0.0 | 0.0 | 0.0 | 0.0 | 0.0 | 0.0 | 0.0 |
| Aqp4 | 0.0 | 0.0 | 0.0 | 0.0 | 0.0 | 0.0 | 0.0 | 0.0 | 0.0 | 0.0 | 0.0 | 0.0 |
| Aqp5 | 0.3 | 0.0 | 0.0 | 0.2 | 0.1 | 0.0 | 0.1 | 0.4 | 0.3 | 2.6 | 0.8 | 0.9 |
| Aqp7 | 0.0 | 0.0 | 0.0 | 0.0 | 0.0 | 0.1 | 0.0 | 0.0 | 0.0 | 0.0 | 0.0 | 0.0 |
| Aqp9 | 0.2 | 0.1 | 0.0 | 0.2 | 0.7 | 0.0 | 0.3 | 0.0 | 0.0 | 0.0 | 0.0 | 0.1 |
| Aqr | 12.8 | 11.9 | 13.7 | 13.6 | 12.2 | 12.6 | 12.4 | 13.0 | 10.8 | 11.6 | 12.0 | 11.8 |
| Ar | 1.3 | 2.1 | 2.2 | 1.6 | 1.6 | 2.1 | 1.5 | 1.7 | 1.2 | 1.1 | 1.7 | 1.1 |
| Araf | 42.7 | 43.8 | 37.9 | 43.9 | 36.8 | 44.5 | 40.7 | 30.9 | 39.1 | 39.4 | 43.2 | 40.7 |
| Arap1 | 13.3 | 16.9 | 16.7 | 13.5 | 17.3 | 13.1 | 20.8 | 29.9 | 24.4 | 12.5 | 16.6 | 15.1 |
| Arap2 | 6.2 | 5.7 | 5.7 | 4.5 | 5.3 | 4.2 | 4.0 | 2.8 | 3.5 | 7.6 | 5.5 | 6.2 |
| Arap3 | 0.7 | 0.4 | 0.6 | 2.4 | 4.9 | 0.2 | 0.8 | 0.5 | 0.6 | 0.6 | 0.3 | 0.2 |
| Arc | 0.2 | 0.4 | 0.2 | 0.1 | 0.3 | 0.2 | 0.1 | 0.1 | 0.3 | 2.7 | 0.5 | 0.5 |
| Arcn1 | 78.6 | 75.5 | 73.1 | 73.7 | 68.8 | 70.8 | 76.7 | 77.6 | 72.0 | 74.1 | 82.4 | 82.5 |
| Areg | 0.1 | 0.0 | 0.0 | 0.0 | 0.4 | 0.0 | 0.0 | 0.1 | 0.0 | 0.1 | 0.0 | 0.2 |
| Arf1 | 142.0 | 144.4 | 129.2 | 157.5 | 148.9 | 141.0 | 149.5 | 155.4 | 148.1 | 151.4 | 165.3 | 152.3 |
| Arf2 | 12.6 | 9.8 | 11.8 | 12.8 | 12.9 | 9.9 | 15.1 | 16.7 | 17.3 | 14.2 | 15.6 | 12.8 |
| Arf3 | 19.8 | 17.4 | 16.6 | 21.5 | 20.1 | 17.8 | 18.6 | 17.4 | 16.8 | 20.5 | 18.7 | 16.9 |
| Arf4 | 109.7 | 113.1 | 102.9 | 124.8 | 123.2 | 119.6 | 121.1 | 118.1 | 124.3 | 130.3 | 139.0 | 128.1 |
| Arf5 | 36.6 | 36.1 | 36.4 | 34.5 | 38.0 | 27.6 | 35.7 | 49.4 | 46.8 | 42.8 | 36.3 | 38.8 |
| Arf6 | 21.5 | 19.9 | 24.7 | 26.2 | 29.7 | 23.5 | 22.4 | 26.6 | 22.3 | 25.3 | 24.1 | 25.2 |
| Arfgap1 | 18.8 | 18.2 | 17.7 | 16.5 | 16.5 | 17.1 | 19.3 | 21.7 | 20.0 | 22.4 | 21.1 | 18.6 |
| Arfgap2 | 10.5 | 13.0 | 10.9 | 9.2 | 10.5 | 10.0 | 11.5 | 12.1 | 11.5 | 11.3 | 9.9 | 11.1 |
| Arfgap3 | 28.2 | 26.5 | 26.9 | 32.1 | 26.3 | 32.8 | 31.7 | 28.9 | 26.3 | 22.0 | 28.4 | 28.8 |
| Arfgef1 | 8.8 | 11.0 | 9.0 | 9.0 | 8.8 | 9.0 | 8.2 | 7.6 | 8.4 | 8.7 | 9.4 | 8.9 |
| Arfgef2 | 11.7 | 13.4 | 12.8 | 11.5 | 11.9 | 11.4 | 13.2 | 11.1 | 12.5 | 11.6 | 11.4 | 13.7 |
| Arfip1 | 42.0 | 38.7 | 36.9 | 52.1 | 38.3 | 47.6 | 40.7 | 34.6 | 37.3 | 40.7 | 47.5 | 45.5 |
| Arfip2 | 14.8 | 12.4 | 10.4 | 17.9 | 12.4 | 17.3 | 13.2 | 13.0 | 11.4 | 12.7 | 14.8 | 11.3 |
| Arfrp1 | 6.4 | 5.6 | 4.0 | 6.3 | 5.4 | 5.9 | 5.0 | 3.9 | 5.4 | 6.0 | 5.8 | 5.5 |
| Arg1 | 0.0 | 0.1 | 0.0 | 0.0 | 0.0 | 0.0 | 0.0 | 0.1 | 0.0 | 0.0 | 0.0 | 0.0 |
| Arg2 | 0.1 | 0.1 | 0.1 | 0.1 | 0.2 | 0.2 | 0.0 | 0.1 | 0.1 | 0.3 | 0.2 | 0.1 |
| Arglu1 | 22.2 | 20.2 | 26.2 | 24.6 | 19.7 | 19.2 | 23.1 | 24.7 | 21.8 | 21.4 | 24.7 | 25.6 |
| Arhgap1 | 28.2 | 25.7 | 24.3 | 31.8 | 27.8 | 27.0 | 33.1 | 29.7 | 31.7 | 37.6 | 35.4 | 31.2 |
| Arhgap10 | 17.1 | 17.2 | 16.3 | 16.8 | 13.8 | 22.0 | 13.8 | 11.9 | 11.0 | 14.5 | 16.9 | 15.9 |
| Arhgap11a | 3.3 | 4.4 | 5.7 | 2.4 | 7.6 | 2.7 | 6.4 | 2.2 | 4.6 | 3.3 | 4.2 | 3.6 |
| Arhgap12 | 8.0 | 7.9 | 8.0 | 7.5 | 7.3 | 7.1 | 7.2 | 6.7 | 7.4 | 7.8 | 8.3 | 8.4 |
| Arhgap15 | 0.1 | 0.1 | 0.1 | 0.2 | 0.7 | 0.0 | 0.2 | 0.0 | 0.1 | 0.1 | 0.1 | 0.0 |
| Arhgap17 | 3.7 | 3.0 | 2.9 | 3.6 | 4.4 | 3.2 | 4.0 | 3.7 | 3.7 | 3.6 | 3.2 | 2.9 |
| Arhgap18 | 1.7 | 1.3 | 1.4 | 3.0 | 3.3 | 0.6 | 1.3 | 0.8 | 0.9 | 2.2 | 1.3 | 1.3 |
| Arhgap19 | 0.4 | 0.4 | 0.6 | 0.7 | 0.8 | 0.1 | 0.6 | 0.4 | 0.6 | 0.4 | 0.6 | 0.5 |
| Arhgap20 | 2.6 | 3.7 | 2.5 | 2.4 | 3.3 | 2.0 | 3.6 | 1.5 | 3.0 | 1.7 | 2.7 | 2.0 |
| Arhgap21 | 13.4 | 15.5 | 15.1 | 13.8 | 10.5 | 12.9 | 11.6 | 12.8 | 11.4 | 9.4 | 13.0 | 11.1 |
| Arhgap22 | 1.0 | 0.3 | 0.3 | 2.9 | 4.5 | 1.2 | 0.7 | 0.1 | 0.3 | 0.6 | 0.3 | 0.4 |
| Arhgap23 | 7.7 | 10.2 | 10.7 | 7.3 | 9.3 | 6.2 | 13.8 | 12.2 | 14.8 | 10.8 | 11.8 | 10.2 |
| Arhgap24 | 21.3 | 24.8 | 25.8 | 30.0 | 20.4 | 17.1 | 21.6 | 18.0 | 20.3 | 16.6 | 19.2 | 21.3 |
| Arhgap25 | 0.3 | 0.0 | 0.0 | 3.3 | 3.5 | 0.0 | 0.5 | 0.2 | 0.1 | 0.3 | 0.0 | 0.2 |
| Arhgap26 | 0.4 | 0.4 | 0.3 | 0.5 | 0.5 | 0.5 | 0.3 | 0.3 | 0.2 | 0.3 | 0.2 | 0.2 |
| Arhgap27 | 2.2 | 2.2 | 2.2 | 3.4 | 4.6 | 3.2 | 2.5 | 1.5 | 1.8 | 1.7 | 1.7 | 1.5 |

| | | | | | | | | | | | | |
|---|---|---|---|---|---|---|---|---|---|---|---|---|
| Arhgap28 | 1.6 | 1.3 | 1.2 | 2.1 | 1.6 | 2.1 | 1.6 | 1.4 | 0.9 | 1.4 | 1.3 | 0.7 |
| Arhgap29 | 23.2 | 21.7 | 24.5 | 17.6 | 16.8 | 16.9 | 22.3 | 28.0 | 26.1 | 38.1 | 36.8 | 31.4 |
| Arhgap30 | 0.6 | 0.0 | 0.1 | 5.8 | 9.3 | 0.0 | 1.0 | 0.1 | 0.1 | 0.6 | 0.1 | 0.7 |
| Arhgap31 | 7.0 | 8.2 | 8.3 | 8.2 | 7.8 | 9.7 | 9.8 | 8.7 | 8.1 | 5.6 | 7.2 | 7.3 |
| Arhgap32 | 1.7 | 2.5 | 2.6 | 1.8 | 2.1 | 1.8 | 2.7 | 2.1 | 2.4 | 2.5 | 2.2 | 1.8 |
| Arhgap33 | 0.1 | 0.1 | 0.0 | 0.1 | 0.1 | 0.1 | 0.1 | 0.1 | 0.1 | 0.1 | 0.0 | 0.0 |
| Arhgap39 | 1.5 | 1.5 | 1.4 | 1.8 | 1.7 | 1.7 | 1.5 | 1.3 | 1.4 | 1.6 | 1.3 | 1.4 |
| Arhgap4 | 0.3 | 0.1 | 0.1 | 2.0 | 4.3 | 0.2 | 0.7 | 0.1 | 0.3 | 0.2 | 0.2 | 0.4 |
| Arhgap42 | 2.0 | 4.0 | 4.7 | 2.1 | 2.8 | 1.9 | 2.6 | 2.9 | 3.0 | 2.5 | 3.1 | 3.2 |
| Arhgap44 | 5.4 | 5.1 | 4.1 | 5.4 | 3.2 | 6.4 | 3.9 | 3.4 | 3.5 | 5.4 | 4.7 | 3.6 |
| Arhgap5 | 23.5 | 28.0 | 26.5 | 26.2 | 25.4 | 22.5 | 24.8 | 30.4 | 31.1 | 26.0 | 30.5 | 32.2 |
| Arhgap6 | 3.5 | 5.8 | 5.7 | 3.1 | 3.0 | 3.6 | 3.3 | 3.0 | 3.7 | 2.6 | 3.2 | 4.7 |
| Arhgap8 | 0.2 | 0.1 | 0.0 | 0.0 | 0.1 | 0.0 | 0.1 | 0.1 | 0.1 | 0.1 | 0.0 | 0.1 |
| Arhgap9 | 0.5 | 0.1 | 0.1 | 2.9 | 4.9 | 0.1 | 0.6 | 0.1 | 0.2 | 0.2 | 0.4 | 0.3 |
| Arhgdia | 85.7 | 79.7 | 70.3 | 118.6 | 98.3 | 95.2 | 104.9 | 102.0 | 87.5 | 79.8 | 91.5 | 79.0 |
| Arhgdib | 37.6 | 18.6 | 17.7 | 75.9 | 86.7 | 41.5 | 28.9 | 15.4 | 13.5 | 42.8 | 29.9 | 24.0 |
| Arhgdig | 3.3 | 2.4 | 1.2 | 5.6 | 2.4 | 4.5 | 3.4 | 1.6 | 2.1 | 3.4 | 2.4 | 2.5 |
| Arhgef1 | 11.2 | 14.1 | 13.3 | 13.4 | 15.2 | 10.9 | 13.0 | 11.8 | 12.0 | 11.8 | 10.6 | 10.4 |
| Arhgef10 | 14.2 | 12.3 | 12.1 | 14.3 | 12.4 | 13.5 | 14.5 | 14.9 | 13.7 | 11.1 | 13.7 | 12.5 |
| Arhgef10l | 5.4 | 5.6 | 5.6 | 4.9 | 5.1 | 6.3 | 5.4 | 5.6 | 6.7 | 5.5 | 5.0 | 6.1 |
| Arhgef11 | 8.9 | 8.7 | 8.2 | 8.6 | 8.2 | 8.8 | 8.6 | 7.2 | 8.0 | 6.6 | 7.1 | 6.8 |
| Arhgef12 | 22.5 | 24.6 | 23.5 | 23.6 | 19.8 | 23.2 | 21.8 | 21.7 | 22.8 | 21.3 | 21.6 | 23.0 |
| Arhgef15 | 0.1 | 0.0 | 0.0 | 0.0 | 0.0 | 0.0 | 0.1 | 0.0 | 0.0 | 0.0 | 0.1 | 0.0 |
| Arhgef16 | 0.1 | 0.0 | 0.1 | 0.1 | 0.1 | 0.0 | 0.0 | 0.0 | 0.1 | 0.0 | 0.0 | 0.0 |
| Arhgef17 | 11.9 | 13.0 | 12.8 | 11.2 | 10.8 | 13.1 | 13.8 | 10.6 | 12.8 | 12.8 | 12.2 | 11.8 |
| Arhgef18 | 13.8 | 16.0 | 13.0 | 10.9 | 10.7 | 13.7 | 12.3 | 10.5 | 13.2 | 11.5 | 12.5 | 11.8 |
| Arhgef19 | 1.7 | 2.0 | 2.0 | 1.2 | 2.3 | 1.4 | 2.1 | 3.0 | 2.4 | 1.9 | 1.8 | 2.1 |
| Arhgef2 | 34.0 | 25.0 | 22.6 | 37.3 | 27.8 | 35.9 | 29.0 | 38.2 | 32.5 | 23.8 | 31.0 | 23.5 |
| Arhgef25 | 31.7 | 35.6 | 34.3 | 26.3 | 26.5 | 29.2 | 32.3 | 36.0 | 39.3 | 31.7 | 33.1 | 30.2 |
| Arhgef26 | 0.6 | 1.2 | 1.2 | 0.6 | 1.2 | 0.3 | 1.7 | 1.7 | 1.6 | 1.5 | 1.3 | 1.4 |
| Arhgef28 | 5.7 | 5.6 | 6.1 | 5.2 | 4.8 | 4.9 | 3.6 | 4.8 | 2.7 | 4.2 | 4.4 | 3.5 |
| Arhgef3 | 5.9 | 6.5 | 7.5 | 5.7 | 6.7 | 4.5 | 4.0 | 5.1 | 3.1 | 3.9 | 4.2 | 5.1 |
| Arhgef33 | 0.0 | 0.0 | 0.0 | 0.0 | 0.0 | 0.0 | 0.0 | 0.0 | 0.0 | 0.1 | 0.0 | 0.0 |
| Arhgef37 | 0.6 | 0.3 | 0.2 | 0.8 | 0.1 | 1.1 | 0.1 | 0.1 | 0.1 | 0.2 | 0.2 | 0.1 |
| Arhgef39 | 0.3 | 0.9 | 0.7 | 0.4 | 0.7 | 0.3 | 1.6 | 0.5 | 1.0 | 0.5 | 0.8 | 0.4 |
| Arhgef4 | 1.5 | 1.5 | 1.3 | 1.5 | 1.3 | 1.9 | 1.3 | 0.8 | 0.7 | 0.9 | 1.1 | 1.0 |
| Arhgef40 | 13.0 | 17.7 | 21.4 | 14.1 | 18.2 | 15.4 | 22.7 | 23.8 | 22.6 | 18.2 | 16.3 | 18.2 |
| Arhgef5 | 17.7 | 12.7 | 11.3 | 14.3 | 9.4 | 14.7 | 11.2 | 8.5 | 8.6 | 16.3 | 15.0 | 14.3 |
| Arhgef6 | 9.9 | 9.0 | 10.2 | 12.6 | 13.1 | 10.8 | 7.1 | 6.8 | 5.4 | 4.3 | 5.2 | 6.3 |
| Arhgef7 | 6.5 | 7.6 | 7.9 | 7.9 | 11.1 | 6.2 | 8.3 | 8.6 | 8.4 | 8.2 | 7.9 | 8.5 |
| Arhgef9 | 1.3 | 1.4 | 2.0 | 1.2 | 0.9 | 1.6 | 0.9 | 1.2 | 0.9 | 0.9 | 1.2 | 1.4 |
| Arid1a | 1.5 | 1.9 | 2.1 | 1.6 | 1.9 | 1.5 | 2.3 | 1.9 | 1.9 | 1.8 | 1.7 | 1.6 |
| Arid1b | 4.1 | 5.2 | 5.1 | 4.8 | 4.3 | 4.5 | 5.3 | 4.9 | 4.7 | 4.4 | 4.6 | 4.6 |
| Arid2 | 3.2 | 4.1 | 3.8 | 3.2 | 3.5 | 3.3 | 3.7 | 4.1 | 3.9 | 4.0 | 4.2 | 3.6 |
| Arid3a | 0.6 | 0.7 | 0.5 | 0.7 | 1.1 | 0.5 | 1.2 | 0.6 | 1.0 | 1.3 | 0.8 | 0.9 |
| Arid3b | 0.2 | 0.2 | 0.3 | 0.3 | 0.5 | 0.2 | 0.3 | 0.1 | 0.2 | 0.2 | 0.2 | 0.2 |
| Arid4a | 9.0 | 8.5 | 9.8 | 12.8 | 9.8 | 9.6 | 10.5 | 6.7 | 7.1 | 8.3 | 11.3 | 11.1 |
| Arid4b | 8.8 | 9.7 | 11.3 | 9.8 | 8.3 | 8.6 | 8.2 | 9.4 | 8.3 | 10.9 | 10.6 | 11.5 |
| Arid5a | 1.8 | 2.3 | 2.9 | 1.1 | 2.3 | 0.8 | 2.0 | 1.9 | 1.6 | 2.1 | 1.7 | 2.0 |
| Arid5b | 22.5 | 30.2 | 34.6 | 20.3 | 18.6 | 21.6 | 20.2 | 24.9 | 19.2 | 25.1 | 24.1 | 23.5 |
| Arih1 | 25.0 | 25.3 | 22.2 | 25.4 | 24.1 | 27.7 | 23.2 | 20.3 | 22.5 | 28.5 | 29.6 | 26.3 |
| Arih2 | 12.7 | 12.3 | 11.6 | 13.0 | 11.4 | 11.4 | 12.7 | 12.3 | 12.3 | 12.3 | 12.6 | 12.0 |
| Arl1 | 171.9 | 168.5 | 173.8 | 173.9 | 162.8 | 173.1 | 194.4 | 175.0 | 168.0 | 175.4 | 208.5 | 206.9 |
| Arl10 | 5.4 | 6.1 | 4.5 | 5.5 | 5.6 | 5.0 | 6.3 | 6.2 | 7.1 | 6.0 | 4.5 | 4.9 |
| Arl11 | 0.5 | 0.0 | 0.1 | 2.8 | 6.0 | 0.1 | 0.8 | 0.1 | 0.1 | 0.3 | 0.1 | 0.2 |
| Arl13b | 6.1 | 6.8 | 8.7 | 6.7 | 6.2 | 6.6 | 6.9 | 6.4 | 6.3 | 6.6 | 7.3 | 6.9 |
| Arl14ep | 9.6 | 7.9 | 8.0 | 9.7 | 8.5 | 9.4 | 9.1 | 10.1 | 10.1 | 9.3 | 11.5 | 10.3 |

| | | | | | | | | | | | | |
|---|---|---|---|---|---|---|---|---|---|---|---|---|
| Arl14epl | 0.1 | 0.0 | 0.1 | 0.0 | 0.0 | 0.0 | 0.0 | 0.0 | 0.1 | 0.1 | 0.1 | 0.1 |
| Arl15 | 5.4 | 7.0 | 7.4 | 6.7 | 7.1 | 6.2 | 5.8 | 6.5 | 5.4 | 6.2 | 6.0 | 6.5 |
| Arl16 | 9.2 | 7.9 | 7.8 | 11.2 | 8.1 | 8.9 | 9.4 | 7.5 | 7.4 | 8.4 | 9.0 | 7.8 |
| Arl2 | 31.1 | 24.2 | 23.8 | 35.7 | 22.9 | 43.4 | 28.1 | 30.2 | 28.3 | 26.4 | 25.8 | 25.7 |
| Arl2bp | 45.3 | 40.1 | 37.4 | 48.9 | 34.7 | 43.1 | 41.8 | 38.8 | 39.3 | 30.3 | 40.4 | 33.3 |
| Arl3 | 42.6 | 38.7 | 43.2 | 50.6 | 37.4 | 49.0 | 41.1 | 42.1 | 36.4 | 41.5 | 45.3 | 47.0 |
| Arl4a | 1.3 | 1.2 | 1.4 | 1.1 | 1.4 | 1.3 | 1.6 | 1.6 | 1.7 | 2.0 | 1.8 | 1.8 |
| Arl4c | 2.9 | 1.2 | 1.3 | 4.9 | 6.0 | 3.1 | 2.2 | 1.3 | 2.1 | 4.7 | 2.8 | 2.0 |
| Arl4d | 0.7 | 0.7 | 0.6 | 0.2 | 0.6 | 0.8 | 0.8 | 2.0 | 1.1 | 5.5 | 4.1 | 1.6 |
| Arl5a | 42.6 | 42.1 | 38.0 | 48.9 | 38.5 | 44.6 | 43.5 | 30.8 | 48.1 | 41.7 | 51.8 | 49.2 |
| Arl5b | 2.5 | 2.7 | 2.1 | 2.5 | 2.4 | 1.9 | 1.9 | 2.1 | 2.4 | 4.0 | 3.2 | 2.8 |
| Arl5c | 0.1 | 0.2 | 0.1 | 0.9 | 1.1 | 0.3 | 0.3 | 0.2 | 0.2 | 0.2 | 0.1 | 0.2 |
| Arl6 | 8.8 | 8.2 | 8.4 | 9.1 | 7.4 | 10.4 | 7.2 | 7.6 | 7.6 | 8.1 | 9.3 | 8.8 |
| Arl6ip1 | 64.6 | 63.1 | 56.4 | 63.9 | 75.7 | 66.8 | 66.6 | 46.3 | 70.0 | 72.5 | 67.2 | 62.4 |
| Arl6ip4 | 22.7 | 18.3 | 18.3 | 21.0 | 18.7 | 20.2 | 21.9 | 22.8 | 18.1 | 22.4 | 20.3 | 22.5 |
| Arl6ip5 | 73.7 | 56.9 | 60.7 | 77.2 | 69.2 | 65.8 | 69.5 | 61.1 | 61.6 | 69.3 | 74.2 | 76.7 |
| Arl6ip6 | 2.7 | 4.0 | 4.9 | 1.8 | 3.7 | 2.9 | 3.3 | 4.1 | 4.4 | 4.1 | 3.9 | 3.7 |
| Arl8a | 25.1 | 23.0 | 21.9 | 29.3 | 23.9 | 29.2 | 27.2 | 20.7 | 19.5 | 23.1 | 24.1 | 22.3 |
| Arl8b | 33.7 | 32.8 | 32.7 | 36.1 | 35.0 | 37.2 | 29.6 | 29.4 | 28.9 | 33.2 | 33.6 | 36.7 |
| Armc1 | 12.4 | 11.3 | 11.7 | 10.8 | 14.0 | 12.6 | 12.0 | 11.4 | 13.9 | 13.7 | 12.9 | 13.2 |
| Armc10 | 12.8 | 11.3 | 11.6 | 13.0 | 10.6 | 12.8 | 9.4 | 12.7 | 10.6 | 12.5 | 11.8 | 11.4 |
| Armc2 | 0.1 | 0.1 | 0.1 | 0.0 | 0.1 | 0.0 | 0.0 | 0.0 | 0.0 | 0.1 | 0.0 | 0.1 |
| Armc5 | 3.4 | 4.1 | 3.2 | 3.4 | 4.1 | 3.8 | 4.3 | 4.6 | 4.9 | 4.2 | 3.3 | 3.8 |
| Armc6 | 3.3 | 2.4 | 1.5 | 2.8 | 3.2 | 2.8 | 3.5 | 3.2 | 3.3 | 3.9 | 3.1 | 2.8 |
| Armc7 | 2.3 | 1.9 | 1.6 | 3.1 | 2.9 | 1.9 | 2.0 | 1.8 | 1.7 | 1.6 | 1.7 | 1.7 |
| Armc8 | 5.8 | 5.7 | 5.1 | 6.4 | 5.1 | 5.7 | 5.4 | 5.8 | 5.6 | 5.1 | 5.7 | 5.4 |
| Armc9 | 4.8 | 4.1 | 4.2 | 4.2 | 3.4 | 4.7 | 3.5 | 3.9 | 3.0 | 4.3 | 3.9 | 3.8 |
| Armcx1 | 16.4 | 13.4 | 15.7 | 20.3 | 13.0 | 20.3 | 13.4 | 13.5 | 14.0 | 13.7 | 15.6 | 15.7 |
| Armcx2 | 31.2 | 32.2 | 31.2 | 32.4 | 24.4 | 38.4 | 26.2 | 28.8 | 32.0 | 41.9 | 37.7 | 33.8 |
| Armcx3 | 31.9 | 29.5 | 30.0 | 33.7 | 27.9 | 35.6 | 28.6 | 30.0 | 34.1 | 41.4 | 41.6 | 39.4 |
| Armcx4 | 1.1 | 1.2 | 1.4 | 1.3 | 1.5 | 1.5 | 1.8 | 1.4 | 1.4 | 1.0 | 1.2 | 1.4 |
| Armcx5 | 3.8 | 3.0 | 3.5 | 3.7 | 3.4 | 3.8 | 3.7 | 3.2 | 3.5 | 3.4 | 4.0 | 4.0 |
| Armcx6 | 5.1 | 5.3 | 5.5 | 5.4 | 3.8 | 6.9 | 4.6 | 4.0 | 5.8 | 7.1 | 6.7 | 6.2 |
| Arnt | 8.3 | 11.3 | 14.6 | 7.3 | 10.2 | 7.8 | 10.8 | 13.9 | 10.7 | 10.6 | 10.2 | 12.6 |
| Arnt2 | 7.0 | 5.8 | 3.7 | 5.4 | 3.9 | 5.7 | 3.6 | 3.8 | 2.7 | 3.3 | 4.3 | 3.1 |
| Arntl | 4.3 | 6.7 | 5.3 | 4.6 | 5.3 | 5.3 | 4.7 | 5.9 | 5.4 | 4.6 | 5.4 | 5.1 |
| Arntl2 | 5.0 | 4.3 | 3.7 | 3.1 | 4.0 | 3.9 | 3.5 | 3.0 | 2.8 | 4.2 | 4.0 | 4.0 |
| Arpc1a | 43.7 | 46.1 | 37.0 | 41.3 | 37.6 | 40.1 | 36.7 | 38.8 | 38.7 | 44.3 | 42.2 | 41.0 |
| Arpc1b | 192.7 | 168.4 | 157.3 | 247.5 | 271.2 | 204.8 | 215.6 | 204.4 | 197.4 | 196.3 | 201.3 | 174.3 |
| Arpc2 | 139.7 | 120.9 | 123.7 | 169.3 | 138.6 | 144.3 | 131.6 | 124.6 | 114.4 | 124.2 | 142.6 | 147.6 |
| Arpc3 | 177.0 | 134.7 | 141.5 | 205.3 | 185.6 | 164.5 | 159.5 | 149.7 | 134.4 | 158.2 | 157.0 | 182.3 |
| Arpc4 | 38.6 | 34.8 | 27.8 | 40.6 | 53.4 | 33.1 | 42.8 | 35.9 | 41.9 | 42.9 | 39.8 | 37.0 |
| Arpc5 | 49.0 | 46.4 | 46.6 | 57.3 | 74.1 | 42.1 | 52.9 | 51.1 | 52.2 | 53.4 | 60.9 | 57.4 |
| Arpc5l | 43.6 | 35.0 | 32.9 | 38.7 | 35.0 | 38.7 | 31.5 | 32.4 | 32.2 | 37.1 | 38.0 | 36.4 |
| Arpp19 | 13.6 | 13.6 | 13.1 | 15.5 | 13.9 | 15.5 | 12.4 | 13.7 | 11.9 | 14.0 | 16.6 | 15.0 |
| Arpp21 | 0.0 | 0.0 | 0.0 | 0.0 | 0.0 | 0.0 | 0.0 | 0.0 | 0.0 | 0.0 | 0.0 | 0.0 |
| Arrb1 | 3.3 | 3.1 | 3.0 | 6.0 | 7.5 | 3.1 | 3.4 | 2.6 | 2.8 | 2.4 | 2.8 | 2.6 |
| Arrb2 | 3.4 | 2.2 | 2.3 | 12.9 | 21.7 | 2.3 | 4.5 | 3.1 | 2.3 | 2.7 | 1.5 | 2.4 |
| Arrdc1 | 3.5 | 4.0 | 3.7 | 2.8 | 5.2 | 4.0 | 4.5 | 4.2 | 4.2 | 4.0 | 3.5 | 2.9 |
| Arrdc2 | 2.2 | 2.8 | 2.4 | 1.8 | 2.2 | 1.9 | 2.7 | 3.2 | 2.9 | 2.0 | 2.6 | 2.4 |
| Arrdc3 | 4.0 | 4.7 | 5.4 | 3.3 | 3.0 | 6.9 | 2.1 | 3.0 | 2.6 | 4.1 | 3.4 | 3.5 |
| Arrdc4 | 6.8 | 5.9 | 5.6 | 9.7 | 9.1 | 5.7 | 9.0 | 7.5 | 8.5 | 7.4 | 9.6 | 8.9 |
| Arsa | 20.2 | 17.8 | 17.0 | 18.8 | 17.5 | 21.9 | 18.2 | 20.4 | 20.3 | 15.2 | 15.5 | 15.9 |
| Arsb | 4.8 | 5.2 | 5.7 | 4.5 | 4.7 | 6.5 | 4.2 | 4.1 | 3.9 | 3.9 | 4.0 | 4.6 |
| Arsg | 2.2 | 2.4 | 2.5 | 1.5 | 2.1 | 2.3 | 2.5 | 2.8 | 2.3 | 2.1 | 2.0 | 2.2 |
| Arsi | 0.5 | 1.4 | 1.4 | 0.0 | 0.4 | 0.0 | 1.1 | 2.1 | 2.2 | 1.6 | 1.3 | 2.1 |
| Arsj | 1.5 | 2.1 | 2.6 | 1.2 | 2.2 | 1.4 | 2.7 | 2.4 | 2.3 | 1.6 | 1.8 | 1.9 |

| | | | | | | | | | | | | |
|---|---|---|---|---|---|---|---|---|---|---|---|---|
| Arsk | 6.1 | 5.9 | 6.5 | 9.2 | 6.6 | 8.7 | 5.8 | 6.5 | 5.4 | 5.3 | 6.7 | 6.5 |
| Art3 | 1.5 | 0.8 | 1.3 | 1.0 | 0.9 | 0.7 | 0.5 | 0.5 | 0.2 | 0.2 | 0.1 | 0.5 |
| Art4 | 0.8 | 1.1 | 2.5 | 0.4 | 1.5 | 0.3 | 0.7 | 1.0 | 0.8 | 0.7 | 0.7 | 1.3 |
| Art5 | 0.1 | 0.0 | 0.0 | 0.0 | 0.0 | 0.0 | 0.0 | 0.0 | 0.0 | 0.0 | 0.0 | 0.0 |
| Artn | 0.4 | 0.2 | 0.5 | 0.3 | 0.3 | 0.1 | 0.3 | 0.6 | 0.3 | 0.2 | 0.1 | 0.2 |
| Arv1 | 0.8 | 1.0 | 0.8 | 1.2 | 1.2 | 1.1 | 0.8 | 1.0 | 0.9 | 0.8 | 0.8 | 0.8 |
| Arvcf | 9.2 | 16.4 | 17.7 | 7.4 | 10.3 | 9.0 | 13.9 | 18.0 | 21.1 | 9.9 | 12.0 | 13.1 |
| Arx | 0.1 | 0.1 | 0.0 | 0.1 | 0.0 | 0.0 | 0.1 | 0.0 | 0.0 | 0.0 | 0.0 | 0.0 |
| Arxes1 | 1.3 | 1.7 | 1.0 | 2.4 | 1.8 | 1.5 | 2.9 | 1.3 | 3.4 | 1.7 | 1.9 | 1.9 |
| Arxes2 | 5.1 | 6.9 | 8.8 | 11.7 | 9.0 | 8.7 | 13.2 | 7.2 | 15.3 | 10.5 | 10.7 | 16.2 |
| As3mt | 1.9 | 2.1 | 1.8 | 1.8 | 1.7 | 1.7 | 2.6 | 2.3 | 2.1 | 2.3 | 2.0 | 2.0 |
| Asah1 | 246.6 | 193.7 | 169.9 | 247.7 | 191.3 | 244.0 | 196.3 | 231.3 | 222.9 | 166.9 | 252.7 | 243.4 |
| Asah2 | 11.8 | 11.5 | 12.6 | 14.1 | 11.3 | 15.7 | 10.1 | 8.9 | 9.0 | 11.1 | 11.4 | 12.9 |
| Asap1 | 16.2 | 22.3 | 19.3 | 18.9 | 16.0 | 20.5 | 23.6 | 15.6 | 19.3 | 15.3 | 19.2 | 20.5 |
| Asap2 | 2.3 | 2.0 | 1.9 | 1.9 | 1.7 | 1.9 | 1.9 | 1.6 | 1.7 | 2.3 | 2.2 | 1.8 |
| Asap3 | 5.4 | 7.4 | 9.1 | 5.6 | 5.9 | 6.3 | 6.8 | 8.7 | 7.3 | 4.4 | 5.0 | 6.3 |
| Asb1 | 6.5 | 6.5 | 5.7 | 5.7 | 5.8 | 5.9 | 5.3 | 4.4 | 5.8 | 4.9 | 5.3 | 5.2 |
| Asb13 | 1.4 | 2.5 | 2.0 | 1.4 | 1.5 | 1.9 | 1.3 | 1.4 | 1.6 | 1.5 | 1.5 | 1.4 |
| Asb14 | 0.1 | 0.1 | 0.0 | 0.0 | 0.0 | 0.0 | 0.1 | 0.0 | 0.0 | 0.0 | 0.0 | 0.0 |
| Asb15 | 0.0 | 0.0 | 0.0 | 0.0 | 0.0 | 0.0 | 0.0 | 0.0 | 0.0 | 0.0 | 0.0 | 0.0 |
| Asb16 | 0.0 | 0.0 | 0.0 | 0.0 | 0.0 | 0.2 | 0.0 | 0.0 | 0.0 | 0.0 | 0.0 | 0.0 |
| Asb2 | 0.3 | 0.1 | 0.2 | 1.7 | 4.7 | 0.3 | 1.2 | 0.1 | 0.6 | 0.9 | 0.7 | 0.4 |
| Asb3 | 7.0 | 7.8 | 7.3 | 8.7 | 6.5 | 8.2 | 7.5 | 6.7 | 6.9 | 7.2 | 8.1 | 7.2 |
| Asb4 | 0.3 | 0.4 | 0.5 | 0.3 | 0.5 | 0.4 | 0.4 | 0.6 | 0.3 | 0.2 | 0.2 | 0.3 |
| Asb5 | 25.5 | 15.5 | 6.4 | 25.4 | 17.5 | 13.6 | 23.7 | 4.7 | 11.1 | 10.0 | 12.1 | 11.2 |
| Asb6 | 10.9 | 9.9 | 9.7 | 12.8 | 11.0 | 10.7 | 12.1 | 10.7 | 12.7 | 10.7 | 11.1 | 9.8 |
| Asb7 | 3.9 | 4.0 | 4.3 | 4.5 | 3.5 | 4.1 | 3.7 | 4.0 | 3.8 | 4.1 | 4.1 | 4.1 |
| Asb8 | 8.2 | 8.3 | 7.1 | 7.5 | 8.0 | 8.9 | 7.6 | 7.6 | 7.6 | 8.1 | 7.2 | 7.7 |
| Ascc1 | 13.9 | 13.7 | 11.1 | 13.0 | 10.8 | 14.8 | 12.2 | 13.7 | 12.3 | 13.2 | 13.9 | 13.2 |
| Ascc2 | 13.5 | 12.2 | 11.1 | 13.6 | 12.7 | 14.7 | 13.4 | 14.5 | 12.4 | 11.6 | 13.3 | 11.4 |
| Ascc3 | 10.0 | 9.1 | 8.0 | 10.2 | 9.6 | 9.2 | 9.2 | 8.7 | 8.4 | 9.8 | 11.4 | 10.8 |
| Ascl2 | 0.2 | 0.2 | 0.1 | 0.0 | 0.2 | 0.1 | 0.1 | 0.0 | 0.2 | 0.1 | 0.1 | 0.1 |
| Ascl3 | 0.4 | 0.1 | 0.2 | 0.0 | 0.1 | 0.1 | 0.1 | 0.1 | 0.0 | 0.1 | 0.0 | 0.1 |
| Asf1a | 5.0 | 4.8 | 4.9 | 4.1 | 4.0 | 4.4 | 4.0 | 4.8 | 4.5 | 5.2 | 4.4 | 5.2 |
| Asf1b | 1.7 | 3.6 | 4.4 | 1.4 | 3.7 | 0.7 | 4.7 | 2.6 | 3.6 | 1.5 | 3.6 | 2.0 |
| Asgr1 | 0.0 | 0.0 | 0.0 | 0.0 | 0.1 | 0.0 | 0.0 | 0.1 | 0.1 | 0.0 | 0.0 | 0.0 |
| Ash1l | 7.9 | 8.6 | 8.9 | 8.1 | 7.8 | 7.8 | 8.5 | 8.5 | 7.2 | 7.3 | 8.3 | 7.9 |
| Ash2l | 17.4 | 18.5 | 17.2 | 17.7 | 15.8 | 16.5 | 16.8 | 18.3 | 17.1 | 17.8 | 18.2 | 18.5 |
| Asic1 | 0.5 | 0.4 | 0.4 | 0.5 | 0.5 | 0.5 | 0.6 | 0.4 | 0.6 | 0.9 | 0.7 | 0.9 |
| Asic3 | 0.7 | 1.0 | 0.8 | 0.2 | 1.0 | 0.4 | 0.4 | 0.3 | 0.4 | 1.7 | 1.8 | 1.6 |
| Asic5 | 0.0 | 0.0 | 0.0 | 0.0 | 0.0 | 0.0 | 0.0 | 0.0 | 0.0 | 0.0 | 0.1 | 0.1 |
| Asl | 14.1 | 13.7 | 11.6 | 15.4 | 14.0 | 14.4 | 13.6 | 13.3 | 14.1 | 11.5 | 12.6 | 11.4 |
| Asna1 | 29.5 | 27.3 | 25.6 | 35.2 | 29.5 | 31.5 | 25.4 | 30.4 | 24.4 | 29.8 | 31.2 | 28.6 |
| Asns | 69.9 | 45.9 | 34.0 | 69.0 | 38.4 | 58.9 | 53.6 | 68.6 | 65.6 | 41.1 | 65.0 | 40.6 |
| Asnsd1 | 35.7 | 28.4 | 26.9 | 28.2 | 28.0 | 34.1 | 25.4 | 27.3 | 27.7 | 33.1 | 34.4 | 32.2 |
| Aspa | 0.2 | 0.2 | 0.5 | 0.0 | 0.0 | 0.3 | 0.2 | 0.3 | 0.1 | 0.2 | 0.0 | 0.1 |
| Aspg | 0.1 | 0.4 | 0.6 | 0.2 | 0.4 | 0.2 | 0.2 | 0.3 | 0.3 | 0.1 | 0.2 | 0.1 |
| Asph | 37.5 | 33.0 | 37.1 | 43.5 | 35.3 | 44.2 | 36.9 | 33.4 | 29.9 | 31.7 | 38.5 | 36.1 |
| Asphd2 | 1.2 | 1.8 | 1.3 | 0.9 | 0.8 | 2.5 | 1.0 | 0.9 | 1.0 | 1.5 | 1.1 | 0.4 |
| Aspm | 0.8 | 1.3 | 2.2 | 0.7 | 2.3 | 0.2 | 2.8 | 1.1 | 2.6 | 1.2 | 2.2 | 1.3 |
| Aspn | 1.3 | 1.7 | 6.3 | 3.1 | 2.6 | 1.3 | 2.2 | 7.0 | 4.2 | 5.9 | 16.4 | 5.6 |
| Asprv1 | 0.0 | 0.1 | 0.1 | 0.0 | 0.0 | 0.1 | 0.0 | 0.0 | 0.0 | 0.1 | 0.0 | 0.0 |
| Aspscr1 | 13.6 | 15.3 | 12.7 | 15.2 | 14.1 | 13.6 | 13.5 | 14.3 | 13.8 | 12.7 | 13.4 | 12.0 |
| Asrgl1 | 4.9 | 5.7 | 7.0 | 4.9 | 4.1 | 5.7 | 6.6 | 5.6 | 5.0 | 4.9 | 5.5 | 5.5 |
| Ass1 | 48.2 | 31.6 | 24.6 | 69.3 | 58.0 | 73.7 | 30.0 | 19.1 | 28.0 | 49.3 | 30.3 | 28.5 |
| Aste1 | 1.9 | 1.9 | 1.6 | 1.7 | 2.2 | 1.6 | 1.9 | 1.5 | 1.7 | 1.8 | 1.8 | 1.5 |
| Astn1 | 0.0 | 0.0 | 0.0 | 0.0 | 0.0 | 0.0 | 0.0 | 0.0 | 0.0 | 0.0 | 0.0 | 0.0 |

| | | | | | | | | | | | | |
|---|---|---|---|---|---|---|---|---|---|---|---|---|
| Astn2 | 0.1 | 0.3 | 0.1 | 0.1 | 0.3 | 0.1 | 0.2 | 0.2 | 0.2 | 0.0 | 0.1 | 0.0 |
| Asun | 8.6 | 8.8 | 8.7 | 8.9 | 8.5 | 7.7 | 8.6 | 8.3 | 7.7 | 8.7 | 9.2 | 9.0 |
| Asxl1 | 3.4 | 4.6 | 4.6 | 3.2 | 3.9 | 3.6 | 4.0 | 4.4 | 4.5 | 3.8 | 4.4 | 4.1 |
| Asxl2 | 3.0 | 3.2 | 3.1 | 3.6 | 3.1 | 3.0 | 2.9 | 3.2 | 2.7 | 2.7 | 2.9 | 3.1 |
| Asxl3 | 0.0 | 0.0 | 0.0 | 0.0 | 0.0 | 0.0 | 0.0 | 0.0 | 0.0 | 0.0 | 0.0 | 0.0 |
| Asz1 | 0.1 | 0.0 | 0.0 | 0.1 | 0.1 | 0.1 | 0.0 | 0.0 | 0.0 | 0.0 | 0.0 | 0.0 |
| Atad1 | 26.8 | 26.9 | 26.7 | 27.3 | 26.5 | 30.9 | 27.0 | 26.1 | 26.7 | 27.7 | 29.1 | 30.2 |
| Atad2 | 5.9 | 8.4 | 11.6 | 5.4 | 10.0 | 5.2 | 11.2 | 7.5 | 8.0 | 6.1 | 8.9 | 6.3 |
| Atad2b | 2.0 | 2.3 | 2.7 | 1.9 | 2.3 | 2.1 | 2.3 | 2.4 | 2.4 | 2.4 | 2.8 | 3.0 |
| Atad3a | 7.7 | 6.8 | 6.2 | 7.2 | 7.1 | 6.7 | 9.0 | 7.0 | 7.1 | 8.6 | 7.1 | 6.8 |
| Atad5 | 0.6 | 1.1 | 1.3 | 0.5 | 1.1 | 0.5 | 0.8 | 0.9 | 0.7 | 0.7 | 0.7 | 0.8 |
| Atat1 | 11.2 | 12.6 | 12.8 | 12.8 | 9.2 | 11.7 | 12.0 | 10.5 | 10.6 | 12.4 | 13.7 | 11.0 |
| Atcay | 0.0 | 0.0 | 0.0 | 0.0 | 0.0 | 0.0 | 0.0 | 0.0 | 0.0 | 0.0 | 0.0 | 0.0 |
| Ate1 | 16.0 | 16.7 | 14.8 | 17.5 | 16.1 | 16.3 | 17.3 | 11.1 | 15.4 | 16.4 | 17.5 | 17.4 |
| Atf1 | 7.6 | 7.7 | 10.0 | 8.8 | 8.6 | 8.9 | 9.6 | 9.5 | 7.9 | 8.0 | 9.2 | 9.8 |
| Atf2 | 8.3 | 9.3 | 10.5 | 10.1 | 8.7 | 9.8 | 8.9 | 9.4 | 8.5 | 8.5 | 10.2 | 10.0 |
| Atf3 | 4.3 | 1.6 | 1.2 | 11.7 | 13.2 | 1.5 | 4.4 | 0.8 | 2.1 | 7.1 | 3.5 | 2.4 |
| Atf4 | 145.1 | 98.6 | 81.0 | 140.2 | 99.2 | 134.2 | 130.0 | 133.5 | 130.0 | 100.7 | 138.7 | 109.6 |
| Atf5 | 101.4 | 66.9 | 46.1 | 124.9 | 53.4 | 127.0 | 69.7 | 101.9 | 87.4 | 48.2 | 76.9 | 54.3 |
| Atf6 | 13.9 | 11.2 | 10.9 | 16.0 | 11.6 | 13.6 | 10.9 | 11.4 | 10.6 | 12.2 | 11.6 | 12.4 |
| Atf6b | 7.6 | 7.9 | 8.3 | 7.5 | 9.2 | 8.2 | 8.4 | 9.2 | 8.6 | 7.9 | 7.3 | 7.2 |
| Atf7 | 1.4 | 1.9 | 2.4 | 1.4 | 1.6 | 1.8 | 1.9 | 2.4 | 2.0 | 1.4 | 1.6 | 1.8 |
| Atf7ip | 7.1 | 8.1 | 10.4 | 6.5 | 7.6 | 6.1 | 8.3 | 8.5 | 7.2 | 6.7 | 8.3 | 7.3 |
| Atf7ip2 | 0.1 | 0.2 | 0.3 | 0.1 | 0.3 | 0.0 | 0.2 | 0.1 | 0.1 | 0.1 | 0.1 | 0.3 |
| Atg10 | 10.1 | 8.9 | 6.7 | 9.0 | 9.0 | 9.2 | 7.8 | 6.3 | 7.4 | 6.9 | 8.3 | 8.5 |
| Atg12 | 38.9 | 33.0 | 26.3 | 47.8 | 33.5 | 40.4 | 28.5 | 19.8 | 27.1 | 30.2 | 30.4 | 29.3 |
| Atg13 | 5.9 | 6.4 | 6.4 | 6.9 | 6.9 | 6.2 | 6.8 | 6.9 | 6.5 | 6.9 | 6.1 | 7.5 |
| Atg14 | 4.1 | 4.7 | 4.0 | 4.2 | 3.7 | 5.1 | 3.3 | 4.1 | 4.1 | 4.1 | 3.8 | 3.7 |
| Atg16l1 | 9.2 | 8.3 | 7.4 | 8.6 | 8.8 | 8.2 | 8.5 | 7.8 | 9.2 | 9.4 | 9.1 | 8.6 |
| Atg16l2 | 4.2 | 4.9 | 4.8 | 3.4 | 4.3 | 3.9 | 3.8 | 4.9 | 7.0 | 4.6 | 3.2 | 4.6 |
| Atg2a | 4.1 | 4.2 | 3.9 | 4.0 | 4.8 | 4.2 | 4.7 | 3.8 | 4.6 | 5.1 | 3.8 | 2.9 |
| Atg2b | 5.4 | 5.6 | 5.6 | 4.2 | 4.9 | 4.8 | 5.3 | 4.6 | 4.8 | 5.0 | 4.8 | 4.7 |
| Atg3 | 26.0 | 26.9 | 32.5 | 30.4 | 25.3 | 24.3 | 29.0 | 28.2 | 24.1 | 29.3 | 31.9 | 37.6 |
| Atg4a | 14.3 | 12.5 | 12.3 | 15.0 | 11.7 | 13.1 | 12.0 | 10.7 | 10.9 | 10.4 | 12.4 | 13.2 |
| Atg4b | 14.6 | 14.1 | 11.6 | 14.8 | 12.7 | 13.9 | 13.4 | 11.9 | 13.0 | 13.5 | 12.6 | 12.4 |
| Atg4c | 2.2 | 2.5 | 2.9 | 2.9 | 2.8 | 2.8 | 2.8 | 3.0 | 2.2 | 2.4 | 2.0 | 2.5 |
| Atg4d | 2.3 | 2.6 | 2.5 | 2.0 | 2.5 | 1.7 | 2.8 | 2.5 | 2.9 | 2.9 | 2.5 | 2.4 |
| Atg5 | 13.0 | 12.3 | 11.8 | 13.2 | 12.3 | 12.3 | 10.9 | 12.8 | 11.3 | 12.2 | 12.8 | 12.2 |
| Atg7 | 7.6 | 6.3 | 5.5 | 8.3 | 7.6 | 7.9 | 6.1 | 5.9 | 5.8 | 6.0 | 5.8 | 6.1 |
| Atg9a | 7.4 | 8.0 | 7.8 | 7.6 | 7.7 | 7.5 | 8.6 | 9.2 | 8.7 | 6.8 | 7.4 | 6.3 |
| Atg9b | 0.1 | 0.0 | 0.1 | 0.0 | 0.1 | 0.1 | 0.1 | 0.0 | 0.1 | 0.0 | 0.1 | 0.0 |
| Athl1 | 9.1 | 8.6 | 8.1 | 8.9 | 7.5 | 7.8 | 7.6 | 9.1 | 8.7 | 9.6 | 8.1 | 8.9 |
| Atic | 32.9 | 27.8 | 23.5 | 33.0 | 29.4 | 32.2 | 29.1 | 25.9 | 24.7 | 31.2 | 29.9 | 27.5 |
| Atl1 | 7.4 | 7.9 | 6.3 | 8.8 | 6.3 | 10.1 | 7.6 | 4.9 | 6.1 | 5.4 | 6.8 | 6.5 |
| Atl2 | 6.9 | 7.2 | 7.3 | 6.3 | 6.8 | 5.8 | 6.5 | 6.5 | 7.6 | 8.9 | 7.2 | 8.2 |
| Atl3 | 22.8 | 26.2 | 26.8 | 21.1 | 24.7 | 20.1 | 26.9 | 27.6 | 29.1 | 28.6 | 30.0 | 32.6 |
| Atm | 3.7 | 3.5 | 3.4 | 3.4 | 3.2 | 4.0 | 2.6 | 3.3 | 2.5 | 3.6 | 3.1 | 3.1 |
| Atmin | 6.2 | 6.4 | 5.4 | 6.1 | 5.7 | 7.3 | 4.7 | 5.7 | 5.3 | 5.1 | 5.1 | 4.7 |
| Atn1 | 1.2 | 2.2 | 2.7 | 1.7 | 2.4 | 1.8 | 3.5 | 3.7 | 3.6 | 2.1 | 2.2 | 2.0 |
| Atoh8 | 40.5 | 67.9 | 88.7 | 30.9 | 40.6 | 31.1 | 59.8 | 74.2 | 60.2 | 43.1 | 48.9 | 54.8 |
| Atox1 | 216.1 | 160.4 | 115.1 | 185.6 | 219.2 | 166.3 | 132.1 | 109.9 | 143.1 | 181.5 | 133.8 | 142.0 |
| Atp10a | 3.0 | 3.6 | 4.2 | 2.7 | 4.6 | 2.3 | 5.5 | 6.6 | 6.5 | 3.6 | 5.1 | 4.3 |
| Atp10b | 0.0 | 0.0 | 0.0 | 0.0 | 0.0 | 0.0 | 0.0 | 0.0 | 0.0 | 0.0 | 0.0 | 0.0 |
| Atp10d | 3.5 | 4.4 | 3.5 | 2.6 | 2.7 | 2.5 | 2.1 | 2.9 | 2.4 | 2.3 | 2.7 | 2.4 |
| Atp11a | 29.4 | 24.0 | 22.1 | 30.5 | 25.1 | 26.0 | 28.6 | 24.8 | 22.3 | 23.0 | 25.3 | 22.9 |
| Atp11b | 7.2 | 8.2 | 8.4 | 7.5 | 7.8 | 7.0 | 6.9 | 6.6 | 6.9 | 8.8 | 7.6 | 8.5 |
| Atp11c | 1.7 | 2.4 | 3.1 | 2.2 | 2.6 | 1.8 | 2.2 | 2.5 | 2.3 | 2.0 | 2.1 | 2.2 |

| | | | | | | | | | | | | |
|---|---|---|---|---|---|---|---|---|---|---|---|---|
| Atp12a | 0.0 | 0.0 | 0.0 | 0.0 | 0.0 | 0.0 | 0.0 | 0.0 | 0.0 | 0.0 | 0.0 | 0.0 |
| Atp13a1 | 9.3 | 10.0 | 9.2 | 10.5 | 10.5 | 9.8 | 11.5 | 10.9 | 10.4 | 10.8 | 9.1 | 8.5 |
| Atp13a2 | 4.6 | 4.2 | 3.4 | 6.7 | 10.0 | 4.7 | 4.2 | 3.3 | 3.5 | 5.1 | 3.8 | 3.4 |
| Atp13a3 | 22.3 | 24.8 | 21.7 | 17.3 | 23.6 | 17.9 | 23.3 | 21.6 | 26.0 | 24.6 | 27.8 | 25.5 |
| Atp13a5 | 0.5 | 0.1 | 0.1 | 0.6 | 0.3 | 0.2 | 0.4 | 0.0 | 0.1 | 0.2 | 0.1 | 0.1 |
| Atp1a1 | 101.1 | 106.9 | 101.9 | 80.1 | 102.3 | 78.5 | 129.0 | 120.5 | 125.1 | 143.0 | 127.5 | 119.3 |
| Atp1a2 | 0.2 | 0.3 | 1.1 | 0.0 | 0.1 | 0.0 | 0.1 | 0.4 | 0.1 | 0.0 | 0.0 | 0.1 |
| Atp1a3 | 0.1 | 0.0 | 0.0 | 0.4 | 1.0 | 0.0 | 0.1 | 0.0 | 0.0 | 0.1 | 0.1 | 0.1 |
| Atp1a4 | 0.3 | 0.2 | 0.2 | 0.4 | 0.1 | 0.1 | 0.1 | 0.1 | 0.1 | 0.1 | 0.1 | 0.2 |
| Atp1b1 | 8.2 | 4.7 | 4.5 | 6.2 | 3.4 | 4.4 | 1.9 | 2.5 | 3.5 | 31.3 | 9.4 | 9.1 |
| Atp1b2 | 0.0 | 0.0 | 0.0 | 0.0 | 0.0 | 0.0 | 0.0 | 0.0 | 0.0 | 0.1 | 0.0 | 0.0 |
| Atp1b3 | 108.7 | 89.3 | 72.8 | 111.4 | 100.4 | 100.6 | 84.2 | 70.0 | 80.9 | 99.3 | 91.1 | 93.9 |
| Atp1b4 | 0.0 | 0.0 | 0.0 | 0.0 | 0.0 | 0.0 | 0.0 | 0.0 | 0.0 | 0.0 | 0.0 | 0.0 |
| Atp2a1 | 0.0 | 0.0 | 0.0 | 0.0 | 0.0 | 0.0 | 0.0 | 0.0 | 0.0 | 0.0 | 0.0 | 0.0 |
| Atp2a2 | 51.9 | 49.3 | 47.0 | 48.8 | 50.3 | 39.3 | 56.3 | 52.6 | 56.0 | 54.5 | 54.8 | 52.2 |
| Atp2a3 | 0.3 | 0.1 | 0.1 | 1.4 | 2.4 | 0.1 | 0.3 | 0.1 | 0.2 | 0.2 | 0.1 | 0.1 |
| Atp2b1 | 9.0 | 9.6 | 11.7 | 10.4 | 13.2 | 7.3 | 11.5 | 15.2 | 12.2 | 11.2 | 12.6 | 13.6 |
| Atp2b2 | 0.0 | 0.0 | 0.0 | 0.0 | 0.0 | 0.0 | 0.0 | 0.0 | 0.0 | 0.0 | 0.0 | 0.0 |
| Atp2b4 | 0.7 | 0.7 | 1.1 | 0.4 | 1.1 | 0.3 | 1.0 | 1.2 | 1.1 | 3.3 | 2.6 | 1.2 |
| Atp2c1 | 30.9 | 31.6 | 30.3 | 31.2 | 28.3 | 28.9 | 31.9 | 26.8 | 33.7 | 28.1 | 31.8 | 32.9 |
| Atp4a | 0.0 | 0.0 | 0.0 | 0.0 | 0.0 | 0.0 | 0.0 | 0.0 | 0.0 | 0.0 | 0.0 | 0.0 |
| Atp5a1 | 152.3 | 132.7 | 123.2 | 136.1 | 138.8 | 129.3 | 128.7 | 138.7 | 132.6 | 149.9 | 133.2 | 143.0 |
| Atp5b | 428.3 | 365.6 | 299.1 | 417.5 | 360.0 | 368.0 | 357.5 | 342.1 | 336.0 | 375.3 | 380.6 | 342.3 |
| Atp5c1 | 121.4 | 109.7 | 97.4 | 115.3 | 108.4 | 113.6 | 101.2 | 102.9 | 95.6 | 113.7 | 113.4 | 108.0 |
| Atp5d | 98.9 | 86.7 | 84.7 | 89.1 | 88.8 | 95.1 | 93.9 | 97.4 | 94.1 | 98.2 | 89.6 | 87.5 |
| Atp5e | 238.0 | 212.3 | 149.4 | 148.8 | 201.6 | 195.1 | 164.8 | 183.1 | 219.5 | 218.6 | 163.4 | 154.4 |
| Atp5f1 | 100.8 | 89.9 | 91.2 | 109.9 | 93.1 | 93.0 | 98.3 | 95.7 | 88.4 | 99.8 | 112.6 | 113.7 |
| Atp5g1 | 98.7 | 68.5 | 54.5 | 93.9 | 80.6 | 85.1 | 77.3 | 88.8 | 69.4 | 81.6 | 83.0 | 60.8 |
| Atp5g2 | 137.8 | 122.1 | 100.5 | 108.1 | 110.5 | 134.7 | 108.7 | 132.6 | 126.9 | 134.7 | 126.0 | 111.8 |
| Atp5g3 | 100.1 | 108.0 | 75.6 | 90.9 | 101.7 | 90.2 | 82.4 | 87.8 | 103.0 | 109.4 | 86.3 | 88.8 |
| Atp5h | 237.5 | 189.2 | 219.2 | 245.1 | 190.8 | 236.7 | 215.4 | 216.9 | 183.6 | 223.1 | 239.2 | 227.7 |
| Atp5j | 100.9 | 96.7 | 81.6 | 102.9 | 90.7 | 92.4 | 77.0 | 92.7 | 86.9 | 101.2 | 93.8 | 92.7 |
| Atp5j2 | 236.1 | 188.2 | 170.4 | 204.5 | 201.6 | 228.5 | 183.6 | 194.4 | 196.5 | 229.4 | 199.8 | 196.5 |
| Atp5k | 224.9 | 206.2 | 196.3 | 208.3 | 203.0 | 209.0 | 170.0 | 202.6 | 219.0 | 211.0 | 190.2 | 235.6 |
| Atp5l | 246.5 | 230.7 | 184.5 | 213.0 | 249.2 | 230.5 | 195.0 | 205.9 | 221.4 | 258.2 | 226.1 | 231.3 |
| Atp5o | 140.3 | 127.4 | 122.2 | 118.3 | 128.0 | 121.8 | 126.2 | 142.3 | 124.7 | 131.7 | 127.8 | 127.8 |
| Atp5s | 7.0 | 6.8 | 5.9 | 6.3 | 4.9 | 8.4 | 4.5 | 4.7 | 4.8 | 5.7 | 5.6 | 6.3 |
| Atp5sl | 3.6 | 3.1 | 3.2 | 4.4 | 3.1 | 4.4 | 2.7 | 3.4 | 2.7 | 3.6 | 2.7 | 3.4 |
| Atp6ap1 | 141.9 | 114.5 | 95.5 | 163.0 | 124.7 | 154.5 | 90.6 | 101.9 | 89.2 | 100.9 | 101.5 | 106.7 |
| Atp6ap2 | 59.1 | 41.4 | 34.1 | 67.4 | 58.0 | 67.0 | 30.7 | 34.0 | 33.0 | 40.0 | 41.7 | 45.1 |
| Atp6v0a1 | 16.1 | 12.5 | 10.8 | 18.3 | 16.3 | 15.9 | 12.7 | 9.6 | 10.3 | 12.5 | 11.1 | 10.6 |
| Atp6v0a2 | 18.9 | 16.8 | 17.2 | 19.6 | 20.5 | 17.1 | 18.4 | 20.9 | 19.2 | 21.2 | 19.2 | 19.9 |
| Atp6v0a4 | 0.2 | 0.2 | 0.1 | 0.1 | 0.1 | 0.1 | 0.1 | 0.1 | 0.1 | 0.2 | 0.1 | 0.1 |
| Atp6v0b | 87.8 | 71.9 | 57.4 | 106.9 | 99.1 | 91.0 | 74.1 | 64.2 | 76.0 | 92.1 | 71.3 | 77.1 |
| Atp6v0c | 207.5 | 160.5 | 140.0 | 249.0 | 235.9 | 205.1 | 171.7 | 148.0 | 150.1 | 175.5 | 157.1 | 155.6 |
| Atp6v0c-ps2 | 5.1 | 4.5 | 1.1 | 10.5 | 16.1 | 12.2 | 0.0 | 1.1 | 1.5 | 7.8 | 0.0 | 1.8 |
| Atp6v0d1 | 64.5 | 53.7 | 41.0 | 70.0 | 63.6 | 64.4 | 52.9 | 48.6 | 47.5 | 60.2 | 59.6 | 56.1 |
| Atp6v0d2 | 0.2 | 0.1 | 0.1 | 1.6 | 0.4 | 0.1 | 0.0 | 0.0 | 0.0 | 0.1 | 0.1 | 0.2 |
| Atp6v0e | 184.9 | 136.5 | 108.9 | 204.2 | 153.4 | 156.4 | 136.1 | 116.6 | 130.1 | 155.3 | 144.5 | 129.8 |
| Atp6v0e2 | 6.1 | 3.0 | 1.9 | 4.1 | 1.1 | 6.9 | 1.3 | 1.5 | 0.9 | 5.5 | 3.7 | 2.3 |
| Atp6v1a | 78.1 | 63.3 | 56.2 | 86.4 | 77.5 | 81.5 | 51.2 | 52.6 | 52.1 | 66.6 | 60.6 | 64.8 |
| Atp6v1b1 | 0.4 | 0.2 | 0.0 | 0.1 | 0.2 | 0.3 | 0.1 | 0.0 | 0.1 | 0.0 | 0.0 | 0.0 |
| Atp6v1b2 | 30.4 | 28.6 | 22.4 | 32.3 | 41.0 | 26.6 | 25.2 | 23.6 | 25.9 | 30.6 | 26.2 | 26.2 |
| Atp6v1c1 | 49.2 | 41.2 | 32.0 | 51.8 | 44.2 | 47.0 | 35.2 | 30.2 | 33.7 | 39.5 | 38.5 | 38.8 |
| Atp6v1c2 | 0.1 | 0.0 | 0.1 | 0.0 | 0.0 | 0.0 | 0.0 | 0.0 | 0.0 | 0.0 | 0.1 | 0.0 |
| Atp6v1d | 63.2 | 55.8 | 55.8 | 71.7 | 56.7 | 63.7 | 51.7 | 50.2 | 42.9 | 58.9 | 59.9 | 70.1 |
| Atp6v1e1 | 138.9 | 93.0 | 75.9 | 136.5 | 112.8 | 118.0 | 80.2 | 72.0 | 78.6 | 108.3 | 106.0 | 103.9 |

| | | | | | | | | | | | | |
|---|---|---|---|---|---|---|---|---|---|---|---|---|
| Atp6v1e2 | 0.0 | 0.0 | 0.0 | 0.0 | 0.0 | 0.0 | 0.0 | 0.0 | 0.0 | 0.0 | 0.1 | 0.0 |
| Atp6v1f | 155.7 | 109.0 | 93.8 | 172.2 | 114.8 | 148.4 | 96.6 | 91.7 | 90.0 | 122.3 | 107.6 | 105.7 |
| Atp6v1g1 | 104.1 | 89.3 | 70.2 | 126.8 | 91.1 | 116.4 | 74.1 | 77.9 | 80.4 | 92.7 | 88.5 | 86.2 |
| Atp6v1g2 | 0.2 | 0.2 | 0.1 | 0.1 | 0.3 | 0.4 | 0.2 | 0.5 | 0.4 | 0.4 | 0.3 | 0.5 |
| Atp6v1h | 81.8 | 60.9 | 46.4 | 79.2 | 64.7 | 72.1 | 49.3 | 39.5 | 52.0 | 70.5 | 56.6 | 63.3 |
| Atp7a | 6.0 | 7.2 | 7.6 | 5.9 | 7.4 | 4.9 | 5.9 | 7.7 | 5.7 | 6.9 | 6.9 | 6.7 |
| Atp7b | 0.0 | 0.0 | 0.0 | 0.0 | 0.0 | 0.1 | 0.0 | 0.0 | 0.0 | 0.0 | 0.0 | 0.0 |
| Atp8a1 | 0.5 | 0.3 | 0.3 | 1.1 | 1.7 | 0.4 | 0.4 | 0.4 | 0.3 | 1.1 | 0.4 | 0.6 |
| Atp8a2 | 0.1 | 0.1 | 0.0 | 0.0 | 0.1 | 0.1 | 0.0 | 0.0 | 0.0 | 0.0 | 0.1 | 0.1 |
| Atp8b1 | 9.2 | 10.5 | 11.2 | 12.4 | 13.6 | 11.2 | 7.8 | 8.6 | 6.9 | 5.6 | 5.9 | 7.3 |
| Atp8b2 | 32.9 | 37.0 | 33.8 | 33.5 | 32.9 | 32.8 | 38.5 | 38.4 | 39.6 | 30.0 | 32.0 | 33.1 |
| Atp8b3 | 0.0 | 0.0 | 0.0 | 0.0 | 0.1 | 0.0 | 0.0 | 0.0 | 0.0 | 0.0 | 0.0 | 0.0 |
| Atp8b4 | 0.4 | 0.3 | 0.2 | 2.8 | 5.1 | 0.2 | 0.7 | 0.2 | 0.1 | 0.6 | 0.1 | 0.3 |
| Atp8b5 | 0.1 | 0.1 | 0.1 | 0.1 | 0.1 | 0.2 | 0.1 | 0.2 | 0.1 | 0.4 | 0.2 | 0.1 |
| Atp9a | 52.9 | 41.9 | 33.0 | 55.3 | 37.5 | 61.6 | 38.6 | 32.7 | 32.6 | 35.9 | 34.8 | 36.9 |
| Atp9b | 15.8 | 14.1 | 13.0 | 15.6 | 15.3 | 15.2 | 16.9 | 15.0 | 15.8 | 14.5 | 15.2 | 15.1 |
| Atpaf1 | 4.9 | 4.7 | 4.8 | 5.0 | 4.8 | 4.9 | 4.2 | 4.9 | 4.4 | 4.4 | 4.8 | 4.1 |
| Atpaf2 | 9.1 | 9.2 | 7.3 | 11.1 | 8.3 | 9.5 | 9.2 | 8.5 | 7.6 | 7.6 | 6.8 | 7.1 |
| Atpbd4 | 2.1 | 2.0 | 1.6 | 2.3 | 2.4 | 2.4 | 2.6 | 2.2 | 1.6 | 2.1 | 2.6 | 2.2 |
| Atpif1 | 177.5 | 168.0 | 185.4 | 215.1 | 172.7 | 204.1 | 172.8 | 190.5 | 156.3 | 188.0 | 219.3 | 192.5 |
| Atr | 4.4 | 4.4 | 3.6 | 3.8 | 4.3 | 3.7 | 3.4 | 3.4 | 3.3 | 3.8 | 4.1 | 3.5 |
| Atraid | 76.2 | 66.7 | 60.7 | 62.4 | 59.5 | 75.0 | 61.4 | 60.4 | 66.0 | 72.1 | 60.8 | 80.6 |
| Atrip | 4.2 | 5.2 | 4.2 | 4.7 | 3.6 | 3.6 | 3.6 | 3.8 | 4.2 | 4.4 | 4.0 | 3.9 |
| Atrn | 9.7 | 9.5 | 9.6 | 10.6 | 11.4 | 8.5 | 10.1 | 10.7 | 10.5 | 9.3 | 10.3 | 9.6 |
| Atrnl1 | 10.8 | 10.3 | 11.3 | 9.3 | 10.8 | 10.0 | 9.0 | 11.5 | 10.9 | 8.7 | 8.5 | 9.3 |
| Atrx | 16.5 | 16.2 | 20.5 | 17.3 | 16.0 | 15.9 | 15.4 | 18.2 | 13.8 | 17.3 | 19.1 | 19.1 |
| Atxn1 | 4.3 | 6.5 | 7.8 | 4.2 | 6.1 | 3.9 | 6.9 | 7.0 | 6.6 | 6.1 | 6.4 | 6.6 |
| Atxn10 | 55.1 | 47.7 | 47.5 | 48.6 | 44.4 | 45.5 | 45.0 | 49.5 | 41.3 | 44.1 | 46.1 | 49.1 |
| Atxn1l | 6.7 | 8.1 | 7.6 | 7.8 | 7.7 | 6.5 | 7.8 | 7.3 | 8.2 | 6.4 | 7.3 | 7.3 |
| Atxn2 | 3.6 | 5.1 | 5.5 | 4.8 | 4.4 | 5.1 | 6.1 | 4.5 | 4.6 | 3.9 | 5.3 | 4.3 |
| Atxn2l | 5.6 | 7.1 | 7.1 | 6.8 | 7.0 | 7.0 | 8.5 | 6.8 | 6.9 | 5.5 | 6.0 | 4.6 |
| Atxn3 | 11.3 | 10.9 | 10.2 | 13.3 | 9.7 | 11.3 | 8.5 | 7.9 | 8.1 | 10.4 | 9.0 | 9.9 |
| Atxn7 | 3.7 | 4.5 | 4.3 | 3.8 | 4.0 | 3.4 | 4.5 | 3.3 | 4.0 | 3.4 | 4.3 | 4.1 |
| Atxn7l1 | 1.7 | 2.7 | 2.7 | 3.8 | 2.6 | 3.1 | 2.8 | 2.4 | 1.8 | 1.5 | 1.6 | 1.8 |
| Atxn7l2 | 0.3 | 0.2 | 0.4 | 0.3 | 0.3 | 0.2 | 0.2 | 0.4 | 0.3 | 0.4 | 0.3 | 0.5 |
| Atxn7l3 | 9.6 | 11.2 | 10.0 | 9.0 | 10.0 | 10.4 | 10.2 | 11.3 | 12.5 | 11.4 | 10.1 | 10.0 |
| Atxn7l3b | 22.8 | 20.7 | 20.2 | 20.8 | 21.1 | 22.9 | 18.3 | 20.7 | 23.9 | 25.4 | 21.9 | 20.7 |
| AU015228 | 0.1 | 0.1 | 0.0 | 0.0 | 0.1 | 0.0 | 0.1 | 0.0 | 0.0 | 0.0 | 0.0 | 0.0 |
| AU015836 | 0.0 | 0.0 | 0.0 | 0.0 | 0.0 | 0.1 | 0.0 | 0.0 | 0.0 | 0.0 | 0.0 | 0.0 |
| AU019823 | 4.3 | 4.1 | 4.4 | 4.9 | 5.5 | 4.6 | 4.6 | 4.6 | 4.4 | 5.1 | 5.3 | 5.3 |
| AU021063 | 0.5 | 0.6 | 0.3 | 0.2 | 0.2 | 0.4 | 0.4 | 0.7 | 0.5 | 0.1 | 0.1 | 0.7 |
| AU021092 | 1.9 | 0.6 | 0.4 | 1.0 | 0.6 | 0.8 | 0.7 | 0.6 | 0.5 | 0.5 | 0.8 | 0.3 |
| AU022252 | 5.6 | 6.0 | 5.7 | 6.1 | 6.0 | 6.1 | 5.5 | 6.9 | 6.2 | 6.2 | 6.1 | 6.1 |
| AU022793 | 0.0 | 0.0 | 0.0 | 0.1 | 0.2 | 0.0 | 0.1 | 0.0 | 0.0 | 0.0 | 0.0 | 0.0 |
| AU023762 | 0.0 | 0.0 | 0.0 | 0.0 | 0.0 | 0.0 | 0.0 | 0.0 | 0.0 | 0.0 | 0.0 | 0.0 |
| AU023871 | 0.0 | 0.0 | 0.0 | 0.0 | 0.0 | 0.0 | 0.1 | 0.1 | 0.0 | 0.0 | 0.0 | 0.0 |
| AU040320 | 9.0 | 9.7 | 9.6 | 9.5 | 8.9 | 8.3 | 9.6 | 10.8 | 11.0 | 7.8 | 8.9 | 9.2 |
| AU040972 | 0.0 | 0.1 | 0.2 | 0.3 | 0.1 | 0.0 | 0.2 | 0.2 | 0.0 | 0.1 | 0.0 | 0.0 |
| AU041133 | 1.6 | 1.8 | 1.5 | 1.4 | 1.5 | 1.9 | 0.8 | 1.4 | 1.6 | 2.0 | 1.8 | 1.8 |
| Auh | 15.7 | 14.1 | 15.2 | 15.3 | 11.5 | 12.5 | 15.1 | 16.4 | 14.9 | 15.7 | 14.6 | 18.1 |
| Aup1 | 33.7 | 31.7 | 31.3 | 43.1 | 37.9 | 38.3 | 37.8 | 33.3 | 36.8 | 35.8 | 36.4 | 36.8 |
| Aurka | 2.9 | 4.8 | 8.0 | 1.3 | 6.8 | 0.7 | 11.0 | 3.3 | 7.7 | 4.2 | 7.2 | 4.7 |
| Aurkaip1 | 41.8 | 31.1 | 27.2 | 42.1 | 33.5 | 39.0 | 33.7 | 35.2 | 35.6 | 39.8 | 35.0 | 36.3 |
| Aurkb | 1.5 | 3.2 | 5.1 | 1.1 | 5.0 | 1.0 | 6.5 | 2.4 | 4.5 | 2.2 | 2.6 | 2.5 |
| Aurkc | 0.1 | 0.0 | 0.0 | 0.2 | 0.1 | 0.2 | 0.1 | 0.1 | 0.0 | 0.1 | 0.0 | 0.1 |
| Auts2 | 0.4 | 0.6 | 0.8 | 0.2 | 0.4 | 0.4 | 0.3 | 0.6 | 0.4 | 0.6 | 0.3 | 0.4 |
| AV039307 | 0.3 | 0.5 | 0.1 | 0.4 | 0.4 | 1.0 | 0.2 | 0.5 | 0.2 | 0.4 | 0.3 | 0.3 |

| | | | | | | | | | | | | |
|---|---|---|---|---|---|---|---|---|---|---|---|---|
| AV051173 | 0.6 | 1.0 | 0.5 | 2.2 | 1.9 | 0.9 | 0.9 | 0.9 | 0.9 | 1.9 | 1.2 | 0.7 |
| Aven | 6.2 | 5.9 | 5.6 | 5.0 | 6.2 | 3.7 | 6.1 | 7.2 | 5.8 | 6.5 | 5.7 | 5.2 |
| Avil | 0.6 | 0.4 | 0.5 | 0.4 | 0.2 | 0.6 | 0.3 | 0.4 | 0.4 | 0.3 | 0.4 | 0.3 |
| Avl9 | 6.6 | 7.6 | 6.7 | 6.9 | 6.6 | 7.5 | 5.4 | 5.3 | 5.9 | 7.6 | 7.5 | 7.5 |
| Avpi1 | 12.7 | 12.8 | 9.7 | 9.7 | 8.4 | 12.0 | 11.8 | 11.7 | 12.7 | 12.3 | 11.8 | 10.4 |
| Avpr1a | 2.5 | 4.3 | 7.9 | 3.9 | 6.1 | 1.8 | 7.5 | 6.9 | 6.3 | 3.7 | 3.7 | 3.7 |
| Avpr1b | 0.0 | 0.0 | 0.0 | 0.1 | 0.0 | 0.0 | 0.0 | 0.0 | 0.0 | 0.0 | 0.0 | 0.0 |
| AW011738 | 0.5 | 0.4 | 0.4 | 0.4 | 0.6 | 0.6 | 0.6 | 0.6 | 0.5 | 0.5 | 0.3 | 0.5 |
| AW046200 | 0.1 | 0.1 | 0.1 | 0.0 | 0.1 | 0.0 | 0.1 | 0.1 | 0.1 | 0.1 | 0.1 | 0.1 |
| AW112010 | 0.9 | 0.4 | 0.4 | 1.2 | 1.1 | 0.2 | 0.8 | 0.3 | 0.3 | 0.1 | 0.0 | 0.2 |
| AW146154 | 2.2 | 1.9 | 2.2 | 2.5 | 1.8 | 2.5 | 1.7 | 2.1 | 2.0 | 2.1 | 2.4 | 2.5 |
| AW209491 | 8.5 | 6.6 | 5.5 | 6.9 | 5.5 | 7.6 | 5.0 | 5.7 | 5.4 | 6.1 | 6.8 | 6.0 |
| AW549877 | 8.3 | 10.1 | 10.1 | 10.7 | 9.6 | 12.8 | 9.2 | 9.5 | 10.7 | 10.4 | 10.9 | 11.0 |
| AW551984 | 0.0 | 0.0 | 0.0 | 0.0 | 0.0 | 0.0 | 0.0 | 0.1 | 0.0 | 0.0 | 0.0 | 0.0 |
| AW554918 | 1.2 | 1.3 | 1.5 | 1.5 | 1.1 | 1.4 | 1.6 | 1.5 | 1.2 | 1.1 | 1.2 | 1.1 |
| Awat2 | 0.2 | 0.1 | 0.0 | 0.0 | 0.2 | 0.6 | 0.0 | 0.1 | 0.0 | 0.2 | 0.1 | 0.1 |
| Axin1 | 8.3 | 8.9 | 8.2 | 8.1 | 7.6 | 7.6 | 8.4 | 7.9 | 8.5 | 7.3 | 8.6 | 8.1 |
| Axin2 | 0.1 | 0.1 | 0.1 | 0.1 | 0.1 | 0.0 | 0.1 | 0.1 | 0.0 | 0.4 | 0.1 | 0.2 |
| Axl | 123.5 | 129.1 | 99.6 | 126.9 | 130.4 | 148.4 | 154.3 | 130.4 | 136.3 | 141.3 | 141.5 | 125.5 |
| AY074887 | 1.6 | 0.9 | 0.6 | 2.2 | 1.0 | 1.6 | 1.1 | 0.9 | 1.0 | 1.1 | 0.8 | 0.5 |
| AY358078 | 0.0 | 0.0 | 0.1 | 0.2 | 0.0 | 0.1 | 0.0 | 0.0 | 0.0 | 0.0 | 0.1 | 0.0 |
| AY512931 | 0.1 | 0.3 | 0.2 | 0.1 | 0.1 | 0.1 | 0.2 | 0.1 | 0.1 | 0.1 | 0.1 | 0.2 |
| Azi1 | 3.1 | 4.1 | 4.3 | 3.0 | 2.8 | 3.5 | 3.5 | 3.4 | 3.2 | 3.0 | 3.0 | 2.8 |
| Azi2 | 16.1 | 15.3 | 14.5 | 16.9 | 14.8 | 16.0 | 13.2 | 12.8 | 14.5 | 15.2 | 14.8 | 16.2 |
| Azin1 | 23.9 | 24.8 | 21.0 | 26.2 | 26.1 | 23.6 | 22.4 | 17.7 | 22.8 | 22.0 | 22.7 | 23.1 |
| B020004J07Rik | 0.1 | 0.1 | 0.1 | 0.0 | 0.1 | 0.1 | 0.1 | 0.1 | 0.1 | 0.1 | 0.1 | 0.0 |
| B130006D01Rik | 0.1 | 0.2 | 0.2 | 0.1 | 0.2 | 0.2 | 0.1 | 0.2 | 0.1 | 0.1 | 0.1 | 0.2 |
| B130024G19Rik | 0.0 | 0.2 | 0.2 | 0.1 | 0.1 | 0.1 | 0.1 | 0.1 | 0.1 | 0.2 | 0.1 | 0.2 |
| B130034C11Rik | 0.1 | 0.2 | 0.1 | 0.3 | 0.2 | 0.1 | 0.1 | 0.1 | 0.0 | 0.0 | 0.1 | 0.1 |
| B230118H07Rik | 8.3 | 7.4 | 6.8 | 10.0 | 5.2 | 10.8 | 5.2 | 5.3 | 4.2 | 4.9 | 5.9 | 6.1 |
| B230120H23Rik | 26.6 | 21.4 | 19.3 | 23.0 | 22.7 | 21.0 | 20.5 | 15.8 | 14.9 | 22.8 | 22.0 | 19.2 |
| B230206H07Rik | 0.0 | 0.0 | 0.0 | 0.1 | 0.0 | 0.0 | 0.0 | 0.0 | 0.0 | 0.1 | 0.1 | 0.0 |
| B230208H11Rik | 0.2 | 0.1 | 0.1 | 0.1 | 0.2 | 0.0 | 0.0 | 0.0 | 0.1 | 0.2 | 0.0 | 0.2 |
| B230216G23Rik | 0.3 | 0.4 | 0.3 | 0.1 | 0.2 | 0.4 | 0.4 | 0.3 | 0.3 | 0.4 | 0.3 | 0.2 |
| B230216N24Rik | 0.1 | 0.5 | 0.1 | 0.2 | 0.1 | 0.2 | 0.0 | 0.0 | 0.1 | 0.0 | 0.1 | 0.0 |
| B230217C12Rik | 1.1 | 1.0 | 1.2 | 1.1 | 1.2 | 1.1 | 0.9 | 1.0 | 1.0 | 0.6 | 1.0 | 0.8 |
| B230217O12Rik | 1.0 | 0.4 | 0.6 | 0.7 | 0.5 | 0.8 | 0.6 | 1.0 | 0.4 | 0.5 | 0.6 | 0.5 |
| B230219D22Rik | 18.3 | 17.6 | 18.0 | 19.6 | 19.7 | 19.0 | 19.7 | 20.1 | 21.8 | 20.6 | 23.9 | 21.8 |
| B230319C09Rik | 0.2 | 0.4 | 0.2 | 0.2 | 0.3 | 0.3 | 0.2 | 0.3 | 0.2 | 0.2 | 0.3 | 0.3 |
| B230378P21Rik | 0.1 | 0.1 | 0.1 | 0.1 | 0.2 | 0.0 | 0.1 | 0.1 | 0.1 | 0.0 | 0.1 | 0.0 |
| B2m | 1573.3 | 1115.4 | 1584.3 | 2763.8 | 1377.3 | 1827.6 | 904.5 | 753.2 | 592.7 | 612.2 | 739.9 | 954.2 |
| B330016D10Rik | 0.4 | 0.7 | 0.9 | 0.5 | 0.6 | 0.8 | 0.6 | 0.6 | 0.5 | 0.6 | 0.5 | 0.7 |
| B3galnt1 | 2.8 | 4.1 | 4.3 | 2.9 | 2.7 | 4.1 | 6.3 | 3.5 | 3.9 | 5.1 | 6.4 | 4.7 |
| B3galnt2 | 16.2 | 15.7 | 12.6 | 15.7 | 12.7 | 15.3 | 12.9 | 12.7 | 13.6 | 16.4 | 16.1 | 15.3 |
| B3galt1 | 0.4 | 0.8 | 1.6 | 0.6 | 0.9 | 0.4 | 1.0 | 1.7 | 1.6 | 1.0 | 1.2 | 1.3 |
| B3galt2 | 0.0 | 0.1 | 0.1 | 0.0 | 0.0 | 0.1 | 0.0 | 0.1 | 0.1 | 0.1 | 0.1 | 0.1 |
| B3galt4 | 1.5 | 1.4 | 1.4 | 1.7 | 1.7 | 1.7 | 1.5 | 1.8 | 1.8 | 1.4 | 1.5 | 1.5 |
| B3galt5 | 0.0 | 0.0 | 0.0 | 0.0 | 0.0 | 0.0 | 0.0 | 0.0 | 0.0 | 0.0 | 0.0 | 0.0 |
| B3galt6 | 2.0 | 1.8 | 2.1 | 2.0 | 2.2 | 1.9 | 2.2 | 2.3 | 2.8 | 3.0 | 2.6 | 2.5 |
| B3galtl | 4.6 | 4.3 | 4.2 | 6.0 | 6.0 | 6.0 | 4.7 | 5.2 | 3.9 | 3.9 | 4.3 | 4.2 |
| B3gat2 | 0.2 | 0.2 | 0.2 | 0.2 | 0.2 | 0.2 | 0.2 | 0.2 | 0.1 | 0.1 | 0.3 | 0.2 |
| B3gat3 | 8.4 | 6.4 | 6.8 | 10.0 | 9.3 | 8.9 | 9.5 | 8.7 | 10.2 | 9.0 | 8.9 | 9.1 |
| B3gnt1 | 17.4 | 15.4 | 14.2 | 18.1 | 15.0 | 20.8 | 17.4 | 14.5 | 16.8 | 16.8 | 16.2 | 15.8 |
| B3gnt2 | 8.1 | 7.9 | 8.1 | 9.9 | 12.2 | 7.9 | 9.0 | 8.8 | 8.2 | 7.5 | 9.2 | 9.2 |
| B3gnt3 | 1.0 | 1.0 | 0.7 | 1.6 | 2.5 | 1.7 | 1.8 | 1.2 | 1.0 | 0.7 | 0.9 | 0.9 |
| B3gnt4 | 0.2 | 0.2 | 0.3 | 0.2 | 0.3 | 0.1 | 0.3 | 0.2 | 0.3 | 0.1 | 0.3 | 0.4 |
| B3gnt5 | 0.0 | 0.0 | 0.0 | 0.0 | 0.0 | 0.0 | 0.1 | 0.0 | 0.1 | 0.0 | 0.0 | 0.0 |

| | | | | | | | | | | | | |
|---|---|---|---|---|---|---|---|---|---|---|---|---|
| B3gnt6 | 0.1 | 0.0 | 0.0 | 0.0 | 0.0 | 0.0 | 0.0 | 0.0 | 0.0 | 0.1 | 0.0 | 0.0 |
| B3gnt7 | 0.0 | 0.0 | 0.0 | 0.0 | 0.1 | 0.0 | 0.0 | 0.0 | 0.0 | 0.0 | 0.0 | 0.0 |
| B3gnt8 | 0.3 | 0.7 | 1.3 | 0.5 | 1.4 | 0.3 | 0.6 | 1.5 | 0.7 | 0.4 | 0.6 | 0.6 |
| B3gnt9 | 10.9 | 10.3 | 12.7 | 8.8 | 10.4 | 11.9 | 13.3 | 17.1 | 15.6 | 16.7 | 14.1 | 18.5 |
| B3gntl1 | 3.1 | 3.2 | 2.5 | 3.0 | 2.7 | 3.4 | 2.5 | 3.0 | 2.8 | 2.6 | 2.9 | 2.3 |
| B430010I23Rik | 0.2 | 0.1 | 0.2 | 0.1 | 0.1 | 0.0 | 0.0 | 0.2 | 0.1 | 0.1 | 0.3 | 0.1 |
| B430212C06Rik | 0.0 | 0.0 | 0.0 | 0.2 | 0.1 | 0.0 | 0.0 | 0.0 | 0.0 | 0.0 | 0.0 | 0.1 |
| B430306N03Rik | 0.3 | 0.0 | 0.0 | 0.9 | 1.0 | 0.0 | 0.1 | 0.0 | 0.0 | 0.1 | 0.1 | 0.1 |
| B430319G15Rik | 1.5 | 1.7 | 1.4 | 1.4 | 1.0 | 0.9 | 1.3 | 1.2 | 1.3 | 1.4 | 1.7 | 1.6 |
| B4galnt1 | 2.4 | 2.4 | 1.7 | 4.1 | 5.3 | 4.7 | 4.7 | 2.6 | 3.3 | 4.8 | 4.5 | 3.6 |
| B4galnt2 | 0.8 | 0.1 | 0.1 | 0.3 | 0.2 | 0.1 | 0.0 | 0.1 | 0.0 | 0.1 | 0.1 | 0.1 |
| B4galnt3 | 0.0 | 0.0 | 0.0 | 0.0 | 0.0 | 0.0 | 0.0 | 0.0 | 0.0 | 0.0 | 0.0 | 0.0 |
| B4galt1 | 35.2 | 45.9 | 48.1 | 22.0 | 36.7 | 25.0 | 41.0 | 55.6 | 52.7 | 40.5 | 38.4 | 53.1 |
| B4galt2 | 12.1 | 11.7 | 10.6 | 13.5 | 11.8 | 15.4 | 14.2 | 13.3 | 12.5 | 11.2 | 11.1 | 10.5 |
| B4galt3 | 4.1 | 4.1 | 5.4 | 3.7 | 5.9 | 4.8 | 5.6 | 4.1 | 5.9 | 6.1 | 4.9 | 5.1 |
| B4galt4 | 23.1 | 17.4 | 17.2 | 16.5 | 12.0 | 17.4 | 16.3 | 16.9 | 13.9 | 30.9 | 27.0 | 21.8 |
| B4galt5 | 15.8 | 12.8 | 15.9 | 15.4 | 25.2 | 11.6 | 24.4 | 20.2 | 16.3 | 14.7 | 14.6 | 18.3 |
| B4galt6 | 3.9 | 3.8 | 3.6 | 4.1 | 4.0 | 3.9 | 4.3 | 3.2 | 3.3 | 3.9 | 4.5 | 3.6 |
| B4galt7 | 15.4 | 13.5 | 9.9 | 18.2 | 18.7 | 15.7 | 20.5 | 14.7 | 15.2 | 13.1 | 16.7 | 12.3 |
| B630005N14Rik | 5.9 | 5.6 | 6.8 | 6.7 | 7.0 | 6.2 | 5.2 | 5.1 | 5.6 | 6.6 | 7.2 | 6.1 |
| B630019K06Rik | 1.7 | 1.6 | 1.5 | 1.9 | 1.3 | 2.2 | 1.7 | 1.3 | 1.4 | 1.4 | 1.5 | 1.7 |
| B930003M22Rik | 0.1 | 0.1 | 0.1 | 0.0 | 0.2 | 0.1 | 0.1 | 0.1 | 0.1 | 0.1 | 0.0 | 0.1 |
| B930041F14Rik | 0.9 | 0.9 | 1.1 | 0.8 | 0.9 | 1.5 | 1.2 | 1.3 | 1.0 | 1.3 | 0.9 | 1.4 |
| B930059L03Rik | 0.4 | 0.4 | 0.6 | 0.6 | 0.5 | 0.5 | 0.4 | 0.7 | 0.2 | 0.4 | 0.2 | 0.5 |
| B9d1 | 8.3 | 8.9 | 8.8 | 7.7 | 7.8 | 10.6 | 5.8 | 9.2 | 7.4 | 5.8 | 9.6 | 9.5 |
| B9d2 | 1.4 | 1.9 | 1.5 | 1.1 | 1.8 | 1.4 | 1.8 | 1.6 | 1.3 | 1.8 | 1.6 | 1.6 |
| Baalc | 0.4 | 0.1 | 0.1 | 0.3 | 0.2 | 0.1 | 0.1 | 0.0 | 0.1 | 0.4 | 0.1 | 0.3 |
| Baat1 | 0.1 | 0.2 | 0.1 | 0.4 | 0.3 | 0.1 | 0.2 | 0.4 | 0.3 | 0.1 | 0.3 | 0.2 |
| Babam1 | 30.3 | 29.2 | 27.3 | 34.2 | 26.2 | 33.6 | 29.0 | 30.6 | 26.0 | 25.6 | 26.3 | 24.9 |
| Bace1 | 19.1 | 20.8 | 21.3 | 22.9 | 22.2 | 23.2 | 25.9 | 21.6 | 24.8 | 18.0 | 17.8 | 19.5 |
| Bace2 | 2.0 | 2.0 | 2.9 | 2.9 | 2.1 | 2.2 | 1.7 | 2.6 | 1.7 | 1.7 | 1.7 | 1.9 |
| Bach1 | 5.0 | 5.5 | 6.5 | 5.1 | 7.9 | 4.9 | 5.3 | 7.0 | 6.7 | 7.7 | 6.1 | 7.1 |
| Bach2 | 0.0 | 0.0 | 0.0 | 0.0 | 0.0 | 0.0 | 0.0 | 0.0 | 0.0 | 0.2 | 0.1 | 0.0 |
| Bad | 18.5 | 16.4 | 16.8 | 17.3 | 16.7 | 18.9 | 18.8 | 17.1 | 16.5 | 17.2 | 16.9 | 16.0 |
| Bag1 | 180.1 | 122.7 | 103.0 | 188.1 | 102.8 | 164.8 | 114.3 | 102.7 | 91.3 | 111.2 | 133.5 | 128.7 |
| Bag2 | 15.4 | 16.6 | 15.7 | 17.1 | 17.7 | 16.3 | 23.1 | 23.6 | 22.8 | 18.0 | 25.9 | 18.7 |
| Bag3 | 18.5 | 25.4 | 27.2 | 25.3 | 22.1 | 24.0 | 26.9 | 21.2 | 19.0 | 23.5 | 22.0 | 22.8 |
| Bag4 | 3.4 | 3.6 | 4.2 | 3.9 | 3.7 | 4.2 | 3.4 | 3.6 | 3.6 | 3.9 | 3.9 | 3.9 |
| Bag5 | 15.1 | 13.7 | 14.1 | 14.8 | 12.9 | 15.3 | 12.9 | 14.5 | 13.9 | 12.2 | 15.9 | 16.6 |
| Bag6 | 21.3 | 25.8 | 22.0 | 18.4 | 23.7 | 18.9 | 25.3 | 21.4 | 24.7 | 20.9 | 18.9 | 16.9 |
| Bahcc1 | 0.9 | 1.4 | 1.4 | 0.9 | 1.4 | 1.2 | 1.7 | 1.4 | 1.6 | 1.1 | 1.1 | 0.9 |
| Bahd1 | 3.5 | 4.7 | 3.9 | 3.5 | 3.6 | 3.6 | 3.8 | 3.5 | 4.1 | 3.7 | 3.5 | 3.1 |
| Bai1 | 0.1 | 0.0 | 0.0 | 0.1 | 0.0 | 0.0 | 0.1 | 0.0 | 0.0 | 0.0 | 0.0 | 0.0 |
| Bai2 | 0.3 | 0.3 | 0.2 | 0.2 | 0.2 | 0.3 | 0.2 | 0.2 | 0.3 | 0.6 | 0.4 | 0.3 |
| Bai3 | 0.0 | 0.0 | 0.0 | 0.0 | 0.0 | 0.0 | 0.0 | 0.0 | 0.0 | 0.0 | 0.0 | 0.0 |
| Baiap2 | 5.0 | 6.0 | 7.4 | 5.0 | 6.2 | 5.1 | 8.1 | 6.1 | 6.1 | 6.0 | 5.1 | 6.0 |
| Baiap2l1 | 0.8 | 0.9 | 1.3 | 0.7 | 0.5 | 0.5 | 1.3 | 1.0 | 1.3 | 2.4 | 1.7 | 1.5 |
| Baiap2l2 | 0.1 | 0.0 | 0.1 | 0.1 | 0.0 | 0.1 | 0.1 | 0.1 | 0.0 | 0.0 | 0.0 | 0.1 |
| Baiap3 | 0.1 | 0.1 | 0.2 | 0.3 | 0.1 | 0.2 | 0.1 | 0.1 | 0.1 | 0.1 | 0.1 | 0.2 |
| Bak1 | 17.7 | 20.7 | 20.4 | 22.0 | 22.6 | 17.2 | 19.7 | 24.1 | 19.6 | 16.6 | 21.1 | 18.1 |
| Bambi | 0.4 | 0.5 | 0.6 | 0.3 | 0.6 | 0.3 | 0.9 | 0.9 | 0.8 | 0.9 | 0.9 | 0.5 |
| Bambi-ps1 | 0.3 | 0.3 | 0.2 | 0.1 | 0.6 | 0.5 | 0.2 | 0.1 | 0.1 | 0.1 | 0.4 | 0.1 |
| Banf1 | 37.7 | 34.2 | 31.2 | 51.0 | 41.9 | 44.0 | 38.3 | 41.7 | 35.7 | 40.9 | 38.6 | 37.0 |
| Bank1 | 0.1 | 0.0 | 0.0 | 0.1 | 0.0 | 0.0 | 0.0 | 0.1 | 0.0 | 0.1 | 0.0 | 0.1 |
| Banp | 4.5 | 5.3 | 4.7 | 4.5 | 4.9 | 5.8 | 3.7 | 3.7 | 4.6 | 5.2 | 5.4 | 4.5 |
| Bap1 | 10.8 | 12.2 | 13.0 | 11.1 | 12.3 | 12.5 | 10.6 | 12.9 | 12.2 | 11.1 | 9.7 | 10.3 |
| Bard1 | 0.4 | 0.8 | 1.1 | 0.2 | 0.8 | 0.2 | 1.1 | 0.4 | 0.8 | 0.4 | 0.5 | 0.4 |

| | | | | | | | | | | | | |
|---|---|---|---|---|---|---|---|---|---|---|---|---|
| Barhl1 | 0.0 | 0.0 | 0.0 | 0.0 | 0.0 | 0.0 | 0.0 | 0.0 | 0.0 | 0.0 | 0.0 | 0.0 |
| Barx1 | 0.2 | 0.1 | 0.0 | 0.2 | 0.0 | 0.1 | 0.1 | 0.0 | 0.0 | 0.1 | 0.0 | 0.0 |
| Barx2 | 0.0 | 0.0 | 0.0 | 0.1 | 0.1 | 0.0 | 0.0 | 0.0 | 0.0 | 0.0 | 0.0 | 0.0 |
| Basp1 | 83.5 | 56.7 | 49.9 | 107.6 | 88.9 | 61.3 | 120.8 | 75.8 | 81.8 | 97.6 | 115.9 | 86.7 |
| Batf | 0.5 | 0.4 | 0.8 | 0.8 | 1.3 | 0.8 | 0.2 | 0.2 | 0.0 | 0.4 | 0.1 | 0.3 |
| Batf2 | 0.1 | 0.1 | 0.0 | 0.1 | 0.3 | 0.1 | 0.1 | 0.0 | 0.0 | 0.0 | 0.0 | 0.1 |
| Batf3 | 0.0 | 0.0 | 0.0 | 0.1 | 0.2 | 0.0 | 0.1 | 0.1 | 0.1 | 0.0 | 0.0 | 0.0 |
| Bax | 91.8 | 83.1 | 72.3 | 96.8 | 100.7 | 89.2 | 89.7 | 82.8 | 88.0 | 95.8 | 85.2 | 77.7 |
| Baz1a | 7.3 | 8.3 | 9.8 | 7.6 | 9.1 | 5.9 | 9.0 | 8.5 | 8.4 | 8.4 | 9.2 | 9.6 |
| Baz1b | 24.5 | 26.6 | 29.2 | 25.8 | 25.0 | 23.9 | 29.1 | 26.1 | 25.6 | 27.0 | 29.6 | 31.1 |
| Baz2a | 3.2 | 4.6 | 4.3 | 3.8 | 3.9 | 3.3 | 4.5 | 4.2 | 4.3 | 3.6 | 4.4 | 3.7 |
| Baz2b | 4.2 | 4.2 | 4.4 | 4.7 | 3.9 | 3.5 | 3.7 | 3.7 | 3.7 | 4.7 | 4.5 | 4.5 |
| BB287469 | 0.1 | 0.0 | 0.1 | 0.1 | 0.1 | 0.0 | 0.1 | 0.1 | 0.0 | 0.1 | 0.1 | 0.1 |
| Bbc3 | 4.3 | 4.3 | 4.0 | 4.1 | 4.3 | 4.6 | 4.2 | 4.6 | 5.3 | 5.7 | 4.3 | 4.1 |
| Bbip1 | 7.3 | 6.0 | 5.6 | 6.8 | 6.2 | 6.0 | 5.6 | 6.8 | 6.4 | 6.6 | 8.0 | 7.4 |
| Bbox1 | 0.2 | 0.1 | 0.2 | 0.1 | 0.0 | 0.0 | 0.0 | 0.1 | 0.1 | 0.0 | 0.1 | 0.0 |
| Bbs1 | 0.8 | 0.9 | 1.1 | 0.6 | 0.8 | 1.0 | 0.6 | 1.0 | 0.7 | 0.6 | 0.6 | 0.7 |
| Bbs10 | 1.4 | 1.7 | 1.4 | 1.5 | 1.3 | 1.2 | 1.0 | 1.7 | 1.6 | 1.3 | 1.4 | 1.8 |
| Bbs12 | 1.3 | 1.4 | 1.0 | 0.6 | 1.2 | 1.3 | 0.9 | 1.0 | 1.3 | 1.7 | 1.6 | 1.3 |
| Bbs2 | 3.7 | 3.6 | 3.4 | 3.3 | 2.9 | 5.5 | 2.8 | 3.5 | 2.6 | 3.1 | 2.5 | 2.9 |
| Bbs4 | 4.1 | 2.5 | 2.1 | 5.7 | 3.1 | 4.5 | 2.8 | 2.3 | 2.2 | 2.4 | 3.1 | 3.0 |
| Bbs5 | 8.0 | 7.0 | 6.2 | 7.6 | 6.0 | 9.2 | 4.9 | 5.2 | 5.6 | 8.0 | 7.8 | 7.2 |
| Bbs7 | 8.2 | 7.7 | 8.7 | 7.3 | 6.8 | 7.6 | 7.1 | 7.0 | 6.3 | 5.4 | 7.0 | 8.6 |
| Bbs9 | 2.6 | 2.6 | 2.7 | 2.1 | 1.9 | 2.9 | 1.9 | 1.6 | 1.8 | 1.8 | 1.8 | 2.2 |
| Bbx | 10.1 | 10.1 | 10.7 | 12.2 | 9.2 | 12.6 | 8.6 | 9.6 | 8.0 | 11.2 | 11.5 | 10.1 |
| BC002163 | 3.5 | 3.8 | 7.4 | 4.0 | 3.2 | 2.7 | 2.5 | 2.3 | 2.9 | 2.5 | 2.9 | 5.5 |
| BC003331 | 31.8 | 28.4 | 26.2 | 37.4 | 27.0 | 31.8 | 23.5 | 22.6 | 25.2 | 27.5 | 31.6 | 31.2 |
| BC003965 | 4.8 | 4.7 | 3.4 | 5.3 | 4.6 | 4.7 | 3.4 | 3.7 | 4.0 | 4.8 | 4.1 | 3.7 |
| BC004004 | 24.8 | 18.5 | 17.4 | 28.1 | 20.2 | 22.4 | 22.6 | 18.8 | 18.6 | 22.8 | 22.4 | 22.4 |
| BC005537 | 24.3 | 25.7 | 23.0 | 28.1 | 32.0 | 24.1 | 23.4 | 26.3 | 27.4 | 18.7 | 22.1 | 19.9 |
| BC005561 | 1.1 | 1.8 | 2.3 | 1.3 | 1.5 | 1.6 | 1.8 | 1.9 | 1.3 | 1.6 | 1.9 | 1.8 |
| BC005624 | 39.2 | 33.3 | 41.7 | 56.9 | 33.8 | 44.2 | 36.2 | 37.3 | 25.7 | 31.6 | 38.3 | 43.2 |
| BC005764 | 0.9 | 1.1 | 1.0 | 0.6 | 1.2 | 0.7 | 0.9 | 1.1 | 1.2 | 1.2 | 0.7 | 0.9 |
| BC006965 | 0.1 | 0.1 | 0.1 | 0.1 | 0.1 | 0.0 | 0.0 | 0.0 | 0.0 | 0.0 | 0.0 | 0.1 |
| BC016423 | 5.4 | 5.8 | 4.9 | 5.3 | 5.7 | 5.2 | 5.5 | 5.9 | 5.3 | 5.5 | 6.3 | 5.7 |
| BC016579 | 0.0 | 0.1 | 0.0 | 0.2 | 0.0 | 0.0 | 0.1 | 0.0 | 0.0 | 0.1 | 0.1 | 0.0 |
| BC017158 | 6.0 | 5.7 | 5.8 | 6.8 | 5.5 | 7.3 | 6.3 | 6.8 | 5.8 | 4.8 | 5.2 | 5.8 |
| BC017612 | 7.7 | 6.1 | 4.8 | 6.5 | 4.9 | 7.0 | 5.7 | 4.5 | 6.8 | 5.9 | 7.0 | 6.4 |
| BC017643 | 13.3 | 12.5 | 12.4 | 14.1 | 15.0 | 13.5 | 14.1 | 13.4 | 14.1 | 13.6 | 13.5 | 13.4 |
| BC018242 | 2.0 | 2.9 | 2.5 | 2.1 | 2.1 | 2.5 | 2.2 | 2.2 | 2.1 | 1.5 | 1.8 | 1.5 |
| BC018473 | 0.1 | 0.1 | 0.0 | 0.0 | 0.1 | 0.1 | 0.0 | 0.0 | 0.0 | 0.1 | 0.0 | 0.0 |
| BC018507 | 7.8 | 9.1 | 8.6 | 7.9 | 8.9 | 8.5 | 8.7 | 9.5 | 9.5 | 8.4 | 9.2 | 8.5 |
| BC020402 | 0.9 | 0.8 | 0.7 | 0.5 | 0.8 | 0.9 | 1.3 | 1.2 | 0.8 | 0.9 | 0.8 | 1.0 |
| BC021767 | 0.6 | 0.4 | 0.4 | 0.6 | 0.3 | 0.4 | 0.3 | 0.3 | 0.3 | 0.6 | 0.5 | 0.3 |
| BC021785 | 0.2 | 0.2 | 0.3 | 0.3 | 0.4 | 0.6 | 0.2 | 0.4 | 0.2 | 0.2 | 0.2 | 0.3 |
| BC021891 | 0.1 | 0.1 | 0.1 | 0.1 | 0.1 | 0.0 | 0.1 | 0.1 | 0.1 | 0.0 | 0.1 | 0.1 |
| BC022687 | 11.8 | 8.2 | 5.6 | 8.4 | 6.7 | 11.3 | 6.5 | 6.7 | 6.1 | 10.8 | 7.6 | 8.8 |
| BC023829 | 10.6 | 12.6 | 11.7 | 12.4 | 11.8 | 12.1 | 11.8 | 11.5 | 10.9 | 12.3 | 12.1 | 13.7 |
| BC024139 | 0.0 | 0.0 | 0.0 | 0.0 | 0.0 | 0.0 | 0.0 | 0.0 | 0.0 | 0.0 | 0.0 | 0.0 |
| BC024582 | 0.0 | 0.1 | 0.0 | 0.0 | 0.1 | 0.0 | 0.0 | 0.0 | 0.0 | 0.1 | 0.0 | 0.0 |
| BC024978 | 0.8 | 0.6 | 1.0 | 0.9 | 0.8 | 0.9 | 1.0 | 0.7 | 1.0 | 0.9 | 0.9 | 0.6 |
| BC025920 | 0.4 | 0.3 | 0.5 | 0.2 | 0.3 | 0.5 | 0.1 | 0.2 | 0.2 | 0.2 | 0.1 | 0.2 |
| BC026585 | 9.1 | 8.4 | 7.2 | 9.0 | 5.7 | 10.7 | 5.9 | 5.8 | 4.4 | 3.7 | 7.1 | 6.6 |
| BC026590 | 3.3 | 3.3 | 3.1 | 3.7 | 3.3 | 3.7 | 2.8 | 3.5 | 2.7 | 3.2 | 4.1 | 2.8 |
| BC027231 | 4.4 | 5.0 | 4.8 | 4.5 | 4.5 | 4.2 | 5.3 | 4.7 | 4.6 | 4.7 | 5.5 | 5.6 |
| BC028528 | 9.5 | 14.1 | 19.1 | 24.0 | 25.0 | 17.1 | 18.4 | 12.0 | 16.8 | 15.5 | 17.3 | 18.7 |
| BC029214 | 7.1 | 5.7 | 5.5 | 6.6 | 4.2 | 7.0 | 6.1 | 8.3 | 7.6 | 5.4 | 6.0 | 6.0 |

| | | | | | | | | | | | | |
|---|---|---|---|---|---|---|---|---|---|---|---|---|
| BC029722 | 28.3 | 19.5 | 18.4 | 27.1 | 24.8 | 25.4 | 22.8 | 20.3 | 20.5 | 31.0 | 28.5 | 26.5 |
| BC030307 | 0.6 | 0.5 | 0.3 | 0.3 | 0.5 | 0.4 | 0.5 | 0.5 | 0.4 | 0.5 | 0.4 | 0.4 |
| BC030336 | 3.9 | 4.3 | 5.0 | 4.5 | 4.2 | 4.7 | 3.7 | 4.0 | 4.5 | 4.7 | 4.9 | 5.2 |
| BC030476 | 0.0 | 0.1 | 0.0 | 0.0 | 0.0 | 0.0 | 0.0 | 0.0 | 0.0 | 0.0 | 0.0 | 0.0 |
| BC030867 | 0.2 | 0.8 | 0.5 | 0.0 | 0.8 | 0.1 | 0.8 | 0.4 | 0.7 | 0.4 | 0.4 | 0.2 |
| BC031181 | 100.6 | 82.5 | 72.4 | 114.8 | 83.6 | 94.5 | 85.4 | 76.7 | 74.2 | 89.9 | 93.0 | 95.7 |
| BC031361 | 0.5 | 0.4 | 0.3 | 0.4 | 0.4 | 0.5 | 0.3 | 0.5 | 0.4 | 0.7 | 0.5 | 0.5 |
| BC033916 | 0.1 | 0.1 | 0.1 | 0.0 | 0.0 | 0.0 | 0.0 | 0.0 | 0.0 | 0.0 | 0.0 | 0.1 |
| BC035044 | 0.0 | 0.0 | 0.0 | 0.1 | 0.1 | 0.0 | 0.0 | 0.0 | 0.0 | 0.0 | 0.0 | 0.0 |
| BC037032 | 0.1 | 0.1 | 0.1 | 0.1 | 0.0 | 0.1 | 0.1 | 0.1 | 0.1 | 0.2 | 0.0 | 0.2 |
| BC037034 | 3.5 | 3.2 | 2.6 | 4.5 | 4.7 | 2.9 | 3.9 | 2.8 | 3.8 | 5.4 | 2.7 | 2.9 |
| BC037704 | 0.1 | 0.0 | 0.2 | 0.1 | 0.2 | 0.0 | 0.0 | 0.1 | 0.2 | 0.3 | 0.2 | 0.3 |
| BC039771 | 0.1 | 0.3 | 0.5 | 0.1 | 0.1 | 0.1 | 0.1 | 0.1 | 0.2 | 0.1 | 0.2 | 0.2 |
| BC048403 | 1.3 | 1.7 | 1.1 | 1.4 | 1.5 | 1.2 | 1.6 | 1.6 | 1.3 | 1.1 | 1.6 | 1.2 |
| BC048644 | 0.0 | 0.0 | 0.0 | 0.0 | 0.0 | 0.0 | 0.0 | 0.1 | 0.0 | 0.0 | 0.0 | 0.0 |
| BC049715 | 0.4 | 0.1 | 0.1 | 0.3 | 0.5 | 0.2 | 0.0 | 0.0 | 0.0 | 0.2 | 0.0 | 0.0 |
| BC049762 | 0.6 | 0.1 | 0.4 | 0.1 | 0.5 | 0.3 | 0.3 | 0.4 | 0.2 | 0.5 | 0.3 | 0.5 |
| BC051019 | 0.0 | 0.1 | 0.1 | 0.0 | 0.1 | 0.0 | 0.0 | 0.1 | 0.1 | 0.0 | 0.1 | 0.0 |
| BC051142 | 0.6 | 0.5 | 0.5 | 0.8 | 0.4 | 0.6 | 0.3 | 0.5 | 0.2 | 0.7 | 1.0 | 0.5 |
| BC051226 | 2.5 | 2.9 | 2.9 | 2.3 | 1.7 | 4.2 | 2.2 | 2.3 | 2.6 | 1.7 | 3.1 | 1.3 |
| BC051628 | 0.0 | 0.1 | 0.0 | 0.0 | 0.0 | 0.1 | 0.1 | 0.0 | 0.0 | 0.0 | 0.0 | 0.0 |
| BC052040 | 0.5 | 0.7 | 0.8 | 0.4 | 0.6 | 0.7 | 0.8 | 0.9 | 0.8 | 0.5 | 0.6 | 0.7 |
| BC052688 | 0.3 | 0.6 | 0.8 | 0.3 | 0.5 | 0.1 | 0.1 | 0.6 | 0.6 | 0.4 | 0.3 | 0.2 |
| BC053749 | 0.2 | 0.5 | 0.6 | 0.3 | 0.3 | 0.2 | 0.3 | 0.3 | 0.4 | 0.2 | 0.2 | 0.2 |
| BC055111 | 0.0 | 0.0 | 0.0 | 0.1 | 0.0 | 0.0 | 0.0 | 0.0 | 0.1 | 0.0 | 0.1 | 0.0 |
| BC055324 | 1.1 | 1.7 | 2.0 | 0.7 | 1.6 | 1.0 | 1.7 | 1.6 | 1.8 | 1.5 | 1.6 | 1.1 |
| BC056474 | 37.7 | 30.2 | 23.7 | 43.9 | 29.1 | 36.8 | 31.3 | 30.7 | 26.1 | 27.3 | 29.1 | 27.0 |
| BC061194 | 0.1 | 0.0 | 0.1 | 0.0 | 0.0 | 0.2 | 0.0 | 0.0 | 0.1 | 0.0 | 0.0 | 0.0 |
| BC064078 | 0.0 | 0.1 | 0.0 | 0.3 | 0.1 | 0.2 | 0.1 | 0.1 | 0.0 | 0.0 | 0.0 | 0.1 |
| BC065397 | 0.2 | 0.2 | 0.3 | 0.2 | 0.3 | 0.1 | 0.2 | 0.3 | 0.3 | 0.1 | 0.2 | 0.2 |
| BC068157 | 0.1 | 0.1 | 0.1 | 0.1 | 0.1 | 0.1 | 0.1 | 0.1 | 0.2 | 0.1 | 0.1 | 0.1 |
| BC068281 | 2.3 | 2.3 | 2.6 | 1.8 | 1.9 | 2.1 | 2.4 | 2.5 | 1.9 | 2.2 | 2.1 | 2.5 |
| BC090627 | 0.4 | 0.0 | 0.0 | 0.0 | 0.2 | 0.2 | 0.1 | 0.2 | 0.1 | 0.2 | 0.0 | 0.2 |
| BC096441 | 1.9 | 1.1 | 1.1 | 1.8 | 2.9 | 1.0 | 1.2 | 1.5 | 1.7 | 1.8 | 1.3 | 2.2 |
| Bc1 | 2.3 | 0.0 | 1.2 | 0.0 | 1.3 | 0.1 | 0.8 | 0.6 | 1.8 | 1.2 | 0.4 | 0.0 |
| BC100451 | 2.5 | 1.8 | 1.6 | 2.6 | 1.8 | 2.2 | 2.4 | 1.9 | 1.5 | 2.7 | 2.7 | 2.6 |
| BC125332 | 0.0 | 0.0 | 0.0 | 0.0 | 0.0 | 0.0 | 0.0 | 0.0 | 0.0 | 0.0 | 0.0 | 0.0 |
| Bcam | 8.0 | 8.9 | 8.0 | 10.4 | 9.4 | 12.5 | 10.2 | 5.2 | 9.1 | 9.7 | 8.9 | 8.7 |
| Bcan | 0.1 | 0.1 | 0.0 | 0.3 | 0.1 | 0.1 | 0.0 | 0.0 | 0.0 | 0.1 | 0.0 | 0.0 |
| Bcap29 | 10.1 | 11.1 | 11.1 | 13.2 | 12.0 | 13.5 | 10.0 | 8.6 | 8.5 | 9.4 | 13.4 | 11.4 |
| Bcap31 | 155.5 | 137.2 | 123.1 | 166.3 | 141.5 | 167.8 | 143.4 | 138.0 | 131.1 | 148.1 | 166.3 | 155.5 |
| Bcar1 | 11.7 | 13.1 | 11.6 | 11.6 | 13.3 | 11.3 | 16.0 | 13.6 | 15.5 | 14.6 | 15.1 | 13.5 |
| Bcar3 | 0.4 | 0.1 | 0.2 | 0.4 | 1.0 | 0.2 | 0.4 | 0.4 | 0.5 | 1.5 | 1.0 | 0.7 |
| Bcas1 | 0.0 | 0.1 | 0.0 | 0.1 | 0.0 | 0.0 | 0.0 | 0.1 | 0.0 | 0.0 | 0.0 | 0.0 |
| Bcas2 | 22.6 | 19.0 | 21.0 | 25.3 | 19.0 | 23.2 | 18.4 | 21.1 | 17.1 | 19.9 | 18.2 | 19.3 |
| Bcas3 | 8.6 | 7.9 | 7.4 | 8.8 | 7.3 | 9.6 | 7.5 | 8.4 | 6.8 | 7.5 | 7.3 | 7.3 |
| Bcat1 | 3.1 | 2.3 | 2.6 | 3.7 | 3.1 | 3.5 | 7.2 | 10.5 | 7.3 | 4.1 | 7.4 | 4.2 |
| Bcat2 | 7.2 | 7.3 | 7.0 | 7.5 | 8.7 | 9.6 | 6.6 | 7.5 | 7.8 | 7.4 | 6.2 | 6.4 |
| Bccip | 32.6 | 22.3 | 24.9 | 37.9 | 28.5 | 31.9 | 22.9 | 24.4 | 20.9 | 24.5 | 26.9 | 27.5 |
| Bcdin3d | 1.8 | 1.8 | 2.0 | 2.7 | 1.5 | 1.7 | 2.0 | 1.9 | 1.6 | 1.9 | 1.8 | 2.2 |
| Bche | 0.1 | 0.0 | 0.0 | 0.1 | 0.0 | 0.1 | 0.1 | 0.1 | 0.1 | 0.4 | 0.2 | 0.2 |
| Bckdha | 17.9 | 17.0 | 15.0 | 19.2 | 15.4 | 19.6 | 15.5 | 18.5 | 16.5 | 22.6 | 16.9 | 19.3 |
| Bckdhb | 3.9 | 4.6 | 5.1 | 3.6 | 3.8 | 4.6 | 4.3 | 4.5 | 4.0 | 4.8 | 4.0 | 4.3 |
| Bckdk | 12.3 | 12.7 | 13.4 | 12.2 | 10.4 | 12.6 | 12.0 | 13.0 | 12.0 | 11.2 | 11.7 | 11.5 |
| Bcl10 | 28.1 | 23.2 | 25.6 | 22.7 | 26.2 | 18.4 | 27.8 | 24.9 | 26.1 | 33.0 | 30.3 | 28.5 |
| Bcl11b | 0.0 | 0.0 | 0.0 | 0.0 | 0.0 | 0.0 | 0.0 | 0.0 | 0.0 | 0.0 | 0.0 | 0.0 |
| Bcl2 | 2.3 | 2.3 | 2.3 | 3.8 | 4.2 | 3.4 | 1.5 | 1.3 | 1.7 | 2.4 | 1.5 | 1.5 |

| | | | | | | | | | | | | |
|---|---|---|---|---|---|---|---|---|---|---|---|---|
| Bcl2a1a | 0.5 | 0.0 | 0.0 | 2.4 | 4.5 | 0.0 | 1.0 | 0.1 | 0.3 | 0.2 | 0.3 | 0.4 |
| Bcl2a1b | 1.8 | 0.1 | 0.1 | 7.9 | 15.0 | 0.2 | 1.8 | 0.2 | 0.3 | 0.8 | 0.4 | 1.5 |
| Bcl2a1d | 0.8 | 0.1 | 0.0 | 2.9 | 8.0 | 0.1 | 1.6 | 0.2 | 0.4 | 0.5 | 0.4 | 0.6 |
| Bcl2l1 | 38.4 | 41.7 | 32.9 | 42.8 | 44.4 | 41.3 | 38.4 | 30.0 | 35.3 | 39.6 | 38.8 | 33.8 |
| Bcl2l10 | 0.0 | 0.1 | 0.0 | 0.0 | 0.0 | 0.1 | 0.0 | 0.0 | 0.0 | 0.0 | 0.0 | 0.0 |
| Bcl2l11 | 0.7 | 1.1 | 1.7 | 0.7 | 1.8 | 0.9 | 1.1 | 1.4 | 1.5 | 1.9 | 1.1 | 1.3 |
| Bcl2l12 | 3.7 | 3.6 | 3.9 | 3.2 | 3.8 | 3.5 | 4.5 | 4.2 | 4.4 | 3.7 | 3.4 | 2.7 |
| Bcl2l13 | 4.1 | 3.9 | 3.6 | 3.7 | 3.5 | 3.5 | 3.5 | 3.5 | 3.2 | 3.4 | 3.3 | 3.0 |
| Bcl2l14 | 0.0 | 0.0 | 0.1 | 0.0 | 0.1 | 0.0 | 0.0 | 0.0 | 0.0 | 0.1 | 0.0 | 0.1 |
| Bcl2l15 | 0.2 | 0.0 | 0.4 | 0.2 | 0.0 | 0.1 | 0.1 | 0.1 | 0.1 | 0.1 | 0.1 | 0.1 |
| Bcl2l2 | 32.1 | 29.1 | 24.6 | 40.0 | 30.5 | 35.0 | 29.2 | 21.2 | 29.2 | 27.6 | 30.5 | 28.0 |
| Bcl3 | 2.5 | 3.4 | 4.8 | 1.8 | 5.2 | 1.6 | 4.0 | 6.5 | 4.7 | 3.8 | 3.5 | 3.4 |
| Bcl6 | 6.3 | 7.1 | 8.7 | 9.0 | 8.9 | 8.5 | 6.8 | 6.9 | 6.1 | 6.1 | 5.9 | 5.6 |
| Bcl6b | 0.5 | 0.2 | 0.3 | 0.1 | 0.2 | 0.0 | 0.3 | 0.7 | 0.3 | 0.1 | 0.1 | 0.1 |
| Bcl7a | 0.7 | 0.8 | 0.9 | 1.1 | 0.6 | 1.2 | 0.8 | 0.8 | 0.7 | 0.9 | 1.0 | 0.9 |
| Bcl7b | 16.3 | 11.9 | 11.2 | 18.7 | 12.2 | 14.9 | 15.7 | 12.1 | 11.8 | 12.3 | 14.2 | 13.3 |
| Bcl7c | 8.8 | 6.9 | 8.2 | 8.4 | 6.6 | 6.7 | 7.7 | 7.6 | 8.5 | 8.9 | 7.3 | 8.3 |
| Bcl9 | 0.7 | 0.9 | 1.0 | 0.7 | 0.7 | 0.8 | 0.7 | 1.0 | 0.8 | 0.8 | 0.6 | 0.7 |
| Bcl9l | 2.5 | 4.7 | 4.5 | 2.9 | 3.2 | 2.9 | 4.3 | 4.5 | 4.3 | 3.1 | 2.7 | 2.8 |
| Bclaf1 | 20.0 | 17.6 | 23.9 | 22.4 | 19.6 | 16.7 | 26.5 | 25.0 | 21.1 | 22.7 | 28.0 | 27.8 |
| Bcmo1 | 0.3 | 0.2 | 0.1 | 0.2 | 0.1 | 0.3 | 0.2 | 0.2 | 0.1 | 0.1 | 0.1 | 0.1 |
| Bco2 | 0.3 | 0.2 | 0.1 | 0.4 | 0.3 | 0.4 | 0.3 | 0.1 | 0.1 | 0.2 | 0.1 | 0.1 |
| Bcor | 4.2 | 4.6 | 4.9 | 3.7 | 4.2 | 3.8 | 5.1 | 4.1 | 4.3 | 3.7 | 4.0 | 4.0 |
| Bcorl1 | 0.7 | 1.0 | 1.1 | 0.7 | 0.8 | 0.7 | 1.2 | 1.0 | 1.0 | 0.7 | 1.0 | 0.7 |
| Bcr | 9.7 | 11.0 | 11.8 | 7.1 | 8.1 | 9.1 | 10.0 | 10.7 | 8.5 | 10.4 | 9.4 | 9.0 |
| Bcs1l | 6.6 | 6.9 | 7.0 | 6.1 | 4.9 | 6.1 | 5.9 | 6.8 | 6.0 | 7.0 | 7.2 | 6.8 |
| Bdh1 | 0.5 | 0.5 | 0.5 | 0.6 | 0.8 | 0.4 | 0.8 | 1.3 | 1.1 | 0.6 | 1.1 | 0.5 |
| Bdh2 | 7.4 | 6.4 | 5.7 | 4.6 | 3.3 | 7.7 | 2.3 | 6.4 | 3.4 | 3.1 | 3.3 | 3.3 |
| Bdkrb1 | 0.2 | 0.2 | 0.2 | 0.0 | 0.1 | 0.0 | 0.2 | 0.1 | 0.1 | 0.2 | 0.2 | 0.2 |
| Bdnf | 31.0 | 25.7 | 22.4 | 35.1 | 25.8 | 26.4 | 30.6 | 16.5 | 24.2 | 17.7 | 26.2 | 27.8 |
| Bdp1 | 6.6 | 7.1 | 7.6 | 6.9 | 6.7 | 5.7 | 6.6 | 5.5 | 5.8 | 6.1 | 7.4 | 7.3 |
| Bean1 | 1.0 | 1.0 | 0.9 | 1.8 | 0.7 | 2.0 | 0.6 | 0.6 | 0.6 | 0.4 | 0.5 | 0.5 |
| Becn1 | 88.6 | 68.6 | 61.6 | 111.6 | 72.3 | 94.2 | 73.4 | 57.0 | 59.6 | 63.4 | 70.9 | 74.9 |
| Begain | 0.4 | 0.3 | 0.3 | 0.2 | 0.3 | 0.3 | 0.2 | 0.2 | 0.3 | 0.2 | 0.2 | 0.3 |
| Bend3 | 1.0 | 1.3 | 1.1 | 1.3 | 1.1 | 1.1 | 1.7 | 1.6 | 1.5 | 1.1 | 1.3 | 1.2 |
| Bend4 | 1.1 | 1.1 | 0.6 | 1.0 | 1.0 | 1.0 | 0.8 | 0.4 | 0.7 | 1.2 | 1.1 | 0.8 |
| Bend6 | 3.5 | 3.4 | 2.7 | 4.8 | 3.3 | 4.5 | 3.0 | 3.3 | 2.1 | 2.1 | 2.7 | 1.9 |
| Bend7 | 0.2 | 0.1 | 0.0 | 0.1 | 0.1 | 0.2 | 0.1 | 0.0 | 0.0 | 0.2 | 0.0 | 0.1 |
| Best1 | 0.4 | 0.6 | 0.8 | 0.3 | 0.9 | 0.5 | 0.5 | 0.4 | 0.2 | 0.5 | 0.4 | 0.4 |
| Best3 | 0.4 | 0.2 | 0.1 | 0.2 | 0.1 | 0.3 | 0.2 | 0.1 | 0.0 | 0.2 | 0.0 | 0.1 |
| Bet1 | 16.1 | 18.4 | 19.5 | 20.3 | 18.5 | 21.7 | 14.8 | 18.4 | 18.3 | 19.2 | 21.9 | 19.9 |
| Bet1l | 30.8 | 25.0 | 20.1 | 24.4 | 24.4 | 23.6 | 23.2 | 22.1 | 25.6 | 26.3 | 24.6 | 25.2 |
| Bet3l | 0.0 | 0.0 | 0.0 | 0.0 | 0.0 | 0.0 | 0.0 | 0.1 | 0.0 | 0.0 | 0.2 | 0.0 |
| Bex1 | 0.1 | 0.0 | 0.0 | 0.1 | 0.0 | 0.1 | 0.0 | 0.0 | 0.0 | 0.1 | 0.0 | 0.0 |
| Bex2 | 0.5 | 0.7 | 0.7 | 0.3 | 0.4 | 0.5 | 0.6 | 0.5 | 0.6 | 0.7 | 0.6 | 0.3 |
| Bex4 | 0.1 | 0.2 | 0.0 | 0.5 | 0.3 | 0.3 | 0.0 | 0.1 | 0.1 | 0.3 | 0.3 | 0.3 |
| Bfar | 19.2 | 17.8 | 19.9 | 19.1 | 18.4 | 16.6 | 20.0 | 20.6 | 19.3 | 19.7 | 20.4 | 22.0 |
| Bfsp1 | 0.5 | 0.3 | 0.4 | 0.3 | 0.3 | 0.3 | 0.4 | 0.4 | 0.2 | 0.3 | 0.5 | 0.6 |
| Bfsp2 | 0.1 | 0.1 | 0.0 | 0.0 | 0.0 | 0.0 | 0.0 | 0.0 | 0.0 | 0.1 | 0.0 | 0.1 |
| Bglap | 0.2 | 0.3 | 0.4 | 0.8 | 0.3 | 0.2 | 0.1 | 0.1 | 0.0 | 0.4 | 0.0 | 0.2 |
| Bglap2 | 0.2 | 0.5 | 0.3 | 0.4 | 0.5 | 0.2 | 0.1 | 0.1 | 0.0 | 0.3 | 0.0 | 0.1 |
| Bgn | 858.2 | 950.3 | 1109.6 | 1106.3 | 1092.3 | 1188.2 | 1202.8 | 985.2 | 852.7 | 728.8 | 782.9 | 773.0 |
| Bhlha15 | 0.0 | 0.0 | 0.1 | 0.1 | 0.0 | 0.1 | 0.0 | 0.0 | 0.0 | 0.0 | 0.1 | 0.0 |
| Bhlhb9 | 7.9 | 10.8 | 11.5 | 7.1 | 7.7 | 10.8 | 7.8 | 9.9 | 9.4 | 11.0 | 9.4 | 9.1 |
| Bhlhe22 | 0.0 | 0.0 | 0.1 | 0.0 | 0.0 | 0.0 | 0.1 | 0.0 | 0.1 | 0.2 | 0.0 | 0.2 |
| Bhlhe40 | 16.1 | 20.1 | 20.5 | 12.2 | 21.1 | 16.9 | 18.9 | 18.6 | 17.2 | 15.7 | 16.7 | 17.4 |
| Bhlhe41 | 1.9 | 1.8 | 1.8 | 2.8 | 2.0 | 2.4 | 1.0 | 1.5 | 0.9 | 1.8 | 1.3 | 1.3 |

| | | | | | | | | | | | | |
|---|---|---|---|---|---|---|---|---|---|---|---|---|
| Bicc1 | 51.3 | 83.7 | 126.7 | 43.7 | 59.6 | 39.5 | 73.2 | 115.0 | 75.8 | 63.1 | 67.2 | 95.8 |
| Bicd1 | 1.9 | 1.6 | 1.2 | 1.7 | 1.2 | 2.3 | 1.4 | 1.5 | 1.2 | 1.9 | 2.2 | 1.5 |
| Bicd2 | 48.3 | 52.4 | 43.8 | 55.1 | 41.9 | 58.3 | 55.5 | 53.4 | 48.8 | 33.5 | 51.4 | 39.7 |
| Bid | 24.7 | 17.4 | 13.2 | 36.8 | 27.1 | 26.4 | 25.8 | 19.2 | 18.4 | 20.3 | 25.9 | 21.1 |
| Bik | 0.1 | 0.0 | 0.1 | 0.2 | 0.5 | 0.0 | 0.3 | 0.1 | 0.1 | 0.0 | 0.0 | 0.1 |
| Bin1 | 29.2 | 33.0 | 31.3 | 35.3 | 30.2 | 32.9 | 27.4 | 34.2 | 23.8 | 27.4 | 29.3 | 22.2 |
| Bin2 | 0.5 | 0.0 | 0.0 | 3.5 | 6.9 | 0.0 | 0.9 | 0.1 | 0.2 | 0.5 | 0.1 | 0.2 |
| Bin3 | 14.6 | 14.9 | 14.1 | 16.0 | 17.0 | 14.9 | 16.2 | 17.4 | 13.6 | 13.6 | 17.3 | 15.5 |
| Birc2 | 17.7 | 18.0 | 21.9 | 15.0 | 22.6 | 14.7 | 13.3 | 13.5 | 15.5 | 12.3 | 12.1 | 17.8 |
| Birc3 | 7.8 | 6.8 | 7.3 | 9.6 | 13.3 | 6.4 | 9.0 | 6.3 | 6.0 | 4.9 | 5.7 | 6.9 |
| Birc5 | 3.5 | 7.8 | 12.7 | 2.0 | 12.6 | 1.5 | 15.1 | 5.6 | 11.5 | 5.2 | 9.4 | 7.7 |
| Birc6 | 12.7 | 13.6 | 12.1 | 12.7 | 14.3 | 11.4 | 13.5 | 13.8 | 11.9 | 12.4 | 13.5 | 11.6 |
| Birc7 | 0.0 | 0.1 | 0.0 | 0.0 | 0.0 | 0.2 | 0.0 | 0.0 | 0.0 | 0.0 | 0.1 | 0.0 |
| Bivm | 5.7 | 5.8 | 6.0 | 6.7 | 4.3 | 6.7 | 4.6 | 6.1 | 5.5 | 5.2 | 5.6 | 6.0 |
| Blcap | 15.7 | 15.9 | 14.7 | 19.3 | 18.4 | 16.8 | 15.1 | 12.9 | 15.5 | 15.8 | 15.9 | 14.1 |
| Blk | 0.0 | 0.0 | 0.0 | 0.0 | 0.0 | 0.0 | 0.0 | 0.0 | 0.0 | 0.0 | 0.0 | 0.0 |
| Blm | 0.8 | 1.1 | 1.7 | 0.5 | 1.4 | 0.6 | 1.4 | 1.0 | 1.2 | 0.6 | 1.1 | 0.8 |
| Blmh | 14.7 | 16.6 | 19.2 | 15.7 | 15.1 | 15.8 | 13.0 | 17.5 | 17.5 | 15.5 | 14.7 | 16.8 |
| Blnk | 0.4 | 0.0 | 0.0 | 2.6 | 6.1 | 0.0 | 0.8 | 0.1 | 0.1 | 0.3 | 0.1 | 0.2 |
| Bloc1s1 | 75.8 | 65.3 | 66.2 | 72.0 | 62.9 | 78.4 | 69.8 | 87.5 | 73.8 | 77.6 | 78.0 | 75.6 |
| Bloc1s2a | 27.4 | 26.7 | 23.3 | 24.5 | 27.4 | 25.2 | 22.9 | 21.2 | 23.7 | 30.0 | 24.7 | 21.7 |
| Bloc1s3 | 5.5 | 5.2 | 5.1 | 6.3 | 5.6 | 6.2 | 5.6 | 5.4 | 5.6 | 5.7 | 4.9 | 5.2 |
| Bloc1s4 | 9.0 | 7.6 | 7.4 | 8.8 | 8.9 | 8.4 | 8.1 | 7.3 | 9.3 | 9.5 | 8.3 | 9.5 |
| Bloc1s5 | 10.8 | 10.4 | 9.1 | 10.2 | 9.2 | 9.5 | 8.2 | 9.7 | 8.2 | 9.0 | 8.8 | 9.9 |
| Bloc1s6 | 6.8 | 6.9 | 5.9 | 7.2 | 6.8 | 6.8 | 5.1 | 5.7 | 5.7 | 5.9 | 6.1 | 5.6 |
| Blvra | 13.8 | 13.6 | 14.7 | 14.5 | 18.3 | 14.3 | 12.7 | 14.9 | 11.7 | 12.3 | 12.7 | 14.7 |
| Blvrb | 55.2 | 58.1 | 37.3 | 134.3 | 95.2 | 115.1 | 28.8 | 39.4 | 17.6 | 22.5 | 21.9 | 20.4 |
| Blzf1 | 8.0 | 7.9 | 8.1 | 9.4 | 7.4 | 7.7 | 7.6 | 7.4 | 7.9 | 7.2 | 9.7 | 9.4 |
| Bmf | 0.4 | 0.7 | 0.7 | 0.4 | 0.5 | 0.4 | 0.4 | 1.1 | 0.7 | 0.6 | 0.5 | 0.4 |
| Bmi1 | 13.8 | 14.0 | 16.0 | 17.7 | 13.4 | 18.6 | 12.7 | 12.2 | 13.0 | 13.9 | 17.9 | 18.1 |
| Bmp1 | 65.2 | 59.2 | 62.0 | 49.0 | 76.8 | 52.2 | 82.4 | 74.9 | 90.7 | 84.6 | 73.8 | 80.3 |
| Bmp15 | 0.1 | 0.0 | 0.0 | 0.0 | 0.0 | 0.0 | 0.0 | 0.0 | 0.0 | 0.0 | 0.0 | 0.0 |
| Bmp2 | 0.1 | 0.1 | 0.1 | 0.1 | 0.1 | 0.0 | 0.0 | 0.1 | 0.1 | 0.0 | 0.0 | 0.0 |
| Bmp2k | 6.5 | 8.0 | 8.0 | 6.7 | 8.3 | 5.4 | 7.2 | 6.1 | 7.3 | 12.5 | 8.6 | 9.2 |
| Bmp3 | 0.3 | 0.0 | 0.2 | 0.2 | 0.0 | 0.0 | 0.0 | 0.0 | 0.3 | 0.1 | 0.1 | 0.0 |
| Bmp4 | 56.4 | 51.5 | 51.0 | 36.8 | 29.9 | 25.4 | 43.0 | 45.1 | 50.9 | 33.5 | 49.3 | 49.9 |
| Bmp5 | 0.0 | 0.0 | 0.0 | 0.0 | 0.0 | 0.0 | 0.0 | 0.0 | 0.0 | 0.0 | 0.0 | 0.0 |
| Bmp6 | 40.3 | 52.2 | 42.2 | 45.7 | 40.9 | 47.1 | 48.6 | 48.6 | 44.0 | 33.2 | 35.6 | 30.3 |
| Bmp8a | 0.6 | 0.4 | 0.2 | 0.5 | 0.5 | 0.6 | 0.5 | 0.2 | 0.3 | 0.4 | 0.4 | 0.3 |
| Bmp8b | 0.1 | 0.0 | 0.1 | 0.1 | 0.1 | 0.1 | 0.1 | 0.0 | 0.0 | 0.1 | 0.0 | 0.0 |
| Bmper | 12.5 | 14.6 | 15.1 | 10.5 | 13.7 | 9.0 | 25.5 | 28.7 | 36.1 | 43.7 | 32.4 | 42.0 |
| Bmpr1a | 29.4 | 34.0 | 39.5 | 28.3 | 27.9 | 29.6 | 31.4 | 35.9 | 35.3 | 42.8 | 42.0 | 46.1 |
| Bmpr1b | 0.4 | 0.2 | 0.2 | 0.5 | 0.3 | 0.3 | 0.3 | 0.2 | 0.1 | 0.6 | 0.6 | 0.4 |
| Bmpr2 | 14.1 | 16.4 | 18.7 | 15.0 | 13.8 | 15.2 | 16.6 | 14.5 | 12.4 | 13.5 | 14.6 | 15.5 |
| Bms1 | 15.3 | 14.3 | 16.6 | 19.0 | 17.0 | 15.9 | 16.3 | 15.3 | 14.0 | 13.7 | 17.0 | 16.1 |
| Bmx | 5.9 | 10.3 | 24.6 | 1.0 | 3.4 | 0.8 | 8.0 | 19.4 | 7.8 | 6.5 | 5.7 | 10.9 |
| Bmyc | 2.7 | 2.8 | 3.7 | 4.0 | 3.7 | 3.8 | 4.0 | 4.7 | 4.9 | 3.9 | 2.9 | 4.3 |
| Bnc1 | 14.3 | 6.2 | 3.6 | 6.1 | 1.9 | 5.6 | 1.5 | 1.8 | 2.3 | 19.9 | 11.1 | 7.0 |
| Bnc2 | 2.2 | 3.8 | 6.5 | 2.3 | 3.2 | 2.3 | 4.6 | 5.2 | 4.9 | 4.1 | 3.9 | 5.2 |
| Bnip1 | 11.4 | 10.9 | 10.1 | 9.9 | 11.2 | 10.8 | 11.9 | 9.8 | 10.4 | 9.9 | 10.9 | 10.3 |
| Bnip2 | 39.9 | 35.8 | 32.2 | 49.8 | 35.0 | 41.9 | 36.6 | 29.4 | 32.1 | 34.9 | 39.1 | 37.5 |
| Bnip3 | 23.0 | 25.5 | 25.0 | 24.3 | 22.8 | 31.3 | 24.1 | 17.1 | 26.1 | 22.0 | 23.6 | 31.4 |
| Bnip3l | 30.0 | 34.6 | 33.3 | 37.8 | 32.6 | 38.9 | 30.8 | 33.3 | 31.4 | 41.2 | 36.2 | 39.3 |
| Bnipl | 0.1 | 0.0 | 0.0 | 0.0 | 0.1 | 0.1 | 0.2 | 0.1 | 0.1 | 0.1 | 0.0 | 0.1 |
| Boc | 5.9 | 7.1 | 7.9 | 4.8 | 4.9 | 4.7 | 8.8 | 8.5 | 8.6 | 6.4 | 6.3 | 7.4 |
| Bod1 | 39.3 | 36.3 | 26.2 | 43.5 | 26.6 | 47.2 | 28.9 | 28.0 | 29.3 | 28.5 | 29.8 | 27.7 |
| Bod1l | 23.5 | 24.0 | 21.6 | 24.4 | 19.7 | 19.2 | 23.8 | 18.2 | 18.3 | 18.4 | 25.9 | 22.8 |

| | | | | | | | | | | | | |
|---|---|---|---|---|---|---|---|---|---|---|---|---|
| Bok | 4.3 | 4.3 | 3.9 | 4.6 | 8.8 | 4.5 | 7.2 | 5.2 | 7.6 | 7.1 | 5.2 | 5.7 |
| Bola1 | 9.9 | 9.6 | 9.2 | 8.2 | 9.4 | 9.2 | 11.2 | 9.8 | 10.7 | 9.5 | 10.5 | 9.2 |
| Bola2 | 109.9 | 78.9 | 66.8 | 103.3 | 86.2 | 119.1 | 91.8 | 84.1 | 85.8 | 86.3 | 81.6 | 78.8 |
| Bola3 | 64.4 | 46.7 | 50.5 | 88.5 | 51.3 | 74.9 | 56.4 | 46.1 | 40.2 | 47.4 | 55.1 | 53.9 |
| Bop1 | 19.8 | 17.4 | 14.6 | 19.6 | 19.8 | 16.6 | 19.9 | 17.1 | 17.3 | 18.1 | 18.0 | 15.7 |
| Bora | 0.7 | 1.1 | 1.2 | 0.4 | 1.3 | 0.4 | 1.2 | 1.0 | 1.4 | 0.9 | 1.1 | 0.7 |
| Bpgm | 14.4 | 12.2 | 9.8 | 17.3 | 10.7 | 16.9 | 10.9 | 9.0 | 7.5 | 8.3 | 10.3 | 9.7 |
| Bphl | 25.4 | 24.0 | 19.9 | 26.8 | 21.0 | 26.5 | 18.7 | 20.2 | 20.6 | 22.4 | 20.9 | 20.3 |
| Bpnt1 | 24.0 | 22.9 | 20.1 | 25.2 | 21.4 | 24.6 | 22.0 | 16.3 | 18.8 | 22.9 | 25.2 | 21.4 |
| Bptf | 7.6 | 7.7 | 8.1 | 8.5 | 7.5 | 6.9 | 8.5 | 7.5 | 7.1 | 8.4 | 9.2 | 8.7 |
| Braf | 5.6 | 7.5 | 7.1 | 5.8 | 4.8 | 5.6 | 5.6 | 5.0 | 5.7 | 5.6 | 6.2 | 6.1 |
| Brap | 16.0 | 14.6 | 15.8 | 18.1 | 15.7 | 16.4 | 16.2 | 15.4 | 14.7 | 15.8 | 16.8 | 16.8 |
| Brat1 | 5.1 | 5.0 | 4.5 | 3.9 | 5.1 | 5.0 | 4.7 | 4.4 | 5.3 | 4.8 | 3.7 | 4.2 |
| Brca1 | 1.3 | 2.4 | 3.6 | 0.8 | 3.1 | 0.9 | 3.2 | 1.9 | 2.6 | 1.4 | 2.0 | 1.8 |
| Brca2 | 0.6 | 1.0 | 1.3 | 0.3 | 0.7 | 0.5 | 1.0 | 0.8 | 0.7 | 0.5 | 0.8 | 0.5 |
| Brcc3 | 10.3 | 9.5 | 8.2 | 9.4 | 8.2 | 9.8 | 6.1 | 8.3 | 8.0 | 7.6 | 9.4 | 8.8 |
| Brd1 | 9.7 | 11.5 | 11.3 | 9.2 | 10.0 | 11.3 | 10.7 | 10.3 | 10.9 | 9.4 | 11.4 | 10.2 |
| Brd2 | 29.1 | 27.7 | 26.3 | 33.1 | 30.1 | 31.3 | 35.8 | 32.3 | 30.8 | 37.3 | 35.2 | 34.1 |
| Brd3 | 8.2 | 10.5 | 10.7 | 8.8 | 8.8 | 8.9 | 11.6 | 10.4 | 9.2 | 7.9 | 9.2 | 9.7 |
| Brd4 | 1.5 | 1.6 | 2.5 | 1.1 | 2.1 | 1.5 | 3.3 | 2.8 | 2.4 | 2.1 | 2.1 | 2.0 |
| Brd7 | 15.2 | 16.8 | 21.4 | 17.2 | 18.2 | 15.1 | 17.4 | 21.2 | 16.3 | 17.2 | 21.1 | 22.7 |
| Brd8 | 7.7 | 7.7 | 9.0 | 7.9 | 7.5 | 7.0 | 9.1 | 9.4 | 9.1 | 7.5 | 9.1 | 8.8 |
| Brd9 | 12.9 | 13.1 | 16.7 | 15.0 | 12.3 | 14.7 | 17.4 | 15.5 | 13.8 | 11.9 | 15.6 | 15.9 |
| Brdt | 0.2 | 0.3 | 0.4 | 0.3 | 0.3 | 0.3 | 0.3 | 0.2 | 0.2 | 0.3 | 0.2 | 0.3 |
| Bre | 33.8 | 30.8 | 25.6 | 34.8 | 27.5 | 34.6 | 27.9 | 31.3 | 25.7 | 25.4 | 31.3 | 28.3 |
| Brf1 | 9.5 | 10.1 | 9.0 | 9.2 | 9.4 | 10.4 | 10.3 | 10.0 | 9.5 | 8.4 | 8.8 | 9.7 |
| Brf2 | 5.2 | 4.6 | 4.8 | 5.0 | 3.9 | 5.0 | 4.6 | 4.6 | 4.7 | 4.2 | 3.8 | 4.2 |
| Bri3 | 22.8 | 20.7 | 21.5 | 28.7 | 20.0 | 27.3 | 19.1 | 19.8 | 21.5 | 27.1 | 19.6 | 24.2 |
| Bri3bp | 4.0 | 4.2 | 4.2 | 4.1 | 3.5 | 5.1 | 4.0 | 3.4 | 3.4 | 3.8 | 3.2 | 3.6 |
| Bricd5 | 0.8 | 0.8 | 0.6 | 1.3 | 0.9 | 1.0 | 1.5 | 0.5 | 0.8 | 0.7 | 1.4 | 0.5 |
| Brip1 | 0.6 | 1.1 | 1.8 | 0.4 | 1.6 | 0.4 | 1.8 | 0.8 | 1.3 | 0.9 | 1.2 | 1.1 |
| Brix1 | 9.9 | 11.6 | 11.5 | 10.7 | 10.4 | 10.1 | 10.6 | 10.9 | 8.4 | 12.0 | 11.9 | 12.3 |
| Brk1 | 82.2 | 76.6 | 67.4 | 83.5 | 78.6 | 85.1 | 75.9 | 77.5 | 81.4 | 92.9 | 82.2 | 76.7 |
| Brms1 | 7.2 | 6.6 | 7.5 | 6.7 | 9.1 | 5.9 | 6.5 | 8.4 | 7.2 | 7.3 | 7.3 | 7.5 |
| Brms1l | 13.1 | 12.8 | 15.2 | 12.1 | 11.0 | 11.5 | 12.8 | 13.9 | 13.0 | 11.0 | 12.3 | 12.4 |
| Brox | 31.5 | 29.1 | 26.2 | 35.1 | 32.7 | 31.3 | 29.2 | 24.5 | 28.3 | 29.5 | 30.9 | 30.0 |
| Brpf1 | 3.7 | 3.9 | 4.7 | 4.0 | 5.1 | 3.5 | 5.6 | 4.8 | 4.6 | 3.6 | 4.0 | 4.2 |
| Brpf3 | 1.5 | 1.8 | 2.2 | 1.1 | 1.8 | 1.7 | 2.2 | 2.2 | 2.0 | 1.6 | 1.6 | 1.6 |
| Brsk1 | 2.2 | 3.7 | 3.2 | 2.4 | 3.1 | 2.5 | 4.2 | 1.9 | 3.9 | 2.9 | 3.2 | 3.5 |
| Brsk2 | 0.1 | 0.0 | 0.0 | 0.0 | 0.1 | 0.1 | 0.1 | 0.1 | 0.0 | 0.0 | 0.0 | 0.0 |
| Brwd1 | 19.0 | 17.8 | 15.2 | 16.6 | 15.9 | 17.4 | 13.7 | 14.4 | 15.1 | 18.7 | 16.4 | 15.4 |
| Brwd3 | 2.0 | 2.4 | 2.6 | 1.8 | 2.3 | 1.5 | 2.1 | 2.3 | 2.1 | 2.0 | 2.8 | 2.7 |
| Bscl2 | 29.9 | 29.8 | 26.8 | 23.6 | 29.2 | 24.2 | 25.4 | 25.7 | 25.8 | 30.7 | 25.2 | 29.1 |
| Bsdc1 | 30.0 | 26.9 | 22.5 | 32.3 | 26.0 | 28.3 | 24.8 | 22.0 | 23.8 | 26.5 | 27.4 | 26.0 |
| Bsg | 288.1 | 269.8 | 248.6 | 319.5 | 260.5 | 325.4 | 255.4 | 238.3 | 266.5 | 301.3 | 277.4 | 333.5 |
| Bsn | 0.0 | 0.0 | 0.0 | 0.0 | 0.0 | 0.0 | 0.0 | 0.0 | 0.0 | 0.0 | 0.0 | 0.0 |
| Bspry | 0.1 | 0.1 | 0.1 | 0.0 | 0.1 | 0.1 | 0.0 | 0.1 | 0.1 | 0.1 | 0.1 | 0.0 |
| Bst1 | 1.2 | 0.7 | 0.5 | 1.4 | 1.7 | 0.9 | 1.0 | 0.4 | 0.3 | 0.8 | 0.4 | 0.3 |
| Bst2 | 167.8 | 83.3 | 58.8 | 279.6 | 110.3 | 133.5 | 39.5 | 27.9 | 33.0 | 39.6 | 25.5 | 60.7 |
| Btaf1 | 11.2 | 11.9 | 10.8 | 11.5 | 11.5 | 11.5 | 11.3 | 11.2 | 12.0 | 12.5 | 13.1 | 13.0 |
| Btbd1 | 45.6 | 38.4 | 33.6 | 52.3 | 35.5 | 49.6 | 35.6 | 29.8 | 32.3 | 37.8 | 39.4 | 41.4 |
| Btbd10 | 14.8 | 16.0 | 12.5 | 16.8 | 14.0 | 15.0 | 14.5 | 12.6 | 13.4 | 12.1 | 14.8 | 14.3 |
| Btbd11 | 0.6 | 0.3 | 0.3 | 0.4 | 0.3 | 0.6 | 0.3 | 0.2 | 0.2 | 0.2 | 0.2 | 0.2 |
| Btbd16 | 0.0 | 0.0 | 0.1 | 0.0 | 0.0 | 0.0 | 0.0 | 0.0 | 0.0 | 0.0 | 0.0 | 0.1 |
| Btbd19 | 4.4 | 3.1 | 2.5 | 5.1 | 3.5 | 3.8 | 3.6 | 2.5 | 2.8 | 3.4 | 2.7 | 2.3 |
| Btbd2 | 6.6 | 7.7 | 8.6 | 7.0 | 6.4 | 8.3 | 6.5 | 7.9 | 7.6 | 7.7 | 5.9 | 6.1 |
| Btbd3 | 2.5 | 3.7 | 4.4 | 2.4 | 4.1 | 2.8 | 4.5 | 6.3 | 6.6 | 4.5 | 4.8 | 4.0 |

| | | | | | | | | | | | | |
|---|---|---|---|---|---|---|---|---|---|---|---|---|
| Btbd6 | 2.9 | 3.5 | 3.3 | 3.1 | 3.3 | 4.1 | 2.2 | 3.2 | 2.7 | 3.4 | 3.0 | 2.6 |
| Btbd7 | 14.4 | 15.9 | 15.9 | 15.2 | 13.4 | 14.2 | 15.7 | 15.9 | 16.2 | 13.5 | 17.2 | 15.4 |
| Btbd8 | 2.4 | 2.1 | 1.4 | 1.5 | 1.5 | 1.2 | 1.4 | 1.8 | 1.5 | 2.1 | 1.8 | 1.7 |
| Btbd9 | 2.6 | 2.5 | 2.8 | 2.3 | 2.6 | 2.3 | 2.6 | 2.6 | 2.7 | 3.2 | 2.8 | 2.4 |
| Btc | 1.3 | 1.1 | 1.3 | 0.9 | 0.9 | 1.4 | 1.2 | 1.6 | 0.7 | 0.8 | 1.4 | 1.2 |
| Btd | 11.6 | 12.7 | 16.1 | 11.0 | 13.3 | 11.3 | 16.3 | 15.3 | 15.0 | 11.9 | 12.2 | 13.1 |
| Btf3 | 254.4 | 222.5 | 196.0 | 222.6 | 208.1 | 221.6 | 203.3 | 230.3 | 217.1 | 252.7 | 270.5 | 237.8 |
| Btf3l4 | 14.4 | 13.0 | 12.6 | 12.6 | 12.0 | 13.4 | 11.2 | 14.4 | 13.5 | 17.1 | 15.0 | 14.8 |
| Btg1 | 6.2 | 6.9 | 6.9 | 6.7 | 10.9 | 5.3 | 6.7 | 6.5 | 8.3 | 9.6 | 6.7 | 8.4 |
| Btg2 | 16.0 | 16.2 | 15.4 | 14.3 | 16.1 | 13.9 | 11.7 | 9.0 | 13.8 | 48.4 | 17.0 | 17.0 |
| Btg3 | 4.8 | 0.0 | 0.0 | 0.0 | 0.0 | 0.0 | 0.0 | 8.2 | 1.1 | 0.0 | 0.0 | 5.1 |
| Btk | 0.3 | 0.0 | 0.0 | 1.6 | 3.7 | 0.0 | 0.6 | 0.1 | 0.0 | 0.2 | 0.1 | 0.1 |
| Btla | 0.0 | 0.0 | 0.0 | 0.0 | 0.0 | 0.0 | 0.0 | 0.0 | 0.0 | 0.0 | 0.0 | 0.0 |
| Btn2a2 | 0.0 | 0.0 | 0.0 | 0.0 | 0.0 | 0.0 | 0.0 | 0.0 | 0.0 | 0.0 | 0.0 | 0.0 |
| Btnl9 | 0.1 | 0.0 | 0.0 | 0.0 | 0.0 | 0.0 | 0.1 | 0.0 | 0.1 | 0.0 | 0.0 | 0.0 |
| Btrc | 4.3 | 4.4 | 3.6 | 4.5 | 3.2 | 4.2 | 3.8 | 3.6 | 3.4 | 3.7 | 4.1 | 4.1 |
| Bub1 | 0.8 | 2.1 | 3.2 | 0.6 | 3.2 | 0.4 | 3.6 | 1.3 | 3.1 | 1.5 | 2.4 | 1.6 |
| Bub1b | 2.5 | 4.6 | 6.9 | 1.7 | 5.5 | 1.8 | 6.8 | 3.8 | 5.3 | 3.3 | 5.1 | 4.1 |
| Bub3 | 31.6 | 28.5 | 26.5 | 32.2 | 30.3 | 28.3 | 32.4 | 29.5 | 29.9 | 28.6 | 30.8 | 27.1 |
| Bud13 | 3.3 | 2.9 | 3.6 | 3.3 | 3.1 | 3.0 | 3.7 | 3.3 | 3.2 | 2.9 | 3.6 | 3.4 |
| Bud31 | 32.1 | 27.2 | 27.0 | 35.4 | 28.6 | 26.1 | 26.7 | 30.1 | 30.9 | 32.9 | 34.2 | 28.7 |
| Bves | 0.0 | 0.0 | 0.0 | 0.0 | 0.0 | 0.0 | 0.0 | 0.0 | 0.0 | 0.1 | 0.0 | 0.0 |
| Bysl | 9.9 | 8.9 | 8.3 | 9.9 | 9.3 | 8.7 | 11.4 | 9.4 | 9.6 | 9.9 | 9.5 | 9.6 |
| Bzrap1 | 0.0 | 0.0 | 0.0 | 0.0 | 0.1 | 0.0 | 0.0 | 0.0 | 0.0 | 0.0 | 0.0 | 0.0 |
| Bzw1 | 79.4 | 68.8 | 57.9 | 76.2 | 74.3 | 68.4 | 63.1 | 63.8 | 67.8 | 78.0 | 78.0 | 74.7 |
| Bzw2 | 8.9 | 8.1 | 7.7 | 7.0 | 8.3 | 7.4 | 9.1 | 12.8 | 10.2 | 12.1 | 13.6 | 10.2 |
| C030006K11Rik | 5.0 | 5.0 | 3.8 | 6.1 | 3.6 | 5.9 | 4.3 | 6.0 | 5.4 | 5.7 | 4.2 | 5.6 |
| C030016D13Rik | 0.1 | 0.1 | 0.1 | 0.1 | 0.1 | 0.2 | 0.2 | 0.2 | 0.1 | 0.0 | 0.1 | 0.2 |
| C030030A07Rik | 0.0 | 0.0 | 0.0 | 0.1 | 0.0 | 0.1 | 0.0 | 0.0 | 0.1 | 0.1 | 0.0 | 0.0 |
| C030034I22Rik | 0.4 | 0.4 | 0.5 | 0.4 | 0.4 | 0.4 | 0.6 | 0.5 | 0.3 | 0.5 | 0.5 | 0.5 |
| C030034L19Rik | 0.0 | 0.0 | 0.0 | 0.0 | 0.2 | 0.0 | 0.0 | 0.0 | 0.0 | 0.0 | 0.0 | 0.0 |
| C030037D09Rik | 2.5 | 2.1 | 2.2 | 3.5 | 2.7 | 3.2 | 1.9 | 1.7 | 2.1 | 1.5 | 1.4 | 1.7 |
| C030039L03Rik | 0.5 | 0.5 | 0.6 | 0.4 | 0.5 | 0.7 | 0.3 | 0.4 | 0.5 | 0.5 | 0.5 | 0.5 |
| C030046E11Rik | 9.0 | 10.6 | 10.2 | 7.8 | 8.6 | 7.9 | 8.6 | 8.8 | 9.0 | 9.3 | 9.4 | 8.9 |
| C130021I20Rik | 0.0 | 0.0 | 0.0 | 0.1 | 0.0 | 0.0 | 0.0 | 0.0 | 0.0 | 0.0 | 0.0 | 0.0 |
| C130026I21Rik | 0.8 | 1.2 | 1.5 | 0.8 | 1.1 | 0.7 | 0.6 | 0.5 | 0.5 | 0.9 | 0.7 | 0.9 |
| C130026L21Rik | 0.0 | 0.0 | 0.1 | 0.0 | 0.0 | 0.0 | 0.0 | 0.0 | 0.0 | 0.0 | 0.0 | 0.0 |
| C130036L24Rik | 0.4 | 0.5 | 0.2 | 0.4 | 0.2 | 0.5 | 0.6 | 0.4 | 0.5 | 0.3 | 0.2 | 0.2 |
| C130050O18Rik | 0.1 | 0.1 | 0.1 | 0.6 | 1.3 | 0.0 | 0.2 | 0.1 | 0.1 | 0.2 | 0.1 | 0.1 |
| C130060C02Rik | 0.3 | 0.3 | 0.2 | 0.2 | 0.2 | 0.1 | 0.2 | 0.0 | 0.2 | 0.0 | 0.1 | 0.1 |
| C130074G19Rik | 1.7 | 1.3 | 1.2 | 0.7 | 0.4 | 0.5 | 0.8 | 0.8 | 0.7 | 0.7 | 0.9 | 1.1 |
| C130079G13Rik | 0.0 | 0.0 | 0.0 | 0.0 | 0.0 | 0.0 | 0.0 | 0.0 | 0.0 | 0.0 | 0.0 | 0.0 |
| C130083M11Rik | 3.7 | 4.0 | 4.3 | 3.6 | 3.2 | 4.1 | 3.0 | 2.8 | 2.9 | 3.7 | 4.2 | 3.9 |
| C1d | 9.3 | 9.5 | 8.9 | 9.1 | 7.2 | 8.5 | 6.9 | 8.2 | 8.1 | 9.0 | 9.8 | 10.1 |
| C1galt1 | 11.0 | 12.7 | 15.3 | 6.5 | 12.2 | 9.0 | 10.0 | 13.1 | 13.8 | 10.5 | 14.4 | 14.2 |
| C1galt1c1 | 19.7 | 16.1 | 13.1 | 19.4 | 16.9 | 16.2 | 16.3 | 14.2 | 18.3 | 21.4 | 19.9 | 21.7 |
| C1qa | 7.1 | 0.1 | 0.4 | 81.4 | 93.8 | 0.1 | 4.4 | 2.2 | 1.2 | 5.5 | 3.0 | 6.4 |
| C1qb | 9.6 | 0.0 | 0.4 | 91.0 | 137.0 | 0.0 | 10.5 | 2.5 | 1.7 | 7.9 | 2.5 | 7.5 |
| C1qbp | 37.0 | 27.5 | 26.4 | 38.1 | 29.3 | 34.4 | 26.3 | 26.6 | 20.4 | 30.3 | 32.1 | 27.1 |
| C1qc | 9.0 | 0.0 | 0.4 | 88.2 | 117.4 | 0.0 | 10.3 | 2.3 | 1.9 | 6.6 | 2.2 | 5.3 |
| C1ql1 | 0.0 | 0.1 | 0.0 | 0.1 | 0.0 | 0.1 | 0.1 | 0.0 | 0.1 | 0.2 | 0.1 | 0.0 |
| C1ql3 | 4.5 | 3.2 | 2.8 | 6.1 | 3.4 | 5.5 | 4.9 | 3.3 | 4.4 | 4.4 | 5.8 | 5.5 |
| C1qtnf1 | 0.6 | 0.7 | 1.3 | 0.2 | 0.7 | 0.3 | 0.8 | 2.7 | 1.7 | 2.6 | 1.3 | 1.6 |
| C1qtnf2 | 6.0 | 2.2 | 2.5 | 10.5 | 3.5 | 5.9 | 5.3 | 3.8 | 3.4 | 2.4 | 3.3 | 3.8 |
| C1qtnf3 | 0.2 | 0.0 | 0.0 | 0.2 | 0.1 | 0.1 | 0.4 | 0.6 | 0.1 | 1.1 | 2.8 | 0.2 |
| C1qtnf4 | 0.1 | 0.1 | 0.0 | 0.0 | 0.0 | 0.0 | 0.1 | 0.0 | 0.0 | 0.1 | 0.0 | 0.1 |
| C1qtnf5 | 23.0 | 12.5 | 13.7 | 32.0 | 18.5 | 28.7 | 11.7 | 11.7 | 12.0 | 13.0 | 9.2 | 16.5 |

| | | | | | | | | | | | | |
|---|---|---|---|---|---|---|---|---|---|---|---|---|
| C1qtnf6 | 3.4 | 4.7 | 6.1 | 3.4 | 5.3 | 3.1 | 4.6 | 8.7 | 6.2 | 4.3 | 3.6 | 4.4 |
| C1qtnf7 | 5.9 | 6.1 | 11.5 | 10.5 | 4.5 | 6.3 | 3.8 | 14.4 | 4.0 | 4.3 | 4.8 | 3.9 |
| C1qtnf9 | 0.7 | 0.3 | 0.4 | 0.1 | 0.0 | 0.0 | 0.5 | 0.6 | 0.5 | 0.0 | 0.0 | 0.2 |
| C1ra | 24.6 | 24.4 | 42.4 | 20.7 | 22.2 | 22.2 | 17.6 | 28.0 | 22.3 | 20.8 | 17.2 | 24.9 |
| C1rb | 10.4 | 10.6 | 17.4 | 7.6 | 9.7 | 9.9 | 7.1 | 11.7 | 9.3 | 8.7 | 6.9 | 10.5 |
| C1rl | 9.3 | 8.8 | 10.6 | 10.8 | 9.4 | 12.2 | 9.4 | 8.0 | 8.8 | 8.3 | 8.6 | 10.8 |
| C1s | 27.6 | 31.8 | 50.8 | 22.2 | 36.8 | 27.3 | 18.6 | 23.8 | 21.6 | 65.8 | 44.7 | 41.1 |
| C2 | 4.6 | 3.0 | 2.0 | 3.6 | 2.0 | 3.2 | 0.4 | 0.6 | 0.9 | 7.7 | 2.3 | 2.5 |
| C230035I16Rik | 0.1 | 0.0 | 0.1 | 1.0 | 0.2 | 0.2 | 0.0 | 0.1 | 0.1 | 0.0 | 0.1 | 0.2 |
| C230052I12Rik | 2.1 | 1.8 | 2.2 | 2.5 | 2.4 | 2.1 | 2.0 | 2.4 | 2.2 | 2.5 | 2.0 | 2.0 |
| C230081A13Rik | 21.2 | 27.8 | 29.9 | 20.9 | 23.3 | 22.3 | 29.9 | 26.1 | 27.1 | 21.2 | 24.3 | 26.9 |
| C230091D08Rik | 2.2 | 2.8 | 3.5 | 3.2 | 2.8 | 3.2 | 2.7 | 3.0 | 2.6 | 2.6 | 3.1 | 3.2 |
| C2cd2 | 1.6 | 2.1 | 2.7 | 1.2 | 1.4 | 1.3 | 1.6 | 1.9 | 2.0 | 2.0 | 1.7 | 1.9 |
| C2cd2l | 2.3 | 2.0 | 1.8 | 3.1 | 2.5 | 2.7 | 2.7 | 1.9 | 2.5 | 2.9 | 2.4 | 2.6 |
| C2cd3 | 2.1 | 2.4 | 2.4 | 2.2 | 2.4 | 2.6 | 2.3 | 2.6 | 2.1 | 2.0 | 2.6 | 2.1 |
| C2cd4c | 0.0 | 0.0 | 0.0 | 0.0 | 0.0 | 0.0 | 0.0 | 0.0 | 0.0 | 0.0 | 0.0 | 0.0 |
| C2cd5 | 1.8 | 2.2 | 2.1 | 1.9 | 2.2 | 1.6 | 1.8 | 1.7 | 2.0 | 2.0 | 2.3 | 2.0 |
| C3 | 133.4 | 136.7 | 351.0 | 116.0 | 262.7 | 82.3 | 187.7 | 171.4 | 118.1 | 130.3 | 95.3 | 126.8 |
| C330005M16Rik | 0.1 | 0.0 | 0.0 | 0.0 | 0.2 | 0.0 | 0.0 | 0.0 | 0.0 | 0.0 | 0.0 | 0.0 |
| C330006A16Rik | 7.9 | 5.8 | 5.9 | 7.7 | 7.4 | 7.4 | 6.1 | 5.7 | 6.9 | 9.7 | 6.9 | 6.9 |
| C330007P06Rik | 21.2 | 18.7 | 20.8 | 27.3 | 19.0 | 24.9 | 16.4 | 18.5 | 15.2 | 18.0 | 19.9 | 20.4 |
| C330013E15Rik | 0.9 | 0.7 | 1.0 | 1.0 | 1.0 | 1.4 | 0.7 | 0.9 | 1.0 | 1.2 | 1.1 | 0.9 |
| C330013F16Rik | 0.0 | 0.0 | 0.0 | 0.0 | 0.0 | 0.0 | 0.0 | 0.0 | 0.0 | 0.0 | 0.0 | 0.0 |
| C330018D20Rik | 6.8 | 6.8 | 5.2 | 7.0 | 5.2 | 7.5 | 4.8 | 5.0 | 5.3 | 6.0 | 6.2 | 6.3 |
| C330021F23Rik | 2.2 | 2.2 | 2.0 | 2.6 | 2.1 | 1.8 | 2.2 | 2.5 | 1.6 | 2.0 | 2.2 | 2.7 |
| C330024C12Rik | 0.1 | 0.1 | 0.0 | 0.1 | 0.2 | 0.0 | 0.1 | 0.0 | 0.1 | 0.0 | 0.0 | 0.0 |
| C330024D21Rik | 0.1 | 0.2 | 0.1 | 0.2 | 0.2 | 0.1 | 0.1 | 0.2 | 0.1 | 0.1 | 0.1 | 0.2 |
| C330027C09Rik | 1.4 | 2.5 | 4.4 | 0.9 | 3.8 | 1.0 | 4.8 | 2.2 | 4.2 | 2.0 | 3.5 | 2.7 |
| C3ar1 | 1.9 | 0.1 | 0.2 | 17.0 | 29.3 | 0.1 | 3.3 | 0.5 | 0.5 | 1.7 | 0.6 | 1.6 |
| C430002E04Rik | 0.0 | 0.0 | 0.0 | 0.0 | 0.0 | 0.0 | 0.0 | 0.1 | 0.0 | 0.0 | 0.0 | 0.1 |
| C430049B03Rik | 0.1 | 0.1 | 0.1 | 0.0 | 0.3 | 0.0 | 0.1 | 0.2 | 0.4 | 0.6 | 1.0 | 0.4 |
| C4a | 2.8 | 3.2 | 5.2 | 2.4 | 2.6 | 0.8 | 1.1 | 2.6 | 1.4 | 1.7 | 1.3 | 1.5 |
| C4b | 16.7 | 18.6 | 30.3 | 14.9 | 14.3 | 4.4 | 6.0 | 14.8 | 7.5 | 9.1 | 6.8 | 9.3 |
| C4bp | 0.7 | 0.8 | 0.5 | 0.2 | 0.3 | 0.1 | 0.2 | 0.1 | 0.1 | 0.5 | 0.5 | 0.7 |
| C530005A16Rik | 0.5 | 0.4 | 0.4 | 0.6 | 0.5 | 0.6 | 0.4 | 0.3 | 0.4 | 0.3 | 0.6 | 0.4 |
| C530008M17Rik | 0.1 | 0.1 | 0.1 | 0.1 | 0.2 | 0.2 | 0.2 | 0.1 | 0.2 | 0.1 | 0.2 | 0.1 |
| C530044C16Rik | 0.0 | 0.0 | 0.1 | 0.0 | 0.1 | 0.1 | 0.0 | 0.0 | 0.0 | 0.0 | 0.0 | 0.0 |
| C5ar1 | 1.0 | 0.0 | 0.1 | 11.7 | 19.6 | 0.0 | 1.6 | 0.4 | 0.2 | 1.0 | 0.3 | 0.8 |
| C5ar2 | 0.1 | 0.1 | 0.0 | 0.1 | 0.2 | 0.1 | 0.0 | 0.0 | 0.0 | 0.0 | 0.1 | 0.0 |
| C630043F03Rik | 0.1 | 0.0 | 0.0 | 0.0 | 0.1 | 0.1 | 0.0 | 0.1 | 0.1 | 0.1 | 0.0 | 0.1 |
| C7 | 0.0 | 0.2 | 0.4 | 0.0 | 0.1 | 0.0 | 0.1 | 0.1 | 0.0 | 0.0 | 0.0 | 0.0 |
| C730002L08Rik | 0.3 | 0.4 | 0.4 | 0.7 | 0.4 | 0.5 | 0.3 | 0.2 | 0.2 | 0.9 | 0.7 | 0.5 |
| C730027H18Rik | 0.1 | 0.1 | 0.1 | 0.0 | 0.0 | 0.0 | 0.0 | 0.1 | 0.0 | 0.0 | 0.0 | 0.0 |
| C730036E19Rik | 0.0 | 0.0 | 0.0 | 0.0 | 0.0 | 0.0 | 0.0 | 0.0 | 0.0 | 0.0 | 0.0 | 0.0 |
| C77080 | 1.8 | 2.2 | 1.8 | 1.9 | 1.8 | 2.6 | 1.8 | 1.6 | 1.6 | 2.2 | 1.8 | 1.5 |
| C77370 | 0.2 | 0.2 | 0.2 | 0.1 | 0.2 | 0.1 | 0.1 | 0.1 | 0.0 | 0.1 | 0.1 | 0.1 |
| C78339 | 24.2 | 20.2 | 18.3 | 21.7 | 17.7 | 21.3 | 20.2 | 17.7 | 21.1 | 23.1 | 25.0 | 22.4 |
| C87198 | 0.1 | 0.0 | 0.0 | 0.0 | 0.0 | 0.0 | 0.1 | 0.0 | 0.0 | 0.0 | 0.1 | 0.0 |
| C87436 | 4.8 | 5.8 | 5.1 | 4.6 | 4.2 | 5.4 | 4.6 | 4.9 | 4.8 | 4.6 | 4.3 | 4.2 |
| C8g | 0.7 | 0.8 | 0.6 | 0.4 | 0.6 | 1.3 | 0.4 | 0.8 | 0.5 | 0.6 | 0.5 | 0.8 |
| C9 | 0.0 | 0.0 | 0.0 | 0.0 | 0.0 | 0.0 | 0.0 | 0.0 | 0.0 | 0.1 | 0.1 | 0.0 |
| C920006O11Rik | 0.8 | 1.2 | 0.9 | 0.6 | 0.8 | 1.3 | 0.7 | 1.0 | 0.9 | 1.1 | 0.7 | 1.1 |
| C920009B18Rik | 1.0 | 0.5 | 0.4 | 1.1 | 1.1 | 0.5 | 0.6 | 0.2 | 0.2 | 0.3 | 0.5 | 0.4 |
| C920021L13Rik | 0.1 | 0.2 | 0.3 | 0.2 | 0.1 | 0.3 | 0.5 | 0.4 | 0.3 | 0.1 | 0.1 | 0.1 |
| C920025E04Rik | 2.4 | 1.9 | 1.7 | 4.1 | 2.9 | 3.2 | 2.0 | 1.3 | 1.5 | 1.6 | 0.8 | 2.0 |
| Caap1 | 4.3 | 4.1 | 5.0 | 4.6 | 3.8 | 4.4 | 4.6 | 4.5 | 4.0 | 4.6 | 5.1 | 5.5 |
| Cab39 | 33.1 | 32.3 | 33.3 | 42.9 | 36.4 | 36.0 | 35.8 | 31.2 | 33.9 | 34.5 | 39.5 | 40.7 |

| | | | | | | | | | | | | |
|---|---|---|---|---|---|---|---|---|---|---|---|---|
| Cab39l | 18.3 | 16.8 | 16.0 | 19.7 | 14.9 | 19.3 | 15.4 | 13.8 | 13.0 | 12.7 | 16.0 | 13.0 |
| Cabin1 | 3.7 | 4.2 | 4.3 | 3.5 | 4.1 | 4.5 | 4.1 | 4.4 | 4.0 | 3.5 | 3.6 | 3.2 |
| Cables1 | 0.6 | 1.0 | 1.6 | 0.5 | 0.6 | 0.5 | 0.4 | 1.4 | 0.7 | 0.9 | 0.5 | 0.8 |
| Cables2 | 2.5 | 2.6 | 2.5 | 2.0 | 2.6 | 2.5 | 2.9 | 2.6 | 2.5 | 2.7 | 2.4 | 2.2 |
| Cabp1 | 0.7 | 0.4 | 0.4 | 0.6 | 0.5 | 0.2 | 0.4 | 0.7 | 0.4 | 0.4 | 0.7 | 0.5 |
| Cabp4 | 0.1 | 0.1 | 0.0 | 0.0 | 0.1 | 0.1 | 0.0 | 0.1 | 0.1 | 0.0 | 0.1 | 0.2 |
| Cabyr | 0.3 | 0.4 | 0.2 | 0.5 | 0.2 | 0.1 | 0.3 | 0.2 | 0.2 | 0.3 | 0.1 | 0.2 |
| Cacfd1 | 5.4 | 6.0 | 6.3 | 4.9 | 5.8 | 6.6 | 6.3 | 6.0 | 5.0 | 5.8 | 5.5 | 5.5 |
| Cachd1 | 2.9 | 3.3 | 3.4 | 4.2 | 3.6 | 3.8 | 3.7 | 2.7 | 3.1 | 2.8 | 2.9 | 2.7 |
| Cacna1a | 0.7 | 0.7 | 0.8 | 0.8 | 0.9 | 0.9 | 0.8 | 0.8 | 0.7 | 0.7 | 0.7 | 0.5 |
| Cacna1b | 0.1 | 0.0 | 0.0 | 0.1 | 0.0 | 0.1 | 0.1 | 0.0 | 0.1 | 0.0 | 0.1 | 0.0 |
| Cacna1c | 0.4 | 1.0 | 1.6 | 0.3 | 0.9 | 0.5 | 1.0 | 1.3 | 1.1 | 1.1 | 1.1 | 1.0 |
| Cacna1d | 0.1 | 0.1 | 0.2 | 0.1 | 0.2 | 0.0 | 0.1 | 0.1 | 0.1 | 0.1 | 0.0 | 0.1 |
| Cacna1e | 0.1 | 0.0 | 0.0 | 0.0 | 0.0 | 0.0 | 0.0 | 0.0 | 0.0 | 0.0 | 0.0 | 0.0 |
| Cacna1g | 0.5 | 1.6 | 2.3 | 0.2 | 0.8 | 0.3 | 1.1 | 1.4 | 1.3 | 3.9 | 2.4 | 1.3 |
| Cacna1h | 0.0 | 0.0 | 0.0 | 0.0 | 0.0 | 0.0 | 0.0 | 0.0 | 0.0 | 0.1 | 0.1 | 0.1 |
| Cacna1i | 0.0 | 0.0 | 0.0 | 0.0 | 0.0 | 0.0 | 0.0 | 0.0 | 0.0 | 0.0 | 0.0 | 0.0 |
| Cacna1s | 0.0 | 0.0 | 0.0 | 0.0 | 0.0 | 0.0 | 0.1 | 0.1 | 0.0 | 0.0 | 0.0 | 0.0 |
| Cacna2d1 | 4.5 | 4.1 | 4.9 | 3.6 | 4.9 | 4.5 | 4.9 | 5.1 | 4.1 | 4.7 | 5.3 | 5.7 |
| Cacna2d2 | 0.0 | 0.0 | 0.0 | 0.0 | 0.0 | 0.0 | 0.0 | 0.0 | 0.0 | 0.0 | 0.0 | 0.0 |
| Cacna2d3 | 0.1 | 0.0 | 0.0 | 0.0 | 0.0 | 0.0 | 0.0 | 0.0 | 0.0 | 0.0 | 0.0 | 0.0 |
| Cacna2d4 | 0.0 | 0.0 | 0.0 | 0.0 | 0.0 | 0.0 | 0.0 | 0.0 | 0.0 | 0.0 | 0.0 | 0.0 |
| Cacnb1 | 1.9 | 2.4 | 2.7 | 1.8 | 1.5 | 3.1 | 2.2 | 1.4 | 1.9 | 2.2 | 2.1 | 1.7 |
| Cacnb2 | 0.2 | 0.1 | 0.2 | 0.1 | 0.1 | 0.0 | 0.2 | 0.1 | 0.1 | 0.1 | 0.1 | 0.1 |
| Cacnb3 | 14.9 | 18.5 | 17.7 | 15.0 | 16.0 | 20.6 | 16.6 | 16.4 | 16.9 | 20.1 | 18.7 | 15.8 |
| Cacnb4 | 0.3 | 0.4 | 0.4 | 0.3 | 0.2 | 0.4 | 0.3 | 0.3 | 0.2 | 0.2 | 0.3 | 0.3 |
| Cacng7 | 8.1 | 9.4 | 9.3 | 10.0 | 7.8 | 14.8 | 12.1 | 9.0 | 8.0 | 7.0 | 6.8 | 6.9 |
| Cacng8 | 0.0 | 0.1 | 0.1 | 0.3 | 0.1 | 0.1 | 0.2 | 0.1 | 0.2 | 0.3 | 0.0 | 0.2 |
| Cactin | 9.2 | 9.1 | 8.2 | 8.8 | 8.3 | 7.2 | 9.0 | 8.2 | 8.9 | 8.5 | 8.2 | 7.3 |
| Cacul1 | 12.5 | 10.8 | 9.4 | 13.4 | 12.7 | 9.9 | 11.9 | 10.8 | 12.0 | 12.3 | 12.6 | 10.9 |
| Cacybp | 43.5 | 39.5 | 42.0 | 49.8 | 35.6 | 45.5 | 40.3 | 28.2 | 33.0 | 43.7 | 48.2 | 50.1 |
| Cad | 4.8 | 5.2 | 4.3 | 3.9 | 5.4 | 3.8 | 5.3 | 4.9 | 4.7 | 4.6 | 4.2 | 3.7 |
| Cadm1 | 0.7 | 0.2 | 0.2 | 1.6 | 2.6 | 0.2 | 0.5 | 0.2 | 0.0 | 0.2 | 0.1 | 0.1 |
| Cadm2 | 0.0 | 0.0 | 0.0 | 0.0 | 0.0 | 0.0 | 0.0 | 0.0 | 0.0 | 0.0 | 0.0 | 0.0 |
| Cadm3 | 0.1 | 0.1 | 0.1 | 0.1 | 0.1 | 0.0 | 0.1 | 0.2 | 0.1 | 0.1 | 0.1 | 0.1 |
| Cadm4 | 1.1 | 1.0 | 0.5 | 1.4 | 0.7 | 1.5 | 1.3 | 2.0 | 4.4 | 11.3 | 4.2 | 3.9 |
| Cadps | 0.0 | 0.0 | 0.0 | 0.0 | 0.0 | 0.0 | 0.0 | 0.0 | 0.0 | 0.0 | 0.0 | 0.0 |
| Cadps2 | 0.1 | 0.1 | 0.0 | 0.1 | 0.1 | 0.1 | 0.1 | 0.0 | 0.1 | 0.1 | 0.2 | 0.1 |
| Cage1 | 1.6 | 1.5 | 1.4 | 1.3 | 1.1 | 1.6 | 0.9 | 1.4 | 1.1 | 1.1 | 1.3 | 1.2 |
| Calca | 0.0 | 0.0 | 0.0 | 0.0 | 0.0 | 0.0 | 0.0 | 0.4 | 0.1 | 0.1 | 0.2 | 0.1 |
| Calcb | 0.0 | 0.0 | 0.1 | 0.0 | 0.0 | 0.0 | 0.0 | 0.0 | 0.0 | 0.0 | 0.0 | 0.0 |
| Calcoco1 | 15.9 | 15.2 | 17.0 | 19.1 | 12.3 | 20.2 | 14.4 | 16.2 | 12.6 | 17.5 | 16.3 | 16.2 |
| Calcoco2 | 0.0 | 0.0 | 0.0 | 0.0 | 0.0 | 0.0 | 0.0 | 0.0 | 0.1 | 0.0 | 0.0 | 0.0 |
| Calcrl | 1.7 | 1.7 | 2.1 | 3.9 | 5.1 | 2.7 | 2.6 | 2.7 | 2.2 | 1.5 | 2.4 | 1.8 |
| Cald1 | 463.2 | 378.2 | 364.3 | 601.4 | 373.7 | 423.4 | 475.0 | 285.7 | 341.5 | 393.2 | 531.5 | 522.6 |
| Calhm1 | 0.0 | 0.2 | 0.0 | 0.0 | 0.0 | 0.1 | 0.0 | 0.0 | 0.0 | 0.0 | 0.0 | 0.0 |
| Calhm2 | 7.9 | 9.4 | 8.8 | 9.4 | 9.3 | 9.1 | 10.3 | 9.8 | 10.8 | 9.2 | 9.2 | 8.2 |
| Calm1 | 128.1 | 103.5 | 92.8 | 159.0 | 137.6 | 128.0 | 119.5 | 121.1 | 116.8 | 133.5 | 140.8 | 128.0 |
| Calm2 | 258.1 | 257.2 | 312.9 | 302.3 | 266.0 | 253.1 | 333.5 | 289.0 | 258.4 | 274.3 | 349.8 | 347.7 |
| Calm3 | 147.7 | 133.2 | 156.9 | 200.0 | 143.8 | 170.5 | 180.9 | 165.0 | 146.6 | 150.1 | 171.2 | 162.8 |
| Calml4 | 1.2 | 0.7 | 1.5 | 0.6 | 0.2 | 1.0 | 0.9 | 1.1 | 0.8 | 0.2 | 1.2 | 0.7 |
| Caln1 | 0.1 | 0.0 | 0.1 | 0.0 | 0.0 | 0.1 | 0.0 | 0.0 | 0.0 | 0.0 | 0.0 | 0.0 |
| Calr | 841.4 | 652.9 | 695.9 | 966.8 | 658.1 | 669.9 | 907.9 | 764.3 | 658.8 | 784.3 | 919.5 | 994.3 |
| Calr3 | 2.3 | 1.5 | 1.5 | 3.6 | 2.0 | 3.3 | 1.3 | 1.1 | 0.7 | 1.1 | 0.9 | 1.2 |
| Calr4 | 0.4 | 0.5 | 0.3 | 0.3 | 0.3 | 0.3 | 0.7 | 0.4 | 0.6 | 0.3 | 0.5 | 0.5 |
| Calu | 423.1 | 348.8 | 341.7 | 461.6 | 351.7 | 441.4 | 441.1 | 406.9 | 412.9 | 374.0 | 465.2 | 449.8 |
| Caly | 0.1 | 0.0 | 0.0 | 0.2 | 0.0 | 0.2 | 0.0 | 0.0 | 0.0 | 0.0 | 0.1 | 0.1 |

| | | | | | | | | | | | | |
|---|---|---|---|---|---|---|---|---|---|---|---|---|
| Camk1 | 73.3 | 56.4 | 48.3 | 87.9 | 63.2 | 67.2 | 53.7 | 45.1 | 51.3 | 68.0 | 65.5 | 58.8 |
| Camk1d | 0.5 | 0.4 | 0.3 | 1.2 | 1.6 | 0.3 | 0.5 | 0.5 | 0.2 | 0.2 | 0.3 | 0.3 |
| Camk1g | 0.0 | 0.0 | 0.0 | 0.0 | 0.0 | 0.0 | 0.0 | 0.1 | 0.0 | 0.0 | 0.0 | 0.0 |
| Camk2a | 0.8 | 0.7 | 0.7 | 0.9 | 0.4 | 0.7 | 0.6 | 0.7 | 0.5 | 0.3 | 0.6 | 0.5 |
| Camk2b | 0.3 | 0.2 | 0.2 | 0.4 | 0.2 | 0.3 | 0.2 | 0.0 | 0.1 | 0.1 | 0.1 | 0.1 |
| Camk2d | 14.9 | 13.4 | 13.6 | 14.5 | 13.4 | 14.1 | 14.6 | 14.8 | 15.1 | 19.0 | 18.6 | 19.0 |
| Camk2g | 18.1 | 17.5 | 15.9 | 16.5 | 15.8 | 17.0 | 22.2 | 17.8 | 22.2 | 21.3 | 23.2 | 22.1 |
| Camk2n1 | 5.9 | 3.9 | 2.5 | 12.8 | 4.9 | 10.3 | 4.4 | 2.2 | 3.8 | 3.7 | 3.5 | 3.8 |
| Camk2n2 | 2.2 | 2.0 | 2.1 | 3.2 | 2.0 | 2.8 | 2.4 | 1.7 | 1.9 | 2.0 | 1.6 | 1.8 |
| Camk4 | 0.0 | 0.0 | 0.0 | 0.0 | 0.0 | 0.0 | 0.1 | 0.1 | 0.1 | 0.1 | 0.2 | 0.1 |
| Camkk1 | 2.8 | 2.6 | 2.8 | 2.5 | 2.4 | 4.0 | 2.5 | 2.8 | 2.5 | 1.9 | 2.4 | 2.2 |
| Camkk2 | 7.9 | 11.4 | 16.2 | 6.9 | 11.8 | 6.4 | 10.9 | 12.9 | 9.8 | 9.2 | 8.9 | 9.7 |
| Camkmt | 1.8 | 1.9 | 1.4 | 1.5 | 1.6 | 2.0 | 1.0 | 1.7 | 1.0 | 1.4 | 1.4 | 1.2 |
| Caml | 28.5 | 28.2 | 23.8 | 34.2 | 24.2 | 28.9 | 30.3 | 21.2 | 25.8 | 26.1 | 30.1 | 27.7 |
| Camp | 2.4 | 1.5 | 3.4 | 3.6 | 4.7 | 0.6 | 4.0 | 2.9 | 2.6 | 1.2 | 1.7 | 1.8 |
| Camsap1 | 8.0 | 7.9 | 8.1 | 8.0 | 6.5 | 8.2 | 8.2 | 6.6 | 7.6 | 7.9 | 8.0 | 7.8 |
| Camsap2 | 13.5 | 14.6 | 12.9 | 16.9 | 12.6 | 14.1 | 15.2 | 12.3 | 13.1 | 13.5 | 16.6 | 16.4 |
| Camsap3 | 0.9 | 1.0 | 0.9 | 0.8 | 0.8 | 0.5 | 0.7 | 0.7 | 0.8 | 0.4 | 0.4 | 0.5 |
| Camta1 | 0.5 | 0.6 | 0.8 | 0.8 | 0.7 | 0.6 | 0.9 | 0.9 | 0.6 | 0.8 | 0.6 | 0.9 |
| Camta2 | 1.5 | 2.0 | 1.9 | 1.6 | 2.3 | 1.8 | 2.1 | 1.6 | 1.9 | 1.5 | 1.6 | 1.4 |
| Cand1 | 19.7 | 20.3 | 19.0 | 17.8 | 19.0 | 18.4 | 19.9 | 19.1 | 20.0 | 20.8 | 20.9 | 20.4 |
| Cand2 | 1.1 | 1.7 | 1.8 | 0.8 | 1.2 | 1.5 | 1.1 | 1.6 | 1.3 | 1.2 | 1.1 | 1.0 |
| Cant1 | 15.8 | 15.2 | 16.2 | 14.2 | 15.5 | 17.9 | 16.3 | 17.8 | 18.2 | 17.5 | 15.1 | 16.9 |
| Canx | 325.4 | 284.2 | 277.8 | 348.3 | 278.0 | 328.8 | 292.3 | 284.0 | 263.0 | 286.9 | 340.4 | 348.6 |
| Cap1 | 144.2 | 125.7 | 113.3 | 193.8 | 181.4 | 139.9 | 165.4 | 134.6 | 146.7 | 130.9 | 161.4 | 148.9 |
| Cap2 | 1.2 | 0.7 | 0.4 | 1.8 | 0.7 | 2.1 | 0.6 | 0.5 | 0.5 | 1.7 | 1.2 | 1.2 |
| Capg | 92.4 | 75.4 | 67.1 | 142.4 | 170.1 | 104.2 | 81.9 | 71.1 | 67.7 | 77.7 | 74.3 | 80.5 |
| Capn1 | 15.6 | 14.6 | 15.6 | 16.1 | 15.2 | 17.7 | 15.8 | 16.4 | 13.6 | 14.6 | 14.4 | 14.3 |
| Capn10 | 3.6 | 3.9 | 3.6 | 3.6 | 3.4 | 3.5 | 4.7 | 4.0 | 4.4 | 4.1 | 4.3 | 4.3 |
| Capn11 | 0.1 | 0.1 | 0.0 | 0.0 | 0.1 | 0.3 | 0.1 | 0.2 | 0.1 | 0.2 | 0.1 | 0.0 |
| Capn12 | 0.0 | 0.0 | 0.1 | 0.1 | 0.1 | 0.1 | 0.0 | 0.1 | 0.1 | 0.0 | 0.1 | 0.1 |
| Capn2 | 156.6 | 136.6 | 118.8 | 167.2 | 139.4 | 147.9 | 161.8 | 126.8 | 135.9 | 162.8 | 172.4 | 141.0 |
| Capn3 | 0.1 | 0.1 | 0.1 | 0.1 | 0.1 | 0.0 | 0.1 | 0.0 | 0.0 | 0.0 | 0.1 | 0.1 |
| Capn5 | 5.4 | 5.1 | 5.7 | 4.8 | 3.8 | 5.7 | 4.9 | 6.2 | 4.2 | 3.0 | 4.0 | 4.4 |
| Capn6 | 1.1 | 0.2 | 0.4 | 0.0 | 0.1 | 0.1 | 0.1 | 0.6 | 0.3 | 1.3 | 0.4 | 0.6 |
| Capn7 | 28.4 | 23.8 | 21.8 | 28.9 | 22.7 | 25.6 | 21.7 | 20.0 | 21.0 | 22.1 | 25.7 | 26.3 |
| Capn8 | 0.0 | 0.0 | 0.0 | 0.0 | 0.0 | 0.0 | 0.0 | 0.0 | 0.0 | 0.0 | 0.0 | 0.0 |
| Capn9 | 0.0 | 0.1 | 0.1 | 0.0 | 0.0 | 0.0 | 0.0 | 0.1 | 0.1 | 0.0 | 0.0 | 0.0 |
| Capns1 | 176.9 | 136.8 | 141.7 | 177.8 | 147.0 | 146.5 | 156.3 | 190.4 | 145.1 | 145.9 | 175.0 | 157.7 |
| Capns2 | 0.1 | 0.0 | 0.0 | 0.0 | 0.1 | 0.0 | 0.1 | 0.0 | 0.0 | 0.1 | 0.1 | 0.0 |
| Caprin1 | 93.9 | 81.1 | 89.6 | 106.5 | 89.1 | 88.4 | 101.1 | 95.5 | 84.0 | 91.8 | 109.5 | 108.0 |
| Caprin2 | 1.7 | 2.2 | 2.0 | 1.8 | 1.2 | 2.0 | 1.4 | 1.8 | 1.8 | 1.5 | 2.0 | 2.0 |
| Capza1 | 64.7 | 68.4 | 55.8 | 67.2 | 74.2 | 61.8 | 59.3 | 61.1 | 63.2 | 66.5 | 67.2 | 63.8 |
| Capza2 | 75.4 | 72.4 | 60.3 | 81.7 | 77.7 | 71.0 | 58.1 | 61.2 | 73.2 | 70.8 | 79.7 | 73.8 |
| Capzb | 81.6 | 71.8 | 77.6 | 85.1 | 86.9 | 74.3 | 85.7 | 80.3 | 71.6 | 84.0 | 90.1 | 95.0 |
| Car10 | 0.0 | 0.0 | 0.0 | 0.0 | 0.0 | 0.0 | 0.0 | 0.0 | 0.0 | 0.0 | 0.0 | 0.0 |
| Car11 | 2.0 | 3.0 | 2.3 | 3.4 | 2.1 | 5.4 | 2.5 | 1.8 | 1.9 | 1.9 | 1.7 | 2.9 |
| Car12 | 0.3 | 0.1 | 0.2 | 0.3 | 0.4 | 0.2 | 0.2 | 0.1 | 0.1 | 0.2 | 0.1 | 0.2 |
| Car13 | 9.8 | 8.5 | 10.8 | 7.6 | 9.9 | 8.8 | 9.9 | 13.2 | 9.1 | 8.2 | 9.3 | 11.9 |
| Car14 | 0.8 | 0.7 | 0.7 | 0.8 | 0.8 | 0.8 | 0.5 | 0.8 | 0.8 | 1.0 | 0.8 | 0.8 |
| Car15 | 0.3 | 0.1 | 0.3 | 0.3 | 0.5 | 0.6 | 0.3 | 0.6 | 0.2 | 0.2 | 0.4 | 0.3 |
| Car4 | 0.1 | 0.0 | 0.0 | 0.0 | 0.0 | 0.0 | 0.0 | 0.1 | 0.0 | 0.0 | 0.0 | 0.0 |
| Car5a | 0.0 | 0.0 | 0.0 | 0.0 | 0.0 | 0.0 | 0.0 | 0.0 | 0.0 | 0.0 | 0.0 | 0.0 |
| Car5b | 3.4 | 4.0 | 3.7 | 3.6 | 3.4 | 4.6 | 3.3 | 3.4 | 2.7 | 2.8 | 3.5 | 3.3 |
| Car6 | 3.5 | 0.6 | 0.6 | 4.4 | 0.1 | 3.6 | 1.2 | 0.5 | 0.5 | 0.9 | 1.6 | 1.3 |
| Car7 | 0.3 | 0.3 | 0.3 | 0.2 | 0.2 | 0.5 | 0.1 | 0.3 | 0.2 | 0.1 | 0.5 | 0.2 |
| Car8 | 1.4 | 0.6 | 0.7 | 1.7 | 0.6 | 2.1 | 0.6 | 0.3 | 0.2 | 0.4 | 0.3 | 0.4 |

| | | | | | | | | | | | | |
|---|---|---|---|---|---|---|---|---|---|---|---|---|
| Car9 | 0.3 | 0.3 | 0.3 | 0.3 | 0.6 | 0.5 | 0.1 | 0.2 | 0.1 | 0.7 | 0.3 | 0.2 |
| Card10 | 0.2 | 0.4 | 0.5 | 0.1 | 0.3 | 0.1 | 0.5 | 0.9 | 0.9 | 0.4 | 0.3 | 0.4 |
| Card11 | 0.0 | 0.0 | 0.0 | 0.0 | 0.5 | 0.0 | 0.2 | 0.0 | 0.0 | 0.1 | 0.0 | 0.0 |
| Card14 | 0.0 | 0.0 | 0.0 | 0.1 | 0.0 | 0.1 | 0.0 | 0.1 | 0.0 | 0.0 | 0.0 | 0.1 |
| Card6 | 0.7 | 0.7 | 0.8 | 0.8 | 0.7 | 0.8 | 0.8 | 0.4 | 0.5 | 0.6 | 0.6 | 0.6 |
| Card9 | 0.4 | 0.2 | 0.1 | 1.6 | 3.2 | 0.0 | 0.2 | 0.0 | 0.1 | 0.3 | 0.1 | 0.1 |
| Carf | 2.6 | 2.9 | 2.5 | 1.8 | 2.3 | 2.9 | 1.8 | 2.7 | 2.2 | 2.0 | 2.4 | 2.9 |
| Carhsp1 | 49.8 | 38.3 | 30.9 | 44.6 | 43.0 | 49.1 | 40.2 | 35.1 | 37.6 | 41.4 | 40.8 | 32.7 |
| Carkd | 43.6 | 40.5 | 39.4 | 43.8 | 38.3 | 44.6 | 39.2 | 32.6 | 38.9 | 42.4 | 40.1 | 41.6 |
| Carm1 | 7.6 | 8.2 | 8.7 | 7.3 | 7.7 | 8.0 | 9.1 | 10.1 | 9.2 | 7.7 | 8.7 | 6.8 |
| Carns1 | 0.7 | 0.7 | 0.7 | 0.6 | 0.6 | 0.8 | 0.6 | 0.6 | 0.7 | 0.6 | 0.4 | 0.5 |
| Cars | 40.7 | 34.2 | 28.9 | 39.1 | 30.5 | 39.5 | 36.3 | 40.7 | 36.1 | 28.5 | 39.3 | 32.0 |
| Cars2 | 4.9 | 4.9 | 4.4 | 5.3 | 4.9 | 4.0 | 4.4 | 5.1 | 4.6 | 6.2 | 5.2 | 4.2 |
| Casc1 | 0.2 | 0.2 | 0.2 | 0.4 | 0.2 | 0.4 | 0.1 | 0.2 | 0.2 | 0.3 | 0.2 | 0.3 |
| Casc3 | 3.6 | 3.8 | 4.3 | 3.1 | 3.2 | 3.1 | 4.9 | 3.9 | 3.9 | 3.5 | 3.4 | 3.6 |
| Casc4 | 20.4 | 15.9 | 18.2 | 21.9 | 13.7 | 21.4 | 18.6 | 15.9 | 13.6 | 18.2 | 21.5 | 22.3 |
| Casc5 | 0.5 | 1.0 | 1.9 | 0.2 | 1.7 | 0.1 | 1.7 | 0.9 | 1.6 | 0.9 | 1.6 | 1.0 |
| Casd1 | 7.9 | 9.0 | 11.3 | 8.5 | 9.3 | 7.4 | 8.9 | 9.2 | 11.0 | 9.4 | 10.2 | 10.3 |
| Cask | 8.1 | 8.2 | 8.5 | 7.1 | 7.1 | 6.8 | 10.0 | 9.3 | 10.4 | 7.3 | 9.4 | 9.9 |
| Caskin1 | 0.0 | 0.1 | 0.1 | 0.1 | 0.0 | 0.1 | 0.0 | 0.1 | 0.1 | 0.1 | 0.0 | 0.0 |
| Caskin2 | 3.0 | 3.5 | 3.5 | 2.8 | 3.1 | 3.6 | 4.0 | 4.0 | 3.4 | 3.0 | 3.0 | 2.6 |
| Casp1 | 1.6 | 0.8 | 0.9 | 5.6 | 8.8 | 0.7 | 3.0 | 1.3 | 1.4 | 2.0 | 1.3 | 1.9 |
| Casp12 | 12.5 | 12.6 | 15.5 | 8.7 | 12.1 | 8.4 | 11.0 | 14.9 | 14.3 | 14.9 | 13.0 | 18.4 |
| Casp14 | 0.8 | 0.9 | 0.3 | 0.7 | 0.5 | 0.8 | 0.6 | 0.4 | 0.4 | 0.6 | 0.7 | 0.4 |
| Casp2 | 2.4 | 2.9 | 3.4 | 3.1 | 2.9 | 2.5 | 2.8 | 3.2 | 3.0 | 3.0 | 2.9 | 2.7 |
| Casp3 | 17.8 | 18.9 | 16.5 | 16.4 | 15.8 | 16.8 | 14.0 | 12.5 | 14.2 | 15.2 | 15.3 | 12.9 |
| Casp4 | 20.0 | 16.1 | 22.3 | 14.1 | 23.3 | 11.3 | 15.5 | 11.8 | 14.6 | 13.3 | 12.7 | 21.3 |
| Casp6 | 6.8 | 9.2 | 12.3 | 9.5 | 8.6 | 9.0 | 8.1 | 9.3 | 8.1 | 9.4 | 8.9 | 10.0 |
| Casp7 | 4.0 | 4.6 | 5.7 | 3.6 | 6.2 | 4.0 | 4.9 | 4.8 | 4.5 | 4.6 | 4.3 | 5.1 |
| Casp8 | 15.0 | 13.3 | 13.8 | 15.0 | 16.2 | 12.5 | 17.1 | 13.3 | 13.3 | 15.4 | 17.1 | 15.4 |
| Casp8ap2 | 3.5 | 4.6 | 5.3 | 3.1 | 4.4 | 3.2 | 4.8 | 4.5 | 4.3 | 4.2 | 5.5 | 4.9 |
| Casp9 | 3.6 | 4.8 | 4.8 | 3.7 | 5.1 | 3.8 | 4.7 | 6.0 | 4.8 | 3.7 | 3.4 | 3.5 |
| Casq1 | 0.7 | 0.6 | 0.6 | 1.1 | 0.5 | 0.4 | 0.4 | 0.4 | 0.4 | 0.4 | 0.5 | 0.2 |
| Casq2 | 0.5 | 0.4 | 0.1 | 0.8 | 0.4 | 0.4 | 0.4 | 0.4 | 0.4 | 0.2 | 0.6 | 0.2 |
| Cass4 | 0.1 | 0.2 | 0.1 | 0.2 | 0.2 | 0.3 | 0.1 | 0.1 | 0.1 | 0.1 | 0.1 | 0.1 |
| Cast | 73.3 | 60.9 | 62.9 | 69.2 | 60.0 | 64.3 | 61.8 | 60.0 | 51.9 | 52.5 | 68.8 | 65.1 |
| Casz1 | 0.0 | 0.0 | 0.1 | 0.1 | 0.1 | 0.0 | 0.1 | 0.1 | 0.0 | 0.1 | 0.1 | 0.0 |
| Cat | 104.4 | 95.7 | 72.3 | 172.2 | 137.1 | 159.8 | 63.3 | 74.0 | 47.8 | 67.3 | 72.6 | 56.1 |
| Catsper2 | 0.6 | 0.7 | 0.6 | 0.6 | 0.6 | 0.4 | 0.6 | 0.6 | 0.7 | 0.7 | 0.8 | 0.7 |
| Catsper3 | 0.1 | 0.2 | 0.2 | 0.0 | 0.0 | 0.1 | 0.1 | 0.1 | 0.2 | 0.1 | 0.1 | 0.1 |
| Catsperd | 0.3 | 0.1 | 0.1 | 0.0 | 0.1 | 0.2 | 0.1 | 0.1 | 0.1 | 0.1 | 0.1 | 0.1 |
| Catsperg1 | 0.3 | 0.4 | 0.2 | 0.1 | 0.2 | 0.3 | 0.1 | 0.1 | 0.2 | 0.2 | 0.3 | 0.1 |
| Catsperg2 | 0.1 | 0.1 | 0.1 | 0.0 | 0.1 | 0.1 | 0.1 | 0.0 | 0.1 | 0.1 | 0.1 | 0.1 |
| Cav1 | 125.4 | 69.6 | 63.8 | 58.9 | 57.0 | 67.9 | 87.2 | 94.9 | 77.7 | 181.3 | 170.8 | 120.1 |
| Cav2 | 11.6 | 11.3 | 9.8 | 13.8 | 13.7 | 15.8 | 10.0 | 8.4 | 8.9 | 9.6 | 13.3 | 12.1 |
| Cav3 | 4.1 | 4.4 | 4.5 | 3.5 | 2.6 | 2.8 | 3.3 | 4.3 | 3.8 | 1.2 | 2.2 | 2.6 |
| Cbfa2t2 | 5.2 | 6.1 | 5.8 | 4.8 | 5.1 | 4.8 | 6.1 | 5.6 | 6.4 | 5.5 | 5.1 | 5.7 |
| Cbfa2t3 | 0.0 | 0.0 | 0.0 | 0.0 | 0.0 | 0.0 | 0.0 | 0.0 | 0.0 | 0.0 | 0.0 | 0.0 |
| Cbfb | 13.8 | 16.8 | 21.5 | 16.4 | 20.4 | 15.8 | 19.2 | 20.1 | 18.4 | 19.0 | 20.4 | 21.6 |
| Cbl | 2.2 | 2.8 | 2.7 | 2.4 | 3.3 | 2.2 | 3.1 | 2.5 | 2.5 | 2.8 | 2.6 | 2.2 |
| Cblb | 1.7 | 2.7 | 3.3 | 1.7 | 2.6 | 1.7 | 3.0 | 3.9 | 3.4 | 3.3 | 2.9 | 3.5 |
| Cblc | 0.1 | 0.0 | 0.0 | 0.0 | 0.0 | 0.0 | 0.0 | 0.0 | 0.0 | 0.0 | 0.0 | 0.0 |
| Cbll1 | 0.6 | 1.0 | 1.4 | 1.1 | 1.3 | 1.2 | 1.2 | 1.2 | 1.2 | 1.2 | 1.3 | 1.1 |
| Cbln3 | 0.0 | 0.0 | 0.0 | 0.0 | 0.0 | 0.0 | 0.1 | 0.0 | 0.0 | 0.0 | 0.0 | 0.0 |
| Cbr1 | 27.4 | 25.3 | 25.8 | 33.2 | 24.3 | 38.3 | 21.5 | 24.0 | 19.8 | 25.2 | 22.6 | 23.9 |
| Cbr2 | 2.8 | 1.4 | 1.5 | 0.2 | 0.8 | 0.4 | 0.9 | 1.5 | 0.6 | 1.1 | 1.7 | 0.5 |
| Cbr3 | 38.1 | 30.1 | 18.9 | 41.3 | 28.6 | 42.4 | 19.2 | 21.3 | 16.2 | 25.3 | 20.2 | 18.1 |

| | | | | | | | | | | | | |
|---|---|---|---|---|---|---|---|---|---|---|---|---|
| Cbr4 | 12.9 | 14.4 | 13.0 | 11.8 | 11.0 | 14.1 | 12.0 | 12.9 | 12.7 | 13.0 | 13.7 | 13.9 |
| Cbs | 0.6 | 0.8 | 0.8 | 0.2 | 0.7 | 0.4 | 0.5 | 0.5 | 0.5 | 0.5 | 0.4 | 0.6 |
| Cbwd1 | 10.7 | 11.3 | 10.6 | 11.7 | 9.6 | 11.4 | 8.6 | 10.7 | 9.8 | 10.0 | 11.2 | 10.7 |
| Cbx1 | 18.6 | 18.5 | 20.5 | 18.5 | 16.0 | 17.9 | 20.0 | 21.4 | 17.0 | 18.4 | 20.2 | 21.0 |
| Cbx2 | 1.6 | 2.0 | 1.7 | 1.4 | 1.4 | 1.9 | 1.5 | 1.9 | 1.6 | 1.0 | 1.3 | 1.4 |
| Cbx3 | 43.8 | 48.0 | 60.4 | 40.8 | 45.0 | 49.0 | 48.0 | 51.0 | 47.8 | 58.3 | 64.0 | 60.3 |
| Cbx4 | 2.5 | 3.1 | 3.4 | 2.5 | 2.3 | 2.8 | 2.6 | 2.9 | 2.8 | 2.7 | 2.7 | 2.7 |
| Cbx5 | 15.1 | 16.9 | 20.2 | 12.6 | 15.6 | 12.7 | 17.4 | 20.7 | 20.2 | 14.5 | 18.9 | 18.5 |
| Cbx6 | 6.5 | 8.4 | 9.5 | 6.1 | 6.2 | 8.9 | 7.6 | 9.0 | 8.9 | 10.5 | 7.4 | 7.4 |
| Cbx7 | 1.2 | 1.4 | 1.8 | 1.9 | 1.3 | 2.1 | 0.7 | 0.7 | 1.0 | 1.0 | 0.9 | 1.2 |
| Cbx8 | 4.7 | 5.5 | 4.5 | 3.8 | 4.1 | 5.9 | 4.0 | 3.9 | 3.7 | 5.5 | 3.5 | 4.3 |
| Cby1 | 13.3 | 17.0 | 20.1 | 12.3 | 14.3 | 17.8 | 15.8 | 14.4 | 14.8 | 11.6 | 14.0 | 15.0 |
| Cc2d1a | 5.4 | 6.1 | 6.6 | 5.3 | 5.0 | 6.3 | 6.5 | 5.7 | 6.3 | 5.6 | 6.0 | 5.2 |
| Cc2d1b | 15.0 | 13.6 | 11.3 | 14.2 | 11.8 | 14.9 | 12.7 | 12.7 | 12.3 | 11.6 | 12.7 | 12.6 |
| Cc2d2a | 3.3 | 4.2 | 5.3 | 2.7 | 3.3 | 3.8 | 4.1 | 6.0 | 4.0 | 2.9 | 4.1 | 4.0 |
| Ccar1 | 17.6 | 18.1 | 21.0 | 18.3 | 17.7 | 16.1 | 21.0 | 20.9 | 16.2 | 19.7 | 24.1 | 23.8 |
| Ccbe1 | 0.6 | 1.2 | 2.5 | 0.2 | 0.5 | 0.2 | 0.4 | 0.9 | 1.1 | 0.9 | 0.5 | 1.3 |
| Ccbl1 | 1.2 | 1.6 | 1.3 | 1.5 | 1.0 | 1.7 | 1.4 | 1.7 | 1.0 | 1.3 | 1.1 | 0.8 |
| Ccbl2 | 2.0 | 2.5 | 2.7 | 1.4 | 1.8 | 1.8 | 1.8 | 2.5 | 2.5 | 2.2 | 2.1 | 1.8 |
| Ccbp2 | 0.0 | 0.1 | 0.3 | 0.1 | 0.1 | 0.0 | 0.1 | 0.8 | 0.1 | 0.0 | 0.1 | 0.1 |
| Ccdc101 | 3.1 | 3.3 | 3.0 | 4.3 | 3.6 | 3.1 | 2.1 | 2.9 | 2.7 | 2.7 | 2.8 | 2.0 |
| Ccdc102a | 9.8 | 13.1 | 14.4 | 9.8 | 10.8 | 12.0 | 20.3 | 20.3 | 22.1 | 12.4 | 14.7 | 15.1 |
| Ccdc103 | 0.8 | 0.6 | 0.4 | 0.9 | 0.5 | 0.8 | 0.5 | 0.3 | 0.5 | 0.5 | 0.5 | 0.2 |
| Ccdc104 | 43.1 | 36.5 | 41.0 | 52.2 | 35.2 | 41.8 | 38.9 | 32.0 | 29.7 | 38.9 | 47.0 | 45.4 |
| Ccdc105 | 0.1 | 0.1 | 0.1 | 0.1 | 0.1 | 0.1 | 0.0 | 0.0 | 0.1 | 0.0 | 0.0 | 0.1 |
| Ccdc106 | 0.4 | 0.4 | 0.3 | 0.3 | 0.4 | 0.3 | 0.3 | 0.3 | 0.3 | 0.4 | 0.3 | 0.2 |
| Ccdc107 | 28.8 | 24.0 | 21.2 | 27.9 | 24.0 | 27.9 | 26.2 | 20.6 | 27.6 | 28.6 | 25.9 | 26.9 |
| Ccdc109b | 16.2 | 16.2 | 15.4 | 23.5 | 20.3 | 19.2 | 19.2 | 14.8 | 13.7 | 11.9 | 18.1 | 12.9 |
| Ccdc11 | 0.1 | 0.1 | 0.1 | 0.0 | 0.0 | 0.1 | 0.0 | 0.0 | 0.0 | 0.0 | 0.0 | 0.1 |
| Ccdc110 | 0.0 | 0.1 | 0.0 | 0.1 | 0.1 | 0.1 | 0.0 | 0.1 | 0.1 | 0.0 | 0.1 | 0.1 |
| Ccdc111 | 3.2 | 3.6 | 4.0 | 3.3 | 3.1 | 3.1 | 3.6 | 3.1 | 3.1 | 2.5 | 3.3 | 3.3 |
| Ccdc112 | 1.1 | 1.6 | 1.5 | 0.9 | 1.1 | 1.4 | 1.1 | 1.0 | 1.1 | 1.1 | 1.2 | 1.7 |
| Ccdc114 | 0.6 | 0.6 | 0.5 | 0.6 | 0.3 | 0.6 | 0.5 | 0.5 | 0.4 | 0.5 | 0.5 | 0.4 |
| Ccdc115 | 6.7 | 6.6 | 6.5 | 8.5 | 8.2 | 6.4 | 7.5 | 5.9 | 5.9 | 6.5 | 6.9 | 6.4 |
| Ccdc116 | 0.1 | 0.1 | 0.0 | 0.1 | 0.0 | 0.0 | 0.1 | 0.1 | 0.0 | 0.1 | 0.1 | 0.1 |
| Ccdc117 | 6.6 | 8.9 | 7.7 | 6.8 | 7.3 | 6.7 | 6.3 | 5.5 | 6.9 | 7.6 | 6.5 | 7.5 |
| Ccdc12 | 24.8 | 22.6 | 23.3 | 29.0 | 33.5 | 20.4 | 26.8 | 26.3 | 28.7 | 27.4 | 23.8 | 26.2 |
| Ccdc120 | 0.2 | 0.5 | 0.3 | 0.3 | 0.3 | 0.2 | 0.3 | 0.4 | 0.4 | 0.4 | 0.3 | 0.3 |
| Ccdc121 | 0.0 | 0.0 | 0.1 | 0.0 | 0.1 | 0.0 | 0.0 | 0.1 | 0.0 | 0.0 | 0.0 | 0.1 |
| Ccdc122 | 6.1 | 6.6 | 8.3 | 8.1 | 5.9 | 9.4 | 7.5 | 6.2 | 4.8 | 5.0 | 6.8 | 8.3 |
| Ccdc124 | 36.2 | 32.0 | 30.7 | 50.7 | 34.7 | 41.6 | 39.7 | 37.7 | 31.5 | 34.5 | 37.7 | 33.5 |
| Ccdc125 | 0.9 | 0.6 | 0.4 | 0.8 | 0.6 | 0.7 | 0.6 | 0.7 | 0.5 | 0.7 | 0.9 | 0.6 |
| Ccdc126 | 6.0 | 5.5 | 5.0 | 6.1 | 4.8 | 5.8 | 4.5 | 4.0 | 4.5 | 5.2 | 4.9 | 5.7 |
| Ccdc127 | 21.9 | 22.0 | 21.9 | 22.4 | 21.3 | 21.3 | 24.6 | 20.7 | 20.9 | 20.8 | 25.5 | 25.7 |
| Ccdc13 | 0.0 | 0.0 | 0.1 | 0.0 | 0.0 | 0.2 | 0.0 | 0.0 | 0.0 | 0.0 | 0.0 | 0.0 |
| Ccdc130 | 3.9 | 3.4 | 4.0 | 4.5 | 3.3 | 3.8 | 3.7 | 3.3 | 2.8 | 3.1 | 4.0 | 4.0 |
| Ccdc132 | 12.6 | 13.0 | 10.0 | 14.7 | 12.1 | 13.8 | 10.9 | 11.0 | 10.5 | 11.3 | 11.7 | 11.5 |
| Ccdc134 | 5.0 | 4.5 | 3.5 | 5.5 | 4.6 | 4.0 | 5.8 | 6.1 | 5.7 | 4.3 | 5.6 | 4.0 |
| Ccdc135 | 0.0 | 0.0 | 0.0 | 0.1 | 0.0 | 0.0 | 0.0 | 0.0 | 0.0 | 0.0 | 0.0 | 0.0 |
| Ccdc136 | 2.2 | 2.0 | 2.6 | 2.2 | 1.5 | 2.4 | 1.8 | 2.5 | 1.9 | 3.1 | 2.4 | 1.8 |
| Ccdc137 | 7.2 | 5.3 | 5.0 | 9.6 | 5.9 | 6.6 | 7.0 | 5.0 | 5.3 | 6.1 | 7.1 | 7.0 |
| Ccdc138 | 0.4 | 0.9 | 0.8 | 0.5 | 0.6 | 0.8 | 0.4 | 0.6 | 0.5 | 0.5 | 0.7 | 0.7 |
| Ccdc14 | 0.5 | 0.7 | 0.8 | 0.4 | 0.7 | 0.4 | 0.7 | 0.6 | 0.7 | 0.6 | 0.7 | 0.5 |
| Ccdc141 | 0.3 | 0.2 | 0.2 | 0.3 | 0.2 | 0.2 | 0.3 | 0.2 | 0.4 | 1.2 | 0.5 | 0.3 |
| Ccdc142 | 0.9 | 0.7 | 0.8 | 1.1 | 1.2 | 1.2 | 0.9 | 0.7 | 1.0 | 0.7 | 0.7 | 0.8 |
| Ccdc144b | 0.1 | 0.0 | 0.1 | 0.0 | 0.0 | 0.0 | 0.0 | 0.0 | 0.0 | 0.0 | 0.0 | 0.0 |
| Ccdc146 | 0.0 | 0.0 | 0.0 | 0.0 | 0.0 | 0.0 | 0.0 | 0.0 | 0.0 | 0.0 | 0.1 | 0.0 |

| | | | | | | | | | | | | |
|---|---|---|---|---|---|---|---|---|---|---|---|---|
| Ccdc147 | 0.1 | 0.1 | 0.0 | 0.1 | 0.1 | 0.1 | 0.1 | 0.0 | 0.0 | 0.1 | 0.0 | 0.0 |
| Ccdc148 | 0.3 | 0.1 | 0.1 | 0.1 | 0.1 | 0.2 | 0.0 | 0.0 | 0.0 | 0.1 | 0.1 | 0.0 |
| Ccdc149 | 5.4 | 4.6 | 4.2 | 5.1 | 3.5 | 5.4 | 4.3 | 4.3 | 4.5 | 4.8 | 5.6 | 5.5 |
| Ccdc15 | 0.9 | 1.3 | 1.5 | 0.8 | 1.0 | 0.8 | 1.0 | 0.7 | 0.9 | 0.8 | 0.9 | 1.1 |
| Ccdc150 | 0.0 | 0.0 | 0.0 | 0.0 | 0.0 | 0.0 | 0.0 | 0.0 | 0.0 | 0.0 | 0.0 | 0.0 |
| Ccdc151 | 0.2 | 0.3 | 0.4 | 0.3 | 0.3 | 0.5 | 0.1 | 0.2 | 0.2 | 0.2 | 0.2 | 0.2 |
| Ccdc152 | 0.3 | 0.1 | 0.1 | 0.1 | 0.2 | 0.1 | 0.1 | 0.0 | 0.1 | 0.0 | 0.0 | 0.1 |
| Ccdc154 | 0.0 | 0.1 | 0.0 | 0.0 | 0.0 | 0.1 | 0.0 | 0.0 | 0.0 | 0.0 | 0.0 | 0.0 |
| Ccdc155 | 0.0 | 0.1 | 0.1 | 0.0 | 0.1 | 0.2 | 0.0 | 0.0 | 0.1 | 0.1 | 0.1 | 0.0 |
| Ccdc157 | 3.7 | 3.8 | 4.0 | 4.0 | 3.6 | 4.1 | 3.9 | 3.4 | 4.0 | 3.2 | 3.4 | 3.3 |
| Ccdc158 | 0.1 | 0.1 | 0.1 | 0.0 | 0.1 | 0.2 | 0.1 | 0.0 | 0.1 | 0.2 | 0.1 | 0.1 |
| Ccdc159 | 2.1 | 2.8 | 2.8 | 1.8 | 1.8 | 2.9 | 1.9 | 2.6 | 1.8 | 1.4 | 1.8 | 2.2 |
| Ccdc160 | 0.4 | 0.4 | 0.5 | 0.4 | 0.3 | 0.6 | 0.1 | 0.1 | 0.2 | 0.2 | 0.2 | 0.3 |
| Ccdc162 | 0.1 | 0.0 | 0.0 | 0.0 | 0.0 | 0.0 | 0.0 | 0.0 | 0.0 | 0.0 | 0.0 | 0.0 |
| Ccdc163 | 3.3 | 3.4 | 3.2 | 2.8 | 3.4 | 4.0 | 3.1 | 3.7 | 3.3 | 2.2 | 3.6 | 3.1 |
| Ccdc164 | 0.1 | 0.0 | 0.1 | 0.2 | 0.0 | 0.2 | 0.1 | 0.1 | 0.0 | 0.1 | 0.1 | 0.0 |
| Ccdc166 | 0.5 | 0.4 | 0.5 | 0.3 | 0.7 | 0.7 | 0.2 | 0.4 | 0.5 | 0.5 | 0.4 | 0.4 |
| Ccdc167 | 6.6 | 6.8 | 5.8 | 6.2 | 5.7 | 5.8 | 5.2 | 5.6 | 6.0 | 4.8 | 6.0 | 4.9 |
| Ccdc169 | 0.1 | 0.0 | 0.0 | 0.1 | 0.1 | 0.1 | 0.0 | 0.0 | 0.0 | 0.1 | 0.1 | 0.1 |
| Ccdc17 | 1.0 | 1.3 | 1.2 | 0.6 | 0.8 | 0.8 | 1.0 | 1.0 | 1.0 | 0.8 | 1.1 | 0.8 |
| Ccdc170 | 0.1 | 0.1 | 0.2 | 0.1 | 0.2 | 0.2 | 0.1 | 0.1 | 0.2 | 0.1 | 0.1 | 0.1 |
| Ccdc171 | 1.5 | 1.5 | 1.8 | 1.5 | 1.4 | 1.7 | 1.4 | 1.6 | 1.3 | 1.9 | 1.5 | 1.7 |
| Ccdc173 | 1.5 | 1.3 | 1.8 | 1.9 | 1.1 | 1.8 | 0.9 | 1.3 | 0.8 | 0.7 | 1.0 | 1.7 |
| Ccdc174 | 7.5 | 7.8 | 8.5 | 9.2 | 7.4 | 8.6 | 8.2 | 7.1 | 6.5 | 7.2 | 9.2 | 10.3 |
| Ccdc175 | 0.0 | 0.0 | 0.0 | 0.0 | 0.0 | 0.0 | 0.0 | 0.0 | 0.0 | 0.0 | 0.0 | 0.0 |
| Ccdc176 | 0.2 | 0.3 | 0.3 | 0.4 | 0.2 | 0.3 | 0.1 | 0.2 | 0.2 | 0.1 | 0.1 | 0.2 |
| Ccdc18 | 0.4 | 1.0 | 1.6 | 0.2 | 0.8 | 0.4 | 1.1 | 0.6 | 0.8 | 0.6 | 1.2 | 0.8 |
| Ccdc19 | 1.4 | 0.7 | 0.5 | 2.4 | 1.2 | 1.3 | 1.5 | 0.6 | 0.6 | 0.9 | 1.1 | 1.3 |
| Ccdc22 | 6.9 | 6.8 | 6.1 | 7.5 | 8.0 | 7.4 | 5.3 | 6.5 | 6.0 | 6.8 | 5.6 | 5.1 |
| Ccdc23 | 21.8 | 19.7 | 16.1 | 25.9 | 21.3 | 22.8 | 19.0 | 21.6 | 21.6 | 25.3 | 21.8 | 17.8 |
| Ccdc24 | 0.5 | 0.8 | 0.7 | 0.5 | 0.4 | 1.0 | 0.9 | 0.6 | 0.7 | 0.7 | 0.6 | 0.4 |
| Ccdc25 | 17.7 | 16.8 | 16.6 | 19.7 | 14.5 | 17.0 | 16.1 | 17.5 | 14.6 | 13.6 | 18.2 | 17.8 |
| Ccdc27 | 0.0 | 0.0 | 0.0 | 0.1 | 0.0 | 0.0 | 0.0 | 0.1 | 0.0 | 0.0 | 0.0 | 0.0 |
| Ccdc28a | 5.1 | 3.6 | 3.5 | 6.0 | 4.2 | 6.0 | 3.8 | 4.9 | 4.1 | 4.5 | 4.1 | 4.5 |
| Ccdc28b | 5.0 | 6.3 | 8.7 | 5.9 | 5.8 | 7.6 | 7.1 | 5.9 | 7.1 | 4.6 | 5.7 | 6.5 |
| Ccdc3 | 0.1 | 0.0 | 0.1 | 0.1 | 0.2 | 0.1 | 0.2 | 0.1 | 0.0 | 0.1 | 0.1 | 0.0 |
| Ccdc30 | 0.5 | 0.2 | 0.2 | 0.2 | 0.2 | 0.4 | 0.2 | 0.1 | 0.1 | 0.1 | 0.3 | 0.1 |
| Ccdc32 | 8.6 | 9.0 | 8.6 | 10.4 | 8.5 | 9.4 | 9.2 | 8.5 | 8.1 | 9.0 | 9.1 | 9.6 |
| Ccdc34 | 9.3 | 8.2 | 13.2 | 9.9 | 8.5 | 10.0 | 12.4 | 11.4 | 6.8 | 6.1 | 9.9 | 11.4 |
| Ccdc36 | 0.0 | 0.0 | 0.0 | 0.0 | 0.0 | 0.0 | 0.0 | 0.0 | 0.0 | 0.0 | 0.0 | 0.0 |
| Ccdc37 | 1.3 | 1.4 | 1.3 | 1.5 | 0.9 | 2.3 | 1.5 | 1.6 | 1.9 | 1.0 | 1.4 | 1.0 |
| Ccdc38 | 0.0 | 0.1 | 0.0 | 0.0 | 0.1 | 0.1 | 0.1 | 0.1 | 0.1 | 0.2 | 0.2 | 0.1 |
| Ccdc39 | 1.2 | 1.0 | 1.4 | 1.3 | 0.8 | 1.7 | 1.0 | 0.8 | 0.9 | 0.9 | 0.8 | 1.3 |
| Ccdc40 | 0.3 | 0.3 | 0.2 | 0.5 | 0.2 | 0.4 | 0.2 | 0.1 | 0.0 | 0.2 | 0.2 | 0.2 |
| Ccdc41 | 16.7 | 14.3 | 14.6 | 17.6 | 13.8 | 13.0 | 12.8 | 10.8 | 11.0 | 10.2 | 14.2 | 14.2 |
| Ccdc43 | 9.2 | 8.3 | 7.9 | 11.3 | 7.3 | 11.0 | 6.9 | 7.7 | 6.6 | 7.3 | 9.2 | 8.8 |
| Ccdc47 | 45.5 | 38.4 | 40.6 | 48.2 | 32.9 | 42.8 | 40.9 | 36.6 | 33.0 | 35.5 | 43.5 | 45.1 |
| Ccdc50 | 15.2 | 16.2 | 16.6 | 17.4 | 14.9 | 14.4 | 14.1 | 15.5 | 13.6 | 14.7 | 16.7 | 15.3 |
| Ccdc51 | 2.5 | 2.4 | 2.3 | 2.8 | 2.3 | 2.7 | 2.1 | 2.0 | 2.6 | 2.4 | 2.2 | 2.4 |
| Ccdc53 | 41.9 | 44.1 | 41.0 | 38.5 | 31.6 | 36.1 | 31.1 | 35.1 | 35.9 | 37.8 | 42.2 | 41.5 |
| Ccdc55 | 20.8 | 16.1 | 21.0 | 28.8 | 14.9 | 19.0 | 16.7 | 14.4 | 9.4 | 13.8 | 18.1 | 23.0 |
| Ccdc57 | 0.5 | 0.4 | 0.5 | 0.5 | 0.4 | 0.7 | 0.3 | 0.4 | 0.3 | 0.4 | 0.2 | 0.3 |
| Ccdc58 | 18.3 | 16.5 | 15.6 | 13.0 | 14.2 | 18.2 | 14.0 | 12.6 | 14.0 | 15.7 | 19.0 | 16.1 |
| Ccdc59 | 18.2 | 17.6 | 16.1 | 20.2 | 13.9 | 18.8 | 15.3 | 14.0 | 14.4 | 15.3 | 16.4 | 17.4 |
| Ccdc6 | 12.2 | 13.8 | 13.2 | 12.3 | 11.6 | 13.7 | 15.3 | 16.7 | 14.1 | 12.1 | 16.0 | 12.9 |
| Ccdc61 | 2.3 | 2.2 | 2.1 | 2.5 | 2.4 | 2.2 | 3.3 | 2.9 | 2.9 | 2.7 | 2.0 | 2.2 |
| Ccdc62 | 0.3 | 0.3 | 0.3 | 0.3 | 0.3 | 0.6 | 0.4 | 0.4 | 0.4 | 0.2 | 0.5 | 0.4 |

| | | | | | | | | | | | | |
|---|---|---|---|---|---|---|---|---|---|---|---|---|
| Ccdc63 | 0.1 | 0.1 | 0.1 | 0.1 | 0.1 | 0.1 | 0.1 | 0.1 | 0.0 | 0.0 | 0.1 | 0.0 |
| Ccdc64 | 1.2 | 1.2 | 0.8 | 1.3 | 1.0 | 2.1 | 1.1 | 0.9 | 1.0 | 1.2 | 1.0 | 1.0 |
| Ccdc64b | 0.1 | 0.0 | 0.0 | 0.0 | 0.0 | 0.0 | 0.1 | 0.0 | 0.1 | 0.0 | 0.0 | 0.0 |
| Ccdc65 | 1.0 | 0.8 | 0.8 | 1.0 | 0.8 | 1.3 | 0.4 | 0.5 | 0.4 | 0.6 | 0.7 | 0.9 |
| Ccdc66 | 9.0 | 9.2 | 10.9 | 11.7 | 7.9 | 12.0 | 9.7 | 9.5 | 8.4 | 7.2 | 10.6 | 9.7 |
| Ccdc68 | 8.5 | 8.0 | 7.0 | 10.7 | 5.9 | 8.5 | 7.3 | 4.6 | 6.0 | 5.6 | 8.9 | 9.2 |
| Ccdc69 | 0.2 | 0.2 | 0.5 | 0.3 | 0.4 | 0.2 | 0.2 | 0.3 | 0.3 | 0.3 | 0.5 | 0.2 |
| Ccdc71 | 7.7 | 7.3 | 7.4 | 8.8 | 7.5 | 8.1 | 7.9 | 7.6 | 7.8 | 7.5 | 8.0 | 7.6 |
| Ccdc71l | 3.0 | 3.9 | 5.2 | 3.5 | 3.8 | 2.1 | 3.3 | 4.4 | 4.0 | 3.8 | 3.2 | 4.7 |
| Ccdc73 | 0.1 | 0.2 | 0.1 | 0.1 | 0.1 | 0.1 | 0.1 | 0.1 | 0.0 | 0.1 | 0.2 | 0.1 |
| Ccdc74a | 1.0 | 0.8 | 0.8 | 0.8 | 0.8 | 1.6 | 0.8 | 0.8 | 0.3 | 1.0 | 1.0 | 0.7 |
| Ccdc77 | 3.2 | 3.1 | 3.7 | 2.4 | 3.2 | 3.0 | 3.4 | 2.9 | 2.8 | 2.6 | 3.1 | 2.3 |
| Ccdc78 | 0.1 | 0.2 | 0.1 | 0.2 | 0.1 | 0.0 | 0.1 | 0.1 | 0.1 | 0.1 | 0.0 | 0.1 |
| Ccdc79 | 0.3 | 0.5 | 0.3 | 0.4 | 0.3 | 0.4 | 0.2 | 0.2 | 0.3 | 0.3 | 0.3 | 0.3 |
| Ccdc8 | 1.7 | 3.8 | 5.0 | 1.9 | 3.3 | 2.3 | 3.1 | 4.9 | 3.4 | 2.8 | 2.9 | 3.0 |
| Ccdc80 | 354.9 | 369.1 | 544.1 | 317.2 | 333.6 | 251.4 | 525.1 | 762.5 | 634.0 | 446.4 | 525.4 | 591.6 |
| Ccdc81 | 0.1 | 0.0 | 0.1 | 0.0 | 0.0 | 0.0 | 0.0 | 0.0 | 0.0 | 0.1 | 0.0 | 0.0 |
| Ccdc82 | 14.8 | 19.2 | 23.6 | 15.5 | 16.9 | 15.1 | 18.1 | 22.0 | 17.1 | 14.1 | 17.2 | 17.0 |
| Ccdc83 | 0.1 | 0.1 | 0.0 | 0.1 | 0.1 | 0.2 | 0.0 | 0.0 | 0.0 | 0.0 | 0.0 | 0.0 |
| Ccdc84 | 3.5 | 4.2 | 3.6 | 3.6 | 3.5 | 3.3 | 5.1 | 3.8 | 4.1 | 3.6 | 4.8 | 4.6 |
| Ccdc85a | 0.2 | 0.1 | 0.1 | 0.1 | 0.0 | 0.1 | 0.0 | 0.0 | 0.0 | 0.1 | 0.1 | 0.0 |
| Ccdc85b | 2.6 | 2.1 | 1.9 | 3.2 | 2.9 | 3.8 | 2.9 | 2.0 | 2.6 | 4.6 | 2.7 | 2.3 |
| Ccdc85c | 0.5 | 0.5 | 0.9 | 0.3 | 0.4 | 0.4 | 1.2 | 0.8 | 1.0 | 0.7 | 0.4 | 0.6 |
| Ccdc86 | 4.2 | 3.9 | 3.0 | 4.8 | 4.7 | 3.6 | 4.2 | 3.1 | 3.4 | 4.3 | 3.4 | 3.3 |
| Ccdc87 | 0.1 | 0.1 | 0.1 | 0.0 | 0.1 | 0.2 | 0.0 | 0.0 | 0.0 | 0.1 | 0.1 | 0.0 |
| Ccdc88a | 4.4 | 5.2 | 6.7 | 5.4 | 5.6 | 5.8 | 5.4 | 5.4 | 4.8 | 4.8 | 6.2 | 6.1 |
| Ccdc88b | 0.5 | 0.5 | 0.3 | 1.1 | 2.1 | 0.2 | 0.7 | 0.2 | 0.2 | 0.2 | 0.2 | 0.3 |
| Ccdc88c | 0.4 | 0.4 | 0.7 | 0.2 | 0.1 | 0.2 | 0.2 | 0.5 | 0.1 | 0.1 | 0.2 | 0.1 |
| Ccdc89 | 0.7 | 1.0 | 0.7 | 0.9 | 0.5 | 1.1 | 0.6 | 0.8 | 0.7 | 0.4 | 0.5 | 0.6 |
| Ccdc9 | 7.2 | 6.2 | 7.4 | 9.0 | 7.6 | 7.7 | 8.2 | 8.2 | 7.6 | 7.1 | 7.5 | 7.1 |
| Ccdc90a | 4.1 | 3.6 | 3.8 | 4.3 | 4.3 | 3.8 | 3.7 | 4.4 | 3.8 | 3.0 | 3.5 | 3.9 |
| Ccdc90b | 21.3 | 21.9 | 20.5 | 20.8 | 17.4 | 20.6 | 20.8 | 21.7 | 20.3 | 21.6 | 22.6 | 25.9 |
| Ccdc91 | 25.1 | 20.5 | 21.2 | 32.2 | 19.1 | 28.4 | 21.8 | 21.6 | 18.5 | 21.5 | 26.9 | 25.1 |
| Ccdc92 | 1.9 | 1.5 | 1.3 | 1.9 | 1.6 | 1.7 | 1.6 | 1.4 | 1.1 | 2.0 | 1.9 | 1.4 |
| Ccdc93 | 7.5 | 6.7 | 6.9 | 8.2 | 7.4 | 8.1 | 6.5 | 5.9 | 5.8 | 6.7 | 7.0 | 7.0 |
| Ccdc94 | 3.5 | 3.4 | 2.9 | 4.0 | 3.4 | 2.7 | 4.1 | 4.2 | 3.8 | 3.4 | 3.0 | 2.8 |
| Ccdc96 | 0.3 | 0.2 | 0.1 | 0.2 | 0.3 | 0.4 | 0.1 | 0.1 | 0.1 | 0.2 | 0.2 | 0.3 |
| Ccdc97 | 3.2 | 3.7 | 3.9 | 4.2 | 4.2 | 3.6 | 4.4 | 4.3 | 3.5 | 3.8 | 3.5 | 3.5 |
| Cchcr1 | 3.5 | 4.5 | 4.7 | 3.0 | 3.5 | 2.9 | 4.3 | 3.7 | 4.3 | 3.9 | 3.7 | 2.9 |
| Ccin | 0.0 | 0.1 | 0.2 | 0.0 | 0.1 | 0.1 | 0.1 | 0.2 | 0.1 | 0.1 | 0.0 | 0.0 |
| Cck | 1.0 | 0.3 | 0.1 | 1.4 | 0.2 | 1.2 | 0.0 | 0.0 | 0.2 | 3.0 | 2.0 | 0.8 |
| Ccl11 | 0.0 | 0.1 | 0.3 | 0.0 | 0.5 | 0.0 | 0.0 | 0.2 | 0.1 | 0.1 | 0.1 | 0.0 |
| Ccl12 | 1.2 | 0.0 | 0.1 | 5.6 | 8.4 | 0.0 | 0.2 | 0.1 | 0.2 | 0.6 | 0.0 | 1.1 |
| Ccl17 | 19.0 | 12.2 | 5.5 | 40.1 | 22.5 | 23.9 | 17.7 | 6.0 | 8.6 | 18.6 | 12.6 | 11.9 |
| Ccl19 | 0.1 | 0.3 | 0.9 | 0.1 | 0.5 | 0.0 | 0.1 | 1.0 | 0.2 | 0.1 | 0.0 | 0.1 |
| Ccl2 | 310.4 | 124.7 | 185.5 | 564.4 | 808.5 | 232.0 | 358.7 | 127.5 | 138.8 | 161.7 | 97.5 | 161.4 |
| Ccl20 | 6.5 | 1.5 | 1.7 | 1.6 | 3.6 | 0.2 | 2.4 | 2.0 | 3.1 | 0.8 | 0.9 | 1.4 |
| Ccl21a | 0.0 | 0.1 | 0.1 | 0.0 | 0.0 | 0.0 | 0.0 | 0.0 | 0.0 | 0.0 | 0.0 | 0.0 |
| Ccl21b | 0.0 | 0.1 | 0.3 | 0.0 | 0.1 | 0.0 | 0.0 | 0.2 | 0.0 | 0.0 | 0.0 | 0.0 |
| Ccl21c | 0.0 | 0.3 | 0.1 | 0.0 | 0.0 | 0.0 | 0.0 | 0.0 | 0.0 | 0.0 | 0.0 | 0.0 |
| Ccl25 | 4.0 | 3.7 | 4.1 | 4.9 | 2.8 | 3.7 | 3.3 | 3.2 | 2.6 | 3.6 | 4.1 | 3.9 |
| Ccl27a | 5.0 | 3.9 | 3.8 | 4.3 | 3.1 | 5.9 | 2.7 | 5.3 | 3.2 | 4.9 | 5.1 | 3.7 |
| Ccl27b | 0.8 | 0.6 | 0.9 | 0.6 | 0.4 | 0.5 | 0.4 | 0.5 | 0.5 | 0.8 | 0.6 | 0.6 |
| Ccl28 | 0.1 | 0.2 | 0.2 | 0.1 | 0.1 | 0.1 | 0.5 | 0.2 | 0.2 | 0.1 | 0.1 | 0.1 |
| Ccl3 | 4.9 | 0.0 | 0.5 | 34.8 | 86.5 | 0.1 | 18.9 | 0.5 | 2.4 | 7.3 | 2.0 | 7.0 |
| Ccl4 | 1.7 | 0.1 | 0.1 | 16.8 | 39.8 | 0.0 | 5.8 | 0.1 | 1.2 | 3.6 | 0.3 | 2.4 |
| Ccl5 | 3.3 | 1.3 | 0.9 | 14.6 | 11.8 | 8.0 | 1.9 | 0.1 | 0.4 | 0.4 | 0.1 | 0.7 |

| | | | | | | | | | | | | |
|---|---|---|---|---|---|---|---|---|---|---|---|---|
| Ccl6 | 6.6 | 5.3 | 2.6 | 20.4 | 8.5 | 5.5 | 6.5 | 1.5 | 3.0 | 3.2 | 4.8 | 3.0 |
| Ccl7 | 280.1 | 198.4 | 266.5 | 385.4 | 583.3 | 245.2 | 347.6 | 187.7 | 209.6 | 161.9 | 122.7 | 194.3 |
| Ccl8 | 14.9 | 8.3 | 20.2 | 40.9 | 71.5 | 11.6 | 10.6 | 14.9 | 9.2 | 9.1 | 4.3 | 8.3 |
| Ccl9 | 8.2 | 7.8 | 7.9 | 16.1 | 32.3 | 3.2 | 15.2 | 6.0 | 12.3 | 11.3 | 13.9 | 8.7 |
| Ccm2 | 22.3 | 20.2 | 16.6 | 24.9 | 21.1 | 21.6 | 21.4 | 18.1 | 22.3 | 20.0 | 23.1 | 19.1 |
| Ccm2l | 0.0 | 0.0 | 0.0 | 0.0 | 0.0 | 0.0 | 0.0 | 0.0 | 0.0 | 0.0 | 0.0 | 0.0 |
| Ccna1 | 0.0 | 0.1 | 0.1 | 0.0 | 0.0 | 0.0 | 0.0 | 0.0 | 0.0 | 0.0 | 0.0 | 0.0 |
| Ccna2 | 4.2 | 7.4 | 12.3 | 2.3 | 9.2 | 1.8 | 13.3 | 6.1 | 10.8 | 5.1 | 9.3 | 6.1 |
| Ccnb1 | 3.6 | 7.6 | 11.3 | 1.1 | 10.9 | 1.1 | 15.0 | 4.9 | 12.6 | 5.7 | 11.5 | 7.3 |
| Ccnb2 | 3.6 | 6.3 | 9.7 | 0.8 | 9.9 | 0.9 | 11.7 | 4.0 | 8.4 | 4.4 | 6.4 | 5.8 |
| Ccnb3 | 0.0 | 0.0 | 0.0 | 0.0 | 0.0 | 0.0 | 0.0 | 0.0 | 0.0 | 0.0 | 0.0 | 0.0 |
| Ccnc | 10.5 | 11.1 | 11.4 | 13.8 | 10.2 | 13.4 | 10.4 | 11.8 | 10.5 | 10.1 | 13.0 | 12.2 |
| Ccnd1 | 186.1 | 72.8 | 35.1 | 269.1 | 129.8 | 271.0 | 83.1 | 19.1 | 21.1 | 56.9 | 57.7 | 33.9 |
| Ccnd2 | 148.8 | 116.2 | 99.4 | 136.1 | 130.0 | 162.5 | 126.6 | 95.5 | 97.0 | 174.8 | 147.6 | 126.3 |
| Ccnd3 | 28.8 | 32.1 | 27.5 | 26.8 | 27.7 | 29.3 | 29.7 | 33.4 | 32.2 | 32.1 | 30.4 | 25.9 |
| Ccndbp1 | 20.3 | 20.2 | 17.4 | 24.2 | 18.8 | 22.2 | 19.0 | 19.6 | 17.9 | 20.8 | 19.0 | 19.5 |
| Ccne1 | 4.5 | 6.4 | 6.9 | 3.7 | 5.7 | 4.8 | 7.7 | 5.8 | 5.2 | 5.0 | 7.3 | 5.5 |
| Ccne2 | 0.9 | 1.6 | 2.9 | 0.8 | 2.3 | 0.4 | 3.1 | 2.1 | 1.8 | 1.6 | 2.1 | 1.6 |
| Ccnf | 0.8 | 1.7 | 2.3 | 0.8 | 2.6 | 0.7 | 3.2 | 1.4 | 2.5 | 1.3 | 2.0 | 1.6 |
| Ccng1 | 435.6 | 361.8 | 293.7 | 397.4 | 359.4 | 412.0 | 316.6 | 180.7 | 263.8 | 420.2 | 436.6 | 398.8 |
| Ccng2 | 15.2 | 21.6 | 18.8 | 14.1 | 17.6 | 16.4 | 17.1 | 17.0 | 24.3 | 19.9 | 16.3 | 19.2 |
| Ccnh | 10.6 | 9.2 | 9.9 | 10.4 | 8.3 | 10.0 | 9.4 | 9.7 | 7.8 | 13.6 | 12.8 | 12.2 |
| Ccni | 34.4 | 31.5 | 31.5 | 34.2 | 29.0 | 40.2 | 31.7 | 30.8 | 34.4 | 32.7 | 32.0 | 31.8 |
| Ccnj | 1.0 | 0.9 | 1.0 | 1.0 | 0.8 | 1.1 | 0.8 | 1.0 | 1.1 | 1.0 | 0.9 | 1.0 |
| Ccnjl | 0.0 | 0.0 | 0.0 | 0.0 | 0.0 | 0.0 | 0.0 | 0.1 | 0.0 | 0.0 | 0.0 | 0.0 |
| Ccnk | 1.4 | 1.8 | 2.4 | 1.9 | 1.8 | 1.3 | 1.6 | 2.2 | 1.7 | 1.5 | 1.9 | 1.4 |
| Ccnl1 | 11.5 | 10.7 | 12.0 | 10.0 | 11.7 | 7.9 | 10.3 | 11.6 | 11.8 | 13.9 | 12.2 | 13.6 |
| Ccnl2 | 15.8 | 18.4 | 21.3 | 11.8 | 14.9 | 11.6 | 16.2 | 16.5 | 21.6 | 17.5 | 15.1 | 16.3 |
| Ccno | 0.2 | 0.1 | 0.1 | 0.1 | 0.1 | 0.1 | 0.2 | 0.2 | 0.2 | 0.2 | 0.1 | 0.2 |
| Ccnt1 | 10.7 | 10.7 | 11.1 | 12.3 | 11.2 | 10.4 | 10.0 | 9.4 | 8.6 | 10.1 | 10.9 | 9.3 |
| Ccnt2 | 5.9 | 6.7 | 7.4 | 5.7 | 6.5 | 6.8 | 6.6 | 7.0 | 5.9 | 6.8 | 7.1 | 7.1 |
| Ccny | 23.9 | 24.3 | 24.3 | 25.3 | 21.8 | 24.4 | 23.8 | 28.1 | 25.2 | 23.5 | 23.3 | 24.4 |
| Ccnyl1 | 11.8 | 15.4 | 18.1 | 8.8 | 13.3 | 7.7 | 16.4 | 20.4 | 19.2 | 14.0 | 18.4 | 19.4 |
| Ccp110 | 1.8 | 2.4 | 3.2 | 1.8 | 2.1 | 1.9 | 2.1 | 2.3 | 1.9 | 1.6 | 1.8 | 1.8 |
| Ccpg1 | 35.7 | 26.9 | 31.0 | 40.4 | 27.7 | 36.1 | 27.7 | 28.3 | 24.4 | 30.8 | 31.8 | 36.7 |
| Ccr1 | 0.0 | 0.0 | 0.0 | 0.1 | 0.5 | 0.0 | 0.0 | 0.0 | 0.0 | 0.1 | 0.0 | 0.0 |
| Ccr10 | 0.3 | 0.3 | 0.1 | 0.3 | 0.4 | 0.6 | 0.2 | 0.2 | 0.2 | 0.3 | 0.2 | 0.1 |
| Ccr3 | 0.1 | 0.0 | 0.0 | 0.2 | 0.2 | 0.0 | 0.1 | 0.0 | 0.1 | 0.0 | 0.0 | 0.0 |
| Ccr4 | 0.0 | 0.0 | 0.0 | 0.0 | 0.0 | 0.0 | 0.0 | 0.0 | 0.0 | 0.0 | 0.0 | 0.0 |
| Ccr5 | 0.3 | 0.0 | 0.0 | 2.5 | 5.1 | 0.0 | 0.6 | 0.0 | 0.1 | 0.1 | 0.0 | 0.3 |
| Ccr7 | 0.1 | 0.1 | 0.1 | 0.0 | 0.0 | 0.0 | 0.0 | 0.1 | 0.1 | 0.1 | 0.0 | 0.1 |
| Ccr9 | 0.0 | 0.0 | 0.1 | 0.0 | 0.0 | 0.0 | 0.0 | 0.0 | 0.0 | 0.0 | 0.1 | 0.0 |
| Ccrl1 | 3.2 | 4.0 | 4.6 | 1.9 | 2.9 | 1.8 | 2.1 | 3.8 | 1.8 | 4.8 | 5.9 | 3.6 |
| Ccrl2 | 0.3 | 0.2 | 0.3 | 0.5 | 0.5 | 0.3 | 0.2 | 0.2 | 0.2 | 0.4 | 0.0 | 0.3 |
| Ccrn4l | 1.2 | 1.2 | 0.8 | 1.1 | 1.3 | 0.8 | 1.2 | 0.6 | 1.0 | 1.6 | 1.5 | 1.0 |
| Ccs | 23.5 | 20.2 | 17.7 | 22.0 | 16.6 | 20.9 | 17.4 | 16.4 | 18.9 | 19.6 | 19.0 | 19.6 |
| Ccsap | 0.5 | 0.4 | 0.7 | 0.7 | 0.9 | 0.5 | 1.2 | 0.8 | 0.8 | 0.8 | 0.6 | 1.0 |
| Ccser1 | 0.1 | 0.0 | 0.0 | 0.0 | 0.0 | 0.1 | 0.0 | 0.0 | 0.0 | 0.1 | 0.0 | 0.1 |
| Ccser2 | 22.2 | 21.9 | 20.4 | 24.4 | 19.2 | 23.0 | 19.4 | 17.4 | 19.2 | 21.7 | 22.6 | 22.8 |
| Cct2 | 167.5 | 137.8 | 125.2 | 151.7 | 133.7 | 143.7 | 136.9 | 129.6 | 126.6 | 145.9 | 153.5 | 148.2 |
| Cct3 | 108.5 | 108.6 | 82.4 | 105.1 | 95.5 | 88.5 | 95.8 | 84.4 | 93.1 | 106.1 | 100.1 | 93.2 |
| Cct4 | 118.5 | 115.7 | 91.3 | 87.2 | 99.4 | 97.6 | 84.2 | 87.3 | 101.5 | 109.8 | 106.4 | 100.0 |
| Cct5 | 173.1 | 146.3 | 128.8 | 157.8 | 137.5 | 154.6 | 146.3 | 134.0 | 130.2 | 155.1 | 164.6 | 144.1 |
| Cct6a | 128.2 | 125.5 | 97.4 | 132.1 | 103.8 | 109.2 | 113.2 | 96.6 | 105.0 | 113.9 | 132.1 | 119.2 |
| Cct6b | 0.2 | 0.4 | 0.3 | 0.1 | 0.1 | 0.2 | 0.1 | 0.2 | 0.2 | 0.3 | 0.1 | 0.0 |
| Cct7 | 95.4 | 88.8 | 72.0 | 88.7 | 76.3 | 88.3 | 73.1 | 69.4 | 73.6 | 81.9 | 79.3 | 73.8 |
| Cct8 | 117.7 | 106.5 | 90.9 | 114.0 | 98.7 | 103.6 | 95.3 | 84.1 | 90.1 | 107.1 | 105.5 | 102.2 |

| | | | | | | | | | | | | |
|---|---|---|---|---|---|---|---|---|---|---|---|---|
| Cct8l1 | 0.0 | 0.0 | 0.0 | 0.0 | 0.0 | 0.0 | 0.0 | 0.0 | 0.0 | 0.0 | 0.0 | 0.0 |
| Ccz1 | 41.2 | 38.6 | 34.0 | 38.9 | 35.8 | 39.4 | 34.0 | 34.7 | 34.5 | 35.7 | 39.2 | 37.1 |
| Cd101 | 0.0 | 0.0 | 0.0 | 0.0 | 0.1 | 0.0 | 0.0 | 0.0 | 0.0 | 0.0 | 0.1 | 0.0 |
| Cd109 | 9.8 | 7.8 | 8.4 | 3.3 | 6.8 | 7.5 | 6.4 | 7.7 | 6.6 | 4.6 | 6.9 | 7.6 |
| Cd14 | 2.8 | 0.7 | 0.6 | 19.5 | 54.7 | 0.5 | 8.5 | 1.0 | 1.8 | 5.4 | 1.4 | 2.9 |
| Cd151 | 254.0 | 217.1 | 170.2 | 296.5 | 188.0 | 288.9 | 209.7 | 189.3 | 181.4 | 215.1 | 231.2 | 175.4 |
| Cd160 | 0.0 | 0.1 | 0.1 | 0.0 | 0.1 | 0.1 | 0.2 | 0.1 | 0.1 | 0.1 | 0.2 | 0.2 |
| Cd164 | 46.0 | 44.2 | 47.9 | 60.7 | 51.0 | 56.0 | 52.2 | 46.6 | 48.7 | 54.2 | 57.4 | 55.1 |
| Cd164l2 | 0.0 | 0.0 | 0.0 | 0.0 | 0.1 | 0.0 | 0.1 | 0.1 | 0.2 | 0.0 | 0.1 | 0.1 |
| Cd177 | 0.0 | 0.0 | 0.0 | 0.0 | 0.0 | 0.0 | 0.0 | 0.0 | 0.0 | 0.0 | 0.0 | 0.0 |
| Cd180 | 0.7 | 0.5 | 0.7 | 3.8 | 5.8 | 0.6 | 1.4 | 0.6 | 0.9 | 0.7 | 0.4 | 0.7 |
| Cd1d1 | 1.9 | 2.5 | 2.0 | 2.3 | 2.4 | 2.4 | 0.8 | 2.4 | 1.0 | 2.0 | 1.9 | 1.4 |
| Cd1d2 | 0.1 | 0.2 | 0.0 | 0.1 | 0.1 | 0.1 | 0.1 | 0.1 | 0.0 | 0.1 | 0.1 | 0.1 |
| Cd200 | 6.4 | 4.3 | 7.3 | 4.5 | 2.6 | 4.2 | 3.2 | 3.8 | 4.8 | 9.3 | 8.5 | 11.3 |
| Cd200r1 | 2.3 | 1.8 | 2.6 | 5.6 | 3.4 | 1.8 | 3.5 | 2.7 | 3.9 | 2.7 | 2.7 | 3.3 |
| Cd200r2 | 0.0 | 0.0 | 0.0 | 0.0 | 0.0 | 0.0 | 0.0 | 0.0 | 0.0 | 0.0 | 0.0 | 0.0 |
| Cd200r3 | 0.0 | 0.0 | 0.0 | 0.0 | 0.0 | 0.1 | 0.1 | 0.0 | 0.0 | 0.0 | 0.0 | 0.0 |
| Cd200r4 | 0.4 | 0.3 | 0.5 | 1.0 | 1.2 | 0.5 | 0.5 | 0.4 | 0.6 | 0.4 | 0.4 | 0.3 |
| Cd207 | 0.0 | 0.0 | 0.0 | 0.2 | 1.2 | 0.1 | 0.2 | 0.0 | 0.0 | 0.1 | 0.0 | 0.0 |
| Cd209c | 0.6 | 1.0 | 0.7 | 0.5 | 0.9 | 0.6 | 0.9 | 0.5 | 0.4 | 0.8 | 0.3 | 0.6 |
| Cd22 | 0.0 | 0.0 | 0.0 | 0.1 | 0.1 | 0.0 | 0.0 | 0.0 | 0.0 | 0.0 | 0.0 | 0.0 |
| Cd244 | 0.2 | 0.1 | 0.0 | 0.8 | 1.5 | 0.0 | 0.3 | 0.1 | 0.1 | 0.2 | 0.2 | 0.4 |
| Cd247 | 0.0 | 0.0 | 0.1 | 0.0 | 0.0 | 0.0 | 0.0 | 0.0 | 0.0 | 0.0 | 0.0 | 0.1 |
| Cd248 | 8.6 | 8.9 | 9.5 | 7.4 | 10.5 | 11.7 | 25.6 | 28.8 | 30.6 | 23.1 | 27.4 | 17.8 |
| Cd24a | 2.4 | 1.4 | 2.3 | 0.8 | 1.2 | 0.6 | 0.6 | 1.9 | 0.4 | 3.3 | 2.0 | 3.0 |
| Cd27 | 0.0 | 0.2 | 0.1 | 0.0 | 0.0 | 0.0 | 0.0 | 0.2 | 0.0 | 0.1 | 0.1 | 0.0 |
| Cd274 | 1.4 | 1.1 | 1.1 | 1.5 | 0.8 | 1.4 | 0.8 | 0.4 | 0.4 | 0.4 | 0.6 | 0.7 |
| Cd276 | 1.8 | 1.9 | 2.5 | 2.0 | 1.9 | 2.3 | 2.4 | 3.0 | 2.6 | 2.8 | 2.3 | 2.4 |
| Cd28 | 1.2 | 1.4 | 0.6 | 2.0 | 1.1 | 1.5 | 0.4 | 0.1 | 0.3 | 0.1 | 0.2 | 0.1 |
| Cd2ap | 12.1 | 11.4 | 13.7 | 13.0 | 12.0 | 12.2 | 12.7 | 13.4 | 10.8 | 16.0 | 18.3 | 17.0 |
| Cd2bp2 | 20.0 | 17.1 | 16.3 | 20.2 | 17.7 | 20.7 | 18.4 | 17.6 | 17.4 | 18.2 | 18.7 | 19.6 |
| Cd300a | 0.2 | 0.0 | 0.0 | 2.2 | 3.0 | 0.0 | 0.3 | 0.1 | 0.0 | 0.2 | 0.0 | 0.1 |
| Cd300e | 0.1 | 0.0 | 0.0 | 0.5 | 0.6 | 0.0 | 0.5 | 0.0 | 0.1 | 0.1 | 0.0 | 0.2 |
| Cd300lb | 0.4 | 0.0 | 0.1 | 3.5 | 6.7 | 0.0 | 0.5 | 0.1 | 0.1 | 0.3 | 0.0 | 0.3 |
| Cd300ld | 1.9 | 0.0 | 0.1 | 11.4 | 22.9 | 0.0 | 3.3 | 0.1 | 0.6 | 1.7 | 0.8 | 2.0 |
| Cd300lf | 0.2 | 0.0 | 0.0 | 3.1 | 4.7 | 0.0 | 0.7 | 0.0 | 0.1 | 0.2 | 0.2 | 0.4 |
| Cd300lg | 0.1 | 0.0 | 0.0 | 0.0 | 0.0 | 0.0 | 0.1 | 0.0 | 0.0 | 0.1 | 0.0 | 0.0 |
| Cd300lh | 0.2 | 0.0 | 0.0 | 1.5 | 2.6 | 0.0 | 0.4 | 0.0 | 0.1 | 0.2 | 0.0 | 0.1 |
| Cd302 | 46.4 | 47.0 | 61.2 | 53.6 | 51.9 | 49.1 | 45.4 | 46.7 | 43.6 | 46.1 | 44.9 | 58.4 |
| Cd320 | 5.2 | 4.3 | 3.5 | 3.7 | 3.7 | 4.2 | 4.1 | 4.8 | 3.9 | 5.0 | 4.2 | 4.1 |
| Cd33 | 0.1 | 0.0 | 0.0 | 0.8 | 1.0 | 0.0 | 0.1 | 0.0 | 0.0 | 0.0 | 0.0 | 0.0 |
| Cd34 | 22.5 | 10.7 | 21.1 | 8.7 | 5.0 | 14.4 | 6.4 | 27.1 | 10.3 | 11.5 | 11.3 | 15.6 |
| Cd36 | 1.7 | 0.2 | 0.2 | 7.5 | 26.0 | 0.0 | 1.5 | 1.0 | 0.6 | 1.9 | 0.5 | 1.5 |
| Cd37 | 0.4 | 0.0 | 0.0 | 2.9 | 4.6 | 0.0 | 0.5 | 0.1 | 0.0 | 0.1 | 0.0 | 0.1 |
| Cd38 | 2.0 | 2.4 | 2.5 | 2.1 | 2.2 | 2.1 | 1.9 | 2.7 | 2.2 | 1.7 | 2.4 | 2.1 |
| Cd3eap | 11.9 | 8.5 | 8.1 | 15.1 | 10.0 | 9.5 | 12.4 | 8.8 | 7.9 | 11.1 | 13.4 | 12.5 |
| Cd4 | 0.0 | 0.0 | 0.0 | 0.1 | 0.0 | 0.0 | 0.0 | 0.0 | 0.0 | 0.0 | 0.0 | 0.0 |
| Cd40 | 7.0 | 4.1 | 2.8 | 12.2 | 11.1 | 7.6 | 7.9 | 2.4 | 3.7 | 3.7 | 3.2 | 3.4 |
| Cd44 | 22.2 | 15.3 | 18.4 | 35.2 | 41.8 | 24.0 | 37.3 | 18.1 | 18.1 | 29.4 | 26.8 | 24.7 |
| Cd46 | 0.0 | 0.2 | 0.2 | 0.2 | 0.2 | 0.4 | 0.3 | 0.5 | 0.5 | 0.2 | 0.4 | 0.3 |
| Cd47 | 15.6 | 16.7 | 15.7 | 17.2 | 22.7 | 14.1 | 19.2 | 16.8 | 15.5 | 16.3 | 15.1 | 17.1 |
| Cd48 | 1.4 | 0.0 | 0.1 | 11.9 | 16.5 | 0.0 | 2.1 | 0.5 | 0.2 | 1.6 | 0.3 | 0.7 |
| Cd5 | 0.0 | 0.0 | 0.0 | 0.1 | 0.7 | 0.0 | 0.2 | 0.0 | 0.0 | 0.1 | 0.0 | 0.0 |
| Cd52 | 11.6 | 0.0 | 0.8 | 101.0 | 232.0 | 0.1 | 43.1 | 1.7 | 7.2 | 14.9 | 5.9 | 12.9 |
| Cd53 | 2.8 | 0.2 | 0.2 | 14.9 | 22.7 | 0.3 | 2.1 | 0.4 | 0.5 | 2.5 | 0.7 | 1.6 |
| Cd55 | 17.0 | 19.2 | 33.8 | 9.4 | 14.4 | 11.1 | 13.9 | 34.8 | 11.4 | 13.1 | 15.2 | 14.5 |
| Cd59a | 14.4 | 9.1 | 9.2 | 15.9 | 11.8 | 13.6 | 11.4 | 9.2 | 10.0 | 15.4 | 13.5 | 12.5 |

| | | | | | | | | | | | | |
|---|---|---|---|---|---|---|---|---|---|---|---|---|
| Cd59b | 0.7 | 0.9 | 0.3 | 0.6 | 0.3 | 0.5 | 0.3 | 0.1 | 0.7 | 0.5 | 0.5 | 0.5 |
| Cd63 | 208.8 | 193.2 | 183.8 | 232.1 | 246.5 | 210.9 | 168.0 | 217.6 | 176.4 | 208.6 | 175.5 | 229.4 |
| Cd68 | 4.6 | 0.8 | 1.3 | 52.0 | 45.5 | 1.2 | 4.1 | 1.9 | 1.2 | 3.7 | 2.2 | 2.6 |
| Cd69 | 0.1 | 0.0 | 0.0 | 0.4 | 4.7 | 0.0 | 2.1 | 0.1 | 0.4 | 0.4 | 0.3 | 0.9 |
| Cd70 | 0.2 | 0.0 | 0.0 | 0.2 | 0.4 | 0.1 | 0.0 | 0.0 | 0.0 | 0.0 | 0.0 | 0.0 |
| Cd72 | 7.0 | 0.8 | 0.9 | 68.0 | 128.9 | 0.4 | 19.2 | 2.0 | 3.7 | 9.1 | 3.6 | 8.5 |
| Cd74 | 0.5 | 0.1 | 0.2 | 1.6 | 5.7 | 0.2 | 0.7 | 0.3 | 0.3 | 0.4 | 0.2 | 0.2 |
| Cd79a | 0.1 | 0.0 | 0.0 | 0.1 | 0.0 | 0.1 | 0.0 | 0.1 | 0.1 | 0.0 | 0.0 | 0.0 |
| Cd80 | 8.1 | 9.8 | 9.1 | 5.7 | 8.9 | 6.8 | 6.9 | 3.8 | 5.2 | 4.8 | 6.7 | 7.4 |
| Cd81 | 610.7 | 549.0 | 467.2 | 624.6 | 505.5 | 621.8 | 562.7 | 513.0 | 541.5 | 628.0 | 658.3 | 591.1 |
| Cd82 | 15.8 | 15.4 | 14.9 | 13.8 | 13.8 | 15.1 | 15.0 | 19.2 | 11.6 | 14.3 | 13.7 | 15.8 |
| Cd83 | 0.4 | 0.5 | 1.1 | 1.2 | 1.2 | 1.1 | 0.3 | 0.3 | 0.1 | 0.1 | 0.0 | 0.1 |
| Cd84 | 1.1 | 0.1 | 0.2 | 12.4 | 13.5 | 0.0 | 0.7 | 0.3 | 0.1 | 0.6 | 0.3 | 0.5 |
| Cd86 | 0.2 | 0.0 | 0.0 | 1.2 | 2.4 | 0.0 | 0.3 | 0.0 | 0.0 | 0.2 | 0.0 | 0.0 |
| Cd8a | 0.0 | 0.0 | 0.0 | 0.0 | 0.0 | 0.0 | 0.0 | 0.0 | 0.0 | 0.0 | 0.0 | 0.0 |
| Cd8b1 | 0.0 | 0.0 | 0.0 | 0.0 | 0.0 | 0.0 | 0.0 | 0.0 | 0.0 | 0.0 | 0.0 | 0.0 |
| Cd9 | 52.6 | 29.7 | 44.9 | 58.2 | 76.7 | 45.6 | 42.4 | 58.3 | 43.5 | 47.2 | 58.6 | 62.6 |
| Cd93 | 3.2 | 0.8 | 1.7 | 4.2 | 7.3 | 0.4 | 1.9 | 3.7 | 2.9 | 1.0 | 0.7 | 0.9 |
| Cd96 | 0.1 | 0.1 | 0.1 | 0.0 | 0.0 | 0.0 | 0.0 | 0.1 | 0.0 | 0.0 | 0.0 | 0.0 |
| Cd97 | 19.1 | 20.4 | 15.4 | 18.4 | 21.0 | 15.3 | 23.9 | 19.9 | 26.0 | 19.2 | 21.9 | 19.7 |
| Cd99l2 | 24.8 | 23.5 | 22.9 | 22.8 | 19.9 | 28.4 | 20.3 | 25.0 | 21.2 | 20.1 | 21.6 | 23.2 |
| Cda | 1.1 | 1.6 | 1.2 | 1.6 | 1.9 | 1.5 | 2.4 | 1.2 | 2.7 | 1.8 | 1.5 | 2.3 |
| Cdadc1 | 5.6 | 6.8 | 7.3 | 8.5 | 6.6 | 7.7 | 7.5 | 5.9 | 7.5 | 8.2 | 7.0 | 8.1 |
| Cdan1 | 1.6 | 1.8 | 2.0 | 1.3 | 2.0 | 1.4 | 2.3 | 1.8 | 2.0 | 1.6 | 1.5 | 1.7 |
| Cdc123 | 26.9 | 28.2 | 28.0 | 27.0 | 25.6 | 28.2 | 24.9 | 24.6 | 24.8 | 28.1 | 28.3 | 27.3 |
| Cdc14a | 0.6 | 0.5 | 0.8 | 0.6 | 0.7 | 0.5 | 0.6 | 0.7 | 0.4 | 0.5 | 0.6 | 0.5 |
| Cdc14b | 5.3 | 5.7 | 4.5 | 4.6 | 4.5 | 5.2 | 4.9 | 4.5 | 5.6 | 4.8 | 5.3 | 4.7 |
| Cdc16 | 40.7 | 40.9 | 43.1 | 36.4 | 35.8 | 38.2 | 41.9 | 44.7 | 43.9 | 42.0 | 44.3 | 43.2 |
| Cdc20 | 4.3 | 9.0 | 11.9 | 1.5 | 12.0 | 1.2 | 17.0 | 6.2 | 13.2 | 5.3 | 10.4 | 7.0 |
| Cdc23 | 18.3 | 19.8 | 20.0 | 17.1 | 17.7 | 17.9 | 19.1 | 18.1 | 19.7 | 18.2 | 18.8 | 18.0 |
| Cdc25a | 3.8 | 3.6 | 3.6 | 3.9 | 4.1 | 3.7 | 5.0 | 3.2 | 4.0 | 4.2 | 4.2 | 3.8 |
| Cdc25b | 0.7 | 1.3 | 2.0 | 0.3 | 1.5 | 0.5 | 2.1 | 1.0 | 1.7 | 1.0 | 1.1 | 1.2 |
| Cdc25c | 0.8 | 1.1 | 1.8 | 0.3 | 1.7 | 0.4 | 2.1 | 0.8 | 1.8 | 0.6 | 1.5 | 0.8 |
| Cdc26 | 24.4 | 16.3 | 16.1 | 19.2 | 18.3 | 17.7 | 14.1 | 20.5 | 19.5 | 21.0 | 20.4 | 17.1 |
| Cdc27 | 14.7 | 16.0 | 16.1 | 14.0 | 14.0 | 13.5 | 15.1 | 16.5 | 15.6 | 16.1 | 17.7 | 18.4 |
| Cdc34 | 39.8 | 32.5 | 24.1 | 41.6 | 34.2 | 37.9 | 28.6 | 30.3 | 30.1 | 36.3 | 31.5 | 28.5 |
| Cdc37 | 44.7 | 40.4 | 36.7 | 61.3 | 44.5 | 54.1 | 44.1 | 42.6 | 35.7 | 42.4 | 40.3 | 42.8 |
| Cdc37l1 | 12.9 | 15.2 | 16.0 | 13.5 | 12.0 | 13.9 | 12.3 | 14.8 | 12.3 | 12.0 | 11.9 | 13.9 |
| Cdc40 | 5.3 | 5.9 | 6.2 | 5.4 | 4.9 | 5.1 | 5.3 | 5.5 | 4.8 | 6.6 | 5.6 | 6.0 |
| Cdc42 | 199.2 | 193.5 | 176.3 | 223.4 | 226.0 | 191.7 | 191.6 | 195.4 | 204.6 | 205.0 | 224.6 | 206.3 |
| Cdc42bpa | 19.3 | 18.6 | 16.1 | 19.4 | 17.3 | 21.2 | 19.9 | 16.3 | 17.5 | 18.2 | 20.2 | 18.3 |
| Cdc42bpb | 18.3 | 18.6 | 18.1 | 17.8 | 17.7 | 18.0 | 22.8 | 19.6 | 20.8 | 17.5 | 18.5 | 17.9 |
| Cdc42bpg | 7.1 | 6.0 | 5.7 | 4.5 | 5.5 | 6.1 | 5.3 | 4.1 | 5.0 | 9.1 | 6.2 | 6.0 |
| Cdc42ep1 | 20.2 | 28.7 | 26.5 | 19.6 | 24.1 | 17.3 | 32.2 | 34.6 | 35.3 | 23.5 | 24.7 | 21.4 |
| Cdc42ep2 | 18.4 | 20.7 | 18.5 | 12.4 | 18.4 | 12.8 | 30.9 | 44.0 | 32.0 | 20.3 | 29.1 | 19.2 |
| Cdc42ep3 | 8.5 | 6.2 | 7.6 | 7.5 | 6.6 | 5.1 | 3.6 | 4.0 | 5.9 | 14.2 | 10.4 | 8.1 |
| Cdc42ep4 | 10.3 | 12.9 | 12.4 | 10.1 | 12.0 | 9.0 | 11.8 | 13.3 | 13.8 | 11.0 | 9.7 | 10.3 |
| Cdc42ep5 | 10.5 | 9.4 | 12.6 | 12.3 | 11.7 | 10.2 | 14.1 | 16.0 | 10.5 | 7.5 | 8.3 | 8.7 |
| Cdc42se1 | 23.4 | 22.6 | 23.3 | 27.4 | 29.2 | 19.9 | 29.9 | 30.0 | 27.8 | 23.5 | 28.7 | 24.1 |
| Cdc42se2 | 17.1 | 14.6 | 10.9 | 19.4 | 19.8 | 16.3 | 12.8 | 12.6 | 13.1 | 11.4 | 12.6 | 12.0 |
| Cdc45 | 2.6 | 3.8 | 6.9 | 1.9 | 4.9 | 1.4 | 5.9 | 3.1 | 4.7 | 2.4 | 3.7 | 3.8 |
| Cdc5l | 16.1 | 18.2 | 19.5 | 18.1 | 17.6 | 17.3 | 19.1 | 19.0 | 17.3 | 20.6 | 19.1 | 20.6 |
| Cdc6 | 0.6 | 1.0 | 1.9 | 0.2 | 1.7 | 0.3 | 1.7 | 1.2 | 1.4 | 0.9 | 1.3 | 0.7 |
| Cdc7 | 0.6 | 1.0 | 1.1 | 0.7 | 1.4 | 0.7 | 1.0 | 0.9 | 1.2 | 0.8 | 1.2 | 0.6 |
| Cdc73 | 2.6 | 3.1 | 2.8 | 2.4 | 2.9 | 2.9 | 2.9 | 3.2 | 2.6 | 3.5 | 3.0 | 3.7 |
| Cdca2 | 1.0 | 2.3 | 3.6 | 0.6 | 3.2 | 0.6 | 4.1 | 1.9 | 3.2 | 1.4 | 2.6 | 2.2 |
| Cdca3 | 3.9 | 5.9 | 10.6 | 1.7 | 8.8 | 1.0 | 13.1 | 4.9 | 8.6 | 4.1 | 7.5 | 4.6 |

| | | | | | | | | | | | | |
|---|---|---|---|---|---|---|---|---|---|---|---|---|
| Cdca4 | 7.6 | 9.2 | 8.2 | 6.7 | 8.5 | 7.2 | 9.7 | 7.4 | 9.0 | 8.7 | 7.9 | 7.2 |
| Cdca5 | 0.9 | 1.9 | 2.5 | 0.5 | 2.6 | 0.6 | 3.2 | 1.6 | 2.2 | 1.1 | 1.9 | 1.2 |
| Cdca7 | 1.4 | 2.0 | 2.0 | 0.8 | 2.1 | 0.9 | 1.8 | 1.8 | 1.7 | 1.7 | 2.1 | 1.2 |
| Cdca7l | 1.0 | 1.2 | 1.4 | 1.0 | 1.6 | 0.8 | 1.3 | 0.7 | 0.8 | 0.7 | 1.0 | 0.7 |
| Cdca8 | 2.3 | 4.4 | 7.6 | 2.0 | 6.6 | 1.7 | 8.8 | 3.9 | 6.3 | 2.9 | 6.1 | 3.8 |
| Cdcp1 | 0.0 | 0.0 | 0.0 | 0.1 | 0.6 | 0.0 | 0.0 | 0.0 | 0.0 | 0.1 | 0.0 | 0.0 |
| Cdh1 | 0.0 | 0.0 | 0.0 | 0.1 | 0.0 | 0.0 | 0.0 | 0.0 | 0.0 | 0.0 | 0.0 | 0.0 |
| Cdh10 | 0.2 | 0.1 | 0.1 | 0.2 | 0.2 | 0.1 | 0.4 | 0.3 | 0.3 | 0.2 | 0.2 | 0.2 |
| Cdh11 | 18.6 | 9.0 | 14.7 | 7.1 | 10.5 | 6.4 | 14.2 | 17.4 | 18.5 | 63.4 | 41.0 | 38.7 |
| Cdh12 | 0.0 | 0.0 | 0.0 | 0.0 | 0.0 | 0.0 | 0.0 | 0.0 | 0.0 | 0.0 | 0.0 | 0.0 |
| Cdh13 | 2.5 | 0.4 | 1.2 | 1.0 | 0.5 | 1.3 | 0.9 | 2.1 | 1.0 | 0.3 | 0.5 | 0.7 |
| Cdh15 | 0.0 | 0.0 | 0.0 | 0.0 | 0.0 | 0.0 | 0.0 | 0.0 | 0.0 | 0.0 | 0.0 | 0.0 |
| Cdh17 | 0.1 | 0.0 | 0.1 | 0.2 | 0.0 | 0.0 | 0.1 | 0.0 | 0.0 | 0.0 | 0.1 | 0.1 |
| Cdh19 | 0.9 | 1.5 | 2.2 | 0.9 | 1.4 | 1.3 | 1.7 | 2.4 | 1.8 | 0.5 | 1.2 | 1.2 |
| Cdh2 | 41.8 | 31.4 | 39.1 | 36.1 | 35.5 | 45.0 | 49.4 | 65.0 | 59.5 | 54.0 | 60.8 | 80.4 |
| Cdh23 | 0.3 | 0.3 | 0.3 | 0.2 | 0.3 | 0.3 | 0.3 | 0.2 | 0.2 | 0.2 | 0.3 | 0.2 |
| Cdh24 | 0.1 | 0.1 | 0.1 | 0.0 | 0.2 | 0.3 | 0.2 | 0.2 | 0.2 | 0.4 | 0.3 | 0.2 |
| Cdh26 | 1.0 | 0.6 | 0.2 | 1.0 | 0.5 | 0.5 | 0.2 | 0.1 | 0.1 | 0.3 | 0.2 | 0.2 |
| Cdh3 | 1.8 | 0.9 | 0.9 | 0.4 | 0.4 | 0.5 | 1.0 | 1.8 | 2.3 | 14.1 | 8.7 | 5.2 |
| Cdh4 | 0.0 | 0.0 | 0.0 | 0.0 | 0.0 | 0.0 | 0.0 | 0.0 | 0.0 | 0.0 | 0.0 | 0.0 |
| Cdh5 | 2.7 | 0.7 | 2.0 | 0.9 | 0.8 | 0.5 | 1.8 | 3.9 | 1.7 | 0.4 | 0.3 | 0.5 |
| Cdh6 | 0.0 | 0.0 | 0.0 | 0.0 | 0.0 | 0.0 | 0.0 | 0.0 | 0.0 | 0.0 | 0.0 | 0.0 |
| Cdhr1 | 0.4 | 0.2 | 0.2 | 0.3 | 0.3 | 0.5 | 0.3 | 0.1 | 0.1 | 0.4 | 0.3 | 0.2 |
| Cdhr3 | 0.0 | 0.0 | 0.0 | 0.1 | 0.2 | 0.0 | 0.1 | 0.0 | 0.0 | 0.0 | 0.0 | 0.1 |
| Cdip1 | 6.3 | 6.3 | 6.1 | 6.7 | 6.2 | 6.6 | 6.7 | 6.3 | 6.5 | 7.0 | 6.6 | 5.9 |
| Cdipt | 35.4 | 28.2 | 23.5 | 39.5 | 29.6 | 32.9 | 29.9 | 30.0 | 28.9 | 30.7 | 28.6 | 26.4 |
| Cdk1 | 6.3 | 11.6 | 16.6 | 2.7 | 14.6 | 3.6 | 18.4 | 7.6 | 15.7 | 10.1 | 12.9 | 10.3 |
| Cdk10 | 11.4 | 11.1 | 11.5 | 13.9 | 10.6 | 12.4 | 11.4 | 10.6 | 12.6 | 11.3 | 10.4 | 8.7 |
| Cdk11b | 26.0 | 28.8 | 32.0 | 33.5 | 25.0 | 28.9 | 30.4 | 25.4 | 21.2 | 22.7 | 28.1 | 30.4 |
| Cdk12 | 5.8 | 6.9 | 7.0 | 6.8 | 6.4 | 6.4 | 7.0 | 6.9 | 5.8 | 5.7 | 7.2 | 6.7 |
| Cdk13 | 8.0 | 8.0 | 8.2 | 8.1 | 7.3 | 7.7 | 9.0 | 8.4 | 8.1 | 8.7 | 9.7 | 9.3 |
| Cdk14 | 14.4 | 19.4 | 23.2 | 17.4 | 22.7 | 17.1 | 19.0 | 20.9 | 19.2 | 19.9 | 19.3 | 20.7 |
| Cdk15 | 0.7 | 0.6 | 0.4 | 1.2 | 0.6 | 0.8 | 0.7 | 0.5 | 0.6 | 0.3 | 0.7 | 0.3 |
| Cdk16 | 32.2 | 30.4 | 32.1 | 31.5 | 28.1 | 28.8 | 35.7 | 34.6 | 33.9 | 27.3 | 34.1 | 30.2 |
| Cdk17 | 8.1 | 9.7 | 8.7 | 7.7 | 10.2 | 8.0 | 10.3 | 8.6 | 10.7 | 9.7 | 11.8 | 9.6 |
| Cdk18 | 7.1 | 6.1 | 3.8 | 14.8 | 15.3 | 10.9 | 8.1 | 3.4 | 6.7 | 6.3 | 5.7 | 4.4 |
| Cdk19 | 2.6 | 4.1 | 4.9 | 2.9 | 4.0 | 2.8 | 3.8 | 5.6 | 4.8 | 3.7 | 4.0 | 4.5 |
| Cdk2 | 4.2 | 5.7 | 6.5 | 3.4 | 6.5 | 3.5 | 5.9 | 5.7 | 6.4 | 5.2 | 5.2 | 4.2 |
| Cdk20 | 1.5 | 0.9 | 1.2 | 1.1 | 1.7 | 1.6 | 1.2 | 1.1 | 1.2 | 1.2 | 1.0 | 1.2 |
| Cdk2ap1 | 62.4 | 61.0 | 63.5 | 63.0 | 59.1 | 63.2 | 65.0 | 79.0 | 61.0 | 57.8 | 64.4 | 52.4 |
| Cdk2ap2 | 36.6 | 37.0 | 27.6 | 46.6 | 31.8 | 41.2 | 36.0 | 39.3 | 33.1 | 35.2 | 34.0 | 29.4 |
| Cdk3-ps | 0.0 | 0.0 | 0.0 | 0.0 | 0.1 | 0.0 | 0.1 | 0.0 | 0.1 | 0.1 | 0.1 | 0.1 |
| Cdk4 | 98.5 | 88.0 | 77.8 | 83.7 | 82.7 | 93.9 | 79.1 | 87.7 | 81.2 | 102.1 | 90.8 | 81.9 |
| Cdk5 | 14.6 | 11.8 | 10.5 | 14.2 | 12.1 | 15.2 | 10.0 | 11.1 | 11.9 | 13.2 | 13.6 | 13.8 |
| Cdk5r1 | 0.3 | 0.2 | 0.2 | 0.3 | 0.3 | 0.4 | 0.2 | 0.2 | 0.3 | 0.3 | 0.2 | 0.3 |
| Cdk5rap1 | 3.8 | 3.6 | 3.5 | 4.1 | 2.8 | 4.4 | 2.7 | 2.7 | 2.8 | 3.5 | 3.2 | 3.7 |
| Cdk5rap2 | 5.9 | 6.1 | 7.9 | 5.7 | 6.1 | 6.0 | 6.2 | 6.3 | 5.0 | 5.1 | 6.0 | 5.8 |
| Cdk5rap3 | 22.9 | 24.3 | 21.9 | 23.2 | 20.2 | 22.9 | 20.3 | 25.3 | 21.8 | 26.7 | 22.7 | 23.5 |
| Cdk6 | 19.9 | 17.6 | 14.6 | 24.5 | 24.3 | 15.3 | 18.4 | 12.4 | 13.7 | 14.2 | 15.7 | 15.5 |
| Cdk7 | 8.1 | 8.1 | 8.0 | 8.6 | 7.3 | 7.8 | 7.7 | 8.9 | 9.3 | 10.1 | 9.5 | 9.8 |
| Cdk8 | 12.3 | 14.6 | 16.8 | 14.7 | 13.3 | 14.8 | 13.3 | 12.0 | 12.0 | 11.9 | 14.2 | 13.1 |
| Cdk9 | 6.1 | 5.9 | 5.9 | 6.8 | 6.6 | 7.5 | 6.6 | 7.8 | 6.9 | 6.7 | 6.3 | 6.4 |
| Cdkal1 | 8.9 | 8.0 | 7.4 | 10.6 | 7.7 | 9.9 | 7.4 | 7.4 | 6.9 | 6.8 | 7.5 | 7.2 |
| Cdkl1 | 0.8 | 1.2 | 0.6 | 1.1 | 1.1 | 0.7 | 0.8 | 0.6 | 1.3 | 1.2 | 1.1 | 0.8 |
| Cdkl2 | 4.2 | 3.2 | 2.8 | 4.2 | 3.7 | 4.7 | 3.4 | 2.9 | 2.8 | 2.7 | 3.4 | 3.3 |
| Cdkl3 | 4.4 | 3.3 | 3.5 | 4.5 | 3.1 | 4.1 | 3.3 | 2.8 | 2.3 | 3.1 | 3.5 | 3.4 |
| Cdkl4 | 0.1 | 0.2 | 0.1 | 0.1 | 0.1 | 0.1 | 0.1 | 0.1 | 0.1 | 0.1 | 0.0 | 0.1 |

| | | | | | | | | | | | | |
|---|---|---|---|---|---|---|---|---|---|---|---|---|
| Cdkl5 | 1.0 | 1.3 | 1.3 | 0.8 | 0.9 | 1.0 | 0.7 | 1.3 | 0.8 | 0.8 | 0.8 | 0.9 |
| Cdkn1a | 76.6 | 85.4 | 80.0 | 78.8 | 81.7 | 70.0 | 68.6 | 75.5 | 65.2 | 83.6 | 69.8 | 72.9 |
| Cdkn1b | 9.9 | 14.1 | 19.0 | 11.1 | 10.8 | 13.8 | 12.5 | 17.5 | 18.0 | 14.6 | 16.2 | 16.7 |
| Cdkn1c | 0.6 | 0.6 | 0.7 | 1.1 | 0.3 | 0.3 | 0.4 | 0.9 | 0.4 | 0.5 | 0.5 | 0.6 |
| Cdkn2a | 44.2 | 8.9 | 3.6 | 54.9 | 16.8 | 39.4 | 9.9 | 5.2 | 11.0 | 26.3 | 16.5 | 11.4 |
| Cdkn2aip | 4.7 | 4.4 | 5.2 | 3.9 | 3.6 | 4.0 | 4.9 | 4.1 | 4.3 | 4.6 | 4.3 | 5.2 |
| Cdkn2aipnl | 5.6 | 4.1 | 4.7 | 6.0 | 7.4 | 4.2 | 5.7 | 5.2 | 4.0 | 4.8 | 6.7 | 5.1 |
| Cdkn2b | 46.1 | 23.0 | 17.8 | 47.3 | 28.5 | 44.6 | 32.0 | 17.3 | 33.5 | 58.8 | 39.5 | 40.5 |
| Cdkn2c | 6.3 | 8.1 | 8.4 | 5.6 | 7.6 | 4.9 | 7.8 | 9.0 | 9.6 | 8.1 | 8.4 | 7.7 |
| Cdkn2d | 5.2 | 8.1 | 9.0 | 4.3 | 7.2 | 5.6 | 9.8 | 5.4 | 10.0 | 6.7 | 7.1 | 6.5 |
| Cdkn3 | 2.3 | 4.0 | 8.2 | 0.5 | 5.1 | 1.1 | 7.7 | 3.1 | 5.3 | 3.8 | 5.5 | 4.6 |
| Cdnf | 4.3 | 4.1 | 4.3 | 5.4 | 3.1 | 5.4 | 3.6 | 3.2 | 3.1 | 3.5 | 4.7 | 4.7 |
| Cdo1 | 24.1 | 13.4 | 13.8 | 13.2 | 12.0 | 10.2 | 8.8 | 3.1 | 6.0 | 11.2 | 10.1 | 8.7 |
| Cdon | 7.6 | 6.6 | 10.5 | 3.5 | 6.2 | 2.8 | 6.8 | 9.9 | 8.3 | 12.3 | 9.9 | 9.7 |
| Cdpf1 | 13.2 | 11.3 | 6.9 | 13.3 | 8.8 | 13.2 | 9.1 | 9.2 | 9.4 | 12.1 | 10.8 | 11.3 |
| Cdr2 | 33.8 | 28.3 | 21.7 | 37.8 | 28.2 | 31.6 | 30.8 | 25.9 | 26.9 | 27.6 | 32.7 | 27.4 |
| Cdr2l | 14.0 | 13.3 | 11.7 | 15.8 | 12.1 | 15.1 | 14.7 | 13.6 | 14.1 | 13.7 | 15.2 | 12.2 |
| Cdrt4 | 0.8 | 0.1 | 0.3 | 0.9 | 0.5 | 0.6 | 0.2 | 0.1 | 0.4 | 0.2 | 0.0 | 0.1 |
| Cds1 | 0.5 | 0.3 | 0.2 | 0.7 | 0.7 | 0.3 | 0.3 | 0.2 | 0.1 | 0.3 | 0.1 | 0.2 |
| Cds2 | 18.5 | 19.9 | 15.8 | 20.2 | 17.4 | 18.4 | 16.7 | 13.0 | 17.5 | 15.8 | 14.8 | 14.9 |
| Cdsn | 0.0 | 0.1 | 0.1 | 0.1 | 0.1 | 0.1 | 0.1 | 0.1 | 0.0 | 0.2 | 0.2 | 0.1 |
| Cdt1 | 0.7 | 1.6 | 1.9 | 0.5 | 2.3 | 0.5 | 2.2 | 1.4 | 1.6 | 1.5 | 1.3 | 0.9 |
| Cdv3 | 44.5 | 37.6 | 33.5 | 40.2 | 39.8 | 36.6 | 42.1 | 39.3 | 40.1 | 44.4 | 47.6 | 44.4 |
| Cdyl | 4.1 | 4.1 | 5.2 | 3.7 | 4.3 | 2.9 | 4.2 | 4.5 | 4.1 | 3.5 | 4.6 | 4.3 |
| Cdyl2 | 1.6 | 1.9 | 1.5 | 1.8 | 2.1 | 1.9 | 2.0 | 1.7 | 2.0 | 1.8 | 1.6 | 1.8 |
| Ceacam1 | 0.1 | 0.1 | 0.0 | 0.4 | 0.5 | 0.1 | 0.0 | 0.0 | 0.0 | 0.1 | 0.0 | 0.0 |
| Ceacam16 | 0.0 | 0.0 | 0.0 | 0.0 | 0.1 | 0.1 | 0.0 | 0.0 | 0.0 | 0.0 | 0.0 | 0.0 |
| Ceacam19 | 0.1 | 0.0 | 0.0 | 0.1 | 0.2 | 0.0 | 0.0 | 0.0 | 0.0 | 0.0 | 0.0 | 0.0 |
| Ceacam2 | 0.0 | 0.0 | 0.0 | 0.0 | 0.1 | 0.0 | 0.0 | 0.0 | 0.0 | 0.0 | 0.0 | 0.0 |
| Ceacam20 | 0.0 | 0.0 | 0.0 | 0.1 | 0.0 | 0.0 | 0.1 | 0.0 | 0.0 | 0.0 | 0.0 | 0.0 |
| Cebpa | 1.8 | 2.2 | 2.8 | 3.3 | 4.2 | 1.9 | 2.3 | 2.6 | 2.4 | 4.5 | 3.1 | 3.8 |
| Cebpb | 48.2 | 46.0 | 53.9 | 32.5 | 43.4 | 27.9 | 46.1 | 56.3 | 43.2 | 66.5 | 38.5 | 58.6 |
| Cebpd | 73.0 | 87.1 | 100.5 | 45.2 | 60.7 | 41.5 | 80.1 | 90.4 | 85.9 | 67.0 | 65.1 | 73.9 |
| Cebpg | 10.4 | 8.6 | 8.2 | 11.0 | 10.3 | 10.3 | 8.6 | 9.6 | 10.2 | 8.4 | 10.9 | 9.0 |
| Cebpz | 16.4 | 14.8 | 14.6 | 16.0 | 14.6 | 15.5 | 13.9 | 13.3 | 13.1 | 16.3 | 18.5 | 16.3 |
| Cecr2 | 0.0 | 0.0 | 0.0 | 0.0 | 0.0 | 0.0 | 0.0 | 0.0 | 0.0 | 0.0 | 0.0 | 0.0 |
| Cecr5 | 2.4 | 2.7 | 2.5 | 1.6 | 2.4 | 2.0 | 1.7 | 3.1 | 3.4 | 2.2 | 2.1 | 2.5 |
| Cecr6 | 0.0 | 0.0 | 0.0 | 0.0 | 0.0 | 0.0 | 0.0 | 0.0 | 0.0 | 0.0 | 0.0 | 0.0 |
| Cela1 | 1.4 | 0.7 | 0.9 | 1.5 | 1.1 | 1.5 | 0.5 | 1.1 | 0.5 | 0.8 | 0.8 | 0.7 |
| Celf1 | 11.0 | 12.4 | 8.8 | 14.6 | 12.9 | 13.2 | 11.1 | 9.7 | 10.1 | 11.6 | 14.1 | 11.5 |
| Celf2 | 10.7 | 12.5 | 15.1 | 11.8 | 11.9 | 10.6 | 11.0 | 13.0 | 12.5 | 8.4 | 12.1 | 9.4 |
| Celf3 | 0.0 | 0.1 | 0.0 | 0.0 | 0.0 | 0.1 | 0.0 | 0.1 | 0.0 | 0.0 | 0.0 | 0.0 |
| Celf4 | 1.5 | 1.2 | 1.3 | 1.4 | 0.9 | 1.6 | 1.1 | 0.6 | 0.4 | 0.8 | 0.8 | 1.0 |
| Celf5 | 0.7 | 1.0 | 1.0 | 0.9 | 0.9 | 0.9 | 0.7 | 0.3 | 0.5 | 0.6 | 0.5 | 0.6 |
| Celf6 | 0.0 | 0.0 | 0.0 | 0.0 | 0.0 | 0.0 | 0.0 | 0.0 | 0.0 | 0.0 | 0.0 | 0.0 |
| Celsr1 | 0.0 | 0.0 | 0.0 | 0.0 | 0.0 | 0.0 | 0.0 | 0.0 | 0.0 | 0.1 | 0.0 | 0.0 |
| Celsr2 | 0.0 | 0.0 | 0.0 | 0.0 | 0.0 | 0.0 | 0.0 | 0.0 | 0.0 | 0.0 | 0.0 | 0.0 |
| Celsr3 | 0.0 | 0.1 | 0.0 | 0.0 | 0.1 | 0.1 | 0.1 | 0.0 | 0.0 | 0.0 | 0.0 | 0.1 |
| Cend1 | 0.5 | 0.4 | 0.7 | 1.0 | 0.4 | 0.9 | 0.5 | 0.9 | 0.8 | 0.4 | 0.6 | 0.5 |
| Cenpa | 2.6 | 6.3 | 8.0 | 1.1 | 7.5 | 1.3 | 9.0 | 2.3 | 9.1 | 4.1 | 5.7 | 4.3 |
| Cenpb | 76.4 | 53.4 | 45.2 | 85.9 | 53.2 | 80.6 | 57.3 | 45.1 | 47.0 | 48.1 | 55.2 | 52.5 |
| Cenpc1 | 8.7 | 9.8 | 9.7 | 7.2 | 8.8 | 8.3 | 10.4 | 9.0 | 9.1 | 8.6 | 10.8 | 11.1 |
| Cenpe | 1.9 | 3.8 | 6.5 | 0.8 | 5.4 | 0.8 | 6.8 | 2.1 | 4.4 | 2.5 | 4.7 | 3.6 |
| Cenpf | 1.1 | 2.0 | 4.0 | 0.2 | 3.1 | 0.2 | 4.4 | 1.4 | 3.1 | 1.6 | 3.0 | 2.0 |
| Cenph | 1.6 | 3.9 | 4.6 | 0.9 | 4.4 | 0.7 | 4.1 | 1.8 | 2.5 | 2.3 | 2.8 | 2.3 |
| Cenpi | 1.3 | 2.2 | 4.1 | 0.7 | 3.4 | 0.9 | 3.7 | 1.5 | 3.4 | 1.8 | 2.1 | 2.2 |
| Cenpj | 2.5 | 3.5 | 3.9 | 3.3 | 3.5 | 2.9 | 2.9 | 2.8 | 2.8 | 2.8 | 3.3 | 3.5 |

| | | | | | | | | | | | | |
|---|---|---|---|---|---|---|---|---|---|---|---|---|
| Cenpk | 2.2 | 4.8 | 4.9 | 1.3 | 3.7 | 2.2 | 4.1 | 3.9 | 3.7 | 2.9 | 3.6 | 3.2 |
| Cenpl | 1.0 | 1.1 | 1.1 | 1.1 | 1.3 | 0.9 | 1.6 | 1.2 | 1.2 | 0.9 | 1.0 | 1.0 |
| Cenpm | 1.0 | 1.0 | 2.5 | 0.3 | 2.0 | 0.3 | 2.6 | 1.1 | 1.6 | 0.7 | 1.2 | 1.0 |
| Cenpn | 1.5 | 2.8 | 3.8 | 1.0 | 3.7 | 1.4 | 5.9 | 2.6 | 3.5 | 1.7 | 2.8 | 2.4 |
| Cenpo | 1.7 | 2.0 | 2.2 | 1.7 | 2.3 | 1.8 | 2.5 | 2.4 | 2.4 | 2.1 | 1.9 | 2.0 |
| Cenpp | 1.3 | 2.1 | 2.6 | 0.8 | 2.4 | 0.6 | 2.4 | 0.8 | 2.5 | 1.0 | 1.4 | 0.8 |
| Cenpq | 3.5 | 5.6 | 8.6 | 3.5 | 4.9 | 4.0 | 6.9 | 6.2 | 6.3 | 4.2 | 6.7 | 5.5 |
| Cenpt | 5.1 | 5.6 | 5.1 | 4.5 | 5.5 | 3.4 | 5.8 | 4.9 | 6.4 | 3.8 | 4.2 | 3.4 |
| Cenpv | 4.6 | 3.9 | 3.1 | 4.8 | 3.4 | 6.0 | 3.9 | 3.5 | 3.6 | 3.4 | 3.4 | 4.0 |
| Cenpw | 1.0 | 1.5 | 1.8 | 0.5 | 1.6 | 0.3 | 1.1 | 0.7 | 1.6 | 1.3 | 1.0 | 1.1 |
| Cep104 | 6.5 | 7.2 | 6.4 | 6.5 | 6.6 | 7.0 | 6.1 | 5.0 | 5.8 | 5.8 | 5.1 | 5.8 |
| Cep110 | 3.1 | 3.6 | 4.5 | 3.3 | 4.3 | 3.0 | 3.9 | 3.3 | 3.7 | 2.6 | 4.0 | 3.2 |
| Cep112 | 0.4 | 0.8 | 1.0 | 0.5 | 0.3 | 0.8 | 0.7 | 0.7 | 0.6 | 0.4 | 0.7 | 0.6 |
| Cep120 | 8.7 | 9.8 | 9.4 | 9.8 | 8.7 | 8.1 | 9.3 | 8.0 | 8.7 | 8.4 | 9.7 | 8.9 |
| Cep128 | 1.0 | 1.6 | 2.2 | 0.8 | 2.2 | 0.9 | 1.6 | 1.1 | 1.4 | 0.9 | 1.2 | 1.2 |
| Cep135 | 2.0 | 2.2 | 2.0 | 2.2 | 2.0 | 1.6 | 2.1 | 1.7 | 1.8 | 2.3 | 2.4 | 1.9 |
| Cep152 | 1.1 | 1.5 | 1.4 | 1.5 | 1.6 | 1.4 | 1.5 | 1.3 | 1.4 | 1.0 | 1.5 | 1.0 |
| Cep164 | 8.8 | 8.6 | 7.8 | 9.8 | 8.6 | 9.2 | 8.5 | 6.5 | 8.0 | 7.7 | 8.8 | 7.4 |
| Cep170 | 14.5 | 16.0 | 16.0 | 16.3 | 14.7 | 15.6 | 16.6 | 15.3 | 14.5 | 12.3 | 17.1 | 14.9 |
| Cep170b | 12.9 | 15.3 | 13.6 | 12.9 | 13.3 | 14.9 | 13.9 | 10.5 | 11.8 | 13.6 | 11.7 | 11.1 |
| Cep19 | 8.4 | 8.6 | 8.6 | 8.5 | 6.4 | 9.9 | 7.4 | 7.7 | 6.7 | 7.4 | 7.4 | 7.9 |
| Cep192 | 5.7 | 7.6 | 7.0 | 6.1 | 7.6 | 6.8 | 6.8 | 5.2 | 6.0 | 5.5 | 6.5 | 5.4 |
| Cep250 | 6.8 | 6.0 | 5.8 | 10.3 | 9.0 | 8.2 | 8.6 | 5.2 | 5.5 | 5.6 | 6.1 | 6.3 |
| Cep290 | 4.0 | 3.5 | 4.0 | 4.1 | 3.4 | 4.2 | 3.0 | 2.7 | 2.4 | 3.4 | 4.1 | 3.5 |
| Cep350 | 5.1 | 6.0 | 6.5 | 5.5 | 5.8 | 5.4 | 6.3 | 5.8 | 5.5 | 5.1 | 6.0 | 6.4 |
| Cep44 | 5.6 | 6.8 | 7.5 | 4.6 | 6.5 | 6.2 | 5.0 | 5.4 | 6.0 | 5.4 | 6.6 | 6.0 |
| Cep55 | 1.2 | 3.1 | 6.1 | 0.8 | 5.3 | 0.7 | 6.2 | 2.2 | 4.4 | 2.2 | 4.0 | 2.7 |
| Cep57 | 6.8 | 6.5 | 6.9 | 6.5 | 5.7 | 6.6 | 6.2 | 5.4 | 5.4 | 7.3 | 7.4 | 6.7 |
| Cep57l1 | 2.2 | 2.8 | 3.1 | 2.0 | 2.4 | 2.5 | 3.5 | 2.8 | 2.6 | 2.3 | 3.0 | 2.5 |
| Cep63 | 12.4 | 10.3 | 9.9 | 12.1 | 7.9 | 12.6 | 10.7 | 9.1 | 8.1 | 10.0 | 10.7 | 11.2 |
| Cep68 | 4.0 | 4.6 | 4.3 | 3.9 | 4.0 | 4.2 | 4.3 | 4.8 | 4.9 | 5.0 | 4.8 | 4.1 |
| Cep70 | 1.6 | 1.5 | 1.8 | 1.2 | 1.8 | 1.7 | 1.3 | 1.5 | 1.2 | 1.4 | 2.0 | 1.7 |
| Cep72 | 0.7 | 1.0 | 0.9 | 0.6 | 0.8 | 0.5 | 0.9 | 0.6 | 0.8 | 0.5 | 0.7 | 0.5 |
| Cep76 | 1.8 | 2.6 | 2.3 | 2.1 | 2.0 | 1.8 | 2.4 | 1.7 | 1.8 | 2.2 | 2.3 | 2.4 |
| Cep78 | 3.1 | 3.6 | 3.9 | 3.5 | 3.4 | 3.6 | 3.0 | 2.9 | 3.0 | 2.5 | 3.2 | 3.0 |
| Cep85 | 2.1 | 2.6 | 3.6 | 2.0 | 2.8 | 1.8 | 3.1 | 3.0 | 3.1 | 2.5 | 2.9 | 3.0 |
| Cep85l | 1.3 | 1.6 | 1.5 | 1.5 | 1.5 | 1.2 | 1.5 | 1.5 | 1.2 | 1.4 | 1.6 | 1.7 |
| Cep89 | 3.1 | 3.2 | 4.0 | 2.7 | 2.7 | 3.0 | 2.6 | 3.1 | 2.9 | 2.7 | 3.0 | 2.9 |
| Cep95 | 3.6 | 4.2 | 5.2 | 2.3 | 3.2 | 2.9 | 4.0 | 4.4 | 3.6 | 3.7 | 4.8 | 5.1 |
| Cep97 | 2.5 | 3.0 | 2.2 | 2.5 | 1.9 | 2.6 | 1.6 | 2.6 | 1.8 | 1.9 | 2.4 | 2.1 |
| Cept1 | 11.0 | 12.5 | 11.9 | 11.8 | 14.2 | 12.4 | 11.1 | 10.0 | 14.4 | 16.9 | 15.3 | 15.1 |
| Cercam | 12.7 | 14.7 | 16.3 | 13.0 | 11.7 | 18.7 | 12.8 | 16.1 | 13.1 | 11.8 | 10.7 | 12.1 |
| Cerk | 4.0 | 3.1 | 2.5 | 6.3 | 5.9 | 3.2 | 3.8 | 3.3 | 3.6 | 5.0 | 3.6 | 3.3 |
| Cerkl | 2.0 | 2.1 | 2.2 | 1.5 | 1.6 | 1.8 | 1.5 | 2.1 | 1.1 | 0.6 | 1.2 | 1.1 |
| Cers1 | 0.6 | 0.4 | 0.5 | 0.3 | 0.2 | 0.9 | 0.3 | 0.3 | 0.3 | 0.5 | 0.1 | 0.5 |
| Cers2 | 55.1 | 49.5 | 48.1 | 62.1 | 60.8 | 50.5 | 64.0 | 53.3 | 61.6 | 53.6 | 58.8 | 50.9 |
| Cers3 | 0.0 | 0.0 | 0.0 | 0.0 | 0.0 | 0.1 | 0.0 | 0.0 | 0.0 | 0.1 | 0.0 | 0.0 |
| Cers4 | 6.2 | 4.4 | 4.1 | 5.6 | 4.0 | 5.1 | 6.2 | 4.6 | 4.7 | 4.3 | 5.2 | 4.8 |
| Cers5 | 30.6 | 25.9 | 22.8 | 30.0 | 30.2 | 27.1 | 33.9 | 26.2 | 32.6 | 31.7 | 30.9 | 28.7 |
| Cers6 | 28.8 | 35.2 | 35.5 | 28.7 | 33.3 | 26.3 | 37.1 | 29.5 | 33.8 | 26.4 | 28.5 | 31.3 |
| Ces1a | 0.1 | 0.3 | 0.1 | 0.1 | 0.2 | 0.3 | 0.1 | 0.4 | 0.1 | 0.2 | 0.1 | 0.2 |
| Ces1d | 1.3 | 2.0 | 3.8 | 0.7 | 1.2 | 1.1 | 0.2 | 0.8 | 0.2 | 0.1 | 0.4 | 0.1 |
| Ces1f | 0.0 | 0.0 | 0.0 | 0.0 | 0.1 | 0.1 | 0.0 | 0.0 | 0.0 | 0.0 | 0.0 | 0.0 |
| Ces1g | 0.4 | 0.2 | 0.3 | 0.4 | 0.5 | 0.5 | 0.0 | 0.1 | 0.0 | 0.1 | 0.1 | 0.1 |
| Ces2a | 0.0 | 0.0 | 0.0 | 0.0 | 0.0 | 0.0 | 0.0 | 0.0 | 0.0 | 0.0 | 0.0 | 0.0 |
| Ces2b | 0.1 | 0.2 | 0.2 | 0.1 | 0.1 | 0.1 | 0.0 | 0.0 | 0.0 | 0.0 | 0.0 | 0.0 |
| Ces2c | 0.2 | 0.1 | 0.1 | 0.4 | 0.2 | 0.1 | 0.1 | 0.0 | 0.0 | 0.0 | 0.0 | 0.0 |

| | | | | | | | | | | | | |
|---|---|---|---|---|---|---|---|---|---|---|---|---|
| Ces2d-ps | 0.0 | 0.0 | 0.0 | 0.1 | 0.0 | 0.0 | 0.0 | 0.0 | 0.0 | 0.0 | 0.0 | 0.0 |
| Ces2e | 33.5 | 22.0 | 16.2 | 13.0 | 19.8 | 10.5 | 9.0 | 1.8 | 3.2 | 8.0 | 5.8 | 6.3 |
| Ces2f | 0.3 | 0.4 | 0.2 | 0.3 | 0.5 | 0.1 | 0.1 | 0.0 | 0.0 | 0.2 | 0.1 | 0.1 |
| Ces2g | 12.1 | 11.5 | 9.5 | 10.6 | 11.6 | 10.5 | 9.3 | 5.8 | 5.0 | 7.6 | 6.5 | 5.6 |
| Cetn2 | 27.0 | 24.2 | 25.8 | 26.3 | 20.3 | 30.0 | 22.9 | 26.4 | 20.3 | 21.6 | 30.3 | 28.5 |
| Cetn3 | 45.5 | 39.3 | 47.7 | 53.6 | 40.5 | 41.2 | 46.2 | 46.7 | 40.7 | 41.8 | 54.9 | 55.1 |
| Cetn4 | 4.8 | 3.7 | 5.0 | 4.2 | 4.4 | 5.8 | 4.6 | 2.2 | 2.9 | 5.0 | 5.0 | 5.5 |
| Cfb | 1.9 | 1.4 | 2.2 | 2.2 | 3.2 | 0.4 | 0.6 | 0.8 | 0.3 | 1.0 | 0.5 | 0.9 |
| Cfdp1 | 72.7 | 66.1 | 81.4 | 90.8 | 59.4 | 74.8 | 77.8 | 73.3 | 59.4 | 68.9 | 88.7 | 94.8 |
| Cfh | 107.1 | 130.6 | 143.6 | 40.6 | 64.0 | 33.4 | 47.5 | 60.1 | 89.6 | 95.5 | 56.8 | 123.4 |
| Cfhr1 | 0.0 | 0.0 | 0.1 | 0.0 | 0.0 | 0.0 | 0.0 | 0.0 | 0.0 | 0.0 | 0.0 | 0.0 |
| Cfhr2 | 19.4 | 21.6 | 23.1 | 6.2 | 11.0 | 5.8 | 8.1 | 9.6 | 15.8 | 15.2 | 8.6 | 19.1 |
| Cfi | 10.0 | 15.5 | 15.9 | 9.2 | 13.1 | 4.6 | 7.4 | 9.4 | 6.8 | 4.8 | 4.1 | 7.6 |
| Cfl1 | 359.7 | 294.7 | 254.3 | 460.7 | 422.1 | 348.8 | 418.3 | 359.6 | 334.4 | 378.3 | 428.4 | 349.2 |
| Cfl2 | 43.4 | 37.0 | 32.0 | 45.6 | 32.5 | 44.0 | 34.1 | 26.2 | 36.9 | 40.7 | 42.5 | 40.8 |
| Cflar | 49.1 | 40.7 | 35.4 | 66.5 | 44.2 | 62.4 | 43.6 | 24.2 | 27.9 | 36.5 | 39.0 | 37.1 |
| Cfp | 3.5 | 0.7 | 0.9 | 28.3 | 60.5 | 0.5 | 8.7 | 1.2 | 2.2 | 4.8 | 1.8 | 2.9 |
| Cftr | 0.0 | 0.0 | 0.0 | 0.0 | 0.0 | 0.0 | 0.0 | 0.0 | 0.0 | 0.0 | 0.0 | 0.0 |
| Cggbp1 | 16.1 | 18.4 | 18.8 | 18.4 | 19.8 | 16.7 | 19.5 | 20.6 | 21.5 | 19.7 | 22.7 | 20.6 |
| Cgn | 0.2 | 0.2 | 0.1 | 0.1 | 0.1 | 0.1 | 0.1 | 0.1 | 0.2 | 0.8 | 0.6 | 0.2 |
| Cgnl1 | 14.6 | 7.3 | 6.6 | 16.2 | 8.8 | 16.0 | 3.8 | 3.0 | 2.1 | 3.8 | 2.9 | 3.0 |
| Cgref1 | 21.3 | 16.9 | 12.8 | 19.3 | 12.4 | 20.8 | 14.3 | 11.6 | 8.8 | 8.9 | 14.1 | 14.7 |
| Cgrrf1 | 29.4 | 30.9 | 24.9 | 28.9 | 24.6 | 32.6 | 27.8 | 23.8 | 23.1 | 27.4 | 27.5 | 28.8 |
| Ch25h | 6.9 | 4.9 | 6.1 | 12.6 | 28.6 | 4.2 | 9.2 | 3.4 | 5.1 | 10.1 | 4.1 | 8.1 |
| Chac1 | 12.8 | 7.1 | 4.5 | 11.4 | 4.7 | 12.0 | 4.4 | 7.2 | 7.4 | 8.5 | 9.1 | 4.9 |
| Chac2 | 3.1 | 3.5 | 4.0 | 4.6 | 4.1 | 4.0 | 4.3 | 3.8 | 3.9 | 3.4 | 4.7 | 4.2 |
| Chad | 0.0 | 0.1 | 0.1 | 0.2 | 0.1 | 0.1 | 0.1 | 0.2 | 0.0 | 0.0 | 0.1 | 0.0 |
| Chadl | 0.6 | 0.5 | 0.4 | 0.6 | 0.4 | 0.6 | 0.4 | 0.3 | 0.6 | 0.3 | 0.3 | 0.5 |
| Chaf1a | 4.1 | 5.3 | 6.5 | 3.1 | 5.8 | 3.0 | 7.7 | 4.4 | 4.9 | 3.4 | 5.2 | 3.7 |
| Chaf1b | 1.8 | 3.3 | 4.2 | 2.0 | 3.9 | 1.5 | 4.8 | 2.6 | 3.3 | 2.2 | 3.4 | 2.1 |
| Chchd1 | 47.7 | 44.5 | 43.7 | 50.8 | 44.2 | 52.8 | 43.1 | 38.3 | 35.1 | 43.7 | 43.2 | 46.1 |
| Chchd10 | 2.0 | 0.6 | 0.7 | 3.8 | 0.7 | 3.0 | 0.3 | 0.7 | 0.5 | 2.1 | 1.2 | 0.3 |
| Chchd2 | 148.8 | 151.8 | 113.0 | 127.9 | 132.8 | 128.1 | 92.2 | 109.2 | 126.8 | 154.9 | 119.8 | 126.5 |
| Chchd3 | 41.4 | 33.9 | 35.7 | 40.5 | 30.8 | 39.5 | 35.4 | 39.3 | 34.1 | 33.1 | 42.2 | 35.8 |
| Chchd4 | 9.2 | 6.6 | 5.4 | 9.5 | 9.7 | 9.9 | 6.1 | 5.7 | 6.5 | 8.6 | 8.0 | 7.4 |
| Chchd5 | 6.2 | 4.6 | 6.6 | 7.5 | 5.8 | 8.5 | 5.9 | 6.6 | 6.2 | 6.2 | 6.6 | 6.4 |
| Chchd6 | 7.7 | 7.3 | 5.9 | 8.7 | 5.9 | 9.9 | 5.6 | 4.9 | 5.4 | 5.4 | 6.4 | 5.6 |
| Chchd7 | 19.5 | 16.0 | 14.6 | 20.5 | 11.8 | 16.2 | 13.4 | 14.1 | 13.1 | 12.5 | 13.3 | 15.0 |
| Chd1 | 9.3 | 9.9 | 10.9 | 9.0 | 9.7 | 8.2 | 10.7 | 8.9 | 9.3 | 10.5 | 11.0 | 11.0 |
| Chd1l | 2.5 | 2.0 | 2.3 | 2.9 | 2.2 | 2.7 | 2.2 | 2.4 | 1.9 | 2.2 | 2.1 | 1.9 |
| Chd2 | 8.4 | 8.3 | 9.6 | 8.4 | 7.2 | 8.6 | 8.8 | 7.8 | 6.9 | 7.4 | 9.0 | 9.7 |
| Chd3 | 20.9 | 19.6 | 18.2 | 26.1 | 19.8 | 22.7 | 23.2 | 21.1 | 24.3 | 23.1 | 25.6 | 26.6 |
| Chd4 | 35.6 | 36.1 | 38.8 | 34.6 | 35.4 | 32.7 | 42.0 | 39.2 | 38.6 | 37.6 | 40.7 | 41.7 |
| Chd5 | 0.6 | 0.3 | 0.2 | 0.9 | 0.3 | 0.7 | 0.3 | 0.2 | 0.2 | 0.2 | 0.2 | 0.3 |
| Chd6 | 7.4 | 7.3 | 7.0 | 7.8 | 6.1 | 7.3 | 6.7 | 6.0 | 5.9 | 6.1 | 6.7 | 6.9 |
| Chd7 | 0.5 | 0.4 | 0.4 | 0.6 | 0.8 | 0.3 | 0.6 | 0.4 | 0.4 | 0.3 | 0.4 | 0.3 |
| Chd8 | 9.7 | 12.1 | 12.5 | 10.2 | 11.5 | 10.1 | 12.0 | 11.9 | 11.3 | 11.2 | 12.1 | 11.5 |
| Chd9 | 4.1 | 4.6 | 4.8 | 4.2 | 4.4 | 3.9 | 5.0 | 4.7 | 4.3 | 4.3 | 4.8 | 5.3 |
| Chdh | 0.0 | 0.0 | 0.0 | 0.0 | 0.0 | 0.0 | 0.0 | 0.0 | 0.0 | 0.0 | 0.0 | 0.0 |
| Chek1 | 0.6 | 0.9 | 1.1 | 0.4 | 1.1 | 0.4 | 1.0 | 1.0 | 0.9 | 0.7 | 0.9 | 0.8 |
| Chek2 | 6.4 | 6.7 | 7.4 | 5.7 | 7.5 | 5.2 | 4.9 | 4.6 | 5.1 | 5.7 | 5.9 | 5.9 |
| Cherp | 2.6 | 3.5 | 3.7 | 3.2 | 3.8 | 3.2 | 4.2 | 3.6 | 3.6 | 2.9 | 3.0 | 2.7 |
| Chfr | 17.9 | 21.7 | 21.4 | 13.2 | 14.3 | 11.5 | 18.2 | 21.7 | 23.0 | 13.5 | 19.0 | 18.4 |
| Chga | 0.1 | 0.1 | 0.1 | 0.1 | 0.0 | 0.0 | 0.0 | 0.0 | 0.1 | 0.0 | 0.0 | 0.1 |
| Chgb | 0.3 | 0.3 | 0.2 | 0.1 | 0.2 | 0.4 | 0.3 | 0.1 | 0.2 | 0.1 | 0.3 | 0.3 |
| Chi3l1 | 0.3 | 0.3 | 0.8 | 0.0 | 0.1 | 0.1 | 0.0 | 0.9 | 0.2 | 0.3 | 0.5 | 0.5 |
| Chic1 | 0.8 | 0.8 | 1.1 | 0.8 | 0.7 | 1.0 | 0.5 | 0.7 | 0.7 | 0.9 | 0.8 | 0.9 |

| | | | | | | | | | | | | |
|---|---|---|---|---|---|---|---|---|---|---|---|---|
| Chic2 | 24.0 | 23.3 | 22.6 | 23.0 | 26.8 | 21.8 | 27.4 | 21.7 | 24.1 | 33.1 | 27.7 | 27.6 |
| Chid1 | 37.0 | 29.6 | 23.0 | 34.8 | 27.6 | 33.6 | 25.5 | 21.8 | 25.3 | 32.0 | 28.7 | 30.8 |
| Chit1 | 0.1 | 0.1 | 0.0 | 0.0 | 0.0 | 0.0 | 0.3 | 0.0 | 0.0 | 0.0 | 0.0 | 0.0 |
| Chka | 3.2 | 3.5 | 2.8 | 2.6 | 3.2 | 3.0 | 3.5 | 3.0 | 3.3 | 4.0 | 3.2 | 2.5 |
| Chkb | 7.5 | 7.1 | 7.0 | 7.1 | 8.2 | 7.3 | 7.9 | 7.0 | 9.2 | 8.2 | 6.5 | 7.3 |
| Chl1 | 0.1 | 0.0 | 0.0 | 0.0 | 0.0 | 0.0 | 0.0 | 0.0 | 0.0 | 0.0 | 0.0 | 0.0 |
| Chm | 8.5 | 8.9 | 8.8 | 11.9 | 9.2 | 10.7 | 8.8 | 8.7 | 8.8 | 9.4 | 11.4 | 11.0 |
| Chml | 1.4 | 1.3 | 1.4 | 1.3 | 1.3 | 1.9 | 1.5 | 1.5 | 1.4 | 1.3 | 1.7 | 1.8 |
| Chmp1a | 40.9 | 41.5 | 34.2 | 42.6 | 40.3 | 44.4 | 40.8 | 37.2 | 41.7 | 44.1 | 38.1 | 38.1 |
| Chmp1b | 26.1 | 24.7 | 28.0 | 37.5 | 25.0 | 29.2 | 27.3 | 24.8 | 24.3 | 28.6 | 32.9 | 34.4 |
| Chmp2a | 88.0 | 85.9 | 97.4 | 90.9 | 75.3 | 84.5 | 96.8 | 100.9 | 85.0 | 86.1 | 98.3 | 105.6 |
| Chmp2b | 26.6 | 25.3 | 27.1 | 27.0 | 23.6 | 30.2 | 24.9 | 25.7 | 23.1 | 25.9 | 29.8 | 31.1 |
| Chmp3 | 93.5 | 69.4 | 60.3 | 102.6 | 66.0 | 92.1 | 73.6 | 55.3 | 55.8 | 68.2 | 81.2 | 79.2 |
| Chmp4b | 92.0 | 83.8 | 90.2 | 107.8 | 82.2 | 94.3 | 92.9 | 91.3 | 78.2 | 89.0 | 100.8 | 106.4 |
| Chmp4c | 0.8 | 0.2 | 0.4 | 0.4 | 0.2 | 0.2 | 0.2 | 0.1 | 0.1 | 1.0 | 0.4 | 0.4 |
| Chmp5 | 132.1 | 119.6 | 110.7 | 147.4 | 102.4 | 144.2 | 104.0 | 91.2 | 95.1 | 115.8 | 123.9 | 122.8 |
| Chmp6 | 15.0 | 13.3 | 14.7 | 14.5 | 15.6 | 13.0 | 15.2 | 16.8 | 16.9 | 14.0 | 14.5 | 13.7 |
| Chmp7 | 27.5 | 25.0 | 20.3 | 27.9 | 23.8 | 28.6 | 21.1 | 17.9 | 20.8 | 22.6 | 20.9 | 20.5 |
| Chn1 | 6.8 | 5.7 | 4.1 | 9.0 | 5.0 | 7.3 | 4.6 | 3.0 | 3.4 | 2.9 | 3.3 | 3.4 |
| Chn2 | 1.6 | 1.3 | 0.8 | 1.3 | 1.2 | 1.2 | 1.1 | 1.3 | 0.9 | 3.2 | 2.3 | 1.1 |
| Chodl | 0.0 | 0.0 | 0.0 | 0.0 | 0.0 | 0.0 | 0.0 | 0.0 | 0.0 | 0.0 | 0.0 | 0.0 |
| Chordc1 | 30.7 | 34.4 | 34.7 | 36.7 | 31.1 | 34.1 | 32.1 | 26.7 | 28.1 | 37.3 | 42.2 | 39.0 |
| Chp1 | 60.7 | 54.7 | 49.4 | 56.0 | 51.7 | 55.4 | 51.5 | 50.2 | 51.3 | 61.1 | 57.7 | 56.1 |
| Chpf | 28.4 | 17.9 | 14.3 | 27.1 | 26.9 | 34.6 | 32.0 | 23.4 | 26.6 | 28.3 | 23.9 | 23.8 |
| Chpf2 | 36.2 | 31.2 | 24.8 | 37.7 | 36.8 | 35.9 | 35.0 | 29.2 | 35.0 | 29.9 | 27.1 | 27.0 |
| Chpt1 | 22.0 | 20.6 | 21.6 | 22.7 | 17.3 | 29.6 | 19.8 | 16.0 | 18.6 | 20.7 | 22.2 | 22.4 |
| Chrac1 | 18.5 | 17.7 | 18.8 | 19.2 | 19.0 | 18.5 | 20.7 | 19.1 | 20.8 | 19.3 | 19.8 | 18.9 |
| Chrd | 1.0 | 0.7 | 0.3 | 0.5 | 0.4 | 1.0 | 1.0 | 1.0 | 1.1 | 1.1 | 1.5 | 1.1 |
| Chrdl1 | 0.3 | 0.0 | 0.3 | 0.0 | 0.0 | 0.0 | 0.0 | 0.1 | 0.0 | 0.0 | 0.0 | 0.1 |
| Chrm1 | 0.0 | 0.0 | 0.0 | 0.0 | 0.0 | 0.0 | 0.0 | 0.0 | 0.0 | 0.0 | 0.0 | 0.0 |
| Chrm3 | 0.2 | 0.1 | 0.1 | 0.1 | 0.2 | 0.1 | 0.1 | 0.0 | 0.0 | 0.0 | 0.0 | 0.1 |
| Chrm4 | 0.1 | 0.0 | 0.1 | 0.1 | 0.0 | 0.1 | 0.1 | 0.1 | 0.0 | 0.0 | 0.0 | 0.0 |
| Chrna1 | 0.0 | 0.0 | 0.0 | 0.0 | 0.0 | 0.0 | 0.1 | 0.1 | 0.0 | 0.0 | 0.0 | 0.0 |
| Chrna10 | 0.0 | 0.0 | 0.0 | 0.0 | 0.0 | 0.0 | 0.0 | 0.0 | 0.0 | 0.0 | 0.0 | 0.0 |
| Chrna2 | 0.1 | 0.2 | 0.2 | 0.2 | 0.2 | 0.1 | 0.2 | 0.1 | 0.1 | 0.2 | 0.2 | 0.2 |
| Chrna4 | 0.0 | 0.0 | 0.0 | 0.0 | 0.0 | 0.0 | 0.0 | 0.0 | 0.0 | 0.0 | 0.0 | 0.1 |
| Chrna7 | 0.3 | 0.1 | 0.1 | 0.3 | 0.1 | 0.6 | 0.1 | 0.1 | 0.1 | 0.0 | 0.0 | 0.0 |
| Chrna9 | 0.0 | 0.0 | 0.0 | 0.0 | 0.0 | 0.0 | 0.0 | 0.0 | 0.0 | 0.0 | 0.0 | 0.0 |
| Chrnb1 | 2.6 | 2.2 | 2.2 | 3.9 | 2.7 | 2.6 | 3.1 | 3.3 | 3.2 | 2.6 | 2.6 | 2.2 |
| Chrnb2 | 0.2 | 0.2 | 0.0 | 0.1 | 0.1 | 0.2 | 0.1 | 0.1 | 0.0 | 0.1 | 0.0 | 0.0 |
| Chst1 | 0.2 | 0.3 | 0.4 | 0.3 | 0.2 | 0.5 | 0.5 | 0.6 | 0.4 | 0.3 | 0.2 | 0.2 |
| Chst10 | 1.9 | 1.5 | 1.4 | 2.3 | 1.3 | 2.6 | 1.7 | 1.3 | 1.1 | 1.0 | 1.2 | 1.1 |
| Chst11 | 3.6 | 3.3 | 3.6 | 3.3 | 4.3 | 2.9 | 3.9 | 3.9 | 4.9 | 5.5 | 3.5 | 7.0 |
| Chst12 | 15.2 | 14.3 | 14.6 | 22.0 | 19.0 | 18.9 | 21.8 | 16.6 | 16.2 | 17.7 | 22.9 | 15.2 |
| Chst13 | 0.0 | 0.1 | 0.1 | 0.0 | 0.1 | 0.1 | 0.0 | 0.1 | 0.1 | 0.1 | 0.1 | 0.1 |
| Chst14 | 15.0 | 12.3 | 11.6 | 13.1 | 12.3 | 14.2 | 16.2 | 13.1 | 15.0 | 12.1 | 12.1 | 11.4 |
| Chst15 | 0.4 | 0.5 | 0.4 | 0.6 | 0.8 | 0.7 | 0.7 | 0.5 | 0.6 | 1.2 | 1.0 | 0.9 |
| Chst2 | 0.6 | 1.0 | 1.0 | 0.8 | 1.5 | 1.2 | 2.0 | 1.3 | 1.8 | 1.4 | 1.2 | 1.2 |
| Chst3 | 0.0 | 0.0 | 0.0 | 0.0 | 0.2 | 0.0 | 0.0 | 0.1 | 0.0 | 0.0 | 0.0 | 0.0 |
| Chst5 | 0.0 | 0.0 | 0.0 | 0.0 | 0.1 | 0.0 | 0.0 | 0.0 | 0.0 | 0.0 | 0.0 | 0.0 |
| Chst7 | 5.4 | 3.8 | 3.1 | 4.5 | 4.1 | 5.1 | 5.2 | 3.8 | 4.1 | 3.1 | 2.7 | 2.9 |
| Chst8 | 0.1 | 0.0 | 0.0 | 0.0 | 0.0 | 0.0 | 0.0 | 0.0 | 0.0 | 0.0 | 0.0 | 0.0 |
| Chsy1 | 15.1 | 15.1 | 21.9 | 13.1 | 15.8 | 14.0 | 20.7 | 20.8 | 18.8 | 16.4 | 18.6 | 21.8 |
| Chsy3 | 0.5 | 0.8 | 0.9 | 0.5 | 0.9 | 0.3 | 1.0 | 0.9 | 1.2 | 0.8 | 0.7 | 0.9 |
| Chtf18 | 0.8 | 1.5 | 2.0 | 0.6 | 1.7 | 0.7 | 1.9 | 1.1 | 1.5 | 1.0 | 1.6 | 0.8 |
| Chtf8 | 32.4 | 34.2 | 29.5 | 33.7 | 35.5 | 33.6 | 34.5 | 34.3 | 37.4 | 36.0 | 33.4 | 31.2 |
| Chtop | 15.6 | 13.8 | 16.0 | 14.0 | 14.1 | 15.8 | 14.0 | 17.4 | 16.3 | 16.3 | 15.4 | 16.0 |

| | | | | | | | | | | | | |
|---|---|---|---|---|---|---|---|---|---|---|---|---|
| Chuk | 17.9 | 19.8 | 16.6 | 16.1 | 17.5 | 17.4 | 16.8 | 14.4 | 18.2 | 17.7 | 18.9 | 17.3 |
| Churc1 | 28.1 | 19.2 | 15.1 | 21.3 | 18.1 | 23.4 | 17.6 | 14.7 | 17.7 | 22.6 | 21.5 | 18.7 |
| Ciao1 | 18.0 | 17.5 | 15.3 | 21.1 | 14.5 | 17.1 | 14.6 | 12.9 | 12.9 | 15.5 | 13.8 | 15.7 |
| Ciapin1 | 2.3 | 2.1 | 1.7 | 2.3 | 2.4 | 1.6 | 2.4 | 2.2 | 2.5 | 2.0 | 2.1 | 1.9 |
| Cib1 | 26.2 | 25.2 | 20.8 | 26.0 | 31.2 | 24.3 | 27.7 | 27.0 | 27.8 | 27.4 | 25.3 | 26.8 |
| Cib2 | 7.5 | 6.5 | 4.9 | 5.7 | 4.0 | 7.0 | 3.9 | 3.6 | 3.8 | 5.6 | 5.0 | 5.0 |
| Cib3 | 0.1 | 0.2 | 0.1 | 0.0 | 0.2 | 0.0 | 0.1 | 0.0 | 0.0 | 0.1 | 0.0 | 0.1 |
| Cic | 2.8 | 3.4 | 3.6 | 3.3 | 3.9 | 3.2 | 4.0 | 3.5 | 3.5 | 3.0 | 2.5 | 2.3 |
| Cideb | 0.3 | 0.4 | 0.3 | 0.5 | 0.3 | 0.1 | 0.7 | 0.6 | 0.4 | 0.2 | 0.4 | 0.7 |
| Ciita | 0.0 | 0.1 | 0.0 | 0.0 | 0.0 | 0.1 | 0.0 | 0.0 | 0.0 | 0.1 | 0.0 | 0.0 |
| Cilp | 0.1 | 0.4 | 2.4 | 0.0 | 0.2 | 0.1 | 0.3 | 2.3 | 0.7 | 0.5 | 1.0 | 0.5 |
| Cilp2 | 0.1 | 0.0 | 0.0 | 0.1 | 0.0 | 0.0 | 0.0 | 0.0 | 0.0 | 0.2 | 0.0 | 0.1 |
| Cinp | 8.7 | 9.6 | 9.4 | 9.6 | 8.8 | 9.0 | 9.7 | 9.6 | 8.9 | 9.2 | 9.7 | 9.2 |
| Cir1 | 12.9 | 13.0 | 15.2 | 18.7 | 11.1 | 15.4 | 11.1 | 13.0 | 9.8 | 14.2 | 14.7 | 18.2 |
| Cirbp | 4.7 | 3.3 | 5.0 | 3.2 | 3.5 | 4.4 | 4.3 | 6.4 | 5.3 | 5.0 | 3.8 | 5.8 |
| Cirh1a | 15.3 | 15.9 | 12.7 | 16.3 | 14.6 | 15.4 | 12.9 | 14.1 | 11.1 | 14.8 | 14.7 | 13.5 |
| Cisd1 | 35.8 | 29.5 | 23.4 | 29.3 | 28.8 | 31.1 | 26.6 | 22.0 | 25.8 | 30.1 | 26.1 | 26.5 |
| Cisd2 | 8.7 | 8.0 | 10.0 | 10.0 | 9.0 | 8.9 | 9.0 | 9.9 | 9.5 | 8.7 | 10.7 | 9.7 |
| Cisd3 | 15.0 | 13.7 | 14.0 | 15.0 | 13.3 | 13.8 | 11.7 | 13.6 | 12.7 | 11.6 | 13.5 | 11.6 |
| Cish | 0.5 | 0.3 | 0.3 | 0.1 | 0.7 | 0.5 | 0.5 | 0.3 | 0.4 | 0.5 | 0.3 | 0.3 |
| Cit | 0.2 | 0.3 | 0.7 | 0.1 | 0.6 | 0.1 | 0.9 | 0.3 | 0.6 | 0.3 | 0.4 | 0.3 |
| Cited2 | 23.1 | 24.6 | 19.5 | 34.5 | 20.7 | 32.2 | 20.2 | 12.5 | 14.8 | 17.5 | 18.4 | 13.2 |
| Cited4 | 0.2 | 0.2 | 0.1 | 0.0 | 0.1 | 0.1 | 0.2 | 0.2 | 0.1 | 0.1 | 0.1 | 0.0 |
| Ciz1 | 9.2 | 9.9 | 12.2 | 14.0 | 9.3 | 11.2 | 11.8 | 11.7 | 9.5 | 7.8 | 10.6 | 10.5 |
| Ckap2 | 10.0 | 17.5 | 29.0 | 4.0 | 21.5 | 5.6 | 25.8 | 9.3 | 20.3 | 13.9 | 21.0 | 15.2 |
| Ckap2l | 2.1 | 4.4 | 7.6 | 1.0 | 5.7 | 0.6 | 9.8 | 3.1 | 6.7 | 2.7 | 6.1 | 4.4 |
| Ckap4 | 43.3 | 40.4 | 48.2 | 42.8 | 44.0 | 37.8 | 60.8 | 53.1 | 50.3 | 51.8 | 51.3 | 49.2 |
| Ckap5 | 21.6 | 22.9 | 23.3 | 19.4 | 21.9 | 22.3 | 22.1 | 21.5 | 23.2 | 19.8 | 24.0 | 22.8 |
| Ckb | 91.1 | 67.1 | 26.9 | 160.9 | 166.2 | 121.3 | 56.2 | 32.3 | 30.4 | 60.4 | 43.2 | 39.1 |
| Cklf | 9.1 | 10.3 | 6.2 | 10.7 | 14.6 | 6.8 | 8.8 | 5.4 | 10.7 | 8.7 | 8.9 | 7.6 |
| Ckmt1 | 0.7 | 0.3 | 0.2 | 0.1 | 0.0 | 0.1 | 0.0 | 0.0 | 0.1 | 2.7 | 0.9 | 0.7 |
| Cks1b | 28.3 | 25.2 | 31.0 | 21.2 | 29.0 | 20.9 | 38.9 | 30.4 | 32.8 | 26.7 | 36.6 | 23.9 |
| Cks2 | 16.3 | 24.2 | 32.8 | 18.5 | 33.7 | 16.6 | 34.4 | 20.9 | 32.5 | 24.0 | 29.7 | 24.9 |
| Clasp1 | 11.7 | 12.2 | 12.4 | 12.1 | 12.4 | 12.8 | 13.2 | 11.0 | 11.5 | 12.3 | 12.8 | 12.4 |
| Clasp2 | 11.3 | 16.6 | 16.5 | 10.8 | 14.8 | 13.8 | 17.1 | 15.2 | 20.5 | 13.2 | 15.0 | 17.3 |
| Clasrp | 5.5 | 6.1 | 7.7 | 5.9 | 5.1 | 5.6 | 7.9 | 5.7 | 7.0 | 6.8 | 6.5 | 6.4 |
| Clca1 | 26.4 | 31.3 | 44.0 | 18.5 | 53.4 | 14.1 | 54.1 | 59.2 | 65.1 | 45.4 | 36.4 | 49.4 |
| Clca2 | 3.3 | 3.6 | 4.9 | 2.0 | 5.8 | 1.4 | 5.5 | 6.9 | 8.1 | 5.9 | 4.0 | 6.3 |
| Clca4 | 0.1 | 0.1 | 0.1 | 0.0 | 0.2 | 0.0 | 0.1 | 0.1 | 0.2 | 0.1 | 0.1 | 0.2 |
| Clca5 | 0.1 | 0.0 | 0.1 | 0.1 | 0.1 | 0.1 | 0.1 | 0.1 | 0.1 | 0.0 | 0.0 | 0.0 |
| Clca6 | 0.1 | 0.0 | 0.1 | 0.2 | 0.0 | 0.1 | 0.0 | 0.0 | 0.0 | 0.0 | 0.0 | 0.0 |
| Clcc1 | 13.5 | 13.4 | 16.3 | 13.1 | 14.2 | 14.8 | 12.2 | 11.6 | 13.4 | 12.7 | 12.6 | 13.6 |
| Clcf1 | 5.5 | 6.4 | 5.9 | 3.7 | 4.0 | 4.7 | 6.0 | 7.6 | 5.7 | 7.2 | 8.1 | 5.5 |
| Clcn1 | 0.0 | 0.0 | 0.0 | 0.0 | 0.0 | 0.0 | 0.0 | 0.0 | 0.0 | 0.1 | 0.0 | 0.0 |
| Clcn2 | 0.8 | 1.1 | 0.9 | 0.8 | 0.8 | 1.1 | 1.0 | 0.8 | 1.2 | 1.4 | 0.9 | 0.9 |
| Clcn3 | 4.9 | 3.8 | 3.3 | 3.6 | 4.0 | 3.9 | 3.3 | 3.0 | 3.2 | 4.4 | 4.1 | 3.8 |
| Clcn4-2 | 14.7 | 13.0 | 12.5 | 16.8 | 15.8 | 15.5 | 16.7 | 14.4 | 15.4 | 16.1 | 15.3 | 16.5 |
| Clcn5 | 3.4 | 2.6 | 2.6 | 2.5 | 3.0 | 3.2 | 1.8 | 3.2 | 2.3 | 2.8 | 2.1 | 2.4 |
| Clcn6 | 1.5 | 1.8 | 1.8 | 1.6 | 1.2 | 1.2 | 2.0 | 1.7 | 1.9 | 1.0 | 1.3 | 1.1 |
| Clcn7 | 21.4 | 18.0 | 16.4 | 25.1 | 21.9 | 23.6 | 17.4 | 16.4 | 15.7 | 16.3 | 13.5 | 14.9 |
| Clcnkb | 0.0 | 0.0 | 0.0 | 0.0 | 0.0 | 0.0 | 0.0 | 0.0 | 0.0 | 0.0 | 0.0 | 0.0 |
| Cldn1 | 0.1 | 0.2 | 0.1 | 0.0 | 0.0 | 0.1 | 0.0 | 0.1 | 0.1 | 0.0 | 0.1 | 0.1 |
| Cldn10 | 0.1 | 0.1 | 0.0 | 0.0 | 0.0 | 0.0 | 0.0 | 0.0 | 0.0 | 0.1 | 0.1 | 0.1 |
| Cldn11 | 0.0 | 0.0 | 0.0 | 0.1 | 0.0 | 0.0 | 0.0 | 0.0 | 0.0 | 0.0 | 0.1 | 0.0 |
| Cldn12 | 8.5 | 10.4 | 9.8 | 9.4 | 9.4 | 9.4 | 11.4 | 11.2 | 11.4 | 12.3 | 12.3 | 10.8 |
| Cldn15 | 8.3 | 15.8 | 10.9 | 7.9 | 10.4 | 8.6 | 13.3 | 16.3 | 13.1 | 18.8 | 14.0 | 7.4 |
| Cldn19 | 0.0 | 0.0 | 0.0 | 0.0 | 0.0 | 0.0 | 0.0 | 0.0 | 0.0 | 0.0 | 0.0 | 0.0 |

| | | | | | | | | | | | | |
|---|---|---|---|---|---|---|---|---|---|---|---|---|
| Cldn20 | 0.1 | 0.1 | 0.2 | 0.2 | 0.3 | 0.0 | 0.0 | 0.1 | 0.0 | 0.0 | 0.0 | 0.0 |
| Cldn22 | 0.8 | 0.4 | 0.4 | 0.3 | 0.2 | 0.5 | 0.7 | 0.3 | 0.2 | 0.3 | 0.3 | 0.5 |
| Cldn23 | 0.0 | 0.0 | 0.0 | 0.0 | 0.0 | 0.0 | 0.0 | 0.0 | 0.0 | 0.0 | 0.0 | 0.0 |
| Cldn24 | 0.1 | 0.0 | 0.3 | 0.0 | 0.2 | 0.0 | 0.0 | 0.1 | 0.1 | 0.2 | 0.1 | 0.1 |
| Cldn25 | 12.9 | 13.8 | 13.5 | 12.9 | 12.3 | 15.0 | 13.5 | 13.3 | 15.4 | 15.0 | 14.8 | 14.9 |
| Cldn3 | 0.2 | 0.1 | 0.0 | 0.1 | 0.0 | 0.0 | 0.1 | 0.0 | 0.0 | 0.0 | 0.1 | 0.0 |
| Cldn5 | 0.1 | 0.1 | 0.4 | 0.0 | 0.1 | 0.0 | 0.3 | 0.7 | 0.3 | 0.1 | 0.0 | 0.0 |
| Cldn7 | 0.0 | 0.2 | 0.0 | 0.0 | 0.0 | 0.1 | 0.0 | 0.1 | 0.1 | 0.1 | 0.0 | 0.0 |
| Cldnd2 | 0.0 | 0.2 | 0.2 | 0.0 | 0.0 | 0.0 | 0.1 | 0.0 | 0.0 | 0.0 | 0.0 | 0.0 |
| Clec10a | 0.0 | 0.0 | 0.0 | 0.1 | 0.1 | 0.0 | 0.0 | 0.0 | 0.0 | 0.1 | 0.0 | 0.0 |
| Clec11a | 5.9 | 5.7 | 7.2 | 3.8 | 5.5 | 5.2 | 6.6 | 4.8 | 5.7 | 6.2 | 6.5 | 7.8 |
| Clec12a | 1.1 | 0.0 | 0.0 | 8.7 | 12.6 | 0.0 | 0.8 | 0.2 | 0.3 | 1.0 | 0.2 | 0.7 |
| Clec14a | 0.1 | 0.0 | 0.1 | 0.1 | 0.0 | 0.0 | 0.0 | 0.1 | 0.0 | 0.1 | 0.2 | 0.0 |
| Clec16a | 3.6 | 4.1 | 4.3 | 4.5 | 3.6 | 4.1 | 3.4 | 3.3 | 3.1 | 3.3 | 3.3 | 3.4 |
| Clec18a | 0.0 | 0.0 | 0.0 | 0.1 | 0.0 | 0.0 | 0.0 | 0.0 | 0.0 | 0.0 | 0.0 | 0.0 |
| Clec1a | 0.1 | 0.0 | 0.1 | 0.0 | 0.0 | 0.0 | 0.1 | 0.3 | 0.2 | 0.0 | 0.1 | 0.0 |
| Clec2d | 48.4 | 69.6 | 113.1 | 40.3 | 65.0 | 40.6 | 76.2 | 113.4 | 83.5 | 63.4 | 72.5 | 105.0 |
| Clec2e | 0.0 | 0.0 | 0.1 | 0.0 | 0.0 | 0.0 | 0.0 | 0.0 | 0.1 | 0.2 | 0.1 | 0.1 |
| Clec2g | 0.1 | 0.2 | 0.1 | 0.0 | 0.1 | 0.1 | 0.1 | 0.1 | 0.1 | 0.2 | 0.4 | 0.1 |
| Clec2h | 0.0 | 0.0 | 0.0 | 0.0 | 0.0 | 0.0 | 0.0 | 0.0 | 0.0 | 0.0 | 0.0 | 0.0 |
| Clec2i | 0.0 | 0.2 | 0.0 | 0.1 | 0.0 | 0.0 | 0.0 | 0.0 | 0.1 | 0.0 | 0.0 | 0.1 |
| Clec2l | 0.0 | 0.1 | 0.1 | 0.0 | 0.1 | 0.2 | 0.2 | 0.2 | 0.3 | 0.1 | 0.0 | 0.0 |
| Clec3b | 0.1 | 0.3 | 0.8 | 0.0 | 0.0 | 0.0 | 0.3 | 1.0 | 0.4 | 0.1 | 0.2 | 0.2 |
| Clec4a1 | 0.4 | 0.0 | 0.0 | 1.7 | 9.0 | 0.0 | 1.1 | 0.2 | 0.4 | 0.5 | 0.2 | 0.3 |
| Clec4a2 | 0.1 | 0.0 | 0.0 | 0.6 | 2.2 | 0.0 | 0.3 | 0.1 | 0.1 | 0.1 | 0.1 | 0.1 |
| Clec4a3 | 0.1 | 0.0 | 0.0 | 0.4 | 0.5 | 0.0 | 0.3 | 0.0 | 0.1 | 0.1 | 0.1 | 0.1 |
| Clec4a4 | 0.2 | 0.0 | 0.0 | 0.3 | 0.4 | 0.0 | 0.0 | 0.0 | 0.0 | 0.0 | 0.0 | 0.0 |
| Clec4d | 4.8 | 0.1 | 0.2 | 42.2 | 65.5 | 0.0 | 5.9 | 0.9 | 0.9 | 4.6 | 1.1 | 2.5 |
| Clec4e | 0.3 | 0.0 | 0.1 | 3.1 | 4.1 | 0.0 | 0.4 | 0.0 | 0.1 | 0.4 | 0.1 | 0.3 |
| Clec4f | 0.0 | 0.0 | 0.0 | 0.0 | 0.0 | 0.0 | 0.0 | 0.0 | 0.0 | 0.0 | 0.0 | 0.0 |
| Clec4n | 7.7 | 0.1 | 0.7 | 26.1 | 51.3 | 0.0 | 2.1 | 0.7 | 0.3 | 1.8 | 0.7 | 0.5 |
| Clec5a | 0.4 | 0.0 | 0.0 | 3.3 | 6.2 | 0.0 | 0.7 | 0.1 | 0.1 | 0.4 | 0.2 | 0.4 |
| Clec7a | 0.4 | 0.0 | 0.0 | 2.0 | 1.5 | 0.0 | 0.3 | 0.1 | 0.0 | 0.2 | 0.3 | 0.1 |
| Clec9a | 0.0 | 0.0 | 0.0 | 0.0 | 0.0 | 0.0 | 0.0 | 0.1 | 0.0 | 0.0 | 0.0 | 0.0 |
| Clgn | 0.0 | 0.0 | 0.0 | 0.0 | 0.1 | 0.0 | 0.0 | 0.0 | 0.0 | 0.0 | 0.0 | 0.0 |
| Clhc1 | 0.4 | 0.7 | 1.0 | 0.4 | 1.2 | 0.3 | 1.7 | 0.9 | 1.0 | 0.7 | 0.8 | 0.7 |
| Clic1 | 129.3 | 107.9 | 109.2 | 147.3 | 151.2 | 121.7 | 128.4 | 117.7 | 117.1 | 130.8 | 132.7 | 132.7 |
| Clic3 | 0.4 | 0.3 | 0.0 | 0.0 | 0.2 | 0.4 | 0.2 | 0.5 | 0.2 | 0.1 | 0.2 | 0.2 |
| Clic4 | 162.7 | 153.7 | 140.7 | 172.8 | 153.6 | 138.6 | 145.7 | 138.0 | 135.6 | 129.6 | 154.2 | 153.4 |
| Clic5 | 0.3 | 0.1 | 0.0 | 0.2 | 0.1 | 0.2 | 0.1 | 0.1 | 0.1 | 0.1 | 0.1 | 0.1 |
| Clic6 | 0.1 | 0.0 | 0.0 | 0.0 | 0.0 | 0.0 | 0.0 | 0.0 | 0.0 | 0.0 | 0.0 | 0.0 |
| Clint1 | 37.5 | 36.5 | 37.5 | 41.1 | 36.5 | 35.6 | 48.2 | 40.8 | 39.2 | 38.4 | 47.1 | 47.3 |
| Clip1 | 63.5 | 59.2 | 56.8 | 64.1 | 51.5 | 50.8 | 54.8 | 49.4 | 40.7 | 45.3 | 54.9 | 58.9 |
| Clip2 | 9.8 | 8.9 | 7.6 | 12.5 | 11.4 | 10.7 | 11.6 | 7.8 | 10.3 | 9.4 | 9.5 | 9.8 |
| Clip3 | 2.5 | 3.0 | 3.4 | 2.6 | 2.6 | 3.7 | 2.7 | 3.3 | 2.8 | 3.1 | 3.7 | 3.3 |
| Clip4 | 0.8 | 0.8 | 1.0 | 1.1 | 0.4 | 1.1 | 0.7 | 0.7 | 0.3 | 0.6 | 0.8 | 0.8 |
| Clk1 | 29.0 | 33.0 | 37.8 | 28.3 | 31.9 | 31.1 | 32.7 | 32.9 | 30.4 | 32.9 | 33.1 | 36.2 |
| Clk2 | 6.5 | 7.0 | 8.5 | 8.2 | 7.3 | 6.8 | 8.2 | 7.8 | 7.5 | 7.1 | 7.7 | 7.7 |
| Clk3 | 14.9 | 16.4 | 17.4 | 16.0 | 15.7 | 15.3 | 18.8 | 16.3 | 16.9 | 15.4 | 16.4 | 16.5 |
| Clk4 | 10.8 | 12.8 | 14.5 | 12.9 | 10.3 | 11.2 | 12.8 | 12.5 | 11.7 | 11.2 | 13.6 | 12.4 |
| Clmn | 0.1 | 0.2 | 0.2 | 0.1 | 0.0 | 0.1 | 0.0 | 0.1 | 0.0 | 0.2 | 0.2 | 0.1 |
| Clmp | 14.5 | 26.8 | 52.9 | 15.2 | 30.9 | 13.7 | 32.8 | 52.2 | 28.6 | 19.9 | 24.6 | 28.7 |
| Cln3 | 0.5 | 0.8 | 0.6 | 1.7 | 2.2 | 0.7 | 0.7 | 0.4 | 0.6 | 1.1 | 0.7 | 0.7 |
| Cln5 | 31.5 | 24.3 | 23.5 | 36.6 | 33.4 | 32.1 | 27.1 | 24.9 | 25.9 | 31.2 | 30.7 | 34.3 |
| Cln6 | 2.3 | 3.0 | 3.1 | 2.4 | 3.5 | 2.6 | 2.2 | 3.9 | 3.3 | 2.6 | 3.0 | 1.9 |
| Cln8 | 3.8 | 3.0 | 2.8 | 4.4 | 4.6 | 3.4 | 2.4 | 2.5 | 2.5 | 3.3 | 2.7 | 2.5 |
| Clnk | 0.0 | 0.0 | 0.0 | 0.1 | 0.1 | 0.0 | 0.0 | 0.1 | 0.0 | 0.0 | 0.1 | 0.0 |

| | | | | | | | | | | | | |
|---|---|---|---|---|---|---|---|---|---|---|---|---|
| Clns1a | 12.2 | 11.9 | 10.1 | 11.1 | 11.7 | 12.1 | 10.9 | 11.6 | 11.2 | 12.4 | 11.7 | 10.0 |
| Clock | 11.0 | 12.0 | 12.3 | 12.2 | 10.5 | 11.1 | 10.2 | 11.8 | 10.9 | 10.1 | 13.6 | 12.3 |
| Clp1 | 4.6 | 4.3 | 3.2 | 3.6 | 4.5 | 3.8 | 3.9 | 3.7 | 3.6 | 3.8 | 3.0 | 3.9 |
| Clpb | 8.0 | 6.9 | 7.3 | 9.0 | 6.1 | 8.2 | 7.2 | 7.4 | 6.0 | 6.1 | 6.8 | 6.4 |
| Clpp | 19.4 | 17.3 | 14.6 | 25.3 | 15.5 | 21.4 | 17.9 | 18.7 | 13.9 | 20.1 | 21.2 | 16.4 |
| Clptm1 | 24.5 | 24.6 | 23.2 | 28.7 | 27.3 | 25.2 | 28.4 | 27.4 | 26.4 | 26.9 | 24.5 | 24.8 |
| Clptm1l | 70.3 | 63.5 | 55.5 | 81.2 | 67.6 | 75.5 | 63.9 | 56.5 | 60.1 | 63.5 | 65.7 | 61.8 |
| Clpx | 10.9 | 12.3 | 12.1 | 8.8 | 9.2 | 9.0 | 8.7 | 10.7 | 10.8 | 11.4 | 10.7 | 11.8 |
| Clspn | 1.2 | 2.2 | 3.8 | 0.5 | 2.9 | 0.5 | 4.2 | 2.1 | 2.3 | 1.4 | 2.2 | 1.8 |
| Clstn1 | 43.6 | 42.2 | 39.3 | 44.3 | 38.8 | 48.2 | 46.2 | 46.8 | 40.0 | 35.3 | 38.6 | 37.2 |
| Clstn2 | 0.1 | 0.0 | 0.1 | 0.2 | 0.0 | 0.0 | 0.1 | 0.1 | 0.0 | 0.1 | 0.0 | 0.1 |
| Clstn3 | 0.0 | 0.0 | 0.0 | 0.0 | 0.0 | 0.0 | 0.0 | 0.0 | 0.0 | 0.0 | 0.0 | 0.0 |
| Clta | 183.8 | 143.1 | 160.5 | 187.1 | 157.5 | 152.1 | 157.9 | 147.8 | 132.9 | 171.1 | 174.5 | 178.5 |
| Cltb | 90.5 | 83.5 | 81.7 | 110.7 | 73.7 | 79.3 | 108.8 | 91.7 | 80.9 | 85.2 | 109.2 | 88.8 |
| Cltc | 89.9 | 85.9 | 74.4 | 86.4 | 93.0 | 86.1 | 78.4 | 91.1 | 83.4 | 88.5 | 89.5 | 82.8 |
| Clu | 10.2 | 12.7 | 23.8 | 6.0 | 7.8 | 7.7 | 5.5 | 14.7 | 7.2 | 32.2 | 16.6 | 14.0 |
| Cluap1 | 9.8 | 9.9 | 8.5 | 7.7 | 6.4 | 9.8 | 5.6 | 7.9 | 7.1 | 8.3 | 7.7 | 7.8 |
| Cluh | 7.1 | 5.5 | 5.0 | 6.9 | 7.0 | 6.9 | 7.0 | 6.2 | 5.4 | 5.4 | 5.5 | 4.8 |
| Clvs1 | 0.1 | 0.1 | 0.0 | 0.0 | 0.0 | 0.0 | 0.1 | 0.1 | 0.1 | 0.0 | 0.0 | 0.0 |
| Clybl | 7.2 | 8.3 | 9.9 | 6.4 | 6.8 | 9.2 | 7.3 | 8.1 | 6.5 | 5.5 | 6.9 | 6.4 |
| Cma1 | 0.0 | 0.0 | 0.0 | 0.1 | 0.0 | 0.0 | 0.1 | 0.0 | 0.0 | 0.0 | 0.0 | 0.0 |
| Cmah | 0.4 | 0.5 | 0.5 | 0.6 | 0.8 | 0.2 | 1.0 | 0.9 | 0.9 | 0.7 | 1.3 | 1.1 |
| Cmas | 28.1 | 22.0 | 18.2 | 30.3 | 22.8 | 27.8 | 22.8 | 19.5 | 20.4 | 24.4 | 27.2 | 23.6 |
| Cmbl | 2.1 | 0.6 | 0.5 | 2.1 | 0.7 | 2.5 | 1.2 | 0.6 | 0.9 | 1.1 | 0.5 | 0.7 |
| Cmc1 | 9.2 | 8.3 | 6.3 | 7.7 | 8.7 | 8.1 | 5.9 | 6.0 | 7.3 | 9.2 | 8.0 | 9.8 |
| Cmc2 | 7.6 | 5.6 | 7.1 | 6.2 | 6.8 | 6.1 | 6.3 | 5.3 | 5.1 | 6.9 | 5.5 | 6.7 |
| Cmip | 3.0 | 3.9 | 4.4 | 3.4 | 4.9 | 3.4 | 4.2 | 3.1 | 3.1 | 2.8 | 3.2 | 3.1 |
| Cmklr1 | 2.8 | 2.8 | 3.6 | 2.6 | 3.1 | 0.9 | 4.4 | 8.4 | 5.1 | 2.0 | 4.3 | 3.4 |
| Cml1 | 1.3 | 0.8 | 1.3 | 1.3 | 1.1 | 0.9 | 0.5 | 0.6 | 0.6 | 1.5 | 0.7 | 0.6 |
| Cml2 | 0.0 | 0.0 | 0.0 | 0.0 | 0.0 | 0.0 | 0.0 | 0.0 | 0.0 | 0.0 | 0.0 | 0.0 |
| Cml3 | 0.0 | 0.1 | 0.3 | 0.0 | 0.1 | 0.0 | 0.0 | 0.2 | 0.0 | 0.0 | 0.0 | 0.1 |
| Cmpk1 | 35.6 | 34.5 | 35.0 | 43.1 | 36.9 | 31.9 | 29.8 | 28.3 | 30.2 | 36.4 | 37.6 | 38.1 |
| Cmpk2 | 1.8 | 0.4 | 0.4 | 2.8 | 0.5 | 1.0 | 0.3 | 0.4 | 0.3 | 0.2 | 0.5 | 1.0 |
| Cmss1 | 4.1 | 3.5 | 3.1 | 3.2 | 2.5 | 6.0 | 2.3 | 3.9 | 2.6 | 3.0 | 3.7 | 3.1 |
| Cmtm3 | 17.7 | 19.5 | 19.6 | 26.8 | 24.2 | 29.0 | 24.2 | 24.1 | 19.9 | 23.0 | 22.9 | 22.2 |
| Cmtm4 | 15.3 | 14.2 | 11.9 | 16.8 | 14.5 | 20.5 | 14.1 | 9.4 | 13.3 | 13.2 | 13.0 | 13.9 |
| Cmtm5 | 0.1 | 0.4 | 0.1 | 0.2 | 0.1 | 0.1 | 0.0 | 0.1 | 0.1 | 0.3 | 0.4 | 0.2 |
| Cmtm6 | 26.8 | 25.5 | 20.9 | 30.1 | 26.2 | 24.0 | 25.0 | 18.1 | 25.5 | 23.0 | 24.3 | 22.3 |
| Cmtm7 | 30.2 | 25.8 | 20.9 | 33.0 | 31.3 | 27.0 | 26.7 | 24.9 | 25.6 | 30.7 | 28.8 | 24.3 |
| Cmtm8 | 0.2 | 0.0 | 0.2 | 0.4 | 0.2 | 0.0 | 0.2 | 0.2 | 0.2 | 0.1 | 0.1 | 0.1 |
| Cmya5 | 0.0 | 0.0 | 0.1 | 0.0 | 0.1 | 0.1 | 0.0 | 0.1 | 0.1 | 0.1 | 0.0 | 0.1 |
| Cnbd2 | 1.0 | 1.1 | 1.2 | 1.5 | 1.0 | 1.1 | 0.7 | 0.9 | 0.5 | 0.8 | 1.1 | 0.8 |
| Cnbp | 85.9 | 75.6 | 74.5 | 93.4 | 81.4 | 91.8 | 71.5 | 72.9 | 76.5 | 89.8 | 83.6 | 81.3 |
| Cndp2 | 55.1 | 45.5 | 36.5 | 76.9 | 77.2 | 59.5 | 50.7 | 39.8 | 47.2 | 43.4 | 44.5 | 49.4 |
| Cnep1r1 | 11.3 | 12.0 | 12.0 | 10.4 | 12.1 | 10.7 | 11.3 | 10.5 | 13.2 | 11.8 | 13.6 | 13.4 |
| Cnga2 | 0.0 | 0.0 | 0.0 | 0.0 | 0.0 | 0.0 | 0.0 | 0.0 | 0.0 | 0.0 | 0.0 | 0.0 |
| Cnga3 | 0.1 | 0.0 | 0.0 | 0.0 | 0.1 | 0.0 | 0.0 | 0.0 | 0.0 | 0.0 | 0.0 | 0.0 |
| Cngb1 | 0.1 | 0.1 | 0.2 | 0.2 | 0.1 | 0.3 | 0.1 | 0.2 | 0.1 | 0.2 | 0.3 | 0.1 |
| Cngb3 | 0.0 | 0.0 | 0.0 | 0.1 | 0.1 | 0.1 | 0.0 | 0.0 | 0.0 | 0.0 | 0.0 | 0.0 |
| Cnih | 64.0 | 60.1 | 58.1 | 72.8 | 61.4 | 73.4 | 72.7 | 62.0 | 70.1 | 71.7 | 82.1 | 72.5 |
| Cnih2 | 0.3 | 0.4 | 0.4 | 0.1 | 0.2 | 0.5 | 0.4 | 0.5 | 0.3 | 0.5 | 0.4 | 0.6 |
| Cnih4 | 10.4 | 9.4 | 8.8 | 11.9 | 9.5 | 10.2 | 10.1 | 8.4 | 9.0 | 12.4 | 10.9 | 11.1 |
| Cnksr1 | 0.4 | 0.2 | 0.4 | 0.2 | 0.3 | 0.2 | 0.0 | 0.1 | 0.1 | 0.1 | 0.2 | 0.1 |
| Cnksr3 | 1.9 | 1.9 | 2.4 | 1.8 | 1.9 | 1.8 | 1.4 | 1.6 | 1.6 | 2.7 | 1.7 | 2.0 |
| Cnn1 | 4.1 | 2.0 | 1.0 | 5.4 | 3.9 | 3.0 | 8.8 | 2.9 | 10.3 | 18.7 | 28.9 | 12.3 |
| Cnn2 | 173.6 | 169.5 | 140.8 | 182.5 | 186.1 | 146.8 | 282.4 | 203.4 | 254.4 | 194.7 | 268.8 | 208.6 |
| Cnn3 | 128.9 | 135.8 | 155.3 | 127.0 | 127.3 | 124.6 | 173.1 | 200.5 | 178.3 | 161.2 | 202.2 | 193.5 |

| | | | | | | | | | | | | |
|---|---|---|---|---|---|---|---|---|---|---|---|---|
| Cnnm1 | 0.0 | 0.0 | 0.0 | 0.0 | 0.0 | 0.0 | 0.0 | 0.0 | 0.0 | 0.0 | 0.0 | 0.0 |
| Cnnm2 | 3.0 | 2.6 | 2.7 | 3.0 | 2.2 | 2.4 | 2.1 | 2.5 | 2.3 | 2.4 | 2.2 | 2.4 |
| Cnnm3 | 1.5 | 1.8 | 1.7 | 1.4 | 1.4 | 1.6 | 1.7 | 1.9 | 1.7 | 1.4 | 1.4 | 1.6 |
| Cnnm4 | 1.3 | 1.4 | 1.3 | 1.2 | 1.5 | 1.4 | 1.4 | 1.2 | 1.2 | 1.5 | 1.3 | 1.0 |
| Cnot1 | 13.8 | 15.3 | 17.0 | 13.5 | 16.2 | 13.4 | 14.8 | 18.0 | 14.4 | 14.0 | 15.2 | 14.3 |
| Cnot10 | 7.6 | 8.5 | 8.0 | 7.1 | 7.0 | 8.0 | 7.6 | 7.7 | 7.8 | 8.4 | 7.9 | 7.7 |
| Cnot11 | 5.2 | 5.3 | 6.4 | 5.7 | 5.8 | 5.2 | 5.7 | 6.3 | 6.6 | 6.8 | 5.9 | 6.5 |
| Cnot2 | 17.9 | 18.3 | 21.4 | 18.1 | 15.9 | 17.6 | 18.2 | 18.8 | 17.5 | 18.2 | 20.3 | 20.5 |
| Cnot3 | 2.1 | 2.8 | 3.4 | 2.0 | 2.9 | 2.0 | 3.2 | 3.8 | 3.2 | 2.9 | 3.5 | 3.0 |
| Cnot4 | 5.4 | 5.7 | 6.0 | 7.6 | 5.8 | 6.6 | 5.8 | 5.6 | 4.6 | 4.7 | 6.1 | 5.9 |
| Cnot6 | 5.6 | 7.1 | 8.6 | 3.6 | 7.9 | 4.6 | 6.6 | 8.0 | 8.6 | 8.3 | 7.2 | 8.0 |
| Cnot6l | 2.6 | 3.7 | 4.6 | 3.8 | 3.9 | 2.7 | 3.4 | 3.2 | 3.5 | 4.0 | 3.6 | 3.6 |
| Cnot7 | 11.9 | 14.3 | 14.7 | 11.7 | 13.5 | 12.0 | 12.2 | 12.7 | 13.2 | 14.4 | 16.5 | 15.0 |
| Cnot8 | 25.6 | 25.4 | 24.4 | 27.1 | 23.7 | 26.1 | 26.3 | 25.1 | 23.9 | 26.5 | 25.8 | 25.5 |
| Cnp | 6.3 | 7.0 | 7.0 | 6.8 | 8.1 | 7.3 | 5.6 | 4.4 | 6.0 | 5.1 | 4.8 | 4.9 |
| Cnppd1 | 12.9 | 11.5 | 11.5 | 13.3 | 11.3 | 12.7 | 12.9 | 12.1 | 14.4 | 13.6 | 11.3 | 13.2 |
| Cnpy2 | 61.2 | 52.5 | 64.5 | 57.6 | 50.9 | 56.4 | 59.7 | 64.8 | 61.0 | 70.6 | 59.9 | 74.0 |
| Cnpy3 | 27.1 | 23.6 | 24.3 | 31.2 | 27.2 | 34.2 | 25.7 | 27.4 | 24.7 | 24.0 | 22.8 | 25.4 |
| Cnpy4 | 23.2 | 22.1 | 22.5 | 28.9 | 20.9 | 26.0 | 24.3 | 22.3 | 20.5 | 17.9 | 23.6 | 22.7 |
| Cnr1 | 0.1 | 0.0 | 0.0 | 0.1 | 0.0 | 0.1 | 0.1 | 0.0 | 0.1 | 0.0 | 0.0 | 0.0 |
| Cnr2 | 0.1 | 0.0 | 0.0 | 0.8 | 1.3 | 0.0 | 0.1 | 0.0 | 0.0 | 0.1 | 0.0 | 0.0 |
| Cnrip1 | 16.2 | 15.3 | 16.4 | 19.0 | 13.3 | 16.2 | 15.7 | 13.4 | 14.3 | 12.3 | 14.8 | 15.3 |
| Cnst | 3.6 | 4.0 | 3.8 | 3.9 | 3.9 | 3.4 | 4.4 | 4.1 | 4.8 | 3.9 | 4.5 | 4.3 |
| Cntd1 | 0.1 | 0.1 | 0.6 | 0.1 | 0.3 | 0.2 | 0.2 | 0.1 | 0.2 | 0.2 | 0.1 | 0.1 |
| Cntf | 0.1 | 0.0 | 0.3 | 0.1 | 0.3 | 0.5 | 0.3 | 0.4 | 0.6 | 0.0 | 0.0 | 0.5 |
| Cntfr | 0.2 | 0.2 | 0.5 | 0.2 | 0.3 | 0.3 | 0.1 | 0.1 | 0.2 | 0.2 | 0.2 | 0.3 |
| Cntln | 4.6 | 5.5 | 6.3 | 4.4 | 3.8 | 5.1 | 4.5 | 5.5 | 3.9 | 3.8 | 5.4 | 5.3 |
| Cntn1 | 0.0 | 0.0 | 0.0 | 0.0 | 0.0 | 0.0 | 0.0 | 0.0 | 0.0 | 0.0 | 0.0 | 0.0 |
| Cntn2 | 4.9 | 8.5 | 8.4 | 5.2 | 4.2 | 5.1 | 4.3 | 4.2 | 4.5 | 2.0 | 2.4 | 1.4 |
| Cntn4 | 0.2 | 0.1 | 0.0 | 0.3 | 0.0 | 0.1 | 0.1 | 0.0 | 0.1 | 0.0 | 0.1 | 0.1 |
| Cntn5 | 0.0 | 0.1 | 0.0 | 0.0 | 0.0 | 0.0 | 0.0 | 0.0 | 0.0 | 0.0 | 0.1 | 0.0 |
| Cntnap1 | 0.3 | 0.3 | 0.4 | 0.3 | 0.3 | 0.3 | 0.3 | 0.4 | 0.3 | 0.4 | 0.2 | 0.2 |
| Cntnap2 | 0.0 | 0.0 | 0.0 | 0.0 | 0.0 | 0.0 | 0.0 | 0.0 | 0.0 | 0.0 | 0.0 | 0.0 |
| Cntnap3 | 0.0 | 0.0 | 0.0 | 0.0 | 0.0 | 0.0 | 0.0 | 0.0 | 0.0 | 0.0 | 0.0 | 0.0 |
| Cntnap5a | 0.0 | 0.0 | 0.0 | 0.0 | 0.0 | 0.0 | 0.0 | 0.0 | 0.0 | 0.0 | 0.0 | 0.0 |
| Cntnap5b | 0.0 | 0.0 | 0.0 | 0.0 | 0.0 | 0.0 | 0.0 | 0.0 | 0.0 | 0.0 | 0.0 | 0.0 |
| Cntrob | 1.9 | 1.9 | 2.4 | 1.5 | 1.8 | 2.0 | 2.4 | 2.3 | 2.5 | 2.1 | 1.8 | 1.9 |
| Coa3 | 46.1 | 46.0 | 39.9 | 54.5 | 42.4 | 57.1 | 43.4 | 45.6 | 44.8 | 42.3 | 37.6 | 43.9 |
| Coa4 | 6.1 | 5.2 | 4.9 | 5.8 | 6.4 | 7.9 | 4.3 | 5.5 | 3.7 | 5.9 | 5.3 | 5.8 |
| Coa5 | 27.4 | 24.0 | 20.2 | 28.9 | 24.4 | 27.1 | 21.8 | 20.8 | 20.4 | 23.6 | 24.9 | 22.2 |
| Coa6 | 11.3 | 10.7 | 6.4 | 11.0 | 10.3 | 13.9 | 9.4 | 8.2 | 8.8 | 11.6 | 9.0 | 7.2 |
| Coasy | 8.6 | 6.1 | 5.1 | 8.7 | 6.5 | 8.7 | 7.1 | 5.9 | 6.8 | 8.7 | 5.7 | 7.3 |
| Cobl | 5.1 | 2.1 | 0.9 | 4.8 | 1.4 | 3.1 | 1.5 | 0.3 | 0.5 | 1.9 | 2.4 | 1.0 |
| Cobll1 | 5.9 | 4.8 | 5.3 | 3.0 | 2.6 | 3.0 | 3.1 | 3.7 | 2.6 | 8.1 | 6.3 | 5.7 |
| Coch | 0.3 | 0.4 | 0.3 | 0.0 | 0.3 | 0.3 | 0.3 | 0.1 | 0.2 | 0.2 | 0.3 | 0.1 |
| Cog1 | 11.1 | 10.7 | 10.2 | 10.8 | 11.1 | 10.8 | 11.5 | 11.3 | 11.1 | 9.4 | 10.4 | 10.1 |
| Cog2 | 10.1 | 11.1 | 10.3 | 10.7 | 10.5 | 11.9 | 10.7 | 12.4 | 11.6 | 11.9 | 10.9 | 10.5 |
| Cog3 | 11.8 | 11.6 | 10.7 | 11.6 | 10.3 | 11.6 | 10.8 | 11.0 | 12.2 | 10.9 | 12.8 | 12.7 |
| Cog4 | 37.4 | 38.4 | 37.0 | 37.4 | 39.4 | 45.6 | 37.5 | 38.7 | 34.3 | 39.3 | 38.1 | 35.5 |
| Cog5 | 11.1 | 11.1 | 8.4 | 9.7 | 9.9 | 10.5 | 7.2 | 9.6 | 8.6 | 8.7 | 8.2 | 8.7 |
| Cog6 | 15.9 | 15.1 | 13.0 | 13.7 | 13.9 | 15.4 | 12.8 | 12.6 | 14.7 | 15.5 | 17.7 | 16.2 |
| Cog7 | 12.6 | 12.1 | 11.3 | 13.4 | 10.4 | 13.1 | 9.1 | 8.7 | 9.6 | 8.2 | 9.0 | 10.0 |
| Cog8 | 13.8 | 13.5 | 12.9 | 12.3 | 12.0 | 12.2 | 11.3 | 14.2 | 12.8 | 14.0 | 13.4 | 12.1 |
| Coil | 2.4 | 2.3 | 2.4 | 2.6 | 2.0 | 2.4 | 2.2 | 2.6 | 2.3 | 2.6 | 2.5 | 2.4 |
| Col10a1 | 0.1 | 0.1 | 0.1 | 0.1 | 0.2 | 0.1 | 0.2 | 0.1 | 0.1 | 0.2 | 0.2 | 0.1 |
| Col11a1 | 7.7 | 12.4 | 16.8 | 3.6 | 17.4 | 5.3 | 20.2 | 24.7 | 34.4 | 8.0 | 11.6 | 11.9 |
| Col11a2 | 0.3 | 0.3 | 0.2 | 0.4 | 0.3 | 0.5 | 0.4 | 0.1 | 0.4 | 0.4 | 0.2 | 0.2 |

| | | | | | | | | | | | | |
|---|---|---|---|---|---|---|---|---|---|---|---|---|
| Col12a1 | 89.9 | 94.3 | 86.6 | 66.7 | 83.6 | 58.7 | 72.8 | 89.7 | 87.7 | 75.5 | 87.0 | 72.2 |
| Col13a1 | 0.1 | 0.1 | 0.1 | 0.0 | 0.0 | 0.1 | 0.0 | 0.0 | 0.0 | 0.0 | 0.0 | 0.0 |
| Col14a1 | 16.8 | 53.6 | 103.2 | 12.3 | 54.1 | 12.7 | 35.1 | 79.4 | 79.8 | 27.5 | 22.6 | 63.2 |
| Col15a1 | 0.6 | 0.5 | 1.3 | 0.2 | 0.8 | 0.2 | 1.5 | 1.3 | 2.0 | 2.0 | 0.7 | 2.0 |
| Col16a1 | 32.5 | 33.2 | 36.1 | 27.3 | 30.6 | 36.6 | 41.2 | 40.0 | 42.2 | 40.2 | 34.4 | 37.3 |
| Col17a1 | 0.1 | 0.1 | 0.1 | 0.1 | 0.1 | 0.1 | 0.1 | 0.1 | 0.1 | 0.1 | 0.1 | 0.0 |
| Col18a1 | 7.8 | 8.5 | 10.9 | 4.8 | 7.1 | 5.8 | 6.6 | 8.3 | 6.4 | 4.4 | 4.0 | 4.5 |
| Col19a1 | 0.0 | 0.0 | 0.0 | 0.0 | 0.0 | 0.0 | 0.0 | 0.0 | 0.0 | 0.0 | 0.0 | 0.0 |
| Col1a1 | 120.6 | 200.1 | 239.2 | 78.3 | 159.3 | 95.1 | 169.7 | 353.2 | 281.8 | 216.3 | 173.5 | 134.1 |
| Col1a2 | 309.7 | 440.3 | 716.6 | 131.1 | 378.0 | 256.5 | 531.0 | 1042.3 | 978.3 | 662.3 | 522.0 | 545.8 |
| Col20a1 | 0.4 | 0.4 | 0.3 | 0.5 | 0.3 | 0.9 | 0.2 | 0.2 | 0.3 | 0.3 | 0.4 | 0.3 |
| Col22a1 | 0.5 | 0.2 | 0.2 | 0.2 | 0.5 | 0.1 | 0.2 | 0.1 | 0.1 | 0.1 | 0.0 | 0.1 |
| Col23a1 | 0.3 | 0.2 | 0.2 | 0.3 | 0.1 | 0.3 | 0.2 | 0.2 | 0.2 | 0.2 | 0.2 | 0.2 |
| Col24a1 | 0.0 | 0.0 | 0.0 | 0.0 | 0.0 | 0.0 | 0.0 | 0.1 | 0.1 | 0.1 | 0.1 | 0.1 |
| Col25a1 | 0.0 | 0.0 | 0.0 | 0.0 | 0.0 | 0.0 | 0.0 | 0.0 | 0.0 | 0.0 | 0.0 | 0.0 |
| Col26a1 | 0.0 | 0.1 | 0.0 | 0.0 | 0.0 | 0.0 | 0.0 | 0.1 | 0.0 | 0.0 | 0.0 | 0.0 |
| Col27a1 | 0.4 | 0.3 | 0.5 | 0.3 | 0.5 | 0.2 | 0.5 | 0.7 | 0.7 | 1.0 | 0.6 | 0.5 |
| Col28a1 | 8.4 | 14.5 | 28.7 | 5.5 | 21.6 | 1.3 | 38.9 | 26.5 | 49.5 | 10.8 | 18.2 | 20.5 |
| Col2a1 | 0.2 | 0.0 | 0.2 | 0.1 | 0.2 | 0.4 | 1.2 | 0.9 | 0.8 | 1.3 | 2.5 | 0.5 |
| Col3a1 | 1324.3 | 1836.6 | 2732.5 | 738.9 | 1244.5 | 544.6 | 1525.1 | 3090.8 | 2898.3 | 1828.3 | 1646.4 | 2457.6 |
| Col4a1 | 465.5 | 612.7 | 615.7 | 413.6 | 560.6 | 562.7 | 567.2 | 473.7 | 624.0 | 527.5 | 465.7 | 516.7 |
| Col4a2 | 235.0 | 295.8 | 331.8 | 233.5 | 284.1 | 285.5 | 311.9 | 269.8 | 311.6 | 246.0 | 221.3 | 257.4 |
| Col4a3 | 0.5 | 0.3 | 0.4 | 0.3 | 0.1 | 0.3 | 0.1 | 0.2 | 0.1 | 0.1 | 0.1 | 0.1 |
| Col4a3bp | 11.7 | 7.7 | 6.4 | 12.8 | 8.7 | 9.6 | 9.2 | 7.0 | 8.2 | 8.9 | 11.4 | 11.2 |
| Col4a4 | 0.4 | 0.2 | 0.4 | 0.1 | 0.1 | 0.3 | 0.1 | 0.2 | 0.1 | 0.0 | 0.0 | 0.1 |
| Col4a5 | 123.0 | 127.7 | 119.0 | 96.4 | 108.6 | 111.7 | 105.2 | 118.3 | 150.4 | 119.9 | 107.1 | 111.2 |
| Col4a6 | 17.2 | 13.8 | 11.9 | 13.6 | 12.8 | 13.2 | 13.5 | 12.8 | 22.0 | 29.1 | 21.1 | 21.1 |
| Col5a1 | 76.4 | 87.5 | 90.9 | 59.7 | 81.0 | 64.4 | 110.8 | 155.5 | 161.6 | 83.9 | 86.6 | 78.3 |
| Col5a2 | 1028.7 | 963.4 | 921.2 | 731.1 | 729.4 | 671.2 | 802.7 | 951.2 | 1069.1 | 743.2 | 785.0 | 816.5 |
| Col5a3 | 2.0 | 2.6 | 3.2 | 1.8 | 2.8 | 2.0 | 1.7 | 4.6 | 2.4 | 2.3 | 2.3 | 1.4 |
| Col6a1 | 156.9 | 210.9 | 356.2 | 110.8 | 263.1 | 95.4 | 301.1 | 316.2 | 313.2 | 252.6 | 270.0 | 358.6 |
| Col6a2 | 46.6 | 76.9 | 138.7 | 23.0 | 84.1 | 20.0 | 100.8 | 123.9 | 106.2 | 90.3 | 91.3 | 131.8 |
| Col6a3 | 1.3 | 1.9 | 3.9 | 0.8 | 2.9 | 1.6 | 2.1 | 5.0 | 3.3 | 4.8 | 4.6 | 3.5 |
| Col6a4 | 0.1 | 0.1 | 0.0 | 0.0 | 0.1 | 0.0 | 0.0 | 0.0 | 0.0 | 0.0 | 0.0 | 0.0 |
| Col6a5 | 0.0 | 0.0 | 0.0 | 0.0 | 0.0 | 0.0 | 0.0 | 0.0 | 0.0 | 0.0 | 0.0 | 0.0 |
| Col6a6 | 0.0 | 0.0 | 0.0 | 0.0 | 0.0 | 0.0 | 0.0 | 0.0 | 0.0 | 0.0 | 0.0 | 0.0 |
| Col7a1 | 0.6 | 0.8 | 0.6 | 1.0 | 0.8 | 1.1 | 0.7 | 0.3 | 0.6 | 0.5 | 0.3 | 0.3 |
| Col8a1 | 361.4 | 427.0 | 552.1 | 333.8 | 433.8 | 362.4 | 522.5 | 556.4 | 626.3 | 444.6 | 526.2 | 550.8 |
| Col8a2 | 0.9 | 1.3 | 2.2 | 0.3 | 0.6 | 0.6 | 0.9 | 2.5 | 1.7 | 2.8 | 4.1 | 1.8 |
| Col9a2 | 0.1 | 0.1 | 0.1 | 0.1 | 0.1 | 0.0 | 0.0 | 0.1 | 0.1 | 0.1 | 0.0 | 0.1 |
| Col9a3 | 0.2 | 0.3 | 0.2 | 0.2 | 0.3 | 0.4 | 0.2 | 0.1 | 0.2 | 0.3 | 0.2 | 0.2 |
| Colec10 | 0.0 | 0.0 | 0.0 | 0.1 | 0.0 | 0.1 | 0.0 | 0.0 | 0.0 | 0.0 | 0.0 | 0.0 |
| Colec11 | 3.3 | 4.7 | 5.9 | 2.9 | 4.1 | 2.6 | 3.7 | 2.7 | 3.3 | 8.5 | 3.3 | 6.3 |
| Colec12 | 38.1 | 55.2 | 81.3 | 33.0 | 45.3 | 38.6 | 41.7 | 54.2 | 56.6 | 68.1 | 49.6 | 66.4 |
| Colq | 0.3 | 0.2 | 0.3 | 0.3 | 0.5 | 0.1 | 0.3 | 0.1 | 0.1 | 0.1 | 0.1 | 0.1 |
| Commd1 | 46.8 | 35.9 | 39.8 | 49.0 | 39.3 | 47.6 | 42.6 | 43.0 | 39.7 | 39.1 | 42.8 | 36.1 |
| Commd10 | 13.1 | 11.0 | 10.3 | 14.7 | 11.2 | 12.7 | 12.3 | 13.0 | 13.0 | 10.0 | 12.5 | 9.8 |
| Commd2 | 4.4 | 4.4 | 3.8 | 4.8 | 4.9 | 3.6 | 3.8 | 4.4 | 4.8 | 4.0 | 4.8 | 3.9 |
| Commd3 | 126.4 | 113.4 | 106.6 | 124.0 | 102.5 | 125.9 | 102.0 | 108.8 | 100.6 | 120.4 | 127.2 | 119.0 |
| Commd4 | 27.8 | 26.2 | 19.3 | 30.3 | 27.1 | 29.9 | 25.0 | 23.6 | 27.7 | 26.9 | 21.9 | 21.4 |
| Commd5 | 13.8 | 13.6 | 11.2 | 14.2 | 12.7 | 17.8 | 13.4 | 13.3 | 14.8 | 15.3 | 12.5 | 13.1 |
| Commd6 | 25.2 | 25.2 | 18.0 | 21.4 | 22.3 | 23.5 | 18.2 | 20.1 | 23.5 | 27.0 | 23.8 | 22.0 |
| Commd7 | 11.4 | 9.6 | 9.0 | 12.7 | 11.8 | 10.8 | 10.7 | 10.0 | 11.1 | 10.9 | 12.2 | 10.5 |
| Commd8 | 13.1 | 14.4 | 13.7 | 15.0 | 14.2 | 15.2 | 11.7 | 12.8 | 12.1 | 15.6 | 15.3 | 15.1 |
| Commd9 | 17.1 | 14.9 | 11.3 | 20.4 | 16.5 | 17.1 | 15.0 | 14.7 | 15.4 | 16.4 | 17.1 | 14.0 |
| Comp | 3.0 | 4.1 | 15.6 | 0.6 | 2.0 | 0.7 | 5.4 | 21.7 | 8.1 | 4.3 | 7.5 | 15.0 |
| Comt | 71.9 | 79.5 | 83.5 | 76.4 | 71.9 | 66.6 | 91.8 | 109.6 | 90.9 | 63.0 | 79.0 | 73.4 |

| | | | | | | | | | | | | |
|---|---|---|---|---|---|---|---|---|---|---|---|---|
| Comtd1 | 7.8 | 5.7 | 3.9 | 7.7 | 6.1 | 9.2 | 5.0 | 5.0 | 5.3 | 5.9 | 5.2 | 5.1 |
| Copa | 127.7 | 114.6 | 107.1 | 126.9 | 110.0 | 122.4 | 105.6 | 119.9 | 102.0 | 106.3 | 118.3 | 112.4 |
| Copb1 | 56.5 | 58.4 | 50.7 | 52.5 | 53.0 | 49.5 | 47.9 | 54.3 | 57.3 | 58.3 | 60.4 | 57.8 |
| Copb2 | 139.4 | 123.9 | 120.1 | 140.9 | 117.6 | 134.9 | 123.1 | 120.8 | 108.7 | 126.7 | 136.4 | 145.1 |
| Cope | 68.8 | 62.8 | 58.1 | 77.1 | 68.9 | 67.0 | 63.1 | 73.7 | 63.0 | 62.9 | 67.4 | 63.1 |
| Copg1 | 69.2 | 60.7 | 55.5 | 71.1 | 63.0 | 65.8 | 66.5 | 67.0 | 58.2 | 62.8 | 68.6 | 62.6 |
| Copg2 | 25.1 | 21.5 | 16.1 | 20.2 | 17.7 | 23.5 | 14.3 | 17.7 | 17.3 | 17.0 | 19.4 | 16.3 |
| Coprs | 19.0 | 18.4 | 11.6 | 17.2 | 14.4 | 21.8 | 14.7 | 12.2 | 14.3 | 17.1 | 13.7 | 14.7 |
| Cops2 | 33.1 | 40.4 | 43.6 | 44.0 | 40.5 | 44.9 | 38.5 | 39.9 | 39.7 | 45.0 | 52.0 | 50.1 |
| Cops3 | 44.3 | 41.4 | 40.7 | 43.5 | 35.8 | 39.8 | 35.2 | 37.1 | 35.9 | 42.1 | 47.3 | 42.7 |
| Cops4 | 40.9 | 36.6 | 34.2 | 42.5 | 34.8 | 38.3 | 37.9 | 34.0 | 33.5 | 41.9 | 41.7 | 41.1 |
| Cops5 | 51.7 | 44.4 | 39.7 | 43.3 | 43.4 | 41.3 | 40.9 | 42.2 | 47.0 | 46.3 | 47.5 | 42.8 |
| Cops6 | 45.5 | 46.3 | 41.5 | 42.5 | 41.9 | 42.0 | 39.6 | 45.4 | 41.9 | 45.9 | 41.6 | 42.7 |
| Cops7a | 31.1 | 30.9 | 28.7 | 36.2 | 31.0 | 32.3 | 35.2 | 33.2 | 29.3 | 29.9 | 30.2 | 25.7 |
| Cops7b | 4.6 | 4.9 | 3.6 | 4.4 | 4.5 | 5.3 | 5.0 | 5.4 | 4.3 | 4.2 | 5.3 | 3.9 |
| Cops8 | 30.6 | 29.7 | 22.7 | 29.2 | 27.0 | 28.7 | 25.4 | 26.2 | 26.1 | 26.2 | 26.9 | 25.1 |
| Copz1 | 32.0 | 33.5 | 28.4 | 32.2 | 31.5 | 32.1 | 29.5 | 35.9 | 29.4 | 34.3 | 29.9 | 29.7 |
| Copz2 | 116.2 | 88.6 | 92.3 | 127.5 | 84.0 | 120.0 | 113.5 | 103.6 | 98.2 | 95.6 | 113.0 | 114.4 |
| Coq10a | 4.7 | 4.1 | 3.5 | 4.2 | 3.6 | 5.7 | 3.2 | 3.8 | 3.7 | 3.7 | 3.7 | 4.1 |
| Coq10b | 35.1 | 37.7 | 30.0 | 30.9 | 32.1 | 31.3 | 31.7 | 32.1 | 40.9 | 39.0 | 39.3 | 36.3 |
| Coq2 | 24.8 | 22.9 | 18.8 | 26.8 | 21.9 | 26.9 | 23.3 | 18.2 | 23.2 | 22.6 | 24.1 | 20.5 |
| Coq3 | 6.7 | 6.2 | 4.9 | 6.0 | 5.1 | 6.8 | 5.3 | 4.9 | 4.3 | 4.9 | 5.9 | 5.5 |
| Coq4 | 2.6 | 2.0 | 2.3 | 3.0 | 2.4 | 3.2 | 2.4 | 2.6 | 2.1 | 2.4 | 2.3 | 1.8 |
| Coq5 | 11.3 | 10.2 | 9.3 | 11.0 | 9.8 | 12.4 | 8.7 | 9.0 | 8.8 | 8.6 | 8.8 | 10.6 |
| Coq6 | 7.1 | 5.9 | 4.7 | 6.6 | 5.6 | 6.1 | 4.8 | 5.4 | 5.9 | 5.5 | 5.1 | 5.5 |
| Coq7 | 13.6 | 11.5 | 11.3 | 13.9 | 10.6 | 14.2 | 10.0 | 10.2 | 8.1 | 9.3 | 9.2 | 10.8 |
| Coq9 | 15.0 | 13.5 | 12.7 | 14.5 | 11.9 | 15.1 | 12.1 | 14.7 | 12.3 | 13.2 | 12.2 | 12.7 |
| Corin | 0.1 | 0.1 | 0.1 | 0.0 | 0.0 | 0.0 | 0.0 | 0.0 | 0.0 | 0.1 | 0.0 | 0.0 |
| Coro1a | 2.6 | 0.1 | 0.1 | 21.2 | 50.2 | 0.2 | 7.2 | 0.6 | 1.5 | 3.5 | 0.9 | 2.0 |
| Coro1b | 57.6 | 54.6 | 53.6 | 64.1 | 70.4 | 61.8 | 55.1 | 62.7 | 56.5 | 59.2 | 55.3 | 61.2 |
| Coro1c | 51.9 | 42.4 | 35.1 | 67.7 | 55.3 | 48.2 | 60.3 | 42.6 | 48.8 | 41.8 | 53.4 | 45.4 |
| Coro2a | 0.1 | 0.0 | 0.0 | 1.4 | 2.9 | 0.0 | 0.4 | 0.1 | 0.1 | 0.1 | 0.1 | 0.2 |
| Coro2b | 0.5 | 0.6 | 1.2 | 0.6 | 0.5 | 0.9 | 0.6 | 0.9 | 0.4 | 0.5 | 0.9 | 0.5 |
| Coro6 | 0.2 | 0.2 | 0.1 | 0.1 | 0.2 | 0.3 | 0.2 | 0.1 | 0.1 | 0.2 | 0.5 | 0.1 |
| Coro7 | 3.5 | 3.3 | 2.8 | 3.8 | 4.6 | 3.2 | 3.4 | 2.9 | 3.3 | 2.5 | 2.7 | 2.5 |
| Cotl1 | 10.5 | 10.2 | 10.8 | 23.5 | 39.1 | 10.8 | 11.6 | 8.6 | 9.5 | 12.3 | 9.3 | 7.8 |
| Cox10 | 2.5 | 2.3 | 1.9 | 3.0 | 2.4 | 2.4 | 2.2 | 2.3 | 2.5 | 2.2 | 2.4 | 1.7 |
| Cox11 | 2.1 | 2.5 | 2.0 | 1.7 | 2.3 | 2.5 | 2.1 | 2.3 | 2.4 | 2.7 | 2.7 | 2.0 |
| Cox14 | 34.6 | 32.2 | 21.7 | 30.6 | 34.1 | 34.5 | 24.7 | 31.3 | 33.1 | 39.9 | 26.1 | 27.8 |
| Cox15 | 4.6 | 4.1 | 4.3 | 4.1 | 4.0 | 4.5 | 3.7 | 4.9 | 4.7 | 3.5 | 4.1 | 4.0 |
| Cox16 | 6.2 | 6.2 | 6.5 | 7.9 | 6.8 | 6.8 | 6.8 | 7.0 | 6.3 | 6.3 | 7.7 | 7.9 |
| Cox17 | 87.1 | 72.4 | 51.8 | 77.9 | 72.3 | 96.6 | 62.5 | 53.4 | 56.1 | 74.0 | 60.0 | 66.8 |
| Cox18 | 8.3 | 8.7 | 6.8 | 7.8 | 7.9 | 8.1 | 9.0 | 8.0 | 7.3 | 7.8 | 8.4 | 7.6 |
| Cox19 | 32.0 | 33.0 | 21.8 | 28.5 | 25.0 | 27.9 | 25.4 | 23.3 | 22.7 | 30.2 | 24.5 | 26.7 |
| Cox20 | 38.3 | 35.5 | 39.7 | 44.4 | 32.7 | 41.8 | 39.3 | 39.6 | 34.7 | 41.0 | 46.2 | 41.0 |
| Cox4i1 | 418.0 | 320.0 | 277.1 | 354.0 | 334.2 | 368.8 | 281.5 | 302.3 | 291.9 | 355.4 | 311.5 | 303.5 |
| Cox4i2 | 0.4 | 1.6 | 2.3 | 0.3 | 0.5 | 0.5 | 0.7 | 0.6 | 0.5 | 0.3 | 0.8 | 0.5 |
| Cox5a | 96.0 | 70.2 | 50.8 | 67.1 | 83.7 | 71.1 | 56.0 | 64.5 | 69.4 | 74.6 | 66.8 | 68.1 |
| Cox5b | 163.6 | 130.8 | 122.9 | 146.1 | 124.5 | 130.8 | 133.7 | 141.4 | 128.7 | 146.0 | 152.9 | 148.6 |
| Cox6a1 | 294.8 | 257.2 | 206.0 | 289.9 | 255.5 | 242.9 | 242.9 | 232.7 | 245.8 | 259.4 | 262.8 | 243.8 |
| Cox6a2 | 3.5 | 1.7 | 1.2 | 6.1 | 0.6 | 3.5 | 1.0 | 2.4 | 1.4 | 0.9 | 1.7 | 1.0 |
| Cox6b1 | 358.4 | 282.8 | 262.5 | 358.8 | 278.6 | 327.9 | 291.9 | 280.9 | 257.7 | 298.5 | 322.3 | 319.2 |
| Cox6b2 | 6.0 | 7.3 | 6.0 | 1.7 | 4.2 | 2.1 | 3.5 | 1.9 | 3.9 | 10.0 | 4.4 | 2.5 |
| Cox6c | 292.9 | 236.2 | 231.6 | 256.5 | 244.9 | 285.5 | 201.8 | 207.6 | 222.0 | 269.2 | 253.8 | 254.2 |
| Cox7a1 | 1.8 | 1.0 | 0.9 | 0.9 | 2.1 | 0.7 | 0.5 | 0.2 | 1.2 | 2.5 | 2.0 | 2.9 |
| Cox7a2 | 129.1 | 110.9 | 90.8 | 108.7 | 110.7 | 109.9 | 95.8 | 86.3 | 99.2 | 114.7 | 109.7 | 103.6 |
| Cox7a2l | 203.7 | 141.5 | 156.5 | 183.4 | 162.8 | 204.1 | 155.9 | 167.3 | 149.5 | 175.8 | 180.9 | 176.5 |

| | | | | | | | | | | | | |
|---|---|---|---|---|---|---|---|---|---|---|---|---|
| Cox7b | 95.2 | 79.7 | 69.0 | 89.6 | 79.6 | 84.0 | 74.1 | 83.1 | 71.5 | 85.2 | 96.6 | 86.8 |
| Cox7c | 169.3 | 148.1 | 128.8 | 160.7 | 142.6 | 164.9 | 136.2 | 134.8 | 143.3 | 163.3 | 143.9 | 145.7 |
| Cox8a | 312.7 | 256.5 | 216.9 | 286.6 | 265.2 | 278.1 | 233.9 | 223.7 | 245.4 | 306.8 | 260.4 | 238.0 |
| Cp | 17.6 | 15.8 | 25.0 | 19.8 | 19.4 | 17.2 | 15.7 | 15.7 | 13.4 | 10.0 | 9.3 | 11.6 |
| Cpa1 | 0.0 | 0.0 | 0.0 | 0.0 | 0.0 | 0.0 | 0.1 | 0.0 | 0.0 | 0.0 | 0.2 | 0.0 |
| Cpa2 | 0.1 | 0.0 | 0.0 | 0.2 | 0.0 | 0.2 | 0.0 | 0.0 | 0.1 | 0.1 | 0.0 | 0.0 |
| Cpa3 | 0.1 | 0.2 | 0.1 | 0.0 | 0.1 | 0.0 | 0.1 | 0.1 | 0.1 | 0.2 | 0.0 | 0.0 |
| Cpa4 | 0.1 | 0.0 | 0.0 | 0.1 | 0.0 | 0.0 | 0.0 | 0.0 | 0.0 | 0.0 | 0.1 | 0.0 |
| Cpa6 | 0.3 | 0.1 | 0.0 | 0.1 | 0.3 | 0.3 | 0.3 | 0.2 | 0.3 | 0.0 | 0.1 | 0.2 |
| Cpb1 | 0.0 | 0.1 | 0.1 | 0.1 | 0.0 | 0.0 | 0.0 | 0.0 | 0.0 | 0.0 | 0.0 | 0.0 |
| Cpb2 | 0.0 | 0.0 | 0.1 | 0.0 | 0.0 | 0.0 | 0.0 | 0.0 | 0.0 | 0.0 | 0.0 | 0.0 |
| Cpd | 50.3 | 70.0 | 58.6 | 51.6 | 69.1 | 35.2 | 56.3 | 46.9 | 52.5 | 45.4 | 48.4 | 53.5 |
| Cpe | 255.9 | 204.5 | 193.4 | 122.6 | 168.5 | 244.8 | 172.9 | 206.1 | 183.0 | 294.6 | 286.5 | 249.0 |
| Cpeb1 | 14.4 | 12.0 | 9.4 | 20.1 | 11.6 | 20.4 | 10.8 | 8.0 | 8.1 | 9.6 | 9.3 | 9.3 |
| Cpeb2 | 0.8 | 0.8 | 0.8 | 1.0 | 1.4 | 0.8 | 0.9 | 1.0 | 0.8 | 1.0 | 0.9 | 0.8 |
| Cpeb3 | 0.7 | 0.9 | 0.9 | 1.0 | 1.0 | 0.9 | 0.8 | 0.7 | 0.6 | 0.5 | 0.8 | 0.7 |
| Cpeb4 | 6.6 | 7.5 | 6.4 | 9.3 | 8.7 | 6.2 | 6.3 | 5.3 | 6.7 | 7.0 | 6.3 | 7.1 |
| Cped1 | 20.9 | 19.1 | 20.7 | 16.7 | 13.6 | 20.1 | 13.5 | 17.0 | 16.4 | 12.2 | 14.8 | 20.4 |
| Cphx1 | 0.1 | 0.0 | 0.0 | 0.1 | 0.0 | 0.1 | 0.0 | 0.0 | 0.0 | 0.0 | 0.1 | 0.0 |
| Cphx2 | 0.0 | 0.0 | 0.0 | 0.0 | 0.0 | 0.0 | 0.0 | 0.0 | 0.0 | 0.0 | 0.0 | 0.0 |
| Cplx2 | 0.9 | 0.7 | 0.8 | 0.9 | 0.4 | 0.8 | 0.8 | 0.6 | 0.6 | 1.3 | 1.4 | 1.1 |
| Cplx3 | 0.1 | 0.1 | 0.1 | 0.1 | 0.1 | 0.1 | 0.1 | 0.0 | 0.0 | 0.0 | 0.1 | 0.1 |
| Cpm | 0.2 | 0.2 | 0.2 | 0.2 | 0.3 | 0.1 | 0.2 | 0.1 | 0.1 | 0.4 | 0.1 | 0.1 |
| Cpne1 | 17.1 | 20.1 | 23.3 | 17.4 | 21.3 | 17.5 | 22.4 | 24.5 | 23.2 | 21.1 | 20.1 | 23.2 |
| Cpne2 | 9.3 | 9.4 | 9.7 | 12.4 | 9.4 | 11.1 | 8.7 | 8.7 | 8.2 | 6.5 | 7.4 | 7.9 |
| Cpne3 | 23.2 | 22.8 | 21.7 | 25.3 | 22.6 | 24.9 | 21.5 | 20.0 | 20.7 | 22.3 | 26.3 | 22.4 |
| Cpne4 | 0.0 | 0.0 | 0.0 | 0.0 | 0.0 | 0.0 | 0.0 | 0.0 | 0.0 | 0.0 | 0.0 | 0.0 |
| Cpne5 | 0.8 | 0.6 | 0.3 | 1.2 | 0.7 | 1.4 | 0.6 | 0.2 | 0.4 | 0.5 | 0.4 | 0.3 |
| Cpne7 | 0.0 | 0.0 | 0.0 | 0.0 | 0.0 | 0.1 | 0.0 | 0.0 | 0.0 | 0.1 | 0.0 | 0.0 |
| Cpne8 | 44.8 | 44.9 | 39.7 | 43.1 | 42.8 | 37.0 | 41.6 | 31.6 | 46.3 | 42.8 | 44.8 | 47.2 |
| Cpne9 | 0.0 | 0.0 | 0.0 | 0.0 | 0.0 | 0.0 | 0.0 | 0.0 | 0.0 | 0.0 | 0.0 | 0.0 |
| Cpox | 10.4 | 9.3 | 10.6 | 8.0 | 7.5 | 9.5 | 8.0 | 10.0 | 8.1 | 8.1 | 9.7 | 8.3 |
| Cpped1 | 14.7 | 15.6 | 15.2 | 14.2 | 14.2 | 14.4 | 14.6 | 12.8 | 12.6 | 15.9 | 14.7 | 14.9 |
| Cpq | 120.0 | 115.6 | 98.6 | 94.3 | 78.3 | 122.0 | 68.4 | 61.1 | 72.0 | 83.7 | 80.3 | 71.3 |
| Cps1 | 0.0 | 0.0 | 0.0 | 0.0 | 0.0 | 0.0 | 0.0 | 0.0 | 0.0 | 0.0 | 0.0 | 0.0 |
| Cpsf1 | 8.6 | 8.9 | 7.2 | 9.0 | 9.9 | 9.9 | 7.8 | 8.5 | 7.8 | 7.7 | 7.8 | 7.0 |
| Cpsf2 | 12.0 | 12.0 | 12.0 | 11.5 | 11.6 | 11.4 | 11.9 | 11.7 | 11.8 | 11.7 | 12.4 | 12.8 |
| Cpsf3 | 24.8 | 23.2 | 21.6 | 26.2 | 22.8 | 25.9 | 20.1 | 22.5 | 17.0 | 20.7 | 22.3 | 21.1 |
| Cpsf3l | 12.4 | 12.3 | 11.2 | 12.4 | 12.2 | 11.8 | 12.4 | 11.7 | 12.1 | 11.2 | 11.3 | 10.8 |
| Cpsf4 | 3.8 | 4.1 | 3.7 | 4.2 | 4.9 | 4.6 | 4.7 | 4.9 | 4.3 | 4.2 | 4.1 | 3.8 |
| Cpsf4l | 0.0 | 0.1 | 0.1 | 0.0 | 0.1 | 0.0 | 0.3 | 0.1 | 0.1 | 0.1 | 0.1 | 0.1 |
| Cpsf6 | 5.9 | 7.3 | 8.5 | 6.5 | 6.8 | 7.2 | 7.1 | 7.8 | 6.4 | 7.2 | 7.9 | 8.0 |
| Cpsf7 | 5.2 | 5.6 | 6.2 | 5.8 | 5.2 | 5.2 | 6.3 | 6.9 | 5.3 | 5.2 | 5.9 | 5.8 |
| Cpt1a | 18.0 | 11.7 | 11.9 | 26.3 | 19.6 | 19.8 | 15.1 | 18.3 | 13.8 | 21.7 | 14.5 | 18.9 |
| Cpt1b | 0.0 | 0.3 | 0.1 | 0.5 | 0.1 | 0.2 | 0.2 | 0.2 | 0.3 | 0.3 | 0.2 | 0.1 |
| Cpt1c | 17.2 | 17.3 | 17.6 | 14.2 | 14.9 | 18.5 | 15.6 | 14.7 | 16.6 | 20.5 | 14.9 | 14.3 |
| Cpt2 | 10.0 | 15.0 | 13.8 | 10.3 | 10.6 | 10.2 | 10.4 | 17.7 | 13.1 | 13.6 | 13.1 | 11.4 |
| Cpxm1 | 6.2 | 16.1 | 28.1 | 4.1 | 13.8 | 2.2 | 4.6 | 13.9 | 10.4 | 25.1 | 7.3 | 14.6 |
| Cpxm2 | 3.7 | 3.2 | 7.3 | 0.7 | 2.0 | 0.6 | 4.1 | 9.6 | 6.0 | 6.8 | 10.3 | 13.5 |
| Cpz | 0.4 | 0.4 | 0.5 | 0.4 | 0.5 | 0.0 | 0.3 | 0.5 | 0.9 | 8.2 | 2.6 | 2.3 |
| Cr1l | 28.7 | 24.6 | 26.5 | 34.0 | 26.0 | 24.6 | 28.0 | 28.2 | 27.6 | 25.9 | 27.6 | 29.8 |
| Cr2 | 0.0 | 0.0 | 0.0 | 0.0 | 0.0 | 0.0 | 0.0 | 0.0 | 0.0 | 0.0 | 0.0 | 0.1 |
| Crabp1 | 1.3 | 0.7 | 2.4 | 0.3 | 0.5 | 0.3 | 0.6 | 2.7 | 0.8 | 0.2 | 0.8 | 1.1 |
| Crabp2 | 2.9 | 0.7 | 0.2 | 0.7 | 0.4 | 0.7 | 0.3 | 1.4 | 0.9 | 1.7 | 2.7 | 2.4 |
| Cradd | 3.6 | 3.2 | 3.2 | 2.9 | 3.3 | 3.1 | 3.0 | 3.0 | 2.7 | 2.9 | 1.5 | 3.1 |
| Cramp1l | 1.9 | 2.4 | 2.3 | 1.7 | 2.2 | 1.8 | 2.2 | 2.0 | 2.3 | 2.0 | 1.9 | 1.7 |
| Crat | 9.5 | 9.2 | 9.3 | 10.2 | 10.2 | 9.8 | 12.1 | 10.3 | 10.8 | 8.5 | 10.3 | 10.0 |

| | | | | | | | | | | | | |
|---|---|---|---|---|---|---|---|---|---|---|---|---|
| Crb2 | 0.7 | 0.3 | 0.3 | 0.4 | 0.4 | 0.3 | 0.6 | 0.4 | 1.1 | 3.3 | 1.5 | 1.1 |
| Crbn | 12.4 | 12.3 | 11.7 | 16.1 | 13.1 | 14.5 | 12.3 | 10.8 | 11.2 | 12.6 | 13.4 | 13.9 |
| Crcp | 16.6 | 11.3 | 10.5 | 18.0 | 15.2 | 17.7 | 17.0 | 14.3 | 14.6 | 13.9 | 19.6 | 15.2 |
| Crct1 | 12.3 | 2.9 | 0.7 | 3.4 | 1.1 | 3.1 | 1.7 | 0.2 | 0.3 | 2.0 | 2.6 | 2.0 |
| Creb1 | 4.2 | 5.0 | 6.2 | 4.3 | 5.0 | 4.6 | 4.8 | 5.5 | 4.9 | 4.8 | 5.8 | 5.5 |
| Creb3 | 67.9 | 51.0 | 44.8 | 67.1 | 51.7 | 54.0 | 67.3 | 45.4 | 57.3 | 66.9 | 66.7 | 63.9 |
| Creb3l1 | 2.7 | 3.0 | 3.6 | 2.7 | 3.5 | 2.3 | 4.6 | 7.2 | 6.5 | 4.9 | 5.9 | 4.8 |
| Creb3l2 | 13.0 | 14.7 | 14.4 | 13.8 | 16.2 | 13.1 | 19.6 | 14.7 | 15.8 | 15.8 | 15.7 | 15.5 |
| Creb3l3 | 0.3 | 0.2 | 0.3 | 0.1 | 0.1 | 0.1 | 0.3 | 0.4 | 0.2 | 0.3 | 0.2 | 0.3 |
| Creb3l4 | 1.5 | 1.4 | 1.5 | 1.7 | 1.3 | 1.5 | 1.1 | 1.2 | 1.1 | 0.7 | 1.7 | 1.3 |
| Creb5 | 0.1 | 0.0 | 0.0 | 0.2 | 0.6 | 0.0 | 0.1 | 0.0 | 0.0 | 0.1 | 0.1 | 0.0 |
| Crebbp | 4.9 | 6.5 | 7.4 | 5.8 | 6.2 | 5.0 | 7.4 | 6.8 | 6.3 | 5.3 | 5.8 | 5.0 |
| Crebl2 | 17.8 | 13.9 | 10.8 | 20.5 | 11.7 | 19.7 | 10.4 | 7.8 | 10.5 | 13.3 | 11.5 | 12.9 |
| Crebrf | 12.2 | 12.3 | 12.9 | 15.0 | 10.4 | 14.9 | 9.3 | 9.6 | 10.0 | 12.6 | 11.9 | 14.3 |
| Crebzf | 11.6 | 8.6 | 8.9 | 9.4 | 8.1 | 8.8 | 8.2 | 9.5 | 11.8 | 11.0 | 8.9 | 10.4 |
| Creg1 | 68.6 | 60.1 | 53.6 | 125.3 | 119.0 | 84.2 | 43.2 | 45.1 | 36.0 | 59.9 | 46.1 | 54.6 |
| Creg2 | 0.0 | 0.0 | 0.0 | 0.4 | 0.6 | 0.0 | 0.1 | 0.0 | 0.0 | 0.0 | 0.0 | 0.0 |
| Creld1 | 25.0 | 23.5 | 22.4 | 27.5 | 23.5 | 28.9 | 22.2 | 24.5 | 24.1 | 24.4 | 23.7 | 20.6 |
| Creld2 | 47.0 | 36.3 | 29.7 | 56.5 | 35.9 | 38.8 | 37.1 | 35.2 | 33.8 | 39.0 | 36.9 | 38.1 |
| Crem | 3.4 | 2.6 | 2.3 | 4.7 | 3.2 | 3.4 | 2.6 | 2.0 | 2.1 | 3.1 | 2.2 | 1.9 |
| Crhbp | 0.1 | 0.0 | 0.1 | 0.1 | 0.1 | 0.1 | 0.1 | 0.2 | 0.1 | 0.0 | 0.0 | 0.0 |
| Crim1 | 74.7 | 72.0 | 73.1 | 69.6 | 54.9 | 70.9 | 80.4 | 51.3 | 84.1 | 68.9 | 70.0 | 74.0 |
| Crip1 | 155.4 | 161.5 | 150.3 | 82.7 | 155.2 | 117.2 | 135.4 | 181.0 | 168.0 | 451.4 | 258.6 | 234.9 |
| Crip2 | 131.4 | 136.2 | 121.4 | 167.1 | 136.9 | 154.1 | 149.3 | 180.1 | 119.5 | 97.6 | 139.5 | 106.8 |
| Cript | 32.6 | 31.1 | 25.8 | 32.7 | 34.5 | 31.4 | 26.9 | 26.6 | 32.5 | 33.5 | 33.1 | 32.8 |
| Crisp2 | 0.1 | 0.2 | 0.0 | 0.1 | 0.1 | 0.0 | 0.2 | 0.1 | 0.0 | 0.3 | 0.1 | 0.1 |
| Crispld1 | 0.1 | 0.0 | 0.2 | 0.0 | 0.0 | 0.0 | 0.1 | 0.0 | 0.0 | 0.0 | 0.0 | 0.1 |
| Crispld2 | 4.4 | 8.6 | 13.0 | 3.7 | 5.7 | 2.8 | 4.7 | 8.5 | 9.8 | 3.9 | 4.6 | 6.3 |
| Crk | 40.4 | 46.5 | 43.1 | 42.2 | 46.6 | 40.2 | 49.7 | 44.5 | 51.9 | 49.8 | 52.7 | 53.0 |
| Crkl | 10.0 | 11.3 | 9.7 | 10.2 | 11.0 | 9.1 | 10.2 | 10.7 | 11.7 | 10.8 | 10.5 | 10.2 |
| Crlf1 | 56.0 | 49.6 | 34.0 | 38.4 | 43.9 | 89.8 | 111.6 | 84.1 | 101.4 | 68.2 | 71.9 | 79.2 |
| Crlf2 | 12.1 | 9.5 | 9.4 | 11.6 | 13.2 | 10.5 | 12.2 | 11.5 | 10.6 | 11.2 | 9.7 | 10.6 |
| Crlf3 | 7.8 | 8.2 | 6.7 | 7.9 | 9.6 | 7.9 | 8.2 | 6.4 | 7.6 | 7.2 | 8.9 | 7.7 |
| Crls1 | 22.1 | 20.0 | 16.6 | 23.8 | 17.6 | 20.7 | 17.8 | 19.1 | 20.6 | 29.9 | 29.0 | 26.5 |
| Crmp1 | 0.5 | 0.2 | 0.2 | 0.4 | 0.2 | 0.3 | 0.5 | 0.3 | 0.3 | 0.0 | 0.1 | 0.1 |
| Crnkl1 | 16.0 | 16.6 | 16.4 | 15.7 | 14.6 | 15.1 | 15.3 | 17.4 | 13.4 | 15.7 | 16.2 | 18.7 |
| Crocc | 1.2 | 1.4 | 1.6 | 0.9 | 1.0 | 1.6 | 1.2 | 1.3 | 1.4 | 1.3 | 1.3 | 1.1 |
| Crot | 28.0 | 27.6 | 26.0 | 26.4 | 27.1 | 25.0 | 22.6 | 23.5 | 24.2 | 34.0 | 27.2 | 29.4 |
| Crtac1 | 0.0 | 0.0 | 0.0 | 0.0 | 0.0 | 0.0 | 0.0 | 0.0 | 0.0 | 0.0 | 0.0 | 0.0 |
| Crtap | 87.6 | 75.5 | 82.1 | 80.9 | 76.2 | 83.6 | 91.9 | 78.1 | 80.5 | 87.2 | 76.1 | 88.3 |
| Crtc1 | 0.6 | 0.7 | 0.7 | 0.7 | 0.7 | 0.7 | 0.8 | 1.0 | 0.7 | 0.7 | 0.5 | 0.4 |
| Crtc2 | 1.9 | 2.6 | 2.2 | 2.4 | 2.4 | 2.0 | 2.7 | 2.6 | 2.2 | 2.0 | 1.7 | 1.7 |
| Crtc3 | 4.4 | 6.5 | 7.9 | 4.2 | 4.7 | 4.1 | 5.8 | 6.2 | 6.6 | 4.4 | 4.4 | 5.2 |
| Crx | 0.0 | 0.0 | 0.0 | 0.0 | 0.0 | 0.1 | 0.0 | 0.0 | 0.0 | 0.0 | 0.0 | 0.0 |
| Cry1 | 1.2 | 1.3 | 1.3 | 0.9 | 1.2 | 1.5 | 1.4 | 1.5 | 1.7 | 1.2 | 1.6 | 1.4 |
| Cry2 | 5.1 | 5.5 | 4.8 | 5.2 | 4.5 | 5.8 | 4.6 | 4.6 | 4.3 | 3.7 | 4.2 | 3.4 |
| Cryab | 268.5 | 328.7 | 273.3 | 324.1 | 205.7 | 341.6 | 226.4 | 275.3 | 221.9 | 293.9 | 294.0 | 268.6 |
| Cryba1 | 0.1 | 0.2 | 0.1 | 0.2 | 0.1 | 0.1 | 0.0 | 0.1 | 0.1 | 0.0 | 0.1 | 0.0 |
| Cryba4 | 0.2 | 0.0 | 0.1 | 0.0 | 0.2 | 0.1 | 0.0 | 0.0 | 0.1 | 0.1 | 0.2 | 0.0 |
| Crybb1 | 0.1 | 0.1 | 0.1 | 0.0 | 0.0 | 0.0 | 0.0 | 0.0 | 0.0 | 0.1 | 0.0 | 0.0 |
| Crybb3 | 0.1 | 0.1 | 0.0 | 0.1 | 0.1 | 0.2 | 0.2 | 0.1 | 0.2 | 0.3 | 0.2 | 0.1 |
| Crygf | 0.0 | 0.0 | 0.0 | 0.0 | 0.0 | 0.0 | 0.0 | 0.0 | 0.0 | 0.0 | 0.0 | 0.0 |
| Crygn | 0.3 | 0.1 | 0.0 | 0.0 | 0.2 | 0.1 | 0.0 | 0.1 | 0.1 | 0.2 | 0.1 | 0.1 |
| Crygs | 0.1 | 0.5 | 0.3 | 0.2 | 0.1 | 0.1 | 0.4 | 0.1 | 0.3 | 0.0 | 0.5 | 0.3 |
| Cryl1 | 4.5 | 6.6 | 4.9 | 5.2 | 4.7 | 5.3 | 3.8 | 4.6 | 3.6 | 4.3 | 3.6 | 3.4 |
| Crym | 2.3 | 1.6 | 1.8 | 1.0 | 1.7 | 0.8 | 0.8 | 1.2 | 0.5 | 0.7 | 0.6 | 0.6 |
| Cryz | 4.5 | 4.3 | 4.5 | 3.6 | 3.1 | 6.2 | 2.2 | 3.7 | 2.6 | 3.7 | 2.9 | 3.4 |

| | | | | | | | | | | | | |
|---|---|---|---|---|---|---|---|---|---|---|---|---|
| Cryzl1 | 12.4 | 13.6 | 13.2 | 13.8 | 10.6 | 13.7 | 11.1 | 12.2 | 12.3 | 12.8 | 12.7 | 14.1 |
| Cs | 58.3 | 56.0 | 50.1 | 57.8 | 58.0 | 56.3 | 59.2 | 59.2 | 56.0 | 51.0 | 61.5 | 53.8 |
| Csad | 14.0 | 16.6 | 16.2 | 9.7 | 12.1 | 13.8 | 10.7 | 12.8 | 15.4 | 13.7 | 11.4 | 13.4 |
| Csda | 62.4 | 50.2 | 46.2 | 64.7 | 45.0 | 62.9 | 64.6 | 65.3 | 48.7 | 50.0 | 66.0 | 47.9 |
| Csdc2 | 1.5 | 2.4 | 2.8 | 1.5 | 1.7 | 1.6 | 2.5 | 3.8 | 2.4 | 2.5 | 2.3 | 2.1 |
| Csde1 | 270.5 | 191.5 | 170.3 | 289.3 | 187.8 | 280.2 | 155.6 | 156.1 | 139.7 | 166.8 | 197.7 | 182.8 |
| Cse1l | 12.3 | 14.0 | 14.5 | 12.8 | 15.4 | 12.5 | 15.5 | 15.1 | 15.4 | 15.1 | 15.3 | 13.2 |
| Csf1 | 408.2 | 364.8 | 331.7 | 453.8 | 437.3 | 491.7 | 547.6 | 256.4 | 351.7 | 374.7 | 360.3 | 343.8 |
| Csf1r | 6.6 | 2.3 | 1.7 | 35.2 | 61.2 | 1.7 | 7.4 | 2.0 | 2.1 | 5.0 | 2.9 | 4.4 |
| Csf2 | 0.1 | 0.2 | 0.2 | 0.0 | 0.2 | 0.2 | 0.3 | 0.0 | 0.1 | 0.0 | 0.0 | 0.1 |
| Csf2ra | 3.4 | 2.4 | 3.2 | 9.3 | 12.5 | 3.0 | 3.7 | 2.8 | 3.1 | 2.5 | 2.3 | 3.0 |
| Csf2rb | 0.1 | 0.0 | 0.1 | 1.6 | 1.7 | 0.0 | 0.1 | 0.2 | 0.0 | 0.1 | 0.0 | 0.1 |
| Csf2rb2 | 0.1 | 0.0 | 0.1 | 0.4 | 0.5 | 0.0 | 0.1 | 0.2 | 0.1 | 0.0 | 0.0 | 0.1 |
| Csf3r | 0.0 | 0.0 | 0.0 | 0.3 | 0.2 | 0.0 | 0.0 | 0.0 | 0.0 | 0.0 | 0.0 | 0.0 |
| Csgalnact1 | 16.2 | 22.3 | 30.9 | 18.7 | 31.4 | 12.6 | 40.6 | 33.4 | 38.9 | 20.4 | 22.7 | 31.1 |
| Csgalnact2 | 20.6 | 15.6 | 15.5 | 21.4 | 15.1 | 22.3 | 15.9 | 13.0 | 13.8 | 16.7 | 18.4 | 19.3 |
| Csk | 13.6 | 17.1 | 13.7 | 20.1 | 23.8 | 16.9 | 18.2 | 13.6 | 16.4 | 14.4 | 14.9 | 12.9 |
| Csl | 0.1 | 0.2 | 0.2 | 0.4 | 0.1 | 0.2 | 0.2 | 0.2 | 0.2 | 0.2 | 0.2 | 0.2 |
| Csmd1 | 0.0 | 0.0 | 0.0 | 0.0 | 0.1 | 0.0 | 0.0 | 0.0 | 0.0 | 0.0 | 0.0 | 0.0 |
| Csmd3 | 0.0 | 0.0 | 0.0 | 0.0 | 0.0 | 0.0 | 0.0 | 0.0 | 0.0 | 0.0 | 0.0 | 0.0 |
| Csnk1a1 | 93.1 | 101.5 | 118.0 | 95.9 | 92.3 | 108.8 | 90.4 | 112.7 | 95.5 | 109.0 | 109.3 | 110.8 |
| Csnk1d | 22.8 | 25.9 | 24.6 | 20.7 | 23.2 | 20.8 | 25.3 | 25.6 | 25.1 | 25.0 | 24.4 | 22.9 |
| Csnk1e | 17.3 | 18.7 | 16.6 | 17.1 | 15.2 | 19.1 | 18.0 | 14.7 | 16.8 | 14.5 | 17.5 | 15.2 |
| Csnk1g1 | 5.2 | 6.3 | 6.5 | 5.7 | 6.2 | 6.0 | 6.5 | 6.4 | 6.3 | 6.3 | 6.5 | 6.4 |
| Csnk1g2 | 47.1 | 47.4 | 46.0 | 63.6 | 45.1 | 63.0 | 56.6 | 51.3 | 48.8 | 44.9 | 47.4 | 44.1 |
| Csnk1g3 | 8.7 | 9.7 | 10.4 | 9.7 | 9.3 | 9.1 | 10.7 | 10.6 | 9.1 | 10.2 | 10.8 | 12.1 |
| Csnk2a1 | 40.7 | 39.3 | 38.2 | 40.7 | 37.1 | 42.3 | 37.0 | 35.9 | 38.3 | 43.0 | 42.9 | 41.7 |
| Csnk2a2 | 4.5 | 4.7 | 4.7 | 4.8 | 4.7 | 5.6 | 5.3 | 5.1 | 5.0 | 5.1 | 5.3 | 5.1 |
| Csnk2b | 43.1 | 40.6 | 43.1 | 49.4 | 42.4 | 47.2 | 47.7 | 52.2 | 40.8 | 44.3 | 53.0 | 48.5 |
| Cspg4 | 0.5 | 0.3 | 0.5 | 0.1 | 0.1 | 0.2 | 0.6 | 0.5 | 0.4 | 0.4 | 0.5 | 0.4 |
| Cspg5 | 0.1 | 0.1 | 0.1 | 0.1 | 0.2 | 0.2 | 0.3 | 0.1 | 0.2 | 0.2 | 0.2 | 0.1 |
| Cspp1 | 4.6 | 4.8 | 6.1 | 4.6 | 4.1 | 4.2 | 5.9 | 5.1 | 4.5 | 4.4 | 5.7 | 5.8 |
| Csrnp1 | 3.1 | 3.3 | 2.3 | 2.6 | 2.7 | 1.7 | 3.4 | 2.3 | 3.4 | 5.6 | 3.5 | 3.2 |
| Csrnp2 | 2.5 | 2.9 | 2.7 | 3.2 | 2.8 | 3.1 | 2.8 | 2.4 | 2.5 | 2.7 | 2.7 | 2.5 |
| Csrp1 | 109.9 | 103.7 | 99.6 | 103.9 | 120.3 | 94.3 | 141.0 | 112.4 | 122.8 | 115.8 | 177.5 | 120.6 |
| Csrp2 | 22.6 | 35.8 | 57.7 | 28.1 | 54.4 | 21.4 | 49.4 | 99.2 | 76.1 | 36.9 | 39.4 | 61.1 |
| Csrp2bp | 5.4 | 4.9 | 5.7 | 6.7 | 6.4 | 6.9 | 6.2 | 6.1 | 5.5 | 5.0 | 5.4 | 5.3 |
| Cst3 | 522.8 | 425.6 | 370.8 | 630.9 | 426.0 | 598.0 | 343.0 | 323.6 | 379.8 | 377.9 | 319.9 | 414.9 |
| Cst6 | 9.5 | 6.4 | 2.6 | 13.4 | 5.5 | 15.2 | 2.6 | 3.4 | 1.1 | 2.2 | 3.1 | 2.7 |
| Cst7 | 0.2 | 0.0 | 0.0 | 1.0 | 1.3 | 0.0 | 0.4 | 0.0 | 0.0 | 0.1 | 0.0 | 0.0 |
| Cst9 | 0.2 | 0.0 | 0.0 | 0.0 | 0.0 | 0.0 | 0.0 | 0.0 | 0.0 | 0.3 | 0.1 | 0.0 |
| Cstad | 1.2 | 1.0 | 1.9 | 0.6 | 0.6 | 0.9 | 0.8 | 1.2 | 1.1 | 1.4 | 1.2 | 0.9 |
| Cstb | 238.3 | 154.2 | 125.4 | 283.3 | 226.1 | 225.1 | 147.1 | 125.1 | 134.9 | 174.2 | 155.0 | 172.0 |
| Cstf1 | 9.1 | 8.5 | 7.6 | 8.4 | 9.2 | 8.3 | 9.7 | 8.4 | 8.6 | 8.2 | 9.8 | 8.0 |
| Cstf2 | 4.0 | 4.1 | 5.4 | 5.0 | 4.5 | 4.2 | 4.6 | 5.6 | 3.9 | 5.0 | 5.7 | 4.9 |
| Cstf2t | 15.4 | 18.5 | 19.1 | 16.1 | 15.6 | 15.3 | 16.4 | 16.1 | 17.6 | 17.2 | 17.5 | 18.3 |
| Cstf3 | 3.7 | 4.4 | 4.2 | 3.3 | 4.9 | 3.5 | 3.3 | 4.3 | 4.4 | 4.8 | 5.2 | 4.8 |
| Cstl1 | 0.1 | 0.1 | 0.0 | 0.1 | 0.0 | 0.0 | 0.0 | 0.1 | 0.0 | 0.0 | 0.0 | 0.0 |
| Ctage5 | 26.1 | 22.3 | 20.5 | 28.2 | 21.5 | 23.7 | 23.5 | 20.9 | 22.2 | 23.5 | 25.7 | 23.5 |
| Ctbp1 | 34.8 | 34.0 | 29.7 | 37.4 | 35.7 | 36.6 | 37.8 | 39.1 | 40.1 | 34.6 | 36.8 | 33.2 |
| Ctbp2 | 16.1 | 17.0 | 17.3 | 15.4 | 16.5 | 14.8 | 16.5 | 17.6 | 15.9 | 15.4 | 16.0 | 16.5 |
| Ctbs | 6.5 | 5.8 | 6.4 | 6.3 | 6.0 | 7.0 | 6.1 | 4.5 | 5.1 | 4.9 | 6.3 | 6.5 |
| Ctc1 | 5.9 | 6.4 | 6.6 | 4.9 | 5.5 | 5.7 | 5.4 | 4.0 | 5.3 | 5.1 | 4.2 | 4.1 |
| Ctcf | 9.3 | 9.4 | 11.0 | 9.2 | 9.9 | 7.9 | 10.6 | 12.2 | 10.2 | 11.3 | 11.3 | 12.2 |
| Ctcfl | 0.0 | 0.0 | 0.0 | 0.0 | 0.0 | 0.0 | 0.0 | 0.0 | 0.0 | 0.0 | 0.0 | 0.0 |
| Ctdnep1 | 10.5 | 9.8 | 10.2 | 10.4 | 10.8 | 10.8 | 10.7 | 11.6 | 10.3 | 10.3 | 8.9 | 7.8 |
| Ctdp1 | 7.5 | 6.3 | 5.6 | 7.1 | 7.6 | 5.4 | 9.7 | 7.2 | 6.9 | 7.1 | 8.8 | 7.1 |

| | | | | | | | | | | | | |
|---|---|---|---|---|---|---|---|---|---|---|---|---|
| Ctdsp1 | 10.1 | 11.9 | 10.9 | 11.7 | 11.6 | 11.8 | 12.8 | 14.0 | 12.7 | 12.4 | 10.9 | 11.4 |
| Ctdsp2 | 23.7 | 33.5 | 34.3 | 20.2 | 22.0 | 25.9 | 26.9 | 36.7 | 31.6 | 24.7 | 23.8 | 24.5 |
| Ctdspl | 15.3 | 18.9 | 18.0 | 12.9 | 10.0 | 16.2 | 14.7 | 13.3 | 14.2 | 17.2 | 16.5 | 15.3 |
| Ctdspl2 | 4.0 | 4.4 | 5.3 | 4.5 | 4.6 | 4.5 | 4.0 | 4.6 | 4.5 | 4.5 | 5.2 | 4.7 |
| Ctf1 | 0.7 | 0.7 | 0.6 | 0.2 | 0.1 | 0.3 | 0.5 | 0.6 | 0.7 | 3.2 | 1.3 | 1.0 |
| Ctgf | 855.7 | 737.3 | 510.7 | 1119.1 | 857.9 | 1079.6 | 1308.6 | 385.7 | 816.9 | 624.5 | 808.9 | 690.5 |
| Cth | 3.7 | 1.8 | 1.3 | 2.2 | 0.8 | 1.6 | 1.2 | 1.2 | 1.3 | 1.4 | 1.6 | 0.9 |
| Cthrc1 | 32.0 | 33.0 | 41.1 | 21.7 | 25.2 | 27.9 | 28.1 | 50.1 | 53.7 | 61.4 | 47.5 | 73.0 |
| Ctif | 2.1 | 2.6 | 2.7 | 2.0 | 2.2 | 1.9 | 2.6 | 3.4 | 3.0 | 2.7 | 2.7 | 2.7 |
| Ctla2a | 53.2 | 8.0 | 6.2 | 39.9 | 11.6 | 43.8 | 6.2 | 6.9 | 3.6 | 5.4 | 5.7 | 5.5 |
| Ctla2b | 6.3 | 2.4 | 1.5 | 8.7 | 6.6 | 6.9 | 2.1 | 1.3 | 1.0 | 1.4 | 1.0 | 1.7 |
| Ctla4 | 0.1 | 0.0 | 0.0 | 0.0 | 0.0 | 0.0 | 0.0 | 0.0 | 0.0 | 0.0 | 0.0 | 0.0 |
| Ctnna1 | 102.7 | 90.9 | 89.2 | 102.5 | 91.4 | 103.5 | 103.9 | 97.5 | 95.3 | 101.6 | 117.4 | 107.4 |
| Ctnna2 | 0.6 | 0.3 | 0.5 | 0.1 | 0.3 | 0.1 | 0.2 | 0.2 | 0.1 | 0.1 | 0.6 | 0.2 |
| Ctnna3 | 0.0 | 0.0 | 0.0 | 0.2 | 0.0 | 0.0 | 0.1 | 0.0 | 0.0 | 0.0 | 0.0 | 0.0 |
| Ctnnal1 | 6.4 | 5.7 | 8.1 | 4.3 | 4.7 | 5.9 | 4.7 | 6.1 | 5.7 | 8.5 | 6.8 | 5.4 |
| Ctnnb1 | 157.7 | 142.3 | 117.0 | 187.8 | 149.6 | 181.9 | 132.1 | 126.2 | 133.4 | 161.2 | 147.2 | 136.9 |
| Ctnnbip1 | 7.7 | 6.1 | 7.1 | 8.7 | 6.9 | 6.2 | 6.6 | 6.5 | 7.3 | 8.4 | 7.7 | 7.5 |
| Ctnnbl1 | 10.0 | 9.1 | 8.4 | 10.2 | 8.8 | 10.4 | 9.0 | 9.5 | 8.2 | 9.0 | 8.9 | 9.8 |
| Ctnnd1 | 46.6 | 50.5 | 36.7 | 62.6 | 60.0 | 72.2 | 58.3 | 39.3 | 57.1 | 57.3 | 60.5 | 56.7 |
| Ctnnd2 | 0.5 | 0.2 | 0.2 | 0.3 | 0.1 | 0.3 | 0.3 | 0.1 | 0.1 | 0.2 | 0.2 | 0.1 |
| Ctns | 5.3 | 5.2 | 4.8 | 5.8 | 4.4 | 6.5 | 4.1 | 5.1 | 4.9 | 4.5 | 4.7 | 4.5 |
| Ctps | 136.5 | 112.8 | 108.2 | 109.6 | 99.7 | 83.4 | 105.0 | 101.8 | 102.7 | 95.5 | 102.5 | 102.8 |
| Ctps2 | 12.0 | 12.0 | 11.6 | 10.8 | 10.2 | 10.1 | 10.6 | 10.8 | 10.3 | 9.9 | 10.6 | 11.1 |
| Ctr9 | 16.5 | 17.3 | 18.0 | 17.0 | 15.8 | 17.4 | 17.4 | 17.4 | 14.2 | 18.4 | 19.9 | 19.9 |
| Ctrb1 | 0.0 | 0.0 | 0.0 | 0.0 | 0.0 | 0.1 | 0.1 | 0.0 | 0.0 | 0.0 | 0.0 | 0.0 |
| Ctrl | 0.0 | 0.0 | 0.0 | 0.0 | 0.1 | 0.1 | 0.1 | 0.0 | 0.0 | 0.0 | 0.2 | 0.1 |
| Ctsa | 80.8 | 54.8 | 53.6 | 106.6 | 90.3 | 83.4 | 53.7 | 63.5 | 52.5 | 65.6 | 58.9 | 70.3 |
| Ctsb | 134.0 | 97.0 | 93.3 | 214.9 | 219.1 | 114.4 | 77.3 | 84.2 | 81.2 | 109.6 | 85.9 | 99.6 |
| Ctsc | 1.5 | 0.3 | 0.4 | 7.8 | 17.6 | 0.2 | 3.3 | 1.6 | 1.3 | 2.4 | 1.4 | 2.2 |
| Ctsd | 1438.7 | 1236.2 | 1192.5 | 1748.3 | 1698.3 | 1649.1 | 1054.7 | 1271.2 | 1155.5 | 1110.3 | 1058.4 | 1219.8 |
| Ctsf | 34.3 | 33.5 | 29.1 | 31.9 | 27.0 | 36.4 | 22.4 | 22.2 | 22.7 | 31.4 | 26.2 | 28.5 |
| Ctsh | 49.6 | 38.1 | 45.2 | 72.4 | 74.7 | 64.4 | 55.8 | 55.0 | 48.1 | 49.9 | 58.9 | 66.4 |
| Ctsk | 33.2 | 17.4 | 20.0 | 31.9 | 23.8 | 27.0 | 21.5 | 33.7 | 24.4 | 23.5 | 22.1 | 28.9 |
| Ctsl | 536.0 | 372.4 | 379.0 | 566.6 | 368.6 | 562.5 | 291.7 | 333.9 | 301.4 | 355.4 | 374.6 | 406.0 |
| Ctsll3 | 0.0 | 0.0 | 0.0 | 0.0 | 0.0 | 0.0 | 0.0 | 0.0 | 0.0 | 0.0 | 0.0 | 0.1 |
| Ctso | 6.4 | 6.8 | 8.8 | 6.9 | 8.9 | 7.0 | 7.4 | 7.6 | 8.4 | 7.6 | 7.1 | 8.1 |
| Ctss | 8.6 | 0.5 | 1.1 | 87.8 | 113.7 | 0.2 | 7.5 | 5.2 | 1.3 | 6.1 | 3.7 | 5.1 |
| Ctsw | 1.2 | 0.4 | 0.3 | 2.0 | 1.2 | 0.8 | 0.9 | 0.1 | 0.5 | 0.4 | 0.5 | 0.4 |
| Ctsz | 291.4 | 195.4 | 188.4 | 430.0 | 306.6 | 323.1 | 189.7 | 147.1 | 151.8 | 184.3 | 182.0 | 213.3 |
| Cttn | 87.7 | 85.2 | 91.8 | 90.8 | 77.5 | 84.4 | 98.6 | 101.9 | 91.3 | 87.6 | 100.7 | 107.0 |
| Cttnbp2 | 0.0 | 0.0 | 0.0 | 0.0 | 0.0 | 0.0 | 0.0 | 0.1 | 0.0 | 0.1 | 0.1 | 0.1 |
| Cttnbp2nl | 19.1 | 18.1 | 14.2 | 21.5 | 19.1 | 16.4 | 17.7 | 13.8 | 13.7 | 16.7 | 18.0 | 15.0 |
| Ctu1 | 2.6 | 2.5 | 2.0 | 2.1 | 2.5 | 1.8 | 2.4 | 1.9 | 2.9 | 2.6 | 2.7 | 2.3 |
| Ctu2 | 3.2 | 3.5 | 2.9 | 3.6 | 3.1 | 3.7 | 3.7 | 3.7 | 3.8 | 2.7 | 3.0 | 2.8 |
| Ctxn1 | 0.7 | 0.7 | 0.6 | 1.5 | 1.4 | 1.1 | 1.0 | 0.8 | 1.5 | 3.6 | 2.3 | 1.4 |
| Cubn | 0.7 | 0.4 | 0.5 | 0.4 | 0.5 | 0.5 | 0.4 | 0.4 | 0.3 | 0.2 | 0.3 | 0.2 |
| Cuedc1 | 7.1 | 7.5 | 6.8 | 8.5 | 7.5 | 8.6 | 8.4 | 6.9 | 7.6 | 6.7 | 7.3 | 7.5 |
| Cuedc2 | 13.1 | 10.7 | 12.0 | 15.5 | 12.1 | 14.0 | 10.0 | 12.4 | 9.6 | 12.7 | 12.2 | 12.8 |
| Cul1 | 32.8 | 32.2 | 31.0 | 34.4 | 36.8 | 31.5 | 33.4 | 34.0 | 35.0 | 38.1 | 38.0 | 38.9 |
| Cul2 | 10.9 | 11.3 | 10.4 | 10.0 | 10.1 | 12.2 | 9.6 | 10.0 | 10.4 | 9.8 | 11.3 | 10.7 |
| Cul3 | 33.8 | 38.9 | 40.2 | 36.2 | 35.6 | 33.7 | 38.0 | 38.2 | 38.7 | 36.3 | 45.4 | 42.8 |
| Cul4a | 49.0 | 41.2 | 33.8 | 46.5 | 38.4 | 46.1 | 40.6 | 33.3 | 37.3 | 41.6 | 43.1 | 42.5 |
| Cul4b | 21.7 | 21.9 | 20.2 | 21.4 | 20.6 | 20.5 | 19.9 | 17.5 | 20.0 | 21.5 | 24.1 | 23.0 |
| Cul5 | 12.7 | 13.3 | 12.5 | 12.6 | 10.7 | 14.3 | 12.1 | 11.6 | 12.1 | 12.2 | 14.7 | 13.2 |
| Cul7 | 15.5 | 15.5 | 15.9 | 13.1 | 11.8 | 17.0 | 14.7 | 16.5 | 14.9 | 16.4 | 14.0 | 14.8 |
| Cul9 | 4.2 | 4.9 | 4.7 | 4.0 | 3.9 | 5.5 | 3.5 | 4.1 | 3.9 | 3.1 | 2.7 | 3.3 |

| | | | | | | | | | | | | |
|---|---|---|---|---|---|---|---|---|---|---|---|---|
| Cuta | 34.2 | 33.4 | 35.5 | 38.3 | 31.3 | 36.3 | 33.5 | 38.4 | 35.4 | 33.4 | 30.4 | 29.0 |
| Cutc | 1.7 | 1.3 | 1.4 | 1.7 | 1.5 | 0.8 | 0.8 | 1.0 | 1.4 | 1.3 | 1.5 | 1.3 |
| Cux1 | 31.6 | 29.7 | 27.6 | 37.2 | 37.9 | 32.8 | 42.6 | 35.3 | 38.3 | 35.7 | 43.3 | 43.0 |
| Cux2 | 0.0 | 0.0 | 0.0 | 0.0 | 0.0 | 0.0 | 0.0 | 0.1 | 0.0 | 0.0 | 0.0 | 0.0 |
| Cwc15 | 70.5 | 60.8 | 71.0 | 95.2 | 60.9 | 78.4 | 75.6 | 67.1 | 54.8 | 59.6 | 80.2 | 76.0 |
| Cwc22 | 9.3 | 8.8 | 10.5 | 9.7 | 8.9 | 8.8 | 9.6 | 9.4 | 8.0 | 7.9 | 10.1 | 10.5 |
| Cwc25 | 4.9 | 4.9 | 6.4 | 5.9 | 5.6 | 5.0 | 5.6 | 6.4 | 5.4 | 5.2 | 6.0 | 6.1 |
| Cwc27 | 10.4 | 11.2 | 12.3 | 14.3 | 9.5 | 16.5 | 10.4 | 9.8 | 8.4 | 8.8 | 11.0 | 11.8 |
| Cwf19l1 | 1.9 | 2.3 | 2.0 | 2.3 | 2.1 | 1.9 | 1.9 | 2.0 | 1.7 | 2.1 | 2.1 | 1.9 |
| Cwf19l2 | 6.4 | 6.8 | 7.1 | 7.3 | 5.9 | 7.3 | 6.1 | 5.6 | 5.3 | 5.7 | 6.8 | 7.6 |
| Cx3cl1 | 9.1 | 8.8 | 9.0 | 18.7 | 24.4 | 16.9 | 19.3 | 7.5 | 11.1 | 18.1 | 11.4 | 11.8 |
| Cx3cr1 | 1.0 | 0.2 | 0.2 | 5.4 | 20.3 | 0.2 | 2.8 | 0.3 | 0.6 | 1.2 | 0.4 | 1.2 |
| Cxadr | 2.1 | 1.2 | 1.3 | 0.6 | 0.7 | 1.0 | 1.0 | 2.1 | 3.3 | 24.1 | 9.6 | 6.6 |
| Cxcl1 | 17.7 | 15.9 | 59.6 | 26.1 | 30.6 | 8.5 | 21.0 | 27.4 | 15.3 | 14.9 | 7.3 | 15.5 |
| Cxcl10 | 2.0 | 0.2 | 0.6 | 6.1 | 1.0 | 1.5 | 0.4 | 0.1 | 0.2 | 0.6 | 0.6 | 1.1 |
| Cxcl12 | 26.7 | 30.9 | 82.8 | 26.3 | 54.4 | 50.9 | 67.1 | 104.5 | 129.3 | 145.9 | 106.1 | 161.8 |
| Cxcl14 | 1.6 | 0.9 | 2.1 | 2.0 | 2.5 | 0.7 | 4.7 | 2.5 | 3.1 | 5.8 | 4.8 | 6.6 |
| Cxcl16 | 14.5 | 6.7 | 11.3 | 33.6 | 47.5 | 16.3 | 18.5 | 9.3 | 9.8 | 12.4 | 7.2 | 13.1 |
| Cxcl2 | 0.5 | 0.2 | 0.2 | 1.9 | 4.2 | 0.1 | 1.3 | 0.1 | 0.3 | 0.4 | 0.1 | 0.2 |
| Cxcl3 | 0.3 | 0.3 | 0.1 | 0.6 | 0.5 | 0.1 | 0.3 | 0.2 | 0.0 | 0.0 | 0.1 | 0.2 |
| Cxcl5 | 9.2 | 12.1 | 63.7 | 8.8 | 40.3 | 2.7 | 24.6 | 28.1 | 17.1 | 2.8 | 3.0 | 9.4 |
| Cxcl9 | 0.0 | 0.0 | 0.0 | 0.0 | 0.0 | 0.0 | 0.0 | 0.0 | 0.0 | 0.0 | 0.0 | 0.0 |
| Cxcr1 | 0.0 | 0.0 | 0.1 | 0.0 | 0.0 | 0.0 | 0.0 | 0.0 | 0.0 | 0.0 | 0.0 | 0.0 |
| Cxcr2 | 0.0 | 0.1 | 0.1 | 0.0 | 0.1 | 0.1 | 0.0 | 0.0 | 0.0 | 0.1 | 0.1 | 0.0 |
| Cxcr3 | 0.0 | 0.0 | 0.0 | 0.6 | 2.2 | 0.0 | 0.3 | 0.1 | 0.1 | 0.1 | 0.1 | 0.0 |
| Cxcr4 | 1.5 | 0.7 | 1.2 | 2.5 | 2.7 | 0.2 | 1.5 | 2.2 | 1.2 | 0.2 | 0.5 | 0.6 |
| Cxcr5 | 0.0 | 0.1 | 0.0 | 0.1 | 0.1 | 0.0 | 0.1 | 0.1 | 0.0 | 0.2 | 0.1 | 0.0 |
| Cxcr6 | 0.1 | 0.0 | 0.1 | 0.2 | 0.2 | 0.2 | 0.1 | 0.2 | 0.0 | 0.2 | 0.1 | 0.2 |
| Cxcr7 | 11.4 | 17.1 | 13.6 | 6.8 | 10.7 | 9.7 | 17.4 | 14.1 | 15.3 | 20.3 | 21.7 | 15.9 |
| Cxx1a | 7.0 | 6.7 | 6.3 | 9.0 | 6.7 | 8.1 | 8.1 | 7.4 | 7.1 | 7.1 | 7.8 | 6.4 |
| Cxx1b | 10.4 | 9.6 | 8.9 | 12.6 | 11.3 | 12.1 | 10.6 | 11.2 | 11.5 | 10.7 | 11.6 | 10.0 |
| Cxx1c | 12.8 | 14.6 | 14.5 | 16.3 | 15.3 | 18.4 | 18.2 | 13.9 | 16.4 | 18.5 | 14.5 | 14.0 |
| Cxxc1 | 5.2 | 5.3 | 6.6 | 6.2 | 5.5 | 5.2 | 6.6 | 7.2 | 6.1 | 6.4 | 6.2 | 7.3 |
| Cxxc4 | 0.0 | 0.0 | 0.0 | 0.0 | 0.0 | 0.0 | 0.0 | 0.0 | 0.0 | 0.0 | 0.0 | 0.0 |
| Cxxc5 | 5.7 | 7.1 | 7.0 | 5.3 | 5.1 | 6.7 | 7.3 | 9.3 | 8.7 | 7.9 | 9.3 | 7.9 |
| Cyb5 | 607.5 | 645.5 | 579.7 | 745.2 | 580.8 | 657.0 | 459.1 | 429.8 | 350.3 | 479.0 | 481.2 | 454.9 |
| Cyb561 | 0.5 | 0.3 | 0.4 | 0.2 | 0.3 | 0.6 | 0.2 | 0.6 | 0.2 | 0.3 | 0.3 | 0.3 |
| Cyb561d1 | 0.9 | 0.8 | 0.9 | 0.9 | 1.0 | 0.7 | 0.8 | 0.8 | 1.1 | 0.9 | 0.7 | 0.9 |
| Cyb561d2 | 7.7 | 6.9 | 5.7 | 6.9 | 6.9 | 6.7 | 7.1 | 6.5 | 7.9 | 8.4 | 6.3 | 7.0 |
| Cyb5b | 42.3 | 42.3 | 41.8 | 41.6 | 37.0 | 38.1 | 47.4 | 48.7 | 41.4 | 33.8 | 46.5 | 36.7 |
| Cyb5d1 | 31.7 | 25.4 | 21.6 | 31.9 | 22.0 | 26.9 | 22.4 | 16.7 | 17.9 | 23.3 | 22.1 | 25.0 |
| Cyb5d2 | 8.3 | 7.8 | 5.4 | 9.4 | 6.6 | 8.2 | 6.2 | 5.3 | 5.9 | 6.3 | 6.5 | 6.6 |
| Cyb5r1 | 48.1 | 31.5 | 22.7 | 63.4 | 28.0 | 63.3 | 36.1 | 46.2 | 42.0 | 31.8 | 42.6 | 30.0 |
| Cyb5r2 | 1.0 | 0.4 | 0.4 | 0.9 | 0.3 | 0.9 | 0.2 | 0.4 | 0.4 | 0.1 | 0.4 | 0.4 |
| Cyb5r3 | 255.1 | 211.3 | 203.9 | 246.0 | 191.1 | 238.7 | 239.7 | 253.3 | 209.3 | 257.9 | 298.0 | 259.2 |
| Cyb5r4 | 18.6 | 19.0 | 19.1 | 20.2 | 20.9 | 16.9 | 16.8 | 18.7 | 18.3 | 20.2 | 21.3 | 20.7 |
| Cyb5rl | 3.1 | 2.2 | 1.3 | 3.1 | 2.3 | 2.9 | 1.9 | 1.6 | 1.9 | 2.7 | 2.4 | 2.1 |
| Cyba | 107.0 | 77.0 | 63.8 | 142.2 | 158.4 | 125.1 | 83.9 | 60.6 | 70.4 | 100.4 | 67.8 | 78.2 |
| Cybasc3 | 8.6 | 8.0 | 7.4 | 8.3 | 9.6 | 9.8 | 6.8 | 6.5 | 8.6 | 9.4 | 8.2 | 6.8 |
| Cybb | 2.0 | 0.1 | 0.1 | 17.7 | 23.9 | 0.0 | 2.2 | 0.4 | 0.4 | 1.0 | 0.3 | 0.9 |
| Cybrd1 | 0.0 | 0.0 | 0.0 | 0.0 | 0.0 | 0.0 | 0.0 | 0.0 | 0.0 | 0.2 | 0.1 | 0.0 |
| Cyc1 | 26.8 | 28.4 | 21.7 | 24.9 | 26.7 | 26.1 | 24.6 | 26.3 | 26.4 | 27.7 | 23.9 | 21.6 |
| Cycs | 25.2 | 19.4 | 20.6 | 30.7 | 24.3 | 23.9 | 23.3 | 17.9 | 20.8 | 23.9 | 28.3 | 26.9 |
| Cyct | 0.2 | 0.2 | 0.2 | 0.0 | 0.3 | 0.1 | 0.5 | 0.2 | 0.0 | 0.0 | 0.0 | 0.0 |
| Cyfip1 | 29.9 | 25.6 | 23.3 | 30.4 | 34.9 | 24.3 | 26.1 | 28.2 | 24.4 | 26.7 | 29.9 | 25.3 |
| Cyfip2 | 0.8 | 0.5 | 0.3 | 2.1 | 3.4 | 0.6 | 0.7 | 0.1 | 0.2 | 0.4 | 0.3 | 0.4 |
| Cygb | 19.5 | 37.1 | 56.9 | 24.0 | 33.3 | 33.8 | 28.0 | 61.7 | 43.0 | 20.7 | 19.4 | 40.8 |

| | | | | | | | | | | | | |
|---|---|---|---|---|---|---|---|---|---|---|---|---|
| Cyhr1 | 34.1 | 33.7 | 29.6 | 38.6 | 31.3 | 39.8 | 31.5 | 30.6 | 33.5 | 34.3 | 30.5 | 29.0 |
| Cyld | 14.3 | 11.6 | 9.7 | 13.3 | 11.0 | 12.5 | 8.6 | 7.7 | 7.9 | 12.5 | 11.7 | 10.8 |
| Cyp11b1 | 0.1 | 0.0 | 0.0 | 0.0 | 0.0 | 0.1 | 0.0 | 0.0 | 0.0 | 0.0 | 0.1 | 0.0 |
| Cyp17a1 | 0.0 | 0.0 | 0.0 | 0.0 | 0.0 | 0.0 | 0.0 | 0.0 | 0.0 | 0.0 | 0.0 | 0.0 |
| Cyp1a1 | 0.2 | 0.1 | 0.0 | 0.4 | 0.0 | 0.3 | 0.0 | 0.0 | 0.0 | 0.0 | 0.0 | 0.0 |
| Cyp1b1 | 45.0 | 57.8 | 94.7 | 55.5 | 96.9 | 51.1 | 92.4 | 139.3 | 112.5 | 93.6 | 91.0 | 108.6 |
| Cyp20a1 | 32.1 | 27.9 | 22.0 | 29.4 | 27.6 | 31.0 | 34.5 | 32.6 | 33.7 | 25.8 | 34.2 | 31.1 |
| Cyp26b1 | 0.6 | 0.4 | 0.2 | 0.8 | 0.2 | 1.1 | 0.3 | 0.0 | 0.1 | 0.3 | 0.5 | 0.2 |
| Cyp26c1 | 0.0 | 0.0 | 0.0 | 0.1 | 0.1 | 0.1 | 0.0 | 0.0 | 0.0 | 0.0 | 0.0 | 0.0 |
| Cyp27a1 | 2.5 | 2.9 | 3.4 | 3.9 | 1.8 | 4.2 | 0.9 | 2.1 | 1.7 | 1.0 | 1.3 | 1.6 |
| Cyp27b1 | 0.1 | 0.0 | 0.0 | 0.0 | 0.0 | 0.0 | 0.0 | 0.0 | 0.0 | 0.0 | 0.0 | 0.0 |
| Cyp2ab1 | 0.0 | 0.0 | 0.0 | 0.0 | 0.0 | 0.0 | 0.0 | 0.0 | 0.0 | 0.0 | 0.0 | 0.0 |
| Cyp2b10 | 0.0 | 0.0 | 0.0 | 0.0 | 0.0 | 0.0 | 0.0 | 0.0 | 0.0 | 0.0 | 0.0 | 0.0 |
| Cyp2c44 | 0.0 | 0.0 | 0.0 | 0.0 | 0.0 | 0.0 | 0.0 | 0.0 | 0.0 | 0.1 | 0.0 | 0.0 |
| Cyp2c55 | 0.0 | 0.0 | 0.1 | 0.0 | 0.0 | 0.0 | 0.0 | 0.0 | 0.1 | 0.0 | 0.0 | 0.0 |
| Cyp2d22 | 2.4 | 3.5 | 3.2 | 1.3 | 2.3 | 2.2 | 3.4 | 4.4 | 4.9 | 2.6 | 3.2 | 2.8 |
| Cyp2d26 | 0.0 | 0.1 | 0.0 | 0.0 | 0.1 | 0.0 | 0.0 | 0.0 | 0.1 | 0.0 | 0.1 | 0.0 |
| Cyp2d9 | 0.0 | 0.1 | 0.0 | 0.1 | 0.0 | 0.0 | 0.0 | 0.0 | 0.0 | 0.0 | 0.0 | 0.0 |
| Cyp2j13 | 0.0 | 0.0 | 0.0 | 0.0 | 0.0 | 0.0 | 0.0 | 0.0 | 0.0 | 0.0 | 0.0 | 0.0 |
| Cyp2j6 | 8.0 | 6.8 | 7.6 | 8.4 | 7.1 | 10.3 | 8.1 | 7.2 | 7.0 | 7.5 | 7.9 | 8.6 |
| Cyp2j9 | 2.4 | 2.1 | 2.9 | 3.0 | 2.4 | 4.0 | 2.9 | 1.9 | 1.7 | 1.5 | 2.0 | 2.3 |
| Cyp2r1 | 1.1 | 1.9 | 2.0 | 0.8 | 1.8 | 1.0 | 1.7 | 1.5 | 2.4 | 1.6 | 1.6 | 1.3 |
| Cyp2s1 | 0.8 | 0.2 | 0.2 | 0.1 | 0.0 | 0.2 | 0.1 | 0.4 | 0.3 | 7.2 | 2.5 | 1.3 |
| Cyp2t4 | 0.0 | 0.1 | 0.0 | 0.0 | 0.0 | 0.0 | 0.0 | 0.0 | 0.0 | 0.0 | 0.0 | 0.0 |
| Cyp2u1 | 7.6 | 6.3 | 5.2 | 8.2 | 7.1 | 9.3 | 8.1 | 5.0 | 5.6 | 4.6 | 5.2 | 5.3 |
| Cyp39a1 | 1.1 | 1.8 | 2.6 | 0.6 | 0.9 | 0.7 | 1.1 | 2.1 | 1.7 | 1.2 | 1.4 | 1.3 |
| Cyp3a13 | 0.3 | 0.0 | 0.1 | 0.1 | 0.1 | 0.0 | 0.0 | 0.0 | 0.0 | 0.0 | 0.1 | 0.0 |
| Cyp46a1 | 0.3 | 0.1 | 0.4 | 0.1 | 0.2 | 0.3 | 0.2 | 0.2 | 0.2 | 0.2 | 0.1 | 0.1 |
| Cyp4a12b | 0.2 | 0.0 | 0.0 | 0.2 | 0.0 | 0.1 | 0.1 | 0.0 | 0.0 | 0.0 | 0.0 | 0.0 |
| Cyp4b1 | 0.3 | 0.4 | 0.3 | 0.1 | 0.3 | 0.1 | 0.3 | 0.8 | 0.4 | 0.1 | 0.1 | 0.0 |
| Cyp4f13 | 6.0 | 6.0 | 5.5 | 6.0 | 5.1 | 7.2 | 5.1 | 4.6 | 5.4 | 6.1 | 5.4 | 5.9 |
| Cyp4f14 | 0.3 | 0.3 | 0.4 | 0.2 | 0.1 | 0.4 | 0.1 | 0.2 | 0.1 | 0.1 | 0.2 | 0.3 |
| Cyp4f15 | 0.0 | 0.0 | 0.0 | 0.0 | 0.0 | 0.0 | 0.0 | 0.0 | 0.0 | 0.0 | 0.0 | 0.0 |
| Cyp4f16 | 4.6 | 4.1 | 3.3 | 5.3 | 6.1 | 5.1 | 4.7 | 4.7 | 4.5 | 4.3 | 4.7 | 4.0 |
| Cyp4f17 | 7.5 | 6.5 | 5.7 | 6.7 | 5.9 | 6.6 | 6.2 | 5.2 | 5.7 | 7.4 | 6.3 | 5.9 |
| Cyp4f18 | 0.2 | 0.0 | 0.0 | 0.5 | 1.3 | 0.0 | 0.1 | 0.0 | 0.0 | 0.1 | 0.0 | 0.0 |
| Cyp4f41-ps | 0.3 | 0.2 | 0.2 | 0.5 | 0.2 | 0.2 | 0.2 | 0.4 | 0.2 | 0.2 | 0.2 | 0.2 |
| Cyp4v3 | 9.5 | 8.4 | 11.8 | 10.8 | 11.3 | 12.1 | 8.5 | 9.2 | 7.4 | 5.5 | 5.8 | 10.4 |
| Cyp51 | 22.6 | 28.8 | 29.1 | 9.0 | 18.6 | 12.5 | 50.9 | 64.4 | 46.8 | 17.4 | 51.5 | 22.9 |
| Cyp7b1 | 0.4 | 0.5 | 1.8 | 0.0 | 0.6 | 0.3 | 0.5 | 1.5 | 0.7 | 1.0 | 0.5 | 1.3 |
| Cyr61 | 294.1 | 313.3 | 334.2 | 228.7 | 254.3 | 200.4 | 379.8 | 263.8 | 379.2 | 346.2 | 312.9 | 323.6 |
| Cys1 | 3.1 | 3.3 | 3.0 | 2.7 | 2.4 | 3.2 | 2.1 | 2.7 | 2.9 | 3.2 | 2.8 | 3.2 |
| Cysltr1 | 0.1 | 0.0 | 0.0 | 0.3 | 1.0 | 0.1 | 0.1 | 0.0 | 0.0 | 0.1 | 0.0 | 0.1 |
| Cystm1 | 17.1 | 7.9 | 5.5 | 23.3 | 6.9 | 22.2 | 5.4 | 5.1 | 5.6 | 7.1 | 9.6 | 5.5 |
| Cyth1 | 2.7 | 3.1 | 3.0 | 4.6 | 5.9 | 2.5 | 2.8 | 2.6 | 2.6 | 2.6 | 2.4 | 2.9 |
| Cyth2 | 9.2 | 9.3 | 9.8 | 9.3 | 9.5 | 8.7 | 12.1 | 11.0 | 12.3 | 12.7 | 12.0 | 11.6 |
| Cyth3 | 60.6 | 92.5 | 98.1 | 60.0 | 61.7 | 67.3 | 77.8 | 73.6 | 79.9 | 45.7 | 62.1 | 69.4 |
| Cyth4 | 1.6 | 0.0 | 0.2 | 12.7 | 20.9 | 0.0 | 2.4 | 0.3 | 0.5 | 1.1 | 0.3 | 1.0 |
| Cytip | 0.0 | 0.0 | 0.0 | 0.2 | 0.4 | 0.0 | 0.0 | 0.0 | 0.0 | 0.0 | 0.0 | 0.0 |
| D030018L15Rik | 0.0 | 0.0 | 0.0 | 0.0 | 0.0 | 0.0 | 0.0 | 0.0 | 0.0 | 0.0 | 0.0 | 0.0 |
| D030025P21Rik | 0.6 | 0.5 | 0.7 | 0.4 | 1.6 | 0.9 | 3.9 | 1.5 | 2.9 | 6.3 | 2.8 | 2.5 |
| D030028A08Rik | 0.7 | 0.8 | 0.7 | 0.5 | 0.8 | 0.3 | 0.8 | 0.8 | 0.6 | 1.0 | 0.6 | 0.8 |
| D030040B21Rik | 0.0 | 0.0 | 0.0 | 0.0 | 0.0 | 0.0 | 0.0 | 0.0 | 0.0 | 0.0 | 0.0 | 0.0 |
| D030047H15Rik | 0.1 | 0.0 | 0.1 | 0.0 | 0.1 | 0.1 | 0.1 | 0.1 | 0.0 | 0.0 | 0.0 | 0.1 |
| D030056L22Rik | 8.8 | 9.7 | 8.9 | 5.3 | 9.9 | 7.9 | 6.7 | 6.2 | 9.3 | 11.6 | 9.1 | 7.7 |
| D10Bwg1379e | 0.2 | 0.1 | 0.1 | 0.3 | 0.3 | 0.2 | 0.1 | 0.1 | 0.0 | 0.1 | 0.1 | 0.1 |
| D10Jhu81e | 22.5 | 20.7 | 21.4 | 19.4 | 18.1 | 20.6 | 17.8 | 22.8 | 18.9 | 20.9 | 17.6 | 22.4 |

| | | | | | | | | | | | | |
|---|---|---|---|---|---|---|---|---|---|---|---|---|
| D10Wsu102e | 6.4 | 5.6 | 5.0 | 5.9 | 6.4 | 5.6 | 7.0 | 6.2 | 5.5 | 6.6 | 6.3 | 6.2 |
| D10Wsu52e | 67.1 | 56.0 | 51.1 | 70.5 | 57.3 | 64.1 | 56.7 | 57.6 | 53.6 | 63.2 | 61.3 | 59.7 |
| D11Wsu47e | 3.6 | 3.6 | 3.5 | 3.9 | 3.7 | 2.8 | 3.5 | 4.5 | 4.7 | 4.1 | 3.9 | 3.7 |
| D130009I18Rik | 0.0 | 0.0 | 0.0 | 0.0 | 0.0 | 0.0 | 0.0 | 0.0 | 0.0 | 0.0 | 0.0 | 0.0 |
| D130017N08Rik | 0.4 | 0.6 | 0.5 | 0.3 | 0.5 | 0.8 | 0.3 | 0.6 | 0.3 | 0.5 | 0.3 | 0.6 |
| D130020L05Rik | 2.4 | 2.4 | 2.4 | 2.2 | 2.4 | 2.2 | 3.2 | 2.9 | 3.2 | 2.6 | 1.9 | 2.8 |
| D130040H23Rik | 0.5 | 1.1 | 1.5 | 0.6 | 1.0 | 1.1 | 0.6 | 1.0 | 0.8 | 0.8 | 0.8 | 0.9 |
| D130043K22Rik | 0.0 | 0.0 | 0.0 | 0.0 | 0.0 | 0.0 | 0.0 | 0.0 | 0.0 | 0.0 | 0.1 | 0.0 |
| D14Abb1e | 4.6 | 5.7 | 6.8 | 4.7 | 5.7 | 5.0 | 4.7 | 5.8 | 5.4 | 5.9 | 6.6 | 6.8 |
| D15Ertd621e | 33.4 | 34.1 | 29.6 | 33.2 | 35.5 | 34.0 | 33.6 | 30.6 | 33.4 | 35.4 | 36.5 | 38.4 |
| D16Ertd472e | 1.7 | 1.4 | 1.8 | 1.9 | 1.7 | 1.8 | 1.2 | 1.6 | 1.3 | 2.5 | 2.1 | 1.6 |
| D17Ertd648e | 0.6 | 0.5 | 0.4 | 0.4 | 0.7 | 0.4 | 0.7 | 0.5 | 0.3 | 0.5 | 0.2 | 0.3 |
| D17H6S53E | 6.0 | 6.4 | 6.5 | 7.3 | 6.6 | 7.6 | 7.1 | 6.9 | 7.6 | 6.4 | 6.0 | 8.0 |
| D17Wsu104e | 51.3 | 46.9 | 43.5 | 44.2 | 52.0 | 48.8 | 51.1 | 50.6 | 55.3 | 53.4 | 45.4 | 47.6 |
| D17Wsu92e | 29.3 | 26.7 | 22.5 | 36.3 | 26.2 | 34.1 | 24.2 | 19.2 | 20.5 | 21.2 | 21.7 | 20.5 |
| D19Bwg1357e | 19.7 | 15.0 | 14.5 | 23.6 | 16.8 | 18.7 | 18.3 | 17.1 | 13.7 | 14.8 | 18.9 | 17.4 |
| D19Ertd737e | 8.5 | 6.2 | 6.9 | 9.9 | 5.2 | 8.8 | 7.4 | 5.8 | 6.1 | 6.2 | 8.1 | 8.2 |
| D1Ertd622e | 2.5 | 1.8 | 1.6 | 4.7 | 5.4 | 2.4 | 2.3 | 2.1 | 1.7 | 2.8 | 2.5 | 2.5 |
| D1Pas1 | 0.0 | 0.0 | 0.0 | 0.0 | 0.0 | 0.0 | 0.0 | 0.0 | 0.0 | 0.0 | 0.0 | 0.0 |
| D230025D16Rik | 6.4 | 6.9 | 7.7 | 5.8 | 6.6 | 6.2 | 6.5 | 7.0 | 6.6 | 6.7 | 6.0 | 6.7 |
| D2hgdh | 4.2 | 4.1 | 4.5 | 4.5 | 3.9 | 5.0 | 3.8 | 4.8 | 4.0 | 3.4 | 3.0 | 3.6 |
| D2Wsu81e | 9.0 | 8.8 | 10.6 | 10.6 | 8.5 | 10.3 | 10.2 | 9.2 | 9.0 | 10.1 | 10.1 | 8.9 |
| D330022K07Rik | 0.3 | 0.6 | 0.6 | 0.3 | 0.5 | 0.6 | 0.2 | 0.3 | 0.2 | 0.1 | 0.0 | 0.2 |
| D330023K18Rik | 3.3 | 3.1 | 2.9 | 5.2 | 3.8 | 3.7 | 3.2 | 2.4 | 3.3 | 4.6 | 2.7 | 3.2 |
| D330041H03Rik | 3.4 | 2.7 | 2.6 | 2.2 | 2.3 | 2.9 | 2.4 | 2.6 | 2.1 | 2.8 | 2.7 | 2.4 |
| D330045A20Rik | 0.2 | 0.0 | 0.1 | 0.5 | 1.5 | 0.1 | 0.2 | 0.0 | 0.1 | 0.2 | 0.1 | 0.1 |
| D330050G23Rik | 0.4 | 0.4 | 0.2 | 0.2 | 0.6 | 0.4 | 0.2 | 0.3 | 0.1 | 0.3 | 0.2 | 0.2 |
| D330050I16Rik | 3.2 | 2.6 | 3.6 | 2.8 | 2.8 | 2.3 | 3.6 | 2.7 | 3.3 | 2.8 | 2.6 | 3.4 |
| D3Bwg0562e | 0.0 | 0.0 | 0.0 | 0.0 | 0.0 | 0.0 | 0.0 | 0.0 | 0.0 | 0.0 | 0.0 | 0.0 |
| D3Ertd254e | 3.1 | 3.0 | 3.7 | 3.1 | 3.3 | 3.6 | 3.4 | 3.5 | 3.2 | 3.6 | 4.2 | 3.9 |
| D3Ertd751e | 3.8 | 3.5 | 4.8 | 5.4 | 3.4 | 5.8 | 3.9 | 3.0 | 3.3 | 4.5 | 4.8 | 5.4 |
| D430020J02Rik | 0.5 | 0.8 | 1.0 | 0.2 | 0.8 | 0.4 | 1.5 | 0.7 | 0.9 | 0.7 | 0.9 | 0.6 |
| D430041D05Rik | 0.0 | 0.0 | 0.0 | 0.0 | 0.0 | 0.0 | 0.0 | 0.0 | 0.0 | 0.0 | 0.0 | 0.0 |
| D430042O09Rik | 7.3 | 7.9 | 6.9 | 7.5 | 7.1 | 8.2 | 6.1 | 5.6 | 5.3 | 5.7 | 5.7 | 6.0 |
| D4Ertd617e | 0.0 | 0.0 | 0.0 | 0.0 | 0.0 | 0.0 | 0.0 | 0.0 | 0.0 | 0.0 | 0.0 | 0.1 |
| D4Wsu53e | 60.8 | 61.5 | 64.3 | 46.5 | 61.6 | 48.4 | 53.5 | 60.5 | 80.3 | 58.7 | 54.9 | 63.7 |
| D5Ertd579e | 13.3 | 10.3 | 9.2 | 13.4 | 10.8 | 14.2 | 11.4 | 10.7 | 10.7 | 10.9 | 12.4 | 11.0 |
| D5Ertd605e | 3.2 | 2.6 | 1.5 | 2.4 | 2.4 | 2.8 | 3.4 | 1.7 | 1.8 | 1.2 | 1.4 | 2.0 |
| D630003M21Rik | 0.2 | 0.2 | 0.2 | 0.1 | 0.3 | 0.2 | 0.2 | 0.3 | 0.3 | 0.2 | 0.2 | 0.1 |
| D630010B17Rik | 0.4 | 0.5 | 0.8 | 0.3 | 0.8 | 0.2 | 1.0 | 1.4 | 1.6 | 1.5 | 0.9 | 0.7 |
| D630023F18Rik | 0.0 | 0.0 | 0.0 | 0.0 | 0.0 | 0.0 | 0.0 | 0.0 | 0.0 | 0.0 | 0.0 | 0.0 |
| D630029K05Rik | 0.1 | 0.2 | 0.2 | 0.1 | 0.2 | 0.3 | 0.1 | 0.1 | 0.1 | 0.0 | 0.0 | 0.1 |
| D630032N06Rik | 0.5 | 0.5 | 0.6 | 1.5 | 1.0 | 1.0 | 0.4 | 0.7 | 0.9 | 0.2 | 1.1 | 0.7 |
| D630033O11Rik | 0.1 | 0.1 | 0.2 | 0.0 | 0.1 | 0.0 | 0.0 | 0.1 | 0.0 | 0.0 | 0.1 | 0.0 |
| D630037F22Rik | 2.1 | 2.4 | 2.3 | 2.0 | 1.9 | 2.9 | 1.7 | 2.0 | 1.6 | 1.8 | 1.9 | 2.0 |
| D630039A03Rik | 0.0 | 0.0 | 0.0 | 0.0 | 0.0 | 0.1 | 0.1 | 0.0 | 0.0 | 0.1 | 0.0 | 0.0 |
| D630041G03Rik | 0.1 | 0.1 | 0.1 | 0.2 | 0.1 | 0.2 | 0.1 | 0.1 | 0.1 | 0.1 | 0.1 | 0.1 |
| D630045J12Rik | 0.4 | 0.6 | 0.8 | 0.4 | 0.4 | 0.8 | 0.5 | 0.4 | 0.4 | 0.3 | 0.3 | 0.3 |
| D630045M09Rik | 1.2 | 0.6 | 0.5 | 1.3 | 0.5 | 0.7 | 1.0 | 0.4 | 0.5 | 0.6 | 1.4 | 0.3 |
| D6Ertd527e | 0.0 | 0.0 | 0.1 | 0.1 | 0.0 | 0.0 | 0.0 | 0.1 | 0.0 | 0.0 | 0.1 | 0.1 |
| D6Wsu163e | 11.6 | 11.5 | 9.7 | 14.1 | 11.0 | 11.9 | 8.7 | 8.4 | 8.9 | 9.9 | 10.3 | 11.7 |
| D730001G18Rik | 0.1 | 0.0 | 0.1 | 0.0 | 0.0 | 0.0 | 0.0 | 0.0 | 0.0 | 0.0 | 0.0 | 0.0 |
| D730005E14Rik | 0.1 | 0.1 | 0.3 | 0.0 | 0.2 | 0.1 | 0.2 | 0.3 | 0.2 | 0.1 | 0.2 | 0.3 |
| D730039F16Rik | 0.4 | 0.3 | 0.1 | 0.1 | 0.1 | 0.2 | 0.0 | 0.0 | 0.1 | 0.1 | 0.1 | 0.1 |
| D7Ertd443e | 0.0 | 0.1 | 0.0 | 0.1 | 0.0 | 0.0 | 0.0 | 0.1 | 0.1 | 0.0 | 0.1 | 0.0 |
| D7Ertd715e | 0.4 | 0.6 | 0.8 | 0.7 | 0.5 | 0.7 | 0.7 | 0.4 | 0.7 | 0.4 | 1.0 | 1.0 |
| D830005E20Rik | 0.0 | 0.0 | 0.0 | 0.0 | 0.0 | 0.0 | 0.0 | 0.0 | 0.0 | 0.0 | 0.0 | 0.0 |

| | | | | | | | | | | | | |
|---|---|---|---|---|---|---|---|---|---|---|---|---|
| D830030K20Rik | 0.1 | 0.1 | 0.2 | 0.1 | 0.2 | 0.2 | 0.1 | 0.1 | 0.1 | 0.1 | 0.1 | 0.0 |
| D830031N03Rik | 15.4 | 9.3 | 6.5 | 8.1 | 5.9 | 9.2 | 8.3 | 7.6 | 7.9 | 11.8 | 13.6 | 9.6 |
| D830046C22Rik | 0.0 | 0.0 | 0.0 | 0.0 | 0.1 | 0.0 | 0.1 | 0.0 | 0.0 | 0.0 | 0.0 | 0.0 |
| D8Ertd738e | 87.4 | 76.5 | 64.8 | 95.5 | 80.5 | 86.4 | 75.9 | 93.4 | 79.9 | 86.8 | 84.9 | 79.6 |
| D8Ertd82e | 1.1 | 1.3 | 2.2 | 1.3 | 2.6 | 0.6 | 2.6 | 2.7 | 3.8 | 1.7 | 1.9 | 2.0 |
| D930015E06Rik | 1.5 | 2.3 | 2.2 | 1.4 | 2.1 | 1.4 | 1.3 | 1.9 | 1.4 | 1.3 | 1.3 | 1.0 |
| D930015M05Rik | 0.3 | 0.4 | 0.3 | 0.2 | 0.2 | 0.2 | 0.3 | 0.1 | 0.2 | 0.3 | 0.3 | 0.2 |
| D930016D06Rik | 0.8 | 0.9 | 0.9 | 0.8 | 0.7 | 0.6 | 0.8 | 0.9 | 0.7 | 0.7 | 0.9 | 0.9 |
| D930048N14Rik | 0.1 | 0.1 | 0.1 | 0.0 | 0.2 | 0.3 | 0.2 | 0.1 | 0.1 | 0.0 | 0.0 | 0.1 |
| Daam1 | 16.6 | 16.7 | 18.3 | 15.6 | 18.3 | 12.5 | 16.7 | 16.4 | 13.7 | 13.8 | 16.9 | 16.6 |
| Daam2 | 0.2 | 0.3 | 0.3 | 0.2 | 0.3 | 0.4 | 0.3 | 0.3 | 0.4 | 1.2 | 0.8 | 0.6 |
| Dab1 | 0.1 | 0.0 | 0.0 | 0.1 | 0.0 | 0.0 | 0.1 | 0.0 | 0.0 | 0.0 | 0.0 | 0.0 |
| Dab2 | 9.0 | 8.5 | 10.8 | 12.2 | 14.0 | 6.7 | 7.2 | 8.4 | 5.7 | 10.5 | 8.7 | 8.0 |
| Dab2ip | 12.6 | 14.9 | 13.5 | 12.9 | 13.3 | 14.9 | 15.2 | 15.3 | 14.7 | 11.5 | 13.3 | 12.2 |
| Dach1 | 0.1 | 0.0 | 0.0 | 0.0 | 0.0 | 0.0 | 0.0 | 0.0 | 0.0 | 0.0 | 0.0 | 0.0 |
| Dach2 | 0.2 | 0.0 | 0.0 | 0.1 | 0.0 | 0.0 | 0.0 | 0.0 | 0.0 | 0.0 | 0.0 | 0.0 |
| Dact1 | 4.0 | 8.2 | 10.7 | 3.1 | 5.2 | 2.4 | 8.1 | 21.3 | 14.7 | 5.9 | 6.6 | 9.7 |
| Dact2 | 0.1 | 0.1 | 0.1 | 0.1 | 0.1 | 0.2 | 0.1 | 0.1 | 0.1 | 0.2 | 0.1 | 0.1 |
| Dact3 | 12.8 | 17.5 | 26.1 | 9.7 | 12.4 | 10.1 | 18.8 | 26.1 | 23.4 | 14.4 | 13.7 | 17.3 |
| Dad1 | 133.9 | 113.8 | 108.6 | 176.9 | 118.3 | 136.9 | 139.1 | 112.0 | 107.8 | 123.8 | 136.8 | 118.1 |
| Daf2 | 0.4 | 0.7 | 0.8 | 0.3 | 0.4 | 0.3 | 0.5 | 1.1 | 0.3 | 0.3 | 0.5 | 0.5 |
| Dag1 | 26.2 | 25.7 | 29.1 | 27.7 | 25.8 | 30.7 | 35.5 | 36.5 | 38.0 | 36.6 | 34.5 | 32.8 |
| Dagla | 0.7 | 0.7 | 0.7 | 0.7 | 0.8 | 0.8 | 0.8 | 0.8 | 1.0 | 0.5 | 0.6 | 0.6 |
| Daglb | 9.9 | 8.3 | 7.9 | 10.5 | 9.7 | 10.9 | 8.6 | 10.6 | 7.5 | 6.7 | 7.5 | 7.3 |
| Dak | 5.3 | 5.5 | 4.4 | 3.7 | 4.6 | 5.9 | 4.8 | 5.0 | 5.8 | 11.8 | 6.4 | 5.2 |
| Dalrd3 | 6.9 | 6.3 | 5.0 | 7.7 | 6.4 | 7.0 | 5.8 | 6.4 | 7.0 | 8.1 | 5.8 | 5.3 |
| Dancr | 1.3 | 1.5 | 1.0 | 2.2 | 1.6 | 0.7 | 1.3 | 1.4 | 1.8 | 1.7 | 1.5 | 1.5 |
| Dand5 | 2.3 | 1.9 | 2.1 | 2.8 | 2.2 | 2.4 | 2.1 | 2.5 | 2.0 | 2.1 | 2.6 | 1.9 |
| Dap | 91.9 | 91.8 | 75.5 | 114.1 | 87.3 | 116.5 | 95.4 | 84.8 | 70.3 | 68.4 | 91.1 | 77.8 |
| Dap3 | 8.5 | 8.2 | 6.8 | 7.4 | 8.0 | 7.9 | 7.7 | 8.5 | 7.3 | 7.9 | 8.0 | 7.7 |
| Dapk1 | 7.2 | 10.1 | 17.0 | 5.0 | 8.0 | 4.4 | 5.9 | 9.7 | 7.4 | 7.9 | 6.5 | 8.6 |
| Dapk2 | 1.5 | 0.4 | 0.5 | 0.6 | 0.5 | 0.2 | 0.4 | 0.9 | 0.7 | 0.3 | 0.2 | 0.2 |
| Dapk3 | 15.4 | 13.7 | 11.2 | 13.0 | 12.3 | 12.7 | 13.2 | 14.1 | 13.2 | 15.2 | 13.4 | 13.5 |
| Dapp1 | 3.3 | 3.7 | 4.2 | 3.1 | 3.8 | 3.3 | 3.4 | 4.7 | 3.8 | 3.5 | 3.7 | 4.7 |
| Dars | 52.5 | 52.1 | 41.9 | 51.9 | 47.7 | 50.1 | 39.6 | 41.8 | 42.0 | 51.0 | 51.2 | 48.7 |
| Dars2 | 5.2 | 5.8 | 5.6 | 3.9 | 4.4 | 4.9 | 4.3 | 4.6 | 5.1 | 4.5 | 5.0 | 4.6 |
| Daxx | 13.2 | 14.4 | 13.3 | 15.2 | 12.7 | 11.3 | 13.3 | 12.4 | 12.7 | 10.4 | 12.4 | 13.5 |
| Dazap1 | 11.5 | 12.9 | 15.9 | 12.6 | 14.2 | 12.1 | 15.6 | 16.1 | 15.2 | 14.8 | 14.9 | 13.3 |
| Dazap2 | 44.0 | 43.2 | 43.9 | 52.0 | 50.2 | 43.6 | 41.2 | 47.6 | 44.8 | 45.1 | 42.3 | 44.1 |
| Dazl | 0.0 | 0.0 | 0.0 | 0.0 | 0.0 | 0.0 | 0.0 | 0.1 | 0.0 | 0.0 | 0.0 | 0.0 |
| Dbc1 | 0.5 | 0.4 | 0.4 | 0.2 | 0.3 | 0.2 | 0.5 | 0.6 | 0.3 | 0.7 | 0.8 | 0.7 |
| Dbf4 | 6.5 | 8.3 | 9.3 | 5.5 | 8.6 | 5.6 | 9.5 | 5.6 | 9.1 | 6.1 | 9.2 | 7.2 |
| Dbh | 0.0 | 0.0 | 0.0 | 0.0 | 0.0 | 0.0 | 0.0 | 0.0 | 0.0 | 0.0 | 0.0 | 0.0 |
| Dbi | 375.9 | 459.0 | 423.2 | 355.8 | 306.7 | 323.0 | 454.8 | 565.6 | 438.4 | 284.5 | 498.4 | 379.9 |
| Dbil5 | 0.0 | 0.0 | 0.3 | 0.0 | 0.3 | 0.4 | 0.1 | 0.3 | 0.2 | 0.1 | 0.0 | 0.1 |
| Dbn1 | 62.5 | 55.5 | 43.4 | 75.1 | 53.4 | 64.9 | 65.1 | 52.4 | 56.7 | 55.3 | 61.2 | 50.0 |
| Dbndd1 | 0.8 | 0.6 | 0.7 | 0.6 | 0.5 | 1.1 | 0.4 | 1.2 | 0.4 | 0.9 | 0.4 | 0.5 |
| Dbndd2 | 6.2 | 6.5 | 5.7 | 3.8 | 5.0 | 2.8 | 4.5 | 6.8 | 6.4 | 7.3 | 6.9 | 6.1 |
| Dbnl | 27.1 | 24.9 | 25.4 | 29.6 | 27.0 | 25.1 | 28.8 | 27.1 | 27.2 | 27.9 | 27.5 | 30.5 |
| Dbp | 16.2 | 17.3 | 20.9 | 13.5 | 9.2 | 23.0 | 9.9 | 14.9 | 8.5 | 11.4 | 9.2 | 12.6 |
| Dbr1 | 6.0 | 5.2 | 6.7 | 5.4 | 5.6 | 5.8 | 6.4 | 5.3 | 5.5 | 6.0 | 6.0 | 5.9 |
| Dbt | 8.7 | 8.2 | 9.4 | 8.6 | 7.4 | 8.8 | 7.0 | 6.5 | 6.2 | 7.2 | 8.2 | 7.9 |
| Dcaf10 | 4.6 | 5.4 | 4.6 | 4.4 | 4.2 | 4.5 | 4.2 | 4.7 | 5.2 | 5.0 | 4.9 | 4.9 |
| Dcaf11 | 13.7 | 13.8 | 12.0 | 14.0 | 13.9 | 12.7 | 12.0 | 10.9 | 12.3 | 11.1 | 11.8 | 11.8 |
| Dcaf12 | 13.1 | 14.0 | 13.2 | 13.1 | 15.3 | 13.3 | 13.3 | 12.6 | 14.6 | 14.1 | 14.2 | 12.7 |
| Dcaf12l1 | 0.1 | 0.1 | 0.1 | 0.1 | 0.0 | 0.1 | 0.1 | 0.0 | 0.1 | 1.8 | 0.8 | 0.4 |
| Dcaf12l2 | 0.0 | 0.0 | 0.0 | 0.0 | 0.0 | 0.0 | 0.0 | 0.0 | 0.1 | 2.1 | 0.6 | 0.3 |

| | | | | | | | | | | | | |
|---|---|---|---|---|---|---|---|---|---|---|---|---|
| Dcaf13 | 30.6 | 26.8 | 23.4 | 31.4 | 24.3 | 24.1 | 26.7 | 25.4 | 23.9 | 27.1 | 28.8 | 28.9 |
| Dcaf15 | 3.4 | 4.1 | 4.0 | 3.2 | 3.9 | 2.9 | 5.0 | 4.1 | 5.1 | 3.4 | 3.4 | 2.9 |
| Dcaf17 | 3.5 | 3.6 | 3.8 | 2.7 | 2.9 | 2.5 | 2.8 | 3.9 | 3.1 | 2.9 | 2.8 | 2.8 |
| Dcaf4 | 11.2 | 11.7 | 11.1 | 10.1 | 9.1 | 10.9 | 9.7 | 9.0 | 9.5 | 11.1 | 10.6 | 9.9 |
| Dcaf5 | 12.5 | 12.8 | 11.5 | 14.7 | 12.1 | 13.9 | 12.7 | 9.8 | 10.8 | 10.6 | 10.8 | 12.6 |
| Dcaf6 | 21.7 | 19.8 | 21.0 | 20.0 | 18.5 | 22.2 | 17.8 | 20.5 | 18.3 | 17.7 | 22.1 | 19.8 |
| Dcaf7 | 19.6 | 20.9 | 19.6 | 19.0 | 20.3 | 19.6 | 19.5 | 20.3 | 20.6 | 21.7 | 18.8 | 18.3 |
| Dcaf8 | 36.4 | 37.5 | 41.5 | 38.8 | 29.1 | 37.6 | 32.9 | 36.6 | 33.1 | 34.5 | 36.1 | 38.7 |
| Dcakd | 6.4 | 7.8 | 8.4 | 8.8 | 8.4 | 7.3 | 7.9 | 9.5 | 7.2 | 7.2 | 7.3 | 6.4 |
| Dcbld1 | 1.4 | 1.5 | 1.7 | 1.2 | 1.4 | 1.2 | 1.5 | 1.4 | 1.4 | 1.3 | 1.0 | 1.5 |
| Dcbld2 | 53.3 | 53.0 | 43.7 | 43.5 | 43.6 | 44.3 | 56.5 | 40.6 | 57.6 | 47.5 | 52.5 | 52.6 |
| Dcc | 0.0 | 0.0 | 0.0 | 0.0 | 0.0 | 0.0 | 0.0 | 0.0 | 0.0 | 0.0 | 0.0 | 0.0 |
| Dcdc2a | 0.0 | 0.0 | 0.0 | 0.0 | 0.0 | 0.0 | 0.0 | 0.0 | 0.0 | 0.0 | 0.0 | 0.0 |
| Dcdc2b | 0.2 | 0.4 | 0.2 | 0.2 | 0.1 | 0.5 | 0.0 | 0.1 | 0.0 | 0.1 | 0.3 | 0.2 |
| Dcdc2c | 5.7 | 7.0 | 5.1 | 4.6 | 6.8 | 4.6 | 5.9 | 4.7 | 4.9 | 5.1 | 4.1 | 4.4 |
| Dchs1 | 0.1 | 0.1 | 0.1 | 0.1 | 0.0 | 0.0 | 0.0 | 0.1 | 0.1 | 0.0 | 0.0 | 0.0 |
| Dck | 2.3 | 2.3 | 2.9 | 2.2 | 5.2 | 1.6 | 4.0 | 2.4 | 2.8 | 1.7 | 2.9 | 1.8 |
| Dclk1 | 12.0 | 16.9 | 35.7 | 15.0 | 33.6 | 10.8 | 34.0 | 54.1 | 42.6 | 17.5 | 22.8 | 35.8 |
| Dclk2 | 0.9 | 0.6 | 1.0 | 0.9 | 0.6 | 0.9 | 0.5 | 0.6 | 0.5 | 0.5 | 0.6 | 0.5 |
| Dclk3 | 1.9 | 2.4 | 6.2 | 1.0 | 1.4 | 0.6 | 0.5 | 3.0 | 1.7 | 0.9 | 0.7 | 2.1 |
| Dclre1a | 5.9 | 5.3 | 5.3 | 6.4 | 4.0 | 6.7 | 4.2 | 4.6 | 3.7 | 3.2 | 3.8 | 3.6 |
| Dclre1b | 1.1 | 1.6 | 1.6 | 1.6 | 1.6 | 1.0 | 1.3 | 1.2 | 1.3 | 1.2 | 1.8 | 1.5 |
| Dclre1c | 1.7 | 1.6 | 1.8 | 1.8 | 2.1 | 2.0 | 1.8 | 1.7 | 1.8 | 1.3 | 1.5 | 1.4 |
| Dcn | 442.4 | 536.9 | 758.9 | 374.0 | 411.1 | 220.9 | 245.8 | 524.1 | 341.8 | 355.9 | 244.3 | 418.5 |
| Dcp1a | 3.1 | 3.9 | 3.8 | 3.2 | 3.7 | 3.1 | 3.7 | 3.9 | 3.9 | 3.3 | 3.9 | 3.5 |
| Dcp1b | 0.8 | 0.6 | 0.8 | 1.0 | 0.7 | 1.1 | 0.7 | 0.7 | 0.5 | 0.9 | 0.8 | 0.5 |
| Dcp2 | 5.1 | 6.1 | 6.0 | 4.7 | 5.9 | 4.9 | 4.5 | 4.7 | 5.2 | 5.3 | 5.2 | 5.4 |
| Dcps | 5.8 | 5.7 | 6.5 | 6.4 | 6.7 | 6.3 | 8.6 | 8.2 | 7.3 | 5.6 | 6.0 | 6.2 |
| Dcst1 | 0.0 | 0.1 | 0.1 | 0.0 | 0.0 | 0.0 | 0.1 | 0.1 | 0.1 | 0.1 | 0.0 | 0.0 |
| Dct | 0.0 | 0.0 | 0.0 | 0.0 | 0.0 | 0.0 | 0.0 | 0.0 | 0.0 | 0.0 | 0.0 | 0.0 |
| Dctd | 4.1 | 3.4 | 2.8 | 4.2 | 3.1 | 3.8 | 2.9 | 3.3 | 2.1 | 3.8 | 3.4 | 2.6 |
| Dctn1 | 28.5 | 29.7 | 29.5 | 30.6 | 30.1 | 27.9 | 32.2 | 28.0 | 30.0 | 27.7 | 29.7 | 28.5 |
| Dctn2 | 82.7 | 77.1 | 69.7 | 76.5 | 68.5 | 85.3 | 77.3 | 80.3 | 84.3 | 72.6 | 76.8 | 76.4 |
| Dctn3 | 49.4 | 48.7 | 42.4 | 54.4 | 45.0 | 47.7 | 40.0 | 45.8 | 43.4 | 45.7 | 42.1 | 43.8 |
| Dctn4 | 23.6 | 24.4 | 26.4 | 24.8 | 23.7 | 25.6 | 26.7 | 28.7 | 27.1 | 26.4 | 28.8 | 27.6 |
| Dctn5 | 34.8 | 30.3 | 29.7 | 40.1 | 30.7 | 41.1 | 30.4 | 28.7 | 28.0 | 27.6 | 32.7 | 30.6 |
| Dctn6 | 42.3 | 43.6 | 48.4 | 62.1 | 44.9 | 50.5 | 54.3 | 47.9 | 45.4 | 52.8 | 64.5 | 60.0 |
| Dctpp1 | 10.5 | 8.5 | 10.2 | 7.0 | 11.0 | 9.7 | 9.2 | 9.6 | 9.6 | 9.1 | 7.7 | 5.7 |
| Dcun1d1 | 8.5 | 8.6 | 10.4 | 10.0 | 8.9 | 11.4 | 8.4 | 9.1 | 8.7 | 11.6 | 12.4 | 12.2 |
| Dcun1d2 | 7.9 | 6.6 | 7.0 | 9.9 | 8.6 | 8.8 | 7.2 | 7.9 | 7.1 | 8.3 | 10.1 | 8.2 |
| Dcun1d3 | 7.6 | 9.0 | 8.3 | 8.0 | 8.0 | 9.1 | 7.9 | 8.2 | 7.8 | 8.1 | 9.8 | 7.2 |
| Dcun1d4 | 6.5 | 7.2 | 6.7 | 5.7 | 5.5 | 5.1 | 5.7 | 6.9 | 6.2 | 5.9 | 6.9 | 5.2 |
| Dcun1d5 | 36.9 | 35.0 | 30.5 | 28.6 | 32.5 | 29.4 | 28.4 | 32.3 | 38.6 | 38.5 | 32.0 | 36.8 |
| Dcxr | 62.7 | 59.1 | 58.5 | 35.7 | 47.3 | 54.1 | 40.0 | 31.3 | 42.9 | 55.9 | 42.9 | 45.7 |
| Dda1 | 27.7 | 26.3 | 23.0 | 30.0 | 24.9 | 28.4 | 31.1 | 28.1 | 27.7 | 25.8 | 28.4 | 26.1 |
| Ddah1 | 27.1 | 25.6 | 33.1 | 22.0 | 28.8 | 22.9 | 50.8 | 46.5 | 43.7 | 52.8 | 57.3 | 57.0 |
| Ddah2 | 20.8 | 17.1 | 15.7 | 21.4 | 13.9 | 24.1 | 21.1 | 24.3 | 29.6 | 18.2 | 19.7 | 17.1 |
| Ddb1 | 264.2 | 235.6 | 210.3 | 235.0 | 221.3 | 245.9 | 218.9 | 217.6 | 213.2 | 253.9 | 240.5 | 234.2 |
| Ddb2 | 2.6 | 2.7 | 3.3 | 4.1 | 3.2 | 3.9 | 3.5 | 3.8 | 3.1 | 2.4 | 3.0 | 3.1 |
| Ddc | 0.4 | 0.2 | 0.1 | 0.2 | 0.1 | 0.2 | 0.0 | 0.1 | 0.1 | 0.7 | 0.5 | 0.1 |
| Ddhd1 | 3.4 | 3.3 | 3.4 | 3.1 | 3.0 | 2.8 | 3.4 | 2.7 | 2.8 | 3.7 | 3.1 | 3.5 |
| Ddhd2 | 11.7 | 10.6 | 9.7 | 12.3 | 9.8 | 11.6 | 9.3 | 9.5 | 8.6 | 9.1 | 9.9 | 9.6 |
| Ddi1 | 0.1 | 0.2 | 0.1 | 0.0 | 0.0 | 0.1 | 0.0 | 0.0 | 0.0 | 0.1 | 0.1 | 0.0 |
| Ddi2 | 14.8 | 14.0 | 16.9 | 16.7 | 16.3 | 13.8 | 14.7 | 14.5 | 12.5 | 12.7 | 13.3 | 13.6 |
| Ddit3 | 107.6 | 46.4 | 32.1 | 183.9 | 46.3 | 124.0 | 45.8 | 38.1 | 41.8 | 45.4 | 56.7 | 55.7 |
| Ddit4 | 8.4 | 12.6 | 13.1 | 6.8 | 12.1 | 10.4 | 12.6 | 15.3 | 17.7 | 15.8 | 11.8 | 12.2 |
| Ddit4l | 7.4 | 7.2 | 6.3 | 6.6 | 6.2 | 7.7 | 5.7 | 7.1 | 8.0 | 15.2 | 8.9 | 10.1 |

| | | | | | | | | | | | | |
|---|---|---|---|---|---|---|---|---|---|---|---|---|
| Ddn | 0.0 | 0.0 | 0.0 | 0.0 | 0.0 | 0.1 | 0.0 | 0.0 | 0.0 | 0.0 | 0.0 | 0.0 |
| Ddo | 1.4 | 0.4 | 0.5 | 0.9 | 0.3 | 1.1 | 0.0 | 0.1 | 0.2 | 0.3 | 0.2 | 0.3 |
| Ddost | 113.6 | 96.9 | 101.0 | 124.0 | 98.6 | 123.3 | 106.0 | 107.8 | 97.0 | 104.6 | 101.3 | 107.5 |
| Ddr1 | 42.6 | 47.9 | 41.8 | 34.3 | 37.1 | 35.1 | 46.2 | 39.6 | 47.6 | 48.8 | 38.4 | 38.5 |
| Ddr2 | 54.4 | 47.8 | 45.1 | 56.2 | 39.9 | 58.0 | 36.1 | 38.8 | 34.9 | 36.6 | 43.5 | 32.8 |
| Ddrgk1 | 43.1 | 33.7 | 38.5 | 67.6 | 33.9 | 50.0 | 47.8 | 34.6 | 31.3 | 36.1 | 41.4 | 48.9 |
| Ddt | 17.2 | 18.1 | 18.8 | 16.8 | 14.6 | 19.7 | 15.3 | 18.2 | 16.0 | 16.5 | 13.3 | 14.8 |
| Ddx1 | 79.8 | 73.6 | 69.9 | 79.2 | 63.8 | 77.6 | 65.2 | 64.6 | 56.8 | 63.1 | 71.2 | 69.7 |
| Ddx10 | 8.9 | 8.8 | 9.4 | 9.9 | 8.3 | 7.8 | 9.2 | 8.6 | 7.1 | 8.3 | 9.8 | 9.2 |
| Ddx11 | 0.7 | 1.0 | 1.5 | 0.6 | 1.0 | 0.7 | 1.4 | 1.1 | 1.1 | 0.9 | 0.8 | 0.8 |
| Ddx17 | 61.8 | 72.8 | 70.5 | 53.9 | 62.3 | 63.1 | 57.9 | 64.1 | 69.5 | 61.7 | 61.3 | 63.3 |
| Ddx18 | 18.6 | 19.0 | 16.2 | 18.9 | 18.2 | 19.9 | 17.3 | 16.8 | 14.7 | 18.6 | 18.6 | 16.4 |
| Ddx19a | 6.4 | 6.8 | 7.4 | 7.7 | 6.9 | 6.4 | 5.8 | 6.8 | 5.9 | 6.2 | 6.5 | 5.9 |
| Ddx19b | 2.6 | 2.6 | 2.2 | 2.7 | 2.6 | 2.6 | 2.2 | 2.2 | 2.1 | 2.3 | 2.4 | 2.3 |
| Ddx20 | 4.8 | 5.0 | 5.1 | 4.5 | 4.8 | 4.6 | 4.4 | 3.9 | 3.7 | 4.9 | 4.4 | 4.0 |
| Ddx21 | 23.3 | 21.6 | 19.1 | 26.2 | 23.9 | 19.9 | 27.8 | 23.3 | 20.4 | 22.6 | 26.2 | 23.5 |
| Ddx23 | 32.0 | 29.1 | 28.8 | 37.4 | 28.9 | 30.9 | 32.7 | 27.9 | 26.4 | 27.1 | 32.6 | 32.1 |
| Ddx24 | 49.2 | 39.4 | 38.8 | 52.8 | 42.0 | 49.0 | 43.6 | 36.6 | 34.9 | 39.3 | 41.3 | 47.1 |
| Ddx25 | 0.0 | 0.0 | 0.0 | 0.0 | 0.0 | 0.0 | 0.0 | 0.1 | 0.1 | 0.0 | 0.0 | 0.0 |
| Ddx26b | 0.6 | 0.7 | 0.8 | 0.9 | 1.0 | 0.9 | 0.4 | 0.7 | 0.6 | 0.5 | 0.7 | 0.5 |
| Ddx27 | 12.3 | 11.6 | 11.4 | 16.2 | 11.1 | 13.1 | 14.2 | 11.8 | 10.6 | 10.0 | 13.6 | 12.5 |
| Ddx28 | 5.1 | 5.1 | 3.7 | 4.0 | 5.1 | 4.5 | 4.6 | 5.5 | 4.9 | 4.2 | 4.3 | 5.4 |
| Ddx31 | 4.1 | 3.6 | 3.0 | 4.2 | 4.2 | 4.2 | 3.6 | 3.2 | 3.0 | 3.3 | 3.6 | 2.6 |
| Ddx39 | 14.5 | 9.5 | 7.6 | 11.7 | 12.9 | 9.5 | 13.4 | 10.4 | 10.9 | 11.5 | 13.3 | 9.2 |
| Ddx39b | 49.5 | 46.6 | 49.5 | 43.5 | 45.0 | 47.7 | 51.5 | 53.2 | 48.3 | 49.0 | 47.3 | 43.8 |
| Ddx3x | 63.7 | 69.8 | 78.9 | 73.2 | 70.0 | 66.5 | 63.6 | 67.8 | 63.1 | 66.8 | 72.2 | 75.2 |
| Ddx3y | 33.3 | 27.7 | 31.5 | 41.0 | 29.1 | 36.4 | 26.3 | 26.4 | 21.1 | 29.2 | 33.8 | 37.6 |
| Ddx4 | 0.3 | 0.3 | 0.3 | 0.0 | 0.2 | 0.1 | 0.1 | 0.2 | 0.2 | 0.5 | 0.3 | 0.2 |
| Ddx41 | 7.8 | 8.4 | 7.3 | 8.1 | 9.0 | 9.6 | 8.0 | 7.5 | 7.1 | 9.8 | 8.0 | 8.0 |
| Ddx42 | 20.5 | 20.7 | 22.4 | 22.1 | 19.9 | 19.2 | 21.3 | 21.7 | 20.4 | 19.8 | 23.4 | 24.8 |
| Ddx43 | 0.0 | 0.0 | 0.0 | 0.1 | 0.0 | 0.0 | 0.0 | 0.0 | 0.0 | 0.0 | 0.0 | 0.0 |
| Ddx46 | 11.2 | 9.6 | 10.7 | 14.1 | 11.2 | 10.8 | 12.8 | 10.2 | 9.4 | 9.4 | 11.5 | 11.5 |
| Ddx47 | 18.9 | 19.6 | 19.4 | 20.1 | 19.3 | 20.4 | 17.6 | 19.7 | 18.3 | 21.9 | 19.4 | 19.8 |
| Ddx49 | 17.0 | 15.3 | 12.0 | 15.9 | 15.8 | 17.4 | 16.0 | 15.4 | 15.8 | 15.8 | 16.2 | 14.2 |
| Ddx5 | 148.8 | 151.7 | 163.1 | 144.6 | 146.5 | 137.3 | 146.4 | 152.0 | 163.3 | 166.0 | 164.4 | 177.6 |
| Ddx50 | 49.7 | 49.5 | 46.6 | 47.5 | 38.6 | 46.0 | 43.5 | 40.5 | 40.3 | 46.2 | 50.4 | 51.4 |
| Ddx51 | 1.6 | 1.7 | 1.6 | 1.3 | 1.6 | 1.5 | 1.6 | 1.6 | 1.4 | 1.6 | 1.3 | 1.4 |
| Ddx52 | 12.5 | 11.2 | 11.5 | 9.9 | 10.5 | 11.8 | 8.6 | 9.9 | 9.6 | 10.6 | 9.9 | 10.1 |
| Ddx54 | 13.0 | 13.3 | 12.5 | 14.0 | 14.5 | 14.0 | 14.7 | 14.6 | 13.3 | 13.0 | 14.0 | 12.4 |
| Ddx55 | 2.9 | 2.6 | 3.2 | 3.5 | 2.7 | 3.1 | 2.6 | 2.8 | 2.6 | 2.4 | 3.3 | 2.8 |
| Ddx56 | 9.0 | 7.1 | 5.5 | 7.8 | 7.3 | 9.4 | 7.4 | 6.6 | 6.3 | 6.9 | 6.7 | 6.1 |
| Ddx58 | 19.4 | 12.3 | 16.8 | 21.9 | 16.6 | 15.0 | 14.3 | 11.2 | 12.5 | 12.7 | 12.9 | 20.9 |
| Ddx59 | 3.4 | 3.3 | 2.8 | 2.9 | 2.5 | 3.0 | 2.9 | 3.2 | 2.5 | 3.1 | 3.7 | 3.3 |
| Ddx6 | 17.9 | 23.6 | 26.7 | 18.3 | 23.4 | 21.0 | 20.2 | 24.6 | 21.0 | 24.5 | 25.3 | 26.3 |
| Ddx60 | 0.8 | 0.5 | 0.8 | 0.6 | 0.7 | 0.4 | 0.4 | 0.4 | 0.5 | 0.2 | 0.4 | 0.6 |
| Deaf1 | 2.9 | 3.1 | 2.8 | 2.6 | 2.4 | 2.4 | 3.2 | 2.8 | 2.4 | 3.3 | 2.8 | 2.5 |
| Dear1 | 0.9 | 0.1 | 0.1 | 0.3 | 0.4 | 0.4 | 0.5 | 0.2 | 0.2 | 0.1 | 0.4 | 0.6 |
| Deb1 | 19.3 | 16.1 | 15.2 | 15.4 | 16.0 | 17.4 | 14.4 | 21.6 | 14.5 | 16.5 | 24.2 | 17.8 |
| Decr1 | 21.7 | 19.1 | 16.3 | 17.4 | 15.8 | 17.6 | 16.7 | 17.6 | 18.0 | 19.8 | 17.4 | 19.7 |
| Decr2 | 20.3 | 19.3 | 16.8 | 16.5 | 16.5 | 19.6 | 17.0 | 18.5 | 19.7 | 17.8 | 18.2 | 17.5 |
| Dedd | 7.2 | 7.4 | 6.5 | 6.4 | 6.8 | 7.0 | 7.1 | 7.1 | 6.5 | 8.0 | 8.3 | 8.1 |
| Dedd2 | 2.5 | 2.0 | 1.8 | 3.2 | 2.2 | 2.2 | 1.7 | 1.8 | 1.8 | 2.3 | 2.1 | 1.9 |
| Def6 | 7.0 | 6.4 | 7.4 | 8.2 | 9.5 | 6.0 | 5.1 | 5.5 | 4.8 | 6.5 | 6.1 | 5.0 |
| Def8 | 11.2 | 11.6 | 10.1 | 10.1 | 10.0 | 11.0 | 10.0 | 11.2 | 9.1 | 9.3 | 9.7 | 9.4 |
| Defb25 | 3.1 | 2.8 | 1.4 | 2.9 | 1.9 | 2.9 | 0.9 | 0.2 | 1.1 | 0.8 | 0.7 | 1.3 |
| Degs1 | 144.3 | 93.1 | 76.5 | 156.4 | 126.1 | 130.5 | 106.3 | 91.2 | 91.9 | 114.4 | 116.3 | 106.3 |
| Degs2 | 0.4 | 0.5 | 0.3 | 1.1 | 1.4 | 0.5 | 0.4 | 0.4 | 0.4 | 0.5 | 0.5 | 0.6 |

| | | | | | | | | | | | | |
|---|---|---|---|---|---|---|---|---|---|---|---|---|
| Dek | 11.5 | 14.0 | 22.6 | 11.3 | 16.6 | 9.9 | 18.9 | 16.8 | 15.3 | 15.2 | 20.0 | 19.8 |
| Dennd1a | 3.3 | 3.5 | 3.4 | 3.8 | 3.1 | 4.1 | 3.8 | 3.1 | 2.9 | 3.0 | 2.9 | 3.0 |
| Dennd1b | 2.8 | 3.3 | 3.1 | 3.2 | 3.2 | 3.1 | 2.9 | 2.8 | 2.7 | 3.0 | 2.9 | 3.0 |
| Dennd1c | 0.1 | 0.0 | 0.0 | 0.8 | 1.3 | 0.0 | 0.3 | 0.0 | 0.0 | 0.1 | 0.0 | 0.1 |
| Dennd2a | 10.0 | 10.0 | 8.1 | 8.9 | 8.5 | 10.0 | 9.9 | 9.8 | 9.7 | 7.4 | 9.7 | 7.7 |
| Dennd2c | 2.1 | 2.3 | 2.1 | 1.6 | 2.9 | 2.0 | 1.7 | 1.2 | 1.7 | 2.3 | 2.2 | 1.8 |
| Dennd2d | 0.2 | 0.2 | 0.1 | 0.2 | 0.3 | 0.2 | 0.2 | 0.1 | 0.2 | 0.3 | 0.3 | 0.2 |
| Dennd3 | 0.1 | 0.1 | 0.1 | 0.1 | 0.1 | 0.1 | 0.0 | 0.1 | 0.1 | 0.2 | 0.1 | 0.1 |
| Dennd4a | 6.5 | 7.1 | 7.8 | 7.5 | 5.8 | 6.9 | 6.6 | 8.5 | 7.0 | 4.6 | 6.5 | 7.0 |
| Dennd4b | 0.8 | 0.8 | 0.9 | 1.0 | 1.7 | 0.8 | 0.9 | 1.0 | 1.0 | 0.8 | 0.7 | 0.7 |
| Dennd4c | 9.1 | 9.6 | 9.8 | 10.3 | 9.3 | 10.4 | 8.5 | 8.6 | 8.7 | 9.8 | 9.7 | 9.5 |
| Dennd5a | 77.9 | 60.8 | 49.3 | 93.4 | 57.8 | 84.9 | 59.5 | 40.3 | 49.2 | 51.6 | 56.2 | 54.1 |
| Dennd5b | 1.7 | 2.3 | 2.1 | 1.6 | 1.8 | 1.5 | 2.1 | 1.6 | 2.1 | 2.2 | 2.3 | 2.7 |
| Dennd6a | 4.6 | 5.8 | 6.7 | 5.6 | 5.8 | 4.9 | 5.5 | 5.5 | 5.7 | 5.9 | 6.1 | 6.2 |
| Dennd6b | 0.5 | 0.5 | 0.4 | 0.4 | 0.4 | 0.5 | 0.3 | 0.4 | 0.4 | 0.3 | 0.4 | 0.3 |
| Denr | 17.7 | 16.2 | 17.1 | 19.7 | 15.3 | 15.1 | 20.3 | 19.3 | 14.0 | 16.4 | 19.7 | 20.7 |
| Depdc1a | 0.7 | 1.5 | 3.4 | 0.1 | 2.8 | 0.3 | 2.8 | 1.2 | 3.2 | 1.4 | 3.5 | 2.1 |
| Depdc1b | 0.2 | 0.4 | 0.5 | 0.0 | 0.3 | 0.1 | 0.3 | 0.1 | 0.4 | 0.3 | 0.4 | 0.2 |
| Depdc5 | 3.4 | 4.0 | 4.4 | 2.9 | 2.8 | 3.7 | 2.9 | 3.4 | 3.1 | 2.7 | 3.1 | 3.5 |
| Depdc7 | 2.2 | 2.3 | 2.6 | 2.0 | 2.5 | 0.8 | 1.3 | 1.4 | 1.2 | 1.1 | 1.3 | 1.2 |
| Deptor | 0.6 | 1.2 | 2.6 | 0.6 | 1.1 | 0.3 | 0.8 | 1.7 | 0.9 | 0.8 | 0.4 | 1.0 |
| Dera | 6.8 | 7.6 | 6.2 | 6.1 | 7.7 | 4.8 | 6.8 | 7.8 | 7.2 | 6.2 | 7.2 | 6.9 |
| Derl1 | 72.3 | 67.7 | 54.8 | 66.9 | 62.0 | 72.1 | 61.1 | 68.2 | 71.6 | 70.5 | 66.3 | 69.3 |
| Derl2 | 13.8 | 14.7 | 14.7 | 14.1 | 14.3 | 15.1 | 14.8 | 15.6 | 15.7 | 13.5 | 14.7 | 15.4 |
| Derl3 | 0.6 | 0.8 | 0.6 | 1.0 | 0.3 | 0.8 | 0.3 | 0.3 | 0.2 | 1.4 | 0.9 | 0.7 |
| Des | 1.6 | 0.9 | 0.6 | 1.6 | 1.1 | 1.9 | 0.7 | 1.0 | 2.6 | 36.3 | 18.2 | 10.4 |
| Desi1 | 5.6 | 6.5 | 5.9 | 5.6 | 6.9 | 4.8 | 7.1 | 7.5 | 6.2 | 6.1 | 6.1 | 5.7 |
| Desi2 | 8.5 | 9.6 | 9.0 | 8.8 | 9.0 | 9.3 | 7.9 | 9.6 | 10.4 | 10.1 | 10.8 | 9.7 |
| Det1 | 5.1 | 5.0 | 4.2 | 5.0 | 5.2 | 5.8 | 4.1 | 4.7 | 4.0 | 4.9 | 5.1 | 3.9 |
| Dexi | 6.5 | 7.7 | 6.5 | 6.2 | 6.0 | 7.3 | 5.6 | 6.7 | 6.9 | 6.6 | 5.4 | 6.1 |
| Dffa | 3.2 | 4.1 | 3.8 | 3.6 | 4.5 | 3.0 | 3.8 | 4.2 | 4.2 | 3.3 | 4.1 | 3.6 |
| Dffb | 1.0 | 0.9 | 0.9 | 0.5 | 1.0 | 0.9 | 0.9 | 0.8 | 0.9 | 0.8 | 0.8 | 1.0 |
| Dfna5 | 1.4 | 1.6 | 1.4 | 2.3 | 1.3 | 2.3 | 0.8 | 1.2 | 0.9 | 1.1 | 1.2 | 1.1 |
| Dfnb59 | 0.0 | 0.1 | 0.1 | 0.0 | 0.1 | 0.0 | 0.1 | 0.0 | 0.0 | 0.0 | 0.0 | 0.1 |
| Dgat1 | 7.0 | 6.5 | 5.8 | 7.2 | 5.9 | 7.7 | 6.2 | 6.4 | 7.4 | 5.8 | 5.8 | 6.3 |
| Dgat2 | 1.8 | 2.2 | 2.7 | 1.8 | 1.8 | 1.9 | 2.3 | 1.7 | 1.9 | 2.0 | 1.6 | 1.4 |
| Dgcr14 | 4.8 | 5.3 | 5.5 | 5.1 | 5.2 | 5.7 | 5.3 | 5.3 | 4.2 | 4.3 | 4.7 | 4.3 |
| Dgcr2 | 14.2 | 16.0 | 16.0 | 12.9 | 15.9 | 14.7 | 19.7 | 23.0 | 19.0 | 14.7 | 15.5 | 13.2 |
| Dgcr6 | 10.1 | 10.2 | 8.6 | 10.0 | 7.3 | 10.1 | 8.2 | 10.4 | 8.8 | 8.0 | 9.5 | 9.1 |
| Dgcr8 | 7.2 | 6.6 | 6.1 | 6.8 | 7.0 | 5.9 | 9.3 | 7.6 | 7.8 | 7.6 | 8.5 | 8.0 |
| Dgka | 3.3 | 4.1 | 4.3 | 3.0 | 2.7 | 3.3 | 3.1 | 4.0 | 3.8 | 3.2 | 3.0 | 3.3 |
| Dgkd | 6.7 | 5.5 | 4.9 | 8.9 | 13.2 | 5.2 | 6.4 | 6.6 | 7.6 | 7.8 | 7.3 | 6.9 |
| Dgke | 2.0 | 1.9 | 1.5 | 2.0 | 1.6 | 2.1 | 1.7 | 1.5 | 1.5 | 1.6 | 1.4 | 1.5 |
| Dgkg | 0.3 | 0.1 | 0.0 | 0.2 | 0.3 | 0.4 | 0.0 | 0.0 | 0.0 | 0.1 | 0.1 | 0.1 |
| Dgkh | 1.9 | 1.8 | 1.3 | 2.3 | 3.2 | 1.8 | 1.7 | 1.4 | 1.2 | 1.7 | 1.7 | 1.4 |
| Dgki | 0.0 | 0.0 | 0.0 | 0.1 | 0.0 | 0.1 | 0.0 | 0.0 | 0.0 | 0.0 | 0.0 | 0.0 |
| Dgkq | 5.1 | 5.1 | 4.2 | 4.8 | 4.5 | 5.1 | 5.0 | 3.9 | 5.2 | 4.3 | 4.2 | 4.6 |
| Dgkz | 5.9 | 5.9 | 5.2 | 8.2 | 10.3 | 5.5 | 6.9 | 5.4 | 5.6 | 6.6 | 5.7 | 5.0 |
| Dguok | 21.5 | 22.7 | 18.6 | 19.5 | 18.8 | 22.9 | 17.8 | 17.9 | 17.8 | 17.8 | 19.3 | 17.9 |
| Dhcr24 | 9.8 | 9.1 | 8.4 | 4.0 | 7.7 | 5.2 | 22.6 | 24.1 | 19.6 | 6.6 | 17.5 | 8.3 |
| Dhcr7 | 2.5 | 4.5 | 4.2 | 2.2 | 3.0 | 2.6 | 4.5 | 6.0 | 4.6 | 1.9 | 4.5 | 1.5 |
| Dhdds | 8.9 | 9.4 | 8.7 | 10.7 | 8.9 | 10.2 | 8.6 | 10.1 | 7.9 | 7.8 | 8.4 | 7.3 |
| Dhdh | 5.7 | 5.7 | 5.7 | 8.4 | 4.6 | 7.4 | 3.5 | 2.4 | 1.8 | 1.8 | 2.3 | 3.0 |
| Dhfr | 1.2 | 1.3 | 1.9 | 0.7 | 1.6 | 1.0 | 2.1 | 1.1 | 1.4 | 1.1 | 1.6 | 1.4 |
| Dhh | 0.0 | 0.1 | 0.1 | 0.0 | 0.0 | 0.0 | 0.1 | 0.0 | 0.1 | 0.0 | 0.0 | 0.0 |
| Dhodh | 2.4 | 2.0 | 2.1 | 1.7 | 2.2 | 2.3 | 2.1 | 2.4 | 2.2 | 2.4 | 2.4 | 1.8 |
| Dhps | 13.9 | 12.5 | 9.1 | 12.8 | 9.8 | 14.4 | 11.2 | 10.1 | 11.6 | 10.6 | 11.3 | 12.7 |

| | | | | | | | | | | | | |
|---|---|---|---|---|---|---|---|---|---|---|---|---|
| Dhrs1 | 27.6 | 26.1 | 27.5 | 33.1 | 27.5 | 28.6 | 27.3 | 30.7 | 25.6 | 25.5 | 27.5 | 25.8 |
| Dhrs11 | 2.3 | 2.6 | 2.2 | 2.4 | 2.8 | 2.2 | 2.5 | 2.9 | 2.3 | 2.5 | 1.9 | 1.8 |
| Dhrs13 | 1.9 | 1.5 | 2.0 | 0.8 | 1.2 | 1.8 | 1.9 | 2.0 | 2.0 | 2.4 | 1.1 | 1.5 |
| Dhrs3 | 12.5 | 15.5 | 19.2 | 24.8 | 31.1 | 11.3 | 31.0 | 32.2 | 53.0 | 21.4 | 30.1 | 41.8 |
| Dhrs4 | 11.9 | 12.0 | 13.4 | 13.8 | 12.5 | 10.6 | 14.4 | 15.3 | 13.9 | 17.0 | 17.4 | 16.7 |
| Dhrs7 | 15.1 | 15.8 | 17.9 | 16.6 | 13.8 | 16.2 | 17.2 | 19.8 | 17.6 | 11.9 | 12.7 | 16.4 |
| Dhrs7b | 10.7 | 9.5 | 7.6 | 10.1 | 9.1 | 9.7 | 10.1 | 9.5 | 9.9 | 9.0 | 9.8 | 9.3 |
| Dhrs9 | 0.6 | 0.1 | 0.1 | 0.6 | 0.5 | 0.2 | 0.2 | 0.1 | 0.1 | 0.7 | 0.9 | 0.2 |
| Dhtkd1 | 1.5 | 1.3 | 1.1 | 1.4 | 1.1 | 1.3 | 1.2 | 1.0 | 0.8 | 1.0 | 0.9 | 1.0 |
| Dhx15 | 47.3 | 42.3 | 44.8 | 47.8 | 48.3 | 40.1 | 48.0 | 46.2 | 45.7 | 48.0 | 53.3 | 52.7 |
| Dhx16 | 11.6 | 11.4 | 13.0 | 11.4 | 11.5 | 11.9 | 10.9 | 11.7 | 10.8 | 11.8 | 10.4 | 11.1 |
| Dhx29 | 13.8 | 13.0 | 12.7 | 13.8 | 12.1 | 13.8 | 12.0 | 10.6 | 10.5 | 11.4 | 12.1 | 11.8 |
| Dhx30 | 12.7 | 11.9 | 10.4 | 12.6 | 11.6 | 12.5 | 11.2 | 10.9 | 11.1 | 11.7 | 11.8 | 10.8 |
| Dhx32 | 15.0 | 12.9 | 11.2 | 13.0 | 11.8 | 12.2 | 12.1 | 11.3 | 11.1 | 11.9 | 11.7 | 10.5 |
| Dhx33 | 9.1 | 11.3 | 14.3 | 8.9 | 10.1 | 9.1 | 10.7 | 11.9 | 12.6 | 10.4 | 9.9 | 10.4 |
| Dhx34 | 2.1 | 2.2 | 2.3 | 2.0 | 2.0 | 2.4 | 2.0 | 2.9 | 2.2 | 2.5 | 2.0 | 2.3 |
| Dhx35 | 3.2 | 3.2 | 3.6 | 2.4 | 3.2 | 3.6 | 2.7 | 2.9 | 3.4 | 3.3 | 2.6 | 3.1 |
| Dhx36 | 14.3 | 15.6 | 14.1 | 13.9 | 13.9 | 14.7 | 13.1 | 14.3 | 14.3 | 15.4 | 17.3 | 15.4 |
| Dhx37 | 4.0 | 4.2 | 3.2 | 3.9 | 4.2 | 3.6 | 3.9 | 3.2 | 2.9 | 3.0 | 3.0 | 2.7 |
| Dhx38 | 8.2 | 7.9 | 9.9 | 10.6 | 10.4 | 8.9 | 10.8 | 10.2 | 8.6 | 9.8 | 9.6 | 10.6 |
| Dhx40 | 15.4 | 15.1 | 15.7 | 14.4 | 15.1 | 16.5 | 15.3 | 16.4 | 15.3 | 19.4 | 19.0 | 18.4 |
| Dhx57 | 8.5 | 7.6 | 7.5 | 9.6 | 7.5 | 10.0 | 7.5 | 7.1 | 5.9 | 7.0 | 7.6 | 7.6 |
| Dhx58 | 3.5 | 2.2 | 3.3 | 4.2 | 3.1 | 2.7 | 2.1 | 2.1 | 1.8 | 1.1 | 1.5 | 3.9 |
| Dhx8 | 11.3 | 9.8 | 9.8 | 11.2 | 10.5 | 10.6 | 8.7 | 9.6 | 7.5 | 9.0 | 10.5 | 9.5 |
| Dhx9 | 8.5 | 11.1 | 10.6 | 9.2 | 13.4 | 7.9 | 11.4 | 12.4 | 11.5 | 13.8 | 12.7 | 12.8 |
| Diablo | 16.1 | 14.2 | 14.8 | 15.9 | 13.9 | 16.0 | 16.6 | 17.1 | 14.4 | 14.6 | 15.2 | 16.1 |
| Diap1 | 5.0 | 4.8 | 4.2 | 7.4 | 8.2 | 4.8 | 6.1 | 4.1 | 4.4 | 5.2 | 4.9 | 4.4 |
| Diap2 | 1.8 | 2.0 | 2.2 | 1.4 | 2.3 | 1.4 | 1.8 | 1.6 | 1.6 | 1.8 | 1.7 | 1.8 |
| Diap3 | 4.9 | 7.0 | 9.9 | 2.2 | 8.1 | 2.2 | 10.9 | 4.4 | 8.2 | 4.8 | 7.7 | 6.3 |
| Dicer1 | 4.2 | 4.7 | 4.9 | 3.7 | 4.3 | 3.4 | 4.6 | 4.9 | 4.5 | 4.3 | 4.7 | 4.8 |
| Dido1 | 9.3 | 9.8 | 11.0 | 9.6 | 10.0 | 8.7 | 12.1 | 11.4 | 10.4 | 9.0 | 9.9 | 10.7 |
| Diexf | 1.7 | 1.6 | 1.4 | 2.2 | 1.9 | 2.1 | 2.0 | 1.9 | 1.4 | 1.8 | 1.7 | 1.8 |
| Dimt1 | 4.7 | 5.0 | 4.5 | 4.4 | 4.3 | 4.2 | 4.5 | 4.6 | 4.5 | 4.9 | 5.1 | 4.0 |
| Dio2 | 0.1 | 0.1 | 0.2 | 0.1 | 0.1 | 0.1 | 0.1 | 0.1 | 0.0 | 0.1 | 0.1 | 0.0 |
| Dio3 | 1.2 | 3.2 | 6.1 | 1.3 | 6.5 | 0.3 | 17.6 | 11.8 | 20.8 | 8.9 | 8.5 | 7.7 |
| Dio3os | 0.4 | 1.8 | 1.6 | 0.2 | 1.5 | 0.3 | 2.7 | 3.1 | 6.2 | 1.9 | 1.8 | 1.7 |
| Dip2a | 7.7 | 8.4 | 7.5 | 7.2 | 8.3 | 8.1 | 8.4 | 7.8 | 8.6 | 6.6 | 7.5 | 7.6 |
| Dip2b | 5.0 | 5.0 | 5.0 | 4.3 | 5.0 | 4.3 | 4.8 | 4.5 | 4.7 | 4.3 | 4.6 | 4.5 |
| Dip2c | 7.3 | 6.3 | 5.5 | 7.5 | 5.6 | 7.1 | 6.0 | 4.7 | 5.8 | 5.7 | 6.4 | 6.2 |
| Diras1 | 0.0 | 0.0 | 0.0 | 0.0 | 0.0 | 0.0 | 0.0 | 0.1 | 0.0 | 0.0 | 0.0 | 0.0 |
| Diras2 | 0.1 | 0.2 | 0.2 | 0.1 | 0.2 | 0.2 | 0.2 | 0.2 | 0.1 | 0.1 | 0.1 | 0.1 |
| Dirc2 | 12.8 | 11.1 | 10.0 | 12.7 | 10.4 | 13.2 | 12.6 | 10.5 | 13.1 | 12.8 | 12.8 | 12.3 |
| Dis3 | 4.8 | 4.3 | 4.9 | 4.7 | 4.5 | 4.5 | 5.1 | 5.0 | 4.2 | 4.3 | 5.1 | 4.7 |
| Dis3l | 5.4 | 5.3 | 6.0 | 5.8 | 5.9 | 7.0 | 5.1 | 6.4 | 5.3 | 5.4 | 6.3 | 6.3 |
| Dis3l2 | 3.3 | 3.0 | 3.4 | 3.6 | 3.1 | 3.1 | 3.1 | 3.9 | 2.8 | 3.1 | 2.9 | 2.9 |
| Disc1 | 0.1 | 0.2 | 0.2 | 0.3 | 0.5 | 0.1 | 0.5 | 0.1 | 0.4 | 0.1 | 0.2 | 0.1 |
| Disp1 | 14.6 | 16.3 | 12.0 | 13.3 | 12.6 | 19.9 | 16.8 | 14.5 | 14.2 | 15.9 | 18.8 | 13.0 |
| Disp2 | 0.4 | 0.5 | 0.4 | 0.3 | 0.3 | 0.3 | 0.5 | 0.5 | 0.6 | 0.4 | 0.4 | 0.3 |
| Dixdc1 | 1.4 | 2.0 | 3.0 | 1.3 | 1.4 | 2.0 | 1.5 | 2.1 | 1.9 | 1.6 | 1.7 | 2.3 |
| Dkc1 | 5.3 | 5.3 | 5.6 | 6.1 | 5.9 | 4.6 | 5.8 | 6.3 | 5.1 | 5.6 | 7.2 | 4.9 |
| Dkk2 | 0.5 | 0.1 | 0.1 | 0.9 | 0.2 | 0.4 | 0.6 | 0.0 | 0.4 | 0.2 | 0.3 | 0.2 |
| Dkk3 | 25.0 | 21.2 | 19.7 | 25.1 | 17.9 | 17.9 | 15.0 | 7.1 | 10.3 | 25.5 | 21.2 | 22.4 |
| Dkk4 | 0.0 | 0.0 | 0.0 | 0.1 | 0.0 | 0.0 | 0.0 | 0.0 | 0.0 | 0.0 | 0.0 | 0.0 |
| Dkkl1 | 0.1 | 0.0 | 0.2 | 0.1 | 0.1 | 0.1 | 0.0 | 0.0 | 0.2 | 0.1 | 0.0 | 0.0 |
| Dlat | 14.8 | 13.8 | 12.0 | 15.0 | 11.8 | 13.4 | 13.1 | 12.8 | 12.1 | 12.6 | 14.3 | 13.4 |
| Dlc1 | 33.4 | 37.8 | 26.3 | 35.1 | 26.6 | 28.3 | 31.6 | 18.0 | 22.7 | 27.1 | 28.9 | 21.5 |
| Dld | 36.6 | 36.9 | 37.9 | 44.6 | 37.2 | 37.4 | 37.1 | 34.5 | 34.4 | 38.2 | 38.8 | 44.8 |

| | | | | | | | | | | | | |
|---|---|---|---|---|---|---|---|---|---|---|---|---|
| Dlec1 | 0.2 | 0.2 | 0.2 | 0.2 | 0.2 | 0.3 | 0.1 | 0.2 | 0.1 | 0.2 | 0.1 | 0.1 |
| Dleu2 | 1.2 | 1.0 | 1.4 | 1.7 | 2.4 | 0.7 | 1.2 | 1.3 | 1.1 | 1.9 | 1.2 | 1.5 |
| Dlg1 | 62.1 | 63.3 | 58.1 | 65.7 | 51.0 | 66.9 | 69.3 | 71.6 | 76.3 | 66.2 | 87.3 | 78.7 |
| Dlg2 | 0.6 | 0.8 | 2.4 | 0.8 | 0.7 | 0.8 | 1.1 | 2.2 | 1.6 | 1.1 | 1.3 | 2.1 |
| Dlg3 | 15.2 | 18.6 | 19.0 | 12.7 | 12.1 | 10.7 | 18.0 | 12.9 | 17.0 | 27.8 | 25.0 | 24.9 |
| Dlg4 | 9.8 | 12.1 | 12.0 | 9.2 | 10.0 | 10.9 | 12.2 | 10.1 | 10.8 | 10.0 | 10.8 | 10.1 |
| Dlg5 | 6.7 | 8.0 | 7.6 | 8.1 | 7.3 | 9.0 | 9.1 | 6.2 | 5.9 | 6.7 | 6.8 | 6.3 |
| Dlgap1 | 0.0 | 0.0 | 0.1 | 0.0 | 0.0 | 0.0 | 0.0 | 0.0 | 0.0 | 0.0 | 0.0 | 0.0 |
| Dlgap2 | 0.0 | 0.0 | 0.0 | 0.0 | 0.0 | 0.0 | 0.0 | 0.0 | 0.0 | 0.0 | 0.0 | 0.0 |
| Dlgap3 | 0.0 | 0.0 | 0.1 | 0.0 | 0.0 | 0.0 | 0.0 | 0.0 | 0.0 | 0.0 | 0.0 | 0.0 |
| Dlgap4 | 7.0 | 6.4 | 6.5 | 7.9 | 7.0 | 6.8 | 7.8 | 5.9 | 6.7 | 6.8 | 6.7 | 5.7 |
| Dlgap5 | 2.5 | 5.3 | 8.0 | 0.8 | 7.3 | 1.0 | 9.6 | 3.3 | 7.4 | 3.1 | 7.3 | 4.9 |
| Dlk1 | 0.0 | 0.0 | 0.0 | 0.0 | 0.0 | 0.0 | 0.1 | 0.0 | 0.0 | 0.0 | 0.1 | 0.0 |
| Dlk2 | 0.0 | 0.2 | 0.1 | 0.0 | 0.1 | 0.0 | 0.3 | 0.1 | 0.1 | 0.3 | 0.2 | 0.1 |
| Dll1 | 0.1 | 0.1 | 0.0 | 0.0 | 0.1 | 0.0 | 0.0 | 0.1 | 0.1 | 0.0 | 0.0 | 0.0 |
| Dll4 | 0.1 | 0.1 | 0.3 | 0.1 | 0.0 | 0.0 | 0.3 | 0.4 | 0.3 | 0.0 | 0.0 | 0.1 |
| Dlst | 32.5 | 29.9 | 29.3 | 30.0 | 30.7 | 27.6 | 35.1 | 33.3 | 33.3 | 29.5 | 36.8 | 31.7 |
| Dlx2 | 0.3 | 0.2 | 0.1 | 0.2 | 0.1 | 0.3 | 0.2 | 0.2 | 0.1 | 0.2 | 0.2 | 0.1 |
| Dlx4 | 0.4 | 0.3 | 0.2 | 0.4 | 0.3 | 0.3 | 0.3 | 0.2 | 0.2 | 0.5 | 0.2 | 0.3 |
| Dlx5 | 0.0 | 0.0 | 0.0 | 0.0 | 0.0 | 0.0 | 0.0 | 0.0 | 0.0 | 0.0 | 0.1 | 0.1 |
| Dlx6as1 | 0.0 | 0.0 | 0.0 | 0.0 | 0.0 | 0.0 | 0.0 | 0.0 | 0.0 | 0.0 | 0.0 | 0.0 |
| Dmap1 | 7.5 | 8.6 | 8.4 | 6.9 | 6.8 | 9.7 | 8.0 | 6.8 | 8.0 | 8.2 | 7.1 | 7.7 |
| Dmc1 | 0.0 | 0.0 | 0.0 | 0.0 | 0.1 | 0.0 | 0.1 | 0.0 | 0.0 | 0.0 | 0.0 | 0.1 |
| Dmd | 1.8 | 2.0 | 2.3 | 2.0 | 1.9 | 2.0 | 1.9 | 1.7 | 1.9 | 1.2 | 2.0 | 1.8 |
| Dmkn | 0.0 | 0.0 | 0.1 | 0.0 | 0.0 | 0.1 | 0.2 | 0.1 | 0.1 | 0.4 | 0.9 | 0.1 |
| Dmp1 | 0.1 | 0.0 | 0.0 | 0.0 | 0.0 | 0.0 | 0.0 | 0.0 | 0.0 | 0.0 | 0.0 | 0.0 |
| Dmpk | 28.1 | 16.7 | 15.7 | 39.0 | 19.4 | 27.8 | 27.5 | 16.1 | 23.4 | 19.9 | 27.0 | 21.9 |
| Dmrt2 | 0.8 | 0.4 | 0.4 | 1.1 | 0.5 | 0.6 | 0.5 | 0.2 | 0.2 | 0.1 | 0.4 | 0.2 |
| Dmrta2 | 0.6 | 0.6 | 0.6 | 0.4 | 0.6 | 0.5 | 0.9 | 0.7 | 0.8 | 0.7 | 0.6 | 0.6 |
| Dmrtc1a | 0.8 | 0.7 | 1.0 | 0.4 | 0.3 | 1.1 | 0.4 | 0.3 | 0.3 | 0.5 | 0.6 | 0.8 |
| Dmrtc1c1 | 0.0 | 0.0 | 0.0 | 0.0 | 0.0 | 0.0 | 0.0 | 0.0 | 0.0 | 0.0 | 0.0 | 0.0 |
| Dmrtc1c2 | 0.0 | 0.0 | 0.0 | 0.0 | 0.1 | 0.0 | 0.0 | 0.0 | 0.0 | 0.0 | 0.0 | 0.0 |
| Dmtf1 | 12.8 | 13.7 | 14.7 | 12.4 | 11.9 | 13.1 | 13.9 | 14.5 | 14.1 | 14.4 | 16.1 | 16.4 |
| Dmwd | 13.4 | 15.0 | 12.2 | 13.7 | 11.4 | 16.0 | 13.1 | 11.6 | 14.0 | 11.7 | 12.0 | 11.3 |
| Dmxl1 | 6.2 | 7.0 | 6.2 | 5.8 | 6.4 | 6.2 | 5.3 | 5.8 | 6.2 | 7.1 | 7.7 | 6.9 |
| Dmxl2 | 2.1 | 1.9 | 1.5 | 2.5 | 1.8 | 2.5 | 1.5 | 1.3 | 1.4 | 1.4 | 1.5 | 1.7 |
| Dna2 | 0.2 | 0.3 | 0.3 | 0.2 | 0.6 | 0.1 | 0.5 | 0.3 | 0.3 | 0.2 | 0.4 | 0.3 |
| Dnaaf1 | 0.0 | 0.0 | 0.0 | 0.0 | 0.0 | 0.0 | 0.0 | 0.0 | 0.0 | 0.0 | 0.0 | 0.0 |
| Dnaaf2 | 1.5 | 2.0 | 2.0 | 1.3 | 2.1 | 1.6 | 2.2 | 1.6 | 1.9 | 1.9 | 1.8 | 1.5 |
| Dnaaf3 | 0.3 | 0.5 | 0.5 | 0.5 | 0.3 | 0.4 | 0.4 | 0.5 | 0.7 | 0.4 | 0.4 | 0.5 |
| Dnahc1 | 0.0 | 0.0 | 0.0 | 0.0 | 0.0 | 0.0 | 0.0 | 0.0 | 0.0 | 0.0 | 0.0 | 0.0 |
| Dnahc10 | 0.2 | 0.1 | 0.1 | 0.1 | 0.1 | 0.1 | 0.1 | 0.1 | 0.1 | 0.1 | 0.1 | 0.1 |
| Dnahc11 | 0.0 | 0.0 | 0.1 | 0.1 | 0.1 | 0.0 | 0.1 | 0.0 | 0.0 | 0.0 | 0.1 | 0.0 |
| Dnahc17 | 0.0 | 0.1 | 0.0 | 0.0 | 0.0 | 0.0 | 0.0 | 0.0 | 0.0 | 0.0 | 0.0 | 0.1 |
| Dnahc2 | 0.0 | 0.0 | 0.0 | 0.0 | 0.0 | 0.0 | 0.0 | 0.0 | 0.0 | 0.0 | 0.0 | 0.0 |
| Dnahc5 | 0.0 | 0.0 | 0.0 | 0.0 | 0.0 | 0.0 | 0.0 | 0.0 | 0.0 | 0.0 | 0.0 | 0.0 |
| Dnahc6 | 0.0 | 0.0 | 0.0 | 0.0 | 0.0 | 0.0 | 0.0 | 0.0 | 0.0 | 0.0 | 0.0 | 0.0 |
| Dnahc7a | 0.1 | 0.1 | 0.1 | 0.1 | 0.0 | 0.1 | 0.1 | 0.0 | 0.0 | 0.0 | 0.0 | 0.0 |
| Dnahc7b | 0.4 | 0.3 | 0.4 | 0.1 | 0.2 | 0.3 | 0.2 | 0.3 | 0.1 | 0.2 | 0.2 | 0.2 |
| Dnahc8 | 0.4 | 0.4 | 0.4 | 0.3 | 0.2 | 0.3 | 0.1 | 0.1 | 0.1 | 0.3 | 0.3 | 0.2 |
| Dnahc9 | 0.0 | 0.0 | 0.1 | 0.0 | 0.0 | 0.0 | 0.0 | 0.1 | 0.0 | 0.0 | 0.0 | 0.1 |
| Dnaic1 | 0.1 | 0.1 | 0.1 | 0.3 | 0.0 | 0.1 | 0.0 | 0.0 | 0.0 | 0.0 | 0.0 | 0.0 |
| Dnaic2 | 2.2 | 2.0 | 1.7 | 2.5 | 1.4 | 1.9 | 1.1 | 0.7 | 0.7 | 0.6 | 1.0 | 1.2 |
| Dnaja1 | 40.0 | 43.3 | 45.5 | 37.3 | 34.0 | 30.8 | 36.5 | 35.2 | 40.1 | 48.8 | 49.4 | 55.5 |
| Dnaja2 | 67.1 | 63.0 | 66.1 | 73.8 | 57.4 | 66.0 | 57.9 | 58.9 | 52.0 | 64.5 | 70.3 | 66.9 |
| Dnaja3 | 19.1 | 18.7 | 16.8 | 19.8 | 16.0 | 21.2 | 15.9 | 17.4 | 16.5 | 15.6 | 17.7 | 15.5 |
| Dnaja4 | 8.3 | 8.6 | 7.3 | 8.9 | 6.9 | 9.1 | 7.6 | 5.8 | 7.9 | 8.7 | 8.4 | 9.2 |

| | | | | | | | | | | | | |
|---|---|---|---|---|---|---|---|---|---|---|---|---|
| Dnajb1 | 28.2 | 32.0 | 22.4 | 27.4 | 28.4 | 24.1 | 30.5 | 18.2 | 25.0 | 35.1 | 30.2 | 25.3 |
| Dnajb11 | 85.7 | 70.9 | 66.8 | 91.9 | 75.0 | 70.2 | 83.4 | 78.2 | 70.8 | 72.8 | 81.1 | 82.4 |
| Dnajb12 | 17.7 | 18.8 | 19.6 | 17.0 | 17.9 | 16.6 | 23.0 | 21.0 | 19.2 | 18.8 | 21.4 | 21.8 |
| Dnajb13 | 0.6 | 0.6 | 0.3 | 0.3 | 0.2 | 0.3 | 0.2 | 0.1 | 0.2 | 0.1 | 0.1 | 0.1 |
| Dnajb14 | 10.0 | 10.8 | 9.7 | 10.0 | 9.1 | 11.2 | 10.4 | 8.5 | 8.9 | 10.6 | 10.1 | 11.5 |
| Dnajb2 | 19.2 | 24.4 | 22.6 | 20.0 | 17.8 | 22.7 | 21.8 | 18.3 | 21.8 | 17.5 | 19.6 | 20.6 |
| Dnajb3 | 0.9 | 1.3 | 1.4 | 1.5 | 1.5 | 1.1 | 1.4 | 1.2 | 1.5 | 1.0 | 1.4 | 2.2 |
| Dnajb4 | 32.0 | 33.6 | 30.0 | 34.8 | 27.9 | 31.4 | 21.3 | 18.4 | 21.4 | 27.4 | 29.0 | 29.4 |
| Dnajb5 | 3.6 | 3.2 | 4.8 | 3.0 | 3.8 | 2.8 | 4.3 | 3.9 | 3.7 | 2.7 | 4.2 | 3.4 |
| Dnajb6 | 79.3 | 89.5 | 101.6 | 85.6 | 81.7 | 80.8 | 87.6 | 94.1 | 91.3 | 90.5 | 96.7 | 119.4 |
| Dnajb7 | 0.0 | 0.1 | 0.0 | 0.2 | 0.1 | 0.0 | 0.0 | 0.0 | 0.1 | 0.0 | 0.0 | 0.1 |
| Dnajb9 | 15.0 | 13.8 | 14.6 | 22.9 | 17.4 | 15.4 | 17.1 | 16.4 | 17.6 | 22.6 | 22.3 | 23.3 |
| Dnajc1 | 18.5 | 19.5 | 22.6 | 18.8 | 15.8 | 17.6 | 19.4 | 19.0 | 17.8 | 18.7 | 20.2 | 23.1 |
| Dnajc10 | 70.6 | 67.4 | 60.1 | 78.7 | 76.9 | 78.6 | 61.7 | 58.9 | 54.7 | 61.7 | 63.2 | 59.5 |
| Dnajc11 | 13.7 | 10.1 | 10.2 | 14.8 | 11.5 | 13.7 | 12.4 | 12.6 | 10.7 | 12.6 | 13.4 | 11.6 |
| Dnajc12 | 1.8 | 1.8 | 1.3 | 2.5 | 2.0 | 2.6 | 1.5 | 1.1 | 1.6 | 2.3 | 1.6 | 1.3 |
| Dnajc13 | 12.4 | 11.5 | 12.1 | 11.7 | 12.8 | 12.5 | 11.7 | 12.8 | 11.1 | 12.0 | 12.1 | 13.3 |
| Dnajc14 | 14.0 | 12.7 | 13.2 | 15.4 | 13.7 | 13.3 | 14.2 | 13.7 | 13.4 | 13.8 | 14.7 | 15.6 |
| Dnajc15 | 25.5 | 17.5 | 18.8 | 26.2 | 22.0 | 22.8 | 16.9 | 19.5 | 14.6 | 17.3 | 17.4 | 18.7 |
| Dnajc16 | 3.7 | 4.1 | 4.1 | 3.1 | 4.0 | 3.8 | 4.3 | 3.6 | 4.1 | 3.3 | 4.0 | 3.5 |
| Dnajc17 | 6.5 | 4.3 | 4.7 | 8.9 | 4.7 | 5.9 | 5.7 | 4.7 | 3.7 | 4.3 | 4.8 | 5.8 |
| Dnajc18 | 13.7 | 13.2 | 12.2 | 14.8 | 12.2 | 14.7 | 12.9 | 13.2 | 12.9 | 13.6 | 14.3 | 14.4 |
| Dnajc19 | 26.2 | 21.8 | 23.1 | 28.7 | 24.4 | 28.4 | 24.5 | 27.2 | 25.0 | 21.9 | 23.8 | 22.5 |
| Dnajc2 | 23.6 | 19.0 | 19.2 | 26.5 | 17.3 | 20.9 | 22.3 | 18.5 | 14.4 | 17.3 | 25.1 | 22.3 |
| Dnajc21 | 12.2 | 11.2 | 12.8 | 11.9 | 9.2 | 10.5 | 10.6 | 11.0 | 10.9 | 10.0 | 14.1 | 13.4 |
| Dnajc24 | 6.4 | 7.2 | 6.2 | 6.9 | 5.6 | 7.6 | 5.1 | 5.8 | 6.1 | 5.8 | 5.1 | 5.4 |
| Dnajc25 | 11.1 | 9.0 | 11.3 | 12.9 | 8.7 | 10.8 | 14.2 | 14.0 | 11.9 | 11.0 | 12.6 | 13.6 |
| Dnajc27 | 2.4 | 2.2 | 2.0 | 2.2 | 1.6 | 2.2 | 1.9 | 1.7 | 1.8 | 1.7 | 1.8 | 1.7 |
| Dnajc28 | 2.3 | 2.3 | 2.2 | 2.7 | 1.4 | 2.6 | 1.5 | 1.6 | 1.5 | 1.7 | 1.5 | 2.4 |
| Dnajc3 | 54.1 | 49.8 | 53.1 | 64.0 | 51.2 | 48.2 | 61.5 | 56.7 | 57.3 | 59.5 | 62.9 | 71.1 |
| Dnajc30 | 9.2 | 9.1 | 8.1 | 9.4 | 7.9 | 8.9 | 9.3 | 9.1 | 8.8 | 9.2 | 8.9 | 7.7 |
| Dnajc4 | 7.8 | 8.0 | 11.1 | 11.5 | 7.8 | 10.0 | 11.6 | 10.5 | 9.9 | 9.4 | 11.0 | 8.7 |
| Dnajc5 | 23.6 | 22.6 | 18.7 | 22.8 | 23.3 | 22.7 | 22.2 | 21.4 | 21.4 | 22.7 | 23.0 | 21.8 |
| Dnajc5b | 0.0 | 0.2 | 0.0 | 0.0 | 0.0 | 0.0 | 0.0 | 0.2 | 0.0 | 0.0 | 0.0 | 0.0 |
| Dnajc6 | 0.2 | 0.2 | 0.1 | 0.2 | 0.2 | 0.3 | 0.0 | 0.1 | 0.1 | 0.2 | 0.2 | 0.1 |
| Dnajc7 | 20.4 | 20.1 | 19.8 | 23.5 | 20.3 | 20.1 | 21.2 | 20.5 | 18.9 | 19.6 | 22.2 | 23.2 |
| Dnajc8 | 94.8 | 89.0 | 108.7 | 124.4 | 75.3 | 105.1 | 99.7 | 86.7 | 60.7 | 73.4 | 96.8 | 102.0 |
| Dnajc9 | 16.3 | 19.2 | 24.1 | 11.9 | 23.2 | 14.4 | 21.3 | 13.6 | 16.6 | 16.9 | 20.7 | 17.9 |
| Dnalc1 | 5.0 | 4.8 | 4.7 | 5.5 | 4.7 | 5.8 | 4.4 | 3.3 | 4.1 | 4.9 | 5.1 | 4.7 |
| Dnalc4 | 8.4 | 8.1 | 8.0 | 10.1 | 8.0 | 10.9 | 8.4 | 8.9 | 8.4 | 8.8 | 9.0 | 8.8 |
| Dnali1 | 0.2 | 0.2 | 0.3 | 0.3 | 0.1 | 0.2 | 0.1 | 0.1 | 0.1 | 0.3 | 0.2 | 0.2 |
| Dnase1 | 0.1 | 0.1 | 0.1 | 0.1 | 0.1 | 0.0 | 0.1 | 0.2 | 0.1 | 0.2 | 0.0 | 0.1 |
| Dnase1l1 | 4.9 | 4.3 | 5.7 | 10.6 | 11.0 | 8.5 | 6.0 | 5.9 | 6.6 | 5.1 | 6.2 | 6.0 |
| Dnase1l2 | 0.3 | 0.6 | 0.5 | 0.2 | 0.5 | 0.3 | 0.5 | 0.5 | 0.9 | 0.3 | 0.4 | 0.8 |
| Dnase2a | 8.8 | 7.7 | 8.6 | 13.1 | 13.3 | 10.2 | 6.9 | 8.9 | 7.0 | 7.7 | 6.5 | 9.6 |
| Dnd1 | 0.3 | 0.4 | 0.5 | 0.4 | 0.5 | 0.4 | 0.3 | 0.5 | 0.5 | 0.4 | 0.2 | 0.7 |
| Dner | 0.1 | 0.1 | 0.1 | 0.2 | 0.1 | 0.1 | 0.1 | 0.1 | 0.1 | 0.1 | 0.1 | 0.0 |
| Dnlz | 9.2 | 6.7 | 6.1 | 9.3 | 7.0 | 9.3 | 7.2 | 8.4 | 7.6 | 8.9 | 8.2 | 7.2 |
| Dnm1 | 0.4 | 0.6 | 1.3 | 0.2 | 0.4 | 0.9 | 0.4 | 1.3 | 0.4 | 0.7 | 0.8 | 0.7 |
| Dnm1l | 24.6 | 25.7 | 25.7 | 27.2 | 25.6 | 26.5 | 26.3 | 26.3 | 26.6 | 27.3 | 30.6 | 29.5 |
| Dnm2 | 36.1 | 35.7 | 36.7 | 41.0 | 37.6 | 36.7 | 41.9 | 43.3 | 39.8 | 33.6 | 40.8 | 39.6 |
| Dnm3 | 0.3 | 0.4 | 0.4 | 0.4 | 0.2 | 0.3 | 0.2 | 0.3 | 0.2 | 0.2 | 0.2 | 0.2 |
| Dnm3os | 1.6 | 2.7 | 4.8 | 2.2 | 3.4 | 1.8 | 2.9 | 5.3 | 5.3 | 3.4 | 3.5 | 5.0 |
| Dnmbp | 3.2 | 3.6 | 3.9 | 3.3 | 3.3 | 3.8 | 3.6 | 3.6 | 3.7 | 3.3 | 3.6 | 4.0 |
| Dnmt1 | 7.1 | 9.3 | 13.1 | 7.2 | 12.5 | 5.9 | 14.4 | 10.6 | 10.3 | 7.9 | 10.7 | 8.4 |
| Dnmt3a | 2.9 | 3.5 | 3.5 | 2.6 | 3.5 | 2.2 | 2.7 | 3.1 | 2.9 | 3.1 | 2.6 | 2.9 |
| Dnmt3b | 0.2 | 0.1 | 0.2 | 0.1 | 0.2 | 0.2 | 0.1 | 0.4 | 0.2 | 0.2 | 0.2 | 0.3 |

| | | | | | | | | | | | | |
|---|---|---|---|---|---|---|---|---|---|---|---|---|
| Dnmt3l | 3.8 | 4.0 | 3.2 | 3.9 | 4.4 | 3.5 | 4.0 | 3.2 | 2.2 | 1.5 | 1.4 | 2.3 |
| Dnpep | 64.3 | 77.1 | 83.3 | 59.0 | 66.2 | 52.5 | 78.7 | 80.2 | 90.4 | 63.4 | 67.1 | 78.7 |
| Dnph1 | 1.9 | 1.6 | 0.9 | 1.4 | 1.5 | 1.4 | 1.0 | 1.2 | 1.1 | 1.4 | 2.1 | 1.5 |
| Dntt | 0.1 | 0.1 | 0.0 | 0.0 | 0.0 | 0.0 | 0.1 | 0.1 | 0.1 | 0.0 | 0.0 | 0.0 |
| Dnttip1 | 7.3 | 8.2 | 9.7 | 8.4 | 8.4 | 9.4 | 9.8 | 9.6 | 9.8 | 9.3 | 9.3 | 10.0 |
| Dnttip2 | 25.4 | 24.5 | 24.0 | 24.9 | 21.3 | 22.9 | 21.4 | 22.6 | 21.3 | 21.9 | 26.9 | 27.2 |
| Doc2a | 0.5 | 0.8 | 0.6 | 0.7 | 0.7 | 0.6 | 0.7 | 0.5 | 0.5 | 0.5 | 0.4 | 0.5 |
| Doc2b | 0.0 | 0.1 | 0.0 | 0.0 | 0.0 | 0.2 | 0.0 | 0.1 | 0.1 | 0.0 | 0.1 | 0.1 |
| Doc2g | 0.4 | 0.7 | 0.5 | 0.5 | 0.5 | 0.8 | 0.5 | 0.5 | 0.9 | 0.8 | 0.3 | 0.5 |
| Dock1 | 19.1 | 18.2 | 18.5 | 18.7 | 16.7 | 21.4 | 18.6 | 17.0 | 16.7 | 15.7 | 17.2 | 17.1 |
| Dock10 | 0.6 | 0.3 | 0.6 | 2.6 | 4.6 | 0.1 | 1.0 | 0.8 | 0.5 | 0.6 | 0.4 | 0.7 |
| Dock11 | 3.7 | 3.2 | 2.8 | 4.6 | 4.3 | 4.1 | 2.6 | 2.1 | 2.3 | 2.5 | 2.6 | 2.4 |
| Dock2 | 0.5 | 0.0 | 0.1 | 5.3 | 9.0 | 0.0 | 1.1 | 0.2 | 0.1 | 0.4 | 0.1 | 0.4 |
| Dock3 | 0.1 | 0.1 | 0.1 | 0.1 | 0.1 | 0.1 | 0.1 | 0.1 | 0.0 | 0.1 | 0.1 | 0.0 |
| Dock4 | 1.8 | 2.1 | 2.0 | 2.4 | 3.9 | 1.7 | 1.5 | 1.1 | 1.1 | 1.9 | 1.5 | 1.1 |
| Dock5 | 3.9 | 2.4 | 1.6 | 4.2 | 4.0 | 3.4 | 4.0 | 1.8 | 1.9 | 5.9 | 4.0 | 3.3 |
| Dock6 | 1.4 | 1.7 | 1.5 | 1.2 | 1.3 | 1.4 | 1.6 | 1.5 | 1.6 | 1.1 | 1.2 | 1.1 |
| Dock7 | 20.7 | 21.5 | 21.2 | 17.6 | 20.1 | 17.9 | 21.1 | 20.2 | 21.1 | 19.9 | 23.1 | 20.1 |
| Dock8 | 0.6 | 0.1 | 0.1 | 4.2 | 7.3 | 0.1 | 0.7 | 0.2 | 0.2 | 1.3 | 0.6 | 0.5 |
| Dock9 | 2.6 | 2.0 | 2.0 | 2.2 | 1.8 | 2.5 | 1.8 | 2.2 | 1.6 | 2.7 | 2.4 | 1.9 |
| Dohh | 18.2 | 14.8 | 14.9 | 20.4 | 16.6 | 20.3 | 18.1 | 13.8 | 16.8 | 18.6 | 15.5 | 14.3 |
| Dok1 | 16.3 | 15.6 | 14.4 | 17.7 | 21.8 | 16.3 | 22.2 | 14.8 | 25.1 | 26.2 | 20.2 | 22.6 |
| Dok2 | 0.3 | 0.2 | 0.0 | 1.7 | 4.8 | 0.1 | 0.5 | 0.2 | 0.2 | 0.3 | 0.1 | 0.4 |
| Dok3 | 0.9 | 0.3 | 0.1 | 3.0 | 6.9 | 0.2 | 1.5 | 0.2 | 0.4 | 0.4 | 0.4 | 0.5 |
| Dok4 | 4.0 | 4.0 | 3.9 | 5.0 | 4.1 | 4.4 | 4.9 | 5.4 | 5.9 | 4.7 | 4.1 | 4.5 |
| Dok5 | 3.5 | 3.1 | 2.3 | 3.0 | 3.3 | 3.2 | 3.2 | 3.1 | 2.5 | 3.1 | 3.8 | 3.6 |
| Dok6 | 0.5 | 0.9 | 0.7 | 1.1 | 1.2 | 0.8 | 1.5 | 1.6 | 1.5 | 1.1 | 1.8 | 1.1 |
| Dok7 | 0.0 | 0.0 | 0.1 | 0.0 | 0.0 | 0.0 | 0.0 | 0.0 | 0.0 | 0.0 | 0.1 | 0.0 |
| Dolk | 9.2 | 9.5 | 6.8 | 10.4 | 9.1 | 9.2 | 9.0 | 7.8 | 8.4 | 8.8 | 7.9 | 9.2 |
| Dolpp1 | 3.3 | 4.5 | 4.2 | 3.8 | 4.3 | 3.2 | 4.6 | 5.4 | 5.5 | 4.0 | 4.8 | 3.8 |
| Dom3z | 5.7 | 6.1 | 5.2 | 5.2 | 5.8 | 4.6 | 6.5 | 5.6 | 5.6 | 5.6 | 6.6 | 5.3 |
| Donson | 2.9 | 4.4 | 5.0 | 2.5 | 4.9 | 2.3 | 5.5 | 4.3 | 5.2 | 3.5 | 4.9 | 4.4 |
| Dopey1 | 4.0 | 4.2 | 3.8 | 3.2 | 3.4 | 3.5 | 3.2 | 3.3 | 3.8 | 3.7 | 3.8 | 3.5 |
| Dopey2 | 3.8 | 4.6 | 4.1 | 5.0 | 4.3 | 4.0 | 4.3 | 4.3 | 4.2 | 2.9 | 4.0 | 3.3 |
| Dos | 2.9 | 2.7 | 2.7 | 3.9 | 2.8 | 4.5 | 3.2 | 2.2 | 2.8 | 3.4 | 3.2 | 2.9 |
| Dot1l | 0.6 | 1.0 | 0.9 | 0.6 | 1.6 | 0.7 | 1.1 | 1.0 | 1.1 | 1.1 | 0.9 | 0.7 |
| Dpagt1 | 13.2 | 12.8 | 11.0 | 14.3 | 12.2 | 14.7 | 15.4 | 14.4 | 12.2 | 12.5 | 13.3 | 11.3 |
| Dpcd | 11.7 | 10.0 | 9.3 | 13.0 | 9.3 | 12.1 | 9.3 | 9.2 | 8.0 | 9.1 | 9.5 | 9.9 |
| Dpcr1 | 0.3 | 0.2 | 0.3 | 0.1 | 0.1 | 0.1 | 0.1 | 0.1 | 0.1 | 0.2 | 0.4 | 0.2 |
| Dpep1 | 10.9 | 25.5 | 57.9 | 19.2 | 28.5 | 10.2 | 20.7 | 51.2 | 29.1 | 13.0 | 9.3 | 18.8 |
| Dpep2 | 0.5 | 0.3 | 0.3 | 4.7 | 6.9 | 0.2 | 0.8 | 0.2 | 0.3 | 0.4 | 0.1 | 0.4 |
| Dpf1 | 0.8 | 1.2 | 1.5 | 1.0 | 0.6 | 1.4 | 1.1 | 0.6 | 0.7 | 0.8 | 0.9 | 0.7 |
| Dpf2 | 9.8 | 12.2 | 10.7 | 9.8 | 9.5 | 10.8 | 10.2 | 12.8 | 10.3 | 10.8 | 10.0 | 9.6 |
| Dpf3 | 0.0 | 0.0 | 0.0 | 0.0 | 0.0 | 0.0 | 0.1 | 0.1 | 0.0 | 0.1 | 0.0 | 0.0 |
| Dph1 | 3.4 | 2.6 | 2.2 | 3.2 | 3.6 | 3.4 | 3.0 | 3.4 | 3.3 | 3.6 | 2.6 | 2.7 |
| Dph2 | 2.9 | 2.4 | 1.6 | 3.3 | 2.8 | 2.1 | 2.8 | 2.4 | 2.6 | 2.4 | 2.0 | 1.9 |
| Dph3 | 7.4 | 5.0 | 4.2 | 5.7 | 5.7 | 5.3 | 6.4 | 6.7 | 5.7 | 7.5 | 6.5 | 5.8 |
| Dph5 | 5.9 | 4.4 | 4.3 | 6.4 | 5.2 | 5.0 | 5.0 | 5.3 | 4.4 | 5.4 | 6.2 | 4.7 |
| Dpm1 | 16.7 | 18.5 | 19.6 | 15.3 | 15.3 | 18.8 | 12.9 | 13.3 | 13.6 | 17.6 | 15.1 | 18.1 |
| Dpm2 | 31.1 | 25.8 | 22.1 | 30.3 | 28.6 | 24.6 | 31.5 | 27.1 | 28.6 | 29.8 | 25.2 | 27.0 |
| Dpm3 | 46.3 | 46.4 | 36.7 | 36.2 | 40.7 | 42.4 | 42.0 | 41.0 | 45.9 | 51.2 | 43.9 | 40.2 |
| Dpp10 | 0.0 | 0.0 | 0.0 | 0.0 | 0.0 | 0.0 | 0.0 | 0.0 | 0.0 | 0.0 | 0.0 | 0.0 |
| Dpp3 | 21.1 | 18.1 | 16.4 | 21.4 | 21.1 | 20.5 | 20.2 | 20.3 | 19.5 | 20.4 | 18.9 | 19.3 |
| Dpp4 | 1.6 | 2.6 | 2.2 | 0.5 | 0.4 | 0.3 | 0.3 | 0.8 | 1.3 | 3.0 | 2.4 | 1.0 |
| Dpp7 | 45.4 | 34.6 | 30.7 | 45.1 | 40.5 | 54.0 | 22.5 | 27.4 | 27.6 | 30.8 | 26.3 | 32.3 |
| Dpp8 | 42.9 | 39.3 | 37.7 | 48.5 | 39.3 | 49.7 | 38.3 | 35.9 | 35.8 | 38.8 | 44.0 | 42.7 |
| Dpp9 | 10.9 | 11.0 | 10.0 | 12.6 | 13.4 | 11.1 | 14.3 | 12.4 | 12.9 | 11.5 | 11.9 | 10.8 |

| | | | | | | | | | | | | |
|---|---|---|---|---|---|---|---|---|---|---|---|---|
| Dppa2 | 0.1 | 0.0 | 0.0 | 0.3 | 0.0 | 0.1 | 0.0 | 0.0 | 0.0 | 0.1 | 0.2 | 0.0 |
| Dpt | 22.9 | 47.1 | 124.1 | 5.6 | 16.0 | 2.4 | 12.3 | 50.6 | 21.9 | 11.2 | 6.4 | 25.2 |
| Dpy19l1 | 22.1 | 20.4 | 19.2 | 26.6 | 23.8 | 25.6 | 26.9 | 25.5 | 28.8 | 20.1 | 24.9 | 25.1 |
| Dpy19l2 | 0.0 | 0.0 | 0.0 | 0.0 | 0.1 | 0.0 | 0.0 | 0.0 | 0.0 | 0.0 | 0.0 | 0.0 |
| Dpy19l3 | 2.4 | 2.7 | 2.7 | 2.2 | 2.2 | 2.5 | 3.0 | 2.8 | 2.7 | 3.1 | 2.7 | 2.6 |
| Dpy19l4 | 9.8 | 9.8 | 9.3 | 8.1 | 10.5 | 12.0 | 8.9 | 10.5 | 10.4 | 9.8 | 10.7 | 9.8 |
| Dpy30 | 24.7 | 19.7 | 20.7 | 24.0 | 19.4 | 28.0 | 21.2 | 20.3 | 16.3 | 17.4 | 24.1 | 18.5 |
| Dpyd | 0.9 | 0.8 | 0.8 | 0.7 | 0.6 | 0.8 | 0.5 | 0.5 | 0.2 | 0.3 | 0.4 | 0.7 |
| Dpysl2 | 128.5 | 153.5 | 140.8 | 107.0 | 127.7 | 111.3 | 150.0 | 153.8 | 137.6 | 107.9 | 138.2 | 126.3 |
| Dpysl3 | 23.6 | 17.1 | 22.8 | 18.7 | 17.5 | 28.2 | 20.0 | 26.7 | 19.6 | 22.2 | 25.6 | 24.5 |
| Dpysl4 | 0.7 | 0.2 | 0.4 | 0.4 | 0.2 | 0.7 | 0.2 | 0.2 | 0.3 | 0.3 | 0.2 | 0.3 |
| Dpysl5 | 0.1 | 0.1 | 0.1 | 0.1 | 0.1 | 0.1 | 0.1 | 0.1 | 0.1 | 0.3 | 0.1 | 0.1 |
| Dqx1 | 0.3 | 0.3 | 0.2 | 0.4 | 0.2 | 0.5 | 0.2 | 0.2 | 0.3 | 0.2 | 0.2 | 0.1 |
| Dr1 | 8.9 | 8.4 | 11.3 | 10.9 | 10.7 | 10.0 | 9.7 | 10.1 | 9.7 | 10.1 | 11.5 | 10.1 |
| Dram1 | 9.6 | 13.8 | 23.2 | 10.2 | 20.1 | 7.9 | 16.0 | 16.7 | 12.4 | 10.1 | 10.0 | 14.2 |
| Dram2 | 7.6 | 7.7 | 9.1 | 9.1 | 9.6 | 8.7 | 6.9 | 8.2 | 9.1 | 10.2 | 10.7 | 10.1 |
| Drap1 | 82.2 | 67.8 | 67.1 | 106.1 | 69.9 | 101.3 | 84.8 | 71.4 | 66.2 | 75.7 | 80.2 | 81.3 |
| Draxin | 0.0 | 0.0 | 0.0 | 0.0 | 0.0 | 0.0 | 0.0 | 0.0 | 0.0 | 0.0 | 0.1 | 0.0 |
| Drd1a | 0.0 | 0.0 | 0.0 | 0.1 | 0.0 | 0.0 | 0.0 | 0.0 | 0.0 | 0.0 | 0.0 | 0.0 |
| Drd3 | 0.0 | 0.0 | 0.1 | 0.0 | 0.0 | 0.0 | 0.0 | 0.0 | 0.0 | 0.0 | 0.0 | 0.0 |
| Drd4 | 0.0 | 0.0 | 0.0 | 0.1 | 0.0 | 0.0 | 0.2 | 0.0 | 0.1 | 0.3 | 0.0 | 0.1 |
| Drg1 | 44.2 | 42.6 | 34.9 | 40.4 | 37.6 | 37.7 | 36.2 | 35.7 | 37.6 | 41.2 | 36.1 | 37.7 |
| Drg2 | 25.7 | 24.3 | 20.3 | 29.0 | 19.8 | 25.3 | 22.0 | 21.1 | 19.3 | 24.4 | 22.9 | 19.0 |
| Drosha | 5.4 | 5.2 | 5.5 | 6.4 | 4.2 | 5.4 | 5.1 | 5.7 | 4.1 | 4.6 | 5.3 | 4.8 |
| Drp2 | 1.3 | 1.9 | 2.1 | 0.9 | 1.1 | 1.2 | 1.0 | 1.3 | 0.9 | 0.8 | 0.8 | 1.1 |
| Dsc2 | 0.1 | 0.0 | 0.0 | 0.0 | 0.1 | 0.1 | 0.0 | 0.0 | 0.0 | 0.0 | 0.0 | 0.0 |
| Dsc3 | 0.0 | 0.0 | 0.0 | 0.0 | 0.0 | 0.0 | 0.0 | 0.0 | 0.0 | 0.0 | 0.0 | 0.0 |
| Dscam | 0.0 | 0.0 | 0.0 | 0.1 | 0.0 | 0.0 | 0.0 | 0.0 | 0.0 | 0.0 | 0.0 | 0.0 |
| Dscaml1 | 0.0 | 0.0 | 0.0 | 0.0 | 0.0 | 0.0 | 0.0 | 0.0 | 0.0 | 0.0 | 0.0 | 0.0 |
| Dscc1 | 0.5 | 0.9 | 1.4 | 0.2 | 0.9 | 0.2 | 1.5 | 0.7 | 0.9 | 0.4 | 0.8 | 0.6 |
| Dscr3 | 36.5 | 33.6 | 30.3 | 33.0 | 33.9 | 35.3 | 29.3 | 28.3 | 30.3 | 29.4 | 30.0 | 30.2 |
| Dse | 58.4 | 67.2 | 69.9 | 61.7 | 57.0 | 60.7 | 63.9 | 61.1 | 64.0 | 48.9 | 56.2 | 66.9 |
| Dsel | 3.8 | 4.1 | 6.2 | 4.4 | 5.2 | 5.3 | 5.2 | 6.9 | 5.9 | 6.2 | 6.9 | 8.0 |
| Dsg2 | 0.0 | 0.0 | 0.0 | 0.0 | 0.0 | 0.0 | 0.0 | 0.0 | 0.0 | 0.0 | 0.0 | 0.0 |
| Dsn1 | 2.9 | 3.7 | 3.6 | 2.6 | 3.1 | 2.5 | 3.0 | 2.9 | 3.6 | 4.0 | 3.6 | 2.8 |
| Dsp | 1.3 | 0.5 | 0.4 | 0.5 | 0.3 | 0.4 | 0.1 | 0.4 | 0.1 | 0.4 | 0.4 | 0.7 |
| Dst | 48.6 | 48.2 | 46.2 | 46.0 | 47.8 | 44.4 | 54.9 | 59.1 | 43.4 | 35.2 | 46.5 | 44.8 |
| Dstn | 269.9 | 201.7 | 178.3 | 294.9 | 209.5 | 276.6 | 216.5 | 175.7 | 204.6 | 269.7 | 329.0 | 254.5 |
| Dstyk | 9.5 | 9.1 | 8.5 | 8.7 | 8.4 | 9.5 | 9.0 | 8.5 | 9.5 | 9.1 | 8.7 | 10.3 |
| Dtd1 | 12.0 | 8.7 | 9.1 | 13.2 | 7.8 | 11.3 | 9.7 | 10.9 | 6.8 | 10.5 | 11.0 | 9.6 |
| Dtd2 | 4.0 | 3.7 | 3.1 | 4.0 | 2.8 | 3.6 | 2.6 | 3.9 | 2.7 | 3.2 | 3.5 | 3.3 |
| Dtl | 0.8 | 2.0 | 3.1 | 0.6 | 2.6 | 0.5 | 2.9 | 1.6 | 2.2 | 1.3 | 1.7 | 1.2 |
| Dtna | 3.1 | 2.3 | 1.4 | 2.4 | 1.9 | 2.4 | 1.6 | 1.4 | 1.4 | 2.2 | 3.0 | 1.9 |
| Dtnb | 3.4 | 4.1 | 3.8 | 3.1 | 3.3 | 4.4 | 4.2 | 4.5 | 3.7 | 5.2 | 4.9 | 3.5 |
| Dtnbp1 | 26.0 | 25.3 | 26.1 | 26.6 | 24.9 | 20.9 | 31.5 | 33.1 | 33.8 | 31.1 | 29.3 | 35.9 |
| Dtwd1 | 4.5 | 5.4 | 6.4 | 4.4 | 4.0 | 5.6 | 5.3 | 4.9 | 5.6 | 5.1 | 5.7 | 5.7 |
| Dtwd2 | 0.7 | 0.6 | 0.9 | 1.0 | 0.8 | 0.8 | 0.6 | 0.7 | 0.8 | 0.7 | 0.6 | 0.7 |
| Dtx1 | 0.2 | 0.3 | 0.1 | 0.1 | 0.1 | 0.5 | 0.1 | 0.0 | 0.0 | 0.1 | 0.1 | 0.1 |
| Dtx2 | 3.7 | 3.7 | 3.6 | 3.9 | 3.1 | 3.4 | 4.1 | 4.3 | 4.4 | 5.2 | 4.3 | 3.8 |
| Dtx3 | 8.2 | 9.4 | 9.1 | 8.6 | 7.1 | 9.3 | 7.4 | 9.4 | 11.8 | 8.1 | 8.2 | 8.1 |
| Dtx3l | 4.5 | 4.2 | 5.9 | 5.7 | 5.0 | 4.4 | 4.0 | 4.7 | 3.8 | 3.3 | 3.7 | 5.6 |
| Dtx4 | 10.6 | 15.8 | 15.4 | 9.6 | 14.0 | 4.1 | 8.0 | 11.7 | 12.6 | 9.7 | 7.7 | 13.4 |
| Dtymk | 16.2 | 18.9 | 16.1 | 12.6 | 16.7 | 13.2 | 20.4 | 15.3 | 18.7 | 16.9 | 17.8 | 14.2 |
| Duox1 | 0.1 | 0.1 | 0.1 | 0.0 | 0.1 | 0.0 | 0.1 | 0.1 | 0.0 | 0.0 | 0.1 | 0.0 |
| Duox2 | 0.0 | 0.0 | 0.0 | 0.0 | 0.0 | 0.0 | 0.0 | 0.0 | 0.0 | 0.0 | 0.0 | 0.0 |
| Duoxa1 | 0.1 | 0.1 | 0.2 | 0.3 | 0.1 | 0.3 | 0.1 | 0.1 | 0.1 | 0.1 | 0.0 | 0.0 |
| Dupd1 | 0.0 | 0.0 | 0.2 | 0.0 | 0.0 | 0.0 | 0.0 | 0.2 | 0.0 | 0.0 | 0.0 | 0.0 |

| | | | | | | | | | | | | |
|---|---|---|---|---|---|---|---|---|---|---|---|---|
| Dus1l | 8.6 | 8.5 | 8.0 | 7.4 | 8.3 | 6.3 | 7.5 | 8.8 | 7.7 | 7.4 | 7.7 | 7.5 |
| Dus2l | 5.2 | 4.5 | 3.6 | 4.8 | 4.2 | 5.8 | 4.5 | 3.7 | 4.1 | 4.3 | 4.5 | 3.7 |
| Dus3l | 6.2 | 6.3 | 5.8 | 6.4 | 6.5 | 5.4 | 7.0 | 7.6 | 7.1 | 7.0 | 7.9 | 5.8 |
| Dus4l | 2.0 | 1.6 | 1.0 | 2.0 | 1.7 | 1.4 | 1.2 | 2.0 | 1.6 | 1.3 | 1.4 | 1.8 |
| Dusp1 | 20.4 | 21.0 | 15.6 | 23.8 | 16.0 | 21.1 | 22.2 | 11.2 | 20.9 | 26.0 | 19.9 | 15.3 |
| Dusp10 | 3.1 | 2.5 | 1.8 | 4.5 | 2.2 | 5.0 | 2.2 | 0.8 | 1.0 | 1.5 | 1.6 | 1.5 |
| Dusp11 | 8.0 | 8.8 | 9.6 | 7.5 | 8.5 | 7.1 | 8.9 | 11.1 | 10.9 | 10.3 | 9.8 | 9.4 |
| Dusp12 | 9.3 | 9.3 | 7.3 | 11.4 | 9.1 | 10.5 | 8.0 | 6.9 | 9.4 | 10.0 | 9.1 | 7.9 |
| Dusp13 | 0.3 | 0.1 | 0.1 | 0.1 | 0.3 | 0.1 | 0.1 | 0.1 | 0.0 | 0.1 | 0.1 | 0.1 |
| Dusp14 | 0.7 | 0.6 | 0.2 | 0.8 | 0.5 | 0.9 | 0.3 | 0.5 | 0.5 | 1.7 | 1.1 | 0.5 |
| Dusp15 | 0.4 | 0.1 | 0.3 | 0.4 | 0.3 | 0.3 | 0.3 | 0.2 | 0.1 | 0.3 | 0.4 | 0.5 |
| Dusp16 | 4.1 | 4.0 | 3.3 | 4.0 | 4.6 | 3.9 | 3.5 | 2.9 | 3.0 | 3.8 | 3.4 | 2.8 |
| Dusp18 | 2.6 | 2.2 | 1.6 | 3.2 | 1.9 | 3.4 | 1.7 | 2.1 | 1.5 | 1.4 | 1.8 | 1.4 |
| Dusp19 | 12.8 | 13.0 | 10.8 | 14.7 | 11.6 | 13.9 | 10.9 | 7.5 | 10.1 | 11.5 | 10.6 | 11.0 |
| Dusp2 | 0.0 | 0.0 | 0.0 | 0.0 | 0.0 | 0.1 | 0.1 | 0.0 | 0.1 | 0.0 | 0.0 | 0.0 |
| Dusp22 | 7.4 | 8.5 | 7.9 | 6.4 | 5.6 | 6.5 | 4.5 | 5.6 | 5.7 | 5.6 | 5.8 | 6.2 |
| Dusp23 | 1.2 | 1.5 | 2.1 | 1.3 | 1.6 | 1.3 | 1.5 | 2.5 | 2.0 | 1.3 | 2.3 | 1.5 |
| Dusp27 | 0.5 | 0.5 | 0.4 | 1.4 | 0.4 | 0.5 | 0.6 | 0.5 | 0.7 | 0.5 | 0.4 | 0.9 |
| Dusp28 | 1.4 | 0.6 | 0.6 | 1.0 | 0.8 | 0.7 | 0.9 | 0.9 | 1.0 | 1.1 | 0.9 | 0.9 |
| Dusp3 | 30.6 | 18.4 | 13.2 | 40.9 | 26.7 | 28.4 | 18.0 | 13.4 | 14.4 | 21.7 | 21.0 | 19.4 |
| Dusp4 | 29.9 | 20.5 | 10.2 | 29.8 | 28.6 | 40.7 | 26.8 | 6.8 | 12.6 | 46.6 | 25.9 | 20.6 |
| Dusp5 | 0.3 | 0.4 | 0.5 | 0.3 | 0.7 | 0.3 | 1.1 | 0.7 | 0.7 | 5.1 | 1.6 | 1.5 |
| Dusp6 | 6.6 | 6.4 | 5.3 | 9.2 | 11.6 | 6.5 | 6.7 | 4.4 | 5.3 | 24.9 | 9.2 | 8.5 |
| Dusp7 | 5.1 | 5.1 | 5.7 | 6.0 | 6.3 | 5.7 | 5.5 | 6.2 | 5.3 | 4.7 | 4.9 | 4.4 |
| Dusp8 | 1.8 | 1.3 | 0.7 | 1.4 | 1.4 | 1.3 | 1.4 | 1.1 | 1.0 | 1.0 | 1.2 | 0.8 |
| Dusp9 | 0.0 | 0.0 | 0.0 | 0.1 | 0.1 | 0.1 | 0.2 | 0.1 | 0.0 | 0.1 | 0.0 | 0.1 |
| Dut | 5.3 | 5.7 | 6.4 | 3.4 | 5.1 | 4.7 | 6.9 | 5.7 | 6.1 | 6.1 | 5.8 | 6.2 |
| Duxbl1 | 0.1 | 0.0 | 0.0 | 0.2 | 0.0 | 0.1 | 0.1 | 0.0 | 0.1 | 0.0 | 0.0 | 0.0 |
| Duxbl2 | 0.1 | 0.1 | 0.3 | 0.0 | 0.2 | 0.1 | 0.1 | 0.3 | 0.1 | 0.1 | 0.0 | 0.2 |
| Duxbl3 | 0.1 | 0.1 | 0.3 | 0.1 | 0.1 | 0.2 | 0.1 | 0.3 | 0.1 | 0.1 | 0.1 | 0.2 |
| Dvl1 | 18.5 | 18.3 | 15.5 | 18.9 | 15.9 | 18.2 | 19.4 | 16.7 | 21.6 | 18.7 | 19.8 | 17.5 |
| Dvl2 | 5.2 | 6.8 | 6.9 | 4.5 | 5.3 | 5.5 | 7.0 | 6.9 | 7.4 | 6.2 | 6.2 | 6.4 |
| Dvl3 | 1.8 | 2.6 | 2.6 | 2.1 | 2.2 | 2.1 | 3.1 | 2.8 | 2.6 | 2.2 | 2.1 | 1.8 |
| Dydc2 | 0.5 | 0.3 | 0.4 | 0.8 | 0.2 | 0.1 | 0.0 | 0.2 | 0.1 | 0.3 | 0.1 | 0.5 |
| Dym | 11.8 | 11.2 | 10.2 | 13.3 | 9.7 | 15.4 | 10.4 | 10.6 | 9.0 | 8.2 | 11.1 | 8.0 |
| Dync1h1 | 93.6 | 77.7 | 67.2 | 101.6 | 81.7 | 99.4 | 81.1 | 67.8 | 69.1 | 66.4 | 71.4 | 69.6 |
| Dync1i1 | 0.8 | 0.2 | 0.2 | 0.4 | 0.1 | 0.4 | 0.2 | 0.2 | 0.1 | 1.3 | 0.9 | 0.5 |
| Dync1i2 | 81.4 | 72.5 | 67.8 | 89.7 | 72.8 | 79.8 | 71.3 | 66.1 | 65.9 | 80.6 | 86.8 | 84.8 |
| Dync1li1 | 41.3 | 37.9 | 30.3 | 43.2 | 36.6 | 37.1 | 40.2 | 34.3 | 36.1 | 38.2 | 41.7 | 37.0 |
| Dync1li2 | 76.0 | 60.3 | 48.6 | 84.1 | 61.9 | 82.3 | 60.4 | 41.7 | 53.3 | 55.2 | 61.9 | 62.9 |
| Dync2h1 | 1.6 | 1.9 | 2.1 | 1.1 | 1.4 | 1.8 | 1.3 | 1.7 | 1.5 | 1.5 | 1.4 | 1.3 |
| Dync2li1 | 13.2 | 12.9 | 16.2 | 14.9 | 9.3 | 18.4 | 10.4 | 11.2 | 9.0 | 12.1 | 10.8 | 12.9 |
| Dynll1 | 56.8 | 52.7 | 45.7 | 51.8 | 55.8 | 39.5 | 62.7 | 63.9 | 72.5 | 83.0 | 80.7 | 66.1 |
| Dynll2 | 39.7 | 43.0 | 34.8 | 48.9 | 41.6 | 58.0 | 35.7 | 32.2 | 30.1 | 38.1 | 35.9 | 35.0 |
| Dynlrb1 | 174.8 | 167.5 | 165.4 | 225.0 | 158.5 | 162.0 | 185.2 | 197.8 | 148.7 | 150.3 | 209.6 | 165.9 |
| Dynlrb2 | 0.0 | 0.0 | 0.4 | 0.0 | 0.0 | 0.1 | 0.1 | 0.1 | 0.2 | 0.0 | 0.0 | 0.0 |
| Dynlt1a | 33.9 | 33.6 | 29.8 | 31.2 | 33.0 | 31.6 | 29.5 | 31.1 | 28.4 | 30.2 | 31.6 | 26.4 |
| Dynlt1b | 35.5 | 36.9 | 35.7 | 35.9 | 36.6 | 36.3 | 32.4 | 35.9 | 32.3 | 34.1 | 33.9 | 29.3 |
| Dynlt1c | 25.2 | 24.5 | 24.2 | 22.3 | 25.1 | 24.7 | 21.7 | 23.3 | 21.5 | 22.3 | 23.3 | 20.2 |
| Dynlt1f | 3.2 | 3.7 | 2.9 | 4.5 | 3.3 | 4.1 | 3.3 | 3.4 | 3.8 | 2.2 | 2.9 | 2.9 |
| Dynlt3 | 90.0 | 77.8 | 82.8 | 112.0 | 75.4 | 109.8 | 79.8 | 67.3 | 75.4 | 85.6 | 105.5 | 104.5 |
| Dyrk1a | 5.9 | 5.5 | 5.7 | 5.8 | 5.9 | 5.6 | 5.6 | 5.8 | 5.9 | 5.9 | 6.4 | 5.9 |
| Dyrk1b | 3.0 | 4.2 | 3.7 | 3.3 | 2.8 | 3.7 | 3.2 | 5.0 | 4.3 | 3.3 | 3.4 | 2.6 |
| Dyrk2 | 8.2 | 7.9 | 8.3 | 8.7 | 8.6 | 8.1 | 8.8 | 8.8 | 9.5 | 7.5 | 8.8 | 7.3 |
| Dyrk3 | 0.7 | 0.4 | 0.2 | 0.4 | 0.4 | 0.3 | 0.4 | 0.1 | 0.1 | 0.7 | 0.5 | 0.4 |
| Dyrk4 | 0.1 | 0.1 | 0.0 | 0.1 | 0.1 | 0.1 | 0.0 | 0.0 | 0.0 | 0.0 | 0.0 | 0.0 |
| Dysf | 6.5 | 4.6 | 3.2 | 7.6 | 5.1 | 7.4 | 7.3 | 4.8 | 6.4 | 5.0 | 6.8 | 5.1 |

| | | | | | | | | | | | | |
|---|---|---|---|---|---|---|---|---|---|---|---|---|
| Dyx1c1 | 1.5 | 1.2 | 1.5 | 0.9 | 0.8 | 2.2 | 0.7 | 0.8 | 0.9 | 0.5 | 1.0 | 1.3 |
| Dzank1 | 0.3 | 0.4 | 0.3 | 0.4 | 0.3 | 0.4 | 0.2 | 0.2 | 0.2 | 0.3 | 0.2 | 0.2 |
| Dzip1 | 26.6 | 20.2 | 18.2 | 25.9 | 17.7 | 30.6 | 16.4 | 10.6 | 11.3 | 15.0 | 16.4 | 15.2 |
| Dzip1l | 8.3 | 7.5 | 6.9 | 6.5 | 7.7 | 5.8 | 11.9 | 7.3 | 11.6 | 10.9 | 9.7 | 9.7 |
| Dzip3 | 7.0 | 8.8 | 9.2 | 7.1 | 6.3 | 7.4 | 6.1 | 6.1 | 6.8 | 7.9 | 8.8 | 8.0 |
| E030003E18Rik | 35.1 | 36.2 | 43.6 | 48.5 | 20.9 | 43.5 | 27.6 | 31.6 | 30.8 | 18.3 | 29.1 | 37.5 |
| E030011O05Rik | 17.8 | 19.8 | 14.0 | 19.5 | 21.2 | 19.1 | 23.9 | 17.2 | 20.0 | 16.5 | 18.5 | 17.4 |
| E030018B13Rik | 0.0 | 0.1 | 0.1 | 0.3 | 0.0 | 0.0 | 0.2 | 0.1 | 0.1 | 0.1 | 0.0 | 0.1 |
| E030024N20Rik | 1.7 | 1.7 | 1.5 | 2.6 | 1.9 | 2.2 | 2.0 | 2.1 | 1.7 | 1.8 | 1.9 | 1.8 |
| E030030I06Rik | 2.7 | 1.8 | 2.2 | 2.2 | 3.2 | 1.9 | 1.9 | 1.3 | 1.4 | 2.5 | 2.1 | 1.6 |
| E130003G02Rik | 0.0 | 0.0 | 0.0 | 0.0 | 0.0 | 0.0 | 0.0 | 0.0 | 0.0 | 0.0 | 0.0 | 0.0 |
| E130012A19Rik | 0.2 | 0.4 | 0.3 | 0.1 | 0.2 | 0.1 | 0.3 | 0.2 | 0.4 | 0.2 | 0.2 | 0.2 |
| E130018N17Rik | 0.0 | 0.0 | 0.0 | 0.0 | 0.0 | 0.0 | 0.0 | 0.0 | 0.0 | 0.0 | 0.0 | 0.0 |
| E130102H24Rik | 0.7 | 1.8 | 1.8 | 1.4 | 0.6 | 1.9 | 0.9 | 1.7 | 1.0 | 1.0 | 0.6 | 1.7 |
| E130112N10Rik | 0.3 | 0.2 | 0.1 | 0.2 | 0.2 | 0.2 | 0.1 | 0.1 | 0.2 | 0.1 | 0.1 | 0.1 |
| E130114P18Rik | 0.1 | 0.2 | 0.4 | 0.1 | 0.0 | 0.0 | 0.1 | 0.2 | 0.1 | 0.1 | 0.1 | 0.2 |
| E130201H02Rik | 0.1 | 0.1 | 0.1 | 0.1 | 0.2 | 0.2 | 0.1 | 0.1 | 0.0 | 0.1 | 0.2 | 0.3 |
| E130218I03Rik | 1.5 | 1.3 | 1.8 | 1.8 | 1.6 | 1.4 | 2.4 | 1.0 | 1.3 | 1.9 | 0.6 | 1.2 |
| E130304I02Rik | 0.8 | 0.6 | 0.7 | 0.3 | 0.3 | 0.4 | 0.2 | 0.3 | 0.2 | 0.5 | 0.7 | 0.4 |
| E130307A14Rik | 1.2 | 1.0 | 0.8 | 0.7 | 0.9 | 0.7 | 1.0 | 1.0 | 0.9 | 1.0 | 0.8 | 0.9 |
| E130308A19Rik | 1.9 | 2.2 | 1.5 | 2.1 | 1.9 | 2.6 | 2.6 | 2.5 | 2.9 | 2.2 | 2.4 | 2.5 |
| E130309D02Rik | 4.5 | 4.3 | 3.9 | 4.3 | 5.0 | 4.5 | 5.9 | 3.9 | 5.0 | 5.2 | 3.5 | 4.0 |
| E130309D14Rik | 0.1 | 0.1 | 0.0 | 0.2 | 0.0 | 0.1 | 0.2 | 0.1 | 0.0 | 0.2 | 0.0 | 0.1 |
| E130310I04Rik | 0.1 | 0.2 | 0.1 | 0.4 | 0.2 | 0.1 | 0.7 | 0.1 | 0.2 | 0.1 | 0.2 | 0.1 |
| E130311K13Rik | 8.4 | 8.2 | 9.5 | 7.0 | 5.8 | 7.9 | 6.6 | 6.7 | 7.2 | 7.5 | 7.6 | 9.6 |
| E130317F20Rik | 1.3 | 0.8 | 0.9 | 1.0 | 0.9 | 1.0 | 1.2 | 1.2 | 1.4 | 1.0 | 1.4 | 1.2 |
| E230016K23Rik | 0.4 | 0.6 | 0.7 | 0.2 | 0.5 | 0.5 | 0.6 | 0.6 | 0.6 | 0.3 | 0.5 | 0.4 |
| E230016M11Rik | 2.1 | 1.0 | 1.4 | 2.0 | 1.4 | 3.2 | 1.3 | 1.1 | 0.8 | 1.8 | 1.1 | 1.1 |
| E230025N22Rik | 0.2 | 0.1 | 0.2 | 0.1 | 0.1 | 0.1 | 0.1 | 0.1 | 0.0 | 0.1 | 0.2 | 0.1 |
| E2f1 | 9.2 | 7.9 | 6.6 | 9.7 | 7.1 | 11.0 | 7.4 | 4.9 | 6.0 | 6.4 | 6.4 | 5.4 |
| E2f2 | 0.2 | 0.0 | 0.1 | 0.2 | 0.9 | 0.0 | 0.2 | 0.2 | 0.1 | 0.3 | 0.2 | 0.2 |
| E2f3 | 5.2 | 5.5 | 5.4 | 5.2 | 4.5 | 5.4 | 5.2 | 4.3 | 4.9 | 4.2 | 4.7 | 4.7 |
| E2f4 | 6.1 | 7.5 | 6.6 | 6.3 | 7.0 | 6.4 | 8.3 | 7.9 | 7.5 | 6.9 | 6.5 | 6.1 |
| E2f5 | 3.4 | 3.3 | 3.2 | 3.0 | 3.3 | 3.1 | 3.8 | 3.2 | 3.4 | 3.0 | 3.8 | 3.3 |
| E2f6 | 12.9 | 14.1 | 9.9 | 16.7 | 13.5 | 14.2 | 12.3 | 9.5 | 10.2 | 11.7 | 14.4 | 10.8 |
| E2f7 | 0.2 | 0.3 | 0.5 | 0.1 | 0.5 | 0.1 | 0.8 | 0.3 | 0.5 | 0.2 | 0.3 | 0.1 |
| E2f8 | 0.4 | 0.6 | 1.1 | 0.2 | 1.0 | 0.1 | 1.1 | 0.7 | 0.7 | 0.4 | 0.8 | 0.4 |
| E330009J07Rik | 0.5 | 0.6 | 0.9 | 0.3 | 0.7 | 0.9 | 0.4 | 0.6 | 0.6 | 0.6 | 0.7 | 1.7 |
| E330011O21Rik | 0.4 | 0.2 | 0.5 | 0.1 | 0.3 | 0.2 | 0.1 | 0.5 | 0.1 | 0.8 | 0.6 | 0.3 |
| E330013P04Rik | 1.2 | 0.2 | 0.2 | 0.3 | 0.3 | 0.5 | 0.0 | 0.3 | 0.1 | 0.7 | 0.2 | 0.1 |
| E330020D12Rik | 0.2 | 0.3 | 0.6 | 0.7 | 0.4 | 0.4 | 0.3 | 0.4 | 0.3 | 0.2 | 0.3 | 0.3 |
| E330023G01Rik | 0.2 | 0.0 | 0.1 | 0.0 | 0.2 | 0.1 | 0.2 | 0.1 | 0.0 | 0.0 | 0.1 | 0.1 |
| E330033B04Rik | 0.2 | 0.3 | 0.3 | 0.1 | 0.2 | 0.2 | 0.3 | 0.2 | 0.2 | 0.2 | 0.3 | 0.1 |
| E330034G19Rik | 0.1 | 0.0 | 0.1 | 0.0 | 0.1 | 0.1 | 0.0 | 0.0 | 0.1 | 0.0 | 0.1 | 0.0 |
| E430018J23Rik | 2.5 | 2.2 | 2.5 | 2.7 | 2.0 | 2.7 | 2.2 | 2.4 | 2.3 | 2.9 | 2.2 | 2.8 |
| E430025E21Rik | 39.1 | 31.9 | 27.1 | 42.1 | 33.8 | 42.1 | 28.4 | 28.4 | 25.5 | 30.2 | 32.2 | 31.0 |
| E4f1 | 3.4 | 3.3 | 4.0 | 4.4 | 3.3 | 3.0 | 3.7 | 4.0 | 4.1 | 4.0 | 3.5 | 3.8 |
| E530011L22Rik | 1.0 | 0.8 | 1.0 | 0.9 | 0.6 | 0.9 | 0.7 | 0.9 | 0.8 | 0.8 | 0.8 | 1.2 |
| Eaf1 | 14.3 | 15.4 | 11.5 | 18.3 | 17.1 | 14.6 | 14.9 | 11.6 | 13.2 | 13.7 | 13.5 | 12.0 |
| Eaf2 | 0.3 | 0.2 | 0.2 | 0.2 | 0.3 | 0.3 | 0.3 | 0.4 | 0.2 | 0.2 | 0.2 | 0.2 |
| Eapp | 30.5 | 27.1 | 28.5 | 27.6 | 23.6 | 32.3 | 30.6 | 31.0 | 26.1 | 28.1 | 33.3 | 34.2 |
| Ears2 | 5.3 | 3.6 | 2.9 | 4.2 | 3.8 | 4.7 | 3.3 | 3.8 | 4.1 | 4.2 | 3.1 | 3.4 |
| Ebag9 | 7.6 | 6.7 | 8.2 | 8.1 | 6.1 | 7.7 | 8.0 | 8.3 | 7.0 | 7.6 | 9.4 | 9.5 |
| Ebf1 | 13.1 | 22.2 | 31.9 | 16.3 | 15.1 | 11.2 | 22.8 | 29.8 | 24.2 | 11.1 | 18.8 | 17.6 |
| Ebf2 | 2.2 | 3.4 | 4.1 | 3.1 | 2.6 | 4.0 | 3.4 | 4.5 | 3.4 | 2.7 | 3.0 | 3.3 |
| Ebf3 | 2.1 | 4.4 | 4.4 | 4.4 | 3.6 | 3.9 | 6.5 | 4.9 | 5.4 | 2.8 | 4.1 | 3.8 |
| Ebf4 | 0.2 | 0.7 | 0.6 | 0.2 | 0.6 | 0.7 | 0.6 | 0.2 | 0.6 | 0.7 | 0.7 | 0.6 |

| | | | | | | | | | | | | |
|---|---|---|---|---|---|---|---|---|---|---|---|---|
| Ebi3 | 0.6 | 0.0 | 0.1 | 2.6 | 4.7 | 0.0 | 0.8 | 0.1 | 0.3 | 0.5 | 0.2 | 0.1 |
| Ebna1bp2 | 14.3 | 12.7 | 10.4 | 16.7 | 12.7 | 13.6 | 13.5 | 12.0 | 11.1 | 13.3 | 14.4 | 13.5 |
| Ebp | 12.5 | 12.9 | 13.1 | 12.5 | 12.1 | 11.4 | 16.5 | 18.1 | 14.4 | 12.8 | 18.2 | 13.7 |
| Ebpl | 6.7 | 6.1 | 5.2 | 5.3 | 5.8 | 5.4 | 5.7 | 5.8 | 6.0 | 6.0 | 5.7 | 5.3 |
| Ecd | 11.7 | 10.8 | 10.7 | 13.0 | 10.1 | 11.5 | 10.5 | 10.9 | 9.4 | 10.5 | 10.9 | 10.0 |
| Ece1 | 54.1 | 49.0 | 49.4 | 56.1 | 46.3 | 54.0 | 49.2 | 45.3 | 37.6 | 33.3 | 39.4 | 38.7 |
| Ece2 | 18.7 | 14.8 | 12.3 | 16.9 | 17.0 | 20.5 | 14.9 | 13.6 | 15.0 | 14.2 | 13.1 | 16.0 |
| Ecel1 | 0.1 | 0.0 | 0.0 | 0.0 | 0.0 | 0.1 | 0.0 | 0.0 | 0.0 | 0.0 | 0.0 | 0.0 |
| Ech1 | 36.7 | 30.7 | 31.3 | 32.8 | 32.9 | 38.7 | 32.6 | 38.7 | 33.1 | 37.1 | 33.6 | 33.8 |
| Echdc1 | 4.1 | 4.3 | 4.4 | 4.0 | 3.8 | 4.2 | 4.6 | 5.2 | 3.7 | 3.7 | 5.4 | 4.1 |
| Echdc2 | 9.5 | 12.1 | 14.5 | 5.8 | 6.1 | 8.7 | 7.0 | 11.5 | 9.0 | 9.9 | 8.1 | 9.1 |
| Echdc3 | 5.6 | 4.5 | 4.3 | 4.3 | 2.7 | 6.7 | 3.5 | 3.1 | 3.1 | 2.6 | 2.7 | 3.2 |
| Echs1 | 31.0 | 33.9 | 35.0 | 16.2 | 22.8 | 19.2 | 26.7 | 44.2 | 35.7 | 28.7 | 31.4 | 28.0 |
| Eci1 | 26.3 | 20.4 | 15.3 | 19.5 | 16.5 | 22.7 | 16.1 | 16.8 | 18.9 | 22.6 | 19.1 | 18.8 |
| Eci2 | 32.5 | 25.9 | 26.3 | 26.5 | 24.1 | 28.5 | 23.0 | 27.9 | 23.6 | 24.9 | 21.6 | 24.5 |
| Eci3 | 0.1 | 0.3 | 0.4 | 0.1 | 0.2 | 0.2 | 0.0 | 0.1 | 0.2 | 0.2 | 0.4 | 0.5 |
| Ecm1 | 97.9 | 109.6 | 103.2 | 119.6 | 128.0 | 164.6 | 146.2 | 125.9 | 108.9 | 102.8 | 117.1 | 95.4 |
| Ecm2 | 0.6 | 0.8 | 1.1 | 1.0 | 0.8 | 0.8 | 0.6 | 1.0 | 0.6 | 0.6 | 0.6 | 0.8 |
| Ecscr | 9.7 | 6.7 | 8.3 | 9.4 | 12.5 | 5.3 | 10.8 | 9.5 | 7.1 | 5.1 | 5.9 | 6.6 |
| Ecsit | 7.7 | 7.2 | 6.4 | 6.6 | 6.3 | 8.6 | 6.3 | 7.0 | 7.4 | 6.2 | 6.9 | 6.4 |
| Ect2 | 1.4 | 2.8 | 4.6 | 0.9 | 4.0 | 0.8 | 5.2 | 1.9 | 4.3 | 2.8 | 3.9 | 2.8 |
| Ect2l | 0.0 | 0.0 | 0.0 | 0.0 | 0.0 | 0.1 | 0.0 | 0.1 | 0.0 | 0.0 | 0.0 | 0.0 |
| Eda | 0.8 | 1.1 | 1.1 | 0.5 | 0.7 | 0.5 | 0.9 | 0.9 | 1.0 | 0.5 | 0.6 | 0.7 |
| Eda2r | 20.5 | 20.9 | 20.5 | 19.7 | 21.1 | 18.5 | 18.8 | 14.8 | 20.9 | 30.7 | 26.0 | 24.8 |
| Edar | 0.1 | 0.0 | 0.0 | 0.0 | 0.1 | 0.0 | 0.0 | 0.0 | 0.0 | 0.0 | 0.0 | 0.0 |
| Edaradd | 0.1 | 0.1 | 0.0 | 0.1 | 0.0 | 0.0 | 0.0 | 0.1 | 0.0 | 0.0 | 0.0 | 0.0 |
| Edc3 | 1.2 | 1.7 | 2.1 | 1.4 | 2.0 | 1.4 | 1.9 | 1.7 | 1.7 | 1.6 | 1.7 | 1.4 |
| Edc4 | 9.3 | 11.0 | 9.7 | 8.7 | 10.1 | 9.7 | 11.0 | 9.9 | 10.0 | 9.0 | 8.6 | 8.0 |
| Edem1 | 21.6 | 21.6 | 20.0 | 24.1 | 25.7 | 20.4 | 21.8 | 22.1 | 23.4 | 19.8 | 21.2 | 21.6 |
| Edem2 | 12.8 | 11.3 | 11.7 | 14.3 | 15.5 | 11.3 | 12.8 | 13.1 | 12.6 | 12.8 | 10.8 | 11.5 |
| Edem3 | 19.6 | 18.6 | 18.4 | 21.1 | 19.4 | 18.7 | 19.3 | 17.5 | 18.6 | 17.6 | 20.2 | 21.8 |
| Edf1 | 172.1 | 125.8 | 145.6 | 154.4 | 149.5 | 136.2 | 160.3 | 163.6 | 156.7 | 156.7 | 157.2 | 179.6 |
| Edil3 | 0.1 | 0.0 | 0.0 | 0.2 | 0.6 | 0.0 | 0.1 | 0.1 | 0.0 | 0.1 | 0.1 | 0.1 |
| Edn1 | 8.1 | 5.1 | 4.1 | 5.6 | 3.3 | 3.8 | 2.7 | 2.3 | 2.3 | 4.8 | 3.1 | 2.3 |
| Ednra | 0.1 | 0.1 | 0.2 | 0.0 | 0.1 | 0.0 | 0.2 | 0.5 | 0.3 | 0.6 | 0.6 | 0.7 |
| Ednrb | 0.1 | 0.1 | 0.1 | 0.3 | 0.5 | 0.2 | 0.1 | 0.1 | 0.1 | 0.1 | 0.1 | 0.0 |
| Eea1 | 12.6 | 10.0 | 9.4 | 15.8 | 12.9 | 13.9 | 11.2 | 7.6 | 8.6 | 11.4 | 12.9 | 13.1 |
| Eed | 13.0 | 13.8 | 15.4 | 13.6 | 13.3 | 13.1 | 12.6 | 11.5 | 13.0 | 14.6 | 13.9 | 13.4 |
| Eef1a1 | 4875.7 | 3820.5 | 3815.1 | 3914.7 | 3706.4 | 4497.8 | 3663.6 | 5103.2 | 4069.7 | 4452.1 | 5121.7 | 4572.8 |
| Eef1a2 | 2.7 | 3.7 | 6.4 | 3.2 | 2.8 | 3.6 | 0.8 | 3.3 | 0.5 | 1.0 | 1.2 | 1.4 |
| Eef1b2 | 263.6 | 210.2 | 205.7 | 193.0 | 195.7 | 217.9 | 191.6 | 237.3 | 185.3 | 249.6 | 239.7 | 220.6 |
| Eef1d | 101.8 | 80.1 | 81.0 | 89.5 | 72.1 | 96.4 | 88.7 | 83.9 | 70.3 | 85.5 | 96.1 | 89.0 |
| Eef1e1 | 23.5 | 21.0 | 16.1 | 27.8 | 19.3 | 21.0 | 21.3 | 17.3 | 16.0 | 22.1 | 23.5 | 19.5 |
| Eef1g | 580.1 | 551.9 | 561.0 | 493.6 | 469.4 | 540.5 | 563.0 | 628.1 | 531.8 | 512.4 | 624.7 | 565.9 |
| Eef2 | 766.9 | 611.3 | 638.8 | 591.2 | 648.4 | 717.1 | 649.8 | 816.0 | 693.4 | 792.4 | 758.3 | 748.1 |
| Eef2k | 3.3 | 3.9 | 4.2 | 3.4 | 2.8 | 3.8 | 4.1 | 6.2 | 4.3 | 3.4 | 4.4 | 3.7 |
| Eefsec | 5.0 | 5.3 | 5.0 | 4.9 | 4.2 | 5.8 | 5.2 | 4.7 | 3.4 | 3.9 | 4.0 | 4.3 |
| Eepd1 | 0.2 | 0.2 | 0.3 | 0.1 | 0.5 | 0.1 | 0.2 | 0.3 | 0.1 | 0.1 | 0.1 | 0.1 |
| Efcab1 | 0.1 | 0.1 | 0.1 | 0.0 | 0.0 | 0.0 | 0.0 | 0.0 | 0.0 | 0.1 | 0.0 | 0.1 |
| Efcab11 | 0.4 | 0.5 | 0.9 | 0.1 | 0.5 | 0.3 | 1.3 | 0.6 | 1.1 | 0.3 | 0.9 | 0.3 |
| Efcab12 | 0.1 | 0.1 | 0.3 | 0.1 | 0.0 | 0.1 | 0.1 | 0.2 | 0.0 | 0.1 | 0.1 | 0.0 |
| Efcab14 | 9.9 | 8.8 | 9.8 | 10.4 | 9.3 | 10.2 | 8.7 | 8.1 | 8.3 | 7.8 | 8.1 | 8.1 |
| Efcab2 | 0.8 | 0.7 | 0.6 | 0.9 | 0.4 | 0.8 | 0.6 | 0.5 | 0.6 | 0.6 | 0.7 | 0.7 |
| Efcab3 | 0.0 | 0.1 | 0.0 | 0.1 | 0.1 | 0.0 | 0.1 | 0.0 | 0.2 | 0.0 | 0.0 | 0.1 |
| Efcab4a | 1.8 | 1.4 | 1.2 | 1.9 | 0.9 | 2.2 | 1.2 | 0.9 | 1.0 | 1.0 | 0.7 | 1.0 |
| Efcab4b | 0.6 | 0.3 | 0.8 | 0.3 | 1.0 | 0.7 | 0.4 | 0.7 | 0.2 | 0.3 | 0.5 | 0.5 |
| Efcab5 | 0.2 | 0.2 | 0.2 | 0.4 | 0.2 | 0.2 | 0.1 | 0.1 | 0.1 | 0.1 | 0.1 | 0.1 |

| | | | | | | | | | | | | |
|---|---|---|---|---|---|---|---|---|---|---|---|---|
| Efcab6 | 0.0 | 0.0 | 0.0 | 0.0 | 0.0 | 0.0 | 0.0 | 0.0 | 0.0 | 0.0 | 0.0 | 0.0 |
| Efcab7 | 1.7 | 1.4 | 1.6 | 1.5 | 1.3 | 2.3 | 1.2 | 1.4 | 1.3 | 1.5 | 1.4 | 1.3 |
| Efcab8 | 0.0 | 0.1 | 0.0 | 0.0 | 0.0 | 0.1 | 0.0 | 0.0 | 0.2 | 0.0 | 0.0 | 0.0 |
| Efcab9 | 0.1 | 0.2 | 0.1 | 0.0 | 0.3 | 0.3 | 0.2 | 0.1 | 0.2 | 0.2 | 0.3 | 0.2 |
| Efcc1 | 0.1 | 0.0 | 0.1 | 0.1 | 0.1 | 0.1 | 0.1 | 0.1 | 0.0 | 0.0 | 0.0 | 0.0 |
| Efemp1 | 346.4 | 234.1 | 249.4 | 109.7 | 92.7 | 48.8 | 152.9 | 155.9 | 159.4 | 215.2 | 219.7 | 204.5 |
| Efemp2 | 174.3 | 149.0 | 147.4 | 197.9 | 170.6 | 182.9 | 211.7 | 184.6 | 196.5 | 177.5 | 180.2 | 191.7 |
| Efha1 | 37.0 | 33.2 | 33.1 | 37.5 | 30.9 | 36.1 | 33.3 | 34.1 | 35.4 | 38.4 | 39.4 | 40.3 |
| Efha2 | 1.8 | 2.1 | 2.1 | 2.6 | 2.1 | 1.9 | 2.5 | 2.3 | 2.1 | 2.6 | 2.9 | 2.6 |
| Efhb | 0.1 | 0.4 | 0.3 | 0.1 | 0.1 | 0.2 | 0.2 | 0.1 | 0.1 | 0.1 | 0.1 | 0.2 |
| Efhc1 | 3.2 | 3.2 | 3.2 | 2.7 | 2.1 | 3.3 | 2.4 | 2.5 | 2.1 | 2.8 | 2.7 | 2.4 |
| Efhc2 | 0.0 | 0.0 | 0.0 | 0.0 | 0.0 | 0.0 | 0.0 | 0.0 | 0.0 | 0.0 | 0.0 | 0.0 |
| Efhd1 | 0.2 | 0.2 | 0.2 | 0.1 | 0.1 | 0.0 | 0.0 | 0.2 | 0.1 | 0.1 | 0.0 | 0.1 |
| Efhd2 | 12.9 | 12.1 | 12.5 | 22.0 | 32.1 | 11.1 | 15.6 | 12.8 | 12.2 | 15.6 | 13.3 | 13.9 |
| Efna1 | 0.8 | 1.0 | 1.8 | 0.6 | 0.9 | 0.4 | 0.8 | 2.9 | 1.6 | 2.0 | 1.7 | 1.2 |
| Efna2 | 0.7 | 1.3 | 1.7 | 0.5 | 0.6 | 0.7 | 0.9 | 1.1 | 1.1 | 1.5 | 1.4 | 1.2 |
| Efna3 | 2.1 | 2.9 | 1.9 | 3.0 | 1.5 | 2.7 | 1.7 | 1.4 | 1.2 | 1.7 | 1.7 | 1.6 |
| Efna4 | 3.4 | 3.1 | 3.0 | 3.1 | 2.4 | 3.6 | 3.9 | 3.4 | 3.7 | 4.2 | 4.5 | 3.1 |
| Efna5 | 1.5 | 1.7 | 2.3 | 1.1 | 1.8 | 1.4 | 2.1 | 2.0 | 1.7 | 2.4 | 2.0 | 1.6 |
| Efnb1 | 6.1 | 5.7 | 5.9 | 6.5 | 6.1 | 5.5 | 9.1 | 7.5 | 12.3 | 15.3 | 13.8 | 12.0 |
| Efnb2 | 4.1 | 3.5 | 3.4 | 3.4 | 3.8 | 3.3 | 5.0 | 3.3 | 4.6 | 7.0 | 5.3 | 4.1 |
| Efnb3 | 0.1 | 0.1 | 0.0 | 0.1 | 0.1 | 0.1 | 0.0 | 0.0 | 0.1 | 0.0 | 0.0 | 0.0 |
| Efr3a | 23.3 | 22.4 | 20.0 | 22.7 | 19.1 | 23.4 | 19.1 | 19.1 | 19.5 | 20.7 | 22.6 | 20.5 |
| Efr3b | 2.4 | 2.6 | 2.7 | 2.4 | 2.2 | 2.9 | 1.9 | 2.4 | 1.9 | 1.4 | 1.6 | 2.2 |
| Efs | 2.1 | 3.8 | 4.3 | 0.6 | 1.8 | 1.9 | 2.3 | 6.0 | 4.1 | 2.2 | 3.1 | 2.9 |
| Eftud1 | 6.7 | 6.3 | 6.3 | 7.1 | 5.8 | 8.1 | 5.6 | 6.5 | 5.2 | 6.6 | 6.3 | 5.7 |
| Eftud2 | 16.8 | 15.5 | 16.8 | 20.0 | 19.1 | 17.4 | 17.8 | 17.7 | 14.2 | 15.5 | 15.9 | 16.5 |
| Egf | 0.2 | 0.1 | 0.1 | 0.2 | 0.1 | 0.2 | 0.1 | 0.1 | 0.1 | 0.2 | 0.2 | 0.1 |
| Egfl6 | 0.0 | 0.0 | 0.1 | 0.0 | 0.0 | 0.0 | 0.0 | 0.0 | 0.0 | 0.0 | 0.1 | 0.0 |
| Egfl7 | 3.4 | 1.5 | 2.0 | 1.7 | 1.7 | 2.6 | 2.2 | 2.8 | 2.7 | 1.6 | 0.6 | 1.4 |
| Egfl8 | 0.2 | 0.1 | 0.4 | 0.2 | 0.1 | 0.4 | 0.4 | 0.4 | 0.3 | 0.3 | 0.3 | 0.1 |
| Egflam | 0.1 | 0.1 | 0.3 | 0.1 | 0.1 | 0.0 | 0.2 | 0.3 | 0.2 | 0.1 | 0.1 | 0.1 |
| Egfr | 12.7 | 14.6 | 19.3 | 8.9 | 12.3 | 8.6 | 11.9 | 16.6 | 15.3 | 30.3 | 20.4 | 19.6 |
| Egln1 | 24.3 | 21.2 | 15.8 | 29.3 | 23.7 | 31.6 | 23.2 | 13.4 | 19.6 | 19.5 | 19.8 | 19.9 |
| Egln2 | 15.4 | 14.7 | 13.9 | 17.0 | 15.6 | 16.9 | 18.1 | 18.8 | 17.6 | 18.7 | 16.3 | 16.5 |
| Egln3 | 1.6 | 2.3 | 1.3 | 2.0 | 1.9 | 2.9 | 1.7 | 0.7 | 1.0 | 2.3 | 1.5 | 1.0 |
| Egr1 | 0.6 | 1.0 | 1.1 | 0.4 | 1.1 | 0.5 | 0.8 | 0.4 | 1.1 | 8.1 | 1.8 | 1.4 |
| Egr2 | 0.2 | 0.2 | 0.4 | 0.6 | 0.9 | 0.1 | 0.7 | 0.2 | 1.0 | 3.4 | 0.9 | 1.3 |
| Egr3 | 0.0 | 0.0 | 0.0 | 0.0 | 0.1 | 0.0 | 0.1 | 0.1 | 0.2 | 0.7 | 0.1 | 0.2 |
| Ehbp1 | 9.3 | 11.8 | 13.2 | 7.8 | 8.0 | 8.1 | 10.6 | 9.8 | 10.1 | 9.2 | 10.8 | 10.8 |
| Ehbp1l1 | 22.3 | 18.1 | 15.2 | 28.1 | 28.6 | 19.8 | 33.0 | 18.6 | 26.5 | 21.7 | 25.3 | 22.3 |
| Ehd1 | 39.3 | 30.1 | 27.4 | 33.9 | 35.7 | 29.3 | 40.8 | 42.6 | 37.1 | 39.2 | 45.0 | 35.8 |
| Ehd2 | 60.4 | 69.4 | 71.5 | 57.1 | 68.8 | 55.9 | 80.5 | 108.2 | 84.2 | 57.0 | 74.6 | 68.2 |
| Ehd3 | 16.2 | 16.9 | 17.3 | 21.0 | 13.4 | 14.8 | 16.0 | 13.0 | 19.2 | 12.4 | 15.9 | 15.5 |
| Ehd4 | 6.9 | 4.4 | 3.8 | 12.4 | 12.1 | 6.4 | 5.4 | 3.3 | 3.6 | 5.1 | 5.0 | 4.2 |
| Ehf | 0.0 | 0.0 | 0.0 | 0.0 | 0.0 | 0.0 | 0.0 | 0.0 | 0.0 | 0.0 | 0.0 | 0.0 |
| Ehhadh | 1.8 | 2.2 | 1.9 | 1.7 | 1.7 | 1.8 | 2.3 | 1.8 | 1.8 | 1.4 | 1.8 | 2.0 |
| Ehmt1 | 6.5 | 8.1 | 8.7 | 7.3 | 7.5 | 6.8 | 7.2 | 8.3 | 7.2 | 7.3 | 7.9 | 7.9 |
| Ehmt2 | 25.9 | 24.3 | 24.7 | 22.4 | 22.5 | 24.3 | 23.6 | 22.7 | 24.3 | 29.5 | 23.7 | 23.3 |
| Ei24 | 42.4 | 44.4 | 41.9 | 41.2 | 45.7 | 36.5 | 41.6 | 33.7 | 41.0 | 42.3 | 42.9 | 39.2 |
| Eid1 | 21.0 | 17.1 | 21.0 | 30.0 | 25.5 | 26.1 | 26.9 | 22.2 | 23.5 | 32.8 | 32.9 | 35.2 |
| Eid2 | 8.4 | 7.5 | 8.3 | 7.8 | 6.5 | 8.7 | 8.8 | 8.7 | 8.3 | 9.5 | 8.1 | 9.4 |
| Eid2b | 1.9 | 2.5 | 2.3 | 2.2 | 2.0 | 2.5 | 2.5 | 2.0 | 2.3 | 2.9 | 1.7 | 2.4 |
| Eid3 | 1.3 | 0.7 | 0.4 | 1.7 | 1.3 | 1.6 | 1.0 | 0.5 | 0.5 | 1.7 | 0.5 | 0.8 |
| Eif1 | 36.6 | 38.5 | 36.8 | 33.1 | 47.5 | 37.6 | 38.6 | 46.7 | 47.0 | 48.9 | 43.9 | 43.5 |
| Eif1a | 43.1 | 33.8 | 32.5 | 50.4 | 43.6 | 41.2 | 43.3 | 39.2 | 39.5 | 45.4 | 55.3 | 47.2 |
| Eif1ad | 5.5 | 4.8 | 5.2 | 5.6 | 5.9 | 5.3 | 4.9 | 5.8 | 5.3 | 4.7 | 5.8 | 4.6 |

| | | | | | | | | | | | | |
|---|---|---|---|---|---|---|---|---|---|---|---|---|
| Eif1ax | 64.1 | 56.1 | 52.5 | 66.0 | 57.0 | 62.5 | 53.6 | 60.8 | 58.3 | 62.5 | 71.7 | 62.9 |
| Eif1b | 24.7 | 21.4 | 24.6 | 33.6 | 22.5 | 33.9 | 23.7 | 24.9 | 18.7 | 22.1 | 22.2 | 24.2 |
| Eif2a | 28.9 | 24.6 | 24.4 | 23.9 | 21.3 | 26.3 | 22.0 | 24.0 | 20.9 | 24.0 | 27.7 | 27.2 |
| Eif2ak1 | 12.9 | 14.9 | 15.4 | 15.7 | 12.8 | 13.4 | 14.6 | 14.1 | 12.9 | 11.7 | 14.4 | 14.6 |
| Eif2ak2 | 24.9 | 18.2 | 23.0 | 27.2 | 20.5 | 16.6 | 18.5 | 13.5 | 14.7 | 13.9 | 17.4 | 25.1 |
| Eif2ak3 | 11.0 | 10.1 | 9.4 | 10.3 | 10.6 | 10.2 | 11.8 | 10.0 | 10.6 | 8.6 | 10.4 | 10.7 |
| Eif2ak4 | 4.5 | 4.9 | 4.6 | 3.8 | 5.2 | 4.2 | 4.9 | 4.3 | 4.9 | 5.1 | 5.0 | 5.0 |
| Eif2b1 | 14.5 | 14.1 | 11.4 | 14.1 | 12.6 | 13.0 | 13.7 | 13.3 | 13.1 | 14.4 | 13.6 | 12.1 |
| Eif2b2 | 17.9 | 17.4 | 15.5 | 20.7 | 16.4 | 18.0 | 17.6 | 17.5 | 15.8 | 16.8 | 17.5 | 16.0 |
| Eif2b3 | 11.9 | 10.8 | 8.7 | 12.6 | 10.7 | 11.4 | 11.0 | 9.8 | 9.9 | 11.2 | 12.2 | 11.8 |
| Eif2b4 | 9.0 | 9.3 | 8.7 | 10.0 | 8.6 | 8.2 | 9.4 | 9.3 | 8.2 | 8.1 | 8.7 | 9.7 |
| Eif2b5 | 32.0 | 27.3 | 24.0 | 30.4 | 24.3 | 30.6 | 25.0 | 26.4 | 23.9 | 25.1 | 25.7 | 27.1 |
| Eif2c1 | 5.4 | 6.3 | 5.6 | 5.1 | 4.7 | 6.5 | 4.5 | 5.4 | 4.6 | 4.4 | 4.3 | 4.1 |
| Eif2c2 | 5.5 | 6.6 | 5.1 | 5.8 | 6.8 | 6.6 | 6.4 | 4.8 | 5.9 | 5.1 | 5.9 | 5.0 |
| Eif2c3 | 4.5 | 5.1 | 4.7 | 4.4 | 4.6 | 5.4 | 3.4 | 3.9 | 3.1 | 4.2 | 4.0 | 3.7 |
| Eif2c4 | 1.3 | 1.6 | 1.7 | 1.7 | 1.2 | 1.0 | 1.1 | 1.1 | 1.3 | 1.1 | 1.4 | 1.4 |
| Eif2d | 18.3 | 18.2 | 18.0 | 18.6 | 16.3 | 18.7 | 15.5 | 16.8 | 15.4 | 16.0 | 17.8 | 16.0 |
| Eif2s1 | 32.9 | 33.3 | 30.4 | 34.0 | 33.6 | 28.8 | 34.6 | 31.8 | 30.2 | 35.4 | 38.6 | 36.3 |
| Eif2s2 | 87.7 | 71.8 | 69.3 | 76.7 | 61.7 | 72.2 | 75.2 | 78.1 | 72.5 | 75.7 | 94.0 | 87.5 |
| Eif2s3x | 64.5 | 51.2 | 47.8 | 64.1 | 52.6 | 62.1 | 52.7 | 49.7 | 47.3 | 53.7 | 61.7 | 56.5 |
| Eif2s3y | 25.7 | 24.2 | 30.2 | 28.4 | 24.9 | 25.9 | 24.9 | 27.2 | 24.6 | 26.1 | 23.4 | 31.8 |
| Eif3a | 114.6 | 100.3 | 114.6 | 140.3 | 111.2 | 111.5 | 126.9 | 105.2 | 88.8 | 104.3 | 135.8 | 137.8 |
| Eif3b | 53.6 | 53.2 | 47.1 | 59.7 | 59.5 | 57.5 | 61.7 | 56.8 | 51.0 | 56.7 | 56.7 | 55.6 |
| Eif3c | 121.5 | 85.9 | 86.8 | 121.4 | 85.6 | 116.5 | 91.0 | 105.4 | 80.1 | 90.4 | 112.0 | 95.4 |
| Eif3d | 59.7 | 59.8 | 57.1 | 65.2 | 53.2 | 57.7 | 60.9 | 63.2 | 53.6 | 57.9 | 61.1 | 58.7 |
| Eif3e | 126.4 | 99.7 | 97.2 | 103.2 | 101.3 | 111.9 | 91.8 | 100.7 | 94.1 | 119.9 | 123.5 | 110.4 |
| Eif3f | 87.0 | 77.1 | 67.6 | 61.4 | 67.5 | 75.7 | 66.7 | 81.3 | 65.3 | 70.9 | 79.1 | 68.4 |
| Eif3g | 67.0 | 73.7 | 68.3 | 69.2 | 60.9 | 71.8 | 66.6 | 69.2 | 59.0 | 71.8 | 71.6 | 70.0 |
| Eif3h | 124.9 | 109.9 | 111.6 | 116.1 | 104.7 | 120.3 | 103.3 | 125.5 | 100.0 | 110.6 | 122.4 | 114.6 |
| Eif3i | 107.0 | 87.7 | 74.8 | 88.3 | 84.3 | 91.7 | 76.8 | 92.4 | 85.5 | 92.2 | 93.8 | 78.9 |
| Eif3j1 | 13.0 | 11.4 | 14.6 | 21.3 | 16.8 | 12.2 | 14.2 | 12.9 | 16.3 | 24.0 | 18.4 | 20.6 |
| Eif3j2 | 9.3 | 11.2 | 7.8 | 4.7 | 5.9 | 8.5 | 11.1 | 8.8 | 6.2 | 2.8 | 10.9 | 7.6 |
| Eif3k | 150.6 | 125.6 | 119.2 | 144.0 | 116.1 | 149.8 | 125.9 | 146.7 | 113.5 | 135.4 | 141.2 | 127.8 |
| Eif3l | 81.6 | 71.3 | 65.1 | 79.4 | 68.3 | 76.0 | 77.0 | 79.0 | 67.9 | 76.9 | 81.8 | 74.3 |
| Eif3m | 87.7 | 74.9 | 58.6 | 67.5 | 70.9 | 71.5 | 58.9 | 63.0 | 74.2 | 95.7 | 77.1 | 78.3 |
| Eif4a1 | 288.3 | 255.0 | 229.3 | 275.6 | 295.0 | 230.8 | 277.9 | 288.1 | 260.9 | 293.3 | 304.1 | 278.6 |
| Eif4a2 | 160.1 | 162.3 | 170.6 | 126.9 | 124.7 | 168.0 | 123.0 | 115.7 | 159.0 | 187.8 | 176.4 | 196.5 |
| Eif4a3 | 41.2 | 43.8 | 41.7 | 40.3 | 41.7 | 36.8 | 47.6 | 46.5 | 44.4 | 41.9 | 46.0 | 46.4 |
| Eif4b | 98.8 | 86.2 | 97.1 | 85.5 | 81.5 | 94.9 | 90.8 | 104.4 | 86.9 | 97.9 | 109.2 | 110.1 |
| Eif4e | 17.4 | 15.9 | 14.7 | 18.6 | 16.7 | 15.4 | 18.2 | 16.6 | 16.1 | 19.8 | 21.0 | 18.4 |
| Eif4e2 | 27.5 | 20.9 | 25.4 | 27.6 | 25.0 | 22.6 | 28.9 | 31.3 | 26.1 | 26.3 | 31.4 | 30.0 |
| Eif4e3 | 4.2 | 4.8 | 5.1 | 7.9 | 5.4 | 4.9 | 2.8 | 2.5 | 2.0 | 1.7 | 2.0 | 2.4 |
| Eif4ebp1 | 71.5 | 48.9 | 37.3 | 66.2 | 46.1 | 72.2 | 53.7 | 82.3 | 77.1 | 43.9 | 69.0 | 45.8 |
| Eif4ebp2 | 2.9 | 4.6 | 5.1 | 3.8 | 4.6 | 3.6 | 4.5 | 4.1 | 4.7 | 3.9 | 4.0 | 3.8 |
| Eif4ebp3 | 2.2 | 2.4 | 2.3 | 2.1 | 2.0 | 2.6 | 1.6 | 2.2 | 1.9 | 2.0 | 1.5 | 2.1 |
| Eif4enif1 | 6.9 | 8.2 | 8.8 | 7.4 | 6.1 | 7.2 | 8.1 | 6.8 | 6.9 | 6.0 | 7.1 | 7.7 |
| Eif4g1 | 124.7 | 107.8 | 99.0 | 135.3 | 106.5 | 118.2 | 127.8 | 104.1 | 100.2 | 101.7 | 117.7 | 105.1 |
| Eif4g2 | 77.4 | 74.5 | 67.2 | 80.6 | 79.6 | 78.2 | 74.0 | 76.9 | 77.0 | 79.7 | 85.7 | 73.5 |
| Eif4g3 | 11.6 | 12.4 | 13.0 | 12.3 | 11.2 | 13.0 | 14.9 | 10.2 | 9.5 | 10.8 | 11.8 | 10.6 |
| Eif4h | 78.8 | 71.5 | 61.0 | 79.7 | 66.9 | 77.7 | 69.0 | 67.0 | 71.9 | 75.9 | 69.2 | 69.1 |
| Eif5 | 45.4 | 37.5 | 36.1 | 48.7 | 37.6 | 45.0 | 36.5 | 33.4 | 38.7 | 41.6 | 43.0 | 40.6 |
| Eif5a | 183.3 | 150.3 | 142.9 | 196.4 | 187.1 | 173.1 | 193.3 | 177.0 | 153.8 | 170.7 | 174.2 | 152.2 |
| Eif5a2 | 4.7 | 5.2 | 6.3 | 6.7 | 5.2 | 6.6 | 5.6 | 5.5 | 5.8 | 5.6 | 5.9 | 6.8 |
| Eif5b | 12.6 | 13.1 | 15.9 | 17.9 | 13.8 | 13.9 | 15.4 | 14.1 | 9.7 | 12.1 | 14.5 | 16.2 |
| Eif6 | 32.6 | 27.1 | 21.7 | 36.0 | 33.1 | 27.9 | 30.2 | 26.6 | 29.7 | 31.2 | 28.4 | 24.9 |
| Elac1 | 2.0 | 2.1 | 2.5 | 2.4 | 2.0 | 2.7 | 2.5 | 2.2 | 2.5 | 2.1 | 2.0 | 2.5 |
| Elac2 | 6.8 | 7.1 | 6.0 | 5.8 | 5.6 | 5.9 | 6.3 | 6.1 | 4.9 | 6.3 | 6.2 | 5.7 |

| | | | | | | | | | | | | |
|---|---|---|---|---|---|---|---|---|---|---|---|---|
| Elavl1 | 10.2 | 10.4 | 10.3 | 12.1 | 11.8 | 11.1 | 11.9 | 11.0 | 10.3 | 10.6 | 13.1 | 10.8 |
| Elavl2 | 0.1 | 0.0 | 0.0 | 0.1 | 0.0 | 0.0 | 0.0 | 0.0 | 0.0 | 0.1 | 0.0 | 0.0 |
| Elavl3 | 0.0 | 0.0 | 0.0 | 0.0 | 0.0 | 0.0 | 0.0 | 0.0 | 0.0 | 0.0 | 0.0 | 0.0 |
| Elavl4 | 0.0 | 0.0 | 0.0 | 0.0 | 0.0 | 0.0 | 0.0 | 0.0 | 0.0 | 0.0 | 0.0 | 0.0 |
| Elf1 | 11.2 | 11.8 | 15.5 | 10.9 | 12.3 | 9.8 | 12.8 | 13.0 | 13.5 | 10.6 | 12.4 | 14.0 |
| Elf2 | 17.3 | 18.8 | 20.0 | 18.5 | 19.3 | 19.0 | 15.8 | 17.1 | 17.5 | 15.3 | 17.4 | 17.7 |
| Elf4 | 1.9 | 2.8 | 3.2 | 2.2 | 3.0 | 1.5 | 3.1 | 3.2 | 3.0 | 2.8 | 3.0 | 2.6 |
| Elf5 | 0.0 | 0.1 | 0.0 | 0.1 | 0.1 | 0.2 | 0.0 | 0.0 | 0.0 | 0.1 | 0.0 | 0.0 |
| Elfn1 | 0.4 | 0.5 | 0.7 | 0.6 | 0.6 | 0.4 | 0.8 | 1.0 | 0.8 | 0.6 | 0.7 | 0.7 |
| Elfn2 | 0.0 | 0.0 | 0.0 | 0.0 | 0.0 | 0.0 | 0.0 | 0.0 | 0.0 | 0.0 | 0.0 | 0.0 |
| Elk1 | 4.4 | 4.4 | 5.1 | 4.5 | 4.5 | 4.3 | 5.0 | 5.8 | 5.0 | 5.0 | 5.6 | 4.3 |
| Elk3 | 11.7 | 10.6 | 11.0 | 13.8 | 13.6 | 12.8 | 14.9 | 11.0 | 10.6 | 10.5 | 11.2 | 10.3 |
| Elk4 | 7.4 | 9.0 | 9.8 | 8.4 | 8.4 | 7.4 | 9.4 | 9.3 | 7.9 | 7.3 | 8.0 | 8.2 |
| Ell | 4.0 | 4.8 | 4.7 | 4.6 | 4.8 | 4.7 | 4.9 | 3.8 | 4.2 | 5.0 | 4.5 | 3.8 |
| Ell2 | 12.4 | 10.3 | 10.6 | 15.8 | 18.6 | 10.2 | 13.2 | 9.6 | 10.1 | 11.2 | 12.2 | 14.7 |
| Ell3 | 0.2 | 0.2 | 0.4 | 0.5 | 0.3 | 0.3 | 0.2 | 0.4 | 0.3 | 0.2 | 0.3 | 0.3 |
| Elmo1 | 1.1 | 1.0 | 1.1 | 2.3 | 3.6 | 0.8 | 1.0 | 0.9 | 0.7 | 0.5 | 0.6 | 0.4 |
| Elmo2 | 15.3 | 15.2 | 13.3 | 13.7 | 14.1 | 16.5 | 14.3 | 12.4 | 14.1 | 14.7 | 13.0 | 12.1 |
| Elmo3 | 0.5 | 0.6 | 1.0 | 0.3 | 0.4 | 0.6 | 0.3 | 0.4 | 0.6 | 0.8 | 0.8 | 0.7 |
| Elmod1 | 0.2 | 0.0 | 0.0 | 0.2 | 0.0 | 0.0 | 0.0 | 0.0 | 0.0 | 0.0 | 0.0 | 0.0 |
| Elmod2 | 6.1 | 6.6 | 6.7 | 6.0 | 6.3 | 6.3 | 5.3 | 6.4 | 6.3 | 7.9 | 7.3 | 7.8 |
| Elmod3 | 2.7 | 3.3 | 2.8 | 2.8 | 2.4 | 2.8 | 2.2 | 3.1 | 2.8 | 2.8 | 2.1 | 2.4 |
| Elmsan1 | 2.9 | 3.6 | 3.9 | 2.7 | 3.0 | 2.5 | 3.8 | 3.9 | 3.4 | 3.0 | 3.3 | 3.5 |
| Eln | 1.4 | 1.2 | 3.9 | 0.2 | 0.2 | 0.1 | 0.4 | 8.7 | 1.2 | 0.5 | 0.7 | 1.6 |
| Elof1 | 21.9 | 20.6 | 21.1 | 24.0 | 22.6 | 23.6 | 20.7 | 22.2 | 20.6 | 21.9 | 25.6 | 22.1 |
| Elovl1 | 28.1 | 27.7 | 27.2 | 31.7 | 29.3 | 24.3 | 38.2 | 43.4 | 40.8 | 26.4 | 35.0 | 29.6 |
| Elovl2 | 0.0 | 0.0 | 0.0 | 0.0 | 0.0 | 0.0 | 0.0 | 0.0 | 0.0 | 0.0 | 0.0 | 0.0 |
| Elovl4 | 0.6 | 0.4 | 0.4 | 0.4 | 0.5 | 0.5 | 0.5 | 0.4 | 0.3 | 0.2 | 0.3 | 0.4 |
| Elovl5 | 25.2 | 28.7 | 31.5 | 23.7 | 25.7 | 30.0 | 28.4 | 28.0 | 27.2 | 23.8 | 31.8 | 21.8 |
| Elovl6 | 3.5 | 4.6 | 4.0 | 1.7 | 2.8 | 1.8 | 7.9 | 9.4 | 6.9 | 2.5 | 8.8 | 2.4 |
| Elovl7 | 0.1 | 0.0 | 0.0 | 0.1 | 0.1 | 0.1 | 0.1 | 0.0 | 0.0 | 0.1 | 0.1 | 0.0 |
| Elp2 | 27.2 | 27.3 | 22.9 | 25.0 | 23.0 | 28.4 | 22.9 | 23.0 | 21.3 | 25.6 | 25.4 | 22.3 |
| Elp3 | 25.2 | 23.6 | 20.9 | 26.3 | 20.0 | 24.6 | 20.9 | 23.0 | 20.7 | 21.8 | 22.1 | 22.1 |
| Elp4 | 3.6 | 3.9 | 3.6 | 3.1 | 3.2 | 3.1 | 2.8 | 3.2 | 3.3 | 3.5 | 3.4 | 3.2 |
| Elp5 | 26.6 | 24.8 | 21.3 | 25.1 | 22.8 | 28.7 | 22.7 | 25.7 | 23.0 | 24.4 | 27.2 | 22.8 |
| Elp6 | 2.5 | 1.5 | 1.3 | 1.7 | 1.9 | 1.8 | 1.6 | 1.7 | 1.6 | 1.5 | 1.7 | 2.0 |
| Eltd1 | 1.0 | 0.2 | 0.7 | 0.5 | 0.2 | 0.2 | 0.8 | 1.4 | 1.0 | 0.2 | 0.3 | 0.2 |
| Emb | 3.1 | 9.2 | 15.0 | 5.7 | 8.6 | 0.8 | 6.0 | 7.3 | 8.9 | 10.5 | 7.6 | 15.0 |
| Emc1 | 13.7 | 13.3 | 12.3 | 15.7 | 13.3 | 15.1 | 12.9 | 11.9 | 11.9 | 11.5 | 11.1 | 9.9 |
| Emc10 | 25.3 | 21.8 | 23.8 | 28.6 | 25.7 | 26.9 | 25.7 | 25.6 | 26.9 | 22.6 | 21.4 | 20.5 |
| Emc2 | 41.8 | 40.3 | 43.1 | 48.8 | 39.0 | 39.1 | 39.0 | 40.7 | 36.9 | 44.9 | 48.2 | 49.0 |
| Emc3 | 54.9 | 50.4 | 47.7 | 59.9 | 51.1 | 52.6 | 54.6 | 55.3 | 54.0 | 52.8 | 56.5 | 55.5 |
| Emc4 | 39.9 | 35.9 | 32.0 | 45.3 | 38.9 | 46.2 | 37.9 | 38.8 | 38.8 | 44.3 | 38.4 | 35.7 |
| Emc6 | 19.5 | 17.1 | 14.4 | 17.7 | 19.0 | 19.8 | 17.0 | 17.1 | 17.6 | 20.9 | 19.3 | 15.8 |
| Emc7 | 82.8 | 72.4 | 63.8 | 96.2 | 69.1 | 84.2 | 69.3 | 57.7 | 69.8 | 67.4 | 69.3 | 76.4 |
| Emc8 | 7.0 | 5.4 | 5.2 | 7.7 | 6.4 | 5.6 | 6.9 | 5.9 | 5.7 | 6.2 | 6.4 | 6.3 |
| Emc9 | 11.2 | 10.0 | 10.1 | 9.0 | 8.3 | 10.9 | 9.3 | 7.9 | 10.7 | 8.3 | 9.4 | 11.7 |
| Emcn | 7.6 | 1.5 | 2.2 | 6.0 | 2.7 | 5.5 | 2.1 | 3.7 | 1.4 | 0.7 | 1.8 | 0.8 |
| Emd | 24.7 | 25.0 | 23.9 | 21.7 | 22.6 | 25.2 | 24.5 | 21.5 | 22.4 | 21.2 | 26.3 | 22.5 |
| Eme1 | 3.3 | 3.4 | 3.3 | 3.1 | 3.5 | 2.8 | 4.4 | 3.7 | 3.8 | 3.2 | 4.0 | 3.6 |
| Eme2 | 6.8 | 7.0 | 6.3 | 7.0 | 5.0 | 7.8 | 5.1 | 4.6 | 5.0 | 5.7 | 5.8 | 4.9 |
| Emg1 | 28.7 | 25.5 | 19.6 | 24.7 | 23.0 | 26.8 | 20.2 | 18.9 | 23.1 | 25.8 | 21.5 | 20.7 |
| Emid1 | 0.1 | 0.2 | 0.3 | 0.1 | 0.2 | 0.1 | 0.1 | 0.2 | 0.1 | 0.2 | 0.1 | 0.1 |
| Emilin1 | 58.6 | 72.5 | 87.5 | 72.5 | 107.6 | 75.5 | 107.0 | 82.5 | 98.3 | 69.3 | 54.6 | 83.3 |
| Emilin2 | 1.7 | 2.0 | 2.2 | 2.7 | 7.2 | 0.6 | 3.1 | 2.7 | 2.5 | 7.8 | 5.4 | 4.4 |
| Emilin3 | 0.1 | 0.0 | 0.0 | 0.1 | 0.0 | 0.0 | 0.0 | 0.0 | 0.0 | 0.0 | 0.0 | 0.0 |
| Eml1 | 7.6 | 7.7 | 7.6 | 6.5 | 6.3 | 9.9 | 7.4 | 8.1 | 8.3 | 12.0 | 9.5 | 9.9 |

| | | | | | | | | | | | | |
|---|---|---|---|---|---|---|---|---|---|---|---|---|
| Eml2 | 7.7 | 6.9 | 6.6 | 8.7 | 7.3 | 9.5 | 5.2 | 4.9 | 5.3 | 9.5 | 5.5 | 7.0 |
| Eml3 | 8.1 | 9.8 | 9.3 | 7.8 | 8.2 | 8.2 | 9.4 | 8.3 | 9.7 | 8.9 | 7.1 | 8.4 |
| Eml4 | 7.8 | 11.2 | 11.9 | 7.7 | 11.8 | 6.7 | 12.4 | 12.1 | 10.9 | 9.4 | 10.0 | 9.7 |
| Eml5 | 0.8 | 0.9 | 1.8 | 1.5 | 1.0 | 1.6 | 1.3 | 1.0 | 1.0 | 1.2 | 1.6 | 1.9 |
| Eml6 | 1.4 | 1.1 | 1.2 | 1.2 | 1.0 | 1.4 | 0.9 | 1.0 | 0.8 | 0.9 | 1.0 | 1.2 |
| Emp1 | 154.3 | 135.0 | 173.3 | 88.5 | 188.1 | 109.6 | 272.5 | 232.2 | 282.2 | 182.4 | 240.4 | 275.2 |
| Emp2 | 28.8 | 23.0 | 21.9 | 37.4 | 26.0 | 32.9 | 27.5 | 20.6 | 24.2 | 25.0 | 26.8 | 23.8 |
| Emp3 | 133.4 | 109.3 | 113.0 | 171.2 | 143.4 | 139.3 | 174.4 | 178.7 | 151.5 | 140.9 | 155.4 | 138.2 |
| Emr1 | 6.0 | 0.0 | 0.3 | 42.8 | 78.6 | 0.1 | 8.2 | 1.3 | 1.6 | 4.6 | 1.6 | 3.5 |
| Emr4 | 0.0 | 0.0 | 0.0 | 0.0 | 0.0 | 0.0 | 0.0 | 0.0 | 0.0 | 0.0 | 0.0 | 0.0 |
| Emx2 | 3.4 | 0.6 | 0.8 | 4.1 | 1.4 | 4.7 | 1.0 | 0.4 | 1.0 | 1.9 | 1.8 | 0.8 |
| Emx2os | 0.6 | 0.2 | 0.3 | 0.7 | 0.5 | 0.6 | 0.4 | 0.3 | 0.4 | 0.5 | 0.4 | 0.3 |
| En1 | 0.4 | 0.3 | 0.1 | 0.8 | 0.1 | 0.5 | 0.5 | 0.3 | 0.5 | 0.4 | 0.6 | 0.4 |
| En2 | 0.1 | 0.0 | 0.0 | 0.2 | 0.0 | 0.0 | 0.1 | 0.0 | 0.0 | 0.0 | 0.0 | 0.0 |
| Enah | 4.7 | 4.1 | 5.2 | 4.1 | 3.2 | 3.6 | 4.6 | 3.7 | 3.3 | 6.9 | 7.1 | 6.3 |
| Enc1 | 43.1 | 47.4 | 55.5 | 32.7 | 45.3 | 27.0 | 36.5 | 60.1 | 36.5 | 50.6 | 37.3 | 47.3 |
| Endod1 | 14.4 | 7.4 | 6.1 | 20.0 | 19.6 | 13.5 | 16.3 | 6.1 | 9.6 | 11.4 | 12.1 | 12.0 |
| Endog | 1.5 | 1.7 | 1.3 | 2.1 | 2.1 | 1.6 | 2.1 | 2.2 | 2.8 | 2.7 | 1.5 | 1.8 |
| Endou | 0.8 | 0.8 | 1.0 | 0.7 | 0.7 | 1.2 | 1.2 | 0.9 | 0.6 | 0.7 | 0.8 | 1.2 |
| Endov | 1.5 | 1.5 | 1.6 | 2.2 | 1.7 | 2.1 | 1.7 | 1.8 | 1.7 | 1.3 | 1.7 | 1.3 |
| Eng | 45.8 | 51.5 | 49.1 | 64.9 | 62.0 | 62.9 | 72.0 | 45.0 | 51.3 | 31.9 | 38.0 | 34.6 |
| Engase | 0.7 | 0.8 | 1.0 | 0.8 | 1.0 | 0.7 | 0.6 | 0.9 | 0.9 | 0.6 | 0.7 | 0.4 |
| Enho | 7.1 | 5.5 | 3.3 | 11.8 | 4.8 | 11.2 | 3.9 | 1.8 | 2.6 | 4.0 | 2.4 | 4.8 |
| Enkd1 | 2.5 | 2.5 | 2.8 | 2.2 | 2.3 | 3.1 | 3.0 | 2.5 | 1.9 | 1.9 | 2.2 | 1.4 |
| Enkur | 0.1 | 0.0 | 0.1 | 0.3 | 0.2 | 0.1 | 0.2 | 0.1 | 0.1 | 0.0 | 0.1 | 0.1 |
| Eno1 | 168.6 | 149.9 | 108.5 | 186.9 | 190.6 | 172.1 | 174.7 | 159.6 | 177.9 | 169.7 | 183.2 | 155.7 |
| Eno2 | 2.0 | 2.9 | 3.0 | 1.4 | 2.2 | 2.9 | 2.0 | 2.0 | 2.5 | 4.1 | 2.6 | 2.8 |
| Eno3 | 1.4 | 1.5 | 1.4 | 1.9 | 1.3 | 1.5 | 1.7 | 1.6 | 2.0 | 2.7 | 2.0 | 1.6 |
| Eno4 | 0.0 | 0.0 | 0.0 | 0.1 | 0.1 | 0.0 | 0.0 | 0.0 | 0.0 | 0.0 | 0.0 | 0.0 |
| Enoph1 | 8.2 | 7.4 | 7.2 | 8.2 | 7.7 | 9.5 | 8.8 | 7.2 | 8.4 | 8.3 | 9.1 | 9.0 |
| Enox1 | 0.6 | 0.8 | 1.2 | 0.7 | 0.9 | 0.8 | 1.0 | 1.0 | 0.7 | 0.8 | 0.6 | 0.9 |
| Enox2 | 6.4 | 6.9 | 6.7 | 6.5 | 6.0 | 6.1 | 5.4 | 6.1 | 6.2 | 4.8 | 6.7 | 6.6 |
| Enpep | 0.1 | 0.1 | 0.1 | 0.0 | 0.1 | 0.1 | 0.1 | 0.5 | 0.2 | 0.6 | 0.4 | 0.3 |
| Enpp1 | 7.8 | 10.1 | 11.2 | 6.5 | 8.4 | 9.1 | 10.6 | 5.4 | 11.4 | 13.0 | 7.4 | 16.3 |
| Enpp2 | 0.7 | 0.3 | 0.3 | 0.8 | 0.7 | 0.8 | 0.1 | 0.2 | 0.0 | 0.1 | 0.1 | 0.2 |
| Enpp3 | 0.1 | 0.2 | 0.4 | 0.1 | 0.2 | 0.0 | 0.4 | 0.9 | 0.4 | 0.1 | 0.3 | 0.3 |
| Enpp4 | 3.0 | 2.3 | 1.8 | 3.0 | 2.0 | 3.2 | 1.4 | 2.2 | 1.4 | 2.5 | 2.2 | 1.8 |
| Enpp5 | 43.6 | 34.6 | 31.5 | 41.9 | 24.3 | 51.4 | 20.9 | 17.0 | 15.9 | 30.1 | 26.9 | 29.4 |
| Enpp6 | 0.3 | 0.2 | 0.3 | 0.1 | 0.1 | 0.0 | 0.1 | 0.1 | 0.0 | 0.2 | 0.1 | 0.1 |
| Ensa | 66.6 | 59.4 | 50.8 | 74.1 | 59.4 | 62.8 | 58.9 | 50.2 | 55.3 | 59.1 | 66.1 | 59.2 |
| Enthd1 | 0.0 | 0.0 | 0.0 | 0.0 | 0.0 | 0.0 | 0.1 | 0.0 | 0.0 | 0.0 | 0.0 | 0.0 |
| Enthd2 | 3.7 | 3.8 | 3.5 | 3.6 | 4.0 | 4.1 | 3.5 | 3.3 | 3.8 | 3.2 | 3.6 | 3.2 |
| Entpd1 | 0.7 | 0.2 | 0.2 | 1.3 | 0.7 | 0.4 | 0.2 | 0.2 | 0.1 | 0.2 | 0.2 | 0.1 |
| Entpd2 | 3.1 | 5.5 | 11.6 | 2.2 | 4.3 | 2.4 | 3.5 | 20.2 | 5.8 | 2.4 | 2.5 | 3.3 |
| Entpd3 | 0.0 | 0.1 | 0.0 | 0.1 | 0.0 | 0.0 | 0.0 | 0.0 | 0.0 | 0.0 | 0.0 | 0.0 |
| Entpd4 | 14.6 | 13.2 | 12.6 | 15.7 | 13.2 | 14.1 | 12.3 | 13.4 | 12.1 | 15.0 | 14.1 | 14.1 |
| Entpd5 | 8.0 | 7.4 | 6.4 | 8.6 | 8.0 | 9.7 | 6.2 | 6.6 | 5.8 | 6.9 | 6.9 | 6.7 |
| Entpd6 | 3.0 | 3.6 | 3.7 | 3.7 | 3.7 | 3.2 | 2.9 | 4.1 | 3.8 | 3.5 | 3.3 | 3.2 |
| Entpd7 | 1.7 | 1.8 | 1.5 | 1.4 | 2.4 | 1.3 | 1.9 | 2.0 | 2.0 | 1.8 | 1.7 | 1.5 |
| Eny2 | 11.0 | 8.6 | 8.8 | 9.3 | 8.1 | 8.4 | 9.6 | 11.4 | 9.2 | 12.1 | 12.3 | 9.3 |
| Eogt | 11.9 | 12.5 | 11.8 | 11.2 | 11.5 | 12.8 | 12.7 | 13.6 | 13.5 | 8.9 | 14.2 | 11.5 |
| Eomes | 0.0 | 0.0 | 0.0 | 0.1 | 0.0 | 0.0 | 0.0 | 0.0 | 0.0 | 0.0 | 0.1 | 0.0 |
| Ep300 | 2.8 | 3.1 | 3.2 | 3.1 | 3.1 | 3.0 | 3.2 | 2.7 | 2.9 | 2.5 | 2.7 | 2.6 |
| Ep400 | 3.4 | 4.1 | 4.4 | 3.4 | 4.0 | 3.3 | 4.7 | 3.8 | 4.0 | 3.4 | 3.6 | 3.0 |
| Epas1 | 23.1 | 27.6 | 28.9 | 32.2 | 35.1 | 22.1 | 33.0 | 17.7 | 22.3 | 16.8 | 17.8 | 21.9 |
| Epb4.1 | 5.0 | 4.3 | 4.4 | 7.6 | 5.2 | 5.7 | 4.0 | 4.4 | 3.2 | 4.5 | 4.6 | 4.2 |
| Epb4.1l1 | 11.2 | 10.3 | 9.6 | 12.3 | 8.4 | 13.7 | 12.2 | 7.6 | 8.9 | 7.7 | 9.8 | 9.3 |

| | | | | | | | | | | | | |
|---|---|---|---|---|---|---|---|---|---|---|---|---|
| Epb4.1l2 | 84.0 | 81.5 | 90.3 | 105.1 | 88.8 | 85.1 | 108.7 | 77.2 | 88.5 | 66.0 | 84.7 | 98.4 |
| Epb4.1l3 | 15.5 | 13.4 | 12.0 | 19.8 | 12.6 | 25.3 | 17.4 | 15.3 | 16.7 | 13.6 | 18.9 | 16.9 |
| Epb4.1l4a | 0.7 | 0.3 | 0.3 | 0.2 | 0.2 | 0.1 | 0.3 | 0.4 | 0.2 | 1.0 | 1.1 | 0.5 |
| Epb4.1l4b | 1.1 | 0.7 | 1.1 | 1.1 | 0.9 | 0.9 | 1.2 | 1.2 | 2.3 | 1.4 | 1.8 | 1.1 |
| Epb4.1l5 | 1.8 | 1.8 | 1.4 | 1.1 | 1.0 | 1.5 | 1.7 | 1.3 | 1.8 | 2.6 | 2.1 | 2.0 |
| Epb4.2 | 0.0 | 0.1 | 0.0 | 0.0 | 0.0 | 0.0 | 0.0 | 0.0 | 0.0 | 0.0 | 0.0 | 0.0 |
| Epb4.9 | 1.1 | 0.8 | 0.7 | 1.6 | 0.5 | 1.4 | 0.7 | 0.4 | 0.4 | 0.3 | 0.5 | 0.5 |
| Epc1 | 4.8 | 4.8 | 5.1 | 4.4 | 5.3 | 5.2 | 5.8 | 5.9 | 5.4 | 5.4 | 6.2 | 5.8 |
| Epc2 | 5.1 | 5.2 | 7.6 | 5.3 | 5.8 | 5.3 | 5.8 | 6.9 | 6.0 | 5.8 | 7.3 | 7.2 |
| Epdr1 | 37.2 | 28.5 | 23.8 | 30.4 | 24.0 | 34.9 | 23.5 | 22.7 | 22.7 | 26.0 | 27.5 | 26.4 |
| Epg5 | 6.2 | 7.0 | 6.1 | 6.0 | 5.9 | 7.1 | 5.6 | 4.8 | 5.4 | 5.4 | 5.5 | 5.4 |
| Epgn | 0.0 | 0.0 | 0.0 | 0.0 | 0.0 | 0.1 | 0.0 | 0.1 | 0.0 | 0.0 | 0.0 | 0.0 |
| Epha1 | 0.5 | 0.4 | 0.3 | 0.2 | 0.3 | 0.3 | 0.5 | 0.6 | 0.6 | 5.8 | 2.5 | 0.8 |
| Epha2 | 11.3 | 7.0 | 4.7 | 9.6 | 8.4 | 8.6 | 10.3 | 3.3 | 6.9 | 23.4 | 14.2 | 8.6 |
| Epha3 | 0.3 | 0.3 | 0.4 | 0.0 | 0.1 | 0.0 | 0.1 | 0.4 | 0.3 | 1.2 | 1.0 | 0.6 |
| Epha4 | 2.2 | 2.3 | 4.0 | 3.1 | 4.8 | 6.2 | 5.2 | 4.9 | 5.0 | 4.3 | 7.2 | 4.0 |
| Epha5 | 0.0 | 0.0 | 0.0 | 0.0 | 0.0 | 0.0 | 0.0 | 0.0 | 0.0 | 0.0 | 0.0 | 0.0 |
| Epha6 | 0.0 | 0.0 | 0.0 | 0.0 | 0.0 | 0.0 | 0.0 | 0.0 | 0.0 | 0.0 | 0.0 | 0.0 |
| Epha7 | 0.6 | 0.7 | 1.7 | 0.3 | 0.8 | 0.3 | 1.2 | 1.5 | 1.1 | 1.1 | 1.1 | 1.7 |
| Ephb1 | 0.0 | 0.0 | 0.0 | 0.0 | 0.0 | 0.0 | 0.0 | 0.1 | 0.0 | 0.0 | 0.0 | 0.0 |
| Ephb2 | 2.2 | 3.1 | 4.0 | 1.9 | 3.0 | 2.2 | 3.7 | 4.5 | 4.8 | 6.5 | 5.6 | 4.6 |
| Ephb3 | 3.8 | 5.6 | 6.7 | 3.5 | 3.9 | 4.3 | 4.0 | 8.7 | 6.8 | 6.6 | 5.6 | 4.7 |
| Ephb4 | 7.3 | 9.4 | 9.5 | 7.2 | 9.6 | 9.7 | 12.7 | 12.0 | 12.4 | 13.1 | 11.1 | 9.8 |
| Ephb6 | 3.0 | 2.4 | 2.4 | 2.2 | 2.2 | 3.0 | 3.2 | 3.4 | 2.8 | 3.1 | 2.4 | 2.8 |
| Ephx1 | 354.3 | 307.6 | 235.2 | 416.4 | 273.0 | 464.3 | 299.3 | 225.1 | 214.1 | 315.2 | 309.7 | 293.3 |
| Ephx2 | 14.0 | 14.4 | 14.8 | 15.3 | 12.9 | 21.7 | 10.2 | 13.8 | 11.7 | 16.0 | 18.6 | 21.2 |
| Ephx3 | 0.0 | 0.0 | 0.0 | 0.0 | 0.0 | 0.1 | 0.0 | 0.0 | 0.0 | 0.0 | 0.1 | 0.0 |
| Ephx4 | 0.1 | 0.2 | 0.0 | 0.1 | 0.1 | 0.1 | 0.0 | 0.2 | 0.1 | 0.0 | 0.0 | 0.1 |
| Epm2a | 4.2 | 3.2 | 2.5 | 3.6 | 2.8 | 4.2 | 3.7 | 1.8 | 3.2 | 2.4 | 2.8 | 3.1 |
| Epm2aip1 | 5.2 | 5.9 | 7.1 | 5.4 | 6.0 | 5.3 | 6.2 | 7.1 | 5.9 | 6.7 | 6.9 | 6.9 |
| Epn1 | 22.6 | 22.3 | 19.5 | 24.8 | 22.0 | 22.1 | 20.3 | 19.2 | 18.0 | 16.1 | 16.9 | 16.3 |
| Epn2 | 14.5 | 18.1 | 19.1 | 13.6 | 13.1 | 15.2 | 15.7 | 15.8 | 18.4 | 14.5 | 15.4 | 16.7 |
| Epn3 | 0.1 | 0.0 | 0.1 | 0.0 | 0.0 | 0.1 | 0.0 | 0.0 | 0.0 | 0.1 | 0.0 | 0.0 |
| Epor | 0.3 | 0.3 | 0.3 | 0.0 | 0.1 | 0.6 | 0.1 | 0.2 | 0.1 | 0.1 | 0.1 | 0.1 |
| Eppk1 | 0.0 | 0.1 | 0.0 | 0.1 | 0.0 | 0.1 | 0.1 | 0.1 | 0.0 | 0.1 | 0.0 | 0.0 |
| Eprs | 92.9 | 70.5 | 66.0 | 93.1 | 60.3 | 91.0 | 65.4 | 90.5 | 66.2 | 60.0 | 84.4 | 68.5 |
| Eps15 | 10.6 | 11.5 | 12.9 | 11.6 | 12.8 | 10.3 | 10.8 | 15.6 | 12.0 | 10.5 | 12.9 | 13.0 |
| Eps15l1 | 11.3 | 11.9 | 12.3 | 12.5 | 12.3 | 10.5 | 13.5 | 13.3 | 12.0 | 10.7 | 12.4 | 11.6 |
| Eps8 | 7.6 | 7.8 | 9.1 | 5.2 | 6.8 | 4.5 | 8.3 | 9.6 | 8.5 | 17.2 | 11.3 | 12.7 |
| Eps8l1 | 0.9 | 0.7 | 0.5 | 0.9 | 0.5 | 0.8 | 0.7 | 0.8 | 0.6 | 0.3 | 0.8 | 0.5 |
| Eps8l2 | 0.6 | 0.6 | 0.3 | 0.5 | 0.2 | 1.2 | 0.6 | 0.2 | 0.4 | 2.5 | 1.5 | 1.3 |
| Eps8l3 | 0.1 | 0.0 | 0.0 | 0.0 | 0.0 | 0.0 | 0.0 | 0.0 | 0.0 | 0.0 | 0.0 | 0.0 |
| Epsti1 | 0.2 | 0.0 | 0.1 | 1.0 | 1.3 | 0.0 | 0.2 | 0.1 | 0.1 | 0.1 | 0.0 | 0.0 |
| Ept1 | 7.4 | 6.4 | 6.0 | 7.6 | 6.3 | 7.0 | 7.3 | 6.4 | 9.0 | 6.8 | 7.9 | 6.5 |
| Epyc | 0.2 | 0.3 | 1.6 | 0.0 | 0.0 | 0.0 | 0.0 | 1.1 | 0.2 | 0.2 | 0.2 | 0.4 |
| Eqtn | 0.1 | 0.0 | 0.0 | 0.0 | 0.0 | 0.1 | 0.0 | 0.0 | 0.2 | 0.0 | 0.0 | 0.0 |
| Eral1 | 6.2 | 5.8 | 5.6 | 6.7 | 5.6 | 6.4 | 4.8 | 6.4 | 5.9 | 5.7 | 4.8 | 5.0 |
| Erap1 | 9.1 | 6.2 | 7.2 | 12.3 | 13.5 | 10.2 | 6.3 | 7.0 | 6.1 | 6.8 | 6.4 | 7.6 |
| Erbb2 | 2.3 | 2.6 | 2.9 | 2.1 | 2.3 | 2.5 | 3.3 | 3.5 | 3.9 | 2.9 | 2.5 | 2.5 |
| Erbb2ip | 22.6 | 24.7 | 25.1 | 25.8 | 22.9 | 23.5 | 21.9 | 23.3 | 24.3 | 25.5 | 26.9 | 29.1 |
| Erbb3 | 0.1 | 0.2 | 0.1 | 0.3 | 0.2 | 0.2 | 0.1 | 0.1 | 0.1 | 0.1 | 0.2 | 0.1 |
| Erbb4 | 0.1 | 0.1 | 0.0 | 0.0 | 0.0 | 0.0 | 0.0 | 0.0 | 0.0 | 0.3 | 0.1 | 0.1 |
| Erc1 | 5.4 | 6.5 | 6.7 | 4.6 | 5.9 | 4.2 | 6.5 | 6.2 | 5.5 | 4.9 | 6.1 | 6.9 |
| Erc2 | 0.2 | 0.0 | 0.1 | 0.3 | 0.1 | 0.4 | 0.0 | 0.0 | 0.0 | 0.3 | 0.2 | 0.1 |
| Ercc1 | 15.7 | 13.4 | 12.8 | 15.2 | 11.9 | 14.6 | 17.4 | 16.1 | 15.6 | 15.8 | 17.2 | 15.6 |
| Ercc2 | 3.4 | 3.5 | 3.3 | 4.3 | 4.0 | 3.6 | 3.7 | 3.9 | 3.8 | 3.8 | 3.3 | 3.2 |
| Ercc3 | 17.8 | 16.6 | 16.3 | 18.3 | 17.2 | 17.3 | 18.1 | 17.9 | 15.5 | 16.0 | 18.2 | 18.8 |

| | | | | | | | | | | | | |
|---|---|---|---|---|---|---|---|---|---|---|---|---|
| Ercc4 | 2.1 | 2.2 | 2.1 | 2.1 | 2.0 | 1.8 | 2.0 | 2.2 | 1.8 | 2.2 | 2.0 | 2.1 |
| Ercc5 | 13.4 | 13.9 | 12.5 | 11.4 | 10.3 | 12.1 | 11.2 | 8.8 | 10.2 | 13.4 | 12.7 | 13.0 |
| Ercc6 | 3.1 | 2.7 | 2.6 | 4.4 | 3.8 | 3.6 | 3.2 | 2.8 | 2.8 | 2.8 | 3.4 | 3.0 |
| Ercc6l | 0.7 | 1.4 | 2.3 | 0.3 | 1.9 | 0.2 | 2.2 | 1.3 | 1.7 | 1.0 | 1.7 | 1.2 |
| Ercc6l2 | 5.5 | 5.1 | 5.0 | 4.3 | 4.7 | 4.8 | 4.2 | 4.6 | 4.2 | 4.3 | 5.6 | 5.5 |
| Ercc8 | 1.0 | 1.1 | 1.0 | 0.8 | 1.1 | 1.0 | 1.1 | 0.8 | 1.0 | 1.0 | 1.1 | 1.2 |
| Erdr1 | 14.5 | 13.0 | 19.4 | 12.2 | 18.6 | 10.3 | 26.3 | 14.1 | 21.0 | 14.2 | 18.2 | 6.8 |
| Ereg | 12.3 | 10.3 | 8.8 | 13.2 | 16.9 | 7.3 | 21.9 | 11.9 | 15.6 | 15.6 | 20.2 | 16.5 |
| Erf | 8.0 | 9.1 | 9.4 | 7.6 | 9.5 | 8.1 | 12.1 | 12.5 | 11.2 | 7.9 | 8.4 | 8.3 |
| Erg | 1.0 | 0.2 | 0.3 | 0.3 | 0.4 | 0.2 | 0.5 | 0.9 | 0.6 | 0.1 | 0.2 | 0.3 |
| Ergic1 | 74.6 | 59.4 | 45.6 | 82.5 | 56.6 | 77.5 | 69.8 | 44.0 | 51.0 | 59.4 | 67.7 | 52.9 |
| Ergic2 | 24.0 | 25.0 | 24.1 | 22.8 | 24.2 | 20.1 | 24.6 | 25.8 | 25.4 | 25.1 | 27.5 | 26.2 |
| Ergic3 | 83.8 | 76.0 | 81.2 | 92.6 | 78.8 | 92.4 | 78.1 | 80.6 | 71.2 | 83.0 | 73.0 | 81.3 |
| Erh | 69.1 | 60.5 | 60.4 | 70.5 | 66.5 | 66.0 | 63.0 | 68.1 | 61.3 | 71.5 | 76.0 | 67.8 |
| Eri1 | 4.6 | 5.8 | 6.5 | 4.0 | 6.1 | 4.8 | 5.6 | 5.2 | 5.3 | 5.3 | 5.4 | 5.2 |
| Eri2 | 2.1 | 2.9 | 3.2 | 1.6 | 3.2 | 1.9 | 3.1 | 2.6 | 2.8 | 2.4 | 2.6 | 2.4 |
| Eri3 | 26.1 | 24.9 | 24.0 | 26.3 | 22.8 | 27.4 | 26.4 | 28.9 | 26.6 | 27.3 | 26.3 | 26.3 |
| Erich1 | 10.5 | 9.5 | 11.0 | 11.8 | 8.5 | 11.1 | 9.7 | 7.4 | 7.4 | 8.3 | 10.1 | 10.7 |
| Erlec1 | 7.4 | 6.2 | 6.9 | 9.6 | 7.4 | 7.7 | 7.8 | 8.0 | 6.4 | 8.7 | 7.4 | 10.4 |
| Erlin1 | 7.9 | 8.0 | 7.7 | 8.2 | 8.6 | 8.5 | 8.0 | 6.6 | 7.3 | 6.2 | 7.0 | 7.4 |
| Erlin2 | 27.2 | 25.0 | 22.3 | 30.6 | 24.6 | 30.0 | 24.5 | 22.8 | 24.2 | 22.7 | 24.2 | 24.6 |
| Ermap | 0.0 | 0.0 | 0.0 | 0.1 | 0.0 | 0.0 | 0.0 | 0.0 | 0.0 | 0.0 | 0.1 | 0.0 |
| Ermp1 | 2.4 | 2.1 | 3.2 | 1.9 | 2.6 | 2.5 | 2.1 | 2.3 | 2.5 | 2.5 | 2.3 | 2.5 |
| Ern1 | 8.2 | 8.9 | 7.8 | 9.0 | 10.4 | 9.1 | 13.1 | 7.5 | 8.4 | 8.4 | 8.5 | 9.6 |
| Ero1l | 44.7 | 32.5 | 22.8 | 55.6 | 32.8 | 56.8 | 33.1 | 23.3 | 31.0 | 39.2 | 39.6 | 34.5 |
| Ero1lb | 2.9 | 2.6 | 2.8 | 3.2 | 3.7 | 3.0 | 2.6 | 2.4 | 2.4 | 2.9 | 2.6 | 2.9 |
| Erp27 | 0.1 | 0.0 | 0.0 | 0.0 | 0.1 | 0.0 | 0.0 | 0.0 | 0.0 | 0.1 | 0.0 | 0.0 |
| Erp29 | 85.5 | 72.8 | 75.3 | 105.9 | 85.3 | 81.2 | 82.7 | 82.4 | 77.0 | 78.3 | 79.8 | 81.4 |
| Erp44 | 38.5 | 37.1 | 40.6 | 44.3 | 39.5 | 37.0 | 41.8 | 40.2 | 41.7 | 40.8 | 43.0 | 47.3 |
| Errfi1 | 4.6 | 5.9 | 5.3 | 3.4 | 5.3 | 4.2 | 5.8 | 4.4 | 4.8 | 8.1 | 6.6 | 5.8 |
| Erv3 | 0.0 | 0.0 | 0.0 | 0.0 | 0.0 | 0.1 | 0.0 | 0.0 | 0.0 | 0.0 | 0.0 | 0.0 |
| Esam | 1.2 | 0.5 | 0.6 | 0.5 | 0.6 | 0.2 | 1.2 | 1.6 | 1.2 | 2.0 | 1.5 | 0.7 |
| Esco1 | 4.3 | 4.9 | 4.3 | 4.9 | 5.4 | 4.0 | 3.9 | 4.6 | 4.7 | 5.3 | 5.1 | 5.1 |
| Esco2 | 0.8 | 2.4 | 4.8 | 0.3 | 2.6 | 0.2 | 2.9 | 1.6 | 2.3 | 1.3 | 2.2 | 1.6 |
| Esd | 496.9 | 421.5 | 238.4 | 553.6 | 382.8 | 568.8 | 253.0 | 229.7 | 190.5 | 296.2 | 268.6 | 232.6 |
| Esf1 | 11.0 | 12.1 | 11.3 | 13.3 | 10.0 | 10.8 | 10.9 | 9.3 | 8.3 | 10.3 | 13.2 | 12.6 |
| Esm1 | 4.5 | 2.6 | 2.0 | 1.9 | 0.4 | 5.5 | 1.4 | 1.1 | 1.6 | 0.6 | 0.3 | 0.8 |
| Espl1 | 0.7 | 1.0 | 1.3 | 0.3 | 1.2 | 0.5 | 1.4 | 0.8 | 1.1 | 0.5 | 0.9 | 0.6 |
| Espn | 0.6 | 0.6 | 0.6 | 0.2 | 0.6 | 0.3 | 0.5 | 0.4 | 0.4 | 0.7 | 0.7 | 0.3 |
| Espnl | 0.0 | 0.0 | 0.0 | 0.1 | 0.0 | 0.0 | 0.0 | 0.0 | 0.0 | 0.0 | 0.0 | 0.0 |
| Esr1 | 0.7 | 0.6 | 1.3 | 0.7 | 0.8 | 0.7 | 0.7 | 0.8 | 0.7 | 0.6 | 0.6 | 0.8 |
| Esr2 | 0.1 | 0.1 | 0.0 | 0.1 | 0.0 | 0.0 | 0.1 | 0.0 | 0.0 | 0.0 | 0.0 | 0.0 |
| Esrp2 | 1.2 | 1.3 | 0.5 | 1.0 | 1.2 | 0.8 | 2.2 | 1.2 | 2.1 | 2.6 | 2.1 | 2.2 |
| Esrra | 7.7 | 7.3 | 5.6 | 9.5 | 8.1 | 8.8 | 7.1 | 4.9 | 7.5 | 8.1 | 6.6 | 6.4 |
| Esrrb | 0.0 | 0.0 | 0.0 | 0.0 | 0.0 | 0.0 | 0.0 | 0.0 | 0.0 | 0.0 | 0.0 | 0.0 |
| Esrrg | 0.0 | 0.0 | 0.0 | 0.0 | 0.0 | 0.0 | 0.0 | 0.0 | 0.0 | 0.0 | 0.0 | 0.0 |
| Esyt1 | 16.1 | 16.4 | 18.8 | 24.9 | 31.5 | 16.9 | 19.7 | 23.4 | 22.2 | 17.8 | 18.0 | 15.5 |
| Esyt2 | 22.7 | 21.8 | 18.7 | 20.2 | 22.5 | 21.1 | 27.8 | 20.6 | 28.4 | 27.7 | 31.3 | 26.5 |
| Esyt3 | 0.0 | 0.0 | 0.0 | 0.0 | 0.0 | 0.1 | 0.0 | 0.0 | 0.1 | 0.3 | 0.2 | 0.2 |
| Etaa1 | 1.2 | 2.0 | 2.2 | 0.9 | 1.8 | 1.4 | 1.6 | 1.8 | 2.0 | 1.7 | 1.5 | 1.7 |
| Etf1 | 55.0 | 49.7 | 47.0 | 53.8 | 48.4 | 49.4 | 50.6 | 44.0 | 47.5 | 52.9 | 56.6 | 57.5 |
| Etfa | 25.6 | 25.3 | 25.0 | 21.6 | 24.5 | 25.1 | 19.9 | 24.2 | 20.6 | 23.5 | 21.2 | 25.0 |
| Etfb | 68.8 | 69.8 | 63.0 | 74.4 | 64.5 | 62.1 | 63.4 | 62.0 | 66.4 | 83.8 | 72.7 | 70.5 |
| Etfdh | 19.5 | 18.5 | 17.2 | 20.8 | 16.2 | 19.2 | 16.1 | 16.9 | 16.6 | 18.9 | 18.1 | 19.4 |
| Ethe1 | 4.3 | 5.3 | 4.9 | 5.4 | 4.0 | 6.1 | 4.5 | 5.0 | 3.8 | 4.3 | 4.6 | 3.5 |
| Etl4 | 0.9 | 0.8 | 0.9 | 0.9 | 0.8 | 1.0 | 1.1 | 0.8 | 1.2 | 0.6 | 0.9 | 1.1 |
| Etnk1 | 8.7 | 9.5 | 8.6 | 10.5 | 9.3 | 10.3 | 9.3 | 8.1 | 9.0 | 9.8 | 10.5 | 9.3 |

| | | | | | | | | | | | | |
|---|---|---|---|---|---|---|---|---|---|---|---|---|
| Etnk2 | 0.1 | 0.6 | 0.4 | 0.2 | 0.2 | 0.2 | 0.1 | 0.2 | 0.2 | 0.1 | 0.1 | 0.0 |
| Etohd2 | 0.5 | 0.4 | 0.5 | 0.5 | 0.4 | 0.5 | 0.3 | 0.4 | 0.3 | 0.5 | 0.4 | 0.4 |
| Etohi1 | 11.0 | 11.2 | 13.9 | 13.1 | 12.9 | 13.2 | 10.9 | 13.8 | 11.9 | 12.8 | 15.9 | 14.6 |
| Ets1 | 22.1 | 22.7 | 18.6 | 25.8 | 26.3 | 21.5 | 28.8 | 19.1 | 23.4 | 16.3 | 19.8 | 18.4 |
| Ets2 | 4.4 | 2.8 | 3.6 | 10.3 | 20.7 | 3.5 | 6.0 | 3.7 | 4.0 | 5.1 | 3.9 | 4.0 |
| Etv1 | 1.0 | 1.3 | 1.3 | 2.3 | 1.1 | 1.5 | 1.2 | 1.2 | 1.1 | 0.9 | 0.9 | 1.1 |
| Etv3 | 3.8 | 4.2 | 3.6 | 5.2 | 5.7 | 3.9 | 4.3 | 3.9 | 3.8 | 4.0 | 4.4 | 4.0 |
| Etv4 | 0.9 | 0.8 | 0.6 | 0.9 | 1.2 | 1.3 | 0.8 | 0.5 | 0.5 | 2.8 | 0.9 | 0.8 |
| Etv5 | 6.3 | 5.7 | 5.1 | 6.3 | 7.6 | 7.0 | 6.9 | 3.6 | 4.2 | 9.1 | 6.6 | 4.7 |
| Etv6 | 4.9 | 6.7 | 7.2 | 6.0 | 6.7 | 4.8 | 8.9 | 7.9 | 8.7 | 5.7 | 7.5 | 6.6 |
| EU599041 | 0.0 | 0.0 | 0.1 | 0.0 | 0.0 | 0.0 | 0.0 | 0.1 | 0.1 | 0.0 | 0.0 | 0.1 |
| Eva1a | 0.1 | 0.1 | 0.0 | 0.0 | 0.0 | 0.0 | 0.0 | 0.1 | 0.1 | 0.0 | 0.0 | 0.0 |
| Eva1b | 18.5 | 20.0 | 16.4 | 16.5 | 16.4 | 17.4 | 20.2 | 23.3 | 25.5 | 25.0 | 18.8 | 18.2 |
| Eva1c | 1.9 | 1.3 | 2.4 | 0.5 | 1.1 | 0.9 | 1.3 | 1.5 | 1.1 | 1.9 | 1.7 | 1.6 |
| Evc | 10.2 | 10.0 | 12.4 | 8.8 | 8.4 | 10.1 | 10.8 | 10.7 | 9.1 | 8.9 | 8.6 | 9.7 |
| Evc2 | 11.7 | 13.5 | 13.4 | 12.4 | 9.6 | 13.6 | 13.1 | 12.4 | 12.1 | 11.3 | 11.9 | 11.6 |
| Evi2a | 2.6 | 1.3 | 1.2 | 11.2 | 11.9 | 3.3 | 2.8 | 0.8 | 1.1 | 1.9 | 1.5 | 1.6 |
| Evi2b | 0.3 | 0.1 | 0.1 | 2.4 | 3.2 | 0.1 | 0.7 | 0.1 | 0.1 | 0.4 | 0.2 | 0.2 |
| Evi5 | 13.4 | 12.9 | 12.2 | 14.3 | 12.1 | 13.9 | 13.0 | 11.8 | 11.9 | 12.6 | 14.2 | 14.1 |
| Evi5l | 6.1 | 7.6 | 6.8 | 7.1 | 4.7 | 6.1 | 6.9 | 5.4 | 5.8 | 5.7 | 4.8 | 5.7 |
| Evl | 3.3 | 2.5 | 2.5 | 7.9 | 9.2 | 3.8 | 4.9 | 3.0 | 3.4 | 3.7 | 4.2 | 3.2 |
| Evpl | 0.1 | 0.2 | 0.1 | 0.1 | 0.1 | 0.3 | 0.1 | 0.1 | 0.0 | 0.3 | 0.2 | 0.1 |
| Ewsr1 | 43.6 | 44.3 | 45.0 | 58.9 | 44.3 | 45.5 | 53.0 | 48.7 | 44.5 | 44.4 | 48.6 | 48.3 |
| Exd1 | 1.1 | 1.2 | 1.0 | 0.9 | 0.7 | 1.0 | 1.1 | 0.6 | 1.0 | 0.7 | 1.1 | 0.7 |
| Exd2 | 4.2 | 4.1 | 3.4 | 4.8 | 3.5 | 5.4 | 4.4 | 3.7 | 4.1 | 3.5 | 4.3 | 3.6 |
| Exo1 | 0.4 | 0.9 | 1.4 | 0.2 | 1.0 | 0.1 | 1.1 | 0.9 | 0.8 | 0.5 | 0.9 | 0.6 |
| Exo5 | 5.9 | 5.6 | 6.0 | 7.7 | 6.2 | 7.6 | 5.6 | 5.7 | 6.7 | 5.7 | 5.6 | 7.6 |
| Exoc1 | 11.6 | 12.4 | 10.3 | 11.6 | 11.2 | 12.7 | 10.9 | 10.6 | 11.8 | 10.2 | 11.3 | 10.6 |
| Exoc2 | 20.6 | 19.9 | 16.6 | 19.1 | 19.5 | 20.6 | 17.4 | 14.3 | 16.8 | 18.3 | 17.7 | 17.3 |
| Exoc3 | 20.1 | 17.1 | 15.4 | 21.1 | 15.7 | 17.3 | 16.0 | 13.3 | 14.4 | 16.4 | 17.9 | 16.4 |
| Exoc3l | 0.6 | 0.6 | 0.4 | 0.6 | 0.3 | 0.6 | 0.5 | 0.7 | 0.4 | 0.3 | 0.3 | 0.5 |
| Exoc3l4 | 2.1 | 3.2 | 3.8 | 1.9 | 2.7 | 2.7 | 2.2 | 1.7 | 1.6 | 1.9 | 1.5 | 1.8 |
| Exoc4 | 73.2 | 70.9 | 70.9 | 59.9 | 64.6 | 65.7 | 62.6 | 64.7 | 60.6 | 75.1 | 75.6 | 76.0 |
| Exoc5 | 20.9 | 22.7 | 21.0 | 23.9 | 23.0 | 22.7 | 22.9 | 19.3 | 23.7 | 24.5 | 27.2 | 27.6 |
| Exoc6 | 3.3 | 1.5 | 1.2 | 3.7 | 2.5 | 2.8 | 1.1 | 1.5 | 1.1 | 2.9 | 1.8 | 2.0 |
| Exoc6b | 13.1 | 13.0 | 11.3 | 14.0 | 12.6 | 12.8 | 13.0 | 10.8 | 10.8 | 12.6 | 13.4 | 12.7 |
| Exoc7 | 19.7 | 19.2 | 16.8 | 19.1 | 16.6 | 20.4 | 15.9 | 15.2 | 14.5 | 15.9 | 16.1 | 15.7 |
| Exoc8 | 6.3 | 6.4 | 6.3 | 7.2 | 6.6 | 7.0 | 7.1 | 6.3 | 7.1 | 7.0 | 6.2 | 6.9 |
| Exog | 1.6 | 1.7 | 1.9 | 1.6 | 1.6 | 1.9 | 1.4 | 1.4 | 1.4 | 1.6 | 1.5 | 1.5 |
| Exosc1 | 8.7 | 10.2 | 9.1 | 11.1 | 8.8 | 10.7 | 9.7 | 10.3 | 8.6 | 7.9 | 11.2 | 9.2 |
| Exosc10 | 14.9 | 13.5 | 14.6 | 16.3 | 13.6 | 14.4 | 15.8 | 14.7 | 13.3 | 13.9 | 14.9 | 14.5 |
| Exosc2 | 7.0 | 6.5 | 6.2 | 7.8 | 6.2 | 6.0 | 8.7 | 5.6 | 5.7 | 7.1 | 6.4 | 6.4 |
| Exosc3 | 11.8 | 9.3 | 11.9 | 11.3 | 11.1 | 11.5 | 11.3 | 11.5 | 9.2 | 12.4 | 12.8 | 12.8 |
| Exosc4 | 8.8 | 7.9 | 6.3 | 7.5 | 7.2 | 7.8 | 6.6 | 7.1 | 6.9 | 8.9 | 7.5 | 5.4 |
| Exosc5 | 9.0 | 7.8 | 6.0 | 9.2 | 7.9 | 10.4 | 6.6 | 7.6 | 7.3 | 8.9 | 7.0 | 6.0 |
| Exosc6 | 4.9 | 5.9 | 4.6 | 4.6 | 6.2 | 4.5 | 8.4 | 6.0 | 5.8 | 7.4 | 7.5 | 6.6 |
| Exosc7 | 10.1 | 11.2 | 10.2 | 10.1 | 8.3 | 9.9 | 8.4 | 9.1 | 8.4 | 9.9 | 9.2 | 7.6 |
| Exosc8 | 8.1 | 11.2 | 10.7 | 8.7 | 9.1 | 8.4 | 9.5 | 10.1 | 10.6 | 8.3 | 10.1 | 7.7 |
| Exosc9 | 30.3 | 26.5 | 22.2 | 29.3 | 20.7 | 26.0 | 22.6 | 20.5 | 21.4 | 21.5 | 25.1 | 25.3 |
| Exph5 | 0.0 | 0.0 | 0.0 | 0.0 | 0.0 | 0.0 | 0.0 | 0.0 | 0.0 | 0.0 | 0.0 | 0.0 |
| Ext1 | 70.0 | 60.7 | 57.0 | 75.7 | 79.3 | 65.9 | 84.6 | 66.5 | 80.8 | 72.1 | 74.5 | 83.1 |
| Ext2 | 37.5 | 34.4 | 30.7 | 35.0 | 30.9 | 37.9 | 33.6 | 31.8 | 31.7 | 29.8 | 28.7 | 28.0 |
| Extl1 | 0.8 | 0.4 | 0.1 | 0.9 | 0.2 | 1.1 | 0.3 | 0.3 | 0.2 | 0.3 | 0.4 | 0.2 |
| Extl2 | 11.2 | 11.2 | 11.5 | 13.3 | 9.6 | 13.5 | 11.2 | 11.0 | 9.4 | 12.1 | 11.8 | 12.2 |
| Extl3 | 15.9 | 18.1 | 18.4 | 16.8 | 24.9 | 14.6 | 22.7 | 15.6 | 19.5 | 19.7 | 15.5 | 18.3 |
| Eya1 | 0.1 | 0.2 | 0.1 | 0.3 | 0.1 | 0.1 | 0.1 | 0.1 | 0.1 | 0.0 | 0.1 | 0.0 |
| Eya2 | 1.4 | 3.2 | 3.9 | 1.1 | 2.0 | 1.4 | 1.7 | 2.2 | 1.5 | 1.7 | 2.0 | 1.9 |

| | | | | | | | | | | | | |
|---|---|---|---|---|---|---|---|---|---|---|---|---|
| Eya3 | 6.2 | 7.3 | 7.7 | 7.6 | 8.2 | 6.9 | 7.4 | 8.3 | 7.0 | 6.2 | 7.1 | 6.3 |
| Eya4 | 7.0 | 7.1 | 7.7 | 7.0 | 6.1 | 10.6 | 9.5 | 9.6 | 9.5 | 6.6 | 11.8 | 10.1 |
| Ezh1 | 10.2 | 12.9 | 10.4 | 10.0 | 8.9 | 12.0 | 7.8 | 9.8 | 9.0 | 8.3 | 8.6 | 9.6 |
| Ezh2 | 1.9 | 2.5 | 3.7 | 1.4 | 3.6 | 1.5 | 3.4 | 3.6 | 3.1 | 3.0 | 3.1 | 3.4 |
| Ezr | 66.7 | 26.5 | 17.2 | 36.3 | 21.4 | 20.3 | 13.3 | 13.6 | 13.3 | 152.1 | 81.1 | 49.7 |
| F11r | 2.7 | 0.6 | 0.8 | 1.0 | 1.4 | 0.7 | 0.8 | 1.2 | 1.3 | 10.0 | 5.2 | 3.1 |
| F13a1 | 0.2 | 0.0 | 0.0 | 0.3 | 0.1 | 0.1 | 0.0 | 0.0 | 0.0 | 0.0 | 0.0 | 0.0 |
| F2r | 98.0 | 101.2 | 103.9 | 124.2 | 94.5 | 116.5 | 125.4 | 105.2 | 119.6 | 128.9 | 113.6 | 126.1 |
| F2rl1 | 0.4 | 0.3 | 0.4 | 0.1 | 0.3 | 0.1 | 0.3 | 0.4 | 0.3 | 0.1 | 0.3 | 0.1 |
| F2rl2 | 0.1 | 0.0 | 0.0 | 0.1 | 0.1 | 0.0 | 0.0 | 0.0 | 0.0 | 0.0 | 0.1 | 0.0 |
| F2rl3 | 0.2 | 0.2 | 0.2 | 0.0 | 0.2 | 0.0 | 0.2 | 0.2 | 0.2 | 0.2 | 0.1 | 0.1 |
| F3 | 95.1 | 145.3 | 159.1 | 96.4 | 112.2 | 80.4 | 86.5 | 75.9 | 70.3 | 113.9 | 132.3 | 90.4 |
| F420014N23Rik | 0.5 | 0.6 | 0.5 | 0.7 | 0.4 | 0.4 | 0.6 | 0.6 | 0.4 | 0.4 | 0.4 | 0.5 |
| F5 | 0.1 | 0.1 | 0.1 | 0.0 | 0.0 | 0.0 | 0.0 | 0.0 | 0.0 | 0.0 | 0.0 | 0.0 |
| F630028O10Rik | 0.2 | 0.0 | 0.0 | 2.0 | 2.4 | 0.0 | 0.2 | 0.0 | 0.0 | 0.1 | 0.1 | 0.1 |
| F630042J09Rik | 0.0 | 0.0 | 0.0 | 0.0 | 0.0 | 0.0 | 0.0 | 0.0 | 0.0 | 0.0 | 0.0 | 0.0 |
| F630111L10Rik | 0.0 | 0.0 | 0.0 | 0.0 | 0.0 | 0.0 | 0.0 | 0.0 | 0.0 | 0.0 | 0.0 | 0.0 |
| F630206G17Rik | 0.0 | 0.0 | 0.0 | 0.0 | 0.0 | 0.0 | 0.0 | 0.0 | 0.0 | 0.0 | 0.1 | 0.0 |
| F730035M05Rik | 0.1 | 0.0 | 0.0 | 0.1 | 0.1 | 0.0 | 0.1 | 0.0 | 0.0 | 0.0 | 0.0 | 0.0 |
| F730043M19Rik | 4.2 | 6.7 | 5.8 | 6.9 | 7.1 | 8.8 | 10.0 | 5.0 | 5.5 | 4.3 | 5.7 | 5.6 |
| F8 | 1.0 | 1.1 | 1.0 | 0.7 | 0.7 | 0.8 | 0.9 | 1.1 | 0.7 | 1.0 | 1.2 | 1.0 |
| F830016B08Rik | 0.6 | 0.3 | 1.0 | 1.1 | 0.8 | 0.4 | 0.3 | 0.2 | 0.2 | 0.2 | 0.3 | 0.9 |
| F8a | 1.9 | 1.8 | 1.5 | 1.9 | 1.4 | 1.5 | 1.5 | 1.8 | 1.7 | 1.8 | 1.8 | 1.5 |
| F9 | 0.0 | 0.0 | 0.0 | 0.0 | 0.2 | 0.0 | 0.0 | 0.0 | 0.0 | 0.0 | 0.0 | 0.0 |
| F930015N05Rik | 0.0 | 0.0 | 0.0 | 0.0 | 0.1 | 0.1 | 0.1 | 0.0 | 0.0 | 0.0 | 0.0 | 0.0 |
| Faah | 0.3 | 0.4 | 0.2 | 0.4 | 0.3 | 0.7 | 0.2 | 0.3 | 0.3 | 0.2 | 0.4 | 0.2 |
| Fabp3 | 8.1 | 3.4 | 2.0 | 5.1 | 2.2 | 4.8 | 2.7 | 3.4 | 1.4 | 6.7 | 5.3 | 7.2 |
| Fabp4 | 6.6 | 2.4 | 2.8 | 8.2 | 2.4 | 2.0 | 3.2 | 5.3 | 6.3 | 4.4 | 2.0 | 3.6 |
| Fabp5 | 96.4 | 139.3 | 125.2 | 65.1 | 72.4 | 51.0 | 134.6 | 160.7 | 227.7 | 72.4 | 160.7 | 105.2 |
| Fabp7 | 0.5 | 0.3 | 0.4 | 0.5 | 0.3 | 0.4 | 0.1 | 0.3 | 0.2 | 0.2 | 0.2 | 0.4 |
| Fadd | 2.4 | 2.2 | 2.0 | 2.9 | 2.3 | 2.0 | 2.4 | 2.7 | 2.5 | 2.4 | 2.4 | 2.3 |
| Fads1 | 44.9 | 52.5 | 45.8 | 39.4 | 38.4 | 46.7 | 56.8 | 61.9 | 63.9 | 28.9 | 56.5 | 36.4 |
| Fads2 | 63.7 | 86.7 | 81.5 | 46.5 | 66.9 | 54.0 | 105.0 | 97.5 | 91.2 | 54.1 | 94.8 | 56.8 |
| Fads3 | 59.0 | 63.7 | 47.4 | 64.3 | 56.9 | 56.1 | 79.8 | 73.7 | 85.0 | 44.6 | 64.0 | 54.5 |
| Fads6 | 11.6 | 14.9 | 14.1 | 8.0 | 9.1 | 6.6 | 6.4 | 14.5 | 12.0 | 7.5 | 7.1 | 13.4 |
| Faf1 | 19.2 | 18.6 | 15.5 | 15.4 | 15.1 | 16.7 | 16.6 | 16.0 | 17.7 | 16.9 | 19.4 | 16.4 |
| Faf2 | 22.1 | 21.4 | 22.3 | 26.3 | 21.5 | 22.6 | 25.2 | 21.8 | 21.4 | 20.8 | 22.7 | 23.2 |
| Fah | 27.6 | 27.6 | 29.4 | 22.0 | 19.8 | 23.3 | 22.3 | 20.9 | 18.0 | 26.7 | 25.4 | 23.8 |
| Fahd1 | 3.6 | 3.4 | 3.6 | 3.5 | 3.3 | 4.8 | 2.7 | 3.6 | 3.5 | 3.1 | 3.7 | 2.5 |
| Fahd2a | 3.2 | 3.8 | 3.1 | 2.7 | 1.9 | 5.4 | 2.1 | 2.3 | 1.9 | 2.3 | 2.0 | 2.9 |
| Faim | 22.2 | 20.8 | 26.5 | 29.3 | 18.7 | 24.3 | 26.0 | 23.3 | 19.1 | 20.4 | 23.6 | 27.0 |
| Faim2 | 0.0 | 0.1 | 0.1 | 0.0 | 0.0 | 0.0 | 0.0 | 0.1 | 0.0 | 0.0 | 0.0 | 0.1 |
| Fam101a | 0.1 | 0.3 | 0.2 | 0.2 | 0.1 | 0.2 | 0.3 | 0.3 | 0.2 | 0.1 | 0.0 | 0.2 |
| Fam101b | 20.9 | 21.9 | 26.6 | 20.4 | 28.8 | 24.5 | 42.2 | 45.0 | 44.1 | 31.4 | 42.5 | 47.1 |
| Fam102a | 1.5 | 1.4 | 1.9 | 1.9 | 1.7 | 1.7 | 1.1 | 1.9 | 1.1 | 1.9 | 1.1 | 1.5 |
| Fam102b | 12.7 | 14.7 | 15.0 | 11.6 | 15.8 | 10.5 | 16.3 | 15.1 | 15.8 | 12.3 | 13.6 | 12.9 |
| Fam103a1 | 25.2 | 22.8 | 28.3 | 29.9 | 22.2 | 28.5 | 24.4 | 25.1 | 20.5 | 23.7 | 31.9 | 32.3 |
| Fam104a | 10.8 | 11.5 | 11.4 | 14.7 | 12.5 | 12.9 | 14.3 | 12.7 | 12.4 | 12.3 | 13.1 | 12.6 |
| Fam105a | 0.6 | 0.2 | 0.2 | 3.4 | 6.0 | 0.2 | 0.7 | 0.1 | 0.2 | 0.5 | 0.3 | 0.6 |
| Fam105b | 13.8 | 11.3 | 11.4 | 16.6 | 19.2 | 10.9 | 13.6 | 12.8 | 11.5 | 12.7 | 14.2 | 14.8 |
| Fam107a | 0.0 | 0.0 | 0.0 | 0.1 | 0.0 | 0.0 | 0.0 | 0.0 | 0.0 | 0.0 | 0.0 | 0.0 |
| Fam107b | 24.3 | 15.2 | 10.8 | 19.8 | 16.1 | 15.9 | 14.8 | 6.9 | 10.2 | 15.2 | 15.5 | 15.4 |
| Fam108a | 31.5 | 25.7 | 22.2 | 25.3 | 27.5 | 28.8 | 27.1 | 28.2 | 28.1 | 33.0 | 26.1 | 21.9 |
| Fam108b | 6.3 | 6.5 | 6.3 | 5.3 | 5.0 | 4.6 | 5.0 | 6.1 | 7.3 | 6.1 | 7.2 | 6.8 |
| Fam108c | 19.7 | 20.1 | 18.7 | 11.8 | 12.7 | 11.3 | 13.0 | 16.8 | 17.2 | 14.2 | 16.4 | 17.1 |
| Fam109a | 3.1 | 3.4 | 2.7 | 2.6 | 2.7 | 2.7 | 3.0 | 3.1 | 3.4 | 3.1 | 2.6 | 2.4 |
| Fam109b | 0.5 | 0.5 | 0.6 | 0.5 | 0.3 | 0.5 | 0.7 | 0.7 | 0.6 | 0.8 | 0.3 | 0.6 |

| | | | | | | | | | | | | |
|---|---|---|---|---|---|---|---|---|---|---|---|---|
| Fam110a | 2.6 | 2.8 | 2.7 | 2.3 | 3.0 | 2.6 | 2.3 | 1.4 | 2.2 | 3.8 | 2.5 | 2.2 |
| Fam110b | 2.7 | 2.9 | 2.4 | 2.8 | 2.3 | 2.9 | 2.3 | 3.1 | 2.5 | 2.6 | 2.6 | 2.0 |
| Fam110c | 4.2 | 1.5 | 0.7 | 1.2 | 0.5 | 1.0 | 0.6 | 0.8 | 1.7 | 18.8 | 7.7 | 5.3 |
| Fam111a | 7.9 | 11.4 | 15.2 | 8.2 | 20.7 | 4.3 | 16.6 | 10.9 | 12.0 | 10.2 | 14.6 | 9.5 |
| Fam114a1 | 104.3 | 99.5 | 104.0 | 117.8 | 82.7 | 111.6 | 113.5 | 106.6 | 97.1 | 92.6 | 122.3 | 121.1 |
| Fam114a2 | 27.5 | 29.0 | 30.9 | 29.8 | 24.8 | 28.7 | 26.4 | 28.0 | 26.1 | 27.6 | 30.2 | 29.8 |
| Fam115a | 25.9 | 26.5 | 22.4 | 20.3 | 20.4 | 22.0 | 23.0 | 19.6 | 24.3 | 23.2 | 22.6 | 21.8 |
| Fam115c | 5.5 | 5.8 | 7.0 | 4.8 | 6.2 | 5.3 | 5.5 | 6.2 | 5.4 | 6.9 | 8.1 | 5.2 |
| Fam115e | 0.0 | 0.0 | 0.0 | 0.0 | 0.0 | 0.0 | 0.0 | 0.0 | 0.0 | 0.0 | 0.0 | 0.0 |
| Fam117a | 3.1 | 4.0 | 3.4 | 2.1 | 2.6 | 3.1 | 2.5 | 2.8 | 3.3 | 3.3 | 2.8 | 2.9 |
| Fam117b | 4.2 | 4.7 | 4.3 | 3.6 | 3.9 | 4.7 | 3.8 | 4.2 | 4.0 | 4.3 | 5.2 | 4.5 |
| Fam118a | 8.1 | 7.7 | 5.9 | 6.6 | 8.1 | 6.7 | 6.7 | 5.0 | 5.7 | 7.1 | 6.3 | 6.6 |
| Fam118b | 13.5 | 10.4 | 9.7 | 10.3 | 10.2 | 10.0 | 8.7 | 9.7 | 9.1 | 12.7 | 11.8 | 10.5 |
| Fam120a | 63.2 | 68.7 | 64.0 | 66.2 | 68.0 | 67.3 | 70.5 | 61.5 | 64.4 | 61.1 | 71.5 | 60.7 |
| Fam120aos | 1.9 | 1.4 | 2.0 | 1.6 | 2.0 | 1.9 | 1.3 | 1.6 | 1.7 | 1.9 | 2.8 | 2.3 |
| Fam120b | 12.7 | 11.5 | 11.8 | 11.8 | 11.4 | 13.8 | 11.3 | 9.9 | 10.1 | 12.5 | 13.0 | 12.7 |
| Fam120c | 3.5 | 3.5 | 3.4 | 3.4 | 3.0 | 3.4 | 3.0 | 3.4 | 2.4 | 3.3 | 3.3 | 3.0 |
| Fam122a | 3.8 | 3.6 | 3.9 | 3.9 | 3.4 | 3.1 | 3.8 | 4.2 | 3.8 | 3.8 | 4.2 | 3.5 |
| Fam122b | 0.5 | 0.6 | 0.9 | 0.8 | 0.7 | 1.0 | 0.7 | 0.5 | 0.5 | 0.4 | 0.4 | 0.5 |
| Fam124a | 8.0 | 7.0 | 4.8 | 10.2 | 5.0 | 8.5 | 4.8 | 3.3 | 2.8 | 2.1 | 2.9 | 3.1 |
| Fam126a | 4.7 | 6.3 | 5.7 | 5.3 | 6.1 | 4.2 | 5.4 | 5.3 | 5.9 | 5.8 | 6.5 | 5.3 |
| Fam126b | 1.5 | 2.0 | 1.6 | 1.7 | 1.5 | 1.9 | 1.3 | 1.2 | 1.5 | 1.9 | 1.9 | 1.7 |
| Fam129a | 44.7 | 34.9 | 41.8 | 55.5 | 39.4 | 51.4 | 35.8 | 43.7 | 34.4 | 24.6 | 37.2 | 32.9 |
| Fam129b | 252.4 | 223.5 | 158.2 | 303.9 | 226.1 | 302.2 | 237.1 | 153.3 | 179.8 | 192.1 | 202.5 | 182.5 |
| Fam129c | 0.8 | 0.5 | 0.4 | 0.6 | 0.4 | 0.6 | 0.4 | 0.4 | 0.5 | 0.5 | 0.6 | 0.6 |
| Fam131a | 2.9 | 3.7 | 3.0 | 3.1 | 1.9 | 4.9 | 2.6 | 2.2 | 3.0 | 3.8 | 3.2 | 3.1 |
| Fam131b | 0.5 | 0.8 | 1.0 | 0.2 | 0.7 | 1.0 | 1.7 | 2.5 | 1.9 | 1.4 | 2.1 | 1.1 |
| Fam131c | 0.1 | 0.2 | 0.1 | 0.2 | 0.1 | 0.3 | 0.2 | 0.1 | 0.0 | 0.1 | 0.2 | 0.0 |
| Fam132a | 7.4 | 9.6 | 9.1 | 4.4 | 7.6 | 8.8 | 4.5 | 8.0 | 8.5 | 10.0 | 5.6 | 7.7 |
| Fam132b | 1.1 | 2.0 | 1.2 | 0.4 | 1.5 | 0.7 | 1.9 | 0.7 | 1.9 | 0.7 | 1.2 | 1.3 |
| Fam133b | 5.7 | 5.5 | 6.9 | 6.7 | 6.4 | 5.8 | 6.9 | 6.7 | 5.6 | 6.6 | 7.4 | 7.6 |
| Fam134a | 11.4 | 12.1 | 12.2 | 14.4 | 12.5 | 15.2 | 13.3 | 12.8 | 13.5 | 13.4 | 10.8 | 11.8 |
| Fam134b | 2.5 | 1.1 | 0.9 | 5.1 | 5.9 | 2.6 | 1.2 | 0.5 | 0.5 | 1.9 | 1.6 | 1.4 |
| Fam134c | 12.2 | 14.1 | 14.5 | 12.3 | 14.5 | 12.0 | 13.8 | 15.6 | 15.7 | 14.8 | 13.6 | 13.2 |
| Fam135a | 4.2 | 4.8 | 4.1 | 4.4 | 3.6 | 4.1 | 3.5 | 3.4 | 3.7 | 3.6 | 4.4 | 4.3 |
| Fam136a | 16.5 | 14.7 | 13.0 | 14.7 | 13.2 | 16.1 | 11.2 | 12.5 | 13.5 | 14.2 | 14.7 | 13.0 |
| Fam13a | 0.3 | 0.4 | 0.4 | 0.8 | 0.4 | 0.4 | 0.1 | 0.1 | 0.1 | 0.1 | 0.0 | 0.0 |
| Fam13b | 31.0 | 25.4 | 19.5 | 34.3 | 27.3 | 27.6 | 27.8 | 18.8 | 26.0 | 29.0 | 32.9 | 31.3 |
| Fam13c | 3.7 | 9.1 | 15.8 | 2.9 | 5.6 | 4.1 | 7.4 | 16.6 | 11.4 | 5.3 | 7.4 | 8.8 |
| Fam149a | 11.0 | 10.5 | 10.7 | 12.4 | 8.6 | 12.8 | 9.7 | 9.4 | 8.4 | 8.0 | 10.1 | 9.6 |
| Fam149b | 15.4 | 13.5 | 13.7 | 16.9 | 12.7 | 17.8 | 13.8 | 13.7 | 11.1 | 12.6 | 16.2 | 14.6 |
| Fam150a | 0.0 | 0.1 | 0.0 | 0.1 | 0.1 | 0.0 | 0.0 | 0.0 | 0.0 | 0.1 | 0.3 | 0.0 |
| Fam151b | 2.8 | 2.0 | 1.8 | 2.8 | 2.1 | 2.4 | 2.1 | 2.5 | 1.8 | 2.0 | 1.4 | 2.1 |
| Fam154b | 0.2 | 0.2 | 0.2 | 0.1 | 0.1 | 0.2 | 0.0 | 0.1 | 0.1 | 0.1 | 0.2 | 0.2 |
| Fam155a | 0.0 | 0.0 | 0.0 | 0.0 | 0.0 | 0.0 | 0.0 | 0.0 | 0.0 | 0.0 | 0.0 | 0.0 |
| Fam159b | 0.1 | 0.1 | 0.1 | 0.2 | 0.1 | 0.1 | 0.0 | 0.0 | 0.0 | 0.0 | 0.0 | 0.0 |
| Fam160a1 | 6.1 | 5.8 | 5.6 | 5.6 | 5.5 | 5.9 | 6.5 | 4.7 | 4.8 | 5.2 | 6.1 | 5.6 |
| Fam160a2 | 1.2 | 1.5 | 1.3 | 1.1 | 1.2 | 1.2 | 1.3 | 1.1 | 1.4 | 1.5 | 1.2 | 1.2 |
| Fam160b1 | 7.4 | 7.7 | 7.1 | 6.1 | 7.0 | 7.3 | 7.2 | 7.1 | 7.5 | 8.1 | 8.3 | 7.7 |
| Fam160b2 | 1.8 | 1.8 | 2.1 | 1.7 | 2.1 | 2.2 | 2.2 | 2.4 | 1.8 | 1.9 | 2.0 | 1.8 |
| Fam161a | 1.2 | 1.5 | 2.1 | 1.8 | 1.4 | 1.7 | 1.3 | 1.7 | 1.7 | 1.1 | 1.6 | 2.2 |
| Fam161b | 0.8 | 0.8 | 0.8 | 0.9 | 0.5 | 1.0 | 0.5 | 0.4 | 0.6 | 0.6 | 0.4 | 0.6 |
| Fam162a | 77.9 | 67.7 | 63.4 | 64.4 | 64.1 | 73.1 | 62.0 | 52.5 | 58.9 | 80.9 | 72.5 | 84.4 |
| Fam163a | 0.0 | 0.0 | 0.0 | 0.0 | 0.0 | 0.0 | 0.0 | 0.0 | 0.0 | 0.0 | 0.0 | 0.0 |
| Fam163b | 0.0 | 0.1 | 0.1 | 0.0 | 0.0 | 0.1 | 0.0 | 0.1 | 0.0 | 0.0 | 0.0 | 0.0 |
| Fam166a | 0.2 | 0.0 | 0.1 | 0.0 | 0.2 | 0.0 | 0.0 | 0.0 | 0.3 | 0.1 | 0.1 | 0.2 |
| Fam166b | 0.2 | 0.2 | 0.4 | 0.1 | 0.1 | 0.2 | 0.0 | 0.1 | 0.2 | 0.2 | 0.2 | 0.1 |

| | | | | | | | | | | | | |
|---|---|---|---|---|---|---|---|---|---|---|---|---|
| Fam167a | 0.1 | 0.0 | 0.0 | 0.0 | 0.0 | 0.0 | 0.0 | 0.0 | 0.0 | 0.0 | 0.1 | 0.0 |
| Fam167b | 0.2 | 0.1 | 0.4 | 0.1 | 0.0 | 0.0 | 0.2 | 0.1 | 0.3 | 0.0 | 0.1 | 0.2 |
| Fam168a | 4.5 | 5.8 | 6.5 | 4.3 | 5.4 | 4.3 | 5.4 | 6.1 | 5.5 | 5.7 | 5.2 | 5.7 |
| Fam168b | 24.7 | 25.6 | 22.0 | 24.8 | 26.0 | 22.3 | 24.6 | 20.2 | 24.7 | 23.6 | 22.0 | 23.2 |
| Fam169a | 0.1 | 0.0 | 0.1 | 0.2 | 0.1 | 0.1 | 0.1 | 0.1 | 0.1 | 0.1 | 0.1 | 0.2 |
| Fam169b | 1.6 | 2.2 | 1.2 | 1.9 | 1.4 | 1.6 | 1.5 | 2.0 | 1.3 | 0.9 | 1.2 | 0.6 |
| Fam171a1 | 12.8 | 15.4 | 14.9 | 12.4 | 13.3 | 15.1 | 17.0 | 13.1 | 13.6 | 12.6 | 13.8 | 14.0 |
| Fam171a2 | 3.2 | 3.3 | 3.0 | 2.6 | 2.0 | 3.3 | 2.3 | 3.7 | 3.9 | 3.3 | 3.2 | 2.9 |
| Fam171b | 2.0 | 2.1 | 2.2 | 2.0 | 1.7 | 3.1 | 1.8 | 2.9 | 1.8 | 1.6 | 2.4 | 2.3 |
| Fam172a | 5.9 | 7.0 | 6.9 | 6.0 | 6.0 | 5.8 | 6.3 | 6.4 | 7.3 | 6.4 | 6.9 | 6.9 |
| Fam173a | 25.1 | 24.5 | 20.0 | 19.2 | 19.8 | 25.0 | 17.2 | 19.5 | 24.3 | 25.7 | 18.0 | 18.3 |
| Fam173b | 9.1 | 8.3 | 7.1 | 9.5 | 8.0 | 9.1 | 9.4 | 6.9 | 8.1 | 8.0 | 9.0 | 8.3 |
| Fam174a | 11.3 | 10.9 | 10.2 | 12.5 | 10.1 | 13.5 | 10.9 | 9.6 | 11.1 | 12.3 | 10.9 | 12.6 |
| Fam174b | 5.7 | 3.3 | 1.6 | 8.7 | 2.1 | 5.8 | 3.3 | 1.6 | 1.4 | 2.2 | 2.7 | 1.5 |
| Fam175a | 2.5 | 2.5 | 3.1 | 2.4 | 2.7 | 3.2 | 3.0 | 2.9 | 3.1 | 2.3 | 3.6 | 2.7 |
| Fam175b | 16.1 | 18.1 | 18.8 | 16.4 | 17.2 | 14.7 | 17.1 | 17.8 | 18.9 | 17.0 | 18.2 | 20.8 |
| Fam177a | 25.9 | 20.9 | 23.6 | 30.6 | 24.4 | 24.4 | 29.4 | 21.4 | 25.9 | 20.9 | 28.5 | 30.8 |
| Fam178a | 13.9 | 19.0 | 18.0 | 13.8 | 13.8 | 13.3 | 13.4 | 10.6 | 12.6 | 13.1 | 13.2 | 15.7 |
| Fam178b | 0.6 | 0.9 | 1.5 | 0.3 | 0.6 | 0.9 | 0.5 | 0.9 | 0.7 | 1.0 | 0.5 | 0.7 |
| Fam179a | 0.0 | 0.0 | 0.0 | 0.2 | 0.0 | 0.0 | 0.0 | 0.0 | 0.0 | 0.0 | 0.0 | 0.0 |
| Fam179b | 6.9 | 6.9 | 7.0 | 6.3 | 6.1 | 7.4 | 5.4 | 6.2 | 6.4 | 6.7 | 7.1 | 6.6 |
| Fam180a | 2.7 | 1.8 | 1.0 | 0.4 | 0.5 | 0.6 | 1.3 | 2.4 | 1.5 | 8.1 | 2.7 | 3.2 |
| Fam184a | 0.4 | 0.2 | 0.3 | 0.2 | 0.2 | 0.3 | 0.1 | 0.2 | 0.1 | 2.0 | 1.2 | 0.5 |
| Fam184b | 0.0 | 0.0 | 0.0 | 0.0 | 0.0 | 0.0 | 0.0 | 0.0 | 0.0 | 0.0 | 0.1 | 0.0 |
| Fam185a | 1.3 | 1.4 | 1.4 | 1.3 | 1.2 | 1.9 | 1.2 | 1.1 | 1.4 | 1.7 | 1.5 | 1.3 |
| Fam186b | 0.1 | 0.1 | 0.3 | 0.1 | 0.1 | 0.2 | 0.1 | 0.0 | 0.1 | 0.1 | 0.2 | 0.0 |
| Fam188a | 15.9 | 14.1 | 12.4 | 15.9 | 14.6 | 13.1 | 13.0 | 10.9 | 13.5 | 15.3 | 15.5 | 14.6 |
| Fam188b | 1.9 | 2.0 | 1.7 | 1.6 | 1.6 | 1.9 | 1.4 | 1.7 | 1.5 | 1.9 | 1.7 | 1.8 |
| Fam189a2 | 0.5 | 0.4 | 0.4 | 0.5 | 0.3 | 0.6 | 0.6 | 0.3 | 0.9 | 0.6 | 0.7 | 0.7 |
| Fam189b | 2.3 | 3.0 | 2.7 | 2.3 | 3.0 | 3.1 | 3.8 | 2.7 | 3.2 | 2.4 | 2.8 | 1.7 |
| Fam192a | 20.9 | 19.1 | 19.0 | 27.5 | 18.7 | 21.6 | 19.3 | 18.7 | 15.8 | 17.6 | 20.9 | 22.2 |
| Fam193a | 2.9 | 4.1 | 4.2 | 3.5 | 3.3 | 3.3 | 4.3 | 5.0 | 4.3 | 3.3 | 4.6 | 4.1 |
| Fam193b | 3.3 | 3.6 | 3.2 | 3.1 | 3.1 | 4.0 | 3.6 | 3.0 | 3.9 | 3.0 | 2.7 | 2.7 |
| Fam194a | 0.1 | 0.1 | 0.0 | 0.0 | 0.0 | 0.1 | 0.0 | 0.1 | 0.0 | 0.1 | 0.0 | 0.0 |
| Fam195a | 3.9 | 2.7 | 2.7 | 4.1 | 2.7 | 3.7 | 2.6 | 2.2 | 3.8 | 3.0 | 2.9 | 2.3 |
| Fam195b | 33.2 | 28.0 | 26.0 | 27.1 | 26.0 | 28.9 | 25.1 | 29.1 | 29.7 | 33.0 | 27.7 | 26.5 |
| Fam196a | 0.4 | 0.4 | 0.3 | 0.1 | 0.4 | 0.2 | 0.2 | 0.3 | 0.2 | 0.4 | 0.3 | 0.2 |
| Fam196b | 0.0 | 0.0 | 0.0 | 0.1 | 0.1 | 0.0 | 0.0 | 0.0 | 0.0 | 0.0 | 0.0 | 0.0 |
| Fam198a | 0.1 | 0.1 | 0.2 | 0.1 | 0.1 | 0.1 | 0.1 | 0.2 | 0.2 | 0.1 | 0.0 | 0.0 |
| Fam198b | 75.8 | 67.9 | 69.2 | 93.4 | 75.0 | 106.6 | 102.9 | 73.0 | 87.2 | 73.0 | 93.8 | 88.0 |
| Fam199x | 1.7 | 2.3 | 2.8 | 2.2 | 2.0 | 2.0 | 2.0 | 2.3 | 1.9 | 2.3 | 2.3 | 2.4 |
| Fam19a1 | 0.0 | 0.0 | 0.0 | 0.0 | 0.0 | 0.0 | 0.0 | 0.0 | 0.0 | 0.0 | 0.0 | 0.0 |
| Fam19a2 | 0.0 | 0.0 | 0.0 | 0.0 | 0.0 | 0.0 | 0.0 | 0.0 | 0.0 | 0.0 | 0.0 | 0.0 |
| Fam19a3 | 0.0 | 0.0 | 0.0 | 0.0 | 0.1 | 0.0 | 0.0 | 0.0 | 0.0 | 0.0 | 0.0 | 0.0 |
| Fam19a5 | 1.9 | 1.7 | 0.9 | 1.3 | 1.3 | 2.4 | 3.1 | 2.3 | 2.2 | 2.0 | 2.5 | 2.3 |
| Fam203a | 2.7 | 2.3 | 2.1 | 2.5 | 2.7 | 3.7 | 3.2 | 2.6 | 2.8 | 2.9 | 2.6 | 1.8 |
| Fam207a | 8.9 | 9.1 | 8.1 | 9.3 | 9.7 | 9.6 | 8.5 | 9.3 | 9.4 | 10.6 | 8.3 | 9.1 |
| Fam209 | 0.0 | 0.3 | 0.1 | 0.0 | 0.2 | 0.2 | 0.1 | 0.1 | 0.0 | 0.4 | 0.0 | 0.2 |
| Fam20a | 6.8 | 11.3 | 17.8 | 4.2 | 12.5 | 3.9 | 20.8 | 21.5 | 30.2 | 16.8 | 15.7 | 24.5 |
| Fam20b | 28.3 | 27.1 | 23.9 | 31.7 | 24.0 | 30.9 | 25.5 | 20.1 | 23.6 | 23.2 | 25.5 | 25.9 |
| Fam20c | 21.0 | 18.1 | 17.1 | 22.6 | 24.1 | 20.8 | 26.3 | 25.9 | 22.0 | 17.8 | 23.2 | 19.9 |
| Fam21 | 28.6 | 24.5 | 23.3 | 29.4 | 22.9 | 26.3 | 21.0 | 22.4 | 18.6 | 21.9 | 23.8 | 24.5 |
| Fam210a | 4.0 | 4.3 | 4.3 | 3.6 | 3.8 | 4.3 | 3.9 | 3.8 | 4.3 | 5.3 | 4.5 | 5.0 |
| Fam210b | 3.6 | 3.8 | 3.2 | 3.8 | 3.6 | 3.8 | 3.8 | 3.2 | 3.0 | 3.9 | 3.9 | 3.5 |
| Fam211a | 18.4 | 18.4 | 13.7 | 19.2 | 12.3 | 21.9 | 16.8 | 13.1 | 16.0 | 12.3 | 17.1 | 11.6 |
| Fam211b | 2.8 | 1.9 | 1.8 | 3.3 | 1.4 | 3.4 | 1.2 | 1.1 | 0.8 | 1.0 | 1.2 | 0.8 |
| Fam212a | 4.9 | 6.2 | 5.2 | 6.5 | 6.0 | 5.9 | 7.1 | 6.9 | 5.5 | 4.2 | 5.2 | 4.0 |

| | | | | | | | | | | | | |
|---|---|---|---|---|---|---|---|---|---|---|---|---|
| Fam212b | 6.5 | 5.4 | 4.0 | 6.5 | 5.7 | 6.2 | 5.7 | 2.8 | 4.0 | 6.3 | 5.9 | 5.5 |
| Fam213a | 8.1 | 5.4 | 4.7 | 8.4 | 4.3 | 8.9 | 3.7 | 4.5 | 3.0 | 5.7 | 5.7 | 5.5 |
| Fam213b | 19.0 | 18.7 | 12.4 | 15.3 | 13.0 | 19.0 | 9.3 | 14.0 | 12.9 | 13.9 | 13.9 | 12.0 |
| Fam214a | 9.0 | 10.9 | 10.9 | 7.2 | 8.4 | 8.7 | 7.0 | 7.0 | 8.3 | 9.0 | 8.2 | 8.1 |
| Fam214b | 20.3 | 19.6 | 14.9 | 20.7 | 20.2 | 18.2 | 21.3 | 16.9 | 19.6 | 17.3 | 19.2 | 17.6 |
| Fam216a | 21.4 | 22.0 | 21.3 | 24.1 | 18.4 | 22.3 | 19.3 | 18.3 | 16.5 | 24.1 | 20.4 | 18.7 |
| Fam217a | 0.1 | 0.1 | 0.1 | 0.0 | 0.0 | 0.0 | 0.0 | 0.1 | 0.0 | 0.0 | 0.1 | 0.1 |
| Fam217b | 0.9 | 0.2 | 0.4 | 0.8 | 0.9 | 0.6 | 0.5 | 0.4 | 0.4 | 1.0 | 0.7 | 0.9 |
| Fam219a | 5.4 | 4.8 | 3.2 | 6.4 | 4.1 | 4.9 | 5.5 | 3.5 | 4.0 | 3.5 | 4.4 | 3.1 |
| Fam219b | 13.2 | 11.7 | 9.1 | 14.2 | 11.3 | 13.6 | 12.1 | 8.3 | 10.1 | 10.7 | 9.9 | 10.9 |
| Fam220a | 17.3 | 18.5 | 15.3 | 17.0 | 13.4 | 22.9 | 13.9 | 12.4 | 12.8 | 13.4 | 16.5 | 13.7 |
| Fam221a | 0.5 | 0.8 | 0.8 | 0.3 | 0.6 | 0.8 | 0.5 | 0.4 | 0.3 | 0.4 | 0.3 | 0.4 |
| Fam221b | 0.1 | 0.1 | 0.1 | 0.1 | 0.0 | 0.2 | 0.0 | 0.0 | 0.0 | 0.0 | 0.1 | 0.1 |
| Fam222a | 0.0 | 0.0 | 0.0 | 0.0 | 0.0 | 0.0 | 0.0 | 0.0 | 0.0 | 0.0 | 0.0 | 0.0 |
| Fam222b | 2.8 | 2.8 | 3.1 | 3.4 | 3.4 | 2.8 | 3.5 | 2.8 | 3.0 | 3.0 | 2.9 | 2.7 |
| Fam227a | 0.2 | 0.3 | 0.2 | 0.1 | 0.2 | 0.2 | 0.2 | 0.2 | 0.1 | 0.2 | 0.2 | 0.2 |
| Fam227b | 0.0 | 0.0 | 0.1 | 0.0 | 0.0 | 0.0 | 0.0 | 0.1 | 0.0 | 0.1 | 0.1 | 0.0 |
| Fam228a | 0.1 | 0.0 | 0.1 | 0.1 | 0.1 | 0.1 | 0.0 | 0.0 | 0.0 | 0.0 | 0.0 | 0.0 |
| Fam228b | 0.2 | 0.2 | 0.3 | 0.3 | 0.2 | 0.5 | 0.2 | 0.1 | 0.3 | 0.3 | 0.2 | 0.2 |
| Fam229b | 5.3 | 4.6 | 4.3 | 3.9 | 3.0 | 4.9 | 3.1 | 4.7 | 3.8 | 3.9 | 4.0 | 3.8 |
| Fam25c | 0.4 | 0.3 | 0.5 | 0.0 | 0.4 | 0.5 | 0.6 | 1.1 | 0.1 | 0.2 | 1.0 | 0.2 |
| Fam26d | 0.1 | 0.0 | 0.0 | 0.0 | 0.0 | 0.0 | 0.0 | 0.0 | 0.0 | 0.0 | 0.0 | 0.0 |
| Fam26e | 26.8 | 25.5 | 23.7 | 19.8 | 16.0 | 22.1 | 15.1 | 13.1 | 16.1 | 14.7 | 19.6 | 22.0 |
| Fam26f | 0.4 | 0.7 | 1.1 | 0.8 | 0.8 | 0.7 | 0.4 | 0.5 | 0.7 | 0.4 | 0.1 | 0.7 |
| Fam32a | 43.6 | 37.7 | 35.2 | 55.2 | 36.0 | 46.3 | 40.4 | 33.4 | 30.4 | 35.5 | 39.3 | 43.3 |
| Fam35a | 3.2 | 3.5 | 3.6 | 2.3 | 3.0 | 3.0 | 2.7 | 3.0 | 3.4 | 3.3 | 3.1 | 3.7 |
| Fam3a | 13.6 | 12.1 | 12.0 | 13.8 | 12.8 | 14.3 | 13.9 | 14.1 | 15.3 | 14.3 | 12.7 | 13.2 |
| Fam3c | 23.8 | 18.9 | 22.9 | 28.2 | 23.4 | 24.3 | 27.1 | 20.3 | 19.2 | 24.8 | 26.6 | 28.4 |
| Fam43a | 6.9 | 5.9 | 6.6 | 3.5 | 3.3 | 3.5 | 3.1 | 4.0 | 3.0 | 4.3 | 3.7 | 4.2 |
| Fam43b | 0.0 | 0.0 | 0.0 | 0.1 | 0.0 | 0.0 | 0.0 | 0.0 | 0.0 | 0.0 | 0.0 | 0.0 |
| Fam45a | 13.4 | 13.5 | 13.6 | 18.6 | 15.2 | 16.2 | 12.9 | 14.1 | 12.9 | 14.2 | 17.5 | 14.7 |
| Fam46a | 1.9 | 2.8 | 6.3 | 1.6 | 2.8 | 1.4 | 2.3 | 5.4 | 3.1 | 3.4 | 1.9 | 4.6 |
| Fam46b | 3.2 | 3.3 | 2.5 | 1.3 | 2.1 | 1.5 | 3.6 | 3.7 | 4.6 | 4.2 | 5.3 | 3.3 |
| Fam46c | 0.5 | 0.4 | 0.3 | 0.6 | 1.2 | 0.2 | 0.4 | 0.2 | 0.5 | 3.1 | 0.7 | 0.7 |
| Fam49a | 9.3 | 8.9 | 9.6 | 11.2 | 11.1 | 7.6 | 9.0 | 7.4 | 7.8 | 13.4 | 11.1 | 9.2 |
| Fam49b | 4.7 | 4.0 | 3.8 | 5.7 | 8.2 | 4.4 | 4.7 | 4.1 | 4.1 | 5.0 | 5.3 | 4.7 |
| Fam50a | 56.5 | 39.3 | 33.2 | 89.3 | 33.3 | 72.1 | 31.3 | 27.3 | 24.0 | 31.4 | 37.4 | 39.7 |
| Fam53a | 8.9 | 9.1 | 8.2 | 10.5 | 7.6 | 10.8 | 8.1 | 8.7 | 9.1 | 8.4 | 9.1 | 8.3 |
| Fam53b | 4.2 | 6.2 | 6.2 | 4.3 | 5.6 | 5.1 | 4.2 | 4.0 | 4.1 | 3.0 | 3.3 | 3.2 |
| Fam53c | 5.5 | 6.1 | 5.1 | 6.3 | 6.5 | 5.6 | 5.6 | 5.2 | 5.4 | 5.0 | 4.6 | 4.2 |
| Fam57a | 2.1 | 2.1 | 2.9 | 2.9 | 2.8 | 2.3 | 5.0 | 3.8 | 3.5 | 2.9 | 3.1 | 2.8 |
| Fam57b | 0.3 | 0.3 | 0.2 | 0.2 | 0.1 | 0.3 | 0.0 | 0.2 | 0.2 | 0.2 | 0.4 | 0.1 |
| Fam58b | 9.6 | 8.8 | 7.8 | 9.0 | 8.2 | 8.9 | 8.1 | 10.0 | 8.3 | 8.8 | 6.7 | 8.3 |
| Fam5b | 0.0 | 0.0 | 0.1 | 0.0 | 0.0 | 0.0 | 0.0 | 0.1 | 0.0 | 0.0 | 0.0 | 0.0 |
| Fam5c | 0.0 | 0.0 | 0.0 | 0.0 | 0.0 | 0.0 | 0.0 | 0.0 | 0.0 | 0.0 | 0.0 | 0.0 |
| Fam60a | 2.8 | 2.7 | 2.0 | 4.0 | 4.0 | 3.1 | 3.0 | 2.2 | 3.2 | 4.7 | 4.4 | 3.0 |
| Fam63a | 11.0 | 11.0 | 10.2 | 11.5 | 11.4 | 8.3 | 13.2 | 14.8 | 11.8 | 11.9 | 11.9 | 11.8 |
| Fam63b | 14.5 | 17.1 | 17.1 | 13.6 | 15.9 | 13.7 | 16.3 | 16.7 | 17.3 | 12.4 | 17.9 | 18.2 |
| Fam64a | 2.6 | 4.8 | 6.1 | 0.6 | 6.3 | 0.7 | 11.3 | 2.5 | 7.4 | 3.2 | 6.2 | 2.9 |
| Fam65a | 2.4 | 3.1 | 2.7 | 2.7 | 2.6 | 3.0 | 2.9 | 2.7 | 2.6 | 3.2 | 2.3 | 2.4 |
| Fam65b | 1.6 | 2.4 | 1.8 | 1.3 | 2.0 | 3.8 | 3.7 | 4.3 | 4.5 | 1.4 | 2.7 | 1.9 |
| Fam65c | 1.6 | 5.1 | 6.3 | 1.1 | 2.0 | 1.1 | 1.9 | 4.6 | 3.9 | 1.2 | 1.3 | 1.8 |
| Fam69a | 10.2 | 10.4 | 10.0 | 9.5 | 11.0 | 7.9 | 7.9 | 8.8 | 9.2 | 11.9 | 11.5 | 9.7 |
| Fam69b | 9.0 | 7.4 | 5.7 | 8.3 | 5.8 | 13.2 | 6.3 | 5.1 | 5.9 | 7.4 | 5.3 | 6.3 |
| Fam69c | 0.0 | 0.1 | 0.0 | 0.0 | 0.1 | 0.1 | 0.0 | 0.0 | 0.0 | 0.0 | 0.0 | 0.0 |
| Fam71d | 0.0 | 0.0 | 0.0 | 0.1 | 0.0 | 0.0 | 0.0 | 0.0 | 0.0 | 0.0 | 0.0 | 0.0 |
| Fam71e1 | 0.3 | 0.5 | 0.4 | 0.3 | 0.3 | 0.7 | 0.3 | 0.4 | 0.5 | 0.4 | 0.2 | 0.2 |

| | | | | | | | | | | | | |
|---|---|---|---|---|---|---|---|---|---|---|---|---|
| Fam71f1 | 0.3 | 0.3 | 0.1 | 0.4 | 0.3 | 0.2 | 0.1 | 0.1 | 0.1 | 0.2 | 0.1 | 0.1 |
| Fam71f2 | 0.1 | 0.1 | 0.1 | 0.2 | 0.0 | 0.0 | 0.1 | 0.1 | 0.2 | 0.1 | 0.0 | 0.1 |
| Fam72a | 1.6 | 2.1 | 1.3 | 1.0 | 1.4 | 1.5 | 1.4 | 1.9 | 2.1 | 1.4 | 1.6 | 1.2 |
| Fam73a | 0.8 | 0.6 | 0.6 | 0.7 | 0.4 | 0.9 | 0.5 | 0.4 | 0.4 | 1.1 | 0.5 | 0.6 |
| Fam73b | 7.7 | 7.9 | 6.3 | 7.0 | 7.4 | 6.7 | 7.5 | 6.4 | 7.8 | 7.6 | 7.0 | 5.9 |
| Fam76a | 11.7 | 13.8 | 14.6 | 11.1 | 12.1 | 13.2 | 11.8 | 14.9 | 13.5 | 12.5 | 13.8 | 14.6 |
| Fam76b | 2.5 | 2.9 | 4.5 | 3.3 | 3.6 | 2.8 | 3.6 | 3.8 | 3.2 | 2.8 | 3.4 | 3.4 |
| Fam78a | 0.1 | 0.1 | 0.0 | 0.1 | 0.3 | 0.0 | 0.1 | 0.1 | 0.0 | 0.0 | 0.1 | 0.0 |
| Fam78b | 0.4 | 0.2 | 0.3 | 0.9 | 0.6 | 0.8 | 0.5 | 0.3 | 0.4 | 0.5 | 0.4 | 0.4 |
| Fam81a | 0.4 | 0.3 | 0.5 | 0.4 | 0.4 | 0.2 | 0.4 | 0.0 | 0.2 | 0.2 | 0.3 | 0.2 |
| Fam83a | 0.5 | 0.5 | 1.2 | 0.1 | 0.3 | 0.1 | 0.1 | 1.2 | 0.4 | 0.8 | 0.8 | 1.1 |
| Fam83c | 0.0 | 0.1 | 0.1 | 0.3 | 0.1 | 0.0 | 0.2 | 0.0 | 0.1 | 0.1 | 0.1 | 0.0 |
| Fam83d | 2.5 | 3.2 | 5.7 | 0.9 | 4.2 | 1.0 | 5.8 | 4.5 | 5.1 | 3.2 | 3.8 | 4.0 |
| Fam83e | 0.0 | 0.0 | 0.0 | 0.0 | 0.0 | 0.1 | 0.0 | 0.0 | 0.1 | 0.0 | 0.0 | 0.0 |
| Fam83f | 0.0 | 0.0 | 0.0 | 0.2 | 0.4 | 0.1 | 0.0 | 0.0 | 0.0 | 0.0 | 0.0 | 0.0 |
| Fam83g | 0.2 | 0.2 | 0.1 | 0.1 | 0.1 | 0.1 | 0.1 | 0.1 | 0.1 | 0.2 | 0.2 | 0.1 |
| Fam83h | 1.9 | 1.6 | 1.7 | 1.5 | 1.8 | 1.9 | 1.5 | 1.6 | 1.9 | 1.9 | 1.5 | 1.5 |
| Fam84a | 0.1 | 0.1 | 0.1 | 0.0 | 0.0 | 0.0 | 0.1 | 0.1 | 0.2 | 1.6 | 0.7 | 0.3 |
| Fam84b | 1.2 | 0.8 | 1.0 | 1.0 | 0.9 | 1.0 | 0.6 | 0.9 | 0.8 | 1.6 | 1.2 | 1.2 |
| Fam86 | 7.1 | 6.8 | 5.4 | 6.9 | 6.6 | 6.9 | 6.1 | 6.7 | 7.0 | 6.7 | 7.5 | 5.8 |
| Fam89a | 0.2 | 0.1 | 0.1 | 0.1 | 0.2 | 0.1 | 0.0 | 0.1 | 0.2 | 0.1 | 0.0 | 0.1 |
| Fam89b | 17.4 | 14.3 | 13.5 | 20.7 | 17.9 | 16.9 | 18.8 | 17.2 | 19.9 | 20.8 | 17.2 | 15.6 |
| Fam92a | 37.1 | 32.7 | 36.8 | 35.5 | 23.9 | 41.9 | 29.4 | 28.7 | 24.5 | 31.1 | 38.8 | 37.8 |
| Fam96a | 18.2 | 16.3 | 15.9 | 18.9 | 25.7 | 15.8 | 15.8 | 15.5 | 16.3 | 16.4 | 17.4 | 17.5 |
| Fam96b | 29.1 | 25.8 | 28.7 | 31.8 | 25.5 | 31.8 | 30.1 | 26.6 | 27.7 | 24.5 | 26.2 | 30.2 |
| Fam98a | 17.1 | 15.3 | 15.9 | 21.0 | 15.7 | 16.1 | 16.5 | 14.2 | 12.2 | 14.0 | 16.9 | 16.8 |
| Fam98b | 2.5 | 2.2 | 3.1 | 2.3 | 2.7 | 2.6 | 2.9 | 2.5 | 2.8 | 3.1 | 2.5 | 2.9 |
| Fam98c | 4.2 | 3.1 | 2.9 | 2.7 | 3.1 | 3.1 | 3.6 | 3.3 | 3.6 | 3.6 | 2.2 | 3.2 |
| Fan1 | 3.5 | 3.1 | 2.9 | 2.9 | 2.9 | 3.7 | 2.8 | 2.8 | 2.1 | 2.7 | 2.8 | 2.3 |
| Fanca | 0.9 | 1.2 | 1.3 | 0.6 | 1.3 | 0.4 | 1.8 | 0.9 | 1.4 | 0.9 | 1.3 | 0.8 |
| Fancb | 0.9 | 1.5 | 1.6 | 0.6 | 1.3 | 0.8 | 1.6 | 0.8 | 1.3 | 0.9 | 1.5 | 1.3 |
| Fancc | 2.1 | 2.7 | 3.0 | 1.4 | 2.7 | 1.9 | 2.6 | 3.6 | 3.5 | 2.4 | 2.2 | 2.7 |
| Fancd2 | 0.6 | 1.2 | 1.6 | 0.1 | 1.3 | 0.2 | 1.7 | 0.6 | 1.3 | 0.6 | 0.9 | 0.8 |
| Fance | 3.3 | 4.3 | 4.2 | 3.0 | 3.3 | 3.8 | 3.3 | 4.4 | 4.1 | 4.6 | 4.0 | 4.2 |
| Fancf | 1.0 | 1.3 | 1.2 | 1.3 | 1.1 | 1.5 | 1.4 | 1.8 | 1.4 | 1.2 | 1.1 | 1.2 |
| Fancg | 1.7 | 2.3 | 2.3 | 1.5 | 2.2 | 1.4 | 1.8 | 1.8 | 2.4 | 1.6 | 1.9 | 1.7 |
| Fanci | 0.5 | 0.8 | 1.5 | 0.3 | 1.3 | 0.2 | 1.2 | 0.7 | 1.0 | 0.5 | 0.9 | 0.7 |
| Fancl | 2.2 | 2.3 | 3.4 | 1.9 | 2.4 | 2.2 | 2.6 | 2.3 | 2.2 | 2.2 | 2.0 | 2.0 |
| Fancm | 1.7 | 2.3 | 2.3 | 1.4 | 1.8 | 1.4 | 2.0 | 1.8 | 2.0 | 1.8 | 2.0 | 1.7 |
| Fank1 | 0.1 | 0.1 | 0.1 | 0.0 | 0.1 | 0.1 | 0.1 | 0.1 | 0.0 | 0.1 | 0.0 | 0.0 |
| Fap | 0.2 | 0.6 | 1.5 | 0.2 | 0.6 | 0.0 | 0.5 | 1.1 | 1.3 | 1.0 | 0.4 | 0.9 |
| Far1 | 15.6 | 16.5 | 17.0 | 18.3 | 18.0 | 16.2 | 19.0 | 18.4 | 20.0 | 16.4 | 21.9 | 19.8 |
| Far2 | 0.1 | 0.1 | 0.1 | 0.1 | 0.1 | 0.1 | 0.3 | 0.1 | 0.2 | 0.1 | 0.2 | 0.2 |
| Farp1 | 16.1 | 14.8 | 12.4 | 12.3 | 13.3 | 14.8 | 16.1 | 13.5 | 14.8 | 16.7 | 15.2 | 13.2 |
| Farp2 | 2.4 | 2.3 | 2.3 | 2.3 | 2.3 | 1.8 | 2.3 | 2.5 | 2.1 | 2.9 | 2.8 | 2.2 |
| Fars2 | 7.3 | 6.7 | 5.1 | 6.4 | 6.0 | 6.7 | 5.3 | 5.7 | 5.8 | 6.5 | 6.4 | 5.7 |
| Farsa | 14.7 | 14.2 | 12.4 | 15.5 | 14.6 | 13.9 | 15.7 | 15.5 | 14.6 | 13.8 | 13.5 | 12.8 |
| Farsb | 25.5 | 24.5 | 17.6 | 29.0 | 23.6 | 23.1 | 19.1 | 19.9 | 20.2 | 22.4 | 24.3 | 19.4 |
| Fas | 54.7 | 51.5 | 51.2 | 59.1 | 70.5 | 43.9 | 41.0 | 40.5 | 39.6 | 36.6 | 44.6 | 49.4 |
| Fasl | 0.0 | 0.0 | 0.0 | 0.0 | 0.0 | 0.0 | 0.0 | 0.0 | 0.0 | 0.0 | 0.0 | 0.0 |
| Fasn | 15.6 | 16.0 | 12.5 | 10.7 | 15.1 | 10.5 | 28.3 | 22.1 | 21.9 | 12.2 | 19.1 | 10.4 |
| Fastk | 16.4 | 15.8 | 13.8 | 18.2 | 14.5 | 19.1 | 14.7 | 16.0 | 15.1 | 13.2 | 15.1 | 12.6 |
| Fastkd1 | 2.2 | 2.3 | 1.9 | 1.9 | 1.9 | 2.3 | 2.0 | 2.1 | 2.1 | 2.0 | 1.7 | 2.2 |
| Fastkd2 | 4.3 | 3.7 | 2.8 | 6.1 | 4.2 | 5.5 | 3.6 | 3.7 | 3.5 | 4.5 | 4.9 | 3.9 |
| Fastkd3 | 2.3 | 2.8 | 2.6 | 2.7 | 4.5 | 2.1 | 2.4 | 2.6 | 3.0 | 2.5 | 2.0 | 2.8 |
| Fastkd5 | 3.6 | 3.3 | 2.7 | 3.6 | 3.4 | 3.1 | 3.5 | 3.3 | 3.3 | 3.6 | 3.6 | 3.2 |
| Fat1 | 117.9 | 134.6 | 135.8 | 100.6 | 109.8 | 114.6 | 182.1 | 125.3 | 151.2 | 101.3 | 119.5 | 123.9 |

| | | | | | | | | | | | | |
|---|---|---|---|---|---|---|---|---|---|---|---|---|
| Fat2 | 0.1 | 0.1 | 0.1 | 0.0 | 0.1 | 0.0 | 0.0 | 0.0 | 0.0 | 0.0 | 0.0 | 0.0 |
| Fat3 | 0.1 | 0.0 | 0.0 | 0.9 | 1.3 | 0.0 | 0.1 | 0.0 | 0.0 | 0.0 | 0.0 | 0.1 |
| Fat4 | 1.4 | 2.1 | 2.6 | 1.4 | 1.7 | 1.3 | 3.0 | 2.7 | 3.3 | 1.9 | 2.4 | 2.4 |
| Fau | 504.2 | 438.2 | 446.7 | 464.0 | 453.3 | 463.5 | 435.8 | 564.4 | 450.9 | 491.7 | 502.7 | 474.3 |
| Faxc | 2.2 | 2.1 | 2.0 | 2.6 | 1.9 | 3.1 | 2.1 | 1.5 | 1.7 | 1.7 | 2.0 | 1.7 |
| Fbf1 | 9.0 | 8.8 | 8.1 | 9.9 | 7.2 | 11.8 | 8.8 | 6.8 | 7.1 | 8.8 | 8.5 | 8.2 |
| Fbl | 17.4 | 13.8 | 14.1 | 14.3 | 17.3 | 15.8 | 16.8 | 15.4 | 12.7 | 17.3 | 15.2 | 15.6 |
| Fblim1 | 10.5 | 9.6 | 9.4 | 16.8 | 15.7 | 11.8 | 13.8 | 9.5 | 12.6 | 9.5 | 10.6 | 8.1 |
| Fbll1 | 0.0 | 0.0 | 0.0 | 0.0 | 0.0 | 0.0 | 0.0 | 0.0 | 0.0 | 0.0 | 0.1 | 0.0 |
| Fbln1 | 3.0 | 8.6 | 30.2 | 1.2 | 5.4 | 2.2 | 5.6 | 17.0 | 9.5 | 6.5 | 5.1 | 8.9 |
| Fbln2 | 1000.8 | 930.2 | 905.6 | 687.1 | 959.0 | 795.5 | 1128.7 | 1590.9 | 1269.1 | 822.8 | 964.5 | 953.7 |
| Fbln5 | 25.1 | 23.8 | 28.1 | 23.2 | 19.2 | 21.6 | 22.9 | 30.0 | 22.6 | 39.0 | 34.3 | 27.1 |
| Fbln7 | 0.8 | 1.0 | 0.9 | 0.7 | 0.6 | 0.6 | 0.6 | 0.3 | 1.0 | 0.9 | 0.7 | 1.1 |
| Fbn1 | 167.6 | 214.1 | 289.4 | 126.7 | 213.2 | 144.3 | 257.8 | 454.6 | 312.7 | 189.5 | 230.6 | 243.4 |
| Fbn2 | 0.0 | 0.1 | 0.2 | 0.0 | 0.1 | 0.0 | 0.0 | 0.2 | 0.1 | 0.1 | 0.2 | 0.1 |
| Fbrs | 1.9 | 2.7 | 2.3 | 2.1 | 2.4 | 2.0 | 3.1 | 2.1 | 3.0 | 1.9 | 2.3 | 1.8 |
| Fbrsl1 | 1.0 | 1.3 | 1.2 | 1.0 | 1.5 | 1.0 | 1.2 | 1.2 | 0.9 | 1.0 | 1.1 | 0.8 |
| Fbxl12 | 2.1 | 2.0 | 2.0 | 2.1 | 2.3 | 2.4 | 1.7 | 2.4 | 2.5 | 2.1 | 2.1 | 1.5 |
| Fbxl13 | 0.0 | 0.1 | 0.2 | 0.0 | 0.1 | 0.0 | 0.1 | 0.4 | 0.1 | 0.0 | 0.1 | 0.0 |
| Fbxl14 | 5.7 | 5.1 | 5.4 | 6.1 | 5.1 | 5.5 | 5.3 | 5.0 | 5.5 | 5.3 | 4.8 | 4.9 |
| Fbxl15 | 1.4 | 0.9 | 1.0 | 0.9 | 0.9 | 1.1 | 1.2 | 1.7 | 1.7 | 2.1 | 1.7 | 1.2 |
| Fbxl16 | 1.0 | 1.2 | 1.1 | 1.9 | 0.8 | 1.5 | 1.3 | 0.9 | 1.4 | 1.8 | 1.4 | 1.3 |
| Fbxl17 | 1.1 | 1.5 | 1.1 | 1.1 | 1.1 | 1.2 | 1.0 | 1.0 | 1.1 | 1.1 | 1.1 | 1.0 |
| Fbxl18 | 2.7 | 3.3 | 3.2 | 3.1 | 3.1 | 3.0 | 3.4 | 3.3 | 3.0 | 2.5 | 2.4 | 2.1 |
| Fbxl19 | 3.1 | 3.7 | 3.7 | 3.0 | 3.0 | 3.1 | 3.5 | 3.3 | 3.9 | 3.5 | 2.7 | 3.1 |
| Fbxl2 | 2.6 | 1.8 | 1.3 | 3.6 | 1.6 | 3.2 | 1.9 | 1.3 | 1.4 | 1.0 | 1.4 | 1.6 |
| Fbxl20 | 9.1 | 9.3 | 9.2 | 8.1 | 8.1 | 9.1 | 8.4 | 8.4 | 8.5 | 9.2 | 9.0 | 9.2 |
| Fbxl21 | 1.5 | 1.2 | 1.0 | 1.0 | 1.0 | 1.6 | 1.1 | 0.7 | 0.6 | 0.9 | 0.6 | 0.8 |
| Fbxl22 | 0.3 | 0.4 | 0.5 | 0.5 | 0.5 | 0.3 | 0.5 | 0.4 | 0.5 | 0.5 | 1.0 | 0.6 |
| Fbxl3 | 14.7 | 15.3 | 15.5 | 16.1 | 13.8 | 17.3 | 12.5 | 14.5 | 14.3 | 13.6 | 15.3 | 15.6 |
| Fbxl4 | 11.2 | 8.7 | 6.5 | 10.3 | 7.6 | 11.0 | 8.0 | 7.0 | 7.4 | 7.6 | 8.2 | 7.5 |
| Fbxl5 | 14.0 | 15.6 | 15.6 | 12.9 | 14.0 | 13.6 | 14.7 | 14.8 | 16.0 | 15.8 | 17.3 | 17.4 |
| Fbxl6 | 8.3 | 7.6 | 7.2 | 5.4 | 7.3 | 6.9 | 7.2 | 8.6 | 8.8 | 7.8 | 6.1 | 6.0 |
| Fbxl7 | 1.1 | 0.9 | 0.8 | 1.0 | 1.1 | 1.2 | 1.2 | 1.3 | 1.4 | 1.1 | 1.2 | 1.2 |
| Fbxl8 | 3.2 | 2.9 | 2.9 | 2.3 | 2.8 | 3.1 | 3.1 | 3.8 | 3.9 | 2.9 | 2.8 | 2.9 |
| Fbxo10 | 3.2 | 3.5 | 4.4 | 3.2 | 2.7 | 3.2 | 3.8 | 4.2 | 3.8 | 3.1 | 3.6 | 4.1 |
| Fbxo11 | 5.1 | 6.8 | 7.0 | 6.7 | 5.8 | 6.7 | 5.7 | 6.2 | 5.5 | 5.9 | 6.8 | 6.2 |
| Fbxo15 | 0.1 | 0.1 | 0.2 | 0.2 | 0.1 | 0.2 | 0.0 | 0.0 | 0.0 | 0.1 | 0.0 | 0.1 |
| Fbxo16 | 0.3 | 0.3 | 0.1 | 0.2 | 0.2 | 0.2 | 0.1 | 0.1 | 0.1 | 0.4 | 0.4 | 0.1 |
| Fbxo17 | 5.4 | 3.9 | 3.0 | 4.3 | 3.6 | 3.6 | 4.7 | 3.8 | 3.8 | 3.1 | 3.2 | 3.3 |
| Fbxo18 | 29.8 | 29.7 | 27.9 | 26.8 | 25.8 | 29.1 | 28.5 | 28.4 | 29.6 | 28.3 | 29.2 | 30.5 |
| Fbxo2 | 0.3 | 0.3 | 0.2 | 0.3 | 0.3 | 0.3 | 0.3 | 0.4 | 0.4 | 1.1 | 0.7 | 0.3 |
| Fbxo21 | 10.7 | 10.3 | 9.6 | 10.6 | 8.3 | 13.0 | 9.3 | 9.3 | 9.1 | 8.7 | 8.2 | 8.5 |
| Fbxo22 | 13.9 | 14.9 | 15.6 | 18.7 | 15.1 | 16.6 | 16.3 | 16.0 | 14.8 | 14.9 | 16.5 | 15.5 |
| Fbxo24 | 0.4 | 0.1 | 0.2 | 0.5 | 0.2 | 0.4 | 0.3 | 0.3 | 0.4 | 0.2 | 0.1 | 0.2 |
| Fbxo25 | 30.6 | 25.4 | 20.6 | 28.7 | 23.7 | 28.6 | 26.4 | 26.1 | 25.3 | 26.6 | 25.9 | 23.4 |
| Fbxo27 | 0.1 | 0.1 | 0.0 | 0.0 | 0.1 | 0.1 | 0.0 | 0.2 | 0.2 | 0.1 | 0.2 | 0.1 |
| Fbxo28 | 14.2 | 13.1 | 11.1 | 15.0 | 10.2 | 13.1 | 10.1 | 9.4 | 9.8 | 11.6 | 11.2 | 11.5 |
| Fbxo3 | 35.1 | 34.6 | 33.6 | 31.0 | 29.2 | 34.5 | 30.3 | 27.7 | 29.2 | 32.7 | 32.3 | 31.6 |
| Fbxo30 | 4.7 | 5.2 | 5.1 | 4.7 | 4.9 | 5.7 | 4.7 | 4.6 | 5.6 | 5.3 | 5.7 | 5.1 |
| Fbxo31 | 3.3 | 3.0 | 3.0 | 3.1 | 2.3 | 3.1 | 2.8 | 3.1 | 2.8 | 2.8 | 2.9 | 2.5 |
| Fbxo32 | 5.7 | 5.4 | 4.7 | 5.4 | 10.0 | 4.1 | 8.3 | 5.2 | 8.2 | 9.1 | 8.9 | 12.3 |
| Fbxo33 | 6.2 | 6.1 | 5.2 | 7.0 | 6.7 | 6.5 | 5.7 | 5.1 | 5.9 | 7.2 | 6.8 | 5.9 |
| Fbxo34 | 6.0 | 6.4 | 5.9 | 6.2 | 6.5 | 5.6 | 5.9 | 5.0 | 5.8 | 6.0 | 5.8 | 5.8 |
| Fbxo36 | 4.3 | 5.5 | 4.7 | 3.1 | 3.7 | 6.8 | 4.2 | 5.9 | 4.6 | 4.2 | 4.6 | 5.1 |
| Fbxo38 | 15.5 | 16.0 | 16.4 | 15.7 | 14.0 | 15.0 | 14.2 | 15.9 | 15.0 | 16.5 | 15.5 | 18.3 |
| Fbxo4 | 7.5 | 7.1 | 6.9 | 6.2 | 5.3 | 6.6 | 6.3 | 7.1 | 6.3 | 5.0 | 6.5 | 6.3 |

| | | | | | | | | | | | | |
|---|---|---|---|---|---|---|---|---|---|---|---|---|
| Fbxo40 | 0.0 | 0.0 | 0.0 | 0.1 | 0.1 | 0.0 | 0.0 | 0.0 | 0.0 | 0.0 | 0.0 | 0.1 |
| Fbxo41 | 0.3 | 0.4 | 0.2 | 0.3 | 0.2 | 0.2 | 0.2 | 0.2 | 0.2 | 0.1 | 0.2 | 0.1 |
| Fbxo42 | 4.3 | 4.6 | 4.2 | 4.0 | 4.4 | 4.5 | 4.5 | 3.7 | 4.3 | 4.0 | 4.4 | 3.6 |
| Fbxo43 | 0.0 | 0.0 | 0.0 | 0.0 | 0.0 | 0.0 | 0.0 | 0.0 | 0.0 | 0.0 | 0.0 | 0.0 |
| Fbxo44 | 2.5 | 3.1 | 2.0 | 3.3 | 1.7 | 3.6 | 1.6 | 1.4 | 1.8 | 2.9 | 2.6 | 2.3 |
| Fbxo45 | 3.7 | 4.4 | 4.2 | 4.0 | 4.2 | 3.9 | 3.6 | 3.6 | 3.8 | 4.0 | 4.7 | 4.1 |
| Fbxo46 | 2.0 | 2.0 | 2.1 | 1.8 | 2.1 | 1.6 | 2.3 | 2.2 | 2.5 | 2.4 | 2.1 | 2.1 |
| Fbxo47 | 0.3 | 0.3 | 0.4 | 0.4 | 0.3 | 0.3 | 0.3 | 0.4 | 0.2 | 0.3 | 0.3 | 0.2 |
| Fbxo48 | 0.2 | 0.4 | 0.6 | 0.0 | 0.6 | 0.0 | 0.5 | 0.1 | 0.4 | 0.2 | 0.4 | 0.1 |
| Fbxo5 | 1.5 | 2.4 | 3.6 | 0.8 | 3.2 | 0.8 | 5.1 | 2.4 | 3.1 | 1.7 | 3.9 | 2.1 |
| Fbxo6 | 30.9 | 27.3 | 21.4 | 30.8 | 21.6 | 30.7 | 22.6 | 24.4 | 24.8 | 24.3 | 26.6 | 23.1 |
| Fbxo7 | 7.4 | 7.2 | 7.8 | 5.6 | 7.3 | 6.5 | 7.2 | 8.8 | 8.1 | 8.5 | 8.0 | 8.5 |
| Fbxo8 | 7.2 | 7.6 | 7.5 | 8.6 | 6.9 | 7.8 | 7.2 | 6.4 | 7.4 | 7.3 | 8.8 | 8.6 |
| Fbxo9 | 11.8 | 8.6 | 8.9 | 12.9 | 10.6 | 8.0 | 13.0 | 12.2 | 13.9 | 12.0 | 12.1 | 12.7 |
| Fbxw10 | 0.6 | 0.8 | 1.1 | 0.7 | 0.5 | 0.6 | 0.7 | 0.6 | 0.4 | 0.4 | 0.7 | 0.9 |
| Fbxw11 | 20.2 | 20.6 | 18.2 | 20.1 | 19.8 | 19.8 | 18.3 | 16.8 | 18.6 | 18.0 | 19.8 | 18.4 |
| Fbxw15 | 0.0 | 0.1 | 0.0 | 0.0 | 0.1 | 0.0 | 0.0 | 0.0 | 0.0 | 0.2 | 0.1 | 0.0 |
| Fbxw16 | 0.0 | 0.1 | 0.1 | 0.0 | 0.0 | 0.0 | 0.1 | 0.0 | 0.0 | 0.0 | 0.1 | 0.0 |
| Fbxw17 | 7.9 | 4.9 | 6.1 | 6.6 | 5.7 | 7.6 | 6.0 | 5.1 | 6.7 | 5.8 | 5.4 | 5.0 |
| Fbxw18 | 0.5 | 0.2 | 0.0 | 0.6 | 0.3 | 0.3 | 0.7 | 0.1 | 0.3 | 0.4 | 0.4 | 0.1 |
| Fbxw19 | 0.0 | 0.1 | 0.0 | 0.0 | 0.0 | 0.0 | 0.0 | 0.0 | 0.0 | 0.0 | 0.0 | 0.0 |
| Fbxw2 | 23.0 | 20.4 | 20.3 | 25.5 | 19.7 | 24.7 | 21.8 | 20.4 | 20.9 | 21.1 | 21.0 | 22.0 |
| Fbxw21 | 0.0 | 0.0 | 0.0 | 0.0 | 0.0 | 0.1 | 0.0 | 0.0 | 0.0 | 0.0 | 0.0 | 0.0 |
| Fbxw24 | 0.1 | 0.1 | 0.0 | 0.0 | 0.1 | 0.1 | 0.2 | 0.1 | 0.1 | 0.2 | 0.2 | 0.0 |
| Fbxw26 | 0.1 | 0.0 | 0.0 | 0.1 | 0.0 | 0.0 | 0.1 | 0.0 | 0.1 | 0.0 | 0.0 | 0.1 |
| Fbxw4 | 6.7 | 6.5 | 5.3 | 5.4 | 5.8 | 5.7 | 3.8 | 4.8 | 4.3 | 5.3 | 4.8 | 4.8 |
| Fbxw5 | 11.6 | 11.0 | 9.8 | 11.1 | 11.3 | 12.1 | 10.7 | 11.6 | 12.1 | 11.9 | 10.7 | 9.5 |
| Fbxw7 | 6.9 | 6.0 | 5.7 | 6.5 | 6.7 | 5.4 | 6.1 | 4.4 | 5.0 | 6.6 | 6.6 | 5.8 |
| Fbxw8 | 24.3 | 23.2 | 19.0 | 22.0 | 20.9 | 21.5 | 21.5 | 21.6 | 23.5 | 22.5 | 21.5 | 21.3 |
| Fbxw9 | 10.2 | 8.8 | 7.6 | 9.1 | 7.0 | 10.3 | 8.6 | 8.4 | 9.2 | 9.7 | 7.3 | 6.3 |
| Fcer1g | 8.0 | 0.1 | 0.3 | 87.5 | 127.5 | 0.1 | 18.1 | 1.5 | 1.8 | 9.7 | 2.2 | 6.0 |
| Fcer2a | 0.0 | 0.0 | 0.0 | 0.1 | 0.1 | 0.0 | 0.1 | 0.0 | 0.1 | 0.0 | 0.0 | 0.1 |
| Fcf1 | 40.2 | 39.2 | 35.0 | 50.2 | 33.0 | 36.5 | 38.3 | 39.2 | 32.1 | 36.0 | 42.2 | 39.7 |
| Fcgbp | 0.0 | 0.0 | 0.0 | 0.0 | 0.0 | 0.0 | 0.0 | 0.0 | 0.0 | 0.0 | 0.0 | 0.0 |
| Fcgr1 | 0.1 | 0.0 | 0.0 | 0.6 | 0.8 | 0.1 | 0.1 | 0.0 | 0.0 | 0.1 | 0.0 | 0.0 |
| Fcgr2b | 1.0 | 0.0 | 0.1 | 4.8 | 9.7 | 0.0 | 1.1 | 0.2 | 0.2 | 0.2 | 0.2 | 0.4 |
| Fcgr3 | 3.2 | 0.2 | 0.5 | 23.9 | 27.4 | 0.0 | 1.5 | 1.1 | 0.4 | 1.1 | 1.1 | 1.4 |
| Fcgr4 | 0.2 | 0.1 | 0.1 | 1.3 | 1.6 | 0.1 | 0.2 | 0.2 | 0.1 | 0.8 | 0.2 | 0.7 |
| Fcgrt | 68.5 | 67.4 | 71.2 | 71.4 | 57.6 | 91.6 | 68.4 | 74.7 | 74.5 | 71.3 | 59.9 | 75.7 |
| Fcho1 | 0.0 | 0.0 | 0.1 | 0.1 | 0.1 | 0.0 | 0.0 | 0.0 | 0.0 | 0.0 | 0.0 | 0.0 |
| Fcho2 | 2.4 | 3.4 | 3.7 | 2.9 | 3.3 | 3.3 | 3.7 | 3.8 | 3.2 | 3.7 | 4.1 | 5.2 |
| Fchsd1 | 2.5 | 3.0 | 2.6 | 2.9 | 2.2 | 3.3 | 2.7 | 2.4 | 2.7 | 2.9 | 2.4 | 2.6 |
| Fchsd2 | 9.3 | 10.8 | 12.7 | 8.9 | 8.4 | 9.4 | 9.7 | 11.9 | 9.7 | 8.8 | 9.0 | 10.0 |
| Fcna | 0.5 | 0.2 | 0.1 | 0.8 | 0.5 | 0.5 | 0.2 | 0.0 | 0.1 | 0.1 | 0.1 | 0.2 |
| Fcrl1 | 0.0 | 0.0 | 0.0 | 0.3 | 0.8 | 0.0 | 0.0 | 0.0 | 0.0 | 0.1 | 0.0 | 0.0 |
| Fcrls | 1.6 | 0.0 | 0.0 | 15.4 | 28.8 | 0.0 | 2.1 | 0.0 | 0.1 | 1.2 | 0.5 | 0.9 |
| Fdft1 | 19.0 | 21.9 | 20.8 | 9.1 | 14.6 | 12.2 | 33.3 | 39.8 | 29.0 | 13.1 | 34.6 | 15.9 |
| Fdps | 98.1 | 115.5 | 105.6 | 38.3 | 61.7 | 57.8 | 172.6 | 211.5 | 174.0 | 52.2 | 165.8 | 78.2 |
| Fdx1 | 15.3 | 12.0 | 12.5 | 13.1 | 11.6 | 12.7 | 13.4 | 11.8 | 11.7 | 14.1 | 13.7 | 13.5 |
| Fdx1l | 25.5 | 20.3 | 16.6 | 29.0 | 21.8 | 31.3 | 22.7 | 19.7 | 19.8 | 21.5 | 21.4 | 20.3 |
| Fdxacb1 | 2.1 | 2.1 | 1.7 | 2.6 | 1.6 | 1.7 | 1.5 | 1.7 | 2.2 | 1.9 | 1.4 | 1.8 |
| Fdxr | 8.3 | 12.2 | 13.6 | 6.8 | 8.9 | 7.4 | 7.5 | 17.1 | 15.0 | 12.9 | 8.6 | 13.3 |
| Fech | 32.3 | 33.0 | 30.0 | 36.7 | 26.3 | 36.0 | 29.3 | 31.6 | 25.8 | 32.3 | 33.4 | 29.9 |
| Fem1a | 4.9 | 4.9 | 4.2 | 4.8 | 5.1 | 5.1 | 4.3 | 4.5 | 4.8 | 4.8 | 4.6 | 4.3 |
| Fem1b | 9.9 | 12.4 | 11.7 | 10.6 | 12.3 | 10.4 | 10.2 | 12.0 | 11.6 | 12.9 | 12.5 | 11.7 |
| Fem1c | 4.2 | 4.1 | 4.2 | 3.6 | 4.8 | 3.7 | 3.9 | 4.1 | 4.5 | 5.2 | 4.5 | 4.5 |
| Fen1 | 5.4 | 6.9 | 6.7 | 5.4 | 9.7 | 3.5 | 8.5 | 5.8 | 6.9 | 5.1 | 5.3 | 4.6 |

| | | | | | | | | | | | | |
|---|---|---|---|---|---|---|---|---|---|---|---|---|
| Fendrr | 0.1 | 0.0 | 0.1 | 0.1 | 0.0 | 0.0 | 0.1 | 0.0 | 0.0 | 0.0 | 0.0 | 0.0 |
| Fer1l4 | 0.0 | 0.0 | 0.0 | 0.0 | 0.0 | 0.0 | 0.0 | 0.0 | 0.0 | 0.0 | 0.0 | 0.0 |
| Fermt1 | 0.0 | 0.0 | 0.0 | 0.1 | 0.1 | 0.0 | 0.0 | 0.0 | 0.0 | 0.0 | 0.0 | 0.0 |
| Fermt2 | 45.7 | 50.7 | 48.1 | 47.5 | 47.7 | 40.6 | 55.6 | 43.5 | 55.7 | 53.2 | 61.9 | 57.6 |
| Fermt3 | 1.5 | 0.2 | 0.2 | 9.0 | 16.4 | 0.1 | 2.4 | 0.2 | 0.4 | 0.9 | 0.5 | 0.9 |
| Fert2 | 23.0 | 26.5 | 32.1 | 24.5 | 22.5 | 27.6 | 29.7 | 31.6 | 26.5 | 22.2 | 28.6 | 32.2 |
| Fes | 4.5 | 4.5 | 5.7 | 12.2 | 13.3 | 6.0 | 7.3 | 6.1 | 6.2 | 3.9 | 4.3 | 4.3 |
| Fetub | 1.3 | 1.7 | 1.4 | 1.3 | 1.6 | 2.2 | 1.3 | 1.0 | 0.3 | 0.7 | 0.8 | 0.4 |
| Fez1 | 8.8 | 4.1 | 4.0 | 7.5 | 3.2 | 11.6 | 6.8 | 3.3 | 3.2 | 7.2 | 6.5 | 5.4 |
| Fez2 | 49.3 | 31.3 | 24.2 | 63.3 | 30.4 | 60.1 | 36.9 | 23.4 | 28.9 | 41.6 | 39.3 | 34.3 |
| Fgd1 | 1.5 | 2.1 | 2.2 | 1.2 | 1.7 | 1.8 | 2.0 | 2.6 | 2.5 | 2.0 | 1.8 | 1.6 |
| Fgd2 | 0.1 | 0.0 | 0.0 | 0.6 | 1.3 | 0.0 | 0.1 | 0.0 | 0.0 | 0.2 | 0.0 | 0.1 |
| Fgd3 | 58.9 | 41.5 | 17.9 | 79.7 | 48.7 | 83.9 | 31.9 | 15.6 | 18.8 | 15.9 | 26.9 | 14.2 |
| Fgd4 | 4.8 | 3.9 | 3.6 | 6.2 | 3.8 | 4.9 | 3.1 | 3.5 | 2.6 | 3.5 | 3.3 | 3.7 |
| Fgd5 | 0.1 | 0.1 | 0.1 | 0.0 | 0.0 | 0.0 | 0.1 | 0.2 | 0.2 | 0.0 | 0.0 | 0.0 |
| Fgd6 | 0.5 | 0.7 | 0.6 | 0.7 | 1.2 | 0.5 | 0.9 | 0.7 | 0.7 | 1.2 | 1.1 | 0.9 |
| Fgf1 | 0.2 | 0.2 | 0.1 | 0.7 | 0.3 | 0.3 | 0.3 | 0.2 | 0.1 | 0.4 | 0.3 | 0.2 |
| Fgf10 | 0.0 | 0.0 | 0.0 | 0.0 | 0.0 | 0.1 | 0.0 | 0.0 | 0.0 | 0.1 | 0.0 | 0.0 |
| Fgf11 | 0.3 | 0.6 | 1.4 | 0.6 | 0.6 | 0.4 | 0.6 | 0.9 | 1.1 | 0.8 | 0.6 | 1.0 |
| Fgf12 | 0.0 | 0.0 | 0.0 | 0.0 | 0.0 | 0.0 | 0.0 | 0.0 | 0.0 | 0.0 | 0.0 | 0.0 |
| Fgf13 | 0.2 | 0.1 | 0.1 | 0.1 | 0.4 | 0.2 | 0.1 | 0.0 | 0.1 | 0.1 | 0.1 | 0.2 |
| Fgf14 | 0.3 | 0.1 | 0.2 | 0.5 | 0.1 | 0.3 | 0.1 | 0.2 | 0.1 | 0.1 | 0.2 | 0.2 |
| Fgf16 | 0.1 | 0.0 | 0.0 | 0.0 | 0.0 | 0.0 | 0.0 | 0.1 | 0.0 | 0.0 | 0.0 | 0.0 |
| Fgf17 | 0.0 | 0.0 | 0.1 | 0.0 | 0.0 | 0.0 | 0.0 | 0.0 | 0.0 | 0.0 | 0.0 | 0.1 |
| Fgf18 | 6.0 | 2.6 | 1.8 | 2.4 | 0.8 | 3.2 | 2.0 | 3.2 | 3.6 | 4.5 | 3.7 | 3.6 |
| Fgf2 | 16.5 | 18.7 | 20.5 | 11.1 | 13.1 | 10.4 | 17.7 | 10.4 | 15.9 | 35.5 | 21.0 | 25.7 |
| Fgf20 | 0.0 | 0.1 | 0.0 | 0.0 | 0.0 | 0.0 | 0.0 | 0.0 | 0.0 | 0.0 | 0.0 | 0.0 |
| Fgf21 | 0.2 | 0.2 | 0.1 | 0.6 | 0.0 | 0.6 | 0.0 | 0.1 | 0.2 | 0.4 | 0.1 | 0.4 |
| Fgf23 | 0.0 | 0.1 | 0.0 | 0.0 | 0.0 | 0.0 | 0.0 | 0.0 | 0.0 | 0.1 | 0.0 | 0.0 |
| Fgf5 | 0.0 | 0.0 | 0.0 | 0.0 | 0.0 | 0.0 | 0.0 | 0.0 | 0.0 | 0.0 | 0.0 | 0.0 |
| Fgf7 | 10.6 | 15.6 | 25.2 | 14.6 | 11.0 | 18.1 | 14.4 | 13.0 | 11.6 | 17.0 | 18.2 | 23.3 |
| Fgf9 | 2.2 | 4.4 | 5.6 | 2.7 | 2.7 | 1.3 | 2.6 | 3.6 | 3.9 | 1.7 | 2.0 | 2.5 |
| Fgfbp3 | 2.9 | 2.8 | 3.3 | 3.1 | 3.0 | 4.8 | 2.8 | 3.1 | 3.0 | 4.5 | 3.9 | 3.4 |
| Fgfr1 | 25.1 | 26.5 | 26.1 | 27.9 | 26.9 | 31.5 | 31.5 | 32.4 | 25.9 | 23.4 | 21.7 | 24.5 |
| Fgfr1op | 5.4 | 5.2 | 5.7 | 4.2 | 4.5 | 4.8 | 4.6 | 4.1 | 4.1 | 4.5 | 4.4 | 4.6 |
| Fgfr1op2 | 22.4 | 23.3 | 24.5 | 28.3 | 22.0 | 23.3 | 23.3 | 24.3 | 22.9 | 21.0 | 25.9 | 24.6 |
| Fgfr2 | 7.1 | 7.4 | 9.6 | 3.3 | 3.8 | 3.6 | 6.2 | 7.9 | 7.2 | 13.2 | 10.4 | 13.7 |
| Fgfr3 | 0.8 | 1.3 | 1.5 | 0.2 | 0.6 | 0.2 | 0.6 | 1.0 | 1.0 | 1.4 | 1.2 | 1.0 |
| Fgfr4 | 0.0 | 0.0 | 0.0 | 0.0 | 0.0 | 0.0 | 0.0 | 0.0 | 0.0 | 0.0 | 0.0 | 0.0 |
| Fgfrl1 | 24.1 | 23.2 | 26.3 | 20.7 | 19.6 | 20.9 | 29.1 | 30.4 | 34.6 | 22.6 | 28.9 | 26.2 |
| Fggy | 17.0 | 14.5 | 11.9 | 16.5 | 11.0 | 16.1 | 10.7 | 9.6 | 10.2 | 10.9 | 11.0 | 11.5 |
| Fgl1 | 0.3 | 0.3 | 0.8 | 0.4 | 0.2 | 0.3 | 0.5 | 0.4 | 0.2 | 0.3 | 0.4 | 0.8 |
| Fgl2 | 0.2 | 0.4 | 1.6 | 0.1 | 0.3 | 0.0 | 0.3 | 0.8 | 0.4 | 0.2 | 0.4 | 0.7 |
| Fgr | 0.1 | 0.0 | 0.0 | 0.3 | 1.5 | 0.0 | 0.1 | 0.0 | 0.0 | 0.0 | 0.0 | 0.0 |
| Fh1 | 32.3 | 30.2 | 25.6 | 27.4 | 26.6 | 25.1 | 25.1 | 24.9 | 24.5 | 27.2 | 28.4 | 26.7 |
| Fhad1 | 0.0 | 0.1 | 0.0 | 0.0 | 0.1 | 0.0 | 0.1 | 0.0 | 0.0 | 0.0 | 0.1 | 0.1 |
| Fhdc1 | 8.8 | 7.3 | 6.6 | 8.0 | 5.8 | 10.4 | 7.0 | 5.8 | 6.0 | 11.9 | 10.1 | 8.0 |
| Fhit | 1.8 | 1.6 | 1.4 | 0.7 | 0.9 | 1.3 | 0.4 | 1.3 | 0.6 | 1.2 | 0.7 | 1.1 |
| Fhl1 | 92.3 | 84.9 | 55.8 | 84.2 | 53.9 | 82.1 | 58.5 | 35.3 | 48.2 | 61.7 | 86.4 | 59.5 |
| Fhl2 | 130.1 | 133.6 | 86.8 | 210.7 | 129.9 | 193.0 | 157.2 | 103.2 | 170.1 | 81.1 | 112.0 | 114.2 |
| Fhl3 | 2.6 | 3.4 | 2.1 | 4.9 | 8.5 | 3.1 | 5.7 | 3.4 | 4.1 | 4.3 | 5.1 | 2.7 |
| Fhl4 | 0.6 | 1.0 | 1.2 | 0.4 | 0.9 | 0.6 | 0.6 | 0.6 | 0.7 | 0.8 | 0.7 | 0.5 |
| Fhod1 | 1.7 | 2.0 | 1.4 | 1.5 | 2.0 | 1.7 | 2.1 | 1.7 | 2.4 | 2.4 | 1.4 | 1.5 |
| Fhod3 | 0.2 | 0.1 | 0.2 | 0.1 | 0.1 | 0.1 | 0.1 | 0.1 | 0.1 | 0.2 | 0.2 | 0.2 |
| Fibcd1 | 0.1 | 0.0 | 0.0 | 0.2 | 0.0 | 0.1 | 0.0 | 0.0 | 0.0 | 0.0 | 0.1 | 0.0 |
| Fibin | 16.0 | 12.7 | 20.1 | 9.8 | 8.8 | 4.8 | 9.5 | 16.9 | 18.4 | 6.9 | 9.4 | 14.9 |
| Fibp | 16.5 | 16.6 | 17.4 | 16.5 | 15.0 | 16.1 | 17.3 | 18.9 | 19.3 | 15.9 | 14.2 | 16.5 |

| | | | | | | | | | | | | |
|---|---|---|---|---|---|---|---|---|---|---|---|---|
| Ficd | 7.3 | 6.9 | 6.5 | 7.6 | 5.8 | 8.2 | 7.2 | 5.3 | 7.0 | 5.7 | 7.0 | 6.4 |
| Fig4 | 10.8 | 10.8 | 9.6 | 10.4 | 9.2 | 11.1 | 10.1 | 10.9 | 9.5 | 10.1 | 11.7 | 12.7 |
| Figf | 27.5 | 47.1 | 106.5 | 14.5 | 31.1 | 13.1 | 56.4 | 121.4 | 83.0 | 44.8 | 56.4 | 70.6 |
| Fign | 0.1 | 0.1 | 0.1 | 0.0 | 0.0 | 0.0 | 0.1 | 0.1 | 0.1 | 0.1 | 0.1 | 0.1 |
| Fignl1 | 1.6 | 3.0 | 4.1 | 0.7 | 3.7 | 0.7 | 4.1 | 2.0 | 3.0 | 1.8 | 2.7 | 2.6 |
| Fignl2 | 0.9 | 1.1 | 1.1 | 0.9 | 0.7 | 0.8 | 1.0 | 1.0 | 0.8 | 1.0 | 0.8 | 0.7 |
| Filip1 | 0.8 | 1.0 | 1.0 | 0.8 | 0.8 | 0.7 | 0.6 | 0.5 | 0.3 | 0.4 | 0.3 | 0.3 |
| Filip1l | 42.2 | 44.4 | 47.4 | 58.1 | 45.2 | 38.9 | 48.5 | 28.6 | 38.4 | 36.4 | 50.6 | 48.3 |
| Fip1l1 | 15.0 | 15.3 | 18.4 | 19.2 | 13.2 | 18.2 | 18.2 | 14.8 | 11.2 | 14.9 | 17.4 | 17.2 |
| Fis1 | 101.9 | 86.3 | 71.7 | 96.9 | 84.0 | 83.8 | 91.4 | 94.6 | 98.6 | 104.8 | 94.0 | 90.6 |
| Fitm2 | 6.5 | 6.2 | 5.4 | 5.4 | 5.3 | 5.4 | 6.5 | 5.4 | 5.9 | 5.5 | 5.9 | 4.6 |
| Fiz1 | 5.1 | 4.7 | 4.3 | 5.1 | 4.3 | 4.9 | 4.5 | 4.7 | 4.9 | 4.8 | 4.1 | 4.2 |
| Fjx1 | 1.0 | 0.7 | 0.6 | 1.1 | 0.9 | 0.7 | 1.0 | 1.1 | 1.2 | 1.7 | 1.1 | 1.0 |
| Fkbp10 | 88.9 | 71.3 | 67.5 | 78.5 | 72.6 | 70.4 | 85.2 | 81.7 | 82.3 | 75.9 | 83.1 | 72.2 |
| Fkbp11 | 35.7 | 23.3 | 21.8 | 37.7 | 24.9 | 40.4 | 33.6 | 20.7 | 23.0 | 31.2 | 28.3 | 26.7 |
| Fkbp14 | 33.7 | 30.6 | 30.0 | 36.7 | 31.8 | 30.3 | 40.1 | 31.2 | 35.8 | 36.2 | 37.4 | 39.3 |
| Fkbp15 | 12.6 | 13.1 | 14.2 | 13.9 | 14.5 | 12.6 | 14.3 | 14.6 | 13.7 | 12.6 | 13.8 | 15.1 |
| Fkbp1a | 34.6 | 27.2 | 27.7 | 41.5 | 31.5 | 35.1 | 36.5 | 33.7 | 30.9 | 40.2 | 38.3 | 35.9 |
| Fkbp1b | 1.1 | 0.7 | 1.9 | 0.9 | 1.8 | 0.7 | 1.1 | 1.8 | 1.0 | 0.9 | 1.0 | 1.4 |
| Fkbp2 | 18.9 | 19.4 | 20.6 | 35.0 | 17.1 | 24.2 | 27.1 | 28.4 | 25.7 | 21.5 | 26.4 | 29.9 |
| Fkbp3 | 99.9 | 84.2 | 84.4 | 99.8 | 71.1 | 114.7 | 76.5 | 72.7 | 68.0 | 81.2 | 91.7 | 89.2 |
| Fkbp4 | 30.2 | 30.6 | 29.8 | 32.4 | 28.9 | 32.8 | 30.5 | 27.3 | 29.0 | 35.4 | 33.3 | 28.0 |
| Fkbp5 | 1.8 | 2.6 | 3.9 | 1.9 | 4.1 | 1.8 | 3.6 | 4.2 | 3.0 | 3.1 | 3.6 | 3.3 |
| Fkbp6 | 0.0 | 0.1 | 0.0 | 0.0 | 0.0 | 0.2 | 0.0 | 0.1 | 0.0 | 0.0 | 0.1 | 0.0 |
| Fkbp7 | 63.9 | 58.2 | 66.5 | 77.5 | 54.1 | 64.2 | 59.4 | 67.4 | 59.8 | 72.4 | 70.7 | 77.1 |
| Fkbp8 | 71.0 | 67.0 | 57.3 | 72.4 | 65.0 | 68.3 | 68.6 | 70.3 | 71.9 | 76.8 | 69.5 | 61.4 |
| Fkbp9 | 176.3 | 155.9 | 159.1 | 148.8 | 140.9 | 156.3 | 140.9 | 137.9 | 133.1 | 153.2 | 143.8 | 155.0 |
| Fkbpl | 6.0 | 5.2 | 6.0 | 6.4 | 5.6 | 5.7 | 5.7 | 5.0 | 4.0 | 5.6 | 6.1 | 6.1 |
| Fkrp | 9.5 | 8.6 | 8.1 | 9.0 | 9.6 | 8.5 | 11.7 | 10.4 | 10.9 | 9.2 | 8.9 | 9.1 |
| Fktn | 8.5 | 8.3 | 7.5 | 7.5 | 7.4 | 7.4 | 8.0 | 6.5 | 7.9 | 6.3 | 7.8 | 8.2 |
| Flad1 | 6.5 | 6.0 | 4.9 | 6.5 | 5.5 | 6.2 | 5.3 | 5.3 | 5.8 | 5.3 | 4.6 | 5.2 |
| Flcn | 8.8 | 7.9 | 7.7 | 8.8 | 8.4 | 10.3 | 6.2 | 7.9 | 8.5 | 10.2 | 7.0 | 8.1 |
| Fli1 | 3.6 | 3.8 | 5.2 | 6.3 | 7.6 | 3.6 | 5.0 | 5.0 | 3.9 | 3.6 | 4.8 | 5.1 |
| Flii | 52.0 | 46.0 | 45.6 | 61.3 | 51.9 | 51.7 | 55.0 | 49.1 | 48.5 | 44.2 | 51.7 | 51.7 |
| Flna | 211.5 | 173.8 | 146.2 | 221.8 | 242.9 | 190.7 | 270.1 | 215.5 | 258.5 | 234.4 | 283.9 | 215.2 |
| Flnb | 89.9 | 64.8 | 51.5 | 87.3 | 75.8 | 84.0 | 96.0 | 64.6 | 65.1 | 72.4 | 86.0 | 63.6 |
| Flnc | 27.5 | 25.5 | 20.1 | 21.4 | 19.2 | 23.8 | 27.3 | 23.2 | 25.4 | 38.9 | 28.0 | 21.3 |
| Flot1 | 60.6 | 77.3 | 95.0 | 48.8 | 58.0 | 64.4 | 84.4 | 100.3 | 95.9 | 65.7 | 82.5 | 77.0 |
| Flot2 | 26.5 | 23.4 | 24.4 | 32.6 | 23.4 | 32.4 | 27.6 | 23.4 | 23.3 | 21.0 | 25.2 | 22.7 |
| Flrt1 | 0.0 | 0.0 | 0.1 | 0.0 | 0.0 | 0.0 | 0.0 | 0.1 | 0.0 | 0.1 | 0.0 | 0.0 |
| Flrt2 | 3.8 | 2.5 | 2.9 | 2.3 | 2.8 | 2.5 | 4.9 | 9.4 | 6.0 | 14.0 | 10.9 | 7.2 |
| Flrt3 | 1.3 | 0.5 | 0.6 | 1.7 | 1.0 | 0.7 | 0.7 | 0.4 | 0.3 | 1.1 | 1.3 | 0.5 |
| Flt1 | 0.2 | 0.3 | 0.2 | 0.3 | 0.2 | 0.2 | 0.2 | 0.2 | 0.2 | 1.9 | 2.0 | 1.0 |
| Flt3 | 0.0 | 0.0 | 0.0 | 0.0 | 0.0 | 0.0 | 0.0 | 0.0 | 0.0 | 0.0 | 0.0 | 0.0 |
| Flt3l | 1.7 | 2.4 | 3.2 | 2.3 | 1.3 | 2.1 | 1.6 | 1.8 | 1.7 | 1.6 | 1.9 | 2.1 |
| Flt4 | 0.7 | 0.2 | 0.3 | 1.0 | 0.6 | 0.8 | 0.8 | 0.5 | 0.5 | 1.7 | 0.8 | 0.9 |
| Flywch1 | 16.6 | 17.0 | 15.0 | 20.7 | 14.7 | 20.7 | 15.4 | 14.2 | 14.3 | 14.7 | 15.2 | 13.9 |
| Flywch2 | 0.4 | 0.5 | 0.3 | 0.1 | 0.3 | 0.1 | 0.3 | 0.3 | 0.2 | 1.0 | 0.3 | 0.3 |
| Fmn1 | 7.1 | 6.4 | 5.0 | 8.5 | 6.1 | 7.8 | 7.6 | 4.9 | 6.5 | 5.4 | 7.2 | 6.5 |
| Fmn2 | 0.0 | 0.0 | 0.0 | 0.0 | 0.0 | 0.0 | 0.1 | 0.0 | 0.0 | 0.0 | 0.0 | 0.0 |
| Fmnl1 | 0.6 | 0.5 | 0.5 | 1.8 | 4.3 | 0.1 | 0.9 | 0.7 | 0.3 | 0.5 | 0.3 | 0.2 |
| Fmnl2 | 0.8 | 1.2 | 1.7 | 1.1 | 2.0 | 0.8 | 1.1 | 1.7 | 1.2 | 1.9 | 1.2 | 1.6 |
| Fmnl3 | 4.9 | 6.5 | 6.1 | 6.1 | 9.2 | 5.0 | 9.5 | 8.6 | 9.7 | 6.7 | 5.6 | 7.7 |
| Fmo1 | 1.3 | 2.8 | 12.6 | 1.1 | 4.2 | 0.9 | 3.1 | 15.0 | 4.8 | 1.5 | 1.5 | 7.3 |
| Fmo2 | 3.3 | 8.8 | 16.0 | 1.5 | 4.2 | 1.0 | 2.9 | 6.5 | 4.5 | 3.9 | 3.0 | 10.9 |
| Fmo3 | 0.0 | 0.0 | 0.1 | 0.1 | 0.0 | 0.0 | 0.1 | 0.1 | 0.2 | 0.1 | 0.1 | 0.3 |
| Fmo4 | 0.1 | 0.1 | 0.3 | 0.0 | 0.1 | 0.2 | 0.1 | 0.4 | 0.1 | 0.1 | 0.1 | 0.1 |

| | | | | | | | | | | | | |
|---|---|---|---|---|---|---|---|---|---|---|---|---|
| Fmo5 | 1.6 | 1.9 | 2.0 | 1.8 | 1.1 | 2.4 | 1.6 | 1.6 | 1.4 | 1.0 | 1.2 | 1.4 |
| Fmod | 37.1 | 35.5 | 61.6 | 12.4 | 22.1 | 18.9 | 50.1 | 35.5 | 34.2 | 30.8 | 41.5 | 53.4 |
| Fmr1 | 9.7 | 14.3 | 20.3 | 10.2 | 12.5 | 10.1 | 15.5 | 16.5 | 18.5 | 23.6 | 21.3 | 26.9 |
| Fmr1nb | 2.8 | 1.7 | 2.0 | 1.6 | 1.7 | 1.4 | 2.7 | 0.7 | 1.1 | 1.3 | 0.6 | 1.0 |
| Fn1 | 475.3 | 550.1 | 692.1 | 453.5 | 812.9 | 555.7 | 790.1 | 819.5 | 799.9 | 858.6 | 686.5 | 741.0 |
| Fn3k | 0.0 | 0.1 | 0.1 | 0.2 | 0.1 | 0.0 | 0.0 | 0.0 | 0.0 | 0.0 | 0.1 | 0.0 |
| Fn3krp | 7.5 | 8.7 | 7.8 | 9.6 | 5.7 | 8.8 | 7.1 | 7.2 | 7.4 | 6.6 | 7.0 | 6.5 |
| Fnbp1 | 11.7 | 14.0 | 16.1 | 16.3 | 18.3 | 15.6 | 14.1 | 11.2 | 10.4 | 11.3 | 10.5 | 12.5 |
| Fnbp1l | 0.4 | 0.6 | 1.1 | 0.8 | 1.8 | 0.5 | 1.4 | 1.6 | 1.6 | 2.0 | 1.5 | 1.6 |
| Fnbp4 | 4.2 | 4.4 | 4.7 | 4.4 | 4.4 | 4.1 | 4.9 | 4.7 | 4.6 | 5.3 | 4.6 | 4.8 |
| Fndc1 | 4.3 | 12.4 | 26.1 | 4.9 | 20.6 | 4.0 | 23.0 | 33.2 | 26.7 | 10.4 | 10.8 | 12.6 |
| Fndc3a | 19.5 | 26.5 | 27.6 | 22.8 | 20.1 | 26.8 | 22.3 | 21.1 | 21.2 | 18.4 | 20.1 | 26.1 |
| Fndc3b | 38.1 | 45.7 | 46.6 | 40.9 | 46.2 | 38.1 | 52.3 | 50.5 | 55.1 | 40.5 | 50.5 | 50.2 |
| Fndc4 | 0.6 | 0.7 | 1.4 | 0.4 | 0.4 | 1.0 | 1.6 | 1.2 | 1.2 | 1.2 | 1.2 | 1.1 |
| Fndc5 | 0.3 | 0.2 | 0.3 | 0.2 | 0.2 | 0.2 | 0.3 | 0.5 | 0.4 | 0.5 | 0.3 | 0.3 |
| Fndc7 | 0.0 | 0.0 | 0.0 | 0.0 | 0.0 | 0.0 | 0.0 | 0.0 | 0.0 | 0.1 | 0.0 | 0.1 |
| Fndc8 | 0.1 | 0.0 | 0.0 | 0.0 | 0.1 | 0.1 | 0.1 | 0.1 | 0.0 | 0.0 | 0.1 | 0.1 |
| Fndc9 | 0.0 | 0.0 | 0.0 | 0.1 | 0.2 | 0.0 | 0.1 | 0.0 | 0.0 | 0.0 | 0.1 | 0.0 |
| Fnip1 | 10.6 | 11.9 | 11.8 | 11.1 | 10.4 | 12.5 | 9.5 | 9.4 | 9.4 | 10.7 | 11.1 | 13.2 |
| Fnip2 | 4.6 | 4.5 | 4.1 | 7.4 | 7.6 | 5.3 | 4.4 | 3.0 | 3.4 | 3.2 | 3.4 | 3.9 |
| Fnta | 50.0 | 43.9 | 45.0 | 40.4 | 39.4 | 40.8 | 42.4 | 41.9 | 44.1 | 51.6 | 47.7 | 51.2 |
| Fntb | 3.7 | 3.3 | 3.0 | 3.5 | 2.8 | 3.4 | 3.6 | 3.1 | 3.3 | 2.8 | 3.1 | 2.6 |
| Focad | 6.9 | 6.0 | 5.3 | 5.6 | 5.1 | 5.6 | 4.4 | 4.9 | 4.7 | 5.3 | 5.2 | 5.2 |
| Folh1 | 0.0 | 0.0 | 0.0 | 0.0 | 0.0 | 0.1 | 0.0 | 0.0 | 0.0 | 0.1 | 0.1 | 0.0 |
| Folr1 | 3.7 | 3.4 | 2.2 | 4.4 | 2.7 | 4.4 | 3.0 | 1.5 | 2.8 | 9.9 | 5.5 | 4.6 |
| Folr2 | 1.2 | 0.4 | 0.1 | 5.5 | 3.9 | 1.2 | 0.5 | 0.7 | 0.4 | 0.9 | 1.0 | 0.5 |
| Fopnl | 26.4 | 25.1 | 21.4 | 26.0 | 23.7 | 25.8 | 24.8 | 24.1 | 26.5 | 27.6 | 28.0 | 24.1 |
| Fos | 4.9 | 6.0 | 4.0 | 2.7 | 4.9 | 1.6 | 3.3 | 3.4 | 3.7 | 21.4 | 5.8 | 4.1 |
| Fosb | 0.2 | 0.2 | 0.2 | 0.2 | 0.2 | 0.2 | 0.3 | 0.1 | 0.1 | 0.7 | 0.1 | 0.2 |
| Fosl1 | 0.4 | 0.3 | 0.2 | 0.5 | 0.9 | 0.5 | 0.8 | 0.2 | 0.2 | 0.6 | 0.4 | 0.3 |
| Fosl2 | 25.4 | 33.4 | 33.3 | 22.1 | 23.4 | 27.1 | 25.3 | 28.0 | 26.9 | 25.0 | 23.7 | 25.2 |
| Foxa2 | 0.0 | 0.0 | 0.0 | 0.0 | 0.0 | 0.0 | 0.0 | 0.0 | 0.0 | 0.5 | 0.2 | 0.1 |
| Foxc1 | 2.2 | 2.6 | 3.7 | 2.2 | 1.9 | 1.7 | 2.4 | 2.1 | 2.6 | 2.9 | 2.6 | 2.8 |
| Foxc2 | 2.0 | 1.8 | 1.6 | 1.7 | 2.1 | 1.4 | 3.1 | 1.8 | 1.9 | 2.9 | 2.8 | 2.8 |
| Foxd1 | 0.1 | 0.1 | 0.1 | 0.1 | 0.1 | 0.1 | 0.0 | 0.1 | 0.1 | 0.1 | 0.0 | 0.1 |
| Foxd2 | 0.3 | 0.2 | 0.2 | 0.1 | 0.1 | 0.1 | 0.2 | 0.1 | 0.3 | 0.4 | 0.1 | 0.2 |
| Foxe1 | 0.0 | 0.0 | 0.4 | 0.0 | 0.3 | 0.2 | 0.2 | 0.4 | 0.0 | 0.0 | 0.0 | 0.4 |
| Foxf1 | 2.0 | 1.2 | 1.0 | 2.8 | 2.5 | 2.1 | 1.8 | 1.3 | 1.8 | 1.5 | 1.9 | 1.7 |
| Foxf2 | 1.3 | 1.4 | 0.8 | 2.5 | 1.8 | 1.4 | 1.7 | 1.2 | 1.2 | 0.9 | 0.7 | 0.5 |
| Foxg1 | 0.1 | 0.2 | 0.6 | 0.8 | 0.6 | 0.7 | 0.5 | 0.7 | 0.4 | 0.4 | 0.4 | 0.3 |
| Foxj1 | 0.0 | 0.0 | 0.1 | 0.0 | 0.0 | 0.0 | 0.0 | 0.0 | 0.0 | 0.0 | 0.1 | 0.0 |
| Foxj2 | 2.3 | 3.4 | 3.8 | 3.7 | 3.0 | 3.0 | 3.2 | 3.5 | 3.3 | 2.5 | 2.7 | 2.6 |
| Foxj3 | 6.6 | 6.6 | 7.1 | 7.2 | 5.9 | 7.0 | 6.8 | 6.6 | 6.0 | 5.7 | 7.0 | 6.6 |
| Foxk1 | 1.3 | 1.7 | 1.6 | 1.6 | 1.7 | 1.3 | 2.0 | 1.6 | 1.6 | 1.6 | 1.9 | 1.5 |
| Foxk2 | 12.2 | 14.8 | 13.7 | 10.1 | 12.2 | 12.9 | 15.7 | 15.0 | 13.9 | 12.0 | 14.9 | 11.7 |
| Foxl1 | 0.1 | 0.1 | 0.0 | 0.1 | 0.0 | 0.2 | 0.1 | 0.1 | 0.1 | 0.1 | 0.0 | 0.1 |
| Foxl2 | 0.1 | 0.0 | 0.1 | 0.1 | 0.0 | 0.2 | 0.0 | 0.0 | 0.0 | 0.1 | 0.0 | 0.0 |
| Foxl2os | 0.2 | 0.1 | 0.0 | 0.2 | 0.1 | 0.1 | 0.1 | 0.0 | 0.0 | 0.0 | 0.0 | 0.0 |
| Foxm1 | 0.8 | 1.4 | 2.1 | 0.4 | 1.9 | 0.5 | 3.2 | 1.0 | 2.1 | 0.9 | 1.4 | 1.2 |
| Foxn1 | 0.0 | 0.0 | 0.0 | 0.0 | 0.0 | 0.0 | 0.0 | 0.0 | 0.0 | 0.0 | 0.0 | 0.0 |
| Foxn2 | 4.7 | 5.1 | 4.8 | 5.5 | 5.7 | 3.6 | 4.3 | 5.0 | 4.5 | 4.6 | 5.8 | 5.1 |
| Foxn3 | 13.3 | 14.2 | 16.4 | 11.7 | 11.1 | 11.8 | 12.9 | 18.4 | 14.0 | 11.4 | 14.1 | 13.5 |
| Foxo1 | 1.8 | 1.9 | 2.4 | 1.6 | 1.5 | 1.6 | 1.5 | 1.8 | 1.3 | 1.2 | 1.1 | 1.6 |
| Foxo3 | 9.5 | 11.4 | 11.7 | 8.9 | 9.9 | 10.1 | 10.6 | 10.6 | 9.7 | 11.3 | 9.7 | 9.3 |
| Foxo4 | 6.2 | 6.1 | 5.2 | 5.5 | 5.2 | 7.2 | 4.3 | 5.2 | 5.3 | 6.5 | 5.7 | 5.1 |
| Foxo6 | 0.2 | 0.1 | 0.0 | 0.1 | 0.0 | 0.1 | 0.0 | 0.2 | 0.1 | 0.6 | 0.3 | 0.1 |
| Foxp1 | 5.6 | 6.7 | 7.3 | 6.4 | 7.1 | 5.6 | 8.8 | 8.5 | 8.2 | 8.8 | 9.3 | 9.2 |

| | | | | | | | | | | | | |
|---|---|---|---|---|---|---|---|---|---|---|---|---|
| Foxp2 | 3.0 | 4.5 | 5.6 | 3.2 | 2.8 | 2.4 | 3.3 | 5.4 | 4.6 | 2.2 | 2.8 | 4.4 |
| Foxp3 | 0.1 | 0.1 | 0.0 | 0.0 | 0.0 | 0.0 | 0.0 | 0.0 | 0.0 | 0.0 | 0.0 | 0.1 |
| Foxp4 | 2.0 | 2.2 | 2.2 | 2.1 | 2.5 | 1.8 | 2.9 | 3.0 | 2.7 | 2.5 | 2.3 | 2.1 |
| Foxq1 | 0.0 | 0.0 | 0.1 | 0.2 | 0.1 | 0.0 | 0.1 | 0.0 | 0.0 | 0.1 | 0.0 | 0.0 |
| Foxred1 | 8.3 | 8.6 | 7.6 | 6.8 | 6.1 | 7.8 | 7.5 | 7.3 | 7.3 | 5.5 | 6.9 | 6.0 |
| Foxred2 | 0.4 | 0.4 | 0.4 | 0.6 | 0.6 | 0.8 | 0.5 | 0.4 | 0.5 | 0.8 | 0.4 | 0.5 |
| Foxs1 | 3.3 | 3.6 | 5.4 | 2.9 | 7.1 | 2.6 | 7.8 | 5.2 | 8.1 | 12.9 | 6.9 | 7.7 |
| Fpgs | 6.0 | 5.7 | 4.9 | 6.7 | 6.5 | 5.9 | 8.9 | 8.8 | 8.8 | 7.9 | 6.9 | 6.8 |
| Fpgt | 7.1 | 7.5 | 7.6 | 8.0 | 8.1 | 7.8 | 7.7 | 6.4 | 7.9 | 8.0 | 8.0 | 8.0 |
| Fpr1 | 1.3 | 0.0 | 0.0 | 4.4 | 13.6 | 0.0 | 2.6 | 0.4 | 0.5 | 0.8 | 0.4 | 1.2 |
| Fpr2 | 0.3 | 0.0 | 0.1 | 1.1 | 4.1 | 0.0 | 0.7 | 0.1 | 0.3 | 0.1 | 0.0 | 0.8 |
| Fra10ac1 | 8.4 | 7.8 | 9.0 | 9.1 | 6.5 | 9.5 | 7.6 | 6.4 | 6.6 | 7.5 | 8.4 | 8.5 |
| Fras1 | 0.3 | 0.2 | 0.2 | 0.1 | 0.1 | 0.2 | 0.1 | 0.2 | 0.2 | 1.0 | 0.7 | 0.4 |
| Frat1 | 0.1 | 0.2 | 0.1 | 0.0 | 0.0 | 0.0 | 0.1 | 0.1 | 0.2 | 0.1 | 0.1 | 0.1 |
| Frat2 | 0.3 | 0.3 | 0.4 | 0.1 | 0.3 | 0.1 | 0.2 | 0.4 | 0.4 | 0.6 | 0.3 | 0.2 |
| Frem1 | 0.4 | 0.7 | 0.7 | 0.5 | 0.8 | 0.8 | 0.5 | 0.4 | 0.3 | 0.5 | 0.5 | 0.3 |
| Frem2 | 0.0 | 0.0 | 0.0 | 0.0 | 0.0 | 0.0 | 0.0 | 0.0 | 0.0 | 0.0 | 0.0 | 0.0 |
| Frem3 | 0.0 | 0.0 | 0.0 | 0.0 | 0.0 | 0.0 | 0.0 | 0.0 | 0.0 | 0.0 | 0.0 | 0.0 |
| Frg1 | 26.4 | 27.6 | 28.4 | 30.4 | 25.5 | 28.8 | 29.3 | 28.5 | 23.8 | 25.6 | 33.6 | 33.5 |
| Frk | 4.1 | 3.6 | 3.6 | 5.3 | 4.0 | 4.4 | 4.1 | 3.2 | 3.8 | 6.2 | 5.3 | 5.0 |
| Frmd3 | 1.7 | 1.8 | 0.9 | 1.4 | 0.7 | 0.7 | 0.9 | 0.9 | 0.5 | 0.7 | 0.8 | 1.2 |
| Frmd4a | 10.6 | 13.3 | 11.8 | 12.6 | 11.7 | 11.6 | 17.3 | 11.1 | 14.0 | 11.0 | 12.9 | 13.2 |
| Frmd4b | 2.4 | 1.9 | 1.6 | 2.7 | 2.5 | 1.5 | 0.8 | 1.6 | 0.8 | 7.1 | 3.4 | 2.1 |
| Frmd5 | 0.1 | 0.0 | 0.1 | 0.0 | 0.0 | 0.1 | 0.0 | 0.0 | 0.1 | 0.1 | 0.1 | 0.0 |
| Frmd6 | 19.6 | 23.8 | 22.6 | 21.8 | 24.8 | 26.6 | 29.6 | 20.5 | 26.0 | 28.9 | 26.2 | 24.6 |
| Frmd7 | 0.0 | 0.0 | 0.0 | 0.0 | 0.0 | 0.0 | 0.0 | 0.1 | 0.0 | 0.6 | 0.5 | 0.2 |
| Frmd8 | 12.5 | 13.7 | 10.6 | 10.8 | 13.9 | 11.6 | 12.2 | 10.8 | 13.2 | 14.5 | 12.3 | 9.8 |
| Frmpd1 | 1.1 | 0.2 | 0.1 | 0.7 | 0.2 | 0.4 | 0.5 | 0.2 | 0.3 | 0.7 | 0.8 | 0.6 |
| Frmpd4 | 0.0 | 0.0 | 0.0 | 0.0 | 0.0 | 0.0 | 0.0 | 0.0 | 0.0 | 0.0 | 0.0 | 0.0 |
| Frrs1 | 9.3 | 6.7 | 6.7 | 9.1 | 10.3 | 6.6 | 6.7 | 6.2 | 6.8 | 7.8 | 8.1 | 7.9 |
| Frrs1l | 0.1 | 0.0 | 0.1 | 0.0 | 0.1 | 0.2 | 0.0 | 0.0 | 0.0 | 0.0 | 0.1 | 0.0 |
| Frs2 | 4.5 | 6.1 | 7.2 | 4.3 | 5.6 | 4.6 | 6.6 | 6.1 | 6.8 | 6.7 | 7.0 | 7.7 |
| Frs3 | 1.1 | 1.5 | 0.9 | 0.6 | 0.7 | 1.7 | 1.4 | 0.8 | 1.3 | 0.9 | 0.9 | 0.9 |
| Fry | 0.2 | 0.2 | 0.2 | 0.3 | 0.2 | 0.1 | 0.2 | 0.2 | 0.1 | 0.2 | 0.2 | 0.1 |
| Fryl | 3.8 | 4.9 | 4.8 | 3.0 | 4.3 | 3.1 | 4.6 | 3.4 | 4.1 | 4.1 | 3.7 | 3.6 |
| Frzb | 70.7 | 74.1 | 84.8 | 78.4 | 106.4 | 86.1 | 73.3 | 54.8 | 66.0 | 64.6 | 64.2 | 67.9 |
| Fsbp | 0.3 | 0.8 | 1.0 | 0.4 | 0.5 | 0.7 | 0.6 | 0.7 | 0.4 | 0.5 | 0.5 | 0.4 |
| Fscn1 | 38.7 | 41.4 | 42.3 | 43.1 | 48.4 | 36.9 | 53.1 | 39.7 | 56.2 | 60.3 | 54.0 | 34.2 |
| Fscn2 | 0.0 | 0.0 | 0.0 | 0.1 | 0.0 | 0.0 | 0.0 | 0.1 | 0.1 | 0.1 | 0.0 | 0.0 |
| Fsd1 | 1.4 | 1.9 | 1.7 | 1.1 | 0.7 | 1.7 | 1.7 | 1.7 | 1.0 | 1.1 | 1.4 | 1.1 |
| Fsd1l | 0.9 | 1.3 | 1.1 | 1.7 | 0.9 | 1.3 | 1.0 | 0.8 | 0.9 | 0.8 | 1.2 | 0.8 |
| Fsd2 | 0.1 | 0.1 | 0.2 | 0.2 | 0.2 | 0.2 | 0.2 | 0.1 | 0.0 | 0.1 | 0.1 | 0.1 |
| Fsip1 | 0.3 | 0.2 | 0.1 | 0.3 | 0.4 | 0.3 | 0.2 | 0.3 | 0.2 | 0.2 | 0.1 | 0.1 |
| Fst | 21.2 | 19.9 | 28.9 | 10.4 | 18.0 | 13.7 | 19.7 | 15.0 | 21.2 | 65.5 | 43.8 | 47.9 |
| Fstl1 | 497.3 | 523.2 | 639.5 | 435.8 | 604.7 | 467.8 | 710.7 | 868.7 | 792.9 | 531.7 | 660.1 | 682.0 |
| Fstl3 | 9.9 | 7.1 | 4.2 | 10.5 | 7.7 | 10.3 | 10.5 | 6.3 | 8.3 | 7.2 | 8.1 | 7.1 |
| Fstl4 | 0.0 | 0.0 | 0.0 | 0.0 | 0.0 | 0.0 | 0.0 | 0.0 | 0.0 | 0.1 | 0.1 | 0.0 |
| Fstl5 | 0.0 | 0.0 | 0.0 | 0.0 | 0.0 | 0.1 | 0.0 | 0.0 | 0.0 | 0.0 | 0.0 | 0.0 |
| Fth1 | 1653.0 | 1693.9 | 1925.0 | 3377.4 | 4068.0 | 2505.3 | 1212.7 | 1384.7 | 697.6 | 1494.1 | 1203.7 | 1338.2 |
| Ftl1 | 6728.5 | 5479.7 | 2871.3 | 9390.9 | 6918.7 | 10883.5 | 3034.4 | 2878.7 | 2423.1 | 4218.3 | 3402.5 | 3164.2 |
| Fto | 15.7 | 16.6 | 16.1 | 16.0 | 14.3 | 16.8 | 14.6 | 15.7 | 14.1 | 15.5 | 14.5 | 14.1 |
| Ftsj1 | 3.7 | 3.2 | 2.5 | 2.9 | 2.9 | 2.5 | 3.0 | 2.9 | 2.8 | 2.9 | 3.2 | 2.4 |
| Ftsj2 | 3.4 | 3.3 | 3.1 | 3.7 | 3.7 | 3.6 | 2.4 | 2.8 | 2.1 | 3.5 | 3.6 | 2.7 |
| Ftsj3 | 33.0 | 28.1 | 25.9 | 34.4 | 25.5 | 27.5 | 30.1 | 24.1 | 21.1 | 25.9 | 31.2 | 27.5 |
| Ftsjd1 | 2.3 | 1.8 | 1.5 | 2.8 | 2.3 | 2.6 | 2.0 | 1.6 | 1.9 | 2.4 | 1.8 | 1.6 |
| Ftsjd2 | 25.7 | 23.4 | 20.8 | 27.7 | 22.1 | 26.3 | 21.2 | 19.1 | 20.9 | 19.7 | 19.1 | 23.4 |
| Ftx | 0.3 | 0.3 | 0.4 | 0.3 | 0.4 | 0.4 | 0.3 | 0.4 | 0.4 | 0.6 | 0.6 | 0.4 |

| | | | | | | | | | | | | |
|---|---|---|---|---|---|---|---|---|---|---|---|---|
| Fubp1 | 12.8 | 12.4 | 13.2 | 16.1 | 13.8 | 14.3 | 14.1 | 12.3 | 11.6 | 11.4 | 15.1 | 13.6 |
| Fubp3 | 11.3 | 10.9 | 12.2 | 12.8 | 11.3 | 12.3 | 13.0 | 12.2 | 10.5 | 11.7 | 13.8 | 13.3 |
| Fuca1 | 50.1 | 36.9 | 35.5 | 54.8 | 47.5 | 53.0 | 34.4 | 28.1 | 27.4 | 40.1 | 31.5 | 39.6 |
| Fuca2 | 14.7 | 10.9 | 9.8 | 16.2 | 12.3 | 13.9 | 8.3 | 7.7 | 7.2 | 9.7 | 9.6 | 13.0 |
| Fuk | 3.1 | 2.9 | 2.5 | 2.5 | 2.3 | 3.3 | 2.1 | 2.2 | 2.6 | 2.6 | 2.5 | 1.8 |
| Fundc1 | 15.1 | 13.6 | 13.9 | 15.8 | 14.8 | 14.6 | 13.9 | 14.4 | 12.4 | 13.7 | 17.0 | 14.5 |
| Fundc2 | 14.9 | 11.4 | 10.5 | 15.3 | 12.4 | 14.1 | 9.8 | 9.7 | 10.0 | 12.9 | 12.5 | 11.8 |
| Fuom | 6.9 | 7.0 | 7.1 | 8.1 | 5.6 | 6.4 | 6.0 | 6.3 | 6.1 | 7.2 | 6.4 | 7.1 |
| Furin | 10.8 | 13.7 | 14.6 | 13.3 | 18.3 | 11.3 | 16.8 | 16.2 | 17.6 | 14.5 | 12.9 | 12.6 |
| Fus | 2.7 | 5.4 | 6.9 | 4.1 | 5.5 | 3.5 | 6.9 | 6.4 | 6.5 | 6.4 | 6.5 | 5.6 |
| Fut10 | 7.9 | 7.4 | 7.7 | 7.3 | 6.8 | 9.0 | 7.2 | 6.0 | 6.6 | 6.5 | 6.8 | 6.6 |
| Fut11 | 2.7 | 4.1 | 6.2 | 2.2 | 3.8 | 3.5 | 4.0 | 6.9 | 6.5 | 4.8 | 3.6 | 5.1 |
| Fut4 | 0.5 | 0.7 | 0.6 | 0.6 | 1.0 | 0.9 | 0.8 | 0.7 | 0.8 | 0.8 | 0.6 | 0.7 |
| Fut7 | 0.0 | 0.0 | 0.1 | 0.6 | 0.3 | 0.1 | 0.1 | 0.0 | 0.0 | 0.0 | 0.0 | 0.1 |
| Fut8 | 12.3 | 14.2 | 16.4 | 12.2 | 11.8 | 10.8 | 13.2 | 12.9 | 11.3 | 13.4 | 12.9 | 13.4 |
| Fuz | 4.1 | 5.9 | 5.8 | 4.4 | 4.0 | 5.6 | 3.9 | 3.5 | 4.0 | 5.2 | 4.1 | 4.9 |
| Fv1 | 2.1 | 2.1 | 2.7 | 2.3 | 2.4 | 3.3 | 2.5 | 2.0 | 1.8 | 2.8 | 2.3 | 3.2 |
| Fxn | 1.9 | 1.9 | 1.4 | 1.5 | 1.8 | 1.2 | 2.1 | 1.7 | 2.4 | 1.6 | 2.6 | 2.0 |
| Fxr1 | 69.8 | 68.9 | 60.3 | 66.3 | 56.6 | 62.0 | 57.8 | 55.2 | 57.8 | 68.4 | 73.6 | 70.8 |
| Fxr2 | 29.8 | 29.7 | 25.2 | 31.4 | 25.0 | 31.4 | 29.3 | 24.6 | 28.0 | 28.4 | 31.5 | 30.1 |
| Fxyd1 | 5.8 | 16.8 | 32.8 | 4.5 | 6.2 | 4.1 | 4.8 | 15.7 | 6.9 | 4.0 | 4.1 | 6.2 |
| Fxyd2 | 0.2 | 0.4 | 0.8 | 0.9 | 0.7 | 0.3 | 0.2 | 0.2 | 0.4 | 0.4 | 0.8 | 0.4 |
| Fxyd3 | 0.0 | 0.0 | 0.5 | 0.0 | 0.0 | 0.2 | 0.0 | 0.6 | 0.1 | 0.1 | 0.0 | 0.2 |
| Fxyd5 | 29.0 | 13.4 | 16.3 | 85.6 | 70.2 | 29.8 | 29.8 | 11.2 | 7.9 | 14.5 | 15.1 | 15.7 |
| Fxyd6 | 4.0 | 5.4 | 12.3 | 2.8 | 5.6 | 2.5 | 4.4 | 23.4 | 10.3 | 9.7 | 6.7 | 10.8 |
| Fxyd7 | 0.1 | 0.1 | 0.0 | 0.0 | 0.1 | 0.0 | 0.0 | 0.3 | 0.0 | 0.0 | 0.0 | 0.0 |
| Fyb | 1.4 | 0.0 | 0.3 | 13.5 | 22.9 | 0.2 | 3.1 | 0.3 | 0.5 | 1.6 | 0.5 | 1.4 |
| Fyco1 | 7.1 | 7.6 | 7.5 | 8.0 | 6.7 | 9.5 | 7.1 | 7.2 | 5.8 | 6.1 | 5.7 | 6.7 |
| Fyn | 15.8 | 13.6 | 12.2 | 17.8 | 14.1 | 18.0 | 18.2 | 14.5 | 14.0 | 11.8 | 16.4 | 11.9 |
| Fyttd1 | 15.1 | 15.4 | 14.9 | 13.2 | 14.1 | 14.9 | 13.6 | 14.5 | 14.9 | 16.8 | 15.7 | 16.2 |
| Fzd1 | 6.0 | 8.7 | 14.3 | 5.2 | 7.8 | 4.8 | 10.5 | 14.1 | 14.5 | 22.1 | 13.0 | 15.8 |
| Fzd10 | 0.1 | 0.1 | 0.1 | 0.2 | 0.1 | 0.1 | 0.0 | 0.0 | 0.1 | 0.0 | 0.1 | 0.0 |
| Fzd2 | 19.2 | 22.1 | 20.3 | 15.7 | 16.0 | 16.7 | 20.7 | 28.8 | 27.9 | 15.0 | 15.8 | 18.1 |
| Fzd3 | 0.8 | 0.8 | 0.7 | 0.7 | 0.7 | 0.8 | 0.7 | 0.8 | 0.7 | 1.4 | 1.2 | 1.0 |
| Fzd4 | 4.8 | 10.9 | 15.9 | 2.8 | 5.9 | 3.1 | 7.7 | 12.3 | 9.3 | 5.3 | 6.2 | 6.1 |
| Fzd5 | 7.1 | 8.1 | 11.8 | 6.1 | 9.0 | 5.4 | 14.3 | 13.3 | 18.2 | 6.6 | 10.8 | 11.3 |
| Fzd6 | 4.2 | 1.7 | 1.7 | 2.3 | 1.3 | 1.9 | 1.3 | 1.9 | 2.0 | 4.2 | 3.4 | 3.8 |
| Fzd7 | 7.9 | 10.0 | 11.0 | 7.4 | 8.2 | 7.5 | 10.2 | 10.6 | 11.0 | 11.7 | 10.7 | 10.9 |
| Fzd8 | 7.4 | 8.9 | 11.6 | 6.9 | 7.2 | 6.3 | 11.3 | 14.7 | 17.1 | 8.1 | 8.3 | 9.2 |
| Fzd9 | 0.2 | 0.1 | 0.0 | 0.1 | 0.1 | 0.2 | 0.1 | 0.2 | 0.1 | 0.1 | 0.1 | 0.1 |
| Fzr1 | 3.7 | 4.5 | 5.3 | 4.6 | 6.1 | 4.0 | 5.9 | 5.3 | 6.2 | 5.4 | 4.0 | 5.1 |
| G0s2 | 43.4 | 79.3 | 96.7 | 43.9 | 42.9 | 92.7 | 44.7 | 83.5 | 69.9 | 23.0 | 28.8 | 43.7 |
| G2e3 | 0.8 | 1.4 | 2.0 | 0.8 | 1.8 | 0.8 | 2.2 | 1.5 | 2.1 | 2.0 | 2.0 | 2.0 |
| G3bp1 | 46.7 | 45.7 | 43.0 | 46.3 | 51.3 | 45.6 | 52.2 | 46.1 | 45.7 | 50.2 | 54.0 | 46.7 |
| G3bp2 | 33.3 | 32.3 | 32.7 | 39.9 | 34.0 | 34.3 | 31.5 | 29.0 | 28.5 | 37.3 | 36.6 | 37.5 |
| G530011O06Rik | 1.2 | 2.6 | 2.8 | 1.9 | 3.7 | 1.8 | 1.6 | 1.5 | 1.8 | 1.7 | 1.6 | 1.2 |
| G630025P09Rik | 0.4 | 0.8 | 0.7 | 0.4 | 0.5 | 0.5 | 0.5 | 0.9 | 0.6 | 0.4 | 0.4 | 0.8 |
| G630071F17Rik | 0.3 | 0.4 | 0.2 | 0.4 | 0.5 | 0.3 | 0.5 | 0.6 | 0.6 | 0.3 | 0.5 | 0.7 |
| G630090E17Rik | 1.2 | 1.5 | 1.5 | 1.3 | 1.5 | 1.2 | 1.0 | 1.5 | 1.2 | 1.3 | 1.5 | 1.2 |
| G6pc3 | 14.5 | 13.3 | 10.8 | 15.3 | 12.6 | 19.2 | 13.5 | 12.9 | 12.4 | 12.8 | 10.4 | 10.6 |
| G6pd2 | 0.0 | 0.1 | 0.0 | 0.1 | 0.0 | 0.1 | 0.0 | 0.1 | 0.0 | 0.0 | 0.0 | 0.0 |
| G6pdx | 30.6 | 34.6 | 32.2 | 29.3 | 41.2 | 22.7 | 25.4 | 47.2 | 23.4 | 25.7 | 30.2 | 24.8 |
| G730013B05Rik | 0.0 | 0.0 | 0.0 | 0.0 | 0.1 | 0.1 | 0.1 | 0.0 | 0.1 | 0.0 | 0.0 | 0.0 |
| Gaa | 50.4 | 48.2 | 53.6 | 52.0 | 47.8 | 56.5 | 39.9 | 48.2 | 41.4 | 49.7 | 39.5 | 42.7 |
| Gab1 | 7.8 | 10.7 | 14.1 | 6.2 | 6.7 | 6.5 | 8.5 | 11.8 | 11.3 | 8.0 | 8.8 | 9.9 |
| Gab2 | 8.7 | 9.6 | 10.1 | 9.6 | 9.7 | 6.5 | 10.0 | 9.7 | 8.7 | 7.3 | 9.2 | 11.1 |
| Gab3 | 0.2 | 0.3 | 0.2 | 0.4 | 0.5 | 0.1 | 0.3 | 0.2 | 0.3 | 0.2 | 0.3 | 0.2 |

| | | | | | | | | | | | | |
|---|---|---|---|---|---|---|---|---|---|---|---|---|
| Gabarap | 277.7 | 220.4 | 210.3 | 286.5 | 225.2 | 276.2 | 234.6 | 266.6 | 220.9 | 243.8 | 269.5 | 260.1 |
| Gabarapl1 | 110.1 | 80.4 | 73.1 | 133.8 | 80.2 | 129.0 | 84.5 | 69.3 | 79.3 | 131.0 | 98.9 | 106.3 |
| Gabarapl2 | 78.8 | 68.3 | 54.9 | 74.9 | 65.4 | 71.7 | 56.7 | 55.5 | 68.4 | 74.0 | 70.4 | 69.7 |
| Gabbr1 | 1.8 | 1.8 | 1.8 | 1.7 | 1.4 | 1.7 | 1.8 | 1.8 | 2.1 | 1.5 | 1.7 | 1.9 |
| Gabbr2 | 0.0 | 0.0 | 0.0 | 0.0 | 0.0 | 0.0 | 0.0 | 0.0 | 0.0 | 0.0 | 0.0 | 0.0 |
| Gabpa | 11.5 | 10.5 | 12.3 | 12.0 | 11.8 | 11.7 | 12.7 | 12.4 | 11.0 | 14.1 | 14.5 | 16.3 |
| Gabpb1 | 6.8 | 7.2 | 7.2 | 6.2 | 6.6 | 7.3 | 6.9 | 7.4 | 7.1 | 6.9 | 7.1 | 7.1 |
| Gabpb2 | 3.4 | 4.1 | 4.6 | 3.2 | 4.1 | 3.1 | 4.2 | 4.7 | 4.0 | 3.9 | 4.3 | 4.8 |
| Gabra1 | 0.3 | 0.1 | 0.0 | 0.1 | 0.0 | 0.1 | 0.1 | 0.1 | 0.0 | 0.1 | 0.2 | 0.1 |
| Gabra3 | 0.5 | 0.7 | 0.6 | 0.2 | 0.3 | 0.1 | 0.4 | 0.4 | 1.0 | 0.9 | 0.6 | 1.3 |
| Gabra4 | 0.0 | 0.0 | 0.0 | 0.0 | 0.0 | 0.0 | 0.0 | 0.1 | 0.1 | 1.7 | 1.0 | 0.4 |
| Gabrb1 | 1.1 | 0.4 | 0.1 | 0.5 | 0.2 | 0.2 | 0.2 | 0.2 | 0.4 | 3.9 | 2.2 | 1.1 |
| Gabrb2 | 0.0 | 0.0 | 0.0 | 0.0 | 0.0 | 0.0 | 0.0 | 0.0 | 0.0 | 0.0 | 0.0 | 0.0 |
| Gabrb3 | 0.1 | 0.0 | 0.0 | 0.1 | 0.0 | 0.1 | 0.0 | 0.0 | 0.0 | 0.2 | 0.1 | 0.1 |
| Gabrd | 0.2 | 0.1 | 0.1 | 0.0 | 0.1 | 0.0 | 0.1 | 0.0 | 0.1 | 0.1 | 0.1 | 0.1 |
| Gabre | 0.1 | 0.2 | 0.2 | 0.0 | 0.1 | 0.0 | 0.2 | 0.1 | 0.2 | 0.2 | 0.1 | 0.1 |
| Gabrp | 0.0 | 0.0 | 0.0 | 0.0 | 0.0 | 0.0 | 0.0 | 0.0 | 0.0 | 0.0 | 0.0 | 0.0 |
| Gabrr2 | 0.0 | 0.0 | 0.0 | 0.0 | 0.0 | 0.0 | 0.0 | 0.0 | 0.1 | 0.0 | 0.0 | 0.0 |
| Gadd45a | 20.4 | 11.4 | 11.9 | 23.1 | 15.4 | 31.1 | 13.1 | 10.8 | 8.8 | 15.4 | 15.0 | 13.2 |
| Gadd45b | 26.0 | 22.1 | 21.2 | 29.1 | 26.3 | 22.7 | 30.2 | 34.8 | 30.1 | 19.3 | 23.2 | 17.9 |
| Gadd45g | 103.0 | 100.7 | 104.4 | 136.6 | 90.5 | 71.2 | 128.8 | 87.2 | 142.3 | 91.9 | 103.2 | 131.8 |
| Gadd45gip1 | 12.4 | 10.0 | 9.8 | 11.9 | 9.1 | 12.4 | 12.7 | 12.0 | 10.8 | 12.6 | 11.6 | 9.9 |
| Gadl1 | 0.0 | 0.0 | 0.0 | 0.0 | 0.0 | 0.0 | 0.0 | 0.0 | 0.0 | 0.0 | 0.0 | 0.0 |
| Gak | 22.8 | 21.1 | 19.0 | 26.1 | 23.6 | 23.5 | 23.7 | 21.2 | 21.3 | 19.0 | 20.8 | 20.9 |
| Gal | 0.4 | 0.4 | 0.6 | 0.1 | 0.6 | 0.7 | 0.3 | 0.1 | 0.0 | 0.1 | 0.1 | 0.1 |
| Gal3st1 | 1.1 | 0.8 | 0.8 | 1.1 | 0.6 | 1.3 | 0.7 | 1.3 | 1.2 | 0.6 | 0.9 | 0.6 |
| Gal3st2 | 13.4 | 10.1 | 10.0 | 11.1 | 12.0 | 3.7 | 16.1 | 9.9 | 11.1 | 8.1 | 11.2 | 8.4 |
| Gal3st3 | 0.0 | 0.0 | 0.0 | 0.1 | 0.0 | 0.0 | 0.1 | 0.0 | 0.0 | 0.1 | 0.0 | 0.0 |
| Gal3st4 | 0.7 | 0.9 | 1.2 | 1.0 | 1.0 | 1.2 | 1.2 | 1.3 | 1.1 | 1.2 | 0.7 | 0.9 |
| Galc | 2.4 | 2.2 | 2.3 | 4.0 | 3.6 | 3.2 | 2.7 | 2.0 | 2.3 | 2.6 | 2.5 | 2.7 |
| Gale | 5.4 | 5.2 | 3.4 | 3.9 | 4.2 | 3.4 | 5.9 | 6.2 | 5.0 | 4.8 | 5.1 | 3.2 |
| Galk1 | 16.9 | 17.4 | 13.6 | 12.7 | 15.0 | 15.9 | 18.6 | 19.4 | 18.8 | 17.1 | 15.2 | 15.4 |
| Galk2 | 23.4 | 25.3 | 23.2 | 24.4 | 22.1 | 29.4 | 22.0 | 21.3 | 20.2 | 17.1 | 18.7 | 20.1 |
| Galm | 14.0 | 11.7 | 9.0 | 15.8 | 9.1 | 16.0 | 8.1 | 6.1 | 7.2 | 8.3 | 8.7 | 8.9 |
| Galns | 8.2 | 7.3 | 7.8 | 10.7 | 10.0 | 9.9 | 9.2 | 6.9 | 7.3 | 7.5 | 8.2 | 8.1 |
| Galnt1 | 36.8 | 31.6 | 32.0 | 39.3 | 36.6 | 37.1 | 36.0 | 31.4 | 34.0 | 36.5 | 39.0 | 40.5 |
| Galnt10 | 19.0 | 16.1 | 16.8 | 26.7 | 23.6 | 30.4 | 27.2 | 22.9 | 24.1 | 20.7 | 20.8 | 22.0 |
| Galnt11 | 15.1 | 15.9 | 13.6 | 13.6 | 13.6 | 13.7 | 14.9 | 12.7 | 13.7 | 14.6 | 13.8 | 15.9 |
| Galnt12 | 1.1 | 1.1 | 1.9 | 1.5 | 2.1 | 0.5 | 1.0 | 1.2 | 1.1 | 1.0 | 0.9 | 1.1 |
| Galnt14 | 0.0 | 0.1 | 0.0 | 0.0 | 0.0 | 0.0 | 0.1 | 0.0 | 0.0 | 0.0 | 0.0 | 0.0 |
| Galnt15 | 0.1 | 0.1 | 0.3 | 0.0 | 0.0 | 0.1 | 0.1 | 0.0 | 0.0 | 0.2 | 0.0 | 0.0 |
| Galnt16 | 1.2 | 1.1 | 1.9 | 1.4 | 0.9 | 1.8 | 1.2 | 2.6 | 2.1 | 0.9 | 1.3 | 1.5 |
| Galnt18 | 6.2 | 2.9 | 2.3 | 7.0 | 5.0 | 6.0 | 6.1 | 4.8 | 4.9 | 8.3 | 11.2 | 4.0 |
| Galnt2 | 41.2 | 38.7 | 36.8 | 37.9 | 37.8 | 44.0 | 44.6 | 46.7 | 44.9 | 41.3 | 41.4 | 46.9 |
| Galnt3 | 0.1 | 0.0 | 0.0 | 0.2 | 0.9 | 0.0 | 0.1 | 0.1 | 0.0 | 0.1 | 0.1 | 0.1 |
| Galnt4 | 6.6 | 6.4 | 8.6 | 5.1 | 5.7 | 5.7 | 6.8 | 10.3 | 7.7 | 8.3 | 8.8 | 9.2 |
| Galnt5 | 0.0 | 0.0 | 0.0 | 0.0 | 0.0 | 0.0 | 0.1 | 0.0 | 0.0 | 0.0 | 0.1 | 0.0 |
| Galnt6 | 0.1 | 0.0 | 0.0 | 1.3 | 1.4 | 0.0 | 0.1 | 0.0 | 0.0 | 0.1 | 0.0 | 0.1 |
| Galnt7 | 5.6 | 6.7 | 6.3 | 7.1 | 6.8 | 5.7 | 5.4 | 5.1 | 4.1 | 4.3 | 5.0 | 5.6 |
| Galnt9 | 0.0 | 0.0 | 0.0 | 0.1 | 0.2 | 0.0 | 0.1 | 0.0 | 0.0 | 0.0 | 0.0 | 0.0 |
| Galr2 | 0.2 | 0.1 | 0.1 | 0.2 | 0.2 | 0.1 | 0.1 | 0.2 | 0.1 | 0.1 | 0.3 | 0.1 |
| Galt | 4.2 | 4.3 | 5.1 | 4.6 | 4.2 | 4.3 | 5.1 | 6.3 | 4.7 | 3.6 | 4.7 | 4.7 |
| Gamt | 6.8 | 7.7 | 5.1 | 8.9 | 6.2 | 7.4 | 5.0 | 8.3 | 8.3 | 5.7 | 5.3 | 5.5 |
| Gan | 3.2 | 3.1 | 3.4 | 3.8 | 3.7 | 3.2 | 4.1 | 3.1 | 3.4 | 2.6 | 3.0 | 3.3 |
| Ganab | 62.5 | 62.8 | 57.2 | 59.4 | 59.0 | 65.3 | 58.0 | 63.9 | 60.6 | 61.3 | 57.1 | 56.6 |
| Ganc | 4.6 | 4.8 | 5.0 | 4.4 | 3.7 | 4.3 | 4.1 | 4.5 | 3.7 | 4.5 | 4.6 | 4.6 |
| Gap43 | 0.1 | 0.0 | 0.2 | 0.1 | 0.2 | 0.0 | 0.2 | 0.2 | 0.1 | 0.3 | 0.2 | 0.2 |

| | | | | | | | | | | | | |
|---|---|---|---|---|---|---|---|---|---|---|---|---|
| Gapdh | 231.0 | 192.0 | 186.8 | 328.8 | 246.0 | 249.6 | 250.8 | 229.3 | 174.9 | 200.4 | 238.4 | 213.9 |
| Gapdhs | 0.0 | 0.1 | 0.1 | 0.0 | 0.1 | 0.0 | 0.0 | 0.0 | 0.1 | 0.0 | 0.0 | 0.0 |
| Gapvd1 | 11.9 | 11.2 | 10.1 | 11.1 | 12.4 | 11.0 | 11.1 | 10.9 | 11.3 | 10.9 | 11.8 | 10.9 |
| Gar1 | 2.2 | 2.1 | 3.3 | 4.3 | 4.0 | 4.1 | 3.7 | 3.0 | 3.8 | 4.2 | 4.8 | 4.2 |
| Garem | 1.0 | 0.8 | 0.8 | 0.6 | 0.4 | 0.8 | 0.5 | 0.6 | 0.5 | 1.0 | 0.8 | 0.5 |
| Gareml | 4.8 | 9.2 | 8.9 | 4.5 | 9.4 | 4.4 | 10.1 | 10.4 | 12.2 | 7.8 | 5.6 | 8.0 |
| Garnl3 | 4.0 | 4.0 | 3.5 | 3.5 | 3.9 | 3.4 | 4.0 | 4.4 | 3.5 | 3.3 | 3.7 | 3.4 |
| Gars | 129.3 | 97.3 | 78.6 | 105.3 | 92.4 | 99.7 | 100.8 | 118.7 | 103.2 | 82.5 | 117.9 | 86.9 |
| Gart | 15.8 | 13.4 | 11.8 | 14.0 | 15.7 | 15.3 | 13.6 | 14.1 | 12.4 | 14.3 | 15.1 | 11.9 |
| Gas1 | 2.2 | 2.3 | 9.0 | 0.7 | 0.9 | 0.4 | 1.8 | 6.6 | 4.6 | 12.3 | 3.8 | 6.0 |
| Gas2 | 1.0 | 0.9 | 1.4 | 0.8 | 1.0 | 1.2 | 1.0 | 1.9 | 1.4 | 1.0 | 1.5 | 1.0 |
| Gas2l1 | 18.4 | 16.3 | 11.4 | 17.9 | 15.9 | 17.1 | 20.0 | 15.1 | 18.6 | 16.2 | 15.9 | 14.4 |
| Gas2l3 | 0.7 | 1.0 | 1.7 | 0.9 | 2.2 | 0.6 | 2.0 | 0.7 | 1.6 | 0.8 | 1.3 | 0.9 |
| Gas5 | 28.8 | 25.6 | 25.2 | 22.5 | 26.9 | 24.1 | 22.6 | 29.4 | 32.1 | 31.8 | 32.1 | 32.5 |
| Gas6 | 303.8 | 361.2 | 551.0 | 311.6 | 366.1 | 287.1 | 335.3 | 424.0 | 387.0 | 450.4 | 435.4 | 665.1 |
| Gas7 | 4.0 | 5.6 | 8.8 | 3.4 | 7.7 | 3.7 | 5.5 | 15.1 | 4.3 | 3.1 | 3.9 | 3.5 |
| Gas8 | 10.1 | 7.7 | 10.9 | 13.5 | 8.0 | 11.9 | 12.2 | 9.9 | 8.8 | 10.2 | 12.2 | 12.1 |
| Gata2 | 1.4 | 1.1 | 0.7 | 2.2 | 1.1 | 1.8 | 1.2 | 0.5 | 0.9 | 0.9 | 0.8 | 0.8 |
| Gata3 | 0.4 | 0.6 | 0.3 | 0.5 | 0.4 | 0.3 | 0.5 | 0.4 | 0.5 | 0.4 | 0.3 | 0.4 |
| Gata4 | 20.1 | 23.5 | 19.9 | 20.7 | 14.4 | 20.8 | 16.0 | 16.0 | 16.5 | 12.4 | 13.2 | 17.1 |
| Gata5 | 0.0 | 0.0 | 0.0 | 0.0 | 0.0 | 0.0 | 0.0 | 0.0 | 0.1 | 1.3 | 0.6 | 0.2 |
| Gata6 | 6.4 | 6.3 | 6.4 | 5.2 | 3.9 | 4.9 | 5.2 | 6.4 | 5.7 | 14.0 | 7.8 | 7.5 |
| Gatad1 | 48.2 | 41.5 | 36.1 | 45.3 | 37.5 | 44.4 | 40.3 | 33.5 | 37.7 | 40.3 | 42.3 | 41.7 |
| Gatad2a | 13.1 | 12.7 | 12.1 | 15.5 | 13.8 | 14.2 | 15.9 | 14.7 | 13.4 | 11.9 | 14.4 | 12.2 |
| Gatad2b | 2.4 | 2.9 | 4.4 | 2.9 | 2.9 | 3.1 | 2.9 | 3.9 | 3.2 | 2.9 | 3.3 | 2.9 |
| Gatc | 5.2 | 6.4 | 6.0 | 7.2 | 6.3 | 7.9 | 6.0 | 6.7 | 5.6 | 4.8 | 5.9 | 5.2 |
| Gatm | 0.3 | 0.1 | 0.2 | 0.4 | 0.9 | 0.3 | 0.5 | 0.5 | 1.0 | 4.5 | 1.9 | 1.3 |
| Gatsl2 | 2.5 | 3.7 | 4.7 | 3.0 | 3.3 | 3.2 | 3.1 | 3.9 | 3.5 | 3.0 | 3.1 | 3.2 |
| Gatsl3 | 17.0 | 15.5 | 15.4 | 14.6 | 14.9 | 16.9 | 17.1 | 17.1 | 17.9 | 14.1 | 12.3 | 14.0 |
| Gba | 78.2 | 58.1 | 59.3 | 79.1 | 69.0 | 75.6 | 54.9 | 57.5 | 51.0 | 56.5 | 52.3 | 69.3 |
| Gba2 | 2.0 | 2.4 | 2.4 | 1.9 | 2.6 | 2.2 | 2.3 | 2.6 | 2.9 | 2.9 | 2.5 | 2.7 |
| Gbas | 16.6 | 15.4 | 15.6 | 17.4 | 14.4 | 15.9 | 14.4 | 17.9 | 14.8 | 13.7 | 15.9 | 17.8 |
| Gbe1 | 18.9 | 15.0 | 8.6 | 29.6 | 17.3 | 35.3 | 6.7 | 7.2 | 4.9 | 7.2 | 6.7 | 6.3 |
| Gbf1 | 12.2 | 14.0 | 14.7 | 10.9 | 13.0 | 11.1 | 13.9 | 14.4 | 13.2 | 11.4 | 12.3 | 12.1 |
| Gbgt1 | 1.2 | 1.0 | 0.9 | 0.7 | 0.5 | 0.8 | 0.4 | 0.4 | 0.2 | 0.8 | 0.6 | 0.7 |
| Gbp1 | 0.0 | 0.0 | 0.0 | 0.0 | 0.0 | 0.0 | 0.1 | 0.0 | 0.0 | 0.0 | 0.0 | 0.0 |
| Gbp10 | 1.5 | 1.2 | 2.4 | 3.1 | 1.9 | 1.5 | 0.9 | 1.0 | 0.6 | 0.8 | 0.8 | 1.4 |
| Gbp11 | 0.2 | 0.1 | 0.2 | 0.2 | 0.2 | 0.1 | 0.1 | 0.1 | 0.0 | 0.0 | 0.0 | 0.1 |
| Gbp2 | 13.9 | 8.1 | 12.3 | 20.9 | 14.4 | 8.7 | 12.7 | 13.4 | 11.3 | 9.5 | 11.5 | 17.3 |
| Gbp3 | 10.2 | 5.4 | 9.1 | 19.6 | 8.5 | 7.8 | 5.0 | 3.1 | 4.1 | 2.9 | 2.8 | 9.5 |
| Gbp4 | 0.3 | 0.2 | 0.1 | 0.7 | 0.4 | 0.2 | 0.1 | 0.0 | 0.0 | 0.1 | 0.0 | 0.1 |
| Gbp5 | 1.1 | 0.6 | 1.9 | 1.7 | 1.9 | 0.9 | 1.1 | 0.9 | 0.7 | 0.4 | 0.5 | 1.6 |
| Gbp6 | 3.5 | 2.0 | 4.3 | 5.3 | 3.4 | 2.7 | 1.7 | 1.9 | 1.2 | 1.4 | 1.2 | 3.0 |
| Gbp7 | 2.3 | 2.3 | 3.7 | 3.3 | 2.5 | 2.4 | 2.3 | 2.1 | 2.2 | 1.7 | 2.1 | 3.9 |
| Gbp8 | 0.5 | 0.6 | 0.3 | 0.6 | 0.5 | 0.4 | 0.2 | 0.1 | 0.0 | 0.1 | 0.2 | 0.2 |
| Gbp9 | 2.1 | 1.5 | 2.4 | 4.9 | 2.3 | 2.6 | 1.3 | 1.0 | 1.0 | 0.7 | 0.9 | 1.5 |
| Gbx1 | 0.1 | 0.1 | 0.1 | 0.2 | 0.1 | 0.1 | 0.2 | 0.1 | 0.1 | 0.1 | 0.1 | 0.0 |
| Gbx2 | 0.1 | 0.6 | 1.7 | 0.1 | 1.4 | 0.0 | 1.9 | 2.4 | 3.0 | 1.6 | 1.2 | 1.8 |
| Gca | 2.6 | 2.7 | 2.4 | 3.1 | 2.2 | 3.2 | 2.4 | 1.9 | 2.2 | 1.7 | 2.1 | 2.0 |
| Gcat | 2.1 | 2.5 | 2.4 | 1.4 | 1.8 | 2.4 | 3.1 | 3.4 | 3.0 | 1.5 | 2.2 | 1.5 |
| Gcc1 | 9.6 | 9.1 | 8.0 | 10.8 | 7.6 | 10.0 | 8.4 | 9.2 | 9.6 | 8.0 | 9.0 | 8.3 |
| Gcc2 | 10.1 | 12.0 | 12.3 | 10.3 | 9.2 | 10.6 | 8.7 | 9.5 | 8.7 | 9.1 | 10.9 | 10.6 |
| Gcdh | 9.7 | 9.8 | 10.6 | 7.9 | 8.2 | 9.3 | 6.9 | 8.5 | 8.5 | 9.8 | 8.8 | 9.1 |
| Gcfc2 | 1.8 | 1.9 | 1.9 | 2.0 | 1.8 | 2.1 | 1.8 | 1.8 | 1.5 | 1.8 | 2.0 | 2.0 |
| Gcgr | 0.0 | 0.0 | 0.1 | 0.0 | 0.0 | 0.0 | 0.0 | 0.0 | 0.0 | 0.1 | 0.0 | 0.0 |
| Gch1 | 3.3 | 2.1 | 1.8 | 3.2 | 4.3 | 1.9 | 2.4 | 1.1 | 1.2 | 3.3 | 1.9 | 1.5 |
| Gchfr | 0.6 | 0.3 | 0.4 | 0.1 | 0.2 | 0.5 | 0.0 | 0.1 | 0.2 | 0.1 | 0.1 | 0.2 |

| | | | | | | | | | | | | |
|---|---|---|---|---|---|---|---|---|---|---|---|---|
| Gck | 0.0 | 0.1 | 0.1 | 0.0 | 0.0 | 0.1 | 0.1 | 0.0 | 0.0 | 0.1 | 0.0 | 0.1 |
| Gckr | 0.0 | 0.0 | 0.0 | 0.0 | 0.0 | 0.0 | 0.0 | 0.0 | 0.0 | 0.0 | 0.0 | 0.0 |
| Gclc | 19.7 | 14.5 | 11.9 | 25.1 | 18.0 | 23.8 | 12.3 | 9.9 | 7.6 | 13.6 | 13.4 | 10.6 |
| Gclm | 70.7 | 61.0 | 31.8 | 131.1 | 73.6 | 118.1 | 37.0 | 32.9 | 21.3 | 36.4 | 35.2 | 28.0 |
| Gcm1 | 0.1 | 0.1 | 0.1 | 0.1 | 0.1 | 0.2 | 0.1 | 0.0 | 0.0 | 0.0 | 0.1 | 0.0 |
| Gcn1l1 | 14.8 | 16.1 | 14.4 | 14.1 | 15.2 | 15.8 | 14.9 | 15.3 | 15.4 | 14.4 | 12.5 | 12.3 |
| Gcnt1 | 1.0 | 0.4 | 0.4 | 3.5 | 6.2 | 0.5 | 0.7 | 0.7 | 0.7 | 3.6 | 1.5 | 1.6 |
| Gcnt2 | 7.3 | 7.3 | 6.2 | 7.8 | 8.4 | 5.1 | 5.9 | 5.0 | 5.8 | 10.0 | 8.0 | 6.6 |
| Gcnt4 | 0.1 | 0.3 | 1.0 | 0.1 | 0.5 | 0.2 | 0.6 | 1.3 | 1.4 | 1.1 | 1.0 | 1.4 |
| Gcnt7 | 0.3 | 0.2 | 0.4 | 0.6 | 0.4 | 0.3 | 0.3 | 0.3 | 0.1 | 0.2 | 0.2 | 0.3 |
| Gcsh | 41.2 | 30.7 | 25.5 | 45.9 | 35.7 | 39.4 | 33.1 | 31.0 | 31.0 | 32.4 | 37.2 | 32.8 |
| Gda | 4.4 | 4.7 | 5.3 | 3.8 | 4.7 | 2.0 | 4.3 | 5.3 | 4.7 | 5.7 | 4.7 | 7.7 |
| Gdap1 | 0.7 | 0.4 | 0.3 | 0.7 | 0.3 | 0.9 | 0.6 | 0.4 | 0.4 | 0.4 | 0.5 | 0.4 |
| Gdap10 | 0.4 | 0.8 | 0.5 | 0.5 | 0.7 | 0.6 | 0.3 | 0.4 | 0.2 | 0.3 | 0.2 | 0.3 |
| Gdap1l1 | 0.1 | 0.0 | 0.1 | 0.2 | 0.1 | 0.2 | 0.0 | 0.0 | 0.1 | 0.0 | 0.1 | 0.0 |
| Gdap2 | 8.1 | 8.9 | 8.2 | 8.6 | 8.5 | 8.8 | 7.8 | 7.9 | 8.3 | 7.6 | 8.6 | 8.8 |
| Gde1 | 30.5 | 24.4 | 20.9 | 35.2 | 29.8 | 31.2 | 25.9 | 20.7 | 21.5 | 30.9 | 27.3 | 25.1 |
| Gdf1 | 0.1 | 0.3 | 0.4 | 0.1 | 0.1 | 0.1 | 0.1 | 0.1 | 0.1 | 0.1 | 0.1 | 0.1 |
| Gdf10 | 0.0 | 0.0 | 0.4 | 0.0 | 0.0 | 0.0 | 0.1 | 0.7 | 0.1 | 0.0 | 0.0 | 0.0 |
| Gdf11 | 6.0 | 7.7 | 7.1 | 6.6 | 6.7 | 8.0 | 9.1 | 6.4 | 7.2 | 6.7 | 9.0 | 7.8 |
| Gdf15 | 23.4 | 20.7 | 13.3 | 26.1 | 22.5 | 34.2 | 13.3 | 6.3 | 9.9 | 13.1 | 9.8 | 6.3 |
| Gdf2 | 0.0 | 0.0 | 0.0 | 0.0 | 0.0 | 0.0 | 0.0 | 0.1 | 0.0 | 0.0 | 0.0 | 0.0 |
| Gdf3 | 0.0 | 0.0 | 0.0 | 0.1 | 0.1 | 0.1 | 0.1 | 0.0 | 0.0 | 0.1 | 0.0 | 0.1 |
| Gdf5 | 0.1 | 0.0 | 0.0 | 0.0 | 0.1 | 0.1 | 0.0 | 0.0 | 0.0 | 0.0 | 0.0 | 0.1 |
| Gdf6 | 139.2 | 130.3 | 118.4 | 115.5 | 118.3 | 100.8 | 125.4 | 55.7 | 93.4 | 102.9 | 86.6 | 113.5 |
| Gdf7 | 0.0 | 0.0 | 0.0 | 0.0 | 0.1 | 0.0 | 0.0 | 0.1 | 0.1 | 0.0 | 0.1 | 0.2 |
| Gdf9 | 0.4 | 0.5 | 0.4 | 0.7 | 0.4 | 0.4 | 0.6 | 0.8 | 0.5 | 0.4 | 0.5 | 0.7 |
| Gdi1 | 27.2 | 30.6 | 32.3 | 32.7 | 24.6 | 31.9 | 30.6 | 30.8 | 31.0 | 26.4 | 30.1 | 27.8 |
| Gdi2 | 167.0 | 143.1 | 137.7 | 165.5 | 155.3 | 163.5 | 140.2 | 142.7 | 136.6 | 155.0 | 167.7 | 163.0 |
| Gdnf | 0.7 | 0.3 | 0.2 | 0.7 | 0.7 | 0.5 | 1.3 | 0.3 | 0.9 | 0.7 | 0.8 | 0.8 |
| Gdpd1 | 5.9 | 3.4 | 3.1 | 5.4 | 5.5 | 6.2 | 2.5 | 2.7 | 2.2 | 5.5 | 3.9 | 3.9 |
| Gdpd2 | 0.0 | 0.0 | 0.0 | 0.0 | 0.0 | 0.0 | 0.0 | 0.1 | 0.2 | 1.4 | 0.4 | 0.5 |
| Gdpd3 | 0.2 | 0.6 | 0.2 | 0.1 | 0.5 | 0.1 | 0.2 | 0.4 | 0.2 | 0.4 | 0.5 | 0.3 |
| Gdpd4 | 0.0 | 0.0 | 0.1 | 0.1 | 0.0 | 0.0 | 0.0 | 0.0 | 0.0 | 0.1 | 0.0 | 0.1 |
| Gdpd5 | 1.2 | 0.9 | 0.9 | 1.8 | 2.0 | 0.9 | 0.9 | 1.1 | 1.0 | 1.2 | 1.1 | 0.8 |
| Gdpgp1 | 0.9 | 1.1 | 0.9 | 1.4 | 1.0 | 1.2 | 1.2 | 1.1 | 1.0 | 1.0 | 1.2 | 0.8 |
| Gem | 0.6 | 1.0 | 1.9 | 0.6 | 1.1 | 0.7 | 0.7 | 1.5 | 0.7 | 1.1 | 1.0 | 0.5 |
| Gemin2 | 2.5 | 3.2 | 2.6 | 2.3 | 2.7 | 3.0 | 2.6 | 2.3 | 3.2 | 2.3 | 2.8 | 2.6 |
| Gemin4 | 5.4 | 5.4 | 4.4 | 5.2 | 5.3 | 4.8 | 5.3 | 5.1 | 4.3 | 4.8 | 5.4 | 4.4 |
| Gemin5 | 4.2 | 4.1 | 3.5 | 4.1 | 4.3 | 3.8 | 4.0 | 4.2 | 3.6 | 4.1 | 4.1 | 3.9 |
| Gemin6 | 7.8 | 7.5 | 6.2 | 6.9 | 8.1 | 5.5 | 7.3 | 4.9 | 6.6 | 5.6 | 7.2 | 5.1 |
| Gemin7 | 42.8 | 37.5 | 29.7 | 33.1 | 36.6 | 39.9 | 33.7 | 34.8 | 38.2 | 38.2 | 37.2 | 32.5 |
| Gemin8 | 1.9 | 2.8 | 3.0 | 1.7 | 2.5 | 1.9 | 2.1 | 2.4 | 1.8 | 1.9 | 2.1 | 2.0 |
| Gen1 | 0.5 | 1.2 | 1.7 | 0.3 | 1.3 | 0.4 | 1.8 | 1.0 | 1.2 | 0.8 | 1.3 | 1.0 |
| Get4 | 13.0 | 13.1 | 11.0 | 11.2 | 12.7 | 14.2 | 13.3 | 13.0 | 14.3 | 15.9 | 14.0 | 13.7 |
| Gfap | 0.0 | 0.0 | 0.0 | 0.0 | 0.0 | 0.0 | 0.0 | 0.0 | 0.0 | 0.1 | 0.0 | 0.1 |
| Gfer | 10.7 | 9.5 | 11.3 | 9.2 | 9.1 | 10.4 | 10.8 | 9.6 | 9.7 | 12.7 | 11.3 | 11.3 |
| Gfi1 | 0.0 | 0.0 | 0.0 | 0.0 | 0.0 | 0.0 | 0.0 | 0.0 | 0.0 | 0.0 | 0.0 | 0.0 |
| Gfm1 | 13.6 | 13.0 | 12.7 | 13.7 | 13.3 | 13.4 | 14.1 | 14.1 | 12.1 | 12.5 | 14.6 | 12.6 |
| Gfm2 | 7.3 | 7.8 | 7.2 | 6.3 | 7.1 | 6.2 | 5.9 | 7.3 | 6.3 | 6.5 | 7.3 | 6.7 |
| Gfod1 | 0.7 | 0.8 | 1.1 | 0.6 | 0.6 | 0.5 | 1.2 | 1.3 | 1.8 | 2.1 | 1.6 | 1.6 |
| Gfod2 | 2.2 | 2.7 | 2.2 | 2.9 | 2.5 | 2.4 | 2.5 | 2.4 | 2.6 | 3.7 | 2.9 | 2.5 |
| Gfpt1 | 14.6 | 17.2 | 16.5 | 15.5 | 16.8 | 15.6 | 17.5 | 17.7 | 17.9 | 20.4 | 21.1 | 21.1 |
| Gfpt2 | 49.4 | 108.3 | 145.0 | 28.9 | 86.1 | 23.7 | 129.1 | 150.2 | 103.6 | 53.9 | 83.3 | 92.9 |
| Gfra1 | 0.5 | 0.9 | 3.9 | 0.1 | 0.6 | 0.2 | 0.4 | 8.8 | 1.3 | 0.7 | 0.7 | 1.0 |
| Gfra2 | 0.7 | 0.7 | 0.9 | 0.4 | 0.8 | 0.6 | 0.4 | 0.7 | 0.2 | 0.2 | 0.4 | 0.3 |
| Gfra4 | 0.9 | 0.9 | 0.6 | 1.2 | 0.5 | 1.1 | 1.1 | 0.6 | 1.0 | 1.4 | 1.0 | 1.0 |

| | | | | | | | | | | | | |
|---|---|---|---|---|---|---|---|---|---|---|---|---|
| Gga1 | 8.5 | 9.4 | 7.6 | 8.0 | 8.7 | 8.9 | 10.1 | 7.7 | 9.9 | 8.1 | 7.1 | 7.6 |
| Gga2 | 10.4 | 10.6 | 8.6 | 10.0 | 10.7 | 12.3 | 8.6 | 10.4 | 10.1 | 9.8 | 9.8 | 10.0 |
| Gga3 | 2.0 | 2.1 | 2.1 | 2.4 | 2.9 | 2.2 | 2.7 | 1.8 | 2.7 | 1.6 | 2.1 | 1.5 |
| Ggact | 10.9 | 9.8 | 10.0 | 11.6 | 12.3 | 10.4 | 10.0 | 10.8 | 8.9 | 13.1 | 10.3 | 11.1 |
| Ggct | 13.7 | 12.0 | 13.2 | 10.7 | 9.7 | 8.3 | 12.3 | 14.1 | 11.1 | 16.7 | 16.1 | 15.9 |
| Ggcx | 31.8 | 32.9 | 32.9 | 30.2 | 31.1 | 34.7 | 27.8 | 35.4 | 28.4 | 29.6 | 28.0 | 27.9 |
| Ggh | 31.3 | 27.5 | 27.1 | 34.5 | 30.8 | 37.4 | 27.5 | 27.3 | 31.2 | 28.2 | 28.7 | 34.3 |
| Ggn | 0.7 | 0.7 | 0.9 | 0.3 | 0.6 | 0.6 | 0.8 | 0.7 | 0.8 | 0.5 | 0.5 | 0.6 |
| Ggnbp1 | 0.7 | 0.7 | 0.7 | 0.7 | 0.6 | 0.7 | 0.7 | 0.5 | 0.6 | 0.8 | 0.3 | 0.7 |
| Ggnbp2 | 26.4 | 27.9 | 27.7 | 25.7 | 25.9 | 26.0 | 26.5 | 27.9 | 27.0 | 26.1 | 31.2 | 29.0 |
| Ggps1 | 29.3 | 25.8 | 23.4 | 30.6 | 23.6 | 28.6 | 21.5 | 21.0 | 21.6 | 26.3 | 26.3 | 26.9 |
| Ggt1 | 2.4 | 1.1 | 0.7 | 6.0 | 1.9 | 5.2 | 0.2 | 0.3 | 0.0 | 0.1 | 0.2 | 0.1 |
| Ggt5 | 1.5 | 1.9 | 2.8 | 2.5 | 2.9 | 2.3 | 1.5 | 2.5 | 2.5 | 1.2 | 0.9 | 1.3 |
| Ggt6 | 0.1 | 0.0 | 0.1 | 0.1 | 0.0 | 0.1 | 0.0 | 0.1 | 0.1 | 0.0 | 0.0 | 0.0 |
| Ggt7 | 3.7 | 5.4 | 5.1 | 4.4 | 4.9 | 4.5 | 6.1 | 7.3 | 6.3 | 3.9 | 4.3 | 3.5 |
| Ggta1 | 30.8 | 30.5 | 33.7 | 30.7 | 33.8 | 32.6 | 30.2 | 26.4 | 29.4 | 30.7 | 33.3 | 33.0 |
| Ghdc | 9.7 | 8.3 | 6.1 | 9.3 | 7.8 | 9.2 | 7.9 | 6.2 | 8.1 | 7.8 | 7.6 | 6.8 |
| Ghitm | 102.2 | 71.8 | 56.9 | 110.0 | 71.2 | 103.9 | 60.1 | 75.0 | 65.8 | 72.8 | 78.6 | 70.2 |
| Ghr | 199.7 | 160.2 | 121.8 | 192.9 | 138.5 | 210.8 | 124.6 | 92.8 | 118.6 | 78.1 | 111.1 | 128.2 |
| Ghrl | 0.1 | 0.0 | 0.0 | 0.0 | 0.1 | 0.5 | 0.2 | 0.1 | 0.1 | 0.2 | 0.0 | 0.2 |
| Gid4 | 9.3 | 7.0 | 6.6 | 9.7 | 6.4 | 10.7 | 6.0 | 6.0 | 5.9 | 6.5 | 7.3 | 7.1 |
| Gid8 | 11.7 | 11.9 | 13.0 | 11.7 | 9.3 | 11.3 | 11.9 | 12.3 | 10.0 | 11.0 | 12.1 | 12.1 |
| Gigyf1 | 1.4 | 1.5 | 1.9 | 1.4 | 1.6 | 1.8 | 1.7 | 1.6 | 1.6 | 1.4 | 1.3 | 1.2 |
| Gigyf2 | 10.4 | 10.1 | 11.7 | 11.0 | 10.2 | 10.8 | 11.4 | 10.9 | 9.5 | 9.9 | 11.8 | 12.8 |
| Gimap1 | 0.5 | 0.3 | 0.4 | 0.4 | 0.5 | 0.4 | 0.5 | 0.9 | 0.4 | 0.6 | 0.5 | 0.5 |
| Gimap4 | 0.1 | 0.1 | 0.1 | 0.1 | 0.1 | 0.0 | 0.3 | 0.4 | 0.4 | 0.0 | 0.0 | 0.1 |
| Gimap5 | 0.1 | 0.0 | 0.0 | 0.0 | 0.0 | 0.0 | 0.0 | 0.0 | 0.0 | 0.0 | 0.0 | 0.0 |
| Gimap6 | 1.9 | 1.2 | 1.6 | 2.3 | 1.3 | 0.9 | 1.7 | 1.9 | 1.3 | 0.5 | 0.8 | 0.6 |
| Gimap8 | 0.1 | 0.1 | 0.1 | 0.0 | 0.0 | 0.0 | 0.1 | 0.2 | 0.1 | 0.0 | 0.0 | 0.0 |
| Gimap9 | 2.9 | 2.5 | 2.8 | 1.6 | 2.2 | 2.7 | 1.9 | 2.2 | 1.6 | 2.2 | 2.8 | 2.9 |
| Gin1 | 1.6 | 1.9 | 1.9 | 2.2 | 1.6 | 2.2 | 1.8 | 2.3 | 1.3 | 1.5 | 2.4 | 1.7 |
| Ginm1 | 49.2 | 44.6 | 46.3 | 49.9 | 42.7 | 51.7 | 46.3 | 43.1 | 44.3 | 42.6 | 46.5 | 53.7 |
| Gins1 | 0.9 | 1.6 | 1.2 | 1.2 | 2.1 | 0.6 | 2.4 | 1.3 | 1.9 | 1.2 | 1.3 | 0.9 |
| Gins2 | 7.0 | 9.0 | 9.9 | 5.1 | 9.6 | 5.4 | 12.4 | 7.9 | 8.8 | 6.4 | 9.0 | 6.4 |
| Gins3 | 1.7 | 1.7 | 2.6 | 2.1 | 2.4 | 1.7 | 3.1 | 1.9 | 2.0 | 1.6 | 2.3 | 2.2 |
| Gins4 | 14.4 | 17.4 | 23.6 | 17.0 | 16.7 | 14.8 | 19.5 | 25.1 | 22.2 | 19.4 | 21.0 | 20.6 |
| Gipc1 | 30.4 | 27.3 | 23.7 | 32.2 | 29.8 | 31.4 | 31.1 | 26.1 | 30.1 | 28.2 | 29.4 | 25.6 |
| Gipc2 | 0.3 | 0.1 | 0.1 | 0.0 | 0.2 | 0.2 | 0.1 | 0.0 | 0.0 | 0.2 | 0.2 | 0.0 |
| Gipr | 0.0 | 0.0 | 0.1 | 0.0 | 0.1 | 0.0 | 0.0 | 0.0 | 0.0 | 0.0 | 0.0 | 0.0 |
| Git1 | 7.2 | 7.4 | 6.7 | 7.4 | 7.0 | 9.0 | 7.2 | 6.7 | 7.0 | 6.4 | 6.7 | 5.5 |
| Git2 | 5.3 | 5.6 | 6.1 | 5.0 | 6.2 | 5.7 | 6.7 | 6.2 | 5.4 | 5.5 | 6.2 | 5.4 |
| Gja1 | 8.1 | 6.6 | 8.6 | 4.3 | 8.5 | 4.7 | 9.1 | 7.9 | 6.0 | 17.6 | 11.9 | 8.1 |
| Gja3 | 0.3 | 0.2 | 0.2 | 0.3 | 0.1 | 0.5 | 0.1 | 0.1 | 0.0 | 0.0 | 0.1 | 0.1 |
| Gja4 | 0.4 | 1.0 | 1.6 | 0.2 | 0.4 | 0.4 | 0.6 | 1.6 | 0.4 | 0.8 | 0.3 | 1.1 |
| Gja5 | 0.0 | 0.0 | 0.1 | 0.0 | 0.1 | 0.1 | 0.0 | 0.2 | 0.0 | 0.0 | 0.0 | 0.1 |
| Gja6 | 0.0 | 0.0 | 0.0 | 0.0 | 0.0 | 0.0 | 0.1 | 0.0 | 0.0 | 0.0 | 0.1 | 0.1 |
| Gja8 | 0.0 | 0.0 | 0.0 | 0.0 | 0.0 | 0.0 | 0.0 | 0.0 | 0.0 | 0.0 | 0.0 | 0.0 |
| Gjb3 | 3.4 | 3.4 | 3.5 | 0.5 | 2.5 | 1.8 | 5.0 | 5.2 | 2.2 | 6.9 | 6.8 | 5.2 |
| Gjb4 | 1.9 | 2.8 | 3.0 | 1.4 | 1.9 | 1.4 | 3.2 | 4.7 | 2.1 | 3.7 | 4.2 | 3.1 |
| Gjb5 | 9.0 | 5.6 | 6.8 | 3.7 | 6.5 | 2.9 | 7.7 | 6.8 | 4.8 | 12.5 | 10.7 | 10.6 |
| Gjc1 | 3.3 | 3.2 | 4.2 | 2.9 | 3.8 | 3.4 | 5.2 | 4.6 | 4.0 | 3.2 | 4.1 | 3.4 |
| Gjc2 | 1.7 | 1.8 | 1.1 | 1.3 | 1.4 | 1.8 | 1.6 | 1.2 | 1.6 | 1.5 | 1.0 | 1.2 |
| Gjc3 | 0.0 | 0.1 | 0.1 | 0.0 | 0.1 | 0.0 | 0.1 | 0.1 | 0.1 | 0.0 | 0.1 | 0.1 |
| Gk5 | 2.1 | 3.7 | 4.0 | 1.7 | 2.8 | 1.8 | 2.5 | 2.3 | 2.0 | 1.8 | 1.9 | 2.3 |
| Gkap1 | 22.8 | 23.6 | 25.2 | 24.1 | 16.3 | 26.9 | 20.2 | 18.7 | 17.3 | 19.0 | 21.5 | 25.5 |
| Gla | 11.1 | 8.0 | 6.4 | 15.8 | 12.9 | 9.7 | 6.8 | 6.8 | 6.1 | 9.6 | 7.7 | 9.2 |
| Glb1 | 40.7 | 32.4 | 34.1 | 38.0 | 37.4 | 39.6 | 31.4 | 34.9 | 31.2 | 36.4 | 32.5 | 33.0 |

| | | | | | | | | | | | | |
|---|---|---|---|---|---|---|---|---|---|---|---|---|
| Glb1l | 6.3 | 6.0 | 6.5 | 6.8 | 6.1 | 6.5 | 6.2 | 6.6 | 7.1 | 5.9 | 6.0 | 7.3 |
| Glb1l2 | 0.1 | 0.1 | 0.2 | 0.1 | 0.0 | 0.1 | 0.1 | 0.1 | 0.1 | 0.1 | 0.0 | 0.1 |
| Glcci1 | 1.0 | 1.5 | 2.1 | 0.8 | 1.2 | 0.8 | 1.3 | 1.7 | 1.2 | 0.8 | 0.9 | 0.9 |
| Glce | 10.0 | 8.4 | 8.6 | 10.6 | 8.1 | 10.8 | 9.7 | 11.6 | 10.5 | 8.7 | 10.5 | 8.9 |
| Gldc | 6.4 | 6.3 | 4.9 | 6.9 | 3.6 | 4.2 | 2.6 | 4.0 | 2.9 | 2.0 | 1.9 | 1.7 |
| Gldn | 0.1 | 0.0 | 0.0 | 0.3 | 0.0 | 0.1 | 0.0 | 0.0 | 0.0 | 0.0 | 0.0 | 0.1 |
| Gle1 | 12.7 | 11.2 | 11.1 | 14.5 | 12.5 | 11.4 | 12.7 | 11.0 | 10.4 | 11.4 | 12.0 | 11.3 |
| Glg1 | 43.4 | 43.7 | 42.6 | 41.7 | 47.6 | 46.9 | 44.2 | 46.2 | 44.5 | 41.1 | 40.1 | 38.8 |
| Gli1 | 0.0 | 0.0 | 0.0 | 0.0 | 0.0 | 0.0 | 0.0 | 0.1 | 0.1 | 0.0 | 0.0 | 0.0 |
| Gli2 | 0.1 | 0.1 | 0.3 | 0.0 | 0.1 | 0.0 | 0.1 | 0.1 | 0.1 | 0.2 | 0.1 | 0.1 |
| Gli3 | 3.0 | 3.7 | 4.3 | 2.9 | 3.0 | 3.4 | 4.2 | 3.6 | 3.2 | 3.2 | 3.0 | 3.4 |
| Glipr1 | 36.6 | 18.5 | 12.2 | 73.2 | 108.6 | 20.5 | 38.6 | 13.4 | 18.7 | 56.7 | 59.5 | 49.7 |
| Glipr2 | 21.7 | 24.0 | 33.0 | 19.7 | 32.7 | 21.7 | 31.0 | 43.4 | 35.6 | 30.1 | 36.4 | 27.9 |
| Glis1 | 0.1 | 0.2 | 0.2 | 0.0 | 0.0 | 0.1 | 0.2 | 0.2 | 0.2 | 0.1 | 0.2 | 0.1 |
| Glis2 | 4.6 | 4.2 | 4.5 | 3.2 | 4.0 | 4.2 | 5.2 | 8.4 | 5.7 | 4.2 | 5.8 | 3.5 |
| Glis3 | 2.9 | 5.1 | 4.8 | 2.9 | 3.2 | 3.3 | 4.4 | 4.3 | 3.9 | 3.0 | 4.0 | 3.7 |
| Glmn | 3.3 | 3.5 | 3.7 | 3.8 | 3.3 | 3.6 | 3.2 | 4.0 | 3.9 | 3.7 | 3.5 | 3.9 |
| Glo1 | 70.6 | 56.1 | 48.9 | 72.4 | 51.1 | 71.9 | 57.0 | 49.4 | 49.0 | 64.0 | 64.9 | 64.6 |
| Glod4 | 32.5 | 30.8 | 28.4 | 26.7 | 27.0 | 31.7 | 22.8 | 23.4 | 23.7 | 29.5 | 26.7 | 28.7 |
| Glp1r | 0.0 | 0.0 | 0.0 | 0.0 | 0.1 | 0.1 | 0.1 | 0.0 | 0.0 | 0.1 | 0.0 | 0.0 |
| Glp2r | 0.3 | 0.3 | 0.2 | 0.8 | 0.2 | 0.6 | 0.0 | 0.1 | 0.0 | 0.0 | 0.0 | 0.0 |
| Glra1 | 0.0 | 0.1 | 0.0 | 0.1 | 0.0 | 0.1 | 0.1 | 0.0 | 0.0 | 0.0 | 0.0 | 0.0 |
| Glra3 | 0.0 | 0.0 | 0.0 | 0.0 | 0.0 | 0.0 | 0.0 | 0.0 | 0.0 | 0.0 | 0.0 | 0.0 |
| Glrb | 0.3 | 0.2 | 0.3 | 0.2 | 0.1 | 0.6 | 0.1 | 0.2 | 0.0 | 0.2 | 0.4 | 0.3 |
| Glrx | 11.6 | 11.1 | 12.6 | 24.4 | 42.1 | 11.1 | 15.6 | 8.6 | 9.5 | 14.6 | 8.8 | 13.2 |
| Glrx2 | 3.9 | 3.7 | 3.8 | 3.4 | 3.2 | 3.1 | 3.5 | 3.9 | 3.3 | 4.2 | 4.7 | 4.3 |
| Glrx3 | 90.8 | 69.3 | 41.5 | 62.7 | 52.8 | 62.6 | 39.1 | 41.1 | 48.8 | 67.5 | 53.9 | 51.3 |
| Glrx5 | 36.4 | 30.2 | 21.1 | 31.3 | 29.8 | 31.7 | 24.3 | 24.4 | 29.7 | 35.0 | 24.4 | 25.0 |
| Gls | 38.1 | 37.5 | 29.3 | 39.5 | 38.3 | 32.7 | 39.3 | 32.9 | 44.9 | 40.6 | 48.8 | 43.5 |
| Gls2 | 0.1 | 0.1 | 0.1 | 0.2 | 0.3 | 0.2 | 0.3 | 0.3 | 0.2 | 0.2 | 0.1 | 0.1 |
| Glt25d1 | 59.1 | 49.2 | 48.2 | 73.3 | 63.2 | 68.2 | 58.5 | 59.3 | 57.9 | 47.6 | 52.6 | 51.8 |
| Glt25d2 | 0.1 | 0.2 | 0.2 | 0.2 | 0.1 | 0.2 | 0.1 | 0.4 | 0.2 | 0.2 | 0.1 | 0.1 |
| Glt28d2 | 1.1 | 0.8 | 0.9 | 0.8 | 0.9 | 1.1 | 1.0 | 1.1 | 1.0 | 1.3 | 1.0 | 1.0 |
| Glt8d1 | 24.4 | 25.0 | 21.6 | 15.6 | 19.5 | 19.7 | 18.4 | 20.0 | 24.9 | 22.1 | 21.6 | 22.1 |
| Glt8d2 | 12.5 | 9.9 | 8.8 | 11.2 | 10.0 | 11.7 | 7.4 | 4.2 | 6.5 | 4.4 | 6.2 | 5.8 |
| Gltp | 13.9 | 13.5 | 13.3 | 17.4 | 22.0 | 14.1 | 15.0 | 14.7 | 13.1 | 11.3 | 15.7 | 10.4 |
| Gltpd1 | 5.8 | 5.7 | 4.5 | 6.0 | 5.8 | 5.3 | 6.2 | 4.5 | 5.4 | 5.3 | 6.0 | 3.8 |
| Gltpd2 | 0.7 | 0.4 | 0.3 | 0.4 | 0.2 | 0.6 | 0.7 | 0.8 | 0.4 | 0.3 | 0.3 | 0.6 |
| Gltscr1 | 0.2 | 0.3 | 0.3 | 0.3 | 0.3 | 0.4 | 0.4 | 0.4 | 0.5 | 0.3 | 0.2 | 0.2 |
| Gltscr1l | 1.7 | 2.5 | 2.9 | 2.0 | 2.2 | 2.4 | 2.2 | 2.3 | 2.2 | 1.7 | 2.0 | 2.0 |
| Gltscr2 | 52.2 | 46.3 | 54.8 | 37.1 | 43.5 | 43.4 | 48.2 | 57.2 | 51.1 | 50.7 | 56.7 | 56.1 |
| Glud1 | 77.7 | 65.7 | 50.5 | 73.4 | 67.1 | 70.4 | 65.1 | 59.7 | 61.1 | 66.9 | 72.7 | 62.5 |
| Glul | 15.3 | 30.9 | 57.6 | 17.7 | 28.8 | 16.9 | 28.7 | 44.2 | 24.8 | 39.7 | 28.3 | 35.2 |
| Glyat | 0.2 | 0.0 | 0.0 | 0.0 | 0.1 | 0.1 | 0.0 | 0.0 | 0.0 | 0.0 | 0.0 | 0.0 |
| Glyctk | 0.3 | 0.3 | 0.2 | 0.6 | 0.4 | 0.4 | 0.4 | 0.3 | 0.4 | 0.2 | 0.4 | 0.3 |
| Glyr1 | 30.2 | 33.9 | 32.8 | 33.2 | 31.6 | 31.9 | 31.5 | 31.7 | 30.2 | 30.2 | 32.9 | 32.1 |
| Gm10012 | 3.4 | 2.9 | 3.7 | 4.4 | 4.9 | 5.1 | 3.5 | 3.6 | 3.6 | 6.2 | 2.9 | 4.6 |
| Gm10033 | 0.6 | 1.8 | 2.2 | 1.2 | 1.2 | 1.2 | 0.9 | 1.8 | 1.2 | 1.3 | 2.3 | 1.3 |
| Gm10046 | 0.2 | 0.4 | 0.4 | 0.6 | 0.3 | 0.3 | 0.2 | 0.3 | 0.4 | 0.1 | 0.5 | 0.3 |
| Gm10052 | 40.4 | 35.2 | 47.9 | 42.7 | 48.4 | 34.7 | 61.1 | 66.2 | 56.4 | 52.8 | 60.1 | 52.5 |
| Gm10069 | 0.3 | 0.4 | 0.4 | 0.2 | 0.4 | 0.3 | 0.3 | 0.3 | 0.4 | 0.2 | 0.4 | 0.3 |
| Gm10094 | 314.4 | 221.1 | 172.7 | 294.1 | 178.5 | 259.7 | 170.7 | 115.7 | 146.4 | 266.1 | 247.9 | 194.6 |
| Gm101 | 0.0 | 0.0 | 0.0 | 0.0 | 0.0 | 0.0 | 0.0 | 0.0 | 0.0 | 0.0 | 0.0 | 0.0 |
| Gm10125 | 0.1 | 0.0 | 0.1 | 0.1 | 0.1 | 0.0 | 0.1 | 0.1 | 0.1 | 0.1 | 0.1 | 0.1 |
| Gm10190 | 0.2 | 0.1 | 0.0 | 0.1 | 0.1 | 0.1 | 0.0 | 0.1 | 0.2 | 0.1 | 0.1 | 0.1 |
| Gm10220 | 0.1 | 0.2 | 0.2 | 0.0 | 0.2 | 0.2 | 0.1 | 0.1 | 0.1 | 0.3 | 0.1 | 0.1 |
| Gm10336 | 1.3 | 1.7 | 1.9 | 1.6 | 1.7 | 1.8 | 1.4 | 1.6 | 1.5 | 1.8 | 2.0 | 1.7 |

| | | | | | | | | | | | | |
|---|---|---|---|---|---|---|---|---|---|---|---|---|
| Gm10364 | 0.0 | 0.0 | 0.0 | 0.2 | 0.0 | 0.0 | 0.1 | 0.0 | 0.0 | 0.0 | 0.0 | 0.0 |
| Gm10389 | 0.3 | 0.5 | 0.4 | 0.3 | 0.4 | 0.3 | 0.4 | 0.3 | 0.2 | 0.4 | 0.3 | 0.3 |
| Gm10390 | 0.1 | 0.3 | 0.3 | 0.4 | 0.3 | 0.3 | 0.2 | 0.3 | 0.2 | 0.3 | 0.4 | 0.5 |
| Gm10400 | 0.0 | 0.1 | 0.1 | 0.0 | 0.1 | 0.0 | 0.1 | 0.1 | 0.0 | 0.1 | 0.1 | 0.1 |
| Gm10406 | 0.3 | 0.5 | 0.5 | 0.2 | 0.2 | 0.3 | 0.4 | 0.4 | 0.5 | 0.3 | 0.4 | 0.4 |
| Gm10408 | 0.1 | 0.1 | 0.1 | 0.1 | 0.1 | 0.1 | 0.0 | 0.1 | 0.1 | 0.0 | 0.1 | 0.0 |
| Gm10409 | 0.2 | 0.3 | 0.3 | 0.2 | 0.3 | 0.5 | 0.3 | 0.3 | 0.4 | 0.3 | 0.2 | 0.2 |
| Gm10413 | 0.0 | 0.0 | 0.0 | 0.0 | 0.0 | 0.0 | 0.0 | 0.0 | 0.0 | 0.0 | 0.0 | 0.0 |
| Gm10416 | 0.4 | 0.8 | 0.5 | 0.1 | 0.0 | 0.6 | 0.4 | 0.3 | 0.4 | 0.5 | 0.5 | 0.4 |
| Gm10432 | 0.0 | 0.0 | 0.0 | 0.0 | 0.0 | 0.0 | 0.0 | 0.0 | 0.0 | 0.0 | 0.0 | 0.0 |
| Gm10433 | 0.0 | 0.1 | 0.0 | 0.0 | 0.0 | 0.0 | 0.1 | 0.0 | 0.1 | 0.0 | 0.0 | 0.0 |
| Gm1045 | 0.0 | 0.1 | 0.0 | 0.0 | 0.0 | 0.1 | 0.0 | 0.0 | 0.1 | 0.1 | 0.2 | 0.0 |
| Gm10451 | 2.8 | 1.7 | 1.4 | 2.0 | 1.7 | 1.8 | 0.8 | 1.3 | 1.1 | 0.9 | 1.8 | 1.2 |
| Gm10471 | 0.3 | 0.3 | 0.4 | 0.2 | 0.3 | 0.2 | 0.1 | 0.1 | 0.1 | 0.4 | 0.3 | 0.1 |
| Gm10474 | 0.0 | 0.1 | 0.0 | 0.1 | 0.0 | 0.0 | 0.0 | 0.0 | 0.0 | 0.0 | 0.0 | 0.0 |
| Gm10485 | 0.1 | 0.0 | 0.0 | 0.1 | 0.0 | 0.0 | 0.0 | 0.1 | 0.1 | 0.0 | 0.1 | 0.1 |
| Gm10509 | 1.9 | 2.8 | 2.3 | 2.0 | 2.3 | 3.1 | 1.7 | 2.8 | 2.3 | 2.8 | 2.8 | 2.8 |
| Gm10510 | 0.0 | 0.0 | 0.0 | 0.0 | 0.0 | 0.0 | 0.0 | 0.0 | 0.0 | 0.0 | 0.0 | 0.0 |
| Gm10512 | 0.0 | 0.0 | 0.0 | 0.0 | 0.0 | 0.0 | 0.0 | 0.0 | 0.0 | 0.1 | 0.1 | 0.0 |
| Gm10516 | 1.4 | 1.1 | 1.7 | 2.0 | 0.9 | 1.8 | 0.9 | 1.3 | 1.2 | 1.2 | 1.3 | 1.5 |
| Gm10532 | 0.8 | 1.2 | 1.3 | 1.0 | 0.9 | 1.2 | 1.8 | 1.4 | 1.3 | 1.1 | 1.2 | 1.1 |
| Gm10536 | 0.0 | 0.0 | 0.1 | 0.0 | 0.1 | 0.1 | 0.0 | 0.0 | 0.0 | 0.0 | 0.0 | 0.1 |
| Gm10538 | 0.0 | 0.1 | 0.0 | 0.0 | 0.0 | 0.0 | 0.0 | 0.0 | 0.0 | 0.0 | 0.0 | 0.0 |
| Gm10548 | 0.2 | 0.1 | 0.2 | 0.2 | 0.1 | 0.1 | 0.2 | 0.1 | 0.1 | 0.1 | 0.1 | 0.2 |
| Gm10560 | 0.3 | 0.7 | 0.8 | 0.3 | 0.5 | 0.8 | 0.5 | 0.7 | 0.4 | 0.5 | 0.2 | 0.3 |
| Gm10578 | 0.1 | 0.1 | 0.1 | 0.1 | 0.0 | 0.0 | 0.1 | 0.1 | 0.0 | 0.0 | 0.0 | 0.0 |
| Gm10584 | 0.0 | 0.0 | 0.0 | 0.0 | 0.0 | 0.0 | 0.0 | 0.0 | 0.1 | 0.1 | 0.1 | 0.1 |
| Gm10635 | 0.0 | 0.0 | 0.0 | 0.0 | 0.0 | 0.0 | 0.0 | 0.0 | 0.0 | 0.0 | 0.0 | 0.0 |
| Gm10636 | 0.0 | 0.0 | 0.0 | 0.0 | 0.0 | 0.0 | 0.0 | 0.0 | 0.0 | 0.0 | 0.0 | 0.0 |
| Gm10638 | 1.3 | 0.8 | 0.9 | 0.5 | 0.9 | 0.8 | 0.9 | 0.9 | 1.5 | 1.0 | 0.8 | 1.2 |
| Gm10639 | 0.4 | 0.2 | 0.0 | 0.3 | 0.2 | 0.4 | 0.0 | 0.1 | 0.0 | 0.0 | 0.0 | 0.1 |
| Gm10640 | 0.1 | 0.1 | 0.1 | 0.4 | 0.1 | 0.1 | 0.1 | 0.1 | 0.1 | 0.0 | 0.1 | 0.1 |
| Gm10653 | 2.1 | 1.9 | 2.0 | 2.1 | 1.8 | 2.0 | 2.0 | 2.2 | 1.7 | 1.8 | 2.0 | 2.1 |
| Gm10658 | 0.1 | 0.3 | 0.2 | 0.2 | 0.0 | 0.2 | 0.2 | 0.1 | 0.2 | 0.1 | 0.3 | 0.2 |
| Gm10677 | 0.0 | 0.0 | 0.0 | 0.2 | 0.1 | 0.0 | 0.0 | 0.0 | 0.0 | 0.0 | 0.0 | 0.0 |
| Gm10681 | 0.1 | 0.0 | 0.0 | 0.1 | 0.0 | 0.0 | 0.0 | 0.0 | 0.0 | 0.0 | 0.0 | 0.0 |
| Gm10684 | 0.1 | 0.1 | 0.0 | 0.0 | 0.0 | 0.1 | 0.0 | 0.0 | 0.0 | 0.1 | 0.0 | 0.0 |
| Gm10731 | 0.0 | 0.0 | 0.0 | 0.0 | 0.0 | 0.0 | 0.0 | 0.0 | 0.0 | 0.0 | 0.1 | 0.0 |
| Gm10754 | 0.0 | 0.0 | 0.1 | 0.0 | 0.0 | 0.0 | 0.0 | 0.0 | 0.0 | 0.0 | 0.0 | 0.0 |
| Gm10767 | 0.9 | 1.0 | 1.2 | 1.5 | 0.8 | 1.0 | 0.6 | 0.8 | 0.6 | 0.7 | 0.8 | 1.5 |
| Gm10768 | 0.0 | 0.0 | 0.0 | 0.0 | 0.0 | 0.0 | 0.0 | 0.0 | 0.0 | 0.0 | 0.0 | 0.0 |
| Gm10778 | 0.3 | 0.5 | 0.4 | 0.4 | 0.4 | 0.4 | 0.3 | 0.4 | 0.4 | 0.4 | 0.3 | 0.5 |
| Gm1078 | 0.2 | 0.2 | 0.1 | 0.0 | 0.1 | 0.1 | 0.0 | 0.1 | 0.1 | 0.1 | 0.1 | 0.1 |
| Gm10785 | 0.4 | 0.6 | 0.8 | 1.0 | 0.4 | 0.7 | 0.5 | 0.6 | 0.4 | 0.5 | 0.6 | 0.5 |
| Gm10790 | 0.1 | 0.1 | 0.3 | 0.0 | 0.2 | 0.2 | 0.0 | 0.1 | 0.1 | 0.0 | 0.1 | 0.1 |
| Gm10791 | 0.1 | 0.2 | 0.2 | 0.2 | 0.3 | 0.2 | 0.4 | 0.3 | 0.2 | 0.3 | 0.4 | 0.1 |
| Gm10804 | 0.1 | 0.2 | 0.0 | 0.3 | 0.2 | 0.1 | 0.0 | 0.2 | 0.0 | 0.2 | 0.2 | 0.0 |
| Gm10814 | 0.3 | 0.3 | 0.4 | 0.2 | 0.3 | 0.4 | 0.2 | 0.1 | 0.3 | 0.2 | 0.2 | 0.3 |
| Gm10818 | 0.1 | 0.1 | 0.1 | 0.4 | 0.2 | 0.0 | 0.1 | 0.3 | 0.1 | 0.0 | 0.2 | 0.2 |
| Gm10825 | 0.2 | 0.2 | 0.2 | 0.1 | 0.1 | 0.3 | 0.1 | 0.1 | 0.1 | 0.1 | 0.1 | 0.1 |
| Gm10845 | 5.1 | 5.9 | 5.9 | 7.1 | 5.3 | 5.5 | 5.9 | 5.2 | 4.1 | 3.8 | 5.3 | 6.2 |
| Gm10865 | 0.3 | 0.9 | 0.1 | 1.1 | 0.2 | 0.5 | 0.2 | 0.3 | 0.6 | 0.2 | 0.3 | 0.5 |
| Gm10872 | 0.0 | 0.1 | 0.0 | 0.0 | 0.0 | 0.0 | 0.0 | 0.0 | 0.0 | 0.0 | 0.0 | 0.0 |
| Gm10941 | 0.2 | 0.2 | 0.6 | 0.9 | 0.3 | 0.5 | 0.2 | 0.7 | 0.3 | 0.1 | 0.0 | 0.4 |
| Gm11110 | 1.1 | 1.6 | 0.9 | 1.0 | 1.3 | 1.7 | 0.8 | 0.7 | 0.9 | 0.6 | 0.8 | 0.9 |
| Gm11127 | 3.9 | 3.1 | 2.7 | 4.2 | 3.7 | 3.1 | 1.9 | 2.1 | 2.1 | 1.9 | 1.5 | 1.8 |
| Gm11128 | 0.0 | 0.0 | 0.1 | 0.0 | 0.0 | 0.0 | 0.0 | 0.0 | 0.0 | 0.0 | 0.0 | 0.0 |

| | | | | | | | | | | | | |
|---|---|---|---|---|---|---|---|---|---|---|---|---|
| Gm11149 | 0.6 | 0.2 | 0.3 | 0.3 | 0.3 | 0.2 | 0.7 | 0.1 | 0.8 | 0.9 | 1.0 | 1.0 |
| Gm11166 | 0.0 | 0.0 | 0.0 | 0.0 | 0.0 | 0.0 | 0.0 | 0.0 | 0.0 | 0.0 | 0.0 | 0.0 |
| Gm11201 | 0.2 | 0.3 | 0.4 | 0.3 | 0.4 | 0.3 | 0.4 | 0.3 | 0.1 | 0.3 | 0.2 | 0.3 |
| Gm11202 | 0.0 | 0.0 | 0.0 | 0.0 | 0.0 | 0.0 | 0.0 | 0.0 | 0.1 | 0.0 | 0.0 | 0.0 |
| Gm1123 | 0.0 | 0.0 | 0.0 | 0.0 | 0.1 | 0.0 | 0.0 | 0.0 | 0.0 | 0.1 | 0.0 | 0.0 |
| Gm11346 | 0.0 | 0.0 | 0.0 | 0.0 | 0.0 | 0.2 | 0.0 | 0.0 | 0.0 | 0.0 | 0.0 | 0.0 |
| Gm11351 | 4.5 | 1.0 | 0.3 | 0.9 | 0.6 | 0.6 | 0.2 | 0.7 | 0.2 | 0.9 | 0.4 | 0.4 |
| Gm1141 | 0.0 | 0.0 | 0.0 | 0.1 | 0.1 | 0.0 | 0.0 | 0.0 | 0.0 | 0.1 | 0.1 | 0.1 |
| Gm11413 | 0.5 | 0.1 | 0.4 | 0.3 | 0.5 | 0.1 | 0.5 | 0.3 | 0.1 | 0.1 | 0.3 | 0.3 |
| Gm11435 | 0.0 | 0.0 | 0.0 | 0.1 | 0.0 | 0.2 | 0.0 | 0.0 | 0.0 | 0.0 | 0.0 | 0.0 |
| Gm11517 | 0.3 | 0.9 | 0.7 | 0.3 | 0.5 | 0.8 | 1.4 | 0.8 | 0.6 | 0.7 | 0.5 | 0.7 |
| Gm11545 | 0.0 | 0.0 | 0.0 | 0.1 | 0.5 | 0.0 | 0.1 | 0.0 | 0.0 | 0.0 | 0.0 | 0.0 |
| Gm11602 | 0.5 | 0.8 | 0.5 | 0.4 | 0.5 | 0.3 | 0.4 | 0.4 | 0.4 | 0.3 | 0.6 | 0.4 |
| Gm11627 | 2.9 | 2.8 | 2.6 | 2.0 | 2.0 | 1.5 | 1.5 | 2.4 | 2.4 | 1.4 | 2.7 | 2.9 |
| Gm11648 | 0.0 | 0.0 | 0.1 | 0.0 | 0.1 | 0.0 | 0.1 | 0.0 | 0.0 | 0.1 | 0.0 | 0.0 |
| Gm11696 | 0.1 | 0.2 | 0.2 | 0.2 | 0.3 | 0.2 | 0.1 | 0.2 | 0.1 | 0.1 | 0.2 | 0.1 |
| Gm11711 | 0.0 | 0.0 | 0.0 | 0.4 | 0.4 | 0.0 | 0.1 | 0.0 | 0.0 | 0.0 | 0.0 | 0.0 |
| Gm11744 | 1.8 | 2.7 | 1.4 | 1.7 | 2.0 | 2.2 | 1.7 | 2.2 | 2.2 | 1.4 | 1.1 | 1.8 |
| Gm11747 | 0.4 | 0.4 | 0.7 | 0.2 | 0.6 | 0.1 | 0.3 | 1.2 | 0.7 | 1.2 | 0.4 | 1.0 |
| Gm11837 | 0.2 | 0.2 | 0.5 | 0.1 | 0.1 | 0.1 | 0.1 | 0.3 | 0.3 | 0.1 | 0.0 | 0.1 |
| Gm11944 | 0.0 | 0.1 | 0.0 | 0.1 | 0.0 | 0.0 | 0.2 | 0.2 | 0.1 | 0.1 | 0.3 | 0.2 |
| Gm11974 | 11.7 | 10.3 | 7.5 | 13.4 | 9.6 | 10.1 | 7.3 | 8.3 | 7.6 | 11.1 | 9.0 | 7.4 |
| Gm11992 | 0.1 | 0.1 | 0.0 | 0.2 | 0.0 | 0.0 | 0.1 | 0.0 | 0.1 | 0.0 | 0.1 | 0.0 |
| Gm12060 | 0.1 | 0.1 | 0.0 | 0.1 | 0.0 | 0.3 | 0.0 | 0.1 | 0.0 | 0.1 | 0.1 | 0.2 |
| Gm12070 | 38.0 | 33.6 | 30.4 | 53.9 | 42.5 | 41.3 | 41.6 | 37.8 | 28.8 | 33.7 | 38.1 | 38.4 |
| Gm12185 | 1.1 | 1.0 | 0.6 | 1.2 | 0.9 | 0.9 | 0.8 | 0.3 | 0.5 | 0.5 | 0.6 | 0.6 |
| Gm12191 | 753.5 | 615.6 | 603.1 | 613.9 | 555.3 | 716.7 | 532.0 | 655.8 | 582.2 | 620.8 | 650.9 | 680.5 |
| Gm12216 | 0.1 | 0.1 | 0.1 | 0.1 | 0.1 | 0.0 | 0.0 | 0.0 | 0.0 | 0.1 | 0.1 | 0.2 |
| Gm12238 | 0.0 | 3.5 | 4.8 | 11.3 | 4.9 | 0.0 | 0.0 | 0.0 | 5.9 | 3.3 | 0.0 | 0.0 |
| Gm12250 | 0.4 | 0.1 | 0.2 | 0.9 | 0.3 | 0.2 | 0.1 | 0.0 | 0.1 | 0.0 | 0.1 | 0.3 |
| Gm12295 | 0.0 | 0.0 | 0.0 | 0.0 | 0.0 | 0.0 | 0.0 | 0.0 | 0.0 | 0.0 | 0.0 | 0.0 |
| Gm12359 | 0.4 | 0.8 | 0.7 | 0.7 | 0.6 | 0.8 | 0.9 | 0.9 | 0.5 | 0.4 | 0.6 | 0.6 |
| Gm12504 | 0.3 | 0.4 | 0.6 | 0.4 | 0.5 | 0.3 | 0.5 | 0.5 | 0.4 | 0.5 | 0.4 | 0.6 |
| Gm12505 | 0.8 | 1.1 | 1.2 | 1.0 | 1.2 | 0.8 | 1.7 | 0.2 | 1.2 | 3.4 | 0.6 | 2.1 |
| Gm12522 | 0.0 | 0.2 | 0.3 | 0.0 | 0.1 | 0.2 | 0.0 | 0.1 | 0.1 | 0.1 | 0.2 | 0.1 |
| Gm12530 | 0.0 | 0.1 | 0.0 | 0.0 | 0.1 | 0.1 | 0.0 | 0.0 | 0.1 | 0.0 | 0.0 | 0.0 |
| Gm12603 | 0.9 | 0.3 | 0.2 | 0.4 | 0.0 | 1.6 | 0.6 | 0.1 | 0.3 | 0.2 | 0.7 | 0.3 |
| Gm12657 | 5.6 | 5.2 | 5.3 | 4.5 | 6.5 | 5.4 | 4.2 | 6.0 | 5.9 | 7.3 | 5.5 | 6.7 |
| Gm12669 | 114.8 | 92.0 | 100.9 | 122.0 | 93.2 | 131.7 | 93.2 | 98.4 | 78.5 | 108.4 | 127.4 | 105.5 |
| Gm128 | 0.1 | 0.3 | 0.2 | 0.1 | 0.1 | 0.3 | 0.2 | 0.3 | 0.3 | 0.2 | 0.2 | 0.2 |
| Gm129 | 2.3 | 2.0 | 1.9 | 2.4 | 1.5 | 3.0 | 1.7 | 2.1 | 1.5 | 1.7 | 1.6 | 1.3 |
| Gm12942 | 5.6 | 7.0 | 5.4 | 8.2 | 5.0 | 8.8 | 5.3 | 6.5 | 5.3 | 5.8 | 6.5 | 5.4 |
| Gm13032 | 0.0 | 0.1 | 0.0 | 0.0 | 0.0 | 0.1 | 0.0 | 0.0 | 0.0 | 0.0 | 0.0 | 0.0 |
| Gm13034 | 0.2 | 0.5 | 0.3 | 0.5 | 0.4 | 0.4 | 0.6 | 0.3 | 0.4 | 0.4 | 0.3 | 0.3 |
| Gm13051 | 0.1 | 0.0 | 0.0 | 0.0 | 0.0 | 0.0 | 0.0 | 0.1 | 0.0 | 0.0 | 0.0 | 0.0 |
| Gm13139 | 9.3 | 11.9 | 14.1 | 13.1 | 14.3 | 15.5 | 9.0 | 11.1 | 10.7 | 11.2 | 16.8 | 14.6 |
| Gm13152 | 8.0 | 10.2 | 10.2 | 9.7 | 7.2 | 11.5 | 5.7 | 6.8 | 5.6 | 6.5 | 8.1 | 9.7 |
| Gm13154 | 0.2 | 0.2 | 0.2 | 0.5 | 0.4 | 0.4 | 0.2 | 0.2 | 0.2 | 0.3 | 0.3 | 0.3 |
| Gm13157 | 10.4 | 11.7 | 14.7 | 13.9 | 13.6 | 16.2 | 8.4 | 10.5 | 9.7 | 13.4 | 13.9 | 14.3 |
| Gm13212 | 6.9 | 8.9 | 10.7 | 9.3 | 10.5 | 11.7 | 5.9 | 7.8 | 6.6 | 9.9 | 10.6 | 10.6 |
| Gm13238 | 0.9 | 1.2 | 1.2 | 1.0 | 0.7 | 1.5 | 0.7 | 0.9 | 0.6 | 1.3 | 1.1 | 1.1 |
| Gm13242 | 0.2 | 0.2 | 0.1 | 0.2 | 0.1 | 0.2 | 0.1 | 0.1 | 0.1 | 0.1 | 0.1 | 0.1 |
| Gm13247 | 0.1 | 0.1 | 0.3 | 0.1 | 0.2 | 0.3 | 0.1 | 0.2 | 0.1 | 0.1 | 0.2 | 0.1 |
| Gm13251 | 3.6 | 4.8 | 4.9 | 5.2 | 4.2 | 6.4 | 3.9 | 4.6 | 3.3 | 3.6 | 5.6 | 4.6 |
| Gm13271 | 0.1 | 0.4 | 0.1 | 0.1 | 0.1 | 0.3 | 0.4 | 0.5 | 0.4 | 0.2 | 0.3 | 0.2 |
| Gm13272 | 0.1 | 0.0 | 0.2 | 0.0 | 0.2 | 0.3 | 0.2 | 0.1 | 0.3 | 0.1 | 0.4 | 0.0 |
| Gm13275 | 0.2 | 0.1 | 0.1 | 0.1 | 0.3 | 0.4 | 0.2 | 0.3 | 0.2 | 0.2 | 0.1 | 0.3 |

| | | | | | | | | | | | | |
|---|---|---|---|---|---|---|---|---|---|---|---|---|
| Gm13276 | 0.2 | 0.1 | 0.1 | 0.1 | 0.2 | 0.2 | 0.3 | 0.2 | 0.1 | 0.2 | 0.2 | 0.2 |
| Gm13277 | 0.2 | 0.2 | 0.1 | 0.1 | 0.2 | 0.1 | 0.3 | 0.2 | 0.2 | 0.1 | 0.2 | 0.2 |
| Gm13278 | 0.1 | 0.1 | 0.1 | 0.1 | 0.2 | 0.2 | 0.2 | 0.2 | 0.2 | 0.2 | 0.2 | 0.2 |
| Gm13279 | 0.0 | 0.0 | 0.0 | 0.0 | 0.0 | 0.0 | 0.0 | 0.0 | 0.0 | 0.0 | 0.0 | 0.0 |
| Gm13283 | 0.2 | 0.2 | 0.2 | 0.0 | 0.2 | 0.4 | 0.3 | 0.2 | 0.1 | 0.5 | 0.1 | 0.3 |
| Gm13285 | 0.0 | 0.0 | 0.0 | 0.0 | 0.0 | 0.0 | 0.0 | 0.0 | 0.0 | 0.0 | 0.1 | 0.0 |
| Gm13286 | 0.0 | 0.1 | 0.1 | 0.0 | 0.1 | 0.0 | 0.0 | 0.1 | 0.0 | 0.1 | 0.0 | 0.1 |
| Gm13288 | 0.1 | 0.2 | 0.2 | 0.1 | 0.1 | 0.2 | 0.2 | 0.1 | 0.2 | 0.1 | 0.1 | 0.2 |
| Gm13289 | 0.1 | 0.0 | 0.1 | 0.1 | 0.1 | 0.1 | 0.2 | 0.1 | 0.1 | 0.1 | 0.1 | 0.0 |
| Gm13290 | 0.0 | 0.1 | 0.0 | 0.1 | 0.1 | 0.1 | 0.1 | 0.1 | 0.1 | 0.1 | 0.1 | 0.0 |
| Gm13293 | 2.1 | 1.1 | 1.2 | 1.1 | 1.1 | 0.9 | 1.5 | 2.0 | 1.3 | 1.9 | 3.1 | 1.9 |
| Gm13298 | 0.6 | 1.0 | 1.2 | 0.4 | 0.4 | 0.4 | 0.5 | 0.7 | 0.3 | 0.3 | 0.4 | 0.5 |
| Gm13305 | 4.7 | 5.5 | 6.4 | 4.5 | 3.9 | 3.8 | 4.1 | 6.3 | 4.4 | 3.7 | 3.1 | 4.1 |
| Gm13306 | 0.0 | 0.0 | 0.0 | 0.0 | 0.0 | 0.0 | 0.0 | 0.0 | 0.0 | 0.0 | 0.0 | 0.0 |
| Gm13308 | 0.8 | 0.2 | 0.4 | 0.4 | 0.2 | 0.9 | 0.5 | 1.6 | 0.3 | 0.3 | 0.0 | 0.1 |
| Gm13315 | 0.0 | 0.0 | 0.0 | 0.0 | 0.0 | 0.0 | 0.0 | 0.0 | 0.0 | 0.0 | 0.0 | 0.1 |
| Gm13363 | 41.1 | 43.0 | 39.0 | 50.4 | 47.8 | 57.0 | 41.1 | 35.3 | 34.3 | 39.5 | 44.0 | 41.1 |
| Gm13375 | 2.2 | 2.1 | 1.7 | 2.2 | 1.3 | 1.8 | 2.1 | 1.9 | 1.6 | 1.5 | 2.0 | 1.4 |
| Gm13399 | 0.2 | 0.3 | 0.3 | 0.3 | 0.3 | 0.2 | 0.2 | 0.3 | 0.2 | 0.2 | 0.1 | 0.2 |
| Gm13446 | 0.3 | 0.2 | 0.3 | 0.3 | 0.3 | 0.6 | 0.4 | 0.2 | 0.3 | 0.3 | 0.3 | 0.2 |
| Gm13476 | 3.7 | 4.1 | 4.6 | 5.1 | 5.5 | 3.9 | 4.2 | 6.3 | 3.3 | 2.7 | 3.6 | 4.7 |
| Gm13483 | 0.6 | 0.7 | 0.6 | 0.5 | 0.6 | 0.4 | 0.4 | 0.5 | 0.7 | 0.7 | 0.8 | 0.6 |
| Gm13497 | 0.0 | 0.0 | 0.0 | 0.0 | 0.0 | 0.0 | 0.0 | 0.0 | 0.0 | 0.0 | 0.0 | 0.0 |
| Gm13498 | 0.2 | 0.3 | 0.2 | 0.4 | 0.3 | 0.2 | 0.2 | 0.1 | 0.2 | 0.3 | 0.3 | 0.2 |
| Gm13547 | 1.9 | 1.2 | 3.0 | 0.6 | 1.6 | 1.8 | 3.4 | 2.0 | 1.9 | 1.2 | 0.9 | 1.2 |
| Gm13582 | 0.1 | 0.2 | 0.3 | 0.1 | 0.2 | 0.2 | 0.2 | 0.1 | 0.2 | 0.1 | 0.2 | 0.1 |
| Gm13704 | 0.2 | 0.3 | 0.2 | 0.2 | 0.2 | 0.1 | 0.3 | 0.2 | 0.2 | 0.3 | 0.1 | 0.3 |
| Gm13710 | 0.1 | 0.1 | 0.4 | 0.2 | 0.3 | 0.3 | 0.1 | 0.0 | 0.1 | 0.2 | 0.0 | 0.1 |
| Gm13718 | 0.0 | 0.0 | 0.1 | 0.0 | 0.0 | 0.0 | 0.1 | 0.1 | 0.0 | 0.0 | 0.1 | 0.1 |
| Gm13749 | 0.1 | 0.1 | 0.3 | 0.0 | 0.1 | 0.1 | 0.1 | 0.2 | 0.1 | 0.1 | 0.1 | 0.2 |
| Gm13807 | 0.1 | 0.0 | 0.3 | 0.2 | 0.3 | 0.1 | 0.1 | 0.1 | 0.1 | 0.1 | 0.1 | 0.1 |
| Gm13826 | 8.9 | 7.7 | 6.8 | 6.2 | 8.2 | 7.4 | 6.6 | 8.1 | 8.0 | 8.2 | 7.7 | 8.0 |
| Gm13845 | 0.8 | 1.1 | 1.1 | 0.4 | 0.6 | 0.6 | 0.7 | 0.8 | 0.7 | 0.5 | 0.8 | 0.5 |
| Gm13889 | 0.3 | 0.6 | 0.6 | 0.3 | 1.1 | 0.1 | 0.6 | 0.4 | 0.6 | 0.2 | 0.4 | 0.3 |
| Gm13939 | 0.4 | 0.4 | 0.2 | 0.2 | 0.2 | 0.2 | 0.2 | 0.1 | 0.2 | 0.3 | 0.0 | 0.1 |
| Gm14005 | 19.2 | 18.3 | 12.7 | 23.2 | 24.4 | 14.6 | 24.1 | 17.4 | 19.1 | 17.0 | 25.0 | 16.8 |
| Gm14023 | 0.1 | 0.0 | 0.1 | 0.1 | 0.1 | 0.1 | 0.0 | 0.0 | 0.0 | 0.1 | 0.0 | 0.1 |
| Gm14057 | 0.1 | 0.2 | 0.1 | 0.2 | 0.3 | 0.3 | 0.3 | 0.3 | 0.2 | 0.3 | 0.3 | 0.2 |
| Gm14085 | 0.0 | 0.0 | 0.0 | 0.0 | 0.1 | 0.0 | 0.0 | 0.0 | 0.0 | 0.0 | 0.0 | 0.0 |
| Gm14124 | 0.1 | 0.1 | 0.1 | 0.1 | 0.1 | 0.1 | 0.1 | 0.1 | 0.1 | 0.1 | 0.1 | 0.1 |
| Gm14137 | 0.0 | 0.1 | 0.1 | 0.0 | 0.0 | 0.0 | 0.1 | 0.1 | 0.1 | 0.0 | 0.0 | 0.0 |
| Gm14139 | 0.0 | 0.0 | 0.0 | 0.2 | 0.0 | 0.1 | 0.0 | 0.1 | 0.0 | 0.1 | 0.0 | 0.0 |
| Gm14288 | 12.3 | 11.5 | 11.3 | 9.7 | 10.9 | 11.0 | 10.2 | 12.2 | 11.6 | 14.6 | 13.5 | 10.8 |
| Gm14295 | 19.2 | 20.3 | 24.6 | 25.9 | 25.2 | 28.5 | 20.6 | 26.4 | 24.0 | 26.4 | 30.1 | 25.1 |
| Gm14305 | 9.3 | 7.3 | 6.4 | 8.9 | 6.5 | 8.4 | 6.2 | 6.4 | 6.1 | 8.2 | 8.2 | 8.7 |
| Gm14306 | 2.5 | 2.3 | 1.7 | 1.1 | 2.6 | 1.8 | 1.3 | 2.1 | 2.4 | 3.1 | 2.2 | 2.7 |
| Gm14308 | 3.5 | 1.5 | 0.8 | 1.4 | 0.8 | 1.5 | 1.5 | 1.2 | 0.9 | 0.9 | 1.0 | 1.0 |
| Gm14322 | 3.0 | 2.3 | 2.5 | 3.9 | 2.5 | 3.7 | 2.6 | 2.4 | 2.7 | 3.6 | 3.0 | 3.6 |
| Gm14325 | 9.2 | 7.8 | 7.9 | 9.8 | 7.6 | 10.3 | 6.6 | 7.5 | 7.4 | 10.0 | 10.6 | 10.4 |
| Gm14326 | 8.8 | 7.6 | 6.7 | 8.7 | 7.4 | 9.9 | 6.3 | 6.9 | 6.8 | 8.8 | 9.8 | 9.6 |
| Gm14327 | 0.4 | 1.1 | 0.7 | 0.6 | 0.7 | 1.7 | 0.6 | 0.9 | 0.9 | 1.1 | 0.6 | 1.1 |
| Gm14378 | 0.0 | 0.0 | 0.0 | 0.0 | 0.1 | 0.0 | 0.2 | 0.0 | 0.1 | 0.1 | 0.1 | 0.1 |
| Gm14379 | 0.2 | 0.3 | 0.1 | 0.3 | 0.3 | 0.6 | 0.1 | 0.4 | 0.2 | 0.2 | 0.2 | 0.2 |
| Gm14391 | 4.8 | 3.8 | 2.9 | 3.1 | 2.7 | 3.4 | 3.0 | 2.7 | 2.7 | 3.9 | 3.8 | 3.6 |
| Gm14393 | 10.8 | 8.3 | 7.6 | 10.1 | 8.4 | 8.5 | 8.4 | 5.6 | 7.7 | 8.7 | 9.2 | 8.1 |
| Gm14403 | 2.3 | 1.9 | 2.3 | 2.5 | 1.7 | 2.8 | 1.8 | 2.1 | 2.4 | 3.8 | 3.2 | 3.9 |
| Gm14405 | 0.8 | 0.7 | 0.5 | 0.7 | 0.7 | 0.8 | 0.8 | 0.5 | 0.7 | 0.7 | 0.7 | 0.8 |

| | | | | | | | | | | | | |
|---|---|---|---|---|---|---|---|---|---|---|---|---|
| Gm14420 | 8.0 | 6.8 | 6.9 | 9.6 | 7.4 | 8.8 | 7.4 | 7.8 | 7.9 | 8.2 | 8.7 | 9.4 |
| Gm14430 | 3.0 | 2.6 | 2.6 | 3.2 | 2.8 | 3.8 | 2.7 | 2.6 | 2.5 | 3.5 | 3.6 | 3.2 |
| Gm14431 | 3.6 | 3.1 | 3.2 | 4.1 | 3.6 | 3.8 | 2.9 | 3.2 | 2.9 | 3.6 | 3.9 | 4.0 |
| Gm14434 | 4.4 | 3.9 | 3.8 | 4.3 | 4.0 | 5.5 | 3.8 | 3.6 | 3.7 | 5.1 | 5.0 | 4.8 |
| Gm14436 | 8.7 | 7.1 | 7.6 | 9.5 | 8.4 | 8.9 | 7.4 | 8.6 | 6.9 | 7.5 | 9.2 | 8.7 |
| Gm14440 | 3.6 | 3.3 | 3.9 | 4.7 | 4.1 | 4.5 | 3.9 | 4.2 | 3.6 | 4.5 | 4.9 | 5.0 |
| Gm14446 | 1.7 | 1.4 | 1.0 | 1.5 | 0.8 | 1.1 | 0.5 | 0.5 | 0.5 | 0.8 | 0.9 | 1.2 |
| Gm14492 | 0.6 | 0.9 | 1.2 | 0.9 | 1.7 | 1.8 | 1.4 | 1.7 | 1.5 | 0.4 | 0.9 | 1.2 |
| Gm14548 | 0.2 | 0.0 | 0.0 | 0.4 | 0.8 | 0.0 | 0.0 | 0.1 | 0.0 | 0.0 | 0.0 | 0.0 |
| Gm14634 | 0.0 | 0.0 | 0.0 | 0.1 | 0.0 | 0.0 | 0.0 | 0.0 | 0.0 | 0.1 | 0.0 | 0.0 |
| Gm14827 | 0.0 | 0.0 | 0.0 | 0.0 | 0.0 | 0.0 | 0.0 | 0.0 | 0.0 | 0.0 | 0.0 | 0.0 |
| Gm14872 | 2.7 | 2.5 | 2.5 | 2.6 | 2.4 | 2.6 | 2.4 | 2.5 | 2.0 | 3.8 | 2.4 | 2.3 |
| Gm14873 | 0.3 | 0.1 | 0.1 | 0.1 | 0.1 | 0.3 | 0.2 | 0.0 | 0.2 | 0.0 | 0.0 | 0.0 |
| Gm15070 | 0.4 | 0.3 | 0.4 | 0.6 | 0.6 | 0.1 | 0.3 | 0.1 | 0.2 | 0.4 | 0.3 | 0.3 |
| Gm15085 | 0.0 | 0.0 | 0.0 | 0.0 | 0.0 | 0.0 | 0.0 | 0.0 | 0.0 | 0.0 | 0.0 | 0.0 |
| Gm15133 | 0.4 | 0.5 | 0.3 | 0.6 | 0.2 | 0.1 | 0.4 | 0.2 | 0.1 | 0.5 | 0.5 | 0.6 |
| Gm15217 | 0.5 | 0.1 | 0.3 | 0.5 | 0.2 | 0.0 | 0.4 | 0.5 | 0.3 | 0.1 | 0.3 | 0.3 |
| Gm15319 | 0.2 | 0.3 | 0.2 | 0.1 | 0.1 | 0.2 | 0.2 | 0.1 | 0.1 | 0.2 | 0.1 | 0.2 |
| Gm15328 | 0.1 | 0.1 | 0.1 | 0.1 | 0.1 | 0.1 | 0.2 | 0.1 | 0.1 | 0.1 | 0.1 | 0.2 |
| Gm15350 | 1.6 | 0.6 | 0.3 | 1.6 | 1.3 | 1.5 | 1.0 | 1.2 | 0.7 | 1.2 | 0.9 | 0.6 |
| Gm15401 | 0.2 | 0.2 | 0.0 | 0.1 | 0.1 | 0.1 | 0.1 | 0.0 | 0.2 | 0.0 | 0.0 | 0.0 |
| Gm15408 | 0.1 | 0.0 | 0.3 | 0.4 | 0.1 | 0.1 | 0.2 | 0.3 | 0.2 | 0.1 | 0.1 | 0.2 |
| Gm15413 | 0.1 | 0.0 | 0.1 | 0.1 | 0.0 | 0.1 | 0.1 | 0.0 | 0.1 | 0.1 | 0.0 | 0.0 |
| Gm15417 | 2.2 | 2.0 | 1.0 | 2.0 | 1.9 | 2.8 | 1.3 | 1.8 | 2.1 | 1.3 | 1.6 | 2.2 |
| Gm15421 | 0.9 | 1.5 | 0.7 | 0.9 | 1.2 | 0.2 | 0.4 | 0.9 | 1.0 | 1.0 | 0.6 | 1.2 |
| Gm15441 | 0.1 | 0.0 | 0.1 | 0.3 | 0.2 | 0.1 | 0.1 | 0.3 | 0.1 | 0.1 | 0.1 | 0.2 |
| Gm15446 | 1.0 | 0.9 | 0.9 | 1.0 | 0.9 | 1.1 | 1.2 | 1.0 | 0.8 | 1.0 | 1.1 | 1.4 |
| Gm15455 | 0.1 | 0.1 | 0.1 | 0.1 | 0.1 | 0.1 | 0.1 | 0.1 | 0.1 | 0.1 | 0.1 | 0.1 |
| Gm15471 | 0.5 | 0.1 | 0.1 | 0.1 | 0.1 | 0.1 | 0.1 | 0.1 | 0.1 | 0.0 | 0.2 | 0.0 |
| Gm15545 | 0.8 | 0.7 | 0.8 | 0.5 | 1.1 | 0.5 | 0.7 | 0.9 | 0.8 | 0.4 | 0.5 | 0.6 |
| Gm15612 | 0.2 | 0.2 | 0.5 | 0.4 | 0.3 | 0.4 | 0.2 | 0.4 | 0.3 | 0.3 | 0.2 | 0.1 |
| Gm1564 | 0.0 | 0.0 | 0.0 | 0.0 | 0.0 | 0.0 | 0.0 | 0.0 | 0.0 | 0.0 | 0.0 | 0.0 |
| Gm15645 | 0.0 | 0.1 | 0.1 | 0.0 | 0.0 | 0.2 | 0.1 | 0.0 | 0.1 | 0.1 | 0.1 | 0.1 |
| Gm15663 | 0.3 | 0.1 | 0.1 | 0.2 | 0.2 | 0.2 | 0.2 | 0.2 | 0.2 | 0.2 | 0.3 | 0.2 |
| Gm15698 | 0.0 | 0.0 | 0.0 | 0.0 | 0.1 | 0.0 | 0.0 | 0.1 | 0.0 | 0.8 | 0.6 | 0.4 |
| Gm15706 | 0.2 | 0.4 | 0.0 | 0.3 | 0.2 | 0.3 | 0.2 | 0.5 | 0.5 | 0.3 | 0.5 | 0.3 |
| Gm15708 | 0.2 | 0.1 | 0.3 | 0.1 | 0.1 | 0.1 | 0.1 | 0.1 | 0.0 | 0.1 | 0.2 | 0.1 |
| Gm15713 | 0.2 | 0.2 | 0.2 | 0.0 | 0.1 | 0.2 | 0.2 | 0.1 | 0.2 | 0.2 | 0.0 | 0.1 |
| Gm15760 | 0.3 | 0.4 | 0.3 | 0.3 | 0.4 | 0.3 | 0.2 | 0.4 | 0.3 | 0.2 | 0.4 | 0.2 |
| Gm15772 | 1404.5 | 1116.0 | 1221.9 | 1201.7 | 1031.5 | 1350.7 | 1087.2 | 1323.0 | 974.2 | 1158.9 | 1310.1 | 1301.4 |
| Gm15787 | 0.6 | 0.5 | 0.5 | 0.5 | 0.4 | 0.2 | 0.6 | 0.6 | 0.4 | 0.4 | 0.4 | 0.4 |
| Gm15800 | 2.2 | 2.7 | 2.5 | 2.0 | 2.6 | 2.3 | 2.8 | 2.3 | 2.5 | 2.0 | 2.2 | 1.9 |
| Gm15880 | 0.1 | 0.1 | 0.1 | 0.0 | 0.1 | 0.1 | 0.1 | 0.0 | 0.0 | 0.1 | 0.0 | 0.1 |
| Gm15910 | 0.3 | 0.6 | 0.5 | 0.2 | 0.4 | 0.5 | 0.7 | 0.3 | 0.2 | 0.4 | 0.3 | 0.3 |
| Gm15915 | 0.0 | 0.1 | 0.2 | 0.1 | 0.1 | 0.1 | 0.0 | 0.0 | 0.0 | 0.0 | 0.0 | 0.1 |
| Gm15987 | 2.8 | 0.9 | 1.5 | 1.7 | 2.2 | 0.8 | 1.7 | 0.9 | 0.2 | 0.5 | 1.3 | 0.5 |
| Gm16023 | 0.9 | 1.2 | 1.0 | 1.1 | 0.8 | 1.0 | 1.3 | 1.1 | 0.9 | 0.7 | 0.9 | 0.6 |
| Gm16039 | 3.9 | 4.8 | 4.6 | 3.1 | 4.4 | 3.7 | 4.8 | 4.1 | 4.2 | 3.8 | 4.3 | 4.0 |
| Gm16062 | 1.6 | 1.0 | 0.8 | 0.6 | 1.6 | 1.2 | 1.4 | 0.7 | 1.1 | 2.1 | 0.8 | 1.2 |
| Gm16119 | 1.0 | 0.9 | 0.9 | 0.9 | 1.5 | 1.1 | 1.2 | 1.1 | 1.0 | 1.4 | 0.7 | 1.0 |
| Gm16157 | 0.3 | 0.1 | 0.4 | 0.1 | 0.3 | 0.3 | 0.3 | 0.2 | 0.2 | 0.1 | 0.3 | 0.0 |
| Gm16197 | 2.2 | 2.0 | 1.6 | 2.6 | 1.7 | 2.2 | 1.3 | 2.0 | 1.7 | 1.0 | 1.7 | 1.3 |
| Gm16287 | 0.0 | 0.0 | 0.0 | 0.1 | 0.2 | 0.1 | 0.0 | 0.0 | 0.0 | 0.0 | 0.1 | 0.0 |
| Gm16386 | 2.4 | 1.6 | 1.7 | 1.5 | 1.9 | 2.6 | 2.2 | 2.1 | 1.4 | 1.6 | 1.5 | 1.5 |
| Gm16432 | 0.2 | 0.2 | 0.3 | 0.2 | 0.2 | 0.3 | 0.2 | 0.3 | 0.3 | 0.2 | 0.0 | 0.1 |
| Gm16515 | 4.4 | 4.5 | 4.0 | 4.4 | 4.5 | 4.4 | 3.9 | 4.0 | 4.1 | 4.3 | 3.4 | 3.3 |
| Gm16516 | 2.0 | 1.5 | 1.9 | 3.2 | 2.3 | 1.0 | 2.0 | 1.9 | 1.8 | 1.6 | 2.9 | 2.1 |

| | | | | | | | | | | | | |
|---|---|---|---|---|---|---|---|---|---|---|---|---|
| Gm16523 | 0.0 | 0.1 | 0.0 | 0.0 | 0.0 | 0.0 | 0.0 | 0.0 | 0.0 | 0.0 | 0.0 | 0.0 |
| Gm16532 | 0.1 | 0.0 | 0.0 | 0.0 | 0.1 | 0.1 | 0.0 | 0.0 | 0.0 | 0.0 | 0.0 | 0.0 |
| Gm16548 | 0.0 | 0.0 | 0.0 | 0.0 | 0.0 | 0.0 | 0.0 | 0.0 | 0.0 | 0.0 | 0.1 | 0.0 |
| Gm16576 | 0.4 | 0.3 | 0.3 | 0.4 | 0.4 | 0.8 | 0.3 | 0.1 | 0.2 | 0.3 | 0.2 | 0.2 |
| Gm16596 | 0.1 | 0.3 | 0.2 | 0.1 | 0.2 | 0.2 | 0.1 | 0.2 | 0.3 | 0.2 | 0.3 | 0.3 |
| Gm166 | 3.0 | 3.6 | 3.4 | 3.9 | 3.6 | 4.5 | 3.8 | 3.3 | 3.3 | 3.2 | 3.0 | 2.9 |
| Gm1661 | 0.2 | 0.5 | 0.6 | 0.1 | 0.3 | 0.3 | 0.1 | 0.3 | 0.3 | 0.2 | 0.2 | 0.3 |
| Gm16617 | 0.6 | 1.2 | 1.8 | 1.4 | 1.1 | 1.3 | 1.7 | 1.4 | 1.0 | 1.0 | 1.1 | 1.2 |
| Gm16675 | 0.7 | 1.1 | 0.8 | 0.9 | 1.0 | 0.5 | 0.9 | 0.8 | 0.8 | 0.4 | 0.5 | 0.7 |
| Gm16677 | 0.2 | 0.1 | 0.2 | 0.1 | 0.1 | 0.0 | 0.0 | 0.2 | 0.1 | 0.1 | 0.2 | 0.1 |
| Gm16702 | 0.6 | 0.7 | 0.4 | 0.5 | 0.6 | 0.5 | 0.7 | 0.6 | 0.4 | 0.8 | 0.5 | 0.5 |
| Gm1673 | 3.4 | 3.9 | 3.6 | 2.6 | 2.9 | 3.7 | 3.4 | 4.8 | 3.9 | 4.1 | 3.5 | 5.6 |
| Gm16793 | 0.9 | 0.6 | 0.5 | 1.2 | 0.7 | 0.6 | 0.8 | 0.5 | 0.6 | 0.5 | 0.6 | 0.4 |
| Gm16796 | 0.0 | 0.0 | 0.1 | 0.0 | 0.1 | 0.2 | 0.0 | 0.0 | 0.0 | 0.0 | 0.1 | 0.1 |
| Gm16845 | 0.8 | 0.6 | 0.4 | 0.7 | 0.7 | 1.1 | 0.8 | 0.9 | 0.4 | 0.7 | 0.9 | 1.0 |
| Gm16853 | 0.1 | 0.1 | 0.0 | 0.0 | 0.1 | 0.0 | 0.1 | 0.0 | 0.0 | 0.1 | 0.1 | 0.0 |
| Gm16861 | 0.5 | 0.4 | 0.6 | 0.8 | 0.4 | 0.8 | 0.4 | 0.7 | 0.3 | 0.4 | 0.7 | 0.3 |
| Gm16880 | 0.4 | 0.4 | 0.6 | 0.2 | 0.5 | 0.2 | 0.4 | 0.4 | 0.2 | 0.3 | 0.4 | 0.6 |
| Gm16894 | 0.0 | 0.1 | 0.3 | 0.0 | 0.0 | 0.1 | 0.3 | 0.1 | 0.0 | 0.1 | 0.1 | 0.1 |
| Gm16897 | 0.6 | 0.3 | 0.5 | 0.4 | 0.3 | 0.5 | 0.3 | 0.9 | 0.3 | 0.4 | 0.4 | 0.5 |
| Gm16907 | 3.4 | 3.5 | 2.9 | 2.9 | 2.9 | 2.8 | 2.9 | 2.4 | 2.4 | 2.1 | 2.4 | 2.4 |
| Gm16938 | 0.5 | 0.4 | 0.5 | 0.6 | 0.4 | 0.6 | 0.5 | 0.3 | 0.3 | 0.5 | 0.6 | 0.5 |
| Gm16973 | 0.9 | 1.6 | 1.2 | 0.9 | 1.6 | 0.8 | 1.3 | 1.1 | 1.5 | 1.3 | 1.4 | 1.4 |
| Gm16982 | 1.4 | 1.2 | 1.0 | 1.1 | 1.1 | 0.9 | 1.3 | 1.7 | 1.9 | 1.2 | 1.5 | 1.4 |
| Gm16998 | 0.4 | 0.5 | 0.2 | 0.2 | 0.7 | 0.3 | 0.4 | 0.8 | 0.5 | 0.4 | 0.4 | 0.5 |
| Gm17019 | 0.0 | 0.0 | 0.0 | 0.0 | 0.0 | 0.0 | 0.0 | 0.0 | 0.0 | 0.0 | 0.0 | 0.0 |
| Gm17066 | 1.0 | 0.9 | 1.5 | 1.0 | 1.2 | 1.4 | 1.4 | 1.4 | 1.1 | 1.0 | 1.4 | 1.5 |
| Gm17296 | 0.8 | 1.4 | 1.3 | 0.8 | 1.3 | 1.1 | 1.3 | 1.0 | 1.1 | 1.1 | 1.1 | 1.0 |
| Gm17384 | 0.1 | 0.0 | 0.0 | 0.3 | 0.1 | 0.1 | 0.0 | 0.0 | 0.0 | 0.0 | 0.0 | 0.0 |
| Gm17455 | 0.9 | 0.4 | 0.7 | 0.2 | 0.1 | 0.5 | 0.0 | 0.4 | 0.3 | 0.3 | 0.6 | 0.4 |
| Gm17644 | 2.7 | 4.4 | 4.1 | 2.7 | 3.2 | 3.0 | 4.6 | 2.9 | 1.9 | 2.9 | 2.4 | 3.2 |
| Gm17757 | 0.5 | 0.4 | 0.4 | 0.7 | 0.6 | 0.1 | 0.3 | 0.1 | 0.3 | 0.2 | 0.2 | 0.4 |
| Gm17762 | 1.0 | 0.9 | 1.0 | 0.9 | 1.1 | 1.0 | 1.2 | 1.2 | 1.2 | 0.7 | 0.9 | 0.9 |
| Gm17769 | 0.2 | 0.2 | 0.4 | 0.1 | 0.2 | 0.1 | 0.2 | 0.4 | 0.2 | 0.1 | 0.1 | 0.1 |
| Gm17801 | 0.4 | 0.2 | 0.5 | 0.9 | 0.6 | 0.2 | 0.4 | 0.5 | 0.7 | 0.6 | 0.7 | 0.5 |
| Gm17821 | 1.6 | 2.0 | 1.6 | 1.4 | 1.6 | 1.4 | 1.7 | 1.5 | 1.1 | 1.3 | 1.4 | 1.2 |
| Gm1821 | 72.4 | 73.3 | 70.4 | 101.9 | 79.5 | 87.0 | 89.8 | 83.1 | 71.8 | 74.8 | 85.0 | 79.2 |
| Gm18853 | 0.0 | 0.0 | 0.0 | 0.0 | 0.0 | 0.0 | 0.0 | 0.0 | 0.0 | 0.0 | 0.0 | 0.0 |
| Gm19276 | 0.0 | 0.1 | 0.0 | 0.0 | 0.2 | 0.0 | 0.1 | 0.0 | 0.1 | 0.3 | 0.1 | 0.2 |
| Gm19299 | 0.0 | 0.1 | 0.0 | 0.0 | 0.1 | 0.1 | 0.0 | 0.0 | 0.1 | 0.1 | 0.0 | 0.0 |
| Gm19345 | 0.0 | 0.0 | 0.0 | 0.0 | 0.1 | 0.0 | 0.0 | 0.0 | 0.0 | 0.0 | 0.0 | 0.0 |
| Gm1943 | 11.1 | 7.9 | 7.4 | 10.7 | 9.2 | 9.6 | 7.7 | 8.0 | 8.8 | 9.4 | 9.3 | 8.7 |
| Gm19434 | 0.0 | 0.1 | 0.0 | 0.0 | 0.1 | 0.0 | 0.0 | 0.0 | 0.0 | 0.0 | 0.0 | 0.0 |
| Gm19461 | 0.0 | 0.0 | 0.1 | 0.0 | 0.0 | 0.0 | 0.0 | 0.0 | 0.0 | 0.0 | 0.0 | 0.0 |
| Gm19466 | 0.1 | 0.1 | 0.1 | 0.0 | 0.1 | 0.1 | 0.1 | 0.0 | 0.0 | 0.0 | 0.0 | 0.0 |
| Gm19510 | 0.1 | 0.0 | 0.1 | 0.1 | 0.1 | 0.0 | 0.0 | 0.7 | 0.0 | 0.2 | 0.2 | 0.1 |
| Gm19522 | 0.7 | 2.6 | 5.7 | 1.0 | 1.0 | 1.1 | 1.7 | 2.2 | 1.6 | 1.0 | 1.1 | 1.6 |
| Gm19557 | 0.2 | 0.3 | 0.2 | 0.5 | 0.1 | 0.3 | 0.2 | 0.3 | 0.1 | 0.3 | 0.2 | 0.3 |
| Gm19583 | 0.0 | 0.1 | 0.0 | 0.0 | 0.0 | 0.1 | 0.0 | 0.1 | 0.0 | 0.0 | 0.0 | 0.0 |
| Gm19589 | 0.0 | 0.0 | 0.0 | 0.0 | 0.0 | 0.0 | 0.0 | 0.0 | 0.0 | 0.0 | 0.0 | 0.0 |
| Gm19619 | 0.1 | 0.0 | 0.0 | 0.0 | 0.0 | 0.0 | 0.0 | 0.0 | 0.0 | 0.0 | 0.0 | 0.0 |
| Gm19705 | 3.7 | 2.8 | 3.6 | 3.4 | 4.2 | 2.1 | 2.9 | 1.3 | 2.4 | 4.6 | 2.7 | 2.3 |
| Gm19710 | 0.0 | 0.4 | 0.1 | 0.2 | 0.2 | 0.2 | 0.1 | 0.1 | 0.1 | 0.6 | 0.2 | 0.2 |
| Gm19757 | 1.6 | 2.4 | 1.4 | 1.5 | 1.8 | 1.8 | 1.6 | 1.7 | 1.2 | 1.3 | 1.2 | 1.5 |
| Gm1976 | 1.7 | 2.6 | 1.8 | 1.6 | 2.0 | 2.4 | 1.6 | 2.1 | 1.5 | 2.0 | 1.9 | 1.7 |
| Gm1987 | 0.0 | 0.1 | 0.0 | 0.0 | 0.0 | 0.0 | 0.0 | 0.0 | 0.0 | 0.0 | 0.0 | 0.0 |
| Gm19897 | 0.3 | 0.2 | 0.1 | 0.2 | 0.3 | 0.2 | 0.3 | 0.1 | 0.2 | 0.3 | 0.1 | 0.2 |

| | | | | | | | | | | | | |
|---|---|---|---|---|---|---|---|---|---|---|---|---|
| Gm2002 | 0.1 | 0.0 | 0.0 | 0.0 | 0.0 | 0.1 | 0.0 | 0.1 | 0.1 | 0.0 | 0.0 | 0.0 |
| Gm2011 | 0.7 | 0.7 | 0.8 | 0.3 | 0.5 | 0.7 | 0.6 | 0.5 | 0.3 | 0.5 | 0.6 | 0.9 |
| Gm20110 | 0.0 | 0.0 | 0.0 | 0.0 | 0.0 | 0.0 | 0.0 | 0.0 | 0.0 | 0.0 | 0.0 | 0.1 |
| Gm20139 | 0.0 | 0.0 | 0.0 | 0.1 | 0.0 | 0.0 | 0.0 | 0.0 | 0.0 | 0.0 | 0.0 | 0.0 |
| Gm2016 | 0.1 | 0.1 | 0.1 | 0.1 | 0.1 | 0.1 | 0.2 | 0.1 | 0.1 | 0.1 | 0.1 | 0.1 |
| Gm20199 | 0.3 | 0.5 | 0.3 | 0.4 | 0.5 | 0.4 | 0.6 | 0.3 | 0.3 | 0.5 | 0.4 | 0.3 |
| Gm2022 | 0.1 | 0.1 | 0.1 | 0.1 | 0.1 | 0.1 | 0.1 | 0.1 | 0.1 | 0.1 | 0.1 | 0.1 |
| Gm20257 | 0.9 | 1.1 | 0.6 | 1.0 | 1.1 | 0.8 | 0.7 | 1.0 | 0.6 | 1.0 | 0.8 | 1.2 |
| Gm2027 | 0.0 | 0.1 | 0.1 | 0.1 | 0.1 | 0.0 | 0.0 | 0.0 | 0.1 | 0.0 | 0.1 | 0.1 |
| Gm20300 | 2.9 | 3.2 | 3.7 | 3.7 | 2.8 | 4.4 | 2.6 | 3.0 | 2.6 | 3.0 | 4.0 | 3.4 |
| Gm20324 | 0.2 | 0.5 | 0.1 | 0.0 | 0.2 | 0.0 | 0.1 | 0.2 | 0.0 | 0.1 | 0.5 | 0.1 |
| Gm20337 | 0.3 | 0.4 | 0.3 | 0.7 | 0.4 | 0.4 | 0.1 | 0.5 | 0.3 | 0.3 | 0.2 | 0.3 |
| Gm20362 | 0.0 | 0.0 | 0.1 | 0.1 | 0.1 | 0.0 | 0.0 | 0.0 | 0.1 | 0.1 | 0.1 | 0.1 |
| Gm20554 | 0.0 | 0.0 | 0.0 | 0.1 | 0.0 | 0.1 | 0.1 | 0.0 | 0.0 | 0.1 | 0.1 | 0.1 |
| Gm20594 | 0.0 | 0.0 | 0.0 | 1.0 | 0.0 | 0.2 | 2.1 | 0.0 | 0.3 | 0.0 | 0.0 | 0.0 |
| Gm20597 | 0.0 | 0.0 | 0.0 | 0.0 | 0.0 | 0.0 | 0.0 | 0.0 | 0.0 | 0.0 | 0.0 | 0.0 |
| Gm20604 | 2.5 | 2.1 | 1.7 | 2.0 | 2.6 | 1.9 | 2.3 | 2.0 | 2.2 | 1.8 | 2.2 | 1.4 |
| Gm20605 | 0.9 | 0.6 | 0.4 | 0.7 | 1.2 | 0.6 | 1.0 | 0.5 | 0.9 | 0.1 | 0.3 | 0.2 |
| Gm20735 | 0.3 | 0.1 | 0.5 | 0.1 | 0.2 | 0.0 | 0.2 | 0.0 | 0.2 | 0.2 | 0.0 | 0.3 |
| Gm20736 | 0.0 | 0.0 | 0.0 | 0.0 | 0.0 | 0.0 | 0.0 | 0.0 | 0.0 | 0.0 | 0.0 | 0.0 |
| Gm20744 | 1.7 | 0.8 | 0.5 | 1.5 | 0.8 | 1.5 | 1.0 | 0.5 | 0.8 | 2.8 | 2.9 | 1.2 |
| Gm20748 | 5.4 | 3.9 | 3.5 | 6.2 | 4.3 | 5.6 | 5.6 | 4.1 | 3.9 | 4.5 | 4.0 | 2.7 |
| Gm20752 | 0.0 | 0.0 | 0.0 | 0.0 | 0.0 | 0.0 | 0.0 | 0.0 | 0.0 | 0.0 | 0.0 | 0.0 |
| Gm20755 | 0.1 | 0.0 | 0.1 | 0.0 | 0.0 | 0.0 | 0.0 | 0.0 | 0.0 | 0.1 | 0.1 | 0.0 |
| Gm20765 | 0.0 | 0.0 | 0.0 | 0.0 | 0.0 | 0.0 | 0.0 | 0.0 | 0.0 | 0.0 | 0.0 | 0.0 |
| Gm20767 | 0.3 | 0.3 | 0.1 | 0.1 | 0.2 | 0.2 | 0.3 | 0.2 | 0.0 | 0.2 | 0.2 | 0.2 |
| Gm20878 | 0.0 | 0.5 | 0.5 | 0.5 | 0.2 | 0.0 | 0.0 | 0.0 | 0.3 | 0.3 | 0.2 | 0.3 |
| Gm20939 | 0.8 | 1.4 | 1.7 | 0.7 | 0.9 | 1.2 | 1.0 | 1.2 | 1.2 | 0.9 | 1.1 | 1.1 |
| Gm21119 | 0.1 | 0.3 | 0.3 | 0.1 | 0.1 | 0.3 | 0.2 | 0.1 | 0.1 | 0.4 | 0.1 | 0.2 |
| Gm2115 | 3.3 | 1.6 | 1.0 | 3.2 | 2.6 | 2.9 | 5.3 | 0.9 | 1.4 | 2.8 | 3.1 | 2.3 |
| Gm21293 | 0.0 | 0.0 | 0.0 | 0.0 | 0.0 | 0.0 | 0.0 | 0.0 | 0.0 | 0.0 | 0.0 | 0.0 |
| Gm21304 | 0.0 | 0.0 | 0.0 | 0.0 | 0.0 | 0.0 | 0.0 | 0.0 | 0.0 | 0.0 | 0.0 | 0.0 |
| Gm21312 | 0.0 | 0.0 | 0.0 | 0.0 | 0.0 | 0.0 | 0.0 | 0.0 | 0.0 | 0.0 | 0.0 | 0.0 |
| Gm21319 | 0.0 | 0.0 | 0.0 | 0.0 | 0.0 | 0.0 | 0.0 | 0.0 | 0.0 | 0.0 | 0.0 | 0.0 |
| Gm216 | 0.6 | 0.7 | 0.9 | 1.2 | 0.5 | 1.1 | 0.9 | 0.6 | 0.5 | 0.5 | 0.5 | 0.7 |
| Gm21949 | 0.0 | 0.0 | 0.0 | 0.0 | 0.2 | 0.0 | 0.0 | 0.0 | 0.0 | 0.0 | 0.0 | 0.0 |
| Gm2381 | 0.0 | 0.1 | 0.1 | 0.1 | 0.1 | 0.1 | 0.0 | 0.0 | 0.0 | 0.1 | 0.0 | 0.0 |
| Gm2382 | 7.6 | 8.8 | 8.4 | 7.8 | 8.3 | 6.9 | 8.5 | 8.7 | 9.4 | 7.2 | 8.3 | 8.8 |
| Gm2518 | 1.1 | 1.1 | 1.3 | 1.1 | 1.2 | 1.0 | 1.2 | 1.3 | 1.3 | 1.1 | 1.1 | 1.0 |
| Gm266 | 0.0 | 0.0 | 0.0 | 0.0 | 0.0 | 0.0 | 0.0 | 0.1 | 0.1 | 0.0 | 0.0 | 0.1 |
| Gm2897 | 0.7 | 1.2 | 1.0 | 0.4 | 0.5 | 0.8 | 0.9 | 1.1 | 0.8 | 0.7 | 1.0 | 0.8 |
| Gm2a | 27.5 | 24.5 | 21.4 | 28.0 | 23.4 | 28.2 | 19.0 | 15.5 | 14.6 | 27.1 | 22.3 | 21.7 |
| Gm3002 | 0.2 | 0.4 | 0.6 | 0.0 | 0.2 | 0.1 | 0.2 | 0.6 | 0.0 | 0.7 | 0.3 | 0.2 |
| Gm3086 | 0.1 | 0.2 | 0.1 | 0.0 | 0.3 | 0.1 | 0.1 | 0.1 | 0.1 | 0.2 | 0.5 | 0.3 |
| Gm3219 | 1.3 | 2.0 | 1.7 | 1.1 | 1.7 | 1.5 | 1.5 | 2.1 | 1.6 | 2.3 | 1.0 | 1.8 |
| Gm3230 | 0.2 | 0.1 | 0.2 | 0.2 | 0.2 | 0.2 | 0.1 | 0.1 | 0.2 | 0.1 | 0.1 | 0.2 |
| Gm3258 | 0.6 | 0.2 | 0.2 | 0.2 | 0.2 | 0.5 | 0.4 | 0.3 | 0.3 | 0.2 | 0.2 | 0.5 |
| Gm3264 | 0.4 | 1.0 | 0.9 | 0.3 | 0.3 | 0.2 | 0.3 | 0.7 | 1.0 | 0.7 | 0.9 | 1.0 |
| Gm3317 | 0.3 | 0.5 | 0.5 | 0.1 | 0.2 | 0.3 | 0.4 | 0.5 | 0.4 | 0.4 | 0.3 | 0.4 |
| Gm3383 | 0.4 | 0.5 | 0.4 | 0.4 | 0.1 | 0.5 | 0.4 | 0.5 | 0.2 | 0.4 | 0.5 | 0.5 |
| Gm3414 | 0.5 | 0.5 | 0.4 | 0.5 | 0.4 | 0.3 | 0.4 | 0.2 | 0.4 | 0.4 | 0.5 | 0.4 |
| Gm3417 | 0.0 | 0.1 | 0.0 | 0.1 | 0.0 | 0.0 | 0.0 | 0.0 | 0.0 | 0.0 | 0.0 | 0.0 |
| Gm3435 | 0.5 | 0.7 | 0.6 | 0.4 | 0.6 | 0.6 | 0.5 | 0.3 | 0.4 | 0.4 | 0.5 | 0.5 |
| Gm3494 | 0.3 | 0.5 | 0.5 | 0.1 | 0.3 | 0.3 | 0.4 | 0.5 | 0.5 | 0.4 | 0.3 | 0.4 |
| Gm3500 | 0.2 | 0.6 | 0.5 | 0.3 | 0.2 | 0.4 | 0.4 | 0.5 | 0.4 | 0.3 | 0.4 | 0.4 |
| Gm3558 | 0.5 | 0.7 | 0.7 | 0.4 | 0.3 | 0.6 | 0.6 | 0.8 | 0.6 | 0.7 | 0.6 | 0.6 |
| Gm3604 | 0.6 | 1.3 | 1.5 | 0.6 | 1.3 | 1.1 | 1.0 | 1.1 | 0.9 | 0.8 | 1.0 | 1.1 |

| | | | | | | | | | | | | |
|---|---|---|---|---|---|---|---|---|---|---|---|---|
| Gm3696 | 0.3 | 0.6 | 0.6 | 0.3 | 0.3 | 0.5 | 0.5 | 0.6 | 0.4 | 0.4 | 0.6 | 0.5 |
| Gm3716 | 0.9 | 0.7 | 0.6 | 0.5 | 0.7 | 0.5 | 0.5 | 0.3 | 0.4 | 0.4 | 0.5 | 0.3 |
| Gm3776 | 0.7 | 0.7 | 0.5 | 0.9 | 0.6 | 1.3 | 0.4 | 0.5 | 0.0 | 0.1 | 0.1 | 0.2 |
| Gm3893 | 2.0 | 3.7 | 3.7 | 1.5 | 1.7 | 1.4 | 1.7 | 3.2 | 1.2 | 0.9 | 1.3 | 1.4 |
| Gm4013 | 0.6 | 1.4 | 1.1 | 0.4 | 0.5 | 1.6 | 0.7 | 0.7 | 0.2 | 1.7 | 0.4 | 0.4 |
| Gm4027 | 0.3 | 0.2 | 0.3 | 0.4 | 0.4 | 0.2 | 0.5 | 0.3 | 0.3 | 0.3 | 0.4 | 0.4 |
| Gm4070 | 13.8 | 6.9 | 4.4 | 15.1 | 7.6 | 6.1 | 4.5 | 4.2 | 4.6 | 4.9 | 3.4 | 10.0 |
| Gm4262 | 1.2 | 1.1 | 0.9 | 1.2 | 1.2 | 1.0 | 0.9 | 1.2 | 1.1 | 0.7 | 0.8 | 0.8 |
| Gm4285 | 0.7 | 0.9 | 0.7 | 1.0 | 0.9 | 0.4 | 0.6 | 0.7 | 0.7 | 0.9 | 0.5 | 1.0 |
| Gm4340 | 0.0 | 0.0 | 0.0 | 0.0 | 0.0 | 0.0 | 0.0 | 0.0 | 0.0 | 0.0 | 0.0 | 0.0 |
| Gm4349 | 1.0 | 1.4 | 1.0 | 1.1 | 1.3 | 1.1 | 1.0 | 0.9 | 1.0 | 0.8 | 1.3 | 1.0 |
| Gm4477 | 0.0 | 0.1 | 0.3 | 0.0 | 0.1 | 0.0 | 0.0 | 0.1 | 0.0 | 0.0 | 0.0 | 0.0 |
| Gm4532 | 0.9 | 1.0 | 1.5 | 1.3 | 0.8 | 1.3 | 1.5 | 1.4 | 0.6 | 0.7 | 0.5 | 0.8 |
| Gm4636 | 0.4 | 0.6 | 0.5 | 0.2 | 0.5 | 0.3 | 0.4 | 0.4 | 0.5 | 0.5 | 0.6 | 0.4 |
| Gm4724 | 4.2 | 3.7 | 3.6 | 4.2 | 3.9 | 5.2 | 3.6 | 3.5 | 3.5 | 4.8 | 4.8 | 4.6 |
| Gm4736 | 0.1 | 0.1 | 0.2 | 0.1 | 0.2 | 0.1 | 0.1 | 0.1 | 0.1 | 0.0 | 0.1 | 0.0 |
| Gm4759 | 0.1 | 0.0 | 0.0 | 0.0 | 0.1 | 0.0 | 0.0 | 0.0 | 0.0 | 0.0 | 0.0 | 0.0 |
| Gm4787 | 0.1 | 0.1 | 0.1 | 0.2 | 0.2 | 0.2 | 0.2 | 0.1 | 0.2 | 0.1 | 0.0 | 0.2 |
| Gm4788 | 5.5 | 6.3 | 5.4 | 2.0 | 3.4 | 1.8 | 2.5 | 3.0 | 4.8 | 4.8 | 2.4 | 5.3 |
| Gm4832 | 0.1 | 0.2 | 0.1 | 0.0 | 0.1 | 0.0 | 0.2 | 0.3 | 0.0 | 0.1 | 0.1 | 0.5 |
| Gm4841 | 0.3 | 0.1 | 0.3 | 0.4 | 0.2 | 0.1 | 0.0 | 0.1 | 0.1 | 0.1 | 0.0 | 0.4 |
| Gm4884 | 0.0 | 0.0 | 0.0 | 0.0 | 0.0 | 0.0 | 0.0 | 0.0 | 0.0 | 0.0 | 0.0 | 0.0 |
| Gm4890 | 0.5 | 0.3 | 0.3 | 0.5 | 0.4 | 0.5 | 0.3 | 0.3 | 0.3 | 0.3 | 0.3 | 0.4 |
| Gm4925 | 0.1 | 0.3 | 0.4 | 0.4 | 0.3 | 0.4 | 0.4 | 0.5 | 0.5 | 0.3 | 0.5 | 0.2 |
| Gm4944 | 2.9 | 4.2 | 4.8 | 4.6 | 4.7 | 5.3 | 3.7 | 4.4 | 3.9 | 4.5 | 6.1 | 5.6 |
| Gm4951 | 1.3 | 0.6 | 1.5 | 1.3 | 0.7 | 0.8 | 0.5 | 0.3 | 0.4 | 0.2 | 0.6 | 1.5 |
| Gm4952 | 0.0 | 0.1 | 0.0 | 0.0 | 0.0 | 0.0 | 0.0 | 0.0 | 0.0 | 0.0 | 0.0 | 0.0 |
| Gm4961 | 3.5 | 3.6 | 2.7 | 3.8 | 3.6 | 2.7 | 3.2 | 2.5 | 3.1 | 1.5 | 2.1 | 2.5 |
| Gm4980 | 0.1 | 0.1 | 0.2 | 0.0 | 0.2 | 0.1 | 0.4 | 0.3 | 0.3 | 0.1 | 0.1 | 0.1 |
| Gm4984 | 0.1 | 0.0 | 0.0 | 0.0 | 0.0 | 0.0 | 0.2 | 0.0 | 0.1 | 0.0 | 0.0 | 0.2 |
| Gm5039 | 0.2 | 0.1 | 0.1 | 0.2 | 0.2 | 0.0 | 0.2 | 0.1 | 0.1 | 0.1 | 0.1 | 0.1 |
| Gm5069 | 0.5 | 0.3 | 0.5 | 0.9 | 0.5 | 0.7 | 0.7 | 0.5 | 0.4 | 0.5 | 0.5 | 0.6 |
| Gm5077 | 3.1 | 3.5 | 5.4 | 2.4 | 4.6 | 2.9 | 1.8 | 2.8 | 2.5 | 8.0 | 4.4 | 4.7 |
| Gm5082 | 0.0 | 0.0 | 0.0 | 0.1 | 0.1 | 0.1 | 0.0 | 0.0 | 0.0 | 0.0 | 0.0 | 0.0 |
| Gm5086 | 0.2 | 0.1 | 0.1 | 0.0 | 0.1 | 0.2 | 0.1 | 0.1 | 0.1 | 0.1 | 0.0 | 0.1 |
| Gm5088 | 2.1 | 1.5 | 1.5 | 2.0 | 1.8 | 2.1 | 1.7 | 1.9 | 1.2 | 1.7 | 2.0 | 1.9 |
| Gm5105 | 0.0 | 0.0 | 0.0 | 0.1 | 0.0 | 0.1 | 0.0 | 0.0 | 0.0 | 0.0 | 0.0 | 0.0 |
| Gm5113 | 0.8 | 1.0 | 1.0 | 1.2 | 0.7 | 1.1 | 1.1 | 1.0 | 1.1 | 0.8 | 1.0 | 1.4 |
| Gm5114 | 0.0 | 0.0 | 0.0 | 0.0 | 0.0 | 0.0 | 0.0 | 0.0 | 0.0 | 0.0 | 0.0 | 0.0 |
| Gm5122 | 0.0 | 0.0 | 0.0 | 0.0 | 0.0 | 0.0 | 0.0 | 0.0 | 0.0 | 0.0 | 0.0 | 0.0 |
| Gm5124 | 0.1 | 0.2 | 0.0 | 0.1 | 0.1 | 0.4 | 0.1 | 0.2 | 0.0 | 0.1 | 0.1 | 0.1 |
| Gm5126 | 0.1 | 0.2 | 0.1 | 0.1 | 0.1 | 0.1 | 0.1 | 0.1 | 0.1 | 0.3 | 0.1 | 0.1 |
| Gm5127 | 0.3 | 0.0 | 0.1 | 0.1 | 0.1 | 0.0 | 0.0 | 0.0 | 0.0 | 0.0 | 0.0 | 0.1 |
| Gm5129 | 0.7 | 1.1 | 1.5 | 1.0 | 1.8 | 0.3 | 2.0 | 1.8 | 1.5 | 1.1 | 0.9 | 2.3 |
| Gm514 | 0.0 | 0.1 | 0.0 | 0.0 | 0.0 | 0.1 | 0.0 | 0.1 | 0.0 | 0.0 | 0.0 | 0.0 |
| Gm5141 | 0.6 | 0.8 | 1.2 | 0.8 | 1.0 | 1.2 | 0.7 | 1.0 | 0.7 | 0.9 | 0.8 | 0.8 |
| Gm5148 | 11.2 | 8.8 | 9.4 | 9.6 | 9.1 | 9.7 | 9.7 | 10.5 | 8.9 | 9.3 | 10.4 | 10.8 |
| Gm5150 | 0.0 | 0.0 | 0.0 | 0.1 | 0.6 | 0.0 | 0.0 | 0.0 | 0.0 | 0.1 | 0.0 | 0.1 |
| Gm5176 | 0.4 | 1.0 | 1.1 | 0.2 | 0.8 | 0.4 | 1.4 | 0.4 | 0.4 | 0.7 | 0.6 | 0.4 |
| Gm5177 | 0.1 | 0.0 | 0.1 | 0.2 | 0.1 | 0.1 | 0.1 | 0.1 | 0.0 | 0.1 | 0.2 | 0.1 |
| Gm527 | 0.8 | 1.4 | 1.1 | 1.0 | 1.0 | 1.1 | 1.2 | 1.6 | 1.6 | 1.4 | 1.8 | 1.8 |
| Gm53 | 0.9 | 0.1 | 0.3 | 2.3 | 0.5 | 0.4 | 0.1 | 0.0 | 0.1 | 0.1 | 0.4 | 0.1 |
| Gm5415 | 0.0 | 0.0 | 0.0 | 0.0 | 0.0 | 0.0 | 0.0 | 0.0 | 0.0 | 0.0 | 0.0 | 0.0 |
| Gm5424 | 232.0 | 130.5 | 100.6 | 352.6 | 234.7 | 325.5 | 140.8 | 65.0 | 55.6 | 114.8 | 110.9 | 120.0 |
| Gm5431 | 9.6 | 6.7 | 4.2 | 9.4 | 5.5 | 7.9 | 6.9 | 2.5 | 3.6 | 3.6 | 5.4 | 5.3 |
| Gm5434 | 0.1 | 0.0 | 0.0 | 0.0 | 0.0 | 0.1 | 0.1 | 0.0 | 0.0 | 0.0 | 0.1 | 0.0 |
| Gm5458 | 0.0 | 0.0 | 0.0 | 0.0 | 0.0 | 0.0 | 0.0 | 0.0 | 0.0 | 0.1 | 0.0 | 0.0 |

| | | | | | | | | | | | | |
|---|---|---|---|---|---|---|---|---|---|---|---|---|
| Gm5464 | 0.6 | 0.4 | 0.6 | 0.2 | 0.4 | 0.4 | 0.6 | 0.7 | 0.5 | 0.2 | 0.4 | 0.5 |
| Gm5468 | 0.0 | 0.0 | 0.0 | 0.0 | 0.0 | 0.0 | 0.0 | 0.0 | 0.0 | 0.0 | 0.0 | 0.0 |
| Gm5475 | 0.0 | 0.0 | 0.0 | 0.0 | 0.0 | 0.0 | 0.0 | 0.0 | 0.0 | 0.0 | 0.0 | 0.0 |
| Gm5506 | 8.8 | 7.7 | 6.4 | 11.7 | 10.5 | 9.3 | 10.9 | 9.2 | 9.8 | 9.8 | 9.7 | 11.6 |
| Gm5512 | 0.2 | 0.2 | 0.6 | 0.3 | 0.3 | 0.2 | 0.1 | 0.2 | 0.4 | 0.2 | 0.3 | 0.3 |
| Gm5523 | 1.4 | 1.1 | 1.5 | 2.9 | 1.9 | 1.7 | 1.9 | 1.5 | 1.6 | 1.3 | 1.2 | 1.7 |
| Gm5531 | 0.0 | 0.1 | 0.0 | 0.0 | 0.0 | 0.0 | 0.0 | 0.0 | 0.0 | 0.0 | 0.0 | 0.0 |
| Gm5538 | 0.3 | 0.1 | 0.1 | 0.2 | 0.1 | 0.2 | 0.0 | 0.0 | 0.0 | 0.0 | 0.1 | 0.0 |
| Gm5544 | 0.0 | 0.0 | 0.0 | 0.3 | 0.0 | 0.0 | 0.1 | 0.0 | 0.0 | 0.1 | 0.0 | 0.3 |
| Gm5577 | 0.3 | 0.5 | 0.4 | 0.0 | 0.3 | 0.2 | 0.2 | 0.2 | 0.6 | 0.1 | 0.3 | 0.5 |
| Gm5595 | 1.0 | 1.4 | 1.3 | 1.0 | 0.9 | 1.2 | 0.9 | 1.2 | 1.3 | 1.8 | 1.3 | 1.2 |
| Gm5607 | 0.0 | 0.0 | 0.0 | 0.0 | 0.0 | 0.0 | 0.0 | 0.0 | 0.0 | 0.0 | 0.0 | 0.0 |
| Gm561 | 17.2 | 15.4 | 10.3 | 16.0 | 13.4 | 16.1 | 14.3 | 9.8 | 15.7 | 17.6 | 13.3 | 13.7 |
| Gm5617 | 5.1 | 5.3 | 5.8 | 5.4 | 5.0 | 5.3 | 4.2 | 6.0 | 6.1 | 7.1 | 6.4 | 5.0 |
| Gm5627 | 0.1 | 0.0 | 0.2 | 0.1 | 0.2 | 0.0 | 0.1 | 0.4 | 0.0 | 0.0 | 0.0 | 0.0 |
| Gm5643 | 0.5 | 0.6 | 0.9 | 0.8 | 0.8 | 0.6 | 1.0 | 1.2 | 1.2 | 1.0 | 1.2 | 1.2 |
| Gm5662 | 0.0 | 0.0 | 0.0 | 0.0 | 0.0 | 0.0 | 0.0 | 0.0 | 0.0 | 0.0 | 0.0 | 0.0 |
| Gm5766 | 0.0 | 0.0 | 0.0 | 0.0 | 0.0 | 0.0 | 0.0 | 0.0 | 0.0 | 0.1 | 0.0 | 0.0 |
| Gm5779 | 0.1 | 0.0 | 0.1 | 0.0 | 0.1 | 0.1 | 0.2 | 0.0 | 0.2 | 0.0 | 0.1 | 0.3 |
| Gm5795 | 0.0 | 0.0 | 0.0 | 0.0 | 0.0 | 0.0 | 0.0 | 0.0 | 0.0 | 0.0 | 0.0 | 0.0 |
| Gm5796 | 0.3 | 0.4 | 0.4 | 0.3 | 0.3 | 0.4 | 0.4 | 0.6 | 0.4 | 0.3 | 0.5 | 0.5 |
| Gm5801 | 6.7 | 6.9 | 7.5 | 6.2 | 7.6 | 6.8 | 6.9 | 6.9 | 6.7 | 7.0 | 6.8 | 7.0 |
| Gm5803 | 6.1 | 5.5 | 7.4 | 6.0 | 6.2 | 4.8 | 8.1 | 7.9 | 8.3 | 8.2 | 8.3 | 7.9 |
| Gm5820 | 0.5 | 0.2 | 0.2 | 0.4 | 0.3 | 0.5 | 0.3 | 0.1 | 0.2 | 0.5 | 0.4 | 0.2 |
| Gm5860 | 0.2 | 0.1 | 0.1 | 0.2 | 0.1 | 0.1 | 0.0 | 0.1 | 0.1 | 0.2 | 0.1 | 0.1 |
| Gm5878 | 0.0 | 0.0 | 0.0 | 0.0 | 0.0 | 0.0 | 0.0 | 0.0 | 0.0 | 0.0 | 0.0 | 0.0 |
| Gm5901 | 0.4 | 0.1 | 0.4 | 0.5 | 0.3 | 0.2 | 0.4 | 0.4 | 0.4 | 0.1 | 0.6 | 0.3 |
| Gm5918 | 10.0 | 10.0 | 9.3 | 12.4 | 7.4 | 12.8 | 7.8 | 6.5 | 5.9 | 7.5 | 8.9 | 10.0 |
| Gm595 | 0.0 | 0.1 | 0.0 | 0.0 | 0.0 | 0.0 | 0.0 | 0.0 | 0.0 | 0.0 | 0.0 | 0.0 |
| Gm6034 | 0.1 | 0.0 | 0.0 | 0.0 | 0.0 | 0.0 | 0.1 | 0.0 | 0.1 | 0.0 | 0.0 | 0.1 |
| Gm608 | 2.5 | 2.7 | 2.9 | 2.4 | 2.2 | 2.3 | 2.3 | 2.4 | 2.2 | 2.0 | 2.2 | 2.1 |
| Gm6086 | 1.5 | 1.2 | 1.2 | 1.3 | 1.6 | 0.5 | 2.0 | 1.5 | 1.3 | 0.9 | 1.2 | 1.1 |
| Gm6225 | 0.1 | 0.0 | 0.3 | 0.1 | 0.1 | 0.2 | 0.2 | 0.2 | 0.2 | 0.3 | 0.1 | 0.2 |
| Gm6251 | 11.8 | 8.2 | 10.3 | 9.8 | 10.0 | 10.2 | 12.1 | 9.0 | 8.7 | 11.8 | 9.2 | 11.4 |
| Gm6277 | 0.1 | 0.1 | 0.2 | 0.2 | 0.0 | 0.0 | 0.0 | 0.1 | 0.0 | 0.1 | 0.0 | 0.1 |
| Gm6297 | 0.4 | 0.3 | 0.3 | 0.1 | 0.4 | 0.6 | 0.4 | 0.3 | 0.3 | 0.2 | 0.3 | 0.4 |
| Gm6313 | 0.0 | 0.1 | 0.1 | 0.2 | 0.0 | 0.1 | 0.1 | 0.1 | 0.1 | 0.1 | 0.1 | 0.1 |
| Gm6377 | 0.1 | 0.0 | 0.0 | 0.2 | 0.7 | 0.0 | 0.1 | 0.1 | 0.1 | 0.2 | 0.0 | 0.1 |
| Gm6402 | 1.3 | 1.0 | 0.9 | 1.6 | 1.3 | 1.2 | 1.4 | 1.1 | 1.2 | 1.3 | 1.2 | 1.3 |
| Gm6432 | 0.0 | 0.1 | 0.1 | 0.0 | 0.0 | 0.2 | 0.0 | 0.1 | 0.1 | 0.1 | 0.0 | 0.0 |
| Gm6455 | 0.3 | 0.0 | 0.0 | 0.1 | 0.1 | 0.1 | 0.1 | 0.1 | 0.0 | 0.0 | 0.0 | 0.0 |
| Gm6460 | 0.2 | 0.1 | 0.0 | 0.1 | 0.2 | 0.1 | 0.2 | 0.2 | 0.0 | 0.0 | 0.1 | 0.1 |
| Gm6484 | 0.7 | 1.1 | 1.0 | 0.3 | 0.6 | 0.1 | 1.2 | 2.4 | 1.4 | 0.9 | 1.6 | 1.2 |
| Gm6498 | 0.1 | 0.1 | 0.1 | 0.1 | 0.2 | 0.0 | 0.3 | 0.2 | 0.0 | 0.0 | 0.1 | 0.1 |
| Gm6524 | 0.0 | 0.0 | 0.0 | 0.0 | 0.0 | 0.0 | 0.0 | 0.0 | 0.0 | 0.1 | 0.0 | 0.0 |
| Gm6525 | 0.0 | 0.1 | 0.3 | 0.0 | 0.1 | 0.0 | 0.1 | 0.0 | 0.1 | 0.1 | 0.0 | 0.0 |
| Gm6537 | 1.3 | 1.1 | 1.3 | 1.8 | 0.5 | 0.6 | 0.8 | 1.8 | 1.0 | 0.7 | 1.4 | 0.9 |
| Gm6548 | 16.6 | 14.1 | 15.1 | 18.5 | 15.4 | 12.5 | 12.3 | 10.8 | 11.7 | 12.2 | 11.9 | 17.6 |
| Gm6568 | 0.1 | 0.2 | 0.3 | 0.5 | 0.1 | 0.1 | 0.3 | 0.3 | 0.2 | 0.2 | 0.2 | 0.5 |
| Gm6578 | 0.1 | 0.1 | 0.0 | 0.0 | 0.1 | 0.0 | 0.1 | 0.0 | 0.0 | 0.1 | 0.0 | 0.0 |
| Gm6583 | 0.1 | 0.1 | 0.0 | 0.2 | 0.1 | 0.2 | 0.1 | 0.1 | 0.1 | 0.1 | 0.1 | 0.0 |
| Gm6607 | 0.0 | 0.1 | 0.1 | 0.0 | 0.1 | 0.0 | 0.1 | 0.1 | 0.1 | 0.0 | 0.0 | 0.1 |
| Gm6623 | 0.5 | 0.5 | 0.5 | 0.8 | 0.7 | 0.8 | 0.6 | 0.4 | 0.4 | 0.7 | 0.9 | 0.7 |
| Gm6634 | 1.1 | 0.4 | 0.3 | 0.6 | 0.7 | 0.6 | 0.4 | 0.3 | 0.2 | 0.4 | 0.6 | 0.5 |
| Gm6642 | 0.3 | 0.1 | 0.5 | 0.0 | 0.1 | 0.0 | 0.2 | 0.2 | 0.2 | 0.2 | 0.0 | 0.1 |
| Gm6644 | 100.8 | 73.0 | 57.3 | 112.6 | 88.3 | 82.0 | 66.6 | 62.3 | 56.0 | 75.1 | 78.6 | 61.8 |
| Gm6654 | 1.3 | 0.9 | 1.3 | 1.9 | 1.4 | 1.1 | 1.0 | 1.7 | 1.3 | 1.0 | 1.1 | 1.3 |

| | | | | | | | | | | | | |
|---|---|---|---|---|---|---|---|---|---|---|---|---|
| Gm6682 | 5.6 | 5.6 | 7.0 | 9.3 | 6.4 | 6.1 | 9.8 | 7.9 | 7.1 | 4.8 | 7.7 | 5.2 |
| Gm6710 | 3.4 | 3.3 | 3.7 | 5.4 | 3.7 | 4.8 | 3.3 | 3.6 | 3.3 | 4.5 | 4.9 | 4.8 |
| Gm6756 | 0.0 | 0.0 | 0.0 | 0.0 | 0.0 | 0.0 | 0.0 | 0.0 | 0.0 | 0.0 | 0.0 | 0.0 |
| Gm6787 | 0.1 | 0.1 | 0.0 | 0.0 | 0.0 | 0.0 | 0.1 | 0.0 | 0.0 | 0.1 | 0.0 | 0.0 |
| Gm6793 | 23.2 | 25.4 | 34.7 | 28.8 | 32.1 | 25.4 | 31.4 | 34.1 | 26.0 | 27.1 | 33.7 | 38.5 |
| Gm684 | 1.9 | 2.5 | 2.7 | 2.8 | 2.1 | 3.3 | 2.3 | 3.0 | 2.1 | 1.8 | 2.6 | 2.8 |
| Gm6878 | 0.0 | 0.1 | 0.0 | 0.0 | 0.1 | 0.0 | 0.2 | 0.1 | 0.0 | 0.0 | 0.0 | 0.0 |
| Gm6880 | 0.1 | 0.0 | 0.2 | 0.1 | 0.0 | 0.2 | 0.0 | 0.0 | 0.0 | 0.0 | 0.0 | 0.0 |
| Gm6904 | 0.7 | 0.5 | 0.3 | 0.9 | 0.5 | 0.5 | 0.0 | 0.1 | 0.0 | 0.6 | 0.2 | 0.3 |
| Gm6981 | 0.4 | 0.4 | 0.7 | 0.5 | 0.6 | 0.4 | 0.5 | 0.5 | 0.3 | 0.4 | 0.5 | 0.5 |
| Gm7030 | 8.2 | 5.0 | 5.7 | 9.0 | 7.6 | 6.6 | 3.0 | 3.0 | 2.9 | 3.1 | 1.9 | 2.8 |
| Gm7102 | 2.8 | 3.1 | 2.6 | 2.0 | 3.2 | 2.1 | 3.0 | 2.6 | 2.5 | 2.6 | 1.9 | 2.1 |
| Gm711 | 0.0 | 0.0 | 0.0 | 0.0 | 0.1 | 0.1 | 0.0 | 0.0 | 0.1 | 0.0 | 0.0 | 0.0 |
| Gm7120 | 8.6 | 8.5 | 11.5 | 9.3 | 8.7 | 6.4 | 13.3 | 9.2 | 11.6 | 10.8 | 9.3 | 10.4 |
| Gm715 | 1.4 | 1.7 | 1.8 | 1.7 | 1.1 | 0.7 | 1.3 | 0.9 | 0.8 | 1.1 | 1.3 | 0.8 |
| Gm7168 | 0.1 | 0.0 | 0.0 | 0.0 | 0.0 | 0.0 | 0.0 | 0.0 | 0.0 | 0.0 | 0.2 | 0.1 |
| Gm7173 | 0.0 | 0.1 | 0.1 | 0.1 | 0.0 | 0.0 | 0.1 | 0.1 | 0.1 | 0.2 | 0.1 | 0.1 |
| Gm7334 | 37.2 | 44.3 | 39.4 | 39.8 | 32.8 | 43.4 | 41.7 | 25.0 | 33.6 | 53.5 | 49.8 | 41.3 |
| Gm7367 | 2.4 | 6.9 | 10.6 | 11.9 | 8.1 | 7.7 | 9.6 | 8.8 | 3.9 | 7.8 | 6.3 | 6.5 |
| Gm7444 | 0.0 | 0.1 | 0.2 | 0.1 | 0.1 | 0.1 | 0.0 | 0.1 | 0.0 | 0.1 | 0.1 | 0.0 |
| Gm7609 | 4.1 | 3.9 | 3.6 | 7.0 | 4.1 | 4.3 | 3.1 | 1.9 | 2.0 | 1.8 | 1.9 | 3.3 |
| Gm7694 | 0.2 | 0.3 | 0.3 | 0.2 | 0.2 | 0.2 | 0.2 | 0.1 | 0.2 | 0.3 | 0.2 | 0.1 |
| Gm7854 | 0.2 | 0.0 | 0.0 | 0.1 | 0.1 | 0.0 | 0.1 | 0.0 | 0.0 | 0.1 | 0.0 | 0.1 |
| Gm7904 | 0.1 | 0.0 | 0.0 | 0.0 | 0.0 | 0.0 | 0.0 | 0.0 | 0.0 | 0.0 | 0.0 | 0.0 |
| Gm7977 | 0.2 | 0.2 | 0.2 | 0.6 | 0.3 | 0.2 | 0.2 | 0.2 | 0.2 | 0.1 | 0.2 | 0.2 |
| Gm8096 | 0.0 | 0.0 | 0.0 | 0.1 | 0.0 | 0.0 | 0.1 | 0.0 | 0.0 | 0.0 | 0.1 | 0.0 |
| Gm8234 | 0.6 | 0.4 | 0.8 | 0.6 | 0.6 | 0.7 | 0.6 | 0.5 | 0.5 | 0.5 | 0.5 | 0.5 |
| Gm8300 | 0.0 | 0.0 | 0.0 | 0.0 | 0.0 | 0.0 | 0.0 | 0.0 | 0.0 | 0.0 | 0.0 | 0.0 |
| Gm8369 | 0.0 | 0.0 | 0.1 | 0.0 | 0.0 | 0.0 | 0.0 | 0.0 | 0.0 | 0.0 | 0.0 | 0.0 |
| Gm8579 | 0.0 | 0.0 | 0.0 | 0.0 | 0.1 | 0.1 | 0.0 | 0.0 | 0.0 | 0.1 | 0.0 | 0.1 |
| Gm8580 | 0.2 | 0.2 | 0.4 | 0.2 | 0.4 | 0.6 | 0.5 | 1.0 | 0.2 | 0.5 | 0.4 | 0.3 |
| Gm8615 | 0.4 | 0.3 | 0.3 | 0.4 | 0.5 | 0.5 | 0.3 | 0.3 | 0.2 | 0.4 | 0.3 | 0.3 |
| Gm8764 | 0.0 | 0.0 | 0.0 | 0.0 | 0.0 | 0.0 | 0.0 | 0.0 | 0.0 | 0.0 | 0.0 | 0.0 |
| Gm8765 | 0.0 | 0.0 | 0.0 | 0.0 | 0.0 | 0.0 | 0.0 | 0.0 | 0.0 | 0.0 | 0.0 | 0.0 |
| Gm8773 | 0.3 | 0.5 | 0.3 | 0.0 | 0.7 | 0.5 | 0.5 | 0.1 | 0.4 | 0.5 | 0.5 | 0.6 |
| Gm8787 | 0.1 | 0.1 | 0.0 | 0.0 | 0.0 | 0.1 | 0.0 | 0.0 | 0.2 | 0.1 | 0.0 | 0.0 |
| Gm8801 | 0.2 | 0.2 | 0.2 | 0.3 | 0.3 | 0.1 | 0.1 | 0.2 | 0.3 | 0.2 | 0.1 | 0.2 |
| Gm8817 | 0.0 | 0.0 | 0.0 | 0.0 | 0.1 | 0.3 | 0.0 | 0.2 | 0.1 | 0.1 | 0.0 | 0.1 |
| Gm8883 | 0.0 | 0.0 | 0.0 | 0.1 | 0.0 | 0.1 | 0.1 | 0.1 | 0.0 | 0.0 | 0.1 | 0.1 |
| Gm8898 | 4.7 | 4.0 | 4.1 | 5.5 | 4.4 | 5.0 | 3.8 | 4.3 | 3.6 | 4.5 | 5.1 | 4.8 |
| Gm8909 | 2.7 | 1.8 | 1.8 | 2.7 | 2.7 | 2.1 | 1.3 | 1.0 | 1.0 | 1.1 | 0.7 | 1.1 |
| Gm8979 | 3.9 | 2.0 | 2.6 | 6.0 | 3.7 | 1.8 | 2.7 | 1.2 | 2.2 | 1.2 | 2.3 | 3.4 |
| Gm8989 | 1.2 | 0.6 | 0.9 | 2.5 | 1.2 | 0.7 | 1.0 | 0.5 | 0.8 | 0.4 | 0.9 | 1.3 |
| Gm8994 | 0.0 | 0.1 | 0.1 | 0.1 | 0.1 | 0.1 | 0.0 | 0.0 | 0.1 | 0.1 | 0.1 | 0.1 |
| Gm9054 | 0.0 | 0.1 | 0.1 | 0.1 | 0.4 | 0.2 | 0.0 | 0.1 | 0.2 | 0.3 | 0.3 | 0.1 |
| Gm906 | 0.0 | 0.0 | 0.0 | 0.0 | 0.0 | 0.0 | 0.0 | 0.0 | 0.0 | 0.0 | 0.0 | 0.0 |
| Gm9079 | 0.6 | 0.5 | 0.7 | 1.0 | 0.8 | 0.7 | 0.9 | 0.7 | 1.1 | 0.9 | 0.8 | 1.1 |
| Gm9159 | 0.7 | 0.9 | 1.3 | 1.0 | 0.6 | 0.8 | 1.1 | 0.7 | 0.8 | 0.5 | 0.8 | 0.7 |
| Gm9199 | 0.1 | 0.1 | 0.1 | 0.4 | 0.1 | 0.1 | 0.1 | 0.1 | 0.0 | 0.1 | 0.2 | 0.3 |
| Gm9268 | 0.0 | 0.0 | 0.0 | 0.0 | 0.0 | 0.0 | 0.0 | 0.0 | 0.0 | 0.0 | 0.0 | 0.0 |
| Gm960 | 0.1 | 0.1 | 0.0 | 0.0 | 0.1 | 0.1 | 0.0 | 0.1 | 0.0 | 0.0 | 0.0 | 0.0 |
| Gm9696 | 0.3 | 0.7 | 1.2 | 0.6 | 0.5 | 0.2 | 0.4 | 0.2 | 0.1 | 0.2 | 0.1 | 0.3 |
| Gm9705 | 0.4 | 0.4 | 0.3 | 0.7 | 0.8 | 0.4 | 0.6 | 0.6 | 0.5 | 0.5 | 0.7 | 0.3 |
| Gm973 | 0.1 | 0.3 | 0.0 | 0.1 | 0.0 | 0.0 | 0.0 | 0.0 | 0.0 | 0.0 | 0.0 | 0.0 |
| Gm9767 | 0.1 | 0.1 | 0.3 | 0.3 | 0.3 | 0.1 | 1.0 | 0.3 | 0.7 | 0.7 | 0.3 | 0.3 |
| Gm9776 | 0.9 | 1.2 | 0.8 | 0.8 | 0.9 | 1.0 | 0.9 | 1.1 | 1.0 | 0.8 | 0.9 | 0.9 |
| Gm9833 | 1.1 | 1.3 | 1.8 | 0.9 | 1.3 | 1.1 | 1.8 | 1.4 | 1.4 | 1.4 | 1.7 | 1.9 |

| | | | | | | | | | | | | |
|---|---|---|---|---|---|---|---|---|---|---|---|---|
| Gm9846 | 1299.3 | 1141.1 | 1048.5 | 912.1 | 1030.3 | 1124.1 | 918.1 | 1276.5 | 1166.6 | 1282.5 | 1163.1 | 1140.7 |
| Gm9855 | 4.6 | 5.9 | 4.8 | 3.6 | 5.0 | 3.1 | 5.4 | 6.1 | 6.5 | 4.8 | 5.7 | 3.7 |
| Gm9895 | 0.2 | 0.1 | 0.4 | 0.0 | 0.1 | 0.2 | 0.0 | 0.2 | 0.3 | 0.2 | 0.2 | 0.0 |
| Gm9897 | 1.3 | 1.3 | 1.2 | 1.6 | 1.1 | 1.4 | 1.0 | 1.2 | 1.1 | 1.6 | 1.7 | 1.2 |
| Gm9920 | 0.0 | 0.2 | 0.1 | 0.1 | 0.0 | 0.1 | 0.0 | 0.2 | 0.0 | 0.4 | 0.1 | 0.1 |
| Gm9958 | 0.6 | 0.5 | 0.7 | 0.5 | 0.5 | 0.9 | 0.7 | 0.6 | 0.3 | 0.3 | 0.3 | 0.5 |
| Gm996 | 0.0 | 0.0 | 0.0 | 0.0 | 0.0 | 0.0 | 0.0 | 0.0 | 0.0 | 0.1 | 0.0 | 0.0 |
| Gm9961 | 0.0 | 0.0 | 0.1 | 0.0 | 0.0 | 0.0 | 0.0 | 0.2 | 0.1 | 0.0 | 0.0 | 0.0 |
| Gm9992 | 0.0 | 0.1 | 0.0 | 0.0 | 0.0 | 0.0 | 0.0 | 0.0 | 0.1 | 0.1 | 0.0 | 0.0 |
| Gm9994 | 6.2 | 5.0 | 4.5 | 5.5 | 5.1 | 1.5 | 7.2 | 4.7 | 5.2 | 3.8 | 5.2 | 3.4 |
| Gmcl1 | 6.3 | 7.1 | 7.3 | 5.0 | 6.8 | 6.6 | 5.3 | 6.7 | 6.3 | 6.3 | 6.2 | 6.5 |
| Gmds | 14.4 | 13.9 | 12.3 | 10.4 | 10.8 | 9.7 | 11.2 | 8.8 | 11.0 | 10.9 | 11.5 | 10.3 |
| Gmeb1 | 1.7 | 1.7 | 1.6 | 1.7 | 1.7 | 1.5 | 1.8 | 2.0 | 1.9 | 1.8 | 1.9 | 1.9 |
| Gmeb2 | 2.4 | 2.6 | 2.5 | 2.2 | 2.5 | 2.4 | 3.2 | 2.9 | 2.7 | 2.8 | 2.3 | 2.6 |
| Gmfb | 48.2 | 44.2 | 35.5 | 65.3 | 44.7 | 49.5 | 38.2 | 29.6 | 36.7 | 48.6 | 46.1 | 43.0 |
| Gmfg | 1.0 | 0.3 | 0.2 | 4.1 | 6.1 | 0.1 | 1.3 | 0.1 | 0.2 | 0.8 | 0.2 | 0.8 |
| Gmip | 4.6 | 4.4 | 3.6 | 5.8 | 7.2 | 3.4 | 4.2 | 3.4 | 3.9 | 4.4 | 3.8 | 4.0 |
| Gmnn | 4.4 | 7.9 | 9.8 | 4.8 | 9.9 | 3.8 | 10.2 | 4.9 | 8.7 | 5.8 | 6.5 | 5.2 |
| Gmppa | 31.6 | 30.9 | 32.3 | 31.8 | 28.3 | 29.9 | 30.1 | 33.9 | 30.6 | 27.3 | 31.8 | 29.6 |
| Gmppb | 16.6 | 15.4 | 12.0 | 15.4 | 15.6 | 14.2 | 15.1 | 15.9 | 16.4 | 14.6 | 16.2 | 16.8 |
| Gmpr | 4.7 | 4.7 | 2.8 | 6.1 | 3.5 | 6.0 | 2.3 | 2.4 | 2.6 | 4.4 | 3.2 | 2.8 |
| Gmpr2 | 18.5 | 15.1 | 13.2 | 18.2 | 17.7 | 18.2 | 16.3 | 13.9 | 15.1 | 15.1 | 15.9 | 16.7 |
| Gmps | 13.3 | 14.1 | 11.8 | 9.3 | 11.7 | 11.8 | 11.5 | 10.0 | 12.5 | 14.7 | 13.3 | 13.1 |
| Gna11 | 27.9 | 24.6 | 22.7 | 28.8 | 24.1 | 28.6 | 27.5 | 23.7 | 24.5 | 23.6 | 24.8 | 21.1 |
| Gna12 | 34.2 | 35.9 | 29.8 | 36.1 | 35.0 | 36.8 | 39.8 | 30.6 | 35.4 | 30.0 | 35.6 | 30.4 |
| Gna13 | 13.9 | 14.1 | 11.1 | 15.7 | 15.4 | 15.8 | 11.4 | 10.6 | 12.6 | 11.5 | 11.2 | 11.0 |
| Gna14 | 0.1 | 0.1 | 0.1 | 0.1 | 0.1 | 0.2 | 0.1 | 0.1 | 0.0 | 0.1 | 0.0 | 0.1 |
| Gna15 | 0.4 | 0.4 | 0.3 | 1.3 | 2.3 | 0.2 | 0.5 | 0.1 | 0.2 | 0.3 | 0.3 | 0.3 |
| Gnai1 | 5.2 | 5.1 | 5.7 | 5.0 | 5.2 | 3.0 | 6.6 | 4.4 | 7.2 | 6.6 | 6.5 | 7.3 |
| Gnai2 | 168.9 | 142.6 | 138.9 | 217.2 | 175.9 | 185.2 | 177.6 | 151.8 | 148.6 | 139.9 | 159.1 | 148.3 |
| Gnai3 | 52.5 | 55.9 | 50.6 | 53.1 | 52.1 | 53.1 | 49.3 | 49.3 | 56.3 | 50.9 | 58.4 | 56.4 |
| Gnal | 0.7 | 0.6 | 0.6 | 0.7 | 0.5 | 0.7 | 0.7 | 0.5 | 0.6 | 0.6 | 0.4 | 0.7 |
| Gnao1 | 1.1 | 1.9 | 2.6 | 1.2 | 1.6 | 1.2 | 2.5 | 2.5 | 2.8 | 1.3 | 1.6 | 1.8 |
| Gnaq | 21.6 | 22.0 | 20.9 | 24.8 | 22.5 | 21.1 | 24.2 | 18.2 | 21.0 | 19.2 | 24.3 | 25.8 |
| Gnas | 511.8 | 472.8 | 434.1 | 562.9 | 451.8 | 586.6 | 505.8 | 516.4 | 474.5 | 423.6 | 529.3 | 442.0 |
| Gnat1 | 0.0 | 0.0 | 0.0 | 0.0 | 0.0 | 0.0 | 0.0 | 0.0 | 0.0 | 0.0 | 0.0 | 0.0 |
| Gnat2 | 1.8 | 0.9 | 0.7 | 1.9 | 0.9 | 1.5 | 0.9 | 0.6 | 0.5 | 1.3 | 1.4 | 0.9 |
| Gnaz | 0.6 | 0.8 | 0.5 | 1.1 | 0.4 | 1.2 | 0.4 | 0.5 | 0.3 | 0.2 | 0.4 | 0.2 |
| Gnb1 | 100.1 | 102.2 | 87.8 | 120.3 | 105.9 | 112.4 | 111.8 | 96.5 | 99.2 | 94.7 | 110.4 | 99.6 |
| Gnb1l | 0.8 | 0.8 | 0.7 | 0.7 | 0.5 | 0.7 | 0.4 | 0.5 | 0.4 | 0.7 | 0.5 | 0.7 |
| Gnb2 | 80.9 | 85.2 | 78.1 | 75.9 | 97.9 | 76.3 | 100.1 | 93.0 | 105.8 | 96.0 | 91.9 | 82.4 |
| Gnb2l1 | 697.0 | 585.2 | 514.7 | 488.8 | 563.6 | 574.7 | 531.0 | 686.0 | 605.8 | 640.6 | 636.3 | 568.2 |
| Gnb3 | 0.0 | 0.0 | 0.0 | 0.0 | 0.1 | 0.0 | 0.0 | 0.0 | 0.0 | 0.1 | 0.1 | 0.1 |
| Gnb4 | 3.4 | 2.2 | 2.7 | 4.0 | 3.3 | 3.7 | 2.7 | 2.4 | 2.8 | 3.0 | 2.3 | 2.6 |
| Gnb5 | 9.5 | 8.9 | 7.2 | 9.5 | 7.4 | 10.9 | 7.5 | 7.3 | 6.6 | 7.0 | 6.4 | 6.6 |
| Gne | 16.8 | 18.7 | 16.8 | 15.2 | 16.0 | 17.2 | 17.9 | 15.0 | 15.4 | 15.8 | 16.6 | 16.8 |
| Gng10 | 41.2 | 41.3 | 35.3 | 49.4 | 40.0 | 53.9 | 38.6 | 41.1 | 36.1 | 30.8 | 45.4 | 41.0 |
| Gng11 | 20.9 | 14.6 | 12.8 | 19.4 | 17.6 | 24.7 | 16.0 | 14.9 | 16.3 | 17.9 | 14.5 | 18.9 |
| Gng12 | 52.8 | 49.1 | 51.2 | 56.6 | 52.6 | 51.4 | 49.1 | 46.6 | 46.4 | 47.4 | 57.0 | 54.1 |
| Gng13 | 0.9 | 1.0 | 1.7 | 0.0 | 0.2 | 1.0 | 0.7 | 0.3 | 0.1 | 0.8 | 0.0 | 0.1 |
| Gng2 | 1.6 | 1.0 | 1.2 | 5.6 | 5.8 | 2.9 | 1.3 | 0.7 | 0.7 | 0.6 | 0.7 | 0.6 |
| Gng3 | 0.3 | 0.1 | 0.0 | 0.1 | 0.1 | 0.2 | 0.2 | 0.2 | 0.1 | 0.2 | 0.1 | 0.1 |
| Gng4 | 0.1 | 0.2 | 0.1 | 0.2 | 0.1 | 0.5 | 0.2 | 0.2 | 0.1 | 0.1 | 0.1 | 0.2 |
| Gng5 | 120.0 | 138.1 | 166.8 | 212.1 | 184.7 | 195.6 | 167.4 | 187.3 | 189.5 | 212.0 | 230.9 | 197.5 |
| Gng7 | 0.3 | 0.4 | 0.4 | 0.1 | 0.2 | 0.2 | 0.2 | 0.2 | 0.3 | 0.3 | 0.3 | 0.3 |
| Gng8 | 5.4 | 8.5 | 19.1 | 7.1 | 8.2 | 6.6 | 11.2 | 16.9 | 11.3 | 5.6 | 7.0 | 7.6 |
| Gngt2 | 3.5 | 2.0 | 2.2 | 13.7 | 19.5 | 0.8 | 3.6 | 2.1 | 2.6 | 3.1 | 2.7 | 3.3 |

| | | | | | | | | | | | | |
|---|---|---|---|---|---|---|---|---|---|---|---|---|
| Gnl1 | 18.3 | 20.1 | 17.5 | 19.7 | 16.1 | 22.6 | 17.8 | 15.6 | 14.8 | 14.0 | 15.7 | 14.8 |
| Gnl2 | 21.2 | 16.1 | 17.3 | 21.6 | 17.1 | 18.0 | 19.6 | 17.9 | 16.6 | 32.9 | 26.3 | 25.1 |
| Gnl3 | 29.6 | 24.0 | 18.5 | 31.3 | 22.9 | 26.9 | 21.1 | 17.9 | 17.8 | 24.2 | 25.9 | 25.1 |
| Gnl3l | 19.8 | 17.3 | 17.2 | 19.1 | 16.6 | 18.9 | 18.2 | 16.6 | 15.4 | 19.2 | 20.1 | 18.7 |
| Gnpat | 15.4 | 14.8 | 12.9 | 11.1 | 14.4 | 14.6 | 12.7 | 15.5 | 14.9 | 14.9 | 15.2 | 13.8 |
| Gnpda1 | 8.0 | 6.1 | 5.0 | 8.6 | 7.6 | 10.3 | 4.3 | 5.8 | 4.5 | 7.7 | 5.9 | 6.6 |
| Gnpda2 | 19.3 | 17.8 | 17.7 | 23.3 | 19.8 | 25.7 | 17.3 | 17.4 | 15.0 | 15.8 | 21.7 | 20.5 |
| Gnpnat1 | 13.5 | 14.9 | 15.3 | 15.9 | 12.7 | 15.3 | 12.0 | 12.9 | 14.0 | 14.9 | 16.1 | 15.9 |
| Gnptab | 9.2 | 7.8 | 11.6 | 8.0 | 11.3 | 8.2 | 12.0 | 12.5 | 9.0 | 11.9 | 12.2 | 11.6 |
| Gnptg | 15.1 | 10.3 | 10.4 | 17.5 | 12.3 | 17.1 | 10.2 | 11.0 | 9.7 | 12.5 | 10.8 | 11.2 |
| Gnrh1 | 0.4 | 0.1 | 0.2 | 0.3 | 0.2 | 0.6 | 0.1 | 0.4 | 0.3 | 0.1 | 0.1 | 0.1 |
| Gns | 103.3 | 72.9 | 64.6 | 124.1 | 102.9 | 114.6 | 73.8 | 60.7 | 62.1 | 76.1 | 68.7 | 80.3 |
| Golga1 | 6.4 | 6.2 | 6.3 | 5.4 | 5.3 | 5.0 | 6.0 | 6.2 | 5.9 | 5.6 | 7.2 | 6.3 |
| Golga2 | 25.6 | 25.5 | 25.3 | 25.6 | 22.8 | 25.6 | 30.1 | 27.8 | 27.3 | 22.5 | 28.3 | 27.1 |
| Golga3 | 24.7 | 23.4 | 22.6 | 24.3 | 21.3 | 23.2 | 23.8 | 21.2 | 21.3 | 20.4 | 26.2 | 26.1 |
| Golga4 | 34.7 | 28.4 | 24.1 | 44.1 | 27.9 | 42.7 | 26.4 | 19.9 | 19.4 | 24.2 | 29.3 | 28.4 |
| Golga5 | 28.5 | 27.4 | 26.6 | 29.7 | 25.2 | 26.3 | 27.2 | 27.5 | 25.1 | 23.9 | 30.5 | 29.7 |
| Golga7 | 51.6 | 53.5 | 60.0 | 54.9 | 54.0 | 52.0 | 52.3 | 61.3 | 66.4 | 64.3 | 65.3 | 65.2 |
| Golgb1 | 39.1 | 35.8 | 33.7 | 45.0 | 32.8 | 44.4 | 34.8 | 28.9 | 26.9 | 31.0 | 38.9 | 38.4 |
| Golim4 | 72.8 | 68.4 | 71.1 | 88.0 | 59.0 | 79.5 | 68.6 | 48.9 | 50.4 | 58.4 | 69.4 | 79.4 |
| Golm1 | 9.7 | 9.1 | 10.1 | 11.4 | 12.9 | 8.0 | 10.7 | 11.1 | 9.7 | 11.7 | 9.6 | 9.8 |
| Golph3 | 51.6 | 51.5 | 53.0 | 53.1 | 49.2 | 52.6 | 51.6 | 51.6 | 49.7 | 52.5 | 59.8 | 61.5 |
| Golph3l | 11.0 | 11.4 | 11.7 | 13.7 | 10.0 | 11.8 | 8.9 | 10.8 | 9.5 | 10.8 | 10.8 | 12.5 |
| Golt1b | 32.4 | 30.5 | 24.1 | 26.5 | 31.5 | 30.8 | 32.1 | 28.7 | 35.8 | 34.7 | 37.5 | 31.1 |
| Gon4l | 5.5 | 6.7 | 6.6 | 5.7 | 5.9 | 6.1 | 6.0 | 6.7 | 5.6 | 6.0 | 6.1 | 6.1 |
| Gopc | 7.4 | 8.9 | 10.2 | 7.3 | 8.6 | 7.0 | 10.1 | 8.6 | 8.7 | 8.3 | 8.9 | 9.6 |
| Gorab | 4.9 | 5.6 | 5.5 | 5.4 | 4.7 | 5.7 | 5.7 | 5.4 | 5.4 | 5.3 | 7.3 | 6.4 |
| Gorasp1 | 8.7 | 8.1 | 7.5 | 10.4 | 8.8 | 10.0 | 8.3 | 7.3 | 8.1 | 9.3 | 8.4 | 7.9 |
| Gorasp2 | 58.2 | 56.9 | 54.7 | 68.0 | 56.1 | 57.2 | 69.3 | 57.7 | 59.0 | 57.1 | 62.0 | 65.4 |
| Gosr1 | 10.5 | 11.8 | 11.6 | 12.0 | 10.9 | 10.3 | 11.1 | 12.1 | 11.4 | 11.5 | 11.5 | 12.1 |
| Gosr2 | 47.3 | 46.0 | 41.9 | 50.0 | 47.3 | 42.6 | 43.8 | 51.2 | 44.9 | 43.9 | 47.3 | 46.7 |
| Got1 | 16.6 | 13.8 | 10.3 | 18.5 | 13.7 | 19.7 | 15.3 | 15.8 | 14.3 | 15.2 | 17.1 | 12.5 |
| Got1l1 | 0.2 | 0.1 | 0.3 | 0.1 | 0.1 | 0.0 | 0.0 | 0.1 | 0.0 | 0.2 | 0.2 | 0.0 |
| Got2 | 51.8 | 43.0 | 36.0 | 53.6 | 46.1 | 48.2 | 41.9 | 38.8 | 32.6 | 45.3 | 45.7 | 37.9 |
| Gp1ba | 0.5 | 0.5 | 0.4 | 0.4 | 0.4 | 0.8 | 0.4 | 0.3 | 0.3 | 0.6 | 0.5 | 0.3 |
| Gp1bb | 0.7 | 0.6 | 0.8 | 0.6 | 0.7 | 0.6 | 1.3 | 1.5 | 1.8 | 0.7 | 1.1 | 0.8 |
| Gp49a | 2.9 | 0.1 | 0.1 | 36.4 | 51.4 | 0.0 | 4.4 | 1.0 | 0.6 | 2.2 | 1.2 | 2.5 |
| Gp5 | 0.0 | 0.1 | 0.0 | 0.0 | 0.0 | 0.1 | 0.1 | 0.0 | 0.0 | 0.1 | 0.0 | 0.0 |
| Gpaa1 | 10.7 | 11.8 | 8.9 | 9.9 | 10.9 | 10.8 | 11.8 | 12.8 | 11.9 | 9.8 | 9.0 | 7.8 |
| Gpam | 12.0 | 22.9 | 45.5 | 10.1 | 19.0 | 9.8 | 13.6 | 23.7 | 14.1 | 12.8 | 11.3 | 14.4 |
| Gpank1 | 4.9 | 5.8 | 5.5 | 6.7 | 4.6 | 6.9 | 5.2 | 5.3 | 4.9 | 4.5 | 5.3 | 4.7 |
| Gpat2 | 0.1 | 0.0 | 0.1 | 0.1 | 0.1 | 0.3 | 0.2 | 0.0 | 0.1 | 0.1 | 0.1 | 0.1 |
| Gpatch1 | 5.9 | 5.5 | 6.5 | 5.8 | 4.5 | 5.7 | 6.2 | 5.9 | 4.3 | 5.2 | 5.9 | 6.4 |
| Gpatch11 | 3.8 | 3.4 | 3.8 | 3.8 | 2.8 | 3.5 | 3.7 | 3.8 | 3.5 | 3.9 | 4.5 | 4.2 |
| Gpatch2 | 4.7 | 4.4 | 4.2 | 4.3 | 3.9 | 4.6 | 4.5 | 3.9 | 3.8 | 4.2 | 4.8 | 4.3 |
| Gpatch2l | 6.0 | 6.3 | 5.4 | 6.2 | 5.8 | 6.1 | 6.4 | 5.4 | 5.5 | 5.6 | 5.8 | 6.1 |
| Gpatch3 | 2.5 | 2.0 | 2.0 | 2.8 | 2.9 | 2.7 | 2.2 | 2.4 | 2.0 | 2.7 | 2.2 | 2.5 |
| Gpatch4 | 9.3 | 6.1 | 6.7 | 14.6 | 7.4 | 9.1 | 9.2 | 6.2 | 3.8 | 5.5 | 8.1 | 7.1 |
| Gpatch8 | 4.8 | 5.4 | 6.1 | 5.3 | 4.8 | 4.4 | 5.5 | 5.3 | 4.9 | 4.6 | 4.8 | 4.9 |
| Gpbar1 | 0.1 | 0.1 | 0.5 | 0.4 | 0.1 | 0.1 | 0.4 | 0.5 | 0.3 | 0.3 | 0.3 | 0.1 |
| Gpbp1 | 19.6 | 21.0 | 23.1 | 21.8 | 20.7 | 22.2 | 17.5 | 19.7 | 19.9 | 22.9 | 24.5 | 23.8 |
| Gpbp1l1 | 12.2 | 12.9 | 13.2 | 14.8 | 12.7 | 13.4 | 11.4 | 13.1 | 11.9 | 15.8 | 14.4 | 14.0 |
| Gpc1 | 5.1 | 3.6 | 3.1 | 4.3 | 3.3 | 5.9 | 4.7 | 6.2 | 5.0 | 6.5 | 6.2 | 6.2 |
| Gpc2 | 1.0 | 1.2 | 1.0 | 0.8 | 0.7 | 1.0 | 0.8 | 1.1 | 1.3 | 1.6 | 1.0 | 0.7 |
| Gpc3 | 0.3 | 0.6 | 2.7 | 0.1 | 0.4 | 0.1 | 0.9 | 2.6 | 4.3 | 16.6 | 8.0 | 6.7 |
| Gpc4 | 55.0 | 47.8 | 39.7 | 48.6 | 44.7 | 40.9 | 52.5 | 46.1 | 59.2 | 42.3 | 53.7 | 39.1 |
| Gpc5 | 0.0 | 0.0 | 0.1 | 0.0 | 0.0 | 0.0 | 0.0 | 0.0 | 0.0 | 0.0 | 0.0 | 0.0 |

| | | | | | | | | | | | | |
|---|---|---|---|---|---|---|---|---|---|---|---|---|
| Gpc6 | 5.8 | 7.3 | 10.7 | 5.2 | 8.7 | 5.4 | 10.2 | 13.9 | 12.2 | 7.9 | 9.0 | 9.7 |
| Gpcpd1 | 6.8 | 11.8 | 13.3 | 6.2 | 7.1 | 6.6 | 6.0 | 10.0 | 6.6 | 6.8 | 6.9 | 8.0 |
| Gpd1 | 0.2 | 0.2 | 0.1 | 0.2 | 0.1 | 0.2 | 0.1 | 0.2 | 0.2 | 0.1 | 0.2 | 0.1 |
| Gpd1l | 8.4 | 8.3 | 8.2 | 6.8 | 7.2 | 7.5 | 7.2 | 7.4 | 8.2 | 8.7 | 8.6 | 7.9 |
| Gpd2 | 5.4 | 5.1 | 5.0 | 4.9 | 4.6 | 5.0 | 4.9 | 4.7 | 4.0 | 5.0 | 5.6 | 5.2 |
| Gpha2 | 0.3 | 0.2 | 0.0 | 0.0 | 0.1 | 0.3 | 0.2 | 0.0 | 0.4 | 0.2 | 0.3 | 0.4 |
| Gphn | 2.6 | 3.3 | 4.5 | 2.0 | 2.6 | 2.1 | 2.4 | 4.1 | 2.6 | 3.2 | 2.9 | 3.2 |
| Gpi1 | 107.4 | 92.8 | 83.7 | 126.5 | 105.4 | 116.3 | 104.0 | 102.1 | 93.1 | 106.7 | 105.2 | 100.3 |
| Gpihbp1 | 0.0 | 0.1 | 0.3 | 0.0 | 0.0 | 0.0 | 0.3 | 1.0 | 0.6 | 0.0 | 0.0 | 0.0 |
| Gpkow | 7.9 | 9.5 | 10.3 | 11.0 | 8.4 | 10.5 | 8.4 | 7.7 | 7.1 | 9.1 | 8.9 | 9.1 |
| Gpld1 | 2.6 | 3.0 | 2.3 | 2.8 | 2.5 | 2.4 | 2.4 | 2.2 | 2.3 | 1.9 | 2.1 | 2.4 |
| Gpm6a | 16.2 | 5.9 | 2.9 | 3.8 | 0.8 | 2.0 | 0.5 | 2.5 | 4.2 | 70.1 | 33.5 | 16.9 |
| Gpm6b | 10.7 | 20.9 | 36.9 | 14.7 | 24.2 | 12.0 | 16.7 | 26.1 | 16.4 | 9.9 | 9.2 | 15.3 |
| Gpn1 | 11.7 | 10.1 | 8.3 | 11.5 | 9.7 | 11.4 | 9.0 | 9.5 | 9.1 | 10.3 | 10.4 | 9.7 |
| Gpn2 | 5.2 | 4.3 | 4.4 | 6.1 | 6.7 | 4.3 | 6.5 | 6.8 | 6.8 | 5.6 | 6.1 | 5.3 |
| Gpn3 | 7.9 | 8.9 | 8.7 | 8.7 | 7.3 | 8.0 | 7.7 | 8.6 | 7.1 | 8.9 | 8.2 | 7.2 |
| Gpnmb | 69.5 | 28.5 | 20.4 | 116.5 | 37.8 | 60.9 | 11.1 | 13.1 | 8.2 | 17.0 | 18.3 | 23.3 |
| Gpr1 | 0.0 | 0.2 | 0.0 | 0.0 | 0.1 | 0.0 | 0.1 | 0.1 | 0.1 | 0.1 | 0.0 | 0.0 |
| Gpr107 | 24.4 | 22.7 | 21.2 | 25.2 | 21.9 | 24.8 | 20.8 | 21.2 | 22.7 | 21.6 | 21.3 | 21.8 |
| Gpr108 | 20.9 | 21.3 | 18.9 | 24.3 | 20.6 | 25.1 | 22.5 | 21.2 | 20.4 | 20.8 | 20.2 | 19.5 |
| Gpr110 | 0.0 | 0.0 | 0.0 | 0.0 | 0.0 | 0.0 | 0.0 | 0.0 | 0.0 | 0.0 | 0.0 | 0.0 |
| Gpr111 | 0.0 | 0.0 | 0.0 | 0.0 | 0.0 | 0.0 | 0.0 | 0.0 | 0.0 | 0.0 | 0.1 | 0.0 |
| Gpr115 | 0.0 | 0.0 | 0.1 | 0.0 | 0.0 | 0.0 | 0.0 | 0.0 | 0.0 | 0.1 | 0.0 | 0.1 |
| Gpr116 | 1.3 | 0.3 | 0.6 | 0.6 | 0.4 | 0.2 | 0.7 | 1.0 | 0.8 | 0.4 | 0.5 | 0.3 |
| Gpr123 | 0.1 | 0.1 | 0.1 | 0.1 | 0.1 | 0.1 | 0.1 | 0.0 | 0.0 | 0.1 | 0.0 | 0.0 |
| Gpr124 | 1.1 | 1.5 | 2.5 | 0.9 | 1.2 | 1.2 | 2.5 | 3.9 | 4.3 | 3.3 | 2.5 | 2.2 |
| Gpr125 | 14.7 | 11.2 | 9.1 | 13.7 | 9.4 | 12.9 | 12.4 | 9.0 | 11.1 | 12.9 | 12.7 | 11.2 |
| Gpr126 | 1.8 | 0.7 | 0.8 | 2.1 | 3.3 | 1.2 | 1.7 | 1.4 | 1.9 | 1.6 | 1.3 | 1.7 |
| Gpr128 | 0.0 | 0.0 | 0.0 | 0.0 | 0.0 | 0.0 | 0.0 | 0.0 | 0.0 | 0.0 | 0.0 | 0.0 |
| Gpr132 | 0.0 | 0.0 | 0.1 | 0.2 | 0.7 | 0.1 | 0.1 | 0.0 | 0.0 | 0.1 | 0.0 | 0.0 |
| Gpr133 | 0.9 | 3.1 | 6.5 | 0.6 | 1.3 | 0.5 | 3.3 | 16.6 | 10.6 | 9.3 | 4.9 | 5.8 |
| Gpr135 | 0.2 | 0.1 | 0.2 | 0.1 | 0.2 | 0.2 | 0.3 | 0.3 | 0.2 | 0.3 | 0.1 | 0.4 |
| Gpr137 | 12.5 | 11.9 | 11.9 | 13.9 | 12.7 | 15.2 | 12.1 | 13.8 | 13.4 | 12.5 | 9.9 | 9.1 |
| Gpr137b | 12.2 | 9.4 | 8.5 | 18.1 | 14.7 | 16.8 | 11.8 | 11.0 | 9.3 | 7.7 | 9.3 | 8.5 |
| Gpr137b-ps | 12.6 | 10.2 | 9.3 | 17.2 | 15.5 | 15.4 | 12.7 | 11.8 | 9.7 | 7.9 | 9.3 | 8.5 |
| Gpr137c | 0.6 | 0.5 | 0.3 | 0.3 | 0.6 | 0.6 | 0.5 | 0.2 | 0.4 | 0.5 | 0.5 | 0.4 |
| Gpr141 | 0.6 | 0.4 | 0.1 | 1.5 | 0.4 | 0.8 | 0.4 | 0.1 | 0.4 | 0.1 | 0.3 | 0.1 |
| Gpr143 | 0.4 | 0.1 | 0.1 | 0.2 | 0.1 | 0.1 | 0.0 | 0.0 | 0.0 | 0.0 | 0.0 | 0.1 |
| Gpr146 | 2.0 | 1.9 | 2.0 | 1.7 | 1.8 | 1.8 | 1.3 | 1.6 | 1.3 | 2.1 | 1.6 | 1.7 |
| Gpr151 | 0.1 | 0.1 | 0.3 | 0.1 | 0.3 | 0.0 | 0.2 | 0.1 | 0.2 | 0.2 | 0.1 | 0.2 |
| Gpr152 | 0.0 | 0.0 | 0.0 | 0.0 | 0.0 | 0.0 | 0.0 | 0.0 | 0.0 | 0.1 | 0.0 | 0.0 |
| Gpr153 | 4.7 | 4.4 | 5.6 | 3.0 | 5.8 | 4.3 | 10.5 | 10.2 | 10.2 | 7.9 | 7.7 | 7.3 |
| Gpr155 | 0.6 | 1.0 | 1.2 | 1.0 | 0.9 | 0.8 | 0.6 | 0.9 | 0.5 | 0.5 | 0.4 | 0.6 |
| Gpr156 | 0.1 | 0.1 | 0.2 | 0.1 | 0.1 | 0.1 | 0.1 | 0.2 | 0.2 | 0.1 | 0.1 | 0.1 |
| Gpr157 | 0.5 | 0.6 | 0.5 | 0.4 | 0.4 | 0.4 | 0.4 | 0.5 | 0.5 | 0.3 | 0.5 | 0.4 |
| Gpr160 | 0.1 | 0.1 | 0.1 | 0.0 | 0.2 | 0.2 | 0.1 | 0.1 | 0.0 | 0.1 | 0.0 | 0.2 |
| Gpr161 | 3.6 | 3.4 | 3.3 | 3.7 | 3.4 | 4.7 | 3.9 | 4.1 | 3.5 | 2.6 | 3.7 | 2.7 |
| Gpr162 | 0.8 | 0.7 | 0.9 | 0.9 | 1.0 | 1.0 | 0.4 | 0.9 | 0.8 | 0.4 | 0.6 | 0.8 |
| Gpr165 | 0.0 | 0.0 | 0.0 | 0.0 | 0.0 | 0.0 | 0.0 | 0.0 | 0.0 | 0.0 | 0.0 | 0.0 |
| Gpr17 | 0.1 | 0.2 | 0.1 | 0.1 | 0.1 | 0.1 | 0.3 | 0.1 | 0.2 | 0.1 | 0.1 | 0.2 |
| Gpr173 | 0.2 | 0.3 | 0.3 | 0.1 | 0.2 | 0.2 | 0.2 | 0.2 | 0.2 | 0.1 | 0.0 | 0.1 |
| Gpr176 | 15.8 | 16.0 | 12.9 | 19.1 | 18.2 | 18.2 | 18.8 | 14.7 | 21.1 | 18.0 | 15.8 | 17.6 |
| Gpr179 | 0.0 | 0.0 | 0.0 | 0.0 | 0.0 | 0.0 | 0.0 | 0.0 | 0.0 | 0.0 | 0.0 | 0.0 |
| Gpr18 | 0.1 | 0.0 | 0.0 | 0.5 | 0.9 | 0.1 | 0.2 | 0.0 | 0.4 | 0.1 | 0.0 | 0.0 |
| Gpr180 | 15.7 | 14.7 | 12.7 | 13.5 | 13.9 | 13.9 | 16.2 | 15.2 | 15.1 | 16.3 | 15.2 | 15.2 |
| Gpr182 | 0.3 | 0.1 | 0.2 | 0.2 | 0.2 | 0.4 | 0.3 | 0.1 | 0.1 | 0.1 | 0.1 | 0.1 |
| Gpr183 | 0.0 | 0.0 | 0.1 | 0.2 | 0.8 | 0.0 | 0.1 | 0.0 | 0.0 | 0.1 | 0.0 | 0.1 |

| | | | | | | | | | | | | |
|---|---|---|---|---|---|---|---|---|---|---|---|---|
| Gpr19 | 1.6 | 1.7 | 1.9 | 1.4 | 1.2 | 2.1 | 1.7 | 1.5 | 1.4 | 1.2 | 1.6 | 1.4 |
| Gpr20 | 0.0 | 0.1 | 0.0 | 0.0 | 0.0 | 0.0 | 0.0 | 0.0 | 0.0 | 0.0 | 0.1 | 0.0 |
| Gpr21 | 0.1 | 0.2 | 0.2 | 0.1 | 0.2 | 0.2 | 0.2 | 0.2 | 0.1 | 0.2 | 0.1 | 0.2 |
| Gpr22 | 0.1 | 0.0 | 0.1 | 0.0 | 0.0 | 0.0 | 0.0 | 0.1 | 0.0 | 0.0 | 0.0 | 0.1 |
| Gpr27 | 0.4 | 0.2 | 0.1 | 0.5 | 0.2 | 0.5 | 0.2 | 0.1 | 0.2 | 0.1 | 0.1 | 0.3 |
| Gpr3 | 0.0 | 0.0 | 0.0 | 0.0 | 0.0 | 0.0 | 0.0 | 0.1 | 0.0 | 0.1 | 0.1 | 0.0 |
| Gpr30 | 0.0 | 0.1 | 0.0 | 0.0 | 0.1 | 0.0 | 0.0 | 0.1 | 0.0 | 0.2 | 0.1 | 0.0 |
| Gpr31b | 0.1 | 0.1 | 0.0 | 0.6 | 2.0 | 0.1 | 0.6 | 0.0 | 0.1 | 0.2 | 0.1 | 0.0 |
| Gpr34 | 0.0 | 0.0 | 0.0 | 0.0 | 0.1 | 0.0 | 0.1 | 0.0 | 0.0 | 0.1 | 0.1 | 0.0 |
| Gpr35 | 1.7 | 0.9 | 0.3 | 3.1 | 2.1 | 3.7 | 2.3 | 0.4 | 0.7 | 0.8 | 0.9 | 1.0 |
| Gpr37 | 0.0 | 0.0 | 0.0 | 0.0 | 0.0 | 0.0 | 0.0 | 0.1 | 0.0 | 0.0 | 0.1 | 0.0 |
| Gpr39 | 25.6 | 20.8 | 20.4 | 23.7 | 21.2 | 19.6 | 32.3 | 22.1 | 30.2 | 35.3 | 31.9 | 38.2 |
| Gpr4 | 0.2 | 0.3 | 0.4 | 0.1 | 0.3 | 0.3 | 0.3 | 0.5 | 0.1 | 0.3 | 0.1 | 0.2 |
| Gpr44 | 0.0 | 0.1 | 0.0 | 0.0 | 0.0 | 0.1 | 0.0 | 0.1 | 0.1 | 0.1 | 0.0 | 0.0 |
| Gpr45 | 0.1 | 0.0 | 0.0 | 0.0 | 0.0 | 0.1 | 0.0 | 0.0 | 0.1 | 0.0 | 0.0 | 0.0 |
| Gpr52 | 0.1 | 0.0 | 0.1 | 0.1 | 0.1 | 0.0 | 0.0 | 0.1 | 0.1 | 0.1 | 0.1 | 0.1 |
| Gpr55 | 0.0 | 0.0 | 0.0 | 0.1 | 0.0 | 0.0 | 0.0 | 0.0 | 0.0 | 0.0 | 0.0 | 0.0 |
| Gpr56 | 0.2 | 0.2 | 0.2 | 0.2 | 0.1 | 0.1 | 0.3 | 0.3 | 0.3 | 1.0 | 0.5 | 0.3 |
| Gpr61 | 0.0 | 0.1 | 0.1 | 0.2 | 0.0 | 0.1 | 0.0 | 0.0 | 0.0 | 0.0 | 0.0 | 0.1 |
| Gpr62 | 0.1 | 0.2 | 0.1 | 0.1 | 0.1 | 0.1 | 0.1 | 0.1 | 0.0 | 0.1 | 0.2 | 0.1 |
| Gpr63 | 0.2 | 0.3 | 0.2 | 0.1 | 0.2 | 0.4 | 0.1 | 0.5 | 0.3 | 0.4 | 0.3 | 0.4 |
| Gpr64 | 1.3 | 1.0 | 1.4 | 1.2 | 1.3 | 1.1 | 1.7 | 1.2 | 1.2 | 1.2 | 1.2 | 1.5 |
| Gpr65 | 0.1 | 0.0 | 0.0 | 1.3 | 1.4 | 0.0 | 0.1 | 0.0 | 0.0 | 0.1 | 0.1 | 0.1 |
| Gpr68 | 0.7 | 0.5 | 0.2 | 0.6 | 0.6 | 0.5 | 0.4 | 0.4 | 0.3 | 1.0 | 0.7 | 0.5 |
| Gpr75 | 0.4 | 0.5 | 0.3 | 0.4 | 0.3 | 0.4 | 0.2 | 0.2 | 0.1 | 0.3 | 0.2 | 0.3 |
| Gpr81 | 0.0 | 0.0 | 0.0 | 0.0 | 0.0 | 0.0 | 0.0 | 0.0 | 0.1 | 0.1 | 0.0 | 0.1 |
| Gpr82 | 0.0 | 0.1 | 0.0 | 0.0 | 0.1 | 0.0 | 0.0 | 0.0 | 0.1 | 0.0 | 0.0 | 0.0 |
| Gpr83 | 0.0 | 0.0 | 0.0 | 0.0 | 0.0 | 0.0 | 0.0 | 0.0 | 0.0 | 0.0 | 0.0 | 0.0 |
| Gpr84 | 0.1 | 0.1 | 0.0 | 1.6 | 3.5 | 0.0 | 0.7 | 0.0 | 0.2 | 0.3 | 0.0 | 0.3 |
| Gpr85 | 0.3 | 0.1 | 0.2 | 0.4 | 0.6 | 0.4 | 0.6 | 0.2 | 0.2 | 0.6 | 0.2 | 0.5 |
| Gpr88 | 0.2 | 0.3 | 0.9 | 0.1 | 0.2 | 0.1 | 0.2 | 1.3 | 1.2 | 4.3 | 1.8 | 2.1 |
| Gpr89 | 11.4 | 11.8 | 9.7 | 11.3 | 10.0 | 11.9 | 9.7 | 11.2 | 9.3 | 10.4 | 11.0 | 9.2 |
| Gpr97 | 0.0 | 0.0 | 0.0 | 0.0 | 0.0 | 0.1 | 0.0 | 0.0 | 0.0 | 0.0 | 0.0 | 0.0 |
| Gpr98 | 0.0 | 0.0 | 0.0 | 0.0 | 0.0 | 0.0 | 0.0 | 0.0 | 0.0 | 0.0 | 0.0 | 0.0 |
| Gprasp1 | 6.5 | 7.9 | 9.3 | 5.9 | 5.0 | 8.0 | 5.2 | 5.8 | 6.2 | 7.0 | 5.9 | 7.2 |
| Gprasp2 | 0.5 | 0.8 | 1.1 | 0.5 | 0.3 | 0.6 | 0.2 | 0.4 | 0.5 | 0.7 | 0.2 | 0.5 |
| Gprc5a | 2.7 | 1.3 | 0.6 | 3.1 | 1.4 | 1.7 | 1.5 | 0.9 | 1.0 | 1.5 | 1.8 | 0.7 |
| Gprc5b | 7.2 | 8.4 | 10.1 | 4.4 | 8.5 | 8.5 | 11.5 | 12.2 | 9.6 | 10.6 | 11.1 | 8.8 |
| Gprc5c | 0.1 | 0.1 | 0.0 | 0.3 | 0.1 | 0.2 | 0.1 | 0.0 | 0.0 | 0.1 | 0.0 | 0.0 |
| Gprc6a | 0.4 | 0.3 | 0.1 | 0.5 | 0.2 | 0.2 | 0.2 | 0.1 | 0.1 | 0.1 | 0.3 | 0.1 |
| Gprin1 | 0.1 | 0.0 | 0.0 | 0.2 | 0.1 | 0.1 | 0.1 | 0.1 | 0.1 | 0.1 | 0.1 | 0.1 |
| Gprin2 | 0.2 | 0.1 | 0.1 | 0.1 | 0.1 | 0.1 | 0.0 | 0.2 | 0.1 | 0.1 | 0.0 | 0.1 |
| Gprin3 | 0.1 | 0.1 | 0.2 | 0.0 | 0.0 | 0.0 | 0.0 | 0.1 | 0.1 | 0.3 | 0.3 | 0.3 |
| Gps1 | 51.2 | 48.1 | 42.0 | 50.8 | 44.3 | 48.5 | 49.3 | 44.4 | 44.8 | 49.5 | 49.3 | 47.8 |
| Gps2 | 7.1 | 7.4 | 9.6 | 10.1 | 8.5 | 7.2 | 10.4 | 10.0 | 10.0 | 7.1 | 9.2 | 9.6 |
| Gpsm1 | 9.6 | 12.1 | 11.1 | 10.2 | 9.4 | 8.9 | 13.8 | 11.0 | 13.5 | 10.9 | 9.8 | 9.9 |
| Gpsm2 | 2.6 | 3.3 | 4.3 | 1.6 | 2.9 | 1.9 | 3.4 | 3.5 | 3.3 | 3.8 | 3.5 | 3.6 |
| Gpsm3 | 4.8 | 5.1 | 5.2 | 12.7 | 12.2 | 7.5 | 5.8 | 4.5 | 4.4 | 4.7 | 5.4 | 4.5 |
| Gpt | 0.6 | 0.7 | 0.8 | 0.6 | 0.7 | 0.6 | 0.3 | 0.6 | 0.4 | 0.8 | 0.5 | 0.4 |
| Gpt2 | 9.3 | 7.0 | 5.8 | 8.6 | 5.5 | 9.5 | 5.6 | 7.7 | 6.9 | 8.0 | 7.6 | 6.3 |
| Gpx1 | 146.0 | 100.9 | 89.3 | 140.8 | 105.6 | 132.6 | 105.1 | 93.9 | 86.6 | 147.9 | 111.9 | 114.4 |
| Gpx3 | 72.6 | 85.0 | 99.1 | 80.2 | 81.2 | 65.2 | 78.0 | 67.0 | 90.2 | 63.9 | 56.3 | 95.9 |
| Gpx4 | 422.5 | 338.4 | 274.1 | 449.7 | 308.7 | 498.9 | 280.5 | 299.5 | 286.3 | 316.1 | 301.8 | 310.0 |
| Gpx7 | 23.8 | 30.6 | 37.4 | 16.8 | 26.3 | 18.7 | 31.1 | 38.8 | 41.1 | 26.7 | 26.7 | 32.8 |
| Gpx8 | 203.4 | 151.7 | 149.2 | 225.0 | 149.6 | 201.1 | 148.3 | 143.7 | 127.5 | 186.4 | 180.7 | 192.9 |
| Gramd1a | 16.7 | 15.9 | 15.5 | 14.1 | 16.1 | 15.9 | 17.2 | 16.2 | 18.2 | 14.1 | 14.4 | 13.6 |
| Gramd1b | 3.0 | 2.1 | 1.6 | 4.2 | 2.9 | 4.0 | 2.2 | 2.3 | 1.7 | 1.9 | 2.3 | 2.1 |

| | | | | | | | | | | | | |
|---|---|---|---|---|---|---|---|---|---|---|---|---|
| Gramd1c | 0.3 | 0.5 | 0.8 | 0.3 | 0.3 | 0.5 | 0.3 | 0.5 | 0.4 | 0.4 | 0.3 | 0.3 |
| Gramd2 | 0.1 | 0.5 | 0.3 | 0.0 | 0.1 | 0.1 | 0.2 | 0.2 | 0.2 | 0.0 | 0.0 | 0.0 |
| Gramd3 | 6.6 | 7.1 | 6.2 | 6.1 | 6.1 | 6.7 | 5.8 | 7.6 | 6.3 | 6.0 | 7.1 | 6.0 |
| Gramd4 | 8.9 | 6.9 | 5.5 | 10.6 | 7.2 | 8.6 | 9.8 | 6.6 | 8.0 | 7.2 | 6.9 | 6.6 |
| Grap | 3.8 | 3.3 | 1.8 | 5.4 | 2.4 | 5.0 | 1.9 | 1.8 | 1.8 | 1.0 | 1.1 | 0.9 |
| Grap2 | 0.0 | 0.0 | 0.0 | 0.0 | 0.0 | 0.0 | 0.0 | 0.0 | 0.0 | 0.0 | 0.0 | 0.0 |
| Grasp | 3.3 | 3.4 | 4.1 | 2.9 | 3.2 | 4.1 | 3.9 | 3.9 | 4.3 | 3.2 | 3.4 | 3.8 |
| Grb10 | 40.0 | 35.0 | 33.4 | 35.1 | 25.2 | 40.0 | 27.3 | 43.0 | 34.2 | 55.6 | 57.5 | 31.2 |
| Grb14 | 29.9 | 34.2 | 51.3 | 18.5 | 23.2 | 12.5 | 23.7 | 38.3 | 25.9 | 21.1 | 30.1 | 36.5 |
| Grb2 | 10.0 | 12.2 | 14.1 | 11.1 | 14.6 | 11.8 | 11.6 | 13.0 | 11.8 | 12.8 | 12.2 | 13.1 |
| Grcc10 | 57.4 | 58.0 | 54.2 | 52.8 | 55.1 | 70.2 | 37.9 | 54.1 | 48.6 | 67.2 | 54.5 | 46.8 |
| Greb1 | 0.2 | 0.4 | 0.9 | 0.1 | 0.5 | 0.1 | 0.5 | 0.5 | 0.4 | 0.4 | 0.4 | 0.5 |
| Greb1l | 0.6 | 1.2 | 1.6 | 0.4 | 0.8 | 0.5 | 0.9 | 1.2 | 1.0 | 0.7 | 0.5 | 0.8 |
| Grem1 | 0.1 | 0.1 | 0.0 | 0.1 | 0.1 | 0.4 | 0.4 | 0.1 | 0.4 | 10.0 | 8.3 | 4.5 |
| Grem2 | 0.1 | 0.1 | 0.0 | 0.1 | 0.1 | 0.1 | 0.6 | 0.1 | 0.3 | 2.0 | 1.3 | 1.4 |
| Grhl1 | 0.2 | 0.3 | 0.2 | 0.1 | 0.1 | 0.1 | 0.1 | 0.1 | 0.1 | 0.3 | 0.2 | 0.3 |
| Grhl3 | 0.2 | 0.2 | 0.2 | 0.4 | 0.2 | 0.1 | 0.2 | 0.0 | 0.0 | 0.2 | 0.1 | 0.1 |
| Grhpr | 9.7 | 10.5 | 10.0 | 7.8 | 9.1 | 9.5 | 8.4 | 8.1 | 8.6 | 10.4 | 8.8 | 7.3 |
| Gria1 | 0.0 | 0.0 | 0.0 | 0.0 | 0.0 | 0.0 | 0.0 | 0.0 | 0.0 | 0.0 | 0.0 | 0.0 |
| Gria3 | 8.2 | 7.4 | 9.3 | 5.8 | 8.1 | 7.2 | 7.9 | 6.6 | 6.5 | 11.3 | 9.7 | 8.7 |
| Gria4 | 3.9 | 1.4 | 0.7 | 0.8 | 1.0 | 0.4 | 1.2 | 0.3 | 1.8 | 2.8 | 1.5 | 1.9 |
| Grid2ip | 0.1 | 0.0 | 0.0 | 0.0 | 0.0 | 0.0 | 0.0 | 0.0 | 0.0 | 0.0 | 0.0 | 0.0 |
| Grik1 | 0.0 | 0.0 | 0.0 | 0.0 | 0.0 | 0.0 | 0.0 | 0.0 | 0.0 | 0.0 | 0.0 | 0.0 |
| Grik2 | 0.1 | 0.2 | 0.5 | 0.1 | 0.5 | 0.1 | 0.5 | 0.6 | 0.4 | 0.1 | 0.2 | 0.1 |
| Grik3 | 0.0 | 0.0 | 0.0 | 0.0 | 0.0 | 0.0 | 0.0 | 0.0 | 0.0 | 0.0 | 0.0 | 0.0 |
| Grik4 | 0.0 | 0.1 | 0.0 | 0.1 | 0.1 | 0.0 | 0.1 | 0.0 | 0.0 | 0.0 | 0.0 | 0.0 |
| Grik5 | 0.9 | 0.9 | 1.1 | 0.4 | 0.4 | 0.9 | 0.5 | 0.6 | 0.6 | 0.7 | 0.7 | 0.6 |
| Grin1 | 0.5 | 0.6 | 0.6 | 0.3 | 0.4 | 0.5 | 1.0 | 0.9 | 0.6 | 0.3 | 0.6 | 0.4 |
| Grin2b | 0.0 | 0.0 | 0.0 | 0.0 | 0.0 | 0.0 | 0.0 | 0.0 | 0.0 | 0.0 | 0.0 | 0.0 |
| Grin2c | 0.0 | 0.0 | 0.0 | 0.0 | 0.0 | 0.0 | 0.0 | 0.0 | 0.0 | 0.0 | 0.0 | 0.0 |
| Grin2d | 0.1 | 0.1 | 0.1 | 0.2 | 0.1 | 0.1 | 0.2 | 0.2 | 0.1 | 0.1 | 0.1 | 0.1 |
| Grin3a | 0.1 | 0.1 | 0.1 | 0.1 | 0.1 | 0.1 | 0.1 | 0.1 | 0.0 | 0.1 | 0.1 | 0.1 |
| Grin3b | 0.2 | 0.1 | 0.1 | 0.1 | 0.0 | 0.1 | 0.0 | 0.0 | 0.1 | 0.1 | 0.1 | 0.1 |
| Grina | 108.2 | 87.7 | 65.1 | 135.7 | 82.5 | 116.8 | 63.9 | 59.1 | 60.8 | 75.2 | 71.7 | 65.9 |
| Grip1 | 0.1 | 0.0 | 0.0 | 0.1 | 0.0 | 0.1 | 0.0 | 0.0 | 0.0 | 0.0 | 0.1 | 0.0 |
| Grip2 | 0.0 | 0.0 | 0.0 | 0.0 | 0.0 | 0.0 | 0.0 | 0.0 | 0.0 | 0.0 | 0.0 | 0.0 |
| Gripap1 | 7.0 | 6.0 | 6.7 | 8.9 | 7.2 | 6.8 | 7.0 | 7.1 | 6.5 | 7.0 | 7.5 | 7.4 |
| Grk1 | 0.3 | 0.3 | 0.3 | 0.2 | 0.2 | 0.2 | 0.3 | 0.3 | 0.3 | 0.2 | 0.2 | 0.2 |
| Grk4 | 2.4 | 3.6 | 3.1 | 2.0 | 2.2 | 2.2 | 2.8 | 2.8 | 2.6 | 2.5 | 2.7 | 2.8 |
| Grk5 | 32.9 | 31.5 | 31.0 | 23.9 | 27.0 | 29.9 | 29.5 | 26.1 | 29.0 | 20.7 | 25.6 | 31.8 |
| Grk6 | 8.4 | 9.2 | 8.0 | 9.1 | 10.2 | 8.8 | 12.4 | 9.1 | 11.9 | 10.3 | 10.3 | 10.5 |
| Grlf1 | 23.3 | 25.7 | 24.5 | 22.7 | 20.7 | 22.8 | 22.3 | 23.1 | 20.8 | 21.8 | 23.0 | 22.7 |
| Grm1 | 0.0 | 0.0 | 0.0 | 0.1 | 0.0 | 0.0 | 0.0 | 0.0 | 0.0 | 0.0 | 0.0 | 0.0 |
| Grm3 | 0.0 | 0.0 | 0.0 | 0.0 | 0.0 | 0.0 | 0.1 | 0.0 | 0.0 | 0.1 | 0.1 | 0.0 |
| Grm5 | 0.0 | 0.0 | 0.0 | 0.0 | 0.0 | 0.0 | 0.0 | 0.0 | 0.0 | 0.0 | 0.0 | 0.0 |
| Grm7 | 0.0 | 0.0 | 0.0 | 0.0 | 0.0 | 0.0 | 0.0 | 0.0 | 0.0 | 0.0 | 0.0 | 0.0 |
| Grn | 91.2 | 79.6 | 77.9 | 116.3 | 134.4 | 88.8 | 70.8 | 72.9 | 75.4 | 61.4 | 59.7 | 61.0 |
| Grpel1 | 16.5 | 14.6 | 12.5 | 15.7 | 14.0 | 16.1 | 13.9 | 13.1 | 13.2 | 14.3 | 16.4 | 13.7 |
| Grpel2 | 5.2 | 4.0 | 3.4 | 5.3 | 4.3 | 5.1 | 4.4 | 3.5 | 3.6 | 4.0 | 3.9 | 4.2 |
| Grrp1 | 0.0 | 0.0 | 0.0 | 0.0 | 0.0 | 0.0 | 0.1 | 0.0 | 0.1 | 0.0 | 0.0 | 0.0 |
| Grsf1 | 28.4 | 29.3 | 26.8 | 24.0 | 23.4 | 25.3 | 24.9 | 23.5 | 25.4 | 27.3 | 27.7 | 29.3 |
| Grtp1 | 1.0 | 0.8 | 0.7 | 0.7 | 0.3 | 0.9 | 0.6 | 0.7 | 0.5 | 1.0 | 0.7 | 1.1 |
| Grwd1 | 6.7 | 5.7 | 4.5 | 6.6 | 6.6 | 5.9 | 5.9 | 5.3 | 5.1 | 5.7 | 5.1 | 3.7 |
| Gsc | 0.0 | 0.0 | 0.1 | 0.0 | 0.0 | 0.1 | 0.0 | 0.1 | 0.2 | 2.1 | 1.8 | 0.7 |
| Gsdma | 0.0 | 0.0 | 0.0 | 0.0 | 0.0 | 0.0 | 0.0 | 0.0 | 0.0 | 0.0 | 0.0 | 0.0 |
| Gsdmc4 | 0.1 | 0.0 | 0.0 | 0.0 | 0.0 | 0.0 | 0.0 | 0.0 | 0.0 | 0.0 | 0.0 | 0.0 |
| Gsdmcl-ps | 0.0 | 0.1 | 0.0 | 0.0 | 0.1 | 0.0 | 0.0 | 0.0 | 0.0 | 0.0 | 0.0 | 0.1 |

| | | | | | | | | | | | | |
|---|---|---|---|---|---|---|---|---|---|---|---|---|
| Gsdmd | 10.9 | 12.9 | 14.4 | 16.6 | 14.4 | 12.1 | 12.3 | 12.3 | 13.3 | 11.0 | 10.7 | 11.6 |
| Gse1 | 1.0 | 1.6 | 1.5 | 1.3 | 1.6 | 1.2 | 1.7 | 1.2 | 1.1 | 1.3 | 1.2 | 1.0 |
| Gsg1 | 0.1 | 0.0 | 0.3 | 0.3 | 0.0 | 0.0 | 0.2 | 0.2 | 0.0 | 0.0 | 0.1 | 0.1 |
| Gsg1l | 0.0 | 0.0 | 0.0 | 0.0 | 0.0 | 0.0 | 0.0 | 0.0 | 0.0 | 0.0 | 0.0 | 0.0 |
| Gsg2 | 0.7 | 1.0 | 1.3 | 0.5 | 1.0 | 0.3 | 1.5 | 0.6 | 1.3 | 0.7 | 0.8 | 0.8 |
| Gsk3a | 12.9 | 13.3 | 12.0 | 15.0 | 11.8 | 13.2 | 13.2 | 11.9 | 12.3 | 11.6 | 11.4 | 10.7 |
| Gsk3b | 9.0 | 10.7 | 10.7 | 8.7 | 10.1 | 10.0 | 10.5 | 10.6 | 9.7 | 9.0 | 10.2 | 8.6 |
| Gskip | 12.4 | 11.3 | 9.4 | 10.2 | 10.5 | 9.2 | 9.3 | 8.9 | 10.3 | 10.8 | 9.4 | 11.3 |
| Gsn | 215.4 | 290.6 | 353.2 | 192.5 | 237.1 | 234.6 | 250.7 | 276.3 | 253.0 | 233.1 | 227.8 | 257.2 |
| Gspt1 | 16.8 | 16.1 | 17.0 | 17.5 | 16.2 | 14.6 | 17.6 | 15.4 | 15.5 | 20.4 | 18.6 | 19.3 |
| Gspt2 | 3.4 | 3.3 | 3.2 | 2.6 | 2.2 | 3.8 | 3.2 | 3.1 | 2.7 | 3.0 | 3.3 | 3.3 |
| Gsr | 17.1 | 13.5 | 13.8 | 24.4 | 22.3 | 20.5 | 15.0 | 16.1 | 13.1 | 12.8 | 12.9 | 15.0 |
| Gss | 19.7 | 15.4 | 9.2 | 22.2 | 18.2 | 24.0 | 11.7 | 10.1 | 9.4 | 15.4 | 13.9 | 11.5 |
| Gsta1 | 0.9 | 0.5 | 0.5 | 0.8 | 0.7 | 1.1 | 0.2 | 0.6 | 0.1 | 0.1 | 0.1 | 0.2 |
| Gsta2 | 0.5 | 0.2 | 0.2 | 0.5 | 0.5 | 0.4 | 0.2 | 0.4 | 0.1 | 0.1 | 0.2 | 0.1 |
| Gsta3 | 76.4 | 73.1 | 54.0 | 134.0 | 66.7 | 104.7 | 17.9 | 46.2 | 12.5 | 40.6 | 26.8 | 28.4 |
| Gsta4 | 4.2 | 7.3 | 8.5 | 5.5 | 9.7 | 15.0 | 2.8 | 20.9 | 4.8 | 47.8 | 23.9 | 12.2 |
| Gstcd | 1.7 | 2.4 | 1.9 | 1.5 | 2.0 | 1.9 | 2.2 | 2.1 | 2.2 | 1.5 | 2.6 | 2.1 |
| Gstk1 | 8.8 | 10.9 | 10.3 | 7.8 | 8.3 | 10.9 | 9.0 | 8.3 | 8.1 | 6.9 | 8.4 | 7.3 |
| Gstm1 | 465.0 | 539.6 | 305.3 | 812.7 | 406.7 | 949.2 | 185.8 | 200.8 | 111.9 | 158.5 | 176.7 | 129.0 |
| Gstm2 | 257.5 | 266.4 | 285.4 | 330.9 | 222.4 | 281.7 | 244.0 | 277.4 | 206.5 | 196.8 | 236.5 | 218.5 |
| Gstm3 | 2.6 | 2.4 | 1.7 | 4.7 | 2.7 | 4.1 | 0.9 | 1.1 | 0.4 | 0.7 | 1.2 | 0.9 |
| Gstm4 | 15.3 | 16.9 | 13.6 | 16.4 | 10.6 | 20.1 | 10.5 | 9.8 | 11.3 | 8.7 | 10.5 | 9.8 |
| Gstm5 | 78.5 | 68.2 | 75.7 | 65.0 | 60.9 | 73.0 | 59.0 | 72.7 | 56.8 | 55.5 | 69.9 | 55.0 |
| Gstm6 | 6.1 | 5.8 | 3.5 | 6.2 | 3.2 | 5.3 | 1.9 | 1.9 | 1.7 | 2.0 | 3.7 | 2.2 |
| Gstm7 | 5.3 | 5.4 | 4.3 | 6.1 | 4.3 | 6.8 | 3.7 | 2.6 | 3.0 | 2.9 | 2.5 | 2.4 |
| Gsto1 | 162.2 | 85.7 | 54.0 | 174.8 | 118.3 | 139.9 | 120.4 | 62.3 | 65.8 | 148.0 | 125.3 | 90.1 |
| Gsto2 | 1.6 | 1.5 | 0.9 | 2.8 | 1.0 | 2.7 | 1.1 | 1.1 | 0.8 | 1.4 | 1.2 | 1.4 |
| Gstp1 | 105.1 | 97.7 | 82.0 | 120.5 | 80.6 | 162.3 | 57.9 | 77.5 | 59.1 | 75.8 | 67.1 | 64.5 |
| Gstp2 | 32.3 | 30.3 | 25.2 | 39.0 | 24.2 | 54.2 | 18.2 | 22.8 | 15.1 | 21.3 | 20.6 | 17.9 |
| Gstt1 | 11.1 | 15.5 | 27.4 | 6.9 | 10.3 | 10.4 | 12.6 | 25.7 | 15.6 | 10.6 | 12.3 | 13.4 |
| Gstt2 | 2.4 | 1.8 | 1.5 | 1.5 | 1.3 | 4.2 | 0.9 | 0.9 | 1.1 | 1.5 | 1.2 | 0.8 |
| Gstt3 | 4.5 | 6.0 | 6.5 | 3.4 | 3.6 | 4.4 | 3.6 | 4.6 | 3.3 | 5.1 | 5.7 | 4.8 |
| Gstz1 | 11.8 | 10.9 | 10.4 | 9.5 | 9.1 | 11.0 | 8.3 | 11.7 | 9.1 | 9.0 | 11.4 | 9.4 |
| Gt(ROSA)26Sor | 2.5 | 3.4 | 2.9 | 2.8 | 2.8 | 2.8 | 2.6 | 3.2 | 3.8 | 3.2 | 5.0 | 4.8 |
| Gtdc1 | 2.9 | 2.8 | 2.4 | 3.0 | 2.6 | 2.9 | 2.2 | 2.6 | 2.5 | 2.6 | 2.7 | 2.1 |
| Gtdc2 | 4.5 | 4.3 | 3.4 | 6.1 | 4.5 | 6.3 | 4.9 | 3.1 | 4.7 | 5.2 | 3.4 | 4.1 |
| Gtf2a1 | 6.1 | 7.1 | 7.3 | 9.4 | 7.9 | 7.7 | 6.8 | 7.5 | 6.5 | 6.9 | 7.7 | 8.1 |
| Gtf2a1l | 0.0 | 0.0 | 0.0 | 0.0 | 0.0 | 0.1 | 0.0 | 0.0 | 0.0 | 0.0 | 0.1 | 0.0 |
| Gtf2a2 | 23.9 | 26.5 | 24.0 | 23.1 | 24.6 | 26.2 | 23.8 | 26.0 | 25.0 | 27.0 | 27.0 | 27.0 |
| Gtf2b | 41.6 | 41.6 | 35.1 | 40.6 | 34.5 | 33.3 | 35.3 | 34.6 | 34.3 | 48.9 | 41.8 | 40.0 |
| Gtf2e1 | 10.2 | 12.4 | 9.5 | 11.7 | 10.8 | 10.6 | 9.5 | 9.5 | 9.7 | 9.6 | 11.5 | 9.7 |
| Gtf2e2 | 14.4 | 14.6 | 14.0 | 11.5 | 13.1 | 11.9 | 12.1 | 13.8 | 14.4 | 16.5 | 16.3 | 15.5 |
| Gtf2f1 | 37.9 | 38.0 | 42.8 | 47.6 | 31.8 | 34.1 | 42.7 | 38.7 | 30.5 | 30.2 | 41.5 | 43.6 |
| Gtf2f2 | 15.9 | 15.8 | 21.9 | 16.1 | 17.5 | 14.8 | 17.1 | 19.0 | 16.6 | 15.3 | 20.3 | 18.9 |
| Gtf2h1 | 32.9 | 29.3 | 27.3 | 34.5 | 28.5 | 32.3 | 25.9 | 27.1 | 24.5 | 26.0 | 31.7 | 28.2 |
| Gtf2h2 | 5.5 | 5.7 | 5.4 | 6.4 | 6.0 | 5.7 | 5.4 | 6.2 | 5.9 | 5.5 | 6.0 | 6.2 |
| Gtf2h3 | 7.5 | 7.8 | 7.0 | 6.2 | 6.4 | 6.6 | 5.5 | 7.0 | 6.7 | 8.1 | 7.8 | 7.0 |
| Gtf2h4 | 6.7 | 6.7 | 6.3 | 5.2 | 5.5 | 8.4 | 6.4 | 6.0 | 6.2 | 5.3 | 5.6 | 5.0 |
| Gtf2h5 | 43.9 | 37.3 | 35.8 | 39.1 | 41.0 | 39.4 | 38.7 | 37.9 | 37.8 | 41.7 | 40.8 | 39.7 |
| Gtf2i | 13.4 | 15.0 | 15.3 | 11.6 | 12.2 | 14.0 | 13.3 | 14.9 | 13.7 | 15.6 | 14.0 | 14.2 |
| Gtf2ird1 | 5.4 | 6.8 | 6.0 | 5.7 | 5.2 | 6.2 | 6.0 | 4.9 | 5.7 | 5.7 | 5.4 | 5.1 |
| Gtf2ird2 | 1.3 | 1.4 | 1.8 | 1.5 | 1.6 | 1.4 | 1.3 | 1.5 | 1.6 | 1.9 | 1.7 | 1.7 |
| Gtf3a | 16.1 | 13.1 | 13.2 | 13.1 | 11.2 | 14.2 | 11.3 | 12.8 | 11.3 | 13.5 | 11.6 | 11.0 |
| Gtf3c1 | 20.3 | 18.5 | 17.7 | 18.2 | 16.7 | 18.6 | 18.5 | 16.3 | 16.6 | 22.0 | 18.6 | 17.2 |
| Gtf3c2 | 4.7 | 5.5 | 6.1 | 4.7 | 5.5 | 4.8 | 5.3 | 6.6 | 6.3 | 5.4 | 5.8 | 5.0 |
| Gtf3c3 | 13.8 | 15.6 | 14.5 | 12.8 | 12.7 | 14.5 | 11.6 | 14.5 | 12.7 | 13.5 | 14.3 | 13.9 |

| | | | | | | | | | | | | |
|---|---|---|---|---|---|---|---|---|---|---|---|---|
| Gtf3c4 | 7.4 | 7.8 | 7.2 | 7.3 | 7.0 | 7.5 | 6.9 | 6.6 | 7.0 | 7.0 | 7.3 | 6.9 |
| Gtf3c5 | 4.2 | 5.3 | 5.2 | 4.0 | 5.5 | 4.3 | 5.6 | 5.9 | 5.0 | 4.6 | 5.0 | 4.6 |
| Gtf3c6 | 16.4 | 12.8 | 14.5 | 22.2 | 15.2 | 15.8 | 16.4 | 16.8 | 13.0 | 16.2 | 18.2 | 18.0 |
| Gtl3 | 13.9 | 13.6 | 14.9 | 15.5 | 13.2 | 16.3 | 16.0 | 17.1 | 14.7 | 16.5 | 15.6 | 15.0 |
| Gtpbp1 | 7.2 | 7.0 | 6.1 | 7.5 | 6.4 | 7.4 | 6.4 | 7.4 | 7.6 | 6.1 | 7.1 | 6.7 |
| Gtpbp10 | 7.5 | 7.9 | 7.3 | 8.0 | 6.5 | 8.2 | 6.6 | 5.9 | 5.6 | 6.5 | 7.0 | 6.4 |
| Gtpbp2 | 9.0 | 9.4 | 10.0 | 8.8 | 9.0 | 9.5 | 8.2 | 10.0 | 11.2 | 8.5 | 9.2 | 7.9 |
| Gtpbp3 | 3.4 | 3.9 | 3.4 | 3.7 | 3.9 | 3.4 | 3.4 | 3.7 | 3.4 | 3.5 | 3.2 | 3.2 |
| Gtpbp4 | 35.5 | 33.7 | 30.8 | 35.8 | 30.4 | 34.1 | 31.2 | 25.7 | 24.8 | 32.8 | 34.5 | 33.9 |
| Gtpbp5 | 6.5 | 6.1 | 5.3 | 7.1 | 5.2 | 7.5 | 5.0 | 5.0 | 5.2 | 6.0 | 6.4 | 5.5 |
| Gtpbp6 | 4.2 | 4.4 | 3.8 | 4.1 | 3.7 | 3.6 | 4.2 | 4.1 | 5.1 | 4.1 | 3.4 | 3.6 |
| Gtpbp8 | 9.8 | 10.2 | 10.5 | 10.0 | 9.0 | 8.5 | 8.2 | 10.4 | 11.3 | 10.2 | 12.1 | 11.7 |
| Gtse1 | 12.4 | 13.8 | 13.8 | 12.5 | 15.4 | 13.7 | 15.9 | 7.6 | 12.0 | 13.3 | 12.8 | 10.7 |
| Guca1a | 2.3 | 0.8 | 0.6 | 2.3 | 1.6 | 1.9 | 1.0 | 1.1 | 0.5 | 0.9 | 1.3 | 1.4 |
| Guca1b | 0.3 | 0.1 | 0.2 | 0.6 | 0.4 | 0.2 | 0.1 | 0.1 | 0.2 | 0.2 | 0.2 | 0.2 |
| Gucd1 | 5.5 | 6.8 | 6.7 | 5.3 | 6.0 | 4.5 | 6.0 | 7.8 | 7.4 | 5.7 | 5.8 | 6.1 |
| Gucy1a2 | 2.9 | 4.4 | 4.3 | 3.3 | 4.2 | 2.7 | 5.5 | 2.7 | 4.6 | 4.8 | 6.1 | 4.8 |
| Gucy1a3 | 2.0 | 6.1 | 11.7 | 1.6 | 2.8 | 1.0 | 3.5 | 9.1 | 14.8 | 5.4 | 4.9 | 14.9 |
| Gucy1b3 | 6.7 | 10.4 | 16.7 | 7.4 | 8.5 | 8.0 | 12.5 | 11.7 | 24.4 | 7.2 | 9.8 | 18.8 |
| Gucy2c | 0.2 | 0.2 | 0.1 | 0.0 | 0.1 | 0.1 | 0.0 | 0.0 | 0.0 | 0.1 | 0.1 | 0.1 |
| Gucy2e | 0.0 | 0.0 | 0.0 | 0.0 | 0.0 | 0.0 | 0.0 | 0.0 | 0.0 | 0.0 | 0.0 | 0.0 |
| Gucy2g | 0.0 | 0.0 | 0.0 | 0.0 | 0.0 | 0.0 | 0.0 | 0.0 | 0.0 | 0.1 | 0.0 | 0.0 |
| Guf1 | 2.8 | 3.3 | 3.5 | 3.1 | 2.8 | 2.9 | 3.3 | 3.5 | 3.2 | 2.9 | 3.2 | 3.1 |
| Guk1 | 44.7 | 31.7 | 23.9 | 47.5 | 28.5 | 45.2 | 27.6 | 29.5 | 29.2 | 31.6 | 29.2 | 27.4 |
| Gulp1 | 7.6 | 8.4 | 11.8 | 8.5 | 5.2 | 8.1 | 8.7 | 7.9 | 8.0 | 8.1 | 12.9 | 9.6 |
| Gusb | 40.6 | 29.4 | 24.0 | 64.3 | 88.4 | 41.2 | 34.2 | 24.8 | 25.4 | 34.4 | 26.6 | 29.6 |
| Gvin1 | 2.8 | 1.6 | 6.2 | 9.5 | 7.6 | 1.3 | 7.2 | 0.8 | 4.5 | 1.1 | 5.9 | 4.4 |
| Gxylt1 | 5.7 | 6.2 | 7.0 | 5.0 | 6.9 | 3.8 | 7.0 | 6.3 | 6.2 | 5.1 | 7.0 | 7.1 |
| Gxylt2 | 3.8 | 3.2 | 5.6 | 2.3 | 2.9 | 2.3 | 1.7 | 5.8 | 2.7 | 4.6 | 2.9 | 4.1 |
| Gyg | 92.1 | 85.1 | 66.3 | 76.5 | 65.1 | 57.6 | 56.5 | 53.0 | 81.0 | 51.1 | 58.7 | 77.0 |
| Gyk | 2.0 | 1.5 | 1.2 | 2.6 | 3.5 | 1.8 | 2.1 | 1.2 | 1.4 | 1.9 | 1.8 | 1.7 |
| Gyltl1b | 0.0 | 0.0 | 0.0 | 0.0 | 0.0 | 0.0 | 0.0 | 0.0 | 0.1 | 0.0 | 0.0 | 0.0 |
| Gypc | 14.3 | 11.9 | 12.8 | 13.5 | 10.2 | 16.8 | 12.5 | 12.0 | 11.7 | 10.0 | 14.0 | 12.4 |
| Gys1 | 8.2 | 9.2 | 9.9 | 7.1 | 8.8 | 8.1 | 11.6 | 10.6 | 10.7 | 9.6 | 9.8 | 9.6 |
| Gys2 | 0.0 | 0.0 | 0.0 | 0.0 | 0.0 | 0.1 | 0.0 | 0.0 | 0.0 | 0.0 | 0.0 | 0.0 |
| Gzf1 | 10.1 | 10.8 | 9.3 | 10.0 | 8.8 | 9.9 | 7.9 | 8.1 | 8.1 | 9.7 | 9.3 | 10.2 |
| Gzmc | 0.2 | 0.2 | 0.1 | 0.3 | 0.2 | 0.2 | 0.4 | 0.2 | 0.1 | 0.3 | 0.1 | 0.6 |
| Gzme | 0.0 | 0.0 | 0.0 | 0.3 | 0.0 | 0.0 | 0.0 | 0.1 | 0.0 | 0.1 | 0.6 | 0.2 |
| Gzmk | 0.0 | 0.0 | 0.0 | 0.0 | 0.0 | 0.1 | 0.0 | 0.0 | 0.0 | 0.0 | 0.0 | 0.0 |
| Gzmm | 0.2 | 0.2 | 0.1 | 0.2 | 0.3 | 0.3 | 0.2 | 0.2 | 0.3 | 0.3 | 0.2 | 0.2 |
| H13 | 40.0 | 32.8 | 28.4 | 50.8 | 37.9 | 37.9 | 44.6 | 38.2 | 32.7 | 46.5 | 41.5 | 41.5 |
| H19 | 0.3 | 0.6 | 2.0 | 0.1 | 1.0 | 0.1 | 1.5 | 1.2 | 2.0 | 1.2 | 1.4 | 0.9 |
| H1f0 | 30.1 | 31.3 | 33.4 | 27.5 | 25.4 | 34.5 | 29.8 | 38.0 | 35.6 | 30.0 | 33.8 | 34.5 |
| H1fx | 0.5 | 0.6 | 0.8 | 0.6 | 0.8 | 0.5 | 0.5 | 1.3 | 1.6 | 1.2 | 0.6 | 1.1 |
| H2-Aa | 0.4 | 0.1 | 0.1 | 0.2 | 0.1 | 0.1 | 0.0 | 0.0 | 0.0 | 0.1 | 0.1 | 0.0 |
| H2-Ab1 | 0.5 | 0.4 | 0.1 | 0.9 | 0.8 | 2.0 | 0.8 | 0.1 | 0.2 | 0.5 | 0.3 | 0.3 |
| H2afj | 21.5 | 18.6 | 17.0 | 18.0 | 21.4 | 16.7 | 20.4 | 19.2 | 18.4 | 27.9 | 21.0 | 20.0 |
| H2afv | 8.9 | 10.7 | 14.3 | 8.1 | 10.5 | 11.2 | 11.5 | 12.1 | 11.7 | 11.8 | 12.1 | 11.6 |
| H2afx | 6.9 | 6.7 | 7.8 | 4.1 | 9.0 | 5.8 | 10.5 | 8.5 | 9.2 | 8.2 | 8.2 | 6.6 |
| H2afy | 31.8 | 30.2 | 23.6 | 33.5 | 31.1 | 32.4 | 30.0 | 25.8 | 25.7 | 28.5 | 29.5 | 28.6 |
| H2afy2 | 4.5 | 4.8 | 7.6 | 3.6 | 3.6 | 5.1 | 4.6 | 6.1 | 5.1 | 4.1 | 4.5 | 4.8 |
| H2afy3 | 0.2 | 0.3 | 0.1 | 0.1 | 0.3 | 0.2 | 0.2 | 0.1 | 0.1 | 0.3 | 0.2 | 0.1 |
| H2afz | 39.5 | 45.8 | 44.8 | 35.0 | 57.9 | 42.8 | 46.7 | 43.2 | 57.3 | 48.0 | 45.6 | 37.6 |
| H2-Bl | 0.4 | 0.2 | 0.2 | 0.6 | 0.4 | 0.4 | 0.4 | 0.2 | 0.3 | 0.2 | 0.1 | 0.2 |
| H2-D1 | 196.8 | 124.2 | 175.1 | 332.2 | 220.4 | 177.1 | 94.8 | 79.6 | 62.3 | 52.7 | 48.6 | 75.6 |
| H2-DMa | 1.8 | 2.0 | 1.4 | 1.9 | 2.6 | 1.9 | 1.9 | 1.3 | 1.0 | 1.5 | 1.5 | 1.0 |
| H2-DMb1 | 3.2 | 3.3 | 2.7 | 3.9 | 3.7 | 2.8 | 1.4 | 1.6 | 2.2 | 6.0 | 3.4 | 3.4 |

| | | | | | | | | | | | | |
|---|---|---|---|---|---|---|---|---|---|---|---|---|
| H2-DMb2 | 0.2 | 0.1 | 0.1 | 0.4 | 0.3 | 0.1 | 0.1 | 0.0 | 0.1 | 0.4 | 0.5 | 0.2 |
| H2-Eb1 | 0.6 | 0.4 | 0.7 | 0.6 | 0.4 | 0.3 | 0.3 | 0.4 | 0.5 | 0.2 | 0.4 | 0.4 |
| H2-K1 | 203.3 | 136.6 | 186.8 | 348.6 | 237.6 | 219.7 | 126.7 | 100.4 | 82.8 | 54.5 | 63.1 | 85.5 |
| H2-K2 | 2.4 | 2.4 | 2.1 | 3.5 | 2.3 | 2.2 | 1.1 | 1.2 | 1.7 | 1.4 | 1.0 | 1.7 |
| H2-Ke2 | 26.0 | 26.0 | 25.3 | 32.9 | 29.1 | 28.9 | 26.3 | 28.3 | 23.1 | 26.0 | 28.5 | 29.0 |
| H2-Ke6 | 10.8 | 9.3 | 11.6 | 8.6 | 6.8 | 13.0 | 9.5 | 13.1 | 9.9 | 10.9 | 10.9 | 10.7 |
| H2-L | 7.0 | 8.5 | 16.2 | 24.2 | 14.6 | 16.4 | 9.0 | 5.9 | 1.2 | 4.3 | 3.1 | 9.2 |
| H2-M3 | 4.3 | 4.5 | 4.6 | 3.2 | 4.5 | 4.3 | 4.1 | 4.1 | 4.4 | 3.8 | 3.6 | 3.7 |
| H2-M5 | 0.1 | 0.1 | 0.1 | 0.2 | 0.0 | 0.2 | 0.1 | 0.0 | 0.1 | 0.0 | 0.1 | 0.1 |
| H2-Q1 | 3.5 | 2.5 | 2.7 | 4.6 | 4.1 | 3.2 | 1.7 | 1.6 | 1.6 | 1.4 | 0.9 | 1.6 |
| H2-Q10 | 1.0 | 0.9 | 0.7 | 1.9 | 1.1 | 1.0 | 0.8 | 0.9 | 0.5 | 0.4 | 0.2 | 0.6 |
| H2-Q2 | 0.2 | 0.0 | 0.2 | 0.2 | 0.1 | 0.1 | 0.1 | 0.1 | 0.1 | 0.1 | 0.0 | 0.1 |
| H2-Q4 | 14.9 | 10.3 | 13.2 | 31.7 | 19.8 | 15.8 | 11.9 | 6.1 | 6.2 | 5.3 | 6.0 | 7.6 |
| H2-Q5 | 1.3 | 0.8 | 0.8 | 2.0 | 1.6 | 1.2 | 0.8 | 0.4 | 0.4 | 0.5 | 0.1 | 0.5 |
| H2-Q6 | 0.0 | 3.0 | 3.2 | 0.2 | 4.3 | 4.3 | 2.2 | 1.9 | 1.8 | 1.4 | 1.1 | 1.7 |
| H2-Q7 | 4.2 | 2.1 | 2.5 | 5.5 | 3.6 | 3.5 | 1.5 | 1.5 | 1.2 | 0.9 | 0.8 | 1.4 |
| H2-Q8 | 5.5 | 0.0 | 1.0 | 6.6 | 1.0 | 0.0 | 0.0 | 0.0 | 0.0 | 0.0 | 0.0 | 0.0 |
| H2-Q9 | 0.0 | 0.2 | 0.9 | 0.0 | 0.2 | 0.0 | 0.4 | 0.0 | 0.0 | 0.2 | 0.0 | 0.0 |
| H2-T10 | 4.6 | 4.5 | 4.1 | 5.7 | 4.4 | 4.5 | 4.1 | 3.7 | 4.0 | 3.3 | 3.3 | 4.9 |
| H2-T22 | 17.1 | 16.2 | 12.6 | 16.1 | 16.1 | 16.3 | 11.4 | 15.2 | 16.0 | 14.9 | 16.3 | 15.9 |
| H2-T23 | 34.4 | 25.4 | 28.4 | 44.3 | 38.0 | 33.1 | 29.0 | 27.8 | 30.0 | 29.5 | 23.5 | 39.1 |
| H2-T24 | 1.7 | 1.3 | 1.3 | 2.0 | 1.8 | 1.5 | 2.3 | 1.4 | 1.3 | 1.2 | 1.6 | 1.4 |
| H2-T3 | 0.0 | 0.0 | 0.0 | 0.0 | 0.0 | 0.0 | 0.0 | 0.0 | 0.0 | 0.0 | 0.0 | 0.0 |
| H2-T9 | 24.1 | 18.6 | 19.4 | 31.0 | 19.6 | 21.8 | 19.6 | 15.9 | 18.5 | 16.9 | 15.8 | 22.8 |
| H3f3a | 104.7 | 108.0 | 141.8 | 130.0 | 125.3 | 117.6 | 99.9 | 144.3 | 115.0 | 133.0 | 133.6 | 130.1 |
| H3f3b | 64.6 | 67.6 | 81.8 | 74.9 | 81.8 | 62.4 | 84.5 | 90.8 | 93.7 | 122.9 | 85.4 | 98.9 |
| H60b | 0.4 | 0.2 | 0.3 | 0.6 | 0.3 | 0.1 | 0.1 | 0.0 | 0.0 | 0.3 | 0.3 | 0.0 |
| H60c | 0.0 | 0.0 | 0.0 | 0.0 | 0.0 | 0.0 | 0.0 | 0.0 | 0.0 | 0.0 | 0.0 | 0.0 |
| H6pd | 45.9 | 53.0 | 59.1 | 39.6 | 67.7 | 31.6 | 63.5 | 54.4 | 59.2 | 80.6 | 52.1 | 62.3 |
| Haao | 0.0 | 0.0 | 0.0 | 0.5 | 0.7 | 0.1 | 0.0 | 0.0 | 0.1 | 0.0 | 0.1 | 0.0 |
| Habp2 | 0.0 | 0.1 | 0.1 | 0.0 | 0.1 | 0.0 | 0.0 | 0.0 | 0.0 | 0.1 | 0.0 | 0.1 |
| Habp4 | 10.8 | 9.0 | 7.9 | 12.6 | 7.1 | 11.3 | 7.9 | 8.2 | 7.6 | 8.2 | 9.7 | 9.4 |
| Hace1 | 3.6 | 4.0 | 3.5 | 3.0 | 3.2 | 3.7 | 3.8 | 3.9 | 4.1 | 4.3 | 4.3 | 3.7 |
| Hacl1 | 7.0 | 8.4 | 7.9 | 5.0 | 5.8 | 7.9 | 5.3 | 6.7 | 6.1 | 5.0 | 5.8 | 6.2 |
| Hadh | 23.8 | 21.3 | 20.0 | 28.5 | 19.0 | 24.9 | 22.3 | 19.8 | 19.8 | 24.5 | 24.6 | 24.5 |
| Hadha | 38.1 | 35.2 | 35.5 | 35.7 | 35.7 | 33.0 | 37.0 | 39.2 | 35.4 | 36.3 | 38.0 | 40.2 |
| Hadhb | 61.3 | 62.6 | 47.3 | 54.8 | 57.5 | 52.2 | 46.3 | 44.0 | 51.4 | 56.4 | 52.3 | 54.2 |
| Hagh | 22.1 | 18.8 | 15.4 | 23.9 | 17.1 | 24.5 | 16.8 | 17.2 | 18.1 | 18.8 | 24.0 | 20.3 |
| Haghl | 9.3 | 10.5 | 10.8 | 9.4 | 7.8 | 10.6 | 10.5 | 10.9 | 10.8 | 11.1 | 9.4 | 10.6 |
| Hal | 0.1 | 0.0 | 0.0 | 0.0 | 0.0 | 0.0 | 0.0 | 0.0 | 0.0 | 0.2 | 0.2 | 0.1 |
| Hand2 | 11.3 | 9.5 | 13.2 | 7.8 | 7.5 | 5.9 | 10.0 | 9.6 | 8.2 | 6.2 | 8.6 | 11.5 |
| Hap1 | 0.3 | 0.1 | 0.2 | 0.3 | 0.1 | 0.1 | 0.0 | 0.1 | 0.0 | 0.1 | 0.0 | 0.1 |
| Hapln1 | 0.0 | 0.0 | 0.0 | 0.0 | 0.0 | 0.0 | 0.0 | 0.0 | 0.0 | 0.0 | 0.0 | 0.0 |
| Hapln3 | 0.0 | 0.1 | 0.3 | 0.3 | 0.2 | 0.2 | 0.1 | 0.0 | 0.1 | 0.1 | 0.0 | 0.2 |
| Hapln4 | 0.2 | 0.1 | 0.1 | 0.3 | 0.1 | 0.3 | 0.1 | 0.1 | 0.1 | 0.1 | 0.1 | 0.1 |
| Harbi1 | 3.1 | 3.7 | 3.0 | 2.7 | 2.7 | 3.1 | 2.6 | 2.9 | 3.5 | 4.1 | 2.9 | 3.3 |
| Hars | 40.9 | 37.8 | 33.6 | 48.8 | 34.8 | 39.7 | 43.7 | 34.1 | 34.5 | 38.1 | 40.4 | 36.2 |
| Hars2 | 5.0 | 5.4 | 6.0 | 4.9 | 5.0 | 5.4 | 4.8 | 5.3 | 5.6 | 5.4 | 5.1 | 5.8 |
| Has1 | 0.2 | 0.2 | 0.3 | 0.1 | 0.2 | 0.1 | 0.3 | 0.4 | 0.2 | 0.7 | 0.4 | 0.4 |
| Has2 | 2.2 | 1.6 | 1.9 | 2.0 | 4.3 | 0.7 | 4.2 | 4.0 | 6.6 | 3.8 | 4.0 | 4.6 |
| Has2as | 0.9 | 0.3 | 0.4 | 1.0 | 1.0 | 0.0 | 0.8 | 0.7 | 1.5 | 0.9 | 1.3 | 0.1 |
| Has3 | 0.2 | 0.3 | 0.3 | 0.1 | 0.3 | 0.2 | 0.3 | 0.3 | 0.3 | 0.2 | 0.3 | 0.3 |
| Hat1 | 10.3 | 14.7 | 17.2 | 7.8 | 16.1 | 8.4 | 14.6 | 11.2 | 14.4 | 11.9 | 12.9 | 12.3 |
| Haus1 | 12.7 | 11.3 | 10.5 | 8.4 | 9.7 | 11.3 | 8.9 | 10.0 | 8.7 | 8.9 | 11.0 | 9.8 |
| Haus2 | 7.1 | 7.0 | 5.9 | 6.9 | 5.9 | 6.2 | 5.3 | 5.5 | 5.7 | 5.5 | 6.1 | 4.8 |
| Haus3 | 5.6 | 9.2 | 10.6 | 6.9 | 9.1 | 6.7 | 10.7 | 8.4 | 9.7 | 9.7 | 10.1 | 10.0 |
| Haus4 | 2.5 | 3.4 | 4.1 | 2.4 | 3.4 | 2.5 | 5.1 | 3.4 | 3.4 | 2.6 | 3.5 | 2.4 |

| | | | | | | | | | | | | |
|---|---|---|---|---|---|---|---|---|---|---|---|---|
| Haus5 | 1.4 | 2.1 | 2.3 | 1.4 | 2.1 | 1.3 | 2.0 | 1.9 | 2.1 | 1.3 | 1.7 | 1.3 |
| Haus6 | 3.7 | 4.9 | 4.3 | 2.5 | 5.5 | 2.5 | 4.1 | 3.2 | 3.9 | 3.2 | 4.1 | 3.4 |
| Haus7 | 13.5 | 13.4 | 13.1 | 11.0 | 10.8 | 10.5 | 13.8 | 14.5 | 14.3 | 8.6 | 12.9 | 11.7 |
| Haus8 | 6.0 | 5.5 | 5.6 | 8.1 | 9.7 | 6.3 | 6.1 | 5.2 | 5.9 | 5.1 | 5.9 | 4.5 |
| Havcr2 | 0.4 | 0.0 | 0.1 | 1.6 | 3.9 | 0.1 | 0.7 | 0.1 | 0.1 | 0.2 | 0.1 | 0.3 |
| Hax1 | 67.8 | 59.8 | 57.0 | 71.0 | 53.0 | 65.4 | 52.8 | 65.1 | 56.7 | 49.1 | 55.9 | 54.1 |
| Hbegf | 12.4 | 6.4 | 5.8 | 10.0 | 8.8 | 8.3 | 10.7 | 4.4 | 7.6 | 11.9 | 9.9 | 10.4 |
| Hbp1 | 40.0 | 44.6 | 48.4 | 40.0 | 38.5 | 39.3 | 35.9 | 45.2 | 43.0 | 48.5 | 44.1 | 51.0 |
| Hbs1l | 42.7 | 36.3 | 28.0 | 35.4 | 28.0 | 40.6 | 26.9 | 25.9 | 29.0 | 31.1 | 32.8 | 29.8 |
| Hc | 0.3 | 0.2 | 0.3 | 0.1 | 0.1 | 0.2 | 0.1 | 0.2 | 0.2 | 0.1 | 0.2 | 0.2 |
| Hccs | 10.5 | 11.4 | 12.8 | 11.2 | 9.4 | 8.6 | 9.6 | 10.2 | 8.9 | 6.9 | 10.1 | 9.7 |
| Hcfc1 | 6.3 | 7.5 | 7.7 | 6.5 | 7.1 | 6.2 | 8.6 | 8.2 | 7.8 | 6.3 | 6.6 | 6.1 |
| Hcfc1r1 | 25.5 | 28.8 | 36.6 | 28.3 | 19.2 | 36.4 | 27.5 | 47.0 | 30.6 | 30.7 | 34.4 | 33.3 |
| Hcfc2 | 11.1 | 11.9 | 10.6 | 10.4 | 9.8 | 9.6 | 9.7 | 9.9 | 11.6 | 9.5 | 10.1 | 10.9 |
| Hck | 3.2 | 1.5 | 1.2 | 6.8 | 11.8 | 0.7 | 2.7 | 0.6 | 1.2 | 2.3 | 1.5 | 2.3 |
| Hcls1 | 3.9 | 0.4 | 0.5 | 25.1 | 58.3 | 0.4 | 9.1 | 1.0 | 2.0 | 4.3 | 1.2 | 3.1 |
| Hcn1 | 0.0 | 0.0 | 0.0 | 0.0 | 0.0 | 0.0 | 0.0 | 0.0 | 0.0 | 0.0 | 0.0 | 0.0 |
| Hcn2 | 1.5 | 1.3 | 1.0 | 1.2 | 1.0 | 2.3 | 1.6 | 0.8 | 1.3 | 1.1 | 0.9 | 1.3 |
| Hcn3 | 0.0 | 0.0 | 0.0 | 0.0 | 0.0 | 0.0 | 0.0 | 0.0 | 0.0 | 0.1 | 0.0 | 0.1 |
| Hcrtr1 | 0.0 | 0.1 | 0.0 | 0.0 | 0.0 | 0.0 | 0.0 | 0.0 | 0.0 | 0.0 | 0.1 | 0.0 |
| Hcst | 0.0 | 0.0 | 0.0 | 0.5 | 0.7 | 0.0 | 0.2 | 0.0 | 0.1 | 0.0 | 0.0 | 0.0 |
| Hdac1 | 17.0 | 18.6 | 24.8 | 16.2 | 20.2 | 16.1 | 20.9 | 24.6 | 20.2 | 20.5 | 20.6 | 23.9 |
| Hdac10 | 2.3 | 2.7 | 2.8 | 2.2 | 2.3 | 2.0 | 2.6 | 2.7 | 3.0 | 2.5 | 2.8 | 2.0 |
| Hdac11 | 7.1 | 5.5 | 6.7 | 7.3 | 4.1 | 9.4 | 4.7 | 4.4 | 4.2 | 5.1 | 4.7 | 5.2 |
| Hdac2 | 45.8 | 44.1 | 44.4 | 41.0 | 36.1 | 40.0 | 45.5 | 42.5 | 37.8 | 42.6 | 51.2 | 47.5 |
| Hdac3 | 25.7 | 23.7 | 24.0 | 23.2 | 20.1 | 25.9 | 22.6 | 25.0 | 21.5 | 20.0 | 23.1 | 23.6 |
| Hdac4 | 3.3 | 4.0 | 3.6 | 4.0 | 3.6 | 3.3 | 3.0 | 3.2 | 3.2 | 3.2 | 3.0 | 2.8 |
| Hdac5 | 12.4 | 15.3 | 13.3 | 13.7 | 10.4 | 14.2 | 10.3 | 13.8 | 13.0 | 10.6 | 10.7 | 12.0 |
| Hdac6 | 3.3 | 3.8 | 4.1 | 3.3 | 3.4 | 3.2 | 3.4 | 4.4 | 3.8 | 4.7 | 3.4 | 3.8 |
| Hdac7 | 8.1 | 12.4 | 11.6 | 6.1 | 8.7 | 8.5 | 11.7 | 13.4 | 12.5 | 9.0 | 9.9 | 7.9 |
| Hdac8 | 5.3 | 5.1 | 5.9 | 5.4 | 4.8 | 4.9 | 4.1 | 6.2 | 4.0 | 6.3 | 6.5 | 5.2 |
| Hdac9 | 0.1 | 0.1 | 0.0 | 0.2 | 0.5 | 0.1 | 0.2 | 0.1 | 0.0 | 0.1 | 0.1 | 0.1 |
| Hdc | 0.1 | 0.1 | 0.1 | 0.1 | 0.1 | 0.1 | 0.0 | 0.0 | 0.0 | 0.1 | 0.1 | 0.0 |
| Hddc2 | 14.0 | 8.6 | 10.1 | 12.3 | 8.7 | 14.2 | 12.5 | 7.5 | 7.2 | 9.8 | 11.7 | 8.1 |
| Hddc3 | 4.0 | 5.0 | 4.9 | 3.4 | 2.5 | 5.9 | 3.6 | 5.8 | 4.2 | 4.3 | 4.3 | 4.4 |
| Hdgf | 83.7 | 79.9 | 81.0 | 94.5 | 72.5 | 80.5 | 81.8 | 88.1 | 68.3 | 70.4 | 87.1 | 69.7 |
| Hdgfrp2 | 22.3 | 26.3 | 27.9 | 25.2 | 20.6 | 24.3 | 26.0 | 22.9 | 21.9 | 21.4 | 24.4 | 25.4 |
| Hdgfrp3 | 3.5 | 4.6 | 7.0 | 3.4 | 3.6 | 4.4 | 4.8 | 5.2 | 3.9 | 4.4 | 5.1 | 5.5 |
| Hdhd2 | 20.3 | 18.4 | 17.2 | 22.9 | 18.1 | 21.9 | 18.3 | 16.6 | 17.1 | 18.4 | 20.2 | 18.8 |
| Hdhd3 | 1.6 | 1.8 | 2.1 | 0.7 | 2.2 | 1.7 | 1.5 | 1.8 | 1.8 | 3.1 | 1.4 | 1.3 |
| Hdlbp | 119.7 | 109.3 | 116.4 | 116.5 | 108.0 | 107.4 | 115.0 | 135.2 | 105.9 | 110.2 | 136.9 | 136.7 |
| Hdx | 0.4 | 0.5 | 0.6 | 0.2 | 0.5 | 0.4 | 0.5 | 0.6 | 0.5 | 0.6 | 0.7 | 0.5 |
| Heatr1 | 5.9 | 5.7 | 4.8 | 5.5 | 6.9 | 6.2 | 5.1 | 5.5 | 5.0 | 5.2 | 5.7 | 4.6 |
| Heatr2 | 3.7 | 3.3 | 3.1 | 4.1 | 3.0 | 4.0 | 3.6 | 3.1 | 2.6 | 2.6 | 3.0 | 2.5 |
| Heatr3 | 11.6 | 10.2 | 9.1 | 11.3 | 10.1 | 9.0 | 11.6 | 11.2 | 10.7 | 12.5 | 11.2 | 10.6 |
| Heatr5a | 12.5 | 14.0 | 12.5 | 13.2 | 14.0 | 12.9 | 13.0 | 15.0 | 13.8 | 11.9 | 14.0 | 12.5 |
| Heatr5b | 9.6 | 10.6 | 9.6 | 9.6 | 8.5 | 10.3 | 8.7 | 8.9 | 8.4 | 9.4 | 9.0 | 8.6 |
| Heatr6 | 5.8 | 6.1 | 5.2 | 5.2 | 6.2 | 5.4 | 5.6 | 4.9 | 5.4 | 4.9 | 5.7 | 4.8 |
| Heatr8 | 0.0 | 0.0 | 0.0 | 0.0 | 0.0 | 0.0 | 0.0 | 0.0 | 0.0 | 0.0 | 0.0 | 0.0 |
| Hebp1 | 17.5 | 14.0 | 15.4 | 14.1 | 11.9 | 16.2 | 13.8 | 12.0 | 14.8 | 11.4 | 15.7 | 17.3 |
| Hebp2 | 12.4 | 10.1 | 9.1 | 10.5 | 6.7 | 11.3 | 7.7 | 9.3 | 9.1 | 9.8 | 9.3 | 9.0 |
| Heca | 10.5 | 10.5 | 9.7 | 10.6 | 10.1 | 12.5 | 10.8 | 8.4 | 9.5 | 11.8 | 12.0 | 11.9 |
| Hectd1 | 29.5 | 30.7 | 30.6 | 25.8 | 27.8 | 25.9 | 28.8 | 33.3 | 31.1 | 33.0 | 32.3 | 34.3 |
| Hectd2 | 0.5 | 1.0 | 0.9 | 0.5 | 0.9 | 1.0 | 1.0 | 0.7 | 0.7 | 0.7 | 0.9 | 1.0 |
| Hectd3 | 8.2 | 7.8 | 7.9 | 9.1 | 7.4 | 10.3 | 6.8 | 7.0 | 7.5 | 7.8 | 7.5 | 7.9 |
| Hecw1 | 0.4 | 0.4 | 0.4 | 0.4 | 0.4 | 0.7 | 0.8 | 0.6 | 0.7 | 0.5 | 0.8 | 0.5 |
| Hecw2 | 0.4 | 0.2 | 0.4 | 0.4 | 0.5 | 0.3 | 0.3 | 0.2 | 0.2 | 0.6 | 0.5 | 0.7 |

| | | | | | | | | | | | | |
|---|---|---|---|---|---|---|---|---|---|---|---|---|
| Heg1 | 99.6 | 135.6 | 161.3 | 54.6 | 108.9 | 40.8 | 146.9 | 122.1 | 106.2 | 86.3 | 133.2 | 129.0 |
| Helb | 2.6 | 3.1 | 3.6 | 2.9 | 3.3 | 2.7 | 2.5 | 3.3 | 2.7 | 2.6 | 2.8 | 2.9 |
| Hells | 2.2 | 3.5 | 5.4 | 1.1 | 4.2 | 1.2 | 5.0 | 3.3 | 4.0 | 2.6 | 4.0 | 3.2 |
| Helq | 2.1 | 2.2 | 2.1 | 2.1 | 1.9 | 1.8 | 1.8 | 1.7 | 1.7 | 2.1 | 2.1 | 1.7 |
| Helz | 3.0 | 4.0 | 3.9 | 3.3 | 3.0 | 3.2 | 3.7 | 3.3 | 3.2 | 3.0 | 3.2 | 2.9 |
| Helz2 | 3.2 | 3.3 | 4.1 | 3.1 | 3.7 | 2.8 | 3.2 | 2.6 | 3.0 | 2.3 | 2.2 | 2.7 |
| Hemk1 | 1.9 | 1.9 | 1.7 | 1.5 | 1.3 | 2.0 | 1.3 | 1.2 | 2.5 | 1.9 | 1.2 | 1.7 |
| Henmt1 | 0.0 | 0.1 | 0.0 | 0.0 | 0.0 | 0.0 | 0.0 | 0.0 | 0.1 | 0.1 | 0.0 | 0.1 |
| Hepacam | 0.0 | 0.0 | 0.0 | 0.0 | 0.0 | 0.1 | 0.0 | 0.0 | 0.0 | 0.0 | 0.0 | 0.0 |
| Heph | 5.2 | 9.5 | 13.7 | 3.8 | 7.7 | 3.9 | 2.7 | 11.4 | 2.4 | 2.2 | 2.2 | 3.5 |
| Hephl1 | 1.1 | 2.0 | 1.6 | 0.4 | 1.3 | 0.3 | 3.0 | 5.8 | 1.9 | 1.3 | 4.6 | 1.9 |
| Herc1 | 6.9 | 6.8 | 6.1 | 6.8 | 7.1 | 5.9 | 6.2 | 6.0 | 5.9 | 5.4 | 6.1 | 5.7 |
| Herc2 | 15.1 | 15.1 | 13.7 | 14.0 | 14.8 | 14.2 | 15.4 | 13.9 | 13.7 | 11.9 | 14.0 | 13.4 |
| Herc3 | 0.7 | 0.6 | 0.4 | 1.0 | 1.6 | 0.8 | 0.7 | 0.4 | 0.6 | 0.5 | 0.3 | 0.7 |
| Herc4 | 8.7 | 9.5 | 9.5 | 8.5 | 8.9 | 8.4 | 8.6 | 8.1 | 9.5 | 9.3 | 9.3 | 8.3 |
| Herc6 | 3.9 | 3.9 | 4.1 | 4.3 | 4.1 | 3.7 | 3.4 | 2.3 | 3.0 | 2.8 | 3.7 | 4.0 |
| Herpud1 | 54.5 | 43.0 | 38.4 | 60.6 | 43.0 | 55.0 | 41.1 | 54.6 | 49.3 | 41.1 | 52.6 | 40.1 |
| Herpud2 | 9.1 | 11.3 | 9.6 | 10.0 | 9.6 | 10.2 | 11.2 | 9.4 | 11.4 | 8.7 | 11.0 | 10.3 |
| Hes1 | 17.3 | 17.7 | 22.7 | 13.4 | 16.6 | 12.4 | 20.7 | 27.5 | 26.0 | 21.0 | 24.7 | 25.9 |
| Hes3 | 0.0 | 0.0 | 0.0 | 0.0 | 0.0 | 0.0 | 0.0 | 0.0 | 0.0 | 0.0 | 0.0 | 0.0 |
| Hes6 | 6.9 | 7.3 | 6.6 | 5.9 | 6.2 | 5.6 | 7.7 | 7.8 | 9.2 | 9.6 | 7.4 | 8.6 |
| Hes7 | 0.0 | 0.0 | 0.1 | 0.0 | 0.0 | 0.1 | 0.1 | 0.2 | 0.1 | 0.0 | 0.2 | 0.1 |
| Hexa | 151.3 | 94.2 | 92.4 | 223.8 | 168.6 | 212.5 | 102.4 | 88.2 | 92.5 | 136.3 | 105.8 | 134.8 |
| Hexb | 54.1 | 43.1 | 42.7 | 70.5 | 90.5 | 58.3 | 35.3 | 40.4 | 42.9 | 43.7 | 39.3 | 49.8 |
| Hexdc | 1.2 | 0.8 | 0.7 | 0.7 | 0.6 | 1.2 | 0.7 | 1.0 | 0.7 | 1.2 | 0.8 | 0.8 |
| Hexim1 | 5.9 | 6.6 | 8.2 | 7.1 | 7.1 | 7.4 | 6.9 | 7.5 | 7.2 | 8.3 | 6.2 | 7.2 |
| Hexim2 | 0.7 | 0.9 | 0.9 | 0.6 | 0.4 | 0.7 | 0.5 | 0.7 | 0.7 | 0.4 | 0.4 | 0.5 |
| Hey1 | 0.4 | 1.1 | 1.3 | 0.3 | 0.4 | 0.2 | 0.3 | 0.5 | 0.5 | 0.5 | 0.5 | 0.5 |
| Hey2 | 1.0 | 1.4 | 2.1 | 0.5 | 1.2 | 0.4 | 2.2 | 2.2 | 2.6 | 1.3 | 2.6 | 1.7 |
| Heyl | 0.1 | 0.2 | 0.4 | 0.1 | 0.2 | 0.1 | 0.2 | 0.8 | 0.8 | 0.3 | 0.2 | 0.5 |
| Hfe | 4.0 | 3.1 | 3.2 | 6.6 | 4.1 | 5.5 | 1.2 | 3.8 | 1.6 | 2.0 | 1.7 | 1.9 |
| Hfm1 | 0.1 | 0.0 | 0.0 | 0.0 | 0.0 | 0.0 | 0.0 | 0.0 | 0.0 | 0.0 | 0.0 | 0.0 |
| Hgd | 0.0 | 0.0 | 0.0 | 0.1 | 0.1 | 0.0 | 0.0 | 0.1 | 0.0 | 0.1 | 0.0 | 0.0 |
| Hgf | 0.8 | 0.9 | 1.7 | 0.5 | 1.1 | 0.2 | 0.9 | 0.9 | 0.9 | 2.2 | 1.4 | 2.2 |
| Hgfac | 0.1 | 0.0 | 0.1 | 0.2 | 0.1 | 0.1 | 0.3 | 0.0 | 0.0 | 0.1 | 0.1 | 0.1 |
| Hgs | 22.6 | 23.3 | 20.6 | 24.0 | 22.2 | 23.4 | 27.1 | 22.6 | 22.7 | 21.9 | 23.5 | 20.2 |
| Hgsnat | 28.2 | 26.7 | 22.6 | 28.3 | 38.2 | 28.9 | 26.1 | 24.3 | 24.2 | 26.2 | 23.3 | 24.0 |
| Hhat | 1.6 | 1.6 | 1.4 | 1.3 | 1.6 | 1.3 | 2.2 | 2.8 | 2.5 | 2.0 | 2.2 | 1.8 |
| Hhatl | 0.0 | 0.1 | 0.0 | 0.0 | 0.0 | 0.0 | 0.0 | 0.0 | 0.0 | 0.1 | 0.0 | 0.0 |
| Hhex | 0.1 | 0.0 | 0.0 | 0.8 | 0.8 | 0.0 | 0.1 | 0.1 | 0.0 | 0.2 | 0.1 | 0.0 |
| Hhipl1 | 1.3 | 1.8 | 1.6 | 1.6 | 1.4 | 2.2 | 2.0 | 2.3 | 2.3 | 1.7 | 1.8 | 2.0 |
| Hhipl2 | 0.1 | 0.1 | 0.1 | 0.0 | 0.0 | 0.0 | 0.1 | 0.0 | 0.0 | 0.0 | 0.0 | 0.0 |
| Hiat1 | 13.6 | 15.1 | 14.7 | 14.1 | 16.0 | 13.2 | 16.8 | 15.8 | 18.6 | 15.3 | 17.0 | 16.4 |
| Hiatl1 | 19.9 | 19.2 | 17.2 | 20.3 | 20.4 | 19.1 | 19.8 | 16.9 | 19.2 | 18.0 | 21.1 | 19.8 |
| Hibadh | 33.0 | 35.5 | 29.9 | 34.9 | 31.2 | 37.1 | 27.3 | 29.8 | 31.7 | 33.1 | 31.2 | 31.2 |
| Hibch | 14.4 | 13.9 | 12.8 | 11.5 | 9.6 | 13.5 | 9.9 | 11.0 | 10.2 | 11.6 | 11.9 | 12.4 |
| Hic1 | 4.4 | 5.8 | 7.0 | 3.4 | 6.5 | 4.4 | 9.1 | 10.1 | 11.4 | 6.5 | 5.4 | 7.1 |
| Hic2 | 0.1 | 0.2 | 0.2 | 0.1 | 0.3 | 0.1 | 0.2 | 0.3 | 0.2 | 0.3 | 0.2 | 0.2 |
| Hid1 | 1.5 | 0.5 | 0.6 | 1.0 | 0.3 | 1.3 | 0.4 | 0.9 | 0.6 | 1.5 | 1.0 | 1.0 |
| Hif1a | 38.3 | 37.6 | 50.8 | 37.8 | 43.8 | 31.1 | 48.6 | 54.7 | 51.6 | 45.3 | 50.8 | 68.7 |
| Hif1an | 4.9 | 5.5 | 4.9 | 4.8 | 5.2 | 4.1 | 4.6 | 5.2 | 5.4 | 5.0 | 4.6 | 4.9 |
| Hif3a | 0.0 | 0.0 | 0.0 | 0.0 | 0.0 | 0.0 | 0.0 | 0.0 | 0.0 | 0.0 | 0.0 | 0.0 |
| Higd1a | 24.3 | 19.2 | 16.8 | 22.1 | 19.7 | 21.8 | 19.6 | 15.0 | 21.0 | 20.8 | 18.6 | 18.4 |
| Higd2a | 90.9 | 86.4 | 80.2 | 107.8 | 91.4 | 92.8 | 89.3 | 86.1 | 80.3 | 97.1 | 98.1 | 86.8 |
| Hilpda | 7.8 | 6.6 | 4.3 | 8.1 | 6.7 | 6.2 | 6.2 | 6.1 | 6.1 | 9.6 | 5.9 | 8.0 |
| Hils1 | 0.1 | 0.1 | 0.0 | 0.0 | 0.1 | 0.1 | 0.0 | 0.1 | 0.0 | 0.0 | 0.0 | 0.2 |
| Hinfp | 4.1 | 4.2 | 3.8 | 3.8 | 4.3 | 3.7 | 5.0 | 4.1 | 4.6 | 4.6 | 4.5 | 4.3 |

| | | | | | | | | | | | | |
|---|---|---|---|---|---|---|---|---|---|---|---|---|
| Hint1 | 194.3 | 170.1 | 142.9 | 199.6 | 176.6 | 217.4 | 173.5 | 168.4 | 160.7 | 175.7 | 191.7 | 167.5 |
| Hint2 | 18.6 | 14.1 | 11.1 | 16.3 | 12.4 | 20.7 | 13.1 | 10.3 | 14.5 | 12.2 | 12.2 | 13.1 |
| Hint3 | 14.3 | 14.4 | 11.1 | 7.8 | 12.5 | 14.2 | 7.2 | 11.2 | 12.8 | 12.3 | 11.3 | 10.8 |
| Hip1 | 14.9 | 20.3 | 20.8 | 12.5 | 15.2 | 12.3 | 17.2 | 21.7 | 16.8 | 12.6 | 17.1 | 16.5 |
| Hip1r | 2.3 | 2.2 | 2.0 | 1.9 | 1.7 | 2.1 | 2.6 | 2.2 | 1.9 | 2.1 | 2.2 | 2.1 |
| Hipk1 | 22.1 | 24.4 | 23.0 | 29.1 | 24.9 | 26.9 | 22.0 | 16.6 | 18.2 | 18.0 | 19.4 | 18.6 |
| Hipk2 | 3.7 | 3.1 | 3.2 | 6.5 | 3.4 | 4.0 | 3.2 | 2.1 | 1.8 | 2.6 | 3.0 | 2.3 |
| Hipk3 | 38.5 | 43.1 | 40.1 | 51.5 | 38.2 | 44.6 | 38.4 | 32.3 | 34.7 | 31.6 | 38.0 | 37.3 |
| Hipk4 | 0.1 | 0.1 | 0.1 | 0.0 | 0.1 | 0.0 | 0.1 | 0.1 | 0.1 | 0.2 | 0.0 | 0.1 |
| Hira | 2.3 | 2.0 | 2.3 | 2.1 | 2.3 | 2.1 | 2.1 | 2.4 | 2.3 | 2.2 | 2.3 | 2.2 |
| Hirip3 | 1.8 | 2.3 | 3.9 | 1.8 | 2.2 | 2.3 | 3.5 | 2.6 | 2.1 | 1.6 | 2.4 | 2.2 |
| Hist1h1a | 3.8 | 4.9 | 10.1 | 2.2 | 7.5 | 1.3 | 15.6 | 12.3 | 11.4 | 12.9 | 10.6 | 7.6 |
| Hist1h1b | 4.4 | 3.8 | 8.2 | 3.4 | 6.7 | 4.3 | 12.5 | 8.7 | 10.1 | 11.9 | 7.0 | 7.6 |
| Hist1h1c | 99.5 | 121.6 | 95.2 | 87.8 | 82.1 | 98.1 | 80.3 | 85.6 | 99.9 | 138.6 | 89.8 | 105.9 |
| Hist1h1d | 5.5 | 5.9 | 6.6 | 4.1 | 4.4 | 3.7 | 6.9 | 7.6 | 10.6 | 18.3 | 8.0 | 8.6 |
| Hist1h1e | 19.2 | 17.3 | 20.6 | 16.0 | 15.7 | 16.3 | 20.1 | 23.3 | 24.8 | 35.3 | 20.1 | 21.6 |
| Hist1h1t | 0.4 | 0.2 | 0.6 | 0.0 | 0.1 | 0.3 | 0.3 | 0.4 | 0.7 | 1.1 | 0.1 | 0.3 |
| Hist1h2ab | 9.1 | 7.4 | 13.7 | 3.8 | 11.7 | 4.1 | 22.9 | 20.3 | 21.9 | 20.9 | 17.0 | 13.2 |
| Hist1h2ac | 1.5 | 1.4 | 2.4 | 1.2 | 1.9 | 0.9 | 3.6 | 3.1 | 3.6 | 5.1 | 2.6 | 2.6 |
| Hist1h2ad | 8.1 | 7.0 | 12.9 | 4.3 | 9.7 | 2.8 | 20.9 | 18.5 | 17.8 | 19.4 | 14.0 | 11.1 |
| Hist1h2ae | 3.9 | 3.1 | 6.5 | 2.2 | 5.1 | 1.5 | 9.2 | 10.1 | 8.6 | 10.7 | 7.7 | 5.5 |
| Hist1h2af | 6.9 | 5.5 | 11.6 | 3.4 | 8.9 | 2.2 | 15.3 | 13.4 | 12.6 | 13.5 | 9.8 | 7.8 |
| Hist1h2ag | 9.2 | 5.9 | 12.3 | 4.6 | 9.6 | 2.6 | 21.8 | 20.6 | 18.5 | 20.3 | 14.0 | 10.7 |
| Hist1h2ah | 8.4 | 7.2 | 13.9 | 4.5 | 10.3 | 2.9 | 20.6 | 19.7 | 19.5 | 20.8 | 14.8 | 11.6 |
| Hist1h2ai | 8.2 | 7.1 | 12.3 | 4.5 | 10.4 | 2.9 | 20.9 | 18.1 | 18.0 | 18.9 | 14.0 | 11.6 |
| Hist1h2ak | 5.2 | 2.3 | 8.2 | 2.2 | 5.9 | 0.7 | 11.1 | 12.2 | 10.9 | 9.8 | 10.1 | 6.1 |
| Hist1h2an | 6.6 | 6.0 | 10.5 | 3.1 | 8.1 | 2.0 | 15.5 | 14.7 | 16.0 | 15.9 | 10.3 | 9.4 |
| Hist1h2ao | 3.8 | 5.0 | 10.3 | 3.3 | 7.7 | 2.1 | 14.5 | 15.5 | 14.2 | 16.8 | 11.6 | 8.9 |
| Hist1h2ap | 3.2 | 0.0 | 0.2 | 0.0 | 0.2 | 0.5 | 3.1 | 0.0 | 1.4 | 0.2 | 0.0 | 0.0 |
| Hist1h2ba | 29.7 | 26.9 | 22.6 | 20.1 | 27.2 | 19.6 | 25.3 | 26.4 | 29.0 | 36.2 | 25.5 | 20.9 |
| Hist1h2bb | 21.7 | 21.5 | 31.5 | 10.4 | 30.3 | 9.0 | 43.4 | 39.3 | 46.7 | 41.1 | 32.2 | 26.4 |
| Hist1h2bc | 255.4 | 246.3 | 217.0 | 252.6 | 228.3 | 211.1 | 202.4 | 204.9 | 211.5 | 262.0 | 247.7 | 248.8 |
| Hist1h2be | 6.7 | 7.1 | 6.9 | 7.5 | 8.3 | 5.8 | 8.2 | 8.0 | 8.8 | 12.1 | 8.5 | 7.5 |
| Hist1h2bf | 37.0 | 38.6 | 31.4 | 35.3 | 42.5 | 38.9 | 34.2 | 36.5 | 48.4 | 50.9 | 36.9 | 36.7 |
| Hist1h2bg | 43.7 | 51.2 | 47.7 | 39.0 | 50.9 | 37.0 | 49.2 | 50.2 | 57.5 | 69.5 | 51.2 | 46.6 |
| Hist1h2bh | 37.9 | 42.0 | 37.4 | 28.7 | 39.9 | 26.3 | 33.3 | 34.1 | 41.6 | 42.2 | 38.0 | 33.1 |
| Hist1h2bj | 41.1 | 35.5 | 36.4 | 25.8 | 41.7 | 25.5 | 41.1 | 41.7 | 46.2 | 52.1 | 36.5 | 31.8 |
| Hist1h2bk | 39.4 | 36.6 | 36.6 | 27.6 | 46.4 | 23.4 | 47.0 | 46.5 | 56.8 | 57.4 | 41.3 | 35.1 |
| Hist1h2bl | 30.8 | 29.1 | 27.2 | 23.1 | 33.0 | 21.6 | 31.5 | 32.4 | 34.8 | 38.5 | 30.2 | 25.3 |
| Hist1h2bm | 8.8 | 9.6 | 10.3 | 6.5 | 12.1 | 6.5 | 12.2 | 12.2 | 11.8 | 15.9 | 12.4 | 8.7 |
| Hist1h2bn | 27.8 | 27.6 | 30.2 | 19.7 | 33.4 | 14.9 | 41.1 | 37.4 | 43.4 | 46.9 | 31.9 | 25.6 |
| Hist1h2bp | 14.0 | 14.4 | 13.6 | 10.1 | 16.5 | 9.7 | 15.7 | 15.6 | 17.8 | 19.1 | 14.7 | 12.1 |
| Hist1h2bq | 7.0 | 6.5 | 6.5 | 5.5 | 7.0 | 5.0 | 7.2 | 7.1 | 8.0 | 9.7 | 6.6 | 5.8 |
| Hist1h2br | 7.0 | 6.5 | 6.2 | 5.3 | 6.8 | 5.0 | 6.9 | 6.9 | 7.8 | 9.2 | 6.7 | 5.6 |
| Hist1h3a | 6.0 | 6.8 | 12.3 | 2.8 | 9.0 | 1.8 | 15.4 | 15.4 | 16.4 | 17.7 | 11.4 | 9.1 |
| Hist1h3b | 10.8 | 10.9 | 23.1 | 6.6 | 18.2 | 5.4 | 31.3 | 31.8 | 33.3 | 33.5 | 21.5 | 18.5 |
| Hist1h3c | 8.7 | 8.0 | 16.1 | 4.4 | 13.2 | 2.2 | 23.8 | 20.7 | 22.5 | 17.7 | 16.5 | 11.4 |
| Hist1h3d | 10.2 | 9.3 | 18.3 | 5.8 | 13.8 | 4.8 | 22.2 | 26.7 | 22.2 | 24.1 | 17.4 | 16.2 |
| Hist1h3e | 7.8 | 6.6 | 13.0 | 5.9 | 9.7 | 3.9 | 19.7 | 19.3 | 18.2 | 28.3 | 13.7 | 11.7 |
| Hist1h3f | 5.4 | 7.0 | 11.6 | 4.2 | 9.7 | 2.9 | 16.8 | 15.5 | 14.4 | 15.0 | 11.5 | 10.7 |
| Hist1h3g | 5.1 | 6.0 | 10.3 | 3.4 | 8.2 | 2.0 | 17.0 | 16.0 | 14.7 | 15.4 | 12.3 | 10.9 |
| Hist1h3h | 11.2 | 11.5 | 22.0 | 6.2 | 17.6 | 4.8 | 27.8 | 31.0 | 31.0 | 34.9 | 21.2 | 20.6 |
| Hist1h3i | 11.7 | 11.6 | 23.6 | 6.7 | 16.7 | 4.5 | 31.1 | 32.1 | 31.8 | 38.6 | 19.9 | 23.0 |
| Hist1h4a | 31.4 | 40.3 | 58.4 | 19.3 | 50.1 | 23.2 | 61.7 | 63.7 | 65.4 | 66.5 | 50.1 | 47.0 |
| Hist1h4b | 28.6 | 37.7 | 55.8 | 19.5 | 46.4 | 23.2 | 64.9 | 59.8 | 68.1 | 60.5 | 49.3 | 46.1 |
| Hist1h4c | 52.6 | 69.9 | 92.9 | 39.2 | 75.2 | 35.8 | 98.0 | 102.1 | 126.9 | 110.5 | 82.4 | 89.2 |
| Hist1h4d | 49.5 | 61.8 | 94.3 | 31.4 | 63.0 | 44.6 | 96.9 | 126.4 | 116.3 | 103.0 | 78.1 | 77.9 |

| | | | | | | | | | | | | |
|---|---|---|---|---|---|---|---|---|---|---|---|---|
| Hist1h4f | 18.4 | 21.2 | 38.1 | 13.2 | 30.4 | 7.7 | 47.3 | 46.8 | 43.5 | 40.0 | 31.9 | 24.6 |
| Hist1h4h | 49.7 | 65.1 | 85.5 | 38.8 | 72.1 | 44.2 | 83.1 | 97.9 | 104.9 | 90.4 | 78.9 | 70.4 |
| Hist1h4i | 22.5 | 26.7 | 34.8 | 22.4 | 30.2 | 18.3 | 33.4 | 36.3 | 39.1 | 45.9 | 31.3 | 35.6 |
| Hist1h4j | 29.5 | 40.3 | 55.2 | 21.2 | 43.0 | 22.4 | 60.0 | 55.6 | 68.4 | 59.3 | 47.4 | 42.4 |
| Hist1h4k | 29.5 | 39.3 | 54.9 | 18.6 | 44.1 | 21.9 | 58.2 | 59.2 | 66.0 | 58.9 | 45.8 | 40.8 |
| Hist1h4m | 43.4 | 44.3 | 48.8 | 30.3 | 49.6 | 33.4 | 46.7 | 79.4 | 72.0 | 82.8 | 54.2 | 64.1 |
| Hist1h4n | 15.3 | 15.2 | 19.3 | 12.7 | 23.0 | 12.9 | 21.3 | 27.4 | 32.1 | 29.1 | 21.8 | 27.3 |
| Hist2h2aa1 | 13.7 | 11.4 | 11.8 | 10.2 | 13.0 | 9.2 | 16.1 | 17.5 | 18.8 | 22.7 | 16.5 | 15.6 |
| Hist2h2aa2 | 0.2 | 0.5 | 0.2 | 0.7 | 0.5 | 0.3 | 1.9 | 0.5 | 0.4 | 0.8 | 0.8 | 1.3 |
| Hist2h2ab | 0.9 | 0.5 | 1.2 | 0.4 | 1.2 | 0.8 | 1.2 | 1.6 | 1.8 | 2.0 | 1.6 | 1.3 |
| Hist2h2ac | 3.2 | 2.1 | 6.5 | 6.5 | 7.6 | 1.1 | 9.5 | 8.2 | 10.3 | 11.1 | 6.1 | 5.6 |
| Hist2h2bb | 7.0 | 7.0 | 14.2 | 3.5 | 14.5 | 4.1 | 24.2 | 22.7 | 23.8 | 17.3 | 21.0 | 14.1 |
| Hist2h2be | 0.7 | 0.7 | 0.5 | 0.9 | 0.6 | 0.7 | 0.6 | 0.5 | 0.5 | 0.8 | 0.8 | 0.7 |
| Hist2h3b | 1.9 | 1.3 | 3.4 | 2.3 | 3.1 | 2.0 | 5.3 | 5.0 | 3.6 | 4.4 | 3.5 | 2.2 |
| Hist2h3c1 | 2.3 | 2.0 | 3.6 | 3.1 | 4.0 | 3.1 | 4.9 | 5.3 | 4.7 | 4.9 | 5.2 | 4.7 |
| Hist2h3c2 | 3.0 | 3.5 | 3.2 | 3.7 | 3.9 | 2.5 | 5.1 | 5.5 | 5.4 | 4.8 | 5.5 | 4.8 |
| Hist2h4 | 6.9 | 5.5 | 10.0 | 5.5 | 7.6 | 2.5 | 8.7 | 13.9 | 12.7 | 12.5 | 10.9 | 9.4 |
| Hist3h2a | 6.3 | 4.6 | 5.1 | 7.0 | 6.0 | 6.8 | 4.3 | 5.5 | 4.9 | 6.8 | 6.1 | 6.8 |
| Hist3h2bb-ps | 0.2 | 0.0 | 0.0 | 0.0 | 0.1 | 0.0 | 0.1 | 0.0 | 0.1 | 0.0 | 0.0 | 0.1 |
| Hist4h4 | 0.7 | 1.1 | 1.3 | 0.9 | 0.8 | 0.7 | 1.1 | 1.6 | 2.2 | 2.1 | 1.2 | 1.6 |
| Hivep1 | 8.6 | 8.6 | 7.7 | 12.9 | 8.7 | 11.5 | 8.3 | 4.9 | 5.5 | 5.4 | 7.1 | 6.1 |
| Hivep2 | 4.8 | 5.7 | 6.4 | 4.7 | 6.1 | 5.3 | 5.8 | 5.3 | 4.9 | 4.3 | 4.6 | 4.8 |
| Hivep3 | 0.2 | 0.3 | 0.4 | 0.2 | 0.3 | 0.1 | 0.5 | 0.3 | 0.3 | 0.2 | 0.3 | 0.3 |
| Hjurp | 1.3 | 1.7 | 2.2 | 0.8 | 1.9 | 1.1 | 2.1 | 1.6 | 2.4 | 1.6 | 2.2 | 1.7 |
| Hk1 | 23.9 | 20.8 | 21.1 | 29.6 | 24.0 | 29.3 | 27.6 | 19.6 | 19.9 | 19.2 | 20.7 | 20.4 |
| Hk2 | 6.5 | 5.4 | 5.1 | 5.3 | 7.2 | 3.9 | 5.8 | 4.4 | 5.4 | 6.8 | 6.3 | 6.5 |
| Hk3 | 0.7 | 0.1 | 0.1 | 6.6 | 8.4 | 0.0 | 0.8 | 0.1 | 0.1 | 0.5 | 0.1 | 0.2 |
| Hlcs | 2.9 | 3.0 | 3.0 | 2.7 | 2.8 | 3.2 | 2.5 | 2.8 | 2.4 | 3.1 | 2.9 | 2.4 |
| Hlf | 0.0 | 0.1 | 0.1 | 0.0 | 0.1 | 0.0 | 0.0 | 0.0 | 0.0 | 0.0 | 0.0 | 0.0 |
| Hltf | 13.7 | 13.9 | 13.8 | 13.7 | 12.9 | 13.6 | 11.7 | 11.4 | 11.8 | 13.8 | 14.2 | 16.0 |
| Hlx | 0.7 | 0.9 | 1.4 | 1.9 | 2.7 | 1.3 | 1.3 | 1.3 | 1.8 | 2.4 | 1.3 | 1.1 |
| Hmbox1 | 6.1 | 6.5 | 5.7 | 5.6 | 5.2 | 5.2 | 5.2 | 4.8 | 4.7 | 5.5 | 5.5 | 4.9 |
| Hmbs | 12.5 | 13.1 | 12.2 | 11.4 | 11.9 | 12.0 | 11.8 | 15.6 | 12.5 | 13.3 | 14.0 | 11.8 |
| Hmcn1 | 0.0 | 0.0 | 0.1 | 0.0 | 0.1 | 0.0 | 0.0 | 0.1 | 0.0 | 0.1 | 0.1 | 0.0 |
| Hmg20a | 9.0 | 10.4 | 10.3 | 9.5 | 8.0 | 10.7 | 9.4 | 9.8 | 8.8 | 8.3 | 9.2 | 8.4 |
| Hmg20b | 12.3 | 15.7 | 13.5 | 13.3 | 13.4 | 13.9 | 14.5 | 14.4 | 14.5 | 15.0 | 15.1 | 13.1 |
| Hmga1 | 3.1 | 3.6 | 0.9 | 7.3 | 8.7 | 6.6 | 6.5 | 4.3 | 4.5 | 5.5 | 2.2 | 5.2 |
| Hmga1-rs1 | 10.5 | 3.2 | 4.4 | 12.0 | 12.0 | 12.5 | 6.9 | 2.4 | 1.1 | 6.2 | 10.1 | 2.7 |
| Hmga2 | 0.5 | 0.3 | 0.3 | 0.5 | 0.4 | 0.6 | 0.6 | 0.5 | 0.4 | 2.0 | 1.4 | 1.1 |
| Hmga2-ps1 | 0.8 | 0.3 | 0.6 | 1.5 | 2.0 | 0.3 | 0.6 | 0.5 | 0.2 | 0.2 | 0.1 | 0.5 |
| Hmgb1 | 66.3 | 71.9 | 109.1 | 86.5 | 68.6 | 73.1 | 81.5 | 81.0 | 68.0 | 75.5 | 82.8 | 94.6 |
| Hmgb1-rs17 | 0.4 | 0.7 | 1.1 | 0.7 | 0.5 | 0.7 | 0.9 | 0.8 | 0.5 | 0.8 | 0.6 | 1.0 |
| Hmgb2 | 2.5 | 4.3 | 9.2 | 1.5 | 5.4 | 1.7 | 8.6 | 4.9 | 5.7 | 4.0 | 6.2 | 5.2 |
| Hmgb3 | 1.1 | 1.5 | 2.0 | 1.0 | 1.7 | 1.0 | 1.5 | 1.7 | 1.2 | 1.8 | 2.1 | 1.3 |
| Hmgcl | 9.1 | 8.8 | 7.8 | 7.4 | 10.7 | 8.5 | 7.9 | 7.9 | 7.7 | 11.0 | 8.5 | 7.0 |
| Hmgcll1 | 0.1 | 0.1 | 0.0 | 0.0 | 0.0 | 0.1 | 0.0 | 0.0 | 0.0 | 0.2 | 0.1 | 0.0 |
| Hmgcr | 14.2 | 17.2 | 14.8 | 7.7 | 14.2 | 8.4 | 23.0 | 27.4 | 23.4 | 11.4 | 22.3 | 12.5 |
| Hmgcs1 | 30.1 | 44.0 | 47.0 | 14.6 | 24.7 | 21.7 | 66.7 | 96.1 | 64.1 | 19.9 | 67.0 | 30.2 |
| Hmgcs2 | 1.0 | 0.8 | 1.6 | 0.6 | 0.6 | 1.0 | 0.3 | 0.9 | 0.2 | 0.8 | 0.5 | 0.9 |
| Hmgn1 | 156.0 | 151.9 | 151.8 | 140.0 | 114.4 | 152.7 | 125.9 | 133.5 | 135.9 | 185.8 | 160.8 | 153.7 |
| Hmgn2 | 24.1 | 31.9 | 47.7 | 21.9 | 28.4 | 24.5 | 31.5 | 42.4 | 33.5 | 30.7 | 31.4 | 33.0 |
| Hmgn3 | 32.9 | 33.2 | 45.9 | 36.4 | 31.1 | 37.8 | 43.0 | 40.2 | 32.7 | 30.3 | 44.3 | 42.1 |
| Hmgn5 | 3.2 | 4.8 | 8.6 | 5.0 | 3.3 | 2.4 | 4.9 | 4.7 | 2.1 | 2.1 | 4.9 | 4.0 |
| Hmgxb3 | 7.2 | 6.8 | 5.9 | 6.1 | 6.8 | 7.0 | 7.3 | 6.9 | 7.0 | 7.0 | 6.6 | 6.0 |
| Hmgxb4 | 6.1 | 7.0 | 7.8 | 5.8 | 5.8 | 6.2 | 5.9 | 6.7 | 6.4 | 6.3 | 7.3 | 7.7 |
| Hmha1 | 1.9 | 2.1 | 1.8 | 5.0 | 7.4 | 1.3 | 1.6 | 0.9 | 1.0 | 3.9 | 2.4 | 1.8 |
| Hmmr | 3.0 | 6.7 | 12.3 | 1.1 | 8.9 | 1.2 | 11.7 | 3.9 | 9.2 | 4.3 | 8.7 | 6.8 |

| | | | | | | | | | | | | |
|---|---|---|---|---|---|---|---|---|---|---|---|---|
| Hmox1 | 332.1 | 258.9 | 150.3 | 617.9 | 326.1 | 686.4 | 181.4 | 132.0 | 97.8 | 293.1 | 194.0 | 159.2 |
| Hmox2 | 18.0 | 18.8 | 20.3 | 19.5 | 21.1 | 17.7 | 20.3 | 17.8 | 18.2 | 20.2 | 20.1 | 24.7 |
| Hn1 | 47.9 | 39.6 | 33.9 | 46.1 | 48.3 | 43.9 | 46.5 | 38.2 | 45.6 | 42.7 | 45.9 | 36.3 |
| Hn1l | 15.3 | 15.2 | 15.7 | 17.9 | 21.0 | 15.8 | 20.1 | 15.7 | 17.0 | 15.8 | 20.0 | 14.6 |
| Hnf1a | 0.0 | 0.0 | 0.1 | 0.1 | 0.1 | 0.0 | 0.0 | 0.0 | 0.0 | 0.0 | 0.0 | 0.0 |
| Hnmt | 0.9 | 0.5 | 0.7 | 0.9 | 1.8 | 0.5 | 0.6 | 0.4 | 0.2 | 0.3 | 0.2 | 0.3 |
| Hnrnpa0 | 17.8 | 15.3 | 18.1 | 14.1 | 18.7 | 17.0 | 19.9 | 21.4 | 23.7 | 26.1 | 18.0 | 21.5 |
| Hnrnpa1 | 5.0 | 7.3 | 7.9 | 5.7 | 8.9 | 6.4 | 7.5 | 9.2 | 14.1 | 12.6 | 11.7 | 9.1 |
| Hnrnpa2b1 | 70.5 | 73.9 | 94.5 | 75.6 | 81.8 | 64.6 | 71.5 | 80.3 | 69.0 | 76.5 | 77.1 | 88.4 |
| Hnrnpa3 | 9.8 | 11.7 | 11.4 | 9.2 | 11.2 | 9.9 | 9.6 | 8.6 | 10.7 | 10.5 | 10.7 | 11.9 |
| Hnrnpab | 91.7 | 76.3 | 84.7 | 97.4 | 77.0 | 77.8 | 92.7 | 84.6 | 76.6 | 88.2 | 92.7 | 93.9 |
| Hnrnpc | 58.8 | 68.9 | 75.4 | 58.4 | 61.7 | 67.3 | 57.6 | 64.6 | 53.1 | 67.6 | 69.4 | 69.2 |
| Hnrnpd | 0.8 | 1.1 | 1.3 | 0.9 | 1.3 | 0.8 | 1.3 | 1.1 | 1.1 | 1.6 | 1.3 | 1.2 |
| Hnrnpf | 100.6 | 94.0 | 96.6 | 111.1 | 101.6 | 101.9 | 103.3 | 97.1 | 90.3 | 97.6 | 106.9 | 102.3 |
| Hnrnph1 | 40.1 | 47.7 | 63.1 | 46.3 | 46.9 | 36.5 | 61.9 | 61.4 | 62.4 | 54.0 | 63.0 | 60.0 |
| Hnrnph2 | 32.8 | 33.4 | 41.7 | 41.7 | 33.7 | 34.3 | 34.9 | 34.1 | 32.4 | 35.6 | 39.6 | 46.5 |
| Hnrnph3 | 4.1 | 5.1 | 7.0 | 5.5 | 3.7 | 6.5 | 4.9 | 4.5 | 4.3 | 4.4 | 4.6 | 6.2 |
| Hnrnpk | 228.1 | 216.7 | 208.6 | 218.8 | 216.6 | 212.2 | 209.6 | 217.4 | 199.7 | 214.2 | 233.6 | 222.2 |
| Hnrnpl | 47.8 | 52.0 | 54.1 | 44.2 | 51.5 | 41.7 | 54.4 | 68.0 | 57.5 | 57.5 | 54.9 | 55.9 |
| Hnrnpm | 33.6 | 34.0 | 41.2 | 41.6 | 38.4 | 33.7 | 39.6 | 40.9 | 32.9 | 35.4 | 40.0 | 42.0 |
| Hnrnpr | 21.6 | 20.9 | 28.4 | 22.5 | 20.2 | 21.7 | 26.3 | 30.4 | 23.8 | 23.1 | 26.6 | 29.9 |
| Hnrnpu | 73.4 | 76.5 | 86.6 | 73.4 | 80.6 | 63.8 | 85.2 | 89.5 | 78.3 | 86.6 | 91.9 | 97.6 |
| Hnrnpul1 | 34.6 | 32.9 | 38.3 | 35.0 | 30.4 | 32.3 | 38.5 | 39.8 | 33.0 | 35.5 | 38.8 | 39.0 |
| Hnrnpul2 | 10.5 | 11.6 | 13.6 | 10.4 | 11.4 | 10.1 | 12.3 | 12.9 | 11.0 | 12.7 | 12.5 | 14.4 |
| Hnrpdl | 11.3 | 12.9 | 17.9 | 13.7 | 12.7 | 12.9 | 20.1 | 15.3 | 17.9 | 16.7 | 18.7 | 18.7 |
| Hnrpll | 12.3 | 13.8 | 15.0 | 11.7 | 11.7 | 12.8 | 12.6 | 13.4 | 14.0 | 13.4 | 13.6 | 14.5 |
| Hoga1 | 1.1 | 1.0 | 1.4 | 1.2 | 0.9 | 1.0 | 1.0 | 1.0 | 1.0 | 0.5 | 0.7 | 0.9 |
| Homer1 | 2.6 | 2.2 | 2.2 | 2.0 | 2.6 | 2.6 | 3.2 | 2.4 | 2.6 | 4.5 | 3.6 | 3.0 |
| Homer2 | 0.1 | 0.1 | 0.3 | 0.1 | 0.1 | 0.1 | 0.1 | 0.2 | 0.1 | 0.1 | 0.1 | 0.1 |
| Homer3 | 17.2 | 14.5 | 12.8 | 19.0 | 16.3 | 18.3 | 16.9 | 11.6 | 13.8 | 17.9 | 14.5 | 15.6 |
| Homez | 1.4 | 1.6 | 1.4 | 1.0 | 1.0 | 1.2 | 1.0 | 1.2 | 1.1 | 1.1 | 1.1 | 1.1 |
| Hook1 | 0.1 | 0.2 | 0.2 | 0.0 | 0.2 | 0.2 | 0.1 | 0.1 | 0.0 | 0.2 | 0.0 | 0.2 |
| Hook2 | 3.8 | 4.4 | 3.9 | 2.8 | 4.0 | 3.7 | 3.8 | 3.1 | 3.5 | 4.0 | 3.0 | 2.9 |
| Hook3 | 10.9 | 12.8 | 13.5 | 12.3 | 12.0 | 11.3 | 11.5 | 11.6 | 10.9 | 10.9 | 13.8 | 13.6 |
| Hopx | 0.2 | 0.6 | 0.2 | 0.1 | 0.1 | 0.3 | 0.2 | 0.7 | 0.3 | 1.4 | 0.7 | 0.6 |
| Hormad2 | 0.2 | 0.0 | 0.1 | 0.0 | 0.0 | 0.1 | 0.0 | 0.0 | 0.1 | 0.1 | 0.0 | 0.0 |
| Hoxa1 | 0.6 | 0.5 | 0.2 | 0.6 | 0.5 | 0.3 | 0.3 | 0.4 | 0.4 | 1.1 | 0.5 | 0.5 |
| Hoxa10 | 0.1 | 0.0 | 0.0 | 0.2 | 0.0 | 0.1 | 0.0 | 0.0 | 0.0 | 0.0 | 0.0 | 0.0 |
| Hoxa2 | 0.3 | 0.1 | 0.2 | 0.1 | 0.1 | 0.3 | 0.1 | 0.3 | 0.2 | 1.1 | 0.8 | 0.4 |
| Hoxa3 | 0.0 | 0.1 | 0.0 | 0.0 | 0.0 | 0.0 | 0.0 | 0.1 | 0.0 | 0.6 | 0.2 | 0.2 |
| Hoxa4 | 0.0 | 0.0 | 0.1 | 0.1 | 0.0 | 0.2 | 0.0 | 0.1 | 0.0 | 0.9 | 0.3 | 0.1 |
| Hoxa5 | 0.1 | 0.0 | 0.0 | 0.3 | 0.0 | 0.0 | 0.1 | 0.1 | 0.2 | 5.4 | 2.8 | 1.6 |
| Hoxa6 | 0.0 | 0.0 | 0.0 | 0.0 | 0.0 | 0.0 | 0.0 | 0.0 | 0.1 | 0.1 | 0.5 | 0.1 |
| Hoxa7 | 0.1 | 0.0 | 0.0 | 0.1 | 0.0 | 0.0 | 0.0 | 0.0 | 0.0 | 0.5 | 0.6 | 0.1 |
| Hoxb13 | 0.1 | 0.0 | 0.0 | 0.0 | 0.0 | 0.0 | 0.0 | 0.0 | 0.0 | 0.1 | 0.0 | 0.0 |
| Hoxb2 | 0.9 | 1.2 | 1.7 | 0.7 | 1.6 | 1.0 | 1.3 | 1.5 | 3.7 | 4.1 | 2.5 | 2.5 |
| Hoxb3 | 0.4 | 0.4 | 0.6 | 0.4 | 0.5 | 0.7 | 0.4 | 0.6 | 0.8 | 1.3 | 1.3 | 1.0 |
| Hoxb4 | 1.0 | 1.0 | 0.7 | 1.3 | 0.9 | 1.3 | 0.9 | 0.6 | 1.3 | 2.9 | 1.6 | 1.8 |
| Hoxb5 | 0.0 | 0.0 | 0.0 | 0.0 | 0.0 | 0.0 | 0.0 | 0.0 | 0.1 | 0.2 | 0.1 | 0.0 |
| Hoxb6 | 0.2 | 0.2 | 0.0 | 0.8 | 0.0 | 0.6 | 0.0 | 0.0 | 0.1 | 0.4 | 0.4 | 0.4 |
| Hoxb7 | 0.1 | 0.0 | 0.0 | 0.4 | 0.0 | 0.1 | 0.1 | 0.1 | 0.1 | 0.8 | 1.0 | 0.1 |
| Hoxb8 | 0.0 | 0.0 | 0.0 | 0.0 | 0.0 | 0.0 | 0.0 | 0.0 | 0.0 | 0.0 | 0.1 | 0.0 |
| Hoxb9 | 0.4 | 0.0 | 0.0 | 1.1 | 0.1 | 0.2 | 0.0 | 0.0 | 0.0 | 0.0 | 0.3 | 0.0 |
| Hoxc10 | 0.0 | 0.0 | 0.0 | 0.1 | 0.0 | 0.0 | 0.0 | 0.0 | 0.0 | 0.0 | 0.0 | 0.0 |
| Hoxc13 | 1.9 | 1.0 | 0.8 | 1.9 | 0.9 | 1.0 | 0.7 | 0.1 | 0.3 | 0.7 | 0.7 | 0.4 |
| Hoxc4 | 0.0 | 0.0 | 0.0 | 0.2 | 0.0 | 0.0 | 0.0 | 0.0 | 0.1 | 0.7 | 0.7 | 0.5 |
| Hoxc5 | 0.0 | 0.0 | 0.0 | 0.1 | 0.0 | 0.1 | 0.0 | 0.0 | 0.1 | 0.8 | 0.5 | 0.4 |

| | | | | | | | | | | | | |
|---|---|---|---|---|---|---|---|---|---|---|---|---|
| Hoxc6 | 0.2 | 0.2 | 0.1 | 0.2 | 0.1 | 0.1 | 0.1 | 0.0 | 0.2 | 1.7 | 2.5 | 0.6 |
| Hoxc8 | 0.1 | 0.0 | 0.0 | 0.0 | 0.0 | 0.0 | 0.0 | 0.0 | 0.0 | 0.1 | 0.4 | 0.1 |
| Hoxc9 | 0.0 | 0.0 | 0.0 | 0.1 | 0.0 | 0.0 | 0.0 | 0.0 | 0.0 | 0.0 | 0.1 | 0.0 |
| Hoxd10 | 0.1 | 0.0 | 0.0 | 0.1 | 0.1 | 0.0 | 0.0 | 0.0 | 0.0 | 0.0 | 0.0 | 0.0 |
| Hoxd3 | 0.0 | 0.0 | 0.0 | 0.1 | 0.0 | 0.0 | 0.1 | 0.0 | 0.1 | 0.1 | 0.1 | 0.1 |
| Hoxd4 | 0.2 | 0.2 | 0.1 | 0.4 | 0.1 | 0.1 | 0.1 | 0.1 | 0.2 | 0.2 | 0.2 | 0.2 |
| Hoxd8 | 2.7 | 2.3 | 3.1 | 4.2 | 2.8 | 2.3 | 3.3 | 4.0 | 3.8 | 3.5 | 2.9 | 3.2 |
| Hoxd9 | 0.9 | 0.5 | 0.6 | 1.0 | 0.7 | 0.9 | 0.5 | 0.4 | 0.5 | 0.6 | 0.5 | 0.5 |
| Hp | 0.9 | 3.0 | 11.7 | 2.4 | 23.5 | 1.0 | 12.3 | 7.6 | 7.4 | 20.0 | 4.2 | 12.8 |
| Hp1bp3 | 43.9 | 49.8 | 53.1 | 46.1 | 41.4 | 47.6 | 48.5 | 46.7 | 47.5 | 46.1 | 49.6 | 53.0 |
| Hpcal1 | 15.3 | 11.6 | 9.9 | 15.7 | 18.1 | 12.6 | 11.6 | 7.2 | 8.3 | 16.2 | 11.6 | 11.3 |
| Hpcal4 | 0.0 | 0.0 | 0.0 | 0.0 | 0.0 | 0.0 | 0.0 | 0.0 | 0.0 | 0.0 | 0.0 | 0.0 |
| Hpd | 0.0 | 0.0 | 0.1 | 0.0 | 0.0 | 0.0 | 0.0 | 0.0 | 0.0 | 0.0 | 0.0 | 0.0 |
| Hpdl | 0.2 | 0.1 | 0.1 | 0.1 | 0.0 | 0.1 | 0.1 | 0.0 | 0.2 | 0.2 | 0.1 | 0.1 |
| Hpgd | 12.4 | 11.5 | 15.3 | 4.3 | 3.3 | 0.7 | 2.0 | 4.5 | 2.1 | 2.1 | 2.2 | 2.2 |
| Hpgds | 0.4 | 0.1 | 0.1 | 3.9 | 5.1 | 0.0 | 0.7 | 0.1 | 0.1 | 0.4 | 0.1 | 0.3 |
| Hpn | 0.1 | 0.2 | 0.1 | 0.2 | 0.2 | 0.0 | 0.1 | 0.1 | 0.2 | 0.1 | 0.1 | 0.1 |
| Hprt | 61.6 | 50.9 | 41.0 | 62.0 | 61.2 | 54.2 | 41.4 | 35.2 | 41.2 | 61.0 | 50.6 | 51.4 |
| Hps1 | 9.7 | 8.6 | 7.7 | 8.3 | 8.5 | 9.0 | 8.1 | 8.3 | 9.8 | 8.9 | 8.0 | 7.8 |
| Hps3 | 5.9 | 5.5 | 4.9 | 5.4 | 5.9 | 5.4 | 4.9 | 4.3 | 5.8 | 4.7 | 5.1 | 5.3 |
| Hps4 | 4.6 | 4.9 | 4.3 | 4.0 | 4.4 | 4.4 | 4.0 | 3.6 | 3.9 | 3.5 | 3.2 | 3.2 |
| Hps5 | 9.1 | 8.6 | 7.9 | 9.4 | 8.9 | 9.3 | 8.3 | 7.8 | 8.0 | 7.9 | 8.1 | 8.4 |
| Hps6 | 3.5 | 3.3 | 3.5 | 3.5 | 3.5 | 3.9 | 3.7 | 3.1 | 3.8 | 4.5 | 3.3 | 3.2 |
| Hpse | 1.3 | 1.3 | 1.2 | 1.2 | 1.3 | 1.5 | 1.0 | 0.7 | 0.7 | 0.9 | 0.6 | 1.2 |
| Hpx | 0.4 | 0.9 | 0.5 | 0.2 | 0.4 | 0.6 | 0.3 | 0.4 | 0.4 | 0.4 | 0.6 | 0.4 |
| Hr | 1.5 | 1.5 | 1.1 | 1.6 | 0.8 | 2.0 | 1.0 | 1.1 | 0.6 | 0.6 | 0.8 | 0.6 |
| Hras1 | 26.4 | 20.5 | 17.8 | 25.2 | 23.0 | 24.1 | 23.6 | 20.1 | 22.3 | 23.2 | 27.0 | 22.6 |
| Hrasls | 0.6 | 0.3 | 0.2 | 0.7 | 0.2 | 0.8 | 0.2 | 0.1 | 0.1 | 0.2 | 0.2 | 0.2 |
| Hrc | 0.0 | 0.0 | 0.0 | 0.0 | 0.0 | 0.0 | 0.0 | 0.0 | 0.0 | 0.0 | 0.0 | 0.0 |
| Hrct1 | 9.1 | 8.4 | 6.3 | 13.3 | 7.1 | 10.1 | 8.5 | 3.6 | 4.6 | 5.3 | 5.9 | 6.6 |
| Hrh1 | 0.1 | 0.1 | 0.1 | 0.3 | 0.2 | 0.1 | 0.1 | 0.1 | 0.1 | 0.2 | 0.1 | 0.1 |
| Hrh2 | 0.0 | 0.1 | 0.0 | 0.0 | 0.0 | 0.0 | 0.1 | 0.0 | 0.0 | 0.0 | 0.0 | 0.0 |
| Hrk | 0.0 | 0.0 | 0.0 | 0.0 | 0.0 | 0.0 | 0.0 | 0.0 | 0.0 | 0.0 | 0.0 | 0.0 |
| Hrsp12 | 26.7 | 24.7 | 29.3 | 34.0 | 29.9 | 37.7 | 20.8 | 27.1 | 16.0 | 23.2 | 24.2 | 23.6 |
| Hs1bp3 | 8.0 | 8.6 | 7.3 | 8.8 | 8.2 | 10.0 | 7.5 | 6.1 | 7.1 | 8.6 | 6.6 | 7.0 |
| Hs2st1 | 9.5 | 10.1 | 10.2 | 9.7 | 11.1 | 10.9 | 10.2 | 9.2 | 10.7 | 15.3 | 13.0 | 12.4 |
| Hs3st1 | 0.5 | 0.6 | 1.8 | 0.6 | 1.1 | 0.2 | 1.1 | 1.4 | 1.0 | 1.1 | 1.1 | 1.1 |
| Hs3st3a1 | 0.6 | 0.5 | 0.6 | 0.4 | 0.6 | 0.5 | 0.5 | 0.4 | 0.3 | 0.6 | 0.4 | 0.2 |
| Hs3st3b1 | 0.4 | 0.3 | 0.3 | 0.4 | 0.4 | 0.2 | 0.3 | 0.2 | 0.3 | 0.4 | 0.3 | 0.3 |
| Hs3st4 | 0.1 | 0.0 | 0.0 | 0.0 | 0.0 | 0.0 | 0.1 | 0.0 | 0.0 | 0.0 | 0.0 | 0.0 |
| Hs3st6 | 0.3 | 0.3 | 0.1 | 0.2 | 0.3 | 0.2 | 0.0 | 0.0 | 0.1 | 0.1 | 0.1 | 0.0 |
| Hs6st1 | 3.8 | 2.5 | 3.1 | 3.6 | 3.3 | 2.7 | 3.7 | 2.5 | 2.9 | 3.5 | 4.0 | 3.0 |
| Hs6st2 | 35.0 | 35.2 | 36.7 | 37.7 | 43.2 | 37.3 | 50.7 | 40.7 | 64.7 | 55.0 | 61.0 | 65.8 |
| Hs6st3 | 0.0 | 0.1 | 0.1 | 0.0 | 0.0 | 0.0 | 0.1 | 0.0 | 0.0 | 0.0 | 0.1 | 0.0 |
| Hsbp1 | 106.8 | 87.6 | 84.1 | 118.2 | 86.6 | 104.4 | 91.6 | 89.2 | 77.6 | 99.3 | 103.6 | 100.7 |
| Hscb | 21.4 | 19.1 | 14.0 | 21.1 | 12.7 | 24.3 | 9.0 | 13.8 | 12.1 | 14.2 | 14.5 | 16.4 |
| Hsd11b1 | 216.9 | 265.8 | 246.4 | 320.4 | 227.9 | 232.7 | 101.7 | 116.0 | 83.8 | 67.5 | 69.1 | 72.4 |
| Hsd11b2 | 0.1 | 0.0 | 0.0 | 0.0 | 0.1 | 0.0 | 0.0 | 0.0 | 0.0 | 0.0 | 0.1 | 0.1 |
| Hsd17b1 | 0.3 | 0.3 | 0.1 | 0.2 | 0.2 | 0.5 | 0.2 | 0.0 | 0.1 | 0.2 | 0.2 | 0.3 |
| Hsd17b10 | 31.1 | 30.8 | 28.3 | 34.0 | 25.5 | 29.9 | 27.1 | 36.4 | 30.2 | 27.3 | 31.6 | 25.6 |
| Hsd17b11 | 7.4 | 5.5 | 8.0 | 7.5 | 7.5 | 9.4 | 6.6 | 8.2 | 5.6 | 7.3 | 7.1 | 8.5 |
| Hsd17b12 | 57.2 | 49.4 | 39.0 | 58.3 | 54.1 | 49.5 | 55.2 | 54.0 | 47.0 | 53.2 | 58.9 | 45.0 |
| Hsd17b13 | 0.0 | 0.0 | 0.0 | 0.0 | 0.1 | 0.0 | 0.1 | 0.0 | 0.0 | 0.0 | 0.1 | 0.0 |
| Hsd17b4 | 43.9 | 36.3 | 35.8 | 45.8 | 36.5 | 46.0 | 31.5 | 32.8 | 30.4 | 34.5 | 35.8 | 36.6 |
| Hsd17b6 | 0.0 | 0.0 | 0.0 | 0.0 | 0.0 | 0.0 | 0.0 | 0.0 | 0.0 | 0.0 | 0.0 | 0.0 |
| Hsd17b7 | 12.0 | 15.7 | 14.9 | 7.5 | 9.7 | 6.9 | 20.1 | 23.0 | 18.1 | 7.7 | 20.7 | 8.7 |
| Hsd3b1 | 0.0 | 0.0 | 0.0 | 0.1 | 0.0 | 0.0 | 0.0 | 0.0 | 0.0 | 0.0 | 0.0 | 0.0 |

| | | | | | | | | | | | | |
|---|---|---|---|---|---|---|---|---|---|---|---|---|
| Hsd3b2 | 0.0 | 0.0 | 0.0 | 0.0 | 0.0 | 0.0 | 0.0 | 0.0 | 0.0 | 0.0 | 0.0 | 0.0 |
| Hsd3b3 | 0.0 | 0.1 | 0.1 | 0.0 | 0.0 | 0.0 | 0.0 | 0.0 | 0.1 | 0.0 | 0.0 | 0.1 |
| Hsd3b4 | 0.0 | 0.0 | 0.0 | 0.0 | 0.0 | 0.0 | 0.1 | 0.0 | 0.0 | 0.0 | 0.0 | 0.0 |
| Hsd3b7 | 16.4 | 14.0 | 12.6 | 17.0 | 14.7 | 20.9 | 12.7 | 16.4 | 14.5 | 11.7 | 10.9 | 13.2 |
| Hsdl1 | 5.4 | 5.1 | 4.2 | 8.2 | 5.4 | 6.9 | 5.0 | 4.7 | 4.2 | 4.8 | 3.7 | 5.5 |
| Hsdl2 | 8.9 | 7.4 | 8.7 | 9.8 | 7.1 | 10.4 | 7.2 | 8.0 | 7.7 | 7.9 | 8.5 | 9.9 |
| Hsf1 | 12.4 | 13.0 | 11.7 | 12.4 | 11.1 | 13.3 | 13.3 | 12.5 | 13.1 | 10.8 | 12.8 | 9.9 |
| Hsf2 | 11.9 | 10.4 | 8.8 | 11.7 | 8.4 | 9.6 | 9.3 | 7.5 | 8.3 | 10.1 | 11.3 | 9.4 |
| Hsf2bp | 0.0 | 0.0 | 0.1 | 0.0 | 0.0 | 0.0 | 0.0 | 0.0 | 0.0 | 0.0 | 0.1 | 0.0 |
| Hsf3 | 0.1 | 0.1 | 0.2 | 0.0 | 0.1 | 0.2 | 0.0 | 0.1 | 0.0 | 0.0 | 0.0 | 0.0 |
| Hsf4 | 1.6 | 2.6 | 2.3 | 2.1 | 1.7 | 2.1 | 1.9 | 1.7 | 1.6 | 2.7 | 2.3 | 1.3 |
| Hsf5 | 0.0 | 0.0 | 0.0 | 0.0 | 0.0 | 0.0 | 0.0 | 0.0 | 0.0 | 0.0 | 0.0 | 0.0 |
| Hsp90aa1 | 671.6 | 621.3 | 500.3 | 759.3 | 472.5 | 623.2 | 453.6 | 291.8 | 347.9 | 532.7 | 564.2 | 549.3 |
| Hsp90ab1 | 596.7 | 583.6 | 615.3 | 685.4 | 531.1 | 579.0 | 575.9 | 554.8 | 456.5 | 557.6 | 651.7 | 620.1 |
| Hsp90b1 | 838.4 | 735.1 | 727.5 | 846.8 | 631.0 | 660.8 | 744.3 | 776.1 | 697.0 | 780.4 | 910.4 | 915.7 |
| Hspa12a | 4.0 | 3.1 | 3.2 | 3.8 | 3.2 | 3.7 | 3.2 | 2.9 | 2.4 | 2.5 | 3.2 | 2.9 |
| Hspa12b | 3.3 | 3.5 | 2.9 | 3.5 | 2.1 | 3.2 | 2.6 | 2.0 | 2.3 | 1.9 | 1.6 | 1.4 |
| Hspa13 | 22.9 | 21.7 | 23.6 | 26.8 | 24.3 | 25.6 | 25.3 | 22.1 | 25.2 | 26.3 | 25.8 | 28.7 |
| Hspa14 | 25.1 | 30.1 | 27.0 | 21.5 | 23.1 | 21.0 | 23.7 | 20.9 | 24.0 | 23.8 | 23.9 | 24.7 |
| Hspa1a | 5.1 | 7.2 | 7.3 | 3.6 | 7.0 | 3.7 | 11.3 | 5.0 | 7.8 | 14.8 | 10.1 | 8.2 |
| Hspa1b | 9.7 | 18.4 | 15.2 | 6.7 | 13.5 | 5.4 | 21.1 | 10.1 | 18.5 | 25.6 | 21.1 | 14.8 |
| Hspa1l | 0.8 | 0.9 | 0.7 | 0.5 | 0.8 | 0.6 | 1.1 | 0.6 | 0.8 | 1.1 | 1.1 | 0.8 |
| Hspa2 | 7.2 | 7.6 | 9.1 | 6.7 | 7.1 | 6.8 | 9.6 | 9.9 | 9.2 | 12.2 | 11.4 | 12.4 |
| Hspa4 | 65.0 | 56.3 | 54.1 | 68.4 | 58.7 | 60.2 | 54.2 | 48.5 | 47.3 | 59.9 | 62.4 | 61.7 |
| Hspa4l | 15.5 | 18.1 | 14.0 | 16.1 | 12.1 | 15.8 | 10.4 | 8.0 | 10.1 | 13.2 | 14.6 | 11.2 |
| Hspa5 | 646.4 | 464.4 | 391.1 | 655.0 | 423.0 | 505.2 | 377.9 | 499.5 | 460.6 | 471.9 | 501.4 | 475.8 |
| Hspa8 | 1564.8 | 1453.9 | 1318.9 | 1432.8 | 1268.3 | 1313.4 | 1367.4 | 1362.2 | 1258.7 | 1513.0 | 1698.0 | 1513.4 |
| Hspa9 | 157.7 | 100.5 | 85.9 | 161.4 | 94.1 | 159.7 | 99.8 | 118.3 | 92.5 | 105.5 | 125.4 | 105.8 |
| Hspb1 | 385.4 | 534.1 | 421.3 | 461.7 | 304.4 | 423.7 | 378.5 | 360.9 | 441.4 | 515.1 | 430.4 | 408.6 |
| Hspb11 | 34.8 | 32.7 | 45.6 | 24.1 | 24.4 | 31.2 | 31.1 | 29.9 | 29.5 | 31.8 | 37.6 | 44.6 |
| Hspb2 | 7.1 | 6.1 | 7.0 | 3.4 | 3.2 | 5.2 | 3.5 | 7.5 | 6.3 | 10.1 | 4.3 | 6.4 |
| Hspb6 | 2.6 | 2.9 | 2.7 | 2.6 | 2.0 | 2.4 | 2.9 | 2.4 | 1.9 | 1.7 | 2.1 | 2.6 |
| Hspb7 | 5.2 | 5.2 | 3.3 | 7.2 | 3.4 | 6.9 | 5.7 | 4.7 | 3.9 | 2.8 | 3.3 | 3.7 |
| Hspb8 | 71.7 | 54.6 | 40.9 | 86.0 | 44.5 | 89.7 | 49.0 | 35.8 | 44.8 | 60.0 | 55.1 | 45.9 |
| Hspb9 | 0.2 | 0.3 | 0.0 | 0.0 | 0.0 | 0.1 | 0.1 | 0.2 | 0.1 | 0.2 | 0.0 | 0.1 |
| Hspbap1 | 2.3 | 2.9 | 2.1 | 2.0 | 2.0 | 1.9 | 2.1 | 2.1 | 2.3 | 2.5 | 2.3 | 2.3 |
| Hspbp1 | 6.4 | 5.8 | 3.9 | 7.2 | 6.1 | 7.7 | 5.0 | 5.3 | 5.1 | 5.1 | 5.5 | 4.8 |
| Hspd1 | 156.9 | 139.1 | 109.0 | 153.7 | 119.4 | 147.7 | 121.4 | 98.2 | 105.5 | 134.6 | 152.6 | 132.2 |
| Hspe1 | 176.3 | 169.7 | 151.1 | 194.6 | 135.4 | 190.1 | 125.3 | 131.5 | 132.1 | 165.7 | 176.4 | 164.1 |
| Hspg2 | 16.6 | 26.7 | 26.9 | 12.0 | 23.2 | 16.3 | 31.7 | 21.6 | 25.8 | 19.3 | 15.3 | 11.1 |
| Hsph1 | 37.3 | 53.7 | 48.1 | 46.0 | 41.4 | 42.6 | 30.3 | 27.1 | 24.7 | 40.5 | 36.7 | 35.1 |
| Htatip2 | 33.0 | 30.9 | 25.6 | 33.7 | 30.9 | 44.4 | 25.6 | 28.1 | 28.0 | 29.2 | 27.5 | 30.3 |
| Htatsf1 | 25.0 | 21.4 | 23.3 | 26.6 | 18.7 | 23.0 | 22.4 | 20.0 | 17.8 | 19.4 | 24.3 | 23.7 |
| Htr1d | 0.0 | 0.1 | 0.1 | 0.1 | 0.1 | 0.2 | 0.0 | 0.0 | 0.1 | 0.1 | 0.0 | 0.1 |
| Htr2a | 1.2 | 0.4 | 0.4 | 0.7 | 0.6 | 0.6 | 0.7 | 0.4 | 0.6 | 1.6 | 1.2 | 1.1 |
| Htr2b | 0.2 | 0.2 | 0.2 | 0.5 | 1.2 | 0.3 | 0.3 | 0.2 | 0.1 | 0.3 | 0.2 | 0.3 |
| Htr4 | 0.1 | 0.1 | 0.0 | 0.0 | 0.0 | 0.0 | 0.0 | 0.0 | 0.0 | 0.0 | 0.0 | 0.0 |
| Htr5b | 0.0 | 0.1 | 0.0 | 0.0 | 0.0 | 0.0 | 0.0 | 0.0 | 0.0 | 0.0 | 0.0 | 0.0 |
| Htr7 | 0.1 | 0.1 | 0.0 | 0.0 | 0.0 | 0.1 | 0.0 | 0.0 | 0.0 | 0.0 | 0.0 | 0.1 |
| Htra1 | 53.3 | 38.2 | 28.9 | 73.5 | 42.9 | 82.4 | 48.0 | 42.8 | 59.5 | 112.7 | 93.8 | 62.9 |
| Htra2 | 35.6 | 30.6 | 23.9 | 31.3 | 28.6 | 38.1 | 31.9 | 24.4 | 31.3 | 26.2 | 29.1 | 29.1 |
| Htra3 | 0.4 | 1.0 | 1.6 | 0.3 | 0.6 | 0.6 | 0.5 | 1.1 | 1.0 | 0.7 | 0.5 | 0.8 |
| Htra4 | 3.3 | 2.2 | 2.8 | 1.3 | 2.3 | 2.0 | 1.6 | 0.5 | 0.8 | 1.0 | 0.6 | 1.5 |
| Htt | 4.2 | 4.1 | 3.5 | 3.9 | 4.6 | 3.8 | 3.9 | 3.8 | 4.1 | 3.9 | 3.8 | 3.3 |
| Hunk | 0.1 | 0.1 | 0.1 | 0.0 | 0.1 | 0.0 | 0.0 | 0.0 | 0.0 | 0.2 | 0.0 | 0.1 |
| Hus1 | 2.5 | 2.7 | 2.3 | 2.6 | 2.3 | 2.4 | 2.1 | 2.5 | 2.3 | 2.0 | 2.8 | 2.3 |
| Hus1b | 0.1 | 0.0 | 0.2 | 0.0 | 0.1 | 0.0 | 0.0 | 0.0 | 0.0 | 0.0 | 0.1 | 0.0 |

| | | | | | | | | | | | | |
|---|---|---|---|---|---|---|---|---|---|---|---|---|
| Huwe1 | 16.8 | 16.9 | 15.4 | 18.4 | 16.7 | 17.5 | 18.0 | 16.8 | 14.2 | 14.3 | 15.8 | 14.3 |
| Hvcn1 | 13.3 | 9.7 | 7.5 | 29.8 | 16.8 | 28.9 | 5.8 | 6.5 | 4.9 | 4.6 | 3.4 | 5.4 |
| Hyal1 | 9.5 | 7.2 | 7.2 | 7.4 | 7.9 | 9.9 | 5.9 | 4.3 | 4.0 | 6.1 | 5.1 | 4.9 |
| Hyal2 | 8.8 | 9.7 | 8.7 | 9.9 | 9.8 | 10.7 | 9.8 | 8.4 | 8.5 | 12.6 | 9.7 | 8.7 |
| Hyal3 | 0.1 | 0.3 | 0.2 | 0.2 | 0.1 | 0.0 | 0.3 | 0.2 | 0.1 | 0.2 | 0.1 | 0.2 |
| Hydin | 0.0 | 0.0 | 0.0 | 0.0 | 0.0 | 0.0 | 0.0 | 0.0 | 0.0 | 0.0 | 0.0 | 0.0 |
| Hyi | 20.3 | 18.5 | 14.4 | 19.3 | 13.1 | 24.3 | 17.9 | 13.0 | 15.1 | 17.6 | 16.7 | 16.0 |
| Hyls1 | 2.9 | 3.4 | 4.2 | 1.5 | 3.5 | 1.3 | 4.4 | 2.9 | 3.5 | 3.2 | 3.9 | 3.0 |
| Hyou1 | 71.3 | 64.0 | 54.2 | 85.7 | 60.0 | 75.5 | 67.7 | 66.5 | 59.8 | 61.2 | 65.0 | 56.7 |
| Hypk | 91.1 | 74.6 | 96.9 | 108.4 | 69.6 | 89.2 | 97.7 | 96.9 | 73.5 | 76.6 | 84.3 | 99.9 |
| I730028E13Rik | 0.1 | 0.1 | 0.0 | 0.1 | 0.0 | 0.1 | 0.0 | 0.0 | 0.0 | 0.0 | 0.0 | 0.0 |
| I830012O16Rik | 23.0 | 12.0 | 8.4 | 31.0 | 6.0 | 24.5 | 4.6 | 2.9 | 2.5 | 2.6 | 5.3 | 10.4 |
| I830077J02Rik | 0.2 | 0.0 | 0.0 | 0.2 | 0.2 | 0.0 | 0.0 | 0.0 | 0.0 | 0.1 | 0.1 | 0.0 |
| Iah1 | 37.8 | 30.3 | 26.7 | 30.7 | 31.9 | 34.8 | 31.9 | 43.2 | 47.6 | 39.8 | 38.4 | 39.3 |
| Iars | 24.9 | 18.8 | 15.0 | 23.3 | 19.4 | 21.8 | 19.9 | 20.6 | 19.1 | 18.4 | 22.8 | 15.8 |
| Iars2 | 31.3 | 28.3 | 23.6 | 27.3 | 25.2 | 28.5 | 27.1 | 24.6 | 25.4 | 27.4 | 27.3 | 27.2 |
| Iba57 | 1.0 | 1.0 | 0.7 | 1.7 | 1.3 | 1.1 | 1.1 | 1.1 | 1.2 | 1.3 | 1.0 | 1.1 |
| Ibtk | 13.5 | 11.2 | 9.8 | 12.1 | 12.2 | 9.8 | 11.2 | 10.8 | 10.6 | 12.3 | 13.4 | 11.6 |
| Ica1 | 12.9 | 11.0 | 7.8 | 13.2 | 8.4 | 12.5 | 8.2 | 6.8 | 5.9 | 8.8 | 9.0 | 7.2 |
| Ica1l | 1.0 | 0.3 | 0.2 | 1.0 | 0.5 | 0.6 | 0.6 | 0.1 | 0.4 | 0.5 | 0.5 | 0.4 |
| Icam1 | 77.9 | 69.0 | 71.0 | 77.8 | 92.2 | 63.7 | 41.2 | 33.0 | 21.5 | 35.8 | 23.9 | 37.1 |
| Icam2 | 0.8 | 0.2 | 0.6 | 0.3 | 0.3 | 0.2 | 0.4 | 0.9 | 0.5 | 0.0 | 0.1 | 0.2 |
| Icam4 | 0.1 | 0.2 | 0.2 | 0.0 | 0.2 | 0.8 | 0.2 | 0.1 | 0.0 | 0.1 | 0.1 | 0.0 |
| Icam5 | 0.2 | 0.2 | 0.2 | 0.1 | 0.3 | 0.4 | 0.1 | 0.2 | 0.2 | 0.3 | 0.1 | 0.1 |
| Ick | 4.1 | 4.3 | 4.5 | 4.4 | 3.9 | 4.6 | 4.4 | 4.8 | 4.4 | 4.1 | 5.2 | 4.6 |
| Icmt | 18.8 | 16.1 | 13.5 | 19.7 | 17.6 | 19.4 | 17.9 | 13.4 | 16.5 | 15.8 | 15.5 | 15.3 |
| Icos | 0.1 | 0.0 | 0.0 | 0.0 | 0.0 | 0.1 | 0.0 | 0.0 | 0.0 | 0.0 | 0.1 | 0.1 |
| Icosl | 0.4 | 1.1 | 1.9 | 0.8 | 2.0 | 0.3 | 0.9 | 0.7 | 0.6 | 0.3 | 0.3 | 0.6 |
| Ict1 | 23.9 | 17.2 | 17.1 | 26.5 | 18.3 | 21.6 | 20.2 | 22.3 | 17.9 | 18.0 | 21.8 | 19.1 |
| Id1 | 44.4 | 50.9 | 59.9 | 26.3 | 35.8 | 24.2 | 33.0 | 42.8 | 42.2 | 89.2 | 41.9 | 44.3 |
| Id2 | 22.8 | 24.7 | 41.4 | 16.7 | 24.4 | 12.8 | 21.7 | 28.7 | 25.6 | 29.0 | 23.9 | 29.8 |
| Id3 | 138.1 | 215.8 | 254.8 | 83.0 | 158.8 | 106.1 | 159.4 | 227.7 | 229.1 | 232.7 | 138.7 | 192.0 |
| Id4 | 1.1 | 1.8 | 3.3 | 1.2 | 1.6 | 0.7 | 2.4 | 2.3 | 2.4 | 3.3 | 2.9 | 3.7 |
| Ide | 39.9 | 32.9 | 32.2 | 40.1 | 32.3 | 42.2 | 31.0 | 36.7 | 31.1 | 39.7 | 43.8 | 39.5 |
| Idh1 | 96.4 | 97.2 | 88.7 | 86.1 | 73.9 | 78.3 | 88.4 | 121.5 | 93.7 | 63.1 | 104.9 | 78.8 |
| Idh2 | 9.3 | 11.3 | 14.8 | 7.0 | 8.0 | 11.3 | 10.7 | 13.4 | 12.5 | 10.3 | 10.5 | 10.7 |
| Idh3a | 20.6 | 18.8 | 16.4 | 23.3 | 19.0 | 22.5 | 18.5 | 17.0 | 17.1 | 21.1 | 23.1 | 18.1 |
| Idh3b | 45.1 | 43.3 | 40.5 | 48.6 | 45.2 | 45.9 | 42.5 | 40.5 | 41.2 | 47.0 | 47.6 | 46.7 |
| Idh3g | 30.4 | 28.1 | 29.3 | 35.1 | 28.2 | 27.9 | 29.6 | 32.3 | 29.2 | 25.2 | 32.6 | 29.3 |
| Idi1 | 20.1 | 24.5 | 25.5 | 5.6 | 15.0 | 9.1 | 46.7 | 57.6 | 40.8 | 11.0 | 53.3 | 18.9 |
| Idi2 | 0.1 | 0.1 | 0.0 | 0.0 | 0.0 | 0.0 | 0.0 | 0.0 | 0.0 | 0.0 | 0.0 | 0.0 |
| Idnk | 5.8 | 5.2 | 4.9 | 6.2 | 6.1 | 7.0 | 4.4 | 6.4 | 5.7 | 6.5 | 5.2 | 5.5 |
| Ido1 | 0.1 | 0.0 | 0.1 | 0.1 | 0.1 | 0.0 | 0.1 | 0.1 | 0.0 | 0.1 | 0.0 | 0.1 |
| Ido2 | 0.0 | 0.1 | 0.1 | 0.0 | 0.0 | 0.0 | 0.0 | 0.0 | 0.0 | 0.0 | 0.0 | 0.0 |
| Ids | 10.0 | 9.1 | 10.0 | 11.3 | 10.7 | 12.5 | 11.1 | 9.5 | 8.4 | 9.1 | 9.9 | 9.8 |
| Idua | 6.8 | 6.5 | 6.7 | 9.5 | 7.8 | 9.8 | 6.3 | 7.3 | 7.3 | 5.9 | 6.3 | 5.8 |
| Ier2 | 3.6 | 3.4 | 3.0 | 3.1 | 3.9 | 2.8 | 4.3 | 3.1 | 4.8 | 20.5 | 5.0 | 3.6 |
| Ier3 | 56.2 | 47.0 | 43.5 | 53.8 | 40.6 | 42.9 | 47.1 | 38.3 | 47.3 | 72.2 | 40.7 | 45.9 |
| Ier3ip1 | 35.9 | 34.8 | 33.2 | 34.8 | 35.7 | 36.8 | 35.3 | 33.8 | 37.0 | 38.0 | 42.8 | 43.7 |
| Ier5 | 10.4 | 8.9 | 7.2 | 10.0 | 12.9 | 7.2 | 8.2 | 5.4 | 7.4 | 15.8 | 10.8 | 10.3 |
| Ier5l | 11.9 | 15.0 | 18.6 | 6.0 | 14.9 | 8.3 | 19.8 | 18.2 | 21.1 | 18.8 | 11.8 | 15.6 |
| Iffo1 | 9.6 | 10.6 | 9.2 | 8.9 | 8.9 | 9.6 | 9.1 | 6.9 | 8.4 | 7.1 | 8.1 | 8.1 |
| Iffo2 | 6.3 | 6.3 | 5.8 | 6.0 | 7.3 | 4.6 | 7.2 | 6.0 | 6.9 | 7.2 | 7.0 | 6.4 |
| Ifi203 | 1.5 | 1.1 | 1.9 | 3.0 | 1.6 | 1.4 | 1.0 | 0.7 | 1.0 | 0.5 | 0.9 | 1.8 |
| Ifi204 | 8.7 | 7.0 | 10.7 | 12.1 | 7.2 | 5.8 | 6.4 | 7.4 | 5.4 | 9.8 | 7.5 | 11.6 |
| Ifi205 | 13.5 | 12.6 | 25.6 | 18.0 | 16.4 | 8.9 | 14.0 | 18.2 | 12.4 | 7.6 | 9.1 | 18.6 |
| Ifi27l1 | 211.0 | 181.9 | 136.5 | 186.7 | 147.2 | 154.9 | 115.8 | 94.8 | 133.3 | 127.3 | 100.5 | 131.1 |

| | | | | | | | | | | | | |
|---|---|---|---|---|---|---|---|---|---|---|---|---|
| Ifi27l2a | 6.8 | 12.1 | 25.0 | 9.5 | 10.3 | 3.1 | 10.8 | 17.4 | 9.8 | 11.4 | 5.8 | 8.3 |
| Ifi27l2b | 1.1 | 0.6 | 0.3 | 1.4 | 0.6 | 0.7 | 0.6 | 0.2 | 0.2 | 1.0 | 0.9 | 0.8 |
| Ifi30 | 31.0 | 19.5 | 23.4 | 42.0 | 45.5 | 43.8 | 21.2 | 21.7 | 14.9 | 21.8 | 24.1 | 19.2 |
| Ifi35 | 21.3 | 17.6 | 17.9 | 23.6 | 19.2 | 18.2 | 19.1 | 16.5 | 19.6 | 16.8 | 14.7 | 21.6 |
| Ifi44 | 2.5 | 0.9 | 1.6 | 4.8 | 1.9 | 2.3 | 0.9 | 0.2 | 0.5 | 0.4 | 0.9 | 1.9 |
| Ifi47 | 7.3 | 6.5 | 3.5 | 10.3 | 9.5 | 5.9 | 5.3 | 2.1 | 3.9 | 7.8 | 4.4 | 5.1 |
| Ifih1 | 7.1 | 5.0 | 6.1 | 8.3 | 6.3 | 6.3 | 5.5 | 4.2 | 4.3 | 4.0 | 4.0 | 7.2 |
| Ifit1 | 22.2 | 9.5 | 13.1 | 31.6 | 7.3 | 15.2 | 5.9 | 2.7 | 4.5 | 2.9 | 9.5 | 24.0 |
| Ifit2 | 15.1 | 9.0 | 6.8 | 21.5 | 6.1 | 17.5 | 5.8 | 3.6 | 3.9 | 3.8 | 5.3 | 6.9 |
| Ifit3 | 153.0 | 66.0 | 39.4 | 173.9 | 27.2 | 164.5 | 15.8 | 7.4 | 7.9 | 11.5 | 20.9 | 37.4 |
| Ifitm1 | 3.3 | 6.9 | 6.8 | 3.5 | 3.5 | 6.7 | 3.2 | 3.5 | 4.2 | 7.7 | 2.4 | 3.9 |
| Ifitm10 | 0.3 | 0.4 | 0.5 | 0.4 | 0.7 | 0.6 | 0.6 | 0.7 | 0.5 | 0.8 | 0.3 | 0.6 |
| Ifitm2 | 316.1 | 335.1 | 392.2 | 317.4 | 322.9 | 307.0 | 326.7 | 348.7 | 317.6 | 395.2 | 346.0 | 336.6 |
| Ifitm3 | 482.1 | 405.5 | 440.4 | 710.4 | 511.0 | 407.9 | 394.5 | 316.8 | 311.9 | 369.2 | 338.5 | 404.4 |
| Ifitm5 | 0.4 | 0.5 | 0.5 | 0.3 | 0.8 | 0.6 | 0.7 | 0.4 | 0.3 | 0.3 | 0.5 | 0.4 |
| Ifitm7 | 0.0 | 0.0 | 0.0 | 0.0 | 0.0 | 0.0 | 0.1 | 0.0 | 0.0 | 0.0 | 0.0 | 0.0 |
| Ifnar1 | 12.1 | 12.4 | 13.7 | 12.0 | 14.4 | 10.4 | 13.0 | 13.8 | 13.2 | 12.1 | 11.8 | 12.5 |
| Ifnar2 | 70.5 | 73.8 | 91.9 | 80.7 | 90.1 | 61.8 | 72.0 | 67.3 | 60.8 | 59.5 | 59.4 | 69.9 |
| Ifngr1 | 46.8 | 59.1 | 80.4 | 51.5 | 58.5 | 54.5 | 56.4 | 48.6 | 53.2 | 40.3 | 37.7 | 49.9 |
| Ifngr2 | 57.3 | 38.4 | 34.2 | 62.3 | 58.5 | 54.7 | 41.1 | 27.8 | 28.7 | 40.8 | 37.3 | 33.3 |
| Ifnlr1 | 0.3 | 0.3 | 0.2 | 0.2 | 0.1 | 0.3 | 0.1 | 0.1 | 0.2 | 0.2 | 0.1 | 0.4 |
| Ifnz | 0.2 | 0.1 | 0.2 | 0.4 | 0.3 | 0.1 | 0.3 | 0.2 | 0.3 | 0.4 | 0.1 | 0.4 |
| Ifrd1 | 21.7 | 14.7 | 10.2 | 21.1 | 18.2 | 17.5 | 16.9 | 15.3 | 16.2 | 14.9 | 17.3 | 11.3 |
| Ifrd2 | 3.9 | 3.7 | 3.9 | 3.8 | 3.9 | 3.7 | 4.3 | 4.8 | 4.5 | 3.7 | 4.4 | 3.8 |
| Ift122 | 6.5 | 5.7 | 5.6 | 5.8 | 5.6 | 7.5 | 5.3 | 6.3 | 5.1 | 6.2 | 5.9 | 5.8 |
| Ift140 | 3.4 | 4.6 | 4.4 | 3.4 | 3.6 | 4.4 | 3.9 | 4.0 | 4.1 | 3.6 | 4.1 | 4.1 |
| Ift172 | 8.4 | 9.0 | 9.2 | 9.5 | 7.8 | 10.7 | 7.1 | 7.8 | 7.3 | 6.7 | 7.1 | 7.6 |
| Ift20 | 49.0 | 48.7 | 49.4 | 49.7 | 44.0 | 46.4 | 53.7 | 58.0 | 49.7 | 47.9 | 56.9 | 54.9 |
| Ift27 | 13.9 | 13.4 | 14.5 | 11.1 | 12.4 | 11.4 | 12.4 | 14.1 | 12.5 | 15.2 | 12.1 | 11.9 |
| Ift43 | 41.9 | 39.8 | 40.2 | 32.0 | 34.8 | 34.4 | 37.0 | 43.1 | 43.2 | 40.3 | 42.6 | 46.6 |
| Ift46 | 12.0 | 11.4 | 11.7 | 11.1 | 8.9 | 12.4 | 11.7 | 11.4 | 10.6 | 11.2 | 11.9 | 12.1 |
| Ift52 | 14.8 | 15.6 | 16.8 | 16.8 | 15.1 | 16.2 | 15.5 | 14.5 | 14.5 | 14.1 | 15.1 | 15.5 |
| Ift57 | 8.3 | 7.0 | 7.5 | 10.2 | 6.8 | 10.0 | 6.6 | 6.8 | 5.3 | 7.1 | 8.8 | 8.3 |
| Ift74 | 12.5 | 12.7 | 14.0 | 12.4 | 10.0 | 12.6 | 12.8 | 12.8 | 10.5 | 12.8 | 14.7 | 15.3 |
| Ift80 | 6.2 | 6.8 | 8.3 | 5.9 | 7.1 | 6.8 | 5.9 | 7.0 | 5.9 | 6.6 | 6.2 | 7.1 |
| Ift81 | 8.6 | 9.1 | 9.0 | 7.7 | 6.4 | 10.0 | 7.6 | 7.6 | 7.2 | 7.4 | 8.6 | 9.4 |
| Ift88 | 5.3 | 3.9 | 4.9 | 5.7 | 3.6 | 5.5 | 4.2 | 4.4 | 3.7 | 4.3 | 4.7 | 5.1 |
| Igbp1 | 29.1 | 25.6 | 28.6 | 26.8 | 24.5 | 31.2 | 25.3 | 28.5 | 22.2 | 29.8 | 30.3 | 29.8 |
| Igdcc3 | 0.0 | 0.0 | 0.0 | 0.0 | 0.0 | 0.0 | 0.0 | 0.0 | 0.0 | 0.0 | 0.0 | 0.0 |
| Igdcc4 | 4.3 | 5.5 | 5.6 | 3.7 | 4.3 | 6.2 | 4.8 | 4.4 | 4.3 | 3.9 | 3.8 | 3.6 |
| Igf1 | 15.0 | 9.2 | 17.7 | 39.0 | 30.1 | 8.5 | 10.7 | 15.8 | 9.8 | 16.8 | 12.5 | 14.2 |
| Igf1r | 17.9 | 20.9 | 16.6 | 20.5 | 19.4 | 23.2 | 26.1 | 17.6 | 18.7 | 15.0 | 18.7 | 13.5 |
| Igf2 | 0.2 | 0.3 | 0.4 | 0.4 | 0.2 | 0.1 | 0.1 | 0.1 | 0.2 | 0.3 | 0.2 | 0.2 |
| Igf2bp1 | 0.3 | 0.1 | 0.1 | 0.3 | 0.2 | 0.3 | 0.1 | 0.1 | 0.1 | 0.1 | 0.1 | 0.1 |
| Igf2bp2 | 2.9 | 3.0 | 3.1 | 3.2 | 3.7 | 3.0 | 3.4 | 3.5 | 2.9 | 4.8 | 4.5 | 3.9 |
| Igf2bp3 | 3.0 | 2.0 | 1.1 | 2.4 | 1.5 | 2.1 | 1.1 | 0.8 | 0.9 | 0.6 | 1.1 | 0.8 |
| Igf2r | 53.5 | 51.5 | 56.4 | 36.3 | 52.7 | 40.9 | 39.4 | 60.9 | 48.5 | 38.2 | 41.8 | 43.8 |
| Igfals | 0.1 | 0.1 | 0.0 | 0.0 | 0.0 | 0.1 | 0.0 | 0.1 | 0.1 | 0.1 | 0.1 | 0.1 |
| Igfbp2 | 570.4 | 167.1 | 223.0 | 701.9 | 187.6 | 503.0 | 141.3 | 138.0 | 133.7 | 606.1 | 586.5 | 324.4 |
| Igfbp3 | 54.1 | 74.5 | 61.0 | 113.7 | 60.5 | 39.2 | 15.6 | 17.4 | 38.2 | 198.3 | 55.3 | 57.6 |
| Igfbp4 | 129.3 | 106.7 | 297.0 | 14.3 | 20.1 | 11.5 | 28.7 | 167.8 | 64.4 | 127.5 | 107.9 | 155.2 |
| Igfbp5 | 0.3 | 0.1 | 0.8 | 0.2 | 2.7 | 0.1 | 0.9 | 1.6 | 1.0 | 0.9 | 1.1 | 1.2 |
| Igfbp6 | 13.5 | 10.7 | 7.2 | 7.8 | 4.5 | 13.4 | 9.4 | 14.7 | 8.0 | 50.6 | 35.5 | 16.3 |
| Igfbp7 | 1348.9 | 1187.8 | 1265.6 | 1592.1 | 1333.2 | 1847.7 | 1386.5 | 1114.7 | 1195.4 | 1051.3 | 1067.3 | 1238.1 |
| Igflr1 | 0.4 | 0.4 | 0.4 | 0.5 | 0.7 | 0.3 | 0.4 | 0.2 | 0.9 | 0.4 | 0.3 | 0.1 |
| Igfn1 | 0.0 | 0.0 | 0.0 | 0.0 | 0.0 | 0.0 | 0.0 | 0.0 | 0.0 | 0.0 | 0.0 | 0.0 |
| Ighmbp2 | 1.2 | 1.2 | 1.4 | 1.3 | 1.3 | 1.5 | 1.4 | 1.5 | 1.6 | 1.5 | 1.2 | 1.3 |

| | | | | | | | | | | | | |
|---|---|---|---|---|---|---|---|---|---|---|---|---|
| Iglon5 | 0.1 | 0.0 | 0.1 | 0.0 | 0.0 | 0.0 | 0.1 | 0.1 | 0.1 | 0.1 | 0.0 | 0.1 |
| Igsf10 | 0.2 | 0.3 | 0.6 | 0.1 | 0.2 | 0.2 | 0.2 | 0.9 | 0.2 | 0.2 | 0.3 | 0.2 |
| Igsf11 | 0.3 | 0.1 | 0.0 | 0.4 | 0.2 | 0.3 | 0.1 | 0.1 | 0.0 | 0.1 | 0.1 | 0.1 |
| Igsf21 | 0.0 | 0.1 | 0.1 | 0.0 | 0.0 | 0.0 | 0.0 | 0.0 | 0.0 | 0.0 | 0.0 | 0.0 |
| Igsf23 | 0.4 | 0.1 | 0.3 | 0.4 | 0.2 | 0.2 | 0.2 | 0.1 | 0.1 | 0.2 | 0.3 | 0.2 |
| Igsf3 | 3.5 | 4.5 | 6.0 | 3.6 | 4.5 | 4.2 | 5.1 | 7.7 | 5.3 | 6.7 | 5.2 | 5.8 |
| Igsf6 | 0.3 | 0.1 | 0.1 | 2.4 | 3.4 | 0.1 | 0.6 | 0.1 | 0.1 | 0.2 | 0.2 | 0.3 |
| Igsf8 | 37.8 | 49.1 | 47.2 | 38.8 | 39.6 | 40.7 | 45.2 | 46.6 | 52.7 | 34.5 | 38.2 | 37.7 |
| Igsf9 | 0.1 | 0.0 | 0.0 | 0.3 | 0.8 | 0.1 | 0.2 | 0.0 | 0.1 | 0.2 | 0.0 | 0.1 |
| Igsf9b | 2.6 | 1.9 | 1.1 | 2.0 | 1.4 | 1.9 | 1.2 | 0.4 | 0.9 | 1.6 | 1.5 | 0.8 |
| Igtp | 4.8 | 3.8 | 4.3 | 8.1 | 4.3 | 3.9 | 2.5 | 2.0 | 2.4 | 2.5 | 2.9 | 4.3 |
| Iigp1 | 3.8 | 1.5 | 3.9 | 4.1 | 1.9 | 1.2 | 1.2 | 0.6 | 0.7 | 0.5 | 2.2 | 3.3 |
| Ik | 78.3 | 71.7 | 82.0 | 95.9 | 63.3 | 75.0 | 70.3 | 65.9 | 51.7 | 66.0 | 71.7 | 82.2 |
| Ikbip | 68.8 | 61.8 | 66.2 | 69.3 | 57.9 | 68.4 | 68.0 | 60.2 | 61.2 | 66.5 | 71.2 | 75.0 |
| Ikbkap | 7.8 | 6.4 | 5.9 | 6.5 | 7.3 | 6.5 | 6.6 | 6.8 | 6.7 | 6.5 | 7.1 | 6.2 |
| Ikbkb | 12.4 | 11.6 | 10.9 | 15.0 | 13.3 | 12.5 | 13.7 | 11.9 | 11.3 | 10.4 | 11.8 | 12.5 |
| Ikbke | 7.5 | 7.3 | 8.4 | 8.4 | 11.4 | 5.7 | 8.1 | 5.8 | 6.9 | 5.6 | 5.1 | 5.8 |
| Ikbkg | 28.6 | 25.1 | 16.9 | 41.3 | 24.0 | 41.7 | 25.2 | 14.9 | 20.3 | 21.2 | 22.9 | 22.0 |
| Ikzf1 | 0.1 | 0.0 | 0.0 | 0.9 | 2.0 | 0.0 | 0.3 | 0.0 | 0.0 | 0.2 | 0.0 | 0.1 |
| Ikzf2 | 1.4 | 1.5 | 1.6 | 1.5 | 1.2 | 1.1 | 1.4 | 1.2 | 1.6 | 1.7 | 2.0 | 1.7 |
| Ikzf4 | 0.0 | 0.1 | 0.0 | 0.0 | 0.0 | 0.0 | 0.0 | 0.0 | 0.0 | 0.1 | 0.0 | 0.0 |
| Ikzf5 | 3.8 | 4.9 | 5.3 | 5.1 | 4.5 | 4.7 | 6.4 | 5.5 | 5.2 | 4.3 | 6.5 | 6.1 |
| Il10 | 0.0 | 0.0 | 0.0 | 0.1 | 0.0 | 0.0 | 0.1 | 0.0 | 0.0 | 0.0 | 0.0 | 0.0 |
| Il10ra | 0.4 | 0.0 | 0.0 | 3.6 | 7.3 | 0.0 | 0.9 | 0.1 | 0.2 | 0.5 | 0.2 | 0.3 |
| Il10rb | 16.6 | 15.8 | 14.1 | 19.9 | 24.9 | 11.9 | 15.4 | 14.0 | 15.8 | 21.0 | 16.3 | 16.4 |
| Il11 | 0.3 | 0.5 | 0.5 | 0.2 | 0.2 | 0.3 | 0.6 | 0.4 | 0.6 | 0.4 | 0.4 | 0.6 |
| Il11ra1 | 44.7 | 47.7 | 62.5 | 42.5 | 34.9 | 37.5 | 38.1 | 62.3 | 39.8 | 32.0 | 30.0 | 38.9 |
| Il12a | 0.1 | 0.1 | 0.1 | 0.0 | 0.0 | 0.1 | 0.2 | 0.0 | 0.1 | 0.0 | 0.0 | 0.0 |
| Il12rb1 | 0.0 | 0.0 | 0.0 | 0.0 | 0.0 | 0.0 | 0.0 | 0.0 | 0.0 | 0.0 | 0.0 | 0.0 |
| Il12rb2 | 0.1 | 0.1 | 0.1 | 0.4 | 0.1 | 0.0 | 0.2 | 0.0 | 0.1 | 0.2 | 0.0 | 0.0 |
| Il13ra1 | 3.4 | 3.7 | 4.5 | 3.2 | 5.1 | 2.4 | 3.5 | 4.2 | 3.5 | 3.5 | 2.7 | 3.9 |
| Il13ra2 | 0.3 | 0.0 | 0.0 | 0.4 | 0.2 | 0.0 | 0.1 | 0.0 | 0.1 | 0.0 | 0.0 | 0.0 |
| Il15 | 2.5 | 2.1 | 2.9 | 2.5 | 3.5 | 1.5 | 2.0 | 1.3 | 3.5 | 1.9 | 1.6 | 2.3 |
| Il15ra | 0.6 | 0.5 | 0.9 | 0.6 | 0.7 | 1.0 | 0.6 | 0.5 | 0.3 | 1.0 | 1.2 | 0.4 |
| Il16 | 16.3 | 13.7 | 17.4 | 8.8 | 8.3 | 8.9 | 8.9 | 9.7 | 4.9 | 8.4 | 9.4 | 10.2 |
| Il17b | 0.1 | 0.1 | 0.1 | 0.0 | 0.1 | 0.1 | 0.0 | 0.1 | 0.0 | 0.0 | 0.0 | 0.0 |
| Il17c | 0.0 | 0.0 | 0.2 | 0.0 | 0.1 | 0.0 | 0.1 | 0.1 | 0.1 | 0.0 | 0.1 | 0.0 |
| Il17d | 1.8 | 1.5 | 2.0 | 2.9 | 1.5 | 2.2 | 2.7 | 2.2 | 2.2 | 1.4 | 1.7 | 1.4 |
| Il17ra | 14.2 | 16.0 | 14.7 | 15.4 | 13.8 | 13.1 | 16.5 | 14.3 | 13.3 | 10.7 | 11.9 | 11.6 |
| Il17rb | 0.1 | 0.1 | 0.1 | 0.1 | 0.1 | 0.2 | 0.2 | 0.2 | 0.1 | 0.0 | 0.0 | 0.1 |
| Il17rc | 11.5 | 11.5 | 10.3 | 8.1 | 8.9 | 9.7 | 11.5 | 12.0 | 13.0 | 10.5 | 9.4 | 9.3 |
| Il17rd | 2.0 | 2.3 | 1.7 | 2.0 | 2.0 | 2.6 | 1.9 | 1.9 | 2.0 | 2.4 | 2.0 | 1.9 |
| Il17re | 0.7 | 0.4 | 0.4 | 0.1 | 0.1 | 0.0 | 0.1 | 0.2 | 0.1 | 1.7 | 1.0 | 0.6 |
| Il18 | 3.1 | 1.9 | 1.6 | 1.1 | 1.6 | 1.6 | 2.6 | 1.7 | 1.9 | 12.7 | 7.1 | 3.2 |
| Il18bp | 0.9 | 0.8 | 1.2 | 1.4 | 1.5 | 1.0 | 1.1 | 0.9 | 0.6 | 0.8 | 0.4 | 0.9 |
| Il18r1 | 0.0 | 0.0 | 0.0 | 0.0 | 0.0 | 0.0 | 0.0 | 0.0 | 0.0 | 0.2 | 0.1 | 0.0 |
| Il18rap | 0.2 | 0.0 | 0.0 | 0.1 | 0.1 | 0.1 | 0.2 | 0.0 | 0.1 | 0.2 | 0.2 | 0.2 |
| Il1a | 0.1 | 0.2 | 0.1 | 0.2 | 0.0 | 0.1 | 0.1 | 0.0 | 0.0 | 0.0 | 0.1 | 0.0 |
| Il1b | 0.1 | 0.1 | 0.0 | 0.2 | 0.5 | 0.0 | 0.1 | 0.0 | 0.0 | 0.1 | 0.0 | 0.0 |
| Il1f10 | 0.0 | 0.0 | 0.0 | 0.0 | 0.0 | 0.0 | 0.0 | 0.0 | 0.0 | 0.2 | 0.3 | 0.1 |
| Il1f9 | 0.7 | 0.8 | 0.5 | 0.9 | 1.7 | 0.5 | 0.6 | 0.2 | 0.3 | 0.4 | 0.0 | 0.2 |
| Il1r1 | 7.9 | 18.8 | 41.6 | 7.8 | 15.3 | 4.9 | 14.2 | 33.3 | 20.3 | 26.7 | 15.4 | 19.7 |
| Il1r2 | 0.2 | 0.4 | 1.1 | 0.0 | 0.4 | 0.2 | 0.3 | 0.5 | 0.3 | 0.2 | 0.1 | 0.5 |
| Il1rap | 24.8 | 34.0 | 44.9 | 21.8 | 33.9 | 15.4 | 34.4 | 26.7 | 35.6 | 19.3 | 24.0 | 36.1 |
| Il1rapl1 | 0.1 | 0.1 | 0.1 | 0.0 | 0.1 | 0.1 | 0.0 | 0.2 | 0.1 | 0.1 | 0.1 | 0.2 |
| Il1rapl2 | 0.0 | 0.0 | 0.0 | 0.0 | 0.0 | 0.0 | 0.0 | 0.0 | 0.0 | 0.0 | 0.0 | 0.0 |
| Il1rl1 | 0.2 | 0.2 | 0.2 | 0.1 | 0.3 | 0.0 | 0.3 | 0.1 | 0.2 | 0.5 | 0.4 | 0.3 |

| | | | | | | | | | | | | |
|---|---|---|---|---|---|---|---|---|---|---|---|---|
| Il1rl2 | 3.2 | 3.3 | 4.2 | 2.9 | 3.9 | 1.7 | 4.5 | 5.5 | 3.9 | 4.0 | 3.6 | 4.4 |
| Il1rn | 0.1 | 0.0 | 0.0 | 1.9 | 1.4 | 0.0 | 0.2 | 0.1 | 0.0 | 1.0 | 0.6 | 0.5 |
| Il20ra | 0.0 | 0.0 | 0.0 | 0.0 | 0.0 | 0.0 | 0.0 | 0.0 | 0.0 | 0.1 | 0.1 | 0.1 |
| Il20rb | 0.2 | 0.3 | 0.2 | 0.1 | 0.3 | 0.2 | 0.2 | 0.3 | 0.3 | 0.2 | 0.4 | 0.3 |
| Il21r | 0.3 | 0.0 | 0.0 | 1.5 | 2.9 | 0.0 | 0.3 | 0.0 | 0.1 | 0.2 | 0.1 | 0.2 |
| Il22ra1 | 0.0 | 0.0 | 0.0 | 0.0 | 0.0 | 0.0 | 0.0 | 0.1 | 0.0 | 0.2 | 0.1 | 0.0 |
| Il22ra2 | 0.0 | 0.0 | 0.1 | 0.0 | 0.0 | 0.0 | 0.0 | 0.0 | 0.0 | 0.0 | 0.0 | 0.0 |
| Il23a | 0.1 | 0.2 | 0.1 | 0.2 | 0.2 | 0.2 | 0.1 | 0.0 | 0.1 | 0.2 | 0.1 | 0.1 |
| Il23r | 0.0 | 0.0 | 0.0 | 0.0 | 0.1 | 0.1 | 0.0 | 0.0 | 0.0 | 0.0 | 0.0 | 0.0 |
| Il27ra | 3.8 | 4.3 | 5.3 | 4.3 | 5.9 | 5.1 | 7.7 | 5.1 | 8.1 | 4.0 | 3.6 | 5.5 |
| Il2ra | 0.0 | 0.0 | 0.0 | 0.0 | 0.0 | 0.0 | 0.0 | 0.0 | 0.0 | 0.0 | 0.0 | 0.0 |
| Il2rb | 0.3 | 0.2 | 0.2 | 0.1 | 0.1 | 0.3 | 0.1 | 0.2 | 0.1 | 0.0 | 0.2 | 0.1 |
| Il2rg | 1.4 | 0.4 | 0.3 | 6.9 | 11.0 | 0.6 | 3.2 | 1.0 | 0.8 | 0.9 | 0.4 | 0.6 |
| Il31ra | 0.1 | 0.1 | 0.1 | 0.0 | 0.0 | 0.0 | 0.0 | 0.0 | 0.0 | 0.1 | 0.0 | 0.0 |
| Il33 | 0.2 | 1.0 | 8.2 | 0.2 | 2.2 | 0.1 | 2.0 | 12.7 | 13.4 | 15.4 | 5.0 | 24.5 |
| Il34 | 12.4 | 16.3 | 15.9 | 12.3 | 20.5 | 14.6 | 17.4 | 11.9 | 12.3 | 10.6 | 11.1 | 8.6 |
| Il3ra | 13.3 | 10.1 | 11.3 | 11.3 | 11.9 | 11.8 | 10.6 | 11.6 | 13.3 | 10.3 | 7.9 | 8.1 |
| Il4i1 | 0.0 | 0.0 | 0.0 | 0.0 | 0.0 | 0.0 | 0.0 | 0.0 | 0.0 | 0.1 | 0.0 | 0.0 |
| Il4ra | 27.0 | 28.0 | 32.2 | 33.9 | 46.1 | 23.3 | 73.3 | 45.1 | 51.4 | 53.2 | 46.2 | 58.4 |
| Il5ra | 0.0 | 0.0 | 0.0 | 0.0 | 0.0 | 0.0 | 0.0 | 0.0 | 0.0 | 0.0 | 0.0 | 0.0 |
| Il6 | 80.2 | 107.5 | 102.9 | 46.8 | 120.3 | 27.4 | 91.5 | 75.8 | 89.6 | 60.2 | 67.6 | 91.2 |
| Il6ra | 1.1 | 1.9 | 2.5 | 1.3 | 2.1 | 0.9 | 1.7 | 2.6 | 1.7 | 1.2 | 1.5 | 1.8 |
| Il6st | 191.1 | 244.8 | 364.8 | 153.2 | 176.5 | 151.0 | 212.6 | 280.8 | 251.9 | 129.9 | 162.1 | 218.7 |
| Il7 | 1.0 | 1.1 | 0.8 | 1.8 | 1.7 | 1.4 | 1.5 | 0.4 | 0.6 | 0.4 | 0.7 | 1.0 |
| Il7r | 0.1 | 0.0 | 0.0 | 2.7 | 4.5 | 0.0 | 0.4 | 0.0 | 0.1 | 0.2 | 0.0 | 0.2 |
| Il8 | 0.0 | 0.0 | 0.1 | 0.0 | 0.0 | 0.0 | 0.0 | 0.0 | 0.0 | 0.0 | 0.0 | 0.0 |
| Ildr2 | 3.3 | 1.4 | 1.4 | 1.7 | 0.9 | 1.7 | 1.7 | 1.6 | 2.5 | 10.5 | 5.7 | 5.7 |
| Ilf2 | 19.9 | 18.9 | 23.4 | 17.3 | 19.3 | 16.9 | 22.3 | 20.2 | 21.1 | 21.9 | 24.3 | 22.4 |
| Ilf3 | 11.9 | 12.8 | 13.8 | 12.0 | 11.7 | 11.9 | 13.5 | 15.0 | 12.4 | 14.0 | 13.5 | 13.2 |
| Ilk | 57.3 | 53.2 | 47.9 | 64.8 | 53.5 | 48.7 | 58.8 | 56.0 | 58.7 | 52.4 | 65.5 | 55.3 |
| Ilkap | 20.1 | 20.5 | 21.1 | 21.6 | 18.5 | 22.9 | 21.7 | 17.9 | 21.0 | 22.7 | 20.4 | 23.1 |
| Ilvbl | 30.0 | 25.5 | 18.9 | 26.1 | 23.2 | 23.5 | 30.1 | 24.6 | 28.5 | 32.0 | 29.0 | 28.7 |
| Immp1l | 26.5 | 27.3 | 20.7 | 24.7 | 21.4 | 24.9 | 18.2 | 21.9 | 26.5 | 25.8 | 27.2 | 23.9 |
| Immp2l | 1.4 | 1.6 | 1.6 | 2.7 | 1.1 | 1.7 | 1.3 | 1.3 | 1.4 | 1.5 | 1.1 | 0.7 |
| Immt | 38.7 | 32.8 | 30.1 | 42.8 | 35.9 | 36.2 | 38.1 | 38.6 | 32.4 | 33.7 | 39.3 | 33.8 |
| Imp3 | 30.8 | 25.6 | 23.0 | 24.4 | 27.6 | 25.1 | 24.0 | 26.5 | 27.1 | 38.1 | 27.1 | 25.5 |
| Imp4 | 9.2 | 8.2 | 8.4 | 10.2 | 10.7 | 8.5 | 11.0 | 9.8 | 8.9 | 8.7 | 9.8 | 8.9 |
| Impa1 | 13.5 | 12.8 | 11.0 | 11.1 | 13.1 | 14.4 | 13.6 | 11.2 | 12.9 | 13.3 | 13.9 | 13.4 |
| Impa2 | 1.4 | 1.7 | 2.5 | 1.5 | 2.6 | 1.9 | 2.9 | 2.2 | 2.7 | 2.3 | 3.0 | 2.0 |
| Impact | 82.5 | 62.4 | 54.6 | 108.6 | 63.0 | 99.2 | 63.0 | 44.5 | 48.9 | 62.5 | 66.9 | 70.4 |
| Impad1 | 25.6 | 23.4 | 25.2 | 22.9 | 24.9 | 25.5 | 25.6 | 24.5 | 26.1 | 28.3 | 28.5 | 28.1 |
| Impdh1 | 11.7 | 11.9 | 11.0 | 14.3 | 13.8 | 11.9 | 12.6 | 12.9 | 13.3 | 9.8 | 11.6 | 9.5 |
| Impdh2 | 56.3 | 50.5 | 53.2 | 40.2 | 49.3 | 50.3 | 52.2 | 47.9 | 51.1 | 58.4 | 53.8 | 54.4 |
| Impg1 | 0.0 | 0.0 | 0.0 | 0.0 | 0.0 | 0.0 | 0.0 | 0.0 | 0.0 | 0.0 | 0.0 | 0.0 |
| Impg2 | 0.1 | 0.1 | 0.1 | 0.0 | 0.1 | 0.0 | 0.0 | 0.1 | 0.1 | 0.1 | 0.1 | 0.1 |
| Ina | 0.1 | 0.0 | 0.0 | 0.0 | 0.1 | 0.0 | 0.1 | 0.0 | 0.0 | 0.0 | 0.1 | 0.1 |
| Inadl | 0.9 | 0.6 | 0.6 | 0.6 | 0.6 | 0.6 | 1.0 | 0.8 | 0.6 | 1.2 | 1.0 | 1.0 |
| Inca1 | 1.5 | 1.2 | 1.7 | 0.9 | 1.3 | 2.1 | 0.8 | 2.2 | 1.1 | 1.2 | 0.7 | 1.4 |
| Incenp | 3.1 | 6.0 | 9.9 | 4.2 | 8.0 | 2.7 | 11.0 | 5.4 | 6.4 | 3.7 | 6.7 | 5.1 |
| Inf2 | 4.0 | 4.3 | 2.9 | 4.2 | 4.9 | 4.6 | 5.8 | 3.3 | 4.4 | 7.6 | 5.1 | 4.4 |
| Ing1 | 6.7 | 7.4 | 7.7 | 6.6 | 7.2 | 6.7 | 8.3 | 8.4 | 8.6 | 8.2 | 8.8 | 8.7 |
| Ing2 | 2.2 | 2.5 | 2.5 | 1.7 | 2.3 | 2.1 | 2.0 | 2.2 | 1.6 | 2.1 | 2.4 | 2.1 |
| Ing3 | 1.9 | 2.3 | 2.4 | 2.1 | 1.7 | 1.7 | 2.1 | 2.2 | 1.9 | 2.0 | 2.6 | 2.5 |
| Ing4 | 19.4 | 18.2 | 19.7 | 18.3 | 17.6 | 16.9 | 17.0 | 17.5 | 18.3 | 21.0 | 17.1 | 18.8 |
| Ing5 | 3.5 | 3.8 | 3.8 | 2.8 | 3.6 | 3.3 | 3.9 | 3.9 | 3.9 | 3.9 | 4.2 | 3.8 |
| Inha | 0.3 | 0.4 | 0.3 | 0.3 | 0.0 | 0.6 | 0.6 | 0.4 | 0.5 | 0.4 | 0.3 | 0.4 |
| Inhba | 37.0 | 36.4 | 28.7 | 48.6 | 30.8 | 46.1 | 56.5 | 24.9 | 36.0 | 32.9 | 50.3 | 41.0 |

| | | | | | | | | | | | | |
|---|---|---|---|---|---|---|---|---|---|---|---|---|
| Inhbb | 21.5 | 38.2 | 50.7 | 15.0 | 38.9 | 16.4 | 51.1 | 30.4 | 46.9 | 43.8 | 37.2 | 61.0 |
| Inip | 3.6 | 3.1 | 3.8 | 4.4 | 3.7 | 3.3 | 3.4 | 3.0 | 3.4 | 3.0 | 3.1 | 3.4 |
| Inmt | 0.1 | 0.0 | 0.2 | 0.1 | 0.0 | 0.0 | 0.1 | 0.1 | 0.0 | 0.0 | 0.0 | 0.1 |
| Ino80 | 5.4 | 5.1 | 5.3 | 6.1 | 5.5 | 5.4 | 5.9 | 5.7 | 5.8 | 5.7 | 5.6 | 5.4 |
| Ino80b | 8.5 | 7.4 | 7.7 | 6.3 | 6.5 | 7.2 | 7.5 | 7.9 | 6.5 | 7.5 | 6.6 | 8.2 |
| Ino80c | 9.5 | 7.4 | 6.8 | 10.3 | 7.9 | 7.6 | 8.2 | 7.7 | 7.1 | 6.8 | 8.4 | 9.4 |
| Ino80d | 1.2 | 1.6 | 1.7 | 1.2 | 1.6 | 1.4 | 1.4 | 1.5 | 1.4 | 1.4 | 1.3 | 1.4 |
| Ino80e | 8.7 | 7.9 | 8.9 | 11.7 | 11.0 | 11.9 | 11.1 | 9.8 | 8.0 | 10.5 | 10.9 | 8.5 |
| Inpp1 | 11.1 | 9.7 | 7.9 | 9.7 | 9.1 | 10.7 | 9.1 | 6.9 | 7.3 | 9.4 | 9.2 | 8.2 |
| Inpp4a | 1.5 | 1.8 | 1.9 | 1.4 | 1.8 | 1.8 | 1.7 | 1.7 | 1.9 | 1.9 | 1.7 | 1.7 |
| Inpp4b | 0.1 | 0.0 | 0.0 | 0.9 | 1.8 | 0.0 | 0.4 | 0.1 | 0.1 | 0.2 | 0.1 | 0.1 |
| Inpp5a | 12.2 | 13.9 | 13.2 | 9.6 | 10.3 | 11.1 | 11.5 | 11.1 | 13.1 | 11.2 | 12.8 | 13.9 |
| Inpp5b | 8.6 | 9.3 | 7.8 | 9.7 | 11.3 | 8.3 | 8.7 | 9.8 | 10.1 | 8.3 | 8.8 | 8.7 |
| Inpp5d | 0.7 | 0.1 | 0.1 | 5.1 | 8.8 | 0.0 | 1.1 | 0.2 | 0.2 | 0.5 | 0.1 | 0.4 |
| Inpp5e | 8.0 | 8.6 | 8.3 | 7.3 | 8.0 | 8.7 | 8.7 | 7.7 | 8.4 | 7.7 | 7.5 | 7.9 |
| Inpp5f | 7.4 | 6.8 | 7.0 | 6.8 | 5.5 | 7.3 | 6.4 | 5.4 | 5.8 | 6.2 | 7.5 | 7.3 |
| Inpp5j | 0.0 | 0.0 | 0.0 | 0.0 | 0.0 | 0.0 | 0.0 | 0.0 | 0.0 | 0.0 | 0.0 | 0.0 |
| Inpp5k | 6.9 | 6.2 | 6.2 | 6.5 | 5.6 | 6.8 | 6.9 | 8.1 | 6.9 | 8.0 | 7.9 | 6.9 |
| Inppl1 | 23.6 | 26.6 | 22.4 | 24.7 | 26.4 | 27.8 | 24.4 | 21.3 | 26.7 | 29.8 | 24.3 | 28.1 |
| Ins2 | 0.0 | 0.0 | 0.0 | 0.2 | 0.0 | 0.0 | 0.2 | 0.0 | 0.0 | 0.2 | 0.2 | 0.0 |
| Insc | 1.6 | 0.4 | 0.2 | 0.3 | 0.1 | 0.2 | 0.4 | 0.4 | 0.4 | 0.9 | 0.9 | 0.6 |
| Insig1 | 50.1 | 75.5 | 60.4 | 25.1 | 41.5 | 44.9 | 98.7 | 102.9 | 94.4 | 27.7 | 87.7 | 39.9 |
| Insig2 | 13.9 | 15.9 | 14.2 | 14.6 | 15.3 | 16.9 | 12.0 | 12.1 | 13.4 | 16.2 | 16.5 | 14.9 |
| Insl3 | 0.0 | 0.0 | 0.0 | 0.0 | 0.0 | 0.1 | 0.0 | 0.0 | 0.0 | 0.0 | 0.1 | 0.0 |
| Insl6 | 2.4 | 1.4 | 1.3 | 2.7 | 2.0 | 3.5 | 0.4 | 0.6 | 0.9 | 1.0 | 0.9 | 0.7 |
| Insm2 | 0.0 | 0.0 | 0.0 | 0.1 | 0.0 | 0.0 | 0.0 | 0.0 | 0.0 | 0.0 | 0.1 | 0.0 |
| Insr | 4.1 | 4.3 | 4.3 | 3.6 | 3.6 | 4.4 | 4.0 | 3.6 | 4.3 | 4.0 | 3.8 | 4.1 |
| Insrr | 0.0 | 0.0 | 0.0 | 0.0 | 0.0 | 0.0 | 0.0 | 0.0 | 0.0 | 0.0 | 0.0 | 0.0 |
| Ints1 | 5.8 | 6.2 | 5.3 | 5.5 | 6.1 | 6.2 | 6.4 | 6.3 | 6.9 | 5.6 | 5.6 | 4.6 |
| Ints10 | 10.3 | 11.4 | 11.2 | 9.7 | 11.0 | 10.9 | 11.9 | 11.2 | 10.1 | 10.1 | 10.8 | 9.9 |
| Ints12 | 4.6 | 4.5 | 4.6 | 4.9 | 5.0 | 4.5 | 4.3 | 4.6 | 4.3 | 4.5 | 5.3 | 4.7 |
| Ints2 | 5.9 | 6.2 | 5.1 | 6.1 | 5.9 | 5.5 | 5.0 | 4.9 | 4.8 | 5.9 | 5.4 | 5.0 |
| Ints3 | 8.4 | 10.3 | 9.5 | 8.9 | 9.7 | 10.4 | 10.0 | 11.3 | 10.3 | 9.7 | 10.2 | 8.9 |
| Ints4 | 12.6 | 11.9 | 11.8 | 13.3 | 11.8 | 12.9 | 11.8 | 13.0 | 11.1 | 11.1 | 12.2 | 10.8 |
| Ints5 | 3.9 | 5.0 | 4.9 | 3.9 | 4.6 | 3.9 | 5.3 | 5.0 | 5.4 | 4.4 | 4.3 | 3.7 |
| Ints6 | 6.5 | 8.8 | 7.2 | 5.5 | 5.7 | 5.8 | 5.0 | 5.8 | 5.7 | 5.6 | 6.6 | 6.6 |
| Ints7 | 2.7 | 3.8 | 4.9 | 2.6 | 3.9 | 2.1 | 4.3 | 5.2 | 4.0 | 3.4 | 3.8 | 3.1 |
| Ints8 | 18.1 | 19.7 | 18.8 | 15.8 | 18.7 | 16.8 | 17.2 | 17.4 | 19.3 | 21.5 | 21.8 | 21.3 |
| Ints9 | 4.2 | 4.8 | 4.7 | 4.5 | 4.6 | 4.7 | 4.9 | 4.7 | 3.5 | 3.9 | 4.1 | 4.1 |
| Intu | 1.4 | 1.4 | 1.3 | 1.1 | 1.3 | 1.6 | 1.2 | 1.2 | 1.1 | 1.2 | 1.3 | 1.2 |
| Invs | 5.0 | 4.6 | 4.9 | 5.4 | 4.3 | 5.6 | 4.6 | 5.2 | 4.7 | 4.5 | 5.4 | 4.3 |
| Ip6k1 | 14.4 | 17.1 | 16.2 | 12.3 | 14.3 | 16.2 | 12.8 | 14.6 | 17.4 | 16.3 | 13.5 | 13.9 |
| Ip6k2 | 8.2 | 8.2 | 6.8 | 7.5 | 6.7 | 8.0 | 6.4 | 6.2 | 6.6 | 8.9 | 7.3 | 7.1 |
| Ip6k3 | 0.4 | 0.4 | 0.4 | 0.8 | 0.6 | 0.6 | 0.1 | 0.3 | 0.1 | 0.1 | 0.1 | 0.3 |
| Ipmk | 5.0 | 6.2 | 7.6 | 4.5 | 5.7 | 4.7 | 6.1 | 6.8 | 5.6 | 5.2 | 5.4 | 6.1 |
| Ipo11 | 11.1 | 11.3 | 10.7 | 9.7 | 10.7 | 11.2 | 9.1 | 9.6 | 9.6 | 9.7 | 10.8 | 10.4 |
| Ipo13 | 12.7 | 12.6 | 10.7 | 17.2 | 16.1 | 16.7 | 14.0 | 12.6 | 12.0 | 11.6 | 10.0 | 9.9 |
| Ipo4 | 28.4 | 20.7 | 19.8 | 25.1 | 22.6 | 27.2 | 22.1 | 20.5 | 20.6 | 22.4 | 22.0 | 21.1 |
| Ipo5 | 42.9 | 30.1 | 24.7 | 40.6 | 33.6 | 38.7 | 32.7 | 29.9 | 27.9 | 32.5 | 34.8 | 31.2 |
| Ipo7 | 61.6 | 53.6 | 43.1 | 54.9 | 52.8 | 55.4 | 48.0 | 39.8 | 49.3 | 60.6 | 63.2 | 60.9 |
| Ipo8 | 12.6 | 12.0 | 10.8 | 14.6 | 11.6 | 13.6 | 10.9 | 10.4 | 10.6 | 11.6 | 11.6 | 11.4 |
| Ipo9 | 13.3 | 15.1 | 15.9 | 13.2 | 15.5 | 12.2 | 15.5 | 16.6 | 15.9 | 14.4 | 15.6 | 13.8 |
| Ipp | 5.3 | 5.4 | 4.0 | 4.7 | 3.8 | 5.3 | 3.2 | 3.9 | 4.1 | 5.2 | 4.1 | 4.3 |
| Ippk | 4.0 | 4.4 | 3.5 | 4.7 | 3.6 | 3.9 | 3.5 | 3.8 | 3.9 | 4.0 | 4.2 | 4.3 |
| Ipw | 0.5 | 0.1 | 0.0 | 0.0 | 0.0 | 0.1 | 0.0 | 0.0 | 0.0 | 0.1 | 0.2 | 0.0 |
| Iqcb1 | 2.7 | 3.2 | 2.7 | 2.4 | 2.2 | 3.4 | 2.8 | 2.6 | 2.6 | 3.4 | 3.0 | 3.2 |
| Iqcc | 6.2 | 6.1 | 7.2 | 6.9 | 6.0 | 6.6 | 7.0 | 8.3 | 7.1 | 6.6 | 6.6 | 7.7 |

| | | | | | | | | | | | | |
|---|---|---|---|---|---|---|---|---|---|---|---|---|
| Iqcd | 0.2 | 0.2 | 0.4 | 0.4 | 0.3 | 0.3 | 0.2 | 0.3 | 0.1 | 0.4 | 0.2 | 0.2 |
| Iqce | 2.2 | 2.5 | 2.3 | 2.5 | 2.1 | 2.5 | 2.4 | 2.0 | 1.9 | 2.0 | 2.0 | 2.2 |
| Iqcg | 2.9 | 2.9 | 2.6 | 2.6 | 2.9 | 2.7 | 2.9 | 2.7 | 2.7 | 2.5 | 2.8 | 2.8 |
| Iqch | 0.1 | 0.1 | 0.0 | 0.0 | 0.1 | 0.1 | 0.1 | 0.0 | 0.0 | 0.1 | 0.1 | 0.0 |
| Iqck | 0.9 | 0.7 | 0.4 | 1.0 | 0.5 | 0.9 | 0.6 | 0.5 | 0.3 | 0.8 | 0.7 | 0.6 |
| Iqgap1 | 65.5 | 57.0 | 55.8 | 86.7 | 94.7 | 73.0 | 78.7 | 59.1 | 64.2 | 58.9 | 69.7 | 67.6 |
| Iqgap2 | 0.6 | 0.4 | 0.6 | 1.5 | 1.2 | 0.2 | 0.5 | 0.3 | 0.3 | 0.6 | 0.7 | 0.7 |
| Iqgap3 | 1.4 | 2.1 | 3.0 | 0.5 | 3.7 | 0.4 | 5.2 | 1.2 | 3.0 | 1.2 | 2.0 | 1.4 |
| Iqsec1 | 5.9 | 6.7 | 5.6 | 6.0 | 5.2 | 5.8 | 6.7 | 5.9 | 6.3 | 5.8 | 5.6 | 5.3 |
| Iqsec2 | 1.8 | 2.4 | 2.3 | 1.9 | 1.9 | 1.7 | 2.3 | 2.0 | 1.9 | 1.6 | 1.7 | 1.5 |
| Iqub | 0.1 | 0.0 | 0.0 | 0.0 | 0.0 | 0.0 | 0.0 | 0.0 | 0.0 | 0.0 | 0.0 | 0.0 |
| Irak1 | 21.5 | 21.5 | 19.1 | 23.4 | 19.1 | 24.3 | 21.4 | 18.3 | 18.2 | 16.7 | 18.2 | 18.3 |
| Irak1bp1 | 6.8 | 5.6 | 5.6 | 8.9 | 4.5 | 7.2 | 5.9 | 4.3 | 4.4 | 4.8 | 6.0 | 6.2 |
| Irak2 | 5.3 | 4.0 | 3.3 | 7.0 | 6.7 | 4.4 | 6.0 | 3.4 | 5.3 | 5.7 | 5.9 | 5.7 |
| Irak3 | 5.4 | 7.1 | 9.6 | 5.5 | 7.9 | 3.8 | 8.2 | 10.9 | 7.7 | 4.2 | 6.6 | 7.7 |
| Irak4 | 4.3 | 4.2 | 5.3 | 5.3 | 5.4 | 4.2 | 4.6 | 5.0 | 4.4 | 3.9 | 5.0 | 4.2 |
| Ireb2 | 3.5 | 5.2 | 4.7 | 2.7 | 4.5 | 3.5 | 4.0 | 4.2 | 4.3 | 4.9 | 4.6 | 4.5 |
| Irf1 | 9.7 | 9.1 | 11.1 | 11.7 | 18.4 | 12.2 | 11.9 | 11.2 | 10.6 | 11.5 | 9.7 | 12.3 |
| Irf2 | 2.2 | 3.3 | 4.0 | 3.1 | 3.6 | 2.8 | 3.4 | 2.8 | 2.6 | 2.8 | 2.2 | 2.7 |
| Irf2bp1 | 3.8 | 4.4 | 3.9 | 4.1 | 4.0 | 4.6 | 5.0 | 4.7 | 4.9 | 4.6 | 4.0 | 4.2 |
| Irf2bp2 | 13.0 | 14.7 | 16.8 | 12.6 | 16.4 | 14.9 | 13.3 | 13.2 | 14.7 | 16.9 | 12.9 | 13.0 |
| Irf2bpl | 7.9 | 9.0 | 6.8 | 7.3 | 7.9 | 7.1 | 6.9 | 8.0 | 8.7 | 10.3 | 6.7 | 6.7 |
| Irf3 | 16.4 | 15.4 | 13.8 | 15.8 | 14.7 | 16.6 | 17.3 | 14.6 | 17.1 | 18.4 | 16.8 | 15.9 |
| Irf4 | 0.0 | 0.0 | 0.0 | 0.0 | 0.0 | 0.0 | 0.0 | 0.0 | 0.0 | 0.0 | 0.0 | 0.0 |
| Irf5 | 3.5 | 2.8 | 2.8 | 7.9 | 14.1 | 2.6 | 3.7 | 1.2 | 2.4 | 4.3 | 2.3 | 3.2 |
| Irf6 | 0.3 | 0.3 | 0.3 | 0.2 | 0.3 | 0.3 | 0.3 | 0.3 | 0.2 | 0.1 | 0.2 | 0.3 |
| Irf7 | 14.5 | 8.1 | 10.0 | 27.6 | 8.5 | 12.5 | 4.9 | 3.0 | 4.2 | 3.3 | 3.6 | 8.4 |
| Irf8 | 0.9 | 0.2 | 0.3 | 4.8 | 7.1 | 0.2 | 0.8 | 0.2 | 0.2 | 0.3 | 0.1 | 0.3 |
| Irf9 | 12.8 | 9.2 | 12.3 | 13.9 | 10.7 | 10.1 | 9.3 | 8.7 | 9.6 | 7.6 | 7.7 | 15.7 |
| Irg1 | 0.5 | 0.1 | 0.1 | 0.9 | 8.5 | 0.1 | 1.7 | 0.1 | 0.3 | 0.4 | 0.1 | 0.3 |
| Irgm1 | 44.4 | 26.4 | 21.5 | 55.4 | 32.8 | 38.9 | 27.8 | 16.2 | 26.0 | 29.5 | 26.7 | 41.7 |
| Irgm2 | 9.3 | 5.7 | 9.1 | 11.9 | 8.1 | 5.9 | 6.1 | 5.3 | 6.6 | 4.9 | 6.2 | 12.0 |
| Irgq | 6.7 | 5.7 | 4.5 | 6.2 | 5.8 | 5.8 | 5.8 | 4.7 | 5.5 | 6.2 | 5.5 | 5.2 |
| Irs1 | 6.5 | 8.2 | 7.8 | 6.9 | 8.1 | 6.0 | 8.4 | 9.2 | 8.9 | 8.9 | 9.1 | 6.9 |
| Irs2 | 1.6 | 1.8 | 1.9 | 1.3 | 2.5 | 2.0 | 2.6 | 2.0 | 2.6 | 3.2 | 1.6 | 2.2 |
| Irs3 | 0.2 | 0.6 | 0.5 | 0.7 | 0.4 | 0.5 | 0.2 | 0.2 | 0.3 | 0.4 | 0.4 | 0.3 |
| Irs4 | 0.0 | 0.0 | 0.0 | 0.0 | 0.0 | 0.0 | 0.0 | 0.0 | 0.0 | 0.0 | 0.0 | 0.0 |
| Irx1 | 0.3 | 0.2 | 0.2 | 0.4 | 0.0 | 0.2 | 0.1 | 0.1 | 0.2 | 1.0 | 1.0 | 0.6 |
| Irx2 | 0.5 | 0.2 | 0.2 | 1.1 | 0.3 | 0.6 | 0.8 | 0.2 | 0.3 | 0.8 | 0.7 | 0.4 |
| Irx3 | 0.6 | 0.8 | 1.0 | 0.5 | 0.7 | 0.8 | 0.7 | 0.7 | 0.6 | 0.4 | 0.6 | 0.5 |
| Irx5 | 1.2 | 0.9 | 0.4 | 1.6 | 0.8 | 1.3 | 0.7 | 0.2 | 0.5 | 0.5 | 0.4 | 0.4 |
| Isca1 | 31.5 | 28.6 | 28.0 | 27.0 | 27.5 | 27.9 | 27.4 | 30.0 | 31.1 | 36.9 | 34.6 | 33.1 |
| Isca2 | 22.0 | 24.2 | 22.1 | 21.5 | 17.8 | 23.5 | 20.5 | 21.9 | 23.5 | 20.7 | 23.4 | 20.0 |
| Iscu | 87.6 | 63.2 | 56.8 | 100.2 | 64.2 | 88.6 | 64.6 | 64.8 | 59.8 | 69.5 | 76.5 | 74.4 |
| Isg15 | 19.0 | 7.7 | 15.7 | 29.6 | 14.7 | 16.1 | 10.7 | 6.8 | 10.3 | 7.2 | 10.9 | 27.2 |
| Isg20 | 10.7 | 11.4 | 11.3 | 14.9 | 8.2 | 11.7 | 12.3 | 13.6 | 12.4 | 8.0 | 14.2 | 11.9 |
| Isg20l2 | 7.5 | 7.7 | 8.3 | 6.6 | 7.5 | 6.9 | 7.5 | 9.2 | 8.4 | 7.7 | 7.3 | 7.4 |
| Isl1 | 0.0 | 0.0 | 0.0 | 0.0 | 0.0 | 0.0 | 0.0 | 0.0 | 0.1 | 0.4 | 0.1 | 0.0 |
| Isl2 | 0.1 | 0.0 | 0.0 | 0.1 | 0.0 | 0.0 | 0.1 | 0.0 | 0.1 | 0.0 | 0.1 | 0.0 |
| Islr | 11.3 | 16.2 | 28.3 | 12.2 | 16.0 | 14.5 | 13.2 | 18.4 | 15.2 | 13.2 | 10.9 | 13.2 |
| Islr2 | 0.0 | 0.0 | 0.0 | 0.0 | 0.0 | 0.0 | 0.0 | 0.0 | 0.0 | 0.0 | 0.0 | 0.0 |
| Ism1 | 1.1 | 2.5 | 8.3 | 0.3 | 1.4 | 0.4 | 2.3 | 9.4 | 7.4 | 2.5 | 1.9 | 7.9 |
| Isoc1 | 5.0 | 5.1 | 4.6 | 6.3 | 5.8 | 4.7 | 4.0 | 4.3 | 4.2 | 7.0 | 6.6 | 5.0 |
| Isoc2a | 9.9 | 9.6 | 8.5 | 8.0 | 10.1 | 10.5 | 9.1 | 9.2 | 10.2 | 9.5 | 8.3 | 9.4 |
| Isoc2b | 10.8 | 9.9 | 10.2 | 10.1 | 8.0 | 10.6 | 8.3 | 10.2 | 8.2 | 10.6 | 8.9 | 11.0 |
| Ispd | 1.8 | 1.8 | 2.0 | 1.2 | 1.8 | 1.8 | 1.6 | 2.0 | 2.0 | 2.3 | 2.2 | 1.8 |
| Ist1 | 17.1 | 19.4 | 17.6 | 15.8 | 16.9 | 15.5 | 19.5 | 18.4 | 19.2 | 19.9 | 18.3 | 19.4 |

| | | | | | | | | | | | | |
|---|---|---|---|---|---|---|---|---|---|---|---|---|
| Isy1 | 13.0 | 11.1 | 15.0 | 13.7 | 13.4 | 13.2 | 13.9 | 13.6 | 12.4 | 14.0 | 13.5 | 17.7 |
| Isyna1 | 4.2 | 5.1 | 6.4 | 4.6 | 7.2 | 3.0 | 2.6 | 5.6 | 3.9 | 15.9 | 6.9 | 6.5 |
| Itch | 16.7 | 17.7 | 17.1 | 17.4 | 16.6 | 17.6 | 15.9 | 16.9 | 16.9 | 18.2 | 19.4 | 19.1 |
| Itfg1 | 67.2 | 65.2 | 58.8 | 68.4 | 63.9 | 78.7 | 60.5 | 59.4 | 60.8 | 61.0 | 64.5 | 66.6 |
| Itfg2 | 2.5 | 2.1 | 1.9 | 2.9 | 2.5 | 2.8 | 2.8 | 2.7 | 2.4 | 3.3 | 2.5 | 3.1 |
| Itfg3 | 20.8 | 20.6 | 17.4 | 19.3 | 18.5 | 20.3 | 19.2 | 19.9 | 19.2 | 20.7 | 17.8 | 15.4 |
| Itga1 | 7.5 | 9.5 | 15.5 | 4.4 | 10.3 | 3.1 | 11.2 | 15.2 | 17.6 | 15.5 | 16.1 | 15.3 |
| Itga10 | 0.6 | 0.7 | 0.5 | 0.4 | 0.5 | 0.6 | 0.7 | 0.3 | 0.7 | 0.7 | 0.6 | 0.5 |
| Itga11 | 8.7 | 17.6 | 27.7 | 9.4 | 18.7 | 14.0 | 27.2 | 27.0 | 36.2 | 35.8 | 40.9 | 39.0 |
| Itga2 | 0.8 | 0.7 | 1.0 | 0.4 | 0.9 | 0.3 | 1.2 | 0.7 | 0.6 | 0.3 | 0.5 | 0.5 |
| Itga2b | 0.3 | 0.5 | 0.5 | 0.4 | 0.4 | 0.4 | 0.4 | 0.4 | 0.3 | 0.3 | 0.3 | 0.3 |
| Itga3 | 3.1 | 1.3 | 1.4 | 1.5 | 1.0 | 1.8 | 1.3 | 1.9 | 2.2 | 9.6 | 4.0 | 3.1 |
| Itga4 | 2.0 | 1.8 | 2.0 | 1.7 | 1.8 | 1.8 | 1.7 | 3.9 | 1.6 | 1.6 | 2.1 | 2.4 |
| Itga5 | 8.7 | 8.5 | 7.5 | 8.8 | 15.5 | 9.1 | 12.3 | 10.2 | 10.2 | 10.5 | 10.3 | 8.3 |
| Itga6 | 4.4 | 1.8 | 1.6 | 4.5 | 5.5 | 3.6 | 1.7 | 1.2 | 1.3 | 2.4 | 2.2 | 1.4 |
| Itga7 | 4.2 | 2.3 | 1.8 | 1.9 | 1.3 | 1.6 | 1.3 | 1.2 | 1.4 | 1.8 | 1.6 | 1.8 |
| Itga8 | 0.5 | 0.4 | 0.7 | 0.5 | 0.6 | 0.5 | 0.2 | 0.5 | 0.4 | 0.4 | 0.4 | 0.3 |
| Itga9 | 7.8 | 7.7 | 8.5 | 7.8 | 9.4 | 6.9 | 8.7 | 7.3 | 9.2 | 8.3 | 7.4 | 10.3 |
| Itgad | 0.0 | 0.0 | 0.0 | 0.3 | 0.7 | 0.0 | 0.1 | 0.0 | 0.0 | 0.0 | 0.0 | 0.0 |
| Itgae | 0.0 | 0.0 | 0.0 | 0.0 | 0.1 | 0.0 | 0.0 | 0.0 | 0.0 | 0.0 | 0.0 | 0.0 |
| Itgal | 0.2 | 0.1 | 0.0 | 1.0 | 4.1 | 0.1 | 0.8 | 0.1 | 0.2 | 0.3 | 0.1 | 0.2 |
| Itgam | 2.3 | 0.0 | 0.2 | 13.7 | 33.1 | 0.0 | 3.1 | 0.5 | 0.5 | 1.6 | 0.6 | 1.3 |
| Itgav | 46.0 | 47.0 | 48.5 | 37.4 | 52.8 | 45.0 | 49.1 | 40.4 | 38.6 | 43.7 | 42.8 | 39.3 |
| Itgax | 0.7 | 0.0 | 0.0 | 7.1 | 14.2 | 0.0 | 1.0 | 0.1 | 0.1 | 0.4 | 0.1 | 0.3 |
| Itgb1 | 372.2 | 342.9 | 323.3 | 363.8 | 359.8 | 359.2 | 418.9 | 368.5 | 437.3 | 359.8 | 439.2 | 448.3 |
| Itgb1bp1 | 17.9 | 15.0 | 13.8 | 18.4 | 14.2 | 18.5 | 13.5 | 13.1 | 13.2 | 15.4 | 16.2 | 16.4 |
| Itgb1bp2 | 0.1 | 0.0 | 0.1 | 0.1 | 0.0 | 0.0 | 0.2 | 0.0 | 0.1 | 0.2 | 0.0 | 0.0 |
| Itgb2 | 4.1 | 0.3 | 0.4 | 32.1 | 61.1 | 0.1 | 5.4 | 0.8 | 0.7 | 3.5 | 1.0 | 2.2 |
| Itgb3 | 1.0 | 0.8 | 1.0 | 0.9 | 0.8 | 0.9 | 1.0 | 2.0 | 1.7 | 1.5 | 1.7 | 1.6 |
| Itgb3bp | 1.2 | 1.0 | 1.3 | 1.0 | 0.9 | 1.3 | 0.8 | 1.7 | 0.7 | 1.0 | 1.3 | 1.3 |
| Itgb4 | 0.2 | 0.2 | 0.2 | 0.2 | 0.2 | 0.3 | 0.2 | 0.2 | 0.2 | 0.4 | 0.2 | 0.2 |
| Itgb5 | 259.8 | 298.4 | 289.9 | 289.3 | 298.5 | 383.1 | 373.8 | 275.9 | 391.4 | 295.3 | 264.0 | 391.9 |
| Itgb6 | 0.2 | 0.1 | 0.1 | 0.0 | 0.1 | 0.1 | 0.1 | 0.1 | 0.1 | 0.1 | 0.1 | 0.1 |
| Itgb7 | 0.0 | 0.0 | 0.0 | 0.1 | 0.4 | 0.0 | 0.1 | 0.0 | 0.0 | 0.1 | 0.0 | 0.0 |
| Itgb8 | 1.7 | 3.6 | 5.3 | 1.3 | 3.6 | 1.9 | 3.6 | 6.0 | 6.9 | 3.3 | 3.0 | 5.6 |
| Itgbl1 | 61.5 | 19.4 | 20.1 | 39.5 | 15.3 | 48.4 | 13.7 | 33.7 | 19.4 | 14.2 | 22.9 | 29.6 |
| Itih1 | 0.0 | 0.1 | 0.0 | 0.1 | 0.0 | 0.0 | 0.0 | 0.1 | 0.0 | 0.0 | 0.0 | 0.0 |
| Itih2 | 1.9 | 1.3 | 1.3 | 0.4 | 0.8 | 1.0 | 0.5 | 1.0 | 0.4 | 0.6 | 0.7 | 1.0 |
| Itih3 | 0.0 | 0.1 | 0.2 | 0.0 | 0.0 | 0.0 | 0.0 | 0.2 | 0.0 | 0.0 | 0.0 | 0.1 |
| Itih4 | 0.1 | 0.0 | 0.1 | 0.0 | 0.1 | 0.0 | 0.1 | 0.0 | 0.0 | 0.1 | 0.0 | 0.0 |
| Itih5 | 0.1 | 0.1 | 0.2 | 0.1 | 0.1 | 0.0 | 0.0 | 0.1 | 0.0 | 0.0 | 0.1 | 0.0 |
| Itm2a | 2.9 | 2.6 | 4.9 | 0.6 | 1.2 | 1.2 | 1.1 | 3.8 | 2.5 | 3.6 | 2.8 | 3.2 |
| Itm2b | 463.3 | 408.6 | 481.2 | 602.3 | 592.4 | 493.0 | 396.0 | 376.3 | 372.4 | 444.4 | 364.6 | 495.6 |
| Itm2c | 153.9 | 126.1 | 116.7 | 191.4 | 142.4 | 173.3 | 121.8 | 110.0 | 111.9 | 144.6 | 108.8 | 123.7 |
| Itpa | 18.0 | 16.2 | 16.0 | 17.6 | 18.1 | 15.6 | 13.1 | 16.3 | 18.1 | 17.6 | 15.7 | 13.2 |
| Itpk1 | 4.7 | 6.0 | 4.2 | 5.0 | 3.7 | 4.3 | 5.7 | 6.1 | 5.5 | 4.3 | 5.1 | 4.2 |
| Itpka | 0.0 | 0.1 | 0.1 | 0.1 | 0.1 | 0.2 | 0.0 | 0.0 | 0.0 | 0.0 | 0.0 | 0.0 |
| Itpkb | 4.1 | 4.2 | 5.1 | 5.0 | 6.2 | 4.2 | 4.4 | 4.9 | 4.3 | 3.9 | 3.8 | 4.4 |
| Itpkc | 3.6 | 4.2 | 4.2 | 3.1 | 4.1 | 4.1 | 4.4 | 4.5 | 4.3 | 4.3 | 3.7 | 4.3 |
| Itpr1 | 15.1 | 15.7 | 16.3 | 17.7 | 16.8 | 18.1 | 17.1 | 13.0 | 15.8 | 16.6 | 16.2 | 14.9 |
| Itpr2 | 8.3 | 7.3 | 7.6 | 9.5 | 7.7 | 9.8 | 7.3 | 6.5 | 6.1 | 4.9 | 5.8 | 6.8 |
| Itpr3 | 2.3 | 1.7 | 1.8 | 1.7 | 2.6 | 2.0 | 1.7 | 2.3 | 1.8 | 1.9 | 2.0 | 1.4 |
| Itprip | 7.7 | 7.0 | 4.7 | 7.4 | 10.8 | 7.0 | 12.1 | 4.7 | 8.3 | 7.4 | 7.7 | 6.0 |
| Itpripl1 | 2.8 | 3.0 | 3.1 | 3.0 | 3.6 | 2.6 | 3.1 | 2.5 | 2.5 | 2.0 | 2.1 | 2.0 |
| Itpripl2 | 75.8 | 62.9 | 54.8 | 89.8 | 83.6 | 92.5 | 64.2 | 48.4 | 56.1 | 55.0 | 52.5 | 57.1 |
| Itsn1 | 36.9 | 52.5 | 65.4 | 49.7 | 45.5 | 40.5 | 63.9 | 70.3 | 62.3 | 32.0 | 43.2 | 51.0 |
| Itsn2 | 18.2 | 18.6 | 16.9 | 20.2 | 20.5 | 16.0 | 20.4 | 16.6 | 16.6 | 16.9 | 21.1 | 22.1 |

| | | | | | | | | | | | | |
|---|---|---|---|---|---|---|---|---|---|---|---|---|
| Ivd | 13.6 | 16.2 | 18.4 | 11.1 | 11.0 | 12.1 | 10.6 | 14.7 | 13.5 | 13.3 | 14.1 | 12.5 |
| Ivl | 2.3 | 0.4 | 0.2 | 0.8 | 0.5 | 0.2 | 0.6 | 0.1 | 0.2 | 7.5 | 2.5 | 1.6 |
| Ivns1abp | 18.4 | 17.0 | 16.4 | 23.9 | 20.8 | 20.9 | 22.5 | 17.2 | 21.5 | 21.7 | 24.9 | 17.6 |
| Iws1 | 4.2 | 4.0 | 4.1 | 4.5 | 4.0 | 3.9 | 4.8 | 4.2 | 3.9 | 3.9 | 4.8 | 4.6 |
| Izumo4 | 9.2 | 10.4 | 11.4 | 7.9 | 8.8 | 9.5 | 9.7 | 8.5 | 13.5 | 7.3 | 9.1 | 11.2 |
| Jag1 | 37.7 | 43.6 | 42.7 | 36.5 | 42.3 | 32.5 | 43.8 | 42.6 | 57.1 | 26.8 | 38.8 | 34.2 |
| Jag2 | 0.1 | 0.1 | 0.1 | 0.1 | 0.2 | 0.0 | 0.0 | 0.0 | 0.1 | 0.1 | 0.0 | 0.0 |
| Jagn1 | 23.3 | 20.5 | 16.5 | 23.8 | 19.4 | 20.2 | 19.9 | 21.3 | 19.8 | 22.3 | 21.8 | 20.5 |
| Jak1 | 53.4 | 55.1 | 57.5 | 54.7 | 63.0 | 54.2 | 65.2 | 60.3 | 58.0 | 56.8 | 60.5 | 70.2 |
| Jak2 | 22.4 | 31.0 | 31.6 | 22.9 | 32.2 | 22.4 | 30.7 | 25.0 | 27.0 | 31.0 | 31.1 | 32.2 |
| Jak3 | 0.8 | 1.4 | 1.3 | 0.9 | 1.3 | 0.8 | 1.5 | 0.7 | 1.3 | 1.0 | 0.8 | 0.8 |
| Jakmip2 | 0.0 | 0.0 | 0.0 | 0.0 | 0.0 | 0.0 | 0.1 | 0.1 | 0.0 | 0.0 | 0.0 | 0.0 |
| Jakmip3 | 0.5 | 0.5 | 0.4 | 0.6 | 0.4 | 0.9 | 0.3 | 0.3 | 0.2 | 0.2 | 0.2 | 0.4 |
| Jam2 | 25.3 | 23.4 | 21.8 | 18.0 | 21.0 | 16.2 | 19.4 | 19.0 | 13.5 | 22.3 | 22.7 | 23.0 |
| Jam3 | 35.8 | 30.7 | 26.4 | 34.5 | 26.7 | 36.8 | 26.3 | 27.8 | 28.9 | 30.9 | 31.3 | 28.6 |
| Jarid2 | 1.1 | 1.4 | 1.5 | 0.9 | 1.6 | 1.0 | 1.3 | 1.5 | 1.3 | 1.6 | 1.7 | 1.4 |
| Jazf1 | 2.1 | 2.2 | 2.7 | 2.4 | 1.9 | 2.5 | 1.7 | 1.9 | 1.6 | 2.5 | 1.8 | 1.9 |
| Jdp2 | 36.0 | 30.5 | 31.7 | 44.6 | 28.4 | 38.2 | 38.1 | 38.6 | 36.4 | 31.4 | 40.3 | 34.4 |
| Jhdm1d | 2.3 | 2.5 | 2.7 | 2.3 | 3.1 | 1.9 | 1.6 | 1.8 | 1.8 | 2.3 | 1.8 | 2.4 |
| Jkamp | 20.2 | 18.9 | 17.9 | 21.0 | 19.2 | 20.6 | 18.8 | 19.2 | 20.1 | 22.7 | 21.7 | 20.4 |
| Jmjd1c | 7.2 | 7.8 | 7.4 | 8.1 | 8.5 | 7.9 | 7.4 | 6.7 | 6.0 | 6.8 | 6.6 | 7.5 |
| Jmjd4 | 2.1 | 2.2 | 2.5 | 1.7 | 2.6 | 2.3 | 2.2 | 2.1 | 2.9 | 2.7 | 2.5 | 2.2 |
| Jmjd6 | 17.7 | 15.7 | 15.4 | 15.3 | 17.1 | 14.2 | 19.9 | 15.7 | 18.1 | 18.0 | 19.0 | 17.7 |
| Jmjd7 | 2.1 | 1.7 | 1.5 | 1.6 | 1.3 | 1.7 | 2.0 | 2.7 | 1.1 | 1.6 | 1.4 | 1.3 |
| Jmjd8 | 8.0 | 8.5 | 10.4 | 10.0 | 8.8 | 10.2 | 8.9 | 11.6 | 9.1 | 10.0 | 8.1 | 10.3 |
| Jmy | 4.2 | 4.4 | 4.3 | 4.5 | 3.8 | 4.6 | 3.9 | 3.9 | 4.0 | 5.2 | 4.8 | 4.9 |
| Josd1 | 13.1 | 12.9 | 11.3 | 12.4 | 11.8 | 13.1 | 10.6 | 11.8 | 11.9 | 13.0 | 10.8 | 11.2 |
| Josd2 | 25.1 | 23.2 | 17.2 | 25.2 | 25.8 | 25.1 | 20.7 | 21.2 | 25.7 | 21.6 | 22.3 | 17.7 |
| Jph1 | 0.2 | 0.1 | 0.1 | 0.2 | 0.1 | 0.2 | 0.2 | 0.1 | 0.2 | 0.5 | 0.5 | 0.2 |
| Jph2 | 33.9 | 37.5 | 35.7 | 34.4 | 32.6 | 36.4 | 50.2 | 35.5 | 51.3 | 29.5 | 35.4 | 38.6 |
| Jph3 | 0.2 | 0.1 | 0.1 | 0.3 | 0.2 | 0.2 | 0.2 | 0.1 | 0.1 | 0.1 | 0.2 | 0.1 |
| Jph4 | 0.1 | 0.2 | 0.1 | 0.1 | 0.1 | 0.1 | 0.1 | 0.1 | 0.1 | 0.3 | 0.2 | 0.2 |
| Jpx | 0.0 | 0.0 | 0.1 | 0.0 | 0.0 | 0.0 | 0.1 | 0.1 | 0.0 | 0.1 | 0.0 | 0.1 |
| Jrk | 1.6 | 1.4 | 1.5 | 1.1 | 1.2 | 1.7 | 1.4 | 1.4 | 1.4 | 1.2 | 1.2 | 1.5 |
| Jrkl | 4.7 | 4.7 | 5.0 | 4.1 | 3.9 | 4.3 | 3.7 | 4.6 | 5.7 | 4.9 | 6.2 | 6.3 |
| Jsrp1 | 0.2 | 0.2 | 0.1 | 0.0 | 0.1 | 0.2 | 0.0 | 0.0 | 0.0 | 0.1 | 0.0 | 0.2 |
| Jtb | 28.7 | 25.1 | 22.2 | 26.3 | 27.1 | 23.1 | 24.7 | 26.2 | 26.3 | 27.9 | 25.1 | 28.3 |
| Jun | 9.1 | 5.7 | 4.1 | 9.8 | 10.8 | 5.1 | 7.1 | 4.9 | 5.2 | 14.7 | 7.6 | 6.0 |
| Junb | 34.8 | 40.7 | 44.6 | 34.6 | 57.1 | 26.5 | 48.4 | 42.4 | 52.8 | 77.6 | 38.6 | 47.6 |
| Jund | 23.7 | 14.6 | 10.6 | 19.0 | 15.6 | 19.8 | 15.5 | 17.5 | 15.6 | 17.3 | 13.3 | 13.6 |
| Jup | 13.0 | 12.1 | 6.8 | 13.3 | 12.0 | 12.4 | 14.7 | 11.8 | 14.4 | 13.5 | 17.9 | 9.4 |
| Kalrn | 3.5 | 4.3 | 5.6 | 3.2 | 5.3 | 2.4 | 5.3 | 4.7 | 6.2 | 8.0 | 6.7 | 8.3 |
| Kank1 | 12.8 | 11.1 | 12.0 | 10.9 | 7.3 | 10.7 | 4.8 | 5.4 | 5.0 | 20.6 | 11.1 | 10.8 |
| Kank2 | 52.7 | 53.2 | 44.9 | 55.5 | 42.1 | 53.4 | 56.6 | 53.7 | 50.1 | 43.9 | 51.6 | 48.0 |
| Kank3 | 2.2 | 1.7 | 1.7 | 1.2 | 1.7 | 2.0 | 1.2 | 0.8 | 1.4 | 2.0 | 1.3 | 1.5 |
| Kank4 | 0.1 | 0.0 | 0.0 | 0.2 | 0.0 | 0.0 | 0.1 | 0.1 | 0.1 | 0.3 | 0.1 | 0.1 |
| Kansl1 | 8.5 | 9.3 | 9.2 | 8.9 | 8.9 | 9.0 | 8.4 | 8.7 | 8.9 | 8.3 | 8.6 | 8.2 |
| Kansl1l | 3.7 | 4.7 | 5.4 | 3.7 | 4.6 | 4.1 | 4.0 | 5.2 | 4.4 | 5.3 | 5.3 | 5.5 |
| Kansl2 | 36.6 | 38.2 | 29.4 | 37.4 | 31.4 | 30.9 | 33.3 | 31.8 | 32.7 | 33.9 | 33.6 | 31.0 |
| Kansl3 | 10.1 | 9.6 | 8.8 | 10.5 | 9.1 | 10.4 | 10.8 | 7.6 | 9.6 | 6.8 | 8.5 | 8.1 |
| Kars | 47.3 | 41.5 | 38.1 | 53.8 | 41.9 | 48.8 | 40.7 | 43.5 | 34.1 | 37.8 | 43.5 | 39.9 |
| Kat2a | 5.3 | 5.5 | 5.8 | 5.1 | 5.7 | 5.2 | 5.0 | 6.2 | 4.7 | 5.3 | 5.3 | 3.9 |
| Kat2b | 19.7 | 24.3 | 26.2 | 16.3 | 15.7 | 15.7 | 14.8 | 21.2 | 22.0 | 17.7 | 20.7 | 23.0 |
| Kat5 | 9.5 | 9.8 | 10.0 | 11.3 | 9.2 | 10.5 | 12.2 | 11.5 | 10.3 | 10.1 | 10.2 | 10.3 |
| Kat6a | 2.9 | 3.9 | 4.8 | 3.4 | 4.0 | 3.2 | 4.3 | 4.4 | 3.8 | 4.0 | 4.5 | 4.2 |
| Kat6b | 3.1 | 3.8 | 4.2 | 3.2 | 3.0 | 3.4 | 3.8 | 4.9 | 3.7 | 4.2 | 4.4 | 4.5 |
| Kat7 | 7.4 | 8.8 | 8.2 | 7.4 | 7.5 | 7.8 | 8.6 | 8.5 | 7.7 | 7.3 | 7.6 | 7.8 |

| | | | | | | | | | | | | |
|---|---|---|---|---|---|---|---|---|---|---|---|---|
| Kat8 | 11.9 | 12.3 | 12.5 | 11.0 | 10.5 | 11.4 | 9.4 | 11.6 | 11.0 | 10.7 | 11.8 | 9.5 |
| Katna1 | 18.7 | 17.7 | 15.9 | 16.1 | 14.9 | 15.5 | 14.0 | 14.8 | 13.7 | 16.7 | 15.8 | 15.6 |
| Katnal1 | 7.4 | 7.0 | 7.2 | 9.2 | 5.5 | 7.9 | 6.1 | 4.7 | 5.6 | 5.2 | 6.3 | 6.7 |
| Katnal2 | 0.3 | 0.6 | 0.7 | 0.5 | 0.5 | 0.6 | 0.6 | 0.3 | 0.4 | 0.4 | 0.6 | 0.5 |
| Katnb1 | 3.7 | 4.1 | 4.0 | 3.7 | 3.8 | 4.4 | 3.6 | 3.4 | 3.5 | 3.7 | 4.0 | 3.3 |
| Katnbl1 | 6.0 | 7.7 | 6.6 | 6.9 | 6.6 | 7.0 | 6.8 | 6.0 | 7.5 | 6.5 | 8.8 | 6.8 |
| Kazald1 | 2.6 | 2.5 | 2.6 | 2.6 | 1.5 | 6.8 | 3.5 | 2.6 | 3.5 | 4.0 | 3.9 | 3.7 |
| Kazn | 3.1 | 3.7 | 4.1 | 2.5 | 3.7 | 2.8 | 4.0 | 5.7 | 4.2 | 5.2 | 6.1 | 3.4 |
| Kbtbd11 | 0.0 | 0.0 | 0.1 | 0.0 | 0.0 | 0.0 | 0.0 | 0.1 | 0.0 | 0.1 | 0.0 | 0.1 |
| Kbtbd2 | 8.8 | 9.0 | 8.6 | 8.4 | 9.0 | 8.1 | 9.2 | 8.1 | 10.1 | 10.3 | 9.5 | 9.6 |
| Kbtbd3 | 0.7 | 0.7 | 0.8 | 1.3 | 0.7 | 1.1 | 0.6 | 0.7 | 1.1 | 0.9 | 1.2 | 1.1 |
| Kbtbd4 | 6.4 | 7.4 | 6.2 | 5.7 | 7.2 | 6.5 | 6.1 | 6.3 | 6.9 | 7.1 | 6.9 | 6.4 |
| Kbtbd7 | 1.2 | 1.6 | 2.1 | 1.5 | 1.3 | 1.4 | 1.4 | 1.8 | 1.9 | 1.8 | 1.5 | 1.9 |
| Kbtbd8 | 0.3 | 0.4 | 0.3 | 0.3 | 0.5 | 0.2 | 0.2 | 0.3 | 0.3 | 0.5 | 0.3 | 0.4 |
| Kcmf1 | 23.4 | 21.1 | 19.4 | 24.5 | 18.4 | 26.1 | 20.0 | 17.8 | 19.1 | 19.5 | 21.6 | 20.0 |
| Kcna1 | 0.0 | 0.0 | 0.0 | 0.0 | 0.0 | 0.0 | 0.0 | 0.0 | 0.0 | 0.0 | 0.0 | 0.0 |
| Kcna2 | 0.0 | 0.0 | 0.0 | 0.0 | 0.0 | 0.0 | 0.0 | 0.0 | 0.0 | 0.0 | 0.0 | 0.0 |
| Kcna3 | 0.0 | 0.0 | 0.0 | 0.2 | 0.3 | 0.0 | 0.1 | 0.0 | 0.0 | 0.1 | 0.0 | 0.0 |
| Kcna4 | 0.0 | 0.0 | 0.0 | 0.0 | 0.0 | 0.0 | 0.0 | 0.0 | 0.0 | 0.0 | 0.0 | 0.0 |
| Kcna5 | 0.0 | 0.0 | 0.1 | 0.0 | 0.0 | 0.0 | 0.0 | 0.1 | 0.1 | 0.0 | 0.0 | 0.0 |
| Kcna6 | 0.0 | 0.0 | 0.0 | 0.0 | 0.0 | 0.0 | 0.0 | 0.0 | 0.0 | 0.0 | 0.0 | 0.0 |
| Kcnab1 | 24.1 | 30.6 | 25.8 | 31.1 | 20.0 | 42.3 | 26.0 | 14.6 | 27.9 | 23.8 | 21.7 | 16.6 |
| Kcnab2 | 1.3 | 0.7 | 0.8 | 4.5 | 6.3 | 0.8 | 1.1 | 0.6 | 0.5 | 0.7 | 0.5 | 0.7 |
| Kcnab3 | 0.0 | 0.0 | 0.1 | 0.1 | 0.1 | 0.0 | 0.1 | 0.1 | 0.1 | 0.1 | 0.1 | 0.1 |
| Kcnb1 | 0.6 | 1.2 | 1.3 | 0.6 | 0.6 | 0.9 | 0.9 | 1.4 | 0.9 | 0.8 | 0.6 | 0.9 |
| Kcnc2 | 0.0 | 0.0 | 0.0 | 0.0 | 0.0 | 0.0 | 0.0 | 0.0 | 0.0 | 0.0 | 0.0 | 0.0 |
| Kcnc3 | 0.0 | 0.0 | 0.0 | 0.0 | 0.0 | 0.0 | 0.0 | 0.0 | 0.0 | 0.0 | 0.0 | 0.0 |
| Kcnd1 | 0.4 | 1.5 | 1.1 | 0.1 | 0.7 | 0.7 | 1.0 | 1.2 | 0.8 | 0.6 | 0.6 | 0.5 |
| Kcnd2 | 0.0 | 0.0 | 0.0 | 0.0 | 0.0 | 0.0 | 0.0 | 0.0 | 0.0 | 0.0 | 0.0 | 0.0 |
| Kcnd3 | 0.1 | 0.0 | 0.1 | 0.0 | 0.0 | 0.2 | 0.0 | 0.1 | 0.0 | 0.1 | 0.1 | 0.0 |
| Kcne1 | 0.0 | 0.0 | 0.0 | 0.0 | 0.0 | 0.0 | 0.0 | 0.0 | 0.0 | 0.0 | 0.0 | 0.0 |
| Kcne1l | 0.2 | 0.3 | 0.4 | 0.0 | 0.5 | 0.2 | 0.1 | 0.3 | 0.2 | 0.0 | 0.1 | 0.2 |
| Kcne2 | 0.1 | 0.0 | 0.0 | 0.0 | 0.0 | 0.0 | 0.0 | 0.0 | 0.0 | 0.0 | 0.0 | 0.0 |
| Kcne3 | 0.1 | 0.0 | 0.0 | 0.4 | 0.2 | 0.0 | 0.0 | 0.1 | 0.1 | 0.0 | 0.0 | 0.0 |
| Kcne4 | 78.7 | 128.2 | 125.7 | 85.7 | 78.5 | 107.5 | 87.1 | 132.9 | 136.6 | 39.8 | 45.5 | 100.4 |
| Kcnf1 | 0.0 | 0.0 | 0.0 | 0.0 | 0.0 | 0.1 | 0.0 | 0.0 | 0.0 | 0.0 | 0.0 | 0.0 |
| Kcng2 | 0.1 | 0.1 | 0.0 | 0.0 | 0.0 | 0.0 | 0.1 | 0.1 | 0.0 | 0.0 | 0.0 | 0.0 |
| Kcng4 | 0.0 | 0.0 | 0.0 | 0.1 | 0.1 | 0.1 | 0.1 | 0.0 | 0.0 | 0.0 | 0.0 | 0.0 |
| Kcnh1 | 0.2 | 0.2 | 0.4 | 0.1 | 0.2 | 0.2 | 0.1 | 0.2 | 0.2 | 0.1 | 0.1 | 0.2 |
| Kcnh2 | 0.2 | 0.1 | 0.1 | 0.1 | 0.1 | 0.1 | 0.2 | 0.2 | 0.2 | 0.1 | 0.2 | 0.1 |
| Kcnh3 | 0.0 | 0.0 | 0.0 | 0.0 | 0.0 | 0.0 | 0.0 | 0.0 | 0.0 | 0.0 | 0.0 | 0.0 |
| Kcnh4 | 0.0 | 0.0 | 0.0 | 0.0 | 0.0 | 0.0 | 0.0 | 0.0 | 0.0 | 0.0 | 0.0 | 0.0 |
| Kcnh5 | 0.1 | 0.0 | 0.1 | 0.0 | 0.1 | 0.1 | 0.0 | 0.0 | 0.0 | 0.0 | 0.0 | 0.0 |
| Kcnip1 | 0.0 | 0.0 | 0.0 | 0.0 | 0.0 | 0.0 | 0.0 | 0.0 | 0.0 | 0.0 | 0.0 | 0.0 |
| Kcnip2 | 0.1 | 0.0 | 0.0 | 0.1 | 0.0 | 0.0 | 0.0 | 0.0 | 0.1 | 0.0 | 0.0 | 0.0 |
| Kcnip3 | 0.4 | 0.3 | 0.6 | 0.6 | 0.6 | 0.4 | 0.5 | 0.9 | 0.5 | 0.3 | 0.3 | 0.4 |
| Kcnip4 | 0.1 | 0.2 | 0.0 | 0.2 | 0.1 | 0.3 | 0.2 | 0.2 | 0.3 | 0.2 | 0.1 | 0.2 |
| Kcnj10 | 0.0 | 0.0 | 0.0 | 0.0 | 0.0 | 0.0 | 0.0 | 0.0 | 0.0 | 0.0 | 0.0 | 0.0 |
| Kcnj12 | 0.0 | 0.0 | 0.0 | 0.0 | 0.0 | 0.0 | 0.0 | 0.0 | 0.0 | 0.0 | 0.0 | 0.0 |
| Kcnj13 | 0.1 | 0.2 | 0.2 | 0.0 | 0.0 | 0.1 | 0.1 | 0.1 | 0.1 | 0.2 | 0.2 | 0.1 |
| Kcnj14 | 0.0 | 0.0 | 0.1 | 0.0 | 0.1 | 0.0 | 0.0 | 0.0 | 0.0 | 0.1 | 0.0 | 0.0 |
| Kcnj15 | 3.6 | 2.6 | 3.3 | 3.7 | 4.4 | 3.6 | 5.3 | 2.8 | 3.2 | 5.4 | 4.5 | 7.1 |
| Kcnj2 | 0.2 | 0.1 | 0.1 | 1.7 | 2.4 | 0.0 | 0.3 | 0.3 | 0.2 | 0.2 | 0.3 | 0.3 |
| Kcnj4 | 1.9 | 1.8 | 1.2 | 1.5 | 1.6 | 2.3 | 1.1 | 0.8 | 0.8 | 1.4 | 0.8 | 1.3 |
| Kcnj5 | 0.0 | 0.1 | 0.1 | 0.0 | 0.0 | 0.0 | 0.0 | 0.0 | 0.0 | 0.0 | 0.0 | 0.0 |
| Kcnj6 | 0.0 | 0.0 | 0.1 | 0.0 | 0.0 | 0.1 | 0.0 | 0.0 | 0.0 | 0.0 | 0.0 | 0.0 |
| Kcnj8 | 0.0 | 0.1 | 0.1 | 0.0 | 0.0 | 0.0 | 0.0 | 0.1 | 0.2 | 0.1 | 0.1 | 0.1 |

| | | | | | | | | | | | | |
|---|---|---|---|---|---|---|---|---|---|---|---|---|
| Kcnj9 | 0.0 | 0.0 | 0.0 | 0.0 | 0.0 | 0.1 | 0.0 | 0.0 | 0.0 | 0.0 | 0.0 | 0.0 |
| Kcnk1 | 0.0 | 0.0 | 0.0 | 0.0 | 0.0 | 0.0 | 0.0 | 0.0 | 0.0 | 0.0 | 0.0 | 0.0 |
| Kcnk12 | 0.0 | 0.0 | 0.0 | 0.1 | 0.0 | 0.0 | 0.0 | 0.0 | 0.0 | 0.0 | 0.0 | 0.0 |
| Kcnk13 | 0.2 | 0.0 | 0.0 | 1.3 | 2.9 | 0.0 | 0.3 | 0.0 | 0.1 | 0.3 | 0.1 | 0.1 |
| Kcnk15 | 0.0 | 0.0 | 0.1 | 0.0 | 0.0 | 0.0 | 0.0 | 0.1 | 0.0 | 0.0 | 0.1 | 0.0 |
| Kcnk2 | 1.4 | 1.2 | 0.6 | 2.5 | 1.3 | 1.8 | 1.2 | 0.8 | 0.8 | 0.5 | 0.6 | 0.5 |
| Kcnk5 | 0.1 | 0.1 | 0.2 | 0.1 | 0.2 | 0.1 | 0.2 | 0.2 | 0.2 | 0.0 | 0.3 | 0.1 |
| Kcnk6 | 0.2 | 0.2 | 0.1 | 0.9 | 1.0 | 0.1 | 0.1 | 0.2 | 0.1 | 0.2 | 0.1 | 0.2 |
| Kcnk7 | 0.1 | 0.0 | 0.1 | 0.1 | 0.3 | 0.0 | 0.2 | 0.1 | 0.1 | 0.2 | 0.2 | 0.0 |
| Kcnma1 | 0.4 | 0.3 | 0.2 | 0.8 | 0.3 | 0.8 | 0.2 | 0.4 | 0.3 | 0.5 | 0.9 | 0.4 |
| Kcnmb1 | 0.2 | 0.0 | 0.0 | 0.3 | 0.1 | 0.3 | 0.1 | 0.0 | 0.1 | 0.0 | 0.0 | 0.0 |
| Kcnmb3 | 0.1 | 0.1 | 0.2 | 0.0 | 0.1 | 0.0 | 0.0 | 0.0 | 0.0 | 0.0 | 0.1 | 0.0 |
| Kcnmb4 | 0.3 | 0.0 | 0.0 | 0.5 | 0.0 | 0.1 | 0.0 | 0.1 | 0.0 | 0.0 | 0.0 | 0.0 |
| Kcnn1 | 1.0 | 0.8 | 1.0 | 0.9 | 0.6 | 1.4 | 0.7 | 0.6 | 0.7 | 0.8 | 0.8 | 0.8 |
| Kcnn3 | 0.5 | 0.3 | 0.3 | 0.8 | 0.6 | 0.8 | 0.5 | 0.4 | 0.2 | 0.1 | 0.2 | 0.1 |
| Kcnn4 | 1.0 | 0.3 | 0.2 | 7.7 | 13.5 | 0.2 | 1.5 | 0.2 | 0.2 | 0.9 | 0.2 | 0.7 |
| Kcnq1 | 0.0 | 0.0 | 0.1 | 0.2 | 0.0 | 0.0 | 0.0 | 0.1 | 0.0 | 0.0 | 0.0 | 0.0 |
| Kcnq1ot1 | 0.4 | 0.7 | 0.5 | 0.4 | 0.5 | 0.5 | 0.7 | 0.5 | 0.3 | 0.5 | 0.5 | 0.4 |
| Kcnq3 | 0.0 | 0.0 | 0.0 | 0.0 | 0.0 | 0.0 | 0.0 | 0.0 | 0.0 | 0.0 | 0.0 | 0.0 |
| Kcnq4 | 0.3 | 0.4 | 0.5 | 0.3 | 0.1 | 0.5 | 0.2 | 0.2 | 0.3 | 0.2 | 0.1 | 0.1 |
| Kcnq5 | 0.2 | 0.2 | 0.1 | 0.2 | 0.1 | 0.3 | 0.0 | 0.0 | 0.0 | 0.1 | 0.0 | 0.1 |
| Kcnrg | 0.1 | 0.2 | 0.1 | 0.1 | 0.4 | 0.1 | 0.1 | 0.2 | 0.1 | 0.1 | 0.1 | 0.1 |
| Kcns1 | 0.1 | 0.1 | 0.1 | 0.1 | 0.1 | 0.1 | 0.0 | 0.2 | 0.3 | 0.1 | 0.1 | 0.1 |
| Kcns3 | 0.1 | 0.1 | 0.1 | 0.0 | 0.2 | 0.0 | 0.1 | 0.1 | 0.6 | 0.3 | 0.1 | 0.1 |
| Kcnt1 | 0.0 | 0.0 | 0.0 | 0.0 | 0.0 | 0.0 | 0.0 | 0.0 | 0.0 | 0.0 | 0.0 | 0.0 |
| Kcnt2 | 3.3 | 4.7 | 5.5 | 2.1 | 2.2 | 2.5 | 2.3 | 4.9 | 3.3 | 2.1 | 2.7 | 2.6 |
| Kcnu1 | 0.2 | 0.2 | 0.2 | 0.3 | 0.2 | 0.2 | 0.1 | 0.0 | 0.1 | 0.2 | 0.1 | 0.1 |
| Kcnv2 | 0.0 | 0.0 | 0.1 | 0.0 | 0.0 | 0.0 | 0.0 | 0.0 | 0.0 | 0.0 | 0.0 | 0.0 |
| Kcp | 0.2 | 0.8 | 0.8 | 0.1 | 0.3 | 0.1 | 0.1 | 0.4 | 0.5 | 0.2 | 0.1 | 0.2 |
| Kctd1 | 1.6 | 1.7 | 1.5 | 2.2 | 1.6 | 1.9 | 1.5 | 1.6 | 1.4 | 2.9 | 2.2 | 2.0 |
| Kctd10 | 96.7 | 84.3 | 57.5 | 112.3 | 75.8 | 106.3 | 96.3 | 56.8 | 79.9 | 91.2 | 82.4 | 77.4 |
| Kctd11 | 9.5 | 11.5 | 12.6 | 9.0 | 16.1 | 7.1 | 16.2 | 20.8 | 17.7 | 14.9 | 14.3 | 14.9 |
| Kctd12 | 1.3 | 0.5 | 0.8 | 3.3 | 14.2 | 0.4 | 2.9 | 1.1 | 0.8 | 2.2 | 1.2 | 1.8 |
| Kctd12b | 0.2 | 0.4 | 0.7 | 0.4 | 0.5 | 0.3 | 0.4 | 0.4 | 0.3 | 0.2 | 0.2 | 0.4 |
| Kctd13 | 4.6 | 4.3 | 3.7 | 4.4 | 4.8 | 4.7 | 3.9 | 3.8 | 3.8 | 4.1 | 3.9 | 3.8 |
| Kctd14 | 0.1 | 0.0 | 0.1 | 0.0 | 0.0 | 0.0 | 0.0 | 0.2 | 0.1 | 0.0 | 0.0 | 0.1 |
| Kctd15 | 3.4 | 3.5 | 5.8 | 2.0 | 3.5 | 1.9 | 5.4 | 9.4 | 7.1 | 7.0 | 7.8 | 4.6 |
| Kctd16 | 0.1 | 0.0 | 0.0 | 0.2 | 0.0 | 0.1 | 0.0 | 0.1 | 0.0 | 0.0 | 0.0 | 0.0 |
| Kctd17 | 11.6 | 12.7 | 14.2 | 14.6 | 11.8 | 14.0 | 15.8 | 13.8 | 13.9 | 12.7 | 13.1 | 12.0 |
| Kctd18 | 5.5 | 4.7 | 3.6 | 5.9 | 4.2 | 5.2 | 4.2 | 4.2 | 4.5 | 4.8 | 4.3 | 5.5 |
| Kctd2 | 14.8 | 10.5 | 10.5 | 14.6 | 10.6 | 15.4 | 11.3 | 9.2 | 10.9 | 9.3 | 13.0 | 11.3 |
| Kctd20 | 23.2 | 22.7 | 19.9 | 23.6 | 22.0 | 21.6 | 22.5 | 20.2 | 21.0 | 20.0 | 21.8 | 18.4 |
| Kctd21 | 3.9 | 3.9 | 3.0 | 4.2 | 4.2 | 4.0 | 3.5 | 3.3 | 3.8 | 3.7 | 4.5 | 3.4 |
| Kctd3 | 9.5 | 9.3 | 9.4 | 10.0 | 8.5 | 10.7 | 8.8 | 9.2 | 9.6 | 8.6 | 9.3 | 8.6 |
| Kctd4 | 0.1 | 0.1 | 0.1 | 0.0 | 0.1 | 0.1 | 0.0 | 0.0 | 0.0 | 0.0 | 0.1 | 0.1 |
| Kctd5 | 28.2 | 30.2 | 27.8 | 22.2 | 28.4 | 20.6 | 29.7 | 28.4 | 36.6 | 28.1 | 31.0 | 30.7 |
| Kctd6 | 3.6 | 2.9 | 3.6 | 3.3 | 3.8 | 3.7 | 3.1 | 3.6 | 3.3 | 3.0 | 3.2 | 3.6 |
| Kctd7 | 2.1 | 3.0 | 2.5 | 2.6 | 3.1 | 2.6 | 3.4 | 3.1 | 3.5 | 2.3 | 2.8 | 2.7 |
| Kctd8 | 0.0 | 0.0 | 0.0 | 0.0 | 0.0 | 0.0 | 0.0 | 0.0 | 0.0 | 0.0 | 0.0 | 0.0 |
| Kctd9 | 6.8 | 7.3 | 7.3 | 5.8 | 6.9 | 5.4 | 9.6 | 8.5 | 9.8 | 6.6 | 8.7 | 7.4 |
| Kdelc1 | 10.5 | 13.3 | 13.0 | 8.2 | 11.4 | 11.6 | 12.3 | 15.0 | 13.4 | 14.0 | 13.8 | 12.9 |
| Kdelc2 | 12.0 | 12.9 | 17.7 | 11.9 | 13.6 | 13.1 | 17.6 | 20.0 | 18.1 | 15.1 | 17.0 | 19.0 |
| Kdelr1 | 63.5 | 59.2 | 56.4 | 65.7 | 57.7 | 69.1 | 68.0 | 74.4 | 65.5 | 67.2 | 65.5 | 63.4 |
| Kdelr2 | 119.9 | 106.9 | 109.3 | 125.4 | 113.6 | 118.0 | 140.8 | 145.9 | 126.0 | 120.3 | 139.8 | 130.9 |
| Kdelr3 | 58.4 | 53.7 | 44.4 | 55.3 | 53.3 | 51.3 | 57.4 | 60.7 | 60.1 | 48.9 | 60.4 | 55.2 |
| Kdm1a | 14.9 | 15.4 | 16.1 | 13.4 | 13.3 | 16.0 | 15.5 | 18.2 | 17.1 | 19.1 | 16.9 | 18.2 |
| Kdm1b | 4.3 | 3.8 | 3.7 | 5.3 | 3.6 | 5.4 | 3.4 | 3.0 | 3.2 | 3.6 | 3.8 | 3.5 |

| | | | | | | | | | | | | |
|---|---|---|---|---|---|---|---|---|---|---|---|---|
| Kdm2a | 10.7 | 11.4 | 12.3 | 12.2 | 11.5 | 11.2 | 12.5 | 12.6 | 10.9 | 10.8 | 11.9 | 11.4 |
| Kdm2b | 4.2 | 4.7 | 4.3 | 3.9 | 4.3 | 4.4 | 4.5 | 4.1 | 4.2 | 4.2 | 4.6 | 4.2 |
| Kdm3a | 7.7 | 7.5 | 6.9 | 6.0 | 8.0 | 6.6 | 6.9 | 6.4 | 7.9 | 9.5 | 7.5 | 8.8 |
| Kdm3b | 6.1 | 7.0 | 7.4 | 6.8 | 6.3 | 6.2 | 8.1 | 6.9 | 6.9 | 6.8 | 7.1 | 7.0 |
| Kdm4a | 19.9 | 18.6 | 18.5 | 18.8 | 17.9 | 17.8 | 19.9 | 21.2 | 21.3 | 18.2 | 19.6 | 18.1 |
| Kdm4b | 4.4 | 5.3 | 5.2 | 3.1 | 4.3 | 4.3 | 4.8 | 5.6 | 6.1 | 4.8 | 4.8 | 5.0 |
| Kdm4c | 9.2 | 8.4 | 8.3 | 7.2 | 7.1 | 6.8 | 7.1 | 6.9 | 8.1 | 9.3 | 8.9 | 9.2 |
| Kdm4d | 0.2 | 0.2 | 0.2 | 0.2 | 0.3 | 0.2 | 0.2 | 0.1 | 0.1 | 0.1 | 0.0 | 0.2 |
| Kdm5a | 11.0 | 11.6 | 10.7 | 10.4 | 9.6 | 10.4 | 9.8 | 9.5 | 9.1 | 10.5 | 10.4 | 11.1 |
| Kdm5b | 11.7 | 13.8 | 11.8 | 11.4 | 10.9 | 13.3 | 10.2 | 12.2 | 12.8 | 12.8 | 12.2 | 12.6 |
| Kdm5c | 11.5 | 12.4 | 12.3 | 11.9 | 12.0 | 10.7 | 13.1 | 13.6 | 13.8 | 13.4 | 12.7 | 12.8 |
| Kdm5d | 7.8 | 6.4 | 7.3 | 5.4 | 5.5 | 7.2 | 6.1 | 7.3 | 6.2 | 6.8 | 7.3 | 6.8 |
| Kdm6a | 2.6 | 3.1 | 3.3 | 2.3 | 2.6 | 2.5 | 2.2 | 3.0 | 3.0 | 2.8 | 2.5 | 2.8 |
| Kdm6b | 0.4 | 0.6 | 0.5 | 0.4 | 0.4 | 0.5 | 0.4 | 0.7 | 0.8 | 0.7 | 0.4 | 0.4 |
| Kdm8 | 0.9 | 1.2 | 0.8 | 1.3 | 1.1 | 0.7 | 1.2 | 1.1 | 0.9 | 1.2 | 1.3 | 1.2 |
| Kdr | 0.3 | 0.1 | 0.3 | 0.1 | 0.1 | 0.0 | 0.5 | 0.7 | 0.6 | 0.6 | 0.2 | 0.2 |
| Kdsr | 11.1 | 7.0 | 5.4 | 12.6 | 9.3 | 9.9 | 9.7 | 7.3 | 8.1 | 9.7 | 11.2 | 8.9 |
| Keap1 | 15.0 | 13.6 | 14.0 | 17.0 | 14.0 | 17.5 | 16.4 | 14.5 | 15.7 | 16.8 | 14.8 | 15.5 |
| Khdc1b | 0.0 | 0.0 | 0.0 | 0.0 | 0.0 | 0.0 | 0.0 | 0.1 | 0.0 | 0.0 | 0.1 | 0.0 |
| Khdrbs1 | 14.4 | 15.3 | 17.3 | 13.8 | 16.2 | 14.8 | 16.7 | 17.8 | 15.5 | 17.6 | 17.0 | 15.3 |
| Khdrbs3 | 16.6 | 22.0 | 30.3 | 16.9 | 18.8 | 15.2 | 28.1 | 33.3 | 24.5 | 17.0 | 25.8 | 24.4 |
| Khk | 0.5 | 1.2 | 0.9 | 1.1 | 1.0 | 0.9 | 0.6 | 0.9 | 0.6 | 2.2 | 1.4 | 0.8 |
| Khnyn | 5.2 | 7.4 | 7.2 | 4.4 | 5.7 | 6.5 | 6.0 | 6.1 | 7.1 | 5.9 | 4.7 | 6.2 |
| Khsrp | 8.7 | 9.8 | 10.5 | 8.3 | 10.2 | 9.3 | 11.7 | 11.4 | 11.4 | 11.5 | 10.3 | 8.7 |
| Kidins220 | 33.1 | 28.0 | 27.7 | 33.5 | 29.3 | 30.3 | 30.8 | 26.7 | 27.9 | 33.1 | 36.0 | 34.7 |
| Kif11 | 1.7 | 3.3 | 5.1 | 0.4 | 5.0 | 0.6 | 6.5 | 2.6 | 5.2 | 2.5 | 4.7 | 2.7 |
| Kif13a | 8.5 | 7.7 | 7.5 | 8.3 | 7.7 | 8.3 | 9.2 | 8.1 | 8.0 | 7.9 | 8.7 | 8.4 |
| Kif13b | 10.1 | 11.4 | 11.3 | 10.6 | 9.7 | 9.3 | 11.3 | 10.1 | 10.4 | 12.2 | 9.9 | 11.7 |
| Kif14 | 0.2 | 0.4 | 0.8 | 0.1 | 0.6 | 0.0 | 0.9 | 0.3 | 0.6 | 0.3 | 0.5 | 0.4 |
| Kif15 | 0.6 | 1.5 | 2.2 | 0.3 | 1.9 | 0.3 | 2.4 | 1.0 | 1.6 | 0.7 | 1.1 | 1.0 |
| Kif16b | 6.6 | 6.2 | 6.6 | 7.8 | 6.4 | 6.9 | 5.8 | 6.0 | 5.7 | 5.7 | 6.6 | 7.1 |
| Kif17 | 0.1 | 0.0 | 0.1 | 0.0 | 0.0 | 0.1 | 0.0 | 0.0 | 0.1 | 0.2 | 0.1 | 0.0 |
| Kif18a | 1.0 | 1.6 | 2.6 | 0.7 | 1.9 | 0.9 | 2.5 | 1.1 | 1.7 | 1.1 | 1.9 | 1.4 |
| Kif18b | 0.7 | 2.0 | 3.3 | 0.5 | 2.9 | 0.3 | 4.6 | 1.3 | 2.9 | 1.1 | 2.2 | 1.3 |
| Kif19a | 0.1 | 0.0 | 0.1 | 0.2 | 0.1 | 0.0 | 0.0 | 0.0 | 0.0 | 0.1 | 0.1 | 0.0 |
| Kif1a | 38.5 | 34.3 | 22.2 | 39.1 | 25.4 | 44.1 | 27.6 | 17.6 | 17.6 | 16.8 | 22.8 | 21.1 |
| Kif1b | 27.7 | 28.9 | 26.0 | 29.8 | 25.4 | 30.0 | 27.1 | 24.9 | 23.5 | 23.9 | 29.0 | 26.3 |
| Kif1c | 72.1 | 58.2 | 50.2 | 82.0 | 52.4 | 88.5 | 66.8 | 50.3 | 56.1 | 54.9 | 65.2 | 57.8 |
| Kif20a | 4.4 | 8.0 | 13.4 | 1.9 | 11.1 | 1.7 | 15.2 | 6.0 | 11.6 | 5.8 | 10.6 | 7.0 |
| Kif20b | 1.2 | 2.8 | 5.1 | 0.7 | 3.9 | 0.5 | 5.0 | 1.8 | 3.7 | 1.7 | 3.2 | 2.3 |
| Kif21a | 3.8 | 3.0 | 3.9 | 3.5 | 2.6 | 3.4 | 2.6 | 3.3 | 2.6 | 4.3 | 3.3 | 4.0 |
| Kif21b | 1.0 | 1.4 | 1.2 | 1.5 | 1.8 | 1.4 | 1.1 | 1.5 | 1.1 | 0.6 | 0.8 | 0.6 |
| Kif22 | 1.6 | 3.6 | 5.2 | 0.9 | 4.2 | 0.6 | 6.7 | 3.0 | 5.3 | 2.3 | 4.5 | 2.3 |
| Kif23 | 1.3 | 2.1 | 3.8 | 0.7 | 3.8 | 0.3 | 4.4 | 1.9 | 2.8 | 1.5 | 3.0 | 2.0 |
| Kif24 | 0.3 | 0.4 | 0.5 | 0.3 | 0.4 | 0.2 | 0.5 | 0.4 | 0.4 | 0.2 | 0.2 | 0.3 |
| Kif26a | 0.2 | 0.3 | 0.3 | 0.1 | 0.2 | 0.2 | 0.3 | 0.2 | 0.2 | 0.1 | 0.1 | 0.1 |
| Kif26b | 7.3 | 12.6 | 18.7 | 6.1 | 14.1 | 4.8 | 17.7 | 22.9 | 23.9 | 16.9 | 15.1 | 18.8 |
| Kif27 | 0.1 | 0.1 | 0.2 | 0.0 | 0.1 | 0.2 | 0.1 | 0.1 | 0.1 | 0.1 | 0.1 | 0.0 |
| Kif2a | 5.6 | 5.3 | 4.7 | 6.8 | 4.6 | 6.9 | 5.3 | 4.3 | 4.5 | 4.7 | 5.2 | 4.6 |
| Kif2c | 1.7 | 3.7 | 6.1 | 0.5 | 4.8 | 0.5 | 7.5 | 2.3 | 5.1 | 2.4 | 4.2 | 3.1 |
| Kif3a | 7.9 | 7.7 | 8.1 | 9.0 | 6.5 | 9.1 | 7.1 | 6.5 | 6.2 | 6.8 | 8.3 | 8.5 |
| Kif3b | 14.9 | 12.6 | 11.1 | 15.7 | 13.0 | 15.1 | 11.9 | 10.7 | 10.6 | 10.9 | 12.3 | 13.1 |
| Kif3c | 5.5 | 5.5 | 5.0 | 6.6 | 5.2 | 7.3 | 5.9 | 4.0 | 5.1 | 4.6 | 5.4 | 5.1 |
| Kif4 | 1.4 | 2.6 | 4.0 | 0.6 | 3.8 | 0.5 | 5.0 | 1.9 | 3.7 | 2.0 | 3.7 | 2.3 |
| Kif5a | 0.9 | 0.8 | 0.5 | 0.8 | 0.6 | 0.9 | 0.6 | 0.5 | 0.4 | 0.6 | 0.5 | 0.5 |
| Kif5b | 83.4 | 66.2 | 53.4 | 94.0 | 65.0 | 81.6 | 65.8 | 56.1 | 72.5 | 62.0 | 85.1 | 79.2 |
| Kif5c | 1.8 | 1.6 | 1.6 | 1.9 | 1.5 | 1.4 | 1.7 | 1.0 | 0.9 | 1.2 | 1.1 | 1.1 |

| | | | | | | | | | | | | |
|---|---|---|---|---|---|---|---|---|---|---|---|---|
| Kif6 | 0.1 | 0.1 | 0.1 | 0.2 | 0.1 | 0.2 | 0.0 | 0.1 | 0.0 | 0.1 | 0.2 | 0.1 |
| Kif7 | 3.3 | 4.9 | 4.4 | 3.0 | 2.5 | 3.1 | 3.5 | 4.7 | 3.7 | 3.4 | 3.4 | 3.4 |
| Kif9 | 1.6 | 1.3 | 1.4 | 1.6 | 1.1 | 1.8 | 1.1 | 1.1 | 0.8 | 1.1 | 1.2 | 1.2 |
| Kifap3 | 14.4 | 14.2 | 14.3 | 14.3 | 12.9 | 16.3 | 13.3 | 12.7 | 14.4 | 14.1 | 15.9 | 17.0 |
| Kifc1 | 3.7 | 5.1 | 6.3 | 2.0 | 6.3 | 2.0 | 7.1 | 2.6 | 6.0 | 4.4 | 5.1 | 3.7 |
| Kifc2 | 0.7 | 0.8 | 1.0 | 0.8 | 0.5 | 0.8 | 0.6 | 0.8 | 1.0 | 0.7 | 0.3 | 0.6 |
| Kifc3 | 14.0 | 11.3 | 9.2 | 12.8 | 10.6 | 10.9 | 13.5 | 10.4 | 12.6 | 16.4 | 14.0 | 11.6 |
| Kifc5b | 1.7 | 2.9 | 3.5 | 0.9 | 3.6 | 0.8 | 3.7 | 1.4 | 2.8 | 1.8 | 2.7 | 1.8 |
| Kin | 7.8 | 8.8 | 9.8 | 8.6 | 6.9 | 7.3 | 8.7 | 8.0 | 7.4 | 9.7 | 10.5 | 10.4 |
| Kirrel | 16.8 | 25.2 | 28.4 | 16.4 | 19.5 | 19.3 | 32.6 | 28.8 | 33.8 | 21.5 | 22.4 | 21.9 |
| Kirrel3 | 0.1 | 0.0 | 0.1 | 0.1 | 0.1 | 0.1 | 0.1 | 0.1 | 0.1 | 0.1 | 0.1 | 0.1 |
| Kiss1r | 0.5 | 0.5 | 0.5 | 0.4 | 0.3 | 0.5 | 0.5 | 0.4 | 0.5 | 0.4 | 0.4 | 0.4 |
| Kit | 0.3 | 0.2 | 0.3 | 0.2 | 0.1 | 0.1 | 0.2 | 0.6 | 0.4 | 0.1 | 0.1 | 0.0 |
| Kitl | 20.1 | 18.3 | 10.3 | 30.6 | 14.3 | 33.7 | 9.7 | 7.9 | 7.8 | 8.6 | 8.0 | 8.1 |
| Klb | 0.0 | 0.0 | 0.0 | 0.1 | 0.0 | 0.0 | 0.1 | 0.0 | 0.0 | 0.0 | 0.0 | 0.0 |
| Klc1 | 73.2 | 62.6 | 60.2 | 76.9 | 56.4 | 72.2 | 77.2 | 61.9 | 61.8 | 57.3 | 72.1 | 66.4 |
| Klc2 | 5.1 | 4.6 | 4.7 | 6.4 | 4.7 | 6.1 | 5.4 | 4.6 | 4.9 | 4.8 | 5.9 | 4.9 |
| Klc3 | 2.9 | 2.3 | 2.3 | 1.5 | 1.6 | 3.2 | 0.8 | 1.0 | 1.4 | 4.3 | 2.6 | 2.0 |
| Klc4 | 7.4 | 7.5 | 6.6 | 10.0 | 7.8 | 7.3 | 6.6 | 7.2 | 7.1 | 8.4 | 7.2 | 6.6 |
| Klf1 | 0.1 | 0.2 | 0.1 | 0.2 | 0.1 | 0.1 | 0.0 | 0.1 | 0.0 | 0.1 | 0.0 | 0.0 |
| Klf10 | 2.5 | 2.1 | 2.7 | 2.4 | 4.7 | 1.2 | 3.9 | 5.9 | 4.4 | 5.0 | 4.1 | 3.6 |
| Klf11 | 1.8 | 2.2 | 2.1 | 2.5 | 2.3 | 2.6 | 2.2 | 2.3 | 2.0 | 2.6 | 1.9 | 1.9 |
| Klf12 | 0.7 | 0.9 | 1.1 | 0.4 | 0.5 | 0.8 | 0.7 | 0.9 | 0.8 | 0.4 | 0.7 | 0.4 |
| Klf13 | 4.4 | 4.9 | 4.5 | 4.2 | 5.0 | 4.1 | 4.9 | 6.5 | 5.1 | 3.4 | 4.1 | 4.1 |
| Klf15 | 5.4 | 4.8 | 5.0 | 3.9 | 2.2 | 7.0 | 2.7 | 3.3 | 3.8 | 2.6 | 2.4 | 3.2 |
| Klf16 | 4.1 | 4.1 | 3.7 | 3.8 | 3.8 | 3.3 | 4.8 | 4.5 | 5.3 | 3.5 | 4.0 | 3.6 |
| Klf17 | 0.0 | 0.0 | 0.0 | 0.0 | 0.0 | 0.0 | 0.0 | 0.0 | 0.0 | 0.0 | 0.0 | 0.0 |
| Klf2 | 0.4 | 0.6 | 0.2 | 1.2 | 0.9 | 1.0 | 1.8 | 2.3 | 2.3 | 1.9 | 1.3 | 1.0 |
| Klf3 | 18.1 | 20.9 | 24.7 | 19.9 | 16.4 | 20.2 | 27.0 | 29.6 | 24.5 | 19.3 | 25.8 | 23.4 |
| Klf4 | 21.5 | 24.0 | 22.4 | 15.8 | 16.3 | 19.5 | 28.9 | 35.8 | 27.6 | 17.1 | 29.9 | 22.3 |
| Klf5 | 0.6 | 0.3 | 0.3 | 0.3 | 0.3 | 0.4 | 0.5 | 0.5 | 0.6 | 0.4 | 0.7 | 0.3 |
| Klf6 | 31.4 | 33.4 | 32.2 | 36.9 | 39.1 | 32.9 | 44.6 | 35.3 | 47.2 | 33.7 | 43.2 | 38.5 |
| Klf7 | 2.7 | 3.2 | 3.8 | 3.6 | 3.3 | 3.7 | 3.6 | 4.2 | 3.3 | 2.7 | 2.5 | 2.5 |
| Klf8 | 0.0 | 0.0 | 0.1 | 0.1 | 0.2 | 0.1 | 0.0 | 0.1 | 0.0 | 0.1 | 0.0 | 0.0 |
| Klf9 | 8.5 | 8.2 | 9.6 | 8.2 | 6.3 | 10.1 | 8.7 | 10.8 | 9.0 | 12.1 | 10.0 | 12.1 |
| Klhdc1 | 2.8 | 3.3 | 3.5 | 3.0 | 2.1 | 3.7 | 2.3 | 2.5 | 2.0 | 2.7 | 2.8 | 3.6 |
| Klhdc10 | 5.8 | 5.8 | 5.9 | 6.1 | 5.6 | 6.2 | 5.4 | 5.2 | 5.2 | 5.3 | 5.2 | 5.4 |
| Klhdc2 | 34.9 | 35.7 | 30.5 | 31.9 | 26.8 | 40.8 | 29.2 | 27.0 | 30.4 | 33.9 | 32.1 | 31.0 |
| Klhdc3 | 9.2 | 11.4 | 11.2 | 10.5 | 12.4 | 10.6 | 12.3 | 11.5 | 12.0 | 13.3 | 10.6 | 9.5 |
| Klhdc4 | 11.1 | 10.6 | 10.0 | 13.0 | 11.4 | 11.0 | 11.9 | 11.0 | 10.2 | 12.3 | 14.0 | 12.2 |
| Klhdc5 | 6.9 | 6.9 | 6.9 | 6.1 | 6.3 | 6.2 | 6.0 | 6.7 | 6.4 | 6.9 | 7.1 | 6.8 |
| Klhdc7a | 2.2 | 1.6 | 1.6 | 1.1 | 1.4 | 1.0 | 0.9 | 0.7 | 0.7 | 1.4 | 1.1 | 1.3 |
| Klhdc8a | 3.1 | 2.3 | 1.0 | 4.7 | 2.6 | 3.3 | 2.4 | 1.4 | 2.3 | 3.7 | 4.4 | 1.9 |
| Klhdc8b | 2.4 | 2.6 | 2.8 | 1.7 | 1.7 | 1.9 | 1.6 | 1.6 | 2.9 | 1.8 | 1.4 | 1.7 |
| Klhdc9 | 1.1 | 0.6 | 0.9 | 0.7 | 1.0 | 1.0 | 0.9 | 0.9 | 0.6 | 1.4 | 0.9 | 0.9 |
| Klhl10 | 0.3 | 0.4 | 0.4 | 0.1 | 0.4 | 0.4 | 0.3 | 0.3 | 0.2 | 0.2 | 0.2 | 0.2 |
| Klhl11 | 10.2 | 12.2 | 10.4 | 11.0 | 9.4 | 10.3 | 9.6 | 9.5 | 9.7 | 11.5 | 10.8 | 11.1 |
| Klhl12 | 8.6 | 8.0 | 7.2 | 6.1 | 6.3 | 9.2 | 6.0 | 5.8 | 6.1 | 6.5 | 5.8 | 6.6 |
| Klhl13 | 27.4 | 22.5 | 13.6 | 18.7 | 16.9 | 11.8 | 27.3 | 16.0 | 43.4 | 28.7 | 40.0 | 44.1 |
| Klhl15 | 1.8 | 2.2 | 1.7 | 1.5 | 2.1 | 1.4 | 2.2 | 1.9 | 1.8 | 2.0 | 1.9 | 2.1 |
| Klhl17 | 3.7 | 4.3 | 3.4 | 4.3 | 3.0 | 6.4 | 3.0 | 2.9 | 3.5 | 3.6 | 3.1 | 3.2 |
| Klhl18 | 3.4 | 3.5 | 3.2 | 3.4 | 3.2 | 3.0 | 3.7 | 3.6 | 3.3 | 3.2 | 3.3 | 3.1 |
| Klhl2 | 10.8 | 9.1 | 8.9 | 12.2 | 9.7 | 8.7 | 11.2 | 8.0 | 8.8 | 11.0 | 12.1 | 11.4 |
| Klhl20 | 7.1 | 7.9 | 8.4 | 8.7 | 7.7 | 8.1 | 8.0 | 7.8 | 7.5 | 7.8 | 8.0 | 8.7 |
| Klhl21 | 9.6 | 9.2 | 7.6 | 8.3 | 9.3 | 11.4 | 9.6 | 7.7 | 9.5 | 11.8 | 8.6 | 8.3 |
| Klhl22 | 25.9 | 23.1 | 18.7 | 24.4 | 19.7 | 25.1 | 19.3 | 19.1 | 19.3 | 24.4 | 21.8 | 19.4 |
| Klhl23 | 1.6 | 1.5 | 1.3 | 1.0 | 0.9 | 2.3 | 1.4 | 1.2 | 1.5 | 1.5 | 1.7 | 1.6 |

| | | | | | | | | | | | | |
|---|---|---|---|---|---|---|---|---|---|---|---|---|
| Klhl24 | 20.5 | 23.4 | 21.6 | 21.3 | 20.8 | 22.0 | 16.3 | 17.2 | 20.0 | 25.6 | 23.4 | 26.5 |
| Klhl25 | 5.8 | 5.0 | 4.5 | 4.9 | 6.3 | 4.9 | 5.7 | 5.3 | 5.7 | 4.4 | 4.6 | 5.5 |
| Klhl26 | 11.9 | 13.0 | 11.5 | 11.4 | 12.2 | 14.6 | 15.8 | 12.3 | 13.9 | 15.0 | 14.7 | 13.9 |
| Klhl28 | 4.7 | 6.4 | 5.7 | 5.5 | 5.4 | 5.9 | 4.8 | 3.9 | 5.9 | 5.5 | 4.8 | 5.6 |
| Klhl29 | 0.5 | 1.1 | 1.5 | 0.5 | 0.9 | 0.6 | 1.2 | 0.7 | 1.0 | 1.0 | 0.7 | 0.9 |
| Klhl3 | 0.0 | 0.0 | 0.2 | 0.1 | 0.0 | 0.1 | 0.1 | 0.1 | 0.1 | 0.1 | 0.0 | 0.0 |
| Klhl30 | 0.3 | 0.3 | 0.1 | 1.0 | 0.2 | 0.6 | 0.2 | 0.1 | 0.3 | 0.3 | 0.1 | 0.1 |
| Klhl31 | 0.0 | 0.0 | 0.0 | 0.0 | 0.0 | 0.0 | 0.0 | 0.0 | 0.0 | 0.0 | 0.0 | 0.0 |
| Klhl32 | 0.1 | 0.0 | 0.1 | 0.1 | 0.0 | 0.2 | 0.0 | 0.0 | 0.0 | 0.0 | 0.0 | 0.0 |
| Klhl33 | 0.1 | 0.0 | 0.2 | 0.3 | 0.2 | 0.1 | 0.2 | 0.2 | 0.2 | 0.2 | 0.2 | 0.1 |
| Klhl35 | 0.1 | 0.1 | 0.0 | 0.1 | 0.0 | 0.0 | 0.1 | 0.0 | 0.1 | 0.1 | 0.1 | 0.1 |
| Klhl36 | 3.6 | 4.2 | 3.9 | 4.3 | 3.0 | 4.2 | 3.4 | 3.9 | 3.6 | 3.2 | 2.3 | 2.9 |
| Klhl38 | 0.0 | 0.0 | 0.0 | 0.0 | 0.2 | 0.1 | 0.1 | 0.1 | 0.1 | 0.2 | 0.1 | 0.1 |
| Klhl4 | 0.1 | 0.0 | 0.1 | 0.0 | 0.0 | 0.0 | 0.1 | 0.1 | 0.1 | 0.0 | 0.0 | 0.0 |
| Klhl41 | 0.2 | 0.1 | 0.0 | 0.1 | 0.1 | 0.2 | 0.1 | 0.0 | 0.2 | 0.2 | 0.1 | 0.1 |
| Klhl5 | 33.8 | 33.7 | 33.1 | 34.0 | 32.6 | 32.0 | 38.7 | 25.5 | 27.9 | 29.5 | 34.7 | 33.6 |
| Klhl6 | 9.7 | 11.2 | 10.8 | 11.7 | 14.5 | 10.0 | 10.1 | 4.5 | 6.7 | 9.9 | 7.6 | 9.0 |
| Klhl7 | 9.9 | 8.3 | 8.0 | 9.0 | 6.8 | 9.7 | 7.3 | 7.4 | 7.7 | 9.2 | 9.4 | 10.7 |
| Klhl8 | 3.7 | 3.6 | 3.4 | 3.5 | 2.5 | 4.4 | 2.7 | 2.2 | 2.6 | 2.6 | 3.3 | 2.7 |
| Klhl9 | 33.7 | 34.4 | 37.5 | 32.8 | 35.0 | 36.9 | 33.9 | 39.9 | 38.5 | 45.0 | 43.6 | 44.6 |
| Klk10 | 0.1 | 0.1 | 0.0 | 0.2 | 0.1 | 0.0 | 0.0 | 0.0 | 0.0 | 0.1 | 0.0 | 0.1 |
| Klk8 | 2.9 | 0.5 | 0.2 | 2.8 | 0.3 | 3.1 | 0.5 | 0.1 | 0.3 | 0.3 | 0.5 | 0.6 |
| Klkb1 | 0.1 | 0.0 | 0.1 | 0.0 | 0.1 | 0.1 | 0.0 | 0.0 | 0.0 | 0.0 | 0.0 | 0.0 |
| Klra18 | 0.0 | 0.1 | 0.0 | 0.0 | 0.0 | 0.1 | 0.0 | 0.0 | 0.0 | 0.0 | 0.1 | 0.0 |
| Klra2 | 2.7 | 1.9 | 1.1 | 2.8 | 4.5 | 1.6 | 2.0 | 0.5 | 0.8 | 0.8 | 2.0 | 1.5 |
| Klra22 | 0.0 | 0.0 | 0.0 | 0.0 | 0.0 | 0.0 | 0.0 | 0.0 | 0.0 | 0.0 | 0.0 | 0.0 |
| Klra33 | 0.2 | 0.0 | 0.0 | 0.0 | 0.0 | 0.0 | 0.0 | 0.0 | 0.0 | 0.0 | 0.0 | 0.1 |
| Klra4 | 0.0 | 0.0 | 0.0 | 0.0 | 0.0 | 0.0 | 0.0 | 0.0 | 0.0 | 0.2 | 0.0 | 0.0 |
| Klrb1c | 0.0 | 0.0 | 0.0 | 0.0 | 0.0 | 0.0 | 0.0 | 0.0 | 0.0 | 0.0 | 0.0 | 0.0 |
| Klrg2 | 0.7 | 0.5 | 0.3 | 0.4 | 0.3 | 0.3 | 0.5 | 0.9 | 0.7 | 0.7 | 0.6 | 0.6 |
| Kndc1 | 0.0 | 0.0 | 0.0 | 0.0 | 0.0 | 0.0 | 0.0 | 0.0 | 0.0 | 0.0 | 0.0 | 0.0 |
| Kng1 | 0.0 | 0.0 | 0.0 | 0.0 | 0.0 | 0.0 | 0.0 | 0.0 | 0.0 | 0.1 | 0.1 | 0.1 |
| Kng2 | 0.0 | 0.0 | 0.1 | 0.1 | 0.1 | 0.1 | 0.0 | 0.0 | 0.1 | 0.5 | 0.3 | 0.1 |
| Knstrn | 1.6 | 2.7 | 4.8 | 0.8 | 4.1 | 0.7 | 6.2 | 2.3 | 4.3 | 2.0 | 3.9 | 2.6 |
| Kntc1 | 1.2 | 2.0 | 3.2 | 0.5 | 2.9 | 0.3 | 3.1 | 1.3 | 2.8 | 1.2 | 1.8 | 1.4 |
| Kpna1 | 17.2 | 17.8 | 15.0 | 18.5 | 16.8 | 17.7 | 15.1 | 13.4 | 15.0 | 16.2 | 17.0 | 16.2 |
| Kpna2 | 37.1 | 40.8 | 49.4 | 30.2 | 47.0 | 27.3 | 54.4 | 36.3 | 43.0 | 37.1 | 48.1 | 34.9 |
| Kpna3 | 10.6 | 10.9 | 11.1 | 12.7 | 12.8 | 11.4 | 11.5 | 10.2 | 10.0 | 11.0 | 12.8 | 12.2 |
| Kpna4 | 34.3 | 34.4 | 31.0 | 41.9 | 36.2 | 36.3 | 35.1 | 29.1 | 31.6 | 30.8 | 40.7 | 36.6 |
| Kpna6 | 20.8 | 18.0 | 16.3 | 24.7 | 18.3 | 22.0 | 18.5 | 17.1 | 16.7 | 18.8 | 19.2 | 17.5 |
| Kpna7 | 0.0 | 0.0 | 0.0 | 0.0 | 0.0 | 0.0 | 0.0 | 0.0 | 0.0 | 0.0 | 0.0 | 0.0 |
| Kpnb1 | 54.3 | 48.6 | 48.4 | 50.5 | 53.9 | 47.5 | 63.2 | 58.0 | 59.3 | 51.4 | 62.2 | 52.1 |
| Kptn | 3.4 | 2.9 | 3.3 | 2.8 | 2.4 | 2.3 | 3.8 | 4.6 | 4.1 | 3.7 | 3.4 | 3.4 |
| Kras | 6.1 | 6.1 | 6.6 | 9.1 | 7.4 | 8.6 | 9.7 | 8.1 | 8.2 | 6.5 | 9.7 | 9.1 |
| Krba1 | 1.5 | 1.2 | 1.2 | 1.0 | 1.0 | 1.4 | 1.2 | 1.2 | 1.2 | 1.6 | 1.2 | 1.1 |
| Krcc1 | 70.8 | 57.4 | 62.6 | 82.7 | 49.5 | 74.6 | 64.5 | 54.7 | 45.1 | 46.7 | 80.0 | 72.1 |
| Kremen1 | 7.7 | 8.8 | 8.6 | 7.9 | 9.1 | 7.9 | 8.8 | 8.9 | 10.0 | 8.4 | 8.2 | 8.7 |
| Kri1 | 9.5 | 8.2 | 10.3 | 11.3 | 10.1 | 10.4 | 11.6 | 10.6 | 9.6 | 8.1 | 10.8 | 10.4 |
| Krit1 | 10.2 | 11.9 | 11.6 | 10.3 | 9.3 | 10.4 | 9.5 | 9.6 | 11.0 | 10.2 | 11.5 | 11.4 |
| Krr1 | 5.2 | 5.0 | 4.2 | 5.9 | 5.2 | 6.3 | 4.6 | 4.5 | 4.1 | 4.8 | 5.3 | 5.7 |
| Krt10 | 0.2 | 0.4 | 0.3 | 0.4 | 0.3 | 0.3 | 0.3 | 0.4 | 0.3 | 0.1 | 0.3 | 0.3 |
| Krt14 | 0.0 | 0.0 | 0.0 | 0.0 | 0.0 | 0.0 | 0.0 | 0.2 | 0.1 | 1.6 | 1.1 | 0.1 |
| Krt17 | 0.0 | 0.0 | 0.0 | 0.0 | 0.0 | 0.0 | 0.0 | 0.0 | 0.0 | 0.1 | 0.0 | 0.0 |
| Krt18 | 0.4 | 0.0 | 0.0 | 0.0 | 0.0 | 0.2 | 0.0 | 0.5 | 0.2 | 3.0 | 0.5 | 1.0 |
| Krt19 | 0.6 | 0.1 | 0.2 | 0.1 | 0.0 | 0.1 | 0.2 | 0.1 | 0.2 | 0.9 | 0.5 | 0.2 |
| Krt20 | 0.0 | 0.0 | 0.0 | 0.1 | 0.0 | 0.1 | 0.1 | 0.0 | 0.1 | 0.0 | 0.0 | 0.0 |
| Krt222 | 0.1 | 0.1 | 0.1 | 0.0 | 0.1 | 0.1 | 0.1 | 0.1 | 0.1 | 0.0 | 0.1 | 0.0 |

| | | | | | | | | | | | | |
|---|---|---|---|---|---|---|---|---|---|---|---|---|
| Krt23 | 0.0 | 0.0 | 0.0 | 0.0 | 0.0 | 0.0 | 0.0 | 0.0 | 0.0 | 0.0 | 0.0 | 0.0 |
| Krt24 | 0.2 | 0.0 | 0.0 | 0.3 | 0.1 | 0.1 | 0.1 | 0.0 | 0.0 | 0.0 | 0.0 | 0.0 |
| Krt5 | 0.0 | 0.0 | 0.0 | 0.0 | 0.0 | 0.0 | 0.0 | 0.0 | 0.0 | 0.0 | 0.0 | 0.0 |
| Krt7 | 0.2 | 0.2 | 0.3 | 0.2 | 0.3 | 0.2 | 0.2 | 0.5 | 0.2 | 4.2 | 1.9 | 0.6 |
| Krt78 | 0.0 | 0.0 | 0.0 | 0.0 | 0.0 | 0.0 | 0.0 | 0.0 | 0.0 | 0.0 | 0.0 | 0.1 |
| Krt8 | 0.0 | 0.0 | 0.0 | 0.0 | 0.0 | 0.0 | 0.0 | 0.4 | 0.2 | 1.7 | 0.8 | 0.6 |
| Krt80 | 0.7 | 0.1 | 0.0 | 0.8 | 0.3 | 0.3 | 0.1 | 0.2 | 0.1 | 0.1 | 0.1 | 0.1 |
| Krtcap2 | 130.9 | 122.7 | 113.2 | 139.4 | 113.2 | 150.1 | 128.5 | 151.8 | 142.4 | 113.9 | 122.8 | 121.6 |
| Krtcap3 | 0.1 | 0.1 | 0.2 | 0.5 | 0.3 | 0.1 | 0.9 | 0.3 | 0.2 | 0.2 | 0.4 | 0.5 |
| Ksr1 | 2.8 | 4.6 | 5.4 | 3.5 | 4.4 | 3.2 | 3.4 | 3.7 | 3.1 | 2.7 | 2.8 | 2.7 |
| Ksr2 | 0.0 | 0.0 | 0.0 | 0.0 | 0.0 | 0.0 | 0.0 | 0.0 | 0.0 | 0.0 | 0.0 | 0.0 |
| Kti12 | 5.9 | 6.8 | 5.2 | 6.6 | 7.3 | 5.7 | 5.9 | 6.0 | 6.0 | 8.6 | 6.3 | 6.0 |
| Ktn1 | 29.3 | 25.7 | 25.3 | 29.2 | 23.1 | 30.6 | 24.5 | 21.2 | 21.7 | 25.2 | 28.4 | 26.4 |
| Kxd1 | 18.8 | 18.6 | 16.0 | 18.2 | 14.7 | 21.0 | 16.4 | 16.9 | 15.7 | 16.9 | 17.2 | 18.8 |
| Kynu | 0.0 | 0.1 | 0.0 | 0.0 | 0.0 | 0.0 | 0.0 | 0.0 | 0.0 | 0.0 | 0.0 | 0.0 |
| L1cam | 0.4 | 0.1 | 0.1 | 2.0 | 4.0 | 0.3 | 0.9 | 0.2 | 0.2 | 0.3 | 0.3 | 0.3 |
| L2hgdh | 2.9 | 3.6 | 3.7 | 2.1 | 3.0 | 2.5 | 3.2 | 3.0 | 3.5 | 3.8 | 3.7 | 3.1 |
| L3hypdh | 7.3 | 8.1 | 6.9 | 6.6 | 6.6 | 7.8 | 7.1 | 6.6 | 5.1 | 10.0 | 8.3 | 7.9 |
| L3mbtl1 | 0.0 | 0.0 | 0.0 | 0.0 | 0.0 | 0.0 | 0.0 | 0.0 | 0.0 | 0.0 | 0.0 | 0.0 |
| L3mbtl2 | 2.8 | 3.0 | 2.9 | 3.3 | 3.6 | 3.7 | 3.3 | 3.2 | 3.3 | 2.9 | 2.8 | 2.6 |
| L3mbtl3 | 8.5 | 8.4 | 7.8 | 7.4 | 6.7 | 6.0 | 5.3 | 5.9 | 5.8 | 7.5 | 6.7 | 7.1 |
| l7Rn6 | 20.0 | 21.8 | 18.1 | 21.9 | 18.5 | 20.4 | 18.1 | 15.7 | 20.3 | 21.2 | 21.4 | 20.0 |
| Lacc1 | 5.0 | 4.1 | 3.7 | 5.8 | 8.1 | 5.0 | 4.2 | 3.3 | 3.3 | 4.7 | 3.9 | 4.4 |
| Lace1 | 5.2 | 4.3 | 4.0 | 4.5 | 3.9 | 5.0 | 3.9 | 3.7 | 3.9 | 4.3 | 3.7 | 4.2 |
| Lactb | 4.5 | 4.0 | 5.8 | 5.9 | 7.4 | 4.8 | 4.6 | 4.7 | 5.7 | 5.7 | 5.6 | 6.1 |
| Lactb2 | 16.3 | 18.0 | 16.2 | 21.0 | 15.7 | 22.5 | 17.3 | 17.4 | 14.5 | 12.8 | 19.7 | 17.0 |
| Lag3 | 0.1 | 0.1 | 0.2 | 0.0 | 0.1 | 0.0 | 0.1 | 0.1 | 0.1 | 0.0 | 0.1 | 0.1 |
| Lage3 | 28.7 | 25.8 | 21.4 | 30.5 | 20.6 | 29.0 | 22.3 | 23.1 | 21.2 | 22.6 | 19.7 | 18.5 |
| Lair1 | 0.4 | 0.0 | 0.0 | 5.0 | 7.0 | 0.0 | 1.2 | 0.2 | 0.1 | 0.6 | 0.2 | 0.4 |
| Lama1 | 0.6 | 0.5 | 0.7 | 0.4 | 1.7 | 0.3 | 1.9 | 1.7 | 2.2 | 2.5 | 1.6 | 1.7 |
| Lama2 | 32.0 | 33.4 | 32.5 | 27.4 | 25.3 | 30.2 | 22.7 | 40.4 | 24.7 | 8.8 | 16.0 | 12.4 |
| Lama3 | 0.0 | 0.0 | 0.0 | 0.0 | 0.1 | 0.1 | 0.0 | 0.0 | 0.0 | 0.0 | 0.0 | 0.0 |
| Lama4 | 62.4 | 93.0 | 136.9 | 33.7 | 93.3 | 46.3 | 96.1 | 131.0 | 103.0 | 66.4 | 75.9 | 85.7 |
| Lama5 | 0.5 | 0.5 | 0.6 | 0.1 | 0.3 | 0.2 | 0.3 | 1.0 | 1.3 | 2.6 | 1.2 | 1.1 |
| Lamb1 | 55.4 | 47.4 | 60.6 | 56.5 | 93.9 | 66.6 | 97.9 | 66.7 | 76.9 | 88.1 | 76.1 | 63.4 |
| Lamb2 | 69.9 | 83.3 | 98.9 | 49.6 | 59.9 | 55.0 | 72.7 | 104.7 | 101.8 | 48.3 | 55.0 | 62.8 |
| Lamb3 | 1.1 | 0.8 | 0.8 | 0.6 | 0.8 | 0.7 | 0.9 | 1.1 | 0.8 | 0.3 | 0.3 | 0.3 |
| Lamc1 | 91.0 | 104.0 | 135.4 | 60.5 | 88.9 | 65.4 | 91.5 | 155.8 | 106.8 | 61.8 | 79.7 | 77.9 |
| Lamc2 | 3.0 | 2.7 | 2.4 | 2.5 | 2.1 | 1.3 | 2.9 | 4.1 | 2.7 | 1.8 | 3.5 | 2.4 |
| Lamc3 | 0.0 | 0.0 | 0.1 | 0.0 | 0.0 | 0.0 | 0.0 | 0.1 | 0.0 | 0.1 | 0.1 | 0.1 |
| Lamp1 | 503.8 | 420.8 | 405.3 | 563.0 | 484.9 | 538.0 | 386.4 | 413.2 | 378.1 | 389.2 | 427.7 | 474.2 |
| Lamp2 | 170.2 | 164.0 | 172.2 | 205.3 | 192.8 | 199.9 | 125.5 | 175.4 | 145.7 | 164.1 | 157.1 | 183.2 |
| Lamp3 | 0.0 | 0.0 | 0.0 | 0.0 | 0.0 | 0.0 | 0.0 | 0.0 | 0.0 | 0.0 | 0.0 | 0.0 |
| Lamtor1 | 58.2 | 51.7 | 45.1 | 63.8 | 56.8 | 56.3 | 54.5 | 56.0 | 51.8 | 57.1 | 53.0 | 56.5 |
| Lamtor2 | 55.0 | 49.1 | 48.0 | 63.5 | 57.1 | 67.7 | 50.9 | 52.4 | 51.2 | 53.9 | 50.2 | 54.7 |
| Lamtor3 | 37.3 | 37.9 | 35.0 | 42.1 | 33.2 | 40.9 | 32.8 | 29.0 | 32.8 | 34.9 | 35.8 | 38.6 |
| Lamtor4 | 25.4 | 20.6 | 19.2 | 23.7 | 24.0 | 23.8 | 17.6 | 25.5 | 21.2 | 26.2 | 18.8 | 19.8 |
| Lamtor5 | 39.5 | 38.7 | 35.8 | 41.9 | 38.8 | 43.4 | 36.0 | 35.2 | 40.9 | 46.9 | 39.7 | 40.7 |
| Lancl1 | 8.2 | 8.0 | 6.3 | 7.1 | 5.8 | 8.0 | 5.7 | 5.2 | 6.3 | 7.2 | 5.4 | 5.9 |
| Lancl2 | 2.5 | 2.8 | 2.8 | 2.3 | 2.3 | 2.2 | 2.4 | 2.8 | 2.6 | 2.3 | 2.4 | 1.9 |
| Lancl3 | 0.3 | 0.1 | 0.1 | 0.6 | 0.2 | 0.5 | 0.1 | 0.0 | 0.1 | 0.1 | 0.0 | 0.1 |
| Lap3 | 28.7 | 25.1 | 23.0 | 27.4 | 22.3 | 25.8 | 25.9 | 24.4 | 25.4 | 28.3 | 30.6 | 27.1 |
| Laptm4a | 249.4 | 218.9 | 203.7 | 246.8 | 228.3 | 228.8 | 210.2 | 233.1 | 242.5 | 385.1 | 296.7 | 297.9 |
| Laptm4b | 21.6 | 21.0 | 20.4 | 17.8 | 20.8 | 20.0 | 15.7 | 20.2 | 17.4 | 25.0 | 17.0 | 20.6 |
| Laptm5 | 3.1 | 0.2 | 0.2 | 28.9 | 52.6 | 0.1 | 5.3 | 0.7 | 0.8 | 2.6 | 0.7 | 1.9 |
| Large | 26.7 | 24.6 | 21.8 | 24.5 | 22.4 | 25.6 | 27.6 | 20.0 | 32.2 | 24.7 | 25.6 | 30.6 |
| Larp1 | 14.8 | 16.3 | 15.6 | 16.3 | 14.2 | 14.7 | 18.3 | 15.1 | 15.1 | 13.1 | 16.4 | 14.7 |

| | | | | | | | | | | | | |
|---|---|---|---|---|---|---|---|---|---|---|---|---|
| Larp1b | 8.0 | 7.1 | 7.4 | 7.5 | 6.3 | 5.9 | 7.0 | 4.9 | 7.3 | 6.7 | 10.6 | 9.9 |
| Larp4 | 9.9 | 11.7 | 13.1 | 10.5 | 11.6 | 9.9 | 11.9 | 12.7 | 11.5 | 12.7 | 15.8 | 15.4 |
| Larp4b | 17.8 | 17.3 | 16.7 | 17.0 | 17.1 | 17.0 | 16.3 | 14.7 | 15.4 | 15.4 | 17.2 | 16.7 |
| Larp6 | 3.0 | 3.9 | 4.3 | 3.0 | 2.6 | 3.2 | 2.8 | 2.9 | 2.5 | 2.5 | 2.1 | 2.6 |
| Larp7 | 11.6 | 10.3 | 14.4 | 11.8 | 12.4 | 7.9 | 14.9 | 11.4 | 10.5 | 11.2 | 14.3 | 12.7 |
| Lars | 48.6 | 34.3 | 27.6 | 46.1 | 33.0 | 41.4 | 35.7 | 37.6 | 33.5 | 32.4 | 40.0 | 31.1 |
| Lars2 | 0.5 | 0.6 | 0.6 | 0.8 | 0.8 | 0.6 | 0.5 | 0.8 | 0.7 | 0.7 | 0.7 | 0.5 |
| Las1l | 15.6 | 12.6 | 13.4 | 19.0 | 13.8 | 16.0 | 15.8 | 11.5 | 10.4 | 14.5 | 14.9 | 16.1 |
| Lasp1 | 49.6 | 54.3 | 46.8 | 57.3 | 65.3 | 48.2 | 70.9 | 65.9 | 64.4 | 43.6 | 57.6 | 45.9 |
| Lat | 0.0 | 0.1 | 0.0 | 0.0 | 0.1 | 0.1 | 0.0 | 0.1 | 0.1 | 0.1 | 0.0 | 0.0 |
| Lat2 | 1.4 | 0.5 | 0.4 | 15.0 | 23.0 | 0.6 | 3.2 | 0.6 | 0.8 | 1.6 | 0.8 | 1.5 |
| Lats1 | 9.5 | 9.8 | 10.1 | 10.7 | 9.0 | 10.0 | 10.3 | 10.6 | 9.7 | 9.3 | 11.2 | 9.9 |
| Lats2 | 48.2 | 42.8 | 34.7 | 54.4 | 34.2 | 49.2 | 42.6 | 30.3 | 33.8 | 45.3 | 44.8 | 34.7 |
| Lax1 | 0.0 | 0.0 | 0.0 | 0.0 | 0.0 | 0.0 | 0.0 | 0.0 | 0.1 | 0.0 | 0.0 | 0.0 |
| Layn | 37.0 | 35.6 | 37.5 | 56.9 | 45.5 | 50.6 | 41.5 | 33.0 | 29.8 | 29.1 | 32.2 | 34.7 |
| Lbh | 113.0 | 120.2 | 97.2 | 112.7 | 95.5 | 118.2 | 124.0 | 106.6 | 167.2 | 95.2 | 113.6 | 125.9 |
| Lbp | 5.6 | 5.3 | 15.2 | 4.0 | 10.1 | 2.1 | 6.8 | 11.2 | 5.7 | 4.3 | 3.7 | 6.1 |
| Lbr | 6.1 | 6.3 | 7.1 | 4.3 | 6.6 | 4.3 | 9.2 | 7.2 | 7.4 | 5.6 | 8.2 | 5.5 |
| Lbx1 | 0.2 | 0.1 | 0.0 | 0.2 | 0.1 | 0.2 | 0.0 | 0.0 | 0.0 | 0.0 | 0.0 | 0.0 |
| Lbx2 | 0.4 | 0.1 | 0.2 | 0.3 | 0.2 | 1.1 | 0.1 | 0.1 | 0.1 | 0.2 | 0.1 | 0.1 |
| Lca5 | 1.8 | 2.0 | 3.3 | 1.5 | 2.4 | 1.8 | 2.9 | 2.5 | 1.9 | 1.2 | 2.2 | 2.1 |
| Lca5l | 0.2 | 0.2 | 0.4 | 0.2 | 0.3 | 0.2 | 0.2 | 0.3 | 0.3 | 0.3 | 0.4 | 0.3 |
| Lcat | 1.7 | 1.4 | 1.6 | 0.9 | 1.3 | 1.2 | 1.5 | 0.9 | 0.9 | 1.4 | 0.8 | 1.2 |
| Lce1g | 0.3 | 0.0 | 0.0 | 0.8 | 0.1 | 0.7 | 0.0 | 0.0 | 0.0 | 0.0 | 0.0 | 0.1 |
| Lce1m | 0.0 | 0.0 | 0.0 | 0.0 | 0.0 | 0.0 | 0.0 | 0.0 | 0.0 | 0.0 | 0.0 | 0.0 |
| Lck | 0.0 | 0.0 | 0.0 | 0.0 | 0.0 | 0.0 | 0.0 | 0.0 | 0.0 | 0.0 | 0.1 | 0.0 |
| Lclat1 | 16.8 | 20.2 | 19.2 | 17.8 | 16.7 | 17.2 | 26.4 | 18.2 | 31.6 | 16.4 | 23.7 | 24.8 |
| Lcmt1 | 18.6 | 14.9 | 14.3 | 16.4 | 12.8 | 19.2 | 13.4 | 13.1 | 12.3 | 13.5 | 14.7 | 12.8 |
| Lcmt2 | 1.9 | 2.3 | 1.7 | 1.5 | 2.1 | 2.5 | 1.6 | 2.2 | 2.2 | 2.7 | 1.9 | 2.1 |
| Lcn2 | 6.3 | 3.1 | 8.4 | 4.7 | 2.1 | 1.5 | 1.2 | 2.0 | 0.6 | 0.5 | 0.5 | 0.9 |
| Lcor | 0.6 | 0.8 | 0.7 | 0.9 | 0.7 | 0.7 | 0.8 | 0.7 | 0.5 | 0.6 | 0.7 | 0.8 |
| Lcorl | 1.2 | 1.3 | 1.6 | 1.3 | 1.3 | 1.0 | 1.1 | 0.7 | 1.2 | 1.1 | 1.3 | 1.3 |
| Lcp1 | 9.8 | 0.5 | 1.0 | 64.3 | 119.4 | 0.6 | 16.6 | 1.9 | 2.8 | 8.9 | 3.3 | 8.0 |
| Lcp2 | 0.5 | 0.1 | 0.1 | 2.4 | 3.9 | 0.0 | 0.9 | 0.3 | 0.2 | 0.3 | 0.3 | 0.2 |
| Lct | 0.0 | 0.0 | 0.0 | 0.0 | 0.0 | 0.0 | 0.0 | 0.0 | 0.0 | 0.0 | 0.0 | 0.0 |
| Lctl | 0.1 | 0.0 | 0.0 | 0.0 | 0.0 | 0.1 | 0.0 | 0.0 | 0.0 | 0.1 | 0.1 | 0.0 |
| Ldb1 | 21.4 | 20.9 | 22.2 | 25.4 | 20.8 | 23.0 | 24.9 | 22.2 | 23.8 | 22.0 | 21.5 | 20.6 |
| Ldb2 | 0.0 | 0.0 | 0.0 | 0.0 | 0.0 | 0.0 | 0.0 | 0.2 | 0.1 | 0.5 | 0.5 | 0.2 |
| Ldb3 | 1.4 | 1.0 | 0.7 | 1.5 | 1.2 | 1.1 | 1.8 | 1.1 | 1.5 | 1.0 | 1.8 | 1.3 |
| Ldha | 229.5 | 227.4 | 185.8 | 216.9 | 258.0 | 207.0 | 240.4 | 189.8 | 229.4 | 232.7 | 204.1 | 222.2 |
| Ldhal6b | 0.2 | 0.1 | 0.2 | 0.1 | 0.0 | 0.1 | 0.1 | 0.1 | 0.1 | 0.0 | 0.3 | 0.1 |
| Ldhb | 18.2 | 24.4 | 28.7 | 15.1 | 16.2 | 22.7 | 17.0 | 16.8 | 14.1 | 79.6 | 30.3 | 31.6 |
| Ldhd | 0.4 | 0.3 | 0.4 | 0.2 | 0.1 | 0.2 | 0.1 | 0.4 | 0.2 | 1.1 | 0.3 | 0.2 |
| Ldlr | 36.0 | 54.1 | 47.6 | 19.2 | 37.0 | 23.8 | 89.9 | 79.4 | 82.9 | 21.4 | 57.1 | 30.1 |
| Ldlrad1 | 0.0 | 0.0 | 0.0 | 0.0 | 0.0 | 0.1 | 0.0 | 0.0 | 0.0 | 0.0 | 0.1 | 0.0 |
| Ldlrad2 | 0.1 | 0.0 | 0.0 | 0.0 | 0.0 | 0.1 | 0.1 | 0.1 | 0.1 | 0.0 | 0.1 | 0.0 |
| Ldlrad3 | 1.8 | 1.7 | 2.3 | 2.1 | 1.7 | 2.0 | 1.9 | 2.5 | 2.0 | 1.9 | 2.1 | 2.0 |
| Ldlrad4 | 19.7 | 19.3 | 20.8 | 23.6 | 24.4 | 21.5 | 30.4 | 24.1 | 23.6 | 19.7 | 23.8 | 25.5 |
| Ldlrap1 | 10.8 | 11.4 | 8.9 | 12.3 | 12.4 | 11.6 | 10.2 | 8.8 | 10.4 | 9.8 | 9.1 | 7.9 |
| Ldoc1l | 2.8 | 2.2 | 2.2 | 2.4 | 2.0 | 3.5 | 2.3 | 2.3 | 2.3 | 2.5 | 2.4 | 2.5 |
| Leap2 | 0.0 | 0.3 | 0.0 | 0.0 | 0.2 | 0.0 | 0.0 | 0.0 | 0.1 | 0.3 | 0.8 | 0.1 |
| Lef1 | 0.1 | 0.0 | 0.0 | 0.0 | 0.0 | 0.0 | 0.0 | 0.0 | 0.0 | 0.0 | 0.1 | 0.0 |
| Lefty1 | 6.5 | 6.2 | 4.4 | 5.9 | 4.1 | 6.5 | 4.8 | 4.9 | 5.0 | 5.6 | 5.2 | 5.4 |
| Lefty2 | 0.1 | 0.1 | 0.1 | 0.0 | 0.0 | 0.1 | 0.1 | 0.0 | 0.0 | 0.1 | 0.0 | 0.1 |
| Lekr1 | 0.9 | 0.9 | 0.8 | 1.0 | 0.8 | 1.2 | 0.7 | 0.6 | 0.4 | 0.9 | 0.6 | 0.9 |
| Lemd1 | 0.3 | 0.0 | 0.1 | 0.0 | 0.1 | 0.0 | 0.1 | 0.1 | 0.0 | 0.1 | 0.1 | 0.0 |
| Lemd2 | 12.1 | 11.5 | 10.5 | 12.5 | 11.0 | 13.5 | 12.3 | 11.0 | 12.6 | 13.8 | 11.9 | 12.3 |

| | | | | | | | | | | | | |
|---|---|---|---|---|---|---|---|---|---|---|---|---|
| Lemd3 | 2.9 | 3.7 | 3.2 | 2.9 | 3.5 | 3.2 | 3.6 | 3.7 | 3.4 | 3.5 | 3.7 | 3.8 |
| Lenep | 1.2 | 0.9 | 1.2 | 0.8 | 1.0 | 1.6 | 1.3 | 1.2 | 0.7 | 0.6 | 0.3 | 1.0 |
| Leng1 | 9.3 | 9.9 | 9.8 | 11.9 | 8.1 | 9.7 | 10.6 | 8.8 | 9.5 | 7.9 | 10.2 | 10.8 |
| Leng8 | 5.1 | 6.4 | 6.1 | 5.3 | 5.3 | 5.0 | 6.5 | 5.1 | 7.2 | 4.8 | 4.4 | 4.1 |
| Leng9 | 2.4 | 2.3 | 3.0 | 2.1 | 2.7 | 2.3 | 2.2 | 2.7 | 2.4 | 3.0 | 3.6 | 3.0 |
| Leo1 | 16.2 | 18.3 | 17.4 | 17.4 | 15.7 | 15.6 | 17.7 | 14.9 | 14.5 | 13.2 | 15.7 | 16.7 |
| Lep | 0.0 | 0.0 | 0.0 | 0.1 | 0.0 | 0.0 | 0.0 | 0.0 | 0.0 | 0.0 | 0.0 | 0.0 |
| Lepr | 0.1 | 0.1 | 0.1 | 0.1 | 0.1 | 0.1 | 0.1 | 0.1 | 0.1 | 0.1 | 0.1 | 0.0 |
| Lepre1 | 51.3 | 42.9 | 42.4 | 58.3 | 47.1 | 49.5 | 55.9 | 61.6 | 49.5 | 47.8 | 54.1 | 42.5 |
| Leprel1 | 0.6 | 0.6 | 0.9 | 0.3 | 0.4 | 0.2 | 0.5 | 0.2 | 0.2 | 0.2 | 0.4 | 0.2 |
| Leprel2 | 39.0 | 35.0 | 42.0 | 35.1 | 38.8 | 46.0 | 47.5 | 41.0 | 45.5 | 36.4 | 34.7 | 40.7 |
| Leprel4 | 71.7 | 57.8 | 52.1 | 45.2 | 47.9 | 49.2 | 65.8 | 75.6 | 70.0 | 90.4 | 77.4 | 73.5 |
| Leprot | 66.0 | 58.6 | 53.4 | 71.8 | 65.9 | 62.0 | 68.9 | 58.1 | 69.1 | 59.2 | 64.0 | 66.5 |
| Leprotl1 | 18.3 | 16.5 | 13.0 | 25.1 | 16.9 | 20.5 | 16.6 | 15.0 | 18.2 | 19.0 | 20.3 | 18.5 |
| Letm1 | 9.1 | 7.5 | 8.7 | 8.3 | 8.2 | 6.9 | 9.0 | 9.7 | 8.2 | 7.5 | 8.8 | 8.2 |
| Letm2 | 1.2 | 1.3 | 1.3 | 1.7 | 0.9 | 1.6 | 1.0 | 1.3 | 1.0 | 0.8 | 1.4 | 1.0 |
| Letmd1 | 6.6 | 5.6 | 4.8 | 7.4 | 6.1 | 6.2 | 6.1 | 6.7 | 6.0 | 5.9 | 6.4 | 6.0 |
| Lfng | 0.4 | 0.2 | 0.3 | 1.9 | 3.1 | 0.1 | 0.8 | 1.5 | 1.3 | 3.4 | 1.8 | 1.3 |
| Lgals1 | 1073.2 | 817.0 | 740.4 | 1211.2 | 994.6 | 1106.1 | 1112.4 | 997.8 | 941.4 | 936.3 | 1081.2 | 919.1 |
| Lgals2 | 1.2 | 0.7 | 0.1 | 0.1 | 0.6 | 0.4 | 0.7 | 0.6 | 1.1 | 0.9 | 0.9 | 0.6 |
| Lgals3 | 32.5 | 7.3 | 5.1 | 185.7 | 189.8 | 22.6 | 16.1 | 9.8 | 5.9 | 24.1 | 16.6 | 14.4 |
| Lgals3bp | 81.5 | 60.5 | 72.2 | 132.4 | 87.5 | 84.8 | 45.4 | 35.8 | 30.3 | 33.1 | 23.6 | 41.7 |
| Lgals4 | 0.2 | 0.1 | 0.2 | 0.1 | 0.1 | 0.3 | 0.3 | 0.1 | 0.4 | 0.1 | 0.1 | 0.0 |
| Lgals6 | 0.1 | 0.4 | 0.1 | 0.0 | 0.2 | 0.1 | 0.0 | 0.0 | 0.0 | 0.4 | 0.0 | 0.4 |
| Lgals7 | 0.0 | 0.0 | 0.0 | 0.0 | 0.1 | 0.0 | 0.0 | 0.0 | 0.2 | 0.1 | 0.0 | 0.1 |
| Lgals8 | 22.1 | 19.1 | 18.7 | 28.8 | 19.5 | 24.9 | 17.1 | 17.9 | 16.0 | 19.3 | 18.4 | 19.4 |
| Lgals9 | 49.7 | 39.2 | 30.5 | 64.9 | 43.5 | 42.2 | 37.1 | 26.8 | 23.2 | 24.2 | 28.7 | 23.2 |
| Lgalsl | 6.1 | 6.6 | 7.6 | 6.7 | 4.9 | 7.5 | 5.1 | 6.5 | 6.5 | 5.8 | 6.6 | 6.8 |
| Lgi2 | 3.0 | 5.5 | 14.5 | 2.4 | 3.4 | 3.4 | 3.8 | 16.0 | 7.3 | 5.2 | 2.9 | 7.7 |
| Lgi3 | 0.1 | 0.1 | 0.1 | 0.0 | 0.1 | 0.0 | 0.1 | 0.2 | 0.1 | 0.2 | 0.1 | 0.2 |
| Lgi4 | 0.8 | 1.1 | 1.6 | 0.3 | 0.6 | 0.3 | 0.8 | 1.1 | 0.5 | 0.4 | 0.5 | 0.7 |
| Lgmn | 51.0 | 51.9 | 68.1 | 58.6 | 70.9 | 50.0 | 53.8 | 78.5 | 60.6 | 68.6 | 64.5 | 67.5 |
| Lgr4 | 3.7 | 4.2 | 4.0 | 2.8 | 3.7 | 4.0 | 4.6 | 10.1 | 7.3 | 4.0 | 5.9 | 4.6 |
| Lgr5 | 0.0 | 0.0 | 0.0 | 0.0 | 0.0 | 0.0 | 0.0 | 0.0 | 0.0 | 0.0 | 0.0 | 0.0 |
| Lgr6 | 0.0 | 0.0 | 0.0 | 0.1 | 0.1 | 0.1 | 0.1 | 0.0 | 0.0 | 0.0 | 0.0 | 0.0 |
| Lhfp | 273.8 | 240.2 | 235.2 | 314.5 | 218.3 | 256.6 | 265.5 | 178.1 | 249.5 | 227.3 | 251.2 | 280.4 |
| Lhfpl1 | 0.1 | 0.9 | 0.3 | 0.1 | 0.1 | 0.2 | 0.0 | 0.1 | 0.1 | 0.1 | 0.3 | 0.2 |
| Lhfpl2 | 10.1 | 9.2 | 13.5 | 14.5 | 24.2 | 9.1 | 15.5 | 12.6 | 13.4 | 20.6 | 16.8 | 18.1 |
| Lhfpl3 | 0.3 | 0.1 | 0.1 | 0.1 | 0.0 | 0.2 | 0.1 | 0.0 | 0.0 | 0.1 | 0.0 | 0.0 |
| Lhfpl4 | 0.3 | 0.4 | 0.3 | 0.5 | 0.3 | 0.7 | 0.6 | 0.4 | 0.3 | 0.1 | 0.2 | 0.2 |
| Lhpp | 5.9 | 5.5 | 4.8 | 6.8 | 6.0 | 6.1 | 5.0 | 4.9 | 3.9 | 5.9 | 5.4 | 4.5 |
| Lhx2 | 0.2 | 0.1 | 0.1 | 0.1 | 0.1 | 0.1 | 0.0 | 0.0 | 0.1 | 0.0 | 0.0 | 0.0 |
| Lhx4 | 0.0 | 0.0 | 0.0 | 0.0 | 0.0 | 0.0 | 0.0 | 0.0 | 0.0 | 0.0 | 0.1 | 0.0 |
| Lhx5 | 0.0 | 0.0 | 0.0 | 0.0 | 0.0 | 0.0 | 0.0 | 0.0 | 0.0 | 0.0 | 0.0 | 0.0 |
| Lhx6 | 0.3 | 0.0 | 0.1 | 0.3 | 0.0 | 0.2 | 0.2 | 0.2 | 0.2 | 0.3 | 0.4 | 0.1 |
| Lhx8 | 0.0 | 0.0 | 0.0 | 0.0 | 0.0 | 0.0 | 0.0 | 0.1 | 0.0 | 0.0 | 0.1 | 0.0 |
| Lhx9 | 0.3 | 0.1 | 0.1 | 0.2 | 0.1 | 0.3 | 0.2 | 0.2 | 0.2 | 0.2 | 0.2 | 0.0 |
| Lias | 13.4 | 14.0 | 11.7 | 14.9 | 11.5 | 14.2 | 10.6 | 12.7 | 11.1 | 12.2 | 12.0 | 13.6 |
| Lif | 8.1 | 6.7 | 5.5 | 7.4 | 9.0 | 6.7 | 9.4 | 4.7 | 6.5 | 11.0 | 6.7 | 6.7 |
| Lifr | 6.2 | 7.9 | 10.5 | 6.1 | 10.1 | 5.0 | 10.7 | 10.1 | 8.0 | 9.8 | 7.8 | 9.7 |
| Lig1 | 2.4 | 3.9 | 5.4 | 1.8 | 5.0 | 1.7 | 6.1 | 3.9 | 4.0 | 2.5 | 3.7 | 2.8 |
| Lig3 | 6.0 | 6.2 | 5.5 | 5.4 | 5.1 | 5.1 | 4.9 | 5.7 | 5.0 | 5.2 | 5.7 | 4.6 |
| Lig4 | 2.3 | 2.2 | 2.4 | 2.0 | 2.1 | 2.2 | 1.8 | 2.3 | 2.1 | 2.1 | 2.5 | 3.0 |
| Lilra5 | 0.0 | 0.0 | 0.0 | 0.0 | 0.0 | 0.0 | 0.0 | 0.0 | 0.0 | 0.1 | 0.0 | 0.0 |
| Lilra6 | 0.1 | 0.0 | 0.0 | 0.9 | 1.4 | 0.0 | 0.2 | 0.0 | 0.0 | 0.1 | 0.0 | 0.0 |
| Lilrb3 | 1.3 | 0.0 | 0.1 | 11.0 | 16.0 | 0.0 | 2.0 | 0.2 | 0.2 | 1.2 | 0.2 | 0.9 |
| Lilrb4 | 10.6 | 0.0 | 0.8 | 90.8 | 119.0 | 0.1 | 11.2 | 1.5 | 1.7 | 7.9 | 2.5 | 6.2 |

| | | | | | | | | | | | | |
|---|---|---|---|---|---|---|---|---|---|---|---|---|
| Lima1 | 33.2 | 36.7 | 43.9 | 27.2 | 35.2 | 23.2 | 39.0 | 37.1 | 34.9 | 56.1 | 55.2 | 50.0 |
| Limch1 | 14.7 | 17.8 | 24.1 | 17.7 | 14.9 | 9.6 | 20.6 | 27.2 | 20.3 | 11.3 | 22.0 | 15.3 |
| Limd1 | 12.5 | 14.1 | 12.0 | 11.5 | 12.8 | 11.3 | 12.1 | 13.7 | 14.5 | 14.4 | 11.4 | 12.8 |
| Limd2 | 5.8 | 6.4 | 6.1 | 8.5 | 11.4 | 6.3 | 7.2 | 6.4 | 7.0 | 6.6 | 6.4 | 5.0 |
| Lime1 | 3.0 | 3.1 | 2.6 | 2.8 | 2.9 | 3.3 | 3.6 | 2.6 | 4.2 | 2.6 | 3.3 | 2.3 |
| Limk1 | 5.4 | 6.7 | 5.5 | 6.2 | 6.3 | 5.1 | 7.5 | 6.0 | 5.1 | 4.4 | 6.3 | 4.8 |
| Limk2 | 12.1 | 11.5 | 9.3 | 9.9 | 9.0 | 10.7 | 10.2 | 7.9 | 10.6 | 11.6 | 11.1 | 10.3 |
| Lims1 | 32.6 | 36.9 | 37.8 | 39.2 | 38.8 | 37.8 | 34.8 | 35.9 | 38.2 | 37.5 | 43.4 | 44.1 |
| Lims2 | 6.5 | 5.2 | 3.0 | 7.8 | 4.8 | 5.6 | 7.3 | 4.0 | 9.1 | 6.0 | 10.6 | 6.9 |
| Lin37 | 5.4 | 6.7 | 6.6 | 7.2 | 5.1 | 4.8 | 6.1 | 5.7 | 5.0 | 5.9 | 6.0 | 4.9 |
| Lin52 | 7.6 | 6.5 | 7.6 | 8.3 | 6.4 | 7.5 | 6.7 | 6.0 | 7.4 | 7.4 | 7.0 | 7.6 |
| Lin54 | 3.4 | 4.4 | 5.3 | 3.5 | 4.6 | 3.2 | 5.4 | 3.9 | 4.5 | 4.0 | 5.5 | 5.2 |
| Lin7a | 0.0 | 0.0 | 0.0 | 0.0 | 0.0 | 0.0 | 0.0 | 0.0 | 0.0 | 0.0 | 0.0 | 0.0 |
| Lin7b | 0.4 | 0.3 | 0.5 | 0.1 | 0.3 | 1.0 | 0.2 | 0.3 | 0.4 | 0.2 | 0.5 | 0.4 |
| Lin7c | 21.4 | 23.5 | 25.5 | 28.8 | 19.2 | 28.6 | 21.8 | 17.2 | 18.8 | 20.3 | 26.3 | 25.2 |
| Lin9 | 1.2 | 1.8 | 2.6 | 1.4 | 2.0 | 1.3 | 2.0 | 1.6 | 1.8 | 1.4 | 2.5 | 1.7 |
| Lingo1 | 1.6 | 2.7 | 3.0 | 1.7 | 2.1 | 2.9 | 3.6 | 3.4 | 4.1 | 1.9 | 2.8 | 2.4 |
| Lingo4 | 0.0 | 0.0 | 0.0 | 0.0 | 0.0 | 0.0 | 0.0 | 0.0 | 0.0 | 0.1 | 0.0 | 0.0 |
| Lins | 1.8 | 1.5 | 1.3 | 2.0 | 1.4 | 2.3 | 1.6 | 1.9 | 1.5 | 1.6 | 1.6 | 1.3 |
| Lipa | 44.1 | 33.6 | 33.4 | 72.3 | 87.7 | 37.6 | 28.9 | 31.9 | 30.9 | 29.5 | 27.8 | 34.8 |
| Lipc | 0.1 | 0.0 | 0.1 | 0.1 | 0.1 | 0.1 | 0.0 | 0.0 | 0.1 | 0.0 | 0.1 | 0.0 |
| Lipe | 1.9 | 2.4 | 2.5 | 2.3 | 2.3 | 1.9 | 1.6 | 2.0 | 1.3 | 1.8 | 1.7 | 2.0 |
| Lipg | 1.0 | 0.9 | 0.3 | 0.6 | 0.2 | 0.5 | 0.7 | 0.7 | 0.7 | 0.8 | 1.3 | 0.6 |
| Liph | 0.1 | 0.1 | 0.0 | 0.0 | 0.1 | 0.0 | 0.1 | 0.0 | 0.0 | 0.0 | 0.0 | 0.0 |
| Lipn | 0.1 | 0.0 | 0.0 | 0.0 | 0.0 | 0.1 | 0.0 | 0.0 | 0.0 | 0.0 | 0.0 | 0.0 |
| Lipo1 | 8.1 | 7.1 | 7.4 | 11.6 | 8.5 | 9.0 | 9.5 | 7.7 | 8.3 | 7.8 | 10.6 | 9.5 |
| Lipt1 | 2.5 | 2.0 | 1.6 | 3.5 | 2.3 | 2.6 | 2.2 | 2.3 | 2.2 | 2.3 | 2.0 | 2.0 |
| Lipt2 | 2.2 | 1.7 | 1.3 | 2.0 | 1.2 | 0.9 | 1.5 | 2.4 | 1.7 | 1.7 | 2.5 | 1.7 |
| Litaf | 56.8 | 45.4 | 40.9 | 61.1 | 75.1 | 43.4 | 54.5 | 44.4 | 47.1 | 51.4 | 49.2 | 48.0 |
| Lix1 | 0.8 | 0.6 | 0.7 | 1.1 | 1.8 | 1.0 | 0.5 | 0.4 | 0.2 | 0.4 | 0.5 | 0.5 |
| Lix1l | 24.0 | 28.5 | 32.6 | 25.0 | 23.5 | 27.9 | 28.4 | 29.9 | 29.2 | 29.7 | 29.5 | 31.4 |
| Llgl1 | 4.6 | 5.1 | 6.2 | 4.6 | 5.1 | 4.9 | 5.5 | 7.1 | 6.3 | 5.6 | 4.9 | 5.1 |
| Llgl2 | 4.2 | 4.2 | 3.0 | 3.2 | 3.5 | 3.2 | 4.0 | 3.1 | 3.9 | 3.7 | 3.5 | 3.2 |
| Llph | 51.5 | 35.5 | 39.1 | 62.6 | 35.3 | 53.9 | 38.3 | 26.3 | 24.5 | 37.7 | 46.0 | 41.8 |
| Lman1 | 114.8 | 104.5 | 122.4 | 133.3 | 94.2 | 110.3 | 120.0 | 123.5 | 96.3 | 96.5 | 117.7 | 122.2 |
| Lman1l | 0.1 | 0.0 | 0.0 | 0.1 | 0.1 | 0.1 | 0.1 | 0.0 | 0.1 | 0.0 | 0.0 | 0.1 |
| Lman2 | 46.1 | 42.1 | 38.3 | 55.3 | 48.0 | 47.4 | 47.0 | 41.9 | 44.0 | 49.6 | 44.1 | 43.5 |
| Lman2l | 3.6 | 3.7 | 3.3 | 4.3 | 3.7 | 3.7 | 2.7 | 3.1 | 3.7 | 4.0 | 3.0 | 4.1 |
| Lmbr1 | 6.3 | 6.3 | 6.5 | 4.6 | 4.8 | 4.8 | 5.3 | 7.2 | 6.4 | 5.4 | 6.3 | 6.0 |
| Lmbr1l | 1.9 | 2.2 | 1.9 | 1.4 | 2.1 | 1.4 | 1.7 | 2.0 | 2.8 | 2.4 | 1.8 | 2.0 |
| Lmbrd1 | 9.4 | 10.4 | 9.2 | 12.2 | 10.5 | 11.8 | 9.8 | 8.4 | 9.0 | 10.4 | 9.9 | 9.6 |
| Lmbrd2 | 5.6 | 5.3 | 5.3 | 6.1 | 5.3 | 5.3 | 5.7 | 4.2 | 4.1 | 6.1 | 6.6 | 5.4 |
| Lmcd1 | 13.0 | 12.0 | 12.1 | 9.8 | 7.2 | 6.1 | 7.1 | 9.6 | 9.7 | 8.9 | 10.5 | 13.3 |
| Lmf1 | 11.0 | 10.3 | 9.4 | 13.1 | 11.0 | 12.9 | 11.0 | 13.4 | 11.1 | 10.8 | 10.3 | 11.0 |
| Lmf2 | 8.3 | 10.1 | 11.0 | 8.6 | 10.3 | 9.6 | 12.1 | 10.8 | 11.9 | 9.3 | 9.6 | 8.6 |
| Lmln | 0.7 | 0.5 | 0.4 | 0.7 | 0.4 | 0.9 | 0.5 | 0.9 | 0.7 | 0.7 | 0.8 | 0.6 |
| Lmna | 172.4 | 152.8 | 138.9 | 183.4 | 149.1 | 157.7 | 189.6 | 192.5 | 163.1 | 160.9 | 182.4 | 159.5 |
| Lmnb1 | 1.6 | 1.8 | 2.9 | 0.6 | 2.5 | 0.7 | 3.8 | 2.5 | 2.8 | 1.9 | 2.3 | 2.0 |
| Lmnb2 | 1.8 | 2.7 | 2.8 | 1.7 | 2.9 | 1.7 | 4.3 | 3.6 | 3.6 | 2.2 | 2.8 | 2.1 |
| Lmo1 | 0.5 | 0.2 | 0.2 | 0.1 | 0.1 | 0.1 | 0.2 | 0.2 | 0.1 | 0.1 | 0.1 | 0.2 |
| Lmo2 | 1.3 | 0.3 | 0.4 | 7.3 | 8.0 | 0.3 | 1.0 | 0.4 | 0.3 | 0.7 | 0.2 | 0.7 |
| Lmo4 | 27.5 | 22.3 | 32.6 | 22.3 | 19.6 | 19.0 | 23.4 | 29.1 | 26.1 | 37.9 | 37.4 | 32.8 |
| Lmo7 | 1.4 | 0.5 | 0.6 | 1.2 | 0.5 | 0.8 | 0.8 | 0.7 | 0.6 | 0.5 | 0.6 | 0.5 |
| Lmod1 | 4.0 | 2.4 | 2.5 | 6.3 | 3.9 | 3.0 | 6.1 | 2.0 | 4.6 | 6.0 | 12.3 | 7.4 |
| Lmod2 | 0.1 | 0.0 | 0.1 | 0.1 | 0.0 | 0.0 | 0.1 | 0.0 | 0.0 | 0.0 | 0.0 | 0.0 |
| Lmod3 | 0.0 | 0.0 | 0.1 | 0.0 | 0.0 | 0.0 | 0.0 | 0.1 | 0.0 | 0.0 | 0.0 | 0.0 |
| Lmtk2 | 5.7 | 5.6 | 4.8 | 4.7 | 4.9 | 5.3 | 5.1 | 3.6 | 4.8 | 4.5 | 4.1 | 4.0 |

| | | | | | | | | | | | | |
|---|---|---|---|---|---|---|---|---|---|---|---|---|
| Lmtk3 | 0.0 | 0.0 | 0.0 | 0.0 | 0.0 | 0.0 | 0.0 | 0.0 | 0.0 | 0.0 | 0.0 | 0.0 |
| Lmx1b | 0.0 | 0.0 | 0.1 | 0.0 | 0.0 | 0.1 | 0.0 | 0.0 | 0.0 | 0.0 | 0.0 | 0.0 |
| Lnp | 2.9 | 3.8 | 4.4 | 4.4 | 3.5 | 4.4 | 4.3 | 3.8 | 3.6 | 3.2 | 4.5 | 4.2 |
| Lnpep | 16.4 | 19.7 | 21.5 | 18.2 | 22.2 | 18.3 | 18.9 | 19.4 | 18.6 | 19.8 | 22.6 | 21.3 |
| Lnx1 | 3.4 | 2.3 | 2.8 | 1.9 | 0.8 | 1.6 | 0.7 | 1.0 | 0.4 | 1.9 | 1.0 | 1.5 |
| Lnx2 | 1.9 | 2.1 | 2.1 | 2.1 | 2.0 | 1.8 | 2.0 | 2.6 | 2.4 | 2.8 | 2.5 | 2.2 |
| LOC100038947 | 0.5 | 0.0 | 0.0 | 0.5 | 5.2 | 0.0 | 0.3 | 0.1 | 0.0 | 0.1 | 0.0 | 0.0 |
| LOC100503496 | 0.1 | 0.2 | 0.1 | 0.2 | 0.2 | 0.2 | 0.1 | 0.4 | 0.2 | 0.2 | 0.2 | 0.1 |
| LOC100504608 | 0.7 | 0.6 | 1.0 | 0.8 | 1.0 | 1.1 | 1.1 | 1.4 | 0.7 | 0.5 | 0.8 | 0.8 |
| LOC100504703 | 2.6 | 3.7 | 4.0 | 2.9 | 3.6 | 2.8 | 1.1 | 3.9 | 2.0 | 3.5 | 2.7 | 4.7 |
| LOC100861615 | 0.3 | 0.6 | 0.6 | 0.7 | 0.5 | 0.6 | 0.8 | 0.7 | 0.6 | 0.6 | 0.6 | 0.6 |
| LOC100861978 | 0.0 | 0.0 | 0.0 | 0.0 | 0.0 | 0.0 | 0.0 | 0.0 | 0.0 | 0.0 | 0.0 | 0.0 |
| LOC106740 | 3.3 | 3.2 | 3.8 | 4.0 | 3.0 | 4.2 | 3.4 | 2.7 | 3.5 | 3.2 | 3.2 | 3.1 |
| LOC171588 | 0.1 | 0.0 | 0.1 | 0.0 | 0.2 | 0.1 | 0.1 | 0.2 | 0.0 | 0.1 | 0.1 | 0.1 |
| Loh12cr1 | 3.6 | 3.7 | 3.6 | 3.4 | 2.7 | 4.7 | 2.8 | 3.0 | 3.1 | 3.4 | 3.0 | 2.9 |
| Lonp1 | 35.3 | 26.7 | 22.6 | 43.0 | 22.6 | 37.7 | 25.8 | 29.6 | 23.5 | 23.2 | 28.5 | 25.0 |
| Lonp2 | 43.5 | 38.8 | 35.6 | 39.6 | 32.2 | 36.7 | 34.0 | 30.6 | 31.1 | 38.1 | 37.7 | 39.1 |
| Lonrf1 | 1.6 | 1.8 | 1.7 | 0.6 | 1.3 | 1.7 | 1.5 | 2.1 | 2.1 | 1.2 | 1.8 | 1.2 |
| Lonrf2 | 2.8 | 2.3 | 1.4 | 3.7 | 1.6 | 3.7 | 2.2 | 1.1 | 1.4 | 1.3 | 1.5 | 1.6 |
| Lonrf3 | 0.6 | 0.9 | 1.1 | 1.3 | 2.4 | 0.4 | 0.8 | 0.5 | 0.6 | 0.5 | 0.3 | 0.4 |
| Lor | 0.1 | 0.0 | 0.0 | 0.1 | 0.0 | 0.0 | 0.0 | 0.0 | 0.0 | 0.0 | 0.0 | 0.0 |
| Lox | 825.0 | 871.5 | 807.1 | 726.0 | 640.3 | 839.1 | 1050.3 | 1240.2 | 974.2 | 651.1 | 992.2 | 878.5 |
| Loxl1 | 51.5 | 57.7 | 69.8 | 46.6 | 63.2 | 44.5 | 79.7 | 76.1 | 79.3 | 64.0 | 52.1 | 60.1 |
| Loxl2 | 194.9 | 200.3 | 152.8 | 208.5 | 219.9 | 219.9 | 195.8 | 149.0 | 188.0 | 152.6 | 152.6 | 152.9 |
| Loxl3 | 49.3 | 45.7 | 40.8 | 59.8 | 70.6 | 61.8 | 103.5 | 39.0 | 73.0 | 75.8 | 72.2 | 84.5 |
| Loxl4 | 3.9 | 3.3 | 3.8 | 3.9 | 4.0 | 4.2 | 5.0 | 6.7 | 6.6 | 2.6 | 4.0 | 4.0 |
| Lpar1 | 39.2 | 45.8 | 54.5 | 55.4 | 46.5 | 50.6 | 48.8 | 47.0 | 33.9 | 37.9 | 44.7 | 41.5 |
| Lpar2 | 0.7 | 0.6 | 0.3 | 0.3 | 0.3 | 0.7 | 0.2 | 0.5 | 0.5 | 1.3 | 0.5 | 0.5 |
| Lpar3 | 0.1 | 0.0 | 0.3 | 0.0 | 0.1 | 0.1 | 0.1 | 0.3 | 0.2 | 0.1 | 0.0 | 0.2 |
| Lpar4 | 0.2 | 0.3 | 0.3 | 0.3 | 0.2 | 0.2 | 0.3 | 0.8 | 0.6 | 0.3 | 0.3 | 0.2 |
| Lpar5 | 0.0 | 0.0 | 0.0 | 0.2 | 0.2 | 0.1 | 0.0 | 0.1 | 0.0 | 0.0 | 0.0 | 0.1 |
| Lpar6 | 3.7 | 3.5 | 2.7 | 3.6 | 3.4 | 2.7 | 2.6 | 2.5 | 2.8 | 4.8 | 3.9 | 3.6 |
| Lpcat1 | 4.7 | 3.6 | 3.6 | 6.5 | 5.7 | 4.6 | 6.0 | 4.5 | 4.7 | 4.8 | 5.3 | 4.5 |
| Lpcat2 | 1.1 | 0.2 | 0.2 | 4.1 | 5.5 | 0.3 | 0.9 | 0.3 | 0.4 | 2.4 | 1.7 | 1.1 |
| Lpcat3 | 8.4 | 11.9 | 13.8 | 8.5 | 10.5 | 8.5 | 13.9 | 18.6 | 14.7 | 9.7 | 13.1 | 10.8 |
| Lpcat4 | 3.9 | 4.9 | 5.4 | 3.5 | 5.4 | 3.2 | 5.7 | 5.7 | 4.6 | 6.2 | 5.8 | 4.5 |
| Lpgat1 | 14.8 | 17.2 | 25.5 | 13.9 | 18.4 | 12.4 | 20.0 | 23.6 | 19.3 | 15.7 | 16.6 | 25.4 |
| Lphn1 | 1.5 | 2.3 | 3.0 | 1.0 | 2.1 | 1.5 | 2.9 | 2.7 | 2.8 | 3.8 | 2.5 | 2.2 |
| Lphn2 | 1.5 | 1.1 | 1.9 | 1.4 | 1.9 | 1.3 | 2.0 | 1.8 | 2.2 | 2.7 | 2.2 | 2.2 |
| Lphn3 | 0.1 | 0.1 | 0.1 | 0.0 | 0.1 | 0.0 | 0.1 | 0.1 | 0.1 | 0.1 | 0.1 | 0.1 |
| Lpin1 | 10.0 | 9.6 | 8.0 | 9.5 | 6.7 | 12.9 | 5.9 | 4.6 | 5.1 | 7.8 | 6.8 | 6.8 |
| Lpin2 | 6.2 | 6.5 | 5.5 | 6.7 | 8.2 | 7.6 | 5.9 | 4.5 | 4.8 | 6.0 | 5.3 | 5.6 |
| Lpin3 | 5.6 | 4.7 | 4.3 | 6.5 | 5.9 | 6.3 | 4.7 | 4.0 | 4.0 | 3.2 | 3.7 | 3.7 |
| Lpl | 7.7 | 12.6 | 21.0 | 32.4 | 60.3 | 5.5 | 7.7 | 10.5 | 4.5 | 5.6 | 2.6 | 4.0 |
| Lpp | 10.8 | 11.6 | 11.1 | 10.2 | 12.3 | 9.4 | 11.8 | 10.3 | 10.9 | 11.4 | 13.4 | 10.6 |
| Lpxn | 0.5 | 0.1 | 0.1 | 6.9 | 12.7 | 0.2 | 1.8 | 0.3 | 0.4 | 0.6 | 0.4 | 0.8 |
| Lrat | 0.0 | 0.0 | 0.0 | 0.0 | 0.0 | 0.0 | 0.0 | 0.0 | 0.0 | 0.0 | 0.0 | 0.0 |
| Lrba | 3.8 | 3.7 | 3.7 | 3.6 | 3.4 | 4.3 | 2.7 | 3.6 | 3.1 | 3.7 | 3.7 | 3.5 |
| Lrch1 | 5.3 | 8.1 | 8.3 | 6.6 | 7.7 | 5.1 | 8.1 | 8.0 | 8.4 | 5.1 | 7.6 | 8.2 |
| Lrch2 | 0.4 | 0.6 | 0.7 | 0.4 | 0.4 | 0.8 | 0.6 | 0.5 | 0.6 | 0.4 | 0.5 | 0.5 |
| Lrch3 | 6.6 | 7.0 | 7.6 | 7.2 | 7.5 | 6.4 | 7.6 | 7.5 | 7.1 | 6.7 | 7.4 | 7.2 |
| Lrch4 | 5.2 | 5.4 | 5.6 | 8.3 | 10.4 | 6.8 | 6.6 | 5.0 | 5.4 | 4.3 | 4.9 | 4.2 |
| Lrdd | 1.4 | 1.6 | 1.6 | 1.2 | 1.8 | 1.5 | 1.9 | 0.8 | 1.4 | 1.5 | 1.4 | 1.1 |
| Lrfn1 | 0.3 | 0.5 | 0.4 | 0.1 | 0.5 | 0.5 | 0.4 | 0.5 | 0.3 | 0.3 | 0.4 | 0.3 |
| Lrfn3 | 1.0 | 1.4 | 1.1 | 1.1 | 1.0 | 1.3 | 0.8 | 1.0 | 1.3 | 1.0 | 0.9 | 1.2 |
| Lrfn4 | 3.0 | 2.6 | 3.6 | 2.7 | 2.9 | 2.7 | 2.5 | 5.1 | 3.2 | 2.9 | 1.9 | 2.9 |
| Lrg1 | 0.1 | 0.3 | 0.1 | 0.2 | 0.2 | 0.1 | 0.1 | 0.0 | 0.1 | 0.0 | 0.0 | 0.1 |

| | | | | | | | | | | | | |
|---|---|---|---|---|---|---|---|---|---|---|---|---|
| Lrguk | 0.1 | 0.1 | 0.1 | 0.1 | 0.0 | 0.1 | 0.0 | 0.0 | 0.0 | 0.0 | 0.0 | 0.0 |
| Lrif1 | 3.5 | 3.3 | 4.9 | 4.9 | 3.5 | 3.7 | 3.2 | 3.8 | 3.0 | 4.8 | 4.5 | 4.7 |
| Lrig1 | 10.6 | 8.2 | 8.9 | 10.6 | 10.2 | 10.5 | 11.5 | 6.9 | 8.6 | 10.3 | 8.4 | 9.4 |
| Lrig2 | 2.7 | 3.0 | 2.7 | 2.4 | 2.5 | 2.7 | 2.2 | 2.4 | 2.9 | 3.1 | 2.8 | 2.6 |
| Lrig3 | 3.4 | 3.5 | 4.7 | 2.4 | 3.0 | 2.7 | 3.5 | 4.6 | 3.7 | 5.4 | 4.5 | 4.1 |
| Lrmp | 0.1 | 0.0 | 0.0 | 2.0 | 2.9 | 0.1 | 0.4 | 0.1 | 0.1 | 0.2 | 0.1 | 0.2 |
| Lrp1 | 52.7 | 77.4 | 78.7 | 47.6 | 81.6 | 58.4 | 96.3 | 75.5 | 86.8 | 59.0 | 51.3 | 47.8 |
| Lrp10 | 25.3 | 23.0 | 20.8 | 27.0 | 26.4 | 29.8 | 28.8 | 23.1 | 28.4 | 27.5 | 23.7 | 21.9 |
| Lrp11 | 10.2 | 7.2 | 5.3 | 11.0 | 6.8 | 12.1 | 8.4 | 5.6 | 6.2 | 7.2 | 8.6 | 7.5 |
| Lrp12 | 5.6 | 5.2 | 5.7 | 6.0 | 5.7 | 4.5 | 5.7 | 5.7 | 5.9 | 4.5 | 4.9 | 5.7 |
| Lrp1b | 0.0 | 0.0 | 0.0 | 0.0 | 0.0 | 0.0 | 0.0 | 0.0 | 0.0 | 0.0 | 0.0 | 0.0 |
| Lrp2 | 1.9 | 0.5 | 0.3 | 0.7 | 0.1 | 0.6 | 0.2 | 0.8 | 1.0 | 23.6 | 9.4 | 3.2 |
| Lrp2bp | 0.0 | 0.1 | 0.0 | 0.1 | 0.1 | 0.0 | 0.1 | 0.1 | 0.1 | 0.0 | 0.1 | 0.1 |
| Lrp3 | 3.5 | 3.5 | 3.2 | 4.1 | 2.7 | 4.9 | 4.0 | 3.1 | 3.0 | 2.4 | 3.2 | 2.7 |
| Lrp4 | 0.8 | 1.2 | 1.4 | 0.7 | 0.8 | 0.9 | 1.0 | 1.8 | 1.5 | 0.5 | 0.8 | 0.9 |
| Lrp5 | 10.7 | 12.8 | 12.8 | 12.0 | 11.6 | 12.5 | 13.7 | 10.3 | 11.9 | 9.0 | 8.2 | 9.4 |
| Lrp6 | 16.9 | 20.4 | 24.5 | 15.9 | 16.1 | 16.9 | 18.9 | 20.3 | 18.9 | 18.6 | 20.3 | 21.5 |
| Lrp8 | 1.2 | 0.9 | 0.9 | 2.1 | 1.5 | 1.6 | 1.5 | 1.3 | 0.7 | 0.6 | 1.0 | 0.6 |
| Lrpap1 | 53.1 | 45.3 | 47.2 | 65.0 | 49.2 | 60.6 | 45.4 | 42.3 | 41.4 | 45.6 | 43.4 | 48.4 |
| Lrpprc | 14.8 | 10.3 | 8.3 | 14.0 | 12.6 | 14.0 | 9.9 | 10.0 | 9.0 | 11.9 | 11.8 | 10.3 |
| Lrr1 | 0.1 | 0.4 | 0.8 | 0.1 | 0.7 | 0.2 | 0.8 | 0.3 | 0.5 | 0.3 | 0.3 | 0.2 |
| Lrrc1 | 1.5 | 1.7 | 1.5 | 1.5 | 1.2 | 1.8 | 1.0 | 1.5 | 1.6 | 3.4 | 2.5 | 1.8 |
| Lrrc10b | 0.0 | 0.0 | 0.0 | 0.0 | 0.0 | 0.0 | 0.0 | 0.0 | 0.0 | 0.1 | 0.0 | 0.1 |
| Lrrc14 | 7.5 | 6.3 | 5.4 | 6.4 | 6.9 | 7.7 | 6.7 | 6.5 | 7.7 | 6.2 | 5.7 | 6.3 |
| Lrrc14b | 1.5 | 1.2 | 1.4 | 1.7 | 1.2 | 1.3 | 1.0 | 1.1 | 1.1 | 1.7 | 1.5 | 1.2 |
| Lrrc15 | 0.0 | 0.0 | 0.0 | 0.0 | 0.0 | 0.1 | 0.0 | 0.0 | 0.0 | 0.0 | 0.0 | 0.0 |
| Lrrc16a | 4.6 | 4.4 | 5.8 | 4.5 | 4.4 | 4.0 | 6.2 | 5.0 | 4.4 | 3.6 | 3.7 | 4.9 |
| Lrrc16b | 0.1 | 0.0 | 0.1 | 0.1 | 0.0 | 0.0 | 0.2 | 0.2 | 0.1 | 0.1 | 0.1 | 0.1 |
| Lrrc17 | 0.2 | 0.4 | 0.4 | 0.2 | 0.1 | 0.1 | 0.2 | 4.1 | 0.6 | 0.5 | 1.1 | 0.3 |
| Lrrc18 | 0.0 | 0.0 | 0.0 | 0.1 | 0.0 | 0.0 | 0.1 | 0.0 | 0.0 | 0.0 | 0.0 | 0.0 |
| Lrrc19 | 0.0 | 0.0 | 0.1 | 0.2 | 0.0 | 0.0 | 0.1 | 0.1 | 0.1 | 0.1 | 0.0 | 0.1 |
| Lrrc2 | 0.0 | 0.0 | 0.0 | 0.0 | 0.0 | 0.0 | 0.0 | 0.0 | 0.0 | 0.0 | 0.0 | 0.0 |
| Lrrc20 | 3.6 | 3.1 | 3.2 | 3.4 | 4.2 | 3.2 | 3.7 | 3.7 | 3.3 | 2.7 | 3.6 | 2.7 |
| Lrrc23 | 0.0 | 0.0 | 0.1 | 0.1 | 0.0 | 0.0 | 0.0 | 0.0 | 0.0 | 0.1 | 0.1 | 0.0 |
| Lrrc24 | 0.5 | 0.3 | 0.4 | 0.1 | 0.7 | 0.6 | 0.4 | 0.3 | 0.5 | 0.6 | 0.5 | 0.6 |
| Lrrc25 | 0.6 | 0.0 | 0.0 | 6.8 | 13.4 | 0.0 | 1.8 | 0.2 | 0.1 | 0.9 | 0.3 | 0.8 |
| Lrrc26 | 0.1 | 0.0 | 0.0 | 0.0 | 0.0 | 0.0 | 0.1 | 0.1 | 0.0 | 0.0 | 0.0 | 0.0 |
| Lrrc27 | 11.7 | 9.6 | 7.7 | 14.1 | 8.1 | 10.8 | 9.3 | 7.0 | 5.6 | 4.6 | 9.7 | 6.0 |
| Lrrc28 | 5.2 | 4.6 | 4.4 | 5.1 | 4.0 | 5.7 | 4.2 | 5.3 | 3.8 | 4.6 | 4.9 | 3.5 |
| Lrrc29 | 0.2 | 0.3 | 0.4 | 0.1 | 0.2 | 0.3 | 0.3 | 0.1 | 0.3 | 0.4 | 0.4 | 0.2 |
| Lrrc3 | 0.6 | 0.7 | 0.7 | 0.4 | 0.5 | 0.6 | 0.5 | 0.3 | 0.3 | 0.2 | 0.2 | 0.3 |
| Lrrc32 | 23.7 | 20.0 | 18.7 | 29.2 | 30.3 | 33.1 | 47.7 | 18.8 | 47.7 | 22.0 | 21.7 | 38.8 |
| Lrrc33 | 0.6 | 0.2 | 0.2 | 3.7 | 5.3 | 0.1 | 0.4 | 0.3 | 0.1 | 0.5 | 0.2 | 0.2 |
| Lrrc34 | 0.1 | 0.0 | 0.0 | 0.2 | 0.0 | 0.0 | 0.0 | 0.0 | 0.0 | 0.1 | 0.0 | 0.0 |
| Lrrc36 | 0.0 | 0.0 | 0.1 | 0.0 | 0.0 | 0.0 | 0.1 | 0.0 | 0.0 | 0.0 | 0.1 | 0.0 |
| Lrrc39 | 0.3 | 0.3 | 0.3 | 0.5 | 0.4 | 0.2 | 0.2 | 0.1 | 0.2 | 0.2 | 0.2 | 0.2 |
| Lrrc4 | 0.1 | 0.2 | 0.2 | 0.0 | 0.1 | 0.1 | 0.2 | 0.1 | 0.1 | 0.3 | 0.2 | 0.2 |
| Lrrc40 | 4.3 | 5.2 | 5.6 | 5.1 | 4.3 | 4.5 | 4.3 | 4.7 | 4.4 | 3.7 | 5.0 | 4.5 |
| Lrrc41 | 19.7 | 22.8 | 18.8 | 19.7 | 18.1 | 20.4 | 21.4 | 21.6 | 20.9 | 20.8 | 18.9 | 18.2 |
| Lrrc42 | 8.4 | 11.0 | 10.8 | 7.4 | 8.7 | 8.6 | 9.9 | 12.2 | 12.1 | 11.2 | 10.7 | 9.9 |
| Lrrc45 | 7.3 | 7.5 | 7.8 | 6.6 | 6.2 | 6.7 | 7.1 | 6.6 | 6.8 | 6.9 | 6.5 | 5.8 |
| Lrrc46 | 0.2 | 0.3 | 0.4 | 0.2 | 0.0 | 0.1 | 0.0 | 0.1 | 0.1 | 0.2 | 0.2 | 0.1 |
| Lrrc47 | 3.3 | 3.8 | 3.4 | 3.2 | 4.0 | 3.3 | 3.9 | 4.1 | 3.1 | 3.8 | 3.4 | 3.4 |
| Lrrc48 | 0.7 | 0.7 | 0.5 | 0.7 | 0.3 | 1.0 | 0.4 | 0.4 | 0.3 | 0.4 | 0.5 | 0.4 |
| Lrrc49 | 4.0 | 3.3 | 3.9 | 3.8 | 3.6 | 4.4 | 4.3 | 4.5 | 3.9 | 4.9 | 5.3 | 4.4 |
| Lrrc4b | 0.0 | 0.0 | 0.0 | 0.0 | 0.0 | 0.0 | 0.0 | 0.0 | 0.0 | 0.1 | 0.0 | 0.0 |
| Lrrc4c | 0.2 | 0.2 | 0.0 | 0.2 | 0.1 | 0.1 | 0.4 | 0.2 | 0.2 | 0.8 | 0.7 | 0.4 |

| | | | | | | | | | | | | |
|---|---|---|---|---|---|---|---|---|---|---|---|---|
| Lrrc51 | 15.8 | 16.2 | 13.4 | 15.9 | 9.4 | 16.6 | 8.2 | 9.8 | 8.2 | 11.1 | 10.0 | 10.4 |
| Lrrc55 | 0.0 | 0.1 | 0.0 | 0.0 | 0.0 | 0.0 | 0.1 | 0.0 | 0.0 | 0.0 | 0.1 | 0.1 |
| Lrrc56 | 0.9 | 0.9 | 1.0 | 0.7 | 0.8 | 0.7 | 1.0 | 1.4 | 0.9 | 0.8 | 0.9 | 1.0 |
| Lrrc57 | 12.8 | 12.5 | 10.2 | 11.5 | 10.0 | 14.7 | 9.9 | 9.1 | 8.8 | 10.3 | 10.6 | 10.6 |
| Lrrc58 | 35.6 | 39.6 | 42.4 | 43.0 | 44.8 | 46.9 | 48.0 | 43.3 | 45.4 | 44.0 | 47.8 | 49.0 |
| Lrrc59 | 61.1 | 53.3 | 50.0 | 63.8 | 61.8 | 54.1 | 72.3 | 71.8 | 60.7 | 59.3 | 70.0 | 59.1 |
| Lrrc61 | 1.6 | 1.4 | 1.5 | 2.1 | 1.8 | 1.9 | 1.7 | 1.9 | 1.8 | 1.4 | 1.7 | 1.8 |
| Lrrc69 | 0.0 | 0.0 | 0.1 | 0.0 | 0.0 | 0.0 | 0.0 | 0.0 | 0.0 | 0.0 | 0.1 | 0.0 |
| Lrrc71 | 2.0 | 2.3 | 2.0 | 2.0 | 2.0 | 2.2 | 2.4 | 1.9 | 2.1 | 1.6 | 1.9 | 1.5 |
| Lrrc73 | 1.0 | 1.2 | 0.9 | 1.9 | 1.1 | 2.0 | 0.6 | 0.8 | 1.6 | 1.8 | 0.9 | 1.1 |
| Lrrc8a | 9.7 | 11.0 | 12.3 | 10.6 | 11.5 | 11.9 | 13.9 | 16.0 | 15.4 | 12.0 | 12.8 | 11.9 |
| Lrrc8b | 3.1 | 2.5 | 2.9 | 2.9 | 2.9 | 3.6 | 2.6 | 3.0 | 2.2 | 1.8 | 2.4 | 1.8 |
| Lrrc8c | 9.1 | 11.4 | 13.4 | 6.3 | 10.6 | 8.4 | 12.2 | 10.0 | 9.4 | 9.2 | 9.7 | 8.0 |
| Lrrc8d | 3.4 | 3.3 | 2.8 | 4.4 | 3.8 | 4.0 | 3.0 | 2.2 | 1.4 | 2.2 | 2.2 | 1.6 |
| Lrrc8e | 0.4 | 0.3 | 0.3 | 0.3 | 0.2 | 0.5 | 0.3 | 0.4 | 0.5 | 0.4 | 0.4 | 0.6 |
| Lrrc9 | 0.3 | 0.5 | 0.4 | 0.2 | 0.3 | 0.4 | 0.3 | 0.1 | 0.1 | 0.1 | 0.1 | 0.2 |
| Lrrcc1 | 6.4 | 7.8 | 9.2 | 5.8 | 6.4 | 8.0 | 6.3 | 7.3 | 6.9 | 6.6 | 8.2 | 8.0 |
| Lrrd1 | 0.0 | 0.0 | 0.0 | 0.0 | 0.0 | 0.0 | 0.0 | 0.0 | 0.0 | 0.0 | 0.0 | 0.0 |
| Lrrfip1 | 67.9 | 47.6 | 42.0 | 61.2 | 49.1 | 53.3 | 54.7 | 36.9 | 38.2 | 55.9 | 62.6 | 58.8 |
| Lrrfip2 | 15.3 | 12.1 | 12.5 | 18.2 | 12.2 | 15.7 | 13.7 | 13.2 | 11.5 | 10.9 | 15.4 | 13.5 |
| Lrriq1 | 0.1 | 0.0 | 0.1 | 0.1 | 0.0 | 0.1 | 0.1 | 0.0 | 0.0 | 0.0 | 0.0 | 0.0 |
| Lrriq3 | 0.7 | 0.7 | 0.6 | 0.6 | 0.6 | 0.6 | 0.6 | 0.4 | 0.5 | 0.5 | 0.5 | 0.7 |
| Lrrk1 | 23.6 | 23.3 | 20.7 | 22.9 | 20.8 | 24.3 | 23.6 | 19.2 | 19.4 | 15.3 | 17.5 | 17.6 |
| Lrrk2 | 10.7 | 9.8 | 9.0 | 11.5 | 9.3 | 13.1 | 10.4 | 7.9 | 9.0 | 9.6 | 10.8 | 10.6 |
| Lrrn1 | 0.0 | 0.0 | 0.0 | 0.0 | 0.0 | 0.0 | 0.1 | 0.0 | 0.1 | 0.0 | 0.0 | 0.0 |
| Lrrn2 | 1.2 | 1.4 | 1.3 | 1.2 | 0.9 | 2.0 | 1.2 | 0.8 | 1.2 | 1.3 | 0.8 | 1.0 |
| Lrrn3 | 0.2 | 0.1 | 0.1 | 0.1 | 0.1 | 0.3 | 0.1 | 0.1 | 0.2 | 0.2 | 0.1 | 0.1 |
| Lrrn4 | 6.3 | 2.8 | 1.5 | 1.3 | 0.8 | 0.6 | 0.3 | 3.5 | 3.9 | 52.9 | 20.6 | 12.9 |
| Lrrn4cl | 7.1 | 6.0 | 5.1 | 6.9 | 3.1 | 11.8 | 4.0 | 4.6 | 4.9 | 5.3 | 8.4 | 6.0 |
| Lrrtm2 | 0.1 | 0.2 | 0.2 | 0.2 | 0.1 | 0.2 | 0.2 | 0.1 | 0.1 | 0.1 | 0.3 | 0.1 |
| Lrrtm3 | 0.0 | 0.0 | 0.0 | 0.0 | 0.0 | 0.0 | 0.0 | 0.0 | 0.0 | 0.0 | 0.0 | 0.0 |
| Lrsam1 | 6.4 | 5.3 | 4.8 | 7.0 | 4.7 | 6.8 | 4.9 | 4.9 | 5.8 | 5.8 | 5.4 | 5.7 |
| Lrtm1 | 0.0 | 0.0 | 0.0 | 0.0 | 0.0 | 0.0 | 0.0 | 0.0 | 0.0 | 0.0 | 0.0 | 0.0 |
| Lrtm2 | 0.2 | 0.0 | 0.0 | 0.0 | 0.0 | 0.0 | 0.0 | 0.1 | 0.0 | 0.2 | 0.1 | 0.0 |
| Lrwd1 | 6.4 | 6.8 | 7.1 | 6.0 | 6.6 | 6.1 | 7.6 | 6.8 | 6.8 | 7.4 | 6.5 | 6.2 |
| Lsamp | 0.1 | 0.1 | 0.1 | 0.1 | 0.0 | 0.0 | 0.0 | 0.0 | 0.0 | 0.0 | 0.0 | 0.0 |
| Lsg1 | 13.6 | 11.8 | 11.3 | 15.7 | 11.6 | 11.5 | 12.5 | 10.5 | 9.7 | 12.5 | 14.1 | 13.2 |
| Lsm1 | 7.5 | 7.5 | 7.2 | 7.5 | 6.9 | 7.1 | 6.7 | 6.4 | 6.9 | 7.0 | 7.6 | 6.9 |
| Lsm10 | 17.9 | 16.6 | 16.8 | 20.3 | 14.8 | 20.9 | 14.5 | 17.2 | 14.8 | 14.8 | 16.0 | 15.6 |
| Lsm11 | 0.9 | 1.0 | 1.0 | 0.8 | 0.9 | 0.7 | 1.0 | 0.9 | 1.1 | 1.0 | 0.9 | 0.9 |
| Lsm12 | 16.9 | 16.3 | 17.2 | 20.2 | 17.6 | 18.6 | 17.8 | 19.3 | 16.0 | 15.1 | 21.0 | 17.9 |
| Lsm14a | 19.0 | 24.2 | 23.3 | 20.6 | 20.2 | 21.9 | 20.1 | 23.1 | 22.3 | 22.5 | 22.6 | 24.7 |
| Lsm14b | 12.9 | 12.5 | 12.3 | 10.7 | 10.6 | 11.7 | 13.2 | 12.8 | 12.8 | 11.8 | 12.7 | 11.7 |
| Lsm2 | 5.0 | 3.9 | 5.6 | 2.6 | 6.6 | 3.5 | 4.8 | 5.3 | 5.8 | 5.5 | 5.8 | 4.5 |
| Lsm3 | 26.3 | 29.9 | 33.6 | 29.1 | 29.0 | 21.2 | 31.9 | 34.2 | 28.5 | 29.3 | 28.0 | 29.1 |
| Lsm4 | 17.7 | 17.1 | 15.4 | 20.7 | 18.3 | 17.6 | 24.5 | 23.1 | 21.0 | 22.1 | 21.7 | 21.3 |
| Lsm5 | 11.5 | 12.4 | 17.4 | 12.8 | 14.4 | 13.7 | 10.4 | 12.6 | 13.7 | 15.3 | 16.8 | 15.1 |
| Lsm6 | 3.3 | 3.3 | 3.7 | 4.4 | 3.6 | 3.2 | 4.0 | 3.6 | 3.3 | 3.6 | 4.4 | 3.8 |
| Lsm7 | 41.4 | 32.0 | 35.0 | 36.4 | 33.0 | 34.4 | 30.9 | 35.6 | 28.2 | 45.3 | 31.2 | 31.7 |
| Lsmd1 | 37.7 | 29.4 | 28.4 | 36.4 | 32.3 | 36.5 | 35.3 | 32.3 | 29.4 | 34.4 | 34.1 | 33.7 |
| Lsp1 | 20.9 | 17.5 | 22.9 | 42.9 | 50.1 | 27.0 | 34.3 | 29.5 | 30.0 | 23.6 | 28.5 | 29.4 |
| Lsr | 0.3 | 0.4 | 0.3 | 0.1 | 0.4 | 0.7 | 0.4 | 0.3 | 0.3 | 1.0 | 0.2 | 0.4 |
| Lss | 12.5 | 15.0 | 13.2 | 6.2 | 8.0 | 8.4 | 19.7 | 19.6 | 16.8 | 5.6 | 18.1 | 7.2 |
| Lst1 | 3.1 | 0.3 | 0.8 | 12.9 | 31.4 | 0.6 | 8.1 | 0.4 | 2.0 | 4.0 | 0.6 | 2.4 |
| Lta | 0.0 | 0.0 | 0.0 | 0.0 | 0.0 | 0.0 | 0.0 | 0.0 | 0.0 | 0.1 | 0.0 | 0.0 |
| Lta4h | 33.5 | 29.6 | 28.1 | 28.1 | 30.8 | 29.6 | 30.2 | 32.5 | 30.7 | 33.7 | 32.9 | 35.0 |
| Ltb4r1 | 0.0 | 0.1 | 0.0 | 0.0 | 0.1 | 0.2 | 0.0 | 0.0 | 0.0 | 0.0 | 0.0 | 0.0 |

| | | | | | | | | | | | | |
|---|---|---|---|---|---|---|---|---|---|---|---|---|
| Ltb4r2 | 0.1 | 0.1 | 0.0 | 0.1 | 0.1 | 0.1 | 0.0 | 0.0 | 0.1 | 0.3 | 0.1 | 0.0 |
| Ltbp1 | 4.1 | 2.7 | 3.1 | 2.8 | 2.8 | 3.7 | 2.7 | 3.8 | 2.0 | 2.4 | 3.0 | 2.2 |
| Ltbp2 | 3.5 | 2.7 | 4.7 | 2.9 | 8.4 | 5.8 | 29.6 | 10.6 | 19.9 | 39.2 | 20.8 | 17.6 |
| Ltbp3 | 8.1 | 9.0 | 8.7 | 5.5 | 8.5 | 7.7 | 9.9 | 7.4 | 10.1 | 9.8 | 6.7 | 6.7 |
| Ltbp4 | 2.1 | 2.0 | 3.3 | 1.2 | 1.5 | 2.1 | 2.3 | 5.9 | 2.1 | 1.7 | 1.7 | 1.3 |
| Ltbr | 39.8 | 34.7 | 37.3 | 35.4 | 38.2 | 36.2 | 45.7 | 42.7 | 43.4 | 40.5 | 41.2 | 38.1 |
| Ltc4s | 0.9 | 0.2 | 0.2 | 1.9 | 1.8 | 0.6 | 0.2 | 0.5 | 0.5 | 0.8 | 0.4 | 0.4 |
| Ltf | 0.1 | 0.0 | 0.1 | 0.0 | 0.0 | 0.0 | 0.0 | 0.0 | 0.0 | 0.0 | 0.0 | 0.0 |
| Ltk | 0.2 | 0.0 | 0.1 | 0.0 | 0.0 | 0.1 | 0.0 | 0.0 | 0.0 | 0.1 | 0.0 | 0.1 |
| Ltn1 | 9.3 | 9.0 | 8.4 | 9.1 | 9.2 | 9.5 | 8.1 | 8.3 | 8.5 | 9.0 | 9.1 | 9.4 |
| Ltv1 | 14.8 | 13.2 | 12.7 | 17.5 | 12.7 | 13.4 | 15.5 | 13.6 | 11.5 | 14.3 | 16.8 | 14.4 |
| Luc7l | 11.1 | 12.2 | 11.4 | 10.3 | 10.6 | 10.9 | 10.8 | 9.9 | 11.5 | 13.2 | 12.0 | 11.0 |
| Luc7l2 | 20.3 | 20.6 | 26.5 | 23.1 | 19.7 | 21.4 | 25.6 | 23.9 | 19.1 | 19.1 | 26.1 | 24.7 |
| Luc7l3 | 20.0 | 19.7 | 26.2 | 21.2 | 18.7 | 17.5 | 22.8 | 21.3 | 17.5 | 17.1 | 22.5 | 28.1 |
| Lum | 3.0 | 19.0 | 67.5 | 0.5 | 6.7 | 0.3 | 9.4 | 78.1 | 43.7 | 19.1 | 8.6 | 42.8 |
| Lurap1 | 1.5 | 1.6 | 1.0 | 1.5 | 1.0 | 1.6 | 1.1 | 1.1 | 1.0 | 1.6 | 0.7 | 1.4 |
| Lurap1l | 38.4 | 22.4 | 14.1 | 65.4 | 54.8 | 51.9 | 32.5 | 18.3 | 26.2 | 15.1 | 23.4 | 13.0 |
| Luzp1 | 26.2 | 31.5 | 29.7 | 27.3 | 29.9 | 24.0 | 30.7 | 27.8 | 30.4 | 28.8 | 32.2 | 33.4 |
| Luzp2 | 0.0 | 0.1 | 0.0 | 0.0 | 0.0 | 0.1 | 0.0 | 0.0 | 0.0 | 0.0 | 0.0 | 0.0 |
| Lxn | 36.3 | 35.2 | 39.4 | 36.3 | 33.8 | 30.3 | 34.9 | 29.2 | 30.2 | 41.2 | 40.7 | 36.3 |
| Ly6a | 97.9 | 87.1 | 85.2 | 91.7 | 68.0 | 70.4 | 67.6 | 162.8 | 59.3 | 36.0 | 68.9 | 53.4 |
| Ly6c1 | 3.8 | 2.0 | 1.9 | 4.0 | 2.8 | 2.0 | 4.2 | 5.7 | 3.7 | 2.1 | 4.8 | 3.6 |
| Ly6c2 | 0.5 | 0.5 | 0.1 | 0.4 | 0.3 | 0.5 | 1.2 | 0.7 | 0.7 | 0.3 | 0.8 | 0.5 |
| Ly6d | 0.1 | 0.3 | 0.2 | 0.1 | 0.1 | 0.0 | 0.0 | 0.3 | 0.1 | 0.0 | 0.0 | 0.2 |
| Ly6e | 22.3 | 24.2 | 28.2 | 45.7 | 27.0 | 26.0 | 22.4 | 22.3 | 15.5 | 15.5 | 14.3 | 18.5 |
| Ly6f | 0.2 | 0.1 | 0.0 | 0.0 | 0.1 | 0.1 | 0.0 | 0.1 | 0.0 | 0.1 | 0.4 | 0.0 |
| Ly6g5b | 0.0 | 0.1 | 0.1 | 0.0 | 0.1 | 0.1 | 0.0 | 0.3 | 0.1 | 0.2 | 0.0 | 0.0 |
| Ly6i | 0.0 | 0.1 | 0.1 | 0.1 | 0.1 | 0.0 | 0.1 | 0.2 | 0.1 | 0.1 | 0.0 | 0.1 |
| Ly6k | 3.0 | 2.2 | 2.9 | 1.6 | 0.8 | 0.6 | 1.5 | 1.4 | 1.2 | 3.5 | 2.8 | 2.2 |
| Ly75 | 0.6 | 0.7 | 0.8 | 0.4 | 0.6 | 0.5 | 0.9 | 0.6 | 0.8 | 1.2 | 1.4 | 0.8 |
| Ly86 | 0.8 | 0.0 | 0.1 | 4.6 | 7.8 | 0.0 | 1.0 | 0.1 | 0.1 | 0.1 | 0.2 | 0.8 |
| Ly9 | 0.7 | 0.5 | 0.2 | 3.9 | 6.1 | 0.3 | 0.8 | 0.3 | 0.3 | 1.0 | 0.9 | 0.6 |
| Ly96 | 7.9 | 8.9 | 7.9 | 12.1 | 9.8 | 8.3 | 5.6 | 5.6 | 7.5 | 10.0 | 7.1 | 9.1 |
| Lyar | 11.7 | 10.5 | 12.9 | 14.1 | 10.7 | 11.3 | 13.7 | 8.9 | 7.8 | 9.7 | 12.1 | 12.4 |
| Lyl1 | 0.3 | 0.1 | 0.0 | 0.5 | 0.5 | 0.0 | 0.1 | 0.1 | 0.0 | 0.2 | 0.1 | 0.0 |
| Lyn | 7.5 | 5.9 | 4.7 | 15.3 | 21.9 | 5.6 | 11.6 | 5.9 | 9.4 | 8.1 | 8.5 | 9.4 |
| Lynx1 | 24.1 | 19.4 | 17.8 | 25.3 | 17.9 | 28.3 | 20.1 | 16.4 | 16.3 | 16.7 | 18.6 | 19.8 |
| Lypd1 | 33.0 | 28.1 | 29.3 | 32.9 | 26.3 | 29.0 | 44.6 | 32.3 | 39.5 | 47.1 | 45.7 | 53.3 |
| Lypd6 | 0.0 | 0.0 | 0.0 | 0.0 | 0.0 | 0.0 | 0.0 | 0.0 | 0.0 | 0.0 | 0.0 | 0.0 |
| Lypd6b | 0.0 | 0.0 | 0.0 | 0.0 | 0.0 | 0.0 | 0.0 | 0.1 | 0.0 | 0.0 | 0.0 | 0.0 |
| Lypla1 | 19.9 | 20.0 | 25.4 | 26.7 | 22.3 | 25.8 | 23.0 | 22.8 | 19.7 | 21.9 | 29.8 | 27.4 |
| Lypla2 | 6.7 | 6.3 | 5.9 | 8.6 | 9.8 | 6.5 | 7.5 | 8.5 | 8.4 | 7.6 | 6.0 | 5.9 |
| Lyplal1 | 7.2 | 5.8 | 4.7 | 6.1 | 4.0 | 8.0 | 3.6 | 4.7 | 3.6 | 4.7 | 4.7 | 5.5 |
| Lyrm1 | 3.8 | 4.0 | 3.5 | 5.1 | 3.3 | 4.8 | 3.6 | 3.4 | 3.6 | 3.9 | 3.7 | 4.6 |
| Lyrm2 | 18.5 | 12.8 | 13.7 | 16.3 | 11.7 | 18.9 | 11.9 | 13.4 | 11.5 | 15.9 | 16.3 | 15.9 |
| Lyrm4 | 4.8 | 4.3 | 4.1 | 5.3 | 3.7 | 5.2 | 4.3 | 4.6 | 3.8 | 4.7 | 4.7 | 4.6 |
| Lyrm5 | 20.1 | 21.2 | 18.4 | 23.1 | 19.0 | 25.2 | 17.9 | 19.3 | 18.3 | 20.5 | 22.8 | 22.0 |
| Lyrm7 | 1.0 | 0.9 | 1.0 | 1.4 | 0.7 | 1.5 | 1.0 | 0.9 | 0.7 | 1.0 | 1.1 | 0.9 |
| Lyrm9 | 1.8 | 1.3 | 0.7 | 1.9 | 1.3 | 2.0 | 0.8 | 0.8 | 0.7 | 0.9 | 1.2 | 1.0 |
| Lysmd1 | 4.8 | 4.4 | 4.3 | 4.7 | 4.0 | 4.4 | 3.7 | 3.8 | 3.5 | 4.2 | 4.3 | 3.7 |
| Lysmd2 | 7.2 | 7.2 | 8.5 | 7.4 | 5.5 | 8.0 | 6.4 | 7.6 | 7.3 | 6.1 | 5.0 | 6.9 |
| Lysmd3 | 4.7 | 6.0 | 7.3 | 6.1 | 5.6 | 4.4 | 6.1 | 7.3 | 6.8 | 6.9 | 8.8 | 7.9 |
| Lysmd4 | 3.8 | 4.1 | 4.3 | 4.0 | 3.5 | 4.1 | 3.6 | 4.4 | 4.3 | 3.9 | 4.2 | 3.8 |
| Lyst | 6.3 | 7.7 | 6.2 | 5.2 | 6.6 | 5.2 | 5.2 | 5.5 | 7.4 | 7.5 | 6.1 | 9.7 |
| Lyve1 | 0.0 | 0.1 | 0.0 | 0.1 | 0.0 | 0.2 | 0.0 | 0.0 | 0.0 | 0.1 | 0.2 | 0.1 |
| Lyz1 | 5.7 | 6.1 | 5.0 | 31.3 | 62.9 | 5.6 | 6.5 | 3.3 | 4.1 | 5.9 | 3.9 | 5.3 |
| Lyz2 | 269.3 | 62.0 | 60.2 | 1261.6 | 2077.8 | 62.4 | 216.6 | 86.4 | 72.6 | 166.1 | 94.0 | 147.1 |

| | | | | | | | | | | | | |
|---|---|---|---|---|---|---|---|---|---|---|---|---|
| Lyzl4 | 0.0 | 0.0 | 0.0 | 0.1 | 0.2 | 0.0 | 0.0 | 0.0 | 0.0 | 0.0 | 0.0 | 0.0 |
| Lzic | 18.4 | 16.9 | 15.6 | 14.0 | 14.4 | 16.7 | 13.5 | 11.6 | 15.6 | 17.4 | 14.5 | 13.5 |
| Lztfl1 | 9.6 | 10.2 | 10.6 | 10.9 | 10.0 | 12.5 | 9.0 | 10.0 | 9.0 | 10.7 | 12.8 | 12.2 |
| Lztr1 | 24.3 | 20.9 | 19.8 | 24.1 | 22.3 | 27.9 | 20.5 | 22.8 | 23.8 | 25.5 | 23.0 | 21.3 |
| Lzts1 | 0.4 | 0.3 | 0.7 | 0.3 | 0.2 | 0.2 | 0.1 | 0.2 | 0.1 | 0.2 | 0.1 | 0.3 |
| Lzts2 | 24.0 | 29.8 | 26.7 | 19.5 | 19.9 | 23.9 | 25.2 | 32.1 | 32.8 | 26.9 | 25.1 | 24.0 |
| M1ap | 0.4 | 0.4 | 0.4 | 0.2 | 0.5 | 0.1 | 0.5 | 0.3 | 0.4 | 0.4 | 0.3 | 0.6 |
| M6pr | 77.4 | 72.4 | 70.4 | 97.4 | 96.3 | 80.1 | 85.3 | 59.4 | 76.5 | 76.4 | 79.1 | 73.4 |
| Maats1 | 0.2 | 0.0 | 0.1 | 0.1 | 0.0 | 0.1 | 0.0 | 0.0 | 0.0 | 0.1 | 0.2 | 0.0 |
| Mab21l1 | 0.1 | 0.3 | 0.1 | 0.1 | 0.2 | 0.3 | 0.2 | 0.2 | 0.2 | 0.1 | 0.2 | 0.1 |
| Mab21l2 | 0.0 | 0.0 | 0.0 | 0.0 | 0.0 | 0.0 | 0.0 | 0.0 | 0.0 | 0.0 | 0.0 | 0.0 |
| Mab21l3 | 2.8 | 2.0 | 1.7 | 0.7 | 1.5 | 1.1 | 1.1 | 0.7 | 0.9 | 3.6 | 2.4 | 1.4 |
| Macc1 | 0.0 | 0.1 | 0.1 | 0.1 | 0.1 | 0.0 | 0.1 | 0.0 | 0.1 | 0.1 | 0.1 | 0.1 |
| Macf1 | 46.6 | 44.3 | 38.1 | 43.7 | 43.1 | 40.8 | 47.2 | 43.6 | 42.4 | 36.5 | 45.6 | 41.4 |
| Macrod1 | 2.2 | 2.1 | 2.9 | 2.2 | 1.7 | 2.6 | 2.2 | 3.0 | 2.2 | 2.3 | 1.5 | 2.2 |
| Macrod2 | 0.5 | 0.3 | 0.4 | 0.9 | 0.3 | 0.5 | 0.6 | 0.3 | 0.3 | 0.3 | 0.4 | 0.5 |
| Mad1l1 | 5.9 | 7.3 | 8.1 | 6.0 | 6.8 | 5.9 | 8.4 | 9.1 | 6.4 | 5.9 | 6.7 | 6.3 |
| Mad2l1 | 4.8 | 7.1 | 12.1 | 3.2 | 9.4 | 3.5 | 10.6 | 6.5 | 9.0 | 6.4 | 8.9 | 6.8 |
| Mad2l1bp | 5.1 | 4.8 | 5.0 | 4.7 | 6.6 | 4.0 | 5.1 | 5.2 | 6.6 | 5.5 | 5.0 | 5.9 |
| Mad2l2 | 4.3 | 4.5 | 4.4 | 4.8 | 3.7 | 4.2 | 2.8 | 4.5 | 3.6 | 3.4 | 2.9 | 3.0 |
| Madcam1 | 0.0 | 0.1 | 0.0 | 0.2 | 0.0 | 0.1 | 0.0 | 0.0 | 0.0 | 0.0 | 0.0 | 0.0 |
| Madd | 1.0 | 1.0 | 0.6 | 1.9 | 2.6 | 1.0 | 1.2 | 0.8 | 0.9 | 1.1 | 1.0 | 0.9 |
| Maea | 87.2 | 91.0 | 77.0 | 82.4 | 80.4 | 86.2 | 100.1 | 91.6 | 97.6 | 82.3 | 90.3 | 81.3 |
| Mael | 0.1 | 0.0 | 0.1 | 0.0 | 0.0 | 0.1 | 0.0 | 0.0 | 0.0 | 0.1 | 0.2 | 0.0 |
| Maf | 1.5 | 1.0 | 1.4 | 3.3 | 2.8 | 1.8 | 1.3 | 1.5 | 1.9 | 11.0 | 6.1 | 4.8 |
| Maf1 | 22.9 | 22.9 | 19.6 | 21.8 | 21.7 | 24.0 | 20.0 | 22.4 | 22.8 | 24.5 | 20.4 | 20.4 |
| Mafa | 0.1 | 0.0 | 0.0 | 0.3 | 0.1 | 0.1 | 0.2 | 0.1 | 0.0 | 0.2 | 0.0 | 0.3 |
| Mafb | 0.2 | 0.2 | 0.3 | 2.0 | 3.3 | 0.2 | 0.4 | 0.3 | 0.3 | 0.5 | 0.5 | 0.4 |
| Maff | 76.1 | 44.8 | 29.1 | 103.3 | 42.4 | 79.7 | 40.4 | 19.6 | 19.5 | 31.2 | 35.0 | 32.0 |
| Mafg | 8.0 | 7.6 | 7.0 | 11.8 | 11.2 | 10.0 | 9.3 | 6.3 | 7.4 | 9.0 | 8.7 | 7.5 |
| Mafk | 57.1 | 52.0 | 34.8 | 71.4 | 44.3 | 52.3 | 54.4 | 29.8 | 37.2 | 38.1 | 50.6 | 36.8 |
| Mag | 0.1 | 0.0 | 0.0 | 0.2 | 0.1 | 0.0 | 0.0 | 0.0 | 0.0 | 0.0 | 0.0 | 0.0 |
| Mageb16 | 0.0 | 0.0 | 0.0 | 0.1 | 0.0 | 0.0 | 0.0 | 0.0 | 0.0 | 0.0 | 0.0 | 0.0 |
| Mageb16-ps1 | 0.0 | 0.0 | 0.0 | 0.0 | 0.0 | 0.0 | 0.1 | 0.0 | 0.0 | 0.0 | 0.0 | 0.0 |
| Maged1 | 165.0 | 174.2 | 188.2 | 151.2 | 150.8 | 151.3 | 165.8 | 213.9 | 180.4 | 191.0 | 184.0 | 181.8 |
| Maged2 | 46.5 | 48.6 | 49.0 | 41.1 | 44.8 | 40.6 | 46.7 | 62.1 | 63.3 | 50.8 | 48.4 | 50.5 |
| Magee1 | 8.5 | 9.4 | 8.7 | 6.9 | 7.3 | 7.3 | 8.9 | 6.7 | 8.2 | 8.0 | 7.6 | 8.7 |
| Magee2 | 0.1 | 0.1 | 0.1 | 0.1 | 0.0 | 0.0 | 0.0 | 0.0 | 0.0 | 0.0 | 0.0 | 0.0 |
| Mageh1 | 6.5 | 4.4 | 4.4 | 7.5 | 5.4 | 7.4 | 5.8 | 4.7 | 4.7 | 6.1 | 5.6 | 5.5 |
| Magi1 | 3.0 | 4.1 | 5.5 | 3.5 | 3.4 | 3.3 | 5.1 | 6.6 | 7.0 | 3.5 | 5.1 | 4.9 |
| Magi2 | 0.9 | 0.9 | 0.9 | 0.9 | 0.6 | 1.0 | 1.0 | 0.7 | 0.8 | 0.7 | 0.9 | 1.0 |
| Magi3 | 15.2 | 15.5 | 13.7 | 14.8 | 11.3 | 10.6 | 12.5 | 9.4 | 10.5 | 11.3 | 14.6 | 11.3 |
| Magix | 0.1 | 0.0 | 0.1 | 0.0 | 0.0 | 0.1 | 0.0 | 0.1 | 0.0 | 0.0 | 0.0 | 0.1 |
| Magoh | 42.1 | 39.8 | 48.4 | 39.2 | 39.2 | 44.7 | 38.0 | 40.6 | 38.4 | 49.1 | 44.1 | 46.4 |
| Magohb | 11.9 | 9.5 | 8.2 | 12.5 | 9.7 | 14.6 | 12.1 | 9.9 | 10.5 | 14.0 | 15.2 | 11.2 |
| Magt1 | 36.4 | 35.0 | 30.8 | 41.5 | 35.1 | 37.8 | 35.0 | 31.5 | 34.7 | 35.0 | 43.9 | 39.5 |
| Mak | 0.0 | 0.0 | 0.0 | 0.1 | 0.0 | 0.0 | 0.0 | 0.0 | 0.0 | 0.0 | 0.0 | 0.0 |
| Mak16 | 38.0 | 29.9 | 29.6 | 34.6 | 32.0 | 28.0 | 31.8 | 25.0 | 22.5 | 33.3 | 36.5 | 33.5 |
| Mal | 1.1 | 0.6 | 0.5 | 0.7 | 0.3 | 0.3 | 0.7 | 0.6 | 0.2 | 0.3 | 0.6 | 0.5 |
| Mal2 | 0.2 | 0.1 | 0.1 | 0.1 | 0.0 | 0.0 | 0.1 | 0.1 | 0.1 | 0.3 | 0.3 | 0.3 |
| Malat1 | 2420.3 | 3440.8 | 4115.1 | 2466.4 | 2501.0 | 3203.0 | 2968.2 | 3293.6 | 2910.4 | 2853.3 | 2923.0 | 3476.9 |
| Mall | 0.0 | 0.1 | 0.2 | 0.1 | 0.0 | 0.1 | 0.1 | 0.1 | 0.1 | 0.0 | 0.0 | 0.1 |
| Malsu1 | 14.4 | 12.8 | 14.3 | 13.1 | 13.0 | 14.0 | 12.2 | 15.0 | 12.6 | 15.8 | 14.3 | 13.2 |
| Malt1 | 1.7 | 1.8 | 1.7 | 1.8 | 2.1 | 1.7 | 1.7 | 1.7 | 1.3 | 1.7 | 1.6 | 1.9 |
| Mamdc2 | 0.4 | 0.7 | 1.4 | 0.4 | 0.7 | 0.5 | 1.5 | 2.5 | 4.0 | 1.5 | 1.3 | 2.3 |
| Mamdc4 | 0.1 | 0.1 | 0.2 | 0.1 | 0.2 | 0.1 | 0.2 | 0.2 | 0.2 | 0.1 | 0.1 | 0.1 |
| Maml1 | 2.8 | 3.7 | 3.5 | 3.1 | 3.0 | 3.1 | 3.1 | 3.8 | 3.5 | 3.2 | 3.0 | 3.4 |

| | | | | | | | | | | | | |
|---|---|---|---|---|---|---|---|---|---|---|---|---|
| Maml2 | 7.5 | 10.7 | 10.9 | 8.1 | 7.0 | 9.1 | 8.8 | 10.3 | 8.6 | 6.8 | 8.4 | 8.0 |
| Maml3 | 0.3 | 0.3 | 0.5 | 0.4 | 0.4 | 0.3 | 0.3 | 0.5 | 0.4 | 0.4 | 0.4 | 0.2 |
| Mamld1 | 1.0 | 1.2 | 1.0 | 1.1 | 0.8 | 1.6 | 1.1 | 1.1 | 0.7 | 0.5 | 0.7 | 0.5 |
| Mamstr | 0.1 | 0.3 | 0.2 | 0.0 | 0.1 | 0.3 | 0.3 | 0.5 | 0.3 | 0.2 | 0.2 | 0.2 |
| Man1a | 21.6 | 20.7 | 22.0 | 17.0 | 22.9 | 15.8 | 18.9 | 20.2 | 21.2 | 26.6 | 22.2 | 22.5 |
| Man1a2 | 17.5 | 14.6 | 15.7 | 20.4 | 17.8 | 19.0 | 17.5 | 17.0 | 17.0 | 18.3 | 20.8 | 21.8 |
| Man1b1 | 24.7 | 24.0 | 22.8 | 30.3 | 25.4 | 25.6 | 26.0 | 22.7 | 27.0 | 22.8 | 23.9 | 24.8 |
| Man1c1 | 10.0 | 11.1 | 9.0 | 10.4 | 9.8 | 8.2 | 10.5 | 11.5 | 12.0 | 11.4 | 9.9 | 10.5 |
| Man2a1 | 27.2 | 29.0 | 29.0 | 26.9 | 31.5 | 33.8 | 33.9 | 31.9 | 41.2 | 34.0 | 34.6 | 39.3 |
| Man2a2 | 13.7 | 18.9 | 24.5 | 14.4 | 20.5 | 18.5 | 22.8 | 24.7 | 23.4 | 17.2 | 18.4 | 21.6 |
| Man2b1 | 21.4 | 16.1 | 17.9 | 24.1 | 32.4 | 18.4 | 12.8 | 15.4 | 14.3 | 19.5 | 15.1 | 16.0 |
| Man2b2 | 24.4 | 17.7 | 20.2 | 27.0 | 24.0 | 26.0 | 18.6 | 19.6 | 17.2 | 19.2 | 17.6 | 18.7 |
| Man2c1 | 12.8 | 11.6 | 11.8 | 13.1 | 11.6 | 14.6 | 12.1 | 11.6 | 11.4 | 10.3 | 10.4 | 10.2 |
| Manba | 13.7 | 14.3 | 15.0 | 9.8 | 14.7 | 13.3 | 10.3 | 17.0 | 14.9 | 13.6 | 12.5 | 13.3 |
| Manbal | 17.3 | 12.2 | 13.0 | 16.6 | 13.0 | 16.1 | 15.1 | 15.0 | 16.1 | 14.2 | 16.7 | 15.0 |
| Manea | 12.2 | 11.4 | 12.1 | 14.1 | 10.3 | 13.8 | 11.5 | 10.6 | 10.9 | 10.1 | 13.5 | 12.2 |
| Maneal | 0.4 | 0.2 | 0.1 | 0.2 | 0.2 | 0.5 | 0.3 | 0.3 | 0.2 | 0.2 | 0.2 | 0.2 |
| Manf | 77.2 | 60.4 | 54.8 | 74.0 | 60.5 | 53.0 | 72.4 | 71.7 | 67.9 | 71.6 | 78.4 | 72.8 |
| Mansc1 | 0.7 | 1.0 | 1.0 | 0.5 | 0.9 | 1.5 | 0.9 | 0.9 | 0.7 | 0.6 | 0.8 | 0.7 |
| Mansc4 | 0.2 | 0.2 | 0.1 | 0.1 | 0.1 | 0.2 | 0.3 | 0.1 | 0.1 | 0.2 | 0.0 | 0.1 |
| Maoa | 61.1 | 38.9 | 25.2 | 43.2 | 19.0 | 41.4 | 12.2 | 17.2 | 12.8 | 18.5 | 20.3 | 23.1 |
| Maob | 14.8 | 16.1 | 17.8 | 14.0 | 13.5 | 13.6 | 15.9 | 13.4 | 13.4 | 9.1 | 12.5 | 16.2 |
| Map1a | 8.9 | 10.0 | 9.1 | 9.1 | 7.9 | 7.9 | 9.5 | 7.8 | 7.0 | 7.3 | 8.3 | 5.4 |
| Map1b | 18.5 | 13.1 | 12.0 | 27.2 | 14.8 | 19.2 | 23.4 | 18.8 | 16.2 | 17.4 | 27.1 | 15.1 |
| Map1lc3a | 46.0 | 43.4 | 36.8 | 40.2 | 35.7 | 41.1 | 41.8 | 50.7 | 44.6 | 45.7 | 40.6 | 38.3 |
| Map1lc3b | 149.2 | 128.0 | 102.0 | 159.0 | 129.1 | 155.4 | 117.9 | 117.1 | 117.8 | 140.8 | 131.3 | 135.8 |
| Map1s | 6.0 | 6.8 | 5.6 | 5.2 | 6.3 | 5.4 | 8.3 | 6.0 | 8.4 | 6.3 | 5.0 | 4.6 |
| Map2 | 7.1 | 5.6 | 6.0 | 8.8 | 7.2 | 7.7 | 8.7 | 9.0 | 13.1 | 4.1 | 13.2 | 9.4 |
| Map2k1 | 57.9 | 57.0 | 45.0 | 56.6 | 56.9 | 54.1 | 56.7 | 46.7 | 55.6 | 55.2 | 56.0 | 53.2 |
| Map2k2 | 33.8 | 29.0 | 22.6 | 31.0 | 28.7 | 32.9 | 29.9 | 31.7 | 30.7 | 30.4 | 27.5 | 24.1 |
| Map2k3 | 23.4 | 31.9 | 25.8 | 22.4 | 29.6 | 19.5 | 27.6 | 27.6 | 27.2 | 29.0 | 24.7 | 21.9 |
| Map2k4 | 16.9 | 16.9 | 14.3 | 14.1 | 14.0 | 13.6 | 14.4 | 13.7 | 14.6 | 15.3 | 15.8 | 14.9 |
| Map2k5 | 6.7 | 6.7 | 6.4 | 6.9 | 6.5 | 7.6 | 6.6 | 7.1 | 6.6 | 6.8 | 6.0 | 7.0 |
| Map2k6 | 0.3 | 0.5 | 0.6 | 0.4 | 0.4 | 0.8 | 0.2 | 0.3 | 0.4 | 0.4 | 0.3 | 0.2 |
| Map2k7 | 13.8 | 11.1 | 8.9 | 15.6 | 11.6 | 12.4 | 12.8 | 9.0 | 9.3 | 11.2 | 12.6 | 9.7 |
| Map3k1 | 1.1 | 1.5 | 1.6 | 1.4 | 1.8 | 0.9 | 1.3 | 1.3 | 1.3 | 1.6 | 1.3 | 1.3 |
| Map3k10 | 6.4 | 6.0 | 5.6 | 5.9 | 5.5 | 5.5 | 5.6 | 5.9 | 5.9 | 6.5 | 4.9 | 5.1 |
| Map3k11 | 5.8 | 5.8 | 4.5 | 5.5 | 6.7 | 5.1 | 5.7 | 4.6 | 5.5 | 6.0 | 5.2 | 4.4 |
| Map3k12 | 3.4 | 4.8 | 4.8 | 3.7 | 3.8 | 4.2 | 3.8 | 3.7 | 4.2 | 4.7 | 3.8 | 4.0 |
| Map3k13 | 0.3 | 0.4 | 0.3 | 0.3 | 0.3 | 0.3 | 0.2 | 0.2 | 0.1 | 0.2 | 0.3 | 0.2 |
| Map3k14 | 3.7 | 3.9 | 3.3 | 4.7 | 5.2 | 3.0 | 4.8 | 4.3 | 3.6 | 3.5 | 3.4 | 3.2 |
| Map3k15 | 0.5 | 0.2 | 0.1 | 0.4 | 0.2 | 0.4 | 0.2 | 0.2 | 0.2 | 0.3 | 0.3 | 0.4 |
| Map3k19 | 0.1 | 0.1 | 0.0 | 0.1 | 0.0 | 0.1 | 0.1 | 0.0 | 0.0 | 0.0 | 0.1 | 0.0 |
| Map3k2 | 3.9 | 4.5 | 4.6 | 4.0 | 4.1 | 4.6 | 3.7 | 4.0 | 3.8 | 4.3 | 4.4 | 4.6 |
| Map3k3 | 19.5 | 22.3 | 23.4 | 18.7 | 22.5 | 18.2 | 24.1 | 21.4 | 25.2 | 19.4 | 21.2 | 21.8 |
| Map3k4 | 7.3 | 7.2 | 6.7 | 6.1 | 6.3 | 7.1 | 5.9 | 5.2 | 5.5 | 5.7 | 5.4 | 5.9 |
| Map3k5 | 1.2 | 3.3 | 4.3 | 1.2 | 2.3 | 0.7 | 1.8 | 1.6 | 1.9 | 1.9 | 1.4 | 2.2 |
| Map3k6 | 2.9 | 3.8 | 3.4 | 3.1 | 4.0 | 2.6 | 5.1 | 4.3 | 4.2 | 3.4 | 4.0 | 3.4 |
| Map3k7 | 18.6 | 17.0 | 15.4 | 18.8 | 16.4 | 18.4 | 17.2 | 14.9 | 15.1 | 15.6 | 17.0 | 16.8 |
| Map3k8 | 3.8 | 4.5 | 5.6 | 3.5 | 4.3 | 2.8 | 3.4 | 4.4 | 3.3 | 2.3 | 2.6 | 3.2 |
| Map3k9 | 0.1 | 0.0 | 0.0 | 0.3 | 0.5 | 0.0 | 0.0 | 0.0 | 0.0 | 0.1 | 0.2 | 0.1 |
| Map4 | 57.5 | 46.9 | 40.3 | 66.0 | 47.7 | 62.5 | 50.6 | 40.2 | 43.2 | 34.6 | 45.5 | 42.1 |
| Map4k1 | 0.5 | 0.6 | 0.5 | 0.4 | 0.7 | 0.3 | 0.4 | 0.2 | 0.3 | 0.3 | 0.1 | 0.3 |
| Map4k2 | 3.3 | 3.5 | 3.1 | 2.2 | 3.8 | 2.8 | 3.8 | 2.5 | 2.7 | 2.6 | 2.9 | 3.0 |
| Map4k3 | 40.2 | 58.8 | 73.2 | 44.3 | 43.3 | 33.4 | 44.8 | 53.0 | 56.0 | 36.7 | 47.3 | 56.8 |
| Map4k4 | 83.9 | 81.3 | 90.5 | 91.1 | 74.6 | 88.3 | 70.0 | 59.5 | 55.4 | 80.2 | 71.4 | 84.5 |
| Map4k5 | 20.5 | 23.8 | 23.3 | 25.3 | 20.1 | 23.8 | 28.9 | 20.9 | 26.6 | 27.4 | 27.0 | 30.6 |

| | | | | | | | | | | | | |
|---|---|---|---|---|---|---|---|---|---|---|---|---|
| Map6 | 51.3 | 40.7 | 35.4 | 52.7 | 39.4 | 47.8 | 62.7 | 52.6 | 48.5 | 42.1 | 62.7 | 50.1 |
| Map6d1 | 0.1 | 0.1 | 0.2 | 0.1 | 0.1 | 0.2 | 0.1 | 0.2 | 0.1 | 0.1 | 0.1 | 0.1 |
| Map7 | 2.7 | 2.1 | 1.8 | 3.0 | 1.3 | 2.6 | 1.6 | 0.9 | 1.1 | 1.4 | 1.7 | 1.4 |
| Map7d1 | 24.1 | 30.0 | 31.9 | 26.6 | 28.1 | 23.3 | 41.9 | 30.3 | 31.2 | 27.5 | 31.2 | 27.7 |
| Map7d2 | 1.9 | 0.6 | 0.5 | 6.2 | 1.8 | 4.3 | 0.3 | 0.3 | 0.1 | 0.3 | 0.3 | 0.3 |
| Map9 | 1.9 | 2.0 | 1.7 | 2.0 | 1.5 | 2.3 | 2.3 | 1.7 | 1.5 | 1.4 | 2.2 | 2.0 |
| Mapk1 | 43.6 | 39.4 | 40.4 | 40.0 | 40.8 | 39.7 | 40.7 | 39.9 | 38.7 | 39.7 | 44.8 | 45.2 |
| Mapk10 | 0.0 | 0.0 | 0.0 | 0.0 | 0.0 | 0.0 | 0.0 | 0.0 | 0.0 | 0.1 | 0.1 | 0.0 |
| Mapk11 | 3.2 | 3.3 | 2.8 | 4.2 | 3.2 | 4.5 | 4.6 | 2.9 | 3.4 | 2.6 | 3.1 | 2.0 |
| Mapk12 | 3.0 | 2.4 | 2.0 | 2.7 | 2.4 | 4.5 | 2.2 | 2.9 | 2.8 | 1.9 | 1.7 | 1.7 |
| Mapk13 | 5.7 | 1.9 | 1.4 | 2.0 | 1.2 | 3.5 | 0.5 | 0.3 | 0.4 | 2.7 | 1.2 | 2.0 |
| Mapk14 | 20.4 | 22.1 | 20.1 | 18.3 | 23.8 | 17.9 | 20.2 | 18.6 | 20.0 | 18.9 | 20.6 | 19.0 |
| Mapk15 | 0.0 | 0.0 | 0.0 | 0.0 | 0.0 | 0.0 | 0.0 | 0.0 | 0.0 | 0.0 | 0.0 | 0.0 |
| Mapk1ip1 | 3.6 | 3.3 | 3.9 | 5.6 | 2.9 | 5.1 | 3.6 | 3.1 | 3.4 | 4.2 | 4.2 | 4.0 |
| Mapk1ip1l | 16.0 | 18.7 | 18.4 | 15.9 | 17.7 | 15.9 | 19.5 | 17.8 | 17.9 | 16.6 | 19.5 | 17.2 |
| Mapk3 | 35.5 | 30.6 | 30.6 | 41.7 | 34.6 | 37.1 | 32.2 | 30.0 | 26.1 | 28.5 | 31.5 | 31.1 |
| Mapk4 | 0.1 | 0.1 | 0.0 | 0.0 | 0.0 | 0.1 | 0.0 | 0.0 | 0.1 | 0.2 | 0.1 | 0.0 |
| Mapk6 | 24.9 | 25.0 | 22.9 | 27.3 | 23.9 | 27.7 | 26.6 | 26.9 | 20.2 | 18.0 | 25.2 | 21.3 |
| Mapk7 | 4.3 | 4.9 | 7.7 | 4.5 | 5.5 | 4.4 | 7.8 | 8.6 | 7.0 | 4.2 | 5.5 | 5.2 |
| Mapk8 | 4.0 | 4.2 | 4.0 | 4.0 | 3.6 | 4.0 | 4.0 | 3.6 | 3.8 | 4.1 | 4.3 | 4.2 |
| Mapk8ip1 | 5.2 | 5.5 | 4.6 | 5.1 | 4.3 | 6.4 | 5.4 | 8.8 | 5.1 | 6.6 | 6.3 | 3.9 |
| Mapk8ip2 | 0.0 | 0.0 | 0.0 | 0.0 | 0.0 | 0.0 | 0.0 | 0.0 | 0.0 | 0.0 | 0.0 | 0.0 |
| Mapk8ip3 | 7.6 | 8.6 | 7.6 | 5.7 | 7.1 | 8.5 | 6.9 | 6.1 | 6.9 | 6.7 | 5.9 | 5.9 |
| Mapk9 | 18.9 | 20.2 | 18.8 | 21.5 | 17.6 | 20.4 | 17.4 | 17.4 | 16.5 | 16.0 | 18.6 | 16.9 |
| Mapkap1 | 19.4 | 20.5 | 18.8 | 19.2 | 17.6 | 18.7 | 17.4 | 19.7 | 18.3 | 17.1 | 18.6 | 18.7 |
| Mapkapk2 | 42.4 | 42.9 | 35.4 | 43.1 | 48.1 | 38.2 | 41.2 | 37.2 | 41.8 | 45.3 | 40.1 | 39.3 |
| Mapkapk3 | 5.4 | 5.2 | 4.2 | 5.9 | 9.3 | 4.2 | 4.9 | 3.8 | 4.5 | 7.3 | 6.7 | 5.9 |
| Mapkapk5 | 14.5 | 13.3 | 13.6 | 16.8 | 13.1 | 16.8 | 15.0 | 12.8 | 13.6 | 10.6 | 14.0 | 12.6 |
| Mapkbp1 | 4.1 | 4.2 | 3.7 | 3.3 | 3.8 | 3.4 | 3.7 | 3.3 | 3.3 | 3.4 | 3.7 | 3.3 |
| Mapre1 | 25.4 | 22.0 | 20.4 | 28.1 | 24.2 | 24.7 | 22.8 | 18.6 | 21.0 | 22.5 | 24.5 | 23.8 |
| Mapre2 | 5.8 | 5.5 | 4.7 | 5.6 | 5.5 | 6.4 | 4.0 | 4.6 | 4.2 | 5.4 | 5.6 | 4.2 |
| Mapre3 | 6.0 | 7.0 | 6.2 | 5.6 | 5.8 | 6.9 | 6.2 | 5.4 | 5.6 | 4.9 | 5.8 | 6.0 |
| Mapt | 0.1 | 0.2 | 0.1 | 0.1 | 0.2 | 0.2 | 0.1 | 0.1 | 0.1 | 0.1 | 0.1 | 0.1 |
| Marcks | 39.5 | 44.3 | 69.0 | 45.5 | 51.7 | 41.3 | 59.3 | 76.7 | 72.2 | 62.2 | 61.9 | 71.4 |
| Marcksl1 | 34.3 | 47.6 | 65.1 | 31.7 | 71.4 | 26.6 | 64.9 | 59.4 | 62.5 | 76.9 | 61.0 | 69.2 |
| Marcksl1-ps4 | 0.0 | 0.0 | 0.0 | 0.0 | 0.0 | 0.0 | 0.0 | 0.0 | 0.0 | 0.0 | 0.0 | 0.1 |
| Marco | 0.3 | 0.0 | 0.0 | 0.3 | 2.1 | 0.0 | 0.3 | 0.0 | 0.0 | 0.0 | 0.0 | 0.1 |
| Marf1 | 16.4 | 20.1 | 18.1 | 15.7 | 17.8 | 14.9 | 15.3 | 15.4 | 16.0 | 14.7 | 16.0 | 15.2 |
| Mark1 | 6.7 | 9.3 | 8.4 | 6.8 | 6.9 | 7.2 | 7.4 | 5.1 | 7.0 | 7.5 | 7.1 | 7.1 |
| Mark2 | 4.1 | 4.9 | 4.6 | 4.4 | 5.2 | 3.9 | 4.9 | 4.9 | 5.3 | 5.4 | 4.7 | 4.1 |
| Mark3 | 19.7 | 22.2 | 20.2 | 19.9 | 18.6 | 20.3 | 18.4 | 21.5 | 21.3 | 21.5 | 22.6 | 21.5 |
| Mark4 | 4.8 | 5.5 | 5.4 | 4.1 | 4.5 | 4.3 | 5.9 | 4.8 | 5.5 | 4.9 | 4.9 | 5.5 |
| Mars | 33.0 | 27.1 | 23.8 | 28.1 | 25.5 | 29.6 | 28.4 | 31.7 | 27.4 | 21.9 | 26.5 | 22.8 |
| Mars2 | 2.1 | 1.9 | 2.0 | 2.5 | 2.8 | 1.8 | 2.4 | 1.8 | 2.4 | 1.9 | 1.8 | 2.3 |
| Marveld1 | 14.1 | 16.0 | 14.6 | 14.7 | 16.2 | 18.1 | 15.0 | 17.6 | 19.1 | 15.7 | 13.7 | 13.0 |
| Marveld2 | 0.0 | 0.0 | 0.0 | 0.0 | 0.0 | 0.0 | 0.0 | 0.0 | 0.0 | 0.0 | 0.0 | 0.0 |
| Mas1 | 0.0 | 0.0 | 0.1 | 0.0 | 0.0 | 0.0 | 0.1 | 0.0 | 0.0 | 0.0 | 0.0 | 0.0 |
| Masp1 | 0.9 | 0.4 | 0.2 | 1.4 | 0.4 | 0.7 | 0.3 | 0.1 | 0.4 | 0.5 | 0.3 | 0.4 |
| Masp2 | 0.2 | 0.4 | 0.3 | 0.0 | 0.2 | 0.5 | 0.2 | 0.3 | 0.1 | 0.1 | 0.2 | 0.1 |
| Mast1 | 0.0 | 0.1 | 0.1 | 0.1 | 0.1 | 0.1 | 0.0 | 0.1 | 0.0 | 0.1 | 0.1 | 0.1 |
| Mast2 | 19.6 | 16.7 | 12.3 | 17.1 | 15.0 | 17.5 | 17.2 | 11.4 | 14.1 | 15.1 | 16.7 | 12.4 |
| Mast3 | 1.7 | 2.2 | 2.1 | 2.0 | 2.8 | 1.8 | 2.1 | 1.4 | 1.9 | 2.0 | 1.6 | 1.4 |
| Mast4 | 1.9 | 2.9 | 3.8 | 1.6 | 2.6 | 1.6 | 3.3 | 3.4 | 3.2 | 4.7 | 3.5 | 3.9 |
| Mastl | 1.6 | 3.5 | 5.0 | 1.0 | 4.3 | 1.1 | 5.1 | 1.8 | 3.4 | 1.6 | 3.2 | 2.3 |
| Mat1a | 0.0 | 0.0 | 0.1 | 0.0 | 0.0 | 0.0 | 0.0 | 0.0 | 0.0 | 0.0 | 0.0 | 0.0 |
| Mat2a | 32.4 | 32.3 | 26.9 | 30.2 | 34.7 | 30.1 | 31.4 | 33.0 | 36.7 | 35.7 | 34.4 | 28.0 |
| Mat2b | 25.7 | 27.1 | 24.6 | 25.6 | 27.7 | 22.4 | 23.9 | 25.5 | 28.7 | 29.0 | 28.0 | 27.4 |

| | | | | | | | | | | | | |
|---|---|---|---|---|---|---|---|---|---|---|---|---|
| Matk | 0.1 | 0.1 | 0.1 | 0.2 | 0.3 | 0.0 | 0.2 | 0.0 | 0.2 | 0.3 | 0.2 | 0.1 |
| Matn2 | 5.9 | 10.6 | 25.6 | 4.5 | 11.6 | 6.8 | 13.9 | 51.8 | 22.1 | 10.2 | 11.9 | 14.9 |
| Matn3 | 0.1 | 0.2 | 0.3 | 0.0 | 0.2 | 0.1 | 0.1 | 0.1 | 0.5 | 0.1 | 0.3 | 0.3 |
| Matn4 | 0.9 | 1.9 | 2.9 | 1.0 | 2.3 | 1.8 | 2.2 | 5.4 | 4.9 | 2.5 | 1.5 | 3.2 |
| Matr3 | 34.4 | 33.0 | 38.1 | 34.4 | 31.4 | 33.8 | 34.2 | 38.4 | 35.6 | 47.9 | 44.4 | 48.6 |
| Mau2 | 10.0 | 11.1 | 9.4 | 9.0 | 10.6 | 8.6 | 9.4 | 9.8 | 11.0 | 10.5 | 10.1 | 8.9 |
| Mavs | 11.4 | 11.7 | 10.8 | 12.0 | 10.0 | 13.3 | 10.8 | 9.5 | 9.7 | 10.4 | 10.5 | 9.5 |
| Max | 19.3 | 19.1 | 19.8 | 26.1 | 21.4 | 20.4 | 24.6 | 21.2 | 17.2 | 17.2 | 22.6 | 21.3 |
| Maz | 13.0 | 14.2 | 16.8 | 13.4 | 13.4 | 13.9 | 14.8 | 21.8 | 16.5 | 13.5 | 15.8 | 12.9 |
| Mb21d1 | 0.7 | 1.0 | 1.3 | 0.5 | 1.2 | 0.3 | 1.2 | 0.7 | 1.0 | 0.7 | 1.1 | 1.0 |
| Mb21d2 | 1.4 | 1.0 | 1.0 | 1.5 | 1.2 | 1.8 | 1.0 | 1.2 | 1.0 | 1.6 | 1.3 | 0.9 |
| Mbd1 | 7.4 | 6.9 | 7.0 | 8.4 | 9.2 | 6.1 | 9.0 | 7.2 | 10.5 | 11.3 | 10.4 | 13.9 |
| Mbd2 | 38.9 | 39.5 | 40.3 | 49.5 | 36.9 | 42.7 | 41.1 | 31.9 | 35.9 | 35.1 | 40.1 | 42.4 |
| Mbd3 | 12.9 | 12.0 | 11.2 | 14.3 | 13.5 | 14.1 | 14.1 | 13.9 | 12.7 | 15.4 | 13.2 | 13.1 |
| Mbd4 | 0.4 | 1.1 | 1.4 | 0.5 | 0.9 | 0.6 | 0.9 | 1.0 | 0.9 | 0.8 | 0.7 | 0.8 |
| Mbd5 | 2.8 | 3.0 | 3.4 | 2.6 | 2.6 | 2.7 | 2.6 | 3.0 | 2.4 | 2.4 | 2.8 | 2.7 |
| Mbd6 | 0.4 | 0.5 | 0.6 | 0.4 | 0.6 | 0.4 | 0.5 | 0.7 | 0.7 | 0.5 | 0.5 | 0.5 |
| Mbip | 6.2 | 6.6 | 7.2 | 5.3 | 6.2 | 5.2 | 5.7 | 6.1 | 6.7 | 7.3 | 6.8 | 7.3 |
| Mblac1 | 1.3 | 0.9 | 0.6 | 0.6 | 1.1 | 0.7 | 1.0 | 1.3 | 1.0 | 1.4 | 1.5 | 0.6 |
| Mblac2 | 1.5 | 1.7 | 1.8 | 2.1 | 1.2 | 2.6 | 1.1 | 1.6 | 1.3 | 1.3 | 1.8 | 1.6 |
| Mbnl1 | 52.3 | 56.0 | 65.2 | 87.6 | 64.3 | 80.8 | 65.1 | 60.7 | 56.0 | 51.5 | 76.0 | 61.7 |
| Mbnl2 | 74.1 | 79.5 | 80.3 | 93.6 | 69.8 | 89.3 | 79.8 | 66.4 | 66.9 | 66.4 | 88.5 | 83.5 |
| Mbnl3 | 0.7 | 0.7 | 0.8 | 1.3 | 0.9 | 0.6 | 0.8 | 0.7 | 1.1 | 0.6 | 0.8 | 0.7 |
| Mboat1 | 1.3 | 2.0 | 2.7 | 0.8 | 1.6 | 0.9 | 1.4 | 1.7 | 1.4 | 1.0 | 1.0 | 0.9 |
| Mboat2 | 2.2 | 3.4 | 3.2 | 1.8 | 2.6 | 1.5 | 2.1 | 2.7 | 2.8 | 2.0 | 1.8 | 2.5 |
| Mboat4 | 0.1 | 0.1 | 0.0 | 0.1 | 0.0 | 0.0 | 0.0 | 0.0 | 0.1 | 0.2 | 0.0 | 0.1 |
| Mboat7 | 11.0 | 12.9 | 10.3 | 8.9 | 11.5 | 9.2 | 13.2 | 8.7 | 11.5 | 17.4 | 12.7 | 10.4 |
| Mbp | 2.5 | 3.5 | 3.4 | 1.9 | 2.6 | 2.0 | 0.9 | 3.3 | 1.6 | 11.9 | 7.1 | 3.7 |
| Mbtd1 | 1.8 | 2.5 | 2.6 | 1.7 | 2.4 | 1.9 | 2.2 | 2.2 | 2.7 | 2.5 | 2.7 | 2.3 |
| Mbtps1 | 38.8 | 36.1 | 37.8 | 41.5 | 40.0 | 41.2 | 43.0 | 43.7 | 40.7 | 39.8 | 39.7 | 41.6 |
| Mbtps2 | 2.8 | 3.2 | 2.6 | 2.4 | 2.6 | 3.4 | 2.3 | 2.9 | 2.6 | 2.7 | 2.7 | 2.5 |
| Mc1r | 0.1 | 0.1 | 0.1 | 0.1 | 0.1 | 0.1 | 0.2 | 0.2 | 0.1 | 0.1 | 0.1 | 0.0 |
| Mc2r | 0.0 | 0.0 | 0.1 | 0.1 | 0.0 | 0.0 | 0.0 | 0.0 | 0.0 | 0.0 | 0.0 | 0.0 |
| Mc5r | 0.0 | 0.0 | 0.0 | 0.0 | 0.0 | 0.0 | 0.1 | 0.0 | 0.0 | 0.1 | 0.1 | 0.1 |
| Mcam | 3.9 | 1.2 | 1.5 | 3.6 | 2.3 | 3.7 | 3.3 | 3.7 | 4.2 | 4.9 | 4.9 | 2.5 |
| Mcat | 7.6 | 7.1 | 5.1 | 5.9 | 5.9 | 5.6 | 5.8 | 5.8 | 5.5 | 7.5 | 5.6 | 5.2 |
| Mcc | 3.8 | 3.7 | 3.5 | 4.1 | 3.3 | 4.4 | 3.0 | 3.5 | 2.0 | 2.4 | 2.6 | 1.9 |
| Mccc1 | 4.8 | 4.3 | 4.7 | 5.2 | 4.8 | 5.4 | 3.8 | 4.8 | 3.7 | 5.1 | 3.5 | 4.9 |
| Mccc2 | 6.8 | 7.5 | 7.9 | 5.3 | 5.4 | 9.1 | 6.7 | 7.7 | 7.3 | 7.6 | 8.3 | 8.5 |
| Mcee | 19.3 | 15.0 | 15.8 | 19.5 | 16.5 | 18.3 | 16.1 | 13.9 | 14.2 | 18.2 | 17.8 | 16.7 |
| Mcf2l | 0.1 | 0.2 | 0.0 | 0.2 | 0.1 | 0.3 | 0.2 | 0.1 | 0.2 | 0.2 | 0.1 | 0.1 |
| Mcfd2 | 327.9 | 291.0 | 250.9 | 304.3 | 280.5 | 264.2 | 290.0 | 204.7 | 253.3 | 263.0 | 283.6 | 333.0 |
| Mchr1 | 0.0 | 0.0 | 0.0 | 0.0 | 0.0 | 0.0 | 0.0 | 0.0 | 0.0 | 0.0 | 0.1 | 0.0 |
| Mcl1 | 43.7 | 41.0 | 43.9 | 42.6 | 47.1 | 39.3 | 43.5 | 44.1 | 41.7 | 50.5 | 45.5 | 48.8 |
| Mcm10 | 0.6 | 1.3 | 2.2 | 0.5 | 2.2 | 0.4 | 2.6 | 1.4 | 1.9 | 0.9 | 1.9 | 1.1 |
| Mcm2 | 4.7 | 6.3 | 7.4 | 4.7 | 8.2 | 4.6 | 9.4 | 6.3 | 7.1 | 5.7 | 6.1 | 4.9 |
| Mcm3 | 5.0 | 7.4 | 10.2 | 3.7 | 10.7 | 3.2 | 11.8 | 7.0 | 7.9 | 5.0 | 6.7 | 5.4 |
| Mcm3ap | 7.2 | 8.9 | 8.6 | 7.8 | 8.9 | 8.0 | 8.9 | 9.0 | 8.6 | 8.5 | 8.1 | 8.1 |
| Mcm4 | 6.4 | 10.4 | 12.8 | 5.1 | 12.1 | 4.7 | 12.5 | 9.4 | 10.8 | 8.0 | 11.4 | 8.3 |
| Mcm5 | 2.7 | 4.3 | 6.0 | 1.7 | 6.8 | 1.1 | 7.0 | 4.4 | 5.3 | 3.2 | 4.7 | 3.4 |
| Mcm6 | 6.6 | 9.9 | 13.9 | 6.1 | 14.8 | 3.9 | 14.5 | 10.9 | 11.0 | 8.4 | 9.8 | 7.4 |
| Mcm7 | 8.8 | 10.3 | 13.0 | 6.2 | 13.0 | 6.1 | 15.0 | 9.7 | 12.3 | 10.3 | 12.4 | 10.0 |
| Mcm8 | 1.1 | 1.6 | 1.8 | 0.8 | 1.5 | 1.0 | 1.7 | 1.5 | 1.2 | 1.0 | 1.3 | 1.2 |
| Mcm9 | 0.8 | 0.9 | 1.0 | 0.9 | 1.3 | 0.8 | 1.0 | 1.0 | 0.8 | 1.0 | 0.7 | 0.8 |
| Mcmbp | 8.9 | 9.7 | 8.8 | 8.2 | 9.7 | 7.3 | 9.4 | 8.9 | 10.6 | 10.6 | 10.9 | 8.9 |
| Mcmdc2 | 0.5 | 0.4 | 0.4 | 0.2 | 0.5 | 0.3 | 0.6 | 0.4 | 0.3 | 0.3 | 0.3 | 0.5 |
| Mcoln1 | 15.4 | 14.6 | 12.9 | 18.4 | 13.9 | 20.0 | 16.1 | 14.3 | 14.8 | 13.3 | 13.6 | 14.0 |

| | | | | | | | | | | | | |
|---|---|---|---|---|---|---|---|---|---|---|---|---|
| Mcoln2 | 0.5 | 0.2 | 0.3 | 3.3 | 3.0 | 0.3 | 0.3 | 0.1 | 0.2 | 0.6 | 0.3 | 0.3 |
| Mcph1 | 2.7 | 3.4 | 3.3 | 2.7 | 3.0 | 3.4 | 3.4 | 3.0 | 3.1 | 2.8 | 3.2 | 3.2 |
| Mcpt4 | 0.1 | 0.0 | 0.2 | 0.0 | 0.1 | 0.0 | 0.1 | 0.0 | 0.2 | 0.1 | 0.1 | 0.2 |
| Mcpt8 | 0.0 | 0.0 | 0.2 | 0.0 | 0.0 | 0.0 | 0.0 | 0.0 | 0.0 | 0.1 | 0.0 | 0.2 |
| Mcrs1 | 6.8 | 7.1 | 6.2 | 7.9 | 8.1 | 7.3 | 8.0 | 8.3 | 9.7 | 9.3 | 9.0 | 7.8 |
| Mctp1 | 0.2 | 0.0 | 0.0 | 1.7 | 2.4 | 0.1 | 0.2 | 0.0 | 0.0 | 0.2 | 0.1 | 0.2 |
| Mctp2 | 0.2 | 0.3 | 0.3 | 0.1 | 0.1 | 0.1 | 0.1 | 0.1 | 0.1 | 0.1 | 0.1 | 0.1 |
| Mcts1 | 32.0 | 32.1 | 25.8 | 30.2 | 30.1 | 33.2 | 20.0 | 23.0 | 26.5 | 33.1 | 27.0 | 25.8 |
| Mcts2 | 6.7 | 7.1 | 5.7 | 5.3 | 7.2 | 6.9 | 7.3 | 8.3 | 6.6 | 7.1 | 8.6 | 6.7 |
| Mcu | 8.1 | 8.0 | 8.4 | 8.0 | 6.9 | 8.4 | 8.3 | 8.5 | 8.3 | 6.9 | 7.0 | 7.2 |
| Mdc1 | 1.7 | 3.0 | 4.2 | 1.9 | 3.2 | 1.7 | 4.2 | 3.1 | 3.0 | 2.1 | 2.9 | 2.5 |
| Mdfi | 2.4 | 2.8 | 3.3 | 1.5 | 2.5 | 1.5 | 2.2 | 2.9 | 3.1 | 2.9 | 2.0 | 2.8 |
| Mdfic | 21.3 | 26.5 | 33.0 | 25.1 | 24.7 | 23.1 | 27.4 | 29.3 | 32.6 | 20.3 | 27.1 | 31.8 |
| Mdga1 | 0.5 | 0.5 | 0.7 | 0.5 | 0.9 | 0.7 | 1.3 | 0.9 | 1.0 | 1.0 | 0.9 | 0.9 |
| Mdga2 | 0.0 | 0.0 | 0.0 | 0.0 | 0.0 | 0.0 | 0.0 | 0.0 | 0.0 | 0.0 | 0.0 | 0.0 |
| Mdh1 | 70.6 | 68.8 | 61.1 | 64.7 | 58.9 | 63.8 | 52.7 | 57.6 | 53.0 | 64.9 | 56.8 | 57.0 |
| Mdh1b | 0.1 | 0.0 | 0.1 | 0.1 | 0.0 | 0.1 | 0.0 | 0.0 | 0.0 | 0.0 | 0.0 | 0.0 |
| Mdh2 | 104.5 | 93.2 | 81.0 | 101.7 | 98.7 | 96.2 | 98.0 | 95.7 | 83.3 | 100.6 | 100.9 | 88.2 |
| Mdk | 3.9 | 5.8 | 8.9 | 0.8 | 2.2 | 2.0 | 2.6 | 5.1 | 3.6 | 8.3 | 5.6 | 6.8 |
| Mdm1 | 1.2 | 1.8 | 1.8 | 1.2 | 1.5 | 1.5 | 1.7 | 1.0 | 1.2 | 1.2 | 1.9 | 1.3 |
| Mdm2 | 79.6 | 77.4 | 63.3 | 74.1 | 85.8 | 71.2 | 69.6 | 36.3 | 53.2 | 85.2 | 76.8 | 70.0 |
| Mdm4 | 6.1 | 7.8 | 10.7 | 5.5 | 7.0 | 5.0 | 6.6 | 7.9 | 7.1 | 6.6 | 6.8 | 7.1 |
| Mdn1 | 4.6 | 4.1 | 3.3 | 4.8 | 5.0 | 4.2 | 4.3 | 3.5 | 3.1 | 4.5 | 4.0 | 3.6 |
| Mdp1 | 14.7 | 12.5 | 12.8 | 15.0 | 11.3 | 14.0 | 14.7 | 13.8 | 14.6 | 13.0 | 13.9 | 16.3 |
| Me1 | 38.8 | 37.5 | 34.4 | 40.8 | 35.9 | 42.6 | 36.2 | 38.1 | 33.2 | 31.8 | 41.7 | 35.1 |
| Me2 | 14.9 | 11.4 | 10.8 | 13.3 | 12.4 | 13.4 | 13.1 | 11.3 | 11.8 | 15.1 | 13.5 | 12.7 |
| Me3 | 1.6 | 0.9 | 0.3 | 0.7 | 0.5 | 1.1 | 0.5 | 0.4 | 0.6 | 0.7 | 0.8 | 0.5 |
| Mea1 | 60.6 | 53.0 | 53.3 | 75.1 | 52.8 | 68.5 | 65.1 | 62.3 | 52.9 | 58.8 | 70.2 | 65.1 |
| Meaf6 | 4.3 | 4.6 | 6.6 | 5.3 | 6.6 | 3.5 | 6.0 | 6.0 | 7.1 | 6.0 | 7.1 | 7.9 |
| Mecom | 2.5 | 2.3 | 3.4 | 1.3 | 1.7 | 0.8 | 1.9 | 3.6 | 2.6 | 1.5 | 1.4 | 2.8 |
| Mecp2 | 2.3 | 2.6 | 2.6 | 2.5 | 2.6 | 2.5 | 2.4 | 2.6 | 2.2 | 2.2 | 2.2 | 2.3 |
| Mecr | 12.4 | 10.0 | 7.5 | 11.9 | 11.9 | 10.0 | 9.9 | 9.7 | 7.9 | 7.7 | 7.8 | 7.3 |
| Med1 | 11.6 | 13.3 | 13.9 | 13.2 | 12.5 | 11.2 | 13.3 | 14.1 | 13.6 | 11.8 | 12.8 | 12.3 |
| Med10 | 27.3 | 27.1 | 25.9 | 32.0 | 25.8 | 27.1 | 30.8 | 30.8 | 24.8 | 32.0 | 31.4 | 27.0 |
| Med11 | 8.9 | 8.2 | 8.8 | 11.6 | 11.4 | 9.3 | 10.3 | 10.4 | 10.0 | 11.3 | 10.2 | 9.7 |
| Med12 | 3.3 | 3.9 | 4.5 | 3.3 | 4.2 | 3.4 | 4.6 | 4.9 | 4.2 | 3.2 | 3.6 | 3.4 |
| Med12l | 0.1 | 0.0 | 0.0 | 0.1 | 0.0 | 0.1 | 0.0 | 0.0 | 0.0 | 0.0 | 0.0 | 0.0 |
| Med13 | 5.7 | 7.4 | 7.4 | 5.7 | 5.9 | 6.0 | 5.3 | 6.9 | 5.8 | 6.3 | 6.5 | 6.2 |
| Med13l | 4.9 | 7.0 | 8.2 | 5.1 | 6.4 | 5.9 | 7.0 | 7.6 | 7.7 | 6.7 | 7.1 | 6.5 |
| Med14 | 7.0 | 8.8 | 9.8 | 6.8 | 8.3 | 6.5 | 10.3 | 10.4 | 9.2 | 6.7 | 8.0 | 8.1 |
| Med15 | 5.0 | 5.9 | 7.1 | 6.6 | 7.4 | 5.3 | 8.0 | 7.1 | 6.6 | 5.5 | 6.3 | 5.3 |
| Med16 | 4.3 | 4.4 | 3.9 | 5.0 | 5.1 | 5.0 | 5.9 | 5.4 | 5.3 | 4.9 | 4.6 | 4.2 |
| Med17 | 7.0 | 7.5 | 5.6 | 6.8 | 6.3 | 7.5 | 6.7 | 6.0 | 5.8 | 6.5 | 6.4 | 7.1 |
| Med18 | 3.0 | 2.7 | 2.7 | 2.2 | 3.6 | 2.4 | 3.1 | 2.8 | 2.7 | 2.5 | 2.9 | 2.0 |
| Med19 | 9.2 | 9.7 | 9.6 | 11.5 | 8.3 | 10.4 | 8.1 | 9.1 | 7.1 | 8.0 | 9.7 | 9.6 |
| Med20 | 11.3 | 9.4 | 9.7 | 15.0 | 11.4 | 12.4 | 12.4 | 11.5 | 11.7 | 11.0 | 10.8 | 10.3 |
| Med21 | 39.2 | 40.2 | 31.8 | 46.6 | 36.5 | 43.9 | 34.7 | 38.0 | 39.1 | 38.2 | 47.1 | 34.1 |
| Med22 | 10.9 | 9.5 | 8.4 | 10.2 | 10.1 | 9.6 | 9.8 | 10.1 | 10.9 | 11.8 | 10.2 | 8.6 |
| Med23 | 6.4 | 7.4 | 6.9 | 6.0 | 6.5 | 7.1 | 6.6 | 7.4 | 7.0 | 6.8 | 7.0 | 6.5 |
| Med24 | 8.1 | 9.0 | 9.6 | 9.1 | 8.7 | 8.5 | 8.4 | 8.8 | 8.1 | 7.0 | 7.9 | 7.2 |
| Med25 | 8.8 | 10.6 | 9.4 | 8.6 | 8.7 | 8.7 | 9.2 | 9.5 | 9.4 | 8.7 | 9.2 | 6.9 |
| Med26 | 1.6 | 2.1 | 2.0 | 2.0 | 2.1 | 2.0 | 2.0 | 1.9 | 2.3 | 2.2 | 2.0 | 1.7 |
| Med27 | 12.9 | 13.3 | 11.5 | 13.9 | 10.4 | 11.6 | 12.9 | 13.3 | 12.4 | 12.4 | 12.6 | 11.6 |
| Med28 | 11.4 | 10.1 | 7.8 | 10.9 | 10.7 | 11.1 | 8.2 | 8.3 | 9.0 | 10.3 | 8.8 | 9.0 |
| Med29 | 7.7 | 6.2 | 5.8 | 7.6 | 6.3 | 7.1 | 7.4 | 5.9 | 5.8 | 6.9 | 6.7 | 5.4 |
| Med30 | 17.7 | 18.2 | 15.2 | 16.9 | 16.2 | 15.0 | 18.5 | 16.4 | 18.1 | 20.1 | 17.3 | 21.2 |
| Med31 | 5.4 | 6.7 | 6.1 | 5.6 | 6.6 | 5.7 | 6.0 | 4.3 | 4.6 | 5.6 | 8.3 | 5.6 |

| | | | | | | | | | | | | |
|---|---|---|---|---|---|---|---|---|---|---|---|---|
| Med4 | 8.9 | 11.2 | 12.0 | 7.9 | 11.7 | 7.3 | 10.9 | 10.7 | 11.2 | 8.9 | 10.4 | 11.5 |
| Med6 | 16.7 | 19.0 | 17.9 | 16.9 | 17.0 | 18.1 | 18.1 | 18.3 | 18.5 | 17.4 | 17.2 | 19.6 |
| Med7 | 7.3 | 7.7 | 7.3 | 7.4 | 7.2 | 7.2 | 6.7 | 6.8 | 6.2 | 8.3 | 8.1 | 8.5 |
| Med8 | 13.6 | 13.7 | 14.2 | 14.8 | 13.1 | 10.2 | 13.8 | 14.2 | 13.7 | 13.2 | 14.7 | 14.1 |
| Med9 | 5.0 | 4.9 | 3.9 | 4.7 | 4.2 | 4.8 | 4.4 | 4.4 | 4.7 | 4.6 | 4.7 | 4.0 |
| Medag | 55.2 | 51.2 | 47.8 | 69.0 | 58.7 | 79.9 | 63.9 | 53.3 | 58.6 | 46.1 | 45.6 | 61.6 |
| Mef2a | 10.3 | 8.1 | 9.5 | 11.6 | 13.8 | 9.1 | 10.7 | 8.3 | 8.6 | 13.0 | 10.3 | 11.3 |
| Mef2b | 0.1 | 0.0 | 0.0 | 0.1 | 0.0 | 0.1 | 0.0 | 0.0 | 0.0 | 0.1 | 0.0 | 0.0 |
| Mef2c | 0.4 | 0.5 | 1.0 | 0.8 | 1.1 | 0.4 | 0.5 | 0.8 | 0.6 | 0.4 | 0.5 | 0.7 |
| Mef2d | 3.8 | 4.9 | 4.3 | 4.8 | 4.6 | 5.0 | 5.2 | 4.7 | 4.1 | 3.8 | 4.2 | 3.9 |
| Mefv | 0.0 | 0.0 | 0.0 | 0.0 | 0.0 | 0.0 | 0.0 | 0.0 | 0.0 | 0.0 | 0.0 | 0.0 |
| Meg3 | 20.6 | 21.0 | 21.1 | 22.4 | 19.1 | 24.9 | 30.5 | 22.5 | 45.3 | 29.3 | 30.6 | 20.6 |
| Megf10 | 0.7 | 0.8 | 1.4 | 0.6 | 1.9 | 1.1 | 1.3 | 1.2 | 1.5 | 1.2 | 1.3 | 1.0 |
| Megf11 | 0.3 | 0.1 | 0.1 | 0.1 | 0.2 | 0.2 | 0.2 | 0.1 | 0.2 | 0.3 | 0.2 | 0.2 |
| Megf6 | 0.0 | 0.0 | 0.0 | 0.0 | 0.0 | 0.0 | 0.0 | 0.2 | 0.3 | 0.7 | 0.2 | 0.2 |
| Megf8 | 13.8 | 12.6 | 9.8 | 11.0 | 13.1 | 12.9 | 14.5 | 10.1 | 13.0 | 13.0 | 10.1 | 9.3 |
| Megf9 | 10.8 | 12.2 | 10.1 | 11.9 | 9.8 | 10.1 | 8.8 | 10.2 | 8.9 | 8.9 | 9.0 | 7.4 |
| Meiob | 0.2 | 0.1 | 0.1 | 0.2 | 0.1 | 0.3 | 0.1 | 0.1 | 0.0 | 0.0 | 0.0 | 0.1 |
| Meis1 | 4.2 | 5.9 | 6.3 | 4.0 | 4.2 | 4.8 | 4.5 | 6.5 | 6.3 | 10.4 | 7.6 | 7.1 |
| Meis2 | 5.4 | 4.3 | 5.1 | 4.2 | 2.4 | 3.4 | 3.8 | 4.6 | 3.9 | 7.4 | 6.9 | 6.3 |
| Meis3 | 6.5 | 6.7 | 6.5 | 7.1 | 6.7 | 6.8 | 7.7 | 6.8 | 8.2 | 6.8 | 6.9 | 7.2 |
| Melk | 1.6 | 3.0 | 4.5 | 1.1 | 4.5 | 0.7 | 4.6 | 2.2 | 4.0 | 1.7 | 2.8 | 2.2 |
| Memo1 | 26.3 | 25.5 | 23.7 | 28.7 | 28.4 | 25.3 | 28.2 | 27.2 | 27.0 | 28.9 | 33.7 | 28.9 |
| Men1 | 6.6 | 7.9 | 8.1 | 6.7 | 7.5 | 6.7 | 9.3 | 9.2 | 8.8 | 7.7 | 8.9 | 7.3 |
| Meox1 | 1.2 | 1.1 | 1.9 | 0.8 | 1.4 | 2.0 | 0.6 | 1.2 | 0.8 | 0.5 | 0.3 | 0.4 |
| Meox2 | 0.3 | 0.2 | 0.3 | 0.4 | 0.2 | 0.1 | 0.2 | 1.0 | 0.4 | 0.2 | 0.1 | 0.2 |
| Mep1a | 0.0 | 0.0 | 0.0 | 0.0 | 0.0 | 0.0 | 0.0 | 0.0 | 0.0 | 0.0 | 0.0 | 0.0 |
| Mepce | 5.5 | 6.0 | 6.1 | 5.3 | 6.0 | 5.8 | 6.1 | 6.0 | 5.9 | 6.7 | 5.4 | 5.8 |
| Mertk | 5.3 | 3.8 | 3.0 | 6.8 | 4.1 | 4.6 | 3.2 | 1.3 | 1.6 | 3.4 | 2.6 | 2.2 |
| Mesdc1 | 4.0 | 4.2 | 4.4 | 3.1 | 4.7 | 2.7 | 4.7 | 5.7 | 5.6 | 5.8 | 4.0 | 3.8 |
| Mesdc2 | 25.7 | 23.2 | 22.4 | 30.2 | 21.2 | 28.4 | 22.3 | 20.4 | 20.1 | 21.7 | 21.5 | 24.5 |
| Mesp2 | 0.0 | 0.0 | 0.0 | 0.1 | 0.0 | 0.0 | 0.0 | 0.0 | 0.0 | 0.0 | 0.0 | 0.0 |
| Mest | 1.6 | 0.9 | 0.9 | 1.6 | 1.7 | 1.1 | 1.5 | 2.4 | 1.6 | 2.6 | 3.7 | 1.6 |
| Met | 5.0 | 3.4 | 2.2 | 4.8 | 2.6 | 5.2 | 1.6 | 1.5 | 1.8 | 8.7 | 4.9 | 3.4 |
| Metap1 | 15.8 | 14.4 | 14.9 | 15.2 | 13.0 | 15.4 | 14.8 | 14.5 | 14.2 | 15.3 | 17.7 | 16.0 |
| Metap1d | 12.1 | 9.4 | 8.6 | 9.9 | 8.0 | 8.7 | 6.2 | 9.2 | 6.7 | 7.0 | 8.7 | 7.2 |
| Metap2 | 25.6 | 25.1 | 24.7 | 29.5 | 23.3 | 26.3 | 23.6 | 22.4 | 21.1 | 26.4 | 28.0 | 28.5 |
| Metrn | 10.1 | 8.2 | 5.9 | 9.2 | 7.1 | 11.7 | 7.9 | 4.9 | 7.7 | 7.2 | 7.1 | 5.9 |
| Metrnl | 9.0 | 7.8 | 6.9 | 14.0 | 22.0 | 6.3 | 12.8 | 9.1 | 12.9 | 11.2 | 12.3 | 10.2 |
| Mettl1 | 7.6 | 5.7 | 4.7 | 9.7 | 7.7 | 7.7 | 6.9 | 6.8 | 5.9 | 6.0 | 5.9 | 6.2 |
| Mettl10 | 15.5 | 13.6 | 14.2 | 14.4 | 12.3 | 14.9 | 10.9 | 14.6 | 15.3 | 12.4 | 14.9 | 15.1 |
| Mettl13 | 2.9 | 2.3 | 2.2 | 2.2 | 2.3 | 3.0 | 2.0 | 2.1 | 1.8 | 2.3 | 2.0 | 1.9 |
| Mettl14 | 3.9 | 3.7 | 3.4 | 5.0 | 3.9 | 4.3 | 4.0 | 3.1 | 3.0 | 3.7 | 4.9 | 3.8 |
| Mettl15 | 2.4 | 2.5 | 2.8 | 2.8 | 1.7 | 2.4 | 1.9 | 2.3 | 2.0 | 1.5 | 2.6 | 2.0 |
| Mettl16 | 16.0 | 14.1 | 13.8 | 17.1 | 13.1 | 14.2 | 13.6 | 13.1 | 13.3 | 16.6 | 17.6 | 16.0 |
| Mettl17 | 3.1 | 3.2 | 2.9 | 2.4 | 2.7 | 3.3 | 2.7 | 2.9 | 3.3 | 4.0 | 2.5 | 2.7 |
| Mettl18 | 2.3 | 2.7 | 2.2 | 3.0 | 2.5 | 2.7 | 1.8 | 2.4 | 2.7 | 2.4 | 2.3 | 2.1 |
| Mettl2 | 4.8 | 5.2 | 4.6 | 5.6 | 4.1 | 6.0 | 4.7 | 4.9 | 5.0 | 5.1 | 5.5 | 5.3 |
| Mettl20 | 2.1 | 2.7 | 2.1 | 2.2 | 1.5 | 2.5 | 1.3 | 1.5 | 2.8 | 3.3 | 2.5 | 3.7 |
| Mettl21a | 11.8 | 10.9 | 9.9 | 8.9 | 9.4 | 9.9 | 8.6 | 9.4 | 9.6 | 10.8 | 10.8 | 10.8 |
| Mettl21c | 0.1 | 0.1 | 0.2 | 0.1 | 0.1 | 0.2 | 0.0 | 0.0 | 0.1 | 0.1 | 0.1 | 0.1 |
| Mettl21d | 4.0 | 3.4 | 4.2 | 4.7 | 3.8 | 4.9 | 3.7 | 2.5 | 2.8 | 3.5 | 2.6 | 3.9 |
| Mettl21e | 0.0 | 0.1 | 0.1 | 0.0 | 0.0 | 0.0 | 0.1 | 0.1 | 0.1 | 0.0 | 0.0 | 0.1 |
| Mettl22 | 3.5 | 3.0 | 2.5 | 5.2 | 2.3 | 4.8 | 2.4 | 2.3 | 2.7 | 2.8 | 3.5 | 2.6 |
| Mettl23 | 11.8 | 10.2 | 7.9 | 7.3 | 9.7 | 9.9 | 7.6 | 9.2 | 10.8 | 11.1 | 10.1 | 8.5 |
| Mettl24 | 0.9 | 0.5 | 0.3 | 0.6 | 0.4 | 0.7 | 0.4 | 0.3 | 0.3 | 0.6 | 0.7 | 0.8 |
| Mettl25 | 3.1 | 2.7 | 2.7 | 3.4 | 3.0 | 3.3 | 3.1 | 1.9 | 2.4 | 2.6 | 3.3 | 2.6 |

| | | | | | | | | | | | | |
|---|---|---|---|---|---|---|---|---|---|---|---|---|
| Mettl3 | 10.8 | 10.3 | 9.2 | 11.5 | 10.6 | 10.8 | 11.2 | 10.4 | 10.0 | 10.4 | 11.0 | 10.6 |
| Mettl4 | 5.8 | 6.8 | 6.1 | 3.9 | 5.2 | 6.7 | 4.7 | 5.2 | 5.0 | 5.5 | 5.6 | 5.6 |
| Mettl5 | 6.8 | 9.1 | 7.1 | 7.9 | 5.9 | 9.8 | 6.0 | 7.1 | 5.8 | 7.1 | 7.1 | 7.9 |
| Mettl6 | 10.7 | 11.4 | 10.2 | 8.3 | 11.5 | 10.5 | 10.4 | 9.9 | 9.2 | 12.3 | 11.9 | 10.9 |
| Mettl7a1 | 22.6 | 21.1 | 28.0 | 20.9 | 12.9 | 22.3 | 11.4 | 16.0 | 13.5 | 15.1 | 15.0 | 16.7 |
| Mettl7a2 | 0.4 | 0.3 | 0.4 | 0.5 | 0.1 | 0.4 | 0.1 | 0.3 | 0.2 | 0.3 | 0.2 | 0.3 |
| Mettl7a3 | 0.1 | 0.0 | 0.1 | 0.0 | 0.0 | 0.1 | 0.0 | 0.1 | 0.0 | 0.1 | 0.0 | 0.0 |
| Mettl7b | 0.6 | 0.3 | 0.1 | 0.0 | 0.1 | 0.1 | 0.3 | 0.3 | 0.3 | 0.2 | 0.3 | 0.3 |
| Mettl8 | 2.4 | 2.3 | 2.7 | 1.9 | 1.9 | 2.1 | 2.2 | 2.5 | 1.7 | 2.0 | 2.4 | 2.1 |
| Mettl9 | 26.4 | 25.9 | 26.0 | 22.8 | 21.8 | 22.8 | 25.2 | 27.4 | 32.4 | 32.5 | 32.6 | 31.8 |
| Mex3a | 1.2 | 1.1 | 1.0 | 1.0 | 0.8 | 1.1 | 0.8 | 0.9 | 0.7 | 1.4 | 1.3 | 1.0 |
| Mex3b | 0.5 | 0.4 | 0.5 | 0.6 | 0.6 | 0.8 | 0.6 | 0.7 | 0.4 | 0.5 | 0.7 | 0.4 |
| Mex3c | 19.8 | 20.5 | 17.9 | 18.5 | 19.6 | 18.1 | 19.4 | 18.0 | 21.2 | 23.0 | 24.5 | 21.7 |
| Mex3d | 4.7 | 5.0 | 4.4 | 4.5 | 4.3 | 4.5 | 4.7 | 4.9 | 5.6 | 4.7 | 4.7 | 3.8 |
| Mfap1a | 9.4 | 9.7 | 11.8 | 12.5 | 9.8 | 10.8 | 9.2 | 10.5 | 9.8 | 11.6 | 13.2 | 14.1 |
| Mfap1b | 7.8 | 7.2 | 9.3 | 10.1 | 6.8 | 8.0 | 8.0 | 7.5 | 7.6 | 8.5 | 9.3 | 11.4 |
| Mfap2 | 0.1 | 0.5 | 1.1 | 0.0 | 0.3 | 0.1 | 0.2 | 0.2 | 0.4 | 0.9 | 0.5 | 0.5 |
| Mfap3 | 5.8 | 6.7 | 6.7 | 6.3 | 6.7 | 6.3 | 6.4 | 6.8 | 6.1 | 5.8 | 5.5 | 6.1 |
| Mfap3l | 3.7 | 2.7 | 2.0 | 5.0 | 2.7 | 4.0 | 2.2 | 1.4 | 1.5 | 1.2 | 1.8 | 1.5 |
| Mfap4 | 32.3 | 76.0 | 200.9 | 38.7 | 69.9 | 35.9 | 44.4 | 223.6 | 64.0 | 23.8 | 31.7 | 51.7 |
| Mfap5 | 80.3 | 98.6 | 129.2 | 83.6 | 77.2 | 73.7 | 138.2 | 190.5 | 148.1 | 93.9 | 112.9 | 124.0 |
| Mff | 37.8 | 34.6 | 29.1 | 35.8 | 32.4 | 36.8 | 31.1 | 30.8 | 32.4 | 33.1 | 35.3 | 33.8 |
| Mfge8 | 153.6 | 112.0 | 89.6 | 190.2 | 128.4 | 189.5 | 120.6 | 136.6 | 135.1 | 105.9 | 113.6 | 103.8 |
| Mfhas1 | 6.4 | 6.7 | 5.5 | 5.9 | 6.2 | 5.7 | 6.3 | 6.2 | 7.4 | 5.2 | 5.8 | 5.0 |
| Mfi2 | 0.0 | 0.0 | 0.0 | 0.0 | 0.0 | 0.0 | 0.0 | 0.0 | 0.0 | 0.0 | 0.0 | 0.0 |
| Mfn1 | 10.4 | 10.2 | 9.3 | 9.4 | 8.7 | 10.8 | 8.5 | 8.6 | 8.4 | 9.3 | 10.2 | 10.2 |
| Mfn2 | 25.0 | 22.8 | 18.5 | 27.8 | 19.9 | 25.9 | 19.3 | 17.7 | 17.5 | 18.2 | 18.9 | 18.2 |
| Mfng | 0.1 | 0.0 | 0.0 | 0.3 | 0.5 | 0.0 | 0.1 | 0.2 | 0.1 | 0.1 | 0.0 | 0.0 |
| Mfsd1 | 48.1 | 37.7 | 30.6 | 52.3 | 50.4 | 46.3 | 33.7 | 34.4 | 36.0 | 41.9 | 36.7 | 40.0 |
| Mfsd10 | 8.5 | 9.1 | 9.4 | 7.7 | 9.5 | 9.7 | 10.0 | 10.6 | 10.9 | 9.1 | 7.4 | 7.0 |
| Mfsd11 | 7.4 | 7.5 | 6.5 | 8.6 | 9.9 | 9.8 | 7.2 | 7.4 | 8.8 | 8.7 | 6.7 | 7.8 |
| Mfsd12 | 2.7 | 2.6 | 2.5 | 4.2 | 4.8 | 2.8 | 2.3 | 2.3 | 3.0 | 2.4 | 2.1 | 2.6 |
| Mfsd2a | 1.3 | 1.8 | 1.4 | 0.8 | 2.2 | 0.9 | 3.3 | 5.1 | 3.7 | 2.1 | 4.0 | 1.6 |
| Mfsd2b | 0.1 | 0.0 | 0.0 | 0.1 | 0.1 | 0.1 | 0.0 | 0.0 | 0.0 | 0.1 | 0.0 | 0.1 |
| Mfsd3 | 6.3 | 5.7 | 4.5 | 4.7 | 5.0 | 6.0 | 5.2 | 5.8 | 6.2 | 6.1 | 4.2 | 4.8 |
| Mfsd4 | 2.0 | 2.4 | 2.7 | 1.5 | 2.4 | 2.2 | 1.9 | 2.4 | 2.1 | 2.2 | 2.5 | 2.3 |
| Mfsd5 | 19.6 | 19.3 | 16.4 | 26.2 | 20.1 | 22.5 | 22.1 | 19.0 | 19.5 | 17.5 | 19.1 | 16.0 |
| Mfsd6 | 9.6 | 6.7 | 5.4 | 16.4 | 11.4 | 11.9 | 6.6 | 5.0 | 4.0 | 5.3 | 6.8 | 5.6 |
| Mfsd7a | 1.1 | 1.1 | 1.4 | 1.2 | 1.1 | 1.9 | 1.4 | 1.7 | 1.4 | 0.8 | 1.0 | 1.0 |
| Mfsd7b | 1.8 | 2.1 | 1.9 | 2.7 | 3.6 | 1.8 | 2.3 | 1.5 | 1.9 | 2.2 | 2.1 | 1.8 |
| Mfsd7c | 0.1 | 0.2 | 0.1 | 0.1 | 0.1 | 0.0 | 0.1 | 0.2 | 0.2 | 0.2 | 0.2 | 0.1 |
| Mfsd8 | 4.6 | 4.0 | 3.4 | 4.2 | 3.7 | 4.6 | 3.2 | 3.3 | 3.9 | 4.2 | 3.3 | 4.1 |
| Mfsd9 | 1.7 | 2.0 | 2.3 | 1.5 | 2.0 | 1.9 | 2.0 | 2.5 | 2.1 | 1.8 | 1.9 | 2.0 |
| Mga | 6.0 | 6.8 | 7.3 | 6.1 | 6.5 | 5.9 | 6.3 | 6.8 | 6.2 | 7.3 | 7.7 | 7.5 |
| Mgam | 0.3 | 0.4 | 0.4 | 0.1 | 0.7 | 0.1 | 1.1 | 2.2 | 1.0 | 0.4 | 1.1 | 0.7 |
| Mgarp | 0.2 | 0.5 | 0.3 | 0.3 | 0.4 | 0.8 | 0.5 | 0.1 | 0.4 | 0.3 | 0.2 | 0.2 |
| Mgat1 | 27.3 | 25.3 | 23.5 | 28.4 | 28.5 | 28.7 | 30.8 | 28.0 | 31.9 | 25.3 | 23.4 | 25.5 |
| Mgat2 | 40.1 | 37.6 | 37.3 | 38.1 | 43.0 | 40.1 | 49.0 | 40.0 | 46.2 | 45.4 | 47.6 | 47.2 |
| Mgat3 | 4.7 | 7.4 | 7.7 | 4.1 | 5.8 | 7.4 | 6.5 | 6.1 | 8.3 | 7.5 | 6.1 | 7.6 |
| Mgat4a | 2.3 | 2.9 | 2.9 | 3.3 | 4.4 | 2.6 | 2.4 | 2.2 | 2.0 | 2.0 | 1.9 | 2.4 |
| Mgat4b | 14.6 | 14.1 | 11.9 | 16.1 | 13.5 | 21.2 | 12.9 | 9.2 | 11.6 | 10.4 | 9.8 | 9.9 |
| Mgat5 | 4.1 | 3.2 | 3.4 | 5.2 | 5.8 | 4.6 | 4.3 | 3.6 | 2.9 | 3.6 | 3.8 | 3.6 |
| Mgat5b | 0.0 | 0.0 | 0.0 | 0.0 | 0.0 | 0.0 | 0.0 | 0.0 | 0.0 | 0.0 | 0.0 | 0.0 |
| Mgea5 | 36.0 | 36.6 | 30.5 | 36.5 | 32.1 | 35.8 | 26.2 | 29.4 | 28.6 | 25.5 | 32.5 | 28.7 |
| Mgl2 | 0.3 | 0.0 | 0.0 | 0.1 | 0.1 | 0.2 | 0.0 | 0.1 | 0.1 | 0.0 | 0.0 | 0.1 |
| Mgll | 13.5 | 9.2 | 8.7 | 8.4 | 7.6 | 10.2 | 7.4 | 12.0 | 6.9 | 9.5 | 11.9 | 7.7 |
| Mgme1 | 3.8 | 3.9 | 3.7 | 4.4 | 4.1 | 5.3 | 4.2 | 3.1 | 3.5 | 3.1 | 3.9 | 3.4 |

| | | | | | | | | | | | | |
|---|---|---|---|---|---|---|---|---|---|---|---|---|
| Mgmt | 52.6 | 43.9 | 38.1 | 51.8 | 35.3 | 58.3 | 30.4 | 29.1 | 27.8 | 49.5 | 42.8 | 35.9 |
| Mgp | 55.5 | 66.3 | 202.2 | 9.5 | 37.3 | 17.2 | 46.5 | 158.1 | 103.0 | 771.2 | 227.1 | 478.1 |
| Mgrn1 | 21.2 | 18.6 | 18.1 | 21.2 | 19.6 | 19.1 | 20.4 | 16.7 | 18.2 | 20.1 | 19.6 | 18.9 |
| Mgst1 | 191.4 | 230.3 | 244.9 | 194.0 | 208.4 | 185.8 | 187.6 | 239.7 | 192.8 | 264.8 | 214.8 | 236.8 |
| Mgst2 | 1.3 | 0.8 | 0.7 | 1.2 | 2.0 | 0.7 | 0.2 | 0.4 | 0.4 | 1.2 | 0.3 | 0.2 |
| Mgst3 | 7.4 | 6.2 | 5.3 | 16.4 | 12.0 | 12.1 | 7.4 | 6.7 | 4.5 | 5.1 | 3.4 | 5.2 |
| Mia | 0.2 | 0.2 | 0.2 | 0.0 | 0.1 | 0.0 | 0.0 | 0.2 | 0.1 | 0.2 | 0.0 | 0.0 |
| Mia3 | 37.9 | 38.0 | 43.7 | 41.9 | 33.6 | 43.1 | 41.5 | 39.3 | 34.1 | 31.5 | 40.7 | 39.3 |
| Miat | 0.0 | 0.0 | 0.0 | 0.0 | 0.0 | 0.0 | 0.0 | 0.0 | 0.0 | 0.0 | 0.0 | 0.0 |
| Mib1 | 20.7 | 18.7 | 15.0 | 17.2 | 17.4 | 18.2 | 16.8 | 16.5 | 15.1 | 15.7 | 16.7 | 15.6 |
| Mib2 | 9.7 | 11.0 | 10.4 | 9.2 | 6.9 | 11.9 | 8.4 | 8.3 | 8.4 | 8.5 | 8.3 | 7.7 |
| Mical1 | 12.4 | 12.5 | 13.0 | 16.0 | 14.9 | 14.5 | 13.1 | 12.8 | 12.0 | 8.5 | 10.7 | 11.0 |
| Mical2 | 53.0 | 64.9 | 60.8 | 43.4 | 48.0 | 49.9 | 62.3 | 58.0 | 60.5 | 66.1 | 66.5 | 65.2 |
| Mical3 | 2.6 | 3.0 | 3.2 | 2.4 | 2.9 | 2.3 | 3.9 | 3.8 | 3.3 | 3.6 | 3.7 | 3.7 |
| Micalcl | 0.5 | 0.8 | 0.8 | 0.6 | 0.6 | 0.4 | 0.6 | 0.5 | 0.5 | 0.9 | 0.7 | 1.0 |
| Micall1 | 5.3 | 4.7 | 4.5 | 4.9 | 4.4 | 4.2 | 5.5 | 4.3 | 4.6 | 6.2 | 5.9 | 5.2 |
| Micall2 | 8.7 | 12.0 | 11.9 | 7.9 | 11.8 | 7.5 | 11.7 | 9.0 | 9.3 | 6.4 | 7.7 | 7.9 |
| Micu1 | 29.3 | 26.5 | 23.2 | 32.4 | 23.4 | 32.4 | 21.3 | 26.1 | 20.1 | 21.5 | 21.4 | 23.5 |
| Mid1 | 5.7 | 8.4 | 9.1 | 5.1 | 6.3 | 4.7 | 6.9 | 6.9 | 6.2 | 4.2 | 5.1 | 4.3 |
| Mid1ip1 | 17.5 | 15.6 | 15.3 | 15.0 | 15.8 | 14.3 | 21.1 | 25.3 | 19.8 | 14.3 | 20.2 | 12.8 |
| Mid2 | 3.7 | 4.3 | 4.3 | 4.6 | 3.2 | 4.1 | 3.8 | 4.4 | 4.3 | 3.6 | 5.0 | 4.6 |
| Midn | 5.9 | 7.3 | 7.4 | 6.6 | 7.9 | 6.5 | 6.9 | 8.2 | 9.0 | 8.1 | 6.7 | 6.1 |
| Mien1 | 64.8 | 60.8 | 48.0 | 64.6 | 56.9 | 68.0 | 55.3 | 56.1 | 49.6 | 58.7 | 60.0 | 55.1 |
| Mier1 | 8.9 | 9.8 | 11.6 | 10.9 | 9.4 | 9.6 | 8.9 | 8.9 | 8.6 | 9.5 | 10.5 | 11.2 |
| Mier2 | 6.5 | 7.4 | 6.0 | 6.4 | 6.9 | 6.7 | 8.5 | 6.4 | 7.8 | 6.5 | 6.4 | 6.2 |
| Mier3 | 5.7 | 6.6 | 7.9 | 6.3 | 5.1 | 6.6 | 7.1 | 7.3 | 6.8 | 6.7 | 7.4 | 8.5 |
| Mif | 186.0 | 145.1 | 127.5 | 188.4 | 160.5 | 221.6 | 132.9 | 138.2 | 121.7 | 161.1 | 133.1 | 135.2 |
| Mif4gd | 5.5 | 4.1 | 6.0 | 5.9 | 5.8 | 5.5 | 6.4 | 7.7 | 6.4 | 6.1 | 5.3 | 6.9 |
| Miip | 5.4 | 5.9 | 5.4 | 4.9 | 5.4 | 4.8 | 5.3 | 4.2 | 6.4 | 5.0 | 5.5 | 5.0 |
| Mill2 | 5.3 | 7.1 | 8.9 | 6.2 | 6.3 | 7.3 | 7.0 | 6.6 | 4.1 | 4.1 | 4.1 | 5.4 |
| Milr1 | 0.9 | 0.1 | 0.2 | 4.6 | 10.2 | 0.1 | 1.3 | 0.2 | 0.1 | 0.9 | 0.1 | 1.1 |
| Mina | 6.4 | 4.9 | 4.9 | 8.7 | 6.9 | 6.7 | 7.2 | 5.0 | 5.2 | 6.1 | 7.2 | 6.6 |
| Mink1 | 7.0 | 6.3 | 6.1 | 8.6 | 6.5 | 8.6 | 5.6 | 4.8 | 5.0 | 5.8 | 5.5 | 5.0 |
| Minos1 | 16.0 | 14.3 | 16.7 | 16.0 | 15.4 | 15.8 | 18.1 | 14.2 | 14.4 | 18.2 | 17.0 | 18.3 |
| Minpp1 | 14.7 | 13.5 | 11.9 | 16.0 | 14.7 | 13.6 | 14.1 | 13.3 | 13.8 | 14.8 | 14.2 | 13.0 |
| Mios | 5.5 | 6.2 | 6.0 | 4.9 | 5.3 | 4.0 | 5.8 | 6.7 | 7.8 | 5.6 | 6.4 | 6.5 |
| Mip | 0.1 | 0.0 | 0.0 | 0.0 | 0.0 | 0.0 | 0.0 | 0.1 | 0.0 | 0.0 | 0.0 | 0.0 |
| Mipep | 5.4 | 4.3 | 4.9 | 6.3 | 4.6 | 8.0 | 4.4 | 4.2 | 3.5 | 4.8 | 4.4 | 4.1 |
| Mipol1 | 4.4 | 4.5 | 4.1 | 2.7 | 3.1 | 4.3 | 3.4 | 3.3 | 2.2 | 3.3 | 2.9 | 3.4 |
| Mir143hg | 3.4 | 4.4 | 5.2 | 3.0 | 3.9 | 3.3 | 6.0 | 4.9 | 5.3 | 3.3 | 5.4 | 4.6 |
| Mir17hg | 0.3 | 0.3 | 0.3 | 0.3 | 0.3 | 0.1 | 0.3 | 0.2 | 0.2 | 0.2 | 0.4 | 0.3 |
| Mir1940 | 14.4 | 0.0 | 11.3 | 0.0 | 0.0 | 9.0 | 22.3 | 26.9 | 14.4 | 8.1 | 11.9 | 6.8 |
| Mir1983 | 0.0 | 0.0 | 0.0 | 11.6 | 3.7 | 0.0 | 2.2 | 4.5 | 2.2 | 0.0 | 0.0 | 4.4 |
| Mir2137 | 17453.8 | 60445.9 | 11669.9 | 86708.1 | 30537.4 | 16613.9 | 33465.8 | 19729.2 | 19462.4 | 23332.1 | 46996.7 | 58902.4 |
| Mir22 | 0.0 | 0.0 | 0.0 | 0.0 | 0.0 | 0.0 | 0.0 | 0.0 | 0.0 | 0.0 | 0.0 | 0.0 |
| Mir22hg | 14.2 | 13.8 | 10.9 | 14.4 | 15.7 | 13.5 | 16.1 | 11.7 | 17.4 | 18.4 | 15.7 | 16.1 |
| Mir3069 | 0.0 | 0.0 | 0.0 | 0.0 | 0.0 | 0.0 | 0.0 | 0.0 | 0.0 | 0.0 | 0.0 | 0.0 |
| Mir546 | 0.0 | 0.0 | 1.4 | 0.0 | 0.0 | 0.0 | 0.0 | 0.0 | 0.0 | 0.0 | 0.0 | 0.0 |
| Mir671 | 0.0 | 0.0 | 0.0 | 0.0 | 0.0 | 0.0 | 0.0 | 0.0 | 0.0 | 0.0 | 0.0 | 0.0 |
| Mir678 | 0.0 | 0.0 | 0.0 | 0.0 | 0.0 | 0.0 | 0.0 | 0.0 | 0.0 | 0.0 | 0.0 | 0.0 |
| Mir682 | 0.0 | 19.0 | 0.0 | 18.2 | 18.0 | 7.1 | 0.0 | 8.6 | 7.6 | 1.9 | 8.5 | 0.0 |
| Mir684-1 | 0.0 | 23.7 | 13.3 | 3.0 | 18.2 | 4.3 | 12.2 | 16.9 | 7.0 | 18.8 | 6.6 | 21.1 |
| Mir686 | 0.8 | 0.0 | 0.0 | 0.0 | 0.0 | 0.0 | 0.0 | 0.0 | 0.0 | 0.0 | 0.0 | 0.0 |
| Mir692-1 | 3.3 | 2.3 | 1.1 | 18.0 | 1.8 | 6.9 | 1.1 | 0.0 | 2.8 | 0.0 | 0.5 | 0.0 |
| Mir703 | 12.3 | 7.6 | 97.6 | 49.6 | 42.3 | 67.4 | 15.9 | 60.7 | 39.7 | 88.5 | 9.5 | 101.7 |
| Mir719 | 0.6 | 0.0 | 0.0 | 2.7 | 0.0 | 0.0 | 0.0 | 0.0 | 10.5 | 11.2 | 4.3 | 0.0 |
| Mira | 0.0 | 0.0 | 0.0 | 0.2 | 0.0 | 0.1 | 0.0 | 0.0 | 0.0 | 0.9 | 1.2 | 0.2 |

| | | | | | | | | | | | | |
|---|---|---|---|---|---|---|---|---|---|---|---|---|
| Mirg | 1.7 | 0.9 | 0.9 | 1.0 | 1.3 | 1.2 | 1.7 | 1.8 | 3.0 | 2.8 | 3.7 | 1.8 |
| Mis12 | 13.8 | 15.7 | 15.1 | 13.7 | 14.1 | 13.0 | 13.8 | 13.1 | 12.6 | 13.7 | 14.8 | 15.5 |
| Mis18a | 2.9 | 3.7 | 4.6 | 2.4 | 4.9 | 2.1 | 3.9 | 3.7 | 4.4 | 3.7 | 3.3 | 2.7 |
| Mis18bp1 | 0.9 | 1.5 | 2.0 | 0.6 | 1.9 | 0.5 | 2.3 | 1.1 | 1.8 | 1.1 | 1.6 | 1.4 |
| Mitd1 | 11.7 | 10.1 | 11.3 | 13.9 | 10.4 | 9.3 | 9.6 | 10.1 | 9.2 | 11.1 | 11.3 | 13.2 |
| Mitf | 1.6 | 1.7 | 2.0 | 3.6 | 3.1 | 1.6 | 1.5 | 1.4 | 1.2 | 1.9 | 2.1 | 2.2 |
| Mixl1 | 0.0 | 0.0 | 0.0 | 0.0 | 0.0 | 0.0 | 0.0 | 0.0 | 0.0 | 0.0 | 0.0 | 0.0 |
| Mki67 | 3.5 | 5.9 | 11.1 | 1.2 | 9.0 | 0.9 | 12.6 | 4.7 | 7.5 | 3.8 | 7.7 | 5.5 |
| Mki67ip | 20.5 | 14.9 | 14.7 | 20.9 | 17.8 | 16.2 | 16.9 | 15.3 | 14.3 | 19.3 | 19.4 | 16.3 |
| Mkks | 9.8 | 9.3 | 8.3 | 9.4 | 9.7 | 9.8 | 8.2 | 8.9 | 9.6 | 9.5 | 9.4 | 9.7 |
| Mkl1 | 2.6 | 3.9 | 3.5 | 3.2 | 4.6 | 3.3 | 5.5 | 2.9 | 4.6 | 4.5 | 3.5 | 2.8 |
| Mkl2 | 2.9 | 3.2 | 2.9 | 3.2 | 3.2 | 3.6 | 3.3 | 2.5 | 2.4 | 2.8 | 2.6 | 2.5 |
| Mkln1 | 17.7 | 19.1 | 18.0 | 17.0 | 15.6 | 17.2 | 16.1 | 16.6 | 16.5 | 17.2 | 17.6 | 18.5 |
| Mknk1 | 7.9 | 6.4 | 5.9 | 7.3 | 8.8 | 5.9 | 7.1 | 7.4 | 7.0 | 6.9 | 7.1 | 6.5 |
| Mknk2 | 14.3 | 19.0 | 15.0 | 14.1 | 16.7 | 15.2 | 14.0 | 13.3 | 15.4 | 16.8 | 14.2 | 13.9 |
| Mkrn1 | 12.6 | 12.4 | 11.4 | 13.6 | 13.0 | 12.9 | 9.5 | 11.3 | 10.4 | 10.7 | 11.0 | 11.4 |
| Mkrn2 | 9.1 | 9.0 | 8.2 | 8.5 | 7.0 | 9.9 | 7.1 | 8.5 | 6.9 | 7.0 | 7.9 | 6.9 |
| Mkrn3 | 0.1 | 0.2 | 0.2 | 0.2 | 0.2 | 0.3 | 0.2 | 0.3 | 0.2 | 0.2 | 0.3 | 0.4 |
| Mks1 | 2.1 | 1.4 | 2.0 | 1.9 | 1.5 | 2.0 | 1.8 | 2.0 | 2.0 | 1.8 | 1.3 | 1.7 |
| Mkx | 0.5 | 0.4 | 0.3 | 0.6 | 0.6 | 0.6 | 0.8 | 0.3 | 0.3 | 0.3 | 0.5 | 0.4 |
| Mlana | 7.0 | 1.6 | 0.8 | 3.1 | 0.8 | 1.7 | 0.0 | 0.2 | 0.1 | 1.3 | 0.2 | 1.1 |
| Mlec | 102.4 | 82.7 | 73.7 | 89.8 | 87.7 | 86.4 | 85.0 | 77.3 | 76.4 | 96.6 | 94.6 | 88.9 |
| Mlf1 | 0.4 | 0.7 | 1.4 | 0.3 | 0.7 | 0.5 | 1.3 | 0.7 | 0.8 | 0.6 | 0.8 | 0.6 |
| Mlf1ip | 0.3 | 0.4 | 0.7 | 0.1 | 0.4 | 0.2 | 0.7 | 0.4 | 0.5 | 0.3 | 0.5 | 0.5 |
| Mlf2 | 77.3 | 68.3 | 63.2 | 81.6 | 72.2 | 79.1 | 80.8 | 69.7 | 72.9 | 73.0 | 75.3 | 68.8 |
| Mlh1 | 5.3 | 5.3 | 4.5 | 3.6 | 4.2 | 5.1 | 4.2 | 5.2 | 4.8 | 4.4 | 4.7 | 3.7 |
| Mlh3 | 5.5 | 5.6 | 4.8 | 5.5 | 4.7 | 7.2 | 4.8 | 4.8 | 4.6 | 4.9 | 5.6 | 6.0 |
| Mlkl | 21.7 | 17.9 | 19.4 | 26.3 | 20.8 | 24.0 | 18.8 | 17.5 | 15.1 | 13.7 | 17.3 | 19.4 |
| Mll1 | 1.7 | 2.4 | 2.4 | 1.8 | 2.2 | 2.0 | 2.7 | 1.9 | 1.8 | 1.8 | 1.9 | 1.6 |
| Mll2 | 0.5 | 1.0 | 1.0 | 0.6 | 1.0 | 0.7 | 1.2 | 0.9 | 1.1 | 1.0 | 0.9 | 0.7 |
| Mll3 | 2.2 | 2.6 | 2.6 | 2.1 | 2.3 | 2.2 | 2.2 | 2.3 | 2.1 | 2.2 | 2.0 | 2.2 |
| Mll5 | 3.1 | 4.2 | 5.0 | 3.9 | 4.2 | 4.0 | 4.6 | 4.4 | 4.1 | 4.2 | 5.0 | 4.8 |
| Mllt1 | 6.7 | 7.3 | 6.8 | 6.2 | 6.5 | 7.0 | 6.9 | 7.4 | 6.8 | 6.2 | 7.1 | 6.1 |
| Mllt10 | 3.4 | 5.0 | 5.0 | 3.6 | 3.9 | 3.7 | 4.8 | 5.0 | 4.7 | 4.2 | 4.2 | 4.4 |
| Mllt11 | 8.5 | 8.4 | 7.7 | 11.2 | 9.0 | 9.9 | 9.2 | 8.1 | 9.8 | 7.7 | 10.2 | 8.2 |
| Mllt3 | 12.3 | 15.3 | 15.4 | 9.3 | 8.6 | 13.0 | 11.0 | 10.5 | 11.6 | 9.5 | 12.5 | 14.1 |
| Mllt4 | 10.7 | 10.3 | 10.3 | 10.5 | 8.6 | 9.8 | 10.1 | 9.3 | 9.5 | 10.1 | 11.4 | 9.7 |
| Mllt6 | 3.7 | 4.7 | 5.5 | 4.7 | 4.9 | 4.2 | 4.9 | 5.3 | 5.7 | 5.2 | 4.1 | 5.5 |
| Mlph | 0.1 | 0.1 | 0.0 | 0.2 | 0.1 | 0.3 | 0.0 | 0.1 | 0.0 | 0.0 | 0.1 | 0.0 |
| Mlst8 | 2.2 | 1.7 | 1.9 | 2.6 | 2.5 | 2.3 | 3.0 | 3.4 | 3.2 | 2.5 | 2.9 | 2.0 |
| Mlx | 10.3 | 10.1 | 10.2 | 9.2 | 11.0 | 7.8 | 8.4 | 10.2 | 9.8 | 10.8 | 11.2 | 10.9 |
| Mlxip | 1.9 | 3.0 | 3.2 | 1.9 | 2.8 | 1.6 | 2.4 | 2.5 | 2.7 | 1.8 | 2.1 | 2.2 |
| Mlxipl | 0.1 | 0.1 | 0.0 | 0.0 | 0.0 | 0.0 | 0.0 | 0.0 | 0.0 | 0.1 | 0.0 | 0.0 |
| Mlycd | 6.7 | 5.5 | 3.8 | 6.9 | 4.8 | 5.4 | 4.5 | 5.1 | 5.3 | 6.0 | 4.8 | 4.8 |
| Mmaa | 2.8 | 2.6 | 2.6 | 2.8 | 2.4 | 3.1 | 1.9 | 2.1 | 1.8 | 2.5 | 2.4 | 2.9 |
| Mmab | 7.8 | 9.5 | 9.8 | 6.5 | 7.2 | 7.7 | 11.4 | 12.9 | 10.1 | 5.7 | 11.5 | 7.5 |
| Mmachc | 6.9 | 6.3 | 5.7 | 8.0 | 6.1 | 7.1 | 6.7 | 7.3 | 5.9 | 5.7 | 7.9 | 6.1 |
| Mmadhc | 60.6 | 47.8 | 37.3 | 49.9 | 47.1 | 48.1 | 41.7 | 43.7 | 50.9 | 59.1 | 54.3 | 52.8 |
| Mmd | 11.7 | 8.9 | 8.8 | 27.3 | 15.9 | 22.6 | 8.2 | 5.2 | 6.0 | 22.9 | 12.5 | 10.3 |
| Mme | 0.3 | 0.4 | 0.7 | 0.2 | 0.5 | 0.2 | 0.3 | 0.8 | 0.6 | 0.5 | 0.4 | 0.5 |
| Mmel1 | 0.3 | 0.1 | 0.1 | 0.1 | 0.1 | 0.1 | 0.1 | 0.1 | 0.1 | 0.1 | 0.2 | 0.2 |
| Mmgt1 | 9.1 | 10.8 | 10.3 | 9.6 | 10.5 | 10.1 | 10.6 | 10.1 | 10.8 | 11.5 | 12.5 | 11.5 |
| Mmgt2 | 7.6 | 7.2 | 6.5 | 9.5 | 6.8 | 7.4 | 7.1 | 5.8 | 6.9 | 7.8 | 8.0 | 6.5 |
| Mmp10 | 3.7 | 2.3 | 4.3 | 7.3 | 3.7 | 4.3 | 1.3 | 0.2 | 0.7 | 1.7 | 0.3 | 1.8 |
| Mmp11 | 8.8 | 11.0 | 13.5 | 8.7 | 6.7 | 11.8 | 7.7 | 14.0 | 13.2 | 12.3 | 9.9 | 10.2 |
| Mmp12 | 10.2 | 0.1 | 0.5 | 82.6 | 102.1 | 0.0 | 4.8 | 1.9 | 0.9 | 4.1 | 1.7 | 2.9 |
| Mmp13 | 0.2 | 0.1 | 0.0 | 0.5 | 0.7 | 0.0 | 0.1 | 0.1 | 0.1 | 0.2 | 0.2 | 0.1 |

| | | | | | | | | | | | | |
|---|---|---|---|---|---|---|---|---|---|---|---|---|
| Mmp14 | 55.4 | 44.4 | 51.5 | 45.3 | 62.3 | 41.1 | 63.0 | 77.6 | 76.6 | 87.7 | 75.4 | 80.8 |
| Mmp15 | 0.8 | 0.6 | 0.4 | 0.5 | 0.7 | 0.5 | 0.5 | 0.3 | 0.4 | 0.3 | 0.5 | 0.4 |
| Mmp16 | 8.0 | 6.1 | 5.4 | 4.3 | 6.1 | 4.0 | 8.4 | 8.0 | 7.8 | 3.8 | 7.0 | 5.5 |
| Mmp17 | 0.4 | 0.4 | 1.0 | 0.4 | 0.6 | 0.3 | 1.8 | 2.5 | 3.5 | 2.5 | 1.4 | 1.6 |
| Mmp19 | 14.5 | 15.7 | 20.2 | 15.7 | 15.2 | 14.5 | 15.4 | 16.9 | 15.4 | 16.1 | 13.9 | 19.0 |
| Mmp1a | 0.0 | 0.0 | 0.0 | 0.0 | 0.0 | 0.0 | 0.1 | 0.0 | 0.0 | 0.0 | 0.0 | 0.0 |
| Mmp1b | 0.0 | 0.0 | 0.0 | 0.0 | 0.2 | 0.1 | 0.0 | 0.1 | 0.2 | 0.1 | 0.0 | 0.2 |
| Mmp2 | 74.0 | 72.3 | 118.3 | 46.6 | 58.2 | 53.7 | 47.9 | 83.5 | 53.1 | 100.0 | 82.6 | 89.2 |
| Mmp21 | 0.0 | 0.0 | 0.0 | 0.0 | 0.0 | 0.1 | 0.0 | 0.0 | 0.0 | 0.0 | 0.0 | 0.0 |
| Mmp23 | 20.4 | 27.3 | 36.4 | 20.8 | 25.9 | 31.5 | 33.7 | 64.1 | 53.2 | 43.1 | 31.4 | 45.3 |
| Mmp24 | 0.2 | 0.1 | 0.1 | 0.2 | 0.2 | 0.2 | 0.1 | 0.1 | 0.1 | 0.2 | 0.1 | 0.1 |
| Mmp25 | 0.0 | 0.0 | 0.0 | 0.0 | 0.1 | 0.0 | 0.0 | 0.0 | 0.0 | 0.0 | 0.0 | 0.0 |
| Mmp27 | 1.1 | 1.0 | 1.5 | 0.9 | 0.8 | 0.5 | 0.9 | 0.6 | 0.8 | 0.9 | 0.7 | 1.2 |
| Mmp28 | 1.1 | 1.0 | 1.1 | 0.8 | 0.7 | 2.1 | 1.1 | 1.0 | 1.2 | 0.9 | 1.0 | 0.8 |
| Mmp3 | 116.8 | 98.6 | 180.6 | 196.1 | 95.5 | 121.3 | 21.8 | 10.3 | 31.4 | 52.0 | 15.2 | 73.4 |
| Mmp8 | 3.8 | 6.1 | 11.7 | 7.5 | 14.4 | 1.5 | 9.6 | 6.2 | 8.6 | 11.5 | 8.0 | 15.9 |
| Mmp9 | 0.1 | 0.0 | 0.0 | 0.0 | 0.1 | 0.0 | 0.0 | 0.0 | 0.0 | 0.0 | 0.0 | 0.0 |
| Mmrn1 | 0.0 | 0.0 | 0.0 | 0.0 | 0.0 | 0.0 | 0.0 | 0.0 | 0.0 | 0.1 | 0.0 | 0.0 |
| Mmrn2 | 0.9 | 0.3 | 0.5 | 0.1 | 0.3 | 0.4 | 0.5 | 1.0 | 0.5 | 0.2 | 0.1 | 0.3 |
| Mms19 | 10.9 | 9.9 | 8.5 | 9.5 | 9.0 | 11.0 | 8.7 | 9.8 | 10.0 | 9.8 | 9.8 | 8.8 |
| Mms22l | 1.3 | 1.9 | 2.0 | 0.9 | 1.9 | 1.1 | 2.2 | 1.4 | 2.1 | 1.3 | 1.4 | 1.2 |
| Mn1 | 6.3 | 9.8 | 9.6 | 5.0 | 6.6 | 6.7 | 9.6 | 11.9 | 10.7 | 6.0 | 6.3 | 7.1 |
| Mnat1 | 11.0 | 10.6 | 10.3 | 10.9 | 8.7 | 12.0 | 9.7 | 10.2 | 9.7 | 11.4 | 11.6 | 12.6 |
| Mnd1 | 0.5 | 0.4 | 0.6 | 0.6 | 0.3 | 0.6 | 0.6 | 0.5 | 0.5 | 0.3 | 0.5 | 0.5 |
| Mnda | 21.4 | 17.2 | 28.3 | 22.8 | 21.8 | 13.3 | 20.6 | 22.3 | 16.0 | 13.9 | 17.8 | 26.1 |
| Mndal | 5.8 | 4.2 | 8.4 | 9.0 | 6.4 | 5.5 | 4.7 | 3.5 | 3.7 | 2.9 | 3.5 | 7.5 |
| Mnf1 | 124.7 | 111.7 | 87.5 | 131.9 | 96.2 | 132.8 | 100.1 | 132.8 | 108.3 | 105.6 | 123.6 | 97.2 |
| Mns1 | 1.3 | 1.9 | 3.1 | 1.2 | 2.4 | 0.6 | 3.7 | 1.3 | 1.6 | 0.8 | 1.7 | 2.0 |
| Mnt | 1.0 | 1.2 | 1.2 | 1.3 | 1.2 | 1.5 | 1.1 | 1.5 | 1.2 | 1.2 | 0.9 | 1.0 |
| Moap1 | 0.5 | 1.2 | 1.9 | 0.9 | 0.6 | 1.2 | 1.3 | 0.5 | 0.9 | 1.4 | 0.7 | 1.7 |
| Mob1a | 56.1 | 55.9 | 51.3 | 53.9 | 60.3 | 52.4 | 53.7 | 53.2 | 61.1 | 58.8 | 61.3 | 57.2 |
| Mob1b | 6.9 | 6.4 | 6.6 | 8.3 | 7.8 | 8.3 | 6.7 | 6.2 | 5.9 | 7.5 | 8.7 | 7.1 |
| Mob2 | 13.2 | 12.4 | 9.6 | 13.3 | 12.8 | 11.4 | 12.9 | 11.1 | 10.8 | 12.5 | 12.3 | 12.9 |
| Mob3a | 13.0 | 12.8 | 11.1 | 15.8 | 17.8 | 12.2 | 18.5 | 14.0 | 15.0 | 14.4 | 18.2 | 14.3 |
| Mob3b | 0.1 | 0.1 | 0.0 | 0.1 | 0.1 | 0.2 | 0.1 | 0.1 | 0.1 | 0.1 | 0.1 | 0.1 |
| Mob3c | 4.5 | 3.6 | 3.9 | 5.0 | 4.5 | 3.8 | 4.4 | 3.5 | 3.7 | 4.2 | 4.4 | 3.8 |
| Mob4 | 18.8 | 21.3 | 18.4 | 21.8 | 19.4 | 20.3 | 19.6 | 20.6 | 22.6 | 23.5 | 25.6 | 24.7 |
| Mocos | 9.2 | 5.6 | 4.9 | 10.9 | 7.4 | 7.9 | 5.4 | 4.3 | 4.2 | 5.7 | 4.7 | 5.2 |
| Mocs1 | 9.6 | 8.1 | 7.9 | 9.4 | 9.7 | 9.4 | 9.8 | 9.3 | 9.0 | 10.1 | 8.1 | 9.5 |
| Mocs2 | 24.7 | 18.8 | 16.8 | 21.4 | 16.3 | 19.3 | 18.8 | 15.2 | 17.5 | 18.9 | 18.8 | 19.8 |
| Mocs3 | 4.4 | 3.2 | 3.2 | 3.0 | 2.8 | 4.0 | 3.5 | 3.4 | 4.2 | 4.1 | 3.2 | 4.0 |
| Mog | 0.1 | 0.0 | 0.1 | 0.0 | 0.0 | 0.1 | 0.0 | 0.0 | 0.0 | 0.0 | 0.0 | 0.0 |
| Mogat1 | 0.1 | 0.1 | 0.0 | 0.2 | 0.0 | 0.0 | 0.0 | 0.0 | 0.0 | 0.1 | 0.0 | 0.0 |
| Mogat2 | 2.0 | 0.9 | 1.3 | 1.2 | 0.7 | 0.7 | 0.4 | 0.8 | 0.8 | 2.1 | 1.1 | 0.8 |
| Mogs | 9.9 | 10.1 | 9.6 | 11.0 | 11.3 | 11.3 | 10.5 | 10.9 | 13.9 | 12.8 | 10.5 | 10.3 |
| Mon1a | 4.8 | 5.6 | 5.9 | 6.1 | 5.3 | 6.7 | 5.2 | 5.3 | 5.9 | 5.5 | 3.5 | 4.6 |
| Mon1b | 4.3 | 4.3 | 4.7 | 4.9 | 4.4 | 4.4 | 4.8 | 4.6 | 4.4 | 4.4 | 4.3 | 4.1 |
| Mon2 | 13.6 | 14.4 | 13.4 | 12.8 | 13.8 | 13.6 | 14.0 | 13.3 | 13.2 | 12.0 | 13.1 | 12.3 |
| Morc1 | 0.2 | 0.1 | 0.1 | 0.1 | 0.1 | 0.4 | 0.1 | 0.0 | 0.1 | 0.1 | 0.1 | 0.0 |
| Morc2a | 7.3 | 6.9 | 7.3 | 7.9 | 6.2 | 6.1 | 7.0 | 7.4 | 7.5 | 6.7 | 8.1 | 7.4 |
| Morc2b | 0.0 | 0.1 | 0.0 | 0.1 | 0.0 | 0.1 | 0.0 | 0.0 | 0.0 | 0.0 | 0.0 | 0.0 |
| Morc3 | 10.3 | 10.7 | 10.6 | 9.8 | 11.4 | 8.5 | 9.8 | 8.4 | 11.3 | 12.3 | 12.5 | 12.6 |
| Morc4 | 3.6 | 3.5 | 3.0 | 4.3 | 2.6 | 5.2 | 3.4 | 2.9 | 3.5 | 3.7 | 4.3 | 4.1 |
| Morf4l1 | 56.3 | 57.2 | 64.7 | 63.1 | 56.5 | 58.9 | 59.0 | 65.2 | 54.5 | 59.8 | 63.7 | 65.9 |
| Morf4l2 | 175.5 | 160.0 | 168.2 | 186.8 | 162.1 | 182.0 | 192.0 | 196.9 | 178.2 | 196.2 | 212.6 | 216.8 |
| Morn1 | 0.6 | 0.8 | 0.9 | 0.8 | 0.4 | 1.0 | 0.7 | 0.9 | 0.5 | 0.6 | 1.0 | 0.5 |
| Morn2 | 10.8 | 6.4 | 6.4 | 9.9 | 7.1 | 10.7 | 6.1 | 6.7 | 4.1 | 8.8 | 8.8 | 6.7 |

| | | | | | | | | | | | | |
|---|---|---|---|---|---|---|---|---|---|---|---|---|
| Morn3 | 0.0 | 0.1 | 0.0 | 0.0 | 0.0 | 0.0 | 0.1 | 0.1 | 0.0 | 0.0 | 0.0 | 0.0 |
| Morn4 | 4.4 | 2.9 | 2.5 | 4.3 | 2.7 | 5.6 | 2.4 | 1.3 | 1.6 | 2.0 | 2.6 | 2.3 |
| Morn5 | 0.6 | 0.4 | 0.9 | 0.5 | 0.2 | 0.6 | 0.1 | 0.2 | 0.1 | 0.1 | 0.3 | 0.0 |
| Mospd1 | 10.0 | 8.8 | 7.2 | 9.2 | 7.5 | 8.7 | 6.2 | 6.5 | 6.6 | 8.7 | 8.1 | 8.3 |
| Mospd2 | 21.6 | 18.8 | 15.9 | 16.9 | 17.5 | 15.9 | 17.3 | 16.7 | 18.5 | 15.5 | 21.9 | 16.9 |
| Mospd3 | 0.2 | 0.4 | 0.5 | 0.2 | 0.6 | 0.9 | 0.5 | 0.8 | 0.6 | 0.7 | 0.3 | 0.5 |
| Mov10 | 5.3 | 6.4 | 6.9 | 5.7 | 6.1 | 5.2 | 5.5 | 5.9 | 6.0 | 3.9 | 4.0 | 5.3 |
| Mov10l1 | 0.0 | 0.0 | 0.0 | 0.0 | 0.0 | 0.0 | 0.0 | 0.0 | 0.0 | 0.0 | 0.0 | 0.0 |
| Moxd1 | 0.2 | 0.0 | 0.0 | 0.1 | 0.0 | 0.1 | 0.0 | 0.1 | 0.0 | 0.1 | 0.1 | 0.0 |
| Mpc1 | 39.5 | 30.0 | 39.0 | 40.8 | 31.0 | 45.1 | 32.8 | 32.8 | 34.1 | 26.9 | 38.1 | 42.0 |
| Mpc2 | 38.6 | 35.4 | 29.6 | 29.8 | 30.3 | 33.3 | 26.9 | 29.3 | 38.5 | 40.7 | 30.2 | 33.6 |
| Mpdu1 | 5.3 | 5.1 | 5.1 | 7.1 | 7.2 | 5.3 | 6.0 | 7.5 | 6.4 | 6.4 | 6.2 | 6.9 |
| Mpdz | 25.7 | 29.2 | 29.1 | 31.9 | 22.1 | 32.9 | 26.9 | 27.6 | 23.0 | 22.6 | 28.0 | 22.9 |
| Mpeg1 | 5.7 | 0.3 | 0.7 | 39.9 | 72.1 | 0.2 | 6.6 | 1.2 | 1.3 | 4.4 | 1.6 | 3.4 |
| Mpg | 10.7 | 8.5 | 7.2 | 8.5 | 8.6 | 7.7 | 9.0 | 8.8 | 8.7 | 10.1 | 8.8 | 8.1 |
| Mphosph10 | 16.5 | 15.0 | 15.7 | 19.8 | 14.7 | 15.2 | 15.8 | 13.4 | 12.2 | 14.0 | 18.4 | 16.2 |
| Mphosph6 | 11.0 | 8.1 | 12.3 | 13.2 | 11.7 | 10.3 | 12.6 | 12.3 | 11.2 | 10.5 | 14.0 | 12.1 |
| Mphosph8 | 14.0 | 13.5 | 14.0 | 15.0 | 12.8 | 13.4 | 13.6 | 12.6 | 11.7 | 12.3 | 15.9 | 16.1 |
| Mphosph9 | 0.7 | 0.9 | 1.1 | 0.8 | 1.1 | 0.7 | 0.9 | 1.0 | 0.9 | 0.9 | 1.1 | 0.8 |
| Mpi | 9.3 | 9.1 | 8.6 | 9.0 | 8.1 | 8.8 | 8.7 | 8.4 | 7.0 | 7.8 | 8.8 | 7.5 |
| Mplkip | 2.0 | 2.0 | 2.2 | 2.7 | 2.5 | 2.4 | 2.4 | 2.5 | 2.2 | 2.9 | 2.6 | 3.1 |
| Mpnd | 20.5 | 20.7 | 20.5 | 18.0 | 15.3 | 23.7 | 17.0 | 20.2 | 20.7 | 16.8 | 14.5 | 18.2 |
| Mpo | 0.0 | 0.0 | 0.1 | 0.0 | 0.0 | 0.0 | 0.0 | 0.0 | 0.0 | 0.1 | 0.0 | 0.0 |
| Mpp1 | 44.5 | 42.3 | 39.2 | 43.4 | 37.8 | 40.1 | 31.2 | 28.6 | 34.1 | 34.9 | 38.2 | 36.8 |
| Mpp2 | 2.2 | 2.8 | 3.0 | 1.3 | 1.6 | 1.4 | 2.0 | 1.9 | 1.4 | 4.0 | 2.4 | 2.3 |
| Mpp3 | 0.0 | 0.0 | 0.0 | 0.0 | 0.0 | 0.0 | 0.0 | 0.0 | 0.0 | 0.0 | 0.0 | 0.0 |
| Mpp4 | 0.1 | 0.0 | 0.1 | 0.1 | 0.1 | 0.2 | 0.0 | 0.1 | 0.1 | 0.1 | 0.2 | 0.1 |
| Mpp5 | 14.5 | 16.8 | 18.2 | 14.6 | 12.1 | 13.9 | 14.4 | 13.7 | 13.2 | 17.0 | 19.0 | 19.3 |
| Mpp6 | 8.7 | 7.2 | 7.2 | 9.0 | 9.3 | 7.7 | 7.2 | 5.7 | 5.6 | 8.8 | 8.1 | 7.9 |
| Mpp7 | 3.6 | 4.0 | 3.8 | 3.1 | 2.9 | 2.7 | 2.8 | 3.0 | 2.5 | 2.8 | 3.3 | 2.9 |
| Mppe1 | 6.4 | 5.8 | 6.2 | 6.3 | 5.2 | 6.2 | 7.5 | 6.8 | 6.5 | 5.8 | 5.6 | 7.6 |
| Mpped1 | 0.0 | 0.0 | 0.0 | 0.1 | 0.0 | 0.0 | 0.0 | 0.0 | 0.0 | 0.0 | 0.0 | 0.0 |
| Mpped2 | 0.1 | 0.1 | 0.1 | 0.3 | 0.1 | 0.4 | 0.2 | 0.2 | 0.1 | 0.1 | 0.1 | 0.1 |
| Mprip | 57.8 | 54.2 | 46.6 | 57.3 | 53.1 | 59.1 | 61.1 | 54.9 | 59.1 | 58.3 | 65.7 | 59.6 |
| Mpst | 13.0 | 13.9 | 15.4 | 8.9 | 11.7 | 13.5 | 10.4 | 15.8 | 13.3 | 13.2 | 13.3 | 11.3 |
| Mpv17 | 30.6 | 22.7 | 23.2 | 30.8 | 23.9 | 28.7 | 27.3 | 23.7 | 23.9 | 27.6 | 29.4 | 27.5 |
| Mpv17l | 2.1 | 2.1 | 2.5 | 2.8 | 2.2 | 2.3 | 2.5 | 2.3 | 1.8 | 1.9 | 2.5 | 3.0 |
| Mpv17l2 | 20.6 | 15.1 | 12.7 | 17.3 | 14.1 | 18.3 | 16.1 | 15.7 | 18.5 | 15.6 | 18.2 | 16.6 |
| Mpz | 0.0 | 0.0 | 0.0 | 0.0 | 0.1 | 0.0 | 0.0 | 0.0 | 0.0 | 0.0 | 0.0 | 0.0 |
| Mpzl1 | 5.5 | 9.2 | 9.7 | 4.7 | 8.8 | 7.1 | 10.4 | 12.0 | 11.8 | 7.8 | 7.7 | 7.5 |
| Mpzl2 | 0.2 | 0.1 | 0.1 | 0.1 | 0.1 | 0.0 | 0.0 | 0.1 | 0.1 | 0.6 | 0.3 | 0.3 |
| Mpzl3 | 3.1 | 2.0 | 1.8 | 2.5 | 1.2 | 2.0 | 2.6 | 1.5 | 1.9 | 2.2 | 2.0 | 1.7 |
| Mr1 | 11.8 | 10.7 | 11.6 | 11.7 | 8.0 | 15.3 | 9.0 | 9.3 | 7.9 | 8.1 | 11.3 | 9.7 |
| Mrap | 5.0 | 5.8 | 8.1 | 4.5 | 4.4 | 6.3 | 4.4 | 4.8 | 3.1 | 3.8 | 3.9 | 5.2 |
| Mras | 13.4 | 10.1 | 9.1 | 12.6 | 8.0 | 12.2 | 11.7 | 8.9 | 9.7 | 15.2 | 16.3 | 12.6 |
| Mrc1 | 0.4 | 0.1 | 0.1 | 1.8 | 0.8 | 0.0 | 0.1 | 0.2 | 0.1 | 0.0 | 0.1 | 0.1 |
| Mrc2 | 52.3 | 55.2 | 48.2 | 56.7 | 63.5 | 64.1 | 81.3 | 50.7 | 82.1 | 52.8 | 47.9 | 65.7 |
| Mre11a | 6.9 | 7.4 | 7.2 | 7.4 | 7.6 | 7.0 | 8.1 | 6.5 | 6.5 | 6.7 | 8.2 | 6.3 |
| Mreg | 1.0 | 1.0 | 1.2 | 0.8 | 1.3 | 0.6 | 0.6 | 0.2 | 0.3 | 0.3 | 0.2 | 0.3 |
| Mrfap1 | 154.2 | 138.4 | 148.9 | 142.0 | 139.5 | 151.0 | 149.4 | 154.5 | 148.4 | 172.1 | 156.0 | 169.7 |
| Mrgbp | 7.0 | 5.8 | 6.0 | 5.9 | 5.1 | 5.9 | 6.8 | 6.5 | 4.6 | 5.1 | 5.5 | 6.1 |
| Mrgpre | 2.5 | 1.8 | 1.4 | 5.0 | 2.8 | 4.1 | 3.0 | 1.6 | 2.0 | 1.5 | 1.8 | 2.0 |
| Mrgprf | 5.8 | 8.7 | 10.3 | 4.3 | 5.9 | 3.3 | 9.6 | 16.6 | 11.8 | 5.5 | 9.1 | 6.4 |
| Mri1 | 8.5 | 6.3 | 5.5 | 7.7 | 6.3 | 6.5 | 5.8 | 5.4 | 4.7 | 5.4 | 5.6 | 5.3 |
| Mrm1 | 4.4 | 3.9 | 3.5 | 4.5 | 3.3 | 3.6 | 4.0 | 4.0 | 4.2 | 2.9 | 3.6 | 3.9 |
| Mro | 0.0 | 0.0 | 0.2 | 0.0 | 0.0 | 0.0 | 0.0 | 0.1 | 0.0 | 0.2 | 0.0 | 0.0 |
| Mroh1 | 21.1 | 14.2 | 9.8 | 22.9 | 17.2 | 23.6 | 12.6 | 11.0 | 12.1 | 15.4 | 13.2 | 12.7 |

| | | | | | | | | | | | | |
|---|---|---|---|---|---|---|---|---|---|---|---|---|
| Mroh2a | 0.0 | 0.0 | 0.0 | 0.1 | 0.0 | 0.0 | 0.0 | 0.0 | 0.0 | 0.2 | 0.1 | 0.0 |
| Mroh8 | 0.0 | 0.1 | 0.1 | 0.2 | 0.0 | 0.1 | 0.1 | 0.1 | 0.1 | 0.1 | 0.0 | 0.0 |
| Mrp63 | 10.6 | 8.5 | 7.4 | 9.6 | 8.7 | 8.2 | 7.5 | 7.7 | 8.2 | 9.1 | 9.4 | 7.8 |
| Mrpl1 | 6.3 | 5.5 | 4.9 | 7.3 | 4.7 | 6.0 | 3.9 | 4.1 | 3.8 | 5.6 | 4.6 | 4.5 |
| Mrpl10 | 23.4 | 23.7 | 20.7 | 23.5 | 21.2 | 25.7 | 20.5 | 22.3 | 22.0 | 21.8 | 23.7 | 19.1 |
| Mrpl11 | 6.3 | 5.3 | 5.6 | 6.3 | 4.7 | 6.4 | 4.5 | 5.4 | 5.5 | 5.9 | 5.2 | 5.8 |
| Mrpl12 | 30.3 | 29.3 | 23.6 | 28.3 | 28.6 | 31.7 | 31.7 | 28.7 | 26.2 | 32.6 | 30.2 | 28.4 |
| Mrpl13 | 69.5 | 61.8 | 53.2 | 69.8 | 57.8 | 69.3 | 57.8 | 51.6 | 49.0 | 53.9 | 56.0 | 56.5 |
| Mrpl14 | 15.6 | 17.0 | 15.6 | 16.7 | 19.1 | 18.4 | 14.2 | 14.2 | 16.8 | 19.9 | 14.8 | 18.4 |
| Mrpl15 | 9.5 | 8.9 | 8.5 | 9.9 | 8.5 | 9.6 | 8.3 | 9.7 | 8.7 | 8.8 | 9.4 | 8.6 |
| Mrpl16 | 10.3 | 10.5 | 9.6 | 10.0 | 10.3 | 13.2 | 9.8 | 10.2 | 9.5 | 12.0 | 9.9 | 10.3 |
| Mrpl17 | 5.5 | 4.6 | 4.2 | 4.3 | 4.3 | 4.5 | 4.0 | 4.4 | 3.9 | 5.2 | 4.5 | 4.6 |
| Mrpl18 | 38.7 | 37.6 | 39.9 | 36.4 | 36.0 | 42.9 | 38.1 | 31.2 | 34.1 | 39.3 | 40.0 | 41.0 |
| Mrpl19 | 3.9 | 3.4 | 3.0 | 4.0 | 3.4 | 3.5 | 3.6 | 3.1 | 3.0 | 3.5 | 3.4 | 3.3 |
| Mrpl2 | 21.4 | 19.7 | 17.7 | 26.1 | 16.6 | 21.6 | 20.4 | 18.4 | 18.2 | 18.8 | 18.2 | 18.1 |
| Mrpl20 | 61.3 | 59.3 | 51.1 | 64.9 | 63.5 | 59.4 | 61.4 | 56.8 | 67.7 | 62.0 | 60.6 | 57.5 |
| Mrpl21 | 14.0 | 13.4 | 12.6 | 13.6 | 11.2 | 15.7 | 12.2 | 12.2 | 12.2 | 13.7 | 14.7 | 12.5 |
| Mrpl22 | 17.6 | 18.7 | 17.5 | 19.9 | 16.7 | 21.4 | 14.9 | 14.7 | 15.0 | 18.5 | 17.0 | 16.8 |
| Mrpl23 | 33.6 | 29.7 | 29.4 | 38.2 | 33.0 | 44.7 | 32.9 | 37.8 | 33.0 | 33.4 | 34.3 | 35.0 |
| Mrpl24 | 33.0 | 28.8 | 30.3 | 33.6 | 26.3 | 37.7 | 30.0 | 33.1 | 28.6 | 34.0 | 32.8 | 35.9 |
| Mrpl27 | 34.6 | 28.2 | 25.5 | 34.4 | 28.1 | 28.9 | 28.1 | 26.1 | 25.2 | 28.8 | 28.7 | 31.0 |
| Mrpl28 | 26.7 | 26.6 | 19.7 | 29.8 | 24.3 | 27.5 | 23.5 | 23.6 | 23.5 | 26.9 | 22.1 | 19.4 |
| Mrpl3 | 15.9 | 12.1 | 13.9 | 15.4 | 14.3 | 13.4 | 13.5 | 15.9 | 14.9 | 15.2 | 16.7 | 15.3 |
| Mrpl30 | 66.6 | 58.2 | 49.9 | 56.6 | 48.2 | 61.9 | 38.3 | 52.4 | 43.8 | 55.1 | 53.3 | 49.0 |
| Mrpl32 | 30.2 | 29.4 | 34.9 | 33.8 | 27.5 | 31.6 | 33.4 | 31.8 | 25.7 | 27.8 | 37.5 | 36.1 |
| Mrpl33 | 164.2 | 122.8 | 85.9 | 138.4 | 123.0 | 140.0 | 103.2 | 103.2 | 116.5 | 104.3 | 120.8 | 115.9 |
| Mrpl34 | 30.0 | 23.4 | 25.2 | 29.0 | 26.8 | 32.7 | 26.8 | 28.4 | 26.0 | 32.9 | 26.1 | 27.1 |
| Mrpl35 | 5.8 | 4.5 | 3.7 | 6.2 | 4.5 | 5.6 | 4.1 | 4.2 | 3.6 | 4.5 | 4.9 | 4.0 |
| Mrpl36 | 22.2 | 19.9 | 16.7 | 25.6 | 23.5 | 22.8 | 20.1 | 20.5 | 23.9 | 27.2 | 22.8 | 21.2 |
| Mrpl37 | 13.8 | 13.0 | 11.7 | 11.0 | 12.2 | 11.5 | 12.1 | 12.9 | 12.3 | 10.2 | 10.9 | 11.0 |
| Mrpl38 | 15.1 | 13.5 | 12.3 | 15.6 | 12.9 | 17.7 | 13.4 | 11.9 | 10.7 | 15.4 | 15.3 | 11.7 |
| Mrpl39 | 18.7 | 17.1 | 17.0 | 18.8 | 15.4 | 17.5 | 15.5 | 15.9 | 13.9 | 16.4 | 16.9 | 17.8 |
| Mrpl4 | 16.8 | 17.7 | 17.3 | 21.8 | 17.8 | 18.7 | 16.7 | 18.5 | 16.1 | 17.5 | 14.7 | 14.7 |
| Mrpl40 | 16.5 | 15.3 | 15.6 | 19.1 | 16.0 | 16.1 | 13.9 | 15.5 | 14.8 | 13.2 | 13.4 | 14.6 |
| Mrpl41 | 7.7 | 7.0 | 6.6 | 10.6 | 5.9 | 9.1 | 7.1 | 7.0 | 5.7 | 6.9 | 8.3 | 7.9 |
| Mrpl42 | 60.6 | 45.9 | 51.9 | 75.5 | 48.2 | 69.5 | 48.5 | 52.6 | 42.5 | 49.3 | 54.9 | 51.2 |
| Mrpl43 | 22.8 | 21.5 | 21.5 | 23.1 | 22.0 | 23.7 | 20.8 | 23.7 | 22.1 | 20.6 | 23.8 | 23.5 |
| Mrpl44 | 12.4 | 13.7 | 12.3 | 11.9 | 11.9 | 11.5 | 10.6 | 11.3 | 11.9 | 11.8 | 11.8 | 10.3 |
| Mrpl45 | 9.2 | 7.9 | 8.2 | 8.4 | 8.4 | 9.3 | 8.4 | 8.6 | 8.7 | 9.6 | 8.5 | 9.2 |
| Mrpl46 | 7.8 | 9.3 | 10.2 | 9.5 | 8.6 | 8.6 | 7.5 | 10.6 | 8.7 | 9.5 | 9.1 | 7.9 |
| Mrpl47 | 10.9 | 10.6 | 5.7 | 9.8 | 8.4 | 11.4 | 7.9 | 6.9 | 8.4 | 9.6 | 10.1 | 9.0 |
| Mrpl48 | 30.6 | 24.1 | 22.1 | 31.8 | 24.3 | 25.6 | 27.2 | 27.9 | 24.8 | 31.1 | 32.0 | 29.7 |
| Mrpl49 | 14.1 | 12.2 | 12.7 | 14.8 | 12.1 | 13.1 | 14.0 | 13.1 | 13.6 | 14.7 | 15.1 | 12.9 |
| Mrpl50 | 8.5 | 8.8 | 8.5 | 9.4 | 9.3 | 9.0 | 8.6 | 8.6 | 9.5 | 9.9 | 10.3 | 9.4 |
| Mrpl51 | 32.8 | 28.1 | 28.3 | 35.4 | 26.4 | 35.1 | 33.3 | 27.0 | 26.4 | 27.6 | 30.2 | 33.5 |
| Mrpl52 | 105.2 | 83.3 | 69.7 | 96.4 | 99.6 | 98.5 | 95.0 | 83.6 | 92.5 | 106.5 | 100.0 | 83.3 |
| Mrpl53 | 42.8 | 39.0 | 33.3 | 40.9 | 37.1 | 32.9 | 36.3 | 37.1 | 37.9 | 42.7 | 40.3 | 38.1 |
| Mrpl54 | 67.6 | 52.1 | 46.4 | 59.0 | 52.1 | 54.7 | 69.8 | 69.0 | 58.5 | 68.4 | 72.3 | 67.4 |
| Mrpl55 | 16.3 | 13.6 | 10.9 | 16.3 | 12.5 | 16.9 | 11.5 | 12.1 | 11.3 | 11.1 | 9.6 | 10.2 |
| Mrpl9 | 17.7 | 12.9 | 11.3 | 14.2 | 13.0 | 16.7 | 13.7 | 12.8 | 13.0 | 15.9 | 16.2 | 15.5 |
| Mrps10 | 21.8 | 18.7 | 17.4 | 23.9 | 17.3 | 21.5 | 17.4 | 15.4 | 14.4 | 17.1 | 17.7 | 16.0 |
| Mrps11 | 8.7 | 7.9 | 7.9 | 8.3 | 8.6 | 7.4 | 6.3 | 9.6 | 7.7 | 8.6 | 8.2 | 7.9 |
| Mrps12 | 58.2 | 58.5 | 47.7 | 58.5 | 58.3 | 47.8 | 62.1 | 66.8 | 61.0 | 70.1 | 64.8 | 57.4 |
| Mrps14 | 14.2 | 12.5 | 9.2 | 10.7 | 11.7 | 12.0 | 8.8 | 9.5 | 11.2 | 15.1 | 9.9 | 10.7 |
| Mrps15 | 25.2 | 23.8 | 24.2 | 27.2 | 21.8 | 28.8 | 26.4 | 29.5 | 23.9 | 25.8 | 31.9 | 24.3 |
| Mrps16 | 45.7 | 40.5 | 43.1 | 39.0 | 43.7 | 41.4 | 42.4 | 43.0 | 46.1 | 43.6 | 40.5 | 41.0 |
| Mrps17 | 18.2 | 17.8 | 15.5 | 17.4 | 15.5 | 16.3 | 15.5 | 17.7 | 17.5 | 18.8 | 17.4 | 17.0 |

| | | | | | | | | | | | | |
|---|---|---|---|---|---|---|---|---|---|---|---|---|
| Mrps18a | 24.1 | 18.5 | 17.7 | 25.7 | 19.4 | 28.4 | 18.1 | 17.6 | 19.9 | 21.0 | 17.9 | 17.7 |
| Mrps18b | 21.8 | 18.1 | 15.5 | 26.9 | 17.7 | 20.1 | 17.1 | 18.3 | 14.7 | 17.6 | 20.3 | 17.0 |
| Mrps18c | 43.1 | 39.7 | 33.2 | 41.3 | 37.7 | 39.3 | 25.8 | 34.7 | 35.6 | 42.4 | 35.2 | 34.7 |
| Mrps2 | 9.0 | 8.3 | 8.2 | 11.3 | 9.0 | 9.6 | 9.3 | 8.5 | 7.0 | 7.7 | 8.3 | 8.4 |
| Mrps21 | 153.3 | 146.5 | 145.7 | 157.5 | 136.0 | 176.7 | 147.3 | 161.9 | 129.0 | 149.9 | 154.7 | 156.1 |
| Mrps22 | 12.4 | 13.6 | 10.8 | 14.7 | 10.4 | 10.7 | 11.6 | 10.7 | 10.5 | 11.1 | 11.7 | 10.1 |
| Mrps23 | 25.5 | 22.6 | 23.3 | 26.3 | 25.3 | 26.2 | 27.0 | 21.1 | 21.0 | 25.1 | 28.4 | 23.5 |
| Mrps24 | 22.1 | 19.9 | 17.6 | 15.0 | 17.2 | 19.2 | 17.7 | 20.3 | 20.3 | 22.0 | 17.2 | 19.2 |
| Mrps25 | 6.7 | 6.3 | 5.9 | 7.3 | 6.2 | 6.5 | 5.7 | 6.2 | 6.6 | 6.4 | 6.8 | 6.7 |
| Mrps26 | 20.4 | 18.7 | 16.6 | 17.9 | 17.3 | 18.0 | 17.5 | 17.3 | 18.4 | 20.4 | 17.8 | 16.8 |
| Mrps27 | 6.8 | 6.5 | 6.9 | 6.9 | 6.4 | 7.6 | 7.2 | 7.2 | 5.4 | 6.7 | 6.5 | 5.7 |
| Mrps28 | 17.8 | 16.5 | 14.2 | 19.6 | 17.6 | 15.0 | 18.2 | 19.1 | 18.2 | 17.9 | 17.7 | 16.7 |
| Mrps30 | 14.3 | 13.0 | 12.0 | 11.2 | 10.2 | 12.1 | 13.9 | 12.2 | 12.4 | 13.2 | 11.6 | 12.6 |
| Mrps31 | 8.8 | 9.9 | 10.2 | 9.1 | 8.3 | 8.9 | 9.0 | 8.4 | 7.9 | 8.1 | 9.7 | 9.2 |
| Mrps33 | 33.6 | 25.7 | 23.8 | 34.0 | 29.0 | 34.2 | 28.6 | 27.6 | 24.5 | 26.8 | 30.5 | 29.6 |
| Mrps34 | 20.1 | 19.0 | 19.2 | 20.0 | 16.8 | 22.4 | 19.9 | 21.6 | 17.0 | 25.6 | 18.2 | 20.1 |
| Mrps35 | 11.1 | 10.3 | 10.7 | 10.5 | 8.7 | 10.2 | 10.0 | 11.3 | 8.1 | 9.4 | 12.0 | 10.6 |
| Mrps36 | 22.2 | 20.7 | 19.1 | 25.9 | 21.9 | 29.3 | 22.7 | 26.9 | 24.7 | 23.5 | 28.1 | 25.0 |
| Mrps5 | 13.5 | 13.4 | 14.0 | 17.6 | 11.5 | 14.8 | 14.1 | 13.5 | 10.2 | 11.4 | 13.1 | 12.8 |
| Mrps6 | 37.1 | 37.4 | 38.3 | 35.7 | 31.5 | 39.5 | 28.8 | 38.9 | 26.9 | 30.8 | 32.4 | 28.2 |
| Mrps7 | 23.2 | 18.7 | 18.8 | 22.9 | 21.0 | 22.2 | 20.9 | 19.2 | 20.7 | 27.6 | 24.9 | 21.2 |
| Mrps9 | 16.9 | 14.4 | 13.1 | 17.6 | 11.6 | 18.1 | 12.8 | 12.4 | 11.3 | 13.6 | 14.0 | 13.4 |
| Mrrf | 14.1 | 12.2 | 9.6 | 14.4 | 11.1 | 12.7 | 11.4 | 11.1 | 10.3 | 13.8 | 15.1 | 13.6 |
| Mrs2 | 10.0 | 7.7 | 7.9 | 8.1 | 8.5 | 7.7 | 8.4 | 7.5 | 7.8 | 6.9 | 7.0 | 7.4 |
| Mrto4 | 24.1 | 18.7 | 14.4 | 23.9 | 22.4 | 23.6 | 20.1 | 18.4 | 16.5 | 22.2 | 21.8 | 17.6 |
| Mrvi1 | 1.5 | 1.2 | 1.0 | 3.1 | 2.3 | 2.1 | 1.8 | 0.6 | 1.3 | 2.3 | 2.2 | 1.3 |
| Ms4a2 | 0.0 | 0.0 | 0.0 | 0.0 | 0.0 | 0.0 | 0.0 | 0.0 | 0.0 | 0.1 | 0.1 | 0.0 |
| Ms4a4b | 0.1 | 0.0 | 0.1 | 0.0 | 0.0 | 0.0 | 0.3 | 0.0 | 0.0 | 0.1 | 0.0 | 0.0 |
| Ms4a4c | 0.0 | 0.0 | 0.1 | 0.0 | 0.1 | 0.0 | 0.1 | 0.0 | 0.0 | 0.1 | 0.0 | 0.0 |
| Ms4a4d | 17.5 | 24.8 | 40.1 | 18.9 | 33.7 | 7.4 | 39.0 | 40.7 | 34.8 | 15.4 | 21.1 | 28.3 |
| Ms4a6b | 0.3 | 0.0 | 0.0 | 2.1 | 3.2 | 0.1 | 0.6 | 0.2 | 0.2 | 0.3 | 0.0 | 0.1 |
| Ms4a6c | 0.8 | 0.1 | 0.1 | 4.2 | 6.0 | 0.3 | 2.0 | 0.5 | 0.5 | 0.7 | 0.3 | 0.6 |
| Ms4a6d | 2.3 | 0.2 | 0.1 | 16.8 | 29.4 | 0.0 | 3.7 | 0.9 | 0.7 | 1.8 | 0.9 | 1.5 |
| Ms4a7 | 5.0 | 0.0 | 0.3 | 45.9 | 68.4 | 0.0 | 6.9 | 1.1 | 0.9 | 4.9 | 1.6 | 2.6 |
| Msantd1 | 0.3 | 0.3 | 0.2 | 0.2 | 0.2 | 0.2 | 0.3 | 0.3 | 0.3 | 0.2 | 0.1 | 0.2 |
| Msantd2 | 2.5 | 2.6 | 3.2 | 1.8 | 2.1 | 2.2 | 2.4 | 2.2 | 2.2 | 1.9 | 1.7 | 2.2 |
| Msantd3 | 10.0 | 9.3 | 8.8 | 13.8 | 8.6 | 11.1 | 12.5 | 8.7 | 7.9 | 7.8 | 10.5 | 8.5 |
| Msantd4 | 29.8 | 27.2 | 25.6 | 32.2 | 25.4 | 29.5 | 28.3 | 22.6 | 26.0 | 28.5 | 32.5 | 31.8 |
| Msc | 0.3 | 0.4 | 0.7 | 0.3 | 0.8 | 0.2 | 0.4 | 1.0 | 1.0 | 0.1 | 0.3 | 0.4 |
| Msh2 | 3.7 | 4.5 | 5.5 | 3.5 | 3.9 | 4.2 | 3.5 | 4.7 | 3.7 | 3.9 | 4.5 | 4.0 |
| Msh3 | 1.9 | 1.6 | 1.7 | 1.9 | 1.6 | 1.8 | 1.4 | 1.6 | 1.2 | 1.9 | 1.6 | 1.6 |
| Msh4 | 0.0 | 0.0 | 0.0 | 0.1 | 0.1 | 0.1 | 0.0 | 0.1 | 0.1 | 0.0 | 0.1 | 0.0 |
| Msh5 | 0.1 | 0.2 | 0.1 | 0.1 | 0.2 | 0.1 | 0.1 | 0.1 | 0.1 | 0.1 | 0.1 | 0.1 |
| Msh6 | 11.9 | 13.3 | 14.8 | 7.7 | 11.4 | 11.0 | 11.7 | 10.5 | 10.0 | 14.1 | 12.7 | 11.9 |
| Msi1 | 0.3 | 0.3 | 0.2 | 0.1 | 0.1 | 0.1 | 0.2 | 0.2 | 0.4 | 0.4 | 0.5 | 0.3 |
| Msi2 | 21.8 | 19.9 | 21.9 | 26.5 | 21.8 | 22.1 | 21.7 | 16.1 | 19.1 | 17.8 | 22.4 | 24.4 |
| Msl1 | 11.4 | 13.6 | 13.9 | 12.1 | 11.9 | 12.1 | 11.0 | 12.0 | 11.6 | 10.3 | 11.6 | 11.7 |
| Msl2 | 2.4 | 2.8 | 3.8 | 3.0 | 3.1 | 2.7 | 3.0 | 2.6 | 2.9 | 3.6 | 3.3 | 3.4 |
| Msl3 | 16.2 | 16.0 | 14.9 | 15.8 | 15.0 | 13.2 | 14.9 | 15.3 | 13.3 | 15.9 | 16.9 | 16.4 |
| Msl3l2 | 1.4 | 1.5 | 1.9 | 1.0 | 1.5 | 1.3 | 1.2 | 1.5 | 1.6 | 1.5 | 1.6 | 1.4 |
| Msln | 8.4 | 0.9 | 0.2 | 0.7 | 0.2 | 0.5 | 0.3 | 1.8 | 0.9 | 23.3 | 13.3 | 4.3 |
| Mslnl | 0.1 | 0.0 | 0.0 | 0.0 | 0.0 | 0.1 | 0.0 | 0.0 | 0.0 | 0.1 | 0.0 | 0.0 |
| Msmp | 3.2 | 3.6 | 2.5 | 3.3 | 3.7 | 4.1 | 4.3 | 2.8 | 3.2 | 3.1 | 3.7 | 2.0 |
| Msn | 114.2 | 95.2 | 78.5 | 172.0 | 165.6 | 138.0 | 115.3 | 87.2 | 81.3 | 103.1 | 115.2 | 100.3 |
| Msr1 | 2.6 | 0.1 | 0.2 | 19.1 | 29.7 | 0.1 | 2.2 | 0.7 | 0.5 | 1.1 | 0.7 | 1.5 |
| Msra | 3.4 | 2.0 | 2.3 | 4.2 | 5.0 | 3.1 | 3.4 | 3.5 | 2.6 | 2.9 | 3.6 | 2.6 |
| Msrb1 | 41.0 | 35.3 | 36.9 | 49.2 | 54.0 | 31.9 | 53.4 | 52.9 | 55.3 | 48.9 | 64.6 | 47.0 |

| | | | | | | | | | | | | |
|---|---|---|---|---|---|---|---|---|---|---|---|---|
| Msrb2 | 17.4 | 12.3 | 12.1 | 18.1 | 13.1 | 12.0 | 15.1 | 12.4 | 12.2 | 10.7 | 13.8 | 15.0 |
| Msrb3 | 60.7 | 51.8 | 52.0 | 64.2 | 55.5 | 52.9 | 68.1 | 49.1 | 62.2 | 62.1 | 76.1 | 69.7 |
| Mss51 | 0.1 | 0.2 | 0.1 | 0.1 | 0.0 | 0.1 | 0.1 | 0.1 | 0.3 | 0.2 | 0.2 | 0.1 |
| Mst1 | 0.4 | 0.2 | 0.2 | 0.2 | 0.2 | 0.2 | 0.3 | 0.1 | 0.1 | 0.1 | 0.2 | 0.1 |
| Mst1r | 6.8 | 9.7 | 8.5 | 5.3 | 6.4 | 5.4 | 7.0 | 7.6 | 6.8 | 8.6 | 7.2 | 7.0 |
| Msto1 | 13.1 | 11.3 | 12.5 | 15.8 | 12.6 | 12.8 | 14.2 | 14.0 | 13.0 | 13.4 | 15.3 | 12.1 |
| Msx1 | 1.2 | 1.1 | 0.9 | 0.8 | 0.7 | 0.9 | 0.8 | 0.8 | 0.7 | 0.9 | 0.7 | 0.7 |
| Msx1as | 1.7 | 1.5 | 1.8 | 1.6 | 1.0 | 1.4 | 1.6 | 1.4 | 1.1 | 1.3 | 1.2 | 1.3 |
| Msx2 | 0.2 | 0.1 | 0.1 | 0.2 | 0.1 | 0.1 | 0.0 | 0.0 | 0.0 | 0.1 | 0.1 | 0.1 |
| Mt1 | 301.6 | 237.2 | 232.8 | 294.0 | 235.8 | 383.9 | 208.3 | 280.6 | 201.4 | 439.3 | 297.6 | 384.5 |
| Mt2 | 155.9 | 117.9 | 105.7 | 155.7 | 138.8 | 211.3 | 112.8 | 140.0 | 89.8 | 202.2 | 137.1 | 157.3 |
| Mt3 | 0.0 | 0.0 | 0.0 | 0.1 | 0.1 | 0.0 | 0.0 | 0.1 | 0.0 | 0.2 | 0.0 | 0.0 |
| Mta1 | 14.4 | 17.1 | 15.0 | 14.1 | 12.0 | 19.5 | 14.1 | 14.8 | 12.2 | 11.1 | 14.7 | 10.9 |
| Mta2 | 27.9 | 28.4 | 30.4 | 26.2 | 28.4 | 27.2 | 32.3 | 30.0 | 30.5 | 30.2 | 32.2 | 30.8 |
| Mta3 | 17.4 | 17.8 | 19.7 | 17.8 | 13.9 | 19.2 | 19.7 | 19.7 | 15.1 | 12.3 | 16.9 | 17.9 |
| Mtag2 | 0.1 | 0.2 | 0.2 | 0.3 | 0.0 | 0.2 | 0.1 | 0.1 | 0.2 | 0.1 | 0.1 | 0.1 |
| Mtap | 31.3 | 23.7 | 19.3 | 33.1 | 23.2 | 35.3 | 26.8 | 21.2 | 24.1 | 31.6 | 31.1 | 29.3 |
| Mtap7d3 | 1.1 | 1.5 | 1.9 | 1.4 | 1.2 | 1.8 | 1.1 | 1.4 | 1.2 | 0.9 | 1.2 | 0.8 |
| Mtbp | 1.9 | 2.1 | 3.1 | 1.7 | 2.4 | 1.5 | 3.0 | 3.2 | 2.7 | 1.9 | 2.9 | 2.3 |
| Mtch1 | 76.6 | 65.4 | 74.0 | 70.0 | 70.4 | 61.6 | 81.1 | 102.0 | 89.8 | 82.4 | 77.8 | 88.9 |
| Mtch2 | 47.8 | 43.1 | 37.0 | 42.6 | 38.2 | 38.8 | 36.8 | 44.2 | 42.4 | 41.9 | 45.4 | 43.0 |
| Mtcp1 | 3.8 | 4.9 | 2.9 | 2.2 | 3.4 | 4.4 | 4.5 | 4.6 | 3.5 | 3.3 | 4.0 | 3.5 |
| Mtdh | 31.0 | 25.7 | 28.6 | 33.9 | 25.0 | 30.6 | 29.3 | 29.8 | 25.1 | 27.3 | 34.0 | 33.5 |
| Mterf | 1.9 | 1.6 | 1.7 | 2.2 | 1.6 | 2.3 | 2.0 | 1.6 | 2.0 | 2.6 | 2.5 | 1.5 |
| Mterfd1 | 7.6 | 7.9 | 8.5 | 7.3 | 8.3 | 8.5 | 7.8 | 9.6 | 8.5 | 9.4 | 10.9 | 9.2 |
| Mterfd2 | 3.6 | 3.8 | 3.7 | 3.5 | 3.2 | 3.4 | 4.0 | 4.2 | 3.9 | 4.8 | 5.2 | 4.5 |
| Mterfd3 | 4.7 | 4.4 | 5.1 | 4.0 | 3.6 | 4.4 | 4.6 | 4.5 | 4.3 | 4.1 | 4.8 | 4.9 |
| Mtf1 | 3.1 | 3.6 | 3.4 | 3.4 | 3.8 | 3.1 | 4.0 | 3.1 | 3.2 | 3.1 | 3.4 | 3.2 |
| Mtf2 | 2.5 | 3.2 | 3.5 | 2.4 | 3.2 | 2.2 | 3.1 | 3.2 | 2.7 | 3.2 | 2.8 | 2.9 |
| Mtfmt | 5.0 | 4.4 | 4.3 | 4.2 | 3.8 | 4.4 | 4.4 | 4.2 | 4.2 | 4.7 | 5.1 | 4.8 |
| Mtfp1 | 0.3 | 0.6 | 0.3 | 0.3 | 0.4 | 0.4 | 0.3 | 0.5 | 0.3 | 0.2 | 0.4 | 0.3 |
| Mtfr1 | 18.3 | 17.9 | 13.8 | 17.9 | 13.5 | 17.0 | 15.3 | 13.8 | 16.2 | 15.5 | 19.8 | 16.1 |
| Mtfr1l | 44.0 | 37.9 | 30.9 | 46.4 | 36.8 | 44.7 | 30.9 | 31.8 | 33.1 | 36.3 | 32.8 | 33.8 |
| Mtfr2 | 0.3 | 0.6 | 0.6 | 0.1 | 1.1 | 0.3 | 0.9 | 0.4 | 0.5 | 0.5 | 0.5 | 0.4 |
| Mtg1 | 4.4 | 3.9 | 2.8 | 5.4 | 3.3 | 4.2 | 3.6 | 3.8 | 4.1 | 3.6 | 4.2 | 3.3 |
| Mthfd1 | 23.0 | 19.9 | 19.4 | 17.4 | 18.1 | 18.5 | 19.3 | 16.9 | 16.3 | 22.3 | 20.5 | 20.9 |
| Mthfd1l | 5.5 | 3.9 | 3.7 | 5.0 | 4.8 | 5.3 | 5.6 | 5.8 | 4.3 | 3.3 | 4.7 | 3.3 |
| Mthfd2 | 25.7 | 18.3 | 14.4 | 25.2 | 21.5 | 25.7 | 22.7 | 30.1 | 28.9 | 16.3 | 27.9 | 15.6 |
| Mthfd2l | 2.7 | 2.2 | 2.7 | 4.5 | 3.0 | 3.3 | 3.3 | 2.5 | 2.7 | 2.6 | 2.6 | 3.6 |
| Mthfr | 12.9 | 12.4 | 11.2 | 12.1 | 12.8 | 12.0 | 13.3 | 12.0 | 14.3 | 14.7 | 12.3 | 11.7 |
| Mthfs | 7.1 | 7.6 | 6.8 | 6.6 | 8.0 | 6.9 | 6.5 | 7.1 | 7.9 | 6.3 | 6.6 | 7.7 |
| Mthfsd | 7.0 | 7.0 | 7.2 | 9.6 | 6.6 | 7.4 | 6.8 | 8.4 | 8.5 | 8.0 | 8.7 | 6.6 |
| Mtif2 | 10.4 | 9.5 | 8.0 | 9.3 | 8.4 | 9.5 | 8.3 | 8.5 | 7.9 | 9.2 | 9.6 | 9.7 |
| Mtif3 | 3.8 | 3.2 | 3.5 | 4.1 | 3.2 | 4.4 | 3.0 | 3.3 | 2.9 | 3.6 | 4.0 | 3.7 |
| Mtl5 | 0.0 | 0.1 | 0.2 | 0.1 | 0.1 | 0.1 | 0.0 | 0.1 | 0.1 | 0.1 | 0.1 | 0.2 |
| Mtm1 | 3.8 | 3.9 | 3.1 | 3.6 | 3.3 | 5.3 | 2.5 | 3.1 | 3.6 | 3.3 | 3.2 | 3.6 |
| Mtmr1 | 5.6 | 5.2 | 5.5 | 5.4 | 4.7 | 5.2 | 4.5 | 3.9 | 4.7 | 4.5 | 5.2 | 5.0 |
| Mtmr10 | 7.5 | 6.2 | 5.1 | 8.9 | 6.3 | 7.3 | 6.7 | 5.2 | 5.1 | 6.7 | 6.3 | 6.4 |
| Mtmr11 | 7.8 | 8.7 | 8.5 | 7.8 | 7.0 | 10.1 | 8.7 | 6.5 | 7.6 | 6.9 | 6.7 | 6.3 |
| Mtmr12 | 5.6 | 5.9 | 5.6 | 4.7 | 5.1 | 5.4 | 5.9 | 5.9 | 5.3 | 5.1 | 6.0 | 5.9 |
| Mtmr14 | 5.6 | 4.9 | 4.5 | 5.2 | 6.3 | 3.7 | 5.9 | 5.2 | 5.9 | 5.6 | 5.0 | 5.1 |
| Mtmr2 | 34.9 | 29.5 | 28.7 | 34.3 | 27.5 | 34.6 | 32.3 | 27.4 | 28.0 | 26.5 | 33.3 | 30.6 |
| Mtmr3 | 15.0 | 17.8 | 16.1 | 14.7 | 15.0 | 16.4 | 14.4 | 15.1 | 16.8 | 14.6 | 14.4 | 14.6 |
| Mtmr4 | 9.0 | 9.1 | 8.7 | 8.0 | 8.3 | 8.8 | 8.8 | 8.9 | 9.9 | 9.0 | 8.8 | 8.2 |
| Mtmr6 | 28.2 | 29.6 | 28.0 | 31.5 | 29.0 | 29.1 | 27.7 | 24.5 | 30.0 | 28.9 | 31.5 | 32.8 |
| Mtmr7 | 0.7 | 0.8 | 0.9 | 0.9 | 0.8 | 1.0 | 0.7 | 0.7 | 0.6 | 0.9 | 1.1 | 1.1 |
| Mtmr9 | 18.4 | 15.5 | 15.4 | 19.9 | 14.4 | 17.1 | 14.7 | 16.0 | 14.4 | 14.5 | 15.3 | 14.8 |

| | | | | | | | | | | | | |
|---|---|---|---|---|---|---|---|---|---|---|---|---|
| Mtnr1a | 0.1 | 0.1 | 0.1 | 0.0 | 0.1 | 0.0 | 0.4 | 0.2 | 0.4 | 0.1 | 0.2 | 0.2 |
| Mto1 | 5.0 | 5.2 | 4.6 | 4.3 | 5.0 | 5.4 | 4.3 | 4.7 | 4.7 | 5.5 | 5.7 | 4.6 |
| Mtor | 7.3 | 7.4 | 6.7 | 6.5 | 6.5 | 7.8 | 6.9 | 6.7 | 7.1 | 6.7 | 6.9 | 6.6 |
| Mtpap | 7.0 | 6.7 | 7.7 | 7.0 | 6.8 | 8.5 | 7.0 | 6.8 | 6.7 | 6.6 | 7.5 | 7.6 |
| Mtpn | 66.3 | 57.5 | 52.1 | 84.2 | 65.2 | 77.6 | 56.7 | 48.1 | 53.3 | 56.0 | 71.0 | 64.1 |
| Mtr | 3.0 | 3.2 | 3.5 | 3.7 | 3.8 | 3.4 | 3.5 | 3.9 | 3.5 | 3.9 | 3.7 | 3.7 |
| Mtrf1 | 3.6 | 3.4 | 3.2 | 3.5 | 2.5 | 4.0 | 3.0 | 2.8 | 3.0 | 3.0 | 2.9 | 3.3 |
| Mtrf1l | 2.4 | 3.0 | 3.5 | 2.2 | 2.4 | 2.2 | 2.5 | 3.8 | 2.9 | 3.3 | 2.6 | 3.3 |
| Mtrr | 5.5 | 4.4 | 5.0 | 6.0 | 5.8 | 5.0 | 5.2 | 4.8 | 4.5 | 4.5 | 5.2 | 4.4 |
| Mtss1 | 4.7 | 5.3 | 4.6 | 8.1 | 6.7 | 5.1 | 5.5 | 3.3 | 8.1 | 14.9 | 10.2 | 10.1 |
| Mtss1l | 2.4 | 2.9 | 2.8 | 3.6 | 2.8 | 4.2 | 2.8 | 2.5 | 2.5 | 1.8 | 2.5 | 2.3 |
| Mttp | 2.3 | 2.2 | 2.6 | 1.3 | 1.4 | 1.7 | 1.6 | 2.2 | 1.5 | 1.3 | 1.6 | 1.8 |
| Mtus1 | 2.1 | 2.3 | 3.4 | 2.4 | 2.1 | 2.1 | 2.2 | 3.2 | 2.2 | 2.9 | 3.1 | 2.5 |
| Mtus2 | 0.8 | 0.5 | 0.6 | 0.7 | 0.4 | 0.8 | 0.5 | 0.5 | 0.4 | 0.4 | 0.4 | 0.6 |
| Mtx1 | 15.2 | 14.0 | 12.0 | 14.9 | 12.9 | 13.4 | 13.9 | 14.4 | 13.5 | 12.5 | 13.1 | 12.8 |
| Mtx2 | 39.5 | 36.3 | 31.5 | 31.5 | 30.1 | 33.0 | 28.9 | 29.9 | 31.0 | 36.6 | 32.2 | 34.9 |
| Mtx3 | 3.4 | 3.4 | 3.0 | 3.1 | 2.6 | 3.6 | 2.8 | 2.7 | 2.7 | 2.8 | 2.8 | 2.7 |
| Muc1 | 0.3 | 0.2 | 0.4 | 0.2 | 0.2 | 0.2 | 0.3 | 0.3 | 0.2 | 0.2 | 0.2 | 0.2 |
| Muc13 | 0.0 | 0.0 | 0.0 | 0.0 | 0.0 | 0.1 | 0.0 | 0.0 | 0.0 | 0.0 | 0.0 | 0.0 |
| Muc15 | 0.1 | 0.2 | 0.1 | 0.0 | 0.1 | 0.1 | 0.1 | 0.1 | 0.0 | 0.0 | 0.0 | 0.0 |
| Muc19 | 0.0 | 0.0 | 0.0 | 0.0 | 0.0 | 0.0 | 0.0 | 0.0 | 0.0 | 0.0 | 0.0 | 0.0 |
| Muc2 | 0.0 | 0.0 | 0.0 | 0.0 | 0.0 | 0.0 | 0.0 | 0.0 | 0.0 | 0.0 | 0.0 | 0.0 |
| Muc5b | 0.0 | 0.0 | 0.0 | 0.0 | 0.0 | 0.0 | 0.0 | 0.0 | 0.0 | 0.0 | 0.0 | 0.0 |
| Muc6 | 0.0 | 0.0 | 0.0 | 0.0 | 0.0 | 0.0 | 0.1 | 0.1 | 0.1 | 0.0 | 0.0 | 0.0 |
| Mucl1 | 0.0 | 0.0 | 0.0 | 0.1 | 0.0 | 0.0 | 0.0 | 0.0 | 0.0 | 0.0 | 0.0 | 0.0 |
| Mul1 | 4.4 | 4.5 | 3.7 | 4.9 | 4.0 | 4.3 | 4.1 | 3.8 | 4.2 | 4.7 | 4.1 | 3.3 |
| Mum1 | 5.8 | 6.1 | 5.6 | 4.8 | 5.8 | 6.0 | 6.6 | 5.4 | 6.7 | 5.8 | 5.3 | 5.0 |
| Mum1l1 | 11.1 | 8.7 | 5.6 | 12.0 | 5.9 | 9.9 | 6.3 | 3.6 | 5.2 | 6.3 | 8.0 | 5.9 |
| Murc | 0.2 | 0.1 | 0.1 | 0.2 | 0.2 | 0.1 | 0.1 | 0.1 | 0.1 | 0.2 | 0.2 | 0.3 |
| Mus81 | 5.2 | 4.6 | 4.4 | 4.0 | 5.0 | 5.3 | 5.2 | 4.9 | 6.1 | 4.8 | 5.8 | 4.9 |
| Musk | 0.2 | 0.3 | 0.6 | 0.1 | 0.2 | 0.1 | 0.2 | 0.3 | 0.1 | 0.3 | 0.0 | 0.1 |
| Mustn1 | 15.4 | 11.0 | 8.8 | 15.2 | 8.4 | 11.4 | 12.6 | 13.6 | 7.9 | 11.1 | 16.0 | 10.4 |
| Mut | 18.2 | 15.3 | 15.1 | 15.9 | 12.2 | 16.9 | 13.8 | 12.9 | 13.0 | 13.7 | 15.8 | 14.4 |
| Mutyh | 0.6 | 0.4 | 0.7 | 0.8 | 0.4 | 0.5 | 0.4 | 0.5 | 0.8 | 0.5 | 0.5 | 0.8 |
| Mvb12a | 28.3 | 22.8 | 20.7 | 30.0 | 21.7 | 28.1 | 20.7 | 23.8 | 21.1 | 23.0 | 20.8 | 21.3 |
| Mvb12b | 6.8 | 6.2 | 6.1 | 7.4 | 6.2 | 7.7 | 6.0 | 4.7 | 4.7 | 4.6 | 4.9 | 4.3 |
| Mvd | 8.0 | 12.0 | 9.2 | 3.7 | 6.4 | 6.0 | 16.3 | 19.8 | 13.9 | 5.7 | 14.1 | 5.4 |
| Mvk | 14.1 | 17.3 | 15.4 | 8.0 | 11.8 | 10.5 | 27.4 | 29.7 | 26.3 | 11.8 | 20.5 | 12.7 |
| Mvp | 83.2 | 78.9 | 73.1 | 101.3 | 89.7 | 83.7 | 88.7 | 85.7 | 80.4 | 64.7 | 76.5 | 79.7 |
| Mx1 | 0.4 | 0.1 | 0.1 | 0.6 | 0.2 | 0.2 | 0.1 | 0.0 | 0.1 | 0.0 | 0.1 | 0.4 |
| Mx2 | 1.3 | 0.5 | 1.0 | 1.7 | 0.5 | 1.0 | 0.2 | 0.3 | 0.3 | 0.2 | 0.4 | 1.0 |
| Mxd1 | 1.3 | 2.2 | 2.2 | 1.5 | 1.5 | 1.5 | 1.7 | 1.7 | 2.0 | 1.8 | 1.7 | 1.6 |
| Mxd3 | 2.9 | 3.8 | 4.2 | 2.6 | 4.7 | 2.4 | 5.6 | 3.6 | 5.7 | 3.3 | 4.4 | 3.0 |
| Mxd4 | 49.3 | 49.6 | 50.0 | 46.0 | 46.6 | 44.6 | 55.2 | 72.1 | 61.7 | 56.2 | 53.9 | 50.7 |
| Mxi1 | 4.6 | 6.1 | 5.3 | 6.0 | 3.9 | 7.6 | 5.3 | 4.2 | 3.7 | 4.1 | 5.0 | 4.4 |
| Mxra7 | 64.4 | 59.3 | 67.3 | 80.4 | 52.7 | 84.7 | 65.8 | 58.6 | 50.1 | 59.0 | 62.9 | 71.5 |
| Mxra8 | 114.1 | 106.6 | 120.5 | 142.7 | 129.9 | 151.7 | 148.9 | 144.0 | 152.3 | 158.9 | 128.5 | 148.5 |
| Myadm | 63.0 | 53.6 | 50.8 | 74.4 | 71.7 | 62.1 | 93.8 | 74.1 | 67.9 | 39.7 | 65.6 | 49.1 |
| Myb | 0.0 | 0.0 | 0.1 | 0.0 | 0.0 | 0.0 | 0.1 | 0.0 | 0.0 | 0.0 | 0.0 | 0.0 |
| Mybbp1a | 23.4 | 20.1 | 17.8 | 26.6 | 21.7 | 24.2 | 22.3 | 19.7 | 17.1 | 22.5 | 21.3 | 20.0 |
| Mybl1 | 4.0 | 5.4 | 6.3 | 2.9 | 5.5 | 2.9 | 4.7 | 1.8 | 3.5 | 4.4 | 5.0 | 4.2 |
| Mybl2 | 0.4 | 0.8 | 1.2 | 0.2 | 1.0 | 0.2 | 1.4 | 0.7 | 0.9 | 0.7 | 1.0 | 0.4 |
| Mybpc2 | 0.0 | 0.0 | 0.0 | 0.2 | 0.0 | 0.0 | 0.0 | 0.0 | 0.0 | 0.0 | 0.0 | 0.0 |
| Mybpc3 | 0.1 | 0.0 | 0.0 | 0.1 | 0.0 | 0.0 | 0.0 | 0.0 | 0.0 | 0.0 | 0.0 | 0.0 |
| Mybph | 0.0 | 0.0 | 0.0 | 0.0 | 0.0 | 0.0 | 0.0 | 0.0 | 0.0 | 0.0 | 0.0 | 0.0 |
| Myc | 4.9 | 5.1 | 3.8 | 7.8 | 7.0 | 7.6 | 7.2 | 5.5 | 4.7 | 4.8 | 5.7 | 4.6 |
| Mycbp | 8.7 | 10.2 | 9.0 | 11.5 | 10.1 | 10.2 | 9.4 | 8.9 | 10.3 | 10.8 | 12.7 | 11.3 |

| | | | | | | | | | | | | |
|---|---|---|---|---|---|---|---|---|---|---|---|---|
| Mycbp2 | 19.1 | 29.5 | 29.0 | 20.1 | 24.4 | 15.6 | 23.7 | 21.6 | 20.4 | 15.9 | 18.1 | 18.2 |
| Mycbpap | 0.7 | 1.3 | 0.9 | 0.5 | 0.5 | 0.9 | 0.6 | 0.6 | 0.5 | 0.3 | 0.5 | 0.3 |
| Mycl1 | 0.2 | 0.0 | 0.0 | 0.3 | 0.2 | 0.1 | 0.1 | 0.2 | 0.2 | 0.5 | 0.5 | 0.1 |
| Mycn | 3.6 | 5.4 | 4.9 | 1.3 | 3.2 | 2.0 | 4.4 | 4.0 | 2.7 | 6.3 | 5.4 | 2.4 |
| Myct1 | 0.6 | 0.1 | 0.3 | 0.1 | 0.1 | 0.2 | 0.3 | 0.4 | 0.2 | 0.1 | 0.1 | 0.1 |
| Myd88 | 9.4 | 9.6 | 11.6 | 10.2 | 12.7 | 7.6 | 9.7 | 11.3 | 9.7 | 12.6 | 8.9 | 11.3 |
| Myef2 | 9.3 | 11.0 | 13.5 | 9.9 | 10.2 | 9.4 | 14.2 | 11.0 | 11.4 | 11.8 | 13.9 | 14.1 |
| Myeov2 | 245.5 | 185.6 | 145.4 | 200.0 | 178.4 | 211.2 | 175.9 | 168.0 | 174.2 | 190.1 | 198.5 | 196.0 |
| Myg1 | 8.4 | 8.7 | 7.9 | 8.9 | 7.6 | 8.5 | 9.4 | 10.0 | 9.3 | 8.7 | 9.5 | 8.4 |
| Myh1 | 1.9 | 1.0 | 0.7 | 0.8 | 0.3 | 0.2 | 0.4 | 0.5 | 0.5 | 0.7 | 0.9 | 0.6 |
| Myh10 | 15.2 | 12.1 | 15.2 | 11.9 | 12.8 | 14.5 | 13.5 | 11.7 | 13.6 | 22.6 | 21.5 | 18.3 |
| Myh11 | 2.0 | 0.5 | 0.2 | 2.4 | 1.0 | 1.6 | 1.9 | 1.0 | 1.6 | 4.4 | 5.9 | 2.5 |
| Myh13 | 0.0 | 0.0 | 0.0 | 0.0 | 0.0 | 0.0 | 0.0 | 0.0 | 0.0 | 0.0 | 0.0 | 0.0 |
| Myh14 | 0.6 | 0.5 | 0.4 | 0.2 | 0.4 | 0.3 | 0.3 | 0.2 | 0.2 | 0.2 | 0.3 | 0.2 |
| Myh15 | 0.0 | 0.0 | 0.0 | 0.0 | 0.0 | 0.0 | 0.0 | 0.0 | 0.0 | 0.0 | 0.0 | 0.0 |
| Myh2 | 1.2 | 0.6 | 0.5 | 1.1 | 0.2 | 0.4 | 0.1 | 0.3 | 0.1 | 0.4 | 0.4 | 0.3 |
| Myh3 | 0.0 | 0.0 | 0.0 | 0.0 | 0.0 | 0.0 | 0.0 | 0.0 | 0.0 | 0.0 | 0.0 | 0.0 |
| Myh4 | 0.0 | 0.0 | 0.0 | 0.0 | 0.0 | 0.0 | 0.0 | 0.0 | 0.0 | 0.0 | 0.0 | 0.0 |
| Myh6 | 0.0 | 0.0 | 0.0 | 0.0 | 0.0 | 0.0 | 0.0 | 0.0 | 0.0 | 0.0 | 0.0 | 0.0 |
| Myh7b | 0.0 | 0.0 | 0.0 | 0.0 | 0.0 | 0.0 | 0.0 | 0.0 | 0.0 | 0.0 | 0.0 | 0.0 |
| Myh8 | 0.1 | 0.1 | 0.1 | 0.1 | 0.0 | 0.0 | 0.0 | 0.0 | 0.0 | 0.1 | 0.1 | 0.1 |
| Myh9 | 212.9 | 179.8 | 196.2 | 214.5 | 250.8 | 195.2 | 278.3 | 233.7 | 236.7 | 251.6 | 277.7 | 285.9 |
| Myl1 | 0.0 | 0.0 | 0.1 | 0.0 | 0.0 | 0.0 | 0.0 | 0.0 | 0.1 | 0.1 | 0.0 | 0.2 |
| Myl10 | 0.2 | 0.0 | 0.1 | 0.0 | 0.1 | 0.2 | 0.3 | 0.1 | 0.3 | 0.2 | 0.1 | 0.3 |
| Myl12a | 342.0 | 252.3 | 203.2 | 392.1 | 252.2 | 302.3 | 292.0 | 206.6 | 252.1 | 273.7 | 297.9 | 286.6 |
| Myl12b | 207.7 | 166.1 | 186.3 | 262.7 | 156.3 | 236.2 | 162.6 | 170.4 | 140.5 | 142.5 | 164.4 | 171.9 |
| Myl2 | 0.2 | 0.2 | 0.1 | 0.0 | 0.1 | 0.3 | 0.0 | 0.1 | 0.1 | 0.0 | 0.5 | 0.1 |
| Myl3 | 0.0 | 0.0 | 0.0 | 0.0 | 0.0 | 0.0 | 0.1 | 0.0 | 0.0 | 0.0 | 0.0 | 0.0 |
| Myl4 | 10.9 | 11.9 | 8.2 | 8.9 | 6.4 | 4.4 | 13.5 | 14.9 | 16.3 | 6.1 | 10.1 | 8.5 |
| Myl6 | 998.0 | 795.3 | 815.5 | 1104.1 | 880.7 | 860.2 | 1041.0 | 1017.1 | 1033.6 | 940.7 | 1310.1 | 1087.9 |
| Myl6b | 0.6 | 0.5 | 0.5 | 0.6 | 0.8 | 1.1 | 0.9 | 0.7 | 0.8 | 0.4 | 0.6 | 0.6 |
| Myl7 | 0.4 | 0.1 | 0.3 | 0.0 | 0.1 | 0.0 | 0.0 | 0.2 | 0.0 | 0.2 | 0.5 | 0.1 |
| Myl9 | 75.9 | 47.0 | 45.9 | 96.6 | 96.8 | 50.7 | 173.7 | 72.3 | 147.7 | 140.8 | 241.3 | 156.5 |
| Mylip | 6.9 | 8.5 | 10.3 | 9.3 | 10.6 | 5.9 | 7.4 | 7.6 | 8.4 | 8.2 | 7.1 | 8.6 |
| Mylk | 0.8 | 0.6 | 1.3 | 0.7 | 1.3 | 0.6 | 1.3 | 1.4 | 2.2 | 3.9 | 3.3 | 3.4 |
| Mylk2 | 0.3 | 0.2 | 0.2 | 0.0 | 0.1 | 0.2 | 0.2 | 0.2 | 0.1 | 0.3 | 0.2 | 0.2 |
| Mylk3 | 0.7 | 0.4 | 0.3 | 0.3 | 0.6 | 0.3 | 0.3 | 0.1 | 0.4 | 1.6 | 0.7 | 0.6 |
| Mylpf | 1.3 | 1.0 | 1.1 | 0.8 | 1.1 | 1.3 | 0.8 | 0.7 | 0.9 | 0.6 | 0.6 | 1.2 |
| Mynn | 4.7 | 4.8 | 4.7 | 4.1 | 4.6 | 5.2 | 3.8 | 4.5 | 4.3 | 4.7 | 4.7 | 5.5 |
| Myo10 | 21.9 | 33.5 | 38.6 | 26.4 | 29.1 | 24.5 | 29.5 | 23.0 | 25.6 | 24.6 | 23.1 | 34.8 |
| Myo15 | 0.0 | 0.0 | 0.0 | 0.1 | 0.0 | 0.0 | 0.0 | 0.0 | 0.0 | 0.0 | 0.0 | 0.0 |
| Myo16 | 0.0 | 0.0 | 0.0 | 0.1 | 0.0 | 0.0 | 0.0 | 0.0 | 0.0 | 0.0 | 0.0 | 0.0 |
| Myo18a | 8.1 | 5.8 | 6.0 | 8.1 | 7.4 | 7.0 | 7.0 | 5.5 | 5.9 | 7.5 | 8.4 | 7.8 |
| Myo18b | 0.1 | 0.2 | 0.1 | 0.1 | 0.1 | 0.2 | 0.1 | 0.1 | 0.0 | 0.0 | 0.0 | 0.0 |
| Myo19 | 2.1 | 2.5 | 2.6 | 2.2 | 2.6 | 1.9 | 3.3 | 3.0 | 2.9 | 2.0 | 3.2 | 2.1 |
| Myo1a | 0.1 | 0.0 | 0.1 | 0.0 | 0.1 | 0.1 | 0.1 | 0.1 | 0.1 | 0.0 | 0.1 | 0.1 |
| Myo1b | 13.7 | 19.8 | 16.8 | 15.7 | 21.5 | 18.3 | 21.4 | 14.9 | 20.4 | 17.6 | 21.1 | 16.2 |
| Myo1c | 134.2 | 111.3 | 86.0 | 135.7 | 95.0 | 120.8 | 120.5 | 112.6 | 108.6 | 113.0 | 129.2 | 105.9 |
| Myo1d | 11.5 | 11.5 | 11.4 | 9.5 | 11.1 | 9.8 | 13.0 | 10.4 | 12.1 | 12.2 | 12.2 | 13.4 |
| Myo1e | 5.6 | 3.5 | 3.9 | 8.3 | 11.7 | 5.0 | 7.4 | 5.0 | 5.0 | 5.9 | 6.9 | 5.9 |
| Myo1f | 1.4 | 0.0 | 0.0 | 15.1 | 21.3 | 0.0 | 2.0 | 0.2 | 0.3 | 1.4 | 0.3 | 0.9 |
| Myo1g | 0.3 | 0.0 | 0.0 | 2.4 | 6.0 | 0.0 | 0.7 | 0.1 | 0.2 | 0.5 | 0.2 | 0.2 |
| Myo1h | 0.5 | 0.4 | 0.5 | 0.6 | 0.4 | 0.3 | 0.5 | 0.6 | 0.2 | 0.4 | 0.4 | 0.6 |
| Myo3b | 0.0 | 0.0 | 0.0 | 0.0 | 0.0 | 0.0 | 0.0 | 0.0 | 0.0 | 0.0 | 0.0 | 0.0 |
| Myo5a | 15.4 | 11.4 | 9.4 | 17.7 | 15.3 | 16.1 | 10.2 | 8.6 | 7.3 | 9.8 | 11.7 | 10.6 |
| Myo5b | 0.1 | 0.1 | 0.0 | 0.1 | 0.0 | 0.0 | 0.1 | 0.1 | 0.4 | 2.4 | 0.6 | 0.5 |
| Myo5c | 0.0 | 0.0 | 0.0 | 0.0 | 0.0 | 0.0 | 0.0 | 0.0 | 0.0 | 0.0 | 0.0 | 0.0 |

| | | | | | | | | | | | | |
|---|---|---|---|---|---|---|---|---|---|---|---|---|
| Myo6 | 10.2 | 8.7 | 7.6 | 11.5 | 7.8 | 11.9 | 7.3 | 6.2 | 6.4 | 7.3 | 8.2 | 8.7 |
| Myo7a | 10.3 | 12.9 | 14.9 | 10.6 | 14.4 | 8.4 | 12.2 | 13.2 | 13.7 | 8.7 | 7.9 | 11.7 |
| Myo9a | 4.5 | 4.9 | 5.0 | 3.8 | 4.2 | 3.9 | 4.9 | 6.0 | 5.0 | 4.3 | 5.7 | 5.2 |
| Myo9b | 16.2 | 17.5 | 17.0 | 17.3 | 19.0 | 16.3 | 20.1 | 17.0 | 18.8 | 16.8 | 17.4 | 18.0 |
| Myoc | 0.0 | 0.1 | 0.6 | 0.1 | 0.0 | 0.0 | 0.0 | 0.1 | 0.0 | 0.0 | 0.0 | 0.0 |
| Myocd | 0.0 | 0.0 | 0.1 | 0.0 | 0.0 | 0.0 | 0.1 | 0.0 | 0.0 | 0.1 | 0.0 | 0.0 |
| Myof | 35.8 | 35.4 | 39.8 | 37.5 | 44.5 | 31.9 | 47.2 | 40.5 | 43.6 | 42.4 | 45.3 | 49.2 |
| Myom1 | 0.7 | 0.4 | 0.4 | 0.3 | 0.4 | 0.5 | 0.4 | 0.3 | 0.6 | 0.4 | 0.7 | 0.5 |
| Myom2 | 0.6 | 0.1 | 0.1 | 0.4 | 0.1 | 0.1 | 0.0 | 0.1 | 0.0 | 0.0 | 0.0 | 0.0 |
| Myom3 | 0.0 | 0.0 | 0.0 | 0.0 | 0.0 | 0.0 | 0.0 | 0.0 | 0.0 | 0.0 | 0.0 | 0.0 |
| Myot | 0.0 | 0.1 | 0.0 | 0.0 | 0.1 | 0.0 | 0.0 | 0.0 | 0.0 | 0.0 | 0.0 | 0.0 |
| Myoz1 | 0.1 | 0.1 | 0.1 | 0.2 | 0.3 | 0.0 | 0.1 | 0.1 | 0.1 | 0.2 | 0.0 | 0.0 |
| Myoz2 | 0.3 | 0.0 | 0.0 | 0.0 | 0.0 | 0.0 | 0.0 | 0.0 | 0.0 | 0.2 | 0.2 | 0.2 |
| Myoz3 | 0.1 | 0.0 | 0.1 | 0.1 | 0.1 | 0.1 | 0.1 | 0.1 | 0.1 | 0.0 | 0.0 | 0.1 |
| Mypn | 0.0 | 0.0 | 0.0 | 0.0 | 0.0 | 0.0 | 0.0 | 0.0 | 0.0 | 0.0 | 0.0 | 0.0 |
| Mypop | 1.4 | 2.0 | 1.8 | 1.5 | 1.6 | 1.6 | 1.8 | 2.7 | 1.8 | 1.6 | 1.6 | 1.6 |
| Myrf | 6.9 | 7.8 | 6.9 | 5.7 | 5.6 | 4.8 | 8.8 | 8.0 | 6.8 | 8.6 | 7.9 | 5.8 |
| Myrfl | 0.1 | 0.0 | 0.1 | 0.1 | 0.1 | 0.1 | 0.1 | 0.1 | 0.1 | 0.1 | 0.1 | 0.0 |
| Myrip | 0.0 | 0.0 | 0.0 | 0.0 | 0.0 | 0.0 | 0.0 | 0.0 | 0.0 | 0.0 | 0.0 | 0.0 |
| Mysm1 | 3.9 | 4.2 | 4.1 | 4.1 | 4.0 | 4.2 | 3.3 | 3.5 | 3.4 | 3.5 | 4.5 | 4.3 |
| Myt1 | 0.0 | 0.0 | 0.0 | 0.0 | 0.0 | 0.0 | 0.0 | 0.0 | 0.0 | 0.0 | 0.0 | 0.0 |
| Myt1l | 0.0 | 0.0 | 0.0 | 0.0 | 0.0 | 0.0 | 0.0 | 0.0 | 0.0 | 0.0 | 0.0 | 0.0 |
| Myzap | 0.9 | 0.9 | 1.0 | 0.4 | 0.6 | 0.9 | 0.7 | 0.8 | 0.6 | 0.4 | 0.3 | 0.5 |
| Mzf1 | 0.3 | 0.2 | 0.3 | 0.2 | 0.3 | 0.4 | 0.2 | 0.4 | 0.2 | 0.2 | 0.1 | 0.1 |
| Mzt1 | 12.7 | 11.9 | 14.8 | 15.7 | 12.9 | 14.9 | 14.8 | 12.8 | 14.2 | 13.9 | 17.6 | 17.3 |
| Mzt2 | 7.3 | 7.3 | 6.4 | 8.3 | 6.5 | 8.3 | 5.2 | 9.2 | 8.4 | 8.3 | 8.6 | 5.7 |
| N28178 | 0.1 | 0.2 | 0.3 | 0.2 | 0.1 | 0.1 | 0.2 | 0.2 | 0.1 | 0.1 | 0.1 | 0.0 |
| N4bp1 | 7.8 | 9.0 | 8.5 | 7.2 | 7.7 | 8.2 | 7.8 | 7.1 | 7.5 | 8.2 | 8.0 | 8.1 |
| N4bp2 | 1.3 | 1.6 | 1.8 | 1.4 | 1.6 | 1.1 | 1.3 | 2.3 | 1.7 | 1.7 | 1.6 | 1.8 |
| N4bp2l1 | 2.0 | 2.0 | 2.3 | 1.6 | 1.6 | 1.6 | 1.6 | 1.6 | 1.4 | 1.8 | 1.7 | 2.3 |
| N4bp2l2 | 8.0 | 7.3 | 6.8 | 7.2 | 7.0 | 7.1 | 6.5 | 6.3 | 6.4 | 7.8 | 7.8 | 7.5 |
| N4bp3 | 0.6 | 0.3 | 0.7 | 0.3 | 0.6 | 0.3 | 0.9 | 1.0 | 0.8 | 0.5 | 0.4 | 0.3 |
| N6amt1 | 4.9 | 4.2 | 3.5 | 4.9 | 3.9 | 3.8 | 5.2 | 4.3 | 4.2 | 4.5 | 4.5 | 4.9 |
| N6amt2 | 18.6 | 17.6 | 16.5 | 18.3 | 16.2 | 17.7 | 17.0 | 20.2 | 16.9 | 17.3 | 18.4 | 13.1 |
| Naa10 | 9.0 | 8.8 | 8.8 | 8.2 | 8.2 | 7.1 | 9.0 | 10.2 | 10.4 | 11.6 | 9.9 | 7.9 |
| Naa15 | 5.4 | 6.8 | 6.1 | 6.6 | 7.6 | 5.7 | 6.8 | 6.9 | 5.7 | 7.3 | 7.3 | 7.2 |
| Naa16 | 1.2 | 2.0 | 2.1 | 1.4 | 1.7 | 1.3 | 1.7 | 1.5 | 1.4 | 1.8 | 1.9 | 1.5 |
| Naa20 | 26.3 | 25.4 | 23.9 | 26.2 | 23.7 | 24.3 | 26.7 | 24.1 | 24.6 | 29.3 | 28.5 | 30.0 |
| Naa25 | 11.6 | 11.0 | 9.1 | 12.4 | 10.6 | 10.3 | 11.8 | 11.3 | 10.5 | 11.0 | 12.2 | 10.3 |
| Naa30 | 3.3 | 4.1 | 4.7 | 3.7 | 4.1 | 4.5 | 3.4 | 4.3 | 4.1 | 4.9 | 4.8 | 4.9 |
| Naa35 | 8.3 | 7.6 | 7.6 | 7.1 | 6.1 | 6.2 | 6.5 | 6.3 | 6.8 | 8.2 | 8.0 | 8.1 |
| Naa38 | 16.6 | 16.7 | 16.9 | 16.8 | 16.3 | 15.9 | 17.7 | 14.5 | 18.2 | 18.7 | 18.7 | 16.3 |
| Naa40 | 5.0 | 5.9 | 5.5 | 4.2 | 5.4 | 4.3 | 4.4 | 3.9 | 4.7 | 4.9 | 4.5 | 3.9 |
| Naa50 | 8.6 | 9.3 | 11.1 | 13.3 | 12.4 | 12.5 | 10.7 | 11.3 | 9.8 | 11.2 | 13.6 | 11.3 |
| Naa60 | 14.0 | 15.4 | 18.2 | 16.7 | 17.0 | 15.9 | 15.8 | 17.5 | 14.7 | 12.3 | 15.0 | 15.3 |
| Naaa | 6.1 | 4.6 | 4.6 | 4.8 | 4.4 | 6.6 | 4.2 | 5.9 | 3.9 | 4.3 | 5.6 | 4.0 |
| Naalad2 | 31.5 | 33.5 | 37.4 | 30.7 | 34.4 | 41.0 | 32.1 | 35.5 | 34.9 | 25.3 | 28.7 | 32.8 |
| Naaladl1 | 0.0 | 0.0 | 0.0 | 0.0 | 0.1 | 0.1 | 0.1 | 0.0 | 0.0 | 0.0 | 0.0 | 0.0 |
| Nab1 | 20.8 | 21.4 | 23.3 | 24.9 | 21.7 | 20.8 | 22.6 | 20.7 | 18.9 | 18.7 | 24.4 | 23.3 |
| Nab2 | 9.8 | 12.3 | 11.2 | 8.0 | 10.0 | 8.9 | 11.9 | 13.9 | 15.5 | 17.8 | 13.4 | 11.4 |
| Nabp1 | 5.0 | 4.2 | 5.5 | 4.9 | 7.1 | 4.7 | 4.5 | 3.8 | 4.6 | 4.8 | 5.4 | 7.0 |
| Nabp2 | 16.5 | 13.3 | 14.8 | 18.9 | 11.6 | 17.8 | 14.0 | 14.5 | 11.9 | 10.8 | 15.6 | 13.1 |
| Naca | 291.5 | 236.7 | 232.2 | 251.7 | 224.0 | 256.0 | 233.8 | 254.9 | 245.4 | 287.0 | 311.0 | 286.2 |
| Nacad | 1.0 | 1.6 | 1.4 | 1.6 | 1.1 | 1.6 | 2.1 | 1.5 | 1.7 | 1.1 | 1.6 | 1.2 |
| Nacc1 | 10.9 | 11.6 | 11.5 | 11.9 | 11.4 | 13.0 | 13.1 | 11.5 | 11.3 | 10.5 | 11.9 | 10.1 |
| Nacc2 | 15.8 | 14.4 | 11.0 | 18.6 | 14.8 | 17.1 | 16.9 | 12.2 | 14.7 | 18.5 | 16.6 | 15.3 |
| Nadk | 59.4 | 49.3 | 46.6 | 56.5 | 61.1 | 45.1 | 64.6 | 53.2 | 48.9 | 45.6 | 55.9 | 46.9 |

| | | | | | | | | | | | | |
|---|---|---|---|---|---|---|---|---|---|---|---|---|
| Nadkd1 | 3.8 | 4.6 | 5.1 | 4.3 | 3.9 | 6.6 | 3.8 | 5.2 | 4.4 | 4.4 | 4.6 | 4.6 |
| Nadsyn1 | 1.7 | 1.4 | 1.4 | 1.6 | 1.2 | 1.9 | 1.4 | 1.3 | 1.3 | 1.3 | 1.0 | 1.6 |
| Nae1 | 26.2 | 28.4 | 22.6 | 25.6 | 23.4 | 28.0 | 24.2 | 21.1 | 28.1 | 24.8 | 28.6 | 27.7 |
| Naf1 | 1.8 | 2.4 | 2.0 | 1.4 | 1.5 | 1.5 | 1.9 | 1.5 | 1.6 | 2.2 | 1.4 | 1.5 |
| Naga | 16.4 | 13.9 | 16.6 | 19.2 | 16.0 | 25.0 | 12.1 | 16.7 | 13.6 | 13.6 | 11.8 | 14.8 |
| Nagk | 31.9 | 24.0 | 23.9 | 37.8 | 29.4 | 32.2 | 28.7 | 26.2 | 30.8 | 28.5 | 32.1 | 28.8 |
| Naglu | 15.4 | 12.5 | 11.8 | 20.5 | 13.7 | 22.0 | 9.9 | 10.8 | 12.3 | 13.9 | 9.8 | 12.3 |
| Nagpa | 8.0 | 6.3 | 5.9 | 10.5 | 10.4 | 10.3 | 7.0 | 7.1 | 6.5 | 8.1 | 6.0 | 7.5 |
| Naif1 | 1.6 | 1.4 | 1.1 | 1.3 | 1.4 | 1.2 | 1.6 | 1.5 | 1.3 | 1.3 | 1.3 | 1.4 |
| Naip1 | 0.1 | 0.0 | 0.0 | 0.2 | 0.2 | 0.0 | 0.1 | 0.0 | 0.0 | 0.0 | 0.0 | 0.0 |
| Naip2 | 1.8 | 0.8 | 0.5 | 3.2 | 4.9 | 0.8 | 1.1 | 0.5 | 0.4 | 0.5 | 0.5 | 0.6 |
| Naip5 | 0.1 | 0.1 | 0.0 | 0.4 | 0.8 | 0.0 | 0.1 | 0.0 | 0.0 | 0.1 | 0.1 | 0.0 |
| Naip6 | 0.0 | 0.0 | 0.0 | 0.2 | 0.4 | 0.0 | 0.0 | 0.0 | 0.0 | 0.0 | 0.1 | 0.1 |
| Naip7 | 0.1 | 0.1 | 0.0 | 0.3 | 0.1 | 0.1 | 0.1 | 0.1 | 0.0 | 0.0 | 0.0 | 0.0 |
| Nalcn | 0.2 | 0.1 | 0.3 | 0.2 | 0.1 | 0.2 | 0.1 | 0.2 | 0.1 | 0.1 | 0.1 | 0.1 |
| Nampt | 10.6 | 9.8 | 9.5 | 12.1 | 8.6 | 11.5 | 6.4 | 7.7 | 6.2 | 6.5 | 8.1 | 7.9 |
| Nanog | 0.0 | 0.1 | 0.0 | 0.0 | 0.0 | 0.0 | 0.1 | 0.0 | 0.0 | 0.1 | 0.1 | 0.1 |
| Nanos1 | 1.5 | 2.3 | 2.1 | 1.5 | 3.4 | 1.6 | 2.5 | 1.5 | 1.9 | 2.3 | 2.4 | 1.9 |
| Nanp | 7.0 | 7.4 | 7.7 | 7.0 | 6.5 | 6.7 | 5.7 | 8.1 | 6.5 | 7.9 | 7.0 | 6.9 |
| Nans | 25.3 | 21.9 | 20.3 | 25.4 | 26.1 | 23.8 | 25.3 | 19.2 | 19.8 | 19.8 | 24.1 | 21.1 |
| Nap1l1 | 180.5 | 154.6 | 155.0 | 204.5 | 171.6 | 177.1 | 200.1 | 146.0 | 136.2 | 168.0 | 198.0 | 199.9 |
| Nap1l2 | 0.4 | 0.2 | 0.3 | 0.6 | 1.1 | 0.8 | 0.7 | 0.2 | 0.2 | 0.3 | 0.3 | 0.4 |
| Nap1l3 | 2.0 | 1.7 | 1.8 | 1.4 | 1.0 | 2.0 | 1.4 | 1.4 | 1.3 | 1.8 | 1.6 | 1.6 |
| Nap1l4 | 36.9 | 35.5 | 34.4 | 37.9 | 32.4 | 34.4 | 33.3 | 32.0 | 32.7 | 37.8 | 36.2 | 36.4 |
| Nap1l5 | 0.1 | 0.3 | 0.3 | 0.2 | 0.3 | 0.2 | 0.2 | 0.2 | 0.2 | 0.1 | 0.2 | 0.0 |
| Napa | 58.7 | 47.1 | 37.5 | 65.7 | 52.7 | 64.4 | 48.5 | 43.8 | 41.9 | 46.2 | 49.5 | 44.0 |
| Napb | 1.3 | 1.1 | 0.8 | 1.7 | 1.0 | 1.5 | 1.0 | 0.9 | 0.8 | 0.8 | 1.1 | 0.9 |
| Napepld | 2.0 | 2.4 | 3.0 | 1.8 | 1.8 | 1.9 | 2.3 | 2.6 | 2.0 | 1.8 | 2.1 | 2.3 |
| Napg | 7.9 | 8.4 | 8.5 | 10.0 | 10.8 | 9.4 | 8.3 | 7.7 | 9.2 | 7.7 | 9.4 | 9.3 |
| Naprt1 | 2.7 | 3.9 | 4.2 | 2.0 | 2.9 | 2.8 | 3.8 | 5.2 | 5.3 | 3.5 | 3.6 | 4.0 |
| Napsa | 0.0 | 0.0 | 0.0 | 0.4 | 0.8 | 0.0 | 0.1 | 0.1 | 0.0 | 0.1 | 0.0 | 0.0 |
| Narf | 6.7 | 6.8 | 6.1 | 6.4 | 5.4 | 7.1 | 5.4 | 6.2 | 6.6 | 6.5 | 6.0 | 6.8 |
| Narfl | 5.5 | 5.6 | 3.9 | 5.7 | 5.5 | 5.3 | 4.1 | 4.1 | 4.9 | 6.3 | 4.6 | 4.8 |
| Narg2 | 3.7 | 3.3 | 3.7 | 3.4 | 3.6 | 4.2 | 3.2 | 3.0 | 3.4 | 3.7 | 4.0 | 3.7 |
| Nars | 164.7 | 107.6 | 89.7 | 203.8 | 112.1 | 161.5 | 118.6 | 116.0 | 101.5 | 104.4 | 135.6 | 112.1 |
| Nars2 | 2.2 | 1.7 | 1.5 | 1.7 | 1.6 | 1.7 | 1.4 | 1.1 | 1.1 | 1.6 | 1.4 | 1.5 |
| Nasp | 8.1 | 8.2 | 12.3 | 7.6 | 10.5 | 5.8 | 11.5 | 11.1 | 9.7 | 8.6 | 11.4 | 9.6 |
| Nat1 | 0.0 | 0.0 | 0.0 | 0.0 | 0.0 | 0.0 | 0.0 | 0.0 | 0.0 | 0.1 | 0.0 | 0.0 |
| Nat10 | 6.3 | 4.3 | 5.0 | 6.8 | 5.1 | 5.6 | 5.7 | 5.4 | 4.5 | 4.9 | 5.2 | 4.7 |
| Nat14 | 2.4 | 2.4 | 2.2 | 1.9 | 1.7 | 2.9 | 1.6 | 1.5 | 1.2 | 2.2 | 1.3 | 2.0 |
| Nat2 | 5.0 | 5.0 | 5.9 | 3.7 | 5.1 | 4.4 | 4.8 | 5.3 | 6.4 | 5.8 | 6.1 | 7.2 |
| Nat3 | 0.0 | 0.0 | 0.0 | 0.1 | 0.0 | 0.0 | 0.0 | 0.0 | 0.0 | 0.0 | 0.0 | 0.0 |
| Nat6 | 10.6 | 9.7 | 9.2 | 11.9 | 9.6 | 12.4 | 8.8 | 8.5 | 7.8 | 8.0 | 8.3 | 8.8 |
| Nat8l | 0.2 | 0.1 | 0.1 | 0.1 | 0.1 | 0.0 | 0.4 | 0.4 | 0.4 | 0.6 | 0.6 | 0.3 |
| Nat9 | 24.1 | 20.0 | 16.2 | 24.4 | 15.6 | 25.3 | 14.4 | 13.1 | 11.6 | 15.1 | 17.1 | 15.8 |
| Nav1 | 4.3 | 8.4 | 8.7 | 4.3 | 6.1 | 4.6 | 8.8 | 9.4 | 7.9 | 5.2 | 7.3 | 6.7 |
| Nav2 | 0.4 | 0.4 | 0.4 | 0.6 | 0.8 | 0.4 | 0.5 | 0.2 | 0.3 | 0.4 | 0.4 | 0.3 |
| Nav3 | 0.8 | 0.8 | 0.8 | 0.8 | 1.1 | 0.5 | 1.4 | 0.7 | 0.7 | 0.8 | 0.8 | 0.6 |
| Nbas | 14.9 | 13.9 | 13.0 | 12.7 | 12.4 | 13.4 | 11.0 | 15.0 | 12.7 | 12.1 | 13.0 | 12.6 |
| Nbea | 7.7 | 8.0 | 7.5 | 6.2 | 8.3 | 6.2 | 8.4 | 7.8 | 8.6 | 7.9 | 8.2 | 8.3 |
| Nbeal1 | 8.9 | 9.1 | 7.9 | 9.9 | 8.6 | 10.3 | 8.2 | 7.2 | 7.9 | 9.2 | 10.4 | 9.2 |
| Nbeal2 | 0.4 | 0.2 | 0.2 | 1.6 | 2.9 | 0.4 | 0.6 | 0.2 | 0.4 | 0.5 | 0.3 | 0.4 |
| Nbl1 | 19.5 | 14.5 | 11.8 | 22.9 | 17.3 | 32.7 | 25.6 | 18.9 | 22.4 | 37.9 | 30.8 | 25.6 |
| Nbn | 7.7 | 7.6 | 8.3 | 7.7 | 8.9 | 6.6 | 9.0 | 8.3 | 8.9 | 8.7 | 10.1 | 9.7 |
| Nbr1 | 44.1 | 43.2 | 44.2 | 42.5 | 42.6 | 45.8 | 37.2 | 40.2 | 36.7 | 46.4 | 43.4 | 49.1 |
| Ncald | 1.6 | 1.0 | 0.8 | 2.7 | 1.2 | 1.5 | 0.8 | 0.4 | 0.5 | 0.7 | 0.8 | 0.5 |
| Ncam1 | 7.1 | 4.6 | 6.1 | 5.3 | 10.3 | 6.2 | 17.2 | 7.8 | 19.6 | 21.1 | 17.6 | 22.3 |

| | | | | | | | | | | | | |
|---|---|---|---|---|---|---|---|---|---|---|---|---|
| Ncam2 | 0.0 | 0.1 | 0.0 | 0.0 | 0.0 | 0.0 | 0.0 | 0.0 | 0.0 | 0.0 | 0.0 | 0.1 |
| Ncapd2 | 2.0 | 4.4 | 7.1 | 1.8 | 5.4 | 1.9 | 7.1 | 4.4 | 5.7 | 2.9 | 4.4 | 3.8 |
| Ncapd3 | 3.2 | 3.8 | 5.3 | 2.7 | 3.8 | 2.6 | 4.5 | 3.6 | 3.3 | 2.9 | 4.3 | 2.9 |
| Ncapg | 1.4 | 3.6 | 5.4 | 0.7 | 5.0 | 0.6 | 4.3 | 1.9 | 4.1 | 2.4 | 3.9 | 2.5 |
| Ncapg2 | 1.4 | 2.5 | 4.1 | 1.4 | 3.8 | 0.7 | 4.4 | 2.2 | 3.0 | 1.8 | 2.8 | 1.8 |
| Ncaph | 2.7 | 4.4 | 7.6 | 1.1 | 6.2 | 1.2 | 6.7 | 4.0 | 6.1 | 3.2 | 4.9 | 3.4 |
| Ncaph2 | 18.3 | 20.6 | 20.1 | 18.6 | 18.0 | 20.7 | 19.9 | 19.1 | 20.2 | 18.5 | 18.4 | 17.6 |
| Ncbp1 | 17.0 | 15.3 | 15.4 | 17.8 | 15.0 | 15.8 | 16.3 | 16.1 | 13.0 | 14.3 | 16.4 | 15.7 |
| Ncbp2 | 30.5 | 30.2 | 26.8 | 33.1 | 33.2 | 29.5 | 31.1 | 29.2 | 30.1 | 34.5 | 34.3 | 34.2 |
| Ncdn | 5.9 | 5.9 | 4.6 | 7.1 | 6.3 | 5.8 | 6.9 | 6.3 | 6.6 | 6.1 | 5.3 | 5.5 |
| Nceh1 | 6.3 | 4.2 | 3.3 | 12.9 | 10.1 | 8.2 | 4.3 | 3.1 | 2.9 | 6.4 | 5.8 | 4.2 |
| Ncf1 | 1.6 | 0.1 | 0.1 | 14.0 | 24.5 | 0.0 | 2.5 | 0.4 | 0.5 | 1.6 | 0.3 | 1.1 |
| Ncf2 | 2.5 | 0.4 | 0.4 | 12.9 | 25.0 | 0.5 | 2.8 | 0.6 | 0.5 | 1.9 | 0.6 | 1.7 |
| Ncf4 | 0.8 | 0.0 | 0.1 | 8.0 | 13.6 | 0.0 | 1.4 | 0.1 | 0.4 | 0.6 | 0.1 | 0.6 |
| Nck1 | 24.8 | 28.0 | 28.2 | 24.7 | 28.7 | 20.9 | 24.1 | 25.2 | 24.0 | 23.6 | 27.3 | 28.6 |
| Nck2 | 11.2 | 11.0 | 11.1 | 10.7 | 10.2 | 11.2 | 11.1 | 10.1 | 12.0 | 11.1 | 12.2 | 10.7 |
| Nckap1 | 96.1 | 92.6 | 77.0 | 92.7 | 77.6 | 97.5 | 79.7 | 76.1 | 76.4 | 81.1 | 91.0 | 82.9 |
| Nckap1l | 1.7 | 0.1 | 0.1 | 13.2 | 25.3 | 0.1 | 2.9 | 0.5 | 0.5 | 1.6 | 0.5 | 1.0 |
| Nckap5 | 0.3 | 0.2 | 0.4 | 0.2 | 0.1 | 0.1 | 0.1 | 0.5 | 0.1 | 0.3 | 0.3 | 0.3 |
| Nckap5l | 1.1 | 1.6 | 1.3 | 0.9 | 1.3 | 1.0 | 1.3 | 1.7 | 1.9 | 1.2 | 1.0 | 1.0 |
| Nckipsd | 1.9 | 2.1 | 2.3 | 2.0 | 2.0 | 2.2 | 2.3 | 2.7 | 2.8 | 2.0 | 2.2 | 2.5 |
| Ncl | 43.2 | 31.1 | 34.0 | 52.3 | 35.3 | 39.7 | 37.6 | 30.7 | 19.1 | 27.8 | 34.6 | 35.7 |
| Ncln | 16.3 | 14.5 | 11.5 | 18.2 | 18.5 | 13.8 | 20.1 | 16.7 | 19.3 | 17.6 | 16.3 | 12.7 |
| Ncmap | 0.1 | 0.0 | 0.0 | 0.0 | 0.0 | 0.1 | 0.0 | 0.1 | 0.0 | 0.0 | 0.1 | 0.0 |
| Ncoa1 | 2.8 | 3.4 | 4.3 | 3.3 | 2.9 | 3.3 | 3.1 | 4.0 | 3.0 | 2.5 | 2.6 | 2.9 |
| Ncoa2 | 6.4 | 7.9 | 8.1 | 6.7 | 6.3 | 7.2 | 6.8 | 7.2 | 6.5 | 4.9 | 6.7 | 6.3 |
| Ncoa3 | 5.5 | 8.3 | 8.8 | 6.2 | 7.7 | 5.4 | 6.7 | 6.9 | 7.1 | 4.7 | 5.4 | 6.2 |
| Ncoa4 | 52.9 | 48.1 | 40.1 | 52.7 | 46.2 | 47.2 | 38.8 | 37.6 | 40.1 | 52.8 | 51.0 | 48.4 |
| Ncoa5 | 6.8 | 7.3 | 7.6 | 7.3 | 7.2 | 7.5 | 7.4 | 8.5 | 8.0 | 8.3 | 7.8 | 7.4 |
| Ncoa6 | 1.6 | 2.4 | 2.5 | 1.9 | 2.3 | 1.7 | 2.7 | 2.7 | 2.4 | 1.9 | 2.1 | 1.9 |
| Ncoa7 | 2.2 | 3.0 | 3.2 | 2.8 | 3.2 | 1.9 | 3.1 | 2.6 | 2.5 | 1.9 | 2.3 | 3.1 |
| Ncor1 | 18.3 | 19.8 | 23.4 | 21.7 | 19.0 | 21.9 | 20.0 | 22.1 | 17.7 | 19.0 | 22.6 | 21.7 |
| Ncor2 | 1.3 | 2.1 | 2.0 | 1.6 | 2.3 | 1.7 | 2.9 | 2.6 | 2.5 | 2.2 | 2.1 | 1.9 |
| Ncrna00085 | 0.9 | 0.6 | 0.7 | 0.3 | 0.6 | 0.5 | 0.7 | 0.9 | 0.5 | 0.4 | 0.5 | 1.2 |
| Ncrna00086 | 0.4 | 0.3 | 0.2 | 0.3 | 0.4 | 0.5 | 0.4 | 0.2 | 0.3 | 0.2 | 0.2 | 0.1 |
| Ncs1 | 15.3 | 14.8 | 12.1 | 13.8 | 14.3 | 16.3 | 17.5 | 13.3 | 17.3 | 12.8 | 15.0 | 13.2 |
| Ncstn | 40.7 | 37.6 | 37.9 | 41.4 | 43.3 | 41.0 | 37.7 | 40.3 | 37.4 | 33.2 | 31.1 | 32.8 |
| Ndc80 | 2.1 | 4.8 | 6.9 | 0.9 | 6.7 | 0.6 | 7.6 | 2.4 | 5.8 | 2.7 | 5.8 | 4.0 |
| Nde1 | 29.3 | 31.6 | 35.0 | 27.9 | 30.0 | 28.0 | 36.1 | 33.2 | 30.1 | 31.1 | 35.7 | 33.6 |
| Ndel1 | 60.9 | 54.4 | 47.8 | 62.4 | 49.8 | 51.9 | 57.7 | 49.3 | 47.3 | 45.9 | 55.7 | 56.7 |
| Ndfip1 | 143.3 | 114.3 | 89.8 | 121.8 | 120.3 | 123.3 | 112.7 | 92.3 | 123.1 | 141.3 | 127.7 | 128.2 |
| Ndfip2 | 18.2 | 15.4 | 11.8 | 17.1 | 14.8 | 15.8 | 14.7 | 12.6 | 16.4 | 17.2 | 16.0 | 16.7 |
| Ndn | 48.8 | 45.0 | 40.6 | 54.5 | 44.3 | 53.4 | 53.4 | 46.0 | 50.4 | 46.7 | 46.7 | 46.1 |
| Ndnf | 0.7 | 2.1 | 4.5 | 0.5 | 0.9 | 0.2 | 2.6 | 2.7 | 3.4 | 1.6 | 1.5 | 3.9 |
| Ndnl2 | 11.6 | 10.1 | 11.9 | 9.4 | 10.3 | 11.7 | 13.4 | 11.8 | 11.3 | 11.9 | 12.3 | 11.8 |
| Ndor1 | 4.1 | 4.2 | 3.1 | 3.9 | 4.4 | 4.5 | 4.0 | 3.6 | 3.8 | 3.5 | 2.9 | 2.8 |
| Ndrg1 | 27.0 | 23.1 | 25.6 | 21.9 | 24.2 | 23.9 | 49.7 | 96.8 | 69.6 | 30.5 | 40.9 | 43.4 |
| Ndrg2 | 7.7 | 11.9 | 22.0 | 8.0 | 8.9 | 13.4 | 5.4 | 15.3 | 7.2 | 6.1 | 5.2 | 7.4 |
| Ndrg3 | 25.2 | 23.1 | 21.2 | 28.3 | 19.3 | 28.9 | 17.1 | 19.2 | 16.8 | 19.9 | 21.8 | 20.4 |
| Ndrg4 | 35.5 | 32.6 | 25.6 | 45.6 | 31.3 | 43.8 | 37.4 | 35.4 | 33.6 | 23.9 | 37.9 | 30.2 |
| Ndst1 | 16.1 | 18.0 | 17.3 | 15.7 | 16.3 | 15.7 | 22.4 | 18.1 | 19.6 | 15.9 | 17.2 | 17.2 |
| Ndst2 | 3.8 | 4.1 | 3.8 | 4.0 | 4.7 | 3.9 | 4.1 | 4.5 | 4.9 | 4.6 | 3.6 | 3.7 |
| Ndst3 | 0.4 | 0.4 | 0.2 | 0.4 | 0.5 | 0.2 | 0.3 | 0.2 | 0.1 | 0.1 | 0.1 | 0.1 |
| Ndst4 | 0.0 | 0.0 | 0.0 | 0.0 | 0.0 | 0.0 | 0.0 | 0.0 | 0.0 | 0.0 | 0.0 | 0.0 |
| Ndufa1 | 97.7 | 79.6 | 83.9 | 105.2 | 83.5 | 106.2 | 83.5 | 74.7 | 81.7 | 95.8 | 78.1 | 93.2 |
| Ndufa10 | 57.7 | 46.7 | 43.6 | 60.7 | 43.8 | 55.2 | 42.5 | 50.0 | 40.7 | 46.8 | 52.1 | 48.0 |
| Ndufa11 | 14.1 | 12.0 | 10.3 | 13.2 | 12.0 | 12.7 | 12.5 | 11.3 | 12.0 | 11.3 | 9.8 | 10.0 |

| | | | | | | | | | | | | |
|---|---|---|---|---|---|---|---|---|---|---|---|---|
| Ndufa12 | 79.8 | 72.2 | 78.9 | 107.9 | 63.9 | 88.1 | 74.0 | 77.9 | 58.6 | 72.1 | 84.4 | 75.7 |
| Ndufa13 | 99.1 | 84.1 | 78.8 | 108.5 | 81.7 | 102.4 | 90.2 | 88.9 | 78.8 | 96.0 | 87.7 | 96.0 |
| Ndufa2 | 119.9 | 105.2 | 78.8 | 108.9 | 102.6 | 100.0 | 90.3 | 102.7 | 98.6 | 101.3 | 86.7 | 83.5 |
| Ndufa3 | 210.9 | 178.0 | 162.9 | 205.2 | 178.0 | 203.7 | 178.7 | 190.6 | 205.4 | 208.5 | 201.3 | 180.4 |
| Ndufa4 | 267.6 | 208.9 | 170.2 | 236.9 | 211.9 | 229.1 | 185.6 | 213.7 | 200.1 | 268.3 | 238.8 | 204.1 |
| Ndufa4l2 | 5.4 | 12.1 | 13.4 | 7.5 | 9.5 | 15.0 | 4.9 | 3.0 | 4.1 | 2.8 | 2.9 | 6.1 |
| Ndufa5 | 61.4 | 53.4 | 57.2 | 57.1 | 46.7 | 51.5 | 51.1 | 55.3 | 44.6 | 50.6 | 56.6 | 55.2 |
| Ndufa6 | 121.7 | 104.5 | 88.0 | 99.0 | 96.6 | 106.2 | 88.2 | 94.4 | 95.4 | 116.8 | 112.9 | 109.3 |
| Ndufa7 | 217.9 | 180.7 | 131.7 | 188.9 | 176.1 | 211.8 | 147.4 | 158.2 | 167.9 | 182.8 | 169.9 | 151.3 |
| Ndufa8 | 60.8 | 56.2 | 50.1 | 59.8 | 56.3 | 59.1 | 59.4 | 55.3 | 53.4 | 67.1 | 58.1 | 59.1 |
| Ndufa9 | 38.6 | 37.5 | 31.6 | 39.4 | 35.6 | 35.9 | 28.0 | 31.2 | 30.1 | 32.8 | 30.2 | 30.3 |
| Ndufab1 | 37.7 | 33.4 | 27.5 | 29.3 | 33.7 | 31.3 | 28.4 | 26.3 | 30.1 | 38.2 | 28.6 | 31.7 |
| Ndufaf1 | 12.4 | 11.6 | 9.8 | 12.5 | 8.7 | 13.3 | 8.3 | 9.0 | 8.9 | 12.1 | 10.9 | 12.5 |
| Ndufaf2 | 25.7 | 26.2 | 33.5 | 31.9 | 23.9 | 28.8 | 30.6 | 27.7 | 24.5 | 25.2 | 31.1 | 33.7 |
| Ndufaf3 | 12.8 | 13.2 | 10.2 | 14.9 | 12.1 | 17.8 | 9.1 | 13.7 | 12.7 | 12.3 | 12.3 | 12.7 |
| Ndufaf4 | 3.0 | 3.2 | 3.0 | 3.8 | 3.0 | 3.7 | 2.9 | 2.8 | 3.1 | 3.6 | 3.9 | 3.8 |
| Ndufaf5 | 3.1 | 2.4 | 2.3 | 2.7 | 2.4 | 2.5 | 2.3 | 2.1 | 1.9 | 2.0 | 2.6 | 2.1 |
| Ndufaf6 | 2.8 | 2.4 | 2.5 | 2.4 | 2.3 | 3.5 | 2.4 | 2.0 | 2.6 | 3.4 | 2.8 | 2.4 |
| Ndufaf7 | 7.4 | 6.5 | 6.2 | 6.1 | 5.8 | 7.8 | 6.3 | 6.9 | 5.6 | 6.4 | 6.5 | 6.9 |
| Ndufb10 | 88.8 | 73.9 | 78.6 | 84.2 | 67.0 | 82.3 | 81.4 | 78.9 | 73.5 | 85.3 | 90.7 | 98.9 |
| Ndufb11 | 58.2 | 52.9 | 51.3 | 54.7 | 50.1 | 54.4 | 49.7 | 51.3 | 54.3 | 69.5 | 54.4 | 60.1 |
| Ndufb2 | 55.4 | 42.7 | 34.2 | 43.3 | 43.8 | 48.6 | 37.4 | 43.0 | 43.0 | 51.1 | 38.8 | 38.8 |
| Ndufb3 | 73.6 | 59.1 | 64.9 | 66.1 | 59.5 | 76.5 | 55.2 | 55.7 | 53.5 | 70.5 | 62.1 | 68.6 |
| Ndufb4 | 143.2 | 130.5 | 124.7 | 132.0 | 113.9 | 147.8 | 121.4 | 126.9 | 117.3 | 128.8 | 142.1 | 134.0 |
| Ndufb5 | 49.1 | 42.6 | 38.9 | 42.5 | 42.5 | 47.7 | 33.3 | 41.9 | 38.2 | 49.1 | 40.8 | 41.3 |
| Ndufb6 | 89.5 | 83.1 | 61.7 | 75.9 | 75.7 | 76.9 | 50.7 | 46.3 | 69.1 | 74.5 | 61.8 | 61.2 |
| Ndufb7 | 86.8 | 74.6 | 69.4 | 95.4 | 81.7 | 86.9 | 85.5 | 76.0 | 70.6 | 81.3 | 77.2 | 80.6 |
| Ndufb8 | 106.5 | 99.2 | 84.6 | 102.9 | 95.5 | 102.2 | 92.2 | 78.0 | 78.4 | 84.5 | 88.6 | 81.4 |
| Ndufb9 | 99.3 | 93.9 | 86.3 | 93.2 | 79.7 | 89.4 | 82.3 | 92.6 | 77.3 | 89.8 | 87.7 | 85.0 |
| Ndufc1 | 98.1 | 93.3 | 64.6 | 77.8 | 89.6 | 86.5 | 60.5 | 69.4 | 82.1 | 93.5 | 75.0 | 76.2 |
| Ndufc2 | 51.6 | 56.4 | 49.5 | 51.9 | 56.1 | 53.6 | 46.8 | 45.2 | 51.9 | 62.0 | 59.2 | 52.2 |
| Ndufs1 | 31.7 | 31.1 | 28.5 | 30.3 | 28.5 | 29.9 | 27.5 | 28.1 | 27.3 | 30.2 | 31.0 | 31.0 |
| Ndufs2 | 79.3 | 76.0 | 68.5 | 74.8 | 72.0 | 73.5 | 68.1 | 72.9 | 72.0 | 85.5 | 79.8 | 77.0 |
| Ndufs3 | 56.3 | 52.5 | 43.2 | 45.5 | 48.0 | 47.8 | 43.8 | 43.8 | 46.1 | 49.8 | 43.5 | 43.2 |
| Ndufs4 | 32.1 | 29.9 | 24.2 | 23.7 | 26.3 | 25.4 | 21.8 | 25.8 | 27.4 | 31.1 | 25.5 | 28.6 |
| Ndufs5 | 47.2 | 45.0 | 33.5 | 54.1 | 44.0 | 47.1 | 45.1 | 42.9 | 43.4 | 44.0 | 39.3 | 42.4 |
| Ndufs6 | 63.4 | 57.5 | 59.4 | 71.1 | 56.4 | 62.0 | 58.2 | 67.2 | 55.4 | 64.8 | 58.5 | 60.2 |
| Ndufs7 | 36.1 | 36.1 | 27.5 | 35.4 | 30.6 | 40.1 | 30.7 | 36.1 | 36.9 | 33.3 | 32.1 | 27.3 |
| Ndufs8 | 26.6 | 17.9 | 18.2 | 26.5 | 23.3 | 24.0 | 24.8 | 32.1 | 24.0 | 25.4 | 32.2 | 23.9 |
| Ndufv1 | 33.4 | 32.6 | 28.5 | 33.4 | 30.1 | 29.9 | 30.1 | 29.6 | 30.6 | 31.5 | 26.6 | 27.6 |
| Ndufv2 | 34.3 | 35.9 | 31.2 | 32.7 | 30.4 | 33.7 | 29.8 | 36.2 | 30.2 | 36.3 | 38.2 | 32.9 |
| Ndufv3 | 98.5 | 91.5 | 92.6 | 98.1 | 85.3 | 95.7 | 87.6 | 113.8 | 88.9 | 102.3 | 103.1 | 104.7 |
| Neat1 | 62.8 | 101.6 | 99.0 | 60.7 | 76.8 | 84.3 | 81.9 | 79.1 | 88.3 | 60.9 | 53.4 | 74.1 |
| Neb | 0.4 | 0.6 | 0.6 | 0.4 | 0.4 | 0.5 | 0.4 | 0.3 | 0.3 | 0.3 | 0.4 | 0.3 |
| Nebl | 0.1 | 0.1 | 0.0 | 0.1 | 0.0 | 0.2 | 0.1 | 0.0 | 0.0 | 0.2 | 0.1 | 0.1 |
| Necab1 | 0.0 | 0.0 | 0.0 | 0.0 | 0.0 | 0.1 | 0.1 | 0.0 | 0.0 | 0.0 | 0.0 | 0.0 |
| Necab2 | 0.0 | 0.0 | 0.0 | 0.0 | 0.0 | 0.0 | 0.0 | 0.1 | 0.0 | 0.0 | 0.0 | 0.1 |
| Necab3 | 0.2 | 0.2 | 0.2 | 0.1 | 0.2 | 0.0 | 0.3 | 0.1 | 0.1 | 0.1 | 0.1 | 0.1 |
| Necap1 | 17.2 | 16.6 | 14.7 | 19.7 | 16.1 | 17.0 | 15.4 | 13.7 | 13.5 | 13.9 | 16.1 | 13.8 |
| Necap2 | 45.2 | 38.9 | 32.7 | 47.6 | 44.1 | 50.2 | 41.3 | 34.1 | 37.5 | 36.1 | 37.2 | 35.0 |
| Nedd1 | 6.8 | 6.9 | 7.5 | 5.6 | 7.2 | 6.2 | 6.5 | 6.6 | 7.4 | 7.4 | 7.7 | 6.8 |
| Nedd4 | 296.0 | 301.2 | 348.6 | 259.7 | 292.7 | 292.2 | 329.2 | 404.4 | 355.4 | 368.7 | 380.4 | 392.9 |
| Nedd4l | 12.1 | 12.3 | 10.9 | 17.3 | 12.9 | 16.9 | 9.8 | 6.6 | 5.7 | 8.2 | 8.0 | 7.2 |
| Nedd8 | 154.2 | 132.1 | 124.7 | 188.0 | 143.6 | 155.1 | 150.6 | 146.6 | 132.6 | 147.2 | 154.3 | 147.6 |
| Nedd9 | 30.1 | 34.3 | 39.9 | 41.1 | 37.3 | 28.8 | 44.7 | 38.3 | 50.7 | 28.9 | 37.3 | 52.0 |
| Nefh | 0.0 | 0.0 | 0.0 | 0.0 | 0.0 | 0.0 | 0.0 | 0.0 | 0.0 | 0.0 | 0.0 | 0.0 |
| Nefl | 0.0 | 0.0 | 0.0 | 0.0 | 0.0 | 0.1 | 0.0 | 0.0 | 0.0 | 0.0 | 0.0 | 0.0 |

| | | | | | | | | | | | | |
|---|---|---|---|---|---|---|---|---|---|---|---|---|
| Nefm | 0.1 | 0.1 | 0.0 | 0.1 | 0.0 | 0.1 | 0.3 | 0.1 | 0.1 | 0.0 | 0.1 | 0.1 |
| Negr1 | 0.2 | 0.4 | 0.6 | 0.1 | 0.3 | 0.4 | 0.2 | 0.5 | 0.2 | 0.1 | 0.2 | 0.2 |
| Neil1 | 2.0 | 2.7 | 2.9 | 1.9 | 2.3 | 2.6 | 1.9 | 2.0 | 2.1 | 2.0 | 2.3 | 2.6 |
| Neil2 | 1.6 | 1.7 | 1.5 | 1.0 | 1.1 | 2.2 | 0.6 | 0.7 | 0.6 | 0.9 | 0.6 | 0.9 |
| Neil3 | 0.6 | 1.9 | 3.1 | 0.4 | 2.6 | 0.2 | 2.4 | 0.8 | 1.5 | 0.7 | 1.5 | 1.0 |
| Nek1 | 6.5 | 6.8 | 7.7 | 6.1 | 6.1 | 7.3 | 6.0 | 6.3 | 5.5 | 5.6 | 7.3 | 6.6 |
| Nek10 | 0.0 | 0.0 | 0.0 | 0.0 | 0.0 | 0.0 | 0.0 | 0.0 | 0.0 | 0.1 | 0.0 | 0.0 |
| Nek11 | 0.1 | 0.1 | 0.2 | 0.3 | 0.0 | 0.1 | 0.1 | 0.2 | 0.1 | 0.0 | 0.0 | 0.1 |
| Nek2 | 1.4 | 2.4 | 3.4 | 0.4 | 3.7 | 0.9 | 4.6 | 1.6 | 3.6 | 1.4 | 3.2 | 1.8 |
| Nek3 | 1.4 | 1.6 | 2.1 | 1.8 | 1.7 | 1.6 | 2.4 | 1.6 | 1.7 | 1.9 | 1.9 | 1.6 |
| Nek4 | 5.6 | 5.8 | 5.1 | 6.1 | 4.3 | 6.2 | 4.6 | 4.3 | 4.1 | 4.5 | 5.4 | 6.0 |
| Nek5 | 0.2 | 0.1 | 0.1 | 0.2 | 0.1 | 0.3 | 0.2 | 0.1 | 0.0 | 0.1 | 0.1 | 0.1 |
| Nek6 | 26.0 | 34.0 | 35.8 | 27.7 | 32.9 | 25.8 | 32.5 | 32.5 | 36.4 | 36.2 | 29.5 | 37.4 |
| Nek7 | 45.2 | 37.3 | 33.8 | 46.8 | 36.1 | 48.6 | 39.8 | 28.9 | 35.3 | 38.7 | 43.9 | 41.8 |
| Nek8 | 4.0 | 3.8 | 2.9 | 4.1 | 3.2 | 4.3 | 3.6 | 4.3 | 5.0 | 3.4 | 3.5 | 3.1 |
| Nek9 | 35.6 | 29.8 | 25.2 | 40.5 | 28.5 | 38.4 | 22.9 | 19.9 | 22.6 | 28.1 | 23.8 | 26.9 |
| Nelfa | 5.6 | 5.7 | 6.5 | 4.4 | 5.4 | 4.8 | 6.2 | 6.7 | 5.9 | 5.0 | 6.2 | 5.4 |
| Nelfb | 21.4 | 25.1 | 21.9 | 18.2 | 20.4 | 21.6 | 20.7 | 19.2 | 23.6 | 18.6 | 18.8 | 18.3 |
| Nelfcd | 7.5 | 8.9 | 9.2 | 8.1 | 8.2 | 7.7 | 8.8 | 8.4 | 8.1 | 6.8 | 6.5 | 6.7 |
| Nelfe | 17.2 | 18.1 | 16.6 | 16.0 | 14.4 | 15.6 | 15.9 | 15.3 | 14.6 | 14.1 | 15.3 | 15.2 |
| Nell2 | 0.1 | 0.0 | 0.0 | 0.1 | 0.0 | 0.1 | 0.0 | 0.0 | 0.0 | 0.1 | 0.1 | 0.0 |
| Nemf | 23.0 | 19.9 | 22.8 | 22.5 | 17.5 | 22.4 | 20.2 | 17.9 | 16.3 | 19.3 | 23.1 | 23.8 |
| Nenf | 100.0 | 81.6 | 83.0 | 84.4 | 66.8 | 96.5 | 86.6 | 83.1 | 89.0 | 87.5 | 79.3 | 96.0 |
| Neo1 | 16.6 | 17.4 | 19.3 | 12.1 | 15.1 | 13.6 | 19.7 | 22.1 | 23.9 | 21.9 | 20.8 | 23.3 |
| Nepn | 0.0 | 0.0 | 0.0 | 0.0 | 0.0 | 0.0 | 0.0 | 0.0 | 0.0 | 0.0 | 0.0 | 0.0 |
| Nes | 3.9 | 1.4 | 1.4 | 5.2 | 10.2 | 6.3 | 5.4 | 2.1 | 2.7 | 3.3 | 3.3 | 3.1 |
| Nespas | 0.4 | 0.3 | 0.2 | 0.3 | 0.3 | 0.5 | 0.2 | 0.5 | 0.3 | 0.2 | 0.5 | 0.1 |
| Net1 | 1.3 | 1.1 | 1.5 | 0.7 | 1.2 | 0.8 | 1.4 | 0.8 | 1.1 | 7.6 | 3.1 | 2.2 |
| Neto1 | 0.0 | 0.0 | 0.0 | 0.0 | 0.0 | 0.0 | 0.0 | 0.0 | 0.0 | 0.0 | 0.0 | 0.0 |
| Neto2 | 0.6 | 0.4 | 0.6 | 0.6 | 0.6 | 0.6 | 0.5 | 0.4 | 0.3 | 0.8 | 0.7 | 0.6 |
| Neu1 | 8.1 | 7.6 | 6.2 | 9.5 | 8.7 | 10.4 | 5.5 | 5.5 | 5.4 | 6.2 | 5.2 | 6.7 |
| Neu3 | 1.3 | 1.7 | 1.6 | 1.1 | 1.3 | 1.4 | 1.8 | 1.7 | 1.9 | 2.1 | 1.9 | 2.2 |
| Neurl1a | 0.5 | 0.4 | 0.3 | 0.4 | 0.3 | 0.5 | 0.3 | 0.4 | 0.3 | 0.4 | 0.5 | 0.2 |
| Neurl1b | 0.5 | 1.2 | 1.9 | 0.2 | 1.6 | 0.3 | 1.6 | 0.8 | 1.6 | 1.1 | 1.2 | 0.8 |
| Neurl2 | 1.4 | 0.9 | 1.0 | 1.5 | 1.1 | 2.0 | 1.4 | 0.9 | 1.0 | 1.6 | 1.3 | 1.0 |
| Neurl3 | 1.0 | 0.2 | 0.4 | 4.3 | 10.5 | 0.7 | 1.3 | 0.3 | 0.4 | 1.2 | 0.5 | 0.6 |
| Neurl4 | 3.5 | 3.9 | 4.1 | 4.0 | 3.6 | 3.3 | 3.8 | 4.0 | 4.4 | 3.9 | 3.6 | 3.4 |
| Nexn | 10.6 | 7.5 | 9.7 | 16.4 | 8.6 | 13.6 | 14.3 | 8.1 | 7.9 | 7.0 | 14.0 | 13.6 |
| Nf1 | 4.2 | 5.2 | 4.6 | 4.2 | 4.7 | 4.9 | 4.3 | 4.4 | 4.1 | 4.8 | 4.6 | 4.4 |
| Nf2 | 24.3 | 21.4 | 21.0 | 28.3 | 19.2 | 24.6 | 21.6 | 14.2 | 14.0 | 18.1 | 22.4 | 19.9 |
| Nfam1 | 0.3 | 0.1 | 0.2 | 2.4 | 4.2 | 0.1 | 0.5 | 0.1 | 0.0 | 0.2 | 0.1 | 0.3 |
| Nfasc | 0.2 | 0.3 | 0.5 | 0.1 | 0.3 | 0.1 | 0.2 | 0.5 | 0.1 | 0.2 | 0.1 | 0.2 |
| Nfat5 | 3.4 | 4.0 | 3.9 | 4.5 | 3.7 | 4.1 | 4.4 | 3.4 | 3.4 | 3.2 | 3.1 | 3.0 |
| Nfatc1 | 1.6 | 2.1 | 2.1 | 2.7 | 2.8 | 2.5 | 2.8 | 2.3 | 2.0 | 1.6 | 1.6 | 2.0 |
| Nfatc2 | 0.1 | 0.1 | 0.0 | 0.2 | 0.4 | 0.0 | 0.0 | 0.0 | 0.0 | 0.0 | 0.0 | 0.0 |
| Nfatc2ip | 0.5 | 0.5 | 0.7 | 0.5 | 0.5 | 0.5 | 0.5 | 0.5 | 0.4 | 0.5 | 0.5 | 0.7 |
| Nfatc3 | 8.2 | 9.8 | 9.1 | 7.3 | 7.8 | 8.8 | 8.8 | 8.9 | 8.6 | 8.4 | 8.9 | 8.2 |
| Nfatc4 | 3.1 | 3.8 | 4.3 | 3.0 | 2.7 | 3.9 | 3.7 | 4.9 | 4.4 | 4.8 | 3.5 | 4.4 |
| Nfe2 | 0.1 | 0.0 | 0.2 | 0.0 | 0.2 | 0.1 | 0.3 | 0.1 | 0.1 | 0.1 | 0.0 | 0.1 |
| Nfe2l1 | 126.1 | 86.4 | 83.8 | 165.7 | 92.4 | 167.4 | 96.8 | 101.5 | 80.2 | 69.0 | 88.0 | 85.4 |
| Nfe2l2 | 61.6 | 54.8 | 57.3 | 78.9 | 69.1 | 60.9 | 65.8 | 54.0 | 50.9 | 65.3 | 66.6 | 71.2 |
| Nfe2l3 | 0.6 | 0.5 | 0.5 | 0.5 | 0.4 | 0.3 | 0.5 | 0.6 | 0.5 | 2.4 | 1.4 | 0.7 |
| Nfia | 6.3 | 9.2 | 11.6 | 6.2 | 6.1 | 6.2 | 6.7 | 8.9 | 6.2 | 4.4 | 5.9 | 6.3 |
| Nfib | 11.0 | 13.3 | 14.5 | 13.4 | 10.9 | 14.2 | 12.1 | 13.6 | 9.4 | 12.4 | 11.2 | 10.8 |
| Nfic | 3.9 | 5.0 | 5.6 | 5.0 | 5.6 | 4.9 | 6.6 | 6.3 | 6.1 | 5.0 | 5.2 | 4.6 |
| Nfil3 | 4.8 | 4.6 | 6.0 | 2.9 | 5.7 | 2.3 | 8.0 | 9.0 | 8.0 | 6.4 | 6.4 | 6.7 |
| Nfix | 10.6 | 14.2 | 16.3 | 10.9 | 11.7 | 12.6 | 14.0 | 17.6 | 15.0 | 11.4 | 12.0 | 12.1 |

| | | | | | | | | | | | | |
|---|---|---|---|---|---|---|---|---|---|---|---|---|
| Nfkb1 | 20.2 | 23.7 | 26.1 | 21.8 | 27.0 | 19.8 | 28.1 | 29.4 | 27.9 | 18.1 | 22.1 | 21.5 |
| Nfkb2 | 15.9 | 14.5 | 16.6 | 16.0 | 23.5 | 15.1 | 24.1 | 16.2 | 18.6 | 16.0 | 16.0 | 16.8 |
| Nfkbia | 20.7 | 18.9 | 30.2 | 25.6 | 44.2 | 15.5 | 32.3 | 25.4 | 23.1 | 25.0 | 21.3 | 26.9 |
| Nfkbib | 6.9 | 6.7 | 5.8 | 8.0 | 7.5 | 6.0 | 8.7 | 8.5 | 7.1 | 6.2 | 6.4 | 6.4 |
| Nfkbid | 0.2 | 0.1 | 0.2 | 0.3 | 0.8 | 0.1 | 0.2 | 0.2 | 0.1 | 0.2 | 0.2 | 0.1 |
| Nfkbie | 4.6 | 2.8 | 3.7 | 7.2 | 9.8 | 6.8 | 6.7 | 4.0 | 3.8 | 3.4 | 3.5 | 3.7 |
| Nfkbil1 | 5.7 | 4.6 | 5.5 | 4.9 | 4.4 | 5.5 | 4.1 | 5.0 | 3.8 | 4.7 | 5.1 | 4.9 |
| Nfkbiz | 9.0 | 8.1 | 8.4 | 7.4 | 11.9 | 6.8 | 11.8 | 10.8 | 8.4 | 12.3 | 9.8 | 8.4 |
| Nfrkb | 2.7 | 2.4 | 2.8 | 2.5 | 3.0 | 2.6 | 3.2 | 2.9 | 2.5 | 2.7 | 2.8 | 2.0 |
| Nfs1 | 18.5 | 19.4 | 17.3 | 16.8 | 17.3 | 18.0 | 15.4 | 17.0 | 16.9 | 16.4 | 17.6 | 16.4 |
| Nfu1 | 35.0 | 31.5 | 28.7 | 43.2 | 29.9 | 37.3 | 29.5 | 27.3 | 30.6 | 29.4 | 29.9 | 30.8 |
| Nfx1 | 13.7 | 13.6 | 13.6 | 12.4 | 12.0 | 13.3 | 12.4 | 12.3 | 11.6 | 12.3 | 13.9 | 13.3 |
| Nfxl1 | 3.4 | 3.8 | 2.9 | 3.2 | 3.6 | 2.6 | 2.8 | 3.8 | 3.9 | 3.9 | 3.9 | 3.6 |
| Nfya | 3.7 | 4.3 | 4.9 | 3.9 | 6.1 | 3.8 | 4.4 | 5.0 | 4.9 | 4.4 | 4.5 | 4.2 |
| Nfyb | 10.8 | 9.3 | 8.8 | 10.4 | 7.9 | 10.5 | 9.7 | 7.1 | 7.2 | 8.9 | 9.5 | 11.9 |
| Nfyc | 7.3 | 7.7 | 8.0 | 7.7 | 7.7 | 6.7 | 9.1 | 11.1 | 8.9 | 8.3 | 8.7 | 8.0 |
| Ngb | 0.1 | 0.2 | 0.1 | 0.1 | 0.0 | 0.1 | 0.1 | 0.1 | 0.1 | 0.1 | 0.0 | 0.0 |
| Ngdn | 15.8 | 14.4 | 11.5 | 14.5 | 14.1 | 13.0 | 14.0 | 13.5 | 14.2 | 15.7 | 15.3 | 14.8 |
| Ngef | 0.1 | 0.1 | 0.2 | 0.0 | 0.1 | 0.1 | 0.1 | 0.1 | 0.1 | 0.2 | 0.1 | 0.2 |
| Ngf | 190.9 | 185.3 | 137.9 | 302.9 | 167.3 | 273.0 | 151.4 | 77.6 | 80.0 | 102.6 | 104.3 | 120.8 |
| Ngfr | 0.2 | 0.1 | 0.1 | 0.1 | 0.0 | 0.1 | 0.0 | 0.1 | 0.0 | 0.0 | 0.0 | 0.1 |
| Ngfrap1 | 37.8 | 24.8 | 28.6 | 39.2 | 22.5 | 44.8 | 34.2 | 25.2 | 21.8 | 35.5 | 37.6 | 30.9 |
| Ngly1 | 11.0 | 9.8 | 10.3 | 8.3 | 8.9 | 8.8 | 9.2 | 9.4 | 9.8 | 11.4 | 11.6 | 11.4 |
| Ngrn | 8.7 | 8.5 | 8.1 | 9.4 | 7.6 | 12.6 | 6.0 | 6.3 | 7.9 | 9.3 | 8.4 | 9.4 |
| Nhej1 | 3.6 | 4.0 | 1.8 | 4.1 | 3.3 | 4.2 | 3.6 | 4.9 | 3.7 | 3.9 | 3.1 | 3.1 |
| Nhlh1 | 0.0 | 0.1 | 0.0 | 0.0 | 0.0 | 0.0 | 0.0 | 0.0 | 0.0 | 0.0 | 0.0 | 0.0 |
| Nhlrc1 | 1.1 | 0.8 | 0.5 | 1.5 | 0.9 | 1.5 | 0.8 | 0.6 | 0.8 | 1.0 | 0.9 | 1.3 |
| Nhlrc2 | 11.3 | 10.4 | 9.8 | 12.1 | 9.8 | 13.1 | 9.0 | 8.3 | 7.7 | 8.4 | 8.7 | 7.9 |
| Nhlrc3 | 12.5 | 10.3 | 8.2 | 16.0 | 11.2 | 18.1 | 8.8 | 9.1 | 8.7 | 8.3 | 10.9 | 9.8 |
| Nhlrc4 | 0.1 | 0.1 | 0.1 | 0.0 | 0.1 | 0.1 | 0.0 | 0.0 | 0.0 | 0.0 | 0.0 | 0.0 |
| Nhp2 | 19.8 | 15.9 | 15.5 | 19.7 | 18.8 | 20.2 | 20.8 | 17.5 | 16.0 | 21.5 | 18.8 | 19.2 |
| Nhp2l1 | 45.4 | 45.3 | 40.8 | 46.4 | 45.1 | 44.3 | 46.3 | 43.9 | 42.0 | 52.1 | 47.8 | 44.5 |
| Nhs | 3.4 | 2.9 | 3.6 | 3.3 | 3.8 | 1.4 | 4.8 | 2.5 | 3.4 | 3.1 | 3.6 | 4.5 |
| Nhsl1 | 1.1 | 1.3 | 1.4 | 0.8 | 0.9 | 0.6 | 1.4 | 2.5 | 1.4 | 1.5 | 1.4 | 1.2 |
| Nhsl2 | 0.1 | 0.0 | 0.1 | 0.1 | 0.1 | 0.1 | 0.0 | 0.1 | 0.1 | 0.1 | 0.1 | 0.1 |
| Niacr1 | 0.0 | 0.0 | 0.0 | 0.4 | 0.8 | 0.0 | 0.2 | 0.0 | 0.0 | 0.1 | 0.0 | 0.0 |
| Nicn1 | 14.6 | 12.4 | 12.5 | 13.9 | 9.8 | 17.0 | 11.4 | 11.7 | 11.1 | 10.4 | 11.4 | 10.5 |
| Nid1 | 46.2 | 57.9 | 79.3 | 41.6 | 59.9 | 66.3 | 62.7 | 101.8 | 77.1 | 51.4 | 53.3 | 49.8 |
| Nid2 | 13.4 | 17.3 | 30.0 | 4.9 | 12.8 | 2.3 | 17.6 | 22.2 | 20.7 | 10.8 | 7.7 | 10.6 |
| Nif3l1 | 11.8 | 10.0 | 9.1 | 12.8 | 10.2 | 12.8 | 10.5 | 9.7 | 8.8 | 10.2 | 11.7 | 9.5 |
| Nim1 | 0.7 | 0.8 | 1.1 | 0.6 | 0.6 | 0.9 | 1.1 | 1.2 | 1.2 | 0.6 | 0.9 | 0.8 |
| Nin | 7.1 | 8.0 | 9.6 | 8.3 | 8.5 | 7.0 | 9.6 | 8.5 | 8.4 | 6.0 | 8.3 | 8.1 |
| Ninj1 | 47.8 | 39.9 | 33.2 | 65.1 | 53.8 | 47.4 | 38.2 | 33.2 | 30.0 | 43.2 | 37.9 | 32.6 |
| Ninl | 16.1 | 16.8 | 15.7 | 15.2 | 13.7 | 15.1 | 10.5 | 6.2 | 8.7 | 12.3 | 9.9 | 12.4 |
| Nip7 | 7.6 | 5.6 | 4.9 | 7.3 | 6.8 | 7.1 | 5.9 | 6.7 | 5.6 | 7.2 | 6.4 | 5.5 |
| Nipa1 | 9.4 | 9.1 | 9.3 | 9.2 | 8.4 | 9.2 | 9.8 | 5.5 | 8.6 | 8.9 | 9.9 | 10.1 |
| Nipa2 | 8.4 | 9.7 | 8.9 | 8.4 | 11.0 | 8.2 | 8.2 | 8.8 | 9.9 | 11.2 | 10.4 | 9.9 |
| Nipal1 | 0.1 | 0.1 | 0.1 | 0.0 | 0.2 | 0.1 | 0.3 | 0.2 | 0.2 | 0.3 | 0.2 | 0.3 |
| Nipal2 | 0.1 | 0.1 | 0.1 | 0.0 | 0.1 | 0.1 | 0.1 | 0.0 | 0.0 | 0.1 | 0.1 | 0.1 |
| Nipal3 | 2.4 | 1.5 | 1.1 | 2.8 | 1.4 | 2.7 | 1.3 | 1.5 | 1.2 | 1.8 | 2.0 | 1.5 |
| Nipal4 | 0.9 | 1.4 | 1.6 | 0.8 | 1.5 | 0.4 | 1.3 | 0.8 | 0.8 | 0.7 | 1.1 | 0.8 |
| Nipbl | 8.0 | 9.3 | 9.6 | 7.5 | 8.3 | 8.2 | 8.1 | 9.3 | 8.1 | 8.4 | 8.8 | 9.1 |
| Nipsnap1 | 3.7 | 1.9 | 1.5 | 1.5 | 1.2 | 3.4 | 1.1 | 0.9 | 0.7 | 2.3 | 1.8 | 1.4 |
| Nipsnap3b | 12.3 | 12.0 | 13.5 | 12.4 | 13.2 | 10.6 | 12.8 | 12.8 | 16.1 | 11.2 | 14.9 | 13.4 |
| Nisch | 53.0 | 56.0 | 56.5 | 47.3 | 51.4 | 48.0 | 56.3 | 62.7 | 55.1 | 54.1 | 50.3 | 50.2 |
| Nit1 | 29.7 | 31.7 | 24.7 | 27.4 | 23.3 | 34.3 | 23.1 | 24.2 | 23.9 | 28.2 | 27.0 | 24.2 |
| Nit2 | 13.0 | 13.2 | 9.5 | 11.9 | 9.1 | 14.5 | 7.0 | 9.1 | 9.0 | 12.1 | 9.6 | 10.0 |

| | | | | | | | | | | | | |
|---|---|---|---|---|---|---|---|---|---|---|---|---|
| Nkain1 | 7.8 | 4.1 | 3.1 | 7.9 | 4.7 | 9.7 | 4.6 | 3.2 | 2.8 | 4.4 | 3.8 | 4.2 |
| Nkain2 | 0.7 | 0.8 | 0.7 | 0.8 | 0.5 | 1.2 | 0.6 | 0.5 | 0.4 | 0.4 | 0.6 | 0.4 |
| Nkain3 | 0.3 | 0.1 | 0.1 | 0.3 | 0.3 | 0.1 | 0.2 | 0.3 | 0.4 | 0.6 | 0.3 | 0.4 |
| Nkain4 | 41.8 | 20.6 | 10.8 | 29.9 | 12.3 | 25.2 | 17.0 | 16.4 | 11.3 | 19.9 | 17.0 | 14.7 |
| Nkap | 5.8 | 5.9 | 6.9 | 5.1 | 4.8 | 5.9 | 5.5 | 5.6 | 5.4 | 5.4 | 5.8 | 6.0 |
| Nkapl | 0.4 | 0.6 | 0.6 | 0.3 | 0.3 | 0.3 | 0.5 | 0.4 | 0.2 | 0.4 | 0.6 | 0.7 |
| Nkd1 | 0.8 | 1.4 | 1.1 | 0.5 | 0.4 | 0.5 | 0.9 | 1.1 | 1.1 | 3.0 | 1.6 | 2.0 |
| Nkd2 | 17.6 | 24.4 | 26.1 | 26.0 | 32.1 | 22.8 | 55.4 | 40.6 | 42.6 | 37.5 | 37.9 | 40.9 |
| Nkiras1 | 3.1 | 2.6 | 2.5 | 3.8 | 2.3 | 3.4 | 2.3 | 1.8 | 2.1 | 2.1 | 2.8 | 2.4 |
| Nkiras2 | 4.8 | 4.7 | 4.5 | 5.8 | 4.9 | 5.0 | 4.7 | 4.8 | 5.3 | 4.2 | 5.0 | 4.3 |
| Nkpd1 | 0.0 | 0.0 | 0.0 | 0.0 | 0.0 | 0.1 | 0.0 | 0.0 | 0.0 | 0.0 | 0.0 | 0.0 |
| Nkrf | 1.5 | 1.6 | 1.3 | 1.4 | 1.7 | 1.6 | 1.7 | 1.4 | 1.7 | 1.6 | 1.8 | 1.4 |
| Nktr | 12.1 | 12.7 | 12.0 | 12.2 | 12.3 | 11.2 | 13.1 | 11.9 | 11.8 | 11.3 | 13.0 | 13.4 |
| Nkx2-1 | 0.0 | 0.0 | 0.0 | 0.0 | 0.0 | 0.0 | 0.0 | 0.0 | 0.0 | 0.0 | 0.0 | 0.0 |
| Nkx2-5 | 0.2 | 0.0 | 0.1 | 0.0 | 0.0 | 0.0 | 0.0 | 0.3 | 0.0 | 0.0 | 0.0 | 0.0 |
| Nkx2-9 | 0.3 | 0.3 | 0.1 | 0.1 | 0.3 | 0.1 | 0.1 | 0.0 | 0.1 | 0.1 | 0.2 | 0.0 |
| Nkx6-2 | 0.6 | 0.6 | 0.5 | 0.6 | 0.6 | 0.8 | 0.6 | 0.3 | 0.8 | 0.4 | 0.4 | 0.5 |
| Nle1 | 2.4 | 1.8 | 1.2 | 2.8 | 2.4 | 2.2 | 2.3 | 1.9 | 2.3 | 2.4 | 2.5 | 1.9 |
| Nlgn1 | 0.1 | 0.0 | 0.1 | 0.0 | 0.0 | 0.1 | 0.1 | 0.0 | 0.0 | 0.0 | 0.0 | 0.0 |
| Nlgn2 | 4.0 | 6.1 | 7.9 | 3.7 | 4.8 | 4.1 | 6.3 | 7.3 | 6.8 | 5.4 | 4.3 | 4.7 |
| Nlgn3 | 0.0 | 0.0 | 0.1 | 0.0 | 0.0 | 0.0 | 0.0 | 0.0 | 0.0 | 0.0 | 0.0 | 0.0 |
| Nlk | 4.9 | 4.8 | 4.3 | 6.0 | 6.1 | 5.7 | 4.9 | 4.0 | 4.8 | 5.1 | 5.2 | 4.6 |
| Nln | 12.1 | 9.6 | 8.2 | 12.6 | 10.2 | 12.4 | 9.7 | 8.9 | 9.2 | 9.8 | 9.5 | 9.7 |
| Nlrc3 | 1.3 | 0.5 | 0.4 | 1.3 | 0.6 | 0.9 | 0.3 | 0.3 | 0.3 | 0.6 | 0.3 | 0.4 |
| Nlrc4 | 0.1 | 0.0 | 0.0 | 0.3 | 0.7 | 0.0 | 0.1 | 0.0 | 0.0 | 0.1 | 0.0 | 0.1 |
| Nlrc5 | 1.2 | 1.0 | 0.9 | 1.6 | 1.3 | 0.7 | 0.5 | 0.4 | 0.2 | 0.2 | 0.4 | 0.5 |
| Nlrp10 | 0.1 | 0.0 | 0.0 | 0.1 | 0.0 | 0.0 | 0.0 | 0.0 | 0.0 | 0.0 | 0.1 | 0.0 |
| Nlrp12 | 0.1 | 0.0 | 0.0 | 0.1 | 0.0 | 0.1 | 0.0 | 0.0 | 0.0 | 0.0 | 0.0 | 0.0 |
| Nlrp1a | 0.3 | 0.5 | 0.3 | 0.5 | 1.2 | 0.4 | 0.4 | 0.4 | 0.3 | 0.3 | 0.2 | 0.4 |
| Nlrp1b | 0.1 | 0.0 | 0.0 | 0.5 | 1.3 | 0.0 | 0.1 | 0.0 | 0.0 | 0.0 | 0.0 | 0.0 |
| Nlrp1c-ps | 0.0 | 0.0 | 0.0 | 0.2 | 0.4 | 0.0 | 0.1 | 0.0 | 0.0 | 0.0 | 0.0 | 0.0 |
| Nlrp3 | 0.3 | 0.1 | 0.0 | 1.5 | 3.7 | 0.0 | 0.5 | 0.0 | 0.2 | 0.2 | 0.1 | 0.2 |
| Nlrp4f | 0.1 | 0.0 | 0.0 | 0.0 | 0.0 | 0.0 | 0.0 | 0.0 | 0.0 | 0.0 | 0.0 | 0.0 |
| Nlrp5 | 0.2 | 0.2 | 0.3 | 0.2 | 0.1 | 0.2 | 0.1 | 0.2 | 0.2 | 0.2 | 0.2 | 0.1 |
| Nlrp5-ps | 2.0 | 2.5 | 3.4 | 2.3 | 1.8 | 3.2 | 1.9 | 2.2 | 2.2 | 1.6 | 1.8 | 1.9 |
| Nlrp9b | 0.0 | 0.0 | 0.0 | 0.0 | 0.0 | 0.0 | 0.0 | 0.0 | 0.0 | 0.0 | 0.0 | 0.0 |
| Nlrx1 | 3.6 | 3.4 | 3.6 | 2.9 | 2.9 | 3.8 | 3.3 | 3.1 | 3.3 | 2.8 | 2.6 | 3.1 |
| Nmb | 0.3 | 1.3 | 1.2 | 0.7 | 1.0 | 1.1 | 0.6 | 0.5 | 1.0 | 0.5 | 0.4 | 0.5 |
| Nmd3 | 18.0 | 17.2 | 15.8 | 17.9 | 16.7 | 16.6 | 16.2 | 17.1 | 16.9 | 18.5 | 21.7 | 17.4 |
| Nme1 | 76.1 | 54.4 | 39.2 | 78.1 | 59.0 | 65.1 | 51.8 | 43.2 | 42.1 | 63.3 | 57.5 | 49.1 |
| Nme2 | 806.0 | 551.8 | 415.8 | 619.6 | 504.6 | 640.6 | 461.8 | 503.9 | 507.0 | 601.7 | 568.5 | 548.3 |
| Nme3 | 4.3 | 3.9 | 3.8 | 4.4 | 4.7 | 6.2 | 2.5 | 3.9 | 4.7 | 7.2 | 4.2 | 4.9 |
| Nme4 | 7.6 | 5.6 | 5.0 | 7.4 | 4.4 | 8.8 | 5.5 | 4.8 | 4.6 | 8.5 | 5.2 | 5.7 |
| Nme5 | 1.6 | 0.8 | 1.1 | 1.3 | 0.8 | 2.5 | 1.0 | 1.2 | 0.4 | 1.5 | 1.1 | 0.5 |
| Nme6 | 5.3 | 3.9 | 3.9 | 5.4 | 4.6 | 5.1 | 5.5 | 4.0 | 5.0 | 3.7 | 4.3 | 4.0 |
| Nme7 | 9.7 | 9.7 | 9.2 | 8.6 | 8.9 | 11.4 | 8.4 | 9.0 | 7.7 | 12.6 | 9.4 | 10.3 |
| Nmi | 5.4 | 4.3 | 5.4 | 7.8 | 5.5 | 4.1 | 4.4 | 4.4 | 3.3 | 3.6 | 4.9 | 5.5 |
| Nmnat1 | 4.8 | 5.1 | 3.8 | 4.1 | 5.1 | 4.3 | 4.4 | 4.3 | 5.1 | 4.6 | 4.1 | 4.8 |
| Nmnat2 | 1.4 | 1.1 | 1.3 | 1.1 | 1.0 | 0.9 | 1.9 | 2.1 | 1.2 | 1.0 | 1.6 | 1.1 |
| Nmnat3 | 2.4 | 2.1 | 1.6 | 3.2 | 2.0 | 2.6 | 1.5 | 1.6 | 1.0 | 2.5 | 1.8 | 1.7 |
| Nmral1 | 1.7 | 2.3 | 3.7 | 1.5 | 2.5 | 2.6 | 2.8 | 2.9 | 3.0 | 2.1 | 2.9 | 2.7 |
| Nmrk1 | 3.3 | 3.5 | 3.4 | 4.0 | 2.5 | 3.0 | 2.5 | 3.6 | 2.8 | 2.6 | 3.1 | 3.5 |
| Nmt1 | 67.8 | 62.9 | 67.5 | 78.0 | 70.2 | 68.3 | 77.6 | 72.3 | 65.3 | 64.9 | 71.2 | 72.1 |
| Nmt2 | 19.6 | 20.3 | 16.9 | 18.8 | 16.3 | 19.6 | 18.6 | 13.8 | 16.1 | 17.5 | 19.9 | 18.0 |
| Nnat | 1.1 | 0.7 | 0.6 | 1.2 | 0.7 | 1.1 | 1.1 | 0.6 | 0.8 | 1.3 | 1.6 | 0.9 |
| Nnmt | 44.1 | 40.4 | 38.1 | 48.7 | 38.8 | 38.6 | 39.8 | 44.5 | 39.0 | 38.6 | 35.7 | 31.2 |
| Nnt | 10.5 | 8.4 | 7.7 | 10.3 | 10.1 | 8.6 | 9.4 | 9.6 | 9.0 | 10.6 | 12.2 | 10.1 |

| | | | | | | | | | | | | |
|---|---|---|---|---|---|---|---|---|---|---|---|---|
| Noa1 | 3.5 | 3.2 | 3.3 | 3.8 | 3.7 | 2.8 | 3.0 | 3.8 | 3.1 | 3.3 | 2.9 | 3.4 |
| Nob1 | 6.3 | 6.4 | 5.6 | 6.1 | 7.2 | 7.5 | 7.4 | 6.7 | 7.1 | 9.9 | 8.4 | 5.8 |
| Noc2l | 13.9 | 11.3 | 11.0 | 16.5 | 12.9 | 15.5 | 13.6 | 12.6 | 9.8 | 13.2 | 13.3 | 11.7 |
| Noc3l | 7.7 | 6.5 | 6.1 | 8.4 | 6.7 | 6.8 | 7.2 | 6.1 | 5.7 | 7.0 | 8.1 | 7.7 |
| Noc4l | 5.3 | 5.4 | 4.2 | 4.8 | 4.8 | 4.2 | 5.3 | 5.3 | 4.6 | 4.2 | 3.9 | 4.1 |
| Nod1 | 20.8 | 26.1 | 27.8 | 13.5 | 20.7 | 12.3 | 24.4 | 25.2 | 23.1 | 28.3 | 23.7 | 25.6 |
| Nod2 | 0.1 | 0.0 | 0.1 | 0.1 | 0.3 | 0.0 | 0.1 | 0.0 | 0.0 | 0.0 | 0.0 | 0.0 |
| Nodal | 0.0 | 0.1 | 0.0 | 0.0 | 0.0 | 0.0 | 0.0 | 0.0 | 0.0 | 0.0 | 0.0 | 0.0 |
| Nog | 1.2 | 0.7 | 0.9 | 0.7 | 0.6 | 0.3 | 0.5 | 0.3 | 0.1 | 0.4 | 0.4 | 0.3 |
| Nol10 | 4.0 | 4.2 | 4.2 | 4.1 | 4.2 | 3.9 | 4.7 | 4.1 | 3.9 | 3.6 | 4.8 | 4.0 |
| Nol11 | 9.3 | 7.8 | 8.0 | 9.4 | 8.7 | 9.2 | 8.4 | 9.0 | 8.1 | 9.2 | 9.2 | 9.1 |
| Nol12 | 6.8 | 7.4 | 5.8 | 7.2 | 5.8 | 5.8 | 6.6 | 7.8 | 6.2 | 7.1 | 8.8 | 7.4 |
| Nol3 | 3.7 | 4.1 | 5.4 | 4.6 | 4.1 | 5.2 | 5.1 | 6.0 | 5.0 | 3.7 | 4.4 | 5.9 |
| Nol4 | 0.0 | 0.0 | 0.0 | 0.0 | 0.0 | 0.0 | 0.0 | 0.0 | 0.0 | 0.0 | 0.0 | 0.0 |
| Nol6 | 7.0 | 6.2 | 5.2 | 7.9 | 7.4 | 6.8 | 6.5 | 6.2 | 5.0 | 6.6 | 5.4 | 4.9 |
| Nol7 | 9.8 | 7.7 | 7.9 | 11.6 | 8.7 | 10.5 | 8.8 | 9.2 | 8.0 | 9.7 | 12.1 | 9.7 |
| Nol8 | 9.8 | 9.3 | 7.7 | 11.4 | 8.3 | 9.8 | 7.8 | 6.9 | 6.9 | 8.7 | 9.5 | 9.1 |
| Nol9 | 6.8 | 7.0 | 7.4 | 6.7 | 7.7 | 6.4 | 8.8 | 9.0 | 7.0 | 6.9 | 7.0 | 8.4 |
| Nolc1 | 15.3 | 11.9 | 13.3 | 18.4 | 14.1 | 13.5 | 14.8 | 14.0 | 10.7 | 12.6 | 14.4 | 13.4 |
| Nom1 | 11.1 | 9.5 | 9.2 | 10.8 | 9.3 | 10.2 | 9.8 | 8.7 | 8.6 | 9.4 | 9.4 | 10.3 |
| Nomo1 | 46.5 | 44.7 | 39.0 | 39.8 | 41.1 | 44.7 | 40.2 | 35.5 | 34.4 | 34.9 | 32.8 | 30.7 |
| Nono | 69.8 | 67.3 | 80.7 | 85.0 | 66.9 | 72.9 | 86.6 | 85.5 | 72.3 | 80.9 | 91.3 | 86.9 |
| Nop10 | 46.9 | 37.8 | 33.5 | 52.8 | 43.0 | 48.1 | 37.9 | 41.0 | 40.8 | 48.5 | 44.9 | 37.9 |
| Nop14 | 17.1 | 14.9 | 15.0 | 21.1 | 13.9 | 17.1 | 17.9 | 14.7 | 11.7 | 15.1 | 18.8 | 19.1 |
| Nop16 | 12.6 | 9.5 | 9.5 | 12.4 | 10.5 | 11.0 | 10.2 | 9.1 | 9.1 | 12.4 | 11.6 | 10.2 |
| Nop2 | 17.8 | 17.2 | 13.5 | 19.6 | 16.3 | 18.8 | 18.9 | 14.3 | 13.3 | 17.4 | 18.9 | 15.2 |
| Nop56 | 33.4 | 29.9 | 27.0 | 32.5 | 30.3 | 28.6 | 37.9 | 30.5 | 28.1 | 30.4 | 36.3 | 30.0 |
| Nop58 | 29.0 | 25.2 | 22.6 | 27.9 | 28.6 | 19.6 | 29.7 | 25.7 | 25.8 | 31.4 | 35.7 | 28.4 |
| Nop9 | 5.2 | 5.1 | 4.4 | 4.0 | 5.0 | 4.8 | 4.4 | 4.3 | 5.2 | 5.1 | 4.1 | 4.0 |
| Nos1 | 0.0 | 0.1 | 0.0 | 0.0 | 0.0 | 0.0 | 0.0 | 0.0 | 0.0 | 0.0 | 0.0 | 0.0 |
| Nos1ap | 0.1 | 0.1 | 0.3 | 0.1 | 0.3 | 0.1 | 0.1 | 0.1 | 0.0 | 0.3 | 0.1 | 0.2 |
| Nos2 | 0.1 | 0.0 | 0.1 | 0.0 | 0.0 | 0.0 | 0.0 | 0.2 | 0.2 | 0.0 | 0.0 | 0.0 |
| Nos3 | 0.2 | 0.1 | 0.1 | 0.1 | 0.0 | 0.1 | 0.1 | 0.3 | 0.2 | 0.0 | 0.0 | 0.0 |
| Nosip | 10.3 | 10.1 | 9.3 | 11.1 | 9.0 | 10.8 | 10.0 | 10.7 | 9.9 | 8.9 | 9.9 | 10.4 |
| Nostrin | 0.5 | 0.1 | 0.1 | 1.9 | 2.6 | 0.3 | 0.2 | 0.1 | 0.2 | 0.3 | 0.1 | 0.2 |
| Notch1 | 0.9 | 1.8 | 2.4 | 0.9 | 1.9 | 1.0 | 2.3 | 2.9 | 2.3 | 1.5 | 1.5 | 1.3 |
| Notch2 | 13.6 | 18.8 | 20.6 | 12.4 | 20.4 | 12.0 | 25.6 | 24.3 | 23.1 | 16.7 | 17.2 | 17.5 |
| Notch3 | 2.9 | 5.9 | 5.8 | 1.9 | 4.0 | 2.7 | 4.1 | 3.4 | 5.3 | 3.2 | 1.9 | 2.8 |
| Notch4 | 1.2 | 0.7 | 0.7 | 2.0 | 2.5 | 1.2 | 2.6 | 1.4 | 1.7 | 1.1 | 0.8 | 0.9 |
| Notum | 0.0 | 0.1 | 0.1 | 0.0 | 0.0 | 0.1 | 0.0 | 0.0 | 0.0 | 0.1 | 0.0 | 0.0 |
| Nov | 0.8 | 0.5 | 0.8 | 0.6 | 0.6 | 0.5 | 0.5 | 2.4 | 2.2 | 8.8 | 21.1 | 3.5 |
| Nova1 | 0.0 | 0.1 | 0.1 | 0.0 | 0.1 | 0.0 | 0.1 | 0.1 | 0.0 | 0.1 | 0.1 | 0.1 |
| Nova2 | 0.0 | 0.0 | 0.0 | 0.1 | 0.0 | 0.1 | 0.0 | 0.0 | 0.0 | 0.0 | 0.0 | 0.0 |
| Nox1 | 0.0 | 0.0 | 0.0 | 0.7 | 0.8 | 0.0 | 0.3 | 0.0 | 0.0 | 0.1 | 0.0 | 0.0 |
| Nox4 | 4.5 | 3.1 | 3.6 | 2.8 | 3.0 | 3.3 | 3.6 | 3.4 | 4.0 | 2.8 | 3.3 | 3.8 |
| Noxo1 | 0.5 | 0.6 | 0.5 | 0.5 | 0.6 | 0.8 | 0.6 | 0.7 | 0.7 | 0.4 | 0.4 | 0.5 |
| Noxred1 | 0.3 | 0.2 | 0.3 | 0.7 | 0.2 | 0.2 | 0.0 | 0.3 | 0.3 | 0.3 | 0.2 | 0.2 |
| Npas1 | 0.1 | 0.2 | 0.1 | 0.2 | 0.2 | 0.1 | 0.1 | 0.1 | 0.3 | 0.2 | 0.0 | 0.1 |
| Npas2 | 0.1 | 0.2 | 0.4 | 0.0 | 0.2 | 0.1 | 0.1 | 0.3 | 0.3 | 0.1 | 0.1 | 0.1 |
| Npas3 | 0.9 | 0.8 | 0.8 | 0.9 | 0.7 | 0.6 | 0.8 | 0.8 | 0.6 | 1.4 | 1.1 | 0.9 |
| Npas4 | 0.0 | 0.0 | 0.0 | 0.0 | 0.0 | 0.0 | 0.0 | 0.0 | 0.0 | 0.1 | 0.1 | 0.0 |
| Npat | 3.9 | 4.8 | 4.7 | 3.7 | 3.9 | 3.9 | 4.1 | 4.3 | 4.2 | 3.9 | 4.9 | 4.5 |
| Npb | 0.1 | 0.0 | 0.1 | 0.0 | 0.0 | 0.0 | 0.0 | 0.0 | 0.0 | 0.0 | 0.3 | 0.0 |
| Npc1 | 8.5 | 9.8 | 9.7 | 11.2 | 13.4 | 10.7 | 10.2 | 12.1 | 11.1 | 10.2 | 9.0 | 10.4 |
| Npc2 | 157.3 | 123.7 | 111.0 | 168.7 | 133.3 | 171.5 | 111.0 | 102.7 | 111.0 | 163.0 | 136.8 | 143.4 |
| Npcd | 0.2 | 0.5 | 0.5 | 0.5 | 0.5 | 0.6 | 0.5 | 0.2 | 0.2 | 0.2 | 0.2 | 0.4 |
| Npdc1 | 70.8 | 65.2 | 59.6 | 76.7 | 59.0 | 84.8 | 64.6 | 68.8 | 63.4 | 60.3 | 56.3 | 56.3 |

| | | | | | | | | | | | | |
|---|---|---|---|---|---|---|---|---|---|---|---|---|
| Npepl1 | 14.0 | 13.8 | 13.5 | 12.5 | 13.9 | 13.3 | 14.5 | 14.0 | 15.9 | 15.7 | 14.2 | 13.5 |
| Npepps | 12.0 | 11.4 | 10.8 | 10.2 | 10.4 | 12.8 | 10.8 | 10.0 | 12.2 | 13.8 | 11.6 | 13.1 |
| Npff | 0.1 | 0.3 | 0.2 | 0.2 | 0.3 | 0.1 | 0.2 | 0.4 | 0.3 | 0.2 | 0.4 | 0.6 |
| Npffr2 | 0.0 | 0.0 | 0.0 | 0.0 | 0.0 | 0.0 | 0.0 | 0.0 | 0.0 | 0.0 | 0.0 | 0.0 |
| Nphp1 | 9.2 | 8.8 | 10.1 | 9.1 | 6.9 | 9.6 | 8.6 | 9.3 | 7.1 | 7.2 | 8.9 | 8.2 |
| Nphp3 | 1.3 | 1.4 | 1.9 | 1.0 | 0.9 | 1.1 | 1.6 | 1.8 | 1.5 | 2.0 | 1.1 | 1.5 |
| Nphp4 | 0.5 | 0.4 | 0.6 | 0.5 | 0.4 | 0.7 | 0.6 | 0.4 | 0.4 | 0.3 | 0.3 | 0.4 |
| Nphs1 | 0.1 | 0.0 | 0.1 | 0.1 | 0.1 | 0.0 | 0.0 | 0.0 | 0.0 | 0.0 | 0.1 | 0.0 |
| Nphs2 | 0.0 | 0.0 | 0.0 | 0.0 | 0.0 | 0.1 | 0.0 | 0.0 | 0.0 | 0.0 | 0.0 | 0.0 |
| Npl | 0.2 | 0.3 | 0.5 | 0.4 | 0.5 | 0.6 | 0.4 | 1.2 | 1.0 | 5.3 | 1.9 | 2.6 |
| Nploc4 | 19.4 | 20.2 | 17.7 | 21.6 | 20.4 | 20.5 | 21.1 | 19.7 | 20.3 | 19.5 | 20.0 | 19.0 |
| Npm1 | 498.1 | 335.8 | 340.2 | 476.0 | 332.0 | 410.7 | 353.1 | 333.7 | 276.0 | 416.4 | 472.9 | 435.1 |
| Npm2 | 0.2 | 0.2 | 0.2 | 0.0 | 0.2 | 0.0 | 0.0 | 0.0 | 0.0 | 0.0 | 0.0 | 0.1 |
| Npm3 | 17.7 | 13.9 | 12.2 | 12.3 | 15.6 | 16.7 | 14.2 | 14.2 | 16.2 | 19.2 | 17.1 | 13.6 |
| Npm3-ps1 | 0.5 | 0.2 | 0.4 | 0.6 | 0.5 | 0.4 | 0.8 | 0.5 | 0.5 | 0.4 | 0.5 | 0.5 |
| Npnt | 2.6 | 1.6 | 2.2 | 0.7 | 1.2 | 1.2 | 2.4 | 1.0 | 1.7 | 3.6 | 3.7 | 2.6 |
| Nppb | 0.7 | 0.1 | 0.1 | 0.5 | 0.3 | 0.2 | 0.2 | 0.0 | 0.3 | 0.4 | 1.7 | 0.0 |
| Npr1 | 0.1 | 0.1 | 0.2 | 0.0 | 0.0 | 0.0 | 0.0 | 0.1 | 0.1 | 0.6 | 0.2 | 0.2 |
| Npr2 | 19.7 | 17.7 | 16.6 | 16.2 | 13.2 | 20.0 | 14.7 | 11.6 | 12.4 | 10.9 | 12.6 | 12.0 |
| Npr3 | 11.2 | 15.7 | 34.2 | 7.1 | 6.8 | 3.5 | 8.2 | 13.2 | 7.4 | 8.6 | 11.1 | 18.4 |
| Nprl2 | 7.8 | 8.7 | 7.2 | 7.4 | 6.4 | 9.0 | 6.5 | 6.4 | 6.4 | 7.6 | 6.1 | 7.1 |
| Nprl3 | 8.2 | 7.8 | 6.1 | 7.0 | 7.0 | 7.9 | 6.6 | 7.3 | 6.0 | 5.7 | 6.9 | 5.7 |
| Nps | 0.0 | 0.0 | 0.0 | 0.0 | 0.0 | 0.1 | 0.0 | 0.1 | 0.0 | 0.0 | 0.0 | 0.0 |
| Npsr1 | 0.1 | 0.0 | 0.0 | 0.1 | 0.1 | 0.1 | 0.1 | 0.0 | 0.0 | 0.0 | 0.0 | 0.0 |
| Nptn | 103.2 | 104.0 | 103.7 | 120.2 | 118.7 | 100.8 | 129.5 | 118.1 | 130.9 | 126.0 | 150.7 | 143.4 |
| Nptx1 | 0.0 | 0.0 | 0.0 | 0.1 | 0.1 | 0.1 | 0.0 | 0.0 | 0.0 | 0.0 | 0.0 | 0.0 |
| Nptx2 | 0.0 | 0.0 | 0.1 | 0.1 | 0.1 | 0.0 | 0.0 | 0.0 | 0.1 | 0.1 | 0.0 | 0.0 |
| Nptxr | 0.2 | 0.1 | 0.0 | 0.1 | 0.2 | 0.3 | 0.4 | 0.1 | 0.2 | 0.4 | 0.2 | 0.0 |
| Npy | 0.4 | 0.0 | 0.1 | 8.1 | 13.5 | 0.0 | 1.6 | 0.2 | 0.2 | 0.9 | 0.3 | 0.7 |
| Npy1r | 2.6 | 2.1 | 1.6 | 2.0 | 0.7 | 1.0 | 1.2 | 0.7 | 1.1 | 0.3 | 0.5 | 0.6 |
| Npy6r | 0.0 | 0.0 | 0.0 | 0.1 | 0.0 | 0.0 | 0.0 | 0.0 | 0.0 | 0.0 | 0.0 | 0.0 |
| Nqo1 | 91.6 | 52.5 | 22.8 | 146.5 | 56.5 | 136.0 | 15.2 | 33.9 | 8.3 | 18.8 | 18.0 | 12.7 |
| Nqo2 | 5.7 | 5.7 | 5.6 | 5.9 | 6.3 | 5.8 | 5.0 | 5.3 | 5.6 | 4.9 | 6.5 | 5.7 |
| Nr1d1 | 5.7 | 9.8 | 10.0 | 7.1 | 6.9 | 11.0 | 5.5 | 6.4 | 4.7 | 5.3 | 3.8 | 4.1 |
| Nr1d2 | 17.3 | 21.2 | 24.9 | 19.0 | 18.3 | 21.3 | 18.2 | 21.6 | 18.8 | 20.5 | 20.3 | 23.8 |
| Nr1h2 | 16.7 | 16.7 | 14.1 | 19.1 | 15.9 | 19.2 | 16.7 | 18.0 | 15.9 | 15.9 | 15.2 | 14.1 |
| Nr1h3 | 4.0 | 5.3 | 4.9 | 3.5 | 4.8 | 3.2 | 5.3 | 7.8 | 7.4 | 5.2 | 5.1 | 5.3 |
| Nr1h4 | 2.1 | 2.0 | 1.2 | 2.8 | 1.7 | 3.0 | 2.6 | 2.3 | 1.7 | 1.9 | 1.4 | 2.1 |
| Nr1h5 | 0.0 | 0.0 | 0.0 | 0.0 | 0.0 | 0.0 | 0.0 | 0.0 | 0.0 | 0.0 | 0.0 | 0.0 |
| Nr1i2 | 0.0 | 0.0 | 0.0 | 0.0 | 0.1 | 0.0 | 0.0 | 0.0 | 0.0 | 0.0 | 0.0 | 0.0 |
| Nr2c1 | 2.6 | 2.7 | 2.5 | 2.9 | 2.5 | 2.6 | 3.0 | 3.1 | 2.7 | 2.9 | 2.6 | 3.0 |
| Nr2c2 | 3.0 | 3.0 | 3.1 | 2.8 | 2.6 | 3.1 | 3.2 | 3.4 | 3.5 | 2.8 | 3.4 | 3.3 |
| Nr2c2ap | 4.2 | 4.6 | 4.1 | 5.5 | 6.3 | 5.6 | 5.9 | 4.5 | 5.1 | 4.5 | 4.5 | 5.5 |
| Nr2f1 | 0.5 | 0.4 | 0.6 | 0.5 | 0.7 | 0.8 | 0.9 | 0.8 | 1.1 | 0.9 | 0.9 | 0.9 |
| Nr2f2 | 1.5 | 2.1 | 2.7 | 1.9 | 1.6 | 2.0 | 1.9 | 1.9 | 2.2 | 2.5 | 2.1 | 2.0 |
| Nr2f6 | 16.0 | 14.5 | 12.2 | 11.9 | 15.0 | 12.8 | 16.5 | 15.2 | 19.1 | 19.9 | 13.3 | 15.6 |
| Nr3c1 | 22.8 | 25.9 | 26.9 | 23.6 | 22.2 | 26.6 | 20.4 | 23.7 | 22.2 | 22.1 | 23.2 | 25.9 |
| Nr3c2 | 0.9 | 0.9 | 1.0 | 0.8 | 0.5 | 1.2 | 0.5 | 0.7 | 0.3 | 0.4 | 0.3 | 0.6 |
| Nr4a1 | 0.7 | 0.7 | 0.6 | 0.4 | 0.6 | 0.7 | 0.5 | 0.3 | 0.7 | 2.8 | 0.8 | 0.5 |
| Nr4a2 | 0.4 | 0.5 | 0.3 | 0.8 | 0.2 | 0.7 | 0.2 | 0.4 | 0.2 | 0.2 | 0.2 | 0.3 |
| Nr4a3 | 0.0 | 0.0 | 0.0 | 0.0 | 0.1 | 0.0 | 0.0 | 0.0 | 0.0 | 0.3 | 0.1 | 0.0 |
| Nr5a2 | 0.0 | 0.0 | 0.1 | 0.0 | 0.0 | 0.0 | 0.1 | 0.0 | 0.0 | 0.0 | 0.0 | 0.0 |
| Nr6a1 | 0.6 | 0.8 | 0.8 | 0.5 | 0.7 | 0.8 | 0.7 | 0.7 | 0.6 | 0.7 | 1.0 | 0.8 |
| Nradd | 13.1 | 13.4 | 7.8 | 11.6 | 8.8 | 16.9 | 7.9 | 7.8 | 9.8 | 18.7 | 10.4 | 10.5 |
| Nrap | 0.0 | 0.0 | 0.0 | 0.0 | 0.0 | 0.0 | 0.0 | 0.0 | 0.0 | 0.0 | 0.0 | 0.0 |
| Nrarp | 0.0 | 0.1 | 0.0 | 0.0 | 0.0 | 0.0 | 0.1 | 0.2 | 0.1 | 0.0 | 0.0 | 0.0 |
| Nras | 20.5 | 19.0 | 15.4 | 21.6 | 19.1 | 18.2 | 17.0 | 19.4 | 17.6 | 16.6 | 20.6 | 16.6 |

| | | | | | | | | | | | | |
|---|---|---|---|---|---|---|---|---|---|---|---|---|
| Nrbf2 | 10.0 | 11.3 | 11.5 | 8.6 | 10.1 | 8.7 | 10.3 | 10.8 | 9.8 | 11.3 | 11.9 | 12.8 |
| Nrbp1 | 38.1 | 35.8 | 35.2 | 40.0 | 34.4 | 37.8 | 37.3 | 38.9 | 37.6 | 41.1 | 40.8 | 40.6 |
| Nrbp2 | 3.2 | 4.2 | 4.4 | 2.5 | 2.2 | 3.4 | 2.9 | 5.5 | 5.3 | 4.4 | 4.5 | 4.0 |
| Nrcam | 0.3 | 0.2 | 0.2 | 0.4 | 0.3 | 0.3 | 0.2 | 0.1 | 0.1 | 0.2 | 0.2 | 0.1 |
| Nrd1 | 40.1 | 33.2 | 32.3 | 39.4 | 35.4 | 37.1 | 33.9 | 31.1 | 27.3 | 29.3 | 33.9 | 34.7 |
| Nrde2 | 3.0 | 2.6 | 3.0 | 2.8 | 2.7 | 2.7 | 2.7 | 3.6 | 3.0 | 3.2 | 3.6 | 2.7 |
| Nrep | 0.5 | 0.1 | 0.3 | 0.7 | 0.4 | 0.3 | 0.4 | 1.3 | 1.4 | 1.2 | 0.7 | 0.7 |
| Nrf1 | 4.2 | 4.9 | 4.9 | 4.5 | 4.8 | 4.9 | 5.1 | 5.3 | 5.3 | 5.2 | 4.4 | 5.0 |
| Nrg1 | 2.8 | 1.2 | 1.1 | 2.2 | 1.5 | 1.8 | 1.0 | 0.6 | 0.5 | 1.1 | 1.6 | 1.1 |
| Nrg2 | 0.1 | 0.0 | 0.0 | 0.0 | 0.0 | 0.1 | 0.0 | 0.0 | 0.0 | 0.0 | 0.0 | 0.1 |
| Nrg3 | 0.0 | 0.0 | 0.0 | 0.0 | 0.0 | 0.0 | 0.0 | 0.0 | 0.0 | 0.0 | 0.0 | 0.0 |
| Nrg4 | 0.6 | 0.8 | 0.4 | 0.4 | 0.4 | 0.4 | 0.4 | 0.4 | 0.7 | 0.6 | 0.4 | 0.7 |
| Nrip1 | 4.1 | 4.8 | 6.5 | 3.3 | 4.1 | 3.4 | 4.8 | 5.6 | 5.0 | 4.0 | 4.2 | 4.5 |
| Nrip2 | 0.5 | 0.3 | 0.3 | 1.0 | 0.1 | 1.2 | 0.2 | 0.3 | 0.2 | 0.5 | 0.5 | 0.2 |
| Nrip3 | 0.2 | 0.3 | 0.2 | 0.3 | 0.2 | 0.1 | 0.2 | 0.2 | 0.2 | 0.1 | 0.1 | 0.1 |
| Nrk | 0.1 | 0.0 | 0.1 | 0.0 | 0.0 | 0.0 | 0.0 | 0.0 | 0.0 | 0.0 | 0.0 | 0.0 |
| Nrm | 2.9 | 2.7 | 3.4 | 2.2 | 2.7 | 2.8 | 3.3 | 3.4 | 3.9 | 2.6 | 2.6 | 2.2 |
| Nrn1 | 0.9 | 0.3 | 0.3 | 0.1 | 0.3 | 1.0 | 0.3 | 0.2 | 0.4 | 0.3 | 0.2 | 0.4 |
| Nrn1l | 0.5 | 0.7 | 0.0 | 0.4 | 0.3 | 0.3 | 0.2 | 0.1 | 0.4 | 0.3 | 0.2 | 0.1 |
| Nron | 0.0 | 0.1 | 0.1 | 0.0 | 0.0 | 0.0 | 0.1 | 0.1 | 0.0 | 0.1 | 0.0 | 0.1 |
| Nrp | 0.9 | 0.7 | 0.4 | 0.4 | 0.7 | 0.7 | 1.4 | 1.2 | 0.9 | 0.7 | 1.5 | 0.9 |
| Nrp1 | 10.0 | 12.5 | 24.0 | 16.9 | 30.4 | 6.3 | 12.8 | 20.6 | 17.9 | 21.0 | 16.1 | 20.1 |
| Nrp2 | 8.9 | 6.6 | 8.6 | 22.8 | 22.1 | 22.0 | 13.9 | 10.7 | 9.2 | 10.1 | 9.8 | 9.5 |
| Nrsn2 | 0.0 | 0.0 | 0.0 | 0.0 | 0.0 | 0.0 | 0.0 | 0.0 | 0.0 | 0.0 | 0.0 | 0.0 |
| Nrtn | 0.0 | 0.1 | 0.1 | 0.1 | 0.0 | 0.0 | 0.0 | 0.1 | 0.1 | 0.3 | 0.1 | 0.1 |
| Nrxn1 | 0.0 | 0.0 | 0.1 | 0.0 | 0.0 | 0.0 | 0.0 | 0.0 | 0.0 | 0.0 | 0.0 | 0.0 |
| Nrxn2 | 0.2 | 0.2 | 0.4 | 0.3 | 0.2 | 0.4 | 0.2 | 0.3 | 0.1 | 0.1 | 0.1 | 0.1 |
| Nrxn3 | 0.0 | 0.0 | 0.0 | 0.0 | 0.0 | 0.0 | 0.0 | 0.0 | 0.0 | 0.0 | 0.0 | 0.0 |
| Nsa2 | 50.7 | 41.2 | 52.2 | 45.6 | 41.3 | 45.7 | 45.7 | 55.9 | 41.5 | 49.5 | 56.1 | 61.2 |
| Nsd1 | 10.6 | 11.6 | 11.4 | 9.9 | 10.0 | 10.2 | 9.3 | 10.8 | 9.8 | 8.3 | 9.8 | 9.9 |
| Nsdhl | 12.2 | 14.4 | 14.9 | 4.1 | 7.8 | 8.9 | 23.0 | 27.8 | 21.1 | 6.9 | 21.5 | 9.8 |
| Nsf | 13.6 | 12.2 | 11.4 | 14.2 | 15.4 | 11.4 | 13.2 | 12.4 | 11.8 | 12.0 | 13.1 | 12.4 |
| Nsfl1c | 50.2 | 46.1 | 46.7 | 59.4 | 43.2 | 56.3 | 50.5 | 54.6 | 45.2 | 47.6 | 48.2 | 49.3 |
| Nsg1 | 10.3 | 10.1 | 11.6 | 11.7 | 12.7 | 9.7 | 13.7 | 12.4 | 10.7 | 8.1 | 9.4 | 7.8 |
| Nsl1 | 0.9 | 1.3 | 2.0 | 0.5 | 2.0 | 0.4 | 2.5 | 0.8 | 1.5 | 0.9 | 1.5 | 1.1 |
| Nsmaf | 15.2 | 14.8 | 12.8 | 14.6 | 12.0 | 17.2 | 12.2 | 10.6 | 12.5 | 16.5 | 14.1 | 14.6 |
| Nsmce1 | 21.4 | 20.8 | 20.3 | 18.0 | 16.9 | 19.4 | 18.6 | 17.5 | 18.9 | 19.9 | 20.2 | 21.6 |
| Nsmce2 | 20.1 | 19.7 | 20.1 | 20.8 | 19.4 | 18.7 | 18.2 | 20.3 | 17.0 | 18.3 | 23.7 | 21.1 |
| Nsmce4a | 14.4 | 15.2 | 17.1 | 12.1 | 13.6 | 14.3 | 15.3 | 15.7 | 16.5 | 16.2 | 16.6 | 16.6 |
| Nsmf | 8.6 | 8.8 | 9.1 | 7.7 | 7.6 | 9.5 | 9.6 | 8.4 | 9.5 | 7.9 | 7.9 | 8.5 |
| Nsun2 | 21.6 | 17.7 | 15.7 | 24.2 | 19.5 | 22.1 | 20.9 | 19.4 | 17.3 | 18.6 | 20.8 | 19.0 |
| Nsun3 | 2.2 | 2.3 | 2.4 | 3.1 | 2.4 | 2.1 | 1.7 | 2.2 | 2.3 | 1.8 | 1.8 | 2.2 |
| Nsun4 | 5.2 | 4.6 | 3.5 | 4.6 | 4.0 | 3.7 | 4.6 | 5.0 | 5.0 | 3.7 | 4.1 | 3.9 |
| Nsun5 | 1.6 | 1.9 | 1.5 | 1.9 | 1.8 | 1.9 | 1.7 | 1.8 | 1.9 | 2.0 | 1.9 | 1.5 |
| Nsun6 | 2.4 | 3.0 | 2.8 | 2.3 | 2.3 | 1.9 | 2.4 | 2.5 | 2.7 | 2.4 | 2.6 | 2.8 |
| Nsun7 | 0.4 | 0.5 | 0.3 | 0.5 | 0.5 | 0.7 | 0.3 | 0.4 | 0.3 | 0.4 | 0.2 | 0.5 |
| Nt5c | 35.0 | 32.4 | 35.3 | 39.1 | 30.5 | 35.0 | 39.4 | 43.8 | 32.4 | 44.3 | 41.0 | 37.1 |
| Nt5c1b | 0.0 | 0.2 | 0.0 | 0.1 | 0.0 | 0.0 | 0.0 | 0.1 | 0.0 | 0.1 | 0.1 | 0.0 |
| Nt5c2 | 5.9 | 5.1 | 4.5 | 6.4 | 6.1 | 4.8 | 5.0 | 4.5 | 4.5 | 4.9 | 4.9 | 4.9 |
| Nt5c3 | 8.2 | 8.7 | 7.8 | 8.5 | 7.6 | 8.9 | 7.1 | 6.8 | 7.6 | 10.1 | 8.9 | 7.7 |
| Nt5c3l | 14.3 | 13.8 | 14.1 | 15.6 | 12.3 | 14.9 | 15.7 | 16.7 | 12.2 | 12.4 | 17.5 | 13.3 |
| Nt5dc1 | 4.4 | 4.9 | 3.9 | 5.4 | 5.0 | 4.1 | 4.4 | 4.2 | 4.4 | 4.6 | 5.3 | 5.0 |
| Nt5dc2 | 4.7 | 6.7 | 7.6 | 6.2 | 8.6 | 5.5 | 6.9 | 8.6 | 7.7 | 7.1 | 6.9 | 6.7 |
| Nt5dc3 | 2.5 | 2.3 | 2.1 | 2.4 | 2.6 | 2.5 | 2.4 | 2.1 | 1.8 | 2.1 | 2.6 | 2.3 |
| Nt5e | 2.3 | 1.3 | 2.0 | 1.7 | 2.4 | 0.7 | 1.5 | 2.4 | 1.9 | 2.0 | 2.1 | 2.6 |
| Nt5m | 9.1 | 8.0 | 6.4 | 8.1 | 7.7 | 7.2 | 9.4 | 6.5 | 8.2 | 8.3 | 8.2 | 9.1 |
| Ntan1 | 25.8 | 22.9 | 21.9 | 32.0 | 21.6 | 33.1 | 22.3 | 25.8 | 25.0 | 21.2 | 23.5 | 21.0 |

| | | | | | | | | | | | | |
|---|---|---|---|---|---|---|---|---|---|---|---|---|
| Ntf5 | 0.8 | 1.3 | 1.3 | 0.8 | 0.7 | 0.8 | 0.9 | 1.1 | 0.9 | 1.7 | 1.4 | 1.2 |
| Nthl1 | 4.0 | 3.7 | 3.0 | 5.0 | 3.1 | 6.3 | 2.8 | 2.7 | 3.4 | 4.3 | 3.9 | 3.5 |
| Ntm | 0.2 | 0.1 | 0.0 | 0.0 | 0.0 | 0.1 | 0.0 | 0.0 | 0.0 | 0.0 | 0.1 | 0.0 |
| Ntmt1 | 14.5 | 11.3 | 9.0 | 12.3 | 10.2 | 12.8 | 11.1 | 11.7 | 13.3 | 13.6 | 13.4 | 9.4 |
| Ntn1 | 5.0 | 5.3 | 6.0 | 4.2 | 5.4 | 4.7 | 3.9 | 2.4 | 2.6 | 2.1 | 1.8 | 2.3 |
| Ntn3 | 0.4 | 0.5 | 0.5 | 0.5 | 0.4 | 0.8 | 0.4 | 0.4 | 0.5 | 0.4 | 0.3 | 0.4 |
| Ntn4 | 25.1 | 19.4 | 13.6 | 23.5 | 14.1 | 22.5 | 17.7 | 10.8 | 11.6 | 12.4 | 14.8 | 15.7 |
| Ntng1 | 0.0 | 0.0 | 0.0 | 0.0 | 0.1 | 0.0 | 0.0 | 0.0 | 0.0 | 0.0 | 0.0 | 0.0 |
| Ntng2 | 1.3 | 1.5 | 1.6 | 1.9 | 1.7 | 1.9 | 1.8 | 1.2 | 1.5 | 1.2 | 1.3 | 1.5 |
| Ntpcr | 11.9 | 9.8 | 9.4 | 10.6 | 9.6 | 9.1 | 8.9 | 10.7 | 9.5 | 11.6 | 9.9 | 9.4 |
| Ntrk1 | 0.4 | 0.4 | 0.7 | 0.4 | 0.5 | 0.8 | 0.2 | 0.4 | 0.3 | 0.1 | 0.1 | 0.3 |
| Ntrk2 | 0.0 | 0.0 | 0.0 | 0.0 | 0.0 | 0.0 | 0.0 | 0.0 | 0.0 | 0.0 | 0.0 | 0.0 |
| Ntrk3 | 0.1 | 0.0 | 0.0 | 0.0 | 0.0 | 0.0 | 0.0 | 0.0 | 0.0 | 0.0 | 0.1 | 0.1 |
| Nuak1 | 13.0 | 14.1 | 14.8 | 17.5 | 16.4 | 12.5 | 23.3 | 13.7 | 20.3 | 20.2 | 20.5 | 22.1 |
| Nuak2 | 6.2 | 6.2 | 4.8 | 6.5 | 9.3 | 8.6 | 9.5 | 7.3 | 9.1 | 7.0 | 7.6 | 5.8 |
| Nub1 | 26.5 | 22.3 | 20.6 | 28.6 | 20.4 | 27.8 | 21.5 | 21.9 | 20.1 | 23.7 | 25.3 | 26.0 |
| Nubp1 | 9.2 | 7.4 | 6.7 | 8.3 | 7.3 | 7.1 | 8.3 | 7.2 | 8.1 | 8.4 | 7.0 | 7.8 |
| Nubp2 | 13.7 | 11.4 | 11.0 | 15.9 | 14.4 | 13.2 | 13.6 | 11.9 | 11.6 | 12.5 | 12.0 | 11.5 |
| Nubpl | 8.3 | 6.5 | 6.4 | 4.5 | 6.5 | 7.1 | 5.1 | 5.5 | 4.8 | 4.5 | 5.8 | 4.8 |
| Nucb1 | 93.0 | 94.5 | 116.3 | 100.7 | 84.5 | 107.0 | 96.7 | 117.9 | 84.8 | 83.1 | 88.8 | 87.3 |
| Nucb2 | 76.4 | 52.8 | 65.0 | 96.1 | 52.5 | 72.5 | 68.0 | 63.5 | 43.6 | 49.3 | 67.6 | 77.5 |
| Nucks1 | 19.3 | 20.8 | 25.6 | 20.2 | 21.2 | 18.4 | 21.6 | 23.1 | 21.5 | 20.2 | 23.4 | 23.2 |
| Nudc | 60.0 | 63.9 | 72.1 | 72.9 | 57.2 | 64.2 | 72.3 | 60.9 | 51.4 | 57.1 | 70.1 | 64.5 |
| Nudcd1 | 11.0 | 10.2 | 9.2 | 11.0 | 10.5 | 10.7 | 9.3 | 8.8 | 8.1 | 12.1 | 12.6 | 11.0 |
| Nudcd2 | 22.6 | 22.6 | 21.4 | 25.6 | 23.8 | 22.9 | 22.3 | 20.5 | 20.9 | 25.4 | 26.1 | 24.1 |
| Nudcd3 | 10.4 | 11.1 | 10.1 | 10.6 | 9.8 | 10.9 | 11.9 | 10.0 | 11.8 | 10.8 | 10.0 | 11.1 |
| Nudt1 | 4.6 | 5.6 | 4.9 | 4.2 | 4.7 | 4.1 | 5.8 | 5.8 | 5.1 | 4.5 | 3.9 | 4.6 |
| Nudt10 | 0.4 | 0.1 | 0.1 | 0.1 | 0.1 | 0.2 | 0.1 | 0.1 | 0.1 | 0.2 | 0.2 | 0.1 |
| Nudt11 | 0.2 | 0.2 | 0.1 | 0.5 | 0.1 | 0.3 | 0.1 | 0.1 | 0.1 | 0.3 | 0.1 | 0.1 |
| Nudt12 | 3.2 | 3.4 | 3.0 | 3.0 | 2.1 | 4.3 | 2.5 | 2.5 | 2.3 | 3.3 | 3.3 | 2.7 |
| Nudt13 | 1.9 | 2.0 | 2.1 | 2.5 | 1.9 | 2.0 | 1.3 | 1.2 | 1.6 | 1.4 | 1.2 | 1.4 |
| Nudt14 | 6.0 | 6.3 | 5.0 | 4.2 | 6.4 | 5.6 | 5.7 | 6.8 | 5.7 | 7.0 | 5.7 | 5.4 |
| Nudt15 | 1.1 | 1.0 | 0.6 | 0.7 | 1.3 | 0.8 | 0.8 | 0.8 | 0.9 | 1.2 | 1.2 | 0.9 |
| Nudt16 | 5.7 | 6.2 | 4.9 | 5.4 | 4.5 | 7.0 | 5.4 | 5.0 | 6.0 | 5.4 | 5.6 | 5.9 |
| Nudt16l1 | 10.0 | 10.5 | 9.0 | 8.9 | 9.8 | 13.0 | 8.2 | 9.4 | 9.5 | 11.1 | 8.5 | 9.2 |
| Nudt17 | 1.1 | 1.4 | 1.1 | 0.7 | 0.8 | 1.7 | 0.8 | 0.5 | 0.9 | 1.5 | 1.2 | 1.3 |
| Nudt18 | 3.0 | 2.9 | 2.4 | 2.5 | 2.8 | 3.9 | 2.5 | 2.7 | 2.4 | 2.7 | 2.4 | 2.3 |
| Nudt19 | 17.6 | 13.8 | 12.0 | 15.1 | 12.4 | 13.8 | 11.0 | 11.2 | 11.2 | 12.9 | 11.7 | 11.5 |
| Nudt2 | 18.1 | 14.2 | 16.9 | 19.0 | 14.8 | 21.0 | 19.2 | 15.8 | 14.7 | 15.0 | 21.2 | 17.0 |
| Nudt21 | 26.3 | 25.1 | 23.9 | 20.4 | 24.2 | 20.3 | 24.3 | 25.6 | 26.2 | 27.2 | 26.3 | 23.4 |
| Nudt22 | 8.4 | 12.0 | 8.2 | 8.3 | 6.4 | 8.9 | 7.3 | 7.3 | 8.0 | 7.3 | 7.2 | 7.0 |
| Nudt3 | 43.9 | 34.4 | 32.3 | 60.7 | 39.1 | 52.8 | 42.0 | 31.5 | 35.5 | 40.0 | 42.3 | 39.7 |
| Nudt4 | 19.5 | 22.3 | 25.5 | 18.6 | 22.2 | 17.7 | 23.9 | 25.2 | 23.1 | 30.5 | 31.1 | 31.9 |
| Nudt5 | 19.7 | 19.2 | 15.3 | 17.9 | 18.6 | 20.5 | 16.2 | 13.2 | 15.8 | 19.1 | 16.8 | 15.0 |
| Nudt6 | 9.0 | 8.0 | 9.8 | 6.5 | 6.8 | 6.0 | 9.5 | 7.0 | 8.4 | 17.1 | 12.4 | 13.0 |
| Nudt7 | 9.2 | 8.1 | 6.1 | 6.3 | 7.2 | 7.5 | 5.6 | 6.8 | 7.3 | 8.2 | 6.7 | 7.1 |
| Nudt8 | 16.4 | 16.8 | 12.7 | 16.3 | 12.5 | 19.7 | 14.3 | 13.3 | 14.2 | 15.5 | 13.1 | 16.4 |
| Nudt9 | 23.4 | 22.8 | 25.8 | 30.6 | 20.9 | 29.1 | 27.7 | 22.6 | 19.9 | 20.4 | 24.5 | 27.4 |
| Nuf2 | 2.3 | 3.9 | 7.4 | 0.5 | 6.2 | 0.7 | 8.4 | 2.9 | 6.3 | 3.2 | 6.1 | 3.6 |
| Nufip1 | 2.9 | 2.5 | 2.5 | 3.5 | 3.0 | 2.7 | 2.5 | 3.1 | 2.6 | 3.0 | 3.0 | 3.1 |
| Nufip2 | 11.3 | 13.3 | 15.4 | 12.2 | 12.5 | 11.6 | 13.1 | 15.0 | 12.7 | 12.1 | 15.6 | 14.6 |
| Nuggc | 0.3 | 0.4 | 0.2 | 0.4 | 0.2 | 0.1 | 0.2 | 0.3 | 0.1 | 0.1 | 0.3 | 0.3 |
| Numa1 | 20.3 | 21.0 | 22.3 | 22.1 | 21.7 | 21.6 | 25.9 | 23.6 | 23.1 | 20.2 | 20.0 | 21.1 |
| Numb | 10.0 | 12.6 | 12.0 | 12.5 | 10.6 | 9.7 | 12.3 | 10.6 | 11.1 | 10.6 | 12.0 | 11.6 |
| Numbl | 23.0 | 26.5 | 22.2 | 23.0 | 19.2 | 23.6 | 26.4 | 29.8 | 28.6 | 21.6 | 23.4 | 20.2 |
| Nup107 | 8.1 | 10.0 | 9.4 | 7.4 | 10.1 | 6.5 | 10.4 | 9.8 | 9.2 | 8.1 | 10.8 | 8.1 |
| Nup133 | 3.9 | 4.1 | 3.9 | 3.7 | 4.1 | 3.8 | 3.9 | 4.1 | 4.1 | 3.1 | 3.7 | 3.7 |

| | | | | | | | | | | | | |
|---|---|---|---|---|---|---|---|---|---|---|---|---|
| Nup153 | 13.3 | 12.5 | 11.0 | 13.6 | 11.9 | 11.6 | 12.2 | 10.9 | 10.7 | 11.5 | 12.6 | 10.9 |
| Nup155 | 4.7 | 5.5 | 5.5 | 4.1 | 5.9 | 4.7 | 5.4 | 5.1 | 4.9 | 4.6 | 5.2 | 4.2 |
| Nup160 | 2.9 | 3.5 | 3.8 | 3.5 | 4.0 | 3.3 | 3.5 | 3.3 | 3.1 | 3.3 | 3.5 | 3.2 |
| Nup188 | 5.0 | 6.0 | 5.7 | 4.2 | 5.8 | 4.4 | 5.8 | 7.0 | 6.3 | 5.7 | 5.7 | 4.5 |
| Nup205 | 14.1 | 12.8 | 11.8 | 12.2 | 14.1 | 12.9 | 13.1 | 11.5 | 11.1 | 11.6 | 13.0 | 11.3 |
| Nup210 | 0.1 | 0.0 | 0.0 | 0.0 | 0.1 | 0.1 | 0.0 | 0.1 | 0.0 | 0.1 | 0.0 | 0.0 |
| Nup210l | 0.2 | 0.3 | 0.3 | 0.2 | 0.2 | 0.3 | 0.2 | 0.3 | 0.3 | 0.4 | 0.3 | 0.3 |
| Nup214 | 2.9 | 3.7 | 3.9 | 3.7 | 4.1 | 3.1 | 3.8 | 3.9 | 3.1 | 2.9 | 2.6 | 2.5 |
| Nup35 | 12.8 | 11.9 | 9.8 | 11.2 | 11.2 | 7.4 | 11.5 | 8.5 | 11.2 | 11.7 | 12.7 | 9.7 |
| Nup37 | 8.1 | 8.4 | 8.6 | 4.7 | 8.2 | 5.4 | 7.2 | 7.1 | 7.7 | 8.5 | 7.7 | 7.3 |
| Nup43 | 4.8 | 6.2 | 6.4 | 5.0 | 6.0 | 5.4 | 6.4 | 5.4 | 5.8 | 5.3 | 5.9 | 5.3 |
| Nup50 | 10.5 | 10.1 | 11.4 | 11.2 | 9.8 | 10.8 | 10.5 | 10.3 | 9.7 | 11.3 | 11.0 | 10.7 |
| Nup54 | 8.2 | 7.9 | 8.5 | 7.8 | 8.4 | 8.6 | 9.3 | 8.8 | 8.9 | 8.5 | 10.6 | 8.7 |
| Nup62 | 7.3 | 7.2 | 5.9 | 7.7 | 7.2 | 7.8 | 8.5 | 7.4 | 7.2 | 6.8 | 8.1 | 6.3 |
| Nup62cl | 0.2 | 0.2 | 0.1 | 0.2 | 0.2 | 0.1 | 0.1 | 0.1 | 0.2 | 0.2 | 0.2 | 0.1 |
| Nup62-il4i1 | 0.3 | 0.3 | 0.2 | 0.4 | 0.3 | 0.4 | 0.4 | 0.4 | 0.3 | 0.3 | 0.2 | 0.3 |
| Nup85 | 9.9 | 12.1 | 13.4 | 9.5 | 12.3 | 9.6 | 13.7 | 11.6 | 12.5 | 10.5 | 11.0 | 9.9 |
| Nup88 | 30.2 | 27.9 | 25.3 | 30.3 | 26.5 | 30.9 | 27.7 | 25.9 | 27.2 | 26.5 | 29.3 | 27.9 |
| Nup93 | 8.7 | 9.8 | 10.5 | 8.6 | 11.1 | 7.9 | 11.4 | 11.6 | 10.8 | 9.9 | 10.5 | 9.8 |
| Nup98 | 18.4 | 18.3 | 19.4 | 18.4 | 18.7 | 16.2 | 20.6 | 19.6 | 18.1 | 18.5 | 19.0 | 19.6 |
| Nupl1 | 15.8 | 15.7 | 15.1 | 16.3 | 14.9 | 13.7 | 16.7 | 14.7 | 15.7 | 15.9 | 17.5 | 14.8 |
| Nupl2 | 4.3 | 3.9 | 3.8 | 4.8 | 4.2 | 3.7 | 4.0 | 4.1 | 3.5 | 4.1 | 4.3 | 3.8 |
| Nupr1 | 207.6 | 62.4 | 46.4 | 291.4 | 78.0 | 200.1 | 109.9 | 76.7 | 78.1 | 114.8 | 154.8 | 122.2 |
| Nupr1l | 0.9 | 1.0 | 0.9 | 0.1 | 0.4 | 1.2 | 0.1 | 0.7 | 0.6 | 0.9 | 0.5 | 0.4 |
| Nus1 | 20.8 | 19.1 | 15.1 | 22.3 | 21.9 | 20.9 | 20.5 | 15.4 | 20.2 | 24.2 | 23.3 | 22.0 |
| Nusap1 | 2.5 | 4.3 | 8.2 | 0.8 | 5.8 | 1.1 | 8.4 | 2.9 | 5.6 | 2.9 | 5.6 | 3.8 |
| Nutf2 | 5.2 | 5.1 | 4.7 | 8.0 | 4.7 | 4.9 | 3.9 | 4.8 | 4.8 | 5.4 | 5.8 | 4.4 |
| Nutf2-ps1 | 51.6 | 43.5 | 45.6 | 64.8 | 50.5 | 56.5 | 61.5 | 57.0 | 49.7 | 50.3 | 56.3 | 53.1 |
| Nvl | 9.9 | 10.8 | 10.9 | 10.7 | 10.5 | 10.7 | 11.4 | 10.2 | 10.5 | 10.1 | 11.7 | 10.1 |
| Nwd1 | 0.0 | 0.0 | 0.0 | 0.0 | 0.0 | 0.1 | 0.0 | 0.1 | 0.0 | 0.0 | 0.1 | 0.0 |
| Nxf1 | 18.6 | 19.9 | 19.0 | 17.0 | 18.2 | 16.8 | 18.7 | 18.0 | 20.0 | 17.9 | 20.5 | 18.7 |
| Nxf2 | 0.2 | 0.1 | 0.4 | 0.5 | 0.1 | 0.3 | 0.2 | 0.1 | 0.2 | 0.2 | 0.1 | 0.1 |
| Nxf3 | 0.2 | 0.1 | 0.0 | 0.1 | 0.0 | 0.0 | 0.0 | 0.1 | 0.0 | 0.1 | 0.0 | 0.0 |
| Nxf7 | 0.1 | 0.0 | 0.0 | 0.0 | 0.0 | 0.0 | 0.0 | 0.0 | 0.0 | 0.0 | 0.0 | 0.0 |
| Nxn | 29.5 | 29.3 | 32.5 | 18.0 | 27.0 | 23.1 | 27.6 | 31.7 | 30.7 | 61.4 | 42.7 | 44.4 |
| Nxnl1 | 0.0 | 0.0 | 0.0 | 0.0 | 0.0 | 0.0 | 0.0 | 0.0 | 0.0 | 0.0 | 0.0 | 0.0 |
| Nxnl2 | 0.6 | 0.6 | 0.7 | 0.1 | 0.4 | 0.3 | 0.6 | 0.6 | 0.9 | 2.1 | 2.1 | 1.0 |
| Nxpe2 | 0.0 | 0.0 | 0.0 | 0.0 | 0.1 | 0.0 | 0.0 | 0.0 | 0.0 | 0.0 | 0.0 | 0.0 |
| Nxpe3 | 0.4 | 0.6 | 0.5 | 0.4 | 0.4 | 0.5 | 0.3 | 0.4 | 0.5 | 0.3 | 0.5 | 0.5 |
| Nxpe4 | 3.2 | 3.5 | 3.8 | 2.7 | 2.6 | 3.4 | 2.4 | 3.6 | 2.5 | 1.8 | 2.8 | 2.8 |
| Nxpe5 | 0.3 | 0.0 | 0.0 | 1.1 | 1.9 | 0.1 | 0.0 | 0.0 | 0.0 | 0.1 | 0.0 | 0.0 |
| Nxph1 | 0.0 | 0.0 | 0.0 | 0.0 | 0.0 | 0.0 | 0.0 | 0.0 | 0.0 | 0.1 | 0.0 | 0.0 |
| Nxph3 | 0.1 | 0.1 | 0.1 | 0.0 | 0.1 | 0.0 | 0.1 | 0.1 | 0.2 | 0.5 | 0.3 | 0.1 |
| Nxph4 | 0.3 | 0.2 | 0.4 | 0.1 | 0.4 | 0.6 | 0.5 | 0.4 | 0.4 | 0.4 | 0.3 | 0.6 |
| Nxt1 | 8.0 | 8.2 | 9.4 | 8.2 | 10.1 | 7.4 | 10.2 | 8.6 | 9.9 | 8.0 | 9.2 | 8.6 |
| Nxt2 | 7.3 | 8.2 | 9.7 | 9.9 | 8.1 | 9.0 | 8.6 | 7.5 | 8.1 | 7.2 | 9.5 | 9.3 |
| Nyap1 | 2.0 | 2.3 | 2.2 | 1.4 | 1.0 | 2.3 | 1.2 | 1.2 | 1.2 | 1.2 | 1.3 | 1.3 |
| Nyap2 | 0.0 | 0.0 | 0.0 | 0.0 | 0.0 | 0.0 | 0.0 | 0.0 | 0.0 | 0.0 | 0.0 | 0.0 |
| Nynrin | 2.9 | 4.2 | 4.9 | 3.1 | 2.7 | 4.1 | 4.1 | 4.4 | 4.5 | 4.2 | 3.6 | 3.8 |
| Nyx | 0.2 | 0.2 | 0.3 | 0.1 | 0.2 | 0.1 | 0.2 | 0.2 | 0.1 | 0.1 | 0.0 | 0.1 |
| Oacyl | 0.1 | 0.1 | 0.0 | 0.2 | 0.3 | 0.0 | 0.2 | 0.1 | 0.2 | 0.2 | 0.1 | 0.2 |
| Oaf | 23.4 | 20.9 | 17.5 | 27.6 | 27.1 | 36.2 | 32.3 | 24.1 | 35.2 | 26.8 | 27.9 | 24.6 |
| Oard1 | 9.4 | 7.4 | 9.4 | 6.5 | 7.5 | 9.6 | 6.5 | 7.9 | 7.4 | 8.9 | 9.6 | 9.9 |
| Oas1a | 3.7 | 2.3 | 3.1 | 4.7 | 4.2 | 2.2 | 1.2 | 1.2 | 0.8 | 1.1 | 0.5 | 2.4 |
| Oas1b | 1.4 | 0.8 | 1.2 | 1.1 | 0.9 | 0.9 | 0.8 | 0.6 | 0.7 | 0.5 | 0.4 | 0.7 |
| Oas1c | 3.0 | 2.7 | 3.0 | 2.9 | 1.8 | 3.1 | 1.6 | 1.4 | 1.4 | 1.6 | 1.3 | 1.5 |
| Oas1d | 0.1 | 0.0 | 0.0 | 0.1 | 0.1 | 0.1 | 0.0 | 0.1 | 0.0 | 0.1 | 0.0 | 0.1 |

| | | | | | | | | | | | | |
|---|---|---|---|---|---|---|---|---|---|---|---|---|
| Oas1g | 0.4 | 0.4 | 0.4 | 0.8 | 0.7 | 0.3 | 0.3 | 0.2 | 0.2 | 0.1 | 0.2 | 0.4 |
| Oas2 | 1.6 | 1.4 | 1.9 | 1.6 | 1.6 | 0.9 | 0.5 | 0.3 | 0.2 | 0.1 | 0.1 | 0.8 |
| Oas3 | 0.2 | 0.1 | 0.1 | 0.7 | 0.3 | 0.1 | 0.1 | 0.0 | 0.0 | 0.0 | 0.0 | 0.1 |
| Oasl1 | 0.9 | 0.3 | 0.4 | 2.1 | 0.3 | 1.4 | 0.2 | 0.2 | 0.1 | 0.2 | 0.2 | 0.4 |
| Oasl2 | 6.2 | 3.3 | 6.2 | 11.1 | 5.0 | 5.6 | 3.7 | 2.5 | 2.1 | 1.5 | 2.2 | 6.7 |
| Oat | 128.7 | 123.9 | 126.6 | 117.1 | 117.4 | 109.9 | 120.3 | 149.6 | 148.7 | 151.5 | 143.9 | 155.0 |
| Oaz1 | 159.8 | 176.6 | 134.8 | 146.3 | 207.6 | 147.9 | 111.2 | 116.9 | 216.2 | 221.9 | 137.7 | 136.6 |
| Oaz1-ps | 47.0 | 17.0 | 26.9 | 57.8 | 19.4 | 45.9 | 76.8 | 69.8 | 9.2 | 0.1 | 47.7 | 44.6 |
| Oaz2 | 35.3 | 36.0 | 37.0 | 33.3 | 33.9 | 32.6 | 30.3 | 30.0 | 38.7 | 44.0 | 37.0 | 38.7 |
| Oaz3 | 0.1 | 0.0 | 0.0 | 0.0 | 0.0 | 0.0 | 0.2 | 0.0 | 0.1 | 0.0 | 0.1 | 0.0 |
| Obfc1 | 5.1 | 5.2 | 5.2 | 4.1 | 4.3 | 5.6 | 3.5 | 5.3 | 3.3 | 3.8 | 4.2 | 3.9 |
| Obscn | 0.0 | 0.0 | 0.0 | 0.0 | 0.0 | 0.0 | 0.0 | 0.0 | 0.0 | 0.0 | 0.0 | 0.0 |
| Obsl1 | 1.8 | 2.3 | 3.0 | 1.2 | 1.8 | 1.9 | 3.0 | 3.0 | 3.7 | 3.0 | 2.3 | 2.6 |
| Oc90 | 0.0 | 0.0 | 0.0 | 0.0 | 0.0 | 0.0 | 0.0 | 0.0 | 0.0 | 0.0 | 0.0 | 0.0 |
| Oca2 | 0.0 | 0.0 | 0.0 | 0.0 | 0.0 | 0.0 | 0.0 | 0.0 | 0.0 | 0.0 | 0.0 | 0.0 |
| Ocel1 | 2.9 | 2.9 | 2.4 | 2.0 | 2.7 | 2.8 | 3.1 | 3.2 | 3.2 | 2.8 | 2.0 | 2.8 |
| Ociad1 | 79.5 | 74.9 | 66.8 | 80.1 | 69.6 | 71.3 | 72.5 | 69.7 | 70.0 | 79.3 | 78.9 | 80.0 |
| Ociad2 | 1.8 | 1.4 | 1.2 | 1.6 | 0.9 | 1.7 | 0.6 | 0.8 | 0.8 | 1.8 | 1.2 | 1.1 |
| Ocln | 0.0 | 0.0 | 0.0 | 0.0 | 0.0 | 0.0 | 0.0 | 0.0 | 0.0 | 0.0 | 0.0 | 0.0 |
| Ocrl | 6.5 | 7.0 | 5.9 | 7.1 | 5.8 | 7.4 | 6.5 | 5.6 | 6.3 | 5.3 | 5.9 | 5.8 |
| Ocstamp | 0.2 | 0.2 | 0.1 | 0.2 | 0.1 | 0.1 | 0.1 | 0.1 | 0.1 | 0.0 | 0.2 | 0.1 |
| Odc1 | 94.0 | 67.1 | 51.5 | 89.7 | 58.4 | 101.8 | 59.9 | 44.8 | 46.4 | 69.9 | 61.0 | 53.8 |
| Odf1 | 0.0 | 0.0 | 0.1 | 0.1 | 0.0 | 0.0 | 0.0 | 0.0 | 0.0 | 0.0 | 0.0 | 0.0 |
| Odf2 | 16.1 | 16.6 | 19.6 | 16.7 | 15.3 | 16.1 | 20.5 | 18.3 | 17.8 | 14.3 | 19.2 | 17.4 |
| Odf2l | 5.6 | 6.7 | 5.2 | 5.6 | 4.6 | 5.2 | 4.3 | 4.6 | 4.6 | 5.8 | 5.5 | 5.0 |
| Odf3 | 0.1 | 0.0 | 0.0 | 0.0 | 0.0 | 0.0 | 0.0 | 0.0 | 0.1 | 0.0 | 0.0 | 0.0 |
| Odf3b | 0.0 | 0.0 | 0.0 | 0.0 | 0.0 | 0.1 | 0.1 | 0.0 | 0.0 | 0.0 | 0.1 | 0.0 |
| Odf3l2 | 0.0 | 0.0 | 0.0 | 0.0 | 0.0 | 0.0 | 0.0 | 0.0 | 0.0 | 0.0 | 0.1 | 0.0 |
| Ofd1 | 3.7 | 4.5 | 4.3 | 3.2 | 3.3 | 3.0 | 4.6 | 4.0 | 3.7 | 3.4 | 4.3 | 4.7 |
| Ogdh | 14.8 | 14.8 | 14.7 | 16.8 | 16.0 | 15.5 | 16.4 | 15.5 | 13.1 | 13.1 | 14.4 | 12.8 |
| Ogdhl | 15.6 | 12.0 | 8.2 | 11.6 | 9.6 | 19.4 | 5.6 | 4.1 | 4.3 | 10.4 | 6.6 | 7.7 |
| Ogfod1 | 7.2 | 6.2 | 5.6 | 7.2 | 5.9 | 6.7 | 6.5 | 5.1 | 5.6 | 6.3 | 6.0 | 6.7 |
| Ogfod2 | 6.6 | 6.1 | 4.7 | 6.3 | 5.9 | 6.4 | 4.3 | 4.4 | 5.2 | 6.2 | 5.3 | 4.8 |
| Ogfod3 | 2.0 | 1.2 | 2.3 | 2.0 | 1.5 | 1.9 | 2.0 | 3.4 | 1.7 | 1.8 | 2.0 | 1.7 |
| Ogfr | 17.8 | 17.0 | 19.4 | 16.0 | 18.4 | 13.8 | 16.9 | 20.9 | 19.5 | 18.6 | 17.6 | 20.3 |
| Ogfrl1 | 2.2 | 2.6 | 3.6 | 2.8 | 2.4 | 3.1 | 2.3 | 2.1 | 1.4 | 1.2 | 1.9 | 1.9 |
| Ogg1 | 4.0 | 3.8 | 3.3 | 3.9 | 3.9 | 4.3 | 3.5 | 3.8 | 3.3 | 4.1 | 3.0 | 3.3 |
| Ogn | 5.1 | 6.3 | 13.4 | 3.7 | 4.0 | 2.7 | 2.6 | 7.6 | 7.4 | 9.5 | 9.1 | 9.2 |
| Ogt | 16.5 | 18.2 | 16.8 | 15.9 | 17.1 | 18.5 | 14.4 | 16.8 | 17.8 | 15.0 | 16.5 | 14.5 |
| Oip5 | 0.8 | 1.5 | 2.2 | 0.5 | 1.7 | 0.7 | 1.9 | 1.5 | 1.8 | 1.2 | 1.5 | 0.7 |
| Oit3 | 0.2 | 0.1 | 0.1 | 0.6 | 0.9 | 0.5 | 0.1 | 0.1 | 0.0 | 0.1 | 0.1 | 0.1 |
| Ola1 | 36.3 | 28.0 | 30.1 | 34.4 | 29.1 | 34.1 | 29.7 | 27.7 | 27.7 | 29.8 | 34.5 | 33.5 |
| Olfm1 | 7.4 | 7.0 | 7.3 | 11.5 | 10.1 | 11.5 | 8.8 | 5.6 | 5.5 | 4.9 | 7.6 | 6.6 |
| Olfm2 | 0.6 | 0.6 | 0.2 | 0.5 | 0.5 | 0.9 | 0.4 | 0.1 | 0.1 | 0.3 | 0.7 | 0.4 |
| Olfml1 | 1.1 | 4.0 | 10.2 | 0.5 | 1.9 | 0.3 | 2.3 | 8.5 | 3.7 | 1.3 | 1.5 | 2.4 |
| Olfml2a | 0.1 | 0.1 | 0.3 | 0.0 | 0.0 | 0.0 | 0.1 | 0.1 | 0.0 | 0.0 | 0.0 | 0.1 |
| Olfml2b | 6.7 | 6.5 | 11.4 | 3.3 | 6.3 | 5.1 | 9.4 | 17.1 | 12.6 | 19.0 | 17.1 | 15.8 |
| Olfml3 | 34.5 | 69.0 | 182.5 | 31.0 | 83.7 | 20.4 | 82.4 | 125.2 | 96.0 | 56.8 | 51.6 | 94.2 |
| Olfr1033 | 0.3 | 0.3 | 0.3 | 0.4 | 0.3 | 0.4 | 0.4 | 0.3 | 0.4 | 0.9 | 0.6 | 0.6 |
| Olfr1034 | 0.1 | 0.0 | 0.0 | 0.0 | 0.0 | 0.0 | 0.1 | 0.1 | 0.0 | 0.1 | 0.1 | 0.0 |
| Olfr1314 | 0.1 | 0.1 | 0.0 | 0.0 | 0.0 | 0.1 | 0.0 | 0.0 | 0.0 | 0.0 | 0.1 | 0.0 |
| Olfr1317 | 0.1 | 0.0 | 0.0 | 0.2 | 0.1 | 0.0 | 0.1 | 0.0 | 0.0 | 0.0 | 0.1 | 0.1 |
| Olfr1318 | 0.2 | 0.2 | 0.1 | 0.0 | 0.1 | 0.2 | 0.2 | 0.0 | 0.1 | 0.1 | 0.1 | 0.1 |
| Olfr1372-ps1 | 0.1 | 0.1 | 0.0 | 0.2 | 0.1 | 0.1 | 0.0 | 0.0 | 0.0 | 0.0 | 0.1 | 0.1 |
| Olfr1396 | 0.1 | 0.0 | 0.0 | 0.0 | 0.0 | 0.0 | 0.0 | 0.0 | 0.0 | 0.0 | 0.0 | 0.0 |
| Olfr1417 | 0.0 | 0.1 | 0.0 | 0.1 | 0.0 | 0.0 | 0.0 | 0.0 | 0.0 | 0.0 | 0.1 | 0.0 |
| Olfr1420 | 0.0 | 0.0 | 0.0 | 0.1 | 0.0 | 0.1 | 0.0 | 0.0 | 0.0 | 0.0 | 0.0 | 0.0 |

| | | | | | | | | | | | | |
|---|---|---|---|---|---|---|---|---|---|---|---|---|
| Olfr18 | 0.0 | 0.0 | 0.0 | 0.0 | 0.0 | 0.0 | 0.0 | 0.0 | 0.0 | 0.0 | 0.0 | 0.0 |
| Olfr212 | 0.0 | 0.0 | 0.0 | 0.2 | 0.0 | 0.1 | 0.1 | 0.0 | 0.0 | 0.1 | 0.0 | 0.0 |
| Olfr239 | 0.0 | 0.0 | 0.0 | 0.0 | 0.0 | 0.0 | 0.0 | 0.0 | 0.0 | 0.0 | 0.0 | 0.0 |
| Olfr419 | 0.0 | 0.0 | 0.0 | 0.1 | 0.1 | 0.0 | 0.0 | 0.0 | 0.0 | 0.0 | 0.1 | 0.0 |
| Olfr420 | 0.3 | 0.2 | 0.2 | 0.3 | 0.1 | 0.3 | 0.2 | 0.1 | 0.2 | 0.2 | 0.1 | 0.0 |
| Olfr421-ps1 | 0.0 | 0.2 | 0.0 | 0.3 | 0.1 | 0.0 | 0.1 | 0.0 | 0.0 | 0.0 | 0.1 | 0.0 |
| Olfr456 | 0.1 | 0.2 | 0.0 | 0.0 | 0.1 | 0.0 | 0.2 | 0.1 | 0.1 | 0.0 | 0.0 | 0.0 |
| Olfr543 | 0.1 | 0.3 | 0.3 | 0.2 | 0.1 | 0.1 | 0.1 | 0.4 | 0.1 | 0.4 | 0.1 | 0.1 |
| Olfr544 | 0.3 | 0.2 | 0.1 | 0.0 | 0.3 | 0.2 | 0.0 | 0.1 | 0.1 | 0.2 | 0.2 | 0.2 |
| Olfr545 | 0.3 | 0.1 | 0.2 | 0.1 | 0.3 | 0.1 | 0.0 | 0.1 | 0.0 | 0.1 | 0.1 | 0.1 |
| Olfr558 | 0.0 | 0.0 | 0.0 | 0.0 | 0.0 | 0.0 | 0.1 | 0.1 | 0.1 | 0.0 | 0.0 | 0.0 |
| Olfr56 | 0.2 | 0.1 | 0.1 | 0.4 | 0.2 | 0.2 | 0.2 | 0.1 | 0.1 | 0.1 | 0.2 | 0.1 |
| Olfr613 | 2.2 | 3.2 | 2.5 | 1.9 | 2.7 | 1.7 | 2.2 | 1.7 | 1.5 | 2.1 | 2.4 | 1.8 |
| Olfr692 | 0.0 | 0.0 | 0.0 | 0.0 | 0.0 | 0.0 | 0.0 | 0.0 | 0.1 | 0.0 | 0.1 | 0.0 |
| Olfr78 | 0.1 | 0.0 | 0.0 | 0.1 | 0.1 | 0.0 | 0.0 | 0.0 | 0.0 | 0.0 | 0.0 | 0.0 |
| Olfr856-ps1 | 7.5 | 10.0 | 8.5 | 7.4 | 8.3 | 6.5 | 9.1 | 6.5 | 5.4 | 6.8 | 6.7 | 5.6 |
| Olfr920 | 0.0 | 0.1 | 0.1 | 0.3 | 0.1 | 0.0 | 0.0 | 0.0 | 0.0 | 0.1 | 0.2 | 0.1 |
| Olfr99 | 0.0 | 0.0 | 0.0 | 0.1 | 0.1 | 0.1 | 0.0 | 0.0 | 0.0 | 0.0 | 0.0 | 0.1 |
| Olr1 | 1.2 | 0.5 | 0.4 | 0.9 | 2.0 | 0.3 | 2.5 | 0.5 | 1.7 | 2.6 | 1.3 | 2.2 |
| Oma1 | 7.1 | 6.3 | 5.7 | 6.7 | 6.2 | 6.1 | 6.1 | 4.9 | 5.3 | 7.8 | 7.5 | 7.2 |
| Omd | 0.3 | 0.2 | 0.5 | 0.5 | 0.2 | 0.4 | 0.2 | 0.3 | 0.2 | 0.2 | 0.0 | 0.2 |
| Omg | 0.0 | 0.0 | 0.1 | 0.0 | 0.1 | 0.1 | 0.1 | 0.1 | 0.1 | 0.1 | 0.1 | 0.1 |
| Omp | 0.1 | 0.0 | 0.1 | 0.0 | 0.1 | 0.0 | 0.1 | 0.2 | 0.0 | 0.0 | 0.1 | 0.1 |
| Onecut2 | 0.0 | 0.0 | 0.0 | 0.1 | 0.0 | 0.1 | 0.1 | 0.0 | 0.0 | 0.0 | 0.0 | 0.0 |
| Opa1 | 11.9 | 11.7 | 10.5 | 11.7 | 11.1 | 12.6 | 11.0 | 10.3 | 9.5 | 10.7 | 11.3 | 10.3 |
| Opa3 | 8.4 | 7.6 | 7.4 | 9.9 | 8.3 | 8.1 | 9.7 | 8.6 | 8.3 | 7.4 | 9.0 | 8.1 |
| Opcml | 0.0 | 0.0 | 0.0 | 0.0 | 0.0 | 0.1 | 0.0 | 0.0 | 0.0 | 0.0 | 0.0 | 0.0 |
| Ophn1 | 1.8 | 2.1 | 1.9 | 2.1 | 2.6 | 1.7 | 1.8 | 2.7 | 2.3 | 2.3 | 2.3 | 2.1 |
| Oplah | 12.2 | 11.7 | 9.6 | 6.2 | 6.4 | 9.2 | 4.9 | 9.1 | 5.9 | 9.8 | 8.1 | 6.6 |
| Opn1mw | 0.0 | 0.3 | 0.1 | 0.2 | 0.1 | 0.0 | 0.1 | 0.4 | 0.1 | 0.2 | 0.1 | 0.1 |
| Opn1sw | 0.3 | 0.4 | 0.4 | 0.2 | 0.4 | 0.5 | 0.4 | 0.7 | 0.4 | 0.3 | 0.3 | 0.4 |
| Opn3 | 1.4 | 1.1 | 1.3 | 1.4 | 1.1 | 1.0 | 1.6 | 1.2 | 1.4 | 0.8 | 1.3 | 0.9 |
| Opn5 | 0.0 | 0.0 | 0.0 | 0.0 | 0.0 | 0.0 | 0.0 | 0.0 | 0.0 | 0.0 | 0.0 | 0.0 |
| Oprl1 | 0.1 | 0.2 | 0.1 | 0.1 | 0.2 | 0.1 | 0.1 | 0.1 | 0.1 | 0.1 | 0.1 | 0.1 |
| Optc | 1.1 | 1.2 | 0.9 | 0.8 | 0.4 | 0.9 | 0.3 | 0.8 | 0.4 | 1.0 | 0.7 | 1.0 |
| Optn | 13.2 | 14.1 | 13.8 | 17.3 | 11.9 | 14.8 | 13.5 | 12.5 | 10.5 | 8.0 | 12.6 | 12.5 |
| Orai1 | 6.6 | 5.0 | 4.5 | 6.3 | 7.6 | 5.9 | 8.5 | 5.9 | 6.7 | 6.3 | 6.7 | 5.8 |
| Orai2 | 14.9 | 15.1 | 15.3 | 14.2 | 14.0 | 12.5 | 15.3 | 13.6 | 13.2 | 12.6 | 12.5 | 14.4 |
| Orai3 | 10.5 | 11.3 | 11.4 | 13.3 | 10.3 | 14.6 | 11.2 | 11.9 | 11.5 | 10.5 | 10.6 | 10.0 |
| Oraov1 | 13.6 | 13.3 | 12.8 | 15.0 | 12.3 | 11.8 | 12.8 | 12.8 | 14.4 | 9.9 | 14.0 | 13.9 |
| Orc1 | 0.6 | 0.9 | 1.3 | 0.2 | 0.9 | 0.2 | 1.1 | 0.7 | 0.9 | 0.7 | 1.0 | 0.6 |
| Orc2 | 9.4 | 9.6 | 8.7 | 9.8 | 8.4 | 9.1 | 8.3 | 7.2 | 7.4 | 8.9 | 8.9 | 8.5 |
| Orc3 | 7.4 | 8.5 | 8.0 | 7.4 | 7.6 | 8.0 | 7.1 | 7.6 | 6.9 | 7.2 | 8.0 | 7.9 |
| Orc4 | 10.5 | 11.9 | 11.4 | 10.8 | 11.4 | 12.1 | 9.8 | 9.8 | 11.4 | 13.0 | 13.2 | 12.0 |
| Orc5 | 6.0 | 7.8 | 7.2 | 6.8 | 6.8 | 6.1 | 7.0 | 6.8 | 6.7 | 6.0 | 7.7 | 6.6 |
| Orc6 | 8.5 | 11.1 | 10.6 | 5.8 | 8.3 | 6.7 | 9.2 | 9.9 | 10.1 | 10.9 | 9.4 | 9.0 |
| ORF63 | 0.5 | 0.5 | 0.3 | 0.8 | 0.9 | 0.5 | 0.6 | 0.3 | 1.0 | 1.1 | 1.2 | 0.8 |
| Orm1 | 0.0 | 0.1 | 0.1 | 0.0 | 0.0 | 0.0 | 0.1 | 0.0 | 0.1 | 0.1 | 0.0 | 0.0 |
| Orm2 | 0.1 | 0.0 | 0.0 | 0.1 | 0.1 | 0.1 | 0.1 | 0.1 | 0.0 | 0.0 | 0.0 | 0.1 |
| Orm3 | 0.0 | 0.1 | 0.0 | 0.3 | 0.1 | 0.0 | 0.0 | 0.1 | 0.0 | 0.1 | 0.0 | 0.0 |
| Ormdl1 | 4.8 | 4.8 | 4.9 | 5.7 | 6.8 | 7.3 | 4.5 | 3.8 | 4.5 | 6.0 | 5.1 | 5.1 |
| Ormdl2 | 15.0 | 16.1 | 13.3 | 14.9 | 14.8 | 14.0 | 13.5 | 15.4 | 15.3 | 15.1 | 17.1 | 15.6 |
| Ormdl3 | 38.8 | 37.8 | 34.1 | 47.0 | 37.2 | 46.5 | 35.4 | 30.3 | 38.1 | 40.2 | 37.2 | 40.8 |
| Os9 | 37.1 | 32.7 | 41.0 | 48.7 | 37.4 | 44.8 | 39.3 | 43.7 | 35.5 | 33.9 | 37.6 | 41.9 |
| Osbp | 12.6 | 13.1 | 13.0 | 12.5 | 12.2 | 10.4 | 13.5 | 12.4 | 12.8 | 11.0 | 13.2 | 13.8 |
| Osbp2 | 0.1 | 0.0 | 0.1 | 0.2 | 0.1 | 0.2 | 0.1 | 0.0 | 0.0 | 0.1 | 0.1 | 0.1 |
| Osbpl10 | 0.7 | 0.6 | 0.7 | 0.8 | 0.7 | 0.6 | 0.7 | 0.8 | 0.5 | 0.8 | 0.8 | 0.6 |

| | | | | | | | | | | | | |
|---|---|---|---|---|---|---|---|---|---|---|---|---|
| Osbpl11 | 7.5 | 7.1 | 6.8 | 7.3 | 6.9 | 8.2 | 7.1 | 7.8 | 7.4 | 6.9 | 8.2 | 8.0 |
| Osbpl1a | 12.4 | 12.1 | 12.2 | 12.2 | 9.2 | 12.3 | 10.8 | 9.9 | 11.2 | 8.9 | 10.3 | 10.6 |
| Osbpl2 | 18.9 | 16.5 | 16.2 | 22.5 | 14.5 | 20.5 | 16.7 | 17.9 | 14.1 | 17.9 | 20.3 | 20.3 |
| Osbpl3 | 1.3 | 0.6 | 0.4 | 1.0 | 1.1 | 0.8 | 0.8 | 0.5 | 0.4 | 1.1 | 0.8 | 0.7 |
| Osbpl5 | 38.8 | 36.7 | 28.4 | 40.8 | 34.2 | 38.8 | 40.6 | 30.3 | 38.1 | 35.2 | 36.5 | 32.4 |
| Osbpl6 | 0.2 | 0.2 | 0.3 | 0.2 | 0.2 | 0.2 | 0.2 | 0.2 | 0.2 | 0.3 | 0.4 | 0.2 |
| Osbpl7 | 0.9 | 0.9 | 1.1 | 0.6 | 0.9 | 1.0 | 1.0 | 1.1 | 0.8 | 0.9 | 0.6 | 0.8 |
| Osbpl8 | 7.9 | 8.4 | 8.8 | 16.6 | 17.8 | 8.1 | 9.3 | 8.5 | 8.5 | 8.1 | 10.1 | 9.7 |
| Osbpl9 | 58.0 | 53.6 | 50.5 | 61.5 | 45.6 | 58.3 | 55.7 | 48.4 | 49.6 | 50.4 | 56.6 | 56.9 |
| Oscar | 0.1 | 0.1 | 0.1 | 0.1 | 0.1 | 0.1 | 0.0 | 0.2 | 0.1 | 0.1 | 0.1 | 0.1 |
| Oscp1 | 2.0 | 1.7 | 1.9 | 1.6 | 1.3 | 2.2 | 1.6 | 1.7 | 1.7 | 1.5 | 1.7 | 1.8 |
| Osgep | 16.2 | 14.8 | 16.8 | 13.3 | 14.8 | 13.5 | 17.9 | 17.8 | 17.4 | 17.3 | 16.2 | 16.9 |
| Osgepl1 | 2.5 | 2.9 | 2.9 | 2.0 | 2.1 | 2.3 | 2.3 | 2.6 | 2.5 | 2.1 | 2.7 | 2.4 |
| Osgin1 | 5.4 | 5.6 | 3.9 | 8.4 | 6.6 | 7.1 | 5.1 | 3.2 | 2.8 | 8.9 | 4.9 | 4.5 |
| Osgin2 | 2.9 | 3.1 | 2.7 | 2.7 | 5.4 | 2.4 | 2.9 | 2.7 | 3.5 | 3.3 | 3.2 | 3.2 |
| Osm | 0.1 | 0.0 | 0.0 | 0.2 | 0.6 | 0.0 | 0.1 | 0.0 | 0.0 | 0.1 | 0.0 | 0.0 |
| Osmr | 60.6 | 78.8 | 90.3 | 57.0 | 79.7 | 46.2 | 89.3 | 84.0 | 82.2 | 82.2 | 82.1 | 92.3 |
| Osr1 | 2.2 | 1.7 | 2.5 | 1.1 | 1.3 | 2.1 | 2.2 | 4.2 | 3.3 | 6.0 | 4.7 | 3.8 |
| Osr2 | 0.1 | 0.1 | 0.1 | 0.0 | 0.0 | 0.1 | 0.0 | 0.1 | 0.2 | 0.1 | 0.0 | 0.1 |
| Ost4 | 313.8 | 277.8 | 239.1 | 369.1 | 274.7 | 373.5 | 289.8 | 269.3 | 249.4 | 249.6 | 292.0 | 270.2 |
| Ostc | 114.3 | 104.6 | 91.8 | 122.5 | 102.2 | 102.3 | 105.8 | 102.4 | 113.7 | 114.2 | 116.5 | 114.9 |
| Ostf1 | 60.3 | 50.6 | 49.1 | 74.6 | 71.8 | 55.5 | 61.8 | 48.6 | 59.7 | 54.3 | 62.4 | 61.3 |
| Ostm1 | 32.4 | 27.1 | 24.1 | 34.3 | 29.1 | 32.5 | 26.2 | 23.3 | 26.2 | 27.8 | 29.8 | 31.7 |
| Ostn | 0.3 | 0.1 | 0.0 | 0.0 | 0.0 | 0.0 | 0.0 | 0.0 | 0.0 | 0.0 | 0.1 | 0.0 |
| Otoa | 0.1 | 0.1 | 0.0 | 0.1 | 0.2 | 0.0 | 0.0 | 0.0 | 0.0 | 0.0 | 0.0 | 0.0 |
| Otof | 0.0 | 0.0 | 0.0 | 0.0 | 0.0 | 0.0 | 0.0 | 0.0 | 0.0 | 0.0 | 0.0 | 0.0 |
| Otogl | 0.0 | 0.0 | 0.0 | 0.0 | 0.0 | 0.0 | 0.0 | 0.0 | 0.0 | 0.0 | 0.0 | 0.0 |
| Ott | 0.0 | 0.0 | 0.0 | 0.0 | 0.0 | 0.0 | 0.0 | 0.0 | 0.0 | 0.0 | 0.0 | 0.0 |
| )TTMUSG0000001660 | 0.1 | 0.7 | 1.6 | 2.8 | 1.4 | 0.9 | 0.6 | 1.2 | 1.2 | 1.7 | 2.1 | 2.4 |
| Otub1 | 40.1 | 39.9 | 33.0 | 40.7 | 32.8 | 40.5 | 37.9 | 38.3 | 35.7 | 36.3 | 35.5 | 32.9 |
| Otub2 | 4.0 | 3.5 | 3.0 | 4.8 | 2.9 | 4.7 | 2.8 | 3.0 | 2.9 | 2.6 | 3.4 | 2.8 |
| Otud1 | 3.7 | 2.6 | 3.2 | 3.7 | 3.0 | 3.3 | 2.4 | 2.1 | 2.5 | 2.6 | 2.4 | 2.8 |
| Otud3 | 6.1 | 7.7 | 6.4 | 7.0 | 6.6 | 7.0 | 7.5 | 6.7 | 6.2 | 6.5 | 6.8 | 7.5 |
| Otud4 | 8.9 | 9.6 | 9.9 | 11.2 | 9.5 | 9.9 | 9.8 | 8.0 | 7.9 | 9.4 | 10.6 | 9.3 |
| Otud5 | 15.4 | 16.2 | 16.2 | 17.0 | 15.1 | 16.1 | 16.4 | 15.1 | 16.1 | 16.0 | 16.8 | 15.6 |
| Otud6b | 21.2 | 19.4 | 18.7 | 26.7 | 18.4 | 22.9 | 20.6 | 16.9 | 17.0 | 20.2 | 23.4 | 22.3 |
| Otud7a | 0.1 | 0.1 | 0.1 | 0.1 | 0.1 | 0.1 | 0.1 | 0.0 | 0.1 | 0.0 | 0.1 | 0.0 |
| Otud7b | 8.4 | 7.7 | 7.1 | 11.3 | 7.3 | 12.2 | 8.5 | 6.7 | 7.5 | 8.3 | 9.0 | 8.2 |
| Ovca2 | 4.1 | 5.0 | 4.6 | 4.7 | 5.2 | 4.2 | 4.1 | 5.6 | 4.5 | 5.3 | 5.3 | 4.1 |
| Ovgp1 | 0.5 | 0.8 | 0.6 | 0.4 | 0.5 | 0.5 | 0.4 | 0.4 | 0.4 | 0.4 | 0.5 | 0.5 |
| Ovol1 | 0.1 | 0.1 | 0.1 | 0.0 | 0.1 | 0.1 | 0.1 | 0.1 | 0.1 | 0.1 | 0.0 | 0.0 |
| Oxa1l | 8.4 | 7.1 | 7.3 | 9.0 | 7.0 | 7.9 | 7.4 | 9.5 | 7.5 | 8.2 | 8.0 | 7.8 |
| Oxct1 | 36.2 | 33.4 | 39.9 | 26.5 | 25.8 | 35.7 | 29.1 | 33.6 | 33.9 | 33.2 | 39.1 | 37.0 |
| Oxct2a | 0.0 | 0.0 | 0.1 | 0.0 | 0.0 | 0.0 | 0.0 | 0.0 | 0.0 | 0.0 | 0.0 | 0.0 |
| Oxld1 | 4.3 | 5.0 | 4.4 | 4.4 | 4.9 | 4.9 | 3.6 | 4.4 | 4.6 | 4.7 | 4.0 | 4.7 |
| Oxnad1 | 4.3 | 5.2 | 4.2 | 4.3 | 3.6 | 4.8 | 3.1 | 4.3 | 3.6 | 3.9 | 4.7 | 4.0 |
| Oxr1 | 25.8 | 22.9 | 23.7 | 36.0 | 24.7 | 33.5 | 27.5 | 24.6 | 21.0 | 21.3 | 28.7 | 23.4 |
| Oxsm | 2.5 | 2.7 | 3.3 | 2.7 | 2.7 | 2.4 | 2.7 | 2.9 | 2.6 | 2.4 | 2.5 | 2.5 |
| Oxsr1 | 16.4 | 12.4 | 9.4 | 18.6 | 12.3 | 19.1 | 11.2 | 8.8 | 10.0 | 14.1 | 13.9 | 12.1 |
| Oxtr | 0.2 | 0.9 | 3.8 | 0.1 | 0.4 | 0.1 | 0.5 | 1.2 | 0.3 | 0.3 | 0.3 | 0.3 |
| P2rx1 | 0.1 | 0.0 | 0.1 | 0.1 | 0.0 | 0.1 | 0.0 | 0.1 | 0.0 | 0.0 | 0.0 | 0.1 |
| P2rx3 | 0.3 | 0.2 | 0.3 | 0.1 | 0.2 | 0.2 | 0.1 | 0.1 | 0.1 | 0.2 | 0.1 | 0.3 |
| P2rx4 | 18.8 | 16.6 | 22.2 | 28.7 | 29.5 | 20.3 | 23.5 | 22.7 | 20.0 | 20.6 | 18.7 | 25.5 |
| P2rx5 | 0.5 | 0.4 | 0.6 | 0.6 | 0.5 | 0.8 | 0.6 | 0.5 | 0.3 | 0.4 | 0.4 | 0.4 |
| P2rx6 | 0.2 | 0.5 | 0.7 | 0.4 | 0.4 | 0.6 | 0.0 | 0.7 | 0.3 | 0.6 | 0.3 | 0.3 |
| P2rx7 | 4.1 | 4.1 | 4.8 | 4.6 | 5.1 | 4.5 | 2.3 | 2.6 | 1.7 | 3.3 | 2.8 | 2.3 |
| P2ry1 | 0.2 | 0.3 | 0.3 | 0.1 | 0.4 | 0.1 | 0.3 | 0.3 | 0.2 | 0.2 | 0.1 | 0.2 |

| | | | | | | | | | | | | |
|---|---|---|---|---|---|---|---|---|---|---|---|---|
| P2ry12 | 0.0 | 0.0 | 0.0 | 0.1 | 0.2 | 0.0 | 0.0 | 0.0 | 0.0 | 0.0 | 0.0 | 0.0 |
| P2ry13 | 0.0 | 0.0 | 0.0 | 0.1 | 0.0 | 0.0 | 0.0 | 0.0 | 0.0 | 0.0 | 0.0 | 0.0 |
| P2ry14 | 0.1 | 0.2 | 0.1 | 0.2 | 0.1 | 0.1 | 0.1 | 0.0 | 0.1 | 0.0 | 0.0 | 0.1 |
| P2ry2 | 2.6 | 2.8 | 2.8 | 3.4 | 2.9 | 2.8 | 3.0 | 2.6 | 1.6 | 1.6 | 1.5 | 1.5 |
| P2ry4 | 0.0 | 0.0 | 0.0 | 0.0 | 0.0 | 0.0 | 0.0 | 0.0 | 0.0 | 0.0 | 0.0 | 0.0 |
| P2ry6 | 5.0 | 3.8 | 4.0 | 7.5 | 14.0 | 3.0 | 4.4 | 3.0 | 2.5 | 2.2 | 1.7 | 3.0 |
| P4ha1 | 187.9 | 186.2 | 155.2 | 183.9 | 150.3 | 194.0 | 183.8 | 140.0 | 200.2 | 193.2 | 197.9 | 200.3 |
| P4ha2 | 48.1 | 53.8 | 43.8 | 67.4 | 54.3 | 60.1 | 53.2 | 35.5 | 53.0 | 40.3 | 41.1 | 47.1 |
| P4ha3 | 3.7 | 3.8 | 5.3 | 4.2 | 8.0 | 5.4 | 10.3 | 9.5 | 10.7 | 7.3 | 7.0 | 9.3 |
| P4hb | 572.4 | 519.7 | 522.7 | 559.9 | 543.8 | 470.1 | 600.6 | 653.4 | 587.6 | 630.1 | 629.6 | 718.5 |
| P4htm | 4.1 | 3.2 | 3.3 | 4.5 | 2.2 | 6.9 | 2.4 | 2.7 | 2.8 | 2.6 | 2.6 | 3.5 |
| Pa2g4 | 25.0 | 21.9 | 21.1 | 28.7 | 24.4 | 24.2 | 30.2 | 21.2 | 18.7 | 23.5 | 24.8 | 23.3 |
| Pabpc1 | 108.1 | 88.5 | 89.6 | 121.7 | 103.3 | 118.5 | 109.7 | 100.0 | 76.8 | 93.3 | 117.4 | 91.4 |
| Pabpc1l | 0.1 | 0.1 | 0.0 | 0.1 | 0.1 | 0.1 | 0.1 | 0.1 | 0.0 | 0.1 | 0.0 | 0.0 |
| Pabpc4 | 28.4 | 18.5 | 18.0 | 24.1 | 18.0 | 27.4 | 21.7 | 21.5 | 18.1 | 20.4 | 23.6 | 21.1 |
| Pabpc4l | 0.5 | 0.6 | 0.7 | 0.4 | 0.3 | 0.9 | 0.4 | 0.5 | 0.5 | 0.5 | 0.5 | 0.7 |
| Pabpc5 | 0.3 | 0.4 | 0.3 | 0.2 | 0.1 | 0.8 | 0.3 | 0.1 | 0.1 | 0.2 | 0.4 | 0.2 |
| Pabpc6 | 0.0 | 0.0 | 0.0 | 0.0 | 0.0 | 0.0 | 0.0 | 0.0 | 0.0 | 0.0 | 0.0 | 0.0 |
| Pabpn1 | 24.9 | 23.8 | 27.3 | 23.6 | 22.9 | 24.0 | 26.5 | 22.7 | 25.9 | 21.1 | 24.1 | 22.9 |
| Pacrg | 0.2 | 0.3 | 0.5 | 0.2 | 0.0 | 0.6 | 0.3 | 0.4 | 0.1 | 0.4 | 0.2 | 0.3 |
| Pacrgl | 2.7 | 2.7 | 3.3 | 2.5 | 3.0 | 2.6 | 2.3 | 3.6 | 2.5 | 2.9 | 2.6 | 2.7 |
| Pacs1 | 2.9 | 3.4 | 3.0 | 2.7 | 3.0 | 3.4 | 3.7 | 2.6 | 2.8 | 2.6 | 2.7 | 2.8 |
| Pacs2 | 19.5 | 21.2 | 17.5 | 21.3 | 19.2 | 23.1 | 18.0 | 17.6 | 16.4 | 13.9 | 14.4 | 13.8 |
| Pacsin1 | 0.1 | 0.1 | 0.0 | 0.1 | 0.0 | 0.1 | 0.0 | 0.0 | 0.0 | 0.0 | 0.0 | 0.0 |
| Pacsin2 | 34.0 | 33.2 | 28.6 | 39.3 | 32.0 | 34.4 | 34.2 | 29.2 | 28.1 | 38.9 | 42.7 | 31.2 |
| Pacsin3 | 14.0 | 17.2 | 14.6 | 21.5 | 15.6 | 18.5 | 18.6 | 14.8 | 16.3 | 12.2 | 13.2 | 15.1 |
| Padi1 | 0.0 | 0.0 | 0.0 | 0.0 | 0.0 | 0.1 | 0.0 | 0.0 | 0.0 | 0.0 | 0.0 | 0.0 |
| Padi2 | 0.1 | 0.0 | 0.0 | 0.7 | 1.0 | 0.0 | 0.1 | 0.1 | 0.0 | 0.1 | 0.1 | 0.0 |
| Padi3 | 0.0 | 0.0 | 0.0 | 0.0 | 0.0 | 0.0 | 0.0 | 0.0 | 0.0 | 0.0 | 0.0 | 0.1 |
| Padi4 | 0.2 | 0.1 | 0.1 | 0.2 | 0.1 | 0.1 | 0.0 | 0.1 | 0.1 | 0.1 | 0.2 | 0.1 |
| Paf1 | 30.3 | 29.4 | 34.8 | 35.2 | 26.3 | 26.5 | 33.1 | 28.5 | 24.7 | 29.8 | 35.4 | 38.0 |
| Pafah1b1 | 41.4 | 42.7 | 37.8 | 41.6 | 38.5 | 39.6 | 38.6 | 37.6 | 42.6 | 42.9 | 45.7 | 45.0 |
| Pafah1b2 | 30.7 | 31.3 | 33.0 | 34.1 | 29.7 | 32.5 | 34.5 | 30.8 | 29.9 | 31.8 | 38.8 | 35.9 |
| Pafah1b3 | 4.8 | 5.9 | 4.7 | 5.0 | 5.4 | 5.6 | 6.7 | 5.9 | 7.2 | 5.9 | 7.5 | 6.4 |
| Pafah2 | 6.5 | 4.1 | 2.7 | 7.4 | 5.4 | 6.0 | 3.8 | 3.8 | 4.1 | 6.1 | 4.2 | 4.3 |
| Pag1 | 1.0 | 1.4 | 1.9 | 1.2 | 1.5 | 0.7 | 1.6 | 1.5 | 2.0 | 1.5 | 1.2 | 1.4 |
| Pagr1a | 7.6 | 8.7 | 9.5 | 10.7 | 9.2 | 8.5 | 10.1 | 11.8 | 10.9 | 9.3 | 8.9 | 9.9 |
| Paics | 76.9 | 59.7 | 60.2 | 61.8 | 48.8 | 65.4 | 53.3 | 57.0 | 52.3 | 56.6 | 59.6 | 58.3 |
| Paip1 | 16.7 | 17.5 | 15.9 | 14.3 | 15.4 | 15.9 | 14.6 | 13.8 | 16.4 | 18.6 | 17.4 | 18.3 |
| Paip2 | 120.5 | 125.4 | 120.9 | 116.6 | 107.6 | 116.9 | 103.0 | 106.7 | 102.1 | 116.7 | 132.2 | 129.4 |
| Paip2b | 12.8 | 12.3 | 12.0 | 12.1 | 10.2 | 12.0 | 10.0 | 10.6 | 11.1 | 10.3 | 12.9 | 11.1 |
| Pak1 | 5.8 | 5.5 | 5.2 | 7.2 | 7.5 | 8.0 | 6.7 | 5.6 | 4.2 | 5.7 | 5.3 | 4.4 |
| Pak1ip1 | 34.0 | 29.2 | 25.1 | 33.9 | 29.0 | 29.0 | 27.2 | 25.4 | 26.0 | 29.7 | 32.3 | 29.1 |
| Pak2 | 13.8 | 15.0 | 14.4 | 16.0 | 17.3 | 15.6 | 16.2 | 13.8 | 15.0 | 16.2 | 17.8 | 16.5 |
| Pak3 | 7.3 | 7.9 | 6.5 | 7.4 | 5.8 | 7.5 | 5.6 | 4.0 | 6.2 | 7.0 | 8.9 | 6.5 |
| Pak4 | 12.4 | 12.9 | 11.0 | 8.5 | 9.8 | 9.3 | 16.1 | 18.1 | 16.0 | 11.3 | 15.4 | 12.7 |
| Pak6 | 0.1 | 0.1 | 0.1 | 0.0 | 0.0 | 0.0 | 0.0 | 0.1 | 0.0 | 0.0 | 0.0 | 0.0 |
| Pak7 | 0.0 | 0.0 | 0.0 | 0.0 | 0.0 | 0.0 | 0.0 | 0.0 | 0.0 | 0.0 | 0.0 | 0.0 |
| Palb2 | 0.5 | 0.7 | 0.8 | 0.3 | 0.8 | 0.2 | 0.6 | 0.4 | 0.6 | 0.3 | 0.6 | 0.3 |
| Pald1 | 2.3 | 2.7 | 2.8 | 1.9 | 3.0 | 1.6 | 2.4 | 2.6 | 2.3 | 3.1 | 2.3 | 2.4 |
| Palld | 2.8 | 3.0 | 2.5 | 3.4 | 5.0 | 2.4 | 5.0 | 2.9 | 5.2 | 9.1 | 7.5 | 6.1 |
| Palm | 15.5 | 15.2 | 16.4 | 15.4 | 11.1 | 18.8 | 16.5 | 19.1 | 20.3 | 13.2 | 17.1 | 18.7 |
| Palm2 | 2.9 | 2.7 | 3.1 | 3.3 | 2.7 | 3.1 | 3.6 | 2.2 | 2.7 | 2.3 | 2.9 | 3.4 |
| Palm3 | 0.6 | 0.6 | 0.7 | 1.2 | 0.5 | 0.7 | 0.6 | 0.5 | 0.3 | 0.4 | 0.4 | 0.4 |
| Palmd | 0.4 | 0.1 | 0.4 | 0.1 | 0.1 | 0.1 | 0.1 | 0.7 | 0.2 | 2.4 | 1.4 | 0.7 |
| Pam | 126.5 | 107.2 | 121.0 | 108.1 | 91.7 | 109.1 | 76.7 | 76.6 | 69.8 | 60.9 | 65.9 | 84.0 |
| Pam16 | 34.1 | 28.5 | 27.2 | 36.8 | 29.8 | 36.6 | 27.3 | 25.5 | 28.1 | 38.1 | 29.5 | 30.5 |

| | | | | | | | | | | | | |
|---|---|---|---|---|---|---|---|---|---|---|---|---|
| Pamr1 | 19.8 | 13.3 | 9.0 | 18.7 | 13.7 | 17.2 | 3.4 | 8.5 | 4.7 | 17.7 | 13.1 | 11.8 |
| Pan2 | 1.6 | 2.1 | 2.2 | 1.6 | 2.2 | 1.8 | 2.2 | 2.2 | 2.4 | 1.9 | 1.6 | 1.7 |
| Pan3 | 7.3 | 8.6 | 8.8 | 7.5 | 8.0 | 7.0 | 7.8 | 8.1 | 7.9 | 8.1 | 7.6 | 7.5 |
| Pank1 | 1.9 | 2.3 | 2.4 | 1.2 | 2.2 | 2.3 | 2.0 | 2.0 | 2.2 | 1.6 | 2.0 | 1.9 |
| Pank2 | 7.5 | 7.0 | 6.6 | 6.9 | 7.3 | 7.3 | 7.5 | 6.9 | 6.3 | 7.0 | 7.2 | 6.7 |
| Pank3 | 12.4 | 15.0 | 14.4 | 11.7 | 11.9 | 13.1 | 14.6 | 14.3 | 16.0 | 11.8 | 18.2 | 14.4 |
| Pank4 | 6.5 | 7.9 | 5.8 | 6.3 | 6.5 | 7.7 | 6.3 | 6.3 | 6.9 | 6.4 | 5.7 | 5.7 |
| Panx1 | 15.5 | 11.5 | 8.9 | 17.0 | 16.5 | 13.7 | 11.2 | 11.1 | 9.1 | 9.1 | 11.6 | 9.8 |
| Panx2 | 0.1 | 0.1 | 0.0 | 0.0 | 0.0 | 0.0 | 0.0 | 0.1 | 0.0 | 0.0 | 0.1 | 0.0 |
| Paox | 4.4 | 5.1 | 4.6 | 3.0 | 2.8 | 3.5 | 3.7 | 4.6 | 4.4 | 3.9 | 3.4 | 3.3 |
| Papd4 | 13.4 | 12.5 | 12.3 | 12.4 | 12.0 | 13.1 | 10.2 | 10.8 | 11.6 | 10.2 | 12.1 | 12.3 |
| Papd5 | 8.3 | 8.3 | 8.3 | 12.9 | 8.5 | 11.2 | 8.0 | 6.7 | 7.4 | 7.8 | 8.9 | 8.8 |
| Papd7 | 4.5 | 4.5 | 4.6 | 4.7 | 4.7 | 4.5 | 6.4 | 4.8 | 5.4 | 4.9 | 5.2 | 5.2 |
| Papln | 0.1 | 0.1 | 0.0 | 0.0 | 0.0 | 0.0 | 0.0 | 0.0 | 0.0 | 0.2 | 0.0 | 0.1 |
| Papola | 24.8 | 29.3 | 26.9 | 26.3 | 29.6 | 25.1 | 26.2 | 27.3 | 27.6 | 27.7 | 29.6 | 27.3 |
| Papolb | 0.0 | 0.2 | 0.0 | 0.0 | 0.1 | 0.2 | 0.1 | 0.0 | 0.0 | 0.0 | 0.1 | 0.0 |
| Papolg | 4.2 | 4.4 | 4.8 | 3.8 | 4.4 | 3.8 | 4.8 | 4.1 | 4.5 | 5.5 | 5.7 | 5.4 |
| Pappa | 13.4 | 17.6 | 22.1 | 11.6 | 13.4 | 8.7 | 17.7 | 19.3 | 24.5 | 17.2 | 18.4 | 20.5 |
| Pappa2 | 0.2 | 0.1 | 0.2 | 0.1 | 0.1 | 0.1 | 0.2 | 1.3 | 0.5 | 0.3 | 0.4 | 0.1 |
| Papss1 | 21.8 | 20.1 | 17.9 | 16.6 | 18.5 | 16.9 | 22.4 | 25.9 | 22.5 | 21.6 | 25.9 | 19.2 |
| Papss2 | 24.9 | 22.1 | 20.7 | 24.0 | 21.8 | 20.3 | 23.8 | 24.6 | 23.2 | 14.4 | 24.4 | 20.8 |
| Paqr3 | 1.8 | 2.0 | 1.7 | 2.2 | 1.7 | 2.1 | 1.5 | 2.3 | 2.2 | 1.9 | 1.2 | 1.9 |
| Paqr4 | 2.5 | 4.0 | 3.9 | 3.2 | 3.1 | 4.2 | 5.3 | 5.4 | 5.0 | 3.1 | 4.6 | 2.8 |
| Paqr5 | 0.1 | 0.1 | 0.3 | 0.1 | 0.1 | 0.0 | 0.1 | 0.1 | 0.0 | 0.1 | 0.0 | 0.0 |
| Paqr6 | 0.2 | 0.2 | 0.3 | 0.0 | 0.0 | 0.1 | 0.1 | 0.3 | 0.1 | 0.0 | 0.1 | 0.1 |
| Paqr7 | 7.3 | 5.6 | 5.5 | 8.8 | 7.7 | 7.6 | 6.0 | 5.7 | 5.5 | 4.6 | 4.8 | 4.1 |
| Paqr8 | 1.1 | 1.3 | 1.8 | 1.2 | 1.6 | 1.2 | 1.7 | 2.0 | 1.4 | 1.9 | 1.5 | 1.7 |
| Paqr9 | 0.0 | 0.0 | 0.0 | 0.1 | 0.0 | 0.0 | 0.0 | 0.0 | 0.0 | 0.0 | 0.0 | 0.0 |
| Pard3 | 10.1 | 10.9 | 11.3 | 10.1 | 9.2 | 9.9 | 11.0 | 12.2 | 11.1 | 10.2 | 12.1 | 11.7 |
| Pard3b | 2.4 | 2.3 | 2.3 | 2.5 | 1.5 | 3.1 | 2.4 | 2.4 | 1.9 | 1.9 | 2.2 | 2.4 |
| Pard6a | 3.1 | 2.7 | 2.7 | 3.7 | 2.3 | 3.3 | 2.9 | 3.1 | 3.2 | 3.4 | 3.1 | 2.8 |
| Pard6b | 2.3 | 2.9 | 2.3 | 2.3 | 2.9 | 2.8 | 2.9 | 3.1 | 3.1 | 3.5 | 2.8 | 2.3 |
| Pard6g | 5.0 | 5.1 | 4.7 | 3.6 | 4.5 | 4.8 | 4.8 | 4.6 | 4.4 | 4.7 | 5.0 | 4.3 |
| Parg | 16.9 | 16.7 | 15.5 | 15.9 | 15.9 | 16.7 | 13.3 | 13.1 | 13.2 | 14.8 | 14.9 | 15.6 |
| Park2 | 1.1 | 1.0 | 1.3 | 0.9 | 0.6 | 1.1 | 0.7 | 0.8 | 0.8 | 0.5 | 0.7 | 0.7 |
| Park7 | 107.5 | 88.0 | 79.8 | 130.1 | 88.7 | 118.2 | 90.1 | 95.5 | 79.1 | 89.9 | 97.5 | 87.6 |
| Parl | 17.5 | 14.7 | 12.3 | 17.1 | 16.3 | 14.9 | 15.0 | 16.0 | 12.7 | 14.8 | 18.1 | 16.2 |
| Parm1 | 25.0 | 15.1 | 7.9 | 40.2 | 16.3 | 26.0 | 13.2 | 5.2 | 6.8 | 9.0 | 11.3 | 10.6 |
| Parn | 11.6 | 11.0 | 11.4 | 11.4 | 8.9 | 12.8 | 10.5 | 12.2 | 10.3 | 10.1 | 11.1 | 11.6 |
| Parp1 | 12.8 | 12.4 | 13.3 | 11.6 | 13.7 | 10.9 | 13.0 | 14.3 | 10.8 | 13.4 | 12.9 | 12.1 |
| Parp10 | 3.9 | 4.1 | 4.4 | 4.7 | 5.2 | 4.7 | 3.9 | 3.4 | 5.0 | 4.8 | 3.5 | 4.4 |
| Parp11 | 4.8 | 5.1 | 5.2 | 4.8 | 4.9 | 4.2 | 4.5 | 3.9 | 3.9 | 3.8 | 3.9 | 4.1 |
| Parp12 | 8.3 | 6.7 | 8.4 | 9.2 | 8.5 | 8.1 | 7.5 | 8.1 | 7.8 | 6.7 | 6.5 | 8.9 |
| Parp14 | 5.1 | 4.3 | 5.2 | 7.8 | 7.1 | 5.2 | 4.3 | 3.3 | 2.9 | 2.5 | 2.6 | 5.4 |
| Parp16 | 0.5 | 0.6 | 0.8 | 0.5 | 0.5 | 0.8 | 0.2 | 0.8 | 0.6 | 0.7 | 0.5 | 0.5 |
| Parp2 | 10.7 | 11.5 | 13.2 | 11.1 | 10.8 | 11.3 | 11.0 | 10.1 | 9.4 | 8.1 | 10.5 | 10.1 |
| Parp3 | 80.3 | 81.1 | 82.5 | 95.1 | 81.2 | 85.2 | 92.6 | 83.6 | 93.3 | 70.5 | 86.6 | 92.5 |
| Parp4 | 1.5 | 1.7 | 1.9 | 1.4 | 1.7 | 1.7 | 1.9 | 1.4 | 1.6 | 1.5 | 1.5 | 1.6 |
| Parp6 | 7.2 | 6.2 | 5.9 | 7.6 | 4.9 | 8.2 | 5.2 | 4.7 | 5.6 | 5.3 | 5.1 | 4.0 |
| Parp8 | 6.2 | 5.6 | 6.1 | 7.3 | 6.4 | 6.4 | 5.7 | 4.5 | 5.1 | 6.8 | 5.7 | 6.0 |
| Parp9 | 8.4 | 6.3 | 9.3 | 9.6 | 9.3 | 7.1 | 7.9 | 8.1 | 7.8 | 6.5 | 6.9 | 12.6 |
| Parpbp | 0.8 | 1.6 | 2.4 | 0.3 | 2.4 | 0.3 | 2.8 | 1.1 | 2.2 | 1.0 | 1.8 | 1.5 |
| Pars2 | 1.4 | 1.4 | 1.4 | 1.0 | 1.3 | 1.7 | 1.1 | 1.3 | 1.5 | 1.3 | 1.4 | 1.1 |
| Parva | 50.7 | 50.1 | 50.8 | 53.1 | 47.7 | 47.0 | 63.9 | 52.1 | 58.5 | 58.8 | 67.3 | 59.6 |
| Parvb | 0.4 | 0.2 | 0.2 | 0.6 | 0.7 | 0.7 | 0.7 | 0.3 | 0.4 | 0.5 | 0.4 | 0.3 |
| Parvg | 0.1 | 0.0 | 0.0 | 1.0 | 2.3 | 0.0 | 0.2 | 0.1 | 0.0 | 0.3 | 0.0 | 0.1 |
| Pask | 0.9 | 1.2 | 1.6 | 0.6 | 1.3 | 0.7 | 1.5 | 1.5 | 1.4 | 1.1 | 1.3 | 1.0 |

| | | | | | | | | | | | | |
|---|---|---|---|---|---|---|---|---|---|---|---|---|
| Pate2 | 0.0 | 0.0 | 0.0 | 0.0 | 0.0 | 0.0 | 0.0 | 0.1 | 0.0 | 0.0 | 0.0 | 0.1 |
| Patl1 | 1.7 | 2.1 | 2.3 | 2.0 | 2.1 | 1.8 | 2.7 | 1.9 | 1.7 | 1.7 | 2.5 | 1.9 |
| Patl2 | 0.0 | 0.0 | 0.1 | 0.1 | 0.0 | 0.0 | 0.0 | 0.0 | 0.0 | 0.0 | 0.0 | 0.0 |
| Patz1 | 3.4 | 2.7 | 3.0 | 2.4 | 3.1 | 3.3 | 3.0 | 3.0 | 2.4 | 2.3 | 2.2 | 2.5 |
| Pawr | 16.3 | 14.8 | 13.4 | 16.7 | 14.8 | 13.3 | 17.1 | 10.8 | 19.6 | 34.5 | 25.1 | 23.9 |
| Pax2 | 0.0 | 0.0 | 0.0 | 0.0 | 0.0 | 0.0 | 0.0 | 0.0 | 0.0 | 0.0 | 0.0 | 0.0 |
| Pax3 | 1.2 | 0.5 | 0.3 | 2.2 | 1.0 | 1.7 | 0.6 | 0.5 | 0.5 | 0.3 | 0.3 | 0.3 |
| Pax6 | 0.1 | 0.1 | 0.1 | 0.1 | 0.1 | 0.0 | 0.1 | 0.0 | 0.1 | 0.0 | 0.0 | 0.0 |
| Pax9 | 0.0 | 0.0 | 0.0 | 0.2 | 0.0 | 0.0 | 0.0 | 0.0 | 0.0 | 0.0 | 0.0 | 0.0 |
| Paxbp1 | 5.2 | 6.2 | 6.8 | 5.3 | 5.6 | 5.4 | 5.2 | 5.6 | 6.2 | 6.4 | 6.7 | 6.2 |
| Paxip1 | 2.1 | 2.3 | 2.5 | 2.1 | 2.2 | 2.0 | 2.5 | 2.5 | 2.1 | 1.9 | 2.2 | 2.0 |
| Pbdc1 | 5.8 | 6.0 | 6.7 | 6.7 | 7.4 | 6.4 | 7.4 | 6.9 | 5.3 | 6.8 | 7.4 | 6.8 |
| Pbk | 2.5 | 4.2 | 6.0 | 0.9 | 6.2 | 0.6 | 7.2 | 2.5 | 5.5 | 2.7 | 4.4 | 2.9 |
| Pbld1 | 0.1 | 0.0 | 0.0 | 0.1 | 0.0 | 0.0 | 0.0 | 0.0 | 0.0 | 0.0 | 0.1 | 0.0 |
| Pbld2 | 0.8 | 1.4 | 1.0 | 0.7 | 0.7 | 0.9 | 0.7 | 0.9 | 0.6 | 0.7 | 0.9 | 0.8 |
| Pbp2 | 2.0 | 0.6 | 0.4 | 1.1 | 1.2 | 1.6 | 3.0 | 0.4 | 1.8 | 2.0 | 1.2 | 1.6 |
| Pbrm1 | 10.9 | 11.4 | 12.1 | 10.7 | 11.1 | 12.2 | 11.4 | 13.1 | 11.8 | 12.0 | 14.0 | 13.3 |
| Pbx1 | 9.2 | 12.2 | 14.8 | 11.4 | 10.3 | 9.3 | 12.6 | 13.7 | 11.9 | 11.2 | 11.7 | 13.2 |
| Pbx2 | 7.4 | 8.3 | 8.9 | 7.1 | 7.6 | 8.0 | 7.2 | 7.2 | 7.7 | 7.9 | 7.4 | 8.2 |
| Pbx3 | 3.6 | 4.4 | 5.0 | 4.0 | 4.3 | 3.9 | 5.7 | 5.3 | 5.4 | 4.7 | 5.2 | 5.1 |
| Pbx4 | 0.8 | 1.1 | 1.2 | 0.8 | 1.1 | 1.4 | 1.0 | 1.0 | 0.9 | 0.9 | 1.3 | 1.1 |
| Pbxip1 | 31.3 | 28.0 | 32.3 | 27.8 | 32.6 | 29.3 | 34.2 | 35.6 | 38.2 | 31.6 | 33.5 | 34.7 |
| Pcbd1 | 2.5 | 2.3 | 2.2 | 2.2 | 1.2 | 2.3 | 0.6 | 0.9 | 1.2 | 1.4 | 1.6 | 1.6 |
| Pcbd2 | 20.3 | 18.1 | 18.3 | 20.7 | 17.9 | 21.9 | 17.1 | 22.0 | 16.3 | 18.4 | 20.2 | 18.1 |
| Pcbp1 | 20.6 | 21.8 | 21.2 | 24.7 | 24.6 | 23.9 | 24.0 | 25.6 | 22.0 | 26.9 | 22.4 | 22.2 |
| Pcbp2 | 7.3 | 7.2 | 8.4 | 6.8 | 7.9 | 7.5 | 9.3 | 9.1 | 9.4 | 10.2 | 8.4 | 8.6 |
| Pcbp3 | 1.6 | 1.7 | 2.6 | 2.0 | 1.7 | 2.4 | 2.2 | 1.6 | 1.7 | 1.4 | 1.6 | 1.5 |
| Pcbp4 | 23.0 | 22.8 | 18.7 | 22.7 | 19.2 | 23.5 | 22.4 | 23.8 | 23.2 | 17.0 | 20.3 | 17.1 |
| Pcca | 5.5 | 7.9 | 7.2 | 5.3 | 6.0 | 5.3 | 5.4 | 6.5 | 6.1 | 6.2 | 5.8 | 6.0 |
| Pccb | 11.5 | 11.9 | 12.0 | 14.0 | 11.4 | 11.7 | 12.2 | 13.9 | 10.0 | 9.5 | 11.6 | 12.2 |
| Pcdh1 | 1.5 | 1.5 | 1.4 | 1.3 | 1.9 | 2.2 | 3.1 | 1.9 | 2.8 | 1.4 | 1.4 | 1.6 |
| Pcdh10 | 0.0 | 0.0 | 0.0 | 0.0 | 0.0 | 0.1 | 0.0 | 0.0 | 0.0 | 0.0 | 0.0 | 0.0 |
| Pcdh11x | 0.1 | 0.1 | 0.0 | 0.1 | 0.0 | 0.0 | 0.0 | 0.0 | 0.2 | 0.4 | 0.3 | 0.2 |
| Pcdh12 | 0.3 | 0.1 | 0.2 | 0.1 | 0.3 | 0.0 | 0.4 | 0.4 | 0.4 | 0.0 | 0.0 | 0.1 |
| Pcdh15 | 0.0 | 0.0 | 0.0 | 0.0 | 0.0 | 0.0 | 0.0 | 0.0 | 0.0 | 0.0 | 0.0 | 0.0 |
| Pcdh17 | 0.0 | 0.0 | 0.0 | 0.0 | 0.0 | 0.0 | 0.0 | 0.0 | 0.0 | 0.0 | 0.0 | 0.0 |
| Pcdh18 | 9.3 | 8.5 | 7.4 | 10.4 | 9.6 | 8.3 | 14.1 | 12.3 | 13.8 | 6.7 | 11.5 | 8.1 |
| Pcdh19 | 0.5 | 0.9 | 1.5 | 0.4 | 1.4 | 0.6 | 2.8 | 3.3 | 2.8 | 1.0 | 1.3 | 1.2 |
| Pcdh20 | 0.0 | 0.0 | 0.0 | 0.0 | 0.0 | 0.0 | 0.0 | 0.0 | 0.0 | 0.0 | 0.0 | 0.0 |
| Pcdh7 | 13.8 | 13.4 | 13.1 | 12.2 | 16.1 | 8.7 | 20.3 | 14.1 | 21.7 | 24.0 | 20.7 | 22.1 |
| Pcdh8 | 0.0 | 0.0 | 0.0 | 0.0 | 0.0 | 0.0 | 0.0 | 0.0 | 0.0 | 0.0 | 0.0 | 0.0 |
| Pcdh9 | 6.2 | 4.7 | 5.1 | 7.3 | 4.9 | 7.6 | 7.4 | 3.5 | 6.8 | 3.2 | 4.8 | 7.1 |
| Pcdha1 | 0.0 | 0.0 | 0.0 | 0.1 | 0.0 | 0.0 | 0.0 | 0.0 | 0.0 | 0.0 | 0.0 | 0.0 |
| Pcdha10 | 0.3 | 0.1 | 0.1 | 0.5 | 0.2 | 0.5 | 0.2 | 0.2 | 0.4 | 0.2 | 0.3 | 0.2 |
| Pcdha11 | 0.0 | 0.0 | 0.0 | 0.1 | 0.0 | 0.1 | 0.0 | 0.0 | 0.0 | 0.0 | 0.0 | 0.0 |
| Pcdha12 | 0.0 | 0.0 | 0.1 | 0.1 | 0.1 | 0.0 | 0.0 | 0.0 | 0.0 | 0.1 | 0.0 | 0.0 |
| Pcdha2 | 0.0 | 0.0 | 0.0 | 0.0 | 0.0 | 0.0 | 0.0 | 0.0 | 0.0 | 0.0 | 0.0 | 0.0 |
| Pcdha3 | 0.0 | 0.0 | 0.0 | 0.0 | 0.0 | 0.0 | 0.0 | 0.0 | 0.0 | 0.0 | 0.0 | 0.0 |
| Pcdha4 | 0.0 | 0.0 | 0.1 | 0.0 | 0.0 | 0.0 | 0.1 | 0.1 | 0.0 | 0.0 | 0.1 | 0.0 |
| Pcdha4-g | 0.1 | 0.1 | 0.0 | 0.0 | 0.1 | 0.2 | 0.0 | 0.0 | 0.1 | 0.0 | 0.0 | 0.0 |
| Pcdha5 | 0.0 | 0.0 | 0.0 | 0.0 | 0.0 | 0.0 | 0.0 | 0.0 | 0.0 | 0.0 | 0.1 | 0.0 |
| Pcdha6 | 0.0 | 0.0 | 0.0 | 0.0 | 0.0 | 0.0 | 0.0 | 0.0 | 0.0 | 0.0 | 0.0 | 0.0 |
| Pcdha7 | 0.0 | 0.0 | 0.0 | 0.0 | 0.0 | 0.0 | 0.0 | 0.0 | 0.0 | 0.0 | 0.0 | 0.0 |
| Pcdha8 | 0.1 | 0.0 | 0.1 | 0.0 | 0.0 | 0.0 | 0.0 | 0.0 | 0.1 | 0.0 | 0.0 | 0.1 |
| Pcdha9 | 0.0 | 0.1 | 0.0 | 0.0 | 0.1 | 0.0 | 0.0 | 0.1 | 0.0 | 0.0 | 0.0 | 0.0 |
| Pcdhac1 | 0.0 | 0.0 | 0.0 | 0.0 | 0.0 | 0.0 | 0.0 | 0.0 | 0.0 | 0.0 | 0.0 | 0.0 |
| Pcdhac2 | 0.1 | 0.0 | 0.0 | 0.2 | 0.1 | 0.2 | 0.2 | 0.1 | 0.1 | 0.2 | 0.1 | 0.1 |

| | | | | | | | | | | | | |
|---|---|---|---|---|---|---|---|---|---|---|---|---|
| Pcdhb10 | 0.0 | 0.1 | 0.1 | 0.0 | 0.0 | 0.0 | 0.0 | 0.0 | 0.0 | 0.0 | 0.0 | 0.1 |
| Pcdhb11 | 0.1 | 0.2 | 0.1 | 0.1 | 0.1 | 0.0 | 0.1 | 0.2 | 0.2 | 0.1 | 0.2 | 0.1 |
| Pcdhb12 | 0.1 | 0.2 | 0.2 | 0.0 | 0.1 | 0.0 | 0.1 | 0.2 | 0.2 | 0.0 | 0.1 | 0.0 |
| Pcdhb13 | 0.1 | 0.0 | 0.2 | 0.0 | 0.1 | 0.0 | 0.1 | 0.2 | 0.0 | 0.1 | 0.0 | 0.1 |
| Pcdhb14 | 0.6 | 1.0 | 1.6 | 0.8 | 1.0 | 1.0 | 1.2 | 1.8 | 1.5 | 1.0 | 1.1 | 1.2 |
| Pcdhb15 | 0.1 | 0.2 | 0.3 | 0.2 | 0.1 | 0.1 | 0.3 | 0.4 | 0.3 | 0.3 | 0.2 | 0.3 |
| Pcdhb16 | 0.4 | 0.8 | 1.3 | 0.4 | 0.9 | 0.5 | 0.9 | 1.6 | 1.2 | 1.1 | 1.1 | 1.0 |
| Pcdhb17 | 3.4 | 3.5 | 4.4 | 3.2 | 3.4 | 2.6 | 4.0 | 5.3 | 3.8 | 3.3 | 3.8 | 3.7 |
| Pcdhb18 | 0.3 | 0.5 | 0.6 | 0.5 | 0.4 | 0.5 | 0.5 | 0.6 | 0.5 | 0.5 | 0.4 | 0.5 |
| Pcdhb19 | 0.4 | 0.6 | 1.0 | 0.6 | 0.5 | 0.5 | 0.7 | 1.1 | 0.9 | 0.9 | 0.8 | 0.7 |
| Pcdhb2 | 0.1 | 0.3 | 0.3 | 0.1 | 0.1 | 0.1 | 0.3 | 0.3 | 0.3 | 0.1 | 0.2 | 0.3 |
| Pcdhb20 | 0.3 | 0.7 | 0.9 | 0.5 | 0.6 | 0.4 | 0.4 | 1.2 | 0.9 | 0.8 | 0.7 | 0.6 |
| Pcdhb21 | 0.7 | 0.7 | 0.8 | 0.5 | 0.6 | 0.5 | 0.8 | 1.2 | 1.2 | 0.6 | 0.7 | 0.9 |
| Pcdhb22 | 3.7 | 4.3 | 4.1 | 4.3 | 4.3 | 4.2 | 4.3 | 5.5 | 5.7 | 5.5 | 4.9 | 5.1 |
| Pcdhb3 | 0.1 | 0.1 | 0.2 | 0.2 | 0.1 | 0.2 | 0.3 | 0.3 | 0.1 | 0.2 | 0.1 | 0.2 |
| Pcdhb4 | 0.1 | 0.1 | 0.2 | 0.1 | 0.1 | 0.2 | 0.2 | 0.2 | 0.1 | 0.1 | 0.1 | 0.1 |
| Pcdhb5 | 0.4 | 0.4 | 0.7 | 0.3 | 0.5 | 0.2 | 0.6 | 0.8 | 0.8 | 0.6 | 0.5 | 0.6 |
| Pcdhb7 | 0.2 | 0.2 | 0.4 | 0.0 | 0.1 | 0.2 | 0.3 | 0.5 | 0.3 | 0.3 | 0.2 | 0.2 |
| Pcdhb8 | 0.0 | 0.0 | 0.1 | 0.0 | 0.0 | 0.0 | 0.0 | 0.1 | 0.0 | 0.0 | 0.0 | 0.0 |
| Pcdhb9 | 0.1 | 0.2 | 0.3 | 0.0 | 0.1 | 0.1 | 0.3 | 0.4 | 0.3 | 0.2 | 0.0 | 0.2 |
| Pcdhga1 | 0.8 | 0.7 | 0.7 | 0.9 | 0.8 | 1.0 | 0.9 | 0.8 | 0.6 | 0.7 | 0.5 | 0.9 |
| Pcdhga10 | 3.1 | 2.9 | 3.0 | 2.9 | 3.2 | 4.4 | 5.4 | 4.6 | 4.2 | 4.1 | 3.5 | 4.3 |
| Pcdhga11 | 0.9 | 1.0 | 1.2 | 1.0 | 1.1 | 1.1 | 1.4 | 1.5 | 1.0 | 1.0 | 1.1 | 0.9 |
| Pcdhga12 | 0.7 | 0.5 | 0.7 | 0.4 | 0.8 | 0.6 | 0.8 | 0.8 | 0.5 | 0.5 | 0.5 | 0.4 |
| Pcdhga2 | 1.1 | 1.1 | 1.2 | 0.6 | 0.9 | 1.2 | 1.2 | 1.4 | 1.0 | 1.2 | 1.0 | 1.1 |
| Pcdhga3 | 0.7 | 0.9 | 1.2 | 0.7 | 0.7 | 0.8 | 1.2 | 2.3 | 1.4 | 1.2 | 1.3 | 1.1 |
| Pcdhga4 | 0.8 | 1.2 | 1.5 | 0.8 | 1.1 | 1.0 | 1.2 | 1.7 | 1.2 | 1.4 | 0.8 | 1.2 |
| Pcdhga5 | 1.2 | 2.3 | 2.9 | 0.8 | 1.6 | 1.6 | 2.2 | 3.4 | 2.5 | 1.8 | 1.5 | 2.0 |
| Pcdhga6 | 0.7 | 0.9 | 1.5 | 0.7 | 1.1 | 0.8 | 1.3 | 2.1 | 2.1 | 1.2 | 1.2 | 1.6 |
| Pcdhga7 | 2.5 | 3.0 | 3.3 | 2.6 | 2.9 | 2.4 | 3.4 | 3.0 | 3.8 | 3.5 | 3.2 | 3.1 |
| Pcdhga8 | 1.8 | 1.5 | 1.4 | 2.2 | 1.4 | 2.1 | 2.0 | 2.2 | 1.6 | 1.5 | 2.1 | 1.2 |
| Pcdhga9 | 0.6 | 0.4 | 0.6 | 0.6 | 0.5 | 0.5 | 0.7 | 0.7 | 0.4 | 0.7 | 0.6 | 0.5 |
| Pcdhgb1 | 0.5 | 1.1 | 1.1 | 0.6 | 0.6 | 0.8 | 1.2 | 1.6 | 1.0 | 0.8 | 0.9 | 0.8 |
| Pcdhgb2 | 4.1 | 4.6 | 4.9 | 2.9 | 4.2 | 4.6 | 4.2 | 5.2 | 4.8 | 3.6 | 4.2 | 4.8 |
| Pcdhgb4 | 3.5 | 3.6 | 3.2 | 4.3 | 3.4 | 4.8 | 3.8 | 3.1 | 3.8 | 3.0 | 3.7 | 3.3 |
| Pcdhgb5 | 1.1 | 1.4 | 1.6 | 1.0 | 1.2 | 1.0 | 2.1 | 2.3 | 1.6 | 1.3 | 1.7 | 1.7 |
| Pcdhgb6 | 1.6 | 1.8 | 2.9 | 2.0 | 1.8 | 1.5 | 2.6 | 3.9 | 3.1 | 2.8 | 2.4 | 2.3 |
| Pcdhgb7 | 0.6 | 0.8 | 1.0 | 0.7 | 1.0 | 1.0 | 1.3 | 1.4 | 1.0 | 1.1 | 0.8 | 1.0 |
| Pcdhgb8 | 0.4 | 0.3 | 0.3 | 0.2 | 0.3 | 0.3 | 0.5 | 0.4 | 0.2 | 0.2 | 0.4 | 0.3 |
| Pcdhgc3 | 27.2 | 31.1 | 39.8 | 33.5 | 48.4 | 36.0 | 54.3 | 52.0 | 50.0 | 35.4 | 33.1 | 39.5 |
| Pcdhgc4 | 0.4 | 0.7 | 1.0 | 0.3 | 1.1 | 0.4 | 1.0 | 1.4 | 1.0 | 0.7 | 0.5 | 0.6 |
| Pcdhgc5 | 3.7 | 4.2 | 6.3 | 3.6 | 6.4 | 4.2 | 7.6 | 6.3 | 7.3 | 6.9 | 5.0 | 6.2 |
| Pced1a | 4.3 | 5.2 | 5.4 | 5.0 | 5.4 | 5.4 | 5.0 | 4.9 | 5.1 | 5.3 | 4.8 | 4.9 |
| Pced1b | 0.5 | 0.4 | 0.4 | 1.0 | 0.6 | 0.7 | 0.3 | 0.5 | 0.2 | 0.3 | 0.3 | 0.4 |
| Pcf11 | 5.5 | 6.3 | 7.7 | 5.9 | 6.6 | 6.1 | 6.9 | 6.1 | 5.9 | 7.8 | 6.7 | 7.3 |
| Pcgf1 | 5.4 | 4.5 | 4.2 | 3.6 | 4.4 | 4.7 | 3.8 | 3.5 | 4.3 | 3.8 | 4.8 | 4.1 |
| Pcgf2 | 0.5 | 0.9 | 1.3 | 1.0 | 0.5 | 1.2 | 1.0 | 1.4 | 1.1 | 0.6 | 1.0 | 1.1 |
| Pcgf3 | 20.2 | 19.2 | 21.7 | 23.1 | 19.7 | 24.1 | 20.5 | 20.8 | 18.4 | 18.7 | 24.3 | 20.6 |
| Pcgf5 | 11.4 | 11.6 | 8.0 | 12.5 | 9.5 | 10.3 | 7.8 | 5.4 | 6.6 | 7.5 | 9.1 | 9.0 |
| Pcgf6 | 2.8 | 2.9 | 2.4 | 2.7 | 2.2 | 2.7 | 2.9 | 2.7 | 2.3 | 2.6 | 2.8 | 2.3 |
| Pcid2 | 5.5 | 6.6 | 6.4 | 6.3 | 5.8 | 6.5 | 5.9 | 6.4 | 5.8 | 5.9 | 6.1 | 5.9 |
| Pcif1 | 2.9 | 3.0 | 3.7 | 2.8 | 3.1 | 2.6 | 3.5 | 4.5 | 3.7 | 2.8 | 2.6 | 3.0 |
| Pck2 | 23.9 | 17.7 | 16.3 | 19.3 | 14.6 | 24.8 | 20.0 | 29.2 | 26.5 | 17.6 | 22.3 | 15.6 |
| Pclo | 0.0 | 0.0 | 0.0 | 0.0 | 0.0 | 0.0 | 0.0 | 0.0 | 0.0 | 0.0 | 0.0 | 0.0 |
| Pcm1 | 9.3 | 9.8 | 10.9 | 10.0 | 8.5 | 9.4 | 8.4 | 8.9 | 7.9 | 9.0 | 10.6 | 11.0 |
| Pcmt1 | 21.9 | 19.4 | 16.5 | 21.0 | 20.9 | 21.8 | 18.8 | 17.5 | 19.4 | 18.8 | 21.5 | 18.3 |
| Pcmtd1 | 17.1 | 14.5 | 17.3 | 24.1 | 13.5 | 25.6 | 15.6 | 13.3 | 11.6 | 15.6 | 21.0 | 19.7 |

| | | | | | | | | | | | | |
|---|---|---|---|---|---|---|---|---|---|---|---|---|
| Pcmtd2 | 8.2 | 8.9 | 10.3 | 8.6 | 8.0 | 10.2 | 8.2 | 8.7 | 8.7 | 9.6 | 9.4 | 12.2 |
| Pcna | 38.7 | 44.9 | 47.2 | 39.0 | 68.1 | 26.1 | 57.9 | 40.5 | 42.6 | 37.8 | 46.3 | 37.8 |
| Pcnp | 47.1 | 43.1 | 50.4 | 64.9 | 46.9 | 61.8 | 47.8 | 43.1 | 43.4 | 49.4 | 52.9 | 58.4 |
| Pcnt | 2.3 | 2.8 | 3.6 | 2.1 | 3.1 | 2.1 | 3.7 | 2.8 | 2.9 | 2.3 | 2.5 | 2.5 |
| Pcnx | 6.8 | 8.0 | 7.2 | 6.5 | 7.0 | 8.8 | 6.9 | 6.1 | 6.4 | 4.2 | 5.0 | 4.8 |
| Pcnxl2 | 0.0 | 0.0 | 0.0 | 0.0 | 0.0 | 0.0 | 0.0 | 0.0 | 0.0 | 0.0 | 0.0 | 0.0 |
| Pcnxl3 | 3.9 | 4.5 | 4.3 | 3.6 | 4.7 | 4.3 | 4.8 | 5.1 | 5.5 | 4.4 | 3.8 | 3.2 |
| Pcnxl4 | 4.2 | 5.0 | 3.9 | 4.4 | 3.8 | 4.7 | 4.0 | 3.3 | 3.8 | 4.3 | 3.7 | 4.6 |
| Pcolce | 167.6 | 180.9 | 190.5 | 179.7 | 165.7 | 194.0 | 220.8 | 229.7 | 236.3 | 160.5 | 182.5 | 157.8 |
| Pcolce2 | 32.4 | 35.4 | 41.3 | 27.2 | 46.2 | 34.5 | 82.0 | 54.9 | 55.9 | 44.3 | 46.5 | 66.8 |
| Pcp2 | 0.0 | 0.2 | 0.1 | 0.1 | 0.1 | 0.1 | 0.0 | 0.0 | 0.2 | 0.2 | 0.2 | 0.0 |
| Pcp4l1 | 0.0 | 0.0 | 0.0 | 0.5 | 0.6 | 0.0 | 0.1 | 0.2 | 0.1 | 0.1 | 0.0 | 0.0 |
| Pcsk1 | 0.0 | 0.0 | 0.0 | 0.6 | 0.5 | 0.0 | 0.0 | 0.0 | 0.0 | 0.0 | 0.0 | 0.0 |
| Pcsk2 | 0.0 | 0.0 | 0.0 | 0.0 | 0.0 | 0.0 | 0.0 | 0.0 | 0.0 | 0.0 | 0.0 | 0.0 |
| Pcsk4 | 0.1 | 0.0 | 0.0 | 0.1 | 0.1 | 0.2 | 0.0 | 0.0 | 0.0 | 0.0 | 0.0 | 0.0 |
| Pcsk5 | 15.6 | 15.4 | 19.0 | 7.8 | 10.4 | 5.4 | 9.3 | 7.8 | 8.5 | 16.8 | 12.7 | 16.3 |
| Pcsk6 | 97.4 | 137.5 | 122.5 | 105.7 | 86.9 | 154.1 | 87.1 | 81.9 | 78.5 | 44.6 | 51.9 | 40.6 |
| Pcsk7 | 5.9 | 6.9 | 6.1 | 6.1 | 6.6 | 5.4 | 6.4 | 5.8 | 7.0 | 5.3 | 5.5 | 5.6 |
| Pcsk9 | 0.2 | 0.5 | 0.4 | 0.0 | 0.1 | 0.1 | 0.2 | 1.4 | 0.3 | 0.2 | 0.4 | 0.1 |
| Pctp | 2.3 | 1.8 | 1.6 | 3.9 | 4.3 | 1.7 | 1.8 | 1.4 | 1.4 | 2.5 | 1.9 | 1.2 |
| Pcx | 13.5 | 12.2 | 14.0 | 11.0 | 11.9 | 10.8 | 9.0 | 9.3 | 7.7 | 8.9 | 7.3 | 7.0 |
| Pcyox1 | 53.5 | 39.8 | 37.6 | 56.7 | 45.8 | 60.4 | 38.9 | 37.1 | 35.6 | 42.2 | 41.2 | 40.1 |
| Pcyox1l | 2.5 | 3.4 | 3.6 | 2.5 | 3.4 | 2.7 | 3.9 | 2.8 | 3.2 | 2.7 | 3.1 | 2.6 |
| Pcyt1a | 16.6 | 16.6 | 14.9 | 19.8 | 16.3 | 16.5 | 17.0 | 15.9 | 15.7 | 15.2 | 16.2 | 18.3 |
| Pcyt1b | 2.3 | 2.6 | 2.3 | 2.5 | 2.4 | 3.0 | 3.7 | 4.0 | 3.1 | 1.6 | 3.2 | 1.8 |
| Pcyt2 | 11.9 | 14.4 | 12.4 | 7.2 | 10.6 | 10.4 | 16.6 | 17.4 | 15.0 | 9.4 | 17.0 | 8.5 |
| Pdap1 | 39.3 | 33.2 | 40.4 | 51.3 | 35.0 | 41.8 | 47.7 | 41.1 | 31.4 | 36.5 | 48.2 | 44.7 |
| Pdcd10 | 22.9 | 27.3 | 26.7 | 25.9 | 26.4 | 23.2 | 24.9 | 24.9 | 26.0 | 27.1 | 30.6 | 32.2 |
| Pdcd11 | 7.1 | 6.5 | 6.3 | 7.6 | 7.1 | 6.8 | 7.0 | 7.4 | 5.8 | 7.1 | 7.3 | 6.8 |
| Pdcd2 | 14.0 | 9.9 | 9.3 | 13.1 | 8.9 | 13.9 | 9.8 | 9.8 | 9.8 | 13.6 | 11.3 | 11.1 |
| Pdcd2l | 8.5 | 7.0 | 6.7 | 8.3 | 7.3 | 8.0 | 7.1 | 6.6 | 6.9 | 7.0 | 6.1 | 7.2 |
| Pdcd4 | 27.6 | 44.4 | 54.8 | 24.6 | 31.4 | 24.6 | 38.4 | 60.5 | 50.7 | 39.6 | 42.0 | 58.2 |
| Pdcd5 | 85.0 | 80.0 | 79.0 | 92.6 | 60.0 | 83.9 | 74.1 | 84.1 | 66.3 | 70.9 | 85.8 | 81.4 |
| Pdcd6 | 37.3 | 33.4 | 33.2 | 37.6 | 31.9 | 34.9 | 35.0 | 35.6 | 31.2 | 35.0 | 39.2 | 38.2 |
| Pdcd6ip | 21.9 | 23.5 | 23.0 | 24.2 | 26.3 | 23.3 | 26.7 | 23.2 | 25.5 | 24.1 | 27.0 | 24.6 |
| Pdcd7 | 3.0 | 3.3 | 3.5 | 2.5 | 2.7 | 3.1 | 2.9 | 3.0 | 3.4 | 3.4 | 2.9 | 2.6 |
| Pdcl | 10.0 | 10.2 | 12.4 | 13.4 | 10.6 | 12.3 | 12.2 | 12.8 | 11.6 | 12.6 | 16.0 | 14.4 |
| Pdcl3 | 31.8 | 28.3 | 27.5 | 38.2 | 27.8 | 28.8 | 34.4 | 27.0 | 31.0 | 27.6 | 36.2 | 33.1 |
| Pddc1 | 12.2 | 12.6 | 10.4 | 11.7 | 11.8 | 12.3 | 9.9 | 10.9 | 12.6 | 16.3 | 11.8 | 12.0 |
| Pde10a | 2.4 | 2.3 | 2.3 | 2.9 | 2.3 | 2.4 | 2.8 | 2.5 | 2.3 | 2.0 | 2.6 | 2.4 |
| Pde11a | 0.0 | 0.0 | 0.0 | 0.0 | 0.0 | 0.0 | 0.0 | 0.0 | 0.0 | 0.0 | 0.0 | 0.0 |
| Pde12 | 6.6 | 7.4 | 5.6 | 8.1 | 7.2 | 6.6 | 7.2 | 5.8 | 6.8 | 7.1 | 7.1 | 6.8 |
| Pde1a | 16.1 | 43.9 | 65.2 | 13.5 | 26.5 | 14.4 | 35.8 | 64.0 | 40.5 | 20.9 | 21.8 | 30.9 |
| Pde1b | 1.2 | 1.2 | 0.8 | 1.6 | 2.1 | 1.7 | 0.8 | 0.5 | 0.7 | 0.8 | 0.5 | 0.8 |
| Pde1c | 0.2 | 0.3 | 0.1 | 0.3 | 0.2 | 0.2 | 0.2 | 0.1 | 0.1 | 0.1 | 0.1 | 0.1 |
| Pde2a | 3.1 | 2.8 | 2.0 | 4.7 | 3.5 | 4.7 | 3.8 | 1.9 | 3.8 | 2.3 | 1.8 | 2.5 |
| Pde3a | 3.7 | 7.4 | 8.4 | 3.2 | 4.0 | 3.3 | 5.7 | 6.5 | 7.8 | 4.6 | 5.1 | 7.6 |
| Pde3b | 2.2 | 2.6 | 3.6 | 1.3 | 1.7 | 1.1 | 3.4 | 3.6 | 3.7 | 4.0 | 4.5 | 5.4 |
| Pde4a | 1.1 | 1.4 | 1.5 | 1.6 | 1.4 | 1.7 | 1.6 | 1.2 | 1.5 | 1.4 | 1.3 | 1.2 |
| Pde4b | 5.2 | 6.3 | 6.5 | 4.3 | 4.4 | 5.6 | 3.6 | 3.7 | 3.2 | 4.1 | 3.9 | 5.3 |
| Pde4c | 0.0 | 0.0 | 0.0 | 0.0 | 0.1 | 0.1 | 0.0 | 0.0 | 0.0 | 0.0 | 0.0 | 0.0 |
| Pde4d | 0.8 | 1.0 | 1.1 | 1.1 | 0.9 | 1.4 | 1.1 | 1.0 | 0.9 | 1.3 | 1.3 | 1.2 |
| Pde4dip | 27.8 | 22.2 | 18.9 | 27.8 | 23.0 | 26.2 | 24.6 | 19.3 | 20.6 | 24.3 | 27.7 | 26.9 |
| Pde5a | 2.6 | 3.6 | 3.5 | 1.7 | 2.5 | 1.9 | 2.5 | 3.0 | 3.7 | 3.6 | 3.6 | 4.3 |
| Pde6a | 0.0 | 0.0 | 0.0 | 0.0 | 0.0 | 0.0 | 0.0 | 0.0 | 0.0 | 0.0 | 0.0 | 0.0 |
| Pde6b | 0.0 | 0.0 | 0.0 | 0.0 | 0.0 | 0.0 | 0.0 | 0.0 | 0.0 | 0.0 | 0.0 | 0.0 |
| Pde6d | 14.9 | 14.6 | 16.2 | 14.1 | 14.4 | 15.5 | 15.1 | 14.4 | 15.4 | 12.9 | 15.0 | 14.6 |

| | | | | | | | | | | | | |
|---|---|---|---|---|---|---|---|---|---|---|---|---|
| Pde6g | 0.4 | 0.1 | 0.3 | 0.4 | 0.2 | 0.2 | 0.1 | 0.0 | 0.1 | 0.2 | 0.1 | 0.2 |
| Pde6h | 0.3 | 0.3 | 0.1 | 0.0 | 0.0 | 0.0 | 0.0 | 0.0 | 0.0 | 0.2 | 0.1 | 0.1 |
| Pde7a | 6.8 | 7.3 | 8.9 | 5.7 | 7.7 | 6.3 | 8.2 | 10.9 | 10.0 | 6.9 | 8.5 | 8.6 |
| Pde7b | 0.8 | 0.4 | 0.3 | 0.5 | 0.3 | 0.6 | 0.2 | 0.2 | 0.1 | 0.4 | 0.3 | 0.3 |
| Pde8a | 5.3 | 5.8 | 7.4 | 6.6 | 7.5 | 6.0 | 6.9 | 6.0 | 5.3 | 3.6 | 5.5 | 5.3 |
| Pde8b | 0.5 | 0.5 | 0.2 | 0.5 | 0.4 | 0.4 | 0.3 | 0.1 | 0.3 | 0.6 | 0.6 | 0.4 |
| Pde9a | 0.1 | 0.2 | 0.5 | 0.1 | 0.1 | 0.1 | 0.1 | 1.4 | 0.1 | 0.1 | 0.0 | 0.1 |
| Pdf | 6.5 | 6.8 | 5.9 | 8.5 | 7.4 | 8.2 | 5.4 | 6.5 | 7.6 | 8.1 | 6.7 | 7.2 |
| Pdgfa | 40.2 | 34.2 | 26.0 | 33.4 | 25.1 | 43.8 | 29.8 | 28.2 | 30.9 | 30.6 | 36.3 | 27.5 |
| Pdgfb | 1.4 | 0.3 | 0.5 | 2.8 | 7.8 | 0.2 | 1.1 | 1.0 | 0.9 | 0.6 | 0.3 | 0.4 |
| Pdgfc | 1.1 | 0.7 | 0.6 | 1.0 | 0.4 | 1.1 | 0.7 | 0.6 | 1.1 | 1.4 | 1.2 | 1.5 |
| Pdgfd | 26.5 | 18.4 | 16.0 | 25.3 | 13.1 | 19.2 | 12.6 | 6.4 | 8.8 | 9.7 | 10.7 | 11.7 |
| Pdgfra | 28.7 | 51.2 | 86.5 | 31.9 | 58.8 | 42.4 | 54.4 | 49.1 | 43.4 | 46.5 | 35.0 | 49.3 |
| Pdgfrb | 56.6 | 77.8 | 83.8 | 50.8 | 71.3 | 56.6 | 101.9 | 89.3 | 114.4 | 76.2 | 74.4 | 93.2 |
| Pdgfrl | 0.6 | 1.3 | 3.3 | 0.7 | 1.0 | 1.1 | 1.8 | 4.5 | 2.8 | 2.2 | 2.4 | 2.5 |
| Pdha1 | 72.9 | 66.4 | 63.5 | 68.6 | 58.8 | 65.4 | 71.8 | 70.8 | 70.6 | 73.3 | 81.7 | 83.4 |
| Pdhb | 42.3 | 42.1 | 33.0 | 40.1 | 34.1 | 36.7 | 30.8 | 33.7 | 36.7 | 34.6 | 37.3 | 33.3 |
| Pdhx | 7.8 | 8.8 | 8.1 | 7.7 | 7.2 | 6.7 | 7.7 | 7.6 | 7.1 | 7.4 | 7.9 | 6.5 |
| Pdia2 | 0.0 | 0.0 | 0.1 | 0.0 | 0.0 | 0.0 | 0.0 | 0.0 | 0.0 | 0.1 | 0.1 | 0.0 |
| Pdia3 | 296.6 | 257.4 | 258.4 | 304.0 | 258.2 | 260.1 | 248.9 | 260.0 | 244.2 | 276.1 | 304.5 | 309.4 |
| Pdia4 | 181.1 | 158.4 | 157.9 | 193.6 | 136.2 | 167.6 | 161.9 | 173.1 | 152.0 | 178.5 | 189.3 | 176.7 |
| Pdia5 | 11.1 | 8.8 | 10.1 | 10.3 | 11.7 | 10.0 | 12.3 | 12.9 | 11.1 | 11.1 | 12.1 | 13.3 |
| Pdia6 | 237.5 | 221.9 | 207.3 | 241.5 | 199.5 | 214.9 | 213.0 | 234.1 | 226.6 | 220.1 | 227.8 | 216.3 |
| Pdik1l | 2.1 | 2.0 | 2.1 | 2.4 | 2.1 | 2.2 | 2.1 | 1.7 | 1.7 | 1.9 | 1.7 | 2.1 |
| Pdk1 | 7.2 | 7.1 | 6.7 | 6.7 | 7.5 | 8.2 | 6.9 | 4.8 | 6.9 | 6.7 | 5.8 | 8.4 |
| Pdk2 | 2.6 | 3.2 | 3.5 | 2.9 | 2.6 | 3.5 | 3.2 | 3.5 | 3.2 | 3.5 | 2.9 | 3.0 |
| Pdk3 | 16.5 | 20.1 | 18.4 | 12.5 | 16.3 | 13.1 | 17.1 | 20.4 | 19.8 | 19.5 | 21.8 | 18.5 |
| Pdk4 | 5.7 | 6.5 | 7.1 | 8.4 | 7.9 | 8.8 | 7.5 | 2.8 | 5.5 | 7.2 | 6.2 | 7.7 |
| Pdlim1 | 81.9 | 70.3 | 55.4 | 127.5 | 71.1 | 110.9 | 71.1 | 41.9 | 54.0 | 75.2 | 75.6 | 70.6 |
| Pdlim2 | 41.3 | 58.0 | 59.0 | 42.0 | 37.6 | 49.6 | 56.3 | 73.7 | 58.4 | 48.1 | 49.9 | 46.8 |
| Pdlim3 | 15.1 | 52.7 | 93.4 | 7.5 | 38.1 | 3.4 | 28.9 | 64.5 | 51.4 | 43.6 | 31.9 | 73.5 |
| Pdlim4 | 0.3 | 0.1 | 0.2 | 2.1 | 3.3 | 0.4 | 0.3 | 0.1 | 0.3 | 2.6 | 2.5 | 1.4 |
| Pdlim5 | 50.3 | 45.8 | 45.7 | 53.1 | 49.4 | 48.5 | 58.1 | 45.8 | 57.5 | 52.3 | 63.7 | 59.8 |
| Pdlim7 | 18.5 | 19.5 | 16.4 | 26.2 | 25.2 | 21.5 | 25.6 | 19.5 | 23.9 | 22.1 | 26.4 | 19.1 |
| Pdp1 | 2.5 | 2.7 | 3.3 | 3.4 | 3.1 | 3.3 | 2.5 | 4.4 | 3.3 | 3.2 | 2.8 | 3.4 |
| Pdp2 | 1.9 | 2.1 | 2.0 | 1.2 | 2.1 | 1.6 | 2.5 | 2.1 | 1.9 | 1.4 | 2.1 | 1.7 |
| Pdpk1 | 13.5 | 14.7 | 13.8 | 15.9 | 11.1 | 13.4 | 13.7 | 11.7 | 13.0 | 11.2 | 14.4 | 14.0 |
| Pdpn | 233.4 | 171.4 | 148.1 | 273.9 | 226.2 | 180.3 | 223.1 | 194.0 | 143.2 | 299.5 | 295.5 | 203.6 |
| Pdpr | 4.5 | 5.5 | 5.2 | 4.8 | 4.8 | 5.5 | 5.1 | 5.5 | 4.8 | 4.2 | 5.5 | 5.5 |
| Pdrg1 | 94.0 | 67.7 | 60.5 | 88.2 | 73.1 | 77.4 | 72.5 | 63.5 | 68.4 | 91.5 | 91.7 | 76.6 |
| Pds5a | 22.9 | 24.5 | 21.8 | 21.2 | 21.4 | 22.5 | 19.9 | 20.9 | 18.7 | 20.7 | 22.5 | 21.4 |
| Pds5b | 2.1 | 2.6 | 3.6 | 2.0 | 2.6 | 2.5 | 2.8 | 3.3 | 2.7 | 2.9 | 3.0 | 3.1 |
| Pdss1 | 0.8 | 0.8 | 0.6 | 0.9 | 0.7 | 0.3 | 0.9 | 1.0 | 1.1 | 1.0 | 0.9 | 0.7 |
| Pdss2 | 3.1 | 3.3 | 2.4 | 3.4 | 2.8 | 3.2 | 2.4 | 3.0 | 3.0 | 3.3 | 2.5 | 2.4 |
| Pdxdc1 | 68.6 | 57.0 | 54.5 | 70.8 | 53.1 | 63.8 | 55.2 | 56.5 | 49.4 | 61.4 | 65.2 | 65.8 |
| Pdxk | 6.0 | 6.2 | 6.3 | 6.5 | 6.1 | 5.6 | 5.0 | 6.3 | 5.2 | 5.3 | 4.8 | 4.5 |
| Pdxk-ps | 0.0 | 0.0 | 0.0 | 0.0 | 0.0 | 0.0 | 0.0 | 0.0 | 0.0 | 0.0 | 0.0 | 0.0 |
| Pdxp | 0.4 | 1.0 | 0.7 | 0.7 | 1.1 | 0.6 | 0.9 | 0.6 | 1.0 | 1.1 | 0.9 | 0.9 |
| Pdzd11 | 37.5 | 31.4 | 31.9 | 40.8 | 32.6 | 35.5 | 34.6 | 34.3 | 36.1 | 38.3 | 41.9 | 40.9 |
| Pdzd2 | 2.4 | 3.8 | 5.7 | 1.4 | 2.0 | 1.5 | 2.0 | 2.8 | 1.5 | 1.5 | 1.6 | 1.0 |
| Pdzd3 | 0.0 | 0.1 | 0.0 | 0.0 | 0.0 | 0.1 | 0.0 | 0.0 | 0.0 | 0.0 | 0.1 | 0.0 |
| Pdzd4 | 1.8 | 2.0 | 2.7 | 1.9 | 2.1 | 2.6 | 3.1 | 3.0 | 2.9 | 2.3 | 3.0 | 2.5 |
| Pdzd7 | 0.7 | 0.8 | 1.0 | 0.6 | 0.6 | 1.1 | 0.8 | 0.9 | 0.6 | 1.0 | 0.9 | 1.0 |
| Pdzd8 | 13.0 | 12.2 | 13.4 | 13.4 | 11.2 | 11.7 | 14.2 | 13.5 | 13.5 | 11.8 | 15.1 | 14.2 |
| Pdzd9 | 0.2 | 0.1 | 0.1 | 0.1 | 0.1 | 0.1 | 0.1 | 0.2 | 0.1 | 0.1 | 0.3 | 0.3 |
| Pdzk1 | 1.1 | 0.8 | 0.4 | 1.0 | 0.8 | 0.8 | 0.9 | 0.8 | 0.9 | 0.7 | 0.6 | 0.8 |
| Pdzk1ip1 | 0.3 | 0.1 | 0.1 | 0.1 | 0.0 | 0.1 | 0.2 | 0.4 | 0.0 | 0.0 | 0.2 | 0.1 |

| | | | | | | | | | | | | |
|---|---|---|---|---|---|---|---|---|---|---|---|---|
| Pdzrn3 | 24.6 | 33.9 | 40.7 | 25.1 | 32.8 | 25.1 | 60.0 | 55.3 | 59.8 | 55.0 | 61.7 | 72.0 |
| Pdzrn4 | 0.1 | 0.1 | 0.0 | 0.1 | 0.1 | 0.1 | 0.2 | 0.1 | 0.0 | 0.0 | 0.0 | 0.1 |
| Pea15a | 211.6 | 196.4 | 156.5 | 308.9 | 198.9 | 240.5 | 213.8 | 140.8 | 188.4 | 174.7 | 205.3 | 178.0 |
| Pear1 | 5.9 | 6.5 | 6.2 | 6.4 | 7.1 | 8.0 | 8.1 | 5.7 | 7.4 | 8.1 | 5.2 | 5.5 |
| Pebp1 | 184.3 | 137.7 | 109.2 | 160.2 | 136.0 | 170.6 | 117.4 | 120.2 | 129.4 | 157.5 | 133.5 | 132.3 |
| Pecam1 | 4.0 | 0.9 | 2.3 | 1.2 | 1.1 | 1.0 | 2.4 | 4.5 | 2.9 | 0.4 | 0.5 | 0.7 |
| Pecr | 7.5 | 5.6 | 4.2 | 8.3 | 4.5 | 8.0 | 5.3 | 4.4 | 3.5 | 4.2 | 4.6 | 4.6 |
| Pef1 | 29.2 | 24.3 | 19.0 | 29.1 | 23.2 | 24.7 | 25.0 | 26.6 | 28.1 | 29.9 | 30.6 | 24.7 |
| Peg10 | 56.3 | 198.8 | 177.8 | 35.5 | 80.5 | 29.1 | 67.9 | 88.2 | 87.5 | 63.3 | 71.4 | 77.2 |
| Peg12 | 1.0 | 0.9 | 1.0 | 0.8 | 0.8 | 1.5 | 1.4 | 1.1 | 1.3 | 0.9 | 1.0 | 0.9 |
| Peg13 | 4.7 | 3.5 | 3.9 | 5.3 | 4.0 | 5.3 | 4.8 | 4.3 | 4.8 | 4.8 | 4.3 | 4.1 |
| Peg3 | 1.6 | 1.7 | 1.7 | 1.6 | 1.1 | 1.3 | 1.2 | 1.8 | 1.2 | 2.2 | 2.7 | 1.7 |
| Peg3as | 2.1 | 1.9 | 1.3 | 1.7 | 0.9 | 1.1 | 0.9 | 1.7 | 1.4 | 2.5 | 2.9 | 2.6 |
| Peli1 | 3.2 | 4.1 | 4.8 | 2.7 | 3.7 | 3.0 | 3.7 | 4.7 | 3.9 | 4.1 | 4.0 | 4.6 |
| Peli2 | 1.0 | 1.1 | 1.5 | 2.0 | 0.9 | 1.3 | 1.0 | 1.7 | 1.7 | 1.1 | 1.0 | 1.0 |
| Peli3 | 1.6 | 1.2 | 1.2 | 1.0 | 0.9 | 1.6 | 1.0 | 0.4 | 0.8 | 1.1 | 0.7 | 0.9 |
| Pelo | 8.4 | 7.6 | 7.9 | 6.1 | 6.7 | 7.2 | 6.7 | 9.0 | 7.3 | 9.4 | 8.8 | 7.1 |
| Pelp1 | 3.9 | 4.5 | 4.5 | 4.0 | 4.8 | 4.3 | 4.7 | 3.6 | 4.1 | 4.4 | 3.7 | 3.7 |
| Pemt | 2.8 | 0.8 | 0.9 | 0.9 | 1.3 | 1.3 | 2.1 | 1.3 | 1.6 | 2.2 | 1.9 | 1.1 |
| Peo1 | 3.1 | 3.2 | 2.9 | 3.1 | 2.3 | 3.4 | 2.9 | 2.9 | 2.4 | 3.4 | 3.4 | 3.1 |
| Pepd | 15.7 | 15.9 | 11.6 | 18.0 | 15.7 | 16.8 | 12.8 | 13.2 | 12.4 | 14.3 | 12.9 | 13.4 |
| Per1 | 1.1 | 1.7 | 1.4 | 1.4 | 1.1 | 1.5 | 1.2 | 1.0 | 1.4 | 1.6 | 1.2 | 1.0 |
| Per2 | 0.6 | 0.7 | 0.8 | 0.7 | 0.7 | 0.9 | 0.9 | 0.6 | 0.7 | 1.2 | 0.9 | 0.9 |
| Per3 | 5.2 | 7.5 | 8.5 | 4.0 | 4.8 | 5.6 | 5.0 | 7.5 | 5.8 | 4.1 | 4.2 | 4.6 |
| Perp | 11.2 | 4.7 | 2.7 | 3.4 | 2.4 | 4.8 | 1.8 | 1.0 | 1.0 | 8.5 | 4.5 | 3.5 |
| Pes1 | 26.5 | 21.9 | 18.8 | 32.3 | 23.6 | 27.6 | 24.9 | 21.1 | 19.4 | 23.3 | 24.1 | 23.5 |
| Pet100 | 15.6 | 16.6 | 19.8 | 20.4 | 16.7 | 19.5 | 20.5 | 17.3 | 14.7 | 15.2 | 18.0 | 20.3 |
| Pet112 | 4.0 | 4.4 | 4.0 | 3.3 | 3.1 | 4.4 | 2.6 | 3.9 | 3.2 | 3.2 | 3.4 | 3.1 |
| Pet117 | 0.0 | 0.3 | 0.6 | 0.9 | 0.0 | 1.7 | 0.6 | 2.2 | 0.2 | 2.2 | 0.7 | 1.8 |
| Pet2 | 0.0 | 0.0 | 0.0 | 0.0 | 0.0 | 0.0 | 0.0 | 0.0 | 0.0 | 0.0 | 0.0 | 0.0 |
| Pex1 | 2.2 | 2.1 | 2.3 | 2.1 | 2.3 | 2.2 | 1.7 | 1.7 | 1.6 | 2.1 | 1.8 | 1.9 |
| Pex10 | 1.6 | 1.9 | 1.8 | 1.5 | 1.4 | 1.6 | 1.4 | 2.0 | 1.5 | 1.7 | 1.8 | 1.4 |
| Pex11a | 1.2 | 1.1 | 0.6 | 1.0 | 0.8 | 1.6 | 0.9 | 0.7 | 0.4 | 1.8 | 0.8 | 1.1 |
| Pex11b | 13.7 | 12.5 | 12.1 | 10.8 | 12.2 | 15.0 | 12.8 | 12.4 | 12.2 | 13.7 | 10.7 | 12.2 |
| Pex11c | 2.4 | 2.7 | 2.5 | 2.5 | 2.2 | 2.9 | 2.5 | 3.3 | 3.2 | 3.2 | 2.4 | 1.8 |
| Pex12 | 2.8 | 3.4 | 3.7 | 4.2 | 3.0 | 4.9 | 3.2 | 3.0 | 2.8 | 3.5 | 2.7 | 3.3 |
| Pex13 | 8.5 | 9.0 | 7.6 | 7.5 | 8.1 | 8.6 | 7.2 | 7.4 | 8.9 | 10.9 | 9.9 | 10.5 |
| Pex14 | 19.2 | 21.1 | 18.5 | 20.8 | 17.3 | 17.9 | 21.8 | 19.1 | 19.7 | 18.0 | 19.5 | 18.3 |
| Pex16 | 6.1 | 7.8 | 6.3 | 8.0 | 6.0 | 7.5 | 8.4 | 6.6 | 7.5 | 6.2 | 5.7 | 6.4 |
| Pex19 | 41.4 | 48.0 | 44.2 | 43.2 | 38.2 | 39.0 | 39.7 | 42.5 | 45.5 | 36.1 | 38.8 | 38.7 |
| Pex2 | 14.6 | 12.6 | 11.8 | 11.0 | 12.0 | 10.2 | 12.0 | 13.1 | 15.5 | 14.1 | 14.3 | 13.8 |
| Pex26 | 1.4 | 1.3 | 1.1 | 1.5 | 1.3 | 1.5 | 1.2 | 1.2 | 1.3 | 1.5 | 1.2 | 1.2 |
| Pex3 | 11.7 | 12.0 | 10.3 | 10.0 | 10.1 | 12.2 | 10.2 | 9.3 | 9.9 | 10.2 | 10.2 | 11.4 |
| Pex5 | 17.1 | 15.0 | 13.5 | 13.1 | 14.1 | 14.8 | 12.6 | 12.6 | 14.4 | 13.9 | 14.0 | 11.4 |
| Pex5l | 1.6 | 0.5 | 0.1 | 1.3 | 0.1 | 0.2 | 0.2 | 0.0 | 0.2 | 0.1 | 0.3 | 0.2 |
| Pex6 | 8.7 | 10.7 | 9.2 | 6.6 | 8.2 | 9.2 | 8.0 | 8.5 | 9.4 | 9.9 | 6.9 | 7.4 |
| Pex7 | 15.2 | 15.1 | 11.8 | 13.0 | 10.8 | 11.4 | 11.1 | 10.1 | 9.6 | 12.2 | 11.9 | 12.0 |
| Pf4 | 3.6 | 3.0 | 2.7 | 6.5 | 9.6 | 3.0 | 2.6 | 1.4 | 1.2 | 1.4 | 0.5 | 1.0 |
| Pfas | 4.1 | 4.0 | 3.5 | 4.5 | 4.3 | 3.9 | 3.9 | 4.2 | 3.8 | 3.2 | 3.5 | 3.1 |
| Pfdn1 | 42.5 | 32.2 | 32.0 | 43.7 | 34.7 | 39.6 | 34.1 | 33.3 | 29.2 | 39.3 | 40.3 | 40.3 |
| Pfdn2 | 11.9 | 12.2 | 13.3 | 14.6 | 13.4 | 15.3 | 12.3 | 11.4 | 9.4 | 15.6 | 14.1 | 14.6 |
| Pfdn4 | 32.1 | 25.9 | 25.3 | 25.9 | 22.7 | 25.5 | 22.4 | 22.4 | 25.0 | 26.2 | 31.5 | 27.4 |
| Pfdn5 | 170.9 | 141.4 | 162.3 | 177.3 | 134.4 | 181.2 | 169.1 | 172.7 | 132.9 | 160.1 | 165.9 | 179.9 |
| Pfkfb1 | 0.3 | 0.7 | 0.8 | 0.3 | 0.4 | 0.4 | 0.8 | 0.4 | 0.7 | 0.5 | 0.4 | 0.4 |
| Pfkfb2 | 6.2 | 5.7 | 5.2 | 6.1 | 4.3 | 4.9 | 4.2 | 4.2 | 3.9 | 5.4 | 5.5 | 5.4 |
| Pfkfb3 | 4.8 | 4.4 | 2.7 | 5.3 | 5.1 | 4.6 | 5.2 | 3.0 | 4.9 | 5.0 | 5.1 | 4.6 |
| Pfkfb4 | 1.8 | 1.9 | 1.7 | 2.0 | 2.6 | 2.2 | 2.4 | 2.1 | 2.1 | 1.6 | 2.0 | 1.6 |

| | | | | | | | | | | | | |
|---|---|---|---|---|---|---|---|---|---|---|---|---|
| Pfkl | 21.1 | 21.7 | 22.6 | 17.5 | 27.1 | 21.8 | 27.8 | 19.7 | 28.5 | 26.5 | 22.2 | 32.4 |
| Pfkm | 15.5 | 16.1 | 16.2 | 16.9 | 14.2 | 19.3 | 15.3 | 14.3 | 12.9 | 15.0 | 13.7 | 14.8 |
| Pfkp | 17.8 | 17.6 | 15.1 | 21.1 | 23.4 | 19.7 | 16.5 | 14.2 | 14.3 | 11.4 | 15.0 | 13.5 |
| Pfn1 | 264.7 | 204.9 | 204.4 | 339.9 | 348.3 | 243.0 | 305.8 | 272.8 | 282.7 | 278.9 | 276.0 | 232.4 |
| Pfn2 | 37.3 | 32.8 | 30.5 | 44.7 | 30.7 | 47.4 | 33.8 | 32.9 | 30.0 | 38.3 | 38.9 | 40.3 |
| Pfn4 | 0.2 | 0.4 | 0.4 | 0.3 | 0.1 | 0.4 | 0.1 | 0.3 | 0.1 | 0.3 | 0.3 | 0.5 |
| Pgam1 | 207.9 | 175.6 | 126.6 | 224.3 | 220.6 | 212.4 | 190.6 | 155.2 | 177.0 | 181.4 | 179.1 | 166.0 |
| Pgam2 | 3.2 | 3.3 | 5.5 | 1.3 | 2.0 | 2.4 | 1.7 | 2.5 | 2.9 | 1.9 | 2.1 | 4.6 |
| Pgam5 | 16.5 | 12.7 | 10.6 | 13.3 | 12.0 | 15.0 | 12.8 | 11.3 | 12.3 | 14.4 | 12.6 | 11.6 |
| Pgap1 | 0.5 | 0.7 | 0.7 | 0.8 | 0.9 | 0.7 | 0.6 | 0.9 | 0.9 | 1.1 | 1.0 | 0.9 |
| Pgap2 | 20.7 | 20.3 | 16.2 | 20.0 | 17.4 | 22.1 | 16.5 | 17.0 | 15.9 | 16.6 | 15.7 | 16.0 |
| Pgap3 | 0.7 | 0.7 | 0.9 | 0.8 | 0.8 | 0.8 | 0.8 | 0.9 | 0.9 | 0.9 | 0.6 | 0.5 |
| Pgbd1 | 0.3 | 0.3 | 0.2 | 0.2 | 0.1 | 0.2 | 0.2 | 0.3 | 0.1 | 0.3 | 0.2 | 0.3 |
| Pgbd5 | 0.1 | 0.2 | 0.3 | 0.2 | 0.2 | 0.1 | 0.2 | 0.2 | 0.1 | 0.0 | 0.2 | 0.2 |
| Pgd | 61.6 | 66.2 | 47.0 | 109.9 | 99.5 | 96.2 | 61.5 | 66.9 | 41.3 | 53.9 | 53.6 | 45.4 |
| Pgf | 4.5 | 1.5 | 3.5 | 0.9 | 0.8 | 0.8 | 1.8 | 5.7 | 3.3 | 0.7 | 0.9 | 1.5 |
| Pggt1b | 7.6 | 7.8 | 8.5 | 7.8 | 8.6 | 8.6 | 7.4 | 6.7 | 7.9 | 7.2 | 7.6 | 8.3 |
| Pgk1 | 198.8 | 157.4 | 114.3 | 195.4 | 250.7 | 157.3 | 160.9 | 133.7 | 173.3 | 158.5 | 153.3 | 162.2 |
| Pgls | 54.9 | 39.7 | 31.8 | 44.8 | 39.3 | 41.4 | 42.1 | 39.9 | 39.6 | 49.4 | 36.5 | 37.5 |
| Pglyrp1 | 0.1 | 0.1 | 0.0 | 0.0 | 0.0 | 0.1 | 0.3 | 0.0 | 0.1 | 0.1 | 0.0 | 0.0 |
| Pglyrp3 | 0.3 | 0.1 | 0.0 | 0.3 | 0.1 | 0.0 | 0.1 | 0.1 | 0.1 | 0.3 | 0.2 | 0.1 |
| Pgm1 | 7.2 | 8.0 | 7.7 | 9.3 | 8.5 | 8.0 | 8.4 | 8.4 | 7.7 | 8.8 | 8.8 | 9.5 |
| Pgm2 | 36.3 | 34.6 | 30.4 | 40.4 | 32.9 | 45.0 | 33.7 | 33.9 | 29.5 | 33.8 | 33.4 | 33.8 |
| Pgm2l1 | 0.7 | 0.6 | 0.8 | 2.0 | 2.4 | 0.8 | 0.7 | 0.8 | 0.6 | 0.5 | 0.7 | 0.6 |
| Pgm3 | 10.3 | 9.3 | 8.5 | 11.2 | 9.4 | 9.8 | 10.1 | 9.3 | 8.9 | 9.6 | 11.3 | 11.0 |
| Pgm5 | 0.1 | 0.1 | 0.1 | 0.2 | 0.1 | 0.1 | 0.1 | 0.2 | 0.0 | 0.1 | 0.0 | 0.1 |
| Pgp | 15.2 | 13.2 | 9.5 | 9.7 | 12.1 | 10.3 | 15.9 | 12.8 | 14.8 | 14.6 | 12.4 | 11.5 |
| Pgpep1 | 9.6 | 9.1 | 7.1 | 8.4 | 6.7 | 10.4 | 7.0 | 7.0 | 7.3 | 8.2 | 8.5 | 8.6 |
| Pgpep1l | 3.3 | 2.8 | 1.4 | 2.7 | 2.8 | 4.7 | 2.0 | 3.3 | 2.3 | 2.2 | 2.2 | 1.1 |
| Pgr | 0.0 | 0.0 | 0.0 | 0.0 | 0.0 | 0.0 | 0.0 | 0.0 | 0.0 | 0.0 | 0.0 | 0.0 |
| Pgrmc1 | 98.8 | 94.3 | 76.1 | 110.1 | 76.3 | 122.4 | 68.6 | 67.5 | 75.1 | 112.4 | 96.5 | 93.0 |
| Pgrmc2 | 18.3 | 17.4 | 17.8 | 17.8 | 13.7 | 15.8 | 17.6 | 15.1 | 17.7 | 18.7 | 19.3 | 19.4 |
| Pgs1 | 15.9 | 17.7 | 14.6 | 15.5 | 18.3 | 12.7 | 20.2 | 16.8 | 18.5 | 17.0 | 17.0 | 16.2 |
| Phactr1 | 3.7 | 4.7 | 5.1 | 5.3 | 4.0 | 5.1 | 4.4 | 4.5 | 3.8 | 2.5 | 3.0 | 3.4 |
| Phactr2 | 6.6 | 5.3 | 4.8 | 9.8 | 4.7 | 7.6 | 5.6 | 4.0 | 4.1 | 3.6 | 4.8 | 4.8 |
| Phactr3 | 0.0 | 0.0 | 0.0 | 0.0 | 0.0 | 0.0 | 0.0 | 0.0 | 0.0 | 0.0 | 0.0 | 0.0 |
| Phactr4 | 1.6 | 2.1 | 2.1 | 2.1 | 2.0 | 2.0 | 2.9 | 2.1 | 1.9 | 1.5 | 1.6 | 1.4 |
| Phax | 26.3 | 23.7 | 26.8 | 26.4 | 22.6 | 24.6 | 25.6 | 25.6 | 22.3 | 28.3 | 30.3 | 31.4 |
| Phb | 58.5 | 51.4 | 41.8 | 61.3 | 48.0 | 51.6 | 54.0 | 47.0 | 48.0 | 53.0 | 52.4 | 44.3 |
| Phb2 | 50.3 | 44.1 | 40.4 | 47.9 | 45.1 | 46.9 | 47.5 | 49.0 | 44.7 | 44.9 | 50.0 | 42.1 |
| Phc1 | 1.9 | 1.9 | 2.1 | 1.8 | 1.7 | 2.2 | 2.5 | 2.5 | 2.2 | 1.8 | 1.9 | 1.8 |
| Phc2 | 10.5 | 10.4 | 9.6 | 11.5 | 14.2 | 9.5 | 15.2 | 11.5 | 13.5 | 14.9 | 12.5 | 12.4 |
| Phc3 | 4.5 | 5.4 | 5.3 | 4.7 | 4.6 | 4.7 | 4.6 | 4.7 | 4.0 | 4.3 | 4.5 | 4.5 |
| Phex | 0.0 | 0.0 | 0.0 | 0.0 | 0.0 | 0.0 | 0.0 | 0.0 | 0.0 | 0.0 | 0.0 | 0.0 |
| Phf1 | 7.6 | 10.6 | 10.0 | 8.6 | 7.6 | 9.0 | 9.0 | 7.6 | 10.1 | 8.8 | 6.7 | 8.2 |
| Phf10 | 36.2 | 38.4 | 41.3 | 33.2 | 27.4 | 32.2 | 29.4 | 38.6 | 33.3 | 31.6 | 35.3 | 38.2 |
| Phf11a | 1.5 | 0.7 | 1.6 | 2.2 | 1.3 | 1.1 | 0.8 | 0.7 | 0.6 | 0.4 | 0.3 | 0.9 |
| Phf11b | 2.3 | 1.2 | 1.6 | 4.9 | 2.2 | 1.4 | 0.6 | 0.4 | 0.5 | 0.5 | 0.5 | 0.8 |
| Phf11c | 0.8 | 0.6 | 0.6 | 0.8 | 0.7 | 0.6 | 0.4 | 0.6 | 0.4 | 0.5 | 0.2 | 0.4 |
| Phf11d | 7.8 | 4.9 | 8.9 | 13.3 | 5.2 | 6.9 | 3.7 | 3.0 | 2.5 | 1.4 | 2.4 | 6.0 |
| Phf12 | 1.8 | 3.0 | 3.1 | 2.2 | 2.6 | 2.1 | 2.3 | 3.0 | 2.4 | 1.9 | 2.6 | 2.0 |
| Phf13 | 4.0 | 5.5 | 4.8 | 2.5 | 4.3 | 4.6 | 5.2 | 3.7 | 5.1 | 4.8 | 4.7 | 3.7 |
| Phf14 | 8.8 | 9.1 | 10.3 | 9.3 | 7.6 | 8.0 | 8.5 | 9.6 | 7.8 | 8.7 | 9.1 | 11.0 |
| Phf15 | 3.0 | 2.5 | 3.2 | 4.5 | 4.8 | 2.5 | 2.8 | 2.0 | 1.5 | 3.8 | 2.7 | 2.5 |
| Phf16 | 2.4 | 1.8 | 1.4 | 1.8 | 1.8 | 2.2 | 1.4 | 1.4 | 1.2 | 2.3 | 2.1 | 2.0 |
| Phf17 | 21.3 | 26.3 | 26.4 | 20.2 | 17.9 | 21.7 | 20.5 | 23.6 | 25.9 | 26.1 | 31.6 | 32.3 |
| Phf19 | 0.3 | 0.4 | 0.7 | 0.3 | 0.7 | 0.3 | 1.0 | 0.4 | 0.7 | 0.3 | 0.4 | 0.5 |

| | | | | | | | | | | | | |
|---|---|---|---|---|---|---|---|---|---|---|---|---|
| Phf2 | 4.0 | 5.4 | 5.9 | 4.7 | 4.6 | 5.4 | 5.3 | 5.3 | 5.0 | 4.7 | 5.0 | 4.3 |
| Phf20 | 5.8 | 7.0 | 6.8 | 5.4 | 6.2 | 6.9 | 6.7 | 6.5 | 6.7 | 6.8 | 6.7 | 7.8 |
| Phf20l1 | 5.4 | 5.7 | 6.2 | 6.0 | 5.8 | 5.4 | 6.6 | 6.9 | 5.8 | 6.9 | 7.5 | 6.9 |
| Phf21a | 4.2 | 4.4 | 5.4 | 4.3 | 3.8 | 4.6 | 4.0 | 4.9 | 3.7 | 3.7 | 4.0 | 4.0 |
| Phf21b | 0.1 | 0.1 | 0.1 | 0.0 | 0.0 | 0.1 | 0.1 | 0.1 | 0.0 | 0.0 | 0.0 | 0.2 |
| Phf23 | 18.8 | 19.4 | 19.2 | 19.6 | 20.8 | 17.9 | 22.4 | 21.9 | 22.7 | 21.5 | 20.3 | 22.6 |
| Phf3 | 20.1 | 22.1 | 22.6 | 19.4 | 17.2 | 19.4 | 21.3 | 20.2 | 18.0 | 19.2 | 23.1 | 24.2 |
| Phf5a | 11.5 | 10.4 | 8.9 | 11.2 | 10.8 | 9.2 | 10.1 | 10.2 | 11.0 | 11.8 | 8.5 | 9.5 |
| Phf6 | 3.5 | 4.6 | 5.0 | 4.6 | 4.4 | 4.0 | 3.9 | 4.2 | 3.7 | 4.5 | 5.5 | 4.5 |
| Phf7 | 1.6 | 2.0 | 1.9 | 1.3 | 1.5 | 1.8 | 1.6 | 1.7 | 1.4 | 1.6 | 1.6 | 1.7 |
| Phf8 | 11.3 | 11.0 | 9.7 | 9.2 | 9.2 | 6.7 | 10.9 | 8.3 | 10.9 | 8.9 | 11.0 | 9.9 |
| Phgdh | 65.8 | 42.5 | 28.7 | 52.2 | 30.4 | 55.9 | 46.1 | 73.6 | 57.3 | 51.1 | 60.6 | 33.1 |
| Phip | 5.1 | 6.3 | 7.2 | 5.2 | 7.1 | 5.3 | 5.6 | 6.3 | 5.9 | 6.7 | 6.9 | 7.1 |
| Phka1 | 3.6 | 4.0 | 3.9 | 3.8 | 3.5 | 4.2 | 3.6 | 3.7 | 3.4 | 2.8 | 3.6 | 3.4 |
| Phka2 | 2.5 | 2.7 | 3.1 | 2.6 | 4.1 | 2.2 | 2.9 | 3.1 | 2.9 | 2.8 | 2.7 | 2.6 |
| Phkb | 12.4 | 12.0 | 10.7 | 12.8 | 10.5 | 14.1 | 10.9 | 12.9 | 11.4 | 11.1 | 12.2 | 11.3 |
| Phkg1 | 0.6 | 0.6 | 1.2 | 0.7 | 0.5 | 1.3 | 0.4 | 1.0 | 0.5 | 0.5 | 0.5 | 0.8 |
| Phkg2 | 6.9 | 5.5 | 4.4 | 6.3 | 5.6 | 6.4 | 5.6 | 4.9 | 6.2 | 5.6 | 5.5 | 4.8 |
| Phlda1 | 1.5 | 1.3 | 0.8 | 1.3 | 2.6 | 1.0 | 1.2 | 0.5 | 1.0 | 2.3 | 1.4 | 1.0 |
| Phlda2 | 0.3 | 0.1 | 0.0 | 0.1 | 0.2 | 0.0 | 0.1 | 0.0 | 0.0 | 0.1 | 0.4 | 0.0 |
| Phlda3 | 116.9 | 129.0 | 114.8 | 88.7 | 105.2 | 94.2 | 124.1 | 150.8 | 157.1 | 146.1 | 130.3 | 135.9 |
| Phldb1 | 29.6 | 29.3 | 26.0 | 30.2 | 27.5 | 30.5 | 39.1 | 34.3 | 30.9 | 28.9 | 34.7 | 28.1 |
| Phldb2 | 94.8 | 126.4 | 142.5 | 79.2 | 106.1 | 74.6 | 132.8 | 131.1 | 144.8 | 111.4 | 136.6 | 168.7 |
| Phldb3 | 0.6 | 0.3 | 0.3 | 1.1 | 0.6 | 0.5 | 0.6 | 0.2 | 0.3 | 1.1 | 0.8 | 0.4 |
| Phlpp1 | 1.8 | 2.1 | 1.7 | 2.1 | 1.7 | 2.0 | 2.1 | 1.6 | 2.5 | 2.1 | 2.3 | 2.0 |
| Phlpp2 | 1.3 | 1.8 | 1.6 | 1.2 | 1.5 | 1.2 | 1.8 | 1.7 | 1.9 | 1.6 | 1.7 | 1.4 |
| Phospho1 | 0.1 | 0.2 | 0.1 | 0.3 | 0.2 | 0.3 | 0.1 | 0.2 | 0.2 | 0.1 | 0.1 | 0.2 |
| Phospho2 | 11.9 | 12.9 | 10.9 | 13.6 | 11.4 | 12.2 | 8.9 | 10.3 | 10.7 | 12.3 | 11.3 | 13.2 |
| Phox2a | 0.0 | 0.0 | 0.0 | 0.0 | 0.1 | 0.2 | 0.0 | 0.0 | 0.0 | 0.1 | 0.1 | 0.0 |
| Phpt1 | 10.0 | 9.6 | 10.6 | 8.5 | 6.9 | 9.2 | 7.3 | 12.7 | 8.4 | 13.5 | 10.6 | 10.2 |
| Phrf1 | 7.0 | 8.1 | 8.0 | 8.0 | 8.0 | 7.4 | 9.2 | 8.7 | 7.7 | 7.4 | 7.3 | 7.9 |
| Phtf1 | 29.1 | 27.4 | 25.5 | 32.0 | 26.2 | 34.9 | 29.9 | 23.8 | 25.4 | 26.3 | 32.0 | 29.4 |
| Phtf2 | 3.2 | 2.9 | 3.0 | 2.8 | 3.3 | 2.6 | 2.8 | 2.5 | 3.0 | 3.2 | 3.2 | 3.0 |
| Phxr4 | 0.3 | 1.0 | 0.9 | 0.7 | 0.5 | 0.4 | 0.9 | 0.6 | 0.3 | 0.5 | 0.7 | 0.3 |
| Phyh | 10.8 | 10.9 | 15.0 | 10.8 | 8.8 | 12.9 | 8.6 | 14.9 | 8.6 | 6.8 | 8.3 | 10.7 |
| Phyhd1 | 14.4 | 9.6 | 9.2 | 11.1 | 7.2 | 15.5 | 11.2 | 22.6 | 19.1 | 13.4 | 14.0 | 13.8 |
| Phyhip | 0.6 | 0.8 | 0.9 | 0.4 | 0.5 | 0.3 | 0.5 | 0.6 | 0.3 | 0.6 | 0.4 | 0.6 |
| Phyhipl | 0.8 | 2.0 | 4.6 | 1.0 | 1.5 | 1.3 | 2.0 | 4.0 | 3.2 | 1.8 | 2.1 | 2.4 |
| Pi15 | 0.4 | 0.5 | 2.5 | 0.3 | 0.7 | 0.3 | 0.9 | 3.8 | 2.2 | 0.6 | 0.8 | 1.3 |
| Pi16 | 0.7 | 1.0 | 2.7 | 0.3 | 0.4 | 0.1 | 0.3 | 2.7 | 0.4 | 0.3 | 0.6 | 0.5 |
| Pi4k2a | 10.1 | 10.3 | 8.6 | 9.6 | 10.6 | 10.4 | 10.0 | 8.8 | 9.2 | 8.7 | 9.0 | 8.5 |
| Pi4k2b | 14.9 | 16.5 | 15.6 | 15.0 | 13.6 | 14.5 | 15.0 | 12.9 | 13.7 | 13.1 | 14.5 | 15.6 |
| Pi4ka | 16.0 | 14.8 | 13.6 | 18.9 | 16.3 | 16.9 | 16.5 | 14.1 | 13.9 | 14.0 | 14.9 | 14.6 |
| Pi4kb | 14.8 | 15.2 | 15.0 | 14.4 | 13.1 | 14.9 | 13.3 | 15.2 | 14.2 | 14.2 | 14.1 | 14.4 |
| Pianp | 0.0 | 0.0 | 0.1 | 0.0 | 0.0 | 0.0 | 0.1 | 0.0 | 0.0 | 0.1 | 0.0 | 0.1 |
| Pias1 | 5.5 | 7.0 | 7.1 | 6.0 | 5.3 | 5.7 | 6.3 | 6.6 | 6.0 | 5.9 | 6.3 | 6.4 |
| Pias2 | 9.4 | 9.7 | 10.2 | 9.8 | 9.8 | 9.5 | 9.5 | 8.5 | 7.9 | 9.8 | 9.7 | 10.0 |
| Pias3 | 6.6 | 7.1 | 7.0 | 5.8 | 6.0 | 6.1 | 7.1 | 6.8 | 7.2 | 6.4 | 6.7 | 6.5 |
| Pias4 | 6.4 | 7.9 | 6.0 | 6.3 | 7.7 | 7.0 | 5.7 | 7.5 | 6.9 | 7.3 | 5.9 | 6.1 |
| Pibf1 | 5.5 | 5.1 | 5.8 | 5.3 | 5.2 | 5.6 | 4.7 | 4.6 | 4.8 | 5.8 | 6.1 | 5.7 |
| Picalm | 54.6 | 63.4 | 79.9 | 64.3 | 79.6 | 57.8 | 65.0 | 76.6 | 67.3 | 69.7 | 81.7 | 83.6 |
| Pick1 | 3.0 | 2.2 | 2.4 | 3.1 | 2.2 | 3.0 | 2.4 | 3.2 | 2.0 | 2.7 | 2.4 | 2.2 |
| Pid1 | 10.1 | 8.7 | 9.3 | 13.0 | 18.2 | 6.7 | 10.4 | 8.0 | 11.3 | 8.7 | 9.0 | 9.4 |
| Piezo1 | 23.8 | 19.6 | 15.9 | 19.1 | 19.4 | 19.0 | 24.5 | 15.0 | 18.4 | 31.8 | 25.2 | 17.2 |
| Piezo2 | 1.8 | 3.2 | 3.5 | 1.5 | 3.5 | 1.7 | 6.8 | 5.1 | 7.5 | 9.3 | 8.2 | 9.9 |
| Pif1 | 0.3 | 0.6 | 0.8 | 0.0 | 0.6 | 0.1 | 1.0 | 0.4 | 1.2 | 0.3 | 0.7 | 0.4 |
| Piga | 1.2 | 1.6 | 2.0 | 1.0 | 1.5 | 1.0 | 1.5 | 1.8 | 2.1 | 1.8 | 1.4 | 1.6 |

| | | | | | | | | | | | | |
|---|---|---|---|---|---|---|---|---|---|---|---|---|
| Pigb | 3.5 | 3.3 | 3.1 | 3.5 | 2.7 | 3.4 | 3.1 | 2.9 | 2.8 | 2.9 | 2.7 | 2.5 |
| Pigc | 5.6 | 6.8 | 6.3 | 5.6 | 6.1 | 6.6 | 5.8 | 6.5 | 7.7 | 6.6 | 6.5 | 7.3 |
| Pigf | 23.3 | 24.5 | 23.0 | 22.4 | 24.2 | 25.5 | 24.3 | 25.1 | 23.0 | 23.8 | 29.4 | 25.9 |
| Pigg | 7.1 | 8.0 | 6.6 | 7.4 | 6.2 | 7.9 | 6.1 | 6.0 | 7.1 | 6.4 | 6.7 | 6.7 |
| Pigh | 7.8 | 8.2 | 8.2 | 9.0 | 7.4 | 9.3 | 9.6 | 8.7 | 9.0 | 9.2 | 10.5 | 9.0 |
| Pigk | 34.4 | 30.4 | 27.6 | 32.4 | 28.8 | 36.4 | 27.6 | 26.8 | 28.3 | 27.6 | 28.6 | 29.5 |
| Pigl | 1.5 | 1.8 | 1.5 | 1.9 | 1.7 | 2.1 | 1.1 | 1.8 | 1.4 | 1.5 | 1.9 | 1.3 |
| Pigm | 2.6 | 2.7 | 2.2 | 2.8 | 2.9 | 2.6 | 2.6 | 2.4 | 2.7 | 2.6 | 2.4 | 2.3 |
| Pign | 9.5 | 10.6 | 10.9 | 9.2 | 10.8 | 8.3 | 10.5 | 10.9 | 11.2 | 11.1 | 11.2 | 11.1 |
| Pigo | 0.8 | 1.3 | 1.0 | 0.8 | 1.2 | 0.8 | 1.3 | 1.8 | 1.8 | 1.2 | 0.8 | 1.0 |
| Pigp | 33.5 | 34.9 | 31.2 | 27.7 | 28.9 | 28.0 | 28.1 | 30.5 | 36.0 | 35.3 | 31.6 | 35.0 |
| Pigq | 8.7 | 7.9 | 7.5 | 10.8 | 8.4 | 9.5 | 7.7 | 8.5 | 7.5 | 6.9 | 7.3 | 7.4 |
| Pigs | 22.1 | 18.1 | 16.7 | 21.0 | 23.1 | 23.5 | 21.4 | 23.9 | 21.5 | 19.2 | 18.0 | 17.7 |
| Pigt | 6.7 | 6.7 | 8.4 | 8.1 | 8.3 | 8.3 | 8.9 | 8.2 | 6.8 | 6.7 | 6.8 | 6.7 |
| Pigu | 8.2 | 7.1 | 8.9 | 7.9 | 9.5 | 8.4 | 10.2 | 11.9 | 9.5 | 8.3 | 9.9 | 8.7 |
| Pigv | 3.5 | 2.9 | 2.1 | 3.8 | 3.3 | 2.8 | 3.1 | 3.1 | 3.8 | 3.3 | 3.1 | 3.0 |
| Pigw | 0.5 | 0.7 | 0.7 | 1.0 | 0.9 | 0.9 | 0.8 | 1.0 | 0.8 | 0.9 | 0.9 | 0.9 |
| Pigx | 13.5 | 11.2 | 12.1 | 13.7 | 9.6 | 12.8 | 9.4 | 10.5 | 12.3 | 11.4 | 12.8 | 12.7 |
| Pigyl | 16.0 | 17.5 | 17.5 | 12.8 | 14.1 | 15.7 | 16.4 | 15.9 | 21.2 | 27.1 | 19.4 | 19.3 |
| Pigz | 0.1 | 0.2 | 0.2 | 0.4 | 0.4 | 0.0 | 0.4 | 0.3 | 0.1 | 0.1 | 0.2 | 0.2 |
| Pih1d1 | 19.0 | 15.6 | 17.7 | 17.4 | 17.0 | 18.0 | 17.5 | 19.1 | 19.0 | 14.9 | 16.0 | 14.8 |
| Pih1d2 | 0.7 | 0.9 | 0.6 | 0.8 | 0.8 | 1.3 | 0.7 | 0.7 | 0.7 | 0.7 | 0.9 | 0.5 |
| Pik3ap1 | 1.6 | 0.0 | 0.0 | 8.9 | 18.4 | 0.0 | 2.7 | 0.3 | 0.4 | 1.5 | 0.4 | 1.2 |
| Pik3c2a | 17.5 | 17.1 | 15.2 | 18.1 | 20.1 | 15.9 | 16.9 | 15.6 | 16.4 | 17.5 | 21.4 | 18.7 |
| Pik3c2b | 0.2 | 0.2 | 0.2 | 0.3 | 0.1 | 0.3 | 0.2 | 0.1 | 0.1 | 0.1 | 0.1 | 0.1 |
| Pik3c3 | 11.6 | 13.0 | 13.4 | 10.3 | 11.9 | 11.2 | 9.8 | 11.5 | 10.7 | 11.4 | 11.8 | 11.5 |
| Pik3ca | 14.1 | 14.9 | 13.4 | 14.4 | 14.0 | 15.3 | 12.3 | 12.0 | 13.7 | 15.0 | 15.8 | 15.6 |
| Pik3cb | 4.2 | 3.0 | 2.5 | 4.5 | 5.7 | 3.8 | 3.5 | 2.9 | 4.1 | 4.1 | 5.1 | 3.7 |
| Pik3cd | 1.4 | 1.6 | 1.3 | 2.2 | 3.2 | 1.6 | 1.6 | 1.2 | 1.1 | 1.3 | 1.2 | 1.1 |
| Pik3cg | 0.3 | 0.0 | 0.0 | 1.5 | 3.2 | 0.0 | 0.3 | 0.1 | 0.1 | 0.2 | 0.1 | 0.2 |
| Pik3ip1 | 8.4 | 9.0 | 11.1 | 7.7 | 4.9 | 13.2 | 4.1 | 9.5 | 6.4 | 9.0 | 6.2 | 7.2 |
| Pik3r1 | 15.4 | 19.5 | 26.8 | 17.7 | 23.8 | 15.2 | 22.6 | 24.1 | 21.6 | 16.6 | 19.4 | 23.8 |
| Pik3r2 | 14.3 | 14.1 | 12.3 | 14.5 | 11.7 | 15.2 | 15.6 | 16.3 | 14.6 | 16.1 | 14.0 | 14.4 |
| Pik3r3 | 9.3 | 8.6 | 11.1 | 10.5 | 12.6 | 7.2 | 12.7 | 13.7 | 12.9 | 16.4 | 16.9 | 17.5 |
| Pik3r4 | 10.3 | 10.9 | 11.4 | 9.8 | 9.7 | 10.0 | 9.7 | 10.2 | 9.7 | 8.9 | 8.6 | 9.5 |
| Pik3r5 | 0.6 | 0.2 | 0.1 | 2.5 | 4.8 | 0.3 | 0.8 | 0.2 | 0.2 | 0.4 | 0.2 | 0.5 |
| Pik3r6 | 0.1 | 0.0 | 0.0 | 0.3 | 0.8 | 0.0 | 0.1 | 0.1 | 0.0 | 0.0 | 0.0 | 0.0 |
| Pikfyve | 6.9 | 7.3 | 6.7 | 6.6 | 6.3 | 6.8 | 5.7 | 6.5 | 6.1 | 6.3 | 6.6 | 6.7 |
| Pilra | 1.2 | 0.1 | 0.2 | 8.0 | 18.4 | 0.1 | 2.2 | 0.2 | 0.7 | 1.3 | 0.5 | 1.2 |
| Pilrb1 | 0.5 | 0.0 | 0.0 | 5.3 | 9.7 | 0.0 | 0.8 | 0.0 | 0.1 | 0.8 | 0.1 | 0.6 |
| Pilrb2 | 0.3 | 0.0 | 0.0 | 3.0 | 5.0 | 0.0 | 0.5 | 0.1 | 0.1 | 0.3 | 0.0 | 0.3 |
| Pim1 | 7.2 | 8.1 | 9.5 | 7.9 | 10.4 | 6.1 | 6.7 | 8.8 | 7.4 | 7.8 | 6.0 | 7.7 |
| Pim2 | 1.0 | 1.2 | 1.0 | 1.1 | 1.3 | 1.1 | 1.6 | 0.9 | 1.0 | 1.0 | 1.0 | 0.7 |
| Pim3 | 6.5 | 8.0 | 6.4 | 3.6 | 4.3 | 5.1 | 5.6 | 6.9 | 8.5 | 9.2 | 6.8 | 7.4 |
| Pin1 | 12.6 | 10.9 | 7.8 | 12.3 | 10.4 | 10.1 | 10.4 | 8.8 | 9.8 | 10.8 | 10.2 | 8.6 |
| Pin1-ps1 | 0.0 | 0.3 | 0.0 | 0.1 | 0.0 | 0.0 | 0.0 | 0.0 | 0.1 | 0.1 | 0.0 | 0.0 |
| Pin4 | 60.3 | 49.2 | 50.5 | 63.3 | 47.5 | 59.3 | 44.7 | 39.5 | 35.0 | 49.7 | 54.6 | 53.3 |
| Pink1 | 49.0 | 41.4 | 35.0 | 53.7 | 34.8 | 63.8 | 43.1 | 35.6 | 41.1 | 42.7 | 40.2 | 44.7 |
| Pinx1 | 8.2 | 6.1 | 7.5 | 12.0 | 7.5 | 8.2 | 8.0 | 7.1 | 4.9 | 6.5 | 8.6 | 7.9 |
| Pion | 1.4 | 1.0 | 1.2 | 2.6 | 6.1 | 1.7 | 1.4 | 0.7 | 0.9 | 1.1 | 1.0 | 1.1 |
| Pip4k2a | 13.1 | 14.3 | 14.2 | 19.8 | 15.4 | 15.6 | 15.0 | 12.0 | 14.0 | 12.5 | 15.6 | 15.8 |
| Pip4k2b | 17.6 | 18.2 | 16.7 | 19.9 | 17.0 | 18.9 | 17.7 | 14.3 | 14.8 | 15.7 | 17.0 | 15.1 |
| Pip4k2c | 13.0 | 11.6 | 11.7 | 15.4 | 11.7 | 16.5 | 11.7 | 9.5 | 10.9 | 10.9 | 9.7 | 11.2 |
| Pip5k1a | 16.2 | 15.1 | 16.4 | 15.6 | 14.0 | 14.3 | 16.8 | 16.5 | 17.1 | 16.6 | 15.8 | 17.3 |
| Pip5k1b | 0.0 | 0.0 | 0.0 | 0.1 | 0.0 | 0.0 | 0.1 | 0.1 | 0.0 | 0.0 | 0.0 | 0.0 |
| Pip5k1c | 14.2 | 13.3 | 10.8 | 19.0 | 15.7 | 16.6 | 15.7 | 11.8 | 12.4 | 11.3 | 13.0 | 10.8 |
| Pipox | 0.1 | 0.0 | 0.0 | 0.0 | 0.0 | 0.0 | 0.0 | 0.0 | 0.0 | 0.0 | 0.0 | 0.0 |

| | | | | | | | | | | | | |
|---|---|---|---|---|---|---|---|---|---|---|---|---|
| Pir | 21.2 | 23.2 | 22.9 | 21.6 | 15.4 | 24.3 | 19.9 | 23.0 | 19.1 | 13.3 | 15.6 | 17.9 |
| Pira1 | 0.0 | 0.0 | 0.0 | 0.2 | 0.1 | 0.0 | 0.0 | 0.0 | 0.0 | 0.0 | 0.0 | 0.0 |
| Pira11 | 0.0 | 0.0 | 0.0 | 2.0 | 2.8 | 0.0 | 0.4 | 0.0 | 0.1 | 0.2 | 0.1 | 0.2 |
| Pira2 | 0.2 | 0.0 | 0.0 | 1.8 | 2.9 | 0.0 | 0.3 | 0.1 | 0.1 | 0.2 | 0.0 | 0.2 |
| Pira4 | 0.2 | 0.0 | 0.0 | 1.6 | 0.0 | 0.0 | 0.2 | 0.0 | 0.0 | 0.1 | 0.1 | 0.1 |
| Pira6 | 0.2 | 0.0 | 0.0 | 1.6 | 2.1 | 0.0 | 0.2 | 0.0 | 0.0 | 0.2 | 0.1 | 0.2 |
| Pira7 | 0.0 | 0.0 | 0.0 | 0.0 | 1.9 | 0.0 | 0.0 | 0.0 | 0.0 | 0.0 | 0.0 | 0.0 |
| Pisd | 20.1 | 16.8 | 15.5 | 23.7 | 20.8 | 19.3 | 18.4 | 16.9 | 19.2 | 18.2 | 17.8 | 17.1 |
| Pisd-ps1 | 35.9 | 37.1 | 30.4 | 31.8 | 29.4 | 40.5 | 32.0 | 22.5 | 33.5 | 29.9 | 29.9 | 26.7 |
| Pisd-ps2 | 8.2 | 9.1 | 6.9 | 7.8 | 7.4 | 11.0 | 6.5 | 5.7 | 9.7 | 6.6 | 5.8 | 7.1 |
| Pithd1 | 8.7 | 9.8 | 8.7 | 9.9 | 8.4 | 10.7 | 9.4 | 9.4 | 9.4 | 7.8 | 9.2 | 9.1 |
| Pitpna | 30.8 | 24.7 | 24.2 | 39.8 | 38.0 | 28.8 | 35.6 | 27.1 | 27.1 | 30.4 | 35.2 | 34.0 |
| Pitpnb | 10.7 | 13.1 | 13.3 | 12.3 | 12.8 | 12.5 | 14.7 | 14.1 | 13.8 | 13.1 | 14.3 | 12.7 |
| Pitpnc1 | 1.4 | 1.4 | 1.4 | 1.7 | 1.9 | 1.3 | 1.0 | 0.8 | 0.9 | 1.4 | 1.4 | 1.0 |
| Pitpnm1 | 6.9 | 6.8 | 6.0 | 7.9 | 7.3 | 8.7 | 6.4 | 5.1 | 5.9 | 5.0 | 5.5 | 4.9 |
| Pitpnm2 | 1.7 | 2.1 | 2.0 | 1.6 | 1.7 | 2.1 | 1.8 | 1.9 | 1.6 | 1.7 | 1.5 | 1.4 |
| Pitpnm3 | 0.0 | 0.0 | 0.1 | 0.0 | 0.0 | 0.0 | 0.0 | 0.0 | 0.0 | 0.0 | 0.0 | 0.0 |
| Pitrm1 | 22.6 | 21.2 | 22.9 | 25.1 | 23.0 | 27.1 | 23.0 | 22.1 | 21.5 | 18.6 | 24.8 | 21.1 |
| Pitx1 | 0.0 | 0.0 | 0.0 | 0.0 | 0.0 | 0.0 | 0.0 | 0.0 | 0.1 | 0.0 | 0.0 | 0.0 |
| Pitx2 | 0.1 | 0.1 | 0.0 | 0.2 | 0.0 | 0.3 | 0.1 | 0.1 | 0.1 | 0.3 | 0.3 | 0.3 |
| Piwil2 | 0.4 | 0.3 | 0.2 | 0.3 | 0.2 | 0.2 | 0.2 | 0.3 | 0.1 | 0.3 | 0.2 | 0.2 |
| Piwil4 | 0.2 | 0.2 | 0.1 | 0.4 | 0.2 | 0.2 | 0.2 | 0.1 | 0.1 | 0.2 | 0.1 | 0.1 |
| Pja1 | 23.3 | 21.2 | 20.3 | 24.7 | 18.4 | 25.0 | 20.9 | 19.6 | 19.7 | 25.0 | 24.7 | 22.3 |
| Pja2 | 39.1 | 43.2 | 41.4 | 44.6 | 38.0 | 45.3 | 44.1 | 38.1 | 37.7 | 41.0 | 46.1 | 47.1 |
| Pkd1 | 11.2 | 11.7 | 11.0 | 10.2 | 12.3 | 12.1 | 16.3 | 15.1 | 15.9 | 10.5 | 10.6 | 10.3 |
| Pkd1l2 | 0.0 | 0.0 | 0.0 | 0.0 | 0.1 | 0.1 | 0.0 | 0.0 | 0.0 | 0.0 | 0.0 | 0.0 |
| Pkd1l3 | 0.0 | 0.0 | 0.0 | 0.0 | 0.0 | 0.0 | 0.0 | 0.0 | 0.0 | 0.0 | 0.0 | 0.0 |
| Pkd2 | 43.8 | 38.9 | 41.9 | 47.9 | 41.6 | 49.8 | 51.3 | 40.1 | 43.3 | 34.5 | 42.3 | 45.1 |
| Pkd2l2 | 0.2 | 0.3 | 0.2 | 0.1 | 0.4 | 0.2 | 0.2 | 0.2 | 0.2 | 0.5 | 0.3 | 0.3 |
| Pkdcc | 1.7 | 2.9 | 2.6 | 1.6 | 2.1 | 1.7 | 2.4 | 3.0 | 3.8 | 7.4 | 3.1 | 3.3 |
| Pkdrej | 0.1 | 0.2 | 0.1 | 0.1 | 0.1 | 0.2 | 0.0 | 0.1 | 0.1 | 0.1 | 0.1 | 0.1 |
| Pkhd1 | 0.1 | 0.0 | 0.0 | 0.1 | 0.0 | 0.0 | 0.0 | 0.0 | 0.0 | 0.0 | 0.0 | 0.0 |
| Pkhd1l1 | 9.7 | 2.8 | 1.4 | 0.6 | 0.5 | 0.5 | 0.3 | 1.5 | 1.1 | 20.7 | 9.5 | 4.2 |
| Pkia | 13.8 | 11.4 | 11.6 | 10.9 | 8.8 | 10.6 | 10.1 | 8.8 | 12.1 | 12.8 | 13.1 | 16.6 |
| Pkib | 0.2 | 0.0 | 0.0 | 0.2 | 0.2 | 0.0 | 0.1 | 0.0 | 0.0 | 0.0 | 0.0 | 0.0 |
| Pkig | 34.3 | 42.4 | 39.6 | 30.8 | 31.8 | 27.5 | 33.1 | 30.7 | 44.3 | 28.1 | 28.4 | 35.2 |
| Pkm | 432.4 | 382.5 | 327.7 | 588.7 | 614.5 | 425.3 | 421.9 | 405.2 | 310.0 | 398.1 | 382.0 | 351.3 |
| Pkmyt1 | 0.5 | 1.1 | 1.6 | 0.6 | 1.2 | 0.8 | 2.7 | 1.9 | 2.4 | 0.8 | 1.1 | 0.7 |
| Pkn1 | 20.4 | 15.2 | 11.6 | 20.2 | 16.7 | 17.7 | 16.2 | 12.8 | 14.3 | 14.6 | 14.6 | 13.7 |
| Pkn2 | 20.1 | 22.5 | 20.9 | 18.5 | 16.3 | 20.5 | 17.6 | 17.9 | 19.1 | 18.8 | 22.8 | 21.2 |
| Pkn3 | 0.6 | 0.8 | 1.2 | 0.4 | 0.8 | 0.6 | 1.0 | 0.5 | 0.8 | 0.5 | 0.6 | 0.5 |
| Pknox1 | 3.4 | 4.0 | 3.6 | 3.9 | 3.8 | 3.7 | 3.1 | 3.3 | 3.6 | 4.9 | 4.1 | 3.9 |
| Pknox2 | 3.8 | 5.2 | 8.7 | 3.5 | 5.1 | 2.5 | 5.9 | 8.9 | 5.9 | 3.0 | 3.7 | 4.6 |
| Pkp1 | 0.4 | 0.0 | 0.1 | 0.0 | 0.0 | 0.0 | 0.0 | 0.2 | 0.0 | 0.2 | 0.0 | 0.1 |
| Pkp2 | 2.1 | 0.6 | 0.3 | 1.9 | 0.8 | 1.5 | 0.5 | 0.3 | 0.6 | 3.1 | 1.7 | 0.9 |
| Pkp3 | 0.5 | 0.4 | 0.2 | 0.2 | 0.4 | 0.3 | 0.1 | 0.1 | 0.2 | 0.2 | 0.2 | 0.2 |
| Pkp4 | 9.0 | 9.3 | 9.3 | 10.6 | 10.1 | 10.3 | 11.2 | 7.6 | 8.5 | 8.4 | 11.6 | 9.3 |
| Pla1a | 20.9 | 17.6 | 12.4 | 27.2 | 18.9 | 28.4 | 14.9 | 3.8 | 9.5 | 17.0 | 13.8 | 17.2 |
| Pla2g12a | 16.1 | 11.7 | 9.3 | 17.1 | 10.5 | 17.4 | 10.1 | 9.2 | 8.9 | 11.9 | 10.7 | 10.7 |
| Pla2g15 | 11.9 | 10.8 | 11.8 | 12.2 | 14.3 | 12.4 | 14.2 | 11.9 | 15.9 | 17.6 | 14.4 | 18.8 |
| Pla2g16 | 6.3 | 6.0 | 7.1 | 7.2 | 6.1 | 7.2 | 5.9 | 7.6 | 6.5 | 6.0 | 6.3 | 7.7 |
| Pla2g2c | 0.0 | 0.0 | 0.0 | 0.0 | 0.0 | 0.0 | 0.0 | 0.0 | 0.0 | 0.0 | 0.0 | 0.0 |
| Pla2g2e | 1.8 | 1.2 | 0.4 | 1.3 | 1.3 | 2.4 | 0.6 | 0.8 | 0.2 | 0.4 | 0.5 | 0.2 |
| Pla2g3 | 0.1 | 0.0 | 0.0 | 0.0 | 0.0 | 0.0 | 0.0 | 0.1 | 0.0 | 0.0 | 0.0 | 0.0 |
| Pla2g4a | 18.6 | 12.4 | 11.2 | 18.5 | 22.3 | 14.8 | 9.6 | 10.3 | 8.5 | 18.9 | 15.5 | 14.6 |
| Pla2g4b | 0.3 | 0.2 | 0.4 | 0.3 | 0.2 | 0.2 | 0.4 | 0.3 | 0.4 | 0.3 | 0.3 | 0.2 |
| Pla2g4c | 0.0 | 0.0 | 0.0 | 0.0 | 0.1 | 0.0 | 0.1 | 0.1 | 0.0 | 0.1 | 0.1 | 0.1 |

| | | | | | | | | | | | | |
|---|---|---|---|---|---|---|---|---|---|---|---|---|
| Pla2g4f | 0.0 | 0.0 | 0.0 | 0.0 | 0.0 | 0.0 | 0.0 | 0.0 | 0.0 | 0.0 | 0.1 | 0.0 |
| Pla2g5 | 1.1 | 0.4 | 0.3 | 0.8 | 0.5 | 0.6 | 0.1 | 0.2 | 0.1 | 0.2 | 0.1 | 0.3 |
| Pla2g6 | 4.5 | 4.7 | 3.4 | 5.2 | 4.0 | 4.5 | 4.6 | 3.8 | 4.3 | 4.5 | 4.7 | 3.7 |
| Pla2g7 | 2.7 | 0.1 | 0.3 | 5.8 | 9.8 | 0.1 | 0.6 | 0.8 | 0.4 | 0.7 | 0.3 | 0.5 |
| Pla2r1 | 9.6 | 8.8 | 8.1 | 9.1 | 8.4 | 12.0 | 9.1 | 7.9 | 8.8 | 6.3 | 7.8 | 7.7 |
| Plaa | 16.2 | 14.1 | 13.7 | 17.1 | 14.5 | 15.6 | 15.6 | 13.4 | 13.4 | 14.4 | 17.1 | 16.6 |
| Plac8 | 6.6 | 16.5 | 32.6 | 5.3 | 15.2 | 11.6 | 69.8 | 35.0 | 62.6 | 93.6 | 87.0 | 109.2 |
| Plac9a | 103.1 | 75.1 | 59.8 | 106.2 | 55.7 | 95.5 | 67.3 | 58.5 | 52.9 | 31.4 | 57.4 | 42.6 |
| Plac9b | 33.2 | 20.3 | 17.6 | 37.2 | 19.2 | 28.9 | 20.0 | 33.0 | 16.6 | 8.7 | 21.3 | 12.8 |
| Plag1 | 0.5 | 0.2 | 0.1 | 0.4 | 0.2 | 0.6 | 0.3 | 0.3 | 0.3 | 0.4 | 0.7 | 0.6 |
| Plagl1 | 0.1 | 0.1 | 0.1 | 0.2 | 0.2 | 0.1 | 0.2 | 0.5 | 0.6 | 0.7 | 0.7 | 0.6 |
| Plagl2 | 0.5 | 0.7 | 0.8 | 0.6 | 1.2 | 0.7 | 0.8 | 0.9 | 0.7 | 0.5 | 0.8 | 0.6 |
| Plat | 14.7 | 10.1 | 12.7 | 8.4 | 9.5 | 8.5 | 8.5 | 14.6 | 7.6 | 18.2 | 10.5 | 14.6 |
| Plau | 6.8 | 7.0 | 5.5 | 10.3 | 16.2 | 5.0 | 5.5 | 3.4 | 4.4 | 12.5 | 6.2 | 3.8 |
| Plaur | 6.0 | 2.5 | 1.5 | 8.8 | 11.9 | 4.3 | 7.1 | 2.9 | 4.4 | 10.6 | 7.7 | 5.1 |
| Plb1 | 0.2 | 0.1 | 0.2 | 0.4 | 0.1 | 0.1 | 0.2 | 0.1 | 0.1 | 0.2 | 0.1 | 0.1 |
| Plbd1 | 0.1 | 0.0 | 0.0 | 0.1 | 1.3 | 0.0 | 0.3 | 0.1 | 0.1 | 0.0 | 0.0 | 0.1 |
| Plbd2 | 14.8 | 13.0 | 12.8 | 13.8 | 14.8 | 14.4 | 14.1 | 14.0 | 13.7 | 11.6 | 10.8 | 12.0 |
| Plcb1 | 1.5 | 1.3 | 1.4 | 1.1 | 0.9 | 1.0 | 1.2 | 1.3 | 1.1 | 1.2 | 1.7 | 1.5 |
| Plcb2 | 0.1 | 0.0 | 0.0 | 1.3 | 1.8 | 0.0 | 0.1 | 0.0 | 0.0 | 0.1 | 0.0 | 0.1 |
| Plcb3 | 19.6 | 21.2 | 22.5 | 21.5 | 20.2 | 21.3 | 22.5 | 18.0 | 18.1 | 16.0 | 20.1 | 18.4 |
| Plcb4 | 22.1 | 21.2 | 24.1 | 17.4 | 18.2 | 18.4 | 22.3 | 24.8 | 24.6 | 15.3 | 27.7 | 22.7 |
| Plcd1 | 16.3 | 14.3 | 15.4 | 15.0 | 11.7 | 17.2 | 15.5 | 14.7 | 15.0 | 14.0 | 13.5 | 14.4 |
| Plcd3 | 2.8 | 3.2 | 2.1 | 1.6 | 2.0 | 2.4 | 2.0 | 2.1 | 2.1 | 2.2 | 1.7 | 1.8 |
| Plcd4 | 0.0 | 0.1 | 0.0 | 0.0 | 0.1 | 0.0 | 0.0 | 0.1 | 0.0 | 0.3 | 0.1 | 0.1 |
| Plce1 | 1.7 | 2.3 | 3.4 | 1.1 | 1.5 | 1.5 | 2.1 | 3.4 | 3.2 | 4.4 | 3.9 | 4.4 |
| Plcg1 | 15.1 | 13.4 | 11.9 | 13.9 | 9.9 | 14.9 | 12.1 | 12.1 | 11.4 | 10.5 | 11.8 | 11.3 |
| Plcg2 | 5.7 | 3.5 | 3.2 | 11.7 | 14.1 | 5.5 | 5.6 | 2.1 | 2.5 | 4.6 | 3.9 | 4.3 |
| Plch1 | 0.0 | 0.0 | 0.0 | 0.0 | 0.0 | 0.0 | 0.1 | 0.0 | 0.0 | 0.2 | 0.0 | 0.0 |
| Plch2 | 0.1 | 0.2 | 0.2 | 0.1 | 0.1 | 0.2 | 0.1 | 0.1 | 0.2 | 0.1 | 0.1 | 0.2 |
| Plcl1 | 2.8 | 3.4 | 4.5 | 2.2 | 2.7 | 2.4 | 2.8 | 4.7 | 3.9 | 6.0 | 4.7 | 4.6 |
| Plcl2 | 1.4 | 2.2 | 2.5 | 2.7 | 2.6 | 2.0 | 1.5 | 2.3 | 1.3 | 1.1 | 1.3 | 1.4 |
| Plcxd1 | 0.5 | 0.4 | 0.4 | 0.3 | 0.3 | 0.5 | 0.5 | 0.2 | 0.5 | 0.3 | 0.4 | 0.3 |
| Plcxd2 | 2.8 | 3.9 | 3.5 | 2.6 | 3.3 | 2.4 | 4.7 | 3.3 | 6.2 | 4.3 | 4.7 | 5.3 |
| Plcxd3 | 0.0 | 0.0 | 0.0 | 0.0 | 0.0 | 0.0 | 0.0 | 0.0 | 0.0 | 0.0 | 0.0 | 0.0 |
| Pld1 | 5.2 | 5.1 | 5.2 | 3.5 | 4.5 | 4.2 | 4.2 | 4.4 | 4.3 | 5.2 | 5.7 | 5.0 |
| Pld2 | 4.7 | 5.5 | 5.8 | 4.6 | 6.1 | 5.2 | 5.6 | 7.1 | 6.9 | 5.8 | 5.9 | 5.6 |
| Pld3 | 62.2 | 57.2 | 64.1 | 71.5 | 66.3 | 72.8 | 53.4 | 65.0 | 49.3 | 51.1 | 47.7 | 56.3 |
| Pld4 | 2.4 | 0.1 | 0.2 | 20.3 | 40.6 | 0.1 | 5.5 | 0.6 | 0.7 | 2.3 | 0.7 | 1.1 |
| Pld6 | 0.1 | 0.3 | 0.1 | 0.1 | 0.1 | 0.2 | 0.3 | 0.2 | 0.1 | 0.1 | 0.2 | 0.1 |
| Plec | 36.4 | 37.8 | 33.6 | 33.3 | 44.2 | 30.7 | 49.5 | 42.6 | 42.3 | 32.5 | 35.5 | 26.9 |
| Plek | 2.6 | 0.8 | 1.2 | 14.3 | 22.6 | 1.1 | 3.9 | 1.1 | 1.4 | 2.7 | 1.6 | 2.3 |
| Plek2 | 0.2 | 0.2 | 0.2 | 0.3 | 0.4 | 0.0 | 0.1 | 0.1 | 0.0 | 0.3 | 0.1 | 0.1 |
| Plekha1 | 7.2 | 6.9 | 7.1 | 8.5 | 7.6 | 7.3 | 7.7 | 7.9 | 8.3 | 7.5 | 9.0 | 9.0 |
| Plekha2 | 16.1 | 17.1 | 14.2 | 20.0 | 19.3 | 18.6 | 17.8 | 11.3 | 19.7 | 15.1 | 16.7 | 16.7 |
| Plekha3 | 20.7 | 18.0 | 18.7 | 27.8 | 19.8 | 22.3 | 23.4 | 17.9 | 18.3 | 18.0 | 22.9 | 20.4 |
| Plekha4 | 1.3 | 1.3 | 1.9 | 1.0 | 1.1 | 1.3 | 0.7 | 1.2 | 1.1 | 1.0 | 0.4 | 1.2 |
| Plekha5 | 2.0 | 2.7 | 3.3 | 2.0 | 1.8 | 2.3 | 2.3 | 2.4 | 2.2 | 2.0 | 2.0 | 2.3 |
| Plekha6 | 0.4 | 0.6 | 1.2 | 0.3 | 0.4 | 0.3 | 0.2 | 0.4 | 0.2 | 0.1 | 0.2 | 0.1 |
| Plekha7 | 9.5 | 11.1 | 11.0 | 5.0 | 7.4 | 4.0 | 11.9 | 12.4 | 10.2 | 9.1 | 12.4 | 11.5 |
| Plekha8 | 2.7 | 3.3 | 3.6 | 2.6 | 2.7 | 2.2 | 2.7 | 3.3 | 3.3 | 3.0 | 3.1 | 3.4 |
| Plekhb1 | 0.3 | 0.3 | 0.6 | 0.1 | 0.4 | 0.1 | 0.3 | 0.2 | 0.5 | 0.9 | 0.4 | 0.6 |
| Plekhb2 | 25.5 | 21.8 | 16.7 | 32.5 | 25.0 | 28.5 | 18.7 | 12.1 | 14.3 | 22.7 | 20.1 | 16.5 |
| Plekhd1 | 0.0 | 0.0 | 0.0 | 0.0 | 0.0 | 0.0 | 0.0 | 0.0 | 0.0 | 0.0 | 0.0 | 0.0 |
| Plekhf1 | 15.9 | 13.4 | 13.5 | 17.1 | 15.2 | 12.4 | 16.6 | 15.8 | 17.1 | 18.3 | 17.3 | 18.8 |
| Plekhf2 | 3.6 | 3.6 | 3.8 | 4.1 | 4.8 | 3.0 | 3.4 | 3.9 | 4.5 | 3.5 | 3.5 | 3.5 |
| Plekhg1 | 1.1 | 1.1 | 1.4 | 1.1 | 1.8 | 0.8 | 1.2 | 1.0 | 0.7 | 0.8 | 1.0 | 0.8 |

| | | | | | | | | | | | | |
|---|---|---|---|---|---|---|---|---|---|---|---|---|
| Plekhg2 | 9.7 | 18.9 | 20.3 | 8.6 | 12.1 | 9.6 | 17.3 | 18.4 | 21.3 | 11.8 | 14.1 | 12.0 |
| Plekhg3 | 5.5 | 6.4 | 6.5 | 4.9 | 6.5 | 5.1 | 9.2 | 7.8 | 9.2 | 12.9 | 11.3 | 9.3 |
| Plekhg4 | 0.0 | 0.0 | 0.1 | 0.0 | 0.1 | 0.1 | 0.1 | 0.1 | 0.1 | 0.2 | 0.2 | 0.1 |
| Plekhg5 | 6.3 | 9.4 | 10.2 | 4.4 | 6.4 | 6.3 | 9.2 | 11.4 | 10.9 | 7.4 | 7.7 | 9.3 |
| Plekhg6 | 0.1 | 0.2 | 0.1 | 0.2 | 0.2 | 0.1 | 0.2 | 0.1 | 0.2 | 0.2 | 0.1 | 0.2 |
| Plekhh1 | 0.2 | 0.3 | 0.2 | 0.2 | 0.2 | 0.2 | 0.2 | 0.2 | 0.2 | 0.2 | 0.2 | 0.1 |
| Plekhh2 | 4.4 | 6.5 | 6.4 | 4.0 | 3.9 | 4.2 | 3.5 | 3.8 | 3.2 | 6.3 | 4.3 | 3.3 |
| Plekhh3 | 2.9 | 2.7 | 2.4 | 2.5 | 2.0 | 2.8 | 3.2 | 4.3 | 4.0 | 3.0 | 3.0 | 2.3 |
| Plekhj1 | 11.9 | 10.4 | 10.2 | 12.4 | 11.2 | 13.2 | 11.0 | 10.9 | 13.0 | 11.9 | 11.8 | 11.5 |
| Plekhm1 | 2.5 | 3.2 | 3.5 | 3.6 | 4.9 | 3.0 | 3.1 | 3.1 | 2.9 | 2.5 | 2.5 | 2.5 |
| Plekhm2 | 6.5 | 6.4 | 6.0 | 7.7 | 6.5 | 7.5 | 7.4 | 6.9 | 6.4 | 6.0 | 6.2 | 6.8 |
| Plekhm3 | 3.2 | 4.2 | 3.9 | 3.9 | 5.0 | 3.1 | 3.6 | 3.6 | 3.5 | 3.1 | 3.1 | 3.4 |
| Plekhn1 | 1.1 | 1.5 | 1.3 | 1.2 | 1.7 | 1.4 | 1.1 | 1.1 | 0.7 | 0.7 | 0.7 | 0.8 |
| Plekho1 | 5.4 | 4.6 | 4.0 | 12.3 | 14.6 | 7.4 | 6.8 | 5.6 | 6.1 | 10.4 | 11.3 | 8.2 |
| Plekho2 | 26.7 | 26.1 | 20.6 | 32.0 | 31.1 | 25.9 | 32.5 | 29.7 | 30.5 | 24.9 | 27.5 | 26.1 |
| Plekhs1 | 0.2 | 0.2 | 0.0 | 0.4 | 0.1 | 0.2 | 0.0 | 0.0 | 0.1 | 0.0 | 0.0 | 0.0 |
| Plgrkt | 33.0 | 30.7 | 27.8 | 29.3 | 25.4 | 32.6 | 28.6 | 23.6 | 28.0 | 27.8 | 28.3 | 27.2 |
| Plin1 | 0.0 | 0.0 | 0.0 | 0.0 | 0.0 | 0.0 | 0.0 | 0.0 | 0.0 | 0.0 | 0.0 | 0.0 |
| Plin2 | 49.4 | 42.7 | 30.8 | 100.5 | 89.0 | 50.7 | 39.5 | 38.1 | 35.7 | 52.0 | 40.9 | 37.6 |
| Plin3 | 48.2 | 43.5 | 35.4 | 53.0 | 36.1 | 55.4 | 33.1 | 35.3 | 33.5 | 34.8 | 37.4 | 34.5 |
| Plin4 | 1.5 | 0.8 | 0.7 | 1.3 | 0.3 | 1.5 | 0.2 | 0.6 | 0.2 | 0.2 | 0.3 | 0.4 |
| Plin5 | 0.0 | 0.1 | 0.0 | 0.0 | 0.0 | 0.0 | 0.0 | 0.0 | 0.0 | 0.0 | 0.0 | 0.0 |
| Plk1 | 1.7 | 2.9 | 6.2 | 0.6 | 5.3 | 0.3 | 7.7 | 2.2 | 5.6 | 1.9 | 3.6 | 3.1 |
| Plk1s1 | 16.5 | 12.8 | 14.9 | 15.3 | 11.2 | 17.4 | 12.5 | 11.5 | 10.6 | 13.3 | 14.7 | 15.0 |
| Plk2 | 47.8 | 23.9 | 19.8 | 47.9 | 42.8 | 34.6 | 34.7 | 18.3 | 24.3 | 55.3 | 42.9 | 30.7 |
| Plk3 | 4.2 | 3.5 | 2.9 | 4.7 | 7.3 | 3.5 | 5.3 | 2.7 | 3.6 | 6.3 | 3.8 | 3.6 |
| Plk4 | 3.0 | 4.4 | 5.4 | 2.5 | 5.0 | 2.7 | 5.6 | 3.2 | 4.9 | 3.1 | 4.2 | 3.5 |
| Plk5 | 0.0 | 0.1 | 0.0 | 0.0 | 0.0 | 0.0 | 0.0 | 0.0 | 0.0 | 0.0 | 0.0 | 0.0 |
| Pllp | 0.3 | 0.4 | 0.1 | 0.2 | 0.1 | 0.2 | 0.1 | 0.1 | 0.1 | 0.1 | 0.1 | 0.2 |
| Pln | 0.1 | 0.1 | 0.1 | 0.0 | 0.0 | 0.0 | 0.1 | 0.1 | 0.0 | 0.0 | 0.0 | 0.1 |
| Plod1 | 25.5 | 24.6 | 24.0 | 24.5 | 27.7 | 24.8 | 22.9 | 25.3 | 24.1 | 22.2 | 19.8 | 19.3 |
| Plod2 | 277.2 | 338.9 | 386.7 | 219.0 | 245.0 | 245.2 | 253.7 | 292.0 | 326.1 | 295.8 | 319.7 | 396.1 |
| Plod3 | 74.1 | 82.7 | 85.8 | 86.8 | 81.9 | 91.2 | 97.7 | 107.1 | 99.5 | 76.1 | 78.1 | 76.5 |
| Plp1 | 0.2 | 0.1 | 0.1 | 0.1 | 0.1 | 0.1 | 0.2 | 0.5 | 0.2 | 0.3 | 0.1 | 0.2 |
| Plp2 | 38.7 | 34.8 | 33.7 | 55.2 | 55.6 | 48.7 | 51.1 | 63.0 | 43.7 | 37.5 | 50.1 | 41.0 |
| Plrg1 | 28.0 | 30.4 | 26.8 | 28.8 | 26.7 | 29.0 | 25.5 | 27.9 | 24.1 | 29.0 | 28.1 | 24.9 |
| Pls1 | 0.3 | 0.2 | 0.2 | 0.0 | 0.2 | 0.2 | 0.0 | 0.0 | 0.0 | 0.1 | 0.1 | 0.1 |
| Pls3 | 123.9 | 104.3 | 88.5 | 98.9 | 94.9 | 97.2 | 94.4 | 90.6 | 111.7 | 113.1 | 120.8 | 137.1 |
| Plscr1 | 13.2 | 11.7 | 12.4 | 9.6 | 14.5 | 10.1 | 12.7 | 10.7 | 12.8 | 20.0 | 15.7 | 16.8 |
| Plscr2 | 31.0 | 30.5 | 26.8 | 28.0 | 33.3 | 20.5 | 30.7 | 23.5 | 34.4 | 50.8 | 42.1 | 40.3 |
| Plscr3 | 8.6 | 8.8 | 8.8 | 9.9 | 9.0 | 8.6 | 9.9 | 12.8 | 9.7 | 8.6 | 9.3 | 6.9 |
| Plscr4 | 5.4 | 6.6 | 8.0 | 7.6 | 6.2 | 7.9 | 7.3 | 6.6 | 6.2 | 4.9 | 6.3 | 7.4 |
| Pltp | 47.5 | 29.5 | 28.7 | 27.0 | 27.9 | 36.5 | 20.0 | 23.0 | 24.6 | 22.4 | 20.7 | 28.7 |
| Plvap | 0.7 | 0.1 | 0.3 | 0.3 | 0.6 | 0.3 | 0.3 | 0.6 | 0.3 | 0.2 | 0.1 | 0.2 |
| Plxdc1 | 0.1 | 0.0 | 0.0 | 0.7 | 0.8 | 0.0 | 0.1 | 0.0 | 0.0 | 0.0 | 0.1 | 0.0 |
| Plxdc2 | 27.4 | 26.8 | 34.3 | 31.2 | 31.5 | 31.4 | 40.9 | 38.1 | 39.2 | 37.7 | 37.7 | 40.2 |
| Plxna1 | 18.3 | 18.0 | 16.3 | 23.5 | 25.5 | 21.7 | 26.3 | 20.0 | 22.9 | 19.3 | 20.1 | 16.0 |
| Plxna2 | 2.0 | 1.8 | 3.4 | 1.4 | 2.3 | 1.2 | 3.0 | 6.3 | 4.1 | 2.6 | 2.5 | 5.0 |
| Plxna3 | 0.8 | 1.1 | 1.4 | 0.9 | 0.7 | 2.2 | 0.9 | 0.9 | 1.0 | 1.0 | 0.9 | 0.6 |
| Plxna4 | 1.8 | 1.4 | 2.2 | 0.9 | 1.3 | 0.8 | 1.5 | 3.2 | 2.0 | 6.5 | 3.9 | 2.8 |
| Plxnb1 | 0.5 | 0.4 | 0.6 | 0.2 | 0.5 | 0.3 | 0.5 | 0.5 | 0.9 | 1.9 | 1.0 | 0.9 |
| Plxnb2 | 79.8 | 72.7 | 68.8 | 71.6 | 78.6 | 76.1 | 83.6 | 66.5 | 70.9 | 79.0 | 70.2 | 70.9 |
| Plxnb3 | 0.1 | 0.1 | 0.1 | 0.1 | 0.1 | 0.1 | 0.1 | 0.2 | 0.1 | 0.1 | 0.2 | 0.1 |
| Plxnc1 | 0.6 | 0.8 | 1.5 | 2.4 | 3.6 | 0.3 | 0.6 | 1.2 | 0.6 | 0.7 | 0.4 | 0.7 |
| Plxnd1 | 1.6 | 1.7 | 2.6 | 1.8 | 3.6 | 1.3 | 2.0 | 2.7 | 2.7 | 1.8 | 1.3 | 1.9 |
| Pm20d1 | 2.0 | 1.8 | 1.7 | 0.4 | 1.1 | 0.4 | 0.7 | 0.5 | 0.4 | 0.4 | 0.4 | 0.6 |
| Pm20d2 | 2.1 | 1.3 | 1.7 | 0.9 | 0.6 | 1.2 | 0.6 | 0.7 | 0.4 | 0.7 | 0.6 | 0.9 |

| | | | | | | | | | | | | |
|---|---|---|---|---|---|---|---|---|---|---|---|---|
| Pmaip1 | 12.1 | 9.0 | 8.4 | 10.8 | 13.5 | 9.9 | 9.4 | 1.9 | 6.3 | 16.0 | 10.3 | 12.2 |
| Pmel | 0.5 | 0.4 | 0.6 | 0.3 | 0.2 | 0.6 | 0.3 | 0.5 | 0.5 | 0.4 | 0.5 | 0.2 |
| Pmepa1 | 21.6 | 24.2 | 22.8 | 30.7 | 26.7 | 32.0 | 39.9 | 27.7 | 33.1 | 24.4 | 32.4 | 33.1 |
| Pmf1 | 3.0 | 3.3 | 4.5 | 3.7 | 4.2 | 1.5 | 6.3 | 3.7 | 3.9 | 2.6 | 3.5 | 3.2 |
| Pml | 6.0 | 6.8 | 7.4 | 5.9 | 7.5 | 6.3 | 8.4 | 4.9 | 5.8 | 6.4 | 5.3 | 6.2 |
| Pmm1 | 43.1 | 36.9 | 43.3 | 31.3 | 35.2 | 35.3 | 38.0 | 43.6 | 40.8 | 51.4 | 42.7 | 46.3 |
| Pmm2 | 12.0 | 9.5 | 9.7 | 14.5 | 12.4 | 11.3 | 14.9 | 10.3 | 11.3 | 12.1 | 13.2 | 13.4 |
| Pmp2 | 0.1 | 0.1 | 0.0 | 0.0 | 0.0 | 0.0 | 0.2 | 0.2 | 0.3 | 0.4 | 1.0 | 0.2 |
| Pmp22 | 90.8 | 115.2 | 147.3 | 72.9 | 95.1 | 81.0 | 182.2 | 242.6 | 234.4 | 106.7 | 171.4 | 163.3 |
| Pmpca | 19.8 | 18.6 | 17.4 | 22.3 | 19.4 | 19.6 | 21.1 | 17.7 | 17.9 | 17.6 | 20.5 | 18.1 |
| Pmpcb | 45.0 | 37.8 | 39.6 | 42.9 | 36.9 | 42.4 | 37.4 | 38.5 | 36.0 | 39.0 | 43.4 | 40.2 |
| Pms1 | 1.6 | 1.9 | 2.4 | 2.0 | 1.8 | 2.1 | 2.0 | 2.4 | 1.7 | 2.0 | 2.3 | 2.1 |
| Pms2 | 1.9 | 2.3 | 2.3 | 1.8 | 2.0 | 1.6 | 2.4 | 2.3 | 2.0 | 2.2 | 2.5 | 2.1 |
| Pmvk | 8.4 | 12.6 | 13.4 | 6.5 | 7.6 | 8.3 | 17.9 | 21.0 | 17.8 | 6.1 | 16.4 | 8.1 |
| Pnck | 0.7 | 0.4 | 0.3 | 0.0 | 0.3 | 0.8 | 0.6 | 0.2 | 0.2 | 0.8 | 0.7 | 0.5 |
| Pnkd | 22.3 | 21.1 | 17.5 | 16.4 | 17.3 | 23.4 | 18.3 | 18.7 | 19.0 | 22.0 | 20.4 | 19.2 |
| Pnkp | 10.5 | 11.8 | 9.6 | 9.4 | 11.7 | 10.7 | 10.4 | 10.4 | 12.0 | 10.5 | 9.1 | 9.7 |
| Pnldc1 | 0.5 | 0.4 | 0.2 | 0.1 | 0.2 | 0.4 | 0.3 | 0.4 | 0.4 | 0.3 | 0.2 | 0.2 |
| Pnma1 | 0.1 | 0.1 | 0.3 | 0.1 | 0.2 | 0.2 | 0.2 | 0.3 | 0.2 | 0.3 | 0.4 | 0.2 |
| Pnma2 | 0.4 | 0.6 | 0.3 | 0.4 | 0.3 | 0.2 | 0.4 | 0.4 | 0.3 | 0.7 | 0.4 | 0.3 |
| Pnmal2 | 0.1 | 0.3 | 0.4 | 0.1 | 0.2 | 0.2 | 0.2 | 0.4 | 0.2 | 0.4 | 0.2 | 0.3 |
| Pnn | 13.5 | 13.7 | 17.9 | 15.8 | 13.2 | 12.7 | 17.1 | 14.2 | 13.4 | 13.8 | 18.8 | 18.6 |
| Pno1 | 32.1 | 24.3 | 18.5 | 29.7 | 27.6 | 27.9 | 21.1 | 18.9 | 19.5 | 23.7 | 26.4 | 23.0 |
| Pnp | 59.1 | 68.7 | 79.1 | 42.3 | 61.3 | 33.2 | 53.4 | 68.9 | 58.6 | 69.2 | 61.2 | 67.6 |
| Pnp2 | 8.6 | 9.0 | 12.9 | 7.7 | 7.7 | 5.7 | 8.7 | 11.3 | 8.0 | 9.7 | 11.7 | 11.6 |
| Pnpla1 | 0.0 | 0.0 | 0.0 | 0.0 | 0.0 | 0.1 | 0.0 | 0.0 | 0.0 | 0.0 | 0.0 | 0.0 |
| Pnpla2 | 6.9 | 6.4 | 4.7 | 9.2 | 6.3 | 8.9 | 5.4 | 5.0 | 4.4 | 5.0 | 6.4 | 4.2 |
| Pnpla3 | 0.0 | 0.0 | 0.1 | 0.0 | 0.0 | 0.1 | 0.1 | 0.1 | 0.0 | 0.0 | 0.1 | 0.0 |
| Pnpla6 | 3.9 | 3.9 | 3.5 | 3.9 | 3.2 | 4.4 | 3.5 | 3.1 | 3.1 | 3.6 | 3.3 | 2.8 |
| Pnpla7 | 4.0 | 3.4 | 3.2 | 6.2 | 5.2 | 4.0 | 3.1 | 2.4 | 2.4 | 4.3 | 3.5 | 3.1 |
| Pnpla8 | 19.5 | 19.5 | 18.8 | 19.8 | 16.3 | 21.6 | 17.5 | 17.6 | 19.8 | 18.9 | 23.9 | 22.6 |
| Pnpo | 2.8 | 2.9 | 2.4 | 2.1 | 2.7 | 2.2 | 1.9 | 2.5 | 2.2 | 4.1 | 2.6 | 3.4 |
| Pnpt1 | 9.6 | 10.8 | 10.7 | 11.5 | 9.7 | 8.1 | 9.0 | 9.7 | 9.4 | 10.7 | 10.2 | 11.0 |
| Pnrc1 | 23.2 | 27.5 | 30.5 | 26.5 | 28.0 | 27.9 | 26.7 | 24.1 | 24.0 | 28.6 | 25.7 | 24.6 |
| Pnrc2 | 28.9 | 30.0 | 38.1 | 34.8 | 28.8 | 30.7 | 32.7 | 37.5 | 36.1 | 32.2 | 39.3 | 43.1 |
| Poc1a | 1.2 | 1.7 | 2.3 | 1.0 | 1.9 | 0.5 | 2.2 | 1.5 | 1.9 | 1.3 | 1.5 | 1.4 |
| Poc1b | 0.8 | 1.2 | 1.4 | 0.9 | 1.2 | 1.2 | 0.9 | 1.5 | 1.2 | 1.2 | 1.2 | 1.2 |
| Poc5 | 4.7 | 5.5 | 6.1 | 4.2 | 4.6 | 3.9 | 4.8 | 4.1 | 5.3 | 5.3 | 5.3 | 5.0 |
| Podn | 2.5 | 9.8 | 18.1 | 0.8 | 2.7 | 1.3 | 2.4 | 14.5 | 6.6 | 9.2 | 4.9 | 5.3 |
| Podnl1 | 0.3 | 0.4 | 0.3 | 0.3 | 0.3 | 0.4 | 0.5 | 0.6 | 0.7 | 0.3 | 0.3 | 0.4 |
| Podxl | 0.2 | 0.1 | 0.2 | 0.1 | 0.1 | 0.1 | 0.2 | 0.4 | 0.2 | 0.7 | 0.3 | 0.3 |
| Podxl2 | 2.3 | 2.6 | 2.1 | 3.9 | 2.4 | 5.1 | 1.7 | 1.8 | 1.9 | 4.0 | 2.5 | 2.7 |
| Pofut1 | 21.6 | 20.3 | 17.0 | 24.4 | 19.2 | 28.9 | 19.8 | 19.5 | 18.4 | 17.6 | 17.5 | 16.5 |
| Pofut2 | 44.7 | 43.1 | 45.9 | 50.2 | 49.7 | 52.6 | 55.0 | 50.0 | 50.9 | 55.5 | 51.8 | 53.6 |
| Pogk | 6.3 | 5.1 | 4.2 | 7.1 | 5.7 | 7.3 | 4.9 | 4.3 | 4.9 | 4.2 | 5.2 | 4.6 |
| Poglut1 | 31.1 | 26.8 | 23.5 | 30.9 | 25.0 | 30.6 | 25.0 | 23.7 | 23.4 | 25.3 | 26.0 | 27.0 |
| Pogz | 1.1 | 1.3 | 1.5 | 1.0 | 1.3 | 1.2 | 1.4 | 1.5 | 1.3 | 1.2 | 1.3 | 1.0 |
| Pola1 | 2.4 | 3.5 | 3.7 | 1.8 | 4.1 | 1.6 | 4.2 | 2.5 | 2.9 | 2.1 | 3.3 | 2.4 |
| Pola2 | 3.6 | 3.8 | 4.7 | 3.1 | 3.9 | 3.3 | 4.6 | 3.9 | 3.9 | 2.7 | 4.7 | 3.1 |
| Polb | 24.6 | 26.7 | 23.9 | 22.9 | 22.2 | 22.4 | 18.1 | 15.9 | 17.0 | 20.5 | 20.4 | 20.4 |
| Pold1 | 1.8 | 3.0 | 3.4 | 2.0 | 3.8 | 1.9 | 3.6 | 2.3 | 3.3 | 2.3 | 2.5 | 1.9 |
| Pold2 | 6.4 | 8.9 | 9.6 | 7.1 | 6.2 | 8.2 | 10.1 | 10.7 | 8.9 | 5.9 | 7.9 | 6.4 |
| Pold3 | 2.6 | 2.8 | 3.2 | 3.3 | 3.1 | 2.7 | 2.9 | 3.1 | 2.6 | 2.4 | 2.6 | 2.8 |
| Pold4 | 40.2 | 32.8 | 30.8 | 35.0 | 31.6 | 40.5 | 39.9 | 34.5 | 40.3 | 39.5 | 40.6 | 42.5 |
| Poldip2 | 30.0 | 31.7 | 33.1 | 29.9 | 27.4 | 24.9 | 32.2 | 35.6 | 33.2 | 27.7 | 28.9 | 31.8 |
| Poldip3 | 20.9 | 24.5 | 22.1 | 20.9 | 22.9 | 21.1 | 23.8 | 26.9 | 25.0 | 23.6 | 23.8 | 23.8 |
| Pole | 1.2 | 1.6 | 2.5 | 0.9 | 2.3 | 0.7 | 3.0 | 1.6 | 2.0 | 0.9 | 1.7 | 1.2 |

| | | | | | | | | | | | | |
|---|---|---|---|---|---|---|---|---|---|---|---|---|
| Pole2 | 0.9 | 1.8 | 2.9 | 0.5 | 1.9 | 0.5 | 2.1 | 1.8 | 2.4 | 1.7 | 1.9 | 1.6 |
| Pole3 | 46.2 | 42.8 | 51.3 | 60.5 | 34.6 | 39.5 | 48.2 | 41.2 | 29.3 | 37.2 | 53.5 | 47.7 |
| Pole4 | 17.8 | 19.3 | 15.0 | 20.9 | 20.7 | 19.3 | 16.9 | 14.3 | 18.0 | 15.2 | 17.4 | 12.4 |
| Polg | 9.4 | 10.0 | 9.5 | 8.8 | 9.6 | 7.9 | 10.9 | 10.1 | 11.2 | 10.2 | 9.3 | 9.4 |
| Polg2 | 1.5 | 1.2 | 1.5 | 1.1 | 1.5 | 1.5 | 1.1 | 1.1 | 1.4 | 1.7 | 1.3 | 1.3 |
| Polh | 9.0 | 9.4 | 10.8 | 6.9 | 10.6 | 8.5 | 11.7 | 8.8 | 9.7 | 8.5 | 9.9 | 8.2 |
| Poli | 3.3 | 2.9 | 2.9 | 3.5 | 2.3 | 3.4 | 2.7 | 2.2 | 1.9 | 2.4 | 2.7 | 2.3 |
| Polk | 14.6 | 13.8 | 12.5 | 14.7 | 12.4 | 13.2 | 12.1 | 8.4 | 11.2 | 13.2 | 14.8 | 13.1 |
| Poll | 3.4 | 4.1 | 3.5 | 4.2 | 4.0 | 4.2 | 3.3 | 3.6 | 3.9 | 3.6 | 3.3 | 3.1 |
| Polm | 1.3 | 1.5 | 1.5 | 1.0 | 1.3 | 1.3 | 1.4 | 1.3 | 1.2 | 1.6 | 1.1 | 1.4 |
| Poln | 0.1 | 0.3 | 0.2 | 0.2 | 0.1 | 0.4 | 0.2 | 0.3 | 0.1 | 0.2 | 0.2 | 0.2 |
| Polq | 0.3 | 0.4 | 0.7 | 0.2 | 0.6 | 0.1 | 0.9 | 0.4 | 0.7 | 0.4 | 0.6 | 0.3 |
| Polr1a | 7.8 | 6.4 | 5.7 | 9.0 | 7.4 | 7.3 | 7.6 | 6.8 | 5.4 | 7.0 | 7.0 | 6.9 |
| Polr1b | 2.8 | 2.2 | 2.0 | 2.5 | 3.0 | 2.3 | 2.7 | 2.6 | 2.4 | 3.3 | 2.7 | 2.6 |
| Polr1c | 18.8 | 16.6 | 15.6 | 15.0 | 16.4 | 16.0 | 15.0 | 16.1 | 14.8 | 19.1 | 15.4 | 16.2 |
| Polr1d | 97.9 | 77.3 | 73.8 | 90.1 | 77.4 | 90.9 | 73.5 | 69.6 | 72.1 | 95.5 | 90.1 | 85.8 |
| Polr1e | 3.8 | 3.4 | 3.3 | 3.8 | 3.3 | 3.1 | 3.0 | 3.1 | 2.6 | 3.2 | 3.1 | 2.3 |
| Polr2a | 2.5 | 3.0 | 3.2 | 2.1 | 2.9 | 2.3 | 4.0 | 3.1 | 3.1 | 3.3 | 2.6 | 3.1 |
| Polr2b | 20.1 | 19.1 | 20.2 | 18.3 | 17.7 | 20.7 | 16.9 | 21.3 | 17.4 | 19.0 | 19.7 | 20.9 |
| Polr2c | 24.6 | 23.1 | 21.3 | 23.4 | 22.6 | 23.8 | 23.6 | 20.5 | 20.1 | 22.4 | 22.6 | 22.5 |
| Polr2d | 15.2 | 15.6 | 16.3 | 14.8 | 16.7 | 14.4 | 19.1 | 19.1 | 18.1 | 16.9 | 17.9 | 16.4 |
| Polr2e | 30.5 | 28.1 | 25.6 | 30.9 | 25.4 | 33.2 | 26.8 | 22.1 | 20.6 | 25.8 | 25.2 | 26.0 |
| Polr2f | 42.2 | 40.8 | 42.9 | 51.4 | 43.8 | 44.5 | 43.2 | 40.6 | 39.5 | 37.2 | 45.7 | 42.2 |
| Polr2g | 54.1 | 40.4 | 29.7 | 46.9 | 41.9 | 49.8 | 35.3 | 35.9 | 33.3 | 50.2 | 37.9 | 38.2 |
| Polr2h | 16.8 | 13.2 | 13.2 | 13.0 | 13.1 | 15.9 | 13.8 | 12.6 | 14.6 | 17.0 | 14.3 | 14.2 |
| Polr2i | 12.3 | 11.6 | 12.4 | 13.6 | 9.8 | 14.9 | 14.1 | 15.3 | 12.6 | 12.6 | 11.6 | 13.1 |
| Polr2j | 38.5 | 41.4 | 40.6 | 44.1 | 40.3 | 44.5 | 48.3 | 46.7 | 39.7 | 42.0 | 42.6 | 48.2 |
| Polr2k | 42.8 | 37.9 | 33.2 | 42.8 | 32.7 | 48.8 | 29.7 | 29.3 | 33.1 | 38.4 | 45.9 | 43.3 |
| Polr2l | 56.8 | 62.2 | 48.3 | 51.6 | 55.1 | 60.3 | 54.2 | 52.0 | 55.2 | 60.6 | 53.9 | 52.0 |
| Polr2m | 60.9 | 54.6 | 56.0 | 74.3 | 50.9 | 63.6 | 57.8 | 57.6 | 49.6 | 55.4 | 67.8 | 58.8 |
| Polr3a | 5.7 | 4.9 | 4.9 | 6.1 | 5.8 | 5.6 | 5.5 | 4.9 | 4.9 | 5.1 | 5.3 | 4.9 |
| Polr3b | 4.2 | 2.9 | 3.2 | 4.3 | 3.8 | 3.8 | 4.1 | 4.3 | 3.3 | 4.0 | 4.3 | 4.2 |
| Polr3c | 9.2 | 10.2 | 10.5 | 10.8 | 9.5 | 9.5 | 10.9 | 11.3 | 9.5 | 8.5 | 11.1 | 9.0 |
| Polr3d | 14.6 | 14.2 | 14.2 | 14.8 | 14.0 | 12.7 | 17.6 | 15.4 | 15.0 | 14.2 | 17.9 | 17.4 |
| Polr3e | 5.2 | 4.6 | 3.8 | 5.8 | 5.4 | 5.5 | 5.5 | 4.7 | 4.7 | 4.8 | 5.5 | 5.0 |
| Polr3f | 5.1 | 6.0 | 6.7 | 5.7 | 5.1 | 5.3 | 5.2 | 5.7 | 5.3 | 5.1 | 5.1 | 5.5 |
| Polr3g | 2.2 | 2.3 | 2.6 | 1.8 | 1.5 | 2.9 | 1.7 | 2.3 | 1.7 | 1.9 | 1.9 | 1.7 |
| Polr3gl | 8.2 | 9.2 | 12.8 | 8.3 | 6.2 | 8.8 | 6.7 | 8.3 | 6.7 | 6.5 | 8.7 | 10.1 |
| Polr3h | 7.1 | 6.1 | 4.5 | 7.1 | 5.2 | 7.2 | 6.6 | 5.5 | 5.4 | 6.6 | 6.3 | 5.5 |
| Polr3k | 7.6 | 8.1 | 7.1 | 9.7 | 8.6 | 8.8 | 7.0 | 8.2 | 7.9 | 9.7 | 8.8 | 8.4 |
| Polrmt | 3.9 | 3.6 | 3.4 | 3.6 | 3.3 | 3.8 | 3.9 | 3.7 | 3.8 | 3.6 | 3.6 | 3.4 |
| Pom121 | 0.7 | 1.2 | 1.2 | 0.9 | 1.4 | 1.2 | 1.4 | 1.2 | 1.1 | 1.1 | 0.8 | 0.8 |
| Pom121l2 | 0.0 | 0.0 | 0.1 | 0.1 | 0.0 | 0.0 | 0.0 | 0.0 | 0.1 | 0.1 | 0.0 | 0.1 |
| Pomc | 0.9 | 0.6 | 0.6 | 0.2 | 0.8 | 0.5 | 0.7 | 0.4 | 0.8 | 0.4 | 0.3 | 0.5 |
| Pomgnt1 | 27.1 | 24.6 | 21.4 | 27.1 | 22.9 | 26.2 | 25.9 | 20.3 | 26.4 | 26.0 | 24.3 | 22.8 |
| Pomp | 139.1 | 111.0 | 104.0 | 145.5 | 121.2 | 139.5 | 111.7 | 93.9 | 100.6 | 126.8 | 117.3 | 118.8 |
| Pomt1 | 5.8 | 5.9 | 6.5 | 5.8 | 5.4 | 6.8 | 7.3 | 6.8 | 7.0 | 6.2 | 6.9 | 6.2 |
| Pomt2 | 4.1 | 3.6 | 3.2 | 3.1 | 3.5 | 3.6 | 3.8 | 4.3 | 3.5 | 3.9 | 3.3 | 3.2 |
| Pon1 | 0.0 | 0.0 | 0.0 | 0.0 | 0.0 | 0.1 | 0.0 | 0.0 | 0.0 | 0.0 | 0.0 | 0.1 |
| Pon2 | 67.8 | 54.9 | 53.8 | 97.0 | 66.1 | 92.4 | 64.5 | 46.9 | 51.3 | 57.9 | 62.0 | 68.2 |
| Pon3 | 29.3 | 26.4 | 29.9 | 28.7 | 31.0 | 27.9 | 22.6 | 25.4 | 21.6 | 22.1 | 22.8 | 28.9 |
| Pop1 | 4.5 | 5.3 | 4.3 | 4.4 | 4.7 | 5.1 | 4.2 | 3.4 | 3.7 | 4.7 | 4.4 | 4.2 |
| Pop4 | 34.5 | 27.6 | 25.6 | 32.1 | 30.9 | 24.4 | 32.5 | 22.2 | 26.5 | 26.2 | 34.5 | 27.4 |
| Pop5 | 8.2 | 8.1 | 7.2 | 9.7 | 8.5 | 5.6 | 7.1 | 5.6 | 8.8 | 10.3 | 8.1 | 8.1 |
| Pop7 | 9.0 | 9.1 | 6.3 | 10.7 | 9.6 | 8.5 | 10.2 | 10.6 | 9.4 | 10.2 | 7.8 | 9.5 |
| Popdc2 | 2.2 | 1.0 | 0.6 | 2.4 | 1.2 | 1.2 | 1.3 | 0.3 | 1.3 | 1.5 | 1.8 | 1.0 |
| Popdc3 | 0.4 | 0.1 | 0.0 | 0.2 | 0.0 | 0.3 | 0.0 | 0.0 | 0.0 | 0.0 | 0.0 | 0.0 |

| | | | | | | | | | | | | |
|---|---|---|---|---|---|---|---|---|---|---|---|---|
| Por | 13.6 | 14.6 | 14.5 | 18.9 | 19.8 | 16.8 | 20.7 | 16.3 | 16.9 | 16.8 | 15.5 | 16.0 |
| Porcn | 1.3 | 1.0 | 1.3 | 1.6 | 1.1 | 1.7 | 1.5 | 1.3 | 1.6 | 1.2 | 1.3 | 1.7 |
| Postn | 172.2 | 214.7 | 415.7 | 186.5 | 375.7 | 246.2 | 157.8 | 422.7 | 308.4 | 256.1 | 293.9 | 344.5 |
| Pot1a | 3.7 | 4.6 | 4.4 | 2.8 | 3.7 | 3.1 | 3.4 | 3.7 | 4.2 | 3.9 | 4.0 | 3.8 |
| Pot1b | 0.9 | 1.2 | 1.1 | 0.6 | 0.9 | 0.8 | 0.8 | 0.8 | 0.9 | 1.0 | 0.7 | 0.8 |
| Pou2f1 | 0.8 | 0.8 | 0.9 | 0.7 | 0.8 | 0.9 | 0.8 | 0.7 | 0.7 | 0.7 | 0.9 | 0.7 |
| Pou2f2 | 0.1 | 0.0 | 0.0 | 0.8 | 1.9 | 0.0 | 0.3 | 0.0 | 0.1 | 0.1 | 0.1 | 0.1 |
| Pou2f3 | 0.1 | 0.1 | 0.1 | 0.2 | 0.1 | 0.4 | 0.1 | 0.1 | 0.1 | 0.0 | 0.0 | 0.1 |
| Pou3f1 | 0.1 | 0.0 | 0.1 | 0.0 | 0.0 | 0.0 | 0.0 | 0.0 | 0.0 | 0.1 | 0.0 | 0.1 |
| Pou3f3 | 0.0 | 0.1 | 0.0 | 0.0 | 0.0 | 0.1 | 0.0 | 0.0 | 0.0 | 0.0 | 0.0 | 0.0 |
| Pou3f4 | 0.0 | 0.0 | 0.0 | 0.0 | 0.0 | 0.0 | 0.0 | 0.0 | 0.0 | 0.0 | 0.1 | 0.0 |
| Pou4f1 | 0.0 | 0.1 | 0.0 | 0.0 | 0.0 | 0.0 | 0.0 | 0.0 | 0.0 | 0.0 | 0.0 | 0.0 |
| Pou5f1 | 0.0 | 0.0 | 0.1 | 0.0 | 0.1 | 0.0 | 0.1 | 0.0 | 0.0 | 0.0 | 0.0 | 0.0 |
| Pou5f2 | 0.0 | 0.1 | 0.0 | 0.0 | 0.1 | 0.0 | 0.0 | 0.1 | 0.0 | 0.1 | 0.0 | 0.1 |
| Pou6f1 | 3.3 | 3.1 | 3.1 | 3.3 | 2.5 | 3.4 | 3.1 | 2.6 | 2.6 | 2.4 | 2.4 | 2.3 |
| Pp2d1 | 0.3 | 0.8 | 2.1 | 1.9 | 1.0 | 1.6 | 1.4 | 1.7 | 1.4 | 1.8 | 1.9 | 2.4 |
| Ppa1 | 38.7 | 32.7 | 29.4 | 41.2 | 33.9 | 34.1 | 32.2 | 30.9 | 30.0 | 34.0 | 36.8 | 29.3 |
| Ppa2 | 15.5 | 12.9 | 13.8 | 15.6 | 12.4 | 13.7 | 10.8 | 13.4 | 13.2 | 16.5 | 15.9 | 13.0 |
| Ppan | 9.2 | 8.2 | 7.1 | 10.8 | 10.0 | 8.7 | 10.2 | 8.8 | 8.8 | 9.1 | 10.1 | 7.6 |
| Ppap2a | 60.4 | 68.2 | 86.2 | 59.0 | 64.9 | 54.9 | 67.7 | 96.8 | 90.6 | 82.5 | 81.5 | 72.0 |
| Ppap2b | 32.0 | 59.5 | 84.2 | 25.1 | 51.0 | 28.8 | 59.3 | 56.1 | 53.9 | 40.6 | 37.3 | 51.2 |
| Ppap2c | 18.3 | 11.4 | 9.0 | 19.9 | 14.3 | 21.7 | 11.3 | 12.6 | 11.3 | 12.2 | 9.8 | 10.8 |
| Ppapdc1b | 18.1 | 14.2 | 17.5 | 19.9 | 18.2 | 18.0 | 20.5 | 22.9 | 21.4 | 16.8 | 21.4 | 18.8 |
| Ppapdc2 | 1.6 | 1.9 | 1.4 | 1.4 | 1.7 | 1.9 | 1.6 | 2.0 | 2.0 | 1.8 | 2.0 | 2.5 |
| Ppapdc3 | 3.4 | 2.7 | 2.8 | 3.0 | 2.7 | 3.5 | 3.0 | 2.6 | 3.3 | 3.1 | 2.6 | 2.8 |
| Ppara | 0.3 | 0.6 | 0.6 | 0.3 | 0.3 | 0.3 | 0.3 | 0.4 | 0.4 | 0.3 | 0.2 | 0.4 |
| Ppard | 2.7 | 3.2 | 2.6 | 2.5 | 2.7 | 2.5 | 2.8 | 3.1 | 2.3 | 2.8 | 2.6 | 2.6 |
| Pparg | 1.7 | 1.3 | 0.8 | 3.6 | 2.0 | 3.8 | 1.5 | 0.5 | 1.6 | 0.9 | 1.0 | 1.2 |
| Ppargc1a | 0.0 | 0.0 | 0.0 | 0.0 | 0.0 | 0.0 | 0.0 | 0.0 | 0.0 | 0.0 | 0.0 | 0.0 |
| Ppargc1b | 0.0 | 0.0 | 0.0 | 0.1 | 0.0 | 0.0 | 0.1 | 0.0 | 0.0 | 0.0 | 0.0 | 0.0 |
| Ppat | 2.5 | 2.9 | 3.0 | 2.5 | 2.4 | 2.2 | 2.6 | 2.8 | 2.3 | 2.7 | 2.9 | 2.5 |
| Ppcdc | 4.4 | 3.7 | 3.5 | 5.0 | 4.4 | 5.0 | 4.0 | 3.9 | 3.9 | 4.2 | 4.4 | 3.7 |
| Ppcs | 6.6 | 6.1 | 5.2 | 6.2 | 4.9 | 6.3 | 5.1 | 4.8 | 5.2 | 5.8 | 4.9 | 5.7 |
| Ppdpf | 14.4 | 14.6 | 17.5 | 19.8 | 12.0 | 22.0 | 15.8 | 18.8 | 15.0 | 15.6 | 16.3 | 14.1 |
| Ppef1 | 1.7 | 0.5 | 0.3 | 2.3 | 1.2 | 0.7 | 0.7 | 0.6 | 0.4 | 0.4 | 0.4 | 0.3 |
| Ppef2 | 0.0 | 0.0 | 0.0 | 0.1 | 0.0 | 0.1 | 0.0 | 0.0 | 0.0 | 0.0 | 0.0 | 0.0 |
| Ppfia1 | 16.7 | 16.4 | 17.0 | 18.5 | 16.8 | 18.1 | 19.0 | 16.0 | 17.6 | 17.4 | 19.1 | 20.1 |
| Ppfia2 | 0.0 | 0.0 | 0.0 | 0.0 | 0.0 | 0.0 | 0.0 | 0.0 | 0.0 | 0.0 | 0.0 | 0.0 |
| Ppfia3 | 0.2 | 0.1 | 0.1 | 0.1 | 0.2 | 0.1 | 0.2 | 0.1 | 0.2 | 0.1 | 0.1 | 0.1 |
| Ppfia4 | 0.1 | 0.0 | 0.0 | 0.2 | 0.5 | 0.0 | 0.1 | 0.0 | 0.0 | 0.0 | 0.1 | 0.0 |
| Ppfibp1 | 185.3 | 188.0 | 202.3 | 211.4 | 161.2 | 211.6 | 228.9 | 187.5 | 195.4 | 212.4 | 246.9 | 261.2 |
| Ppfibp2 | 2.5 | 1.8 | 1.8 | 5.3 | 9.0 | 1.8 | 2.2 | 1.7 | 1.2 | 1.9 | 1.5 | 2.3 |
| Pphln1 | 6.8 | 7.5 | 7.8 | 7.8 | 7.3 | 6.9 | 6.9 | 7.4 | 6.4 | 6.9 | 7.3 | 7.9 |
| Ppia | 1117.3 | 886.9 | 761.5 | 1181.8 | 978.2 | 1160.5 | 991.2 | 990.9 | 916.2 | 863.4 | 1033.5 | 841.8 |
| Ppib | 213.8 | 182.2 | 188.5 | 213.0 | 182.3 | 181.4 | 197.0 | 187.5 | 175.8 | 203.3 | 188.0 | 218.1 |
| Ppic | 94.6 | 61.3 | 80.9 | 102.4 | 91.4 | 75.7 | 79.5 | 88.7 | 78.1 | 88.0 | 78.1 | 85.5 |
| Ppid | 45.5 | 41.5 | 39.2 | 51.5 | 35.4 | 46.6 | 36.5 | 27.6 | 26.8 | 41.8 | 44.7 | 41.6 |
| Ppie | 13.1 | 10.5 | 9.1 | 13.1 | 9.5 | 11.0 | 8.9 | 11.0 | 11.1 | 11.4 | 10.0 | 10.3 |
| Ppif | 2.5 | 3.1 | 2.1 | 1.6 | 2.2 | 2.0 | 2.2 | 2.7 | 2.2 | 3.0 | 2.7 | 1.8 |
| Ppifos | 0.3 | 0.2 | 0.5 | 0.0 | 0.2 | 0.4 | 0.3 | 0.2 | 0.2 | 0.2 | 0.1 | 0.1 |
| Ppig | 9.9 | 8.8 | 11.7 | 13.5 | 8.5 | 10.6 | 10.5 | 9.6 | 6.6 | 8.5 | 12.0 | 12.8 |
| Ppih | 6.0 | 7.8 | 9.1 | 4.9 | 7.9 | 6.5 | 7.2 | 8.9 | 8.0 | 7.5 | 8.0 | 7.3 |
| Ppil1 | 7.8 | 9.9 | 10.4 | 10.4 | 10.6 | 9.7 | 11.0 | 11.7 | 10.7 | 11.2 | 11.6 | 10.9 |
| Ppil2 | 28.4 | 29.2 | 30.2 | 30.9 | 28.9 | 31.3 | 29.2 | 31.6 | 26.4 | 31.1 | 35.9 | 33.3 |
| Ppil3 | 33.2 | 26.6 | 24.6 | 28.2 | 25.1 | 30.2 | 25.3 | 24.7 | 24.6 | 26.9 | 28.2 | 28.2 |
| Ppil4 | 10.3 | 10.8 | 13.2 | 11.1 | 10.0 | 10.1 | 11.5 | 11.4 | 10.0 | 11.0 | 12.8 | 14.0 |
| Ppil6 | 0.3 | 0.2 | 0.2 | 0.3 | 0.1 | 0.2 | 0.0 | 0.0 | 0.1 | 0.1 | 0.0 | 0.0 |

| | | | | | | | | | | | | |
|---|---|---|---|---|---|---|---|---|---|---|---|---|
| Ppip5k1 | 4.9 | 4.7 | 3.6 | 4.5 | 3.6 | 5.8 | 2.9 | 3.1 | 2.6 | 4.4 | 4.0 | 4.0 |
| Ppip5k2 | 4.3 | 4.3 | 4.3 | 4.9 | 4.8 | 3.9 | 3.8 | 4.8 | 4.9 | 4.8 | 4.9 | 5.0 |
| Ppl | 4.7 | 3.3 | 3.4 | 0.8 | 1.1 | 0.7 | 2.8 | 5.4 | 2.6 | 3.4 | 5.9 | 5.2 |
| Ppm1a | 46.4 | 42.4 | 38.5 | 47.7 | 34.8 | 48.9 | 44.1 | 35.2 | 36.0 | 35.0 | 40.6 | 41.1 |
| Ppm1b | 25.7 | 24.8 | 24.0 | 27.7 | 23.1 | 26.3 | 26.2 | 19.0 | 22.2 | 22.4 | 25.7 | 24.0 |
| Ppm1d | 3.5 | 3.9 | 6.4 | 2.9 | 4.1 | 3.2 | 4.3 | 4.5 | 4.1 | 5.2 | 4.4 | 5.6 |
| Ppm1e | 0.0 | 0.0 | 0.1 | 0.0 | 0.1 | 0.0 | 0.0 | 0.1 | 0.1 | 0.1 | 0.1 | 0.1 |
| Ppm1f | 21.9 | 22.1 | 17.2 | 20.2 | 19.1 | 20.8 | 18.0 | 15.7 | 17.0 | 21.5 | 18.8 | 17.5 |
| Ppm1g | 26.3 | 23.4 | 27.6 | 28.1 | 26.6 | 24.7 | 33.9 | 26.7 | 23.7 | 23.9 | 28.3 | 31.2 |
| Ppm1h | 4.4 | 4.0 | 3.2 | 5.5 | 5.1 | 4.9 | 3.0 | 2.6 | 2.6 | 2.9 | 2.9 | 3.0 |
| Ppm1j | 0.5 | 0.3 | 0.4 | 0.3 | 0.4 | 0.7 | 0.2 | 0.4 | 0.3 | 0.3 | 0.2 | 0.4 |
| Ppm1k | 4.6 | 5.7 | 5.8 | 4.2 | 4.2 | 4.9 | 4.5 | 3.0 | 3.9 | 4.6 | 4.8 | 4.3 |
| Ppm1l | 0.3 | 0.4 | 0.3 | 0.4 | 0.2 | 0.3 | 0.3 | 0.5 | 0.4 | 1.1 | 0.6 | 0.2 |
| Ppm1m | 12.5 | 15.4 | 15.2 | 13.4 | 13.5 | 14.7 | 12.5 | 12.7 | 14.1 | 13.2 | 14.2 | 14.1 |
| Ppme1 | 21.1 | 19.5 | 16.7 | 22.2 | 17.7 | 19.8 | 23.2 | 17.4 | 17.7 | 20.5 | 22.0 | 20.8 |
| Ppox | 7.3 | 9.0 | 6.6 | 6.3 | 6.9 | 7.1 | 6.6 | 7.3 | 8.4 | 7.0 | 6.2 | 5.4 |
| Ppp1ca | 95.3 | 90.7 | 93.9 | 87.9 | 94.6 | 83.5 | 89.4 | 107.0 | 99.5 | 98.7 | 101.4 | 92.8 |
| Ppp1cb | 36.6 | 39.3 | 45.1 | 46.5 | 39.4 | 45.1 | 36.1 | 36.7 | 37.8 | 39.1 | 44.5 | 47.0 |
| Ppp1cc | 58.3 | 71.0 | 68.5 | 52.3 | 65.2 | 54.0 | 65.8 | 83.3 | 81.2 | 79.8 | 77.6 | 75.7 |
| Ppp1r10 | 5.4 | 5.7 | 5.9 | 6.2 | 6.2 | 5.0 | 6.9 | 6.1 | 5.8 | 6.4 | 7.0 | 6.2 |
| Ppp1r11 | 25.4 | 17.3 | 17.8 | 31.2 | 18.6 | 26.8 | 22.1 | 16.9 | 16.0 | 20.8 | 22.5 | 20.3 |
| Ppp1r12a | 28.1 | 26.0 | 29.7 | 30.2 | 22.7 | 25.3 | 29.1 | 26.4 | 25.9 | 30.3 | 36.7 | 35.1 |
| Ppp1r12b | 3.6 | 3.1 | 2.7 | 3.2 | 2.1 | 2.7 | 2.6 | 2.0 | 2.8 | 3.0 | 3.2 | 3.3 |
| Ppp1r12c | 24.2 | 21.7 | 19.2 | 27.0 | 23.7 | 25.7 | 22.1 | 16.6 | 21.0 | 21.6 | 21.0 | 20.4 |
| Ppp1r13b | 2.3 | 3.1 | 2.9 | 2.3 | 3.0 | 2.3 | 2.5 | 2.3 | 2.7 | 2.8 | 2.6 | 2.5 |
| Ppp1r13l | 0.5 | 0.5 | 0.3 | 0.4 | 0.6 | 0.5 | 1.0 | 0.5 | 0.6 | 0.9 | 0.6 | 0.5 |
| Ppp1r14a | 20.8 | 14.5 | 10.5 | 24.1 | 10.1 | 23.2 | 12.8 | 8.7 | 10.6 | 4.5 | 6.5 | 4.4 |
| Ppp1r14b | 74.9 | 64.0 | 55.3 | 71.8 | 72.3 | 66.1 | 67.6 | 93.6 | 72.3 | 82.6 | 76.2 | 65.6 |
| Ppp1r14c | 0.0 | 0.0 | 0.0 | 0.0 | 0.0 | 0.0 | 0.0 | 0.0 | 0.0 | 0.0 | 0.0 | 0.0 |
| Ppp1r15a | 18.4 | 13.8 | 12.0 | 23.9 | 11.5 | 19.3 | 16.0 | 11.6 | 12.6 | 18.3 | 15.2 | 15.2 |
| Ppp1r15b | 10.0 | 9.7 | 9.8 | 7.5 | 9.8 | 9.1 | 10.3 | 10.8 | 10.7 | 9.5 | 9.9 | 8.7 |
| Ppp1r16a | 6.1 | 5.9 | 5.4 | 6.7 | 6.0 | 7.9 | 6.0 | 6.2 | 6.5 | 5.5 | 5.6 | 5.5 |
| Ppp1r16b | 0.3 | 0.2 | 0.2 | 0.1 | 0.1 | 0.1 | 0.2 | 0.3 | 0.3 | 0.0 | 0.1 | 0.0 |
| Ppp1r18 | 14.3 | 14.5 | 13.1 | 19.8 | 19.0 | 14.5 | 17.4 | 14.7 | 15.6 | 15.4 | 14.6 | 15.1 |
| Ppp1r1a | 0.1 | 0.0 | 0.0 | 0.0 | 0.1 | 0.0 | 0.1 | 0.0 | 0.1 | 0.1 | 0.0 | 0.0 |
| Ppp1r1b | 0.1 | 0.0 | 0.0 | 0.0 | 0.0 | 0.0 | 0.0 | 0.0 | 0.1 | 0.0 | 0.0 | 0.1 |
| Ppp1r1c | 0.0 | 0.0 | 0.0 | 0.0 | 0.0 | 0.0 | 0.0 | 0.0 | 0.0 | 0.0 | 0.0 | 0.0 |
| Ppp1r2 | 33.6 | 31.6 | 35.1 | 36.7 | 26.8 | 32.8 | 26.3 | 26.8 | 26.1 | 24.3 | 31.8 | 27.5 |
| Ppp1r21 | 7.1 | 8.1 | 7.7 | 7.4 | 8.9 | 7.3 | 7.7 | 6.8 | 6.9 | 7.9 | 7.8 | 8.1 |
| Ppp1r26 | 1.0 | 1.3 | 1.1 | 1.1 | 0.6 | 1.3 | 0.8 | 0.8 | 0.6 | 0.9 | 0.8 | 0.8 |
| Ppp1r27 | 0.2 | 0.2 | 0.0 | 0.5 | 0.2 | 0.2 | 0.0 | 0.1 | 0.1 | 0.2 | 0.1 | 0.2 |
| Ppp1r2-ps3 | 0.3 | 0.4 | 0.6 | 0.1 | 0.2 | 0.3 | 0.3 | 0.4 | 0.3 | 0.1 | 0.2 | 0.3 |
| Ppp1r32 | 0.0 | 0.1 | 0.0 | 0.0 | 0.0 | 0.1 | 0.0 | 0.0 | 0.0 | 0.1 | 0.0 | 0.0 |
| Ppp1r35 | 6.3 | 7.1 | 4.3 | 5.8 | 5.4 | 5.3 | 5.2 | 6.2 | 6.5 | 7.3 | 6.0 | 5.8 |
| Ppp1r36 | 0.4 | 0.4 | 0.4 | 0.1 | 0.3 | 0.4 | 0.3 | 0.2 | 0.5 | 0.1 | 0.4 | 0.4 |
| Ppp1r37 | 15.4 | 16.7 | 14.5 | 15.4 | 13.7 | 15.4 | 14.8 | 14.9 | 15.4 | 14.1 | 13.3 | 14.6 |
| Ppp1r3a | 0.0 | 0.0 | 0.0 | 0.0 | 0.0 | 0.0 | 0.0 | 0.0 | 0.0 | 0.0 | 0.0 | 0.0 |
| Ppp1r3b | 2.4 | 3.0 | 3.0 | 2.2 | 2.8 | 2.9 | 2.8 | 2.3 | 2.9 | 2.2 | 2.5 | 2.4 |
| Ppp1r3c | 0.7 | 1.3 | 2.7 | 0.6 | 0.6 | 1.3 | 0.6 | 1.2 | 0.9 | 1.2 | 0.6 | 1.1 |
| Ppp1r3d | 0.6 | 0.5 | 0.2 | 1.1 | 0.5 | 0.7 | 0.4 | 0.4 | 0.4 | 1.7 | 0.9 | 0.6 |
| Ppp1r3e | 0.2 | 0.1 | 0.2 | 0.2 | 0.3 | 0.2 | 0.2 | 0.4 | 0.4 | 0.5 | 0.1 | 0.3 |
| Ppp1r3f | 0.5 | 0.6 | 0.6 | 0.7 | 0.4 | 0.4 | 0.5 | 0.5 | 0.5 | 0.8 | 0.6 | 0.6 |
| Ppp1r7 | 9.9 | 9.0 | 9.3 | 10.6 | 8.5 | 8.9 | 8.3 | 10.2 | 9.1 | 7.9 | 9.2 | 9.2 |
| Ppp1r8 | 14.4 | 12.4 | 13.5 | 14.2 | 12.1 | 14.7 | 13.3 | 12.9 | 12.5 | 14.2 | 14.6 | 13.9 |
| Ppp1r9a | 1.5 | 1.6 | 1.6 | 1.1 | 0.7 | 1.8 | 0.9 | 0.8 | 0.7 | 1.1 | 0.9 | 1.0 |
| Ppp1r9b | 21.0 | 22.6 | 23.5 | 25.0 | 21.8 | 22.7 | 22.6 | 20.2 | 19.5 | 19.0 | 20.3 | 20.4 |
| Ppp2ca | 80.1 | 77.4 | 77.5 | 75.7 | 77.5 | 76.7 | 75.6 | 84.6 | 76.4 | 81.9 | 86.5 | 83.4 |

| | | | | | | | | | | | | |
|---|---|---|---|---|---|---|---|---|---|---|---|---|
| Ppp2cb | 40.7 | 39.0 | 29.6 | 38.5 | 34.6 | 38.1 | 35.6 | 32.9 | 38.4 | 37.5 | 39.6 | 37.3 |
| Ppp2r1a | 62.7 | 55.1 | 47.8 | 58.7 | 59.1 | 56.8 | 60.1 | 58.5 | 59.9 | 60.7 | 61.0 | 51.9 |
| Ppp2r1b | 9.2 | 11.2 | 10.3 | 9.5 | 10.7 | 9.9 | 8.6 | 9.1 | 8.5 | 9.2 | 9.0 | 9.3 |
| Ppp2r2a | 3.7 | 4.7 | 4.9 | 4.7 | 4.4 | 3.6 | 5.5 | 4.7 | 4.7 | 4.9 | 5.0 | 5.2 |
| Ppp2r2b | 0.2 | 0.1 | 0.0 | 0.1 | 0.0 | 0.0 | 0.0 | 0.1 | 0.2 | 2.6 | 0.7 | 0.4 |
| Ppp2r2c | 1.6 | 0.8 | 0.6 | 1.3 | 0.9 | 1.4 | 0.4 | 0.1 | 0.1 | 0.2 | 0.3 | 0.3 |
| Ppp2r2d | 14.1 | 13.0 | 13.8 | 13.4 | 12.5 | 13.3 | 14.0 | 13.2 | 13.1 | 11.1 | 14.4 | 14.7 |
| Ppp2r3a | 3.8 | 4.6 | 5.1 | 3.2 | 3.6 | 4.0 | 4.5 | 5.0 | 4.4 | 3.1 | 4.0 | 4.5 |
| Ppp2r3c | 13.5 | 12.0 | 10.6 | 14.3 | 11.9 | 12.9 | 10.2 | 10.2 | 9.7 | 11.2 | 12.1 | 12.3 |
| Ppp2r3d | 0.7 | 0.8 | 0.6 | 0.5 | 1.0 | 0.5 | 0.8 | 0.9 | 0.9 | 0.9 | 0.7 | 0.8 |
| Ppp2r4 | 23.6 | 22.5 | 21.4 | 25.0 | 26.9 | 26.4 | 26.8 | 23.7 | 25.7 | 24.7 | 23.6 | 22.3 |
| Ppp2r5a | 14.5 | 14.5 | 14.4 | 16.4 | 16.1 | 14.7 | 13.9 | 14.7 | 14.5 | 18.8 | 17.0 | 16.4 |
| Ppp2r5b | 16.6 | 16.3 | 13.2 | 15.4 | 13.4 | 16.1 | 15.7 | 12.3 | 16.8 | 18.3 | 15.8 | 12.4 |
| Ppp2r5c | 39.5 | 43.7 | 48.0 | 42.2 | 41.9 | 41.9 | 40.0 | 42.2 | 38.0 | 38.0 | 45.0 | 44.1 |
| Ppp2r5d | 21.3 | 24.1 | 26.1 | 20.3 | 22.3 | 22.0 | 23.6 | 23.5 | 23.5 | 22.6 | 24.5 | 21.9 |
| Ppp2r5e | 5.4 | 6.8 | 6.2 | 5.8 | 5.1 | 5.5 | 6.1 | 5.0 | 5.1 | 5.5 | 6.3 | 5.8 |
| Ppp3ca | 26.0 | 22.0 | 26.4 | 25.2 | 25.6 | 24.6 | 25.3 | 28.0 | 22.8 | 30.5 | 27.8 | 30.3 |
| Ppp3cb | 7.3 | 8.0 | 9.2 | 7.9 | 7.7 | 8.0 | 7.7 | 9.0 | 9.4 | 8.6 | 9.3 | 9.9 |
| Ppp3cc | 7.8 | 8.9 | 8.6 | 6.4 | 6.2 | 5.6 | 7.8 | 7.9 | 7.7 | 8.6 | 8.8 | 10.3 |
| Ppp3r1 | 33.0 | 32.7 | 29.6 | 31.5 | 30.3 | 29.3 | 28.8 | 24.5 | 28.5 | 36.0 | 36.7 | 37.3 |
| Ppp4c | 37.4 | 37.7 | 34.2 | 33.0 | 39.8 | 31.9 | 37.2 | 37.1 | 37.3 | 38.8 | 35.1 | 32.6 |
| Ppp4r1 | 17.3 | 16.9 | 14.0 | 19.6 | 19.9 | 16.9 | 21.4 | 21.3 | 23.1 | 17.6 | 20.0 | 17.0 |
| Ppp4r1l-ps | 0.4 | 0.4 | 0.3 | 0.7 | 0.4 | 0.5 | 0.5 | 0.5 | 0.5 | 0.4 | 0.4 | 0.4 |
| Ppp4r2 | 26.0 | 27.6 | 29.5 | 25.5 | 24.9 | 25.5 | 24.5 | 25.8 | 24.2 | 27.1 | 30.2 | 31.2 |
| Ppp4r4 | 0.4 | 0.6 | 0.5 | 0.4 | 0.4 | 0.7 | 0.4 | 0.5 | 0.5 | 0.3 | 0.5 | 0.4 |
| Ppp5c | 19.2 | 18.0 | 18.1 | 17.9 | 19.2 | 17.4 | 21.9 | 19.3 | 17.2 | 18.8 | 18.0 | 16.9 |
| Ppp6c | 28.6 | 28.6 | 26.3 | 31.7 | 26.3 | 27.6 | 24.4 | 26.2 | 24.9 | 26.2 | 26.2 | 25.2 |
| Ppp6r1 | 18.2 | 17.7 | 18.7 | 20.5 | 21.0 | 17.2 | 19.1 | 18.9 | 18.8 | 17.7 | 17.2 | 16.9 |
| Ppp6r2 | 8.0 | 8.4 | 8.1 | 7.6 | 6.5 | 7.7 | 8.1 | 9.1 | 8.6 | 8.3 | 7.4 | 8.1 |
| Ppp6r3 | 15.9 | 18.7 | 20.5 | 15.5 | 18.0 | 15.5 | 17.8 | 20.6 | 19.9 | 17.8 | 20.5 | 20.1 |
| Pprc1 | 2.3 | 2.7 | 2.7 | 2.3 | 3.2 | 2.8 | 3.7 | 3.2 | 3.3 | 2.6 | 3.0 | 2.1 |
| Ppt1 | 61.7 | 46.5 | 41.7 | 74.2 | 55.0 | 69.4 | 37.3 | 35.8 | 37.6 | 45.6 | 43.7 | 52.1 |
| Ppt2 | 6.9 | 7.1 | 7.0 | 8.7 | 11.5 | 8.0 | 6.2 | 7.3 | 7.0 | 7.1 | 6.1 | 5.9 |
| Pptc7 | 4.6 | 5.0 | 4.4 | 5.6 | 5.0 | 5.1 | 5.3 | 4.7 | 5.2 | 5.4 | 5.2 | 5.7 |
| Ppwd1 | 2.9 | 3.7 | 3.3 | 2.6 | 3.0 | 3.1 | 2.8 | 3.2 | 3.0 | 2.9 | 3.0 | 2.5 |
| Ppyr1 | 0.1 | 0.0 | 0.0 | 0.0 | 0.1 | 0.0 | 0.2 | 0.1 | 0.0 | 0.1 | 0.1 | 0.1 |
| Pqbp1 | 34.7 | 36.6 | 45.0 | 47.1 | 31.1 | 36.8 | 45.1 | 40.4 | 30.1 | 33.8 | 51.3 | 48.3 |
| Pqlc1 | 4.7 | 5.2 | 4.0 | 6.0 | 8.2 | 5.5 | 6.0 | 4.3 | 5.8 | 6.1 | 5.0 | 4.3 |
| Pqlc2 | 2.7 | 2.3 | 2.4 | 3.9 | 3.5 | 2.8 | 3.2 | 2.6 | 2.9 | 2.6 | 2.2 | 1.9 |
| Pqlc3 | 32.1 | 27.2 | 34.7 | 33.4 | 28.3 | 40.2 | 24.9 | 34.3 | 22.2 | 29.1 | 27.9 | 32.9 |
| Pradc1 | 1.6 | 2.2 | 2.3 | 1.6 | 1.8 | 1.6 | 2.3 | 2.2 | 2.4 | 2.1 | 2.4 | 2.0 |
| Praf2 | 58.8 | 52.0 | 53.8 | 72.7 | 50.7 | 67.3 | 60.8 | 63.3 | 49.2 | 48.6 | 63.7 | 54.8 |
| Pram1 | 0.1 | 0.2 | 0.1 | 0.3 | 0.6 | 0.0 | 0.2 | 0.1 | 0.1 | 0.1 | 0.1 | 0.0 |
| Pramef12 | 0.2 | 0.1 | 0.0 | 0.1 | 0.1 | 0.1 | 0.2 | 0.1 | 0.2 | 0.1 | 0.1 | 0.3 |
| Pramef8 | 6.8 | 6.4 | 6.0 | 6.4 | 5.9 | 5.9 | 6.0 | 5.9 | 6.3 | 6.5 | 6.6 | 6.2 |
| Prc1 | 5.2 | 8.9 | 16.0 | 1.5 | 12.6 | 1.8 | 21.3 | 8.2 | 15.9 | 7.9 | 14.1 | 10.2 |
| Prcc | 9.7 | 10.2 | 10.8 | 10.2 | 9.2 | 9.6 | 10.9 | 11.2 | 9.8 | 10.2 | 11.2 | 9.6 |
| Prcp | 6.8 | 7.7 | 8.6 | 6.2 | 8.3 | 6.8 | 8.5 | 12.3 | 9.0 | 8.1 | 9.0 | 9.2 |
| Prdm1 | 0.0 | 0.0 | 0.0 | 0.1 | 0.0 | 0.0 | 0.1 | 0.0 | 0.0 | 0.0 | 0.0 | 0.0 |
| Prdm10 | 0.8 | 1.0 | 1.0 | 0.9 | 1.1 | 0.8 | 1.2 | 1.4 | 1.1 | 0.7 | 1.3 | 1.0 |
| Prdm11 | 0.4 | 0.7 | 0.4 | 0.4 | 0.4 | 0.5 | 0.4 | 0.5 | 0.5 | 0.3 | 0.1 | 0.4 |
| Prdm15 | 1.6 | 2.1 | 1.6 | 1.7 | 2.0 | 2.0 | 2.0 | 1.9 | 1.9 | 2.1 | 2.1 | 2.0 |
| Prdm16 | 0.4 | 0.4 | 0.5 | 0.5 | 0.4 | 0.5 | 0.4 | 0.4 | 0.4 | 0.5 | 0.3 | 0.4 |
| Prdm2 | 6.1 | 7.5 | 6.5 | 6.8 | 6.0 | 5.8 | 6.5 | 6.3 | 6.0 | 5.9 | 6.3 | 6.3 |
| Prdm4 | 8.4 | 8.4 | 8.4 | 7.7 | 8.0 | 7.3 | 8.4 | 8.9 | 8.1 | 7.3 | 8.7 | 7.4 |
| Prdm5 | 11.0 | 10.7 | 14.4 | 9.8 | 9.0 | 12.0 | 10.0 | 12.5 | 11.2 | 10.5 | 12.5 | 13.5 |
| Prdm6 | 0.2 | 0.3 | 0.2 | 0.1 | 0.1 | 0.2 | 0.1 | 0.1 | 0.0 | 0.1 | 0.0 | 0.0 |

| | | | | | | | | | | | | |
|---|---|---|---|---|---|---|---|---|---|---|---|---|
| Prdm8 | 0.1 | 0.2 | 0.1 | 0.2 | 0.1 | 0.1 | 0.1 | 0.1 | 0.1 | 0.1 | 0.0 | 0.0 |
| Prdm9 | 0.3 | 0.4 | 0.6 | 0.5 | 0.3 | 0.6 | 0.4 | 0.4 | 0.3 | 0.4 | 0.4 | 0.4 |
| Prdx1 | 758.4 | 597.9 | 406.4 | 1314.7 | 1874.2 | 807.1 | 471.7 | 403.0 | 396.2 | 665.1 | 484.4 | 487.9 |
| Prdx2 | 312.0 | 250.5 | 237.6 | 273.6 | 264.1 | 273.0 | 256.6 | 247.2 | 219.7 | 288.8 | 271.3 | 273.9 |
| Prdx3 | 43.4 | 36.8 | 30.5 | 38.2 | 33.3 | 39.1 | 31.1 | 38.2 | 37.3 | 35.2 | 34.1 | 36.2 |
| Prdx4 | 117.2 | 118.6 | 116.2 | 122.4 | 146.9 | 135.2 | 117.2 | 134.3 | 132.4 | 112.9 | 109.2 | 115.1 |
| Prdx5 | 297.9 | 286.9 | 271.9 | 322.1 | 293.9 | 264.0 | 307.6 | 252.4 | 249.1 | 249.5 | 250.0 | 274.9 |
| Prdx6 | 77.0 | 58.4 | 40.7 | 105.9 | 75.8 | 96.8 | 41.7 | 48.3 | 39.7 | 65.8 | 50.2 | 40.6 |
| Prdx6b | 0.1 | 0.0 | 0.0 | 0.0 | 0.1 | 0.0 | 0.0 | 0.0 | 0.0 | 0.0 | 0.0 | 0.0 |
| Preb | 6.2 | 7.1 | 6.7 | 6.7 | 7.6 | 7.1 | 7.6 | 6.7 | 6.5 | 6.8 | 7.1 | 6.7 |
| Prelid1 | 158.9 | 135.6 | 128.2 | 168.7 | 154.8 | 154.1 | 177.1 | 173.8 | 157.1 | 141.3 | 179.2 | 157.9 |
| Prelid2 | 5.7 | 6.5 | 4.0 | 3.7 | 3.6 | 7.4 | 5.2 | 3.3 | 4.8 | 3.9 | 4.7 | 4.4 |
| Prelp | 311.4 | 314.1 | 326.6 | 376.7 | 323.0 | 298.6 | 393.0 | 173.1 | 205.6 | 191.1 | 230.9 | 250.7 |
| Prep | 23.4 | 13.9 | 14.2 | 20.9 | 18.4 | 18.4 | 19.1 | 19.0 | 15.1 | 16.8 | 20.2 | 17.0 |
| Prepl | 8.5 | 7.4 | 7.0 | 8.3 | 8.1 | 7.5 | 7.4 | 6.7 | 7.7 | 10.5 | 9.6 | 8.0 |
| Prex1 | 1.2 | 0.9 | 0.8 | 5.0 | 8.0 | 1.4 | 1.5 | 0.6 | 0.5 | 0.8 | 0.6 | 0.7 |
| Prex2 | 12.6 | 15.1 | 14.9 | 12.3 | 7.8 | 12.1 | 11.4 | 7.9 | 13.6 | 7.3 | 11.2 | 11.5 |
| Prg2 | 0.0 | 0.1 | 0.0 | 0.0 | 0.0 | 0.1 | 0.1 | 0.0 | 0.0 | 0.0 | 0.0 | 0.1 |
| Prg4 | 0.5 | 0.4 | 0.2 | 0.2 | 0.3 | 0.2 | 0.1 | 0.4 | 0.3 | 0.3 | 0.2 | 0.2 |
| Prickle1 | 0.9 | 1.6 | 1.9 | 0.5 | 0.6 | 0.4 | 1.1 | 1.9 | 2.4 | 5.4 | 2.8 | 3.4 |
| Prickle2 | 2.2 | 2.5 | 3.0 | 1.5 | 2.0 | 1.7 | 1.8 | 2.1 | 2.0 | 2.3 | 1.8 | 1.9 |
| Prickle3 | 3.8 | 3.7 | 3.9 | 5.1 | 5.3 | 4.0 | 4.4 | 5.1 | 4.7 | 4.1 | 4.4 | 3.6 |
| Prim1 | 2.6 | 4.1 | 5.1 | 1.6 | 4.9 | 2.3 | 5.0 | 2.9 | 4.4 | 2.2 | 3.6 | 2.9 |
| Prim2 | 7.7 | 9.4 | 11.9 | 8.0 | 9.2 | 5.6 | 13.1 | 9.5 | 8.2 | 6.3 | 9.1 | 8.8 |
| Prkaa1 | 10.8 | 11.9 | 13.2 | 12.8 | 11.8 | 12.3 | 12.7 | 13.4 | 12.2 | 12.3 | 15.0 | 14.7 |
| Prkaa2 | 0.5 | 0.6 | 0.5 | 0.4 | 0.3 | 0.6 | 0.2 | 0.2 | 0.1 | 0.3 | 0.2 | 0.3 |
| Prkab1 | 5.5 | 5.6 | 6.3 | 7.0 | 6.8 | 5.5 | 6.9 | 7.9 | 6.1 | 7.8 | 8.0 | 7.8 |
| Prkab2 | 14.2 | 13.6 | 11.2 | 15.7 | 11.8 | 17.7 | 13.4 | 8.4 | 13.9 | 10.9 | 14.0 | 14.0 |
| Prkaca | 31.5 | 33.6 | 32.6 | 32.0 | 28.4 | 34.2 | 31.6 | 28.4 | 28.8 | 27.1 | 28.8 | 28.4 |
| Prkacb | 26.4 | 26.3 | 25.2 | 33.7 | 28.1 | 29.8 | 28.3 | 21.1 | 24.8 | 24.3 | 25.8 | 28.3 |
| Prkag1 | 32.9 | 31.4 | 29.9 | 28.3 | 28.5 | 27.6 | 31.4 | 30.8 | 29.4 | 31.5 | 33.4 | 30.3 |
| Prkag2 | 6.0 | 4.0 | 2.9 | 6.3 | 4.0 | 4.5 | 5.0 | 2.8 | 4.1 | 5.8 | 5.6 | 3.9 |
| Prkag3 | 0.0 | 0.0 | 0.0 | 0.0 | 0.0 | 0.0 | 0.0 | 0.0 | 0.0 | 0.0 | 0.0 | 0.1 |
| Prkar1a | 101.9 | 112.7 | 119.8 | 88.5 | 106.2 | 93.8 | 115.3 | 109.7 | 130.8 | 138.6 | 123.6 | 160.5 |
| Prkar1b | 3.0 | 3.2 | 1.6 | 3.7 | 1.3 | 5.9 | 1.3 | 0.5 | 0.8 | 2.9 | 2.5 | 2.3 |
| Prkar2a | 11.1 | 10.2 | 8.7 | 14.2 | 9.6 | 12.2 | 12.5 | 8.5 | 9.3 | 10.4 | 12.8 | 10.4 |
| Prkar2b | 9.3 | 11.3 | 11.5 | 14.5 | 14.3 | 16.9 | 9.6 | 10.0 | 9.2 | 8.0 | 7.1 | 7.8 |
| Prkca | 3.2 | 3.7 | 4.4 | 3.1 | 3.9 | 2.9 | 3.9 | 3.8 | 3.2 | 4.0 | 4.6 | 3.8 |
| Prkcb | 0.9 | 0.0 | 0.1 | 6.5 | 13.1 | 0.0 | 1.8 | 0.1 | 0.3 | 0.9 | 0.3 | 0.8 |
| Prkcd | 22.1 | 24.2 | 24.1 | 28.4 | 34.3 | 22.6 | 23.2 | 19.3 | 24.0 | 27.0 | 23.9 | 24.7 |
| Prkcdbp | 48.3 | 38.6 | 49.3 | 41.1 | 30.8 | 36.5 | 53.1 | 118.7 | 69.9 | 49.2 | 58.8 | 48.3 |
| Prkce | 2.0 | 2.7 | 2.8 | 2.0 | 1.9 | 1.9 | 2.4 | 2.6 | 2.5 | 2.2 | 2.0 | 2.3 |
| Prkcg | 0.1 | 0.1 | 0.1 | 0.1 | 0.1 | 0.1 | 0.2 | 0.1 | 0.2 | 0.1 | 0.1 | 0.1 |
| Prkch | 0.6 | 0.2 | 0.3 | 2.3 | 3.6 | 0.2 | 0.6 | 0.5 | 0.4 | 0.4 | 0.3 | 0.4 |
| Prkci | 12.8 | 13.8 | 13.5 | 10.7 | 9.1 | 13.8 | 10.5 | 11.7 | 12.4 | 12.3 | 13.5 | 12.1 |
| Prkcq | 0.7 | 0.5 | 0.2 | 0.9 | 0.5 | 0.5 | 0.4 | 0.4 | 0.5 | 0.2 | 0.5 | 0.2 |
| Prkcsh | 91.3 | 77.9 | 74.1 | 98.9 | 84.0 | 95.6 | 83.6 | 83.9 | 76.9 | 85.0 | 79.3 | 74.5 |
| Prkcz | 1.4 | 0.9 | 0.7 | 1.7 | 0.7 | 2.0 | 0.9 | 0.6 | 0.7 | 2.3 | 1.5 | 0.9 |
| Prkd1 | 3.5 | 3.3 | 3.4 | 1.4 | 2.0 | 1.6 | 3.2 | 2.5 | 4.0 | 4.2 | 4.1 | 3.9 |
| Prkd2 | 2.4 | 3.0 | 3.8 | 2.8 | 2.7 | 3.6 | 3.3 | 2.3 | 2.6 | 2.4 | 2.3 | 2.6 |
| Prkd3 | 10.4 | 11.2 | 12.4 | 9.5 | 8.9 | 11.0 | 9.6 | 11.6 | 11.5 | 11.0 | 11.2 | 12.3 |
| Prkdc | 2.6 | 3.0 | 3.0 | 2.3 | 2.6 | 2.8 | 2.2 | 2.9 | 2.3 | 2.0 | 2.5 | 2.2 |
| Prkg1 | 14.5 | 22.6 | 28.0 | 17.3 | 20.0 | 15.4 | 26.5 | 27.0 | 27.7 | 18.3 | 23.9 | 28.4 |
| Prkg2 | 0.2 | 0.1 | 0.1 | 0.2 | 0.3 | 0.2 | 0.2 | 0.1 | 0.2 | 0.8 | 0.5 | 0.4 |
| Prkra | 19.5 | 21.0 | 19.8 | 16.9 | 16.2 | 17.8 | 17.0 | 20.8 | 19.2 | 19.3 | 18.9 | 19.7 |
| Prkrip1 | 10.1 | 9.2 | 9.1 | 12.1 | 8.4 | 9.3 | 10.1 | 9.8 | 8.7 | 10.1 | 11.2 | 11.3 |
| Prkrir | 18.1 | 16.6 | 17.3 | 17.5 | 16.3 | 17.0 | 19.5 | 19.2 | 19.2 | 19.9 | 22.2 | 21.2 |

| | | | | | | | | | | | | |
|---|---|---|---|---|---|---|---|---|---|---|---|---|
| Prkx | 10.9 | 9.9 | 6.9 | 12.9 | 12.4 | 8.8 | 9.5 | 7.2 | 8.9 | 7.4 | 10.2 | 8.7 |
| Prl | 0.4 | 0.1 | 0.0 | 0.0 | 0.0 | 0.0 | 0.0 | 0.1 | 0.1 | 0.1 | 0.1 | 0.1 |
| Prl2c2 | 0.3 | 0.3 | 0.0 | 0.5 | 0.5 | 1.8 | 0.1 | 0.4 | 0.3 | 0.5 | 0.4 | 0.2 |
| Prl2c3 | 0.0 | 0.0 | 0.2 | 0.5 | 0.8 | 1.8 | 0.2 | 0.0 | 0.0 | 0.0 | 0.0 | 0.0 |
| Prl2c4 | 0.2 | 0.3 | 0.0 | 0.0 | 0.0 | 0.0 | 0.0 | 0.5 | 0.3 | 0.4 | 0.6 | 0.1 |
| Prl2c5 | 0.0 | 0.0 | 0.1 | 0.1 | 0.1 | 0.0 | 0.0 | 0.0 | 0.0 | 0.0 | 0.0 | 0.0 |
| Prl3a1 | 2.7 | 1.6 | 0.3 | 1.4 | 0.2 | 1.4 | 0.3 | 0.4 | 0.1 | 0.2 | 0.4 | 0.3 |
| Prl7a1 | 0.8 | 0.4 | 0.4 | 0.5 | 0.4 | 0.7 | 0.0 | 0.5 | 0.0 | 0.2 | 0.0 | 0.2 |
| Prl7a2 | 0.1 | 0.0 | 0.0 | 0.0 | 0.0 | 0.2 | 0.1 | 0.0 | 0.0 | 0.0 | 0.0 | 0.0 |
| Prl7b1 | 0.1 | 0.0 | 0.0 | 0.0 | 0.1 | 0.0 | 0.0 | 0.0 | 0.0 | 0.0 | 0.0 | 0.0 |
| Prl7d1 | 0.1 | 0.0 | 0.1 | 0.0 | 0.1 | 0.0 | 0.0 | 0.0 | 0.0 | 0.0 | 0.1 | 0.0 |
| Prl8a9 | 0.1 | 0.0 | 0.1 | 0.0 | 0.0 | 0.0 | 0.0 | 0.1 | 0.0 | 0.0 | 0.0 | 0.1 |
| Prlr | 0.1 | 0.0 | 0.0 | 0.0 | 0.0 | 0.0 | 0.0 | 0.0 | 0.0 | 0.0 | 0.0 | 0.0 |
| Prmt1 | 22.3 | 21.8 | 21.5 | 19.2 | 22.8 | 22.2 | 22.3 | 23.9 | 20.6 | 23.4 | 23.4 | 20.0 |
| Prmt10 | 6.5 | 6.3 | 5.2 | 6.2 | 5.3 | 6.8 | 4.6 | 5.0 | 4.8 | 5.0 | 5.6 | 6.0 |
| Prmt2 | 20.9 | 18.6 | 15.2 | 22.0 | 19.8 | 21.4 | 20.5 | 17.6 | 19.1 | 17.5 | 20.8 | 18.3 |
| Prmt3 | 11.7 | 10.2 | 8.3 | 11.6 | 9.6 | 10.2 | 11.0 | 9.7 | 10.4 | 11.7 | 12.6 | 10.1 |
| Prmt5 | 15.5 | 12.7 | 11.8 | 15.7 | 13.2 | 16.1 | 14.7 | 13.5 | 12.0 | 15.3 | 16.9 | 13.4 |
| Prmt6 | 2.9 | 2.5 | 2.6 | 3.3 | 3.2 | 3.2 | 3.1 | 3.4 | 3.7 | 4.2 | 3.9 | 3.6 |
| Prmt7 | 9.4 | 9.8 | 8.0 | 9.6 | 8.7 | 9.6 | 9.5 | 8.9 | 9.3 | 8.8 | 8.6 | 9.1 |
| Prnd | 3.9 | 1.1 | 2.1 | 2.0 | 2.0 | 0.7 | 2.6 | 4.6 | 2.9 | 0.5 | 0.8 | 0.7 |
| Prnp | 329.9 | 220.5 | 171.8 | 346.3 | 198.5 | 242.7 | 183.9 | 172.9 | 193.3 | 242.9 | 230.8 | 234.2 |
| Prob1 | 0.1 | 0.2 | 0.2 | 0.1 | 0.1 | 0.4 | 0.1 | 0.2 | 0.1 | 0.1 | 0.1 | 0.1 |
| Proca1 | 0.4 | 0.4 | 0.4 | 0.9 | 0.4 | 0.4 | 0.3 | 0.6 | 0.3 | 0.4 | 0.5 | 0.9 |
| Procr | 19.9 | 12.5 | 8.6 | 48.2 | 49.4 | 36.2 | 11.2 | 6.4 | 4.7 | 12.1 | 8.4 | 7.6 |
| Prodh | 0.7 | 0.8 | 0.4 | 1.5 | 0.5 | 0.9 | 0.5 | 0.3 | 0.3 | 0.3 | 0.2 | 0.3 |
| Prokr1 | 0.0 | 0.1 | 0.0 | 0.1 | 0.2 | 0.0 | 0.0 | 0.0 | 0.0 | 0.0 | 0.0 | 0.0 |
| Prokr2 | 0.0 | 0.1 | 0.0 | 0.0 | 0.0 | 0.0 | 0.1 | 0.1 | 0.0 | 0.0 | 0.0 | 0.0 |
| Prom1 | 0.0 | 0.0 | 0.0 | 0.1 | 0.0 | 0.0 | 0.0 | 0.0 | 0.0 | 0.0 | 0.0 | 0.0 |
| Prorsd1 | 6.7 | 6.1 | 5.7 | 6.6 | 6.3 | 7.6 | 5.1 | 5.4 | 6.0 | 7.4 | 7.0 | 7.1 |
| Pros1 | 26.4 | 29.0 | 36.7 | 21.4 | 34.2 | 21.9 | 27.2 | 26.4 | 28.2 | 33.1 | 26.3 | 33.0 |
| Prosapip1 | 3.2 | 4.0 | 3.4 | 3.9 | 2.9 | 4.4 | 3.3 | 2.6 | 2.4 | 2.3 | 2.6 | 2.3 |
| Prosc | 13.8 | 12.1 | 9.8 | 12.4 | 10.7 | 13.4 | 10.1 | 9.1 | 10.0 | 12.6 | 11.8 | 10.0 |
| Proser1 | 0.4 | 0.3 | 0.7 | 0.2 | 0.6 | 0.5 | 0.7 | 0.5 | 0.5 | 0.3 | 0.4 | 0.3 |
| Proser2 | 0.0 | 0.0 | 0.0 | 0.0 | 0.0 | 0.0 | 0.0 | 0.0 | 0.0 | 0.0 | 0.0 | 0.0 |
| Prox1 | 0.1 | 0.1 | 0.1 | 0.1 | 0.1 | 0.2 | 0.2 | 0.1 | 0.1 | 0.1 | 0.1 | 0.1 |
| Prox2 | 0.5 | 0.7 | 0.7 | 0.5 | 0.4 | 0.6 | 0.5 | 0.7 | 0.6 | 0.4 | 0.5 | 0.7 |
| Proz | 0.2 | 0.1 | 0.1 | 0.0 | 0.1 | 0.1 | 0.1 | 0.1 | 0.1 | 0.1 | 0.1 | 0.1 |
| Prpf18 | 11.3 | 12.2 | 12.8 | 9.7 | 10.3 | 10.1 | 10.0 | 12.3 | 11.5 | 12.5 | 13.4 | 12.8 |
| Prpf19 | 8.4 | 6.9 | 6.2 | 8.1 | 7.1 | 7.6 | 6.3 | 6.3 | 6.3 | 7.6 | 6.9 | 6.9 |
| Prpf3 | 7.0 | 8.4 | 8.8 | 10.3 | 8.0 | 8.3 | 8.5 | 7.9 | 7.0 | 8.1 | 9.3 | 9.2 |
| Prpf31 | 13.2 | 11.8 | 11.9 | 16.3 | 12.7 | 13.1 | 11.9 | 11.8 | 9.8 | 11.2 | 12.9 | 11.9 |
| Prpf38a | 16.2 | 14.4 | 14.5 | 16.5 | 12.6 | 13.7 | 13.1 | 12.9 | 11.7 | 14.1 | 14.4 | 14.8 |
| Prpf38b | 12.4 | 13.2 | 21.1 | 15.6 | 13.8 | 12.2 | 19.7 | 15.9 | 13.4 | 15.1 | 18.5 | 21.8 |
| Prpf39 | 4.9 | 5.4 | 7.4 | 5.8 | 5.9 | 5.2 | 6.0 | 5.8 | 5.2 | 6.7 | 6.8 | 6.7 |
| Prpf4 | 3.3 | 4.0 | 3.8 | 3.5 | 4.1 | 3.7 | 3.9 | 3.7 | 3.4 | 3.6 | 3.9 | 3.7 |
| Prpf40a | 26.2 | 27.2 | 28.4 | 28.8 | 26.1 | 24.6 | 26.7 | 26.1 | 22.5 | 24.5 | 31.3 | 31.1 |
| Prpf40b | 3.9 | 4.0 | 4.7 | 2.4 | 2.8 | 4.8 | 3.1 | 3.7 | 3.7 | 3.3 | 3.1 | 3.5 |
| Prpf4b | 16.5 | 16.7 | 18.8 | 16.3 | 16.5 | 14.6 | 18.0 | 16.9 | 16.8 | 18.4 | 21.0 | 21.3 |
| Prpf6 | 29.9 | 29.4 | 29.4 | 34.2 | 26.6 | 32.4 | 29.4 | 29.4 | 23.6 | 28.7 | 29.6 | 31.4 |
| Prpf8 | 27.6 | 28.6 | 30.7 | 27.2 | 29.8 | 27.7 | 26.9 | 30.4 | 25.4 | 27.1 | 26.6 | 27.2 |
| Prph | 0.1 | 0.2 | 0.1 | 0.1 | 0.4 | 0.1 | 0.1 | 0.1 | 0.1 | 0.5 | 0.3 | 0.2 |
| Prph2 | 0.1 | 0.0 | 0.0 | 0.0 | 0.0 | 0.0 | 0.0 | 0.0 | 0.0 | 0.0 | 0.0 | 0.0 |
| Prps1 | 6.3 | 6.6 | 6.1 | 5.3 | 5.5 | 5.5 | 6.3 | 5.9 | 5.9 | 6.0 | 5.8 | 5.5 |
| Prps1l3 | 6.2 | 6.7 | 6.7 | 5.3 | 5.9 | 5.7 | 6.4 | 6.4 | 6.8 | 7.2 | 8.0 | 6.9 |
| Prps2 | 17.4 | 16.6 | 15.8 | 17.1 | 16.9 | 19.6 | 13.6 | 10.4 | 12.6 | 17.3 | 16.0 | 15.7 |
| Prpsap1 | 25.3 | 24.2 | 20.8 | 21.2 | 20.2 | 20.0 | 21.5 | 21.8 | 21.6 | 21.6 | 19.4 | 19.4 |

| | | | | | | | | | | | | |
|---|---|---|---|---|---|---|---|---|---|---|---|---|
| Prpsap2 | 8.4 | 8.2 | 6.8 | 8.3 | 7.7 | 9.8 | 6.4 | 7.5 | 8.0 | 9.0 | 8.5 | 8.2 |
| Prr11 | 0.5 | 0.9 | 1.7 | 0.3 | 1.0 | 0.2 | 1.5 | 0.5 | 1.2 | 0.6 | 0.9 | 0.7 |
| Prr12 | 0.5 | 1.1 | 1.1 | 0.5 | 0.7 | 0.9 | 1.0 | 0.9 | 1.1 | 0.8 | 0.6 | 0.7 |
| Prr13 | 88.3 | 83.8 | 69.7 | 160.6 | 94.1 | 127.3 | 86.2 | 77.5 | 64.9 | 68.9 | 80.8 | 68.9 |
| Prr14 | 8.7 | 8.8 | 9.5 | 8.4 | 8.2 | 7.3 | 11.0 | 10.0 | 9.8 | 8.2 | 9.2 | 8.3 |
| Prr14l | 5.8 | 6.7 | 5.6 | 4.7 | 5.7 | 4.9 | 5.2 | 5.9 | 6.0 | 6.6 | 6.2 | 6.4 |
| Prr15 | 0.3 | 0.6 | 0.2 | 0.3 | 0.5 | 0.2 | 0.2 | 1.3 | 0.9 | 7.7 | 2.6 | 1.1 |
| Prr15l | 0.1 | 0.1 | 0.0 | 0.0 | 0.1 | 0.0 | 0.1 | 0.1 | 0.1 | 0.4 | 0.3 | 0.1 |
| Prr16 | 0.3 | 0.5 | 0.7 | 0.1 | 0.3 | 0.1 | 0.3 | 0.3 | 0.1 | 0.2 | 0.1 | 0.4 |
| Prr18 | 0.0 | 0.0 | 0.0 | 0.0 | 0.1 | 0.1 | 0.0 | 0.0 | 0.0 | 0.0 | 0.0 | 0.0 |
| Prr22 | 0.0 | 0.1 | 0.0 | 0.0 | 0.0 | 0.0 | 0.1 | 0.1 | 0.1 | 0.1 | 0.0 | 0.1 |
| Prr24 | 3.3 | 3.0 | 2.9 | 4.4 | 2.8 | 3.3 | 3.6 | 3.4 | 4.0 | 3.1 | 3.1 | 3.1 |
| Prr3 | 3.8 | 4.5 | 5.0 | 3.2 | 3.7 | 3.5 | 4.0 | 4.1 | 4.0 | 3.5 | 3.4 | 4.2 |
| Prr5 | 1.9 | 1.8 | 1.7 | 1.3 | 1.4 | 1.5 | 2.5 | 1.7 | 1.7 | 2.1 | 2.5 | 1.4 |
| Prr5l | 0.2 | 0.2 | 0.2 | 0.2 | 0.2 | 0.1 | 0.1 | 0.0 | 0.1 | 0.2 | 0.2 | 0.1 |
| Prr7 | 0.5 | 0.4 | 0.3 | 0.2 | 0.8 | 0.5 | 0.2 | 0.8 | 0.9 | 0.8 | 0.3 | 0.4 |
| Prrc1 | 9.1 | 11.1 | 10.5 | 9.0 | 9.4 | 10.0 | 10.1 | 11.7 | 11.1 | 8.9 | 10.4 | 9.4 |
| Prrc2a | 6.0 | 10.2 | 10.2 | 6.9 | 9.0 | 7.1 | 11.6 | 8.3 | 9.1 | 7.6 | 7.5 | 6.2 |
| Prrc2b | 10.4 | 12.3 | 11.3 | 10.3 | 10.3 | 11.2 | 12.3 | 9.0 | 10.2 | 10.1 | 9.3 | 8.5 |
| Prrc2c | 13.1 | 14.9 | 17.4 | 14.9 | 16.3 | 12.9 | 18.9 | 15.2 | 14.0 | 13.1 | 14.9 | 14.6 |
| Prrg1 | 0.7 | 0.7 | 2.3 | 1.0 | 1.2 | 1.0 | 1.3 | 1.9 | 0.8 | 0.6 | 0.9 | 1.2 |
| Prrg2 | 5.4 | 5.6 | 5.6 | 7.6 | 6.4 | 7.4 | 6.5 | 6.3 | 7.3 | 6.3 | 6.7 | 7.0 |
| Prrg3 | 5.8 | 5.4 | 6.0 | 5.0 | 4.3 | 4.5 | 6.1 | 5.9 | 4.2 | 3.7 | 4.2 | 4.9 |
| Prrg4 | 3.8 | 3.2 | 3.0 | 3.7 | 3.7 | 4.4 | 2.9 | 1.0 | 1.9 | 1.5 | 1.9 | 1.5 |
| Prrt1 | 0.9 | 0.0 | 0.0 | 0.0 | 0.1 | 0.0 | 3.2 | 0.0 | 0.0 | 0.0 | 0.0 | 0.0 |
| Prrt2 | 0.1 | 0.7 | 1.1 | 0.1 | 0.3 | 0.3 | 0.5 | 0.6 | 0.6 | 0.2 | 0.2 | 0.3 |
| Prrt3 | 0.0 | 0.0 | 0.0 | 0.1 | 0.1 | 0.1 | 0.0 | 0.1 | 0.0 | 0.0 | 0.1 | 0.1 |
| Prrt4 | 0.4 | 0.3 | 0.2 | 0.3 | 0.2 | 0.4 | 0.7 | 0.5 | 0.5 | 0.2 | 0.3 | 0.3 |
| Prrx1 | 11.5 | 17.0 | 20.1 | 14.6 | 15.4 | 18.4 | 16.7 | 17.1 | 19.9 | 16.8 | 16.6 | 22.8 |
| Prrx2 | 3.0 | 4.0 | 5.9 | 0.8 | 3.0 | 1.8 | 4.4 | 6.7 | 5.4 | 6.5 | 7.3 | 7.2 |
| Prss12 | 4.5 | 2.4 | 1.8 | 1.1 | 1.5 | 1.2 | 1.6 | 2.6 | 1.8 | 7.0 | 5.3 | 4.5 |
| Prss16 | 0.1 | 0.1 | 0.1 | 0.2 | 0.3 | 0.1 | 0.1 | 0.0 | 0.1 | 0.2 | 0.0 | 0.2 |
| Prss23 | 246.8 | 176.3 | 122.7 | 228.8 | 139.0 | 211.8 | 231.8 | 162.2 | 280.6 | 248.1 | 321.1 | 260.2 |
| Prss27 | 0.3 | 0.2 | 0.4 | 0.1 | 0.3 | 0.2 | 0.1 | 0.2 | 0.2 | 0.2 | 0.1 | 0.2 |
| Prss30 | 0.0 | 0.0 | 0.0 | 0.0 | 0.1 | 0.0 | 0.0 | 0.0 | 0.0 | 0.0 | 0.0 | 0.0 |
| Prss33 | 0.0 | 0.0 | 0.1 | 0.0 | 0.0 | 0.0 | 0.0 | 0.0 | 0.1 | 0.0 | 0.0 | 0.0 |
| Prss35 | 0.1 | 0.1 | 0.2 | 0.0 | 0.0 | 0.0 | 0.1 | 0.1 | 0.1 | 0.3 | 0.2 | 0.4 |
| Prss36 | 1.5 | 1.4 | 1.0 | 1.5 | 0.9 | 2.0 | 1.0 | 0.9 | 0.9 | 1.6 | 1.0 | 1.0 |
| Prss45 | 0.1 | 0.1 | 0.0 | 0.0 | 0.1 | 0.2 | 0.0 | 0.0 | 0.1 | 0.0 | 0.0 | 0.0 |
| Prss46 | 0.1 | 0.0 | 0.1 | 0.3 | 0.2 | 0.1 | 0.1 | 0.0 | 0.1 | 0.2 | 0.2 | 0.2 |
| Prss48 | 0.1 | 0.0 | 0.0 | 0.0 | 0.0 | 0.1 | 0.0 | 0.0 | 0.0 | 0.0 | 0.0 | 0.0 |
| Prss50 | 0.0 | 0.0 | 0.0 | 0.0 | 0.0 | 0.0 | 0.0 | 0.0 | 0.0 | 0.0 | 0.0 | 0.0 |
| Prss53 | 1.1 | 1.0 | 1.2 | 1.1 | 1.2 | 1.4 | 1.0 | 1.3 | 1.2 | 1.1 | 1.1 | 0.9 |
| Prss57 | 13.0 | 8.8 | 8.2 | 19.0 | 10.0 | 13.9 | 17.0 | 9.5 | 12.1 | 9.1 | 12.6 | 15.2 |
| Prss8 | 0.0 | 0.1 | 0.1 | 0.0 | 0.0 | 0.0 | 0.0 | 0.0 | 0.0 | 0.0 | 0.1 | 0.0 |
| Prtg | 0.0 | 0.0 | 0.0 | 0.1 | 0.0 | 0.0 | 0.0 | 0.0 | 0.0 | 0.0 | 0.0 | 0.0 |
| Prtn3 | 0.1 | 0.0 | 0.1 | 0.0 | 0.0 | 0.0 | 0.0 | 0.0 | 0.0 | 0.1 | 0.0 | 0.1 |
| Prune | 7.5 | 8.5 | 8.5 | 7.0 | 6.4 | 9.5 | 8.5 | 9.1 | 8.4 | 7.7 | 8.2 | 8.3 |
| Prune2 | 1.5 | 0.9 | 0.4 | 1.3 | 1.1 | 1.8 | 0.8 | 0.5 | 0.4 | 0.3 | 0.8 | 0.4 |
| Prx | 0.3 | 0.3 | 0.3 | 0.2 | 0.3 | 0.2 | 0.3 | 0.3 | 0.4 | 0.4 | 0.3 | 0.2 |
| Psap | 360.2 | 300.4 | 278.8 | 481.5 | 535.1 | 413.8 | 260.2 | 309.1 | 289.8 | 330.6 | 290.7 | 296.1 |
| Psat1 | 11.0 | 7.3 | 7.4 | 5.0 | 5.0 | 4.1 | 9.3 | 28.3 | 11.6 | 10.9 | 17.3 | 6.9 |
| Psd | 0.3 | 0.3 | 0.3 | 0.3 | 0.4 | 0.3 | 0.4 | 0.4 | 0.5 | 0.5 | 0.4 | 0.4 |
| Psd2 | 1.6 | 1.6 | 0.8 | 1.9 | 1.4 | 2.0 | 0.8 | 0.4 | 0.4 | 0.7 | 0.7 | 0.6 |
| Psd3 | 10.0 | 14.2 | 15.4 | 8.9 | 12.0 | 8.9 | 12.9 | 15.4 | 14.8 | 8.3 | 11.2 | 13.1 |
| Psd4 | 0.3 | 0.0 | 0.0 | 2.3 | 4.4 | 0.0 | 0.5 | 0.1 | 0.1 | 0.3 | 0.2 | 0.3 |
| Psen1 | 25.6 | 26.5 | 24.2 | 31.1 | 26.6 | 25.3 | 32.9 | 28.9 | 26.0 | 28.2 | 29.1 | 27.9 |

| | | | | | | | | | | | | |
|---|---|---|---|---|---|---|---|---|---|---|---|---|
| Psen2 | 12.0 | 12.3 | 10.1 | 17.4 | 15.2 | 15.9 | 13.8 | 11.2 | 11.3 | 16.2 | 12.9 | 12.0 |
| Psenen | 53.7 | 38.7 | 44.4 | 60.8 | 44.4 | 52.1 | 43.0 | 47.0 | 41.5 | 48.5 | 46.3 | 50.2 |
| Psg17 | 0.0 | 0.1 | 0.1 | 0.2 | 0.1 | 0.0 | 0.1 | 0.0 | 0.0 | 0.0 | 0.1 | 0.0 |
| Psg23 | 0.2 | 0.4 | 0.2 | 0.3 | 0.3 | 0.2 | 0.3 | 0.3 | 0.1 | 0.3 | 0.2 | 0.1 |
| Psg29 | 0.0 | 0.0 | 0.0 | 0.0 | 0.0 | 0.0 | 0.0 | 0.0 | 0.0 | 0.0 | 0.0 | 0.0 |
| Psip1 | 12.6 | 15.2 | 24.2 | 12.3 | 12.3 | 15.7 | 20.8 | 21.1 | 15.8 | 19.7 | 23.8 | 24.1 |
| Pskh1 | 8.8 | 9.8 | 10.2 | 8.9 | 9.0 | 10.9 | 9.6 | 9.5 | 9.4 | 11.1 | 10.2 | 9.8 |
| Psma1 | 77.8 | 71.9 | 64.6 | 83.3 | 74.2 | 78.8 | 70.0 | 70.0 | 74.6 | 73.4 | 76.1 | 71.5 |
| Psma2 | 155.5 | 142.2 | 119.0 | 161.1 | 127.2 | 139.9 | 128.7 | 123.8 | 118.7 | 141.6 | 143.6 | 139.3 |
| Psma3 | 111.7 | 98.0 | 86.1 | 115.4 | 95.4 | 101.1 | 87.8 | 94.6 | 92.9 | 100.8 | 108.2 | 101.2 |
| Psma4 | 148.5 | 122.1 | 128.0 | 144.5 | 115.7 | 128.5 | 132.5 | 127.9 | 117.1 | 131.2 | 152.8 | 155.2 |
| Psma5 | 55.9 | 48.4 | 44.8 | 51.5 | 51.7 | 49.8 | 42.3 | 49.9 | 50.7 | 53.0 | 51.7 | 47.4 |
| Psma6 | 87.6 | 75.7 | 68.4 | 81.4 | 83.5 | 70.7 | 71.3 | 70.4 | 71.3 | 76.3 | 71.2 | 70.5 |
| Psma7 | 130.6 | 104.4 | 93.0 | 133.7 | 107.5 | 111.5 | 109.1 | 103.5 | 104.4 | 132.0 | 122.3 | 115.7 |
| Psma8 | 2.0 | 1.2 | 1.0 | 2.1 | 1.4 | 1.4 | 1.5 | 0.7 | 0.8 | 0.7 | 0.7 | 0.6 |
| Psmb1 | 137.8 | 120.5 | 108.6 | 123.9 | 121.0 | 123.9 | 118.3 | 128.0 | 125.9 | 137.6 | 137.9 | 132.0 |
| Psmb10 | 10.3 | 7.9 | 8.3 | 18.4 | 18.2 | 8.2 | 11.7 | 7.7 | 9.8 | 11.1 | 8.4 | 10.9 |
| Psmb11 | 0.0 | 0.0 | 0.0 | 0.0 | 0.1 | 0.0 | 0.0 | 0.0 | 0.0 | 0.0 | 0.0 | 0.0 |
| Psmb2 | 98.2 | 83.1 | 86.7 | 109.0 | 96.1 | 95.4 | 112.8 | 111.4 | 92.9 | 103.0 | 113.7 | 101.3 |
| Psmb3 | 150.0 | 127.3 | 110.5 | 146.5 | 125.1 | 131.9 | 130.9 | 130.7 | 121.1 | 120.4 | 127.8 | 119.0 |
| Psmb4 | 143.6 | 131.0 | 101.9 | 113.6 | 119.6 | 120.3 | 115.6 | 118.7 | 129.6 | 133.2 | 109.6 | 116.4 |
| Psmb5 | 75.5 | 68.8 | 50.8 | 72.6 | 75.2 | 73.2 | 62.4 | 62.9 | 66.2 | 68.8 | 59.1 | 59.0 |
| Psmb6 | 94.8 | 86.8 | 83.4 | 101.1 | 86.1 | 97.9 | 86.8 | 82.9 | 80.4 | 88.1 | 86.6 | 82.6 |
| Psmb7 | 127.2 | 111.0 | 97.7 | 116.3 | 103.2 | 112.3 | 95.1 | 104.8 | 99.0 | 104.3 | 108.3 | 104.8 |
| Psmb8 | 7.3 | 6.9 | 10.7 | 13.5 | 17.8 | 6.6 | 7.0 | 5.1 | 4.8 | 5.8 | 4.4 | 6.7 |
| Psmb9 | 2.4 | 3.9 | 4.9 | 2.7 | 3.8 | 2.3 | 2.1 | 1.5 | 1.4 | 1.7 | 1.4 | 2.7 |
| Psmc1 | 98.4 | 85.5 | 80.5 | 96.8 | 75.4 | 86.8 | 84.1 | 78.1 | 74.5 | 77.2 | 89.8 | 89.2 |
| Psmc2 | 56.0 | 56.6 | 55.6 | 60.7 | 57.1 | 56.5 | 59.9 | 57.2 | 55.5 | 57.4 | 58.0 | 59.7 |
| Psmc3 | 106.3 | 97.1 | 92.2 | 114.0 | 93.2 | 104.3 | 102.7 | 96.8 | 92.9 | 96.1 | 107.9 | 100.4 |
| Psmc3ip | 1.8 | 3.2 | 3.6 | 1.3 | 2.7 | 1.6 | 3.3 | 2.4 | 3.1 | 1.7 | 2.9 | 2.5 |
| Psmc4 | 76.8 | 62.6 | 57.5 | 82.4 | 62.2 | 73.1 | 71.9 | 65.7 | 61.6 | 64.9 | 71.9 | 70.7 |
| Psmc5 | 64.0 | 60.8 | 55.7 | 64.6 | 56.4 | 60.4 | 61.1 | 57.9 | 62.0 | 60.6 | 64.6 | 61.2 |
| Psmc6 | 121.1 | 109.6 | 93.6 | 131.3 | 92.5 | 114.1 | 93.7 | 86.2 | 92.5 | 100.4 | 108.2 | 106.1 |
| Psmd1 | 57.0 | 47.8 | 49.8 | 59.4 | 55.5 | 50.4 | 57.7 | 57.6 | 51.7 | 52.5 | 58.7 | 57.9 |
| Psmd10 | 19.2 | 15.8 | 14.2 | 20.6 | 20.6 | 19.1 | 16.9 | 15.7 | 13.6 | 14.8 | 18.5 | 17.6 |
| Psmd11 | 33.3 | 26.7 | 27.7 | 39.1 | 30.9 | 39.6 | 31.4 | 32.0 | 27.2 | 29.0 | 31.7 | 29.7 |
| Psmd12 | 42.7 | 41.8 | 38.2 | 45.2 | 41.3 | 40.0 | 44.3 | 40.5 | 40.4 | 42.9 | 49.7 | 42.9 |
| Psmd13 | 48.0 | 41.6 | 34.2 | 44.7 | 45.7 | 42.0 | 44.3 | 41.5 | 40.9 | 41.5 | 42.1 | 43.1 |
| Psmd14 | 67.7 | 57.7 | 46.7 | 66.3 | 58.5 | 59.4 | 57.4 | 52.3 | 55.5 | 61.3 | 62.8 | 62.1 |
| Psmd2 | 119.0 | 104.3 | 89.1 | 122.3 | 108.5 | 112.7 | 99.7 | 101.7 | 92.7 | 97.6 | 99.1 | 98.3 |
| Psmd3 | 28.0 | 28.3 | 27.5 | 31.4 | 30.3 | 27.0 | 35.6 | 35.7 | 34.0 | 30.5 | 30.7 | 28.1 |
| Psmd4 | 96.6 | 89.8 | 84.4 | 106.0 | 77.7 | 94.4 | 91.6 | 87.9 | 82.4 | 86.8 | 88.7 | 90.5 |
| Psmd5 | 29.9 | 25.9 | 20.0 | 29.9 | 25.2 | 27.4 | 24.2 | 21.3 | 21.8 | 25.4 | 25.2 | 20.9 |
| Psmd6 | 75.4 | 68.0 | 58.9 | 70.2 | 62.3 | 70.3 | 65.6 | 59.7 | 68.5 | 73.0 | 71.5 | 69.1 |
| Psmd7 | 58.0 | 62.1 | 67.3 | 82.6 | 66.9 | 65.7 | 85.9 | 75.7 | 61.9 | 63.7 | 73.9 | 76.7 |
| Psmd8 | 181.9 | 116.8 | 92.9 | 199.9 | 121.9 | 170.6 | 116.3 | 95.9 | 96.2 | 122.6 | 126.3 | 120.3 |
| Psmd9 | 8.7 | 8.0 | 8.5 | 8.8 | 8.2 | 7.9 | 8.9 | 8.2 | 8.9 | 8.9 | 9.4 | 9.1 |
| Psme1 | 39.0 | 39.8 | 53.6 | 45.2 | 47.0 | 37.5 | 43.6 | 46.2 | 38.6 | 38.9 | 39.7 | 49.3 |
| Psme2 | 87.5 | 74.1 | 65.2 | 92.0 | 87.0 | 80.6 | 74.3 | 55.3 | 69.7 | 70.8 | 57.7 | 75.4 |
| Psme3 | 18.7 | 17.6 | 17.3 | 21.6 | 17.9 | 19.1 | 18.5 | 18.3 | 16.6 | 16.7 | 17.5 | 17.7 |
| Psme4 | 18.9 | 21.6 | 16.6 | 16.5 | 20.8 | 16.5 | 15.4 | 20.3 | 18.7 | 17.6 | 20.4 | 17.9 |
| Psmf1 | 6.9 | 6.5 | 5.4 | 7.8 | 6.0 | 7.6 | 6.5 | 6.2 | 5.9 | 5.9 | 5.9 | 5.8 |
| Psmg1 | 13.9 | 14.3 | 12.3 | 12.8 | 12.5 | 14.7 | 12.6 | 14.7 | 12.5 | 15.0 | 13.7 | 13.3 |
| Psmg2 | 13.9 | 15.1 | 12.9 | 11.5 | 13.0 | 13.6 | 12.0 | 11.0 | 13.2 | 14.5 | 13.3 | 12.3 |
| Psmg3 | 7.0 | 6.8 | 5.6 | 6.6 | 8.6 | 7.1 | 7.1 | 7.9 | 8.6 | 7.5 | 5.7 | 6.0 |
| Psmg4 | 9.5 | 8.9 | 6.5 | 7.8 | 10.9 | 10.1 | 9.0 | 8.4 | 8.2 | 8.5 | 6.8 | 7.3 |
| Pspc1 | 7.1 | 7.5 | 8.8 | 6.6 | 6.6 | 7.8 | 9.7 | 6.8 | 6.0 | 8.1 | 8.3 | 9.5 |

| | | | | | | | | | | | | |
|---|---|---|---|---|---|---|---|---|---|---|---|---|
| Psph | 27.1 | 18.0 | 11.8 | 25.6 | 13.6 | 21.7 | 20.1 | 21.4 | 19.7 | 18.9 | 25.6 | 14.6 |
| Pspn | 1.0 | 0.0 | 0.4 | 0.3 | 0.3 | 0.8 | 0.1 | 0.3 | 0.3 | 0.0 | 0.0 | 0.2 |
| Psrc1 | 17.6 | 19.5 | 22.1 | 13.0 | 15.2 | 20.0 | 14.5 | 13.2 | 14.6 | 31.5 | 18.7 | 19.6 |
| Pstk | 7.8 | 6.9 | 5.6 | 6.5 | 5.7 | 8.8 | 6.8 | 6.0 | 7.6 | 6.8 | 7.2 | 6.0 |
| Pstpip1 | 7.6 | 5.5 | 5.6 | 14.2 | 14.5 | 6.0 | 9.9 | 5.9 | 6.7 | 8.9 | 9.6 | 6.5 |
| Pstpip2 | 0.5 | 0.3 | 0.4 | 0.4 | 0.6 | 0.2 | 0.6 | 0.3 | 0.2 | 0.3 | 0.5 | 0.3 |
| Ptafr | 0.1 | 0.0 | 0.1 | 2.2 | 1.9 | 0.0 | 0.3 | 0.2 | 0.1 | 0.2 | 0.0 | 0.0 |
| Ptar1 | 6.7 | 5.7 | 4.9 | 6.2 | 5.8 | 5.9 | 6.2 | 6.1 | 6.4 | 6.9 | 7.8 | 6.7 |
| Ptbp1 | 39.2 | 38.0 | 34.6 | 46.0 | 48.4 | 36.1 | 51.2 | 45.6 | 42.7 | 43.8 | 48.1 | 37.9 |
| Ptbp2 | 3.4 | 4.7 | 5.6 | 4.2 | 4.1 | 4.2 | 4.1 | 4.3 | 4.2 | 3.9 | 4.5 | 4.8 |
| Ptbp3 | 8.8 | 13.6 | 14.9 | 13.0 | 16.3 | 10.9 | 14.2 | 15.0 | 13.7 | 11.8 | 14.1 | 14.4 |
| Ptcd1 | 7.1 | 5.7 | 5.4 | 6.3 | 5.5 | 6.7 | 6.4 | 6.1 | 6.2 | 6.8 | 6.6 | 7.1 |
| Ptcd2 | 9.7 | 9.0 | 6.9 | 9.9 | 9.5 | 10.4 | 8.3 | 8.0 | 8.1 | 9.2 | 8.7 | 7.2 |
| Ptcd3 | 16.9 | 16.4 | 14.7 | 14.7 | 14.7 | 14.9 | 15.3 | 15.5 | 14.8 | 17.7 | 17.3 | 15.3 |
| Ptch1 | 1.2 | 1.4 | 1.7 | 1.3 | 1.2 | 1.3 | 1.3 | 1.3 | 1.2 | 1.4 | 1.0 | 1.0 |
| Ptch2 | 0.0 | 0.1 | 0.1 | 0.0 | 0.0 | 0.0 | 0.0 | 0.1 | 0.1 | 0.0 | 0.0 | 0.0 |
| Ptchd1 | 0.1 | 0.0 | 0.0 | 0.7 | 1.0 | 0.1 | 0.1 | 0.1 | 0.0 | 0.1 | 0.0 | 0.1 |
| Ptchd2 | 0.0 | 0.0 | 0.0 | 0.0 | 0.0 | 0.0 | 0.0 | 0.0 | 0.0 | 0.0 | 0.0 | 0.0 |
| Ptchd4 | 0.4 | 0.2 | 0.2 | 0.3 | 0.2 | 0.2 | 0.1 | 0.1 | 0.1 | 0.2 | 0.1 | 0.2 |
| Ptcra | 0.0 | 0.1 | 0.1 | 0.2 | 0.0 | 0.0 | 0.1 | 0.0 | 0.0 | 0.0 | 0.0 | 0.0 |
| Ptdss1 | 27.1 | 22.8 | 19.7 | 26.7 | 21.2 | 25.3 | 22.2 | 20.9 | 21.2 | 21.4 | 22.8 | 23.4 |
| Ptdss2 | 6.3 | 7.5 | 7.9 | 5.3 | 7.2 | 8.2 | 7.4 | 8.7 | 7.8 | 9.0 | 7.4 | 8.5 |
| Pten | 23.0 | 25.0 | 26.6 | 24.4 | 23.7 | 24.8 | 20.4 | 24.5 | 22.3 | 24.6 | 25.1 | 26.7 |
| Pter | 7.6 | 6.1 | 5.1 | 6.8 | 4.5 | 5.9 | 5.7 | 5.4 | 5.0 | 5.9 | 7.0 | 6.3 |
| Ptgds | 0.0 | 0.3 | 0.0 | 0.4 | 0.0 | 0.0 | 0.0 | 0.1 | 0.2 | 0.1 | 0.0 | 0.1 |
| Ptger1 | 8.1 | 4.5 | 2.9 | 7.4 | 6.6 | 6.3 | 6.1 | 5.5 | 5.6 | 5.7 | 5.5 | 5.5 |
| Ptger2 | 0.1 | 0.0 | 0.0 | 0.4 | 1.1 | 0.1 | 0.1 | 0.0 | 0.0 | 0.3 | 0.1 | 0.2 |
| Ptger3 | 0.1 | 0.1 | 0.0 | 0.0 | 0.0 | 0.0 | 0.0 | 0.0 | 0.0 | 0.0 | 0.0 | 0.0 |
| Ptger4 | 3.2 | 6.8 | 10.0 | 3.2 | 9.0 | 3.2 | 9.6 | 10.7 | 9.2 | 9.3 | 8.8 | 8.6 |
| Ptges | 1.4 | 1.8 | 2.3 | 1.2 | 1.1 | 1.2 | 1.1 | 1.3 | 0.9 | 2.3 | 1.9 | 2.1 |
| Ptges2 | 7.7 | 7.3 | 6.0 | 6.5 | 6.0 | 5.6 | 6.5 | 7.6 | 6.7 | 7.1 | 6.2 | 6.4 |
| Ptges3 | 83.1 | 85.2 | 79.9 | 94.2 | 87.9 | 96.2 | 80.2 | 77.6 | 80.2 | 97.1 | 95.8 | 95.9 |
| Ptges3l | 5.9 | 4.5 | 4.1 | 4.8 | 5.0 | 5.6 | 5.4 | 4.1 | 5.2 | 5.9 | 6.1 | 5.4 |
| Ptgfr | 0.0 | 0.1 | 0.3 | 0.0 | 0.1 | 0.0 | 0.0 | 0.3 | 0.1 | 0.2 | 0.2 | 0.1 |
| Ptgfrn | 27.3 | 27.9 | 33.2 | 25.4 | 29.8 | 29.3 | 30.1 | 29.5 | 27.4 | 25.1 | 24.9 | 27.9 |
| Ptgir | 3.0 | 5.1 | 6.0 | 7.5 | 8.2 | 4.3 | 4.0 | 4.6 | 3.1 | 2.5 | 2.0 | 2.3 |
| Ptgis | 148.9 | 147.3 | 125.5 | 69.7 | 102.2 | 94.9 | 114.7 | 184.1 | 124.7 | 162.2 | 135.4 | 127.9 |
| Ptgr1 | 24.6 | 25.4 | 22.9 | 22.4 | 24.2 | 26.4 | 17.8 | 19.1 | 20.0 | 18.6 | 19.0 | 18.8 |
| Ptgr2 | 22.1 | 20.5 | 20.3 | 23.7 | 19.5 | 24.4 | 18.8 | 17.6 | 16.9 | 21.3 | 21.1 | 22.5 |
| Ptgs1 | 7.5 | 4.3 | 3.9 | 5.4 | 6.0 | 3.1 | 3.9 | 4.1 | 4.2 | 23.2 | 12.1 | 7.9 |
| Ptgs2 | 9.5 | 3.2 | 2.0 | 7.5 | 9.5 | 4.8 | 7.6 | 1.8 | 4.1 | 15.0 | 12.3 | 8.4 |
| Pth1r | 0.6 | 3.1 | 6.2 | 0.4 | 1.2 | 1.1 | 3.1 | 5.4 | 4.4 | 1.7 | 2.8 | 3.4 |
| Pth2r | 0.0 | 0.0 | 0.0 | 0.0 | 0.0 | 0.0 | 0.0 | 0.0 | 0.0 | 0.0 | 0.0 | 0.0 |
| Pthlh | 0.0 | 0.0 | 0.0 | 0.1 | 0.0 | 0.0 | 0.0 | 0.0 | 0.0 | 0.0 | 0.0 | 0.0 |
| Ptk2 | 23.9 | 19.3 | 16.1 | 20.1 | 18.4 | 21.2 | 19.7 | 17.1 | 18.5 | 18.9 | 20.9 | 19.7 |
| Ptk2b | 17.9 | 17.5 | 13.1 | 22.3 | 25.2 | 19.0 | 17.0 | 11.3 | 14.3 | 29.8 | 21.7 | 17.3 |
| Ptk7 | 27.6 | 37.6 | 38.5 | 21.4 | 33.2 | 28.2 | 42.7 | 33.8 | 42.4 | 43.3 | 32.2 | 32.2 |
| Ptma | 208.7 | 170.6 | 266.6 | 292.0 | 250.1 | 265.1 | 227.4 | 233.4 | 169.2 | 278.9 | 270.6 | 272.4 |
| Ptms | 60.8 | 69.1 | 100.4 | 73.9 | 69.2 | 64.1 | 115.1 | 118.6 | 100.9 | 82.8 | 111.8 | 104.1 |
| Ptn | 0.5 | 0.9 | 2.2 | 0.1 | 0.6 | 0.2 | 1.1 | 7.0 | 5.4 | 1.4 | 3.5 | 3.2 |
| Ptov1 | 16.7 | 19.5 | 19.2 | 15.2 | 15.9 | 16.8 | 18.0 | 21.7 | 22.5 | 19.8 | 19.1 | 17.1 |
| Ptp4a1 | 6.9 | 6.4 | 6.0 | 6.4 | 6.4 | 7.5 | 6.4 | 5.7 | 4.6 | 6.7 | 7.2 | 6.1 |
| Ptp4a2 | 84.5 | 68.9 | 60.5 | 89.9 | 76.0 | 84.1 | 67.9 | 63.8 | 67.2 | 73.4 | 83.0 | 71.3 |
| Ptp4a3 | 7.0 | 6.1 | 5.7 | 7.5 | 5.8 | 8.5 | 4.8 | 4.1 | 4.9 | 6.0 | 4.7 | 4.3 |
| Ptpdc1 | 4.9 | 5.4 | 5.5 | 4.6 | 4.5 | 6.7 | 4.9 | 4.3 | 4.6 | 3.9 | 4.7 | 4.6 |
| Ptpla | 22.5 | 13.6 | 13.7 | 20.9 | 17.6 | 17.1 | 24.5 | 22.8 | 25.2 | 26.4 | 26.0 | 21.5 |
| Ptplad1 | 24.1 | 20.3 | 19.0 | 21.6 | 20.0 | 19.8 | 18.7 | 17.7 | 18.0 | 29.7 | 23.7 | 22.1 |

| | | | | | | | | | | | | |
|---|---|---|---|---|---|---|---|---|---|---|---|---|
| Ptplad2 | 21.8 | 16.2 | 16.0 | 12.7 | 15.1 | 13.8 | 15.9 | 13.3 | 16.5 | 22.9 | 23.0 | 22.2 |
| Ptplb | 2.0 | 2.1 | 2.5 | 2.8 | 3.3 | 2.0 | 2.8 | 2.9 | 3.0 | 3.8 | 3.0 | 3.5 |
| Ptpmt1 | 20.5 | 15.5 | 13.4 | 20.4 | 15.2 | 17.8 | 15.7 | 14.5 | 15.9 | 15.2 | 16.8 | 15.6 |
| Ptpn1 | 6.6 | 7.6 | 9.6 | 8.0 | 11.7 | 5.1 | 8.2 | 9.9 | 9.1 | 9.0 | 7.2 | 9.0 |
| Ptpn11 | 80.0 | 61.3 | 48.2 | 91.6 | 63.9 | 81.2 | 64.5 | 42.3 | 51.7 | 57.0 | 60.6 | 60.4 |
| Ptpn12 | 11.7 | 11.7 | 13.4 | 13.0 | 15.8 | 9.0 | 14.4 | 14.8 | 12.7 | 12.1 | 15.7 | 15.0 |
| Ptpn13 | 2.5 | 3.0 | 3.2 | 1.8 | 2.0 | 1.8 | 2.5 | 3.4 | 3.5 | 26.0 | 13.4 | 6.7 |
| Ptpn14 | 7.0 | 6.3 | 7.0 | 4.4 | 5.2 | 5.4 | 5.9 | 5.7 | 5.2 | 9.6 | 7.2 | 7.1 |
| Ptpn18 | 0.2 | 0.0 | 0.1 | 2.1 | 2.8 | 0.0 | 0.3 | 0.0 | 0.1 | 0.2 | 0.1 | 0.1 |
| Ptpn2 | 12.5 | 12.1 | 15.2 | 14.7 | 13.9 | 11.9 | 17.1 | 12.2 | 12.3 | 13.8 | 15.5 | 16.9 |
| Ptpn21 | 4.9 | 4.8 | 4.4 | 6.7 | 3.9 | 6.3 | 5.9 | 4.3 | 4.3 | 4.3 | 4.9 | 5.0 |
| Ptpn22 | 0.1 | 0.1 | 0.0 | 0.7 | 1.6 | 0.1 | 0.2 | 0.0 | 0.0 | 0.1 | 0.0 | 0.1 |
| Ptpn23 | 3.1 | 3.5 | 3.5 | 3.2 | 3.7 | 3.8 | 4.3 | 3.0 | 3.3 | 3.1 | 3.0 | 2.7 |
| Ptpn3 | 1.2 | 1.0 | 1.1 | 1.0 | 0.9 | 1.4 | 0.9 | 0.8 | 0.7 | 1.5 | 1.2 | 1.1 |
| Ptpn4 | 2.0 | 1.7 | 1.3 | 1.8 | 1.6 | 1.8 | 1.4 | 1.5 | 1.2 | 1.9 | 1.6 | 1.7 |
| Ptpn5 | 0.0 | 0.0 | 0.0 | 0.0 | 0.0 | 0.0 | 0.0 | 0.0 | 0.0 | 0.0 | 0.0 | 0.0 |
| Ptpn6 | 2.6 | 0.4 | 0.4 | 19.0 | 32.2 | 0.3 | 5.2 | 0.6 | 0.9 | 2.1 | 1.4 | 2.2 |
| Ptpn7 | 0.4 | 0.1 | 0.1 | 4.3 | 5.8 | 0.0 | 0.7 | 0.1 | 0.1 | 0.5 | 0.2 | 0.5 |
| Ptpn9 | 25.5 | 26.0 | 23.9 | 21.7 | 24.1 | 23.2 | 27.4 | 22.8 | 26.8 | 30.4 | 25.1 | 26.7 |
| Ptpra | 41.9 | 42.5 | 45.9 | 57.0 | 48.3 | 47.8 | 55.9 | 45.4 | 45.9 | 44.4 | 49.4 | 54.9 |
| Ptprb | 4.0 | 2.0 | 3.7 | 4.0 | 4.9 | 4.1 | 6.2 | 7.5 | 5.7 | 4.0 | 5.1 | 4.2 |
| Ptprc | 1.2 | 0.1 | 0.1 | 10.3 | 17.4 | 0.0 | 2.2 | 0.3 | 0.4 | 1.2 | 0.4 | 1.0 |
| Ptprcap | 0.1 | 0.2 | 0.1 | 0.1 | 0.2 | 0.1 | 0.3 | 0.1 | 0.5 | 0.0 | 0.2 | 0.3 |
| Ptprd | 1.9 | 2.2 | 3.1 | 1.8 | 1.7 | 1.8 | 2.4 | 3.2 | 3.3 | 6.3 | 4.6 | 3.9 |
| Ptpre | 0.8 | 0.1 | 0.2 | 3.1 | 4.2 | 0.4 | 0.9 | 0.3 | 0.3 | 0.4 | 0.4 | 0.4 |
| Ptprf | 16.2 | 18.8 | 18.8 | 7.3 | 13.5 | 9.6 | 21.0 | 17.5 | 20.8 | 18.3 | 16.8 | 14.0 |
| Ptprg | 11.8 | 10.6 | 10.5 | 11.4 | 10.5 | 11.3 | 12.2 | 8.5 | 9.8 | 10.5 | 10.0 | 10.3 |
| Ptprj | 8.8 | 8.1 | 7.7 | 13.6 | 17.1 | 7.7 | 10.4 | 6.4 | 7.4 | 7.8 | 7.4 | 8.4 |
| Ptprk | 6.1 | 2.5 | 2.0 | 3.1 | 1.9 | 1.7 | 2.7 | 1.9 | 2.5 | 4.3 | 4.9 | 3.5 |
| Ptprm | 13.1 | 12.2 | 11.7 | 16.0 | 10.5 | 16.2 | 15.1 | 14.1 | 14.7 | 10.4 | 11.4 | 12.6 |
| Ptprn | 2.1 | 2.6 | 3.0 | 2.7 | 5.2 | 2.8 | 3.9 | 5.4 | 3.0 | 3.4 | 2.2 | 2.1 |
| Ptprn2 | 0.1 | 0.1 | 0.0 | 0.0 | 0.0 | 0.1 | 0.1 | 0.0 | 0.0 | 0.0 | 0.0 | 0.0 |
| Ptpro | 0.1 | 0.0 | 0.0 | 1.4 | 1.9 | 0.0 | 0.2 | 0.0 | 0.0 | 0.1 | 0.1 | 0.1 |
| Ptprq | 8.2 | 2.4 | 1.2 | 5.2 | 1.1 | 2.3 | 1.0 | 0.5 | 0.8 | 6.6 | 4.2 | 2.1 |
| Ptprs | 17.7 | 18.7 | 19.8 | 18.7 | 19.3 | 24.3 | 18.8 | 18.0 | 18.0 | 23.6 | 19.3 | 18.5 |
| Ptprt | 0.1 | 0.0 | 0.0 | 0.0 | 0.0 | 0.0 | 0.0 | 0.0 | 0.0 | 0.0 | 0.0 | 0.0 |
| Ptpru | 0.6 | 0.7 | 0.8 | 0.3 | 0.5 | 0.5 | 0.9 | 0.5 | 0.6 | 0.3 | 0.4 | 0.4 |
| Ptprv | 0.1 | 0.2 | 0.2 | 0.1 | 0.3 | 0.1 | 0.1 | 0.1 | 0.3 | 0.7 | 0.2 | 0.2 |
| Ptprz1 | 1.2 | 0.1 | 0.1 | 1.4 | 0.1 | 0.7 | 0.1 | 0.1 | 0.0 | 0.2 | 0.3 | 0.1 |
| Ptrf | 135.3 | 118.7 | 110.1 | 114.0 | 95.9 | 122.2 | 126.1 | 130.2 | 110.6 | 131.2 | 150.2 | 127.9 |
| Ptrh1 | 31.2 | 18.0 | 12.2 | 21.0 | 20.3 | 24.7 | 22.0 | 15.0 | 19.1 | 21.0 | 18.0 | 20.7 |
| Ptrh2 | 4.6 | 5.1 | 3.9 | 4.4 | 4.4 | 4.1 | 4.7 | 5.2 | 5.1 | 5.5 | 4.7 | 4.6 |
| Ptrhd1 | 1.5 | 1.3 | 1.0 | 1.8 | 1.8 | 1.7 | 1.5 | 1.5 | 1.7 | 1.4 | 1.5 | 1.5 |
| Pts | 19.5 | 17.1 | 15.8 | 13.8 | 16.4 | 14.3 | 20.5 | 19.2 | 17.1 | 17.0 | 21.7 | 19.5 |
| Pttg1 | 37.5 | 34.7 | 33.2 | 34.4 | 25.9 | 39.0 | 25.1 | 25.3 | 25.1 | 23.1 | 25.0 | 28.3 |
| Pttg1ip | 64.8 | 62.4 | 58.2 | 78.3 | 60.0 | 73.8 | 67.4 | 72.7 | 75.9 | 64.5 | 67.9 | 67.1 |
| Ptx3 | 206.3 | 175.3 | 204.9 | 172.6 | 239.7 | 84.5 | 226.8 | 145.4 | 143.1 | 124.2 | 138.6 | 139.0 |
| Puf60 | 46.6 | 47.3 | 40.6 | 47.4 | 42.0 | 40.8 | 49.2 | 48.6 | 48.1 | 49.4 | 49.3 | 46.5 |
| Pum1 | 9.3 | 12.4 | 12.5 | 10.1 | 10.1 | 10.7 | 11.3 | 10.7 | 11.1 | 10.8 | 11.2 | 10.6 |
| Pum2 | 13.2 | 14.6 | 15.5 | 13.1 | 14.7 | 13.1 | 13.8 | 13.1 | 13.7 | 14.0 | 14.7 | 15.6 |
| Pura | 12.6 | 10.4 | 9.9 | 13.1 | 9.5 | 12.1 | 11.6 | 9.5 | 9.4 | 10.0 | 9.0 | 10.3 |
| Purb | 8.2 | 8.3 | 8.0 | 7.7 | 8.0 | 7.3 | 7.3 | 7.0 | 7.6 | 8.8 | 9.1 | 8.5 |
| Purg | 2.8 | 2.9 | 3.0 | 4.1 | 2.3 | 4.4 | 3.0 | 2.8 | 2.9 | 3.4 | 2.3 | 2.7 |
| Pus1 | 9.0 | 8.1 | 6.8 | 8.9 | 9.0 | 7.7 | 8.6 | 7.4 | 8.1 | 8.5 | 8.7 | 7.0 |
| Pus10 | 4.5 | 4.0 | 4.0 | 3.7 | 3.9 | 4.0 | 3.3 | 4.3 | 3.8 | 3.6 | 3.8 | 4.1 |
| Pus3 | 4.8 | 4.5 | 3.2 | 5.3 | 4.1 | 4.9 | 4.1 | 4.0 | 3.8 | 4.1 | 4.1 | 3.9 |
| Pus7 | 11.6 | 10.1 | 8.4 | 10.3 | 9.3 | 11.2 | 9.7 | 8.4 | 9.2 | 10.9 | 10.7 | 10.9 |

| | | | | | | | | | | | | |
|---|---|---|---|---|---|---|---|---|---|---|---|---|
| Pus7l | 1.5 | 1.3 | 1.4 | 1.1 | 1.5 | 0.8 | 1.4 | 1.8 | 1.3 | 1.8 | 1.3 | 1.2 |
| Pusl1 | 10.2 | 7.9 | 6.5 | 13.3 | 9.8 | 12.2 | 11.0 | 7.8 | 9.7 | 8.8 | 9.7 | 9.0 |
| Pvalb | 0.0 | 0.0 | 0.2 | 0.1 | 0.1 | 0.4 | 0.1 | 0.4 | 0.0 | 0.0 | 0.1 | 0.0 |
| Pvr | 10.2 | 8.9 | 7.7 | 9.8 | 9.3 | 8.4 | 11.5 | 8.5 | 7.6 | 8.9 | 12.1 | 8.2 |
| Pvrl1 | 1.6 | 1.9 | 1.9 | 1.5 | 2.1 | 2.1 | 2.8 | 1.8 | 1.9 | 1.6 | 1.5 | 1.3 |
| Pvrl2 | 12.8 | 13.7 | 17.3 | 8.9 | 14.4 | 8.4 | 19.2 | 16.7 | 20.3 | 28.9 | 17.9 | 18.2 |
| Pvrl3 | 12.6 | 12.0 | 11.7 | 8.6 | 11.8 | 11.1 | 15.7 | 13.6 | 15.7 | 12.0 | 15.7 | 14.3 |
| Pvrl4 | 0.1 | 0.0 | 0.1 | 0.2 | 0.1 | 0.0 | 0.0 | 0.0 | 0.0 | 0.1 | 0.1 | 0.0 |
| Pvt1 | 6.9 | 6.3 | 5.1 | 6.9 | 5.2 | 7.8 | 5.2 | 4.7 | 4.7 | 6.0 | 4.8 | 4.4 |
| Pwp1 | 10.5 | 9.2 | 8.9 | 10.7 | 9.8 | 10.0 | 9.5 | 8.8 | 8.2 | 9.5 | 10.0 | 9.2 |
| Pwp2 | 6.3 | 5.2 | 3.4 | 6.6 | 6.2 | 6.7 | 5.7 | 3.9 | 4.8 | 5.3 | 4.2 | 4.4 |
| Pwwp2a | 1.6 | 2.2 | 2.6 | 1.6 | 2.4 | 1.5 | 2.0 | 2.4 | 2.1 | 2.1 | 2.2 | 2.1 |
| Pwwp2b | 4.6 | 4.6 | 3.9 | 4.7 | 4.0 | 5.9 | 3.1 | 2.1 | 2.1 | 3.1 | 3.3 | 2.8 |
| Pxdc1 | 36.7 | 33.4 | 20.3 | 29.8 | 27.2 | 29.7 | 26.8 | 21.7 | 24.0 | 17.5 | 28.2 | 15.4 |
| Pxdn | 12.1 | 11.8 | 15.1 | 8.3 | 13.1 | 8.8 | 14.6 | 26.3 | 20.2 | 16.8 | 14.2 | 13.6 |
| Pxk | 10.2 | 11.7 | 11.5 | 10.4 | 9.8 | 9.2 | 11.5 | 9.3 | 10.8 | 14.7 | 13.0 | 10.9 |
| Pxmp2 | 0.6 | 0.8 | 0.7 | 0.9 | 0.5 | 0.4 | 0.7 | 0.6 | 0.8 | 0.8 | 0.9 | 0.4 |
| Pxmp4 | 9.8 | 9.2 | 9.6 | 9.8 | 7.8 | 8.9 | 8.9 | 9.9 | 9.6 | 8.7 | 10.2 | 10.6 |
| Pxn | 14.8 | 16.9 | 16.2 | 12.6 | 16.1 | 13.8 | 15.4 | 19.4 | 16.6 | 15.9 | 16.7 | 13.9 |
| Pycard | 1.8 | 1.4 | 0.8 | 1.9 | 3.8 | 1.7 | 1.1 | 1.0 | 1.3 | 1.4 | 0.7 | 0.8 |
| Pycr1 | 5.0 | 3.5 | 3.5 | 4.4 | 3.1 | 5.0 | 3.4 | 4.4 | 3.5 | 2.7 | 3.7 | 2.9 |
| Pycr2 | 15.8 | 12.2 | 8.5 | 11.9 | 13.6 | 11.6 | 13.4 | 12.2 | 13.7 | 16.6 | 12.3 | 11.0 |
| Pycrl | 9.3 | 9.0 | 6.9 | 10.8 | 8.5 | 10.1 | 9.2 | 8.4 | 9.7 | 10.5 | 9.5 | 8.8 |
| Pydc3 | 0.0 | 0.0 | 0.0 | 0.2 | 0.0 | 0.0 | 0.0 | 0.0 | 0.0 | 0.0 | 0.0 | 0.0 |
| Pygb | 15.9 | 15.8 | 20.4 | 15.7 | 15.0 | 16.1 | 16.3 | 21.7 | 18.4 | 21.5 | 20.5 | 18.7 |
| Pygl | 9.4 | 15.8 | 15.3 | 10.6 | 15.3 | 13.6 | 7.7 | 6.1 | 7.0 | 12.6 | 9.4 | 11.3 |
| Pygm | 2.0 | 2.9 | 3.5 | 1.9 | 1.6 | 1.9 | 0.9 | 0.4 | 0.6 | 0.5 | 0.4 | 0.5 |
| Pygo1 | 4.8 | 4.4 | 4.8 | 5.1 | 3.0 | 5.9 | 3.9 | 3.5 | 2.8 | 3.3 | 3.4 | 4.5 |
| Pygo2 | 11.1 | 13.1 | 10.2 | 9.4 | 11.3 | 10.2 | 11.7 | 10.7 | 12.7 | 12.6 | 10.4 | 9.9 |
| Pyhin1 | 0.1 | 0.0 | 0.0 | 0.2 | 0.2 | 0.1 | 0.1 | 0.0 | 0.0 | 0.0 | 0.1 | 0.0 |
| Pyroxd1 | 3.5 | 2.6 | 2.5 | 3.5 | 2.7 | 2.3 | 2.3 | 2.8 | 2.7 | 2.7 | 3.0 | 3.2 |
| Pyroxd2 | 1.8 | 2.1 | 2.2 | 1.1 | 1.0 | 1.4 | 1.4 | 1.9 | 2.1 | 1.9 | 1.3 | 1.5 |
| Pyurf | 1.6 | 1.4 | 1.5 | 2.4 | 1.5 | 1.9 | 1.5 | 1.8 | 1.1 | 1.9 | 1.8 | 1.6 |
| Qars | 28.4 | 26.9 | 27.6 | 23.6 | 27.5 | 27.4 | 25.0 | 29.5 | 27.4 | 29.6 | 29.1 | 27.8 |
| Qdpr | 16.7 | 15.6 | 14.7 | 15.9 | 16.9 | 16.0 | 12.5 | 14.7 | 13.1 | 14.6 | 12.6 | 13.0 |
| Qk | 25.8 | 27.1 | 29.8 | 38.7 | 26.8 | 33.0 | 29.7 | 26.0 | 23.0 | 24.2 | 32.7 | 28.7 |
| Qpct | 12.4 | 14.0 | 10.5 | 13.4 | 10.7 | 12.7 | 8.0 | 7.2 | 7.9 | 7.2 | 8.4 | 7.5 |
| Qpctl | 6.0 | 5.6 | 5.8 | 6.0 | 7.1 | 5.6 | 7.4 | 7.6 | 6.6 | 6.9 | 6.8 | 6.8 |
| Qrfp | 0.0 | 0.1 | 0.0 | 0.0 | 0.1 | 0.0 | 0.1 | 0.1 | 0.0 | 0.1 | 0.1 | 0.1 |
| Qrich1 | 28.2 | 30.6 | 31.6 | 33.4 | 28.4 | 31.6 | 33.5 | 36.2 | 31.4 | 29.3 | 34.6 | 31.2 |
| Qrich2 | 0.0 | 0.0 | 0.0 | 0.0 | 0.0 | 0.2 | 0.0 | 0.0 | 0.0 | 0.1 | 0.0 | 0.0 |
| Qrsl1 | 3.5 | 3.0 | 2.8 | 3.0 | 2.9 | 2.8 | 3.1 | 2.4 | 2.4 | 2.8 | 3.1 | 2.5 |
| Qser1 | 2.3 | 2.6 | 2.3 | 2.8 | 2.2 | 2.7 | 2.4 | 1.8 | 1.9 | 2.2 | 2.5 | 2.1 |
| Qsox1 | 55.0 | 44.7 | 42.6 | 48.3 | 48.3 | 53.0 | 45.3 | 44.0 | 47.0 | 50.7 | 45.1 | 48.1 |
| Qsox2 | 6.8 | 6.1 | 6.0 | 7.0 | 5.8 | 7.3 | 6.6 | 7.2 | 7.7 | 5.5 | 6.8 | 5.4 |
| Qtrt1 | 5.3 | 4.0 | 4.1 | 5.0 | 4.4 | 4.3 | 4.3 | 4.8 | 3.9 | 5.3 | 5.1 | 4.0 |
| Qtrtd1 | 2.5 | 2.4 | 2.4 | 2.6 | 2.6 | 2.3 | 2.2 | 2.6 | 2.3 | 2.2 | 3.0 | 2.4 |
| R3hcc1 | 13.4 | 10.7 | 9.3 | 18.7 | 9.3 | 16.4 | 13.3 | 10.9 | 9.3 | 10.9 | 13.8 | 13.5 |
| R3hcc1l | 5.9 | 6.3 | 6.2 | 5.9 | 6.0 | 5.1 | 6.0 | 6.1 | 6.3 | 5.7 | 6.3 | 6.8 |
| R3hdm1 | 3.1 | 3.7 | 4.2 | 4.1 | 3.9 | 3.8 | 3.8 | 4.1 | 3.2 | 3.3 | 4.4 | 3.6 |
| R3hdm2 | 4.6 | 5.6 | 5.9 | 6.0 | 4.5 | 5.3 | 5.9 | 5.1 | 4.2 | 3.5 | 4.8 | 4.7 |
| R3hdm4 | 14.9 | 13.7 | 13.6 | 15.3 | 15.0 | 15.7 | 15.6 | 15.7 | 13.8 | 13.6 | 13.3 | 12.2 |
| R74862 | 2.9 | 2.1 | 1.5 | 2.5 | 1.7 | 1.8 | 1.6 | 1.7 | 1.9 | 2.7 | 2.3 | 1.8 |
| Rab1 | 61.0 | 58.5 | 60.6 | 70.5 | 70.2 | 65.9 | 67.6 | 67.5 | 63.9 | 65.4 | 75.4 | 75.6 |
| Rab10 | 33.9 | 31.1 | 34.9 | 44.1 | 37.8 | 34.1 | 39.0 | 35.8 | 36.9 | 36.6 | 43.2 | 40.4 |
| Rab11a | 34.8 | 31.2 | 26.9 | 35.9 | 33.7 | 31.8 | 30.7 | 33.3 | 30.8 | 37.3 | 40.9 | 34.6 |
| Rab11b | 9.4 | 9.5 | 9.6 | 11.4 | 11.0 | 9.3 | 11.3 | 11.3 | 9.9 | 11.3 | 10.4 | 9.7 |

| | | | | | | | | | | | | |
|---|---|---|---|---|---|---|---|---|---|---|---|---|
| Rab11fip1 | 1.1 | 0.7 | 0.6 | 1.0 | 0.8 | 0.9 | 0.5 | 0.4 | 0.4 | 1.5 | 0.9 | 0.8 |
| Rab11fip2 | 4.1 | 4.3 | 5.0 | 3.1 | 3.3 | 3.9 | 4.3 | 4.6 | 4.9 | 4.9 | 5.3 | 4.7 |
| Rab11fip3 | 5.6 | 6.6 | 7.0 | 6.6 | 5.5 | 6.3 | 7.1 | 5.7 | 5.9 | 5.6 | 5.8 | 6.1 |
| Rab11fip4 | 0.0 | 0.2 | 0.0 | 0.0 | 0.0 | 0.0 | 0.0 | 0.0 | 0.0 | 0.0 | 0.0 | 0.0 |
| Rab11fip5 | 22.7 | 19.4 | 13.7 | 21.4 | 17.3 | 23.6 | 16.5 | 12.7 | 15.0 | 18.1 | 17.5 | 14.9 |
| Rab12 | 35.0 | 32.2 | 34.4 | 37.0 | 31.0 | 37.3 | 34.6 | 32.2 | 31.3 | 31.8 | 33.3 | 36.4 |
| Rab13 | 18.8 | 15.7 | 19.3 | 17.2 | 16.9 | 22.2 | 16.7 | 17.2 | 15.9 | 18.0 | 19.3 | 20.9 |
| Rab14 | 23.4 | 27.2 | 26.8 | 26.5 | 29.1 | 25.8 | 25.9 | 26.0 | 26.8 | 28.6 | 29.4 | 30.9 |
| Rab15 | 0.6 | 1.4 | 1.8 | 0.5 | 1.1 | 0.6 | 1.3 | 0.9 | 1.2 | 0.9 | 0.9 | 1.4 |
| Rab18 | 32.2 | 39.4 | 45.2 | 43.3 | 42.8 | 42.5 | 37.4 | 41.1 | 39.4 | 44.3 | 48.1 | 49.3 |
| Rab19 | 0.3 | 0.2 | 0.3 | 0.2 | 0.5 | 0.0 | 0.2 | 0.1 | 0.1 | 0.0 | 0.0 | 0.1 |
| Rab1b | 37.5 | 33.5 | 28.4 | 43.7 | 41.4 | 32.4 | 38.2 | 36.7 | 35.1 | 35.4 | 36.4 | 31.5 |
| Rab20 | 2.6 | 2.6 | 3.2 | 3.9 | 4.9 | 2.7 | 4.1 | 2.7 | 4.0 | 6.7 | 2.9 | 5.3 |
| Rab21 | 30.9 | 32.2 | 30.9 | 36.6 | 33.4 | 36.2 | 36.0 | 29.8 | 32.8 | 34.8 | 38.2 | 40.4 |
| Rab22a | 24.3 | 19.6 | 18.5 | 26.0 | 20.2 | 24.1 | 20.4 | 19.0 | 21.7 | 22.4 | 21.9 | 22.2 |
| Rab23 | 4.5 | 6.0 | 6.9 | 4.8 | 6.2 | 4.8 | 6.2 | 7.0 | 4.8 | 4.2 | 5.3 | 4.9 |
| Rab24 | 17.9 | 15.8 | 14.3 | 13.1 | 14.7 | 17.7 | 15.6 | 14.2 | 16.7 | 15.3 | 14.5 | 16.0 |
| Rab25 | 0.0 | 0.0 | 0.1 | 0.1 | 0.0 | 0.0 | 0.0 | 0.0 | 0.0 | 0.0 | 0.0 | 0.0 |
| Rab26 | 0.6 | 0.6 | 0.7 | 0.8 | 0.4 | 1.0 | 0.3 | 0.5 | 0.5 | 0.3 | 0.4 | 0.2 |
| Rab27a | 0.3 | 0.2 | 0.5 | 0.3 | 0.3 | 0.3 | 0.4 | 0.4 | 0.4 | 0.9 | 0.5 | 0.5 |
| Rab27b | 0.3 | 0.2 | 0.2 | 0.3 | 0.2 | 0.3 | 0.1 | 0.1 | 0.1 | 0.3 | 0.3 | 0.4 |
| Rab28 | 38.1 | 30.1 | 25.6 | 36.7 | 24.8 | 33.5 | 28.2 | 26.5 | 30.0 | 24.4 | 26.9 | 30.3 |
| Rab2a | 76.4 | 74.4 | 83.7 | 90.3 | 72.1 | 78.0 | 83.7 | 78.2 | 76.2 | 84.7 | 97.0 | 99.0 |
| Rab2b | 10.0 | 7.7 | 7.0 | 9.2 | 8.3 | 10.7 | 9.0 | 8.6 | 7.7 | 7.0 | 8.4 | 8.2 |
| Rab30 | 10.6 | 10.2 | 7.9 | 12.5 | 10.2 | 13.1 | 11.4 | 9.1 | 10.4 | 8.8 | 11.6 | 10.0 |
| Rab31 | 57.7 | 70.6 | 67.2 | 75.4 | 94.0 | 55.2 | 101.3 | 83.2 | 104.6 | 86.3 | 77.6 | 101.0 |
| Rab32 | 9.8 | 9.3 | 9.0 | 11.3 | 11.9 | 9.0 | 11.7 | 14.0 | 10.4 | 15.7 | 15.9 | 11.1 |
| Rab33a | 0.1 | 0.0 | 0.1 | 0.1 | 0.0 | 0.0 | 0.3 | 0.2 | 0.0 | 0.0 | 0.0 | 0.0 |
| Rab33b | 10.3 | 9.8 | 9.7 | 11.6 | 10.2 | 10.2 | 11.7 | 10.4 | 12.2 | 11.5 | 13.9 | 11.1 |
| Rab34 | 53.5 | 54.2 | 49.8 | 40.6 | 40.2 | 47.2 | 49.7 | 57.0 | 58.3 | 57.3 | 52.5 | 51.9 |
| Rab35 | 15.4 | 15.4 | 16.9 | 17.2 | 17.3 | 16.8 | 19.6 | 16.8 | 18.4 | 16.1 | 19.0 | 17.6 |
| Rab36 | 0.7 | 0.7 | 0.7 | 0.7 | 0.5 | 1.1 | 0.7 | 0.6 | 0.6 | 0.4 | 0.5 | 0.4 |
| Rab37 | 0.0 | 0.1 | 0.1 | 0.1 | 0.1 | 0.1 | 0.0 | 0.0 | 0.1 | 0.1 | 0.1 | 0.1 |
| Rab38 | 0.2 | 0.1 | 0.1 | 0.0 | 0.0 | 0.0 | 0.1 | 0.1 | 0.1 | 0.4 | 0.3 | 0.1 |
| Rab39 | 0.0 | 0.0 | 0.0 | 0.0 | 0.1 | 0.0 | 0.1 | 0.0 | 0.0 | 0.1 | 0.0 | 0.0 |
| Rab39b | 0.3 | 0.3 | 0.5 | 0.5 | 0.2 | 0.3 | 0.5 | 0.8 | 0.6 | 0.3 | 0.6 | 0.6 |
| Rab3a | 5.5 | 5.7 | 4.9 | 5.7 | 4.1 | 7.0 | 4.6 | 3.6 | 4.7 | 4.8 | 5.8 | 4.6 |
| Rab3b | 0.1 | 0.1 | 0.0 | 0.3 | 0.1 | 0.2 | 0.0 | 0.0 | 0.1 | 0.2 | 0.1 | 0.1 |
| Rab3c | 0.1 | 0.1 | 0.1 | 0.2 | 0.1 | 0.1 | 0.2 | 0.1 | 0.1 | 0.1 | 0.2 | 0.2 |
| Rab3d | 7.1 | 6.2 | 6.8 | 6.8 | 5.4 | 7.6 | 3.5 | 5.3 | 3.3 | 4.6 | 4.8 | 3.7 |
| Rab3gap1 | 16.0 | 13.7 | 13.8 | 16.0 | 14.0 | 14.4 | 13.0 | 12.2 | 13.7 | 17.5 | 15.8 | 17.0 |
| Rab3gap2 | 13.1 | 11.3 | 9.4 | 14.6 | 13.5 | 13.0 | 11.9 | 9.8 | 10.8 | 11.9 | 12.7 | 11.8 |
| Rab3il1 | 4.2 | 7.0 | 7.1 | 9.7 | 7.8 | 5.8 | 4.8 | 8.4 | 6.2 | 2.5 | 4.2 | 4.0 |
| Rab3ip | 21.1 | 20.6 | 16.9 | 25.4 | 18.5 | 26.4 | 20.0 | 14.9 | 14.5 | 13.8 | 17.0 | 14.4 |
| Rab40b | 2.6 | 4.7 | 5.9 | 1.4 | 2.4 | 2.1 | 3.1 | 3.5 | 3.9 | 5.4 | 3.5 | 4.0 |
| Rab40c | 4.1 | 3.4 | 3.4 | 3.3 | 4.3 | 3.5 | 3.0 | 3.2 | 3.3 | 4.5 | 3.3 | 3.7 |
| Rab42 | 1.3 | 1.6 | 1.2 | 1.2 | 1.5 | 0.9 | 1.0 | 1.2 | 0.9 | 1.3 | 1.0 | 0.7 |
| Rab43 | 10.0 | 8.7 | 8.1 | 13.1 | 9.8 | 12.2 | 9.2 | 7.9 | 8.1 | 9.1 | 9.0 | 8.8 |
| Rab44 | 0.0 | 0.0 | 0.0 | 0.0 | 0.0 | 0.0 | 0.0 | 0.0 | 0.0 | 0.0 | 0.0 | 0.0 |
| Rab4a | 5.1 | 3.1 | 3.7 | 3.7 | 2.7 | 4.4 | 3.9 | 4.0 | 3.9 | 3.5 | 3.9 | 4.3 |
| Rab4b | 17.9 | 18.8 | 18.1 | 18.6 | 19.6 | 16.9 | 21.6 | 23.9 | 22.4 | 18.4 | 18.2 | 20.2 |
| Rab5a | 38.6 | 38.4 | 39.7 | 41.0 | 37.0 | 39.4 | 39.2 | 37.3 | 39.6 | 39.6 | 46.9 | 46.9 |
| Rab5b | 44.8 | 45.8 | 43.3 | 53.2 | 40.5 | 51.0 | 46.4 | 41.8 | 41.8 | 40.4 | 45.0 | 45.0 |
| Rab5c | 78.1 | 69.6 | 66.6 | 93.2 | 86.0 | 85.8 | 79.9 | 71.1 | 68.6 | 74.5 | 75.3 | 73.0 |
| Rab6a | 29.9 | 26.6 | 26.5 | 33.4 | 26.3 | 30.4 | 31.2 | 28.1 | 29.8 | 30.5 | 34.0 | 33.0 |
| Rab6b | 3.4 | 3.1 | 3.0 | 5.0 | 2.4 | 3.9 | 3.8 | 3.0 | 2.9 | 3.3 | 2.7 | 2.8 |
| Rab7 | 155.2 | 129.6 | 121.3 | 177.0 | 146.6 | 163.7 | 138.8 | 118.9 | 111.4 | 133.0 | 140.2 | 150.3 |

| | | | | | | | | | | | | |
|---|---|---|---|---|---|---|---|---|---|---|---|---|
| Rab7l1 | 24.6 | 28.9 | 33.4 | 23.4 | 27.1 | 21.1 | 20.6 | 23.8 | 25.4 | 19.9 | 21.3 | 28.9 |
| Rab8a | 33.7 | 30.3 | 36.0 | 43.0 | 33.1 | 35.9 | 40.7 | 37.3 | 32.0 | 31.7 | 38.2 | 40.2 |
| Rab8b | 9.0 | 12.2 | 16.9 | 15.6 | 20.3 | 8.4 | 19.2 | 19.9 | 16.2 | 12.6 | 13.8 | 17.6 |
| Rab9 | 20.4 | 28.1 | 39.7 | 21.4 | 21.2 | 18.0 | 26.5 | 34.6 | 28.3 | 27.5 | 33.0 | 37.2 |
| Rabac1 | 134.1 | 106.2 | 76.4 | 129.8 | 102.2 | 125.2 | 103.3 | 90.0 | 118.0 | 111.2 | 91.8 | 89.2 |
| Rabep1 | 22.6 | 18.6 | 17.1 | 23.7 | 19.1 | 21.9 | 18.3 | 14.9 | 16.7 | 18.1 | 21.0 | 20.8 |
| Rabep2 | 7.0 | 6.8 | 7.2 | 6.0 | 6.6 | 6.1 | 6.6 | 8.1 | 8.2 | 7.4 | 7.2 | 6.4 |
| Rabepk | 8.5 | 7.7 | 8.1 | 7.9 | 6.7 | 8.4 | 8.2 | 8.0 | 7.1 | 7.7 | 8.0 | 7.3 |
| Rabgap1 | 23.6 | 23.6 | 23.0 | 21.2 | 22.0 | 22.2 | 24.1 | 21.3 | 21.4 | 23.2 | 25.0 | 25.6 |
| Rabgap1l | 13.0 | 11.7 | 12.6 | 11.5 | 9.1 | 12.7 | 11.6 | 9.3 | 10.8 | 11.9 | 13.2 | 14.0 |
| Rabgef1 | 12.3 | 12.7 | 10.1 | 12.5 | 12.0 | 13.1 | 12.9 | 9.5 | 10.7 | 11.2 | 11.6 | 11.1 |
| Rabggta | 4.4 | 3.7 | 2.5 | 5.1 | 3.8 | 4.2 | 4.1 | 2.8 | 4.0 | 3.8 | 3.6 | 3.1 |
| Rabggtb | 39.4 | 32.6 | 31.2 | 34.2 | 30.5 | 36.7 | 31.1 | 28.0 | 29.3 | 31.6 | 31.8 | 33.6 |
| Rabif | 10.6 | 10.6 | 9.0 | 11.0 | 11.1 | 10.5 | 10.1 | 11.6 | 11.6 | 13.3 | 11.8 | 11.6 |
| Rabl2 | 2.3 | 2.6 | 2.5 | 1.7 | 2.3 | 2.6 | 2.9 | 3.1 | 2.7 | 2.5 | 2.5 | 2.5 |
| Rabl3 | 2.3 | 2.3 | 2.8 | 2.8 | 2.8 | 1.9 | 2.0 | 2.6 | 2.7 | 2.4 | 2.4 | 2.1 |
| Rabl5 | 14.7 | 13.0 | 10.7 | 18.1 | 12.2 | 15.4 | 10.2 | 11.3 | 9.2 | 10.5 | 11.3 | 11.5 |
| Rabl6 | 19.6 | 20.0 | 22.9 | 20.2 | 17.6 | 20.1 | 19.2 | 21.7 | 18.0 | 19.8 | 22.3 | 22.6 |
| Rac1 | 106.8 | 104.8 | 106.3 | 121.3 | 116.0 | 112.8 | 121.3 | 123.6 | 120.4 | 111.5 | 133.2 | 120.7 |
| Rac2 | 1.3 | 0.0 | 0.1 | 11.1 | 21.9 | 0.0 | 3.6 | 0.2 | 0.7 | 1.6 | 0.4 | 1.2 |
| Rac3 | 2.1 | 3.0 | 2.6 | 4.0 | 2.4 | 5.0 | 2.1 | 1.7 | 2.3 | 3.2 | 2.5 | 2.1 |
| Racgap1 | 2.9 | 6.1 | 9.1 | 1.9 | 7.6 | 1.9 | 10.7 | 4.6 | 7.4 | 4.6 | 8.1 | 4.9 |
| Rad1 | 6.7 | 7.0 | 5.9 | 5.9 | 6.0 | 6.1 | 5.9 | 5.2 | 6.3 | 6.2 | 6.2 | 6.8 |
| Rad17 | 10.9 | 12.9 | 13.1 | 12.7 | 11.3 | 12.2 | 11.6 | 11.0 | 12.2 | 12.1 | 12.6 | 13.5 |
| Rad18 | 2.9 | 4.3 | 5.6 | 2.1 | 4.1 | 2.6 | 3.9 | 3.9 | 3.7 | 2.9 | 3.6 | 4.0 |
| Rad21 | 12.9 | 14.3 | 19.2 | 13.0 | 16.2 | 13.1 | 19.4 | 15.0 | 16.3 | 15.5 | 17.5 | 17.8 |
| Rad23a | 20.7 | 21.3 | 21.5 | 22.0 | 21.1 | 23.8 | 21.6 | 22.6 | 23.6 | 21.8 | 21.4 | 21.1 |
| Rad23b | 59.7 | 60.2 | 64.5 | 72.2 | 53.9 | 63.9 | 65.1 | 69.2 | 62.8 | 69.6 | 74.5 | 75.2 |
| Rad50 | 12.9 | 12.0 | 14.6 | 15.4 | 12.5 | 13.2 | 13.7 | 11.8 | 9.8 | 9.6 | 13.0 | 13.0 |
| Rad51 | 1.3 | 3.0 | 4.5 | 0.9 | 4.2 | 0.7 | 5.3 | 2.1 | 3.1 | 2.3 | 2.6 | 1.9 |
| Rad51ap1 | 1.3 | 2.6 | 4.1 | 0.8 | 3.8 | 0.3 | 3.7 | 2.0 | 2.9 | 1.3 | 2.7 | 2.0 |
| Rad51b | 1.5 | 1.4 | 1.8 | 0.9 | 1.1 | 0.7 | 0.8 | 0.4 | 0.5 | 0.5 | 0.4 | 0.7 |
| Rad51c | 0.1 | 0.4 | 0.7 | 0.1 | 0.4 | 0.2 | 0.5 | 0.4 | 0.2 | 0.1 | 0.3 | 0.3 |
| Rad51d | 2.3 | 2.0 | 1.9 | 2.0 | 1.7 | 2.5 | 1.6 | 1.8 | 1.9 | 2.1 | 2.1 | 1.9 |
| Rad52 | 4.9 | 5.1 | 4.1 | 4.1 | 4.3 | 5.0 | 4.4 | 4.6 | 4.8 | 4.8 | 5.9 | 5.8 |
| Rad54b | 0.7 | 1.0 | 1.9 | 0.4 | 1.1 | 0.5 | 1.6 | 0.5 | 1.3 | 0.6 | 1.1 | 0.9 |
| Rad54l | 0.5 | 1.1 | 1.6 | 0.2 | 1.4 | 0.2 | 1.8 | 0.8 | 1.4 | 0.6 | 1.2 | 0.7 |
| Rad54l2 | 2.5 | 2.8 | 3.1 | 2.8 | 2.4 | 2.5 | 2.9 | 2.7 | 2.7 | 2.6 | 2.5 | 2.7 |
| Rad9a | 9.1 | 9.1 | 8.0 | 9.7 | 9.0 | 8.2 | 8.4 | 9.2 | 9.3 | 9.2 | 8.8 | 7.3 |
| Rad9b | 0.8 | 1.6 | 0.9 | 0.4 | 0.5 | 0.7 | 0.8 | 0.6 | 0.7 | 0.9 | 0.7 | 0.5 |
| Radil | 0.6 | 0.5 | 0.5 | 0.5 | 0.6 | 1.1 | 0.6 | 0.6 | 0.5 | 0.4 | 0.5 | 0.5 |
| Rae1 | 17.2 | 14.2 | 12.6 | 16.4 | 13.1 | 17.6 | 13.8 | 14.2 | 14.2 | 14.5 | 15.5 | 13.5 |
| Raet1a | 2.1 | 0.8 | 0.8 | 1.6 | 1.4 | 1.8 | 0.7 | 0.2 | 0.2 | 1.0 | 0.7 | 0.5 |
| Raet1b | 0.0 | 0.0 | 0.0 | 0.0 | 0.0 | 0.0 | 0.0 | 0.0 | 0.0 | 0.0 | 0.0 | 0.0 |
| Raet1c | 1.9 | 0.8 | 0.7 | 0.6 | 1.8 | 1.9 | 1.2 | 0.3 | 0.6 | 0.9 | 1.3 | 0.8 |
| Raet1d | 5.7 | 5.1 | 6.4 | 5.9 | 6.4 | 5.1 | 5.1 | 2.7 | 3.6 | 3.0 | 5.1 | 3.9 |
| Raet1e | 8.0 | 3.9 | 5.4 | 6.6 | 6.3 | 5.1 | 3.0 | 1.8 | 2.5 | 4.0 | 3.5 | 4.1 |
| Raf1 | 20.0 | 20.1 | 19.4 | 19.6 | 21.1 | 16.9 | 20.1 | 22.1 | 20.9 | 18.2 | 20.6 | 19.9 |
| Rai1 | 2.0 | 3.0 | 2.5 | 2.1 | 2.5 | 2.5 | 2.9 | 2.5 | 3.0 | 2.7 | 2.4 | 2.1 |
| Rai14 | 61.6 | 62.7 | 55.9 | 67.1 | 62.3 | 40.5 | 61.9 | 33.8 | 60.3 | 67.6 | 74.9 | 101.1 |
| Rai2 | 2.5 | 4.2 | 8.1 | 2.0 | 3.3 | 2.5 | 4.4 | 7.2 | 3.6 | 2.2 | 2.4 | 5.3 |
| Rala | 26.7 | 23.4 | 24.5 | 32.6 | 23.8 | 26.8 | 27.1 | 25.7 | 24.6 | 25.2 | 31.7 | 29.1 |
| Ralb | 24.0 | 22.7 | 22.6 | 24.0 | 31.6 | 21.2 | 28.7 | 30.4 | 31.4 | 28.3 | 27.5 | 29.6 |
| Ralbp1 | 36.5 | 34.3 | 34.8 | 38.2 | 33.7 | 34.2 | 37.5 | 33.7 | 32.7 | 30.3 | 36.4 | 38.5 |
| Ralgapa1 | 7.2 | 7.8 | 6.7 | 6.3 | 6.3 | 7.6 | 5.9 | 6.0 | 6.7 | 7.3 | 7.6 | 6.9 |
| Ralgapa2 | 2.8 | 2.0 | 2.6 | 5.8 | 9.6 | 2.0 | 2.8 | 2.3 | 1.9 | 2.6 | 2.1 | 2.4 |
| Ralgapb | 13.1 | 12.8 | 11.3 | 13.1 | 12.0 | 13.4 | 11.7 | 11.8 | 12.4 | 11.8 | 12.4 | 11.9 |

| | | | | | | | | | | | | |
|---|---|---|---|---|---|---|---|---|---|---|---|---|
| Ralgds | 7.9 | 6.7 | 7.0 | 8.1 | 7.7 | 8.3 | 7.9 | 5.7 | 6.3 | 6.8 | 7.4 | 6.3 |
| Ralgps1 | 1.4 | 1.3 | 1.0 | 1.2 | 1.4 | 1.8 | 1.3 | 1.4 | 1.0 | 0.9 | 1.1 | 1.0 |
| Ralgps2 | 5.1 | 7.7 | 7.7 | 4.7 | 7.1 | 3.1 | 7.4 | 5.8 | 10.0 | 5.7 | 6.4 | 8.0 |
| Raly | 76.1 | 73.3 | 65.6 | 81.3 | 66.6 | 65.6 | 73.5 | 66.4 | 71.9 | 61.2 | 78.5 | 65.7 |
| Ramp1 | 0.1 | 0.1 | 0.2 | 0.3 | 0.2 | 0.1 | 0.2 | 0.1 | 0.4 | 0.5 | 0.4 | 0.5 |
| Ramp2 | 1.5 | 3.6 | 6.2 | 1.2 | 1.6 | 1.0 | 1.7 | 6.4 | 1.5 | 0.6 | 1.1 | 1.8 |
| Ramp3 | 0.0 | 0.0 | 0.1 | 0.0 | 0.0 | 0.0 | 0.1 | 0.0 | 0.1 | 0.0 | 0.1 | 0.0 |
| Ran | 120.5 | 99.2 | 76.7 | 118.3 | 109.2 | 111.0 | 95.6 | 88.4 | 95.4 | 100.2 | 97.3 | 80.1 |
| Ranbp1 | 90.1 | 80.0 | 93.8 | 112.5 | 83.5 | 87.7 | 114.5 | 92.0 | 78.9 | 79.9 | 109.3 | 92.0 |
| Ranbp10 | 4.5 | 4.8 | 4.4 | 4.8 | 4.8 | 4.0 | 5.0 | 5.0 | 4.7 | 4.4 | 4.7 | 4.5 |
| Ranbp17 | 0.5 | 0.5 | 0.4 | 0.5 | 0.4 | 0.4 | 0.6 | 0.5 | 0.5 | 0.4 | 0.6 | 0.4 |
| Ranbp2 | 26.5 | 26.3 | 23.1 | 23.9 | 26.1 | 24.0 | 21.9 | 22.3 | 21.7 | 26.1 | 27.5 | 25.7 |
| Ranbp3 | 20.2 | 20.1 | 18.0 | 20.5 | 19.4 | 22.5 | 19.6 | 20.5 | 19.7 | 19.8 | 19.5 | 19.2 |
| Ranbp3l | 0.0 | 0.0 | 0.0 | 0.1 | 0.0 | 0.0 | 0.0 | 0.0 | 0.0 | 0.0 | 0.0 | 0.0 |
| Ranbp6 | 3.1 | 3.9 | 3.8 | 2.9 | 3.2 | 4.2 | 2.6 | 3.3 | 3.7 | 4.1 | 3.7 | 3.8 |
| Ranbp9 | 16.9 | 17.8 | 18.9 | 18.1 | 14.6 | 21.2 | 15.1 | 14.3 | 13.7 | 14.4 | 16.0 | 16.1 |
| Rangap1 | 17.9 | 19.2 | 21.3 | 19.5 | 24.4 | 17.3 | 25.2 | 18.1 | 21.0 | 18.8 | 20.7 | 18.2 |
| Rangrf | 7.9 | 7.3 | 5.4 | 7.4 | 5.7 | 4.7 | 6.3 | 6.5 | 7.1 | 7.0 | 7.4 | 5.1 |
| Rap1a | 29.7 | 29.6 | 37.0 | 35.6 | 40.5 | 31.7 | 39.9 | 36.9 | 37.5 | 37.8 | 45.1 | 42.7 |
| Rap1b | 55.8 | 57.7 | 59.1 | 73.2 | 83.0 | 58.0 | 64.0 | 59.5 | 56.4 | 59.8 | 73.4 | 68.0 |
| Rap1gap | 1.1 | 1.1 | 0.9 | 0.9 | 0.7 | 1.0 | 0.6 | 0.4 | 0.5 | 0.6 | 0.6 | 0.6 |
| Rap1gap2 | 2.2 | 2.3 | 2.1 | 2.9 | 4.5 | 2.4 | 5.5 | 2.5 | 4.1 | 3.8 | 3.7 | 4.0 |
| Rap1gds1 | 20.1 | 18.8 | 15.3 | 23.0 | 21.8 | 22.1 | 18.8 | 16.0 | 17.2 | 17.2 | 18.7 | 15.9 |
| Rap2a | 10.4 | 12.0 | 10.7 | 11.7 | 14.9 | 8.5 | 10.4 | 9.9 | 11.8 | 15.9 | 12.4 | 11.0 |
| Rap2b | 17.6 | 17.3 | 18.7 | 18.9 | 18.8 | 19.3 | 15.8 | 19.0 | 16.2 | 16.1 | 16.4 | 18.0 |
| Rap2c | 7.9 | 8.7 | 10.7 | 10.8 | 10.4 | 9.8 | 9.4 | 8.7 | 8.7 | 11.5 | 11.0 | 11.5 |
| Rapgef1 | 18.1 | 15.4 | 12.5 | 18.3 | 14.2 | 17.4 | 17.6 | 14.3 | 14.6 | 13.0 | 14.7 | 13.0 |
| Rapgef2 | 7.1 | 7.5 | 7.8 | 7.5 | 7.4 | 6.4 | 8.2 | 7.6 | 7.0 | 7.2 | 7.2 | 7.8 |
| Rapgef3 | 12.4 | 10.1 | 8.2 | 11.8 | 8.9 | 14.0 | 11.0 | 9.0 | 10.1 | 14.0 | 12.2 | 11.9 |
| Rapgef4 | 0.5 | 0.3 | 0.1 | 0.3 | 0.4 | 0.2 | 0.4 | 0.2 | 0.3 | 0.4 | 0.3 | 0.4 |
| Rapgef5 | 2.3 | 1.1 | 1.2 | 4.9 | 6.2 | 0.4 | 1.5 | 0.5 | 0.6 | 0.5 | 0.4 | 0.7 |
| Rapgef6 | 6.3 | 9.2 | 9.3 | 6.0 | 6.4 | 6.2 | 6.8 | 7.4 | 7.9 | 7.6 | 8.0 | 9.7 |
| Rapgefl1 | 0.3 | 0.6 | 0.5 | 0.3 | 0.3 | 0.3 | 0.4 | 0.4 | 0.5 | 0.4 | 0.3 | 0.3 |
| Raph1 | 4.6 | 5.5 | 4.3 | 5.2 | 4.9 | 4.9 | 4.5 | 3.1 | 3.5 | 3.0 | 3.5 | 3.2 |
| Rapsn | 2.4 | 1.5 | 0.9 | 2.7 | 1.5 | 2.7 | 1.4 | 0.5 | 1.0 | 1.2 | 0.9 | 1.5 |
| Rara | 3.3 | 5.8 | 6.3 | 4.5 | 4.5 | 5.1 | 5.4 | 8.3 | 5.9 | 4.4 | 5.0 | 5.2 |
| Rarb | 1.4 | 1.4 | 0.8 | 0.8 | 1.0 | 0.8 | 0.8 | 0.5 | 1.3 | 1.2 | 0.9 | 1.5 |
| Rarg | 12.0 | 14.0 | 13.1 | 12.0 | 11.0 | 11.8 | 12.5 | 13.4 | 14.2 | 12.5 | 12.9 | 13.5 |
| Rarres1 | 0.9 | 0.6 | 1.0 | 0.4 | 0.9 | 0.2 | 0.4 | 0.9 | 0.6 | 1.4 | 0.4 | 1.2 |
| Rarres2 | 4.6 | 4.8 | 12.0 | 2.7 | 6.0 | 2.4 | 3.5 | 4.5 | 5.0 | 3.7 | 2.3 | 3.5 |
| Rars | 76.0 | 58.3 | 50.3 | 88.4 | 59.8 | 76.9 | 58.6 | 49.0 | 44.5 | 54.4 | 67.7 | 56.6 |
| Rars2 | 8.1 | 9.2 | 8.8 | 8.1 | 8.6 | 8.4 | 8.3 | 9.5 | 9.2 | 8.2 | 10.0 | 9.2 |
| Rasa1 | 18.4 | 16.5 | 16.3 | 19.5 | 19.0 | 17.7 | 16.5 | 14.0 | 17.2 | 20.5 | 19.5 | 20.8 |
| Rasa2 | 4.3 | 5.3 | 5.2 | 5.3 | 5.3 | 5.1 | 5.8 | 5.0 | 5.6 | 5.4 | 5.8 | 5.8 |
| Rasa3 | 34.5 | 62.2 | 86.5 | 33.8 | 50.4 | 34.8 | 51.5 | 74.9 | 52.6 | 46.3 | 42.2 | 64.1 |
| Rasa4 | 12.6 | 13.3 | 9.3 | 21.5 | 15.4 | 20.4 | 15.3 | 11.8 | 16.9 | 9.5 | 13.8 | 9.3 |
| Rasal1 | 0.2 | 0.2 | 0.1 | 0.1 | 0.1 | 0.1 | 0.0 | 0.0 | 0.1 | 0.1 | 0.1 | 0.0 |
| Rasal2 | 3.7 | 3.7 | 4.1 | 3.2 | 3.5 | 3.0 | 4.2 | 4.2 | 4.1 | 4.6 | 4.3 | 5.3 |
| Rasal3 | 0.1 | 0.0 | 0.0 | 0.6 | 1.4 | 0.0 | 0.3 | 0.0 | 0.0 | 0.0 | 0.0 | 0.1 |
| Rasd1 | 0.0 | 0.0 | 0.1 | 0.1 | 0.0 | 0.3 | 0.0 | 0.0 | 0.1 | 0.1 | 0.0 | 0.1 |
| Rasd2 | 0.0 | 0.0 | 0.0 | 0.1 | 0.0 | 0.0 | 0.0 | 0.0 | 0.0 | 0.0 | 0.0 | 0.0 |
| Rasef | 0.0 | 0.0 | 0.0 | 0.0 | 0.0 | 0.0 | 0.0 | 0.0 | 0.0 | 0.0 | 0.0 | 0.0 |
| Rasgef1a | 0.3 | 0.1 | 0.1 | 0.4 | 0.1 | 0.3 | 0.1 | 0.1 | 0.0 | 0.0 | 0.1 | 0.1 |
| Rasgef1b | 0.6 | 0.1 | 0.2 | 4.8 | 5.5 | 0.1 | 0.8 | 0.2 | 0.3 | 0.6 | 0.2 | 0.5 |
| Rasgef1c | 0.0 | 0.1 | 0.2 | 0.0 | 0.1 | 0.0 | 0.1 | 0.2 | 0.0 | 0.1 | 0.1 | 0.0 |
| Rasgrf2 | 0.1 | 0.0 | 0.0 | 0.0 | 0.0 | 0.0 | 0.0 | 0.1 | 0.2 | 1.2 | 0.5 | 0.3 |
| Rasgrp1 | 0.0 | 0.0 | 0.0 | 0.0 | 0.0 | 0.0 | 0.0 | 0.0 | 0.0 | 0.0 | 0.1 | 0.0 |

| | | | | | | | | | | | | |
|---|---|---|---|---|---|---|---|---|---|---|---|---|
| Rasgrp2 | 0.4 | 0.4 | 0.4 | 0.5 | 0.2 | 0.2 | 0.8 | 0.5 | 0.8 | 0.2 | 0.4 | 0.2 |
| Rasgrp3 | 3.0 | 1.4 | 1.1 | 2.8 | 1.4 | 2.5 | 1.7 | 2.0 | 2.1 | 2.5 | 2.7 | 1.2 |
| Rasgrp4 | 0.2 | 0.1 | 0.1 | 0.2 | 0.5 | 0.1 | 0.2 | 0.2 | 0.2 | 0.1 | 0.2 | 0.2 |
| Rasip1 | 0.2 | 0.2 | 0.2 | 0.3 | 0.1 | 0.0 | 0.1 | 0.3 | 0.3 | 0.1 | 0.2 | 0.1 |
| Rasl10a | 0.5 | 0.2 | 0.3 | 0.5 | 0.3 | 0.5 | 0.3 | 0.2 | 0.3 | 0.3 | 0.2 | 0.2 |
| Rasl10b | 0.1 | 0.0 | 0.1 | 0.0 | 0.0 | 0.0 | 0.1 | 0.1 | 0.0 | 0.2 | 0.1 | 0.1 |
| Rasl11a | 9.9 | 22.0 | 28.4 | 4.9 | 11.4 | 4.1 | 11.2 | 10.2 | 15.9 | 50.8 | 20.6 | 31.6 |
| Rasl11b | 1.7 | 0.9 | 2.1 | 1.3 | 3.4 | 1.5 | 2.5 | 2.9 | 2.2 | 2.9 | 1.5 | 2.7 |
| Rasl12 | 1.8 | 3.5 | 4.2 | 2.0 | 3.9 | 1.9 | 3.6 | 5.1 | 4.2 | 1.9 | 2.1 | 3.2 |
| Rasl2-9 | 0.0 | 0.1 | 0.0 | 0.1 | 0.0 | 0.1 | 0.0 | 0.1 | 0.0 | 0.1 | 0.0 | 0.1 |
| Rassf1 | 18.9 | 11.9 | 9.9 | 19.5 | 22.1 | 13.4 | 23.4 | 15.7 | 18.6 | 19.4 | 20.8 | 16.5 |
| Rassf10 | 0.0 | 0.0 | 0.0 | 0.0 | 0.0 | 0.0 | 0.0 | 0.0 | 0.0 | 0.0 | 0.0 | 0.0 |
| Rassf2 | 0.4 | 0.2 | 0.5 | 1.9 | 2.6 | 0.1 | 0.5 | 1.0 | 0.4 | 0.3 | 0.2 | 0.2 |
| Rassf3 | 18.7 | 14.1 | 8.8 | 24.2 | 19.0 | 21.9 | 15.6 | 6.1 | 10.4 | 14.0 | 15.8 | 12.6 |
| Rassf4 | 0.8 | 0.1 | 0.1 | 3.8 | 9.3 | 0.1 | 1.3 | 0.4 | 0.3 | 1.0 | 0.4 | 0.5 |
| Rassf5 | 3.7 | 2.5 | 2.2 | 4.0 | 4.7 | 3.5 | 2.8 | 1.7 | 2.1 | 3.3 | 2.8 | 2.3 |
| Rassf6 | 2.5 | 2.1 | 2.1 | 3.5 | 4.0 | 2.6 | 3.6 | 1.3 | 2.0 | 2.0 | 2.7 | 1.5 |
| Rassf7 | 1.8 | 1.1 | 1.1 | 1.6 | 1.2 | 2.0 | 0.7 | 1.0 | 1.5 | 1.3 | 1.7 | 1.1 |
| Rassf8 | 13.9 | 12.6 | 12.0 | 14.5 | 9.8 | 14.0 | 11.8 | 11.6 | 12.0 | 12.3 | 15.9 | 15.6 |
| Rassf9 | 0.0 | 0.0 | 0.1 | 0.0 | 0.0 | 0.0 | 0.0 | 0.1 | 0.1 | 0.1 | 0.1 | 0.0 |
| Raver1 | 1.5 | 2.9 | 3.2 | 2.9 | 3.2 | 1.9 | 3.6 | 3.7 | 3.5 | 2.9 | 2.6 | 2.5 |
| Raver1-fdx1l | 1.3 | 0.6 | 1.2 | 0.4 | 1.1 | 1.2 | 0.9 | 0.7 | 0.7 | 1.5 | 1.0 | 0.8 |
| Raver2 | 0.7 | 0.8 | 0.7 | 0.6 | 0.5 | 1.3 | 0.7 | 0.5 | 0.6 | 0.5 | 0.6 | 0.6 |
| Rax | 0.0 | 0.0 | 0.1 | 0.0 | 0.1 | 0.0 | 0.1 | 0.0 | 0.0 | 0.1 | 0.1 | 0.0 |
| Rb1 | 6.3 | 8.7 | 8.9 | 7.0 | 7.5 | 6.8 | 7.2 | 7.2 | 6.5 | 5.9 | 8.5 | 7.3 |
| Rb1cc1 | 16.0 | 17.0 | 17.3 | 16.4 | 15.4 | 15.4 | 15.0 | 15.6 | 16.2 | 16.1 | 19.6 | 19.2 |
| Rbak | 2.2 | 2.4 | 2.8 | 2.3 | 2.2 | 2.4 | 2.6 | 2.0 | 2.3 | 3.0 | 2.7 | 3.0 |
| Rbbp4 | 30.5 | 30.7 | 28.4 | 28.9 | 27.5 | 30.3 | 26.0 | 26.7 | 28.2 | 31.9 | 31.3 | 29.8 |
| Rbbp5 | 7.0 | 7.3 | 7.7 | 7.8 | 7.4 | 7.8 | 6.6 | 7.5 | 6.5 | 7.2 | 7.5 | 7.0 |
| Rbbp6 | 6.8 | 7.7 | 9.2 | 8.0 | 7.4 | 6.6 | 9.1 | 7.8 | 6.7 | 7.3 | 8.2 | 8.2 |
| Rbbp7 | 47.0 | 51.9 | 48.9 | 40.9 | 46.1 | 38.0 | 49.6 | 46.2 | 51.6 | 62.1 | 61.0 | 55.4 |
| Rbbp8 | 5.3 | 5.4 | 5.4 | 6.5 | 5.8 | 5.1 | 6.6 | 5.1 | 5.7 | 6.1 | 6.0 | 5.3 |
| Rbbp9 | 8.8 | 10.8 | 12.9 | 10.7 | 9.1 | 12.5 | 9.9 | 11.7 | 10.1 | 11.9 | 12.0 | 13.6 |
| Rbck1 | 19.8 | 19.3 | 17.4 | 16.6 | 19.4 | 21.5 | 21.1 | 22.5 | 19.4 | 20.4 | 17.7 | 17.1 |
| Rbfa | 17.1 | 15.6 | 15.1 | 18.2 | 14.1 | 16.1 | 16.9 | 15.4 | 13.7 | 16.7 | 16.6 | 17.0 |
| Rbfox1 | 0.1 | 0.0 | 0.1 | 0.1 | 0.0 | 0.1 | 0.1 | 0.1 | 0.0 | 0.0 | 0.1 | 0.0 |
| Rbfox2 | 17.1 | 19.5 | 19.3 | 15.9 | 18.9 | 16.5 | 22.0 | 23.2 | 20.8 | 21.5 | 21.0 | 19.6 |
| Rbfox3 | 0.0 | 0.0 | 0.0 | 0.0 | 0.0 | 0.0 | 0.0 | 0.0 | 0.0 | 0.0 | 0.0 | 0.0 |
| Rbks | 1.8 | 1.6 | 0.9 | 1.6 | 1.5 | 2.2 | 1.8 | 1.4 | 1.5 | 1.4 | 0.9 | 1.4 |
| Rbl1 | 2.1 | 2.6 | 3.2 | 1.7 | 3.0 | 2.4 | 2.8 | 2.4 | 2.3 | 1.8 | 2.5 | 2.2 |
| Rbl2 | 10.6 | 11.0 | 13.0 | 12.0 | 10.0 | 13.4 | 10.3 | 11.5 | 10.5 | 12.4 | 11.9 | 12.9 |
| Rbm10 | 5.7 | 5.8 | 7.6 | 5.6 | 5.9 | 6.1 | 7.0 | 8.7 | 7.4 | 7.3 | 7.7 | 7.1 |
| Rbm11 | 0.1 | 0.0 | 0.0 | 0.2 | 0.0 | 0.2 | 0.2 | 0.0 | 0.0 | 0.1 | 0.0 | 0.1 |
| Rbm12 | 4.1 | 4.5 | 4.9 | 4.0 | 5.0 | 4.4 | 4.4 | 5.6 | 5.5 | 4.5 | 5.2 | 5.3 |
| Rbm12b1 | 4.1 | 5.7 | 4.9 | 4.3 | 4.7 | 5.3 | 4.2 | 4.4 | 5.1 | 5.3 | 4.6 | 5.2 |
| Rbm12b2 | 4.3 | 5.6 | 5.8 | 4.4 | 5.0 | 5.3 | 4.2 | 4.9 | 5.0 | 5.6 | 5.3 | 5.8 |
| Rbm14 | 3.9 | 4.3 | 4.9 | 4.7 | 5.2 | 4.5 | 5.5 | 5.7 | 5.1 | 5.1 | 4.5 | 3.8 |
| Rbm15 | 4.9 | 5.7 | 5.6 | 4.7 | 6.1 | 4.6 | 6.0 | 5.6 | 5.9 | 6.0 | 5.3 | 5.2 |
| Rbm15b | 9.2 | 9.8 | 10.5 | 8.6 | 8.5 | 11.1 | 9.6 | 11.4 | 11.9 | 10.8 | 10.4 | 10.7 |
| Rbm17 | 52.3 | 46.5 | 53.1 | 56.0 | 43.9 | 53.1 | 56.2 | 48.1 | 39.2 | 46.9 | 55.4 | 57.7 |
| Rbm18 | 10.6 | 10.4 | 11.6 | 12.1 | 12.0 | 10.7 | 11.6 | 11.3 | 12.2 | 13.3 | 12.7 | 13.3 |
| Rbm19 | 4.9 | 3.9 | 3.4 | 5.7 | 4.6 | 4.2 | 4.2 | 4.0 | 3.6 | 4.1 | 3.8 | 3.3 |
| Rbm20 | 0.1 | 0.1 | 0.1 | 0.1 | 0.1 | 0.1 | 0.1 | 0.1 | 0.1 | 0.3 | 0.2 | 0.2 |
| Rbm22 | 10.5 | 11.9 | 13.2 | 11.5 | 10.9 | 11.2 | 12.8 | 13.6 | 12.8 | 12.4 | 13.8 | 14.1 |
| Rbm24 | 0.0 | 0.0 | 0.0 | 0.0 | 0.0 | 0.0 | 0.0 | 0.0 | 0.0 | 0.0 | 0.0 | 0.0 |
| Rbm25 | 22.4 | 23.6 | 28.0 | 26.4 | 21.1 | 20.3 | 29.0 | 24.7 | 19.6 | 21.2 | 30.6 | 29.5 |
| Rbm26 | 5.4 | 6.2 | 8.4 | 5.6 | 6.4 | 5.9 | 7.5 | 6.8 | 5.5 | 7.4 | 7.9 | 7.0 |

| | | | | | | | | | | | | |
|---|---|---|---|---|---|---|---|---|---|---|---|---|
| Rbm27 | 4.4 | 5.1 | 5.5 | 5.2 | 4.5 | 4.2 | 4.9 | 5.1 | 4.2 | 4.6 | 5.0 | 5.5 |
| Rbm28 | 27.8 | 20.9 | 19.4 | 29.1 | 19.2 | 25.9 | 21.2 | 20.2 | 19.2 | 25.8 | 25.7 | 19.4 |
| Rbm3 | 51.0 | 27.3 | 39.8 | 46.9 | 36.0 | 30.1 | 51.4 | 40.0 | 33.4 | 25.3 | 46.3 | 48.6 |
| Rbm33 | 2.4 | 3.2 | 3.2 | 2.7 | 2.6 | 2.5 | 2.9 | 2.6 | 2.5 | 2.6 | 2.6 | 2.7 |
| Rbm34 | 4.2 | 4.8 | 4.6 | 5.1 | 4.4 | 4.9 | 4.4 | 4.3 | 4.2 | 4.5 | 5.3 | 4.6 |
| Rbm38 | 0.5 | 0.6 | 0.5 | 1.0 | 1.2 | 0.6 | 0.7 | 0.5 | 0.4 | 1.1 | 0.7 | 0.4 |
| Rbm39 | 53.7 | 58.5 | 68.9 | 54.7 | 51.0 | 49.6 | 55.9 | 67.1 | 57.2 | 66.8 | 68.0 | 70.6 |
| Rbm4 | 4.4 | 5.0 | 4.4 | 5.4 | 4.9 | 4.2 | 5.5 | 6.4 | 5.3 | 4.8 | 5.1 | 4.1 |
| Rbm41 | 2.4 | 3.2 | 3.6 | 2.4 | 2.6 | 2.0 | 2.7 | 3.4 | 3.0 | 2.5 | 3.7 | 3.8 |
| Rbm42 | 30.9 | 31.9 | 25.9 | 32.9 | 28.6 | 29.1 | 29.7 | 31.6 | 30.5 | 29.9 | 27.9 | 26.5 |
| Rbm43 | 11.6 | 9.0 | 9.5 | 12.1 | 10.5 | 11.6 | 9.3 | 7.2 | 7.6 | 9.0 | 10.6 | 10.2 |
| Rbm44 | 0.1 | 0.3 | 0.2 | 0.1 | 0.2 | 0.1 | 0.2 | 0.1 | 0.1 | 0.1 | 0.1 | 0.1 |
| Rbm45 | 23.8 | 21.7 | 21.4 | 21.3 | 20.5 | 21.1 | 22.5 | 22.3 | 21.5 | 23.4 | 23.4 | 23.2 |
| Rbm46 | 0.3 | 0.4 | 0.4 | 0.1 | 0.2 | 0.0 | 0.2 | 0.4 | 0.4 | 0.2 | 0.3 | 0.2 |
| Rbm47 | 0.7 | 0.7 | 0.5 | 1.2 | 1.3 | 0.7 | 0.5 | 0.3 | 0.3 | 0.5 | 0.5 | 0.6 |
| Rbm48 | 1.9 | 1.9 | 3.0 | 2.1 | 2.1 | 2.1 | 2.0 | 2.0 | 1.6 | 2.4 | 2.2 | 2.4 |
| Rbm4b | 2.8 | 3.6 | 3.8 | 2.9 | 2.5 | 3.3 | 3.0 | 3.7 | 2.7 | 3.4 | 3.8 | 3.5 |
| Rbm5 | 25.7 | 29.2 | 35.1 | 28.3 | 26.5 | 26.3 | 29.3 | 35.0 | 31.8 | 30.2 | 34.3 | 36.1 |
| Rbm6 | 18.8 | 19.1 | 21.1 | 18.4 | 16.2 | 17.6 | 17.2 | 18.8 | 17.7 | 18.6 | 21.0 | 22.4 |
| Rbm7 | 25.4 | 23.7 | 26.9 | 30.7 | 22.0 | 27.5 | 24.1 | 25.8 | 23.5 | 26.1 | 30.3 | 29.4 |
| Rbm8a | 17.5 | 17.1 | 19.5 | 21.0 | 14.1 | 17.5 | 17.8 | 16.9 | 15.4 | 17.8 | 20.1 | 21.5 |
| Rbms1 | 43.7 | 47.0 | 47.5 | 46.6 | 43.3 | 37.9 | 57.8 | 51.2 | 53.5 | 48.7 | 58.0 | 54.8 |
| Rbms2 | 12.6 | 12.0 | 12.3 | 11.4 | 10.2 | 12.0 | 12.3 | 14.2 | 13.0 | 11.6 | 13.5 | 12.8 |
| Rbms3 | 22.7 | 28.6 | 32.7 | 22.8 | 25.2 | 22.5 | 32.5 | 33.3 | 31.5 | 25.6 | 31.0 | 31.2 |
| Rbmx | 6.8 | 8.1 | 9.9 | 6.0 | 7.3 | 5.4 | 8.1 | 9.1 | 8.2 | 5.5 | 7.1 | 7.0 |
| Rbmx2 | 2.3 | 2.6 | 2.7 | 1.6 | 2.9 | 2.1 | 3.7 | 2.7 | 2.7 | 2.8 | 3.2 | 2.6 |
| Rbmxl1 | 16.7 | 15.4 | 17.7 | 16.7 | 15.3 | 16.3 | 18.5 | 15.7 | 16.3 | 20.6 | 18.8 | 19.1 |
| Rbp1 | 33.7 | 23.4 | 29.9 | 36.7 | 36.6 | 27.5 | 57.1 | 20.9 | 58.6 | 67.5 | 51.4 | 73.7 |
| Rbp4 | 0.0 | 0.0 | 0.0 | 0.1 | 0.0 | 0.0 | 0.0 | 0.0 | 0.1 | 0.0 | 0.0 | 0.0 |
| Rbpj | 23.5 | 16.8 | 14.2 | 29.4 | 22.0 | 34.2 | 11.8 | 8.1 | 8.3 | 13.8 | 12.3 | 11.5 |
| Rbpjl | 0.0 | 0.0 | 0.0 | 0.0 | 0.0 | 0.0 | 0.0 | 0.0 | 0.0 | 0.1 | 0.0 | 0.0 |
| Rbpms | 9.1 | 11.1 | 12.1 | 10.2 | 7.3 | 8.9 | 10.8 | 9.6 | 7.8 | 10.9 | 10.2 | 9.4 |
| Rbpms2 | 2.4 | 2.7 | 2.6 | 3.1 | 2.0 | 3.7 | 2.4 | 2.1 | 2.4 | 1.8 | 1.8 | 2.0 |
| Rbx1 | 38.7 | 35.2 | 34.8 | 40.8 | 34.1 | 34.7 | 35.5 | 35.8 | 39.2 | 42.8 | 40.0 | 39.6 |
| Rc3h1 | 4.5 | 5.5 | 5.5 | 4.7 | 5.4 | 5.0 | 4.9 | 5.7 | 4.9 | 5.4 | 5.7 | 5.3 |
| Rc3h2 | 11.0 | 12.8 | 11.4 | 12.8 | 10.8 | 11.9 | 11.1 | 10.1 | 10.4 | 10.7 | 12.7 | 11.9 |
| Rcan1 | 11.5 | 7.3 | 6.2 | 12.9 | 8.9 | 10.4 | 9.8 | 6.9 | 6.9 | 9.7 | 10.4 | 8.3 |
| Rcan2 | 12.4 | 12.1 | 9.2 | 10.1 | 4.5 | 11.3 | 3.1 | 2.9 | 3.3 | 6.3 | 5.4 | 5.4 |
| Rcan3 | 6.5 | 5.8 | 4.8 | 7.4 | 5.6 | 8.5 | 5.9 | 5.1 | 5.8 | 4.3 | 5.2 | 5.5 |
| Rcbtb1 | 12.3 | 11.7 | 10.6 | 10.3 | 9.4 | 13.7 | 8.7 | 8.9 | 9.7 | 10.2 | 11.5 | 10.6 |
| Rcbtb2 | 11.4 | 12.4 | 11.8 | 12.6 | 10.2 | 14.4 | 9.9 | 11.4 | 11.0 | 11.3 | 10.7 | 12.5 |
| Rcc1 | 3.3 | 3.2 | 3.2 | 3.3 | 4.2 | 2.6 | 5.2 | 3.6 | 4.1 | 3.7 | 3.5 | 3.3 |
| Rcc2 | 16.6 | 15.4 | 14.8 | 18.1 | 19.7 | 17.7 | 18.6 | 25.1 | 19.8 | 18.9 | 18.3 | 14.6 |
| Rccd1 | 2.2 | 2.3 | 1.8 | 2.1 | 2.2 | 1.6 | 1.4 | 1.4 | 2.4 | 1.8 | 2.1 | 1.7 |
| Rce1 | 7.1 | 8.3 | 7.3 | 7.9 | 7.9 | 5.8 | 9.0 | 10.3 | 9.9 | 8.0 | 8.8 | 6.9 |
| Rchy1 | 15.0 | 12.6 | 11.6 | 12.9 | 10.9 | 12.0 | 10.4 | 10.4 | 10.8 | 16.6 | 11.3 | 12.7 |
| Rcl1 | 6.8 | 7.1 | 7.3 | 6.7 | 8.0 | 6.6 | 8.2 | 7.9 | 7.7 | 8.3 | 7.4 | 7.0 |
| Rcn1 | 117.6 | 94.5 | 100.9 | 139.0 | 94.0 | 137.6 | 117.2 | 101.0 | 100.6 | 118.5 | 121.6 | 130.4 |
| Rcn2 | 115.6 | 86.3 | 85.2 | 100.3 | 88.2 | 112.5 | 94.1 | 75.6 | 89.3 | 102.7 | 105.4 | 104.9 |
| Rcn3 | 272.8 | 246.5 | 308.6 | 326.7 | 276.3 | 306.0 | 364.5 | 339.6 | 317.6 | 338.4 | 312.6 | 351.8 |
| Rcor1 | 7.3 | 10.5 | 9.7 | 8.0 | 8.2 | 7.5 | 9.6 | 9.2 | 9.0 | 8.5 | 9.4 | 8.2 |
| Rcor2 | 0.6 | 1.0 | 0.8 | 0.5 | 0.3 | 0.5 | 0.4 | 1.3 | 0.6 | 0.5 | 0.6 | 0.3 |
| Rcor3 | 3.5 | 3.6 | 4.9 | 4.0 | 3.6 | 4.2 | 4.4 | 5.9 | 4.8 | 3.4 | 4.1 | 3.8 |
| Rcsd1 | 6.0 | 7.6 | 8.8 | 9.4 | 10.2 | 6.1 | 6.9 | 6.0 | 5.4 | 5.8 | 5.3 | 6.8 |
| Rcvrn | 0.0 | 0.0 | 0.0 | 0.0 | 0.0 | 0.1 | 0.0 | 0.0 | 0.1 | 0.1 | 0.1 | 0.0 |
| Rd3 | 0.1 | 0.0 | 0.0 | 0.0 | 0.0 | 0.0 | 0.0 | 0.0 | 0.1 | 0.0 | 0.1 | 0.0 |
| Rdh1 | 0.0 | 0.0 | 0.0 | 0.0 | 0.0 | 0.0 | 0.0 | 0.0 | 0.0 | 0.0 | 0.0 | 0.0 |

| | | | | | | | | | | | | |
|---|---|---|---|---|---|---|---|---|---|---|---|---|
| Rdh10 | 1.9 | 1.6 | 1.5 | 2.2 | 2.6 | 1.5 | 2.3 | 1.6 | 2.5 | 3.9 | 3.0 | 2.4 |
| Rdh11 | 16.8 | 18.7 | 16.9 | 13.9 | 14.8 | 15.2 | 20.4 | 16.8 | 19.0 | 13.0 | 17.5 | 13.1 |
| Rdh12 | 0.3 | 0.8 | 0.9 | 0.5 | 0.6 | 0.6 | 0.6 | 0.6 | 0.4 | 0.6 | 0.3 | 0.5 |
| Rdh13 | 3.7 | 3.8 | 3.1 | 4.7 | 3.9 | 4.2 | 4.0 | 3.4 | 3.7 | 3.1 | 3.6 | 3.2 |
| Rdh14 | 12.6 | 11.1 | 10.7 | 11.3 | 10.7 | 10.0 | 9.8 | 11.5 | 11.7 | 13.2 | 13.8 | 13.8 |
| Rdh16 | 0.0 | 0.0 | 0.0 | 0.0 | 0.0 | 0.0 | 0.0 | 0.0 | 0.0 | 0.0 | 0.0 | 0.0 |
| Rdh19 | 0.0 | 0.0 | 0.0 | 0.0 | 0.0 | 0.0 | 0.0 | 0.0 | 0.0 | 0.0 | 0.0 | 0.0 |
| Rdh5 | 2.0 | 2.1 | 2.5 | 1.3 | 2.8 | 2.7 | 2.2 | 3.2 | 2.2 | 2.0 | 2.5 | 2.5 |
| Rdh9 | 0.2 | 0.1 | 0.1 | 0.1 | 0.1 | 0.0 | 0.1 | 0.1 | 0.0 | 0.1 | 0.1 | 0.0 |
| Rdm1 | 1.7 | 3.6 | 2.4 | 0.6 | 2.5 | 3.2 | 2.7 | 2.0 | 3.3 | 4.2 | 5.1 | 2.8 |
| Rdx | 43.9 | 47.7 | 57.3 | 50.6 | 40.7 | 46.1 | 46.5 | 47.9 | 42.1 | 45.3 | 56.8 | 59.6 |
| Rec8 | 0.0 | 0.1 | 0.1 | 0.0 | 0.0 | 0.0 | 0.1 | 0.0 | 0.1 | 0.3 | 0.2 | 0.0 |
| Reck | 18.7 | 17.6 | 21.3 | 11.3 | 10.5 | 15.4 | 13.2 | 13.9 | 13.0 | 13.3 | 14.4 | 16.9 |
| Recql | 9.9 | 10.6 | 10.0 | 9.5 | 11.0 | 9.1 | 10.9 | 8.4 | 9.7 | 9.0 | 9.8 | 9.5 |
| Recql4 | 0.3 | 0.4 | 0.6 | 0.2 | 0.4 | 0.2 | 0.8 | 0.5 | 0.4 | 0.3 | 0.3 | 0.3 |
| Recql5 | 3.3 | 3.2 | 3.5 | 2.7 | 3.0 | 2.7 | 3.1 | 3.4 | 3.5 | 3.1 | 2.9 | 3.5 |
| Reep1 | 0.3 | 0.2 | 0.2 | 0.2 | 0.1 | 0.4 | 0.2 | 0.2 | 0.3 | 0.8 | 0.4 | 0.2 |
| Reep2 | 0.6 | 0.3 | 0.5 | 0.3 | 0.6 | 0.6 | 0.3 | 0.1 | 0.3 | 0.5 | 0.4 | 0.4 |
| Reep3 | 11.0 | 9.2 | 9.2 | 14.8 | 11.9 | 14.4 | 10.3 | 11.4 | 8.9 | 8.8 | 11.0 | 9.8 |
| Reep4 | 6.2 | 5.5 | 7.1 | 5.5 | 7.2 | 5.0 | 8.8 | 6.2 | 7.2 | 6.3 | 6.6 | 5.3 |
| Reep5 | 57.9 | 50.4 | 42.4 | 73.4 | 56.7 | 60.6 | 51.7 | 44.1 | 44.6 | 47.7 | 52.4 | 51.7 |
| Reep6 | 0.4 | 0.4 | 0.6 | 0.5 | 0.5 | 0.4 | 0.5 | 0.3 | 0.3 | 0.4 | 0.4 | 0.3 |
| Reg4 | 0.0 | 0.0 | 0.0 | 0.0 | 0.1 | 0.1 | 0.0 | 0.1 | 0.0 | 0.1 | 0.0 | 0.0 |
| Rel | 0.6 | 0.6 | 0.6 | 0.7 | 1.2 | 0.8 | 0.8 | 0.7 | 0.7 | 0.6 | 0.7 | 0.8 |
| Rela | 15.0 | 16.8 | 15.1 | 20.5 | 18.9 | 16.5 | 21.4 | 15.2 | 15.5 | 13.2 | 14.1 | 13.9 |
| Relb | 11.0 | 9.6 | 9.0 | 9.6 | 13.5 | 10.5 | 13.0 | 9.3 | 10.4 | 9.1 | 7.4 | 9.2 |
| Rell1 | 5.4 | 6.4 | 7.0 | 6.3 | 5.2 | 6.7 | 6.1 | 5.8 | 7.6 | 4.9 | 6.6 | 6.8 |
| Rell2 | 0.1 | 0.0 | 0.1 | 0.1 | 0.1 | 0.1 | 0.0 | 0.1 | 0.2 | 0.2 | 0.1 | 0.0 |
| Reln | 0.0 | 0.0 | 0.0 | 0.1 | 0.0 | 0.0 | 0.0 | 0.0 | 0.0 | 0.0 | 0.0 | 0.0 |
| Relt | 0.8 | 0.9 | 1.0 | 1.2 | 1.4 | 1.2 | 1.5 | 1.3 | 1.0 | 1.1 | 1.2 | 0.9 |
| Rem1 | 1.0 | 0.5 | 0.8 | 0.8 | 0.5 | 1.5 | 0.7 | 0.6 | 0.5 | 2.0 | 1.0 | 1.3 |
| Rem2 | 0.0 | 0.0 | 0.0 | 0.0 | 0.0 | 0.0 | 0.0 | 0.0 | 0.0 | 0.0 | 0.0 | 0.0 |
| Renbp | 43.5 | 28.9 | 23.3 | 46.9 | 35.2 | 46.2 | 24.6 | 21.3 | 20.5 | 33.6 | 26.4 | 33.1 |
| Rep15 | 3.1 | 3.6 | 3.7 | 3.6 | 3.1 | 4.9 | 4.9 | 3.9 | 4.7 | 6.3 | 5.6 | 6.3 |
| Repin1 | 5.7 | 5.9 | 5.6 | 5.3 | 5.4 | 7.2 | 4.5 | 5.5 | 5.3 | 4.5 | 4.6 | 4.2 |
| Reps1 | 10.9 | 11.1 | 9.4 | 9.9 | 10.0 | 10.3 | 10.8 | 10.0 | 10.4 | 12.2 | 11.5 | 10.3 |
| Reps2 | 0.6 | 1.1 | 1.8 | 0.8 | 1.2 | 0.4 | 0.8 | 1.4 | 1.0 | 0.8 | 0.6 | 0.9 |
| Rer1 | 100.7 | 84.2 | 74.1 | 95.4 | 85.7 | 87.3 | 89.4 | 75.2 | 85.8 | 91.7 | 95.9 | 98.3 |
| Rere | 2.4 | 3.1 | 3.6 | 2.3 | 2.1 | 2.9 | 3.0 | 2.9 | 2.7 | 3.6 | 3.0 | 3.2 |
| Rerg | 1.8 | 2.2 | 1.8 | 1.7 | 1.4 | 1.4 | 1.3 | 0.9 | 1.2 | 1.5 | 0.8 | 1.7 |
| Rest | 13.0 | 11.4 | 13.3 | 12.9 | 13.1 | 13.2 | 13.6 | 13.1 | 12.0 | 14.6 | 15.0 | 14.5 |
| Ret | 0.1 | 0.1 | 0.1 | 0.1 | 0.1 | 0.1 | 0.1 | 0.2 | 0.1 | 0.1 | 0.0 | 0.1 |
| Retsat | 6.6 | 6.9 | 6.5 | 9.0 | 7.4 | 8.5 | 6.5 | 6.0 | 5.9 | 7.3 | 5.3 | 6.3 |
| Rev1 | 5.6 | 6.1 | 5.8 | 4.5 | 5.1 | 4.8 | 4.9 | 5.8 | 5.1 | 5.8 | 4.9 | 5.4 |
| Rev3l | 2.6 | 3.8 | 4.6 | 2.3 | 2.5 | 2.6 | 2.2 | 3.8 | 2.6 | 2.6 | 2.5 | 2.7 |
| Rex2 | 0.2 | 0.2 | 0.2 | 0.2 | 0.1 | 0.2 | 0.1 | 0.1 | 0.1 | 0.1 | 0.2 | 0.1 |
| Rexo1 | 2.1 | 2.4 | 3.0 | 2.2 | 2.5 | 2.3 | 2.9 | 2.9 | 2.8 | 2.7 | 2.4 | 2.2 |
| Rexo2 | 101.6 | 83.0 | 88.6 | 117.5 | 87.6 | 99.3 | 100.9 | 95.4 | 90.1 | 95.9 | 111.6 | 98.0 |
| Rexo4 | 11.9 | 11.8 | 12.5 | 12.5 | 11.6 | 11.3 | 12.8 | 13.1 | 10.4 | 12.1 | 12.6 | 12.1 |
| Rfc1 | 10.1 | 12.4 | 13.1 | 10.3 | 10.4 | 13.1 | 11.3 | 12.1 | 10.4 | 10.4 | 11.3 | 11.0 |
| Rfc2 | 12.6 | 13.8 | 14.5 | 11.8 | 13.8 | 11.1 | 13.7 | 12.5 | 12.0 | 10.8 | 12.4 | 10.8 |
| Rfc3 | 5.4 | 6.8 | 7.9 | 6.1 | 7.1 | 4.4 | 7.3 | 5.5 | 6.8 | 6.6 | 6.7 | 5.8 |
| Rfc4 | 4.8 | 5.2 | 5.4 | 3.1 | 5.7 | 3.7 | 4.4 | 3.9 | 5.5 | 3.7 | 3.9 | 2.7 |
| Rfc5 | 2.7 | 3.0 | 4.9 | 2.0 | 3.6 | 1.7 | 5.7 | 4.7 | 3.9 | 3.1 | 3.3 | 3.4 |
| Rfesd | 6.2 | 4.8 | 3.8 | 5.6 | 4.1 | 6.8 | 4.1 | 3.8 | 4.6 | 5.9 | 5.9 | 6.3 |
| Rffl | 2.3 | 2.7 | 3.2 | 1.9 | 2.3 | 1.9 | 2.7 | 3.1 | 2.9 | 2.9 | 2.2 | 2.9 |
| Rfk | 23.7 | 18.1 | 15.3 | 27.0 | 18.3 | 27.1 | 15.7 | 14.3 | 14.4 | 18.1 | 19.0 | 18.1 |

| | | | | | | | | | | | | |
|---|---|---|---|---|---|---|---|---|---|---|---|---|
| Rfng | 2.2 | 3.0 | 2.7 | 2.3 | 2.2 | 2.7 | 2.5 | 2.0 | 2.4 | 2.2 | 2.4 | 2.2 |
| Rft1 | 9.7 | 8.5 | 9.6 | 9.8 | 8.5 | 9.3 | 7.8 | 8.0 | 8.7 | 8.3 | 9.3 | 9.6 |
| Rftn1 | 33.3 | 46.2 | 42.4 | 32.7 | 37.3 | 27.9 | 44.8 | 37.3 | 45.9 | 38.5 | 37.7 | 42.9 |
| Rftn2 | 4.2 | 3.3 | 3.5 | 6.5 | 3.4 | 5.0 | 3.9 | 4.4 | 3.8 | 2.9 | 4.2 | 4.5 |
| Rfwd2 | 31.6 | 27.9 | 24.3 | 28.3 | 25.3 | 31.2 | 22.9 | 23.9 | 25.1 | 25.8 | 30.5 | 26.1 |
| Rfwd3 | 8.2 | 9.6 | 9.5 | 7.8 | 9.3 | 7.7 | 8.1 | 8.0 | 8.4 | 7.9 | 8.4 | 7.6 |
| Rfx1 | 1.1 | 1.7 | 1.4 | 1.1 | 1.6 | 1.1 | 1.8 | 1.3 | 1.6 | 1.4 | 1.2 | 0.8 |
| Rfx2 | 0.2 | 0.1 | 0.2 | 0.0 | 0.1 | 0.1 | 0.1 | 0.1 | 0.1 | 0.1 | 0.1 | 0.1 |
| Rfx3 | 0.1 | 0.3 | 0.2 | 0.1 | 0.2 | 0.1 | 0.1 | 0.2 | 0.2 | 0.3 | 0.2 | 0.2 |
| Rfx5 | 4.2 | 5.0 | 7.1 | 5.0 | 5.2 | 5.1 | 5.0 | 5.0 | 5.1 | 4.8 | 4.2 | 4.6 |
| Rfx7 | 2.7 | 3.9 | 3.8 | 2.6 | 3.1 | 3.1 | 3.0 | 3.5 | 3.0 | 3.2 | 3.3 | 2.7 |
| Rfx8 | 0.0 | 0.1 | 0.0 | 0.1 | 0.0 | 0.0 | 0.0 | 0.0 | 0.0 | 0.0 | 0.0 | 0.0 |
| Rfxank | 1.2 | 1.6 | 1.7 | 1.2 | 1.4 | 1.6 | 1.2 | 1.6 | 1.3 | 1.2 | 1.1 | 1.4 |
| Rfxap | 4.0 | 3.1 | 3.2 | 3.9 | 3.0 | 3.8 | 4.1 | 3.1 | 3.6 | 4.2 | 4.8 | 4.5 |
| Rgag1 | 0.1 | 0.1 | 0.1 | 0.1 | 0.0 | 0.1 | 0.1 | 0.1 | 0.0 | 0.0 | 0.0 | 0.1 |
| Rgag4 | 2.1 | 2.0 | 1.7 | 2.3 | 3.2 | 2.9 | 1.8 | 0.9 | 1.6 | 1.3 | 1.7 | 1.4 |
| Rgcc | 0.7 | 2.8 | 5.6 | 0.3 | 0.7 | 0.2 | 1.2 | 4.2 | 3.3 | 2.2 | 1.5 | 2.3 |
| Rgl1 | 21.3 | 32.9 | 34.1 | 21.2 | 21.8 | 18.4 | 21.9 | 23.1 | 23.7 | 23.3 | 23.6 | 27.3 |
| Rgl2 | 7.1 | 6.4 | 5.2 | 8.1 | 8.1 | 6.5 | 6.2 | 5.2 | 6.0 | 5.3 | 5.1 | 4.5 |
| Rgl3 | 1.6 | 1.7 | 1.9 | 1.0 | 1.3 | 1.3 | 1.2 | 1.6 | 2.3 | 1.4 | 1.3 | 1.4 |
| Rgma | 0.7 | 1.7 | 2.4 | 0.8 | 0.9 | 0.8 | 1.1 | 2.4 | 1.4 | 0.8 | 0.9 | 0.9 |
| Rgmb | 4.4 | 5.6 | 5.5 | 4.5 | 4.5 | 5.8 | 5.5 | 5.5 | 5.6 | 6.4 | 4.8 | 5.4 |
| Rgn | 0.1 | 0.1 | 0.0 | 0.0 | 0.0 | 0.1 | 0.0 | 0.0 | 0.1 | 0.2 | 0.1 | 0.2 |
| Rgp1 | 4.6 | 4.9 | 4.5 | 4.4 | 4.9 | 5.1 | 5.0 | 4.5 | 5.0 | 4.4 | 5.0 | 4.4 |
| Rgs1 | 0.4 | 0.0 | 0.1 | 5.8 | 8.4 | 0.1 | 1.4 | 0.2 | 0.3 | 1.5 | 0.3 | 0.8 |
| Rgs10 | 16.4 | 14.9 | 21.0 | 27.2 | 30.4 | 19.8 | 18.1 | 18.6 | 17.4 | 17.9 | 18.5 | 16.8 |
| Rgs11 | 0.5 | 0.3 | 0.4 | 0.7 | 0.7 | 0.4 | 0.7 | 0.3 | 0.7 | 0.9 | 0.5 | 0.4 |
| Rgs12 | 9.9 | 7.8 | 7.2 | 9.9 | 12.1 | 8.1 | 11.4 | 5.8 | 7.5 | 8.4 | 7.5 | 7.4 |
| Rgs14 | 0.1 | 0.0 | 0.0 | 0.6 | 1.8 | 0.0 | 0.1 | 0.0 | 0.0 | 0.1 | 0.0 | 0.1 |
| Rgs16 | 1.4 | 1.7 | 3.7 | 1.5 | 3.9 | 1.1 | 4.1 | 2.6 | 3.1 | 9.1 | 3.6 | 4.8 |
| Rgs17 | 0.3 | 0.6 | 0.7 | 0.4 | 0.7 | 0.4 | 0.5 | 0.4 | 0.8 | 0.3 | 0.4 | 0.6 |
| Rgs18 | 0.0 | 0.0 | 0.0 | 1.4 | 1.7 | 0.0 | 0.1 | 0.0 | 0.0 | 0.1 | 0.0 | 0.0 |
| Rgs19 | 11.9 | 9.6 | 10.6 | 13.8 | 15.4 | 11.3 | 12.4 | 13.3 | 12.0 | 11.3 | 12.9 | 10.1 |
| Rgs2 | 0.7 | 0.3 | 0.3 | 4.8 | 6.9 | 0.2 | 0.8 | 0.3 | 0.4 | 0.9 | 0.2 | 0.6 |
| Rgs20 | 0.0 | 0.0 | 0.1 | 0.1 | 0.1 | 0.2 | 0.4 | 0.0 | 0.0 | 0.0 | 0.2 | 0.1 |
| Rgs22 | 0.0 | 0.0 | 0.0 | 0.0 | 0.0 | 0.0 | 0.0 | 0.0 | 0.0 | 0.0 | 0.0 | 0.0 |
| Rgs3 | 11.9 | 10.2 | 12.6 | 12.4 | 15.3 | 8.6 | 11.8 | 10.4 | 10.6 | 8.1 | 8.5 | 9.9 |
| Rgs4 | 7.3 | 8.9 | 13.6 | 5.5 | 5.4 | 6.8 | 3.3 | 10.1 | 5.9 | 2.7 | 5.5 | 3.8 |
| Rgs5 | 0.6 | 0.6 | 0.8 | 0.9 | 0.9 | 0.1 | 0.2 | 0.6 | 0.5 | 0.4 | 0.6 | 0.5 |
| Rgs6 | 0.2 | 0.1 | 0.2 | 0.2 | 0.1 | 0.2 | 0.0 | 0.1 | 0.1 | 0.0 | 0.0 | 0.1 |
| Rgs7 | 0.0 | 0.1 | 0.1 | 0.0 | 0.0 | 0.0 | 0.0 | 0.0 | 0.0 | 0.0 | 0.0 | 0.0 |
| Rgs7bp | 0.0 | 0.0 | 0.0 | 0.0 | 0.0 | 0.0 | 0.0 | 0.0 | 0.0 | 0.0 | 0.0 | 0.0 |
| Rgs8 | 0.0 | 0.0 | 0.0 | 0.0 | 0.0 | 0.0 | 0.0 | 0.0 | 0.0 | 0.0 | 0.0 | 0.0 |
| Rgs9 | 0.3 | 0.3 | 0.2 | 0.3 | 0.4 | 0.3 | 0.3 | 0.2 | 0.2 | 0.1 | 0.2 | 0.1 |
| Rgs9bp | 0.0 | 0.0 | 0.0 | 0.0 | 0.0 | 0.0 | 0.0 | 0.0 | 0.0 | 0.0 | 0.0 | 0.0 |
| Rgsl1 | 0.0 | 0.0 | 0.1 | 0.0 | 0.0 | 0.0 | 0.0 | 0.0 | 0.0 | 0.0 | 0.0 | 0.0 |
| Rhag | 0.0 | 0.0 | 0.0 | 0.0 | 0.0 | 0.0 | 0.0 | 0.0 | 0.0 | 0.0 | 0.0 | 0.0 |
| Rhbdd1 | 24.4 | 17.8 | 14.1 | 26.8 | 15.6 | 25.2 | 18.3 | 19.2 | 15.9 | 14.9 | 19.3 | 14.6 |
| Rhbdd2 | 2.7 | 2.8 | 2.2 | 2.6 | 2.2 | 3.0 | 2.6 | 1.9 | 2.2 | 2.3 | 2.1 | 2.3 |
| Rhbdd3 | 4.1 | 3.6 | 2.6 | 3.4 | 3.2 | 4.3 | 3.2 | 2.9 | 4.3 | 3.3 | 2.9 | 3.4 |
| Rhbdf1 | 15.3 | 15.2 | 15.1 | 16.8 | 16.2 | 13.9 | 23.8 | 23.0 | 24.0 | 16.7 | 17.9 | 15.1 |
| Rhbdf2 | 4.9 | 5.1 | 5.0 | 4.0 | 6.7 | 3.8 | 3.7 | 3.5 | 2.5 | 4.1 | 3.8 | 3.2 |
| Rhbdl1 | 2.8 | 4.1 | 3.5 | 2.0 | 1.8 | 2.6 | 1.8 | 1.9 | 2.3 | 3.9 | 2.6 | 2.6 |
| Rhbdl2 | 0.5 | 0.3 | 0.8 | 0.4 | 0.8 | 0.4 | 0.2 | 0.1 | 0.2 | 0.2 | 0.2 | 0.2 |
| Rhbdl3 | 0.4 | 0.5 | 0.7 | 0.2 | 0.3 | 0.5 | 0.4 | 0.7 | 0.5 | 1.0 | 0.7 | 0.9 |
| Rhd | 0.2 | 0.1 | 0.1 | 0.1 | 0.2 | 0.1 | 0.2 | 0.1 | 0.1 | 0.1 | 0.0 | 0.0 |
| Rheb | 51.2 | 42.8 | 40.5 | 57.4 | 47.6 | 49.6 | 46.8 | 37.1 | 47.9 | 53.4 | 51.2 | 51.4 |

| | | | | | | | | | | | | |
|---|---|---|---|---|---|---|---|---|---|---|---|---|
| Rhebl1 | 0.7 | 1.0 | 0.9 | 1.0 | 0.9 | 1.1 | 0.5 | 0.7 | 1.3 | 0.9 | 0.9 | 0.8 |
| Rhno1 | 6.1 | 6.1 | 7.2 | 6.6 | 6.5 | 7.2 | 8.4 | 7.6 | 6.5 | 6.7 | 7.5 | 6.7 |
| Rho | 0.1 | 0.1 | 0.0 | 0.0 | 0.0 | 0.1 | 0.0 | 0.1 | 0.1 | 0.1 | 0.1 | 0.1 |
| Rhoa | 148.3 | 127.7 | 126.7 | 196.2 | 146.6 | 165.4 | 182.3 | 167.6 | 157.1 | 146.7 | 197.0 | 168.7 |
| Rhob | 29.0 | 28.3 | 24.5 | 32.3 | 33.9 | 26.1 | 32.4 | 38.5 | 41.0 | 33.7 | 30.0 | 23.9 |
| Rhobtb1 | 1.9 | 3.1 | 4.9 | 1.7 | 1.5 | 1.7 | 1.3 | 3.0 | 1.9 | 1.7 | 1.5 | 2.0 |
| Rhobtb2 | 1.9 | 2.0 | 2.2 | 1.8 | 2.0 | 2.0 | 1.9 | 2.3 | 2.1 | 2.4 | 2.1 | 2.1 |
| Rhobtb3 | 16.3 | 16.4 | 14.1 | 15.2 | 10.3 | 19.2 | 11.6 | 14.1 | 15.9 | 11.6 | 15.0 | 13.4 |
| Rhoc | 163.9 | 121.6 | 105.3 | 184.0 | 135.9 | 174.1 | 160.4 | 131.0 | 126.6 | 144.8 | 159.6 | 148.4 |
| Rhod | 22.5 | 18.5 | 16.8 | 21.2 | 20.4 | 23.0 | 18.5 | 15.9 | 18.9 | 21.2 | 18.0 | 20.4 |
| Rhof | 0.1 | 0.2 | 0.2 | 0.5 | 0.9 | 0.2 | 0.4 | 0.3 | 0.2 | 0.3 | 0.1 | 0.1 |
| Rhog | 16.4 | 17.5 | 15.1 | 21.6 | 26.7 | 19.7 | 17.9 | 16.2 | 15.8 | 17.1 | 15.8 | 15.9 |
| Rhoh | 0.0 | 0.0 | 0.0 | 0.1 | 0.1 | 0.0 | 0.0 | 0.0 | 0.0 | 0.0 | 0.0 | 0.0 |
| Rhoj | 38.1 | 29.5 | 31.7 | 29.7 | 32.0 | 25.6 | 32.9 | 31.1 | 31.0 | 49.7 | 45.6 | 40.5 |
| Rhoq | 44.1 | 41.8 | 40.7 | 50.0 | 45.5 | 50.1 | 41.5 | 43.7 | 41.5 | 37.7 | 45.1 | 46.3 |
| Rhot1 | 13.4 | 13.3 | 11.4 | 13.3 | 12.7 | 13.1 | 12.1 | 13.2 | 13.5 | 14.0 | 13.3 | 13.9 |
| Rhot2 | 5.1 | 5.5 | 5.2 | 5.8 | 4.8 | 6.0 | 4.6 | 4.0 | 5.1 | 4.7 | 4.8 | 4.0 |
| Rhou | 9.6 | 11.8 | 16.7 | 7.2 | 9.2 | 8.4 | 9.5 | 17.2 | 15.7 | 17.6 | 14.4 | 15.7 |
| Rhov | 0.1 | 0.0 | 0.1 | 0.5 | 0.4 | 0.1 | 0.0 | 0.0 | 0.0 | 0.1 | 0.0 | 0.0 |
| Rhox5 | 19.9 | 27.5 | 23.3 | 30.9 | 29.3 | 39.4 | 37.5 | 29.4 | 31.1 | 14.0 | 31.2 | 17.8 |
| Rhpn1 | 0.2 | 0.4 | 0.1 | 0.1 | 0.1 | 0.3 | 0.2 | 0.2 | 0.1 | 0.4 | 0.2 | 0.0 |
| Rhpn2 | 0.8 | 0.5 | 0.6 | 0.3 | 0.2 | 0.2 | 0.2 | 0.7 | 0.3 | 1.2 | 0.8 | 0.5 |
| Rian | 31.4 | 26.6 | 25.4 | 32.6 | 27.3 | 32.8 | 42.3 | 43.3 | 56.4 | 55.3 | 65.6 | 41.9 |
| Ribc1 | 0.8 | 1.0 | 0.8 | 1.2 | 0.9 | 0.8 | 0.7 | 1.1 | 0.7 | 1.0 | 1.0 | 0.9 |
| Ribc2 | 0.1 | 0.1 | 0.1 | 0.0 | 0.0 | 0.2 | 0.0 | 0.1 | 0.0 | 0.0 | 0.1 | 0.1 |
| Ric3 | 1.8 | 0.8 | 0.8 | 1.9 | 0.7 | 1.7 | 1.2 | 0.8 | 0.5 | 0.4 | 0.5 | 0.4 |
| Ric8 | 24.4 | 19.6 | 15.9 | 25.2 | 19.8 | 24.2 | 21.5 | 20.7 | 20.9 | 21.2 | 21.6 | 19.6 |
| Ric8b | 4.0 | 4.1 | 3.8 | 3.7 | 3.7 | 3.8 | 3.8 | 3.8 | 3.6 | 3.6 | 3.7 | 4.0 |
| Rictor | 7.0 | 7.6 | 6.8 | 6.5 | 6.8 | 6.8 | 7.3 | 6.3 | 7.6 | 7.1 | 7.3 | 7.8 |
| Rif1 | 3.2 | 4.1 | 4.7 | 2.3 | 3.2 | 2.9 | 3.0 | 3.4 | 3.3 | 2.8 | 3.5 | 3.5 |
| Riiad1 | 7.6 | 5.7 | 3.3 | 7.9 | 5.4 | 6.4 | 3.9 | 3.1 | 3.9 | 4.0 | 3.4 | 3.1 |
| Rilp | 1.5 | 0.8 | 0.9 | 1.3 | 1.0 | 1.0 | 1.4 | 1.3 | 1.4 | 0.7 | 1.9 | 1.3 |
| Rilpl1 | 17.9 | 16.2 | 17.6 | 19.8 | 12.6 | 21.4 | 16.2 | 16.3 | 17.3 | 16.3 | 16.8 | 18.3 |
| Rilpl2 | 9.7 | 9.4 | 8.6 | 10.6 | 12.0 | 9.3 | 9.3 | 11.3 | 10.0 | 9.7 | 7.3 | 9.1 |
| Rimbp3 | 0.0 | 0.0 | 0.0 | 0.0 | 0.0 | 0.0 | 0.0 | 0.0 | 0.0 | 0.0 | 0.0 | 0.0 |
| Rimkla | 0.0 | 0.0 | 0.0 | 0.0 | 0.0 | 0.0 | 0.0 | 0.0 | 0.0 | 0.0 | 0.0 | 0.0 |
| Rimklb | 0.0 | 0.0 | 0.0 | 0.0 | 0.0 | 0.0 | 0.0 | 0.0 | 0.0 | 0.0 | 0.0 | 0.0 |
| Rims1 | 0.3 | 0.2 | 0.3 | 0.3 | 0.2 | 0.3 | 0.1 | 0.1 | 0.1 | 0.0 | 0.1 | 0.1 |
| Rims2 | 0.0 | 0.0 | 0.0 | 0.0 | 0.0 | 0.0 | 0.0 | 0.0 | 0.0 | 0.0 | 0.0 | 0.0 |
| Rims3 | 0.1 | 0.1 | 0.0 | 0.0 | 0.1 | 0.2 | 0.0 | 0.0 | 0.1 | 0.0 | 0.0 | 0.0 |
| Rims4 | 0.0 | 0.1 | 0.1 | 0.0 | 0.1 | 0.0 | 0.1 | 0.1 | 0.0 | 0.0 | 0.0 | 0.0 |
| Rin1 | 6.3 | 3.2 | 2.3 | 5.2 | 3.4 | 4.9 | 4.3 | 3.5 | 2.7 | 5.4 | 4.9 | 3.3 |
| Rin2 | 4.1 | 4.9 | 7.3 | 4.3 | 4.6 | 4.0 | 3.1 | 5.4 | 3.8 | 6.4 | 4.4 | 6.1 |
| Rin3 | 0.3 | 0.2 | 0.2 | 0.8 | 1.4 | 0.2 | 0.9 | 0.7 | 0.6 | 0.3 | 0.6 | 0.5 |
| Ring1 | 5.9 | 5.2 | 6.1 | 5.8 | 5.2 | 5.6 | 5.5 | 6.5 | 7.4 | 5.8 | 5.0 | 4.1 |
| Rinl | 0.3 | 0.3 | 0.2 | 1.7 | 2.4 | 0.1 | 0.5 | 0.2 | 0.1 | 0.3 | 0.2 | 0.3 |
| Rint1 | 10.2 | 11.0 | 9.0 | 11.1 | 9.3 | 10.6 | 8.8 | 9.4 | 8.8 | 9.8 | 9.7 | 10.0 |
| Riok1 | 8.2 | 7.8 | 9.1 | 7.5 | 6.9 | 7.6 | 8.5 | 8.0 | 7.7 | 8.7 | 9.6 | 9.1 |
| Riok2 | 13.4 | 14.3 | 11.7 | 14.8 | 10.1 | 13.9 | 10.4 | 9.4 | 8.2 | 11.0 | 11.4 | 10.4 |
| Riok3 | 29.9 | 27.2 | 23.0 | 34.0 | 32.6 | 29.9 | 23.1 | 21.5 | 23.3 | 24.1 | 25.4 | 24.2 |
| Ripk1 | 15.0 | 13.7 | 15.0 | 16.0 | 15.0 | 13.6 | 15.3 | 16.1 | 14.8 | 14.0 | 16.5 | 17.8 |
| Ripk2 | 13.1 | 14.0 | 14.5 | 13.9 | 24.0 | 11.5 | 23.7 | 13.7 | 19.3 | 18.6 | 18.7 | 18.8 |
| Ripk3 | 12.7 | 12.6 | 9.2 | 12.8 | 14.0 | 12.8 | 14.2 | 8.5 | 11.5 | 16.1 | 13.0 | 11.8 |
| Ripk4 | 0.0 | 0.0 | 0.0 | 0.0 | 0.0 | 0.0 | 0.0 | 0.0 | 0.0 | 0.0 | 0.0 | 0.0 |
| Ripply3 | 0.1 | 0.3 | 0.2 | 0.1 | 0.1 | 0.0 | 0.1 | 0.1 | 0.1 | 0.0 | 0.0 | 0.0 |
| Rit1 | 18.3 | 15.9 | 15.3 | 22.4 | 16.7 | 20.2 | 19.2 | 15.8 | 17.7 | 18.2 | 18.9 | 17.7 |
| Rit2 | 0.0 | 0.0 | 0.0 | 0.0 | 0.0 | 0.0 | 0.0 | 0.0 | 0.0 | 0.0 | 0.0 | 0.0 |

| | | | | | | | | | | | | |
|---|---|---|---|---|---|---|---|---|---|---|---|---|
| Rlf | 7.1 | 7.8 | 7.2 | 6.3 | 7.2 | 7.0 | 6.6 | 7.0 | 8.2 | 7.2 | 7.6 | 8.5 |
| Rlim | 16.0 | 17.2 | 17.8 | 19.0 | 18.3 | 17.2 | 16.4 | 19.2 | 16.5 | 18.6 | 20.3 | 20.1 |
| Rln1 | 0.2 | 0.5 | 0.5 | 0.1 | 0.4 | 0.3 | 0.0 | 0.1 | 0.1 | 0.0 | 0.3 | 0.0 |
| Rln3 | 0.2 | 0.0 | 0.0 | 0.6 | 0.2 | 0.0 | 0.0 | 0.1 | 0.1 | 0.0 | 0.0 | 0.1 |
| Rltpr | 0.0 | 0.0 | 0.0 | 0.0 | 0.0 | 0.0 | 0.1 | 0.1 | 0.1 | 0.1 | 0.0 | 0.0 |
| Rmdn1 | 5.1 | 5.3 | 3.9 | 4.3 | 3.9 | 5.3 | 3.7 | 3.9 | 3.9 | 5.0 | 4.7 | 4.8 |
| Rmdn2 | 6.0 | 6.1 | 9.6 | 8.1 | 7.3 | 5.4 | 8.9 | 6.1 | 7.4 | 7.3 | 7.9 | 9.5 |
| Rmdn3 | 11.3 | 11.5 | 10.8 | 13.2 | 10.1 | 13.8 | 12.3 | 10.1 | 10.4 | 12.6 | 11.8 | 10.5 |
| Rmi1 | 4.3 | 4.9 | 5.1 | 5.8 | 6.2 | 4.5 | 4.2 | 5.0 | 5.1 | 5.2 | 5.2 | 5.4 |
| Rmi2 | 0.0 | 0.1 | 0.1 | 0.0 | 0.1 | 0.0 | 0.1 | 0.1 | 0.1 | 0.0 | 0.1 | 0.0 |
| Rmnd1 | 5.0 | 4.8 | 4.1 | 5.6 | 4.2 | 5.0 | 4.6 | 4.1 | 4.1 | 4.0 | 5.1 | 5.1 |
| Rmnd5a | 10.9 | 12.6 | 12.6 | 10.3 | 12.1 | 11.3 | 9.9 | 12.0 | 13.4 | 11.3 | 12.4 | 12.4 |
| Rmnd5b | 10.6 | 11.2 | 8.1 | 11.3 | 10.1 | 11.0 | 11.0 | 8.7 | 10.0 | 9.9 | 9.6 | 8.9 |
| Rmrp | 13901.2 | 12873.3 | 13315.8 | 7095.6 | 13618.9 | 8687.1 | 9228.5 | 14497.7 | 21791.7 | 16337.5 | 7835.1 | 9254.6 |
| Rn4.5s | 785.0 | 1010.3 | 1075.9 | 651.9 | 1659.5 | 692.1 | 868.7 | 1406.8 | 3124.0 | 1492.8 | 650.5 | 888.4 |
| Rn45s | 12.1 | 18.1 | 14.5 | 20.3 | 18.2 | 16.1 | 13.5 | 23.7 | 48.6 | 31.2 | 20.5 | 13.8 |
| Rnase1 | 0.2 | 0.0 | 0.1 | 0.0 | 0.0 | 0.0 | 0.1 | 0.0 | 0.1 | 0.0 | 0.1 | 0.0 |
| Rnase10 | 0.6 | 0.8 | 1.2 | 0.6 | 0.6 | 0.4 | 0.4 | 0.4 | 0.6 | 0.7 | 0.7 | 0.5 |
| Rnase4 | 158.6 | 144.0 | 147.7 | 127.1 | 160.1 | 120.9 | 138.8 | 167.1 | 127.2 | 148.7 | 160.5 | 132.7 |
| Rnase6 | 0.1 | 0.0 | 0.0 | 1.1 | 1.9 | 0.0 | 0.1 | 0.1 | 0.1 | 0.0 | 0.0 | 0.2 |
| Rnaseh1 | 8.6 | 8.8 | 10.0 | 10.7 | 8.4 | 8.7 | 10.6 | 8.1 | 8.1 | 8.5 | 10.5 | 9.2 |
| Rnaseh2a | 4.4 | 4.4 | 5.3 | 4.3 | 4.0 | 4.0 | 5.0 | 5.8 | 4.8 | 5.5 | 4.8 | 5.0 |
| Rnaseh2b | 8.6 | 7.9 | 8.9 | 7.8 | 8.8 | 6.2 | 9.1 | 9.3 | 8.6 | 6.7 | 7.9 | 7.6 |
| Rnaseh2c | 23.9 | 27.8 | 20.5 | 21.5 | 25.6 | 25.0 | 20.3 | 20.4 | 25.1 | 25.8 | 19.5 | 20.4 |
| Rnasek | 92.4 | 80.1 | 71.0 | 116.9 | 92.7 | 98.6 | 85.3 | 71.2 | 70.2 | 82.1 | 70.3 | 76.5 |
| Rnasel | 2.0 | 2.0 | 3.3 | 3.1 | 2.2 | 2.5 | 1.5 | 1.7 | 1.6 | 2.2 | 2.2 | 2.3 |
| Rnaset2a | 41.0 | 37.4 | 35.9 | 67.8 | 54.0 | 48.2 | 44.2 | 38.2 | 32.7 | 36.7 | 38.4 | 41.4 |
| Rnaset2b | 27.4 | 18.7 | 18.4 | 32.8 | 25.6 | 25.1 | 22.5 | 19.1 | 17.9 | 19.9 | 19.3 | 20.4 |
| Rnd1 | 30.4 | 20.5 | 13.5 | 29.8 | 18.9 | 27.8 | 17.0 | 6.0 | 12.4 | 21.9 | 18.7 | 16.5 |
| Rnd2 | 5.7 | 8.2 | 5.3 | 5.9 | 4.2 | 6.6 | 5.5 | 5.9 | 4.6 | 3.8 | 4.4 | 4.8 |
| Rnd3 | 71.4 | 55.3 | 46.3 | 85.0 | 52.8 | 67.1 | 57.4 | 46.2 | 57.1 | 100.0 | 83.4 | 65.6 |
| Rnf10 | 29.6 | 29.8 | 24.8 | 29.7 | 25.5 | 31.8 | 28.8 | 26.2 | 27.5 | 24.3 | 26.2 | 23.6 |
| Rnf103 | 10.3 | 9.9 | 9.1 | 10.8 | 9.7 | 10.1 | 9.8 | 8.7 | 10.2 | 9.6 | 9.4 | 10.9 |
| Rnf11 | 66.7 | 62.3 | 54.4 | 86.6 | 63.5 | 79.9 | 62.1 | 52.8 | 57.9 | 59.2 | 73.2 | 66.6 |
| Rnf111 | 7.5 | 8.3 | 7.4 | 7.7 | 8.3 | 6.9 | 9.0 | 7.3 | 8.1 | 7.7 | 9.0 | 7.9 |
| Rnf112 | 0.1 | 0.0 | 0.1 | 0.0 | 0.1 | 0.0 | 0.0 | 0.2 | 0.1 | 0.1 | 0.2 | 0.1 |
| Rnf113a1 | 1.8 | 1.2 | 1.8 | 2.1 | 1.6 | 2.3 | 1.8 | 1.4 | 1.0 | 1.5 | 2.0 | 1.3 |
| Rnf113a2 | 7.1 | 8.3 | 8.3 | 6.8 | 7.9 | 7.2 | 8.6 | 7.1 | 6.9 | 9.1 | 9.1 | 10.8 |
| Rnf114 | 14.9 | 13.5 | 13.9 | 15.9 | 15.2 | 13.7 | 13.2 | 12.9 | 14.9 | 14.9 | 14.5 | 16.1 |
| Rnf115 | 11.3 | 11.3 | 13.2 | 14.6 | 16.6 | 12.3 | 11.7 | 12.6 | 11.7 | 14.2 | 13.9 | 16.3 |
| Rnf121 | 8.2 | 7.9 | 8.2 | 9.0 | 9.7 | 9.4 | 9.0 | 9.9 | 9.3 | 9.1 | 8.8 | 9.4 |
| Rnf122 | 1.2 | 1.4 | 1.6 | 1.2 | 1.2 | 1.3 | 1.1 | 1.5 | 1.3 | 1.8 | 1.2 | 1.5 |
| Rnf123 | 7.3 | 6.1 | 5.5 | 7.1 | 6.5 | 6.9 | 6.7 | 6.2 | 6.8 | 5.7 | 5.7 | 6.1 |
| Rnf125 | 0.7 | 1.1 | 1.5 | 0.7 | 0.9 | 1.2 | 1.3 | 1.6 | 1.0 | 1.0 | 1.0 | 1.7 |
| Rnf126 | 7.6 | 7.7 | 7.2 | 6.6 | 8.4 | 7.9 | 10.7 | 9.1 | 10.7 | 9.3 | 7.5 | 7.3 |
| Rnf128 | 3.5 | 0.8 | 0.6 | 9.4 | 13.4 | 2.2 | 2.4 | 0.9 | 1.7 | 5.7 | 5.1 | 4.0 |
| Rnf13 | 44.4 | 37.8 | 42.0 | 59.1 | 46.7 | 53.9 | 41.8 | 39.9 | 41.3 | 38.5 | 47.8 | 47.3 |
| Rnf130 | 36.7 | 35.9 | 30.9 | 45.8 | 41.9 | 43.5 | 29.6 | 27.2 | 27.8 | 30.4 | 30.0 | 34.4 |
| Rnf135 | 4.5 | 4.7 | 4.4 | 5.5 | 5.4 | 4.8 | 3.9 | 4.2 | 4.3 | 4.1 | 3.8 | 3.5 |
| Rnf138 | 3.8 | 4.2 | 4.3 | 3.4 | 3.2 | 4.3 | 3.1 | 3.5 | 3.7 | 4.6 | 4.2 | 3.9 |
| Rnf139 | 6.2 | 6.5 | 6.9 | 5.3 | 6.5 | 7.2 | 6.5 | 7.0 | 7.5 | 8.0 | 7.9 | 7.5 |
| Rnf14 | 33.9 | 31.3 | 26.9 | 32.3 | 29.1 | 32.3 | 28.0 | 24.2 | 29.4 | 26.8 | 28.2 | 26.9 |
| Rnf141 | 30.2 | 26.0 | 21.1 | 30.9 | 22.7 | 32.4 | 19.5 | 16.4 | 20.0 | 18.4 | 20.5 | 21.3 |
| Rnf144a | 0.2 | 0.1 | 0.3 | 0.2 | 0.2 | 0.0 | 0.2 | 0.5 | 0.4 | 0.2 | 0.1 | 0.1 |
| Rnf144b | 0.3 | 0.1 | 0.1 | 0.3 | 0.1 | 0.1 | 0.0 | 0.0 | 0.1 | 0.2 | 0.2 | 0.1 |
| Rnf145 | 17.9 | 12.7 | 12.2 | 14.8 | 16.2 | 13.4 | 20.3 | 14.5 | 17.7 | 16.9 | 20.4 | 16.0 |
| Rnf146 | 12.7 | 13.3 | 14.3 | 14.3 | 12.2 | 14.4 | 13.4 | 13.6 | 12.4 | 13.1 | 15.0 | 14.1 |

| | | | | | | | | | | | | |
|---|---|---|---|---|---|---|---|---|---|---|---|---|
| Rnf149 | 23.9 | 19.8 | 19.1 | 34.0 | 33.8 | 23.0 | 23.6 | 17.6 | 18.3 | 28.8 | 23.5 | 26.6 |
| Rnf150 | 2.9 | 3.7 | 5.7 | 4.2 | 5.5 | 2.3 | 3.9 | 5.2 | 4.5 | 4.0 | 2.9 | 4.1 |
| Rnf151 | 0.0 | 0.0 | 0.1 | 0.1 | 0.0 | 0.1 | 0.0 | 0.0 | 0.1 | 0.1 | 0.0 | 0.0 |
| Rnf152 | 0.0 | 0.0 | 0.0 | 0.0 | 0.0 | 0.0 | 0.1 | 0.0 | 0.0 | 0.0 | 0.0 | 0.1 |
| Rnf157 | 1.0 | 1.5 | 1.4 | 1.6 | 1.1 | 1.9 | 1.1 | 0.9 | 0.9 | 0.9 | 1.1 | 1.1 |
| Rnf165 | 0.0 | 0.0 | 0.0 | 0.0 | 0.0 | 0.0 | 0.0 | 0.0 | 0.0 | 0.0 | 0.0 | 0.0 |
| Rnf166 | 16.4 | 16.2 | 16.5 | 17.5 | 15.5 | 20.4 | 15.8 | 15.2 | 15.3 | 15.9 | 16.3 | 13.3 |
| Rnf167 | 11.0 | 11.2 | 9.2 | 11.6 | 9.5 | 12.2 | 9.7 | 10.4 | 9.6 | 11.5 | 8.6 | 11.1 |
| Rnf168 | 6.1 | 5.9 | 6.9 | 6.7 | 6.3 | 5.9 | 6.6 | 6.3 | 6.0 | 6.1 | 7.6 | 6.9 |
| Rnf169 | 7.2 | 8.3 | 8.0 | 6.7 | 7.2 | 7.4 | 6.4 | 5.9 | 7.4 | 8.3 | 7.0 | 6.7 |
| Rnf17 | 1.1 | 1.0 | 1.1 | 0.8 | 0.9 | 1.6 | 0.8 | 0.9 | 0.8 | 1.0 | 1.1 | 0.8 |
| Rnf170 | 8.9 | 9.6 | 11.1 | 9.2 | 7.8 | 7.5 | 7.4 | 9.8 | 8.9 | 7.1 | 9.0 | 9.7 |
| Rnf180 | 0.1 | 0.0 | 0.0 | 0.4 | 0.6 | 0.0 | 0.1 | 0.0 | 0.0 | 0.1 | 0.0 | 0.1 |
| Rnf181 | 118.4 | 135.3 | 129.4 | 111.6 | 114.4 | 117.7 | 140.4 | 144.6 | 143.3 | 138.4 | 144.0 | 137.5 |
| Rnf182 | 0.2 | 0.2 | 0.2 | 0.1 | 0.2 | 0.4 | 0.1 | 0.1 | 0.1 | 0.1 | 0.2 | 0.1 |
| Rnf183 | 0.0 | 0.0 | 0.0 | 0.0 | 0.2 | 0.1 | 0.1 | 0.0 | 0.0 | 0.1 | 0.0 | 0.0 |
| Rnf185 | 9.9 | 7.8 | 7.5 | 9.8 | 7.7 | 9.7 | 7.8 | 7.7 | 7.5 | 9.1 | 9.0 | 9.0 |
| Rnf187 | 51.2 | 38.5 | 33.0 | 46.5 | 37.1 | 48.6 | 39.1 | 38.4 | 37.5 | 43.4 | 36.0 | 39.4 |
| Rnf19a | 13.3 | 13.2 | 13.2 | 13.7 | 14.2 | 11.5 | 14.6 | 15.0 | 15.4 | 15.0 | 15.9 | 15.2 |
| Rnf19b | 13.8 | 15.0 | 15.0 | 18.4 | 24.0 | 10.3 | 19.6 | 18.5 | 21.9 | 17.2 | 17.0 | 20.5 |
| Rnf2 | 9.6 | 12.2 | 15.8 | 10.4 | 10.0 | 9.8 | 10.9 | 12.2 | 12.1 | 12.2 | 14.2 | 14.8 |
| Rnf20 | 30.0 | 26.7 | 28.3 | 32.0 | 24.6 | 27.7 | 27.9 | 23.1 | 22.0 | 26.0 | 31.5 | 32.1 |
| Rnf207 | 0.4 | 0.4 | 0.6 | 0.4 | 0.2 | 0.7 | 0.2 | 0.3 | 0.3 | 0.3 | 0.3 | 0.5 |
| Rnf208 | 0.1 | 0.1 | 0.0 | 0.0 | 0.0 | 0.0 | 0.0 | 0.0 | 0.1 | 0.1 | 0.1 | 0.0 |
| Rnf214 | 9.7 | 11.2 | 12.6 | 11.6 | 9.3 | 10.3 | 11.8 | 10.5 | 10.1 | 9.2 | 11.0 | 12.0 |
| Rnf215 | 12.5 | 11.5 | 11.7 | 12.0 | 11.9 | 12.5 | 15.9 | 14.5 | 16.7 | 12.6 | 12.2 | 13.3 |
| Rnf216 | 7.2 | 8.8 | 8.6 | 7.1 | 7.7 | 7.5 | 8.4 | 8.2 | 7.5 | 8.3 | 8.2 | 7.7 |
| Rnf217 | 5.7 | 4.4 | 3.8 | 4.2 | 3.5 | 4.6 | 4.6 | 4.3 | 4.9 | 5.5 | 4.9 | 4.9 |
| Rnf219 | 3.2 | 3.1 | 3.3 | 2.4 | 3.1 | 2.6 | 3.3 | 3.2 | 3.0 | 2.9 | 2.9 | 3.2 |
| Rnf220 | 5.8 | 6.1 | 6.3 | 6.8 | 5.7 | 6.3 | 6.7 | 6.3 | 6.2 | 5.1 | 5.8 | 5.0 |
| Rnf222 | 0.0 | 0.0 | 0.0 | 0.0 | 0.0 | 0.0 | 0.0 | 0.0 | 0.0 | 0.0 | 0.0 | 0.0 |
| Rnf223 | 0.1 | 0.0 | 0.1 | 0.0 | 0.0 | 0.0 | 0.0 | 0.0 | 0.1 | 0.0 | 0.0 | 0.1 |
| Rnf24 | 2.3 | 2.3 | 1.9 | 2.6 | 2.4 | 2.6 | 1.9 | 1.7 | 1.6 | 2.0 | 2.2 | 1.5 |
| Rnf25 | 17.8 | 15.4 | 14.9 | 17.5 | 13.7 | 17.2 | 14.0 | 14.6 | 12.4 | 14.9 | 14.1 | 14.9 |
| Rnf26 | 9.4 | 10.1 | 9.3 | 8.0 | 9.7 | 10.5 | 11.3 | 9.9 | 9.4 | 7.9 | 7.8 | 8.1 |
| Rnf31 | 3.2 | 3.6 | 4.1 | 3.6 | 4.0 | 3.6 | 3.4 | 3.6 | 4.0 | 3.1 | 3.1 | 2.9 |
| Rnf32 | 0.5 | 0.6 | 0.7 | 0.9 | 0.1 | 0.6 | 0.1 | 0.3 | 0.2 | 0.3 | 0.2 | 0.2 |
| Rnf34 | 14.8 | 15.0 | 15.9 | 15.7 | 14.3 | 16.9 | 14.6 | 17.4 | 15.2 | 16.0 | 18.9 | 17.2 |
| Rnf38 | 3.0 | 3.4 | 3.6 | 4.3 | 3.7 | 3.8 | 3.8 | 3.4 | 3.1 | 2.8 | 3.4 | 3.0 |
| Rnf39 | 0.2 | 0.1 | 0.1 | 0.1 | 0.1 | 0.1 | 0.2 | 0.1 | 0.0 | 0.2 | 0.1 | 0.2 |
| Rnf4 | 36.1 | 35.6 | 32.8 | 36.3 | 34.3 | 32.5 | 36.7 | 40.2 | 40.8 | 36.1 | 40.7 | 37.6 |
| Rnf40 | 9.3 | 10.8 | 11.9 | 10.1 | 11.0 | 9.8 | 11.9 | 12.0 | 11.0 | 11.9 | 10.2 | 11.5 |
| Rnf41 | 11.8 | 11.0 | 9.1 | 12.0 | 11.3 | 11.8 | 11.1 | 10.3 | 11.6 | 11.5 | 11.6 | 11.0 |
| Rnf43 | 0.0 | 0.0 | 0.1 | 0.0 | 0.0 | 0.0 | 0.0 | 0.0 | 0.0 | 0.0 | 0.0 | 0.0 |
| Rnf44 | 3.7 | 4.7 | 4.7 | 3.8 | 4.7 | 4.3 | 5.1 | 4.3 | 5.2 | 4.1 | 4.4 | 3.3 |
| Rnf5 | 7.4 | 9.1 | 9.3 | 8.4 | 10.6 | 9.7 | 9.0 | 8.0 | 7.9 | 9.5 | 12.2 | 8.3 |
| Rnf6 | 25.4 | 28.1 | 30.5 | 27.5 | 26.0 | 24.8 | 25.8 | 27.6 | 27.3 | 24.3 | 30.7 | 28.8 |
| Rnf7 | 47.1 | 45.5 | 42.6 | 54.5 | 45.3 | 54.7 | 46.7 | 43.2 | 46.4 | 51.5 | 52.0 | 52.3 |
| Rnf8 | 8.8 | 8.9 | 7.4 | 6.8 | 6.9 | 8.3 | 7.5 | 7.0 | 7.4 | 8.2 | 7.9 | 7.1 |
| Rnft1 | 9.3 | 10.5 | 11.1 | 9.8 | 9.7 | 8.8 | 11.0 | 10.7 | 12.1 | 12.8 | 14.1 | 13.3 |
| Rnft2 | 0.6 | 0.7 | 0.5 | 0.4 | 0.4 | 0.9 | 0.4 | 0.7 | 0.4 | 0.5 | 0.5 | 0.4 |
| Rngtt | 4.9 | 5.6 | 5.7 | 4.9 | 5.4 | 5.6 | 4.0 | 4.7 | 4.9 | 4.9 | 5.3 | 5.3 |
| Rnh1 | 67.6 | 51.4 | 38.7 | 73.4 | 67.2 | 57.4 | 52.0 | 55.4 | 57.1 | 59.1 | 54.7 | 51.7 |
| Rnls | 1.5 | 1.1 | 1.0 | 1.0 | 1.2 | 0.8 | 1.4 | 1.0 | 0.9 | 1.0 | 1.2 | 1.0 |
| Rnmt | 7.6 | 7.5 | 6.4 | 8.4 | 6.8 | 7.5 | 6.0 | 6.0 | 5.9 | 8.1 | 7.7 | 7.3 |
| Rnmtl1 | 3.4 | 3.6 | 2.9 | 4.2 | 2.9 | 4.4 | 3.1 | 3.0 | 2.3 | 3.1 | 2.7 | 2.7 |
| Rnpc3 | 5.2 | 5.6 | 5.4 | 6.3 | 5.8 | 4.9 | 5.0 | 5.5 | 6.0 | 5.3 | 7.7 | 6.8 |

| | | | | | | | | | | | | |
|---|---|---|---|---|---|---|---|---|---|---|---|---|
| Rnpep | 14.8 | 13.9 | 12.0 | 16.1 | 17.9 | 13.7 | 13.8 | 13.2 | 12.1 | 15.5 | 13.0 | 12.5 |
| Rnpepl1 | 1.8 | 2.1 | 2.2 | 2.1 | 2.6 | 1.9 | 2.3 | 2.6 | 2.7 | 2.2 | 2.3 | 1.6 |
| Rnps1 | 30.7 | 32.5 | 30.5 | 32.0 | 30.0 | 32.2 | 36.5 | 32.8 | 29.9 | 28.6 | 34.5 | 28.4 |
| Rnu11 | 40.6 | 14.2 | 41.7 | 14.5 | 42.8 | 43.7 | 44.4 | 70.5 | 52.6 | 78.6 | 56.7 | 66.4 |
| Rnu12 | 214.5 | 261.0 | 685.4 | 193.7 | 319.8 | 167.8 | 212.9 | 367.6 | 491.1 | 255.2 | 204.1 | 463.4 |
| Rnu73b | 441.8 | 1135.7 | 542.2 | 189.5 | 917.6 | 0.0 | 72.6 | 606.3 | 2156.2 | 1242.8 | 0.0 | 977.8 |
| Robo1 | 8.1 | 8.6 | 10.3 | 10.4 | 11.1 | 10.5 | 11.4 | 7.2 | 6.5 | 5.6 | 6.4 | 7.4 |
| Robo2 | 0.3 | 0.1 | 0.1 | 0.2 | 0.2 | 0.1 | 0.2 | 0.1 | 0.1 | 0.1 | 0.1 | 0.1 |
| Robo3 | 0.0 | 0.2 | 0.1 | 0.0 | 0.1 | 0.1 | 0.0 | 0.0 | 0.1 | 0.1 | 0.1 | 0.0 |
| Robo4 | 0.7 | 0.2 | 0.3 | 0.1 | 0.1 | 0.0 | 0.4 | 0.5 | 0.5 | 0.1 | 0.0 | 0.2 |
| Rock1 | 19.1 | 20.6 | 21.6 | 21.8 | 19.6 | 17.4 | 19.6 | 17.1 | 18.7 | 19.3 | 22.5 | 24.5 |
| Rock2 | 71.0 | 69.7 | 55.1 | 90.4 | 58.7 | 83.9 | 62.4 | 44.8 | 50.0 | 51.4 | 72.0 | 58.4 |
| Rogdi | 5.4 | 4.9 | 6.0 | 4.2 | 3.9 | 5.2 | 3.2 | 5.2 | 3.7 | 3.6 | 3.3 | 3.5 |
| Rom1 | 5.5 | 6.2 | 4.9 | 5.2 | 5.9 | 4.8 | 5.0 | 4.0 | 4.7 | 4.5 | 5.4 | 5.3 |
| Romo1 | 99.0 | 92.1 | 80.8 | 90.0 | 91.7 | 101.2 | 78.6 | 90.2 | 101.1 | 98.6 | 87.4 | 93.0 |
| Ropn1l | 4.6 | 4.2 | 2.4 | 4.1 | 3.2 | 4.5 | 2.4 | 3.5 | 3.1 | 3.2 | 3.2 | 3.3 |
| Ror1 | 1.4 | 2.1 | 2.8 | 1.0 | 1.8 | 1.0 | 2.1 | 4.8 | 3.1 | 4.9 | 3.7 | 4.7 |
| Ror2 | 0.9 | 1.5 | 1.6 | 0.9 | 1.2 | 1.1 | 3.0 | 1.8 | 2.2 | 2.1 | 1.7 | 1.9 |
| Rora | 13.8 | 14.2 | 13.2 | 16.5 | 11.8 | 14.3 | 13.0 | 11.1 | 10.9 | 9.7 | 10.6 | 14.1 |
| Rorb | 0.0 | 0.0 | 0.0 | 0.0 | 0.0 | 0.0 | 0.0 | 0.0 | 0.0 | 0.0 | 0.0 | 0.0 |
| Rorc | 0.5 | 0.2 | 0.1 | 0.1 | 0.0 | 0.2 | 0.0 | 0.2 | 0.1 | 0.8 | 0.4 | 0.1 |
| Rp1 | 0.0 | 0.0 | 0.0 | 0.0 | 0.0 | 0.0 | 0.0 | 0.0 | 0.0 | 0.0 | 0.0 | 0.0 |
| Rp1l1 | 0.0 | 0.0 | 0.0 | 0.0 | 0.0 | 0.0 | 0.0 | 0.0 | 0.0 | 0.0 | 0.0 | 0.0 |
| Rp2h | 7.0 | 7.3 | 7.5 | 8.5 | 7.4 | 8.4 | 5.2 | 5.7 | 5.6 | 6.1 | 7.2 | 6.8 |
| Rp9 | 26.6 | 23.4 | 23.3 | 28.5 | 20.0 | 26.6 | 22.1 | 23.2 | 18.0 | 20.5 | 24.4 | 25.2 |
| Rpa1 | 19.5 | 21.1 | 22.0 | 19.0 | 20.0 | 20.2 | 21.8 | 17.0 | 21.3 | 17.1 | 18.5 | 18.5 |
| Rpa2 | 4.9 | 5.7 | 7.2 | 3.1 | 7.3 | 3.9 | 6.9 | 5.8 | 5.9 | 5.0 | 6.4 | 4.5 |
| Rpa3 | 16.4 | 22.7 | 21.7 | 20.6 | 21.9 | 20.4 | 25.5 | 22.7 | 22.1 | 19.9 | 22.7 | 19.7 |
| Rpain | 6.4 | 7.0 | 6.2 | 6.1 | 5.4 | 7.1 | 4.6 | 8.1 | 6.0 | 7.6 | 7.3 | 7.6 |
| Rpap1 | 3.3 | 4.1 | 3.8 | 2.9 | 3.0 | 3.0 | 4.1 | 3.7 | 4.0 | 2.7 | 3.0 | 2.7 |
| Rpap2 | 13.2 | 12.3 | 12.5 | 13.0 | 10.4 | 14.6 | 9.5 | 11.9 | 10.6 | 12.2 | 12.1 | 11.2 |
| Rpap3 | 12.5 | 13.5 | 13.4 | 12.7 | 11.1 | 12.8 | 11.7 | 11.7 | 9.8 | 11.8 | 13.0 | 12.4 |
| Rpe | 19.1 | 17.2 | 19.5 | 21.1 | 20.0 | 20.7 | 18.3 | 17.0 | 15.3 | 18.4 | 21.9 | 23.2 |
| Rpf1 | 13.2 | 12.6 | 13.4 | 15.7 | 11.6 | 11.4 | 13.9 | 13.2 | 11.7 | 13.7 | 14.6 | 12.5 |
| Rpf2 | 10.8 | 8.7 | 7.8 | 11.5 | 8.7 | 9.7 | 8.4 | 8.0 | 7.2 | 9.6 | 9.2 | 10.1 |
| Rpgr | 1.4 | 1.3 | 1.1 | 1.6 | 1.0 | 1.5 | 0.9 | 1.0 | 0.8 | 1.0 | 1.1 | 1.0 |
| Rpgrip1 | 0.1 | 0.1 | 0.2 | 0.1 | 0.1 | 0.1 | 0.1 | 0.1 | 0.1 | 0.1 | 0.1 | 0.1 |
| Rpgrip1l | 4.6 | 5.4 | 5.9 | 5.5 | 4.0 | 5.5 | 3.9 | 3.5 | 3.3 | 4.5 | 5.3 | 5.1 |
| Rph3a | 0.0 | 0.1 | 0.0 | 0.2 | 0.0 | 0.1 | 0.0 | 0.0 | 0.0 | 0.0 | 0.0 | 0.0 |
| Rph3al | 0.0 | 0.0 | 0.2 | 0.0 | 0.1 | 0.3 | 0.1 | 0.2 | 0.0 | 0.1 | 0.0 | 0.1 |
| Rpia | 6.6 | 6.0 | 5.6 | 5.8 | 5.5 | 4.9 | 6.4 | 5.4 | 5.0 | 5.4 | 7.4 | 5.1 |
| Rpl10 | 1256.4 | 1003.7 | 912.8 | 1001.4 | 1006.3 | 1124.7 | 976.6 | 1143.4 | 1130.2 | 1197.2 | 1143.4 | 1107.3 |
| Rpl10a | 438.6 | 348.5 | 354.8 | 331.1 | 347.1 | 335.2 | 338.7 | 507.4 | 383.1 | 444.8 | 475.6 | 396.5 |
| Rpl11 | 786.5 | 627.0 | 666.8 | 649.0 | 656.2 | 728.0 | 640.9 | 715.8 | 628.3 | 738.3 | 690.0 | 683.6 |
| Rpl12 | 1161.9 | 888.5 | 884.4 | 943.2 | 851.2 | 1017.9 | 838.8 | 1100.0 | 829.0 | 1046.1 | 1025.7 | 956.3 |
| Rpl13 | 767.2 | 627.8 | 545.4 | 531.3 | 605.4 | 627.7 | 551.6 | 715.8 | 685.4 | 741.7 | 633.8 | 614.8 |
| Rpl13a | 1000.4 | 777.1 | 758.0 | 803.0 | 718.6 | 845.1 | 756.0 | 933.8 | 772.6 | 910.4 | 856.8 | 865.5 |
| Rpl14 | 493.5 | 372.9 | 374.5 | 434.5 | 372.5 | 472.0 | 374.0 | 473.4 | 354.2 | 435.0 | 504.9 | 440.4 |
| Rpl15 | 275.2 | 223.5 | 208.0 | 198.8 | 220.9 | 236.2 | 209.3 | 257.5 | 245.5 | 264.7 | 256.6 | 235.4 |
| Rpl17 | 937.6 | 745.9 | 919.4 | 842.8 | 713.4 | 891.4 | 826.8 | 949.9 | 695.3 | 858.4 | 1032.3 | 1032.0 |
| Rpl18 | 500.4 | 421.9 | 392.2 | 376.7 | 411.6 | 409.3 | 408.6 | 486.1 | 430.6 | 488.4 | 465.4 | 412.8 |
| Rpl18a | 426.2 | 359.0 | 391.7 | 401.5 | 354.4 | 420.5 | 387.0 | 514.5 | 372.8 | 412.1 | 442.3 | 396.8 |
| Rpl19 | 1117.1 | 886.2 | 887.8 | 1017.6 | 869.1 | 1012.8 | 897.3 | 1055.8 | 832.6 | 1030.4 | 1100.3 | 1003.9 |
| Rpl21 | 334.5 | 263.8 | 310.8 | 297.0 | 240.1 | 312.2 | 287.7 | 336.4 | 247.2 | 295.1 | 363.3 | 353.9 |
| Rpl22 | 112.1 | 87.0 | 88.1 | 100.6 | 88.6 | 113.6 | 87.9 | 106.6 | 83.9 | 99.6 | 110.4 | 106.1 |
| Rpl22l1 | 47.1 | 46.6 | 47.4 | 19.0 | 38.5 | 23.1 | 18.6 | 46.0 | 43.5 | 53.0 | 34.7 | 34.8 |
| Rpl23 | 656.4 | 513.0 | 576.2 | 572.5 | 514.6 | 609.1 | 533.0 | 616.6 | 495.5 | 588.6 | 635.2 | 665.2 |

| | | | | | | | | | | | | |
|---|---|---|---|---|---|---|---|---|---|---|---|---|
| Rpl23a | 1537.8 | 1292.2 | 1349.2 | 1420.3 | 1137.8 | 1409.2 | 1266.2 | 1616.2 | 1187.1 | 1348.1 | 1594.7 | 1412.1 |
| Rpl24 | 668.0 | 544.8 | 522.1 | 501.4 | 470.6 | 546.8 | 477.1 | 552.0 | 471.1 | 562.2 | 590.4 | 564.4 |
| Rpl26 | 114.4 | 95.5 | 65.6 | 115.3 | 62.9 | 84.4 | 84.6 | 84.2 | 67.0 | 86.8 | 109.1 | 62.5 |
| Rpl27 | 358.2 | 318.8 | 272.4 | 268.4 | 286.7 | 331.0 | 258.4 | 321.4 | 302.7 | 328.3 | 317.9 | 289.4 |
| Rpl27a | 547.9 | 445.0 | 468.4 | 446.2 | 428.8 | 516.5 | 463.8 | 578.1 | 472.5 | 540.4 | 547.1 | 555.2 |
| Rpl28 | 714.7 | 594.0 | 702.2 | 717.0 | 597.2 | 755.6 | 613.3 | 778.8 | 525.7 | 650.7 | 708.8 | 668.8 |
| Rpl29 | 410.9 | 351.4 | 368.0 | 353.2 | 346.5 | 361.8 | 334.5 | 477.7 | 343.6 | 362.3 | 433.5 | 361.7 |
| Rpl3 | 515.7 | 391.5 | 395.3 | 385.1 | 394.7 | 378.6 | 388.7 | 506.0 | 431.9 | 493.6 | 466.4 | 454.8 |
| Rpl30 | 25.6 | 19.1 | 19.6 | 17.8 | 32.5 | 22.7 | 14.6 | 16.8 | 16.9 | 30.7 | 13.9 | 12.4 |
| Rpl31 | 332.0 | 290.6 | 302.1 | 298.5 | 271.9 | 327.2 | 278.0 | 378.1 | 287.1 | 301.6 | 340.5 | 305.8 |
| Rpl31-ps12 | 21.9 | 16.5 | 16.5 | 14.8 | 17.5 | 17.0 | 14.9 | 19.4 | 18.4 | 18.8 | 14.1 | 19.8 |
| Rpl32 | 1221.8 | 975.7 | 994.2 | 1018.1 | 916.5 | 1155.2 | 922.5 | 1142.1 | 895.2 | 1046.5 | 1169.8 | 1010.2 |
| Rpl34 | 12.9 | 14.8 | 9.4 | 11.5 | 15.0 | 9.5 | 10.7 | 13.0 | 16.5 | 11.3 | 14.3 | 12.2 |
| Rpl34-ps1 | 734.6 | 639.6 | 579.1 | 531.0 | 618.3 | 678.6 | 533.7 | 717.7 | 666.3 | 716.9 | 648.2 | 619.0 |
| Rpl35 | 1312.6 | 999.2 | 1138.7 | 1257.2 | 1023.5 | 1370.1 | 1124.0 | 1393.8 | 962.5 | 1101.2 | 1315.1 | 1205.1 |
| Rpl35a | 690.6 | 564.3 | 545.0 | 523.2 | 544.7 | 589.9 | 486.5 | 593.7 | 536.9 | 648.8 | 603.7 | 610.2 |
| Rpl36 | 606.3 | 499.2 | 432.2 | 479.5 | 495.9 | 531.9 | 460.7 | 520.5 | 490.4 | 584.4 | 490.6 | 499.1 |
| Rpl36a | 519.0 | 396.6 | 414.3 | 427.8 | 409.6 | 468.5 | 395.0 | 512.8 | 392.0 | 472.4 | 491.2 | 455.2 |
| Rpl36al | 259.3 | 194.7 | 191.6 | 246.5 | 220.2 | 261.4 | 243.7 | 245.5 | 208.4 | 246.7 | 300.2 | 275.5 |
| Rpl37 | 193.4 | 158.7 | 145.0 | 132.8 | 152.5 | 169.2 | 136.2 | 171.6 | 162.2 | 178.1 | 172.5 | 171.7 |
| Rpl37a | 915.1 | 758.0 | 637.7 | 682.2 | 685.6 | 764.5 | 625.3 | 743.4 | 695.5 | 781.7 | 742.1 | 693.9 |
| Rpl38 | 1359.8 | 1068.6 | 880.5 | 1114.8 | 1015.5 | 1214.4 | 922.1 | 1099.6 | 1016.6 | 1197.6 | 1112.5 | 1002.6 |
| Rpl39 | 1403.3 | 1257.6 | 1090.9 | 937.5 | 1112.0 | 1201.3 | 887.5 | 1211.8 | 1192.1 | 1344.2 | 1113.7 | 1193.7 |
| Rpl39l | 2.5 | 0.8 | 1.3 | 0.4 | 0.6 | 0.8 | 0.3 | 0.7 | 0.6 | 1.2 | 1.4 | 0.6 |
| Rpl3l | 0.0 | 0.1 | 0.1 | 0.1 | 0.1 | 0.0 | 0.1 | 0.0 | 0.0 | 0.0 | 0.0 | 0.1 |
| Rpl4 | 780.1 | 580.8 | 626.2 | 672.6 | 596.6 | 731.7 | 628.8 | 746.6 | 572.1 | 681.4 | 738.7 | 697.0 |
| Rpl41 | 1855.5 | 1507.4 | 1491.4 | 1515.2 | 1338.2 | 1706.4 | 1351.4 | 1905.7 | 1447.2 | 1644.2 | 1639.7 | 1533.2 |
| Rpl5 | 399.5 | 302.0 | 307.4 | 338.2 | 300.5 | 351.0 | 303.1 | 369.0 | 300.6 | 353.4 | 398.0 | 371.6 |
| Rpl6 | 604.0 | 475.4 | 431.2 | 463.8 | 465.3 | 524.9 | 459.5 | 527.1 | 464.2 | 541.6 | 557.5 | 470.9 |
| Rpl7 | 1008.3 | 822.0 | 804.5 | 697.7 | 726.3 | 821.4 | 728.0 | 914.4 | 772.1 | 878.0 | 904.7 | 866.3 |
| Rpl7a | 593.4 | 518.7 | 547.7 | 513.4 | 490.7 | 559.8 | 537.6 | 680.4 | 536.1 | 570.0 | 646.8 | 580.9 |
| Rpl7l1 | 23.9 | 23.0 | 21.9 | 24.6 | 21.3 | 24.0 | 22.2 | 23.6 | 20.7 | 22.9 | 23.4 | 22.2 |
| Rpl8 | 581.1 | 494.0 | 456.0 | 465.2 | 492.7 | 504.1 | 481.2 | 584.1 | 509.1 | 550.9 | 512.3 | 496.2 |
| Rpl9 | 944.2 | 883.8 | 755.8 | 592.5 | 720.5 | 717.7 | 604.6 | 728.8 | 804.7 | 856.0 | 735.6 | 796.1 |
| Rplp0 | 1246.1 | 1034.4 | 935.2 | 982.6 | 921.4 | 1174.2 | 941.3 | 1180.9 | 963.0 | 1077.3 | 1021.6 | 977.1 |
| Rplp1 | 2149.8 | 1644.2 | 1227.5 | 1874.6 | 1534.2 | 2165.5 | 1377.5 | 1726.3 | 1523.6 | 1661.1 | 1438.3 | 1297.3 |
| Rplp2 | 509.7 | 406.6 | 345.3 | 389.4 | 405.7 | 450.3 | 361.8 | 415.8 | 383.0 | 437.0 | 378.4 | 364.8 |
| Rplp2-ps1 | 7.6 | 7.0 | 3.6 | 5.0 | 5.4 | 4.2 | 3.6 | 3.9 | 6.0 | 5.5 | 4.4 | 3.1 |
| Rpn1 | 59.0 | 54.8 | 54.7 | 67.9 | 60.9 | 68.0 | 68.1 | 65.3 | 59.8 | 59.0 | 61.0 | 58.9 |
| Rpn2 | 169.2 | 157.7 | 153.0 | 177.1 | 155.6 | 183.2 | 157.8 | 153.8 | 151.8 | 135.9 | 143.0 | 141.4 |
| Rpp14 | 10.2 | 8.0 | 7.0 | 11.9 | 9.3 | 10.1 | 8.4 | 7.1 | 8.3 | 9.6 | 8.9 | 7.8 |
| Rpp21 | 12.4 | 10.2 | 9.3 | 13.1 | 12.2 | 14.9 | 13.5 | 11.5 | 10.9 | 11.4 | 10.4 | 8.3 |
| Rpp25 | 0.2 | 0.0 | 0.3 | 0.1 | 0.2 | 0.2 | 0.3 | 0.7 | 0.3 | 0.4 | 0.4 | 0.4 |
| Rpp25l | 9.7 | 8.1 | 7.1 | 6.9 | 8.8 | 9.2 | 7.6 | 10.5 | 9.4 | 11.0 | 9.1 | 9.2 |
| Rpp30 | 9.1 | 9.6 | 9.3 | 11.5 | 8.3 | 10.8 | 9.1 | 8.0 | 9.3 | 8.5 | 10.7 | 9.7 |
| Rpp38 | 6.8 | 5.6 | 5.2 | 6.6 | 6.1 | 6.4 | 5.7 | 5.4 | 4.7 | 5.4 | 6.5 | 6.3 |
| Rpp40 | 1.4 | 1.8 | 1.6 | 1.5 | 1.5 | 1.7 | 1.4 | 1.5 | 1.3 | 1.5 | 1.5 | 1.3 |
| Rpph1 | 7669.1 | 6363.4 | 7262.8 | 6999.4 | 8444.7 | 7602.4 | 6792.8 | 8908.7 | 7670.9 | 7687.2 | 6646.2 | 5615.8 |
| Rprd1a | 8.6 | 9.1 | 8.9 | 8.1 | 7.3 | 7.6 | 7.4 | 7.5 | 10.3 | 9.6 | 9.4 | 11.0 |
| Rprd1b | 3.8 | 5.1 | 5.8 | 3.9 | 4.2 | 3.9 | 4.9 | 4.8 | 4.5 | 4.2 | 4.7 | 4.2 |
| Rprd2 | 3.1 | 3.5 | 4.1 | 3.1 | 3.5 | 3.4 | 3.4 | 3.5 | 3.2 | 3.3 | 3.1 | 3.3 |
| Rprl1 | 1.9 | 0.3 | 0.6 | 0.0 | 1.0 | 0.1 | 1.3 | 0.3 | 1.5 | 0.6 | 0.2 | 0.5 |
| Rprl2 | 0.5 | 0.9 | 0.3 | 1.4 | 0.3 | 0.1 | 0.0 | 1.0 | 0.2 | 0.5 | 1.6 | 1.2 |
| Rprl3 | 3.2 | 2.6 | 0.8 | 2.0 | 0.8 | 0.6 | 4.2 | 6.6 | 0.8 | 1.2 | 2.7 | 0.0 |
| Rprm | 2.6 | 1.1 | 0.6 | 1.5 | 1.1 | 1.5 | 1.4 | 0.3 | 0.9 | 0.7 | 0.9 | 1.1 |
| Rps10 | 783.9 | 629.7 | 609.7 | 693.9 | 638.7 | 769.2 | 649.8 | 764.2 | 653.7 | 729.8 | 702.0 | 633.5 |
| Rps11 | 802.8 | 606.8 | 613.0 | 672.5 | 600.8 | 669.8 | 644.8 | 753.6 | 633.8 | 695.0 | 705.4 | 653.0 |

| | | | | | | | | | | | | |
|---|---|---|---|---|---|---|---|---|---|---|---|---|
| Rps12 | 1943.4 | 1550.4 | 1603.5 | 1658.3 | 1395.9 | 1833.4 | 1515.7 | 1985.6 | 1484.9 | 1568.7 | 1740.3 | 1664.1 |
| Rps13 | 503.6 | 420.7 | 336.6 | 345.5 | 413.4 | 429.0 | 319.3 | 400.7 | 433.6 | 471.8 | 407.2 | 397.7 |
| Rps14 | 671.2 | 556.9 | 464.2 | 421.9 | 510.7 | 544.7 | 422.3 | 580.1 | 565.4 | 616.2 | 514.3 | 479.3 |
| Rps15 | 918.7 | 825.5 | 834.8 | 789.7 | 722.5 | 883.4 | 776.2 | 965.4 | 770.8 | 861.1 | 840.9 | 775.5 |
| Rps15a | 53.3 | 44.9 | 39.5 | 33.0 | 39.1 | 39.8 | 32.3 | 42.2 | 41.9 | 47.2 | 43.5 | 45.4 |
| Rps15a-ps4 | 1.5 | 1.6 | 1.1 | 1.0 | 1.4 | 0.9 | 1.1 | 1.4 | 1.3 | 2.1 | 0.8 | 1.1 |
| Rps15a-ps6 | 5.5 | 3.8 | 4.2 | 3.8 | 4.0 | 3.8 | 3.3 | 4.9 | 4.2 | 4.5 | 4.6 | 4.1 |
| Rps16 | 559.4 | 548.7 | 580.6 | 495.2 | 537.2 | 564.9 | 582.6 | 761.3 | 591.5 | 585.4 | 611.2 | 589.6 |
| Rps17 | 1005.2 | 820.9 | 760.6 | 723.3 | 714.7 | 811.8 | 704.4 | 817.6 | 756.3 | 886.2 | 866.4 | 771.2 |
| Rps18 | 1221.8 | 992.4 | 887.0 | 838.0 | 880.2 | 942.6 | 813.2 | 1046.2 | 883.3 | 1013.5 | 1057.8 | 951.0 |
| Rps19 | 789.9 | 711.6 | 608.3 | 543.5 | 637.0 | 662.4 | 565.9 | 695.7 | 660.7 | 732.3 | 644.8 | 602.3 |
| Rps19bp1 | 13.9 | 10.5 | 9.1 | 12.0 | 11.8 | 12.6 | 11.3 | 10.3 | 12.2 | 13.9 | 11.8 | 11.9 |
| Rps19-ps3 | 5.6 | 5.0 | 4.0 | 5.9 | 5.2 | 5.7 | 4.9 | 5.0 | 4.9 | 4.9 | 5.3 | 4.7 |
| Rps2 | 1333.4 | 1101.2 | 939.1 | 933.2 | 959.4 | 1016.2 | 1012.1 | 1247.8 | 1046.3 | 1154.4 | 1150.9 | 1041.5 |
| Rps20 | 1511.5 | 1164.2 | 973.3 | 1010.5 | 1060.2 | 1180.0 | 938.3 | 1147.8 | 1219.8 | 1210.5 | 1047.2 | 1057.4 |
| Rps21 | 902.8 | 706.6 | 618.5 | 644.9 | 670.8 | 835.2 | 593.3 | 641.7 | 711.7 | 878.6 | 587.7 | 666.7 |
| Rps23 | 437.2 | 368.0 | 376.2 | 392.1 | 373.8 | 443.4 | 362.0 | 464.4 | 379.8 | 436.3 | 460.5 | 425.4 |
| Rps24 | 1005.7 | 812.9 | 943.6 | 941.4 | 731.0 | 957.6 | 762.4 | 1082.0 | 673.6 | 810.0 | 1076.8 | 923.3 |
| Rps25 | 647.2 | 507.7 | 505.8 | 517.7 | 497.1 | 550.2 | 462.5 | 610.6 | 459.5 | 550.0 | 609.8 | 572.4 |
| Rps26 | 815.2 | 645.9 | 697.7 | 746.3 | 666.2 | 809.1 | 716.7 | 889.8 | 660.0 | 774.0 | 877.6 | 812.8 |
| Rps27 | 5.8 | 4.5 | 3.5 | 7.2 | 4.4 | 5.2 | 5.1 | 6.1 | 2.6 | 43.8 | 5.1 | 6.2 |
| Rps27a | 636.0 | 508.9 | 456.6 | 469.5 | 542.0 | 562.7 | 425.1 | 532.0 | 512.4 | 622.3 | 545.7 | 587.2 |
| Rps27l | 283.9 | 229.2 | 195.5 | 231.3 | 224.0 | 246.9 | 199.4 | 206.8 | 216.0 | 268.2 | 277.2 | 236.9 |
| Rps28 | 904.8 | 761.1 | 625.0 | 679.8 | 726.3 | 842.8 | 612.3 | 800.1 | 748.1 | 881.9 | 698.6 | 616.2 |
| Rps29 | 1065.1 | 946.7 | 744.7 | 761.9 | 959.4 | 990.4 | 703.1 | 948.9 | 1036.0 | 1074.5 | 798.1 | 796.2 |
| Rps3 | 377.9 | 304.3 | 287.2 | 305.3 | 302.8 | 339.8 | 269.6 | 330.2 | 281.4 | 328.4 | 318.2 | 298.9 |
| Rps3a1 | 1121.1 | 928.2 | 952.6 | 842.3 | 839.4 | 946.1 | 853.3 | 1016.2 | 877.2 | 985.4 | 1054.8 | 1035.4 |
| Rps4x | 969.6 | 758.6 | 708.5 | 647.7 | 685.1 | 781.4 | 639.6 | 858.8 | 744.4 | 786.1 | 797.5 | 742.9 |
| Rps4y2 | 27.6 | 30.2 | 29.5 | 33.7 | 28.5 | 28.9 | 36.6 | 35.8 | 29.1 | 30.7 | 36.0 | 30.1 |
| Rps5 | 721.4 | 590.9 | 510.6 | 553.8 | 563.3 | 632.0 | 516.4 | 704.8 | 550.6 | 590.4 | 595.8 | 514.8 |
| Rps6 | 547.1 | 430.0 | 449.7 | 489.5 | 407.7 | 510.4 | 444.2 | 520.1 | 392.0 | 457.6 | 556.7 | 513.6 |
| Rps6ka1 | 1.1 | 0.8 | 1.2 | 4.9 | 8.1 | 0.8 | 1.5 | 0.7 | 0.7 | 1.2 | 0.8 | 1.0 |
| Rps6ka2 | 3.7 | 3.7 | 3.4 | 3.9 | 4.2 | 3.9 | 3.9 | 3.9 | 4.2 | 3.1 | 3.4 | 3.0 |
| Rps6ka3 | 3.7 | 3.7 | 3.6 | 3.7 | 5.0 | 2.9 | 3.5 | 4.2 | 3.8 | 4.8 | 3.4 | 4.0 |
| Rps6ka4 | 17.3 | 16.8 | 16.5 | 18.7 | 18.0 | 16.4 | 21.1 | 20.6 | 19.7 | 16.9 | 17.1 | 16.8 |
| Rps6ka5 | 1.3 | 1.0 | 1.5 | 1.0 | 1.2 | 0.9 | 1.2 | 1.3 | 1.3 | 1.4 | 1.6 | 1.0 |
| Rps6ka6 | 0.0 | 0.0 | 0.0 | 0.0 | 0.0 | 0.0 | 0.1 | 0.1 | 0.0 | 0.0 | 0.1 | 0.1 |
| Rps6kb1 | 10.5 | 9.8 | 13.6 | 11.0 | 10.3 | 9.3 | 12.7 | 12.5 | 11.6 | 10.9 | 12.8 | 13.4 |
| Rps6kb2 | 3.5 | 3.9 | 2.8 | 3.3 | 2.9 | 2.3 | 4.2 | 3.1 | 3.8 | 3.6 | 3.4 | 3.6 |
| Rps6kc1 | 14.1 | 13.4 | 12.5 | 15.6 | 12.1 | 15.1 | 14.9 | 11.7 | 12.4 | 12.4 | 13.3 | 13.4 |
| Rps6kl1 | 0.0 | 0.0 | 0.0 | 0.1 | 0.0 | 0.0 | 0.0 | 0.0 | 0.1 | 0.0 | 0.0 | 0.0 |
| Rps7 | 624.7 | 508.3 | 503.0 | 466.2 | 473.1 | 534.0 | 456.0 | 577.7 | 508.0 | 581.5 | 553.7 | 532.5 |
| Rps8 | 915.2 | 799.8 | 699.2 | 637.4 | 755.4 | 783.6 | 691.9 | 897.2 | 863.0 | 887.1 | 795.0 | 767.5 |
| Rps9 | 775.3 | 581.8 | 559.6 | 638.5 | 635.7 | 691.0 | 624.3 | 719.3 | 612.1 | 693.7 | 650.9 | 624.8 |
| Rpsa | 667.4 | 614.1 | 589.8 | 583.4 | 553.0 | 631.0 | 587.5 | 739.0 | 576.6 | 574.3 | 599.1 | 544.3 |
| Rptor | 4.5 | 4.6 | 4.4 | 5.2 | 4.7 | 4.4 | 5.3 | 4.6 | 4.7 | 3.9 | 4.2 | 3.7 |
| Rpusd1 | 3.3 | 3.2 | 3.6 | 4.1 | 3.0 | 3.4 | 2.7 | 3.5 | 3.1 | 3.6 | 2.8 | 2.6 |
| Rpusd2 | 0.4 | 0.4 | 0.5 | 0.5 | 0.6 | 0.6 | 0.5 | 0.4 | 0.4 | 0.3 | 0.4 | 0.3 |
| Rpusd3 | 3.3 | 2.9 | 2.0 | 2.2 | 2.1 | 3.4 | 3.2 | 2.6 | 2.1 | 1.9 | 2.1 | 2.4 |
| Rpusd4 | 5.6 | 5.1 | 4.5 | 5.5 | 5.0 | 5.2 | 5.3 | 4.6 | 5.3 | 4.8 | 4.7 | 4.3 |
| Rqcd1 | 8.2 | 8.5 | 7.9 | 8.5 | 9.1 | 8.2 | 8.4 | 8.6 | 8.5 | 7.6 | 8.2 | 6.0 |
| Rrad | 0.7 | 0.6 | 0.7 | 1.8 | 1.9 | 0.7 | 1.0 | 1.9 | 0.9 | 0.9 | 0.6 | 0.9 |
| Rraga | 20.8 | 17.3 | 19.9 | 19.9 | 23.4 | 18.8 | 17.5 | 21.2 | 21.2 | 20.3 | 20.3 | 21.1 |
| Rragb | 0.5 | 0.3 | 0.3 | 0.3 | 0.1 | 0.5 | 0.2 | 0.2 | 0.0 | 0.4 | 0.3 | 0.3 |
| Rragc | 38.8 | 34.0 | 30.5 | 42.1 | 38.2 | 40.0 | 35.2 | 30.9 | 33.4 | 32.3 | 36.9 | 37.3 |
| Rragd | 5.9 | 2.6 | 1.2 | 7.1 | 1.9 | 10.8 | 0.7 | 0.7 | 0.7 | 0.9 | 0.8 | 1.0 |
| Rras | 53.3 | 62.2 | 69.9 | 73.2 | 65.1 | 75.5 | 85.7 | 75.9 | 69.6 | 53.0 | 76.9 | 70.2 |

| | | | | | | | | | | | | |
|---|---|---|---|---|---|---|---|---|---|---|---|---|
| Rras2 | 108.7 | 96.3 | 75.1 | 89.1 | 67.8 | 77.3 | 103.9 | 94.2 | 98.9 | 113.8 | 140.5 | 105.7 |
| Rrbp1 | 80.5 | 77.3 | 96.1 | 93.6 | 79.5 | 86.4 | 105.3 | 97.6 | 82.5 | 77.4 | 93.7 | 103.0 |
| Rreb1 | 1.1 | 1.4 | 1.6 | 1.1 | 1.7 | 1.1 | 1.4 | 1.5 | 1.4 | 1.5 | 1.3 | 1.3 |
| Rrm1 | 49.7 | 61.0 | 56.4 | 47.8 | 49.0 | 57.7 | 59.7 | 55.6 | 50.1 | 72.8 | 75.9 | 60.9 |
| Rrm2 | 14.2 | 18.7 | 22.6 | 7.1 | 27.4 | 6.9 | 32.0 | 11.9 | 20.1 | 11.7 | 17.9 | 10.9 |
| Rrm2b | 10.1 | 9.4 | 10.1 | 13.2 | 8.4 | 12.0 | 9.1 | 7.8 | 7.8 | 8.4 | 11.4 | 11.0 |
| Rrn3 | 27.6 | 25.9 | 24.3 | 28.3 | 25.1 | 26.7 | 24.7 | 21.9 | 23.9 | 25.2 | 25.9 | 25.9 |
| Rrnad1 | 4.6 | 5.1 | 4.6 | 3.8 | 4.6 | 4.8 | 4.4 | 4.7 | 5.1 | 5.4 | 4.6 | 4.8 |
| Rrp1 | 57.7 | 52.2 | 53.2 | 69.4 | 51.9 | 61.8 | 58.6 | 55.2 | 47.3 | 57.1 | 59.0 | 60.1 |
| Rrp12 | 4.4 | 3.0 | 2.3 | 4.8 | 3.6 | 3.7 | 3.1 | 2.7 | 2.2 | 3.4 | 3.6 | 2.7 |
| Rrp15 | 15.9 | 13.5 | 11.8 | 19.1 | 12.6 | 14.8 | 17.0 | 13.3 | 13.3 | 12.9 | 14.8 | 12.5 |
| Rrp1b | 1.9 | 2.0 | 2.0 | 1.9 | 2.1 | 2.0 | 2.0 | 2.1 | 1.9 | 1.7 | 1.8 | 1.8 |
| Rrp36 | 17.3 | 15.0 | 12.9 | 17.9 | 15.7 | 15.2 | 16.7 | 15.5 | 14.4 | 16.8 | 17.0 | 14.3 |
| Rrp7a | 32.5 | 28.6 | 28.0 | 36.0 | 31.8 | 31.3 | 37.2 | 34.9 | 32.1 | 32.1 | 36.8 | 30.6 |
| Rrp8 | 6.7 | 6.2 | 4.9 | 6.9 | 5.2 | 5.7 | 5.0 | 5.2 | 5.3 | 6.5 | 6.6 | 6.0 |
| Rrp9 | 14.4 | 13.6 | 10.0 | 15.1 | 12.7 | 12.5 | 15.6 | 11.3 | 11.4 | 12.6 | 14.6 | 11.9 |
| Rrs1 | 7.6 | 7.7 | 7.4 | 8.0 | 7.1 | 8.2 | 8.9 | 7.3 | 7.9 | 10.2 | 7.8 | 9.5 |
| Rs1 | 0.0 | 0.0 | 0.0 | 0.0 | 0.0 | 0.0 | 0.0 | 0.0 | 0.0 | 0.0 | 0.0 | 0.0 |
| Rsad1 | 1.1 | 1.2 | 0.9 | 1.0 | 0.8 | 1.5 | 0.6 | 1.0 | 0.8 | 0.6 | 0.6 | 0.7 |
| Rsad2 | 2.0 | 0.2 | 0.3 | 2.4 | 0.3 | 0.8 | 0.2 | 0.1 | 0.1 | 0.1 | 0.2 | 0.6 |
| Rsbn1 | 1.3 | 1.6 | 1.5 | 1.3 | 1.7 | 1.6 | 1.7 | 1.4 | 1.6 | 2.0 | 1.8 | 1.9 |
| Rsbn1l | 1.4 | 1.7 | 1.9 | 2.1 | 2.1 | 1.9 | 1.6 | 1.7 | 1.4 | 1.6 | 1.8 | 2.1 |
| Rsc1a1 | 0.0 | 2.0 | 0.5 | 0.0 | 0.0 | 0.0 | 0.0 | 0.4 | 0.0 | 0.0 | 0.4 | 0.0 |
| Rsf1 | 3.7 | 4.2 | 5.2 | 4.2 | 3.7 | 4.0 | 3.9 | 4.1 | 3.5 | 4.8 | 4.9 | 4.8 |
| Rsg1 | 0.3 | 0.5 | 0.5 | 0.2 | 0.8 | 0.4 | 0.6 | 0.5 | 0.7 | 0.5 | 0.6 | 0.5 |
| Rsl1 | 2.7 | 2.5 | 2.7 | 3.3 | 3.2 | 2.0 | 2.0 | 2.1 | 2.2 | 2.3 | 1.9 | 2.1 |
| Rsl1d1 | 49.9 | 44.2 | 38.7 | 49.1 | 42.6 | 42.5 | 40.9 | 38.3 | 36.3 | 53.8 | 51.2 | 46.7 |
| Rsl24d1 | 23.4 | 19.7 | 18.5 | 25.2 | 22.3 | 22.6 | 20.3 | 18.4 | 17.9 | 23.6 | 22.3 | 19.7 |
| Rslcan18 | 1.1 | 1.1 | 1.2 | 1.0 | 0.9 | 1.4 | 1.0 | 1.1 | 1.0 | 1.2 | 1.0 | 0.8 |
| Rsph1 | 2.2 | 2.3 | 2.5 | 3.5 | 1.9 | 3.5 | 1.6 | 1.2 | 1.4 | 1.9 | 3.1 | 2.5 |
| Rsph3a | 19.9 | 17.0 | 15.8 | 21.5 | 13.9 | 22.4 | 15.2 | 14.6 | 11.9 | 15.3 | 17.3 | 16.5 |
| Rsph3b | 21.5 | 18.4 | 17.1 | 23.6 | 15.1 | 24.4 | 16.3 | 15.0 | 12.8 | 16.2 | 17.8 | 16.9 |
| Rsph4a | 0.0 | 0.0 | 0.0 | 0.0 | 0.0 | 0.1 | 0.0 | 0.0 | 0.0 | 0.0 | 0.0 | 0.0 |
| Rsph6a | 0.1 | 0.0 | 0.0 | 0.0 | 0.0 | 0.0 | 0.0 | 0.0 | 0.0 | 0.0 | 0.1 | 0.0 |
| Rsph9 | 4.0 | 4.0 | 3.2 | 3.1 | 2.7 | 5.3 | 3.3 | 4.6 | 3.3 | 4.1 | 2.8 | 3.1 |
| Rspo1 | 0.4 | 1.0 | 2.2 | 0.4 | 0.1 | 0.2 | 0.6 | 2.6 | 3.5 | 33.7 | 13.2 | 7.7 |
| Rspo2 | 0.0 | 0.0 | 0.0 | 0.0 | 0.0 | 0.0 | 0.0 | 0.0 | 0.0 | 0.1 | 0.2 | 0.0 |
| Rspo3 | 2.3 | 1.5 | 1.7 | 0.9 | 0.3 | 0.3 | 0.7 | 1.4 | 1.6 | 15.2 | 9.4 | 7.8 |
| Rspry1 | 9.8 | 10.7 | 10.4 | 9.5 | 9.9 | 9.4 | 9.0 | 10.5 | 11.0 | 10.1 | 11.7 | 12.0 |
| Rsrc1 | 12.2 | 10.9 | 13.6 | 12.7 | 10.5 | 12.3 | 13.1 | 13.2 | 10.1 | 10.0 | 13.3 | 13.4 |
| Rsrc2 | 20.9 | 21.7 | 32.6 | 22.0 | 20.3 | 19.5 | 27.5 | 28.7 | 21.3 | 26.9 | 30.8 | 33.6 |
| Rsu1 | 76.9 | 66.2 | 73.3 | 84.9 | 71.8 | 65.0 | 89.8 | 74.7 | 70.0 | 65.4 | 89.9 | 73.1 |
| Rtbdn | 0.0 | 0.2 | 0.1 | 0.1 | 0.1 | 0.1 | 0.0 | 0.1 | 0.2 | 0.1 | 0.0 | 0.3 |
| Rtca | 20.4 | 19.3 | 16.6 | 20.8 | 18.3 | 20.9 | 18.7 | 17.6 | 18.0 | 17.0 | 19.7 | 19.0 |
| Rtel1 | 2.8 | 2.8 | 3.0 | 2.2 | 3.1 | 2.0 | 3.6 | 3.1 | 3.6 | 2.3 | 2.8 | 2.3 |
| Rtf1 | 7.2 | 7.1 | 8.8 | 7.9 | 6.6 | 8.0 | 8.5 | 8.0 | 6.7 | 6.7 | 8.4 | 9.1 |
| Rtfdc1 | 36.2 | 31.9 | 28.9 | 41.4 | 31.1 | 34.3 | 28.7 | 29.2 | 26.2 | 28.8 | 28.8 | 29.5 |
| Rtkn | 6.4 | 6.6 | 6.8 | 5.4 | 6.5 | 5.5 | 8.6 | 9.5 | 8.2 | 6.1 | 7.7 | 6.5 |
| Rtkn2 | 0.8 | 0.8 | 0.8 | 0.7 | 0.7 | 1.2 | 0.4 | 0.5 | 0.3 | 0.2 | 0.5 | 0.3 |
| Rtl1 | 0.7 | 0.6 | 0.4 | 0.4 | 0.6 | 0.6 | 0.8 | 0.7 | 0.9 | 1.0 | 1.1 | 0.7 |
| Rtn1 | 3.8 | 3.4 | 3.7 | 5.0 | 6.2 | 2.3 | 4.7 | 3.4 | 3.2 | 3.1 | 3.6 | 2.3 |
| Rtn2 | 2.9 | 4.3 | 4.6 | 1.3 | 3.8 | 2.1 | 5.1 | 5.7 | 4.7 | 6.4 | 4.9 | 4.6 |
| Rtn3 | 38.1 | 39.7 | 34.9 | 37.9 | 39.4 | 36.6 | 36.0 | 38.7 | 40.2 | 38.7 | 45.1 | 36.2 |
| Rtn4 | 101.5 | 92.0 | 68.2 | 138.6 | 116.5 | 122.5 | 75.1 | 76.2 | 80.3 | 78.8 | 84.6 | 75.1 |
| Rtn4ip1 | 2.1 | 2.9 | 2.5 | 2.6 | 2.3 | 3.1 | 2.1 | 2.3 | 2.0 | 2.3 | 2.0 | 2.1 |
| Rtn4r | 0.9 | 0.8 | 1.1 | 1.5 | 1.4 | 1.8 | 0.8 | 1.4 | 0.9 | 0.6 | 0.6 | 0.7 |
| Rtn4rl1 | 0.1 | 0.4 | 0.5 | 0.5 | 0.5 | 0.2 | 0.5 | 0.5 | 0.7 | 0.3 | 0.4 | 0.4 |

| | | | | | | | | | | | | |
|---|---|---|---|---|---|---|---|---|---|---|---|---|
| Rtn4rl2 | 0.2 | 0.3 | 0.9 | 0.2 | 0.2 | 0.1 | 0.2 | 0.7 | 0.8 | 0.7 | 0.1 | 0.6 |
| Rtp4 | 4.3 | 1.5 | 2.5 | 5.2 | 3.9 | 2.6 | 2.1 | 1.0 | 2.0 | 1.0 | 1.5 | 5.7 |
| Rttn | 0.9 | 1.0 | 1.3 | 0.7 | 1.1 | 0.9 | 0.8 | 1.0 | 1.2 | 0.9 | 0.9 | 0.9 |
| Rufy1 | 9.3 | 7.8 | 8.9 | 8.8 | 6.9 | 8.2 | 7.1 | 8.7 | 8.1 | 7.6 | 8.9 | 10.0 |
| Rufy2 | 2.7 | 2.6 | 3.0 | 2.7 | 2.2 | 3.2 | 2.4 | 2.8 | 2.4 | 2.3 | 3.0 | 2.9 |
| Rufy3 | 2.0 | 2.4 | 2.1 | 2.2 | 2.0 | 2.0 | 1.9 | 2.2 | 1.9 | 1.8 | 1.8 | 2.3 |
| Rufy4 | 0.2 | 0.1 | 0.1 | 0.2 | 0.1 | 0.2 | 0.5 | 0.2 | 0.2 | 0.2 | 0.4 | 0.1 |
| Rundc1 | 3.0 | 3.5 | 3.4 | 3.6 | 3.3 | 3.4 | 3.3 | 3.4 | 3.1 | 3.2 | 2.9 | 3.9 |
| Rundc3a | 0.9 | 0.7 | 0.7 | 0.9 | 1.2 | 1.1 | 0.7 | 1.0 | 0.9 | 1.0 | 0.9 | 0.8 |
| Rundc3b | 0.1 | 0.0 | 0.0 | 0.1 | 0.0 | 0.0 | 0.0 | 0.0 | 0.0 | 0.0 | 0.0 | 0.0 |
| Runx1 | 2.3 | 2.8 | 4.7 | 3.2 | 5.7 | 2.1 | 6.4 | 8.8 | 6.4 | 6.8 | 6.3 | 7.0 |
| Runx1t1 | 1.9 | 2.4 | 2.8 | 1.9 | 1.5 | 2.3 | 1.9 | 2.0 | 2.1 | 1.2 | 2.0 | 2.3 |
| Runx2 | 0.7 | 1.3 | 1.4 | 1.2 | 1.5 | 1.2 | 1.7 | 1.3 | 1.3 | 1.1 | 1.0 | 1.0 |
| Runx3 | 0.0 | 0.0 | 0.0 | 0.1 | 0.2 | 0.0 | 0.0 | 0.0 | 0.0 | 0.0 | 0.0 | 0.0 |
| Rusc1 | 4.8 | 4.5 | 4.0 | 6.5 | 4.4 | 5.7 | 4.2 | 4.6 | 4.4 | 4.0 | 4.2 | 4.4 |
| Rusc2 | 13.8 | 13.0 | 9.4 | 16.2 | 12.0 | 14.9 | 13.5 | 8.0 | 11.2 | 14.3 | 13.9 | 10.3 |
| Ruvbl1 | 25.6 | 21.1 | 19.9 | 26.7 | 19.0 | 24.4 | 21.7 | 18.2 | 17.7 | 22.3 | 22.6 | 20.6 |
| Ruvbl2 | 17.0 | 16.6 | 15.5 | 17.8 | 15.3 | 17.6 | 17.7 | 13.6 | 13.7 | 15.0 | 16.6 | 14.5 |
| Rwdd1 | 57.1 | 48.1 | 47.8 | 53.0 | 42.9 | 52.0 | 50.2 | 47.7 | 43.6 | 48.2 | 60.4 | 54.6 |
| Rwdd2a | 1.1 | 1.1 | 1.0 | 1.5 | 1.1 | 2.3 | 0.7 | 1.1 | 0.7 | 1.1 | 1.1 | 1.1 |
| Rwdd2b | 2.0 | 1.5 | 1.8 | 1.8 | 1.8 | 1.8 | 2.7 | 2.1 | 1.9 | 2.2 | 2.5 | 2.0 |
| Rwdd3 | 1.1 | 1.5 | 1.6 | 2.1 | 1.1 | 2.4 | 1.5 | 1.5 | 1.2 | 1.3 | 1.8 | 1.6 |
| Rwdd4a | 9.3 | 8.5 | 8.7 | 10.5 | 7.8 | 9.9 | 8.8 | 9.6 | 7.1 | 8.8 | 10.4 | 9.3 |
| Rxfp3 | 0.1 | 0.1 | 0.0 | 0.1 | 0.1 | 0.0 | 0.3 | 0.2 | 0.3 | 0.2 | 0.2 | 0.1 |
| Rxfp4 | 0.2 | 0.1 | 0.0 | 0.2 | 0.1 | 0.0 | 0.1 | 0.0 | 0.1 | 0.1 | 0.1 | 0.0 |
| Rxra | 12.3 | 17.8 | 20.2 | 13.3 | 12.9 | 14.5 | 13.3 | 16.1 | 14.8 | 11.9 | 11.6 | 13.1 |
| Rxrb | 6.4 | 7.1 | 6.5 | 6.7 | 6.6 | 8.2 | 6.4 | 8.0 | 8.2 | 9.0 | 6.1 | 6.9 |
| Rxrg | 0.1 | 0.0 | 0.0 | 0.2 | 0.0 | 0.0 | 0.0 | 0.0 | 0.0 | 0.0 | 0.0 | 0.0 |
| Rybp | 3.4 | 3.7 | 4.2 | 3.9 | 4.6 | 3.5 | 3.9 | 3.5 | 4.0 | 4.6 | 4.7 | 4.9 |
| Ryk | 50.5 | 48.0 | 47.8 | 62.7 | 44.7 | 66.9 | 59.6 | 58.3 | 58.3 | 63.2 | 70.9 | 61.8 |
| Ryr1 | 0.0 | 0.0 | 0.0 | 0.0 | 0.0 | 0.0 | 0.0 | 0.0 | 0.0 | 0.0 | 0.0 | 0.0 |
| Ryr2 | 0.0 | 0.0 | 0.0 | 0.0 | 0.0 | 0.0 | 0.0 | 0.0 | 0.0 | 0.0 | 0.0 | 0.0 |
| Ryr3 | 0.0 | 0.0 | 0.0 | 0.0 | 0.0 | 0.0 | 0.0 | 0.0 | 0.0 | 0.0 | 0.0 | 0.0 |
| S100a1 | 22.4 | 18.3 | 22.8 | 30.0 | 24.0 | 23.2 | 26.9 | 16.1 | 15.9 | 91.6 | 61.6 | 49.2 |
| S100a10 | 350.9 | 284.7 | 213.8 | 312.9 | 298.4 | 300.2 | 366.2 | 433.6 | 350.8 | 365.6 | 467.4 | 312.7 |
| S100a11 | 261.0 | 290.8 | 305.7 | 342.4 | 291.1 | 288.5 | 426.4 | 416.7 | 292.8 | 257.8 | 341.8 | 313.1 |
| S100a13 | 69.6 | 62.4 | 53.6 | 80.7 | 67.1 | 74.1 | 73.2 | 47.9 | 59.7 | 60.8 | 60.4 | 68.6 |
| S100a14 | 0.2 | 0.3 | 0.3 | 0.1 | 0.2 | 0.0 | 0.1 | 0.0 | 0.2 | 0.4 | 0.1 | 0.1 |
| S100a16 | 56.9 | 44.7 | 38.5 | 50.8 | 42.6 | 46.6 | 55.1 | 43.1 | 46.5 | 57.2 | 51.1 | 42.4 |
| S100a3 | 2.8 | 2.1 | 1.1 | 3.2 | 1.7 | 3.0 | 1.7 | 0.3 | 0.7 | 1.9 | 1.6 | 0.7 |
| S100a4 | 3342.9 | 2308.5 | 1885.0 | 2696.6 | 2247.6 | 2563.8 | 2422.8 | 1690.1 | 1698.7 | 1774.8 | 2316.4 | 2116.9 |
| S100a5 | 0.2 | 0.0 | 0.1 | 0.2 | 0.1 | 0.1 | 0.1 | 0.0 | 0.1 | 0.3 | 0.4 | 0.0 |
| S100a6 | 2962.2 | 2032.3 | 1333.3 | 2201.0 | 1786.8 | 2529.7 | 1944.5 | 1264.9 | 1658.0 | 2826.6 | 2217.9 | 2017.0 |
| S100a7a | 3.3 | 2.8 | 2.3 | 2.1 | 1.6 | 3.3 | 0.8 | 1.0 | 1.0 | 1.8 | 0.9 | 1.0 |
| S100a8 | 2.7 | 1.8 | 1.7 | 2.0 | 1.1 | 0.5 | 0.3 | 0.0 | 0.1 | 0.9 | 0.2 | 0.5 |
| S100b | 6.3 | 2.2 | 1.9 | 1.7 | 1.1 | 2.3 | 1.1 | 1.6 | 0.8 | 0.9 | 0.9 | 2.1 |
| S100pbp | 8.9 | 9.7 | 10.3 | 6.0 | 7.9 | 7.5 | 9.4 | 10.8 | 10.1 | 9.8 | 9.0 | 9.3 |
| S1pr1 | 1.0 | 0.3 | 0.5 | 2.5 | 4.2 | 0.1 | 0.8 | 1.0 | 0.7 | 0.7 | 0.5 | 0.8 |
| S1pr2 | 12.4 | 13.7 | 13.7 | 17.0 | 19.4 | 16.2 | 16.6 | 18.0 | 15.3 | 11.9 | 13.0 | 9.8 |
| S1pr3 | 0.2 | 0.3 | 0.5 | 0.1 | 0.8 | 0.3 | 1.2 | 1.1 | 0.6 | 4.2 | 3.6 | 2.1 |
| S1pr4 | 0.0 | 0.0 | 0.0 | 0.0 | 0.0 | 0.0 | 0.0 | 0.0 | 0.0 | 0.0 | 0.0 | 0.0 |
| S1pr5 | 0.6 | 0.6 | 0.3 | 0.2 | 0.3 | 0.1 | 0.3 | 0.6 | 0.4 | 0.5 | 0.4 | 0.4 |
| Saa3 | 3.2 | 6.0 | 10.5 | 4.7 | 37.7 | 0.6 | 8.1 | 8.9 | 8.1 | 28.2 | 3.0 | 16.3 |
| Saal1 | 1.7 | 1.8 | 1.7 | 1.8 | 1.8 | 2.1 | 1.8 | 1.6 | 1.6 | 1.5 | 1.7 | 1.7 |
| Sac3d1 | 6.7 | 5.8 | 5.1 | 6.5 | 7.7 | 6.1 | 6.3 | 5.3 | 7.7 | 8.2 | 5.7 | 5.0 |
| Sacm1l | 26.9 | 28.0 | 26.3 | 28.0 | 27.7 | 28.5 | 26.6 | 25.1 | 30.8 | 30.0 | 34.6 | 32.8 |
| Sacs | 1.6 | 1.3 | 0.9 | 0.8 | 1.1 | 1.2 | 1.1 | 1.1 | 1.0 | 1.0 | 1.4 | 1.0 |

| | | | | | | | | | | | | |
|---|---|---|---|---|---|---|---|---|---|---|---|---|
| Sae1 | 43.6 | 42.2 | 42.8 | 41.0 | 42.6 | 43.2 | 44.2 | 41.0 | 46.8 | 37.3 | 43.9 | 43.3 |
| Safb | 9.6 | 10.8 | 13.7 | 11.1 | 11.4 | 11.3 | 14.2 | 12.7 | 10.7 | 10.5 | 12.0 | 13.2 |
| Safb2 | 5.2 | 5.8 | 7.5 | 7.6 | 6.6 | 6.2 | 8.2 | 6.6 | 5.8 | 6.6 | 6.2 | 6.7 |
| Sag | 0.1 | 0.1 | 0.1 | 0.0 | 0.2 | 0.0 | 0.1 | 0.1 | 0.1 | 0.1 | 0.3 | 0.1 |
| Sall2 | 2.9 | 4.0 | 4.0 | 3.0 | 3.1 | 3.4 | 3.4 | 3.7 | 3.3 | 3.8 | 3.6 | 3.5 |
| Sall3 | 0.0 | 0.0 | 0.1 | 0.1 | 0.0 | 0.0 | 0.0 | 0.0 | 0.0 | 0.0 | 0.0 | 0.0 |
| Samd1 | 4.1 | 4.7 | 5.4 | 3.8 | 4.8 | 4.7 | 5.7 | 7.4 | 5.6 | 5.0 | 4.7 | 4.6 |
| Samd10 | 0.9 | 0.4 | 0.2 | 0.4 | 0.5 | 0.6 | 0.2 | 0.4 | 0.3 | 0.4 | 0.4 | 0.3 |
| Samd11 | 2.2 | 2.2 | 1.7 | 3.4 | 2.7 | 2.8 | 2.5 | 2.5 | 1.7 | 2.1 | 2.6 | 1.7 |
| Samd12 | 2.0 | 1.0 | 0.7 | 0.7 | 0.9 | 1.2 | 0.2 | 0.3 | 0.4 | 1.3 | 0.9 | 0.5 |
| Samd14 | 2.1 | 2.8 | 3.0 | 1.7 | 1.9 | 2.0 | 2.5 | 3.1 | 3.6 | 2.1 | 2.2 | 2.1 |
| Samd4 | 31.7 | 23.2 | 14.3 | 40.2 | 21.2 | 34.8 | 22.4 | 10.6 | 14.7 | 18.8 | 20.9 | 18.1 |
| Samd4b | 4.4 | 5.0 | 3.8 | 4.5 | 4.8 | 4.6 | 6.0 | 4.7 | 4.9 | 4.9 | 4.4 | 4.1 |
| Samd5 | 1.8 | 1.8 | 2.5 | 1.3 | 3.4 | 1.5 | 3.8 | 3.5 | 3.4 | 3.5 | 3.8 | 3.2 |
| Samd8 | 7.8 | 8.1 | 7.5 | 7.5 | 7.7 | 7.4 | 7.4 | 7.2 | 7.8 | 7.6 | 8.1 | 8.6 |
| Samd9l | 11.3 | 8.3 | 10.2 | 14.8 | 12.6 | 8.7 | 8.8 | 8.2 | 7.5 | 9.9 | 10.2 | 14.9 |
| Samhd1 | 12.5 | 13.2 | 14.1 | 12.3 | 13.1 | 7.6 | 10.1 | 11.8 | 9.4 | 8.7 | 9.7 | 8.9 |
| Samm50 | 35.8 | 29.8 | 25.3 | 32.2 | 30.8 | 35.1 | 30.9 | 28.3 | 29.7 | 31.8 | 28.6 | 30.5 |
| Samsn1 | 0.3 | 0.0 | 0.0 | 1.9 | 3.7 | 0.0 | 0.6 | 0.1 | 0.1 | 0.4 | 0.1 | 0.3 |
| Sap130 | 3.4 | 4.5 | 5.2 | 4.4 | 4.5 | 3.6 | 5.4 | 5.8 | 4.1 | 3.6 | 4.4 | 3.5 |
| Sap18 | 16.4 | 12.0 | 6.8 | 15.7 | 9.5 | 12.1 | 9.5 | 6.0 | 7.4 | 21.1 | 15.4 | 9.9 |
| Sap25 | 0.0 | 0.1 | 0.3 | 0.6 | 0.1 | 0.1 | 0.5 | 0.1 | 0.5 | 0.5 | 0.4 | 0.3 |
| Sap30 | 3.3 | 3.6 | 2.7 | 5.1 | 6.0 | 3.1 | 3.0 | 1.8 | 2.5 | 4.4 | 3.3 | 3.4 |
| Sap30bp | 14.7 | 12.5 | 14.7 | 17.9 | 12.8 | 14.8 | 16.2 | 15.1 | 11.9 | 13.4 | 15.6 | 15.6 |
| Sap30l | 16.3 | 12.1 | 11.4 | 12.5 | 11.7 | 15.8 | 12.0 | 12.7 | 11.7 | 13.7 | 14.3 | 13.5 |
| Sapcd1 | 0.0 | 0.1 | 0.1 | 0.1 | 0.1 | 0.1 | 0.0 | 0.0 | 0.0 | 0.3 | 0.0 | 0.0 |
| Sapcd2 | 0.1 | 0.2 | 0.3 | 0.1 | 0.3 | 0.1 | 0.4 | 0.2 | 0.6 | 0.2 | 0.1 | 0.2 |
| Sar1a | 95.6 | 82.9 | 69.8 | 94.9 | 83.2 | 81.9 | 91.3 | 84.8 | 90.8 | 85.3 | 92.0 | 91.4 |
| Sar1b | 36.8 | 32.6 | 34.8 | 45.6 | 34.9 | 42.1 | 34.7 | 33.2 | 32.8 | 37.7 | 42.2 | 41.2 |
| Sardh | 10.0 | 11.6 | 12.8 | 6.5 | 6.9 | 6.5 | 7.6 | 12.7 | 8.1 | 8.9 | 7.2 | 8.8 |
| Sarm1 | 0.1 | 0.2 | 0.2 | 0.2 | 0.1 | 0.1 | 0.3 | 0.2 | 0.2 | 0.1 | 0.1 | 0.1 |
| Sarnp | 52.2 | 46.1 | 52.8 | 50.1 | 39.9 | 47.4 | 45.4 | 46.8 | 39.2 | 47.9 | 56.8 | 58.1 |
| Sars | 61.2 | 39.1 | 35.6 | 61.7 | 39.2 | 57.1 | 41.5 | 49.1 | 40.1 | 43.5 | 49.4 | 43.5 |
| Sars2 | 2.9 | 2.8 | 2.5 | 2.4 | 2.7 | 2.2 | 3.5 | 3.5 | 3.3 | 3.4 | 2.9 | 2.6 |
| Sart1 | 11.4 | 10.4 | 10.9 | 13.1 | 10.0 | 12.5 | 12.2 | 10.5 | 9.4 | 10.1 | 11.9 | 10.8 |
| Sart3 | 5.5 | 6.1 | 6.7 | 6.6 | 6.6 | 6.8 | 7.5 | 7.3 | 7.1 | 7.6 | 7.0 | 6.5 |
| Sash1 | 22.9 | 27.6 | 28.5 | 30.2 | 28.0 | 23.0 | 24.7 | 21.9 | 23.3 | 14.7 | 17.7 | 22.8 |
| Sash3 | 0.3 | 0.0 | 0.0 | 1.8 | 2.9 | 0.0 | 0.4 | 0.1 | 0.1 | 0.2 | 0.0 | 0.2 |
| Sass6 | 0.8 | 0.9 | 1.4 | 0.6 | 1.1 | 0.6 | 1.1 | 1.0 | 1.0 | 0.8 | 1.3 | 0.9 |
| Sat1 | 108.3 | 72.2 | 58.8 | 102.2 | 98.1 | 89.0 | 101.3 | 66.3 | 97.3 | 113.3 | 99.0 | 117.3 |
| Sat2 | 4.4 | 5.3 | 7.5 | 4.7 | 3.8 | 4.8 | 4.8 | 5.1 | 5.2 | 3.8 | 3.4 | 5.4 |
| Satb1 | 0.2 | 0.2 | 0.2 | 0.3 | 0.1 | 0.4 | 0.2 | 0.2 | 0.1 | 0.3 | 0.3 | 0.2 |
| Satb2 | 0.1 | 0.1 | 0.2 | 0.2 | 0.1 | 0.1 | 0.1 | 0.1 | 0.1 | 0.1 | 0.1 | 0.1 |
| Sav1 | 28.2 | 28.7 | 31.2 | 32.1 | 29.7 | 33.1 | 32.9 | 27.2 | 29.6 | 23.3 | 31.3 | 30.9 |
| Saysd1 | 4.0 | 3.0 | 3.5 | 3.3 | 3.4 | 3.7 | 3.8 | 3.2 | 3.6 | 3.8 | 3.6 | 3.1 |
| Sbds | 61.9 | 46.8 | 52.3 | 87.9 | 50.4 | 67.5 | 61.2 | 49.9 | 47.7 | 49.5 | 61.8 | 64.9 |
| Sbf1 | 8.9 | 11.7 | 9.4 | 9.2 | 9.8 | 9.7 | 10.7 | 10.3 | 11.5 | 9.4 | 8.8 | 7.8 |
| Sbf2 | 12.7 | 12.6 | 13.2 | 13.2 | 11.3 | 13.6 | 12.6 | 11.8 | 12.1 | 9.4 | 11.6 | 11.7 |
| Sbk1 | 1.8 | 1.8 | 1.7 | 1.3 | 1.7 | 1.3 | 1.4 | 1.5 | 1.5 | 1.7 | 1.1 | 1.3 |
| Sbk2 | 0.2 | 0.1 | 0.1 | 0.1 | 0.1 | 0.2 | 0.0 | 0.0 | 0.0 | 0.0 | 0.1 | 0.0 |
| Sbno1 | 10.1 | 10.3 | 10.0 | 10.8 | 10.9 | 10.7 | 9.8 | 9.5 | 9.8 | 10.4 | 10.7 | 10.8 |
| Sbno2 | 14.6 | 17.2 | 19.1 | 13.2 | 21.1 | 10.2 | 19.1 | 19.0 | 20.9 | 19.0 | 16.3 | 18.6 |
| Sbpl | 0.0 | 0.1 | 0.0 | 0.0 | 0.1 | 0.0 | 0.0 | 0.0 | 0.0 | 0.0 | 0.0 | 0.1 |
| Sbsn | 14.5 | 12.2 | 15.1 | 7.4 | 12.7 | 6.8 | 11.1 | 9.9 | 7.7 | 33.0 | 24.6 | 16.7 |
| Sbspon | 0.0 | 0.1 | 0.0 | 0.0 | 0.0 | 0.1 | 0.0 | 0.0 | 0.0 | 0.0 | 0.0 | 0.0 |
| Sc4mol | 51.8 | 62.4 | 52.3 | 20.0 | 35.4 | 34.9 | 87.2 | 92.7 | 82.2 | 30.0 | 90.2 | 38.4 |
| Sc5d | 40.6 | 49.7 | 53.2 | 25.3 | 32.5 | 31.8 | 70.1 | 71.3 | 66.8 | 29.2 | 69.9 | 40.1 |

| | | | | | | | | | | | | |
|---|---|---|---|---|---|---|---|---|---|---|---|---|
| Scaf1 | 7.4 | 8.2 | 8.2 | 7.7 | 7.7 | 7.4 | 9.5 | 7.8 | 9.0 | 7.7 | 7.7 | 7.2 |
| Scaf11 | 18.1 | 19.4 | 20.5 | 17.5 | 18.2 | 17.6 | 18.8 | 20.5 | 18.4 | 18.4 | 20.5 | 20.5 |
| Scaf4 | 2.1 | 2.4 | 2.5 | 2.4 | 2.8 | 2.0 | 3.3 | 2.0 | 2.2 | 2.1 | 2.3 | 2.0 |
| Scaf8 | 8.6 | 8.2 | 9.0 | 7.9 | 7.9 | 8.3 | 9.3 | 8.9 | 9.2 | 8.5 | 9.6 | 9.1 |
| Scai | 1.0 | 1.2 | 1.4 | 0.9 | 0.8 | 1.1 | 0.9 | 1.3 | 1.0 | 1.2 | 1.3 | 1.3 |
| Scamp1 | 15.9 | 13.6 | 14.6 | 16.2 | 14.6 | 17.5 | 13.4 | 9.9 | 12.8 | 15.8 | 15.2 | 16.1 |
| Scamp2 | 13.1 | 11.6 | 9.0 | 16.1 | 15.6 | 16.2 | 14.0 | 12.8 | 13.0 | 14.9 | 12.6 | 11.1 |
| Scamp3 | 25.2 | 25.7 | 24.6 | 30.8 | 28.1 | 27.2 | 29.7 | 30.5 | 26.1 | 26.2 | 28.4 | 24.8 |
| Scamp4 | 15.4 | 12.9 | 11.9 | 16.2 | 15.4 | 13.9 | 17.9 | 16.3 | 15.6 | 13.9 | 14.6 | 11.2 |
| Scamp5 | 7.9 | 6.2 | 4.6 | 9.1 | 7.3 | 6.7 | 5.8 | 5.6 | 5.2 | 6.7 | 5.5 | 5.2 |
| Scand1 | 30.2 | 18.3 | 19.4 | 15.3 | 18.6 | 14.0 | 21.3 | 26.9 | 24.9 | 34.9 | 20.7 | 25.8 |
| Scap | 15.2 | 15.5 | 15.8 | 14.1 | 15.9 | 15.5 | 16.3 | 13.6 | 13.2 | 14.5 | 11.8 | 11.6 |
| Scaper | 5.5 | 6.3 | 7.1 | 5.5 | 4.8 | 6.5 | 5.4 | 6.0 | 5.4 | 5.3 | 6.2 | 6.1 |
| Scara3 | 6.9 | 9.7 | 13.5 | 4.9 | 6.3 | 7.3 | 8.0 | 11.1 | 8.2 | 8.9 | 8.1 | 10.1 |
| Scara5 | 0.4 | 1.5 | 3.5 | 0.2 | 0.6 | 0.7 | 0.1 | 4.7 | 0.7 | 0.5 | 0.6 | 0.6 |
| Scarb1 | 4.1 | 4.8 | 4.9 | 2.5 | 4.4 | 4.0 | 6.6 | 5.9 | 5.8 | 2.8 | 5.3 | 3.3 |
| Scarb2 | 115.3 | 90.7 | 91.1 | 100.1 | 99.0 | 93.4 | 81.2 | 77.9 | 121.3 | 128.7 | 98.4 | 155.5 |
| Scarf1 | 0.3 | 0.2 | 0.2 | 0.4 | 0.2 | 0.2 | 0.1 | 0.2 | 0.1 | 0.1 | 0.0 | 0.1 |
| Scarf2 | 10.6 | 8.3 | 6.4 | 9.6 | 7.8 | 11.2 | 10.7 | 8.1 | 9.9 | 9.8 | 7.6 | 8.0 |
| Scarna10 | 265.1 | 795.0 | 301.2 | 94.7 | 489.4 | 108.6 | 72.6 | 519.7 | 862.5 | 517.8 | 0.0 | 488.9 |
| Scarna13 | 208.1 | 189.8 | 175.4 | 109.6 | 162.1 | 143.2 | 126.8 | 197.8 | 141.7 | 188.5 | 172.3 | 130.3 |
| Scarna17 | 560.2 | 1804.2 | 0.0 | 0.0 | 0.0 | 692.8 | 0.0 | 538.7 | 0.0 | 0.0 | 738.7 | 514.1 |
| Scarna2 | 1949.5 | 0.0 | 2119.8 | 1342.7 | 2556.4 | 2011.5 | 516.7 | 0.0 | 326.0 | 1120.7 | 1733.1 | 893.0 |
| Scarna3a | 43.8 | 75.6 | 63.0 | 9.4 | 67.6 | 23.1 | 50.5 | 78.8 | 160.1 | 98.9 | 52.5 | 49.7 |
| Scarna3b | 23.0 | 7.0 | 16.8 | 0.0 | 19.6 | 0.0 | 0.0 | 26.8 | 17.7 | 9.8 | 4.6 | 0.0 |
| Scarna6 | 39.7 | 51.6 | 43.2 | 22.4 | 56.0 | 38.2 | 26.7 | 42.2 | 72.6 | 58.6 | 34.5 | 29.2 |
| Sccpdh | 29.1 | 26.4 | 24.1 | 28.0 | 23.2 | 30.3 | 23.1 | 20.4 | 23.8 | 25.1 | 27.1 | 25.3 |
| Scd1 | 18.2 | 34.9 | 42.7 | 12.7 | 13.4 | 19.8 | 33.0 | 81.7 | 42.0 | 11.9 | 37.1 | 15.2 |
| Scd2 | 84.3 | 163.2 | 157.6 | 44.4 | 79.4 | 85.2 | 180.8 | 276.1 | 184.7 | 45.1 | 153.5 | 59.4 |
| Scd3 | 0.9 | 1.9 | 1.9 | 0.6 | 0.8 | 1.1 | 2.2 | 3.7 | 2.2 | 0.4 | 1.5 | 0.6 |
| Scd4 | 0.3 | 0.5 | 0.7 | 0.1 | 0.2 | 0.3 | 0.7 | 1.1 | 0.6 | 0.2 | 0.4 | 0.3 |
| Scel | 90.9 | 132.8 | 166.0 | 87.8 | 87.6 | 70.9 | 133.1 | 155.6 | 117.6 | 90.8 | 136.4 | 145.0 |
| Scfd1 | 41.0 | 41.4 | 36.2 | 36.9 | 35.1 | 37.3 | 36.8 | 39.8 | 39.7 | 41.1 | 43.0 | 43.6 |
| Scfd2 | 3.9 | 3.9 | 3.7 | 3.6 | 3.6 | 3.5 | 3.3 | 2.7 | 3.3 | 3.5 | 3.6 | 3.6 |
| Scg2 | 0.7 | 1.7 | 2.7 | 1.0 | 0.8 | 1.5 | 1.3 | 5.6 | 3.9 | 0.7 | 0.8 | 2.5 |
| Scg3 | 0.1 | 0.1 | 0.0 | 0.0 | 0.0 | 0.0 | 0.1 | 0.0 | 0.1 | 0.0 | 0.1 | 0.0 |
| Scgb1a1 | 0.1 | 0.1 | 0.2 | 0.2 | 0.1 | 0.2 | 0.3 | 0.7 | 0.1 | 0.2 | 0.9 | 0.4 |
| Scgb1b30 | 0.0 | 0.0 | 0.1 | 0.0 | 0.0 | 0.1 | 0.0 | 0.0 | 0.1 | 0.1 | 0.2 | 0.0 |
| Scgb3a1 | 0.2 | 0.3 | 1.1 | 0.7 | 0.5 | 0.3 | 0.0 | 0.3 | 0.2 | 0.2 | 0.2 | 0.2 |
| Scgn | 0.2 | 0.1 | 0.0 | 0.2 | 0.1 | 0.0 | 0.1 | 0.0 | 0.0 | 0.0 | 0.1 | 0.0 |
| Schip1 | 7.8 | 5.4 | 5.1 | 7.3 | 7.0 | 5.7 | 8.2 | 5.5 | 8.2 | 9.3 | 9.6 | 7.3 |
| Scimp | 0.3 | 0.0 | 0.0 | 0.8 | 1.6 | 0.0 | 0.3 | 0.1 | 0.1 | 0.1 | 0.0 | 0.0 |
| Scin | 0.7 | 0.6 | 1.4 | 0.2 | 0.8 | 0.2 | 0.7 | 1.1 | 0.4 | 1.0 | 1.4 | 1.6 |
| Sclt1 | 3.2 | 3.2 | 3.1 | 3.2 | 2.7 | 3.0 | 2.6 | 3.1 | 2.8 | 4.0 | 4.0 | 3.9 |
| Scly | 8.1 | 7.8 | 6.6 | 10.9 | 9.4 | 11.3 | 7.5 | 5.7 | 6.5 | 5.5 | 6.7 | 6.3 |
| Scmh1 | 8.9 | 6.7 | 6.1 | 8.2 | 6.0 | 8.9 | 6.2 | 4.8 | 4.8 | 6.8 | 6.7 | 5.4 |
| Scml2 | 0.3 | 0.7 | 0.9 | 0.1 | 0.8 | 0.3 | 0.7 | 0.4 | 0.5 | 0.3 | 0.5 | 0.3 |
| Scml4 | 0.6 | 0.6 | 0.4 | 1.4 | 0.8 | 0.9 | 0.7 | 0.4 | 0.4 | 0.3 | 0.3 | 0.2 |
| Scn11a | 0.0 | 0.0 | 0.0 | 0.0 | 0.0 | 0.0 | 0.0 | 0.0 | 0.0 | 0.0 | 0.0 | 0.0 |
| Scn1a | 0.0 | 0.0 | 0.0 | 0.0 | 0.0 | 0.0 | 0.0 | 0.0 | 0.0 | 0.0 | 0.0 | 0.0 |
| Scn1b | 31.0 | 30.8 | 29.9 | 30.3 | 29.5 | 31.9 | 24.9 | 21.2 | 23.6 | 23.8 | 20.7 | 25.6 |
| Scn2a1 | 0.8 | 0.9 | 1.3 | 0.8 | 0.7 | 1.2 | 0.9 | 1.0 | 0.9 | 0.9 | 1.2 | 0.9 |
| Scn2b | 0.1 | 0.1 | 0.2 | 0.0 | 0.0 | 0.0 | 0.0 | 0.1 | 0.0 | 0.0 | 0.0 | 0.1 |
| Scn3a | 0.0 | 0.0 | 0.0 | 0.0 | 0.0 | 0.0 | 0.0 | 0.0 | 0.0 | 0.0 | 0.0 | 0.0 |
| Scn3b | 1.3 | 0.7 | 0.7 | 0.7 | 1.6 | 0.4 | 0.9 | 0.5 | 0.4 | 1.5 | 0.8 | 0.6 |
| Scn4a | 0.0 | 0.0 | 0.0 | 0.0 | 0.0 | 0.0 | 0.0 | 0.0 | 0.0 | 0.0 | 0.0 | 0.0 |
| Scn4b | 0.1 | 0.2 | 0.1 | 0.1 | 0.3 | 0.1 | 0.1 | 0.0 | 0.1 | 0.0 | 0.1 | 0.1 |

| | | | | | | | | | | | | |
|---|---|---|---|---|---|---|---|---|---|---|---|---|
| Scn5a | 0.0 | 0.0 | 0.0 | 0.0 | 0.0 | 0.0 | 0.0 | 0.0 | 0.0 | 0.0 | 0.0 | 0.0 |
| Scn7a | 4.8 | 10.4 | 22.9 | 3.0 | 8.4 | 1.8 | 7.3 | 14.0 | 13.3 | 4.2 | 5.1 | 10.8 |
| Scn8a | 0.0 | 0.0 | 0.0 | 0.0 | 0.0 | 0.0 | 0.0 | 0.0 | 0.0 | 0.0 | 0.0 | 0.0 |
| Scn9a | 0.0 | 0.0 | 0.0 | 0.0 | 0.0 | 0.0 | 0.0 | 0.0 | 0.0 | 0.0 | 0.0 | 0.0 |
| Scnm1 | 15.2 | 15.4 | 15.7 | 23.2 | 13.5 | 17.0 | 16.2 | 17.4 | 14.0 | 15.5 | 19.4 | 17.3 |
| Scnn1a | 0.3 | 0.2 | 0.2 | 0.3 | 0.3 | 0.2 | 0.1 | 0.2 | 0.2 | 0.2 | 0.3 | 0.2 |
| Sco1 | 3.5 | 2.8 | 3.0 | 3.8 | 3.3 | 3.0 | 3.2 | 2.8 | 3.0 | 3.5 | 3.6 | 3.3 |
| Sco2 | 10.4 | 7.9 | 6.6 | 12.7 | 9.9 | 10.0 | 11.7 | 11.8 | 10.0 | 10.4 | 10.1 | 8.6 |
| Scoc | 40.2 | 39.0 | 39.9 | 49.2 | 40.8 | 50.2 | 34.6 | 29.8 | 35.9 | 47.4 | 50.8 | 44.7 |
| Scp2 | 49.8 | 59.0 | 71.9 | 44.8 | 44.7 | 42.2 | 37.1 | 64.9 | 48.2 | 53.2 | 49.1 | 54.1 |
| Scpep1 | 80.8 | 72.3 | 69.4 | 94.7 | 67.4 | 119.1 | 53.6 | 79.8 | 61.1 | 64.6 | 82.8 | 65.6 |
| Scrib | 4.5 | 4.9 | 4.7 | 3.8 | 4.4 | 4.6 | 4.6 | 4.2 | 5.0 | 4.5 | 4.3 | 3.9 |
| Scrn1 | 35.5 | 25.2 | 22.5 | 37.4 | 18.9 | 36.6 | 27.7 | 31.4 | 23.6 | 26.5 | 33.0 | 32.4 |
| Scrn2 | 2.8 | 3.4 | 2.5 | 1.9 | 2.0 | 3.2 | 2.1 | 3.3 | 2.6 | 2.9 | 2.6 | 2.9 |
| Scrn3 | 4.2 | 5.1 | 5.1 | 4.2 | 3.3 | 5.3 | 3.8 | 4.5 | 4.2 | 6.0 | 5.7 | 5.3 |
| Sct | 0.1 | 0.2 | 0.5 | 0.0 | 0.1 | 0.0 | 0.4 | 0.0 | 0.0 | 0.1 | 0.0 | 0.2 |
| Sctr | 0.1 | 0.1 | 0.0 | 0.1 | 0.0 | 0.2 | 0.0 | 0.0 | 0.0 | 0.0 | 0.0 | 0.0 |
| Scube1 | 0.0 | 0.1 | 0.0 | 0.0 | 0.0 | 0.1 | 0.1 | 0.0 | 0.0 | 0.1 | 0.0 | 0.0 |
| Scube2 | 0.1 | 0.1 | 0.3 | 0.2 | 0.2 | 0.2 | 0.2 | 0.3 | 0.2 | 0.1 | 0.0 | 0.2 |
| Scube3 | 0.1 | 0.1 | 0.2 | 0.2 | 0.2 | 0.1 | 0.1 | 0.2 | 0.2 | 0.7 | 0.1 | 0.2 |
| Scx | 15.0 | 4.2 | 2.6 | 2.9 | 2.3 | 3.5 | 1.8 | 2.1 | 1.2 | 3.5 | 2.8 | 4.1 |
| Scyl1 | 35.2 | 33.4 | 33.3 | 40.6 | 36.5 | 35.4 | 41.2 | 38.6 | 37.8 | 37.0 | 39.4 | 38.4 |
| Scyl2 | 13.1 | 12.8 | 12.4 | 13.2 | 12.5 | 13.1 | 11.5 | 11.5 | 11.5 | 11.7 | 11.9 | 12.5 |
| Scyl3 | 3.6 | 4.1 | 3.9 | 3.3 | 4.2 | 3.4 | 3.2 | 3.8 | 4.3 | 3.9 | 3.2 | 3.8 |
| Sdad1 | 4.1 | 3.5 | 3.1 | 4.3 | 3.6 | 4.1 | 4.1 | 3.7 | 3.1 | 3.6 | 4.3 | 3.6 |
| Sdc1 | 35.0 | 32.0 | 30.9 | 21.0 | 30.1 | 28.0 | 35.9 | 32.4 | 35.8 | 35.9 | 33.6 | 42.4 |
| Sdc2 | 73.2 | 63.6 | 67.0 | 87.3 | 55.0 | 83.1 | 67.3 | 58.8 | 78.6 | 71.2 | 85.5 | 89.2 |
| Sdc3 | 30.8 | 29.3 | 26.8 | 48.5 | 49.1 | 40.0 | 38.1 | 26.6 | 30.1 | 20.0 | 22.9 | 24.5 |
| Sdc4 | 67.4 | 64.8 | 69.6 | 47.1 | 70.2 | 36.5 | 57.9 | 51.6 | 56.2 | 187.9 | 85.4 | 99.7 |
| Sdcbp | 82.0 | 69.2 | 57.2 | 96.0 | 102.3 | 81.6 | 65.5 | 59.7 | 63.7 | 73.7 | 80.5 | 69.4 |
| Sdcbp2 | 0.5 | 0.2 | 0.6 | 0.2 | 0.3 | 0.4 | 0.1 | 0.3 | 0.2 | 0.2 | 0.0 | 0.3 |
| Sdccag3 | 31.6 | 27.4 | 24.8 | 32.9 | 28.5 | 29.3 | 30.1 | 26.5 | 27.7 | 26.0 | 29.9 | 27.2 |
| Sdccag8 | 11.4 | 11.3 | 10.8 | 11.9 | 9.6 | 11.6 | 9.1 | 10.8 | 8.3 | 8.9 | 10.7 | 10.5 |
| Sde2 | 8.6 | 9.5 | 8.7 | 8.3 | 8.1 | 7.8 | 8.6 | 8.5 | 7.7 | 8.2 | 8.9 | 8.4 |
| Sdf2 | 50.6 | 46.1 | 41.3 | 45.9 | 46.9 | 42.4 | 47.7 | 44.1 | 50.4 | 56.3 | 50.7 | 52.6 |
| Sdf2l1 | 41.6 | 33.8 | 29.8 | 47.6 | 37.8 | 39.9 | 34.8 | 30.9 | 34.7 | 36.4 | 34.8 | 34.0 |
| Sdf4 | 39.3 | 32.2 | 38.6 | 42.3 | 34.1 | 37.5 | 39.6 | 38.9 | 33.7 | 35.7 | 36.3 | 43.2 |
| Sdha | 63.3 | 59.6 | 55.5 | 62.7 | 61.1 | 64.0 | 58.6 | 60.5 | 55.8 | 59.7 | 60.8 | 63.1 |
| Sdhaf1 | 7.6 | 6.8 | 6.5 | 8.1 | 6.5 | 8.5 | 9.2 | 8.0 | 6.3 | 8.6 | 10.6 | 8.3 |
| Sdhaf2 | 14.9 | 13.5 | 12.0 | 16.7 | 12.5 | 15.8 | 12.5 | 11.3 | 11.4 | 14.8 | 16.1 | 14.5 |
| Sdhb | 67.8 | 57.8 | 51.2 | 65.6 | 60.4 | 62.1 | 52.3 | 55.6 | 48.6 | 54.4 | 58.9 | 56.4 |
| Sdhc | 65.8 | 61.1 | 50.7 | 64.4 | 57.5 | 62.3 | 50.1 | 51.7 | 57.7 | 62.5 | 55.8 | 50.4 |
| Sdhd | 51.4 | 44.6 | 41.0 | 42.3 | 50.3 | 44.8 | 44.3 | 41.8 | 49.6 | 50.3 | 45.1 | 45.8 |
| Sdk1 | 1.4 | 1.4 | 1.7 | 1.1 | 1.6 | 1.0 | 1.3 | 0.8 | 1.1 | 2.1 | 1.0 | 1.3 |
| Sdk2 | 0.0 | 0.0 | 0.0 | 0.0 | 0.0 | 0.0 | 0.0 | 0.0 | 0.0 | 0.0 | 0.0 | 0.0 |
| Sdpr | 101.4 | 58.4 | 46.2 | 75.5 | 42.8 | 55.2 | 78.5 | 52.2 | 64.5 | 87.2 | 99.6 | 79.5 |
| Sdr39u1 | 4.7 | 5.4 | 6.0 | 5.0 | 4.0 | 4.8 | 3.6 | 4.4 | 4.4 | 4.4 | 4.4 | 6.0 |
| Sdr42e1 | 2.3 | 2.4 | 2.2 | 2.2 | 2.0 | 2.4 | 2.0 | 1.6 | 1.7 | 1.9 | 1.6 | 2.4 |
| Sds | 0.1 | 0.0 | 0.0 | 0.0 | 0.0 | 0.1 | 0.1 | 0.1 | 0.2 | 0.1 | 0.1 | 0.0 |
| Sdsl | 10.9 | 10.0 | 5.7 | 9.0 | 7.1 | 6.7 | 4.8 | 3.5 | 4.9 | 7.0 | 4.6 | 4.8 |
| Sebox | 0.1 | 0.0 | 0.0 | 0.0 | 0.1 | 0.1 | 0.0 | 0.0 | 0.0 | 0.1 | 0.0 | 0.0 |
| Sec1 | 0.0 | 0.0 | 0.1 | 0.0 | 0.0 | 0.1 | 0.0 | 0.0 | 0.0 | 0.1 | 0.0 | 0.1 |
| Sec11a | 45.1 | 43.4 | 42.7 | 50.3 | 43.2 | 45.5 | 45.7 | 52.0 | 49.9 | 54.0 | 50.7 | 54.1 |
| Sec11c | 78.4 | 53.2 | 45.7 | 85.3 | 62.3 | 67.3 | 56.4 | 37.4 | 41.6 | 59.6 | 57.9 | 63.0 |
| Sec13 | 132.7 | 117.3 | 105.0 | 130.0 | 111.1 | 113.5 | 122.9 | 123.5 | 111.7 | 118.5 | 130.5 | 124.5 |
| Sec14l1 | 18.7 | 16.3 | 15.0 | 17.9 | 16.9 | 19.0 | 17.3 | 17.5 | 15.7 | 14.4 | 15.9 | 12.8 |
| Sec14l2 | 0.7 | 0.3 | 0.3 | 0.6 | 0.5 | 0.7 | 0.8 | 0.8 | 0.6 | 0.2 | 0.6 | 0.5 |

| | | | | | | | | | | | | |
|---|---|---|---|---|---|---|---|---|---|---|---|---|
| Sec14l5 | 0.1 | 0.2 | 0.1 | 0.0 | 0.3 | 0.1 | 0.1 | 0.0 | 0.0 | 0.2 | 0.0 | 0.1 |
| Sec16a | 18.3 | 20.4 | 18.9 | 17.8 | 19.4 | 17.8 | 19.5 | 19.3 | 18.0 | 16.4 | 19.0 | 17.1 |
| Sec16b | 1.9 | 1.2 | 1.8 | 1.6 | 1.3 | 1.2 | 1.8 | 2.1 | 1.8 | 1.8 | 1.8 | 1.6 |
| Sec22a | 5.9 | 5.1 | 5.0 | 5.6 | 5.3 | 5.7 | 4.6 | 4.8 | 5.3 | 5.3 | 7.1 | 5.4 |
| Sec22b | 58.4 | 55.8 | 54.1 | 60.9 | 52.0 | 58.4 | 51.9 | 55.7 | 49.9 | 48.9 | 52.3 | 52.2 |
| Sec22c | 1.1 | 0.9 | 0.8 | 1.0 | 0.9 | 1.0 | 0.9 | 0.8 | 0.9 | 0.8 | 1.0 | 0.8 |
| Sec23a | 61.7 | 53.4 | 47.6 | 69.3 | 52.5 | 65.8 | 57.9 | 52.0 | 53.3 | 51.8 | 68.2 | 60.6 |
| Sec23b | 24.8 | 23.4 | 21.9 | 26.4 | 24.1 | 21.4 | 24.5 | 23.9 | 24.0 | 23.9 | 25.2 | 23.7 |
| Sec23ip | 22.2 | 21.0 | 22.4 | 18.6 | 20.3 | 18.6 | 21.9 | 23.1 | 20.1 | 20.3 | 24.4 | 23.2 |
| Sec24a | 2.3 | 2.8 | 2.5 | 2.4 | 2.8 | 2.6 | 2.8 | 2.7 | 2.4 | 2.6 | 3.0 | 2.3 |
| Sec24b | 4.1 | 5.1 | 4.7 | 4.3 | 4.4 | 4.5 | 5.3 | 5.1 | 4.9 | 4.0 | 5.2 | 3.6 |
| Sec24c | 23.3 | 24.8 | 20.0 | 21.8 | 23.0 | 23.1 | 22.7 | 21.6 | 22.6 | 21.3 | 24.0 | 19.3 |
| Sec24d | 38.6 | 36.6 | 37.7 | 38.3 | 37.7 | 37.5 | 40.3 | 45.7 | 42.5 | 36.4 | 42.1 | 38.8 |
| Sec31a | 74.7 | 74.0 | 71.8 | 78.9 | 70.2 | 74.0 | 81.1 | 87.8 | 75.4 | 72.8 | 82.4 | 78.6 |
| Sec31b | 0.2 | 0.4 | 0.3 | 0.3 | 0.2 | 0.4 | 0.2 | 0.3 | 0.2 | 0.2 | 0.1 | 0.2 |
| Sec61a1 | 63.8 | 57.9 | 54.1 | 64.7 | 64.4 | 57.4 | 66.8 | 76.1 | 69.1 | 69.9 | 66.8 | 66.2 |
| Sec61a2 | 8.9 | 10.0 | 8.6 | 9.8 | 8.3 | 10.2 | 7.6 | 8.3 | 8.0 | 8.4 | 8.3 | 7.4 |
| Sec61b | 121.8 | 98.8 | 90.4 | 109.6 | 104.7 | 108.2 | 95.2 | 109.2 | 112.0 | 121.7 | 111.8 | 101.8 |
| Sec61g | 139.9 | 108.8 | 115.4 | 138.6 | 104.4 | 116.5 | 108.1 | 110.1 | 101.6 | 118.6 | 134.0 | 148.6 |
| Sec62 | 64.8 | 57.6 | 70.7 | 90.1 | 54.7 | 73.0 | 68.8 | 59.4 | 52.4 | 60.1 | 75.9 | 85.5 |
| Sec63 | 14.6 | 13.2 | 13.6 | 15.7 | 14.3 | 12.4 | 16.5 | 13.6 | 13.7 | 15.6 | 17.3 | 17.2 |
| Secisbp2 | 4.9 | 4.8 | 3.4 | 5.4 | 4.6 | 4.5 | 4.3 | 5.2 | 4.9 | 4.5 | 4.8 | 4.6 |
| Secisbp2l | 20.9 | 21.2 | 19.7 | 24.2 | 20.9 | 20.3 | 21.9 | 19.0 | 20.9 | 18.0 | 22.8 | 21.7 |
| Sectm1a | 0.0 | 0.0 | 0.0 | 0.0 | 0.0 | 0.0 | 0.0 | 0.0 | 0.0 | 0.0 | 0.0 | 0.0 |
| Seh1l | 11.6 | 10.9 | 10.7 | 11.8 | 11.5 | 11.9 | 11.6 | 11.2 | 11.0 | 12.9 | 12.5 | 11.4 |
| Sel1l | 43.3 | 43.3 | 43.5 | 45.5 | 41.8 | 42.6 | 44.1 | 46.1 | 45.7 | 37.0 | 43.3 | 40.4 |
| Sel1l3 | 0.0 | 0.0 | 0.0 | 0.0 | 0.1 | 0.0 | 0.0 | 0.0 | 0.0 | 0.0 | 0.0 | 0.0 |
| Sele | 0.2 | 0.2 | 0.1 | 0.4 | 0.4 | 0.1 | 0.3 | 0.1 | 0.1 | 0.2 | 0.2 | 0.2 |
| Selenbp1 | 5.1 | 6.7 | 9.3 | 2.1 | 2.1 | 4.6 | 2.4 | 7.4 | 3.4 | 2.4 | 2.2 | 3.2 |
| Selenbp2 | 1.8 | 2.3 | 3.2 | 0.5 | 0.7 | 1.5 | 0.7 | 2.6 | 1.2 | 0.9 | 0.6 | 0.9 |
| Selk | 82.0 | 57.2 | 51.8 | 93.8 | 63.8 | 83.4 | 55.3 | 49.7 | 50.5 | 67.7 | 70.4 | 75.5 |
| Selm | 94.7 | 59.8 | 50.5 | 91.3 | 57.8 | 88.4 | 82.6 | 74.8 | 68.7 | 77.0 | 77.5 | 75.9 |
| Selp | 0.8 | 0.2 | 0.8 | 0.2 | 0.5 | 0.2 | 0.3 | 0.2 | 0.2 | 0.5 | 0.1 | 0.7 |
| Selplg | 0.6 | 0.0 | 0.0 | 5.2 | 8.9 | 0.0 | 1.6 | 0.1 | 0.2 | 0.5 | 0.2 | 0.4 |
| Selrc1 | 1.3 | 1.0 | 1.0 | 1.4 | 1.2 | 1.0 | 0.9 | 1.5 | 1.1 | 1.3 | 0.8 | 1.1 |
| Sema3a | 0.7 | 0.2 | 0.0 | 0.6 | 0.2 | 0.5 | 0.3 | 0.2 | 0.4 | 2.8 | 2.1 | 0.7 |
| Sema3b | 4.7 | 2.6 | 2.1 | 6.0 | 1.9 | 5.0 | 1.3 | 2.0 | 1.9 | 2.8 | 3.0 | 2.6 |
| Sema3c | 8.0 | 4.6 | 4.2 | 10.9 | 6.1 | 6.8 | 3.5 | 5.4 | 2.4 | 5.9 | 5.3 | 3.2 |
| Sema3d | 25.6 | 16.7 | 21.0 | 20.6 | 16.3 | 12.9 | 19.0 | 26.2 | 19.1 | 16.5 | 25.0 | 23.8 |
| Sema3e | 4.2 | 1.5 | 0.4 | 1.6 | 0.6 | 2.4 | 0.6 | 0.3 | 0.7 | 3.0 | 2.8 | 1.7 |
| Sema3f | 4.2 | 2.4 | 2.5 | 1.0 | 0.4 | 0.7 | 0.9 | 2.8 | 2.2 | 12.2 | 5.6 | 4.1 |
| Sema3g | 0.1 | 0.1 | 0.2 | 0.1 | 0.2 | 0.0 | 0.1 | 0.1 | 0.1 | 0.0 | 0.1 | 0.1 |
| Sema4a | 0.2 | 0.2 | 0.3 | 0.4 | 0.7 | 0.1 | 0.1 | 0.1 | 0.2 | 0.6 | 0.3 | 0.1 |
| Sema4b | 0.9 | 0.9 | 1.0 | 1.1 | 2.5 | 0.6 | 1.3 | 1.1 | 1.3 | 1.8 | 1.0 | 1.2 |
| Sema4c | 3.8 | 4.4 | 4.3 | 3.3 | 4.6 | 3.5 | 4.3 | 4.5 | 4.9 | 4.4 | 3.6 | 3.8 |
| Sema4d | 0.6 | 0.0 | 0.1 | 5.2 | 10.0 | 0.1 | 1.0 | 0.2 | 0.2 | 0.5 | 0.2 | 0.3 |
| Sema4f | 0.2 | 0.1 | 0.0 | 0.1 | 0.0 | 0.1 | 0.0 | 0.1 | 0.0 | 0.0 | 0.2 | 0.1 |
| Sema4g | 0.3 | 0.6 | 0.5 | 0.2 | 0.3 | 0.3 | 0.2 | 0.3 | 0.2 | 0.4 | 0.3 | 0.2 |
| Sema5a | 0.3 | 0.4 | 0.4 | 0.2 | 0.2 | 0.3 | 0.4 | 0.6 | 0.3 | 0.6 | 0.5 | 0.5 |
| Sema5b | 0.0 | 0.0 | 0.0 | 0.0 | 0.0 | 0.0 | 0.0 | 0.0 | 0.0 | 0.0 | 0.0 | 0.0 |
| Sema6a | 0.1 | 0.5 | 0.8 | 0.1 | 0.5 | 0.2 | 0.6 | 0.5 | 0.5 | 0.3 | 0.2 | 0.2 |
| Sema6b | 0.1 | 0.2 | 0.2 | 0.4 | 0.5 | 0.1 | 0.3 | 0.1 | 0.1 | 0.0 | 0.1 | 0.1 |
| Sema6c | 0.5 | 0.6 | 0.5 | 0.2 | 0.3 | 0.4 | 0.5 | 0.5 | 0.6 | 0.5 | 0.5 | 0.5 |
| Sema6d | 4.6 | 9.9 | 12.3 | 3.5 | 5.9 | 3.0 | 5.8 | 7.6 | 7.0 | 3.8 | 4.2 | 5.0 |
| Sema7a | 0.5 | 0.1 | 0.1 | 0.3 | 0.6 | 0.2 | 1.7 | 0.5 | 1.0 | 1.7 | 1.3 | 1.1 |
| Senp1 | 3.2 | 3.2 | 3.1 | 3.4 | 3.0 | 3.1 | 3.6 | 3.3 | 3.2 | 2.3 | 3.0 | 2.9 |
| Senp2 | 10.2 | 10.7 | 10.4 | 11.4 | 9.9 | 12.0 | 9.2 | 8.5 | 9.6 | 8.7 | 9.8 | 9.2 |

| | | | | | | | | | | | | |
|---|---|---|---|---|---|---|---|---|---|---|---|---|
| Senp3 | 28.1 | 27.1 | 25.2 | 25.7 | 25.6 | 27.6 | 25.5 | 24.7 | 24.1 | 26.9 | 25.0 | 23.3 |
| Senp5 | 10.2 | 10.3 | 8.9 | 9.9 | 10.3 | 10.6 | 9.0 | 8.4 | 9.1 | 10.3 | 11.0 | 9.2 |
| Senp6 | 19.7 | 21.4 | 20.3 | 21.8 | 19.2 | 22.0 | 20.1 | 19.4 | 20.4 | 19.8 | 23.9 | 21.9 |
| Senp7 | 2.7 | 2.8 | 3.3 | 2.4 | 2.1 | 2.3 | 2.3 | 3.1 | 2.7 | 3.5 | 3.3 | 3.5 |
| Senp8 | 2.1 | 2.5 | 2.7 | 2.7 | 2.1 | 2.6 | 1.7 | 2.6 | 2.4 | 2.5 | 2.8 | 2.3 |
| Sephs1 | 5.5 | 5.9 | 6.2 | 5.3 | 5.7 | 4.6 | 6.9 | 6.6 | 6.5 | 5.6 | 6.5 | 6.4 |
| Sephs2 | 20.0 | 17.4 | 15.7 | 19.3 | 23.4 | 17.4 | 19.0 | 15.3 | 14.5 | 17.9 | 17.6 | 15.5 |
| Sepn1 | 17.1 | 17.3 | 16.6 | 13.6 | 16.7 | 17.6 | 18.6 | 18.0 | 19.9 | 16.7 | 15.7 | 14.6 |
| Sepp1 | 36.3 | 32.9 | 53.2 | 53.3 | 45.0 | 33.2 | 37.9 | 48.0 | 40.9 | 77.0 | 59.9 | 80.6 |
| Sepsecs | 4.8 | 4.7 | 4.4 | 4.8 | 4.1 | 4.5 | 4.7 | 4.1 | 4.7 | 4.4 | 4.2 | 4.8 |
| Sepw1 | 75.6 | 49.1 | 48.7 | 89.0 | 48.5 | 90.8 | 53.7 | 53.6 | 46.4 | 60.2 | 57.7 | 59.2 |
| Serac1 | 0.9 | 1.0 | 1.1 | 0.8 | 1.1 | 1.2 | 0.9 | 1.0 | 0.8 | 0.9 | 0.8 | 0.8 |
| Serbp1 | 33.6 | 29.1 | 37.3 | 39.5 | 29.1 | 30.7 | 37.2 | 36.2 | 28.2 | 34.2 | 42.5 | 43.1 |
| Serf1 | 40.5 | 34.8 | 41.5 | 51.0 | 32.8 | 46.5 | 35.4 | 38.3 | 30.7 | 37.4 | 45.9 | 46.5 |
| Serf2 | 154.0 | 126.6 | 112.9 | 192.2 | 136.0 | 180.2 | 143.0 | 127.0 | 124.0 | 131.9 | 139.2 | 148.9 |
| Sergef | 3.9 | 3.4 | 3.3 | 3.0 | 3.2 | 3.5 | 3.1 | 3.4 | 2.7 | 3.0 | 3.0 | 2.8 |
| Serhl | 10.8 | 9.1 | 9.2 | 9.7 | 6.9 | 9.4 | 9.3 | 10.5 | 9.0 | 8.8 | 8.8 | 9.4 |
| Serinc1 | 129.3 | 112.4 | 111.2 | 158.6 | 111.9 | 145.1 | 115.1 | 107.1 | 104.9 | 123.0 | 143.9 | 144.3 |
| Serinc2 | 0.5 | 0.4 | 0.2 | 1.2 | 0.4 | 1.8 | 0.2 | 0.2 | 0.1 | 0.2 | 0.3 | 0.1 |
| Serinc3 | 129.7 | 115.5 | 114.5 | 152.2 | 136.0 | 143.1 | 120.5 | 126.3 | 123.0 | 128.8 | 123.8 | 139.0 |
| Serinc4 | 22.0 | 15.7 | 13.0 | 24.5 | 19.3 | 24.4 | 15.0 | 13.5 | 16.2 | 19.1 | 18.4 | 18.8 |
| Serinc5 | 3.1 | 4.1 | 4.6 | 2.4 | 3.8 | 2.7 | 4.0 | 5.7 | 5.5 | 5.4 | 5.1 | 5.2 |
| Serp1 | 103.3 | 92.8 | 92.4 | 112.3 | 109.9 | 101.2 | 93.9 | 99.2 | 108.2 | 124.6 | 131.9 | 125.4 |
| Serp2 | 0.8 | 0.1 | 0.1 | 0.4 | 0.2 | 0.4 | 0.3 | 0.2 | 0.0 | 0.6 | 0.4 | 0.3 |
| Serpina11 | 0.0 | 0.0 | 0.0 | 0.0 | 0.1 | 0.2 | 0.0 | 0.0 | 0.0 | 0.0 | 0.0 | 0.1 |
| Serpina12 | 0.3 | 0.4 | 0.3 | 0.3 | 0.3 | 0.4 | 0.3 | 0.3 | 0.3 | 0.2 | 0.2 | 0.3 |
| Serpina3a | 0.0 | 0.1 | 0.0 | 0.0 | 0.0 | 0.0 | 0.0 | 0.0 | 0.0 | 0.0 | 0.0 | 0.0 |
| Serpina3b | 0.1 | 0.1 | 0.0 | 0.0 | 0.0 | 0.0 | 0.0 | 0.0 | 0.0 | 0.0 | 0.0 | 0.0 |
| Serpina3c | 2.3 | 2.2 | 2.9 | 1.3 | 2.5 | 1.1 | 1.3 | 2.2 | 1.2 | 0.7 | 1.0 | 1.6 |
| Serpina3f | 0.2 | 0.4 | 1.1 | 0.2 | 0.8 | 0.1 | 0.3 | 1.0 | 0.3 | 0.2 | 0.1 | 0.4 |
| Serpina3g | 5.0 | 5.6 | 14.5 | 1.4 | 13.7 | 0.6 | 7.2 | 11.0 | 5.2 | 4.2 | 2.6 | 5.8 |
| Serpina3h | 3.7 | 4.5 | 10.2 | 1.4 | 5.3 | 0.6 | 2.8 | 10.7 | 3.2 | 0.7 | 0.8 | 2.1 |
| Serpina3i | 2.2 | 2.2 | 5.9 | 1.1 | 4.9 | 0.3 | 2.5 | 4.1 | 1.8 | 1.2 | 0.7 | 2.0 |
| Serpina3j | 0.0 | 0.0 | 0.0 | 0.0 | 0.0 | 0.0 | 0.0 | 0.0 | 0.0 | 0.0 | 0.0 | 0.0 |
| Serpina3k | 0.0 | 0.0 | 0.1 | 0.0 | 0.0 | 0.0 | 0.0 | 0.1 | 0.0 | 0.0 | 0.0 | 0.0 |
| Serpina3m | 0.2 | 0.9 | 2.7 | 0.0 | 0.6 | 0.0 | 0.4 | 0.7 | 0.2 | 0.5 | 0.3 | 0.5 |
| Serpina3n | 69.9 | 163.6 | 547.7 | 23.7 | 163.4 | 18.6 | 89.9 | 302.8 | 79.3 | 66.6 | 57.7 | 140.7 |
| Serpina4-ps1 | 0.0 | 0.1 | 0.2 | 0.0 | 0.0 | 0.1 | 0.0 | 0.0 | 0.0 | 0.0 | 0.0 | 0.1 |
| Serpina5 | 0.1 | 0.1 | 0.1 | 0.0 | 0.1 | 0.0 | 0.0 | 0.1 | 0.0 | 0.0 | 0.1 | 0.0 |
| Serpina9 | 2.5 | 2.1 | 1.3 | 0.9 | 1.1 | 1.3 | 1.0 | 2.2 | 1.3 | 1.2 | 1.8 | 0.9 |
| Serpinb12 | 0.1 | 0.0 | 0.0 | 0.2 | 0.2 | 0.0 | 0.0 | 0.0 | 0.0 | 0.0 | 0.0 | 0.0 |
| Serpinb1a | 32.5 | 16.0 | 13.7 | 16.0 | 17.9 | 15.8 | 17.7 | 24.5 | 27.3 | 22.3 | 26.8 | 34.3 |
| Serpinb1b | 1.5 | 1.5 | 1.1 | 1.7 | 1.4 | 1.9 | 0.2 | 0.7 | 0.7 | 0.9 | 0.6 | 0.8 |
| Serpinb1c | 0.9 | 0.4 | 0.1 | 1.5 | 0.2 | 1.1 | 0.0 | 0.1 | 0.0 | 0.2 | 0.2 | 0.1 |
| Serpinb2 | 0.0 | 0.0 | 0.1 | 0.2 | 0.1 | 0.0 | 0.4 | 0.0 | 0.2 | 0.3 | 0.2 | 0.2 |
| Serpinb6a | 387.2 | 283.0 | 230.1 | 356.8 | 277.1 | 365.5 | 270.6 | 245.9 | 274.5 | 243.2 | 304.8 | 326.1 |
| Serpinb6b | 87.8 | 33.5 | 27.1 | 42.3 | 24.1 | 45.0 | 19.2 | 16.0 | 27.0 | 76.4 | 66.5 | 56.3 |
| Serpinb6c | 8.9 | 3.8 | 1.4 | 7.1 | 2.5 | 4.8 | 1.1 | 0.8 | 0.8 | 1.4 | 2.9 | 1.2 |
| Serpinb8 | 4.2 | 3.5 | 4.7 | 1.4 | 3.0 | 1.6 | 3.3 | 3.2 | 3.1 | 3.0 | 4.6 | 4.2 |
| Serpinb9 | 26.4 | 20.6 | 23.8 | 26.4 | 32.3 | 23.2 | 25.9 | 31.7 | 54.5 | 33.0 | 42.6 | 54.6 |
| Serpinb9b | 295.4 | 133.1 | 58.7 | 225.3 | 109.0 | 240.2 | 60.1 | 48.2 | 43.8 | 87.4 | 129.2 | 99.8 |
| Serpinb9c | 0.1 | 0.0 | 0.0 | 0.3 | 0.0 | 0.0 | 0.0 | 0.0 | 0.0 | 0.0 | 0.0 | 0.0 |
| Serpinc1 | 0.1 | 0.0 | 0.1 | 0.1 | 0.0 | 0.0 | 0.0 | 0.1 | 0.0 | 0.0 | 0.0 | 0.0 |
| Serpind1 | 1.5 | 1.2 | 0.8 | 2.1 | 3.7 | 1.2 | 2.8 | 1.4 | 1.8 | 2.3 | 1.4 | 1.4 |
| Serpine1 | 1136.3 | 1360.5 | 912.0 | 1589.6 | 1106.2 | 1667.2 | 1306.9 | 740.8 | 806.3 | 799.2 | 891.7 | 769.0 |
| Serpine2 | 126.0 | 158.0 | 165.5 | 93.6 | 164.6 | 118.0 | 105.7 | 110.5 | 93.2 | 76.0 | 82.0 | 97.7 |
| Serpine3 | 2.4 | 3.5 | 2.0 | 1.3 | 1.4 | 0.6 | 1.4 | 1.8 | 0.6 | 1.4 | 1.1 | 0.8 |

| | | | | | | | | | | | | |
|---|---|---|---|---|---|---|---|---|---|---|---|---|
| Serpinf1 | 183.4 | 207.8 | 306.5 | 244.4 | 188.0 | 277.2 | 250.4 | 355.3 | 299.4 | 238.6 | 261.1 | 238.5 |
| Serpinf2 | 0.0 | 0.1 | 0.1 | 0.1 | 0.0 | 0.0 | 0.0 | 0.0 | 0.1 | 0.1 | 0.0 | 0.0 |
| Serping1 | 173.8 | 240.3 | 355.5 | 178.4 | 215.4 | 137.7 | 190.2 | 261.7 | 189.0 | 141.3 | 124.4 | 183.1 |
| Serpinh1 | 1054.5 | 1201.6 | 1295.3 | 964.9 | 975.8 | 908.3 | 1254.5 | 1671.3 | 1586.9 | 1384.1 | 1463.4 | 1275.9 |
| Serpini1 | 4.5 | 4.7 | 6.2 | 4.2 | 5.6 | 4.7 | 3.6 | 5.0 | 2.8 | 4.0 | 4.3 | 5.8 |
| Sertad1 | 16.3 | 13.7 | 12.2 | 16.4 | 16.4 | 12.9 | 19.3 | 16.8 | 18.0 | 20.2 | 17.5 | 14.1 |
| Sertad2 | 5.8 | 6.2 | 5.1 | 5.8 | 5.8 | 5.9 | 5.9 | 6.4 | 6.3 | 5.2 | 6.4 | 5.4 |
| Sertad3 | 4.0 | 4.2 | 3.8 | 2.6 | 3.0 | 4.0 | 4.3 | 4.8 | 4.0 | 3.1 | 3.1 | 3.8 |
| Sertad4 | 1.7 | 1.8 | 3.4 | 1.4 | 2.9 | 1.7 | 4.4 | 10.6 | 5.3 | 3.2 | 4.2 | 3.4 |
| Sesn1 | 6.5 | 7.5 | 9.2 | 5.4 | 6.3 | 6.9 | 5.2 | 7.8 | 7.7 | 8.1 | 7.9 | 7.1 |
| Sesn2 | 10.3 | 11.5 | 9.1 | 8.0 | 9.6 | 9.2 | 10.3 | 9.2 | 10.5 | 12.0 | 10.0 | 7.1 |
| Sesn3 | 8.7 | 11.9 | 14.5 | 6.8 | 9.0 | 7.2 | 10.1 | 12.6 | 10.8 | 13.7 | 12.6 | 12.2 |
| Sestd1 | 5.6 | 6.0 | 5.4 | 5.4 | 4.9 | 6.2 | 4.9 | 4.1 | 4.3 | 4.4 | 4.9 | 5.1 |
| Set | 63.4 | 57.3 | 67.1 | 76.2 | 61.5 | 62.2 | 72.4 | 64.7 | 58.7 | 68.5 | 75.2 | 70.8 |
| Setbp1 | 2.2 | 3.3 | 4.3 | 2.6 | 2.3 | 2.9 | 3.3 | 4.0 | 3.8 | 3.3 | 2.9 | 4.0 |
| Setd1a | 2.2 | 2.5 | 2.5 | 1.9 | 2.2 | 2.1 | 2.9 | 2.3 | 2.1 | 2.0 | 2.3 | 1.7 |
| Setd1b | 0.1 | 0.3 | 0.4 | 0.3 | 0.3 | 0.1 | 0.4 | 0.4 | 0.3 | 0.3 | 0.2 | 0.2 |
| Setd2 | 9.1 | 10.3 | 11.2 | 7.5 | 8.4 | 7.7 | 9.4 | 10.3 | 8.6 | 8.6 | 10.0 | 9.8 |
| Setd3 | 64.1 | 52.6 | 48.9 | 75.5 | 51.6 | 72.7 | 54.5 | 43.5 | 45.3 | 47.3 | 55.3 | 55.9 |
| Setd4 | 0.7 | 1.1 | 0.8 | 0.5 | 0.9 | 1.1 | 1.1 | 0.8 | 1.2 | 0.7 | 0.7 | 0.8 |
| Setd5 | 9.4 | 12.6 | 14.0 | 8.9 | 10.8 | 10.1 | 13.5 | 14.5 | 12.6 | 9.7 | 10.7 | 11.1 |
| Setd6 | 6.7 | 7.1 | 6.9 | 8.4 | 5.1 | 9.8 | 5.4 | 6.8 | 4.7 | 5.9 | 6.8 | 5.8 |
| Setd7 | 20.5 | 22.4 | 24.2 | 26.9 | 20.7 | 23.1 | 17.7 | 20.1 | 17.8 | 22.1 | 19.6 | 20.9 |
| Setd8 | 28.5 | 29.8 | 31.5 | 34.3 | 25.4 | 33.3 | 31.3 | 29.6 | 23.1 | 22.6 | 32.6 | 28.5 |
| Setdb1 | 6.7 | 7.2 | 7.2 | 5.5 | 7.3 | 6.4 | 6.4 | 7.6 | 6.7 | 6.2 | 6.6 | 6.5 |
| Setdb2 | 2.2 | 2.1 | 3.0 | 3.0 | 2.2 | 1.7 | 2.2 | 2.3 | 2.1 | 1.8 | 2.7 | 2.1 |
| Setmar | 1.1 | 1.2 | 1.5 | 1.1 | 1.1 | 1.6 | 1.3 | 1.4 | 1.1 | 1.2 | 1.5 | 1.2 |
| Setx | 11.4 | 12.2 | 11.2 | 10.5 | 10.6 | 10.0 | 10.9 | 11.7 | 11.0 | 10.4 | 12.7 | 10.9 |
| Sez6l | 0.0 | 0.0 | 0.0 | 0.0 | 0.0 | 0.0 | 0.0 | 0.0 | 0.1 | 0.4 | 0.1 | 0.1 |
| Sez6l2 | 0.0 | 0.0 | 0.0 | 0.2 | 1.1 | 0.0 | 0.3 | 0.1 | 0.1 | 0.1 | 0.0 | 0.1 |
| Sf1 | 8.1 | 9.7 | 9.6 | 9.3 | 9.9 | 9.0 | 10.8 | 9.3 | 9.1 | 8.7 | 9.9 | 8.7 |
| Sf3a1 | 7.8 | 9.1 | 8.6 | 9.7 | 7.9 | 8.3 | 9.7 | 8.5 | 7.7 | 8.0 | 9.7 | 8.4 |
| Sf3a2 | 1.9 | 2.4 | 2.8 | 3.5 | 3.3 | 3.0 | 3.6 | 3.3 | 2.3 | 2.2 | 2.8 | 1.9 |
| Sf3a3 | 28.3 | 23.9 | 23.7 | 27.8 | 23.2 | 23.6 | 22.4 | 23.1 | 19.5 | 21.1 | 22.9 | 22.8 |
| Sf3b1 | 48.7 | 46.1 | 42.7 | 50.1 | 49.0 | 43.6 | 43.9 | 40.4 | 45.2 | 49.8 | 48.6 | 48.4 |
| Sf3b2 | 57.9 | 56.6 | 61.9 | 67.4 | 53.5 | 68.2 | 62.3 | 62.3 | 48.3 | 54.0 | 59.8 | 59.9 |
| Sf3b3 | 26.5 | 26.3 | 28.9 | 27.1 | 30.0 | 26.8 | 32.1 | 33.6 | 28.7 | 29.7 | 29.9 | 26.5 |
| Sf3b4 | 9.6 | 10.3 | 9.6 | 13.4 | 12.2 | 11.2 | 11.7 | 9.9 | 9.9 | 10.8 | 10.2 | 8.6 |
| Sf3b5 | 58.1 | 51.4 | 47.3 | 60.4 | 58.6 | 64.1 | 56.6 | 57.5 | 52.6 | 58.4 | 53.4 | 46.2 |
| Sfi1 | 3.5 | 3.1 | 3.0 | 3.0 | 2.8 | 3.4 | 2.6 | 2.5 | 3.2 | 2.5 | 3.1 | 2.7 |
| Sfmbt1 | 1.5 | 1.7 | 1.7 | 1.7 | 2.1 | 1.7 | 1.7 | 1.4 | 1.7 | 1.8 | 1.7 | 1.8 |
| Sfmbt2 | 0.3 | 0.3 | 0.2 | 0.5 | 0.3 | 0.4 | 0.2 | 0.2 | 0.2 | 0.4 | 0.5 | 0.3 |
| Sfn | 2.0 | 1.2 | 1.0 | 2.3 | 1.7 | 1.4 | 1.4 | 1.3 | 1.2 | 1.4 | 1.3 | 1.3 |
| Sfpi1 | 1.7 | 0.0 | 0.0 | 10.5 | 20.9 | 0.0 | 2.7 | 0.3 | 0.5 | 1.3 | 0.5 | 1.4 |
| Sfpq | 15.2 | 16.6 | 20.5 | 17.2 | 16.3 | 12.7 | 18.8 | 17.3 | 16.0 | 18.5 | 21.5 | 17.1 |
| Sfr1 | 70.4 | 68.5 | 81.5 | 76.8 | 61.5 | 72.5 | 81.9 | 93.8 | 67.2 | 67.9 | 91.8 | 88.6 |
| Sfrp1 | 44.4 | 141.5 | 460.5 | 31.5 | 122.7 | 51.2 | 202.6 | 435.6 | 280.2 | 232.1 | 224.0 | 348.9 |
| Sfrp2 | 4.3 | 4.5 | 18.7 | 1.2 | 2.2 | 0.9 | 2.0 | 25.0 | 6.9 | 4.6 | 3.0 | 5.9 |
| Sfrp4 | 1.6 | 0.7 | 0.3 | 0.5 | 0.1 | 0.5 | 0.1 | 0.1 | 0.1 | 0.5 | 0.6 | 0.3 |
| Sfrp5 | 0.0 | 0.0 | 0.1 | 0.0 | 0.0 | 0.0 | 0.0 | 0.0 | 0.0 | 0.0 | 0.0 | 0.0 |
| Sfrs18 | 11.3 | 12.7 | 17.5 | 12.1 | 10.6 | 11.6 | 13.7 | 13.2 | 12.9 | 11.3 | 12.9 | 13.9 |
| Sfswap | 7.5 | 7.4 | 8.6 | 7.0 | 7.2 | 6.3 | 9.6 | 9.6 | 8.4 | 7.2 | 7.7 | 7.5 |
| Sft2d1 | 15.3 | 16.1 | 12.7 | 16.0 | 18.0 | 16.1 | 14.6 | 13.6 | 22.3 | 19.0 | 15.9 | 14.4 |
| Sft2d2 | 15.6 | 13.0 | 10.7 | 14.6 | 11.2 | 14.9 | 10.1 | 9.4 | 9.9 | 15.0 | 13.1 | 12.6 |
| Sft2d3 | 1.5 | 1.8 | 1.4 | 1.7 | 1.4 | 1.8 | 1.8 | 1.8 | 1.7 | 1.6 | 1.9 | 1.9 |
| Sftpb | 0.0 | 0.1 | 0.0 | 0.0 | 0.0 | 0.0 | 0.0 | 0.0 | 0.0 | 0.0 | 0.0 | 0.0 |
| Sfxn1 | 13.1 | 12.3 | 8.8 | 12.5 | 10.3 | 14.5 | 10.4 | 11.0 | 11.1 | 11.8 | 12.5 | 10.0 |

| | | | | | | | | | | | | |
|---|---|---|---|---|---|---|---|---|---|---|---|---|
| Sfxn2 | 2.3 | 1.7 | 1.4 | 2.3 | 2.4 | 1.6 | 2.1 | 2.4 | 2.4 | 2.0 | 2.3 | 2.4 |
| Sfxn3 | 13.4 | 15.0 | 11.9 | 15.0 | 14.2 | 13.8 | 15.0 | 17.2 | 13.4 | 12.8 | 13.3 | 9.7 |
| Sfxn4 | 1.2 | 0.9 | 1.2 | 1.0 | 0.7 | 1.5 | 0.6 | 0.8 | 0.8 | 0.6 | 0.7 | 1.0 |
| Sfxn5 | 0.9 | 1.0 | 1.2 | 1.4 | 1.6 | 1.2 | 1.1 | 1.0 | 1.1 | 1.5 | 0.7 | 1.0 |
| Sgca | 0.1 | 0.1 | 0.1 | 0.1 | 0.0 | 0.3 | 0.2 | 0.0 | 0.1 | 0.1 | 0.0 | 0.0 |
| Sgcb | 14.2 | 14.8 | 20.1 | 17.5 | 15.0 | 15.6 | 13.3 | 17.0 | 13.6 | 10.9 | 13.2 | 16.5 |
| Sgcd | 6.6 | 4.2 | 6.2 | 4.7 | 4.8 | 6.0 | 4.5 | 3.4 | 4.7 | 5.5 | 6.7 | 4.7 |
| Sgce | 62.0 | 58.5 | 61.9 | 60.5 | 56.5 | 62.8 | 69.7 | 74.9 | 67.0 | 54.0 | 64.3 | 66.9 |
| Sgcg | 0.0 | 0.0 | 0.0 | 0.0 | 0.0 | 0.0 | 0.0 | 0.0 | 0.0 | 0.0 | 0.0 | 0.0 |
| Sgcz | 0.0 | 0.0 | 0.0 | 0.0 | 0.0 | 0.0 | 0.0 | 0.0 | 0.0 | 0.0 | 0.0 | 0.0 |
| Sgip1 | 0.2 | 0.1 | 0.1 | 0.2 | 0.2 | 0.4 | 0.1 | 0.1 | 0.0 | 0.0 | 0.0 | 0.0 |
| Sgk1 | 13.8 | 18.3 | 20.7 | 15.1 | 23.1 | 10.4 | 36.6 | 35.7 | 26.6 | 42.0 | 33.2 | 37.6 |
| Sgk2 | 0.0 | 0.0 | 0.0 | 0.0 | 0.0 | 0.0 | 0.0 | 0.0 | 0.0 | 0.0 | 0.0 | 0.0 |
| Sgk3 | 2.8 | 3.0 | 3.1 | 2.8 | 3.5 | 2.2 | 2.7 | 2.8 | 2.9 | 3.2 | 3.4 | 3.2 |
| Sgms1 | 6.6 | 5.8 | 6.2 | 8.6 | 7.5 | 7.0 | 9.2 | 8.1 | 7.3 | 7.9 | 9.9 | 6.5 |
| Sgms2 | 8.6 | 7.9 | 5.8 | 7.5 | 6.7 | 9.7 | 8.5 | 6.2 | 5.9 | 5.8 | 8.4 | 6.6 |
| Sgol1 | 0.7 | 1.8 | 2.3 | 0.4 | 2.1 | 0.3 | 2.6 | 1.0 | 2.0 | 1.1 | 1.5 | 1.4 |
| Sgol2 | 1.4 | 2.6 | 4.5 | 0.5 | 3.1 | 0.9 | 3.9 | 1.6 | 2.8 | 1.5 | 2.7 | 1.7 |
| Sgpl1 | 48.7 | 44.4 | 33.6 | 54.2 | 52.8 | 51.4 | 37.5 | 30.0 | 35.0 | 46.7 | 39.5 | 41.4 |
| Sgpp1 | 15.6 | 17.8 | 22.7 | 21.0 | 15.5 | 19.6 | 17.7 | 20.2 | 18.2 | 19.7 | 21.4 | 24.1 |
| Sgpp2 | 0.1 | 0.1 | 0.1 | 0.1 | 0.0 | 0.1 | 0.1 | 0.0 | 0.0 | 0.1 | 0.1 | 0.1 |
| Sgsh | 13.9 | 11.9 | 11.2 | 15.2 | 11.7 | 18.3 | 12.0 | 10.0 | 9.9 | 11.5 | 11.1 | 10.5 |
| Sgsm1 | 1.2 | 1.7 | 1.3 | 1.1 | 1.0 | 2.1 | 1.8 | 1.3 | 1.0 | 1.0 | 1.1 | 0.8 |
| Sgsm2 | 3.4 | 3.9 | 3.5 | 3.0 | 3.0 | 4.4 | 2.8 | 3.6 | 3.2 | 3.3 | 3.1 | 3.0 |
| Sgsm3 | 4.8 | 4.2 | 3.4 | 4.4 | 4.1 | 5.5 | 4.3 | 4.4 | 3.9 | 4.1 | 3.7 | 3.8 |
| Sgta | 24.1 | 24.6 | 24.4 | 26.9 | 24.9 | 27.3 | 28.7 | 27.1 | 22.6 | 21.7 | 23.5 | 22.1 |
| Sgtb | 3.1 | 3.1 | 3.0 | 2.9 | 1.9 | 3.5 | 3.1 | 3.3 | 3.2 | 3.3 | 3.4 | 3.5 |
| Sh2b1 | 4.6 | 4.4 | 4.2 | 4.6 | 4.6 | 5.0 | 5.2 | 4.5 | 5.1 | 5.2 | 4.6 | 4.2 |
| Sh2b2 | 1.0 | 1.2 | 1.7 | 1.2 | 2.1 | 1.0 | 2.3 | 1.9 | 2.0 | 1.6 | 1.4 | 1.3 |
| Sh2b3 | 7.2 | 6.6 | 5.9 | 6.0 | 8.4 | 7.1 | 7.3 | 5.9 | 7.1 | 6.7 | 6.3 | 6.1 |
| Sh2d1b1 | 0.2 | 0.0 | 0.0 | 1.2 | 5.1 | 0.0 | 0.7 | 0.0 | 0.1 | 0.6 | 0.2 | 0.6 |
| Sh2d2a | 0.0 | 0.0 | 0.0 | 0.0 | 0.0 | 0.0 | 0.0 | 0.0 | 0.0 | 0.0 | 0.0 | 0.0 |
| Sh2d3c | 0.3 | 0.3 | 0.3 | 1.0 | 1.0 | 0.1 | 0.2 | 0.3 | 0.3 | 0.2 | 0.1 | 0.1 |
| Sh2d4a | 6.5 | 5.1 | 5.5 | 9.1 | 4.9 | 6.4 | 5.5 | 4.5 | 5.1 | 2.2 | 4.4 | 2.7 |
| Sh2d4b | 0.1 | 0.1 | 0.2 | 0.0 | 0.1 | 0.1 | 0.2 | 0.0 | 0.0 | 0.0 | 0.0 | 0.0 |
| Sh2d5 | 0.0 | 0.0 | 0.1 | 0.0 | 0.1 | 0.0 | 0.1 | 0.0 | 0.1 | 0.1 | 0.1 | 0.1 |
| Sh2d7 | 0.1 | 0.0 | 0.1 | 0.1 | 0.1 | 0.1 | 0.0 | 0.0 | 0.1 | 0.1 | 0.0 | 0.0 |
| Sh3bgr | 1.7 | 1.8 | 1.1 | 1.6 | 0.9 | 1.8 | 1.1 | 0.5 | 1.3 | 2.9 | 3.6 | 1.7 |
| Sh3bgrl | 70.9 | 78.2 | 105.2 | 112.3 | 94.3 | 100.9 | 87.8 | 79.3 | 94.8 | 101.1 | 126.4 | 128.0 |
| Sh3bgrl2 | 0.6 | 0.2 | 0.5 | 2.7 | 2.9 | 0.2 | 0.5 | 0.4 | 0.2 | 0.7 | 0.2 | 0.6 |
| Sh3bgrl3 | 155.2 | 129.5 | 116.3 | 233.8 | 260.4 | 177.5 | 179.1 | 128.4 | 122.7 | 131.5 | 143.1 | 134.7 |
| Sh3bp1 | 4.2 | 4.8 | 4.2 | 4.8 | 5.1 | 3.5 | 4.2 | 3.5 | 3.4 | 3.6 | 3.1 | 2.9 |
| Sh3bp2 | 4.4 | 5.0 | 5.1 | 7.1 | 10.8 | 3.7 | 7.2 | 7.5 | 6.2 | 4.5 | 4.8 | 4.5 |
| Sh3bp4 | 3.2 | 3.5 | 2.7 | 3.7 | 3.4 | 3.5 | 3.5 | 3.4 | 3.6 | 5.2 | 4.8 | 2.9 |
| Sh3bp5 | 16.9 | 17.1 | 24.3 | 17.2 | 18.4 | 18.6 | 21.6 | 19.8 | 17.2 | 15.0 | 16.5 | 19.1 |
| Sh3bp5l | 8.6 | 8.1 | 7.8 | 8.2 | 7.9 | 7.3 | 9.1 | 9.3 | 10.0 | 7.8 | 9.0 | 7.7 |
| Sh3d19 | 21.0 | 21.5 | 21.9 | 29.7 | 20.3 | 28.0 | 20.8 | 17.1 | 14.2 | 16.8 | 18.4 | 16.4 |
| Sh3d21 | 0.8 | 0.9 | 0.8 | 0.3 | 0.3 | 1.0 | 0.4 | 1.0 | 0.4 | 1.4 | 1.1 | 1.2 |
| Sh3gl1 | 21.7 | 23.4 | 25.1 | 23.2 | 26.6 | 20.0 | 30.5 | 33.3 | 32.2 | 25.6 | 24.4 | 24.2 |
| Sh3gl3 | 1.4 | 0.8 | 0.4 | 1.5 | 0.8 | 2.3 | 0.7 | 0.3 | 0.5 | 0.6 | 1.5 | 0.9 |
| Sh3glb1 | 48.9 | 49.2 | 47.0 | 60.4 | 48.8 | 52.5 | 53.3 | 47.6 | 50.1 | 46.1 | 63.3 | 58.1 |
| Sh3glb2 | 25.7 | 21.5 | 17.7 | 27.4 | 19.4 | 24.4 | 19.1 | 17.7 | 18.4 | 26.8 | 24.0 | 19.0 |
| Sh3kbp1 | 2.4 | 1.7 | 1.8 | 5.2 | 4.8 | 4.1 | 3.7 | 2.2 | 2.3 | 8.1 | 7.8 | 5.4 |
| Sh3pxd2a | 26.2 | 23.5 | 22.1 | 26.9 | 27.2 | 28.9 | 28.4 | 25.5 | 24.1 | 21.8 | 24.5 | 22.1 |
| Sh3pxd2b | 3.2 | 4.8 | 4.7 | 4.4 | 6.7 | 4.6 | 4.2 | 4.6 | 4.8 | 5.1 | 4.0 | 5.0 |
| Sh3rf1 | 7.2 | 7.2 | 6.4 | 7.6 | 8.0 | 6.4 | 8.9 | 4.7 | 6.7 | 13.9 | 10.0 | 10.5 |
| Sh3rf2 | 0.1 | 0.1 | 0.1 | 0.1 | 0.1 | 0.1 | 0.1 | 0.1 | 0.0 | 0.2 | 0.2 | 0.1 |

| | | | | | | | | | | | | |
|---|---|---|---|---|---|---|---|---|---|---|---|---|
| Sh3rf3 | 0.9 | 1.5 | 1.9 | 1.1 | 1.3 | 0.7 | 1.5 | 2.4 | 1.7 | 1.4 | 1.4 | 1.3 |
| Sh3tc1 | 0.8 | 0.3 | 0.3 | 1.0 | 1.6 | 0.6 | 0.3 | 0.3 | 0.3 | 0.9 | 0.3 | 0.3 |
| Sh3tc2 | 7.4 | 7.0 | 5.4 | 5.5 | 5.3 | 6.1 | 6.0 | 6.8 | 8.0 | 8.7 | 8.6 | 6.5 |
| Sh3yl1 | 4.0 | 4.3 | 4.6 | 2.4 | 3.3 | 3.6 | 3.3 | 3.3 | 3.0 | 3.1 | 3.0 | 2.8 |
| Shank1 | 0.0 | 0.0 | 0.0 | 0.0 | 0.0 | 0.0 | 0.0 | 0.0 | 0.0 | 0.0 | 0.0 | 0.0 |
| Shank2 | 0.1 | 0.1 | 0.2 | 0.1 | 0.1 | 0.3 | 0.1 | 0.2 | 0.1 | 0.0 | 0.1 | 0.1 |
| Shank3 | 0.4 | 0.5 | 0.4 | 0.6 | 0.3 | 0.4 | 0.7 | 0.4 | 0.6 | 0.5 | 0.4 | 0.5 |
| Sharpin | 13.2 | 11.4 | 12.6 | 16.1 | 12.6 | 14.2 | 14.5 | 14.2 | 11.8 | 12.1 | 12.8 | 11.5 |
| Shb | 6.6 | 5.2 | 4.8 | 3.8 | 4.1 | 3.1 | 4.8 | 4.2 | 4.7 | 9.5 | 5.8 | 4.5 |
| Shc1 | 60.0 | 63.7 | 61.9 | 53.7 | 64.6 | 57.3 | 60.5 | 58.3 | 58.8 | 79.9 | 63.2 | 63.3 |
| Shc2 | 17.6 | 13.7 | 11.0 | 24.6 | 11.4 | 28.8 | 10.7 | 9.5 | 8.3 | 9.3 | 11.3 | 11.4 |
| Shc3 | 0.4 | 0.3 | 0.3 | 0.4 | 0.3 | 0.6 | 0.2 | 0.1 | 0.1 | 0.1 | 0.0 | 0.2 |
| Shc4 | 0.6 | 0.4 | 0.3 | 0.6 | 0.5 | 0.8 | 0.5 | 0.4 | 0.5 | 0.8 | 0.8 | 0.8 |
| Shcbp1 | 1.2 | 3.3 | 5.8 | 0.5 | 4.3 | 0.3 | 5.2 | 2.0 | 3.9 | 2.0 | 3.6 | 2.3 |
| Shd | 0.1 | 0.1 | 0.2 | 0.1 | 0.0 | 0.1 | 0.0 | 0.0 | 0.2 | 0.0 | 0.0 | 0.1 |
| She | 0.1 | 0.1 | 0.1 | 0.0 | 0.0 | 0.0 | 0.1 | 0.1 | 0.1 | 0.1 | 0.0 | 0.0 |
| Shf | 1.4 | 2.3 | 2.6 | 1.2 | 1.6 | 1.5 | 2.1 | 3.1 | 2.5 | 2.1 | 2.5 | 2.5 |
| Shfm1 | 261.0 | 201.9 | 236.9 | 260.8 | 204.0 | 237.4 | 248.0 | 223.1 | 194.1 | 238.7 | 250.4 | 277.5 |
| Shisa2 | 0.3 | 0.6 | 0.4 | 0.2 | 0.2 | 0.2 | 0.1 | 0.1 | 0.1 | 0.1 | 0.2 | 0.1 |
| Shisa4 | 20.5 | 18.6 | 18.1 | 24.6 | 17.3 | 32.6 | 17.3 | 19.3 | 17.1 | 23.2 | 24.3 | 14.9 |
| Shisa5 | 98.4 | 91.4 | 85.1 | 129.7 | 102.7 | 112.9 | 101.8 | 96.0 | 93.1 | 81.0 | 83.2 | 91.9 |
| Shisa6 | 0.0 | 0.1 | 0.1 | 0.0 | 0.1 | 0.0 | 0.1 | 0.2 | 0.2 | 0.0 | 0.1 | 0.2 |
| Shisa9 | 0.0 | 0.0 | 0.0 | 0.0 | 0.0 | 0.0 | 0.0 | 0.0 | 0.0 | 0.0 | 0.0 | 0.0 |
| Shkbp1 | 3.0 | 2.6 | 2.6 | 3.8 | 3.0 | 2.6 | 2.6 | 3.0 | 3.0 | 3.3 | 2.4 | 2.7 |
| Shmt1 | 1.6 | 1.6 | 1.6 | 0.6 | 1.3 | 0.9 | 1.5 | 1.0 | 1.1 | 1.4 | 1.2 | 1.6 |
| Shmt2 | 19.2 | 13.5 | 12.8 | 18.7 | 12.9 | 18.7 | 18.6 | 22.4 | 16.5 | 11.9 | 16.5 | 11.3 |
| Shoc2 | 19.1 | 19.0 | 18.9 | 20.7 | 18.6 | 20.6 | 21.3 | 19.6 | 20.6 | 18.7 | 24.4 | 22.3 |
| Shox2 | 2.7 | 1.6 | 1.8 | 3.3 | 1.9 | 1.8 | 2.2 | 2.3 | 2.4 | 2.3 | 1.8 | 1.7 |
| Shpk | 2.1 | 2.3 | 1.8 | 1.7 | 2.3 | 1.8 | 1.8 | 1.6 | 1.8 | 1.6 | 1.5 | 1.6 |
| Shprh | 6.4 | 6.4 | 6.2 | 6.8 | 5.5 | 6.6 | 5.4 | 5.9 | 5.7 | 7.0 | 6.4 | 6.0 |
| Shq1 | 2.8 | 2.4 | 1.9 | 2.4 | 2.4 | 2.5 | 1.8 | 1.8 | 2.0 | 2.3 | 1.9 | 1.9 |
| Shroom1 | 1.3 | 1.2 | 1.6 | 0.7 | 0.9 | 1.2 | 1.1 | 1.4 | 1.3 | 1.1 | 1.5 | 1.1 |
| Shroom2 | 6.8 | 6.0 | 4.9 | 4.1 | 3.9 | 3.6 | 4.0 | 3.1 | 3.4 | 7.8 | 6.5 | 5.4 |
| Shroom3 | 4.6 | 5.4 | 5.5 | 3.4 | 3.6 | 2.5 | 3.5 | 3.8 | 3.7 | 6.6 | 4.3 | 3.8 |
| Shroom4 | 0.9 | 1.0 | 1.0 | 0.5 | 0.6 | 0.7 | 0.8 | 0.7 | 0.8 | 1.3 | 0.8 | 0.8 |
| Siae | 6.8 | 7.1 | 8.1 | 6.1 | 6.8 | 8.3 | 5.8 | 7.6 | 6.9 | 6.2 | 5.6 | 6.7 |
| Siah1a | 5.1 | 5.0 | 5.4 | 5.3 | 4.6 | 5.9 | 4.6 | 4.6 | 6.0 | 5.9 | 5.3 | 5.8 |
| Siah1b | 1.1 | 1.0 | 1.2 | 0.7 | 1.1 | 0.6 | 1.5 | 1.3 | 1.1 | 1.2 | 2.0 | 1.0 |
| Siah2 | 3.5 | 3.6 | 2.8 | 3.5 | 3.4 | 2.8 | 3.4 | 3.1 | 3.7 | 3.2 | 3.9 | 2.8 |
| Sidt1 | 0.0 | 0.0 | 0.0 | 0.0 | 0.0 | 0.0 | 0.0 | 0.0 | 0.0 | 0.0 | 0.0 | 0.0 |
| Sidt2 | 34.7 | 38.2 | 34.8 | 33.2 | 36.2 | 35.1 | 39.6 | 33.8 | 37.1 | 35.9 | 34.4 | 33.2 |
| Sigirr | 0.1 | 0.1 | 0.0 | 0.0 | 0.1 | 0.0 | 0.0 | 0.1 | 0.2 | 0.0 | 0.0 | 0.0 |
| Siglec1 | 0.1 | 0.0 | 0.0 | 0.4 | 0.4 | 0.0 | 0.0 | 0.0 | 0.0 | 0.1 | 0.0 | 0.0 |
| Siglec5 | 0.0 | 0.0 | 0.1 | 0.0 | 0.0 | 0.0 | 0.0 | 0.1 | 0.1 | 0.0 | 0.0 | 0.0 |
| Siglece | 0.2 | 0.0 | 0.0 | 1.4 | 5.7 | 0.0 | 1.3 | 0.1 | 0.1 | 0.4 | 0.2 | 0.2 |
| Siglecg | 16.3 | 15.9 | 7.1 | 15.4 | 11.2 | 12.1 | 15.6 | 5.5 | 8.9 | 5.0 | 9.9 | 5.7 |
| Sigmar1 | 18.9 | 18.2 | 17.0 | 19.7 | 19.8 | 16.2 | 30.1 | 29.6 | 23.9 | 17.7 | 23.4 | 18.7 |
| Sik1 | 3.2 | 4.0 | 3.5 | 2.7 | 2.5 | 3.9 | 2.2 | 3.3 | 3.0 | 3.0 | 2.2 | 2.8 |
| Sik2 | 5.3 | 6.3 | 6.2 | 5.7 | 5.5 | 6.0 | 5.3 | 5.3 | 4.6 | 5.2 | 4.9 | 4.8 |
| Sik3 | 3.1 | 3.5 | 3.3 | 2.7 | 2.7 | 3.6 | 3.3 | 2.8 | 2.9 | 2.9 | 3.1 | 2.6 |
| Sike1 | 10.5 | 9.2 | 9.8 | 9.9 | 9.5 | 9.2 | 8.6 | 9.6 | 10.0 | 9.2 | 9.3 | 9.3 |
| Sil1 | 50.2 | 41.8 | 40.0 | 56.3 | 36.8 | 59.9 | 45.0 | 41.2 | 36.7 | 38.8 | 40.6 | 46.7 |
| Sim2 | 0.0 | 0.0 | 0.0 | 0.0 | 0.0 | 0.0 | 0.0 | 0.0 | 0.0 | 0.3 | 0.2 | 0.0 |
| Simc1 | 3.3 | 3.4 | 3.2 | 3.0 | 2.7 | 3.4 | 4.0 | 3.1 | 3.0 | 3.1 | 4.0 | 3.7 |
| Sin3a | 6.9 | 8.1 | 9.9 | 7.2 | 7.6 | 7.3 | 9.0 | 10.6 | 8.2 | 7.7 | 8.5 | 8.7 |
| Sin3b | 71.2 | 68.9 | 57.7 | 66.1 | 59.2 | 59.1 | 59.1 | 63.0 | 63.2 | 62.5 | 56.0 | 59.1 |
| Sipa1 | 2.7 | 3.7 | 3.6 | 3.6 | 6.1 | 2.7 | 4.4 | 4.0 | 4.5 | 3.9 | 3.5 | 3.1 |

| | | | | | | | | | | | | |
|---|---|---|---|---|---|---|---|---|---|---|---|---|
| Sipa1l1 | 20.6 | 23.4 | 22.7 | 18.7 | 20.0 | 16.4 | 34.1 | 27.9 | 33.4 | 26.6 | 32.1 | 31.1 |
| Sipa1l2 | 2.7 | 3.0 | 3.1 | 2.6 | 4.8 | 1.9 | 4.7 | 3.6 | 3.9 | 5.5 | 4.4 | 4.1 |
| Sipa1l3 | 0.8 | 1.2 | 1.0 | 0.8 | 1.1 | 1.0 | 1.6 | 0.9 | 1.0 | 1.0 | 1.1 | 0.7 |
| Sirpa | 12.7 | 10.5 | 12.1 | 32.7 | 40.4 | 11.7 | 11.4 | 9.8 | 8.1 | 8.5 | 7.2 | 9.5 |
| Sirpb1a | 0.1 | 0.0 | 0.0 | 0.2 | 1.9 | 0.0 | 0.1 | 0.0 | 0.0 | 0.0 | 0.0 | 0.0 |
| Sirpb1b | 0.3 | 0.0 | 0.0 | 0.3 | 3.0 | 0.0 | 0.1 | 0.0 | 0.0 | 0.0 | 0.0 | 0.0 |
| Sirt1 | 3.2 | 2.8 | 2.7 | 2.4 | 2.7 | 2.8 | 2.1 | 2.8 | 2.7 | 3.1 | 2.7 | 3.1 |
| Sirt2 | 43.4 | 38.3 | 35.6 | 50.6 | 40.8 | 49.3 | 40.1 | 39.5 | 33.1 | 36.8 | 39.3 | 43.2 |
| Sirt3 | 4.8 | 4.4 | 3.4 | 5.6 | 3.6 | 5.9 | 3.4 | 3.2 | 4.1 | 3.4 | 3.0 | 3.1 |
| Sirt4 | 2.6 | 3.1 | 3.7 | 2.6 | 3.3 | 3.5 | 2.7 | 3.4 | 3.1 | 2.7 | 2.8 | 2.8 |
| Sirt5 | 3.3 | 2.9 | 3.1 | 4.6 | 2.8 | 4.0 | 4.0 | 3.4 | 3.1 | 2.7 | 2.7 | 3.8 |
| Sirt6 | 3.2 | 3.0 | 3.2 | 3.5 | 3.7 | 3.9 | 3.1 | 3.3 | 3.2 | 3.5 | 2.6 | 2.8 |
| Sirt7 | 6.5 | 7.3 | 5.3 | 7.9 | 7.9 | 6.0 | 6.2 | 6.6 | 7.1 | 6.5 | 7.0 | 6.4 |
| Siva1 | 33.3 | 28.7 | 24.3 | 24.3 | 24.9 | 32.4 | 22.3 | 20.3 | 25.4 | 31.3 | 26.2 | 24.3 |
| Six1 | 1.0 | 1.1 | 0.8 | 1.5 | 1.0 | 1.4 | 1.3 | 0.9 | 1.0 | 1.4 | 1.7 | 1.8 |
| Six2 | 0.2 | 0.1 | 0.0 | 0.4 | 0.2 | 0.2 | 0.3 | 0.1 | 0.1 | 0.1 | 0.1 | 0.0 |
| Six4 | 2.1 | 2.3 | 2.0 | 2.4 | 2.3 | 2.4 | 1.7 | 1.9 | 1.7 | 2.3 | 2.0 | 2.1 |
| Six5 | 1.9 | 2.3 | 2.3 | 1.8 | 1.7 | 2.0 | 2.1 | 2.8 | 2.6 | 2.3 | 2.1 | 1.8 |
| Ska1 | 0.2 | 0.6 | 1.1 | 0.1 | 0.9 | 0.1 | 0.9 | 0.5 | 1.0 | 0.3 | 0.7 | 0.3 |
| Ska2 | 6.7 | 7.7 | 7.9 | 6.2 | 6.1 | 4.6 | 8.6 | 6.0 | 7.2 | 7.7 | 9.3 | 7.1 |
| Ska3 | 1.2 | 2.0 | 2.1 | 0.7 | 2.1 | 0.6 | 2.6 | 1.3 | 2.2 | 1.2 | 1.9 | 1.1 |
| Skap1 | 0.1 | 0.1 | 0.2 | 0.2 | 0.2 | 0.3 | 0.2 | 0.0 | 0.1 | 0.2 | 0.2 | 0.1 |
| Skap2 | 28.0 | 20.9 | 21.6 | 39.1 | 43.3 | 24.1 | 27.1 | 22.7 | 25.7 | 40.3 | 33.8 | 37.0 |
| Ski | 22.9 | 29.8 | 29.3 | 21.3 | 25.6 | 21.7 | 31.9 | 31.4 | 31.3 | 24.6 | 25.1 | 23.7 |
| Skida1 | 0.5 | 0.6 | 0.7 | 0.8 | 0.3 | 0.5 | 0.6 | 1.0 | 0.7 | 0.8 | 0.6 | 0.7 |
| Skil | 11.1 | 10.7 | 10.7 | 12.1 | 13.8 | 9.7 | 12.6 | 11.2 | 12.7 | 11.1 | 12.4 | 13.4 |
| Skiv2l | 10.7 | 11.2 | 10.9 | 9.4 | 10.0 | 10.4 | 10.3 | 12.3 | 10.9 | 10.1 | 8.9 | 9.4 |
| Skiv2l2 | 19.9 | 20.9 | 20.0 | 21.1 | 18.9 | 19.4 | 17.8 | 19.4 | 16.5 | 18.4 | 18.9 | 19.2 |
| Skor1 | 0.0 | 0.0 | 0.1 | 0.0 | 0.0 | 0.0 | 0.0 | 0.0 | 0.0 | 0.0 | 0.0 | 0.0 |
| Skor2 | 0.0 | 0.0 | 0.0 | 0.0 | 0.0 | 0.0 | 0.0 | 0.0 | 0.0 | 0.0 | 0.0 | 0.0 |
| Skp1a | 210.4 | 195.4 | 168.8 | 174.7 | 176.8 | 185.3 | 137.3 | 143.8 | 154.7 | 212.2 | 191.7 | 186.5 |
| Skp2 | 1.3 | 1.7 | 2.5 | 1.2 | 1.8 | 1.2 | 2.2 | 2.2 | 1.9 | 1.7 | 2.4 | 1.5 |
| Sla | 0.5 | 0.4 | 0.2 | 1.6 | 3.1 | 0.2 | 1.8 | 1.0 | 1.2 | 0.5 | 0.4 | 0.9 |
| Sla2 | 14.4 | 12.6 | 9.3 | 12.3 | 14.8 | 10.5 | 12.2 | 11.5 | 14.1 | 16.3 | 13.9 | 13.7 |
| Slain1 | 0.1 | 0.1 | 0.0 | 0.0 | 0.0 | 0.2 | 0.1 | 0.0 | 0.0 | 0.0 | 0.0 | 0.0 |
| Slain2 | 17.9 | 15.3 | 13.0 | 22.6 | 15.4 | 18.5 | 14.9 | 12.7 | 12.8 | 14.4 | 16.6 | 16.0 |
| Slamf6 | 0.0 | 0.0 | 0.0 | 0.0 | 0.0 | 0.0 | 0.0 | 0.0 | 0.0 | 0.0 | 0.0 | 0.0 |
| Slamf7 | 0.1 | 0.0 | 0.1 | 0.8 | 1.1 | 0.0 | 0.3 | 0.1 | 0.1 | 0.1 | 0.0 | 0.1 |
| Slamf8 | 0.2 | 0.1 | 0.2 | 0.5 | 1.3 | 0.2 | 0.5 | 0.1 | 0.2 | 0.3 | 0.1 | 0.1 |
| Slamf9 | 3.0 | 0.3 | 0.2 | 14.2 | 31.3 | 0.1 | 6.8 | 0.5 | 1.3 | 3.4 | 1.1 | 3.8 |
| Slbp | 10.5 | 16.7 | 20.0 | 10.0 | 16.8 | 11.7 | 19.6 | 14.3 | 18.7 | 16.1 | 19.2 | 17.4 |
| Slc10a1 | 0.5 | 0.3 | 0.4 | 0.2 | 0.5 | 0.4 | 0.1 | 0.2 | 0.3 | 0.4 | 0.5 | 0.3 |
| Slc10a3 | 16.2 | 15.9 | 17.4 | 18.7 | 16.5 | 15.9 | 18.3 | 16.5 | 15.2 | 16.6 | 15.7 | 17.4 |
| Slc10a6 | 0.6 | 2.2 | 3.8 | 0.1 | 0.6 | 0.2 | 0.3 | 0.9 | 0.6 | 0.4 | 0.4 | 0.5 |
| Slc10a7 | 4.0 | 4.0 | 4.2 | 4.2 | 4.2 | 4.5 | 4.9 | 5.2 | 4.6 | 4.2 | 5.0 | 4.9 |
| Slc11a1 | 3.0 | 0.4 | 0.3 | 18.4 | 40.1 | 0.6 | 3.4 | 0.6 | 0.9 | 2.1 | 1.4 | 1.6 |
| Slc11a2 | 11.9 | 9.8 | 9.1 | 13.7 | 14.1 | 10.8 | 10.0 | 9.6 | 8.0 | 7.7 | 7.6 | 7.4 |
| Slc12a1 | 0.0 | 0.0 | 0.0 | 0.0 | 0.0 | 0.0 | 0.0 | 0.0 | 0.0 | 0.0 | 0.0 | 0.0 |
| Slc12a2 | 3.6 | 2.8 | 2.9 | 2.8 | 3.0 | 2.4 | 2.7 | 2.9 | 2.6 | 3.1 | 2.8 | 3.0 |
| Slc12a4 | 29.7 | 28.4 | 26.6 | 28.8 | 29.2 | 26.9 | 34.0 | 30.3 | 34.1 | 31.4 | 29.7 | 28.6 |
| Slc12a5 | 0.1 | 0.1 | 0.1 | 0.1 | 0.1 | 0.2 | 0.1 | 0.0 | 0.0 | 0.0 | 0.0 | 0.1 |
| Slc12a6 | 8.4 | 8.5 | 7.9 | 8.7 | 10.5 | 8.4 | 8.7 | 8.2 | 8.5 | 8.0 | 8.8 | 7.7 |
| Slc12a7 | 2.1 | 2.0 | 1.7 | 2.3 | 3.3 | 1.9 | 1.9 | 1.6 | 1.8 | 2.3 | 2.1 | 1.9 |
| Slc12a8 | 1.8 | 1.6 | 1.5 | 0.8 | 1.0 | 0.6 | 1.1 | 1.1 | 0.9 | 1.1 | 1.6 | 1.2 |
| Slc12a9 | 1.5 | 2.2 | 2.3 | 2.0 | 2.7 | 2.2 | 1.9 | 2.6 | 2.6 | 2.3 | 1.8 | 1.9 |
| Slc13a3 | 0.0 | 0.0 | 0.0 | 0.0 | 0.1 | 0.0 | 0.1 | 0.1 | 0.0 | 0.0 | 0.0 | 0.0 |
| Slc13a4 | 0.3 | 0.7 | 0.7 | 0.0 | 0.0 | 0.1 | 0.2 | 0.3 | 0.1 | 0.1 | 0.2 | 0.2 |

| | | | | | | | | | | | | |
|---|---|---|---|---|---|---|---|---|---|---|---|---|
| Slc13a5 | 0.0 | 0.1 | 0.0 | 0.1 | 0.0 | 0.1 | 0.1 | 0.1 | 0.1 | 0.0 | 0.2 | 0.1 |
| Slc14a2 | 0.0 | 0.1 | 0.0 | 0.1 | 0.0 | 0.1 | 0.1 | 0.0 | 0.0 | 0.0 | 0.0 | 0.0 |
| Slc15a1 | 0.1 | 0.0 | 0.0 | 0.1 | 0.1 | 0.0 | 0.1 | 0.0 | 0.0 | 0.0 | 0.1 | 0.1 |
| Slc15a2 | 0.1 | 0.0 | 0.0 | 0.0 | 0.0 | 0.1 | 0.0 | 0.1 | 0.0 | 0.0 | 0.1 | 0.1 |
| Slc15a3 | 2.2 | 0.5 | 0.9 | 12.1 | 25.5 | 1.3 | 4.1 | 0.5 | 0.8 | 1.9 | 0.7 | 1.9 |
| Slc15a4 | 12.0 | 11.9 | 7.6 | 13.3 | 14.2 | 12.8 | 10.5 | 9.6 | 9.5 | 10.4 | 9.0 | 9.9 |
| Slc15a5 | 0.0 | 0.0 | 0.0 | 0.0 | 0.0 | 0.0 | 0.0 | 0.0 | 0.0 | 0.0 | 0.0 | 0.0 |
| Slc16a1 | 9.1 | 9.3 | 11.1 | 5.4 | 4.8 | 5.7 | 4.6 | 5.9 | 5.0 | 9.2 | 7.4 | 7.0 |
| Slc16a10 | 1.5 | 1.4 | 2.0 | 1.5 | 2.5 | 0.9 | 0.9 | 0.8 | 0.7 | 0.7 | 0.6 | 0.8 |
| Slc16a11 | 0.0 | 0.0 | 0.0 | 0.3 | 0.0 | 0.0 | 0.1 | 0.1 | 0.1 | 0.2 | 0.2 | 0.1 |
| Slc16a12 | 0.0 | 0.0 | 0.0 | 0.0 | 0.0 | 0.0 | 0.0 | 0.0 | 0.0 | 0.0 | 0.0 | 0.0 |
| Slc16a13 | 0.8 | 0.7 | 0.6 | 0.9 | 0.6 | 1.3 | 0.7 | 0.7 | 0.6 | 1.2 | 0.8 | 0.8 |
| Slc16a2 | 4.5 | 5.4 | 8.5 | 4.5 | 6.7 | 3.5 | 5.4 | 5.8 | 3.8 | 3.0 | 2.3 | 3.9 |
| Slc16a3 | 3.8 | 4.4 | 2.8 | 4.8 | 12.5 | 4.9 | 7.3 | 2.9 | 6.4 | 5.1 | 4.2 | 5.3 |
| Slc16a4 | 3.0 | 3.5 | 4.6 | 1.6 | 2.6 | 1.4 | 2.0 | 4.2 | 4.3 | 8.8 | 7.1 | 6.3 |
| Slc16a5 | 0.6 | 0.4 | 0.4 | 0.4 | 0.5 | 0.4 | 0.5 | 0.4 | 0.5 | 0.4 | 0.5 | 0.2 |
| Slc16a6 | 0.4 | 0.3 | 0.5 | 0.7 | 0.9 | 0.4 | 0.4 | 0.5 | 0.3 | 0.3 | 0.5 | 0.5 |
| Slc16a7 | 0.2 | 0.2 | 0.3 | 0.3 | 0.4 | 0.0 | 0.3 | 0.3 | 0.2 | 0.3 | 0.2 | 0.2 |
| Slc16a8 | 0.0 | 0.1 | 0.0 | 0.2 | 0.0 | 0.0 | 0.0 | 0.1 | 0.1 | 0.1 | 0.0 | 0.0 |
| Slc16a9 | 2.1 | 2.6 | 3.9 | 2.1 | 2.1 | 3.2 | 2.7 | 2.5 | 2.4 | 2.4 | 2.0 | 2.7 |
| Slc17a5 | 4.4 | 4.9 | 4.9 | 5.1 | 5.0 | 5.7 | 4.0 | 4.9 | 4.2 | 4.7 | 4.7 | 4.7 |
| Slc17a7 | 0.0 | 0.1 | 0.1 | 0.1 | 0.1 | 0.1 | 0.0 | 0.0 | 0.0 | 0.0 | 0.0 | 0.0 |
| Slc17a8 | 0.0 | 0.0 | 0.0 | 0.0 | 0.0 | 0.0 | 0.0 | 0.0 | 0.0 | 0.0 | 0.0 | 0.0 |
| Slc17a9 | 1.7 | 0.9 | 0.6 | 2.2 | 1.0 | 1.0 | 0.9 | 0.5 | 0.7 | 0.7 | 1.1 | 1.0 |
| Slc18a1 | 0.2 | 0.1 | 0.1 | 0.2 | 0.2 | 0.2 | 0.1 | 0.1 | 0.2 | 0.1 | 0.2 | 0.2 |
| Slc18a2 | 0.5 | 1.0 | 0.9 | 0.7 | 0.7 | 0.7 | 1.0 | 1.2 | 0.9 | 0.9 | 1.4 | 0.9 |
| Slc18b1 | 2.5 | 3.2 | 3.0 | 2.9 | 2.9 | 3.7 | 3.0 | 3.0 | 2.7 | 3.3 | 2.7 | 2.9 |
| Slc19a1 | 1.4 | 1.9 | 1.5 | 1.8 | 1.5 | 1.6 | 2.2 | 2.5 | 2.2 | 1.4 | 1.7 | 1.7 |
| Slc19a2 | 14.0 | 13.4 | 11.8 | 11.8 | 12.9 | 15.8 | 10.7 | 9.1 | 11.4 | 16.0 | 12.3 | 11.2 |
| Slc19a3 | 0.5 | 0.4 | 0.3 | 0.3 | 0.2 | 0.4 | 0.3 | 0.2 | 0.3 | 0.1 | 0.3 | 0.2 |
| Slc1a1 | 0.0 | 0.0 | 0.0 | 0.0 | 0.0 | 0.0 | 0.0 | 0.0 | 0.0 | 0.0 | 0.0 | 0.0 |
| Slc1a2 | 0.1 | 0.2 | 0.2 | 0.4 | 0.3 | 0.2 | 0.1 | 0.2 | 0.1 | 0.3 | 0.3 | 0.1 |
| Slc1a3 | 7.4 | 13.7 | 21.9 | 9.2 | 11.8 | 6.4 | 12.9 | 26.9 | 15.1 | 8.9 | 12.1 | 12.1 |
| Slc1a4 | 14.0 | 9.8 | 8.6 | 15.3 | 12.8 | 14.6 | 16.7 | 17.3 | 16.4 | 9.9 | 14.0 | 9.9 |
| Slc1a5 | 19.0 | 22.6 | 29.1 | 11.8 | 13.3 | 18.8 | 21.2 | 38.7 | 33.4 | 11.2 | 15.9 | 15.6 |
| Slc1a6 | 16.8 | 17.3 | 10.0 | 21.4 | 12.2 | 26.7 | 21.8 | 8.6 | 23.3 | 16.5 | 18.1 | 18.4 |
| Slc1a7 | 0.1 | 0.1 | 0.1 | 0.0 | 0.1 | 0.0 | 0.1 | 0.1 | 0.1 | 0.1 | 0.1 | 0.1 |
| Slc20a1 | 13.0 | 10.6 | 8.6 | 17.7 | 14.6 | 13.1 | 14.9 | 13.3 | 15.3 | 13.2 | 11.8 | 11.9 |
| Slc20a2 | 10.8 | 8.3 | 6.4 | 15.3 | 8.7 | 12.3 | 11.8 | 7.8 | 8.9 | 10.7 | 10.1 | 8.8 |
| Slc22a13 | 0.0 | 0.0 | 0.0 | 0.1 | 0.0 | 0.0 | 0.0 | 0.0 | 0.0 | 0.0 | 0.0 | 0.0 |
| Slc22a13b-ps | 0.0 | 0.1 | 0.1 | 0.0 | 0.1 | 0.0 | 0.0 | 0.0 | 0.0 | 0.0 | 0.0 | 0.0 |
| Slc22a14 | 0.1 | 0.0 | 0.0 | 0.1 | 0.0 | 0.0 | 0.0 | 0.0 | 0.0 | 0.1 | 0.0 | 0.0 |
| Slc22a15 | 1.3 | 1.0 | 1.1 | 1.3 | 1.0 | 1.0 | 1.3 | 0.8 | 1.0 | 0.9 | 1.0 | 1.2 |
| Slc22a17 | 2.0 | 2.6 | 2.7 | 1.6 | 1.9 | 2.1 | 2.0 | 2.1 | 2.8 | 3.0 | 1.6 | 1.9 |
| Slc22a18 | 10.5 | 6.8 | 4.9 | 10.2 | 7.7 | 10.6 | 5.2 | 3.8 | 3.5 | 4.3 | 3.3 | 4.7 |
| Slc22a2 | 0.2 | 0.2 | 0.2 | 0.0 | 0.2 | 0.2 | 0.2 | 0.1 | 0.0 | 0.0 | 0.1 | 0.1 |
| Slc22a21 | 0.9 | 0.8 | 0.9 | 1.1 | 0.8 | 0.7 | 0.9 | 0.5 | 0.7 | 0.9 | 0.9 | 1.0 |
| Slc22a23 | 3.5 | 3.1 | 1.5 | 7.0 | 2.8 | 7.1 | 2.5 | 3.1 | 2.1 | 2.1 | 2.2 | 1.2 |
| Slc22a27 | 0.0 | 0.0 | 0.0 | 0.0 | 0.0 | 0.0 | 0.0 | 0.0 | 0.0 | 0.0 | 0.0 | 0.0 |
| Slc22a3 | 0.1 | 0.1 | 0.0 | 0.2 | 0.2 | 0.1 | 0.0 | 0.0 | 0.0 | 0.0 | 0.1 | 0.0 |
| Slc22a4 | 0.9 | 0.4 | 0.2 | 0.9 | 1.5 | 0.9 | 0.5 | 0.3 | 0.3 | 0.4 | 0.2 | 0.6 |
| Slc22a5 | 5.1 | 4.3 | 3.3 | 4.5 | 4.1 | 5.2 | 3.9 | 3.6 | 4.1 | 5.5 | 3.6 | 4.0 |
| Slc23a1 | 0.0 | 0.1 | 0.2 | 0.1 | 0.0 | 0.0 | 0.1 | 0.1 | 0.1 | 0.1 | 0.1 | 0.1 |
| Slc23a2 | 4.7 | 4.5 | 4.4 | 6.2 | 7.3 | 4.6 | 4.5 | 4.2 | 4.5 | 3.9 | 3.6 | 4.1 |
| Slc23a3 | 0.0 | 0.1 | 0.0 | 0.0 | 0.0 | 0.0 | 0.0 | 0.0 | 0.0 | 0.1 | 0.0 | 0.0 |
| Slc24a1 | 0.0 | 0.0 | 0.0 | 0.0 | 0.0 | 0.0 | 0.0 | 0.0 | 0.0 | 0.0 | 0.0 | 0.0 |
| Slc24a2 | 0.0 | 0.0 | 0.0 | 0.0 | 0.0 | 0.0 | 0.0 | 0.0 | 0.0 | 0.0 | 0.0 | 0.0 |

| | | | | | | | | | | | | |
|---|---|---|---|---|---|---|---|---|---|---|---|---|
| Slc24a3 | 3.7 | 3.5 | 3.3 | 3.0 | 2.4 | 3.1 | 2.6 | 2.5 | 3.2 | 3.5 | 3.8 | 3.6 |
| Slc24a4 | 0.0 | 0.0 | 0.0 | 0.0 | 0.0 | 0.0 | 0.0 | 0.0 | 0.0 | 0.0 | 0.0 | 0.0 |
| Slc24a5 | 1.4 | 1.0 | 0.6 | 2.5 | 1.2 | 2.7 | 0.8 | 0.7 | 0.4 | 0.4 | 0.6 | 0.4 |
| Slc24a6 | 5.3 | 4.7 | 3.8 | 6.8 | 6.5 | 6.2 | 4.1 | 2.7 | 3.9 | 3.3 | 2.4 | 3.5 |
| Slc25a1 | 55.5 | 66.2 | 57.5 | 41.8 | 43.9 | 59.8 | 59.1 | 62.5 | 53.6 | 41.5 | 58.4 | 44.7 |
| Slc25a10 | 2.5 | 1.8 | 2.2 | 1.9 | 2.8 | 1.7 | 2.3 | 2.3 | 2.2 | 1.8 | 1.9 | 1.8 |
| Slc25a11 | 31.5 | 29.9 | 26.1 | 29.2 | 31.1 | 28.8 | 28.7 | 29.4 | 30.6 | 31.0 | 27.2 | 27.1 |
| Slc25a12 | 11.3 | 12.2 | 11.6 | 10.6 | 11.6 | 10.9 | 10.2 | 11.2 | 11.0 | 9.6 | 9.3 | 10.1 |
| Slc25a13 | 1.6 | 1.0 | 1.1 | 1.3 | 2.6 | 1.0 | 1.4 | 0.9 | 1.0 | 2.7 | 2.4 | 1.4 |
| Slc25a14 | 3.0 | 2.9 | 2.4 | 3.2 | 2.5 | 2.8 | 2.0 | 1.6 | 2.1 | 2.2 | 2.5 | 3.0 |
| Slc25a15 | 3.8 | 3.8 | 3.7 | 3.0 | 2.9 | 4.0 | 2.9 | 2.5 | 2.7 | 3.3 | 3.0 | 3.6 |
| Slc25a16 | 6.5 | 6.9 | 6.8 | 7.3 | 6.6 | 7.5 | 6.2 | 7.0 | 7.3 | 7.5 | 7.2 | 7.7 |
| Slc25a17 | 22.3 | 23.9 | 23.6 | 21.7 | 23.3 | 20.8 | 25.0 | 21.8 | 24.2 | 22.8 | 27.0 | 25.6 |
| Slc25a18 | 0.1 | 0.0 | 0.1 | 0.0 | 0.1 | 0.1 | 0.1 | 0.0 | 0.1 | 0.2 | 0.0 | 0.0 |
| Slc25a19 | 4.6 | 3.7 | 3.8 | 6.4 | 4.6 | 6.5 | 3.0 | 4.2 | 4.1 | 4.8 | 3.3 | 3.7 |
| Slc25a2 | 0.0 | 0.2 | 0.1 | 0.0 | 0.1 | 0.1 | 0.1 | 0.1 | 0.1 | 0.2 | 0.1 | 0.0 |
| Slc25a20 | 18.3 | 16.2 | 14.2 | 17.8 | 16.6 | 16.5 | 13.2 | 15.7 | 15.1 | 18.5 | 17.7 | 18.0 |
| Slc25a21 | 0.0 | 0.0 | 0.0 | 0.0 | 0.0 | 0.0 | 0.0 | 0.0 | 0.0 | 0.0 | 0.0 | 0.0 |
| Slc25a22 | 2.4 | 3.1 | 2.0 | 2.9 | 3.0 | 3.0 | 2.9 | 2.6 | 2.7 | 2.4 | 2.1 | 2.0 |
| Slc25a23 | 0.7 | 1.1 | 1.2 | 0.8 | 0.7 | 1.3 | 0.6 | 0.8 | 0.7 | 1.7 | 0.7 | 0.8 |
| Slc25a24 | 12.1 | 8.8 | 9.2 | 17.2 | 13.2 | 14.2 | 12.2 | 11.7 | 11.5 | 11.4 | 15.1 | 11.8 |
| Slc25a25 | 5.2 | 4.3 | 3.1 | 4.3 | 5.7 | 3.9 | 4.5 | 3.4 | 4.3 | 5.9 | 5.0 | 4.2 |
| Slc25a26 | 2.2 | 1.8 | 1.7 | 2.3 | 2.2 | 2.6 | 1.5 | 1.7 | 2.5 | 2.7 | 2.2 | 2.0 |
| Slc25a27 | 0.4 | 0.5 | 0.5 | 0.2 | 0.4 | 0.4 | 0.2 | 0.4 | 0.3 | 0.4 | 0.2 | 0.3 |
| Slc25a28 | 3.8 | 3.6 | 4.4 | 4.2 | 3.8 | 4.0 | 4.5 | 3.8 | 4.3 | 5.2 | 4.7 | 5.1 |
| Slc25a29 | 1.2 | 1.0 | 0.9 | 0.5 | 1.1 | 0.7 | 1.0 | 1.0 | 0.9 | 1.0 | 0.8 | 0.8 |
| Slc25a3 | 193.9 | 156.6 | 127.6 | 158.9 | 162.2 | 163.4 | 134.9 | 145.1 | 151.7 | 158.8 | 159.3 | 140.1 |
| Slc25a30 | 8.1 | 8.5 | 9.1 | 8.1 | 10.4 | 9.1 | 13.3 | 10.8 | 13.5 | 11.0 | 13.4 | 11.7 |
| Slc25a31 | 0.1 | 0.1 | 0.1 | 0.0 | 0.0 | 0.2 | 0.3 | 0.0 | 0.0 | 0.1 | 0.0 | 0.1 |
| Slc25a32 | 6.3 | 5.3 | 5.4 | 6.7 | 6.3 | 4.8 | 5.8 | 5.1 | 6.5 | 7.0 | 6.9 | 7.1 |
| Slc25a33 | 3.5 | 2.4 | 1.5 | 3.8 | 2.9 | 3.9 | 1.6 | 1.8 | 2.0 | 2.5 | 2.2 | 1.8 |
| Slc25a34 | 0.1 | 0.0 | 0.0 | 0.0 | 0.1 | 0.0 | 0.1 | 0.1 | 0.0 | 0.0 | 0.0 | 0.0 |
| Slc25a35 | 0.9 | 1.7 | 1.6 | 1.2 | 1.2 | 1.4 | 1.2 | 1.6 | 1.2 | 0.7 | 0.9 | 0.7 |
| Slc25a36 | 12.8 | 13.8 | 14.1 | 15.0 | 14.6 | 15.9 | 12.5 | 13.7 | 13.3 | 16.0 | 15.2 | 15.1 |
| Slc25a37 | 7.2 | 7.6 | 6.0 | 7.6 | 9.1 | 8.9 | 7.7 | 4.4 | 5.5 | 6.4 | 5.9 | 5.7 |
| Slc25a38 | 9.5 | 7.6 | 7.5 | 8.3 | 8.0 | 7.8 | 8.3 | 8.0 | 10.5 | 8.3 | 9.5 | 8.4 |
| Slc25a39 | 67.3 | 57.7 | 46.9 | 64.3 | 55.9 | 68.7 | 51.6 | 55.9 | 51.5 | 55.7 | 54.3 | 45.8 |
| Slc25a4 | 351.7 | 259.3 | 245.8 | 313.6 | 271.5 | 346.7 | 290.5 | 279.9 | 277.4 | 317.9 | 300.8 | 308.2 |
| Slc25a40 | 5.1 | 6.4 | 7.0 | 4.5 | 5.6 | 6.1 | 6.2 | 5.0 | 6.5 | 5.5 | 7.1 | 6.6 |
| Slc25a41 | 0.0 | 0.0 | 0.0 | 0.0 | 0.1 | 0.2 | 0.0 | 0.0 | 0.0 | 0.0 | 0.0 | 0.0 |
| Slc25a42 | 1.0 | 1.1 | 1.1 | 0.7 | 1.0 | 1.0 | 1.0 | 0.8 | 0.9 | 1.0 | 0.8 | 0.9 |
| Slc25a43 | 1.9 | 1.2 | 1.2 | 1.6 | 1.7 | 1.8 | 1.7 | 0.7 | 1.0 | 1.2 | 1.2 | 0.8 |
| Slc25a44 | 6.8 | 7.1 | 7.4 | 6.9 | 6.9 | 7.1 | 7.4 | 7.3 | 7.6 | 6.8 | 7.1 | 7.0 |
| Slc25a45 | 4.0 | 4.0 | 4.2 | 6.2 | 6.2 | 4.3 | 5.2 | 4.4 | 4.2 | 3.7 | 3.5 | 3.0 |
| Slc25a46 | 14.0 | 13.9 | 14.6 | 16.1 | 13.8 | 16.0 | 14.4 | 12.9 | 13.0 | 15.4 | 18.2 | 16.1 |
| Slc25a47 | 0.4 | 0.5 | 0.4 | 0.8 | 0.5 | 0.6 | 0.6 | 0.5 | 0.5 | 0.5 | 0.5 | 0.3 |
| Slc25a5 | 102.1 | 81.8 | 76.4 | 99.3 | 112.4 | 83.1 | 80.1 | 76.8 | 74.8 | 96.9 | 91.2 | 80.1 |
| Slc25a51 | 16.6 | 15.9 | 14.4 | 15.1 | 15.5 | 15.7 | 15.2 | 16.6 | 16.8 | 16.0 | 16.8 | 15.8 |
| Slc25a53 | 0.3 | 0.3 | 0.2 | 0.4 | 0.3 | 0.3 | 0.3 | 0.3 | 0.3 | 0.3 | 0.2 | 0.1 |
| Slc26a1 | 0.5 | 0.5 | 0.5 | 0.5 | 0.5 | 0.6 | 0.6 | 0.7 | 0.5 | 0.5 | 0.4 | 0.4 |
| Slc26a10 | 0.0 | 0.2 | 0.0 | 0.0 | 0.0 | 0.1 | 0.1 | 0.2 | 0.0 | 0.1 | 0.0 | 0.0 |
| Slc26a11 | 4.3 | 4.5 | 4.3 | 3.8 | 3.2 | 4.2 | 3.6 | 3.7 | 4.3 | 4.8 | 4.4 | 4.0 |
| Slc26a2 | 3.1 | 2.9 | 2.7 | 3.0 | 3.5 | 2.7 | 3.2 | 3.2 | 3.2 | 2.9 | 3.0 | 2.8 |
| Slc26a5 | 0.0 | 0.0 | 0.0 | 0.0 | 0.0 | 0.0 | 0.0 | 0.0 | 0.0 | 0.0 | 0.0 | 0.0 |
| Slc26a6 | 0.7 | 0.8 | 0.8 | 1.1 | 0.8 | 0.6 | 1.0 | 1.2 | 1.1 | 0.8 | 0.8 | 0.7 |
| Slc26a7 | 0.0 | 0.0 | 0.0 | 0.0 | 0.0 | 0.0 | 0.0 | 0.0 | 0.0 | 0.0 | 0.0 | 0.0 |
| Slc27a1 | 2.3 | 1.9 | 1.9 | 3.3 | 3.9 | 3.1 | 1.2 | 2.2 | 1.7 | 2.4 | 1.2 | 1.7 |

| | | | | | | | | | | | | |
|---|---|---|---|---|---|---|---|---|---|---|---|---|
| Slc27a3 | 3.5 | 6.5 | 7.3 | 1.8 | 4.7 | 2.7 | 5.7 | 10.1 | 6.8 | 3.5 | 3.6 | 2.9 |
| Slc27a4 | 8.9 | 9.2 | 8.5 | 8.7 | 8.8 | 7.7 | 9.9 | 10.2 | 10.3 | 10.0 | 10.4 | 8.6 |
| Slc27a5 | 0.0 | 0.1 | 0.0 | 0.0 | 0.0 | 0.0 | 0.1 | 0.0 | 0.0 | 0.0 | 0.0 | 0.0 |
| Slc27a6 | 0.1 | 0.3 | 0.1 | 0.1 | 0.0 | 0.1 | 0.0 | 0.1 | 0.1 | 0.1 | 0.3 | 0.1 |
| Slc28a1 | 0.0 | 0.0 | 0.1 | 0.0 | 0.0 | 0.0 | 0.0 | 0.0 | 0.0 | 0.0 | 0.0 | 0.0 |
| Slc28a2 | 0.1 | 0.0 | 0.0 | 0.1 | 0.4 | 0.0 | 0.2 | 0.1 | 0.0 | 0.1 | 0.1 | 0.2 |
| Slc28a3 | 0.0 | 0.0 | 0.0 | 0.0 | 0.0 | 0.0 | 0.0 | 0.0 | 0.0 | 0.0 | 0.0 | 0.0 |
| Slc29a1 | 12.4 | 13.9 | 16.0 | 13.0 | 16.6 | 18.6 | 21.4 | 25.5 | 21.1 | 18.8 | 18.7 | 16.9 |
| Slc29a2 | 0.2 | 0.3 | 0.2 | 0.3 | 0.2 | 0.3 | 0.1 | 0.3 | 0.2 | 0.2 | 0.2 | 0.1 |
| Slc29a3 | 3.5 | 4.3 | 4.3 | 5.0 | 5.0 | 5.0 | 4.7 | 4.7 | 4.2 | 4.1 | 4.1 | 3.5 |
| Slc29a4 | 0.1 | 0.0 | 0.0 | 0.0 | 0.1 | 0.0 | 0.1 | 0.0 | 0.0 | 0.0 | 0.0 | 0.0 |
| Slc2a1 | 11.1 | 11.9 | 11.5 | 12.7 | 17.5 | 16.6 | 16.6 | 9.0 | 13.8 | 14.2 | 12.7 | 11.6 |
| Slc2a10 | 5.7 | 5.2 | 4.6 | 5.1 | 5.4 | 5.3 | 6.1 | 6.3 | 6.0 | 4.1 | 4.9 | 4.6 |
| Slc2a12 | 0.1 | 0.1 | 0.2 | 0.1 | 0.1 | 0.1 | 0.1 | 0.2 | 0.1 | 0.2 | 0.1 | 0.2 |
| Slc2a13 | 1.9 | 1.6 | 1.8 | 1.6 | 1.4 | 1.3 | 2.2 | 1.5 | 1.9 | 2.2 | 2.3 | 2.7 |
| Slc2a2 | 0.3 | 0.3 | 0.3 | 0.8 | 0.3 | 0.3 | 0.4 | 0.5 | 0.3 | 0.4 | 0.4 | 0.5 |
| Slc2a3 | 0.8 | 1.1 | 1.2 | 0.6 | 0.8 | 0.9 | 0.8 | 0.9 | 0.7 | 1.9 | 1.0 | 0.8 |
| Slc2a4 | 0.0 | 0.0 | 0.0 | 0.0 | 0.0 | 0.0 | 0.0 | 0.0 | 0.0 | 0.0 | 0.0 | 0.0 |
| Slc2a4rg-ps | 0.7 | 0.8 | 0.4 | 0.5 | 0.6 | 0.5 | 0.6 | 0.5 | 0.6 | 0.3 | 0.7 | 0.3 |
| Slc2a5 | 0.1 | 0.0 | 0.0 | 0.1 | 0.0 | 0.1 | 0.0 | 0.1 | 0.0 | 0.0 | 0.1 | 0.0 |
| Slc2a6 | 7.0 | 5.8 | 6.5 | 12.6 | 16.2 | 7.1 | 9.1 | 8.7 | 4.8 | 2.8 | 4.8 | 3.5 |
| Slc2a8 | 5.8 | 5.2 | 5.4 | 3.5 | 4.7 | 4.7 | 5.4 | 5.6 | 5.9 | 5.7 | 4.0 | 4.4 |
| Slc2a9 | 1.4 | 1.2 | 1.2 | 2.1 | 2.2 | 1.5 | 1.5 | 0.8 | 1.0 | 1.1 | 1.0 | 1.0 |
| Slc30a1 | 4.4 | 4.0 | 4.7 | 4.6 | 4.9 | 3.8 | 4.5 | 4.1 | 4.5 | 6.0 | 4.9 | 4.9 |
| Slc30a4 | 14.2 | 12.9 | 11.4 | 12.2 | 11.4 | 13.4 | 10.8 | 11.7 | 11.9 | 12.0 | 13.1 | 12.2 |
| Slc30a5 | 20.1 | 18.9 | 18.6 | 18.7 | 20.4 | 18.5 | 19.2 | 20.0 | 20.4 | 21.4 | 21.4 | 21.6 |
| Slc30a6 | 7.3 | 7.9 | 7.0 | 7.4 | 7.1 | 7.1 | 7.6 | 7.5 | 8.5 | 7.9 | 8.6 | 8.6 |
| Slc30a7 | 6.1 | 6.8 | 6.8 | 5.7 | 6.6 | 5.5 | 7.1 | 7.6 | 7.0 | 7.1 | 7.5 | 7.2 |
| Slc30a9 | 30.7 | 28.8 | 23.2 | 29.2 | 24.8 | 26.9 | 25.6 | 20.7 | 24.7 | 26.7 | 28.0 | 27.6 |
| Slc31a1 | 9.0 | 12.3 | 12.2 | 9.3 | 10.8 | 7.6 | 10.0 | 13.3 | 10.5 | 10.3 | 10.4 | 10.7 |
| Slc31a2 | 13.3 | 9.9 | 8.3 | 14.8 | 13.4 | 11.6 | 8.5 | 9.7 | 10.0 | 13.6 | 10.6 | 10.3 |
| Slc33a1 | 20.7 | 18.0 | 15.5 | 17.4 | 19.2 | 18.3 | 15.1 | 13.7 | 19.1 | 20.7 | 18.5 | 18.5 |
| Slc35a1 | 28.9 | 26.2 | 20.1 | 25.7 | 22.4 | 26.7 | 18.4 | 17.6 | 22.5 | 22.9 | 22.1 | 19.3 |
| Slc35a2 | 21.5 | 19.3 | 17.5 | 20.9 | 21.3 | 20.4 | 20.5 | 24.3 | 22.8 | 20.3 | 20.2 | 20.1 |
| Slc35a3 | 6.7 | 7.6 | 7.3 | 6.6 | 7.2 | 6.9 | 6.2 | 6.4 | 7.2 | 6.6 | 7.6 | 8.1 |
| Slc35a4 | 10.5 | 10.2 | 8.8 | 11.3 | 11.9 | 11.5 | 10.7 | 11.2 | 10.5 | 10.5 | 8.6 | 7.8 |
| Slc35a5 | 10.6 | 10.5 | 9.9 | 12.9 | 9.9 | 11.7 | 9.9 | 9.1 | 9.8 | 10.7 | 11.2 | 11.8 |
| Slc35b1 | 60.5 | 50.7 | 44.4 | 63.2 | 46.4 | 55.3 | 51.3 | 51.3 | 51.1 | 48.0 | 51.4 | 50.2 |
| Slc35b2 | 16.2 | 17.3 | 14.7 | 15.0 | 15.7 | 18.2 | 19.8 | 19.0 | 18.7 | 20.1 | 16.2 | 17.8 |
| Slc35b3 | 7.0 | 5.9 | 5.6 | 6.2 | 7.0 | 6.5 | 6.5 | 5.2 | 7.5 | 6.6 | 6.8 | 6.3 |
| Slc35b4 | 6.4 | 6.0 | 6.8 | 6.6 | 7.2 | 6.0 | 7.4 | 9.6 | 8.6 | 8.5 | 7.2 | 6.8 |
| Slc35c1 | 1.2 | 1.4 | 0.9 | 1.5 | 1.9 | 1.2 | 1.3 | 1.6 | 1.1 | 1.3 | 1.3 | 1.0 |
| Slc35c2 | 14.3 | 11.4 | 7.1 | 19.0 | 14.0 | 14.1 | 11.7 | 9.3 | 9.6 | 10.6 | 9.9 | 9.7 |
| Slc35d1 | 0.9 | 0.8 | 1.0 | 1.0 | 0.8 | 0.8 | 1.0 | 0.7 | 0.6 | 1.3 | 1.0 | 1.0 |
| Slc35d2 | 2.5 | 3.0 | 2.4 | 2.5 | 2.6 | 2.4 | 2.5 | 2.2 | 2.7 | 2.0 | 2.8 | 2.6 |
| Slc35d3 | 0.0 | 0.0 | 0.0 | 0.0 | 0.0 | 0.0 | 0.0 | 0.0 | 0.0 | 0.0 | 0.0 | 0.0 |
| Slc35e1 | 12.2 | 11.4 | 11.0 | 11.8 | 11.6 | 11.9 | 11.9 | 10.2 | 9.8 | 9.2 | 10.6 | 9.8 |
| Slc35e2 | 2.5 | 2.3 | 2.7 | 3.0 | 2.5 | 3.2 | 2.8 | 2.5 | 2.4 | 2.8 | 2.5 | 2.6 |
| Slc35e3 | 9.1 | 8.1 | 8.1 | 9.3 | 7.6 | 9.0 | 7.5 | 7.0 | 6.8 | 7.6 | 7.0 | 7.3 |
| Slc35e4 | 7.6 | 4.5 | 3.4 | 9.3 | 6.7 | 8.1 | 5.3 | 5.3 | 5.2 | 6.5 | 5.9 | 4.2 |
| Slc35f1 | 0.2 | 0.1 | 0.2 | 0.3 | 0.5 | 0.4 | 0.3 | 0.1 | 0.2 | 0.1 | 0.1 | 0.1 |
| Slc35f2 | 0.0 | 0.0 | 0.0 | 0.1 | 0.0 | 0.0 | 0.0 | 0.0 | 0.0 | 0.0 | 0.0 | 0.0 |
| Slc35f3 | 0.0 | 0.0 | 0.0 | 0.0 | 0.0 | 0.0 | 0.0 | 0.0 | 0.0 | 0.0 | 0.0 | 0.0 |
| Slc35f5 | 19.1 | 18.9 | 16.4 | 18.7 | 18.3 | 19.3 | 17.6 | 18.1 | 21.2 | 18.5 | 19.0 | 18.7 |
| Slc35f6 | 11.4 | 11.0 | 8.9 | 13.6 | 12.5 | 14.2 | 11.5 | 10.9 | 11.2 | 11.0 | 10.7 | 11.0 |
| Slc35g1 | 0.8 | 0.7 | 0.9 | 0.8 | 0.8 | 0.7 | 0.6 | 0.5 | 0.4 | 0.6 | 0.5 | 0.5 |
| Slc35g2 | 0.4 | 0.7 | 0.5 | 0.4 | 0.5 | 0.5 | 0.5 | 0.7 | 0.8 | 0.4 | 0.3 | 0.5 |

| | | | | | | | | | | | | |
|---|---|---|---|---|---|---|---|---|---|---|---|---|
| Slc35g3 | 0.1 | 0.0 | 0.1 | 0.1 | 0.1 | 0.0 | 0.1 | 0.0 | 0.1 | 0.1 | 0.2 | 0.0 |
| Slc36a1 | 2.2 | 2.7 | 2.3 | 2.8 | 3.4 | 3.0 | 2.6 | 2.4 | 2.7 | 2.2 | 2.3 | 1.8 |
| Slc36a2 | 0.1 | 0.1 | 0.1 | 0.0 | 0.1 | 0.0 | 0.1 | 0.1 | 0.1 | 0.1 | 0.0 | 0.0 |
| Slc36a4 | 7.8 | 7.8 | 10.3 | 8.4 | 8.5 | 6.3 | 9.6 | 8.4 | 8.9 | 7.9 | 9.9 | 8.7 |
| Slc37a1 | 0.0 | 0.0 | 0.0 | 0.2 | 0.2 | 0.0 | 0.1 | 0.0 | 0.0 | 0.0 | 0.0 | 0.0 |
| Slc37a2 | 1.2 | 1.1 | 1.7 | 3.3 | 7.1 | 0.8 | 1.2 | 1.0 | 1.1 | 1.0 | 1.0 | 0.9 |
| Slc37a3 | 7.2 | 6.9 | 7.2 | 7.8 | 7.4 | 7.2 | 6.5 | 7.4 | 6.2 | 6.6 | 6.7 | 6.8 |
| Slc37a4 | 4.5 | 4.2 | 3.7 | 5.3 | 4.0 | 5.8 | 4.0 | 4.3 | 4.6 | 3.7 | 4.4 | 3.9 |
| Slc38a1 | 3.5 | 1.7 | 1.3 | 3.5 | 2.6 | 3.7 | 2.1 | 1.5 | 1.8 | 4.6 | 3.5 | 2.4 |
| Slc38a10 | 57.3 | 46.3 | 45.2 | 69.1 | 57.6 | 67.1 | 62.8 | 49.6 | 50.9 | 55.8 | 52.8 | 59.6 |
| Slc38a11 | 0.0 | 0.0 | 0.0 | 0.0 | 0.0 | 0.0 | 0.0 | 0.0 | 0.0 | 0.0 | 0.0 | 0.0 |
| Slc38a2 | 27.0 | 27.4 | 29.9 | 25.8 | 31.2 | 24.6 | 33.2 | 37.0 | 39.5 | 49.7 | 42.7 | 38.9 |
| Slc38a4 | 0.0 | 0.0 | 0.0 | 0.0 | 0.0 | 0.1 | 0.0 | 0.0 | 0.0 | 0.0 | 0.0 | 0.0 |
| Slc38a5 | 0.0 | 0.0 | 0.0 | 0.0 | 0.0 | 0.0 | 0.0 | 0.0 | 0.0 | 0.0 | 0.0 | 0.0 |
| Slc38a6 | 1.8 | 1.9 | 1.8 | 2.1 | 2.0 | 1.9 | 1.4 | 1.6 | 1.4 | 1.3 | 1.0 | 1.4 |
| Slc38a7 | 5.7 | 4.8 | 3.5 | 7.4 | 4.7 | 6.8 | 5.1 | 5.5 | 4.7 | 4.4 | 5.0 | 4.7 |
| Slc38a8 | 0.0 | 0.0 | 0.0 | 0.0 | 0.1 | 0.0 | 0.0 | 0.0 | 0.0 | 0.0 | 0.0 | 0.0 |
| Slc38a9 | 1.2 | 1.3 | 1.3 | 1.3 | 1.2 | 1.2 | 1.0 | 1.0 | 1.1 | 1.1 | 1.0 | 1.0 |
| Slc39a1 | 73.6 | 70.9 | 80.6 | 84.4 | 82.7 | 66.5 | 96.9 | 104.5 | 94.0 | 89.3 | 92.2 | 97.3 |
| Slc39a10 | 4.9 | 3.8 | 3.8 | 4.8 | 5.6 | 3.5 | 4.9 | 4.9 | 5.4 | 14.0 | 7.7 | 7.3 |
| Slc39a11 | 11.8 | 10.5 | 9.4 | 11.8 | 12.4 | 13.6 | 9.5 | 9.0 | 8.5 | 11.2 | 9.9 | 8.6 |
| Slc39a13 | 34.8 | 32.2 | 27.0 | 44.2 | 36.3 | 35.0 | 46.1 | 32.9 | 38.8 | 30.6 | 36.5 | 32.2 |
| Slc39a14 | 15.3 | 19.5 | 26.2 | 11.9 | 28.5 | 11.9 | 30.5 | 36.6 | 29.8 | 20.6 | 20.5 | 24.3 |
| Slc39a2 | 0.1 | 0.1 | 0.1 | 0.2 | 0.1 | 0.1 | 0.0 | 0.0 | 0.0 | 0.1 | 0.1 | 0.1 |
| Slc39a3 | 4.8 | 5.0 | 4.1 | 4.6 | 5.1 | 5.1 | 5.1 | 6.1 | 6.5 | 5.7 | 5.2 | 5.4 |
| Slc39a4 | 1.2 | 0.2 | 0.3 | 2.4 | 4.3 | 1.5 | 0.5 | 0.0 | 0.0 | 0.3 | 0.1 | 0.1 |
| Slc39a5 | 0.0 | 0.0 | 0.0 | 0.0 | 0.0 | 0.0 | 0.0 | 0.0 | 0.0 | 0.0 | 0.0 | 0.0 |
| Slc39a6 | 28.2 | 25.8 | 21.3 | 28.4 | 28.5 | 26.0 | 31.0 | 22.4 | 25.3 | 25.0 | 26.8 | 27.3 |
| Slc39a7 | 105.4 | 84.4 | 77.3 | 117.7 | 83.2 | 103.6 | 97.8 | 90.0 | 87.5 | 84.6 | 92.3 | 92.1 |
| Slc39a8 | 4.6 | 3.6 | 2.0 | 3.4 | 2.8 | 4.0 | 3.4 | 2.4 | 2.2 | 5.8 | 5.8 | 4.0 |
| Slc39a9 | 4.0 | 4.7 | 4.9 | 5.1 | 5.8 | 4.7 | 5.2 | 6.0 | 5.2 | 5.1 | 4.9 | 5.1 |
| Slc3a1 | 0.1 | 0.1 | 0.0 | 0.3 | 0.2 | 0.1 | 0.1 | 0.1 | 0.1 | 0.1 | 0.1 | 0.2 |
| Slc3a2 | 30.3 | 22.5 | 23.2 | 36.8 | 30.0 | 32.4 | 27.2 | 38.3 | 28.5 | 24.4 | 28.0 | 22.5 |
| Slc40a1 | 0.9 | 0.3 | 0.1 | 6.2 | 8.5 | 0.3 | 0.2 | 0.3 | 0.3 | 0.5 | 0.3 | 0.4 |
| Slc41a1 | 8.8 | 10.3 | 12.7 | 8.6 | 9.1 | 9.7 | 10.4 | 12.3 | 9.8 | 8.0 | 8.3 | 9.4 |
| Slc41a2 | 7.5 | 6.3 | 6.2 | 9.0 | 9.9 | 6.8 | 9.3 | 8.7 | 7.3 | 6.9 | 8.6 | 8.3 |
| Slc41a3 | 3.3 | 2.9 | 3.1 | 4.1 | 3.9 | 4.4 | 3.6 | 3.5 | 4.9 | 3.7 | 3.4 | 3.9 |
| Slc43a1 | 0.1 | 0.2 | 0.6 | 0.1 | 0.2 | 0.2 | 0.4 | 0.7 | 0.8 | 1.0 | 1.0 | 0.5 |
| Slc43a2 | 1.3 | 1.2 | 1.4 | 2.7 | 4.2 | 1.0 | 1.1 | 1.1 | 0.7 | 0.9 | 0.8 | 1.1 |
| Slc43a3 | 14.8 | 24.8 | 40.7 | 9.5 | 21.2 | 11.7 | 24.4 | 27.4 | 36.0 | 21.2 | 23.3 | 44.2 |
| Slc44a1 | 16.4 | 19.1 | 21.8 | 13.0 | 14.6 | 14.1 | 11.6 | 20.5 | 16.1 | 13.6 | 14.8 | 15.6 |
| Slc44a2 | 89.4 | 123.0 | 135.1 | 80.7 | 97.2 | 99.6 | 134.8 | 204.5 | 162.3 | 99.0 | 120.7 | 116.6 |
| Slc44a3 | 0.2 | 0.5 | 0.9 | 0.1 | 0.3 | 0.3 | 0.3 | 0.6 | 0.4 | 0.7 | 0.6 | 0.7 |
| Slc44a4 | 0.1 | 0.0 | 0.0 | 0.0 | 0.0 | 0.1 | 0.0 | 0.0 | 0.0 | 0.0 | 0.0 | 0.0 |
| Slc44a5 | 0.0 | 0.0 | 0.0 | 0.0 | 0.0 | 0.0 | 0.1 | 0.0 | 0.0 | 0.0 | 0.0 | 0.0 |
| Slc45a1 | 0.0 | 0.0 | 0.0 | 0.0 | 0.0 | 0.1 | 0.0 | 0.0 | 0.0 | 0.0 | 0.0 | 0.0 |
| Slc45a2 | 0.0 | 0.0 | 0.0 | 0.0 | 0.0 | 0.0 | 0.0 | 0.0 | 0.0 | 0.0 | 0.0 | 0.0 |
| Slc45a3 | 3.1 | 5.0 | 5.8 | 2.3 | 4.2 | 2.5 | 7.5 | 8.3 | 10.4 | 4.9 | 5.4 | 8.3 |
| Slc45a4 | 2.9 | 3.1 | 3.2 | 2.9 | 3.8 | 2.7 | 2.8 | 3.4 | 3.1 | 2.6 | 3.4 | 2.1 |
| Slc46a1 | 2.2 | 1.8 | 1.4 | 3.2 | 2.0 | 2.6 | 2.0 | 1.9 | 2.1 | 2.3 | 1.7 | 1.5 |
| Slc46a2 | 0.0 | 0.0 | 0.1 | 0.0 | 0.0 | 0.0 | 0.0 | 0.0 | 0.0 | 0.0 | 0.0 | 0.0 |
| Slc46a3 | 1.3 | 1.6 | 1.4 | 1.4 | 0.9 | 1.3 | 0.9 | 1.0 | 0.9 | 0.9 | 0.8 | 1.0 |
| Slc47a1 | 0.1 | 0.0 | 0.0 | 0.0 | 0.0 | 0.0 | 0.0 | 0.1 | 0.0 | 0.0 | 0.0 | 0.0 |
| Slc48a1 | 86.3 | 82.7 | 67.8 | 103.1 | 84.1 | 113.7 | 67.6 | 78.1 | 60.3 | 109.9 | 91.1 | 83.0 |
| Slc4a10 | 0.0 | 0.0 | 0.0 | 0.0 | 0.0 | 0.0 | 0.0 | 0.0 | 0.0 | 0.0 | 0.0 | 0.0 |
| Slc4a11 | 0.5 | 0.6 | 0.5 | 0.6 | 0.4 | 0.8 | 0.5 | 0.5 | 0.4 | 0.3 | 0.3 | 0.4 |
| Slc4a1ap | 11.8 | 12.8 | 13.5 | 13.8 | 11.0 | 13.1 | 12.3 | 11.8 | 10.2 | 10.7 | 12.0 | 15.0 |

| | | | | | | | | | | | | |
|---|---|---|---|---|---|---|---|---|---|---|---|---|
| Slc4a2 | 30.2 | 25.1 | 17.5 | 30.3 | 25.1 | 27.2 | 27.4 | 24.2 | 28.3 | 29.0 | 27.6 | 24.1 |
| Slc4a3 | 3.0 | 3.1 | 2.8 | 2.6 | 2.3 | 3.1 | 3.1 | 3.2 | 3.5 | 2.6 | 2.1 | 2.3 |
| Slc4a4 | 3.6 | 3.6 | 4.8 | 0.7 | 1.4 | 1.0 | 3.1 | 6.5 | 3.0 | 4.6 | 3.8 | 3.9 |
| Slc4a5 | 0.0 | 0.0 | 0.0 | 0.0 | 0.0 | 0.0 | 0.1 | 0.0 | 0.0 | 0.1 | 0.0 | 0.0 |
| Slc4a7 | 3.5 | 3.5 | 3.2 | 3.8 | 4.7 | 3.2 | 3.2 | 2.5 | 3.1 | 3.8 | 4.2 | 3.1 |
| Slc4a8 | 0.0 | 0.0 | 0.0 | 0.0 | 0.0 | 0.0 | 0.0 | 0.0 | 0.0 | 0.0 | 0.0 | 0.0 |
| Slc4a9 | 0.0 | 0.0 | 0.0 | 0.0 | 0.0 | 0.0 | 0.0 | 0.0 | 0.0 | 0.0 | 0.0 | 0.0 |
| Slc50a1 | 25.3 | 26.5 | 29.2 | 27.9 | 22.5 | 29.2 | 30.1 | 38.1 | 31.6 | 28.6 | 28.0 | 29.2 |
| Slc51a | 0.0 | 0.0 | 0.0 | 0.0 | 0.0 | 0.0 | 0.0 | 0.0 | 0.0 | 0.1 | 0.0 | 0.0 |
| Slc51b | 0.0 | 0.0 | 0.0 | 0.0 | 0.1 | 0.0 | 0.0 | 0.0 | 0.1 | 0.2 | 0.2 | 0.0 |
| Slc52a2 | 2.6 | 2.3 | 2.6 | 3.0 | 2.7 | 3.0 | 2.1 | 2.3 | 2.5 | 2.2 | 1.9 | 2.2 |
| Slc52a3 | 0.1 | 0.0 | 0.0 | 0.1 | 0.1 | 0.0 | 0.1 | 0.0 | 0.0 | 0.0 | 0.0 | 0.0 |
| Slc5a1 | 0.1 | 0.0 | 0.0 | 0.0 | 0.0 | 0.0 | 0.0 | 0.0 | 0.0 | 0.0 | 0.0 | 0.0 |
| Slc5a11 | 0.0 | 0.1 | 0.0 | 0.1 | 0.1 | 0.0 | 0.1 | 0.0 | 0.1 | 0.0 | 0.1 | 0.0 |
| Slc5a12 | 0.0 | 0.0 | 0.0 | 0.0 | 0.1 | 0.0 | 0.0 | 0.0 | 0.0 | 0.0 | 0.0 | 0.0 |
| Slc5a2 | 0.0 | 0.0 | 0.0 | 0.0 | 0.0 | 0.0 | 0.0 | 0.0 | 0.0 | 0.0 | 0.0 | 0.0 |
| Slc5a3 | 4.4 | 9.6 | 11.1 | 5.0 | 5.2 | 8.3 | 4.6 | 8.8 | 4.8 | 6.1 | 4.8 | 4.4 |
| Slc5a4b | 0.1 | 0.0 | 0.0 | 0.0 | 0.0 | 0.0 | 0.0 | 0.0 | 0.0 | 0.0 | 0.0 | 0.0 |
| Slc5a5 | 0.0 | 0.0 | 0.0 | 0.0 | 0.0 | 0.0 | 0.0 | 0.0 | 0.0 | 0.0 | 0.0 | 0.0 |
| Slc5a6 | 0.8 | 0.7 | 0.9 | 1.2 | 0.9 | 1.2 | 1.0 | 0.9 | 0.8 | 0.8 | 0.7 | 0.7 |
| Slc5a7 | 0.0 | 0.0 | 0.0 | 0.0 | 0.0 | 0.0 | 0.0 | 0.0 | 0.0 | 0.0 | 0.0 | 0.0 |
| Slc5a8 | 0.2 | 0.2 | 0.1 | 0.2 | 0.3 | 0.1 | 0.1 | 0.0 | 0.1 | 0.2 | 0.2 | 0.2 |
| Slc6a1 | 0.0 | 0.0 | 0.0 | 0.0 | 0.0 | 0.0 | 0.0 | 0.0 | 0.0 | 0.0 | 0.0 | 0.0 |
| Slc6a12 | 0.2 | 0.0 | 0.0 | 0.1 | 0.1 | 0.1 | 0.1 | 0.0 | 0.1 | 0.0 | 0.1 | 0.0 |
| Slc6a17 | 0.1 | 0.1 | 0.1 | 0.2 | 0.1 | 0.2 | 0.1 | 0.0 | 0.1 | 0.4 | 0.3 | 0.3 |
| Slc6a18 | 0.0 | 0.0 | 0.0 | 0.0 | 0.0 | 0.0 | 0.0 | 0.0 | 0.0 | 0.0 | 0.0 | 0.0 |
| Slc6a19 | 0.0 | 0.0 | 0.0 | 0.0 | 0.0 | 0.0 | 0.0 | 0.0 | 0.0 | 0.0 | 0.0 | 0.0 |
| Slc6a2 | 0.0 | 0.0 | 0.0 | 0.0 | 0.0 | 0.0 | 0.0 | 0.0 | 0.0 | 0.0 | 0.0 | 0.0 |
| Slc6a20a | 0.0 | 0.0 | 0.0 | 0.0 | 0.0 | 0.0 | 0.0 | 0.0 | 0.0 | 0.0 | 0.0 | 0.0 |
| Slc6a20b | 0.0 | 0.0 | 0.0 | 0.0 | 0.0 | 0.0 | 0.0 | 0.1 | 0.1 | 0.1 | 0.1 | 0.0 |
| Slc6a4 | 0.0 | 0.0 | 0.0 | 0.2 | 0.0 | 0.0 | 0.0 | 0.0 | 0.0 | 0.0 | 0.0 | 0.0 |
| Slc6a6 | 27.5 | 29.6 | 24.8 | 54.8 | 46.9 | 35.7 | 38.4 | 24.6 | 25.0 | 27.8 | 35.5 | 25.5 |
| Slc6a7 | 0.4 | 0.3 | 0.2 | 0.2 | 0.3 | 0.2 | 0.3 | 0.3 | 0.3 | 0.1 | 0.1 | 0.2 |
| Slc6a8 | 19.4 | 18.5 | 16.8 | 22.2 | 21.2 | 23.6 | 20.0 | 16.1 | 19.0 | 19.2 | 17.5 | 19.3 |
| Slc6a9 | 5.0 | 4.1 | 3.7 | 6.2 | 3.8 | 6.6 | 4.6 | 8.5 | 5.7 | 3.5 | 4.2 | 2.8 |
| Slc7a1 | 8.6 | 7.1 | 5.8 | 9.8 | 8.1 | 9.3 | 9.1 | 9.1 | 8.8 | 5.8 | 8.0 | 5.3 |
| Slc7a10 | 0.0 | 0.0 | 0.1 | 0.3 | 0.0 | 0.1 | 0.0 | 0.1 | 0.1 | 0.1 | 0.0 | 0.1 |
| Slc7a11 | 5.1 | 2.8 | 3.1 | 10.4 | 11.7 | 5.7 | 2.5 | 4.4 | 1.9 | 1.5 | 2.4 | 1.3 |
| Slc7a13 | 0.5 | 0.3 | 0.3 | 0.5 | 0.5 | 0.4 | 0.3 | 0.2 | 0.2 | 0.2 | 0.5 | 0.3 |
| Slc7a14 | 0.1 | 0.2 | 0.2 | 0.2 | 0.2 | 0.1 | 0.1 | 0.2 | 0.2 | 0.2 | 0.2 | 0.2 |
| Slc7a2 | 6.0 | 5.4 | 10.4 | 3.0 | 5.7 | 5.2 | 5.2 | 10.0 | 7.0 | 8.0 | 5.0 | 9.4 |
| Slc7a3 | 0.5 | 0.3 | 0.2 | 0.3 | 0.1 | 0.3 | 0.3 | 0.4 | 0.3 | 0.4 | 0.5 | 0.3 |
| Slc7a4 | 0.3 | 0.2 | 0.3 | 0.4 | 0.3 | 0.5 | 0.2 | 0.1 | 0.2 | 0.3 | 0.2 | 0.2 |
| Slc7a5 | 9.8 | 5.4 | 4.5 | 8.4 | 4.5 | 7.6 | 7.5 | 14.6 | 10.9 | 5.6 | 9.3 | 4.4 |
| Slc7a6 | 12.7 | 15.6 | 15.5 | 11.9 | 15.1 | 9.7 | 17.1 | 19.0 | 18.4 | 14.8 | 15.8 | 15.2 |
| Slc7a6os | 10.8 | 11.2 | 12.6 | 11.2 | 9.9 | 9.5 | 12.1 | 12.5 | 12.7 | 12.7 | 11.7 | 13.5 |
| Slc7a7 | 3.1 | 2.5 | 2.4 | 3.1 | 4.9 | 3.8 | 3.8 | 3.1 | 3.7 | 2.7 | 4.0 | 3.2 |
| Slc7a8 | 0.4 | 0.1 | 0.1 | 0.6 | 1.6 | 0.1 | 0.4 | 0.2 | 0.3 | 0.1 | 0.2 | 0.2 |
| Slc8a1 | 3.0 | 5.9 | 8.7 | 3.6 | 6.1 | 2.9 | 8.2 | 10.4 | 9.8 | 5.9 | 6.6 | 8.4 |
| Slc8a2 | 0.0 | 0.0 | 0.0 | 0.0 | 0.0 | 0.0 | 0.0 | 0.0 | 0.0 | 0.1 | 0.0 | 0.0 |
| Slc8a3 | 0.2 | 0.2 | 0.2 | 0.2 | 0.1 | 0.2 | 0.2 | 0.1 | 0.1 | 0.1 | 0.1 | 0.1 |
| Slc9a1 | 2.5 | 3.2 | 4.2 | 3.3 | 3.8 | 3.2 | 4.5 | 4.1 | 3.9 | 2.9 | 2.6 | 2.6 |
| Slc9a2 | 0.0 | 0.0 | 0.0 | 0.1 | 0.0 | 0.1 | 0.0 | 0.0 | 0.0 | 0.0 | 0.0 | 0.0 |
| Slc9a3r1 | 9.6 | 7.6 | 7.7 | 5.2 | 6.7 | 3.3 | 2.9 | 8.1 | 7.8 | 92.0 | 37.7 | 20.4 |
| Slc9a3r2 | 22.9 | 28.9 | 28.2 | 19.7 | 22.2 | 20.9 | 29.4 | 27.4 | 31.1 | 28.7 | 27.9 | 26.0 |
| Slc9a4 | 0.0 | 0.0 | 0.0 | 0.1 | 0.0 | 0.0 | 0.0 | 0.1 | 0.0 | 0.0 | 0.0 | 0.0 |
| Slc9a5 | 0.5 | 0.8 | 0.8 | 0.5 | 0.8 | 0.7 | 0.8 | 0.5 | 0.6 | 0.6 | 0.5 | 0.4 |

| | | | | | | | | | | | | |
|---|---|---|---|---|---|---|---|---|---|---|---|---|
| Slc9a6 | 4.9 | 5.5 | 4.8 | 5.0 | 5.6 | 5.2 | 4.8 | 4.7 | 5.3 | 5.1 | 5.3 | 5.2 |
| Slc9a7 | 3.7 | 1.8 | 1.6 | 3.5 | 1.7 | 3.3 | 1.3 | 0.9 | 0.7 | 1.1 | 1.5 | 1.1 |
| Slc9a8 | 3.5 | 2.7 | 2.3 | 3.5 | 3.6 | 3.0 | 4.1 | 3.7 | 4.0 | 3.1 | 3.0 | 3.1 |
| Slc9a9 | 2.1 | 2.1 | 1.9 | 2.6 | 2.3 | 1.9 | 2.3 | 2.2 | 2.0 | 1.2 | 1.6 | 1.5 |
| Slc9b1 | 0.1 | 0.1 | 0.1 | 0.0 | 0.0 | 0.1 | 0.0 | 0.1 | 0.2 | 0.0 | 0.1 | 0.1 |
| Slc9b2 | 0.1 | 0.1 | 0.1 | 0.3 | 0.2 | 0.3 | 0.1 | 0.1 | 0.0 | 0.1 | 0.2 | 0.2 |
| Slco1a1 | 0.0 | 0.0 | 0.0 | 0.0 | 0.0 | 0.0 | 0.0 | 0.0 | 0.0 | 0.0 | 0.0 | 0.0 |
| Slco1a4 | 0.0 | 0.0 | 0.0 | 0.1 | 0.0 | 0.0 | 0.0 | 0.0 | 0.0 | 0.0 | 0.0 | 0.0 |
| Slco1a5 | 0.2 | 0.1 | 0.1 | 0.2 | 0.2 | 0.1 | 0.1 | 0.0 | 0.1 | 0.2 | 0.1 | 0.0 |
| Slco1c1 | 0.0 | 0.0 | 0.0 | 0.0 | 0.0 | 0.0 | 0.0 | 0.0 | 0.0 | 0.0 | 0.0 | 0.0 |
| Slco2a1 | 7.1 | 5.2 | 2.6 | 3.8 | 3.1 | 3.9 | 5.0 | 3.3 | 3.5 | 6.8 | 6.7 | 5.3 |
| Slco2b1 | 0.0 | 0.0 | 0.1 | 0.0 | 0.0 | 0.0 | 0.0 | 0.0 | 0.1 | 0.0 | 0.0 | 0.0 |
| Slco3a1 | 7.9 | 6.9 | 5.7 | 10.1 | 12.2 | 6.8 | 10.0 | 5.5 | 5.8 | 6.4 | 7.5 | 6.0 |
| Slco4a1 | 0.2 | 0.0 | 0.0 | 0.8 | 1.3 | 0.0 | 0.2 | 0.1 | 0.1 | 0.1 | 0.0 | 0.1 |
| Slco5a1 | 0.1 | 0.1 | 0.1 | 0.1 | 0.2 | 0.1 | 0.1 | 0.1 | 0.1 | 0.1 | 0.1 | 0.1 |
| Slfn10-ps | 0.5 | 0.3 | 0.5 | 1.0 | 1.5 | 0.4 | 0.6 | 0.2 | 0.3 | 0.3 | 0.5 | 0.4 |
| Slfn14-ps | 0.0 | 0.0 | 0.0 | 0.0 | 0.0 | 0.0 | 0.0 | 0.0 | 0.0 | 0.1 | 0.0 | 0.0 |
| Slfn2 | 3.5 | 0.9 | 1.1 | 9.9 | 11.7 | 2.2 | 4.1 | 1.0 | 1.3 | 2.7 | 1.2 | 3.7 |
| Slfn3 | 0.7 | 0.5 | 0.7 | 0.6 | 1.0 | 0.3 | 0.6 | 0.5 | 0.2 | 0.2 | 0.6 | 0.4 |
| Slfn4 | 0.0 | 0.0 | 0.1 | 0.2 | 0.1 | 0.0 | 0.1 | 0.0 | 0.0 | 0.0 | 0.0 | 0.0 |
| Slfn5 | 16.2 | 20.0 | 32.9 | 24.4 | 18.8 | 16.8 | 16.8 | 30.9 | 17.9 | 8.1 | 11.5 | 18.5 |
| Slfn8 | 3.3 | 2.2 | 4.0 | 4.0 | 2.9 | 2.6 | 2.2 | 1.5 | 1.3 | 0.9 | 1.6 | 3.6 |
| Slfn9 | 2.5 | 3.2 | 5.5 | 1.9 | 5.4 | 1.3 | 6.1 | 3.1 | 3.0 | 1.6 | 3.8 | 3.4 |
| Slfnl1 | 0.1 | 0.1 | 0.1 | 0.0 | 0.0 | 0.0 | 0.0 | 0.0 | 0.0 | 0.1 | 0.0 | 0.0 |
| Slirp | 100.3 | 85.6 | 59.7 | 78.8 | 80.9 | 92.2 | 59.8 | 74.4 | 78.5 | 72.1 | 80.1 | 75.8 |
| Slit1 | 0.4 | 0.4 | 0.3 | 0.2 | 0.4 | 0.4 | 0.3 | 0.1 | 0.1 | 0.2 | 0.1 | 0.1 |
| Slit2 | 17.3 | 14.6 | 12.8 | 16.8 | 19.4 | 14.9 | 23.7 | 25.1 | 26.5 | 33.0 | 33.0 | 24.7 |
| Slit3 | 5.9 | 10.7 | 14.5 | 2.7 | 6.5 | 3.0 | 15.0 | 17.4 | 21.5 | 29.8 | 21.1 | 22.6 |
| Slitrk2 | 0.1 | 0.1 | 0.1 | 0.1 | 0.1 | 0.0 | 0.1 | 0.0 | 0.1 | 0.0 | 0.1 | 0.1 |
| Slitrk5 | 0.0 | 0.0 | 0.0 | 0.0 | 0.0 | 0.0 | 0.0 | 0.0 | 0.0 | 0.0 | 0.0 | 0.0 |
| Slitrk6 | 0.1 | 0.0 | 0.0 | 0.0 | 0.0 | 0.0 | 0.0 | 0.1 | 0.0 | 0.0 | 0.0 | 0.0 |
| Slk | 14.2 | 15.3 | 15.6 | 16.1 | 14.0 | 13.0 | 13.6 | 14.1 | 12.3 | 12.9 | 16.2 | 15.3 |
| Slmap | 11.2 | 12.3 | 12.0 | 14.8 | 13.1 | 12.7 | 13.1 | 10.3 | 10.9 | 11.6 | 13.7 | 13.7 |
| Slmo1 | 0.1 | 0.1 | 0.0 | 0.1 | 0.1 | 0.1 | 0.0 | 0.0 | 0.0 | 0.0 | 0.0 | 0.0 |
| Slmo2 | 33.3 | 30.1 | 31.9 | 40.4 | 30.7 | 33.5 | 38.3 | 32.2 | 28.9 | 32.7 | 40.4 | 34.8 |
| Slpi | 0.4 | 0.3 | 0.3 | 0.5 | 0.3 | 0.1 | 0.6 | 0.7 | 0.5 | 2.5 | 1.3 | 0.9 |
| Sltm | 15.2 | 13.0 | 13.9 | 17.4 | 12.8 | 12.5 | 17.1 | 12.4 | 11.2 | 12.3 | 16.8 | 15.9 |
| Slu7 | 27.0 | 19.8 | 21.5 | 31.5 | 20.6 | 29.0 | 21.2 | 18.1 | 14.7 | 18.3 | 23.1 | 22.7 |
| Slx1b | 1.3 | 1.4 | 1.2 | 1.1 | 0.9 | 1.4 | 1.2 | 1.4 | 1.1 | 1.2 | 1.2 | 0.9 |
| Slx4 | 3.2 | 4.1 | 4.4 | 3.4 | 3.7 | 3.0 | 4.5 | 4.1 | 3.7 | 3.1 | 3.8 | 3.4 |
| Slx4ip | 2.5 | 3.3 | 3.0 | 2.9 | 3.0 | 3.0 | 3.2 | 3.0 | 2.9 | 2.5 | 2.9 | 2.5 |
| Smad1 | 8.4 | 9.9 | 12.8 | 8.2 | 9.5 | 8.1 | 10.7 | 12.0 | 10.5 | 9.1 | 9.9 | 10.8 |
| Smad2 | 8.0 | 8.6 | 8.0 | 7.5 | 7.7 | 7.6 | 8.3 | 7.9 | 8.3 | 6.9 | 8.1 | 7.7 |
| Smad3 | 16.0 | 14.2 | 11.7 | 16.9 | 13.6 | 16.3 | 17.3 | 10.6 | 13.9 | 17.5 | 16.8 | 16.4 |
| Smad4 | 20.1 | 21.7 | 22.2 | 20.8 | 20.3 | 23.6 | 20.6 | 20.5 | 21.6 | 20.2 | 23.9 | 21.6 |
| Smad5 | 8.0 | 8.9 | 8.5 | 6.9 | 7.9 | 7.0 | 8.1 | 8.9 | 8.3 | 7.9 | 8.2 | 8.2 |
| Smad6 | 35.7 | 47.1 | 53.3 | 27.5 | 31.1 | 26.1 | 43.3 | 38.7 | 48.6 | 33.1 | 34.9 | 45.1 |
| Smad7 | 14.6 | 16.4 | 16.8 | 8.7 | 13.0 | 11.0 | 15.7 | 12.3 | 16.1 | 14.7 | 11.7 | 15.3 |
| Smad9 | 1.4 | 1.7 | 2.1 | 1.1 | 1.0 | 1.8 | 1.2 | 1.0 | 1.1 | 1.4 | 1.3 | 1.3 |
| Smagp | 0.4 | 0.1 | 0.0 | 1.4 | 1.8 | 0.2 | 0.3 | 0.1 | 0.3 | 0.4 | 0.0 | 0.2 |
| Smap1 | 28.0 | 25.9 | 23.6 | 28.6 | 22.8 | 27.5 | 23.9 | 24.1 | 21.2 | 23.7 | 28.9 | 26.0 |
| Smap2 | 4.3 | 4.7 | 5.2 | 6.3 | 6.0 | 5.3 | 4.8 | 4.6 | 5.1 | 5.6 | 5.5 | 5.1 |
| Smarca1 | 0.6 | 1.2 | 2.5 | 0.4 | 0.7 | 1.0 | 1.0 | 1.7 | 1.1 | 1.0 | 1.3 | 1.4 |
| Smarca2 | 25.6 | 24.3 | 32.0 | 26.1 | 18.8 | 24.2 | 21.5 | 24.6 | 16.6 | 21.5 | 23.0 | 26.8 |
| Smarca4 | 17.4 | 17.7 | 18.9 | 19.3 | 18.7 | 17.2 | 21.2 | 20.2 | 18.2 | 18.5 | 20.2 | 18.0 |
| Smarca5 | 19.2 | 20.3 | 23.2 | 18.9 | 19.4 | 18.1 | 21.1 | 21.8 | 18.8 | 21.1 | 24.5 | 24.4 |
| Smarca5-ps | 0.6 | 0.4 | 0.4 | 0.7 | 0.6 | 0.5 | 0.5 | 0.5 | 0.3 | 0.5 | 0.4 | 0.7 |

| | | | | | | | | | | | | |
|---|---|---|---|---|---|---|---|---|---|---|---|---|
| Smarcad1 | 7.4 | 8.2 | 8.1 | 6.5 | 7.7 | 7.1 | 7.3 | 8.0 | 7.5 | 8.8 | 8.9 | 9.1 |
| Smarcal1 | 3.4 | 3.4 | 3.0 | 3.4 | 2.8 | 3.9 | 2.7 | 3.2 | 2.6 | 2.7 | 2.7 | 2.9 |
| Smarcb1 | 19.5 | 22.4 | 21.9 | 17.3 | 19.5 | 16.8 | 21.2 | 26.0 | 23.7 | 21.7 | 20.4 | 21.9 |
| Smarcc1 | 10.8 | 11.2 | 12.8 | 11.4 | 10.6 | 10.7 | 12.2 | 11.8 | 10.7 | 12.0 | 12.8 | 12.2 |
| Smarcc2 | 31.5 | 32.1 | 38.0 | 39.5 | 28.5 | 36.4 | 36.6 | 35.8 | 29.8 | 28.2 | 34.5 | 35.8 |
| Smarcd1 | 1.7 | 2.2 | 2.4 | 1.9 | 2.1 | 1.6 | 2.2 | 2.8 | 2.3 | 1.9 | 2.1 | 1.8 |
| Smarcd2 | 16.5 | 21.4 | 23.7 | 16.2 | 19.3 | 15.8 | 20.2 | 26.5 | 22.0 | 17.1 | 18.4 | 19.1 |
| Smarcd3 | 10.2 | 12.6 | 12.4 | 13.8 | 9.9 | 11.7 | 14.5 | 13.6 | 16.0 | 9.8 | 12.6 | 14.0 |
| Smarce1 | 28.6 | 28.5 | 33.8 | 35.3 | 25.2 | 25.1 | 29.2 | 34.7 | 26.9 | 31.2 | 36.4 | 34.5 |
| Smc1a | 27.7 | 27.7 | 34.8 | 29.8 | 25.8 | 24.7 | 31.7 | 28.6 | 22.7 | 24.2 | 30.7 | 32.2 |
| Smc1b | 0.0 | 0.0 | 0.0 | 0.1 | 0.0 | 0.0 | 0.1 | 0.0 | 0.1 | 0.0 | 0.1 | 0.1 |
| Smc2 | 4.8 | 8.0 | 12.7 | 3.3 | 10.1 | 3.1 | 10.4 | 5.8 | 8.3 | 5.3 | 8.9 | 7.4 |
| Smc3 | 14.3 | 17.1 | 20.5 | 12.9 | 14.2 | 12.7 | 17.9 | 17.4 | 15.0 | 15.0 | 19.1 | 17.9 |
| Smc4 | 17.2 | 26.2 | 34.0 | 19.2 | 22.8 | 19.9 | 27.9 | 26.2 | 32.3 | 25.6 | 31.8 | 32.9 |
| Smc5 | 10.6 | 13.7 | 15.2 | 9.7 | 12.4 | 11.2 | 14.3 | 13.5 | 16.3 | 11.7 | 14.5 | 16.3 |
| Smc6 | 18.0 | 20.9 | 21.7 | 17.6 | 15.4 | 16.9 | 20.1 | 19.0 | 20.3 | 17.6 | 24.1 | 22.8 |
| Smchd1 | 8.3 | 9.9 | 11.1 | 7.6 | 9.4 | 8.0 | 9.0 | 10.2 | 9.2 | 9.4 | 10.3 | 10.4 |
| Smcr7 | 4.6 | 3.7 | 3.4 | 4.0 | 3.6 | 4.7 | 3.9 | 4.2 | 4.8 | 3.9 | 3.4 | 3.4 |
| Smcr7l | 6.1 | 6.1 | 5.2 | 7.1 | 6.4 | 6.0 | 5.8 | 5.6 | 6.7 | 6.0 | 5.9 | 5.7 |
| Smcr8 | 6.7 | 7.2 | 7.1 | 8.3 | 9.1 | 7.2 | 7.9 | 7.2 | 7.0 | 7.3 | 7.3 | 7.0 |
| Smek1 | 5.7 | 6.5 | 8.1 | 7.1 | 7.4 | 6.3 | 7.5 | 8.1 | 7.4 | 7.4 | 7.8 | 8.9 |
| Smek2 | 12.3 | 14.4 | 16.9 | 13.5 | 13.1 | 13.7 | 12.0 | 14.9 | 13.6 | 14.2 | 17.0 | 17.3 |
| Smg1 | 6.8 | 7.4 | 6.2 | 6.1 | 6.7 | 6.7 | 6.4 | 6.0 | 6.5 | 6.1 | 6.4 | 5.8 |
| Smg5 | 18.8 | 17.7 | 14.2 | 22.0 | 17.6 | 20.7 | 23.8 | 18.4 | 19.6 | 15.1 | 20.6 | 17.6 |
| Smg6 | 7.0 | 6.8 | 7.8 | 6.6 | 6.1 | 6.3 | 7.8 | 5.9 | 6.3 | 5.1 | 6.2 | 7.1 |
| Smg7 | 8.8 | 10.1 | 10.4 | 9.9 | 9.2 | 10.0 | 11.5 | 10.0 | 8.5 | 7.4 | 10.6 | 8.9 |
| Smg8 | 6.0 | 6.2 | 6.3 | 5.4 | 6.1 | 5.5 | 6.2 | 6.3 | 6.1 | 6.8 | 5.7 | 7.3 |
| Smg9 | 3.0 | 3.1 | 2.2 | 4.3 | 4.3 | 2.7 | 4.2 | 3.5 | 3.9 | 3.1 | 3.2 | 3.4 |
| Smim1 | 16.9 | 17.6 | 15.8 | 14.4 | 12.7 | 18.3 | 18.1 | 18.8 | 18.0 | 25.4 | 22.3 | 16.8 |
| Smim11 | 34.5 | 31.7 | 37.8 | 35.9 | 25.6 | 35.1 | 35.3 | 31.1 | 30.0 | 29.7 | 38.8 | 38.7 |
| Smim12 | 15.7 | 12.6 | 12.9 | 14.5 | 14.5 | 15.5 | 14.7 | 14.9 | 13.3 | 15.8 | 17.6 | 12.9 |
| Smim13 | 5.2 | 5.1 | 4.9 | 5.3 | 5.0 | 5.0 | 4.4 | 5.3 | 5.3 | 4.7 | 5.0 | 5.4 |
| Smim14 | 59.1 | 44.3 | 32.2 | 61.1 | 39.5 | 67.0 | 34.3 | 31.0 | 33.5 | 43.8 | 40.2 | 40.3 |
| Smim15 | 30.2 | 28.7 | 33.4 | 36.2 | 35.9 | 35.0 | 36.6 | 32.5 | 38.3 | 34.9 | 43.3 | 44.4 |
| Smim3 | 4.3 | 3.6 | 3.3 | 4.0 | 6.5 | 3.5 | 7.0 | 5.7 | 6.7 | 8.4 | 7.0 | 7.7 |
| Smim4 | 14.5 | 9.4 | 9.7 | 11.5 | 12.3 | 11.6 | 7.4 | 6.7 | 9.6 | 14.1 | 5.6 | 8.6 |
| Smim5 | 0.1 | 0.1 | 0.1 | 0.1 | 0.2 | 0.2 | 0.3 | 0.1 | 0.2 | 0.1 | 0.4 | 0.2 |
| Smim6 | 0.2 | 0.2 | 0.4 | 0.5 | 0.3 | 0.2 | 0.3 | 0.3 | 0.0 | 0.1 | 0.3 | 0.5 |
| Smim7 | 22.8 | 18.7 | 15.6 | 20.3 | 20.4 | 20.2 | 20.0 | 19.1 | 23.1 | 22.1 | 19.3 | 20.0 |
| Smim8 | 7.7 | 8.6 | 10.3 | 8.3 | 7.0 | 9.1 | 7.8 | 8.6 | 6.7 | 7.8 | 9.6 | 10.3 |
| Smim9 | 0.0 | 0.1 | 0.2 | 0.1 | 0.1 | 0.1 | 0.0 | 0.1 | 0.0 | 0.0 | 0.0 | 0.1 |
| Smlr1 | 0.1 | 0.1 | 0.1 | 0.0 | 0.0 | 0.0 | 0.0 | 0.0 | 0.0 | 0.0 | 0.0 | 0.0 |
| Smn1 | 6.0 | 6.6 | 5.1 | 7.8 | 5.8 | 4.9 | 7.3 | 7.2 | 6.3 | 6.6 | 6.8 | 5.7 |
| Smndc1 | 6.6 | 6.9 | 7.5 | 10.4 | 7.6 | 9.0 | 8.1 | 7.0 | 6.3 | 7.4 | 9.3 | 8.4 |
| Smo | 25.2 | 22.4 | 21.3 | 19.8 | 17.9 | 23.4 | 22.9 | 24.6 | 23.5 | 26.6 | 23.6 | 23.2 |
| Smoc1 | 10.4 | 16.2 | 16.7 | 5.2 | 13.1 | 9.5 | 21.9 | 7.0 | 18.1 | 15.6 | 16.5 | 25.3 |
| Smoc2 | 17.6 | 10.8 | 16.6 | 26.9 | 18.9 | 14.6 | 25.9 | 15.7 | 13.4 | 9.5 | 9.1 | 10.3 |
| Smok4a | 0.0 | 0.1 | 0.0 | 0.0 | 0.0 | 0.0 | 0.0 | 0.0 | 0.0 | 0.0 | 0.0 | 0.0 |
| Smox | 5.8 | 4.9 | 3.9 | 9.0 | 8.6 | 5.9 | 5.7 | 3.9 | 4.0 | 4.6 | 3.7 | 4.3 |
| Smpd1 | 50.3 | 40.6 | 34.4 | 55.3 | 37.1 | 57.2 | 37.0 | 30.3 | 34.5 | 39.1 | 37.7 | 36.5 |
| Smpd2 | 9.2 | 10.9 | 8.0 | 8.4 | 7.9 | 9.6 | 9.4 | 9.5 | 11.2 | 10.5 | 9.9 | 10.8 |
| Smpd3 | 22.5 | 10.7 | 5.9 | 14.0 | 6.5 | 15.1 | 4.5 | 3.0 | 3.3 | 39.8 | 18.7 | 10.0 |
| Smpd4 | 3.5 | 3.5 | 3.7 | 3.9 | 3.9 | 4.0 | 4.1 | 4.0 | 3.7 | 2.9 | 3.7 | 3.1 |
| Smpd5 | 0.3 | 0.2 | 0.2 | 0.5 | 0.5 | 0.4 | 0.3 | 0.2 | 0.3 | 0.6 | 0.5 | 0.2 |
| Smpdl3a | 49.0 | 35.6 | 36.7 | 57.1 | 45.9 | 42.2 | 31.4 | 40.8 | 36.1 | 25.5 | 34.2 | 37.5 |
| Smpdl3b | 0.5 | 0.6 | 0.7 | 1.0 | 2.2 | 0.2 | 0.5 | 0.2 | 0.2 | 1.0 | 0.2 | 0.5 |
| Sms | 4.7 | 3.9 | 3.3 | 5.5 | 5.2 | 4.8 | 4.5 | 2.7 | 3.1 | 3.8 | 3.7 | 3.8 |

| | | | | | | | | | | | | |
|---|---|---|---|---|---|---|---|---|---|---|---|---|
| Smtn | 12.1 | 13.5 | 13.7 | 7.7 | 12.9 | 9.6 | 19.5 | 12.5 | 16.0 | 18.4 | 18.2 | 12.6 |
| Smtnl2 | 0.7 | 2.7 | 4.0 | 0.2 | 0.9 | 0.1 | 1.2 | 5.0 | 2.2 | 0.8 | 0.8 | 1.1 |
| Smu1 | 26.5 | 25.8 | 23.6 | 25.1 | 22.1 | 25.6 | 23.9 | 22.4 | 21.8 | 24.0 | 24.3 | 25.9 |
| Smug1 | 2.1 | 2.1 | 1.8 | 2.2 | 2.2 | 2.1 | 1.8 | 2.1 | 2.2 | 1.9 | 1.9 | 1.6 |
| Smurf1 | 16.8 | 17.4 | 15.7 | 16.1 | 18.4 | 18.9 | 24.4 | 17.7 | 19.9 | 14.4 | 18.0 | 17.4 |
| Smurf2 | 30.5 | 26.8 | 24.2 | 37.3 | 26.7 | 46.4 | 25.3 | 19.5 | 22.0 | 33.3 | 29.0 | 28.0 |
| Smyd1 | 0.2 | 0.1 | 0.1 | 0.2 | 0.1 | 0.1 | 0.1 | 0.1 | 0.1 | 0.3 | 0.1 | 0.1 |
| Smyd2 | 31.9 | 27.6 | 23.5 | 35.8 | 27.3 | 31.8 | 26.9 | 25.9 | 25.3 | 25.8 | 28.1 | 26.6 |
| Smyd3 | 2.4 | 2.2 | 1.9 | 2.6 | 1.7 | 2.9 | 1.3 | 1.8 | 1.5 | 1.7 | 1.9 | 2.0 |
| Smyd4 | 2.8 | 2.6 | 2.0 | 2.8 | 2.5 | 3.4 | 2.3 | 1.6 | 2.1 | 2.1 | 2.1 | 2.1 |
| Smyd5 | 9.1 | 7.2 | 5.2 | 9.8 | 8.9 | 9.3 | 8.6 | 7.4 | 6.2 | 6.5 | 8.3 | 5.8 |
| Snai1 | 5.5 | 11.8 | 16.6 | 5.9 | 11.8 | 7.1 | 14.2 | 17.7 | 14.7 | 8.0 | 9.5 | 9.5 |
| Snai2 | 2.3 | 4.2 | 7.0 | 2.5 | 4.7 | 4.3 | 4.2 | 6.0 | 4.4 | 3.6 | 4.6 | 4.4 |
| Snap23 | 27.3 | 29.1 | 26.3 | 32.9 | 29.1 | 26.8 | 26.4 | 28.0 | 24.7 | 29.4 | 33.7 | 32.1 |
| Snap29 | 20.6 | 20.4 | 17.9 | 22.0 | 19.4 | 18.2 | 25.5 | 18.6 | 20.2 | 18.1 | 26.0 | 22.9 |
| Snap47 | 63.6 | 57.5 | 46.5 | 60.2 | 46.7 | 58.6 | 46.3 | 48.0 | 42.4 | 51.4 | 50.0 | 45.7 |
| Snap91 | 0.2 | 0.1 | 0.0 | 0.2 | 0.0 | 0.2 | 0.2 | 0.0 | 0.0 | 0.1 | 0.1 | 0.1 |
| Snapc1 | 7.6 | 9.4 | 9.3 | 7.9 | 7.9 | 9.5 | 8.6 | 8.9 | 8.8 | 8.5 | 9.4 | 9.5 |
| Snapc2 | 10.3 | 10.8 | 9.4 | 8.3 | 9.0 | 10.2 | 10.4 | 9.2 | 11.1 | 11.0 | 10.1 | 9.0 |
| Snapc3 | 8.8 | 10.1 | 10.9 | 7.8 | 8.0 | 8.1 | 9.7 | 10.3 | 9.8 | 8.7 | 10.2 | 9.9 |
| Snapc4 | 2.6 | 2.2 | 2.3 | 3.3 | 2.5 | 2.7 | 2.9 | 2.2 | 2.4 | 2.1 | 2.0 | 1.7 |
| Snapc5 | 25.8 | 22.5 | 26.3 | 25.1 | 21.7 | 22.8 | 23.0 | 29.1 | 21.6 | 22.6 | 28.1 | 26.6 |
| Snapin | 40.0 | 34.5 | 30.6 | 43.7 | 32.1 | 39.6 | 35.6 | 34.5 | 35.3 | 36.3 | 38.9 | 38.7 |
| Snca | 0.1 | 0.0 | 0.0 | 0.0 | 0.1 | 0.0 | 0.0 | 0.0 | 0.0 | 0.0 | 0.0 | 0.0 |
| Sncaip | 0.2 | 0.1 | 0.4 | 0.1 | 0.2 | 0.1 | 0.3 | 0.5 | 0.4 | 0.1 | 0.2 | 0.1 |
| Sncg | 5.5 | 3.7 | 4.1 | 5.8 | 4.6 | 5.9 | 2.0 | 4.8 | 0.8 | 2.2 | 4.0 | 2.7 |
| Snd1 | 57.6 | 53.3 | 50.2 | 52.0 | 53.0 | 44.1 | 60.8 | 68.2 | 60.4 | 59.1 | 68.0 | 57.6 |
| Sned1 | 0.3 | 0.9 | 1.3 | 0.2 | 0.5 | 0.2 | 1.5 | 1.5 | 2.2 | 2.8 | 2.7 | 2.1 |
| Snf8 | 24.7 | 27.1 | 26.6 | 26.5 | 26.3 | 27.1 | 26.6 | 29.6 | 26.5 | 26.5 | 30.0 | 26.0 |
| Snhg1 | 30.8 | 21.1 | 19.6 | 15.8 | 25.6 | 22.5 | 30.3 | 24.9 | 27.2 | 32.1 | 32.0 | 31.8 |
| Snhg10 | 1.8 | 1.4 | 1.0 | 2.0 | 0.8 | 1.8 | 1.8 | 1.0 | 4.3 | 1.5 | 0.9 | 1.8 |
| Snhg11 | 1.8 | 2.4 | 1.2 | 2.2 | 1.3 | 1.9 | 0.8 | 0.4 | 0.4 | 0.2 | 0.5 | 0.3 |
| Snhg12 | 42.1 | 30.4 | 25.4 | 33.4 | 29.9 | 57.4 | 33.7 | 29.3 | 31.6 | 31.3 | 36.1 | 31.9 |
| Snhg3 | 5.5 | 6.3 | 6.9 | 9.7 | 7.4 | 7.8 | 7.4 | 8.0 | 7.8 | 9.7 | 8.8 | 8.3 |
| Snhg4 | 2.0 | 1.4 | 1.5 | 1.6 | 2.1 | 2.1 | 2.1 | 2.0 | 1.9 | 2.3 | 2.5 | 1.9 |
| Snhg5 | 12.2 | 9.6 | 10.9 | 10.9 | 8.7 | 9.3 | 12.1 | 11.8 | 13.4 | 13.8 | 15.7 | 13.3 |
| Snhg6 | 56.7 | 43.4 | 34.6 | 41.9 | 37.7 | 46.8 | 38.0 | 44.6 | 52.6 | 50.9 | 44.5 | 44.7 |
| Snhg7 | 3.0 | 3.2 | 2.4 | 4.4 | 3.2 | 3.7 | 4.3 | 2.4 | 2.8 | 2.6 | 2.3 | 2.5 |
| Snhg8 | 9.6 | 6.0 | 7.0 | 5.8 | 7.4 | 8.2 | 5.1 | 6.2 | 6.5 | 6.9 | 8.5 | 6.5 |
| Snhg9 | 19.6 | 8.0 | 7.1 | 8.6 | 6.5 | 11.9 | 10.1 | 8.7 | 14.0 | 9.3 | 7.7 | 11.0 |
| Snip1 | 8.5 | 7.7 | 7.9 | 8.3 | 8.5 | 6.7 | 7.3 | 8.2 | 9.1 | 10.2 | 9.3 | 8.8 |
| Snn | 11.3 | 7.0 | 5.1 | 12.7 | 8.0 | 10.5 | 9.2 | 6.8 | 7.9 | 11.3 | 11.9 | 9.5 |
| Snora15 | 4.0 | 4.8 | 0.0 | 0.0 | 16.7 | 10.0 | 0.0 | 12.2 | 16.2 | 9.0 | 6.4 | 7.7 |
| Snora16a | 36.2 | 39.5 | 50.5 | 20.7 | 29.8 | 24.4 | 33.5 | 44.2 | 65.5 | 34.8 | 33.5 | 79.8 |
| Snora17 | 56.3 | 34.3 | 51.5 | 20.4 | 65.5 | 27.7 | 42.1 | 54.5 | 58.4 | 54.2 | 41.5 | 50.4 |
| Snora21 | 21.6 | 21.2 | 33.4 | 6.6 | 53.3 | 3.2 | 12.1 | 13.0 | 87.2 | 47.9 | 28.8 | 47.3 |
| Snora23 | 34.1 | 37.1 | 44.4 | 18.8 | 43.6 | 20.9 | 34.7 | 35.8 | 51.0 | 60.7 | 37.7 | 63.5 |
| Snora24 | 0.0 | 27.6 | 19.1 | 3.6 | 13.0 | 0.0 | 0.0 | 14.0 | 51.6 | 13.7 | 1.5 | 0.0 |
| Snora26 | 43.4 | 48.8 | 71.5 | 3.1 | 40.7 | 6.9 | 17.7 | 44.2 | 102.1 | 55.1 | 3.0 | 54.9 |
| Snora28 | 61.3 | 102.2 | 133.7 | 29.5 | 186.0 | 61.4 | 115.8 | 120.0 | 201.6 | 127.8 | 51.5 | 130.4 |
| Snora3 | 16.8 | 36.9 | 13.9 | 26.2 | 45.5 | 21.3 | 20.0 | 24.3 | 20.6 | 3.8 | 48.3 | 42.9 |
| Snora30 | 12.2 | 22.5 | 9.7 | 38.0 | 34.9 | 15.3 | 5.8 | 6.2 | 43.0 | 13.8 | 20.0 | 58.1 |
| Snora31 | 4.8 | 11.6 | 0.0 | 0.0 | 23.7 | 12.0 | 4.6 | 4.9 | 19.3 | 10.8 | 0.0 | 45.9 |
| Snora33 | 20.4 | 16.7 | 26.8 | 25.2 | 16.6 | 25.5 | 6.4 | 0.0 | 20.4 | 15.4 | 22.4 | 32.2 |
| Snora34 | 62.0 | 53.4 | 12.0 | 0.0 | 39.9 | 23.1 | 18.0 | 22.5 | 70.8 | 70.1 | 11.7 | 10.7 |
| Snora43 | 7.0 | 4.2 | 9.0 | 2.4 | 4.6 | 6.7 | 10.8 | 11.1 | 10.9 | 15.9 | 0.0 | 1.8 |
| Snora44 | 60.9 | 111.4 | 153.0 | 39.3 | 96.0 | 48.2 | 60.0 | 105.5 | 108.7 | 180.6 | 146.3 | 133.7 |

| | | | | | | | | | | | | |
|---|---|---|---|---|---|---|---|---|---|---|---|---|
| Snora47 | 0.0 | 0.0 | 0.0 | 0.0 | 6.8 | 3.4 | 7.3 | 6.3 | 19.2 | 0.0 | 0.0 | 2.7 |
| Snora52 | 6.1 | 12.4 | 19.0 | 2.7 | 14.1 | 5.2 | 8.4 | 6.4 | 4.2 | 11.6 | 9.7 | 10.3 |
| Snora5c | 17.1 | 21.0 | 33.2 | 20.7 | 34.1 | 10.6 | 7.9 | 77.1 | 25.5 | 58.0 | 0.0 | 40.1 |
| Snora62 | 19.9 | 18.1 | 27.2 | 9.9 | 29.8 | 9.5 | 25.2 | 20.7 | 23.1 | 14.1 | 15.8 | 37.3 |
| Snora64 | 15.2 | 9.3 | 20.3 | 4.9 | 22.4 | 28.9 | 18.8 | 7.8 | 38.8 | 64.5 | 18.3 | 18.6 |
| Snora65 | 0.0 | 4.4 | 0.0 | 0.0 | 3.1 | 4.6 | 7.2 | 7.5 | 7.4 | 4.1 | 5.8 | 0.0 |
| Snora68 | 132.7 | 101.0 | 62.7 | 0.0 | 112.9 | 114.2 | 56.2 | 91.9 | 182.4 | 138.9 | 17.4 | 85.7 |
| Snora69 | 0.0 | 7.6 | 0.0 | 0.0 | 5.3 | 7.9 | 0.0 | 3.2 | 6.4 | 10.5 | 0.0 | 0.0 |
| Snora70 | 24.0 | 73.0 | 54.6 | 22.4 | 84.5 | 30.6 | 56.1 | 40.1 | 173.4 | 89.8 | 76.4 | 72.3 |
| Snora74a | 932.0 | 947.3 | 859.3 | 582.1 | 1101.6 | 831.7 | 622.4 | 881.0 | 1352.6 | 1047.6 | 586.4 | 773.5 |
| Snora78 | 410.4 | 359.2 | 244.2 | 211.6 | 312.5 | 226.6 | 196.9 | 210.9 | 451.3 | 423.4 | 147.1 | 195.7 |
| Snora7a | 834.0 | 983.8 | 1207.7 | 498.5 | 1200.2 | 617.8 | 699.9 | 936.8 | 1749.3 | 1333.6 | 504.4 | 966.0 |
| Snora81 | 13.1 | 30.9 | 31.6 | 5.3 | 35.9 | 24.2 | 38.3 | 33.1 | 49.6 | 47.8 | 47.5 | 42.5 |
| Snord100 | 751.9 | 161.5 | 336.8 | 265.1 | 340.8 | 308.2 | 203.6 | 612.0 | 1227.5 | 734.3 | 0.0 | 231.2 |
| Snord104 | 1494.2 | 1733.6 | 2058.4 | 1309.3 | 2392.6 | 1136.3 | 1416.3 | 3215.4 | 3052.0 | 2499.2 | 1433.1 | 3083.9 |
| Snord110 | 2211.4 | 0.0 | 1436.0 | 454.3 | 2588.7 | 0.0 | 0.0 | 1680.3 | 4040.0 | 1508.8 | 0.0 | 3630.5 |
| Snord116 | 1.7 | 0.0 | 0.0 | 0.0 | 0.0 | 0.0 | 0.0 | 2.8 | 1.7 | 1.9 | 0.0 | 1.9 |
| Snord12 | 483.5 | 623.8 | 321.9 | 0.0 | 650.0 | 0.0 | 0.0 | 941.0 | 792.6 | 1130.7 | 0.0 | 1486.2 |
| Snord15a | 202.0 | 216.7 | 230.7 | 88.4 | 228.3 | 136.3 | 146.0 | 196.2 | 413.5 | 235.4 | 166.3 | 263.5 |
| Snord15b | 665.0 | 1047.5 | 960.2 | 251.2 | 899.4 | 538.6 | 550.7 | 707.0 | 1470.9 | 1088.2 | 739.5 | 1148.9 |
| Snord16a | 330.5 | 117.8 | 117.8 | 165.6 | 386.9 | 77.8 | 183.0 | 156.5 | 449.3 | 538.8 | 56.5 | 233.1 |
| Snord17 | 449.8 | 372.3 | 342.8 | 335.5 | 466.2 | 368.2 | 289.1 | 404.1 | 731.6 | 537.0 | 332.6 | 378.0 |
| Snord1c | 87.0 | 0.0 | 92.2 | 0.0 | 62.7 | 0.0 | 36.9 | 42.9 | 127.1 | 202.3 | 0.0 | 0.0 |
| Snord22 | 1253.3 | 1601.5 | 1098.0 | 427.0 | 1388.3 | 689.5 | 584.6 | 921.3 | 2780.0 | 2068.0 | 888.4 | 1750.7 |
| Snord32a | 1401.9 | 1437.3 | 1267.5 | 1123.6 | 1809.1 | 697.7 | 1346.9 | 1516.8 | 2605.0 | 2241.5 | 932.7 | 1999.2 |
| Snord33 | 337.8 | 504.8 | 486.2 | 242.2 | 633.0 | 0.0 | 397.1 | 516.0 | 1229.0 | 922.9 | 242.1 | 565.9 |
| Snord34 | 967.0 | 831.8 | 858.4 | 0.0 | 1733.4 | 198.4 | 1039.7 | 784.2 | 2536.2 | 753.8 | 0.0 | 743.1 |
| Snord35a | 451.1 | 586.7 | 308.0 | 41.9 | 686.9 | 244.9 | 176.1 | 286.7 | 737.2 | 701.8 | 197.8 | 383.5 |
| Snord35b | 22.4 | 0.0 | 0.0 | 0.0 | 67.4 | 82.9 | 0.0 | 0.0 | 22.0 | 0.0 | 0.0 | 0.0 |
| Snord47 | 0.0 | 949.1 | 731.2 | 0.0 | 368.8 | 1586.0 | 0.0 | 1606.6 | 5441.2 | 4077.1 | 3727.6 | 2881.1 |
| Snord49a | 2520.8 | 5773.5 | 2750.2 | 0.0 | 5349.6 | 346.4 | 1339.4 | 4848.5 | 7547.0 | 5176.8 | 2955.0 | 3856.0 |
| Snord49b | 6303.1 | 8126.3 | 6160.1 | 4534.7 | 10908.6 | 1796.5 | 2518.4 | 9124.3 | 21914.8 | 11248.3 | 2526.6 | 9807.5 |
| Snord55 | 173.9 | 221.4 | 122.9 | 48.1 | 658.0 | 53.4 | 147.4 | 128.7 | 508.3 | 455.3 | 180.8 | 160.3 |
| Snord57 | 242.4 | 312.6 | 955.9 | 0.0 | 1123.0 | 0.0 | 774.9 | 1169.8 | 2408.2 | 1687.3 | 1263.3 | 1337.4 |
| Snord67 | 0.0 | 63.1 | 7.8 | 0.0 | 8.1 | 0.0 | 0.0 | 10.2 | 0.0 | 11.6 | 17.4 | 9.5 |
| Snord8 | 0.0 | 59.5 | 29.7 | 11.6 | 68.7 | 0.0 | 17.7 | 0.0 | 19.1 | 65.4 | 16.4 | 63.0 |
| Snord82 | 751.9 | 2261.4 | 926.2 | 0.0 | 1022.6 | 0.0 | 101.8 | 1224.0 | 2700.4 | 1174.9 | 606.6 | 2196.5 |
| Snord83b | 2768.3 | 1946.9 | 2415.6 | 789.1 | 2975.9 | 1113.8 | 1289.1 | 2493.3 | 5439.1 | 3447.7 | 1207.6 | 2023.8 |
| Snord87 | 0.0 | 82.1 | 133.2 | 0.0 | 0.0 | 0.0 | 0.0 | 63.1 | 249.9 | 375.1 | 141.3 | 0.0 |
| Snord89 | 142.7 | 193.4 | 157.1 | 122.7 | 423.8 | 0.0 | 58.7 | 206.9 | 371.6 | 221.4 | 91.0 | 349.3 |
| Snord95 | 0.0 | 0.0 | 0.0 | 0.0 | 0.0 | 0.0 | 0.0 | 122.4 | 245.5 | 0.0 | 0.0 | 0.0 |
| Snord99 | 0.0 | 1848.4 | 471.6 | 0.0 | 0.0 | 0.0 | 573.7 | 0.0 | 0.0 | 1619.3 | 0.0 | 657.8 |
| Snph | 0.0 | 0.0 | 0.0 | 0.0 | 0.0 | 0.1 | 0.0 | 0.0 | 0.0 | 0.1 | 0.0 | 0.0 |
| Snrk | 10.2 | 10.8 | 10.5 | 8.7 | 9.2 | 9.6 | 9.8 | 10.7 | 11.7 | 9.7 | 9.9 | 10.8 |
| Snrnp200 | 22.1 | 21.5 | 21.7 | 23.1 | 21.8 | 23.9 | 20.8 | 21.9 | 18.3 | 20.8 | 21.2 | 20.6 |
| Snrnp25 | 11.8 | 13.2 | 8.4 | 13.0 | 8.7 | 12.6 | 10.6 | 10.2 | 9.6 | 6.4 | 8.7 | 7.9 |
| Snrnp27 | 52.7 | 40.5 | 45.9 | 60.9 | 38.9 | 56.1 | 41.1 | 42.1 | 34.2 | 41.9 | 50.3 | 50.9 |
| Snrnp35 | 4.1 | 3.9 | 4.0 | 5.2 | 3.4 | 4.2 | 3.8 | 3.8 | 4.4 | 4.1 | 4.0 | 4.4 |
| Snrnp40 | 10.0 | 10.4 | 11.2 | 8.0 | 10.0 | 7.8 | 10.5 | 12.4 | 10.7 | 10.2 | 11.7 | 10.3 |
| Snrnp48 | 12.2 | 11.8 | 12.7 | 10.7 | 10.5 | 12.0 | 9.4 | 10.4 | 9.7 | 13.2 | 14.1 | 13.2 |
| Snrnp70 | 49.8 | 53.1 | 64.6 | 59.4 | 55.0 | 50.8 | 75.4 | 64.2 | 57.3 | 53.1 | 65.3 | 58.1 |
| Snrpa | 20.4 | 20.9 | 21.1 | 23.8 | 21.5 | 23.3 | 19.6 | 24.0 | 19.8 | 22.3 | 21.7 | 18.7 |
| Snrpa1 | 23.7 | 18.7 | 16.7 | 30.1 | 21.1 | 22.5 | 25.1 | 14.2 | 15.6 | 16.1 | 18.2 | 15.5 |
| Snrpb | 74.6 | 74.1 | 60.0 | 64.2 | 72.8 | 63.2 | 67.5 | 62.5 | 76.5 | 78.2 | 61.9 | 57.6 |
| Snrpb2 | 20.6 | 21.5 | 21.0 | 23.9 | 21.8 | 23.0 | 20.8 | 20.3 | 19.7 | 27.2 | 21.9 | 24.1 |
| Snrpc | 32.0 | 32.7 | 31.4 | 36.7 | 32.4 | 35.9 | 34.7 | 32.5 | 32.5 | 33.8 | 33.7 | 30.2 |
| Snrpd1 | 34.9 | 32.9 | 38.8 | 35.6 | 31.0 | 34.0 | 35.3 | 32.7 | 29.5 | 41.1 | 46.0 | 40.6 |

| | | | | | | | | | | | | |
|---|---|---|---|---|---|---|---|---|---|---|---|---|
| Snrpd2 | 114.2 | 100.6 | 77.0 | 106.4 | 88.4 | 105.3 | 88.1 | 97.2 | 82.8 | 87.0 | 87.2 | 78.6 |
| Snrpd3 | 30.4 | 29.4 | 30.6 | 34.0 | 35.2 | 34.2 | 28.4 | 33.4 | 24.6 | 31.8 | 34.6 | 39.7 |
| Snrpe | 89.2 | 84.2 | 66.7 | 74.7 | 89.8 | 75.3 | 61.4 | 74.4 | 79.2 | 92.1 | 76.6 | 68.2 |
| Snrpf | 22.6 | 18.5 | 21.5 | 23.5 | 19.2 | 22.3 | 22.6 | 25.9 | 18.1 | 21.5 | 25.2 | 25.6 |
| Snrpg | 99.4 | 83.3 | 82.1 | 79.5 | 83.1 | 77.1 | 68.0 | 78.7 | 79.8 | 89.3 | 92.1 | 71.0 |
| Snrpn | 13.5 | 10.0 | 8.6 | 12.5 | 8.5 | 12.6 | 10.1 | 8.2 | 8.4 | 8.2 | 11.2 | 8.8 |
| Snta1 | 6.8 | 7.3 | 6.3 | 4.4 | 4.4 | 5.0 | 5.8 | 6.6 | 11.4 | 12.5 | 8.9 | 10.0 |
| Sntb1 | 0.4 | 0.7 | 1.5 | 0.1 | 0.9 | 0.3 | 0.7 | 2.1 | 1.0 | 0.3 | 0.5 | 1.0 |
| Sntb2 | 15.3 | 18.7 | 18.1 | 13.1 | 14.9 | 12.7 | 13.6 | 13.2 | 12.6 | 18.6 | 13.2 | 15.9 |
| Sntg1 | 0.4 | 0.2 | 0.0 | 0.8 | 0.1 | 0.4 | 0.1 | 0.0 | 0.1 | 0.1 | 0.1 | 0.0 |
| Sntg2 | 0.6 | 0.6 | 0.9 | 0.1 | 0.0 | 0.1 | 0.0 | 0.5 | 0.2 | 0.3 | 0.3 | 0.4 |
| Snupn | 10.4 | 9.7 | 8.8 | 10.9 | 9.2 | 10.0 | 7.3 | 7.3 | 7.6 | 12.2 | 10.5 | 10.1 |
| Snurf | 0.9 | 0.7 | 0.6 | 1.0 | 0.5 | 0.8 | 0.7 | 0.7 | 0.6 | 0.8 | 1.2 | 0.8 |
| Snw1 | 53.5 | 48.4 | 51.0 | 59.7 | 39.3 | 52.8 | 38.7 | 38.9 | 32.5 | 40.7 | 43.4 | 45.8 |
| Snx1 | 48.3 | 51.2 | 48.5 | 61.3 | 55.2 | 51.8 | 49.6 | 49.4 | 48.6 | 50.1 | 55.4 | 55.7 |
| Snx10 | 25.4 | 20.2 | 19.5 | 28.6 | 25.5 | 22.5 | 19.4 | 19.8 | 17.6 | 22.6 | 26.9 | 21.5 |
| Snx11 | 9.4 | 8.8 | 6.9 | 8.9 | 9.1 | 9.8 | 9.4 | 7.4 | 9.4 | 7.3 | 8.3 | 8.0 |
| Snx12 | 24.5 | 24.2 | 22.3 | 24.1 | 22.8 | 24.7 | 23.7 | 23.8 | 23.8 | 23.1 | 24.8 | 26.6 |
| Snx13 | 10.4 | 11.7 | 12.2 | 10.8 | 10.8 | 10.6 | 11.0 | 11.2 | 12.9 | 11.7 | 13.1 | 14.8 |
| Snx14 | 17.5 | 19.6 | 18.6 | 15.5 | 17.3 | 16.5 | 18.3 | 18.0 | 19.6 | 18.7 | 20.0 | 19.9 |
| Snx15 | 7.2 | 8.0 | 7.0 | 9.5 | 8.4 | 8.6 | 7.5 | 8.3 | 8.3 | 7.2 | 7.0 | 5.6 |
| Snx16 | 4.6 | 5.0 | 4.3 | 5.3 | 4.0 | 5.8 | 3.5 | 3.5 | 3.5 | 4.9 | 5.4 | 4.3 |
| Snx17 | 32.2 | 30.6 | 25.2 | 32.3 | 28.6 | 32.2 | 25.2 | 27.8 | 27.8 | 28.4 | 25.6 | 25.2 |
| Snx18 | 33.0 | 31.1 | 29.7 | 29.3 | 32.1 | 30.7 | 33.4 | 27.7 | 27.9 | 28.5 | 31.5 | 31.8 |
| Snx19 | 12.0 | 11.8 | 11.8 | 11.7 | 10.9 | 11.0 | 12.6 | 11.9 | 12.2 | 11.1 | 10.8 | 11.8 |
| Snx2 | 43.9 | 37.8 | 37.0 | 45.9 | 40.9 | 37.6 | 36.3 | 35.2 | 31.6 | 37.7 | 41.6 | 41.1 |
| Snx20 | 0.6 | 0.1 | 0.1 | 4.9 | 8.3 | 0.1 | 0.7 | 0.1 | 0.1 | 1.0 | 0.2 | 0.7 |
| Snx21 | 9.3 | 9.4 | 8.5 | 9.2 | 7.3 | 10.3 | 8.6 | 9.6 | 10.1 | 9.3 | 8.9 | 8.6 |
| Snx22 | 38.5 | 32.7 | 33.2 | 38.8 | 30.6 | 31.9 | 29.6 | 34.5 | 33.0 | 33.1 | 33.3 | 36.7 |
| Snx24 | 11.4 | 10.4 | 8.3 | 9.8 | 9.4 | 11.3 | 9.2 | 8.4 | 9.0 | 10.8 | 9.7 | 8.6 |
| Snx25 | 9.4 | 12.8 | 9.8 | 8.0 | 9.3 | 11.1 | 10.5 | 8.6 | 10.9 | 8.0 | 9.6 | 10.0 |
| Snx27 | 8.1 | 8.4 | 8.0 | 7.4 | 8.2 | 7.7 | 7.6 | 7.2 | 7.5 | 7.6 | 7.4 | 6.5 |
| Snx29 | 1.8 | 2.0 | 1.9 | 3.1 | 2.4 | 2.1 | 1.5 | 1.9 | 1.9 | 2.6 | 2.3 | 2.1 |
| Snx3 | 65.5 | 59.9 | 53.5 | 73.0 | 60.5 | 66.9 | 63.4 | 57.1 | 53.8 | 61.6 | 65.5 | 63.0 |
| Snx30 | 7.6 | 6.8 | 5.1 | 9.4 | 7.6 | 7.8 | 6.7 | 4.2 | 6.8 | 6.7 | 6.3 | 6.0 |
| Snx31 | 0.0 | 0.1 | 0.1 | 0.1 | 0.1 | 0.1 | 0.1 | 0.0 | 0.1 | 0.1 | 0.1 | 0.0 |
| Snx32 | 7.0 | 6.6 | 6.6 | 6.9 | 4.9 | 9.4 | 4.6 | 5.3 | 5.1 | 5.7 | 5.6 | 6.0 |
| Snx33 | 13.8 | 14.2 | 14.0 | 13.2 | 11.4 | 13.4 | 12.0 | 13.4 | 12.9 | 14.4 | 13.4 | 13.3 |
| Snx4 | 36.7 | 35.4 | 40.4 | 41.0 | 35.1 | 37.1 | 39.8 | 40.6 | 37.0 | 35.7 | 45.9 | 41.8 |
| Snx5 | 39.0 | 29.9 | 26.9 | 36.6 | 34.8 | 36.8 | 24.3 | 33.7 | 27.1 | 32.4 | 34.5 | 31.5 |
| Snx6 | 34.9 | 35.8 | 34.5 | 36.3 | 35.3 | 37.1 | 33.1 | 33.3 | 36.3 | 38.2 | 39.1 | 36.0 |
| Snx7 | 31.5 | 28.2 | 31.3 | 24.7 | 28.5 | 19.8 | 38.9 | 31.1 | 38.9 | 44.8 | 54.0 | 41.6 |
| Snx8 | 6.8 | 6.2 | 4.3 | 10.0 | 7.5 | 6.4 | 5.6 | 4.9 | 5.0 | 5.2 | 4.8 | 5.0 |
| Snx9 | 48.6 | 41.4 | 42.9 | 48.6 | 42.2 | 56.4 | 54.3 | 41.5 | 45.7 | 53.5 | 46.8 | 54.9 |
| Soat1 | 13.6 | 14.3 | 17.0 | 17.0 | 20.2 | 15.2 | 18.0 | 14.3 | 15.3 | 23.7 | 19.0 | 19.6 |
| Soat2 | 0.3 | 0.1 | 0.1 | 0.3 | 0.2 | 0.6 | 0.2 | 0.6 | 0.2 | 0.1 | 0.2 | 0.1 |
| Sobp | 0.8 | 1.3 | 1.2 | 0.8 | 1.1 | 0.8 | 1.1 | 1.0 | 1.0 | 0.6 | 0.7 | 0.9 |
| Socs1 | 0.6 | 0.5 | 0.6 | 0.3 | 0.5 | 0.4 | 0.5 | 1.1 | 1.1 | 0.6 | 0.3 | 0.6 |
| Socs2 | 17.4 | 20.3 | 22.8 | 14.4 | 15.1 | 16.9 | 16.9 | 20.4 | 16.7 | 15.0 | 12.0 | 19.8 |
| Socs3 | 5.2 | 12.4 | 19.1 | 3.8 | 10.7 | 2.0 | 12.6 | 24.9 | 17.9 | 17.9 | 11.9 | 16.2 |
| Socs4 | 8.2 | 7.5 | 7.4 | 7.6 | 8.1 | 7.1 | 7.8 | 6.7 | 6.4 | 5.9 | 7.6 | 7.3 |
| Socs5 | 22.2 | 24.7 | 24.7 | 15.8 | 22.2 | 17.8 | 21.1 | 20.9 | 23.9 | 26.8 | 23.7 | 28.4 |
| Socs6 | 7.8 | 7.4 | 6.2 | 8.9 | 7.3 | 9.1 | 6.7 | 6.7 | 7.1 | 7.5 | 8.0 | 7.3 |
| Socs7 | 3.6 | 3.2 | 2.6 | 3.7 | 3.1 | 3.4 | 2.9 | 2.1 | 2.6 | 3.5 | 3.2 | 3.0 |
| Sod1 | 361.3 | 292.9 | 262.8 | 361.2 | 284.2 | 384.5 | 267.4 | 254.1 | 242.5 | 340.8 | 295.8 | 289.5 |
| Sod2 | 21.1 | 18.7 | 27.7 | 22.9 | 35.1 | 16.2 | 27.2 | 25.9 | 20.4 | 18.3 | 19.3 | 20.6 |
| Sod3 | 66.8 | 63.2 | 96.6 | 107.5 | 81.8 | 52.7 | 98.1 | 90.2 | 69.8 | 45.2 | 43.2 | 68.5 |

| | | | | | | | | | | | | |
|---|---|---|---|---|---|---|---|---|---|---|---|---|
| Soga2 | 1.0 | 1.0 | 0.7 | 1.1 | 0.8 | 1.2 | 0.8 | 0.6 | 0.7 | 0.8 | 0.7 | 0.6 |
| Soga3 | 0.1 | 0.0 | 0.0 | 0.0 | 0.0 | 0.0 | 0.1 | 0.0 | 0.0 | 0.0 | 0.0 | 0.0 |
| Sohlh2 | 0.1 | 0.0 | 0.0 | 0.0 | 0.0 | 0.0 | 0.0 | 0.0 | 0.0 | 0.1 | 0.1 | 0.0 |
| Solh | 0.9 | 1.2 | 1.2 | 1.2 | 1.6 | 1.2 | 1.6 | 1.6 | 1.6 | 1.3 | 1.1 | 1.0 |
| Son | 35.5 | 39.3 | 38.5 | 34.1 | 37.1 | 33.3 | 34.8 | 38.2 | 38.9 | 38.4 | 38.4 | 40.3 |
| Sorbs1 | 10.8 | 12.7 | 12.0 | 11.9 | 10.3 | 14.0 | 16.0 | 9.0 | 18.4 | 11.6 | 15.7 | 14.5 |
| Sorbs2 | 11.1 | 11.6 | 12.0 | 16.3 | 9.9 | 20.0 | 8.7 | 6.4 | 7.6 | 2.4 | 5.5 | 6.1 |
| Sorbs3 | 12.7 | 17.9 | 17.2 | 10.2 | 8.7 | 11.1 | 15.3 | 15.0 | 12.1 | 17.5 | 15.8 | 13.9 |
| Sorcs1 | 0.1 | 0.0 | 0.0 | 0.1 | 0.0 | 0.1 | 0.0 | 0.0 | 0.0 | 0.5 | 0.2 | 0.1 |
| Sorcs2 | 0.2 | 0.2 | 0.3 | 0.1 | 0.3 | 0.2 | 0.3 | 1.1 | 0.6 | 1.4 | 1.2 | 0.7 |
| Sorcs3 | 0.0 | 0.0 | 0.0 | 0.1 | 0.0 | 0.0 | 0.0 | 0.0 | 0.0 | 0.0 | 0.0 | 0.0 |
| Sord | 9.7 | 11.6 | 12.5 | 7.8 | 8.5 | 11.8 | 5.1 | 14.0 | 8.3 | 8.3 | 10.0 | 8.6 |
| Sorl1 | 0.4 | 0.3 | 0.4 | 1.0 | 2.4 | 0.4 | 0.3 | 0.2 | 0.2 | 0.3 | 0.2 | 0.3 |
| Sort1 | 2.3 | 1.7 | 1.5 | 1.5 | 1.1 | 2.2 | 0.6 | 1.5 | 1.1 | 3.5 | 1.9 | 1.2 |
| Sos1 | 2.8 | 3.7 | 3.7 | 3.3 | 3.0 | 3.6 | 3.2 | 2.8 | 3.0 | 2.8 | 3.4 | 3.1 |
| Sos2 | 6.0 | 6.9 | 6.1 | 6.1 | 6.0 | 6.2 | 6.3 | 5.2 | 6.1 | 5.5 | 6.5 | 6.6 |
| Sost | 0.0 | 0.0 | 0.0 | 0.0 | 0.0 | 0.0 | 0.0 | 0.0 | 0.0 | 0.0 | 0.0 | 0.0 |
| Sowahb | 0.0 | 0.0 | 0.0 | 0.0 | 0.0 | 0.0 | 0.0 | 0.0 | 0.0 | 0.0 | 0.0 | 0.0 |
| Sowahc | 1.9 | 2.2 | 3.1 | 2.3 | 2.6 | 2.4 | 2.6 | 2.5 | 2.1 | 2.9 | 2.6 | 2.9 |
| Sowahd | 0.1 | 0.1 | 0.2 | 0.0 | 0.1 | 0.0 | 0.0 | 0.4 | 0.4 | 0.4 | 0.5 | 0.1 |
| Sox10 | 0.1 | 0.0 | 0.0 | 0.0 | 0.0 | 0.0 | 0.0 | 0.0 | 0.0 | 0.0 | 0.0 | 0.0 |
| Sox11 | 0.1 | 0.0 | 0.0 | 0.1 | 0.1 | 0.0 | 0.1 | 0.0 | 0.1 | 0.7 | 1.0 | 0.4 |
| Sox12 | 1.0 | 1.2 | 1.6 | 1.0 | 1.0 | 0.8 | 1.4 | 2.1 | 1.9 | 1.7 | 1.4 | 1.2 |
| Sox13 | 1.2 | 1.5 | 1.4 | 0.8 | 1.0 | 1.3 | 1.1 | 1.1 | 1.3 | 0.9 | 1.0 | 0.9 |
| Sox15 | 0.4 | 0.1 | 1.2 | 0.1 | 1.1 | 0.1 | 0.0 | 0.3 | 0.1 | 1.6 | 0.0 | 1.1 |
| Sox17 | 0.2 | 0.1 | 0.1 | 0.0 | 0.0 | 0.0 | 0.1 | 0.2 | 0.2 | 0.0 | 0.1 | 0.0 |
| Sox18 | 0.3 | 0.0 | 0.2 | 0.1 | 0.0 | 0.1 | 0.2 | 0.4 | 0.4 | 0.1 | 0.2 | 0.2 |
| Sox21 | 0.0 | 0.0 | 0.0 | 0.0 | 0.0 | 0.0 | 0.0 | 0.0 | 0.0 | 0.0 | 0.0 | 0.0 |
| Sox2ot | 0.0 | 0.0 | 0.0 | 0.0 | 0.0 | 0.0 | 0.0 | 0.0 | 0.0 | 0.0 | 0.0 | 0.0 |
| Sox30 | 0.0 | 0.1 | 0.1 | 0.1 | 0.0 | 0.1 | 0.0 | 0.0 | 0.0 | 0.1 | 0.0 | 0.0 |
| Sox4 | 23.8 | 18.4 | 17.3 | 25.6 | 14.2 | 23.5 | 13.4 | 16.1 | 14.5 | 24.7 | 17.8 | 16.7 |
| Sox5 | 1.8 | 2.4 | 2.9 | 1.7 | 2.3 | 1.9 | 2.3 | 2.3 | 2.5 | 1.9 | 2.0 | 2.3 |
| Sox6 | 0.6 | 0.3 | 0.5 | 0.1 | 0.1 | 0.1 | 0.2 | 0.5 | 0.2 | 0.5 | 0.4 | 0.4 |
| Sox7 | 0.3 | 0.2 | 0.3 | 0.4 | 0.4 | 0.2 | 0.2 | 0.2 | 0.3 | 0.1 | 0.1 | 0.1 |
| Sox8 | 0.5 | 0.3 | 0.5 | 0.5 | 0.4 | 0.4 | 0.2 | 0.6 | 0.4 | 0.5 | 0.7 | 0.4 |
| Sox9 | 3.0 | 4.6 | 6.3 | 3.5 | 5.6 | 3.2 | 8.8 | 8.4 | 6.5 | 3.6 | 5.0 | 4.2 |
| Sp1 | 5.5 | 6.8 | 7.4 | 5.4 | 6.9 | 7.0 | 6.7 | 8.2 | 7.3 | 7.7 | 8.0 | 7.8 |
| Sp100 | 17.3 | 17.7 | 23.8 | 31.3 | 22.9 | 21.7 | 16.8 | 14.0 | 14.5 | 15.0 | 14.5 | 20.7 |
| Sp110 | 7.8 | 7.5 | 9.9 | 14.5 | 10.9 | 8.2 | 8.5 | 6.0 | 6.1 | 6.3 | 5.7 | 8.9 |
| Sp140 | 5.8 | 6.4 | 8.2 | 4.7 | 6.6 | 4.7 | 4.0 | 4.9 | 4.3 | 4.8 | 4.6 | 6.0 |
| Sp2 | 0.3 | 0.5 | 0.5 | 0.6 | 0.7 | 0.5 | 0.9 | 0.8 | 0.7 | 0.5 | 0.5 | 0.4 |
| Sp3 | 23.3 | 28.0 | 38.5 | 23.7 | 23.1 | 22.0 | 24.5 | 35.2 | 27.8 | 28.3 | 30.5 | 34.4 |
| Sp4 | 1.1 | 1.4 | 1.7 | 1.2 | 1.2 | 1.0 | 1.1 | 1.5 | 1.2 | 1.2 | 1.3 | 1.5 |
| Sp6 | 0.2 | 0.3 | 0.3 | 0.2 | 0.2 | 0.3 | 0.2 | 0.1 | 0.2 | 0.1 | 0.1 | 0.2 |
| Sp7 | 0.0 | 0.0 | 0.0 | 0.0 | 0.0 | 0.0 | 0.0 | 0.0 | 0.0 | 0.0 | 0.0 | 0.0 |
| Sp9 | 0.2 | 0.2 | 0.1 | 0.1 | 0.1 | 0.1 | 0.1 | 0.0 | 0.0 | 0.1 | 0.1 | 0.0 |
| Spa17 | 2.0 | 1.7 | 2.6 | 3.4 | 1.3 | 3.1 | 1.7 | 1.9 | 1.2 | 0.9 | 0.7 | 2.7 |
| Spaca1 | 3.9 | 1.7 | 1.9 | 1.3 | 1.3 | 1.8 | 2.5 | 0.7 | 1.2 | 1.3 | 2.5 | 2.3 |
| Spaca4 | 0.0 | 0.2 | 0.1 | 0.0 | 0.0 | 0.0 | 0.0 | 0.0 | 0.1 | 0.1 | 0.0 | 0.1 |
| Spag1 | 1.0 | 1.3 | 1.2 | 0.9 | 1.2 | 0.9 | 0.9 | 1.0 | 0.9 | 0.8 | 0.8 | 1.0 |
| Spag17 | 0.0 | 0.0 | 0.0 | 0.0 | 0.0 | 0.0 | 0.0 | 0.0 | 0.0 | 0.0 | 0.0 | 0.0 |
| Spag4 | 1.7 | 1.3 | 1.4 | 1.3 | 1.3 | 2.1 | 1.5 | 0.7 | 1.8 | 1.1 | 1.4 | 0.8 |
| Spag5 | 1.6 | 2.6 | 4.2 | 0.7 | 3.9 | 0.6 | 4.7 | 1.7 | 3.8 | 2.1 | 3.3 | 2.6 |
| Spag7 | 27.1 | 25.9 | 30.1 | 33.2 | 24.1 | 26.4 | 37.8 | 35.3 | 31.0 | 28.6 | 33.9 | 31.5 |
| Spag8 | 0.1 | 0.0 | 0.0 | 0.2 | 0.0 | 0.1 | 0.1 | 0.1 | 0.1 | 0.0 | 0.2 | 0.0 |
| Spag9 | 35.6 | 31.6 | 30.8 | 33.9 | 30.6 | 34.4 | 31.5 | 32.2 | 30.9 | 28.6 | 34.3 | 32.5 |
| Sparc | 2867.6 | 2556.9 | 2779.0 | 2529.8 | 2068.2 | 2430.0 | 2233.5 | 3049.5 | 2577.1 | 2828.8 | 2747.2 | 2734.5 |

| | | | | | | | | | | | | |
|---|---|---|---|---|---|---|---|---|---|---|---|---|
| Sparcl1 | 1.9 | 1.6 | 5.3 | 0.5 | 0.7 | 0.4 | 2.5 | 4.4 | 3.6 | 0.4 | 0.7 | 0.7 |
| Spast | 7.6 | 8.1 | 7.8 | 7.8 | 7.1 | 8.3 | 7.1 | 7.7 | 7.3 | 7.6 | 8.5 | 8.1 |
| Spata1 | 1.3 | 1.0 | 1.4 | 1.7 | 0.9 | 1.8 | 1.4 | 0.9 | 1.3 | 1.0 | 1.8 | 1.2 |
| Spata13 | 2.0 | 1.1 | 0.9 | 2.5 | 2.4 | 1.8 | 1.7 | 1.3 | 1.3 | 1.7 | 1.8 | 1.5 |
| Spata17 | 0.2 | 0.3 | 0.1 | 0.3 | 0.0 | 0.6 | 0.2 | 0.2 | 0.1 | 0.1 | 0.1 | 0.2 |
| Spata18 | 0.0 | 0.1 | 0.0 | 0.0 | 0.0 | 0.0 | 0.0 | 0.0 | 0.0 | 0.0 | 0.0 | 0.0 |
| Spata2 | 2.4 | 2.8 | 2.7 | 1.8 | 3.0 | 2.5 | 3.0 | 3.0 | 2.9 | 2.2 | 2.8 | 2.1 |
| Spata20 | 0.0 | 0.0 | 0.0 | 0.0 | 0.0 | 0.0 | 0.0 | 0.0 | 0.0 | 0.0 | 0.0 | 0.0 |
| Spata24 | 4.2 | 3.2 | 5.1 | 2.5 | 3.1 | 4.6 | 3.9 | 4.5 | 4.2 | 2.4 | 3.3 | 2.6 |
| Spata25 | 0.1 | 0.0 | 0.0 | 0.1 | 0.1 | 0.1 | 0.1 | 0.0 | 0.0 | 0.0 | 0.1 | 0.1 |
| Spata2l | 0.5 | 0.5 | 0.8 | 0.5 | 0.8 | 0.9 | 0.7 | 0.8 | 0.7 | 0.8 | 0.4 | 0.5 |
| Spata31d1d | 0.0 | 0.0 | 0.0 | 0.0 | 0.0 | 0.0 | 0.0 | 0.0 | 0.0 | 0.0 | 0.0 | 0.0 |
| Spata4 | 0.2 | 0.1 | 0.2 | 0.5 | 0.0 | 0.2 | 0.2 | 0.1 | 0.1 | 0.1 | 0.2 | 0.1 |
| Spata5 | 11.6 | 14.8 | 14.3 | 11.3 | 12.1 | 8.3 | 13.5 | 14.4 | 16.3 | 11.1 | 13.1 | 14.5 |
| Spata5l1 | 1.2 | 1.3 | 1.4 | 0.8 | 1.2 | 1.7 | 1.3 | 1.2 | 1.3 | 1.8 | 0.8 | 1.2 |
| Spata6 | 14.0 | 16.2 | 14.1 | 15.5 | 13.0 | 17.6 | 14.8 | 14.2 | 12.7 | 10.9 | 14.1 | 12.2 |
| Spata7 | 2.7 | 2.8 | 3.6 | 3.0 | 2.5 | 4.3 | 2.7 | 2.9 | 2.6 | 2.0 | 2.6 | 3.0 |
| Spata9 | 0.2 | 0.3 | 0.5 | 0.3 | 0.2 | 0.3 | 0.2 | 0.3 | 0.3 | 0.3 | 0.3 | 0.1 |
| Spatc1 | 0.0 | 0.1 | 0.0 | 0.0 | 0.0 | 0.0 | 0.0 | 0.0 | 0.0 | 0.0 | 0.0 | 0.0 |
| Spatc1l | 0.0 | 0.1 | 0.0 | 0.0 | 0.0 | 0.0 | 0.0 | 0.0 | 0.0 | 0.0 | 0.0 | 0.0 |
| Spats1 | 0.4 | 0.1 | 0.4 | 0.1 | 0.2 | 0.3 | 0.1 | 0.1 | 0.1 | 0.1 | 0.2 | 0.1 |
| Spats2 | 8.6 | 9.9 | 9.7 | 8.5 | 9.4 | 8.0 | 9.1 | 9.6 | 9.6 | 9.4 | 10.5 | 10.9 |
| Spats2l | 1.9 | 2.3 | 2.8 | 1.8 | 1.8 | 1.6 | 2.0 | 2.3 | 2.1 | 2.9 | 3.0 | 2.5 |
| Spc24 | 1.1 | 2.0 | 3.4 | 0.5 | 3.1 | 0.5 | 4.5 | 2.1 | 3.9 | 1.3 | 2.4 | 2.2 |
| Spc25 | 7.8 | 11.8 | 16.8 | 6.8 | 16.2 | 5.9 | 15.7 | 5.9 | 12.2 | 9.4 | 12.2 | 8.8 |
| Spcs1 | 110.1 | 94.2 | 85.1 | 125.8 | 104.1 | 119.8 | 96.6 | 98.9 | 106.3 | 125.1 | 103.6 | 113.0 |
| Spcs2 | 26.7 | 25.5 | 23.6 | 30.6 | 26.1 | 26.1 | 24.4 | 27.6 | 27.8 | 29.8 | 29.3 | 29.5 |
| Spcs3 | 27.8 | 23.1 | 25.4 | 24.8 | 26.0 | 22.1 | 23.4 | 24.0 | 26.1 | 23.5 | 25.0 | 29.6 |
| Spdef | 0.0 | 0.0 | 0.0 | 0.0 | 0.0 | 0.0 | 0.0 | 0.0 | 0.0 | 0.0 | 0.0 | 0.0 |
| Spdl1 | 1.4 | 2.1 | 3.6 | 1.7 | 3.3 | 1.1 | 4.0 | 2.0 | 3.2 | 1.5 | 2.4 | 2.3 |
| Spdya | 0.1 | 0.1 | 0.2 | 0.0 | 0.1 | 0.1 | 0.2 | 0.1 | 0.1 | 0.1 | 0.1 | 0.0 |
| Specc1 | 7.7 | 11.8 | 13.1 | 7.8 | 12.8 | 5.2 | 10.9 | 11.9 | 17.4 | 9.8 | 9.1 | 17.7 |
| Specc1l | 21.4 | 22.3 | 24.2 | 21.4 | 21.0 | 22.1 | 25.9 | 24.0 | 23.9 | 18.8 | 23.7 | 25.0 |
| Speer1-ps1 | 0.1 | 0.0 | 0.0 | 0.1 | 0.2 | 0.0 | 0.1 | 0.2 | 0.0 | 0.0 | 0.0 | 0.0 |
| Speer3 | 1.4 | 0.9 | 0.6 | 0.8 | 0.3 | 0.3 | 0.7 | 0.5 | 0.4 | 1.0 | 1.1 | 0.6 |
| Speer4a | 0.3 | 0.1 | 0.2 | 0.0 | 0.1 | 0.1 | 0.0 | 0.1 | 0.0 | 0.1 | 0.2 | 0.1 |
| Speer4b | 0.2 | 0.1 | 0.4 | 0.0 | 0.2 | 0.1 | 0.0 | 0.1 | 0.2 | 0.2 | 0.3 | 0.2 |
| Speer4f | 0.0 | 0.0 | 0.0 | 0.0 | 0.0 | 0.0 | 0.0 | 0.0 | 0.0 | 0.0 | 0.0 | 0.0 |
| Speer7-ps1 | 0.2 | 0.5 | 0.5 | 0.0 | 0.0 | 0.1 | 0.1 | 0.2 | 0.0 | 0.2 | 0.1 | 0.0 |
| Speer8-ps1 | 0.0 | 0.0 | 0.1 | 0.0 | 0.0 | 0.1 | 0.0 | 0.0 | 0.0 | 0.0 | 0.1 | 0.1 |
| Spef1 | 1.4 | 1.2 | 1.0 | 1.0 | 1.3 | 2.1 | 1.1 | 1.2 | 1.4 | 1.3 | 1.3 | 0.9 |
| Spef2 | 0.0 | 0.0 | 0.0 | 0.1 | 0.0 | 0.0 | 0.0 | 0.0 | 0.0 | 0.1 | 0.0 | 0.0 |
| Speg | 3.0 | 2.5 | 2.6 | 7.0 | 8.4 | 2.6 | 3.1 | 2.9 | 3.7 | 6.2 | 4.3 | 4.3 |
| Spen | 1.0 | 1.6 | 1.9 | 1.2 | 1.6 | 1.2 | 2.0 | 1.4 | 1.6 | 1.3 | 1.3 | 1.3 |
| Spg11 | 10.5 | 9.7 | 8.8 | 10.7 | 9.3 | 11.8 | 7.5 | 9.2 | 8.0 | 8.5 | 9.2 | 8.9 |
| Spg20 | 36.1 | 33.7 | 29.6 | 46.5 | 27.9 | 40.5 | 35.0 | 29.3 | 32.2 | 28.6 | 34.6 | 35.9 |
| Spg21 | 29.4 | 20.3 | 18.5 | 29.3 | 21.6 | 29.7 | 21.4 | 21.4 | 20.7 | 24.2 | 22.7 | 23.2 |
| Spg7 | 24.8 | 19.5 | 17.6 | 27.7 | 21.6 | 24.3 | 21.6 | 20.8 | 20.6 | 22.9 | 22.7 | 21.8 |
| Sphk1 | 7.0 | 7.4 | 6.7 | 7.9 | 11.0 | 7.7 | 10.6 | 7.9 | 7.7 | 9.0 | 6.1 | 8.4 |
| Sphk2 | 7.4 | 8.5 | 9.3 | 8.2 | 6.7 | 9.3 | 7.3 | 8.1 | 7.2 | 6.8 | 6.7 | 6.9 |
| Sphkap | 0.0 | 0.0 | 0.1 | 0.0 | 0.0 | 0.0 | 0.0 | 0.0 | 0.0 | 0.0 | 0.0 | 0.0 |
| Spib | 0.0 | 0.0 | 0.1 | 0.0 | 0.1 | 0.0 | 0.1 | 0.0 | 0.0 | 0.0 | 0.0 | 0.0 |
| Spic | 0.0 | 0.0 | 0.0 | 0.0 | 0.0 | 0.0 | 0.0 | 0.0 | 0.0 | 0.0 | 0.0 | 0.0 |
| Spice1 | 1.6 | 2.3 | 2.1 | 1.5 | 1.7 | 2.2 | 1.8 | 2.2 | 1.9 | 1.9 | 2.6 | 1.6 |
| Spin1 | 28.2 | 25.3 | 28.2 | 21.9 | 25.5 | 23.4 | 27.8 | 28.9 | 28.2 | 30.8 | 29.3 | 32.7 |
| Spin2 | 1.2 | 1.2 | 2.0 | 1.2 | 1.0 | 1.4 | 1.4 | 1.2 | 0.9 | 1.1 | 1.6 | 1.3 |
| Spin4 | 0.5 | 0.7 | 1.0 | 0.4 | 0.6 | 0.6 | 0.8 | 0.9 | 0.8 | 0.9 | 0.7 | 1.1 |

| | | | | | | | | | | | | |
|---|---|---|---|---|---|---|---|---|---|---|---|---|
| Spink10 | 0.3 | 0.3 | 0.6 | 0.1 | 0.3 | 0.4 | 0.2 | 0.1 | 0.1 | 0.2 | 0.3 | 0.2 |
| Spink2 | 0.1 | 0.3 | 0.5 | 0.0 | 0.1 | 0.0 | 0.2 | 0.1 | 0.0 | 0.0 | 0.0 | 0.5 |
| Spink5 | 0.0 | 0.0 | 0.0 | 0.1 | 0.0 | 0.0 | 0.0 | 0.0 | 0.0 | 0.0 | 0.0 | 0.0 |
| Spink8 | 0.1 | 0.1 | 0.1 | 0.0 | 0.0 | 0.0 | 0.0 | 0.0 | 0.1 | 0.0 | 0.3 | 0.0 |
| Spint1 | 0.2 | 0.1 | 0.1 | 0.3 | 0.5 | 0.1 | 0.1 | 0.1 | 0.1 | 0.4 | 0.1 | 0.1 |
| Spint2 | 1.0 | 0.4 | 0.2 | 0.5 | 0.2 | 1.1 | 0.3 | 0.5 | 0.5 | 1.4 | 1.5 | 0.5 |
| Spint4 | 0.0 | 0.0 | 0.0 | 0.3 | 0.0 | 0.0 | 0.2 | 0.1 | 0.0 | 0.0 | 0.0 | 0.0 |
| Spire1 | 7.7 | 5.3 | 3.3 | 7.3 | 4.6 | 8.1 | 4.4 | 3.0 | 3.0 | 5.1 | 5.5 | 4.3 |
| Spire2 | 1.0 | 0.6 | 0.6 | 1.3 | 0.8 | 0.7 | 1.4 | 2.3 | 2.2 | 2.1 | 1.5 | 1.3 |
| Spn | 1.5 | 0.2 | 0.4 | 8.4 | 28.4 | 0.1 | 5.6 | 0.3 | 1.3 | 2.9 | 1.2 | 3.5 |
| Spns1 | 11.7 | 11.0 | 9.4 | 11.6 | 11.8 | 12.0 | 10.1 | 10.1 | 10.9 | 11.1 | 8.1 | 9.3 |
| Spns2 | 1.7 | 3.3 | 3.8 | 2.5 | 2.3 | 3.0 | 1.4 | 1.5 | 1.6 | 0.7 | 0.5 | 0.8 |
| Spns3 | 0.3 | 0.2 | 0.2 | 0.5 | 0.4 | 0.6 | 0.2 | 0.4 | 0.3 | 0.0 | 0.1 | 0.2 |
| Spock2 | 0.1 | 0.1 | 0.1 | 0.1 | 0.1 | 0.1 | 0.0 | 0.1 | 0.1 | 0.2 | 0.1 | 0.1 |
| Spock3 | 0.0 | 0.0 | 0.0 | 0.0 | 0.0 | 0.0 | 0.0 | 0.0 | 0.0 | 0.0 | 0.0 | 0.0 |
| Spon1 | 0.5 | 0.7 | 2.0 | 0.5 | 0.5 | 0.2 | 0.8 | 1.6 | 0.4 | 0.5 | 0.4 | 0.7 |
| Spon2 | 8.0 | 6.2 | 4.3 | 6.4 | 5.5 | 4.5 | 6.0 | 4.5 | 4.6 | 3.7 | 4.5 | 2.1 |
| Spop | 24.7 | 23.6 | 22.8 | 27.3 | 25.4 | 25.2 | 23.0 | 21.2 | 19.1 | 21.8 | 22.1 | 22.1 |
| Spopl | 7.3 | 6.5 | 6.2 | 7.1 | 6.4 | 7.9 | 6.1 | 5.5 | 6.1 | 6.1 | 6.6 | 5.9 |
| Spp1 | 71.0 | 1.2 | 3.7 | 445.6 | 485.5 | 1.0 | 35.5 | 12.0 | 9.9 | 50.8 | 23.0 | 47.4 |
| Sppl2a | 16.8 | 16.2 | 16.7 | 22.6 | 22.2 | 21.6 | 18.3 | 16.1 | 16.7 | 17.1 | 19.7 | 18.1 |
| Sppl2b | 4.1 | 4.0 | 3.6 | 4.3 | 4.7 | 3.7 | 5.1 | 5.3 | 4.7 | 4.7 | 4.4 | 3.2 |
| Sppl3 | 9.5 | 10.2 | 9.3 | 9.5 | 10.7 | 10.8 | 11.3 | 10.7 | 12.6 | 11.0 | 10.4 | 10.0 |
| Spr | 21.0 | 16.4 | 13.1 | 20.9 | 19.9 | 20.9 | 16.8 | 13.4 | 16.8 | 19.9 | 16.2 | 15.4 |
| Spred1 | 18.3 | 21.5 | 20.4 | 20.1 | 24.7 | 20.7 | 21.7 | 16.7 | 23.2 | 27.3 | 22.1 | 22.9 |
| Spred2 | 2.1 | 2.7 | 2.3 | 2.9 | 2.7 | 2.9 | 2.2 | 1.8 | 1.6 | 2.4 | 2.3 | 1.8 |
| Spred3 | 2.1 | 2.1 | 1.7 | 2.4 | 2.3 | 2.1 | 1.8 | 1.3 | 1.4 | 1.8 | 1.4 | 1.4 |
| Sprn | 0.1 | 0.3 | 0.6 | 0.0 | 0.0 | 0.1 | 0.1 | 0.2 | 0.1 | 0.1 | 0.1 | 0.2 |
| Sprr1a | 0.2 | 0.1 | 0.5 | 0.3 | 0.4 | 0.2 | 0.4 | 0.7 | 0.3 | 0.4 | 0.2 | 0.1 |
| Sprr2a1 | 0.0 | 0.0 | 0.0 | 0.0 | 0.0 | 0.0 | 0.0 | 0.0 | 0.0 | 0.0 | 0.0 | 0.0 |
| Sprr2a2 | 0.0 | 0.0 | 0.0 | 0.0 | 0.0 | 0.0 | 0.0 | 0.0 | 0.0 | 0.0 | 0.0 | 0.0 |
| Sprr3 | 0.7 | 0.2 | 0.1 | 0.9 | 0.4 | 0.2 | 0.7 | 0.0 | 0.5 | 0.6 | 0.4 | 0.3 |
| Sprtn | 7.6 | 7.7 | 7.6 | 7.5 | 6.7 | 6.7 | 8.1 | 6.8 | 7.1 | 6.9 | 8.4 | 7.7 |
| Spry1 | 15.2 | 25.2 | 30.1 | 19.2 | 21.5 | 15.6 | 26.7 | 32.7 | 39.6 | 20.9 | 22.7 | 26.6 |
| Spry2 | 12.8 | 14.6 | 17.9 | 11.4 | 12.8 | 8.1 | 15.0 | 12.2 | 14.5 | 17.5 | 14.7 | 17.6 |
| Spry4 | 1.9 | 2.2 | 2.2 | 1.7 | 2.3 | 1.8 | 2.0 | 1.5 | 1.6 | 3.3 | 1.8 | 1.6 |
| Spryd3 | 13.7 | 13.2 | 10.6 | 13.8 | 13.7 | 14.1 | 13.1 | 13.7 | 14.0 | 13.3 | 14.3 | 14.7 |
| Spryd4 | 5.5 | 5.7 | 3.8 | 5.1 | 5.2 | 6.1 | 5.2 | 4.5 | 5.5 | 5.0 | 5.9 | 5.6 |
| Spryd7 | 12.8 | 11.0 | 11.2 | 11.3 | 11.9 | 10.8 | 12.6 | 12.8 | 10.8 | 11.3 | 12.2 | 14.0 |
| Spsb1 | 7.7 | 9.6 | 12.3 | 6.6 | 10.3 | 5.6 | 9.7 | 12.0 | 8.9 | 14.6 | 10.9 | 11.6 |
| Spsb2 | 6.7 | 6.4 | 6.2 | 5.4 | 6.6 | 6.2 | 6.1 | 5.7 | 6.7 | 9.1 | 5.6 | 6.8 |
| Spsb3 | 12.0 | 12.5 | 11.5 | 12.1 | 11.8 | 13.5 | 13.4 | 12.4 | 13.5 | 12.3 | 11.8 | 11.9 |
| Spsb4 | 0.2 | 0.2 | 0.1 | 0.1 | 0.3 | 0.1 | 0.5 | 0.1 | 0.3 | 0.3 | 0.2 | 0.0 |
| Spta1 | 8.1 | 6.7 | 6.2 | 8.5 | 6.8 | 8.7 | 6.5 | 8.8 | 4.7 | 2.8 | 5.1 | 3.3 |
| Sptan1 | 170.8 | 144.2 | 122.2 | 153.5 | 131.3 | 146.7 | 155.9 | 172.8 | 147.1 | 134.2 | 174.5 | 145.9 |
| Sptb | 0.2 | 0.2 | 0.1 | 0.1 | 0.1 | 0.1 | 0.1 | 0.1 | 0.1 | 0.2 | 0.2 | 0.1 |
| Sptbn1 | 341.6 | 269.8 | 256.3 | 324.5 | 249.1 | 319.0 | 287.8 | 269.2 | 247.5 | 217.9 | 296.6 | 274.6 |
| Sptbn2 | 0.6 | 0.5 | 0.5 | 1.2 | 0.9 | 0.8 | 1.2 | 0.7 | 0.7 | 0.5 | 0.5 | 0.4 |
| Sptbn4 | 0.0 | 0.1 | 0.0 | 0.0 | 0.1 | 0.0 | 0.0 | 0.0 | 0.0 | 0.1 | 0.1 | 0.0 |
| Sptlc1 | 19.3 | 15.1 | 14.0 | 20.0 | 17.5 | 15.9 | 17.7 | 16.3 | 17.8 | 18.7 | 18.9 | 16.9 |
| Sptlc2 | 23.1 | 26.0 | 23.4 | 23.1 | 26.6 | 17.6 | 25.5 | 28.5 | 27.4 | 31.2 | 29.3 | 26.0 |
| Sptssa | 15.6 | 15.1 | 15.8 | 17.8 | 18.1 | 18.0 | 15.1 | 15.9 | 16.7 | 16.3 | 16.6 | 17.6 |
| Spty2d1 | 8.0 | 7.9 | 8.1 | 8.2 | 8.0 | 7.2 | 8.1 | 7.8 | 7.1 | 7.7 | 8.0 | 8.1 |
| Sqle | 16.9 | 18.9 | 19.0 | 6.8 | 14.7 | 9.5 | 35.7 | 40.1 | 34.1 | 15.8 | 36.4 | 16.4 |
| Sqrdl | 45.9 | 36.2 | 28.2 | 46.1 | 35.1 | 45.8 | 30.3 | 28.2 | 23.4 | 27.1 | 26.9 | 27.1 |
| Sqstm1 | 546.4 | 377.7 | 243.2 | 809.2 | 455.8 | 786.9 | 326.3 | 263.0 | 258.9 | 365.3 | 339.9 | 333.0 |
| Sra1 | 79.4 | 82.3 | 74.9 | 65.2 | 63.5 | 68.6 | 64.2 | 77.0 | 68.2 | 74.6 | 73.0 | 78.6 |

| | | | | | | | | | | | | |
|---|---|---|---|---|---|---|---|---|---|---|---|---|
| Srbd1 | 5.2 | 5.3 | 6.3 | 5.6 | 5.5 | 5.4 | 5.0 | 5.9 | 5.0 | 5.6 | 5.8 | 6.2 |
| Src | 10.8 | 12.1 | 13.2 | 9.2 | 10.6 | 11.3 | 12.4 | 12.0 | 12.6 | 12.1 | 12.0 | 10.9 |
| Srcin1 | 0.0 | 0.0 | 0.0 | 0.0 | 0.0 | 0.0 | 0.0 | 0.0 | 0.0 | 0.0 | 0.0 | 0.0 |
| Srcrb4d | 0.4 | 0.4 | 0.3 | 0.4 | 0.4 | 0.4 | 0.3 | 0.5 | 0.3 | 0.3 | 0.5 | 0.4 |
| Srd5a1 | 0.7 | 0.6 | 0.6 | 0.6 | 0.7 | 0.8 | 0.6 | 0.6 | 0.6 | 0.7 | 0.6 | 0.5 |
| Srd5a3 | 12.6 | 11.1 | 8.8 | 13.2 | 10.5 | 13.8 | 8.6 | 9.3 | 9.8 | 10.3 | 8.1 | 10.2 |
| Srebf1 | 12.1 | 16.3 | 15.0 | 11.1 | 13.6 | 15.4 | 19.3 | 19.4 | 21.3 | 12.8 | 13.4 | 11.7 |
| Srebf2 | 7.8 | 10.5 | 12.3 | 5.6 | 10.4 | 6.9 | 17.0 | 19.3 | 17.0 | 9.2 | 12.9 | 8.6 |
| Srek1 | 8.8 | 11.0 | 12.5 | 9.8 | 9.1 | 8.6 | 10.1 | 10.9 | 7.9 | 8.6 | 12.0 | 11.2 |
| Srek1ip1 | 6.0 | 6.7 | 8.4 | 7.6 | 6.4 | 7.2 | 6.7 | 8.1 | 7.3 | 8.9 | 9.6 | 10.1 |
| Srf | 8.0 | 7.5 | 7.0 | 8.4 | 8.0 | 5.4 | 10.3 | 6.8 | 9.4 | 8.5 | 9.4 | 7.5 |
| Srfbp1 | 11.2 | 11.4 | 9.4 | 12.0 | 8.9 | 9.6 | 8.9 | 9.6 | 8.9 | 9.4 | 11.0 | 11.4 |
| Srgap1 | 0.5 | 0.6 | 0.7 | 0.3 | 0.5 | 0.4 | 0.5 | 0.7 | 0.5 | 0.7 | 0.6 | 0.5 |
| Srgap2 | 7.2 | 9.4 | 8.9 | 5.7 | 7.1 | 6.0 | 7.5 | 9.6 | 9.0 | 7.7 | 9.0 | 7.5 |
| Srgap3 | 4.8 | 6.2 | 4.7 | 6.4 | 5.1 | 9.0 | 4.1 | 2.9 | 2.3 | 3.5 | 3.9 | 3.2 |
| Srgn | 1.4 | 0.8 | 1.4 | 7.6 | 18.8 | 0.3 | 2.0 | 0.6 | 0.6 | 1.7 | 0.6 | 1.2 |
| Sri | 33.9 | 33.7 | 36.8 | 42.0 | 35.6 | 36.5 | 38.8 | 36.6 | 35.7 | 32.3 | 37.6 | 38.4 |
| Srl | 0.4 | 0.5 | 0.5 | 0.3 | 0.4 | 0.4 | 0.2 | 0.5 | 0.4 | 0.3 | 0.3 | 0.2 |
| Srm | 32.7 | 28.9 | 24.8 | 31.3 | 27.3 | 28.2 | 32.3 | 31.9 | 28.1 | 30.9 | 33.6 | 24.1 |
| Srms | 0.0 | 0.0 | 0.0 | 0.0 | 0.0 | 0.0 | 0.0 | 0.0 | 0.0 | 0.0 | 0.0 | 0.0 |
| Srp14 | 196.0 | 153.2 | 138.6 | 223.0 | 159.2 | 206.9 | 144.3 | 145.4 | 138.3 | 170.7 | 173.0 | 175.3 |
| Srp19 | 60.9 | 51.0 | 62.5 | 72.0 | 56.7 | 57.5 | 64.3 | 60.4 | 48.2 | 56.1 | 65.0 | 70.5 |
| Srp54a | 43.2 | 40.1 | 44.8 | 46.1 | 41.7 | 43.7 | 50.0 | 43.7 | 45.1 | 42.3 | 51.7 | 58.0 |
| Srp54b | 30.3 | 24.9 | 29.3 | 29.0 | 28.5 | 28.8 | 30.7 | 27.0 | 30.5 | 28.6 | 31.1 | 35.3 |
| Srp54c | 45.1 | 44.2 | 51.6 | 50.5 | 49.9 | 41.9 | 64.8 | 50.6 | 48.0 | 42.6 | 62.0 | 61.7 |
| Srp68 | 17.7 | 20.4 | 21.2 | 19.4 | 17.4 | 19.5 | 18.1 | 21.4 | 17.4 | 19.6 | 19.2 | 20.0 |
| Srp72 | 41.2 | 37.4 | 36.4 | 38.0 | 37.4 | 37.4 | 33.6 | 38.2 | 32.1 | 41.0 | 42.1 | 38.1 |
| Srp9 | 76.7 | 54.5 | 48.2 | 69.4 | 56.1 | 61.7 | 53.8 | 51.5 | 51.0 | 61.8 | 62.7 | 60.0 |
| Srpk1 | 21.3 | 20.8 | 20.6 | 20.2 | 19.1 | 21.6 | 23.0 | 21.8 | 19.1 | 20.3 | 24.0 | 21.2 |
| Srpk2 | 11.3 | 10.4 | 11.0 | 12.4 | 9.6 | 11.9 | 10.2 | 8.9 | 8.3 | 10.3 | 10.6 | 11.6 |
| Srpk3 | 0.0 | 0.0 | 0.0 | 0.1 | 0.5 | 0.1 | 0.1 | 0.0 | 0.1 | 0.0 | 0.0 | 0.0 |
| Srpr | 51.5 | 46.1 | 43.4 | 55.9 | 46.3 | 46.8 | 49.2 | 47.3 | 46.0 | 47.7 | 49.7 | 49.3 |
| Srprb | 22.6 | 19.8 | 17.3 | 27.4 | 18.6 | 20.3 | 23.9 | 22.6 | 20.8 | 20.7 | 23.2 | 21.6 |
| Srpx | 0.7 | 2.8 | 4.9 | 0.6 | 1.1 | 0.4 | 1.2 | 2.9 | 3.8 | 11.3 | 7.3 | 4.1 |
| Srpx2 | 39.2 | 49.8 | 60.8 | 76.7 | 66.4 | 53.1 | 75.7 | 75.6 | 73.0 | 34.1 | 39.8 | 50.3 |
| Srr | 12.8 | 11.2 | 11.2 | 12.9 | 9.7 | 13.0 | 9.8 | 10.0 | 9.3 | 9.1 | 10.3 | 10.2 |
| Srrd | 6.1 | 5.3 | 4.1 | 4.2 | 5.5 | 6.2 | 4.6 | 5.3 | 5.4 | 6.1 | 5.2 | 5.0 |
| Srrm1 | 5.1 | 5.7 | 7.0 | 6.7 | 5.9 | 6.7 | 8.1 | 6.5 | 5.5 | 5.4 | 6.1 | 6.0 |
| Srrm2 | 12.6 | 16.3 | 18.7 | 11.8 | 14.3 | 12.4 | 18.2 | 16.5 | 15.5 | 14.4 | 13.8 | 13.2 |
| Srrm3 | 0.0 | 0.1 | 0.2 | 0.0 | 0.0 | 0.0 | 0.0 | 0.2 | 0.1 | 0.1 | 0.1 | 0.2 |
| Srrm4 | 0.0 | 0.0 | 0.1 | 0.1 | 0.1 | 0.1 | 0.0 | 0.0 | 0.0 | 0.1 | 0.1 | 0.1 |
| Srrt | 21.9 | 21.2 | 23.9 | 24.4 | 22.0 | 19.6 | 27.6 | 24.7 | 21.9 | 24.0 | 25.1 | 24.5 |
| Srsf1 | 18.7 | 16.6 | 21.5 | 19.7 | 18.7 | 16.8 | 20.8 | 19.3 | 19.2 | 19.7 | 22.5 | 22.3 |
| Srsf10 | 21.2 | 23.2 | 26.7 | 25.0 | 23.4 | 23.3 | 23.3 | 23.6 | 23.6 | 25.3 | 30.0 | 28.6 |
| Srsf11 | 18.8 | 18.5 | 23.3 | 20.3 | 17.2 | 18.6 | 21.3 | 18.6 | 16.1 | 17.1 | 20.9 | 21.8 |
| Srsf12 | 0.2 | 0.2 | 0.2 | 0.1 | 0.1 | 0.1 | 0.0 | 0.0 | 0.1 | 0.1 | 0.0 | 0.1 |
| Srsf2 | 32.0 | 29.7 | 31.2 | 33.1 | 33.2 | 28.2 | 40.2 | 33.5 | 37.7 | 30.9 | 34.6 | 31.5 |
| Srsf3 | 28.6 | 27.2 | 28.5 | 27.5 | 28.9 | 27.3 | 32.4 | 27.8 | 31.4 | 32.5 | 34.1 | 32.3 |
| Srsf4 | 7.7 | 9.1 | 11.7 | 8.2 | 8.3 | 8.3 | 9.2 | 10.2 | 8.6 | 8.3 | 9.9 | 8.5 |
| Srsf5 | 114.9 | 110.6 | 129.8 | 123.6 | 102.1 | 102.7 | 123.3 | 122.1 | 124.7 | 124.9 | 121.5 | 142.1 |
| Srsf6 | 26.6 | 25.7 | 24.3 | 24.6 | 25.4 | 23.9 | 27.2 | 28.1 | 28.1 | 30.4 | 30.2 | 28.9 |
| Srsf7 | 13.1 | 14.0 | 15.5 | 15.2 | 15.7 | 15.5 | 16.1 | 15.5 | 16.0 | 18.3 | 17.6 | 14.7 |
| Srsf9 | 28.0 | 23.1 | 25.7 | 24.0 | 26.9 | 24.5 | 26.7 | 24.9 | 24.6 | 30.7 | 26.7 | 29.5 |
| Srxn1 | 80.0 | 45.5 | 28.2 | 136.8 | 83.7 | 101.1 | 35.8 | 30.6 | 22.4 | 34.8 | 33.0 | 27.5 |
| Ss18 | 26.2 | 27.2 | 29.1 | 30.9 | 32.0 | 25.2 | 37.1 | 27.8 | 33.0 | 30.6 | 37.4 | 31.2 |
| Ss18l1 | 1.5 | 1.7 | 1.8 | 1.6 | 1.7 | 1.7 | 1.5 | 1.4 | 1.7 | 1.4 | 1.9 | 1.6 |
| Ssb | 75.5 | 69.5 | 76.0 | 80.4 | 65.8 | 70.1 | 71.2 | 67.0 | 59.8 | 76.3 | 85.7 | 86.0 |

| | | | | | | | | | | | | |
|---|---|---|---|---|---|---|---|---|---|---|---|---|
| Ssbp1 | 18.9 | 16.1 | 21.1 | 21.1 | 15.6 | 19.3 | 19.1 | 17.2 | 14.9 | 16.7 | 22.4 | 21.0 |
| Ssbp2 | 4.0 | 4.0 | 5.5 | 3.7 | 4.2 | 2.9 | 4.1 | 4.6 | 4.0 | 3.8 | 4.7 | 4.9 |
| Ssbp3 | 8.2 | 7.7 | 8.6 | 7.3 | 6.8 | 7.9 | 6.8 | 9.1 | 7.9 | 13.1 | 11.3 | 8.8 |
| Ssbp4 | 7.6 | 8.2 | 8.3 | 8.0 | 9.1 | 7.0 | 7.8 | 11.0 | 8.7 | 9.6 | 7.2 | 7.4 |
| Ssc5d | 1.2 | 1.5 | 2.2 | 1.2 | 1.1 | 1.4 | 1.2 | 1.8 | 1.2 | 1.4 | 1.0 | 0.9 |
| Ssfa2 | 26.7 | 33.3 | 33.9 | 34.9 | 26.4 | 32.6 | 22.7 | 23.9 | 22.4 | 21.1 | 21.4 | 23.6 |
| Ssh1 | 3.6 | 4.5 | 3.7 | 4.0 | 4.2 | 4.2 | 5.4 | 4.8 | 5.1 | 4.3 | 4.0 | 4.0 |
| Ssh2 | 1.6 | 3.0 | 4.4 | 2.5 | 3.7 | 2.0 | 2.0 | 3.6 | 2.5 | 2.4 | 1.9 | 2.5 |
| Ssh3 | 9.7 | 10.0 | 10.4 | 7.9 | 8.1 | 8.4 | 6.9 | 7.3 | 7.2 | 9.9 | 8.6 | 9.6 |
| Ssna1 | 32.6 | 25.7 | 20.9 | 28.4 | 27.8 | 24.6 | 23.3 | 22.8 | 27.8 | 28.0 | 27.5 | 24.0 |
| Sspn | 13.2 | 14.5 | 16.5 | 14.0 | 9.9 | 17.2 | 12.7 | 12.4 | 12.4 | 12.6 | 13.5 | 14.4 |
| Sspo | 0.0 | 0.0 | 0.0 | 0.0 | 0.0 | 0.0 | 0.0 | 0.0 | 0.0 | 0.0 | 0.0 | 0.0 |
| Ssr1 | 27.4 | 25.3 | 27.2 | 35.3 | 27.8 | 29.6 | 30.2 | 28.1 | 26.0 | 28.6 | 31.7 | 33.7 |
| Ssr2 | 67.7 | 59.7 | 61.6 | 65.5 | 62.1 | 63.6 | 61.5 | 75.9 | 67.5 | 67.4 | 69.2 | 63.4 |
| Ssr3 | 177.9 | 169.7 | 172.5 | 195.1 | 179.2 | 174.6 | 187.9 | 192.2 | 196.3 | 208.5 | 220.0 | 222.4 |
| Ssr4 | 160.7 | 128.9 | 117.6 | 152.4 | 128.6 | 128.6 | 123.4 | 137.1 | 123.4 | 129.9 | 134.9 | 131.4 |
| Ssrp1 | 40.7 | 38.7 | 45.8 | 40.3 | 36.3 | 39.8 | 41.8 | 39.1 | 33.4 | 35.4 | 42.2 | 41.0 |
| Sssca1 | 16.8 | 12.8 | 13.7 | 16.5 | 13.4 | 15.3 | 12.3 | 12.7 | 12.2 | 13.7 | 14.7 | 13.4 |
| Sstr2 | 0.0 | 0.0 | 0.0 | 0.0 | 0.1 | 0.1 | 0.1 | 0.1 | 0.0 | 0.3 | 0.0 | 0.0 |
| Sstr3 | 0.0 | 0.0 | 0.1 | 0.0 | 0.0 | 0.0 | 0.1 | 0.0 | 0.0 | 0.0 | 0.0 | 0.0 |
| Sstr5 | 0.0 | 0.0 | 0.0 | 0.1 | 0.1 | 0.1 | 0.0 | 0.0 | 0.0 | 0.0 | 0.0 | 0.0 |
| Ssu72 | 37.2 | 36.4 | 36.8 | 37.5 | 36.3 | 38.0 | 38.6 | 37.0 | 33.5 | 34.4 | 39.2 | 39.8 |
| Ssx2ip | 4.4 | 4.2 | 4.7 | 3.0 | 3.6 | 4.0 | 3.7 | 3.5 | 3.3 | 5.8 | 4.8 | 3.6 |
| Ssxb1 | 0.0 | 0.0 | 0.0 | 0.0 | 0.0 | 0.0 | 0.0 | 0.0 | 0.0 | 0.0 | 0.0 | 0.0 |
| Ssxb10 | 0.0 | 0.0 | 0.1 | 0.0 | 0.1 | 0.0 | 0.1 | 0.0 | 0.0 | 0.0 | 0.0 | 0.0 |
| Ssxb2 | 0.0 | 0.0 | 0.1 | 0.0 | 0.0 | 0.0 | 0.0 | 0.0 | 0.0 | 0.0 | 0.0 | 0.0 |
| Ssxb3 | 0.0 | 0.0 | 0.1 | 0.0 | 0.0 | 0.0 | 0.0 | 0.0 | 0.0 | 0.0 | 0.0 | 0.0 |
| Ssxb5 | 0.1 | 0.0 | 0.1 | 0.0 | 0.1 | 0.0 | 0.1 | 0.0 | 0.0 | 0.0 | 0.0 | 0.0 |
| Ssxb8 | 0.0 | 0.3 | 0.1 | 0.0 | 0.0 | 0.0 | 0.0 | 0.0 | 0.0 | 0.0 | 0.0 | 0.0 |
| Ssxb9 | 0.0 | 0.0 | 0.0 | 0.0 | 0.0 | 0.0 | 0.0 | 0.0 | 0.0 | 0.0 | 0.0 | 0.0 |
| St13 | 172.7 | 152.5 | 166.7 | 201.0 | 135.0 | 179.5 | 145.0 | 122.7 | 116.6 | 161.1 | 180.1 | 195.4 |
| St14 | 0.8 | 0.6 | 0.5 | 0.6 | 0.7 | 0.7 | 0.4 | 0.2 | 0.1 | 0.3 | 0.2 | 0.3 |
| St18 | 0.1 | 0.0 | 0.0 | 0.3 | 0.7 | 0.0 | 0.1 | 0.0 | 0.0 | 0.0 | 0.0 | 0.0 |
| St3gal1 | 4.9 | 7.9 | 9.9 | 3.8 | 7.4 | 3.1 | 8.2 | 14.3 | 11.5 | 10.4 | 7.5 | 7.4 |
| St3gal2 | 9.2 | 9.7 | 7.8 | 9.5 | 9.4 | 9.9 | 10.6 | 8.7 | 9.2 | 9.2 | 9.1 | 9.0 |
| St3gal3 | 20.3 | 18.3 | 13.9 | 19.9 | 17.1 | 19.4 | 22.9 | 16.2 | 19.9 | 17.1 | 22.0 | 17.8 |
| St3gal4 | 8.6 | 9.2 | 11.1 | 5.4 | 10.2 | 7.6 | 12.0 | 21.6 | 10.9 | 10.1 | 10.4 | 9.6 |
| St3gal5 | 10.1 | 7.5 | 7.7 | 17.0 | 15.6 | 10.2 | 12.4 | 9.6 | 11.4 | 13.6 | 13.7 | 16.2 |
| St3gal6 | 3.0 | 3.1 | 2.4 | 2.9 | 5.1 | 2.0 | 1.6 | 2.0 | 1.7 | 3.5 | 2.3 | 2.0 |
| St5 | 20.5 | 22.3 | 20.0 | 19.1 | 21.4 | 13.4 | 26.4 | 21.8 | 27.1 | 21.8 | 21.1 | 24.6 |
| St6gal1 | 0.5 | 0.2 | 0.5 | 0.7 | 1.5 | 0.3 | 0.6 | 0.9 | 1.2 | 0.6 | 0.5 | 1.1 |
| St6galnac1 | 0.0 | 0.1 | 0.1 | 0.0 | 0.0 | 0.0 | 0.0 | 0.0 | 0.0 | 0.0 | 0.1 | 0.0 |
| St6galnac2 | 0.3 | 0.7 | 1.8 | 0.3 | 0.7 | 0.3 | 0.8 | 0.8 | 0.7 | 0.4 | 0.7 | 0.6 |
| St6galnac3 | 0.1 | 0.0 | 0.0 | 0.0 | 0.0 | 0.0 | 0.0 | 0.0 | 0.0 | 0.0 | 0.0 | 0.0 |
| St6galnac4 | 5.2 | 4.9 | 4.6 | 6.5 | 5.8 | 6.1 | 6.7 | 4.7 | 6.6 | 5.9 | 5.6 | 5.4 |
| St6galnac5 | 0.0 | 0.0 | 0.0 | 0.0 | 0.0 | 0.0 | 0.0 | 0.0 | 0.0 | 0.0 | 0.0 | 0.0 |
| St6galnac6 | 21.0 | 23.7 | 19.8 | 19.3 | 20.1 | 24.3 | 23.5 | 19.3 | 25.1 | 18.4 | 19.7 | 18.8 |
| St7 | 9.6 | 7.1 | 6.3 | 7.5 | 6.3 | 7.4 | 8.5 | 7.5 | 6.7 | 6.4 | 7.7 | 7.1 |
| St7l | 3.3 | 3.6 | 3.1 | 3.5 | 3.6 | 3.5 | 3.2 | 3.1 | 3.3 | 3.7 | 3.8 | 3.8 |
| St8sia1 | 0.0 | 0.0 | 0.0 | 0.0 | 0.1 | 0.0 | 0.0 | 0.0 | 0.0 | 0.0 | 0.0 | 0.0 |
| St8sia2 | 0.0 | 0.0 | 0.0 | 0.0 | 0.0 | 0.0 | 0.0 | 0.1 | 0.1 | 0.0 | 0.0 | 0.0 |
| St8sia4 | 0.4 | 0.0 | 0.2 | 0.5 | 0.4 | 0.0 | 0.0 | 0.1 | 0.2 | 0.0 | 0.0 | 0.0 |
| St8sia6 | 0.1 | 0.0 | 0.0 | 0.1 | 0.0 | 0.0 | 0.1 | 0.0 | 0.0 | 0.0 | 0.0 | 0.0 |
| Stab1 | 1.3 | 0.1 | 0.2 | 5.4 | 9.0 | 0.2 | 0.9 | 0.5 | 0.5 | 0.8 | 0.3 | 0.8 |
| Stab2 | 0.0 | 0.0 | 0.0 | 0.0 | 0.0 | 0.0 | 0.0 | 0.0 | 0.0 | 0.0 | 0.0 | 0.0 |
| Stac | 1.9 | 1.5 | 1.2 | 1.3 | 0.6 | 1.3 | 0.6 | 0.8 | 0.6 | 0.8 | 1.3 | 1.4 |
| Stac2 | 0.0 | 0.0 | 0.1 | 0.1 | 0.1 | 0.1 | 0.0 | 0.0 | 0.0 | 0.0 | 0.0 | 0.0 |

| | | | | | | | | | | | | |
|---|---|---|---|---|---|---|---|---|---|---|---|---|
| Stac3 | 0.1 | 0.1 | 0.2 | 0.0 | 0.0 | 0.0 | 0.2 | 0.0 | 0.1 | 0.2 | 0.1 | 0.2 |
| Stag1 | 7.1 | 7.8 | 8.2 | 6.9 | 7.2 | 6.0 | 8.4 | 9.7 | 8.9 | 9.2 | 9.4 | 9.7 |
| Stag2 | 19.6 | 21.6 | 20.7 | 18.9 | 17.5 | 19.7 | 17.5 | 17.8 | 18.2 | 20.7 | 21.4 | 23.5 |
| Stag3 | 0.2 | 0.3 | 0.2 | 0.2 | 0.2 | 0.2 | 0.2 | 0.2 | 0.2 | 0.2 | 0.1 | 0.1 |
| Stam | 13.0 | 12.6 | 11.1 | 14.4 | 11.5 | 13.7 | 11.6 | 10.3 | 11.3 | 12.5 | 14.3 | 13.2 |
| Stam2 | 14.2 | 12.4 | 11.7 | 14.3 | 13.5 | 13.1 | 12.9 | 13.0 | 12.7 | 13.1 | 14.7 | 14.9 |
| Stambp | 3.6 | 5.7 | 6.1 | 5.1 | 4.6 | 5.2 | 5.1 | 5.1 | 4.3 | 4.6 | 5.0 | 5.1 |
| Stambpl1 | 12.9 | 14.8 | 14.0 | 12.2 | 13.4 | 10.7 | 13.0 | 12.9 | 13.2 | 8.2 | 12.4 | 11.8 |
| Stap1 | 3.0 | 0.0 | 0.2 | 20.5 | 47.8 | 0.0 | 6.4 | 0.4 | 1.4 | 3.8 | 1.3 | 4.2 |
| Stap2 | 1.4 | 0.6 | 0.7 | 0.6 | 0.6 | 0.8 | 0.6 | 0.5 | 0.4 | 1.1 | 0.7 | 0.3 |
| Star | 2.3 | 2.3 | 1.2 | 2.7 | 2.6 | 5.2 | 1.9 | 1.1 | 1.1 | 6.2 | 3.7 | 2.3 |
| Stard10 | 6.4 | 4.8 | 6.9 | 2.5 | 3.2 | 4.5 | 3.6 | 5.7 | 5.1 | 8.5 | 4.4 | 5.8 |
| Stard13 | 7.4 | 8.5 | 7.2 | 7.2 | 6.7 | 7.5 | 9.0 | 10.1 | 11.7 | 9.4 | 9.7 | 9.4 |
| Stard3 | 6.4 | 5.8 | 5.7 | 8.2 | 7.9 | 7.4 | 6.9 | 6.5 | 7.7 | 7.9 | 7.2 | 6.3 |
| Stard3nl | 31.3 | 27.2 | 18.3 | 25.4 | 28.8 | 26.4 | 23.1 | 21.8 | 30.6 | 28.6 | 27.7 | 23.9 |
| Stard4 | 3.5 | 6.3 | 6.5 | 1.2 | 3.9 | 2.0 | 7.0 | 10.6 | 8.2 | 3.4 | 7.0 | 3.7 |
| Stard5 | 7.2 | 7.1 | 7.3 | 6.4 | 6.2 | 5.0 | 7.3 | 7.8 | 6.5 | 6.4 | 6.1 | 6.2 |
| Stard6 | 5.0 | 4.9 | 4.7 | 4.6 | 3.1 | 4.7 | 3.4 | 3.5 | 3.5 | 4.0 | 3.9 | 3.8 |
| Stard7 | 14.1 | 15.6 | 12.7 | 12.6 | 11.7 | 13.6 | 11.0 | 11.6 | 12.9 | 12.9 | 10.7 | 12.5 |
| Stard8 | 4.4 | 6.5 | 6.7 | 4.3 | 5.5 | 4.2 | 5.0 | 4.1 | 5.2 | 3.6 | 4.1 | 4.3 |
| Stat1 | 15.0 | 11.1 | 17.9 | 17.2 | 16.6 | 11.8 | 13.2 | 13.4 | 13.7 | 16.6 | 14.2 | 22.1 |
| Stat2 | 10.6 | 8.7 | 12.7 | 10.8 | 10.1 | 8.4 | 10.5 | 9.8 | 9.4 | 3.9 | 6.7 | 11.3 |
| Stat3 | 49.1 | 66.8 | 72.9 | 40.9 | 61.9 | 34.5 | 66.1 | 69.0 | 64.9 | 59.4 | 59.6 | 60.5 |
| Stat4 | 0.1 | 0.0 | 0.0 | 0.4 | 0.2 | 0.0 | 0.1 | 0.1 | 0.0 | 0.1 | 0.1 | 0.0 |
| Stat5a | 3.9 | 4.1 | 4.3 | 3.4 | 3.5 | 3.6 | 4.5 | 3.8 | 4.0 | 3.1 | 2.8 | 3.7 |
| Stat5b | 8.6 | 9.6 | 10.1 | 8.3 | 8.3 | 8.0 | 9.5 | 8.8 | 9.3 | 7.0 | 7.5 | 8.3 |
| Stat6 | 33.6 | 37.4 | 34.0 | 43.6 | 39.4 | 36.7 | 42.0 | 39.2 | 39.8 | 32.4 | 36.2 | 37.5 |
| Stau1 | 23.3 | 27.4 | 27.8 | 25.6 | 24.7 | 27.6 | 31.1 | 28.5 | 27.2 | 26.3 | 27.5 | 29.3 |
| Stau2 | 7.3 | 6.5 | 5.7 | 6.9 | 6.0 | 7.7 | 4.4 | 4.0 | 4.3 | 5.2 | 5.5 | 5.1 |
| Stbd1 | 11.8 | 7.7 | 5.5 | 9.7 | 8.7 | 11.6 | 13.5 | 5.5 | 24.6 | 22.0 | 22.5 | 26.4 |
| Stc1 | 0.1 | 0.1 | 0.5 | 0.0 | 0.0 | 0.0 | 0.1 | 0.1 | 0.1 | 0.1 | 0.1 | 0.1 |
| Stc2 | 4.2 | 3.2 | 2.4 | 6.1 | 4.2 | 4.1 | 4.0 | 5.4 | 2.9 | 2.5 | 2.8 | 2.3 |
| Steap1 | 10.1 | 9.7 | 8.7 | 9.0 | 11.5 | 7.7 | 12.7 | 8.8 | 8.0 | 14.6 | 10.2 | 10.4 |
| Steap2 | 16.9 | 16.5 | 13.1 | 21.5 | 17.4 | 20.6 | 20.0 | 10.6 | 12.2 | 18.1 | 17.1 | 16.1 |
| Steap3 | 2.4 | 5.8 | 10.4 | 2.7 | 3.8 | 2.6 | 3.0 | 9.6 | 3.8 | 3.0 | 2.6 | 3.2 |
| Steap4 | 1.5 | 0.6 | 2.8 | 0.1 | 0.4 | 0.0 | 0.4 | 3.1 | 1.2 | 3.1 | 1.3 | 1.7 |
| Stil | 0.5 | 0.9 | 1.0 | 0.4 | 1.2 | 0.2 | 1.2 | 0.6 | 0.9 | 0.5 | 0.8 | 0.5 |
| Stim1 | 23.7 | 25.4 | 17.5 | 26.9 | 18.5 | 29.3 | 17.2 | 13.4 | 15.5 | 18.1 | 16.8 | 13.2 |
| Stim2 | 11.7 | 11.0 | 12.1 | 12.2 | 10.5 | 11.7 | 12.9 | 11.6 | 10.6 | 12.8 | 13.7 | 11.9 |
| Stip1 | 64.5 | 68.8 | 65.4 | 70.2 | 54.4 | 54.4 | 62.4 | 57.5 | 55.3 | 66.4 | 71.9 | 66.7 |
| Stk10 | 2.5 | 2.1 | 1.8 | 4.5 | 6.5 | 1.7 | 3.0 | 2.7 | 1.7 | 3.5 | 3.1 | 2.5 |
| Stk11 | 32.3 | 32.0 | 30.0 | 37.5 | 31.9 | 38.4 | 31.4 | 29.6 | 28.9 | 27.8 | 30.1 | 30.5 |
| Stk11ip | 1.9 | 1.7 | 1.7 | 2.4 | 2.4 | 2.1 | 2.2 | 2.0 | 2.0 | 2.1 | 1.7 | 1.7 |
| Stk16 | 10.7 | 9.9 | 9.6 | 10.6 | 9.7 | 11.3 | 10.0 | 10.6 | 9.9 | 9.9 | 9.6 | 9.9 |
| Stk17b | 8.8 | 6.6 | 10.6 | 13.5 | 19.4 | 10.1 | 9.7 | 6.5 | 8.8 | 9.9 | 7.6 | 12.7 |
| Stk19 | 10.1 | 7.5 | 5.5 | 10.6 | 9.2 | 9.2 | 8.1 | 8.8 | 8.6 | 9.0 | 8.9 | 7.5 |
| Stk24 | 19.0 | 22.0 | 20.7 | 21.7 | 19.3 | 19.3 | 21.6 | 16.9 | 19.0 | 20.0 | 20.9 | 20.0 |
| Stk25 | 29.4 | 26.6 | 27.5 | 28.8 | 26.8 | 29.7 | 31.3 | 27.0 | 31.3 | 24.1 | 28.3 | 29.0 |
| Stk3 | 16.4 | 17.3 | 21.9 | 19.3 | 14.2 | 17.1 | 20.3 | 19.3 | 17.5 | 16.1 | 23.8 | 22.7 |
| Stk30 | 1.2 | 0.8 | 0.7 | 0.8 | 0.4 | 1.5 | 0.6 | 0.7 | 0.3 | 0.5 | 0.5 | 0.6 |
| Stk31 | 0.1 | 0.1 | 0.0 | 0.1 | 0.0 | 0.0 | 0.1 | 0.0 | 0.0 | 0.0 | 0.0 | 0.0 |
| Stk32a | 0.0 | 0.0 | 0.1 | 0.0 | 0.0 | 0.0 | 0.0 | 0.0 | 0.0 | 0.0 | 0.0 | 0.0 |
| Stk32b | 0.1 | 0.1 | 0.2 | 0.1 | 0.1 | 0.1 | 0.1 | 0.1 | 0.0 | 0.0 | 0.0 | 0.0 |
| Stk32c | 0.5 | 0.6 | 0.5 | 0.9 | 0.3 | 0.6 | 0.6 | 0.2 | 0.4 | 0.1 | 0.2 | 0.2 |
| Stk33 | 0.1 | 0.1 | 0.2 | 0.1 | 0.1 | 0.1 | 0.2 | 0.2 | 0.1 | 0.0 | 0.1 | 0.0 |
| Stk35 | 2.8 | 3.4 | 3.4 | 2.5 | 2.8 | 3.0 | 2.6 | 3.0 | 2.7 | 2.8 | 2.6 | 2.5 |
| Stk36 | 1.1 | 1.0 | 0.9 | 1.0 | 0.9 | 1.3 | 0.6 | 0.8 | 0.7 | 0.9 | 0.8 | 0.7 |

| | | | | | | | | | | | | |
|---|---|---|---|---|---|---|---|---|---|---|---|---|
| Stk38 | 18.2 | 19.8 | 22.0 | 18.4 | 18.6 | 18.5 | 18.0 | 18.9 | 19.6 | 15.7 | 20.2 | 19.4 |
| Stk38l | 8.6 | 7.3 | 7.5 | 8.4 | 8.7 | 6.8 | 6.0 | 5.5 | 4.8 | 5.4 | 5.6 | 6.0 |
| Stk39 | 19.8 | 12.9 | 10.8 | 19.4 | 11.3 | 16.3 | 13.2 | 8.7 | 12.5 | 20.3 | 18.4 | 16.4 |
| Stk4 | 13.0 | 12.8 | 10.4 | 17.4 | 13.6 | 13.4 | 12.0 | 10.0 | 10.0 | 10.6 | 13.1 | 10.7 |
| Stk40 | 5.3 | 5.5 | 5.3 | 6.2 | 7.0 | 4.1 | 5.4 | 5.2 | 4.7 | 5.7 | 6.0 | 4.1 |
| Stmn1 | 18.0 | 24.6 | 39.4 | 11.3 | 37.7 | 10.9 | 38.6 | 13.3 | 31.1 | 22.6 | 31.8 | 21.3 |
| Stmn1-rs1 | 0.0 | 0.0 | 0.1 | 0.0 | 0.1 | 0.0 | 0.1 | 0.0 | 0.0 | 0.0 | 0.3 | 0.0 |
| Stmn2 | 0.2 | 0.0 | 0.0 | 0.0 | 0.0 | 0.1 | 0.1 | 0.0 | 0.0 | 0.0 | 0.0 | 0.1 |
| Stmn4 | 0.0 | 0.0 | 0.0 | 0.0 | 0.2 | 0.0 | 0.1 | 0.0 | 0.1 | 0.0 | 0.1 | 0.0 |
| Stmnd1 | 0.0 | 0.0 | 0.0 | 0.0 | 0.0 | 0.0 | 0.1 | 0.0 | 0.0 | 0.0 | 0.0 | 0.0 |
| Stom | 24.5 | 22.0 | 18.3 | 35.7 | 36.1 | 29.4 | 22.2 | 15.3 | 19.5 | 29.0 | 24.1 | 22.4 |
| Stoml1 | 14.8 | 13.4 | 11.9 | 15.6 | 12.1 | 17.5 | 13.7 | 11.6 | 14.0 | 10.8 | 11.9 | 13.0 |
| Stoml2 | 19.7 | 21.0 | 19.9 | 16.1 | 19.3 | 19.6 | 17.4 | 23.0 | 17.7 | 21.0 | 21.3 | 20.7 |
| Ston1 | 27.4 | 23.5 | 15.4 | 37.7 | 31.8 | 26.8 | 37.8 | 15.3 | 27.4 | 27.3 | 28.0 | 25.4 |
| Ston2 | 0.6 | 0.8 | 1.1 | 0.3 | 0.6 | 0.4 | 0.8 | 1.7 | 0.7 | 0.5 | 1.1 | 0.6 |
| Stox1 | 0.2 | 0.1 | 0.4 | 0.1 | 0.1 | 0.1 | 0.0 | 0.1 | 0.1 | 0.0 | 0.1 | 0.1 |
| Stox2 | 3.6 | 3.8 | 3.5 | 3.5 | 3.5 | 3.4 | 3.7 | 2.9 | 3.0 | 2.8 | 3.3 | 3.4 |
| Stpg1 | 0.1 | 0.1 | 0.1 | 0.1 | 0.0 | 0.1 | 0.0 | 0.1 | 0.0 | 0.0 | 0.1 | 0.1 |
| Stpg2 | 0.1 | 0.0 | 0.0 | 0.1 | 0.0 | 0.0 | 0.0 | 0.0 | 0.0 | 0.0 | 0.1 | 0.0 |
| Stra13 | 10.5 | 10.2 | 10.6 | 15.0 | 12.6 | 10.6 | 8.9 | 12.0 | 9.8 | 10.7 | 12.6 | 9.4 |
| Stra6 | 0.0 | 0.0 | 0.0 | 0.0 | 0.0 | 0.0 | 0.0 | 0.0 | 0.0 | 0.0 | 0.0 | 0.0 |
| Strada | 5.5 | 5.8 | 6.1 | 5.6 | 5.3 | 5.6 | 6.0 | 6.2 | 5.7 | 5.5 | 6.0 | 4.7 |
| Stradb | 9.9 | 9.0 | 6.7 | 7.9 | 6.9 | 9.7 | 6.3 | 5.2 | 6.7 | 7.1 | 6.9 | 7.1 |
| Strap | 51.7 | 47.3 | 41.6 | 49.6 | 46.2 | 49.8 | 44.7 | 42.2 | 41.2 | 47.9 | 48.2 | 44.8 |
| Strbp | 2.5 | 2.0 | 2.0 | 2.2 | 1.9 | 2.6 | 1.3 | 1.3 | 1.2 | 1.9 | 2.0 | 2.0 |
| Strip1 | 8.6 | 9.3 | 10.1 | 9.4 | 9.1 | 9.3 | 11.9 | 10.3 | 10.4 | 9.4 | 9.3 | 9.5 |
| Strip2 | 0.1 | 0.3 | 0.8 | 0.1 | 0.3 | 0.0 | 0.2 | 0.5 | 0.2 | 0.1 | 0.2 | 0.3 |
| Strn | 7.7 | 8.7 | 9.2 | 8.9 | 7.9 | 7.6 | 8.8 | 8.8 | 8.9 | 9.2 | 10.0 | 10.1 |
| Strn3 | 55.5 | 63.6 | 63.3 | 59.1 | 42.3 | 56.7 | 49.5 | 47.6 | 52.5 | 50.3 | 61.2 | 63.0 |
| Strn4 | 15.9 | 16.1 | 13.9 | 16.7 | 15.1 | 15.1 | 15.2 | 12.4 | 15.4 | 13.2 | 14.4 | 13.4 |
| Stt3a | 95.7 | 81.8 | 84.1 | 93.4 | 87.8 | 88.9 | 89.7 | 99.3 | 88.2 | 95.3 | 96.5 | 95.2 |
| Stt3b | 52.2 | 50.8 | 46.4 | 50.5 | 51.2 | 51.4 | 56.3 | 60.9 | 55.4 | 54.6 | 57.4 | 53.9 |
| Stub1 | 36.4 | 37.2 | 33.3 | 37.7 | 31.5 | 38.9 | 39.9 | 42.4 | 38.9 | 37.1 | 35.5 | 33.2 |
| Stx11 | 1.8 | 1.8 | 1.9 | 1.4 | 2.6 | 2.6 | 2.3 | 2.4 | 2.3 | 2.1 | 2.1 | 1.8 |
| Stx12 | 33.6 | 32.9 | 33.5 | 35.5 | 33.6 | 34.3 | 35.2 | 31.3 | 33.7 | 34.5 | 40.1 | 38.4 |
| Stx16 | 9.0 | 10.4 | 9.9 | 10.7 | 9.6 | 11.3 | 9.1 | 9.1 | 9.3 | 9.4 | 9.4 | 8.8 |
| Stx17 | 5.2 | 4.7 | 4.6 | 5.4 | 4.1 | 5.5 | 5.0 | 5.2 | 5.0 | 4.6 | 5.1 | 4.8 |
| Stx18 | 16.5 | 16.5 | 15.3 | 13.2 | 12.4 | 12.3 | 14.3 | 16.8 | 13.9 | 14.8 | 18.1 | 16.6 |
| Stx19 | 0.0 | 0.2 | 0.0 | 0.0 | 0.2 | 0.3 | 0.1 | 0.2 | 0.1 | 0.0 | 0.0 | 0.0 |
| Stx1a | 0.9 | 1.6 | 1.7 | 1.7 | 1.5 | 1.2 | 1.9 | 1.7 | 2.1 | 1.7 | 1.7 | 1.3 |
| Stx1b | 0.0 | 0.0 | 0.2 | 0.0 | 0.0 | 0.1 | 0.0 | 0.0 | 0.0 | 0.1 | 0.0 | 0.1 |
| Stx2 | 23.3 | 23.7 | 21.3 | 25.9 | 22.6 | 22.7 | 25.3 | 30.6 | 25.5 | 18.6 | 25.3 | 22.6 |
| Stx3 | 5.0 | 3.5 | 3.4 | 5.5 | 6.1 | 5.6 | 4.0 | 2.8 | 3.6 | 5.2 | 4.3 | 4.6 |
| Stx4a | 12.8 | 15.4 | 17.8 | 16.7 | 16.7 | 17.8 | 15.7 | 16.7 | 15.1 | 15.2 | 15.6 | 15.6 |
| Stx5a | 31.3 | 33.2 | 28.6 | 31.6 | 27.6 | 30.9 | 28.4 | 32.1 | 32.3 | 34.1 | 31.2 | 31.1 |
| Stx6 | 12.4 | 11.4 | 11.2 | 10.8 | 11.9 | 10.3 | 11.5 | 11.3 | 10.9 | 12.1 | 11.3 | 10.8 |
| Stx7 | 31.4 | 28.9 | 26.5 | 39.3 | 30.4 | 36.8 | 31.0 | 22.8 | 28.3 | 29.8 | 35.9 | 36.6 |
| Stx8 | 7.9 | 6.6 | 5.8 | 7.9 | 5.4 | 7.6 | 5.9 | 5.7 | 5.3 | 5.5 | 6.0 | 6.5 |
| Stxbp1 | 5.1 | 5.8 | 5.9 | 5.8 | 5.4 | 6.1 | 7.0 | 5.2 | 5.2 | 4.2 | 5.0 | 4.3 |
| Stxbp2 | 7.0 | 5.1 | 5.0 | 6.4 | 7.6 | 5.1 | 3.6 | 2.7 | 2.8 | 7.6 | 4.4 | 4.6 |
| Stxbp3a | 12.0 | 12.1 | 12.6 | 11.7 | 12.8 | 11.1 | 10.9 | 11.0 | 12.4 | 13.8 | 13.8 | 14.7 |
| Stxbp3b | 0.4 | 0.4 | 0.3 | 0.5 | 0.3 | 0.6 | 0.8 | 0.3 | 0.4 | 0.8 | 0.5 | 0.4 |
| Stxbp4 | 3.5 | 3.6 | 3.8 | 3.3 | 2.8 | 4.1 | 3.0 | 3.3 | 3.1 | 3.5 | 3.4 | 3.5 |
| Stxbp5 | 4.9 | 5.1 | 4.2 | 5.3 | 4.7 | 5.3 | 3.8 | 3.6 | 3.5 | 4.0 | 4.1 | 3.7 |
| Stxbp5l | 0.0 | 0.0 | 0.0 | 0.0 | 0.0 | 0.0 | 0.0 | 0.0 | 0.0 | 0.0 | 0.0 | 0.0 |
| Stxbp6 | 0.2 | 0.3 | 0.3 | 0.2 | 0.2 | 0.1 | 0.1 | 0.1 | 0.1 | 0.3 | 0.3 | 0.1 |
| Styk1 | 2.1 | 1.0 | 0.7 | 2.3 | 1.0 | 1.5 | 0.9 | 0.3 | 0.6 | 1.2 | 1.4 | 0.6 |

| | | | | | | | | | | | | |
|---|---|---|---|---|---|---|---|---|---|---|---|---|
| Styx | 5.2 | 5.8 | 5.9 | 7.4 | 5.3 | 6.7 | 4.5 | 4.6 | 5.5 | 5.4 | 6.1 | 6.6 |
| Styxl1 | 0.5 | 0.4 | 0.2 | 0.1 | 0.1 | 0.2 | 0.1 | 0.1 | 0.1 | 0.0 | 0.2 | 0.1 |
| Sub1 | 15.4 | 15.3 | 17.2 | 14.0 | 14.2 | 12.5 | 16.4 | 16.7 | 15.9 | 18.4 | 19.4 | 18.7 |
| Sucla2 | 42.5 | 37.6 | 36.5 | 44.4 | 32.8 | 39.1 | 29.5 | 33.5 | 32.2 | 37.8 | 38.7 | 39.9 |
| Suclg1 | 29.3 | 27.2 | 27.8 | 35.1 | 27.0 | 29.2 | 26.3 | 27.4 | 24.9 | 30.1 | 28.7 | 29.6 |
| Suclg2 | 26.3 | 23.3 | 25.8 | 21.7 | 20.6 | 23.8 | 20.2 | 22.7 | 19.1 | 25.3 | 22.6 | 24.1 |
| Sucnr1 | 3.5 | 5.0 | 3.2 | 6.4 | 1.4 | 4.6 | 1.1 | 0.5 | 0.7 | 0.7 | 0.6 | 0.4 |
| Suco | 12.2 | 12.9 | 12.2 | 11.3 | 13.4 | 11.4 | 14.3 | 11.8 | 15.2 | 13.9 | 16.3 | 15.4 |
| Suds3 | 29.6 | 28.2 | 27.8 | 30.4 | 25.3 | 31.6 | 25.4 | 25.9 | 23.1 | 29.3 | 26.2 | 29.3 |
| Sufu | 1.9 | 2.4 | 2.3 | 2.0 | 2.5 | 1.6 | 3.0 | 2.4 | 2.5 | 2.7 | 1.8 | 1.8 |
| Sugp1 | 9.3 | 11.3 | 11.9 | 12.5 | 9.3 | 10.2 | 10.3 | 10.9 | 9.2 | 9.7 | 11.9 | 10.7 |
| Sugp2 | 6.1 | 6.2 | 5.8 | 5.3 | 5.3 | 5.3 | 5.7 | 5.2 | 6.2 | 5.4 | 5.0 | 4.5 |
| Sugt1 | 39.4 | 37.1 | 32.0 | 33.6 | 30.7 | 34.6 | 32.3 | 30.3 | 32.5 | 36.2 | 37.6 | 39.0 |
| Sulf1 | 111.2 | 83.3 | 71.9 | 105.7 | 92.3 | 88.0 | 94.1 | 95.5 | 67.7 | 131.5 | 111.0 | 95.3 |
| Sulf2 | 29.7 | 26.1 | 31.4 | 26.8 | 26.1 | 31.5 | 36.1 | 41.2 | 27.8 | 34.4 | 36.6 | 33.4 |
| Sult1a1 | 0.7 | 0.2 | 0.1 | 0.8 | 0.3 | 0.5 | 0.1 | 0.1 | 0.1 | 0.2 | 0.0 | 0.0 |
| Sult2b1 | 0.3 | 0.1 | 0.1 | 0.1 | 0.2 | 0.0 | 0.0 | 0.2 | 0.1 | 0.2 | 0.2 | 0.1 |
| Sult4a1 | 3.4 | 1.4 | 0.8 | 3.6 | 1.1 | 2.3 | 1.4 | 0.3 | 0.5 | 0.6 | 1.0 | 0.4 |
| Sult5a1 | 0.0 | 0.1 | 0.3 | 0.1 | 0.0 | 0.1 | 0.1 | 0.1 | 0.0 | 0.0 | 0.1 | 0.0 |
| Sult6b1 | 0.1 | 0.1 | 0.2 | 0.2 | 0.3 | 0.1 | 0.1 | 0.2 | 0.3 | 0.1 | 0.1 | 0.2 |
| Sumf1 | 53.4 | 39.4 | 35.0 | 58.5 | 43.9 | 60.1 | 42.0 | 38.5 | 39.1 | 38.8 | 42.8 | 48.3 |
| Sumf2 | 8.4 | 9.0 | 9.0 | 8.4 | 7.9 | 10.4 | 9.9 | 11.1 | 9.1 | 9.0 | 8.7 | 8.1 |
| Sumo1 | 90.6 | 87.4 | 100.8 | 91.7 | 87.4 | 91.5 | 85.0 | 77.9 | 75.7 | 91.8 | 86.7 | 106.4 |
| Sumo2 | 102.7 | 101.7 | 138.9 | 128.6 | 113.2 | 131.9 | 128.7 | 141.0 | 112.9 | 126.5 | 156.7 | 148.6 |
| Sumo3 | 17.8 | 17.0 | 17.2 | 18.2 | 17.2 | 16.7 | 17.3 | 19.4 | 16.2 | 19.3 | 18.7 | 19.8 |
| Sun1 | 17.6 | 16.1 | 16.1 | 17.3 | 15.3 | 17.6 | 16.4 | 16.7 | 16.5 | 14.5 | 16.6 | 15.1 |
| Sun2 | 17.1 | 15.0 | 14.1 | 20.3 | 18.8 | 21.9 | 18.3 | 11.7 | 16.5 | 15.3 | 14.3 | 15.1 |
| Suox | 9.6 | 9.0 | 8.0 | 10.3 | 6.1 | 9.1 | 7.3 | 5.4 | 6.1 | 7.9 | 7.1 | 6.7 |
| Supt16 | 26.9 | 23.4 | 26.7 | 27.1 | 25.6 | 26.7 | 26.1 | 24.3 | 20.2 | 22.8 | 27.1 | 29.5 |
| Supt20 | 8.7 | 10.8 | 10.9 | 8.0 | 10.0 | 8.7 | 12.5 | 10.8 | 11.3 | 9.1 | 11.9 | 10.0 |
| Supt3 | 2.3 | 3.6 | 3.6 | 2.4 | 2.9 | 3.6 | 3.5 | 3.1 | 3.0 | 2.0 | 2.5 | 3.4 |
| Supt4a | 32.1 | 26.2 | 24.0 | 29.6 | 25.2 | 28.1 | 25.1 | 28.6 | 25.3 | 32.0 | 27.2 | 28.8 |
| Supt5 | 52.3 | 49.8 | 47.9 | 61.2 | 42.4 | 45.6 | 45.8 | 38.7 | 39.0 | 43.4 | 45.6 | 46.5 |
| Supt6 | 17.5 | 17.3 | 20.1 | 20.0 | 18.6 | 18.2 | 20.2 | 20.3 | 17.3 | 20.7 | 21.3 | 24.3 |
| Supt7l | 6.0 | 6.1 | 6.3 | 6.9 | 5.7 | 6.0 | 5.6 | 5.8 | 5.2 | 6.1 | 5.4 | 5.6 |
| Supv3l1 | 6.9 | 6.5 | 6.5 | 5.7 | 6.0 | 7.7 | 6.2 | 6.5 | 6.0 | 6.2 | 7.1 | 6.5 |
| Surf1 | 27.7 | 25.4 | 23.5 | 28.9 | 22.8 | 26.9 | 24.4 | 24.7 | 22.1 | 27.0 | 28.8 | 29.0 |
| Surf2 | 18.6 | 15.4 | 16.0 | 19.4 | 11.7 | 19.4 | 18.1 | 16.5 | 12.6 | 15.1 | 18.2 | 18.2 |
| Surf4 | 136.2 | 124.9 | 114.2 | 148.3 | 137.4 | 123.2 | 153.5 | 147.7 | 144.4 | 143.5 | 152.3 | 138.2 |
| Surf6 | 7.8 | 7.1 | 7.1 | 9.4 | 7.9 | 7.8 | 8.0 | 7.3 | 6.8 | 7.1 | 7.6 | 8.5 |
| Susd1 | 0.7 | 0.6 | 0.4 | 0.6 | 1.1 | 0.7 | 0.4 | 0.4 | 0.2 | 0.4 | 0.3 | 0.3 |
| Susd2 | 3.7 | 4.2 | 4.5 | 5.4 | 7.6 | 5.6 | 7.1 | 3.2 | 4.2 | 1.8 | 2.6 | 1.3 |
| Susd3 | 0.3 | 0.0 | 0.0 | 2.1 | 5.9 | 0.0 | 0.5 | 0.1 | 0.1 | 0.1 | 0.1 | 0.1 |
| Susd4 | 0.2 | 0.2 | 0.1 | 0.0 | 0.0 | 0.2 | 0.0 | 0.1 | 0.1 | 0.3 | 0.1 | 0.1 |
| Susd5 | 0.0 | 0.0 | 0.0 | 0.0 | 0.0 | 0.0 | 0.1 | 0.0 | 0.0 | 0.0 | 0.0 | 0.0 |
| Suv39h1 | 2.5 | 2.5 | 2.6 | 1.8 | 3.2 | 1.7 | 3.3 | 2.7 | 3.3 | 2.5 | 2.3 | 2.1 |
| Suv39h2 | 0.4 | 0.3 | 0.5 | 0.3 | 0.4 | 0.3 | 0.4 | 0.3 | 0.3 | 0.3 | 0.4 | 0.4 |
| Suv420h1 | 5.7 | 6.4 | 7.0 | 6.7 | 7.2 | 5.4 | 7.1 | 8.0 | 7.6 | 7.3 | 8.0 | 7.8 |
| Suv420h2 | 1.3 | 2.3 | 2.0 | 1.2 | 2.0 | 1.5 | 1.2 | 1.4 | 2.2 | 1.8 | 1.5 | 1.4 |
| Suz12 | 5.3 | 5.8 | 6.4 | 4.1 | 5.6 | 5.3 | 4.9 | 4.8 | 4.5 | 5.1 | 6.3 | 5.6 |
| Sv2a | 0.2 | 0.2 | 0.2 | 0.1 | 0.1 | 0.1 | 0.1 | 0.2 | 0.3 | 1.4 | 0.5 | 0.3 |
| Sv2b | 0.1 | 0.0 | 0.0 | 0.1 | 0.0 | 0.0 | 0.0 | 0.0 | 0.0 | 0.0 | 0.0 | 0.0 |
| Sv2c | 0.0 | 0.1 | 0.0 | 0.0 | 0.0 | 0.1 | 0.0 | 0.0 | 0.0 | 0.0 | 0.0 | 0.0 |
| Svep1 | 34.4 | 73.7 | 104.5 | 27.1 | 49.6 | 27.6 | 44.0 | 72.5 | 50.6 | 36.1 | 38.0 | 39.1 |
| Svil | 6.8 | 7.1 | 8.7 | 6.5 | 7.1 | 5.5 | 7.8 | 7.0 | 7.2 | 9.8 | 10.1 | 9.6 |
| Svip | 0.6 | 0.1 | 0.3 | 0.2 | 0.1 | 0.3 | 0.0 | 0.1 | 0.1 | 0.2 | 0.2 | 0.1 |
| Svop | 3.7 | 2.7 | 1.5 | 2.2 | 2.5 | 2.4 | 1.4 | 0.4 | 0.9 | 1.8 | 1.3 | 1.8 |

| | | | | | | | | | | | | |
|---|---|---|---|---|---|---|---|---|---|---|---|---|
| Svopl | 0.0 | 0.0 | 0.0 | 0.0 | 0.0 | 0.0 | 0.1 | 0.0 | 0.0 | 0.0 | 0.0 | 0.0 |
| Swap70 | 9.6 | 10.0 | 9.6 | 16.1 | 14.8 | 11.4 | 8.9 | 8.7 | 9.3 | 9.4 | 7.9 | 9.7 |
| Swi5 | 225.8 | 192.4 | 240.2 | 225.7 | 175.2 | 219.2 | 227.8 | 223.7 | 185.1 | 214.4 | 239.9 | 261.2 |
| Swsap1 | 2.6 | 2.0 | 2.2 | 3.6 | 2.6 | 3.1 | 3.0 | 2.8 | 2.3 | 2.3 | 2.7 | 2.4 |
| Swt1 | 3.4 | 4.2 | 4.5 | 3.8 | 3.6 | 3.6 | 2.7 | 3.4 | 3.0 | 4.3 | 5.2 | 4.2 |
| Syap1 | 29.1 | 26.2 | 29.0 | 33.2 | 29.2 | 31.4 | 26.6 | 27.3 | 26.0 | 30.0 | 34.1 | 34.0 |
| Sybu | 0.1 | 0.1 | 0.2 | 0.0 | 0.0 | 0.0 | 0.1 | 0.1 | 0.1 | 0.1 | 0.1 | 0.1 |
| Syce1 | 0.5 | 0.3 | 0.2 | 0.5 | 0.1 | 0.2 | 0.2 | 0.3 | 0.2 | 0.3 | 0.4 | 0.1 |
| Syce2 | 1.2 | 1.9 | 2.9 | 2.8 | 2.6 | 1.6 | 2.8 | 0.8 | 1.9 | 1.9 | 1.6 | 2.2 |
| Sycp1 | 0.0 | 0.0 | 0.0 | 0.0 | 0.0 | 0.0 | 0.0 | 0.0 | 0.0 | 0.0 | 0.0 | 0.0 |
| Sycp1-ps1 | 0.0 | 0.0 | 0.0 | 0.0 | 0.0 | 0.0 | 0.0 | 0.0 | 0.0 | 0.0 | 0.0 | 0.0 |
| Sycp2 | 0.0 | 0.0 | 0.0 | 0.0 | 0.0 | 0.0 | 0.0 | 0.0 | 0.0 | 0.1 | 0.0 | 0.0 |
| Sycp3 | 0.3 | 1.0 | 0.7 | 0.9 | 0.5 | 1.4 | 0.1 | 0.4 | 0.3 | 0.2 | 0.9 | 1.0 |
| Syde1 | 21.6 | 21.9 | 19.6 | 25.1 | 20.0 | 24.1 | 25.9 | 21.2 | 23.2 | 21.2 | 22.1 | 19.1 |
| Syde2 | 1.1 | 1.1 | 1.1 | 1.2 | 0.8 | 1.2 | 1.0 | 1.2 | 1.4 | 1.6 | 1.4 | 1.4 |
| Syf2 | 51.5 | 52.5 | 52.1 | 64.2 | 39.1 | 50.0 | 46.8 | 47.6 | 43.6 | 46.7 | 54.3 | 56.1 |
| Syk | 2.9 | 2.3 | 1.7 | 6.7 | 12.1 | 3.3 | 4.9 | 2.7 | 2.3 | 1.9 | 2.3 | 2.4 |
| Sympk | 21.6 | 21.3 | 17.7 | 21.2 | 20.3 | 21.9 | 18.1 | 17.3 | 17.9 | 18.3 | 18.1 | 15.0 |
| Syn1 | 0.2 | 0.3 | 0.1 | 0.2 | 0.4 | 0.2 | 0.4 | 0.2 | 0.3 | 0.3 | 0.2 | 0.1 |
| Syn2 | 1.8 | 1.8 | 1.2 | 2.0 | 1.1 | 1.8 | 0.7 | 0.3 | 0.4 | 0.7 | 0.6 | 0.5 |
| Syn3 | 0.2 | 0.2 | 0.1 | 0.2 | 0.1 | 0.2 | 0.2 | 0.2 | 0.1 | 0.2 | 0.2 | 0.2 |
| Syna | 0.1 | 0.1 | 0.1 | 0.2 | 0.1 | 0.2 | 0.1 | 0.0 | 0.0 | 0.0 | 0.0 | 0.1 |
| Synb | 0.0 | 0.0 | 0.0 | 0.1 | 0.0 | 0.0 | 0.0 | 0.0 | 0.0 | 0.0 | 0.0 | 0.0 |
| Sync | 7.6 | 6.4 | 5.6 | 8.4 | 5.7 | 9.7 | 6.7 | 6.1 | 6.6 | 6.8 | 8.5 | 7.0 |
| Syncrip | 21.0 | 18.8 | 20.2 | 21.6 | 22.8 | 17.8 | 23.7 | 24.4 | 19.7 | 23.8 | 27.1 | 25.2 |
| Syndig1 | 0.2 | 0.1 | 0.2 | 0.0 | 0.0 | 0.1 | 0.1 | 0.0 | 0.1 | 0.0 | 0.0 | 0.0 |
| Syne1 | 2.6 | 2.3 | 2.3 | 3.4 | 3.6 | 2.5 | 2.6 | 2.7 | 1.9 | 2.0 | 2.1 | 2.0 |
| Syne2 | 5.7 | 7.4 | 6.5 | 4.1 | 5.3 | 6.0 | 5.4 | 5.2 | 5.8 | 12.4 | 8.7 | 9.8 |
| Syne3 | 2.6 | 2.8 | 3.8 | 2.2 | 3.0 | 2.1 | 2.9 | 5.0 | 2.5 | 1.8 | 2.7 | 2.8 |
| Syne4 | 0.0 | 0.1 | 0.0 | 0.0 | 0.0 | 0.0 | 0.1 | 0.0 | 0.1 | 0.1 | 0.0 | 0.0 |
| Syngr1 | 2.6 | 0.8 | 0.4 | 4.6 | 2.0 | 3.1 | 0.3 | 0.4 | 0.2 | 1.1 | 0.8 | 0.6 |
| Syngr2 | 40.4 | 42.0 | 38.0 | 42.8 | 43.8 | 39.5 | 48.1 | 51.2 | 53.0 | 53.0 | 48.6 | 47.2 |
| Syngr3 | 0.1 | 0.1 | 0.1 | 0.1 | 0.1 | 0.0 | 0.0 | 0.0 | 0.1 | 0.0 | 0.1 | 0.0 |
| Syngr4 | 0.1 | 0.1 | 0.0 | 0.0 | 0.0 | 0.0 | 0.0 | 0.0 | 0.3 | 0.1 | 0.1 | 0.0 |
| Synj1 | 1.7 | 2.3 | 2.4 | 2.0 | 2.6 | 1.9 | 2.0 | 2.1 | 2.2 | 2.3 | 2.0 | 1.9 |
| Synj2 | 3.5 | 3.5 | 5.1 | 3.7 | 4.2 | 4.0 | 4.0 | 5.5 | 4.5 | 5.0 | 4.2 | 5.3 |
| Synj2bp | 7.8 | 6.7 | 6.2 | 11.6 | 7.1 | 9.6 | 7.5 | 5.9 | 6.2 | 7.6 | 9.6 | 8.7 |
| Synm | 1.3 | 1.5 | 1.3 | 1.3 | 1.4 | 1.3 | 1.0 | 0.6 | 0.5 | 0.6 | 0.7 | 0.5 |
| Synpo | 46.5 | 42.8 | 35.5 | 57.0 | 56.1 | 42.3 | 75.4 | 42.6 | 63.6 | 49.6 | 53.9 | 55.1 |
| Synpo2 | 6.2 | 7.6 | 7.8 | 16.4 | 9.4 | 11.0 | 10.6 | 4.9 | 8.2 | 4.8 | 7.7 | 9.4 |
| Synpo2l | 0.1 | 0.1 | 0.0 | 0.1 | 0.0 | 0.1 | 0.1 | 0.1 | 0.1 | 0.1 | 0.0 | 0.1 |
| Synpr | 0.3 | 0.1 | 0.1 | 0.0 | 0.2 | 0.0 | 0.1 | 0.2 | 0.2 | 0.7 | 0.3 | 0.3 |
| Synrg | 5.5 | 4.5 | 3.6 | 7.3 | 4.6 | 5.6 | 4.2 | 3.4 | 3.1 | 4.6 | 4.0 | 3.6 |
| Syp | 0.1 | 0.3 | 0.3 | 0.4 | 0.5 | 0.2 | 0.2 | 0.2 | 0.2 | 0.1 | 0.1 | 0.2 |
| Sypl | 6.1 | 5.7 | 5.5 | 6.5 | 6.2 | 7.0 | 5.5 | 5.5 | 6.1 | 5.9 | 6.5 | 6.1 |
| Sypl2 | 0.0 | 0.0 | 0.0 | 0.0 | 0.0 | 0.0 | 0.0 | 0.0 | 0.0 | 0.0 | 0.0 | 0.0 |
| Sys1 | 11.2 | 9.5 | 8.3 | 10.2 | 9.1 | 9.6 | 9.8 | 9.9 | 10.6 | 12.7 | 9.9 | 11.2 |
| Syt1 | 0.3 | 0.0 | 0.0 | 0.1 | 0.0 | 0.0 | 0.0 | 0.0 | 0.3 | 0.0 | 0.0 | 0.3 |
| Syt11 | 3.6 | 3.4 | 3.3 | 4.2 | 4.6 | 3.7 | 2.9 | 3.7 | 2.7 | 3.6 | 2.9 | 3.5 |
| Syt12 | 0.7 | 0.7 | 0.6 | 0.7 | 0.5 | 0.8 | 0.6 | 0.4 | 0.4 | 0.7 | 0.7 | 0.5 |
| Syt13 | 5.1 | 5.7 | 4.3 | 7.3 | 7.5 | 7.7 | 9.2 | 6.3 | 5.7 | 1.5 | 2.9 | 2.3 |
| Syt14 | 0.5 | 0.2 | 0.1 | 0.8 | 0.1 | 0.3 | 0.3 | 0.1 | 0.1 | 0.1 | 0.1 | 0.1 |
| Syt15 | 0.0 | 0.0 | 0.1 | 0.0 | 0.1 | 0.0 | 0.1 | 0.1 | 0.0 | 0.0 | 0.0 | 0.0 |
| Syt17 | 10.1 | 13.1 | 12.1 | 6.5 | 8.2 | 11.4 | 10.8 | 10.2 | 8.4 | 5.7 | 7.9 | 9.4 |
| Syt3 | 0.1 | 0.1 | 0.1 | 0.1 | 0.1 | 0.2 | 0.1 | 0.1 | 0.1 | 0.1 | 0.0 | 0.1 |
| Syt4 | 0.0 | 0.0 | 0.0 | 0.1 | 0.0 | 0.0 | 0.0 | 0.0 | 0.0 | 0.0 | 0.0 | 0.0 |
| Syt5 | 0.1 | 0.0 | 0.0 | 0.1 | 0.1 | 0.2 | 0.3 | 0.0 | 0.1 | 0.1 | 0.0 | 0.1 |

| | | | | | | | | | | | | |
|---|---|---|---|---|---|---|---|---|---|---|---|---|
| Syt6 | 0.1 | 0.2 | 0.2 | 0.0 | 0.1 | 0.0 | 0.2 | 0.0 | 0.1 | 0.1 | 0.1 | 0.2 |
| Syt7 | 0.0 | 0.0 | 0.0 | 0.0 | 0.0 | 0.0 | 0.0 | 0.0 | 0.0 | 0.0 | 0.0 | 0.0 |
| Syt8 | 0.0 | 0.1 | 0.0 | 0.2 | 0.2 | 0.0 | 0.2 | 0.2 | 0.3 | 0.1 | 0.1 | 0.1 |
| Syt9 | 0.1 | 0.1 | 0.0 | 0.0 | 0.0 | 0.1 | 0.1 | 0.0 | 0.0 | 0.0 | 0.0 | 0.0 |
| Sytl1 | 1.1 | 0.5 | 0.6 | 0.4 | 0.6 | 0.5 | 0.6 | 0.3 | 0.8 | 8.4 | 2.3 | 1.2 |
| Sytl2 | 0.9 | 0.7 | 0.7 | 1.5 | 1.0 | 0.7 | 2.0 | 0.7 | 1.2 | 0.8 | 1.2 | 1.2 |
| Sytl3 | 0.0 | 0.0 | 0.0 | 0.0 | 0.1 | 0.0 | 0.0 | 0.0 | 0.0 | 0.1 | 0.0 | 0.0 |
| Sytl4 | 3.3 | 3.4 | 3.6 | 3.0 | 2.7 | 3.5 | 3.7 | 2.9 | 2.6 | 2.6 | 3.4 | 3.6 |
| Sytl5 | 0.2 | 0.2 | 0.1 | 0.1 | 0.1 | 0.2 | 0.0 | 0.1 | 0.0 | 0.2 | 0.1 | 0.1 |
| Syvn1 | 3.0 | 5.1 | 4.1 | 3.3 | 4.4 | 3.9 | 4.8 | 4.2 | 5.2 | 4.5 | 4.2 | 3.2 |
| Szrd1 | 18.1 | 17.1 | 17.0 | 20.5 | 20.9 | 17.7 | 19.5 | 19.4 | 19.2 | 20.6 | 19.3 | 19.3 |
| Szt2 | 1.2 | 1.6 | 1.6 | 1.1 | 1.5 | 1.5 | 1.6 | 1.4 | 1.7 | 1.3 | 1.3 | 1.0 |
| T2 | 0.0 | 0.0 | 0.0 | 0.1 | 0.1 | 0.0 | 0.0 | 0.0 | 0.0 | 0.0 | 0.1 | 0.1 |
| Tab1 | 4.2 | 3.5 | 3.3 | 4.4 | 3.8 | 4.8 | 3.3 | 4.3 | 3.5 | 3.6 | 3.9 | 3.1 |
| Tab2 | 15.1 | 14.8 | 17.4 | 15.3 | 14.1 | 13.4 | 14.7 | 15.0 | 13.8 | 12.1 | 16.3 | 15.7 |
| Tab3 | 1.4 | 1.8 | 1.8 | 1.8 | 1.4 | 1.6 | 1.8 | 1.6 | 1.3 | 1.4 | 1.6 | 1.8 |
| Tac4 | 0.1 | 0.1 | 0.1 | 0.1 | 0.2 | 0.0 | 0.1 | 0.0 | 0.1 | 0.0 | 0.2 | 0.1 |
| Tacc1 | 18.6 | 23.9 | 28.2 | 22.4 | 27.4 | 19.7 | 25.8 | 24.7 | 24.6 | 21.4 | 22.1 | 27.7 |
| Tacc2 | 4.2 | 4.1 | 4.4 | 4.8 | 3.8 | 5.3 | 4.8 | 4.7 | 3.9 | 5.0 | 4.9 | 3.9 |
| Tacc3 | 3.8 | 6.0 | 8.9 | 2.0 | 9.0 | 2.1 | 11.1 | 4.0 | 8.9 | 4.3 | 7.0 | 4.9 |
| Taco1 | 4.7 | 5.7 | 5.4 | 5.3 | 4.6 | 6.2 | 4.9 | 4.9 | 4.1 | 4.4 | 4.9 | 5.0 |
| Tada1 | 6.8 | 6.6 | 6.7 | 6.7 | 6.2 | 6.2 | 6.9 | 7.2 | 7.1 | 6.8 | 7.4 | 7.6 |
| Tada2a | 4.7 | 4.7 | 5.7 | 3.6 | 3.9 | 4.4 | 4.4 | 5.2 | 4.3 | 5.0 | 5.3 | 5.2 |
| Tada2b | 3.8 | 3.6 | 4.1 | 4.4 | 4.1 | 4.1 | 4.6 | 4.1 | 4.0 | 3.8 | 4.8 | 4.2 |
| Tada3 | 8.9 | 11.3 | 11.2 | 9.5 | 11.0 | 10.7 | 12.9 | 11.5 | 11.0 | 10.2 | 12.9 | 10.8 |
| Taf1 | 10.7 | 10.7 | 11.5 | 10.4 | 9.2 | 10.0 | 10.9 | 9.3 | 9.2 | 9.6 | 11.4 | 10.6 |
| Taf10 | 74.4 | 65.8 | 60.9 | 69.2 | 64.2 | 63.3 | 68.1 | 76.8 | 63.1 | 68.2 | 64.6 | 65.8 |
| Taf11 | 13.8 | 14.8 | 14.2 | 13.0 | 13.5 | 16.2 | 14.9 | 16.2 | 14.2 | 14.3 | 15.2 | 14.8 |
| Taf12 | 18.7 | 18.0 | 19.3 | 21.1 | 18.4 | 18.1 | 21.1 | 20.4 | 17.8 | 19.2 | 23.7 | 20.5 |
| Taf13 | 26.4 | 22.6 | 19.6 | 26.8 | 22.5 | 20.6 | 20.3 | 17.3 | 19.3 | 24.4 | 25.5 | 25.2 |
| Taf15 | 7.0 | 7.4 | 8.2 | 7.9 | 8.3 | 7.1 | 7.4 | 8.3 | 6.8 | 7.6 | 7.9 | 8.8 |
| Taf1a | 2.9 | 2.4 | 2.7 | 3.9 | 2.5 | 3.0 | 2.4 | 2.5 | 2.2 | 2.3 | 2.7 | 2.5 |
| Taf1b | 7.4 | 6.3 | 6.7 | 7.2 | 5.5 | 6.5 | 6.6 | 6.2 | 5.7 | 4.7 | 6.4 | 6.3 |
| Taf1c | 2.0 | 1.9 | 1.8 | 2.3 | 1.9 | 1.9 | 1.9 | 2.1 | 2.3 | 2.0 | 1.7 | 1.7 |
| Taf1d | 15.4 | 14.0 | 17.3 | 12.1 | 15.5 | 13.5 | 17.3 | 15.9 | 13.2 | 17.0 | 18.5 | 16.8 |
| Taf2 | 8.6 | 9.6 | 9.1 | 8.4 | 9.0 | 8.4 | 8.5 | 8.9 | 8.7 | 8.8 | 9.9 | 8.8 |
| Taf3 | 1.6 | 1.6 | 2.2 | 2.4 | 1.7 | 2.1 | 1.6 | 2.0 | 1.2 | 1.5 | 1.9 | 1.5 |
| Taf4a | 1.0 | 1.6 | 1.5 | 1.2 | 1.5 | 1.4 | 1.8 | 1.4 | 1.4 | 1.5 | 1.5 | 1.1 |
| Taf4b | 1.6 | 1.6 | 1.2 | 1.5 | 1.4 | 1.6 | 1.4 | 1.3 | 1.3 | 1.5 | 1.7 | 1.3 |
| Taf5 | 1.3 | 1.4 | 1.3 | 1.2 | 1.2 | 1.2 | 1.6 | 1.4 | 1.4 | 1.6 | 1.4 | 1.4 |
| Taf5l | 4.0 | 3.8 | 3.5 | 3.5 | 4.8 | 3.6 | 4.9 | 4.8 | 3.9 | 4.1 | 4.6 | 3.9 |
| Taf6 | 11.6 | 13.2 | 14.8 | 12.8 | 11.6 | 13.1 | 12.1 | 12.8 | 11.4 | 11.9 | 13.1 | 12.4 |
| Taf6l | 2.2 | 2.5 | 2.7 | 2.9 | 2.6 | 2.5 | 3.4 | 2.8 | 2.0 | 2.3 | 2.4 | 2.3 |
| Taf7 | 1.4 | 1.4 | 1.9 | 1.4 | 1.6 | 1.2 | 1.9 | 1.9 | 2.1 | 2.7 | 2.3 | 2.6 |
| Taf7l | 0.1 | 0.0 | 0.0 | 0.0 | 0.0 | 0.0 | 0.0 | 0.0 | 0.0 | 0.0 | 0.1 | 0.0 |
| Taf8 | 3.4 | 3.6 | 3.7 | 4.1 | 4.0 | 3.7 | 4.1 | 4.6 | 4.1 | 3.5 | 4.2 | 4.1 |
| Taf9 | 65.7 | 56.0 | 54.2 | 63.3 | 59.6 | 56.8 | 57.9 | 57.4 | 52.6 | 58.9 | 64.9 | 57.2 |
| Taf9b | 3.5 | 5.2 | 6.8 | 4.4 | 5.1 | 3.4 | 5.4 | 6.0 | 5.0 | 4.8 | 5.8 | 6.1 |
| Tagap | 3.1 | 3.0 | 2.6 | 2.5 | 2.8 | 3.0 | 3.1 | 2.1 | 2.4 | 2.1 | 2.2 | 2.0 |
| Tagap1 | 6.2 | 6.1 | 5.2 | 5.2 | 5.2 | 6.7 | 6.2 | 4.2 | 5.0 | 4.9 | 5.1 | 4.5 |
| Tagln | 441.1 | 284.2 | 203.2 | 439.2 | 380.3 | 343.0 | 493.8 | 247.2 | 432.6 | 521.8 | 676.2 | 445.3 |
| Tagln2 | 61.0 | 45.9 | 41.0 | 81.1 | 127.3 | 58.6 | 108.9 | 58.2 | 69.7 | 70.5 | 62.2 | 61.6 |
| Tagln3 | 0.2 | 0.1 | 0.1 | 0.0 | 0.2 | 0.3 | 0.3 | 0.1 | 0.3 | 0.1 | 0.1 | 0.2 |
| Tal1 | 0.6 | 0.1 | 0.1 | 0.1 | 0.0 | 0.0 | 0.1 | 0.1 | 0.0 | 0.0 | 0.0 | 0.1 |
| Tal2 | 0.1 | 0.1 | 0.0 | 0.0 | 0.0 | 0.0 | 0.2 | 0.0 | 0.1 | 0.0 | 0.1 | 0.0 |
| Taldo1 | 195.1 | 174.9 | 95.4 | 321.5 | 216.5 | 313.4 | 97.3 | 99.7 | 70.7 | 117.4 | 101.9 | 87.7 |
| Tamm41 | 5.0 | 4.2 | 4.4 | 4.3 | 3.3 | 3.6 | 5.1 | 5.6 | 4.9 | 4.6 | 5.2 | 5.0 |

| | | | | | | | | | | | | |
|---|---|---|---|---|---|---|---|---|---|---|---|---|
| Tanc1 | 8.2 | 7.2 | 6.6 | 8.7 | 7.3 | 8.4 | 8.0 | 7.5 | 7.2 | 10.0 | 10.1 | 8.7 |
| Tanc2 | 1.0 | 1.1 | 1.1 | 1.2 | 1.9 | 0.9 | 1.1 | 0.9 | 1.0 | 1.2 | 1.1 | 1.0 |
| Tango2 | 11.0 | 14.3 | 15.6 | 9.0 | 12.9 | 9.3 | 13.5 | 16.3 | 16.2 | 12.3 | 12.9 | 13.8 |
| Tango6 | 2.1 | 2.0 | 1.7 | 1.9 | 1.8 | 2.1 | 2.1 | 2.0 | 1.9 | 2.2 | 1.6 | 1.9 |
| Tank | 18.2 | 19.1 | 19.1 | 21.2 | 25.0 | 15.2 | 21.4 | 17.7 | 18.1 | 19.8 | 23.0 | 21.8 |
| Taok1 | 15.2 | 17.5 | 18.0 | 15.5 | 16.1 | 15.1 | 14.5 | 15.4 | 14.8 | 15.5 | 17.4 | 17.2 |
| Taok2 | 11.4 | 12.2 | 13.7 | 12.8 | 12.1 | 11.6 | 14.3 | 13.6 | 13.0 | 11.7 | 12.6 | 13.0 |
| Taok3 | 7.5 | 6.7 | 7.3 | 10.6 | 10.3 | 8.3 | 7.8 | 6.2 | 5.7 | 8.0 | 8.0 | 8.3 |
| Tap1 | 12.0 | 12.3 | 11.1 | 10.7 | 11.1 | 10.5 | 8.8 | 7.7 | 8.4 | 8.6 | 7.4 | 10.3 |
| Tap2 | 11.5 | 13.6 | 13.6 | 12.6 | 14.9 | 14.6 | 12.0 | 13.1 | 12.6 | 10.7 | 8.8 | 12.0 |
| Tapbp | 50.7 | 44.2 | 55.5 | 61.9 | 73.1 | 49.4 | 44.9 | 53.3 | 43.3 | 44.8 | 40.0 | 44.6 |
| Tapbpl | 3.3 | 2.4 | 2.8 | 4.2 | 3.7 | 3.3 | 3.4 | 2.6 | 2.5 | 3.0 | 3.2 | 2.6 |
| Tapt1 | 7.1 | 8.4 | 8.6 | 6.9 | 6.7 | 8.0 | 6.7 | 7.8 | 8.9 | 7.1 | 8.7 | 8.4 |
| Tarbp2 | 6.4 | 5.1 | 4.3 | 5.0 | 6.5 | 6.1 | 6.6 | 6.3 | 7.4 | 6.5 | 7.3 | 6.4 |
| Tardbp | 12.9 | 13.6 | 12.2 | 13.6 | 14.9 | 11.7 | 13.4 | 13.3 | 13.7 | 13.6 | 14.7 | 14.0 |
| Tarm1 | 0.1 | 0.0 | 0.0 | 0.0 | 1.2 | 0.0 | 0.0 | 0.0 | 0.0 | 0.0 | 0.1 | 0.0 |
| Tars | 52.3 | 42.8 | 39.1 | 52.3 | 42.1 | 47.8 | 51.8 | 55.9 | 43.7 | 44.0 | 60.5 | 48.9 |
| Tars2 | 9.4 | 8.7 | 7.7 | 9.8 | 7.9 | 9.7 | 8.7 | 7.7 | 7.5 | 7.9 | 7.5 | 7.3 |
| Tarsl2 | 3.9 | 3.5 | 2.7 | 3.5 | 2.4 | 4.5 | 2.5 | 2.0 | 2.6 | 2.5 | 3.0 | 3.2 |
| Tas1r1 | 0.1 | 0.1 | 0.1 | 0.1 | 0.1 | 0.2 | 0.1 | 0.1 | 0.0 | 0.3 | 0.1 | 0.1 |
| Tas1r3 | 0.1 | 0.2 | 0.2 | 0.2 | 0.4 | 0.3 | 0.2 | 0.2 | 0.3 | 0.3 | 0.2 | 0.2 |
| Tas2r108 | 0.1 | 0.1 | 0.0 | 0.0 | 0.0 | 0.1 | 0.0 | 0.0 | 0.1 | 0.1 | 0.0 | 0.1 |
| Tas2r135 | 0.0 | 0.0 | 0.1 | 0.0 | 0.0 | 0.0 | 0.0 | 0.0 | 0.0 | 0.0 | 0.0 | 0.0 |
| Tas2r137 | 0.0 | 0.2 | 0.0 | 0.0 | 0.1 | 0.1 | 0.1 | 0.0 | 0.0 | 0.0 | 0.1 | 0.0 |
| Tasp1 | 3.1 | 3.9 | 5.1 | 3.1 | 3.1 | 3.0 | 3.8 | 4.6 | 4.6 | 4.7 | 3.8 | 4.5 |
| Tatdn1 | 5.3 | 5.7 | 5.2 | 5.2 | 5.3 | 6.1 | 5.0 | 5.1 | 5.7 | 6.2 | 6.1 | 5.8 |
| Tatdn2 | 17.0 | 11.1 | 9.6 | 20.4 | 15.5 | 13.5 | 15.6 | 9.6 | 11.5 | 12.8 | 15.7 | 13.5 |
| Tatdn3 | 4.5 | 4.1 | 3.9 | 2.8 | 3.8 | 4.5 | 3.0 | 4.6 | 4.4 | 3.4 | 3.1 | 4.2 |
| Tax1bp1 | 76.6 | 76.9 | 76.0 | 74.9 | 69.2 | 72.8 | 73.0 | 71.0 | 65.7 | 79.3 | 84.4 | 87.9 |
| Tax1bp3 | 44.7 | 44.3 | 43.3 | 37.3 | 47.5 | 38.6 | 48.8 | 46.0 | 58.6 | 58.9 | 54.8 | 53.1 |
| Taz | 12.6 | 12.4 | 11.5 | 10.5 | 11.1 | 13.8 | 9.3 | 9.1 | 14.1 | 11.3 | 10.8 | 11.4 |
| Tbata | 0.0 | 0.1 | 0.0 | 0.0 | 0.1 | 0.2 | 0.1 | 0.0 | 0.0 | 0.1 | 0.0 | 0.0 |
| Tbc1d1 | 7.7 | 7.4 | 6.7 | 8.5 | 9.4 | 7.4 | 10.3 | 6.7 | 8.3 | 7.8 | 8.9 | 8.4 |
| Tbc1d10a | 17.2 | 16.7 | 12.9 | 13.7 | 18.0 | 15.4 | 19.5 | 15.3 | 19.9 | 20.8 | 18.3 | 16.6 |
| Tbc1d10b | 15.5 | 15.6 | 15.3 | 18.1 | 17.1 | 16.0 | 18.8 | 14.8 | 14.5 | 12.8 | 13.6 | 14.9 |
| Tbc1d10c | 0.1 | 0.1 | 0.1 | 0.1 | 0.1 | 0.2 | 0.1 | 0.1 | 0.0 | 0.1 | 0.1 | 0.1 |
| Tbc1d12 | 4.7 | 4.7 | 4.1 | 4.6 | 4.2 | 5.2 | 4.3 | 3.7 | 4.6 | 4.7 | 4.8 | 5.0 |
| Tbc1d13 | 12.5 | 11.8 | 11.6 | 13.6 | 13.7 | 11.7 | 12.5 | 12.9 | 11.8 | 12.3 | 11.9 | 11.7 |
| Tbc1d14 | 2.5 | 3.6 | 3.2 | 3.2 | 4.1 | 3.2 | 3.0 | 4.2 | 3.6 | 3.6 | 3.0 | 3.2 |
| Tbc1d15 | 6.5 | 7.0 | 7.8 | 8.3 | 8.8 | 8.8 | 7.0 | 6.9 | 6.8 | 9.1 | 8.9 | 9.6 |
| Tbc1d16 | 1.3 | 1.0 | 1.2 | 1.2 | 0.8 | 1.2 | 1.0 | 1.2 | 1.2 | 1.9 | 1.3 | 1.2 |
| Tbc1d17 | 14.9 | 12.1 | 10.9 | 16.7 | 12.4 | 15.4 | 14.2 | 13.2 | 12.7 | 12.8 | 12.1 | 12.8 |
| Tbc1d19 | 10.2 | 9.6 | 7.7 | 10.6 | 8.7 | 9.6 | 8.7 | 7.3 | 8.9 | 8.7 | 8.4 | 8.1 |
| Tbc1d2 | 2.3 | 1.2 | 0.8 | 1.1 | 1.1 | 1.5 | 0.9 | 0.5 | 0.5 | 2.5 | 1.4 | 1.0 |
| Tbc1d20 | 21.1 | 19.7 | 14.7 | 20.8 | 19.9 | 16.8 | 20.5 | 20.6 | 21.6 | 19.9 | 19.0 | 21.5 |
| Tbc1d22a | 10.1 | 8.7 | 7.1 | 10.2 | 9.5 | 11.5 | 8.4 | 8.9 | 9.2 | 9.1 | 9.3 | 8.1 |
| Tbc1d22b | 2.5 | 3.3 | 3.2 | 2.9 | 2.7 | 2.7 | 3.0 | 2.6 | 3.1 | 2.1 | 2.4 | 1.8 |
| Tbc1d23 | 16.3 | 19.9 | 18.4 | 18.1 | 16.2 | 16.7 | 16.7 | 13.6 | 17.6 | 16.0 | 17.2 | 19.1 |
| Tbc1d24 | 3.7 | 3.6 | 3.0 | 4.1 | 3.1 | 4.7 | 2.8 | 2.7 | 2.5 | 2.9 | 2.5 | 2.7 |
| Tbc1d25 | 1.7 | 1.6 | 1.5 | 1.8 | 2.1 | 1.1 | 1.8 | 1.9 | 1.7 | 1.5 | 1.7 | 1.4 |
| Tbc1d2b | 11.7 | 12.5 | 14.6 | 10.4 | 11.7 | 11.7 | 11.7 | 11.6 | 11.0 | 15.4 | 13.7 | 13.1 |
| Tbc1d30 | 0.1 | 0.0 | 0.0 | 0.0 | 0.1 | 0.0 | 0.0 | 0.0 | 0.0 | 0.0 | 0.0 | 0.0 |
| Tbc1d4 | 0.8 | 1.0 | 1.9 | 0.6 | 1.7 | 0.7 | 1.8 | 1.9 | 1.4 | 1.1 | 1.4 | 1.1 |
| Tbc1d5 | 6.0 | 6.4 | 7.3 | 6.2 | 6.2 | 6.3 | 6.6 | 6.8 | 5.8 | 6.0 | 6.4 | 7.0 |
| Tbc1d7 | 10.8 | 12.2 | 10.5 | 10.7 | 8.6 | 11.3 | 11.0 | 10.5 | 10.1 | 8.2 | 10.1 | 10.0 |
| Tbc1d8 | 2.2 | 1.8 | 1.8 | 1.6 | 1.8 | 1.3 | 1.5 | 1.5 | 1.3 | 1.8 | 1.9 | 1.6 |
| Tbc1d8b | 5.3 | 5.3 | 6.1 | 6.7 | 6.2 | 6.1 | 5.8 | 6.7 | 5.9 | 6.1 | 7.9 | 6.9 |

| | | | | | | | | | | | | |
|---|---|---|---|---|---|---|---|---|---|---|---|---|
| Tbc1d9 | 1.7 | 0.6 | 0.5 | 3.6 | 4.4 | 2.0 | 1.3 | 0.7 | 0.8 | 0.8 | 0.9 | 0.7 |
| Tbc1d9b | 25.3 | 22.8 | 23.3 | 28.3 | 23.3 | 31.4 | 27.4 | 22.8 | 23.0 | 23.6 | 22.8 | 24.5 |
| Tbca | 223.2 | 171.6 | 157.1 | 233.3 | 163.3 | 208.0 | 150.3 | 163.8 | 142.2 | 165.6 | 204.4 | 194.8 |
| Tbcb | 41.6 | 40.5 | 34.9 | 42.9 | 38.2 | 40.0 | 39.2 | 47.1 | 41.9 | 41.9 | 45.5 | 40.7 |
| Tbcc | 7.5 | 7.0 | 5.2 | 7.9 | 6.8 | 8.5 | 6.3 | 6.5 | 8.2 | 7.6 | 6.9 | 6.8 |
| Tbccd1 | 6.1 | 5.1 | 4.8 | 5.3 | 4.9 | 5.2 | 4.1 | 4.8 | 5.6 | 4.8 | 4.9 | 5.4 |
| Tbcd | 22.6 | 19.1 | 16.4 | 24.6 | 19.3 | 21.6 | 17.4 | 19.2 | 17.4 | 16.2 | 17.5 | 16.8 |
| Tbce | 12.6 | 11.8 | 10.3 | 11.9 | 11.5 | 10.9 | 12.5 | 11.0 | 12.6 | 10.0 | 12.4 | 11.2 |
| Tbcel | 13.8 | 12.4 | 9.9 | 16.6 | 10.7 | 14.2 | 9.3 | 8.5 | 9.4 | 9.9 | 10.0 | 10.0 |
| Tbck | 7.8 | 7.9 | 8.1 | 7.2 | 7.4 | 8.2 | 6.7 | 7.8 | 7.5 | 7.8 | 8.3 | 7.7 |
| Tbk1 | 15.3 | 16.8 | 17.4 | 18.9 | 19.5 | 16.7 | 16.4 | 15.6 | 16.2 | 16.4 | 18.0 | 18.2 |
| Tbkbp1 | 0.9 | 1.1 | 1.3 | 1.0 | 1.3 | 0.6 | 0.9 | 1.3 | 0.8 | 0.9 | 0.8 | 0.6 |
| Tbl1x | 31.3 | 27.1 | 25.5 | 25.0 | 21.4 | 29.9 | 23.9 | 21.8 | 24.6 | 25.1 | 27.0 | 23.2 |
| Tbl1xr1 | 7.7 | 8.7 | 11.7 | 7.4 | 8.7 | 7.8 | 7.9 | 11.3 | 8.5 | 7.8 | 8.2 | 9.2 |
| Tbl2 | 3.4 | 4.4 | 6.2 | 3.8 | 4.5 | 3.7 | 5.7 | 7.0 | 5.6 | 4.4 | 4.5 | 4.7 |
| Tbl3 | 12.0 | 11.7 | 8.6 | 11.2 | 10.7 | 11.2 | 10.3 | 9.7 | 9.3 | 9.5 | 9.9 | 8.8 |
| Tbp | 6.3 | 7.1 | 8.0 | 6.4 | 7.4 | 7.8 | 6.8 | 7.7 | 6.5 | 7.5 | 8.6 | 7.7 |
| Tbpl1 | 9.4 | 10.0 | 9.4 | 10.1 | 10.9 | 9.8 | 9.5 | 10.8 | 10.2 | 9.3 | 10.8 | 9.6 |
| Tbrg1 | 73.5 | 66.1 | 62.9 | 70.6 | 55.7 | 62.9 | 61.1 | 65.2 | 56.2 | 65.1 | 71.2 | 66.1 |
| Tbrg3 | 0.2 | 0.3 | 0.3 | 0.1 | 0.3 | 0.2 | 0.2 | 0.2 | 0.1 | 0.2 | 0.2 | 0.2 |
| Tbrg4 | 3.8 | 3.6 | 2.8 | 3.5 | 4.0 | 3.6 | 4.6 | 4.4 | 3.7 | 3.7 | 4.0 | 2.4 |
| Tbx1 | 0.0 | 0.0 | 0.0 | 0.0 | 0.0 | 0.0 | 0.0 | 0.0 | 0.0 | 0.0 | 0.0 | 0.0 |
| Tbx10 | 0.0 | 0.0 | 0.0 | 0.1 | 0.1 | 0.0 | 0.0 | 0.0 | 0.1 | 0.1 | 0.2 | 0.0 |
| Tbx15 | 1.6 | 0.6 | 0.5 | 2.3 | 0.8 | 1.3 | 1.0 | 0.4 | 0.4 | 0.4 | 0.7 | 0.5 |
| Tbx18 | 18.5 | 16.4 | 13.1 | 12.8 | 11.8 | 10.2 | 17.6 | 17.9 | 20.4 | 16.8 | 21.8 | 23.6 |
| Tbx2 | 1.0 | 1.3 | 1.2 | 0.7 | 0.9 | 0.8 | 0.9 | 0.9 | 0.9 | 1.5 | 0.8 | 1.0 |
| Tbx20 | 120.3 | 165.8 | 176.8 | 103.5 | 143.6 | 107.0 | 162.7 | 103.0 | 116.9 | 72.7 | 101.6 | 125.8 |
| Tbx3 | 0.2 | 0.1 | 0.1 | 0.1 | 0.1 | 0.1 | 0.2 | 0.1 | 0.2 | 0.7 | 0.5 | 0.3 |
| Tbx4 | 0.5 | 0.2 | 0.3 | 0.1 | 0.1 | 0.2 | 0.1 | 0.1 | 0.1 | 0.5 | 0.1 | 0.2 |
| Tbx5 | 0.7 | 0.3 | 0.4 | 0.0 | 0.0 | 0.1 | 0.1 | 0.3 | 0.1 | 0.6 | 0.5 | 0.6 |
| Tbx6 | 0.1 | 0.3 | 0.3 | 0.4 | 0.3 | 0.3 | 0.4 | 0.0 | 0.4 | 0.3 | 0.1 | 0.2 |
| Tbxa2r | 3.6 | 5.7 | 5.7 | 2.5 | 4.2 | 3.3 | 7.6 | 10.8 | 10.9 | 5.9 | 6.4 | 6.2 |
| Tbxas1 | 0.6 | 0.0 | 0.0 | 4.8 | 7.4 | 0.1 | 0.9 | 0.1 | 0.0 | 0.7 | 0.2 | 0.4 |
| Tc2n | 0.1 | 0.2 | 0.1 | 0.1 | 0.0 | 0.0 | 0.1 | 0.1 | 0.1 | 0.1 | 0.0 | 0.0 |
| Tcaim | 2.1 | 2.5 | 2.3 | 2.9 | 2.2 | 2.6 | 2.5 | 2.1 | 2.8 | 2.8 | 3.3 | 3.3 |
| Tcam1 | 0.1 | 0.1 | 0.1 | 0.1 | 0.0 | 0.2 | 0.0 | 0.0 | 0.0 | 0.0 | 0.0 | 0.1 |
| Tcea1 | 27.1 | 27.4 | 25.8 | 27.9 | 25.0 | 30.2 | 24.7 | 27.3 | 24.7 | 29.1 | 33.2 | 28.4 |
| Tcea2 | 1.6 | 2.9 | 1.9 | 2.3 | 1.5 | 2.3 | 2.2 | 1.2 | 1.9 | 2.1 | 1.5 | 2.2 |
| Tcea3 | 23.5 | 22.3 | 28.5 | 21.2 | 13.6 | 28.5 | 17.5 | 20.3 | 16.7 | 19.8 | 20.2 | 24.8 |
| Tceal1 | 6.7 | 7.4 | 10.4 | 9.4 | 6.0 | 12.1 | 7.2 | 7.6 | 7.7 | 8.6 | 9.6 | 10.1 |
| Tceal3 | 0.8 | 0.5 | 1.2 | 0.4 | 0.2 | 0.8 | 0.3 | 0.4 | 0.3 | 1.8 | 1.5 | 1.2 |
| Tceal5 | 0.1 | 0.2 | 0.1 | 0.0 | 0.2 | 0.1 | 0.1 | 0.0 | 0.0 | 0.4 | 0.2 | 0.1 |
| Tceal6 | 0.1 | 0.1 | 0.1 | 0.0 | 0.1 | 0.0 | 0.1 | 0.0 | 0.0 | 0.3 | 0.1 | 0.1 |
| Tceal8 | 31.0 | 25.7 | 35.1 | 37.0 | 25.8 | 34.9 | 40.1 | 38.7 | 28.5 | 34.0 | 47.0 | 41.3 |
| Tceanc | 1.4 | 2.7 | 3.2 | 1.1 | 1.3 | 1.2 | 1.4 | 1.8 | 1.6 | 1.5 | 1.2 | 1.5 |
| Tceanc2 | 3.5 | 2.8 | 2.7 | 3.4 | 3.0 | 3.3 | 2.7 | 2.7 | 2.7 | 2.9 | 3.0 | 3.1 |
| Tceb1 | 45.4 | 49.1 | 40.4 | 46.5 | 49.0 | 40.1 | 39.7 | 40.7 | 46.4 | 48.2 | 55.7 | 45.9 |
| Tceb2 | 355.5 | 325.5 | 263.7 | 346.3 | 308.4 | 361.3 | 306.9 | 328.1 | 360.6 | 326.3 | 338.3 | 307.9 |
| Tceb3 | 27.3 | 26.0 | 25.5 | 32.4 | 24.5 | 26.5 | 25.5 | 24.0 | 20.6 | 23.3 | 26.5 | 25.8 |
| Tcerg1 | 9.0 | 9.6 | 10.1 | 8.8 | 9.0 | 8.5 | 12.3 | 9.4 | 8.9 | 8.9 | 10.0 | 10.1 |
| Tcerg1l | 0.1 | 0.0 | 0.1 | 0.0 | 0.0 | 0.0 | 0.0 | 0.1 | 0.1 | 0.0 | 0.0 | 0.0 |
| Tcf12 | 20.6 | 21.5 | 22.3 | 19.2 | 17.7 | 19.1 | 19.5 | 22.1 | 20.0 | 18.3 | 20.8 | 18.2 |
| Tcf15 | 0.0 | 0.0 | 0.0 | 0.0 | 0.1 | 0.1 | 0.0 | 0.0 | 0.0 | 0.0 | 0.1 | 0.0 |
| Tcf19 | 3.8 | 5.4 | 7.9 | 2.5 | 7.7 | 2.1 | 8.9 | 5.7 | 5.6 | 4.2 | 5.5 | 3.7 |
| Tcf20 | 30.2 | 30.9 | 25.9 | 26.8 | 25.9 | 32.7 | 33.5 | 34.1 | 33.6 | 31.1 | 39.8 | 33.0 |
| Tcf21 | 80.6 | 164.9 | 230.5 | 63.2 | 120.0 | 61.9 | 106.6 | 129.4 | 104.0 | 70.7 | 67.6 | 111.5 |
| Tcf23 | 0.1 | 0.1 | 0.1 | 0.1 | 0.1 | 0.1 | 0.1 | 0.1 | 0.1 | 0.2 | 0.1 | 0.1 |

| | | | | | | | | | | | | |
|---|---|---|---|---|---|---|---|---|---|---|---|---|
| Tcf25 | 111.0 | 105.5 | 103.9 | 119.9 | 100.8 | 113.4 | 122.3 | 107.0 | 110.3 | 93.4 | 114.5 | 107.9 |
| Tcf3 | 7.3 | 8.5 | 8.8 | 7.2 | 7.3 | 8.9 | 8.4 | 10.1 | 8.4 | 8.0 | 7.0 | 6.9 |
| Tcf4 | 9.0 | 12.4 | 16.0 | 12.4 | 11.5 | 11.0 | 12.3 | 15.9 | 12.3 | 10.5 | 11.1 | 11.7 |
| Tcf7 | 0.0 | 0.2 | 0.0 | 0.0 | 0.1 | 0.0 | 0.0 | 0.1 | 0.0 | 0.1 | 0.1 | 0.1 |
| Tcf7l1 | 0.6 | 1.0 | 1.5 | 0.6 | 0.9 | 0.7 | 0.9 | 1.4 | 1.2 | 0.9 | 1.0 | 0.6 |
| Tcf7l2 | 1.2 | 1.8 | 2.2 | 1.5 | 1.4 | 1.6 | 1.7 | 2.1 | 1.4 | 1.5 | 1.8 | 1.3 |
| Tcfl5 | 0.5 | 0.1 | 0.3 | 0.5 | 0.3 | 0.4 | 0.5 | 0.2 | 0.4 | 0.7 | 1.0 | 0.7 |
| Tchh | 0.2 | 0.1 | 0.1 | 0.3 | 0.1 | 0.3 | 0.2 | 0.1 | 0.2 | 0.4 | 0.2 | 0.2 |
| Tchp | 3.0 | 2.3 | 2.8 | 2.3 | 2.6 | 3.2 | 3.0 | 2.6 | 2.2 | 2.6 | 3.1 | 2.6 |
| Tcirg1 | 11.5 | 10.1 | 9.1 | 18.6 | 25.1 | 7.9 | 12.9 | 8.4 | 12.6 | 10.1 | 8.1 | 9.7 |
| Tcn2 | 91.2 | 80.8 | 78.4 | 98.7 | 84.0 | 107.0 | 70.1 | 67.7 | 62.5 | 72.4 | 71.7 | 75.4 |
| Tcof1 | 3.7 | 4.1 | 4.6 | 3.6 | 5.1 | 3.4 | 5.2 | 4.8 | 4.1 | 3.3 | 4.2 | 3.9 |
| Tcp1 | 114.0 | 98.5 | 85.9 | 100.1 | 95.8 | 97.6 | 98.3 | 91.7 | 99.0 | 105.5 | 113.5 | 100.4 |
| Tcp10a | 0.1 | 0.0 | 0.2 | 0.2 | 0.1 | 0.0 | 0.0 | 0.1 | 0.1 | 0.0 | 0.1 | 0.0 |
| Tcp10b | 0.1 | 0.1 | 0.4 | 0.1 | 0.1 | 0.0 | 0.2 | 0.1 | 0.0 | 0.1 | 0.1 | 0.1 |
| Tcp10c | 0.1 | 0.0 | 0.1 | 0.0 | 0.0 | 0.0 | 0.0 | 0.1 | 0.0 | 0.0 | 0.1 | 0.0 |
| Tcp11 | 0.1 | 0.0 | 0.0 | 0.0 | 0.0 | 0.0 | 0.0 | 0.0 | 0.0 | 0.0 | 0.0 | 0.0 |
| Tcp11l1 | 3.6 | 3.5 | 2.7 | 5.2 | 3.8 | 4.0 | 2.0 | 2.0 | 1.6 | 1.3 | 1.9 | 2.2 |
| Tcp11l2 | 4.7 | 7.3 | 6.6 | 5.0 | 5.0 | 4.8 | 5.1 | 5.5 | 5.7 | 8.9 | 6.4 | 8.0 |
| Tcta | 22.8 | 20.9 | 16.2 | 24.4 | 19.9 | 24.4 | 23.3 | 22.0 | 26.9 | 23.4 | 23.1 | 21.5 |
| Tcte2 | 0.9 | 0.3 | 0.2 | 0.7 | 0.5 | 0.9 | 0.2 | 0.2 | 0.3 | 0.4 | 0.7 | 0.3 |
| Tcte3 | 0.1 | 0.0 | 0.2 | 0.1 | 0.1 | 0.1 | 0.2 | 0.2 | 0.1 | 0.2 | 0.1 | 0.0 |
| Tctex1d2 | 10.3 | 8.9 | 9.0 | 9.3 | 11.0 | 11.8 | 9.3 | 9.6 | 9.5 | 10.4 | 9.1 | 9.0 |
| Tctex1d4 | 0.4 | 0.3 | 0.1 | 0.8 | 0.3 | 0.3 | 0.3 | 0.1 | 0.1 | 0.4 | 0.1 | 0.3 |
| Tctn1 | 4.4 | 5.2 | 4.5 | 5.1 | 4.6 | 6.7 | 4.3 | 4.5 | 3.9 | 3.1 | 3.3 | 3.2 |
| Tctn2 | 3.9 | 3.9 | 4.1 | 4.6 | 3.3 | 5.5 | 3.4 | 4.2 | 3.1 | 4.3 | 3.9 | 3.7 |
| Tctn3 | 2.7 | 2.5 | 2.2 | 2.4 | 2.2 | 3.7 | 2.0 | 2.2 | 2.3 | 2.5 | 1.8 | 2.4 |
| Tdg | 2.4 | 1.6 | 2.0 | 2.6 | 2.2 | 2.8 | 2.2 | 2.0 | 1.1 | 1.8 | 1.4 | 2.3 |
| Tdo2 | 0.0 | 0.0 | 0.0 | 0.0 | 0.0 | 0.0 | 0.1 | 0.1 | 0.0 | 0.0 | 0.0 | 0.0 |
| Tdp1 | 3.5 | 3.6 | 3.8 | 3.5 | 3.5 | 3.9 | 3.1 | 3.1 | 3.4 | 3.8 | 4.3 | 3.3 |
| Tdp2 | 8.8 | 11.2 | 10.5 | 6.7 | 9.9 | 7.8 | 10.2 | 8.7 | 11.7 | 11.1 | 11.5 | 11.7 |
| Tdrd1 | 0.2 | 0.2 | 0.1 | 0.2 | 0.2 | 0.4 | 0.2 | 0.1 | 0.1 | 0.1 | 0.3 | 0.1 |
| Tdrd12 | 0.0 | 0.0 | 0.0 | 0.0 | 0.0 | 0.0 | 0.0 | 0.0 | 0.1 | 0.1 | 0.0 | 0.0 |
| Tdrd3 | 14.0 | 13.5 | 14.2 | 14.9 | 10.2 | 14.2 | 10.6 | 12.3 | 10.6 | 10.3 | 13.1 | 13.1 |
| Tdrd5 | 0.0 | 0.0 | 0.0 | 0.0 | 0.1 | 0.1 | 0.0 | 0.0 | 0.0 | 0.0 | 0.0 | 0.0 |
| Tdrd6 | 0.0 | 0.0 | 0.0 | 0.0 | 0.0 | 0.0 | 0.0 | 0.0 | 0.0 | 0.0 | 0.0 | 0.0 |
| Tdrd7 | 25.3 | 19.5 | 16.8 | 26.9 | 19.1 | 26.8 | 17.4 | 13.7 | 16.1 | 15.7 | 18.2 | 20.4 |
| Tdrd9 | 0.0 | 0.0 | 0.0 | 0.0 | 0.1 | 0.0 | 0.0 | 0.0 | 0.0 | 0.0 | 0.0 | 0.0 |
| Tdrkh | 0.6 | 0.5 | 0.3 | 0.7 | 0.4 | 1.0 | 0.6 | 0.1 | 0.2 | 1.0 | 0.5 | 0.4 |
| Tead1 | 9.4 | 12.4 | 11.8 | 10.1 | 9.2 | 9.6 | 13.3 | 10.5 | 11.8 | 9.6 | 10.6 | 10.5 |
| Tead2 | 12.4 | 13.9 | 14.4 | 9.2 | 8.7 | 12.6 | 14.1 | 15.6 | 14.6 | 11.1 | 11.8 | 11.6 |
| Tead3 | 5.5 | 6.6 | 7.7 | 5.2 | 5.6 | 5.5 | 8.3 | 7.6 | 9.0 | 6.6 | 6.7 | 6.8 |
| Tead4 | 0.7 | 0.3 | 0.2 | 0.5 | 0.4 | 0.3 | 0.6 | 0.4 | 0.4 | 0.6 | 0.6 | 0.4 |
| Tec | 2.4 | 1.2 | 1.2 | 4.1 | 5.8 | 1.0 | 1.5 | 1.0 | 1.4 | 7.3 | 3.2 | 3.5 |
| Tecpr1 | 8.3 | 7.7 | 6.6 | 10.7 | 8.7 | 8.9 | 7.7 | 6.2 | 7.4 | 6.7 | 6.8 | 5.8 |
| Tecpr2 | 1.8 | 2.3 | 1.8 | 1.5 | 2.0 | 1.7 | 1.8 | 1.9 | 2.0 | 1.9 | 2.1 | 1.9 |
| Tecr | 67.9 | 71.9 | 72.7 | 72.1 | 55.0 | 69.4 | 92.6 | 115.2 | 80.4 | 53.4 | 82.2 | 61.2 |
| Tecta | 0.0 | 0.0 | 0.0 | 0.0 | 0.0 | 0.0 | 0.0 | 0.0 | 0.0 | 0.0 | 0.0 | 0.0 |
| Tectb | 0.0 | 0.0 | 0.0 | 0.0 | 0.1 | 0.0 | 0.0 | 0.1 | 0.0 | 0.0 | 0.0 | 0.0 |
| Tef | 20.7 | 23.4 | 24.1 | 18.4 | 16.3 | 22.0 | 17.2 | 22.2 | 19.4 | 17.2 | 15.7 | 17.4 |
| Tefm | 5.2 | 5.5 | 4.6 | 5.9 | 4.5 | 5.9 | 5.4 | 4.5 | 5.3 | 5.6 | 5.4 | 5.4 |
| Tek | 4.1 | 4.8 | 8.9 | 1.4 | 3.8 | 1.1 | 5.0 | 11.8 | 7.1 | 5.4 | 6.0 | 7.3 |
| Tekt2 | 0.3 | 0.2 | 0.5 | 0.4 | 0.3 | 0.4 | 0.5 | 0.4 | 0.5 | 0.6 | 0.3 | 0.2 |
| Tekt5 | 0.0 | 0.1 | 0.1 | 0.0 | 0.0 | 0.0 | 0.0 | 0.0 | 0.0 | 0.0 | 0.0 | 0.0 |
| Telo2 | 2.9 | 3.0 | 2.7 | 3.0 | 2.5 | 3.8 | 2.7 | 3.1 | 3.2 | 2.3 | 2.0 | 1.9 |
| Ten1 | 8.0 | 8.2 | 6.6 | 8.4 | 9.2 | 6.6 | 9.2 | 11.7 | 9.5 | 11.5 | 9.6 | 9.1 |
| Tenc1 | 6.9 | 7.8 | 7.6 | 8.9 | 6.1 | 9.0 | 7.8 | 8.8 | 8.1 | 5.9 | 6.4 | 6.1 |

| | | | | | | | | | | | | |
|---|---|---|---|---|---|---|---|---|---|---|---|---|
| Tenm1 | 0.0 | 0.0 | 0.0 | 0.0 | 0.0 | 0.0 | 0.0 | 0.0 | 0.0 | 0.0 | 0.0 | 0.0 |
| Tenm2 | 0.0 | 0.0 | 0.0 | 0.0 | 0.0 | 0.0 | 0.0 | 0.0 | 0.0 | 0.0 | 0.0 | 0.0 |
| Tenm3 | 1.6 | 3.2 | 5.2 | 1.0 | 3.6 | 0.7 | 4.4 | 8.0 | 7.3 | 4.9 | 4.2 | 4.4 |
| Tenm4 | 0.1 | 0.1 | 0.1 | 0.2 | 0.0 | 0.1 | 0.0 | 0.0 | 0.0 | 0.0 | 0.1 | 0.1 |
| Tep1 | 7.2 | 6.7 | 6.3 | 6.9 | 9.6 | 7.0 | 6.3 | 6.7 | 6.1 | 4.7 | 5.1 | 4.9 |
| Terc | 6.1 | 4.4 | 2.9 | 3.3 | 4.4 | 5.5 | 3.2 | 3.1 | 3.5 | 7.3 | 2.9 | 4.4 |
| Terf1 | 4.6 | 6.6 | 7.2 | 5.2 | 5.8 | 6.1 | 7.4 | 5.2 | 5.5 | 4.8 | 5.4 | 6.1 |
| Terf2 | 5.7 | 5.6 | 6.0 | 5.1 | 5.1 | 5.6 | 5.8 | 5.7 | 5.1 | 5.6 | 5.8 | 5.1 |
| Terf2ip | 6.4 | 5.7 | 6.0 | 6.8 | 5.4 | 6.4 | 6.5 | 6.0 | 6.1 | 7.4 | 6.7 | 7.1 |
| Tert | 1.2 | 1.9 | 1.7 | 0.9 | 1.2 | 1.7 | 1.2 | 1.2 | 1.3 | 1.5 | 1.2 | 1.1 |
| Tes | 8.9 | 3.6 | 1.6 | 12.8 | 10.9 | 10.6 | 7.3 | 2.3 | 4.0 | 17.6 | 13.6 | 6.7 |
| Tesc | 0.2 | 0.1 | 0.0 | 0.2 | 0.0 | 0.1 | 0.1 | 0.0 | 0.1 | 0.6 | 0.7 | 0.0 |
| Tesk1 | 6.6 | 6.4 | 5.3 | 7.2 | 6.5 | 7.5 | 7.6 | 6.2 | 6.7 | 7.2 | 6.7 | 5.7 |
| Tesk2 | 1.8 | 1.8 | 2.2 | 1.2 | 1.4 | 1.8 | 2.1 | 2.4 | 2.2 | 1.7 | 2.4 | 2.1 |
| Tet1 | 0.0 | 0.1 | 0.2 | 0.0 | 0.1 | 0.1 | 0.0 | 0.1 | 0.1 | 0.1 | 0.1 | 0.0 |
| Tet2 | 1.0 | 1.3 | 1.4 | 1.0 | 1.3 | 1.2 | 1.2 | 1.4 | 1.1 | 1.4 | 1.4 | 1.6 |
| Tet3 | 1.4 | 1.9 | 2.0 | 1.3 | 1.8 | 1.4 | 1.6 | 2.2 | 1.8 | 1.8 | 1.6 | 1.5 |
| Tex10 | 6.0 | 6.6 | 5.0 | 6.2 | 6.2 | 5.9 | 6.4 | 5.6 | 5.8 | 7.8 | 6.6 | 5.0 |
| Tex11 | 0.5 | 0.4 | 0.3 | 0.2 | 0.2 | 0.4 | 0.2 | 0.4 | 0.2 | 0.1 | 0.1 | 0.2 |
| Tex14 | 0.1 | 0.0 | 0.0 | 0.0 | 0.0 | 0.1 | 0.1 | 0.0 | 0.0 | 0.0 | 0.0 | 0.1 |
| Tex15 | 0.4 | 0.5 | 0.5 | 0.2 | 0.4 | 0.3 | 0.3 | 0.1 | 0.2 | 0.4 | 0.6 | 0.4 |
| Tex19.1 | 0.0 | 0.0 | 0.0 | 0.1 | 0.0 | 0.0 | 0.0 | 0.0 | 0.0 | 0.0 | 0.0 | 0.0 |
| Tex2 | 14.8 | 15.4 | 12.4 | 16.4 | 13.3 | 14.4 | 12.3 | 9.8 | 10.4 | 12.1 | 12.7 | 11.8 |
| Tex22 | 0.2 | 0.1 | 0.1 | 0.0 | 0.1 | 0.2 | 0.2 | 0.0 | 0.1 | 0.1 | 0.1 | 0.0 |
| Tex26 | 0.0 | 0.1 | 0.2 | 0.1 | 0.1 | 0.3 | 0.1 | 0.2 | 0.1 | 0.2 | 0.0 | 0.1 |
| Tex261 | 32.2 | 29.4 | 26.8 | 36.4 | 29.4 | 34.1 | 30.9 | 27.5 | 26.1 | 26.4 | 25.7 | 23.9 |
| Tex264 | 49.9 | 38.1 | 33.2 | 62.4 | 39.9 | 57.8 | 49.1 | 40.1 | 44.7 | 47.4 | 45.2 | 44.7 |
| Tex30 | 1.4 | 2.3 | 2.1 | 1.3 | 3.0 | 1.2 | 1.8 | 1.8 | 2.6 | 2.8 | 3.6 | 2.1 |
| Tex35 | 0.0 | 0.0 | 0.0 | 0.1 | 0.0 | 0.0 | 0.0 | 0.1 | 0.1 | 0.0 | 0.0 | 0.1 |
| Tex38 | 0.0 | 0.1 | 0.0 | 0.1 | 0.0 | 0.2 | 0.0 | 0.0 | 0.0 | 0.0 | 0.0 | 0.1 |
| Tex9 | 1.1 | 1.0 | 1.4 | 1.0 | 0.8 | 1.1 | 1.2 | 1.0 | 0.9 | 0.7 | 1.2 | 0.9 |
| Tfam | 6.5 | 6.7 | 6.0 | 5.6 | 5.7 | 5.9 | 5.6 | 5.5 | 5.5 | 6.3 | 6.2 | 6.2 |
| Tfap2a | 0.0 | 0.0 | 0.0 | 0.0 | 0.0 | 0.1 | 0.0 | 0.0 | 0.0 | 0.0 | 0.0 | 0.0 |
| Tfap2e | 0.0 | 0.1 | 0.0 | 0.1 | 0.0 | 0.0 | 0.0 | 0.0 | 0.0 | 0.0 | 0.1 | 0.0 |
| Tfap4 | 0.5 | 0.8 | 0.7 | 1.0 | 0.8 | 1.0 | 0.7 | 0.6 | 0.5 | 0.7 | 0.4 | 0.6 |
| Tfb1m | 3.4 | 3.2 | 2.5 | 3.2 | 2.7 | 3.9 | 2.4 | 2.6 | 3.7 | 2.7 | 3.0 | 2.3 |
| Tfb2m | 10.2 | 10.5 | 9.4 | 11.6 | 10.4 | 11.4 | 11.1 | 11.0 | 10.2 | 12.4 | 12.7 | 13.6 |
| Tfcp2 | 4.6 | 4.9 | 4.9 | 4.1 | 4.3 | 4.6 | 4.3 | 4.5 | 4.0 | 4.6 | 4.6 | 4.4 |
| Tfcp2l1 | 0.8 | 0.5 | 0.3 | 0.5 | 0.5 | 0.4 | 0.3 | 0.4 | 0.3 | 0.9 | 0.9 | 0.4 |
| Tfdp1 | 37.3 | 41.0 | 44.8 | 36.3 | 39.0 | 35.8 | 46.6 | 37.2 | 36.0 | 35.6 | 43.6 | 38.8 |
| Tfdp2 | 10.1 | 9.6 | 8.0 | 9.5 | 7.0 | 8.5 | 8.0 | 6.9 | 7.4 | 7.6 | 9.0 | 8.1 |
| Tfe3 | 9.3 | 10.2 | 10.6 | 10.5 | 11.5 | 9.4 | 12.2 | 12.9 | 10.5 | 10.5 | 10.2 | 10.0 |
| Tfeb | 2.8 | 3.6 | 3.3 | 2.0 | 3.2 | 2.4 | 3.7 | 3.6 | 3.6 | 4.1 | 3.2 | 3.6 |
| Tfec | 3.5 | 1.1 | 0.5 | 11.2 | 4.1 | 5.3 | 1.5 | 0.6 | 0.4 | 0.5 | 0.6 | 0.6 |
| Tfg | 32.0 | 37.0 | 35.8 | 35.8 | 33.6 | 31.7 | 40.7 | 44.1 | 38.6 | 35.5 | 43.5 | 40.6 |
| Tfip11 | 8.0 | 10.0 | 9.7 | 9.2 | 9.6 | 9.2 | 11.3 | 10.2 | 8.8 | 9.5 | 9.0 | 8.8 |
| Tfpi | 68.0 | 67.3 | 56.8 | 87.3 | 63.6 | 104.6 | 67.4 | 50.3 | 63.1 | 72.2 | 73.7 | 71.8 |
| Tfpi2 | 3.4 | 3.3 | 3.4 | 3.0 | 3.8 | 3.5 | 2.8 | 2.2 | 1.9 | 1.8 | 2.4 | 2.4 |
| Tfpt | 10.4 | 8.5 | 7.6 | 12.0 | 8.5 | 9.1 | 8.5 | 8.5 | 7.6 | 8.9 | 9.7 | 9.0 |
| Tfr2 | 0.2 | 0.2 | 0.2 | 0.3 | 0.2 | 0.2 | 0.3 | 0.2 | 0.5 | 0.6 | 0.5 | 0.4 |
| Tfrc | 6.4 | 6.3 | 3.9 | 9.5 | 7.5 | 8.2 | 3.9 | 5.0 | 3.3 | 4.9 | 5.3 | 4.1 |
| Tg | 2.1 | 1.6 | 0.9 | 2.5 | 2.5 | 1.3 | 3.0 | 3.3 | 3.4 | 1.2 | 2.0 | 1.5 |
| Tgds | 8.4 | 9.2 | 8.0 | 6.8 | 9.2 | 8.3 | 8.2 | 9.0 | 10.0 | 9.4 | 10.0 | 9.1 |
| Tgfa | 1.6 | 2.0 | 1.6 | 1.3 | 1.1 | 1.6 | 0.7 | 1.2 | 0.7 | 0.7 | 0.9 | 0.7 |
| Tgfb1 | 1.3 | 1.5 | 1.4 | 2.9 | 4.4 | 2.3 | 2.0 | 2.3 | 1.8 | 2.5 | 1.6 | 2.0 |
| Tgfb1i1 | 7.5 | 7.5 | 7.8 | 10.1 | 10.3 | 7.7 | 17.1 | 11.4 | 14.2 | 8.0 | 13.6 | 9.1 |
| Tgfb2 | 14.3 | 13.5 | 16.6 | 14.9 | 11.7 | 10.1 | 9.1 | 10.9 | 10.0 | 7.8 | 8.7 | 11.8 |

| | | | | | | | | | | | | |
|---|---|---|---|---|---|---|---|---|---|---|---|---|
| Tgfb3 | 40.3 | 39.3 | 41.8 | 37.1 | 28.8 | 32.6 | 38.6 | 41.9 | 40.9 | 49.5 | 56.9 | 48.4 |
| Tgfbi | 1.0 | 0.2 | 0.8 | 4.8 | 20.6 | 0.1 | 4.7 | 1.6 | 0.7 | 1.3 | 0.6 | 0.8 |
| Tgfbr1 | 8.1 | 8.1 | 8.4 | 11.0 | 13.4 | 7.3 | 5.9 | 5.3 | 6.3 | 10.5 | 8.8 | 8.9 |
| Tgfbr2 | 24.8 | 23.5 | 25.9 | 25.2 | 27.9 | 21.1 | 26.1 | 27.1 | 25.7 | 30.4 | 24.4 | 26.7 |
| Tgfbr3 | 5.5 | 6.9 | 11.4 | 4.0 | 5.8 | 5.8 | 6.6 | 6.9 | 5.7 | 8.7 | 6.3 | 6.7 |
| Tgfbrap1 | 5.4 | 5.3 | 4.5 | 5.8 | 4.9 | 5.8 | 5.5 | 4.9 | 4.9 | 4.0 | 4.8 | 4.3 |
| Tgif1 | 23.2 | 21.2 | 20.0 | 29.0 | 29.5 | 28.2 | 25.3 | 29.6 | 21.5 | 23.9 | 22.8 | 21.7 |
| Tgif2 | 0.9 | 1.2 | 1.1 | 0.8 | 1.0 | 0.7 | 1.1 | 1.2 | 1.1 | 1.1 | 1.1 | 1.0 |
| Tgm1 | 0.0 | 0.0 | 0.1 | 0.0 | 0.0 | 0.0 | 0.1 | 0.2 | 0.0 | 0.0 | 0.1 | 0.1 |
| Tgm2 | 22.3 | 14.1 | 11.6 | 25.9 | 16.2 | 19.9 | 18.0 | 16.3 | 27.7 | 28.3 | 23.1 | 23.8 |
| Tgm3 | 0.0 | 0.0 | 0.0 | 0.0 | 0.0 | 0.0 | 0.0 | 0.0 | 0.0 | 0.0 | 0.0 | 0.0 |
| Tgm4 | 2.9 | 2.6 | 1.8 | 2.2 | 2.3 | 2.6 | 1.9 | 1.8 | 2.0 | 1.7 | 1.9 | 1.7 |
| Tgoln1 | 61.3 | 51.7 | 43.1 | 76.5 | 56.5 | 64.6 | 43.7 | 37.6 | 36.0 | 44.4 | 42.8 | 45.2 |
| Tgoln2 | 72.2 | 53.8 | 49.5 | 76.7 | 55.0 | 63.5 | 47.6 | 37.2 | 37.3 | 47.6 | 45.2 | 52.3 |
| Tgs1 | 4.1 | 4.7 | 4.9 | 3.9 | 4.9 | 4.3 | 4.3 | 4.1 | 4.0 | 5.0 | 4.7 | 4.6 |
| Tgtp1 | 1.7 | 0.9 | 1.2 | 1.4 | 1.7 | 0.7 | 0.8 | 0.3 | 0.6 | 4.9 | 1.4 | 2.4 |
| Tgtp2 | 1.7 | 1.1 | 1.6 | 1.8 | 2.1 | 1.1 | 1.0 | 0.4 | 0.8 | 4.0 | 1.6 | 2.6 |
| Tha1 | 0.3 | 0.3 | 0.7 | 0.3 | 0.6 | 0.6 | 0.5 | 0.8 | 0.9 | 0.7 | 0.4 | 1.2 |
| Thada | 2.8 | 2.9 | 2.9 | 2.8 | 3.0 | 2.6 | 2.8 | 2.8 | 2.5 | 3.1 | 2.9 | 2.6 |
| Thap1 | 3.2 | 3.6 | 3.4 | 3.0 | 3.3 | 3.0 | 3.4 | 2.9 | 2.7 | 3.4 | 3.9 | 3.6 |
| Thap11 | 10.9 | 12.3 | 9.7 | 8.8 | 10.6 | 11.8 | 9.6 | 12.9 | 11.8 | 12.8 | 10.3 | 10.3 |
| Thap2 | 1.5 | 2.0 | 1.9 | 1.4 | 1.9 | 1.9 | 2.1 | 2.2 | 2.1 | 2.0 | 2.1 | 1.9 |
| Thap3 | 5.5 | 5.4 | 4.7 | 5.0 | 4.6 | 7.0 | 5.1 | 5.7 | 5.6 | 7.3 | 4.4 | 4.6 |
| Thap4 | 15.2 | 15.3 | 12.3 | 13.1 | 11.6 | 14.6 | 13.5 | 11.3 | 11.4 | 13.7 | 13.1 | 11.9 |
| Thap6 | 2.2 | 2.0 | 2.0 | 2.1 | 2.1 | 1.9 | 1.6 | 2.1 | 1.8 | 2.0 | 2.3 | 1.7 |
| Thap7 | 10.9 | 9.3 | 10.3 | 13.5 | 10.8 | 14.0 | 13.8 | 13.0 | 12.1 | 12.8 | 11.5 | 11.5 |
| Thbd | 11.5 | 5.6 | 4.1 | 11.0 | 4.4 | 12.2 | 8.1 | 8.6 | 17.2 | 30.1 | 20.7 | 17.7 |
| Thbs1 | 523.6 | 267.1 | 244.0 | 530.5 | 459.5 | 379.5 | 844.4 | 282.6 | 1068.5 | 988.7 | 1078.9 | 1279.1 |
| Thbs2 | 53.1 | 106.5 | 137.9 | 43.8 | 86.5 | 36.6 | 76.2 | 106.9 | 113.5 | 101.7 | 74.9 | 122.6 |
| Thbs3 | 0.9 | 1.9 | 2.9 | 0.8 | 1.6 | 0.9 | 2.3 | 3.8 | 2.7 | 1.4 | 2.0 | 1.9 |
| Thbs4 | 0.0 | 0.0 | 0.1 | 0.0 | 0.0 | 0.0 | 0.0 | 0.2 | 0.0 | 0.0 | 0.0 | 0.0 |
| Them4 | 4.1 | 4.0 | 3.6 | 4.5 | 3.0 | 4.9 | 3.3 | 3.6 | 4.1 | 3.3 | 5.0 | 3.5 |
| Them6 | 0.6 | 0.6 | 0.8 | 0.6 | 0.8 | 0.5 | 0.7 | 0.9 | 1.0 | 0.5 | 0.4 | 0.7 |
| Themis | 0.0 | 0.0 | 0.0 | 0.0 | 0.0 | 0.0 | 0.0 | 0.0 | 0.0 | 0.0 | 0.0 | 0.0 |
| Themis2 | 0.1 | 0.0 | 0.0 | 1.5 | 2.8 | 0.0 | 0.3 | 0.1 | 0.1 | 0.2 | 0.0 | 0.1 |
| Thg1l | 6.7 | 4.4 | 6.5 | 7.3 | 5.8 | 5.5 | 8.8 | 6.9 | 5.0 | 6.5 | 7.2 | 8.4 |
| Thnsl1 | 2.5 | 2.2 | 2.1 | 2.4 | 2.0 | 3.1 | 1.3 | 1.7 | 2.2 | 2.1 | 2.0 | 2.0 |
| Thnsl2 | 8.1 | 10.5 | 8.9 | 7.6 | 6.4 | 8.5 | 10.4 | 9.8 | 11.4 | 7.9 | 9.2 | 9.8 |
| Thoc1 | 9.3 | 9.0 | 8.9 | 9.8 | 8.1 | 7.7 | 8.1 | 8.5 | 8.4 | 8.0 | 9.2 | 9.7 |
| Thoc2 | 7.5 | 8.6 | 10.6 | 10.0 | 8.2 | 8.5 | 7.9 | 8.8 | 7.7 | 8.8 | 10.6 | 10.5 |
| Thoc3 | 9.5 | 8.3 | 8.4 | 9.6 | 7.8 | 7.9 | 9.4 | 8.2 | 8.5 | 9.9 | 10.1 | 8.0 |
| Thoc5 | 17.4 | 15.0 | 14.1 | 17.3 | 14.5 | 16.8 | 15.5 | 15.3 | 13.5 | 13.1 | 14.4 | 14.6 |
| Thoc6 | 6.9 | 7.3 | 5.7 | 6.9 | 6.9 | 6.0 | 5.8 | 7.4 | 7.6 | 7.1 | 5.9 | 6.4 |
| Thoc7 | 57.5 | 51.8 | 42.1 | 46.9 | 41.0 | 49.7 | 45.9 | 42.4 | 43.8 | 50.9 | 52.5 | 51.0 |
| Thop1 | 9.1 | 8.7 | 7.8 | 7.4 | 7.9 | 10.7 | 9.1 | 10.1 | 7.5 | 8.3 | 8.4 | 7.2 |
| Thpo | 0.3 | 0.2 | 0.2 | 0.2 | 0.0 | 0.1 | 0.2 | 0.3 | 0.2 | 0.1 | 0.1 | 0.1 |
| Thra | 9.2 | 12.0 | 14.4 | 8.6 | 7.3 | 12.0 | 10.0 | 13.1 | 11.2 | 10.3 | 10.1 | 10.0 |
| Thrap3 | 33.3 | 30.3 | 38.0 | 36.1 | 29.3 | 31.4 | 38.1 | 38.7 | 30.1 | 30.8 | 37.6 | 41.3 |
| Thrb | 1.5 | 2.1 | 2.2 | 2.3 | 1.5 | 2.8 | 1.0 | 1.1 | 1.0 | 1.8 | 1.7 | 1.5 |
| Thrsp | 0.1 | 0.0 | 0.1 | 0.0 | 0.0 | 0.2 | 0.1 | 0.1 | 0.0 | 0.0 | 0.1 | 0.0 |
| Thsd1 | 0.3 | 0.3 | 0.5 | 0.3 | 0.4 | 0.2 | 0.3 | 0.5 | 0.3 | 0.4 | 0.2 | 0.3 |
| Thsd4 | 0.1 | 0.1 | 0.0 | 0.1 | 0.0 | 0.1 | 0.1 | 0.1 | 0.1 | 0.5 | 0.2 | 0.1 |
| Thsd7a | 7.1 | 9.4 | 14.0 | 8.5 | 8.9 | 10.0 | 11.4 | 16.3 | 12.0 | 5.8 | 8.2 | 10.7 |
| Thsd7b | 0.8 | 1.5 | 2.1 | 0.5 | 0.6 | 0.3 | 0.4 | 1.1 | 0.3 | 0.3 | 0.5 | 0.4 |
| Thtpa | 4.2 | 3.5 | 3.7 | 4.1 | 3.1 | 4.4 | 3.2 | 4.5 | 3.7 | 4.3 | 3.9 | 4.0 |
| Thumpd1 | 15.4 | 14.5 | 13.2 | 16.1 | 13.6 | 15.3 | 14.2 | 13.7 | 13.1 | 14.8 | 14.6 | 14.1 |
| Thumpd2 | 4.0 | 3.8 | 4.6 | 2.7 | 3.5 | 2.8 | 3.4 | 3.4 | 3.5 | 4.7 | 3.8 | 4.4 |

| | | | | | | | | | | | | |
|---|---|---|---|---|---|---|---|---|---|---|---|---|
| Thumpd3 | 14.7 | 13.7 | 11.9 | 15.5 | 13.8 | 16.7 | 12.4 | 10.3 | 10.3 | 13.7 | 14.6 | 15.8 |
| Thy1 | 11.2 | 11.8 | 14.1 | 4.9 | 7.2 | 17.4 | 6.2 | 9.2 | 5.4 | 15.1 | 18.6 | 10.5 |
| Thyn1 | 41.5 | 37.9 | 34.0 | 37.8 | 31.1 | 38.0 | 29.5 | 23.7 | 26.8 | 36.5 | 35.1 | 30.7 |
| Tia1 | 4.7 | 4.8 | 4.9 | 5.6 | 4.5 | 5.2 | 4.6 | 4.8 | 5.0 | 4.9 | 5.5 | 4.9 |
| Tial1 | 3.4 | 3.6 | 4.0 | 3.4 | 4.3 | 3.8 | 4.9 | 4.6 | 4.7 | 4.4 | 4.5 | 4.3 |
| Tiam1 | 12.1 | 17.8 | 26.1 | 6.9 | 15.9 | 6.6 | 20.3 | 25.4 | 21.2 | 13.7 | 17.1 | 20.3 |
| Tiam2 | 0.7 | 0.7 | 0.7 | 0.4 | 0.7 | 0.7 | 0.7 | 0.7 | 0.6 | 0.6 | 0.9 | 0.5 |
| Ticam1 | 3.8 | 4.2 | 4.3 | 3.8 | 4.5 | 3.5 | 4.2 | 4.1 | 3.4 | 3.8 | 4.0 | 3.8 |
| Ticam2 | 0.1 | 0.0 | 0.0 | 0.3 | 0.4 | 0.0 | 0.1 | 0.0 | 0.0 | 0.1 | 0.1 | 0.1 |
| Ticrr | 0.1 | 0.3 | 0.4 | 0.0 | 0.3 | 0.1 | 0.4 | 0.2 | 0.3 | 0.2 | 0.3 | 0.2 |
| Tie1 | 0.5 | 0.1 | 0.2 | 0.3 | 0.2 | 0.1 | 0.4 | 0.7 | 0.5 | 0.1 | 0.0 | 0.1 |
| Tifa | 6.0 | 9.7 | 11.8 | 6.3 | 10.9 | 4.8 | 6.3 | 6.4 | 7.1 | 6.2 | 6.1 | 8.8 |
| Tifab | 0.1 | 0.0 | 0.0 | 0.5 | 1.4 | 0.0 | 0.2 | 0.0 | 0.0 | 0.0 | 0.0 | 0.0 |
| Tigd2 | 4.9 | 5.7 | 5.4 | 5.8 | 5.1 | 5.0 | 4.7 | 4.3 | 4.8 | 4.4 | 5.3 | 5.1 |
| Tigd3 | 0.1 | 0.1 | 0.1 | 0.0 | 0.1 | 0.1 | 0.1 | 0.1 | 0.1 | 0.2 | 0.1 | 0.1 |
| Tigd4 | 0.0 | 0.1 | 0.0 | 0.0 | 0.0 | 0.0 | 0.0 | 0.0 | 0.0 | 0.1 | 0.0 | 0.0 |
| Tigd5 | 0.9 | 1.1 | 0.9 | 1.0 | 1.0 | 0.8 | 1.0 | 1.0 | 1.0 | 1.0 | 0.7 | 0.8 |
| Tigit | 0.0 | 0.0 | 0.0 | 0.3 | 0.0 | 0.0 | 0.1 | 0.0 | 0.0 | 0.0 | 0.1 | 0.0 |
| Timeless | 0.8 | 1.3 | 2.2 | 0.8 | 1.6 | 0.3 | 1.9 | 1.7 | 1.3 | 0.8 | 1.2 | 1.0 |
| Timm10 | 12.9 | 8.3 | 7.4 | 10.6 | 9.9 | 11.2 | 10.9 | 10.4 | 8.3 | 11.4 | 9.6 | 10.2 |
| Timm10b | 17.8 | 16.8 | 13.7 | 20.3 | 19.0 | 14.0 | 16.7 | 18.4 | 18.2 | 16.2 | 17.4 | 13.3 |
| Timm13 | 18.2 | 15.1 | 12.6 | 17.5 | 17.7 | 18.2 | 18.0 | 16.7 | 16.9 | 18.9 | 18.2 | 13.5 |
| Timm17a | 77.9 | 67.9 | 66.8 | 81.9 | 72.3 | 74.3 | 81.4 | 64.8 | 72.5 | 78.1 | 83.6 | 82.8 |
| Timm17b | 9.5 | 10.3 | 8.4 | 8.3 | 8.5 | 7.7 | 9.3 | 10.7 | 10.2 | 10.8 | 9.3 | 10.6 |
| Timm21 | 15.3 | 13.0 | 15.6 | 14.2 | 11.6 | 18.0 | 12.5 | 14.3 | 14.2 | 13.8 | 15.9 | 15.6 |
| Timm22 | 7.9 | 7.4 | 6.0 | 8.0 | 7.3 | 7.7 | 7.2 | 6.8 | 7.6 | 8.6 | 7.3 | 7.4 |
| Timm23 | 88.1 | 75.7 | 72.1 | 89.5 | 72.5 | 78.1 | 72.9 | 67.0 | 63.4 | 64.6 | 69.2 | 70.9 |
| Timm44 | 18.9 | 17.6 | 17.1 | 17.1 | 15.0 | 16.4 | 15.2 | 14.3 | 15.6 | 16.4 | 16.8 | 15.7 |
| Timm50 | 13.7 | 13.4 | 10.7 | 14.3 | 13.2 | 13.8 | 13.3 | 11.8 | 13.1 | 13.3 | 11.9 | 10.8 |
| Timm8a1 | 10.0 | 6.8 | 6.1 | 10.7 | 9.8 | 7.8 | 7.1 | 7.9 | 6.8 | 9.2 | 7.3 | 7.3 |
| Timm8b | 91.0 | 69.8 | 65.7 | 100.4 | 71.1 | 84.9 | 55.5 | 67.6 | 55.4 | 83.8 | 88.9 | 79.4 |
| Timm9 | 9.2 | 7.6 | 6.1 | 7.8 | 8.6 | 7.2 | 8.1 | 6.9 | 8.1 | 8.6 | 8.7 | 6.9 |
| Timmdc1 | 17.8 | 16.6 | 15.0 | 17.8 | 13.9 | 18.8 | 16.2 | 14.9 | 13.7 | 16.6 | 17.2 | 16.7 |
| Timp1 | 487.0 | 394.3 | 441.0 | 349.8 | 508.2 | 344.9 | 857.5 | 498.7 | 503.2 | 772.2 | 649.1 | 823.3 |
| Timp2 | 310.8 | 182.0 | 156.2 | 326.7 | 200.0 | 333.8 | 218.1 | 174.6 | 166.1 | 266.8 | 238.5 | 233.1 |
| Timp3 | 794.7 | 771.6 | 1044.6 | 487.0 | 729.1 | 418.2 | 1136.4 | 1325.8 | 1241.5 | 1141.2 | 1211.1 | 1457.6 |
| Timp4 | 0.1 | 0.0 | 0.1 | 0.0 | 0.0 | 0.1 | 0.0 | 0.0 | 0.0 | 0.0 | 0.0 | 0.0 |
| Tinagl1 | 78.8 | 29.3 | 13.2 | 25.5 | 12.1 | 23.2 | 18.6 | 17.5 | 26.2 | 106.6 | 73.3 | 55.0 |
| Tinf2 | 3.6 | 5.1 | 5.9 | 2.5 | 4.8 | 3.4 | 5.2 | 5.0 | 5.4 | 4.7 | 4.2 | 3.7 |
| Tiparp | 6.0 | 6.7 | 6.1 | 5.2 | 6.0 | 4.8 | 5.9 | 8.0 | 9.1 | 10.4 | 9.1 | 9.0 |
| Tipin | 15.0 | 19.6 | 22.2 | 14.9 | 17.7 | 14.5 | 21.4 | 15.9 | 16.4 | 14.7 | 20.4 | 18.7 |
| Tiprl | 8.2 | 8.5 | 7.6 | 8.0 | 8.1 | 7.4 | 8.6 | 7.1 | 8.3 | 8.1 | 8.6 | 8.6 |
| Tirap | 3.0 | 2.6 | 3.1 | 2.6 | 3.3 | 2.5 | 3.6 | 3.4 | 3.2 | 2.7 | 3.2 | 2.9 |
| Tjap1 | 6.7 | 7.7 | 6.1 | 6.6 | 6.5 | 6.6 | 7.9 | 6.4 | 6.8 | 6.5 | 6.4 | 5.5 |
| Tjp1 | 11.0 | 11.3 | 12.7 | 10.8 | 9.9 | 10.5 | 13.0 | 15.3 | 12.3 | 14.4 | 14.9 | 15.7 |
| Tjp2 | 5.1 | 3.2 | 3.2 | 5.4 | 4.8 | 4.0 | 3.8 | 3.0 | 2.6 | 6.3 | 5.2 | 4.1 |
| Tjp3 | 0.1 | 0.0 | 0.0 | 0.2 | 0.1 | 0.0 | 0.0 | 0.0 | 0.0 | 0.0 | 0.0 | 0.0 |
| Tk1 | 1.9 | 4.1 | 6.0 | 0.9 | 5.6 | 0.7 | 8.7 | 5.0 | 7.2 | 3.4 | 5.8 | 3.5 |
| Tk2 | 14.2 | 10.9 | 10.1 | 16.8 | 11.4 | 17.3 | 10.8 | 7.5 | 9.8 | 10.6 | 8.5 | 11.7 |
| Tkt | 87.1 | 79.5 | 73.9 | 102.2 | 92.1 | 104.3 | 84.9 | 86.2 | 69.5 | 64.5 | 79.2 | 67.8 |
| Tktl1 | 0.0 | 0.0 | 0.0 | 0.0 | 0.0 | 0.2 | 0.0 | 0.0 | 0.0 | 0.1 | 0.0 | 0.1 |
| Tlcd1 | 3.7 | 5.2 | 5.9 | 2.8 | 2.9 | 4.2 | 5.6 | 7.8 | 8.1 | 4.1 | 5.8 | 4.7 |
| Tlcd2 | 9.5 | 14.2 | 11.5 | 5.4 | 6.9 | 12.8 | 9.4 | 15.2 | 10.1 | 5.4 | 10.4 | 6.2 |
| Tle1 | 2.4 | 1.6 | 1.4 | 2.9 | 2.6 | 2.4 | 1.6 | 2.5 | 1.5 | 3.0 | 2.0 | 1.7 |
| Tle2 | 1.0 | 1.9 | 2.4 | 0.7 | 1.1 | 1.0 | 1.6 | 1.8 | 2.8 | 1.2 | 1.2 | 1.8 |
| Tle3 | 5.3 | 6.7 | 6.0 | 5.4 | 6.0 | 5.1 | 7.2 | 5.3 | 5.9 | 6.3 | 5.9 | 4.7 |
| Tle4 | 1.7 | 1.7 | 1.5 | 2.2 | 2.1 | 1.1 | 1.1 | 0.8 | 1.1 | 0.9 | 1.0 | 0.8 |

| | | | | | | | | | | | | |
|---|---|---|---|---|---|---|---|---|---|---|---|---|
| Tle6 | 10.7 | 12.8 | 10.2 | 12.1 | 8.9 | 12.2 | 11.6 | 11.5 | 11.4 | 6.7 | 9.8 | 8.7 |
| Tlk1 | 12.5 | 12.7 | 12.8 | 17.1 | 12.6 | 14.1 | 13.2 | 12.2 | 11.7 | 11.1 | 14.5 | 12.9 |
| Tlk2 | 10.8 | 10.0 | 9.9 | 11.1 | 8.6 | 10.8 | 10.0 | 10.3 | 8.8 | 9.2 | 10.5 | 11.0 |
| Tll1 | 0.7 | 0.3 | 0.2 | 0.5 | 0.3 | 1.6 | 0.7 | 0.5 | 0.5 | 2.3 | 2.1 | 1.0 |
| Tll2 | 0.0 | 0.0 | 0.0 | 0.0 | 0.0 | 0.0 | 0.0 | 0.0 | 0.0 | 0.0 | 0.0 | 0.0 |
| Tln1 | 80.3 | 68.4 | 60.6 | 80.3 | 90.7 | 60.8 | 82.7 | 67.4 | 77.3 | 78.2 | 75.4 | 67.8 |
| Tln2 | 33.2 | 32.5 | 33.2 | 36.1 | 31.7 | 26.3 | 35.1 | 41.9 | 31.7 | 19.5 | 25.0 | 27.5 |
| Tlr1 | 0.2 | 0.3 | 0.3 | 0.5 | 1.3 | 0.1 | 0.4 | 0.1 | 0.3 | 0.2 | 0.1 | 0.2 |
| Tlr13 | 1.4 | 0.0 | 0.1 | 9.4 | 15.1 | 0.0 | 1.2 | 0.1 | 0.2 | 0.7 | 0.4 | 0.7 |
| Tlr2 | 5.2 | 5.0 | 8.0 | 7.0 | 14.0 | 5.3 | 4.6 | 6.4 | 3.3 | 4.1 | 3.0 | 4.8 |
| Tlr3 | 3.4 | 3.6 | 4.6 | 5.0 | 4.6 | 4.1 | 4.2 | 3.6 | 3.2 | 2.7 | 3.2 | 3.7 |
| Tlr4 | 12.8 | 11.5 | 10.1 | 16.0 | 14.8 | 12.8 | 8.2 | 7.7 | 7.5 | 13.0 | 10.5 | 11.3 |
| Tlr5 | 1.6 | 2.1 | 1.4 | 1.3 | 1.3 | 1.8 | 1.4 | 1.7 | 1.5 | 1.1 | 1.7 | 1.4 |
| Tlr6 | 1.3 | 1.1 | 1.0 | 1.7 | 1.3 | 1.3 | 1.2 | 1.2 | 1.2 | 1.0 | 1.3 | 1.3 |
| Tlr7 | 0.3 | 0.0 | 0.0 | 2.6 | 2.9 | 0.0 | 0.2 | 0.1 | 0.0 | 0.2 | 0.1 | 0.3 |
| Tlr8 | 0.3 | 0.0 | 0.0 | 2.1 | 2.3 | 0.0 | 0.2 | 0.0 | 0.0 | 0.1 | 0.1 | 0.1 |
| Tlr9 | 0.0 | 0.1 | 0.0 | 0.2 | 0.4 | 0.0 | 0.1 | 0.0 | 0.1 | 0.0 | 0.0 | 0.0 |
| Tm2d1 | 20.1 | 22.5 | 20.2 | 22.3 | 23.3 | 22.5 | 21.0 | 22.8 | 25.7 | 22.9 | 25.9 | 23.5 |
| Tm2d2 | 83.2 | 75.5 | 64.8 | 68.4 | 75.6 | 73.1 | 72.5 | 61.5 | 69.9 | 82.6 | 72.6 | 70.6 |
| Tm2d3 | 17.7 | 13.9 | 16.7 | 18.7 | 15.0 | 21.3 | 13.8 | 16.4 | 15.2 | 18.2 | 15.8 | 16.3 |
| Tm4sf1 | 75.7 | 58.2 | 46.2 | 36.5 | 40.7 | 47.0 | 35.2 | 36.9 | 50.3 | 60.7 | 56.5 | 58.1 |
| Tm4sf19 | 0.1 | 0.0 | 0.1 | 0.0 | 0.3 | 0.0 | 0.1 | 0.0 | 0.1 | 0.2 | 0.2 | 0.2 |
| Tm4sf20 | 0.0 | 0.1 | 0.1 | 0.1 | 0.1 | 0.0 | 0.0 | 0.1 | 0.0 | 0.1 | 0.0 | 0.1 |
| Tm4sf4 | 0.1 | 0.1 | 0.0 | 0.1 | 0.1 | 0.0 | 0.0 | 0.1 | 0.1 | 0.1 | 0.2 | 0.0 |
| Tm6sf1 | 0.9 | 0.2 | 0.2 | 3.5 | 6.6 | 0.1 | 1.0 | 0.3 | 0.3 | 0.7 | 0.3 | 0.6 |
| Tm6sf2 | 0.1 | 0.0 | 0.1 | 0.0 | 0.1 | 0.0 | 0.0 | 0.0 | 0.1 | 0.1 | 0.2 | 0.0 |
| Tm7sf2 | 3.6 | 4.8 | 4.8 | 2.2 | 2.2 | 3.0 | 8.2 | 14.6 | 10.5 | 3.6 | 9.0 | 3.9 |
| Tm7sf3 | 15.9 | 15.6 | 14.5 | 16.3 | 12.3 | 22.2 | 11.5 | 12.7 | 13.3 | 14.8 | 13.4 | 14.5 |
| Tm9sf1 | 27.0 | 26.9 | 23.5 | 29.3 | 27.5 | 27.6 | 27.6 | 27.3 | 28.3 | 26.4 | 23.6 | 24.8 |
| Tm9sf2 | 58.4 | 56.4 | 53.5 | 57.4 | 56.6 | 58.4 | 55.2 | 58.9 | 63.7 | 63.2 | 61.3 | 64.6 |
| Tm9sf3 | 51.2 | 46.7 | 50.2 | 65.2 | 57.1 | 58.6 | 56.2 | 50.5 | 54.3 | 65.1 | 63.4 | 65.5 |
| Tm9sf4 | 25.5 | 23.9 | 24.1 | 24.6 | 23.0 | 24.4 | 25.8 | 24.4 | 21.9 | 21.7 | 22.0 | 22.7 |
| Tma16 | 9.1 | 5.7 | 5.4 | 10.5 | 10.4 | 7.0 | 6.4 | 5.2 | 5.4 | 6.7 | 8.1 | 6.8 |
| Tma7 | 170.0 | 156.7 | 165.4 | 191.9 | 150.2 | 160.4 | 152.5 | 157.5 | 144.2 | 169.5 | 195.1 | 171.6 |
| Tmbim1 | 97.7 | 78.6 | 65.5 | 96.4 | 77.8 | 84.5 | 85.1 | 81.6 | 79.3 | 67.5 | 77.8 | 71.5 |
| Tmbim4 | 59.4 | 53.8 | 43.6 | 60.3 | 60.3 | 54.3 | 44.7 | 44.5 | 42.5 | 64.3 | 54.0 | 50.9 |
| Tmbim6 | 159.5 | 152.1 | 141.3 | 175.7 | 168.3 | 151.8 | 169.1 | 168.0 | 178.9 | 169.1 | 162.3 | 158.8 |
| Tmc3 | 0.3 | 0.1 | 0.2 | 0.1 | 0.1 | 0.2 | 0.2 | 0.2 | 0.1 | 0.1 | 0.1 | 0.1 |
| Tmc4 | 0.6 | 0.7 | 1.1 | 0.6 | 0.4 | 0.5 | 0.5 | 0.6 | 0.7 | 0.8 | 0.8 | 0.5 |
| Tmc5 | 0.0 | 0.0 | 0.0 | 0.0 | 0.0 | 0.0 | 0.0 | 0.0 | 0.0 | 0.0 | 0.0 | 0.0 |
| Tmc6 | 11.7 | 11.2 | 9.2 | 10.2 | 9.5 | 10.2 | 9.8 | 13.1 | 11.6 | 13.2 | 10.2 | 10.4 |
| Tmc7 | 1.1 | 1.8 | 1.3 | 1.1 | 1.2 | 1.5 | 0.8 | 1.1 | 0.9 | 0.7 | 0.8 | 0.7 |
| Tmc8 | 0.1 | 0.1 | 0.1 | 0.1 | 0.1 | 0.2 | 0.3 | 0.1 | 0.1 | 0.2 | 0.1 | 0.2 |
| Tmcc1 | 5.1 | 4.9 | 4.2 | 5.5 | 4.4 | 4.5 | 4.5 | 4.5 | 4.4 | 4.5 | 4.9 | 4.7 |
| Tmcc2 | 6.6 | 7.4 | 6.6 | 7.7 | 7.7 | 9.7 | 8.2 | 6.1 | 6.8 | 5.2 | 6.7 | 5.5 |
| Tmcc3 | 3.2 | 4.3 | 4.5 | 3.6 | 3.8 | 3.2 | 3.8 | 3.6 | 3.7 | 2.8 | 2.9 | 3.5 |
| Tmco1 | 16.4 | 15.1 | 14.9 | 18.7 | 15.3 | 15.0 | 14.1 | 12.5 | 14.0 | 19.1 | 17.3 | 18.2 |
| Tmco3 | 14.6 | 11.7 | 10.9 | 16.8 | 13.5 | 16.0 | 14.4 | 10.6 | 12.1 | 12.4 | 13.7 | 13.1 |
| Tmco4 | 2.5 | 2.9 | 3.1 | 3.1 | 2.9 | 3.1 | 2.9 | 2.9 | 3.0 | 3.3 | 3.6 | 3.1 |
| Tmco6 | 3.5 | 3.6 | 3.8 | 2.8 | 3.1 | 3.9 | 3.0 | 4.8 | 4.7 | 3.5 | 3.3 | 3.3 |
| Tmed1 | 10.4 | 14.0 | 15.7 | 10.6 | 12.6 | 10.7 | 13.4 | 16.0 | 17.7 | 16.2 | 11.9 | 15.9 |
| Tmed10 | 44.4 | 40.4 | 43.2 | 54.6 | 50.2 | 52.9 | 47.9 | 46.9 | 48.0 | 48.3 | 48.7 | 51.8 |
| Tmed2 | 207.5 | 169.8 | 172.8 | 241.9 | 188.3 | 200.1 | 223.1 | 195.1 | 191.8 | 211.7 | 239.1 | 234.6 |
| Tmed3 | 139.4 | 119.7 | 124.6 | 145.8 | 123.8 | 125.1 | 142.4 | 118.6 | 126.1 | 137.8 | 143.3 | 153.6 |
| Tmed4 | 39.6 | 34.2 | 29.4 | 43.1 | 32.2 | 47.9 | 28.8 | 30.7 | 30.4 | 36.3 | 34.4 | 32.9 |
| Tmed5 | 14.8 | 14.9 | 15.8 | 24.7 | 16.9 | 19.9 | 16.4 | 13.8 | 12.9 | 14.4 | 22.4 | 19.1 |
| Tmed7 | 38.3 | 36.1 | 38.6 | 44.4 | 37.2 | 48.0 | 36.3 | 37.6 | 37.1 | 41.6 | 46.4 | 48.6 |

| | | | | | | | | | | | | |
|---|---|---|---|---|---|---|---|---|---|---|---|---|
| Tmed8 | 5.3 | 5.6 | 5.0 | 5.5 | 5.5 | 5.1 | 4.5 | 3.8 | 4.2 | 4.6 | 4.2 | 4.2 |
| Tmed9 | 94.0 | 97.0 | 85.7 | 104.8 | 100.5 | 105.0 | 95.0 | 95.0 | 102.6 | 103.7 | 91.0 | 92.9 |
| Tmeff1 | 19.5 | 16.4 | 13.4 | 21.5 | 18.3 | 19.1 | 15.3 | 5.3 | 8.7 | 14.3 | 16.7 | 16.5 |
| Tmeff2 | 5.5 | 2.9 | 2.5 | 2.8 | 2.4 | 2.6 | 4.8 | 3.1 | 3.2 | 9.6 | 8.1 | 7.6 |
| Tmem100 | 0.0 | 0.0 | 0.1 | 0.0 | 0.0 | 0.0 | 0.0 | 0.0 | 0.0 | 0.0 | 0.0 | 0.1 |
| Tmem101 | 5.7 | 5.6 | 4.7 | 6.4 | 5.5 | 6.4 | 4.8 | 6.1 | 5.0 | 6.6 | 5.0 | 5.7 |
| Tmem104 | 4.6 | 4.2 | 3.2 | 5.3 | 4.9 | 5.4 | 4.6 | 3.9 | 3.4 | 4.1 | 3.0 | 2.7 |
| Tmem106a | 3.9 | 4.5 | 4.0 | 9.0 | 8.6 | 6.0 | 4.5 | 3.8 | 4.0 | 2.6 | 2.7 | 3.4 |
| Tmem106b | 30.5 | 30.0 | 36.2 | 33.7 | 27.4 | 36.8 | 32.6 | 35.0 | 30.6 | 30.3 | 36.9 | 37.3 |
| Tmem106c | 15.3 | 15.3 | 12.3 | 16.4 | 12.8 | 22.1 | 11.9 | 12.0 | 11.6 | 13.8 | 13.7 | 14.0 |
| Tmem107 | 2.1 | 3.1 | 2.7 | 2.6 | 3.2 | 3.6 | 2.7 | 3.1 | 3.2 | 2.0 | 2.7 | 3.2 |
| Tmem108 | 1.1 | 0.6 | 0.6 | 0.3 | 0.2 | 0.1 | 0.2 | 0.7 | 0.4 | 6.8 | 3.0 | 1.3 |
| Tmem109 | 15.9 | 14.6 | 14.3 | 17.4 | 18.1 | 17.8 | 17.2 | 16.1 | 16.9 | 16.4 | 15.0 | 12.2 |
| Tmem11 | 11.1 | 9.4 | 10.8 | 16.0 | 10.1 | 13.9 | 14.3 | 12.7 | 12.4 | 12.3 | 13.2 | 14.1 |
| Tmem110 | 3.9 | 3.9 | 4.0 | 3.4 | 3.6 | 4.0 | 4.5 | 4.3 | 4.8 | 3.8 | 4.1 | 4.3 |
| Tmem115 | 19.1 | 17.1 | 13.6 | 18.8 | 18.0 | 19.8 | 19.0 | 16.9 | 19.9 | 18.9 | 18.1 | 16.1 |
| Tmem116 | 0.7 | 0.6 | 0.3 | 0.8 | 0.4 | 0.8 | 0.3 | 0.3 | 0.2 | 0.6 | 0.3 | 0.6 |
| Tmem117 | 2.6 | 0.8 | 0.3 | 0.7 | 0.2 | 1.8 | 0.3 | 0.2 | 0.1 | 0.6 | 0.7 | 0.6 |
| Tmem119 | 8.3 | 9.8 | 11.7 | 8.7 | 6.5 | 4.6 | 10.9 | 13.3 | 16.0 | 9.8 | 7.9 | 11.6 |
| Tmem120a | 13.6 | 11.8 | 11.2 | 14.6 | 13.7 | 13.0 | 13.8 | 15.0 | 13.0 | 14.4 | 14.7 | 13.3 |
| Tmem120b | 2.3 | 1.6 | 1.4 | 2.2 | 1.7 | 1.6 | 2.0 | 2.5 | 2.3 | 1.6 | 1.9 | 1.5 |
| Tmem121 | 0.9 | 0.9 | 1.0 | 0.7 | 1.1 | 0.9 | 1.0 | 0.9 | 1.1 | 0.9 | 0.9 | 1.3 |
| Tmem123 | 25.8 | 27.2 | 38.9 | 34.9 | 42.4 | 30.0 | 41.4 | 40.9 | 50.7 | 36.3 | 42.7 | 57.2 |
| Tmem125 | 0.0 | 0.0 | 0.0 | 0.0 | 0.0 | 0.0 | 0.0 | 0.0 | 0.0 | 0.2 | 0.2 | 0.0 |
| Tmem126a | 59.4 | 58.1 | 40.8 | 68.6 | 52.6 | 68.3 | 45.3 | 41.4 | 54.2 | 46.4 | 53.2 | 44.8 |
| Tmem126b | 22.9 | 21.0 | 19.5 | 25.1 | 19.8 | 21.8 | 19.8 | 13.4 | 21.1 | 22.5 | 21.5 | 23.3 |
| Tmem127 | 25.9 | 24.1 | 24.0 | 26.6 | 24.7 | 22.9 | 25.4 | 23.1 | 23.4 | 25.7 | 25.3 | 26.5 |
| Tmem128 | 31.4 | 33.9 | 28.0 | 28.1 | 31.4 | 28.1 | 32.2 | 31.4 | 37.8 | 30.2 | 32.6 | 30.6 |
| Tmem129 | 16.5 | 15.0 | 13.9 | 15.9 | 13.7 | 16.4 | 16.7 | 12.2 | 16.3 | 12.6 | 14.3 | 13.2 |
| Tmem130 | 0.0 | 0.0 | 0.0 | 0.0 | 0.0 | 0.0 | 0.0 | 0.0 | 0.0 | 0.0 | 0.1 | 0.1 |
| Tmem131 | 4.8 | 5.6 | 6.0 | 5.5 | 6.2 | 5.5 | 6.5 | 5.0 | 4.9 | 4.9 | 5.3 | 4.4 |
| Tmem132a | 9.0 | 7.5 | 7.4 | 10.4 | 10.6 | 11.4 | 9.1 | 8.6 | 9.5 | 10.5 | 7.9 | 6.1 |
| Tmem132b | 0.0 | 0.0 | 0.0 | 0.1 | 0.0 | 0.1 | 0.0 | 0.0 | 0.0 | 0.0 | 0.0 | 0.0 |
| Tmem132c | 0.1 | 0.1 | 0.2 | 0.1 | 0.1 | 0.1 | 0.0 | 0.0 | 0.0 | 0.0 | 0.0 | 0.1 |
| Tmem132d | 0.0 | 0.0 | 0.0 | 0.0 | 0.0 | 0.0 | 0.0 | 0.0 | 0.0 | 0.0 | 0.0 | 0.0 |
| Tmem132e | 0.1 | 0.2 | 0.0 | 0.3 | 0.2 | 0.4 | 0.4 | 0.1 | 0.2 | 0.1 | 0.1 | 0.0 |
| Tmem134 | 32.7 | 27.3 | 24.2 | 30.1 | 30.2 | 36.8 | 27.1 | 28.4 | 27.1 | 30.1 | 25.5 | 26.2 |
| Tmem135 | 9.6 | 10.5 | 10.4 | 7.6 | 9.3 | 7.9 | 8.2 | 9.5 | 9.1 | 8.7 | 9.2 | 9.0 |
| Tmem136 | 0.3 | 0.7 | 0.6 | 0.4 | 0.3 | 0.9 | 0.8 | 0.7 | 0.5 | 0.5 | 0.3 | 0.5 |
| Tmem138 | 5.5 | 4.8 | 2.6 | 2.9 | 4.6 | 5.2 | 3.9 | 3.5 | 4.6 | 5.3 | 3.6 | 3.4 |
| Tmem140 | 0.9 | 0.5 | 0.6 | 1.8 | 1.2 | 1.5 | 0.7 | 0.6 | 0.8 | 0.7 | 0.5 | 1.0 |
| Tmem141 | 10.8 | 8.1 | 7.4 | 11.7 | 9.3 | 11.7 | 7.4 | 7.8 | 7.0 | 8.4 | 9.8 | 9.0 |
| Tmem143 | 6.5 | 6.6 | 5.3 | 7.5 | 5.2 | 8.2 | 5.7 | 6.2 | 6.3 | 6.2 | 5.8 | 5.4 |
| Tmem144 | 1.4 | 1.4 | 1.2 | 1.0 | 1.4 | 1.4 | 0.9 | 0.7 | 0.9 | 1.0 | 1.0 | 0.9 |
| Tmem147 | 41.8 | 36.2 | 30.1 | 37.4 | 36.8 | 37.9 | 38.0 | 40.7 | 35.9 | 41.6 | 37.2 | 31.8 |
| Tmem14a | 4.9 | 5.3 | 4.8 | 4.8 | 5.5 | 7.0 | 4.6 | 4.8 | 5.1 | 6.0 | 6.7 | 6.6 |
| Tmem14c | 57.1 | 58.6 | 60.9 | 58.0 | 64.0 | 56.1 | 64.4 | 62.2 | 66.1 | 60.8 | 60.8 | 65.4 |
| Tmem150a | 10.6 | 13.3 | 15.5 | 10.3 | 12.0 | 12.2 | 16.6 | 20.9 | 21.8 | 16.1 | 15.8 | 16.5 |
| Tmem150b | 0.4 | 0.3 | 0.3 | 0.3 | 0.6 | 0.3 | 0.4 | 0.2 | 0.4 | 0.3 | 0.6 | 0.4 |
| Tmem150c | 0.2 | 0.2 | 0.1 | 0.1 | 0.2 | 0.0 | 0.1 | 0.0 | 0.0 | 0.0 | 0.1 | 0.1 |
| Tmem151a | 16.5 | 11.4 | 9.4 | 13.0 | 8.9 | 12.6 | 14.7 | 13.0 | 10.2 | 15.5 | 15.3 | 13.8 |
| Tmem151b | 0.0 | 0.0 | 0.0 | 0.1 | 0.0 | 0.0 | 0.0 | 0.0 | 0.0 | 0.0 | 0.0 | 0.0 |
| Tmem154 | 3.0 | 1.2 | 1.7 | 1.5 | 1.3 | 1.2 | 1.0 | 1.2 | 0.9 | 2.1 | 1.9 | 1.5 |
| Tmem158 | 0.6 | 0.3 | 0.2 | 0.8 | 0.8 | 0.7 | 0.4 | 0.7 | 0.5 | 0.6 | 0.4 | 0.4 |
| Tmem159 | 35.9 | 36.4 | 39.8 | 44.8 | 42.4 | 45.1 | 51.1 | 50.7 | 46.2 | 38.4 | 49.3 | 42.1 |
| Tmem160 | 57.7 | 59.6 | 51.4 | 50.4 | 49.2 | 43.3 | 57.8 | 45.4 | 56.8 | 53.8 | 53.7 | 64.3 |
| Tmem161a | 11.2 | 9.7 | 10.0 | 11.0 | 8.5 | 12.9 | 10.2 | 10.5 | 10.4 | 9.8 | 9.8 | 9.7 |

| | | | | | | | | | | | | |
|---|---|---|---|---|---|---|---|---|---|---|---|---|
| Tmem161b | 2.8 | 3.1 | 3.2 | 2.4 | 3.4 | 2.9 | 3.1 | 2.9 | 2.8 | 2.4 | 2.4 | 2.5 |
| Tmem163 | 0.1 | 0.0 | 0.0 | 0.0 | 0.1 | 0.0 | 0.0 | 0.0 | 0.0 | 0.0 | 0.0 | 0.0 |
| Tmem164 | 6.7 | 7.7 | 8.1 | 7.3 | 9.3 | 5.6 | 6.3 | 8.9 | 7.3 | 6.1 | 6.2 | 6.8 |
| Tmem165 | 29.2 | 29.4 | 30.7 | 25.9 | 27.3 | 28.3 | 30.0 | 29.8 | 35.8 | 34.0 | 34.8 | 34.7 |
| Tmem167 | 6.9 | 7.1 | 6.2 | 6.8 | 7.5 | 6.4 | 6.3 | 7.0 | 7.8 | 8.1 | 8.5 | 7.4 |
| Tmem167b | 37.6 | 31.0 | 30.9 | 37.8 | 31.3 | 36.6 | 30.1 | 30.5 | 31.3 | 33.6 | 37.7 | 37.3 |
| Tmem168 | 8.7 | 9.3 | 9.9 | 9.8 | 10.9 | 9.6 | 10.8 | 10.9 | 13.2 | 12.1 | 12.6 | 12.8 |
| Tmem169 | 0.1 | 0.2 | 0.4 | 0.2 | 0.1 | 0.1 | 0.1 | 0.1 | 0.1 | 0.0 | 0.0 | 0.1 |
| Tmem17 | 1.6 | 0.7 | 1.5 | 1.5 | 1.1 | 1.4 | 1.9 | 2.1 | 2.5 | 1.0 | 1.6 | 1.8 |
| Tmem170 | 1.1 | 1.7 | 1.6 | 1.4 | 1.1 | 1.5 | 1.2 | 1.1 | 1.5 | 1.3 | 1.5 | 1.0 |
| Tmem170b | 2.1 | 2.9 | 2.7 | 2.1 | 2.4 | 2.1 | 2.1 | 2.1 | 2.2 | 2.0 | 2.0 | 1.9 |
| Tmem171 | 0.2 | 0.0 | 0.0 | 1.7 | 2.3 | 0.1 | 0.1 | 0.0 | 0.1 | 0.2 | 0.0 | 0.1 |
| Tmem173 | 6.7 | 7.1 | 9.3 | 9.7 | 13.8 | 4.8 | 9.3 | 8.6 | 8.1 | 12.4 | 8.9 | 8.3 |
| Tmem175 | 7.5 | 6.5 | 6.5 | 7.6 | 6.7 | 7.1 | 6.8 | 6.9 | 7.2 | 6.4 | 7.3 | 7.1 |
| Tmem176a | 67.1 | 59.5 | 58.3 | 74.0 | 73.0 | 53.7 | 62.0 | 37.0 | 52.0 | 54.3 | 34.1 | 54.4 |
| Tmem176b | 112.1 | 82.1 | 85.5 | 113.7 | 106.2 | 71.3 | 87.3 | 63.6 | 77.9 | 93.2 | 58.8 | 83.8 |
| Tmem177 | 2.2 | 2.0 | 1.8 | 2.1 | 1.9 | 2.0 | 1.7 | 1.7 | 1.8 | 1.7 | 1.8 | 1.8 |
| Tmem178 | 0.2 | 1.0 | 1.4 | 0.2 | 0.7 | 0.1 | 0.4 | 1.0 | 1.8 | 0.3 | 0.3 | 0.9 |
| Tmem178b | 0.0 | 0.0 | 0.0 | 0.0 | 0.0 | 0.1 | 0.0 | 0.0 | 0.0 | 0.0 | 0.0 | 0.0 |
| Tmem179 | 0.4 | 0.1 | 0.1 | 0.9 | 0.1 | 1.2 | 0.1 | 0.1 | 0.1 | 0.4 | 0.2 | 0.1 |
| Tmem179b | 19.5 | 21.5 | 18.4 | 20.7 | 20.8 | 22.0 | 23.0 | 21.0 | 23.5 | 22.0 | 18.2 | 23.9 |
| Tmem18 | 9.4 | 10.2 | 10.5 | 9.6 | 9.6 | 9.3 | 8.6 | 9.2 | 9.1 | 8.7 | 8.6 | 9.8 |
| Tmem180 | 0.2 | 0.1 | 0.1 | 0.3 | 0.5 | 0.2 | 0.4 | 0.2 | 0.1 | 0.1 | 0.1 | 0.1 |
| Tmem181a | 19.0 | 18.0 | 17.0 | 18.6 | 18.0 | 20.9 | 17.8 | 17.2 | 17.4 | 15.6 | 14.8 | 15.6 |
| Tmem181b-ps | 6.0 | 5.9 | 5.1 | 6.0 | 6.4 | 6.6 | 5.9 | 5.5 | 6.0 | 5.0 | 5.3 | 5.1 |
| Tmem181c-ps | 4.7 | 4.7 | 4.2 | 4.0 | 4.9 | 5.3 | 4.4 | 4.3 | 4.5 | 4.1 | 4.0 | 4.3 |
| Tmem183a | 15.5 | 15.3 | 12.8 | 13.0 | 13.6 | 16.0 | 14.4 | 13.0 | 17.0 | 15.1 | 16.3 | 14.9 |
| Tmem184a | 1.7 | 1.3 | 1.4 | 1.3 | 0.8 | 1.2 | 1.1 | 1.7 | 1.2 | 1.1 | 2.0 | 0.7 |
| Tmem184b | 24.1 | 27.1 | 22.2 | 30.5 | 27.7 | 28.0 | 27.4 | 20.2 | 21.1 | 19.5 | 21.1 | 18.6 |
| Tmem184c | 27.2 | 26.8 | 29.9 | 24.8 | 24.8 | 25.1 | 28.7 | 27.1 | 24.7 | 28.7 | 32.4 | 32.8 |
| Tmem185b | 5.5 | 5.3 | 4.9 | 5.2 | 5.9 | 4.9 | 5.3 | 5.9 | 6.2 | 6.5 | 5.9 | 5.6 |
| Tmem186 | 3.5 | 3.2 | 4.1 | 4.8 | 3.8 | 3.4 | 4.2 | 3.3 | 3.4 | 3.5 | 3.9 | 4.1 |
| Tmem189 | 23.2 | 21.4 | 21.2 | 29.9 | 29.0 | 23.0 | 24.5 | 23.3 | 23.0 | 19.1 | 24.2 | 20.9 |
| Tmem19 | 11.4 | 13.0 | 13.9 | 9.6 | 14.1 | 10.7 | 12.9 | 12.3 | 14.4 | 11.9 | 12.4 | 12.5 |
| Tmem191c | 0.2 | 0.1 | 0.1 | 0.1 | 0.2 | 0.3 | 0.1 | 0.3 | 0.2 | 0.1 | 0.0 | 0.2 |
| Tmem192 | 16.9 | 10.8 | 9.7 | 18.3 | 13.6 | 14.9 | 12.5 | 9.7 | 8.5 | 11.1 | 10.9 | 10.8 |
| Tmem194 | 1.1 | 1.4 | 1.8 | 0.8 | 1.7 | 0.9 | 2.0 | 1.2 | 1.5 | 1.1 | 1.6 | 1.5 |
| Tmem194b | 0.6 | 0.9 | 1.1 | 0.4 | 0.9 | 0.7 | 0.6 | 0.7 | 0.8 | 0.6 | 0.5 | 0.7 |
| Tmem198 | 0.4 | 0.4 | 0.3 | 0.5 | 0.4 | 0.4 | 0.4 | 0.4 | 0.4 | 0.2 | 0.3 | 0.2 |
| Tmem198b | 8.0 | 8.2 | 9.2 | 6.4 | 8.1 | 7.1 | 8.8 | 9.4 | 9.4 | 9.2 | 8.4 | 9.7 |
| Tmem199 | 18.9 | 16.2 | 13.8 | 17.0 | 15.1 | 17.9 | 12.8 | 11.1 | 13.0 | 13.2 | 14.3 | 14.8 |
| Tmem2 | 4.5 | 4.4 | 5.4 | 3.2 | 4.8 | 3.1 | 5.4 | 5.7 | 5.5 | 9.7 | 7.1 | 6.0 |
| Tmem200a | 0.6 | 0.8 | 1.2 | 0.6 | 1.2 | 0.9 | 2.7 | 0.7 | 1.9 | 1.8 | 1.8 | 2.0 |
| Tmem200b | 1.1 | 1.0 | 1.3 | 0.5 | 0.4 | 0.7 | 0.9 | 1.1 | 1.5 | 1.2 | 0.9 | 0.6 |
| Tmem200c | 0.0 | 0.0 | 0.0 | 0.0 | 0.0 | 0.0 | 0.0 | 0.0 | 0.0 | 0.0 | 0.0 | 0.0 |
| Tmem201 | 2.1 | 2.7 | 2.4 | 2.4 | 2.3 | 3.0 | 2.7 | 2.4 | 2.6 | 2.5 | 2.0 | 2.1 |
| Tmem202 | 2.8 | 1.8 | 1.4 | 4.7 | 1.7 | 3.3 | 1.7 | 1.5 | 1.6 | 3.7 | 2.2 | 3.1 |
| Tmem203 | 7.4 | 8.0 | 7.0 | 7.8 | 8.3 | 10.7 | 7.3 | 7.5 | 7.6 | 10.5 | 5.3 | 7.4 |
| Tmem204 | 7.7 | 7.8 | 6.2 | 13.0 | 11.7 | 8.5 | 11.5 | 6.5 | 7.8 | 7.4 | 5.2 | 6.3 |
| Tmem205 | 34.7 | 29.4 | 20.3 | 32.9 | 29.6 | 39.6 | 20.8 | 24.7 | 30.2 | 30.4 | 25.1 | 21.8 |
| Tmem206 | 2.2 | 1.9 | 1.6 | 3.8 | 3.3 | 2.5 | 2.1 | 1.5 | 1.8 | 2.2 | 1.9 | 1.8 |
| Tmem208 | 26.6 | 23.0 | 20.3 | 26.2 | 30.1 | 26.1 | 28.8 | 28.7 | 31.0 | 34.0 | 28.4 | 28.9 |
| Tmem209 | 8.8 | 8.0 | 7.4 | 10.7 | 8.4 | 9.3 | 8.4 | 7.9 | 7.9 | 7.1 | 8.4 | 7.2 |
| Tmem214 | 17.5 | 15.1 | 15.4 | 20.1 | 15.5 | 16.6 | 18.4 | 17.9 | 17.0 | 16.3 | 19.1 | 16.9 |
| Tmem215 | 0.0 | 0.1 | 0.1 | 0.0 | 0.0 | 0.0 | 0.1 | 0.0 | 0.1 | 0.1 | 0.1 | 0.1 |
| Tmem216 | 4.4 | 4.4 | 3.9 | 4.9 | 3.9 | 4.3 | 3.2 | 3.7 | 4.1 | 4.0 | 3.9 | 3.1 |
| Tmem217 | 0.1 | 0.3 | 0.0 | 0.0 | 0.0 | 0.1 | 0.0 | 0.0 | 0.1 | 0.1 | 0.1 | 0.1 |

| | | | | | | | | | | | | |
|---|---|---|---|---|---|---|---|---|---|---|---|---|
| Tmem218 | 14.9 | 13.0 | 12.4 | 14.8 | 10.4 | 15.8 | 12.5 | 8.7 | 12.4 | 11.8 | 14.1 | 15.2 |
| Tmem219 | 20.3 | 15.5 | 14.9 | 15.6 | 17.0 | 18.1 | 14.9 | 16.2 | 17.3 | 15.9 | 14.2 | 16.2 |
| Tmem220 | 2.0 | 2.9 | 2.8 | 2.8 | 2.1 | 2.5 | 1.4 | 2.0 | 1.8 | 1.5 | 1.9 | 1.3 |
| Tmem221 | 0.1 | 0.0 | 0.0 | 0.4 | 0.3 | 0.0 | 0.1 | 0.0 | 0.1 | 0.2 | 0.0 | 0.1 |
| Tmem222 | 15.8 | 14.8 | 13.8 | 15.5 | 15.1 | 16.7 | 16.4 | 14.3 | 15.7 | 16.6 | 15.4 | 14.9 |
| Tmem223 | 25.5 | 23.5 | 22.9 | 32.2 | 22.3 | 29.9 | 25.0 | 25.2 | 22.1 | 22.9 | 23.7 | 23.7 |
| Tmem229b | 1.1 | 1.0 | 1.5 | 1.9 | 2.6 | 1.1 | 1.4 | 1.2 | 1.1 | 1.2 | 1.2 | 1.0 |
| Tmem230 | 15.3 | 14.8 | 13.5 | 14.2 | 15.2 | 13.0 | 12.0 | 12.2 | 13.4 | 14.5 | 14.1 | 13.6 |
| Tmem231 | 3.9 | 3.7 | 3.5 | 3.3 | 2.4 | 5.4 | 3.7 | 3.4 | 2.7 | 3.6 | 3.4 | 3.5 |
| Tmem232 | 0.0 | 0.0 | 0.1 | 0.0 | 0.0 | 0.0 | 0.0 | 0.0 | 0.0 | 0.0 | 0.0 | 0.0 |
| Tmem234 | 37.6 | 32.9 | 29.9 | 32.5 | 37.7 | 32.2 | 33.0 | 40.5 | 34.1 | 35.6 | 36.7 | 34.9 |
| Tmem236 | 0.0 | 0.0 | 0.0 | 0.0 | 0.0 | 0.0 | 0.0 | 0.0 | 0.0 | 0.0 | 0.0 | 0.0 |
| Tmem237 | 12.0 | 10.6 | 11.7 | 10.7 | 10.5 | 11.7 | 14.1 | 14.0 | 12.1 | 12.7 | 13.3 | 12.8 |
| Tmem238 | 1.3 | 0.7 | 0.5 | 0.9 | 0.8 | 0.9 | 1.4 | 1.0 | 0.8 | 1.4 | 0.8 | 1.2 |
| Tmem239 | 0.1 | 0.1 | 0.1 | 0.0 | 0.0 | 0.1 | 0.0 | 0.0 | 0.0 | 0.0 | 0.0 | 0.0 |
| Tmem240 | 0.2 | 0.0 | 0.1 | 0.1 | 0.2 | 0.3 | 0.1 | 0.2 | 0.2 | 0.2 | 0.3 | 0.1 |
| Tmem241 | 2.0 | 2.5 | 2.1 | 2.2 | 1.8 | 1.8 | 1.3 | 1.8 | 1.8 | 1.7 | 2.0 | 2.0 |
| Tmem242 | 51.8 | 43.9 | 41.2 | 49.2 | 42.8 | 55.0 | 41.2 | 41.9 | 42.5 | 49.6 | 48.1 | 51.9 |
| Tmem243 | 2.1 | 2.7 | 3.1 | 4.0 | 5.0 | 3.1 | 2.9 | 2.2 | 1.9 | 2.4 | 2.1 | 2.4 |
| Tmem245 | 6.2 | 5.3 | 4.9 | 5.7 | 4.8 | 6.2 | 5.0 | 4.3 | 4.4 | 5.5 | 5.3 | 4.4 |
| Tmem246 | 2.2 | 1.8 | 1.7 | 2.2 | 1.3 | 2.7 | 1.9 | 2.1 | 2.1 | 1.3 | 1.2 | 1.5 |
| Tmem248 | 26.1 | 24.2 | 21.2 | 27.4 | 21.8 | 27.2 | 24.9 | 22.0 | 20.4 | 19.5 | 21.8 | 21.7 |
| Tmem25 | 0.1 | 0.1 | 0.1 | 0.0 | 0.0 | 0.2 | 0.0 | 0.0 | 0.0 | 0.1 | 0.1 | 0.0 |
| Tmem251 | 5.2 | 4.6 | 4.7 | 6.4 | 6.0 | 6.5 | 4.9 | 4.7 | 5.3 | 6.0 | 5.1 | 5.2 |
| Tmem253 | 0.5 | 0.3 | 0.8 | 1.0 | 0.5 | 0.8 | 0.2 | 0.6 | 0.5 | 0.3 | 0.3 | 0.5 |
| Tmem254a | 0.7 | 1.2 | 0.9 | 1.1 | 0.7 | 0.5 | 0.6 | 0.8 | 0.8 | 0.5 | 0.9 | 0.5 |
| Tmem254b | 11.1 | 11.3 | 12.6 | 9.7 | 11.0 | 11.0 | 12.7 | 12.3 | 12.4 | 8.4 | 11.8 | 10.6 |
| Tmem254c | 3.9 | 1.5 | 2.1 | 3.0 | 0.4 | 2.9 | 0.2 | 6.1 | 1.5 | 2.8 | 3.5 | 0.4 |
| Tmem255a | 0.1 | 0.1 | 0.2 | 0.1 | 0.1 | 0.1 | 0.1 | 0.1 | 0.0 | 0.1 | 0.1 | 0.1 |
| Tmem255b | 0.0 | 0.0 | 0.1 | 0.0 | 0.1 | 0.0 | 0.0 | 0.1 | 0.0 | 0.0 | 0.1 | 0.1 |
| Tmem256 | 73.3 | 64.7 | 51.3 | 55.3 | 71.8 | 73.0 | 57.0 | 73.1 | 73.6 | 60.7 | 58.3 | 54.8 |
| Tmem258 | 161.3 | 157.8 | 147.4 | 165.3 | 152.4 | 161.3 | 162.9 | 162.0 | 160.8 | 150.2 | 177.4 | 164.3 |
| Tmem259 | 34.2 | 33.3 | 32.2 | 35.0 | 33.1 | 30.3 | 44.9 | 39.3 | 40.6 | 33.5 | 35.8 | 32.0 |
| Tmem26 | 0.0 | 0.0 | 0.0 | 0.0 | 0.0 | 0.0 | 0.0 | 0.0 | 0.0 | 0.0 | 0.0 | 0.0 |
| Tmem27 | 0.0 | 0.0 | 0.1 | 0.0 | 0.0 | 0.1 | 0.0 | 0.1 | 0.0 | 0.1 | 0.0 | 0.0 |
| Tmem29 | 6.0 | 5.7 | 6.0 | 5.8 | 5.2 | 4.7 | 7.5 | 7.6 | 6.8 | 5.9 | 7.5 | 6.7 |
| Tmem30a | 48.9 | 49.3 | 49.5 | 59.1 | 54.7 | 62.8 | 48.3 | 41.2 | 51.5 | 53.0 | 54.4 | 58.8 |
| Tmem30b | 0.0 | 0.0 | 0.0 | 0.0 | 0.0 | 0.0 | 0.0 | 0.0 | 0.0 | 0.1 | 0.1 | 0.1 |
| Tmem33 | 14.1 | 14.9 | 14.4 | 19.9 | 13.8 | 17.9 | 14.1 | 10.9 | 13.4 | 16.7 | 17.3 | 15.9 |
| Tmem35 | 0.4 | 0.7 | 1.1 | 0.2 | 1.2 | 0.6 | 0.5 | 0.4 | 0.5 | 1.3 | 1.5 | 0.6 |
| Tmem37 | 24.7 | 17.0 | 6.3 | 47.8 | 17.6 | 41.7 | 12.5 | 6.7 | 9.0 | 11.4 | 9.7 | 6.2 |
| Tmem38a | 3.6 | 3.0 | 2.6 | 4.0 | 3.8 | 3.3 | 4.1 | 4.1 | 3.6 | 4.0 | 3.8 | 3.6 |
| Tmem38b | 7.2 | 7.4 | 8.6 | 10.1 | 8.3 | 6.3 | 7.8 | 8.2 | 11.1 | 8.6 | 8.7 | 9.7 |
| Tmem39a | 12.6 | 13.6 | 12.4 | 10.3 | 14.5 | 9.9 | 13.7 | 14.9 | 17.8 | 14.1 | 13.8 | 13.7 |
| Tmem39b | 1.1 | 1.6 | 1.2 | 1.2 | 1.3 | 1.2 | 1.7 | 1.8 | 1.6 | 1.5 | 1.6 | 1.1 |
| Tmem40 | 0.7 | 0.4 | 0.5 | 0.6 | 0.4 | 0.3 | 0.4 | 0.5 | 0.3 | 0.3 | 0.2 | 0.3 |
| Tmem41a | 5.1 | 5.3 | 3.1 | 8.0 | 5.1 | 5.3 | 4.8 | 3.6 | 4.5 | 6.0 | 4.7 | 4.7 |
| Tmem41b | 11.9 | 12.5 | 11.4 | 11.1 | 9.8 | 11.2 | 12.7 | 12.1 | 12.6 | 11.5 | 14.3 | 11.3 |
| Tmem42 | 4.2 | 2.6 | 2.7 | 3.4 | 3.4 | 3.1 | 2.6 | 3.1 | 3.5 | 3.3 | 3.8 | 3.9 |
| Tmem43 | 55.4 | 49.4 | 49.5 | 66.7 | 52.5 | 67.7 | 50.3 | 63.0 | 45.9 | 42.3 | 46.1 | 42.6 |
| Tmem44 | 0.3 | 0.2 | 0.3 | 0.2 | 0.2 | 0.3 | 0.3 | 0.2 | 0.3 | 0.4 | 0.3 | 0.2 |
| Tmem45a | 112.5 | 153.7 | 208.7 | 72.5 | 140.0 | 63.0 | 159.6 | 175.3 | 157.4 | 141.7 | 152.4 | 182.3 |
| Tmem45b | 0.1 | 0.1 | 0.1 | 0.0 | 0.1 | 0.1 | 0.3 | 0.1 | 0.1 | 0.3 | 0.0 | 0.1 |
| Tmem47 | 0.7 | 0.7 | 1.3 | 0.8 | 1.2 | 1.5 | 2.6 | 2.1 | 2.5 | 1.8 | 2.7 | 1.9 |
| Tmem48 | 5.4 | 6.0 | 6.2 | 7.1 | 6.9 | 6.2 | 7.2 | 5.0 | 5.8 | 5.5 | 6.3 | 4.5 |
| Tmem5 | 54.0 | 43.5 | 39.3 | 47.1 | 40.2 | 48.5 | 46.0 | 37.3 | 44.5 | 45.0 | 48.4 | 47.4 |
| Tmem50a | 100.8 | 80.7 | 78.8 | 121.1 | 89.5 | 108.7 | 93.4 | 83.2 | 84.6 | 91.8 | 92.4 | 100.6 |

| | | | | | | | | | | | | |
|---|---|---|---|---|---|---|---|---|---|---|---|---|
| Tmem50b | 11.7 | 9.2 | 8.3 | 11.3 | 11.3 | 8.6 | 11.7 | 9.4 | 10.1 | 10.1 | 12.6 | 9.9 |
| Tmem51 | 0.3 | 0.3 | 0.3 | 0.4 | 0.7 | 0.1 | 0.3 | 0.2 | 0.4 | 0.5 | 0.6 | 0.2 |
| Tmem51as1 | 0.0 | 0.0 | 0.0 | 0.0 | 0.0 | 0.0 | 0.0 | 0.0 | 0.0 | 0.0 | 0.0 | 0.0 |
| Tmem52 | 0.1 | 0.0 | 0.0 | 0.0 | 0.0 | 0.0 | 0.0 | 0.1 | 0.0 | 0.1 | 0.0 | 0.1 |
| Tmem52b | 0.0 | 0.0 | 0.0 | 0.0 | 0.0 | 0.0 | 0.1 | 0.0 | 0.0 | 0.0 | 0.0 | 0.1 |
| Tmem53 | 7.7 | 6.7 | 7.7 | 7.0 | 5.6 | 6.0 | 7.3 | 6.7 | 8.6 | 7.6 | 5.4 | 6.8 |
| Tmem54 | 0.5 | 0.2 | 0.2 | 0.3 | 0.3 | 0.1 | 0.3 | 0.0 | 0.2 | 0.0 | 0.1 | 0.2 |
| Tmem55a | 13.7 | 13.2 | 13.8 | 15.6 | 12.5 | 16.2 | 12.0 | 13.5 | 12.9 | 14.7 | 15.2 | 14.7 |
| Tmem55b | 19.9 | 21.1 | 16.9 | 18.0 | 17.0 | 19.4 | 18.8 | 18.3 | 18.0 | 17.5 | 17.1 | 16.1 |
| Tmem56 | 0.3 | 0.1 | 0.1 | 0.7 | 0.1 | 0.5 | 0.2 | 0.1 | 0.1 | 0.2 | 0.1 | 0.2 |
| Tmem57 | 13.6 | 13.7 | 13.8 | 14.6 | 11.8 | 14.3 | 15.7 | 14.6 | 13.5 | 13.9 | 16.9 | 15.0 |
| Tmem59 | 191.1 | 179.9 | 146.9 | 187.7 | 188.9 | 198.0 | 164.1 | 148.2 | 192.8 | 196.7 | 157.4 | 178.9 |
| Tmem59l | 2.3 | 1.9 | 0.6 | 0.7 | 0.8 | 3.4 | 2.2 | 1.0 | 0.7 | 1.0 | 1.5 | 1.2 |
| Tmem60 | 11.6 | 10.6 | 9.9 | 11.0 | 11.5 | 12.6 | 9.3 | 9.1 | 10.4 | 13.9 | 9.6 | 9.4 |
| Tmem62 | 6.8 | 7.7 | 6.1 | 6.6 | 5.1 | 6.1 | 7.3 | 6.7 | 8.9 | 6.5 | 7.6 | 7.8 |
| Tmem63a | 14.0 | 14.8 | 14.5 | 11.9 | 15.9 | 11.8 | 22.4 | 18.9 | 18.9 | 18.4 | 18.6 | 15.2 |
| Tmem63b | 9.6 | 9.2 | 9.5 | 10.2 | 9.0 | 10.0 | 10.7 | 8.7 | 10.0 | 10.6 | 10.6 | 8.4 |
| Tmem63c | 0.0 | 0.0 | 0.0 | 0.1 | 0.0 | 0.0 | 0.0 | 0.0 | 0.0 | 0.0 | 0.0 | 0.0 |
| Tmem64 | 9.3 | 12.0 | 14.6 | 7.4 | 10.4 | 7.6 | 11.3 | 15.7 | 17.0 | 11.4 | 12.0 | 13.4 |
| Tmem65 | 12.3 | 13.1 | 13.6 | 11.4 | 10.3 | 13.7 | 13.0 | 11.3 | 13.2 | 11.4 | 13.9 | 13.3 |
| Tmem66 | 129.9 | 118.4 | 84.6 | 144.1 | 113.6 | 136.3 | 111.8 | 89.6 | 113.0 | 125.8 | 103.6 | 115.7 |
| Tmem67 | 2.4 | 2.9 | 2.6 | 2.4 | 2.3 | 2.4 | 2.3 | 2.6 | 2.1 | 2.2 | 2.9 | 2.4 |
| Tmem68 | 5.6 | 5.8 | 6.3 | 6.4 | 6.9 | 6.4 | 6.1 | 5.5 | 5.8 | 6.6 | 6.3 | 6.5 |
| Tmem69 | 2.5 | 1.8 | 2.2 | 2.6 | 2.2 | 2.4 | 2.1 | 1.9 | 1.6 | 2.1 | 2.1 | 2.2 |
| Tmem70 | 16.2 | 15.7 | 12.8 | 13.5 | 15.1 | 11.8 | 15.1 | 13.1 | 15.7 | 18.3 | 18.0 | 14.8 |
| Tmem71 | 1.1 | 0.7 | 0.3 | 1.3 | 1.2 | 0.9 | 0.5 | 0.2 | 0.3 | 0.1 | 0.2 | 0.2 |
| Tmem72 | 0.0 | 0.0 | 0.0 | 0.0 | 0.0 | 0.0 | 0.0 | 0.0 | 0.0 | 0.0 | 0.0 | 0.0 |
| Tmem74 | 0.1 | 0.2 | 0.0 | 0.0 | 0.1 | 0.1 | 0.3 | 0.0 | 0.2 | 0.1 | 0.2 | 0.0 |
| Tmem74b | 0.2 | 0.0 | 0.0 | 0.2 | 0.0 | 0.2 | 0.2 | 0.1 | 0.1 | 0.1 | 0.1 | 0.1 |
| Tmem79 | 1.0 | 0.6 | 0.6 | 1.1 | 1.1 | 1.0 | 0.8 | 0.7 | 0.6 | 0.7 | 0.8 | 0.6 |
| Tmem8 | 0.6 | 0.6 | 0.4 | 1.6 | 0.9 | 0.8 | 0.6 | 0.3 | 0.3 | 0.8 | 0.7 | 0.5 |
| Tmem80 | 7.5 | 6.2 | 5.3 | 8.4 | 6.2 | 6.9 | 6.1 | 6.2 | 6.5 | 6.2 | 8.1 | 6.3 |
| Tmem81 | 1.2 | 1.5 | 1.2 | 1.3 | 1.2 | 1.3 | 1.1 | 1.2 | 1.1 | 1.3 | 0.9 | 1.1 |
| Tmem82 | 0.1 | 0.0 | 0.1 | 0.1 | 0.2 | 0.1 | 0.0 | 0.0 | 0.0 | 0.1 | 0.1 | 0.1 |
| Tmem86a | 2.6 | 2.1 | 3.0 | 3.7 | 5.0 | 2.7 | 2.4 | 2.8 | 3.4 | 3.7 | 2.0 | 3.4 |
| Tmem86b | 0.4 | 0.3 | 0.2 | 0.2 | 0.4 | 0.1 | 0.3 | 0.2 | 0.1 | 0.3 | 0.3 | 0.1 |
| Tmem87a | 8.5 | 9.7 | 10.1 | 9.5 | 10.1 | 9.2 | 9.8 | 12.2 | 10.6 | 10.6 | 10.1 | 11.6 |
| Tmem87b | 15.5 | 15.2 | 14.8 | 15.4 | 12.8 | 16.4 | 13.9 | 12.4 | 14.7 | 14.9 | 13.9 | 16.4 |
| Tmem88 | 3.4 | 3.3 | 3.0 | 3.2 | 2.7 | 4.1 | 3.4 | 3.6 | 3.5 | 4.1 | 4.6 | 4.3 |
| Tmem88b | 0.1 | 0.2 | 0.2 | 0.1 | 0.1 | 0.1 | 0.1 | 0.3 | 0.2 | 0.1 | 0.1 | 0.1 |
| Tmem8b | 0.9 | 1.1 | 0.7 | 0.7 | 0.5 | 1.1 | 0.5 | 0.5 | 0.5 | 0.4 | 0.5 | 0.5 |
| Tmem8c | 0.1 | 0.2 | 0.2 | 0.1 | 0.1 | 0.0 | 0.0 | 0.1 | 0.1 | 0.0 | 0.0 | 0.0 |
| Tmem9 | 12.7 | 13.8 | 13.1 | 11.6 | 11.5 | 13.7 | 13.2 | 15.9 | 13.7 | 12.0 | 12.6 | 11.5 |
| Tmem91 | 0.3 | 0.2 | 0.1 | 0.2 | 0.3 | 0.6 | 0.0 | 0.6 | 0.3 | 0.1 | 0.5 | 0.3 |
| Tmem95 | 0.0 | 0.1 | 0.2 | 0.1 | 0.1 | 0.1 | 0.1 | 0.2 | 0.1 | 0.4 | 0.1 | 0.4 |
| Tmem97 | 23.5 | 24.1 | 24.5 | 15.4 | 22.6 | 16.8 | 35.7 | 38.5 | 33.1 | 18.6 | 33.4 | 19.6 |
| Tmem98 | 69.9 | 62.0 | 48.6 | 50.3 | 47.4 | 51.0 | 48.0 | 47.3 | 57.8 | 82.1 | 63.2 | 66.1 |
| Tmem9b | 29.4 | 30.9 | 27.4 | 30.3 | 29.1 | 28.4 | 27.7 | 26.7 | 30.0 | 28.3 | 28.0 | 29.7 |
| Tmf1 | 26.8 | 25.0 | 22.4 | 26.8 | 24.4 | 24.4 | 23.8 | 21.8 | 20.7 | 22.5 | 29.0 | 26.6 |
| Tmie | 0.2 | 0.1 | 0.1 | 0.0 | 0.0 | 0.1 | 0.0 | 0.0 | 0.0 | 0.0 | 0.0 | 0.0 |
| Tmigd1 | 0.0 | 0.2 | 0.0 | 0.1 | 0.1 | 0.0 | 0.0 | 0.0 | 0.0 | 0.0 | 0.1 | 0.0 |
| Tmod1 | 0.3 | 0.1 | 0.1 | 0.1 | 0.1 | 0.3 | 0.1 | 0.0 | 0.0 | 0.0 | 0.1 | 0.1 |
| Tmod2 | 0.8 | 0.9 | 1.2 | 0.8 | 0.7 | 1.2 | 1.0 | 1.4 | 0.8 | 0.6 | 0.9 | 0.8 |
| Tmod3 | 46.3 | 43.7 | 45.5 | 53.5 | 40.8 | 49.0 | 41.2 | 40.1 | 34.8 | 31.7 | 41.1 | 41.9 |
| Tmod4 | 0.0 | 0.2 | 0.0 | 0.0 | 0.0 | 0.0 | 0.1 | 0.1 | 0.1 | 0.1 | 0.1 | 0.0 |
| Tmpo | 20.5 | 19.4 | 22.6 | 13.2 | 12.2 | 14.3 | 18.6 | 22.6 | 18.5 | 11.8 | 19.0 | 12.0 |
| Tmppe | 1.7 | 1.9 | 1.9 | 2.1 | 2.3 | 1.7 | 2.3 | 2.0 | 1.9 | 2.3 | 1.8 | 1.7 |

| | | | | | | | | | | | | |
|---|---|---|---|---|---|---|---|---|---|---|---|---|
| Tmprss11e | 0.0 | 0.0 | 0.1 | 0.0 | 0.0 | 0.0 | 0.0 | 0.0 | 0.0 | 0.0 | 0.0 | 0.0 |
| Tmprss2 | 0.0 | 0.0 | 0.0 | 0.0 | 0.0 | 0.0 | 0.0 | 0.0 | 0.0 | 0.0 | 0.0 | 0.0 |
| Tmprss5 | 0.0 | 0.0 | 0.0 | 0.0 | 0.1 | 0.0 | 0.0 | 0.0 | 0.0 | 0.0 | 0.0 | 0.0 |
| Tmprss6 | 0.1 | 0.0 | 0.1 | 0.1 | 0.0 | 0.1 | 0.0 | 0.1 | 0.0 | 0.0 | 0.0 | 0.0 |
| Tmprss7 | 0.1 | 0.1 | 0.1 | 0.1 | 0.2 | 0.1 | 0.1 | 0.0 | 0.2 | 0.2 | 0.2 | 0.1 |
| Tmprss9 | 0.0 | 0.0 | 0.0 | 0.0 | 0.1 | 0.0 | 0.0 | 0.0 | 0.0 | 0.0 | 0.0 | 0.1 |
| Tmsb10 | 457.4 | 346.0 | 368.8 | 557.8 | 309.2 | 472.3 | 420.9 | 635.3 | 412.3 | 426.2 | 524.3 | 456.4 |
| Tmsb15b1 | 0.7 | 0.2 | 0.4 | 1.1 | 0.8 | 1.3 | 0.0 | 0.5 | 0.2 | 0.7 | 0.9 | 1.2 |
| Tmsb15b2 | 0.0 | 0.0 | 0.1 | 0.0 | 0.0 | 0.0 | 0.1 | 0.0 | 0.0 | 0.0 | 0.0 | 0.0 |
| Tmsb15l | 1.7 | 1.2 | 0.6 | 0.4 | 0.4 | 0.8 | 0.8 | 1.0 | 0.9 | 1.9 | 1.3 | 1.2 |
| Tmsb4x | 1096.7 | 938.6 | 797.3 | 1102.8 | 1919.7 | 725.9 | 1345.9 | 1461.5 | 2466.6 | 2210.3 | 1924.8 | 1995.3 |
| Tmtc1 | 0.7 | 1.7 | 3.1 | 0.9 | 0.8 | 0.8 | 0.7 | 1.7 | 0.9 | 0.6 | 0.9 | 0.9 |
| Tmtc2 | 0.4 | 0.3 | 0.4 | 0.2 | 0.3 | 0.2 | 0.4 | 0.4 | 0.3 | 1.2 | 0.5 | 0.5 |
| Tmtc3 | 10.9 | 12.0 | 11.8 | 11.5 | 12.5 | 10.7 | 12.4 | 9.9 | 11.4 | 11.2 | 12.8 | 11.0 |
| Tmtc4 | 3.1 | 3.4 | 3.4 | 4.0 | 3.1 | 3.6 | 4.4 | 5.1 | 4.7 | 3.0 | 4.6 | 4.4 |
| Tmub1 | 2.4 | 1.7 | 2.0 | 2.7 | 1.6 | 4.0 | 1.7 | 1.9 | 2.0 | 2.8 | 2.2 | 2.0 |
| Tmub2 | 12.8 | 13.4 | 11.9 | 13.3 | 11.9 | 15.2 | 12.4 | 12.8 | 14.4 | 12.4 | 12.4 | 12.8 |
| Tmx1 | 14.4 | 15.7 | 19.4 | 17.4 | 17.4 | 18.9 | 16.4 | 15.8 | 16.9 | 16.4 | 18.3 | 20.4 |
| Tmx2 | 29.4 | 27.9 | 20.4 | 34.7 | 25.3 | 35.2 | 25.5 | 23.9 | 24.2 | 24.8 | 25.5 | 24.5 |
| Tmx3 | 19.5 | 20.5 | 21.2 | 20.9 | 20.0 | 22.0 | 18.1 | 22.4 | 20.0 | 18.9 | 23.5 | 22.6 |
| Tmx4 | 10.0 | 10.5 | 11.0 | 13.4 | 11.5 | 11.8 | 11.5 | 8.6 | 10.2 | 10.0 | 11.5 | 10.6 |
| Tnc | 24.9 | 33.1 | 47.9 | 20.3 | 28.6 | 16.2 | 41.1 | 42.3 | 31.6 | 16.6 | 26.9 | 27.6 |
| Tnf | 0.9 | 0.1 | 0.1 | 6.9 | 13.3 | 0.1 | 2.7 | 0.3 | 0.4 | 0.8 | 0.2 | 0.7 |
| Tnfaip1 | 26.3 | 27.5 | 24.8 | 26.0 | 28.4 | 21.5 | 33.2 | 30.2 | 34.9 | 23.7 | 27.4 | 26.0 |
| Tnfaip2 | 28.0 | 33.4 | 41.0 | 36.2 | 55.4 | 26.0 | 44.0 | 42.5 | 38.6 | 43.5 | 36.0 | 40.4 |
| Tnfaip3 | 4.6 | 2.7 | 2.6 | 4.6 | 6.0 | 6.3 | 2.9 | 1.5 | 2.2 | 2.4 | 2.6 | 2.3 |
| Tnfaip6 | 4.6 | 6.0 | 7.4 | 1.5 | 5.0 | 2.1 | 6.8 | 5.7 | 5.7 | 4.4 | 4.0 | 5.8 |
| Tnfaip8 | 2.1 | 2.0 | 3.4 | 3.4 | 6.6 | 1.4 | 2.6 | 2.6 | 2.6 | 5.7 | 3.2 | 3.3 |
| Tnfaip8l1 | 0.5 | 0.4 | 0.5 | 0.2 | 0.5 | 0.2 | 0.7 | 0.4 | 0.5 | 0.2 | 0.1 | 0.2 |
| Tnfaip8l2 | 1.0 | 0.1 | 0.2 | 5.2 | 7.8 | 0.0 | 1.2 | 0.1 | 0.1 | 0.6 | 0.3 | 0.8 |
| Tnfaip8l3 | 0.0 | 0.0 | 0.0 | 0.0 | 0.0 | 0.0 | 0.0 | 0.0 | 0.0 | 0.0 | 0.0 | 0.0 |
| Tnfrsf10b | 12.3 | 12.6 | 12.4 | 10.8 | 13.1 | 13.2 | 13.8 | 10.6 | 12.6 | 12.7 | 13.6 | 11.4 |
| Tnfrsf11a | 0.1 | 0.0 | 0.0 | 0.8 | 1.5 | 0.0 | 0.1 | 0.1 | 0.1 | 0.1 | 0.1 | 0.1 |
| Tnfrsf11b | 26.7 | 22.3 | 15.3 | 37.5 | 33.3 | 27.0 | 32.3 | 8.1 | 25.0 | 21.8 | 26.1 | 25.6 |
| Tnfrsf12a | 63.6 | 43.0 | 26.6 | 59.1 | 55.4 | 40.7 | 73.1 | 38.7 | 61.1 | 88.4 | 63.7 | 54.9 |
| Tnfrsf13b | 0.0 | 0.0 | 0.0 | 0.1 | 0.4 | 0.0 | 0.0 | 0.0 | 0.2 | 0.0 | 0.0 | 0.0 |
| Tnfrsf13c | 0.0 | 0.0 | 0.0 | 0.0 | 0.0 | 0.0 | 0.0 | 0.0 | 0.0 | 0.0 | 0.0 | 0.0 |
| Tnfrsf14 | 2.4 | 2.2 | 2.7 | 3.8 | 2.1 | 3.1 | 2.5 | 2.8 | 3.0 | 1.6 | 1.9 | 1.9 |
| Tnfrsf18 | 0.6 | 1.7 | 1.7 | 0.5 | 2.5 | 1.5 | 1.1 | 0.5 | 0.7 | 0.6 | 0.8 | 0.7 |
| Tnfrsf19 | 0.0 | 0.0 | 0.1 | 0.1 | 0.0 | 0.0 | 0.0 | 0.0 | 0.0 | 0.0 | 0.0 | 0.0 |
| Tnfrsf1a | 40.6 | 46.9 | 53.4 | 35.3 | 49.4 | 36.3 | 50.8 | 55.5 | 52.8 | 48.0 | 44.2 | 46.7 |
| Tnfrsf1b | 17.9 | 15.0 | 14.4 | 20.7 | 29.5 | 12.4 | 20.9 | 13.9 | 17.9 | 16.1 | 14.3 | 18.4 |
| Tnfrsf21 | 1.1 | 0.6 | 0.6 | 2.0 | 1.9 | 1.1 | 1.0 | 0.6 | 0.6 | 1.2 | 0.8 | 0.6 |
| Tnfrsf22 | 1.8 | 1.6 | 1.1 | 1.9 | 1.6 | 1.8 | 1.8 | 1.5 | 1.7 | 1.3 | 1.9 | 1.7 |
| Tnfrsf23 | 9.6 | 7.6 | 6.2 | 13.3 | 10.4 | 9.5 | 9.8 | 5.7 | 6.1 | 9.5 | 8.7 | 9.2 |
| Tnfrsf25 | 0.6 | 1.4 | 0.8 | 0.6 | 0.7 | 0.7 | 1.3 | 1.9 | 1.6 | 0.8 | 0.9 | 0.9 |
| Tnfrsf26 | 44.0 | 35.8 | 25.5 | 44.1 | 41.7 | 37.8 | 29.2 | 15.2 | 20.2 | 29.3 | 27.2 | 29.0 |
| Tnfrsf4 | 0.2 | 0.1 | 0.1 | 0.2 | 0.1 | 0.2 | 0.1 | 0.0 | 0.2 | 0.0 | 0.1 | 0.1 |
| Tnfrsf8 | 0.1 | 0.1 | 0.0 | 0.0 | 0.1 | 0.1 | 0.2 | 0.2 | 0.0 | 0.1 | 0.1 | 0.1 |
| Tnfrsf9 | 0.1 | 0.1 | 0.1 | 0.2 | 0.2 | 0.3 | 0.0 | 0.0 | 0.1 | 0.1 | 0.0 | 0.0 |
| Tnfsf10 | 0.0 | 0.0 | 0.0 | 0.0 | 0.0 | 0.1 | 0.0 | 0.1 | 0.0 | 0.0 | 0.0 | 0.0 |
| Tnfsf11 | 0.0 | 0.1 | 0.1 | 0.0 | 0.0 | 0.0 | 0.1 | 0.1 | 0.1 | 0.1 | 0.1 | 0.1 |
| Tnfsf12 | 6.9 | 7.2 | 7.0 | 9.0 | 7.2 | 9.1 | 7.5 | 7.6 | 7.4 | 7.5 | 7.2 | 7.0 |
| Tnfsf13 | 1.1 | 2.6 | 1.8 | 3.6 | 2.2 | 1.6 | 1.6 | 2.1 | 1.8 | 3.3 | 2.4 | 2.0 |
| Tnfsf13b | 0.7 | 0.7 | 1.0 | 1.1 | 1.4 | 0.3 | 1.3 | 0.8 | 0.7 | 1.4 | 0.8 | 1.6 |
| Tnfsf14 | 0.1 | 0.0 | 0.0 | 0.3 | 0.9 | 0.0 | 0.0 | 0.0 | 0.0 | 0.0 | 0.0 | 0.1 |
| Tnfsf15 | 0.8 | 0.5 | 0.7 | 0.2 | 0.1 | 0.1 | 0.0 | 0.1 | 0.1 | 0.3 | 0.2 | 0.9 |

| | | | | | | | | | | | | |
|---|---|---|---|---|---|---|---|---|---|---|---|---|
| Tnfsf18 | 2.8 | 0.9 | 0.2 | 2.6 | 1.0 | 1.1 | 0.3 | 0.2 | 0.2 | 0.4 | 0.6 | 0.3 |
| Tnfsf4 | 0.1 | 0.2 | 0.1 | 0.5 | 0.5 | 0.3 | 0.2 | 0.0 | 0.2 | 0.1 | 0.2 | 0.1 |
| Tnfsf8 | 0.0 | 0.0 | 0.0 | 0.2 | 0.2 | 0.0 | 0.1 | 0.0 | 0.0 | 0.0 | 0.1 | 0.0 |
| Tnfsf9 | 1.6 | 2.0 | 2.9 | 2.9 | 4.5 | 2.3 | 1.8 | 2.0 | 1.7 | 1.8 | 1.5 | 2.3 |
| Tnik | 0.3 | 0.7 | 0.8 | 0.3 | 0.3 | 0.4 | 0.2 | 0.4 | 0.3 | 0.2 | 0.2 | 0.4 |
| Tnip1 | 19.7 | 15.6 | 24.0 | 31.4 | 41.4 | 19.8 | 31.4 | 20.5 | 18.4 | 15.9 | 17.5 | 23.5 |
| Tnip2 | 10.4 | 13.0 | 15.1 | 10.7 | 13.2 | 8.7 | 15.9 | 13.4 | 14.5 | 10.8 | 11.5 | 13.9 |
| Tnip3 | 0.3 | 0.0 | 0.0 | 1.8 | 4.2 | 0.0 | 0.6 | 0.1 | 0.1 | 0.4 | 0.2 | 0.4 |
| Tnk1 | 2.3 | 2.6 | 2.6 | 2.7 | 2.1 | 2.7 | 2.8 | 3.7 | 2.7 | 2.5 | 2.1 | 2.1 |
| Tnk2 | 7.9 | 6.9 | 4.9 | 7.7 | 5.8 | 6.8 | 7.5 | 4.9 | 7.4 | 9.4 | 7.9 | 5.2 |
| Tnks | 13.5 | 19.1 | 20.5 | 13.1 | 15.2 | 12.4 | 16.7 | 19.2 | 21.2 | 14.5 | 14.8 | 16.6 |
| Tnks1bp1 | 50.5 | 54.2 | 53.1 | 45.7 | 48.8 | 35.4 | 70.1 | 67.1 | 61.4 | 59.3 | 62.5 | 56.4 |
| Tnks2 | 28.6 | 32.0 | 28.6 | 25.2 | 28.1 | 32.1 | 24.0 | 24.7 | 29.1 | 32.7 | 31.4 | 31.2 |
| Tnmd | 0.1 | 0.0 | 0.1 | 0.0 | 0.1 | 0.3 | 0.2 | 0.3 | 0.2 | 0.5 | 1.2 | 0.3 |
| Tnn | 0.0 | 0.0 | 0.0 | 0.0 | 0.0 | 0.0 | 0.0 | 0.0 | 0.0 | 0.0 | 0.0 | 0.0 |
| Tnnc1 | 2.5 | 2.3 | 2.0 | 1.4 | 1.1 | 2.4 | 1.6 | 1.9 | 0.9 | 1.1 | 1.2 | 1.2 |
| Tnnc2 | 0.2 | 0.0 | 0.3 | 0.0 | 0.2 | 0.1 | 0.1 | 0.1 | 0.1 | 0.1 | 0.0 | 0.0 |
| Tnni1 | 0.2 | 0.2 | 0.0 | 0.2 | 0.1 | 0.1 | 0.1 | 0.3 | 0.1 | 0.1 | 0.2 | 0.2 |
| Tnni3 | 0.2 | 0.4 | 0.3 | 0.1 | 0.1 | 0.1 | 0.6 | 0.4 | 0.2 | 0.2 | 0.6 | 0.2 |
| Tnni3k | 0.2 | 0.1 | 0.0 | 0.2 | 0.2 | 0.1 | 0.1 | 0.2 | 0.1 | 0.1 | 0.1 | 0.1 |
| Tnnt1 | 0.2 | 0.2 | 0.7 | 0.4 | 0.3 | 0.4 | 0.2 | 0.7 | 0.3 | 0.1 | 0.1 | 0.2 |
| Tnnt2 | 8.2 | 7.5 | 8.6 | 2.9 | 2.3 | 3.1 | 2.9 | 14.8 | 2.6 | 6.3 | 9.2 | 11.4 |
| Tnnt3 | 0.5 | 0.5 | 0.6 | 0.3 | 0.6 | 0.7 | 0.2 | 0.2 | 0.4 | 0.3 | 0.7 | 0.4 |
| Tnpo1 | 29.7 | 29.6 | 25.1 | 29.3 | 28.4 | 33.7 | 26.2 | 27.9 | 25.5 | 28.3 | 30.8 | 28.3 |
| Tnpo2 | 34.3 | 29.3 | 24.2 | 33.6 | 31.4 | 32.1 | 32.8 | 25.7 | 30.8 | 28.9 | 32.5 | 29.3 |
| Tnpo3 | 21.5 | 20.0 | 19.8 | 19.0 | 19.6 | 20.1 | 20.6 | 21.5 | 19.0 | 22.1 | 23.5 | 21.2 |
| Tnr | 0.0 | 0.0 | 0.0 | 0.0 | 0.0 | 0.0 | 0.0 | 0.0 | 0.0 | 0.0 | 0.0 | 0.0 |
| Tnrc18 | 3.9 | 7.1 | 7.1 | 3.4 | 4.7 | 2.9 | 7.2 | 6.8 | 8.3 | 7.3 | 5.8 | 5.7 |
| Tnrc6a | 9.2 | 10.8 | 11.2 | 11.1 | 10.0 | 9.5 | 10.5 | 10.3 | 9.7 | 8.8 | 9.9 | 10.1 |
| Tnrc6b | 3.1 | 4.1 | 4.3 | 3.5 | 3.2 | 3.5 | 3.5 | 3.8 | 3.1 | 3.3 | 3.3 | 3.4 |
| Tnrc6c | 3.1 | 3.6 | 4.4 | 3.4 | 3.3 | 3.2 | 4.0 | 3.8 | 3.8 | 3.6 | 3.5 | 3.8 |
| Tns1 | 68.2 | 72.8 | 52.2 | 74.0 | 55.7 | 67.0 | 80.6 | 45.5 | 64.4 | 42.4 | 52.5 | 47.7 |
| Tns3 | 68.3 | 100.0 | 104.4 | 81.5 | 89.7 | 77.3 | 121.0 | 111.4 | 115.4 | 87.7 | 100.5 | 106.2 |
| Tns4 | 0.9 | 0.8 | 1.0 | 0.1 | 0.2 | 0.1 | 0.2 | 0.4 | 0.2 | 0.3 | 0.4 | 0.4 |
| Tnxb | 1.2 | 1.3 | 0.9 | 1.1 | 0.6 | 1.1 | 0.4 | 0.6 | 0.2 | 0.4 | 0.4 | 0.2 |
| Tob1 | 5.1 | 6.0 | 7.3 | 5.8 | 4.9 | 5.3 | 5.3 | 6.8 | 4.8 | 5.5 | 5.7 | 5.5 |
| Tob2 | 3.8 | 4.5 | 4.4 | 4.5 | 4.5 | 4.6 | 5.0 | 4.8 | 4.4 | 3.9 | 4.2 | 3.3 |
| Toe1 | 5.9 | 6.1 | 5.6 | 4.8 | 6.0 | 4.6 | 6.3 | 5.8 | 5.9 | 6.2 | 5.3 | 6.6 |
| Tollip | 27.5 | 25.9 | 22.3 | 28.2 | 25.4 | 27.6 | 25.5 | 21.2 | 24.4 | 24.3 | 23.5 | 23.0 |
| Tom1 | 15.1 | 12.0 | 10.8 | 17.9 | 13.2 | 15.6 | 13.2 | 12.3 | 11.6 | 12.4 | 11.9 | 12.9 |
| Tom1l1 | 5.6 | 5.2 | 5.5 | 4.8 | 3.6 | 5.0 | 3.5 | 4.3 | 3.8 | 4.2 | 5.1 | 5.1 |
| Tom1l2 | 11.7 | 12.6 | 10.8 | 11.7 | 10.5 | 11.6 | 11.5 | 9.6 | 11.1 | 12.9 | 10.0 | 10.2 |
| Tomm20 | 24.3 | 19.2 | 17.3 | 23.2 | 20.7 | 23.4 | 20.7 | 17.9 | 20.7 | 28.5 | 25.2 | 23.6 |
| Tomm22 | 74.5 | 68.7 | 63.7 | 73.8 | 68.2 | 66.7 | 65.7 | 65.2 | 68.9 | 73.3 | 67.4 | 69.4 |
| Tomm34 | 14.7 | 16.0 | 13.2 | 18.0 | 14.8 | 17.9 | 15.4 | 14.7 | 13.5 | 18.4 | 15.9 | 15.4 |
| Tomm40 | 28.0 | 24.8 | 19.2 | 27.0 | 25.7 | 25.2 | 26.8 | 22.9 | 23.1 | 24.3 | 24.7 | 21.2 |
| Tomm40l | 6.3 | 5.7 | 4.4 | 6.5 | 5.9 | 7.0 | 5.9 | 4.9 | 5.9 | 4.7 | 5.5 | 5.1 |
| Tomm5 | 57.5 | 43.5 | 35.6 | 52.9 | 51.0 | 53.2 | 47.1 | 43.5 | 48.2 | 54.7 | 51.8 | 42.0 |
| Tomm6 | 26.6 | 23.9 | 19.3 | 26.9 | 26.3 | 22.2 | 21.4 | 26.9 | 22.9 | 24.8 | 25.2 | 23.2 |
| Tomm7 | 47.3 | 37.3 | 30.7 | 44.4 | 41.4 | 47.2 | 32.9 | 33.2 | 32.4 | 39.1 | 38.7 | 38.1 |
| Tomm70a | 28.7 | 26.2 | 22.7 | 27.7 | 25.3 | 26.8 | 25.4 | 22.7 | 24.3 | 27.6 | 29.0 | 26.6 |
| Tomt | 0.8 | 1.1 | 0.8 | 1.3 | 1.1 | 0.9 | 0.9 | 0.5 | 0.7 | 1.4 | 0.7 | 1.0 |
| Tonsl | 0.5 | 0.7 | 1.4 | 0.4 | 1.3 | 0.6 | 1.4 | 0.8 | 1.4 | 0.8 | 0.9 | 0.9 |
| Top1 | 27.8 | 24.5 | 30.9 | 33.6 | 25.6 | 28.0 | 26.7 | 24.2 | 21.2 | 28.5 | 31.7 | 34.0 |
| Top1mt | 6.3 | 5.0 | 4.3 | 5.8 | 4.3 | 6.4 | 4.9 | 4.5 | 3.9 | 3.5 | 5.6 | 5.1 |
| Top2a | 9.0 | 18.2 | 30.0 | 3.1 | 24.2 | 3.2 | 28.9 | 12.2 | 24.2 | 12.7 | 20.6 | 15.0 |
| Top2b | 29.9 | 32.9 | 33.8 | 29.9 | 28.6 | 28.8 | 28.8 | 29.7 | 29.6 | 30.9 | 34.8 | 33.5 |

| | | | | | | | | | | | | |
|---|---|---|---|---|---|---|---|---|---|---|---|---|
| Top3a | 2.3 | 1.9 | 2.1 | 2.2 | 2.9 | 1.9 | 2.3 | 2.1 | 2.0 | 1.9 | 1.8 | 2.0 |
| Top3b | 15.9 | 16.4 | 14.9 | 15.6 | 13.5 | 14.8 | 13.6 | 13.2 | 13.7 | 15.4 | 14.6 | 14.4 |
| Topbp1 | 10.9 | 14.0 | 15.7 | 8.8 | 14.7 | 9.0 | 15.9 | 11.2 | 13.1 | 10.8 | 13.6 | 11.0 |
| Topors | 17.6 | 18.6 | 21.9 | 19.5 | 15.7 | 16.2 | 17.7 | 17.1 | 15.3 | 18.6 | 20.2 | 21.8 |
| Tor1a | 13.6 | 11.0 | 11.5 | 13.6 | 12.1 | 12.5 | 14.5 | 14.1 | 12.7 | 12.5 | 13.4 | 13.2 |
| Tor1aip1 | 14.7 | 14.2 | 15.1 | 17.3 | 15.8 | 15.2 | 18.9 | 16.8 | 15.9 | 12.2 | 15.3 | 16.7 |
| Tor1aip2 | 75.5 | 65.6 | 58.4 | 76.3 | 69.0 | 83.2 | 75.8 | 65.1 | 77.1 | 73.9 | 76.4 | 79.1 |
| Tor1b | 25.0 | 23.9 | 22.5 | 23.5 | 25.4 | 25.0 | 23.2 | 24.5 | 24.8 | 27.6 | 25.4 | 24.8 |
| Tor2a | 5.9 | 6.2 | 7.9 | 5.2 | 8.1 | 5.2 | 7.6 | 10.2 | 9.9 | 8.8 | 6.3 | 8.6 |
| Tor3a | 15.5 | 11.0 | 12.7 | 22.9 | 16.0 | 19.4 | 12.5 | 11.7 | 10.1 | 9.9 | 11.2 | 14.2 |
| Tor4a | 4.2 | 2.5 | 2.6 | 7.6 | 6.5 | 5.2 | 3.1 | 1.6 | 1.7 | 6.8 | 3.9 | 3.4 |
| Tox | 0.1 | 0.1 | 0.2 | 0.0 | 0.0 | 0.0 | 0.1 | 0.1 | 0.1 | 0.1 | 0.0 | 0.1 |
| Tox4 | 14.9 | 15.8 | 15.6 | 16.1 | 15.3 | 15.0 | 14.7 | 15.4 | 14.2 | 16.1 | 16.1 | 15.8 |
| Tpbg | 0.8 | 1.2 | 1.7 | 0.4 | 0.9 | 0.6 | 1.5 | 3.2 | 2.0 | 2.4 | 2.5 | 1.5 |
| Tpcn1 | 5.5 | 4.9 | 4.7 | 5.7 | 5.5 | 5.3 | 5.5 | 4.6 | 5.1 | 4.1 | 4.5 | 3.7 |
| Tpcn2 | 1.9 | 2.3 | 2.1 | 2.8 | 2.8 | 1.6 | 2.6 | 2.4 | 3.0 | 1.9 | 2.7 | 1.6 |
| Tpd52 | 12.7 | 11.6 | 7.9 | 24.5 | 25.0 | 18.6 | 9.7 | 5.9 | 6.4 | 11.1 | 11.9 | 9.2 |
| Tpd52l1 | 1.4 | 0.5 | 0.5 | 0.7 | 0.6 | 1.1 | 0.9 | 1.2 | 0.6 | 1.2 | 1.2 | 0.9 |
| Tpd52l2 | 37.5 | 36.8 | 33.4 | 36.4 | 31.9 | 38.4 | 37.6 | 35.8 | 34.5 | 37.2 | 41.9 | 37.7 |
| Tpgs1 | 10.4 | 10.5 | 10.1 | 10.5 | 10.4 | 9.5 | 11.0 | 13.8 | 11.0 | 12.6 | 10.1 | 10.0 |
| Tpgs2 | 4.5 | 3.7 | 3.6 | 4.4 | 3.8 | 4.9 | 3.4 | 3.7 | 3.2 | 3.9 | 4.6 | 3.3 |
| Tph2 | 0.1 | 0.0 | 0.0 | 0.0 | 0.0 | 0.0 | 0.1 | 0.1 | 0.0 | 0.0 | 0.1 | 0.1 |
| Tpi1 | 144.0 | 133.2 | 126.8 | 145.2 | 151.8 | 159.5 | 150.8 | 122.3 | 130.9 | 159.8 | 149.0 | 159.6 |
| Tpk1 | 3.7 | 3.3 | 1.8 | 3.8 | 2.8 | 3.7 | 1.9 | 1.8 | 2.0 | 2.5 | 2.5 | 2.0 |
| Tpm1 | 412.9 | 322.5 | 280.7 | 530.8 | 406.1 | 400.8 | 534.9 | 298.6 | 435.2 | 618.2 | 731.7 | 552.7 |
| Tpm2 | 58.3 | 47.1 | 42.0 | 69.7 | 47.1 | 58.2 | 108.5 | 43.5 | 73.7 | 91.0 | 137.8 | 98.9 |
| Tpm3 | 154.6 | 124.6 | 127.2 | 211.5 | 158.0 | 140.8 | 171.5 | 144.8 | 126.0 | 120.8 | 160.0 | 145.7 |
| Tpm4 | 561.8 | 570.7 | 545.1 | 743.4 | 596.8 | 598.5 | 737.8 | 571.8 | 643.2 | 533.4 | 741.6 | 641.7 |
| Tpmt | 2.4 | 2.9 | 3.3 | 2.2 | 2.2 | 2.8 | 2.5 | 2.9 | 2.5 | 1.5 | 2.9 | 2.3 |
| Tpp1 | 42.4 | 37.9 | 31.2 | 46.1 | 39.6 | 51.4 | 25.5 | 29.9 | 21.5 | 31.9 | 28.0 | 28.7 |
| Tpp2 | 23.6 | 24.2 | 22.0 | 25.4 | 29.4 | 21.5 | 22.4 | 24.1 | 23.3 | 25.6 | 26.1 | 24.2 |
| Tppp | 0.2 | 0.2 | 0.3 | 0.3 | 0.1 | 0.1 | 0.1 | 0.2 | 0.1 | 0.3 | 0.4 | 0.2 |
| Tppp3 | 0.7 | 0.5 | 0.6 | 1.2 | 0.8 | 0.6 | 0.4 | 0.5 | 0.5 | 0.3 | 0.4 | 0.7 |
| Tpr | 39.6 | 37.8 | 39.6 | 41.9 | 36.4 | 35.7 | 38.6 | 37.7 | 32.0 | 34.3 | 44.3 | 42.8 |
| Tpra1 | 8.1 | 9.6 | 8.5 | 10.8 | 11.0 | 12.7 | 8.7 | 9.4 | 8.6 | 7.8 | 8.5 | 6.9 |
| Tprgl | 66.4 | 54.5 | 45.7 | 69.4 | 55.3 | 67.2 | 56.0 | 52.8 | 60.6 | 63.5 | 65.1 | 59.6 |
| Tprkb | 12.1 | 9.4 | 9.4 | 11.6 | 8.8 | 10.1 | 9.1 | 9.0 | 8.2 | 9.8 | 10.4 | 9.7 |
| Tprn | 2.6 | 2.4 | 1.9 | 2.3 | 2.0 | 2.7 | 2.7 | 2.0 | 1.7 | 2.9 | 2.2 | 2.1 |
| Tpst1 | 24.6 | 28.7 | 37.5 | 14.1 | 24.3 | 20.1 | 28.0 | 30.7 | 25.6 | 26.5 | 25.5 | 26.1 |
| Tpst2 | 21.6 | 18.1 | 16.9 | 22.5 | 22.3 | 16.2 | 28.9 | 19.3 | 24.3 | 27.6 | 25.0 | 22.8 |
| Tpt1 | 781.8 | 665.3 | 680.5 | 635.9 | 709.2 | 729.3 | 626.0 | 853.7 | 789.5 | 856.6 | 811.8 | 820.5 |
| Tpte | 0.0 | 0.0 | 0.0 | 0.0 | 0.0 | 0.0 | 0.0 | 0.0 | 0.0 | 0.0 | 0.0 | 0.0 |
| Tpx2 | 3.7 | 6.3 | 10.9 | 2.4 | 9.2 | 2.0 | 13.0 | 5.3 | 9.6 | 4.9 | 8.8 | 6.2 |
| Tra2a | 30.0 | 27.9 | 32.8 | 31.9 | 30.3 | 24.2 | 30.6 | 30.6 | 30.4 | 34.6 | 35.7 | 36.7 |
| Tra2b | 20.1 | 20.9 | 28.0 | 27.6 | 24.5 | 21.3 | 25.8 | 27.5 | 23.6 | 25.6 | 29.0 | 28.2 |
| Trabd | 8.1 | 8.3 | 6.9 | 7.8 | 8.9 | 8.0 | 8.5 | 9.0 | 9.7 | 9.6 | 8.5 | 9.5 |
| Trabd2b | 1.0 | 0.9 | 1.5 | 0.7 | 1.5 | 0.8 | 1.6 | 2.3 | 2.4 | 1.6 | 1.7 | 2.4 |
| Tradd | 11.3 | 11.0 | 8.9 | 8.4 | 11.8 | 10.2 | 9.1 | 12.2 | 11.9 | 11.8 | 10.1 | 10.7 |
| Traf1 | 0.4 | 0.1 | 0.1 | 1.5 | 3.5 | 0.5 | 0.6 | 0.1 | 0.2 | 0.4 | 0.1 | 0.1 |
| Traf2 | 8.1 | 8.1 | 9.2 | 9.0 | 9.8 | 8.7 | 9.0 | 10.3 | 9.8 | 6.2 | 7.7 | 8.7 |
| Traf3 | 13.5 | 10.5 | 9.3 | 15.9 | 14.2 | 13.7 | 12.1 | 8.0 | 9.3 | 8.8 | 9.5 | 8.9 |
| Traf3ip1 | 2.4 | 2.5 | 3.2 | 2.6 | 2.2 | 2.5 | 2.9 | 2.8 | 2.2 | 2.8 | 2.6 | 3.1 |
| Traf3ip2 | 6.5 | 7.1 | 6.3 | 4.5 | 4.9 | 4.5 | 6.0 | 6.1 | 5.1 | 5.2 | 6.3 | 4.3 |
| Traf3ip3 | 0.2 | 0.2 | 0.2 | 0.5 | 1.6 | 0.2 | 0.3 | 0.3 | 0.2 | 0.3 | 0.2 | 0.2 |
| Traf4 | 6.7 | 7.9 | 7.2 | 6.9 | 8.1 | 7.0 | 7.7 | 8.2 | 8.2 | 9.1 | 8.3 | 6.1 |
| Traf5 | 2.3 | 2.2 | 1.8 | 2.6 | 3.3 | 2.4 | 3.5 | 2.4 | 3.2 | 3.3 | 4.1 | 3.2 |
| Traf6 | 3.4 | 3.5 | 3.1 | 3.4 | 3.2 | 3.8 | 3.0 | 3.1 | 3.0 | 3.2 | 3.0 | 3.2 |

| | | | | | | | | | | | | |
|---|---|---|---|---|---|---|---|---|---|---|---|---|
| Traf7 | 29.7 | 33.8 | 28.6 | 24.9 | 28.9 | 28.4 | 31.9 | 33.9 | 34.9 | 28.1 | 31.9 | 27.6 |
| Trafd1 | 23.3 | 26.5 | 25.6 | 22.7 | 22.9 | 22.6 | 20.3 | 24.9 | 22.1 | 21.1 | 20.7 | 21.7 |
| Traip | 0.5 | 1.1 | 1.2 | 0.4 | 1.2 | 0.3 | 1.8 | 0.7 | 1.3 | 0.7 | 1.0 | 0.8 |
| Trak1 | 7.6 | 8.7 | 8.7 | 7.7 | 7.6 | 7.4 | 8.1 | 9.3 | 6.9 | 6.4 | 8.8 | 6.2 |
| Trak2 | 23.4 | 19.5 | 17.9 | 24.0 | 20.6 | 21.7 | 21.5 | 16.7 | 19.2 | 16.7 | 20.7 | 19.4 |
| Tram1 | 49.0 | 45.8 | 48.7 | 60.3 | 57.1 | 53.5 | 53.9 | 60.8 | 52.1 | 57.8 | 66.1 | 59.7 |
| Tram1l1 | 1.3 | 1.4 | 1.6 | 1.1 | 1.3 | 1.5 | 1.7 | 1.5 | 1.2 | 1.0 | 1.2 | 1.0 |
| Tram2 | 3.3 | 3.4 | 3.3 | 2.4 | 3.7 | 1.8 | 4.1 | 3.5 | 4.0 | 3.0 | 3.0 | 2.2 |
| Trank1 | 0.1 | 0.2 | 0.1 | 0.1 | 0.1 | 0.1 | 0.0 | 0.1 | 0.1 | 0.1 | 0.1 | 0.0 |
| Trap1 | 36.2 | 32.8 | 30.6 | 39.1 | 34.0 | 41.6 | 30.2 | 29.7 | 27.5 | 28.9 | 32.4 | 28.3 |
| Trappc1 | 30.0 | 29.3 | 29.3 | 30.5 | 29.5 | 27.4 | 31.4 | 36.7 | 30.6 | 29.8 | 38.4 | 32.2 |
| Trappc10 | 10.9 | 10.7 | 10.1 | 11.1 | 10.7 | 11.4 | 9.8 | 10.4 | 10.5 | 9.4 | 10.5 | 9.1 |
| Trappc11 | 13.9 | 14.4 | 13.2 | 15.0 | 14.0 | 14.5 | 11.8 | 12.6 | 13.4 | 13.3 | 13.1 | 14.3 |
| Trappc12 | 11.4 | 11.6 | 11.2 | 12.5 | 11.4 | 10.9 | 11.3 | 11.5 | 10.6 | 10.9 | 10.6 | 11.4 |
| Trappc13 | 18.2 | 19.2 | 16.5 | 18.3 | 18.2 | 19.5 | 17.4 | 18.5 | 19.2 | 20.7 | 19.6 | 19.9 |
| Trappc2 | 22.7 | 22.7 | 17.4 | 23.7 | 20.5 | 23.2 | 16.0 | 14.5 | 17.9 | 19.3 | 20.7 | 20.6 |
| Trappc2l | 56.2 | 47.4 | 35.1 | 51.8 | 41.2 | 53.8 | 38.2 | 37.9 | 42.2 | 50.9 | 46.4 | 41.8 |
| Trappc3 | 44.9 | 40.0 | 35.2 | 46.7 | 41.7 | 41.3 | 38.0 | 38.7 | 38.5 | 43.9 | 41.3 | 39.0 |
| Trappc4 | 46.5 | 40.4 | 26.6 | 45.6 | 37.4 | 37.0 | 35.9 | 33.7 | 40.0 | 37.1 | 36.6 | 32.6 |
| Trappc5 | 9.6 | 9.3 | 7.8 | 11.3 | 8.6 | 11.1 | 10.5 | 9.4 | 10.4 | 10.0 | 10.5 | 10.9 |
| Trappc6a | 36.8 | 27.4 | 18.2 | 31.3 | 25.3 | 31.7 | 18.1 | 21.2 | 23.8 | 24.2 | 24.6 | 21.6 |
| Trappc6b | 51.3 | 45.8 | 41.9 | 54.2 | 41.1 | 47.4 | 44.6 | 32.5 | 42.7 | 40.0 | 46.7 | 53.3 |
| Trappc8 | 16.5 | 17.3 | 14.2 | 16.6 | 17.7 | 16.5 | 14.0 | 13.4 | 16.4 | 15.2 | 16.4 | 16.6 |
| Trappc9 | 17.4 | 17.8 | 14.5 | 15.8 | 15.7 | 18.2 | 15.6 | 14.5 | 13.2 | 13.7 | 14.0 | 13.3 |
| Trcg1 | 0.0 | 0.0 | 0.0 | 0.0 | 0.0 | 0.0 | 0.0 | 0.0 | 0.0 | 0.0 | 0.0 | 0.0 |
| Trdmt1 | 1.2 | 1.2 | 1.5 | 1.8 | 1.2 | 1.8 | 1.2 | 1.4 | 1.2 | 1.2 | 1.5 | 1.0 |
| Trdn | 0.1 | 0.1 | 0.0 | 0.1 | 0.0 | 0.1 | 0.1 | 0.0 | 0.0 | 0.0 | 0.1 | 0.0 |
| Trem1 | 0.2 | 0.0 | 0.0 | 1.3 | 2.7 | 0.0 | 0.2 | 0.0 | 0.0 | 0.1 | 0.1 | 0.2 |
| Trem2 | 3.6 | 0.1 | 0.1 | 35.3 | 37.4 | 0.0 | 2.4 | 0.8 | 0.3 | 2.4 | 0.5 | 1.7 |
| Trem3 | 0.0 | 0.0 | 0.0 | 0.3 | 0.5 | 0.1 | 0.1 | 0.0 | 0.0 | 0.0 | 0.1 | 0.0 |
| Treml4 | 0.4 | 0.0 | 0.0 | 0.6 | 2.6 | 0.0 | 0.3 | 0.0 | 0.0 | 0.2 | 0.0 | 0.0 |
| Trerf1 | 0.2 | 0.4 | 0.6 | 0.3 | 0.9 | 0.4 | 0.4 | 0.6 | 0.3 | 0.4 | 0.3 | 0.2 |
| Trex1 | 8.3 | 7.8 | 7.4 | 9.8 | 10.1 | 7.5 | 9.0 | 11.2 | 9.1 | 8.2 | 7.6 | 8.7 |
| Trf | 77.7 | 57.7 | 74.9 | 35.8 | 47.8 | 29.6 | 45.4 | 38.1 | 50.9 | 186.0 | 85.6 | 82.8 |
| Triap1 | 17.0 | 18.6 | 13.6 | 18.3 | 20.2 | 16.6 | 16.9 | 12.9 | 18.7 | 17.2 | 16.0 | 17.5 |
| Trib1 | 2.8 | 4.2 | 4.4 | 1.4 | 4.0 | 0.9 | 3.1 | 3.6 | 3.3 | 7.1 | 4.2 | 4.3 |
| Trib2 | 15.2 | 12.9 | 8.9 | 17.0 | 12.3 | 25.2 | 7.9 | 4.7 | 6.3 | 11.1 | 7.9 | 7.6 |
| Trib3 | 10.3 | 5.4 | 3.1 | 9.8 | 4.2 | 10.5 | 3.9 | 13.1 | 10.0 | 4.0 | 7.3 | 4.5 |
| Tril | 0.1 | 0.3 | 0.5 | 0.1 | 0.2 | 0.1 | 0.2 | 0.8 | 0.6 | 0.2 | 0.1 | 0.2 |
| Trim10 | 0.0 | 0.0 | 0.0 | 0.0 | 0.0 | 0.0 | 0.0 | 0.0 | 0.0 | 0.0 | 0.0 | 0.0 |
| Trim11 | 16.3 | 16.6 | 15.7 | 13.4 | 13.9 | 14.0 | 16.0 | 13.0 | 15.1 | 16.9 | 15.0 | 13.9 |
| Trim12a | 10.4 | 8.3 | 8.2 | 8.8 | 9.9 | 7.4 | 9.0 | 8.7 | 9.3 | 9.9 | 12.1 | 11.2 |
| Trim12c | 13.5 | 11.8 | 13.0 | 13.1 | 13.5 | 10.1 | 8.7 | 9.7 | 10.6 | 10.5 | 12.4 | 14.2 |
| Trim13 | 4.8 | 4.8 | 4.6 | 4.3 | 4.5 | 5.4 | 5.7 | 5.2 | 4.6 | 4.0 | 4.5 | 5.8 |
| Trim14 | 0.2 | 0.3 | 0.2 | 1.2 | 1.1 | 0.1 | 0.1 | 0.3 | 0.0 | 0.0 | 0.0 | 0.1 |
| Trim15 | 0.1 | 0.1 | 0.2 | 0.1 | 0.1 | 0.1 | 0.0 | 0.2 | 0.1 | 0.1 | 0.0 | 0.0 |
| Trim16 | 24.4 | 24.8 | 15.2 | 29.1 | 26.7 | 20.4 | 27.2 | 18.9 | 22.3 | 20.9 | 24.2 | 20.6 |
| Trim17 | 0.2 | 0.3 | 0.2 | 0.0 | 0.2 | 0.3 | 0.1 | 0.2 | 0.2 | 0.1 | 0.2 | 0.1 |
| Trim2 | 5.6 | 5.0 | 5.8 | 5.9 | 4.6 | 6.6 | 4.9 | 6.2 | 4.4 | 6.6 | 6.4 | 5.5 |
| Trim21 | 4.8 | 3.8 | 4.0 | 5.0 | 5.0 | 4.0 | 3.4 | 3.3 | 3.3 | 4.2 | 3.6 | 5.3 |
| Trim23 | 5.5 | 7.0 | 6.7 | 5.9 | 5.4 | 6.4 | 4.5 | 5.5 | 5.8 | 6.8 | 6.6 | 6.7 |
| Trim24 | 6.4 | 6.5 | 6.2 | 5.8 | 5.9 | 5.3 | 6.1 | 4.5 | 6.6 | 7.1 | 8.0 | 7.9 |
| Trim25 | 5.5 | 5.2 | 6.2 | 6.5 | 7.4 | 5.1 | 5.1 | 4.2 | 4.6 | 4.3 | 4.0 | 5.6 |
| Trim26 | 11.8 | 12.1 | 10.5 | 12.8 | 10.5 | 13.0 | 11.3 | 9.8 | 9.7 | 10.5 | 12.1 | 11.8 |
| Trim27 | 7.6 | 7.1 | 6.5 | 6.6 | 7.1 | 6.5 | 7.9 | 7.5 | 7.3 | 8.1 | 8.2 | 6.3 |
| Trim28 | 26.2 | 31.3 | 27.3 | 22.7 | 25.8 | 25.2 | 28.5 | 31.3 | 31.6 | 32.1 | 29.4 | 26.7 |
| Trim29 | 0.2 | 0.1 | 0.1 | 0.2 | 0.1 | 0.1 | 0.1 | 0.1 | 0.1 | 0.1 | 0.2 | 0.1 |

| | | | | | | | | | | | | |
|---|---|---|---|---|---|---|---|---|---|---|---|---|
| Trim3 | 12.6 | 13.9 | 13.4 | 12.4 | 11.4 | 15.1 | 13.1 | 13.1 | 13.5 | 10.5 | 12.0 | 11.1 |
| Trim30a | 7.1 | 6.5 | 7.7 | 8.6 | 7.3 | 5.8 | 5.7 | 4.5 | 3.9 | 3.0 | 4.0 | 7.8 |
| Trim30b | 0.0 | 0.0 | 0.0 | 0.0 | 0.0 | 0.0 | 0.0 | 0.0 | 0.1 | 0.0 | 0.1 | 0.0 |
| Trim30d | 3.8 | 3.7 | 4.2 | 4.2 | 3.1 | 3.3 | 3.9 | 2.3 | 2.6 | 2.8 | 2.5 | 4.9 |
| Trim32 | 9.4 | 9.9 | 8.6 | 7.1 | 8.4 | 8.3 | 7.2 | 7.7 | 9.5 | 9.6 | 8.1 | 8.0 |
| Trim33 | 4.7 | 5.1 | 5.1 | 4.6 | 4.5 | 5.0 | 4.4 | 4.5 | 4.4 | 5.4 | 5.5 | 5.4 |
| Trim34a | 5.9 | 4.3 | 5.2 | 6.5 | 4.5 | 5.1 | 4.0 | 3.0 | 4.2 | 3.8 | 4.3 | 6.3 |
| Trim34b | 3.0 | 1.9 | 2.2 | 2.2 | 2.2 | 2.3 | 1.9 | 1.4 | 2.2 | 1.6 | 1.8 | 3.2 |
| Trim35 | 52.4 | 50.3 | 42.0 | 56.4 | 47.5 | 49.5 | 45.0 | 55.3 | 50.6 | 50.0 | 49.0 | 41.1 |
| Trim36 | 0.0 | 0.1 | 0.0 | 0.1 | 0.1 | 0.0 | 0.1 | 0.0 | 0.0 | 0.0 | 0.0 | 0.0 |
| Trim37 | 8.0 | 6.6 | 6.1 | 7.5 | 6.5 | 9.3 | 5.6 | 5.3 | 4.6 | 5.2 | 5.7 | 5.3 |
| Trim38 | 0.1 | 0.1 | 0.1 | 0.1 | 0.1 | 0.0 | 0.0 | 0.1 | 0.1 | 0.1 | 0.1 | 0.1 |
| Trim39 | 4.6 | 3.9 | 4.3 | 5.7 | 3.8 | 4.6 | 4.7 | 3.5 | 4.0 | 3.7 | 4.1 | 3.9 |
| Trim41 | 9.3 | 9.0 | 8.8 | 11.4 | 9.1 | 10.0 | 9.6 | 7.7 | 8.9 | 8.7 | 9.3 | 9.9 |
| Trim43a | 0.1 | 0.3 | 0.2 | 0.0 | 0.2 | 0.0 | 0.1 | 0.1 | 0.0 | 0.1 | 0.3 | 0.0 |
| Trim43b | 0.1 | 0.3 | 0.1 | 0.1 | 0.2 | 0.1 | 0.2 | 0.2 | 0.1 | 0.1 | 0.1 | 0.0 |
| Trim43c | 0.3 | 0.4 | 0.2 | 0.2 | 0.2 | 0.1 | 0.2 | 0.2 | 0.2 | 0.1 | 0.4 | 0.1 |
| Trim44 | 22.4 | 21.4 | 22.6 | 23.2 | 19.3 | 21.5 | 21.4 | 20.1 | 20.3 | 22.1 | 24.3 | 26.0 |
| Trim45 | 3.1 | 2.8 | 2.3 | 2.9 | 1.9 | 3.6 | 1.3 | 1.4 | 1.6 | 2.4 | 2.2 | 2.1 |
| Trim46 | 2.9 | 2.5 | 2.2 | 2.9 | 2.3 | 3.3 | 2.8 | 3.5 | 3.6 | 2.2 | 2.3 | 2.0 |
| Trim47 | 16.4 | 16.5 | 17.6 | 19.6 | 21.9 | 19.8 | 16.8 | 23.1 | 21.0 | 16.0 | 14.6 | 14.7 |
| Trim50 | 0.0 | 0.0 | 0.0 | 0.0 | 0.0 | 0.0 | 0.0 | 0.0 | 0.0 | 0.1 | 0.0 | 0.0 |
| Trim54 | 0.2 | 0.0 | 0.0 | 0.1 | 0.0 | 0.1 | 0.0 | 0.0 | 0.0 | 0.0 | 0.0 | 0.0 |
| Trim56 | 10.1 | 8.3 | 8.4 | 13.5 | 10.9 | 10.7 | 9.0 | 7.5 | 7.6 | 7.5 | 8.3 | 8.7 |
| Trim59 | 5.0 | 8.9 | 9.3 | 4.7 | 7.7 | 5.5 | 8.7 | 8.6 | 10.5 | 9.8 | 11.4 | 9.8 |
| Trim6 | 0.3 | 0.2 | 0.4 | 0.3 | 0.2 | 0.2 | 0.4 | 0.1 | 0.3 | 0.3 | 0.2 | 0.2 |
| Trim62 | 0.5 | 0.8 | 1.0 | 0.5 | 0.7 | 0.7 | 0.8 | 0.8 | 0.8 | 0.5 | 0.7 | 0.5 |
| Trim63 | 0.5 | 0.3 | 0.4 | 0.2 | 0.1 | 0.3 | 0.0 | 0.1 | 0.0 | 0.0 | 0.1 | 0.1 |
| Trim65 | 0.6 | 0.6 | 0.5 | 0.8 | 0.6 | 0.6 | 0.7 | 0.4 | 0.6 | 0.8 | 0.4 | 0.6 |
| Trim66 | 0.3 | 0.2 | 0.2 | 0.4 | 0.1 | 0.3 | 0.1 | 0.3 | 0.2 | 0.1 | 0.2 | 0.1 |
| Trim68 | 3.6 | 3.7 | 4.1 | 3.1 | 2.9 | 3.8 | 3.2 | 3.4 | 3.7 | 2.8 | 3.4 | 3.5 |
| Trim69 | 0.1 | 0.3 | 0.1 | 0.3 | 0.2 | 0.1 | 0.2 | 0.0 | 0.1 | 0.2 | 0.2 | 0.1 |
| Trim7 | 2.3 | 3.2 | 3.3 | 1.5 | 2.2 | 3.2 | 3.0 | 2.5 | 2.3 | 1.9 | 2.9 | 2.2 |
| Trim71 | 0.0 | 0.0 | 0.0 | 0.1 | 0.0 | 0.1 | 0.0 | 0.1 | 0.1 | 0.0 | 0.1 | 0.0 |
| Trim72 | 0.1 | 0.0 | 0.0 | 0.0 | 0.1 | 0.0 | 0.0 | 0.0 | 0.0 | 0.0 | 0.0 | 0.1 |
| Trim75 | 0.1 | 0.0 | 0.0 | 0.1 | 0.0 | 0.0 | 0.0 | 0.0 | 0.0 | 0.1 | 0.0 | 0.0 |
| Trim8 | 9.2 | 7.9 | 8.1 | 9.7 | 12.1 | 8.0 | 10.1 | 9.1 | 9.1 | 10.1 | 8.5 | 8.0 |
| Trim9 | 0.0 | 0.0 | 0.0 | 0.0 | 0.0 | 0.0 | 0.0 | 0.0 | 0.0 | 0.0 | 0.0 | 0.0 |
| Trio | 8.1 | 7.7 | 7.0 | 7.5 | 8.3 | 7.4 | 9.1 | 6.6 | 7.2 | 8.7 | 8.2 | 7.3 |
| Triobp | 15.1 | 18.1 | 16.3 | 15.1 | 14.4 | 13.3 | 20.4 | 15.9 | 20.0 | 14.3 | 15.4 | 16.1 |
| Trip10 | 21.2 | 23.9 | 25.6 | 22.4 | 18.9 | 23.4 | 25.6 | 25.5 | 22.4 | 20.0 | 22.5 | 22.2 |
| Trip11 | 12.0 | 12.6 | 13.0 | 11.9 | 12.3 | 10.6 | 12.9 | 12.7 | 12.1 | 12.5 | 14.6 | 14.9 |
| Trip12 | 33.6 | 35.6 | 34.4 | 31.0 | 33.1 | 32.2 | 30.5 | 32.8 | 31.7 | 32.7 | 35.1 | 35.3 |
| Trip13 | 1.3 | 2.5 | 3.5 | 0.9 | 2.7 | 0.8 | 3.7 | 2.7 | 2.6 | 2.1 | 2.2 | 2.0 |
| Trip4 | 8.3 | 8.9 | 8.9 | 7.9 | 7.7 | 8.2 | 7.2 | 8.1 | 7.2 | 8.3 | 7.8 | 8.6 |
| Trip6 | 22.1 | 24.5 | 21.0 | 20.5 | 19.0 | 20.0 | 22.8 | 21.7 | 22.6 | 20.6 | 19.3 | 17.4 |
| Triqk | 5.7 | 6.2 | 4.3 | 5.0 | 5.5 | 7.3 | 5.4 | 5.6 | 6.1 | 5.9 | 6.7 | 6.5 |
| Trit1 | 4.4 | 4.2 | 3.1 | 4.0 | 3.2 | 4.7 | 3.3 | 3.2 | 3.7 | 4.6 | 3.9 | 4.1 |
| Trmt1 | 14.5 | 12.6 | 11.3 | 12.0 | 11.7 | 14.7 | 12.2 | 11.2 | 12.1 | 13.5 | 12.9 | 11.5 |
| Trmt10a | 3.4 | 2.4 | 2.4 | 3.1 | 2.9 | 2.3 | 3.1 | 2.4 | 2.2 | 2.7 | 3.3 | 2.4 |
| Trmt10b | 3.0 | 3.3 | 3.4 | 3.7 | 2.8 | 3.2 | 2.6 | 2.8 | 2.8 | 3.1 | 3.2 | 3.0 |
| Trmt10c | 8.7 | 9.1 | 8.7 | 10.6 | 9.1 | 9.1 | 8.7 | 7.2 | 7.6 | 9.5 | 11.1 | 9.7 |
| Trmt11 | 2.6 | 3.2 | 2.8 | 2.6 | 2.3 | 2.9 | 2.0 | 2.2 | 1.9 | 2.5 | 2.5 | 2.8 |
| Trmt112 | 26.0 | 24.1 | 22.2 | 26.1 | 23.3 | 27.0 | 22.4 | 25.0 | 24.8 | 25.2 | 23.3 | 25.0 |
| Trmt12 | 1.9 | 2.0 | 1.6 | 2.1 | 1.4 | 1.8 | 1.8 | 1.8 | 2.1 | 1.6 | 1.5 | 1.9 |
| Trmt13 | 2.6 | 2.1 | 1.9 | 2.3 | 1.9 | 2.0 | 1.7 | 1.4 | 1.9 | 1.6 | 1.4 | 1.9 |
| Trmt1l | 12.5 | 14.5 | 13.8 | 13.0 | 12.6 | 13.6 | 13.9 | 12.7 | 12.2 | 13.2 | 14.6 | 14.1 |

| | | | | | | | | | | | | |
|---|---|---|---|---|---|---|---|---|---|---|---|---|
| Trmt2a | 8.8 | 9.2 | 8.1 | 10.6 | 8.1 | 9.8 | 9.1 | 8.5 | 8.6 | 8.5 | 9.3 | 8.3 |
| Trmt2b | 7.3 | 6.6 | 7.0 | 7.5 | 6.9 | 6.7 | 6.1 | 6.6 | 6.5 | 7.7 | 6.6 | 9.0 |
| Trmt44 | 2.0 | 1.9 | 1.7 | 2.2 | 2.2 | 2.3 | 1.5 | 1.6 | 1.8 | 2.1 | 1.9 | 1.7 |
| Trmt5 | 8.7 | 8.7 | 6.8 | 7.6 | 6.9 | 7.5 | 7.5 | 6.0 | 6.8 | 6.6 | 7.5 | 7.8 |
| Trmt6 | 7.2 | 6.4 | 7.6 | 9.2 | 6.7 | 7.1 | 7.4 | 7.6 | 6.4 | 7.3 | 8.2 | 8.6 |
| Trmt61a | 4.1 | 2.3 | 1.8 | 3.9 | 3.9 | 3.6 | 3.7 | 2.8 | 2.8 | 3.3 | 2.8 | 2.8 |
| Trmt61b | 2.1 | 2.4 | 1.6 | 2.9 | 2.4 | 2.3 | 2.8 | 2.3 | 2.9 | 3.0 | 2.2 | 1.9 |
| Trmu | 4.0 | 3.1 | 3.3 | 3.3 | 3.3 | 3.3 | 2.9 | 3.5 | 4.1 | 3.5 | 3.3 | 3.4 |
| Trnau1ap | 27.0 | 22.3 | 18.7 | 21.1 | 22.0 | 23.5 | 20.5 | 19.8 | 19.8 | 21.3 | 21.8 | 20.1 |
| Trnp1 | 26.5 | 17.1 | 12.4 | 29.4 | 15.5 | 33.2 | 18.1 | 15.2 | 19.5 | 19.2 | 19.2 | 21.3 |
| Trnt1 | 13.6 | 14.4 | 13.6 | 11.7 | 12.8 | 13.6 | 13.8 | 12.9 | 13.3 | 12.8 | 15.1 | 16.5 |
| Tro | 0.4 | 0.8 | 1.0 | 0.5 | 0.3 | 0.7 | 0.9 | 0.9 | 0.8 | 0.5 | 0.7 | 0.5 |
| Troap | 0.4 | 0.6 | 1.1 | 0.1 | 0.9 | 0.1 | 1.8 | 0.7 | 1.5 | 0.3 | 0.6 | 0.5 |
| Trove2 | 5.2 | 5.2 | 5.9 | 5.9 | 4.7 | 6.6 | 5.7 | 5.7 | 5.0 | 6.3 | 6.9 | 6.6 |
| Trp53 | 21.4 | 20.2 | 19.2 | 25.8 | 28.2 | 23.5 | 27.1 | 22.4 | 19.8 | 18.6 | 20.5 | 17.1 |
| Trp53bp1 | 7.4 | 8.4 | 9.2 | 7.9 | 7.8 | 8.3 | 7.3 | 8.3 | 7.0 | 8.8 | 8.0 | 8.5 |
| Trp53bp2 | 5.2 | 6.3 | 5.6 | 4.6 | 4.1 | 4.3 | 5.0 | 4.9 | 4.9 | 6.0 | 6.7 | 5.4 |
| Trp53i11 | 2.0 | 1.5 | 1.9 | 1.5 | 1.5 | 1.9 | 1.3 | 1.5 | 1.8 | 0.8 | 0.7 | 0.8 |
| Trp53i13 | 4.8 | 5.5 | 4.6 | 6.0 | 5.6 | 6.3 | 5.4 | 5.7 | 5.6 | 5.4 | 4.3 | 4.8 |
| Trp53inp1 | 42.2 | 55.9 | 61.8 | 40.4 | 39.7 | 50.9 | 33.5 | 34.7 | 39.7 | 50.8 | 48.8 | 51.7 |
| Trp53inp2 | 31.1 | 41.7 | 41.0 | 36.5 | 38.6 | 35.8 | 37.9 | 29.4 | 28.8 | 20.9 | 25.3 | 20.3 |
| Trp53rk | 0.8 | 0.9 | 0.7 | 0.6 | 0.9 | 0.8 | 0.9 | 0.8 | 0.8 | 0.9 | 0.9 | 0.9 |
| Trp53tg5 | 0.0 | 0.0 | 0.0 | 0.0 | 0.0 | 0.0 | 0.0 | 0.1 | 0.0 | 0.0 | 0.1 | 0.0 |
| Trp63 | 0.0 | 0.0 | 0.0 | 0.1 | 0.1 | 0.0 | 0.0 | 0.0 | 0.0 | 0.0 | 0.0 | 0.0 |
| Trp73 | 0.0 | 0.0 | 0.0 | 0.0 | 0.0 | 0.0 | 0.0 | 0.0 | 0.0 | 0.0 | 0.0 | 0.0 |
| Trpc1 | 3.6 | 3.6 | 3.2 | 3.5 | 2.9 | 2.7 | 3.3 | 3.0 | 3.9 | 2.7 | 4.3 | 3.1 |
| Trpc2 | 0.5 | 0.4 | 0.7 | 0.4 | 0.5 | 0.6 | 0.5 | 0.6 | 0.5 | 0.6 | 0.4 | 0.3 |
| Trpc4ap | 32.5 | 34.4 | 32.3 | 33.8 | 35.3 | 32.1 | 35.1 | 37.6 | 33.7 | 37.9 | 35.2 | 35.5 |
| Trpc5 | 0.0 | 0.0 | 0.0 | 0.0 | 0.0 | 0.0 | 0.0 | 0.0 | 0.0 | 0.0 | 0.0 | 0.0 |
| Trpc6 | 0.0 | 0.1 | 0.0 | 0.0 | 0.0 | 0.0 | 0.0 | 0.0 | 0.0 | 0.1 | 0.0 | 0.1 |
| Trpc7 | 0.0 | 0.0 | 0.0 | 0.0 | 0.0 | 0.0 | 0.0 | 0.0 | 0.0 | 0.1 | 0.1 | 0.0 |
| Trpm2 | 0.0 | 0.0 | 0.0 | 0.2 | 0.6 | 0.0 | 0.1 | 0.0 | 0.0 | 0.1 | 0.0 | 0.0 |
| Trpm3 | 0.1 | 0.0 | 0.0 | 0.1 | 0.0 | 0.1 | 0.1 | 0.0 | 0.1 | 0.1 | 0.1 | 0.1 |
| Trpm4 | 2.2 | 2.4 | 2.3 | 1.9 | 2.5 | 2.6 | 2.2 | 2.9 | 3.2 | 2.3 | 2.3 | 2.3 |
| Trpm5 | 0.0 | 0.1 | 0.0 | 0.0 | 0.0 | 0.1 | 0.1 | 0.1 | 0.0 | 0.0 | 0.0 | 0.0 |
| Trpm6 | 0.5 | 0.5 | 0.6 | 0.2 | 0.5 | 0.3 | 0.4 | 0.5 | 0.5 | 0.4 | 0.4 | 0.5 |
| Trpm7 | 15.1 | 14.7 | 14.0 | 14.0 | 15.0 | 14.0 | 13.9 | 14.8 | 14.7 | 16.7 | 16.6 | 16.9 |
| Trps1 | 2.5 | 3.0 | 3.9 | 2.7 | 3.3 | 2.6 | 2.4 | 4.1 | 2.5 | 2.7 | 2.4 | 3.4 |
| Trpt1 | 3.5 | 4.7 | 4.9 | 3.8 | 2.7 | 4.5 | 3.7 | 3.5 | 4.3 | 3.4 | 3.5 | 3.4 |
| Trpv1 | 0.0 | 0.0 | 0.0 | 0.1 | 0.0 | 0.0 | 0.0 | 0.0 | 0.0 | 0.0 | 0.0 | 0.0 |
| Trpv2 | 2.0 | 1.5 | 1.2 | 8.1 | 11.0 | 2.0 | 3.2 | 1.4 | 2.0 | 2.1 | 2.8 | 2.0 |
| Trpv3 | 0.0 | 0.0 | 0.0 | 0.0 | 0.0 | 0.0 | 0.0 | 0.0 | 0.0 | 0.0 | 0.0 | 0.0 |
| Trpv4 | 0.2 | 0.3 | 0.7 | 0.1 | 0.4 | 0.2 | 0.2 | 0.4 | 0.3 | 0.2 | 0.2 | 0.2 |
| Trpv6 | 0.0 | 0.0 | 0.0 | 0.0 | 0.0 | 0.0 | 0.0 | 0.0 | 0.0 | 0.0 | 0.0 | 0.0 |
| Trrap | 7.2 | 9.0 | 8.8 | 6.7 | 8.0 | 8.0 | 8.4 | 9.0 | 7.9 | 7.7 | 6.9 | 6.5 |
| Trub1 | 1.9 | 1.7 | 1.5 | 1.6 | 1.7 | 1.9 | 1.8 | 1.3 | 1.6 | 1.5 | 1.8 | 1.4 |
| Trub2 | 5.8 | 4.8 | 3.8 | 6.2 | 4.8 | 5.8 | 4.9 | 4.6 | 4.6 | 4.7 | 4.6 | 4.0 |
| Tsacc | 2.6 | 3.0 | 3.1 | 3.9 | 2.3 | 2.6 | 1.9 | 2.0 | 2.0 | 2.8 | 2.2 | 2.1 |
| Tsc1 | 4.3 | 4.6 | 4.4 | 4.2 | 4.0 | 4.3 | 4.0 | 4.1 | 3.6 | 3.7 | 4.2 | 4.1 |
| Tsc2 | 13.6 | 14.2 | 12.2 | 14.1 | 13.5 | 15.6 | 13.6 | 12.0 | 12.4 | 10.6 | 11.1 | 9.3 |
| Tsc22d1 | 49.7 | 31.3 | 26.5 | 53.5 | 35.1 | 46.3 | 36.6 | 41.7 | 50.4 | 126.1 | 73.6 | 57.2 |
| Tsc22d2 | 15.9 | 16.9 | 13.6 | 16.9 | 11.3 | 18.4 | 14.3 | 9.5 | 12.3 | 12.1 | 15.7 | 12.8 |
| Tsc22d3 | 12.3 | 10.5 | 8.3 | 12.5 | 9.1 | 14.2 | 11.2 | 14.4 | 15.6 | 7.9 | 13.6 | 8.4 |
| Tsc22d4 | 10.2 | 10.9 | 12.6 | 12.7 | 13.2 | 11.6 | 11.8 | 14.0 | 13.6 | 13.6 | 11.1 | 10.8 |
| Tsen15 | 13.0 | 14.0 | 14.4 | 14.7 | 15.2 | 14.9 | 16.2 | 16.6 | 15.4 | 15.5 | 17.7 | 16.2 |
| Tsen2 | 4.5 | 4.4 | 3.6 | 4.3 | 4.0 | 4.9 | 3.7 | 4.1 | 3.3 | 3.6 | 4.0 | 3.0 |
| Tsen34 | 43.0 | 41.1 | 40.8 | 38.3 | 34.6 | 43.3 | 40.9 | 43.0 | 41.7 | 42.6 | 38.4 | 37.5 |

| | | | | | | | | | | | | |
|---|---|---|---|---|---|---|---|---|---|---|---|---|
| Tsen54 | 6.4 | 6.1 | 5.6 | 7.3 | 7.2 | 5.7 | 5.9 | 5.4 | 6.9 | 6.3 | 5.5 | 4.8 |
| Tsfm | 9.8 | 8.2 | 7.8 | 8.4 | 8.7 | 9.6 | 8.9 | 7.0 | 9.7 | 8.4 | 6.8 | 6.5 |
| Tsg101 | 46.9 | 51.8 | 43.9 | 51.1 | 41.7 | 47.0 | 41.5 | 41.2 | 40.6 | 41.8 | 47.8 | 39.9 |
| Tsga10 | 0.9 | 1.2 | 1.1 | 0.7 | 0.9 | 1.1 | 1.0 | 0.8 | 0.7 | 0.8 | 0.8 | 1.2 |
| Tsga14 | 2.2 | 2.4 | 1.9 | 2.6 | 2.4 | 2.7 | 2.0 | 1.9 | 1.9 | 2.4 | 2.4 | 2.1 |
| Tshr | 0.2 | 0.1 | 0.3 | 0.2 | 0.3 | 0.2 | 0.2 | 0.1 | 0.2 | 0.1 | 0.1 | 0.1 |
| Tshz1 | 4.3 | 4.9 | 5.9 | 5.9 | 4.2 | 6.9 | 4.8 | 4.6 | 4.1 | 4.3 | 4.5 | 4.9 |
| Tshz2 | 0.6 | 1.0 | 1.3 | 0.8 | 0.3 | 0.5 | 0.4 | 0.9 | 0.3 | 1.7 | 1.9 | 0.9 |
| Tshz3 | 2.1 | 3.3 | 3.7 | 2.5 | 2.7 | 2.7 | 3.8 | 3.3 | 3.1 | 3.2 | 3.6 | 2.9 |
| Tsks | 0.0 | 0.0 | 0.0 | 0.0 | 0.0 | 0.0 | 0.1 | 0.0 | 0.0 | 0.0 | 0.0 | 0.1 |
| Tsku | 23.3 | 21.2 | 16.2 | 22.7 | 24.4 | 22.1 | 29.7 | 24.7 | 21.3 | 18.6 | 20.0 | 18.1 |
| Tslp | 1.6 | 2.0 | 1.4 | 2.5 | 1.4 | 3.5 | 0.8 | 0.9 | 0.9 | 1.0 | 0.9 | 0.4 |
| Tsn | 27.2 | 25.8 | 22.4 | 27.8 | 25.7 | 28.1 | 22.4 | 23.1 | 23.5 | 25.8 | 24.1 | 23.4 |
| Tsnax | 40.2 | 38.3 | 34.1 | 43.5 | 35.7 | 44.3 | 32.7 | 27.5 | 31.1 | 35.2 | 33.9 | 32.7 |
| Tsnaxip1 | 0.0 | 0.0 | 0.1 | 0.0 | 0.0 | 0.0 | 0.0 | 0.1 | 0.0 | 0.0 | 0.0 | 0.0 |
| Tspan1 | 0.0 | 0.1 | 0.0 | 0.0 | 0.0 | 0.0 | 0.1 | 0.0 | 0.1 | 0.0 | 0.1 | 0.0 |
| Tspan10 | 0.0 | 0.0 | 0.0 | 0.0 | 0.0 | 0.0 | 0.0 | 0.0 | 0.0 | 0.0 | 0.0 | 0.0 |
| Tspan11 | 0.0 | 0.0 | 0.0 | 0.0 | 0.0 | 0.0 | 0.0 | 0.0 | 0.0 | 0.0 | 0.0 | 0.0 |
| Tspan12 | 5.6 | 7.9 | 7.1 | 7.1 | 7.6 | 6.0 | 6.7 | 5.1 | 7.9 | 5.2 | 7.0 | 6.5 |
| Tspan13 | 0.3 | 0.1 | 0.1 | 0.3 | 0.2 | 0.0 | 0.2 | 0.7 | 0.5 | 0.2 | 0.1 | 0.1 |
| Tspan14 | 3.9 | 4.8 | 4.2 | 5.6 | 8.2 | 3.3 | 4.4 | 4.8 | 5.8 | 4.2 | 4.4 | 3.6 |
| Tspan15 | 0.6 | 0.3 | 0.3 | 0.5 | 0.2 | 0.4 | 0.3 | 0.3 | 0.3 | 0.3 | 0.3 | 0.2 |
| Tspan17 | 38.8 | 38.2 | 29.6 | 48.8 | 36.9 | 39.5 | 35.8 | 36.8 | 36.1 | 28.2 | 31.6 | 29.3 |
| Tspan18 | 0.3 | 0.4 | 0.7 | 0.1 | 0.6 | 0.3 | 0.6 | 0.6 | 0.7 | 0.4 | 0.3 | 0.6 |
| Tspan2 | 2.5 | 2.3 | 2.1 | 2.6 | 2.0 | 3.3 | 2.5 | 1.4 | 2.2 | 3.7 | 4.7 | 2.8 |
| Tspan3 | 90.7 | 79.7 | 79.2 | 75.2 | 74.9 | 84.9 | 83.2 | 95.0 | 104.0 | 106.2 | 104.2 | 94.9 |
| Tspan31 | 65.2 | 58.9 | 57.0 | 71.4 | 65.9 | 60.2 | 63.8 | 64.4 | 58.1 | 68.2 | 65.7 | 61.8 |
| Tspan32 | 2.1 | 1.9 | 1.2 | 1.7 | 1.5 | 1.1 | 1.8 | 2.0 | 1.9 | 1.3 | 2.2 | 1.0 |
| Tspan33 | 0.1 | 0.0 | 0.1 | 0.1 | 0.0 | 0.0 | 0.1 | 0.0 | 0.0 | 0.4 | 0.1 | 0.1 |
| Tspan4 | 64.7 | 81.9 | 74.2 | 61.5 | 81.1 | 62.2 | 82.0 | 97.4 | 97.1 | 97.9 | 67.4 | 79.4 |
| Tspan5 | 7.0 | 6.8 | 5.4 | 5.0 | 4.0 | 5.8 | 4.1 | 4.1 | 4.2 | 12.2 | 7.9 | 7.2 |
| Tspan6 | 34.3 | 37.1 | 36.6 | 30.4 | 36.2 | 29.9 | 36.6 | 32.1 | 42.4 | 36.7 | 38.2 | 41.5 |
| Tspan7 | 5.2 | 8.8 | 12.5 | 3.2 | 7.3 | 2.7 | 4.4 | 7.4 | 7.1 | 3.5 | 4.6 | 4.9 |
| Tspan8 | 0.1 | 0.0 | 0.0 | 0.0 | 0.0 | 0.0 | 0.0 | 0.2 | 0.0 | 0.8 | 0.6 | 0.4 |
| Tspan9 | 24.9 | 25.6 | 25.0 | 35.6 | 24.3 | 37.8 | 26.3 | 25.4 | 24.7 | 18.0 | 17.5 | 18.0 |
| Tspo | 113.0 | 84.6 | 65.9 | 123.0 | 123.6 | 120.2 | 77.2 | 82.7 | 87.4 | 83.0 | 62.8 | 75.3 |
| Tspyl1 | 26.2 | 29.1 | 26.2 | 26.5 | 26.3 | 24.5 | 23.2 | 22.7 | 26.7 | 29.5 | 26.6 | 27.2 |
| Tspyl2 | 18.2 | 16.7 | 20.4 | 22.7 | 12.6 | 22.3 | 17.8 | 14.8 | 15.0 | 15.9 | 17.8 | 18.7 |
| Tspyl3 | 1.6 | 2.7 | 2.3 | 2.1 | 2.2 | 2.4 | 1.8 | 2.3 | 2.2 | 2.0 | 1.7 | 2.2 |
| Tspyl4 | 4.0 | 2.4 | 1.8 | 4.6 | 1.9 | 4.4 | 2.2 | 2.6 | 2.2 | 2.0 | 3.2 | 2.2 |
| Tspyl5 | 0.9 | 1.2 | 1.1 | 0.9 | 0.6 | 1.0 | 0.8 | 1.1 | 0.9 | 1.0 | 1.3 | 1.0 |
| Tsr1 | 14.3 | 9.5 | 9.2 | 14.6 | 12.7 | 11.7 | 11.8 | 8.8 | 9.6 | 12.6 | 12.7 | 12.1 |
| Tsr2 | 4.8 | 4.9 | 5.0 | 7.5 | 4.7 | 6.6 | 6.7 | 4.9 | 4.9 | 5.7 | 5.9 | 6.1 |
| Tsr3 | 14.1 | 10.4 | 10.4 | 17.7 | 9.7 | 13.9 | 12.0 | 11.0 | 9.6 | 11.0 | 10.9 | 12.9 |
| Tssc1 | 8.6 | 7.9 | 7.3 | 9.8 | 7.9 | 7.9 | 6.9 | 8.4 | 7.8 | 8.2 | 8.0 | 7.0 |
| Tssc4 | 14.0 | 11.2 | 9.4 | 10.9 | 10.1 | 11.0 | 12.7 | 11.2 | 11.8 | 14.1 | 12.4 | 10.7 |
| Tssk1 | 0.0 | 0.2 | 0.0 | 0.3 | 0.0 | 0.0 | 0.1 | 0.1 | 0.0 | 0.1 | 0.1 | 0.1 |
| Tssk2 | 0.0 | 0.1 | 0.0 | 0.1 | 0.1 | 0.1 | 0.1 | 0.0 | 0.1 | 0.1 | 0.0 | 0.1 |
| Tssk3 | 0.0 | 0.0 | 0.0 | 0.0 | 0.0 | 0.1 | 0.1 | 0.0 | 0.0 | 0.0 | 0.0 | 0.0 |
| Tssk4 | 0.0 | 0.3 | 0.2 | 0.2 | 0.1 | 0.1 | 0.3 | 0.1 | 0.2 | 0.1 | 0.2 | 0.2 |
| Tssk6 | 0.2 | 0.1 | 0.3 | 0.3 | 0.3 | 0.3 | 0.1 | 0.5 | 0.4 | 0.2 | 0.2 | 0.2 |
| Tst | 4.1 | 5.1 | 5.8 | 2.0 | 2.0 | 3.4 | 2.8 | 4.6 | 3.8 | 5.5 | 4.6 | 4.1 |
| Tsta3 | 14.0 | 13.1 | 11.1 | 13.3 | 12.0 | 15.1 | 13.2 | 13.0 | 12.9 | 11.2 | 12.7 | 12.5 |
| Tstd1 | 0.0 | 0.1 | 0.1 | 0.6 | 0.1 | 0.0 | 0.1 | 0.1 | 0.2 | 0.5 | 0.3 | 0.0 |
| Tstd2 | 10.4 | 11.0 | 8.9 | 10.2 | 10.7 | 10.7 | 9.5 | 9.6 | 9.3 | 9.2 | 9.9 | 9.2 |
| Tstd3 | 7.2 | 7.0 | 5.8 | 8.5 | 7.7 | 6.6 | 6.4 | 5.8 | 5.4 | 5.9 | 7.3 | 6.5 |
| Ttbk1 | 0.1 | 0.0 | 0.0 | 0.1 | 0.0 | 0.1 | 0.1 | 0.0 | 0.0 | 0.0 | 0.0 | 0.0 |

| | | | | | | | | | | | | |
|---|---|---|---|---|---|---|---|---|---|---|---|---|
| Ttbk2 | 3.4 | 4.5 | 4.2 | 3.0 | 3.3 | 3.6 | 3.4 | 3.6 | 3.8 | 3.1 | 4.2 | 3.8 |
| Ttc1 | 28.8 | 20.4 | 19.4 | 31.5 | 20.1 | 29.6 | 17.4 | 15.4 | 15.4 | 20.0 | 21.4 | 22.6 |
| Ttc12 | 4.5 | 2.6 | 2.7 | 4.3 | 3.0 | 5.0 | 3.0 | 2.5 | 2.4 | 2.8 | 3.2 | 3.1 |
| Ttc13 | 28.0 | 19.4 | 18.4 | 25.8 | 20.5 | 28.3 | 21.2 | 18.6 | 16.7 | 19.8 | 23.3 | 21.2 |
| Ttc14 | 5.5 | 5.5 | 6.3 | 4.9 | 5.0 | 5.1 | 4.8 | 5.1 | 5.5 | 5.4 | 6.1 | 6.0 |
| Ttc16 | 0.2 | 0.3 | 0.2 | 0.2 | 0.1 | 0.3 | 0.2 | 0.1 | 0.2 | 0.3 | 0.4 | 0.2 |
| Ttc17 | 7.0 | 7.6 | 8.0 | 8.6 | 7.6 | 8.2 | 8.6 | 8.9 | 7.1 | 7.6 | 7.8 | 8.2 |
| Ttc18 | 0.1 | 0.1 | 0.1 | 0.1 | 0.1 | 0.1 | 0.1 | 0.1 | 0.1 | 0.0 | 0.1 | 0.1 |
| Ttc19 | 6.2 | 7.8 | 7.8 | 6.7 | 6.5 | 5.5 | 7.0 | 6.5 | 7.9 | 6.9 | 7.5 | 8.2 |
| Ttc21a | 0.0 | 0.0 | 0.0 | 0.0 | 0.0 | 0.1 | 0.0 | 0.0 | 0.0 | 0.0 | 0.0 | 0.0 |
| Ttc21b | 7.6 | 8.5 | 9.4 | 5.8 | 6.7 | 7.9 | 7.7 | 8.6 | 7.6 | 7.8 | 8.2 | 8.1 |
| Ttc22 | 0.0 | 0.0 | 0.0 | 0.0 | 0.0 | 0.0 | 0.0 | 0.0 | 0.0 | 0.1 | 0.0 | 0.0 |
| Ttc23 | 8.5 | 8.5 | 8.1 | 7.6 | 6.2 | 9.0 | 6.2 | 7.8 | 7.1 | 7.1 | 6.3 | 7.4 |
| Ttc23l | 0.1 | 0.0 | 0.0 | 0.0 | 0.0 | 0.0 | 0.0 | 0.0 | 0.0 | 0.0 | 0.1 | 0.0 |
| Ttc25 | 0.2 | 0.2 | 0.3 | 0.3 | 0.3 | 0.4 | 0.2 | 0.2 | 0.1 | 0.1 | 0.2 | 0.3 |
| Ttc26 | 2.1 | 1.8 | 1.8 | 1.7 | 1.3 | 2.2 | 1.5 | 1.6 | 1.4 | 1.6 | 1.7 | 1.4 |
| Ttc27 | 5.1 | 4.8 | 3.4 | 5.4 | 5.0 | 5.8 | 5.0 | 4.4 | 4.2 | 5.9 | 4.7 | 4.2 |
| Ttc28 | 2.7 | 3.9 | 3.9 | 3.3 | 3.5 | 3.2 | 4.2 | 3.4 | 3.7 | 3.0 | 3.1 | 2.8 |
| Ttc3 | 17.6 | 19.7 | 19.5 | 15.6 | 15.8 | 19.0 | 15.2 | 18.3 | 17.0 | 16.9 | 19.0 | 16.8 |
| Ttc30a1 | 1.1 | 0.8 | 1.2 | 1.1 | 0.8 | 0.9 | 1.0 | 0.9 | 1.0 | 1.0 | 0.8 | 1.0 |
| Ttc30a2 | 0.2 | 0.3 | 0.3 | 0.3 | 0.2 | 0.3 | 0.3 | 0.3 | 0.2 | 0.2 | 0.3 | 0.3 |
| Ttc30b | 4.5 | 4.7 | 5.4 | 4.4 | 3.6 | 5.0 | 3.7 | 4.8 | 4.2 | 5.1 | 4.6 | 5.3 |
| Ttc32 | 1.5 | 1.2 | 1.1 | 2.2 | 2.3 | 1.1 | 1.6 | 1.0 | 1.8 | 1.0 | 1.8 | 1.9 |
| Ttc33 | 19.1 | 16.5 | 18.5 | 22.2 | 17.2 | 22.4 | 15.3 | 15.6 | 15.5 | 18.8 | 16.8 | 20.3 |
| Ttc34 | 0.0 | 0.0 | 0.0 | 0.1 | 0.0 | 0.1 | 0.0 | 0.0 | 0.0 | 0.0 | 0.0 | 0.0 |
| Ttc36 | 0.0 | 0.1 | 0.1 | 0.0 | 0.0 | 0.0 | 0.1 | 0.0 | 0.0 | 0.1 | 0.1 | 0.0 |
| Ttc37 | 13.8 | 12.6 | 9.6 | 13.5 | 11.5 | 12.1 | 9.7 | 10.5 | 9.6 | 10.7 | 12.7 | 10.9 |
| Ttc38 | 3.8 | 4.5 | 4.6 | 3.3 | 3.4 | 4.5 | 3.5 | 4.3 | 3.6 | 3.4 | 3.4 | 3.9 |
| Ttc39a | 0.2 | 0.2 | 0.3 | 0.1 | 0.1 | 0.2 | 0.2 | 0.2 | 0.1 | 0.1 | 0.1 | 0.2 |
| Ttc39b | 11.3 | 13.7 | 11.9 | 16.7 | 14.0 | 15.6 | 17.6 | 12.2 | 13.2 | 12.7 | 15.0 | 13.2 |
| Ttc39c | 4.1 | 4.1 | 3.7 | 3.8 | 3.4 | 5.4 | 3.2 | 4.2 | 3.8 | 4.2 | 3.7 | 4.4 |
| Ttc39d | 0.1 | 0.1 | 0.0 | 0.0 | 0.0 | 0.1 | 0.0 | 0.1 | 0.0 | 0.0 | 0.1 | 0.1 |
| Ttc4 | 9.6 | 9.6 | 8.6 | 9.6 | 8.4 | 9.6 | 8.2 | 8.7 | 8.6 | 10.1 | 9.0 | 9.3 |
| Ttc5 | 14.6 | 12.9 | 11.8 | 14.3 | 12.8 | 16.0 | 11.7 | 12.9 | 12.0 | 13.1 | 10.7 | 12.0 |
| Ttc7 | 7.9 | 9.2 | 9.1 | 6.5 | 8.8 | 7.1 | 8.9 | 7.2 | 8.3 | 6.4 | 6.6 | 7.7 |
| Ttc7b | 6.1 | 5.1 | 5.1 | 7.0 | 5.9 | 6.0 | 5.8 | 4.3 | 5.9 | 5.8 | 5.1 | 6.1 |
| Ttc8 | 8.4 | 8.1 | 9.3 | 8.8 | 6.8 | 8.9 | 9.1 | 7.8 | 8.5 | 8.1 | 8.9 | 8.7 |
| Ttc9 | 11.7 | 9.5 | 8.0 | 14.2 | 8.2 | 17.7 | 11.3 | 8.4 | 9.2 | 11.7 | 12.2 | 13.1 |
| Ttc9c | 8.7 | 8.7 | 9.1 | 8.2 | 8.8 | 8.4 | 8.4 | 8.6 | 8.1 | 8.5 | 10.5 | 8.2 |
| Ttf1 | 6.3 | 6.6 | 7.2 | 6.6 | 5.6 | 6.2 | 5.1 | 5.9 | 5.0 | 6.3 | 6.4 | 7.0 |
| Ttf2 | 7.0 | 7.6 | 7.5 | 7.7 | 7.5 | 7.5 | 7.8 | 6.8 | 7.2 | 7.2 | 7.2 | 6.6 |
| Tti1 | 4.8 | 5.0 | 5.8 | 4.7 | 4.8 | 4.6 | 4.6 | 5.2 | 4.7 | 5.4 | 4.9 | 4.6 |
| Tti2 | 5.0 | 4.3 | 4.2 | 4.6 | 4.0 | 4.7 | 4.2 | 5.0 | 4.3 | 4.3 | 4.8 | 4.4 |
| Ttk | 0.8 | 1.7 | 2.9 | 0.3 | 2.4 | 0.4 | 2.9 | 1.4 | 2.1 | 1.2 | 2.1 | 1.4 |
| Ttl | 5.9 | 4.5 | 4.0 | 6.3 | 4.6 | 6.0 | 6.7 | 4.5 | 5.9 | 4.9 | 5.9 | 5.6 |
| Ttll1 | 7.8 | 7.5 | 7.8 | 7.4 | 7.3 | 8.4 | 6.5 | 7.2 | 5.8 | 7.6 | 7.9 | 6.1 |
| Ttll11 | 0.4 | 0.2 | 0.2 | 0.5 | 0.5 | 0.3 | 0.4 | 0.2 | 0.4 | 0.5 | 0.4 | 0.4 |
| Ttll12 | 11.9 | 10.7 | 8.6 | 12.6 | 9.9 | 12.9 | 10.8 | 9.7 | 9.0 | 10.4 | 9.7 | 8.6 |
| Ttll13 | 0.2 | 0.1 | 0.1 | 0.2 | 0.1 | 0.4 | 0.1 | 0.2 | 0.2 | 0.2 | 0.2 | 0.1 |
| Ttll2 | 0.0 | 0.0 | 0.0 | 0.0 | 0.0 | 0.0 | 0.0 | 0.0 | 0.0 | 0.0 | 0.0 | 0.0 |
| Ttll3 | 0.6 | 0.8 | 0.6 | 0.9 | 1.0 | 0.9 | 0.8 | 0.5 | 1.1 | 1.4 | 1.0 | 0.6 |
| Ttll4 | 2.3 | 2.5 | 2.5 | 2.3 | 2.5 | 1.5 | 3.0 | 2.8 | 2.7 | 2.5 | 2.4 | 1.8 |
| Ttll5 | 2.8 | 3.1 | 3.6 | 3.4 | 3.0 | 3.1 | 3.2 | 4.2 | 2.6 | 2.6 | 3.9 | 3.1 |
| Ttll7 | 6.6 | 5.9 | 5.0 | 8.9 | 4.4 | 8.5 | 5.0 | 4.1 | 4.2 | 6.0 | 5.4 | 5.5 |
| Ttll9 | 0.2 | 0.2 | 0.3 | 0.1 | 0.4 | 0.1 | 0.2 | 0.1 | 0.1 | 0.3 | 0.2 | 0.1 |
| Ttn | 0.0 | 0.0 | 0.0 | 0.0 | 0.0 | 0.0 | 0.0 | 0.0 | 0.0 | 0.0 | 0.0 | 0.0 |
| Ttpa | 0.1 | 0.1 | 0.1 | 0.1 | 0.0 | 0.1 | 0.0 | 0.0 | 0.1 | 0.1 | 0.2 | 0.0 |

| | | | | | | | | | | | | |
|---|---|---|---|---|---|---|---|---|---|---|---|---|
| Ttpal | 13.7 | 14.1 | 12.4 | 11.6 | 12.7 | 11.7 | 11.1 | 12.3 | 12.2 | 12.5 | 11.5 | 12.2 |
| Ttyh1 | 0.2 | 0.1 | 0.1 | 0.1 | 0.1 | 0.1 | 0.1 | 0.1 | 0.1 | 0.1 | 0.1 | 0.1 |
| Ttyh2 | 12.0 | 8.9 | 4.9 | 15.6 | 12.2 | 12.3 | 11.8 | 6.2 | 8.5 | 9.5 | 9.2 | 8.0 |
| Ttyh3 | 1.5 | 2.0 | 1.9 | 1.5 | 1.7 | 1.7 | 1.5 | 2.4 | 2.2 | 1.7 | 1.6 | 1.2 |
| Tub | 0.0 | 0.0 | 0.0 | 0.1 | 0.0 | 0.0 | 0.1 | 0.0 | 0.0 | 0.0 | 0.0 | 0.0 |
| Tuba1a | 392.7 | 397.0 | 343.7 | 506.9 | 326.2 | 381.8 | 495.1 | 483.9 | 454.7 | 240.4 | 426.3 | 252.2 |
| Tuba1b | 138.7 | 132.9 | 137.6 | 169.3 | 163.7 | 127.5 | 216.7 | 158.0 | 187.1 | 136.2 | 164.2 | 121.9 |
| Tuba1c | 244.4 | 225.9 | 193.6 | 290.7 | 260.7 | 231.3 | 348.5 | 257.4 | 295.6 | 184.3 | 236.4 | 152.5 |
| Tuba3b | 0.0 | 0.0 | 0.0 | 0.0 | 0.0 | 0.0 | 0.0 | 0.0 | 0.0 | 0.0 | 0.0 | 0.0 |
| Tuba4a | 17.9 | 12.9 | 9.7 | 29.6 | 19.4 | 19.6 | 18.0 | 9.4 | 9.2 | 18.0 | 15.4 | 11.5 |
| Tuba8 | 0.0 | 0.0 | 0.0 | 0.0 | 0.1 | 0.0 | 0.0 | 0.0 | 0.0 | 0.0 | 0.0 | 0.0 |
| Tubb2a | 128.7 | 106.2 | 81.4 | 158.6 | 137.1 | 123.9 | 134.4 | 117.3 | 134.3 | 128.9 | 139.6 | 110.3 |
| Tubb2a-ps2 | 0.5 | 0.0 | 0.0 | 0.3 | 0.1 | 0.1 | 0.4 | 0.2 | 0.2 | 0.3 | 0.4 | 0.2 |
| Tubb2b | 67.6 | 46.0 | 34.9 | 75.3 | 59.7 | 58.6 | 59.9 | 55.7 | 61.1 | 55.5 | 65.2 | 44.2 |
| Tubb3 | 112.4 | 118.5 | 84.5 | 174.0 | 202.3 | 92.9 | 269.1 | 224.3 | 210.3 | 148.0 | 216.0 | 133.7 |
| Tubb4a | 8.4 | 14.2 | 12.8 | 10.4 | 12.2 | 7.8 | 15.1 | 17.7 | 7.3 | 7.1 | 9.8 | 7.4 |
| Tubb4b | 120.7 | 103.0 | 117.1 | 125.5 | 150.6 | 94.4 | 152.4 | 95.5 | 126.1 | 163.7 | 145.6 | 109.3 |
| Tubb5 | 163.5 | 139.9 | 165.5 | 158.8 | 196.4 | 137.6 | 234.2 | 198.7 | 202.4 | 206.9 | 206.2 | 162.9 |
| Tubb6 | 36.9 | 38.1 | 45.9 | 33.0 | 63.2 | 20.1 | 86.8 | 51.9 | 67.9 | 57.2 | 70.3 | 48.9 |
| Tubd1 | 3.7 | 4.6 | 5.6 | 3.2 | 3.6 | 2.9 | 4.5 | 4.3 | 4.2 | 3.9 | 4.1 | 4.2 |
| Tube1 | 0.3 | 0.6 | 0.5 | 0.3 | 0.4 | 0.5 | 0.4 | 0.2 | 0.3 | 0.2 | 0.2 | 0.3 |
| Tubg1 | 9.8 | 11.6 | 10.7 | 8.8 | 10.8 | 10.4 | 14.1 | 11.0 | 9.9 | 9.6 | 10.8 | 8.4 |
| Tubg2 | 3.0 | 3.2 | 3.0 | 3.9 | 2.4 | 3.7 | 3.0 | 2.1 | 2.3 | 2.1 | 1.8 | 2.2 |
| Tubgcp2 | 12.8 | 12.7 | 13.2 | 13.2 | 12.6 | 11.7 | 13.2 | 12.2 | 10.9 | 10.1 | 12.1 | 11.1 |
| Tubgcp3 | 7.4 | 8.2 | 9.0 | 8.6 | 8.0 | 8.6 | 9.3 | 8.9 | 8.9 | 6.7 | 8.6 | 7.7 |
| Tubgcp4 | 10.0 | 9.9 | 8.8 | 11.2 | 10.8 | 11.7 | 9.2 | 8.4 | 8.9 | 8.8 | 8.8 | 7.9 |
| Tubgcp5 | 6.9 | 7.5 | 6.7 | 6.6 | 6.6 | 6.5 | 6.1 | 5.5 | 5.9 | 6.9 | 6.2 | 6.9 |
| Tubgcp6 | 3.4 | 3.7 | 4.2 | 3.9 | 4.0 | 4.1 | 4.2 | 3.9 | 3.9 | 3.3 | 3.7 | 3.6 |
| Tufm | 26.6 | 27.4 | 25.3 | 26.9 | 28.8 | 27.4 | 28.9 | 29.1 | 25.6 | 29.1 | 30.6 | 26.6 |
| Tuft1 | 9.9 | 6.7 | 4.0 | 5.5 | 5.3 | 5.1 | 5.0 | 3.5 | 4.4 | 9.7 | 8.8 | 5.7 |
| Tug1 | 15.2 | 16.5 | 19.0 | 17.4 | 17.7 | 17.0 | 16.7 | 20.8 | 18.0 | 20.0 | 22.3 | 20.3 |
| Tulp1 | 0.0 | 0.1 | 0.0 | 0.0 | 0.0 | 0.0 | 0.0 | 0.1 | 0.0 | 0.1 | 0.2 | 0.1 |
| Tulp2 | 0.3 | 0.4 | 0.1 | 0.1 | 0.2 | 0.3 | 0.2 | 0.4 | 0.2 | 0.1 | 0.2 | 0.0 |
| Tulp3 | 11.0 | 12.3 | 13.3 | 8.7 | 11.0 | 10.7 | 11.9 | 12.9 | 12.8 | 11.0 | 12.3 | 12.5 |
| Tulp4 | 41.7 | 42.0 | 37.5 | 41.5 | 35.9 | 40.4 | 38.3 | 31.8 | 27.3 | 36.7 | 38.2 | 36.8 |
| Tusc1 | 0.7 | 1.5 | 1.2 | 0.6 | 0.8 | 1.8 | 1.2 | 1.3 | 1.2 | 0.9 | 1.0 | 1.7 |
| Tusc2 | 11.3 | 10.3 | 9.5 | 14.3 | 9.4 | 14.3 | 11.1 | 10.1 | 7.8 | 10.0 | 10.9 | 9.4 |
| Tusc3 | 48.8 | 40.7 | 30.3 | 44.9 | 39.6 | 50.2 | 43.9 | 36.9 | 42.6 | 42.7 | 42.6 | 40.2 |
| Tusc5 | 0.6 | 0.3 | 0.2 | 1.0 | 0.3 | 0.9 | 0.3 | 0.2 | 0.2 | 0.1 | 0.3 | 0.2 |
| Tut1 | 7.5 | 7.0 | 7.4 | 8.5 | 6.8 | 7.5 | 8.4 | 6.3 | 6.4 | 7.3 | 7.5 | 7.3 |
| Tvp23a | 7.1 | 8.1 | 8.0 | 6.7 | 6.5 | 8.9 | 7.8 | 8.1 | 7.5 | 5.9 | 6.9 | 7.4 |
| Tvp23b | 43.5 | 39.6 | 34.8 | 43.9 | 33.9 | 40.3 | 32.0 | 28.0 | 32.5 | 37.9 | 41.6 | 38.3 |
| Twf1 | 45.8 | 44.2 | 46.3 | 52.8 | 47.9 | 51.0 | 49.7 | 48.9 | 46.2 | 45.7 | 52.3 | 51.9 |
| Twf2 | 9.8 | 10.6 | 8.7 | 13.3 | 16.7 | 10.5 | 10.2 | 9.7 | 9.1 | 9.7 | 8.2 | 9.3 |
| Twist1 | 6.6 | 6.4 | 8.2 | 4.8 | 5.6 | 6.4 | 8.5 | 6.5 | 7.8 | 15.5 | 10.9 | 11.6 |
| Twist2 | 2.5 | 1.9 | 2.9 | 1.1 | 1.3 | 0.9 | 2.2 | 2.1 | 2.1 | 2.5 | 2.9 | 2.2 |
| Twistnb | 6.9 | 7.7 | 7.2 | 7.7 | 7.5 | 6.8 | 6.9 | 7.8 | 7.4 | 8.1 | 9.2 | 8.4 |
| Twsg1 | 32.2 | 31.6 | 35.8 | 32.9 | 31.9 | 38.0 | 32.1 | 27.8 | 32.1 | 35.3 | 33.5 | 38.1 |
| Txk | 0.0 | 0.1 | 0.0 | 0.0 | 0.0 | 0.1 | 0.1 | 0.1 | 0.0 | 0.2 | 0.1 | 0.0 |
| Txlna | 20.6 | 21.8 | 21.9 | 21.3 | 21.7 | 19.1 | 22.8 | 22.6 | 20.7 | 21.7 | 23.7 | 21.3 |
| Txlnb | 0.9 | 0.5 | 0.5 | 1.1 | 0.6 | 1.9 | 0.2 | 0.3 | 0.2 | 0.4 | 0.5 | 0.2 |
| Txlng | 2.0 | 2.5 | 2.9 | 2.9 | 2.5 | 2.3 | 2.5 | 2.6 | 2.5 | 3.2 | 3.8 | 3.1 |
| Txn1 | 131.4 | 119.3 | 98.2 | 155.2 | 136.6 | 153.5 | 101.2 | 116.8 | 116.1 | 125.9 | 120.3 | 99.4 |
| Txn2 | 48.9 | 43.2 | 38.6 | 62.3 | 49.1 | 55.5 | 50.6 | 47.2 | 40.4 | 45.4 | 52.6 | 42.0 |
| Txndc11 | 8.4 | 9.2 | 7.3 | 9.1 | 9.1 | 8.7 | 9.4 | 8.8 | 9.3 | 8.1 | 8.5 | 7.7 |
| Txndc12 | 36.6 | 32.4 | 36.4 | 39.9 | 36.6 | 38.9 | 34.6 | 35.1 | 34.6 | 38.6 | 38.4 | 35.0 |
| Txndc15 | 39.9 | 37.9 | 41.8 | 41.9 | 38.3 | 46.1 | 38.4 | 35.8 | 38.3 | 43.6 | 35.8 | 43.7 |

| | | | | | | | | | | | | |
|---|---|---|---|---|---|---|---|---|---|---|---|---|
| Txndc16 | 22.5 | 18.3 | 18.3 | 21.1 | 17.9 | 26.5 | 16.1 | 15.8 | 14.9 | 16.2 | 17.7 | 21.4 |
| Txndc17 | 88.1 | 66.7 | 62.3 | 89.3 | 62.5 | 80.6 | 64.9 | 63.1 | 60.9 | 64.0 | 78.3 | 67.5 |
| Txndc2 | 0.2 | 0.2 | 0.2 | 0.2 | 0.2 | 0.2 | 0.2 | 0.2 | 0.1 | 0.3 | 0.1 | 0.1 |
| Txndc5 | 182.6 | 160.1 | 158.1 | 182.2 | 155.5 | 187.1 | 190.7 | 181.1 | 173.5 | 175.1 | 184.2 | 187.8 |
| Txndc9 | 17.2 | 15.7 | 13.8 | 18.6 | 15.8 | 17.4 | 15.2 | 13.5 | 13.7 | 17.2 | 17.2 | 17.1 |
| Txnip | 19.4 | 21.4 | 24.5 | 48.7 | 59.2 | 25.9 | 53.8 | 51.3 | 54.4 | 57.2 | 47.0 | 60.6 |
| Txnl1 | 39.5 | 40.7 | 36.6 | 42.7 | 40.2 | 45.3 | 41.0 | 36.9 | 41.6 | 43.3 | 43.7 | 44.7 |
| Txnl4a | 61.8 | 45.5 | 39.0 | 60.0 | 53.4 | 60.2 | 51.0 | 45.5 | 48.3 | 51.6 | 52.4 | 43.6 |
| Txnl4b | 4.3 | 4.5 | 4.6 | 6.3 | 4.4 | 4.7 | 3.9 | 4.7 | 3.9 | 3.6 | 4.9 | 4.2 |
| Txnrd1 | 64.6 | 36.3 | 25.4 | 97.7 | 65.4 | 73.1 | 38.0 | 26.7 | 25.5 | 46.2 | 36.9 | 31.5 |
| Txnrd2 | 2.6 | 2.9 | 3.0 | 4.4 | 4.0 | 3.5 | 3.4 | 2.3 | 3.1 | 3.2 | 2.4 | 3.2 |
| Txnrd3 | 2.6 | 3.0 | 2.1 | 2.8 | 2.1 | 3.4 | 3.1 | 4.1 | 3.2 | 2.5 | 3.1 | 3.5 |
| Tyk2 | 3.6 | 4.7 | 4.6 | 3.8 | 5.0 | 4.5 | 3.4 | 4.3 | 4.4 | 4.0 | 3.5 | 4.1 |
| Tymp | 0.4 | 0.3 | 0.1 | 0.3 | 0.3 | 0.2 | 0.4 | 0.1 | 0.4 | 0.5 | 0.1 | 0.3 |
| Tyms | 5.8 | 7.1 | 8.2 | 4.5 | 7.5 | 3.8 | 7.6 | 7.3 | 7.7 | 5.5 | 6.4 | 5.8 |
| Tyms-ps | 0.2 | 0.2 | 0.5 | 0.1 | 0.3 | 0.1 | 0.4 | 0.2 | 0.2 | 0.2 | 0.2 | 0.1 |
| Tyr | 0.0 | 0.0 | 0.0 | 0.0 | 0.0 | 0.0 | 0.0 | 0.0 | 0.0 | 0.0 | 0.0 | 0.0 |
| Tyro3 | 4.6 | 5.6 | 6.0 | 3.7 | 4.1 | 4.6 | 5.2 | 6.1 | 5.6 | 3.8 | 3.9 | 3.7 |
| Tyrobp | 15.1 | 0.4 | 0.6 | 133.1 | 177.6 | 0.1 | 17.5 | 3.2 | 2.5 | 9.8 | 2.4 | 6.7 |
| Tysnd1 | 21.6 | 20.8 | 16.8 | 19.6 | 20.5 | 19.8 | 21.3 | 17.8 | 19.7 | 20.9 | 19.0 | 19.2 |
| Tyw1 | 12.5 | 10.7 | 9.4 | 11.9 | 10.4 | 12.6 | 10.5 | 10.0 | 9.4 | 11.1 | 10.2 | 9.5 |
| Tyw3 | 0.4 | 0.4 | 0.4 | 0.5 | 0.5 | 0.5 | 0.6 | 0.5 | 0.4 | 0.5 | 0.5 | 0.6 |
| Tyw5 | 7.1 | 8.6 | 8.2 | 6.7 | 6.8 | 9.2 | 8.0 | 5.4 | 7.6 | 7.7 | 8.3 | 7.7 |
| U05342 | 1.0 | 0.6 | 0.7 | 2.1 | 0.2 | 0.2 | 0.3 | 0.9 | 0.2 | 1.2 | 0.8 | 0.3 |
| U2af1 | 29.3 | 29.4 | 33.4 | 36.5 | 33.8 | 31.6 | 37.5 | 39.3 | 33.7 | 39.1 | 39.9 | 33.9 |
| U2af1l4 | 16.4 | 12.1 | 13.4 | 21.9 | 12.1 | 19.3 | 17.3 | 14.7 | 13.9 | 14.1 | 13.6 | 12.9 |
| U2af2 | 42.6 | 39.3 | 37.2 | 44.5 | 41.5 | 39.4 | 41.4 | 39.8 | 38.5 | 40.6 | 40.1 | 37.3 |
| U2surp | 9.8 | 9.9 | 11.3 | 10.8 | 9.3 | 10.1 | 10.2 | 9.7 | 8.3 | 9.9 | 11.7 | 11.5 |
| Uaca | 10.1 | 11.1 | 12.6 | 10.4 | 9.6 | 8.1 | 10.5 | 12.5 | 9.3 | 13.4 | 11.1 | 13.5 |
| Uap1 | 43.0 | 43.3 | 38.4 | 39.6 | 36.3 | 38.6 | 37.9 | 35.1 | 33.3 | 29.6 | 38.3 | 34.4 |
| Uap1l1 | 44.0 | 24.2 | 19.4 | 46.5 | 34.8 | 39.4 | 18.0 | 22.3 | 16.3 | 28.4 | 18.1 | 25.4 |
| Uba1 | 83.6 | 76.5 | 69.1 | 86.1 | 85.6 | 80.3 | 80.1 | 76.9 | 69.8 | 74.3 | 78.0 | 75.6 |
| Uba1y | 0.0 | 0.0 | 0.0 | 0.0 | 0.0 | 0.0 | 0.0 | 0.0 | 0.0 | 0.0 | 0.0 | 0.0 |
| Uba2 | 34.0 | 32.9 | 36.3 | 31.9 | 31.9 | 30.2 | 36.9 | 36.8 | 36.1 | 33.4 | 40.0 | 36.1 |
| Uba3 | 41.0 | 39.4 | 36.1 | 43.0 | 33.6 | 43.0 | 36.8 | 37.9 | 35.0 | 37.4 | 45.5 | 39.2 |
| Uba5 | 34.3 | 30.1 | 29.6 | 33.9 | 30.3 | 31.2 | 30.1 | 28.9 | 28.9 | 31.9 | 35.1 | 35.2 |
| Uba52 | 1247.5 | 1027.6 | 1038.9 | 1173.2 | 967.4 | 1134.3 | 1054.0 | 1272.4 | 912.0 | 1077.8 | 1091.7 | 1048.6 |
| Uba6 | 7.6 | 8.4 | 8.0 | 7.8 | 8.2 | 8.7 | 7.0 | 7.2 | 8.3 | 8.6 | 9.7 | 9.1 |
| Uba7 | 5.7 | 5.3 | 6.9 | 6.6 | 7.2 | 6.4 | 4.9 | 4.4 | 5.7 | 4.1 | 3.4 | 4.9 |
| Ubac1 | 9.7 | 8.5 | 7.0 | 7.0 | 7.2 | 8.2 | 8.1 | 7.7 | 7.4 | 8.0 | 8.0 | 8.4 |
| Ubac2 | 10.0 | 9.0 | 8.0 | 10.3 | 9.6 | 9.3 | 7.6 | 9.8 | 9.3 | 9.8 | 9.2 | 7.7 |
| Ubald1 | 5.5 | 6.1 | 5.2 | 7.0 | 7.5 | 5.8 | 7.2 | 5.9 | 6.9 | 6.5 | 6.4 | 6.2 |
| Ubald2 | 12.7 | 7.1 | 5.7 | 4.7 | 10.1 | 7.8 | 6.3 | 7.0 | 10.2 | 8.9 | 10.4 | 8.7 |
| Ubap1 | 27.2 | 25.1 | 21.4 | 32.0 | 23.0 | 29.9 | 20.9 | 18.9 | 18.6 | 18.0 | 21.2 | 20.0 |
| Ubap2 | 8.5 | 9.5 | 10.0 | 9.7 | 8.8 | 8.3 | 10.8 | 8.5 | 7.9 | 6.5 | 8.8 | 6.7 |
| Ubap2l | 22.6 | 24.4 | 27.7 | 26.3 | 23.8 | 22.5 | 28.6 | 27.1 | 20.3 | 21.7 | 25.2 | 23.5 |
| Ubash3b | 1.3 | 1.1 | 1.1 | 2.9 | 4.8 | 1.1 | 2.5 | 1.1 | 1.3 | 2.9 | 1.5 | 1.5 |
| Ubb | 542.5 | 508.0 | 518.0 | 684.7 | 580.9 | 637.9 | 659.1 | 612.9 | 506.9 | 590.8 | 618.4 | 549.3 |
| Ubc | 356.6 | 353.6 | 298.1 | 399.6 | 349.0 | 386.2 | 389.6 | 319.8 | 374.9 | 408.2 | 371.2 | 370.5 |
| Ube2a | 15.9 | 15.8 | 14.1 | 15.0 | 16.4 | 13.6 | 14.6 | 13.7 | 15.7 | 17.7 | 16.9 | 16.8 |
| Ube2b | 29.6 | 32.1 | 34.2 | 29.8 | 33.2 | 33.8 | 30.8 | 27.8 | 31.3 | 40.0 | 37.3 | 36.2 |
| Ube2c | 5.3 | 10.4 | 16.1 | 1.9 | 14.0 | 1.5 | 23.2 | 6.4 | 16.9 | 7.3 | 14.9 | 7.1 |
| Ube2cbp | 0.1 | 0.2 | 0.1 | 0.2 | 0.2 | 0.3 | 0.0 | 0.1 | 0.2 | 0.2 | 0.2 | 0.1 |
| Ube2d1 | 7.4 | 7.8 | 7.9 | 9.6 | 8.8 | 9.1 | 10.1 | 7.7 | 7.4 | 8.3 | 7.6 | 8.4 |
| Ube2d2a | 32.3 | 32.8 | 31.4 | 33.2 | 33.5 | 34.9 | 31.2 | 30.0 | 35.7 | 33.7 | 34.9 | 34.5 |
| Ube2d2b | 0.1 | 0.1 | 0.0 | 0.1 | 0.0 | 0.1 | 0.0 | 0.1 | 0.0 | 0.0 | 0.1 | 0.1 |
| Ube2d3 | 56.8 | 64.1 | 77.9 | 84.7 | 75.2 | 73.3 | 82.6 | 78.7 | 72.4 | 70.8 | 90.4 | 86.6 |

| | | | | | | | | | | | | |
|---|---|---|---|---|---|---|---|---|---|---|---|---|
| Ube2e1 | 28.3 | 22.5 | 22.9 | 32.2 | 23.5 | 29.5 | 24.3 | 19.7 | 20.5 | 27.6 | 28.8 | 29.1 |
| Ube2e2 | 10.6 | 11.9 | 15.0 | 10.4 | 13.8 | 12.7 | 16.4 | 14.3 | 12.4 | 12.1 | 15.2 | 18.5 |
| Ube2e3 | 16.2 | 20.7 | 20.3 | 13.8 | 18.0 | 17.1 | 20.9 | 21.6 | 22.4 | 21.4 | 18.9 | 20.0 |
| Ube2f | 31.5 | 29.4 | 25.5 | 36.0 | 35.8 | 29.6 | 32.4 | 26.2 | 27.0 | 26.8 | 29.2 | 27.7 |
| Ube2g1 | 7.1 | 8.9 | 8.5 | 7.4 | 8.7 | 8.2 | 8.6 | 9.1 | 8.9 | 9.5 | 9.9 | 9.6 |
| Ube2g2 | 29.4 | 23.1 | 21.4 | 32.9 | 27.7 | 26.9 | 31.6 | 26.5 | 26.9 | 26.3 | 30.8 | 25.7 |
| Ube2h | 49.1 | 57.5 | 57.0 | 41.5 | 39.3 | 46.9 | 37.9 | 43.0 | 44.4 | 51.9 | 48.4 | 51.6 |
| Ube2i | 78.6 | 66.6 | 59.3 | 77.1 | 68.6 | 75.4 | 68.1 | 60.2 | 64.3 | 75.5 | 71.2 | 67.5 |
| Ube2j1 | 29.0 | 29.6 | 31.7 | 35.0 | 31.0 | 32.2 | 32.2 | 32.7 | 31.6 | 32.5 | 36.1 | 35.3 |
| Ube2j2 | 3.0 | 2.1 | 2.6 | 2.1 | 3.0 | 2.4 | 2.9 | 3.0 | 3.4 | 2.7 | 2.5 | 2.3 |
| Ube2k | 7.0 | 7.2 | 8.1 | 9.3 | 7.9 | 8.1 | 7.3 | 7.0 | 7.4 | 7.8 | 8.3 | 8.7 |
| Ube2l3 | 49.7 | 43.7 | 39.2 | 57.4 | 46.5 | 49.6 | 42.8 | 43.5 | 40.6 | 47.0 | 50.2 | 43.9 |
| Ube2l6 | 21.8 | 19.1 | 20.5 | 27.2 | 23.8 | 14.9 | 18.6 | 17.5 | 15.4 | 22.5 | 17.2 | 22.5 |
| Ube2m | 59.2 | 51.8 | 45.5 | 60.1 | 53.4 | 60.1 | 57.6 | 55.7 | 53.2 | 49.6 | 56.6 | 49.7 |
| Ube2n | 17.0 | 15.7 | 17.4 | 18.5 | 17.0 | 17.6 | 18.2 | 17.4 | 16.5 | 18.2 | 20.6 | 18.1 |
| Ube2o | 7.7 | 8.5 | 7.9 | 7.8 | 8.1 | 8.0 | 10.2 | 7.0 | 8.7 | 7.8 | 7.8 | 6.6 |
| Ube2q1 | 18.1 | 18.2 | 16.4 | 19.6 | 18.9 | 14.9 | 17.8 | 17.4 | 18.1 | 20.5 | 17.5 | 18.1 |
| Ube2q2 | 12.1 | 10.3 | 12.9 | 13.1 | 11.9 | 14.3 | 11.4 | 11.7 | 11.0 | 14.2 | 13.9 | 14.4 |
| Ube2ql1 | 0.0 | 0.0 | 0.0 | 0.1 | 0.0 | 0.1 | 0.0 | 0.0 | 0.0 | 0.1 | 0.0 | 0.0 |
| Ube2r2 | 27.9 | 25.9 | 26.9 | 31.6 | 24.5 | 30.0 | 27.8 | 27.0 | 25.4 | 24.9 | 31.6 | 28.7 |
| Ube2s | 74.6 | 60.5 | 64.8 | 63.9 | 69.0 | 56.4 | 85.6 | 58.8 | 77.9 | 68.8 | 78.9 | 66.0 |
| Ube2t | 1.2 | 2.7 | 3.3 | 0.3 | 3.7 | 0.6 | 4.1 | 1.2 | 3.7 | 1.5 | 2.8 | 2.2 |
| Ube2u | 0.0 | 0.1 | 0.0 | 0.0 | 0.1 | 0.1 | 0.0 | 0.0 | 0.0 | 0.0 | 0.0 | 0.0 |
| Ube2v1 | 50.4 | 44.5 | 40.2 | 59.1 | 45.0 | 52.7 | 53.5 | 49.2 | 44.0 | 50.5 | 55.2 | 46.4 |
| Ube2v2 | 5.6 | 7.3 | 7.4 | 7.5 | 6.9 | 7.1 | 6.6 | 6.9 | 6.3 | 7.3 | 7.9 | 7.8 |
| Ube2w | 13.7 | 15.7 | 15.2 | 17.4 | 15.6 | 16.6 | 14.4 | 14.6 | 17.3 | 19.5 | 19.2 | 20.7 |
| Ube2z | 21.8 | 22.2 | 20.9 | 22.3 | 22.7 | 22.5 | 27.3 | 24.2 | 27.4 | 24.1 | 24.0 | 24.1 |
| Ube3a | 13.9 | 15.7 | 15.3 | 14.2 | 14.0 | 14.2 | 14.6 | 13.2 | 14.2 | 16.3 | 16.7 | 18.0 |
| Ube3b | 13.9 | 16.1 | 14.6 | 12.2 | 14.6 | 13.2 | 15.9 | 14.6 | 16.2 | 13.1 | 13.7 | 12.0 |
| Ube3c | 16.9 | 15.9 | 14.6 | 16.1 | 15.6 | 15.2 | 16.5 | 17.7 | 17.8 | 17.3 | 17.6 | 18.7 |
| Ube4a | 9.5 | 9.6 | 9.0 | 10.9 | 10.1 | 9.4 | 8.9 | 9.9 | 9.1 | 9.3 | 9.9 | 9.0 |
| Ube4b | 12.5 | 14.8 | 14.6 | 12.1 | 13.7 | 12.1 | 14.9 | 16.1 | 14.4 | 14.2 | 14.5 | 13.5 |
| Ubfd1 | 15.2 | 17.5 | 15.3 | 14.2 | 16.8 | 12.7 | 15.2 | 17.9 | 18.0 | 19.7 | 17.5 | 17.2 |
| Ubiad1 | 4.1 | 4.3 | 4.0 | 3.8 | 4.4 | 4.2 | 4.6 | 5.3 | 4.9 | 4.4 | 4.3 | 3.7 |
| Ubl3 | 19.9 | 16.6 | 15.1 | 28.9 | 22.6 | 26.1 | 16.7 | 15.2 | 15.1 | 17.1 | 16.3 | 17.2 |
| Ubl4 | 9.0 | 8.4 | 8.4 | 7.9 | 8.8 | 7.2 | 8.4 | 10.2 | 9.3 | 7.9 | 8.9 | 7.9 |
| Ubl5 | 36.5 | 31.1 | 32.7 | 36.7 | 29.7 | 34.5 | 29.7 | 32.1 | 30.4 | 31.2 | 32.5 | 30.6 |
| Ubl7 | 18.8 | 20.6 | 18.3 | 20.4 | 16.5 | 21.3 | 17.3 | 18.8 | 16.8 | 14.8 | 16.1 | 15.7 |
| Ublcp1 | 19.8 | 21.6 | 21.0 | 20.4 | 19.2 | 21.4 | 18.2 | 20.4 | 19.6 | 20.5 | 23.3 | 22.9 |
| Ubn1 | 8.9 | 10.1 | 10.2 | 8.3 | 9.0 | 8.6 | 9.1 | 9.2 | 9.3 | 9.6 | 9.2 | 10.2 |
| Ubn2 | 1.3 | 1.8 | 1.9 | 1.4 | 1.6 | 1.3 | 1.5 | 1.7 | 1.5 | 1.5 | 1.5 | 1.5 |
| Ubox5 | 1.4 | 1.7 | 1.6 | 2.0 | 1.7 | 1.6 | 1.4 | 1.4 | 1.5 | 1.8 | 1.4 | 1.3 |
| Ubp1 | 12.5 | 14.3 | 14.2 | 12.9 | 13.1 | 12.5 | 15.1 | 13.7 | 15.0 | 11.3 | 13.8 | 12.4 |
| Ubqln1 | 29.5 | 27.2 | 27.6 | 35.8 | 29.7 | 29.4 | 32.3 | 27.9 | 28.4 | 30.4 | 33.9 | 30.0 |
| Ubqln2 | 2.3 | 2.8 | 2.6 | 3.8 | 2.9 | 3.0 | 4.0 | 2.6 | 2.9 | 2.8 | 3.2 | 2.2 |
| Ubqln4 | 9.4 | 9.1 | 7.8 | 11.3 | 9.6 | 9.9 | 9.8 | 8.2 | 8.5 | 9.0 | 9.1 | 8.2 |
| Ubr1 | 8.7 | 11.8 | 12.3 | 8.4 | 10.6 | 7.9 | 12.5 | 13.9 | 14.3 | 10.9 | 11.4 | 13.1 |
| Ubr2 | 9.8 | 10.6 | 10.3 | 10.0 | 9.8 | 10.2 | 10.7 | 11.0 | 10.3 | 9.8 | 10.4 | 10.1 |
| Ubr3 | 9.8 | 9.2 | 10.4 | 9.6 | 9.6 | 9.2 | 10.0 | 9.6 | 9.2 | 9.9 | 10.3 | 10.6 |
| Ubr4 | 15.4 | 16.8 | 15.1 | 16.4 | 15.8 | 17.3 | 18.4 | 13.7 | 14.9 | 13.8 | 13.1 | 12.1 |
| Ubr5 | 43.2 | 57.1 | 51.6 | 41.3 | 40.4 | 43.3 | 48.9 | 42.6 | 49.6 | 47.6 | 49.7 | 56.1 |
| Ubr7 | 10.5 | 12.3 | 12.3 | 9.4 | 9.9 | 11.1 | 9.2 | 10.2 | 10.7 | 12.2 | 10.9 | 10.6 |
| Ubtd1 | 29.1 | 25.8 | 18.1 | 28.2 | 28.4 | 24.6 | 28.4 | 24.0 | 25.6 | 26.5 | 23.7 | 22.0 |
| Ubtd2 | 8.1 | 9.5 | 11.5 | 7.3 | 8.3 | 7.7 | 9.8 | 15.4 | 12.9 | 10.6 | 11.5 | 11.4 |
| Ubtf | 21.2 | 22.1 | 27.3 | 21.6 | 19.9 | 21.0 | 23.7 | 26.9 | 23.3 | 21.2 | 24.8 | 24.3 |
| Ubxn1 | 107.3 | 88.0 | 95.7 | 135.0 | 78.3 | 129.8 | 89.9 | 86.2 | 65.1 | 76.5 | 98.8 | 99.7 |
| Ubxn10 | 0.0 | 0.0 | 0.0 | 0.1 | 0.0 | 0.0 | 0.0 | 0.0 | 0.0 | 0.0 | 0.0 | 0.0 |

| | | | | | | | | | | | | |
|---|---|---|---|---|---|---|---|---|---|---|---|---|
| Ubxn11 | 1.0 | 0.6 | 0.5 | 0.9 | 0.5 | 2.0 | 0.6 | 0.5 | 0.4 | 0.7 | 0.7 | 0.5 |
| Ubxn2a | 23.7 | 20.1 | 21.5 | 25.9 | 18.0 | 27.6 | 18.6 | 14.8 | 15.6 | 20.5 | 21.5 | 22.9 |
| Ubxn2b | 2.7 | 2.2 | 2.3 | 3.3 | 2.3 | 3.0 | 2.0 | 2.3 | 2.3 | 2.9 | 2.7 | 3.0 |
| Ubxn4 | 42.6 | 37.9 | 37.6 | 51.7 | 36.5 | 44.7 | 41.6 | 35.0 | 35.4 | 42.7 | 48.5 | 49.2 |
| Ubxn6 | 27.6 | 26.6 | 24.3 | 28.0 | 24.4 | 27.0 | 25.6 | 27.1 | 27.9 | 25.9 | 24.8 | 28.1 |
| Ubxn7 | 5.5 | 6.6 | 7.2 | 5.0 | 5.1 | 5.4 | 5.4 | 5.8 | 5.6 | 7.3 | 6.6 | 6.4 |
| Ubxn8 | 5.6 | 4.1 | 5.1 | 7.5 | 5.3 | 5.2 | 7.6 | 6.1 | 5.8 | 6.5 | 7.8 | 7.8 |
| Uchl1 | 42.1 | 20.7 | 15.1 | 55.6 | 54.4 | 60.5 | 17.6 | 22.6 | 10.2 | 14.8 | 17.3 | 12.2 |
| Uchl3 | 12.3 | 10.7 | 11.3 | 12.0 | 12.1 | 13.4 | 10.1 | 10.4 | 9.4 | 9.6 | 11.4 | 12.3 |
| Uchl4 | 0.2 | 0.3 | 0.1 | 0.2 | 0.4 | 0.4 | 0.3 | 0.2 | 0.1 | 0.2 | 0.1 | 0.0 |
| Uchl5 | 6.7 | 7.5 | 6.0 | 9.5 | 8.5 | 9.3 | 6.8 | 6.2 | 5.9 | 7.3 | 6.9 | 7.3 |
| Uck1 | 13.9 | 12.5 | 11.1 | 13.2 | 12.1 | 12.6 | 13.1 | 13.1 | 14.6 | 15.5 | 14.6 | 13.6 |
| Uck2 | 34.4 | 34.4 | 41.4 | 29.5 | 34.0 | 31.8 | 41.8 | 49.7 | 37.0 | 49.5 | 47.6 | 43.8 |
| Uckl1 | 14.4 | 13.9 | 11.0 | 13.5 | 13.4 | 14.0 | 10.9 | 12.7 | 14.8 | 13.6 | 13.7 | 14.0 |
| Ucn2 | 0.1 | 0.0 | 0.0 | 0.0 | 0.1 | 0.0 | 0.0 | 0.1 | 0.0 | 0.0 | 0.0 | 0.1 |
| Ucp2 | 0.6 | 0.1 | 0.2 | 5.1 | 9.8 | 0.1 | 1.3 | 0.4 | 0.3 | 1.0 | 0.5 | 0.6 |
| Uevld | 8.0 | 8.1 | 8.5 | 7.5 | 7.5 | 9.6 | 7.2 | 6.6 | 7.3 | 7.0 | 7.7 | 7.8 |
| Ufc1 | 76.5 | 63.8 | 55.5 | 75.8 | 60.8 | 66.2 | 56.3 | 57.2 | 61.4 | 74.6 | 61.5 | 74.8 |
| Ufd1l | 21.7 | 18.1 | 16.4 | 21.7 | 19.6 | 19.2 | 20.3 | 18.2 | 19.7 | 18.2 | 19.7 | 19.9 |
| Ufl1 | 10.4 | 10.2 | 10.1 | 9.8 | 7.9 | 10.1 | 7.9 | 8.5 | 8.4 | 8.8 | 10.8 | 10.9 |
| Ufm1 | 24.6 | 18.9 | 16.6 | 30.7 | 19.4 | 26.3 | 21.4 | 16.3 | 18.1 | 21.9 | 24.9 | 24.8 |
| Ufsp1 | 3.3 | 2.8 | 2.6 | 2.7 | 2.9 | 3.5 | 2.6 | 1.9 | 2.0 | 3.3 | 1.5 | 2.5 |
| Ufsp2 | 35.5 | 30.9 | 35.9 | 38.5 | 33.3 | 32.5 | 33.1 | 34.5 | 29.9 | 31.6 | 36.6 | 39.3 |
| Ugcg | 41.5 | 62.2 | 82.6 | 49.9 | 65.7 | 49.4 | 82.4 | 80.4 | 73.1 | 37.1 | 52.7 | 63.3 |
| Ugdh | 45.9 | 52.3 | 52.9 | 34.1 | 44.9 | 38.5 | 46.5 | 44.5 | 40.8 | 46.8 | 46.4 | 49.4 |
| Uggt1 | 29.7 | 27.2 | 28.0 | 24.5 | 29.0 | 26.8 | 26.9 | 30.8 | 25.6 | 25.7 | 27.8 | 26.3 |
| Uggt2 | 2.5 | 2.7 | 3.1 | 2.0 | 2.3 | 2.5 | 2.5 | 2.7 | 2.7 | 3.4 | 3.1 | 2.9 |
| Ugp2 | 58.7 | 71.4 | 76.4 | 51.2 | 62.3 | 46.1 | 44.5 | 58.3 | 54.4 | 49.7 | 50.9 | 59.4 |
| Ugt1a1 | 0.0 | 0.0 | 0.0 | 0.0 | 0.0 | 0.0 | 0.0 | 0.0 | 0.1 | 0.2 | 0.0 | 0.0 |
| Ugt1a10 | 0.1 | 0.1 | 0.0 | 0.0 | 0.1 | 0.1 | 0.0 | 0.1 | 0.0 | 0.2 | 0.1 | 0.0 |
| Ugt1a2 | 0.0 | 0.1 | 0.0 | 0.0 | 0.0 | 0.0 | 0.0 | 0.0 | 0.0 | 0.1 | 0.0 | 0.0 |
| Ugt1a5 | 0.0 | 0.0 | 0.0 | 0.0 | 0.0 | 0.0 | 0.0 | 0.1 | 0.0 | 0.2 | 0.0 | 0.0 |
| Ugt1a6a | 9.8 | 11.8 | 16.5 | 9.1 | 13.0 | 10.8 | 7.3 | 20.4 | 9.7 | 18.0 | 16.9 | 10.9 |
| Ugt1a6b | 4.7 | 4.9 | 6.1 | 2.3 | 5.4 | 3.4 | 3.7 | 9.4 | 4.0 | 9.8 | 7.8 | 5.2 |
| Ugt1a7c | 15.5 | 11.6 | 13.5 | 10.1 | 16.2 | 9.4 | 8.7 | 23.2 | 12.4 | 44.0 | 23.2 | 17.8 |
| Ugt1a9 | 0.0 | 0.1 | 0.0 | 0.0 | 0.1 | 0.1 | 0.0 | 0.1 | 0.0 | 0.1 | 0.0 | 0.0 |
| Ugt2b35 | 0.0 | 0.0 | 0.0 | 0.1 | 0.0 | 0.0 | 0.0 | 0.0 | 0.0 | 0.0 | 0.0 | 0.0 |
| Uhmk1 | 17.9 | 18.9 | 13.1 | 19.8 | 20.5 | 15.5 | 14.3 | 13.3 | 14.6 | 17.7 | 15.9 | 16.8 |
| Uhrf1 | 3.4 | 6.9 | 9.7 | 1.7 | 9.9 | 1.8 | 11.7 | 6.5 | 7.5 | 4.2 | 7.1 | 4.3 |
| Uhrf1bp1 | 3.6 | 4.1 | 4.1 | 3.1 | 3.9 | 2.6 | 4.3 | 5.3 | 4.4 | 3.5 | 3.9 | 3.6 |
| Uhrf1bp1l | 11.2 | 10.9 | 11.0 | 11.3 | 12.3 | 10.1 | 12.3 | 11.4 | 11.1 | 14.0 | 13.3 | 14.3 |
| Uhrf2 | 16.6 | 20.3 | 23.9 | 16.6 | 19.2 | 18.5 | 17.9 | 20.0 | 21.9 | 21.5 | 23.2 | 22.3 |
| Uimc1 | 14.2 | 14.2 | 16.7 | 13.0 | 12.3 | 13.8 | 13.9 | 14.6 | 12.3 | 12.2 | 15.3 | 15.4 |
| Ulbp1 | 7.7 | 9.3 | 7.8 | 2.7 | 8.8 | 4.9 | 8.0 | 4.9 | 5.9 | 5.0 | 6.9 | 4.1 |
| Ulk1 | 2.7 | 3.9 | 3.9 | 2.5 | 3.0 | 3.2 | 3.1 | 3.3 | 3.4 | 3.5 | 3.2 | 2.7 |
| Ulk2 | 21.8 | 22.0 | 18.3 | 23.0 | 17.7 | 23.2 | 19.2 | 17.2 | 20.2 | 18.1 | 20.2 | 19.0 |
| Ulk3 | 1.4 | 1.7 | 1.3 | 1.8 | 1.5 | 1.2 | 1.5 | 1.2 | 1.4 | 1.1 | 1.2 | 0.9 |
| Ulk4 | 0.6 | 0.9 | 0.7 | 0.7 | 0.5 | 0.7 | 0.7 | 0.8 | 0.6 | 0.5 | 0.5 | 0.7 |
| Umps | 9.2 | 9.1 | 9.6 | 7.5 | 8.9 | 7.6 | 12.6 | 9.6 | 11.6 | 9.7 | 10.3 | 11.2 |
| Unc119 | 13.8 | 12.2 | 8.8 | 15.8 | 13.5 | 13.4 | 12.7 | 11.8 | 11.0 | 15.2 | 12.2 | 11.6 |
| Unc119b | 9.9 | 9.9 | 9.8 | 8.5 | 9.3 | 9.7 | 10.0 | 9.5 | 10.5 | 10.4 | 8.8 | 9.4 |
| Unc13a | 0.1 | 0.1 | 0.1 | 0.1 | 0.1 | 0.2 | 0.2 | 0.1 | 0.2 | 0.2 | 0.1 | 0.2 |
| Unc13b | 1.0 | 0.7 | 0.4 | 0.7 | 0.5 | 0.7 | 0.4 | 0.6 | 0.6 | 1.4 | 0.9 | 0.7 |
| Unc13c | 0.1 | 0.1 | 0.0 | 0.0 | 0.1 | 0.1 | 0.1 | 0.0 | 0.0 | 0.4 | 0.2 | 0.1 |
| Unc13d | 0.2 | 0.0 | 0.1 | 0.9 | 1.2 | 0.2 | 0.2 | 0.1 | 0.0 | 0.1 | 0.1 | 0.1 |
| Unc45a | 11.2 | 10.6 | 8.9 | 11.6 | 10.6 | 11.3 | 10.8 | 11.0 | 10.3 | 11.5 | 10.1 | 9.5 |
| Unc45b | 0.0 | 0.0 | 0.0 | 0.0 | 0.0 | 0.0 | 0.0 | 0.0 | 0.0 | 0.1 | 0.0 | 0.0 |

| | | | | | | | | | | | | |
|---|---|---|---|---|---|---|---|---|---|---|---|---|
| Unc50 | 29.1 | 29.3 | 22.5 | 28.5 | 27.2 | 29.9 | 24.2 | 25.3 | 30.0 | 27.2 | 29.1 | 25.3 |
| Unc5a | 0.7 | 0.7 | 0.7 | 0.9 | 0.7 | 0.7 | 1.0 | 0.9 | 0.6 | 0.6 | 0.5 | 0.5 |
| Unc5b | 50.5 | 66.3 | 65.8 | 46.8 | 65.9 | 49.6 | 89.6 | 70.5 | 87.6 | 73.8 | 64.4 | 77.7 |
| Unc5c | 0.3 | 0.2 | 0.4 | 0.2 | 0.2 | 0.2 | 0.3 | 0.4 | 0.2 | 0.2 | 0.2 | 0.3 |
| Unc5d | 0.0 | 0.0 | 0.0 | 0.0 | 0.0 | 0.0 | 0.0 | 0.0 | 0.0 | 0.0 | 0.0 | 0.0 |
| Unc79 | 0.1 | 0.3 | 0.4 | 0.1 | 0.1 | 0.1 | 0.1 | 0.1 | 0.1 | 0.0 | 0.1 | 0.1 |
| Unc80 | 0.0 | 0.0 | 0.0 | 0.0 | 0.0 | 0.0 | 0.0 | 0.0 | 0.0 | 0.0 | 0.0 | 0.0 |
| Unc93a | 0.0 | 0.1 | 0.0 | 0.0 | 0.0 | 0.0 | 0.0 | 0.1 | 0.0 | 0.0 | 0.0 | 0.0 |
| Unc93b1 | 12.2 | 17.5 | 16.6 | 26.2 | 36.8 | 16.3 | 15.5 | 15.5 | 13.5 | 9.7 | 8.6 | 9.9 |
| Ung | 0.3 | 0.5 | 0.7 | 0.6 | 0.7 | 0.3 | 0.7 | 0.8 | 0.5 | 0.5 | 0.8 | 0.5 |
| Unk | 1.8 | 1.8 | 1.7 | 1.8 | 1.8 | 1.3 | 2.0 | 2.0 | 1.9 | 1.8 | 1.4 | 1.5 |
| Unkl | 2.2 | 3.3 | 3.3 | 3.0 | 2.3 | 2.6 | 3.0 | 2.5 | 3.1 | 3.1 | 2.8 | 2.8 |
| Upb1 | 0.0 | 0.0 | 0.0 | 0.0 | 0.0 | 0.0 | 0.1 | 0.0 | 0.0 | 0.0 | 0.0 | 0.0 |
| Upf1 | 10.2 | 9.8 | 12.4 | 10.9 | 10.9 | 10.4 | 15.3 | 13.4 | 12.8 | 10.2 | 11.2 | 11.9 |
| Upf2 | 11.0 | 10.0 | 12.0 | 13.7 | 11.2 | 10.0 | 14.2 | 10.7 | 9.4 | 10.5 | 12.8 | 16.0 |
| Upf3a | 32.3 | 32.5 | 39.8 | 38.7 | 26.9 | 31.1 | 35.1 | 30.4 | 26.0 | 28.6 | 35.4 | 38.7 |
| Upf3b | 8.4 | 7.1 | 10.4 | 7.8 | 7.2 | 7.7 | 9.9 | 8.0 | 5.7 | 7.5 | 9.7 | 10.5 |
| Upk1a | 0.1 | 0.3 | 0.1 | 0.0 | 0.1 | 0.1 | 0.1 | 0.1 | 0.1 | 0.0 | 0.1 | 0.0 |
| Upk1b | 64.6 | 13.4 | 3.8 | 15.3 | 6.0 | 8.2 | 4.3 | 7.4 | 11.7 | 233.9 | 116.7 | 51.5 |
| Upk3b | 51.3 | 33.1 | 21.6 | 16.5 | 12.2 | 6.8 | 18.7 | 31.8 | 23.4 | 199.5 | 99.0 | 53.3 |
| Upk3bl | 0.1 | 0.0 | 0.1 | 0.0 | 0.0 | 0.0 | 0.0 | 0.1 | 0.0 | 0.0 | 0.0 | 0.0 |
| Upp1 | 0.3 | 0.1 | 0.2 | 0.4 | 0.3 | 0.1 | 0.2 | 0.2 | 0.3 | 0.1 | 0.2 | 0.1 |
| Upp2 | 0.0 | 0.0 | 0.1 | 0.1 | 0.1 | 0.1 | 0.0 | 0.0 | 0.1 | 0.1 | 0.1 | 0.1 |
| Uprt | 7.1 | 6.2 | 5.8 | 8.5 | 6.6 | 6.8 | 7.0 | 4.8 | 6.2 | 6.3 | 7.9 | 8.2 |
| Uqcc | 8.1 | 7.8 | 8.3 | 9.8 | 8.3 | 9.1 | 8.6 | 7.7 | 8.8 | 9.1 | 8.8 | 9.6 |
| Uqcr10 | 133.9 | 102.0 | 85.7 | 114.1 | 104.0 | 105.7 | 94.3 | 94.9 | 104.3 | 131.6 | 105.3 | 108.9 |
| Uqcr11 | 222.3 | 175.2 | 135.2 | 185.0 | 186.3 | 214.3 | 171.9 | 166.9 | 168.9 | 205.2 | 167.5 | 165.6 |
| Uqcrb | 219.2 | 182.2 | 205.9 | 230.9 | 166.0 | 189.7 | 174.9 | 174.1 | 156.6 | 171.7 | 197.8 | 229.6 |
| Uqcrc1 | 51.6 | 48.4 | 43.8 | 48.8 | 47.3 | 45.9 | 46.1 | 48.1 | 46.5 | 46.9 | 44.6 | 43.8 |
| Uqcrc2 | 47.7 | 44.3 | 41.5 | 45.5 | 41.6 | 43.6 | 39.2 | 43.0 | 43.1 | 48.1 | 48.0 | 44.0 |
| Uqcrfs1 | 41.7 | 33.3 | 29.8 | 30.3 | 32.7 | 28.6 | 29.1 | 32.1 | 34.8 | 41.1 | 29.8 | 33.3 |
| Uqcrh | 274.5 | 217.5 | 225.6 | 227.3 | 189.7 | 236.1 | 210.3 | 242.6 | 201.7 | 253.4 | 253.9 | 268.8 |
| Uqcrq | 100.4 | 72.0 | 71.1 | 79.1 | 84.1 | 78.4 | 71.6 | 99.9 | 84.3 | 83.8 | 85.0 | 75.2 |
| Urb1 | 2.0 | 1.6 | 1.5 | 1.6 | 1.8 | 1.8 | 1.8 | 1.4 | 1.4 | 1.8 | 1.4 | 1.5 |
| Urb2 | 3.4 | 3.3 | 2.6 | 3.0 | 3.8 | 2.8 | 3.3 | 3.1 | 3.1 | 3.2 | 3.5 | 2.9 |
| Urgcp | 8.2 | 8.4 | 7.1 | 7.7 | 7.2 | 7.5 | 8.0 | 7.1 | 8.1 | 8.7 | 6.6 | 7.5 |
| Uri1 | 11.1 | 9.3 | 9.5 | 12.0 | 9.0 | 10.3 | 9.6 | 10.5 | 8.9 | 10.1 | 10.1 | 10.8 |
| Urm1 | 12.6 | 10.8 | 8.9 | 14.5 | 11.1 | 12.8 | 12.3 | 11.0 | 11.1 | 13.2 | 11.8 | 9.8 |
| Uroc1 | 0.0 | 0.0 | 0.0 | 0.0 | 0.0 | 0.0 | 0.0 | 0.0 | 0.0 | 0.0 | 0.0 | 0.0 |
| Urod | 22.9 | 22.6 | 21.2 | 19.7 | 19.3 | 26.2 | 17.4 | 22.3 | 18.5 | 21.2 | 17.3 | 20.7 |
| Uros | 9.0 | 8.7 | 7.0 | 8.5 | 7.3 | 7.7 | 8.2 | 7.8 | 8.5 | 9.0 | 9.8 | 9.3 |
| Usb1 | 7.3 | 6.7 | 6.6 | 7.7 | 8.3 | 6.3 | 7.5 | 6.6 | 6.6 | 6.7 | 7.5 | 6.6 |
| Use1 | 73.4 | 55.4 | 52.7 | 53.3 | 47.7 | 67.6 | 50.9 | 60.0 | 60.0 | 63.1 | 59.0 | 65.8 |
| Usf1 | 9.9 | 8.4 | 9.5 | 9.9 | 10.0 | 10.2 | 10.1 | 9.7 | 9.6 | 12.4 | 9.8 | 10.6 |
| Usf2 | 12.8 | 11.3 | 12.8 | 15.0 | 11.3 | 13.9 | 11.8 | 12.0 | 10.1 | 10.3 | 11.5 | 11.0 |
| Ush1c | 0.0 | 0.0 | 0.0 | 0.1 | 0.0 | 0.1 | 0.0 | 0.0 | 0.0 | 0.0 | 0.0 | 0.0 |
| Ush1g | 0.0 | 0.0 | 0.0 | 0.0 | 0.0 | 0.0 | 0.0 | 0.0 | 0.0 | 0.0 | 0.1 | 0.0 |
| Ush2a | 0.0 | 0.0 | 0.0 | 0.0 | 0.0 | 0.0 | 0.0 | 0.0 | 0.0 | 0.0 | 0.0 | 0.0 |
| Ushbp1 | 0.1 | 0.0 | 0.1 | 0.0 | 0.1 | 0.0 | 0.1 | 0.1 | 0.2 | 0.0 | 0.0 | 0.0 |
| Usmg5 | 184.8 | 191.3 | 158.4 | 175.9 | 167.6 | 179.4 | 142.4 | 159.8 | 164.7 | 198.8 | 204.6 | 183.2 |
| Uso1 | 15.0 | 15.1 | 14.6 | 15.7 | 17.3 | 15.8 | 17.0 | 15.1 | 14.6 | 16.9 | 18.4 | 19.1 |
| Usp1 | 5.7 | 6.2 | 8.9 | 4.7 | 7.0 | 5.1 | 9.6 | 8.1 | 7.0 | 6.3 | 8.7 | 8.0 |
| Usp10 | 7.7 | 7.3 | 6.8 | 7.6 | 7.6 | 7.3 | 9.1 | 6.6 | 7.5 | 6.9 | 7.3 | 6.7 |
| Usp11 | 1.4 | 1.2 | 1.9 | 1.4 | 1.3 | 1.3 | 1.4 | 1.6 | 1.6 | 2.0 | 1.5 | 1.6 |
| Usp12 | 11.5 | 14.0 | 13.5 | 11.0 | 12.6 | 9.0 | 13.2 | 11.0 | 13.7 | 13.7 | 14.1 | 14.4 |
| Usp13 | 1.0 | 1.2 | 1.2 | 0.8 | 0.9 | 1.3 | 1.2 | 1.3 | 1.5 | 2.1 | 1.7 | 1.6 |
| Usp14 | 18.4 | 18.2 | 14.8 | 19.8 | 19.1 | 20.0 | 16.0 | 15.0 | 17.7 | 19.9 | 19.8 | 18.6 |

| | | | | | | | | | | | | |
|---|---|---|---|---|---|---|---|---|---|---|---|---|
| Usp15 | 28.8 | 24.4 | 24.6 | 27.7 | 24.2 | 25.7 | 23.2 | 23.0 | 20.4 | 26.6 | 29.6 | 27.8 |
| Usp16 | 29.1 | 24.7 | 23.7 | 30.1 | 27.1 | 21.3 | 28.6 | 26.9 | 25.1 | 24.1 | 31.8 | 30.1 |
| Usp17la | 0.0 | 0.1 | 0.1 | 0.0 | 0.0 | 0.0 | 0.1 | 0.1 | 0.0 | 0.0 | 0.1 | 0.0 |
| Usp18 | 8.2 | 2.7 | 3.6 | 8.0 | 3.1 | 4.0 | 3.6 | 1.6 | 2.4 | 1.1 | 3.2 | 6.3 |
| Usp19 | 26.4 | 26.4 | 25.1 | 25.6 | 25.0 | 26.0 | 24.2 | 24.6 | 27.4 | 27.8 | 24.6 | 25.3 |
| Usp2 | 1.0 | 1.1 | 1.1 | 0.3 | 0.9 | 0.8 | 0.8 | 0.6 | 0.8 | 0.9 | 0.9 | 0.8 |
| Usp20 | 2.6 | 2.8 | 2.5 | 3.0 | 2.3 | 2.9 | 2.3 | 2.2 | 2.1 | 3.2 | 2.1 | 2.0 |
| Usp21 | 5.5 | 7.4 | 8.2 | 5.4 | 6.9 | 6.2 | 5.7 | 6.9 | 7.7 | 7.5 | 5.5 | 6.5 |
| Usp22 | 17.8 | 18.6 | 15.1 | 18.3 | 15.4 | 20.8 | 16.2 | 16.1 | 14.0 | 15.0 | 16.0 | 12.5 |
| Usp24 | 10.6 | 10.5 | 8.8 | 9.4 | 9.9 | 9.9 | 9.0 | 8.7 | 8.5 | 8.0 | 9.2 | 8.4 |
| Usp25 | 18.1 | 18.2 | 20.2 | 19.7 | 19.0 | 18.0 | 19.9 | 18.5 | 20.1 | 21.5 | 23.8 | 25.0 |
| Usp27x | 1.0 | 0.7 | 1.1 | 1.0 | 0.8 | 0.9 | 0.8 | 0.9 | 0.9 | 1.0 | 0.7 | 0.9 |
| Usp28 | 1.9 | 2.6 | 2.7 | 2.2 | 2.2 | 2.3 | 2.5 | 2.7 | 2.5 | 2.2 | 2.7 | 2.1 |
| Usp29 | 0.0 | 0.0 | 0.0 | 0.0 | 0.0 | 0.0 | 0.0 | 0.0 | 0.0 | 0.0 | 0.0 | 0.0 |
| Usp3 | 8.6 | 7.6 | 9.2 | 7.9 | 7.8 | 8.3 | 7.8 | 8.3 | 6.4 | 8.2 | 9.0 | 8.1 |
| Usp30 | 9.2 | 8.5 | 7.9 | 9.7 | 7.3 | 10.3 | 9.1 | 7.8 | 8.1 | 8.5 | 8.5 | 9.0 |
| Usp31 | 3.7 | 4.4 | 3.8 | 3.8 | 3.7 | 3.5 | 3.4 | 3.2 | 3.8 | 3.7 | 3.9 | 3.2 |
| Usp32 | 10.2 | 11.5 | 10.8 | 9.9 | 10.1 | 9.6 | 9.0 | 8.4 | 8.5 | 9.7 | 10.9 | 10.2 |
| Usp33 | 15.8 | 15.7 | 16.3 | 16.8 | 14.6 | 16.4 | 16.4 | 14.8 | 16.1 | 17.8 | 18.7 | 19.4 |
| Usp34 | 6.5 | 7.3 | 6.2 | 6.5 | 6.8 | 6.7 | 7.0 | 6.4 | 6.3 | 6.8 | 6.9 | 6.4 |
| Usp35 | 2.0 | 1.9 | 1.6 | 1.5 | 1.7 | 1.4 | 1.7 | 1.5 | 1.7 | 1.6 | 1.3 | 1.9 |
| Usp36 | 2.1 | 2.1 | 2.2 | 2.2 | 2.0 | 1.9 | 2.3 | 2.3 | 2.5 | 2.1 | 2.1 | 1.9 |
| Usp37 | 1.7 | 2.5 | 2.8 | 1.8 | 2.4 | 1.8 | 2.4 | 2.4 | 2.3 | 2.4 | 2.7 | 2.2 |
| Usp38 | 6.6 | 6.6 | 5.1 | 6.4 | 6.3 | 6.1 | 6.0 | 6.0 | 6.5 | 6.8 | 7.1 | 7.0 |
| Usp39 | 18.8 | 18.8 | 18.4 | 20.3 | 17.3 | 18.2 | 18.8 | 20.7 | 17.3 | 18.9 | 20.7 | 17.7 |
| Usp4 | 28.6 | 26.0 | 22.8 | 28.7 | 26.4 | 28.0 | 27.6 | 26.3 | 26.9 | 25.8 | 28.5 | 26.4 |
| Usp40 | 8.2 | 8.9 | 9.8 | 7.4 | 7.6 | 8.7 | 8.6 | 10.4 | 9.9 | 10.5 | 9.8 | 9.7 |
| Usp42 | 2.1 | 2.7 | 3.5 | 2.4 | 2.4 | 2.2 | 3.0 | 3.2 | 3.3 | 2.2 | 2.9 | 2.7 |
| Usp44 | 0.0 | 0.0 | 0.0 | 0.0 | 0.0 | 0.0 | 0.0 | 0.0 | 0.0 | 0.0 | 0.0 | 0.0 |
| Usp45 | 5.8 | 7.3 | 7.5 | 6.7 | 6.8 | 6.4 | 6.1 | 6.2 | 6.5 | 6.1 | 7.3 | 6.9 |
| Usp46 | 22.0 | 23.8 | 19.8 | 19.3 | 17.1 | 21.4 | 18.2 | 23.5 | 18.5 | 17.9 | 22.4 | 19.0 |
| Usp47 | 39.9 | 41.6 | 39.5 | 39.5 | 36.3 | 38.2 | 40.4 | 38.4 | 39.1 | 39.1 | 46.0 | 45.1 |
| Usp48 | 7.7 | 7.9 | 8.6 | 8.3 | 8.3 | 8.7 | 7.9 | 8.7 | 8.4 | 7.9 | 8.7 | 8.8 |
| Usp49 | 1.3 | 1.8 | 1.4 | 1.5 | 1.5 | 1.0 | 1.4 | 1.3 | 1.7 | 1.3 | 1.8 | 1.3 |
| Usp5 | 38.2 | 33.6 | 28.6 | 39.7 | 35.2 | 36.6 | 39.2 | 35.3 | 36.4 | 37.3 | 35.1 | 33.6 |
| Usp50 | 0.0 | 0.0 | 0.0 | 0.0 | 0.0 | 0.0 | 0.0 | 0.0 | 0.0 | 0.0 | 0.1 | 0.0 |
| Usp51 | 0.1 | 0.0 | 0.0 | 0.1 | 0.1 | 0.0 | 0.2 | 0.1 | 0.0 | 0.1 | 0.0 | 0.0 |
| Usp53 | 9.4 | 7.6 | 6.0 | 11.3 | 6.8 | 9.0 | 8.5 | 6.7 | 9.4 | 15.6 | 15.8 | 14.6 |
| Usp54 | 3.1 | 2.7 | 2.6 | 3.0 | 2.2 | 2.8 | 2.4 | 2.1 | 1.8 | 2.6 | 2.8 | 2.7 |
| Usp6nl | 3.5 | 3.9 | 3.8 | 3.5 | 3.6 | 3.6 | 4.1 | 3.1 | 3.5 | 3.4 | 3.7 | 4.0 |
| Usp7 | 16.7 | 17.9 | 17.4 | 18.5 | 16.8 | 16.4 | 17.5 | 17.1 | 16.2 | 15.7 | 18.3 | 17.6 |
| Usp8 | 28.9 | 29.4 | 30.4 | 34.5 | 28.3 | 29.4 | 28.1 | 28.5 | 26.1 | 29.9 | 31.8 | 34.3 |
| Usp9x | 45.2 | 53.5 | 49.3 | 44.4 | 47.2 | 48.0 | 44.6 | 49.1 | 51.1 | 47.6 | 52.5 | 50.8 |
| Usp9y | 0.0 | 0.0 | 0.0 | 0.0 | 0.0 | 0.0 | 0.1 | 0.0 | 0.0 | 0.0 | 0.0 | 0.0 |
| Uspl1 | 5.1 | 4.9 | 4.0 | 5.5 | 5.5 | 5.2 | 5.0 | 4.6 | 4.9 | 6.0 | 5.2 | 4.5 |
| Ust | 5.2 | 5.6 | 6.1 | 6.0 | 6.3 | 4.9 | 4.9 | 4.6 | 4.8 | 3.9 | 4.3 | 5.1 |
| Utf1 | 0.0 | 0.0 | 0.0 | 0.0 | 0.0 | 0.0 | 0.0 | 0.0 | 0.0 | 0.0 | 0.0 | 0.0 |
| Utp11l | 33.2 | 33.1 | 35.0 | 38.1 | 32.5 | 32.6 | 39.0 | 32.1 | 29.3 | 33.5 | 40.2 | 38.9 |
| Utp14a | 9.6 | 7.0 | 6.6 | 9.1 | 7.4 | 8.6 | 6.6 | 6.4 | 5.6 | 7.3 | 7.7 | 7.1 |
| Utp14b | 2.5 | 3.3 | 2.9 | 1.6 | 2.4 | 1.9 | 4.2 | 3.9 | 2.9 | 1.5 | 2.8 | 2.0 |
| Utp15 | 3.9 | 3.5 | 3.5 | 3.9 | 4.7 | 4.5 | 3.4 | 3.2 | 3.4 | 3.5 | 3.6 | 3.1 |
| Utp18 | 10.3 | 8.9 | 7.8 | 9.5 | 9.4 | 9.0 | 9.8 | 10.0 | 8.6 | 10.3 | 10.8 | 9.6 |
| Utp20 | 8.9 | 7.6 | 6.8 | 7.5 | 8.5 | 8.4 | 7.2 | 6.5 | 6.3 | 7.4 | 7.4 | 7.2 |
| Utp23 | 2.4 | 2.7 | 2.8 | 2.3 | 2.7 | 2.9 | 2.5 | 2.6 | 2.6 | 2.7 | 2.6 | 3.2 |
| Utp3 | 31.5 | 25.7 | 28.1 | 36.1 | 27.4 | 30.3 | 32.4 | 24.7 | 24.0 | 29.1 | 34.1 | 35.9 |
| Utp6 | 19.0 | 17.1 | 17.9 | 23.7 | 17.8 | 20.9 | 19.2 | 17.6 | 15.3 | 20.1 | 20.1 | 20.7 |
| Utrn | 24.3 | 22.3 | 20.9 | 23.6 | 20.3 | 26.1 | 20.3 | 17.6 | 17.5 | 21.1 | 23.3 | 21.0 |

| | | | | | | | | | | | | |
|---|---|---|---|---|---|---|---|---|---|---|---|---|
| Uty | 5.3 | 5.6 | 6.2 | 5.9 | 5.7 | 6.0 | 4.2 | 5.0 | 4.7 | 6.2 | 5.2 | 5.9 |
| Uvrag | 21.7 | 23.4 | 23.4 | 20.9 | 22.8 | 17.5 | 23.0 | 18.3 | 25.0 | 20.7 | 21.4 | 23.0 |
| Uvssa | 3.2 | 3.9 | 4.7 | 3.2 | 2.8 | 3.9 | 3.3 | 3.8 | 3.0 | 2.6 | 3.3 | 3.8 |
| Uxs1 | 26.8 | 20.4 | 21.1 | 22.7 | 21.2 | 20.7 | 24.1 | 22.1 | 17.8 | 18.6 | 21.7 | 21.1 |
| Uxt | 13.4 | 12.5 | 11.1 | 12.7 | 12.3 | 11.7 | 11.6 | 11.9 | 11.6 | 10.3 | 9.8 | 10.4 |
| Vac14 | 5.5 | 4.4 | 4.3 | 6.4 | 5.4 | 5.4 | 5.5 | 5.2 | 5.2 | 3.8 | 4.2 | 3.9 |
| Vamp1 | 0.3 | 0.2 | 0.2 | 0.2 | 0.3 | 0.3 | 0.2 | 0.3 | 0.2 | 0.2 | 0.2 | 0.1 |
| Vamp2 | 2.6 | 2.4 | 2.8 | 2.2 | 2.3 | 2.7 | 3.4 | 3.3 | 3.0 | 2.4 | 1.8 | 2.8 |
| Vamp3 | 48.9 | 47.4 | 42.6 | 46.0 | 46.4 | 43.0 | 44.3 | 47.3 | 49.8 | 45.8 | 53.0 | 41.3 |
| Vamp4 | 5.1 | 6.5 | 7.2 | 5.6 | 5.3 | 6.5 | 4.5 | 5.3 | 5.6 | 5.5 | 6.4 | 6.8 |
| Vamp5 | 18.5 | 30.4 | 28.6 | 15.4 | 25.9 | 20.1 | 37.0 | 51.6 | 46.2 | 32.5 | 36.8 | 32.2 |
| Vamp8 | 48.5 | 33.9 | 34.6 | 65.8 | 44.7 | 38.8 | 48.5 | 41.7 | 42.5 | 53.9 | 53.1 | 52.8 |
| Vangl1 | 5.7 | 6.0 | 6.5 | 6.4 | 6.1 | 6.1 | 8.6 | 7.7 | 7.6 | 6.0 | 6.8 | 6.7 |
| Vangl2 | 1.2 | 1.5 | 1.2 | 0.6 | 0.7 | 1.1 | 1.2 | 1.9 | 1.7 | 1.1 | 0.9 | 1.0 |
| Vapa | 84.8 | 84.1 | 72.9 | 86.8 | 86.7 | 72.5 | 91.6 | 86.1 | 99.5 | 80.5 | 102.3 | 92.2 |
| Vapb | 18.1 | 15.2 | 12.5 | 18.9 | 15.0 | 17.8 | 14.7 | 13.0 | 12.6 | 15.2 | 15.6 | 14.1 |
| Vars | 15.1 | 13.3 | 12.3 | 13.1 | 12.1 | 14.3 | 15.1 | 14.9 | 14.5 | 12.5 | 13.5 | 10.5 |
| Vars2 | 3.5 | 3.9 | 3.5 | 3.2 | 2.7 | 4.1 | 3.2 | 4.0 | 3.3 | 2.7 | 2.7 | 2.6 |
| Vash1 | 1.4 | 1.2 | 1.7 | 1.1 | 1.2 | 0.8 | 0.9 | 2.4 | 1.4 | 0.6 | 0.4 | 0.6 |
| Vash2 | 0.3 | 0.6 | 1.0 | 0.1 | 0.4 | 0.5 | 0.6 | 1.1 | 0.9 | 0.3 | 0.6 | 0.5 |
| Vasn | 16.8 | 19.0 | 19.3 | 15.7 | 17.3 | 17.7 | 23.6 | 21.0 | 21.0 | 23.8 | 19.6 | 17.8 |
| Vasp | 22.9 | 25.4 | 23.3 | 26.8 | 37.2 | 20.0 | 29.6 | 27.3 | 33.8 | 28.9 | 28.1 | 24.7 |
| Vat1 | 111.0 | 82.3 | 60.6 | 136.1 | 85.9 | 135.8 | 79.5 | 83.0 | 72.1 | 80.1 | 79.0 | 78.8 |
| Vat1l | 4.6 | 0.3 | 0.0 | 1.1 | 0.2 | 0.2 | 0.1 | 0.6 | 0.6 | 28.1 | 14.5 | 3.4 |
| Vaultrc5 | 106.0 | 94.0 | 89.6 | 111.6 | 140.9 | 92.9 | 71.8 | 104.1 | 152.4 | 78.4 | 70.3 | 121.0 |
| Vav1 | 0.6 | 0.0 | 0.1 | 5.7 | 11.1 | 0.0 | 1.6 | 0.1 | 0.2 | 0.7 | 0.2 | 0.7 |
| Vav2 | 11.7 | 14.2 | 12.8 | 8.2 | 10.7 | 7.2 | 17.5 | 19.7 | 15.2 | 15.9 | 18.6 | 16.2 |
| Vav3 | 0.6 | 0.8 | 1.3 | 1.6 | 2.4 | 0.2 | 0.6 | 2.1 | 1.1 | 1.0 | 0.8 | 1.0 |
| Vax2 | 4.1 | 2.3 | 1.0 | 4.2 | 1.9 | 4.5 | 1.4 | 1.1 | 1.1 | 1.3 | 1.1 | 1.1 |
| Vbp1 | 23.5 | 27.6 | 27.4 | 25.3 | 26.8 | 24.7 | 25.0 | 25.7 | 29.4 | 29.8 | 30.4 | 30.9 |
| Vcam1 | 212.8 | 170.3 | 212.1 | 177.1 | 285.3 | 146.5 | 144.1 | 141.5 | 150.9 | 206.9 | 134.6 | 247.3 |
| Vcan | 2.9 | 4.9 | 8.7 | 1.4 | 6.3 | 1.6 | 8.9 | 20.8 | 7.3 | 6.8 | 8.8 | 6.3 |
| Vcl | 94.0 | 101.0 | 84.6 | 86.5 | 86.0 | 73.7 | 104.6 | 69.7 | 104.5 | 106.2 | 117.4 | 98.3 |
| Vcp | 244.6 | 217.7 | 202.7 | 244.3 | 225.5 | 226.8 | 240.3 | 241.5 | 224.9 | 245.7 | 251.3 | 245.7 |
| Vcpip1 | 6.8 | 7.7 | 8.2 | 8.0 | 8.8 | 8.0 | 7.6 | 8.0 | 7.3 | 9.1 | 8.9 | 9.1 |
| Vdac1 | 110.6 | 106.8 | 98.5 | 112.2 | 110.8 | 116.4 | 104.3 | 97.8 | 96.5 | 122.5 | 124.9 | 106.4 |
| Vdac2 | 191.0 | 159.7 | 134.2 | 192.2 | 175.5 | 198.8 | 175.0 | 136.4 | 168.8 | 185.0 | 187.0 | 186.1 |
| Vdac3 | 112.9 | 87.3 | 64.6 | 96.2 | 82.1 | 97.3 | 71.8 | 76.7 | 84.5 | 80.3 | 82.8 | 76.2 |
| Vdr | 0.9 | 1.6 | 2.1 | 1.3 | 2.0 | 0.7 | 3.4 | 3.0 | 3.1 | 3.6 | 3.3 | 2.9 |
| Vegfa | 53.8 | 65.2 | 57.5 | 53.2 | 51.6 | 60.4 | 106.7 | 77.8 | 100.4 | 53.7 | 77.1 | 69.8 |
| Vegfb | 6.2 | 6.1 | 5.3 | 7.2 | 5.0 | 8.8 | 4.6 | 5.0 | 4.8 | 4.1 | 5.5 | 3.9 |
| Vegfc | 16.3 | 23.3 | 23.1 | 10.9 | 22.0 | 7.6 | 19.9 | 15.0 | 22.4 | 14.2 | 14.1 | 17.3 |
| Veph1 | 0.3 | 0.1 | 0.1 | 0.3 | 0.2 | 0.2 | 0.2 | 0.1 | 0.1 | 0.1 | 0.2 | 0.1 |
| Vezf1 | 10.9 | 10.0 | 10.0 | 12.4 | 9.2 | 11.9 | 9.7 | 9.1 | 8.9 | 9.4 | 12.0 | 10.4 |
| Vezt | 6.3 | 5.1 | 5.2 | 5.9 | 5.8 | 6.3 | 6.7 | 5.7 | 6.7 | 6.2 | 6.7 | 7.3 |
| Vgf | 0.3 | 0.5 | 0.4 | 1.0 | 0.3 | 0.7 | 0.5 | 0.1 | 0.4 | 0.5 | 0.4 | 0.1 |
| Vgll3 | 65.7 | 94.8 | 88.7 | 83.6 | 73.2 | 84.8 | 87.0 | 84.7 | 84.4 | 70.5 | 93.3 | 83.4 |
| Vgll4 | 8.9 | 9.3 | 9.6 | 11.9 | 10.0 | 11.2 | 9.5 | 10.9 | 9.9 | 14.0 | 8.8 | 9.6 |
| Vhl | 10.0 | 10.1 | 9.5 | 7.7 | 9.6 | 8.0 | 8.9 | 9.5 | 11.3 | 10.6 | 10.2 | 11.0 |
| Vil1 | 0.0 | 0.0 | 0.0 | 0.0 | 0.0 | 0.0 | 0.0 | 0.0 | 0.0 | 0.1 | 0.0 | 0.0 |
| Vill | 2.0 | 2.3 | 1.8 | 0.7 | 1.9 | 1.6 | 0.9 | 2.0 | 1.2 | 2.3 | 2.3 | 2.4 |
| Vim | 1650.9 | 1202.6 | 1111.5 | 1427.9 | 1324.0 | 1453.6 | 1163.1 | 1246.8 | 1028.0 | 1065.0 | 1197.5 | 1038.4 |
| Vimp | 84.1 | 76.4 | 79.7 | 90.3 | 77.7 | 74.4 | 98.8 | 73.1 | 89.4 | 86.1 | 94.2 | 98.8 |
| Vipas39 | 19.0 | 18.1 | 15.8 | 20.1 | 17.9 | 18.3 | 15.0 | 15.8 | 15.3 | 16.9 | 15.8 | 18.1 |
| Vipr1 | 0.0 | 0.0 | 0.1 | 0.1 | 0.1 | 0.0 | 0.0 | 0.0 | 0.0 | 0.0 | 0.0 | 0.0 |
| Vipr2 | 0.1 | 0.2 | 0.3 | 0.1 | 0.1 | 0.2 | 0.1 | 0.2 | 0.1 | 0.0 | 0.0 | 0.0 |
| Vit | 5.8 | 2.5 | 3.4 | 2.7 | 2.1 | 2.2 | 1.7 | 3.7 | 0.7 | 2.6 | 3.6 | 1.9 |

| | | | | | | | | | | | | |
|---|---|---|---|---|---|---|---|---|---|---|---|---|
| Vkorc1 | 76.3 | 72.0 | 65.8 | 79.7 | 71.3 | 72.7 | 69.3 | 72.4 | 67.5 | 80.5 | 73.2 | 78.1 |
| Vkorc1l1 | 6.7 | 8.7 | 8.5 | 8.6 | 7.5 | 7.1 | 8.2 | 8.4 | 11.3 | 9.1 | 10.6 | 8.3 |
| Vldlr | 3.7 | 4.0 | 3.6 | 3.3 | 2.0 | 3.8 | 2.7 | 3.7 | 4.0 | 4.5 | 4.0 | 3.6 |
| Vma21 | 4.9 | 4.9 | 7.4 | 9.1 | 6.9 | 7.0 | 6.5 | 5.5 | 5.1 | 6.0 | 7.7 | 7.9 |
| Vmac | 8.0 | 7.0 | 6.3 | 8.6 | 7.5 | 9.0 | 7.4 | 6.3 | 5.9 | 7.8 | 7.6 | 7.0 |
| Vmn1r45 | 0.1 | 0.0 | 0.0 | 0.0 | 0.0 | 0.0 | 0.0 | 0.0 | 0.0 | 0.0 | 0.0 | 0.0 |
| Vmn1r51 | 0.0 | 0.0 | 0.0 | 0.1 | 0.0 | 0.0 | 0.0 | 0.1 | 0.0 | 0.0 | 0.1 | 0.0 |
| Vmn1r53 | 0.1 | 0.1 | 0.1 | 0.0 | 0.1 | 0.0 | 0.1 | 0.1 | 0.1 | 0.1 | 0.2 | 0.0 |
| Vmn1r58 | 1.2 | 1.9 | 1.7 | 1.0 | 1.9 | 1.2 | 1.8 | 1.2 | 1.4 | 1.6 | 1.2 | 1.3 |
| Vmn1r65 | 0.0 | 0.0 | 0.0 | 0.1 | 0.0 | 0.0 | 0.0 | 0.0 | 0.0 | 0.0 | 0.0 | 0.0 |
| Vmn1r90 | 0.0 | 0.0 | 0.0 | 0.0 | 0.0 | 0.0 | 0.0 | 0.0 | 0.0 | 0.0 | 0.0 | 0.0 |
| Vmn2r1 | 0.1 | 0.1 | 0.1 | 0.1 | 0.0 | 0.0 | 0.0 | 0.1 | 0.1 | 0.0 | 0.1 | 0.0 |
| Vmn2r2 | 0.0 | 0.0 | 0.0 | 0.0 | 0.0 | 0.0 | 0.0 | 0.0 | 0.0 | 0.0 | 0.0 | 0.0 |
| Vmn2r29 | 0.8 | 1.4 | 1.1 | 0.7 | 1.0 | 1.2 | 0.7 | 0.9 | 0.8 | 1.6 | 1.6 | 1.2 |
| Vmn2r3 | 0.0 | 0.0 | 0.0 | 0.0 | 0.0 | 0.0 | 0.0 | 0.0 | 0.0 | 0.0 | 0.0 | 0.0 |
| Vmn2r30 | 0.0 | 0.0 | 0.0 | 0.0 | 0.0 | 0.0 | 0.0 | 0.0 | 0.0 | 0.0 | 0.0 | 0.0 |
| Vmn2r31 | 0.0 | 0.0 | 0.0 | 0.0 | 0.0 | 0.0 | 0.1 | 0.0 | 0.0 | 0.0 | 0.0 | 0.0 |
| Vmn2r33 | 0.0 | 0.0 | 0.0 | 0.0 | 0.0 | 0.0 | 0.0 | 0.0 | 0.0 | 0.0 | 0.0 | 0.0 |
| Vmn2r34 | 0.0 | 0.0 | 0.0 | 0.0 | 0.0 | 0.0 | 0.0 | 0.0 | 0.0 | 0.0 | 0.0 | 0.0 |
| Vmn2r37 | 0.0 | 0.0 | 0.0 | 0.0 | 0.0 | 0.0 | 0.0 | 0.0 | 0.0 | 0.0 | 0.0 | 0.0 |
| Vmn2r38 | 0.0 | 0.0 | 0.0 | 0.0 | 0.0 | 0.0 | 0.0 | 0.0 | 0.0 | 0.0 | 0.0 | 0.0 |
| Vmn2r39 | 0.0 | 0.0 | 0.0 | 0.0 | 0.0 | 0.0 | 0.0 | 0.0 | 0.0 | 0.0 | 0.0 | 0.0 |
| Vmn2r4 | 0.0 | 0.0 | 0.0 | 0.0 | 0.0 | 0.0 | 0.0 | 0.0 | 0.0 | 0.0 | 0.0 | 0.0 |
| Vmn2r43 | 0.0 | 0.0 | 0.0 | 0.0 | 0.0 | 0.0 | 0.0 | 0.0 | 0.0 | 0.0 | 0.0 | 0.0 |
| Vmn2r44 | 0.0 | 0.0 | 0.0 | 0.0 | 0.0 | 0.0 | 0.0 | 0.0 | 0.0 | 0.0 | 0.0 | 0.0 |
| Vmn2r45 | 0.0 | 0.0 | 0.0 | 0.0 | 0.0 | 0.0 | 0.0 | 0.0 | 0.0 | 0.0 | 0.0 | 0.0 |
| Vmn2r46 | 0.0 | 0.1 | 0.1 | 0.1 | 0.1 | 0.1 | 0.1 | 0.1 | 0.2 | 0.1 | 0.1 | 0.1 |
| Vmn2r47 | 0.0 | 0.0 | 0.0 | 0.0 | 0.0 | 0.0 | 0.0 | 0.0 | 0.0 | 0.0 | 0.0 | 0.0 |
| Vmn2r57 | 0.2 | 0.1 | 0.2 | 0.1 | 0.1 | 0.1 | 0.1 | 0.1 | 0.1 | 0.1 | 0.1 | 0.0 |
| Vmn2r62 | 0.0 | 0.0 | 0.0 | 0.0 | 0.0 | 0.0 | 0.0 | 0.0 | 0.0 | 0.0 | 0.0 | 0.0 |
| Vmn2r63 | 0.0 | 0.0 | 0.0 | 0.0 | 0.0 | 0.0 | 0.0 | 0.0 | 0.0 | 0.0 | 0.0 | 0.0 |
| Vmn2r-ps129 | 0.0 | 0.0 | 0.0 | 0.1 | 0.0 | 0.0 | 0.0 | 0.0 | 0.0 | 0.0 | 0.0 | 0.1 |
| Vmo1 | 1.1 | 1.1 | 0.8 | 2.0 | 0.7 | 1.4 | 1.1 | 1.5 | 0.7 | 0.9 | 1.0 | 1.0 |
| Vmp1 | 50.1 | 58.1 | 60.9 | 57.8 | 55.5 | 56.7 | 54.3 | 63.4 | 64.1 | 52.6 | 62.6 | 63.4 |
| Vnn1 | 8.9 | 4.0 | 3.5 | 5.4 | 3.8 | 4.1 | 2.6 | 1.2 | 1.8 | 9.1 | 5.9 | 3.0 |
| Vnn3 | 0.3 | 0.1 | 0.1 | 0.1 | 0.2 | 0.0 | 0.0 | 0.0 | 0.0 | 0.1 | 0.0 | 0.0 |
| Vopp1 | 36.5 | 27.7 | 22.3 | 41.7 | 28.3 | 38.3 | 28.5 | 16.5 | 24.2 | 28.3 | 27.2 | 27.9 |
| Vprbp | 7.5 | 6.7 | 7.0 | 6.2 | 5.6 | 7.2 | 6.4 | 6.9 | 6.7 | 6.8 | 7.5 | 6.5 |
| Vpreb1 | 0.3 | 0.6 | 0.1 | 0.1 | 0.3 | 0.1 | 0.1 | 0.3 | 0.3 | 0.6 | 0.3 | 0.1 |
| Vpreb2 | 0.4 | 0.3 | 0.1 | 0.0 | 0.4 | 0.1 | 0.1 | 0.3 | 0.1 | 0.3 | 0.1 | 0.0 |
| Vpreb3 | 0.1 | 0.0 | 0.0 | 0.0 | 0.2 | 0.3 | 0.2 | 0.0 | 0.1 | 0.0 | 0.0 | 0.0 |
| Vps11 | 14.2 | 11.4 | 11.1 | 14.7 | 12.5 | 16.2 | 10.5 | 10.9 | 10.2 | 12.5 | 10.5 | 11.6 |
| Vps13a | 5.9 | 5.9 | 5.8 | 6.2 | 5.5 | 6.8 | 4.4 | 5.8 | 4.5 | 6.5 | 7.0 | 5.4 |
| Vps13b | 5.7 | 6.3 | 6.4 | 5.3 | 5.9 | 6.0 | 5.5 | 5.8 | 5.5 | 5.3 | 5.8 | 5.6 |
| Vps13c | 4.1 | 4.5 | 4.4 | 3.8 | 5.0 | 4.2 | 3.3 | 4.0 | 3.2 | 3.6 | 3.3 | 3.7 |
| Vps13d | 10.6 | 11.3 | 10.4 | 9.7 | 10.9 | 9.9 | 11.1 | 11.2 | 11.4 | 10.0 | 10.1 | 11.2 |
| Vps16 | 13.5 | 13.6 | 13.0 | 13.0 | 13.9 | 14.5 | 13.8 | 12.4 | 12.7 | 11.3 | 11.5 | 12.4 |
| Vps18 | 8.0 | 6.9 | 6.3 | 10.4 | 9.6 | 9.4 | 7.7 | 6.2 | 6.4 | 7.6 | 6.1 | 6.6 |
| Vps25 | 30.2 | 28.0 | 28.4 | 31.0 | 24.3 | 30.7 | 22.6 | 29.5 | 24.0 | 27.1 | 25.4 | 26.4 |
| Vps26a | 39.4 | 37.1 | 32.9 | 50.3 | 36.0 | 41.9 | 35.8 | 31.7 | 29.8 | 37.1 | 40.9 | 43.5 |
| Vps26b | 10.2 | 9.9 | 9.5 | 9.7 | 8.4 | 9.3 | 8.9 | 9.4 | 8.9 | 9.2 | 9.1 | 8.9 |
| Vps28 | 49.1 | 47.0 | 46.0 | 60.3 | 49.5 | 61.6 | 50.6 | 50.0 | 46.6 | 46.8 | 49.6 | 49.1 |
| Vps29 | 62.8 | 58.0 | 47.9 | 69.5 | 63.3 | 63.7 | 56.9 | 49.0 | 51.2 | 57.7 | 60.4 | 58.7 |
| Vps33a | 9.1 | 8.6 | 8.3 | 11.5 | 8.5 | 9.2 | 8.4 | 7.9 | 7.9 | 7.3 | 8.0 | 8.4 |
| Vps33b | 5.4 | 5.3 | 4.5 | 4.4 | 4.6 | 5.1 | 4.8 | 4.8 | 4.9 | 5.1 | 4.7 | 4.6 |
| Vps35 | 82.0 | 76.6 | 62.7 | 81.4 | 72.3 | 88.9 | 58.4 | 59.1 | 64.2 | 77.7 | 74.0 | 73.6 |
| Vps36 | 33.5 | 33.4 | 32.0 | 33.3 | 33.2 | 30.3 | 32.0 | 29.3 | 29.9 | 36.5 | 37.1 | 34.0 |

| | | | | | | | | | | | | |
|---|---|---|---|---|---|---|---|---|---|---|---|---|
| Vps37a | 8.2 | 8.4 | 8.1 | 9.0 | 7.9 | 8.3 | 8.0 | 6.9 | 7.5 | 8.9 | 8.8 | 9.0 |
| Vps37b | 2.4 | 1.7 | 1.9 | 2.0 | 2.2 | 1.9 | 2.2 | 2.6 | 2.7 | 3.2 | 2.1 | 2.2 |
| Vps37c | 5.5 | 5.4 | 5.3 | 5.5 | 5.9 | 5.5 | 6.0 | 5.8 | 5.7 | 5.6 | 5.7 | 5.4 |
| Vps37d | 1.6 | 1.7 | 1.6 | 1.0 | 1.1 | 2.1 | 1.4 | 1.9 | 1.9 | 1.8 | 1.5 | 1.9 |
| Vps39 | 14.0 | 13.7 | 12.7 | 15.9 | 12.6 | 15.0 | 11.9 | 12.4 | 10.8 | 10.9 | 12.1 | 11.8 |
| Vps41 | 64.7 | 51.2 | 49.2 | 67.5 | 50.0 | 61.9 | 43.7 | 44.5 | 41.4 | 49.7 | 53.4 | 56.0 |
| Vps45 | 6.7 | 6.9 | 5.5 | 7.7 | 6.5 | 7.8 | 4.8 | 5.3 | 5.6 | 6.5 | 5.4 | 6.5 |
| Vps4a | 29.3 | 30.4 | 28.2 | 30.2 | 26.3 | 29.4 | 31.4 | 29.6 | 28.8 | 28.3 | 35.7 | 31.5 |
| Vps4b | 19.8 | 21.1 | 21.5 | 22.6 | 24.1 | 20.6 | 21.4 | 21.3 | 23.0 | 22.0 | 23.2 | 23.9 |
| Vps51 | 4.9 | 5.1 | 5.2 | 5.3 | 5.3 | 5.5 | 4.9 | 6.5 | 5.9 | 6.2 | 5.2 | 5.1 |
| Vps52 | 16.4 | 16.7 | 16.9 | 15.3 | 16.9 | 16.8 | 16.1 | 16.7 | 17.0 | 16.3 | 13.8 | 15.3 |
| Vps53 | 16.7 | 14.7 | 13.7 | 18.0 | 16.6 | 16.0 | 15.1 | 16.2 | 14.1 | 17.8 | 17.9 | 18.1 |
| Vps54 | 6.4 | 6.9 | 7.3 | 6.2 | 6.0 | 5.0 | 6.0 | 6.7 | 7.8 | 7.2 | 8.1 | 7.8 |
| Vps72 | 9.8 | 9.2 | 10.5 | 11.1 | 9.5 | 12.2 | 10.4 | 11.5 | 10.3 | 10.2 | 11.7 | 10.6 |
| Vps8 | 12.0 | 10.8 | 9.9 | 10.6 | 10.2 | 12.6 | 9.9 | 10.0 | 10.2 | 10.3 | 10.8 | 11.1 |
| Vps9d1 | 2.9 | 3.6 | 3.5 | 2.5 | 3.4 | 3.7 | 3.8 | 3.8 | 3.6 | 2.8 | 2.9 | 3.0 |
| Vrk1 | 2.3 | 2.4 | 2.6 | 1.8 | 2.4 | 2.1 | 2.7 | 1.8 | 2.0 | 1.8 | 2.2 | 2.0 |
| Vrk2 | 9.2 | 8.6 | 7.6 | 8.5 | 8.5 | 9.3 | 6.9 | 7.1 | 8.2 | 7.1 | 8.4 | 8.4 |
| Vrk3 | 5.7 | 5.6 | 6.2 | 7.2 | 6.4 | 5.5 | 6.1 | 7.0 | 5.8 | 5.8 | 4.7 | 5.7 |
| Vsig10 | 1.6 | 2.0 | 2.4 | 2.1 | 1.6 | 1.7 | 2.6 | 1.8 | 1.9 | 1.8 | 1.7 | 2.2 |
| Vsig8 | 0.1 | 0.1 | 0.0 | 0.1 | 0.1 | 0.1 | 0.1 | 0.1 | 0.0 | 0.1 | 0.0 | 0.0 |
| Vstm2a | 0.1 | 0.1 | 0.1 | 0.1 | 0.1 | 0.0 | 0.0 | 0.0 | 0.0 | 0.0 | 0.0 | 0.1 |
| Vstm2b | 0.0 | 0.0 | 0.1 | 0.0 | 0.1 | 0.0 | 0.2 | 0.4 | 0.4 | 0.0 | 0.1 | 0.1 |
| Vstm4 | 0.3 | 0.2 | 0.3 | 0.2 | 0.1 | 0.4 | 0.2 | 0.5 | 0.7 | 0.5 | 0.5 | 0.4 |
| Vstm5 | 1.3 | 0.9 | 0.6 | 0.9 | 0.6 | 1.2 | 0.7 | 0.5 | 0.5 | 0.6 | 0.6 | 0.8 |
| Vta1 | 31.4 | 30.3 | 24.1 | 29.1 | 28.1 | 29.4 | 24.3 | 23.6 | 25.6 | 28.4 | 29.2 | 26.9 |
| Vtcn1 | 0.0 | 0.0 | 0.0 | 0.0 | 0.0 | 0.0 | 0.0 | 0.0 | 0.1 | 0.1 | 0.0 | 0.0 |
| Vti1a | 8.7 | 9.0 | 9.2 | 8.3 | 6.9 | 8.2 | 8.6 | 9.4 | 6.8 | 6.6 | 8.1 | 8.2 |
| Vti1b | 91.1 | 67.0 | 59.9 | 103.0 | 67.5 | 93.4 | 77.0 | 63.9 | 69.7 | 77.1 | 81.0 | 87.1 |
| Vtn | 0.0 | 0.1 | 0.0 | 0.0 | 0.0 | 0.0 | 0.0 | 0.1 | 0.1 | 0.0 | 0.0 | 0.0 |
| Vwa1 | 1.2 | 1.0 | 1.0 | 1.0 | 1.3 | 1.3 | 1.6 | 1.2 | 1.5 | 1.3 | 0.8 | 1.0 |
| Vwa2 | 0.0 | 0.0 | 0.0 | 0.0 | 0.0 | 0.0 | 0.0 | 0.0 | 0.0 | 0.0 | 0.0 | 0.0 |
| Vwa3a | 0.0 | 0.0 | 0.1 | 0.0 | 0.0 | 0.0 | 0.0 | 0.0 | 0.0 | 0.0 | 0.0 | 0.0 |
| Vwa5a | 41.1 | 36.3 | 36.3 | 44.5 | 41.7 | 41.7 | 32.1 | 38.3 | 33.9 | 37.4 | 38.3 | 38.1 |
| Vwa5b2 | 0.0 | 0.0 | 0.0 | 0.0 | 0.0 | 0.0 | 0.0 | 0.0 | 0.0 | 0.0 | 0.0 | 0.0 |
| Vwa7 | 0.2 | 0.1 | 0.1 | 0.0 | 0.1 | 0.1 | 0.1 | 0.0 | 0.0 | 0.2 | 0.0 | 0.1 |
| Vwa8 | 12.8 | 9.8 | 9.1 | 12.3 | 10.1 | 11.9 | 8.3 | 10.3 | 7.5 | 9.7 | 9.2 | 9.2 |
| Vwa9 | 7.3 | 7.7 | 8.6 | 7.3 | 8.3 | 7.5 | 8.5 | 9.5 | 8.4 | 8.2 | 8.6 | 8.0 |
| Vwce | 0.0 | 0.0 | 0.1 | 0.0 | 0.0 | 0.0 | 0.0 | 0.0 | 0.0 | 0.0 | 0.0 | 0.0 |
| Vwf | 0.1 | 0.0 | 0.1 | 0.3 | 0.4 | 0.0 | 0.1 | 0.1 | 0.0 | 0.1 | 0.0 | 0.0 |
| Wac | 14.5 | 15.2 | 18.1 | 16.3 | 13.6 | 14.8 | 16.8 | 16.0 | 14.7 | 14.7 | 17.9 | 17.4 |
| Wapal | 22.3 | 22.0 | 23.3 | 22.1 | 22.2 | 19.1 | 20.0 | 21.0 | 19.5 | 20.4 | 23.8 | 22.8 |
| Wars | 23.7 | 22.7 | 14.6 | 28.4 | 18.2 | 25.0 | 22.8 | 23.5 | 22.0 | 16.4 | 21.5 | 16.4 |
| Wars2 | 1.4 | 1.2 | 1.3 | 1.1 | 1.2 | 1.3 | 1.0 | 1.3 | 1.1 | 1.5 | 1.6 | 1.2 |
| Was | 0.3 | 0.0 | 0.0 | 3.5 | 4.6 | 0.0 | 0.7 | 0.1 | 0.1 | 0.3 | 0.1 | 0.4 |
| Wasf1 | 0.1 | 0.3 | 0.4 | 0.2 | 0.1 | 0.3 | 0.3 | 0.4 | 0.3 | 0.5 | 0.3 | 0.4 |
| Wasf2 | 10.1 | 13.1 | 13.5 | 12.5 | 12.7 | 10.2 | 14.2 | 15.3 | 13.3 | 10.0 | 12.3 | 11.3 |
| Wasf3 | 2.4 | 2.6 | 2.1 | 3.7 | 1.8 | 4.3 | 2.6 | 1.4 | 1.5 | 1.1 | 1.6 | 1.5 |
| Wash | 9.0 | 8.5 | 6.8 | 9.9 | 8.0 | 9.1 | 7.3 | 8.0 | 8.4 | 8.3 | 7.9 | 8.3 |
| Wasl | 3.1 | 4.8 | 5.0 | 4.0 | 3.5 | 4.1 | 4.0 | 3.5 | 3.6 | 4.1 | 4.0 | 4.5 |
| Wbp1 | 27.1 | 29.9 | 27.8 | 26.9 | 25.6 | 33.2 | 27.0 | 27.1 | 28.6 | 27.8 | 26.8 | 23.8 |
| Wbp11 | 8.0 | 9.2 | 10.8 | 10.0 | 10.0 | 9.8 | 11.3 | 10.2 | 8.8 | 8.8 | 10.3 | 9.3 |
| Wbp1l | 22.7 | 24.1 | 24.2 | 19.9 | 22.0 | 21.3 | 23.5 | 23.9 | 23.6 | 22.4 | 21.4 | 22.1 |
| Wbp2 | 24.5 | 22.9 | 19.9 | 28.0 | 23.5 | 30.4 | 23.3 | 21.7 | 19.4 | 22.1 | 23.1 | 20.4 |
| Wbp4 | 19.6 | 19.2 | 17.6 | 19.6 | 15.0 | 20.7 | 16.8 | 16.4 | 16.5 | 16.8 | 20.1 | 18.9 |
| Wbp5 | 110.8 | 81.9 | 105.8 | 131.3 | 92.2 | 113.9 | 98.7 | 88.9 | 78.2 | 118.6 | 137.9 | 135.4 |
| Wbp7 | 0.9 | 1.2 | 1.2 | 0.8 | 1.2 | 1.0 | 1.4 | 1.2 | 1.4 | 1.0 | 1.0 | 1.0 |

| | | | | | | | | | | | | |
|---|---|---|---|---|---|---|---|---|---|---|---|---|
| Wbscr16 | 4.0 | 4.0 | 3.2 | 3.7 | 4.0 | 4.2 | 3.7 | 4.5 | 4.1 | 4.0 | 3.8 | 4.1 |
| Wbscr17 | 0.4 | 0.4 | 0.6 | 0.8 | 0.7 | 0.7 | 1.0 | 1.0 | 1.0 | 0.9 | 0.7 | 0.7 |
| Wbscr22 | 12.7 | 12.4 | 11.9 | 15.3 | 13.2 | 15.8 | 13.1 | 14.6 | 13.3 | 12.7 | 12.8 | 13.3 |
| Wbscr27 | 3.4 | 3.4 | 3.8 | 4.1 | 2.6 | 5.0 | 2.8 | 3.6 | 2.5 | 2.2 | 3.2 | 2.8 |
| Wdfy1 | 5.6 | 4.5 | 3.8 | 4.5 | 4.9 | 5.2 | 5.0 | 3.6 | 4.3 | 5.0 | 4.7 | 4.5 |
| Wdfy2 | 5.3 | 7.1 | 7.0 | 5.6 | 7.3 | 5.1 | 5.7 | 6.8 | 5.2 | 5.4 | 5.2 | 5.0 |
| Wdfy3 | 5.2 | 6.3 | 6.2 | 5.6 | 6.6 | 5.6 | 7.1 | 5.5 | 5.6 | 5.3 | 5.5 | 5.2 |
| Wdfy4 | 0.2 | 0.0 | 0.0 | 1.4 | 2.2 | 0.0 | 0.2 | 0.0 | 0.1 | 0.1 | 0.1 | 0.1 |
| Wdhd1 | 1.1 | 1.7 | 1.9 | 0.7 | 1.7 | 0.6 | 2.3 | 1.5 | 1.7 | 1.1 | 1.6 | 0.9 |
| Wdpcp | 4.9 | 4.1 | 4.6 | 4.7 | 3.7 | 5.4 | 3.2 | 4.2 | 3.3 | 3.8 | 3.6 | 3.5 |
| Wdr1 | 127.6 | 102.0 | 83.0 | 142.6 | 142.7 | 98.2 | 134.4 | 101.7 | 118.0 | 116.1 | 143.3 | 118.8 |
| Wdr11 | 8.3 | 8.3 | 8.4 | 8.7 | 8.9 | 8.6 | 8.4 | 8.8 | 8.4 | 7.7 | 7.7 | 7.8 |
| Wdr12 | 9.4 | 6.6 | 5.3 | 9.5 | 7.4 | 8.9 | 5.9 | 5.0 | 5.8 | 7.4 | 6.9 | 6.7 |
| Wdr13 | 9.3 | 8.3 | 7.8 | 9.9 | 8.3 | 9.3 | 10.0 | 8.0 | 9.5 | 8.9 | 10.6 | 8.8 |
| Wdr16 | 0.0 | 0.0 | 0.1 | 0.0 | 0.0 | 0.0 | 0.0 | 0.0 | 0.0 | 0.0 | 0.0 | 0.0 |
| Wdr17 | 0.3 | 0.1 | 0.1 | 0.1 | 0.0 | 0.3 | 0.0 | 0.0 | 0.0 | 0.0 | 0.0 | 0.0 |
| Wdr18 | 11.5 | 10.7 | 7.3 | 11.9 | 10.9 | 11.4 | 10.0 | 9.2 | 9.7 | 10.4 | 9.7 | 8.9 |
| Wdr19 | 5.0 | 5.5 | 5.7 | 4.6 | 4.0 | 6.9 | 4.1 | 5.1 | 4.2 | 5.0 | 5.0 | 5.2 |
| Wdr20a | 5.4 | 5.0 | 5.8 | 6.0 | 4.9 | 5.7 | 5.7 | 6.1 | 4.8 | 5.1 | 6.6 | 6.3 |
| Wdr20b | 0.6 | 0.5 | 0.5 | 0.8 | 0.5 | 0.6 | 0.5 | 0.5 | 0.6 | 0.5 | 0.4 | 0.5 |
| Wdr24 | 6.4 | 6.4 | 5.7 | 7.4 | 5.6 | 7.7 | 6.0 | 6.9 | 6.3 | 6.0 | 5.6 | 6.1 |
| Wdr25 | 1.3 | 1.5 | 1.2 | 1.1 | 1.3 | 1.4 | 1.1 | 1.0 | 1.1 | 1.2 | 1.1 | 1.4 |
| Wdr26 | 19.4 | 21.4 | 20.3 | 18.6 | 21.1 | 19.1 | 18.1 | 17.3 | 19.0 | 19.8 | 18.6 | 19.7 |
| Wdr27 | 0.0 | 0.0 | 0.0 | 0.1 | 0.0 | 0.1 | 0.0 | 0.0 | 0.0 | 0.0 | 0.0 | 0.0 |
| Wdr3 | 9.5 | 8.7 | 7.6 | 9.2 | 10.2 | 9.3 | 9.3 | 8.5 | 8.7 | 10.2 | 9.9 | 8.5 |
| Wdr31 | 0.2 | 0.2 | 0.4 | 0.1 | 0.2 | 0.6 | 0.2 | 0.2 | 0.2 | 0.2 | 0.1 | 0.3 |
| Wdr33 | 16.0 | 16.0 | 15.2 | 14.0 | 13.5 | 15.1 | 13.3 | 14.3 | 14.4 | 12.7 | 15.1 | 17.9 |
| Wdr34 | 4.0 | 3.0 | 3.6 | 3.2 | 3.0 | 4.2 | 4.2 | 3.2 | 3.0 | 3.6 | 3.8 | 3.8 |
| Wdr35 | 13.5 | 11.5 | 11.9 | 8.9 | 9.9 | 10.8 | 7.7 | 7.9 | 7.1 | 11.8 | 10.4 | 9.5 |
| Wdr36 | 19.5 | 19.4 | 14.6 | 18.8 | 17.3 | 18.7 | 17.2 | 17.2 | 16.5 | 18.9 | 19.0 | 15.0 |
| Wdr37 | 4.6 | 5.2 | 5.2 | 4.8 | 4.9 | 3.8 | 4.7 | 4.8 | 4.5 | 5.5 | 4.7 | 4.5 |
| Wdr38 | 0.2 | 0.0 | 0.0 | 0.2 | 0.1 | 0.0 | 0.2 | 0.1 | 0.2 | 0.0 | 0.1 | 0.1 |
| Wdr4 | 3.0 | 2.3 | 1.9 | 2.1 | 2.8 | 2.5 | 2.7 | 2.3 | 2.5 | 2.6 | 2.3 | 1.9 |
| Wdr41 | 7.5 | 6.7 | 5.8 | 8.0 | 7.5 | 7.0 | 6.9 | 7.4 | 6.4 | 7.4 | 7.1 | 6.4 |
| Wdr43 | 15.9 | 13.2 | 13.6 | 18.0 | 14.3 | 13.9 | 16.4 | 16.0 | 12.9 | 15.1 | 18.2 | 17.0 |
| Wdr44 | 2.9 | 3.0 | 2.8 | 3.0 | 2.9 | 2.3 | 2.9 | 2.7 | 3.4 | 2.9 | 3.8 | 4.2 |
| Wdr45 | 14.9 | 11.0 | 10.5 | 13.3 | 11.7 | 15.3 | 9.5 | 12.5 | 10.6 | 14.6 | 11.6 | 12.1 |
| Wdr45b | 20.2 | 21.5 | 19.2 | 18.9 | 20.0 | 18.4 | 20.5 | 22.4 | 19.5 | 19.7 | 19.9 | 19.1 |
| Wdr46 | 8.9 | 8.0 | 6.6 | 9.3 | 8.7 | 9.7 | 8.2 | 8.0 | 6.7 | 7.2 | 7.5 | 7.5 |
| Wdr47 | 4.5 | 4.6 | 3.4 | 4.6 | 4.3 | 4.4 | 3.8 | 3.1 | 4.6 | 4.4 | 5.4 | 4.3 |
| Wdr48 | 16.2 | 15.8 | 14.8 | 18.5 | 15.3 | 17.4 | 15.3 | 15.4 | 14.4 | 15.6 | 18.1 | 16.6 |
| Wdr5 | 10.2 | 9.9 | 8.9 | 9.8 | 8.9 | 8.2 | 9.3 | 9.5 | 9.5 | 9.0 | 9.3 | 8.9 |
| Wdr52 | 0.0 | 0.0 | 0.0 | 0.0 | 0.0 | 0.0 | 0.0 | 0.0 | 0.0 | 0.0 | 0.0 | 0.0 |
| Wdr53 | 7.3 | 8.1 | 7.1 | 7.2 | 7.0 | 5.5 | 5.1 | 5.8 | 5.8 | 6.6 | 7.1 | 6.4 |
| Wdr54 | 0.8 | 0.7 | 1.3 | 0.4 | 0.6 | 1.2 | 0.5 | 0.7 | 1.1 | 0.8 | 0.7 | 0.6 |
| Wdr55 | 8.9 | 10.4 | 8.1 | 10.3 | 9.6 | 8.4 | 9.1 | 10.4 | 8.1 | 9.5 | 9.8 | 9.0 |
| Wdr59 | 2.1 | 2.6 | 2.2 | 2.0 | 2.3 | 2.0 | 2.5 | 2.5 | 2.3 | 2.2 | 2.1 | 2.3 |
| Wdr5b | 1.8 | 2.7 | 2.9 | 1.7 | 2.5 | 2.3 | 2.1 | 2.8 | 2.5 | 2.1 | 1.7 | 3.0 |
| Wdr6 | 18.8 | 19.3 | 19.5 | 13.7 | 16.9 | 15.6 | 18.3 | 19.3 | 21.1 | 20.6 | 16.1 | 16.9 |
| Wdr60 | 5.7 | 5.9 | 6.6 | 6.9 | 5.0 | 6.5 | 6.9 | 5.7 | 4.7 | 5.3 | 6.3 | 6.8 |
| Wdr61 | 52.8 | 48.4 | 41.7 | 41.5 | 42.5 | 47.3 | 42.0 | 37.3 | 39.9 | 43.4 | 41.2 | 44.6 |
| Wdr62 | 0.8 | 0.7 | 0.9 | 0.8 | 0.9 | 0.2 | 1.3 | 0.8 | 0.9 | 0.4 | 0.6 | 0.5 |
| Wdr63 | 0.1 | 0.0 | 0.0 | 0.0 | 0.0 | 0.0 | 0.0 | 0.0 | 0.0 | 0.0 | 0.1 | 0.1 |
| Wdr65 | 0.1 | 0.1 | 0.1 | 0.2 | 0.0 | 0.1 | 0.0 | 0.0 | 0.0 | 0.0 | 0.0 | 0.0 |
| Wdr67 | 7.2 | 7.5 | 8.8 | 5.7 | 6.7 | 7.9 | 7.4 | 6.3 | 6.7 | 5.4 | 7.3 | 5.7 |
| Wdr69 | 0.1 | 0.3 | 0.1 | 0.1 | 0.1 | 0.0 | 0.0 | 0.1 | 0.1 | 0.0 | 0.0 | 0.1 |
| Wdr7 | 10.3 | 10.0 | 9.1 | 11.7 | 9.0 | 11.4 | 8.1 | 7.7 | 7.6 | 8.4 | 8.8 | 9.6 |

| | | | | | | | | | | | | |
|---|---|---|---|---|---|---|---|---|---|---|---|---|
| Wdr70 | 5.9 | 6.3 | 6.9 | 7.3 | 5.7 | 7.1 | 6.7 | 5.5 | 4.8 | 6.4 | 5.1 | 5.4 |
| Wdr72 | 0.0 | 0.0 | 0.0 | 0.0 | 0.0 | 0.0 | 0.0 | 0.0 | 0.0 | 0.1 | 0.1 | 0.1 |
| Wdr73 | 4.3 | 3.7 | 3.6 | 4.3 | 3.4 | 4.4 | 3.0 | 3.8 | 3.6 | 3.9 | 3.2 | 3.7 |
| Wdr74 | 16.2 | 14.2 | 11.4 | 16.1 | 12.7 | 14.4 | 15.3 | 12.1 | 11.7 | 12.6 | 16.0 | 13.0 |
| Wdr75 | 16.1 | 14.9 | 12.4 | 15.1 | 15.2 | 14.2 | 15.2 | 15.1 | 13.9 | 16.6 | 15.8 | 14.8 |
| Wdr76 | 0.7 | 1.0 | 1.4 | 0.6 | 1.1 | 0.9 | 1.5 | 0.9 | 0.9 | 0.7 | 0.9 | 0.7 |
| Wdr77 | 8.6 | 6.4 | 5.6 | 8.0 | 8.3 | 7.2 | 7.3 | 7.8 | 7.3 | 8.5 | 8.5 | 6.6 |
| Wdr78 | 2.2 | 2.2 | 2.2 | 1.9 | 1.7 | 2.7 | 1.4 | 2.0 | 1.3 | 2.0 | 2.2 | 1.8 |
| Wdr8 | 9.3 | 7.9 | 5.8 | 6.8 | 7.4 | 8.5 | 6.0 | 6.3 | 7.3 | 6.9 | 6.6 | 5.6 |
| Wdr81 | 7.2 | 6.7 | 5.6 | 8.2 | 9.0 | 8.1 | 7.6 | 5.7 | 7.0 | 7.5 | 6.1 | 6.1 |
| Wdr82 | 15.0 | 16.8 | 15.8 | 14.4 | 15.0 | 15.8 | 15.1 | 17.0 | 18.1 | 15.6 | 16.8 | 16.0 |
| Wdr83 | 8.3 | 9.0 | 7.1 | 8.9 | 7.3 | 9.6 | 7.7 | 7.7 | 8.5 | 7.2 | 7.1 | 7.2 |
| Wdr85 | 2.5 | 2.7 | 2.8 | 3.3 | 2.2 | 3.2 | 2.4 | 2.7 | 2.6 | 2.8 | 3.0 | 3.2 |
| Wdr89 | 1.7 | 1.4 | 1.1 | 1.3 | 1.1 | 1.0 | 1.2 | 1.6 | 1.1 | 1.0 | 1.4 | 1.3 |
| Wdr90 | 0.5 | 0.7 | 1.0 | 0.3 | 0.7 | 0.5 | 1.0 | 0.6 | 0.9 | 0.4 | 0.6 | 0.5 |
| Wdr91 | 5.0 | 4.3 | 4.9 | 7.1 | 6.9 | 4.6 | 5.2 | 4.7 | 3.8 | 5.8 | 4.8 | 4.1 |
| Wdr92 | 9.2 | 8.7 | 8.0 | 9.3 | 9.2 | 8.3 | 9.1 | 7.3 | 7.6 | 15.2 | 10.2 | 11.5 |
| Wdr93 | 0.3 | 0.3 | 0.3 | 0.2 | 0.2 | 0.5 | 0.3 | 0.2 | 0.1 | 0.2 | 0.1 | 0.1 |
| Wdr95 | 0.1 | 0.1 | 0.1 | 0.1 | 0.0 | 0.0 | 0.0 | 0.1 | 0.1 | 0.0 | 0.0 | 0.0 |
| Wdr96 | 0.3 | 0.3 | 0.3 | 0.5 | 0.3 | 0.3 | 0.4 | 0.4 | 0.3 | 0.2 | 0.4 | 0.4 |
| Wdsub1 | 6.7 | 6.8 | 7.5 | 6.6 | 5.7 | 6.5 | 5.6 | 7.7 | 7.1 | 6.7 | 7.3 | 7.6 |
| Wdtc1 | 5.6 | 5.3 | 5.2 | 4.8 | 4.4 | 5.6 | 4.6 | 5.4 | 5.0 | 5.6 | 5.2 | 4.1 |
| Wdyhv1 | 7.2 | 5.9 | 5.9 | 6.6 | 6.6 | 6.3 | 5.2 | 5.6 | 5.0 | 4.4 | 4.7 | 4.9 |
| Wee1 | 3.0 | 4.0 | 4.7 | 2.7 | 3.5 | 4.2 | 4.2 | 2.8 | 3.3 | 3.0 | 3.5 | 2.9 |
| Wfdc1 | 0.6 | 0.1 | 0.3 | 0.5 | 0.0 | 0.5 | 0.1 | 0.1 | 0.2 | 0.2 | 0.1 | 0.3 |
| Wfdc12 | 0.0 | 0.1 | 0.4 | 0.0 | 0.1 | 0.0 | 0.1 | 0.4 | 0.1 | 0.1 | 0.2 | 0.2 |
| Wfdc15a | 0.2 | 0.3 | 0.0 | 0.0 | 0.4 | 0.0 | 0.0 | 0.4 | 0.1 | 0.0 | 0.0 | 0.1 |
| Wfdc17 | 7.4 | 0.3 | 1.0 | 42.0 | 36.3 | 0.0 | 8.2 | 2.9 | 0.9 | 2.0 | 1.9 | 2.5 |
| Wfdc18 | 0.1 | 0.2 | 0.0 | 0.0 | 0.3 | 0.1 | 0.0 | 0.2 | 0.2 | 0.4 | 0.1 | 0.1 |
| Wfdc3 | 0.3 | 0.1 | 0.0 | 0.1 | 0.1 | 0.1 | 0.0 | 0.0 | 0.0 | 0.2 | 0.1 | 0.1 |
| Wfdc5 | 0.0 | 0.1 | 0.0 | 0.0 | 0.0 | 0.0 | 0.0 | 0.0 | 0.0 | 0.0 | 0.0 | 0.1 |
| Wfdc6a | 0.1 | 0.1 | 0.1 | 0.2 | 0.1 | 0.1 | 0.0 | 0.0 | 0.0 | 0.0 | 0.0 | 0.0 |
| Wfikkn1 | 0.1 | 0.1 | 0.2 | 0.1 | 0.1 | 0.2 | 0.3 | 0.2 | 0.1 | 0.3 | 0.2 | 0.1 |
| Wfikkn2 | 0.3 | 0.3 | 0.5 | 0.5 | 0.2 | 0.3 | 0.3 | 0.5 | 0.3 | 0.3 | 0.1 | 0.3 |
| Wfs1 | 8.6 | 7.8 | 6.9 | 9.9 | 7.3 | 8.3 | 9.4 | 8.4 | 7.4 | 6.8 | 8.2 | 7.0 |
| Whamm | 4.3 | 4.6 | 4.8 | 6.2 | 4.2 | 5.2 | 4.1 | 3.5 | 4.1 | 4.6 | 4.4 | 4.4 |
| Whrn | 0.5 | 0.6 | 0.4 | 1.5 | 0.5 | 0.6 | 0.6 | 0.7 | 0.8 | 1.4 | 0.3 | 0.6 |
| Whsc1 | 6.1 | 6.3 | 7.1 | 5.0 | 6.9 | 5.5 | 6.9 | 4.9 | 6.3 | 6.2 | 7.1 | 5.9 |
| Whsc1l1 | 8.7 | 11.4 | 11.8 | 10.1 | 9.5 | 9.8 | 11.8 | 9.7 | 9.0 | 10.0 | 11.0 | 11.2 |
| Wibg | 7.8 | 7.7 | 8.5 | 11.7 | 6.2 | 6.9 | 8.1 | 9.0 | 6.6 | 6.9 | 7.3 | 7.8 |
| Wif1 | 0.1 | 0.1 | 0.2 | 0.0 | 0.0 | 0.0 | 0.0 | 0.0 | 0.0 | 0.0 | 0.0 | 0.1 |
| Wipf1 | 5.4 | 5.6 | 5.0 | 8.0 | 7.2 | 5.5 | 5.4 | 3.6 | 4.6 | 4.3 | 4.2 | 4.4 |
| Wipf2 | 0.8 | 1.0 | 1.0 | 1.2 | 0.8 | 0.9 | 1.1 | 1.0 | 0.8 | 0.9 | 0.9 | 0.8 |
| Wipf3 | 0.0 | 0.0 | 0.0 | 0.0 | 0.0 | 0.0 | 0.1 | 0.0 | 0.0 | 0.0 | 0.0 | 0.0 |
| Wipi1 | 37.9 | 47.1 | 50.7 | 34.6 | 44.0 | 36.2 | 57.6 | 57.4 | 52.9 | 42.6 | 51.6 | 53.6 |
| Wipi2 | 28.2 | 24.2 | 21.9 | 27.6 | 22.8 | 28.5 | 24.0 | 25.2 | 23.7 | 24.3 | 23.7 | 23.6 |
| Wisp1 | 91.2 | 74.1 | 55.7 | 110.7 | 113.5 | 88.4 | 139.0 | 132.2 | 169.9 | 109.8 | 137.2 | 134.0 |
| Wisp2 | 889.6 | 848.1 | 849.2 | 916.1 | 791.4 | 853.7 | 954.4 | 1006.2 | 992.8 | 674.4 | 749.7 | 835.5 |
| Wiz | 1.2 | 1.7 | 1.7 | 1.7 | 1.4 | 1.7 | 1.4 | 1.7 | 1.6 | 1.4 | 1.6 | 1.4 |
| Wls | 219.2 | 174.5 | 132.7 | 214.8 | 146.1 | 209.2 | 152.3 | 140.7 | 182.5 | 197.7 | 188.3 | 197.3 |
| Wnk1 | 19.9 | 22.8 | 18.9 | 22.3 | 22.0 | 21.7 | 23.8 | 15.2 | 17.4 | 15.9 | 20.3 | 16.5 |
| Wnk2 | 0.0 | 0.0 | 0.0 | 0.0 | 0.0 | 0.1 | 0.0 | 0.0 | 0.0 | 0.0 | 0.0 | 0.0 |
| Wnk3 | 0.4 | 0.4 | 0.3 | 0.3 | 0.4 | 0.4 | 0.4 | 0.4 | 0.4 | 0.4 | 0.6 | 0.4 |
| Wnk4 | 0.0 | 0.0 | 0.1 | 0.1 | 0.1 | 0.1 | 0.1 | 0.1 | 0.1 | 0.1 | 0.1 | 0.1 |
| Wnt10a | 0.2 | 0.0 | 0.0 | 0.1 | 0.0 | 0.0 | 0.0 | 0.0 | 0.0 | 0.1 | 0.0 | 0.0 |
| Wnt10b | 0.1 | 0.1 | 0.0 | 0.1 | 0.1 | 0.0 | 0.0 | 0.0 | 0.0 | 0.0 | 0.0 | 0.0 |
| Wnt11 | 0.2 | 0.4 | 0.4 | 0.1 | 0.3 | 0.2 | 0.3 | 0.4 | 0.3 | 1.1 | 0.7 | 0.4 |

| | | | | | | | | | | | | |
|---|---|---|---|---|---|---|---|---|---|---|---|---|
| Wnt16 | 0.2 | 0.1 | 0.2 | 0.1 | 0.1 | 0.0 | 0.3 | 0.5 | 0.2 | 0.2 | 0.6 | 0.5 |
| Wnt2 | 0.1 | 0.0 | 0.1 | 0.2 | 0.0 | 0.1 | 0.3 | 0.0 | 0.0 | 0.6 | 0.4 | 0.3 |
| Wnt2b | 0.2 | 0.3 | 0.1 | 0.2 | 0.2 | 0.2 | 0.4 | 0.1 | 0.2 | 0.9 | 0.1 | 0.4 |
| Wnt3 | 0.0 | 0.0 | 0.0 | 0.1 | 0.0 | 0.0 | 0.0 | 0.0 | 0.0 | 0.0 | 0.0 | 0.0 |
| Wnt4 | 0.2 | 0.4 | 0.3 | 0.1 | 0.3 | 0.2 | 0.2 | 0.4 | 0.5 | 2.2 | 1.1 | 1.0 |
| Wnt5a | 3.8 | 2.8 | 4.9 | 4.1 | 4.1 | 3.3 | 4.2 | 5.4 | 4.2 | 3.5 | 3.2 | 3.7 |
| Wnt5b | 0.5 | 0.6 | 0.9 | 0.7 | 0.6 | 0.5 | 0.8 | 0.7 | 0.4 | 0.9 | 0.7 | 0.8 |
| Wnt6 | 0.0 | 0.0 | 0.0 | 0.0 | 0.1 | 0.0 | 0.0 | 0.0 | 0.0 | 0.0 | 0.0 | 0.0 |
| Wnt7b | 0.1 | 0.0 | 0.0 | 0.0 | 0.0 | 0.0 | 0.0 | 0.0 | 0.0 | 0.0 | 0.0 | 0.0 |
| Wnt8a | 0.0 | 0.0 | 0.0 | 0.0 | 0.0 | 0.0 | 0.1 | 0.0 | 0.0 | 0.0 | 0.0 | 0.0 |
| Wnt8b | 0.0 | 0.0 | 0.0 | 0.0 | 0.0 | 0.1 | 0.0 | 0.0 | 0.0 | 0.0 | 0.0 | 0.0 |
| Wnt9a | 6.1 | 4.4 | 3.3 | 3.1 | 2.7 | 3.3 | 3.3 | 3.6 | 2.9 | 5.0 | 4.4 | 3.7 |
| Wrap53 | 3.2 | 3.6 | 4.0 | 3.6 | 3.7 | 3.3 | 3.4 | 3.3 | 3.1 | 2.9 | 2.7 | 2.9 |
| Wrb | 7.1 | 6.4 | 4.9 | 8.2 | 6.2 | 8.1 | 7.0 | 6.2 | 6.9 | 8.4 | 8.2 | 7.3 |
| Wrn | 5.6 | 6.0 | 5.9 | 4.9 | 5.0 | 5.8 | 5.0 | 4.9 | 4.8 | 5.0 | 5.5 | 5.4 |
| Wrnip1 | 11.8 | 12.0 | 10.5 | 7.8 | 9.5 | 10.7 | 11.1 | 10.2 | 12.0 | 12.4 | 11.6 | 12.5 |
| Wsb1 | 24.0 | 20.3 | 19.1 | 20.9 | 26.6 | 17.9 | 22.4 | 18.7 | 23.4 | 26.2 | 24.6 | 22.6 |
| Wsb2 | 87.1 | 70.5 | 51.6 | 85.6 | 69.0 | 75.6 | 78.3 | 59.9 | 68.2 | 75.4 | 77.7 | 67.5 |
| Wscd1 | 0.1 | 0.0 | 0.1 | 0.0 | 0.0 | 0.0 | 0.1 | 0.2 | 0.1 | 0.0 | 0.0 | 0.0 |
| Wscd2 | 0.7 | 0.4 | 0.6 | 0.5 | 0.4 | 0.4 | 0.9 | 1.4 | 0.7 | 0.3 | 0.5 | 0.7 |
| Wt1 | 0.2 | 0.0 | 0.0 | 0.1 | 0.1 | 0.0 | 0.1 | 0.3 | 0.7 | 9.0 | 4.1 | 2.6 |
| Wtap | 21.3 | 19.7 | 21.1 | 20.4 | 22.7 | 17.5 | 21.9 | 24.1 | 23.9 | 24.0 | 26.7 | 24.3 |
| Wtip | 7.2 | 7.0 | 6.0 | 6.6 | 6.2 | 7.0 | 6.7 | 8.6 | 9.6 | 10.3 | 7.5 | 8.0 |
| Wwc1 | 7.6 | 6.4 | 4.4 | 5.2 | 3.2 | 5.2 | 4.2 | 2.8 | 4.0 | 3.7 | 4.8 | 3.2 |
| Wwc2 | 37.3 | 34.9 | 27.6 | 36.8 | 30.5 | 36.0 | 37.1 | 26.7 | 38.0 | 39.1 | 37.8 | 36.2 |
| Wwox | 4.3 | 4.0 | 4.0 | 3.3 | 3.2 | 5.3 | 2.8 | 3.4 | 2.8 | 3.6 | 3.6 | 3.3 |
| Wwp1 | 28.4 | 24.9 | 22.8 | 32.1 | 24.0 | 28.1 | 21.9 | 17.6 | 19.9 | 18.9 | 27.1 | 23.6 |
| Wwp2 | 8.3 | 6.0 | 5.4 | 10.6 | 8.2 | 9.0 | 8.0 | 7.4 | 6.9 | 6.8 | 7.9 | 7.7 |
| Wwtr1 | 52.7 | 47.4 | 47.8 | 61.8 | 46.2 | 50.1 | 51.8 | 38.6 | 41.3 | 50.7 | 58.2 | 54.0 |
| Xab2 | 10.9 | 13.3 | 13.8 | 11.4 | 11.8 | 12.6 | 13.5 | 13.1 | 11.9 | 11.4 | 10.9 | 12.9 |
| Xaf1 | 4.8 | 2.7 | 3.0 | 5.7 | 3.0 | 3.3 | 2.4 | 2.6 | 2.4 | 1.9 | 2.4 | 4.7 |
| Xbp1 | 48.1 | 45.9 | 40.6 | 47.5 | 45.7 | 39.7 | 44.2 | 56.8 | 55.0 | 53.7 | 56.1 | 53.3 |
| Xcr1 | 0.1 | 0.0 | 0.0 | 0.1 | 0.1 | 0.0 | 0.0 | 0.1 | 0.0 | 0.1 | 0.0 | 0.0 |
| Xdh | 33.7 | 26.7 | 34.7 | 35.2 | 27.5 | 30.6 | 16.4 | 25.4 | 16.7 | 33.1 | 19.3 | 30.2 |
| Xiap | 6.1 | 7.1 | 8.4 | 6.4 | 6.5 | 6.5 | 5.7 | 7.0 | 7.2 | 6.6 | 7.4 | 8.3 |
| Xirp1 | 0.0 | 0.0 | 0.0 | 0.0 | 0.0 | 0.0 | 0.0 | 0.0 | 0.0 | 0.0 | 0.0 | 0.0 |
| Xirp2 | 0.0 | 0.0 | 0.0 | 0.0 | 0.0 | 0.0 | 0.0 | 0.0 | 0.0 | 0.0 | 0.0 | 0.0 |
| Xist | 0.0 | 0.0 | 0.0 | 0.0 | 0.0 | 0.0 | 0.0 | 0.0 | 0.0 | 0.0 | 0.0 | 0.0 |
| Xk | 0.4 | 0.2 | 0.3 | 0.3 | 0.3 | 0.5 | 0.3 | 0.3 | 0.3 | 0.5 | 0.3 | 0.3 |
| Xkr4 | 1.0 | 1.0 | 1.2 | 0.9 | 0.9 | 0.7 | 1.2 | 1.9 | 1.9 | 0.7 | 1.0 | 0.8 |
| Xkr5 | 0.1 | 0.3 | 0.6 | 0.1 | 0.3 | 0.0 | 0.6 | 0.4 | 0.4 | 0.1 | 0.3 | 0.2 |
| Xkr6 | 0.2 | 0.4 | 0.2 | 0.2 | 0.2 | 0.3 | 0.1 | 0.2 | 0.2 | 0.4 | 0.2 | 0.2 |
| Xkr8 | 3.8 | 3.3 | 2.7 | 3.7 | 3.1 | 4.1 | 3.2 | 2.4 | 2.1 | 3.2 | 2.9 | 3.3 |
| Xkrx | 0.0 | 0.0 | 0.0 | 0.0 | 0.0 | 0.1 | 0.0 | 0.0 | 0.0 | 0.0 | 0.0 | 0.0 |
| Xlr | 5.6 | 5.9 | 5.8 | 5.4 | 4.0 | 5.6 | 5.2 | 4.9 | 3.5 | 4.1 | 6.7 | 6.4 |
| Xlr3a | 1.3 | 2.1 | 0.7 | 1.8 | 1.0 | 3.2 | 0.6 | 1.1 | 0.8 | 0.4 | 1.1 | 1.0 |
| Xlr3b | 1.8 | 3.2 | 1.3 | 2.0 | 2.5 | 3.7 | 0.9 | 1.7 | 1.7 | 0.9 | 1.2 | 1.4 |
| Xlr3c | 1.0 | 1.8 | 0.7 | 1.1 | 1.2 | 1.8 | 0.6 | 1.0 | 1.0 | 0.5 | 0.7 | 0.8 |
| Xlr4a | 0.5 | 0.7 | 0.4 | 0.5 | 0.7 | 1.4 | 0.3 | 0.9 | 0.8 | 0.0 | 0.5 | 0.4 |
| Xlr4b | 0.2 | 0.3 | 0.2 | 0.2 | 0.4 | 0.8 | 0.2 | 0.4 | 0.3 | 0.1 | 0.3 | 0.2 |
| Xlr4c | 0.1 | 0.3 | 0.1 | 0.2 | 0.2 | 0.2 | 0.1 | 0.2 | 0.1 | 0.0 | 0.1 | 0.2 |
| Xpa | 20.7 | 15.3 | 17.9 | 17.1 | 11.5 | 13.8 | 13.5 | 15.8 | 12.4 | 14.3 | 18.6 | 20.5 |
| Xpc | 10.2 | 8.4 | 8.4 | 15.6 | 8.4 | 12.5 | 7.5 | 6.2 | 5.9 | 7.9 | 7.9 | 8.1 |
| Xpnpep1 | 33.9 | 31.4 | 28.9 | 32.0 | 31.9 | 29.1 | 35.1 | 33.7 | 30.0 | 31.4 | 33.7 | 31.5 |
| Xpnpep2 | 0.1 | 0.0 | 0.0 | 0.0 | 0.0 | 0.0 | 0.0 | 0.0 | 0.0 | 0.0 | 0.1 | 0.0 |
| Xpnpep3 | 3.0 | 2.9 | 2.8 | 3.3 | 2.7 | 3.6 | 2.7 | 3.0 | 2.2 | 2.7 | 2.7 | 2.9 |
| Xpo1 | 15.6 | 24.0 | 25.1 | 18.4 | 20.3 | 23.9 | 17.3 | 18.1 | 19.0 | 18.9 | 20.8 | 19.3 |

| | | | | | | | | | | | | |
|---|---|---|---|---|---|---|---|---|---|---|---|---|
| Xpo4 | 3.8 | 4.5 | 4.6 | 3.3 | 4.5 | 3.3 | 3.8 | 4.1 | 4.3 | 3.5 | 4.3 | 4.2 |
| Xpo5 | 19.7 | 17.8 | 18.0 | 18.3 | 18.3 | 17.7 | 18.7 | 18.1 | 16.1 | 17.0 | 18.2 | 16.5 |
| Xpo6 | 10.1 | 10.5 | 9.8 | 9.5 | 10.3 | 10.2 | 10.7 | 9.8 | 10.0 | 8.0 | 8.9 | 7.8 |
| Xpo7 | 3.4 | 3.2 | 3.0 | 3.5 | 2.9 | 3.6 | 2.9 | 3.0 | 2.9 | 3.1 | 3.1 | 3.2 |
| Xpot | 22.4 | 18.4 | 17.0 | 24.6 | 19.8 | 23.2 | 19.1 | 25.7 | 22.6 | 21.2 | 24.6 | 21.0 |
| Xpr1 | 5.4 | 5.2 | 4.8 | 5.7 | 5.7 | 6.2 | 5.3 | 5.2 | 4.6 | 4.8 | 5.3 | 4.9 |
| Xrcc1 | 7.6 | 8.3 | 10.2 | 7.0 | 8.8 | 7.8 | 8.8 | 7.8 | 7.7 | 7.9 | 8.2 | 7.2 |
| Xrcc2 | 0.9 | 1.4 | 1.2 | 0.8 | 1.6 | 1.1 | 1.4 | 1.0 | 1.3 | 0.9 | 1.2 | 0.8 |
| Xrcc3 | 1.2 | 1.5 | 1.0 | 1.3 | 1.0 | 1.0 | 1.2 | 1.6 | 1.3 | 1.3 | 1.1 | 0.8 |
| Xrcc4 | 6.4 | 5.8 | 5.9 | 7.0 | 6.3 | 6.1 | 7.2 | 7.2 | 6.4 | 7.4 | 8.3 | 8.5 |
| Xrcc5 | 13.4 | 10.8 | 9.3 | 11.9 | 8.6 | 12.6 | 7.6 | 9.0 | 8.5 | 10.6 | 10.1 | 10.0 |
| Xrcc6 | 10.5 | 10.4 | 11.1 | 11.0 | 10.2 | 10.6 | 9.9 | 9.5 | 8.4 | 9.8 | 10.1 | 9.7 |
| Xrcc6bp1 | 2.8 | 2.8 | 3.1 | 2.8 | 3.7 | 3.5 | 3.2 | 2.7 | 2.6 | 2.8 | 2.5 | 1.7 |
| Xrn1 | 3.5 | 4.0 | 4.0 | 3.0 | 3.8 | 3.6 | 3.7 | 3.6 | 2.8 | 3.9 | 4.0 | 4.4 |
| Xrn2 | 30.0 | 26.2 | 26.0 | 28.0 | 26.1 | 27.1 | 29.7 | 27.7 | 24.0 | 28.0 | 31.7 | 31.3 |
| Xrra1 | 0.2 | 0.2 | 0.1 | 0.2 | 0.3 | 0.2 | 0.1 | 0.1 | 0.1 | 0.2 | 0.1 | 0.1 |
| Xylb | 0.3 | 0.2 | 0.2 | 0.7 | 0.2 | 0.4 | 0.1 | 0.1 | 0.1 | 0.3 | 0.1 | 0.1 |
| Xylt1 | 0.4 | 0.4 | 0.3 | 0.8 | 1.3 | 0.4 | 0.7 | 1.0 | 0.6 | 0.8 | 0.5 | 0.4 |
| Xylt2 | 6.1 | 4.7 | 5.6 | 10.8 | 16.9 | 5.1 | 7.6 | 6.3 | 6.0 | 5.5 | 4.6 | 5.4 |
| Yae1d1 | 8.4 | 8.1 | 7.8 | 9.6 | 8.3 | 10.8 | 7.8 | 8.3 | 7.3 | 8.7 | 10.7 | 9.8 |
| Yaf2 | 18.3 | 17.1 | 17.7 | 23.6 | 16.6 | 22.4 | 21.1 | 14.8 | 15.2 | 18.1 | 20.8 | 20.9 |
| Yap1 | 35.9 | 36.2 | 28.7 | 41.6 | 30.4 | 42.1 | 30.7 | 22.8 | 27.9 | 33.0 | 31.6 | 31.5 |
| Yars | 25.0 | 20.3 | 15.5 | 22.9 | 19.2 | 23.2 | 21.4 | 24.2 | 21.7 | 18.6 | 22.6 | 18.0 |
| Yars2 | 7.7 | 5.6 | 4.3 | 5.8 | 6.2 | 6.5 | 5.3 | 5.1 | 5.7 | 8.0 | 6.6 | 5.8 |
| Ybey | 1.0 | 1.0 | 1.0 | 1.3 | 0.9 | 1.3 | 0.8 | 1.3 | 1.1 | 1.0 | 1.1 | 0.9 |
| Ybx1 | 178.5 | 148.7 | 164.3 | 210.6 | 158.8 | 188.4 | 190.4 | 194.0 | 130.5 | 145.0 | 209.2 | 180.2 |
| Ydjc | 3.3 | 3.0 | 1.6 | 3.7 | 2.9 | 4.2 | 3.3 | 2.1 | 3.1 | 2.7 | 2.7 | 2.4 |
| Yeats2 | 6.9 | 8.2 | 9.0 | 5.1 | 7.6 | 6.2 | 7.5 | 8.3 | 8.0 | 7.6 | 7.3 | 7.1 |
| Yeats4 | 14.9 | 13.2 | 14.1 | 16.5 | 15.8 | 13.6 | 16.9 | 16.4 | 16.1 | 14.7 | 16.6 | 15.7 |
| Yes1 | 9.5 | 10.4 | 9.5 | 7.8 | 6.8 | 9.5 | 9.2 | 9.2 | 9.4 | 9.1 | 11.1 | 10.7 |
| Yif1a | 47.2 | 41.8 | 36.8 | 50.1 | 39.6 | 44.8 | 48.8 | 45.0 | 47.0 | 43.0 | 47.1 | 42.6 |
| Yif1b | 42.6 | 39.8 | 31.5 | 44.1 | 34.5 | 38.6 | 39.0 | 45.3 | 45.7 | 30.9 | 36.6 | 36.6 |
| Yipf1 | 23.1 | 19.8 | 18.8 | 22.5 | 20.1 | 22.0 | 19.7 | 21.8 | 22.7 | 23.9 | 23.2 | 21.7 |
| Yipf2 | 10.2 | 10.1 | 10.9 | 9.7 | 9.0 | 11.3 | 9.4 | 11.4 | 9.1 | 10.0 | 11.6 | 10.0 |
| Yipf3 | 39.7 | 36.8 | 34.3 | 36.3 | 38.0 | 36.7 | 41.1 | 39.9 | 39.1 | 42.5 | 40.2 | 37.5 |
| Yipf4 | 33.3 | 30.5 | 30.6 | 34.2 | 31.1 | 30.5 | 34.4 | 31.1 | 35.8 | 40.3 | 37.6 | 40.3 |
| Yipf5 | 28.0 | 32.6 | 37.1 | 37.0 | 35.2 | 34.8 | 36.6 | 41.3 | 35.4 | 36.5 | 39.9 | 40.8 |
| Yipf6 | 5.9 | 6.4 | 6.3 | 8.1 | 7.1 | 6.9 | 5.8 | 5.7 | 5.9 | 6.4 | 7.2 | 6.3 |
| Ykt6 | 38.4 | 35.5 | 34.0 | 40.3 | 37.8 | 36.6 | 35.0 | 40.2 | 37.6 | 36.6 | 35.0 | 36.8 |
| Ylpm1 | 1.8 | 2.3 | 2.4 | 2.4 | 2.1 | 2.2 | 2.7 | 2.1 | 1.7 | 2.0 | 2.3 | 2.4 |
| Yme1l1 | 34.4 | 38.9 | 39.7 | 35.5 | 37.0 | 35.6 | 32.4 | 37.1 | 36.4 | 38.9 | 43.4 | 43.5 |
| Yod1 | 1.8 | 1.7 | 2.3 | 1.7 | 1.7 | 1.8 | 1.6 | 1.9 | 1.7 | 1.5 | 2.0 | 2.3 |
| Ypel1 | 1.5 | 0.8 | 1.2 | 1.2 | 0.9 | 1.5 | 0.9 | 1.3 | 1.1 | 0.8 | 1.1 | 0.9 |
| Ypel2 | 4.3 | 4.4 | 5.4 | 3.2 | 3.8 | 4.6 | 3.3 | 6.0 | 5.2 | 6.3 | 5.2 | 5.8 |
| Ypel3 | 35.2 | 36.6 | 39.5 | 32.8 | 30.1 | 40.5 | 31.0 | 43.1 | 38.5 | 37.3 | 34.1 | 38.8 |
| Ypel4 | 0.7 | 1.3 | 1.1 | 0.6 | 0.5 | 1.2 | 0.2 | 1.4 | 0.9 | 0.6 | 0.8 | 1.4 |
| Ypel5 | 35.8 | 28.8 | 25.6 | 38.6 | 31.3 | 37.0 | 28.6 | 27.2 | 34.9 | 34.2 | 32.9 | 36.6 |
| Yrdc | 9.6 | 8.1 | 6.0 | 9.3 | 7.8 | 6.8 | 8.3 | 7.8 | 10.2 | 9.0 | 8.7 | 8.4 |
| Ythdc1 | 15.1 | 15.0 | 19.6 | 18.7 | 14.7 | 14.4 | 16.8 | 15.0 | 13.8 | 16.0 | 19.3 | 19.7 |
| Ythdc2 | 2.5 | 2.8 | 2.6 | 2.1 | 2.3 | 2.7 | 2.5 | 2.7 | 2.4 | 2.6 | 3.0 | 2.8 |
| Ythdf1 | 11.8 | 12.4 | 12.0 | 12.3 | 12.0 | 11.3 | 11.9 | 11.8 | 11.0 | 12.9 | 11.9 | 11.2 |
| Ythdf2 | 13.0 | 12.5 | 12.4 | 13.1 | 13.3 | 12.1 | 12.5 | 13.5 | 13.8 | 13.8 | 14.6 | 13.8 |
| Ythdf3 | 10.0 | 11.6 | 13.3 | 11.1 | 12.0 | 12.5 | 11.5 | 12.2 | 11.4 | 11.6 | 13.5 | 13.3 |
| Ywhab | 45.4 | 56.0 | 58.8 | 52.9 | 64.1 | 50.8 | 63.4 | 64.8 | 62.0 | 58.6 | 62.2 | 63.6 |
| Ywhae | 87.8 | 105.3 | 108.9 | 121.1 | 102.8 | 112.8 | 128.8 | 125.3 | 101.2 | 100.8 | 127.7 | 109.4 |
| Ywhag | 70.9 | 74.9 | 68.2 | 88.6 | 86.4 | 85.0 | 89.1 | 77.1 | 82.1 | 90.2 | 92.0 | 79.0 |
| Ywhah | 46.1 | 39.5 | 41.6 | 66.6 | 64.1 | 45.8 | 54.4 | 46.6 | 47.2 | 38.3 | 45.0 | 45.0 |

| | | | | | | | | | | | | |
|---|---|---|---|---|---|---|---|---|---|---|---|---|
| Ywhaq | 105.9 | 100.6 | 93.6 | 110.1 | 101.6 | 113.3 | 102.4 | 102.2 | 105.6 | 108.4 | 116.9 | 100.2 |
| Ywhaz | 99.9 | 94.8 | 99.5 | 114.0 | 110.4 | 104.3 | 124.8 | 116.9 | 106.4 | 106.9 | 129.6 | 112.6 |
| Yy1 | 14.9 | 17.4 | 19.2 | 13.3 | 15.6 | 15.9 | 16.5 | 17.3 | 15.8 | 17.9 | 17.5 | 18.2 |
| Yy2 | 0.2 | 0.3 | 0.6 | 0.4 | 0.4 | 0.3 | 0.5 | 0.4 | 0.5 | 0.3 | 0.2 | 0.5 |
| Zadh2 | 3.9 | 4.6 | 4.6 | 4.8 | 4.9 | 5.3 | 4.4 | 5.3 | 4.5 | 5.0 | 4.5 | 4.8 |
| Zan | 0.0 | 0.0 | 0.0 | 0.0 | 0.0 | 0.0 | 0.0 | 0.0 | 0.0 | 0.0 | 0.0 | 0.0 |
| Zap70 | 0.0 | 0.0 | 0.0 | 0.1 | 0.0 | 0.1 | 0.1 | 0.0 | 0.0 | 0.0 | 0.0 | 0.0 |
| Zbed3 | 2.0 | 2.6 | 2.9 | 2.9 | 2.1 | 2.4 | 2.6 | 3.3 | 2.5 | 2.8 | 2.8 | 2.4 |
| Zbed4 | 1.8 | 2.4 | 2.0 | 1.8 | 2.4 | 1.5 | 2.0 | 2.1 | 2.3 | 2.1 | 2.5 | 2.1 |
| Zbed6 | 7.5 | 9.0 | 9.3 | 7.1 | 9.9 | 7.0 | 10.2 | 10.9 | 9.9 | 9.0 | 9.3 | 9.4 |
| Zbp1 | 9.2 | 6.2 | 9.3 | 10.0 | 8.8 | 3.0 | 5.7 | 3.4 | 3.0 | 2.4 | 2.0 | 7.0 |
| Zbtb1 | 8.4 | 9.3 | 9.9 | 9.3 | 8.3 | 7.4 | 10.1 | 10.9 | 10.5 | 10.4 | 11.0 | 11.6 |
| Zbtb10 | 1.6 | 1.8 | 1.9 | 1.3 | 1.5 | 1.6 | 1.5 | 1.5 | 1.6 | 1.6 | 1.9 | 1.6 |
| Zbtb11 | 5.6 | 5.5 | 5.2 | 5.5 | 5.2 | 5.9 | 4.7 | 5.0 | 4.7 | 5.7 | 6.0 | 6.0 |
| Zbtb12 | 1.8 | 1.4 | 1.3 | 1.2 | 1.2 | 1.6 | 0.8 | 1.5 | 0.9 | 1.5 | 1.5 | 1.0 |
| Zbtb14 | 5.6 | 5.4 | 6.6 | 6.8 | 5.8 | 5.8 | 5.9 | 5.9 | 5.6 | 4.7 | 6.5 | 5.8 |
| Zbtb16 | 0.0 | 0.1 | 0.3 | 0.0 | 0.1 | 0.0 | 0.0 | 0.1 | 0.0 | 0.0 | 0.0 | 0.0 |
| Zbtb17 | 3.6 | 3.9 | 3.7 | 4.5 | 4.1 | 4.7 | 3.8 | 4.3 | 3.6 | 4.2 | 3.5 | 3.5 |
| Zbtb18 | 5.8 | 5.8 | 4.8 | 8.2 | 5.8 | 7.8 | 5.6 | 4.3 | 5.3 | 4.4 | 6.9 | 4.8 |
| Zbtb2 | 4.1 | 4.4 | 6.2 | 4.7 | 5.0 | 4.3 | 4.9 | 5.3 | 6.0 | 4.9 | 5.1 | 4.7 |
| Zbtb20 | 8.8 | 10.1 | 9.6 | 8.1 | 5.7 | 8.0 | 6.5 | 7.4 | 5.5 | 4.9 | 6.0 | 5.1 |
| Zbtb21 | 3.1 | 3.3 | 2.8 | 3.7 | 3.4 | 3.8 | 2.9 | 2.7 | 2.4 | 2.9 | 2.7 | 2.5 |
| Zbtb22 | 5.5 | 5.6 | 6.1 | 6.5 | 5.7 | 5.5 | 7.0 | 6.7 | 8.0 | 5.6 | 6.4 | 6.7 |
| Zbtb24 | 5.7 | 5.2 | 5.6 | 4.3 | 4.5 | 5.1 | 4.3 | 4.7 | 4.3 | 5.3 | 5.4 | 5.8 |
| Zbtb25 | 2.7 | 2.6 | 3.0 | 2.9 | 2.5 | 3.4 | 2.7 | 3.1 | 2.5 | 2.6 | 2.8 | 2.4 |
| Zbtb26 | 1.6 | 2.4 | 2.5 | 1.7 | 2.2 | 2.3 | 1.5 | 2.1 | 2.4 | 2.3 | 2.4 | 2.6 |
| Zbtb3 | 0.7 | 1.1 | 1.1 | 0.8 | 1.0 | 0.9 | 1.3 | 1.4 | 0.8 | 1.2 | 1.3 | 1.1 |
| Zbtb32 | 0.3 | 0.2 | 0.3 | 0.7 | 0.3 | 0.4 | 0.3 | 0.3 | 0.2 | 0.1 | 0.1 | 0.3 |
| Zbtb33 | 1.5 | 1.9 | 2.4 | 1.4 | 1.6 | 1.3 | 1.6 | 1.5 | 1.5 | 2.0 | 1.8 | 2.6 |
| Zbtb34 | 0.5 | 0.5 | 0.6 | 0.5 | 0.8 | 0.5 | 0.7 | 0.7 | 0.7 | 0.7 | 0.6 | 0.6 |
| Zbtb37 | 1.8 | 1.8 | 2.0 | 1.6 | 1.8 | 2.0 | 1.5 | 2.1 | 1.9 | 1.8 | 2.0 | 1.8 |
| Zbtb38 | 32.6 | 31.5 | 29.4 | 39.3 | 31.2 | 36.4 | 40.9 | 31.7 | 29.5 | 28.1 | 37.7 | 34.2 |
| Zbtb39 | 0.8 | 0.7 | 1.1 | 0.8 | 1.0 | 0.9 | 1.2 | 0.8 | 1.0 | 1.1 | 0.9 | 1.0 |
| Zbtb4 | 16.3 | 16.9 | 16.2 | 18.3 | 14.1 | 19.5 | 15.6 | 13.6 | 14.2 | 12.2 | 14.0 | 14.5 |
| Zbtb40 | 1.9 | 2.3 | 1.6 | 2.1 | 2.3 | 2.0 | 2.0 | 1.8 | 1.9 | 2.0 | 2.0 | 1.9 |
| Zbtb41 | 8.3 | 8.1 | 7.8 | 9.8 | 7.0 | 9.8 | 6.8 | 7.1 | 7.0 | 8.7 | 10.0 | 9.1 |
| Zbtb42 | 0.9 | 1.0 | 0.9 | 1.3 | 1.3 | 1.2 | 1.0 | 0.9 | 1.1 | 1.2 | 1.3 | 0.7 |
| Zbtb43 | 4.0 | 4.0 | 3.7 | 4.5 | 4.2 | 4.4 | 4.2 | 3.8 | 3.8 | 3.8 | 3.9 | 4.1 |
| Zbtb44 | 3.6 | 3.9 | 4.0 | 3.4 | 3.5 | 3.6 | 3.5 | 3.0 | 3.4 | 3.5 | 3.6 | 3.6 |
| Zbtb45 | 1.2 | 0.9 | 0.8 | 1.3 | 1.3 | 1.2 | 1.0 | 1.4 | 1.2 | 1.1 | 1.2 | 0.9 |
| Zbtb46 | 1.9 | 2.3 | 2.1 | 1.5 | 1.8 | 2.2 | 2.0 | 1.9 | 2.1 | 2.8 | 1.8 | 1.8 |
| Zbtb48 | 1.7 | 1.6 | 1.6 | 1.6 | 1.8 | 1.4 | 1.5 | 1.9 | 1.6 | 1.3 | 1.6 | 1.2 |
| Zbtb49 | 1.2 | 1.3 | 1.3 | 1.2 | 1.6 | 1.4 | 1.5 | 1.6 | 1.4 | 1.4 | 1.2 | 1.2 |
| Zbtb5 | 2.4 | 2.6 | 2.4 | 2.1 | 2.2 | 1.9 | 2.5 | 2.4 | 2.7 | 2.8 | 2.1 | 2.8 |
| Zbtb6 | 3.2 | 4.0 | 4.8 | 3.6 | 4.2 | 4.3 | 3.9 | 4.2 | 4.1 | 4.2 | 4.8 | 5.3 |
| Zbtb7a | 6.9 | 8.5 | 8.0 | 7.5 | 9.0 | 7.8 | 10.3 | 8.2 | 8.5 | 6.9 | 7.6 | 7.8 |
| Zbtb7b | 12.7 | 12.3 | 10.8 | 15.2 | 14.0 | 13.2 | 12.2 | 13.1 | 12.3 | 11.3 | 11.6 | 11.3 |
| Zbtb7c | 5.0 | 5.7 | 6.1 | 6.2 | 5.9 | 6.0 | 6.7 | 6.0 | 5.4 | 6.0 | 4.9 | 5.4 |
| Zbtb8a | 1.2 | 2.2 | 3.3 | 0.5 | 1.4 | 0.9 | 2.2 | 2.8 | 3.0 | 1.7 | 2.3 | 2.2 |
| Zbtb8b | 0.5 | 0.9 | 0.8 | 0.3 | 0.8 | 0.4 | 0.7 | 0.3 | 0.7 | 0.7 | 0.6 | 1.0 |
| Zbtb8os | 27.1 | 31.2 | 32.5 | 28.0 | 24.5 | 33.8 | 27.9 | 28.5 | 26.2 | 30.3 | 34.8 | 31.6 |
| Zbtb9 | 3.5 | 3.8 | 3.8 | 3.2 | 3.7 | 3.2 | 3.5 | 3.6 | 3.6 | 3.9 | 3.7 | 3.6 |
| Zbtbd6 | 0.1 | 0.2 | 0.3 | 0.1 | 0.3 | 0.2 | 0.4 | 0.2 | 0.3 | 0.3 | 0.2 | 0.3 |
| Zc2hc1a | 17.3 | 18.3 | 18.3 | 20.0 | 13.2 | 18.6 | 18.2 | 13.5 | 19.4 | 16.3 | 18.9 | 23.9 |
| Zc2hc1c | 0.4 | 0.4 | 0.4 | 0.3 | 0.3 | 0.5 | 0.3 | 0.3 | 0.4 | 0.5 | 0.5 | 0.5 |
| Zc3h10 | 4.1 | 3.6 | 3.4 | 3.5 | 4.3 | 4.5 | 3.6 | 2.9 | 4.0 | 4.6 | 4.0 | 3.4 |
| Zc3h11a | 21.4 | 24.6 | 23.9 | 18.5 | 22.5 | 20.3 | 23.7 | 25.1 | 23.9 | 23.4 | 23.4 | 24.3 |

| | | | | | | | | | | | | |
|---|---|---|---|---|---|---|---|---|---|---|---|---|
| Zc3h12a | 3.4 | 3.9 | 5.1 | 3.4 | 6.8 | 3.1 | 5.9 | 5.2 | 5.6 | 3.6 | 3.4 | 4.1 |
| Zc3h12b | 1.3 | 1.4 | 1.6 | 1.2 | 1.2 | 1.4 | 1.6 | 1.6 | 1.3 | 1.2 | 1.2 | 1.3 |
| Zc3h12c | 2.6 | 2.6 | 2.9 | 2.5 | 3.7 | 2.3 | 2.8 | 2.5 | 2.2 | 2.4 | 2.2 | 2.6 |
| Zc3h12d | 0.1 | 0.0 | 0.0 | 0.8 | 1.4 | 0.0 | 0.2 | 0.0 | 0.0 | 0.1 | 0.1 | 0.0 |
| Zc3h13 | 12.1 | 10.9 | 13.5 | 14.9 | 10.5 | 12.2 | 13.0 | 10.7 | 8.8 | 10.2 | 13.8 | 14.2 |
| Zc3h14 | 33.1 | 34.8 | 31.8 | 31.5 | 26.2 | 36.1 | 27.8 | 26.4 | 25.3 | 27.4 | 32.1 | 30.0 |
| Zc3h15 | 56.8 | 56.9 | 54.6 | 60.7 | 48.7 | 56.3 | 53.6 | 51.4 | 49.8 | 55.4 | 60.6 | 66.3 |
| Zc3h18 | 15.2 | 16.2 | 17.8 | 19.5 | 15.1 | 16.2 | 20.2 | 16.1 | 14.7 | 16.1 | 17.7 | 18.3 |
| Zc3h3 | 1.5 | 1.9 | 1.5 | 1.8 | 1.6 | 1.7 | 1.7 | 1.5 | 1.3 | 1.5 | 1.3 | 1.3 |
| Zc3h4 | 5.4 | 5.8 | 6.5 | 5.1 | 5.3 | 5.3 | 6.4 | 6.3 | 5.8 | 5.6 | 5.6 | 6.1 |
| Zc3h6 | 2.0 | 2.1 | 2.7 | 1.7 | 1.3 | 2.2 | 1.6 | 2.2 | 1.8 | 1.6 | 2.2 | 2.1 |
| Zc3h7a | 16.2 | 18.3 | 16.3 | 15.9 | 18.1 | 12.8 | 17.4 | 14.7 | 18.7 | 15.8 | 19.2 | 18.6 |
| Zc3h7b | 14.3 | 15.0 | 13.3 | 16.1 | 13.1 | 16.7 | 14.8 | 13.1 | 12.6 | 13.2 | 12.5 | 12.0 |
| Zc3h8 | 1.5 | 1.8 | 1.2 | 1.6 | 1.2 | 1.4 | 1.1 | 1.1 | 1.4 | 1.8 | 1.2 | 1.4 |
| Zc3hav1 | 18.6 | 15.4 | 17.0 | 19.9 | 18.1 | 16.8 | 16.4 | 15.9 | 14.9 | 12.1 | 13.1 | 16.1 |
| Zc3hav1l | 0.7 | 1.1 | 1.1 | 0.8 | 0.7 | 1.0 | 0.9 | 1.0 | 1.0 | 0.8 | 0.8 | 0.7 |
| Zc3hc1 | 6.8 | 6.5 | 7.1 | 6.0 | 6.3 | 7.1 | 5.4 | 6.6 | 6.8 | 6.4 | 6.5 | 5.2 |
| Zc4h2 | 4.1 | 4.0 | 3.4 | 4.3 | 2.8 | 4.3 | 3.0 | 3.6 | 3.9 | 3.3 | 3.6 | 4.0 |
| Zcchc10 | 2.1 | 3.3 | 3.5 | 2.1 | 2.6 | 2.8 | 3.2 | 2.6 | 2.1 | 2.9 | 2.2 | 2.6 |
| Zcchc11 | 2.5 | 3.8 | 3.9 | 1.9 | 2.9 | 2.8 | 2.5 | 3.0 | 2.8 | 3.5 | 3.0 | 2.8 |
| Zcchc14 | 3.9 | 5.6 | 5.3 | 3.8 | 4.8 | 3.9 | 5.8 | 5.6 | 6.1 | 5.7 | 4.8 | 4.9 |
| Zcchc17 | 33.9 | 24.1 | 25.2 | 36.7 | 22.6 | 26.7 | 24.1 | 24.0 | 20.0 | 25.4 | 29.2 | 28.4 |
| Zcchc18 | 0.2 | 0.1 | 0.3 | 0.0 | 0.1 | 0.2 | 0.3 | 0.2 | 0.2 | 0.1 | 0.1 | 0.2 |
| Zcchc2 | 3.5 | 3.6 | 3.4 | 4.2 | 3.9 | 4.0 | 3.6 | 2.6 | 3.1 | 3.6 | 3.3 | 3.3 |
| Zcchc24 | 17.7 | 18.8 | 19.4 | 16.5 | 15.7 | 19.6 | 16.7 | 17.7 | 17.0 | 19.5 | 18.2 | 18.1 |
| Zcchc3 | 1.6 | 1.4 | 1.9 | 0.9 | 1.4 | 0.8 | 1.8 | 2.3 | 2.0 | 3.5 | 2.5 | 2.3 |
| Zcchc4 | 2.9 | 2.9 | 2.6 | 2.9 | 2.7 | 3.1 | 2.2 | 2.4 | 2.4 | 3.0 | 2.6 | 2.9 |
| Zcchc5 | 0.0 | 0.0 | 0.0 | 0.1 | 0.0 | 0.1 | 0.0 | 0.1 | 0.2 | 0.1 | 0.0 | 0.0 |
| Zcchc6 | 20.5 | 19.7 | 21.1 | 23.4 | 19.2 | 21.0 | 19.4 | 16.7 | 15.9 | 17.4 | 19.3 | 20.7 |
| Zcchc7 | 9.3 | 8.7 | 9.8 | 9.3 | 7.1 | 7.3 | 7.4 | 9.0 | 6.8 | 8.4 | 9.2 | 10.8 |
| Zcchc8 | 3.3 | 3.9 | 4.7 | 3.4 | 3.8 | 3.2 | 3.7 | 4.3 | 4.2 | 3.8 | 4.0 | 4.1 |
| Zcchc9 | 15.8 | 14.0 | 12.8 | 14.6 | 12.5 | 13.9 | 12.8 | 13.1 | 12.8 | 13.0 | 15.6 | 13.7 |
| Zcrb1 | 40.1 | 32.3 | 40.8 | 54.6 | 38.9 | 45.0 | 45.0 | 38.1 | 32.0 | 36.3 | 46.3 | 45.7 |
| Zcwpw1 | 0.7 | 0.6 | 1.1 | 0.7 | 0.5 | 1.0 | 0.5 | 0.3 | 0.6 | 0.4 | 0.5 | 0.6 |
| Zdbf2 | 0.0 | 0.0 | 0.0 | 0.0 | 0.0 | 0.0 | 0.0 | 0.0 | 0.0 | 0.1 | 0.1 | 0.0 |
| Zdhhc1 | 5.2 | 5.7 | 5.3 | 6.7 | 5.4 | 6.8 | 6.1 | 6.2 | 4.7 | 5.1 | 4.5 | 6.0 |
| Zdhhc12 | 3.8 | 4.2 | 4.8 | 2.9 | 4.1 | 3.4 | 3.9 | 4.5 | 5.4 | 7.2 | 5.1 | 5.1 |
| Zdhhc13 | 8.1 | 7.0 | 7.7 | 7.1 | 8.5 | 6.2 | 8.8 | 8.3 | 8.8 | 8.3 | 9.8 | 8.0 |
| Zdhhc14 | 1.5 | 1.4 | 1.0 | 2.0 | 1.7 | 1.8 | 1.8 | 1.0 | 1.4 | 1.1 | 1.1 | 1.4 |
| Zdhhc15 | 0.7 | 1.0 | 1.3 | 0.4 | 0.8 | 0.6 | 0.6 | 0.8 | 0.6 | 0.5 | 0.4 | 0.6 |
| Zdhhc16 | 6.4 | 7.3 | 5.3 | 7.4 | 7.2 | 5.1 | 8.9 | 10.4 | 9.8 | 6.6 | 7.9 | 6.9 |
| Zdhhc17 | 6.0 | 6.5 | 6.4 | 5.3 | 5.4 | 5.8 | 5.3 | 6.2 | 6.8 | 6.6 | 6.1 | 7.0 |
| Zdhhc18 | 7.4 | 8.7 | 6.6 | 9.0 | 10.3 | 8.4 | 8.5 | 6.1 | 7.9 | 7.0 | 7.0 | 6.4 |
| Zdhhc2 | 33.4 | 23.3 | 27.2 | 36.1 | 48.5 | 37.9 | 38.3 | 31.7 | 37.6 | 35.0 | 52.0 | 36.7 |
| Zdhhc20 | 12.3 | 14.2 | 15.8 | 14.3 | 14.7 | 13.4 | 14.3 | 14.1 | 14.4 | 15.8 | 16.3 | 17.5 |
| Zdhhc21 | 3.3 | 3.8 | 3.9 | 3.6 | 3.9 | 4.1 | 3.5 | 3.3 | 3.5 | 3.2 | 4.2 | 3.8 |
| Zdhhc24 | 4.3 | 3.9 | 3.6 | 4.7 | 3.9 | 4.2 | 4.0 | 3.5 | 4.3 | 4.5 | 4.1 | 4.2 |
| Zdhhc3 | 7.7 | 6.0 | 5.5 | 7.1 | 6.7 | 6.5 | 6.7 | 6.8 | 6.3 | 6.6 | 6.8 | 6.4 |
| Zdhhc4 | 10.0 | 8.4 | 7.8 | 11.6 | 8.3 | 10.7 | 10.4 | 10.4 | 8.2 | 8.3 | 9.3 | 8.1 |
| Zdhhc5 | 22.2 | 21.6 | 21.9 | 24.1 | 23.9 | 23.0 | 26.4 | 22.9 | 23.3 | 23.7 | 25.8 | 23.9 |
| Zdhhc6 | 21.8 | 22.5 | 23.9 | 23.4 | 22.6 | 24.6 | 22.3 | 23.6 | 19.5 | 21.5 | 24.2 | 22.4 |
| Zdhhc7 | 8.8 | 9.5 | 9.0 | 9.5 | 9.4 | 8.8 | 10.7 | 10.4 | 11.0 | 8.4 | 9.4 | 8.3 |
| Zdhhc8 | 5.0 | 6.3 | 5.7 | 6.0 | 5.3 | 6.3 | 7.8 | 5.9 | 7.0 | 4.4 | 5.3 | 4.4 |
| Zdhhc9 | 5.4 | 4.8 | 4.0 | 5.2 | 4.9 | 4.4 | 5.6 | 4.7 | 4.9 | 4.2 | 5.4 | 3.3 |
| Zeb1 | 17.9 | 17.3 | 19.0 | 24.0 | 15.7 | 22.6 | 18.2 | 17.1 | 15.2 | 13.9 | 17.7 | 18.2 |
| Zeb2 | 26.3 | 25.7 | 28.1 | 44.3 | 31.7 | 31.6 | 31.2 | 26.3 | 23.3 | 21.7 | 28.4 | 30.7 |
| Zer1 | 4.9 | 5.7 | 5.3 | 4.7 | 4.8 | 5.6 | 5.0 | 5.4 | 5.5 | 5.6 | 4.8 | 4.7 |

| | | | | | | | | | | | | |
|---|---|---|---|---|---|---|---|---|---|---|---|---|
| Zfand1 | 8.4 | 8.5 | 7.9 | 6.5 | 5.9 | 8.1 | 6.5 | 6.8 | 5.9 | 7.8 | 7.5 | 9.0 |
| Zfand2a | 17.4 | 13.0 | 9.3 | 24.4 | 18.1 | 17.2 | 16.2 | 8.6 | 13.2 | 14.4 | 14.5 | 13.2 |
| Zfand2b | 7.4 | 8.7 | 6.5 | 7.5 | 7.0 | 8.1 | 8.8 | 9.4 | 10.0 | 7.5 | 9.0 | 7.6 |
| Zfand3 | 40.0 | 37.1 | 31.7 | 42.7 | 31.8 | 48.1 | 36.3 | 30.9 | 33.4 | 32.2 | 36.5 | 33.1 |
| Zfand4 | 0.3 | 0.4 | 0.6 | 0.2 | 0.5 | 0.2 | 0.5 | 0.3 | 0.4 | 0.3 | 0.4 | 0.2 |
| Zfand5 | 5.6 | 5.2 | 4.9 | 6.9 | 6.3 | 5.8 | 5.2 | 5.7 | 6.3 | 7.3 | 6.4 | 6.6 |
| Zfand6 | 18.2 | 20.6 | 17.9 | 19.1 | 20.3 | 21.5 | 15.0 | 18.1 | 16.1 | 20.3 | 19.6 | 20.1 |
| Zfa-ps | 0.2 | 0.3 | 0.2 | 0.3 | 0.3 | 0.2 | 0.2 | 0.2 | 0.2 | 0.3 | 0.2 | 0.3 |
| Zfat | 1.5 | 1.5 | 1.3 | 1.3 | 1.4 | 1.2 | 1.6 | 1.4 | 1.6 | 1.6 | 2.0 | 1.6 |
| Zfc3h1 | 10.8 | 11.7 | 11.0 | 9.6 | 9.8 | 10.5 | 9.4 | 10.2 | 10.2 | 9.8 | 10.9 | 10.9 |
| Zfhx2 | 0.0 | 0.0 | 0.0 | 0.0 | 0.0 | 0.0 | 0.0 | 0.0 | 0.0 | 0.0 | 0.0 | 0.0 |
| Zfhx2as | 0.0 | 0.0 | 0.0 | 0.1 | 0.0 | 0.1 | 0.0 | 0.0 | 0.0 | 0.0 | 0.0 | 0.0 |
| Zfhx3 | 1.4 | 2.2 | 2.0 | 2.0 | 2.2 | 2.0 | 2.8 | 2.1 | 2.3 | 2.0 | 2.3 | 1.7 |
| Zfhx4 | 0.2 | 0.2 | 0.2 | 0.1 | 0.1 | 0.2 | 0.2 | 0.2 | 0.2 | 0.5 | 0.5 | 0.4 |
| Zfml | 15.5 | 15.8 | 14.4 | 14.4 | 14.7 | 13.8 | 12.6 | 13.4 | 14.1 | 17.4 | 17.9 | 15.8 |
| Zfp1 | 2.3 | 2.3 | 2.5 | 1.9 | 2.0 | 2.0 | 2.3 | 3.3 | 2.3 | 2.9 | 2.6 | 3.7 |
| Zfp101 | 2.0 | 1.9 | 2.4 | 1.7 | 2.1 | 1.5 | 2.1 | 2.0 | 2.4 | 2.4 | 2.2 | 2.7 |
| Zfp105 | 4.1 | 3.3 | 3.9 | 3.5 | 3.0 | 3.0 | 3.2 | 3.8 | 3.5 | 3.6 | 4.0 | 4.5 |
| Zfp106 | 31.2 | 24.7 | 22.3 | 36.8 | 23.7 | 35.4 | 23.0 | 20.4 | 17.3 | 19.5 | 23.6 | 23.0 |
| Zfp108 | 1.6 | 1.6 | 2.2 | 1.3 | 1.3 | 2.4 | 1.6 | 1.8 | 1.3 | 1.9 | 1.8 | 1.7 |
| Zfp109 | 1.4 | 1.5 | 2.0 | 1.8 | 1.4 | 1.9 | 1.2 | 1.4 | 1.2 | 1.5 | 1.9 | 1.7 |
| Zfp11 | 1.0 | 1.2 | 1.6 | 1.1 | 1.1 | 1.4 | 1.1 | 1.3 | 1.3 | 1.3 | 1.4 | 1.3 |
| Zfp110 | 10.9 | 9.8 | 9.4 | 9.9 | 8.0 | 10.2 | 7.4 | 10.0 | 9.9 | 10.6 | 10.7 | 11.4 |
| Zfp111 | 1.4 | 1.4 | 1.8 | 1.4 | 1.4 | 1.9 | 1.7 | 1.8 | 1.5 | 1.5 | 1.7 | 1.7 |
| Zfp112 | 1.0 | 1.0 | 1.4 | 0.8 | 0.9 | 1.5 | 1.2 | 1.3 | 1.1 | 1.2 | 1.3 | 1.3 |
| Zfp113 | 1.1 | 1.3 | 1.3 | 1.0 | 0.9 | 1.3 | 0.9 | 1.2 | 1.1 | 1.5 | 1.4 | 1.2 |
| Zfp114 | 0.2 | 0.2 | 0.4 | 0.3 | 0.1 | 0.4 | 0.2 | 0.2 | 0.2 | 0.2 | 0.3 | 0.2 |
| Zfp119a | 1.9 | 2.7 | 2.5 | 1.6 | 2.3 | 2.6 | 1.5 | 2.0 | 2.2 | 2.5 | 2.4 | 2.0 |
| Zfp119b | 1.3 | 1.3 | 1.6 | 1.1 | 1.7 | 1.2 | 1.4 | 1.4 | 1.8 | 1.9 | 1.7 | 1.6 |
| Zfp12 | 3.4 | 3.5 | 3.4 | 3.2 | 3.1 | 3.5 | 3.1 | 3.9 | 3.7 | 4.2 | 3.8 | 4.3 |
| Zfp120 | 3.2 | 3.7 | 4.9 | 3.4 | 4.0 | 3.1 | 3.1 | 3.8 | 4.3 | 3.8 | 3.9 | 4.9 |
| Zfp128 | 1.3 | 0.9 | 1.4 | 1.1 | 0.8 | 1.0 | 1.0 | 0.9 | 1.0 | 1.0 | 0.8 | 1.0 |
| Zfp13 | 1.8 | 2.1 | 2.2 | 2.4 | 1.8 | 2.3 | 2.1 | 2.4 | 1.7 | 1.6 | 2.2 | 1.6 |
| Zfp131 | 17.6 | 16.8 | 16.4 | 20.3 | 14.6 | 17.3 | 15.3 | 15.7 | 15.1 | 14.4 | 18.5 | 16.8 |
| Zfp133-ps | 0.8 | 0.7 | 0.9 | 1.3 | 0.4 | 0.6 | 0.8 | 0.6 | 0.5 | 1.2 | 0.7 | 0.5 |
| Zfp14 | 2.2 | 2.0 | 2.1 | 2.0 | 1.7 | 2.6 | 1.7 | 2.0 | 1.5 | 2.1 | 1.7 | 1.9 |
| Zfp142 | 1.1 | 1.3 | 1.2 | 1.4 | 1.4 | 1.4 | 1.6 | 1.4 | 1.1 | 1.1 | 1.3 | 1.1 |
| Zfp143 | 5.3 | 5.3 | 4.6 | 5.9 | 5.1 | 5.1 | 5.3 | 5.0 | 5.1 | 5.4 | 6.0 | 5.5 |
| Zfp146 | 19.7 | 15.3 | 15.8 | 21.3 | 18.3 | 18.7 | 19.1 | 19.8 | 15.9 | 18.8 | 19.7 | 23.0 |
| Zfp148 | 6.0 | 6.7 | 7.7 | 6.0 | 5.8 | 6.8 | 5.9 | 8.1 | 7.1 | 7.1 | 8.0 | 7.8 |
| Zfp157 | 2.0 | 1.7 | 2.0 | 2.0 | 1.8 | 1.6 | 1.5 | 1.9 | 1.9 | 2.1 | 2.2 | 2.1 |
| Zfp160 | 2.4 | 2.5 | 2.6 | 3.0 | 2.6 | 2.6 | 2.6 | 3.1 | 2.9 | 2.9 | 3.3 | 3.4 |
| Zfp169 | 1.3 | 1.3 | 1.6 | 1.3 | 1.5 | 1.5 | 1.2 | 1.5 | 1.2 | 1.2 | 1.2 | 1.4 |
| Zfp174 | 0.9 | 1.1 | 1.2 | 0.9 | 0.9 | 1.0 | 1.1 | 0.9 | 1.0 | 0.9 | 0.8 | 1.0 |
| Zfp180 | 8.0 | 7.9 | 6.2 | 7.8 | 6.9 | 7.1 | 6.6 | 6.5 | 6.6 | 6.6 | 8.4 | 6.5 |
| Zfp182 | 1.9 | 2.2 | 2.2 | 2.4 | 1.8 | 2.0 | 1.7 | 1.9 | 1.8 | 2.0 | 2.6 | 2.8 |
| Zfp184 | 0.7 | 0.6 | 0.8 | 0.5 | 0.3 | 0.4 | 0.4 | 0.6 | 0.4 | 0.8 | 0.5 | 0.3 |
| Zfp185 | 0.2 | 0.1 | 0.1 | 0.0 | 0.1 | 0.0 | 0.1 | 0.2 | 0.1 | 1.2 | 0.6 | 0.3 |
| Zfp189 | 2.7 | 2.8 | 3.4 | 2.1 | 2.0 | 2.2 | 2.2 | 3.1 | 2.4 | 2.5 | 2.6 | 2.6 |
| Zfp191 | 8.0 | 8.5 | 10.4 | 8.6 | 9.4 | 7.7 | 8.1 | 9.2 | 9.2 | 11.8 | 11.0 | 11.7 |
| Zfp2 | 1.5 | 1.3 | 1.5 | 1.6 | 1.3 | 1.7 | 0.9 | 1.3 | 1.3 | 1.8 | 2.1 | 1.5 |
| Zfp202 | 1.4 | 1.0 | 0.9 | 1.3 | 0.9 | 1.1 | 0.8 | 0.9 | 0.8 | 1.2 | 1.2 | 0.9 |
| Zfp207 | 41.5 | 41.9 | 50.9 | 48.3 | 45.1 | 38.4 | 50.9 | 48.3 | 42.8 | 41.9 | 50.9 | 47.1 |
| Zfp212 | 2.2 | 2.5 | 2.8 | 2.7 | 2.5 | 2.5 | 2.4 | 2.7 | 3.0 | 2.9 | 2.8 | 2.8 |
| Zfp213 | 2.7 | 2.4 | 2.2 | 2.9 | 2.7 | 2.8 | 2.0 | 2.8 | 2.4 | 3.0 | 2.6 | 2.5 |
| Zfp217 | 3.0 | 3.2 | 4.4 | 3.0 | 3.9 | 2.8 | 3.5 | 3.0 | 3.1 | 3.1 | 3.0 | 3.1 |
| Zfp219 | 10.2 | 10.8 | 8.6 | 10.8 | 10.2 | 12.1 | 10.3 | 8.5 | 8.7 | 10.5 | 8.0 | 7.7 |

| | | | | | | | | | | | | |
|---|---|---|---|---|---|---|---|---|---|---|---|---|
| Zfp229 | 2.4 | 2.6 | 2.9 | 2.4 | 2.3 | 2.4 | 1.9 | 2.6 | 2.4 | 2.4 | 2.9 | 2.8 |
| Zfp235 | 1.7 | 2.1 | 2.4 | 2.0 | 1.6 | 1.5 | 1.3 | 2.1 | 1.8 | 1.7 | 2.0 | 1.8 |
| Zfp236 | 2.8 | 3.3 | 3.6 | 2.9 | 3.0 | 3.1 | 3.2 | 3.7 | 3.3 | 3.1 | 3.4 | 3.0 |
| Zfp239 | 0.8 | 0.8 | 0.7 | 0.3 | 0.5 | 1.1 | 0.5 | 0.8 | 0.4 | 0.6 | 0.9 | 0.6 |
| Zfp248 | 1.2 | 1.3 | 1.3 | 0.9 | 1.0 | 1.1 | 0.9 | 1.1 | 1.1 | 1.2 | 1.6 | 1.2 |
| Zfp251 | 8.5 | 7.8 | 8.7 | 8.6 | 7.7 | 10.3 | 7.8 | 8.2 | 8.5 | 8.3 | 8.0 | 9.0 |
| Zfp259 | 11.0 | 8.1 | 7.3 | 10.8 | 9.5 | 9.9 | 8.8 | 8.0 | 8.5 | 8.7 | 8.7 | 8.2 |
| Zfp26 | 2.1 | 2.3 | 2.7 | 2.2 | 2.4 | 2.4 | 2.2 | 2.8 | 2.5 | 2.4 | 3.0 | 2.6 |
| Zfp260 | 15.7 | 17.1 | 18.6 | 17.2 | 16.3 | 17.4 | 15.1 | 19.2 | 19.2 | 19.2 | 20.2 | 22.1 |
| Zfp263 | 5.1 | 5.0 | 4.9 | 5.4 | 7.4 | 5.2 | 5.0 | 4.7 | 4.8 | 6.4 | 5.6 | 5.5 |
| Zfp266 | 13.6 | 13.2 | 13.8 | 11.8 | 11.7 | 14.5 | 11.2 | 14.5 | 11.8 | 13.5 | 15.4 | 13.9 |
| Zfp27 | 3.2 | 3.3 | 3.4 | 3.8 | 2.9 | 3.4 | 3.6 | 3.6 | 2.8 | 3.1 | 3.6 | 3.1 |
| Zfp273 | 1.8 | 1.2 | 1.6 | 1.0 | 1.4 | 1.2 | 1.1 | 1.4 | 1.5 | 1.3 | 1.4 | 1.7 |
| Zfp275 | 10.1 | 9.2 | 10.3 | 9.8 | 11.0 | 9.7 | 10.1 | 10.0 | 9.6 | 10.4 | 10.8 | 10.8 |
| Zfp276 | 2.9 | 2.7 | 2.7 | 2.6 | 3.2 | 2.9 | 3.1 | 3.0 | 3.8 | 3.1 | 2.9 | 2.6 |
| Zfp277 | 10.2 | 10.9 | 10.6 | 8.4 | 9.9 | 10.2 | 9.6 | 9.9 | 8.9 | 10.6 | 10.1 | 9.9 |
| Zfp28 | 1.5 | 1.5 | 1.9 | 1.7 | 1.6 | 1.9 | 1.4 | 1.5 | 1.3 | 1.7 | 1.3 | 1.5 |
| Zfp280b | 3.6 | 4.5 | 3.9 | 3.1 | 3.2 | 3.6 | 4.0 | 3.9 | 4.4 | 4.0 | 4.7 | 3.3 |
| Zfp280c | 2.8 | 3.1 | 3.1 | 2.6 | 2.5 | 3.0 | 3.0 | 3.0 | 2.4 | 2.9 | 3.2 | 2.4 |
| Zfp280d | 3.9 | 4.8 | 5.0 | 4.2 | 4.1 | 4.2 | 4.2 | 5.3 | 4.6 | 5.1 | 5.3 | 5.0 |
| Zfp281 | 6.5 | 7.9 | 11.2 | 5.8 | 8.7 | 4.8 | 8.7 | 10.6 | 9.9 | 11.7 | 9.4 | 10.7 |
| Zfp282 | 2.5 | 2.2 | 2.9 | 2.8 | 2.4 | 2.2 | 2.4 | 3.1 | 3.1 | 2.5 | 2.5 | 2.4 |
| Zfp286 | 1.6 | 1.6 | 1.8 | 1.4 | 1.0 | 1.5 | 1.4 | 1.7 | 1.2 | 1.2 | 1.8 | 1.5 |
| Zfp287 | 1.8 | 1.8 | 2.2 | 1.8 | 1.7 | 2.6 | 1.9 | 1.9 | 1.8 | 1.6 | 2.0 | 2.0 |
| Zfp292 | 2.9 | 3.8 | 3.6 | 2.8 | 3.0 | 4.0 | 2.9 | 3.6 | 3.1 | 3.8 | 4.0 | 4.1 |
| Zfp296 | 1.1 | 1.2 | 0.8 | 1.1 | 1.2 | 1.5 | 0.9 | 0.5 | 0.4 | 0.4 | 0.8 | 0.7 |
| Zfp3 | 1.9 | 1.7 | 2.0 | 2.1 | 1.5 | 2.2 | 1.3 | 1.2 | 1.1 | 1.6 | 1.5 | 1.4 |
| Zfp30 | 1.8 | 1.8 | 2.0 | 1.8 | 1.1 | 2.1 | 1.2 | 1.3 | 1.5 | 1.6 | 1.4 | 1.2 |
| Zfp300 | 0.2 | 0.2 | 0.4 | 0.4 | 0.2 | 0.4 | 0.3 | 0.4 | 0.3 | 0.3 | 0.3 | 0.4 |
| Zfp316 | 2.5 | 2.1 | 2.0 | 1.8 | 1.7 | 1.8 | 1.8 | 1.9 | 1.7 | 1.8 | 2.2 | 2.0 |
| Zfp317 | 8.6 | 8.3 | 8.3 | 8.2 | 7.5 | 9.9 | 6.8 | 8.0 | 7.8 | 8.6 | 9.2 | 8.9 |
| Zfp318 | 3.5 | 3.9 | 4.5 | 3.7 | 3.2 | 3.2 | 3.7 | 3.7 | 3.5 | 3.6 | 3.9 | 3.9 |
| Zfp319 | 2.0 | 1.9 | 2.0 | 2.6 | 2.3 | 2.2 | 2.4 | 2.4 | 2.2 | 2.1 | 1.6 | 1.5 |
| Zfp322a | 6.1 | 6.4 | 7.0 | 5.7 | 5.9 | 6.5 | 5.3 | 6.3 | 5.8 | 6.6 | 7.2 | 7.2 |
| Zfp324 | 2.8 | 3.3 | 3.3 | 2.5 | 2.9 | 3.3 | 3.4 | 3.4 | 3.6 | 3.1 | 3.6 | 3.2 |
| Zfp326 | 3.5 | 4.0 | 4.7 | 4.5 | 3.9 | 3.5 | 5.3 | 4.3 | 3.5 | 4.7 | 4.6 | 4.4 |
| Zfp329 | 2.4 | 2.6 | 2.5 | 2.4 | 2.2 | 2.4 | 2.0 | 2.4 | 2.2 | 2.2 | 2.3 | 2.5 |
| Zfp330 | 25.9 | 21.5 | 20.6 | 21.1 | 21.7 | 22.0 | 20.2 | 16.9 | 19.0 | 23.4 | 21.4 | 23.5 |
| Zfp334 | 4.8 | 5.1 | 5.7 | 4.3 | 4.0 | 5.0 | 4.6 | 5.1 | 4.2 | 5.5 | 5.7 | 5.3 |
| Zfp335 | 3.0 | 3.5 | 3.0 | 3.5 | 3.3 | 2.9 | 4.3 | 3.0 | 4.2 | 3.3 | 3.5 | 3.1 |
| Zfp341 | 0.3 | 0.3 | 0.5 | 0.2 | 0.3 | 0.3 | 0.4 | 0.4 | 0.3 | 0.3 | 0.4 | 0.3 |
| Zfp345 | 0.1 | 0.0 | 0.1 | 0.0 | 0.0 | 0.1 | 0.1 | 0.1 | 0.1 | 0.1 | 0.1 | 0.0 |
| Zfp346 | 5.4 | 5.2 | 5.3 | 5.9 | 4.6 | 5.0 | 4.0 | 4.6 | 4.3 | 4.7 | 4.0 | 4.8 |
| Zfp35 | 5.4 | 5.5 | 5.4 | 5.1 | 5.1 | 5.2 | 5.0 | 5.2 | 5.1 | 5.1 | 5.6 | 5.2 |
| Zfp354a | 1.3 | 1.5 | 1.4 | 1.4 | 1.3 | 1.3 | 1.2 | 1.2 | 1.3 | 1.4 | 1.6 | 1.2 |
| Zfp354b | 0.9 | 1.3 | 1.4 | 1.4 | 1.1 | 1.3 | 1.1 | 1.0 | 0.9 | 1.2 | 1.2 | 1.3 |
| Zfp354c | 1.8 | 1.5 | 2.6 | 1.8 | 1.3 | 2.4 | 1.7 | 2.4 | 1.9 | 2.3 | 2.2 | 2.4 |
| Zfp358 | 6.0 | 5.4 | 6.4 | 6.6 | 4.8 | 8.7 | 6.0 | 6.9 | 5.4 | 7.0 | 7.0 | 5.4 |
| Zfp36 | 6.6 | 10.7 | 10.3 | 6.6 | 8.9 | 6.8 | 9.2 | 10.0 | 12.3 | 20.6 | 10.6 | 9.9 |
| Zfp362 | 0.9 | 1.2 | 1.3 | 1.1 | 1.4 | 1.3 | 1.2 | 1.0 | 1.1 | 1.1 | 1.3 | 0.9 |
| Zfp365 | 12.2 | 11.6 | 10.1 | 12.5 | 9.7 | 14.2 | 7.8 | 7.3 | 6.9 | 8.8 | 9.8 | 8.3 |
| Zfp367 | 5.0 | 6.4 | 8.6 | 6.9 | 7.6 | 7.7 | 7.9 | 4.9 | 6.7 | 4.3 | 7.1 | 6.3 |
| Zfp369 | 3.0 | 3.5 | 3.4 | 2.6 | 2.9 | 3.2 | 2.4 | 3.5 | 2.9 | 2.9 | 2.6 | 2.5 |
| Zfp36l1 | 5.4 | 10.5 | 17.2 | 4.8 | 11.3 | 6.8 | 10.3 | 15.0 | 11.3 | 12.7 | 7.5 | 9.7 |
| Zfp36l2 | 18.6 | 24.5 | 32.0 | 18.7 | 23.0 | 19.1 | 17.6 | 22.2 | 16.6 | 18.1 | 16.0 | 14.7 |
| Zfp37 | 5.4 | 3.7 | 4.0 | 5.3 | 3.0 | 3.5 | 3.5 | 3.4 | 3.3 | 5.2 | 5.8 | 5.6 |
| Zfp382 | 2.1 | 2.6 | 2.7 | 1.9 | 2.1 | 2.4 | 2.9 | 2.4 | 2.6 | 2.1 | 2.6 | 2.1 |

| | | | | | | | | | | | | |
|---|---|---|---|---|---|---|---|---|---|---|---|---|
| Zfp383 | 1.3 | 1.2 | 1.3 | 0.8 | 1.0 | 1.4 | 0.8 | 1.7 | 1.2 | 1.3 | 1.3 | 1.3 |
| Zfp384 | 4.6 | 5.6 | 6.1 | 5.7 | 5.2 | 4.4 | 5.3 | 5.6 | 4.9 | 4.3 | 4.9 | 4.5 |
| Zfp385a | 1.5 | 2.6 | 2.5 | 1.5 | 2.3 | 1.9 | 2.4 | 2.5 | 2.1 | 2.5 | 2.3 | 1.4 |
| Zfp385b | 0.1 | 0.0 | 0.0 | 0.2 | 0.0 | 0.0 | 0.1 | 0.1 | 0.0 | 0.0 | 0.0 | 0.0 |
| Zfp385c | 3.4 | 3.8 | 3.2 | 3.7 | 3.6 | 4.0 | 3.6 | 3.8 | 3.9 | 3.3 | 4.0 | 3.3 |
| Zfp386 | 9.1 | 8.9 | 9.5 | 9.5 | 8.5 | 10.3 | 9.4 | 10.8 | 8.7 | 9.6 | 12.8 | 12.0 |
| Zfp389 | 0.5 | 0.3 | 0.3 | 0.1 | 0.3 | 0.3 | 0.3 | 0.2 | 0.1 | 0.2 | 0.6 | 0.3 |
| Zfp39 | 2.0 | 1.6 | 2.5 | 1.5 | 1.7 | 1.5 | 2.0 | 2.1 | 1.9 | 1.9 | 2.2 | 2.2 |
| Zfp395 | 0.0 | 0.1 | 0.4 | 0.0 | 0.1 | 0.1 | 0.1 | 0.2 | 0.2 | 0.5 | 0.2 | 0.2 |
| Zfp397 | 5.8 | 6.8 | 6.6 | 5.8 | 5.8 | 6.4 | 4.5 | 5.6 | 6.2 | 5.7 | 6.7 | 6.2 |
| Zfp398 | 1.4 | 1.3 | 1.3 | 1.1 | 1.1 | 1.9 | 1.0 | 1.6 | 1.1 | 1.4 | 1.8 | 1.3 |
| Zfp40 | 2.0 | 2.4 | 2.3 | 2.5 | 2.5 | 2.3 | 2.0 | 2.2 | 2.1 | 2.3 | 2.9 | 2.7 |
| Zfp407 | 3.3 | 3.3 | 3.0 | 3.4 | 3.6 | 3.1 | 4.0 | 3.9 | 3.6 | 3.8 | 3.7 | 3.7 |
| Zfp408 | 2.5 | 2.4 | 2.2 | 2.7 | 2.3 | 2.6 | 2.7 | 2.6 | 2.6 | 2.4 | 2.7 | 2.5 |
| Zfp41 | 1.2 | 1.7 | 1.7 | 1.0 | 1.1 | 1.4 | 1.3 | 1.5 | 1.3 | 1.0 | 1.2 | 0.9 |
| Zfp410 | 11.9 | 12.7 | 11.0 | 9.8 | 11.0 | 11.6 | 9.9 | 10.5 | 11.4 | 11.7 | 11.3 | 12.1 |
| Zfp414 | 5.5 | 7.3 | 6.8 | 8.3 | 6.2 | 7.5 | 9.1 | 9.4 | 8.5 | 5.7 | 8.4 | 7.3 |
| Zfp418 | 0.6 | 0.9 | 0.9 | 0.8 | 0.7 | 0.8 | 0.9 | 0.8 | 0.8 | 0.8 | 0.9 | 0.6 |
| Zfp420 | 1.6 | 1.5 | 1.4 | 1.6 | 0.9 | 1.5 | 0.8 | 1.0 | 1.0 | 1.2 | 1.5 | 1.3 |
| Zfp422 | 4.1 | 4.3 | 5.0 | 4.2 | 4.1 | 4.2 | 4.3 | 5.2 | 4.2 | 4.6 | 5.2 | 5.1 |
| Zfp423 | 0.4 | 0.5 | 1.0 | 0.4 | 0.5 | 0.4 | 0.4 | 1.0 | 0.6 | 0.6 | 0.6 | 0.6 |
| Zfp426 | 9.0 | 8.1 | 6.7 | 8.1 | 7.6 | 8.2 | 6.3 | 6.8 | 6.7 | 8.2 | 7.5 | 7.6 |
| Zfp428 | 1.5 | 1.6 | 1.3 | 1.1 | 1.4 | 1.1 | 1.2 | 1.3 | 1.2 | 2.2 | 0.8 | 1.6 |
| Zfp429 | 1.9 | 2.0 | 1.9 | 1.3 | 2.6 | 2.1 | 1.6 | 1.7 | 1.9 | 2.1 | 2.2 | 1.9 |
| Zfp433 | 1.8 | 1.8 | 1.6 | 1.7 | 1.9 | 2.0 | 1.3 | 2.1 | 2.3 | 2.3 | 2.1 | 2.1 |
| Zfp438 | 1.5 | 1.5 | 1.4 | 2.0 | 1.5 | 1.7 | 1.2 | 1.3 | 1.2 | 1.3 | 1.4 | 1.4 |
| Zfp442 | 2.8 | 3.5 | 4.0 | 2.8 | 3.1 | 3.4 | 2.5 | 3.2 | 2.6 | 3.9 | 3.2 | 4.1 |
| Zfp444 | 1.5 | 1.4 | 1.2 | 1.6 | 1.4 | 1.7 | 1.4 | 1.5 | 1.5 | 1.8 | 1.4 | 1.3 |
| Zfp445 | 9.5 | 10.4 | 9.4 | 8.5 | 9.7 | 9.2 | 7.9 | 8.6 | 10.3 | 9.7 | 9.6 | 11.0 |
| Zfp446 | 0.8 | 0.6 | 0.6 | 0.6 | 0.6 | 0.6 | 0.7 | 0.6 | 0.7 | 0.7 | 0.6 | 0.5 |
| Zfp449 | 4.5 | 5.2 | 5.2 | 4.7 | 4.7 | 5.0 | 5.3 | 5.0 | 5.3 | 5.5 | 6.7 | 6.1 |
| Zfp451 | 5.3 | 7.4 | 8.6 | 5.0 | 7.1 | 5.9 | 6.7 | 7.4 | 6.8 | 6.3 | 7.2 | 8.1 |
| Zfp454 | 0.7 | 0.8 | 0.9 | 1.0 | 0.4 | 0.9 | 0.7 | 0.6 | 0.4 | 0.4 | 0.7 | 0.9 |
| Zfp455 | 0.8 | 0.8 | 0.9 | 0.7 | 0.9 | 0.7 | 0.5 | 0.7 | 0.7 | 0.6 | 0.6 | 1.1 |
| Zfp456 | 0.8 | 0.9 | 0.9 | 0.9 | 1.2 | 0.7 | 0.6 | 0.6 | 0.8 | 1.1 | 0.7 | 0.8 |
| Zfp457 | 0.0 | 0.1 | 0.1 | 0.0 | 0.1 | 0.0 | 0.0 | 0.0 | 0.0 | 0.1 | 0.0 | 0.0 |
| Zfp458 | 0.9 | 1.1 | 1.1 | 0.9 | 1.0 | 1.1 | 1.1 | 1.1 | 0.7 | 0.9 | 1.1 | 0.9 |
| Zfp459 | 0.2 | 0.1 | 0.1 | 0.3 | 0.2 | 0.3 | 0.2 | 0.1 | 0.1 | 0.1 | 0.1 | 0.1 |
| Zfp46 | 7.5 | 8.1 | 9.8 | 7.9 | 7.7 | 7.6 | 8.1 | 9.8 | 8.4 | 7.4 | 8.1 | 8.1 |
| Zfp462 | 1.9 | 1.7 | 1.7 | 2.0 | 1.4 | 1.9 | 2.0 | 2.0 | 1.7 | 2.2 | 2.9 | 1.6 |
| Zfp467 | 0.7 | 1.5 | 2.3 | 0.8 | 1.7 | 1.1 | 0.9 | 1.4 | 1.8 | 1.5 | 0.9 | 1.3 |
| Zfp472 | 1.2 | 1.1 | 1.4 | 1.7 | 2.1 | 1.1 | 1.3 | 1.7 | 1.3 | 1.5 | 1.4 | 1.6 |
| Zfp473 | 0.1 | 0.2 | 0.3 | 0.1 | 0.3 | 0.2 | 0.2 | 0.2 | 0.2 | 0.2 | 0.2 | 0.2 |
| Zfp474 | 0.2 | 0.1 | 0.1 | 0.1 | 0.2 | 0.1 | 0.2 | 0.0 | 0.2 | 0.3 | 0.2 | 0.2 |
| Zfp488 | 7.1 | 10.0 | 7.9 | 5.8 | 8.6 | 6.7 | 8.3 | 6.2 | 5.8 | 7.1 | 6.5 | 5.5 |
| Zfp493 | 0.6 | 0.4 | 0.5 | 0.6 | 0.4 | 0.7 | 0.4 | 0.4 | 0.5 | 0.6 | 0.5 | 0.7 |
| Zfp503 | 1.1 | 1.3 | 2.1 | 1.0 | 1.3 | 1.3 | 1.4 | 1.7 | 2.0 | 4.3 | 3.1 | 2.5 |
| Zfp507 | 6.0 | 6.1 | 6.9 | 6.0 | 5.2 | 6.3 | 5.6 | 6.5 | 6.1 | 6.1 | 6.6 | 6.2 |
| Zfp51 | 4.1 | 3.6 | 3.9 | 4.0 | 3.4 | 5.0 | 3.1 | 3.6 | 3.5 | 4.4 | 4.4 | 4.2 |
| Zfp511 | 7.0 | 7.0 | 6.7 | 7.9 | 7.3 | 7.5 | 7.7 | 6.6 | 7.5 | 7.4 | 9.1 | 8.2 |
| Zfp512 | 7.2 | 7.6 | 7.7 | 7.4 | 7.0 | 8.5 | 5.3 | 7.2 | 6.1 | 6.5 | 6.2 | 6.6 |
| Zfp513 | 3.6 | 3.1 | 4.5 | 3.6 | 3.6 | 3.5 | 3.7 | 3.9 | 3.8 | 3.3 | 3.6 | 3.5 |
| Zfp516 | 4.8 | 5.5 | 4.9 | 3.9 | 4.6 | 4.8 | 4.1 | 5.2 | 5.0 | 6.3 | 5.3 | 5.0 |
| Zfp518a | 2.6 | 2.8 | 3.2 | 2.9 | 2.9 | 3.2 | 2.4 | 3.0 | 2.5 | 2.9 | 3.4 | 3.1 |
| Zfp518b | 4.7 | 4.2 | 4.7 | 4.0 | 3.8 | 4.6 | 4.1 | 4.4 | 4.1 | 3.6 | 4.3 | 3.9 |
| Zfp52 | 4.1 | 4.5 | 4.9 | 3.7 | 4.5 | 4.7 | 5.1 | 4.2 | 4.7 | 7.3 | 5.7 | 6.3 |
| Zfp521 | 10.7 | 14.2 | 18.0 | 11.1 | 10.1 | 11.6 | 14.4 | 21.9 | 15.4 | 8.1 | 12.5 | 11.1 |

| | | | | | | | | | | | | |
|---|---|---|---|---|---|---|---|---|---|---|---|---|
| Zfp523 | 5.2 | 5.7 | 5.5 | 4.8 | 4.9 | 5.7 | 5.9 | 5.5 | 5.6 | 4.8 | 4.9 | 5.1 |
| Zfp524 | 3.9 | 4.3 | 4.0 | 3.6 | 2.9 | 3.1 | 4.4 | 5.9 | 3.6 | 4.6 | 5.5 | 4.8 |
| Zfp526 | 2.8 | 3.6 | 3.8 | 3.6 | 3.0 | 3.3 | 4.3 | 4.1 | 3.6 | 2.9 | 4.0 | 3.5 |
| Zfp53 | 2.7 | 2.7 | 2.6 | 2.1 | 2.7 | 2.5 | 2.5 | 2.3 | 3.1 | 3.3 | 3.2 | 3.9 |
| Zfp532 | 4.3 | 5.2 | 6.5 | 3.0 | 3.5 | 3.8 | 4.8 | 5.9 | 5.4 | 6.6 | 5.9 | 5.6 |
| Zfp534 | 0.0 | 0.0 | 0.0 | 0.0 | 0.1 | 0.1 | 0.0 | 0.1 | 0.0 | 0.0 | 0.0 | 0.0 |
| Zfp536 | 0.0 | 0.0 | 0.0 | 0.0 | 0.0 | 0.0 | 0.0 | 0.0 | 0.0 | 0.0 | 0.0 | 0.0 |
| Zfp54 | 1.9 | 3.0 | 3.0 | 1.5 | 2.4 | 2.3 | 1.9 | 1.9 | 2.1 | 2.3 | 1.9 | 2.4 |
| Zfp541 | 0.0 | 0.0 | 0.0 | 0.0 | 0.0 | 0.0 | 0.0 | 0.0 | 0.0 | 0.0 | 0.0 | 0.0 |
| Zfp551 | 1.5 | 1.6 | 1.2 | 1.6 | 1.1 | 1.9 | 1.3 | 1.4 | 1.6 | 1.9 | 1.6 | 1.8 |
| Zfp553 | 3.7 | 3.8 | 4.0 | 3.5 | 3.2 | 3.8 | 3.2 | 3.7 | 3.2 | 4.4 | 4.1 | 3.6 |
| Zfp558 | 0.9 | 0.8 | 1.0 | 0.7 | 0.8 | 0.9 | 0.6 | 0.7 | 0.6 | 0.8 | 0.9 | 0.8 |
| Zfp560 | 1.4 | 1.4 | 1.2 | 1.4 | 1.3 | 1.6 | 1.3 | 1.4 | 1.4 | 1.3 | 1.7 | 1.2 |
| Zfp563 | 0.8 | 0.8 | 0.9 | 0.9 | 0.9 | 1.0 | 0.7 | 0.9 | 1.0 | 1.0 | 1.0 | 1.1 |
| Zfp566 | 0.9 | 0.7 | 0.8 | 1.4 | 0.9 | 1.1 | 0.5 | 0.7 | 0.8 | 0.9 | 1.4 | 1.0 |
| Zfp568 | 2.8 | 2.5 | 2.1 | 2.7 | 2.6 | 2.1 | 2.8 | 2.2 | 3.1 | 2.8 | 2.9 | 2.3 |
| Zfp57 | 4.5 | 3.1 | 2.6 | 2.3 | 2.2 | 2.4 | 1.9 | 3.2 | 3.0 | 4.8 | 3.0 | 2.5 |
| Zfp574 | 2.1 | 2.7 | 2.3 | 1.8 | 2.6 | 2.3 | 3.0 | 3.0 | 3.0 | 2.8 | 2.7 | 2.1 |
| Zfp575 | 0.4 | 0.4 | 0.4 | 0.5 | 0.5 | 0.4 | 0.4 | 0.6 | 0.4 | 0.2 | 0.4 | 0.2 |
| Zfp579 | 4.9 | 7.1 | 5.0 | 2.8 | 3.1 | 3.2 | 4.5 | 5.2 | 4.2 | 7.8 | 6.9 | 4.0 |
| Zfp58 | 2.3 | 2.1 | 2.3 | 2.3 | 2.2 | 2.0 | 1.7 | 2.1 | 2.2 | 2.4 | 1.8 | 2.0 |
| Zfp580 | 0.8 | 0.5 | 1.2 | 1.1 | 0.8 | 0.9 | 0.6 | 1.1 | 1.1 | 1.0 | 1.3 | 0.5 |
| Zfp583 | 1.6 | 1.6 | 2.4 | 2.0 | 1.3 | 2.1 | 1.7 | 1.7 | 1.5 | 1.6 | 2.0 | 1.5 |
| Zfp59 | 2.3 | 2.2 | 2.3 | 1.8 | 1.9 | 1.9 | 1.5 | 1.7 | 1.6 | 2.3 | 2.0 | 2.1 |
| Zfp592 | 3.2 | 3.7 | 4.1 | 2.9 | 3.6 | 3.2 | 3.9 | 4.2 | 3.5 | 2.9 | 3.1 | 2.9 |
| Zfp593 | 3.4 | 3.1 | 2.3 | 2.7 | 3.6 | 4.0 | 3.7 | 3.2 | 3.7 | 3.6 | 4.2 | 3.0 |
| Zfp595 | 1.3 | 1.7 | 1.7 | 1.4 | 1.6 | 1.1 | 1.2 | 1.2 | 1.3 | 1.6 | 1.6 | 1.7 |
| Zfp597 | 2.8 | 3.2 | 3.3 | 2.4 | 3.0 | 2.4 | 2.7 | 2.6 | 3.1 | 3.5 | 3.1 | 3.3 |
| Zfp598 | 6.4 | 7.0 | 6.4 | 6.3 | 6.6 | 7.3 | 7.8 | 7.6 | 8.1 | 6.7 | 6.9 | 6.4 |
| Zfp599 | 0.9 | 0.6 | 0.7 | 0.5 | 0.5 | 0.6 | 0.8 | 0.4 | 0.7 | 0.7 | 1.2 | 0.8 |
| Zfp60 | 4.0 | 4.5 | 4.6 | 4.2 | 3.7 | 4.3 | 3.7 | 5.1 | 4.6 | 5.1 | 5.2 | 5.2 |
| Zfp600 | 0.2 | 0.2 | 0.1 | 0.2 | 0.1 | 0.2 | 0.1 | 0.1 | 0.1 | 0.1 | 0.1 | 0.1 |
| Zfp605 | 2.7 | 2.3 | 2.7 | 1.9 | 1.6 | 2.6 | 1.7 | 2.3 | 1.9 | 2.2 | 2.9 | 2.8 |
| Zfp606 | 2.0 | 2.0 | 2.4 | 1.3 | 2.1 | 2.4 | 1.9 | 2.1 | 2.4 | 2.5 | 2.4 | 2.4 |
| Zfp607 | 1.6 | 1.8 | 1.7 | 1.2 | 2.0 | 1.4 | 1.4 | 1.8 | 1.7 | 1.7 | 1.6 | 1.7 |
| Zfp608 | 1.1 | 1.5 | 2.7 | 1.1 | 1.6 | 0.9 | 1.6 | 2.4 | 2.3 | 2.0 | 2.0 | 2.3 |
| Zfp609 | 1.3 | 1.8 | 1.6 | 1.2 | 1.4 | 1.3 | 1.5 | 1.4 | 1.4 | 1.2 | 1.4 | 1.3 |
| Zfp61 | 2.5 | 2.7 | 3.5 | 2.5 | 2.6 | 2.8 | 2.3 | 2.9 | 2.8 | 3.2 | 2.9 | 2.8 |
| Zfp612 | 0.6 | 0.7 | 0.6 | 0.5 | 0.3 | 1.0 | 0.4 | 0.3 | 0.3 | 1.0 | 0.8 | 0.5 |
| Zfp617 | 7.4 | 6.3 | 7.1 | 5.8 | 6.2 | 6.4 | 5.7 | 6.9 | 6.0 | 6.9 | 7.7 | 7.4 |
| Zfp618 | 0.6 | 0.6 | 0.6 | 0.9 | 1.4 | 0.7 | 0.8 | 0.5 | 0.7 | 0.5 | 0.6 | 0.8 |
| Zfp619 | 0.5 | 0.4 | 0.4 | 0.5 | 0.5 | 0.3 | 0.6 | 0.6 | 0.5 | 0.4 | 0.3 | 0.5 |
| Zfp62 | 8.6 | 10.0 | 11.7 | 9.0 | 8.2 | 9.4 | 8.0 | 10.3 | 9.3 | 10.6 | 11.3 | 11.1 |
| Zfp622 | 10.3 | 9.0 | 8.6 | 9.9 | 8.5 | 9.0 | 9.3 | 10.1 | 9.2 | 12.5 | 10.1 | 11.5 |
| Zfp623 | 8.1 | 8.9 | 8.8 | 8.4 | 7.3 | 9.0 | 8.0 | 8.5 | 7.9 | 8.9 | 8.1 | 8.9 |
| Zfp628 | 0.4 | 0.3 | 0.5 | 0.4 | 0.4 | 0.4 | 0.4 | 0.5 | 0.4 | 0.4 | 0.4 | 0.3 |
| Zfp629 | 2.9 | 3.5 | 3.7 | 2.8 | 2.8 | 3.4 | 3.3 | 3.5 | 3.3 | 3.2 | 2.9 | 3.3 |
| Zfp637 | 21.5 | 18.0 | 17.1 | 16.6 | 15.4 | 16.9 | 16.8 | 21.1 | 19.8 | 21.2 | 21.5 | 18.9 |
| Zfp639 | 10.4 | 11.2 | 9.7 | 12.8 | 10.8 | 11.2 | 8.6 | 10.5 | 11.0 | 9.7 | 12.1 | 11.8 |
| Zfp64 | 6.3 | 6.3 | 5.5 | 7.3 | 6.0 | 6.0 | 6.9 | 6.6 | 5.8 | 5.8 | 6.6 | 5.7 |
| Zfp641 | 0.8 | 0.8 | 0.8 | 1.0 | 0.6 | 0.9 | 0.7 | 0.7 | 0.7 | 0.5 | 0.6 | 0.7 |
| Zfp644 | 11.5 | 11.2 | 11.1 | 11.8 | 10.3 | 11.4 | 11.4 | 12.0 | 12.7 | 12.4 | 15.2 | 13.9 |
| Zfp646 | 5.4 | 5.9 | 6.4 | 5.9 | 5.3 | 6.0 | 6.2 | 6.5 | 5.2 | 4.9 | 5.2 | 6.1 |
| Zfp647 | 1.2 | 1.2 | 1.3 | 1.2 | 1.0 | 1.2 | 1.1 | 1.5 | 1.2 | 1.4 | 1.7 | 1.0 |
| Zfp651 | 6.7 | 4.9 | 4.8 | 5.6 | 4.1 | 6.2 | 5.0 | 5.0 | 4.9 | 6.3 | 5.4 | 5.1 |
| Zfp652 | 16.0 | 14.8 | 14.1 | 21.6 | 14.4 | 19.4 | 14.5 | 12.5 | 10.6 | 11.4 | 14.0 | 13.4 |
| Zfp653 | 1.2 | 1.2 | 1.0 | 1.5 | 1.3 | 1.5 | 1.5 | 1.7 | 1.4 | 1.1 | 1.4 | 1.2 |

| | | | | | | | | | | | | |
|---|---|---|---|---|---|---|---|---|---|---|---|---|
| Zfp654 | 0.9 | 1.4 | 1.8 | 1.2 | 1.5 | 1.1 | 1.1 | 1.3 | 1.5 | 1.9 | 1.7 | 2.0 |
| Zfp655 | 13.5 | 12.7 | 10.7 | 12.1 | 11.6 | 13.2 | 11.4 | 10.6 | 11.3 | 13.8 | 12.5 | 13.8 |
| Zfp658 | 1.0 | 0.9 | 1.1 | 0.8 | 0.8 | 0.7 | 0.9 | 1.2 | 0.9 | 1.1 | 1.0 | 1.1 |
| Zfp661 | 1.2 | 1.3 | 1.4 | 1.3 | 1.3 | 1.4 | 1.4 | 1.3 | 1.0 | 0.9 | 1.5 | 1.1 |
| Zfp664 | 16.0 | 15.9 | 14.7 | 17.9 | 14.8 | 18.7 | 13.7 | 13.8 | 13.0 | 14.9 | 15.8 | 13.5 |
| Zfp667 | 1.0 | 1.2 | 1.0 | 0.8 | 0.9 | 1.4 | 0.9 | 1.3 | 1.1 | 1.2 | 0.9 | 1.0 |
| Zfp668 | 1.6 | 1.7 | 1.8 | 1.7 | 1.7 | 1.9 | 2.2 | 2.0 | 2.1 | 1.8 | 1.9 | 1.8 |
| Zfp672 | 7.6 | 8.3 | 9.1 | 6.7 | 6.8 | 7.7 | 6.0 | 7.2 | 9.4 | 8.1 | 7.0 | 6.1 |
| Zfp677 | 2.5 | 2.9 | 3.0 | 2.8 | 2.7 | 3.3 | 1.9 | 2.6 | 2.2 | 3.4 | 3.5 | 3.7 |
| Zfp68 | 9.8 | 10.4 | 11.6 | 9.3 | 8.7 | 10.1 | 8.5 | 11.0 | 10.8 | 10.0 | 10.7 | 12.0 |
| Zfp687 | 2.7 | 3.1 | 2.7 | 2.4 | 2.6 | 2.7 | 2.9 | 3.1 | 2.5 | 2.5 | 2.6 | 2.1 |
| Zfp688 | 6.6 | 6.5 | 6.8 | 6.3 | 5.6 | 7.1 | 4.7 | 5.3 | 6.4 | 6.1 | 6.8 | 6.0 |
| Zfp689 | 1.1 | 1.0 | 1.1 | 1.0 | 1.1 | 1.1 | 1.5 | 1.3 | 1.4 | 1.0 | 1.1 | 0.7 |
| Zfp69 | 0.3 | 0.2 | 0.4 | 0.2 | 0.3 | 0.4 | 0.3 | 0.4 | 0.2 | 0.2 | 0.2 | 0.3 |
| Zfp691 | 1.6 | 1.5 | 1.4 | 2.1 | 0.9 | 1.6 | 2.0 | 1.5 | 1.6 | 1.8 | 1.9 | 1.4 |
| Zfp692 | 2.8 | 3.2 | 3.2 | 3.1 | 2.5 | 3.1 | 2.9 | 2.4 | 3.1 | 2.2 | 2.4 | 2.2 |
| Zfp697 | 3.6 | 4.1 | 4.0 | 3.8 | 3.8 | 2.7 | 4.2 | 3.3 | 3.5 | 6.0 | 5.3 | 5.6 |
| Zfp7 | 1.7 | 1.1 | 1.7 | 2.2 | 1.9 | 1.7 | 2.3 | 1.6 | 1.8 | 2.3 | 2.4 | 1.8 |
| Zfp703 | 0.3 | 0.3 | 0.2 | 0.5 | 0.6 | 0.5 | 0.4 | 0.5 | 0.4 | 0.5 | 0.3 | 0.1 |
| Zfp704 | 2.8 | 3.1 | 3.1 | 2.6 | 2.7 | 2.5 | 2.5 | 2.2 | 2.1 | 2.5 | 2.4 | 2.4 |
| Zfp706 | 8.3 | 8.9 | 8.1 | 8.0 | 8.6 | 8.1 | 8.1 | 9.5 | 9.2 | 11.4 | 10.1 | 9.8 |
| Zfp707 | 1.2 | 1.4 | 1.2 | 1.1 | 1.2 | 1.3 | 0.8 | 1.3 | 1.3 | 1.4 | 1.3 | 1.1 |
| Zfp708 | 0.9 | 1.0 | 1.2 | 0.8 | 1.3 | 1.5 | 1.1 | 1.1 | 1.2 | 1.4 | 1.5 | 1.5 |
| Zfp709 | 2.0 | 2.4 | 2.3 | 2.4 | 2.2 | 2.3 | 1.6 | 2.2 | 2.2 | 2.6 | 2.2 | 2.8 |
| Zfp710 | 2.9 | 2.6 | 2.9 | 4.7 | 5.6 | 2.6 | 3.4 | 2.6 | 3.0 | 2.6 | 2.8 | 2.6 |
| Zfp712 | 0.7 | 0.5 | 0.7 | 0.6 | 0.8 | 0.8 | 0.6 | 0.6 | 0.8 | 0.7 | 0.8 | 1.0 |
| Zfp715 | 5.9 | 6.1 | 5.6 | 5.4 | 6.9 | 6.0 | 5.8 | 5.0 | 5.4 | 5.7 | 5.6 | 5.9 |
| Zfp719 | 3.8 | 3.5 | 3.5 | 3.3 | 3.6 | 3.7 | 3.2 | 3.7 | 3.9 | 4.0 | 3.9 | 3.7 |
| Zfp71-rs1 | 3.3 | 3.9 | 4.0 | 3.4 | 3.5 | 4.0 | 2.6 | 3.6 | 3.2 | 3.9 | 3.7 | 3.9 |
| Zfp72 | 1.3 | 1.3 | 1.7 | 1.6 | 1.5 | 1.7 | 1.5 | 1.5 | 1.4 | 1.5 | 1.5 | 1.7 |
| Zfp738 | 2.8 | 2.6 | 3.1 | 2.9 | 2.9 | 3.2 | 2.2 | 2.7 | 2.9 | 3.3 | 3.3 | 3.1 |
| Zfp74 | 2.9 | 3.1 | 3.3 | 2.7 | 2.4 | 3.5 | 2.1 | 3.0 | 2.9 | 2.3 | 2.8 | 2.9 |
| Zfp740 | 11.2 | 11.9 | 10.2 | 9.9 | 10.5 | 10.8 | 10.3 | 9.3 | 11.7 | 10.7 | 10.2 | 10.8 |
| Zfp746 | 4.8 | 5.4 | 4.9 | 5.2 | 5.0 | 5.4 | 5.2 | 5.2 | 5.4 | 5.4 | 5.7 | 4.9 |
| Zfp747 | 1.6 | 1.6 | 1.9 | 1.9 | 2.0 | 1.7 | 2.1 | 2.0 | 1.9 | 2.0 | 1.8 | 2.2 |
| Zfp748 | 2.7 | 2.6 | 3.7 | 3.1 | 2.7 | 3.9 | 2.8 | 3.2 | 3.2 | 3.1 | 4.2 | 2.9 |
| Zfp750 | 0.1 | 0.1 | 0.1 | 0.0 | 0.1 | 0.0 | 0.2 | 0.1 | 0.0 | 0.1 | 0.1 | 0.0 |
| Zfp758 | 1.4 | 3.0 | 4.0 | 2.5 | 3.1 | 2.9 | 2.4 | 3.1 | 3.5 | 3.9 | 4.4 | 4.5 |
| Zfp759 | 1.7 | 1.7 | 2.2 | 1.4 | 1.8 | 1.8 | 1.2 | 1.7 | 1.5 | 1.6 | 1.9 | 2.1 |
| Zfp760 | 2.5 | 2.9 | 2.6 | 2.1 | 2.6 | 2.9 | 1.9 | 2.2 | 2.6 | 2.9 | 3.1 | 2.6 |
| Zfp763 | 1.2 | 1.1 | 1.4 | 0.9 | 1.0 | 1.4 | 1.0 | 1.2 | 1.6 | 1.7 | 1.4 | 2.0 |
| Zfp764 | 2.1 | 2.5 | 3.2 | 2.9 | 2.9 | 2.7 | 3.3 | 3.2 | 2.9 | 2.9 | 2.9 | 3.4 |
| Zfp768 | 4.7 | 4.6 | 5.4 | 5.5 | 5.3 | 6.0 | 6.1 | 5.7 | 6.5 | 5.3 | 5.2 | 5.6 |
| Zfp770 | 1.7 | 2.1 | 2.3 | 2.4 | 2.4 | 2.9 | 1.8 | 1.8 | 2.1 | 2.5 | 2.4 | 1.9 |
| Zfp771 | 13.8 | 9.4 | 9.7 | 7.0 | 7.9 | 7.0 | 10.7 | 14.0 | 12.9 | 16.3 | 12.8 | 14.2 |
| Zfp772 | 1.6 | 2.0 | 1.9 | 1.2 | 1.9 | 1.9 | 1.6 | 1.8 | 1.7 | 1.5 | 1.5 | 1.7 |
| Zfp773 | 0.0 | 0.0 | 0.1 | 0.2 | 0.1 | 0.1 | 0.0 | 0.1 | 0.0 | 0.0 | 0.1 | 0.1 |
| Zfp775 | 1.6 | 1.8 | 1.5 | 1.3 | 0.9 | 1.5 | 1.3 | 1.2 | 1.2 | 1.0 | 1.2 | 1.2 |
| Zfp777 | 4.1 | 4.4 | 4.5 | 4.4 | 4.2 | 4.5 | 5.3 | 5.2 | 5.1 | 4.5 | 4.9 | 5.3 |
| Zfp78 | 0.5 | 0.6 | 0.8 | 0.7 | 0.6 | 0.6 | 0.6 | 0.6 | 0.6 | 0.7 | 0.8 | 0.9 |
| Zfp780b | 2.9 | 3.6 | 3.0 | 3.0 | 3.1 | 3.0 | 2.2 | 2.7 | 2.6 | 2.8 | 2.8 | 3.1 |
| Zfp781 | 2.2 | 2.4 | 2.4 | 2.4 | 2.0 | 2.2 | 1.7 | 2.2 | 1.9 | 2.6 | 2.2 | 2.2 |
| Zfp783 | 0.6 | 0.5 | 0.6 | 0.2 | 0.3 | 0.4 | 0.3 | 0.7 | 0.4 | 0.4 | 0.3 | 0.3 |
| Zfp784 | 0.6 | 0.8 | 0.8 | 0.8 | 0.7 | 0.9 | 0.7 | 1.0 | 0.6 | 0.9 | 1.2 | 0.8 |
| Zfp786 | 0.2 | 0.2 | 0.2 | 0.3 | 0.2 | 0.2 | 0.2 | 0.2 | 0.2 | 0.2 | 0.3 | 0.2 |
| Zfp787 | 3.9 | 3.4 | 3.0 | 3.5 | 3.8 | 3.7 | 3.5 | 3.3 | 3.3 | 3.7 | 2.9 | 3.1 |
| Zfp788 | 2.0 | 1.6 | 1.8 | 1.5 | 1.6 | 2.0 | 1.6 | 1.6 | 1.6 | 2.0 | 1.8 | 2.1 |

| | | | | | | | | | | | | |
|---|---|---|---|---|---|---|---|---|---|---|---|---|
| Zfp790 | 4.8 | 5.2 | 5.0 | 5.7 | 4.3 | 4.9 | 4.1 | 4.2 | 4.6 | 5.0 | 5.4 | 5.7 |
| Zfp791 | 0.8 | 0.7 | 0.9 | 0.7 | 0.5 | 0.8 | 0.6 | 0.6 | 0.6 | 0.5 | 0.5 | 0.7 |
| Zfp799 | 2.8 | 2.9 | 3.7 | 2.5 | 2.7 | 3.0 | 2.7 | 3.6 | 3.2 | 3.5 | 3.6 | 3.1 |
| Zfp800 | 3.6 | 3.7 | 3.5 | 4.1 | 3.4 | 3.8 | 3.4 | 3.3 | 3.1 | 3.5 | 4.3 | 3.7 |
| Zfp804b | 0.0 | 0.0 | 0.0 | 0.0 | 0.0 | 0.0 | 0.0 | 0.0 | 0.0 | 0.0 | 0.0 | 0.0 |
| Zfp808 | 1.1 | 1.7 | 2.2 | 1.4 | 1.6 | 1.8 | 1.2 | 1.5 | 1.4 | 1.5 | 2.4 | 1.7 |
| Zfp809 | 18.6 | 13.4 | 14.4 | 12.1 | 13.9 | 11.5 | 18.1 | 12.3 | 22.5 | 20.7 | 22.3 | 27.9 |
| Zfp81 | 1.9 | 3.1 | 3.0 | 2.4 | 2.9 | 2.3 | 2.2 | 2.7 | 2.8 | 3.0 | 3.1 | 2.7 |
| Zfp810 | 4.1 | 4.1 | 4.7 | 3.3 | 3.5 | 2.7 | 3.7 | 4.8 | 4.8 | 4.4 | 5.0 | 5.3 |
| Zfp811 | 0.2 | 0.4 | 0.2 | 0.0 | 0.6 | 0.1 | 0.2 | 0.1 | 0.2 | 0.2 | 0.3 | 0.2 |
| Zfp82 | 1.7 | 2.4 | 2.0 | 2.0 | 2.2 | 2.0 | 1.4 | 2.2 | 2.2 | 1.4 | 1.8 | 1.7 |
| Zfp820 | 1.0 | 1.9 | 2.4 | 1.8 | 1.6 | 2.3 | 1.6 | 2.0 | 1.9 | 1.5 | 2.4 | 2.6 |
| Zfp821 | 2.2 | 2.9 | 3.2 | 2.9 | 2.5 | 2.7 | 2.6 | 3.4 | 3.1 | 2.7 | 2.5 | 2.9 |
| Zfp825 | 6.7 | 9.2 | 9.1 | 6.2 | 7.0 | 7.4 | 5.9 | 7.7 | 6.2 | 7.2 | 6.8 | 7.0 |
| Zfp827 | 0.4 | 0.2 | 0.4 | 0.4 | 0.3 | 0.4 | 0.4 | 0.7 | 0.5 | 0.8 | 0.8 | 0.5 |
| Zfp828 | 7.7 | 6.6 | 7.8 | 8.2 | 7.0 | 7.4 | 7.7 | 8.0 | 6.8 | 8.1 | 8.7 | 7.7 |
| Zfp830 | 3.9 | 4.6 | 4.8 | 4.7 | 4.1 | 4.9 | 4.2 | 4.1 | 3.9 | 4.6 | 4.9 | 5.1 |
| Zfp839 | 2.8 | 3.0 | 2.9 | 2.5 | 2.7 | 2.2 | 3.0 | 2.3 | 2.4 | 2.5 | 2.3 | 2.6 |
| Zfp84 | 2.6 | 2.8 | 3.2 | 3.0 | 3.0 | 2.3 | 3.1 | 3.4 | 3.1 | 3.8 | 3.7 | 3.6 |
| Zfp846 | 2.0 | 1.8 | 1.7 | 2.2 | 1.6 | 2.1 | 1.7 | 1.9 | 1.8 | 1.7 | 1.8 | 2.2 |
| Zfp85-rs1 | 1.2 | 1.1 | 1.8 | 1.0 | 1.1 | 1.7 | 1.4 | 1.5 | 1.2 | 1.2 | 1.0 | 1.8 |
| Zfp862 | 0.8 | 0.8 | 0.9 | 0.6 | 0.7 | 0.6 | 0.7 | 0.9 | 0.8 | 0.6 | 0.6 | 0.8 |
| Zfp865 | 1.3 | 1.5 | 2.0 | 1.9 | 1.3 | 1.8 | 1.9 | 2.0 | 1.8 | 1.4 | 1.6 | 1.3 |
| Zfp866 | 3.6 | 3.5 | 3.9 | 3.5 | 3.5 | 3.0 | 3.4 | 4.0 | 3.5 | 3.2 | 4.0 | 4.0 |
| Zfp867 | 4.0 | 3.4 | 3.9 | 3.0 | 2.9 | 3.1 | 2.4 | 3.5 | 3.4 | 3.3 | 3.6 | 3.9 |
| Zfp868 | 2.4 | 2.6 | 2.7 | 2.8 | 2.7 | 2.3 | 2.5 | 3.1 | 2.8 | 3.6 | 2.7 | 3.3 |
| Zfp869 | 8.1 | 7.0 | 7.9 | 8.3 | 7.0 | 7.3 | 7.1 | 7.2 | 7.3 | 8.6 | 7.9 | 8.0 |
| Zfp87 | 3.5 | 3.5 | 3.5 | 2.9 | 3.8 | 3.8 | 3.6 | 4.1 | 3.3 | 4.0 | 3.5 | 3.9 |
| Zfp870 | 1.7 | 1.2 | 1.6 | 1.2 | 1.4 | 1.6 | 1.5 | 1.5 | 1.3 | 1.5 | 1.5 | 1.6 |
| Zfp871 | 3.3 | 4.1 | 4.5 | 3.9 | 4.0 | 3.8 | 3.4 | 3.6 | 3.4 | 3.8 | 3.9 | 3.9 |
| Zfp872 | 0.2 | 0.1 | 0.1 | 0.1 | 0.1 | 0.1 | 0.2 | 0.1 | 0.1 | 0.1 | 0.1 | 0.1 |
| Zfp873 | 1.1 | 1.2 | 1.0 | 1.7 | 1.0 | 1.1 | 0.9 | 1.1 | 1.1 | 1.2 | 1.4 | 1.1 |
| Zfp874a | 7.6 | 6.9 | 6.8 | 7.4 | 6.4 | 6.4 | 5.0 | 4.5 | 5.5 | 8.5 | 6.8 | 6.6 |
| Zfp874b | 3.8 | 3.4 | 2.7 | 4.0 | 3.6 | 3.8 | 1.9 | 2.3 | 2.4 | 3.6 | 3.0 | 3.0 |
| Zfp879 | 0.3 | 0.2 | 0.3 | 0.2 | 0.2 | 0.6 | 0.2 | 0.3 | 0.1 | 0.3 | 0.4 | 0.3 |
| Zfp882 | 0.5 | 0.5 | 0.6 | 0.5 | 0.5 | 0.6 | 0.6 | 0.7 | 0.6 | 0.5 | 0.7 | 0.7 |
| Zfp9 | 5.3 | 6.0 | 6.3 | 6.2 | 5.5 | 6.0 | 5.8 | 7.3 | 6.7 | 5.7 | 7.7 | 7.3 |
| Zfp90 | 4.2 | 4.0 | 4.1 | 4.2 | 3.9 | 4.4 | 4.1 | 4.6 | 3.8 | 4.6 | 5.2 | 4.8 |
| Zfp91 | 20.9 | 18.7 | 21.0 | 24.9 | 19.7 | 21.2 | 20.7 | 18.1 | 17.6 | 17.9 | 20.2 | 22.0 |
| Zfp92 | 0.0 | 0.0 | 0.0 | 0.0 | 0.0 | 0.0 | 0.0 | 0.0 | 0.0 | 0.0 | 0.0 | 0.0 |
| Zfp93 | 2.7 | 3.2 | 3.1 | 3.0 | 2.4 | 3.2 | 2.4 | 2.7 | 2.8 | 2.7 | 2.9 | 3.2 |
| Zfp930 | 3.2 | 3.2 | 2.8 | 3.0 | 3.1 | 3.7 | 2.1 | 2.8 | 2.3 | 2.8 | 3.7 | 3.4 |
| Zfp931 | 3.1 | 3.0 | 2.2 | 4.0 | 2.7 | 3.5 | 2.8 | 2.3 | 2.6 | 4.3 | 3.9 | 3.5 |
| Zfp932 | 7.0 | 7.9 | 6.7 | 5.6 | 6.2 | 7.9 | 4.7 | 5.5 | 5.3 | 6.4 | 8.6 | 6.4 |
| Zfp933 | 3.9 | 3.3 | 3.5 | 4.9 | 3.3 | 4.1 | 3.0 | 3.2 | 2.6 | 3.1 | 3.4 | 3.3 |
| Zfp934 | 1.3 | 2.0 | 2.6 | 1.2 | 2.0 | 2.2 | 1.7 | 1.9 | 1.6 | 1.8 | 2.5 | 2.0 |
| Zfp935 | 3.6 | 4.2 | 4.5 | 3.7 | 4.5 | 3.8 | 3.1 | 4.5 | 4.4 | 4.4 | 4.6 | 5.2 |
| Zfp936 | 0.0 | 0.0 | 0.0 | 0.0 | 0.0 | 0.0 | 0.0 | 0.0 | 0.0 | 0.0 | 0.0 | 0.0 |
| Zfp937 | 2.2 | 2.4 | 2.9 | 2.1 | 1.9 | 2.4 | 2.1 | 2.8 | 2.2 | 2.6 | 2.7 | 2.8 |
| Zfp938 | 3.0 | 2.8 | 2.7 | 3.9 | 3.2 | 3.0 | 2.6 | 2.6 | 3.1 | 3.7 | 3.5 | 3.7 |
| Zfp939 | 1.1 | 1.0 | 0.9 | 0.9 | 0.9 | 0.9 | 0.5 | 0.9 | 0.7 | 0.8 | 1.1 | 0.8 |
| Zfp94 | 1.1 | 1.1 | 1.0 | 1.8 | 1.0 | 1.9 | 0.9 | 1.2 | 0.9 | 1.2 | 0.9 | 1.2 |
| Zfp940 | 0.3 | 0.5 | 0.5 | 0.5 | 0.4 | 0.4 | 0.3 | 0.4 | 0.3 | 0.4 | 0.5 | 0.6 |
| Zfp941 | 0.4 | 0.2 | 0.1 | 0.3 | 0.2 | 0.3 | 0.3 | 0.2 | 0.1 | 0.4 | 0.3 | 0.2 |
| Zfp942 | 3.3 | 4.4 | 4.9 | 4.6 | 4.9 | 5.3 | 4.0 | 4.1 | 4.3 | 4.7 | 5.1 | 5.5 |
| Zfp943 | 3.3 | 4.1 | 5.1 | 3.5 | 4.5 | 3.9 | 3.5 | 4.3 | 4.2 | 4.3 | 5.8 | 4.9 |
| Zfp944 | 2.9 | 4.3 | 4.3 | 3.7 | 3.8 | 3.7 | 3.3 | 3.5 | 3.3 | 4.3 | 4.1 | 4.1 |

| | | | | | | | | | | | | |
|---|---|---|---|---|---|---|---|---|---|---|---|---|
| Zfp945 | 2.2 | 1.9 | 2.2 | 1.9 | 1.7 | 1.9 | 1.9 | 2.4 | 2.2 | 2.4 | 2.9 | 2.6 |
| Zfp946 | 1.2 | 1.8 | 2.2 | 2.2 | 1.9 | 1.9 | 1.8 | 2.5 | 2.0 | 1.7 | 2.7 | 2.0 |
| Zfp947 | 1.1 | 1.7 | 1.5 | 1.9 | 1.5 | 1.8 | 1.2 | 1.7 | 1.2 | 1.6 | 2.0 | 2.1 |
| Zfp948 | 6.3 | 6.8 | 8.3 | 7.2 | 8.5 | 6.5 | 10.0 | 6.8 | 8.1 | 6.9 | 9.6 | 9.8 |
| Zfp949 | 2.1 | 2.0 | 2.5 | 2.1 | 1.9 | 2.2 | 1.8 | 2.6 | 2.6 | 2.4 | 1.9 | 2.9 |
| Zfp951 | 2.6 | 1.8 | 1.7 | 2.3 | 1.9 | 2.5 | 1.7 | 2.4 | 1.7 | 2.2 | 2.1 | 2.2 |
| Zfp952 | 3.1 | 2.9 | 3.1 | 2.5 | 3.1 | 3.0 | 2.2 | 3.0 | 2.9 | 2.6 | 2.8 | 3.4 |
| Zfp953 | 0.8 | 1.0 | 1.1 | 0.7 | 0.9 | 0.6 | 0.8 | 0.8 | 0.8 | 0.9 | 1.1 | 0.9 |
| Zfp954 | 4.9 | 3.9 | 4.3 | 3.7 | 3.9 | 3.7 | 3.7 | 4.1 | 3.8 | 4.1 | 4.4 | 4.5 |
| Zfp955a | 6.5 | 6.5 | 6.7 | 6.0 | 5.9 | 5.8 | 6.1 | 5.5 | 6.4 | 5.8 | 5.9 | 7.4 |
| Zfp955b | 7.2 | 6.9 | 7.1 | 6.0 | 6.2 | 6.5 | 6.3 | 5.3 | 6.1 | 5.9 | 6.5 | 7.9 |
| Zfp956 | 1.8 | 1.9 | 1.8 | 1.8 | 1.4 | 1.9 | 1.3 | 1.5 | 1.8 | 1.4 | 1.7 | 1.7 |
| Zfp957 | 0.1 | 0.0 | 0.0 | 0.0 | 0.0 | 0.0 | 0.0 | 0.0 | 0.0 | 0.0 | 0.0 | 0.0 |
| Zfp958 | 3.9 | 4.8 | 5.1 | 4.4 | 4.3 | 4.1 | 3.7 | 4.1 | 4.3 | 6.5 | 5.5 | 5.4 |
| Zfp959 | 1.5 | 1.6 | 1.8 | 1.7 | 1.6 | 1.3 | 1.3 | 2.2 | 1.7 | 2.0 | 1.6 | 1.8 |
| Zfp960 | 5.4 | 5.8 | 5.7 | 4.9 | 5.1 | 4.5 | 3.6 | 5.2 | 4.8 | 4.8 | 5.7 | 5.6 |
| Zfp961 | 1.7 | 2.1 | 2.6 | 1.9 | 1.9 | 1.8 | 1.5 | 2.0 | 2.3 | 2.0 | 2.0 | 2.1 |
| Zfp963 | 1.5 | 1.6 | 1.7 | 1.4 | 1.2 | 1.2 | 1.8 | 1.8 | 1.3 | 1.7 | 1.3 | 1.4 |
| Zfp964 | 1.5 | 1.4 | 1.5 | 1.1 | 1.0 | 1.4 | 1.1 | 0.9 | 1.5 | 1.5 | 1.3 | 1.5 |
| Zfp97 | 4.9 | 5.3 | 5.1 | 4.3 | 4.8 | 4.7 | 3.5 | 4.4 | 4.5 | 4.5 | 5.6 | 5.0 |
| Zfpl1 | 15.1 | 14.9 | 13.8 | 16.9 | 12.9 | 15.5 | 14.6 | 16.8 | 15.7 | 13.6 | 13.8 | 15.4 |
| Zfpm1 | 3.6 | 3.9 | 3.6 | 2.5 | 2.4 | 2.8 | 3.4 | 3.2 | 3.1 | 7.7 | 5.4 | 4.9 |
| Zfpm2 | 3.4 | 4.0 | 4.0 | 6.4 | 4.3 | 6.2 | 4.1 | 3.4 | 3.6 | 4.1 | 4.2 | 3.7 |
| Zfr | 29.6 | 30.9 | 30.4 | 33.9 | 28.2 | 31.8 | 30.9 | 33.0 | 26.9 | 33.4 | 39.2 | 36.7 |
| Zfr2 | 1.5 | 1.9 | 2.2 | 1.6 | 0.9 | 2.5 | 1.1 | 1.6 | 1.8 | 1.4 | 1.0 | 1.3 |
| Zfx | 8.6 | 8.3 | 7.2 | 7.6 | 7.3 | 7.9 | 7.0 | 6.5 | 7.5 | 9.0 | 10.0 | 10.1 |
| Zfyve1 | 13.4 | 13.6 | 13.1 | 12.1 | 12.4 | 12.9 | 12.0 | 12.6 | 13.9 | 13.3 | 13.1 | 13.0 |
| Zfyve16 | 10.7 | 9.7 | 8.5 | 9.9 | 9.6 | 9.1 | 8.4 | 8.2 | 9.0 | 11.0 | 11.2 | 10.3 |
| Zfyve19 | 3.4 | 3.9 | 3.7 | 3.9 | 3.9 | 3.8 | 4.2 | 4.3 | 3.9 | 4.0 | 4.5 | 3.9 |
| Zfyve20 | 9.9 | 10.3 | 8.9 | 10.8 | 9.5 | 9.1 | 9.6 | 9.0 | 8.9 | 8.8 | 9.8 | 9.8 |
| Zfyve21 | 12.4 | 15.8 | 12.5 | 9.7 | 12.1 | 13.5 | 12.9 | 15.3 | 16.1 | 14.8 | 12.1 | 13.0 |
| Zfyve26 | 2.6 | 3.2 | 2.9 | 2.8 | 3.0 | 2.9 | 2.5 | 3.0 | 2.7 | 2.3 | 2.2 | 2.1 |
| Zfyve27 | 4.0 | 3.9 | 3.1 | 4.9 | 4.3 | 4.4 | 4.0 | 3.8 | 4.1 | 3.8 | 3.8 | 3.7 |
| Zfyve28 | 0.3 | 0.3 | 0.6 | 0.2 | 0.4 | 0.2 | 0.6 | 1.0 | 1.1 | 0.6 | 0.8 | 0.8 |
| Zfyve9 | 13.3 | 11.4 | 9.8 | 11.9 | 8.8 | 13.4 | 9.3 | 10.5 | 9.3 | 10.1 | 10.8 | 10.7 |
| Zglp1 | 0.1 | 0.0 | 0.1 | 0.0 | 0.1 | 0.1 | 0.1 | 0.1 | 0.0 | 0.1 | 0.0 | 0.0 |
| Zgpat | 10.5 | 9.1 | 8.8 | 9.7 | 8.9 | 10.6 | 7.9 | 8.3 | 10.9 | 11.4 | 8.8 | 8.5 |
| Zhx1 | 24.9 | 24.6 | 22.0 | 30.8 | 21.4 | 25.0 | 22.0 | 19.0 | 19.8 | 21.8 | 24.8 | 25.3 |
| Zhx2 | 6.0 | 8.1 | 9.9 | 6.9 | 6.2 | 6.9 | 6.4 | 8.2 | 7.0 | 4.9 | 5.9 | 6.5 |
| Zhx3 | 4.8 | 5.7 | 6.4 | 5.4 | 4.4 | 4.2 | 5.0 | 5.2 | 4.4 | 4.0 | 4.4 | 5.1 |
| Zic1 | 0.0 | 0.0 | 0.0 | 0.0 | 0.0 | 0.0 | 0.0 | 0.0 | 0.1 | 0.1 | 0.1 | 0.1 |
| Zic3 | 0.2 | 0.1 | 0.0 | 0.0 | 0.0 | 0.0 | 0.0 | 0.0 | 0.0 | 0.0 | 0.0 | 0.0 |
| Zic5 | 0.1 | 0.0 | 0.0 | 0.0 | 0.0 | 0.0 | 0.0 | 0.0 | 0.0 | 0.1 | 0.0 | 0.0 |
| Zik1 | 1.4 | 1.5 | 1.2 | 1.3 | 1.6 | 1.4 | 1.5 | 1.3 | 1.4 | 1.3 | 1.4 | 1.5 |
| Zim1 | 0.0 | 0.0 | 0.0 | 0.0 | 0.0 | 0.0 | 0.0 | 0.0 | 0.0 | 0.2 | 0.2 | 0.1 |
| Zkscan1 | 6.0 | 5.9 | 6.8 | 5.7 | 5.4 | 5.8 | 5.1 | 6.6 | 5.6 | 6.8 | 6.1 | 7.0 |
| Zkscan14 | 3.1 | 3.2 | 2.7 | 3.6 | 3.0 | 4.4 | 2.3 | 2.4 | 2.3 | 3.4 | 3.0 | 3.2 |
| Zkscan16 | 0.0 | 0.1 | 0.0 | 0.0 | 0.0 | 0.1 | 0.0 | 0.1 | 0.0 | 0.0 | 0.0 | 0.0 |
| Zkscan17 | 3.9 | 4.3 | 4.7 | 3.3 | 4.0 | 4.6 | 4.2 | 4.9 | 4.5 | 4.4 | 4.5 | 3.6 |
| Zkscan2 | 0.1 | 0.1 | 0.1 | 0.1 | 0.1 | 0.2 | 0.1 | 0.0 | 0.0 | 0.0 | 0.0 | 0.1 |
| Zkscan3 | 12.3 | 12.7 | 11.8 | 10.0 | 9.6 | 9.9 | 10.0 | 11.8 | 13.3 | 13.9 | 12.4 | 12.4 |
| Zkscan4 | 1.5 | 1.9 | 1.6 | 1.3 | 1.2 | 1.6 | 1.3 | 1.2 | 1.6 | 1.7 | 1.3 | 1.5 |
| Zkscan5 | 3.6 | 3.8 | 4.1 | 4.0 | 3.4 | 3.9 | 4.0 | 4.4 | 3.4 | 4.4 | 4.3 | 4.3 |
| Zkscan6 | 3.6 | 3.5 | 3.4 | 2.6 | 3.9 | 4.3 | 3.5 | 3.5 | 3.5 | 4.9 | 4.2 | 4.2 |
| Zkscan7 | 2.2 | 2.4 | 2.7 | 2.0 | 1.8 | 1.7 | 1.6 | 2.5 | 2.1 | 2.5 | 1.8 | 2.2 |
| Zkscan8 | 3.1 | 3.4 | 3.9 | 3.7 | 3.1 | 3.3 | 3.4 | 3.5 | 3.1 | 3.7 | 3.8 | 3.8 |
| Zmat1 | 1.0 | 1.3 | 1.5 | 1.0 | 0.9 | 1.2 | 1.0 | 1.2 | 1.0 | 1.3 | 1.5 | 1.6 |

| | | | | | | | | | | | | |
|---|---|---|---|---|---|---|---|---|---|---|---|---|
| Zmat2 | 27.7 | 23.2 | 27.6 | 37.6 | 22.7 | 28.7 | 23.6 | 24.4 | 17.9 | 21.4 | 27.3 | 26.8 |
| Zmat3 | 6.1 | 6.6 | 5.7 | 6.5 | 6.7 | 7.5 | 6.4 | 5.3 | 5.9 | 7.1 | 5.7 | 5.2 |
| Zmat4 | 0.0 | 0.0 | 0.0 | 0.0 | 0.0 | 0.0 | 0.0 | 0.0 | 0.0 | 0.0 | 0.0 | 0.1 |
| Zmat5 | 11.3 | 10.6 | 6.8 | 13.0 | 13.4 | 10.3 | 9.2 | 10.9 | 10.1 | 10.0 | 10.8 | 9.3 |
| Zmiz1 | 1.1 | 1.7 | 2.0 | 1.6 | 2.2 | 1.3 | 2.8 | 2.3 | 2.5 | 1.9 | 1.9 | 1.6 |
| Zmiz2 | 1.2 | 2.2 | 1.3 | 1.8 | 2.2 | 1.6 | 1.5 | 1.3 | 1.5 | 1.4 | 1.3 | 0.9 |
| Zmpste24 | 24.1 | 23.8 | 22.1 | 26.3 | 22.9 | 24.2 | 24.0 | 22.3 | 21.3 | 23.4 | 25.2 | 24.6 |
| Zmym1 | 2.5 | 3.4 | 3.6 | 3.4 | 3.6 | 3.3 | 3.6 | 2.9 | 3.0 | 3.2 | 3.4 | 2.9 |
| Zmym2 | 10.9 | 11.8 | 11.8 | 10.9 | 10.8 | 11.3 | 10.3 | 11.3 | 11.7 | 11.6 | 13.0 | 13.4 |
| Zmym3 | 3.5 | 4.5 | 6.4 | 2.5 | 3.9 | 4.3 | 3.9 | 5.3 | 4.7 | 4.6 | 4.7 | 3.8 |
| Zmym4 | 6.0 | 7.0 | 6.5 | 5.5 | 5.3 | 5.4 | 5.8 | 6.1 | 5.8 | 6.4 | 6.3 | 6.8 |
| Zmym5 | 4.5 | 4.9 | 5.4 | 4.4 | 4.3 | 4.5 | 4.4 | 4.7 | 5.0 | 4.8 | 4.7 | 5.3 |
| Zmym6 | 5.5 | 6.3 | 6.1 | 5.1 | 4.9 | 6.5 | 4.2 | 4.8 | 5.1 | 5.7 | 4.9 | 6.0 |
| Zmynd10 | 0.1 | 0.0 | 0.1 | 0.2 | 0.1 | 0.2 | 0.0 | 0.0 | 0.1 | 0.0 | 0.0 | 0.0 |
| Zmynd11 | 23.3 | 21.1 | 22.6 | 26.9 | 19.1 | 24.4 | 22.0 | 22.8 | 21.1 | 21.3 | 24.9 | 25.8 |
| Zmynd12 | 0.3 | 0.2 | 0.1 | 0.6 | 0.2 | 0.5 | 0.1 | 0.2 | 0.1 | 0.2 | 0.1 | 0.5 |
| Zmynd15 | 0.8 | 0.4 | 0.8 | 1.8 | 3.9 | 0.8 | 1.0 | 0.6 | 0.5 | 0.8 | 0.4 | 0.9 |
| Zmynd19 | 11.9 | 9.7 | 9.3 | 9.5 | 10.3 | 9.5 | 10.8 | 10.6 | 10.4 | 9.7 | 11.4 | 8.7 |
| Zmynd8 | 12.1 | 13.5 | 14.3 | 15.8 | 13.5 | 13.5 | 14.0 | 14.2 | 12.5 | 11.8 | 11.9 | 11.0 |
| Znf41-ps | 4.7 | 4.9 | 5.4 | 5.6 | 5.0 | 7.0 | 3.8 | 4.4 | 4.1 | 4.7 | 5.3 | 5.6 |
| Znf512b | 3.2 | 3.6 | 4.0 | 2.5 | 2.9 | 2.5 | 3.6 | 3.1 | 3.9 | 3.4 | 2.9 | 2.8 |
| Znfx1 | 15.7 | 16.5 | 17.4 | 22.8 | 18.3 | 15.7 | 16.4 | 14.6 | 14.2 | 10.7 | 13.6 | 15.2 |
| Znhit1 | 17.4 | 16.6 | 15.1 | 22.8 | 14.9 | 18.4 | 17.0 | 17.6 | 14.7 | 16.7 | 19.7 | 18.5 |
| Znhit2 | 11.6 | 10.2 | 7.7 | 10.9 | 8.4 | 12.0 | 9.6 | 9.4 | 10.3 | 10.6 | 9.3 | 8.6 |
| Znhit3 | 4.8 | 6.2 | 5.3 | 5.5 | 5.5 | 5.1 | 5.0 | 5.1 | 4.7 | 5.0 | 3.7 | 5.0 |
| Znhit6 | 4.3 | 3.7 | 3.4 | 4.3 | 3.6 | 3.9 | 3.1 | 2.3 | 2.8 | 4.0 | 3.7 | 4.1 |
| Znrd1 | 11.5 | 10.5 | 9.6 | 12.3 | 9.5 | 11.9 | 9.9 | 13.4 | 9.7 | 13.5 | 11.0 | 12.8 |
| Znrd1as | 10.5 | 11.5 | 11.3 | 11.2 | 6.9 | 10.7 | 6.8 | 9.2 | 5.0 | 7.0 | 6.9 | 9.1 |
| Znrf1 | 5.8 | 4.6 | 4.3 | 6.0 | 6.2 | 5.8 | 4.4 | 4.0 | 4.4 | 4.3 | 4.3 | 3.4 |
| Znrf2 | 21.1 | 17.2 | 14.4 | 21.8 | 16.3 | 18.3 | 16.0 | 12.6 | 14.8 | 17.4 | 17.5 | 21.6 |
| Znrf3 | 0.9 | 0.8 | 0.8 | 0.7 | 0.7 | 0.8 | 1.0 | 0.7 | 0.7 | 0.8 | 0.7 | 0.8 |
| Zp1 | 0.0 | 0.0 | 0.0 | 0.0 | 0.0 | 0.0 | 0.0 | 0.0 | 0.0 | 0.0 | 0.0 | 0.0 |
| Zp3 | 0.1 | 0.0 | 0.0 | 0.0 | 0.0 | 0.2 | 0.1 | 0.2 | 0.1 | 0.0 | 0.1 | 0.0 |
| Zpbp | 0.1 | 0.1 | 0.0 | 0.1 | 0.0 | 0.1 | 0.1 | 0.1 | 0.1 | 0.1 | 0.1 | 0.1 |
| Zpbp2 | 0.1 | 0.1 | 0.1 | 0.1 | 0.0 | 0.1 | 0.2 | 0.2 | 0.0 | 0.1 | 0.0 | 0.0 |
| Zpld1 | 0.0 | 0.0 | 0.0 | 0.0 | 0.0 | 0.0 | 0.0 | 0.0 | 0.0 | 0.0 | 0.0 | 0.0 |
| Zranb1 | 11.4 | 12.1 | 13.7 | 10.7 | 9.8 | 12.1 | 9.4 | 12.1 | 11.5 | 11.0 | 12.0 | 13.3 |
| Zranb2 | 33.8 | 30.7 | 36.8 | 40.6 | 28.1 | 39.3 | 31.3 | 25.8 | 24.7 | 30.7 | 40.6 | 37.9 |
| Zranb3 | 1.0 | 1.5 | 1.6 | 1.9 | 2.5 | 1.0 | 1.7 | 1.5 | 1.4 | 1.2 | 1.2 | 1.0 |
| Zrsr1 | 6.2 | 5.1 | 6.7 | 5.7 | 4.1 | 7.0 | 4.9 | 5.7 | 4.4 | 5.7 | 5.7 | 7.7 |
| Zrsr2 | 7.7 | 7.2 | 9.9 | 9.5 | 7.1 | 7.6 | 8.5 | 8.8 | 5.5 | 7.7 | 9.1 | 10.8 |
| Zscan12 | 2.7 | 2.4 | 2.6 | 2.2 | 2.2 | 2.3 | 2.0 | 2.5 | 2.2 | 2.3 | 2.5 | 3.1 |
| Zscan18 | 0.8 | 1.0 | 0.5 | 1.0 | 0.6 | 0.9 | 0.5 | 0.4 | 0.5 | 0.8 | 0.7 | 0.6 |
| Zscan2 | 1.1 | 0.9 | 1.5 | 1.3 | 1.2 | 1.1 | 0.8 | 1.4 | 0.9 | 1.2 | 1.0 | 1.0 |
| Zscan20 | 1.6 | 1.4 | 1.4 | 1.5 | 1.3 | 1.5 | 1.2 | 1.2 | 1.4 | 1.3 | 1.4 | 1.1 |
| Zscan21 | 5.7 | 6.0 | 6.1 | 7.2 | 5.7 | 6.4 | 5.4 | 7.2 | 6.1 | 6.4 | 6.8 | 7.9 |
| Zscan22 | 1.2 | 1.3 | 1.3 | 1.5 | 1.1 | 1.1 | 0.9 | 1.1 | 0.7 | 1.3 | 1.4 | 1.3 |
| Zscan25 | 4.1 | 3.9 | 2.9 | 3.6 | 3.8 | 3.3 | 3.9 | 3.5 | 3.6 | 3.8 | 3.3 | 3.2 |
| Zscan26 | 12.1 | 12.3 | 13.0 | 12.7 | 10.5 | 12.9 | 9.5 | 11.3 | 10.7 | 13.2 | 12.3 | 12.6 |
| Zscan29 | 4.0 | 3.8 | 3.6 | 4.7 | 4.1 | 3.9 | 4.1 | 3.4 | 3.5 | 3.4 | 3.9 | 3.8 |
| Zswim1 | 3.0 | 3.2 | 2.7 | 3.0 | 2.8 | 3.4 | 2.9 | 3.3 | 2.6 | 2.6 | 3.0 | 2.6 |
| Zswim3 | 1.7 | 2.1 | 1.8 | 1.7 | 1.9 | 1.5 | 2.0 | 1.9 | 1.7 | 1.7 | 1.8 | 1.9 |
| Zswim4 | 4.2 | 3.9 | 3.6 | 4.0 | 5.5 | 3.6 | 4.7 | 3.7 | 3.6 | 4.6 | 3.7 | 3.0 |
| Zswim5 | 0.1 | 0.0 | 0.0 | 0.1 | 0.0 | 0.0 | 0.1 | 0.0 | 0.0 | 0.0 | 0.0 | 0.0 |
| Zswim6 | 8.0 | 9.0 | 9.4 | 8.3 | 9.9 | 7.7 | 12.5 | 7.7 | 9.7 | 9.4 | 9.9 | 10.6 |
| Zswim7 | 6.1 | 7.2 | 7.3 | 4.0 | 5.2 | 6.1 | 7.8 | 7.9 | 6.7 | 6.8 | 6.9 | 8.0 |
| Zswim8 | 11.9 | 12.3 | 12.5 | 13.0 | 11.6 | 11.5 | 15.3 | 12.8 | 14.2 | 12.5 | 12.9 | 11.8 |

| | | | | | | | | | | | | |
|---|---|---|---|---|---|---|---|---|---|---|---|---|
| Zufsp | 4.5 | 5.7 | 4.8 | 5.5 | 4.6 | 3.7 | 4.0 | 4.0 | 4.0 | 5.0 | 5.3 | 4.6 |
| Zw10 | 6.9 | 6.2 | 5.9 | 6.4 | 7.3 | 7.0 | 6.6 | 5.6 | 6.1 | 6.4 | 6.2 | 5.9 |
| Zwilch | 3.1 | 4.5 | 6.0 | 2.2 | 5.2 | 1.7 | 5.4 | 3.0 | 4.5 | 2.4 | 3.8 | 2.6 |
| Zwint | 30.9 | 25.4 | 21.0 | 41.0 | 26.4 | 33.6 | 27.8 | 17.4 | 17.3 | 23.2 | 23.8 | 23.2 |
| Zxda | 1.2 | 1.5 | 1.4 | 1.2 | 1.2 | 1.5 | 1.4 | 1.2 | 1.3 | 1.4 | 1.4 | 1.2 |
| Zxdb | 1.7 | 2.0 | 2.1 | 1.8 | 1.7 | 2.1 | 2.0 | 1.8 | 2.1 | 2.3 | 2.2 | 1.8 |
| Zxdc | 2.7 | 2.8 | 2.4 | 2.7 | 2.4 | 2.6 | 2.1 | 2.5 | 2.3 | 3.0 | 2.5 | 2.8 |
| Zyg11a | 0.0 | 0.0 | 0.0 | 0.0 | 0.0 | 0.0 | 0.0 | 0.0 | 0.0 | 0.0 | 0.0 | 0.0 |
| Zyg11b | 9.2 | 9.5 | 9.2 | 8.9 | 8.1 | 9.7 | 8.0 | 9.0 | 9.0 | 9.1 | 9.5 | 9.3 |
| Zyx | 33.1 | 35.5 | 34.4 | 28.4 | 36.2 | 30.1 | 48.4 | 47.1 | 45.6 | 59.3 | 66.5 | 30.5 |
| Zzef1 | 7.4 | 8.1 | 7.0 | 7.2 | 7.9 | 6.6 | 8.2 | 6.8 | 8.1 | 8.2 | 7.7 | 7.9 |
| Zzz3 | 12.4 | 11.7 | 11.3 | 11.1 | 10.2 | 12.2 | 11.3 | 10.8 | 10.7 | 12.2 | 13.1 | 12.5 |

| Excluded genes | | | | | | | | | | | | |
|---|---|---|---|---|---|---|---|---|---|---|---|---|
| | 1 | 2 | 3 | 4 | 5 | 6 | 7 | 8 | 9 | 10 | 11 | 12 |
| gene | A D0 | A D0 | A D0 | B D1 | B D1 | B D1 | C D3 | C D3 | C D3 | D D7 | D D7 | D D7 |
| 42433 | 0.0 | 0.0 | 0.0 | 0.0 | 0.0 | 0.0 | 0.0 | 0.0 | 0.0 | 0.0 | 0.0 | 0.0 |
| 42439 | 0.0 | 0.0 | 0.0 | 0.0 | 0.0 | 0.0 | 0.0 | 0.0 | 0.0 | 0.0 | 0.0 | 0.0 |
| 42625 | 0.0 | 0.0 | 0.0 | 0.0 | 0.0 | 0.0 | 0.0 | 0.0 | 0.0 | 0.1 | 0.0 | 0.0 |
| 0610008F07Rik | 0.0 | 0.1 | 0.0 | 0.0 | 0.0 | 0.0 | 0.0 | 0.0 | 0.0 | 0.0 | 0.0 | 0.0 |
| 0610009B14Rik | 0.0 | 0.0 | 0.0 | 0.0 | 0.0 | 0.0 | 0.1 | 0.0 | 0.0 | 0.0 | 0.0 | 0.0 |
| 0610031O16Rik | 0.0 | 0.0 | 0.0 | 0.0 | 0.1 | 0.0 | 0.0 | 0.0 | 0.0 | 0.1 | 0.0 | 0.0 |
| 0610040J01Rik | 0.0 | 0.0 | 0.0 | 0.0 | 0.0 | 0.0 | 0.0 | 0.0 | 0.0 | 0.0 | 0.0 | 0.0 |
| 1110028F18Rik | 0.0 | 0.0 | 0.0 | 0.0 | 0.0 | 0.0 | 0.0 | 0.0 | 0.0 | 0.0 | 0.0 | 0.0 |
| 1110032F04Rik | 0.0 | 0.0 | 0.0 | 0.0 | 0.0 | 0.0 | 0.0 | 0.0 | 0.0 | 0.0 | 0.0 | 0.0 |
| 1300015D01Rik | 0.0 | 0.0 | 0.0 | 0.0 | 0.0 | 0.0 | 0.0 | 0.0 | 0.0 | 0.0 | 0.0 | 0.0 |
| 1500012K07Rik | 0.0 | 0.0 | 0.0 | 0.0 | 0.0 | 0.0 | 0.0 | 0.0 | 0.0 | 0.0 | 0.0 | 0.0 |
| 1600002D24Rik | 0.0 | 0.0 | 0.0 | 0.0 | 0.0 | 0.0 | 0.0 | 0.0 | 0.0 | 0.0 | 0.0 | 0.0 |
| 1600019K03Rik | 0.0 | 0.0 | 0.0 | 0.1 | 0.0 | 0.6 | 0.0 | 0.0 | 0.0 | 0.0 | 0.0 | 0.0 |
| 1600029I14Rik | 0.0 | 0.0 | 0.0 | 0.0 | 0.0 | 0.0 | 0.0 | 0.0 | 0.0 | 0.0 | 0.0 | 0.1 |
| 1600029O15Rik | 0.0 | 0.0 | 0.0 | 0.0 | 0.0 | 0.0 | 0.0 | 0.0 | 0.0 | 0.0 | 0.0 | 0.0 |
| 1700001D01Rik | 0.0 | 0.1 | 0.0 | 0.0 | 0.0 | 0.0 | 0.0 | 0.0 | 0.0 | 0.0 | 0.0 | 0.0 |
| 1700001F09Rik | 0.1 | 0.0 | 0.0 | 0.0 | 0.0 | 0.0 | 0.0 | 0.0 | 0.0 | 0.0 | 0.0 | 0.0 |
| 1700003D09Rik | 0.0 | 0.0 | 0.0 | 0.0 | 0.0 | 0.0 | 0.0 | 0.0 | 0.0 | 0.0 | 0.0 | 0.0 |
| 1700003G18Rik | 0.1 | 0.0 | 0.0 | 0.0 | 0.1 | 0.0 | 0.0 | 0.2 | 0.0 | 0.0 | 0.0 | 0.0 |
| 1700006F04Rik | 0.0 | 0.0 | 0.0 | 0.0 | 0.0 | 0.0 | 0.0 | 0.0 | 0.0 | 0.0 | 0.0 | 0.0 |
| 1700006H21Rik | 0.1 | 0.1 | 0.0 | 0.0 | 0.0 | 0.1 | 0.0 | 0.0 | 0.0 | 0.0 | 0.0 | 0.0 |
| 1700007F19Rik | 0.0 | 0.0 | 0.0 | 0.0 | 0.0 | 0.0 | 0.0 | 0.0 | 0.0 | 0.0 | 0.0 | 0.0 |
| 1700007K09Rik | 0.0 | 0.0 | 0.0 | 0.0 | 0.1 | 0.0 | 0.0 | 0.1 | 0.1 | 0.0 | 0.0 | 0.0 |
| 1700009C05Rik | 0.0 | 0.0 | 0.1 | 0.0 | 0.0 | 0.0 | 0.0 | 0.0 | 0.0 | 0.0 | 0.0 | 0.0 |
| 1700010B08Rik | 0.0 | 0.0 | 0.0 | 0.0 | 0.0 | 0.0 | 0.0 | 0.0 | 0.0 | 0.0 | 0.0 | 0.0 |
| 1700010D01Rik | 0.0 | 0.0 | 0.0 | 0.0 | 0.0 | 0.0 | 0.1 | 0.0 | 0.0 | 0.0 | 0.0 | 0.0 |
| 1700010I02Rik | 0.0 | 0.0 | 0.0 | 0.0 | 0.0 | 0.0 | 0.0 | 0.2 | 0.0 | 0.0 | 0.2 | 0.0 |
| 1700011E24Rik | 0.0 | 0.1 | 0.0 | 0.2 | 0.0 | 0.0 | 0.0 | 0.0 | 0.0 | 0.0 | 0.1 | 0.0 |
| 1700011H14Rik | 0.0 | 0.0 | 0.0 | 0.0 | 0.0 | 0.1 | 0.0 | 0.0 | 0.0 | 0.0 | 0.0 | 0.0 |
| 1700011I03Rik | 0.0 | 0.0 | 0.1 | 0.2 | 0.0 | 0.0 | 0.0 | 0.0 | 0.0 | 0.0 | 0.0 | 0.0 |
| 1700011M02Rik | 0.0 | 0.0 | 0.0 | 0.0 | 0.0 | 0.0 | 0.0 | 0.0 | 0.0 | 0.0 | 0.0 | 0.0 |
| 1700012B07Rik | 0.0 | 0.0 | 0.0 | 0.0 | 0.0 | 0.0 | 0.0 | 0.0 | 0.0 | 0.0 | 0.1 | 0.1 |
| 1700012B09Rik | 0.0 | 0.1 | 0.0 | 0.0 | 0.1 | 0.2 | 0.0 | 0.0 | 0.1 | 0.1 | 0.0 | 0.0 |
| 1700012I11Rik | 0.0 | 0.0 | 0.1 | 0.0 | 0.0 | 0.0 | 0.0 | 0.0 | 0.0 | 0.0 | 0.0 | 0.0 |
| 1700012L04Rik | 0.0 | 0.0 | 0.0 | 0.0 | 0.0 | 0.1 | 0.0 | 0.0 | 0.1 | 0.0 | 0.0 | 0.0 |
| 1700013F07Rik | 0.0 | 0.0 | 0.1 | 0.0 | 0.1 | 0.0 | 0.0 | 0.0 | 0.1 | 0.1 | 0.0 | 0.1 |
| 1700013G24Rik | 0.0 | 0.0 | 0.0 | 0.1 | 0.0 | 0.0 | 0.0 | 0.0 | 0.1 | 0.0 | 0.0 | 0.0 |
| 1700013H16Rik | 0.0 | 0.1 | 0.0 | 0.0 | 0.0 | 0.0 | 0.0 | 0.1 | 0.0 | 0.0 | 0.0 | 0.0 |
| 1700015E13Rik | 0.0 | 0.1 | 0.0 | 0.0 | 0.0 | 0.0 | 0.0 | 0.0 | 0.0 | 0.0 | 0.0 | 0.0 |
| 1700015G11Rik | 0.0 | 0.0 | 0.0 | 0.2 | 0.0 | 0.0 | 0.0 | 0.0 | 0.0 | 0.0 | 0.0 | 0.0 |
| 1700016D06Rik | 0.0 | 0.0 | 0.0 | 0.0 | 0.0 | 0.0 | 0.0 | 0.0 | 0.1 | 0.0 | 0.0 | 0.0 |
| 1700016H13Rik | 0.0 | 0.1 | 0.0 | 0.0 | 0.0 | 0.0 | 0.0 | 0.0 | 0.0 | 0.0 | 0.0 | 0.0 |
| 1700016L21Rik | 0.0 | 0.0 | 0.0 | 0.0 | 0.0 | 0.0 | 0.0 | 0.0 | 0.0 | 0.0 | 0.0 | 0.0 |
| 1700017D01Rik | 0.0 | 0.0 | 0.0 | 0.0 | 0.0 | 0.0 | 0.0 | 0.0 | 0.0 | 0.0 | 0.0 | 0.0 |
| 1700017G19Rik | 0.1 | 0.0 | 0.0 | 0.0 | 0.0 | 0.0 | 0.0 | 0.0 | 0.0 | 0.0 | 0.0 | 0.0 |
| 1700018B08Rik | 0.0 | 0.0 | 0.0 | 0.0 | 0.0 | 0.0 | 0.0 | 0.0 | 0.0 | 0.0 | 0.0 | 0.0 |
| 1700018C11Rik | 0.0 | 0.0 | 0.1 | 0.0 | 0.0 | 0.0 | 0.0 | 0.0 | 0.0 | 0.0 | 0.0 | 0.0 |
| 1700018F24Rik | 0.0 | 0.0 | 0.0 | 0.0 | 0.0 | 0.0 | 0.0 | 0.0 | 0.0 | 0.0 | 0.0 | 0.0 |
| 1700019B03Rik | 0.0 | 0.0 | 0.0 | 0.0 | 0.0 | 0.0 | 0.0 | 0.0 | 0.1 | 0.0 | 0.0 | 0.0 |
| 1700019D03Rik | 0.0 | 0.0 | 0.1 | 0.0 | 0.0 | 0.0 | 0.0 | 0.0 | 0.0 | 0.0 | 0.0 | 0.0 |
| 1700019G24Rik | 0.0 | 0.0 | 0.0 | 0.1 | 0.1 | 0.0 | 0.1 | 0.0 | 0.0 | 0.1 | 0.0 | 0.0 |
| 1700019M22Rik | 0.0 | 0.0 | 0.0 | 0.0 | 0.0 | 0.0 | 0.0 | 0.0 | 0.0 | 0.0 | 0.0 | 0.0 |
| 1700019N19Rik | 0.0 | 0.0 | 0.1 | 0.0 | 0.0 | 0.1 | 0.0 | 0.0 | 0.0 | 0.0 | 0.0 | 0.0 |
| 1700020A23Rik | 0.0 | 0.0 | 0.2 | 0.0 | 0.0 | 0.0 | 0.0 | 0.0 | 0.1 | 0.2 | 0.0 | 0.0 |

Excluded Genes

| | | | | | | | | | | | | |
|---|---|---|---|---|---|---|---|---|---|---|---|---|
| 1700020N01Rik | 0.0 | 0.0 | 0.0 | 0.0 | 0.0 | 0.0 | 0.0 | 0.0 | 0.0 | 0.0 | 0.0 | 0.0 |
| 1700021F07Rik | 0.1 | 0.0 | 0.0 | 0.0 | 0.0 | 0.0 | 0.0 | 0.0 | 0.0 | 0.0 | 0.0 | 0.0 |
| 1700021N21Rik | 0.0 | 0.0 | 0.1 | 0.0 | 0.0 | 0.0 | 0.0 | 0.0 | 0.0 | 0.0 | 0.0 | 0.0 |
| 1700022A21Rik | 0.0 | 0.0 | 0.0 | 0.0 | 0.0 | 0.0 | 0.0 | 0.0 | 0.0 | 0.0 | 0.0 | 0.0 |
| 1700022P22Rik | 0.1 | 0.0 | 0.0 | 0.0 | 0.0 | 0.0 | 0.0 | 0.0 | 0.0 | 0.0 | 0.0 | 0.0 |
| 1700023E05Rik | 0.0 | 0.0 | 0.0 | 0.0 | 0.0 | 0.0 | 0.0 | 0.0 | 0.0 | 0.0 | 0.0 | 0.0 |
| 1700023L04Rik | 0.1 | 0.0 | 0.0 | 0.0 | 0.0 | 0.1 | 0.0 | 0.0 | 0.0 | 0.0 | 0.0 | 0.0 |
| 1700024F13Rik | 0.0 | 0.1 | 0.0 | 0.0 | 0.0 | 0.0 | 0.1 | 0.0 | 0.0 | 0.1 | 0.0 | 0.0 |
| 1700024P03Rik | 0.0 | 0.0 | 0.0 | 0.0 | 0.0 | 0.0 | 0.0 | 0.2 | 0.0 | 0.0 | 0.0 | 0.0 |
| 1700025F22Rik | 0.0 | 0.0 | 0.0 | 0.0 | 0.0 | 0.0 | 0.0 | 0.0 | 0.0 | 0.0 | 0.0 | 0.0 |
| 1700025M24Rik | 0.1 | 0.0 | 0.0 | 0.0 | 0.0 | 0.0 | 0.0 | 0.0 | 0.0 | 0.0 | 0.0 | 0.0 |
| 1700026D11Rik | 0.0 | 0.0 | 0.0 | 0.0 | 0.0 | 0.0 | 0.0 | 0.0 | 0.0 | 0.1 | 0.0 | 0.0 |
| 1700027I24Rik | 0.1 | 0.0 | 0.0 | 0.0 | 0.0 | 0.0 | 0.0 | 0.0 | 0.1 | 0.0 | 0.0 | 0.0 |
| 1700028M03Rik | 0.0 | 0.0 | 0.2 | 0.0 | 0.0 | 0.0 | 0.0 | 0.0 | 0.0 | 0.0 | 0.0 | 0.1 |
| 1700029J03Rik | 0.1 | 0.0 | 0.0 | 0.0 | 0.0 | 0.0 | 0.0 | 0.1 | 0.0 | 0.0 | 0.0 | 0.1 |
| 1700029M20Rik | 0.0 | 0.0 | 0.0 | 0.0 | 0.0 | 0.0 | 0.0 | 0.0 | 0.0 | 0.0 | 0.0 | 0.0 |
| 1700029P11Rik | 0.0 | 0.0 | 0.0 | 0.0 | 0.0 | 0.0 | 0.0 | 0.0 | 0.0 | 0.1 | 0.0 | 0.0 |
| 1700030A11Rik | 0.0 | 0.0 | 0.0 | 0.0 | 0.0 | 0.0 | 0.0 | 0.0 | 0.0 | 0.0 | 0.0 | 0.0 |
| 1700030F04Rik | 0.0 | 0.0 | 0.0 | 0.0 | 0.0 | 0.1 | 0.0 | 0.0 | 0.0 | 0.0 | 0.0 | 0.0 |
| 1700030F18Rik | 0.1 | 0.0 | 0.0 | 0.0 | 0.1 | 0.0 | 0.0 | 0.0 | 0.0 | 0.0 | 0.0 | 0.0 |
| 1700030M09Rik | 0.0 | 0.0 | 0.0 | 0.0 | 0.1 | 0.1 | 0.0 | 0.0 | 0.0 | 0.0 | 0.0 | 0.0 |
| 1700030N03Rik | 0.0 | 0.0 | 0.0 | 0.0 | 0.1 | 0.0 | 0.0 | 0.0 | 0.0 | 0.0 | 0.2 | 0.0 |
| 1700030O20Rik | 0.0 | 0.0 | 0.0 | 0.0 | 0.0 | 0.0 | 0.0 | 0.0 | 0.0 | 0.0 | 0.0 | 0.0 |
| 1700031A10Rik | 0.0 | 0.0 | 0.0 | 0.0 | 0.0 | 0.0 | 0.0 | 0.0 | 0.0 | 0.0 | 0.0 | 0.0 |
| 1700036G14Rik | 0.0 | 0.0 | 0.0 | 0.0 | 0.0 | 0.0 | 0.0 | 0.0 | 0.0 | 0.0 | 0.0 | 0.0 |
| 1700040N02Rik | 0.0 | 0.0 | 0.0 | 0.1 | 0.0 | 0.0 | 0.0 | 0.0 | 0.0 | 0.0 | 0.0 | 0.0 |
| 1700042B14Rik | 0.0 | 0.0 | 0.0 | 0.1 | 0.0 | 0.0 | 0.0 | 0.0 | 0.0 | 0.1 | 0.0 | 0.1 |
| 1700045H11Rik | 0.0 | 0.0 | 0.0 | 0.0 | 0.0 | 0.0 | 0.0 | 0.0 | 0.0 | 0.0 | 0.0 | 0.0 |
| 1700047E10Rik | 0.0 | 0.0 | 0.0 | 0.0 | 0.0 | 0.0 | 0.0 | 0.0 | 0.0 | 0.1 | 0.0 | 0.0 |
| 1700049L16Rik | 0.0 | 0.0 | 0.0 | 0.0 | 0.0 | 0.0 | 0.0 | 0.0 | 0.0 | 0.0 | 0.0 | 0.1 |
| 1700051A21Rik | 0.0 | 0.0 | 0.0 | 0.0 | 0.0 | 0.1 | 0.0 | 0.0 | 0.0 | 0.0 | 0.0 | 0.0 |
| 1700054M17Rik | 0.0 | 0.0 | 0.0 | 0.0 | 0.0 | 0.0 | 0.1 | 0.0 | 0.0 | 0.0 | 0.0 | 0.0 |
| 1700060C20Rik | 0.0 | 0.0 | 0.0 | 0.0 | 0.0 | 0.0 | 0.0 | 0.0 | 0.1 | 0.0 | 0.0 | 0.0 |
| 1700064M15Rik | 0.0 | 0.0 | 0.0 | 0.0 | 0.1 | 0.0 | 0.0 | 0.0 | 0.0 | 0.0 | 0.0 | 0.1 |
| 1700065D16Rik | 0.1 | 0.2 | 0.0 | 0.0 | 0.0 | 0.0 | 0.0 | 0.1 | 0.0 | 0.0 | 0.0 | 0.0 |
| 1700065I17Rik | 0.0 | 0.0 | 0.0 | 0.0 | 0.0 | 0.0 | 0.0 | 0.0 | 0.0 | 0.0 | 0.0 | 0.0 |
| 1700065J11Rik | 0.0 | 0.1 | 0.0 | 0.0 | 0.0 | 0.1 | 0.0 | 0.1 | 0.0 | 0.1 | 0.0 | 0.0 |
| 1700065J18Rik | 0.0 | 0.1 | 0.0 | 0.0 | 0.0 | 0.0 | 0.0 | 0.0 | 0.0 | 0.0 | 0.0 | 0.0 |
| 1700066N21Rik | 0.0 | 0.0 | 0.0 | 0.0 | 0.2 | 0.0 | 0.0 | 0.0 | 0.0 | 0.0 | 0.0 | 0.0 |
| 1700067G17Rik | 0.0 | 0.0 | 0.0 | 0.0 | 0.0 | 0.0 | 0.0 | 0.0 | 0.0 | 0.0 | 0.0 | 0.0 |
| 1700069L16Rik | 0.0 | 0.0 | 0.0 | 0.0 | 0.0 | 0.0 | 0.0 | 0.0 | 0.0 | 0.1 | 0.0 | 0.0 |
| 1700072B07Rik | 0.0 | 0.1 | 0.0 | 0.0 | 0.0 | 0.0 | 0.0 | 0.0 | 0.0 | 0.0 | 0.0 | 0.0 |
| 1700072O05Rik | 0.0 | 0.0 | 0.0 | 0.0 | 0.0 | 0.0 | 0.0 | 0.0 | 0.0 | 0.7 | 0.0 | 0.0 |
| 1700074H08Rik | 0.0 | 0.1 | 0.0 | 0.0 | 0.1 | 0.0 | 0.0 | 0.1 | 0.1 | 0.0 | 0.0 | 0.0 |
| 1700081H04Rik | 0.0 | 0.0 | 0.0 | 0.0 | 0.0 | 0.0 | 0.0 | 0.0 | 0.0 | 0.0 | 0.1 | 0.0 |
| 1700085C21Rik | 0.0 | 0.0 | 0.0 | 0.0 | 0.0 | 0.0 | 0.0 | 0.0 | 0.0 | 0.0 | 0.0 | 0.0 |
| 1700086L19Rik | 0.0 | 0.0 | 0.0 | 0.0 | 0.1 | 0.0 | 0.0 | 0.0 | 0.1 | 0.0 | 0.0 | 0.0 |
| 1700091H14Rik | 0.1 | 0.0 | 0.0 | 0.0 | 0.0 | 0.0 | 0.0 | 0.0 | 0.0 | 0.0 | 0.0 | 0.0 |
| 1700092C02Rik | 0.0 | 0.0 | 0.0 | 0.0 | 0.1 | 0.0 | 0.0 | 0.0 | 0.0 | 0.0 | 0.0 | 0.0 |
| 1700092E19Rik | 0.0 | 0.0 | 0.0 | 0.0 | 0.0 | 0.0 | 0.0 | 0.0 | 0.0 | 0.0 | 0.0 | 0.0 |
| 1700092M07Rik | 0.0 | 0.2 | 0.0 | 0.2 | 0.1 | 0.0 | 0.0 | 0.0 | 0.1 | 0.0 | 0.0 | 0.5 |
| 1700095A21Rik | 0.0 | 0.0 | 0.0 | 0.0 | 0.0 | 0.0 | 0.0 | 0.0 | 0.0 | 0.0 | 0.0 | 0.0 |
| 1700095B10Rik | 0.0 | 0.0 | 0.0 | 0.0 | 0.0 | 0.0 | 0.0 | 0.0 | 0.0 | 0.0 | 0.0 | 0.0 |
| 1700096J18Rik | 0.0 | 0.0 | 0.0 | 0.0 | 0.3 | 0.0 | 0.0 | 0.0 | 0.1 | 0.0 | 0.0 | 0.0 |
| 1700097N02Rik | 0.0 | 0.0 | 0.1 | 0.0 | 0.0 | 0.0 | 0.0 | 0.0 | 0.0 | 0.0 | 0.0 | 0.1 |
| 1700100L14Rik | 0.0 | 0.0 | 0.0 | 0.0 | 0.1 | 0.0 | 0.0 | 0.0 | 0.0 | 0.0 | 0.0 | 0.0 |
| 1700102H20Rik | 0.0 | 0.3 | 0.0 | 0.0 | 0.0 | 0.0 | 0.1 | 0.0 | 0.0 | 0.0 | 0.1 | 0.0 |

| | | | | | | | | | | | | |
|---|---|---|---|---|---|---|---|---|---|---|---|---|
| 1700104A03Rik | 0.0 | 0.0 | 0.0 | 0.0 | 0.0 | 0.0 | 0.0 | 0.0 | 0.0 | 0.0 | 0.0 | 0.0 |
| 1700108J01Rik | 0.0 | 0.0 | 0.0 | 0.0 | 0.0 | 0.0 | 0.0 | 0.0 | 0.0 | 0.0 | 0.0 | 0.0 |
| 1700109G14Rik | 0.0 | 0.0 | 0.0 | 0.0 | 0.0 | 0.0 | 0.0 | 0.0 | 0.0 | 0.0 | 0.0 | 0.0 |
| 1700110I01Rik | 0.0 | 0.0 | 0.0 | 0.0 | 0.0 | 0.0 | 0.0 | 0.0 | 0.0 | 0.0 | 0.0 | 0.0 |
| 1700110K17Rik | 0.1 | 0.0 | 0.0 | 0.1 | 0.0 | 0.0 | 0.0 | 0.0 | 0.0 | 0.0 | 0.0 | 0.0 |
| 1700112H15Rik | 0.0 | 0.0 | 0.0 | 0.0 | 0.0 | 0.0 | 0.0 | 0.0 | 0.0 | 0.0 | 0.0 | 0.0 |
| 1700112J05Rik | 0.0 | 0.1 | 0.0 | 0.0 | 0.0 | 0.0 | 0.0 | 0.0 | 0.0 | 0.0 | 0.2 | 0.0 |
| 1700113H08Rik | 0.0 | 0.1 | 0.0 | 0.0 | 0.0 | 0.0 | 0.0 | 0.0 | 0.0 | 0.0 | 0.0 | 0.0 |
| 1700119H24Rik | 0.0 | 0.0 | 0.1 | 0.0 | 0.0 | 0.0 | 0.0 | 0.0 | 0.1 | 0.0 | 0.0 | 0.0 |
| 1700120E14Rik | 0.0 | 0.0 | 0.0 | 0.0 | 0.0 | 0.0 | 0.0 | 0.0 | 0.0 | 0.0 | 0.0 | 0.0 |
| 1700122O11Rik | 0.0 | 0.0 | 0.0 | 0.0 | 0.0 | 0.0 | 0.0 | 0.0 | 0.0 | 0.0 | 0.0 | 0.0 |
| 1700123K08Rik | 0.0 | 0.0 | 0.0 | 0.0 | 0.0 | 0.0 | 0.0 | 0.0 | 0.0 | 0.0 | 0.0 | 0.0 |
| 1700123O21Rik | 0.0 | 0.0 | 0.0 | 0.0 | 0.1 | 0.0 | 0.0 | 0.0 | 0.0 | 0.0 | 0.0 | 0.0 |
| 1700126H18Rik | 0.0 | 0.0 | 0.0 | 0.0 | 0.0 | 0.0 | 0.0 | 0.0 | 0.0 | 0.0 | 0.0 | 0.0 |
| 1700128A07Rik | 0.0 | 0.1 | 0.0 | 0.0 | 0.0 | 0.0 | 0.0 | 0.0 | 0.0 | 0.0 | 0.2 | 0.0 |
| 1810006J02Rik | 0.0 | 0.0 | 0.0 | 0.0 | 0.0 | 0.0 | 0.0 | 0.0 | 0.0 | 0.0 | 0.0 | 0.0 |
| 1810009J06Rik | 0.0 | 0.0 | 0.0 | 0.0 | 0.0 | 0.0 | 0.0 | 0.0 | 0.0 | 0.0 | 0.0 | 0.0 |
| 1810013A23Rik | 0.0 | 0.0 | 0.1 | 0.0 | 0.1 | 0.0 | 0.0 | 0.0 | 0.0 | 0.1 | 0.0 | 0.0 |
| 1810035I16Rik | 0.0 | 0.0 | 0.0 | 0.0 | 0.0 | 0.0 | 0.0 | 0.0 | 0.0 | 0.0 | 0.0 | 0.0 |
| 1810046K07Rik | 0.0 | 0.0 | 0.0 | 0.0 | 0.0 | 0.0 | 0.0 | 0.0 | 0.0 | 0.0 | 0.0 | 0.0 |
| 1810053B23Rik | 0.0 | 0.0 | 0.0 | 0.0 | 0.0 | 0.0 | 0.0 | 0.0 | 0.1 | 0.0 | 0.0 | 0.0 |
| 2010005H15Rik | 0.0 | 0.0 | 0.0 | 0.0 | 0.1 | 0.0 | 0.0 | 0.1 | 0.0 | 0.1 | 0.0 | 0.0 |
| 2010106C02Rik | 0.1 | 0.0 | 0.0 | 0.2 | 0.0 | 0.2 | 0.7 | 0.0 | 0.1 | 0.0 | 0.0 | 0.0 |
| 2010106E10Rik | 0.1 | 0.0 | 0.0 | 0.0 | 0.0 | 0.0 | 0.0 | 0.0 | 0.0 | 0.0 | 0.0 | 0.0 |
| 2010109I03Rik | 0.0 | 0.0 | 0.0 | 0.0 | 0.0 | 0.0 | 0.0 | 0.0 | 0.0 | 0.0 | 0.0 | 0.0 |
| 2010308F09Rik | 0.0 | 0.0 | 0.0 | 0.3 | 0.0 | 0.0 | 0.0 | 0.0 | 0.0 | 0.2 | 0.0 | 0.0 |
| 2210409E12Rik | 0.0 | 0.0 | 0.0 | 0.0 | 0.0 | 0.0 | 0.0 | 0.0 | 0.0 | 0.0 | 0.2 | 0.0 |
| 2210414B05Rik | 0.0 | 0.0 | 0.0 | 0.0 | 0.0 | 0.0 | 0.0 | 0.0 | 0.0 | 0.0 | 0.0 | 0.0 |
| 2210417A02Rik | 0.0 | 0.0 | 0.0 | 0.1 | 0.0 | 0.0 | 0.0 | 0.0 | 0.0 | 0.0 | 0.0 | 0.0 |
| 2210420H20Rik | 0.0 | 0.0 | 0.0 | 0.0 | 0.0 | 0.0 | 0.0 | 0.0 | 0.0 | 0.0 | 0.0 | 0.0 |
| 2300002M23Rik | 0.0 | 0.0 | 0.0 | 0.0 | 0.0 | 0.0 | 0.0 | 0.0 | 0.0 | 0.0 | 0.0 | 0.0 |
| 2300005B03Rik | 0.1 | 0.0 | 0.0 | 0.0 | 0.0 | 0.0 | 0.0 | 0.0 | 0.0 | 0.1 | 0.0 | 0.1 |
| 2310002D06Rik | 0.2 | 0.0 | 0.0 | 0.0 | 0.0 | 0.0 | 0.1 | 0.0 | 0.0 | 0.0 | 0.0 | 0.0 |
| 2310002J15Rik | 0.0 | 0.0 | 0.0 | 0.0 | 0.0 | 0.0 | 0.0 | 0.0 | 0.0 | 0.0 | 0.0 | 0.0 |
| 2310002L09Rik | 0.0 | 0.0 | 0.0 | 0.0 | 0.0 | 0.0 | 0.0 | 0.0 | 0.0 | 0.0 | 0.0 | 0.0 |
| 2310007L24Rik | 0.0 | 0.0 | 0.0 | 0.0 | 0.0 | 0.0 | 0.0 | 0.0 | 0.0 | 0.0 | 0.0 | 0.0 |
| 2310015B20Rik | 0.0 | 0.0 | 0.0 | 0.0 | 0.1 | 0.0 | 0.0 | 0.0 | 0.0 | 0.0 | 0.4 | 0.3 |
| 2310034O05Rik | 0.1 | 0.0 | 0.0 | 0.0 | 0.0 | 0.0 | 0.0 | 0.0 | 0.0 | 0.0 | 0.0 | 0.0 |
| 2310042E22Rik | 0.1 | 0.0 | 0.0 | 0.0 | 0.0 | 0.0 | 0.0 | 0.0 | 0.0 | 0.0 | 0.0 | 0.0 |
| 2310043O21Rik | 0.0 | 0.0 | 0.0 | 0.0 | 0.0 | 0.0 | 0.0 | 0.0 | 0.0 | 0.0 | 0.0 | 0.0 |
| 2310050B05Rik | 0.0 | 0.0 | 0.0 | 0.6 | 0.0 | 0.0 | 0.0 | 0.0 | 0.0 | 0.0 | 0.0 | 0.0 |
| 2310050C09Rik | 0.0 | 0.0 | 0.0 | 0.0 | 0.0 | 0.0 | 0.0 | 0.0 | 0.0 | 0.0 | 0.0 | 0.0 |
| 2310069G16Rik | 0.1 | 0.1 | 0.0 | 0.0 | 0.0 | 0.0 | 0.0 | 0.0 | 0.0 | 0.1 | 0.0 | 0.0 |
| 2310081J21Rik | 0.0 | 0.0 | 0.0 | 0.0 | 0.0 | 0.0 | 0.0 | 0.1 | 0.0 | 0.0 | 0.0 | 0.0 |
| 2410007B07Rik | 0.0 | 0.0 | 0.0 | 0.0 | 0.0 | 0.0 | 0.0 | 0.0 | 0.0 | 0.0 | 0.0 | 0.0 |
| 2410012E07Rik | 0.0 | 0.0 | 0.0 | 0.0 | 0.0 | 0.0 | 0.0 | 0.0 | 0.0 | 0.0 | 0.0 | 0.0 |
| 2410017I17Rik | 0.0 | 0.0 | 0.0 | 0.0 | 0.0 | 0.0 | 0.0 | 0.0 | 0.0 | 0.0 | 0.0 | 0.0 |
| 2410124H12Rik | 0.0 | 0.0 | 0.0 | 0.0 | 0.1 | 0.1 | 0.0 | 0.0 | 0.0 | 0.0 | 0.0 | 0.0 |
| 2410137F16Rik | 0.0 | 0.0 | 0.0 | 0.0 | 0.0 | 0.0 | 0.0 | 0.0 | 0.0 | 0.0 | 0.0 | 0.0 |
| 2410137M14Rik | 0.0 | 0.0 | 0.0 | 0.0 | 0.0 | 0.0 | 0.0 | 0.0 | 0.0 | 0.0 | 0.0 | 0.0 |
| 2510003D18Rik | 0.0 | 0.0 | 0.1 | 0.0 | 0.0 | 0.0 | 0.0 | 0.0 | 0.0 | 0.1 | 0.0 | 0.0 |
| 2610016A17Rik | 0.0 | 0.2 | 0.0 | 0.0 | 0.2 | 0.0 | 0.0 | 0.0 | 0.0 | 0.0 | 0.0 | 0.0 |
| 2610017I09Rik | 0.0 | 0.0 | 0.0 | 0.0 | 0.0 | 0.0 | 0.0 | 0.0 | 0.0 | 0.0 | 0.0 | 0.0 |
| 2610028E06Rik | 0.0 | 0.0 | 0.0 | 0.0 | 0.0 | 0.0 | 0.0 | 0.0 | 0.0 | 0.0 | 0.0 | 0.0 |
| 2610028H24Rik | 0.0 | 0.0 | 0.0 | 0.0 | 0.0 | 0.0 | 0.0 | 0.0 | 0.0 | 0.0 | 0.0 | 0.0 |
| 2610034M16Rik | 0.0 | 0.0 | 0.0 | 0.0 | 0.0 | 0.0 | 0.0 | 0.0 | 0.0 | 0.0 | 0.0 | 0.1 |
| 2610100L16Rik | 0.0 | 0.0 | 0.0 | 0.0 | 0.0 | 0.0 | 0.0 | 0.0 | 0.0 | 0.0 | 0.0 | 0.0 |

| | | | | | | | | | | | | |
|---|---|---|---|---|---|---|---|---|---|---|---|---|
| 2610206C17Rik | 0.0 | 0.0 | 0.0 | 0.0 | 0.0 | 0.0 | 0.0 | 0.0 | 0.0 | 0.0 | 0.0 | 0.0 |
| 2610316D01Rik | 0.0 | 0.0 | 0.0 | 0.0 | 0.0 | 0.0 | 0.0 | 0.0 | 0.0 | 0.0 | 0.0 | 0.0 |
| 2610528J11Rik | 0.0 | 0.0 | 0.0 | 0.0 | 0.0 | 0.0 | 0.0 | 0.0 | 0.0 | 0.0 | 0.0 | 0.0 |
| 2700046A07Rik | 0.0 | 0.0 | 0.0 | 0.0 | 0.0 | 0.0 | 0.0 | 0.0 | 0.0 | 0.0 | 0.0 | 0.0 |
| 2700086A05Rik | 0.0 | 0.0 | 0.0 | 0.0 | 0.0 | 0.0 | 0.0 | 0.0 | 0.0 | 0.1 | 0.0 | 0.0 |
| 2700089I24Rik | 0.0 | 0.0 | 0.0 | 0.0 | 0.0 | 0.0 | 0.0 | 0.0 | 0.0 | 0.0 | 0.0 | 0.0 |
| 2810011L19Rik | 0.0 | 0.0 | 0.0 | 0.0 | 0.0 | 0.0 | 0.0 | 0.0 | 0.0 | 0.0 | 0.0 | 0.0 |
| 2810404M03Rik | 0.0 | 0.0 | 0.0 | 0.0 | 0.1 | 0.0 | 0.2 | 0.0 | 0.0 | 0.1 | 0.0 | 0.0 |
| 2810471M01Rik | 0.0 | 0.0 | 0.0 | 0.0 | 0.0 | 0.0 | 0.0 | 0.0 | 0.0 | 0.0 | 0.0 | 0.0 |
| 2900052N01Rik | 0.0 | 0.0 | 0.0 | 0.0 | 0.0 | 0.0 | 0.0 | 0.0 | 0.0 | 0.0 | 0.0 | 0.0 |
| 2900055J20Rik | 0.2 | 0.0 | 0.0 | 0.0 | 0.0 | 0.1 | 0.0 | 0.1 | 0.0 | 0.0 | 0.0 | 0.0 |
| 2900079G21Rik | 0.0 | 0.0 | 0.0 | 0.0 | 0.0 | 0.0 | 0.0 | 0.0 | 0.0 | 0.0 | 0.0 | 0.0 |
| 2900092D14Rik | 0.0 | 0.0 | 0.0 | 0.0 | 0.0 | 0.0 | 0.0 | 0.0 | 0.0 | 0.0 | 0.0 | 0.0 |
| 3110039M20Rik | 0.1 | 0.0 | 0.0 | 0.0 | 0.1 | 0.1 | 0.0 | 0.1 | 0.0 | 0.0 | 0.0 | 0.0 |
| 3110079O15Rik | 0.0 | 0.0 | 0.0 | 0.0 | 0.0 | 0.0 | 0.0 | 0.0 | 0.0 | 0.0 | 0.1 | 0.0 |
| 3110099E03Rik | 0.1 | 0.0 | 0.0 | 0.0 | 0.0 | 0.0 | 0.0 | 0.0 | 0.0 | 0.0 | 0.0 | 0.0 |
| 3830417A13Rik | 0.1 | 0.0 | 0.0 | 0.3 | 0.0 | 0.0 | 0.0 | 0.0 | 0.0 | 0.0 | 0.0 | 0.0 |
| 4732456N10Rik | 0.0 | 0.0 | 0.0 | 0.0 | 0.0 | 0.0 | 0.0 | 0.0 | 0.0 | 0.0 | 0.0 | 0.0 |
| 4833423E24Rik | 0.0 | 0.0 | 0.0 | 0.0 | 0.0 | 0.0 | 0.0 | 0.0 | 0.0 | 0.0 | 0.0 | 0.0 |
| 4833424O15Rik | 0.0 | 0.0 | 0.0 | 0.0 | 0.0 | 0.0 | 0.0 | 0.0 | 0.0 | 0.0 | 0.0 | 0.0 |
| 4833428L15Rik | 0.0 | 0.0 | 0.0 | 0.0 | 0.0 | 0.0 | 0.0 | 0.0 | 0.0 | 0.0 | 0.0 | 0.0 |
| 4921508D12Rik | 0.1 | 0.0 | 0.0 | 0.1 | 0.0 | 0.0 | 0.1 | 0.0 | 0.0 | 0.0 | 0.0 | 0.0 |
| 4921509O07Rik | 0.0 | 0.0 | 0.0 | 0.0 | 0.0 | 0.0 | 0.1 | 0.0 | 0.0 | 0.0 | 0.0 | 0.0 |
| 4921511M17Rik | 0.0 | 0.0 | 0.0 | 0.0 | 0.0 | 0.0 | 0.0 | 0.0 | 0.0 | 0.0 | 0.0 | 0.0 |
| 4921515E04Rik | 0.0 | 0.0 | 0.0 | 0.0 | 0.0 | 0.0 | 0.0 | 0.0 | 0.0 | 0.0 | 0.0 | 0.0 |
| 4921524L21Rik | 0.0 | 0.0 | 0.0 | 0.0 | 0.0 | 0.0 | 0.0 | 0.0 | 0.0 | 0.0 | 0.0 | 0.0 |
| 4921531P14Rik | 0.0 | 0.0 | 0.0 | 0.0 | 0.1 | 0.0 | 0.0 | 0.0 | 0.0 | 0.3 | 0.0 | 0.0 |
| 4921533I20Rik | 0.0 | 0.0 | 0.0 | 0.0 | 0.0 | 0.0 | 0.0 | 0.0 | 0.0 | 0.0 | 0.0 | 0.0 |
| 4930401C15Rik | 0.0 | 0.0 | 0.0 | 0.0 | 0.1 | 0.0 | 0.0 | 0.0 | 0.0 | 0.0 | 0.0 | 0.0 |
| 4930402F06Rik | 0.0 | 0.0 | 0.0 | 0.0 | 0.0 | 0.0 | 0.0 | 0.0 | 0.0 | 0.0 | 0.0 | 0.0 |
| 4930405A10Rik | 0.0 | 0.1 | 0.0 | 0.0 | 0.0 | 0.0 | 0.0 | 0.0 | 0.0 | 0.0 | 0.0 | 0.0 |
| 4930405D11Rik | 0.0 | 0.0 | 0.0 | 0.0 | 0.0 | 0.0 | 0.0 | 0.0 | 0.0 | 0.0 | 0.0 | 0.0 |
| 4930407I10Rik | 0.0 | 0.0 | 0.0 | 0.0 | 0.0 | 0.0 | 0.0 | 0.0 | 0.0 | 0.0 | 0.0 | 0.0 |
| 4930408F14Rik | 0.0 | 0.0 | 0.0 | 0.1 | 0.0 | 0.0 | 0.0 | 0.0 | 0.0 | 0.0 | 0.0 | 0.0 |
| 4930412B13Rik | 0.0 | 0.0 | 0.0 | 0.0 | 0.0 | 0.0 | 0.0 | 0.0 | 0.0 | 0.0 | 0.0 | 0.0 |
| 4930412O13Rik | 0.0 | 0.0 | 0.0 | 0.0 | 0.0 | 0.0 | 0.0 | 0.0 | 0.0 | 0.0 | 0.0 | 0.0 |
| 4930413F20Rik | 0.0 | 0.0 | 0.1 | 0.0 | 0.0 | 0.0 | 0.0 | 0.0 | 0.0 | 0.0 | 0.0 | 0.0 |
| 4930415F15Rik | 0.0 | 0.1 | 0.0 | 0.0 | 0.0 | 0.1 | 0.0 | 0.0 | 0.0 | 0.0 | 0.0 | 0.0 |
| 4930417O13Rik | 0.0 | 0.0 | 0.0 | 0.0 | 0.0 | 0.0 | 0.0 | 0.0 | 0.0 | 0.0 | 0.0 | 0.0 |
| 4930419G24Rik | 0.0 | 0.0 | 0.0 | 0.0 | 0.0 | 0.0 | 0.0 | 0.0 | 0.0 | 0.0 | 0.0 | 0.0 |
| 4930426D05Rik | 0.0 | 0.0 | 0.0 | 0.1 | 0.0 | 0.0 | 0.0 | 0.0 | 0.0 | 0.0 | 0.0 | 0.0 |
| 4930428D18Rik | 0.0 | 0.0 | 0.0 | 0.0 | 0.0 | 0.0 | 0.0 | 0.0 | 0.0 | 0.0 | 0.0 | 0.0 |
| 4930429D17Rik | 0.0 | 0.0 | 0.0 | 0.0 | 0.0 | 0.0 | 0.0 | 0.0 | 0.0 | 0.0 | 0.0 | 0.0 |
| 4930429F11Rik | 0.0 | 0.0 | 0.0 | 0.0 | 0.0 | 0.0 | 0.0 | 0.0 | 0.0 | 0.0 | 0.0 | 0.0 |
| 4930430A15Rik | 0.0 | 0.0 | 0.0 | 0.0 | 0.0 | 0.0 | 0.0 | 0.0 | 0.0 | 0.0 | 0.0 | 0.0 |
| 4930430D24Rik | 0.0 | 0.0 | 0.0 | 0.0 | 0.0 | 0.0 | 0.0 | 0.0 | 0.0 | 0.0 | 0.0 | 0.0 |
| 4930430J02Rik | 0.0 | 0.0 | 0.0 | 0.0 | 0.2 | 0.0 | 0.0 | 0.0 | 0.0 | 0.0 | 0.0 | 0.0 |
| 4930432J09Rik | 0.0 | 0.0 | 0.0 | 0.0 | 0.0 | 0.0 | 0.0 | 0.0 | 0.0 | 0.0 | 0.0 | 0.0 |
| 4930432M17Rik | 0.0 | 0.0 | 0.0 | 0.0 | 0.0 | 0.0 | 0.0 | 0.0 | 0.0 | 0.0 | 0.0 | 0.0 |
| 4930433I11Rik | 0.0 | 0.0 | 0.0 | 0.0 | 0.0 | 0.0 | 0.0 | 0.0 | 0.0 | 0.0 | 0.0 | 0.0 |
| 4930433N12Rik | 0.0 | 0.0 | 0.0 | 0.0 | 0.0 | 0.0 | 0.0 | 0.0 | 0.0 | 0.0 | 0.0 | 0.0 |
| 4930438E09Rik | 0.0 | 0.0 | 0.0 | 0.0 | 0.0 | 0.0 | 0.0 | 0.1 | 0.0 | 0.1 | 0.0 | 0.1 |
| 4930444F02Rik | 0.0 | 0.1 | 0.0 | 0.0 | 0.0 | 0.0 | 0.0 | 0.0 | 0.0 | 0.0 | 0.0 | 0.0 |
| 4930444M15Rik | 0.0 | 0.0 | 0.0 | 0.0 | 0.0 | 0.0 | 0.0 | 0.0 | 0.0 | 0.0 | 0.0 | 0.0 |
| 4930444P10Rik | 0.1 | 0.1 | 0.0 | 0.0 | 0.0 | 0.0 | 0.0 | 0.0 | 0.1 | 0.0 | 0.0 | 0.0 |
| 4930447J18Rik | 0.0 | 0.0 | 0.0 | 0.0 | 0.0 | 0.0 | 0.0 | 0.0 | 0.0 | 0.0 | 0.0 | 0.0 |
| 4930448H16Rik | 0.0 | 0.0 | 0.1 | 0.1 | 0.0 | 0.1 | 0.2 | 0.0 | 0.0 | 0.0 | 0.0 | 0.1 |

Excluded Genes

| | | | | | | | | | | | | |
|---|---|---|---|---|---|---|---|---|---|---|---|---|
| 4930448I18Rik | 0.1 | 0.0 | 0.0 | 0.0 | 0.0 | 0.0 | 0.0 | 0.0 | 0.0 | 0.0 | 0.0 | 0.0 |
| 4930449E01Rik | 0.1 | 0.0 | 0.0 | 0.0 | 0.0 | 0.0 | 0.0 | 0.0 | 0.0 | 0.0 | 0.0 | 0.0 |
| 4930451C15Rik | 0.0 | 0.0 | 0.0 | 0.0 | 0.0 | 0.0 | 0.0 | 0.0 | 0.0 | 0.0 | 0.1 | 0.0 |
| 4930455B14Rik | 0.0 | 0.0 | 0.0 | 0.0 | 0.0 | 0.0 | 0.0 | 0.0 | 0.0 | 0.0 | 0.0 | 0.0 |
| 4930455D15Rik | 0.0 | 0.0 | 0.1 | 0.0 | 0.0 | 0.0 | 0.0 | 0.0 | 0.0 | 0.0 | 0.0 | 0.0 |
| 4930455H04Rik | 0.0 | 0.0 | 0.0 | 0.0 | 0.0 | 0.0 | 0.0 | 0.0 | 0.0 | 0.0 | 0.0 | 0.0 |
| 4930456L15Rik | 0.0 | 0.0 | 0.0 | 0.0 | 0.0 | 0.0 | 0.0 | 0.0 | 0.0 | 0.0 | 0.0 | 0.0 |
| 4930459L07Rik | 0.0 | 0.0 | 0.0 | 0.0 | 0.0 | 0.0 | 0.0 | 0.0 | 0.0 | 0.0 | 0.0 | 0.0 |
| 4930461G14Rik | 0.2 | 0.0 | 0.0 | 0.2 | 0.0 | 0.0 | 0.0 | 0.0 | 0.0 | 0.0 | 0.0 | 0.0 |
| 4930467K11Rik | 0.0 | 0.0 | 0.2 | 0.0 | 0.0 | 0.0 | 0.0 | 0.0 | 0.0 | 0.0 | 0.0 | 0.1 |
| 4930468A15Rik | 0.0 | 0.0 | 0.0 | 0.0 | 0.0 | 0.0 | 0.0 | 0.0 | 0.0 | 0.0 | 0.0 | 0.0 |
| 4930469G21Rik | 0.0 | 0.0 | 0.0 | 0.1 | 0.0 | 0.0 | 0.0 | 0.0 | 0.0 | 0.0 | 0.0 | 0.0 |
| 4930471C04Rik | 0.0 | 0.0 | 0.0 | 0.1 | 0.0 | 0.0 | 0.0 | 0.0 | 0.0 | 0.0 | 0.0 | 0.0 |
| 4930471G03Rik | 0.0 | 0.0 | 0.0 | 0.0 | 0.1 | 0.0 | 0.0 | 0.0 | 0.0 | 0.0 | 0.0 | 0.0 |
| 4930474G06Rik | 0.0 | 0.0 | 0.0 | 0.0 | 0.0 | 0.0 | 0.0 | 0.0 | 0.0 | 0.0 | 0.0 | 0.0 |
| 4930474H20Rik | 0.0 | 0.0 | 0.0 | 0.0 | 0.0 | 0.0 | 0.0 | 0.0 | 0.0 | 0.0 | 0.0 | 0.0 |
| 4930474M22Rik | 0.0 | 0.0 | 0.0 | 0.0 | 0.1 | 0.0 | 0.0 | 0.0 | 0.1 | 0.0 | 0.0 | 0.0 |
| 4930474N09Rik | 0.0 | 0.0 | 0.0 | 0.0 | 0.0 | 0.0 | 0.0 | 0.0 | 0.0 | 0.0 | 0.0 | 0.0 |
| 4930483O08Rik | 0.0 | 0.0 | 0.0 | 0.0 | 0.1 | 0.1 | 0.0 | 0.0 | 0.0 | 0.0 | 0.0 | 0.0 |
| 4930486I03Rik | 0.0 | 0.0 | 0.1 | 0.0 | 0.0 | 0.0 | 0.0 | 0.0 | 0.0 | 0.0 | 0.0 | 0.0 |
| 4930500L23Rik | 0.0 | 0.0 | 0.0 | 0.0 | 0.0 | 0.0 | 0.0 | 0.1 | 0.0 | 0.0 | 0.0 | 0.0 |
| 4930503E14Rik | 0.0 | 0.0 | 0.0 | 0.0 | 0.0 | 0.0 | 0.0 | 0.0 | 0.0 | 0.0 | 0.0 | 0.0 |
| 4930503E24Rik | 0.0 | 0.0 | 0.0 | 0.0 | 0.0 | 0.0 | 0.0 | 0.0 | 0.1 | 0.0 | 0.0 | 0.0 |
| 4930503H13Rik | 0.0 | 0.0 | 0.0 | 0.0 | 0.0 | 0.0 | 0.0 | 0.0 | 0.0 | 0.0 | 0.0 | 0.0 |
| 4930505G20Rik | 0.0 | 0.0 | 0.0 | 0.0 | 0.0 | 0.0 | 0.0 | 0.0 | 0.0 | 0.0 | 0.0 | 0.0 |
| 4930511A02Rik | 0.0 | 0.0 | 0.0 | 0.0 | 0.0 | 0.0 | 0.0 | 0.0 | 0.0 | 0.0 | 0.0 | 0.0 |
| 4930511E03Rik | 0.0 | 0.0 | 0.0 | 0.0 | 0.0 | 0.0 | 0.0 | 0.0 | 0.0 | 0.0 | 0.0 | 0.0 |
| 4930513D17Rik | 0.0 | 0.0 | 0.0 | 0.0 | 0.0 | 0.0 | 0.0 | 0.1 | 0.0 | 0.0 | 0.0 | 0.0 |
| 4930513O06Rik | 0.0 | 0.1 | 0.0 | 0.0 | 0.0 | 0.0 | 0.0 | 0.0 | 0.0 | 0.0 | 0.0 | 0.0 |
| 4930517E11Rik | 0.0 | 0.0 | 0.0 | 0.0 | 0.0 | 0.0 | 0.0 | 0.0 | 0.0 | 0.0 | 0.0 | 0.0 |
| 4930519F16Rik | 0.0 | 0.0 | 0.1 | 0.0 | 0.0 | 0.0 | 0.1 | 0.0 | 0.0 | 0.0 | 0.0 | 0.0 |
| 4930520P13Rik | 0.0 | 0.0 | 0.0 | 0.0 | 0.0 | 0.0 | 0.0 | 0.0 | 0.0 | 0.1 | 0.0 | 0.0 |
| 4930522H14Rik | 0.0 | 0.0 | 0.0 | 0.0 | 0.0 | 0.0 | 0.0 | 0.0 | 0.0 | 0.0 | 0.0 | 0.0 |
| 4930522O17Rik | 0.0 | 0.0 | 0.0 | 0.0 | 0.0 | 0.1 | 0.0 | 0.0 | 0.0 | 0.0 | 0.0 | 0.0 |
| 4930524O05Rik | 0.1 | 0.0 | 0.0 | 0.0 | 0.0 | 0.0 | 0.0 | 0.0 | 0.0 | 0.0 | 0.0 | 0.0 |
| 4930524O08Rik | 0.0 | 0.0 | 0.0 | 0.0 | 0.0 | 0.0 | 0.0 | 0.0 | 0.0 | 0.0 | 0.0 | 0.0 |
| 4930525D18Rik | 0.0 | 0.0 | 0.0 | 0.0 | 0.0 | 0.0 | 0.0 | 0.0 | 0.0 | 0.0 | 0.0 | 0.0 |
| 4930526D03Rik | 0.1 | 0.0 | 0.0 | 0.0 | 0.0 | 0.0 | 0.0 | 0.0 | 0.0 | 0.0 | 0.0 | 0.0 |
| 4930526L06Rik | 0.0 | 0.0 | 0.0 | 0.0 | 0.1 | 0.0 | 0.0 | 0.0 | 0.0 | 0.0 | 0.0 | 0.0 |
| 4930527F14Rik | 0.0 | 0.0 | 0.0 | 0.1 | 0.0 | 0.0 | 0.0 | 0.0 | 0.0 | 0.0 | 0.0 | 0.0 |
| 4930527G23Rik | 0.0 | 0.0 | 0.0 | 0.0 | 0.0 | 0.0 | 0.0 | 0.0 | 0.0 | 0.0 | 0.0 | 0.0 |
| 4930529M08Rik | 0.0 | 0.0 | 0.0 | 0.1 | 0.0 | 0.0 | 0.0 | 0.0 | 0.0 | 0.0 | 0.0 | 0.1 |
| 4930533P14Rik | 0.0 | 0.0 | 0.0 | 0.0 | 0.0 | 0.0 | 0.0 | 0.0 | 0.0 | 0.0 | 0.0 | 0.0 |
| 4930539N22Rik | 0.0 | 0.0 | 0.0 | 0.0 | 0.0 | 0.0 | 0.0 | 0.0 | 0.0 | 0.0 | 0.0 | 0.0 |
| 4930542C21Rik | 0.0 | 0.0 | 0.0 | 0.0 | 0.0 | 0.0 | 0.0 | 0.0 | 0.0 | 0.0 | 0.0 | 0.0 |
| 4930542D17Rik | 0.0 | 0.0 | 0.0 | 0.0 | 0.0 | 0.0 | 0.0 | 0.0 | 0.0 | 0.0 | 0.0 | 0.0 |
| 4930542H20Rik | 0.0 | 0.0 | 0.0 | 0.0 | 0.0 | 0.0 | 0.0 | 0.0 | 0.0 | 0.0 | 0.0 | 0.0 |
| 4930543E12Rik | 0.0 | 0.0 | 0.0 | 0.0 | 0.0 | 0.0 | 0.0 | 0.0 | 0.0 | 0.0 | 0.0 | 0.0 |
| 4930544D05Rik | 0.0 | 0.0 | 0.0 | 0.0 | 0.1 | 0.0 | 0.0 | 0.0 | 0.0 | 0.0 | 0.0 | 0.0 |
| 4930544G11Rik | 0.0 | 0.0 | 0.0 | 0.0 | 0.0 | 0.0 | 0.0 | 0.0 | 0.0 | 0.0 | 0.0 | 0.0 |
| 4930544M13Rik | 0.0 | 0.0 | 0.0 | 0.0 | 0.0 | 0.0 | 0.0 | 0.0 | 0.0 | 0.0 | 0.0 | 0.0 |
| 4930545L23Rik | 0.0 | 0.0 | 0.0 | 0.0 | 0.0 | 0.0 | 0.0 | 0.0 | 0.0 | 0.0 | 0.0 | 0.0 |
| 4930546K05Rik | 0.0 | 0.0 | 0.0 | 0.0 | 0.0 | 0.0 | 0.0 | 0.0 | 0.0 | 0.0 | 0.0 | 0.0 |
| 4930547E14Rik | 0.0 | 0.0 | 0.0 | 0.0 | 0.0 | 0.0 | 0.0 | 0.0 | 0.1 | 0.0 | 0.0 | 0.0 |
| 4930548G14Rik | 0.0 | 0.0 | 0.0 | 0.0 | 0.0 | 0.0 | 0.0 | 0.0 | 0.0 | 0.0 | 0.0 | 0.0 |
| 4930548H24Rik | 0.0 | 0.0 | 0.0 | 0.0 | 0.0 | 0.0 | 0.0 | 0.0 | 0.0 | 0.1 | 0.0 | 0.0 |
| 4930550L24Rik | 0.0 | 0.0 | 0.0 | 0.1 | 0.0 | 0.0 | 0.0 | 0.0 | 0.0 | 0.0 | 0.0 | 0.0 |

| | | | | | | | | | | | | |
|---|---|---|---|---|---|---|---|---|---|---|---|---|
| 4930552N02Rik | 0.0 | 0.0 | 0.0 | 0.0 | 0.0 | 0.0 | 0.0 | 0.0 | 0.0 | 0.0 | 0.0 | 0.0 |
| 4930553E22Rik | 0.0 | 0.0 | 0.0 | 0.2 | 0.0 | 0.0 | 0.0 | 0.0 | 0.0 | 0.0 | 0.0 | 0.0 |
| 4930555B11Rik | 0.0 | 0.0 | 0.0 | 0.0 | 0.0 | 0.0 | 0.0 | 0.0 | 0.0 | 0.0 | 0.0 | 0.0 |
| 4930556C24Rik | 0.0 | 0.0 | 0.0 | 0.0 | 0.0 | 0.0 | 0.0 | 0.0 | 0.1 | 0.0 | 0.1 | 0.0 |
| 4930556G01Rik | 0.0 | 0.0 | 0.0 | 0.0 | 0.0 | 0.0 | 0.0 | 0.0 | 0.0 | 0.0 | 0.0 | 0.0 |
| 4930557A04Rik | 0.0 | 0.0 | 0.0 | 0.0 | 0.0 | 0.0 | 0.0 | 0.0 | 0.0 | 0.0 | 0.0 | 0.0 |
| 4930557J02Rik | 0.0 | 0.0 | 0.0 | 0.0 | 0.0 | 0.0 | 0.0 | 0.0 | 0.0 | 0.0 | 0.0 | 0.0 |
| 4930558C23Rik | 0.0 | 0.0 | 0.0 | 0.0 | 0.0 | 0.0 | 0.0 | 0.0 | 0.0 | 0.1 | 0.0 | 0.0 |
| 4930558K02Rik | 0.0 | 0.0 | 0.0 | 0.0 | 0.0 | 0.0 | 0.0 | 0.0 | 0.0 | 0.0 | 0.0 | 0.0 |
| 4930563F08Rik | 0.0 | 0.0 | 0.0 | 0.0 | 0.0 | 0.0 | 0.0 | 0.0 | 0.0 | 0.0 | 0.0 | 0.0 |
| 4930564B18Rik | 0.0 | 0.0 | 0.0 | 0.0 | 0.0 | 0.0 | 0.0 | 0.0 | 0.0 | 0.0 | 0.0 | 0.0 |
| 4930564D02Rik | 0.0 | 0.0 | 0.0 | 0.0 | 0.0 | 0.0 | 0.0 | 0.0 | 0.1 | 0.0 | 0.0 | 0.1 |
| 4930564G21Rik | 0.0 | 0.1 | 0.0 | 0.0 | 0.0 | 0.0 | 0.0 | 0.0 | 0.0 | 0.0 | 0.0 | 0.0 |
| 4930564K09Rik | 0.0 | 0.0 | 0.0 | 0.0 | 0.0 | 0.0 | 0.0 | 0.0 | 0.0 | 0.0 | 0.0 | 0.0 |
| 4930565D16Rik | 0.0 | 0.0 | 0.0 | 0.1 | 0.0 | 0.0 | 0.0 | 0.0 | 0.0 | 0.0 | 0.0 | 0.0 |
| 4930567H12Rik | 0.0 | 0.0 | 0.0 | 0.0 | 0.0 | 0.0 | 0.0 | 0.0 | 0.0 | 0.0 | 0.0 | 0.0 |
| 4930567H17Rik | 0.0 | 0.0 | 0.0 | 0.0 | 0.0 | 0.0 | 0.0 | 0.0 | 0.0 | 0.0 | 0.0 | 0.0 |
| 4930568E12Rik | 0.0 | 0.0 | 0.0 | 0.0 | 0.0 | 0.0 | 0.0 | 0.0 | 0.0 | 0.0 | 0.0 | 0.0 |
| 4930568G15Rik | 0.0 | 0.0 | 0.0 | 0.0 | 0.0 | 0.0 | 0.0 | 0.0 | 0.0 | 0.0 | 0.0 | 0.0 |
| 4930572O03Rik | 0.0 | 0.0 | 0.0 | 0.0 | 0.0 | 0.0 | 0.0 | 0.0 | 0.0 | 0.0 | 0.0 | 0.0 |
| 4930572O13Rik | 0.0 | 0.0 | 0.0 | 0.0 | 0.0 | 0.0 | 0.0 | 0.0 | 0.0 | 0.0 | 0.0 | 0.0 |
| 4930573O16Rik | 0.0 | 0.0 | 0.0 | 0.0 | 0.0 | 0.2 | 0.0 | 0.0 | 0.0 | 0.0 | 0.0 | 0.0 |
| 4930578C19Rik | 0.0 | 0.0 | 0.0 | 0.0 | 0.0 | 0.0 | 0.0 | 0.0 | 0.1 | 0.0 | 0.0 | 0.0 |
| 4930578I06Rik | 0.0 | 0.1 | 0.0 | 0.1 | 0.0 | 0.0 | 0.0 | 0.0 | 0.0 | 0.0 | 0.0 | 0.0 |
| 4930578N18Rik | 0.0 | 0.0 | 0.0 | 0.0 | 0.0 | 0.0 | 0.0 | 0.0 | 0.0 | 0.0 | 0.0 | 0.0 |
| 4930579F01Rik | 0.0 | 0.1 | 0.0 | 0.0 | 0.0 | 0.0 | 0.0 | 0.0 | 0.0 | 0.0 | 0.0 | 0.0 |
| 4930583K01Rik | 0.0 | 0.0 | 0.0 | 0.0 | 0.0 | 0.1 | 0.0 | 0.0 | 0.0 | 0.0 | 0.0 | 0.0 |
| 4930584F24Rik | 0.0 | 0.0 | 0.0 | 0.0 | 0.0 | 0.0 | 0.0 | 0.0 | 0.0 | 0.0 | 0.0 | 0.0 |
| 4930590L20Rik | 0.0 | 0.0 | 0.2 | 0.0 | 0.0 | 0.0 | 0.0 | 0.0 | 0.0 | 0.0 | 0.0 | 0.1 |
| 4930593A02Rik | 0.0 | 0.0 | 0.0 | 0.0 | 0.0 | 0.0 | 0.0 | 0.0 | 0.0 | 0.0 | 0.0 | 0.0 |
| 4930593A02Rik | 0.0 | 0.0 | 0.0 | 0.0 | 0.0 | 0.0 | 0.0 | 0.0 | 0.0 | 0.0 | 0.0 | 0.0 |
| 4931406B18Rik | 0.0 | 0.0 | 0.0 | 0.0 | 0.0 | 0.0 | 0.0 | 0.0 | 0.0 | 0.0 | 0.0 | 0.0 |
| 4931409K22Rik | 0.0 | 0.0 | 0.0 | 0.0 | 0.0 | 0.0 | 0.0 | 0.0 | 0.0 | 0.0 | 0.0 | 0.0 |
| 4931417E11Rik | 0.0 | 0.0 | 0.0 | 0.0 | 0.0 | 0.0 | 0.0 | 0.0 | 0.0 | 0.0 | 0.0 | 0.0 |
| 4931423N10Rik | 0.0 | 0.0 | 0.0 | 0.0 | 0.0 | 0.0 | 0.0 | 0.0 | 0.0 | 0.0 | 0.0 | 0.0 |
| 4931428L18Rik | 0.0 | 0.1 | 0.0 | 0.0 | 0.0 | 0.0 | 0.0 | 0.0 | 0.0 | 0.0 | 0.0 | 0.0 |
| 4931429I11Rik | 0.0 | 0.0 | 0.0 | 0.0 | 0.0 | 0.0 | 0.0 | 0.0 | 0.0 | 0.0 | 0.0 | 0.0 |
| 4931429L15Rik | 0.0 | 0.0 | 0.0 | 0.0 | 0.0 | 0.0 | 0.0 | 0.0 | 0.0 | 0.0 | 0.0 | 0.0 |
| 4931440J10Rik | 0.0 | 0.0 | 0.0 | 0.0 | 0.0 | 0.1 | 0.0 | 0.0 | 0.1 | 0.0 | 0.0 | 0.1 |
| 4932411E22Rik | 0.0 | 0.0 | 0.0 | 0.0 | 0.0 | 0.0 | 0.0 | 0.0 | 0.0 | 0.0 | 0.0 | 0.0 |
| 4932411N23Rik | 0.0 | 0.0 | 0.0 | 0.0 | 0.0 | 0.0 | 0.0 | 0.0 | 0.0 | 0.0 | 0.0 | 0.0 |
| 4932412D23Rik | 0.0 | 0.0 | 0.0 | 0.0 | 0.0 | 0.0 | 0.0 | 0.0 | 0.0 | 0.0 | 0.0 | 0.0 |
| 4932414J04Rik | 0.0 | 0.0 | 0.0 | 0.0 | 0.0 | 0.0 | 0.0 | 0.0 | 0.0 | 0.0 | 0.0 | 0.0 |
| 4932414N04Rik | 0.0 | 0.0 | 0.0 | 0.0 | 0.0 | 0.0 | 0.0 | 0.0 | 0.0 | 0.0 | 0.0 | 0.0 |
| 4932415M13Rik | 0.0 | 0.0 | 0.0 | 0.0 | 0.0 | 0.0 | 0.0 | 0.0 | 0.0 | 0.0 | 0.0 | 0.0 |
| 4932416K20Rik | 0.0 | 0.0 | 0.0 | 0.0 | 0.0 | 0.0 | 0.0 | 0.0 | 0.0 | 0.0 | 0.0 | 0.0 |
| 4932443I19Rik | 0.0 | 0.0 | 0.0 | 0.0 | 0.0 | 0.0 | 0.0 | 0.1 | 0.0 | 0.0 | 0.0 | 0.1 |
| 4933400C23Rik | 0.0 | 0.0 | 0.0 | 0.0 | 0.0 | 0.0 | 0.0 | 0.0 | 0.0 | 0.0 | 0.0 | 0.0 |
| 4933400L20Rik | 0.0 | 0.0 | 0.0 | 0.0 | 0.0 | 0.0 | 0.0 | 0.0 | 0.3 | 0.0 | 0.0 | 0.0 |
| 4933401D09Rik | 0.0 | 0.0 | 0.0 | 0.0 | 0.0 | 0.0 | 0.1 | 0.0 | 0.0 | 0.0 | 0.0 | 0.0 |
| 4933401H06Rik | 0.0 | 0.0 | 0.0 | 0.0 | 0.0 | 0.0 | 0.0 | 0.0 | 0.0 | 0.1 | 0.0 | 0.0 |
| 4933402J07Rik | 0.0 | 0.0 | 0.0 | 0.0 | 0.0 | 0.0 | 0.0 | 0.0 | 0.0 | 0.0 | 0.0 | 0.0 |
| 4933402J15Rik | 0.0 | 0.0 | 0.0 | 0.1 | 0.0 | 0.0 | 0.0 | 0.0 | 0.0 | 0.0 | 0.0 | 0.0 |
| 4933402P03Rik | 0.0 | 0.0 | 0.0 | 0.0 | 0.0 | 0.0 | 0.0 | 0.0 | 0.0 | 0.0 | 0.0 | 0.0 |
| 4933405E24Rik | 0.0 | 0.0 | 0.0 | 0.0 | 0.0 | 0.0 | 0.0 | 0.0 | 0.0 | 0.0 | 0.0 | 0.0 |
| 4933406G16Rik | 0.0 | 0.0 | 0.0 | 0.0 | 0.0 | 0.0 | 0.0 | 0.0 | 0.0 | 0.0 | 0.0 | 0.0 |
| 4933406J08Rik | 0.0 | 0.0 | 0.0 | 0.0 | 0.0 | 0.0 | 0.0 | 0.0 | 0.0 | 0.0 | 0.0 | 0.0 |

| | | | | | | | | | | | | |
|---|---|---|---|---|---|---|---|---|---|---|---|---|
| 4933406J10Rik | 0.0 | 0.0 | 0.0 | 0.0 | 0.0 | 0.0 | 0.0 | 0.0 | 0.0 | 0.0 | 0.1 | 0.0 |
| 4933407G14Rik | 0.0 | 0.0 | 0.0 | 0.0 | 0.0 | 0.0 | 0.0 | 0.0 | 0.0 | 0.0 | 0.0 | 0.0 |
| 4933407I05Rik | 0.0 | 0.0 | 0.0 | 0.0 | 0.0 | 0.0 | 0.0 | 0.0 | 0.0 | 0.0 | 0.0 | 0.0 |
| 4933407L21Rik | 0.0 | 0.0 | 0.0 | 0.0 | 0.0 | 0.0 | 0.0 | 0.0 | 0.0 | 0.0 | 0.0 | 0.0 |
| 4933409G03Rik | 0.0 | 0.0 | 0.0 | 0.1 | 0.0 | 0.0 | 0.0 | 0.0 | 0.0 | 0.0 | 0.0 | 0.0 |
| 4933413G19Rik | 0.0 | 0.0 | 0.0 | 0.0 | 0.0 | 0.0 | 0.0 | 0.0 | 0.0 | 0.0 | 0.0 | 0.0 |
| 4933415F23Rik | 0.0 | 0.0 | 0.0 | 0.0 | 0.0 | 0.0 | 0.0 | 0.0 | 0.0 | 0.0 | 0.0 | 0.0 |
| 4933416C03Rik | 0.0 | 0.0 | 0.0 | 0.0 | 0.0 | 0.0 | 0.0 | 0.0 | 0.0 | 0.0 | 0.0 | 0.0 |
| 4933416E03Rik | 0.0 | 0.0 | 0.0 | 0.0 | 0.0 | 0.0 | 0.0 | 0.0 | 0.0 | 0.0 | 0.0 | 0.0 |
| 4933416I08Rik | 0.0 | 0.0 | 0.0 | 0.0 | 0.0 | 0.1 | 0.0 | 0.0 | 0.0 | 0.0 | 0.0 | 0.0 |
| 4933422H20Rik | 0.0 | 0.0 | 0.0 | 0.1 | 0.0 | 0.1 | 0.0 | 0.0 | 0.0 | 0.0 | 0.0 | 0.0 |
| 4933425B07Rik | 0.0 | 0.0 | 0.0 | 0.0 | 0.0 | 0.0 | 0.0 | 0.0 | 0.0 | 0.0 | 0.0 | 0.0 |
| 4933425L06Rik | 0.0 | 0.0 | 0.0 | 0.0 | 0.0 | 0.0 | 0.0 | 0.0 | 0.0 | 0.0 | 0.0 | 0.0 |
| 4933427I22Rik | 0.0 | 0.0 | 0.0 | 0.0 | 0.0 | 0.0 | 0.0 | 0.0 | 0.0 | 0.0 | 0.0 | 0.0 |
| 4933428C19Rik | 0.0 | 0.0 | 0.0 | 0.0 | 0.0 | 0.0 | 0.0 | 0.0 | 0.0 | 0.0 | 0.0 | 0.0 |
| 4933429O19Rik | 0.0 | 0.0 | 0.0 | 0.0 | 0.0 | 0.0 | 0.0 | 0.0 | 0.0 | 0.0 | 0.0 | 0.0 |
| 4933430M04Rik | 0.0 | 0.0 | 0.0 | 0.0 | 0.0 | 0.0 | 0.0 | 0.0 | 0.0 | 0.1 | 0.0 | 0.1 |
| 4933430N04Rik | 0.0 | 0.0 | 0.0 | 0.0 | 0.0 | 0.0 | 0.0 | 0.0 | 0.0 | 0.0 | 0.0 | 0.0 |
| 4933432I03Rik | 0.0 | 0.0 | 0.0 | 0.0 | 0.0 | 0.0 | 0.0 | 0.0 | 0.0 | 0.0 | 0.0 | 0.0 |
| 4933433C11Rik | 0.0 | 0.0 | 0.1 | 0.0 | 0.0 | 0.0 | 0.0 | 0.0 | 0.0 | 0.0 | 0.0 | 0.1 |
| 4933434I20Rik | 0.0 | 0.0 | 0.0 | 0.0 | 0.0 | 0.0 | 0.0 | 0.0 | 0.0 | 0.0 | 0.0 | 0.0 |
| 4933436H12Rik | 0.0 | 0.0 | 0.0 | 0.0 | 0.0 | 0.1 | 0.0 | 0.0 | 0.0 | 0.0 | 0.0 | 0.0 |
| 4933438K21Rik | 0.0 | 0.1 | 0.0 | 0.0 | 0.0 | 0.0 | 0.0 | 0.0 | 0.0 | 0.0 | 0.0 | 0.0 |
| 5031425F14Rik | 0.0 | 0.0 | 0.0 | 0.0 | 0.1 | 0.0 | 0.0 | 0.0 | 0.0 | 0.0 | 0.0 | 0.0 |
| 5033403H07Rik | 0.0 | 0.0 | 0.0 | 0.0 | 0.0 | 0.2 | 0.0 | 0.0 | 0.0 | 0.0 | 0.0 | 0.0 |
| 5033404E19Rik | 0.0 | 0.0 | 0.0 | 0.1 | 0.0 | 0.0 | 0.0 | 0.0 | 0.0 | 0.0 | 0.0 | 0.0 |
| 5330413P13Rik | 0.0 | 0.0 | 0.0 | 0.0 | 0.0 | 0.0 | 0.0 | 0.0 | 0.0 | 0.0 | 0.0 | 0.0 |
| 5330434G04Rik | 0.0 | 0.0 | 0.0 | 0.0 | 0.0 | 0.0 | 0.0 | 0.0 | 0.0 | 0.0 | 0.0 | 0.0 |
| 5330439B14Rik | 0.0 | 0.0 | 0.0 | 0.0 | 0.0 | 0.0 | 0.0 | 0.0 | 0.0 | 0.0 | 0.0 | 0.0 |
| 5430421N21Rik | 0.0 | 0.0 | 0.0 | 0.0 | 0.0 | 0.0 | 0.0 | 0.0 | 0.0 | 0.0 | 0.0 | 0.0 |
| 5430425J12Rik | 0.0 | 0.0 | 0.0 | 0.0 | 0.0 | 0.0 | 0.0 | 0.0 | 0.0 | 0.0 | 0.0 | 0.0 |
| 5430428K19Rik | 0.0 | 0.0 | 0.0 | 0.0 | 0.0 | 0.0 | 0.1 | 0.0 | 0.0 | 0.0 | 0.0 | 0.0 |
| 5430440P10Rik | 0.0 | 0.0 | 0.0 | 0.0 | 0.0 | 0.0 | 0.0 | 0.0 | 0.0 | 0.0 | 0.0 | 0.0 |
| 5730403I07Rik | 0.0 | 0.0 | 0.0 | 0.0 | 0.0 | 0.0 | 0.0 | 0.0 | 0.0 | 0.0 | 0.0 | 0.0 |
| 5730420D15Rik | 0.0 | 0.1 | 0.0 | 0.0 | 0.0 | 0.0 | 0.0 | 0.1 | 0.0 | 0.0 | 0.0 | 0.0 |
| 5730522E02Rik | 0.0 | 0.0 | 0.0 | 0.0 | 0.0 | 0.0 | 0.0 | 0.0 | 0.0 | 0.0 | 0.0 | 0.0 |
| 5830403M04Rik | 0.0 | 0.0 | 0.0 | 0.0 | 0.0 | 0.0 | 0.0 | 0.0 | 0.0 | 0.0 | 0.0 | 0.0 |
| 5830411N06Rik | 0.0 | 0.0 | 0.0 | 0.0 | 0.0 | 0.0 | 0.0 | 0.0 | 0.0 | 0.0 | 0.0 | 0.0 |
| 5830428M24Rik | 0.0 | 0.1 | 0.0 | 0.0 | 0.0 | 0.0 | 0.0 | 0.0 | 0.0 | 0.0 | 0.0 | 0.0 |
| 5830416I19Rik | 0.0 | 0.0 | 0.0 | 0.0 | 0.0 | 0.0 | 0.0 | 0.0 | 0.0 | 0.0 | 0.0 | 0.0 |
| 5930412G12Rik | 0.0 | 0.0 | 0.0 | 0.1 | 0.0 | 0.0 | 0.0 | 0.0 | 0.0 | 0.0 | 0.0 | 0.0 |
| 5930438M14Rik | 0.0 | 0.0 | 0.0 | 0.0 | 0.0 | 0.0 | 0.0 | 0.0 | 0.0 | 0.0 | 0.0 | 0.0 |
| 6030440G07Rik | 0.0 | 0.0 | 0.0 | 0.0 | 0.1 | 0.0 | 0.0 | 0.0 | 0.0 | 0.0 | 0.0 | 0.0 |
| 6330410L21Rik | 0.0 | 0.0 | 0.0 | 0.0 | 0.0 | 0.0 | 0.0 | 0.0 | 0.0 | 0.0 | 0.0 | 0.0 |
| 6330415B21Rik | 0.0 | 0.0 | 0.0 | 0.0 | 0.0 | 0.0 | 0.0 | 0.0 | 0.0 | 0.0 | 0.0 | 0.0 |
| 7420461P10Rik | 0.0 | 0.0 | 0.0 | 0.0 | 0.0 | 0.1 | 0.0 | 0.1 | 0.0 | 0.0 | 0.0 | 0.0 |
| 7530416G11Rik | 0.0 | 0.0 | 0.0 | 0.1 | 0.0 | 0.0 | 0.0 | 0.0 | 0.0 | 0.0 | 0.0 | 0.0 |
| 8430437L04Rik | 0.0 | 0.0 | 0.0 | 0.0 | 0.0 | 0.0 | 0.0 | 0.0 | 0.0 | 0.0 | 0.0 | 0.0 |
| 9030204H09Rik | 0.0 | 0.0 | 0.0 | 0.0 | 0.0 | 0.2 | 0.0 | 0.0 | 0.0 | 0.0 | 0.1 | 0.0 |
| 9030404E10Rik | 0.0 | 0.0 | 0.0 | 0.0 | 0.0 | 0.0 | 0.0 | 0.0 | 0.0 | 0.0 | 0.0 | 0.0 |
| 9030619P08Rik | 0.0 | 0.0 | 0.0 | 0.0 | 0.0 | 0.0 | 0.0 | 0.0 | 0.0 | 0.0 | 0.0 | 0.0 |
| 9130015L21Rik | 0.0 | 0.0 | 0.0 | 0.0 | 0.0 | 0.0 | 0.0 | 0.0 | 0.0 | 0.0 | 0.0 | 0.0 |
| 9130204L05Rik | 0.0 | 0.1 | 0.0 | 0.0 | 0.0 | 0.0 | 0.0 | 0.0 | 0.0 | 0.0 | 0.0 | 0.0 |
| 9130230L23Rik | 0.0 | 0.0 | 0.0 | 0.0 | 0.0 | 0.0 | 0.0 | 0.0 | 0.0 | 0.0 | 0.0 | 0.0 |
| 9230105E05Rik | 0.0 | 0.0 | 0.0 | 0.0 | 0.0 | 0.0 | 0.0 | 0.0 | 0.0 | 0.0 | 0.0 | 0.0 |
| 9230112D13Rik | 0.0 | 0.0 | 0.0 | 0.0 | 0.0 | 0.0 | 0.0 | 0.0 | 0.0 | 0.0 | 0.0 | 0.1 |
| 9330158H04Rik | 0.0 | 0.0 | 0.0 | 0.0 | 0.0 | 0.0 | 0.0 | 0.0 | 0.0 | 0.0 | 0.0 | 0.0 |

| | | | | | | | | | | | | |
|---|---|---|---|---|---|---|---|---|---|---|---|---|
| 9330178D15Rik | 0.0 | 0.0 | 0.0 | 0.0 | 0.0 | 0.0 | 0.0 | 0.0 | 0.0 | 0.0 | 0.0 | 0.0 |
| 9430019J16Rik | 0.0 | 0.0 | 0.0 | 0.0 | 0.0 | 0.0 | 0.0 | 0.0 | 0.0 | 0.0 | 0.0 | 0.0 |
| 9430021M05Rik | 0.0 | 0.0 | 0.0 | 0.0 | 0.0 | 0.0 | 0.0 | 0.0 | 0.0 | 0.0 | 0.0 | 0.0 |
| 9430069I07Rik | 0.0 | 0.0 | 0.0 | 0.0 | 0.0 | 0.0 | 0.0 | 0.3 | 0.1 | 0.0 | 0.1 | 0.0 |
| 9430076G02Rik | 0.0 | 0.0 | 0.0 | 0.0 | 0.0 | 0.0 | 0.0 | 0.0 | 0.0 | 0.0 | 0.0 | 0.0 |
| 9530003J23Rik | 0.0 | 0.0 | 0.0 | 0.0 | 0.1 | 0.0 | 0.1 | 0.0 | 0.0 | 0.0 | 0.0 | 0.0 |
| 9530036O11Rik | 0.0 | 0.0 | 0.0 | 0.0 | 0.0 | 0.0 | 0.0 | 0.0 | 0.0 | 0.0 | 0.0 | 0.0 |
| 9530048J24Rik | 0.0 | 0.0 | 0.1 | 0.0 | 0.0 | 0.0 | 0.0 | 0.0 | 0.0 | 0.0 | 0.0 | 0.0 |
| 9530080O11Rik | 0.0 | 0.0 | 0.0 | 0.0 | 0.0 | 0.0 | 0.0 | 0.0 | 0.0 | 0.0 | 0.0 | 0.0 |
| 9830107B12Rik | 0.0 | 0.0 | 0.0 | 0.0 | 0.0 | 0.0 | 0.0 | 0.0 | 0.0 | 0.0 | 0.0 | 0.0 |
| 9830166K06Rik | 0.0 | 0.0 | 0.0 | 0.0 | 0.0 | 0.0 | 0.0 | 0.0 | 0.0 | 0.0 | 0.0 | 0.0 |
| a | 0.1 | 0.0 | 0.0 | 0.0 | 0.0 | 0.1 | 0.0 | 0.1 | 0.0 | 0.0 | 0.0 | 0.0 |
| A1cf | 0.0 | 0.0 | 0.0 | 0.0 | 0.0 | 0.0 | 0.0 | 0.0 | 0.0 | 0.0 | 0.0 | 0.0 |
| A230001M10Rik | 0.0 | 0.0 | 0.0 | 0.0 | 0.0 | 0.0 | 0.0 | 0.1 | 0.0 | 0.0 | 0.0 | 0.0 |
| A230077H06Rik | 0.0 | 0.0 | 0.0 | 0.0 | 0.0 | 0.0 | 0.0 | 0.0 | 0.0 | 0.0 | 0.0 | 0.0 |
| A330050F15Rik | 0.0 | 0.0 | 0.0 | 0.0 | 0.0 | 0.0 | 0.0 | 0.0 | 0.0 | 0.0 | 0.0 | 0.0 |
| A330076C08Rik | 0.0 | 0.0 | 0.0 | 0.0 | 0.0 | 0.0 | 0.0 | 0.0 | 0.0 | 0.0 | 0.0 | 0.0 |
| A330093E20Rik | 0.0 | 0.0 | 0.0 | 0.0 | 0.0 | 0.0 | 0.0 | 0.0 | 0.0 | 0.0 | 0.0 | 0.0 |
| A430089I19Rik | 0.0 | 0.0 | 0.0 | 0.0 | 0.0 | 0.0 | 0.0 | 0.0 | 0.0 | 0.0 | 0.0 | 0.0 |
| A430093F15Rik | 0.0 | 0.0 | 0.0 | 0.0 | 0.1 | 0.0 | 0.1 | 0.0 | 0.0 | 0.0 | 0.0 | 0.0 |
| A530065N20Rik | 0.0 | 0.0 | 0.0 | 0.0 | 0.0 | 0.0 | 0.0 | 0.0 | 0.0 | 0.0 | 0.0 | 0.0 |
| A630010A05Rik | 0.0 | 0.0 | 0.0 | 0.0 | 0.0 | 0.0 | 0.0 | 0.0 | 0.0 | 0.0 | 0.0 | 0.0 |
| A630012P03Rik | 0.0 | 0.0 | 0.0 | 0.0 | 0.0 | 0.0 | 0.0 | 0.0 | 0.0 | 0.0 | 0.0 | 0.0 |
| A630073D07Rik | 0.0 | 0.0 | 0.0 | 0.0 | 0.0 | 0.0 | 0.0 | 0.0 | 0.0 | 0.0 | 0.0 | 0.0 |
| A630095N17Rik | 0.0 | 0.0 | 0.1 | 0.0 | 0.0 | 0.2 | 0.0 | 0.0 | 0.0 | 0.0 | 0.0 | 0.0 |
| A730017C20Rik | 0.0 | 0.0 | 0.0 | 0.0 | 0.0 | 0.0 | 0.0 | 0.0 | 0.0 | 0.0 | 0.0 | 0.0 |
| A730018C14Rik | 0.0 | 0.0 | 0.0 | 0.0 | 0.0 | 0.0 | 0.0 | 0.0 | 0.0 | 0.0 | 0.0 | 0.0 |
| A730020E08Rik | 0.0 | 0.0 | 0.0 | 0.0 | 0.0 | 0.0 | 0.0 | 0.1 | 0.0 | 0.0 | 0.0 | 0.0 |
| A730036I17Rik | 0.0 | 0.0 | 0.0 | 0.0 | 0.0 | 0.0 | 0.0 | 0.0 | 0.0 | 0.0 | 0.0 | 0.0 |
| A730046J19Rik | 0.0 | 0.0 | 0.0 | 0.0 | 0.0 | 0.0 | 0.0 | 0.0 | 0.0 | 0.0 | 0.0 | 0.0 |
| A730082K24Rik | 0.1 | 0.0 | 0.0 | 0.0 | 0.0 | 0.0 | 0.0 | 0.0 | 0.0 | 0.0 | 0.0 | 0.0 |
| A730085K08Rik | 0.0 | 0.2 | 0.0 | 0.0 | 0.0 | 0.0 | 0.0 | 0.0 | 0.2 | 0.2 | 0.0 | 0.0 |
| A930001A20Rik | 0.0 | 0.0 | 0.0 | 0.0 | 0.0 | 0.0 | 0.0 | 0.0 | 0.0 | 0.0 | 0.0 | 0.0 |
| A930011O12Rik | 0.0 | 0.0 | 0.0 | 0.0 | 0.0 | 0.0 | 0.0 | 0.0 | 0.0 | 0.0 | 0.0 | 0.0 |
| AA387883 | 0.0 | 0.0 | 0.0 | 0.0 | 0.0 | 0.0 | 0.0 | 0.0 | 0.0 | 0.0 | 0.0 | 0.0 |
| Aadacl2 | 0.0 | 0.0 | 0.0 | 0.0 | 0.0 | 0.0 | 0.0 | 0.0 | 0.0 | 0.0 | 0.0 | 0.0 |
| Abca14 | 0.0 | 0.0 | 0.0 | 0.0 | 0.0 | 0.0 | 0.0 | 0.0 | 0.0 | 0.0 | 0.0 | 0.0 |
| Abca15 | 0.0 | 0.0 | 0.0 | 0.0 | 0.0 | 0.0 | 0.0 | 0.0 | 0.0 | 0.0 | 0.0 | 0.0 |
| Abca16 | 0.0 | 0.0 | 0.0 | 0.0 | 0.0 | 0.0 | 0.0 | 0.0 | 0.0 | 0.0 | 0.0 | 0.0 |
| Abcc8 | 0.0 | 0.0 | 0.0 | 0.0 | 0.0 | 0.0 | 0.0 | 0.0 | 0.0 | 0.0 | 0.0 | 0.0 |
| Abcg5 | 0.0 | 0.0 | 0.0 | 0.0 | 0.0 | 0.0 | 0.0 | 0.0 | 0.0 | 0.0 | 0.0 | 0.0 |
| Abhd12b | 0.0 | 0.0 | 0.0 | 0.0 | 0.0 | 0.1 | 0.0 | 0.0 | 0.0 | 0.0 | 0.0 | 0.0 |
| Accsl | 0.0 | 0.0 | 0.0 | 0.0 | 0.0 | 0.0 | 0.0 | 0.0 | 0.0 | 0.0 | 0.0 | 0.0 |
| Ace3 | 0.0 | 0.0 | 0.0 | 0.0 | 0.0 | 0.0 | 0.0 | 0.0 | 0.0 | 0.0 | 0.0 | 0.0 |
| Acer1 | 0.0 | 0.0 | 0.0 | 0.0 | 0.0 | 0.0 | 0.0 | 0.0 | 0.0 | 0.0 | 0.0 | 0.0 |
| Acpt | 0.0 | 0.0 | 0.0 | 0.0 | 0.0 | 0.0 | 0.0 | 0.0 | 0.0 | 0.0 | 0.0 | 0.0 |
| Acr | 0.0 | 0.1 | 0.0 | 0.0 | 0.0 | 0.0 | 0.0 | 0.0 | 0.0 | 0.0 | 0.0 | 0.0 |
| Acrv1 | 0.0 | 0.0 | 0.0 | 0.0 | 0.0 | 0.0 | 0.0 | 0.0 | 0.0 | 0.0 | 0.0 | 0.0 |
| Acsbg2 | 0.0 | 0.0 | 0.0 | 0.0 | 0.0 | 0.0 | 0.0 | 0.0 | 0.0 | 0.0 | 0.0 | 0.0 |
| Acsm1 | 0.0 | 0.0 | 0.0 | 0.0 | 0.0 | 0.0 | 0.0 | 0.0 | 0.0 | 0.0 | 0.0 | 0.0 |
| Acsm2 | 0.0 | 0.0 | 0.0 | 0.0 | 0.0 | 0.0 | 0.0 | 0.0 | 0.0 | 0.0 | 0.0 | 0.0 |
| Acsm5 | 0.0 | 0.0 | 0.0 | 0.0 | 0.0 | 0.0 | 0.0 | 0.0 | 0.0 | 0.0 | 0.0 | 0.0 |
| Actbl2 | 0.0 | 0.0 | 0.0 | 0.0 | 0.0 | 0.0 | 0.0 | 0.0 | 0.0 | 0.0 | 0.0 | 0.0 |
| Actl6b | 0.0 | 0.0 | 0.0 | 0.0 | 0.0 | 0.0 | 0.0 | 0.0 | 0.0 | 0.0 | 0.0 | 0.0 |
| Adad1 | 0.0 | 0.0 | 0.0 | 0.0 | 0.0 | 0.0 | 0.0 | 0.0 | 0.0 | 0.0 | 0.0 | 0.0 |
| Adad2 | 0.0 | 0.0 | 0.0 | 0.0 | 0.0 | 0.0 | 0.0 | 0.0 | 0.0 | 0.0 | 0.0 | 0.0 |
| Adam2 | 0.0 | 0.0 | 0.0 | 0.0 | 0.0 | 0.0 | 0.0 | 0.0 | 0.0 | 0.0 | 0.0 | 0.0 |

Excluded Genes

| | | | | | | | | | | | | |
|---|---|---|---|---|---|---|---|---|---|---|---|---|
| Adam20 | 0.0 | 0.0 | 0.0 | 0.0 | 0.0 | 0.0 | 0.0 | 0.0 | 0.0 | 0.0 | 0.0 | 0.0 |
| Adam24 | 0.0 | 0.0 | 0.0 | 0.0 | 0.0 | 0.0 | 0.0 | 0.0 | 0.0 | 0.0 | 0.0 | 0.0 |
| Adam25 | 0.0 | 0.0 | 0.0 | 0.0 | 0.0 | 0.0 | 0.0 | 0.0 | 0.0 | 0.0 | 0.0 | 0.0 |
| Adam26a | 0.0 | 0.0 | 0.0 | 0.0 | 0.0 | 0.0 | 0.0 | 0.0 | 0.0 | 0.0 | 0.0 | 0.0 |
| Adam26b | 0.0 | 0.0 | 0.0 | 0.0 | 0.0 | 0.0 | 0.0 | 0.0 | 0.0 | 0.0 | 0.0 | 0.0 |
| Adam30 | 0.0 | 0.0 | 0.0 | 0.0 | 0.0 | 0.0 | 0.0 | 0.0 | 0.0 | 0.0 | 0.0 | 0.0 |
| Adam6a | 0.0 | 0.0 | 0.0 | 0.0 | 0.0 | 0.0 | 0.0 | 0.0 | 0.0 | 0.0 | 0.0 | 0.0 |
| Adam6b | 0.0 | 0.0 | 0.0 | 0.0 | 0.0 | 0.0 | 0.0 | 0.0 | 0.0 | 0.0 | 0.0 | 0.0 |
| Adam7 | 0.0 | 0.0 | 0.0 | 0.1 | 0.0 | 0.0 | 0.0 | 0.0 | 0.0 | 0.0 | 0.0 | 0.0 |
| Adamdec1 | 0.0 | 0.0 | 0.0 | 0.0 | 0.0 | 0.0 | 0.0 | 0.0 | 0.0 | 0.0 | 0.0 | 0.0 |
| Adamts18 | 0.0 | 0.0 | 0.0 | 0.0 | 0.0 | 0.0 | 0.0 | 0.0 | 0.0 | 0.0 | 0.0 | 0.0 |
| Adarb2 | 0.0 | 0.0 | 0.0 | 0.0 | 0.0 | 0.0 | 0.0 | 0.0 | 0.0 | 0.0 | 0.0 | 0.0 |
| Adcyap1 | 0.0 | 0.0 | 0.0 | 0.0 | 0.0 | 0.0 | 0.0 | 0.0 | 0.0 | 0.0 | 0.0 | 0.0 |
| Adh6-ps1 | 0.0 | 0.0 | 0.0 | 0.0 | 0.0 | 0.0 | 0.0 | 0.0 | 0.0 | 0.0 | 0.0 | 0.0 |
| Adig | 0.0 | 0.0 | 0.0 | 0.0 | 0.0 | 0.0 | 0.1 | 0.1 | 0.0 | 0.0 | 0.0 | 0.0 |
| Adipoq | 0.0 | 0.0 | 0.0 | 0.0 | 0.0 | 0.0 | 0.0 | 0.0 | 0.0 | 0.0 | 0.1 | 0.1 |
| Adprhl1 | 0.0 | 0.0 | 0.0 | 0.0 | 0.0 | 0.0 | 0.0 | 0.0 | 0.0 | 0.0 | 0.0 | 0.1 |
| Adra2c | 0.0 | 0.0 | 0.0 | 0.0 | 0.0 | 0.0 | 0.0 | 0.0 | 0.0 | 0.0 | 0.0 | 0.0 |
| AF529169 | 0.0 | 0.0 | 0.0 | 0.0 | 0.0 | 0.0 | 0.0 | 0.0 | 0.0 | 0.0 | 0.0 | 0.0 |
| Agmat | 0.0 | 0.0 | 0.0 | 0.0 | 0.0 | 0.0 | 0.1 | 0.0 | 0.0 | 0.0 | 0.0 | 0.0 |
| Agxt2l1 | 0.0 | 0.0 | 0.0 | 0.0 | 0.0 | 0.0 | 0.0 | 0.0 | 0.0 | 0.0 | 0.0 | 0.0 |
| AI593442 | 0.0 | 0.0 | 0.0 | 0.0 | 0.0 | 0.0 | 0.0 | 0.0 | 0.0 | 0.0 | 0.0 | 0.0 |
| AI606473 | 0.0 | 0.0 | 0.0 | 0.0 | 0.0 | 0.0 | 0.0 | 0.0 | 0.0 | 0.1 | 0.0 | 0.0 |
| AI747448 | 0.0 | 0.0 | 0.0 | 0.0 | 0.0 | 0.0 | 0.0 | 0.0 | 0.0 | 0.0 | 0.0 | 0.0 |
| AI836003 | 0.0 | 0.0 | 0.0 | 0.0 | 0.0 | 0.0 | 0.0 | 0.1 | 0.0 | 0.0 | 0.0 | 0.0 |
| AI847159 | 0.0 | 0.0 | 0.0 | 0.1 | 0.0 | 0.0 | 0.0 | 0.0 | 0.0 | 0.0 | 0.0 | 0.0 |
| AI854517 | 0.0 | 0.0 | 0.0 | 0.0 | 0.0 | 0.0 | 0.0 | 0.0 | 0.0 | 0.0 | 0.0 | 0.0 |
| Aicda | 0.0 | 0.0 | 0.0 | 0.0 | 0.0 | 0.0 | 0.0 | 0.0 | 0.0 | 0.0 | 0.0 | 0.0 |
| Aire | 0.0 | 0.0 | 0.0 | 0.0 | 0.0 | 0.0 | 0.0 | 0.0 | 0.0 | 0.0 | 0.0 | 0.0 |
| Ajap1 | 0.0 | 0.0 | 0.0 | 0.0 | 0.0 | 0.0 | 0.0 | 0.0 | 0.0 | 0.0 | 0.0 | 0.0 |
| Akp3 | 0.0 | 0.0 | 0.0 | 0.0 | 0.0 | 0.1 | 0.0 | 0.0 | 0.0 | 0.0 | 0.0 | 0.0 |
| Akr1c20 | 0.0 | 0.0 | 0.0 | 0.0 | 0.0 | 0.0 | 0.0 | 0.0 | 0.0 | 0.0 | 0.0 | 0.0 |
| Akr1c21 | 0.0 | 0.0 | 0.0 | 0.0 | 0.0 | 0.0 | 0.0 | 0.0 | 0.0 | 0.0 | 0.0 | 0.0 |
| Akr1d1 | 0.0 | 0.0 | 0.0 | 0.0 | 0.0 | 0.0 | 0.0 | 0.0 | 0.0 | 0.0 | 0.0 | 0.0 |
| Alb | 0.0 | 0.0 | 0.0 | 0.0 | 0.0 | 0.0 | 0.0 | 0.0 | 0.0 | 0.0 | 0.0 | 0.0 |
| Alox12b | 0.0 | 0.0 | 0.0 | 0.0 | 0.0 | 0.0 | 0.0 | 0.0 | 0.0 | 0.0 | 0.0 | 0.0 |
| Alox15 | 0.0 | 0.0 | 0.0 | 0.0 | 0.0 | 0.0 | 0.0 | 0.0 | 0.0 | 0.0 | 0.0 | 0.0 |
| Alpi | 0.0 | 0.0 | 0.0 | 0.0 | 0.0 | 0.0 | 0.0 | 0.0 | 0.0 | 0.0 | 0.0 | 0.0 |
| Alppl2 | 0.0 | 0.0 | 0.0 | 0.0 | 0.0 | 0.0 | 0.0 | 0.0 | 0.0 | 0.0 | 0.0 | 0.0 |
| Als2cr11 | 0.0 | 0.0 | 0.0 | 0.0 | 0.0 | 0.0 | 0.0 | 0.1 | 0.0 | 0.0 | 0.0 | 0.0 |
| Amelx | 0.0 | 0.0 | 0.0 | 0.0 | 0.0 | 0.0 | 0.1 | 0.0 | 0.0 | 0.0 | 0.0 | 0.0 |
| Amer2 | 0.0 | 0.0 | 0.0 | 0.0 | 0.0 | 0.0 | 0.0 | 0.0 | 0.0 | 0.0 | 0.0 | 0.0 |
| Amer3 | 0.0 | 0.0 | 0.0 | 0.0 | 0.0 | 0.0 | 0.0 | 0.0 | 0.0 | 0.0 | 0.0 | 0.0 |
| Amy2a5 | 0.0 | 0.0 | 0.0 | 0.0 | 0.0 | 0.0 | 0.0 | 0.0 | 0.0 | 0.0 | 0.0 | 0.0 |
| Ang3 | 0.0 | 0.0 | 0.0 | 0.0 | 0.0 | 0.0 | 0.0 | 0.0 | 0.0 | 0.0 | 0.0 | 0.0 |
| Ang4 | 0.0 | 0.0 | 0.0 | 0.0 | 0.1 | 0.0 | 0.0 | 0.1 | 0.0 | 0.0 | 0.0 | 0.0 |
| Ang5 | 0.0 | 0.0 | 0.0 | 0.0 | 0.0 | 0.0 | 0.0 | 0.0 | 0.0 | 0.0 | 0.0 | 0.0 |
| Ankfn1 | 0.0 | 0.0 | 0.0 | 0.0 | 0.0 | 0.0 | 0.1 | 0.0 | 0.0 | 0.0 | 0.1 | 0.0 |
| Ankrd22 | 0.0 | 0.0 | 0.0 | 0.0 | 0.0 | 0.0 | 0.0 | 0.0 | 0.0 | 0.0 | 0.0 | 0.0 |
| Ankrd34b | 0.0 | 0.0 | 0.0 | 0.0 | 0.0 | 0.0 | 0.0 | 0.0 | 0.0 | 0.0 | 0.0 | 0.0 |
| Ankrd36 | 0.0 | 0.0 | 0.0 | 0.0 | 0.0 | 0.0 | 0.0 | 0.0 | 0.0 | 0.0 | 0.0 | 0.0 |
| Ankrd45 | 0.0 | 0.0 | 0.0 | 0.0 | 0.0 | 0.0 | 0.1 | 0.0 | 0.0 | 0.0 | 0.0 | 0.0 |
| Ankrd63 | 0.0 | 0.0 | 0.0 | 0.0 | 0.0 | 0.0 | 0.0 | 0.0 | 0.0 | 0.0 | 0.0 | 0.0 |
| Ankrd66 | 0.0 | 0.0 | 0.0 | 0.0 | 0.1 | 0.0 | 0.0 | 0.0 | 0.0 | 0.0 | 0.0 | 0.0 |
| Ankrd7 | 0.0 | 0.0 | 0.0 | 0.0 | 0.0 | 0.0 | 0.0 | 0.0 | 0.0 | 0.0 | 0.1 | 0.0 |
| Anks4b | 0.0 | 0.0 | 0.0 | 0.0 | 0.0 | 0.0 | 0.0 | 0.0 | 0.0 | 0.0 | 0.0 | 0.0 |
| Ano9 | 0.0 | 0.0 | 0.0 | 0.0 | 0.0 | 0.0 | 0.0 | 0.0 | 0.0 | 0.0 | 0.0 | 0.0 |

| | | | | | | | | | | | | |
|---|---|---|---|---|---|---|---|---|---|---|---|---|
| Antxrl | 0.0 | 0.0 | 0.0 | 0.0 | 0.1 | 0.0 | 0.0 | 0.0 | 0.0 | 0.0 | 0.0 | 0.0 |
| Anxa10 | 0.0 | 0.0 | 0.0 | 0.0 | 0.0 | 0.0 | 0.0 | 0.0 | 0.0 | 0.0 | 0.0 | 0.0 |
| Anxa13 | 0.0 | 0.0 | 0.0 | 0.0 | 0.1 | 0.0 | 0.0 | 0.0 | 0.0 | 0.0 | 0.0 | 0.0 |
| Apcs | 0.0 | 0.0 | 0.0 | 0.0 | 0.0 | 0.0 | 0.0 | 0.0 | 0.0 | 0.0 | 0.0 | 0.0 |
| Apoa1 | 0.0 | 0.0 | 0.0 | 0.0 | 0.0 | 0.0 | 0.0 | 0.0 | 0.0 | 0.0 | 0.0 | 0.0 |
| Apobec4 | 0.0 | 0.0 | 0.0 | 0.0 | 0.0 | 0.0 | 0.0 | 0.0 | 0.0 | 0.0 | 0.0 | 0.0 |
| Apol10a | 0.0 | 0.0 | 0.0 | 0.0 | 0.0 | 0.0 | 0.0 | 0.0 | 0.0 | 0.0 | 0.0 | 0.0 |
| Apol11a | 0.0 | 0.0 | 0.0 | 0.0 | 0.0 | 0.0 | 0.0 | 0.0 | 0.0 | 0.0 | 0.0 | 0.0 |
| Apon | 0.0 | 0.0 | 0.0 | 0.0 | 0.1 | 0.0 | 0.0 | 0.0 | 0.0 | 0.0 | 0.0 | 0.0 |
| Aqp12 | 0.1 | 0.0 | 0.0 | 0.0 | 0.0 | 0.0 | 0.0 | 0.0 | 0.0 | 0.0 | 0.0 | 0.0 |
| Aqp8 | 0.0 | 0.0 | 0.0 | 0.0 | 0.0 | 0.0 | 0.0 | 0.0 | 0.0 | 0.0 | 0.0 | 0.0 |
| Arhgap36 | 0.0 | 0.0 | 0.0 | 0.0 | 0.0 | 0.0 | 0.0 | 0.0 | 0.0 | 0.0 | 0.0 | 0.0 |
| Arhgap40 | 0.0 | 0.0 | 0.0 | 0.0 | 0.0 | 0.0 | 0.0 | 0.0 | 0.0 | 0.0 | 0.0 | 0.0 |
| Arid3c | 0.0 | 0.0 | 0.0 | 0.0 | 0.0 | 0.0 | 0.0 | 0.0 | 0.0 | 0.0 | 0.0 | 0.0 |
| Arl13a | 0.0 | 0.0 | 0.0 | 0.0 | 0.0 | 0.1 | 0.0 | 0.0 | 0.0 | 0.0 | 0.0 | 0.0 |
| Arl9 | 0.0 | 0.0 | 0.0 | 0.0 | 0.1 | 0.0 | 0.0 | 0.0 | 0.1 | 0.0 | 0.0 | 0.0 |
| Armc3 | 0.0 | 0.0 | 0.0 | 0.0 | 0.0 | 0.0 | 0.0 | 0.0 | 0.0 | 0.0 | 0.0 | 0.0 |
| Armc4 | 0.0 | 0.0 | 0.0 | 0.0 | 0.0 | 0.0 | 0.0 | 0.0 | 0.0 | 0.0 | 0.0 | 0.0 |
| Arrdc5 | 0.0 | 0.0 | 0.0 | 0.0 | 0.0 | 0.0 | 0.0 | 0.0 | 0.0 | 0.0 | 0.0 | 0.1 |
| Art1 | 0.0 | 0.0 | 0.0 | 0.0 | 0.0 | 0.0 | 0.0 | 0.0 | 0.0 | 0.0 | 0.0 | 0.0 |
| Art2a-ps | 0.0 | 0.1 | 0.0 | 0.0 | 0.0 | 0.0 | 0.0 | 0.0 | 0.0 | 0.0 | 0.0 | 0.0 |
| Art2b | 0.0 | 0.0 | 0.0 | 0.0 | 0.0 | 0.0 | 0.0 | 0.0 | 0.0 | 0.0 | 0.0 | 0.0 |
| Asb10 | 0.0 | 0.0 | 0.0 | 0.0 | 0.0 | 0.0 | 0.0 | 0.0 | 0.0 | 0.0 | 0.0 | 0.0 |
| Asb11 | 0.0 | 0.0 | 0.0 | 0.0 | 0.0 | 0.0 | 0.0 | 0.0 | 0.0 | 0.0 | 0.0 | 0.0 |
| Asb12 | 0.0 | 0.0 | 0.0 | 0.0 | 0.0 | 0.0 | 0.0 | 0.0 | 0.0 | 0.0 | 0.0 | 0.0 |
| Asb17 | 0.0 | 0.0 | 0.0 | 0.0 | 0.0 | 0.0 | 0.0 | 0.0 | 0.0 | 0.0 | 0.1 | 0.0 |
| Asb9 | 0.1 | 0.0 | 0.0 | 0.0 | 0.0 | 0.0 | 0.0 | 0.0 | 0.0 | 0.0 | 0.0 | 0.0 |
| Ascl1 | 0.0 | 0.0 | 0.0 | 0.0 | 0.0 | 0.0 | 0.0 | 0.0 | 0.0 | 0.0 | 0.0 | 0.0 |
| Ascl5 | 0.0 | 0.0 | 0.0 | 0.0 | 0.0 | 0.0 | 0.0 | 0.0 | 0.0 | 0.0 | 0.0 | 0.0 |
| Asgr2 | 0.0 | 0.0 | 0.0 | 0.0 | 0.0 | 0.0 | 0.0 | 0.0 | 0.0 | 0.0 | 0.0 | 0.0 |
| Asic2 | 0.0 | 0.0 | 0.0 | 0.1 | 0.0 | 0.0 | 0.0 | 0.0 | 0.0 | 0.0 | 0.0 | 0.0 |
| Asic4 | 0.0 | 0.0 | 0.0 | 0.0 | 0.0 | 0.0 | 0.0 | 0.0 | 0.0 | 0.0 | 0.0 | 0.0 |
| Asmt | 0.0 | 0.0 | 0.0 | 0.0 | 0.0 | 0.0 | 0.0 | 0.0 | 0.0 | 0.0 | 0.0 | 0.0 |
| Aspdh | 0.1 | 0.0 | 0.0 | 0.0 | 0.0 | 0.0 | 0.0 | 0.0 | 0.0 | 0.0 | 0.0 | 0.0 |
| Asphd1 | 0.0 | 0.0 | 0.0 | 0.0 | 0.0 | 0.0 | 0.0 | 0.0 | 0.0 | 0.0 | 0.0 | 0.0 |
| Atoh1 | 0.0 | 0.0 | 0.0 | 0.0 | 0.0 | 0.0 | 0.0 | 0.0 | 0.0 | 0.0 | 0.0 | 0.0 |
| Atp13a4 | 0.0 | 0.0 | 0.0 | 0.0 | 0.0 | 0.0 | 0.0 | 0.0 | 0.0 | 0.0 | 0.0 | 0.0 |
| Atp2b3 | 0.0 | 0.0 | 0.0 | 0.0 | 0.0 | 0.0 | 0.0 | 0.0 | 0.0 | 0.0 | 0.0 | 0.0 |
| Atp2c2 | 0.0 | 0.0 | 0.0 | 0.0 | 0.0 | 0.0 | 0.0 | 0.0 | 0.0 | 0.0 | 0.0 | 0.0 |
| Atp4b | 0.0 | 0.0 | 0.0 | 0.0 | 0.0 | 0.0 | 0.0 | 0.0 | 0.0 | 0.0 | 0.0 | 0.0 |
| AU018091 | 0.0 | 0.0 | 0.0 | 0.0 | 0.0 | 0.0 | 0.0 | 0.0 | 0.0 | 0.0 | 0.0 | 0.0 |
| AU018829 | 0.0 | 0.0 | 0.0 | 0.0 | 0.0 | 0.0 | 0.0 | 0.0 | 0.0 | 0.0 | 0.0 | 0.0 |
| AU019990 | 0.0 | 0.0 | 0.0 | 0.1 | 0.0 | 0.0 | 0.1 | 0.0 | 0.0 | 0.0 | 0.0 | 0.0 |
| AU022751 | 0.0 | 0.0 | 0.0 | 0.0 | 0.0 | 0.0 | 0.0 | 0.0 | 0.0 | 0.0 | 0.0 | 0.0 |
| Avpr2 | 0.0 | 0.0 | 0.0 | 0.0 | 0.1 | 0.0 | 0.0 | 0.1 | 0.0 | 0.0 | 0.0 | 0.0 |
| AW549542 | 0.0 | 0.0 | 0.0 | 0.0 | 0.0 | 0.0 | 0.0 | 0.0 | 0.0 | 0.0 | 0.0 | 0.0 |
| Azgp1 | 0.1 | 0.0 | 0.0 | 0.0 | 0.0 | 0.0 | 0.1 | 0.0 | 0.0 | 0.0 | 0.0 | 0.0 |
| B020004C17Rik | 0.0 | 0.0 | 0.0 | 0.0 | 0.0 | 0.0 | 0.0 | 0.0 | 0.0 | 0.0 | 0.0 | 0.0 |
| B020014A21Rik | 0.0 | 0.0 | 0.0 | 0.0 | 0.0 | 0.0 | 0.0 | 0.0 | 0.0 | 0.0 | 0.1 | 0.0 |
| B230119M05Rik | 0.0 | 0.0 | 0.0 | 0.0 | 0.0 | 0.0 | 0.0 | 0.0 | 0.1 | 0.0 | 0.0 | 0.0 |
| B230209E15Rik | 0.0 | 0.0 | 0.0 | 0.0 | 0.0 | 0.0 | 0.0 | 0.0 | 0.0 | 0.0 | 0.0 | 0.0 |
| B230312C02Rik | 0.0 | 0.0 | 0.0 | 0.0 | 0.0 | 0.0 | 0.0 | 0.0 | 0.0 | 0.0 | 0.0 | 0.0 |
| B230323A14Rik | 0.0 | 0.0 | 0.0 | 0.0 | 0.0 | 0.0 | 0.0 | 0.0 | 0.0 | 0.0 | 0.0 | 0.0 |
| B4galnt4 | 0.0 | 0.0 | 0.0 | 0.0 | 0.0 | 0.0 | 0.0 | 0.0 | 0.0 | 0.0 | 0.0 | 0.0 |
| B830017H08Rik | 0.0 | 0.0 | 0.0 | 0.0 | 0.0 | 0.0 | 0.0 | 0.0 | 0.0 | 0.1 | 0.0 | 0.0 |
| B930018H19Rik | 0.1 | 0.0 | 0.0 | 0.0 | 0.1 | 0.0 | 0.1 | 0.0 | 0.0 | 0.0 | 0.0 | 0.1 |
| B930092H01Rik | 0.0 | 0.0 | 0.0 | 0.0 | 0.0 | 0.0 | 0.0 | 0.0 | 0.0 | 0.0 | 0.0 | 0.0 |

| | | | | | | | | | | | | |
|---|---|---|---|---|---|---|---|---|---|---|---|---|
| Baat | 0.0 | 0.0 | 0.0 | 0.0 | 0.0 | 0.0 | 0.0 | 0.0 | 0.0 | 0.0 | 0.0 | 0.0 |
| Barhl2 | 0.0 | 0.0 | 0.0 | 0.0 | 0.0 | 0.0 | 0.0 | 0.0 | 0.0 | 0.0 | 0.0 | 0.0 |
| BB014433 | 0.0 | 0.0 | 0.0 | 0.0 | 0.0 | 0.0 | 0.0 | 0.0 | 0.0 | 0.0 | 0.0 | 0.0 |
| BB019430 | 0.0 | 0.0 | 0.0 | 0.0 | 0.0 | 0.0 | 0.0 | 0.0 | 0.0 | 0.0 | 0.0 | 0.0 |
| BB123696 | 0.0 | 0.0 | 0.0 | 0.0 | 0.0 | 0.0 | 0.0 | 0.0 | 0.0 | 0.0 | 0.0 | 0.0 |
| BB283400 | 0.0 | 0.0 | 0.0 | 0.0 | 0.0 | 0.0 | 0.0 | 0.0 | 0.0 | 0.0 | 0.0 | 0.0 |
| BB557941 | 0.1 | 0.0 | 0.1 | 0.0 | 0.0 | 0.2 | 0.0 | 0.0 | 0.0 | 0.0 | 0.1 | 0.0 |
| BC021614 | 0.0 | 0.0 | 0.0 | 0.0 | 0.0 | 0.0 | 0.0 | 0.0 | 0.0 | 0.0 | 0.0 | 0.0 |
| BC027072 | 0.0 | 0.0 | 0.0 | 0.0 | 0.0 | 0.0 | 0.0 | 0.0 | 0.0 | 0.0 | 0.0 | 0.0 |
| BC030870 | 0.1 | 0.0 | 0.0 | 0.0 | 0.0 | 0.0 | 0.0 | 0.0 | 0.0 | 0.0 | 0.0 | 0.0 |
| BC039966 | 0.0 | 0.1 | 0.0 | 0.0 | 0.0 | 0.0 | 0.0 | 0.0 | 0.0 | 0.0 | 0.0 | 0.0 |
| BC048502 | 0.0 | 0.0 | 0.0 | 0.0 | 0.0 | 0.0 | 0.0 | 0.0 | 0.0 | 0.0 | 0.0 | 0.0 |
| BC048507 | 0.1 | 0.0 | 0.0 | 0.0 | 0.0 | 0.0 | 0.0 | 0.0 | 0.0 | 0.0 | 0.0 | 0.0 |
| BC048609 | 0.2 | 0.0 | 0.0 | 0.0 | 0.2 | 0.0 | 0.0 | 0.4 | 0.0 | 0.1 | 0.0 | 0.0 |
| BC048679 | 0.0 | 0.0 | 0.0 | 0.0 | 0.0 | 0.0 | 0.0 | 0.0 | 0.0 | 0.0 | 0.0 | 0.0 |
| BC049352 | 0.0 | 0.0 | 0.0 | 0.0 | 0.1 | 0.0 | 0.0 | 0.0 | 0.0 | 0.0 | 0.0 | 0.0 |
| BC049635 | 0.0 | 0.0 | 0.1 | 0.0 | 0.0 | 0.0 | 0.0 | 0.1 | 0.0 | 0.0 | 0.0 | 0.0 |
| BC080695 | 0.0 | 0.0 | 0.0 | 0.0 | 0.0 | 0.0 | 0.0 | 0.0 | 0.0 | 0.0 | 0.0 | 0.0 |
| BC089491 | 0.0 | 0.0 | 0.0 | 0.0 | 0.0 | 0.3 | 0.0 | 0.0 | 0.0 | 0.0 | 0.0 | 0.0 |
| BC089597 | 0.0 | 0.1 | 0.0 | 0.0 | 0.0 | 0.0 | 0.0 | 0.0 | 0.0 | 0.0 | 0.0 | 0.0 |
| BC094916 | 0.0 | 0.0 | 0.0 | 0.0 | 0.0 | 0.0 | 0.0 | 0.0 | 0.0 | 0.0 | 0.0 | 0.0 |
| BC107364 | 0.0 | 0.0 | 0.0 | 0.2 | 0.0 | 0.0 | 0.0 | 0.1 | 0.0 | 0.0 | 0.0 | 0.0 |
| BC147527 | 0.0 | 0.0 | 0.0 | 0.0 | 0.0 | 0.0 | 0.0 | 0.0 | 0.0 | 0.0 | 0.0 | 0.0 |
| Bcl11a | 0.0 | 0.0 | 0.0 | 0.0 | 0.0 | 0.0 | 0.0 | 0.0 | 0.0 | 0.0 | 0.0 | 0.0 |
| Bcl2a1c | 0.0 | 0.0 | 0.0 | 0.0 | 0.4 | 0.0 | 0.0 | 0.0 | 0.0 | 0.0 | 0.0 | 0.0 |
| Bdkrb2 | 0.0 | 0.0 | 0.0 | 0.0 | 0.0 | 0.0 | 0.0 | 0.0 | 0.0 | 0.0 | 0.0 | 0.0 |
| Bend5 | 0.0 | 0.0 | 0.0 | 0.0 | 0.0 | 0.0 | 0.0 | 0.0 | 0.0 | 0.0 | 0.0 | 0.0 |
| Best2 | 0.0 | 0.0 | 0.0 | 0.0 | 0.0 | 0.0 | 0.0 | 0.0 | 0.0 | 0.0 | 0.0 | 0.0 |
| Bglap3 | 0.0 | 0.0 | 0.0 | 0.1 | 0.1 | 0.0 | 0.0 | 0.0 | 0.0 | 0.1 | 0.0 | 0.0 |
| Bhmt | 0.0 | 0.0 | 0.0 | 0.0 | 0.0 | 0.0 | 0.0 | 0.0 | 0.0 | 0.0 | 0.0 | 0.0 |
| Bmp7 | 0.0 | 0.0 | 0.0 | 0.0 | 0.0 | 0.0 | 0.0 | 0.0 | 0.0 | 0.0 | 0.1 | 0.0 |
| Boll | 0.0 | 0.0 | 0.0 | 0.0 | 0.0 | 0.0 | 0.0 | 0.0 | 0.0 | 0.0 | 0.0 | 0.0 |
| Bpifa6 | 0.0 | 0.0 | 0.0 | 0.0 | 0.0 | 0.1 | 0.0 | 0.0 | 0.0 | 0.0 | 0.0 | 0.0 |
| Bpifb1 | 0.0 | 0.0 | 0.0 | 0.0 | 0.0 | 0.0 | 0.0 | 0.0 | 0.0 | 0.0 | 0.0 | 0.0 |
| Bpifb2 | 0.0 | 0.0 | 0.0 | 0.0 | 0.0 | 0.0 | 0.0 | 0.0 | 0.0 | 0.0 | 0.0 | 0.0 |
| Bpifb4 | 0.0 | 0.0 | 0.0 | 0.0 | 0.0 | 0.0 | 0.0 | 0.0 | 0.0 | 0.0 | 0.0 | 0.0 |
| Bpifb5 | 0.0 | 0.0 | 0.0 | 0.1 | 0.0 | 0.0 | 0.0 | 0.0 | 0.0 | 0.0 | 0.0 | 0.0 |
| Bpifb6 | 0.0 | 0.0 | 0.0 | 0.0 | 0.0 | 0.1 | 0.0 | 0.0 | 0.0 | 0.0 | 0.0 | 0.0 |
| Bpifb9a | 0.0 | 0.0 | 0.0 | 0.0 | 0.0 | 0.0 | 0.0 | 0.0 | 0.0 | 0.0 | 0.0 | 0.0 |
| Bpifb9b | 0.0 | 0.0 | 0.0 | 0.0 | 0.0 | 0.0 | 0.0 | 0.0 | 0.0 | 0.0 | 0.0 | 0.0 |
| Bpifc | 0.0 | 0.0 | 0.0 | 0.0 | 0.0 | 0.0 | 0.0 | 0.0 | 0.0 | 0.0 | 0.0 | 0.0 |
| Brs3 | 0.0 | 0.0 | 0.0 | 0.0 | 0.0 | 0.0 | 0.0 | 0.0 | 0.0 | 0.0 | 0.0 | 0.0 |
| Bsx | 0.0 | 0.1 | 0.0 | 0.0 | 0.0 | 0.0 | 0.0 | 0.0 | 0.0 | 0.0 | 0.0 | 0.0 |
| Btbd17 | 0.0 | 0.0 | 0.0 | 0.0 | 0.0 | 0.0 | 0.0 | 0.0 | 0.0 | 0.0 | 0.0 | 0.0 |
| Btg4 | 0.0 | 0.0 | 0.0 | 0.0 | 0.0 | 0.0 | 0.0 | 0.0 | 0.0 | 0.0 | 0.0 | 0.0 |
| Btn1a1 | 0.0 | 0.0 | 0.0 | 0.0 | 0.0 | 0.0 | 0.0 | 0.0 | 0.0 | 0.0 | 0.0 | 0.0 |
| Btnl1 | 0.0 | 0.0 | 0.0 | 0.0 | 0.0 | 0.0 | 0.0 | 0.0 | 0.0 | 0.0 | 0.0 | 0.0 |
| Btnl10 | 0.0 | 0.0 | 0.0 | 0.0 | 0.0 | 0.0 | 0.0 | 0.0 | 0.0 | 0.0 | 0.0 | 0.0 |
| Btnl5 | 0.0 | 0.0 | 0.0 | 0.0 | 0.0 | 0.0 | 0.0 | 0.0 | 0.0 | 0.0 | 0.0 | 0.0 |
| C030018K13Rik | 0.0 | 0.0 | 0.0 | 0.0 | 0.0 | 0.0 | 0.0 | 0.0 | 0.0 | 0.0 | 0.1 | 0.0 |
| C130030K03Rik | 0.0 | 0.0 | 0.0 | 0.0 | 0.0 | 0.0 | 0.0 | 0.0 | 0.0 | 0.0 | 0.0 | 0.0 |
| C130060K24Rik | 0.0 | 0.0 | 0.0 | 0.0 | 0.0 | 0.0 | 0.0 | 0.0 | 0.0 | 0.0 | 0.0 | 0.0 |
| C130071C03Rik | 0.0 | 0.0 | 0.0 | 0.0 | 0.0 | 0.0 | 0.0 | 0.0 | 0.0 | 0.0 | 0.0 | 0.0 |
| C130080G10Rik | 0.0 | 0.0 | 0.0 | 0.0 | 0.0 | 0.0 | 0.0 | 0.0 | 0.0 | 0.0 | 0.0 | 0.0 |
| C1ql2 | 0.0 | 0.0 | 0.0 | 0.0 | 0.0 | 0.0 | 0.0 | 0.0 | 0.0 | 0.0 | 0.0 | 0.0 |
| C1ql4 | 0.1 | 0.0 | 0.0 | 0.1 | 0.0 | 0.0 | 0.0 | 0.0 | 0.0 | 0.0 | 0.0 | 0.0 |
| C230004F18Rik | 0.0 | 0.0 | 0.0 | 0.0 | 0.0 | 0.0 | 0.0 | 0.0 | 0.0 | 0.0 | 0.0 | 0.0 |

| | | | | | | | | | | | | |
|---|---|---|---|---|---|---|---|---|---|---|---|---|
| C230024C17Rik | 0.0 | 0.0 | 0.0 | 0.0 | 0.0 | 0.0 | 0.0 | 0.0 | 0.0 | 0.0 | 0.0 | 0.0 |
| C230079O03Rik | 0.0 | 0.0 | 0.0 | 0.0 | 0.0 | 0.0 | 0.0 | 0.0 | 0.0 | 0.0 | 0.0 | 0.0 |
| C2cd4a | 0.0 | 0.0 | 0.0 | 0.0 | 0.0 | 0.0 | 0.0 | 0.0 | 0.0 | 0.0 | 0.0 | 0.0 |
| C2cd4d | 0.0 | 0.0 | 0.0 | 0.0 | 0.0 | 0.0 | 0.0 | 0.0 | 0.0 | 0.0 | 0.0 | 0.0 |
| C330011F03Rik | 0.0 | 0.0 | 0.0 | 0.0 | 0.0 | 0.0 | 0.0 | 0.0 | 0.0 | 0.0 | 0.0 | 0.0 |
| C330022C24Rik | 0.0 | 0.0 | 0.0 | 0.0 | 0.0 | 0.0 | 0.0 | 0.0 | 0.1 | 0.0 | 0.0 | 0.0 |
| C330046G13Rik | 0.0 | 0.0 | 0.0 | 0.0 | 0.0 | 0.0 | 0.0 | 0.0 | 0.0 | 0.0 | 0.0 | 0.0 |
| C4bp-ps1 | 0.0 | 0.1 | 0.1 | 0.1 | 0.1 | 0.0 | 0.0 | 0.0 | 0.0 | 0.0 | 0.0 | 0.0 |
| C6 | 0.0 | 0.0 | 0.0 | 0.0 | 0.0 | 0.0 | 0.0 | 0.0 | 0.0 | 0.1 | 0.0 | 0.0 |
| C630020P19Rik | 0.0 | 0.0 | 0.0 | 0.0 | 0.0 | 0.0 | 0.0 | 0.0 | 0.0 | 0.0 | 0.0 | 0.0 |
| C630028M04Rik | 0.0 | 0.0 | 0.0 | 0.0 | 0.0 | 0.0 | 0.0 | 0.0 | 0.0 | 0.0 | 0.0 | 0.0 |
| C630031E19Rik | 0.0 | 0.0 | 0.0 | 0.0 | 0.0 | 0.0 | 0.0 | 0.0 | 0.0 | 0.0 | 0.0 | 0.0 |
| C86187 | 0.0 | 0.0 | 0.0 | 0.0 | 0.0 | 0.0 | 0.0 | 0.0 | 0.0 | 0.0 | 0.0 | 0.0 |
| C87414 | 0.0 | 0.0 | 0.0 | 0.0 | 0.0 | 0.0 | 0.0 | 0.0 | 0.0 | 0.0 | 0.0 | 0.0 |
| C87977 | 0.0 | 0.0 | 0.0 | 0.0 | 0.0 | 0.0 | 0.0 | 0.0 | 0.0 | 0.0 | 0.0 | 0.0 |
| C8a | 0.0 | 0.0 | 0.0 | 0.0 | 0.0 | 0.0 | 0.0 | 0.0 | 0.0 | 0.0 | 0.0 | 0.0 |
| Cabp5 | 0.0 | 0.0 | 0.0 | 0.0 | 0.0 | 0.0 | 0.0 | 0.0 | 0.0 | 0.0 | 0.0 | 0.0 |
| Cacna1f | 0.0 | 0.0 | 0.0 | 0.0 | 0.0 | 0.0 | 0.0 | 0.0 | 0.0 | 0.0 | 0.0 | 0.0 |
| Cacng1 | 0.0 | 0.1 | 0.0 | 0.0 | 0.0 | 0.0 | 0.0 | 0.0 | 0.0 | 0.0 | 0.1 | 0.0 |
| Cacng2 | 0.0 | 0.0 | 0.0 | 0.0 | 0.0 | 0.0 | 0.0 | 0.0 | 0.0 | 0.0 | 0.0 | 0.0 |
| Cacng3 | 0.0 | 0.0 | 0.0 | 0.0 | 0.0 | 0.0 | 0.0 | 0.0 | 0.0 | 0.0 | 0.0 | 0.0 |
| Cacng4 | 0.0 | 0.0 | 0.0 | 0.0 | 0.0 | 0.0 | 0.0 | 0.0 | 0.0 | 0.0 | 0.0 | 0.0 |
| Cacng5 | 0.0 | 0.0 | 0.0 | 0.0 | 0.0 | 0.0 | 0.0 | 0.0 | 0.0 | 0.0 | 0.0 | 0.0 |
| Cacng6 | 0.0 | 0.0 | 0.0 | 0.0 | 0.0 | 0.0 | 0.0 | 0.0 | 0.0 | 0.0 | 0.0 | 0.0 |
| Calb1 | 0.0 | 0.0 | 0.0 | 0.0 | 0.0 | 0.0 | 0.1 | 0.0 | 0.0 | 0.0 | 0.0 | 0.0 |
| Calb2 | 0.1 | 0.0 | 0.0 | 0.0 | 0.0 | 0.0 | 0.1 | 0.0 | 0.0 | 0.0 | 0.0 | 0.1 |
| Calcr | 0.0 | 0.0 | 0.0 | 0.0 | 0.0 | 0.0 | 0.0 | 0.0 | 0.0 | 0.0 | 0.0 | 0.0 |
| Calml3 | 0.0 | 0.0 | 0.0 | 0.0 | 0.0 | 0.0 | 0.0 | 0.0 | 0.0 | 0.0 | 0.0 | 0.0 |
| Camkv | 0.0 | 0.0 | 0.0 | 0.0 | 0.0 | 0.0 | 0.0 | 0.0 | 0.0 | 0.0 | 0.0 | 0.0 |
| Capn13 | 0.0 | 0.0 | 0.0 | 0.0 | 0.0 | 0.0 | 0.0 | 0.0 | 0.0 | 0.0 | 0.0 | 0.0 |
| Caps2 | 0.0 | 0.0 | 0.0 | 0.0 | 0.0 | 0.0 | 0.0 | 0.0 | 0.0 | 0.0 | 0.0 | 0.0 |
| Capsl | 0.0 | 0.0 | 0.0 | 0.0 | 0.1 | 0.2 | 0.0 | 0.0 | 0.0 | 0.0 | 0.0 | 0.0 |
| Capza3 | 0.0 | 0.0 | 0.0 | 0.0 | 0.0 | 0.0 | 0.0 | 0.0 | 0.0 | 0.0 | 0.0 | 0.0 |
| Car1 | 0.0 | 0.0 | 0.0 | 0.0 | 0.0 | 0.0 | 0.0 | 0.0 | 0.0 | 0.0 | 0.0 | 0.0 |
| Car2 | 0.0 | 0.0 | 0.0 | 0.0 | 0.0 | 0.0 | 0.0 | 0.0 | 0.0 | 0.0 | 0.0 | 0.0 |
| Car3 | 0.0 | 0.0 | 0.0 | 0.1 | 0.0 | 0.0 | 0.0 | 0.0 | 0.0 | 0.0 | 0.0 | 0.1 |
| Casr | 0.0 | 0.0 | 0.0 | 0.0 | 0.0 | 0.0 | 0.0 | 0.0 | 0.0 | 0.0 | 0.0 | 0.0 |
| Catsper1 | 0.0 | 0.0 | 0.0 | 0.0 | 0.0 | 0.0 | 0.0 | 0.0 | 0.0 | 0.0 | 0.0 | 0.0 |
| Catsper4 | 0.0 | 0.0 | 0.0 | 0.0 | 0.0 | 0.0 | 0.0 | 0.0 | 0.0 | 0.0 | 0.0 | 0.0 |
| Catsperb | 0.0 | 0.0 | 0.0 | 0.0 | 0.0 | 0.0 | 0.0 | 0.0 | 0.0 | 0.0 | 0.0 | 0.0 |
| Cbln4 | 0.0 | 0.0 | 0.0 | 0.0 | 0.0 | 0.0 | 0.0 | 0.0 | 0.0 | 0.0 | 0.0 | 0.0 |
| Ccdc108 | 0.0 | 0.0 | 0.0 | 0.0 | 0.0 | 0.0 | 0.0 | 0.0 | 0.0 | 0.0 | 0.0 | 0.0 |
| Ccdc113 | 0.0 | 0.0 | 0.0 | 0.0 | 0.0 | 0.0 | 0.0 | 0.0 | 0.0 | 0.0 | 0.0 | 0.0 |
| Ccdc153 | 0.0 | 0.0 | 0.1 | 0.0 | 0.0 | 0.0 | 0.0 | 0.0 | 0.0 | 0.0 | 0.0 | 0.0 |
| Ccdc172 | 0.0 | 0.0 | 0.0 | 0.1 | 0.0 | 0.0 | 0.0 | 0.0 | 0.0 | 0.0 | 0.0 | 0.0 |
| Ccdc177 | 0.0 | 0.0 | 0.0 | 0.0 | 0.0 | 0.0 | 0.0 | 0.0 | 0.0 | 0.0 | 0.0 | 0.0 |
| Ccdc178 | 0.0 | 0.0 | 0.0 | 0.0 | 0.0 | 0.0 | 0.0 | 0.0 | 0.0 | 0.0 | 0.0 | 0.0 |
| Ccdc33 | 0.0 | 0.0 | 0.0 | 0.0 | 0.0 | 0.0 | 0.0 | 0.0 | 0.0 | 0.0 | 0.0 | 0.0 |
| Ccdc42b | 0.0 | 0.0 | 0.0 | 0.0 | 0.0 | 0.0 | 0.0 | 0.1 | 0.0 | 0.0 | 0.0 | 0.0 |
| Ccdc54 | 0.0 | 0.1 | 0.0 | 0.0 | 0.0 | 0.0 | 0.0 | 0.0 | 0.0 | 0.0 | 0.0 | 0.0 |
| Ccdc60 | 0.1 | 0.0 | 0.0 | 0.0 | 0.0 | 0.0 | 0.0 | 0.0 | 0.0 | 0.0 | 0.0 | 0.1 |
| Ccdc67 | 0.0 | 0.0 | 0.0 | 0.0 | 0.0 | 0.0 | 0.0 | 0.0 | 0.0 | 0.0 | 0.0 | 0.0 |
| Ccdc7 | 0.0 | 0.0 | 0.0 | 0.0 | 0.0 | 0.0 | 0.0 | 0.0 | 0.0 | 0.0 | 0.0 | 0.0 |
| Ccer1 | 0.0 | 0.0 | 0.0 | 0.1 | 0.0 | 0.0 | 0.0 | 0.0 | 0.0 | 0.0 | 0.0 | 0.0 |
| Cckar | 0.0 | 0.0 | 0.0 | 0.0 | 0.0 | 0.0 | 0.0 | 0.0 | 0.0 | 0.0 | 0.0 | 0.0 |
| Cckbr | 0.0 | 0.0 | 0.0 | 0.0 | 0.0 | 0.0 | 0.0 | 0.0 | 0.0 | 0.0 | 0.0 | 0.0 |
| Ccl22 | 0.0 | 0.0 | 0.0 | 0.0 | 0.0 | 0.0 | 0.0 | 0.0 | 0.0 | 0.0 | 0.0 | 0.0 |

| | | | | | | | | | | | | |
|---|---|---|---|---|---|---|---|---|---|---|---|---|
| Ccl24 | 0.0 | 0.0 | 0.0 | 0.0 | 0.0 | 0.0 | 0.1 | 0.0 | 0.0 | 0.0 | 0.0 | 0.0 |
| Ccnb1ip1 | 0.0 | 0.1 | 0.0 | 0.0 | 0.0 | 0.0 | 0.0 | 0.0 | 0.0 | 0.0 | 0.0 | 0.0 |
| Ccr1l1 | 0.0 | 0.0 | 0.0 | 0.0 | 0.0 | 0.0 | 0.0 | 0.0 | 0.0 | 0.0 | 0.0 | 0.0 |
| Ccr2 | 0.0 | 0.0 | 0.0 | 0.0 | 0.1 | 0.0 | 0.0 | 0.0 | 0.0 | 0.0 | 0.0 | 0.0 |
| Ccr6 | 0.0 | 0.0 | 0.0 | 0.0 | 0.0 | 0.0 | 0.0 | 0.0 | 0.0 | 0.0 | 0.0 | 0.0 |
| Cd163 | 0.0 | 0.0 | 0.0 | 0.0 | 0.0 | 0.0 | 0.0 | 0.0 | 0.0 | 0.0 | 0.0 | 0.0 |
| Cd19 | 0.0 | 0.0 | 0.0 | 0.0 | 0.0 | 0.0 | 0.0 | 0.0 | 0.0 | 0.0 | 0.0 | 0.0 |
| Cd2 | 0.0 | 0.0 | 0.0 | 0.0 | 0.1 | 0.0 | 0.0 | 0.0 | 0.0 | 0.0 | 0.0 | 0.0 |
| Cd209a | 0.0 | 0.0 | 0.0 | 0.0 | 0.0 | 0.0 | 0.0 | 0.0 | 0.0 | 0.0 | 0.0 | 0.0 |
| Cd209d | 0.0 | 0.0 | 0.0 | 0.0 | 0.0 | 0.0 | 0.0 | 0.0 | 0.0 | 0.0 | 0.0 | 0.1 |
| Cd209f | 0.0 | 0.0 | 0.0 | 0.0 | 0.0 | 0.0 | 0.0 | 0.0 | 0.0 | 0.0 | 0.0 | 0.1 |
| Cd226 | 0.0 | 0.0 | 0.0 | 0.0 | 0.0 | 0.0 | 0.0 | 0.0 | 0.0 | 0.0 | 0.0 | 0.0 |
| Cd3d | 0.0 | 0.0 | 0.0 | 0.0 | 0.0 | 0.0 | 0.0 | 0.0 | 0.0 | 0.0 | 0.0 | 0.0 |
| Cd3g | 0.0 | 0.0 | 0.0 | 0.0 | 0.0 | 0.0 | 0.0 | 0.0 | 0.0 | 0.0 | 0.0 | 0.0 |
| Cd40lg | 0.0 | 0.0 | 0.0 | 0.0 | 0.0 | 0.0 | 0.0 | 0.0 | 0.0 | 0.0 | 0.0 | 0.0 |
| Cd5l | 0.0 | 0.0 | 0.0 | 0.5 | 0.1 | 0.0 | 0.0 | 0.0 | 0.0 | 0.0 | 0.0 | 0.0 |
| Cd6 | 0.0 | 0.0 | 0.0 | 0.0 | 0.0 | 0.0 | 0.0 | 0.0 | 0.0 | 0.0 | 0.1 | 0.0 |
| Cd7 | 0.0 | 0.0 | 0.0 | 0.0 | 0.0 | 0.0 | 0.0 | 0.0 | 0.0 | 0.0 | 0.0 | 0.0 |
| Cd79b | 0.0 | 0.0 | 0.0 | 0.0 | 0.0 | 0.0 | 0.0 | 0.0 | 0.0 | 0.0 | 0.0 | 0.0 |
| Cdcp2 | 0.0 | 0.0 | 0.0 | 0.0 | 0.0 | 0.0 | 0.0 | 0.0 | 0.0 | 0.0 | 0.0 | 0.0 |
| Cdh16 | 0.0 | 0.0 | 0.0 | 0.0 | 0.0 | 0.0 | 0.0 | 0.0 | 0.0 | 0.0 | 0.0 | 0.0 |
| Cdh18 | 0.0 | 0.0 | 0.0 | 0.0 | 0.0 | 0.0 | 0.0 | 0.0 | 0.0 | 0.0 | 0.0 | 0.0 |
| Cdh20 | 0.0 | 0.0 | 0.0 | 0.0 | 0.0 | 0.0 | 0.0 | 0.0 | 0.0 | 0.0 | 0.0 | 0.0 |
| Cdh22 | 0.0 | 0.0 | 0.0 | 0.0 | 0.0 | 0.0 | 0.0 | 0.0 | 0.0 | 0.0 | 0.0 | 0.0 |
| Cdh8 | 0.0 | 0.0 | 0.0 | 0.0 | 0.0 | 0.0 | 0.0 | 0.0 | 0.0 | 0.0 | 0.0 | 0.0 |
| Cdh9 | 0.0 | 0.0 | 0.0 | 0.0 | 0.0 | 0.0 | 0.0 | 0.0 | 0.0 | 0.0 | 0.0 | 0.0 |
| Cdhr2 | 0.0 | 0.0 | 0.0 | 0.0 | 0.0 | 0.0 | 0.0 | 0.0 | 0.0 | 0.0 | 0.0 | 0.0 |
| Cdhr5 | 0.0 | 0.0 | 0.0 | 0.0 | 0.0 | 0.0 | 0.0 | 0.0 | 0.0 | 0.0 | 0.0 | 0.0 |
| Cdk5r2 | 0.0 | 0.0 | 0.0 | 0.0 | 0.0 | 0.0 | 0.0 | 0.0 | 0.0 | 0.0 | 0.0 | 0.0 |
| Cdr1 | 0.0 | 0.0 | 0.0 | 0.0 | 0.0 | 0.0 | 0.0 | 0.0 | 0.0 | 0.0 | 0.0 | 0.0 |
| Cdx1 | 0.0 | 0.0 | 0.0 | 0.0 | 0.0 | 0.0 | 0.0 | 0.0 | 0.0 | 0.0 | 0.0 | 0.0 |
| Ceacam10 | 0.0 | 0.0 | 0.0 | 0.0 | 0.0 | 0.0 | 0.0 | 0.0 | 0.0 | 0.0 | 0.0 | 0.0 |
| Ceacam13 | 0.0 | 0.0 | 0.0 | 0.0 | 0.0 | 0.0 | 0.0 | 0.0 | 0.0 | 0.0 | 0.0 | 0.0 |
| Ceacam15 | 0.0 | 0.0 | 0.0 | 0.0 | 0.0 | 0.0 | 0.0 | 0.0 | 0.0 | 0.0 | 0.0 | 0.0 |
| Ceacam18 | 0.0 | 0.0 | 0.0 | 0.1 | 0.0 | 0.0 | 0.0 | 0.0 | 0.0 | 0.0 | 0.0 | 0.0 |
| Ceacam9 | 0.0 | 0.0 | 0.0 | 0.0 | 0.0 | 0.0 | 0.0 | 0.0 | 0.0 | 0.0 | 0.0 | 0.0 |
| Cebpe | 0.0 | 0.0 | 0.0 | 0.0 | 0.0 | 0.0 | 0.0 | 0.0 | 0.0 | 0.0 | 0.0 | 0.0 |
| Cel | 0.0 | 0.0 | 0.0 | 0.0 | 0.0 | 0.0 | 0.0 | 0.0 | 0.0 | 0.0 | 0.0 | 0.0 |
| Cer1 | 0.0 | 0.0 | 0.0 | 0.0 | 0.0 | 0.0 | 0.0 | 0.0 | 0.0 | 0.0 | 0.0 | 0.0 |
| Ces1b | 0.0 | 0.0 | 0.0 | 0.0 | 0.0 | 0.0 | 0.0 | 0.0 | 0.0 | 0.0 | 0.0 | 0.0 |
| Ces1c | 0.0 | 0.0 | 0.0 | 0.0 | 0.0 | 0.0 | 0.0 | 0.0 | 0.0 | 0.0 | 0.0 | 0.0 |
| Ces2h | 0.0 | 0.0 | 0.0 | 0.0 | 0.0 | 0.0 | 0.0 | 0.0 | 0.0 | 0.0 | 0.0 | 0.0 |
| Ces3b | 0.0 | 0.0 | 0.0 | 0.0 | 0.0 | 0.0 | 0.0 | 0.0 | 0.0 | 0.0 | 0.0 | 0.0 |
| Ces4a | 0.0 | 0.0 | 0.0 | 0.0 | 0.0 | 0.0 | 0.0 | 0.0 | 0.0 | 0.0 | 0.0 | 0.0 |
| Ces5a | 0.0 | 0.0 | 0.0 | 0.1 | 0.0 | 0.0 | 0.0 | 0.0 | 0.0 | 0.0 | 0.0 | 0.0 |
| Cfd | 0.0 | 0.0 | 0.0 | 0.0 | 0.0 | 0.0 | 0.0 | 0.0 | 0.1 | 0.0 | 0.0 | 0.0 |
| Cga | 0.1 | 0.0 | 0.0 | 0.0 | 0.0 | 0.0 | 0.0 | 0.0 | 0.0 | 0.0 | 0.0 | 0.0 |
| Chat | 0.0 | 0.0 | 0.0 | 0.0 | 0.0 | 0.0 | 0.0 | 0.0 | 0.0 | 0.0 | 0.0 | 0.0 |
| Chi3l3 | 0.0 | 0.0 | 0.1 | 0.2 | 0.0 | 0.0 | 0.1 | 0.0 | 0.0 | 0.0 | 0.0 | 0.0 |
| Chi3l4 | 0.0 | 0.0 | 0.0 | 0.0 | 0.0 | 0.0 | 0.0 | 0.0 | 0.0 | 0.0 | 0.0 | 0.0 |
| Chia | 0.0 | 0.0 | 0.0 | 0.0 | 0.0 | 0.5 | 0.0 | 0.0 | 0.0 | 0.0 | 0.0 | 0.0 |
| Chp2 | 0.0 | 0.0 | 0.0 | 0.0 | 0.0 | 0.0 | 0.0 | 0.0 | 0.0 | 0.0 | 0.0 | 0.0 |
| Chrdl2 | 0.0 | 0.1 | 0.0 | 0.0 | 0.0 | 0.0 | 0.0 | 0.0 | 0.0 | 0.0 | 0.0 | 0.0 |
| Chrm5 | 0.0 | 0.0 | 0.0 | 0.0 | 0.0 | 0.0 | 0.0 | 0.0 | 0.0 | 0.0 | 0.0 | 0.0 |
| Chrna3 | 0.0 | 0.0 | 0.0 | 0.0 | 0.0 | 0.0 | 0.0 | 0.0 | 0.0 | 0.0 | 0.0 | 0.0 |
| Chrna6 | 0.0 | 0.0 | 0.0 | 0.0 | 0.0 | 0.0 | 0.0 | 0.0 | 0.0 | 0.0 | 0.0 | 0.0 |
| Chrnb3 | 0.0 | 0.0 | 0.0 | 0.0 | 0.0 | 0.0 | 0.0 | 0.0 | 0.0 | 0.0 | 0.0 | 0.0 |

| | | | | | | | | | | | | |
|---|---|---|---|---|---|---|---|---|---|---|---|---|
| Chrnb4 | 0.0 | 0.0 | 0.0 | 0.0 | 0.0 | 0.0 | 0.0 | 0.0 | 0.0 | 0.0 | 0.0 | 0.0 |
| Chrnd | 0.0 | 0.0 | 0.0 | 0.0 | 0.0 | 0.0 | 0.0 | 0.0 | 0.0 | 0.0 | 0.0 | 0.0 |
| Chrne | 0.0 | 0.0 | 0.0 | 0.1 | 0.0 | 0.0 | 0.0 | 0.0 | 0.0 | 0.0 | 0.0 | 0.0 |
| Chrng | 0.0 | 0.0 | 0.0 | 0.0 | 0.0 | 0.0 | 0.0 | 0.0 | 0.0 | 0.0 | 0.0 | 0.0 |
| Chst9 | 0.0 | 0.0 | 0.0 | 0.0 | 0.0 | 0.0 | 0.0 | 0.0 | 0.0 | 0.0 | 0.0 | 0.0 |
| Cib4 | 0.0 | 0.0 | 0.0 | 0.0 | 0.0 | 0.0 | 0.0 | 0.0 | 0.0 | 0.0 | 0.0 | 0.0 |
| Cidea | 0.0 | 0.0 | 0.0 | 0.0 | 0.0 | 0.0 | 0.0 | 0.0 | 0.0 | 0.0 | 0.0 | 0.0 |
| Cidec | 0.0 | 0.0 | 0.0 | 0.0 | 0.0 | 0.0 | 0.0 | 0.0 | 0.0 | 0.0 | 0.0 | 0.0 |
| Cited1 | 0.0 | 0.0 | 0.0 | 0.0 | 0.0 | 0.1 | 0.0 | 0.0 | 0.0 | 0.0 | 0.0 | 0.2 |
| Ckm | 0.0 | 0.1 | 0.0 | 0.0 | 0.0 | 0.0 | 0.0 | 0.0 | 0.0 | 0.0 | 0.0 | 0.0 |
| Clca3 | 0.0 | 0.0 | 0.0 | 0.0 | 0.0 | 0.0 | 0.0 | 0.0 | 0.0 | 0.0 | 0.0 | 0.0 |
| Clcnka | 0.0 | 0.0 | 0.0 | 0.0 | 0.0 | 0.1 | 0.0 | 0.0 | 0.0 | 0.0 | 0.0 | 0.0 |
| Cldn13 | 0.0 | 0.0 | 0.0 | 0.0 | 0.0 | 0.0 | 0.0 | 0.0 | 0.0 | 0.0 | 0.0 | 0.0 |
| Cldn14 | 0.0 | 0.0 | 0.0 | 0.0 | 0.0 | 0.0 | 0.0 | 0.0 | 0.0 | 0.0 | 0.0 | 0.0 |
| Cldn18 | 0.0 | 0.0 | 0.0 | 0.0 | 0.0 | 0.0 | 0.0 | 0.0 | 0.0 | 0.0 | 0.0 | 0.0 |
| Cldn2 | 0.0 | 0.0 | 0.0 | 0.0 | 0.0 | 0.0 | 0.0 | 0.0 | 0.0 | 0.0 | 0.0 | 0.0 |
| Cldn26 | 0.0 | 0.0 | 0.0 | 0.0 | 0.0 | 0.0 | 0.0 | 0.0 | 0.0 | 0.0 | 0.0 | 0.0 |
| Cldn4 | 0.0 | 0.0 | 0.0 | 0.1 | 0.0 | 0.0 | 0.0 | 0.0 | 0.0 | 0.0 | 0.0 | 0.0 |
| Cldn6 | 0.0 | 0.0 | 0.0 | 0.0 | 0.0 | 0.0 | 0.0 | 0.0 | 0.0 | 0.0 | 0.0 | 0.0 |
| Cldn9 | 0.0 | 0.0 | 0.0 | 0.0 | 0.0 | 0.0 | 0.0 | 0.0 | 0.0 | 0.1 | 0.0 | 0.0 |
| Clec12b | 0.0 | 0.0 | 0.0 | 0.0 | 0.0 | 0.0 | 0.0 | 0.0 | 0.0 | 0.0 | 0.0 | 0.0 |
| Clec1b | 0.0 | 0.0 | 0.0 | 0.0 | 0.0 | 0.0 | 0.0 | 0.0 | 0.0 | 0.0 | 0.0 | 0.1 |
| Clec3a | 0.0 | 0.0 | 0.0 | 0.0 | 0.0 | 0.0 | 0.0 | 0.0 | 0.0 | 0.0 | 0.0 | 0.0 |
| Clec4b1 | 0.1 | 0.0 | 0.0 | 0.0 | 0.2 | 0.0 | 0.0 | 0.0 | 0.0 | 0.0 | 0.0 | 0.0 |
| Clrn1 | 0.0 | 0.0 | 0.0 | 0.0 | 0.0 | 0.0 | 0.0 | 0.0 | 0.0 | 0.0 | 0.0 | 0.0 |
| Clrn3 | 0.0 | 0.0 | 0.0 | 0.0 | 0.0 | 0.0 | 0.0 | 0.0 | 0.0 | 0.0 | 0.0 | 0.0 |
| Clvs2 | 0.0 | 0.0 | 0.0 | 0.0 | 0.0 | 0.0 | 0.0 | 0.0 | 0.0 | 0.0 | 0.0 | 0.0 |
| Cma2 | 0.0 | 0.0 | 0.0 | 0.0 | 0.0 | 0.0 | 0.0 | 0.0 | 0.1 | 0.0 | 0.0 | 0.0 |
| Cml5 | 0.0 | 0.0 | 0.0 | 0.0 | 0.0 | 0.1 | 0.0 | 0.0 | 0.0 | 0.0 | 0.0 | 0.0 |
| Cmtm1 | 0.0 | 0.0 | 0.0 | 0.1 | 0.0 | 0.1 | 0.0 | 0.0 | 0.0 | 0.0 | 0.0 | 0.0 |
| Cmtm2a | 0.0 | 0.0 | 0.0 | 0.0 | 0.1 | 0.0 | 0.0 | 0.0 | 0.0 | 0.0 | 0.0 | 0.0 |
| Cnfn | 0.0 | 0.0 | 0.0 | 0.0 | 0.0 | 0.0 | 0.1 | 0.0 | 0.0 | 0.0 | 0.0 | 0.0 |
| Cnga1 | 0.0 | 0.0 | 0.0 | 0.0 | 0.0 | 0.0 | 0.0 | 0.0 | 0.0 | 0.0 | 0.0 | 0.0 |
| Cnga4 | 0.0 | 0.0 | 0.1 | 0.0 | 0.0 | 0.0 | 0.0 | 0.0 | 0.0 | 0.0 | 0.0 | 0.0 |
| Cnih3 | 0.0 | 0.0 | 0.0 | 0.0 | 0.0 | 0.0 | 0.0 | 0.0 | 0.0 | 0.0 | 0.0 | 0.0 |
| Cnksr2 | 0.0 | 0.0 | 0.0 | 0.0 | 0.0 | 0.0 | 0.0 | 0.0 | 0.0 | 0.0 | 0.0 | 0.0 |
| Cnpy1 | 0.0 | 0.0 | 0.0 | 0.0 | 0.0 | 0.0 | 0.0 | 0.0 | 0.0 | 0.0 | 0.0 | 0.0 |
| Cntn3 | 0.0 | 0.0 | 0.0 | 0.0 | 0.0 | 0.0 | 0.0 | 0.0 | 0.0 | 0.0 | 0.0 | 0.0 |
| Cntn6 | 0.0 | 0.0 | 0.0 | 0.0 | 0.0 | 0.0 | 0.0 | 0.0 | 0.0 | 0.0 | 0.0 | 0.0 |
| Cntnap4 | 0.0 | 0.0 | 0.0 | 0.0 | 0.0 | 0.0 | 0.0 | 0.0 | 0.0 | 0.0 | 0.0 | 0.0 |
| Cntnap5c | 0.0 | 0.0 | 0.0 | 0.0 | 0.0 | 0.0 | 0.0 | 0.0 | 0.0 | 0.0 | 0.0 | 0.0 |
| Col9a1 | 0.0 | 0.0 | 0.0 | 0.0 | 0.0 | 0.0 | 0.0 | 0.0 | 0.0 | 0.0 | 0.0 | 0.0 |
| Cort | 0.0 | 0.0 | 0.0 | 0.0 | 0.0 | 0.0 | 0.0 | 0.0 | 0.0 | 0.0 | 0.0 | 0.0 |
| Cox7b2 | 0.1 | 0.0 | 0.0 | 0.0 | 0.0 | 0.1 | 0.0 | 0.2 | 0.0 | 0.0 | 0.0 | 0.0 |
| Cox8b | 0.6 | 0.0 | 0.1 | 0.0 | 0.0 | 0.0 | 0.0 | 0.2 | 0.0 | 0.0 | 0.0 | 0.2 |
| Cpa5 | 0.0 | 0.0 | 0.0 | 0.0 | 0.0 | 0.0 | 0.0 | 0.0 | 0.0 | 0.0 | 0.0 | 0.0 |
| Cplx4 | 0.0 | 0.0 | 0.0 | 0.0 | 0.0 | 0.0 | 0.0 | 0.0 | 0.0 | 0.0 | 0.0 | 0.1 |
| Cpn1 | 0.0 | 0.0 | 0.0 | 0.0 | 0.0 | 0.0 | 0.0 | 0.0 | 0.0 | 0.0 | 0.0 | 0.0 |
| Cpn2 | 0.0 | 0.0 | 0.0 | 0.0 | 0.0 | 0.0 | 0.0 | 0.0 | 0.0 | 0.0 | 0.0 | 0.0 |
| Cpvl | 0.0 | 0.0 | 0.0 | 0.0 | 0.0 | 0.0 | 0.0 | 0.0 | 0.0 | 0.0 | 0.0 | 0.1 |
| Crb1 | 0.0 | 0.0 | 0.0 | 0.0 | 0.0 | 0.0 | 0.0 | 0.0 | 0.0 | 0.0 | 0.0 | 0.0 |
| Crb3 | 0.0 | 0.0 | 0.0 | 0.0 | 0.0 | 0.0 | 0.0 | 0.0 | 0.0 | 0.0 | 0.0 | 0.0 |
| Crhr2 | 0.0 | 0.0 | 0.0 | 0.0 | 0.0 | 0.0 | 0.0 | 0.0 | 0.0 | 0.0 | 0.0 | 0.0 |
| Crip3 | 0.0 | 0.0 | 0.0 | 0.0 | 0.0 | 0.0 | 0.2 | 0.0 | 0.0 | 0.0 | 0.0 | 0.0 |
| Crisp1 | 0.0 | 0.0 | 0.0 | 0.0 | 0.0 | 0.0 | 0.0 | 0.0 | 0.0 | 0.0 | 0.0 | 0.0 |
| Crisp4 | 0.0 | 0.0 | 0.0 | 0.0 | 0.0 | 0.0 | 0.0 | 0.0 | 0.0 | 0.0 | 0.1 | 0.0 |
| Crnn | 0.0 | 0.0 | 0.0 | 0.0 | 0.0 | 0.0 | 0.0 | 0.0 | 0.0 | 0.0 | 0.0 | 0.0 |

Excluded Genes

| | | | | | | | | | | | | |
|---|---|---|---|---|---|---|---|---|---|---|---|---|
| Crp | 0.0 | 0.0 | 0.0 | 0.0 | 0.0 | 0.0 | 0.0 | 0.0 | 0.0 | 0.0 | 0.0 | 0.0 |
| Crtam | 0.0 | 0.0 | 0.0 | 0.0 | 0.0 | 0.0 | 0.0 | 0.0 | 0.0 | 0.0 | 0.0 | 0.0 |
| Crxos1 | 0.1 | 0.0 | 0.0 | 0.0 | 0.1 | 0.0 | 0.0 | 0.0 | 0.0 | 0.0 | 0.0 | 0.0 |
| Cryaa | 0.0 | 0.0 | 0.0 | 0.1 | 0.0 | 0.0 | 0.0 | 0.0 | 0.0 | 0.0 | 0.0 | 0.0 |
| Crybb2 | 0.0 | 0.0 | 0.0 | 0.0 | 0.0 | 0.0 | 0.0 | 0.0 | 0.0 | 0.0 | 0.0 | 0.0 |
| Crygf | 0.0 | 0.0 | 0.0 | 0.0 | 0.0 | 0.0 | 0.0 | 0.0 | 0.0 | 0.0 | 0.0 | 0.0 |
| Csf3 | 0.0 | 0.0 | 0.0 | 0.0 | 0.0 | 0.0 | 0.0 | 0.0 | 0.0 | 0.0 | 0.0 | 0.0 |
| Csn1s1 | 0.0 | 0.0 | 0.0 | 0.0 | 0.0 | 0.0 | 0.0 | 0.0 | 0.0 | 0.0 | 0.0 | 0.0 |
| Csn1s2a | 0.0 | 0.0 | 0.0 | 0.0 | 0.0 | 0.0 | 0.0 | 0.0 | 0.0 | 0.0 | 0.0 | 0.0 |
| Csn1s2b | 0.0 | 0.0 | 0.0 | 0.0 | 0.0 | 0.0 | 0.0 | 0.0 | 0.0 | 0.0 | 0.0 | 0.0 |
| Csn3 | 0.0 | 0.0 | 0.0 | 0.0 | 0.0 | 0.0 | 0.0 | 0.0 | 0.0 | 0.0 | 0.0 | 0.0 |
| Csnka2ip | 0.0 | 0.0 | 0.0 | 0.0 | 0.0 | 0.0 | 0.0 | 0.0 | 0.0 | 0.0 | 0.0 | 0.0 |
| Csrnp3 | 0.0 | 0.0 | 0.0 | 0.0 | 0.0 | 0.0 | 0.0 | 0.0 | 0.0 | 0.0 | 0.0 | 0.0 |
| Csrp3 | 0.0 | 0.0 | 0.0 | 0.0 | 0.0 | 0.0 | 0.0 | 0.0 | 0.0 | 0.0 | 0.0 | 0.0 |
| Cst13 | 0.0 | 0.0 | 0.0 | 0.0 | 0.0 | 0.0 | 0.0 | 0.0 | 0.0 | 0.0 | 0.0 | 0.0 |
| Cst8 | 0.0 | 0.0 | 0.1 | 0.0 | 0.0 | 0.0 | 0.0 | 0.0 | 0.0 | 0.0 | 0.0 | 0.0 |
| Csta | 0.0 | 0.0 | 0.0 | 0.0 | 0.0 | 0.0 | 0.0 | 0.0 | 0.0 | 0.0 | 0.0 | 0.0 |
| Ctag2 | 0.0 | 0.0 | 0.0 | 0.0 | 0.0 | 0.0 | 0.0 | 0.0 | 0.0 | 0.0 | 0.0 | 0.0 |
| Ctf2 | 0.0 | 0.0 | 0.1 | 0.0 | 0.0 | 0.0 | 0.0 | 0.0 | 0.0 | 0.0 | 0.0 | 0.0 |
| Ctrc | 0.0 | 0.0 | 0.0 | 0.0 | 0.0 | 0.0 | 0.0 | 0.0 | 0.0 | 0.0 | 0.0 | 0.0 |
| Cts7 | 0.0 | 0.0 | 0.0 | 0.0 | 0.0 | 0.0 | 0.0 | 0.0 | 0.0 | 0.0 | 0.1 | 0.0 |
| Cts8 | 0.0 | 0.0 | 0.0 | 0.0 | 0.0 | 0.0 | 0.0 | 0.0 | 0.0 | 0.0 | 0.0 | 0.0 |
| Cts8-ps | 0.0 | 0.0 | 0.0 | 0.0 | 0.0 | 0.0 | 0.0 | 0.0 | 0.0 | 0.0 | 0.0 | 0.0 |
| Ctse | 0.0 | 0.0 | 0.0 | 0.0 | 0.0 | 0.0 | 0.0 | 0.0 | 0.0 | 0.0 | 0.0 | 0.0 |
| Ctsq | 0.0 | 0.0 | 0.0 | 0.0 | 0.0 | 0.0 | 0.0 | 0.0 | 0.0 | 0.0 | 0.0 | 0.0 |
| Ctxn2 | 0.0 | 0.0 | 0.0 | 0.0 | 0.0 | 0.0 | 0.0 | 0.1 | 0.0 | 0.0 | 0.0 | 0.0 |
| Ctxn3 | 0.0 | 0.0 | 0.0 | 0.0 | 0.0 | 0.0 | 0.1 | 0.0 | 0.0 | 0.0 | 0.0 | 0.0 |
| Cuzd1 | 0.0 | 0.0 | 0.0 | 0.0 | 0.0 | 0.0 | 0.0 | 0.0 | 0.0 | 0.0 | 0.0 | 0.0 |
| Cwh43 | 0.0 | 0.0 | 0.0 | 0.0 | 0.0 | 0.0 | 0.0 | 0.0 | 0.0 | 0.0 | 0.0 | 0.0 |
| Cxcl13 | 0.0 | 0.0 | 0.0 | 0.0 | 0.0 | 0.0 | 0.0 | 0.1 | 0.0 | 0.1 | 0.0 | 0.0 |
| Cxcl17 | 0.1 | 0.0 | 0.3 | 0.0 | 0.0 | 0.0 | 0.1 | 0.1 | 0.0 | 0.1 | 0.0 | 0.2 |
| Cylc2 | 0.0 | 0.0 | 0.0 | 0.0 | 0.0 | 0.0 | 0.0 | 0.0 | 0.0 | 0.0 | 0.0 | 0.0 |
| Cym | 0.0 | 0.0 | 0.0 | 0.0 | 0.0 | 0.0 | 0.0 | 0.0 | 0.0 | 0.0 | 0.0 | 0.0 |
| Cyp11a1 | 0.0 | 0.0 | 0.0 | 0.0 | 0.0 | 0.0 | 0.0 | 0.0 | 0.0 | 0.0 | 0.0 | 0.0 |
| Cyp11b2 | 0.0 | 0.0 | 0.0 | 0.0 | 0.0 | 0.0 | 0.0 | 0.0 | 0.0 | 0.0 | 0.0 | 0.0 |
| Cyp19a1 | 0.0 | 0.0 | 0.0 | 0.0 | 0.0 | 0.0 | 0.0 | 0.0 | 0.0 | 0.0 | 0.0 | 0.0 |
| Cyp1a2 | 0.0 | 0.0 | 0.0 | 0.0 | 0.0 | 0.0 | 0.0 | 0.0 | 0.0 | 0.0 | 0.0 | 0.0 |
| Cyp21a1 | 0.0 | 0.0 | 0.0 | 0.1 | 0.0 | 0.0 | 0.0 | 0.0 | 0.0 | 0.0 | 0.0 | 0.0 |
| Cyp24a1 | 0.0 | 0.0 | 0.0 | 0.0 | 0.0 | 0.0 | 0.0 | 0.0 | 0.0 | 0.0 | 0.0 | 0.0 |
| Cyp26a1 | 0.0 | 0.0 | 0.0 | 0.0 | 0.0 | 0.0 | 0.0 | 0.1 | 0.0 | 0.0 | 0.0 | 0.0 |
| Cyp2a4 | 0.0 | 0.0 | 0.0 | 0.0 | 0.0 | 0.0 | 0.0 | 0.0 | 0.0 | 0.0 | 0.0 | 0.0 |
| Cyp2b13 | 0.0 | 0.0 | 0.0 | 0.0 | 0.0 | 0.0 | 0.0 | 0.0 | 0.0 | 0.0 | 0.0 | 0.0 |
| Cyp2b9 | 0.0 | 0.0 | 0.0 | 0.0 | 0.0 | 0.0 | 0.0 | 0.0 | 0.0 | 0.0 | 0.0 | 0.0 |
| Cyp2c29 | 0.0 | 0.0 | 0.0 | 0.0 | 0.0 | 0.0 | 0.0 | 0.0 | 0.0 | 0.0 | 0.0 | 0.0 |
| Cyp2c38 | 0.0 | 0.0 | 0.0 | 0.0 | 0.0 | 0.0 | 0.0 | 0.0 | 0.0 | 0.0 | 0.0 | 0.0 |
| Cyp2c39 | 0.0 | 0.0 | 0.0 | 0.0 | 0.0 | 0.0 | 0.0 | 0.0 | 0.0 | 0.0 | 0.0 | 0.0 |
| Cyp2c40 | 0.0 | 0.0 | 0.0 | 0.0 | 0.0 | 0.0 | 0.0 | 0.0 | 0.0 | 0.0 | 0.0 | 0.0 |
| Cyp2c50 | 0.0 | 0.0 | 0.0 | 0.0 | 0.0 | 0.0 | 0.0 | 0.0 | 0.0 | 0.0 | 0.0 | 0.0 |
| Cyp2c53-ps | 0.0 | 0.0 | 0.0 | 0.0 | 0.0 | 0.0 | 0.0 | 0.0 | 0.0 | 0.0 | 0.0 | 0.0 |
| Cyp2c65 | 0.0 | 0.0 | 0.0 | 0.0 | 0.0 | 0.0 | 0.0 | 0.0 | 0.0 | 0.0 | 0.0 | 0.0 |
| Cyp2c66 | 0.0 | 0.0 | 0.0 | 0.0 | 0.0 | 0.0 | 0.0 | 0.0 | 0.0 | 0.0 | 0.0 | 0.0 |
| Cyp2c67 | 0.0 | 0.0 | 0.0 | 0.0 | 0.0 | 0.0 | 0.0 | 0.0 | 0.0 | 0.0 | 0.0 | 0.0 |
| Cyp2c68 | 0.0 | 0.0 | 0.0 | 0.0 | 0.0 | 0.0 | 0.0 | 0.0 | 0.0 | 0.0 | 0.0 | 0.0 |
| Cyp2c69 | 0.0 | 0.0 | 0.0 | 0.0 | 0.0 | 0.0 | 0.0 | 0.0 | 0.0 | 0.0 | 0.0 | 0.0 |
| Cyp2d10 | 0.0 | 0.0 | 0.0 | 0.0 | 0.0 | 0.0 | 0.0 | 0.0 | 0.0 | 0.0 | 0.0 | 0.0 |
| Cyp2d11 | 0.0 | 0.0 | 0.0 | 0.0 | 0.0 | 0.0 | 0.0 | 0.0 | 0.0 | 0.0 | 0.0 | 0.0 |
| Cyp2d13 | 0.0 | 0.0 | 0.0 | 0.0 | 0.0 | 0.0 | 0.0 | 0.0 | 0.0 | 0.0 | 0.0 | 0.0 |

Excluded Genes

| | | | | | | | | | | | | |
|---|---|---|---|---|---|---|---|---|---|---|---|---|
| Cyp2d34 | 0.0 | 0.0 | 0.0 | 0.0 | 0.0 | 0.0 | 0.0 | 0.0 | 0.0 | 0.0 | 0.0 | 0.0 |
| Cyp2d37-ps | 0.0 | 0.0 | 0.0 | 0.0 | 0.0 | 0.0 | 0.0 | 0.0 | 0.0 | 0.0 | 0.0 | 0.0 |
| Cyp2d40 | 0.0 | 0.0 | 0.0 | 0.0 | 0.0 | 0.0 | 0.0 | 0.0 | 0.0 | 0.0 | 0.0 | 0.0 |
| Cyp2e1 | 0.0 | 0.0 | 0.0 | 0.0 | 0.0 | 0.0 | 0.0 | 0.0 | 0.0 | 0.0 | 0.0 | 0.0 |
| Cyp2f2 | 0.0 | 0.0 | 0.0 | 0.0 | 0.0 | 0.0 | 0.0 | 0.0 | 0.0 | 0.0 | 0.0 | 0.0 |
| Cyp2j11 | 0.0 | 0.0 | 0.0 | 0.0 | 0.0 | 0.0 | 0.0 | 0.0 | 0.0 | 0.0 | 0.0 | 0.0 |
| Cyp2j12 | 0.0 | 0.0 | 0.0 | 0.0 | 0.0 | 0.0 | 0.0 | 0.0 | 0.0 | 0.0 | 0.0 | 0.0 |
| Cyp2j5 | 0.0 | 0.0 | 0.0 | 0.0 | 0.0 | 0.0 | 0.0 | 0.0 | 0.0 | 0.0 | 0.0 | 0.0 |
| Cyp2j8 | 0.0 | 0.0 | 0.0 | 0.1 | 0.0 | 0.0 | 0.0 | 0.0 | 0.0 | 0.0 | 0.0 | 0.0 |
| Cyp3a16 | 0.0 | 0.0 | 0.0 | 0.0 | 0.0 | 0.0 | 0.0 | 0.0 | 0.0 | 0.0 | 0.0 | 0.0 |
| Cyp3a41a | 0.0 | 0.0 | 0.0 | 0.0 | 0.0 | 0.0 | 0.0 | 0.0 | 0.0 | 0.0 | 0.0 | 0.0 |
| Cyp3a44 | 0.0 | 0.0 | 0.0 | 0.0 | 0.0 | 0.0 | 0.0 | 0.0 | 0.0 | 0.0 | 0.0 | 0.0 |
| Cyp3a57 | 0.0 | 0.0 | 0.0 | 0.0 | 0.0 | 0.0 | 0.0 | 0.0 | 0.0 | 0.0 | 0.0 | 0.0 |
| Cyp3a59 | 0.0 | 0.0 | 0.0 | 0.1 | 0.0 | 0.0 | 0.0 | 0.0 | 0.0 | 0.0 | 0.0 | 0.0 |
| Cyp4a10 | 0.0 | 0.0 | 0.0 | 0.0 | 0.0 | 0.0 | 0.0 | 0.0 | 0.0 | 0.0 | 0.0 | 0.0 |
| Cyp4a12a | 0.1 | 0.0 | 0.0 | 0.1 | 0.0 | 0.0 | 0.0 | 0.0 | 0.0 | 0.0 | 0.0 | 0.0 |
| Cyp4a14 | 0.0 | 0.0 | 0.0 | 0.0 | 0.0 | 0.0 | 0.0 | 0.0 | 0.0 | 0.0 | 0.0 | 0.0 |
| Cyp4a30b-ps | 0.0 | 0.0 | 0.0 | 0.0 | 0.0 | 0.0 | 0.0 | 0.0 | 0.0 | 0.0 | 0.0 | 0.0 |
| Cyp4a31 | 0.0 | 0.0 | 0.0 | 0.0 | 0.0 | 0.0 | 0.0 | 0.0 | 0.0 | 0.0 | 0.0 | 0.0 |
| Cyp4a32 | 0.0 | 0.0 | 0.0 | 0.0 | 0.0 | 0.0 | 0.0 | 0.0 | 0.0 | 0.0 | 0.0 | 0.0 |
| Cyp4f39 | 0.0 | 0.0 | 0.0 | 0.0 | 0.0 | 0.0 | 0.0 | 0.0 | 0.0 | 0.0 | 0.0 | 0.0 |
| Cyp4f40 | 0.0 | 0.0 | 0.0 | 0.0 | 0.0 | 0.0 | 0.0 | 0.0 | 0.0 | 0.0 | 0.0 | 0.0 |
| Cyp8b1 | 0.0 | 0.0 | 0.0 | 0.0 | 0.0 | 0.0 | 0.0 | 0.0 | 0.0 | 0.0 | 0.0 | 0.0 |
| Cypt1 | 0.0 | 0.0 | 0.0 | 0.0 | 0.0 | 0.0 | 0.0 | 0.0 | 0.0 | 0.0 | 0.0 | 0.0 |
| Cypt2 | 0.0 | 0.0 | 0.1 | 0.0 | 0.0 | 0.0 | 0.0 | 0.0 | 0.0 | 0.1 | 0.0 | 0.0 |
| Cypt3 | 0.0 | 0.0 | 0.0 | 0.0 | 0.0 | 0.0 | 0.0 | 0.0 | 0.0 | 0.0 | 0.0 | 0.0 |
| Cypt7 | 0.0 | 0.0 | 0.0 | 0.0 | 0.0 | 0.0 | 0.0 | 0.0 | 0.0 | 0.0 | 0.0 | 0.0 |
| Cypt8 | 0.0 | 0.0 | 0.0 | 0.0 | 0.0 | 0.0 | 0.0 | 0.0 | 0.0 | 0.0 | 0.0 | 0.0 |
| Cysltr2 | 0.0 | 0.0 | 0.0 | 0.1 | 0.0 | 0.0 | 0.0 | 0.0 | 0.0 | 0.0 | 0.0 | 0.0 |
| Cytl1 | 0.0 | 0.0 | 0.0 | 0.0 | 0.0 | 0.0 | 0.0 | 0.0 | 0.0 | 0.0 | 0.0 | 0.0 |
| Cyyr1 | 0.0 | 0.1 | 0.0 | 0.0 | 0.0 | 0.0 | 0.0 | 0.0 | 0.0 | 0.0 | 0.0 | 0.0 |
| D030045P18Rik | 0.0 | 0.0 | 0.0 | 0.0 | 0.0 | 0.0 | 0.0 | 0.0 | 0.0 | 0.0 | 0.0 | 0.0 |
| D130058E03 | 0.0 | 0.0 | 0.0 | 0.0 | 0.0 | 0.0 | 0.0 | 0.0 | 0.0 | 0.0 | 0.0 | 0.0 |
| D430019H16Rik | 0.0 | 0.0 | 0.0 | 0.0 | 0.0 | 0.0 | 0.0 | 0.0 | 0.0 | 0.0 | 0.0 | 0.0 |
| D5Ertd577e | 0.0 | 0.0 | 0.0 | 0.0 | 0.0 | 0.0 | 0.0 | 0.0 | 0.0 | 0.0 | 0.0 | 0.0 |
| D630013N20Rik | 0.0 | 0.0 | 0.0 | 0.0 | 0.0 | 0.0 | 0.0 | 0.0 | 0.0 | 0.0 | 0.0 | 0.0 |
| D6Ertd474e | 0.0 | 0.0 | 0.0 | 0.0 | 0.0 | 0.0 | 0.0 | 0.0 | 0.0 | 0.0 | 0.0 | 0.0 |
| D7Ertd143e | 0.0 | 0.0 | 0.0 | 0.0 | 0.0 | 0.0 | 0.0 | 0.0 | 0.0 | 0.0 | 0.0 | 0.0 |
| D830013O20Rik | 0.0 | 0.0 | 0.0 | 0.0 | 0.0 | 0.0 | 0.0 | 0.0 | 0.0 | 0.0 | 0.0 | 0.0 |
| D930007P13Rik | 0.0 | 0.0 | 0.0 | 0.0 | 0.0 | 0.0 | 0.0 | 0.0 | 0.0 | 0.0 | 0.0 | 0.0 |
| D930020B18Rik | 0.0 | 0.0 | 0.0 | 0.0 | 0.0 | 0.0 | 0.0 | 0.0 | 0.0 | 0.0 | 0.0 | 0.0 |
| D930032P07Rik | 0.0 | 0.0 | 0.1 | 0.0 | 0.0 | 0.0 | 0.0 | 0.0 | 0.0 | 0.0 | 0.0 | 0.1 |
| Dao | 0.0 | 0.0 | 0.0 | 0.0 | 0.0 | 0.0 | 0.0 | 0.0 | 0.0 | 0.0 | 0.0 | 0.0 |
| Darc | 0.0 | 0.0 | 0.0 | 0.0 | 0.0 | 0.0 | 0.1 | 0.0 | 0.0 | 0.0 | 0.0 | 0.0 |
| Dbpht2 | 0.0 | 0.0 | 0.0 | 0.0 | 0.0 | 0.0 | 0.0 | 0.0 | 0.0 | 0.0 | 0.0 | 0.0 |
| Dbx2 | 0.0 | 0.0 | 0.0 | 0.0 | 0.0 | 0.0 | 0.0 | 0.0 | 0.0 | 0.0 | 0.0 | 0.0 |
| Dcstamp | 0.0 | 0.0 | 0.0 | 0.0 | 0.3 | 0.0 | 0.0 | 0.0 | 0.0 | 0.0 | 0.0 | 0.0 |
| Dcx | 0.0 | 0.0 | 0.0 | 0.0 | 0.0 | 0.0 | 0.0 | 0.0 | 0.0 | 0.0 | 0.0 | 0.0 |
| Defb1 | 0.0 | 0.0 | 0.0 | 0.0 | 0.0 | 0.0 | 0.0 | 0.0 | 0.0 | 0.0 | 0.0 | 0.0 |
| Defb18 | 0.0 | 0.0 | 0.0 | 0.0 | 0.0 | 0.0 | 0.0 | 0.0 | 0.0 | 0.0 | 0.0 | 0.0 |
| Defb19 | 0.0 | 0.0 | 0.0 | 0.0 | 0.0 | 0.0 | 0.0 | 0.0 | 0.0 | 0.0 | 0.1 | 0.0 |
| Defb20 | 0.0 | 0.0 | 0.0 | 0.0 | 0.0 | 0.0 | 0.0 | 0.0 | 0.0 | 0.0 | 0.0 | 0.0 |
| Defb26 | 0.0 | 0.0 | 0.0 | 0.0 | 0.0 | 0.0 | 0.0 | 0.0 | 0.0 | 0.0 | 0.0 | 0.2 |
| Defb28 | 0.0 | 0.0 | 0.2 | 0.0 | 0.0 | 0.0 | 0.0 | 0.0 | 0.0 | 0.0 | 0.0 | 0.0 |
| Defb30 | 0.0 | 0.0 | 0.0 | 0.0 | 0.0 | 0.0 | 0.0 | 0.0 | 0.0 | 0.0 | 0.0 | 0.0 |
| Defb42 | 0.1 | 0.0 | 0.1 | 0.0 | 0.0 | 0.0 | 0.0 | 0.0 | 0.0 | 0.0 | 0.0 | 0.0 |
| Defb48 | 0.0 | 0.0 | 0.0 | 0.0 | 0.0 | 0.0 | 0.0 | 0.0 | 0.0 | 0.1 | 0.0 | 0.0 |

| | | | | | | | | | | | | |
|---|---|---|---|---|---|---|---|---|---|---|---|---|
| Defb7 | 0.2 | 0.0 | 0.0 | 0.0 | 0.0 | 0.0 | 0.0 | 0.0 | 0.0 | 0.0 | 0.0 | 0.0 |
| Dgat2l6 | 0.0 | 0.0 | 0.0 | 0.0 | 0.0 | 0.0 | 0.0 | 0.0 | 0.0 | 0.0 | 0.0 | 0.0 |
| Dgkb | 0.0 | 0.0 | 0.0 | 0.0 | 0.0 | 0.0 | 0.0 | 0.0 | 0.0 | 0.0 | 0.0 | 0.0 |
| Dgkk | 0.0 | 0.0 | 0.0 | 0.0 | 0.0 | 0.0 | 0.0 | 0.0 | 0.0 | 0.0 | 0.0 | 0.0 |
| Dhrs2 | 0.0 | 0.0 | 0.0 | 0.0 | 0.0 | 0.0 | 0.0 | 0.0 | 0.0 | 0.0 | 0.0 | 0.0 |
| Dio1 | 0.0 | 0.0 | 0.0 | 0.0 | 0.0 | 0.0 | 0.0 | 0.0 | 0.0 | 0.0 | 0.0 | 0.0 |
| Dkk1 | 0.0 | 0.0 | 0.0 | 0.0 | 0.0 | 0.0 | 0.0 | 0.0 | 0.0 | 0.0 | 0.0 | 0.0 |
| Dleu7 | 0.0 | 0.0 | 0.0 | 0.0 | 0.0 | 0.0 | 0.0 | 0.0 | 0.0 | 0.0 | 0.0 | 0.0 |
| Dll3 | 0.0 | 0.0 | 0.0 | 0.0 | 0.0 | 0.0 | 0.0 | 0.0 | 0.0 | 0.0 | 0.0 | 0.0 |
| Dlx1 | 0.0 | 0.0 | 0.0 | 0.0 | 0.0 | 0.0 | 0.1 | 0.0 | 0.0 | 0.0 | 0.0 | 0.0 |
| Dlx1as | 0.0 | 0.0 | 0.0 | 0.0 | 0.0 | 0.0 | 0.0 | 0.0 | 0.0 | 0.0 | 0.0 | 0.0 |
| Dlx3 | 0.0 | 0.0 | 0.0 | 0.0 | 0.0 | 0.0 | 0.0 | 0.0 | 0.0 | 0.0 | 0.0 | 0.0 |
| Dlx6 | 0.0 | 0.0 | 0.0 | 0.0 | 0.0 | 0.0 | 0.0 | 0.0 | 0.0 | 0.0 | 0.0 | 0.0 |
| Dmbt1 | 0.0 | 0.0 | 0.0 | 0.0 | 0.0 | 0.0 | 0.0 | 0.0 | 0.0 | 0.0 | 0.0 | 0.0 |
| Dmbx1 | 0.0 | 0.0 | 0.0 | 0.0 | 0.0 | 0.0 | 0.0 | 0.0 | 0.0 | 0.0 | 0.0 | 0.0 |
| Dmgdh | 0.1 | 0.0 | 0.0 | 0.0 | 0.0 | 0.1 | 0.0 | 0.0 | 0.0 | 0.0 | 0.0 | 0.0 |
| Dmrt1 | 0.0 | 0.0 | 0.0 | 0.0 | 0.0 | 0.0 | 0.0 | 0.0 | 0.0 | 0.0 | 0.0 | 0.0 |
| Dmrta1 | 0.1 | 0.0 | 0.0 | 0.0 | 0.0 | 0.0 | 0.0 | 0.0 | 0.0 | 0.0 | 0.0 | 0.0 |
| Dmrtb1 | 0.1 | 0.0 | 0.0 | 0.0 | 0.0 | 0.0 | 0.0 | 0.0 | 0.0 | 0.0 | 0.0 | 0.0 |
| Dmrtc1b | 0.0 | 0.0 | 0.0 | 0.0 | 0.0 | 0.0 | 0.0 | 0.0 | 0.0 | 0.0 | 0.0 | 0.0 |
| Dmrtc2 | 0.0 | 0.0 | 0.2 | 0.0 | 0.0 | 0.0 | 0.0 | 0.0 | 0.0 | 0.0 | 0.1 | 0.0 |
| Dnajc22 | 0.0 | 0.1 | 0.0 | 0.0 | 0.0 | 0.0 | 0.0 | 0.0 | 0.0 | 0.0 | 0.0 | 0.0 |
| Dnase1l3 | 0.0 | 0.0 | 0.0 | 0.0 | 0.0 | 0.0 | 0.0 | 0.0 | 0.0 | 0.0 | 0.0 | 0.0 |
| Doxl2 | 0.0 | 0.0 | 0.0 | 0.0 | 0.0 | 0.0 | 0.0 | 0.0 | 0.0 | 0.0 | 0.0 | 0.0 |
| Dpep3 | 0.0 | 0.0 | 0.0 | 0.0 | 0.0 | 0.0 | 0.0 | 0.0 | 0.0 | 0.0 | 0.0 | 0.0 |
| Dpp6 | 0.1 | 0.0 | 0.0 | 0.0 | 0.0 | 0.0 | 0.0 | 0.0 | 0.0 | 0.0 | 0.0 | 0.0 |
| Dppa1 | 0.0 | 0.0 | 0.0 | 0.0 | 0.0 | 0.0 | 0.0 | 0.0 | 0.0 | 0.0 | 0.0 | 0.0 |
| Dppa3 | 0.0 | 0.0 | 0.0 | 0.0 | 0.0 | 0.0 | 0.0 | 0.0 | 0.0 | 0.1 | 0.0 | 0.1 |
| Dppa4 | 0.0 | 0.0 | 0.0 | 0.0 | 0.0 | 0.1 | 0.0 | 0.0 | 0.0 | 0.1 | 0.0 | 0.0 |
| Dppa5a | 0.1 | 0.0 | 0.0 | 0.0 | 0.1 | 0.0 | 0.0 | 0.1 | 0.0 | 0.1 | 0.0 | 0.0 |
| Dpys | 0.0 | 0.0 | 0.0 | 0.1 | 0.1 | 0.0 | 0.0 | 0.0 | 0.0 | 0.1 | 0.0 | 0.0 |
| DQ267102 | 0.0 | 0.0 | 60.2 | 0.0 | 0.0 | 108.6 | 0.0 | 0.0 | 0.0 | 310.7 | 0.0 | 81.5 |
| Drd2 | 0.0 | 0.0 | 0.0 | 0.0 | 0.0 | 0.0 | 0.0 | 0.0 | 0.0 | 0.0 | 0.0 | 0.0 |
| Drd5 | 0.0 | 0.0 | 0.0 | 0.0 | 0.0 | 0.0 | 0.0 | 0.0 | 0.0 | 0.0 | 0.0 | 0.0 |
| Dsc1 | 0.0 | 0.0 | 0.0 | 0.0 | 0.0 | 0.0 | 0.0 | 0.0 | 0.0 | 0.0 | 0.0 | 0.0 |
| Dsg1a | 0.0 | 0.0 | 0.0 | 0.0 | 0.0 | 0.0 | 0.0 | 0.0 | 0.0 | 0.0 | 0.0 | 0.0 |
| Dsg1b | 0.0 | 0.0 | 0.0 | 0.1 | 0.0 | 0.0 | 0.0 | 0.0 | 0.0 | 0.0 | 0.0 | 0.0 |
| Dsg1c | 0.0 | 0.0 | 0.0 | 0.0 | 0.0 | 0.0 | 0.0 | 0.0 | 0.0 | 0.0 | 0.0 | 0.0 |
| Dsg3 | 0.0 | 0.0 | 0.0 | 0.0 | 0.0 | 0.0 | 0.0 | 0.0 | 0.0 | 0.0 | 0.0 | 0.0 |
| Dspp | 0.0 | 0.0 | 0.0 | 0.0 | 0.0 | 0.0 | 0.0 | 0.0 | 0.0 | 0.0 | 0.0 | 0.0 |
| Dusp26 | 0.0 | 0.0 | 0.0 | 0.0 | 0.0 | 0.0 | 0.0 | 0.0 | 0.0 | 0.0 | 0.0 | 0.0 |
| Dux | 0.0 | 0.0 | 0.0 | 0.0 | 0.0 | 0.0 | 0.0 | 0.0 | 0.0 | 0.0 | 0.0 | 0.0 |
| DXBay18 | 0.0 | 0.0 | 0.0 | 0.0 | 0.0 | 0.0 | 0.0 | 0.0 | 0.0 | 0.0 | 0.0 | 0.0 |
| Dydc1 | 0.1 | 0.0 | 0.0 | 0.1 | 0.0 | 0.0 | 0.0 | 0.0 | 0.0 | 0.0 | 0.0 | 0.0 |
| E030013I19Rik | 0.0 | 0.0 | 0.0 | 0.0 | 0.0 | 0.0 | 0.0 | 0.0 | 0.0 | 0.0 | 0.0 | 0.0 |
| E030025P04Rik | 0.0 | 0.0 | 0.0 | 0.0 | 0.0 | 0.0 | 0.0 | 0.0 | 0.0 | 0.0 | 0.0 | 0.0 |
| E030044B06Rik | 0.0 | 0.1 | 0.0 | 0.0 | 0.0 | 0.0 | 0.0 | 0.0 | 0.0 | 0.0 | 0.0 | 0.0 |
| E130006D01Rik | 0.2 | 0.0 | 0.1 | 0.0 | 0.1 | 0.0 | 0.0 | 0.0 | 0.0 | 0.0 | 0.0 | 0.2 |
| E130008D07Rik | 0.0 | 0.0 | 0.0 | 0.0 | 0.0 | 0.0 | 0.0 | 0.0 | 0.0 | 0.0 | 0.0 | 0.0 |
| E130215H24Rik | 0.0 | 0.0 | 0.0 | 0.2 | 0.1 | 0.0 | 0.0 | 0.0 | 0.0 | 0.0 | 0.0 | 0.0 |
| E130309F12Rik | 0.0 | 0.0 | 0.0 | 0.0 | 0.0 | 0.0 | 0.0 | 0.0 | 0.0 | 0.0 | 0.0 | 0.0 |
| E230008N13Rik | 0.0 | 0.0 | 0.0 | 0.0 | 0.0 | 0.0 | 0.0 | 0.0 | 0.0 | 0.0 | 0.0 | 0.0 |
| E230019M04Rik | 0.0 | 0.0 | 0.0 | 0.0 | 0.0 | 0.0 | 0.0 | 0.0 | 0.0 | 0.0 | 0.0 | 0.0 |
| E230029C05Rik | 0.1 | 0.0 | 0.0 | 0.0 | 0.0 | 0.0 | 0.0 | 0.0 | 0.0 | 0.0 | 0.0 | 0.0 |
| E330014E10Rik | 0.0 | 0.0 | 0.0 | 0.0 | 0.0 | 0.0 | 0.0 | 0.0 | 0.0 | 0.0 | 0.0 | 0.0 |
| E330021D16Rik | 0.0 | 0.0 | 0.0 | 0.0 | 0.0 | 0.0 | 0.0 | 0.0 | 0.0 | 0.0 | 0.0 | 0.0 |
| E430016F16Rik | 0.0 | 0.0 | 0.0 | 0.0 | 0.0 | 0.0 | 0.0 | 0.0 | 0.0 | 0.0 | 0.0 | 0.0 |

| | | | | | | | | | | | | |
|---|---|---|---|---|---|---|---|---|---|---|---|---|
| E530001F21Rik | 0.0 | 0.0 | 0.0 | 0.0 | 0.0 | 0.0 | 0.0 | 0.0 | 0.0 | 0.0 | 0.0 | 0.0 |
| Ear10 | 0.0 | 0.0 | 0.0 | 0.0 | 0.0 | 0.0 | 0.0 | 0.0 | 0.0 | 0.1 | 0.0 | 0.0 |
| Ear12 | 0.0 | 0.0 | 0.0 | 0.0 | 0.0 | 0.0 | 0.0 | 0.0 | 0.0 | 0.2 | 0.0 | 0.1 |
| Ear2 | 0.0 | 0.0 | 0.0 | 0.0 | 0.0 | 0.0 | 0.0 | 0.1 | 0.0 | 0.0 | 0.0 | 0.0 |
| Ear5 | 0.0 | 0.0 | 0.0 | 0.0 | 0.1 | 0.0 | 0.0 | 0.0 | 0.0 | 0.0 | 0.0 | 0.0 |
| Eddm3b | 0.0 | 0.0 | 0.0 | 0.0 | 0.0 | 0.0 | 0.0 | 0.0 | 0.0 | 0.0 | 0.0 | 0.0 |
| Edn2 | 0.0 | 0.0 | 0.0 | 0.0 | 0.0 | 0.0 | 0.0 | 0.0 | 0.0 | 0.2 | 0.1 | 0.0 |
| Edn3 | 0.0 | 0.0 | 0.0 | 0.0 | 0.0 | 0.0 | 0.0 | 0.0 | 0.0 | 0.0 | 0.0 | 0.0 |
| Efcab10 | 0.1 | 0.0 | 0.0 | 0.0 | 0.0 | 0.0 | 0.0 | 0.0 | 0.0 | 0.0 | 0.0 | 0.0 |
| Egfem1 | 0.0 | 0.0 | 0.0 | 0.0 | 0.0 | 0.0 | 0.0 | 0.0 | 0.0 | 0.0 | 0.0 | 0.0 |
| Egr4 | 0.0 | 0.0 | 0.0 | 0.1 | 0.0 | 0.0 | 0.0 | 0.0 | 0.0 | 0.0 | 0.0 | 0.0 |
| Eif4e1b | 0.0 | 0.0 | 0.0 | 0.0 | 0.0 | 0.0 | 0.0 | 0.0 | 0.0 | 0.0 | 0.0 | 0.0 |
| Elf3 | 0.0 | 0.0 | 0.0 | 0.0 | 0.0 | 0.0 | 0.0 | 0.0 | 0.0 | 0.0 | 0.0 | 0.0 |
| Elovl3 | 0.0 | 0.0 | 0.0 | 0.0 | 0.0 | 0.0 | 0.0 | 0.0 | 0.0 | 0.0 | 0.0 | 0.0 |
| Emx1 | 0.0 | 0.0 | 0.0 | 0.0 | 0.0 | 0.0 | 0.0 | 0.1 | 0.0 | 0.0 | 0.0 | 0.0 |
| Enam | 0.0 | 0.0 | 0.0 | 0.0 | 0.0 | 0.0 | 0.0 | 0.0 | 0.0 | 0.0 | 0.0 | 0.0 |
| Entpd8 | 0.0 | 0.0 | 0.0 | 0.0 | 0.0 | 0.0 | 0.0 | 0.0 | 0.0 | 0.0 | 0.0 | 0.0 |
| Epcam | 0.0 | 0.0 | 0.0 | 0.0 | 0.0 | 0.0 | 0.0 | 0.0 | 0.0 | 0.0 | 0.0 | 0.0 |
| Epha10 | 0.0 | 0.0 | 0.0 | 0.0 | 0.0 | 0.0 | 0.0 | 0.0 | 0.0 | 0.0 | 0.0 | 0.0 |
| Epha8 | 0.0 | 0.0 | 0.0 | 0.0 | 0.0 | 0.0 | 0.0 | 0.0 | 0.0 | 0.0 | 0.0 | 0.0 |
| Epo | 0.0 | 0.1 | 0.0 | 0.0 | 0.0 | 0.0 | 0.0 | 0.0 | 0.0 | 0.0 | 0.0 | 0.0 |
| Epx | 0.0 | 0.0 | 0.0 | 0.0 | 0.0 | 0.0 | 0.0 | 0.0 | 0.0 | 0.0 | 0.0 | 0.0 |
| Erich2 | 0.0 | 0.0 | 0.0 | 0.1 | 0.0 | 0.1 | 0.0 | 0.0 | 0.0 | 0.0 | 0.0 | 0.0 |
| Ern2 | 0.0 | 0.0 | 0.0 | 0.0 | 0.0 | 0.0 | 0.0 | 0.0 | 0.0 | 0.0 | 0.0 | 0.0 |
| Esrp1 | 0.0 | 0.0 | 0.0 | 0.0 | 0.0 | 0.0 | 0.0 | 0.0 | 0.0 | 0.0 | 0.0 | 0.0 |
| Esx1 | 0.0 | 0.0 | 0.0 | 0.0 | 0.0 | 0.0 | 0.0 | 0.0 | 0.0 | 0.0 | 0.0 | 0.0 |
| Etd | 0.1 | 0.0 | 0.0 | 0.0 | 0.0 | 0.0 | 0.0 | 0.0 | 0.0 | 0.0 | 0.0 | 0.0 |
| Evx2 | 0.0 | 0.0 | 0.0 | 0.0 | 0.0 | 0.0 | 0.0 | 0.0 | 0.0 | 0.0 | 0.0 | 0.0 |
| F11 | 0.0 | 0.0 | 0.0 | 0.0 | 0.0 | 0.0 | 0.0 | 0.0 | 0.0 | 0.0 | 0.0 | 0.0 |
| F10 | 0.0 | 0.0 | 0.0 | 0.0 | 0.0 | 0.0 | 0.0 | 0.0 | 0.0 | 0.0 | 0.0 | 0.0 |
| F12 | 0.0 | 0.0 | 0.0 | 0.0 | 0.0 | 0.0 | 0.0 | 0.0 | 0.0 | 0.0 | 0.0 | 0.0 |
| F13b | 0.0 | 0.0 | 0.0 | 0.0 | 0.0 | 0.0 | 0.0 | 0.0 | 0.0 | 0.0 | 0.0 | 0.0 |
| F2 | 0.0 | 0.0 | 0.0 | 0.0 | 0.0 | 0.0 | 0.0 | 0.0 | 0.0 | 0.0 | 0.0 | 0.0 |
| F7 | 0.0 | 0.0 | 0.0 | 0.0 | 0.0 | 0.0 | 0.0 | 0.0 | 0.0 | 0.0 | 0.0 | 0.0 |
| F830045P16Rik | 0.0 | 0.0 | 0.0 | 0.0 | 0.0 | 0.0 | 0.0 | 0.0 | 0.0 | 0.0 | 0.0 | 0.0 |
| Fa2h | 0.0 | 0.0 | 0.0 | 0.0 | 0.0 | 0.0 | 0.0 | 0.0 | 0.0 | 0.0 | 0.0 | 0.0 |
| Fabp1 | 0.0 | 0.0 | 0.0 | 0.0 | 0.0 | 0.0 | 0.0 | 0.0 | 0.0 | 0.0 | 0.2 | 0.0 |
| Fabp12 | 0.0 | 0.0 | 0.0 | 0.0 | 0.0 | 0.0 | 0.0 | 0.0 | 0.0 | 0.0 | 0.0 | 0.0 |
| Fabp2 | 0.0 | 0.1 | 0.0 | 0.0 | 0.0 | 0.0 | 0.0 | 0.0 | 0.0 | 0.0 | 0.0 | 0.0 |
| Fam124b | 0.0 | 0.0 | 0.0 | 0.0 | 0.0 | 0.0 | 0.0 | 0.0 | 0.0 | 0.0 | 0.0 | 0.0 |
| Fam135b | 0.0 | 0.0 | 0.0 | 0.0 | 0.0 | 0.0 | 0.0 | 0.0 | 0.0 | 0.0 | 0.0 | 0.0 |
| Fam151a | 0.0 | 0.0 | 0.0 | 0.0 | 0.0 | 0.0 | 0.0 | 0.0 | 0.0 | 0.0 | 0.1 | 0.0 |
| Fam154a | 0.0 | 0.0 | 0.0 | 0.0 | 0.0 | 0.1 | 0.0 | 0.0 | 0.0 | 0.0 | 0.0 | 0.0 |
| Fam159a | 0.0 | 0.0 | 0.0 | 0.0 | 0.0 | 0.0 | 0.0 | 0.0 | 0.0 | 0.0 | 0.0 | 0.0 |
| Fam170a | 0.0 | 0.0 | 0.0 | 0.0 | 0.0 | 0.0 | 0.0 | 0.0 | 0.0 | 0.0 | 0.0 | 0.0 |
| Fam170b | 0.0 | 0.0 | 0.0 | 0.0 | 0.0 | 0.0 | 0.0 | 0.0 | 0.0 | 0.0 | 0.0 | 0.0 |
| Fam181a | 0.0 | 0.0 | 0.0 | 0.0 | 0.0 | 0.0 | 0.0 | 0.0 | 0.0 | 0.0 | 0.0 | 0.0 |
| Fam181b | 0.0 | 0.0 | 0.0 | 0.0 | 0.0 | 0.0 | 0.0 | 0.0 | 0.0 | 0.0 | 0.0 | 0.0 |
| Fam187a | 0.0 | 0.0 | 0.0 | 0.0 | 0.1 | 0.0 | 0.1 | 0.0 | 0.0 | 0.0 | 0.0 | 0.0 |
| Fam187b | 0.0 | 0.0 | 0.0 | 0.0 | 0.0 | 0.0 | 0.0 | 0.0 | 0.0 | 0.0 | 0.0 | 0.0 |
| Fam189a1 | 0.0 | 0.0 | 0.0 | 0.0 | 0.0 | 0.0 | 0.0 | 0.0 | 0.0 | 0.0 | 0.0 | 0.0 |
| Fam19a4 | 0.0 | 0.0 | 0.0 | 0.0 | 0.0 | 0.0 | 0.0 | 0.0 | 0.0 | 0.0 | 0.0 | 0.0 |
| Fam216b | 0.0 | 0.0 | 0.0 | 0.0 | 0.0 | 0.1 | 0.0 | 0.0 | 0.0 | 0.0 | 0.0 | 0.0 |
| Fam229a | 0.1 | 0.0 | 0.0 | 0.0 | 0.0 | 0.0 | 0.0 | 0.0 | 0.0 | 0.0 | 0.0 | 0.0 |
| Fam24a | 0.0 | 0.0 | 0.0 | 0.6 | 0.0 | 0.2 | 0.0 | 0.0 | 0.0 | 0.0 | 0.0 | 0.2 |
| Fam3b | 0.0 | 0.0 | 0.0 | 0.0 | 0.0 | 0.0 | 0.0 | 0.0 | 0.0 | 0.0 | 0.0 | 0.0 |
| Fam46d | 0.0 | 0.0 | 0.0 | 0.0 | 0.0 | 0.0 | 0.0 | 0.0 | 0.0 | 0.0 | 0.0 | 0.0 |

| | | | | | | | | | | | | |
|---|---|---|---|---|---|---|---|---|---|---|---|---|
| Fam71a | 0.0 | 0.0 | 0.0 | 0.0 | 0.0 | 0.0 | 0.0 | 0.0 | 0.0 | 0.1 | 0.0 | 0.0 |
| Fam71e2 | 0.0 | 0.0 | 0.0 | 0.0 | 0.0 | 0.0 | 0.0 | 0.0 | 0.0 | 0.0 | 0.0 | 0.0 |
| Fam83b | 0.0 | 0.0 | 0.0 | 0.0 | 0.0 | 0.0 | 0.0 | 0.0 | 0.0 | 0.0 | 0.0 | 0.0 |
| Fam92b | 0.0 | 0.0 | 0.0 | 0.0 | 0.0 | 0.0 | 0.0 | 0.0 | 0.0 | 0.0 | 0.0 | 0.0 |
| Fasl | 0.0 | 0.0 | 0.0 | 0.0 | 0.0 | 0.0 | 0.0 | 0.0 | 0.0 | 0.0 | 0.0 | 0.0 |
| Fate1 | 0.0 | 0.2 | 0.0 | 0.0 | 0.0 | 0.0 | 0.0 | 0.0 | 0.0 | 0.0 | 0.0 | 0.0 |
| Fbp1 | 0.0 | 0.0 | 0.0 | 0.0 | 0.0 | 0.0 | 0.0 | 0.0 | 0.0 | 0.0 | 0.0 | 0.0 |
| Fbp2 | 0.0 | 0.0 | 0.0 | 0.0 | 0.0 | 0.0 | 0.0 | 0.0 | 0.0 | 0.0 | 0.0 | 0.0 |
| Fbxo39 | 0.1 | 0.0 | 0.0 | 0.0 | 0.1 | 0.0 | 0.0 | 0.0 | 0.0 | 0.0 | 0.0 | 0.1 |
| Fbxw13 | 0.0 | 0.0 | 0.0 | 0.0 | 0.0 | 0.0 | 0.0 | 0.0 | 0.0 | 0.0 | 0.0 | 0.0 |
| Fbxw14 | 0.0 | 0.0 | 0.0 | 0.0 | 0.0 | 0.0 | 0.0 | 0.0 | 0.0 | 0.0 | 0.0 | 0.0 |
| Fbxw20 | 0.0 | 0.0 | 0.0 | 0.0 | 0.0 | 0.0 | 0.0 | 0.0 | 0.0 | 0.0 | 0.0 | 0.0 |
| Fbxw22 | 0.0 | 0.0 | 0.0 | 0.0 | 0.0 | 0.0 | 0.0 | 0.0 | 0.0 | 0.0 | 0.0 | 0.0 |
| Fbxw28 | 0.0 | 0.0 | 0.0 | 0.0 | 0.0 | 0.0 | 0.0 | 0.0 | 0.0 | 0.0 | 0.0 | 0.0 |
| Fcamr | 0.0 | 0.0 | 0.0 | 0.0 | 0.0 | 0.0 | 0.0 | 0.0 | 0.0 | 0.0 | 0.0 | 0.0 |
| Fcrl5 | 0.0 | 0.0 | 0.0 | 0.0 | 0.1 | 0.0 | 0.0 | 0.0 | 0.0 | 0.0 | 0.0 | 0.0 |
| Fcrl6 | 0.0 | 0.0 | 0.0 | 0.0 | 0.0 | 0.0 | 0.0 | 0.0 | 0.0 | 0.0 | 0.0 | 0.0 |
| Fcrla | 0.0 | 0.0 | 0.0 | 0.0 | 0.0 | 0.0 | 0.0 | 0.0 | 0.0 | 0.0 | 0.0 | 0.0 |
| Fcrlb | 0.0 | 0.0 | 0.1 | 0.0 | 0.0 | 0.0 | 0.0 | 0.0 | 0.1 | 0.0 | 0.0 | 0.0 |
| Ffar4 | 0.0 | 0.0 | 0.0 | 0.0 | 0.0 | 0.0 | 0.0 | 0.0 | 0.0 | 0.0 | 0.0 | 0.0 |
| Fga | 0.0 | 0.0 | 0.0 | 0.0 | 0.0 | 0.0 | 0.0 | 0.0 | 0.0 | 0.0 | 0.0 | 0.0 |
| Fgb | 0.0 | 0.0 | 0.0 | 0.0 | 0.0 | 0.0 | 0.0 | 0.0 | 0.0 | 0.0 | 0.0 | 0.0 |
| Fgf15 | 0.0 | 0.0 | 0.0 | 0.0 | 0.0 | 0.0 | 0.0 | 0.0 | 0.0 | 0.0 | 0.0 | 0.0 |
| Fgf22 | 0.2 | 0.0 | 0.1 | 0.1 | 0.0 | 0.0 | 0.6 | 0.0 | 0.1 | 0.1 | 0.0 | 0.3 |
| Fgf8 | 0.0 | 0.0 | 0.0 | 0.0 | 0.0 | 0.0 | 0.0 | 0.0 | 0.0 | 0.1 | 0.0 | 0.0 |
| Fgfbp1 | 0.0 | 0.0 | 0.0 | 0.0 | 0.0 | 0.0 | 0.0 | 0.0 | 0.0 | 0.0 | 0.0 | 0.0 |
| Fhl5 | 0.0 | 0.0 | 0.0 | 0.0 | 0.0 | 0.0 | 0.0 | 0.0 | 0.0 | 0.0 | 0.0 | 0.0 |
| Fitm1 | 0.0 | 0.0 | 0.0 | 0.0 | 0.0 | 0.0 | 0.0 | 0.0 | 0.0 | 0.0 | 0.0 | 0.0 |
| Flg2 | 0.0 | 0.0 | 0.0 | 0.0 | 0.0 | 0.0 | 0.0 | 0.0 | 0.0 | 0.0 | 0.0 | 0.0 |
| Fmo6 | 0.0 | 0.0 | 0.0 | 0.0 | 0.0 | 0.0 | 0.0 | 0.0 | 0.0 | 0.0 | 0.0 | 0.0 |
| Fmo9 | 0.0 | 0.0 | 0.0 | 0.0 | 0.0 | 0.0 | 0.0 | 0.0 | 0.0 | 0.0 | 0.0 | 0.0 |
| Fnd3c2 | 0.0 | 0.0 | 0.0 | 0.0 | 0.0 | 0.0 | 0.0 | 0.0 | 0.0 | 0.0 | 0.0 | 0.0 |
| Fndc3c1 | 0.0 | 0.0 | 0.0 | 0.0 | 0.0 | 0.0 | 0.0 | 0.0 | 0.0 | 0.0 | 0.0 | 0.0 |
| Folr4 | 0.0 | 0.0 | 0.0 | 0.0 | 0.0 | 0.0 | 0.0 | 0.0 | 0.0 | 0.0 | 0.0 | 0.0 |
| Foxa1 | 0.0 | 0.0 | 0.0 | 0.0 | 0.0 | 0.0 | 0.0 | 0.0 | 0.0 | 0.0 | 0.0 | 0.0 |
| Foxa3 | 0.0 | 0.0 | 0.0 | 0.0 | 0.0 | 0.0 | 0.0 | 0.0 | 0.0 | 0.0 | 0.0 | 0.0 |
| Foxb1 | 0.0 | 0.0 | 0.0 | 0.0 | 0.0 | 0.0 | 0.0 | 0.0 | 0.0 | 0.0 | 0.0 | 0.0 |
| Foxb2 | 0.0 | 0.0 | 0.0 | 0.0 | 0.0 | 0.0 | 0.0 | 0.0 | 0.0 | 0.0 | 0.0 | 0.0 |
| Foxd3 | 0.0 | 0.0 | 0.0 | 0.0 | 0.0 | 0.0 | 0.0 | 0.0 | 0.0 | 0.0 | 0.0 | 0.0 |
| Foxe3 | 0.0 | 0.0 | 0.0 | 0.0 | 0.0 | 0.0 | 0.0 | 0.1 | 0.0 | 0.0 | 0.0 | 0.1 |
| Foxh1 | 0.0 | 0.0 | 0.0 | 0.0 | 0.0 | 0.0 | 0.0 | 0.0 | 0.0 | 0.1 | 0.0 | 0.0 |
| Foxi1 | 0.0 | 0.0 | 0.0 | 0.0 | 0.0 | 0.0 | 0.0 | 0.0 | 0.0 | 0.0 | 0.0 | 0.0 |
| Foxi2 | 0.0 | 0.0 | 0.0 | 0.0 | 0.0 | 0.0 | 0.0 | 0.0 | 0.0 | 0.0 | 0.0 | 0.0 |
| Foxr1 | 0.0 | 0.0 | 0.0 | 0.1 | 0.0 | 0.0 | 0.0 | 0.0 | 0.0 | 0.0 | 0.0 | 0.0 |
| Foxr2 | 0.0 | 0.0 | 0.0 | 0.0 | 0.0 | 0.0 | 0.0 | 0.0 | 0.0 | 0.0 | 0.0 | 0.0 |
| Fpr3 | 0.0 | 0.0 | 0.0 | 0.0 | 0.0 | 0.0 | 0.0 | 0.0 | 0.0 | 0.0 | 0.0 | 0.0 |
| Fpr-rs3 | 0.0 | 0.0 | 0.0 | 0.0 | 0.0 | 0.0 | 0.0 | 0.0 | 0.0 | 0.0 | 0.0 | 0.0 |
| Frmpd3 | 0.0 | 0.0 | 0.0 | 0.0 | 0.0 | 0.0 | 0.0 | 0.0 | 0.0 | 0.0 | 0.0 | 0.0 |
| Fscn3 | 0.0 | 0.0 | 0.0 | 0.0 | 0.1 | 0.0 | 0.0 | 0.0 | 0.0 | 0.0 | 0.0 | 0.0 |
| Fshb | 0.0 | 0.0 | 0.0 | 0.0 | 0.0 | 0.0 | 0.0 | 0.0 | 0.0 | 0.0 | 0.0 | 0.0 |
| Fshr | 0.0 | 0.0 | 0.0 | 0.0 | 0.0 | 0.0 | 0.0 | 0.0 | 0.0 | 0.0 | 0.0 | 0.0 |
| Ftcd | 0.0 | 0.0 | 0.0 | 0.0 | 0.0 | 0.0 | 0.0 | 0.0 | 0.0 | 0.0 | 0.0 | 0.0 |
| Ftmt | 0.0 | 0.0 | 0.0 | 0.0 | 0.0 | 0.0 | 0.0 | 0.0 | 0.0 | 0.0 | 0.0 | 0.0 |
| Fut1 | 0.0 | 0.0 | 0.1 | 0.1 | 0.0 | 0.0 | 0.0 | 0.0 | 0.0 | 0.0 | 0.0 | 0.0 |
| Fut2 | 0.0 | 0.0 | 0.0 | 0.0 | 0.0 | 0.0 | 0.0 | 0.1 | 0.0 | 0.0 | 0.0 | 0.0 |
| Fut9 | 0.0 | 0.0 | 0.0 | 0.0 | 0.0 | 0.0 | 0.0 | 0.0 | 0.0 | 0.0 | 0.0 | 0.0 |
| Fxyd4 | 0.0 | 0.0 | 0.1 | 0.7 | 0.0 | 0.0 | 0.1 | 0.3 | 0.0 | 0.0 | 0.0 | 0.2 |

| | | | | | | | | | | | | |
|---|---|---|---|---|---|---|---|---|---|---|---|---|
| G630055G22Rik | 0.0 | 0.0 | 0.0 | 0.0 | 0.0 | 0.0 | 0.1 | 0.0 | 0.0 | 0.0 | 0.0 | 0.0 |
| G630093K05Rik | 0.0 | 0.0 | 0.0 | 0.0 | 0.0 | 0.0 | 0.0 | 0.0 | 0.0 | 0.0 | 0.0 | 0.0 |
| G6b | 0.0 | 0.0 | 0.0 | 0.0 | 0.0 | 0.0 | 0.0 | 0.0 | 0.0 | 0.0 | 0.0 | 0.0 |
| G6pc | 0.0 | 0.0 | 0.0 | 0.0 | 0.0 | 0.0 | 0.0 | 0.0 | 0.0 | 0.0 | 0.0 | 0.0 |
| Gabra2 | 0.0 | 0.0 | 0.0 | 0.0 | 0.0 | 0.0 | 0.0 | 0.0 | 0.0 | 0.0 | 0.0 | 0.0 |
| Gabra5 | 0.0 | 0.0 | 0.0 | 0.0 | 0.0 | 0.0 | 0.0 | 0.0 | 0.0 | 0.0 | 0.0 | 0.0 |
| Gabrg1 | 0.0 | 0.0 | 0.0 | 0.0 | 0.0 | 0.0 | 0.0 | 0.0 | 0.0 | 0.0 | 0.0 | 0.0 |
| Gabrg2 | 0.0 | 0.0 | 0.0 | 0.0 | 0.0 | 0.0 | 0.0 | 0.0 | 0.0 | 0.0 | 0.0 | 0.0 |
| Gabrg3 | 0.0 | 0.0 | 0.0 | 0.0 | 0.0 | 0.0 | 0.0 | 0.0 | 0.0 | 0.0 | 0.0 | 0.0 |
| Gabrq | 0.0 | 0.0 | 0.0 | 0.0 | 0.0 | 0.0 | 0.0 | 0.0 | 0.0 | 0.0 | 0.0 | 0.0 |
| Gabrr1 | 0.0 | 0.0 | 0.0 | 0.0 | 0.0 | 0.0 | 0.0 | 0.0 | 0.0 | 0.0 | 0.0 | 0.0 |
| Gabrr3 | 0.0 | 0.0 | 0.0 | 0.0 | 0.0 | 0.0 | 0.0 | 0.0 | 0.0 | 0.0 | 0.0 | 0.0 |
| Gad1 | 0.0 | 0.0 | 0.0 | 0.0 | 0.0 | 0.0 | 0.0 | 0.0 | 0.0 | 0.0 | 0.0 | 0.0 |
| Gad2 | 0.0 | 0.0 | 0.0 | 0.0 | 0.0 | 0.0 | 0.0 | 0.0 | 0.0 | 0.0 | 0.0 | 0.0 |
| Galnt13 | 0.0 | 0.0 | 0.0 | 0.0 | 0.0 | 0.0 | 0.0 | 0.0 | 0.0 | 0.0 | 0.0 | 0.0 |
| Galntl5 | 0.0 | 0.0 | 0.0 | 0.0 | 0.0 | 0.0 | 0.0 | 0.0 | 0.0 | 0.0 | 0.0 | 0.0 |
| Galntl6 | 0.0 | 0.0 | 0.0 | 0.0 | 0.0 | 0.0 | 0.0 | 0.0 | 0.0 | 0.0 | 0.0 | 0.0 |
| Galp | 0.0 | 0.1 | 0.0 | 0.0 | 0.0 | 0.0 | 0.0 | 0.0 | 0.0 | 0.0 | 0.0 | 0.0 |
| Galr1 | 0.0 | 0.0 | 0.0 | 0.0 | 0.0 | 0.0 | 0.0 | 0.0 | 0.0 | 0.0 | 0.0 | 0.0 |
| Galr3 | 0.0 | 0.0 | 0.0 | 0.0 | 0.0 | 0.0 | 0.0 | 0.0 | 0.0 | 0.0 | 0.0 | 0.0 |
| Gapt | 0.0 | 0.0 | 0.0 | 0.0 | 0.0 | 0.0 | 0.0 | 0.0 | 0.0 | 0.0 | 0.0 | 0.0 |
| Gas2l2 | 0.0 | 0.0 | 0.0 | 0.0 | 0.0 | 0.0 | 0.0 | 0.0 | 0.0 | 0.0 | 0.0 | 0.0 |
| Gc | 0.0 | 0.0 | 0.0 | 0.0 | 0.0 | 0.0 | 0.0 | 0.0 | 0.0 | 0.0 | 0.0 | 0.0 |
| Gcm2 | 0.0 | 0.0 | 0.0 | 0.1 | 0.1 | 0.0 | 0.0 | 0.0 | 0.0 | 0.0 | 0.0 | 0.0 |
| Gcnt3 | 0.0 | 0.0 | 0.0 | 0.0 | 0.0 | 0.0 | 0.0 | 0.0 | 0.0 | 0.0 | 0.0 | 0.0 |
| Gcsam | 0.0 | 0.0 | 0.0 | 0.1 | 0.0 | 0.0 | 0.0 | 0.0 | 0.0 | 0.0 | 0.0 | 0.0 |
| Gfra3 | 0.0 | 0.0 | 0.0 | 0.0 | 0.0 | 0.0 | 0.0 | 0.1 | 0.0 | 0.0 | 0.0 | 0.1 |
| Gfral | 0.0 | 0.0 | 0.0 | 0.0 | 0.0 | 0.0 | 0.0 | 0.0 | 0.0 | 0.0 | 0.0 | 0.0 |
| Gh | 0.0 | 0.0 | 0.0 | 0.0 | 0.0 | 0.0 | 0.0 | 0.0 | 0.0 | 0.0 | 0.0 | 0.0 |
| Ghrh | 0.0 | 0.0 | 0.0 | 0.0 | 0.0 | 0.0 | 0.2 | 0.1 | 0.0 | 0.0 | 0.0 | 0.0 |
| Ghsr | 0.0 | 0.0 | 0.0 | 0.0 | 0.0 | 0.0 | 0.0 | 0.0 | 0.0 | 0.0 | 0.0 | 0.0 |
| Gip | 0.1 | 0.0 | 0.0 | 0.1 | 0.0 | 0.0 | 0.0 | 0.0 | 0.0 | 0.0 | 0.0 | 0.0 |
| Gipc3 | 0.0 | 0.0 | 0.0 | 0.0 | 0.0 | 0.0 | 0.0 | 0.0 | 0.0 | 0.0 | 0.1 | 0.0 |
| Gja10 | 0.0 | 0.0 | 0.0 | 0.0 | 0.0 | 0.0 | 0.0 | 0.0 | 0.0 | 0.0 | 0.0 | 0.0 |
| Gjb1 | 0.0 | 0.0 | 0.0 | 0.0 | 0.0 | 0.0 | 0.0 | 0.0 | 0.0 | 0.0 | 0.0 | 0.0 |
| Gjb2 | 0.0 | 0.0 | 0.0 | 0.0 | 0.0 | 0.0 | 0.0 | 0.0 | 0.0 | 0.0 | 0.0 | 0.0 |
| Gjd3 | 0.0 | 0.0 | 0.0 | 0.0 | 0.0 | 0.0 | 0.0 | 0.0 | 0.0 | 0.1 | 0.0 | 0.0 |
| Gjd4 | 0.0 | 0.0 | 0.0 | 0.0 | 0.0 | 0.0 | 0.0 | 0.0 | 0.0 | 0.1 | 0.0 | 0.0 |
| Gje1 | 0.0 | 0.0 | 0.0 | 0.0 | 0.0 | 0.0 | 0.0 | 0.0 | 0.0 | 0.0 | 0.0 | 0.0 |
| Gk2 | 0.0 | 0.0 | 0.0 | 0.0 | 0.0 | 0.0 | 0.0 | 0.0 | 0.0 | 0.0 | 0.0 | 0.0 |
| Gkn3 | 0.0 | 0.0 | 0.0 | 0.0 | 0.0 | 0.0 | 0.0 | 0.0 | 0.0 | 0.0 | 0.0 | 0.0 |
| Glb1l3 | 0.0 | 0.0 | 0.0 | 0.0 | 0.0 | 0.0 | 0.0 | 0.0 | 0.0 | 0.0 | 0.0 | 0.0 |
| Glipr1l1 | 0.0 | 0.0 | 0.0 | 0.0 | 0.0 | 0.0 | 0.0 | 0.0 | 0.0 | 0.0 | 0.0 | 0.0 |
| Glipr1l2 | 0.0 | 0.0 | 0.0 | 0.0 | 0.0 | 0.0 | 0.0 | 0.0 | 0.0 | 0.0 | 0.0 | 0.0 |
| Glra2 | 0.0 | 0.0 | 0.0 | 0.0 | 0.0 | 0.0 | 0.0 | 0.0 | 0.0 | 0.0 | 0.0 | 0.0 |
| Glra4 | 0.0 | 0.0 | 0.0 | 0.0 | 0.0 | 0.0 | 0.0 | 0.0 | 0.0 | 0.0 | 0.0 | 0.0 |
| Glrp1 | 0.0 | 0.0 | 0.0 | 0.0 | 0.0 | 0.0 | 0.1 | 0.0 | 0.0 | 0.0 | 0.0 | 0.0 |
| Glt1d1 | 0.0 | 0.5 | 0.0 | 0.0 | 0.0 | 0.0 | 0.0 | 0.0 | 0.0 | 0.1 | 0.0 | 0.0 |
| Glt6d1 | 0.0 | 0.0 | 0.0 | 0.0 | 0.0 | 0.0 | 0.0 | 0.0 | 0.0 | 0.0 | 0.0 | 0.0 |
| Gm10007 | 0.0 | 0.0 | 0.1 | 0.0 | 0.0 | 0.0 | 0.0 | 0.1 | 0.0 | 0.0 | 0.0 | 0.0 |
| Gm10057 | 0.0 | 0.0 | 0.0 | 0.0 | 0.0 | 0.0 | 0.0 | 0.0 | 0.0 | 0.0 | 0.0 | 0.0 |
| Gm10248 | 0.0 | 0.0 | 0.0 | 0.0 | 0.0 | 0.0 | 0.0 | 0.0 | 0.0 | 0.0 | 0.0 | 0.0 |
| Gm10280 | 0.0 | 0.0 | 0.0 | 0.0 | 0.0 | 0.0 | 0.0 | 0.0 | 0.0 | 0.0 | 0.0 | 0.0 |
| Gm10318 | 0.0 | 0.0 | 0.0 | 0.0 | 0.0 | 0.0 | 0.0 | 0.1 | 0.0 | 0.0 | 0.0 | 0.0 |
| Gm10324 | 0.0 | 0.0 | 0.0 | 0.0 | 0.0 | 0.0 | 0.0 | 0.0 | 0.0 | 0.0 | 0.0 | 0.0 |
| Gm10373 | 0.0 | 0.0 | 0.0 | 0.0 | 0.0 | 0.0 | 0.3 | 0.0 | 0.0 | 0.0 | 0.0 | 0.0 |
| Gm10375 | 0.1 | 0.0 | 0.0 | 0.0 | 0.0 | 0.0 | 0.0 | 0.0 | 0.0 | 0.0 | 0.0 | 0.0 |

| | | | | | | | | | | | | |
|---|---|---|---|---|---|---|---|---|---|---|---|---|
| Gm10377 | 0.1 | 0.0 | 0.0 | 0.0 | 0.0 | 0.0 | 0.0 | 0.0 | 0.0 | 0.0 | 0.0 | 0.0 |
| Gm10415 | 0.0 | 0.0 | 0.0 | 0.0 | 0.0 | 0.0 | 0.0 | 0.0 | 0.0 | 0.0 | 0.0 | 0.0 |
| Gm10436 | 0.0 | 0.0 | 0.0 | 0.0 | 0.0 | 0.0 | 0.0 | 0.0 | 0.0 | 0.0 | 0.0 | 0.0 |
| Gm10439 | 0.0 | 0.0 | 0.0 | 0.0 | 0.0 | 0.0 | 0.0 | 0.0 | 0.0 | 0.0 | 0.0 | 0.0 |
| Gm10440 | 0.0 | 0.0 | 0.0 | 0.0 | 0.0 | 0.0 | 0.0 | 0.0 | 0.0 | 0.0 | 0.0 | 0.0 |
| Gm10466 | 0.0 | 0.0 | 0.0 | 0.0 | 0.0 | 0.0 | 0.0 | 0.0 | 0.0 | 0.0 | 0.0 | 0.0 |
| Gm10494 | 0.0 | 0.0 | 0.0 | 0.0 | 0.0 | 0.0 | 0.0 | 0.0 | 0.0 | 0.0 | 0.0 | 0.0 |
| Gm10510 | 0.0 | 0.0 | 0.0 | 0.0 | 0.0 | 0.0 | 0.0 | 0.0 | 0.0 | 0.0 | 0.0 | 0.0 |
| Gm10549 | 0.0 | 0.0 | 0.0 | 0.0 | 0.0 | 0.0 | 0.0 | 0.0 | 0.0 | 0.0 | 0.0 | 0.0 |
| Gm10662 | 0.0 | 0.0 | 0.0 | 0.0 | 0.0 | 0.0 | 0.0 | 0.0 | 0.0 | 0.0 | 0.0 | 0.0 |
| Gm10664 | 0.0 | 0.0 | 0.0 | 0.0 | 0.0 | 0.0 | 0.0 | 0.0 | 0.0 | 0.0 | 0.0 | 0.0 |
| Gm10697 | 0.0 | 0.0 | 0.0 | 0.0 | 0.0 | 0.0 | 0.0 | 0.0 | 0.0 | 0.0 | 0.0 | 0.0 |
| Gm10714 | 0.0 | 0.1 | 0.0 | 0.0 | 0.0 | 0.0 | 0.0 | 0.0 | 0.2 | 0.1 | 0.0 | 0.0 |
| Gm10789 | 0.0 | 0.0 | 0.0 | 0.0 | 0.0 | 0.0 | 0.0 | 0.0 | 0.0 | 0.0 | 0.0 | 0.0 |
| Gm1082 | 0.0 | 0.0 | 0.0 | 0.0 | 0.0 | 0.0 | 0.0 | 0.0 | 0.0 | 0.0 | 0.0 | 0.0 |
| Gm10823 | 0.0 | 0.0 | 0.0 | 0.0 | 0.0 | 0.0 | 0.0 | 0.0 | 0.0 | 0.0 | 0.0 | 0.0 |
| Gm10857 | 0.0 | 0.0 | 0.0 | 0.0 | 0.0 | 0.0 | 0.0 | 0.0 | 0.0 | 0.0 | 0.0 | 0.0 |
| Gm10863 | 0.0 | 0.0 | 0.0 | 0.0 | 0.0 | 0.0 | 0.0 | 0.0 | 0.0 | 0.0 | 0.1 | 0.0 |
| Gm10921 | 0.0 | 0.0 | 0.0 | 0.0 | 0.0 | 0.0 | 0.0 | 0.0 | 0.0 | 0.0 | 0.0 | 0.0 |
| Gm10922 | 0.0 | 0.0 | 0.0 | 0.0 | 0.0 | 0.0 | 0.0 | 0.0 | 0.0 | 0.0 | 0.0 | 0.0 |
| Gm1110 | 0.0 | 0.0 | 0.0 | 0.0 | 0.0 | 0.0 | 0.0 | 0.0 | 0.0 | 0.0 | 0.0 | 0.0 |
| Gm11190 | 0.0 | 0.0 | 0.0 | 0.0 | 0.0 | 0.0 | 0.0 | 0.0 | 0.0 | 0.0 | 0.0 | 0.0 |
| Gm11194 | 0.0 | 0.0 | 0.0 | 0.0 | 0.0 | 0.0 | 0.0 | 0.0 | 0.0 | 0.0 | 0.0 | 0.0 |
| Gm11213 | 0.0 | 0.0 | 0.0 | 0.0 | 0.0 | 0.0 | 0.1 | 0.0 | 0.0 | 0.0 | 0.0 | 0.0 |
| Gm11437 | 0.0 | 0.1 | 0.0 | 0.0 | 0.0 | 0.0 | 0.0 | 0.0 | 0.0 | 0.0 | 0.0 | 0.0 |
| Gm11487 | 0.0 | 0.0 | 0.0 | 0.0 | 0.0 | 0.0 | 0.0 | 0.0 | 0.0 | 0.0 | 0.0 | 0.0 |
| Gm11529 | 0.0 | 0.0 | 0.0 | 0.0 | 0.0 | 0.0 | 0.0 | 0.0 | 0.0 | 0.0 | 0.0 | 0.0 |
| Gm11541 | 0.0 | 0.0 | 0.0 | 0.0 | 0.0 | 0.0 | 0.0 | 0.0 | 0.0 | 0.0 | 0.0 | 0.0 |
| Gm11544 | 0.0 | 0.0 | 0.0 | 0.0 | 0.0 | 0.0 | 0.0 | 0.0 | 0.0 | 0.0 | 0.0 | 0.0 |
| Gm11548 | 0.0 | 0.0 | 0.0 | 0.0 | 0.0 | 0.0 | 0.0 | 0.0 | 0.0 | 0.0 | 0.0 | 0.0 |
| Gm11549 | 0.0 | 0.0 | 0.0 | 0.0 | 0.0 | 0.0 | 0.0 | 0.0 | 0.0 | 0.0 | 0.0 | 0.0 |
| Gm11565 | 0.0 | 0.0 | 0.0 | 0.0 | 0.0 | 0.0 | 0.0 | 0.0 | 0.0 | 0.0 | 0.0 | 0.0 |
| Gm11762 | 0.0 | 0.0 | 0.0 | 0.0 | 0.0 | 0.0 | 0.0 | 0.0 | 0.0 | 0.0 | 0.0 | 0.0 |
| Gm11961 | 0.0 | 0.1 | 0.0 | 0.2 | 0.0 | 0.0 | 0.0 | 0.0 | 0.1 | 0.1 | 0.0 | 0.0 |
| Gm11978 | 0.0 | 0.0 | 0.0 | 0.0 | 0.0 | 0.0 | 0.0 | 0.0 | 0.0 | 0.0 | 0.0 | 0.0 |
| Gm12130 | 0.0 | 0.0 | 0.0 | 0.0 | 0.0 | 0.0 | 0.0 | 0.0 | 0.0 | 0.0 | 0.0 | 0.0 |
| Gm12159 | 0.0 | 0.0 | 0.0 | 0.0 | 0.0 | 0.0 | 0.0 | 0.0 | 0.0 | 0.0 | 0.0 | 0.0 |
| Gm12253 | 0.0 | 0.0 | 0.0 | 0.0 | 0.0 | 0.0 | 0.0 | 0.0 | 0.0 | 0.0 | 0.0 | 0.0 |
| Gm12360 | 0.0 | 0.0 | 0.0 | 0.0 | 0.0 | 0.0 | 0.0 | 0.0 | 0.0 | 0.0 | 0.0 | 0.0 |
| Gm12409 | 0.0 | 0.1 | 0.1 | 0.0 | 0.1 | 0.0 | 0.0 | 0.0 | 0.0 | 0.1 | 0.0 | 0.0 |
| Gm12597 | 0.0 | 0.0 | 0.0 | 0.0 | 0.0 | 0.0 | 0.0 | 0.0 | 0.0 | 0.0 | 0.0 | 0.0 |
| Gm12695 | 0.0 | 0.0 | 0.0 | 0.0 | 0.0 | 0.0 | 0.0 | 0.0 | 0.0 | 0.0 | 0.0 | 0.0 |
| Gm12709 | 0.0 | 0.0 | 0.0 | 0.0 | 0.0 | 0.0 | 0.0 | 0.0 | 0.0 | 0.0 | 0.0 | 0.0 |
| Gm12718 | 0.0 | 0.0 | 0.0 | 0.0 | 0.0 | 0.0 | 0.0 | 0.0 | 0.0 | 0.0 | 0.1 | 0.0 |
| Gm12794 | 0.0 | 0.0 | 0.0 | 0.0 | 0.0 | 0.0 | 0.0 | 0.0 | 0.0 | 0.0 | 0.0 | 0.0 |
| Gm12830 | 0.0 | 0.0 | 0.0 | 0.0 | 0.0 | 0.0 | 0.0 | 0.0 | 0.0 | 0.0 | 0.0 | 0.0 |
| Gm12839 | 0.0 | 0.0 | 0.0 | 0.0 | 0.0 | 0.0 | 0.0 | 0.0 | 0.0 | 0.0 | 0.1 | 0.0 |
| Gm13003 | 0.0 | 0.0 | 0.0 | 0.0 | 0.0 | 0.0 | 0.0 | 0.0 | 0.0 | 0.0 | 0.0 | 0.0 |
| Gm13011 | 0.0 | 0.0 | 0.0 | 0.0 | 0.0 | 0.0 | 0.0 | 0.0 | 0.0 | 0.0 | 0.0 | 0.0 |
| Gm13031 | 0.0 | 0.0 | 0.0 | 0.5 | 0.0 | 0.0 | 0.0 | 0.0 | 0.0 | 0.0 | 0.0 | 0.0 |
| Gm13043 | 0.0 | 0.0 | 0.0 | 0.0 | 0.0 | 0.0 | 0.0 | 0.0 | 0.0 | 0.0 | 0.0 | 0.0 |
| Gm13119 | 0.0 | 0.0 | 0.0 | 0.0 | 0.0 | 0.0 | 0.0 | 0.0 | 0.0 | 0.0 | 0.0 | 0.0 |
| Gm13124 | 0.0 | 0.0 | 0.0 | 0.0 | 0.0 | 0.0 | 0.0 | 0.0 | 0.0 | 0.0 | 0.0 | 0.0 |
| Gm13128 | 0.0 | 0.0 | 0.1 | 0.0 | 0.0 | 0.0 | 0.0 | 0.0 | 0.0 | 0.0 | 0.0 | 0.0 |
| Gm13177 | 0.0 | 0.0 | 0.0 | 0.0 | 0.0 | 0.0 | 0.0 | 0.1 | 0.0 | 0.0 | 0.0 | 0.0 |
| Gm13178 | 0.0 | 0.0 | 0.0 | 0.1 | 0.0 | 0.0 | 0.1 | 0.0 | 0.0 | 0.0 | 0.0 | 0.0 |
| Gm1332 | 0.0 | 0.0 | 0.0 | 0.0 | 0.0 | 0.0 | 0.0 | 0.1 | 0.0 | 0.1 | 0.0 | 0.0 |

| | | | | | | | | | | | | |
|---|---|---|---|---|---|---|---|---|---|---|---|---|
| Gm13490 | 0.0 | 0.0 | 0.0 | 0.0 | 0.0 | 0.0 | 0.0 | 0.0 | 0.0 | 0.0 | 0.0 | 0.0 |
| Gm13544 | 0.0 | 0.0 | 0.0 | 0.0 | 0.0 | 0.0 | 0.0 | 0.0 | 0.0 | 0.0 | 0.0 | 0.0 |
| Gm13546 | 0.0 | 0.0 | 0.0 | 0.0 | 0.0 | 0.0 | 0.0 | 0.0 | 0.0 | 0.0 | 0.0 | 0.0 |
| Gm13580 | 0.0 | 0.0 | 0.0 | 0.0 | 0.0 | 0.0 | 0.0 | 0.0 | 0.0 | 0.0 | 0.0 | 0.0 |
| Gm136 | 0.0 | 0.0 | 0.0 | 0.0 | 0.0 | 0.0 | 0.0 | 0.0 | 0.0 | 0.0 | 0.0 | 0.0 |
| Gm13629 | 0.0 | 0.0 | 0.0 | 0.0 | 0.0 | 0.0 | 0.0 | 0.0 | 0.0 | 0.0 | 0.0 | 0.0 |
| Gm13752 | 0.0 | 0.0 | 0.0 | 0.0 | 0.0 | 0.0 | 0.0 | 0.0 | 0.0 | 0.0 | 0.0 | 0.0 |
| Gm13769 | 0.0 | 0.0 | 0.0 | 0.0 | 0.0 | 0.0 | 0.0 | 0.0 | 0.0 | 0.0 | 0.0 | 0.0 |
| Gm13944 | 0.0 | 0.0 | 0.0 | 0.1 | 0.0 | 0.0 | 0.0 | 0.0 | 0.0 | 0.0 | 0.0 | 0.0 |
| Gm14092 | 0.0 | 0.0 | 0.0 | 0.0 | 0.0 | 0.0 | 0.1 | 0.0 | 0.0 | 0.0 | 0.0 | 0.0 |
| Gm14164 | 0.0 | 0.0 | 0.0 | 0.0 | 0.0 | 0.0 | 0.0 | 0.0 | 0.0 | 0.0 | 0.0 | 0.0 |
| Gm14169 | 0.0 | 0.0 | 0.0 | 0.0 | 0.0 | 0.0 | 0.0 | 0.0 | 0.0 | 0.0 | 0.0 | 0.0 |
| Gm14204 | 0.1 | 0.0 | 0.0 | 0.0 | 0.0 | 0.0 | 0.0 | 0.0 | 0.0 | 0.0 | 0.0 | 0.0 |
| Gm14207 | 0.0 | 0.0 | 0.0 | 0.0 | 0.0 | 0.0 | 0.1 | 0.0 | 0.0 | 0.0 | 0.0 | 0.0 |
| Gm14318 | 0.0 | 0.0 | 0.0 | 0.0 | 0.0 | 0.0 | 0.0 | 0.0 | 0.0 | 0.0 | 0.0 | 0.0 |
| Gm14345 | 0.0 | 0.0 | 0.0 | 0.0 | 0.0 | 0.0 | 0.0 | 0.0 | 0.0 | 0.0 | 0.0 | 0.0 |
| Gm14346 | 0.0 | 0.0 | 0.0 | 0.0 | 0.0 | 0.0 | 0.0 | 0.0 | 0.0 | 0.0 | 0.0 | 0.0 |
| Gm14351 | 0.0 | 0.0 | 0.0 | 0.0 | 0.0 | 0.0 | 0.0 | 0.0 | 0.0 | 0.0 | 0.0 | 0.0 |
| Gm14354 | 0.0 | 0.0 | 0.0 | 0.1 | 0.0 | 0.0 | 0.0 | 0.0 | 0.0 | 0.0 | 0.0 | 0.0 |
| Gm14461 | 0.0 | 0.0 | 0.0 | 0.2 | 0.0 | 0.0 | 0.0 | 0.0 | 0.0 | 0.0 | 0.0 | 0.0 |
| Gm14474 | 0.0 | 0.0 | 0.0 | 0.0 | 0.0 | 0.0 | 0.0 | 0.0 | 0.0 | 0.0 | 0.0 | 0.0 |
| Gm14478 | 0.0 | 0.0 | 0.0 | 0.0 | 0.0 | 0.0 | 0.0 | 0.0 | 0.0 | 0.0 | 0.0 | 0.0 |
| Gm14482 | 0.0 | 0.0 | 0.0 | 0.0 | 0.0 | 0.0 | 0.0 | 0.0 | 0.0 | 0.0 | 0.0 | 0.0 |
| Gm14483 | 0.0 | 0.0 | 0.0 | 0.0 | 0.0 | 0.0 | 0.0 | 0.0 | 0.0 | 0.0 | 0.0 | 0.0 |
| Gm14484 | 0.0 | 0.0 | 0.0 | 0.0 | 0.0 | 0.0 | 0.0 | 0.0 | 0.0 | 0.0 | 0.0 | 0.0 |
| Gm14496 | 0.0 | 0.0 | 0.0 | 0.0 | 0.0 | 0.0 | 0.0 | 0.0 | 0.0 | 0.0 | 0.0 | 0.0 |
| Gm14501 | 0.0 | 0.0 | 0.0 | 0.0 | 0.0 | 0.1 | 0.0 | 0.0 | 0.1 | 0.0 | 0.0 | 0.0 |
| Gm14525 | 0.0 | 0.0 | 0.0 | 0.0 | 0.0 | 0.0 | 0.0 | 0.0 | 0.0 | 0.0 | 0.0 | 0.0 |
| Gm14625 | 0.0 | 0.0 | 0.0 | 0.0 | 0.0 | 0.0 | 0.0 | 0.0 | 0.0 | 0.0 | 0.0 | 0.0 |
| Gm14635 | 0.0 | 0.0 | 0.0 | 0.0 | 0.0 | 0.0 | 0.0 | 0.0 | 0.0 | 0.0 | 0.0 | 0.0 |
| Gm14685 | 0.0 | 0.0 | 0.0 | 0.0 | 0.0 | 0.0 | 0.0 | 0.0 | 0.0 | 0.0 | 0.0 | 0.0 |
| Gm14692 | 0.0 | 0.0 | 0.0 | 0.0 | 0.0 | 0.0 | 0.0 | 0.0 | 0.0 | 0.0 | 0.0 | 0.0 |
| Gm14718 | 0.0 | 0.0 | 0.0 | 0.0 | 0.0 | 0.0 | 0.0 | 0.0 | 0.0 | 0.0 | 0.0 | 0.1 |
| Gm14725 | 0.0 | 0.0 | 0.0 | 0.0 | 0.0 | 0.0 | 0.0 | 0.0 | 0.0 | 0.0 | 0.0 | 0.0 |
| Gm14812 | 0.0 | 0.0 | 0.0 | 0.0 | 0.0 | 0.0 | 0.0 | 0.0 | 0.0 | 0.0 | 0.1 | 0.0 |
| Gm14858 | 0.0 | 0.0 | 0.0 | 0.0 | 0.0 | 0.0 | 0.0 | 0.0 | 0.0 | 0.0 | 0.0 | 0.0 |
| Gm15008 | 0.0 | 0.0 | 0.0 | 0.0 | 0.0 | 0.0 | 0.0 | 0.0 | 0.0 | 0.0 | 0.4 | 0.0 |
| Gm15023 | 0.0 | 0.0 | 0.0 | 0.0 | 0.0 | 0.0 | 0.0 | 0.0 | 0.0 | 0.0 | 0.0 | 0.0 |
| Gm15056 | 0.0 | 0.0 | 0.0 | 0.0 | 0.0 | 0.0 | 0.0 | 0.0 | 0.0 | 0.2 | 0.0 | 0.0 |
| Gm15091 | 0.0 | 0.0 | 0.0 | 0.0 | 0.0 | 0.0 | 0.0 | 0.0 | 0.0 | 0.0 | 0.0 | 0.0 |
| Gm15093 | 0.0 | 0.0 | 0.0 | 0.0 | 0.0 | 0.0 | 0.0 | 0.0 | 0.0 | 0.0 | 0.0 | 0.0 |
| Gm15097 | 0.0 | 0.0 | 0.0 | 0.0 | 0.0 | 0.0 | 0.0 | 0.0 | 0.0 | 0.0 | 0.0 | 0.0 |
| Gm15104 | 0.1 | 0.3 | 0.0 | 0.0 | 0.2 | 0.1 | 0.0 | 0.1 | 0.1 | 0.0 | 0.0 | 0.0 |
| Gm15107 | 0.0 | 0.0 | 0.0 | 0.0 | 0.0 | 0.0 | 0.0 | 0.0 | 0.0 | 0.0 | 0.0 | 0.0 |
| Gm15114 | 0.0 | 0.0 | 0.0 | 0.0 | 0.0 | 0.0 | 0.0 | 0.0 | 0.0 | 0.0 | 0.0 | 0.0 |
| Gm15127 | 0.0 | 0.0 | 0.0 | 0.0 | 0.0 | 0.0 | 0.0 | 0.0 | 0.0 | 0.0 | 0.0 | 0.0 |
| Gm15179 | 0.0 | 0.0 | 0.0 | 0.0 | 0.0 | 0.0 | 0.0 | 0.0 | 0.0 | 0.1 | 0.0 | 0.0 |
| Gm15850 | 0.0 | 0.0 | 0.0 | 0.0 | 0.0 | 0.0 | 0.0 | 0.0 | 0.0 | 0.0 | 0.0 | 0.0 |
| Gm15941 | 0.0 | 0.0 | 0.0 | 0.0 | 0.0 | 0.0 | 0.0 | 0.0 | 0.0 | 0.0 | 0.0 | 0.0 |
| Gm1604b | 0.0 | 0.0 | 0.0 | 0.0 | 0.0 | 0.0 | 0.0 | 0.0 | 0.0 | 0.0 | 0.0 | 0.0 |
| Gm16065 | 0.0 | 0.0 | 0.0 | 0.0 | 0.0 | 0.0 | 0.0 | 0.0 | 0.0 | 0.0 | 0.0 | 0.0 |
| Gm16294 | 0.0 | 0.0 | 0.0 | 0.0 | 0.0 | 0.0 | 0.0 | 0.0 | 0.0 | 0.0 | 0.0 | 0.0 |
| Gm1631 | 0.0 | 0.0 | 0.0 | 0.0 | 0.1 | 0.0 | 0.0 | 0.0 | 0.1 | 0.0 | 0.0 | 0.0 |
| Gm16325 | 0.0 | 0.0 | 0.0 | 0.0 | 0.0 | 0.0 | 0.0 | 0.0 | 0.0 | 0.0 | 0.0 | 0.0 |
| Gm16336 | 0.0 | 0.0 | 0.0 | 0.0 | 0.0 | 0.0 | 0.0 | 0.0 | 0.0 | 0.0 | 0.2 | 0.0 |
| Gm16367 | 0.0 | 0.0 | 0.0 | 0.0 | 0.0 | 0.0 | 0.0 | 0.0 | 0.0 | 0.0 | 0.0 | 0.0 |
| Gm16381 | 0.0 | 0.0 | 0.0 | 0.0 | 0.0 | 0.0 | 0.0 | 0.0 | 0.0 | 0.0 | 0.0 | 0.0 |

| | | | | | | | | | | | | |
|---|---|---|---|---|---|---|---|---|---|---|---|---|
| Gm16390 | 0.0 | 0.0 | 0.0 | 0.0 | 0.0 | 0.0 | 0.0 | 0.0 | 0.0 | 0.1 | 0.0 | 0.0 |
| Gm16404 | 0.0 | 0.0 | 0.0 | 0.0 | 0.0 | 0.0 | 0.0 | 0.0 | 0.0 | 0.0 | 0.0 | 0.0 |
| Gm16405 | 0.0 | 0.0 | 0.0 | 0.0 | 0.0 | 0.0 | 0.0 | 0.0 | 0.0 | 0.0 | 0.0 | 0.0 |
| Gm16445 | 0.0 | 0.0 | 0.0 | 0.0 | 0.1 | 0.0 | 0.0 | 0.0 | 0.0 | 0.0 | 0.0 | 0.0 |
| Gm1647 | 0.0 | 0.0 | 0.0 | 0.0 | 0.0 | 0.1 | 0.0 | 0.0 | 0.0 | 0.0 | 0.0 | 0.0 |
| Gm16497 | 0.0 | 0.0 | 0.0 | 0.0 | 0.0 | 0.0 | 0.0 | 0.0 | 0.0 | 0.0 | 0.0 | 0.0 |
| Gm1653 | 0.0 | 0.0 | 0.0 | 0.1 | 0.0 | 0.0 | 0.0 | 0.0 | 0.0 | 0.0 | 0.0 | 0.0 |
| Gm16701 | 0.0 | 0.0 | 0.0 | 0.0 | 0.0 | 0.2 | 0.1 | 0.0 | 0.0 | 0.0 | 0.1 | 0.0 |
| Gm16833 | 0.0 | 0.0 | 0.0 | 0.0 | 0.1 | 0.0 | 0.0 | 0.0 | 0.1 | 0.0 | 0.0 | 0.0 |
| Gm16863 | 0.0 | 0.0 | 0.0 | 0.0 | 0.0 | 0.0 | 0.0 | 0.0 | 0.0 | 0.0 | 0.0 | 0.0 |
| Gm1720 | 0.0 | 0.0 | 0.0 | 0.0 | 0.1 | 0.0 | 0.0 | 0.0 | 0.0 | 0.0 | 0.0 | 0.0 |
| Gm17359 | 0.0 | 0.0 | 0.0 | 0.0 | 0.0 | 0.0 | 0.0 | 0.0 | 0.0 | 0.0 | 0.0 | 0.0 |
| Gm17365 | 0.0 | 0.0 | 0.0 | 0.0 | 0.0 | 0.0 | 0.0 | 0.0 | 0.0 | 0.1 | 0.0 | 0.0 |
| Gm17660 | 0.0 | 0.0 | 0.0 | 0.0 | 0.0 | 0.0 | 0.0 | 0.0 | 0.0 | 0.0 | 0.0 | 0.0 |
| Gm17745 | 0.0 | 0.0 | 0.0 | 0.0 | 0.0 | 0.1 | 0.0 | 0.0 | 0.0 | 0.0 | 0.0 | 0.0 |
| Gm17746 | 0.0 | 0.0 | 0.0 | 0.0 | 0.0 | 0.0 | 0.0 | 0.0 | 0.0 | 0.0 | 0.0 | 0.0 |
| Gm17751 | 0.0 | 0.0 | 0.0 | 0.0 | 0.0 | 0.0 | 0.0 | 0.0 | 0.0 | 0.0 | 0.0 | 0.0 |
| Gm18409 | 0.0 | 0.0 | 0.0 | 0.0 | 0.0 | 0.0 | 0.0 | 0.0 | 0.0 | 0.0 | 0.0 | 0.0 |
| Gm19277 | 0.0 | 0.0 | 0.1 | 0.0 | 0.0 | 0.0 | 0.0 | 0.1 | 0.0 | 0.0 | 0.0 | 0.0 |
| Gm19395 | 0.1 | 0.1 | 0.0 | 0.0 | 0.0 | 0.0 | 0.0 | 0.1 | 0.1 | 0.0 | 0.0 | 0.0 |
| Gm19424 | 0.0 | 0.0 | 0.0 | 0.0 | 0.0 | 0.0 | 0.0 | 0.0 | 0.0 | 0.0 | 0.0 | 0.0 |
| Gm1968 | 0.0 | 0.0 | 0.0 | 0.0 | 0.0 | 0.0 | 0.0 | 0.0 | 0.0 | 0.0 | 0.0 | 0.0 |
| Gm19689 | 0.0 | 0.0 | 0.0 | 0.1 | 0.0 | 0.0 | 0.0 | 0.0 | 0.0 | 0.0 | 0.0 | 0.0 |
| Gm19782 | 0.0 | 0.0 | 0.0 | 0.0 | 0.0 | 0.0 | 0.0 | 0.0 | 0.0 | 0.0 | 0.0 | 0.0 |
| Gm1993 | 0.0 | 0.0 | 0.0 | 0.0 | 0.0 | 0.0 | 0.0 | 0.0 | 0.0 | 0.0 | 0.0 | 0.0 |
| Gm1995 | 0.0 | 0.0 | 0.0 | 0.0 | 0.0 | 0.0 | 0.0 | 0.0 | 0.0 | 0.0 | 0.0 | 0.0 |
| Gm20063 | 0.1 | 0.0 | 0.0 | 0.0 | 0.0 | 0.0 | 0.0 | 0.0 | 0.0 | 0.0 | 0.0 | 0.0 |
| Gm20098 | 0.0 | 0.0 | 0.0 | 0.0 | 0.0 | 0.0 | 0.0 | 0.0 | 0.0 | 0.0 | 0.0 | 0.0 |
| Gm2012 | 0.0 | 0.0 | 0.0 | 0.0 | 0.0 | 0.0 | 0.0 | 0.0 | 0.0 | 0.0 | 0.0 | 0.0 |
| Gm20125 | 0.0 | 0.0 | 0.0 | 0.0 | 0.0 | 0.0 | 0.0 | 0.0 | 0.0 | 0.0 | 0.0 | 0.0 |
| Gm20172 | 0.0 | 0.0 | 0.0 | 0.0 | 0.0 | 0.0 | 0.0 | 0.0 | 0.0 | 0.0 | 0.0 | 0.0 |
| Gm20187 | 0.0 | 0.0 | 0.0 | 0.0 | 0.0 | 0.0 | 0.0 | 0.0 | 0.0 | 0.0 | 0.0 | 0.0 |
| Gm2030 | 0.0 | 0.0 | 0.0 | 0.0 | 0.0 | 0.0 | 0.0 | 0.0 | 0.0 | 0.0 | 0.0 | 0.0 |
| Gm2042 | 0.0 | 0.0 | 0.0 | 0.0 | 0.0 | 0.0 | 0.0 | 0.0 | 0.0 | 0.0 | 0.0 | 0.0 |
| Gm20556 | 0.0 | 0.0 | 0.0 | 0.0 | 0.1 | 0.0 | 0.0 | 0.0 | 0.0 | 0.0 | 0.0 | 0.0 |
| Gm2061 | 0.0 | 0.0 | 0.0 | 0.0 | 0.0 | 0.0 | 0.0 | 0.0 | 0.0 | 0.0 | 0.0 | 0.0 |
| Gm20738 | 0.0 | 0.0 | 0.0 | 0.0 | 0.0 | 0.0 | 0.0 | 0.0 | 0.0 | 0.0 | 0.0 | 0.0 |
| Gm20740 | 0.2 | 0.0 | 0.0 | 0.0 | 0.0 | 0.0 | 0.1 | 0.0 | 0.0 | 0.0 | 0.0 | 0.0 |
| Gm20743 | 0.0 | 0.1 | 0.0 | 0.0 | 0.0 | 0.0 | 0.1 | 0.0 | 0.0 | 0.0 | 0.0 | 0.0 |
| Gm20745 | 0.0 | 0.0 | 0.0 | 0.0 | 0.0 | 0.0 | 0.0 | 0.0 | 0.0 | 0.0 | 0.0 | 0.0 |
| Gm20747 | 0.0 | 0.0 | 0.0 | 0.0 | 0.0 | 0.0 | 0.0 | 0.0 | 0.0 | 0.0 | 0.0 | 0.0 |
| Gm20751 | 0.0 | 0.0 | 0.0 | 0.0 | 0.0 | 0.0 | 0.1 | 0.0 | 0.0 | 0.0 | 0.0 | 0.0 |
| Gm20753 | 0.0 | 0.0 | 0.0 | 0.0 | 0.0 | 0.0 | 0.0 | 0.0 | 0.3 | 0.0 | 0.0 | 0.0 |
| Gm20754 | 0.0 | 0.1 | 0.0 | 0.0 | 0.0 | 0.0 | 0.0 | 0.0 | 0.0 | 0.0 | 0.0 | 0.0 |
| Gm20756 | 0.0 | 0.0 | 0.0 | 0.0 | 0.0 | 0.0 | 0.0 | 0.0 | 0.0 | 0.0 | 0.0 | 0.0 |
| Gm20757 | 0.0 | 0.0 | 0.0 | 0.0 | 0.0 | 0.0 | 0.0 | 0.0 | 0.0 | 0.0 | 0.0 | 0.0 |
| Gm20758 | 0.0 | 0.0 | 0.0 | 0.0 | 0.0 | 0.0 | 0.0 | 0.0 | 0.0 | 0.0 | 0.0 | 0.0 |
| Gm20806 | 0.0 | 0.0 | 0.0 | 0.0 | 0.0 | 0.0 | 0.0 | 0.0 | 0.0 | 0.0 | 0.0 | 0.0 |
| Gm20809 | 0.0 | 0.0 | 0.0 | 0.0 | 0.0 | 0.0 | 0.0 | 0.0 | 0.0 | 0.0 | 0.0 | 0.0 |
| Gm20815 | 0.0 | 0.0 | 0.0 | 0.0 | 0.0 | 0.0 | 0.0 | 0.0 | 0.0 | 0.0 | 0.0 | 0.0 |
| Gm20816 | 0.0 | 0.0 | 0.0 | 0.0 | 0.0 | 0.0 | 0.0 | 0.0 | 0.0 | 0.0 | 0.0 | 0.0 |
| Gm20823 | 0.0 | 0.0 | 0.0 | 0.0 | 0.0 | 0.0 | 0.0 | 0.0 | 0.0 | 0.0 | 0.0 | 0.0 |
| Gm2083 | 0.0 | 0.0 | 0.0 | 0.0 | 0.0 | 0.0 | 0.0 | 0.0 | 0.0 | 0.0 | 0.0 | 0.0 |
| Gm20831 | 0.0 | 0.0 | 0.0 | 0.0 | 0.0 | 0.0 | 0.0 | 0.0 | 0.0 | 0.0 | 0.0 | 0.0 |
| Gm20854 | 0.0 | 0.0 | 0.0 | 0.0 | 0.0 | 0.0 | 0.0 | 0.0 | 0.0 | 0.0 | 0.0 | 0.0 |
| Gm20857 | 0.0 | 0.0 | 0.0 | 0.0 | 0.0 | 0.0 | 0.0 | 0.0 | 0.0 | 0.0 | 0.0 | 0.0 |
| Gm20865 | 0.0 | 0.0 | 0.0 | 0.0 | 0.0 | 0.0 | 0.0 | 0.0 | 0.0 | 0.0 | 0.0 | 0.0 |

| | | | | | | | | | | | | |
|---|---|---|---|---|---|---|---|---|---|---|---|---|
| Gm20867 | 0.0 | 0.0 | 0.0 | 0.0 | 0.0 | 0.0 | 0.0 | 0.0 | 0.0 | 0.0 | 0.0 | 0.0 |
| Gm20871 | 0.0 | 0.0 | 0.0 | 0.0 | 0.0 | 0.0 | 0.0 | 0.0 | 0.0 | 0.0 | 0.0 | 0.0 |
| Gm20917 | 0.0 | 0.0 | 0.0 | 0.0 | 0.0 | 0.0 | 0.0 | 0.0 | 0.0 | 0.0 | 0.0 | 0.0 |
| Gm21057 | 0.0 | 0.3 | 0.0 | 0.0 | 0.1 | 0.0 | 0.0 | 0.0 | 0.0 | 0.3 | 0.0 | 0.0 |
| Gm2109 | 0.0 | 0.0 | 0.0 | 0.0 | 0.0 | 0.0 | 0.0 | 0.0 | 0.0 | 0.0 | 0.0 | 0.0 |
| Gm21637 | 0.0 | 0.0 | 0.0 | 0.1 | 0.0 | 0.0 | 0.0 | 0.0 | 0.0 | 0.0 | 0.0 | 0.0 |
| Gm21693 | 0.0 | 0.0 | 0.0 | 0.0 | 0.0 | 0.0 | 0.0 | 0.0 | 0.0 | 0.0 | 0.0 | 0.0 |
| Gm21708 | 0.0 | 0.0 | 0.0 | 0.0 | 0.0 | 0.0 | 0.0 | 0.0 | 0.0 | 0.0 | 0.0 | 0.0 |
| Gm21943 | 0.0 | 0.0 | 0.0 | 0.0 | 0.0 | 0.0 | 0.0 | 0.0 | 0.0 | 0.0 | 0.0 | 0.0 |
| Gm21944 | 0.0 | 0.0 | 0.0 | 0.0 | 0.5 | 0.0 | 0.0 | 0.0 | 0.0 | 0.0 | 0.0 | 0.0 |
| Gm21947 | 0.0 | 0.0 | 0.0 | 0.0 | 0.1 | 0.0 | 0.0 | 0.0 | 0.0 | 0.0 | 0.0 | 0.0 |
| Gm2447 | 0.0 | 0.0 | 0.0 | 0.0 | 0.0 | 0.0 | 0.0 | 0.0 | 0.0 | 0.0 | 0.0 | 0.0 |
| Gm2663 | 0.0 | 0.0 | 0.0 | 0.0 | 0.0 | 0.0 | 0.0 | 0.0 | 0.0 | 0.0 | 0.0 | 0.0 |
| Gm2762 | 0.1 | 0.0 | 0.0 | 0.0 | 0.0 | 0.0 | 0.0 | 0.0 | 0.0 | 0.0 | 0.1 | 0.0 |
| Gm2799 | 0.0 | 0.0 | 0.0 | 0.0 | 0.0 | 0.0 | 0.0 | 0.0 | 0.0 | 0.0 | 0.0 | 0.0 |
| Gm2837 | 0.0 | 0.0 | 0.0 | 0.2 | 0.0 | 0.0 | 0.0 | 0.0 | 0.0 | 0.0 | 0.0 | 0.0 |
| Gm2848 | 0.0 | 0.0 | 0.0 | 0.0 | 0.0 | 0.0 | 0.0 | 0.0 | 0.0 | 0.0 | 0.0 | 0.0 |
| Gm2913 | 0.0 | 0.0 | 0.0 | 0.0 | 0.0 | 0.0 | 0.0 | 0.0 | 0.0 | 0.0 | 0.0 | 0.0 |
| Gm2933 | 0.0 | 0.0 | 0.0 | 0.0 | 0.0 | 0.0 | 0.0 | 0.0 | 0.0 | 0.0 | 0.0 | 0.0 |
| Gm3139 | 0.0 | 0.0 | 0.0 | 0.0 | 0.0 | 0.0 | 0.0 | 0.0 | 0.0 | 0.0 | 0.0 | 0.0 |
| Gm3143 | 0.0 | 0.0 | 0.0 | 0.0 | 0.0 | 0.0 | 0.0 | 0.2 | 0.0 | 0.0 | 0.0 | 0.0 |
| Gm3259 | 0.0 | 0.0 | 0.0 | 0.0 | 0.0 | 0.0 | 0.0 | 0.0 | 0.0 | 0.0 | 0.0 | 0.0 |
| Gm3286 | 0.0 | 0.0 | 0.0 | 0.0 | 0.0 | 0.0 | 0.0 | 0.0 | 0.0 | 0.0 | 0.0 | 0.0 |
| Gm3336 | 0.0 | 0.0 | 0.0 | 0.0 | 0.1 | 0.0 | 0.0 | 0.0 | 0.0 | 0.1 | 0.0 | 0.0 |
| Gm3404 | 0.0 | 0.0 | 0.0 | 0.0 | 0.0 | 0.0 | 0.0 | 0.0 | 0.0 | 0.0 | 0.0 | 0.0 |
| Gm364 | 0.0 | 0.0 | 0.0 | 0.0 | 0.0 | 0.0 | 0.0 | 0.0 | 0.0 | 0.0 | 0.0 | 0.0 |
| Gm3706 | 0.0 | 0.0 | 0.0 | 0.0 | 0.0 | 0.0 | 0.0 | 0.0 | 0.0 | 0.0 | 0.0 | 0.0 |
| Gm3750 | 0.0 | 0.0 | 0.0 | 0.0 | 0.0 | 0.0 | 0.0 | 0.0 | 0.0 | 0.0 | 0.0 | 0.0 |
| Gm3763 | 0.0 | 0.0 | 0.0 | 0.0 | 0.0 | 0.0 | 0.0 | 0.0 | 0.0 | 0.0 | 0.0 | 0.0 |
| Gm3833 | 0.0 | 0.0 | 0.0 | 0.0 | 0.0 | 0.0 | 0.0 | 0.0 | 0.0 | 0.0 | 0.0 | 0.0 |
| Gm3925 | 0.1 | 0.0 | 0.0 | 0.0 | 0.0 | 0.0 | 0.0 | 0.0 | 0.0 | 0.0 | 0.0 | 0.0 |
| Gm4064 | 0.0 | 0.0 | 0.0 | 0.0 | 0.0 | 0.0 | 0.0 | 0.0 | 0.0 | 0.0 | 0.0 | 0.0 |
| Gm410 | 0.0 | 0.0 | 0.0 | 0.0 | 0.0 | 0.0 | 0.0 | 0.0 | 0.0 | 0.0 | 0.0 | 0.0 |
| Gm4133 | 0.0 | 0.0 | 0.0 | 0.0 | 0.0 | 0.0 | 0.0 | 0.0 | 0.0 | 0.0 | 0.0 | 0.0 |
| Gm4187 | 0.0 | 0.0 | 0.0 | 0.0 | 0.0 | 0.0 | 0.0 | 0.0 | 0.0 | 0.0 | 0.0 | 0.0 |
| Gm4224 | 0.0 | 0.0 | 0.0 | 0.0 | 0.0 | 0.0 | 0.0 | 0.0 | 0.0 | 0.0 | 0.0 | 0.0 |
| Gm4278 | 0.0 | 0.0 | 0.0 | 0.0 | 0.0 | 0.0 | 0.0 | 0.0 | 0.0 | 0.0 | 0.0 | 0.0 |
| Gm428 | 0.0 | 0.0 | 0.0 | 0.0 | 0.0 | 0.0 | 0.0 | 0.0 | 0.0 | 0.0 | 0.0 | 0.0 |
| Gm4297 | 0.0 | 0.0 | 0.0 | 0.0 | 0.0 | 0.0 | 0.0 | 0.0 | 0.0 | 0.0 | 0.0 | 0.0 |
| Gm4301 | 0.0 | 0.0 | 0.0 | 0.0 | 0.0 | 0.0 | 0.0 | 0.0 | 0.0 | 0.0 | 0.0 | 0.0 |
| Gm4302 | 0.0 | 0.0 | 0.0 | 0.0 | 0.0 | 0.0 | 0.0 | 0.0 | 0.0 | 0.0 | 0.0 | 0.0 |
| Gm4303 | 0.0 | 0.0 | 0.0 | 0.0 | 0.0 | 0.0 | 0.0 | 0.0 | 0.0 | 0.0 | 0.0 | 0.0 |
| Gm4312 | 0.0 | 0.0 | 0.0 | 0.0 | 0.0 | 0.0 | 0.0 | 0.0 | 0.0 | 0.0 | 0.0 | 0.0 |
| Gm4371 | 0.0 | 0.0 | 0.0 | 0.0 | 0.0 | 0.0 | 0.0 | 0.0 | 0.0 | 0.0 | 0.0 | 0.0 |
| Gm438 | 0.0 | 0.1 | 0.0 | 0.0 | 0.0 | 0.0 | 0.0 | 0.1 | 0.0 | 0.0 | 0.0 | 0.0 |
| Gm4461 | 0.1 | 0.0 | 0.0 | 0.0 | 0.0 | 0.1 | 0.0 | 0.0 | 0.0 | 0.0 | 0.0 | 0.0 |
| Gm4489 | 0.0 | 0.0 | 0.2 | 0.3 | 0.0 | 0.0 | 0.0 | 0.0 | 0.0 | 0.0 | 0.0 | 0.0 |
| Gm4567 | 0.0 | 0.0 | 0.0 | 0.0 | 0.0 | 0.0 | 0.0 | 0.0 | 0.0 | 0.0 | 0.0 | 0.0 |
| Gm4598 | 0.0 | 0.0 | 0.0 | 0.1 | 0.0 | 0.0 | 0.0 | 0.0 | 0.0 | 0.0 | 0.0 | 0.0 |
| Gm4745 | 0.0 | 0.0 | 0.0 | 0.0 | 0.0 | 0.0 | 0.0 | 0.0 | 0.0 | 0.0 | 0.0 | 0.0 |
| Gm4776 | 0.0 | 0.0 | 0.0 | 0.0 | 0.0 | 0.0 | 0.0 | 0.0 | 0.0 | 0.0 | 0.0 | 0.0 |
| Gm4791 | 0.0 | 0.0 | 0.1 | 0.0 | 0.0 | 0.0 | 0.0 | 0.0 | 0.0 | 0.0 | 0.0 | 0.0 |
| Gm4792 | 0.0 | 0.0 | 0.0 | 0.0 | 0.0 | 0.1 | 0.0 | 0.0 | 0.0 | 0.0 | 0.0 | 0.0 |
| Gm4814 | 0.0 | 0.0 | 0.0 | 0.0 | 0.0 | 0.0 | 0.0 | 0.0 | 0.0 | 0.0 | 0.0 | 0.0 |
| Gm4827 | 0.0 | 0.0 | 0.0 | 0.0 | 0.0 | 0.1 | 0.0 | 0.0 | 0.0 | 0.0 | 0.0 | 0.0 |
| Gm4847 | 0.0 | 0.0 | 0.0 | 0.0 | 0.0 | 0.0 | 0.0 | 0.0 | 0.0 | 0.0 | 0.0 | 0.0 |
| Gm4850 | 0.0 | 0.0 | 0.0 | 0.0 | 0.0 | 0.1 | 0.0 | 0.0 | 0.0 | 0.0 | 0.0 | 0.0 |

Excluded Genes

| | | | | | | | | | | | | |
|---|---|---|---|---|---|---|---|---|---|---|---|---|
| Gm4858 | 0.1 | 0.1 | 0.0 | 0.1 | 0.0 | 0.0 | 0.0 | 0.0 | 0.0 | 0.0 | 0.0 | 0.0 |
| Gm4861 | 0.0 | 0.0 | 0.0 | 0.0 | 0.0 | 0.0 | 0.2 | 0.0 | 0.1 | 0.0 | 0.0 | 0.0 |
| Gm4871 | 0.0 | 0.0 | 0.0 | 0.0 | 0.0 | 0.0 | 0.0 | 0.0 | 0.0 | 0.0 | 0.0 | 0.0 |
| Gm4894 | 0.0 | 0.0 | 0.0 | 0.0 | 0.0 | 0.0 | 0.0 | 0.0 | 0.0 | 0.0 | 0.0 | 0.0 |
| Gm4922 | 0.0 | 0.0 | 0.0 | 0.0 | 0.0 | 0.0 | 0.0 | 0.0 | 0.0 | 0.0 | 0.0 | 0.0 |
| Gm4937 | 0.0 | 0.0 | 0.0 | 0.0 | 0.0 | 0.0 | 0.0 | 0.0 | 0.0 | 0.0 | 0.0 | 0.0 |
| Gm4956 | 0.0 | 0.0 | 0.0 | 0.0 | 0.1 | 0.0 | 0.1 | 0.0 | 0.0 | 0.0 | 0.0 | 0.0 |
| Gm4975 | 0.0 | 0.0 | 0.0 | 0.0 | 0.0 | 0.0 | 0.0 | 0.0 | 0.0 | 0.0 | 0.0 | 0.0 |
| Gm4981 | 0.0 | 0.0 | 0.0 | 0.0 | 0.0 | 0.0 | 0.0 | 0.0 | 0.0 | 0.0 | 0.0 | 0.0 |
| Gm5065 | 0.0 | 0.0 | 0.0 | 0.0 | 0.0 | 0.0 | 0.0 | 0.0 | 0.0 | 0.0 | 0.0 | 0.0 |
| Gm5083 | 0.0 | 0.0 | 0.0 | 0.0 | 0.0 | 0.0 | 0.0 | 0.0 | 0.0 | 0.0 | 0.0 | 0.0 |
| Gm5084 | 0.0 | 0.0 | 0.0 | 0.0 | 0.0 | 0.0 | 0.0 | 0.0 | 0.0 | 0.0 | 0.0 | 0.0 |
| Gm5089 | 0.0 | 0.0 | 0.0 | 0.0 | 0.1 | 0.0 | 0.0 | 0.0 | 0.0 | 0.0 | 0.0 | 0.0 |
| Gm5091 | 0.0 | 0.0 | 0.0 | 0.0 | 0.0 | 0.0 | 0.0 | 0.0 | 0.0 | 0.0 | 0.0 | 0.0 |
| Gm5108 | 0.0 | 0.0 | 0.0 | 0.0 | 0.0 | 0.1 | 0.0 | 0.0 | 0.0 | 0.0 | 0.0 | 0.0 |
| Gm5134 | 0.0 | 0.0 | 0.0 | 0.0 | 0.0 | 0.0 | 0.1 | 0.0 | 0.0 | 0.0 | 0.0 | 0.0 |
| Gm5168 | 0.0 | 0.0 | 0.0 | 0.0 | 0.0 | 0.0 | 0.0 | 0.0 | 0.0 | 0.0 | 0.0 | 0.0 |
| Gm5169 | 0.0 | 0.0 | 0.0 | 0.0 | 0.0 | 0.0 | 0.0 | 0.0 | 0.0 | 0.0 | 0.0 | 0.0 |
| Gm525 | 0.0 | 0.0 | 0.0 | 0.0 | 0.0 | 0.0 | 0.1 | 0.0 | 0.0 | 0.0 | 0.0 | 0.0 |
| Gm5294 | 0.0 | 0.0 | 0.1 | 0.0 | 0.0 | 0.0 | 0.0 | 0.0 | 0.0 | 0.0 | 0.0 | 0.0 |
| Gm5382 | 0.0 | 0.0 | 0.0 | 0.0 | 0.0 | 0.0 | 0.0 | 0.0 | 0.0 | 0.0 | 0.3 | 0.0 |
| Gm5420 | 0.0 | 0.0 | 0.0 | 0.0 | 0.0 | 0.0 | 0.0 | 0.0 | 0.0 | 0.0 | 0.0 | 0.0 |
| Gm5460 | 0.0 | 0.0 | 0.0 | 0.0 | 0.0 | 0.0 | 0.0 | 0.0 | 0.0 | 0.0 | 0.0 | 0.0 |
| Gm5483 | 0.0 | 0.0 | 0.0 | 0.0 | 0.1 | 0.0 | 0.0 | 0.0 | 0.0 | 0.0 | 0.0 | 0.0 |
| Gm5535 | 0.0 | 0.0 | 0.0 | 0.0 | 0.0 | 0.0 | 0.0 | 0.0 | 0.0 | 0.0 | 0.0 | 0.0 |
| Gm5547 | 0.0 | 0.0 | 0.0 | 0.0 | 0.2 | 0.0 | 0.0 | 0.0 | 0.0 | 0.2 | 0.0 | 0.0 |
| Gm5591 | 0.0 | 0.0 | 0.0 | 0.0 | 0.0 | 0.0 | 0.0 | 0.0 | 0.0 | 0.0 | 0.0 | 0.0 |
| Gm5592 | 0.0 | 0.0 | 0.0 | 0.0 | 0.0 | 0.0 | 0.0 | 0.0 | 0.0 | 0.0 | 0.0 | 0.0 |
| Gm5615 | 0.0 | 0.0 | 0.0 | 0.0 | 0.0 | 0.0 | 0.0 | 0.0 | 0.0 | 0.0 | 0.0 | 0.0 |
| Gm5622 | 0.0 | 0.0 | 0.0 | 0.0 | 0.0 | 0.0 | 0.0 | 0.0 | 0.0 | 0.0 | 0.0 | 0.0 |
| Gm5640 | 0.0 | 0.0 | 0.0 | 0.0 | 0.0 | 0.0 | 0.0 | 0.0 | 0.0 | 0.0 | 0.0 | 0.0 |
| Gm5725 | 0.0 | 0.0 | 0.0 | 0.0 | 0.0 | 0.0 | 0.0 | 0.0 | 0.0 | 0.0 | 0.0 | 0.0 |
| Gm5728 | 0.0 | 0.0 | 0.0 | 0.0 | 0.0 | 0.0 | 0.0 | 0.0 | 0.0 | 0.0 | 0.0 | 0.0 |
| Gm5741 | 0.0 | 0.0 | 0.0 | 0.0 | 0.0 | 0.0 | 0.0 | 0.0 | 0.0 | 0.0 | 0.0 | 0.0 |
| Gm5797 | 0.0 | 0.0 | 0.0 | 0.0 | 0.0 | 0.0 | 0.0 | 0.0 | 0.0 | 0.0 | 0.1 | 0.0 |
| Gm5833 | 0.0 | 0.0 | 0.0 | 0.0 | 0.0 | 0.0 | 0.0 | 0.0 | 0.0 | 0.0 | 0.0 | 0.0 |
| Gm5886 | 0.0 | 0.0 | 0.0 | 0.0 | 0.0 | 0.0 | 0.1 | 0.0 | 0.0 | 0.0 | 0.0 | 0.0 |
| Gm5891 | 0.0 | 0.0 | 0.0 | 0.0 | 0.0 | 0.0 | 0.0 | 0.0 | 0.0 | 0.0 | 0.0 | 0.0 |
| Gm5934 | 0.0 | 0.0 | 0.0 | 0.1 | 0.0 | 0.0 | 0.0 | 0.0 | 0.0 | 0.0 | 0.0 | 0.0 |
| Gm5935 | 0.0 | 0.0 | 0.0 | 0.0 | 0.0 | 0.0 | 0.0 | 0.0 | 0.0 | 0.0 | 0.0 | 0.0 |
| Gm5936 | 0.0 | 0.0 | 0.0 | 0.0 | 0.0 | 0.0 | 0.0 | 0.0 | 0.0 | 0.0 | 0.0 | 0.0 |
| Gm5941 | 0.0 | 0.0 | 0.0 | 0.0 | 0.0 | 0.0 | 0.0 | 0.0 | 0.0 | 0.0 | 0.0 | 0.1 |
| Gm597 | 0.0 | 0.0 | 0.0 | 0.0 | 0.0 | 0.0 | 0.0 | 0.0 | 0.0 | 0.0 | 0.0 | 0.0 |
| Gm609 | 0.0 | 0.0 | 0.0 | 0.0 | 0.0 | 0.0 | 0.0 | 0.0 | 0.0 | 0.0 | 0.0 | 0.0 |
| Gm6116 | 0.1 | 0.0 | 0.0 | 0.0 | 0.0 | 0.1 | 0.1 | 0.1 | 0.0 | 0.1 | 0.0 | 0.0 |
| Gm6121 | 0.0 | 0.0 | 0.0 | 0.0 | 0.0 | 0.0 | 0.0 | 0.0 | 0.0 | 0.0 | 0.0 | 0.0 |
| Gm614 | 0.0 | 0.0 | 0.0 | 0.1 | 0.0 | 0.0 | 0.1 | 0.0 | 0.0 | 0.0 | 0.0 | 0.0 |
| Gm6164 | 0.0 | 0.0 | 0.0 | 0.0 | 0.0 | 0.0 | 0.0 | 0.0 | 0.0 | 0.0 | 0.0 | 0.0 |
| Gm6249 | 0.0 | 0.0 | 0.0 | 0.0 | 0.0 | 0.0 | 0.0 | 0.0 | 0.0 | 0.0 | 0.0 | 0.0 |
| Gm6289 | 0.0 | 0.0 | 0.0 | 0.0 | 0.0 | 0.0 | 0.0 | 0.0 | 0.0 | 0.0 | 0.0 | 0.0 |
| Gm6300 | 0.0 | 0.0 | 0.0 | 0.0 | 0.0 | 0.0 | 0.0 | 0.0 | 0.0 | 0.0 | 0.0 | 0.0 |
| Gm6307 | 0.0 | 0.0 | 0.0 | 0.1 | 0.0 | 0.0 | 0.0 | 0.0 | 0.0 | 0.0 | 0.0 | 0.0 |
| Gm6329 | 0.0 | 0.1 | 0.0 | 0.0 | 0.1 | 0.0 | 0.1 | 0.0 | 0.0 | 0.0 | 0.0 | 0.0 |
| Gm6367 | 0.0 | 0.0 | 0.0 | 0.0 | 0.0 | 0.0 | 0.0 | 0.0 | 0.0 | 0.0 | 0.0 | 0.0 |
| Gm6408 | 0.0 | 0.0 | 0.0 | 0.0 | 0.0 | 0.0 | 0.0 | 0.0 | 0.0 | 0.0 | 0.0 | 0.0 |
| Gm6416 | 0.0 | 0.0 | 0.0 | 0.0 | 0.0 | 0.0 | 0.0 | 0.0 | 0.0 | 0.0 | 0.0 | 0.0 |
| Gm648 | 0.0 | 0.0 | 0.0 | 0.0 | 0.0 | 0.0 | 0.0 | 0.0 | 0.0 | 0.0 | 0.0 | 0.0 |

Excluded Genes

| | | | | | | | | | | | | |
|---|---|---|---|---|---|---|---|---|---|---|---|---|
| Gm6531 | 0.0 | 0.0 | 0.0 | 0.0 | 0.0 | 0.0 | 0.0 | 0.0 | 0.0 | 0.0 | 0.0 | 0.0 |
| Gm6588 | 0.0 | 0.0 | 0.0 | 0.0 | 0.0 | 0.0 | 0.0 | 0.0 | 0.0 | 0.0 | 0.0 | 0.0 |
| Gm6592 | 0.0 | 0.0 | 0.0 | 0.0 | 0.0 | 0.0 | 0.0 | 0.0 | 0.0 | 0.0 | 0.0 | 0.0 |
| Gm6602 | 0.0 | 0.0 | 0.0 | 0.0 | 0.0 | 0.0 | 0.0 | 0.0 | 0.0 | 0.0 | 0.0 | 0.0 |
| Gm6614 | 0.0 | 0.0 | 0.0 | 0.0 | 0.0 | 0.0 | 0.0 | 0.0 | 0.0 | 0.0 | 0.0 | 0.0 |
| Gm6772 | 0.0 | 0.0 | 0.0 | 0.0 | 0.0 | 0.0 | 0.0 | 0.0 | 0.0 | 0.0 | 0.0 | 0.0 |
| Gm6792 | 0.0 | 0.1 | 0.0 | 0.0 | 0.0 | 0.0 | 0.0 | 0.0 | 0.0 | 0.0 | 0.0 | 0.0 |
| Gm6890 | 0.0 | 0.0 | 0.0 | 0.0 | 0.0 | 0.0 | 0.0 | 0.0 | 0.0 | 0.0 | 0.0 | 0.0 |
| Gm6902 | 0.0 | 0.0 | 0.0 | 0.0 | 0.0 | 0.0 | 0.0 | 0.0 | 0.0 | 0.0 | 0.0 | 0.0 |
| Gm6994 | 0.0 | 0.0 | 0.0 | 0.0 | 0.0 | 0.0 | 0.0 | 0.0 | 0.0 | 0.0 | 0.0 | 0.0 |
| Gm7008 | 0.0 | 0.0 | 0.0 | 0.0 | 0.0 | 0.2 | 0.0 | 0.1 | 0.0 | 0.0 | 0.0 | 0.2 |
| Gm7104 | 0.0 | 0.0 | 0.0 | 0.0 | 0.0 | 0.0 | 0.0 | 0.0 | 0.0 | 0.0 | 0.0 | 0.0 |
| Gm7271 | 0.0 | 0.0 | 0.0 | 0.0 | 0.0 | 0.0 | 0.0 | 0.0 | 0.0 | 0.0 | 0.0 | 0.0 |
| Gm7325 | 0.0 | 0.0 | 0.0 | 0.0 | 0.0 | 0.0 | 0.0 | 0.1 | 0.0 | 0.0 | 0.0 | 0.0 |
| Gm7337 | 0.0 | 0.0 | 0.0 | 0.0 | 0.0 | 0.0 | 0.0 | 0.0 | 0.0 | 0.0 | 0.0 | 0.0 |
| Gm7457 | 0.0 | 0.0 | 0.0 | 0.0 | 0.0 | 0.0 | 0.0 | 0.0 | 0.0 | 0.0 | 0.0 | 0.0 |
| Gm7534 | 0.0 | 0.0 | 0.0 | 0.0 | 0.0 | 0.0 | 0.0 | 0.0 | 0.0 | 0.0 | 0.0 | 0.0 |
| Gm7550 | 0.0 | 0.0 | 0.0 | 0.0 | 0.0 | 0.0 | 0.0 | 0.1 | 0.0 | 0.0 | 0.0 | 0.0 |
| Gm757 | 0.0 | 0.0 | 0.0 | 0.0 | 0.0 | 0.1 | 0.0 | 0.0 | 0.0 | 0.0 | 0.0 | 0.0 |
| Gm765 | 0.0 | 0.0 | 0.0 | 0.0 | 0.0 | 0.0 | 0.0 | 0.1 | 0.0 | 0.0 | 0.0 | 0.0 |
| Gm7714 | 0.0 | 0.0 | 0.0 | 0.0 | 0.0 | 0.1 | 0.0 | 0.0 | 0.0 | 0.0 | 0.0 | 0.0 |
| Gm7903 | 0.0 | 0.0 | 0.0 | 0.0 | 0.0 | 0.0 | 0.0 | 0.0 | 0.0 | 0.0 | 0.0 | 0.0 |
| Gm7978 | 0.0 | 0.0 | 0.0 | 0.0 | 0.0 | 0.0 | 0.0 | 0.0 | 0.0 | 0.0 | 0.0 | 0.0 |
| Gm805 | 0.0 | 0.0 | 0.0 | 0.0 | 0.0 | 0.0 | 0.0 | 0.0 | 0.0 | 0.0 | 0.0 | 0.0 |
| Gm8179 | 0.0 | 0.0 | 0.0 | 0.0 | 0.0 | 0.0 | 0.0 | 0.0 | 0.0 | 0.0 | 0.0 | 0.0 |
| Gm8221 | 0.0 | 0.0 | 0.0 | 0.0 | 0.0 | 0.0 | 0.0 | 0.0 | 0.0 | 0.0 | 0.0 | 0.0 |
| Gm826 | 0.0 | 0.0 | 0.0 | 0.0 | 0.0 | 0.1 | 0.0 | 0.0 | 0.0 | 0.0 | 0.0 | 0.0 |
| Gm8267 | 0.0 | 0.0 | 0.0 | 0.0 | 0.0 | 0.0 | 0.0 | 0.0 | 0.0 | 0.0 | 0.0 | 0.0 |
| Gm833 | 0.0 | 0.0 | 0.0 | 0.0 | 0.0 | 0.0 | 0.0 | 0.0 | 0.0 | 0.0 | 0.0 | 0.0 |
| Gm8363 | 0.0 | 0.0 | 0.0 | 0.0 | 0.0 | 0.0 | 0.0 | 0.0 | 0.0 | 0.0 | 0.0 | 0.0 |
| Gm8439 | 0.0 | 0.0 | 0.0 | 0.0 | 0.0 | 0.0 | 0.0 | 0.0 | 0.0 | 0.0 | 0.0 | 0.0 |
| Gm8693 | 0.0 | 0.0 | 0.0 | 0.0 | 0.0 | 0.0 | 0.0 | 0.0 | 0.0 | 0.0 | 0.0 | 0.0 |
| Gm8709 | 0.0 | 0.0 | 0.1 | 0.0 | 0.0 | 0.0 | 0.0 | 0.0 | 0.0 | 0.0 | 0.0 | 0.0 |
| Gm872 | 0.0 | 0.0 | 0.0 | 0.0 | 0.0 | 0.0 | 0.0 | 0.0 | 0.0 | 0.0 | 0.0 | 0.0 |
| Gm8720 | 0.0 | 0.0 | 0.0 | 0.0 | 0.0 | 0.0 | 0.0 | 0.0 | 0.0 | 0.0 | 0.0 | 0.0 |
| Gm884 | 0.0 | 0.0 | 0.0 | 0.0 | 0.0 | 0.0 | 0.0 | 0.0 | 0.0 | 0.0 | 0.0 | 0.0 |
| Gm8884 | 0.3 | 0.0 | 0.1 | 0.0 | 0.0 | 0.0 | 0.0 | 0.0 | 0.0 | 0.0 | 0.0 | 0.0 |
| Gm889 | 0.1 | 0.1 | 0.0 | 0.0 | 0.0 | 0.0 | 0.0 | 0.0 | 0.0 | 0.1 | 0.0 | 0.0 |
| Gm9047 | 0.0 | 0.0 | 0.0 | 0.2 | 0.0 | 0.0 | 0.0 | 0.0 | 0.0 | 0.0 | 0.0 | 0.1 |
| Gm9125 | 0.0 | 0.0 | 0.0 | 0.0 | 0.0 | 0.0 | 0.0 | 0.0 | 0.0 | 0.0 | 0.0 | 0.0 |
| Gm9376 | 0.0 | 0.0 | 0.0 | 0.0 | 0.0 | 0.0 | 0.0 | 0.0 | 0.0 | 0.0 | 0.0 | 0.0 |
| Gm9573 | 0.0 | 0.0 | 0.0 | 0.0 | 0.0 | 0.0 | 0.0 | 0.0 | 0.0 | 0.0 | 0.0 | 0.0 |
| Gm9758 | 0.0 | 0.0 | 0.0 | 0.0 | 0.0 | 0.0 | 0.0 | 0.0 | 0.0 | 0.0 | 0.0 | 0.0 |
| Gm9839 | 0.0 | 0.0 | 0.0 | 0.0 | 0.0 | 0.0 | 0.0 | 0.0 | 0.0 | 0.0 | 0.0 | 0.0 |
| Gm9926 | 0.0 | 0.0 | 0.0 | 0.0 | 0.0 | 0.0 | 0.0 | 0.0 | 0.0 | 0.0 | 0.0 | 0.0 |
| Gm9962 | 0.0 | 0.0 | 0.0 | 0.0 | 0.0 | 0.0 | 0.0 | 0.0 | 0.0 | 0.0 | 0.0 | 0.0 |
| Gm9999 | 0.0 | 0.0 | 0.0 | 0.0 | 0.0 | 0.0 | 0.0 | 0.1 | 0.0 | 0.0 | 0.0 | 0.0 |
| Gmcl1l | 0.0 | 0.0 | 0.0 | 0.0 | 0.0 | 0.0 | 0.0 | 0.0 | 0.0 | 0.0 | 0.0 | 0.0 |
| Gml | 0.0 | 0.0 | 0.0 | 0.0 | 0.0 | 0.2 | 0.1 | 0.0 | 0.0 | 0.0 | 0.0 | 0.0 |
| Gmnc | 0.0 | 0.0 | 0.0 | 0.0 | 0.0 | 0.0 | 0.0 | 0.0 | 0.0 | 0.0 | 0.0 | 0.0 |
| Gnat3 | 0.0 | 0.0 | 0.0 | 0.0 | 0.0 | 0.0 | 0.0 | 0.0 | 0.0 | 0.0 | 0.0 | 0.0 |
| Gngt1 | 0.0 | 0.0 | 0.0 | 0.0 | 0.0 | 0.0 | 0.0 | 0.0 | 0.0 | 0.0 | 0.0 | 0.0 |
| Gnmt | 0.0 | 0.0 | 0.0 | 0.0 | 0.1 | 0.1 | 0.0 | 0.0 | 0.2 | 0.0 | 0.0 | 0.0 |
| Gnrhr | 0.0 | 0.0 | 0.0 | 0.0 | 0.0 | 0.0 | 0.0 | 0.0 | 0.0 | 0.0 | 0.0 | 0.0 |
| Golga7b | 0.0 | 0.0 | 0.0 | 0.0 | 0.0 | 0.0 | 0.0 | 0.1 | 0.0 | 0.0 | 0.0 | 0.0 |
| Gp2 | 0.0 | 0.0 | 0.0 | 0.0 | 0.0 | 0.0 | 0.0 | 0.0 | 0.0 | 0.0 | 0.0 | 0.0 |
| Gp9 | 0.0 | 0.1 | 0.0 | 0.4 | 0.0 | 0.0 | 0.0 | 0.1 | 0.0 | 0.0 | 0.0 | 0.0 |

| | | | | | | | | | | | | |
|---|---|---|---|---|---|---|---|---|---|---|---|---|
| Gpa33 | 0.0 | 0.0 | 0.0 | 0.0 | 0.0 | 0.0 | 0.0 | 0.0 | 0.0 | 0.0 | 0.0 | 0.0 |
| Gphb5 | 0.0 | 0.0 | 0.0 | 0.0 | 0.0 | 0.0 | 0.0 | 0.0 | 0.0 | 0.0 | 0.0 | 0.0 |
| Gpr101 | 0.0 | 0.0 | 0.0 | 0.0 | 0.0 | 0.0 | 0.0 | 0.0 | 0.0 | 0.0 | 0.0 | 0.0 |
| Gpr113 | 0.0 | 0.0 | 0.0 | 0.0 | 0.0 | 0.0 | 0.0 | 0.0 | 0.0 | 0.0 | 0.0 | 0.0 |
| Gpr114 | 0.0 | 0.0 | 0.0 | 0.0 | 0.0 | 0.0 | 0.0 | 0.0 | 0.0 | 0.0 | 0.0 | 0.0 |
| Gpr119 | 0.0 | 0.0 | 0.0 | 0.0 | 0.0 | 0.0 | 0.0 | 0.0 | 0.0 | 0.0 | 0.0 | 0.0 |
| Gpr139 | 0.0 | 0.0 | 0.0 | 0.0 | 0.0 | 0.0 | 0.0 | 0.0 | 0.0 | 0.0 | 0.0 | 0.0 |
| Gpr149 | 0.0 | 0.0 | 0.0 | 0.0 | 0.0 | 0.0 | 0.0 | 0.0 | 0.0 | 0.0 | 0.0 | 0.0 |
| Gpr15 | 0.0 | 0.0 | 0.1 | 0.0 | 0.0 | 0.1 | 0.0 | 0.0 | 0.0 | 0.0 | 0.0 | 0.0 |
| Gpr150 | 0.0 | 0.0 | 0.0 | 0.0 | 0.0 | 0.0 | 0.0 | 0.0 | 0.0 | 0.0 | 0.0 | 0.0 |
| Gpr158 | 0.0 | 0.0 | 0.0 | 0.0 | 0.0 | 0.0 | 0.0 | 0.0 | 0.0 | 0.0 | 0.0 | 0.0 |
| Gpr171 | 0.0 | 0.0 | 0.0 | 0.0 | 0.0 | 0.0 | 0.0 | 0.0 | 0.0 | 0.0 | 0.0 | 0.0 |
| Gpr174 | 0.0 | 0.0 | 0.0 | 0.0 | 0.0 | 0.0 | 0.0 | 0.0 | 0.0 | 0.0 | 0.0 | 0.0 |
| Gpr25 | 0.0 | 0.0 | 0.0 | 0.0 | 0.0 | 0.0 | 0.0 | 0.0 | 0.0 | 0.0 | 0.0 | 0.0 |
| Gpr26 | 0.0 | 0.0 | 0.0 | 0.0 | 0.0 | 0.0 | 0.0 | 0.0 | 0.0 | 0.0 | 0.0 | 0.0 |
| Gpr33 | 0.0 | 0.0 | 0.0 | 0.0 | 0.0 | 0.0 | 0.0 | 0.0 | 0.0 | 0.0 | 0.0 | 0.0 |
| Gpr37l1 | 0.0 | 0.0 | 0.0 | 0.0 | 0.0 | 0.0 | 0.0 | 0.0 | 0.0 | 0.0 | 0.0 | 0.0 |
| Gpr50 | 0.0 | 0.0 | 0.0 | 0.0 | 0.0 | 0.0 | 0.0 | 0.0 | 0.0 | 0.0 | 0.0 | 0.0 |
| Gpr87 | 0.0 | 0.0 | 0.0 | 0.0 | 0.0 | 0.0 | 0.0 | 0.0 | 0.0 | 0.0 | 0.0 | 0.0 |
| Gprc5d | 0.0 | 0.0 | 0.0 | 0.0 | 0.0 | 0.0 | 0.0 | 0.0 | 0.0 | 0.0 | 0.0 | 0.0 |
| Gpx2-ps1 | 0.0 | 0.0 | 0.0 | 0.1 | 0.1 | 0.0 | 0.0 | 0.0 | 0.0 | 0.2 | 0.0 | 0.0 |
| Gpx5 | 0.0 | 0.0 | 0.0 | 0.1 | 0.0 | 0.1 | 0.0 | 0.0 | 0.0 | 0.0 | 0.0 | 0.0 |
| Gpx6 | 0.0 | 0.0 | 0.0 | 0.0 | 0.0 | 0.0 | 0.0 | 0.0 | 0.0 | 0.0 | 0.0 | 0.0 |
| Grb7 | 0.0 | 0.0 | 0.0 | 0.0 | 0.0 | 0.0 | 0.0 | 0.0 | 0.0 | 0.0 | 0.0 | 0.0 |
| Grhl2 | 0.0 | 0.0 | 0.0 | 0.0 | 0.0 | 0.0 | 0.0 | 0.0 | 0.0 | 0.0 | 0.0 | 0.0 |
| Gria2 | 0.0 | 0.0 | 0.0 | 0.0 | 0.0 | 0.0 | 0.0 | 0.0 | 0.0 | 0.0 | 0.0 | 0.0 |
| Grid1 | 0.0 | 0.0 | 0.0 | 0.0 | 0.0 | 0.0 | 0.0 | 0.0 | 0.0 | 0.0 | 0.0 | 0.0 |
| Grid2 | 0.0 | 0.0 | 0.0 | 0.0 | 0.0 | 0.0 | 0.0 | 0.0 | 0.0 | 0.0 | 0.0 | 0.0 |
| Grin2a | 0.0 | 0.0 | 0.0 | 0.0 | 0.0 | 0.0 | 0.0 | 0.0 | 0.0 | 0.0 | 0.0 | 0.0 |
| Grm4 | 0.0 | 0.0 | 0.0 | 0.0 | 0.0 | 0.0 | 0.0 | 0.0 | 0.0 | 0.0 | 0.0 | 0.0 |
| Grm6 | 0.0 | 0.0 | 0.0 | 0.0 | 0.0 | 0.0 | 0.0 | 0.0 | 0.0 | 0.0 | 0.0 | 0.0 |
| Grm8 | 0.0 | 0.0 | 0.0 | 0.0 | 0.0 | 0.0 | 0.0 | 0.0 | 0.0 | 0.0 | 0.0 | 0.0 |
| Grpr | 0.0 | 0.0 | 0.0 | 0.0 | 0.0 | 0.0 | 0.0 | 0.0 | 0.0 | 0.0 | 0.0 | 0.0 |
| Gsc2 | 0.0 | 0.0 | 0.1 | 0.0 | 0.0 | 0.0 | 0.0 | 0.0 | 0.0 | 0.0 | 0.0 | 0.0 |
| Gsdma3 | 0.0 | 0.0 | 0.0 | 0.0 | 0.0 | 0.1 | 0.0 | 0.0 | 0.0 | 0.0 | 0.0 | 0.0 |
| Gsdmc | 0.0 | 0.0 | 0.0 | 0.0 | 0.0 | 0.0 | 0.0 | 0.0 | 0.0 | 0.0 | 0.0 | 0.0 |
| Gsdmc2 | 0.0 | 0.0 | 0.0 | 0.0 | 0.0 | 0.0 | 0.0 | 0.0 | 0.0 | 0.0 | 0.0 | 0.0 |
| Gsdmc3 | 0.0 | 0.0 | 0.0 | 0.0 | 0.0 | 0.0 | 0.0 | 0.0 | 0.0 | 0.0 | 0.0 | 0.0 |
| Gsdmcl1 | 0.0 | 0.0 | 0.0 | 0.0 | 0.0 | 0.0 | 0.0 | 0.0 | 0.0 | 0.0 | 0.0 | 0.0 |
| Gstt4 | 0.0 | 0.0 | 0.0 | 0.0 | 0.0 | 0.0 | 0.0 | 0.0 | 0.0 | 0.0 | 0.0 | 0.0 |
| Gtsf1 | 0.0 | 0.1 | 0.0 | 0.0 | 0.0 | 0.0 | 0.0 | 0.0 | 0.0 | 0.0 | 0.0 | 0.0 |
| Gtsf1l | 0.0 | 0.0 | 0.0 | 0.0 | 0.0 | 0.0 | 0.0 | 0.0 | 0.0 | 0.0 | 0.0 | 0.0 |
| Guca2a | 0.0 | 0.0 | 0.0 | 0.0 | 0.0 | 0.0 | 0.0 | 0.0 | 0.0 | 0.0 | 0.0 | 0.1 |
| Guca2b | 0.1 | 0.0 | 0.0 | 0.0 | 0.0 | 0.0 | 0.0 | 0.0 | 0.0 | 0.0 | 0.0 | 0.0 |
| Gucy1b2 | 0.0 | 0.0 | 0.0 | 0.0 | 0.0 | 0.0 | 0.0 | 0.0 | 0.0 | 0.0 | 0.0 | 0.0 |
| Gucy2d | 0.0 | 0.0 | 0.0 | 0.0 | 0.0 | 0.0 | 0.0 | 0.0 | 0.0 | 0.0 | 0.0 | 0.0 |
| Gucy2f | 0.0 | 0.0 | 0.0 | 0.0 | 0.0 | 0.0 | 0.0 | 0.0 | 0.0 | 0.0 | 0.0 | 0.0 |
| Gulo | 0.0 | 0.0 | 0.0 | 0.0 | 0.0 | 0.0 | 0.0 | 0.0 | 0.0 | 0.0 | 0.0 | 0.0 |
| Gykl1 | 0.0 | 0.0 | 0.0 | 0.0 | 0.0 | 0.0 | 0.0 | 0.0 | 0.0 | 0.0 | 0.0 | 0.0 |
| Gypa | 0.0 | 0.0 | 0.0 | 0.0 | 0.0 | 0.0 | 0.0 | 0.0 | 0.0 | 0.0 | 0.0 | 0.0 |
| Gzmb | 0.0 | 0.0 | 0.0 | 0.0 | 0.0 | 0.0 | 0.0 | 0.0 | 0.0 | 0.0 | 0.0 | 0.0 |
| Gzmd | 0.0 | 0.0 | 0.0 | 0.0 | 0.0 | 0.0 | 0.0 | 0.0 | 0.0 | 0.0 | 0.1 | 0.0 |
| Gzmn | 0.0 | 0.0 | 0.0 | 0.0 | 0.0 | 0.0 | 0.0 | 0.0 | 0.0 | 0.0 | 0.0 | 0.0 |
| H1foo | 0.0 | 0.0 | 0.0 | 0.0 | 0.0 | 0.0 | 0.0 | 0.0 | 0.0 | 0.0 | 0.0 | 0.0 |
| H2-Eb2 | 0.0 | 0.0 | 0.0 | 0.0 | 0.0 | 0.0 | 0.0 | 0.0 | 0.0 | 0.0 | 0.0 | 0.0 |
| H2-M1 | 0.0 | 0.0 | 0.0 | 0.0 | 0.0 | 0.0 | 0.0 | 0.0 | 0.0 | 0.0 | 0.0 | 0.0 |
| H2-M10.1 | 0.0 | 0.0 | 0.0 | 0.1 | 0.0 | 0.0 | 0.0 | 0.0 | 0.0 | 0.0 | 0.0 | 0.0 |

| | | | | | | | | | | | | |
|---|---|---|---|---|---|---|---|---|---|---|---|---|
| H2-M10.2 | 0.0 | 0.0 | 0.0 | 0.1 | 0.0 | 0.0 | 0.0 | 0.0 | 0.0 | 0.0 | 0.0 | 0.0 |
| H2-M10.3 | 0.0 | 0.0 | 0.0 | 0.0 | 0.0 | 0.0 | 0.0 | 0.0 | 0.0 | 0.0 | 0.0 | 0.0 |
| H2-M10.4 | 0.0 | 0.0 | 0.0 | 0.0 | 0.0 | 0.0 | 0.0 | 0.0 | 0.0 | 0.0 | 0.0 | 0.0 |
| H2-M10.5 | 0.0 | 0.0 | 0.0 | 0.0 | 0.0 | 0.0 | 0.0 | 0.0 | 0.0 | 0.0 | 0.0 | 0.0 |
| H2-M10.6 | 0.0 | 0.0 | 0.0 | 0.0 | 0.0 | 0.0 | 0.0 | 0.0 | 0.0 | 0.0 | 0.0 | 0.0 |
| H2-M11 | 0.0 | 0.0 | 0.0 | 0.0 | 0.0 | 0.0 | 0.0 | 0.0 | 0.0 | 0.0 | 0.0 | 0.0 |
| H2-M2 | 0.0 | 0.0 | 0.0 | 0.0 | 0.0 | 0.0 | 0.1 | 0.0 | 0.0 | 0.0 | 0.0 | 0.0 |
| H2-Oa | 0.0 | 0.0 | 0.0 | 0.0 | 0.0 | 0.0 | 0.0 | 0.0 | 0.0 | 0.0 | 0.0 | 0.0 |
| H2-Ob | 0.0 | 0.0 | 0.0 | 0.0 | 0.1 | 0.0 | 0.0 | 0.0 | 0.0 | 0.0 | 0.0 | 0.0 |
| Hamp | 0.0 | 0.0 | 0.0 | 0.0 | 0.1 | 0.0 | 0.0 | 0.0 | 0.0 | 0.0 | 0.0 | 0.0 |
| Hand1 | 0.0 | 0.0 | 0.0 | 0.0 | 0.0 | 0.0 | 0.0 | 0.0 | 0.0 | 0.0 | 0.0 | 0.1 |
| Hao1 | 0.0 | 0.0 | 0.0 | 0.1 | 0.1 | 0.0 | 0.1 | 0.0 | 0.0 | 0.1 | 0.0 | 0.0 |
| Hapln2 | 0.0 | 0.0 | 0.0 | 0.0 | 0.0 | 0.0 | 0.0 | 0.0 | 0.0 | 0.0 | 0.0 | 0.0 |
| Havcr1 | 0.0 | 0.0 | 0.0 | 0.0 | 0.0 | 0.0 | 0.0 | 0.0 | 0.0 | 0.0 | 0.0 | 0.0 |
| Hba-a1 | 0.0 | 0.0 | 0.0 | 0.0 | 0.0 | 0.0 | 0.0 | 0.0 | 0.0 | 0.0 | 0.0 | 0.0 |
| Hba-a2 | 0.0 | 0.0 | 0.0 | 0.0 | 0.0 | 0.0 | 0.0 | 0.0 | 0.0 | 0.0 | 0.0 | 0.0 |
| Hcn4 | 0.0 | 0.0 | 0.0 | 0.0 | 0.0 | 0.0 | 0.0 | 0.0 | 0.0 | 0.1 | 0.0 | 0.0 |
| Hcrt | 0.0 | 0.0 | 0.0 | 0.0 | 0.0 | 0.0 | 0.0 | 0.0 | 0.0 | 0.0 | 0.0 | 0.0 |
| Hcrtr2 | 0.0 | 0.0 | 0.0 | 0.0 | 0.0 | 0.0 | 0.0 | 0.0 | 0.0 | 0.0 | 0.0 | 0.0 |
| Hdhd1a | 0.0 | 0.0 | 0.0 | 0.1 | 0.0 | 0.0 | 0.0 | 0.0 | 0.0 | 0.0 | 0.0 | 0.0 |
| Hemgn | 0.0 | 0.0 | 0.0 | 0.0 | 0.0 | 0.0 | 0.0 | 0.0 | 0.0 | 0.0 | 0.0 | 0.0 |
| Hemt1 | 0.0 | 0.0 | 0.0 | 0.0 | 0.1 | 0.1 | 0.0 | 0.0 | 0.0 | 0.0 | 0.0 | 0.0 |
| Hepacam2 | 0.0 | 0.0 | 0.0 | 0.0 | 0.0 | 0.0 | 0.0 | 0.0 | 0.0 | 0.0 | 0.0 | 0.0 |
| Hes2 | 0.0 | 0.0 | 0.0 | 0.0 | 0.0 | 0.0 | 0.0 | 0.0 | 0.0 | 0.0 | 0.0 | 0.0 |
| Hes5 | 0.0 | 0.0 | 0.0 | 0.0 | 0.0 | 0.0 | 0.0 | 0.0 | 0.0 | 0.0 | 0.0 | 0.0 |
| Hesx1 | 0.0 | 0.0 | 0.0 | 0.0 | 0.1 | 0.0 | 0.1 | 0.0 | 0.0 | 0.0 | 0.0 | 0.0 |
| Hfe2 | 0.0 | 0.0 | 0.0 | 0.1 | 0.0 | 0.0 | 0.0 | 0.0 | 0.0 | 0.0 | 0.0 | 0.0 |
| Hhip | 0.0 | 0.0 | 0.0 | 0.0 | 0.0 | 0.0 | 0.0 | 0.0 | 0.0 | 0.0 | 0.0 | 0.0 |
| Higd1b | 0.0 | 0.0 | 0.0 | 0.0 | 0.0 | 0.0 | 0.0 | 0.1 | 0.0 | 0.0 | 0.0 | 0.1 |
| Hist1h2aa | 0.0 | 0.0 | 0.0 | 0.0 | 0.1 | 0.0 | 0.0 | 0.1 | 0.0 | 0.0 | 0.0 | 0.0 |
| Hist3h2ba | 0.1 | 0.0 | 0.0 | 0.0 | 0.0 | 0.0 | 0.0 | 0.0 | 0.0 | 0.0 | 0.0 | 0.0 |
| Hkdc1 | 0.0 | 0.0 | 0.0 | 0.0 | 0.0 | 0.0 | 0.0 | 0.0 | 0.0 | 0.0 | 0.0 | 0.0 |
| Hmx1 | 0.0 | 0.0 | 0.0 | 0.0 | 0.0 | 0.0 | 0.0 | 0.0 | 0.0 | 0.0 | 0.0 | 0.0 |
| Hmx2 | 0.0 | 0.0 | 0.0 | 0.0 | 0.0 | 0.0 | 0.0 | 0.0 | 0.0 | 0.0 | 0.0 | 0.0 |
| Hnf1b | 0.0 | 0.0 | 0.0 | 0.0 | 0.0 | 0.0 | 0.0 | 0.0 | 0.0 | 0.0 | 0.0 | 0.0 |
| Hormad1 | 0.0 | 0.0 | 0.0 | 0.0 | 0.0 | 0.0 | 0.0 | 0.0 | 0.0 | 0.0 | 0.0 | 0.0 |
| Hoxa11 | 0.0 | 0.0 | 0.0 | 0.0 | 0.0 | 0.0 | 0.0 | 0.0 | 0.0 | 0.0 | 0.0 | 0.0 |
| Hoxa11as | 0.0 | 0.0 | 0.0 | 0.0 | 0.0 | 0.0 | 0.0 | 0.0 | 0.0 | 0.0 | 0.0 | 0.0 |
| Hoxa13 | 0.1 | 0.0 | 0.0 | 0.0 | 0.0 | 0.0 | 0.0 | 0.0 | 0.0 | 0.0 | 0.0 | 0.0 |
| Hoxa9 | 0.0 | 0.0 | 0.0 | 0.0 | 0.0 | 0.0 | 0.0 | 0.0 | 0.0 | 0.0 | 0.0 | 0.0 |
| Hoxc11 | 0.0 | 0.0 | 0.0 | 0.0 | 0.0 | 0.0 | 0.0 | 0.0 | 0.0 | 0.0 | 0.0 | 0.1 |
| Hoxc12 | 0.1 | 0.0 | 0.0 | 0.0 | 0.0 | 0.0 | 0.0 | 0.0 | 0.0 | 0.0 | 0.0 | 0.0 |
| Hoxd1 | 0.0 | 0.0 | 0.0 | 0.0 | 0.0 | 0.0 | 0.0 | 0.0 | 0.0 | 0.0 | 0.0 | 0.0 |
| Hoxd13 | 0.0 | 0.0 | 0.0 | 0.0 | 0.0 | 0.0 | 0.0 | 0.0 | 0.0 | 0.0 | 0.0 | 0.0 |
| Hpca | 0.0 | 0.0 | 0.0 | 0.0 | 0.0 | 0.0 | 0.0 | 0.0 | 0.0 | 0.0 | 0.0 | 0.0 |
| Hpse2 | 0.0 | 0.0 | 0.0 | 0.0 | 0.0 | 0.0 | 0.0 | 0.0 | 0.0 | 0.0 | 0.0 | 0.0 |
| Hrasls5 | 0.1 | 0.0 | 0.0 | 0.0 | 0.0 | 0.0 | 0.0 | 0.0 | 0.0 | 0.0 | 0.0 | 0.0 |
| Hrh3 | 0.0 | 0.0 | 0.0 | 0.0 | 0.0 | 0.0 | 0.0 | 0.0 | 0.0 | 0.0 | 0.0 | 0.0 |
| Hs3st2 | 0.0 | 0.0 | 0.0 | 0.0 | 0.0 | 0.0 | 0.0 | 0.0 | 0.0 | 0.0 | 0.0 | 0.0 |
| Hs3st5 | 0.0 | 0.0 | 0.0 | 0.0 | 0.0 | 0.0 | 0.0 | 0.0 | 0.0 | 0.0 | 0.0 | 0.0 |
| Hsbp1l1 | 0.1 | 0.0 | 0.0 | 0.0 | 0.0 | 0.0 | 0.0 | 0.0 | 0.1 | 0.0 | 0.0 | 0.0 |
| Hsd17b14 | 0.0 | 0.0 | 0.1 | 0.0 | 0.0 | 0.0 | 0.0 | 0.0 | 0.0 | 0.0 | 0.1 | 0.0 |
| Hsd17b2 | 0.0 | 0.0 | 0.0 | 0.0 | 0.0 | 0.0 | 0.0 | 0.0 | 0.0 | 0.0 | 0.0 | 0.0 |
| Hsd17b3 | 0.0 | 0.0 | 0.0 | 0.0 | 0.0 | 0.0 | 0.0 | 0.0 | 0.0 | 0.0 | 0.0 | 0.0 |
| Hsd3b5 | 0.0 | 0.0 | 0.0 | 0.0 | 0.0 | 0.0 | 0.0 | 0.1 | 0.0 | 0.0 | 0.0 | 0.0 |
| Hsd3b6 | 0.0 | 0.0 | 0.0 | 0.0 | 0.0 | 0.0 | 0.0 | 0.0 | 0.0 | 0.0 | 0.0 | 0.0 |
| Hsh2d | 0.0 | 0.0 | 0.0 | 0.0 | 0.0 | 0.0 | 0.0 | 0.0 | 0.0 | 0.0 | 0.0 | 0.0 |

| | | | | | | | | | | | | |
|---|---|---|---|---|---|---|---|---|---|---|---|---|
| Hspb3 | 0.0 | 0.1 | 0.0 | 0.0 | 0.0 | 0.2 | 0.0 | 0.0 | 0.0 | 0.0 | 0.0 | 0.0 |
| Htr1b | 0.1 | 0.0 | 0.0 | 0.0 | 0.0 | 0.0 | 0.0 | 0.0 | 0.0 | 0.0 | 0.0 | 0.0 |
| Htr1f | 0.0 | 0.0 | 0.0 | 0.0 | 0.0 | 0.0 | 0.0 | 0.0 | 0.0 | 0.0 | 0.0 | 0.0 |
| Htr2c | 0.0 | 0.0 | 0.0 | 0.0 | 0.0 | 0.0 | 0.0 | 0.0 | 0.0 | 0.0 | 0.0 | 0.0 |
| Htr3a | 0.0 | 0.0 | 0.0 | 0.0 | 0.0 | 0.0 | 0.0 | 0.0 | 0.0 | 0.0 | 0.0 | 0.0 |
| Htr3b | 0.0 | 0.0 | 0.0 | 0.0 | 0.0 | 0.0 | 0.0 | 0.0 | 0.0 | 0.0 | 0.0 | 0.0 |
| Htr5a | 0.0 | 0.0 | 0.0 | 0.0 | 0.0 | 0.0 | 0.0 | 0.0 | 0.0 | 0.0 | 0.0 | 0.0 |
| Htr6 | 0.0 | 0.0 | 0.0 | 0.0 | 0.0 | 0.0 | 0.0 | 0.0 | 0.0 | 0.0 | 0.0 | 0.0 |
| Hyal5 | 0.0 | 0.0 | 0.0 | 0.0 | 0.0 | 0.1 | 0.0 | 0.0 | 0.0 | 0.0 | 0.0 | 0.0 |
| Hyal6 | 0.0 | 0.0 | 0.0 | 0.0 | 0.0 | 0.0 | 0.0 | 0.0 | 0.0 | 0.0 | 0.0 | 0.0 |
| Iapp | 0.0 | 0.0 | 0.0 | 0.0 | 0.1 | 0.0 | 0.0 | 0.0 | 0.0 | 0.0 | 0.0 | 0.0 |
| Ibsp | 0.0 | 0.0 | 0.0 | 0.1 | 0.0 | 0.0 | 0.0 | 0.0 | 0.0 | 0.0 | 0.1 | 0.0 |
| Ifi202b | 0.0 | 0.0 | 0.0 | 0.0 | 0.0 | 0.0 | 0.0 | 0.0 | 0.0 | 0.0 | 0.0 | 0.0 |
| Ifi44l | 0.0 | 0.0 | 0.0 | 0.0 | 0.0 | 0.0 | 0.0 | 0.0 | 0.0 | 0.0 | 0.0 | 0.0 |
| Ifitm6 | 0.2 | 0.0 | 0.1 | 1.0 | 1.0 | 0.0 | 0.0 | 0.0 | 0.0 | 0.2 | 0.0 | 0.0 |
| Ifltd1 | 0.0 | 0.0 | 0.0 | 0.0 | 0.0 | 0.0 | 0.0 | 0.0 | 0.0 | 0.0 | 0.0 | 0.0 |
| Ifna13 | 0.2 | 0.0 | 0.0 | 0.0 | 0.0 | 0.1 | 0.0 | 0.0 | 0.0 | 0.0 | 0.0 | 0.0 |
| Ifna6 | 0.0 | 0.0 | 0.0 | 0.0 | 0.0 | 0.0 | 0.0 | 0.0 | 0.0 | 0.0 | 0.0 | 0.0 |
| Ifnab | 0.0 | 0.0 | 0.0 | 0.0 | 0.0 | 0.0 | 0.0 | 0.0 | 0.0 | 0.0 | 0.0 | 0.3 |
| Ifnb1 | 0.0 | 0.0 | 0.0 | 0.0 | 0.0 | 0.5 | 0.0 | 0.0 | 0.0 | 0.1 | 0.0 | 0.1 |
| Ifne | 0.0 | 0.0 | 0.0 | 0.0 | 0.0 | 0.0 | 0.0 | 0.0 | 0.0 | 0.0 | 0.0 | 0.0 |
| Ifng | 0.0 | 0.0 | 0.0 | 0.0 | 0.0 | 0.0 | 0.0 | 0.0 | 0.0 | 0.0 | 0.0 | 0.0 |
| Ifnk | 0.0 | 0.0 | 0.0 | 0.0 | 0.0 | 0.0 | 0.0 | 0.0 | 0.0 | 0.0 | 0.0 | 0.0 |
| Igf2as | 0.0 | 0.0 | 0.0 | 0.0 | 0.0 | 0.0 | 0.0 | 0.0 | 0.0 | 0.0 | 0.0 | 0.0 |
| Igfbpl1 | 0.0 | 0.0 | 0.0 | 0.1 | 0.0 | 0.0 | 0.0 | 0.0 | 0.0 | 0.0 | 0.0 | 0.0 |
| Igj | 0.0 | 0.0 | 0.0 | 0.0 | 0.0 | 0.0 | 0.0 | 0.0 | 0.0 | 0.0 | 0.0 | 0.0 |
| Igll1 | 0.0 | 0.0 | 0.0 | 0.0 | 0.0 | 0.0 | 0.0 | 0.0 | 0.0 | 0.0 | 0.2 | 0.0 |
| Igsf1 | 0.0 | 0.0 | 0.0 | 0.0 | 0.0 | 0.1 | 0.0 | 0.0 | 0.0 | 0.0 | 0.0 | 0.0 |
| Igsf5 | 0.0 | 0.0 | 0.0 | 0.0 | 0.0 | 0.0 | 0.0 | 0.0 | 0.0 | 0.0 | 0.0 | 0.0 |
| Ihh | 0.0 | 0.0 | 0.0 | 0.0 | 0.0 | 0.0 | 0.0 | 0.0 | 0.0 | 0.0 | 0.0 | 0.0 |
| Ikzf3 | 0.0 | 0.0 | 0.0 | 0.0 | 0.0 | 0.0 | 0.0 | 0.0 | 0.0 | 0.0 | 0.0 | 0.0 |
| Il12b | 0.0 | 0.0 | 0.0 | 0.0 | 0.0 | 0.0 | 0.0 | 0.0 | 0.0 | 0.0 | 0.0 | 0.0 |
| Il17a | 0.0 | 0.0 | 0.0 | 0.0 | 0.1 | 0.0 | 0.0 | 0.0 | 0.0 | 0.0 | 0.0 | 0.0 |
| Il19 | 0.1 | 0.2 | 0.0 | 0.0 | 0.0 | 0.1 | 0.0 | 0.0 | 0.0 | 0.0 | 0.0 | 0.0 |
| Il1f5 | 0.0 | 0.0 | 0.0 | 0.0 | 0.0 | 0.0 | 0.0 | 0.0 | 0.0 | 0.0 | 0.0 | 0.0 |
| Il1f8 | 0.0 | 0.0 | 0.0 | 0.0 | 0.0 | 0.1 | 0.0 | 0.0 | 0.0 | 0.0 | 0.0 | 0.0 |
| Il21 | 0.0 | 0.0 | 0.0 | 0.0 | 0.0 | 0.0 | 0.0 | 0.0 | 0.0 | 0.0 | 0.0 | 0.0 |
| Il24 | 0.0 | 0.0 | 0.0 | 0.2 | 0.0 | 0.0 | 0.0 | 0.0 | 0.0 | 0.0 | 0.0 | 0.0 |
| Il25 | 0.0 | 0.0 | 0.0 | 0.0 | 0.0 | 0.0 | 0.0 | 0.0 | 0.0 | 0.0 | 0.1 | 0.0 |
| Il27 | 0.0 | 0.0 | 0.0 | 0.0 | 0.1 | 0.0 | 0.0 | 0.1 | 0.0 | 0.0 | 0.0 | 0.0 |
| Il31 | 0.0 | 0.0 | 0.0 | 0.0 | 0.0 | 0.0 | 0.0 | 0.0 | 0.0 | 0.0 | 0.0 | 0.0 |
| Il4 | 0.0 | 0.0 | 0.1 | 0.0 | 0.0 | 0.2 | 0.0 | 0.2 | 0.0 | 0.0 | 0.0 | 0.0 |
| Il5 | 0.0 | 0.1 | 0.0 | 0.0 | 0.0 | 0.0 | 0.0 | 0.0 | 0.0 | 0.1 | 0.1 | 0.0 |
| Il9r | 0.0 | 0.0 | 0.0 | 0.0 | 0.0 | 0.0 | 0.0 | 0.0 | 0.0 | 0.0 | 0.0 | 0.0 |
| Ildr1 | 0.0 | 0.0 | 0.0 | 0.0 | 0.0 | 0.0 | 0.0 | 0.0 | 0.0 | 0.0 | 0.0 | 0.0 |
| Iltifb | 0.0 | 0.0 | 0.0 | 0.0 | 0.0 | 0.0 | 0.0 | 0.0 | 0.0 | 0.0 | 0.0 | 0.0 |
| Inhbc | 0.1 | 0.0 | 0.0 | 0.0 | 0.0 | 0.0 | 0.0 | 0.0 | 0.0 | 0.0 | 0.0 | 0.0 |
| Inhbe | 0.0 | 0.0 | 0.0 | 0.0 | 0.0 | 0.0 | 0.0 | 0.0 | 0.0 | 0.0 | 0.0 | 0.0 |
| Insl5 | 0.0 | 0.1 | 0.0 | 0.0 | 0.0 | 0.0 | 0.1 | 0.0 | 0.0 | 0.0 | 0.0 | 0.0 |
| Ipcef1 | 0.0 | 0.0 | 0.0 | 0.0 | 0.0 | 0.0 | 0.0 | 0.0 | 0.0 | 0.0 | 0.0 | 0.0 |
| Iqca | 0.0 | 0.0 | 0.0 | 0.0 | 0.0 | 0.0 | 0.0 | 0.0 | 0.0 | 0.0 | 0.0 | 0.0 |
| Iqcf5 | 0.0 | 0.0 | 0.0 | 0.0 | 0.0 | 0.3 | 0.0 | 0.0 | 0.0 | 0.0 | 0.0 | 0.0 |
| Iqcj | 0.0 | 0.0 | 0.0 | 0.0 | 0.0 | 0.0 | 0.0 | 0.0 | 0.0 | 0.0 | 0.0 | 0.0 |
| Iqsec3 | 0.0 | 0.0 | 0.0 | 0.0 | 0.0 | 0.0 | 0.0 | 0.0 | 0.0 | 0.0 | 0.0 | 0.0 |
| Irgc1 | 0.0 | 0.0 | 0.0 | 0.0 | 0.0 | 0.0 | 0.0 | 0.0 | 0.0 | 0.0 | 0.0 | 0.0 |
| Irx4 | 0.0 | 0.0 | 0.0 | 0.0 | 0.0 | 0.0 | 0.0 | 0.0 | 0.0 | 0.0 | 0.0 | 0.0 |
| Irx6 | 0.0 | 0.0 | 0.0 | 0.0 | 0.0 | 0.0 | 0.0 | 0.0 | 0.0 | 0.0 | 0.0 | 0.0 |

| | | | | | | | | | | | |
|---|---|---|---|---|---|---|---|---|---|---|---|
| Itk | 0.0 | 0.0 | 0.0 | 0.0 | 0.0 | 0.0 | 0.0 | 0.0 | 0.0 | 0.0 | 0.0 | 0.0 |
| Itln1 | 0.0 | 0.0 | 0.1 | 0.0 | 0.0 | 0.0 | 0.0 | 0.0 | 0.0 | 0.0 | 0.0 | 0.0 |
| Izumo1 | 0.0 | 0.1 | 0.0 | 0.1 | 0.0 | 0.1 | 0.0 | 0.0 | 0.0 | 0.0 | 0.0 | 0.0 |
| Kbtbd13 | 0.0 | 0.0 | 0.0 | 0.0 | 0.0 | 0.0 | 0.0 | 0.0 | 0.0 | 0.0 | 0.0 | 0.0 |
| Kcna7 | 0.0 | 0.0 | 0.0 | 0.1 | 0.0 | 0.0 | 0.0 | 0.0 | 0.0 | 0.1 | 0.0 | 0.0 |
| Kcnb2 | 0.0 | 0.0 | 0.0 | 0.0 | 0.0 | 0.0 | 0.0 | 0.0 | 0.0 | 0.0 | 0.0 | 0.0 |
| Kcnc1 | 0.0 | 0.0 | 0.0 | 0.0 | 0.0 | 0.0 | 0.0 | 0.0 | 0.0 | 0.0 | 0.0 | 0.0 |
| Kcnc4 | 0.0 | 0.0 | 0.0 | 0.0 | 0.0 | 0.0 | 0.0 | 0.0 | 0.0 | 0.0 | 0.0 | 0.0 |
| Kcng1 | 0.0 | 0.0 | 0.0 | 0.0 | 0.0 | 0.0 | 0.0 | 0.0 | 0.0 | 0.0 | 0.0 | 0.0 |
| Kcng3 | 0.0 | 0.0 | 0.0 | 0.0 | 0.0 | 0.0 | 0.0 | 0.0 | 0.0 | 0.0 | 0.0 | 0.0 |
| Kcnh6 | 0.0 | 0.0 | 0.0 | 0.0 | 0.0 | 0.0 | 0.0 | 0.0 | 0.0 | 0.0 | 0.0 | 0.0 |
| Kcnh7 | 0.0 | 0.0 | 0.0 | 0.0 | 0.0 | 0.0 | 0.0 | 0.0 | 0.0 | 0.0 | 0.0 | 0.0 |
| Kcnh8 | 0.0 | 0.0 | 0.0 | 0.0 | 0.0 | 0.0 | 0.0 | 0.0 | 0.0 | 0.0 | 0.0 | 0.0 |
| Kcnj1 | 0.0 | 0.0 | 0.0 | 0.1 | 0.0 | 0.0 | 0.0 | 0.0 | 0.0 | 0.0 | 0.0 | 0.0 |
| Kcnj16 | 0.0 | 0.0 | 0.0 | 0.0 | 0.0 | 0.0 | 0.0 | 0.0 | 0.0 | 0.0 | 0.0 | 0.0 |
| Kcnj3 | 0.0 | 0.0 | 0.0 | 0.0 | 0.0 | 0.0 | 0.0 | 0.0 | 0.0 | 0.0 | 0.0 | 0.0 |
| Kcnk10 | 0.0 | 0.0 | 0.0 | 0.1 | 0.0 | 0.0 | 0.0 | 0.0 | 0.0 | 0.0 | 0.0 | 0.0 |
| Kcnk16 | 0.0 | 0.0 | 0.0 | 0.0 | 0.0 | 0.0 | 0.0 | 0.0 | 0.0 | 0.0 | 0.0 | 0.0 |
| Kcnk18 | 0.0 | 0.0 | 0.0 | 0.0 | 0.0 | 0.0 | 0.0 | 0.0 | 0.0 | 0.0 | 0.0 | 0.0 |
| Kcnk3 | 0.0 | 0.0 | 0.0 | 0.0 | 0.0 | 0.0 | 0.0 | 0.0 | 0.0 | 0.0 | 0.0 | 0.0 |
| Kcnk4 | 0.0 | 0.0 | 0.1 | 0.1 | 0.0 | 0.0 | 0.0 | 0.0 | 0.0 | 0.0 | 0.0 | 0.0 |
| Kcnk9 | 0.0 | 0.0 | 0.0 | 0.0 | 0.0 | 0.0 | 0.0 | 0.0 | 0.0 | 0.0 | 0.0 | 0.0 |
| Kcnmb2 | 0.0 | 0.0 | 0.0 | 0.0 | 0.0 | 0.0 | 0.0 | 0.0 | 0.0 | 0.0 | 0.0 | 0.0 |
| Kcnq2 | 0.0 | 0.0 | 0.0 | 0.0 | 0.0 | 0.0 | 0.0 | 0.0 | 0.0 | 0.0 | 0.0 | 0.0 |
| Kcns2 | 0.0 | 0.0 | 0.0 | 0.0 | 0.0 | 0.0 | 0.0 | 0.0 | 0.0 | 0.0 | 0.0 | 0.0 |
| Kcnv1 | 0.0 | 0.0 | 0.0 | 0.0 | 0.0 | 0.0 | 0.0 | 0.0 | 0.0 | 0.0 | 0.0 | 0.0 |
| Kctd19 | 0.0 | 0.0 | 0.0 | 0.0 | 0.0 | 0.0 | 0.0 | 0.0 | 0.0 | 0.0 | 0.0 | 0.0 |
| Keg1 | 0.1 | 0.0 | 0.0 | 0.0 | 0.0 | 0.0 | 0.0 | 0.0 | 0.0 | 0.0 | 0.0 | 0.0 |
| Khdrbs2 | 0.0 | 0.0 | 0.0 | 0.0 | 0.0 | 0.0 | 0.0 | 0.0 | 0.0 | 0.0 | 0.0 | 0.0 |
| Kif12 | 0.0 | 0.0 | 0.0 | 0.0 | 0.0 | 0.0 | 0.0 | 0.0 | 0.0 | 0.0 | 0.0 | 0.0 |
| Kirrel2 | 0.0 | 0.0 | 0.0 | 0.0 | 0.0 | 0.0 | 0.0 | 0.0 | 0.0 | 0.0 | 0.0 | 0.0 |
| Kl | 0.0 | 0.0 | 0.0 | 0.0 | 0.0 | 0.0 | 0.0 | 0.0 | 0.0 | 0.0 | 0.0 | 0.0 |
| Klhl1 | 0.0 | 0.0 | 0.0 | 0.0 | 0.0 | 0.0 | 0.0 | 0.0 | 0.0 | 0.0 | 0.0 | 0.0 |
| Klhl14 | 0.0 | 0.0 | 0.0 | 0.0 | 0.0 | 0.0 | 0.0 | 0.0 | 0.0 | 0.0 | 0.0 | 0.0 |
| Klhl40 | 0.0 | 0.0 | 0.0 | 0.0 | 0.0 | 0.0 | 0.0 | 0.0 | 0.0 | 0.0 | 0.0 | 0.0 |
| Klk14 | 0.0 | 0.0 | 0.0 | 0.0 | 0.0 | 0.0 | 0.0 | 0.0 | 0.0 | 0.0 | 0.0 | 0.0 |
| Klk1b27 | 0.0 | 0.0 | 0.0 | 0.0 | 0.0 | 0.1 | 0.0 | 0.0 | 0.0 | 0.0 | 0.0 | 0.0 |
| Klk6 | 0.0 | 0.0 | 0.0 | 0.0 | 0.0 | 0.0 | 0.0 | 0.0 | 0.0 | 0.0 | 0.0 | 0.0 |
| Klk7 | 0.0 | 0.0 | 0.0 | 0.0 | 0.0 | 0.0 | 0.0 | 0.0 | 0.0 | 0.0 | 0.0 | 0.0 |
| Klk9 | 0.0 | 0.0 | 0.0 | 0.0 | 0.0 | 0.0 | 0.0 | 0.0 | 0.0 | 0.0 | 0.0 | 0.0 |
| Klra1 | 0.0 | 0.0 | 0.0 | 0.0 | 0.0 | 0.0 | 0.0 | 0.0 | 0.0 | 0.0 | 0.0 | 0.0 |
| Klra12 | 0.0 | 0.0 | 0.0 | 0.0 | 0.0 | 0.0 | 0.0 | 0.0 | 0.0 | 0.0 | 0.0 | 0.0 |
| Klra15 | 0.0 | 0.0 | 0.0 | 0.0 | 0.0 | 0.0 | 0.0 | 0.0 | 0.0 | 0.0 | 0.0 | 0.0 |
| Klra17 | 0.0 | 0.0 | 0.0 | 0.0 | 0.0 | 0.0 | 0.0 | 0.0 | 0.0 | 0.0 | 0.0 | 0.0 |
| Klra3 | 0.0 | 0.0 | 0.0 | 0.0 | 0.0 | 0.0 | 0.0 | 0.0 | 0.0 | 0.0 | 0.0 | 0.0 |
| Klra7 | 0.0 | 0.0 | 0.0 | 0.0 | 0.0 | 0.0 | 0.0 | 0.0 | 0.0 | 0.0 | 0.0 | 0.0 |
| Klrb1a | 0.1 | 0.0 | 0.0 | 0.0 | 0.0 | 0.0 | 0.0 | 0.0 | 0.0 | 0.0 | 0.0 | 0.0 |
| Klrb1-ps1 | 0.0 | 0.0 | 0.1 | 0.1 | 0.1 | 0.0 | 0.0 | 0.1 | 0.0 | 0.0 | 0.1 | 0.0 |
| Klre1 | 0.0 | 0.0 | 0.0 | 0.1 | 0.0 | 0.0 | 0.0 | 0.0 | 0.1 | 0.0 | 0.0 | 0.1 |
| Klri2 | 0.0 | 0.0 | 0.0 | 0.0 | 0.0 | 0.0 | 0.0 | 0.0 | 0.0 | 0.0 | 0.0 | 0.0 |
| Klrk1 | 0.0 | 0.0 | 0.0 | 0.0 | 0.0 | 0.0 | 0.0 | 0.0 | 0.0 | 0.0 | 0.0 | 0.0 |
| Kncn | 0.0 | 0.0 | 0.0 | 0.0 | 0.0 | 0.0 | 0.0 | 0.0 | 0.0 | 0.0 | 0.0 | 0.0 |
| Kprp | 0.0 | 0.0 | 0.0 | 0.1 | 0.0 | 0.0 | 0.0 | 0.0 | 0.0 | 0.0 | 0.0 | 0.0 |
| Kremen2 | 0.0 | 0.0 | 0.0 | 0.0 | 0.0 | 0.0 | 0.0 | 0.0 | 0.0 | 0.0 | 0.0 | 0.0 |
| Krt1 | 0.0 | 0.0 | 0.0 | 0.0 | 0.0 | 0.0 | 0.0 | 0.0 | 0.0 | 0.0 | 0.0 | 0.0 |
| Krt12 | 0.0 | 0.0 | 0.0 | 0.0 | 0.0 | 0.0 | 0.0 | 0.0 | 0.0 | 0.0 | 0.0 | 0.0 |
| Krt13 | 0.1 | 0.0 | 0.1 | 0.1 | 0.1 | 0.0 | 0.0 | 0.0 | 0.0 | 0.0 | 0.0 | 0.0 |

| | | | | | | | | | | | | |
|---|---|---|---|---|---|---|---|---|---|---|---|---|
| Krt15 | 0.0 | 0.0 | 0.0 | 0.0 | 0.0 | 0.0 | 0.0 | 0.0 | 0.0 | 0.0 | 0.0 | 0.0 |
| Krt16 | 0.0 | 0.0 | 0.0 | 0.0 | 0.0 | 0.0 | 0.0 | 0.0 | 0.0 | 0.0 | 0.0 | 0.0 |
| Krt25 | 0.0 | 0.0 | 0.0 | 0.0 | 0.0 | 0.0 | 0.0 | 0.0 | 0.0 | 0.0 | 0.0 | 0.0 |
| Krt28 | 0.0 | 0.0 | 0.0 | 0.0 | 0.0 | 0.0 | 0.0 | 0.0 | 0.0 | 0.0 | 0.0 | 0.0 |
| Krt31 | 0.0 | 0.0 | 0.0 | 0.0 | 0.0 | 0.0 | 0.0 | 0.0 | 0.0 | 0.0 | 0.0 | 0.0 |
| Krt32 | 0.0 | 0.0 | 0.0 | 0.1 | 0.0 | 0.0 | 0.0 | 0.0 | 0.0 | 0.0 | 0.0 | 0.0 |
| Krt33a | 0.1 | 0.0 | 0.0 | 0.0 | 0.0 | 0.0 | 0.0 | 0.0 | 0.0 | 0.0 | 0.0 | 0.0 |
| Krt36 | 0.0 | 0.0 | 0.0 | 0.0 | 0.0 | 0.0 | 0.0 | 0.0 | 0.0 | 0.0 | 0.0 | 0.0 |
| Krt4 | 0.0 | 0.0 | 0.0 | 0.0 | 0.0 | 0.0 | 0.0 | 0.0 | 0.0 | 0.0 | 0.0 | 0.0 |
| Krt6a | 0.0 | 0.0 | 0.0 | 0.0 | 0.0 | 0.0 | 0.0 | 0.0 | 0.0 | 0.0 | 0.0 | 0.0 |
| Krt6b | 0.0 | 0.0 | 0.0 | 0.0 | 0.0 | 0.0 | 0.0 | 0.0 | 0.0 | 0.0 | 0.0 | 0.0 |
| Krt71 | 0.0 | 0.0 | 0.0 | 0.0 | 0.0 | 0.0 | 0.0 | 0.0 | 0.0 | 0.0 | 0.0 | 0.0 |
| Krt72 | 0.0 | 0.0 | 0.0 | 0.0 | 0.0 | 0.0 | 0.0 | 0.0 | 0.0 | 0.0 | 0.0 | 0.0 |
| Krt73 | 0.0 | 0.0 | 0.0 | 0.0 | 0.0 | 0.0 | 0.0 | 0.0 | 0.0 | 0.0 | 0.0 | 0.0 |
| Krt74 | 0.0 | 0.0 | 0.0 | 0.0 | 0.0 | 0.0 | 0.0 | 0.0 | 0.0 | 0.0 | 0.0 | 0.0 |
| Krt75 | 0.0 | 0.0 | 0.0 | 0.0 | 0.0 | 0.0 | 0.0 | 0.0 | 0.0 | 0.0 | 0.0 | 0.0 |
| Krt76 | 0.0 | 0.0 | 0.0 | 0.0 | 0.0 | 0.0 | 0.0 | 0.0 | 0.0 | 0.0 | 0.0 | 0.0 |
| Krt79 | 0.0 | 0.0 | 0.0 | 0.0 | 0.0 | 0.0 | 0.0 | 0.0 | 0.0 | 0.0 | 0.0 | 0.0 |
| Krt81 | 0.0 | 0.0 | 0.0 | 0.0 | 0.0 | 0.0 | 0.0 | 0.0 | 0.0 | 0.0 | 0.0 | 0.0 |
| Krt83 | 0.0 | 0.0 | 0.0 | 0.0 | 0.0 | 0.0 | 0.0 | 0.0 | 0.0 | 0.0 | 0.0 | 0.0 |
| Krt85 | 0.0 | 0.0 | 0.0 | 0.0 | 0.0 | 0.0 | 0.0 | 0.0 | 0.0 | 0.0 | 0.0 | 0.0 |
| Krt86 | 0.0 | 0.0 | 0.0 | 0.0 | 0.0 | 0.0 | 0.0 | 0.0 | 0.0 | 0.0 | 0.0 | 0.0 |
| Krt9 | 0.0 | 0.0 | 0.0 | 0.0 | 0.0 | 0.0 | 0.0 | 0.0 | 0.0 | 0.0 | 0.0 | 0.0 |
| Krtap11-1 | 0.0 | 0.0 | 0.0 | 0.0 | 0.0 | 0.0 | 0.0 | 0.0 | 0.0 | 0.0 | 0.0 | 0.0 |
| Krtap1-5 | 0.0 | 0.0 | 0.0 | 0.1 | 0.0 | 0.0 | 0.0 | 0.0 | 0.0 | 0.0 | 0.0 | 0.0 |
| Krtap17-1 | 0.0 | 0.1 | 0.0 | 0.0 | 0.0 | 0.0 | 0.0 | 0.0 | 0.0 | 0.0 | 0.0 | 0.0 |
| Krtap2-4 | 0.0 | 0.0 | 0.0 | 0.0 | 0.0 | 0.0 | 0.0 | 0.0 | 0.0 | 0.0 | 0.0 | 0.0 |
| Krtap9-5 | 0.0 | 0.0 | 0.0 | 0.0 | 0.0 | 0.0 | 0.0 | 0.0 | 0.0 | 0.0 | 0.0 | 0.0 |
| Ky | 0.0 | 0.0 | 0.0 | 0.0 | 0.0 | 0.0 | 0.0 | 0.0 | 0.0 | 0.0 | 0.0 | 0.0 |
| L1td1 | 0.0 | 0.0 | 0.0 | 0.0 | 0.0 | 0.0 | 0.0 | 0.0 | 0.0 | 0.0 | 0.0 | 0.0 |
| L3mbtl4 | 0.0 | 0.0 | 0.0 | 0.0 | 0.0 | 0.0 | 0.0 | 0.0 | 0.0 | 0.0 | 0.0 | 0.0 |
| L3mbtl4 | 0.0 | 0.0 | 0.0 | 0.0 | 0.0 | 0.0 | 0.0 | 0.0 | 0.0 | 0.0 | 0.0 | 0.0 |
| Lad1 | 0.0 | 0.0 | 0.0 | 0.0 | 0.0 | 0.0 | 0.1 | 0.0 | 0.0 | 0.0 | 0.0 | 0.0 |
| Lao1 | 0.0 | 0.0 | 0.0 | 0.0 | 0.0 | 0.0 | 0.0 | 0.0 | 0.0 | 0.0 | 0.0 | 0.0 |
| Lce1a1 | 0.0 | 0.0 | 0.0 | 0.0 | 0.1 | 0.0 | 0.0 | 0.0 | 0.0 | 0.0 | 0.0 | 0.0 |
| Lce1a2 | 0.0 | 0.0 | 0.0 | 0.0 | 0.0 | 0.0 | 0.0 | 0.0 | 0.0 | 0.0 | 0.0 | 0.0 |
| Lce1c | 0.0 | 0.0 | 0.0 | 0.0 | 0.0 | 0.0 | 0.0 | 0.0 | 0.0 | 0.0 | 0.0 | 0.0 |
| Lce1e | 0.0 | 0.0 | 0.0 | 0.0 | 0.0 | 0.0 | 0.0 | 0.0 | 0.0 | 0.0 | 0.0 | 0.0 |
| Lce1f | 0.1 | 0.0 | 0.0 | 0.0 | 0.0 | 0.0 | 0.0 | 0.0 | 0.0 | 0.0 | 0.0 | 0.0 |
| Lce1i | 0.0 | 0.0 | 0.0 | 0.0 | 0.0 | 0.0 | 0.0 | 0.0 | 0.0 | 0.0 | 0.0 | 0.0 |
| Lce6a | 0.0 | 0.0 | 0.0 | 0.0 | 0.0 | 0.0 | 0.0 | 0.0 | 0.0 | 0.0 | 0.0 | 0.0 |
| Lcn8 | 0.0 | 0.0 | 0.0 | 0.0 | 0.0 | 0.0 | 0.0 | 0.0 | 0.0 | 0.0 | 0.0 | 0.0 |
| Ldhc | 0.0 | 0.0 | 0.0 | 0.0 | 0.0 | 0.0 | 0.0 | 0.0 | 0.0 | 0.0 | 0.0 | 0.0 |
| Lect1 | 0.0 | 0.0 | 0.1 | 0.0 | 0.0 | 0.0 | 0.0 | 0.0 | 0.0 | 0.0 | 0.0 | 0.0 |
| Lect2 | 0.0 | 0.1 | 0.0 | 0.1 | 0.0 | 0.0 | 0.1 | 0.0 | 0.0 | 0.0 | 0.0 | 0.1 |
| Lgals12 | 0.0 | 0.0 | 0.0 | 0.0 | 0.0 | 0.0 | 0.0 | 0.0 | 0.0 | 0.0 | 0.0 | 0.0 |
| Lgsn | 0.0 | 0.0 | 0.0 | 0.0 | 0.0 | 0.0 | 0.0 | 0.0 | 0.0 | 0.0 | 0.0 | 0.0 |
| Lhb | 0.0 | 0.0 | 0.0 | 0.0 | 0.0 | 0.0 | 0.0 | 0.0 | 0.3 | 0.0 | 0.0 | 0.0 |
| Lhcgr | 0.0 | 0.0 | 0.0 | 0.0 | 0.0 | 0.0 | 0.0 | 0.0 | 0.0 | 0.0 | 0.0 | 0.0 |
| Lhfpl5 | 0.0 | 0.0 | 0.0 | 0.0 | 0.0 | 0.0 | 0.0 | 0.0 | 0.1 | 0.0 | 0.0 | 0.0 |
| Lhx1 | 0.0 | 0.0 | 0.0 | 0.0 | 0.0 | 0.0 | 0.0 | 0.0 | 0.0 | 0.0 | 0.0 | 0.0 |
| Lhx3 | 0.0 | 0.0 | 0.0 | 0.0 | 0.0 | 0.0 | 0.0 | 0.0 | 0.0 | 0.0 | 0.0 | 0.0 |
| Lin28a | 0.0 | 0.0 | 0.0 | 0.0 | 0.0 | 0.0 | 0.0 | 0.0 | 0.0 | 0.0 | 0.0 | 0.0 |
| Lin28b | 0.0 | 0.0 | 0.0 | 0.0 | 0.0 | 0.0 | 0.0 | 0.0 | 0.0 | 0.0 | 0.0 | 0.0 |
| Lingo2 | 0.0 | 0.0 | 0.0 | 0.0 | 0.0 | 0.0 | 0.0 | 0.0 | 0.0 | 0.0 | 0.0 | 0.0 |
| Lingo3 | 0.0 | 0.0 | 0.0 | 0.0 | 0.0 | 0.0 | 0.0 | 0.0 | 0.0 | 0.0 | 0.0 | 0.0 |
| Lipf | 0.0 | 0.0 | 0.0 | 0.0 | 0.0 | 0.0 | 0.0 | 0.0 | 0.0 | 0.0 | 0.0 | 0.0 |

| | | | | | | | | | | | | |
|---|---|---|---|---|---|---|---|---|---|---|---|---|
| Lipi | 0.0 | 0.0 | 0.0 | 0.0 | 0.0 | 0.0 | 0.0 | 0.0 | 0.0 | 0.0 | 0.0 | 0.0 |
| Lipk | 0.0 | 0.0 | 0.0 | 0.0 | 0.0 | 0.0 | 0.0 | 0.0 | 0.0 | 0.0 | 0.1 | 0.0 |
| Lipm | 0.0 | 0.0 | 0.0 | 0.0 | 0.0 | 0.0 | 0.0 | 0.0 | 0.0 | 0.0 | 0.0 | 0.0 |
| Lmo3 | 0.0 | 0.0 | 0.0 | 0.0 | 0.0 | 0.0 | 0.0 | 0.0 | 0.0 | 0.0 | 0.0 | 0.0 |
| Lmx1a | 0.0 | 0.0 | 0.0 | 0.0 | 0.0 | 0.0 | 0.0 | 0.0 | 0.0 | 0.0 | 0.0 | 0.0 |
| LOC100040786 | 0.0 | 0.0 | 0.0 | 0.0 | 0.0 | 0.0 | 0.0 | 0.0 | 0.0 | 0.0 | 0.0 | 0.0 |
| LOC100048884 | 0.0 | 0.0 | 0.0 | 0.0 | 0.0 | 0.0 | 0.0 | 0.0 | 0.0 | 0.0 | 0.0 | 0.0 |
| LOC100502924 | 0.0 | 0.0 | 0.0 | 0.0 | 0.0 | 0.0 | 0.0 | 0.0 | 0.0 | 0.0 | 0.0 | 0.0 |
| LOC100503280 | 0.0 | 0.0 | 0.0 | 0.0 | 0.0 | 0.0 | 0.0 | 0.0 | 0.0 | 0.0 | 0.0 | 0.0 |
| Loxhd1 | 0.0 | 0.0 | 0.0 | 0.0 | 0.0 | 0.0 | 0.0 | 0.0 | 0.0 | 0.0 | 0.0 | 0.0 |
| Lpcat2b | 0.0 | 0.0 | 0.0 | 0.0 | 0.0 | 0.0 | 0.0 | 0.0 | 0.0 | 0.0 | 0.0 | 0.0 |
| Lpo | 0.0 | 0.0 | 0.0 | 0.0 | 0.0 | 0.0 | 0.0 | 0.0 | 0.0 | 0.0 | 0.0 | 0.0 |
| Lrcol1 | 0.0 | 0.0 | 0.0 | 0.0 | 0.0 | 0.0 | 0.0 | 0.1 | 0.0 | 0.0 | 0.0 | 0.0 |
| Lrfn2 | 0.0 | 0.0 | 0.0 | 0.0 | 0.0 | 0.0 | 0.0 | 0.0 | 0.0 | 0.0 | 0.0 | 0.0 |
| Lrfn5 | 0.0 | 0.0 | 0.0 | 0.0 | 0.0 | 0.0 | 0.0 | 0.0 | 0.0 | 0.0 | 0.0 | 0.0 |
| Lrit1 | 0.0 | 0.0 | 0.0 | 0.0 | 0.0 | 0.0 | 0.0 | 0.0 | 0.0 | 0.0 | 0.0 | 0.0 |
| Lrit3 | 0.0 | 0.0 | 0.0 | 0.0 | 0.0 | 0.0 | 0.0 | 0.0 | 0.0 | 0.0 | 0.0 | 0.0 |
| Lrrc10 | 0.0 | 0.0 | 0.0 | 0.1 | 0.1 | 0.0 | 0.0 | 0.0 | 0.0 | 0.0 | 0.0 | 0.0 |
| Lrrc30 | 0.0 | 0.0 | 0.0 | 0.0 | 0.0 | 0.0 | 0.0 | 0.0 | 0.0 | 0.0 | 0.0 | 0.0 |
| Lrrc38 | 0.0 | 0.0 | 0.0 | 0.0 | 0.0 | 0.0 | 0.0 | 0.0 | 0.0 | 0.0 | 0.0 | 0.0 |
| Lrrc3b | 0.1 | 0.0 | 0.0 | 0.1 | 0.0 | 0.0 | 0.0 | 0.0 | 0.0 | 0.0 | 0.0 | 0.1 |
| Lrrc43 | 0.0 | 0.0 | 0.0 | 0.0 | 0.0 | 0.0 | 0.0 | 0.0 | 0.0 | 0.0 | 0.0 | 0.0 |
| Lrrc52 | 0.1 | 0.0 | 0.0 | 0.0 | 0.0 | 0.0 | 0.1 | 0.0 | 0.0 | 0.0 | 0.0 | 0.0 |
| Lrrc6 | 0.1 | 0.0 | 0.0 | 0.0 | 0.0 | 0.0 | 0.0 | 0.0 | 0.0 | 0.0 | 0.0 | 0.0 |
| Lrrc63 | 0.0 | 0.0 | 0.0 | 0.0 | 0.0 | 0.0 | 0.0 | 0.0 | 0.0 | 0.0 | 0.0 | 0.0 |
| Lrrc66 | 0.0 | 0.0 | 0.0 | 0.0 | 0.0 | 0.0 | 0.0 | 0.0 | 0.0 | 0.0 | 0.0 | 0.0 |
| Lrrc7 | 0.0 | 0.0 | 0.0 | 0.0 | 0.0 | 0.0 | 0.0 | 0.0 | 0.0 | 0.0 | 0.0 | 0.0 |
| Lrrtm1 | 0.0 | 0.0 | 0.0 | 0.0 | 0.0 | 0.0 | 0.0 | 0.0 | 0.0 | 0.0 | 0.0 | 0.0 |
| Ltb | 0.1 | 0.0 | 0.0 | 0.2 | 0.0 | 0.0 | 0.1 | 0.0 | 0.0 | 0.0 | 0.0 | 0.0 |
| Luzp4 | 0.0 | 0.0 | 0.0 | 0.0 | 0.0 | 0.0 | 0.0 | 0.0 | 0.0 | 0.0 | 0.0 | 0.0 |
| Ly6g6c | 0.0 | 0.0 | 0.0 | 0.0 | 0.0 | 0.0 | 0.0 | 0.0 | 0.0 | 0.0 | 0.0 | 0.0 |
| Ly6g6d | 0.0 | 0.0 | 0.1 | 0.3 | 0.1 | 0.0 | 0.0 | 0.0 | 0.0 | 0.0 | 0.0 | 0.0 |
| Ly6g6f | 0.0 | 0.0 | 0.0 | 0.0 | 0.0 | 0.0 | 0.0 | 0.0 | 0.0 | 0.0 | 0.0 | 0.0 |
| Ly6h | 0.0 | 0.1 | 0.0 | 0.0 | 0.0 | 0.0 | 0.0 | 0.0 | 0.0 | 0.0 | 0.0 | 0.0 |
| Lyg2 | 0.0 | 0.0 | 0.0 | 0.0 | 0.0 | 0.0 | 0.0 | 0.0 | 0.0 | 0.0 | 0.0 | 0.0 |
| Lypd3 | 0.0 | 0.0 | 0.0 | 0.0 | 0.0 | 0.0 | 0.0 | 0.0 | 0.0 | 0.0 | 0.0 | 0.0 |
| Lypd4 | 0.0 | 0.0 | 0.0 | 0.0 | 0.0 | 0.0 | 0.0 | 0.0 | 0.0 | 0.0 | 0.0 | 0.0 |
| Lypd8 | 0.0 | 0.0 | 0.0 | 0.0 | 0.0 | 0.0 | 0.0 | 0.0 | 0.0 | 0.0 | 0.0 | 0.0 |
| Magea5 | 0.0 | 0.0 | 0.0 | 0.0 | 0.0 | 0.0 | 0.0 | 0.0 | 0.0 | 0.0 | 0.0 | 0.0 |
| Magea6 | 0.0 | 0.0 | 0.0 | 0.0 | 0.0 | 0.0 | 0.0 | 0.0 | 0.0 | 0.0 | 0.0 | 0.0 |
| Magea8 | 0.0 | 0.0 | 0.0 | 0.0 | 0.0 | 0.0 | 0.0 | 0.0 | 0.0 | 0.0 | 0.0 | 0.0 |
| Mageb1 | 0.0 | 0.0 | 0.0 | 0.0 | 0.0 | 0.0 | 0.0 | 0.0 | 0.0 | 0.0 | 0.0 | 0.0 |
| Mageb18 | 0.0 | 0.0 | 0.0 | 0.0 | 0.0 | 0.0 | 0.0 | 0.0 | 0.0 | 0.0 | 0.0 | 0.0 |
| Mageb3 | 0.0 | 0.0 | 0.0 | 0.0 | 0.0 | 0.0 | 0.0 | 0.0 | 0.0 | 0.0 | 0.0 | 0.0 |
| Magel2 | 0.0 | 0.0 | 0.0 | 0.0 | 0.0 | 0.0 | 0.0 | 0.0 | 0.0 | 0.0 | 0.0 | 0.0 |
| Marveld3 | 0.0 | 0.0 | 0.0 | 0.1 | 0.0 | 0.0 | 0.0 | 0.0 | 0.0 | 0.0 | 0.0 | 0.0 |
| Matn1 | 0.0 | 0.0 | 0.0 | 0.0 | 0.1 | 0.0 | 0.0 | 0.0 | 0.0 | 0.0 | 0.0 | 0.0 |
| Mb | 0.0 | 0.0 | 0.1 | 0.0 | 0.0 | 0.0 | 0.0 | 0.0 | 0.0 | 0.0 | 0.2 | 0.0 |
| Mbd3l1 | 0.0 | 0.0 | 0.0 | 0.0 | 0.0 | 0.0 | 0.0 | 0.0 | 0.0 | 0.1 | 0.0 | 0.0 |
| Mbd3l2 | 0.0 | 0.0 | 0.0 | 0.0 | 0.0 | 0.0 | 0.0 | 0.0 | 0.0 | 0.0 | 0.0 | 0.0 |
| Mbl1 | 0.0 | 0.0 | 0.0 | 0.0 | 0.0 | 0.0 | 0.0 | 0.0 | 0.0 | 0.0 | 0.0 | 0.0 |
| Mbl2 | 0.0 | 0.0 | 0.0 | 0.0 | 0.0 | 0.0 | 0.1 | 0.2 | 0.0 | 0.1 | 0.0 | 0.1 |
| Mcf2 | 0.0 | 0.0 | 0.0 | 0.0 | 0.0 | 0.0 | 0.0 | 0.0 | 0.0 | 0.0 | 0.0 | 0.0 |
| Mcin | 0.0 | 0.0 | 0.0 | 0.0 | 0.0 | 0.0 | 0.0 | 0.0 | 0.0 | 0.1 | 0.0 | 0.0 |
| Mcoln3 | 0.0 | 0.1 | 0.0 | 0.0 | 0.0 | 0.0 | 0.0 | 0.0 | 0.0 | 0.0 | 0.0 | 0.0 |
| Mcpt1 | 0.0 | 0.1 | 0.0 | 0.0 | 0.0 | 0.0 | 0.0 | 0.0 | 0.0 | 0.0 | 0.0 | 0.1 |
| Mcpt2 | 0.0 | 0.0 | 0.0 | 0.0 | 0.0 | 0.0 | 0.0 | 0.0 | 0.0 | 0.0 | 0.0 | 0.0 |

| | | | | | | | | | | | | |
|---|---|---|---|---|---|---|---|---|---|---|---|---|
| Mcpt9 | 0.0 | 0.0 | 0.0 | 0.0 | 0.0 | 0.0 | 0.0 | 0.0 | 0.0 | 0.0 | 0.0 | 0.0 |
| Mcpt-ps1 | 0.0 | 0.0 | 0.0 | 0.0 | 0.0 | 0.0 | 0.0 | 0.0 | 0.0 | 0.0 | 0.1 | 0.1 |
| Mei4 | 0.0 | 0.0 | 0.0 | 0.0 | 0.0 | 0.0 | 0.0 | 0.0 | 0.0 | 0.1 | 0.0 | 0.0 |
| Meig1 | 0.2 | 0.0 | 0.0 | 1.0 | 0.0 | 0.0 | 0.0 | 0.1 | 0.1 | 0.0 | 0.0 | 0.1 |
| Mep1b | 0.0 | 0.0 | 0.0 | 0.0 | 0.0 | 0.0 | 0.0 | 0.0 | 0.0 | 0.0 | 0.0 | 0.0 |
| Mepe | 0.0 | 0.0 | 0.0 | 0.0 | 0.0 | 0.0 | 0.0 | 0.0 | 0.0 | 0.0 | 0.0 | 0.0 |
| Mesp1 | 0.0 | 0.0 | 0.0 | 0.0 | 0.0 | 0.0 | 0.0 | 0.0 | 0.0 | 0.0 | 0.1 | 0.0 |
| Mettl11b | 0.0 | 0.0 | 0.0 | 0.0 | 0.0 | 0.0 | 0.0 | 0.0 | 0.0 | 0.0 | 0.0 | 0.0 |
| Mfsd6l | 0.0 | 0.0 | 0.0 | 0.0 | 0.0 | 0.0 | 0.0 | 0.0 | 0.0 | 0.0 | 0.0 | 0.0 |
| Mia2 | 0.0 | 0.0 | 0.0 | 0.0 | 0.0 | 0.0 | 0.0 | 0.0 | 0.0 | 0.0 | 0.0 | 0.0 |
| Mill1 | 0.0 | 0.0 | 0.0 | 0.0 | 0.0 | 0.0 | 0.0 | 0.0 | 0.0 | 0.0 | 0.0 | 0.0 |
| Miox | 0.1 | 0.0 | 0.0 | 0.0 | 0.0 | 0.0 | 0.0 | 0.0 | 0.1 | 0.0 | 0.1 | 0.0 |
| Mir10a | 0.0 | 0.0 | 0.0 | 0.0 | 0.0 | 0.0 | 0.0 | 0.0 | 0.0 | 0.0 | 0.0 | 5.0 |
| Mir1186 | 0.0 | 0.0 | 0.0 | 0.0 | 0.0 | 0.0 | 0.0 | 0.0 | 0.0 | 3.5 | 0.0 | 0.0 |
| Mir1188 | 0.0 | 12.3 | 0.0 | 0.0 | 0.0 | 0.0 | 0.0 | 0.0 | 0.0 | 0.0 | 0.0 | 0.0 |
| Mir1192 | 0.0 | 0.0 | 0.0 | 0.0 | 0.0 | 0.0 | 0.0 | 0.0 | 0.0 | 0.0 | 0.0 | 0.0 |
| Mir1193 | 0.0 | 0.0 | 0.0 | 0.0 | 0.0 | 0.0 | 0.0 | 0.0 | 0.0 | 0.0 | 0.0 | 0.0 |
| Mir1197 | 0.0 | 0.0 | 0.0 | 0.0 | 0.0 | 0.0 | 0.0 | 0.0 | 0.0 | 0.0 | 5.4 | 0.0 |
| Mir1247 | 0.0 | 0.0 | 0.0 | 0.0 | 0.0 | 0.0 | 0.0 | 30.4 | 0.0 | 0.0 | 0.0 | 0.0 |
| Mir1843b | 0.0 | 0.0 | 0.0 | 0.0 | 0.0 | 0.1 | 0.0 | 0.0 | 0.0 | 0.0 | 0.0 | 11.9 |
| Mir1892 | 0.0 | 0.0 | 0.0 | 0.0 | 0.0 | 0.0 | 0.0 | 0.0 | 0.0 | 0.0 | 0.0 | 0.0 |
| Mir1894 | 0.0 | 0.0 | 0.0 | 0.0 | 0.0 | 0.0 | 0.0 | 0.0 | 0.0 | 0.0 | 0.0 | 30.8 |
| Mir1896 | 0.0 | 0.0 | 47.8 | 0.0 | 0.0 | 0.0 | 0.0 | 0.0 | 0.0 | 0.0 | 0.0 | 0.0 |
| Mir1903 | 0.0 | 0.0 | 0.0 | 0.0 | 0.0 | 0.0 | 0.0 | 0.0 | 0.0 | 0.0 | 0.0 | 0.0 |
| Mir1905 | 0.0 | 0.0 | 0.0 | 0.0 | 22.6 | 0.0 | 0.0 | 0.0 | 0.0 | 35.5 | 0.0 | 0.0 |
| Mir1906-1 | 0.0 | 0.0 | 0.0 | 10.2 | 0.0 | 0.0 | 0.0 | 0.0 | 0.0 | 10.5 | 0.0 | 0.0 |
| Mir1938 | 0.0 | 0.0 | 0.0 | 11.0 | 0.0 | 0.0 | 0.0 | 0.0 | 9.0 | 0.0 | 0.0 | 0.0 |
| Mir194-1 | 0.0 | 0.0 | 0.0 | 0.0 | 95.9 | 0.0 | 0.0 | 0.0 | 0.0 | 0.0 | 0.0 | 0.0 |
| Mir1946a | 0.0 | 0.0 | 0.0 | 0.0 | 0.0 | 0.0 | 0.0 | 0.0 | 0.0 | 0.0 | 0.0 | 0.0 |
| Mir1949 | 0.0 | 0.0 | 0.0 | 0.0 | 0.0 | 0.0 | 0.0 | 96.9 | 0.0 | 0.0 | 0.0 | 0.0 |
| Mir1955 | 0.0 | 0.0 | 0.0 | 0.0 | 0.0 | 0.0 | 0.0 | 0.0 | 0.0 | 0.0 | 0.0 | 0.0 |
| Mir1966 | 0.0 | 0.0 | 0.0 | 0.0 | 0.0 | 0.0 | 0.0 | 0.0 | 0.0 | 0.0 | 0.0 | 0.0 |
| Mir199a-2 | 0.0 | 0.0 | 0.0 | 0.0 | 0.0 | 0.0 | 0.0 | 0.0 | 0.0 | 0.0 | 0.0 | 0.0 |
| Mir207 | 0.0 | 0.0 | 0.0 | 0.0 | 0.0 | 0.0 | 0.0 | 0.0 | 0.0 | 0.0 | 0.0 | 0.0 |
| Mir214 | 0.0 | 0.0 | 0.0 | 0.0 | 0.0 | 0.0 | 0.0 | 0.0 | 0.0 | 0.0 | 0.0 | 0.0 |
| Mir219-1 | 0.0 | 0.0 | 0.0 | 0.0 | 0.0 | 0.0 | 5.1 | 0.0 | 5.3 | 5.9 | 0.0 | 0.0 |
| Mir219-2 | 0.0 | 0.0 | 0.0 | 0.0 | 0.0 | 0.0 | 0.0 | 0.0 | 0.0 | 0.0 | 0.0 | 0.0 |
| Mir221 | 0.0 | 0.0 | 0.0 | 0.0 | 0.0 | 0.0 | 0.0 | 0.0 | 0.0 | 0.0 | 0.0 | 0.0 |
| Mir222 | 0.0 | 0.0 | 0.0 | 0.0 | 0.0 | 0.0 | 0.0 | 0.0 | 0.0 | 0.0 | 0.0 | 0.0 |
| Mir223 | 0.0 | 0.0 | 0.0 | 0.0 | 0.0 | 0.0 | 0.0 | 0.0 | 0.0 | 0.0 | 0.0 | 0.0 |
| Mir23b | 0.0 | 13.7 | 0.0 | 0.0 | 0.0 | 0.0 | 0.0 | 0.0 | 0.0 | 0.0 | 0.0 | 0.0 |
| Mir24-1 | 0.0 | 0.0 | 0.0 | 0.0 | 0.0 | 0.0 | 0.0 | 0.0 | 122.7 | 0.0 | 0.0 | 0.0 |
| Mir27a | 0.0 | 0.0 | 0.0 | 0.0 | 0.0 | 0.0 | 55.0 | 0.0 | 0.0 | 0.0 | 0.0 | 0.0 |
| Mir2861 | 0.0 | 0.0 | 22.1 | 0.0 | 22.6 | 0.0 | 0.0 | 0.0 | 0.0 | 0.0 | 0.0 | 0.0 |
| Mir3064 | 0.0 | 0.0 | 0.0 | 896.3 | 0.0 | 0.0 | 0.0 | 0.0 | 0.0 | 0.0 | 0.0 | 0.0 |
| Mir3065 | 0.0 | 0.0 | 0.0 | 0.0 | 0.0 | 0.0 | 0.0 | 0.0 | 0.0 | 0.0 | 0.0 | 0.0 |
| Mir3066 | 0.0 | 0.0 | 0.0 | 0.0 | 0.0 | 0.0 | 0.0 | 0.0 | 0.0 | 0.0 | 0.0 | 0.0 |
| Mir3067 | 0.0 | 0.0 | 0.0 | 0.0 | 0.0 | 0.0 | 0.0 | 0.0 | 0.0 | 0.0 | 0.0 | 0.0 |
| Mir3068 | 0.0 | 0.0 | 0.0 | 0.0 | 0.0 | 0.0 | 0.0 | 0.0 | 0.0 | 0.0 | 0.0 | 0.0 |
| Mir3070a | 0.0 | 0.0 | 0.0 | 0.0 | 0.0 | 0.0 | 0.0 | 0.0 | 0.0 | 0.0 | 0.0 | 8.3 |
| Mir3070b | 0.0 | 0.0 | 0.0 | 0.0 | 0.0 | 0.0 | 0.0 | 0.0 | 0.0 | 0.0 | 0.0 | 8.3 |
| Mir3096 | 0.0 | 0.0 | 0.0 | 0.0 | 0.0 | 0.0 | 0.0 | 0.0 | 8.5 | 0.0 | 0.0 | 0.0 |
| Mir3096b | 0.0 | 0.0 | 0.0 | 0.0 | 0.0 | 0.0 | 14.1 | 0.0 | 0.0 | 0.0 | 0.0 | 0.0 |
| Mir3101 | 0.0 | 0.0 | 0.0 | 0.0 | 0.0 | 0.0 | 0.0 | 0.0 | 0.0 | 0.0 | 0.0 | 0.0 |
| Mir3109 | 0.0 | 0.0 | 0.0 | 0.0 | 0.0 | 0.0 | 0.0 | 0.0 | 0.0 | 0.0 | 0.0 | 0.0 |
| Mir320 | 0.0 | 0.0 | 0.0 | 0.0 | 0.0 | 0.0 | 1756.0 | 0.0 | 0.0 | 0.0 | 0.0 | 0.0 |
| Mir324 | 0.0 | 0.0 | 0.0 | 0.0 | 0.0 | 0.0 | 0.0 | 0.0 | 17.7 | 0.0 | 0.0 | 0.0 |

| | | | | | | | | | | | |
|---|---|---|---|---|---|---|---|---|---|---|---|
| Mir331 | 0.0 | 0.0 | 0.0 | 0.0 | 0.0 | 0.0 | 0.0 | 11.5 | 0.0 | 0.0 | 0.0 | 0.0 |
| Mir343 | 0.0 | 0.0 | 0.0 | 0.0 | 0.0 | 0.0 | 0.0 | 0.0 | 0.0 | 0.0 | 0.0 | 0.0 |
| Mir3470b | 0.0 | 12.8 | 0.0 | 0.0 | 0.0 | 0.0 | 0.0 | 0.0 | 0.0 | 0.0 | 0.0 | 0.0 |
| Mir3471-1 | 0.0 | 35.3 | 11.3 | 1.8 | 0.0 | 0.0 | 1.0 | 0.0 | 0.0 | 0.0 | 0.0 | 0.4 |
| Mir3473 | 0.0 | 0.0 | 0.0 | 0.0 | 0.0 | 0.0 | 0.0 | 0.0 | 0.0 | 0.0 | 0.0 | 0.0 |
| Mir34b | 0.0 | 0.0 | 0.0 | 29.7 | 0.0 | 0.0 | 0.0 | 0.0 | 0.0 | 0.0 | 0.0 | 0.0 |
| Mir425 | 0.0 | 0.0 | 0.0 | 0.0 | 0.0 | 0.0 | 0.0 | 0.0 | 0.0 | 0.0 | 0.0 | 0.0 |
| Mir429 | 0.0 | 0.0 | 0.0 | 0.0 | 0.0 | 0.0 | 0.0 | 0.0 | 0.0 | 0.0 | 0.0 | 0.0 |
| Mir431 | 0.0 | 0.0 | 0.0 | 0.0 | 0.0 | 0.0 | 0.0 | 0.0 | 0.0 | 0.0 | 0.0 | 0.0 |
| Mir432 | 0.0 | 0.0 | 0.0 | 0.0 | 0.0 | 0.0 | 0.0 | 0.0 | 0.0 | 0.0 | 0.0 | 0.0 |
| Mir433 | 0.0 | 0.0 | 0.0 | 0.0 | 0.6 | 0.0 | 0.0 | 0.0 | 0.1 | 0.0 | 2.6 | 0.0 |
| Mir5046 | 0.0 | 0.0 | 0.0 | 0.0 | 0.0 | 0.0 | 0.0 | 0.0 | 0.0 | 0.0 | 0.0 | 0.0 |
| Mir5109 | 0.0 | 0.0 | 0.0 | 0.0 | 0.0 | 0.0 | 3.7 | 0.0 | 0.0 | 0.0 | 0.0 | 0.0 |
| Mir5113 | 0.0 | 0.0 | 0.0 | 0.0 | 0.0 | 0.0 | 0.0 | 0.0 | 0.0 | 0.0 | 0.0 | 0.0 |
| Mir5114 | 0.0 | 0.0 | 0.0 | 0.0 | 0.0 | 0.0 | 0.0 | 0.0 | 0.0 | 0.0 | 0.0 | 0.0 |
| Mir5117 | 0.0 | 0.0 | 0.0 | 0.0 | 0.0 | 0.0 | 0.0 | 0.0 | 0.0 | 0.0 | 0.0 | 0.0 |
| Mir5121 | 0.0 | 0.0 | 0.0 | 0.0 | 0.0 | 0.0 | 0.0 | 0.0 | 62.5 | 0.0 | 0.0 | 0.0 |
| Mir5122 | 0.0 | 0.0 | 0.0 | 0.0 | 0.0 | 0.0 | 0.0 | 0.0 | 0.0 | 0.0 | 0.0 | 0.0 |
| Mir5130 | 0.0 | 0.0 | 0.0 | 0.0 | 0.0 | 0.0 | 0.0 | 0.0 | 0.0 | 0.0 | 0.0 | 0.0 |
| Mir5131 | 0.0 | 0.0 | 0.0 | 0.0 | 0.0 | 0.0 | 0.0 | 1.8 | 0.0 | 0.0 | 0.0 | 0.0 |
| Mir5133 | 0.0 | 60.8 | 0.0 | 0.0 | 0.0 | 58.6 | 0.0 | 0.0 | 0.0 | 0.0 | 0.0 | 0.0 |
| Mir5136 | 0.0 | 0.0 | 0.0 | 0.0 | 0.0 | 0.0 | 0.0 | 0.0 | 0.0 | 0.0 | 0.0 | 0.0 |
| Mir667 | 0.0 | 0.0 | 0.0 | 0.0 | 0.0 | 0.0 | 0.0 | 0.0 | 0.0 | 0.0 | 0.0 | 0.0 |
| Mir677 | 0.0 | 0.0 | 0.0 | 0.0 | 0.0 | 0.0 | 0.0 | 0.0 | 0.0 | 50.6 | 0.0 | 0.0 |
| Mir684-2 | 0.0 | 0.0 | 0.0 | 0.0 | 0.0 | 0.0 | 0.0 | 0.0 | 0.0 | 8.0 | 0.0 | 8.3 |
| Mir690 | 0.0 | 0.0 | 0.0 | 0.0 | 0.0 | 0.0 | 0.0 | 2.8 | 0.0 | 0.0 | 0.0 | 0.0 |
| Mir705 | 0.0 | 0.0 | 0.0 | 0.0 | 0.0 | 0.0 | 0.0 | 0.0 | 0.0 | 0.0 | 0.0 | 0.0 |
| Mir7-1 | 0.0 | 0.0 | 0.0 | 7.3 | 0.0 | 0.0 | 0.0 | 0.0 | 0.0 | 6.5 | 0.0 | 0.0 |
| Mir760 | 0.0 | 0.0 | 0.0 | 0.0 | 3.0 | 0.0 | 0.0 | 0.0 | 0.0 | 0.0 | 0.0 | 0.0 |
| Mir762 | 0.0 | 0.0 | 0.0 | 0.0 | 0.0 | 0.0 | 44.1 | 0.0 | 0.0 | 0.0 | 224.8 | 0.0 |
| Mir877 | 0.0 | 0.0 | 0.0 | 0.0 | 0.0 | 0.0 | 0.0 | 0.0 | 0.0 | 0.0 | 0.0 | 0.0 |
| Mir99b | 0.0 | 0.0 | 0.0 | 0.0 | 0.0 | 121.6 | 0.0 | 0.0 | 0.0 | 0.0 | 0.0 | 0.0 |
| Mirlet7a-1 | 0.0 | 0.0 | 0.0 | 0.0 | 0.0 | 0.0 | 0.0 | 0.0 | 0.0 | 14.8 | 0.0 | 0.0 |
| Mirlet7a-2 | 0.0 | 0.0 | 0.0 | 0.0 | 0.0 | 0.0 | 0.0 | 0.0 | 0.0 | 0.0 | 0.0 | 0.0 |
| Mirlet7f-1 | 0.0 | 0.0 | 0.0 | 0.0 | 0.0 | 0.0 | 0.0 | 0.0 | 0.0 | 0.0 | 0.0 | 0.0 |
| Mirlet7i | 0.0 | 0.0 | 0.0 | 0.0 | 0.0 | 0.0 | 0.0 | 0.0 | 23.7 | 0.0 | 0.0 | 0.0 |
| Mlc1 | 0.0 | 0.0 | 0.0 | 0.0 | 0.0 | 0.0 | 0.0 | 0.0 | 0.0 | 0.0 | 0.0 | 0.0 |
| Mlip | 0.1 | 0.0 | 0.0 | 0.0 | 0.0 | 0.0 | 0.0 | 0.0 | 0.0 | 0.0 | 0.0 | 0.0 |
| Mmd2 | 0.0 | 0.0 | 0.0 | 0.0 | 0.0 | 0.0 | 0.0 | 0.0 | 0.0 | 0.0 | 0.0 | 0.0 |
| Mnx1 | 0.0 | 0.0 | 0.0 | 0.0 | 0.0 | 0.0 | 0.0 | 0.0 | 0.0 | 0.0 | 0.0 | 0.0 |
| Mobp | 0.0 | 0.0 | 0.0 | 0.0 | 0.0 | 0.0 | 0.0 | 0.0 | 0.0 | 0.0 | 0.0 | 0.0 |
| Mos | 0.0 | 0.0 | 0.0 | 0.0 | 0.0 | 0.0 | 0.0 | 0.0 | 0.0 | 0.0 | 0.0 | 0.0 |
| Mospd4 | 0.0 | 0.1 | 0.0 | 0.0 | 0.0 | 0.0 | 0.0 | 0.0 | 0.0 | 0.2 | 0.1 | 0.0 |
| Mptx1 | 0.0 | 0.0 | 0.0 | 0.0 | 0.0 | 0.0 | 0.1 | 0.0 | 0.0 | 0.1 | 0.0 | 0.0 |
| Mptx2 | 0.0 | 0.0 | 0.0 | 0.0 | 0.0 | 0.0 | 0.1 | 0.0 | 0.0 | 0.0 | 0.0 | 0.0 |
| Mrap2 | 0.0 | 0.0 | 0.0 | 0.0 | 0.0 | 0.0 | 0.0 | 0.0 | 0.0 | 0.0 | 0.0 | 0.0 |
| Mrgpra1 | 0.0 | 0.0 | 0.0 | 0.0 | 0.0 | 0.0 | 0.0 | 0.0 | 0.0 | 0.0 | 0.0 | 0.0 |
| Mrgpra2a | 0.0 | 0.0 | 0.0 | 0.0 | 0.0 | 0.0 | 0.0 | 0.0 | 0.0 | 0.0 | 0.0 | 0.0 |
| Mrgpra3 | 0.0 | 0.0 | 0.0 | 0.0 | 0.0 | 0.0 | 0.0 | 0.0 | 0.0 | 0.0 | 0.0 | 0.0 |
| Mrgpra4 | 0.0 | 0.0 | 0.0 | 0.0 | 0.0 | 0.0 | 0.0 | 0.0 | 0.0 | 0.0 | 0.0 | 0.0 |
| Mrgpra6 | 0.0 | 0.0 | 0.0 | 0.0 | 0.0 | 0.0 | 0.0 | 0.0 | 0.0 | 0.0 | 0.0 | 0.0 |
| Mrgpra9 | 0.0 | 0.0 | 0.0 | 0.0 | 0.0 | 0.0 | 0.0 | 0.0 | 0.0 | 0.0 | 0.0 | 0.0 |
| Mrgprb1 | 0.0 | 0.0 | 0.0 | 0.0 | 0.0 | 0.0 | 0.0 | 0.0 | 0.0 | 0.0 | 0.0 | 0.0 |
| Mrgprb2 | 0.0 | 0.0 | 0.0 | 0.0 | 0.0 | 0.0 | 0.0 | 0.0 | 0.0 | 0.0 | 0.0 | 0.0 |
| Mrgprb8 | 0.0 | 0.0 | 0.0 | 0.0 | 0.0 | 0.0 | 0.0 | 0.0 | 0.0 | 0.0 | 0.0 | 0.0 |
| Mrgprd | 0.0 | 0.0 | 0.0 | 0.0 | 0.0 | 0.0 | 0.0 | 0.0 | 0.0 | 0.0 | 0.1 | 0.0 |
| Mrgprg | 0.0 | 0.0 | 0.0 | 0.0 | 0.0 | 0.0 | 0.1 | 0.0 | 0.0 | 0.0 | 0.0 | 0.0 |

| | | | | | | | | | | | | |
|---|---|---|---|---|---|---|---|---|---|---|---|---|
| Mrgprh | 0.0 | 0.0 | 0.0 | 0.0 | 0.0 | 0.0 | 0.0 | 0.0 | 0.0 | 0.0 | 0.0 | 0.0 |
| Mroh4 | 0.0 | 0.0 | 0.0 | 0.1 | 0.0 | 0.0 | 0.0 | 0.0 | 0.0 | 0.0 | 0.0 | 0.0 |
| Mroh5 | 0.0 | 0.0 | 0.0 | 0.2 | 0.0 | 0.0 | 0.0 | 0.0 | 0.0 | 0.0 | 0.0 | 0.0 |
| Mroh9 | 0.0 | 0.0 | 0.0 | 0.0 | 0.0 | 0.0 | 0.0 | 0.0 | 0.0 | 0.0 | 0.0 | 0.0 |
| Ms4a1 | 0.0 | 0.0 | 0.0 | 0.0 | 0.0 | 0.0 | 0.0 | 0.0 | 0.0 | 0.0 | 0.1 | 0.0 |
| Ms4a10 | 0.0 | 0.0 | 0.0 | 0.0 | 0.0 | 0.0 | 0.0 | 0.0 | 0.0 | 0.0 | 0.0 | 0.0 |
| Ms4a13 | 0.0 | 0.0 | 0.0 | 0.0 | 0.0 | 0.0 | 0.0 | 0.0 | 0.0 | 0.0 | 0.0 | 0.0 |
| Ms4a15 | 0.0 | 0.0 | 0.0 | 0.0 | 0.0 | 0.0 | 0.0 | 0.0 | 0.0 | 0.0 | 0.0 | 0.0 |
| Ms4a3 | 0.0 | 0.0 | 0.0 | 0.0 | 0.0 | 0.0 | 0.0 | 0.0 | 0.0 | 0.0 | 0.0 | 0.0 |
| Ms4a5 | 0.0 | 0.0 | 0.0 | 0.0 | 0.0 | 0.0 | 0.0 | 0.0 | 0.0 | 0.0 | 0.0 | 0.0 |
| Msmb | 0.1 | 0.1 | 0.0 | 0.0 | 0.0 | 0.0 | 0.0 | 0.0 | 0.0 | 0.1 | 0.1 | 0.0 |
| Msx3 | 0.0 | 0.0 | 0.0 | 0.0 | 0.0 | 0.0 | 0.0 | 0.0 | 0.0 | 0.0 | 0.0 | 0.0 |
| Muc20 | 0.0 | 0.0 | 0.0 | 0.0 | 0.0 | 0.0 | 0.0 | 0.0 | 0.0 | 0.0 | 0.0 | 0.0 |
| Muc4 | 0.0 | 0.0 | 0.0 | 0.0 | 0.0 | 0.0 | 0.0 | 0.0 | 0.0 | 0.0 | 0.0 | 0.0 |
| Muc5ac | 0.0 | 0.0 | 0.0 | 0.0 | 0.0 | 0.0 | 0.0 | 0.0 | 0.0 | 0.0 | 0.0 | 0.0 |
| Mug1 | 0.0 | 0.0 | 0.0 | 0.0 | 0.0 | 0.0 | 0.0 | 0.0 | 0.0 | 0.0 | 0.0 | 0.0 |
| Mup1 | 0.0 | 0.0 | 0.0 | 0.0 | 0.0 | 0.0 | 0.0 | 0.0 | 0.0 | 0.0 | 0.0 | 0.0 |
| Mup10 | 0.0 | 0.0 | 0.0 | 0.0 | 0.0 | 0.0 | 0.0 | 0.0 | 0.0 | 0.0 | 0.0 | 0.0 |
| Mup11 | 0.0 | 0.0 | 0.0 | 0.0 | 0.0 | 0.0 | 0.0 | 0.0 | 0.0 | 0.0 | 0.0 | 0.0 |
| Mup12 | 0.0 | 0.0 | 0.0 | 0.0 | 0.0 | 0.0 | 0.0 | 0.0 | 0.0 | 0.0 | 0.0 | 0.0 |
| Mup13 | 0.0 | 0.0 | 0.0 | 0.0 | 0.0 | 0.0 | 0.0 | 0.0 | 0.0 | 0.0 | 0.0 | 0.0 |
| Mup14 | 0.0 | 0.0 | 0.0 | 0.0 | 0.0 | 0.0 | 0.0 | 0.0 | 0.0 | 0.0 | 0.0 | 0.0 |
| Mup15 | 0.0 | 0.0 | 0.0 | 0.0 | 0.0 | 0.0 | 0.0 | 0.0 | 0.0 | 0.0 | 0.0 | 0.0 |
| Mup16 | 0.0 | 0.0 | 0.0 | 0.0 | 0.0 | 0.0 | 0.0 | 0.0 | 0.0 | 0.0 | 0.0 | 0.0 |
| Mup19 | 0.0 | 0.0 | 0.0 | 0.0 | 0.0 | 0.0 | 0.0 | 0.0 | 0.0 | 0.0 | 0.0 | 0.0 |
| Mup2 | 0.0 | 0.0 | 0.0 | 0.0 | 0.0 | 0.0 | 0.0 | 0.0 | 0.0 | 0.0 | 0.0 | 0.0 |
| Mup7 | 0.0 | 0.0 | 0.0 | 0.0 | 0.0 | 0.0 | 0.0 | 0.0 | 0.0 | 0.0 | 0.0 | 0.0 |
| Mup8 | 0.0 | 0.0 | 0.0 | 0.0 | 0.0 | 0.0 | 0.0 | 0.0 | 0.0 | 0.0 | 0.0 | 0.0 |
| Mup9 | 0.0 | 0.0 | 0.0 | 0.0 | 0.0 | 0.0 | 0.0 | 0.0 | 0.0 | 0.0 | 0.0 | 0.0 |
| Myadml2 | 0.0 | 0.0 | 0.0 | 0.0 | 0.0 | 0.0 | 0.0 | 0.0 | 0.0 | 0.0 | 0.0 | 0.0 |
| Mybpc1 | 0.0 | 0.0 | 0.0 | 0.0 | 0.0 | 0.0 | 0.0 | 0.0 | 0.0 | 0.0 | 0.0 | 0.0 |
| Mybphl | 0.0 | 0.0 | 0.0 | 0.0 | 0.0 | 0.0 | 0.0 | 0.0 | 0.0 | 0.0 | 0.0 | 0.0 |
| Myf5 | 0.0 | 0.0 | 0.0 | 0.0 | 0.0 | 0.0 | 0.0 | 0.0 | 0.0 | 0.0 | 0.0 | 0.0 |
| Myh7 | 0.0 | 0.0 | 0.0 | 0.0 | 0.0 | 0.0 | 0.0 | 0.0 | 0.0 | 0.0 | 0.0 | 0.0 |
| Mylk4 | 0.0 | 0.0 | 0.0 | 0.0 | 0.0 | 0.0 | 0.0 | 0.0 | 0.0 | 0.0 | 0.0 | 0.0 |
| Myo3a | 0.0 | 0.0 | 0.0 | 0.0 | 0.0 | 0.0 | 0.0 | 0.0 | 0.0 | 0.0 | 0.0 | 0.0 |
| Myo7b | 0.0 | 0.0 | 0.0 | 0.0 | 0.0 | 0.0 | 0.0 | 0.0 | 0.0 | 0.0 | 0.0 | 0.0 |
| Myod1 | 0.0 | 0.0 | 0.0 | 0.0 | 0.0 | 0.0 | 0.0 | 0.0 | 0.0 | 0.0 | 0.0 | 0.0 |
| Myog | 0.1 | 0.0 | 0.0 | 0.0 | 0.0 | 0.0 | 0.0 | 0.0 | 0.0 | 0.0 | 0.0 | 0.0 |
| Mzb1 | 0.0 | 0.0 | 0.0 | 0.0 | 0.0 | 0.0 | 0.0 | 0.0 | 0.0 | 0.0 | 0.1 | 0.0 |
| Naa11 | 0.0 | 0.0 | 0.0 | 0.0 | 0.0 | 0.0 | 0.0 | 0.0 | 0.0 | 0.0 | 0.0 | 0.0 |
| Nags | 0.0 | 0.0 | 0.0 | 0.0 | 0.0 | 0.0 | 0.0 | 0.0 | 0.0 | 0.0 | 0.0 | 0.0 |
| Nanos2 | 0.0 | 0.0 | 0.0 | 0.0 | 0.0 | 0.1 | 0.0 | 0.0 | 0.0 | 0.0 | 0.1 | 0.0 |
| Nanos3 | 0.1 | 0.0 | 0.0 | 0.0 | 0.0 | 0.1 | 0.0 | 0.1 | 0.2 | 0.0 | 0.1 | 0.0 |
| Nat8 | 0.0 | 0.0 | 0.0 | 0.0 | 0.0 | 0.0 | 0.0 | 0.1 | 0.0 | 0.0 | 0.1 | 0.0 |
| Ncan | 0.0 | 0.0 | 0.0 | 0.0 | 0.0 | 0.0 | 0.0 | 0.0 | 0.0 | 0.0 | 0.0 | 0.0 |
| Nccrp1 | 0.0 | 0.0 | 0.0 | 0.0 | 0.0 | 0.0 | 0.0 | 0.0 | 0.0 | 0.1 | 0.0 | 0.0 |
| Ncr1 | 0.0 | 0.0 | 0.0 | 0.0 | 0.0 | 0.0 | 0.0 | 0.0 | 0.0 | 0.0 | 0.0 | 0.0 |
| Nctc1 | 0.0 | 0.0 | 0.0 | 0.0 | 0.0 | 0.0 | 0.0 | 0.0 | 0.0 | 0.0 | 0.0 | 0.0 |
| Nell1 | 0.0 | 0.0 | 0.0 | 0.0 | 0.0 | 0.0 | 0.0 | 0.0 | 0.0 | 0.0 | 0.0 | 0.0 |
| Neu2 | 0.0 | 0.0 | 0.0 | 0.0 | 0.0 | 0.0 | 0.0 | 0.0 | 0.0 | 0.0 | 0.0 | 0.0 |
| Neu4 | 0.0 | 0.0 | 0.0 | 0.0 | 0.0 | 0.0 | 0.0 | 0.0 | 0.0 | 0.0 | 0.0 | 0.0 |
| Neurod1 | 0.0 | 0.0 | 0.0 | 0.0 | 0.0 | 0.0 | 0.0 | 0.0 | 0.0 | 0.0 | 0.0 | 0.0 |
| Neurod2 | 0.0 | 0.0 | 0.0 | 0.0 | 0.0 | 0.0 | 0.0 | 0.0 | 0.0 | 0.0 | 0.0 | 0.0 |
| Neurod4 | 0.0 | 0.0 | 0.0 | 0.0 | 0.0 | 0.0 | 0.0 | 0.0 | 0.0 | 0.0 | 0.0 | 0.0 |
| Neurod6 | 0.0 | 0.0 | 0.0 | 0.0 | 0.0 | 0.0 | 0.0 | 0.0 | 0.0 | 0.0 | 0.0 | 0.0 |
| Neurog2 | 0.0 | 0.0 | 0.0 | 0.0 | 0.0 | 0.0 | 0.0 | 0.0 | 0.0 | 0.0 | 0.0 | 0.0 |

| | | | | | | | | | | | | |
|---|---|---|---|---|---|---|---|---|---|---|---|---|
| Ngp | 0.0 | 0.0 | 0.0 | 0.1 | 0.0 | 0.0 | 0.0 | 0.0 | 0.0 | 0.1 | 0.0 | 0.1 |
| Nhlh2 | 0.0 | 0.0 | 0.0 | 0.0 | 0.0 | 0.0 | 0.0 | 0.0 | 0.0 | 0.0 | 0.0 | 0.0 |
| Ninj2 | 0.0 | 0.1 | 0.0 | 0.0 | 0.0 | 0.2 | 0.0 | 0.1 | 0.1 | 0.0 | 0.0 | 0.0 |
| Nkx1-2 | 0.0 | 0.0 | 0.0 | 0.0 | 0.0 | 0.0 | 0.0 | 0.0 | 0.0 | 0.0 | 0.0 | 0.0 |
| Nkx2-2 | 0.0 | 0.0 | 0.0 | 0.0 | 0.0 | 0.0 | 0.0 | 0.0 | 0.0 | 0.0 | 0.0 | 0.0 |
| Nkx2-2as | 0.0 | 0.0 | 0.0 | 0.0 | 0.0 | 0.0 | 0.0 | 0.0 | 0.0 | 0.0 | 0.0 | 0.0 |
| Nkx2-3 | 0.0 | 0.0 | 0.0 | 0.0 | 0.0 | 0.1 | 0.0 | 0.0 | 0.0 | 0.0 | 0.0 | 0.0 |
| Nkx2-4 | 0.0 | 0.0 | 0.0 | 0.0 | 0.0 | 0.0 | 0.0 | 0.0 | 0.0 | 0.0 | 0.0 | 0.0 |
| Nkx2-6 | 0.0 | 0.0 | 0.0 | 0.0 | 0.0 | 0.0 | 0.0 | 0.0 | 0.0 | 0.0 | 0.0 | 0.0 |
| Nkx3-1 | 0.0 | 0.0 | 0.0 | 0.0 | 0.0 | 0.0 | 0.0 | 0.0 | 0.0 | 0.0 | 0.0 | 0.0 |
| Nkx3-2 | 0.0 | 0.0 | 0.0 | 0.0 | 0.0 | 0.0 | 0.0 | 0.0 | 0.0 | 0.0 | 0.0 | 0.0 |
| Nkx6-1 | 0.0 | 0.0 | 0.0 | 0.0 | 0.0 | 0.0 | 0.0 | 0.0 | 0.0 | 0.0 | 0.0 | 0.0 |
| Nkx6-3 | 0.0 | 0.0 | 0.0 | 0.0 | 0.0 | 0.0 | 0.0 | 0.0 | 0.0 | 0.0 | 0.0 | 0.0 |
| Nlrp14 | 0.0 | 0.0 | 0.0 | 0.0 | 0.0 | 0.0 | 0.0 | 0.0 | 0.0 | 0.0 | 0.0 | 0.0 |
| Nlrp2 | 0.0 | 0.0 | 0.0 | 0.0 | 0.0 | 0.0 | 0.0 | 0.0 | 0.0 | 0.0 | 0.0 | 0.0 |
| Nlrp4c | 0.0 | 0.0 | 0.0 | 0.0 | 0.0 | 0.0 | 0.0 | 0.0 | 0.0 | 0.0 | 0.0 | 0.0 |
| Nlrp4e | 0.0 | 0.0 | 0.0 | 0.0 | 0.0 | 0.0 | 0.0 | 0.0 | 0.0 | 0.0 | 0.0 | 0.0 |
| Nlrp4g | 0.0 | 0.0 | 0.0 | 0.0 | 0.0 | 0.0 | 0.0 | 0.0 | 0.0 | 0.0 | 0.0 | 0.0 |
| Nlrp6 | 0.0 | 0.0 | 0.0 | 0.0 | 0.0 | 0.0 | 0.0 | 0.0 | 0.0 | 0.0 | 0.0 | 0.0 |
| Nlrp9a | 0.0 | 0.0 | 0.0 | 0.0 | 0.0 | 0.0 | 0.0 | 0.0 | 0.0 | 0.0 | 0.0 | 0.0 |
| Nmbr | 0.0 | 0.0 | 0.0 | 0.0 | 0.0 | 0.0 | 0.0 | 0.0 | 0.0 | 0.0 | 0.0 | 0.0 |
| Nme8 | 0.0 | 0.0 | 0.0 | 0.0 | 0.0 | 0.0 | 0.0 | 0.0 | 0.0 | 0.0 | 0.0 | 0.0 |
| Nmrk2 | 0.0 | 0.0 | 0.0 | 0.0 | 0.0 | 0.0 | 0.0 | 0.0 | 0.0 | 0.0 | 0.0 | 0.0 |
| Nms | 0.0 | 0.0 | 0.0 | 0.0 | 0.0 | 0.0 | 0.0 | 0.0 | 0.0 | 0.0 | 0.0 | 0.0 |
| Nmu | 0.0 | 0.0 | 0.0 | 0.0 | 0.0 | 0.0 | 0.0 | 0.0 | 0.0 | 0.0 | 0.0 | 0.0 |
| Nmur1 | 0.0 | 0.0 | 0.0 | 0.0 | 0.0 | 0.0 | 0.0 | 0.0 | 0.0 | 0.0 | 0.0 | 0.0 |
| Nmur2 | 0.0 | 0.0 | 0.0 | 0.0 | 0.0 | 0.0 | 0.0 | 0.0 | 0.0 | 0.0 | 0.0 | 0.0 |
| Nobox | 0.0 | 0.0 | 0.0 | 0.0 | 0.0 | 0.0 | 0.0 | 0.0 | 0.0 | 0.0 | 0.0 | 0.0 |
| Noto | 0.0 | 0.0 | 0.0 | 0.0 | 0.0 | 0.0 | 0.0 | 0.0 | 0.0 | 0.0 | 0.0 | 0.0 |
| Nox3 | 0.0 | 0.0 | 0.0 | 0.0 | 0.1 | 0.0 | 0.0 | 0.0 | 0.0 | 0.0 | 0.1 | 0.0 |
| Npc1l1 | 0.0 | 0.0 | 0.0 | 0.0 | 0.0 | 0.0 | 0.0 | 0.0 | 0.0 | 0.0 | 0.0 | 0.0 |
| Nppa | 0.0 | 0.0 | 0.0 | 0.0 | 0.0 | 0.1 | 0.0 | 0.1 | 0.0 | 0.2 | 0.0 | 0.0 |
| Nppc | 0.0 | 0.0 | 0.0 | 0.0 | 0.0 | 0.0 | 0.0 | 0.0 | 0.0 | 0.1 | 0.0 | 0.1 |
| Npw | 0.0 | 0.0 | 0.0 | 0.0 | 0.0 | 0.0 | 0.0 | 0.0 | 0.0 | 0.0 | 0.0 | 0.0 |
| Npy5r | 0.0 | 0.0 | 0.0 | 0.0 | 0.0 | 0.0 | 0.0 | 0.0 | 0.0 | 0.0 | 0.0 | 0.0 |
| Nr0b1 | 0.0 | 0.0 | 0.0 | 0.0 | 0.0 | 0.0 | 0.0 | 0.0 | 0.0 | 0.0 | 0.0 | 0.0 |
| Nr0b2 | 0.0 | 0.0 | 0.0 | 0.1 | 0.0 | 0.0 | 0.0 | 0.0 | 0.0 | 0.0 | 0.0 | 0.0 |
| Nr1i3 | 0.0 | 0.0 | 0.0 | 0.0 | 0.0 | 0.0 | 0.0 | 0.0 | 0.0 | 0.0 | 0.0 | 0.0 |
| Nr2e1 | 0.0 | 0.0 | 0.0 | 0.1 | 0.0 | 0.0 | 0.0 | 0.0 | 0.0 | 0.0 | 0.0 | 0.0 |
| Nr5a1 | 0.0 | 0.0 | 0.0 | 0.0 | 0.0 | 0.0 | 0.0 | 0.0 | 0.0 | 0.0 | 0.0 | 0.0 |
| Nrgn | 0.0 | 0.1 | 0.0 | 0.0 | 0.0 | 0.0 | 0.0 | 0.0 | 0.0 | 0.0 | 0.0 | 0.0 |
| Nrl | 0.0 | 0.0 | 0.0 | 0.0 | 0.0 | 0.0 | 0.0 | 0.0 | 0.0 | 0.0 | 0.0 | 0.0 |
| Nrsn1 | 0.0 | 0.0 | 0.0 | 0.0 | 0.0 | 0.0 | 0.0 | 0.0 | 0.0 | 0.0 | 0.0 | 0.0 |
| Nsg2 | 0.0 | 0.0 | 0.0 | 0.0 | 0.0 | 0.0 | 0.0 | 0.0 | 0.0 | 0.0 | 0.0 | 0.0 |
| Nt5c1a | 0.0 | 0.0 | 0.0 | 0.0 | 0.0 | 0.0 | 0.0 | 0.0 | 0.0 | 0.0 | 0.0 | 0.0 |
| Ntf3 | 0.0 | 0.0 | 0.0 | 0.0 | 0.0 | 0.0 | 0.0 | 0.0 | 0.1 | 0.0 | 0.0 | 0.0 |
| Ntn5 | 0.0 | 0.0 | 0.0 | 0.0 | 0.0 | 0.0 | 0.0 | 0.0 | 0.0 | 0.0 | 0.0 | 0.0 |
| Nts | 0.0 | 0.0 | 0.0 | 0.0 | 0.1 | 0.0 | 0.0 | 0.1 | 0.0 | 0.0 | 0.0 | 0.0 |
| Ntsr1 | 0.0 | 0.0 | 0.0 | 0.0 | 0.0 | 0.0 | 0.0 | 0.0 | 0.0 | 0.0 | 0.0 | 0.0 |
| Ntsr2 | 0.0 | 0.0 | 0.0 | 0.0 | 0.0 | 0.0 | 0.0 | 0.0 | 0.0 | 0.0 | 0.0 | 0.0 |
| Nxph2 | 0.0 | 0.0 | 0.0 | 0.0 | 0.0 | 0.0 | 0.0 | 0.0 | 0.0 | 0.0 | 0.0 | 0.0 |
| Oas1e | 0.0 | 0.1 | 0.0 | 0.0 | 0.0 | 0.0 | 0.0 | 0.0 | 0.0 | 0.0 | 0.0 | 0.0 |
| Oas1f | 0.0 | 0.0 | 0.0 | 0.0 | 0.0 | 0.0 | 0.0 | 0.1 | 0.0 | 0.0 | 0.0 | 0.0 |
| Oas1h | 0.0 | 0.0 | 0.0 | 0.0 | 0.0 | 0.0 | 0.0 | 0.0 | 0.0 | 0.0 | 0.0 | 0.0 |
| Obox3 | 0.0 | 0.0 | 0.0 | 0.1 | 0.0 | 0.0 | 0.0 | 0.0 | 0.0 | 0.0 | 0.0 | 0.0 |
| Obox6 | 0.0 | 0.0 | 0.0 | 0.0 | 0.0 | 0.0 | 0.0 | 0.0 | 0.0 | 0.0 | 0.0 | 0.0 |
| Ocm | 0.0 | 0.0 | 0.0 | 0.0 | 0.0 | 0.0 | 0.0 | 0.0 | 0.0 | 0.0 | 0.0 | 0.0 |

| | | | | | | | | | | | | |
|---|---|---|---|---|---|---|---|---|---|---|---|---|
| Odam | 0.0 | 0.0 | 0.0 | 0.0 | 0.0 | 0.0 | 0.0 | 0.0 | 0.0 | 0.0 | 0.0 | 0.0 |
| Odf3l1 | 0.0 | 0.0 | 0.0 | 0.0 | 0.0 | 0.2 | 0.0 | 0.1 | 0.0 | 0.0 | 0.0 | 0.0 |
| Odf4 | 0.0 | 0.0 | 0.0 | 0.0 | 0.0 | 0.0 | 0.0 | 0.0 | 0.0 | 0.0 | 0.0 | 0.0 |
| Ofcc1 | 0.0 | 0.0 | 0.0 | 0.0 | 0.0 | 0.0 | 0.0 | 0.0 | 0.0 | 0.0 | 0.0 | 0.0 |
| Oit1 | 0.0 | 0.0 | 0.0 | 0.0 | 0.0 | 0.0 | 0.0 | 0.0 | 0.0 | 0.0 | 0.0 | 0.0 |
| Olfm3 | 0.0 | 0.0 | 0.0 | 0.1 | 0.0 | 0.0 | 0.0 | 0.0 | 0.0 | 0.0 | 0.0 | 0.0 |
| Olfm4 | 0.0 | 0.0 | 0.0 | 0.0 | 0.0 | 0.0 | 0.0 | 0.0 | 0.0 | 0.0 | 0.0 | 0.0 |
| Olfr10 | 0.0 | 0.0 | 0.0 | 0.0 | 0.0 | 0.0 | 0.0 | 0.0 | 0.0 | 0.0 | 0.0 | 0.0 |
| Olfr1002 | 0.0 | 0.0 | 0.0 | 0.0 | 0.0 | 0.0 | 0.0 | 0.0 | 0.0 | 0.0 | 0.0 | 0.0 |
| Olfr1006 | 0.0 | 0.0 | 0.0 | 0.0 | 0.0 | 0.0 | 0.0 | 0.0 | 0.0 | 0.0 | 0.0 | 0.0 |
| Olfr101 | 0.0 | 0.0 | 0.0 | 0.0 | 0.0 | 0.0 | 0.0 | 0.0 | 0.0 | 0.0 | 0.0 | 0.0 |
| Olfr1016 | 0.0 | 0.0 | 0.0 | 0.0 | 0.0 | 0.0 | 0.0 | 0.0 | 0.0 | 0.0 | 0.0 | 0.0 |
| Olfr103 | 0.0 | 0.0 | 0.0 | 0.0 | 0.0 | 0.0 | 0.2 | 0.0 | 0.0 | 0.0 | 0.0 | 0.0 |
| Olfr1032 | 0.0 | 0.0 | 0.0 | 0.0 | 0.0 | 0.0 | 0.0 | 0.0 | 0.0 | 0.0 | 0.0 | 0.0 |
| Olfr1079 | 0.0 | 0.1 | 0.0 | 0.0 | 0.0 | 0.0 | 0.0 | 0.0 | 0.0 | 0.0 | 0.0 | 0.0 |
| Olfr1085 | 0.0 | 0.0 | 0.0 | 0.0 | 0.0 | 0.0 | 0.0 | 0.0 | 0.0 | 0.0 | 0.0 | 0.0 |
| Olfr110 | 0.0 | 0.0 | 0.0 | 0.1 | 0.2 | 0.0 | 0.0 | 0.0 | 0.0 | 0.0 | 0.0 | 0.0 |
| Olfr111 | 0.0 | 0.0 | 0.0 | 0.1 | 0.2 | 0.0 | 0.0 | 0.0 | 0.0 | 0.0 | 0.0 | 0.0 |
| Olfr112 | 0.0 | 0.0 | 0.0 | 0.0 | 0.0 | 0.0 | 0.0 | 0.0 | 0.0 | 0.0 | 0.0 | 0.0 |
| Olfr1123 | 0.0 | 0.0 | 0.0 | 0.0 | 0.0 | 0.0 | 0.0 | 0.0 | 0.0 | 0.0 | 0.0 | 0.0 |
| Olfr1132 | 0.0 | 0.0 | 0.0 | 0.0 | 0.0 | 0.0 | 0.0 | 0.0 | 0.0 | 0.0 | 0.0 | 0.0 |
| Olfr1133 | 0.0 | 0.0 | 0.0 | 0.0 | 0.0 | 0.0 | 0.0 | 0.0 | 0.0 | 0.0 | 0.0 | 0.0 |
| Olfr114 | 0.0 | 0.0 | 0.0 | 0.0 | 0.0 | 0.0 | 0.0 | 0.0 | 0.0 | 0.0 | 0.0 | 0.0 |
| Olfr115 | 0.0 | 0.0 | 0.0 | 0.1 | 0.0 | 0.0 | 0.0 | 0.0 | 0.0 | 0.0 | 0.0 | 0.0 |
| Olfr1158 | 0.0 | 0.0 | 0.0 | 0.1 | 0.0 | 0.0 | 0.0 | 0.0 | 0.0 | 0.0 | 0.0 | 0.0 |
| Olfr1166 | 0.0 | 0.0 | 0.0 | 0.0 | 0.0 | 0.0 | 0.0 | 0.0 | 0.0 | 0.0 | 0.0 | 0.0 |
| Olfr1167 | 0.0 | 0.0 | 0.0 | 0.0 | 0.0 | 0.0 | 0.0 | 0.0 | 0.0 | 0.0 | 0.0 | 0.0 |
| Olfr1189 | 0.0 | 0.0 | 0.0 | 0.0 | 0.0 | 0.0 | 0.0 | 0.0 | 0.0 | 0.0 | 0.0 | 0.0 |
| Olfr12 | 0.0 | 0.0 | 0.0 | 0.0 | 0.0 | 0.0 | 0.0 | 0.0 | 0.0 | 0.0 | 0.0 | 0.0 |
| Olfr1208 | 0.0 | 0.0 | 0.0 | 0.0 | 0.0 | 0.0 | 0.0 | 0.0 | 0.0 | 0.0 | 0.0 | 0.0 |
| Olfr1231 | 0.0 | 0.0 | 0.0 | 0.0 | 0.0 | 0.0 | 0.0 | 0.0 | 0.0 | 0.0 | 0.0 | 0.0 |
| Olfr1233 | 0.0 | 0.0 | 0.0 | 0.3 | 0.0 | 0.0 | 0.0 | 0.0 | 0.0 | 0.0 | 0.0 | 0.0 |
| Olfr1247 | 0.0 | 0.0 | 0.0 | 0.0 | 0.0 | 0.0 | 0.0 | 0.0 | 0.0 | 0.0 | 0.0 | 0.0 |
| Olfr1261 | 0.0 | 0.0 | 0.0 | 0.0 | 0.0 | 0.0 | 0.0 | 0.0 | 0.0 | 0.0 | 0.1 | 0.0 |
| Olfr127 | 0.0 | 0.1 | 0.0 | 0.0 | 0.0 | 0.0 | 0.0 | 0.0 | 0.0 | 0.0 | 0.0 | 0.0 |
| Olfr1270 | 0.0 | 0.0 | 0.0 | 0.0 | 0.0 | 0.0 | 0.0 | 0.0 | 0.0 | 0.0 | 0.0 | 0.0 |
| Olfr1274-ps | 0.0 | 0.0 | 0.0 | 0.0 | 0.0 | 0.0 | 0.0 | 0.0 | 0.0 | 0.0 | 0.0 | 0.0 |
| Olfr1280 | 0.0 | 0.0 | 0.0 | 0.0 | 0.0 | 0.0 | 0.0 | 0.0 | 0.0 | 0.0 | 0.0 | 0.0 |
| Olfr1281 | 0.0 | 0.0 | 0.0 | 0.0 | 0.0 | 0.0 | 0.0 | 0.0 | 0.0 | 0.0 | 0.0 | 0.0 |
| Olfr129 | 0.0 | 0.0 | 0.0 | 0.0 | 0.0 | 0.0 | 0.0 | 0.0 | 0.0 | 0.0 | 0.0 | 0.0 |
| Olfr1297 | 0.0 | 0.0 | 0.0 | 0.0 | 0.0 | 0.0 | 0.0 | 0.0 | 0.0 | 0.0 | 0.0 | 0.0 |
| Olfr1299 | 0.0 | 0.0 | 0.0 | 0.0 | 0.0 | 0.0 | 0.0 | 0.0 | 0.0 | 0.0 | 0.1 | 0.0 |
| Olfr1306 | 0.0 | 0.0 | 0.0 | 0.0 | 0.0 | 0.0 | 0.0 | 0.0 | 0.0 | 0.0 | 0.0 | 0.0 |
| Olfr1307 | 0.0 | 0.0 | 0.0 | 0.0 | 0.0 | 0.0 | 0.0 | 0.0 | 0.0 | 0.0 | 0.0 | 0.0 |
| Olfr1309 | 0.0 | 0.0 | 0.1 | 0.0 | 0.0 | 0.0 | 0.0 | 0.0 | 0.0 | 0.0 | 0.0 | 0.0 |
| Olfr1310 | 0.0 | 0.0 | 0.0 | 0.0 | 0.1 | 0.0 | 0.0 | 0.0 | 0.0 | 0.0 | 0.0 | 0.0 |
| Olfr1313 | 0.0 | 0.0 | 0.0 | 0.0 | 0.0 | 0.0 | 0.0 | 0.0 | 0.0 | 0.0 | 0.0 | 0.0 |
| Olfr1316 | 0.0 | 0.0 | 0.0 | 0.0 | 0.0 | 0.0 | 0.0 | 0.0 | 0.0 | 0.0 | 0.1 | 0.0 |
| Olfr1338 | 0.0 | 0.0 | 0.0 | 0.1 | 0.0 | 0.0 | 0.0 | 0.0 | 0.0 | 0.0 | 0.0 | 0.0 |
| Olfr1344 | 0.0 | 0.1 | 0.0 | 0.0 | 0.0 | 0.0 | 0.0 | 0.0 | 0.0 | 0.0 | 0.0 | 0.0 |
| Olfr1352 | 0.0 | 0.1 | 0.0 | 0.0 | 0.0 | 0.0 | 0.0 | 0.0 | 0.0 | 0.0 | 0.0 | 0.0 |
| Olfr1353 | 0.0 | 0.0 | 0.0 | 0.0 | 0.0 | 0.0 | 0.0 | 0.0 | 0.0 | 0.0 | 0.0 | 0.0 |
| Olfr1355 | 0.0 | 0.0 | 0.0 | 0.0 | 0.0 | 0.0 | 0.0 | 0.0 | 0.0 | 0.0 | 0.0 | 0.0 |
| Olfr1356 | 0.0 | 0.0 | 0.0 | 0.0 | 0.0 | 0.0 | 0.0 | 0.0 | 0.0 | 0.0 | 0.0 | 0.0 |
| Olfr1364 | 0.0 | 0.0 | 0.0 | 0.0 | 0.0 | 0.0 | 0.0 | 0.0 | 0.0 | 0.0 | 0.0 | 0.0 |
| Olfr1366 | 0.0 | 0.0 | 0.0 | 0.0 | 0.0 | 0.0 | 0.0 | 0.0 | 0.0 | 0.0 | 0.0 | 0.0 |
| Olfr1370 | 0.0 | 0.0 | 0.0 | 0.0 | 0.0 | 0.0 | 0.0 | 0.0 | 0.0 | 0.0 | 0.1 | 0.0 |

| | | | | | | | | | | | | |
|---|---|---|---|---|---|---|---|---|---|---|---|---|
| Olfr1378 | 0.0 | 0.0 | 0.0 | 0.0 | 0.0 | 0.0 | 0.1 | 0.0 | 0.0 | 0.0 | 0.0 | 0.0 |
| Olfr1383 | 0.0 | 0.0 | 0.0 | 0.0 | 0.0 | 0.0 | 0.0 | 0.0 | 0.0 | 0.0 | 0.0 | 0.0 |
| Olfr1389 | 0.0 | 0.0 | 0.0 | 0.1 | 0.0 | 0.0 | 0.0 | 0.0 | 0.0 | 0.0 | 0.0 | 0.0 |
| Olfr1392 | 0.0 | 0.0 | 0.0 | 0.0 | 0.0 | 0.0 | 0.0 | 0.0 | 0.0 | 0.0 | 0.0 | 0.0 |
| Olfr1393 | 0.0 | 0.0 | 0.0 | 0.0 | 0.0 | 0.0 | 0.0 | 0.0 | 0.0 | 0.0 | 0.0 | 0.0 |
| Olfr1418 | 0.0 | 0.0 | 0.0 | 0.0 | 0.0 | 0.0 | 0.0 | 0.0 | 0.0 | 0.0 | 0.0 | 0.0 |
| Olfr142 | 0.0 | 0.0 | 0.0 | 0.0 | 0.0 | 0.0 | 0.0 | 0.0 | 0.0 | 0.0 | 0.0 | 0.0 |
| Olfr1431 | 0.0 | 0.0 | 0.0 | 0.0 | 0.0 | 0.0 | 0.0 | 0.0 | 0.0 | 0.0 | 0.0 | 0.0 |
| Olfr1444 | 0.0 | 0.0 | 0.0 | 0.0 | 0.0 | 0.0 | 0.0 | 0.0 | 0.0 | 0.0 | 0.0 | 0.0 |
| Olfr1480 | 0.0 | 0.0 | 0.0 | 0.0 | 0.0 | 0.0 | 0.0 | 0.0 | 0.0 | 0.0 | 0.0 | 0.0 |
| Olfr1487 | 0.0 | 0.0 | 0.0 | 0.0 | 0.0 | 0.0 | 0.0 | 0.0 | 0.0 | 0.0 | 0.0 | 0.0 |
| Olfr15 | 0.0 | 0.0 | 0.0 | 0.0 | 0.0 | 0.0 | 0.0 | 0.0 | 0.0 | 0.0 | 0.0 | 0.0 |
| Olfr1511 | 0.0 | 0.0 | 0.0 | 0.0 | 0.0 | 0.0 | 0.0 | 0.0 | 0.0 | 0.0 | 0.0 | 0.0 |
| Olfr1512 | 0.0 | 0.0 | 0.0 | 0.0 | 0.0 | 0.0 | 0.0 | 0.0 | 0.0 | 0.0 | 0.0 | 0.0 |
| Olfr1532-ps1 | 0.0 | 0.0 | 0.0 | 0.0 | 0.0 | 0.0 | 0.0 | 0.0 | 0.0 | 0.0 | 0.0 | 0.0 |
| Olfr1535 | 0.0 | 0.0 | 0.0 | 0.0 | 0.0 | 0.0 | 0.0 | 0.0 | 0.0 | 0.0 | 0.0 | 0.0 |
| Olfr154 | 0.0 | 0.0 | 0.0 | 0.0 | 0.0 | 0.0 | 0.0 | 0.0 | 0.0 | 0.0 | 0.0 | 0.0 |
| Olfr156 | 0.1 | 0.0 | 0.0 | 0.0 | 0.0 | 0.0 | 0.0 | 0.0 | 0.0 | 0.0 | 0.0 | 0.0 |
| Olfr157 | 0.0 | 0.0 | 0.0 | 0.0 | 0.0 | 0.0 | 0.0 | 0.0 | 0.0 | 0.0 | 0.0 | 0.0 |
| Olfr159 | 0.1 | 0.0 | 0.1 | 0.0 | 0.0 | 0.0 | 0.0 | 0.0 | 0.0 | 0.0 | 0.0 | 0.0 |
| Olfr16 | 0.0 | 0.0 | 0.0 | 0.0 | 0.0 | 0.0 | 0.0 | 0.0 | 0.0 | 0.0 | 0.1 | 0.0 |
| Olfr165 | 0.0 | 0.0 | 0.0 | 0.0 | 0.0 | 0.0 | 0.0 | 0.0 | 0.0 | 0.0 | 0.0 | 0.0 |
| Olfr172 | 0.0 | 0.0 | 0.0 | 0.0 | 0.0 | 0.0 | 0.0 | 0.0 | 0.0 | 0.0 | 0.1 | 0.0 |
| Olfr173 | 0.0 | 0.0 | 0.0 | 0.0 | 0.0 | 0.0 | 0.0 | 0.0 | 0.0 | 0.0 | 0.1 | 0.0 |
| Olfr175-ps1 | 0.0 | 0.0 | 0.0 | 0.0 | 0.0 | 0.0 | 0.0 | 0.0 | 0.0 | 0.0 | 0.0 | 0.0 |
| Olfr176 | 0.0 | 0.0 | 0.0 | 0.0 | 0.0 | 0.0 | 0.0 | 0.0 | 0.0 | 0.0 | 0.0 | 0.0 |
| Olfr177 | 0.0 | 0.0 | 0.0 | 0.0 | 0.0 | 0.0 | 0.0 | 0.0 | 0.0 | 0.0 | 0.0 | 0.0 |
| Olfr178 | 0.0 | 0.0 | 0.0 | 0.0 | 0.0 | 0.0 | 0.0 | 0.0 | 0.0 | 0.0 | 0.0 | 0.0 |
| Olfr179 | 0.0 | 0.0 | 0.0 | 0.0 | 0.0 | 0.0 | 0.0 | 0.0 | 0.0 | 0.0 | 0.0 | 0.0 |
| Olfr180 | 0.0 | 0.0 | 0.0 | 0.0 | 0.0 | 0.0 | 0.0 | 0.0 | 0.0 | 0.0 | 0.0 | 0.0 |
| Olfr191 | 0.0 | 0.0 | 0.0 | 0.0 | 0.0 | 0.0 | 0.0 | 0.0 | 0.0 | 0.0 | 0.0 | 0.0 |
| Olfr193 | 0.0 | 0.0 | 0.0 | 0.0 | 0.0 | 0.0 | 0.0 | 0.0 | 0.0 | 0.0 | 0.0 | 0.0 |
| Olfr195 | 0.0 | 0.0 | 0.0 | 0.0 | 0.0 | 0.0 | 0.0 | 0.0 | 0.0 | 0.0 | 0.0 | 0.0 |
| Olfr203 | 0.0 | 0.0 | 0.0 | 0.0 | 0.0 | 0.0 | 0.0 | 0.0 | 0.0 | 0.0 | 0.0 | 0.0 |
| Olfr221 | 0.0 | 0.0 | 0.0 | 0.0 | 0.0 | 0.0 | 0.0 | 0.0 | 0.0 | 0.0 | 0.0 | 0.0 |
| Olfr223 | 0.0 | 0.0 | 0.0 | 0.0 | 0.0 | 0.0 | 0.0 | 0.0 | 0.0 | 0.0 | 0.0 | 0.0 |
| Olfr231 | 0.0 | 0.0 | 0.0 | 0.0 | 0.0 | 0.0 | 0.0 | 0.0 | 0.0 | 0.0 | 0.0 | 0.0 |
| Olfr267 | 0.0 | 0.0 | 0.0 | 0.0 | 0.0 | 0.0 | 0.2 | 0.0 | 0.0 | 0.0 | 0.0 | 0.0 |
| Olfr275 | 0.0 | 0.0 | 0.0 | 0.0 | 0.0 | 0.0 | 0.0 | 0.0 | 0.0 | 0.0 | 0.0 | 0.0 |
| Olfr283 | 0.0 | 0.0 | 0.0 | 0.0 | 0.0 | 0.2 | 0.0 | 0.0 | 0.0 | 0.0 | 0.0 | 0.0 |
| Olfr286 | 0.0 | 0.1 | 0.0 | 0.0 | 0.0 | 0.0 | 0.0 | 0.0 | 0.0 | 0.0 | 0.0 | 0.0 |
| Olfr287 | 0.0 | 0.0 | 0.0 | 0.0 | 0.0 | 0.0 | 0.0 | 0.0 | 0.0 | 0.0 | 0.0 | 0.0 |
| Olfr288 | 0.0 | 0.0 | 0.0 | 0.0 | 0.0 | 0.0 | 0.0 | 0.0 | 0.0 | 0.0 | 0.0 | 0.0 |
| Olfr290 | 0.0 | 0.0 | 0.0 | 0.0 | 0.0 | 0.0 | 0.0 | 0.0 | 0.0 | 0.0 | 0.0 | 0.0 |
| Olfr291 | 0.0 | 0.0 | 0.0 | 0.0 | 0.0 | 0.0 | 0.0 | 0.0 | 0.0 | 0.0 | 0.0 | 0.0 |
| Olfr292 | 0.0 | 0.0 | 0.0 | 0.0 | 0.0 | 0.0 | 0.1 | 0.0 | 0.0 | 0.0 | 0.0 | 0.0 |
| Olfr298 | 0.0 | 0.0 | 0.0 | 0.0 | 0.0 | 0.0 | 0.0 | 0.0 | 0.0 | 0.0 | 0.0 | 0.0 |
| Olfr299 | 0.0 | 0.0 | 0.0 | 0.0 | 0.0 | 0.0 | 0.0 | 0.0 | 0.0 | 0.0 | 0.0 | 0.0 |
| Olfr303 | 0.0 | 0.0 | 0.0 | 0.0 | 0.0 | 0.0 | 0.0 | 0.0 | 0.0 | 0.0 | 0.0 | 0.0 |
| Olfr307 | 0.0 | 0.1 | 0.0 | 0.0 | 0.0 | 0.0 | 0.0 | 0.0 | 0.0 | 0.0 | 0.0 | 0.0 |
| Olfr308 | 0.0 | 0.0 | 0.0 | 0.0 | 0.0 | 0.0 | 0.0 | 0.0 | 0.0 | 0.0 | 0.0 | 0.0 |
| Olfr31 | 0.1 | 0.0 | 0.0 | 0.0 | 0.1 | 0.0 | 0.0 | 0.0 | 0.0 | 0.0 | 0.0 | 0.0 |
| Olfr311 | 0.0 | 0.0 | 0.0 | 0.0 | 0.0 | 0.0 | 0.0 | 0.0 | 0.0 | 0.0 | 0.0 | 0.0 |
| Olfr330 | 0.0 | 0.0 | 0.0 | 0.0 | 0.0 | 0.0 | 0.0 | 0.0 | 0.0 | 0.0 | 0.0 | 0.0 |
| Olfr366 | 0.0 | 0.0 | 0.0 | 0.0 | 0.0 | 0.0 | 0.0 | 0.0 | 0.0 | 0.0 | 0.0 | 0.0 |
| Olfr368 | 0.0 | 0.0 | 0.0 | 0.0 | 0.0 | 0.0 | 0.0 | 0.0 | 0.0 | 0.0 | 0.0 | 0.0 |
| Olfr370 | 0.0 | 0.0 | 0.0 | 0.0 | 0.0 | 0.0 | 0.0 | 0.0 | 0.0 | 0.0 | 0.0 | 0.0 |

| | | | | | | | | | | | |
|---|---|---|---|---|---|---|---|---|---|---|---|
| Olfr372 | 0.0 | 0.0 | 0.0 | 0.0 | 0.0 | 0.0 | 0.0 | 0.0 | 0.0 | 0.0 | 0.0 | 0.0 |
| Olfr373 | 0.0 | 0.0 | 0.0 | 0.0 | 0.0 | 0.0 | 0.0 | 0.0 | 0.0 | 0.0 | 0.0 | 0.0 |
| Olfr374 | 0.0 | 0.0 | 0.0 | 0.0 | 0.0 | 0.0 | 0.0 | 0.0 | 0.0 | 0.0 | 0.0 | 0.0 |
| Olfr378 | 0.0 | 0.0 | 0.0 | 0.0 | 0.0 | 0.0 | 0.0 | 0.0 | 0.0 | 0.0 | 0.0 | 0.0 |
| Olfr39 | 0.0 | 0.0 | 0.0 | 0.0 | 0.0 | 0.0 | 0.0 | 0.0 | 0.1 | 0.0 | 0.0 | 0.0 |
| Olfr411 | 0.0 | 0.0 | 0.0 | 0.0 | 0.0 | 0.0 | 0.0 | 0.0 | 0.0 | 0.2 | 0.0 | 0.0 |
| Olfr424 | 0.0 | 0.0 | 0.0 | 0.0 | 0.0 | 0.0 | 0.0 | 0.0 | 0.0 | 0.0 | 0.0 | 0.0 |
| Olfr435 | 0.0 | 0.0 | 0.0 | 0.0 | 0.0 | 0.0 | 0.0 | 0.0 | 0.0 | 0.0 | 0.0 | 0.0 |
| Olfr446 | 0.0 | 0.0 | 0.0 | 0.0 | 0.0 | 0.0 | 0.0 | 0.0 | 0.0 | 0.0 | 0.0 | 0.0 |
| Olfr448 | 0.0 | 0.0 | 0.0 | 0.0 | 0.0 | 0.0 | 0.0 | 0.0 | 0.0 | 0.0 | 0.1 | 0.0 |
| Olfr455 | 0.0 | 0.0 | 0.0 | 0.0 | 0.0 | 0.1 | 0.0 | 0.0 | 0.0 | 0.1 | 0.0 | 0.0 |
| Olfr457 | 0.0 | 0.0 | 0.0 | 0.0 | 0.0 | 0.0 | 0.0 | 0.0 | 0.0 | 0.0 | 0.0 | 0.0 |
| Olfr458 | 0.1 | 0.2 | 0.0 | 0.0 | 0.0 | 0.0 | 0.0 | 0.0 | 0.0 | 0.0 | 0.0 | 0.0 |
| Olfr461 | 0.0 | 0.1 | 0.0 | 0.0 | 0.0 | 0.1 | 0.0 | 0.0 | 0.0 | 0.0 | 0.0 | 0.0 |
| Olfr466 | 0.0 | 0.0 | 0.0 | 0.0 | 0.0 | 0.0 | 0.0 | 0.0 | 0.0 | 0.0 | 0.0 | 0.0 |
| Olfr483 | 0.0 | 0.0 | 0.0 | 0.0 | 0.0 | 0.0 | 0.0 | 0.0 | 0.0 | 0.0 | 0.0 | 0.0 |
| Olfr486 | 0.0 | 0.0 | 0.0 | 0.0 | 0.0 | 0.0 | 0.0 | 0.0 | 0.0 | 0.0 | 0.0 | 0.1 |
| Olfr49 | 0.1 | 0.0 | 0.0 | 0.0 | 0.0 | 0.0 | 0.0 | 0.0 | 0.1 | 0.0 | 0.1 | 0.0 |
| Olfr493 | 0.0 | 0.0 | 0.0 | 0.0 | 0.0 | 0.0 | 0.0 | 0.0 | 0.0 | 0.0 | 0.0 | 0.0 |
| Olfr498 | 0.0 | 0.0 | 0.0 | 0.0 | 0.0 | 0.0 | 0.0 | 0.0 | 0.0 | 0.0 | 0.0 | 0.0 |
| Olfr502 | 0.0 | 0.0 | 0.0 | 0.0 | 0.0 | 0.0 | 0.0 | 0.0 | 0.0 | 0.0 | 0.0 | 0.0 |
| Olfr507 | 0.0 | 0.0 | 0.0 | 0.0 | 0.0 | 0.0 | 0.0 | 0.0 | 0.0 | 0.0 | 0.0 | 0.0 |
| Olfr513 | 0.0 | 0.0 | 0.0 | 0.0 | 0.0 | 0.0 | 0.0 | 0.0 | 0.0 | 0.0 | 0.0 | 0.0 |
| Olfr514 | 0.0 | 0.0 | 0.0 | 0.0 | 0.0 | 0.0 | 0.0 | 0.0 | 0.0 | 0.0 | 0.0 | 0.0 |
| Olfr516 | 0.0 | 0.0 | 0.0 | 0.0 | 0.0 | 0.0 | 0.0 | 0.0 | 0.0 | 0.0 | 0.0 | 0.0 |
| Olfr536 | 0.0 | 0.0 | 0.0 | 0.0 | 0.0 | 0.0 | 0.0 | 0.0 | 0.0 | 0.0 | 0.0 | 0.0 |
| Olfr547 | 0.0 | 0.0 | 0.0 | 0.0 | 0.0 | 0.0 | 0.0 | 0.0 | 0.0 | 0.0 | 0.0 | 0.0 |
| Olfr549 | 0.0 | 0.0 | 0.0 | 0.0 | 0.0 | 0.0 | 0.0 | 0.0 | 0.0 | 0.0 | 0.0 | 0.0 |
| Olfr55 | 0.0 | 0.0 | 0.1 | 0.0 | 0.0 | 0.1 | 0.0 | 0.0 | 0.0 | 0.0 | 0.0 | 0.0 |
| Olfr550 | 0.1 | 0.0 | 0.0 | 0.0 | 0.0 | 0.0 | 0.0 | 0.0 | 0.0 | 0.0 | 0.0 | 0.0 |
| Olfr553 | 0.0 | 0.0 | 0.0 | 0.0 | 0.0 | 0.0 | 0.0 | 0.0 | 0.0 | 0.0 | 0.0 | 0.0 |
| Olfr559 | 0.0 | 0.0 | 0.0 | 0.0 | 0.0 | 0.0 | 0.0 | 0.0 | 0.0 | 0.0 | 0.0 | 0.0 |
| Olfr569 | 0.0 | 0.0 | 0.0 | 0.0 | 0.0 | 0.0 | 0.0 | 0.0 | 0.0 | 0.0 | 0.0 | 0.0 |
| Olfr57 | 0.0 | 0.3 | 0.1 | 0.0 | 0.0 | 0.0 | 0.0 | 0.0 | 0.0 | 0.0 | 0.1 | 0.0 |
| Olfr570 | 0.0 | 0.0 | 0.0 | 0.0 | 0.0 | 0.0 | 0.0 | 0.0 | 0.0 | 0.0 | 0.0 | 0.1 |
| Olfr575 | 0.0 | 0.0 | 0.0 | 0.0 | 0.0 | 0.0 | 0.0 | 0.0 | 0.0 | 0.0 | 0.0 | 0.0 |
| Olfr594 | 0.0 | 0.0 | 0.0 | 0.0 | 0.0 | 0.0 | 0.0 | 0.0 | 0.0 | 0.0 | 0.1 | 0.0 |
| Olfr603 | 0.0 | 0.0 | 0.0 | 0.0 | 0.0 | 0.0 | 0.0 | 0.0 | 0.0 | 0.0 | 0.0 | 0.0 |
| Olfr606 | 0.1 | 0.0 | 0.0 | 0.0 | 0.0 | 0.0 | 0.0 | 0.0 | 0.0 | 0.0 | 0.0 | 0.0 |
| Olfr615 | 0.0 | 0.0 | 0.0 | 0.0 | 0.0 | 0.0 | 0.0 | 0.0 | 0.0 | 0.0 | 0.0 | 0.0 |
| Olfr635 | 0.0 | 0.0 | 0.0 | 0.0 | 0.0 | 0.0 | 0.0 | 0.0 | 0.0 | 0.0 | 0.0 | 0.0 |
| Olfr643 | 0.0 | 0.0 | 0.0 | 0.0 | 0.0 | 0.1 | 0.0 | 0.0 | 0.0 | 0.0 | 0.0 | 0.0 |
| Olfr658 | 0.0 | 0.0 | 0.0 | 0.0 | 0.0 | 0.0 | 0.0 | 0.0 | 0.0 | 0.0 | 0.0 | 0.0 |
| Olfr668 | 0.0 | 0.0 | 0.0 | 0.0 | 0.0 | 0.0 | 0.0 | 0.0 | 0.0 | 0.0 | 0.0 | 0.0 |
| Olfr678 | 0.0 | 0.0 | 0.0 | 0.0 | 0.0 | 0.0 | 0.0 | 0.0 | 0.0 | 0.0 | 0.0 | 0.0 |
| Olfr679 | 0.0 | 0.0 | 0.1 | 0.0 | 0.0 | 0.0 | 0.0 | 0.0 | 0.0 | 0.0 | 0.0 | 0.0 |
| Olfr689 | 0.0 | 0.0 | 0.0 | 0.0 | 0.0 | 0.0 | 0.0 | 0.0 | 0.0 | 0.0 | 0.0 | 0.0 |
| Olfr690 | 0.0 | 0.0 | 0.0 | 0.0 | 0.0 | 0.0 | 0.0 | 0.0 | 0.0 | 0.0 | 0.0 | 0.0 |
| Olfr70 | 0.0 | 0.0 | 0.0 | 0.0 | 0.0 | 0.0 | 0.0 | 0.0 | 0.0 | 0.0 | 0.0 | 0.0 |
| Olfr702 | 0.0 | 0.0 | 0.0 | 0.0 | 0.0 | 0.0 | 0.0 | 0.0 | 0.0 | 0.0 | 0.0 | 0.0 |
| Olfr707 | 0.0 | 0.0 | 0.0 | 0.0 | 0.0 | 0.0 | 0.0 | 0.0 | 0.0 | 0.0 | 0.0 | 0.0 |
| Olfr713 | 0.0 | 0.0 | 0.0 | 0.0 | 0.0 | 0.0 | 0.0 | 0.0 | 0.0 | 0.0 | 0.0 | 0.0 |
| Olfr735 | 0.0 | 0.0 | 0.0 | 0.0 | 0.0 | 0.0 | 0.0 | 0.0 | 0.0 | 0.0 | 0.0 | 0.0 |
| Olfr74 | 0.0 | 0.0 | 0.0 | 0.0 | 0.0 | 0.0 | 0.0 | 0.0 | 0.0 | 0.0 | 0.0 | 0.0 |
| Olfr743 | 0.0 | 0.0 | 0.0 | 0.0 | 0.0 | 0.0 | 0.0 | 0.0 | 0.0 | 0.0 | 0.0 | 0.0 |
| Olfr750 | 0.0 | 0.0 | 0.0 | 0.0 | 0.0 | 0.0 | 0.0 | 0.0 | 0.0 | 0.0 | 0.0 | 0.1 |
| Olfr763 | 0.0 | 0.0 | 0.0 | 0.0 | 0.0 | 0.0 | 0.0 | 0.0 | 0.0 | 0.0 | 0.0 | 0.0 |

Excluded Genes

| | | | | | | | | | | | | |
|---|---|---|---|---|---|---|---|---|---|---|---|---|
| Olfr790 | 0.0 | 0.0 | 0.0 | 0.0 | 0.0 | 0.0 | 0.0 | 0.0 | 0.0 | 0.0 | 0.0 | 0.0 |
| Olfr8 | 0.0 | 0.0 | 0.0 | 0.0 | 0.0 | 0.0 | 0.0 | 0.0 | 0.0 | 0.0 | 0.0 | 0.0 |
| Olfr811 | 0.0 | 0.0 | 0.0 | 0.0 | 0.0 | 0.0 | 0.0 | 0.0 | 0.0 | 0.0 | 0.0 | 0.0 |
| Olfr827 | 0.0 | 0.0 | 0.0 | 0.0 | 0.0 | 0.0 | 0.0 | 0.0 | 0.1 | 0.0 | 0.0 | 0.0 |
| Olfr847 | 0.0 | 0.0 | 0.0 | 0.0 | 0.0 | 0.0 | 0.0 | 0.0 | 0.0 | 0.0 | 0.0 | 0.0 |
| Olfr857 | 0.0 | 0.0 | 0.0 | 0.0 | 0.0 | 0.0 | 0.0 | 0.0 | 0.0 | 0.0 | 0.0 | 0.0 |
| Olfr862 | 0.0 | 0.0 | 0.0 | 0.0 | 0.0 | 0.0 | 0.0 | 0.0 | 0.0 | 0.0 | 0.0 | 0.0 |
| Olfr877 | 0.0 | 0.0 | 0.0 | 0.0 | 0.0 | 0.0 | 0.0 | 0.0 | 0.0 | 0.0 | 0.0 | 0.0 |
| Olfr9 | 0.0 | 0.0 | 0.0 | 0.0 | 0.0 | 0.0 | 0.0 | 0.0 | 0.0 | 0.0 | 0.0 | 0.0 |
| Olfr90 | 0.0 | 0.0 | 0.0 | 0.0 | 0.0 | 0.1 | 0.0 | 0.0 | 0.0 | 0.0 | 0.0 | 0.0 |
| Olfr91 | 0.0 | 0.0 | 0.0 | 0.0 | 0.0 | 0.0 | 0.0 | 0.0 | 0.0 | 0.0 | 0.0 | 0.0 |
| Olfr922 | 0.0 | 0.0 | 0.0 | 0.0 | 0.0 | 0.0 | 0.0 | 0.0 | 0.0 | 0.0 | 0.0 | 0.0 |
| Olfr923 | 0.0 | 0.0 | 0.0 | 0.2 | 0.0 | 0.0 | 0.0 | 0.0 | 0.0 | 0.0 | 0.0 | 0.0 |
| Olfr93 | 0.0 | 0.0 | 0.0 | 0.0 | 0.0 | 0.0 | 0.0 | 0.0 | 0.0 | 0.0 | 0.0 | 0.0 |
| Olfr943 | 0.1 | 0.0 | 0.0 | 0.0 | 0.0 | 0.0 | 0.0 | 0.0 | 0.0 | 0.0 | 0.0 | 0.0 |
| Olfr97 | 0.0 | 0.0 | 0.0 | 0.0 | 0.0 | 0.0 | 0.0 | 0.0 | 0.0 | 0.0 | 0.0 | 0.0 |
| Olfr971 | 0.0 | 0.0 | 0.0 | 0.0 | 0.0 | 0.0 | 0.0 | 0.0 | 0.0 | 0.0 | 0.0 | 0.0 |
| Olfr98 | 0.0 | 0.0 | 0.0 | 0.0 | 0.0 | 0.0 | 0.0 | 0.0 | 0.0 | 0.0 | 0.0 | 0.0 |
| Olfr982 | 0.0 | 0.0 | 0.0 | 0.0 | 0.0 | 0.0 | 0.0 | 0.0 | 0.0 | 0.0 | 0.0 | 0.0 |
| Olfr988 | 0.0 | 0.0 | 0.0 | 0.0 | 0.0 | 0.0 | 0.0 | 0.0 | 0.0 | 0.0 | 0.0 | 0.0 |
| Olfr994 | 0.0 | 0.1 | 0.0 | 0.0 | 0.0 | 0.0 | 0.0 | 0.0 | 0.0 | 0.0 | 0.0 | 0.0 |
| Olig2 | 0.0 | 0.0 | 0.0 | 0.0 | 0.0 | 0.0 | 0.0 | 0.0 | 0.0 | 0.0 | 0.0 | 0.0 |
| Olig3 | 0.0 | 0.0 | 0.0 | 0.0 | 0.0 | 0.0 | 0.0 | 0.0 | 0.0 | 0.0 | 0.0 | 0.0 |
| Onecut1 | 0.0 | 0.0 | 0.0 | 0.0 | 0.0 | 0.0 | 0.0 | 0.0 | 0.0 | 0.0 | 0.0 | 0.0 |
| Onecut3 | 0.0 | 0.0 | 0.0 | 0.0 | 0.0 | 0.0 | 0.0 | 0.0 | 0.0 | 0.0 | 0.0 | 0.0 |
| Oog1 | 0.0 | 0.0 | 0.0 | 0.0 | 0.0 | 0.0 | 0.0 | 0.0 | 0.0 | 0.0 | 0.0 | 0.0 |
| Oog2 | 0.0 | 0.0 | 0.0 | 0.0 | 0.0 | 0.0 | 0.0 | 0.0 | 0.0 | 0.0 | 0.0 | 0.0 |
| Oog3 | 0.0 | 0.0 | 0.0 | 0.0 | 0.0 | 0.0 | 0.0 | 0.0 | 0.0 | 0.0 | 0.0 | 0.0 |
| Oosp1 | 0.0 | 0.0 | 0.0 | 0.0 | 0.0 | 0.0 | 0.0 | 0.0 | 0.0 | 0.0 | 0.0 | 0.0 |
| Opalin | 0.0 | 0.0 | 0.0 | 0.0 | 0.0 | 0.0 | 0.0 | 0.0 | 0.0 | 0.0 | 0.0 | 0.1 |
| Opn4 | 0.0 | 0.0 | 0.0 | 0.0 | 0.0 | 0.0 | 0.0 | 0.0 | 0.0 | 0.0 | 0.0 | 0.0 |
| Oprd1 | 0.0 | 0.0 | 0.0 | 0.0 | 0.0 | 0.0 | 0.0 | 0.0 | 0.0 | 0.0 | 0.0 | 0.0 |
| Oprk1 | 0.0 | 0.0 | 0.0 | 0.0 | 0.0 | 0.0 | 0.0 | 0.0 | 0.0 | 0.0 | 0.0 | 0.0 |
| Oprm1 | 0.0 | 0.0 | 0.0 | 0.0 | 0.0 | 0.0 | 0.0 | 0.0 | 0.0 | 0.0 | 0.0 | 0.0 |
| Otc | 0.0 | 0.0 | 0.0 | 0.0 | 0.0 | 0.0 | 0.0 | 0.0 | 0.0 | 0.0 | 0.0 | 0.0 |
| Otog | 0.0 | 0.0 | 0.0 | 0.0 | 0.0 | 0.0 | 0.0 | 0.0 | 0.0 | 0.0 | 0.0 | 0.0 |
| Otop1 | 0.0 | 0.0 | 0.0 | 0.0 | 0.0 | 0.0 | 0.0 | 0.0 | 0.0 | 0.0 | 0.0 | 0.0 |
| Otop2 | 0.0 | 0.0 | 0.0 | 0.0 | 0.0 | 0.0 | 0.0 | 0.0 | 0.0 | 0.0 | 0.0 | 0.0 |
| Otos | 0.1 | 0.0 | 0.0 | 0.0 | 0.0 | 0.0 | 0.0 | 0.0 | 0.0 | 0.0 | 0.0 | 0.0 |
| Otud6a | 0.0 | 0.0 | 0.0 | 0.0 | 0.0 | 0.0 | 0.0 | 0.0 | 0.0 | 0.0 | 0.0 | 0.0 |
| Otx1 | 0.0 | 0.0 | 0.0 | 0.0 | 0.0 | 0.0 | 0.0 | 0.0 | 0.0 | 0.0 | 0.0 | 0.0 |
| Ovch2 | 0.1 | 0.0 | 0.0 | 0.0 | 0.0 | 0.0 | 0.0 | 0.0 | 0.0 | 0.0 | 0.0 | 0.0 |
| Ovol2 | 0.0 | 0.0 | 0.0 | 0.0 | 0.0 | 0.0 | 0.0 | 0.0 | 0.0 | 0.0 | 0.0 | 0.0 |
| Oxct2b | 0.0 | 0.0 | 0.0 | 0.0 | 0.0 | 0.0 | 0.0 | 0.0 | 0.0 | 0.0 | 0.0 | 0.0 |
| Oxgr1 | 0.0 | 0.0 | 0.0 | 0.0 | 0.0 | 0.0 | 0.0 | 0.0 | 0.0 | 0.0 | 0.0 | 0.0 |
| P2rx2 | 0.0 | 0.0 | 0.0 | 0.0 | 0.0 | 0.0 | 0.1 | 0.0 | 0.0 | 0.0 | 0.0 | 0.1 |
| P2ry10 | 0.0 | 0.0 | 0.0 | 0.0 | 0.0 | 0.0 | 0.0 | 0.0 | 0.0 | 0.0 | 0.0 | 0.0 |
| Pabpc2 | 0.0 | 0.0 | 0.0 | 0.0 | 0.0 | 0.0 | 0.0 | 0.0 | 0.0 | 0.0 | 0.0 | 0.0 |
| Pabpn1l | 0.0 | 0.0 | 0.0 | 0.0 | 0.0 | 0.0 | 0.0 | 0.0 | 0.0 | 0.0 | 0.0 | 0.0 |
| Padi6 | 0.0 | 0.0 | 0.0 | 0.0 | 0.0 | 0.0 | 0.0 | 0.0 | 0.0 | 0.0 | 0.0 | 0.0 |
| Pah | 0.0 | 0.0 | 0.0 | 0.0 | 0.0 | 0.0 | 0.0 | 0.0 | 0.0 | 0.0 | 0.0 | 0.0 |
| Panx3 | 0.0 | 0.0 | 0.0 | 0.0 | 0.0 | 0.0 | 0.0 | 0.0 | 0.0 | 0.0 | 0.0 | 0.0 |
| Pate4 | 0.0 | 0.0 | 0.0 | 0.0 | 0.0 | 0.0 | 0.0 | 0.0 | 0.0 | 0.0 | 0.0 | 0.0 |
| Pax1 | 0.0 | 0.0 | 0.0 | 0.0 | 0.0 | 0.0 | 0.0 | 0.0 | 0.0 | 0.0 | 0.0 | 0.0 |
| Pax4 | 0.1 | 0.0 | 0.0 | 0.0 | 0.0 | 0.0 | 0.0 | 0.0 | 0.0 | 0.0 | 0.0 | 0.0 |
| Pax5 | 0.0 | 0.0 | 0.0 | 0.0 | 0.1 | 0.0 | 0.0 | 0.0 | 0.0 | 0.0 | 0.0 | 0.0 |
| Pax6os1 | 0.0 | 0.0 | 0.0 | 0.0 | 0.0 | 0.0 | 0.0 | 0.0 | 0.0 | 0.0 | 0.0 | 0.0 |

| | | | | | | | | | | | | |
|---|---|---|---|---|---|---|---|---|---|---|---|---|
| Pax7 | 0.0 | 0.0 | 0.0 | 0.0 | 0.0 | 0.0 | 0.0 | 0.0 | 0.0 | 0.0 | 0.0 | 0.0 |
| Pax8 | 0.0 | 0.0 | 0.0 | 0.0 | 0.0 | 0.0 | 0.0 | 0.0 | 0.0 | 0.0 | 0.0 | 0.0 |
| Pcdhb1 | 0.0 | 0.0 | 0.0 | 0.0 | 0.0 | 0.0 | 0.0 | 0.0 | 0.0 | 0.0 | 0.0 | 0.0 |
| Pcdhb6 | 0.0 | 0.0 | 0.0 | 0.0 | 0.0 | 0.1 | 0.0 | 0.0 | 0.0 | 0.0 | 0.0 | 0.0 |
| Pcsk1n | 0.0 | 0.0 | 0.0 | 0.0 | 0.0 | 0.0 | 0.0 | 0.0 | 0.0 | 0.0 | 0.0 | 0.0 |
| Pdcd1 | 0.0 | 0.0 | 0.0 | 0.0 | 0.0 | 0.0 | 0.0 | 0.0 | 0.0 | 0.0 | 0.0 | 0.0 |
| Pdcd1lg2 | 0.0 | 0.0 | 0.0 | 0.0 | 0.0 | 0.0 | 0.0 | 0.0 | 0.0 | 0.0 | 0.0 | 0.0 |
| Pdcl2 | 0.0 | 0.0 | 0.0 | 0.0 | 0.0 | 0.0 | 0.0 | 0.0 | 0.0 | 0.0 | 0.0 | 0.0 |
| Pde6c | 0.0 | 0.0 | 0.0 | 0.0 | 0.0 | 0.0 | 0.0 | 0.0 | 0.0 | 0.0 | 0.0 | 0.0 |
| Pdyn | 0.0 | 0.0 | 0.0 | 0.0 | 0.0 | 0.0 | 0.1 | 0.0 | 0.0 | 0.0 | 0.0 | 0.0 |
| Pea15b | 0.0 | 0.0 | 0.0 | 0.0 | 0.0 | 0.0 | 0.0 | 0.0 | 0.0 | 0.0 | 0.0 | 0.0 |
| Pebp4 | 0.0 | 0.1 | 0.0 | 0.0 | 0.0 | 0.0 | 0.0 | 0.0 | 0.0 | 0.0 | 0.0 | 0.0 |
| Penk | 0.0 | 0.0 | 0.0 | 0.0 | 0.0 | 0.1 | 0.0 | 0.0 | 0.0 | 0.0 | 0.0 | 0.1 |
| Pfpl | 0.0 | 0.0 | 0.0 | 0.0 | 0.0 | 0.0 | 0.0 | 0.0 | 0.0 | 0.0 | 0.0 | 0.0 |
| Pga5 | 0.0 | 0.0 | 0.0 | 0.0 | 0.0 | 0.0 | 0.0 | 0.1 | 0.0 | 0.0 | 0.1 | 0.0 |
| Pglyrp2 | 0.0 | 0.0 | 0.0 | 0.0 | 0.0 | 0.0 | 0.0 | 0.0 | 0.0 | 0.0 | 0.0 | 0.0 |
| Pglyrp4 | 0.0 | 0.0 | 0.0 | 0.0 | 0.0 | 0.0 | 0.0 | 0.0 | 0.0 | 0.0 | 0.0 | 0.0 |
| Phgr1 | 0.1 | 0.0 | 0.0 | 0.0 | 0.1 | 0.0 | 0.0 | 0.0 | 0.0 | 0.0 | 0.3 | 0.0 |
| Pigr | 0.0 | 0.0 | 0.0 | 0.0 | 0.0 | 0.0 | 0.0 | 0.0 | 0.0 | 0.0 | 0.0 | 0.0 |
| Pik3c2g | 0.0 | 0.0 | 0.0 | 0.0 | 0.0 | 0.0 | 0.0 | 0.0 | 0.0 | 0.0 | 0.0 | 0.0 |
| Pinlyp | 0.0 | 0.0 | 0.0 | 0.0 | 0.1 | 0.0 | 0.0 | 0.0 | 0.1 | 0.1 | 0.0 | 0.0 |
| Pip5kl1 | 0.0 | 0.0 | 0.0 | 0.0 | 0.0 | 0.0 | 0.0 | 0.0 | 0.0 | 0.1 | 0.0 | 0.0 |
| Pirt | 0.0 | 0.0 | 0.0 | 0.0 | 0.0 | 0.0 | 0.0 | 0.0 | 0.0 | 0.0 | 0.0 | 0.0 |
| Pitx3 | 0.0 | 0.0 | 0.0 | 0.0 | 0.0 | 0.0 | 0.0 | 0.0 | 0.0 | 0.0 | 0.0 | 0.0 |
| Piwil1 | 0.0 | 0.0 | 0.0 | 0.0 | 0.0 | 0.0 | 0.0 | 0.0 | 0.0 | 0.0 | 0.0 | 0.0 |
| Pkd2l1 | 0.0 | 0.0 | 0.0 | 0.0 | 0.0 | 0.0 | 0.0 | 0.0 | 0.0 | 0.0 | 0.0 | 0.0 |
| Pklr | 0.0 | 0.0 | 0.0 | 0.0 | 0.0 | 0.0 | 0.0 | 0.0 | 0.0 | 0.0 | 0.0 | 0.0 |
| Pla2g10 | 0.0 | 0.1 | 0.0 | 0.0 | 0.0 | 0.0 | 0.0 | 0.0 | 0.0 | 0.0 | 0.0 | 0.0 |
| Pla2g12b | 0.0 | 0.0 | 0.0 | 0.0 | 0.0 | 0.0 | 0.0 | 0.0 | 0.0 | 0.0 | 0.0 | 0.0 |
| Pla2g1b | 0.0 | 0.0 | 0.0 | 0.0 | 0.0 | 0.0 | 0.1 | 0.0 | 0.0 | 0.0 | 0.0 | 0.0 |
| Pla2g2d | 0.0 | 0.0 | 0.0 | 0.0 | 0.0 | 0.0 | 0.0 | 0.0 | 0.0 | 0.0 | 0.0 | 0.0 |
| Pla2g4e | 0.0 | 0.0 | 0.0 | 0.0 | 0.0 | 0.0 | 0.0 | 0.0 | 0.0 | 0.0 | 0.0 | 0.0 |
| Plac1 | 0.0 | 0.0 | 0.1 | 0.0 | 0.0 | 0.0 | 0.1 | 0.0 | 0.0 | 0.0 | 0.0 | 0.0 |
| Plac8l1 | 0.0 | 0.0 | 0.0 | 0.0 | 0.0 | 0.1 | 0.0 | 0.0 | 0.0 | 0.0 | 0.0 | 0.0 |
| Plcz1 | 0.0 | 0.0 | 0.0 | 0.1 | 0.0 | 0.0 | 0.0 | 0.0 | 0.0 | 0.0 | 0.0 | 0.0 |
| Pld5 | 0.0 | 0.0 | 0.0 | 0.0 | 0.0 | 0.0 | 0.0 | 0.0 | 0.0 | 0.0 | 0.0 | 0.0 |
| Plscr5 | 0.1 | 0.0 | 0.0 | 0.0 | 0.0 | 0.0 | 0.0 | 0.0 | 0.0 | 0.0 | 0.0 | 0.0 |
| Pmch | 0.0 | 0.0 | 0.0 | 0.0 | 0.1 | 0.0 | 0.0 | 0.1 | 0.1 | 0.0 | 0.0 | 0.0 |
| Pmfbp1 | 0.0 | 0.0 | 0.0 | 0.0 | 0.0 | 0.0 | 0.0 | 0.0 | 0.0 | 0.0 | 0.0 | 0.0 |
| Pnlip | 0.0 | 0.0 | 0.0 | 0.0 | 0.0 | 0.0 | 0.0 | 0.0 | 0.0 | 0.0 | 0.0 | 0.0 |
| Pnliprp1 | 0.0 | 0.0 | 0.0 | 0.0 | 0.0 | 0.0 | 0.0 | 0.0 | 0.0 | 0.0 | 0.0 | 0.0 |
| Pnliprp2 | 0.0 | 0.0 | 0.1 | 0.0 | 0.1 | 0.0 | 0.1 | 0.0 | 0.0 | 0.0 | 0.0 | 0.0 |
| Pnma3 | 0.0 | 0.0 | 0.0 | 0.0 | 0.0 | 0.0 | 0.0 | 0.0 | 0.0 | 0.0 | 0.0 | 0.0 |
| Pnma5 | 0.0 | 0.0 | 0.0 | 0.0 | 0.1 | 0.0 | 0.0 | 0.0 | 0.0 | 0.0 | 0.0 | 0.0 |
| Pnmal1 | 0.0 | 0.0 | 0.0 | 0.0 | 0.0 | 0.0 | 0.0 | 0.0 | 0.0 | 0.0 | 0.0 | 0.0 |
| Pnmt | 0.0 | 0.0 | 0.0 | 0.0 | 0.0 | 0.0 | 0.0 | 0.0 | 0.0 | 0.1 | 0.0 | 0.0 |
| Pnoc | 0.0 | 0.0 | 0.0 | 0.0 | 0.0 | 0.0 | 0.0 | 0.0 | 0.0 | 0.1 | 0.0 | 0.0 |
| Pof1b | 0.0 | 0.0 | 0.0 | 0.0 | 0.0 | 0.0 | 0.0 | 0.0 | 0.0 | 0.0 | 0.0 | 0.0 |
| Pou2af1 | 0.0 | 0.0 | 0.0 | 0.0 | 0.0 | 0.0 | 0.0 | 0.0 | 0.0 | 0.0 | 0.0 | 0.0 |
| Pou3f2 | 0.0 | 0.0 | 0.0 | 0.0 | 0.0 | 0.0 | 0.0 | 0.0 | 0.0 | 0.0 | 0.0 | 0.0 |
| Pou4f3 | 0.0 | 0.0 | 0.0 | 0.0 | 0.0 | 0.0 | 0.0 | 0.0 | 0.0 | 0.0 | 0.0 | 0.0 |
| Pou6f2 | 0.0 | 0.0 | 0.0 | 0.0 | 0.0 | 0.0 | 0.0 | 0.0 | 0.0 | 0.0 | 0.0 | 0.0 |
| Ppapdc1a | 0.0 | 0.0 | 0.0 | 0.0 | 0.0 | 0.0 | 0.0 | 0.0 | 0.0 | 0.0 | 0.1 | 0.0 |
| Ppbp | 0.0 | 0.0 | 0.0 | 0.1 | 0.0 | 0.0 | 0.0 | 0.0 | 0.0 | 0.0 | 0.0 | 0.0 |
| Ppm1n | 0.0 | 0.0 | 0.0 | 0.0 | 0.0 | 0.0 | 0.1 | 0.0 | 0.0 | 0.0 | 0.0 | 0.0 |
| Ppp1r17 | 0.0 | 0.0 | 0.0 | 0.0 | 0.0 | 0.0 | 0.0 | 0.0 | 0.0 | 0.0 | 0.0 | 0.0 |
| Ppp1r3g | 0.0 | 0.0 | 0.0 | 0.0 | 0.0 | 0.0 | 0.0 | 0.0 | 0.0 | 0.0 | 0.0 | 0.0 |

| | | | | | | | | | | | | |
|---|---|---|---|---|---|---|---|---|---|---|---|---|
| Ppp1r42 | 0.0 | 0.0 | 0.2 | 0.0 | 0.1 | 0.0 | 0.0 | 0.0 | 0.0 | 0.0 | 0.0 | 0.0 |
| Pramef17 | 0.0 | 0.0 | 0.1 | 0.0 | 0.0 | 0.0 | 0.0 | 0.0 | 0.0 | 0.0 | 0.0 | 0.0 |
| Prdm12 | 0.0 | 0.0 | 0.0 | 0.0 | 0.0 | 0.0 | 0.0 | 0.0 | 0.0 | 0.0 | 0.0 | 0.0 |
| Prdm13 | 0.0 | 0.0 | 0.0 | 0.0 | 0.0 | 0.0 | 0.0 | 0.0 | 0.0 | 0.0 | 0.0 | 0.0 |
| Prdm14 | 0.0 | 0.0 | 0.0 | 0.0 | 0.0 | 0.0 | 0.0 | 0.0 | 0.0 | 0.0 | 0.0 | 0.0 |
| Prf1 | 0.0 | 0.0 | 0.0 | 0.0 | 0.0 | 0.0 | 0.0 | 0.0 | 0.0 | 0.0 | 0.0 | 0.0 |
| Prima1 | 0.0 | 0.1 | 0.0 | 0.0 | 0.0 | 0.0 | 0.0 | 0.0 | 0.0 | 0.0 | 0.0 | 0.0 |
| Prl2a1 | 0.0 | 0.0 | 0.0 | 0.0 | 0.0 | 0.1 | 0.0 | 0.0 | 0.0 | 0.0 | 0.0 | 0.0 |
| Prl2b1 | 0.0 | 0.1 | 0.0 | 0.0 | 0.0 | 0.0 | 0.0 | 0.0 | 0.0 | 0.0 | 0.0 | 0.0 |
| Prl2c1 | 0.0 | 0.0 | 0.0 | 0.0 | 0.0 | 0.0 | 0.0 | 0.0 | 0.0 | 0.0 | 0.0 | 0.0 |
| Prl3d1 | 0.0 | 0.1 | 0.0 | 0.0 | 0.0 | 0.0 | 0.0 | 0.0 | 0.0 | 0.1 | 0.0 | 0.0 |
| Prl3d2 | 0.0 | 0.0 | 0.0 | 0.0 | 0.0 | 0.0 | 0.1 | 0.1 | 0.0 | 0.2 | 0.0 | 0.0 |
| Prl3d3 | 0.0 | 0.0 | 0.0 | 0.0 | 0.0 | 0.0 | 0.0 | 0.0 | 0.0 | 0.0 | 0.0 | 0.0 |
| Prl4a1 | 0.0 | 0.0 | 0.0 | 0.0 | 0.0 | 0.0 | 0.0 | 0.0 | 0.0 | 0.0 | 0.0 | 0.0 |
| Prl6a1 | 0.0 | 0.0 | 0.0 | 0.1 | 0.0 | 0.0 | 0.0 | 0.0 | 0.0 | 0.0 | 0.0 | 0.0 |
| Prl8a2 | 0.0 | 0.0 | 0.0 | 0.0 | 0.0 | 0.0 | 0.0 | 0.0 | 0.0 | 0.0 | 0.0 | 0.0 |
| Prlh | 0.3 | 0.0 | 0.0 | 0.0 | 0.0 | 0.0 | 0.0 | 0.0 | 0.0 | 0.0 | 0.0 | 0.0 |
| Prlhr | 0.0 | 0.1 | 0.0 | 0.0 | 0.0 | 0.0 | 0.0 | 0.0 | 0.0 | 0.0 | 0.0 | 0.0 |
| Prm1 | 0.0 | 0.0 | 0.0 | 0.0 | 0.0 | 0.0 | 0.0 | 0.0 | 0.0 | 0.1 | 0.0 | 0.0 |
| Prm2 | 0.0 | 0.0 | 0.0 | 0.0 | 0.0 | 0.0 | 0.0 | 0.1 | 0.0 | 0.0 | 0.0 | 0.0 |
| Prmt8 | 0.0 | 0.0 | 0.0 | 0.0 | 0.0 | 0.0 | 0.0 | 0.0 | 0.0 | 0.0 | 0.0 | 0.0 |
| Proc | 0.0 | 0.0 | 0.0 | 0.0 | 0.0 | 0.0 | 0.0 | 0.0 | 0.0 | 0.0 | 0.0 | 0.0 |
| Prok2 | 0.0 | 0.0 | 0.0 | 0.0 | 0.0 | 0.0 | 0.0 | 0.0 | 0.0 | 0.0 | 0.0 | 0.0 |
| Prom2 | 0.0 | 0.0 | 0.0 | 0.0 | 0.0 | 0.0 | 0.0 | 0.0 | 0.0 | 0.0 | 0.0 | 0.0 |
| Prp2 | 0.0 | 0.0 | 0.0 | 0.0 | 0.0 | 0.0 | 0.0 | 0.0 | 0.0 | 0.0 | 0.0 | 0.0 |
| Prps1l1 | 0.0 | 0.1 | 0.0 | 0.0 | 0.0 | 0.0 | 0.0 | 0.1 | 0.0 | 0.0 | 0.0 | 0.0 |
| Prr19 | 0.0 | 0.0 | 0.0 | 0.0 | 0.0 | 0.0 | 0.0 | 0.0 | 0.0 | 0.0 | 0.0 | 0.0 |
| Prr9 | 0.0 | 0.0 | 0.0 | 0.0 | 0.0 | 0.1 | 0.0 | 0.0 | 0.0 | 0.0 | 0.1 | 0.0 |
| Prrxl1 | 0.0 | 0.0 | 0.0 | 0.0 | 0.0 | 0.0 | 0.0 | 0.0 | 0.0 | 0.0 | 0.0 | 0.0 |
| Prss2 | 0.0 | 0.1 | 0.0 | 0.0 | 0.0 | 0.0 | 0.0 | 0.0 | 0.0 | 0.0 | 0.1 | 0.1 |
| Prss22 | 0.0 | 0.1 | 0.0 | 0.0 | 0.0 | 0.0 | 0.0 | 0.0 | 0.0 | 0.0 | 0.0 | 0.0 |
| Prss32 | 0.0 | 0.0 | 0.0 | 0.0 | 0.0 | 0.0 | 0.0 | 0.0 | 0.0 | 0.0 | 0.0 | 0.0 |
| Prss34 | 0.0 | 0.0 | 0.0 | 0.0 | 0.0 | 0.0 | 0.0 | 0.0 | 0.0 | 0.0 | 0.0 | 0.0 |
| Prss37 | 0.0 | 0.0 | 0.0 | 0.0 | 0.0 | 0.0 | 0.1 | 0.0 | 0.0 | 0.0 | 0.1 | 0.0 |
| Prss38 | 0.0 | 0.0 | 0.0 | 0.0 | 0.0 | 0.1 | 0.0 | 0.0 | 0.0 | 0.0 | 0.0 | 0.0 |
| Prss39 | 0.0 | 0.0 | 0.0 | 0.0 | 0.0 | 0.0 | 0.1 | 0.0 | 0.0 | 0.0 | 0.0 | 0.0 |
| Prss41 | 0.0 | 0.0 | 0.1 | 0.0 | 0.0 | 0.0 | 0.0 | 0.0 | 0.0 | 0.0 | 0.0 | 0.0 |
| Prss42 | 0.0 | 0.0 | 0.0 | 0.0 | 0.0 | 0.0 | 0.0 | 0.0 | 0.0 | 0.0 | 0.0 | 0.0 |
| Prss43 | 0.0 | 0.0 | 0.0 | 0.0 | 0.0 | 0.0 | 0.0 | 0.0 | 0.0 | 0.0 | 0.0 | 0.0 |
| Prss51 | 0.0 | 0.0 | 0.0 | 0.0 | 0.0 | 0.0 | 0.0 | 0.0 | 0.0 | 0.0 | 0.0 | 0.0 |
| Prss52 | 0.1 | 0.0 | 0.0 | 0.0 | 0.0 | 0.0 | 0.0 | 0.0 | 0.0 | 0.0 | 0.0 | 0.0 |
| Prss54 | 0.0 | 0.0 | 0.0 | 0.0 | 0.0 | 0.0 | 0.0 | 0.0 | 0.0 | 0.0 | 0.0 | 0.0 |
| Prss55 | 0.0 | 0.0 | 0.0 | 0.0 | 0.0 | 0.0 | 0.0 | 0.0 | 0.0 | 0.0 | 0.0 | 0.0 |
| Prss56 | 0.0 | 0.0 | 0.0 | 0.0 | 0.0 | 0.0 | 0.1 | 0.0 | 0.0 | 0.0 | 0.1 | 0.0 |
| Prss58 | 0.0 | 0.0 | 0.0 | 0.1 | 0.0 | 0.0 | 0.0 | 0.0 | 0.0 | 0.0 | 0.0 | 0.0 |
| Psapl1 | 0.0 | 0.0 | 0.0 | 0.0 | 0.0 | 0.0 | 0.0 | 0.0 | 0.0 | 0.0 | 0.0 | 0.0 |
| Psca | 0.0 | 0.0 | 0.0 | 0.0 | 0.0 | 0.0 | 0.0 | 0.0 | 0.0 | 0.0 | 0.0 | 0.0 |
| Psg16 | 0.1 | 0.0 | 0.0 | 0.0 | 0.0 | 0.0 | 0.0 | 0.0 | 0.0 | 0.0 | 0.0 | 0.0 |
| Psg19 | 0.0 | 0.0 | 0.0 | 0.1 | 0.0 | 0.0 | 0.0 | 0.0 | 0.0 | 0.0 | 0.0 | 0.0 |
| Psg21 | 0.0 | 0.0 | 0.0 | 0.0 | 0.0 | 0.0 | 0.0 | 0.0 | 0.0 | 0.0 | 0.0 | 0.0 |
| Psg22 | 0.0 | 0.0 | 0.0 | 0.0 | 0.0 | 0.0 | 0.0 | 0.0 | 0.0 | 0.0 | 0.0 | 0.0 |
| Psg28 | 0.0 | 0.0 | 0.0 | 0.0 | 0.0 | 0.0 | 0.0 | 0.0 | 0.0 | 0.0 | 0.0 | 0.0 |
| Psors1c2 | 0.0 | 0.1 | 0.0 | 0.0 | 0.0 | 0.0 | 0.0 | 0.1 | 0.0 | 0.0 | 0.0 | 0.0 |
| Ptgdr | 0.0 | 0.0 | 0.0 | 0.0 | 0.0 | 0.0 | 0.0 | 0.0 | 0.0 | 0.0 | 0.0 | 0.0 |
| Ptk6 | 0.0 | 0.0 | 0.0 | 0.0 | 0.0 | 0.0 | 0.0 | 0.0 | 0.0 | 0.0 | 0.0 | 0.0 |
| Ptpn20 | 0.0 | 0.0 | 0.0 | 0.0 | 0.0 | 0.0 | 0.0 | 0.0 | 0.0 | 0.0 | 0.0 | 0.0 |
| Ptprh | 0.0 | 0.0 | 0.0 | 0.0 | 0.0 | 0.0 | 0.0 | 0.0 | 0.0 | 0.0 | 0.0 | 0.0 |

| | | | | | | | | | | | | |
|---|---|---|---|---|---|---|---|---|---|---|---|---|
| Ptprr | 0.0 | 0.0 | 0.0 | 0.0 | 0.0 | 0.0 | 0.0 | 0.0 | 0.0 | 0.0 | 0.0 | 0.0 |
| Ptx4 | 0.0 | 0.0 | 0.0 | 0.0 | 0.0 | 0.0 | 0.0 | 0.0 | 0.0 | 0.0 | 0.0 | 0.0 |
| Pxt1 | 0.0 | 0.0 | 0.0 | 0.0 | 0.0 | 0.0 | 0.0 | 0.0 | 0.0 | 0.0 | 0.0 | 0.0 |
| Pydc4 | 0.0 | 0.0 | 0.0 | 0.0 | 0.0 | 0.0 | 0.0 | 0.0 | 0.0 | 0.0 | 0.0 | 0.0 |
| Pzp | 0.0 | 0.0 | 0.0 | 0.0 | 0.0 | 0.0 | 0.0 | 0.0 | 0.0 | 0.0 | 0.0 | 0.0 |
| Qprt | 0.1 | 0.0 | 0.0 | 0.0 | 0.1 | 0.0 | 0.1 | 0.0 | 0.0 | 0.0 | 0.1 | 0.0 |
| Qrfpr | 0.0 | 0.0 | 0.0 | 0.0 | 0.0 | 0.0 | 0.0 | 0.0 | 0.0 | 0.0 | 0.0 | 0.0 |
| R3hdml | 0.0 | 0.0 | 0.1 | 0.0 | 0.0 | 0.0 | 0.0 | 0.0 | 0.0 | 0.0 | 0.0 | 0.0 |
| Rab17 | 0.1 | 0.0 | 0.0 | 0.0 | 0.0 | 0.0 | 0.0 | 0.0 | 0.0 | 0.0 | 0.0 | 0.0 |
| Rab9b | 0.0 | 0.0 | 0.0 | 0.0 | 0.0 | 0.0 | 0.0 | 0.0 | 0.0 | 0.0 | 0.0 | 0.0 |
| Rad51ap2 | 0.0 | 0.0 | 0.0 | 0.0 | 0.0 | 0.0 | 0.0 | 0.0 | 0.0 | 0.0 | 0.0 | 0.0 |
| Rag1 | 0.0 | 0.0 | 0.0 | 0.0 | 0.0 | 0.0 | 0.0 | 0.0 | 0.0 | 0.0 | 0.0 | 0.0 |
| Rag2 | 0.0 | 0.0 | 0.0 | 0.0 | 0.0 | 0.0 | 0.0 | 0.0 | 0.0 | 0.0 | 0.0 | 0.0 |
| Ralyl | 0.0 | 0.0 | 0.0 | 0.0 | 0.0 | 0.0 | 0.0 | 0.0 | 0.0 | 0.0 | 0.1 | 0.0 |
| Rasgrf1 | 0.0 | 0.0 | 0.0 | 0.0 | 0.0 | 0.0 | 0.0 | 0.0 | 0.0 | 0.0 | 0.0 | 0.0 |
| Rbbp8nl | 0.0 | 0.0 | 0.0 | 0.0 | 0.0 | 0.0 | 0.0 | 0.0 | 0.0 | 0.0 | 0.0 | 0.0 |
| Rbmy | 0.0 | 0.0 | 0.0 | 0.0 | 0.0 | 0.0 | 0.0 | 0.0 | 0.0 | 0.0 | 0.0 | 0.0 |
| Rbp2 | 0.0 | 0.0 | 0.0 | 0.0 | 0.0 | 0.0 | 0.0 | 0.0 | 0.1 | 0.0 | 0.0 | 0.0 |
| Rbp3 | 0.0 | 0.0 | 0.0 | 0.0 | 0.0 | 0.0 | 0.0 | 0.0 | 0.0 | 0.0 | 0.0 | 0.0 |
| Rbp7 | 0.0 | 0.0 | 0.0 | 0.0 | 0.1 | 0.0 | 0.0 | 0.0 | 0.0 | 0.2 | 0.0 | 0.0 |
| Rdh18-ps | 0.0 | 0.0 | 0.0 | 0.0 | 0.0 | 0.0 | 0.0 | 0.0 | 0.0 | 0.0 | 0.0 | 0.0 |
| Rdh7 | 0.0 | 0.0 | 0.0 | 0.0 | 0.0 | 0.0 | 0.0 | 0.0 | 0.0 | 0.0 | 0.0 | 0.0 |
| Rdh8 | 0.0 | 0.0 | 0.0 | 0.1 | 0.0 | 0.0 | 0.0 | 0.0 | 0.0 | 0.0 | 0.0 | 0.0 |
| Reg3b | 0.3 | 0.0 | 0.0 | 0.0 | 0.0 | 0.0 | 0.0 | 0.0 | 0.0 | 0.0 | 0.0 | 0.0 |
| Resp18 | 0.1 | 0.0 | 0.0 | 0.0 | 0.0 | 0.0 | 0.0 | 0.0 | 0.0 | 0.0 | 0.0 | 0.0 |
| Retn | 0.0 | 0.0 | 0.1 | 0.0 | 0.0 | 0.1 | 0.0 | 0.0 | 0.0 | 0.0 | 0.0 | 0.0 |
| Retnla | 0.1 | 0.0 | 0.0 | 0.0 | 0.0 | 0.1 | 0.0 | 0.0 | 0.0 | 0.0 | 0.0 | 0.0 |
| Rfpl3s | 0.0 | 0.1 | 0.0 | 0.0 | 0.0 | 0.0 | 0.0 | 0.0 | 0.0 | 0.0 | 0.0 | 0.0 |
| Rfpl4 | 0.0 | 0.0 | 0.0 | 0.0 | 0.0 | 0.0 | 0.1 | 0.0 | 0.0 | 0.0 | 0.0 | 0.0 |
| Rfx4 | 0.0 | 0.0 | 0.0 | 0.0 | 0.0 | 0.0 | 0.0 | 0.0 | 0.0 | 0.0 | 0.0 | 0.0 |
| Rfx6 | 0.0 | 0.0 | 0.0 | 0.0 | 0.0 | 0.0 | 0.0 | 0.0 | 0.0 | 0.0 | 0.0 | 0.0 |
| Rhbg | 0.0 | 0.0 | 0.0 | 0.0 | 0.0 | 0.0 | 0.0 | 0.0 | 0.0 | 0.0 | 0.0 | 0.0 |
| Rhcg | 0.0 | 0.0 | 0.0 | 0.0 | 0.0 | 0.0 | 0.0 | 0.0 | 0.0 | 0.0 | 0.0 | 0.0 |
| Rhox2a | 0.0 | 0.0 | 0.0 | 0.0 | 0.0 | 0.0 | 0.0 | 0.0 | 0.0 | 0.0 | 0.0 | 0.0 |
| Rhox2b | 0.0 | 0.0 | 0.0 | 0.0 | 0.0 | 0.0 | 0.0 | 0.0 | 0.0 | 0.0 | 0.0 | 0.0 |
| Rhox2c | 0.0 | 0.0 | 0.0 | 0.0 | 0.0 | 0.0 | 0.0 | 0.0 | 0.0 | 0.0 | 0.0 | 0.0 |
| Rhox2d | 0.0 | 0.0 | 0.0 | 0.0 | 0.0 | 0.0 | 0.0 | 0.0 | 0.0 | 0.0 | 0.0 | 0.0 |
| Rhox2e | 0.0 | 0.0 | 0.0 | 0.0 | 0.0 | 0.0 | 0.0 | 0.0 | 0.0 | 0.0 | 0.0 | 0.0 |
| Rhox2h | 0.0 | 0.0 | 0.0 | 0.0 | 0.0 | 0.0 | 0.0 | 0.0 | 0.0 | 0.0 | 0.0 | 0.0 |
| Rhox3a | 0.0 | 0.0 | 0.0 | 0.0 | 0.0 | 0.0 | 0.0 | 0.0 | 0.0 | 0.0 | 0.0 | 0.0 |
| Rhox3c | 0.0 | 0.0 | 0.0 | 0.0 | 0.0 | 0.0 | 0.0 | 0.0 | 0.0 | 0.0 | 0.0 | 0.0 |
| Rhox3e | 0.0 | 0.0 | 0.0 | 0.0 | 0.0 | 0.0 | 0.0 | 0.0 | 0.0 | 0.0 | 0.0 | 0.0 |
| Rhox3f | 0.0 | 0.0 | 0.0 | 0.0 | 0.0 | 0.0 | 0.0 | 0.0 | 0.0 | 0.0 | 0.0 | 0.0 |
| Rhox3h | 0.0 | 0.0 | 0.0 | 0.0 | 0.0 | 0.0 | 0.0 | 0.0 | 0.0 | 0.0 | 0.0 | 0.0 |
| Rhox7 | 0.0 | 0.0 | 0.1 | 0.1 | 0.1 | 0.0 | 0.1 | 0.2 | 0.0 | 0.0 | 0.0 | 0.1 |
| Rhox8 | 0.0 | 0.0 | 0.0 | 0.0 | 0.0 | 0.0 | 0.0 | 0.0 | 0.1 | 0.1 | 0.0 | 0.0 |
| Rimbp2 | 0.0 | 0.0 | 0.0 | 0.0 | 0.0 | 0.0 | 0.0 | 0.0 | 0.0 | 0.0 | 0.0 | 0.0 |
| Ripply1 | 0.0 | 0.0 | 0.0 | 0.0 | 0.0 | 0.0 | 0.0 | 0.0 | 0.0 | 0.0 | 0.0 | 0.0 |
| Rlbp1 | 0.0 | 0.0 | 0.0 | 0.0 | 0.0 | 0.0 | 0.0 | 0.0 | 0.0 | 0.0 | 0.0 | 0.0 |
| Rmst | 0.0 | 0.0 | 0.0 | 0.0 | 0.0 | 0.0 | 0.0 | 0.0 | 0.0 | 0.0 | 0.0 | 0.0 |
| Rnf133 | 0.0 | 0.0 | 0.0 | 0.0 | 0.0 | 0.0 | 0.0 | 0.0 | 0.0 | 0.0 | 0.0 | 0.0 |
| Rnf186 | 0.0 | 0.0 | 0.0 | 0.0 | 0.0 | 0.0 | 0.0 | 0.0 | 0.0 | 0.0 | 0.0 | 0.0 |
| Rnu6 | 0.0 | 0.0 | 0.0 | 0.0 | 0.0 | ######## | ####### | 0.0 | 0.0 | 0.0 | ######## | ######## |
| Ros1 | 0.0 | 0.0 | 0.0 | 0.0 | 0.0 | 0.0 | 0.0 | 0.0 | 0.0 | 0.0 | 0.0 | 0.0 |
| Rpl10l | 0.0 | 0.0 | 0.0 | 0.0 | 0.0 | 0.0 | 0.0 | 0.0 | 0.0 | 0.0 | 0.0 | 0.0 |
| Rprml | 0.0 | 0.0 | 0.0 | 0.1 | 0.0 | 0.0 | 0.0 | 0.0 | 0.0 | 0.0 | 0.0 | 0.0 |
| Rrh | 0.0 | 0.0 | 0.0 | 0.0 | 0.0 | 0.0 | 0.0 | 0.0 | 0.0 | 0.0 | 0.0 | 0.0 |

| | | | | | | | | | | | | |
|---|---|---|---|---|---|---|---|---|---|---|---|---|
| Rspo4 | 0.0 | 0.0 | 0.0 | 0.0 | 0.0 | 0.0 | 0.0 | 0.0 | 0.0 | 0.0 | 0.0 | 0.0 |
| Rtdr1 | 0.0 | 0.0 | 0.0 | 0.0 | 0.0 | 0.0 | 0.0 | 0.0 | 0.0 | 0.0 | 0.1 | 0.0 |
| Rtp1 | 0.0 | 0.0 | 0.0 | 0.0 | 0.0 | 0.0 | 0.0 | 0.0 | 0.0 | 0.0 | 0.0 | 0.0 |
| Rtp2 | 0.0 | 0.0 | 0.0 | 0.0 | 0.0 | 0.0 | 0.0 | 0.0 | 0.0 | 0.0 | 0.0 | 0.0 |
| Rtp3 | 0.0 | 0.0 | 0.1 | 0.0 | 0.0 | 0.0 | 0.0 | 0.0 | 0.0 | 0.0 | 0.0 | 0.0 |
| Rubie | 0.0 | 0.0 | 0.0 | 0.0 | 0.0 | 0.0 | 0.0 | 0.0 | 0.0 | 0.0 | 0.0 | 0.0 |
| Rxfp1 | 0.0 | 0.0 | 0.0 | 0.0 | 0.0 | 0.0 | 0.0 | 0.0 | 0.0 | 0.0 | 0.0 | 0.0 |
| Rxfp2 | 0.0 | 0.0 | 0.0 | 0.0 | 0.0 | 0.0 | 0.0 | 0.0 | 0.0 | 0.0 | 0.0 | 0.0 |
| S100a2 | 0.0 | 0.0 | 0.3 | 0.0 | 0.3 | 0.0 | 0.0 | 0.0 | 0.0 | 0.0 | 0.0 | 0.0 |
| S100a9 | 0.0 | 0.0 | 0.1 | 0.1 | 0.0 | 0.0 | 0.0 | 0.0 | 0.0 | 0.0 | 0.0 | 0.0 |
| S100g | 0.0 | 0.1 | 0.0 | 0.0 | 0.0 | 0.0 | 0.0 | 0.0 | 0.0 | 0.0 | 0.3 | 0.0 |
| Saa2 | 0.0 | 0.0 | 0.0 | 0.0 | 0.1 | 0.0 | 0.0 | 0.0 | 0.0 | 0.0 | 0.0 | 0.0 |
| Saa4 | 0.0 | 0.0 | 0.0 | 0.0 | 0.0 | 0.0 | 0.0 | 0.0 | 0.0 | 0.0 | 0.0 | 0.0 |
| Sall1 | 0.0 | 0.0 | 0.0 | 0.0 | 0.0 | 0.0 | 0.0 | 0.0 | 0.0 | 0.0 | 0.0 | 0.0 |
| Sall4 | 0.0 | 0.0 | 0.0 | 0.0 | 0.0 | 0.0 | 0.0 | 0.0 | 0.0 | 0.0 | 0.0 | 0.0 |
| Samd3 | 0.1 | 0.0 | 0.0 | 0.0 | 0.0 | 0.0 | 0.0 | 0.0 | 0.0 | 0.0 | 0.0 | 0.0 |
| Satl1 | 0.0 | 0.0 | 0.0 | 0.0 | 0.0 | 0.0 | 0.0 | 0.0 | 0.0 | 0.0 | 0.0 | 0.0 |
| Scarna8 | 3.6 | 4.4 | 0.0 | 0.0 | 0.0 | 0.0 | 14.4 | 0.0 | 0.0 | 0.0 | 17.5 | 0.0 |
| Scg5 | 0.0 | 0.0 | 0.0 | 0.0 | 0.0 | 0.0 | 0.0 | 0.0 | 0.0 | 0.0 | 0.0 | 0.0 |
| Scgb2b20 | 0.0 | 0.0 | 0.0 | 0.1 | 0.0 | 0.0 | 0.0 | 0.0 | 0.0 | 0.0 | 0.0 | 0.0 |
| Scgb2b27 | 0.0 | 0.0 | 0.0 | 0.0 | 0.0 | 0.0 | 0.0 | 0.0 | 0.0 | 0.0 | 0.0 | 0.0 |
| Scgb2b7 | 0.0 | 0.0 | 0.0 | 0.1 | 0.0 | 0.0 | 0.0 | 0.0 | 0.0 | 0.0 | 0.0 | 0.0 |
| Scn10a | 0.0 | 0.0 | 0.0 | 0.0 | 0.0 | 0.0 | 0.0 | 0.0 | 0.0 | 0.0 | 0.0 | 0.0 |
| Scnn1b | 0.0 | 0.0 | 0.0 | 0.0 | 0.0 | 0.0 | 0.0 | 0.0 | 0.0 | 0.0 | 0.0 | 0.0 |
| Scrg1 | 0.0 | 0.0 | 0.0 | 0.0 | 0.0 | 0.0 | 0.1 | 0.0 | 0.0 | 0.0 | 0.0 | 0.0 |
| Scrt1 | 0.0 | 0.0 | 0.0 | 0.0 | 0.0 | 0.0 | 0.0 | 0.0 | 0.0 | 0.0 | 0.0 | 0.0 |
| Scrt2 | 0.0 | 0.0 | 0.0 | 0.0 | 0.0 | 0.0 | 0.0 | 0.0 | 0.0 | 0.0 | 0.0 | 0.0 |
| Sdr9c7 | 0.0 | 0.0 | 0.0 | 0.0 | 0.0 | 0.0 | 0.0 | 0.0 | 0.0 | 0.0 | 0.0 | 0.0 |
| Sec14l3 | 0.0 | 0.0 | 0.0 | 0.0 | 0.0 | 0.0 | 0.0 | 0.0 | 0.0 | 0.0 | 0.0 | 0.0 |
| Sec14l4 | 0.0 | 0.0 | 0.0 | 0.0 | 0.0 | 0.0 | 0.0 | 0.0 | 0.0 | 0.0 | 0.0 | 0.0 |
| Sel1l2 | 0.0 | 0.0 | 0.0 | 0.0 | 0.0 | 0.0 | 0.0 | 0.0 | 0.0 | 0.0 | 0.0 | 0.0 |
| Sell | 0.0 | 0.0 | 0.0 | 0.0 | 0.0 | 0.0 | 0.0 | 0.0 | 0.0 | 0.0 | 0.0 | 0.0 |
| Serpina10 | 0.0 | 0.0 | 0.0 | 0.0 | 0.0 | 0.0 | 0.0 | 0.0 | 0.0 | 0.0 | 0.0 | 0.0 |
| Serpina1a | 0.0 | 0.0 | 0.0 | 0.0 | 0.0 | 0.0 | 0.0 | 0.0 | 0.0 | 0.0 | 0.0 | 0.0 |
| Serpina1b | 0.0 | 0.0 | 0.0 | 0.0 | 0.0 | 0.0 | 0.0 | 0.0 | 0.0 | 0.0 | 0.0 | 0.0 |
| Serpina1c | 0.0 | 0.0 | 0.0 | 0.0 | 0.0 | 0.0 | 0.0 | 0.0 | 0.0 | 0.0 | 0.0 | 0.0 |
| Serpina1d | 0.0 | 0.0 | 0.0 | 0.0 | 0.0 | 0.0 | 0.0 | 0.0 | 0.0 | 0.0 | 0.0 | 0.0 |
| Serpina1e | 0.0 | 0.0 | 0.0 | 0.0 | 0.0 | 0.0 | 0.0 | 0.0 | 0.0 | 0.0 | 0.0 | 0.0 |
| Serpina1f | 0.0 | 0.0 | 0.0 | 0.0 | 0.0 | 0.0 | 0.0 | 0.0 | 0.0 | 0.0 | 0.0 | 0.0 |
| Serpina6 | 0.0 | 0.0 | 0.0 | 0.0 | 0.0 | 0.0 | 0.0 | 0.0 | 0.0 | 0.0 | 0.0 | 0.0 |
| Serpina7 | 0.0 | 0.0 | 0.0 | 0.0 | 0.0 | 0.0 | 0.0 | 0.0 | 0.0 | 0.1 | 0.0 | 0.0 |
| Serpinb10 | 0.0 | 0.0 | 0.0 | 0.0 | 0.0 | 0.0 | 0.0 | 0.0 | 0.0 | 0.0 | 0.0 | 0.0 |
| Serpinb11 | 0.0 | 0.0 | 0.0 | 0.0 | 0.0 | 0.0 | 0.0 | 0.0 | 0.0 | 0.0 | 0.0 | 0.0 |
| Serpinb3d | 0.0 | 0.0 | 0.0 | 0.0 | 0.0 | 0.0 | 0.0 | 0.0 | 0.0 | 0.0 | 0.0 | 0.0 |
| Serpinb5 | 0.0 | 0.0 | 0.0 | 0.0 | 0.0 | 0.0 | 0.0 | 0.0 | 0.0 | 0.0 | 0.0 | 0.0 |
| Serpinb6d | 0.0 | 0.0 | 0.0 | 0.0 | 0.0 | 0.0 | 0.0 | 0.0 | 0.0 | 0.0 | 0.0 | 0.0 |
| Serpinb6e | 0.0 | 0.1 | 0.0 | 0.0 | 0.0 | 0.0 | 0.0 | 0.0 | 0.0 | 0.0 | 0.0 | 0.0 |
| Serpinb7 | 0.0 | 0.0 | 0.0 | 0.0 | 0.0 | 0.0 | 0.0 | 0.0 | 0.0 | 0.0 | 0.0 | 0.0 |
| Serpinb9d | 0.0 | 0.0 | 0.0 | 0.1 | 0.1 | 0.0 | 0.0 | 0.0 | 0.0 | 0.0 | 0.0 | 0.0 |
| Serpinb9e | 0.1 | 0.0 | 0.0 | 0.0 | 0.0 | 0.0 | 0.0 | 0.0 | 0.0 | 0.0 | 0.0 | 0.0 |
| Serpinb9f | 0.0 | 0.0 | 0.0 | 0.0 | 0.0 | 0.0 | 0.0 | 0.0 | 0.0 | 0.0 | 0.0 | 0.0 |
| Serpinb9g | 0.0 | 0.0 | 0.0 | 0.0 | 0.0 | 0.0 | 0.0 | 0.0 | 0.0 | 0.0 | 0.0 | 0.0 |
| Sertm1 | 0.0 | 0.0 | 0.0 | 0.0 | 0.0 | 0.0 | 0.0 | 0.0 | 0.0 | 0.0 | 0.0 | 0.0 |
| Sez6 | 0.0 | 0.0 | 0.0 | 0.0 | 0.0 | 0.0 | 0.0 | 0.0 | 0.0 | 0.0 | 0.0 | 0.0 |
| Sfta2 | 0.0 | 0.0 | 0.0 | 0.0 | 0.0 | 0.0 | 0.0 | 0.0 | 0.0 | 0.1 | 0.0 | 0.0 |
| Sftpa1 | 0.0 | 0.0 | 0.0 | 0.0 | 0.0 | 0.0 | 0.0 | 0.0 | 0.0 | 0.0 | 0.0 | 0.0 |
| Sftpc | 0.0 | 0.1 | 0.0 | 0.0 | 0.0 | 0.0 | 0.0 | 0.0 | 0.0 | 0.0 | 0.0 | 0.0 |

| | | | | | | | | | | | | |
|---|---|---|---|---|---|---|---|---|---|---|---|---|
| Sh2d1a | 0.0 | 0.0 | 0.0 | 0.0 | 0.0 | 0.0 | 0.0 | 0.0 | 0.0 | 0.0 | 0.0 | 0.0 |
| Sh2d1b2 | 0.0 | 0.0 | 0.0 | 0.0 | 0.1 | 0.0 | 0.0 | 0.0 | 0.0 | 0.1 | 0.0 | 0.0 |
| Sh3gl2 | 0.0 | 0.0 | 0.0 | 0.0 | 0.0 | 0.0 | 0.0 | 0.0 | 0.0 | 0.0 | 0.0 | 0.0 |
| Shbg | 0.0 | 0.0 | 0.0 | 0.0 | 0.0 | 0.0 | 0.0 | 0.0 | 0.0 | 0.0 | 0.0 | 0.0 |
| Shcbp1l | 0.0 | 0.0 | 0.0 | 0.0 | 0.0 | 0.0 | 0.0 | 0.0 | 0.0 | 0.0 | 0.1 | 0.0 |
| Shh | 0.0 | 0.0 | 0.0 | 0.0 | 0.0 | 0.0 | 0.0 | 0.0 | 0.0 | 0.0 | 0.0 | 0.0 |
| Shisa3 | 0.0 | 0.0 | 0.0 | 0.0 | 0.0 | 0.0 | 0.0 | 0.0 | 0.0 | 0.0 | 0.0 | 0.0 |
| Shisa7 | 0.0 | 0.0 | 0.0 | 0.0 | 0.0 | 0.0 | 0.0 | 0.0 | 0.0 | 0.0 | 0.0 | 0.0 |
| Siah3 | 0.0 | 0.0 | 0.0 | 0.0 | 0.0 | 0.0 | 0.0 | 0.0 | 0.0 | 0.0 | 0.0 | 0.0 |
| Siglec15 | 0.0 | 0.0 | 0.0 | 0.1 | 0.0 | 0.0 | 0.0 | 0.0 | 0.0 | 0.0 | 0.0 | 0.0 |
| Sim1 | 0.0 | 0.0 | 0.0 | 0.0 | 0.0 | 0.0 | 0.0 | 0.0 | 0.0 | 0.0 | 0.0 | 0.0 |
| Sis | 0.0 | 0.0 | 0.0 | 0.0 | 0.0 | 0.0 | 0.0 | 0.0 | 0.0 | 0.0 | 0.0 | 0.0 |
| Sit1 | 0.1 | 0.0 | 0.0 | 0.0 | 0.0 | 0.0 | 0.0 | 0.0 | 0.0 | 0.0 | 0.0 | 0.0 |
| Six3 | 0.0 | 0.0 | 0.0 | 0.0 | 0.0 | 0.0 | 0.0 | 0.0 | 0.0 | 0.0 | 0.0 | 0.0 |
| Six3os1 | 0.0 | 0.0 | 0.0 | 0.0 | 0.0 | 0.1 | 0.0 | 0.0 | 0.0 | 0.0 | 0.0 | 0.0 |
| Skint1 | 0.0 | 0.0 | 0.0 | 0.0 | 0.0 | 0.1 | 0.0 | 0.0 | 0.0 | 0.0 | 0.0 | 0.0 |
| Skint10 | 0.0 | 0.0 | 0.0 | 0.0 | 0.0 | 0.0 | 0.0 | 0.0 | 0.0 | 0.0 | 0.0 | 0.0 |
| Skint3 | 0.0 | 0.0 | 0.0 | 0.0 | 0.1 | 0.0 | 0.0 | 0.0 | 0.0 | 0.0 | 0.0 | 0.0 |
| Skint4 | 0.0 | 0.0 | 0.0 | 0.0 | 0.0 | 0.0 | 0.0 | 0.0 | 0.0 | 0.0 | 0.0 | 0.0 |
| Skint5 | 0.0 | 0.0 | 0.0 | 0.0 | 0.0 | 0.0 | 0.0 | 0.0 | 0.0 | 0.0 | 0.0 | 0.0 |
| Skint6 | 0.0 | 0.0 | 0.0 | 0.0 | 0.0 | 0.0 | 0.0 | 0.0 | 0.0 | 0.0 | 0.0 | 0.0 |
| Skint9 | 0.0 | 0.0 | 0.0 | 0.0 | 0.0 | 0.1 | 0.0 | 0.0 | 0.0 | 0.0 | 0.0 | 0.0 |
| Slamf1 | 0.0 | 0.0 | 0.0 | 0.0 | 0.0 | 0.0 | 0.0 | 0.0 | 0.0 | 0.0 | 0.0 | 0.0 |
| Slc10a4 | 0.0 | 0.0 | 0.0 | 0.0 | 0.0 | 0.0 | 0.0 | 0.0 | 0.0 | 0.0 | 0.0 | 0.0 |
| Slc10a5 | 0.0 | 0.0 | 0.0 | 0.0 | 0.0 | 0.0 | 0.0 | 0.0 | 0.0 | 0.0 | 0.0 | 0.0 |
| Slc12a3 | 0.0 | 0.0 | 0.0 | 0.0 | 0.0 | 0.0 | 0.0 | 0.0 | 0.0 | 0.0 | 0.0 | 0.0 |
| Slc13a1 | 0.0 | 0.0 | 0.0 | 0.0 | 0.0 | 0.0 | 0.0 | 0.0 | 0.0 | 0.0 | 0.0 | 0.0 |
| Slc13a2 | 0.0 | 0.0 | 0.0 | 0.0 | 0.0 | 0.0 | 0.0 | 0.0 | 0.0 | 0.0 | 0.0 | 0.0 |
| Slc14a1 | 0.0 | 0.0 | 0.0 | 0.0 | 0.0 | 0.0 | 0.0 | 0.0 | 0.0 | 0.0 | 0.0 | 0.0 |
| Slc16a14 | 0.0 | 0.0 | 0.0 | 0.0 | 0.0 | 0.0 | 0.0 | 0.0 | 0.0 | 0.0 | 0.0 | 0.0 |
| Slc17a2 | 0.0 | 0.0 | 0.0 | 0.0 | 0.0 | 0.0 | 0.0 | 0.0 | 0.0 | 0.0 | 0.1 | 0.0 |
| Slc17a3 | 0.0 | 0.0 | 0.0 | 0.0 | 0.0 | 0.0 | 0.0 | 0.0 | 0.0 | 0.0 | 0.0 | 0.0 |
| Slc17a4 | 0.0 | 0.0 | 0.0 | 0.0 | 0.0 | 0.0 | 0.0 | 0.0 | 0.0 | 0.0 | 0.0 | 0.0 |
| Slc18a3 | 0.0 | 0.0 | 0.0 | 0.0 | 0.0 | 0.0 | 0.0 | 0.0 | 0.0 | 0.0 | 0.0 | 0.0 |
| Slc22a1 | 0.0 | 0.0 | 0.0 | 0.0 | 0.0 | 0.1 | 0.0 | 0.0 | 0.0 | 0.0 | 0.0 | 0.0 |
| Slc22a12 | 0.0 | 0.0 | 0.0 | 0.0 | 0.0 | 0.1 | 0.0 | 0.0 | 0.0 | 0.0 | 0.0 | 0.0 |
| Slc22a16 | 0.0 | 0.0 | 0.0 | 0.0 | 0.0 | 0.0 | 0.0 | 0.0 | 0.0 | 0.0 | 0.0 | 0.0 |
| Slc22a19 | 0.0 | 0.0 | 0.0 | 0.0 | 0.0 | 0.0 | 0.0 | 0.0 | 0.0 | 0.0 | 0.0 | 0.0 |
| Slc22a22 | 0.0 | 0.0 | 0.0 | 0.0 | 0.0 | 0.0 | 0.0 | 0.0 | 0.0 | 0.0 | 0.0 | 0.0 |
| Slc22a26 | 0.0 | 0.0 | 0.0 | 0.0 | 0.0 | 0.0 | 0.0 | 0.0 | 0.0 | 0.0 | 0.0 | 0.0 |
| Slc22a28 | 0.0 | 0.0 | 0.1 | 0.0 | 0.0 | 0.0 | 0.0 | 0.0 | 0.0 | 0.0 | 0.0 | 0.0 |
| Slc22a29 | 0.0 | 0.0 | 0.0 | 0.0 | 0.0 | 0.0 | 0.0 | 0.0 | 0.0 | 0.0 | 0.0 | 0.0 |
| Slc22a30 | 0.0 | 0.0 | 0.1 | 0.0 | 0.0 | 0.0 | 0.0 | 0.0 | 0.0 | 0.0 | 0.0 | 0.0 |
| Slc22a7 | 0.0 | 0.0 | 0.0 | 0.0 | 0.0 | 0.0 | 0.0 | 0.0 | 0.0 | 0.0 | 0.0 | 0.0 |
| Slc25a48 | 0.0 | 0.0 | 0.0 | 0.0 | 0.0 | 0.0 | 0.0 | 0.0 | 0.0 | 0.0 | 0.0 | 0.0 |
| Slc25a54 | 0.0 | 0.0 | 0.0 | 0.0 | 0.0 | 0.0 | 0.0 | 0.0 | 0.0 | 0.0 | 0.0 | 0.0 |
| Slc26a3 | 0.0 | 0.0 | 0.0 | 0.0 | 0.0 | 0.0 | 0.0 | 0.0 | 0.0 | 0.0 | 0.0 | 0.0 |
| Slc26a4 | 0.0 | 0.0 | 0.0 | 0.0 | 0.0 | 0.0 | 0.0 | 0.0 | 0.0 | 0.0 | 0.0 | 0.0 |
| Slc26a9 | 0.0 | 0.0 | 0.0 | 0.0 | 0.0 | 0.0 | 0.0 | 0.0 | 0.0 | 0.0 | 0.0 | 0.0 |
| Slc27a2 | 0.0 | 0.0 | 0.0 | 0.0 | 0.0 | 0.0 | 0.0 | 0.0 | 0.0 | 0.0 | 0.0 | 0.0 |
| Slc2a7 | 0.0 | 0.0 | 0.0 | 0.0 | 0.0 | 0.0 | 0.0 | 0.0 | 0.0 | 0.0 | 0.0 | 0.0 |
| Slc30a10 | 0.0 | 0.0 | 0.0 | 0.0 | 0.0 | 0.0 | 0.0 | 0.0 | 0.0 | 0.0 | 0.0 | 0.0 |
| Slc30a2 | 0.0 | 0.0 | 0.0 | 0.0 | 0.0 | 0.0 | 0.0 | 0.0 | 0.0 | 0.0 | 0.0 | 0.0 |
| Slc30a3 | 0.0 | 0.0 | 0.0 | 0.0 | 0.0 | 0.0 | 0.0 | 0.0 | 0.0 | 0.0 | 0.0 | 0.0 |
| Slc32a1 | 0.0 | 0.0 | 0.0 | 0.0 | 0.0 | 0.0 | 0.0 | 0.0 | 0.0 | 0.0 | 0.0 | 0.0 |
| Slc34a1 | 0.0 | 0.0 | 0.0 | 0.0 | 0.0 | 0.0 | 0.0 | 0.0 | 0.0 | 0.0 | 0.0 | 0.0 |
| Slc34a2 | 0.0 | 0.0 | 0.0 | 0.0 | 0.0 | 0.0 | 0.0 | 0.0 | 0.0 | 0.0 | 0.0 | 0.0 |

| | | | | | | | | | | | |
|---|---|---|---|---|---|---|---|---|---|---|---|
| Slc35f4 | 0.0 | 0.0 | 0.0 | 0.0 | 0.0 | 0.0 | 0.0 | 0.0 | 0.0 | 0.0 | 0.0 | 0.0 |
| Slc36a3 | 0.1 | 0.0 | 0.0 | 0.0 | 0.0 | 0.0 | 0.0 | 0.0 | 0.0 | 0.0 | 0.0 | 0.0 |
| Slc38a3 | 0.0 | 0.0 | 0.0 | 0.1 | 0.0 | 0.0 | 0.0 | 0.0 | 0.0 | 0.0 | 0.0 | 0.0 |
| Slc39a12 | 0.0 | 0.0 | 0.0 | 0.0 | 0.0 | 0.0 | 0.0 | 0.0 | 0.0 | 0.0 | 0.0 | 0.0 |
| Slc47a2 | 0.0 | 0.0 | 0.0 | 0.0 | 0.0 | 0.0 | 0.0 | 0.0 | 0.0 | 0.0 | 0.0 | 0.0 |
| Slc4a1 | 0.0 | 0.0 | 0.0 | 0.0 | 0.0 | 0.0 | 0.0 | 0.0 | 0.0 | 0.0 | 0.0 | 0.0 |
| Slc5a10 | 0.0 | 0.0 | 0.0 | 0.0 | 0.0 | 0.0 | 0.0 | 0.0 | 0.0 | 0.0 | 0.0 | 0.0 |
| Slc5a4a | 0.0 | 0.0 | 0.0 | 0.0 | 0.0 | 0.0 | 0.0 | 0.0 | 0.0 | 0.0 | 0.0 | 0.0 |
| Slc5a9 | 0.0 | 0.0 | 0.0 | 0.0 | 0.0 | 0.0 | 0.0 | 0.0 | 0.0 | 0.0 | 0.0 | 0.0 |
| Slc6a11 | 0.0 | 0.0 | 0.0 | 0.0 | 0.0 | 0.0 | 0.0 | 0.0 | 0.0 | 0.0 | 0.0 | 0.0 |
| Slc6a13 | 0.0 | 0.0 | 0.1 | 0.0 | 0.1 | 0.0 | 0.0 | 0.0 | 0.0 | 0.0 | 0.0 | 0.0 |
| Slc6a14 | 0.0 | 0.0 | 0.0 | 0.0 | 0.0 | 0.0 | 0.0 | 0.0 | 0.0 | 0.0 | 0.0 | 0.0 |
| Slc6a15 | 0.0 | 0.0 | 0.0 | 0.0 | 0.0 | 0.0 | 0.0 | 0.0 | 0.0 | 0.0 | 0.0 | 0.0 |
| Slc6a3 | 0.0 | 0.0 | 0.0 | 0.0 | 0.0 | 0.0 | 0.0 | 0.0 | 0.0 | 0.0 | 0.0 | 0.0 |
| Slc7a15 | 0.0 | 0.0 | 0.0 | 0.0 | 0.0 | 0.1 | 0.0 | 0.0 | 0.0 | 0.0 | 0.0 | 0.0 |
| Slc7a9 | 0.0 | 0.0 | 0.0 | 0.0 | 0.0 | 0.0 | 0.0 | 0.0 | 0.0 | 0.0 | 0.0 | 0.0 |
| Slc9a3 | 0.0 | 0.0 | 0.0 | 0.0 | 0.0 | 0.0 | 0.0 | 0.0 | 0.0 | 0.0 | 0.0 | 0.0 |
| Slc9c1 | 0.0 | 0.0 | 0.0 | 0.0 | 0.0 | 0.0 | 0.0 | 0.0 | 0.0 | 0.0 | 0.0 | 0.0 |
| Slco1a6 | 0.0 | 0.0 | 0.0 | 0.0 | 0.0 | 0.0 | 0.0 | 0.0 | 0.0 | 0.0 | 0.0 | 0.0 |
| Slco1b2 | 0.0 | 0.0 | 0.0 | 0.0 | 0.0 | 0.0 | 0.0 | 0.0 | 0.0 | 0.0 | 0.0 | 0.0 |
| Slco4c1 | 0.0 | 0.0 | 0.0 | 0.0 | 0.0 | 0.0 | 0.0 | 0.0 | 0.0 | 0.0 | 0.0 | 0.0 |
| Slco6c1 | 0.0 | 0.0 | 0.0 | 0.0 | 0.0 | 0.0 | 0.0 | 0.0 | 0.0 | 0.0 | 0.0 | 0.0 |
| Slco6d1 | 0.0 | 0.0 | 0.0 | 0.0 | 0.0 | 0.0 | 0.0 | 0.0 | 0.0 | 0.0 | 0.0 | 0.0 |
| Slfn1 | 0.0 | 0.0 | 0.0 | 0.0 | 0.0 | 0.0 | 0.0 | 0.0 | 0.0 | 0.0 | 0.0 | 0.0 |
| Slitrk3 | 0.0 | 0.0 | 0.0 | 0.0 | 0.0 | 0.0 | 0.0 | 0.0 | 0.0 | 0.0 | 0.0 | 0.0 |
| Slurp1 | 0.0 | 0.0 | 0.0 | 0.3 | 0.0 | 0.0 | 0.0 | 0.1 | 0.0 | 0.0 | 0.0 | 0.2 |
| Slx | 0.0 | 0.0 | 0.0 | 0.0 | 0.0 | 0.0 | 0.0 | 0.0 | 0.0 | 0.0 | 0.0 | 0.0 |
| Slxl1 | 0.0 | 0.0 | 0.0 | 0.0 | 0.0 | 0.0 | 0.0 | 0.0 | 0.0 | 0.0 | 0.0 | 0.0 |
| Sly | 0.0 | 0.0 | 0.0 | 0.0 | 0.0 | 0.0 | 0.0 | 0.0 | 0.0 | 0.0 | 0.0 | 0.0 |
| Smim18 | 0.0 | 0.1 | 0.0 | 0.0 | 0.1 | 0.0 | 0.0 | 0.1 | 0.0 | 0.3 | 0.0 | 0.1 |
| Smok2a | 0.0 | 0.0 | 0.0 | 0.0 | 0.0 | 0.0 | 0.0 | 0.0 | 0.0 | 0.0 | 0.0 | 0.0 |
| Smok3a | 0.0 | 0.0 | 0.0 | 0.0 | 0.0 | 0.0 | 0.0 | 0.0 | 0.0 | 0.0 | 0.0 | 0.0 |
| Smpx | 0.0 | 0.0 | 0.0 | 0.0 | 0.0 | 0.0 | 0.0 | 0.0 | 0.0 | 0.0 | 0.0 | 0.0 |
| Smr3a | 0.1 | 0.0 | 0.0 | 0.0 | 0.0 | 0.0 | 0.0 | 0.0 | 0.0 | 0.0 | 0.0 | 0.0 |
| Smtnl1 | 0.0 | 0.0 | 0.0 | 0.0 | 0.0 | 0.0 | 0.0 | 0.0 | 0.0 | 0.0 | 0.0 | 0.0 |
| Snai3 | 0.0 | 0.0 | 0.1 | 0.0 | 0.1 | 0.0 | 0.0 | 0.0 | 0.0 | 0.0 | 0.0 | 0.1 |
| Snap25 | 0.0 | 0.0 | 0.0 | 0.0 | 0.0 | 0.0 | 0.0 | 0.0 | 0.0 | 0.0 | 0.0 | 0.0 |
| Snora2b | 0.0 | 5.8 | 0.0 | 0.0 | 0.0 | 0.0 | 0.0 | 0.0 | 0.0 | 0.0 | 0.0 | 0.0 |
| Snora41 | 0.0 | 0.0 | 0.0 | 0.0 | 0.0 | 0.0 | 0.0 | 9.3 | 0.0 | 0.0 | 7.3 | 0.0 |
| Snora61 | 0.0 | 0.0 | 0.0 | 0.0 | 0.0 | 0.0 | 0.0 | 0.0 | 279.5 | 323.6 | 0.0 | 0.0 |
| Snora75 | 7.9 | 0.0 | 9.8 | 0.0 | 3.3 | 0.0 | 0.0 | 0.0 | 4.0 | 0.0 | 0.0 | 3.9 |
| Snord11 | 0.0 | 0.0 | 0.0 | 0.0 | 0.0 | 0.0 | 0.0 | 0.0 | 0.0 | 0.0 | 0.0 | 222.9 |
| Snord116l2 | 1.7 | 0.0 | 0.0 | 0.0 | 0.0 | 0.0 | 0.0 | 0.0 | 1.4 | 1.9 | 0.0 | 0.0 |
| Snord123 | 0.0 | 0.0 | 0.0 | 0.0 | 0.0 | 0.0 | 0.0 | 0.0 | 19.0 | 0.0 | 0.0 | 17.9 |
| Snord19 | 0.0 | 0.0 | 0.0 | 0.0 | 0.0 | 0.0 | 0.0 | 0.0 | 0.0 | 0.0 | 0.0 | 937.2 |
| Snord1b | 0.0 | 182.5 | 67.1 | 0.0 | 102.6 | 0.0 | 0.0 | 141.1 | 139.5 | 0.0 | 100.6 | 0.0 |
| Snord2 | 0.0 | 0.0 | 0.0 | 0.0 | 0.0 | 308.2 | 0.0 | 0.0 | 0.0 | 0.0 | 0.0 | 0.0 |
| Snord23 | 0.0 | 11.9 | 0.0 | 0.0 | 7.6 | 0.0 | 0.0 | 0.0 | 0.0 | 0.0 | 16.4 | 9.0 |
| Snord42b | 0.0 | 1099.4 | 0.0 | 0.0 | 0.0 | 0.0 | 0.0 | 0.0 | 0.0 | 0.0 | 0.0 | 0.0 |
| Snord43 | 3708.9 | 0.0 | 0.0 | 0.0 | 0.0 | 0.0 | 0.0 | 0.0 | 0.0 | 0.0 | 0.0 | 0.0 |
| Snord53 | 0.0 | 0.0 | 0.0 | 0.0 | 0.0 | 0.0 | 0.0 | 0.0 | 0.0 | 146.9 | 0.0 | 0.0 |
| Snord66 | 0.0 | 0.0 | 0.0 | 0.0 | 0.0 | 0.0 | 0.0 | 490.9 | 0.0 | 0.0 | 0.0 | 0.0 |
| Snord69 | 0.0 | 0.0 | 0.0 | 0.0 | 0.0 | 0.0 | 0.0 | 420.1 | 0.0 | 1005.9 | 0.0 | 0.0 |
| Snord71 | 0.0 | 271.8 | 0.0 | 0.0 | 0.0 | 0.0 | 0.0 | 0.0 | 0.0 | 0.0 | 0.0 | 0.0 |
| Snord88c | 0.0 | 0.0 | 0.0 | 0.0 | 38.9 | 0.0 | 0.0 | 0.0 | 0.0 | 0.0 | 0.0 | 0.0 |
| Snord96a | 0.0 | 0.0 | 0.0 | 105.2 | 42.8 | 0.0 | 0.0 | 0.0 | 0.0 | 0.0 | 0.0 | 35.2 |
| Sntn | 0.0 | 0.0 | 0.0 | 0.0 | 0.0 | 0.1 | 0.1 | 0.0 | 0.0 | 0.0 | 0.0 | 0.0 |

| | | | | | | | | | | | |
|---|---|---|---|---|---|---|---|---|---|---|---|
| Sostdc1 | 0.0 | 0.0 | 0.0 | 0.0 | 0.0 | 0.0 | 0.0 | 0.0 | 0.0 | 0.0 | 0.0 | 0.0 |
| Sowaha | 0.0 | 0.0 | 0.0 | 0.0 | 0.0 | 0.0 | 0.0 | 0.0 | 0.0 | 0.0 | 0.0 | 0.0 |
| Sox1 | 0.0 | 0.0 | 0.0 | 0.0 | 0.0 | 0.0 | 0.0 | 0.0 | 0.0 | 0.0 | 0.0 | 0.0 |
| Sox2 | 0.0 | 0.0 | 0.0 | 0.0 | 0.0 | 0.0 | 0.0 | 0.0 | 0.0 | 0.0 | 0.0 | 0.0 |
| Sox3 | 0.0 | 0.0 | 0.0 | 0.0 | 0.0 | 0.0 | 0.0 | 0.0 | 0.0 | 0.0 | 0.0 | 0.0 |
| Sp5 | 0.0 | 0.0 | 0.0 | 0.0 | 0.0 | 0.1 | 0.0 | 0.1 | 0.0 | 0.0 | 0.0 | 0.0 |
| Sp8 | 0.0 | 0.0 | 0.0 | 0.0 | 0.0 | 0.0 | 0.0 | 0.0 | 0.0 | 0.0 | 0.0 | 0.0 |
| Spaca3 | 0.0 | 0.0 | 0.0 | 0.0 | 0.0 | 0.0 | 0.0 | 0.0 | 0.0 | 0.1 | 0.0 | 0.0 |
| Spaca5 | 0.0 | 0.0 | 0.0 | 0.0 | 0.1 | 0.0 | 0.0 | 0.0 | 0.0 | 0.0 | 0.0 | 0.0 |
| Spag11b | 0.0 | 0.0 | 0.0 | 0.0 | 0.0 | 0.0 | 0.0 | 0.0 | 0.0 | 0.0 | 0.0 | 0.0 |
| Spag16 | 0.0 | 0.0 | 0.0 | 0.0 | 0.0 | 0.0 | 0.0 | 0.0 | 0.0 | 0.0 | 0.0 | 0.0 |
| Spag6 | 0.0 | 0.0 | 0.0 | 0.0 | 0.0 | 0.0 | 0.0 | 0.0 | 0.0 | 0.0 | 0.0 | 0.0 |
| Spata16 | 0.0 | 0.0 | 0.0 | 0.0 | 0.0 | 0.0 | 0.0 | 0.0 | 0.0 | 0.0 | 0.0 | 0.0 |
| Spata19 | 0.1 | 0.0 | 0.0 | 0.2 | 0.0 | 0.0 | 0.0 | 0.0 | 0.0 | 0.0 | 0.0 | 0.0 |
| Spata21 | 0.0 | 0.0 | 0.0 | 0.0 | 0.0 | 0.0 | 0.0 | 0.0 | 0.0 | 0.0 | 0.0 | 0.0 |
| Spata3 | 0.0 | 0.1 | 0.0 | 0.0 | 0.0 | 0.1 | 0.0 | 0.0 | 0.1 | 0.1 | 0.0 | 0.0 |
| Spata31d1b | 0.0 | 0.0 | 0.0 | 0.0 | 0.0 | 0.0 | 0.0 | 0.0 | 0.0 | 0.0 | 0.0 | 0.0 |
| Spata32 | 0.0 | 0.0 | 0.0 | 0.3 | 0.0 | 0.0 | 0.0 | 0.0 | 0.0 | 0.0 | 0.0 | 0.0 |
| Spdyb | 0.0 | 0.0 | 0.0 | 0.0 | 0.0 | 0.0 | 0.0 | 0.0 | 0.0 | 0.0 | 0.0 | 0.0 |
| Speer2 | 0.0 | 0.0 | 0.0 | 0.0 | 0.0 | 0.0 | 0.0 | 0.0 | 0.0 | 0.0 | 0.0 | 0.0 |
| Speer4d | 0.0 | 0.0 | 0.0 | 0.0 | 0.0 | 0.0 | 0.0 | 0.0 | 0.0 | 0.0 | 0.0 | 0.0 |
| Speer4e | 0.0 | 0.0 | 0.0 | 0.0 | 0.0 | 0.0 | 0.0 | 0.0 | 0.0 | 0.0 | 0.0 | 0.0 |
| Spem1 | 0.1 | 0.0 | 0.1 | 0.0 | 0.0 | 0.0 | 0.0 | 0.0 | 0.0 | 0.0 | 0.0 | 0.0 |
| Spesp1 | 0.0 | 0.0 | 0.0 | 0.0 | 0.0 | 0.0 | 0.0 | 0.0 | 0.0 | 0.0 | 0.0 | 0.0 |
| Spink13 | 0.0 | 0.0 | 0.0 | 0.0 | 0.0 | 0.0 | 0.0 | 0.1 | 0.0 | 0.0 | 0.0 | 0.0 |
| Spink4 | 0.0 | 0.0 | 0.0 | 0.0 | 0.0 | 0.0 | 0.0 | 0.1 | 0.0 | 0.3 | 0.0 | 0.0 |
| Spn-ps | 0.0 | 0.0 | 0.0 | 0.0 | 0.0 | 0.0 | 0.1 | 0.0 | 0.0 | 0.0 | 0.0 | 0.0 |
| Spock1 | 0.0 | 0.0 | 0.0 | 0.0 | 0.0 | 0.0 | 0.0 | 0.0 | 0.0 | 0.0 | 0.0 | 0.0 |
| Sppl2c | 0.0 | 0.0 | 0.0 | 0.0 | 0.0 | 0.0 | 0.0 | 0.0 | 0.0 | 0.0 | 0.0 | 0.0 |
| Sprr2b | 0.0 | 0.0 | 0.0 | 0.3 | 0.0 | 0.0 | 0.0 | 0.0 | 0.0 | 0.0 | 0.0 | 0.0 |
| Sprr2g | 0.1 | 0.0 | 0.0 | 0.0 | 0.1 | 0.0 | 0.0 | 0.0 | 0.0 | 0.1 | 0.0 | 0.0 |
| Sprr2j-ps | 0.0 | 0.0 | 0.2 | 0.0 | 0.0 | 0.0 | 0.0 | 0.0 | 0.0 | 0.0 | 0.0 | 0.0 |
| Sprr2k | 0.1 | 0.0 | 0.0 | 0.0 | 0.0 | 0.0 | 0.0 | 0.1 | 0.0 | 0.0 | 0.2 | 0.0 |
| Sptlc3 | 0.0 | 0.0 | 0.0 | 0.0 | 0.0 | 0.0 | 0.0 | 0.0 | 0.0 | 0.0 | 0.0 | 0.0 |
| Srd5a2 | 0.3 | 0.0 | 0.0 | 0.0 | 0.0 | 0.0 | 0.0 | 0.0 | 0.0 | 0.1 | 0.0 | 0.0 |
| Sstr1 | 0.0 | 0.0 | 0.0 | 0.0 | 0.0 | 0.0 | 0.0 | 0.0 | 0.0 | 0.0 | 0.0 | 0.0 |
| Sstr4 | 0.0 | 0.0 | 0.0 | 0.1 | 0.0 | 0.0 | 0.0 | 0.0 | 0.1 | 0.2 | 0.0 | 0.1 |
| Ssty1 | 0.0 | 0.0 | 0.0 | 0.0 | 0.0 | 0.0 | 0.0 | 0.0 | 0.0 | 0.0 | 0.0 | 0.0 |
| Ssty2 | 0.0 | 0.0 | 0.0 | 0.0 | 0.0 | 0.0 | 0.0 | 0.0 | 0.0 | 0.0 | 0.0 | 0.0 |
| Ssx9 | 0.0 | 0.0 | 0.0 | 0.0 | 0.0 | 0.0 | 0.0 | 0.0 | 0.0 | 0.0 | 0.0 | 0.0 |
| Ssxb6 | 0.0 | 0.0 | 0.0 | 0.0 | 0.0 | 0.0 | 0.0 | 0.0 | 0.0 | 0.0 | 0.0 | 0.0 |
| St6gal2 | 0.0 | 0.0 | 0.0 | 0.0 | 0.0 | 0.0 | 0.0 | 0.0 | 0.0 | 0.0 | 0.0 | 0.0 |
| St8sia3 | 0.0 | 0.0 | 0.0 | 0.0 | 0.0 | 0.0 | 0.0 | 0.0 | 0.0 | 0.0 | 0.0 | 0.0 |
| St8sia5 | 0.0 | 0.0 | 0.0 | 0.0 | 0.0 | 0.0 | 0.0 | 0.0 | 0.0 | 0.0 | 0.0 | 0.0 |
| Stfa1 | 0.0 | 0.0 | 0.0 | 0.0 | 0.0 | 0.2 | 0.0 | 0.0 | 0.0 | 0.0 | 0.0 | 0.0 |
| Stfa2 | 0.0 | 0.0 | 0.0 | 0.0 | 0.0 | 0.0 | 0.0 | 0.0 | 0.0 | 0.0 | 0.0 | 0.1 |
| Stmn3 | 0.0 | 0.0 | 0.0 | 0.0 | 0.0 | 0.0 | 0.1 | 0.0 | 0.0 | 0.0 | 0.1 | 0.0 |
| Stoml3 | 0.0 | 0.0 | 0.0 | 0.0 | 0.0 | 0.0 | 0.0 | 0.0 | 0.0 | 0.0 | 0.0 | 0.0 |
| Stra8 | 0.0 | 0.0 | 0.0 | 0.0 | 0.0 | 0.1 | 0.0 | 0.0 | 0.0 | 0.0 | 0.0 | 0.0 |
| Strc | 0.0 | 0.0 | 0.0 | 0.0 | 0.0 | 0.0 | 0.0 | 0.0 | 0.0 | 0.0 | 0.0 | 0.0 |
| Sult1b1 | 0.0 | 0.0 | 0.0 | 0.0 | 0.0 | 0.0 | 0.0 | 0.0 | 0.0 | 0.0 | 0.0 | 0.0 |
| Sult1c1 | 0.0 | 0.0 | 0.0 | 0.0 | 0.0 | 0.0 | 0.0 | 0.0 | 0.0 | 0.0 | 0.0 | 0.0 |
| Sult1d1 | 0.0 | 0.0 | 0.0 | 0.0 | 0.0 | 0.0 | 0.0 | 0.0 | 0.0 | 0.0 | 0.0 | 0.0 |
| Sult1e1 | 0.0 | 0.0 | 0.0 | 0.0 | 0.0 | 0.0 | 0.0 | 0.0 | 0.1 | 0.0 | 0.0 | 0.0 |
| Sult2a5 | 0.0 | 0.0 | 0.0 | 0.0 | 0.0 | 0.0 | 0.0 | 0.0 | 0.0 | 0.0 | 0.0 | 0.0 |
| Sult2a6 | 0.0 | 0.0 | 0.1 | 0.0 | 0.0 | 0.1 | 0.0 | 0.0 | 0.0 | 0.0 | 0.0 | 0.1 |
| Sult2a7 | 0.0 | 0.0 | 0.0 | 0.0 | 0.0 | 0.1 | 0.0 | 0.0 | 0.0 | 0.0 | 0.0 | 0.0 |

| | | | | | | | | | | | | |
|---|---|---|---|---|---|---|---|---|---|---|---|---|
| Sun3 | 0.0 | 0.0 | 0.0 | 0.0 | 0.0 | 0.0 | 0.0 | 0.0 | 0.0 | 0.1 | 0.0 | 0.0 |
| Svs1 | 0.0 | 0.0 | 0.0 | 0.0 | 0.0 | 0.0 | 0.0 | 0.0 | 0.0 | 0.0 | 0.0 | 0.0 |
| Svs2 | 0.0 | 0.0 | 0.0 | 0.0 | 0.0 | 0.0 | 0.0 | 0.0 | 0.0 | 0.0 | 0.0 | 0.0 |
| Svs3b | 0.0 | 0.0 | 0.0 | 0.0 | 0.0 | 0.0 | 0.0 | 0.0 | 0.0 | 0.0 | 0.0 | 0.0 |
| Syce1l | 0.0 | 0.0 | 0.0 | 0.0 | 0.0 | 0.0 | 0.0 | 0.0 | 0.0 | 0.1 | 0.0 | 0.0 |
| Syt10 | 0.1 | 0.0 | 0.0 | 0.0 | 0.0 | 0.0 | 0.0 | 0.0 | 0.0 | 0.0 | 0.0 | 0.0 |
| Syt16 | 0.0 | 0.0 | 0.0 | 0.0 | 0.0 | 0.0 | 0.0 | 0.0 | 0.0 | 0.0 | 0.0 | 0.0 |
| Syt2 | 0.0 | 0.0 | 0.0 | 0.0 | 0.0 | 0.0 | 0.0 | 0.0 | 0.0 | 0.0 | 0.0 | 0.0 |
| T | 0.0 | 0.0 | 0.0 | 0.0 | 0.0 | 0.0 | 0.0 | 0.0 | 0.0 | 0.0 | 0.0 | 0.0 |
| Taar2 | 0.0 | 0.0 | 0.0 | 0.0 | 0.0 | 0.0 | 0.0 | 0.0 | 0.0 | 0.0 | 0.0 | 0.0 |
| Taar5 | 0.0 | 0.0 | 0.0 | 0.0 | 0.0 | 0.0 | 0.0 | 0.0 | 0.0 | 0.0 | 0.0 | 0.0 |
| Taar8b | 0.0 | 0.0 | 0.0 | 0.0 | 0.0 | 0.0 | 0.0 | 0.0 | 0.0 | 0.0 | 0.0 | 0.0 |
| Taar8c | 0.0 | 0.0 | 0.0 | 0.0 | 0.0 | 0.0 | 0.0 | 0.0 | 0.0 | 0.0 | 0.0 | 0.0 |
| Tac1 | 0.0 | 0.0 | 0.0 | 0.0 | 0.0 | 0.0 | 0.0 | 0.1 | 0.0 | 0.0 | 0.0 | 0.0 |
| Tac2 | 0.0 | 0.0 | 0.1 | 0.0 | 0.0 | 0.0 | 0.0 | 0.0 | 0.0 | 0.0 | 0.0 | 0.0 |
| Tacr1 | 0.0 | 0.0 | 0.0 | 0.0 | 0.0 | 0.0 | 0.0 | 0.0 | 0.0 | 0.0 | 0.0 | 0.0 |
| Tacr2 | 0.0 | 0.0 | 0.0 | 0.0 | 0.0 | 0.0 | 0.0 | 0.0 | 0.0 | 0.0 | 0.0 | 0.0 |
| Tacr3 | 0.0 | 0.0 | 0.0 | 0.0 | 0.0 | 0.0 | 0.0 | 0.0 | 0.0 | 0.0 | 0.0 | 0.0 |
| Tacstd2 | 0.0 | 0.0 | 0.0 | 0.0 | 0.0 | 0.0 | 0.0 | 0.0 | 0.0 | 0.0 | 0.0 | 0.0 |
| Tas1r2 | 0.0 | 0.0 | 0.0 | 0.0 | 0.0 | 0.0 | 0.0 | 0.0 | 0.0 | 0.1 | 0.0 | 0.0 |
| Tas2r124 | 0.0 | 0.0 | 0.0 | 0.0 | 0.0 | 0.0 | 0.0 | 0.0 | 0.0 | 0.0 | 0.0 | 0.0 |
| Tas2r126 | 0.0 | 0.0 | 0.0 | 0.0 | 0.0 | 0.0 | 0.0 | 0.0 | 0.0 | 0.1 | 0.1 | 0.0 |
| Tas2r138 | 0.0 | 0.0 | 0.0 | 0.0 | 0.0 | 0.1 | 0.0 | 0.0 | 0.0 | 0.0 | 0.0 | 0.0 |
| Tas2r143 | 0.0 | 0.0 | 0.0 | 0.0 | 0.0 | 0.0 | 0.1 | 0.0 | 0.0 | 0.0 | 0.1 | 0.0 |
| Tas2r144 | 0.0 | 0.0 | 0.0 | 0.0 | 0.0 | 0.0 | 0.0 | 0.0 | 0.0 | 0.0 | 0.0 | 0.0 |
| Tbc1d21 | 0.0 | 0.0 | 0.0 | 0.0 | 0.0 | 0.0 | 0.0 | 0.0 | 0.0 | 0.0 | 0.0 | 0.0 |
| Tbpl2 | 0.0 | 0.0 | 0.0 | 0.0 | 0.0 | 0.0 | 0.0 | 0.0 | 0.0 | 0.0 | 0.0 | 0.0 |
| Tbr1 | 0.0 | 0.0 | 0.0 | 0.0 | 0.0 | 0.0 | 0.0 | 0.0 | 0.0 | 0.0 | 0.0 | 0.0 |
| Tbx19 | 0.0 | 0.0 | 0.0 | 0.0 | 0.0 | 0.0 | 0.0 | 0.0 | 0.0 | 0.0 | 0.0 | 0.0 |
| Tcap | 0.0 | 0.0 | 0.0 | 0.0 | 0.0 | 0.0 | 0.0 | 0.0 | 0.0 | 0.0 | 0.0 | 0.0 |
| Tdgf1 | 0.0 | 0.0 | 0.0 | 0.0 | 0.0 | 0.0 | 0.0 | 0.0 | 0.0 | 0.0 | 0.0 | 0.0 |
| Tdh | 0.0 | 0.0 | 0.0 | 0.0 | 0.0 | 0.0 | 0.0 | 0.0 | 0.0 | 0.0 | 0.0 | 0.0 |
| Tdpoz1 | 0.0 | 0.0 | 0.0 | 0.0 | 0.0 | 0.0 | 0.0 | 0.0 | 0.0 | 0.0 | 0.0 | 0.0 |
| Tdpoz3 | 0.1 | 0.0 | 0.0 | 0.0 | 0.0 | 0.0 | 0.0 | 0.0 | 0.0 | 0.0 | 0.0 | 0.0 |
| Tecrl | 0.0 | 0.0 | 0.0 | 0.0 | 0.0 | 0.0 | 0.0 | 0.0 | 0.0 | 0.0 | 0.0 | 0.0 |
| Teddm1 | 0.0 | 0.0 | 0.0 | 0.0 | 0.0 | 0.0 | 0.0 | 0.0 | 0.0 | 0.0 | 0.0 | 0.0 |
| Tekt1 | 0.0 | 0.0 | 0.0 | 0.0 | 0.0 | 0.0 | 0.1 | 0.0 | 0.0 | 0.0 | 0.0 | 0.0 |
| Tekt3 | 0.0 | 0.0 | 0.0 | 0.1 | 0.0 | 0.0 | 0.0 | 0.0 | 0.0 | 0.0 | 0.0 | 0.0 |
| Tepp | 0.1 | 0.0 | 0.0 | 0.0 | 0.0 | 0.0 | 0.0 | 0.1 | 0.0 | 0.0 | 0.0 | 0.0 |
| Tescl | 0.0 | 0.0 | 0.0 | 0.0 | 0.0 | 0.0 | 0.0 | 0.0 | 0.0 | 0.0 | 0.0 | 0.0 |
| Tex101 | 0.0 | 0.0 | 0.0 | 0.0 | 0.0 | 0.0 | 0.0 | 0.0 | 0.0 | 0.0 | 0.0 | 0.0 |
| Tex12 | 0.1 | 0.0 | 0.0 | 0.0 | 0.1 | 0.0 | 0.0 | 0.0 | 0.0 | 0.0 | 0.0 | 0.1 |
| Tex13 | 0.0 | 0.0 | 0.0 | 0.0 | 0.0 | 0.0 | 0.0 | 0.0 | 0.0 | 0.0 | 0.0 | 0.0 |
| Tex16 | 0.0 | 0.0 | 0.0 | 0.0 | 0.0 | 0.0 | 0.0 | 0.0 | 0.0 | 0.0 | 0.0 | 0.0 |
| Tex21 | 0.0 | 0.0 | 0.0 | 0.0 | 0.0 | 0.0 | 0.0 | 0.0 | 0.0 | 0.0 | 0.0 | 0.1 |
| Tex24 | 0.0 | 0.0 | 0.0 | 0.0 | 0.0 | 0.0 | 0.0 | 0.0 | 0.0 | 0.0 | 0.0 | 0.0 |
| Tex29 | 0.0 | 0.0 | 0.0 | 0.0 | 0.0 | 0.0 | 0.0 | 0.0 | 0.1 | 0.1 | 0.0 | 0.0 |
| Tex33 | 0.0 | 0.0 | 0.0 | 0.0 | 0.0 | 0.0 | 0.0 | 0.0 | 0.0 | 0.0 | 0.0 | 0.0 |
| Tex37 | 0.0 | 0.0 | 0.0 | 0.0 | 0.0 | 0.0 | 0.0 | 0.0 | 0.0 | 0.0 | 0.0 | 0.0 |
| Tex40 | 0.1 | 0.0 | 0.0 | 0.0 | 0.0 | 0.0 | 0.0 | 0.0 | 0.0 | 0.1 | 0.0 | 0.0 |
| Tfap2b | 0.0 | 0.0 | 0.0 | 0.0 | 0.0 | 0.0 | 0.0 | 0.0 | 0.0 | 0.0 | 0.0 | 0.0 |
| Tfap2c | 0.0 | 0.0 | 0.0 | 0.0 | 0.0 | 0.0 | 0.0 | 0.0 | 0.0 | 0.0 | 0.0 | 0.0 |
| Tff1 | 0.0 | 0.0 | 0.1 | 0.0 | 0.0 | 0.0 | 0.0 | 0.0 | 0.0 | 0.0 | 0.0 | 0.0 |
| Tgm5 | 0.0 | 0.0 | 0.0 | 0.0 | 0.0 | 0.0 | 0.0 | 0.0 | 0.0 | 0.0 | 0.0 | 0.0 |
| Tgm6 | 0.0 | 0.0 | 0.0 | 0.0 | 0.0 | 0.0 | 0.0 | 0.0 | 0.0 | 0.0 | 0.0 | 0.0 |
| Tgm7 | 0.0 | 0.0 | 0.0 | 0.0 | 0.0 | 0.0 | 0.0 | 0.0 | 0.0 | 0.0 | 0.0 | 0.0 |
| Th | 0.0 | 0.0 | 0.0 | 0.0 | 0.0 | 0.0 | 0.0 | 0.0 | 0.0 | 0.0 | 0.0 | 0.0 |

| | | | | | | | | | | | | |
|---|---|---|---|---|---|---|---|---|---|---|---|---|
| Theg | 0.0 | 0.0 | 0.0 | 0.0 | 0.0 | 0.0 | 0.0 | 0.0 | 0.0 | 0.0 | 0.0 | 0.0 |
| Them5 | 0.1 | 0.0 | 0.0 | 0.0 | 0.0 | 0.0 | 0.0 | 0.0 | 0.0 | 0.0 | 0.0 | 0.0 |
| Themis3 | 0.0 | 0.0 | 0.0 | 0.0 | 0.0 | 0.0 | 0.0 | 0.0 | 0.0 | 0.0 | 0.0 | 0.0 |
| Timd2 | 0.0 | 0.0 | 0.0 | 0.0 | 0.0 | 0.0 | 0.0 | 0.0 | 0.0 | 0.0 | 0.0 | 0.0 |
| Timd4 | 0.0 | 0.0 | 0.0 | 0.0 | 0.0 | 0.0 | 0.0 | 0.0 | 0.0 | 0.0 | 0.0 | 0.0 |
| Timm8a2 | 0.0 | 0.0 | 0.0 | 0.0 | 0.0 | 0.0 | 0.0 | 0.0 | 0.0 | 0.0 | 0.1 | 0.0 |
| Tinag | 0.0 | 0.0 | 0.0 | 0.0 | 0.0 | 0.0 | 0.0 | 0.0 | 0.0 | 0.0 | 0.0 | 0.0 |
| Tktl2 | 0.0 | 0.0 | 0.0 | 0.0 | 0.0 | 0.0 | 0.0 | 0.0 | 0.0 | 0.0 | 0.0 | 0.0 |
| Tlx1 | 0.0 | 0.0 | 0.0 | 0.1 | 0.0 | 0.0 | 0.0 | 0.0 | 0.0 | 0.0 | 0.0 | 0.0 |
| Tlx2 | 0.1 | 0.0 | 0.0 | 0.0 | 0.0 | 0.0 | 0.0 | 0.0 | 0.0 | 0.0 | 0.0 | 0.0 |
| Tlx3 | 0.0 | 0.0 | 0.0 | 0.0 | 0.0 | 0.0 | 0.0 | 0.0 | 0.0 | 0.0 | 0.0 | 0.0 |
| Tm4sf5 | 0.0 | 0.0 | 0.0 | 0.0 | 0.1 | 0.1 | 0.0 | 0.0 | 0.0 | 0.0 | 0.0 | 0.0 |
| Tmc1 | 0.0 | 0.0 | 0.0 | 0.0 | 0.0 | 0.0 | 0.0 | 0.0 | 0.0 | 0.0 | 0.0 | 0.0 |
| Tmc2 | 0.0 | 0.0 | 0.0 | 0.0 | 0.0 | 0.0 | 0.0 | 0.0 | 0.0 | 0.0 | 0.0 | 0.0 |
| Tmco2 | 0.0 | 0.0 | 0.0 | 0.0 | 0.0 | 0.1 | 0.0 | 0.0 | 0.0 | 0.0 | 0.0 | 0.0 |
| Tmed6 | 0.0 | 0.1 | 0.0 | 0.0 | 0.0 | 0.0 | 0.0 | 0.0 | 0.0 | 0.0 | 0.0 | 0.0 |
| Tmem102 | 0.0 | 0.0 | 0.0 | 0.0 | 0.0 | 0.0 | 0.0 | 0.0 | 0.0 | 0.0 | 0.0 | 0.0 |
| Tmem139 | 0.0 | 0.0 | 0.1 | 0.0 | 0.0 | 0.0 | 0.0 | 0.0 | 0.0 | 0.0 | 0.0 | 0.0 |
| Tmem182 | 0.0 | 0.0 | 0.0 | 0.0 | 0.0 | 0.0 | 0.0 | 0.0 | 0.0 | 0.0 | 0.0 | 0.0 |
| Tmem190 | 0.0 | 0.0 | 0.0 | 0.0 | 0.0 | 0.0 | 0.0 | 0.1 | 0.0 | 0.1 | 0.0 | 0.1 |
| Tmem196 | 0.0 | 0.0 | 0.0 | 0.0 | 0.0 | 0.0 | 0.1 | 0.0 | 0.0 | 0.0 | 0.0 | 0.0 |
| Tmem210 | 0.0 | 0.0 | 0.0 | 0.0 | 0.1 | 0.0 | 0.0 | 0.0 | 0.0 | 0.2 | 0.0 | 0.0 |
| Tmem212 | 0.1 | 0.0 | 0.0 | 0.0 | 0.0 | 0.2 | 0.0 | 0.1 | 0.0 | 0.0 | 0.0 | 0.0 |
| Tmem233 | 0.0 | 0.1 | 0.0 | 0.0 | 0.0 | 0.0 | 0.0 | 0.0 | 0.0 | 0.0 | 0.0 | 0.0 |
| Tmem235 | 0.0 | 0.0 | 0.0 | 0.0 | 0.0 | 0.0 | 0.0 | 0.0 | 0.0 | 0.0 | 0.0 | 0.0 |
| Tmem247 | 0.0 | 0.1 | 0.0 | 0.0 | 0.0 | 0.0 | 0.0 | 0.0 | 0.0 | 0.0 | 0.0 | 0.0 |
| Tmem252 | 0.0 | 0.0 | 0.0 | 0.0 | 0.0 | 0.0 | 0.0 | 0.0 | 0.0 | 0.0 | 0.0 | 0.0 |
| Tmem28 | 0.1 | 0.0 | 0.0 | 0.0 | 0.0 | 0.0 | 0.0 | 0.0 | 0.0 | 0.0 | 0.0 | 0.0 |
| Tmem30c | 0.0 | 0.0 | 0.0 | 0.0 | 0.0 | 0.0 | 0.0 | 0.0 | 0.0 | 0.0 | 0.0 | 0.0 |
| Tmprss11a | 0.0 | 0.0 | 0.0 | 0.0 | 0.0 | 0.0 | 0.0 | 0.0 | 0.0 | 0.0 | 0.0 | 0.0 |
| Tmprss11c | 0.0 | 0.0 | 0.0 | 0.0 | 0.0 | 0.0 | 0.0 | 0.0 | 0.0 | 0.0 | 0.0 | 0.0 |
| Tmprss11d | 0.0 | 0.0 | 0.0 | 0.0 | 0.0 | 0.0 | 0.0 | 0.0 | 0.0 | 0.0 | 0.0 | 0.0 |
| Tmprss11g | 0.0 | 0.0 | 0.0 | 0.0 | 0.0 | 0.0 | 0.0 | 0.0 | 0.0 | 0.0 | 0.0 | 0.0 |
| Tmprss12 | 0.0 | 0.0 | 0.0 | 0.0 | 0.0 | 0.0 | 0.0 | 0.0 | 0.0 | 0.0 | 0.0 | 0.0 |
| Tmprss13 | 0.0 | 0.0 | 0.0 | 0.0 | 0.0 | 0.0 | 0.0 | 0.0 | 0.0 | 0.0 | 0.0 | 0.0 |
| Tmprss15 | 0.0 | 0.0 | 0.0 | 0.0 | 0.0 | 0.0 | 0.0 | 0.0 | 0.0 | 0.1 | 0.0 | 0.0 |
| Tmsb15a | 0.0 | 0.7 | 0.0 | 0.0 | 0.0 | 0.0 | 0.0 | 0.0 | 0.4 | 0.0 | 0.0 | 0.0 |
| Tnni2 | 0.1 | 0.0 | 0.1 | 0.2 | 0.3 | 0.0 | 0.0 | 0.1 | 0.0 | 0.1 | 0.0 | 0.0 |
| Tnp2 | 0.0 | 0.0 | 0.1 | 0.0 | 0.0 | 0.0 | 0.0 | 0.0 | 0.0 | 0.0 | 0.0 | 0.0 |
| Topaz1 | 0.0 | 0.0 | 0.0 | 0.0 | 0.0 | 0.0 | 0.0 | 0.0 | 0.0 | 0.0 | 0.0 | 0.0 |
| Tox2 | 0.0 | 0.0 | 0.0 | 0.0 | 0.0 | 0.0 | 0.0 | 0.0 | 0.1 | 0.0 | 0.0 | 0.0 |
| Tox3 | 0.0 | 0.0 | 0.0 | 0.1 | 0.0 | 0.0 | 0.0 | 0.0 | 0.0 | 0.0 | 0.0 | 0.0 |
| Tph1 | 0.0 | 0.1 | 0.0 | 0.0 | 0.0 | 0.0 | 0.0 | 0.0 | 0.0 | 0.0 | 0.0 | 0.0 |
| Tpo | 0.0 | 0.0 | 0.0 | 0.0 | 0.0 | 0.0 | 0.0 | 0.0 | 0.0 | 0.0 | 0.0 | 0.0 |
| Tppp2 | 0.0 | 0.0 | 0.1 | 0.0 | 0.0 | 0.0 | 0.0 | 0.0 | 0.0 | 0.0 | 0.0 | 0.0 |
| Tprg | 0.0 | 0.0 | 0.0 | 0.0 | 0.0 | 0.0 | 0.0 | 0.0 | 0.0 | 0.0 | 0.0 | 0.0 |
| Tpsg1 | 0.0 | 0.0 | 0.0 | 0.0 | 0.0 | 0.0 | 0.0 | 0.0 | 0.0 | 0.0 | 0.0 | 0.0 |
| Trap1a | 0.0 | 0.0 | 0.0 | 0.0 | 0.0 | 0.0 | 0.0 | 0.0 | 0.0 | 0.0 | 0.1 | 0.0 |
| Trat1 | 0.0 | 0.1 | 0.0 | 0.0 | 0.0 | 0.0 | 0.0 | 0.0 | 0.0 | 0.0 | 0.0 | 0.0 |
| Treh | 0.0 | 0.0 | 0.0 | 0.0 | 0.0 | 0.0 | 0.0 | 0.0 | 0.0 | 0.0 | 0.0 | 0.0 |
| Treml1 | 0.0 | 0.0 | 0.0 | 0.0 | 0.0 | 0.0 | 0.0 | 0.0 | 0.0 | 0.0 | 0.1 | 0.0 |
| Treml2 | 0.0 | 0.0 | 0.0 | 0.0 | 0.0 | 0.0 | 0.0 | 0.0 | 0.0 | 0.0 | 0.0 | 0.0 |
| Trex2 | 0.0 | 0.0 | 0.0 | 0.0 | 0.0 | 0.0 | 0.0 | 0.0 | 0.0 | 0.0 | 0.0 | 0.0 |
| Trh | 0.0 | 0.0 | 0.0 | 0.0 | 0.0 | 0.0 | 0.0 | 0.0 | 0.0 | 0.0 | 0.0 | 0.0 |
| Trhde | 0.0 | 0.0 | 0.0 | 0.0 | 0.0 | 0.0 | 0.0 | 0.0 | 0.0 | 0.0 | 0.0 | 0.0 |
| Trhr | 0.0 | 0.0 | 0.0 | 0.0 | 0.0 | 0.0 | 0.0 | 0.0 | 0.0 | 0.0 | 0.0 | 0.0 |
| Trhr2 | 0.1 | 0.0 | 0.0 | 0.0 | 0.0 | 0.0 | 0.0 | 0.0 | 0.0 | 0.0 | 0.0 | 0.0 |

| | | | | | | | | | | | | |
|---|---|---|---|---|---|---|---|---|---|---|---|---|
| Trim30e-ps1 | 0.0 | 0.0 | 0.0 | 0.0 | 0.0 | 0.0 | 0.0 | 0.0 | 0.0 | 0.0 | 0.0 | 0.0 |
| Trim40 | 0.0 | 0.0 | 0.0 | 0.1 | 0.0 | 0.0 | 0.0 | 0.0 | 0.0 | 0.0 | 0.0 | 0.0 |
| Trim52 | 0.0 | 0.1 | 0.0 | 0.2 | 0.0 | 0.0 | 0.0 | 0.1 | 0.0 | 0.0 | 0.0 | 0.0 |
| Trim67 | 0.0 | 0.0 | 0.0 | 0.0 | 0.0 | 0.0 | 0.0 | 0.0 | 0.0 | 0.0 | 0.0 | 0.0 |
| Triml1 | 0.0 | 0.0 | 0.0 | 0.0 | 0.0 | 0.0 | 0.0 | 0.0 | 0.0 | 0.0 | 0.0 | 0.0 |
| Triml2 | 0.0 | 0.0 | 0.0 | 0.0 | 0.0 | 0.1 | 0.0 | 0.0 | 0.0 | 0.0 | 0.0 | 0.0 |
| Trpa1 | 0.0 | 0.0 | 0.0 | 0.0 | 0.0 | 0.0 | 0.0 | 0.0 | 0.0 | 0.0 | 0.0 | 0.0 |
| Trpc3 | 0.0 | 0.0 | 0.0 | 0.0 | 0.0 | 0.0 | 0.0 | 0.0 | 0.0 | 0.0 | 0.0 | 0.0 |
| Trpc4 | 0.0 | 0.0 | 0.0 | 0.0 | 0.0 | 0.0 | 0.0 | 0.0 | 0.0 | 0.0 | 0.0 | 0.0 |
| Trpd52l3 | 0.0 | 0.0 | 0.0 | 0.0 | 0.0 | 0.0 | 0.0 | 0.0 | 0.0 | 0.0 | 0.0 | 0.0 |
| Trpm1 | 0.0 | 0.0 | 0.0 | 0.0 | 0.0 | 0.0 | 0.0 | 0.0 | 0.0 | 0.0 | 0.0 | 0.0 |
| Trpm8 | 0.0 | 0.0 | 0.0 | 0.0 | 0.0 | 0.0 | 0.0 | 0.0 | 0.0 | 0.0 | 0.0 | 0.0 |
| Try4 | 0.0 | 0.0 | 0.0 | 0.0 | 0.0 | 0.0 | 0.0 | 0.0 | 0.0 | 0.0 | 0.0 | 0.0 |
| Try5 | 0.0 | 0.0 | 0.0 | 0.0 | 0.0 | 0.0 | 0.0 | 0.0 | 0.0 | 0.0 | 0.0 | 0.0 |
| Tsga13 | 0.0 | 0.0 | 0.0 | 0.0 | 0.0 | 0.0 | 0.0 | 0.0 | 0.0 | 0.0 | 0.0 | 0.0 |
| Tshb | 0.0 | 0.0 | 0.0 | 0.0 | 0.0 | 0.0 | 0.0 | 0.0 | 0.0 | 0.0 | 0.0 | 0.0 |
| Tsix | 0.0 | 0.0 | 0.0 | 0.0 | 0.0 | 0.0 | 0.0 | 0.0 | 0.0 | 0.0 | 0.0 | 0.0 |
| Tssk5 | 0.0 | 0.0 | 0.0 | 0.0 | 0.0 | 0.0 | 0.0 | 0.0 | 0.0 | 0.0 | 0.0 | 0.0 |
| Tsx | 0.0 | 0.0 | 0.0 | 0.0 | 0.0 | 0.0 | 0.0 | 0.0 | 0.0 | 0.0 | 0.3 | 0.0 |
| Ttc24 | 0.0 | 0.0 | 0.0 | 0.0 | 0.0 | 0.0 | 0.0 | 0.0 | 0.0 | 0.0 | 0.0 | 0.0 |
| Ttc29 | 0.0 | 0.0 | 0.0 | 0.0 | 0.0 | 0.0 | 0.0 | 0.0 | 0.0 | 0.0 | 0.0 | 0.0 |
| Ttc9b | 0.0 | 0.0 | 0.0 | 0.1 | 0.0 | 0.0 | 0.0 | 0.0 | 0.0 | 0.0 | 0.0 | 0.0 |
| Ttll10 | 0.0 | 0.0 | 0.0 | 0.0 | 0.0 | 0.0 | 0.0 | 0.0 | 0.0 | 0.0 | 0.0 | 0.0 |
| Ttll6 | 0.0 | 0.0 | 0.0 | 0.0 | 0.0 | 0.0 | 0.1 | 0.0 | 0.0 | 0.0 | 0.0 | 0.0 |
| Ttll8 | 0.0 | 0.0 | 0.0 | 0.0 | 0.0 | 0.0 | 0.0 | 0.0 | 0.0 | 0.0 | 0.0 | 0.0 |
| Ttr | 0.0 | 0.0 | 0.0 | 0.0 | 0.0 | 0.0 | 0.0 | 0.0 | 0.0 | 0.0 | 0.0 | 0.0 |
| Tuba3a | 0.0 | 0.0 | 0.0 | 0.0 | 0.0 | 0.0 | 0.0 | 0.0 | 0.0 | 0.0 | 0.0 | 0.0 |
| Tubb1 | 0.1 | 0.0 | 0.0 | 0.2 | 0.0 | 0.0 | 0.0 | 0.0 | 0.0 | 0.0 | 0.0 | 0.0 |
| Tyrp1 | 0.0 | 0.0 | 0.0 | 0.0 | 0.0 | 0.0 | 0.0 | 0.0 | 0.0 | 0.0 | 0.0 | 0.0 |
| U90926 | 0.1 | 0.0 | 0.0 | 0.0 | 0.0 | 0.0 | 0.0 | 0.0 | 0.1 | 0.0 | 0.0 | 0.0 |
| Ubd | 0.0 | 0.0 | 0.0 | 0.0 | 0.0 | 0.0 | 0.0 | 0.0 | 0.0 | 0.0 | 0.0 | 0.0 |
| Ube2dnl2 | 0.0 | 0.0 | 0.0 | 0.0 | 0.0 | 0.0 | 0.0 | 0.0 | 0.0 | 0.0 | 0.2 | 0.0 |
| Ubqln3 | 0.0 | 0.0 | 0.0 | 0.0 | 0.0 | 0.0 | 0.0 | 0.0 | 0.0 | 0.0 | 0.0 | 0.0 |
| Ubqlnl | 0.0 | 0.0 | 0.0 | 0.0 | 0.0 | 0.0 | 0.0 | 0.0 | 0.0 | 0.0 | 0.0 | 0.0 |
| Ucma | 0.0 | 0.0 | 0.1 | 0.0 | 0.0 | 0.1 | 0.0 | 0.1 | 0.0 | 0.0 | 0.0 | 0.0 |
| Ucn | 0.0 | 0.0 | 0.0 | 0.0 | 0.0 | 0.0 | 0.1 | 0.0 | 0.0 | 0.0 | 0.0 | 0.0 |
| Ucp3 | 0.0 | 0.0 | 0.0 | 0.0 | 0.0 | 0.0 | 0.0 | 0.0 | 0.0 | 0.0 | 0.0 | 0.0 |
| Ugt2b1 | 0.0 | 0.0 | 0.0 | 0.0 | 0.0 | 0.0 | 0.0 | 0.0 | 0.0 | 0.0 | 0.0 | 0.0 |
| Ugt2b34 | 0.0 | 0.0 | 0.0 | 0.0 | 0.0 | 0.0 | 0.0 | 0.0 | 0.0 | 0.0 | 0.0 | 0.0 |
| Ugt2b38 | 0.0 | 0.0 | 0.0 | 0.0 | 0.0 | 0.0 | 0.0 | 0.0 | 0.0 | 0.0 | 0.0 | 0.0 |
| Ugt2b5 | 0.0 | 0.0 | 0.0 | 0.0 | 0.0 | 0.0 | 0.0 | 0.0 | 0.0 | 0.0 | 0.0 | 0.0 |
| Ugt3a1 | 0.0 | 0.0 | 0.0 | 0.0 | 0.0 | 0.0 | 0.0 | 0.0 | 0.0 | 0.0 | 0.0 | 0.0 |
| Ugt3a2 | 0.0 | 0.0 | 0.0 | 0.0 | 0.0 | 0.0 | 0.0 | 0.0 | 0.0 | 0.0 | 0.0 | 0.0 |
| Ugt8a | 0.0 | 0.0 | 0.0 | 0.0 | 0.0 | 0.0 | 0.0 | 0.0 | 0.0 | 0.0 | 0.0 | 0.0 |
| Umod | 0.0 | 0.0 | 0.0 | 0.0 | 0.0 | 0.0 | 0.0 | 0.0 | 0.0 | 0.0 | 0.0 | 0.0 |
| Umodl1 | 0.0 | 0.0 | 0.0 | 0.0 | 0.0 | 0.0 | 0.0 | 0.0 | 0.0 | 0.0 | 0.0 | 0.0 |
| Uox | 0.0 | 0.0 | 0.0 | 0.0 | 0.0 | 0.0 | 0.0 | 0.0 | 0.0 | 0.0 | 0.0 | 0.0 |
| Upk2 | 0.0 | 0.0 | 0.0 | 0.0 | 0.1 | 0.0 | 0.0 | 0.0 | 0.0 | 0.0 | 0.0 | 0.0 |
| Upk3a | 0.0 | 0.0 | 0.0 | 0.1 | 0.0 | 0.0 | 0.0 | 0.0 | 0.0 | 0.0 | 0.0 | 0.0 |
| Usp17lb | 0.0 | 0.0 | 0.0 | 0.0 | 0.0 | 0.0 | 0.0 | 0.0 | 0.0 | 0.0 | 0.0 | 0.0 |
| Usp17lc | 0.0 | 0.0 | 0.0 | 0.0 | 0.0 | 0.0 | 0.0 | 0.0 | 0.0 | 0.0 | 0.0 | 0.0 |
| Usp17ld | 0.0 | 0.0 | 0.0 | 0.0 | 0.0 | 0.0 | 0.0 | 0.0 | 0.0 | 0.0 | 0.0 | 0.0 |
| Usp26 | 0.0 | 0.0 | 0.0 | 0.0 | 0.0 | 0.0 | 0.0 | 0.0 | 0.0 | 0.0 | 0.0 | 0.0 |
| Usp43 | 0.0 | 0.0 | 0.0 | 0.0 | 0.0 | 0.0 | 0.0 | 0.0 | 0.0 | 0.0 | 0.0 | 0.0 |
| Uts2 | 0.0 | 0.0 | 0.0 | 0.0 | 0.0 | 0.0 | 0.0 | 0.0 | 0.0 | 0.1 | 0.0 | 0.0 |
| Uts2d | 0.0 | 0.0 | 0.0 | 0.0 | 0.0 | 0.0 | 0.0 | 0.0 | 0.0 | 0.0 | 0.0 | 0.0 |
| V1ra8 | 0.0 | 0.0 | 0.0 | 0.0 | 0.0 | 0.0 | 0.0 | 0.0 | 0.0 | 0.0 | 0.0 | 0.0 |

| | | | | | | | | | | | | |
|---|---|---|---|---|---|---|---|---|---|---|---|---|
| Vax2os | 0.0 | 0.0 | 0.0 | 0.0 | 0.0 | 0.1 | 0.0 | 0.0 | 0.0 | 0.0 | 0.0 | 0.0 |
| Vmn1r1 | 0.0 | 0.0 | 0.0 | 0.0 | 0.0 | 0.2 | 0.0 | 0.0 | 0.0 | 0.0 | 0.0 | 0.0 |
| Vmn1r103 | 0.0 | 0.0 | 0.0 | 0.0 | 0.0 | 0.0 | 0.0 | 0.0 | 0.0 | 0.0 | 0.0 | 0.0 |
| Vmn1r104 | 0.0 | 0.0 | 0.0 | 0.0 | 0.0 | 0.0 | 0.0 | 0.0 | 0.0 | 0.0 | 0.0 | 0.0 |
| Vmn1r11 | 0.0 | 0.0 | 0.0 | 0.1 | 0.0 | 0.0 | 0.0 | 0.0 | 0.0 | 0.0 | 0.0 | 0.0 |
| Vmn1r116 | 0.0 | 0.0 | 0.0 | 0.0 | 0.0 | 0.0 | 0.0 | 0.0 | 0.0 | 0.0 | 0.0 | 0.0 |
| Vmn1r117 | 0.0 | 0.0 | 0.0 | 0.0 | 0.0 | 0.0 | 0.0 | 0.0 | 0.0 | 0.0 | 0.0 | 0.0 |
| Vmn1r119 | 0.0 | 0.0 | 0.0 | 0.0 | 0.0 | 0.0 | 0.0 | 0.0 | 0.0 | 0.0 | 0.0 | 0.0 |
| Vmn1r122 | 0.0 | 0.0 | 0.0 | 0.0 | 0.0 | 0.0 | 0.0 | 0.0 | 0.0 | 0.0 | 0.0 | 0.0 |
| Vmn1r128 | 0.0 | 0.0 | 0.0 | 0.0 | 0.0 | 0.0 | 0.0 | 0.0 | 0.0 | 0.0 | 0.0 | 0.0 |
| Vmn1r129 | 0.0 | 0.0 | 0.0 | 0.0 | 0.0 | 0.0 | 0.0 | 0.0 | 0.0 | 0.0 | 0.0 | 0.0 |
| Vmn1r130 | 0.0 | 0.0 | 0.0 | 0.0 | 0.0 | 0.0 | 0.0 | 0.0 | 0.0 | 0.0 | 0.0 | 0.0 |
| Vmn1r135 | 0.0 | 0.0 | 0.0 | 0.0 | 0.0 | 0.0 | 0.0 | 0.0 | 0.0 | 0.0 | 0.0 | 0.0 |
| Vmn1r137 | 0.0 | 0.0 | 0.0 | 0.0 | 0.0 | 0.0 | 0.0 | 0.0 | 0.0 | 0.0 | 0.0 | 0.0 |
| Vmn1r14 | 0.0 | 0.0 | 0.0 | 0.0 | 0.0 | 0.0 | 0.0 | 0.0 | 0.0 | 0.0 | 0.0 | 0.0 |
| Vmn1r143 | 0.0 | 0.0 | 0.0 | 0.0 | 0.0 | 0.0 | 0.0 | 0.0 | 0.0 | 0.0 | 0.0 | 0.0 |
| Vmn1r148 | 0.0 | 0.0 | 0.0 | 0.0 | 0.0 | 0.0 | 0.0 | 0.0 | 0.0 | 0.0 | 0.0 | 0.0 |
| Vmn1r151 | 0.0 | 0.0 | 0.0 | 0.0 | 0.0 | 0.0 | 0.0 | 0.0 | 0.0 | 0.0 | 0.0 | 0.0 |
| Vmn1r152 | 0.0 | 0.0 | 0.0 | 0.0 | 0.0 | 0.0 | 0.0 | 0.0 | 0.0 | 0.0 | 0.0 | 0.0 |
| Vmn1r155 | 0.0 | 0.0 | 0.0 | 0.0 | 0.0 | 0.0 | 0.0 | 0.0 | 0.0 | 0.0 | 0.0 | 0.0 |
| Vmn1r159 | 0.0 | 0.0 | 0.0 | 0.0 | 0.0 | 0.0 | 0.0 | 0.0 | 0.0 | 0.0 | 0.0 | 0.0 |
| Vmn1r163 | 0.0 | 0.0 | 0.0 | 0.0 | 0.0 | 0.0 | 0.0 | 0.0 | 0.0 | 0.0 | 0.0 | 0.0 |
| Vmn1r165 | 0.0 | 0.0 | 0.0 | 0.0 | 0.0 | 0.0 | 0.0 | 0.0 | 0.0 | 0.0 | 0.0 | 0.0 |
| Vmn1r17 | 0.0 | 0.1 | 0.0 | 0.0 | 0.0 | 0.0 | 0.0 | 0.0 | 0.0 | 0.0 | 0.0 | 0.0 |
| Vmn1r177 | 0.0 | 0.0 | 0.0 | 0.0 | 0.0 | 0.0 | 0.0 | 0.0 | 0.0 | 0.0 | 0.0 | 0.0 |
| Vmn1r181 | 0.0 | 0.0 | 0.0 | 0.0 | 0.0 | 0.0 | 0.0 | 0.0 | 0.0 | 0.0 | 0.0 | 0.0 |
| Vmn1r183 | 0.0 | 0.0 | 0.0 | 0.1 | 0.0 | 0.0 | 0.0 | 0.0 | 0.0 | 0.0 | 0.0 | 0.1 |
| Vmn1r186 | 0.0 | 0.0 | 0.0 | 0.0 | 0.0 | 0.0 | 0.0 | 0.0 | 0.0 | 0.0 | 0.0 | 0.0 |
| Vmn1r188 | 0.0 | 0.0 | 0.0 | 0.0 | 0.0 | 0.1 | 0.0 | 0.0 | 0.0 | 0.0 | 0.0 | 0.0 |
| Vmn1r196 | 0.0 | 0.0 | 0.0 | 0.0 | 0.0 | 0.0 | 0.0 | 0.0 | 0.0 | 0.0 | 0.0 | 0.0 |
| Vmn1r21 | 0.0 | 0.0 | 0.0 | 0.0 | 0.0 | 0.0 | 0.0 | 0.0 | 0.0 | 0.0 | 0.0 | 0.0 |
| Vmn1r211 | 0.0 | 0.0 | 0.0 | 0.0 | 0.0 | 0.0 | 0.0 | 0.0 | 0.0 | 0.0 | 0.0 | 0.0 |
| Vmn1r223 | 0.0 | 0.0 | 0.0 | 0.0 | 0.0 | 0.0 | 0.0 | 0.0 | 0.0 | 0.0 | 0.0 | 0.0 |
| Vmn1r224 | 0.0 | 0.0 | 0.0 | 0.0 | 0.0 | 0.0 | 0.0 | 0.0 | 0.0 | 0.0 | 0.0 | 0.0 |
| Vmn1r226 | 0.0 | 0.0 | 0.0 | 0.0 | 0.0 | 0.0 | 0.0 | 0.0 | 0.0 | 0.0 | 0.0 | 0.0 |
| Vmn1r231 | 0.0 | 0.0 | 0.0 | 0.0 | 0.0 | 0.0 | 0.0 | 0.0 | 0.0 | 0.0 | 0.0 | 0.0 |
| Vmn1r235 | 0.0 | 0.0 | 0.0 | 0.0 | 0.0 | 0.0 | 0.0 | 0.0 | 0.0 | 0.0 | 0.0 | 0.0 |
| Vmn1r32 | 0.0 | 0.0 | 0.0 | 0.0 | 0.0 | 0.0 | 0.0 | 0.0 | 0.0 | 0.0 | 0.0 | 0.0 |
| Vmn1r33 | 0.0 | 0.0 | 0.0 | 0.0 | 0.0 | 0.0 | 0.0 | 0.0 | 0.0 | 0.0 | 0.0 | 0.0 |
| Vmn1r35 | 0.0 | 0.0 | 0.0 | 0.0 | 0.0 | 0.0 | 0.0 | 0.0 | 0.0 | 0.0 | 0.0 | 0.0 |
| Vmn1r4 | 0.0 | 0.0 | 0.0 | 0.0 | 0.0 | 0.0 | 0.0 | 0.0 | 0.0 | 0.0 | 0.0 | 0.0 |
| Vmn1r40 | 0.0 | 0.0 | 0.0 | 0.1 | 0.1 | 0.0 | 0.0 | 0.0 | 0.1 | 0.1 | 0.0 | 0.0 |
| Vmn1r41 | 0.0 | 0.0 | 0.0 | 0.0 | 0.0 | 0.0 | 0.0 | 0.0 | 0.0 | 0.0 | 0.0 | 0.0 |
| Vmn1r42 | 0.0 | 0.0 | 0.0 | 0.0 | 0.0 | 0.0 | 0.0 | 0.0 | 0.0 | 0.0 | 0.0 | 0.0 |
| Vmn1r43 | 0.0 | 0.0 | 0.0 | 0.0 | 0.0 | 0.0 | 0.0 | 0.0 | 0.0 | 0.0 | 0.0 | 0.0 |
| Vmn1r44 | 0.0 | 0.0 | 0.0 | 0.0 | 0.0 | 0.0 | 0.0 | 0.0 | 0.0 | 0.0 | 0.0 | 0.0 |
| Vmn1r46 | 0.0 | 0.0 | 0.0 | 0.0 | 0.0 | 0.0 | 0.0 | 0.0 | 0.0 | 0.0 | 0.0 | 0.0 |
| Vmn1r47 | 0.0 | 0.0 | 0.0 | 0.0 | 0.0 | 0.0 | 0.0 | 0.0 | 0.0 | 0.0 | 0.0 | 0.0 |
| Vmn1r48 | 0.0 | 0.0 | 0.0 | 0.0 | 0.0 | 0.1 | 0.0 | 0.0 | 0.0 | 0.0 | 0.0 | 0.0 |
| Vmn1r5 | 0.0 | 0.0 | 0.1 | 0.0 | 0.0 | 0.0 | 0.0 | 0.0 | 0.0 | 0.0 | 0.0 | 0.0 |
| Vmn1r50 | 0.0 | 0.1 | 0.0 | 0.0 | 0.0 | 0.0 | 0.0 | 0.0 | 0.0 | 0.0 | 0.0 | 0.0 |
| Vmn1r52 | 0.0 | 0.0 | 0.0 | 0.0 | 0.0 | 0.0 | 0.0 | 0.0 | 0.0 | 0.0 | 0.0 | 0.0 |
| Vmn1r54 | 0.0 | 0.0 | 0.0 | 0.0 | 0.0 | 0.0 | 0.0 | 0.0 | 0.0 | 0.0 | 0.0 | 0.0 |
| Vmn1r60 | 0.0 | 0.0 | 0.0 | 0.0 | 0.0 | 0.0 | 0.0 | 0.0 | 0.0 | 0.0 | 0.0 | 0.0 |
| Vmn1r88 | 0.0 | 0.0 | 0.0 | 0.0 | 0.0 | 0.0 | 0.0 | 0.0 | 0.0 | 0.0 | 0.0 | 0.0 |
| Vmn1r95 | 0.0 | 0.0 | 0.0 | 0.0 | 0.0 | 0.0 | 0.0 | 0.0 | 0.0 | 0.0 | 0.0 | 0.0 |
| Vmn1r-ps79 | 0.0 | 0.0 | 0.0 | 0.0 | 0.0 | 0.0 | 0.0 | 0.0 | 0.0 | 0.0 | 0.0 | 0.0 |

| | | | | | | | | | | | | |
|---|---|---|---|---|---|---|---|---|---|---|---|---|
| Vmn2r103 | 0.0 | 0.0 | 0.0 | 0.0 | 0.0 | 0.0 | 0.0 | 0.0 | 0.0 | 0.0 | 0.0 | 0.0 |
| Vmn2r106 | 0.0 | 0.0 | 0.0 | 0.0 | 0.0 | 0.0 | 0.0 | 0.0 | 0.0 | 0.0 | 0.0 | 0.0 |
| Vmn2r109 | 0.0 | 0.0 | 0.0 | 0.0 | 0.0 | 0.0 | 0.0 | 0.0 | 0.0 | 0.0 | 0.0 | 0.0 |
| Vmn2r115 | 0.0 | 0.0 | 0.0 | 0.0 | 0.0 | 0.0 | 0.0 | 0.0 | 0.0 | 0.0 | 0.0 | 0.0 |
| Vmn2r118 | 0.0 | 0.0 | 0.0 | 0.0 | 0.0 | 0.0 | 0.0 | 0.0 | 0.0 | 0.0 | 0.0 | 0.0 |
| Vmn2r12 | 0.0 | 0.0 | 0.0 | 0.0 | 0.0 | 0.0 | 0.0 | 0.0 | 0.0 | 0.0 | 0.0 | 0.0 |
| Vmn2r123 | 0.0 | 0.0 | 0.0 | 0.0 | 0.0 | 0.0 | 0.0 | 0.0 | 0.0 | 0.0 | 0.0 | 0.0 |
| Vmn2r14 | 0.0 | 0.0 | 0.0 | 0.0 | 0.0 | 0.0 | 0.0 | 0.0 | 0.0 | 0.0 | 0.0 | 0.0 |
| Vmn2r15 | 0.0 | 0.0 | 0.0 | 0.0 | 0.0 | 0.0 | 0.0 | 0.0 | 0.0 | 0.0 | 0.0 | 0.0 |
| Vmn2r16 | 0.0 | 0.0 | 0.0 | 0.0 | 0.0 | 0.0 | 0.0 | 0.0 | 0.0 | 0.0 | 0.0 | 0.0 |
| Vmn2r18 | 0.0 | 0.0 | 0.0 | 0.0 | 0.0 | 0.0 | 0.0 | 0.0 | 0.0 | 0.0 | 0.0 | 0.0 |
| Vmn2r22 | 0.0 | 0.0 | 0.0 | 0.0 | 0.0 | 0.0 | 0.0 | 0.0 | 0.0 | 0.0 | 0.0 | 0.0 |
| Vmn2r24 | 0.0 | 0.0 | 0.0 | 0.0 | 0.0 | 0.0 | 0.0 | 0.0 | 0.0 | 0.0 | 0.0 | 0.0 |
| Vmn2r28 | 0.0 | 0.0 | 0.0 | 0.0 | 0.0 | 0.0 | 0.0 | 0.0 | 0.0 | 0.0 | 0.0 | 0.0 |
| Vmn2r32 | 0.0 | 0.0 | 0.0 | 0.0 | 0.0 | 0.0 | 0.0 | 0.0 | 0.0 | 0.0 | 0.0 | 0.0 |
| Vmn2r35 | 0.0 | 0.0 | 0.0 | 0.0 | 0.0 | 0.0 | 0.0 | 0.0 | 0.0 | 0.0 | 0.0 | 0.0 |
| Vmn2r36 | 0.0 | 0.0 | 0.0 | 0.0 | 0.0 | 0.0 | 0.0 | 0.0 | 0.0 | 0.0 | 0.0 | 0.0 |
| Vmn2r40 | 0.0 | 0.0 | 0.0 | 0.0 | 0.0 | 0.0 | 0.0 | 0.0 | 0.0 | 0.0 | 0.0 | 0.0 |
| Vmn2r41 | 0.0 | 0.0 | 0.0 | 0.0 | 0.0 | 0.0 | 0.0 | 0.0 | 0.0 | 0.0 | 0.0 | 0.0 |
| Vmn2r42 | 0.0 | 0.0 | 0.0 | 0.0 | 0.0 | 0.0 | 0.0 | 0.0 | 0.0 | 0.0 | 0.0 | 0.0 |
| Vmn2r48 | 0.0 | 0.0 | 0.0 | 0.0 | 0.0 | 0.0 | 0.0 | 0.0 | 0.0 | 0.0 | 0.0 | 0.0 |
| Vmn2r49 | 0.0 | 0.0 | 0.0 | 0.0 | 0.0 | 0.0 | 0.0 | 0.0 | 0.0 | 0.0 | 0.0 | 0.0 |
| Vmn2r5 | 0.0 | 0.0 | 0.0 | 0.0 | 0.0 | 0.0 | 0.0 | 0.0 | 0.0 | 0.0 | 0.0 | 0.0 |
| Vmn2r50 | 0.0 | 0.0 | 0.0 | 0.0 | 0.0 | 0.0 | 0.0 | 0.0 | 0.0 | 0.0 | 0.0 | 0.0 |
| Vmn2r51 | 0.0 | 0.0 | 0.0 | 0.0 | 0.0 | 0.0 | 0.0 | 0.0 | 0.0 | 0.0 | 0.0 | 0.0 |
| Vmn2r52 | 0.0 | 0.0 | 0.0 | 0.0 | 0.0 | 0.0 | 0.0 | 0.0 | 0.0 | 0.0 | 0.0 | 0.0 |
| Vmn2r53 | 0.0 | 0.0 | 0.0 | 0.0 | 0.0 | 0.0 | 0.0 | 0.0 | 0.0 | 0.0 | 0.0 | 0.0 |
| Vmn2r54 | 0.0 | 0.0 | 0.0 | 0.0 | 0.0 | 0.0 | 0.0 | 0.0 | 0.0 | 0.0 | 0.0 | 0.0 |
| Vmn2r55 | 0.0 | 0.0 | 0.0 | 0.0 | 0.0 | 0.1 | 0.0 | 0.0 | 0.0 | 0.0 | 0.0 | 0.0 |
| Vmn2r59 | 0.0 | 0.0 | 0.0 | 0.0 | 0.0 | 0.0 | 0.0 | 0.0 | 0.0 | 0.0 | 0.0 | 0.0 |
| Vmn2r6 | 0.0 | 0.0 | 0.0 | 0.0 | 0.0 | 0.0 | 0.0 | 0.0 | 0.0 | 0.0 | 0.0 | 0.0 |
| Vmn2r60 | 0.0 | 0.0 | 0.0 | 0.0 | 0.0 | 0.0 | 0.0 | 0.0 | 0.0 | 0.0 | 0.0 | 0.0 |
| Vmn2r61 | 0.0 | 0.0 | 0.0 | 0.0 | 0.0 | 0.0 | 0.0 | 0.0 | 0.0 | 0.0 | 0.0 | 0.0 |
| Vmn2r65 | 0.0 | 0.0 | 0.0 | 0.0 | 0.0 | 0.0 | 0.0 | 0.0 | 0.0 | 0.0 | 0.0 | 0.0 |
| Vmn2r66 | 0.0 | 0.0 | 0.0 | 0.0 | 0.0 | 0.0 | 0.0 | 0.0 | 0.0 | 0.0 | 0.0 | 0.0 |
| Vmn2r68 | 0.0 | 0.0 | 0.0 | 0.0 | 0.0 | 0.0 | 0.0 | 0.0 | 0.0 | 0.0 | 0.0 | 0.0 |
| Vmn2r69 | 0.0 | 0.0 | 0.0 | 0.2 | 0.0 | 0.0 | 0.0 | 0.0 | 0.0 | 0.0 | 0.0 | 0.0 |
| Vmn2r7 | 0.0 | 0.0 | 0.0 | 0.0 | 0.0 | 0.0 | 0.0 | 0.0 | 0.0 | 0.0 | 0.0 | 0.0 |
| Vmn2r70 | 0.0 | 0.0 | 0.0 | 0.0 | 0.0 | 0.0 | 0.0 | 0.0 | 0.0 | 0.0 | 0.0 | 0.0 |
| Vmn2r74 | 0.0 | 0.0 | 0.0 | 0.0 | 0.0 | 0.0 | 0.0 | 0.0 | 0.0 | 0.0 | 0.0 | 0.0 |
| Vmn2r77 | 0.0 | 0.0 | 0.0 | 0.0 | 0.0 | 0.0 | 0.0 | 0.0 | 0.0 | 0.0 | 0.0 | 0.0 |
| Vmn2r78 | 0.0 | 0.0 | 0.0 | 0.0 | 0.0 | 0.0 | 0.0 | 0.0 | 0.0 | 0.0 | 0.0 | 0.0 |
| Vmn2r79 | 0.0 | 0.0 | 0.0 | 0.0 | 0.0 | 0.0 | 0.0 | 0.0 | 0.0 | 0.0 | 0.0 | 0.0 |
| Vmn2r8 | 0.0 | 0.0 | 0.0 | 0.0 | 0.0 | 0.0 | 0.0 | 0.0 | 0.0 | 0.0 | 0.0 | 0.0 |
| Vmn2r80 | 0.0 | 0.0 | 0.0 | 0.0 | 0.0 | 0.0 | 0.0 | 0.0 | 0.0 | 0.0 | 0.0 | 0.0 |
| Vmn2r81 | 0.0 | 0.0 | 0.0 | 0.0 | 0.0 | 0.0 | 0.0 | 0.0 | 0.0 | 0.0 | 0.0 | 0.0 |
| Vmn2r82 | 0.0 | 0.0 | 0.0 | 0.0 | 0.0 | 0.0 | 0.0 | 0.0 | 0.0 | 0.0 | 0.0 | 0.0 |
| Vmn2r84 | 0.0 | 0.0 | 0.0 | 0.0 | 0.0 | 0.0 | 0.0 | 0.0 | 0.0 | 0.0 | 0.0 | 0.0 |
| Vmn2r85 | 0.0 | 0.0 | 0.0 | 0.0 | 0.0 | 0.0 | 0.0 | 0.0 | 0.0 | 0.0 | 0.0 | 0.0 |
| Vmn2r86 | 0.0 | 0.0 | 0.0 | 0.0 | 0.0 | 0.0 | 0.0 | 0.0 | 0.0 | 0.0 | 0.0 | 0.0 |
| Vmn2r87 | 0.0 | 0.0 | 0.0 | 0.0 | 0.0 | 0.0 | 0.0 | 0.0 | 0.0 | 0.0 | 0.0 | 0.0 |
| Vmn2r9 | 0.0 | 0.0 | 0.0 | 0.0 | 0.0 | 0.0 | 0.0 | 0.0 | 0.0 | 0.0 | 0.0 | 0.0 |
| Vmn2r90 | 0.0 | 0.0 | 0.0 | 0.0 | 0.0 | 0.0 | 0.0 | 0.0 | 0.0 | 0.0 | 0.0 | 0.0 |
| Vmn2r91 | 0.0 | 0.0 | 0.0 | 0.0 | 0.0 | 0.0 | 0.0 | 0.0 | 0.0 | 0.0 | 0.0 | 0.0 |
| Vmn2r92 | 0.0 | 0.0 | 0.0 | 0.0 | 0.0 | 0.0 | 0.0 | 0.0 | 0.0 | 0.0 | 0.0 | 0.0 |
| Vmn2r93 | 0.0 | 0.0 | 0.0 | 0.0 | 0.0 | 0.0 | 0.0 | 0.0 | 0.0 | 0.0 | 0.0 | 0.0 |
| Vmn2r94 | 0.0 | 0.0 | 0.0 | 0.0 | 0.0 | 0.0 | 0.0 | 0.0 | 0.0 | 0.0 | 0.0 | 0.0 |

| | | | | | | | | | | | |
|---|---|---|---|---|---|---|---|---|---|---|---|
| Vmn2r95 | 0.0 | 0.0 | 0.0 | 0.0 | 0.0 | 0.0 | 0.0 | 0.0 | 0.0 | 0.0 | 0.0 | 0.0 |
| Vmn2r-ps11 | 0.0 | 0.0 | 0.0 | 0.0 | 0.0 | 0.0 | 0.0 | 0.0 | 0.0 | 0.0 | 0.0 | 0.0 |
| Vmn2r-ps159 | 0.0 | 0.0 | 0.0 | 0.0 | 0.0 | 0.0 | 0.0 | 0.0 | 0.0 | 0.0 | 0.0 | 0.0 |
| Vmn2r-ps54 | 0.0 | 0.0 | 0.0 | 0.0 | 0.0 | 0.0 | 0.0 | 0.0 | 0.0 | 0.0 | 0.0 | 0.0 |
| Vsig1 | 0.0 | 0.0 | 0.0 | 0.0 | 0.0 | 0.0 | 0.0 | 0.0 | 0.0 | 0.0 | 0.0 | 0.0 |
| Vsig2 | 0.0 | 0.0 | 0.0 | 0.0 | 0.0 | 0.0 | 0.0 | 0.0 | 0.1 | 0.0 | 0.0 | 0.0 |
| Vsig4 | 0.0 | 0.0 | 0.0 | 0.0 | 0.0 | 0.0 | 0.0 | 0.0 | 0.0 | 0.0 | 0.0 | 0.0 |
| Vsnl1 | 0.0 | 0.0 | 0.0 | 0.0 | 0.0 | 0.0 | 0.0 | 0.0 | 0.0 | 0.0 | 0.0 | 0.0 |
| Vstm2l | 0.0 | 0.0 | 0.0 | 0.0 | 0.0 | 0.0 | 0.0 | 0.0 | 0.0 | 0.0 | 0.0 | 0.0 |
| Vsx2 | 0.0 | 0.0 | 0.0 | 0.0 | 0.0 | 0.0 | 0.0 | 0.0 | 0.0 | 0.0 | 0.0 | 0.0 |
| Vwa5b1 | 0.0 | 0.0 | 0.0 | 0.0 | 0.0 | 0.0 | 0.0 | 0.0 | 0.0 | 0.0 | 0.0 | 0.0 |
| Vwc2 | 0.0 | 0.0 | 0.0 | 0.0 | 0.0 | 0.0 | 0.0 | 0.0 | 0.0 | 0.0 | 0.0 | 0.0 |
| Vwc2l | 0.0 | 0.0 | 0.0 | 0.0 | 0.0 | 0.0 | 0.0 | 0.0 | 0.0 | 0.0 | 0.0 | 0.0 |
| Vwde | 0.0 | 0.0 | 0.0 | 0.0 | 0.0 | 0.0 | 0.0 | 0.0 | 0.0 | 0.0 | 0.0 | 0.0 |
| Wap | 0.0 | 0.0 | 0.0 | 0.0 | 0.0 | 0.0 | 0.0 | 0.0 | 0.0 | 0.0 | 0.0 | 0.0 |
| Wbp2nl | 0.0 | 0.0 | 0.0 | 0.0 | 0.0 | 0.0 | 0.0 | 0.0 | 0.0 | 0.0 | 0.0 | 0.0 |
| Wbscr25 | 0.0 | 0.0 | 0.0 | 0.0 | 0.0 | 0.0 | 0.0 | 0.0 | 0.0 | 0.0 | 0.0 | 0.0 |
| Wdr64 | 0.0 | 0.0 | 0.0 | 0.0 | 0.0 | 0.0 | 0.0 | 0.0 | 0.0 | 0.0 | 0.0 | 0.0 |
| Wdr86 | 0.0 | 0.0 | 0.0 | 0.0 | 0.0 | 0.0 | 0.0 | 0.0 | 0.0 | 0.0 | 0.0 | 0.0 |
| Wee2 | 0.0 | 0.0 | 0.0 | 0.0 | 0.0 | 0.0 | 0.0 | 0.0 | 0.0 | 0.0 | 0.0 | 0.0 |
| Wfdc13 | 0.0 | 0.0 | 0.0 | 0.0 | 0.0 | 0.0 | 0.0 | 0.0 | 0.0 | 0.0 | 0.0 | 0.0 |
| Wfdc16 | 0.0 | 0.0 | 0.0 | 0.0 | 0.0 | 0.0 | 0.0 | 0.0 | 0.0 | 0.0 | 0.0 | 0.0 |
| Wfdc2 | 0.0 | 0.0 | 0.0 | 0.0 | 0.0 | 0.0 | 0.0 | 0.0 | 0.0 | 0.0 | 0.0 | 0.0 |
| Wfdc6b | 0.0 | 0.0 | 0.0 | 0.0 | 0.0 | 0.0 | 0.0 | 0.0 | 0.0 | 0.0 | 0.0 | 0.0 |
| Wfdc8 | 0.0 | 0.0 | 0.0 | 0.0 | 0.0 | 0.0 | 0.0 | 0.0 | 0.0 | 0.0 | 0.0 | 0.0 |
| Wisp3 | 0.0 | 0.0 | 0.0 | 0.0 | 0.0 | 0.0 | 0.0 | 0.0 | 0.0 | 0.1 | 0.0 | 0.0 |
| Wnt1 | 0.0 | 0.0 | 0.0 | 0.0 | 0.0 | 0.0 | 0.0 | 0.0 | 0.0 | 0.0 | 0.0 | 0.0 |
| Wnt3a | 0.0 | 0.0 | 0.0 | 0.0 | 0.0 | 0.0 | 0.0 | 0.0 | 0.0 | 0.0 | 0.0 | 0.0 |
| Wnt7a | 0.1 | 0.0 | 0.0 | 0.3 | 0.0 | 0.1 | 0.1 | 0.0 | 0.0 | 0.1 | 0.0 | 0.0 |
| Wnt9b | 0.0 | 0.0 | 0.0 | 0.0 | 0.0 | 0.0 | 0.0 | 0.0 | 0.0 | 0.0 | 0.0 | 0.0 |
| Xkr7 | 0.0 | 0.0 | 0.0 | 0.0 | 0.0 | 0.0 | 0.0 | 0.0 | 0.0 | 0.0 | 0.0 | 0.0 |
| Xkr9 | 0.0 | 0.0 | 0.0 | 0.0 | 0.0 | 0.0 | 0.0 | 0.0 | 0.0 | 0.0 | 0.0 | 0.0 |
| Ybx2 | 0.1 | 0.0 | 0.1 | 0.0 | 0.0 | 0.0 | 0.0 | 0.0 | 0.0 | 0.0 | 0.0 | 0.0 |
| Yipf7 | 0.0 | 0.0 | 0.0 | 0.0 | 0.0 | 0.0 | 0.0 | 0.0 | 0.0 | 0.0 | 0.0 | 0.0 |
| Zar1 | 0.0 | 0.0 | 0.0 | 0.0 | 0.0 | 0.0 | 0.0 | 0.0 | 0.0 | 0.0 | 0.0 | 0.0 |
| Zar1l | 0.0 | 0.0 | 0.0 | 0.0 | 0.0 | 0.0 | 0.0 | 0.0 | 0.0 | 0.0 | 0.0 | 0.0 |
| Zbbx | 0.0 | 0.0 | 0.0 | 0.0 | 0.0 | 0.0 | 0.0 | 0.0 | 0.0 | 0.0 | 0.0 | 0.0 |
| Zcchc13 | 0.0 | 0.0 | 0.0 | 0.0 | 0.0 | 0.0 | 0.0 | 0.0 | 0.0 | 0.0 | 0.0 | 0.0 |
| Zcchc16 | 0.0 | 0.0 | 0.0 | 0.0 | 0.0 | 0.0 | 0.0 | 0.0 | 0.0 | 0.0 | 0.0 | 0.0 |
| Zdhhc19 | 0.0 | 0.0 | 0.0 | 0.0 | 0.0 | 0.0 | 0.0 | 0.0 | 0.0 | 0.0 | 0.0 | 0.0 |
| Zdhhc22 | 0.0 | 0.0 | 0.0 | 0.0 | 0.1 | 0.0 | 0.0 | 0.0 | 0.0 | 0.0 | 0.1 | 0.0 |
| Zdhhc23 | 0.1 | 0.0 | 0.0 | 0.0 | 0.0 | 0.0 | 0.0 | 0.0 | 0.0 | 0.0 | 0.0 | 0.0 |
| Zfp352 | 0.0 | 0.0 | 0.0 | 0.0 | 0.0 | 0.0 | 0.0 | 0.0 | 0.0 | 0.0 | 0.0 | 0.0 |
| Zfp366 | 0.0 | 0.0 | 0.0 | 0.0 | 0.0 | 0.0 | 0.0 | 0.0 | 0.0 | 0.0 | 0.0 | 0.0 |
| Zfp36l3 | 0.0 | 0.0 | 0.0 | 0.0 | 0.0 | 0.0 | 0.0 | 0.0 | 0.0 | 0.0 | 0.0 | 0.0 |
| Zfp572 | 0.0 | 0.0 | 0.0 | 0.0 | 0.0 | 0.0 | 0.0 | 0.0 | 0.0 | 0.0 | 0.0 | 0.0 |
| Zfp648 | 0.0 | 0.0 | 0.0 | 0.0 | 0.0 | 0.0 | 0.0 | 0.0 | 0.0 | 0.0 | 0.0 | 0.0 |
| Zfp663 | 0.0 | 0.0 | 0.0 | 0.0 | 0.0 | 0.0 | 0.0 | 0.0 | 0.0 | 0.0 | 0.0 | 0.0 |
| Zfp711 | 0.0 | 0.0 | 0.0 | 0.0 | 0.0 | 0.0 | 0.0 | 0.0 | 0.0 | 0.0 | 0.0 | 0.0 |
| Zfp735 | 0.0 | 0.0 | 0.0 | 0.0 | 0.0 | 0.0 | 0.0 | 0.0 | 0.0 | 0.0 | 0.0 | 0.0 |
| Zfp804a | 0.0 | 0.0 | 0.0 | 0.0 | 0.0 | 0.0 | 0.0 | 0.0 | 0.0 | 0.0 | 0.0 | 0.0 |
| Zfp819 | 0.0 | 0.0 | 0.0 | 0.0 | 0.0 | 0.0 | 0.1 | 0.0 | 0.0 | 0.0 | 0.0 | 0.0 |
| Zfp831 | 0.0 | 0.0 | 0.0 | 0.0 | 0.0 | 0.0 | 0.0 | 0.0 | 0.0 | 0.0 | 0.0 | 0.0 |
| Zfy1 | 0.0 | 0.0 | 0.0 | 0.0 | 0.0 | 0.0 | 0.0 | 0.0 | 0.0 | 0.0 | 0.0 | 0.0 |
| Zfy2 | 0.0 | 0.0 | 0.0 | 0.0 | 0.0 | 0.0 | 0.0 | 0.0 | 0.0 | 0.0 | 0.0 | 0.0 |
| Zg16 | 0.0 | 0.0 | 0.0 | 0.2 | 0.0 | 0.0 | 0.0 | 0.0 | 0.0 | 0.0 | 0.0 | 0.0 |
| Zic2 | 0.0 | 0.0 | 0.0 | 0.0 | 0.0 | 0.0 | 0.0 | 0.0 | 0.0 | 0.0 | 0.0 | 0.0 |

| | | | | | | | | | | | | |
|---|---|---|---|---|---|---|---|---|---|---|---|---|
| Zic4 | 0.0 | 0.0 | 0.0 | 0.0 | 0.0 | 0.0 | 0.0 | 0.0 | 0.0 | 0.0 | 0.0 | 0.0 |
| Zim3 | 0.0 | 0.0 | 0.0 | 0.0 | 0.0 | 0.0 | 0.0 | 0.0 | 0.0 | 0.0 | 0.0 | 0.0 |
| Zp3r | 0.0 | 0.0 | 0.0 | 0.0 | 0.0 | 0.0 | 0.0 | 0.0 | 0.0 | 0.0 | 0.0 | 0.0 |
| Zscan10 | 0.0 | 0.0 | 0.0 | 0.0 | 0.0 | 0.0 | 0.0 | 0.0 | 0.0 | 0.0 | 0.0 | 0.0 |
| Zscan4a | 0.0 | 0.0 | 0.0 | 0.0 | 0.0 | 0.0 | 0.0 | 0.0 | 0.0 | 0.0 | 0.0 | 0.0 |
| Zscan4b | 0.0 | 0.0 | 0.0 | 0.0 | 0.0 | 0.0 | 0.0 | 0.0 | 0.0 | 0.0 | 0.0 | 0.0 |
| Zscan4c | 0.0 | 0.0 | 0.0 | 0.0 | 0.0 | 0.0 | 0.0 | 0.0 | 0.0 | 0.0 | 0.0 | 0.0 |
| Zscan4d | 0.0 | 0.0 | 0.0 | 0.0 | 0.0 | 0.0 | 0.0 | 0.0 | 0.0 | 0.0 | 0.0 | 0.0 |
| Zscan4e | 0.0 | 0.0 | 0.0 | 0.0 | 0.0 | 0.0 | 0.0 | 0.0 | 0.0 | 0.0 | 0.0 | 0.0 |
| Zscan4f | 0.0 | 0.0 | 0.0 | 0.0 | 0.0 | 0.0 | 0.0 | 0.0 | 0.0 | 0.0 | 0.0 | 0.0 |
| Zscan5b | 0.0 | 0.0 | 0.0 | 0.0 | 0.0 | 0.0 | 0.0 | 0.0 | 0.0 | 0.0 | 0.0 | 0.0 |
| 1110015O18Rik | 0.0 | 0.0 | 0.0 | 0.0 | 0.0 | 0.0 | 0.0 | 0.0 | 0.0 | 0.0 | 0.0 | 0.0 |
| 1110025L11Rik | 0.0 | 0.0 | 0.0 | 0.0 | 0.0 | 0.0 | 0.0 | 0.0 | 0.0 | 0.0 | 0.0 | 0.0 |
| 1110028F11Rik | 0.0 | 0.0 | 0.0 | 0.0 | 0.0 | 0.0 | 0.0 | 0.0 | 0.0 | 0.0 | 0.0 | 0.0 |
| 1190003K10Rik | 0.0 | 0.0 | 0.0 | 0.0 | 0.0 | 0.0 | 0.0 | 0.0 | 0.0 | 0.0 | 0.0 | 0.0 |
| 1500009C09Rik | 0.0 | 0.0 | 0.0 | 0.0 | 0.0 | 0.0 | 0.0 | 0.0 | 0.0 | 0.0 | 0.0 | 0.0 |
| 1500016L03Rik | 0.0 | 0.0 | 0.0 | 0.0 | 0.0 | 0.0 | 0.0 | 0.0 | 0.0 | 0.0 | 0.0 | 0.0 |
| 1600014K23Rik | 0.0 | 0.0 | 0.0 | 0.0 | 0.0 | 0.0 | 0.0 | 0.0 | 0.0 | 0.0 | 0.0 | 0.0 |
| 1600015I10Rik | 0.0 | 0.0 | 0.0 | 0.0 | 0.0 | 0.0 | 0.0 | 0.0 | 0.0 | 0.0 | 0.0 | 0.0 |
| 1600025M17Rik | 0.0 | 0.0 | 0.0 | 0.0 | 0.0 | 0.0 | 0.0 | 0.0 | 0.0 | 0.0 | 0.0 | 0.0 |
| 1700001C02Rik | 0.0 | 0.0 | 0.0 | 0.0 | 0.0 | 0.0 | 0.0 | 0.0 | 0.0 | 0.0 | 0.0 | 0.0 |
| 1700003C15Rik | 0.0 | 0.0 | 0.0 | 0.0 | 0.0 | 0.0 | 0.0 | 0.0 | 0.0 | 0.0 | 0.0 | 0.0 |
| 1700003F17Rik | 0.0 | 0.0 | 0.0 | 0.0 | 0.0 | 0.0 | 0.0 | 0.0 | 0.0 | 0.0 | 0.0 | 0.0 |
| 1700003G13Rik | 0.0 | 0.0 | 0.0 | 0.0 | 0.0 | 0.0 | 0.0 | 0.0 | 0.0 | 0.0 | 0.0 | 0.0 |
| 1700003H04Rik | 0.0 | 0.0 | 0.0 | 0.0 | 0.0 | 0.0 | 0.0 | 0.0 | 0.0 | 0.0 | 0.0 | 0.0 |
| 1700003L19Rik | 0.0 | 0.0 | 0.0 | 0.0 | 0.0 | 0.0 | 0.0 | 0.0 | 0.0 | 0.0 | 0.0 | 0.0 |
| 1700003P14Rik | 0.0 | 0.0 | 0.0 | 0.0 | 0.0 | 0.0 | 0.0 | 0.0 | 0.0 | 0.0 | 0.0 | 0.0 |
| 1700006A11Rik | 0.0 | 0.0 | 0.0 | 0.0 | 0.0 | 0.0 | 0.0 | 0.0 | 0.0 | 0.0 | 0.0 | 0.0 |
| 1700006E09Rik | 0.0 | 0.0 | 0.0 | 0.0 | 0.0 | 0.0 | 0.0 | 0.0 | 0.0 | 0.0 | 0.0 | 0.0 |
| 1700008A04Rik | 0.0 | 0.0 | 0.0 | 0.0 | 0.0 | 0.0 | 0.0 | 0.0 | 0.0 | 0.0 | 0.0 | 0.0 |
| 1700008K24Rik | 0.0 | 0.0 | 0.0 | 0.0 | 0.0 | 0.0 | 0.0 | 0.0 | 0.0 | 0.0 | 0.0 | 0.0 |
| 1700008P02Rik | 0.0 | 0.0 | 0.0 | 0.0 | 0.0 | 0.0 | 0.0 | 0.0 | 0.0 | 0.0 | 0.0 | 0.0 |
| 1700009J07Rik | 0.0 | 0.0 | 0.0 | 0.0 | 0.0 | 0.0 | 0.0 | 0.0 | 0.0 | 0.0 | 0.0 | 0.0 |
| 1700010J16Rik | 0.0 | 0.0 | 0.0 | 0.0 | 0.0 | 0.0 | 0.0 | 0.0 | 0.0 | 0.0 | 0.0 | 0.0 |
| 1700010K23Rik | 0.0 | 0.0 | 0.0 | 0.0 | 0.0 | 0.0 | 0.0 | 0.0 | 0.0 | 0.0 | 0.0 | 0.0 |
| 1700011A15Rik | 0.0 | 0.0 | 0.0 | 0.0 | 0.0 | 0.0 | 0.0 | 0.0 | 0.0 | 0.0 | 0.0 | 0.0 |
| 1700011B04Rik | 0.0 | 0.0 | 0.0 | 0.0 | 0.0 | 0.0 | 0.0 | 0.0 | 0.0 | 0.0 | 0.0 | 0.0 |
| 1700011L22Rik | 0.0 | 0.0 | 0.0 | 0.0 | 0.0 | 0.0 | 0.0 | 0.0 | 0.0 | 0.0 | 0.0 | 0.0 |
| 1700012A03Rik | 0.0 | 0.0 | 0.0 | 0.0 | 0.0 | 0.0 | 0.0 | 0.0 | 0.0 | 0.0 | 0.0 | 0.0 |
| 1700012P22Rik | 0.0 | 0.0 | 0.0 | 0.0 | 0.0 | 0.0 | 0.0 | 0.0 | 0.0 | 0.0 | 0.0 | 0.0 |
| 1700013D24Rik | 0.0 | 0.0 | 0.0 | 0.0 | 0.0 | 0.0 | 0.0 | 0.0 | 0.0 | 0.0 | 0.0 | 0.0 |
| 1700016C15Rik | 0.0 | 0.0 | 0.0 | 0.0 | 0.0 | 0.0 | 0.0 | 0.0 | 0.0 | 0.0 | 0.0 | 0.0 |
| 1700016G22Rik | 0.0 | 0.0 | 0.0 | 0.0 | 0.0 | 0.0 | 0.0 | 0.0 | 0.0 | 0.0 | 0.0 | 0.0 |
| 1700016P04Rik | 0.0 | 0.0 | 0.0 | 0.0 | 0.0 | 0.0 | 0.0 | 0.0 | 0.0 | 0.0 | 0.0 | 0.0 |
| 1700017J07Rik | 0.0 | 0.0 | 0.0 | 0.0 | 0.0 | 0.0 | 0.0 | 0.0 | 0.0 | 0.0 | 0.0 | 0.0 |
| 1700017N19Rik | 0.0 | 0.0 | 0.0 | 0.0 | 0.0 | 0.0 | 0.0 | 0.0 | 0.0 | 0.0 | 0.0 | 0.0 |
| 1700018A04Rik | 0.0 | 0.0 | 0.0 | 0.0 | 0.0 | 0.0 | 0.0 | 0.0 | 0.0 | 0.0 | 0.0 | 0.0 |
| 1700018B24Rik | 0.0 | 0.0 | 0.0 | 0.0 | 0.0 | 0.0 | 0.0 | 0.0 | 0.0 | 0.0 | 0.0 | 0.0 |
| 1700018G05Rik | 0.0 | 0.0 | 0.0 | 0.0 | 0.0 | 0.0 | 0.0 | 0.0 | 0.0 | 0.0 | 0.0 | 0.0 |
| 1700019A02Rik | 0.0 | 0.0 | 0.0 | 0.0 | 0.0 | 0.0 | 0.0 | 0.0 | 0.0 | 0.0 | 0.0 | 0.0 |
| 1700019E08Rik | 0.0 | 0.0 | 0.0 | 0.0 | 0.0 | 0.0 | 0.0 | 0.0 | 0.0 | 0.0 | 0.0 | 0.0 |
| 1700019O17Rik | 0.0 | 0.0 | 0.0 | 0.0 | 0.0 | 0.0 | 0.0 | 0.0 | 0.0 | 0.0 | 0.0 | 0.0 |
| 1700020G17Rik | 0.0 | 0.0 | 0.0 | 0.0 | 0.0 | 0.0 | 0.0 | 0.0 | 0.0 | 0.0 | 0.0 | 0.0 |
| 1700020M21Rik | 0.0 | 0.0 | 0.0 | 0.0 | 0.0 | 0.0 | 0.0 | 0.0 | 0.0 | 0.0 | 0.0 | 0.0 |
| 1700020N15Rik | 0.0 | 0.0 | 0.0 | 0.0 | 0.0 | 0.0 | 0.0 | 0.0 | 0.0 | 0.0 | 0.0 | 0.0 |
| 1700020N18Rik | 0.0 | 0.0 | 0.0 | 0.0 | 0.0 | 0.0 | 0.0 | 0.0 | 0.0 | 0.0 | 0.0 | 0.0 |
| 1700022A22Rik | 0.0 | 0.0 | 0.0 | 0.0 | 0.0 | 0.0 | 0.0 | 0.0 | 0.0 | 0.0 | 0.0 | 0.0 |

| | | | | | | | | | | | |
|---|---|---|---|---|---|---|---|---|---|---|---|
| 1700022E09Rik | 0.0 | 0.0 | 0.0 | 0.0 | 0.0 | 0.0 | 0.0 | 0.0 | 0.0 | 0.0 | 0.0 |
| 1700022H16Rik | 0.0 | 0.0 | 0.0 | 0.0 | 0.0 | 0.0 | 0.0 | 0.0 | 0.0 | 0.0 | 0.0 |
| 1700023C21Rik | 0.0 | 0.0 | 0.0 | 0.0 | 0.0 | 0.0 | 0.0 | 0.0 | 0.0 | 0.0 | 0.0 |
| 1700023F02Rik | 0.0 | 0.0 | 0.0 | 0.0 | 0.0 | 0.0 | 0.0 | 0.0 | 0.0 | 0.0 | 0.0 |
| 1700024B18Rik | 0.0 | 0.0 | 0.0 | 0.0 | 0.0 | 0.0 | 0.0 | 0.0 | 0.0 | 0.0 | 0.0 |
| 1700024G13Rik | 0.0 | 0.0 | 0.0 | 0.0 | 0.0 | 0.0 | 0.0 | 0.0 | 0.0 | 0.0 | 0.0 |
| 1700024P04Rik | 0.0 | 0.0 | 0.0 | 0.0 | 0.0 | 0.0 | 0.0 | 0.0 | 0.0 | 0.0 | 0.0 |
| 1700025B11Rik | 0.0 | 0.0 | 0.0 | 0.0 | 0.0 | 0.0 | 0.0 | 0.0 | 0.0 | 0.0 | 0.0 |
| 1700025C18Rik | 0.0 | 0.0 | 0.0 | 0.0 | 0.0 | 0.0 | 0.0 | 0.0 | 0.0 | 0.0 | 0.0 |
| 1700025F24Rik | 0.0 | 0.0 | 0.0 | 0.0 | 0.0 | 0.0 | 0.0 | 0.0 | 0.0 | 0.0 | 0.0 |
| 1700025K24Rik | 0.0 | 0.0 | 0.0 | 0.0 | 0.0 | 0.0 | 0.0 | 0.0 | 0.0 | 0.0 | 0.0 |
| 1700026F02Rik | 0.0 | 0.0 | 0.0 | 0.0 | 0.0 | 0.0 | 0.0 | 0.0 | 0.0 | 0.0 | 0.0 |
| 1700027A15Rik | 0.0 | 0.0 | 0.0 | 0.0 | 0.0 | 0.0 | 0.0 | 0.0 | 0.0 | 0.0 | 0.0 |
| 1700027F09Rik | 0.0 | 0.0 | 0.0 | 0.0 | 0.0 | 0.0 | 0.0 | 0.0 | 0.0 | 0.0 | 0.0 |
| 1700027H10Rik | 0.0 | 0.0 | 0.0 | 0.0 | 0.0 | 0.0 | 0.0 | 0.0 | 0.0 | 0.0 | 0.0 |
| 1700027J07Rik | 0.0 | 0.0 | 0.0 | 0.0 | 0.0 | 0.0 | 0.0 | 0.0 | 0.0 | 0.0 | 0.0 |
| 1700028B04Rik | 0.0 | 0.0 | 0.0 | 0.0 | 0.0 | 0.0 | 0.0 | 0.0 | 0.0 | 0.0 | 0.0 |
| 1700028P15Rik | 0.0 | 0.0 | 0.0 | 0.0 | 0.0 | 0.0 | 0.0 | 0.0 | 0.0 | 0.0 | 0.0 |
| 1700029B22Rik | 0.0 | 0.0 | 0.0 | 0.0 | 0.0 | 0.0 | 0.0 | 0.0 | 0.0 | 0.0 | 0.0 |
| 1700029F12Rik | 0.0 | 0.0 | 0.0 | 0.0 | 0.0 | 0.0 | 0.0 | 0.0 | 0.0 | 0.0 | 0.0 |
| 1700029N11Rik | 0.0 | 0.0 | 0.0 | 0.0 | 0.0 | 0.0 | 0.0 | 0.0 | 0.0 | 0.0 | 0.0 |
| 1700031F05Rik | 0.0 | 0.0 | 0.0 | 0.0 | 0.0 | 0.0 | 0.0 | 0.0 | 0.0 | 0.0 | 0.0 |
| 1700031M16Rik | 0.0 | 0.0 | 0.0 | 0.0 | 0.0 | 0.0 | 0.0 | 0.0 | 0.0 | 0.0 | 0.0 |
| 1700031P21Rik | 0.0 | 0.0 | 0.0 | 0.0 | 0.0 | 0.0 | 0.0 | 0.0 | 0.0 | 0.0 | 0.0 |
| 1700034G24Rik | 0.0 | 0.0 | 0.0 | 0.0 | 0.0 | 0.0 | 0.0 | 0.0 | 0.0 | 0.0 | 0.0 |
| 1700034K08Rik | 0.0 | 0.0 | 0.0 | 0.0 | 0.0 | 0.0 | 0.0 | 0.0 | 0.0 | 0.0 | 0.0 |
| 1700034O15Rik | 0.0 | 0.0 | 0.0 | 0.0 | 0.0 | 0.0 | 0.0 | 0.0 | 0.0 | 0.0 | 0.0 |
| 1700036G14Rik | 0.0 | 0.0 | 0.0 | 0.0 | 0.0 | 0.0 | 0.0 | 0.0 | 0.0 | 0.0 | 0.0 |
| 1700039E15Rik | 0.0 | 0.0 | 0.0 | 0.0 | 0.0 | 0.0 | 0.0 | 0.0 | 0.0 | 0.0 | 0.0 |
| 1700039E22Rik | 0.0 | 0.0 | 0.0 | 0.0 | 0.0 | 0.0 | 0.0 | 0.0 | 0.0 | 0.0 | 0.0 |
| 1700041C23Rik | 0.0 | 0.0 | 0.0 | 0.0 | 0.0 | 0.0 | 0.0 | 0.0 | 0.0 | 0.0 | 0.0 |
| 1700041M19Rik | 0.0 | 0.0 | 0.0 | 0.0 | 0.0 | 0.0 | 0.0 | 0.0 | 0.0 | 0.0 | 0.0 |
| 1700042G07Rik | 0.0 | 0.0 | 0.0 | 0.0 | 0.0 | 0.0 | 0.0 | 0.0 | 0.0 | 0.0 | 0.0 |
| 1700044C05Rik | 0.0 | 0.0 | 0.0 | 0.0 | 0.0 | 0.0 | 0.0 | 0.0 | 0.0 | 0.0 | 0.0 |
| 1700044K03Rik | 0.0 | 0.0 | 0.0 | 0.0 | 0.0 | 0.0 | 0.0 | 0.0 | 0.0 | 0.0 | 0.0 |
| 1700045I19Rik | 0.0 | 0.0 | 0.0 | 0.0 | 0.0 | 0.0 | 0.0 | 0.0 | 0.0 | 0.0 | 0.0 |
| 1700046C09Rik | 0.0 | 0.0 | 0.0 | 0.0 | 0.0 | 0.0 | 0.0 | 0.0 | 0.0 | 0.0 | 0.0 |
| 1700047L14Rik | 0.0 | 0.0 | 0.0 | 0.0 | 0.0 | 0.0 | 0.0 | 0.0 | 0.0 | 0.0 | 0.0 |
| 1700049E15Rik | 0.0 | 0.0 | 0.0 | 0.0 | 0.0 | 0.0 | 0.0 | 0.0 | 0.0 | 0.0 | 0.0 |
| 1700049E22Rik | 0.0 | 0.0 | 0.0 | 0.0 | 0.0 | 0.0 | 0.0 | 0.0 | 0.0 | 0.0 | 0.0 |
| 1700052I22Rik | 0.0 | 0.0 | 0.0 | 0.0 | 0.0 | 0.0 | 0.0 | 0.0 | 0.0 | 0.0 | 0.0 |
| 1700054A03Rik | 0.0 | 0.0 | 0.0 | 0.0 | 0.0 | 0.0 | 0.0 | 0.0 | 0.0 | 0.0 | 0.0 |
| 1700054K19Rik | 0.0 | 0.0 | 0.0 | 0.0 | 0.0 | 0.0 | 0.0 | 0.0 | 0.0 | 0.0 | 0.0 |
| 1700054O13Rik | 0.0 | 0.0 | 0.0 | 0.0 | 0.0 | 0.0 | 0.0 | 0.0 | 0.0 | 0.0 | 0.0 |
| 1700055C04Rik | 0.0 | 0.0 | 0.0 | 0.0 | 0.0 | 0.0 | 0.0 | 0.0 | 0.0 | 0.0 | 0.0 |
| 1700057H15Rik | 0.0 | 0.0 | 0.0 | 0.0 | 0.0 | 0.0 | 0.0 | 0.0 | 0.0 | 0.0 | 0.0 |
| 1700060C16Rik | 0.0 | 0.0 | 0.0 | 0.0 | 0.0 | 0.0 | 0.0 | 0.0 | 0.0 | 0.0 | 0.0 |
| 1700061F12Rik | 0.0 | 0.0 | 0.0 | 0.0 | 0.0 | 0.0 | 0.0 | 0.0 | 0.0 | 0.0 | 0.0 |
| 1700063A18Rik | 0.0 | 0.0 | 0.0 | 0.0 | 0.0 | 0.0 | 0.0 | 0.0 | 0.0 | 0.0 | 0.0 |
| 1700063O14Rik | 0.0 | 0.0 | 0.0 | 0.0 | 0.0 | 0.0 | 0.0 | 0.0 | 0.0 | 0.0 | 0.0 |
| 1700064J06Rik | 0.0 | 0.0 | 0.0 | 0.0 | 0.0 | 0.0 | 0.0 | 0.0 | 0.0 | 0.0 | 0.0 |
| 1700065I16Rik | 0.0 | 0.0 | 0.0 | 0.0 | 0.0 | 0.0 | 0.0 | 0.0 | 0.0 | 0.0 | 0.0 |
| 1700065O20Rik | 0.0 | 0.0 | 0.0 | 0.0 | 0.0 | 0.0 | 0.0 | 0.0 | 0.0 | 0.0 | 0.0 |
| 1700066B17Rik | 0.0 | 0.0 | 0.0 | 0.0 | 0.0 | 0.0 | 0.0 | 0.0 | 0.0 | 0.0 | 0.0 |
| 1700066O22Rik | 0.0 | 0.0 | 0.0 | 0.0 | 0.0 | 0.0 | 0.0 | 0.0 | 0.0 | 0.0 | 0.0 |
| 1700067P10Rik | 0.0 | 0.0 | 0.0 | 0.0 | 0.0 | 0.0 | 0.0 | 0.0 | 0.0 | 0.0 | 0.0 |
| 1700069P05Rik | 0.0 | 0.0 | 0.0 | 0.0 | 0.0 | 0.0 | 0.0 | 0.0 | 0.0 | 0.0 | 0.0 |

Excluded Genes

| | | | | | | | | | | | | |
|---|---|---|---|---|---|---|---|---|---|---|---|---|
| 1700074P13Rik | 0.0 | 0.0 | 0.0 | 0.0 | 0.0 | 0.0 | 0.0 | 0.0 | 0.0 | 0.0 | 0.0 | 0.0 |
| 1700080E11Rik | 0.0 | 0.0 | 0.0 | 0.0 | 0.0 | 0.0 | 0.0 | 0.0 | 0.0 | 0.0 | 0.0 | 0.0 |
| 1700080N15Rik | 0.0 | 0.0 | 0.0 | 0.0 | 0.0 | 0.0 | 0.0 | 0.0 | 0.0 | 0.0 | 0.0 | 0.0 |
| 1700080O16Rik | 0.0 | 0.0 | 0.0 | 0.0 | 0.0 | 0.0 | 0.0 | 0.0 | 0.0 | 0.0 | 0.0 | 0.0 |
| 1700081H22Rik | 0.0 | 0.0 | 0.0 | 0.0 | 0.0 | 0.0 | 0.0 | 0.0 | 0.0 | 0.0 | 0.0 | 0.0 |
| 1700084F23Rik | 0.0 | 0.0 | 0.0 | 0.0 | 0.0 | 0.0 | 0.0 | 0.0 | 0.0 | 0.0 | 0.0 | 0.0 |
| 1700085B03Rik | 0.0 | 0.0 | 0.0 | 0.0 | 0.0 | 0.0 | 0.0 | 0.0 | 0.0 | 0.0 | 0.0 | 0.0 |
| 1700091E21Rik | 0.0 | 0.0 | 0.0 | 0.0 | 0.0 | 0.0 | 0.0 | 0.0 | 0.0 | 0.0 | 0.0 | 0.0 |
| 1700092C10Rik | 0.0 | 0.0 | 0.0 | 0.0 | 0.0 | 0.0 | 0.0 | 0.0 | 0.0 | 0.0 | 0.0 | 0.0 |
| 1700092K14Rik | 0.0 | 0.0 | 0.0 | 0.0 | 0.0 | 0.0 | 0.0 | 0.0 | 0.0 | 0.0 | 0.0 | 0.0 |
| 1700094M24Rik | 0.0 | 0.0 | 0.0 | 0.0 | 0.0 | 0.0 | 0.0 | 0.0 | 0.0 | 0.0 | 0.0 | 0.0 |
| 1700095B22Rik | 0.0 | 0.0 | 0.0 | 0.0 | 0.0 | 0.0 | 0.0 | 0.0 | 0.0 | 0.0 | 0.0 | 0.0 |
| 1700101O22Rik | 0.0 | 0.0 | 0.0 | 0.0 | 0.0 | 0.0 | 0.0 | 0.0 | 0.0 | 0.0 | 0.0 | 0.0 |
| 1700106J16Rik | 0.0 | 0.0 | 0.0 | 0.0 | 0.0 | 0.0 | 0.0 | 0.0 | 0.0 | 0.0 | 0.0 | 0.0 |
| 1700109G15Rik | 0.0 | 0.0 | 0.0 | 0.0 | 0.0 | 0.0 | 0.0 | 0.0 | 0.0 | 0.0 | 0.0 | 0.0 |
| 1700109I08Rik | 0.0 | 0.0 | 0.0 | 0.0 | 0.0 | 0.0 | 0.0 | 0.0 | 0.0 | 0.0 | 0.0 | 0.0 |
| 1700110M21Rik | 0.0 | 0.0 | 0.0 | 0.0 | 0.0 | 0.0 | 0.0 | 0.0 | 0.0 | 0.0 | 0.0 | 0.0 |
| 1700120G07Rik | 0.0 | 0.0 | 0.0 | 0.0 | 0.0 | 0.0 | 0.0 | 0.0 | 0.0 | 0.0 | 0.0 | 0.0 |
| 1700121L16Rik | 0.0 | 0.0 | 0.0 | 0.0 | 0.0 | 0.0 | 0.0 | 0.0 | 0.0 | 0.0 | 0.0 | 0.0 |
| 1700121N20Rik | 0.0 | 0.0 | 0.0 | 0.0 | 0.0 | 0.0 | 0.0 | 0.0 | 0.0 | 0.0 | 0.0 | 0.0 |
| 1700123O12Rik | 0.0 | 0.0 | 0.0 | 0.0 | 0.0 | 0.0 | 0.0 | 0.0 | 0.0 | 0.0 | 0.0 | 0.0 |
| 1700125G02Rik | 0.0 | 0.0 | 0.0 | 0.0 | 0.0 | 0.0 | 0.0 | 0.0 | 0.0 | 0.0 | 0.0 | 0.0 |
| 1700129C05Rik | 0.0 | 0.0 | 0.0 | 0.0 | 0.0 | 0.0 | 0.0 | 0.0 | 0.0 | 0.0 | 0.0 | 0.0 |
| 1810007C17Rik | 0.0 | 0.0 | 0.0 | 0.0 | 0.0 | 0.0 | 0.0 | 0.0 | 0.0 | 0.0 | 0.0 | 0.0 |
| 1810007D17Rik | 0.0 | 0.0 | 0.0 | 0.0 | 0.0 | 0.0 | 0.0 | 0.0 | 0.0 | 0.0 | 0.0 | 0.0 |
| 1810007I06Rik | 0.0 | 0.0 | 0.0 | 0.0 | 0.0 | 0.0 | 0.0 | 0.0 | 0.0 | 0.0 | 0.0 | 0.0 |
| 1810018F18Rik | 0.0 | 0.0 | 0.0 | 0.0 | 0.0 | 0.0 | 0.0 | 0.0 | 0.0 | 0.0 | 0.0 | 0.0 |
| 2200002J24Rik | 0.0 | 0.0 | 0.0 | 0.0 | 0.0 | 0.0 | 0.0 | 0.0 | 0.0 | 0.0 | 0.0 | 0.0 |
| 2210019I11Rik | 0.0 | 0.0 | 0.0 | 0.0 | 0.0 | 0.0 | 0.0 | 0.0 | 0.0 | 0.0 | 0.0 | 0.0 |
| 2210407C18Rik | 0.0 | 0.0 | 0.0 | 0.0 | 0.0 | 0.0 | 0.0 | 0.0 | 0.0 | 0.0 | 0.0 | 0.0 |
| 2210409D07Rik | 0.0 | 0.0 | 0.0 | 0.0 | 0.0 | 0.0 | 0.0 | 0.0 | 0.0 | 0.0 | 0.0 | 0.0 |
| 2300003K06Rik | 0.0 | 0.0 | 0.0 | 0.0 | 0.0 | 0.0 | 0.0 | 0.0 | 0.0 | 0.0 | 0.0 | 0.0 |
| 2310005A03Rik | 0.0 | 0.0 | 0.0 | 0.0 | 0.0 | 0.0 | 0.0 | 0.0 | 0.0 | 0.0 | 0.0 | 0.0 |
| 2310016D03Rik | 0.0 | 0.0 | 0.0 | 0.0 | 0.0 | 0.0 | 0.0 | 0.0 | 0.0 | 0.0 | 0.0 | 0.0 |
| 2310020H05Rik | 0.0 | 0.0 | 0.0 | 0.0 | 0.0 | 0.0 | 0.0 | 0.0 | 0.0 | 0.0 | 0.0 | 0.0 |
| 2310030A07Rik | 0.0 | 0.0 | 0.0 | 0.0 | 0.0 | 0.0 | 0.0 | 0.0 | 0.0 | 0.0 | 0.0 | 0.0 |
| 2310034C09Rik | 0.0 | 0.0 | 0.0 | 0.0 | 0.0 | 0.0 | 0.0 | 0.0 | 0.0 | 0.0 | 0.0 | 0.0 |
| 2310043L19Rik | 0.0 | 0.0 | 0.0 | 0.0 | 0.0 | 0.0 | 0.0 | 0.0 | 0.0 | 0.0 | 0.0 | 0.0 |
| 2310057J18Rik | 0.0 | 0.0 | 0.0 | 0.0 | 0.0 | 0.0 | 0.0 | 0.0 | 0.0 | 0.0 | 0.0 | 0.0 |
| 2310057N15Rik | 0.0 | 0.0 | 0.0 | 0.0 | 0.0 | 0.0 | 0.0 | 0.0 | 0.0 | 0.0 | 0.0 | 0.0 |
| 2310061N02Rik | 0.0 | 0.0 | 0.0 | 0.0 | 0.0 | 0.0 | 0.0 | 0.0 | 0.0 | 0.0 | 0.0 | 0.0 |
| 2310065F04Rik | 0.0 | 0.0 | 0.0 | 0.0 | 0.0 | 0.0 | 0.0 | 0.0 | 0.0 | 0.0 | 0.0 | 0.0 |
| 2310079G19Rik | 0.0 | 0.0 | 0.0 | 0.0 | 0.0 | 0.0 | 0.0 | 0.0 | 0.0 | 0.0 | 0.0 | 0.0 |
| 2410012M07Rik | 0.0 | 0.0 | 0.0 | 0.0 | 0.0 | 0.0 | 0.0 | 0.0 | 0.0 | 0.0 | 0.0 | 0.0 |
| 2410088K16Rik | 0.0 | 0.0 | 0.0 | 0.0 | 0.0 | 0.0 | 0.0 | 0.0 | 0.0 | 0.0 | 0.0 | 0.0 |
| 2410114N07Rik | 0.0 | 0.0 | 0.0 | 0.0 | 0.0 | 0.0 | 0.0 | 0.0 | 0.0 | 0.0 | 0.0 | 0.0 |
| 2410141K09Rik | 0.0 | 0.0 | 0.0 | 0.0 | 0.0 | 0.0 | 0.0 | 0.0 | 0.0 | 0.0 | 0.0 | 0.0 |
| 2610035F20Rik | 0.0 | 0.0 | 0.0 | 0.0 | 0.0 | 0.0 | 0.0 | 0.0 | 0.0 | 0.0 | 0.0 | 0.0 |
| 2810007J24Rik | 0.0 | 0.0 | 0.0 | 0.0 | 0.0 | 0.0 | 0.0 | 0.0 | 0.0 | 0.0 | 0.0 | 0.0 |
| 2810049E08Rik | 0.0 | 0.0 | 0.0 | 0.0 | 0.0 | 0.0 | 0.0 | 0.0 | 0.0 | 0.0 | 0.0 | 0.0 |
| 2900093L17Rik | 0.0 | 0.0 | 0.0 | 0.0 | 0.0 | 0.0 | 0.0 | 0.0 | 0.0 | 0.0 | 0.0 | 0.0 |
| 3110009F21Rik | 0.0 | 0.0 | 0.0 | 0.0 | 0.0 | 0.0 | 0.0 | 0.0 | 0.0 | 0.0 | 0.0 | 0.0 |
| 3110015C05Rik | 0.0 | 0.0 | 0.0 | 0.0 | 0.0 | 0.0 | 0.0 | 0.0 | 0.0 | 0.0 | 0.0 | 0.0 |
| 4732490B19Rik | 0.0 | 0.0 | 0.0 | 0.0 | 0.0 | 0.0 | 0.0 | 0.0 | 0.0 | 0.0 | 0.0 | 0.0 |
| 4921501E09Rik | 0.0 | 0.0 | 0.0 | 0.0 | 0.0 | 0.0 | 0.0 | 0.0 | 0.0 | 0.0 | 0.0 | 0.0 |
| 4921504E06Rik | 0.0 | 0.0 | 0.0 | 0.0 | 0.0 | 0.0 | 0.0 | 0.0 | 0.0 | 0.0 | 0.0 | 0.0 |
| 4921506M07Rik | 0.0 | 0.0 | 0.0 | 0.0 | 0.0 | 0.0 | 0.0 | 0.0 | 0.0 | 0.0 | 0.0 | 0.0 |

| | | | | | | | | | | | | |
|---|---|---|---|---|---|---|---|---|---|---|---|---|
| 4921509C19Rik | 0.0 | 0.0 | 0.0 | 0.0 | 0.0 | 0.0 | 0.0 | 0.0 | 0.0 | 0.0 | 0.0 | 0.0 |
| 4921511C20Rik | 0.0 | 0.0 | 0.0 | 0.0 | 0.0 | 0.0 | 0.0 | 0.0 | 0.0 | 0.0 | 0.0 | 0.0 |
| 4921511H03Rik | 0.0 | 0.0 | 0.0 | 0.0 | 0.0 | 0.0 | 0.0 | 0.0 | 0.0 | 0.0 | 0.0 | 0.0 |
| 4921517D22Rik | 0.0 | 0.0 | 0.0 | 0.0 | 0.0 | 0.0 | 0.0 | 0.0 | 0.0 | 0.0 | 0.0 | 0.0 |
| 4921525B02Rik | 0.0 | 0.0 | 0.0 | 0.0 | 0.0 | 0.0 | 0.0 | 0.0 | 0.0 | 0.0 | 0.0 | 0.0 |
| 4921530L21Rik | 0.0 | 0.0 | 0.0 | 0.0 | 0.0 | 0.0 | 0.0 | 0.0 | 0.0 | 0.0 | 0.0 | 0.0 |
| 4922502D21Rik | 0.0 | 0.0 | 0.0 | 0.0 | 0.0 | 0.0 | 0.0 | 0.0 | 0.0 | 0.0 | 0.0 | 0.0 |
| 4922502H24Rik | 0.0 | 0.0 | 0.0 | 0.0 | 0.0 | 0.0 | 0.0 | 0.0 | 0.0 | 0.0 | 0.0 | 0.0 |
| 4922502N22Rik | 0.0 | 0.0 | 0.0 | 0.0 | 0.0 | 0.0 | 0.0 | 0.0 | 0.0 | 0.0 | 0.0 | 0.0 |
| 4922505E12Rik | 0.0 | 0.0 | 0.0 | 0.0 | 0.0 | 0.0 | 0.0 | 0.0 | 0.0 | 0.0 | 0.0 | 0.0 |
| 4930401O10Rik | 0.0 | 0.0 | 0.0 | 0.0 | 0.0 | 0.0 | 0.0 | 0.0 | 0.0 | 0.0 | 0.0 | 0.0 |
| 4930401O12Rik | 0.0 | 0.0 | 0.0 | 0.0 | 0.0 | 0.0 | 0.0 | 0.0 | 0.0 | 0.0 | 0.0 | 0.0 |
| 4930402F11Rik | 0.0 | 0.0 | 0.0 | 0.0 | 0.0 | 0.0 | 0.0 | 0.0 | 0.0 | 0.0 | 0.0 | 0.0 |
| 4930402K13Rik | 0.0 | 0.0 | 0.0 | 0.0 | 0.0 | 0.0 | 0.0 | 0.0 | 0.0 | 0.0 | 0.0 | 0.0 |
| 4930404A05Rik | 0.0 | 0.0 | 0.0 | 0.0 | 0.0 | 0.0 | 0.0 | 0.0 | 0.0 | 0.0 | 0.0 | 0.0 |
| 4930404A10Rik | 0.0 | 0.0 | 0.0 | 0.0 | 0.0 | 0.0 | 0.0 | 0.0 | 0.0 | 0.0 | 0.0 | 0.0 |
| 4930405J17Rik | 0.0 | 0.0 | 0.0 | 0.0 | 0.0 | 0.0 | 0.0 | 0.0 | 0.0 | 0.0 | 0.0 | 0.0 |
| 4930406D18Rik | 0.0 | 0.0 | 0.0 | 0.0 | 0.0 | 0.0 | 0.0 | 0.0 | 0.0 | 0.0 | 0.0 | 0.0 |
| 4930413E15Rik | 0.0 | 0.0 | 0.0 | 0.0 | 0.0 | 0.0 | 0.0 | 0.0 | 0.0 | 0.0 | 0.0 | 0.0 |
| 4930413M19Rik | 0.0 | 0.0 | 0.0 | 0.0 | 0.0 | 0.0 | 0.0 | 0.0 | 0.0 | 0.0 | 0.0 | 0.0 |
| 4930415L06Rik | 0.0 | 0.0 | 0.0 | 0.0 | 0.0 | 0.0 | 0.0 | 0.0 | 0.0 | 0.0 | 0.0 | 0.0 |
| 4930417O22Rik | 0.0 | 0.0 | 0.0 | 0.0 | 0.0 | 0.0 | 0.0 | 0.0 | 0.0 | 0.0 | 0.0 | 0.0 |
| 4930423M02Rik | 0.0 | 0.0 | 0.0 | 0.0 | 0.0 | 0.0 | 0.0 | 0.0 | 0.0 | 0.0 | 0.0 | 0.0 |
| 4930425K10Rik | 0.0 | 0.0 | 0.0 | 0.0 | 0.0 | 0.0 | 0.0 | 0.0 | 0.0 | 0.0 | 0.0 | 0.0 |
| 4930425O10Rik | 0.0 | 0.0 | 0.0 | 0.0 | 0.0 | 0.0 | 0.0 | 0.0 | 0.0 | 0.0 | 0.0 | 0.0 |
| 4930428E07Rik | 0.0 | 0.0 | 0.0 | 0.0 | 0.0 | 0.0 | 0.0 | 0.0 | 0.0 | 0.0 | 0.0 | 0.0 |
| 4930428G15Rik | 0.0 | 0.0 | 0.0 | 0.0 | 0.0 | 0.0 | 0.0 | 0.0 | 0.0 | 0.0 | 0.0 | 0.0 |
| 4930428O21Rik | 0.0 | 0.0 | 0.0 | 0.0 | 0.0 | 0.0 | 0.0 | 0.0 | 0.0 | 0.0 | 0.0 | 0.0 |
| 4930430F21Rik | 0.0 | 0.0 | 0.0 | 0.0 | 0.0 | 0.0 | 0.0 | 0.0 | 0.0 | 0.0 | 0.0 | 0.0 |
| 4930432K09Rik | 0.0 | 0.0 | 0.0 | 0.0 | 0.0 | 0.0 | 0.0 | 0.0 | 0.0 | 0.0 | 0.0 | 0.0 |
| 4930433B08Rik | 0.0 | 0.0 | 0.0 | 0.0 | 0.0 | 0.0 | 0.0 | 0.0 | 0.0 | 0.0 | 0.0 | 0.0 |
| 4930434J06Rik | 0.0 | 0.0 | 0.0 | 0.0 | 0.0 | 0.0 | 0.0 | 0.0 | 0.0 | 0.0 | 0.0 | 0.0 |
| 4930435E12Rik | 0.0 | 0.0 | 0.0 | 0.0 | 0.0 | 0.0 | 0.0 | 0.0 | 0.0 | 0.0 | 0.0 | 0.0 |
| 4930440C22Rik | 0.0 | 0.0 | 0.0 | 0.0 | 0.0 | 0.0 | 0.0 | 0.0 | 0.0 | 0.0 | 0.0 | 0.0 |
| 4930441J16Rik | 0.0 | 0.0 | 0.0 | 0.0 | 0.0 | 0.0 | 0.0 | 0.0 | 0.0 | 0.0 | 0.0 | 0.0 |
| 4930442J19Rik | 0.0 | 0.0 | 0.0 | 0.0 | 0.0 | 0.0 | 0.0 | 0.0 | 0.0 | 0.0 | 0.0 | 0.0 |
| 4930442L01Rik | 0.0 | 0.0 | 0.0 | 0.0 | 0.0 | 0.0 | 0.0 | 0.0 | 0.0 | 0.0 | 0.0 | 0.0 |
| 4930447A16Rik | 0.0 | 0.0 | 0.0 | 0.0 | 0.0 | 0.0 | 0.0 | 0.0 | 0.0 | 0.0 | 0.0 | 0.0 |
| 4930448C13Rik | 0.0 | 0.0 | 0.0 | 0.0 | 0.0 | 0.0 | 0.0 | 0.0 | 0.0 | 0.0 | 0.0 | 0.0 |
| 4930448F12Rik | 0.0 | 0.0 | 0.0 | 0.0 | 0.0 | 0.0 | 0.0 | 0.0 | 0.0 | 0.0 | 0.0 | 0.0 |
| 4930448I06Rik | 0.0 | 0.0 | 0.0 | 0.0 | 0.0 | 0.0 | 0.0 | 0.0 | 0.0 | 0.0 | 0.0 | 0.0 |
| 4930448K20Rik | 0.0 | 0.0 | 0.0 | 0.0 | 0.0 | 0.0 | 0.0 | 0.0 | 0.0 | 0.0 | 0.0 | 0.0 |
| 4930449E18Rik | 0.0 | 0.0 | 0.0 | 0.0 | 0.0 | 0.0 | 0.0 | 0.0 | 0.0 | 0.0 | 0.0 | 0.0 |
| 4930449I24Rik | 0.0 | 0.0 | 0.0 | 0.0 | 0.0 | 0.0 | 0.0 | 0.0 | 0.0 | 0.0 | 0.0 | 0.0 |
| 4930451I11Rik | 0.0 | 0.0 | 0.0 | 0.0 | 0.0 | 0.0 | 0.0 | 0.0 | 0.0 | 0.0 | 0.0 | 0.0 |
| 4930452A19Rik | 0.0 | 0.0 | 0.0 | 0.0 | 0.0 | 0.0 | 0.0 | 0.0 | 0.0 | 0.0 | 0.0 | 0.0 |
| 4930452G13Rik | 0.0 | 0.0 | 0.0 | 0.0 | 0.0 | 0.0 | 0.0 | 0.0 | 0.0 | 0.0 | 0.0 | 0.0 |
| 4930452N14Rik | 0.0 | 0.0 | 0.0 | 0.0 | 0.0 | 0.0 | 0.0 | 0.0 | 0.0 | 0.0 | 0.0 | 0.0 |
| 4930453H23Rik | 0.0 | 0.0 | 0.0 | 0.0 | 0.0 | 0.0 | 0.0 | 0.0 | 0.0 | 0.0 | 0.0 | 0.0 |
| 4930453L07Rik | 0.0 | 0.0 | 0.0 | 0.0 | 0.0 | 0.0 | 0.0 | 0.0 | 0.0 | 0.0 | 0.0 | 0.0 |
| 4930455B14Rik | 0.0 | 0.0 | 0.0 | 0.0 | 0.0 | 0.0 | 0.0 | 0.0 | 0.0 | 0.0 | 0.0 | 0.0 |
| 4930455F16Rik | 0.0 | 0.0 | 0.0 | 0.0 | 0.0 | 0.0 | 0.0 | 0.0 | 0.0 | 0.0 | 0.0 | 0.0 |
| 4930455J16Rik | 0.0 | 0.0 | 0.0 | 0.0 | 0.0 | 0.0 | 0.0 | 0.0 | 0.0 | 0.0 | 0.0 | 0.0 |
| 4930465A12Rik | 0.0 | 0.0 | 0.0 | 0.0 | 0.0 | 0.0 | 0.0 | 0.0 | 0.0 | 0.0 | 0.0 | 0.0 |
| 4930465M20Rik | 0.0 | 0.0 | 0.0 | 0.0 | 0.0 | 0.0 | 0.0 | 0.0 | 0.0 | 0.0 | 0.0 | 0.0 |
| 4930467D21Rik | 0.0 | 0.0 | 0.0 | 0.0 | 0.0 | 0.0 | 0.0 | 0.0 | 0.0 | 0.0 | 0.0 | 0.0 |
| 4930470P17Rik | 0.0 | 0.0 | 0.0 | 0.0 | 0.0 | 0.0 | 0.0 | 0.0 | 0.0 | 0.0 | 0.0 | 0.0 |

| | | | | | | | | | | | |
|---|---|---|---|---|---|---|---|---|---|---|---|
| 4930473O22Rik | 0.0 | 0.0 | 0.0 | 0.0 | 0.0 | 0.0 | 0.0 | 0.0 | 0.0 | 0.0 | 0.0 | 0.0 |
| 4930474N05Rik | 0.0 | 0.0 | 0.0 | 0.0 | 0.0 | 0.0 | 0.0 | 0.0 | 0.0 | 0.0 | 0.0 | 0.0 |
| 4930478P22Rik | 0.0 | 0.0 | 0.0 | 0.0 | 0.0 | 0.0 | 0.0 | 0.0 | 0.0 | 0.0 | 0.0 | 0.0 |
| 4930479M11Rik | 0.0 | 0.0 | 0.0 | 0.0 | 0.0 | 0.0 | 0.0 | 0.0 | 0.0 | 0.0 | 0.0 | 0.0 |
| 4930480E11Rik | 0.0 | 0.0 | 0.0 | 0.0 | 0.0 | 0.0 | 0.0 | 0.0 | 0.0 | 0.0 | 0.0 | 0.0 |
| 4930480K15Rik | 0.0 | 0.0 | 0.0 | 0.0 | 0.0 | 0.0 | 0.0 | 0.0 | 0.0 | 0.0 | 0.0 | 0.0 |
| 4930480M12Rik | 0.0 | 0.0 | 0.0 | 0.0 | 0.0 | 0.0 | 0.0 | 0.0 | 0.0 | 0.0 | 0.0 | 0.0 |
| 4930486F22Rik | 0.0 | 0.0 | 0.0 | 0.0 | 0.0 | 0.0 | 0.0 | 0.0 | 0.0 | 0.0 | 0.0 | 0.0 |
| 4930487D11Rik | 0.0 | 0.0 | 0.0 | 0.0 | 0.0 | 0.0 | 0.0 | 0.0 | 0.0 | 0.0 | 0.0 | 0.0 |
| 4930488B22Rik | 0.0 | 0.0 | 0.0 | 0.0 | 0.0 | 0.0 | 0.0 | 0.0 | 0.0 | 0.0 | 0.0 | 0.0 |
| 4930500F04Rik | 0.0 | 0.0 | 0.0 | 0.0 | 0.0 | 0.0 | 0.0 | 0.0 | 0.0 | 0.0 | 0.0 | 0.0 |
| 4930503H13Rik | 0.0 | 0.0 | 0.0 | 0.0 | 0.0 | 0.0 | 0.0 | 0.0 | 0.0 | 0.0 | 0.0 | 0.0 |
| 4930503O07Rik | 0.0 | 0.0 | 0.0 | 0.0 | 0.0 | 0.0 | 0.0 | 0.0 | 0.0 | 0.0 | 0.0 | 0.0 |
| 4930504O13Rik | 0.0 | 0.0 | 0.0 | 0.0 | 0.0 | 0.0 | 0.0 | 0.0 | 0.0 | 0.0 | 0.0 | 0.0 |
| 4930509J09Rik | 0.0 | 0.0 | 0.0 | 0.0 | 0.0 | 0.0 | 0.0 | 0.0 | 0.0 | 0.0 | 0.0 | 0.0 |
| 4930509K18Rik | 0.0 | 0.0 | 0.0 | 0.0 | 0.0 | 0.0 | 0.0 | 0.0 | 0.0 | 0.0 | 0.0 | 0.0 |
| 4930515B02Rik | 0.0 | 0.0 | 0.0 | 0.0 | 0.0 | 0.0 | 0.0 | 0.0 | 0.0 | 0.0 | 0.0 | 0.0 |
| 4930515G16Rik | 0.0 | 0.0 | 0.0 | 0.0 | 0.0 | 0.0 | 0.0 | 0.0 | 0.0 | 0.0 | 0.0 | 0.0 |
| 4930515L03Rik | 0.0 | 0.0 | 0.0 | 0.0 | 0.0 | 0.0 | 0.0 | 0.0 | 0.0 | 0.0 | 0.0 | 0.0 |
| 4930515L19Rik | 0.0 | 0.0 | 0.0 | 0.0 | 0.0 | 0.0 | 0.0 | 0.0 | 0.0 | 0.0 | 0.0 | 0.0 |
| 4930518P08Rik | 0.0 | 0.0 | 0.0 | 0.0 | 0.0 | 0.0 | 0.0 | 0.0 | 0.0 | 0.0 | 0.0 | 0.0 |
| 4930519D14Rik | 0.0 | 0.0 | 0.0 | 0.0 | 0.0 | 0.0 | 0.0 | 0.0 | 0.0 | 0.0 | 0.0 | 0.0 |
| 4930519F24Rik | 0.0 | 0.0 | 0.0 | 0.0 | 0.0 | 0.0 | 0.0 | 0.0 | 0.0 | 0.0 | 0.0 | 0.0 |
| 4930519H02Rik | 0.0 | 0.0 | 0.0 | 0.0 | 0.0 | 0.0 | 0.0 | 0.0 | 0.0 | 0.0 | 0.0 | 0.0 |
| 4930523O13Rik | 0.0 | 0.0 | 0.0 | 0.0 | 0.0 | 0.0 | 0.0 | 0.0 | 0.0 | 0.0 | 0.0 | 0.0 |
| 4930524B15Rik | 0.0 | 0.0 | 0.0 | 0.0 | 0.0 | 0.0 | 0.0 | 0.0 | 0.0 | 0.0 | 0.0 | 0.0 |
| 4930524C18Rik | 0.0 | 0.0 | 0.0 | 0.0 | 0.0 | 0.0 | 0.0 | 0.0 | 0.0 | 0.0 | 0.0 | 0.0 |
| 4930524N10Rik | 0.0 | 0.0 | 0.0 | 0.0 | 0.0 | 0.0 | 0.0 | 0.0 | 0.0 | 0.0 | 0.0 | 0.0 |
| 4930525M21Rik | 0.0 | 0.0 | 0.0 | 0.0 | 0.0 | 0.0 | 0.0 | 0.0 | 0.0 | 0.0 | 0.0 | 0.0 |
| 4930528P14Rik | 0.0 | 0.0 | 0.0 | 0.0 | 0.0 | 0.0 | 0.0 | 0.0 | 0.0 | 0.0 | 0.0 | 0.0 |
| 4930529F24Rik | 0.0 | 0.0 | 0.0 | 0.0 | 0.0 | 0.0 | 0.0 | 0.0 | 0.0 | 0.0 | 0.0 | 0.0 |
| 4930529K09Rik | 0.0 | 0.0 | 0.0 | 0.0 | 0.0 | 0.0 | 0.0 | 0.0 | 0.0 | 0.0 | 0.0 | 0.0 |
| 4930529L06Rik | 0.0 | 0.0 | 0.0 | 0.0 | 0.0 | 0.0 | 0.0 | 0.0 | 0.0 | 0.0 | 0.0 | 0.0 |
| 4930539C22Rik | 0.0 | 0.0 | 0.0 | 0.0 | 0.0 | 0.0 | 0.0 | 0.0 | 0.0 | 0.0 | 0.0 | 0.0 |
| 4930539M17Rik | 0.0 | 0.0 | 0.0 | 0.0 | 0.0 | 0.0 | 0.0 | 0.0 | 0.0 | 0.0 | 0.0 | 0.0 |
| 4930540M03Rik | 0.0 | 0.0 | 0.0 | 0.0 | 0.0 | 0.0 | 0.0 | 0.0 | 0.0 | 0.0 | 0.0 | 0.0 |
| 4930545E07Rik | 0.0 | 0.0 | 0.0 | 0.0 | 0.0 | 0.0 | 0.0 | 0.0 | 0.0 | 0.0 | 0.0 | 0.0 |
| 4930545H06Rik | 0.0 | 0.0 | 0.0 | 0.0 | 0.0 | 0.0 | 0.0 | 0.0 | 0.0 | 0.0 | 0.0 | 0.0 |
| 4930546C10Rik | 0.0 | 0.0 | 0.0 | 0.0 | 0.0 | 0.0 | 0.0 | 0.0 | 0.0 | 0.0 | 0.0 | 0.0 |
| 4930547C10Rik | 0.0 | 0.0 | 0.0 | 0.0 | 0.0 | 0.0 | 0.0 | 0.0 | 0.0 | 0.0 | 0.0 | 0.0 |
| 4930547E08Rik | 0.0 | 0.0 | 0.0 | 0.0 | 0.0 | 0.0 | 0.0 | 0.0 | 0.0 | 0.0 | 0.0 | 0.0 |
| 4930548J01Rik | 0.0 | 0.0 | 0.0 | 0.0 | 0.0 | 0.0 | 0.0 | 0.0 | 0.0 | 0.0 | 0.0 | 0.0 |
| 4930548K13Rik | 0.0 | 0.0 | 0.0 | 0.0 | 0.0 | 0.0 | 0.0 | 0.0 | 0.0 | 0.0 | 0.0 | 0.0 |
| 4930549C01Rik | 0.0 | 0.0 | 0.0 | 0.0 | 0.0 | 0.0 | 0.0 | 0.0 | 0.0 | 0.0 | 0.0 | 0.0 |
| 4930554C24Rik | 0.0 | 0.0 | 0.0 | 0.0 | 0.0 | 0.0 | 0.0 | 0.0 | 0.0 | 0.0 | 0.0 | 0.0 |
| 4930556J02Rik | 0.0 | 0.0 | 0.0 | 0.0 | 0.0 | 0.0 | 0.0 | 0.0 | 0.0 | 0.0 | 0.0 | 0.0 |
| 4930556N09Rik | 0.0 | 0.0 | 0.0 | 0.0 | 0.0 | 0.0 | 0.0 | 0.0 | 0.0 | 0.0 | 0.0 | 0.0 |
| 4930558G05Rik | 0.0 | 0.0 | 0.0 | 0.0 | 0.0 | 0.0 | 0.0 | 0.0 | 0.0 | 0.0 | 0.0 | 0.0 |
| 4930563M20Rik | 0.0 | 0.0 | 0.0 | 0.0 | 0.0 | 0.0 | 0.0 | 0.0 | 0.0 | 0.0 | 0.0 | 0.0 |
| 4930567J20Rik | 0.0 | 0.0 | 0.0 | 0.0 | 0.0 | 0.0 | 0.0 | 0.0 | 0.0 | 0.0 | 0.0 | 0.0 |
| 4930567K20Rik | 0.0 | 0.0 | 0.0 | 0.0 | 0.0 | 0.0 | 0.0 | 0.0 | 0.0 | 0.0 | 0.0 | 0.0 |
| 4930568D16Rik | 0.0 | 0.0 | 0.0 | 0.0 | 0.0 | 0.0 | 0.0 | 0.0 | 0.0 | 0.0 | 0.0 | 0.0 |
| 4930571K23Rik | 0.0 | 0.0 | 0.0 | 0.0 | 0.0 | 0.0 | 0.0 | 0.0 | 0.0 | 0.0 | 0.0 | 0.0 |
| 4930572K03Rik | 0.0 | 0.0 | 0.0 | 0.0 | 0.0 | 0.0 | 0.0 | 0.0 | 0.0 | 0.0 | 0.0 | 0.0 |
| 4930578E11Rik | 0.0 | 0.0 | 0.0 | 0.0 | 0.0 | 0.0 | 0.0 | 0.0 | 0.0 | 0.0 | 0.0 | 0.0 |
| 4930583P06Rik | 0.0 | 0.0 | 0.0 | 0.0 | 0.0 | 0.0 | 0.0 | 0.0 | 0.0 | 0.0 | 0.0 | 0.0 |
| 4930591A17Rik | 0.0 | 0.0 | 0.0 | 0.0 | 0.0 | 0.0 | 0.0 | 0.0 | 0.0 | 0.0 | 0.0 | 0.0 |

| | | | | | | | | | | | |
|---|---|---|---|---|---|---|---|---|---|---|---|
| 4930593C16Rik | 0.0 | 0.0 | 0.0 | 0.0 | 0.0 | 0.0 | 0.0 | 0.0 | 0.0 | 0.0 | 0.0 | 0.0 |
| 4930595M18Rik | 0.0 | 0.0 | 0.0 | 0.0 | 0.0 | 0.0 | 0.0 | 0.0 | 0.0 | 0.0 | 0.0 | 0.0 |
| 4930596D02Rik | 0.0 | 0.0 | 0.0 | 0.0 | 0.0 | 0.0 | 0.0 | 0.0 | 0.0 | 0.0 | 0.0 | 0.0 |
| 4930596M17Rik | 0.0 | 0.0 | 0.0 | 0.0 | 0.0 | 0.0 | 0.0 | 0.0 | 0.0 | 0.0 | 0.0 | 0.0 |
| 4930597G03Rik | 0.0 | 0.0 | 0.0 | 0.0 | 0.0 | 0.0 | 0.0 | 0.0 | 0.0 | 0.0 | 0.0 | 0.0 |
| 4930598F16Rik | 0.0 | 0.0 | 0.0 | 0.0 | 0.0 | 0.0 | 0.0 | 0.0 | 0.0 | 0.0 | 0.0 | 0.0 |
| 4931408C20Rik | 0.0 | 0.0 | 0.0 | 0.0 | 0.0 | 0.0 | 0.0 | 0.0 | 0.0 | 0.0 | 0.0 | 0.0 |
| 4931417E11Rik | 0.0 | 0.0 | 0.0 | 0.0 | 0.0 | 0.0 | 0.0 | 0.0 | 0.0 | 0.0 | 0.0 | 0.0 |
| 4931419H13Rik | 0.0 | 0.0 | 0.0 | 0.0 | 0.0 | 0.0 | 0.0 | 0.0 | 0.0 | 0.0 | 0.0 | 0.0 |
| 4931420L22Rik | 0.0 | 0.0 | 0.0 | 0.0 | 0.0 | 0.0 | 0.0 | 0.0 | 0.0 | 0.0 | 0.0 | 0.0 |
| 4931429P17Rik | 0.0 | 0.0 | 0.0 | 0.0 | 0.0 | 0.0 | 0.0 | 0.0 | 0.0 | 0.0 | 0.0 | 0.0 |
| 4931431B13Rik | 0.0 | 0.0 | 0.0 | 0.0 | 0.0 | 0.0 | 0.0 | 0.0 | 0.0 | 0.0 | 0.0 | 0.0 |
| 4931431F19Rik | 0.0 | 0.0 | 0.0 | 0.0 | 0.0 | 0.0 | 0.0 | 0.0 | 0.0 | 0.0 | 0.0 | 0.0 |
| 4932429P05Rik | 0.0 | 0.0 | 0.0 | 0.0 | 0.0 | 0.0 | 0.0 | 0.0 | 0.0 | 0.0 | 0.0 | 0.0 |
| 4932702P03Rik | 0.0 | 0.0 | 0.0 | 0.0 | 0.0 | 0.0 | 0.0 | 0.0 | 0.0 | 0.0 | 0.0 | 0.0 |
| 4933400B14Rik | 0.0 | 0.0 | 0.0 | 0.0 | 0.0 | 0.0 | 0.0 | 0.0 | 0.0 | 0.0 | 0.0 | 0.0 |
| 4933401B06Rik | 0.0 | 0.0 | 0.0 | 0.0 | 0.0 | 0.0 | 0.0 | 0.0 | 0.0 | 0.0 | 0.0 | 0.0 |
| 4933401P06Rik | 0.0 | 0.0 | 0.0 | 0.0 | 0.0 | 0.0 | 0.0 | 0.0 | 0.0 | 0.0 | 0.0 | 0.0 |
| 4933402C06Rik | 0.0 | 0.0 | 0.0 | 0.0 | 0.0 | 0.0 | 0.0 | 0.0 | 0.0 | 0.0 | 0.0 | 0.0 |
| 4933402E13Rik | 0.0 | 0.0 | 0.0 | 0.0 | 0.0 | 0.0 | 0.0 | 0.0 | 0.0 | 0.0 | 0.0 | 0.0 |
| 4933402J10Rik | 0.0 | 0.0 | 0.0 | 0.0 | 0.0 | 0.0 | 0.0 | 0.0 | 0.0 | 0.0 | 0.0 | 0.0 |
| 4933402N03Rik | 0.0 | 0.0 | 0.0 | 0.0 | 0.0 | 0.0 | 0.0 | 0.0 | 0.0 | 0.0 | 0.0 | 0.0 |
| 4933403O08Rik | 0.0 | 0.0 | 0.0 | 0.0 | 0.0 | 0.0 | 0.0 | 0.0 | 0.0 | 0.0 | 0.0 | 0.0 |
| 4933404G15Rik | 0.0 | 0.0 | 0.0 | 0.0 | 0.0 | 0.0 | 0.0 | 0.0 | 0.0 | 0.0 | 0.0 | 0.0 |
| 4933405O20Rik | 0.0 | 0.0 | 0.0 | 0.0 | 0.0 | 0.0 | 0.0 | 0.0 | 0.0 | 0.0 | 0.0 | 0.0 |
| 4933406D12Rik | 0.0 | 0.0 | 0.0 | 0.0 | 0.0 | 0.0 | 0.0 | 0.0 | 0.0 | 0.0 | 0.0 | 0.0 |
| 4933406F09Rik | 0.0 | 0.0 | 0.0 | 0.0 | 0.0 | 0.0 | 0.0 | 0.0 | 0.0 | 0.0 | 0.0 | 0.0 |
| 4933406K04Rik | 0.0 | 0.0 | 0.0 | 0.0 | 0.0 | 0.0 | 0.0 | 0.0 | 0.0 | 0.0 | 0.0 | 0.0 |
| 4933411G06Rik | 0.0 | 0.0 | 0.0 | 0.0 | 0.0 | 0.0 | 0.0 | 0.0 | 0.0 | 0.0 | 0.0 | 0.0 |
| 4933412E24Rik | 0.0 | 0.0 | 0.0 | 0.0 | 0.0 | 0.0 | 0.0 | 0.0 | 0.0 | 0.0 | 0.0 | 0.0 |
| 4933413L06Rik | 0.0 | 0.0 | 0.0 | 0.0 | 0.0 | 0.0 | 0.0 | 0.0 | 0.0 | 0.0 | 0.0 | 0.0 |
| 4933416M06Rik | 0.0 | 0.0 | 0.0 | 0.0 | 0.0 | 0.0 | 0.0 | 0.0 | 0.0 | 0.0 | 0.0 | 0.0 |
| 4933417A18Rik | 0.0 | 0.0 | 0.0 | 0.0 | 0.0 | 0.0 | 0.0 | 0.0 | 0.0 | 0.0 | 0.0 | 0.0 |
| 4933421I07Rik | 0.0 | 0.0 | 0.0 | 0.0 | 0.0 | 0.0 | 0.0 | 0.0 | 0.0 | 0.0 | 0.0 | 0.0 |
| 4933427E13Rik | 0.0 | 0.0 | 0.0 | 0.0 | 0.0 | 0.0 | 0.0 | 0.0 | 0.0 | 0.0 | 0.0 | 0.0 |
| 4933429K18Rik | 0.0 | 0.0 | 0.0 | 0.0 | 0.0 | 0.0 | 0.0 | 0.0 | 0.0 | 0.0 | 0.0 | 0.0 |
| 4933430H16Rik | 0.0 | 0.0 | 0.0 | 0.0 | 0.0 | 0.0 | 0.0 | 0.0 | 0.0 | 0.0 | 0.0 | 0.0 |
| 4933432G23Rik | 0.0 | 0.0 | 0.0 | 0.0 | 0.0 | 0.0 | 0.0 | 0.0 | 0.0 | 0.0 | 0.0 | 0.0 |
| 4933433F19Rik | 0.0 | 0.0 | 0.0 | 0.0 | 0.0 | 0.0 | 0.0 | 0.0 | 0.0 | 0.0 | 0.0 | 0.0 |
| 4933436E23Rik | 0.0 | 0.0 | 0.0 | 0.0 | 0.0 | 0.0 | 0.0 | 0.0 | 0.0 | 0.0 | 0.0 | 0.0 |
| 4933436I01Rik | 0.0 | 0.0 | 0.0 | 0.0 | 0.0 | 0.0 | 0.0 | 0.0 | 0.0 | 0.0 | 0.0 | 0.0 |
| 4933438B17Rik | 0.0 | 0.0 | 0.0 | 0.0 | 0.0 | 0.0 | 0.0 | 0.0 | 0.0 | 0.0 | 0.0 | 0.0 |
| 4933440J02Rik | 0.0 | 0.0 | 0.0 | 0.0 | 0.0 | 0.0 | 0.0 | 0.0 | 0.0 | 0.0 | 0.0 | 0.0 |
| 5430401F13Rik | 0.0 | 0.0 | 0.0 | 0.0 | 0.0 | 0.0 | 0.0 | 0.0 | 0.0 | 0.0 | 0.0 | 0.0 |
| 5430402E10Rik | 0.0 | 0.0 | 0.0 | 0.0 | 0.0 | 0.0 | 0.0 | 0.0 | 0.0 | 0.0 | 0.0 | 0.0 |
| 5430427M07Rik | 0.0 | 0.0 | 0.0 | 0.0 | 0.0 | 0.0 | 0.0 | 0.0 | 0.0 | 0.0 | 0.0 | 0.0 |
| 5430434I15Rik | 0.0 | 0.0 | 0.0 | 0.0 | 0.0 | 0.0 | 0.0 | 0.0 | 0.0 | 0.0 | 0.0 | 0.0 |
| 5530400C23Rik | 0.0 | 0.0 | 0.0 | 0.0 | 0.0 | 0.0 | 0.0 | 0.0 | 0.0 | 0.0 | 0.0 | 0.0 |
| 5530401A14Rik | 0.0 | 0.0 | 0.0 | 0.0 | 0.0 | 0.0 | 0.0 | 0.0 | 0.0 | 0.0 | 0.0 | 0.0 |
| 5730412P04Rik | 0.0 | 0.0 | 0.0 | 0.0 | 0.0 | 0.0 | 0.0 | 0.0 | 0.0 | 0.0 | 0.0 | 0.0 |
| 5730435O14Rik | 0.0 | 0.0 | 0.0 | 0.0 | 0.0 | 0.0 | 0.0 | 0.0 | 0.0 | 0.0 | 0.0 | 0.0 |
| 5730457N03Rik | 0.0 | 0.0 | 0.0 | 0.0 | 0.0 | 0.0 | 0.0 | 0.0 | 0.0 | 0.0 | 0.0 | 0.0 |
| 5730460C07Rik | 0.0 | 0.0 | 0.0 | 0.0 | 0.0 | 0.0 | 0.0 | 0.0 | 0.0 | 0.0 | 0.0 | 0.0 |
| 5730488B01Rik | 0.0 | 0.0 | 0.0 | 0.0 | 0.0 | 0.0 | 0.0 | 0.0 | 0.0 | 0.0 | 0.0 | 0.0 |
| 6530411M01Rik | 0.0 | 0.0 | 0.0 | 0.0 | 0.0 | 0.0 | 0.0 | 0.0 | 0.0 | 0.0 | 0.0 | 0.0 |
| 7420426K07Rik | 0.0 | 0.0 | 0.0 | 0.0 | 0.0 | 0.0 | 0.0 | 0.0 | 0.0 | 0.0 | 0.0 | 0.0 |
| 7420701I03Rik | 0.0 | 0.0 | 0.0 | 0.0 | 0.0 | 0.0 | 0.0 | 0.0 | 0.0 | 0.0 | 0.0 | 0.0 |

| | | | | | | | | | | | | |
|---|---|---|---|---|---|---|---|---|---|---|---|---|
| 7630403G23Rik | 0.0 | 0.0 | 0.0 | 0.0 | 0.0 | 0.0 | 0.0 | 0.0 | 0.0 | 0.0 | 0.0 | 0.0 |
| 8030411F24Rik | 0.0 | 0.0 | 0.0 | 0.0 | 0.0 | 0.0 | 0.0 | 0.0 | 0.0 | 0.0 | 0.0 | 0.0 |
| 8030423J24Rik | 0.0 | 0.0 | 0.0 | 0.0 | 0.0 | 0.0 | 0.0 | 0.0 | 0.0 | 0.0 | 0.0 | 0.0 |
| 8030443G20Rik | 0.0 | 0.0 | 0.0 | 0.0 | 0.0 | 0.0 | 0.0 | 0.0 | 0.0 | 0.0 | 0.0 | 0.0 |
| 8430422H06Rik | 0.0 | 0.0 | 0.0 | 0.0 | 0.0 | 0.0 | 0.0 | 0.0 | 0.0 | 0.0 | 0.0 | 0.0 |
| 9130209A04Rik | 0.0 | 0.0 | 0.0 | 0.0 | 0.0 | 0.0 | 0.0 | 0.0 | 0.0 | 0.0 | 0.0 | 0.0 |
| 9130227L01Rik | 0.0 | 0.0 | 0.0 | 0.0 | 0.0 | 0.0 | 0.0 | 0.0 | 0.0 | 0.0 | 0.0 | 0.0 |
| 9230009I02Rik | 0.0 | 0.0 | 0.0 | 0.0 | 0.0 | 0.0 | 0.0 | 0.0 | 0.0 | 0.0 | 0.0 | 0.0 |
| 9230102K24Rik | 0.0 | 0.0 | 0.0 | 0.0 | 0.0 | 0.0 | 0.0 | 0.0 | 0.0 | 0.0 | 0.0 | 0.0 |
| 9230102O04Rik | 0.0 | 0.0 | 0.0 | 0.0 | 0.0 | 0.0 | 0.0 | 0.0 | 0.0 | 0.0 | 0.0 | 0.0 |
| 9230104L09Rik | 0.0 | 0.0 | 0.0 | 0.0 | 0.0 | 0.0 | 0.0 | 0.0 | 0.0 | 0.0 | 0.0 | 0.0 |
| 9230110F15Rik | 0.0 | 0.0 | 0.0 | 0.0 | 0.0 | 0.0 | 0.0 | 0.0 | 0.0 | 0.0 | 0.0 | 0.0 |
| 9330162B11Rik | 0.0 | 0.0 | 0.0 | 0.0 | 0.0 | 0.0 | 0.0 | 0.0 | 0.0 | 0.0 | 0.0 | 0.0 |
| 9330182O14Rik | 0.0 | 0.0 | 0.0 | 0.0 | 0.0 | 0.0 | 0.0 | 0.0 | 0.0 | 0.0 | 0.0 | 0.0 |
| 9430007A20Rik | 0.0 | 0.0 | 0.0 | 0.0 | 0.0 | 0.0 | 0.0 | 0.0 | 0.0 | 0.0 | 0.0 | 0.0 |
| 9430014N10Rik | 0.0 | 0.0 | 0.0 | 0.0 | 0.0 | 0.0 | 0.0 | 0.0 | 0.0 | 0.0 | 0.0 | 0.0 |
| 9530002B09Rik | 0.0 | 0.0 | 0.0 | 0.0 | 0.0 | 0.0 | 0.0 | 0.0 | 0.0 | 0.0 | 0.0 | 0.0 |
| 9630013A20Rik | 0.0 | 0.0 | 0.0 | 0.0 | 0.0 | 0.0 | 0.0 | 0.0 | 0.0 | 0.0 | 0.0 | 0.0 |
| A1bg | 0.0 | 0.0 | 0.0 | 0.0 | 0.0 | 0.0 | 0.0 | 0.0 | 0.0 | 0.0 | 0.0 | 0.0 |
| A330070K13Rik | 0.0 | 0.0 | 0.0 | 0.0 | 0.0 | 0.0 | 0.0 | 0.0 | 0.0 | 0.0 | 0.0 | 0.0 |
| A530006G24Rik | 0.0 | 0.0 | 0.0 | 0.0 | 0.0 | 0.0 | 0.0 | 0.0 | 0.0 | 0.0 | 0.0 | 0.0 |
| A530050N04Rik | 0.0 | 0.0 | 0.0 | 0.0 | 0.0 | 0.0 | 0.0 | 0.0 | 0.0 | 0.0 | 0.0 | 0.0 |
| A530053G22Rik | 0.0 | 0.0 | 0.0 | 0.0 | 0.0 | 0.0 | 0.0 | 0.0 | 0.0 | 0.0 | 0.0 | 0.0 |
| A530058N18Rik | 0.0 | 0.0 | 0.0 | 0.0 | 0.0 | 0.0 | 0.0 | 0.0 | 0.0 | 0.0 | 0.0 | 0.0 |
| A530099J19Rik | 0.0 | 0.0 | 0.0 | 0.0 | 0.0 | 0.0 | 0.0 | 0.0 | 0.0 | 0.0 | 0.0 | 0.0 |
| A630076J17Rik | 0.0 | 0.0 | 0.0 | 0.0 | 0.0 | 0.0 | 0.0 | 0.0 | 0.0 | 0.0 | 0.0 | 0.0 |
| A630095E13Rik | 0.0 | 0.0 | 0.0 | 0.0 | 0.0 | 0.0 | 0.0 | 0.0 | 0.0 | 0.0 | 0.0 | 0.0 |
| A730043L09Rik | 0.0 | 0.0 | 0.0 | 0.0 | 0.0 | 0.0 | 0.0 | 0.0 | 0.0 | 0.0 | 0.0 | 0.0 |
| A830019L24Rik | 0.0 | 0.0 | 0.0 | 0.0 | 0.0 | 0.0 | 0.0 | 0.0 | 0.0 | 0.0 | 0.0 | 0.0 |
| A930006I01Rik | 0.0 | 0.0 | 0.0 | 0.0 | 0.0 | 0.0 | 0.0 | 0.0 | 0.0 | 0.0 | 0.0 | 0.0 |
| A930009A15Rik | 0.0 | 0.0 | 0.0 | 0.0 | 0.0 | 0.0 | 0.0 | 0.0 | 0.0 | 0.0 | 0.0 | 0.0 |
| AA545190 | 0.0 | 0.0 | 0.0 | 0.0 | 0.0 | 0.0 | 0.0 | 0.0 | 0.0 | 0.0 | 0.0 | 0.0 |
| AA619741 | 0.0 | 0.0 | 0.0 | 0.0 | 0.0 | 0.0 | 0.0 | 0.0 | 0.0 | 0.0 | 0.0 | 0.0 |
| Aadacl3 | 0.0 | 0.0 | 0.0 | 0.0 | 0.0 | 0.0 | 0.0 | 0.0 | 0.0 | 0.0 | 0.0 | 0.0 |
| Abcb5 | 0.0 | 0.0 | 0.0 | 0.0 | 0.0 | 0.0 | 0.0 | 0.0 | 0.0 | 0.0 | 0.0 | 0.0 |
| Abo | 0.0 | 0.0 | 0.0 | 0.0 | 0.0 | 0.0 | 0.0 | 0.0 | 0.0 | 0.0 | 0.0 | 0.0 |
| Acnat2 | 0.0 | 0.0 | 0.0 | 0.0 | 0.0 | 0.0 | 0.0 | 0.0 | 0.0 | 0.0 | 0.0 | 0.0 |
| Actrt1 | 0.0 | 0.0 | 0.0 | 0.0 | 0.0 | 0.0 | 0.0 | 0.0 | 0.0 | 0.0 | 0.0 | 0.0 |
| Actrt2 | 0.0 | 0.0 | 0.0 | 0.0 | 0.0 | 0.0 | 0.0 | 0.0 | 0.0 | 0.0 | 0.0 | 0.0 |
| Actrt3 | 0.0 | 0.0 | 0.0 | 0.0 | 0.0 | 0.0 | 0.0 | 0.0 | 0.0 | 0.0 | 0.0 | 0.0 |
| Adam29 | 0.0 | 0.0 | 0.0 | 0.0 | 0.0 | 0.0 | 0.0 | 0.0 | 0.0 | 0.0 | 0.0 | 0.0 |
| Adam34 | 0.0 | 0.0 | 0.0 | 0.0 | 0.0 | 0.0 | 0.0 | 0.0 | 0.0 | 0.0 | 0.0 | 0.0 |
| Adh4 | 0.0 | 0.0 | 0.0 | 0.0 | 0.0 | 0.0 | 0.0 | 0.0 | 0.0 | 0.0 | 0.0 | 0.0 |
| Adh6a | 0.0 | 0.0 | 0.0 | 0.0 | 0.0 | 0.0 | 0.0 | 0.0 | 0.0 | 0.0 | 0.0 | 0.0 |
| AF067061 | 0.0 | 0.0 | 0.0 | 0.0 | 0.0 | 0.0 | 0.0 | 0.0 | 0.0 | 0.0 | 0.0 | 0.0 |
| AF067063 | 0.0 | 0.0 | 0.0 | 0.0 | 0.0 | 0.0 | 0.0 | 0.0 | 0.0 | 0.0 | 0.0 | 0.0 |
| AF357355 | 0.0 | 0.0 | 0.0 | 0.0 | 0.0 | 0.0 | 0.0 | 0.0 | 0.0 | 0.0 | 0.0 | 0.0 |
| AF357359 | 0.0 | 0.0 | 0.0 | 0.0 | 0.0 | 0.0 | 0.0 | 0.0 | 0.0 | 0.0 | 0.0 | 0.0 |
| AF357399 | 0.0 | 0.0 | 0.0 | 0.0 | 0.0 | 0.0 | 0.0 | 0.0 | 0.0 | 0.0 | 0.0 | 0.0 |
| AF357426 | 0.0 | 0.0 | 0.0 | 0.0 | 0.0 | 0.0 | 0.0 | 0.0 | 0.0 | 0.0 | 0.0 | 0.0 |
| Afm | 0.0 | 0.0 | 0.0 | 0.0 | 0.0 | 0.0 | 0.0 | 0.0 | 0.0 | 0.0 | 0.0 | 0.0 |
| Agr2 | 0.0 | 0.0 | 0.0 | 0.0 | 0.0 | 0.0 | 0.0 | 0.0 | 0.0 | 0.0 | 0.0 | 0.0 |
| Agr3 | 0.0 | 0.0 | 0.0 | 0.0 | 0.0 | 0.0 | 0.0 | 0.0 | 0.0 | 0.0 | 0.0 | 0.0 |
| Agxt2 | 0.0 | 0.0 | 0.0 | 0.0 | 0.0 | 0.0 | 0.0 | 0.0 | 0.0 | 0.0 | 0.0 | 0.0 |
| AI646519 | 0.0 | 0.0 | 0.0 | 0.0 | 0.0 | 0.0 | 0.0 | 0.0 | 0.0 | 0.0 | 0.0 | 0.0 |
| Akap4 | 0.0 | 0.0 | 0.0 | 0.0 | 0.0 | 0.0 | 0.0 | 0.0 | 0.0 | 0.0 | 0.0 | 0.0 |
| Akr1c6 | 0.0 | 0.0 | 0.0 | 0.0 | 0.0 | 0.0 | 0.0 | 0.0 | 0.0 | 0.0 | 0.0 | 0.0 |

Excluded Genes

| | | | | | | | | | | | | |
|---|---|---|---|---|---|---|---|---|---|---|---|---|
| Allc | 0.0 | 0.0 | 0.0 | 0.0 | 0.0 | 0.0 | 0.0 | 0.0 | 0.0 | 0.0 | 0.0 | 0.0 |
| Alox12e | 0.0 | 0.0 | 0.0 | 0.0 | 0.0 | 0.0 | 0.0 | 0.0 | 0.0 | 0.0 | 0.0 | 0.0 |
| Ambn | 0.0 | 0.0 | 0.0 | 0.0 | 0.0 | 0.0 | 0.0 | 0.0 | 0.0 | 0.0 | 0.0 | 0.0 |
| Amtn | 0.0 | 0.0 | 0.0 | 0.0 | 0.0 | 0.0 | 0.0 | 0.0 | 0.0 | 0.0 | 0.0 | 0.0 |
| Amy2b | 0.0 | 0.0 | 0.0 | 0.0 | 0.0 | 0.0 | 0.0 | 0.0 | 0.0 | 0.0 | 0.0 | 0.0 |
| Ang6 | 0.0 | 0.0 | 0.0 | 0.0 | 0.0 | 0.0 | 0.0 | 0.0 | 0.0 | 0.0 | 0.0 | 0.0 |
| Ankrd60 | 0.0 | 0.0 | 0.0 | 0.0 | 0.0 | 0.0 | 0.0 | 0.0 | 0.0 | 0.0 | 0.0 | 0.0 |
| Apoa4 | 0.0 | 0.0 | 0.0 | 0.0 | 0.0 | 0.0 | 0.0 | 0.0 | 0.0 | 0.0 | 0.0 | 0.0 |
| Apoc3 | 0.0 | 0.0 | 0.0 | 0.0 | 0.0 | 0.0 | 0.0 | 0.0 | 0.0 | 0.0 | 0.0 | 0.0 |
| Apoc4 | 0.0 | 0.0 | 0.0 | 0.0 | 0.0 | 0.0 | 0.0 | 0.0 | 0.0 | 0.0 | 0.0 | 0.0 |
| Aqp6 | 0.0 | 0.0 | 0.0 | 0.0 | 0.0 | 0.0 | 0.0 | 0.0 | 0.0 | 0.0 | 0.0 | 0.0 |
| Arhgef38 | 0.0 | 0.0 | 0.0 | 0.0 | 0.0 | 0.0 | 0.0 | 0.0 | 0.0 | 0.0 | 0.0 | 0.0 |
| Arl14 | 0.0 | 0.0 | 0.0 | 0.0 | 0.0 | 0.0 | 0.0 | 0.0 | 0.0 | 0.0 | 0.0 | 0.0 |
| Armc12 | 0.0 | 0.0 | 0.0 | 0.0 | 0.0 | 0.0 | 0.0 | 0.0 | 0.0 | 0.0 | 0.0 | 0.0 |
| Arr3 | 0.0 | 0.0 | 0.0 | 0.0 | 0.0 | 0.0 | 0.0 | 0.0 | 0.0 | 0.0 | 0.0 | 0.0 |
| Asb18 | 0.0 | 0.0 | 0.0 | 0.0 | 0.0 | 0.0 | 0.0 | 0.0 | 0.0 | 0.0 | 0.0 | 0.0 |
| Ascl4 | 0.0 | 0.0 | 0.0 | 0.0 | 0.0 | 0.0 | 0.0 | 0.0 | 0.0 | 0.0 | 0.0 | 0.0 |
| Astl | 0.0 | 0.0 | 0.0 | 0.0 | 0.0 | 0.0 | 0.0 | 0.0 | 0.0 | 0.0 | 0.0 | 0.0 |
| Atoh7 | 0.0 | 0.0 | 0.0 | 0.0 | 0.0 | 0.0 | 0.0 | 0.0 | 0.0 | 0.0 | 0.0 | 0.0 |
| Atp6ap1l | 0.0 | 0.0 | 0.0 | 0.0 | 0.0 | 0.0 | 0.0 | 0.0 | 0.0 | 0.0 | 0.0 | 0.0 |
| Atp6v1g3 | 0.0 | 0.0 | 0.0 | 0.0 | 0.0 | 0.0 | 0.0 | 0.0 | 0.0 | 0.0 | 0.0 | 0.0 |
| AU016765 | 0.0 | 0.0 | 0.0 | 0.0 | 0.0 | 0.0 | 0.0 | 0.0 | 0.0 | 0.0 | 0.0 | 0.0 |
| AU022754 | 0.0 | 0.0 | 0.0 | 0.0 | 0.0 | 0.0 | 0.0 | 0.0 | 0.0 | 0.0 | 0.0 | 0.0 |
| Avp | 0.0 | 0.0 | 0.0 | 0.0 | 0.0 | 0.0 | 0.0 | 0.0 | 0.0 | 0.0 | 0.0 | 0.0 |
| AW495222 | 0.0 | 0.0 | 0.0 | 0.0 | 0.0 | 0.0 | 0.0 | 0.0 | 0.0 | 0.0 | 0.0 | 0.0 |
| Awat1 | 0.0 | 0.0 | 0.0 | 0.0 | 0.0 | 0.0 | 0.0 | 0.0 | 0.0 | 0.0 | 0.0 | 0.0 |
| AY512915 | 0.0 | 0.0 | 0.0 | 0.0 | 0.0 | 0.0 | 0.0 | 0.0 | 0.0 | 0.0 | 0.0 | 0.0 |
| AY761184 | 0.0 | 0.0 | 0.0 | 0.0 | 0.0 | 0.0 | 0.0 | 0.0 | 0.0 | 0.0 | 0.0 | 0.0 |
| AY761185 | 0.0 | 0.0 | 0.0 | 0.0 | 0.0 | 0.0 | 0.0 | 0.0 | 0.0 | 0.0 | 0.0 | 0.0 |
| Aym1 | 0.0 | 0.0 | 0.0 | 0.0 | 0.0 | 0.0 | 0.0 | 0.0 | 0.0 | 0.0 | 0.0 | 0.0 |
| B020018J22Rik | 0.0 | 0.0 | 0.0 | 0.0 | 0.0 | 0.0 | 0.0 | 0.0 | 0.0 | 0.0 | 0.0 | 0.0 |
| B020031M17Rik | 0.0 | 0.0 | 0.0 | 0.0 | 0.0 | 0.0 | 0.0 | 0.0 | 0.0 | 0.0 | 0.0 | 0.0 |
| B230209K01Rik | 0.0 | 0.0 | 0.0 | 0.0 | 0.0 | 0.0 | 0.0 | 0.0 | 0.0 | 0.0 | 0.0 | 0.0 |
| B230214G05Rik | 0.0 | 0.0 | 0.0 | 0.0 | 0.0 | 0.0 | 0.0 | 0.0 | 0.0 | 0.0 | 0.0 | 0.0 |
| B3gat1 | 0.0 | 0.0 | 0.0 | 0.0 | 0.0 | 0.0 | 0.0 | 0.0 | 0.0 | 0.0 | 0.0 | 0.0 |
| B930025P03Rik | 0.0 | 0.0 | 0.0 | 0.0 | 0.0 | 0.0 | 0.0 | 0.0 | 0.0 | 0.0 | 0.0 | 0.0 |
| Banf2 | 0.0 | 0.0 | 0.0 | 0.0 | 0.0 | 0.0 | 0.0 | 0.0 | 0.0 | 0.0 | 0.0 | 0.0 |
| BB031773 | 0.0 | 0.0 | 0.0 | 0.0 | 0.0 | 0.0 | 0.0 | 0.0 | 0.0 | 0.0 | 0.0 | 0.0 |
| BC024386 | 0.0 | 0.0 | 0.0 | 0.0 | 0.0 | 0.0 | 0.0 | 0.0 | 0.0 | 0.0 | 0.0 | 0.0 |
| BC030500 | 0.0 | 0.0 | 0.0 | 0.0 | 0.0 | 0.0 | 0.0 | 0.0 | 0.0 | 0.0 | 0.0 | 0.0 |
| BC048546 | 0.0 | 0.0 | 0.0 | 0.0 | 0.0 | 0.0 | 0.0 | 0.0 | 0.0 | 0.0 | 0.0 | 0.0 |
| BC048562 | 0.0 | 0.0 | 0.0 | 0.0 | 0.0 | 0.0 | 0.0 | 0.0 | 0.0 | 0.0 | 0.0 | 0.0 |
| BC048602 | 0.0 | 0.0 | 0.0 | 0.0 | 0.0 | 0.0 | 0.0 | 0.0 | 0.0 | 0.0 | 0.0 | 0.0 |
| BC048671 | 0.0 | 0.0 | 0.0 | 0.0 | 0.0 | 0.0 | 0.0 | 0.0 | 0.0 | 0.0 | 0.0 | 0.0 |
| BC048943 | 0.0 | 0.0 | 0.0 | 0.0 | 0.0 | 0.0 | 0.0 | 0.0 | 0.0 | 0.0 | 0.0 | 0.0 |
| BC049730 | 0.0 | 0.0 | 0.0 | 0.0 | 0.0 | 0.0 | 0.0 | 0.0 | 0.0 | 0.0 | 0.0 | 0.0 |
| BC051070 | 0.0 | 0.0 | 0.0 | 0.0 | 0.0 | 0.0 | 0.0 | 0.0 | 0.0 | 0.0 | 0.0 | 0.0 |
| BC051537 | 0.0 | 0.0 | 0.0 | 0.0 | 0.0 | 0.0 | 0.0 | 0.0 | 0.0 | 0.0 | 0.0 | 0.0 |
| BC051665 | 0.0 | 0.0 | 0.0 | 0.0 | 0.0 | 0.0 | 0.0 | 0.0 | 0.0 | 0.0 | 0.0 | 0.0 |
| BC053393 | 0.0 | 0.0 | 0.0 | 0.0 | 0.0 | 0.0 | 0.0 | 0.0 | 0.0 | 0.0 | 0.0 | 0.0 |
| BC055402 | 0.0 | 0.0 | 0.0 | 0.0 | 0.0 | 0.0 | 0.0 | 0.0 | 0.0 | 0.0 | 0.0 | 0.0 |
| BC061237 | 0.0 | 0.0 | 0.0 | 0.0 | 0.0 | 0.0 | 0.0 | 0.0 | 0.0 | 0.0 | 0.0 | 0.0 |
| BC100530 | 0.0 | 0.0 | 0.0 | 0.0 | 0.0 | 0.0 | 0.0 | 0.0 | 0.0 | 0.0 | 0.0 | 0.0 |
| BC117090 | 0.0 | 0.0 | 0.0 | 0.0 | 0.0 | 0.0 | 0.0 | 0.0 | 0.0 | 0.0 | 0.0 | 0.0 |
| Beta-s | 0.0 | 0.0 | 0.0 | 0.0 | 0.0 | 0.0 | 0.0 | 0.0 | 0.0 | 0.0 | 0.0 | 0.0 |
| Bex6 | 0.0 | 0.0 | 0.0 | 0.0 | 0.0 | 0.0 | 0.0 | 0.0 | 0.0 | 0.0 | 0.0 | 0.0 |
| Bhlha9 | 0.0 | 0.0 | 0.0 | 0.0 | 0.0 | 0.0 | 0.0 | 0.0 | 0.0 | 0.0 | 0.0 | 0.0 |

Excluded Genes

| | | | | | | | | | | | | |
|---|---|---|---|---|---|---|---|---|---|---|---|---|
| Bhlhe23 | 0.0 | 0.0 | 0.0 | 0.0 | 0.0 | 0.0 | 0.0 | 0.0 | 0.0 | 0.0 | 0.0 | 0.0 |
| Bhmt2 | 0.0 | 0.0 | 0.0 | 0.0 | 0.0 | 0.0 | 0.0 | 0.0 | 0.0 | 0.0 | 0.0 | 0.0 |
| Bmp10 | 0.0 | 0.0 | 0.0 | 0.0 | 0.0 | 0.0 | 0.0 | 0.0 | 0.0 | 0.0 | 0.0 | 0.0 |
| Bpi | 0.0 | 0.0 | 0.0 | 0.0 | 0.0 | 0.0 | 0.0 | 0.0 | 0.0 | 0.0 | 0.0 | 0.0 |
| Bpifa1 | 0.0 | 0.0 | 0.0 | 0.0 | 0.0 | 0.0 | 0.0 | 0.0 | 0.0 | 0.0 | 0.0 | 0.0 |
| Bpifa2 | 0.0 | 0.0 | 0.0 | 0.0 | 0.0 | 0.0 | 0.0 | 0.0 | 0.0 | 0.0 | 0.0 | 0.0 |
| Bpifa3 | 0.0 | 0.0 | 0.0 | 0.0 | 0.0 | 0.0 | 0.0 | 0.0 | 0.0 | 0.0 | 0.0 | 0.0 |
| Bpifa5 | 0.0 | 0.0 | 0.0 | 0.0 | 0.0 | 0.0 | 0.0 | 0.0 | 0.0 | 0.0 | 0.0 | 0.0 |
| Bpifb3 | 0.0 | 0.0 | 0.0 | 0.0 | 0.0 | 0.0 | 0.0 | 0.0 | 0.0 | 0.0 | 0.0 | 0.0 |
| Bsnd | 0.0 | 0.0 | 0.0 | 0.0 | 0.0 | 0.0 | 0.0 | 0.0 | 0.0 | 0.0 | 0.0 | 0.0 |
| Bsph1 | 0.0 | 0.0 | 0.0 | 0.0 | 0.0 | 0.0 | 0.0 | 0.0 | 0.0 | 0.0 | 0.0 | 0.0 |
| Bsph2 | 0.0 | 0.0 | 0.0 | 0.0 | 0.0 | 0.0 | 0.0 | 0.0 | 0.0 | 0.0 | 0.0 | 0.0 |
| Btnl2 | 0.0 | 0.0 | 0.0 | 0.0 | 0.0 | 0.0 | 0.0 | 0.0 | 0.0 | 0.0 | 0.0 | 0.0 |
| Btnl4 | 0.0 | 0.0 | 0.0 | 0.0 | 0.0 | 0.0 | 0.0 | 0.0 | 0.0 | 0.0 | 0.0 | 0.0 |
| Btnl6 | 0.0 | 0.0 | 0.0 | 0.0 | 0.0 | 0.0 | 0.0 | 0.0 | 0.0 | 0.0 | 0.0 | 0.0 |
| C030007H22Rik | 0.0 | 0.0 | 0.0 | 0.0 | 0.0 | 0.0 | 0.0 | 0.0 | 0.0 | 0.0 | 0.0 | 0.0 |
| C030013G03Rik | 0.0 | 0.0 | 0.0 | 0.0 | 0.0 | 0.0 | 0.0 | 0.0 | 0.0 | 0.0 | 0.0 | 0.0 |
| C030023E24Rik | 0.0 | 0.0 | 0.0 | 0.0 | 0.0 | 0.0 | 0.0 | 0.0 | 0.0 | 0.0 | 0.0 | 0.0 |
| C1ql2 | 0.0 | 0.0 | 0.0 | 0.0 | 0.0 | 0.0 | 0.0 | 0.0 | 0.0 | 0.0 | 0.0 | 0.0 |
| C2cd4b | 0.0 | 0.0 | 0.0 | 0.0 | 0.0 | 0.0 | 0.0 | 0.0 | 0.0 | 0.0 | 0.0 | 0.0 |
| C330008G21Rik | 0.0 | 0.0 | 0.0 | 0.0 | 0.0 | 0.0 | 0.0 | 0.0 | 0.0 | 0.0 | 0.0 | 0.0 |
| C86695 | 0.0 | 0.0 | 0.0 | 0.0 | 0.0 | 0.0 | 0.0 | 0.0 | 0.0 | 0.0 | 0.0 | 0.0 |
| C87499 | 0.0 | 0.0 | 0.0 | 0.0 | 0.0 | 0.0 | 0.0 | 0.0 | 0.0 | 0.0 | 0.0 | 0.0 |
| C8b | 0.0 | 0.0 | 0.0 | 0.0 | 0.0 | 0.0 | 0.0 | 0.0 | 0.0 | 0.0 | 0.0 | 0.0 |
| Cabp2 | 0.0 | 0.0 | 0.0 | 0.0 | 0.0 | 0.0 | 0.0 | 0.0 | 0.0 | 0.0 | 0.0 | 0.0 |
| Cabp7 | 0.0 | 0.0 | 0.0 | 0.0 | 0.0 | 0.0 | 0.0 | 0.0 | 0.0 | 0.0 | 0.0 | 0.0 |
| Cabs1 | 0.0 | 0.0 | 0.0 | 0.0 | 0.0 | 0.0 | 0.0 | 0.0 | 0.0 | 0.0 | 0.0 | 0.0 |
| Calm4 | 0.0 | 0.0 | 0.0 | 0.0 | 0.0 | 0.0 | 0.0 | 0.0 | 0.0 | 0.0 | 0.0 | 0.0 |
| Calm5 | 0.0 | 0.0 | 0.0 | 0.0 | 0.0 | 0.0 | 0.0 | 0.0 | 0.0 | 0.0 | 0.0 | 0.0 |
| Capza3 | 0.0 | 0.0 | 0.0 | 0.0 | 0.0 | 0.0 | 0.0 | 0.0 | 0.0 | 0.0 | 0.0 | 0.0 |
| Cartpt | 0.0 | 0.0 | 0.0 | 0.0 | 0.0 | 0.0 | 0.0 | 0.0 | 0.0 | 0.0 | 0.0 | 0.0 |
| Cbln1 | 0.0 | 0.0 | 0.0 | 0.0 | 0.0 | 0.0 | 0.0 | 0.0 | 0.0 | 0.0 | 0.0 | 0.0 |
| Cbln2 | 0.0 | 0.0 | 0.0 | 0.0 | 0.0 | 0.0 | 0.0 | 0.0 | 0.0 | 0.0 | 0.0 | 0.0 |
| Ccdc129 | 0.0 | 0.0 | 0.0 | 0.0 | 0.0 | 0.0 | 0.0 | 0.0 | 0.0 | 0.0 | 0.0 | 0.0 |
| Ccdc42 | 0.0 | 0.0 | 0.0 | 0.0 | 0.0 | 0.0 | 0.0 | 0.0 | 0.0 | 0.0 | 0.0 | 0.0 |
| Ccdc70 | 0.0 | 0.0 | 0.0 | 0.0 | 0.0 | 0.0 | 0.0 | 0.0 | 0.0 | 0.0 | 0.0 | 0.0 |
| Ccl1 | 0.0 | 0.0 | 0.0 | 0.0 | 0.0 | 0.0 | 0.0 | 0.0 | 0.0 | 0.0 | 0.0 | 0.0 |
| Ccl26 | 0.0 | 0.0 | 0.0 | 0.0 | 0.0 | 0.0 | 0.0 | 0.0 | 0.0 | 0.0 | 0.0 | 0.0 |
| Ccr8 | 0.0 | 0.0 | 0.0 | 0.0 | 0.0 | 0.0 | 0.0 | 0.0 | 0.0 | 0.0 | 0.0 | 0.0 |
| Cd163l1 | 0.0 | 0.0 | 0.0 | 0.0 | 0.0 | 0.0 | 0.0 | 0.0 | 0.0 | 0.0 | 0.0 | 0.0 |
| Cd209b | 0.0 | 0.0 | 0.0 | 0.0 | 0.0 | 0.0 | 0.0 | 0.0 | 0.0 | 0.0 | 0.0 | 0.0 |
| Cd209e | 0.0 | 0.0 | 0.0 | 0.0 | 0.0 | 0.0 | 0.0 | 0.0 | 0.0 | 0.0 | 0.0 | 0.0 |
| Cd209g | 0.0 | 0.0 | 0.0 | 0.0 | 0.0 | 0.0 | 0.0 | 0.0 | 0.0 | 0.0 | 0.0 | 0.0 |
| Cd300c | 0.0 | 0.0 | 0.0 | 0.0 | 0.0 | 0.0 | 0.0 | 0.0 | 0.0 | 0.0 | 0.0 | 0.0 |
| Cd3e | 0.0 | 0.0 | 0.0 | 0.0 | 0.0 | 0.0 | 0.0 | 0.0 | 0.0 | 0.0 | 0.0 | 0.0 |
| Cdh7 | 0.0 | 0.0 | 0.0 | 0.0 | 0.0 | 0.0 | 0.0 | 0.0 | 0.0 | 0.0 | 0.0 | 0.0 |
| Cdx2 | 0.0 | 0.0 | 0.0 | 0.0 | 0.0 | 0.0 | 0.0 | 0.0 | 0.0 | 0.0 | 0.0 | 0.0 |
| Cdx4 | 0.0 | 0.0 | 0.0 | 0.0 | 0.0 | 0.0 | 0.0 | 0.0 | 0.0 | 0.0 | 0.0 | 0.0 |
| Ceacam11 | 0.0 | 0.0 | 0.0 | 0.0 | 0.0 | 0.0 | 0.0 | 0.0 | 0.0 | 0.0 | 0.0 | 0.0 |
| Ceacam12 | 0.0 | 0.0 | 0.0 | 0.0 | 0.0 | 0.0 | 0.0 | 0.0 | 0.0 | 0.0 | 0.0 | 0.0 |
| Ceacam14 | 0.0 | 0.0 | 0.0 | 0.0 | 0.0 | 0.0 | 0.0 | 0.0 | 0.0 | 0.0 | 0.0 | 0.0 |
| Ceacam3 | 0.0 | 0.0 | 0.0 | 0.0 | 0.0 | 0.0 | 0.0 | 0.0 | 0.0 | 0.0 | 0.0 | 0.0 |
| Ceacam5 | 0.0 | 0.0 | 0.0 | 0.0 | 0.0 | 0.0 | 0.0 | 0.0 | 0.0 | 0.0 | 0.0 | 0.0 |
| Ceacam-ps1 | 0.0 | 0.0 | 0.0 | 0.0 | 0.0 | 0.0 | 0.0 | 0.0 | 0.0 | 0.0 | 0.0 | 0.0 |
| Cela2a | 0.0 | 0.0 | 0.0 | 0.0 | 0.0 | 0.0 | 0.0 | 0.0 | 0.0 | 0.0 | 0.0 | 0.0 |
| Cela3b | 0.0 | 0.0 | 0.0 | 0.0 | 0.0 | 0.0 | 0.0 | 0.0 | 0.0 | 0.0 | 0.0 | 0.0 |
| Ces1e | 0.0 | 0.0 | 0.0 | 0.0 | 0.0 | 0.0 | 0.0 | 0.0 | 0.0 | 0.0 | 0.0 | 0.0 |

Excluded Genes

| | | | | | | | | | | | | |
|---|---|---|---|---|---|---|---|---|---|---|---|---|
| Ces3a | 0.0 | 0.0 | 0.0 | 0.0 | 0.0 | 0.0 | 0.0 | 0.0 | 0.0 | 0.0 | 0.0 | 0.0 |
| Cetn1 | 0.0 | 0.0 | 0.0 | 0.0 | 0.0 | 0.0 | 0.0 | 0.0 | 0.0 | 0.0 | 0.0 | 0.0 |
| Cfc1 | 0.0 | 0.0 | 0.0 | 0.0 | 0.0 | 0.0 | 0.0 | 0.0 | 0.0 | 0.0 | 0.0 | 0.0 |
| Chrm2 | 0.0 | 0.0 | 0.0 | 0.0 | 0.0 | 0.0 | 0.0 | 0.0 | 0.0 | 0.0 | 0.0 | 0.0 |
| Chrna5 | 0.0 | 0.0 | 0.0 | 0.0 | 0.0 | 0.0 | 0.0 | 0.0 | 0.0 | 0.0 | 0.0 | 0.0 |
| Chst4 | 0.0 | 0.0 | 0.0 | 0.0 | 0.0 | 0.0 | 0.0 | 0.0 | 0.0 | 0.0 | 0.0 | 0.0 |
| CK137956 | 0.0 | 0.0 | 0.0 | 0.0 | 0.0 | 0.0 | 0.0 | 0.0 | 0.0 | 0.0 | 0.0 | 0.0 |
| Ckmt2 | 0.0 | 0.0 | 0.0 | 0.0 | 0.0 | 0.0 | 0.0 | 0.0 | 0.0 | 0.0 | 0.0 | 0.0 |
| Cldn16 | 0.0 | 0.0 | 0.0 | 0.0 | 0.0 | 0.0 | 0.0 | 0.0 | 0.0 | 0.0 | 0.0 | 0.0 |
| Cldn17 | 0.0 | 0.0 | 0.0 | 0.0 | 0.0 | 0.0 | 0.0 | 0.0 | 0.0 | 0.0 | 0.0 | 0.0 |
| Cldn8 | 0.0 | 0.0 | 0.0 | 0.0 | 0.0 | 0.0 | 0.0 | 0.0 | 0.0 | 0.0 | 0.0 | 0.0 |
| Clec4b2 | 0.0 | 0.0 | 0.0 | 0.0 | 0.0 | 0.0 | 0.0 | 0.0 | 0.0 | 0.0 | 0.0 | 0.0 |
| Clec4g | 0.0 | 0.0 | 0.0 | 0.0 | 0.0 | 0.0 | 0.0 | 0.0 | 0.0 | 0.0 | 0.0 | 0.0 |
| Clps | 0.0 | 0.0 | 0.0 | 0.0 | 0.0 | 0.0 | 0.0 | 0.0 | 0.0 | 0.0 | 0.0 | 0.0 |
| Clpsl2 | 0.0 | 0.0 | 0.0 | 0.0 | 0.0 | 0.0 | 0.0 | 0.0 | 0.0 | 0.0 | 0.0 | 0.0 |
| Clrn2 | 0.0 | 0.0 | 0.0 | 0.0 | 0.0 | 0.0 | 0.0 | 0.0 | 0.0 | 0.0 | 0.0 | 0.0 |
| Cmtm2b | 0.0 | 0.0 | 0.0 | 0.0 | 0.0 | 0.0 | 0.0 | 0.0 | 0.0 | 0.0 | 0.0 | 0.0 |
| Cndp1 | 0.0 | 0.0 | 0.0 | 0.0 | 0.0 | 0.0 | 0.0 | 0.0 | 0.0 | 0.0 | 0.0 | 0.0 |
| Cox8c | 0.0 | 0.0 | 0.0 | 0.0 | 0.0 | 0.0 | 0.0 | 0.0 | 0.0 | 0.0 | 0.0 | 0.0 |
| Cplx1 | 0.0 | 0.0 | 0.0 | 0.0 | 0.0 | 0.0 | 0.0 | 0.0 | 0.0 | 0.0 | 0.0 | 0.0 |
| Cpne6 | 0.0 | 0.0 | 0.0 | 0.0 | 0.0 | 0.0 | 0.0 | 0.0 | 0.0 | 0.0 | 0.0 | 0.0 |
| Cpxcr1 | 0.0 | 0.0 | 0.0 | 0.0 | 0.0 | 0.0 | 0.0 | 0.0 | 0.0 | 0.0 | 0.0 | 0.0 |
| Crh | 0.0 | 0.0 | 0.0 | 0.0 | 0.0 | 0.0 | 0.0 | 0.0 | 0.0 | 0.0 | 0.0 | 0.0 |
| Crhr1 | 0.0 | 0.0 | 0.0 | 0.0 | 0.0 | 0.0 | 0.0 | 0.0 | 0.0 | 0.0 | 0.0 | 0.0 |
| Crisp3 | 0.0 | 0.0 | 0.0 | 0.0 | 0.0 | 0.0 | 0.0 | 0.0 | 0.0 | 0.0 | 0.0 | 0.0 |
| Cryba2 | 0.0 | 0.0 | 0.0 | 0.0 | 0.0 | 0.0 | 0.0 | 0.0 | 0.0 | 0.0 | 0.0 | 0.0 |
| Cryga | 0.0 | 0.0 | 0.0 | 0.0 | 0.0 | 0.0 | 0.0 | 0.0 | 0.0 | 0.0 | 0.0 | 0.0 |
| Crygb | 0.0 | 0.0 | 0.0 | 0.0 | 0.0 | 0.0 | 0.0 | 0.0 | 0.0 | 0.0 | 0.0 | 0.0 |
| Crygc | 0.0 | 0.0 | 0.0 | 0.0 | 0.0 | 0.0 | 0.0 | 0.0 | 0.0 | 0.0 | 0.0 | 0.0 |
| Crygd | 0.0 | 0.0 | 0.0 | 0.0 | 0.0 | 0.0 | 0.0 | 0.0 | 0.0 | 0.0 | 0.0 | 0.0 |
| Cryge | 0.0 | 0.0 | 0.0 | 0.0 | 0.0 | 0.0 | 0.0 | 0.0 | 0.0 | 0.0 | 0.0 | 0.0 |
| Csn2 | 0.0 | 0.0 | 0.0 | 0.0 | 0.0 | 0.0 | 0.0 | 0.0 | 0.0 | 0.0 | 0.0 | 0.0 |
| Csprs | 0.0 | 0.0 | 0.0 | 0.0 | 0.0 | 0.0 | 0.0 | 0.0 | 0.0 | 0.0 | 0.0 | 0.0 |
| Cst10 | 0.0 | 0.0 | 0.0 | 0.0 | 0.0 | 0.0 | 0.0 | 0.0 | 0.0 | 0.0 | 0.0 | 0.0 |
| Cst11 | 0.0 | 0.0 | 0.0 | 0.0 | 0.0 | 0.0 | 0.0 | 0.0 | 0.0 | 0.0 | 0.0 | 0.0 |
| Cst12 | 0.0 | 0.0 | 0.0 | 0.0 | 0.0 | 0.0 | 0.0 | 0.0 | 0.0 | 0.0 | 0.0 | 0.0 |
| Cts3 | 0.0 | 0.0 | 0.0 | 0.0 | 0.0 | 0.0 | 0.0 | 0.0 | 0.0 | 0.0 | 0.0 | 0.0 |
| Cts6 | 0.0 | 0.0 | 0.0 | 0.0 | 0.0 | 0.0 | 0.0 | 0.0 | 0.0 | 0.0 | 0.0 | 0.0 |
| Ctsg | 0.0 | 0.0 | 0.0 | 0.0 | 0.0 | 0.0 | 0.0 | 0.0 | 0.0 | 0.0 | 0.0 | 0.0 |
| Ctsj | 0.0 | 0.0 | 0.0 | 0.0 | 0.0 | 0.0 | 0.0 | 0.0 | 0.0 | 0.0 | 0.0 | 0.0 |
| Ctsm | 0.0 | 0.0 | 0.0 | 0.0 | 0.0 | 0.0 | 0.0 | 0.0 | 0.0 | 0.0 | 0.0 | 0.0 |
| Ctsr | 0.0 | 0.0 | 0.0 | 0.0 | 0.0 | 0.0 | 0.0 | 0.0 | 0.0 | 0.0 | 0.0 | 0.0 |
| Cxcl11 | 0.0 | 0.0 | 0.0 | 0.0 | 0.0 | 0.0 | 0.0 | 0.0 | 0.0 | 0.0 | 0.0 | 0.0 |
| Cylc1 | 0.0 | 0.0 | 0.0 | 0.0 | 0.0 | 0.0 | 0.0 | 0.0 | 0.0 | 0.0 | 0.0 | 0.0 |
| Cyp2a12 | 0.0 | 0.0 | 0.0 | 0.0 | 0.0 | 0.0 | 0.0 | 0.0 | 0.0 | 0.0 | 0.0 | 0.0 |
| Cyp2a22 | 0.0 | 0.0 | 0.0 | 0.0 | 0.0 | 0.0 | 0.0 | 0.0 | 0.0 | 0.0 | 0.0 | 0.0 |
| Cyp2a5 | 0.0 | 0.0 | 0.0 | 0.0 | 0.0 | 0.0 | 0.0 | 0.0 | 0.0 | 0.0 | 0.0 | 0.0 |
| Cyp2b19 | 0.0 | 0.0 | 0.0 | 0.0 | 0.0 | 0.0 | 0.0 | 0.0 | 0.0 | 0.0 | 0.0 | 0.0 |
| Cyp2b23 | 0.0 | 0.0 | 0.0 | 0.0 | 0.0 | 0.0 | 0.0 | 0.0 | 0.0 | 0.0 | 0.0 | 0.0 |
| Cyp2c37 | 0.0 | 0.0 | 0.0 | 0.0 | 0.0 | 0.0 | 0.0 | 0.0 | 0.0 | 0.0 | 0.0 | 0.0 |
| Cyp2c54 | 0.0 | 0.0 | 0.0 | 0.0 | 0.0 | 0.0 | 0.0 | 0.0 | 0.0 | 0.0 | 0.0 | 0.0 |
| Cyp2c70 | 0.0 | 0.0 | 0.0 | 0.0 | 0.0 | 0.0 | 0.0 | 0.0 | 0.0 | 0.0 | 0.0 | 0.0 |
| Cyp2d12 | 0.0 | 0.0 | 0.0 | 0.0 | 0.0 | 0.0 | 0.0 | 0.0 | 0.0 | 0.0 | 0.0 | 0.0 |
| Cyp2g1 | 0.0 | 0.0 | 0.0 | 0.0 | 0.0 | 0.0 | 0.0 | 0.0 | 0.0 | 0.0 | 0.0 | 0.0 |
| Cyp2w1 | 0.0 | 0.0 | 0.0 | 0.0 | 0.0 | 0.0 | 0.0 | 0.0 | 0.0 | 0.0 | 0.0 | 0.0 |
| Cyp3a11 | 0.0 | 0.0 | 0.0 | 0.0 | 0.0 | 0.0 | 0.0 | 0.0 | 0.0 | 0.0 | 0.0 | 0.0 |
| Cyp3a25 | 0.0 | 0.0 | 0.0 | 0.0 | 0.0 | 0.0 | 0.0 | 0.0 | 0.0 | 0.0 | 0.0 | 0.0 |

Excluded Genes

| | | | | | | | | | | | | |
|---|---|---|---|---|---|---|---|---|---|---|---|---|
| Cyp4a29-ps | 0.0 | 0.0 | 0.0 | 0.0 | 0.0 | 0.0 | 0.0 | 0.0 | 0.0 | 0.0 | 0.0 | 0.0 |
| Cyp4x1 | 0.0 | 0.0 | 0.0 | 0.0 | 0.0 | 0.0 | 0.0 | 0.0 | 0.0 | 0.0 | 0.0 | 0.0 |
| Cyp7a1 | 0.0 | 0.0 | 0.0 | 0.0 | 0.0 | 0.0 | 0.0 | 0.0 | 0.0 | 0.0 | 0.0 | 0.0 |
| Cypt12 | 0.0 | 0.0 | 0.0 | 0.0 | 0.0 | 0.0 | 0.0 | 0.0 | 0.0 | 0.0 | 0.0 | 0.0 |
| Cypt14 | 0.0 | 0.0 | 0.0 | 0.0 | 0.0 | 0.0 | 0.0 | 0.0 | 0.0 | 0.0 | 0.0 | 0.0 |
| Cypt15 | 0.0 | 0.0 | 0.0 | 0.0 | 0.0 | 0.0 | 0.0 | 0.0 | 0.0 | 0.0 | 0.0 | 0.0 |
| Cypt4 | 0.0 | 0.0 | 0.0 | 0.0 | 0.0 | 0.0 | 0.0 | 0.0 | 0.0 | 0.0 | 0.0 | 0.0 |
| Cypt9 | 0.0 | 0.0 | 0.0 | 0.0 | 0.0 | 0.0 | 0.0 | 0.0 | 0.0 | 0.0 | 0.0 | 0.0 |
| D030024E09Rik | 0.0 | 0.0 | 0.0 | 0.0 | 0.0 | 0.0 | 0.0 | 0.0 | 0.0 | 0.0 | 0.0 | 0.0 |
| D030025E07Rik | 0.0 | 0.0 | 0.0 | 0.0 | 0.0 | 0.0 | 0.0 | 0.0 | 0.0 | 0.0 | 0.0 | 0.0 |
| D16Ertd519e | 0.0 | 0.0 | 0.0 | 0.0 | 0.0 | 0.0 | 0.0 | 0.0 | 0.0 | 0.0 | 0.0 | 0.0 |
| D230030E09Rik | 0.0 | 0.0 | 0.0 | 0.0 | 0.0 | 0.0 | 0.0 | 0.0 | 0.0 | 0.0 | 0.0 | 0.0 |
| D430036J16Rik | 0.0 | 0.0 | 0.0 | 0.0 | 0.0 | 0.0 | 0.0 | 0.0 | 0.0 | 0.0 | 0.0 | 0.0 |
| D530049I02Rik | 0.0 | 0.0 | 0.0 | 0.0 | 0.0 | 0.0 | 0.0 | 0.0 | 0.0 | 0.0 | 0.0 | 0.0 |
| D730045A05Rik | 0.0 | 0.0 | 0.0 | 0.0 | 0.0 | 0.0 | 0.0 | 0.0 | 0.0 | 0.0 | 0.0 | 0.0 |
| D730048I06Rik | 0.0 | 0.0 | 0.0 | 0.0 | 0.0 | 0.0 | 0.0 | 0.0 | 0.0 | 0.0 | 0.0 | 0.0 |
| D730050B12Rik | 0.0 | 0.0 | 0.0 | 0.0 | 0.0 | 0.0 | 0.0 | 0.0 | 0.0 | 0.0 | 0.0 | 0.0 |
| D830015G02Rik | 0.0 | 0.0 | 0.0 | 0.0 | 0.0 | 0.0 | 0.0 | 0.0 | 0.0 | 0.0 | 0.0 | 0.0 |
| D930028M14Rik | 0.0 | 0.0 | 0.0 | 0.0 | 0.0 | 0.0 | 0.0 | 0.0 | 0.0 | 0.0 | 0.0 | 0.0 |
| Dapl1 | 0.0 | 0.0 | 0.0 | 0.0 | 0.0 | 0.0 | 0.0 | 0.0 | 0.0 | 0.0 | 0.0 | 0.0 |
| Dbx1 | 0.0 | 0.0 | 0.0 | 0.0 | 0.0 | 0.0 | 0.0 | 0.0 | 0.0 | 0.0 | 0.0 | 0.0 |
| Dcpp1 | 0.0 | 0.0 | 0.0 | 0.0 | 0.0 | 0.0 | 0.0 | 0.0 | 0.0 | 0.0 | 0.0 | 0.0 |
| Dcpp2 | 0.0 | 0.0 | 0.0 | 0.0 | 0.0 | 0.0 | 0.0 | 0.0 | 0.0 | 0.0 | 0.0 | 0.0 |
| Dcpp3 | 0.0 | 0.0 | 0.0 | 0.0 | 0.0 | 0.0 | 0.0 | 0.0 | 0.0 | 0.0 | 0.0 | 0.0 |
| Defa17 | 0.0 | 0.0 | 0.0 | 0.0 | 0.0 | 0.0 | 0.0 | 0.0 | 0.0 | 0.0 | 0.0 | 0.0 |
| Defa20 | 0.0 | 0.0 | 0.0 | 0.0 | 0.0 | 0.0 | 0.0 | 0.0 | 0.0 | 0.0 | 0.0 | 0.0 |
| Defa21 | 0.0 | 0.0 | 0.0 | 0.0 | 0.0 | 0.0 | 0.0 | 0.0 | 0.0 | 0.0 | 0.0 | 0.0 |
| Defa22 | 0.0 | 0.0 | 0.0 | 0.0 | 0.0 | 0.0 | 0.0 | 0.0 | 0.0 | 0.0 | 0.0 | 0.0 |
| Defa24 | 0.0 | 0.0 | 0.0 | 0.0 | 0.0 | 0.0 | 0.0 | 0.0 | 0.0 | 0.0 | 0.0 | 0.0 |
| Defa25 | 0.0 | 0.0 | 0.0 | 0.0 | 0.0 | 0.0 | 0.0 | 0.0 | 0.0 | 0.0 | 0.0 | 0.0 |
| Defa26 | 0.0 | 0.0 | 0.0 | 0.0 | 0.0 | 0.0 | 0.0 | 0.0 | 0.0 | 0.0 | 0.0 | 0.0 |
| Defa3 | 0.0 | 0.0 | 0.0 | 0.0 | 0.0 | 0.0 | 0.0 | 0.0 | 0.0 | 0.0 | 0.0 | 0.0 |
| Defa4 | 0.0 | 0.0 | 0.0 | 0.0 | 0.0 | 0.0 | 0.0 | 0.0 | 0.0 | 0.0 | 0.0 | 0.0 |
| Defa5 | 0.0 | 0.0 | 0.0 | 0.0 | 0.0 | 0.0 | 0.0 | 0.0 | 0.0 | 0.0 | 0.0 | 0.0 |
| Defa6 | 0.0 | 0.0 | 0.0 | 0.0 | 0.0 | 0.0 | 0.0 | 0.0 | 0.0 | 0.0 | 0.0 | 0.0 |
| Defa-ps1 | 0.0 | 0.0 | 0.0 | 0.0 | 0.0 | 0.0 | 0.0 | 0.0 | 0.0 | 0.0 | 0.0 | 0.0 |
| Defa-ps12 | 0.0 | 0.0 | 0.0 | 0.0 | 0.0 | 0.0 | 0.0 | 0.0 | 0.0 | 0.0 | 0.0 | 0.0 |
| Defa-ps13 | 0.0 | 0.0 | 0.0 | 0.0 | 0.0 | 0.0 | 0.0 | 0.0 | 0.0 | 0.0 | 0.0 | 0.0 |
| Defa-rs1 | 0.0 | 0.0 | 0.0 | 0.0 | 0.0 | 0.0 | 0.0 | 0.0 | 0.0 | 0.0 | 0.0 | 0.0 |
| Defb10 | 0.0 | 0.0 | 0.0 | 0.0 | 0.0 | 0.0 | 0.0 | 0.0 | 0.0 | 0.0 | 0.0 | 0.0 |
| Defb11 | 0.0 | 0.0 | 0.0 | 0.0 | 0.0 | 0.0 | 0.0 | 0.0 | 0.0 | 0.0 | 0.0 | 0.0 |
| Defb12 | 0.0 | 0.0 | 0.0 | 0.0 | 0.0 | 0.0 | 0.0 | 0.0 | 0.0 | 0.0 | 0.0 | 0.0 |
| Defb13 | 0.0 | 0.0 | 0.0 | 0.0 | 0.0 | 0.0 | 0.0 | 0.0 | 0.0 | 0.0 | 0.0 | 0.0 |
| Defb14 | 0.0 | 0.0 | 0.0 | 0.0 | 0.0 | 0.0 | 0.0 | 0.0 | 0.0 | 0.0 | 0.0 | 0.0 |
| Defb15 | 0.0 | 0.0 | 0.0 | 0.0 | 0.0 | 0.0 | 0.0 | 0.0 | 0.0 | 0.0 | 0.0 | 0.0 |
| Defb2 | 0.0 | 0.0 | 0.0 | 0.0 | 0.0 | 0.0 | 0.0 | 0.0 | 0.0 | 0.0 | 0.0 | 0.0 |
| Defb21 | 0.0 | 0.0 | 0.0 | 0.0 | 0.0 | 0.0 | 0.0 | 0.0 | 0.0 | 0.0 | 0.0 | 0.0 |
| Defb22 | 0.0 | 0.0 | 0.0 | 0.0 | 0.0 | 0.0 | 0.0 | 0.0 | 0.0 | 0.0 | 0.0 | 0.0 |
| Defb23 | 0.0 | 0.0 | 0.0 | 0.0 | 0.0 | 0.0 | 0.0 | 0.0 | 0.0 | 0.0 | 0.0 | 0.0 |
| Defb29 | 0.0 | 0.0 | 0.0 | 0.0 | 0.0 | 0.0 | 0.0 | 0.0 | 0.0 | 0.0 | 0.0 | 0.0 |
| Defb3 | 0.0 | 0.0 | 0.0 | 0.0 | 0.0 | 0.0 | 0.0 | 0.0 | 0.0 | 0.0 | 0.0 | 0.0 |
| Defb33 | 0.0 | 0.0 | 0.0 | 0.0 | 0.0 | 0.0 | 0.0 | 0.0 | 0.0 | 0.0 | 0.0 | 0.0 |
| Defb34 | 0.0 | 0.0 | 0.0 | 0.0 | 0.0 | 0.0 | 0.0 | 0.0 | 0.0 | 0.0 | 0.0 | 0.0 |
| Defb35 | 0.0 | 0.0 | 0.0 | 0.0 | 0.0 | 0.0 | 0.0 | 0.0 | 0.0 | 0.0 | 0.0 | 0.0 |
| Defb36 | 0.0 | 0.0 | 0.0 | 0.0 | 0.0 | 0.0 | 0.0 | 0.0 | 0.0 | 0.0 | 0.0 | 0.0 |
| Defb37 | 0.0 | 0.0 | 0.0 | 0.0 | 0.0 | 0.0 | 0.0 | 0.0 | 0.0 | 0.0 | 0.0 | 0.0 |
| Defb38 | 0.0 | 0.0 | 0.0 | 0.0 | 0.0 | 0.0 | 0.0 | 0.0 | 0.0 | 0.0 | 0.0 | 0.0 |

Excluded Genes

| | | | | | | | | | | | |
|---|---|---|---|---|---|---|---|---|---|---|---|
| Defb39 | 0.0 | 0.0 | 0.0 | 0.0 | 0.0 | 0.0 | 0.0 | 0.0 | 0.0 | 0.0 | 0.0 | 0.0 |
| Defb4 | 0.0 | 0.0 | 0.0 | 0.0 | 0.0 | 0.0 | 0.0 | 0.0 | 0.0 | 0.0 | 0.0 | 0.0 |
| Defb40 | 0.0 | 0.0 | 0.0 | 0.0 | 0.0 | 0.0 | 0.0 | 0.0 | 0.0 | 0.0 | 0.0 | 0.0 |
| Defb41 | 0.0 | 0.0 | 0.0 | 0.0 | 0.0 | 0.0 | 0.0 | 0.0 | 0.0 | 0.0 | 0.0 | 0.0 |
| Defb43 | 0.0 | 0.0 | 0.0 | 0.0 | 0.0 | 0.0 | 0.0 | 0.0 | 0.0 | 0.0 | 0.0 | 0.0 |
| Defb44-ps | 0.0 | 0.0 | 0.0 | 0.0 | 0.0 | 0.0 | 0.0 | 0.0 | 0.0 | 0.0 | 0.0 | 0.0 |
| Defb45 | 0.0 | 0.0 | 0.0 | 0.0 | 0.0 | 0.0 | 0.0 | 0.0 | 0.0 | 0.0 | 0.0 | 0.0 |
| Defb46 | 0.0 | 0.0 | 0.0 | 0.0 | 0.0 | 0.0 | 0.0 | 0.0 | 0.0 | 0.0 | 0.0 | 0.0 |
| Defb47 | 0.0 | 0.0 | 0.0 | 0.0 | 0.0 | 0.0 | 0.0 | 0.0 | 0.0 | 0.0 | 0.0 | 0.0 |
| Defb5 | 0.0 | 0.0 | 0.0 | 0.0 | 0.0 | 0.0 | 0.0 | 0.0 | 0.0 | 0.0 | 0.0 | 0.0 |
| Defb50 | 0.0 | 0.0 | 0.0 | 0.0 | 0.0 | 0.0 | 0.0 | 0.0 | 0.0 | 0.0 | 0.0 | 0.0 |
| Defb6 | 0.0 | 0.0 | 0.0 | 0.0 | 0.0 | 0.0 | 0.0 | 0.0 | 0.0 | 0.0 | 0.0 | 0.0 |
| Defb8 | 0.0 | 0.0 | 0.0 | 0.0 | 0.0 | 0.0 | 0.0 | 0.0 | 0.0 | 0.0 | 0.0 | 0.0 |
| Defb9 | 0.0 | 0.0 | 0.0 | 0.0 | 0.0 | 0.0 | 0.0 | 0.0 | 0.0 | 0.0 | 0.0 | 0.0 |
| Dhrs7c | 0.0 | 0.0 | 0.0 | 0.0 | 0.0 | 0.0 | 0.0 | 0.0 | 0.0 | 0.0 | 0.0 | 0.0 |
| Dhrsx | 0.0 | 0.0 | 0.0 | 0.0 | 0.0 | 0.0 | 0.0 | 0.0 | 0.0 | 0.0 | 0.0 | 0.0 |
| Dlx6as2 | 0.0 | 0.0 | 0.0 | 0.0 | 0.0 | 0.0 | 0.0 | 0.0 | 0.0 | 0.0 | 0.0 | 0.0 |
| Dmrt3 | 0.0 | 0.0 | 0.0 | 0.0 | 0.0 | 0.0 | 0.0 | 0.0 | 0.0 | 0.0 | 0.0 | 0.0 |
| Dnajb8 | 0.0 | 0.0 | 0.0 | 0.0 | 0.0 | 0.0 | 0.0 | 0.0 | 0.0 | 0.0 | 0.0 | 0.0 |
| Dnajc5g | 0.0 | 0.0 | 0.0 | 0.0 | 0.0 | 0.0 | 0.0 | 0.0 | 0.0 | 0.0 | 0.0 | 0.0 |
| Dnase2b | 0.0 | 0.0 | 0.0 | 0.0 | 0.0 | 0.0 | 0.0 | 0.0 | 0.0 | 0.0 | 0.0 | 0.0 |
| DQ267100 | 0.0 | 0.0 | 0.0 | 0.0 | 0.0 | 0.0 | 0.0 | 0.0 | 0.0 | 0.0 | 0.0 | 0.0 |
| DQ267101 | 0.0 | 0.0 | 0.0 | 0.0 | 0.0 | 0.0 | 0.0 | 0.0 | 0.0 | 0.0 | 0.0 | 0.0 |
| Dsg4 | 0.0 | 0.0 | 0.0 | 0.0 | 0.0 | 0.0 | 0.0 | 0.0 | 0.0 | 0.0 | 0.0 | 0.0 |
| Duoxa2 | 0.0 | 0.0 | 0.0 | 0.0 | 0.0 | 0.0 | 0.0 | 0.0 | 0.0 | 0.0 | 0.0 | 0.0 |
| Dusp21 | 0.0 | 0.0 | 0.0 | 0.0 | 0.0 | 0.0 | 0.0 | 0.0 | 0.0 | 0.0 | 0.0 | 0.0 |
| Dynap | 0.0 | 0.0 | 0.0 | 0.0 | 0.0 | 0.0 | 0.0 | 0.0 | 0.0 | 0.0 | 0.0 | 0.0 |
| Dytn | 0.0 | 0.0 | 0.0 | 0.0 | 0.0 | 0.0 | 0.0 | 0.0 | 0.0 | 0.0 | 0.0 | 0.0 |
| E030002O03Rik | 0.0 | 0.0 | 0.0 | 0.0 | 0.0 | 0.0 | 0.0 | 0.0 | 0.0 | 0.0 | 0.0 | 0.0 |
| E030019B06Rik | 0.0 | 0.0 | 0.0 | 0.0 | 0.0 | 0.0 | 0.0 | 0.0 | 0.0 | 0.0 | 0.0 | 0.0 |
| E030019B13Rik | 0.0 | 0.0 | 0.0 | 0.0 | 0.0 | 0.0 | 0.0 | 0.0 | 0.0 | 0.0 | 0.0 | 0.0 |
| E330012B07Rik | 0.0 | 0.0 | 0.0 | 0.0 | 0.0 | 0.0 | 0.0 | 0.0 | 0.0 | 0.0 | 0.0 | 0.0 |
| E330017A01Rik | 0.0 | 0.0 | 0.0 | 0.0 | 0.0 | 0.0 | 0.0 | 0.0 | 0.0 | 0.0 | 0.0 | 0.0 |
| E330017L17Rik | 0.0 | 0.0 | 0.0 | 0.0 | 0.0 | 0.0 | 0.0 | 0.0 | 0.0 | 0.0 | 0.0 | 0.0 |
| Ear1 | 0.0 | 0.0 | 0.0 | 0.0 | 0.0 | 0.0 | 0.0 | 0.0 | 0.0 | 0.0 | 0.0 | 0.0 |
| Ear11 | 0.0 | 0.0 | 0.0 | 0.0 | 0.0 | 0.0 | 0.0 | 0.0 | 0.0 | 0.0 | 0.0 | 0.0 |
| Ear14 | 0.0 | 0.0 | 0.0 | 0.0 | 0.0 | 0.0 | 0.0 | 0.0 | 0.0 | 0.0 | 0.0 | 0.0 |
| Ear4 | 0.0 | 0.0 | 0.0 | 0.0 | 0.0 | 0.0 | 0.0 | 0.0 | 0.0 | 0.0 | 0.0 | 0.0 |
| Ear6 | 0.0 | 0.0 | 0.0 | 0.0 | 0.0 | 0.0 | 0.0 | 0.0 | 0.0 | 0.0 | 0.0 | 0.0 |
| Ear7 | 0.0 | 0.0 | 0.0 | 0.0 | 0.0 | 0.0 | 0.0 | 0.0 | 0.0 | 0.0 | 0.0 | 0.0 |
| Egfbp2 | 0.0 | 0.0 | 0.0 | 0.0 | 0.0 | 0.0 | 0.0 | 0.0 | 0.0 | 0.0 | 0.0 | 0.0 |
| Elane | 0.0 | 0.0 | 0.0 | 0.0 | 0.0 | 0.0 | 0.0 | 0.0 | 0.0 | 0.0 | 0.0 | 0.0 |
| Enpp7 | 0.0 | 0.0 | 0.0 | 0.0 | 0.0 | 0.0 | 0.0 | 0.0 | 0.0 | 0.0 | 0.0 | 0.0 |
| Eppin | 0.0 | 0.0 | 0.0 | 0.0 | 0.0 | 0.0 | 0.0 | 0.0 | 0.0 | 0.0 | 0.0 | 0.0 |
| Eras | 0.0 | 0.0 | 0.0 | 0.0 | 0.0 | 0.0 | 0.0 | 0.0 | 0.0 | 0.0 | 0.0 | 0.0 |
| Ermn | 0.0 | 0.0 | 0.0 | 0.0 | 0.0 | 0.0 | 0.0 | 0.0 | 0.0 | 0.0 | 0.0 | 0.0 |
| Etv2 | 0.0 | 0.0 | 0.0 | 0.0 | 0.0 | 0.0 | 0.0 | 0.0 | 0.0 | 0.0 | 0.0 | 0.0 |
| Evx1 | 0.0 | 0.0 | 0.0 | 0.0 | 0.0 | 0.0 | 0.0 | 0.0 | 0.0 | 0.0 | 0.0 | 0.0 |
| F830002L21Rik | 0.0 | 0.0 | 0.0 | 0.0 | 0.0 | 0.0 | 0.0 | 0.0 | 0.0 | 0.0 | 0.0 | 0.0 |
| Fabp6 | 0.0 | 0.0 | 0.0 | 0.0 | 0.0 | 0.0 | 0.0 | 0.0 | 0.0 | 0.0 | 0.0 | 0.0 |
| Fabp9 | 0.0 | 0.0 | 0.0 | 0.0 | 0.0 | 0.0 | 0.0 | 0.0 | 0.0 | 0.0 | 0.0 | 0.0 |
| Faim3 | 0.0 | 0.0 | 0.0 | 0.0 | 0.0 | 0.0 | 0.0 | 0.0 | 0.0 | 0.0 | 0.0 | 0.0 |
| Fam122c | 0.0 | 0.0 | 0.0 | 0.0 | 0.0 | 0.0 | 0.0 | 0.0 | 0.0 | 0.0 | 0.0 | 0.0 |
| Fam150b | 0.0 | 0.0 | 0.0 | 0.0 | 0.0 | 0.0 | 0.0 | 0.0 | 0.0 | 0.0 | 0.0 | 0.0 |
| Fam162b | 0.0 | 0.0 | 0.0 | 0.0 | 0.0 | 0.0 | 0.0 | 0.0 | 0.0 | 0.0 | 0.0 | 0.0 |
| Fam183b | 0.0 | 0.0 | 0.0 | 0.0 | 0.0 | 0.0 | 0.0 | 0.0 | 0.0 | 0.0 | 0.0 | 0.0 |
| Fam47c | 0.0 | 0.0 | 0.0 | 0.0 | 0.0 | 0.0 | 0.0 | 0.0 | 0.0 | 0.0 | 0.0 | 0.0 |

| | | | | | | | | | | | | |
|---|---|---|---|---|---|---|---|---|---|---|---|---|
| Fam47e | 0.0 | 0.0 | 0.0 | 0.0 | 0.0 | 0.0 | 0.0 | 0.0 | 0.0 | 0.0 | 0.0 | 0.0 |
| Fam50b | 0.0 | 0.0 | 0.0 | 0.0 | 0.0 | 0.0 | 0.0 | 0.0 | 0.0 | 0.0 | 0.0 | 0.0 |
| Fam71b | 0.0 | 0.0 | 0.0 | 0.0 | 0.0 | 0.0 | 0.0 | 0.0 | 0.0 | 0.0 | 0.0 | 0.0 |
| Fcer1a | 0.0 | 0.0 | 0.0 | 0.0 | 0.0 | 0.0 | 0.0 | 0.0 | 0.0 | 0.0 | 0.0 | 0.0 |
| Fcnb | 0.0 | 0.0 | 0.0 | 0.0 | 0.0 | 0.0 | 0.0 | 0.0 | 0.0 | 0.0 | 0.0 | 0.0 |
| Ferd3l | 0.0 | 0.0 | 0.0 | 0.0 | 0.0 | 0.0 | 0.0 | 0.0 | 0.0 | 0.0 | 0.0 | 0.0 |
| Fev | 0.0 | 0.0 | 0.0 | 0.0 | 0.0 | 0.0 | 0.0 | 0.0 | 0.0 | 0.0 | 0.0 | 0.0 |
| Fezf1 | 0.0 | 0.0 | 0.0 | 0.0 | 0.0 | 0.0 | 0.0 | 0.0 | 0.0 | 0.0 | 0.0 | 0.0 |
| Fezf2 | 0.0 | 0.0 | 0.0 | 0.0 | 0.0 | 0.0 | 0.0 | 0.0 | 0.0 | 0.0 | 0.0 | 0.0 |
| Ffar1 | 0.0 | 0.0 | 0.0 | 0.0 | 0.0 | 0.0 | 0.0 | 0.0 | 0.0 | 0.0 | 0.0 | 0.0 |
| Ffar2 | 0.0 | 0.0 | 0.0 | 0.0 | 0.0 | 0.0 | 0.0 | 0.0 | 0.0 | 0.0 | 0.0 | 0.0 |
| Ffar3 | 0.0 | 0.0 | 0.0 | 0.0 | 0.0 | 0.0 | 0.0 | 0.0 | 0.0 | 0.0 | 0.0 | 0.0 |
| Fgf3 | 0.0 | 0.0 | 0.0 | 0.0 | 0.0 | 0.0 | 0.0 | 0.0 | 0.0 | 0.0 | 0.0 | 0.0 |
| Fgf4 | 0.0 | 0.0 | 0.0 | 0.0 | 0.0 | 0.0 | 0.0 | 0.0 | 0.0 | 0.0 | 0.0 | 0.0 |
| Fgf6 | 0.0 | 0.0 | 0.0 | 0.0 | 0.0 | 0.0 | 0.0 | 0.0 | 0.0 | 0.0 | 0.0 | 0.0 |
| Fgg | 0.0 | 0.0 | 0.0 | 0.0 | 0.0 | 0.0 | 0.0 | 0.0 | 0.0 | 0.0 | 0.0 | 0.0 |
| Figla | 0.0 | 0.0 | 0.0 | 0.0 | 0.0 | 0.0 | 0.0 | 0.0 | 0.0 | 0.0 | 0.0 | 0.0 |
| Foxd4 | 0.0 | 0.0 | 0.0 | 0.0 | 0.0 | 0.0 | 0.0 | 0.0 | 0.0 | 0.0 | 0.0 | 0.0 |
| Foxi3 | 0.0 | 0.0 | 0.0 | 0.0 | 0.0 | 0.0 | 0.0 | 0.0 | 0.0 | 0.0 | 0.0 | 0.0 |
| Foxn4 | 0.0 | 0.0 | 0.0 | 0.0 | 0.0 | 0.0 | 0.0 | 0.0 | 0.0 | 0.0 | 0.0 | 0.0 |
| Fpr-rs4 | 0.0 | 0.0 | 0.0 | 0.0 | 0.0 | 0.0 | 0.0 | 0.0 | 0.0 | 0.0 | 0.0 | 0.0 |
| Fpr-rs6 | 0.0 | 0.0 | 0.0 | 0.0 | 0.0 | 0.0 | 0.0 | 0.0 | 0.0 | 0.0 | 0.0 | 0.0 |
| Fscb | 0.0 | 0.0 | 0.0 | 0.0 | 0.0 | 0.0 | 0.0 | 0.0 | 0.0 | 0.0 | 0.0 | 0.0 |
| Fthl17 | 0.0 | 0.0 | 0.0 | 0.0 | 0.0 | 0.0 | 0.0 | 0.0 | 0.0 | 0.0 | 0.0 | 0.0 |
| Fut4-ps1 | 0.0 | 0.0 | 0.0 | 0.0 | 0.0 | 0.0 | 0.0 | 0.0 | 0.0 | 0.0 | 0.0 | 0.0 |
| G6pc2 | 0.0 | 0.0 | 0.0 | 0.0 | 0.0 | 0.0 | 0.0 | 0.0 | 0.0 | 0.0 | 0.0 | 0.0 |
| Gabra6 | 0.0 | 0.0 | 0.0 | 0.0 | 0.0 | 0.0 | 0.0 | 0.0 | 0.0 | 0.0 | 0.0 | 0.0 |
| Gast | 0.0 | 0.0 | 0.0 | 0.0 | 0.0 | 0.0 | 0.0 | 0.0 | 0.0 | 0.0 | 0.0 | 0.0 |
| Gata1 | 0.0 | 0.0 | 0.0 | 0.0 | 0.0 | 0.0 | 0.0 | 0.0 | 0.0 | 0.0 | 0.0 | 0.0 |
| Gcg | 0.0 | 0.0 | 0.0 | 0.0 | 0.0 | 0.0 | 0.0 | 0.0 | 0.0 | 0.0 | 0.0 | 0.0 |
| Gfi1b | 0.0 | 0.0 | 0.0 | 0.0 | 0.0 | 0.0 | 0.0 | 0.0 | 0.0 | 0.0 | 0.0 | 0.0 |
| Ghrhr | 0.0 | 0.0 | 0.0 | 0.0 | 0.0 | 0.0 | 0.0 | 0.0 | 0.0 | 0.0 | 0.0 | 0.0 |
| Gif | 0.0 | 0.0 | 0.0 | 0.0 | 0.0 | 0.0 | 0.0 | 0.0 | 0.0 | 0.0 | 0.0 | 0.0 |
| Gimap3 | 0.0 | 0.0 | 0.0 | 0.0 | 0.0 | 0.0 | 0.0 | 0.0 | 0.0 | 0.0 | 0.0 | 0.0 |
| Gimap7 | 0.0 | 0.0 | 0.0 | 0.0 | 0.0 | 0.0 | 0.0 | 0.0 | 0.0 | 0.0 | 0.0 | 0.0 |
| Gjb6 | 0.0 | 0.0 | 0.0 | 0.0 | 0.0 | 0.0 | 0.0 | 0.0 | 0.0 | 0.0 | 0.0 | 0.0 |
| Gjd2 | 0.0 | 0.0 | 0.0 | 0.0 | 0.0 | 0.0 | 0.0 | 0.0 | 0.0 | 0.0 | 0.0 | 0.0 |
| Gkn1 | 0.0 | 0.0 | 0.0 | 0.0 | 0.0 | 0.0 | 0.0 | 0.0 | 0.0 | 0.0 | 0.0 | 0.0 |
| Gkn2 | 0.0 | 0.0 | 0.0 | 0.0 | 0.0 | 0.0 | 0.0 | 0.0 | 0.0 | 0.0 | 0.0 | 0.0 |
| Glod5 | 0.0 | 0.0 | 0.0 | 0.0 | 0.0 | 0.0 | 0.0 | 0.0 | 0.0 | 0.0 | 0.0 | 0.0 |
| Glyatl3 | 0.0 | 0.0 | 0.0 | 0.0 | 0.0 | 0.0 | 0.0 | 0.0 | 0.0 | 0.0 | 0.0 | 0.0 |
| Glycam1 | 0.0 | 0.0 | 0.0 | 0.0 | 0.0 | 0.0 | 0.0 | 0.0 | 0.0 | 0.0 | 0.0 | 0.0 |
| Gm10024 | 0.0 | 0.0 | 0.0 | 0.0 | 0.0 | 0.0 | 0.0 | 0.0 | 0.0 | 0.0 | 0.0 | 0.0 |
| Gm10081 | 0.0 | 0.0 | 0.0 | 0.0 | 0.0 | 0.0 | 0.0 | 0.0 | 0.0 | 0.0 | 0.0 | 0.0 |
| Gm10100 | 0.0 | 0.0 | 0.0 | 0.0 | 0.0 | 0.0 | 0.0 | 0.0 | 0.0 | 0.0 | 0.0 | 0.0 |
| Gm10104 | 0.0 | 0.0 | 0.0 | 0.0 | 0.0 | 0.0 | 0.0 | 0.0 | 0.0 | 0.0 | 0.0 | 0.0 |
| Gm10142 | 0.0 | 0.0 | 0.0 | 0.0 | 0.0 | 0.0 | 0.0 | 0.0 | 0.0 | 0.0 | 0.0 | 0.0 |
| Gm10228 | 0.0 | 0.0 | 0.0 | 0.0 | 0.0 | 0.0 | 0.0 | 0.0 | 0.0 | 0.0 | 0.0 | 0.0 |
| Gm10229 | 0.0 | 0.0 | 0.0 | 0.0 | 0.0 | 0.0 | 0.0 | 0.0 | 0.0 | 0.0 | 0.0 | 0.0 |
| Gm10267 | 0.0 | 0.0 | 0.0 | 0.0 | 0.0 | 0.0 | 0.0 | 0.0 | 0.0 | 0.0 | 0.0 | 0.0 |
| Gm10272 | 0.0 | 0.0 | 0.0 | 0.0 | 0.0 | 0.0 | 0.0 | 0.0 | 0.0 | 0.0 | 0.0 | 0.0 |
| Gm10319 | 0.0 | 0.0 | 0.0 | 0.0 | 0.0 | 0.0 | 0.0 | 0.0 | 0.0 | 0.0 | 0.0 | 0.0 |
| Gm10334 | 0.0 | 0.0 | 0.0 | 0.0 | 0.0 | 0.0 | 0.0 | 0.0 | 0.0 | 0.0 | 0.0 | 0.0 |
| Gm10421 | 0.0 | 0.0 | 0.0 | 0.0 | 0.0 | 0.0 | 0.0 | 0.0 | 0.0 | 0.0 | 0.0 | 0.0 |
| Gm10445 | 0.0 | 0.0 | 0.0 | 0.0 | 0.0 | 0.0 | 0.0 | 0.0 | 0.0 | 0.0 | 0.0 | 0.0 |
| Gm10510 | 0.0 | 0.0 | 0.0 | 0.0 | 0.0 | 0.0 | 0.0 | 0.0 | 0.0 | 0.0 | 0.0 | 0.0 |
| Gm10556 | 0.0 | 0.0 | 0.0 | 0.0 | 0.0 | 0.0 | 0.0 | 0.0 | 0.0 | 0.0 | 0.0 | 0.0 |

| | | | | | | | | | | | | |
|---|---|---|---|---|---|---|---|---|---|---|---|---|
| Gm10619 | 0.0 | 0.0 | 0.0 | 0.0 | 0.0 | 0.0 | 0.0 | 0.0 | 0.0 | 0.0 | 0.0 | 0.0 |
| Gm10637 | 0.0 | 0.0 | 0.0 | 0.0 | 0.0 | 0.0 | 0.0 | 0.0 | 0.0 | 0.0 | 0.0 | 0.0 |
| Gm10649 | 0.0 | 0.0 | 0.0 | 0.0 | 0.0 | 0.0 | 0.0 | 0.0 | 0.0 | 0.0 | 0.0 | 0.0 |
| Gm10665 | 0.0 | 0.0 | 0.0 | 0.0 | 0.0 | 0.0 | 0.0 | 0.0 | 0.0 | 0.0 | 0.0 | 0.0 |
| Gm10670 | 0.0 | 0.0 | 0.0 | 0.0 | 0.0 | 0.0 | 0.0 | 0.0 | 0.0 | 0.0 | 0.0 | 0.0 |
| Gm10696 | 0.0 | 0.0 | 0.0 | 0.0 | 0.0 | 0.0 | 0.0 | 0.0 | 0.0 | 0.0 | 0.0 | 0.0 |
| Gm10745 | 0.0 | 0.0 | 0.0 | 0.0 | 0.0 | 0.0 | 0.0 | 0.0 | 0.0 | 0.0 | 0.0 | 0.0 |
| Gm10782 | 0.0 | 0.0 | 0.0 | 0.0 | 0.0 | 0.0 | 0.0 | 0.0 | 0.0 | 0.0 | 0.0 | 0.0 |
| Gm10787 | 0.0 | 0.0 | 0.0 | 0.0 | 0.0 | 0.0 | 0.0 | 0.0 | 0.0 | 0.0 | 0.0 | 0.0 |
| Gm11186 | 0.0 | 0.0 | 0.0 | 0.0 | 0.0 | 0.0 | 0.0 | 0.0 | 0.0 | 0.0 | 0.0 | 0.0 |
| Gm11240 | 0.0 | 0.0 | 0.0 | 0.0 | 0.0 | 0.0 | 0.0 | 0.0 | 0.0 | 0.0 | 0.0 | 0.0 |
| Gm11426 | 0.0 | 0.0 | 0.0 | 0.0 | 0.0 | 0.0 | 0.0 | 0.0 | 0.0 | 0.0 | 0.0 | 0.0 |
| Gm11468 | 0.0 | 0.0 | 0.0 | 0.0 | 0.0 | 0.0 | 0.0 | 0.0 | 0.0 | 0.0 | 0.0 | 0.0 |
| Gm11559 | 0.0 | 0.0 | 0.0 | 0.0 | 0.0 | 0.0 | 0.0 | 0.0 | 0.0 | 0.0 | 0.0 | 0.0 |
| Gm11562 | 0.0 | 0.0 | 0.0 | 0.0 | 0.0 | 0.0 | 0.0 | 0.0 | 0.0 | 0.0 | 0.0 | 0.0 |
| Gm11563 | 0.0 | 0.0 | 0.0 | 0.0 | 0.0 | 0.0 | 0.0 | 0.0 | 0.0 | 0.0 | 0.0 | 0.0 |
| Gm11564 | 0.0 | 0.0 | 0.0 | 0.0 | 0.0 | 0.0 | 0.0 | 0.0 | 0.0 | 0.0 | 0.0 | 0.0 |
| Gm11567 | 0.0 | 0.0 | 0.0 | 0.0 | 0.0 | 0.0 | 0.0 | 0.0 | 0.0 | 0.0 | 0.0 | 0.0 |
| Gm11568 | 0.0 | 0.0 | 0.0 | 0.0 | 0.0 | 0.0 | 0.0 | 0.0 | 0.0 | 0.0 | 0.0 | 0.0 |
| Gm11569 | 0.0 | 0.0 | 0.0 | 0.0 | 0.0 | 0.0 | 0.0 | 0.0 | 0.0 | 0.0 | 0.0 | 0.0 |
| Gm11570 | 0.0 | 0.0 | 0.0 | 0.0 | 0.0 | 0.0 | 0.0 | 0.0 | 0.0 | 0.0 | 0.0 | 0.0 |
| Gm11595 | 0.0 | 0.0 | 0.0 | 0.0 | 0.0 | 0.0 | 0.0 | 0.0 | 0.0 | 0.0 | 0.0 | 0.0 |
| Gm11596 | 0.0 | 0.0 | 0.0 | 0.0 | 0.0 | 0.0 | 0.0 | 0.0 | 0.0 | 0.0 | 0.0 | 0.0 |
| Gm11756 | 0.0 | 0.0 | 0.0 | 0.0 | 0.0 | 0.0 | 0.0 | 0.0 | 0.0 | 0.0 | 0.0 | 0.0 |
| Gm11937 | 0.0 | 0.0 | 0.0 | 0.0 | 0.0 | 0.0 | 0.0 | 0.0 | 0.0 | 0.0 | 0.0 | 0.0 |
| Gm11938 | 0.0 | 0.0 | 0.0 | 0.0 | 0.0 | 0.0 | 0.0 | 0.0 | 0.0 | 0.0 | 0.0 | 0.0 |
| Gm11981 | 0.0 | 0.0 | 0.0 | 0.0 | 0.0 | 0.0 | 0.0 | 0.0 | 0.0 | 0.0 | 0.0 | 0.0 |
| Gm11985 | 0.0 | 0.0 | 0.0 | 0.0 | 0.0 | 0.0 | 0.0 | 0.0 | 0.0 | 0.0 | 0.0 | 0.0 |
| Gm12 | 0.0 | 0.0 | 0.0 | 0.0 | 0.0 | 0.0 | 0.0 | 0.0 | 0.0 | 0.0 | 0.0 | 0.0 |
| Gm12169 | 0.0 | 0.0 | 0.0 | 0.0 | 0.0 | 0.0 | 0.0 | 0.0 | 0.0 | 0.0 | 0.0 | 0.0 |
| Gm12171 | 0.0 | 0.0 | 0.0 | 0.0 | 0.0 | 0.0 | 0.0 | 0.0 | 0.0 | 0.0 | 0.0 | 0.0 |
| Gm12228 | 0.0 | 0.0 | 0.0 | 0.0 | 0.0 | 0.0 | 0.0 | 0.0 | 0.0 | 0.0 | 0.0 | 0.0 |
| Gm12298 | 0.0 | 0.0 | 0.0 | 0.0 | 0.0 | 0.0 | 0.0 | 0.0 | 0.0 | 0.0 | 0.0 | 0.0 |
| Gm12633 | 0.0 | 0.0 | 0.0 | 0.0 | 0.0 | 0.0 | 0.0 | 0.0 | 0.0 | 0.0 | 0.0 | 0.0 |
| Gm12789 | 0.0 | 0.0 | 0.0 | 0.0 | 0.0 | 0.0 | 0.0 | 0.0 | 0.0 | 0.0 | 0.0 | 0.0 |
| Gm12886 | 0.0 | 0.0 | 0.0 | 0.0 | 0.0 | 0.0 | 0.0 | 0.0 | 0.0 | 0.0 | 0.0 | 0.0 |
| Gm12887 | 0.0 | 0.0 | 0.0 | 0.0 | 0.0 | 0.0 | 0.0 | 0.0 | 0.0 | 0.0 | 0.0 | 0.0 |
| Gm12888 | 0.0 | 0.0 | 0.0 | 0.0 | 0.0 | 0.0 | 0.0 | 0.0 | 0.0 | 0.0 | 0.0 | 0.0 |
| Gm13023 | 0.0 | 0.0 | 0.0 | 0.0 | 0.0 | 0.0 | 0.0 | 0.0 | 0.0 | 0.0 | 0.0 | 0.0 |
| Gm13037 | 0.0 | 0.0 | 0.0 | 0.0 | 0.0 | 0.0 | 0.0 | 0.0 | 0.0 | 0.0 | 0.0 | 0.0 |
| Gm13078 | 0.0 | 0.0 | 0.0 | 0.0 | 0.0 | 0.0 | 0.0 | 0.0 | 0.0 | 0.0 | 0.0 | 0.0 |
| Gm13083 | 0.0 | 0.0 | 0.0 | 0.0 | 0.0 | 0.0 | 0.0 | 0.0 | 0.0 | 0.0 | 0.0 | 0.0 |
| Gm13084 | 0.0 | 0.0 | 0.0 | 0.0 | 0.0 | 0.0 | 0.0 | 0.0 | 0.0 | 0.0 | 0.0 | 0.0 |
| Gm13088 | 0.0 | 0.0 | 0.0 | 0.0 | 0.0 | 0.0 | 0.0 | 0.0 | 0.0 | 0.0 | 0.0 | 0.0 |
| Gm13102 | 0.0 | 0.0 | 0.0 | 0.0 | 0.0 | 0.0 | 0.0 | 0.0 | 0.0 | 0.0 | 0.0 | 0.0 |
| Gm13103 | 0.0 | 0.0 | 0.0 | 0.0 | 0.0 | 0.0 | 0.0 | 0.0 | 0.0 | 0.0 | 0.0 | 0.0 |
| Gm13109 | 0.0 | 0.0 | 0.0 | 0.0 | 0.0 | 0.0 | 0.0 | 0.0 | 0.0 | 0.0 | 0.0 | 0.0 |
| Gm13124 | 0.0 | 0.0 | 0.0 | 0.0 | 0.0 | 0.0 | 0.0 | 0.0 | 0.0 | 0.0 | 0.0 | 0.0 |
| Gm13125 | 0.0 | 0.0 | 0.0 | 0.0 | 0.0 | 0.0 | 0.0 | 0.0 | 0.0 | 0.0 | 0.0 | 0.0 |
| Gm1322 | 0.0 | 0.0 | 0.0 | 0.0 | 0.0 | 0.0 | 0.0 | 0.0 | 0.0 | 0.0 | 0.0 | 0.0 |
| Gm13280 | 0.0 | 0.0 | 0.0 | 0.0 | 0.0 | 0.0 | 0.0 | 0.0 | 0.0 | 0.0 | 0.0 | 0.0 |
| Gm13304 | 0.0 | 0.0 | 0.0 | 0.0 | 0.0 | 0.0 | 0.0 | 0.0 | 0.0 | 0.0 | 0.0 | 0.0 |
| Gm13539 | 0.0 | 0.0 | 0.0 | 0.0 | 0.0 | 0.0 | 0.0 | 0.0 | 0.0 | 0.0 | 0.0 | 0.0 |
| Gm14015 | 0.0 | 0.0 | 0.0 | 0.0 | 0.0 | 0.0 | 0.0 | 0.0 | 0.0 | 0.0 | 0.0 | 0.0 |
| Gm14047 | 0.0 | 0.0 | 0.0 | 0.0 | 0.0 | 0.0 | 0.0 | 0.0 | 0.0 | 0.0 | 0.0 | 0.0 |
| Gm14347 | 0.0 | 0.0 | 0.0 | 0.0 | 0.0 | 0.0 | 0.0 | 0.0 | 0.0 | 0.0 | 0.0 | 0.0 |
| Gm14374 | 0.0 | 0.0 | 0.0 | 0.0 | 0.0 | 0.0 | 0.0 | 0.0 | 0.0 | 0.0 | 0.0 | 0.0 |

Excluded Genes

| | | | | | | | | | | | | |
|---|---|---|---|---|---|---|---|---|---|---|---|---|
| Gm14458 | 0.0 | 0.0 | 0.0 | 0.0 | 0.0 | 0.0 | 0.0 | 0.0 | 0.0 | 0.0 | 0.0 | 0.0 |
| Gm14459 | 0.0 | 0.0 | 0.0 | 0.0 | 0.0 | 0.0 | 0.0 | 0.0 | 0.0 | 0.0 | 0.0 | 0.0 |
| Gm14476 | 0.0 | 0.0 | 0.0 | 0.0 | 0.0 | 0.0 | 0.0 | 0.0 | 0.0 | 0.0 | 0.0 | 0.0 |
| Gm14511 | 0.0 | 0.0 | 0.0 | 0.0 | 0.0 | 0.0 | 0.0 | 0.0 | 0.0 | 0.0 | 0.0 | 0.0 |
| Gm14632 | 0.0 | 0.0 | 0.0 | 0.0 | 0.0 | 0.0 | 0.0 | 0.0 | 0.0 | 0.0 | 0.0 | 0.0 |
| Gm14743 | 0.0 | 0.0 | 0.0 | 0.0 | 0.0 | 0.0 | 0.0 | 0.0 | 0.0 | 0.0 | 0.0 | 0.0 |
| Gm14744 | 0.0 | 0.0 | 0.0 | 0.0 | 0.0 | 0.0 | 0.0 | 0.0 | 0.0 | 0.0 | 0.0 | 0.0 |
| Gm14781 | 0.0 | 0.0 | 0.0 | 0.0 | 0.0 | 0.0 | 0.0 | 0.0 | 0.0 | 0.0 | 0.0 | 0.0 |
| Gm14851 | 0.0 | 0.0 | 0.0 | 0.0 | 0.0 | 0.0 | 0.0 | 0.0 | 0.0 | 0.0 | 0.0 | 0.0 |
| Gm14920 | 0.0 | 0.0 | 0.0 | 0.0 | 0.0 | 0.0 | 0.0 | 0.0 | 0.0 | 0.0 | 0.0 | 0.0 |
| Gm1527 | 0.0 | 0.0 | 0.0 | 0.0 | 0.0 | 0.0 | 0.0 | 0.0 | 0.0 | 0.0 | 0.0 | 0.0 |
| Gm15284 | 0.0 | 0.0 | 0.0 | 0.0 | 0.0 | 0.0 | 0.0 | 0.0 | 0.0 | 0.0 | 0.0 | 0.0 |
| Gm15292 | 0.0 | 0.0 | 0.0 | 0.0 | 0.0 | 0.0 | 0.0 | 0.0 | 0.0 | 0.0 | 0.0 | 0.0 |
| Gm15293 | 0.0 | 0.0 | 0.0 | 0.0 | 0.0 | 0.0 | 0.0 | 0.0 | 0.0 | 0.0 | 0.0 | 0.0 |
| Gm15299 | 0.0 | 0.0 | 0.0 | 0.0 | 0.0 | 0.0 | 0.0 | 0.0 | 0.0 | 0.0 | 0.0 | 0.0 |
| Gm15308 | 0.0 | 0.0 | 0.0 | 0.0 | 0.0 | 0.0 | 0.0 | 0.0 | 0.0 | 0.0 | 0.0 | 0.0 |
| Gm15315 | 0.0 | 0.0 | 0.0 | 0.0 | 0.0 | 0.0 | 0.0 | 0.0 | 0.0 | 0.0 | 0.0 | 0.0 |
| Gm15348 | 0.0 | 0.0 | 0.0 | 0.0 | 0.0 | 0.0 | 0.0 | 0.0 | 0.0 | 0.0 | 0.0 | 0.0 |
| Gm15386 | 0.0 | 0.0 | 0.0 | 0.0 | 0.0 | 0.0 | 0.0 | 0.0 | 0.0 | 0.0 | 0.0 | 0.0 |
| Gm15412 | 0.0 | 0.0 | 0.0 | 0.0 | 0.0 | 0.0 | 0.0 | 0.0 | 0.0 | 0.0 | 0.0 | 0.0 |
| Gm1553 | 0.0 | 0.0 | 0.0 | 0.0 | 0.0 | 0.0 | 0.0 | 0.0 | 0.0 | 0.0 | 0.0 | 0.0 |
| Gm156 | 0.0 | 0.0 | 0.0 | 0.0 | 0.0 | 0.0 | 0.0 | 0.0 | 0.0 | 0.0 | 0.0 | 0.0 |
| Gm1587 | 0.0 | 0.0 | 0.0 | 0.0 | 0.0 | 0.0 | 0.0 | 0.0 | 0.0 | 0.0 | 0.0 | 0.0 |
| Gm15881 | 0.0 | 0.0 | 0.0 | 0.0 | 0.0 | 0.0 | 0.0 | 0.0 | 0.0 | 0.0 | 0.0 | 0.0 |
| Gm15997 | 0.0 | 0.0 | 0.0 | 0.0 | 0.0 | 0.0 | 0.0 | 0.0 | 0.0 | 0.0 | 0.0 | 0.0 |
| Gm16063 | 0.0 | 0.0 | 0.0 | 0.0 | 0.0 | 0.0 | 0.0 | 0.0 | 0.0 | 0.0 | 0.0 | 0.0 |
| Gm16130 | 0.0 | 0.0 | 0.0 | 0.0 | 0.0 | 0.0 | 0.0 | 0.0 | 0.0 | 0.0 | 0.0 | 0.0 |
| Gm16291 | 0.0 | 0.0 | 0.0 | 0.0 | 0.0 | 0.0 | 0.0 | 0.0 | 0.0 | 0.0 | 0.0 | 0.0 |
| Gm16551 | 0.0 | 0.0 | 0.0 | 0.0 | 0.0 | 0.0 | 0.0 | 0.0 | 0.0 | 0.0 | 0.0 | 0.0 |
| Gm1715 | 0.0 | 0.0 | 0.0 | 0.0 | 0.0 | 0.0 | 0.0 | 0.0 | 0.0 | 0.0 | 0.0 | 0.0 |
| Gm17252 | 0.0 | 0.0 | 0.0 | 0.0 | 0.0 | 0.0 | 0.0 | 0.0 | 0.0 | 0.0 | 0.0 | 0.0 |
| Gm17495 | 0.0 | 0.0 | 0.0 | 0.0 | 0.0 | 0.0 | 0.0 | 0.0 | 0.0 | 0.0 | 0.0 | 0.0 |
| Gm17677 | 0.0 | 0.0 | 0.0 | 0.0 | 0.0 | 0.0 | 0.0 | 0.0 | 0.0 | 0.0 | 0.0 | 0.0 |
| Gm17689 | 0.0 | 0.0 | 0.0 | 0.0 | 0.0 | 0.0 | 0.0 | 0.0 | 0.0 | 0.0 | 0.0 | 0.0 |
| Gm17727 | 0.0 | 0.0 | 0.0 | 0.0 | 0.0 | 0.0 | 0.0 | 0.0 | 0.0 | 0.0 | 0.0 | 0.0 |
| Gm17830 | 0.0 | 0.0 | 0.0 | 0.0 | 0.0 | 0.0 | 0.0 | 0.0 | 0.0 | 0.0 | 0.0 | 0.0 |
| Gm19303 | 0.0 | 0.0 | 0.0 | 0.0 | 0.0 | 0.0 | 0.0 | 0.0 | 0.0 | 0.0 | 0.0 | 0.0 |
| Gm19402 | 0.0 | 0.0 | 0.0 | 0.0 | 0.0 | 0.0 | 0.0 | 0.0 | 0.0 | 0.0 | 0.0 | 0.0 |
| Gm1965 | 0.0 | 0.0 | 0.0 | 0.0 | 0.0 | 0.0 | 0.0 | 0.0 | 0.0 | 0.0 | 0.0 | 0.0 |
| Gm19668 | 0.0 | 0.0 | 0.0 | 0.0 | 0.0 | 0.0 | 0.0 | 0.0 | 0.0 | 0.0 | 0.0 | 0.0 |
| Gm19784 | 0.0 | 0.0 | 0.0 | 0.0 | 0.0 | 0.0 | 0.0 | 0.0 | 0.0 | 0.0 | 0.0 | 0.0 |
| Gm19990 | 0.0 | 0.0 | 0.0 | 0.0 | 0.0 | 0.0 | 0.0 | 0.0 | 0.0 | 0.0 | 0.0 | 0.0 |
| Gm20268 | 0.0 | 0.0 | 0.0 | 0.0 | 0.0 | 0.0 | 0.0 | 0.0 | 0.0 | 0.0 | 0.0 | 0.0 |
| Gm20356 | 0.0 | 0.0 | 0.0 | 0.0 | 0.0 | 0.0 | 0.0 | 0.0 | 0.0 | 0.0 | 0.0 | 0.0 |
| Gm20408 | 0.0 | 0.0 | 0.0 | 0.0 | 0.0 | 0.0 | 0.0 | 0.0 | 0.0 | 0.0 | 0.0 | 0.0 |
| Gm20410 | 0.0 | 0.0 | 0.0 | 0.0 | 0.0 | 0.0 | 0.0 | 0.0 | 0.0 | 0.0 | 0.0 | 0.0 |
| Gm20516 | 0.0 | 0.0 | 0.0 | 0.0 | 0.0 | 0.0 | 0.0 | 0.0 | 0.0 | 0.0 | 0.0 | 0.0 |
| Gm20579 | 0.0 | 0.0 | 0.0 | 0.0 | 0.0 | 0.0 | 0.0 | 0.0 | 0.0 | 0.0 | 0.0 | 0.0 |
| Gm20580 | 0.0 | 0.0 | 0.0 | 0.0 | 0.0 | 0.0 | 0.0 | 0.0 | 0.0 | 0.0 | 0.0 | 0.0 |
| Gm20581 | 0.0 | 0.0 | 0.0 | 0.0 | 0.0 | 0.0 | 0.0 | 0.0 | 0.0 | 0.0 | 0.0 | 0.0 |
| Gm20611 | 0.0 | 0.0 | 0.0 | 0.0 | 0.0 | 0.0 | 0.0 | 0.0 | 0.0 | 0.0 | 0.0 | 0.0 |
| Gm20741 | 0.0 | 0.0 | 0.0 | 0.0 | 0.0 | 0.0 | 0.0 | 0.0 | 0.0 | 0.0 | 0.0 | 0.0 |
| Gm20750 | 0.0 | 0.0 | 0.0 | 0.0 | 0.0 | 0.0 | 0.0 | 0.0 | 0.0 | 0.0 | 0.0 | 0.0 |
| Gm20759 | 0.0 | 0.0 | 0.0 | 0.0 | 0.0 | 0.0 | 0.0 | 0.0 | 0.0 | 0.0 | 0.0 | 0.0 |
| Gm20822 | 0.0 | 0.0 | 0.0 | 0.0 | 0.0 | 0.0 | 0.0 | 0.0 | 0.0 | 0.0 | 0.0 | 0.0 |
| Gm20826 | 0.0 | 0.0 | 0.0 | 0.0 | 0.0 | 0.0 | 0.0 | 0.0 | 0.0 | 0.0 | 0.0 | 0.0 |
| Gm21002 | 0.0 | 0.0 | 0.0 | 0.0 | 0.0 | 0.0 | 0.0 | 0.0 | 0.0 | 0.0 | 0.0 | 0.0 |

Excluded Genes

| | | | | | | | | | | | |
|---|---|---|---|---|---|---|---|---|---|---|---|
| Gm21276 | 0.0 | 0.0 | 0.0 | 0.0 | 0.0 | 0.0 | 0.0 | 0.0 | 0.0 | 0.0 | 0.0 | 0.0 |
| Gm21498 | 0.0 | 0.0 | 0.0 | 0.0 | 0.0 | 0.0 | 0.0 | 0.0 | 0.0 | 0.0 | 0.0 | 0.0 |
| Gm21541 | 0.0 | 0.0 | 0.0 | 0.0 | 0.0 | 0.0 | 0.0 | 0.0 | 0.0 | 0.0 | 0.0 | 0.0 |
| Gm2176 | 0.0 | 0.0 | 0.0 | 0.0 | 0.0 | 0.0 | 0.0 | 0.0 | 0.0 | 0.0 | 0.0 | 0.0 |
| Gm21945 | 0.0 | 0.0 | 0.0 | 0.0 | 0.0 | 0.0 | 0.0 | 0.0 | 0.0 | 0.0 | 0.0 | 0.0 |
| Gm21946 | 0.0 | 0.0 | 0.0 | 0.0 | 0.0 | 0.0 | 0.0 | 0.0 | 0.0 | 0.0 | 0.0 | 0.0 |
| Gm21950 | 0.0 | 0.0 | 0.0 | 0.0 | 0.0 | 0.0 | 0.0 | 0.0 | 0.0 | 0.0 | 0.0 | 0.0 |
| Gm21951 | 0.0 | 0.0 | 0.0 | 0.0 | 0.0 | 0.0 | 0.0 | 0.0 | 0.0 | 0.0 | 0.0 | 0.0 |
| Gm2506 | 0.0 | 0.0 | 0.0 | 0.0 | 0.0 | 0.0 | 0.0 | 0.0 | 0.0 | 0.0 | 0.0 | 0.0 |
| Gm2516 | 0.0 | 0.0 | 0.0 | 0.0 | 0.0 | 0.0 | 0.0 | 0.0 | 0.0 | 0.0 | 0.0 | 0.0 |
| Gm2694 | 0.0 | 0.0 | 0.0 | 0.0 | 0.0 | 0.0 | 0.0 | 0.0 | 0.0 | 0.0 | 0.0 | 0.0 |
| Gm2696 | 0.0 | 0.0 | 0.0 | 0.0 | 0.0 | 0.0 | 0.0 | 0.0 | 0.0 | 0.0 | 0.0 | 0.0 |
| Gm2721 | 0.0 | 0.0 | 0.0 | 0.0 | 0.0 | 0.0 | 0.0 | 0.0 | 0.0 | 0.0 | 0.0 | 0.0 |
| Gm2825 | 0.0 | 0.0 | 0.0 | 0.0 | 0.0 | 0.0 | 0.0 | 0.0 | 0.0 | 0.0 | 0.0 | 0.0 |
| Gm2863 | 0.0 | 0.0 | 0.0 | 0.0 | 0.0 | 0.0 | 0.0 | 0.0 | 0.0 | 0.0 | 0.0 | 0.0 |
| Gm3238 | 0.0 | 0.0 | 0.0 | 0.0 | 0.0 | 0.0 | 0.0 | 0.0 | 0.0 | 0.0 | 0.0 | 0.0 |
| Gm3279 | 0.0 | 0.0 | 0.0 | 0.0 | 0.0 | 0.0 | 0.0 | 0.0 | 0.0 | 0.0 | 0.0 | 0.0 |
| Gm3285 | 0.0 | 0.0 | 0.0 | 0.0 | 0.0 | 0.0 | 0.0 | 0.0 | 0.0 | 0.0 | 0.0 | 0.0 |
| Gm3402 | 0.0 | 0.0 | 0.0 | 0.0 | 0.0 | 0.0 | 0.0 | 0.0 | 0.0 | 0.0 | 0.0 | 0.0 |
| Gm3409 | 0.0 | 0.0 | 0.0 | 0.0 | 0.0 | 0.0 | 0.0 | 0.0 | 0.0 | 0.0 | 0.0 | 0.0 |
| Gm3415 | 0.0 | 0.0 | 0.0 | 0.0 | 0.0 | 0.0 | 0.0 | 0.0 | 0.0 | 0.0 | 0.0 | 0.0 |
| Gm3428 | 0.0 | 0.0 | 0.0 | 0.0 | 0.0 | 0.0 | 0.0 | 0.0 | 0.0 | 0.0 | 0.0 | 0.0 |
| Gm3434 | 0.0 | 0.0 | 0.0 | 0.0 | 0.0 | 0.0 | 0.0 | 0.0 | 0.0 | 0.0 | 0.0 | 0.0 |
| Gm362 | 0.0 | 0.0 | 0.0 | 0.0 | 0.0 | 0.0 | 0.0 | 0.0 | 0.0 | 0.0 | 0.0 | 0.0 |
| Gm3646 | 0.0 | 0.0 | 0.0 | 0.0 | 0.0 | 0.0 | 0.0 | 0.0 | 0.0 | 0.0 | 0.0 | 0.0 |
| Gm3701 | 0.0 | 0.0 | 0.0 | 0.0 | 0.0 | 0.0 | 0.0 | 0.0 | 0.0 | 0.0 | 0.0 | 0.0 |
| Gm382 | 0.0 | 0.0 | 0.0 | 0.0 | 0.0 | 0.0 | 0.0 | 0.0 | 0.0 | 0.0 | 0.0 | 0.0 |
| Gm3985 | 0.0 | 0.0 | 0.0 | 0.0 | 0.0 | 0.0 | 0.0 | 0.0 | 0.0 | 0.0 | 0.0 | 0.0 |
| Gm41 | 0.0 | 0.0 | 0.0 | 0.0 | 0.0 | 0.0 | 0.0 | 0.0 | 0.0 | 0.0 | 0.0 | 0.0 |
| Gm4175 | 0.0 | 0.0 | 0.0 | 0.0 | 0.0 | 0.0 | 0.0 | 0.0 | 0.0 | 0.0 | 0.0 | 0.0 |
| Gm4177 | 0.0 | 0.0 | 0.0 | 0.0 | 0.0 | 0.0 | 0.0 | 0.0 | 0.0 | 0.0 | 0.0 | 0.0 |
| Gm4179 | 0.0 | 0.0 | 0.0 | 0.0 | 0.0 | 0.0 | 0.0 | 0.0 | 0.0 | 0.0 | 0.0 | 0.0 |
| Gm4201 | 0.0 | 0.0 | 0.0 | 0.0 | 0.0 | 0.0 | 0.0 | 0.0 | 0.0 | 0.0 | 0.0 | 0.0 |
| Gm4214 | 0.0 | 0.0 | 0.0 | 0.0 | 0.0 | 0.0 | 0.0 | 0.0 | 0.0 | 0.0 | 0.0 | 0.0 |
| Gm4216 | 0.0 | 0.0 | 0.0 | 0.0 | 0.0 | 0.0 | 0.0 | 0.0 | 0.0 | 0.0 | 0.0 | 0.0 |
| Gm4251 | 0.0 | 0.0 | 0.0 | 0.0 | 0.0 | 0.0 | 0.0 | 0.0 | 0.0 | 0.0 | 0.0 | 0.0 |
| Gm4265 | 0.0 | 0.0 | 0.0 | 0.0 | 0.0 | 0.0 | 0.0 | 0.0 | 0.0 | 0.0 | 0.0 | 0.0 |
| Gm4345 | 0.0 | 0.0 | 0.0 | 0.0 | 0.0 | 0.0 | 0.0 | 0.0 | 0.0 | 0.0 | 0.0 | 0.0 |
| Gm436 | 0.0 | 0.0 | 0.0 | 0.0 | 0.0 | 0.0 | 0.0 | 0.0 | 0.0 | 0.0 | 0.0 | 0.0 |
| Gm44 | 0.0 | 0.0 | 0.0 | 0.0 | 0.0 | 0.0 | 0.0 | 0.0 | 0.0 | 0.0 | 0.0 | 0.0 |
| Gm4498 | 0.0 | 0.0 | 0.0 | 0.0 | 0.0 | 0.0 | 0.0 | 0.0 | 0.0 | 0.0 | 0.0 | 0.0 |
| Gm4559 | 0.0 | 0.0 | 0.0 | 0.0 | 0.0 | 0.0 | 0.0 | 0.0 | 0.0 | 0.0 | 0.0 | 0.0 |
| Gm4566 | 0.0 | 0.0 | 0.0 | 0.0 | 0.0 | 0.0 | 0.0 | 0.0 | 0.0 | 0.0 | 0.0 | 0.0 |
| Gm4710 | 0.0 | 0.0 | 0.0 | 0.0 | 0.0 | 0.0 | 0.0 | 0.0 | 0.0 | 0.0 | 0.0 | 0.0 |
| Gm4719 | 0.0 | 0.0 | 0.0 | 0.0 | 0.0 | 0.0 | 0.0 | 0.0 | 0.0 | 0.0 | 0.0 | 0.0 |
| Gm4763 | 0.0 | 0.0 | 0.0 | 0.0 | 0.0 | 0.0 | 0.0 | 0.0 | 0.0 | 0.0 | 0.0 | 0.0 |
| Gm4794 | 0.0 | 0.0 | 0.0 | 0.0 | 0.0 | 0.0 | 0.0 | 0.0 | 0.0 | 0.0 | 0.0 | 0.0 |
| Gm4836 | 0.0 | 0.0 | 0.0 | 0.0 | 0.0 | 0.0 | 0.0 | 0.0 | 0.0 | 0.0 | 0.0 | 0.0 |
| Gm4846 | 0.0 | 0.0 | 0.0 | 0.0 | 0.0 | 0.0 | 0.0 | 0.0 | 0.0 | 0.0 | 0.0 | 0.0 |
| Gm4872 | 0.0 | 0.0 | 0.0 | 0.0 | 0.0 | 0.0 | 0.0 | 0.0 | 0.0 | 0.0 | 0.0 | 0.0 |
| Gm4906 | 0.0 | 0.0 | 0.0 | 0.0 | 0.0 | 0.0 | 0.0 | 0.0 | 0.0 | 0.0 | 0.0 | 0.0 |
| Gm4907 | 0.0 | 0.0 | 0.0 | 0.0 | 0.0 | 0.0 | 0.0 | 0.0 | 0.0 | 0.0 | 0.0 | 0.0 |
| Gm4926 | 0.0 | 0.0 | 0.0 | 0.0 | 0.0 | 0.0 | 0.0 | 0.0 | 0.0 | 0.0 | 0.0 | 0.0 |
| Gm4971 | 0.0 | 0.0 | 0.0 | 0.0 | 0.0 | 0.0 | 0.0 | 0.0 | 0.0 | 0.0 | 0.0 | 0.0 |
| Gm5 | 0.0 | 0.0 | 0.0 | 0.0 | 0.0 | 0.0 | 0.0 | 0.0 | 0.0 | 0.0 | 0.0 | 0.0 |
| Gm5071 | 0.0 | 0.0 | 0.0 | 0.0 | 0.0 | 0.0 | 0.0 | 0.0 | 0.0 | 0.0 | 0.0 | 0.0 |
| Gm5087 | 0.0 | 0.0 | 0.0 | 0.0 | 0.0 | 0.0 | 0.0 | 0.0 | 0.0 | 0.0 | 0.0 | 0.0 |

Excluded Genes

| | | | | | | | | | | | |
|---|---|---|---|---|---|---|---|---|---|---|---|
| Gm5095 | 0.0 | 0.0 | 0.0 | 0.0 | 0.0 | 0.0 | 0.0 | 0.0 | 0.0 | 0.0 | 0.0 | 0.0 |
| Gm5111 | 0.0 | 0.0 | 0.0 | 0.0 | 0.0 | 0.0 | 0.0 | 0.0 | 0.0 | 0.0 | 0.0 | 0.0 |
| Gm5132 | 0.0 | 0.0 | 0.0 | 0.0 | 0.0 | 0.0 | 0.0 | 0.0 | 0.0 | 0.0 | 0.0 | 0.0 |
| Gm5136 | 0.0 | 0.0 | 0.0 | 0.0 | 0.0 | 0.0 | 0.0 | 0.0 | 0.0 | 0.0 | 0.0 | 0.0 |
| Gm5142 | 0.0 | 0.0 | 0.0 | 0.0 | 0.0 | 0.0 | 0.0 | 0.0 | 0.0 | 0.0 | 0.0 | 0.0 |
| Gm5166 | 0.0 | 0.0 | 0.0 | 0.0 | 0.0 | 0.0 | 0.0 | 0.0 | 0.0 | 0.0 | 0.0 | 0.0 |
| Gm5334 | 0.0 | 0.0 | 0.0 | 0.0 | 0.0 | 0.0 | 0.0 | 0.0 | 0.0 | 0.0 | 0.0 | 0.0 |
| Gm5346 | 0.0 | 0.0 | 0.0 | 0.0 | 0.0 | 0.0 | 0.0 | 0.0 | 0.0 | 0.0 | 0.0 | 0.0 |
| Gm5347 | 0.0 | 0.0 | 0.0 | 0.0 | 0.0 | 0.0 | 0.0 | 0.0 | 0.0 | 0.0 | 0.0 | 0.0 |
| Gm5409 | 0.0 | 0.0 | 0.0 | 0.0 | 0.0 | 0.0 | 0.0 | 0.0 | 0.0 | 0.0 | 0.0 | 0.0 |
| Gm5414 | 0.0 | 0.0 | 0.0 | 0.0 | 0.0 | 0.0 | 0.0 | 0.0 | 0.0 | 0.0 | 0.0 | 0.0 |
| Gm5416 | 0.0 | 0.0 | 0.0 | 0.0 | 0.0 | 0.0 | 0.0 | 0.0 | 0.0 | 0.0 | 0.0 | 0.0 |
| Gm5441 | 0.0 | 0.0 | 0.0 | 0.0 | 0.0 | 0.0 | 0.0 | 0.0 | 0.0 | 0.0 | 0.0 | 0.0 |
| Gm5476 | 0.0 | 0.0 | 0.0 | 0.0 | 0.0 | 0.0 | 0.0 | 0.0 | 0.0 | 0.0 | 0.0 | 0.0 |
| Gm5477 | 0.0 | 0.0 | 0.0 | 0.0 | 0.0 | 0.0 | 0.0 | 0.0 | 0.0 | 0.0 | 0.0 | 0.0 |
| Gm5478 | 0.0 | 0.0 | 0.0 | 0.0 | 0.0 | 0.0 | 0.0 | 0.0 | 0.0 | 0.0 | 0.0 | 0.0 |
| Gm5480 | 0.0 | 0.0 | 0.0 | 0.0 | 0.0 | 0.0 | 0.0 | 0.0 | 0.0 | 0.0 | 0.0 | 0.0 |
| Gm5485 | 0.0 | 0.0 | 0.0 | 0.0 | 0.0 | 0.0 | 0.0 | 0.0 | 0.0 | 0.0 | 0.0 | 0.0 |
| Gm5494 | 0.0 | 0.0 | 0.0 | 0.0 | 0.0 | 0.0 | 0.0 | 0.0 | 0.0 | 0.0 | 0.0 | 0.0 |
| Gm5549 | 0.0 | 0.0 | 0.0 | 0.0 | 0.0 | 0.0 | 0.0 | 0.0 | 0.0 | 0.0 | 0.0 | 0.0 |
| Gm5634 | 0.0 | 0.0 | 0.0 | 0.0 | 0.0 | 0.0 | 0.0 | 0.0 | 0.0 | 0.0 | 0.0 | 0.0 |
| Gm5635 | 0.0 | 0.0 | 0.0 | 0.0 | 0.0 | 0.0 | 0.0 | 0.0 | 0.0 | 0.0 | 0.0 | 0.0 |
| Gm5712 | 0.0 | 0.0 | 0.0 | 0.0 | 0.0 | 0.0 | 0.0 | 0.0 | 0.0 | 0.0 | 0.0 | 0.0 |
| Gm572 | 0.0 | 0.0 | 0.0 | 0.0 | 0.0 | 0.0 | 0.0 | 0.0 | 0.0 | 0.0 | 0.0 | 0.0 |
| Gm5771 | 0.0 | 0.0 | 0.0 | 0.0 | 0.0 | 0.0 | 0.0 | 0.0 | 0.0 | 0.0 | 0.0 | 0.0 |
| Gm5800 | 0.0 | 0.0 | 0.0 | 0.0 | 0.0 | 0.0 | 0.0 | 0.0 | 0.0 | 0.0 | 0.0 | 0.0 |
| Gm581 | 0.0 | 0.0 | 0.0 | 0.0 | 0.0 | 0.0 | 0.0 | 0.0 | 0.0 | 0.0 | 0.0 | 0.0 |
| Gm5868 | 0.0 | 0.0 | 0.0 | 0.0 | 0.0 | 0.0 | 0.0 | 0.0 | 0.0 | 0.0 | 0.0 | 0.0 |
| Gm5885 | 0.0 | 0.0 | 0.0 | 0.0 | 0.0 | 0.0 | 0.0 | 0.0 | 0.0 | 0.0 | 0.0 | 0.0 |
| Gm590 | 0.0 | 0.0 | 0.0 | 0.0 | 0.0 | 0.0 | 0.0 | 0.0 | 0.0 | 0.0 | 0.0 | 0.0 |
| Gm5916 | 0.0 | 0.0 | 0.0 | 0.0 | 0.0 | 0.0 | 0.0 | 0.0 | 0.0 | 0.0 | 0.0 | 0.0 |
| Gm5925 | 0.0 | 0.0 | 0.0 | 0.0 | 0.0 | 0.0 | 0.0 | 0.0 | 0.0 | 0.0 | 0.0 | 0.0 |
| Gm5938 | 0.0 | 0.0 | 0.0 | 0.0 | 0.0 | 0.0 | 0.0 | 0.0 | 0.0 | 0.0 | 0.0 | 0.0 |
| Gm6040 | 0.0 | 0.0 | 0.0 | 0.0 | 0.0 | 0.0 | 0.0 | 0.0 | 0.0 | 0.0 | 0.0 | 0.0 |
| Gm6042 | 0.0 | 0.0 | 0.0 | 0.0 | 0.0 | 0.0 | 0.0 | 0.0 | 0.0 | 0.0 | 0.0 | 0.0 |
| Gm6084 | 0.0 | 0.0 | 0.0 | 0.0 | 0.0 | 0.0 | 0.0 | 0.0 | 0.0 | 0.0 | 0.0 | 0.0 |
| Gm6150 | 0.0 | 0.0 | 0.0 | 0.0 | 0.0 | 0.0 | 0.0 | 0.0 | 0.0 | 0.0 | 0.0 | 0.0 |
| Gm6213 | 0.0 | 0.0 | 0.0 | 0.0 | 0.0 | 0.0 | 0.0 | 0.0 | 0.0 | 0.0 | 0.0 | 0.0 |
| Gm6260 | 0.0 | 0.0 | 0.0 | 0.0 | 0.0 | 0.0 | 0.0 | 0.0 | 0.0 | 0.0 | 0.0 | 0.0 |
| Gm6268 | 0.0 | 0.0 | 0.0 | 0.0 | 0.0 | 0.0 | 0.0 | 0.0 | 0.0 | 0.0 | 0.0 | 0.0 |
| Gm6370 | 0.0 | 0.0 | 0.0 | 0.0 | 0.0 | 0.0 | 0.0 | 0.0 | 0.0 | 0.0 | 0.0 | 0.0 |
| Gm6406 | 0.0 | 0.0 | 0.0 | 0.0 | 0.0 | 0.0 | 0.0 | 0.0 | 0.0 | 0.0 | 0.0 | 0.0 |
| Gm6567 | 0.0 | 0.0 | 0.0 | 0.0 | 0.0 | 0.0 | 0.0 | 0.0 | 0.0 | 0.0 | 0.0 | 0.0 |
| Gm6639 | 0.0 | 0.0 | 0.0 | 0.0 | 0.0 | 0.0 | 0.0 | 0.0 | 0.0 | 0.0 | 0.0 | 0.0 |
| Gm6696 | 0.0 | 0.0 | 0.0 | 0.0 | 0.0 | 0.0 | 0.0 | 0.0 | 0.0 | 0.0 | 0.0 | 0.0 |
| Gm6760 | 0.0 | 0.0 | 0.0 | 0.0 | 0.0 | 0.0 | 0.0 | 0.0 | 0.0 | 0.0 | 0.0 | 0.0 |
| Gm6812 | 0.0 | 0.0 | 0.0 | 0.0 | 0.0 | 0.0 | 0.0 | 0.0 | 0.0 | 0.0 | 0.0 | 0.0 |
| Gm6927 | 0.0 | 0.0 | 0.0 | 0.0 | 0.0 | 0.0 | 0.0 | 0.0 | 0.0 | 0.0 | 0.0 | 0.0 |
| Gm6936 | 0.0 | 0.0 | 0.0 | 0.0 | 0.0 | 0.0 | 0.0 | 0.0 | 0.0 | 0.0 | 0.0 | 0.0 |
| Gm6938 | 0.0 | 0.0 | 0.0 | 0.0 | 0.0 | 0.0 | 0.0 | 0.0 | 0.0 | 0.0 | 0.0 | 0.0 |
| Gm694 | 0.0 | 0.0 | 0.0 | 0.0 | 0.0 | 0.0 | 0.0 | 0.0 | 0.0 | 0.0 | 0.0 | 0.0 |
| Gm7056 | 0.0 | 0.0 | 0.0 | 0.0 | 0.0 | 0.0 | 0.0 | 0.0 | 0.0 | 0.0 | 0.0 | 0.0 |
| Gm7073 | 0.0 | 0.0 | 0.0 | 0.0 | 0.0 | 0.0 | 0.0 | 0.0 | 0.0 | 0.0 | 0.0 | 0.0 |
| Gm7092 | 0.0 | 0.0 | 0.0 | 0.0 | 0.0 | 0.0 | 0.0 | 0.0 | 0.0 | 0.0 | 0.0 | 0.0 |
| Gm7134 | 0.0 | 0.0 | 0.0 | 0.0 | 0.0 | 0.0 | 0.0 | 0.0 | 0.0 | 0.0 | 0.0 | 0.0 |
| Gm7157 | 0.0 | 0.0 | 0.0 | 0.0 | 0.0 | 0.0 | 0.0 | 0.0 | 0.0 | 0.0 | 0.0 | 0.0 |
| Gm7244 | 0.0 | 0.0 | 0.0 | 0.0 | 0.0 | 0.0 | 0.0 | 0.0 | 0.0 | 0.0 | 0.0 | 0.0 |

| | | | | | | | | | | | |
|---|---|---|---|---|---|---|---|---|---|---|---|
| Gm7257 | 0.0 | 0.0 | 0.0 | 0.0 | 0.0 | 0.0 | 0.0 | 0.0 | 0.0 | 0.0 | 0.0 |
| Gm732 | 0.0 | 0.0 | 0.0 | 0.0 | 0.0 | 0.0 | 0.0 | 0.0 | 0.0 | 0.0 | 0.0 |
| Gm7538 | 0.0 | 0.0 | 0.0 | 0.0 | 0.0 | 0.0 | 0.0 | 0.0 | 0.0 | 0.0 | 0.0 |
| Gm7616 | 0.0 | 0.0 | 0.0 | 0.0 | 0.0 | 0.0 | 0.0 | 0.0 | 0.0 | 0.0 | 0.0 |
| Gm766 | 0.0 | 0.0 | 0.0 | 0.0 | 0.0 | 0.0 | 0.0 | 0.0 | 0.0 | 0.0 | 0.0 |
| Gm773 | 0.0 | 0.0 | 0.0 | 0.0 | 0.0 | 0.0 | 0.0 | 0.0 | 0.0 | 0.0 | 0.0 |
| Gm806 | 0.0 | 0.0 | 0.0 | 0.0 | 0.0 | 0.0 | 0.0 | 0.0 | 0.0 | 0.0 | 0.0 |
| Gm813 | 0.0 | 0.0 | 0.0 | 0.0 | 0.0 | 0.0 | 0.0 | 0.0 | 0.0 | 0.0 | 0.0 |
| Gm815 | 0.0 | 0.0 | 0.0 | 0.0 | 0.0 | 0.0 | 0.0 | 0.0 | 0.0 | 0.0 | 0.0 |
| Gm829 | 0.0 | 0.0 | 0.0 | 0.0 | 0.0 | 0.0 | 0.0 | 0.0 | 0.0 | 0.0 | 0.0 |
| Gm8298 | 0.0 | 0.0 | 0.0 | 0.0 | 0.0 | 0.0 | 0.0 | 0.0 | 0.0 | 0.0 | 0.0 |
| Gm833 | 0.0 | 0.0 | 0.0 | 0.0 | 0.0 | 0.0 | 0.0 | 0.0 | 0.0 | 0.0 | 0.0 |
| Gm839 | 0.0 | 0.0 | 0.0 | 0.0 | 0.0 | 0.0 | 0.0 | 0.0 | 0.0 | 0.0 | 0.0 |
| Gm853 | 0.0 | 0.0 | 0.0 | 0.0 | 0.0 | 0.0 | 0.0 | 0.0 | 0.0 | 0.0 | 0.0 |
| Gm8633 | 0.0 | 0.0 | 0.0 | 0.0 | 0.0 | 0.0 | 0.0 | 0.0 | 0.0 | 0.0 | 0.0 |
| Gm8693 | 0.0 | 0.0 | 0.0 | 0.0 | 0.0 | 0.0 | 0.0 | 0.0 | 0.0 | 0.0 | 0.0 |
| Gm8882 | 0.0 | 0.0 | 0.0 | 0.0 | 0.0 | 0.0 | 0.0 | 0.0 | 0.0 | 0.0 | 0.0 |
| Gm9 | 0.0 | 0.0 | 0.0 | 0.0 | 0.0 | 0.0 | 0.0 | 0.0 | 0.0 | 0.0 | 0.0 |
| Gm904 | 0.0 | 0.0 | 0.0 | 0.0 | 0.0 | 0.0 | 0.0 | 0.0 | 0.0 | 0.0 | 0.0 |
| Gm9041 | 0.0 | 0.0 | 0.0 | 0.0 | 0.0 | 0.0 | 0.0 | 0.0 | 0.0 | 0.0 | 0.0 |
| Gm9112 | 0.0 | 0.0 | 0.0 | 0.0 | 0.0 | 0.0 | 0.0 | 0.0 | 0.0 | 0.0 | 0.0 |
| Gm933 | 0.0 | 0.0 | 0.0 | 0.0 | 0.0 | 0.0 | 0.0 | 0.0 | 0.0 | 0.0 | 0.0 |
| Gm94 | 0.0 | 0.0 | 0.0 | 0.0 | 0.0 | 0.0 | 0.0 | 0.0 | 0.0 | 0.0 | 0.0 |
| Gm9513 | 0.0 | 0.0 | 0.0 | 0.0 | 0.0 | 0.0 | 0.0 | 0.0 | 0.0 | 0.0 | 0.0 |
| Gm97 | 0.0 | 0.0 | 0.0 | 0.0 | 0.0 | 0.0 | 0.0 | 0.0 | 0.0 | 0.0 | 0.0 |
| Gm9731 | 0.0 | 0.0 | 0.0 | 0.0 | 0.0 | 0.0 | 0.0 | 0.0 | 0.0 | 0.0 | 0.0 |
| Gm9733 | 0.0 | 0.0 | 0.0 | 0.0 | 0.0 | 0.0 | 0.0 | 0.0 | 0.0 | 0.0 | 0.0 |
| Gm9866 | 0.0 | 0.0 | 0.0 | 0.0 | 0.0 | 0.0 | 0.0 | 0.0 | 0.0 | 0.0 | 0.0 |
| Gm9871 | 0.0 | 0.0 | 0.0 | 0.0 | 0.0 | 0.0 | 0.0 | 0.0 | 0.0 | 0.0 | 0.0 |
| Gm9899 | 0.0 | 0.0 | 0.0 | 0.0 | 0.0 | 0.0 | 0.0 | 0.0 | 0.0 | 0.0 | 0.0 |
| Golt1a | 0.0 | 0.0 | 0.0 | 0.0 | 0.0 | 0.0 | 0.0 | 0.0 | 0.0 | 0.0 | 0.0 |
| Gp6 | 0.0 | 0.0 | 0.0 | 0.0 | 0.0 | 0.0 | 0.0 | 0.0 | 0.0 | 0.0 | 0.0 |
| Gpr12 | 0.0 | 0.0 | 0.0 | 0.0 | 0.0 | 0.0 | 0.0 | 0.0 | 0.0 | 0.0 | 0.0 |
| Gpr142 | 0.0 | 0.0 | 0.0 | 0.0 | 0.0 | 0.0 | 0.0 | 0.0 | 0.0 | 0.0 | 0.0 |
| Gpr6 | 0.0 | 0.0 | 0.0 | 0.0 | 0.0 | 0.0 | 0.0 | 0.0 | 0.0 | 0.0 | 0.0 |
| Gpx2 | 0.0 | 0.0 | 0.0 | 0.0 | 0.0 | 0.0 | 0.0 | 0.0 | 0.0 | 0.0 | 0.0 |
| Grifin | 0.0 | 0.0 | 0.0 | 0.0 | 0.0 | 0.0 | 0.0 | 0.0 | 0.0 | 0.0 | 0.0 |
| Grm2 | 0.0 | 0.0 | 0.0 | 0.0 | 0.0 | 0.0 | 0.0 | 0.0 | 0.0 | 0.0 | 0.0 |
| Grp | 0.0 | 0.0 | 0.0 | 0.0 | 0.0 | 0.0 | 0.0 | 0.0 | 0.0 | 0.0 | 0.0 |
| Grxcr1 | 0.0 | 0.0 | 0.0 | 0.0 | 0.0 | 0.0 | 0.0 | 0.0 | 0.0 | 0.0 | 0.0 |
| Grxcr2 | 0.0 | 0.0 | 0.0 | 0.0 | 0.0 | 0.0 | 0.0 | 0.0 | 0.0 | 0.0 | 0.0 |
| Gsdma2 | 0.0 | 0.0 | 0.0 | 0.0 | 0.0 | 0.0 | 0.0 | 0.0 | 0.0 | 0.0 | 0.0 |
| Gsx1 | 0.0 | 0.0 | 0.0 | 0.0 | 0.0 | 0.0 | 0.0 | 0.0 | 0.0 | 0.0 | 0.0 |
| Gsx2 | 0.0 | 0.0 | 0.0 | 0.0 | 0.0 | 0.0 | 0.0 | 0.0 | 0.0 | 0.0 | 0.0 |
| Gzma | 0.0 | 0.0 | 0.0 | 0.0 | 0.0 | 0.0 | 0.0 | 0.0 | 0.0 | 0.0 | 0.0 |
| Gzmf | 0.0 | 0.0 | 0.0 | 0.0 | 0.0 | 0.0 | 0.0 | 0.0 | 0.0 | 0.0 | 0.0 |
| Gzmg | 0.0 | 0.0 | 0.0 | 0.0 | 0.0 | 0.0 | 0.0 | 0.0 | 0.0 | 0.0 | 0.0 |
| H1fnt | 0.0 | 0.0 | 0.0 | 0.0 | 0.0 | 0.0 | 0.0 | 0.0 | 0.0 | 0.0 | 0.0 |
| H2afb1 | 0.0 | 0.0 | 0.0 | 0.0 | 0.0 | 0.0 | 0.0 | 0.0 | 0.0 | 0.0 | 0.0 |
| H2bfm | 0.0 | 0.0 | 0.0 | 0.0 | 0.0 | 0.0 | 0.0 | 0.0 | 0.0 | 0.0 | 0.0 |
| H2-Ea-ps | 0.0 | 0.0 | 0.0 | 0.0 | 0.0 | 0.0 | 0.0 | 0.0 | 0.0 | 0.0 | 0.0 |
| H2-M9 | 0.0 | 0.0 | 0.0 | 0.0 | 0.0 | 0.0 | 0.0 | 0.0 | 0.0 | 0.0 | 0.0 |
| Hamp2 | 0.0 | 0.0 | 0.0 | 0.0 | 0.0 | 0.0 | 0.0 | 0.0 | 0.0 | 0.0 | 0.0 |
| Hao2 | 0.0 | 0.0 | 0.0 | 0.0 | 0.0 | 0.0 | 0.0 | 0.0 | 0.0 | 0.0 | 0.0 |
| Hba-x | 0.0 | 0.0 | 0.0 | 0.0 | 0.0 | 0.0 | 0.0 | 0.0 | 0.0 | 0.0 | 0.0 |
| Hbb-b1 | 0.0 | 0.0 | 0.0 | 0.0 | 0.0 | 0.0 | 0.0 | 0.0 | 0.0 | 0.0 | 0.0 |
| Hbb-b2 | 0.0 | 0.0 | 0.0 | 0.0 | 0.0 | 0.0 | 0.0 | 0.0 | 0.0 | 0.0 | 0.0 |

| | | | | | | | | | | | |
|---|---|---|---|---|---|---|---|---|---|---|---|
| Hbb-bh1 | 0.0 | 0.0 | 0.0 | 0.0 | 0.0 | 0.0 | 0.0 | 0.0 | 0.0 | 0.0 | 0.0 |
| Hbb-bh2 | 0.0 | 0.0 | 0.0 | 0.0 | 0.0 | 0.0 | 0.0 | 0.0 | 0.0 | 0.0 | 0.0 |
| Hbb-y | 0.0 | 0.0 | 0.0 | 0.0 | 0.0 | 0.0 | 0.0 | 0.0 | 0.0 | 0.0 | 0.0 |
| Hbq1a | 0.0 | 0.0 | 0.0 | 0.0 | 0.0 | 0.0 | 0.0 | 0.0 | 0.0 | 0.0 | 0.0 |
| Hbq1b | 0.0 | 0.0 | 0.0 | 0.0 | 0.0 | 0.0 | 0.0 | 0.0 | 0.0 | 0.0 | 0.0 |
| Hdgfl1 | 0.0 | 0.0 | 0.0 | 0.0 | 0.0 | 0.0 | 0.0 | 0.0 | 0.0 | 0.0 | 0.0 |
| Helt | 0.0 | 0.0 | 0.0 | 0.0 | 0.0 | 0.0 | 0.0 | 0.0 | 0.0 | 0.0 | 0.0 |
| Hhla1 | 0.0 | 0.0 | 0.0 | 0.0 | 0.0 | 0.0 | 0.0 | 0.0 | 0.0 | 0.0 | 0.0 |
| Higd1c | 0.0 | 0.0 | 0.0 | 0.0 | 0.0 | 0.0 | 0.0 | 0.0 | 0.0 | 0.0 | 0.0 |
| Hmgb4 | 0.0 | 0.0 | 0.0 | 0.0 | 0.0 | 0.0 | 0.0 | 0.0 | 0.0 | 0.0 | 0.0 |
| Hmx3 | 0.0 | 0.0 | 0.0 | 0.0 | 0.0 | 0.0 | 0.0 | 0.0 | 0.0 | 0.0 | 0.0 |
| Hnf4a | 0.0 | 0.0 | 0.0 | 0.0 | 0.0 | 0.0 | 0.0 | 0.0 | 0.0 | 0.0 | 0.0 |
| Hnf4g | 0.0 | 0.0 | 0.0 | 0.0 | 0.0 | 0.0 | 0.0 | 0.0 | 0.0 | 0.0 | 0.0 |
| Hotair | 0.0 | 0.0 | 0.0 | 0.0 | 0.0 | 0.0 | 0.0 | 0.0 | 0.0 | 0.0 | 0.0 |
| Hoxb1 | 0.0 | 0.0 | 0.0 | 0.0 | 0.0 | 0.0 | 0.0 | 0.0 | 0.0 | 0.0 | 0.0 |
| Hoxd11 | 0.0 | 0.0 | 0.0 | 0.0 | 0.0 | 0.0 | 0.0 | 0.0 | 0.0 | 0.0 | 0.0 |
| Hoxd12 | 0.0 | 0.0 | 0.0 | 0.0 | 0.0 | 0.0 | 0.0 | 0.0 | 0.0 | 0.0 | 0.0 |
| Hrg | 0.0 | 0.0 | 0.0 | 0.0 | 0.0 | 0.0 | 0.0 | 0.0 | 0.0 | 0.0 | 0.0 |
| Hrh4 | 0.0 | 0.0 | 0.0 | 0.0 | 0.0 | 0.0 | 0.0 | 0.0 | 0.0 | 0.0 | 0.0 |
| Hrnr | 0.0 | 0.0 | 0.0 | 0.0 | 0.0 | 0.0 | 0.0 | 0.0 | 0.0 | 0.0 | 0.0 |
| Hsfy2 | 0.0 | 0.0 | 0.0 | 0.0 | 0.0 | 0.0 | 0.0 | 0.0 | 0.0 | 0.0 | 0.0 |
| Htr1a | 0.0 | 0.0 | 0.0 | 0.0 | 0.0 | 0.0 | 0.0 | 0.0 | 0.0 | 0.0 | 0.0 |
| Hyal4 | 0.0 | 0.0 | 0.0 | 0.0 | 0.0 | 0.0 | 0.0 | 0.0 | 0.0 | 0.0 | 0.0 |
| I730030J21Rik | 0.0 | 0.0 | 0.0 | 0.0 | 0.0 | 0.0 | 0.0 | 0.0 | 0.0 | 0.0 | 0.0 |
| Ifna1 | 0.0 | 0.0 | 0.0 | 0.0 | 0.0 | 0.0 | 0.0 | 0.0 | 0.0 | 0.0 | 0.0 |
| Ifna11 | 0.0 | 0.0 | 0.0 | 0.0 | 0.0 | 0.0 | 0.0 | 0.0 | 0.0 | 0.0 | 0.0 |
| Ifna12 | 0.0 | 0.0 | 0.0 | 0.0 | 0.0 | 0.0 | 0.0 | 0.0 | 0.0 | 0.0 | 0.0 |
| Ifna14 | 0.0 | 0.0 | 0.0 | 0.0 | 0.0 | 0.0 | 0.0 | 0.0 | 0.0 | 0.0 | 0.0 |
| Ifna2 | 0.0 | 0.0 | 0.0 | 0.0 | 0.0 | 0.0 | 0.0 | 0.0 | 0.0 | 0.0 | 0.0 |
| Ifna4 | 0.0 | 0.0 | 0.0 | 0.0 | 0.0 | 0.0 | 0.0 | 0.0 | 0.0 | 0.0 | 0.0 |
| Ifna5 | 0.0 | 0.0 | 0.0 | 0.0 | 0.0 | 0.0 | 0.0 | 0.0 | 0.0 | 0.0 | 0.0 |
| Ifna7 | 0.0 | 0.0 | 0.0 | 0.0 | 0.0 | 0.0 | 0.0 | 0.0 | 0.0 | 0.0 | 0.0 |
| Ifna9 | 0.0 | 0.0 | 0.0 | 0.0 | 0.0 | 0.0 | 0.0 | 0.0 | 0.0 | 0.0 | 0.0 |
| Ifnl2 | 0.0 | 0.0 | 0.0 | 0.0 | 0.0 | 0.0 | 0.0 | 0.0 | 0.0 | 0.0 | 0.0 |
| Ifnl3 | 0.0 | 0.0 | 0.0 | 0.0 | 0.0 | 0.0 | 0.0 | 0.0 | 0.0 | 0.0 | 0.0 |
| Igbp1b | 0.0 | 0.0 | 0.0 | 0.0 | 0.0 | 0.0 | 0.0 | 0.0 | 0.0 | 0.0 | 0.0 |
| Igfbp1 | 0.0 | 0.0 | 0.0 | 0.0 | 0.0 | 0.0 | 0.0 | 0.0 | 0.0 | 0.0 | 0.0 |
| Igfl3 | 0.0 | 0.0 | 0.0 | 0.0 | 0.0 | 0.0 | 0.0 | 0.0 | 0.0 | 0.0 | 0.0 |
| Il13 | 0.0 | 0.0 | 0.0 | 0.0 | 0.0 | 0.0 | 0.0 | 0.0 | 0.0 | 0.0 | 0.0 |
| Il17f | 0.0 | 0.0 | 0.0 | 0.0 | 0.0 | 0.0 | 0.0 | 0.0 | 0.0 | 0.0 | 0.0 |
| Il1f6 | 0.0 | 0.0 | 0.0 | 0.0 | 0.0 | 0.0 | 0.0 | 0.0 | 0.0 | 0.0 | 0.0 |
| Il2 | 0.0 | 0.0 | 0.0 | 0.0 | 0.0 | 0.0 | 0.0 | 0.0 | 0.0 | 0.0 | 0.0 |
| Il20 | 0.0 | 0.0 | 0.0 | 0.0 | 0.0 | 0.0 | 0.0 | 0.0 | 0.0 | 0.0 | 0.0 |
| Il22 | 0.0 | 0.0 | 0.0 | 0.0 | 0.0 | 0.0 | 0.0 | 0.0 | 0.0 | 0.0 | 0.0 |
| Il3 | 0.0 | 0.0 | 0.0 | 0.0 | 0.0 | 0.0 | 0.0 | 0.0 | 0.0 | 0.0 | 0.0 |
| Il9 | 0.0 | 0.0 | 0.0 | 0.0 | 0.0 | 0.0 | 0.0 | 0.0 | 0.0 | 0.0 | 0.0 |
| Ins1 | 0.0 | 0.0 | 0.0 | 0.0 | 0.0 | 0.0 | 0.0 | 0.0 | 0.0 | 0.0 | 0.0 |
| Insm1 | 0.0 | 0.0 | 0.0 | 0.0 | 0.0 | 0.0 | 0.0 | 0.0 | 0.0 | 0.0 | 0.0 |
| Iqcf1 | 0.0 | 0.0 | 0.0 | 0.0 | 0.0 | 0.0 | 0.0 | 0.0 | 0.0 | 0.0 | 0.0 |
| Iqcf3 | 0.0 | 0.0 | 0.0 | 0.0 | 0.0 | 0.0 | 0.0 | 0.0 | 0.0 | 0.0 | 0.0 |
| Iqcf4 | 0.0 | 0.0 | 0.0 | 0.0 | 0.0 | 0.0 | 0.0 | 0.0 | 0.0 | 0.0 | 0.0 |
| Iqcf6 | 0.0 | 0.0 | 0.0 | 0.0 | 0.0 | 0.0 | 0.0 | 0.0 | 0.0 | 0.0 | 0.0 |
| Isx | 0.0 | 0.0 | 0.0 | 0.0 | 0.0 | 0.0 | 0.0 | 0.0 | 0.0 | 0.0 | 0.0 |
| Itgb2l | 0.0 | 0.0 | 0.0 | 0.0 | 0.0 | 0.0 | 0.0 | 0.0 | 0.0 | 0.0 | 0.0 |
| Iyd | 0.0 | 0.0 | 0.0 | 0.0 | 0.0 | 0.0 | 0.0 | 0.0 | 0.0 | 0.0 | 0.0 |
| Izumo2 | 0.0 | 0.0 | 0.0 | 0.0 | 0.0 | 0.0 | 0.0 | 0.0 | 0.0 | 0.0 | 0.0 |
| Izumo3 | 0.0 | 0.0 | 0.0 | 0.0 | 0.0 | 0.0 | 0.0 | 0.0 | 0.0 | 0.0 | 0.0 |

| | | | | | | | | | | | | |
|---|---|---|---|---|---|---|---|---|---|---|---|---|
| Jakmip1 | 0.0 | 0.0 | 0.0 | 0.0 | 0.0 | 0.0 | 0.0 | 0.0 | 0.0 | 0.0 | 0.0 | 0.0 |
| Kap | 0.0 | 0.0 | 0.0 | 0.0 | 0.0 | 0.0 | 0.0 | 0.0 | 0.0 | 0.0 | 0.0 | 0.0 |
| Kbtbd12 | 0.0 | 0.0 | 0.0 | 0.0 | 0.0 | 0.0 | 0.0 | 0.0 | 0.0 | 0.0 | 0.0 | 0.0 |
| Kcna10 | 0.0 | 0.0 | 0.0 | 0.0 | 0.0 | 0.0 | 0.0 | 0.0 | 0.0 | 0.0 | 0.0 | 0.0 |
| Kcnj11 | 0.0 | 0.0 | 0.0 | 0.0 | 0.0 | 0.0 | 0.0 | 0.0 | 0.0 | 0.0 | 0.0 | 0.0 |
| Kcnn2 | 0.0 | 0.0 | 0.0 | 0.0 | 0.0 | 0.0 | 0.0 | 0.0 | 0.0 | 0.0 | 0.0 | 0.0 |
| Kel | 0.0 | 0.0 | 0.0 | 0.0 | 0.0 | 0.0 | 0.0 | 0.0 | 0.0 | 0.0 | 0.0 | 0.0 |
| Kera | 0.0 | 0.0 | 0.0 | 0.0 | 0.0 | 0.0 | 0.0 | 0.0 | 0.0 | 0.0 | 0.0 | 0.0 |
| Khdc1a | 0.0 | 0.0 | 0.0 | 0.0 | 0.0 | 0.0 | 0.0 | 0.0 | 0.0 | 0.0 | 0.0 | 0.0 |
| Khdc1c | 0.0 | 0.0 | 0.0 | 0.0 | 0.0 | 0.0 | 0.0 | 0.0 | 0.0 | 0.0 | 0.0 | 0.0 |
| Khdc3 | 0.0 | 0.0 | 0.0 | 0.0 | 0.0 | 0.0 | 0.0 | 0.0 | 0.0 | 0.0 | 0.0 | 0.0 |
| Kif2b | 0.0 | 0.0 | 0.0 | 0.0 | 0.0 | 0.0 | 0.0 | 0.0 | 0.0 | 0.0 | 0.0 | 0.0 |
| Kif4-ps | 0.0 | 0.0 | 0.0 | 0.0 | 0.0 | 0.0 | 0.0 | 0.0 | 0.0 | 0.0 | 0.0 | 0.0 |
| Kir3dl1 | 0.0 | 0.0 | 0.0 | 0.0 | 0.0 | 0.0 | 0.0 | 0.0 | 0.0 | 0.0 | 0.0 | 0.0 |
| Kir3dl2 | 0.0 | 0.0 | 0.0 | 0.0 | 0.0 | 0.0 | 0.0 | 0.0 | 0.0 | 0.0 | 0.0 | 0.0 |
| Kis2 | 0.0 | 0.0 | 0.0 | 0.0 | 0.0 | 0.0 | 0.0 | 0.0 | 0.0 | 0.0 | 0.0 | 0.0 |
| Kiss1 | 0.0 | 0.0 | 0.0 | 0.0 | 0.0 | 0.0 | 0.0 | 0.0 | 0.0 | 0.0 | 0.0 | 0.0 |
| Klf14 | 0.0 | 0.0 | 0.0 | 0.0 | 0.0 | 0.0 | 0.0 | 0.0 | 0.0 | 0.0 | 0.0 | 0.0 |
| Klhdc7b | 0.0 | 0.0 | 0.0 | 0.0 | 0.0 | 0.0 | 0.0 | 0.0 | 0.0 | 0.0 | 0.0 | 0.0 |
| Klhl34 | 0.0 | 0.0 | 0.0 | 0.0 | 0.0 | 0.0 | 0.0 | 0.0 | 0.0 | 0.0 | 0.0 | 0.0 |
| Klk1 | 0.0 | 0.0 | 0.0 | 0.0 | 0.0 | 0.0 | 0.0 | 0.0 | 0.0 | 0.0 | 0.0 | 0.0 |
| Klk11 | 0.0 | 0.0 | 0.0 | 0.0 | 0.0 | 0.0 | 0.0 | 0.0 | 0.0 | 0.0 | 0.0 | 0.0 |
| Klk12 | 0.0 | 0.0 | 0.0 | 0.0 | 0.0 | 0.0 | 0.0 | 0.0 | 0.0 | 0.0 | 0.0 | 0.0 |
| Klk13 | 0.0 | 0.0 | 0.0 | 0.0 | 0.0 | 0.0 | 0.0 | 0.0 | 0.0 | 0.0 | 0.0 | 0.0 |
| Klk15 | 0.0 | 0.0 | 0.0 | 0.0 | 0.0 | 0.0 | 0.0 | 0.0 | 0.0 | 0.0 | 0.0 | 0.0 |
| Klk1b1 | 0.0 | 0.0 | 0.0 | 0.0 | 0.0 | 0.0 | 0.0 | 0.0 | 0.0 | 0.0 | 0.0 | 0.0 |
| Klk1b11 | 0.0 | 0.0 | 0.0 | 0.0 | 0.0 | 0.0 | 0.0 | 0.0 | 0.0 | 0.0 | 0.0 | 0.0 |
| Klk1b16 | 0.0 | 0.0 | 0.0 | 0.0 | 0.0 | 0.0 | 0.0 | 0.0 | 0.0 | 0.0 | 0.0 | 0.0 |
| Klk1b21 | 0.0 | 0.0 | 0.0 | 0.0 | 0.0 | 0.0 | 0.0 | 0.0 | 0.0 | 0.0 | 0.0 | 0.0 |
| Klk1b22 | 0.0 | 0.0 | 0.0 | 0.0 | 0.0 | 0.0 | 0.0 | 0.0 | 0.0 | 0.0 | 0.0 | 0.0 |
| Klk1b24 | 0.0 | 0.0 | 0.0 | 0.0 | 0.0 | 0.0 | 0.0 | 0.0 | 0.0 | 0.0 | 0.0 | 0.0 |
| Klk1b26 | 0.0 | 0.0 | 0.0 | 0.0 | 0.0 | 0.0 | 0.0 | 0.0 | 0.0 | 0.0 | 0.0 | 0.0 |
| Klk1b3 | 0.0 | 0.0 | 0.0 | 0.0 | 0.0 | 0.0 | 0.0 | 0.0 | 0.0 | 0.0 | 0.0 | 0.0 |
| Klk1b4 | 0.0 | 0.0 | 0.0 | 0.0 | 0.0 | 0.0 | 0.0 | 0.0 | 0.0 | 0.0 | 0.0 | 0.0 |
| Klk1b5 | 0.0 | 0.0 | 0.0 | 0.0 | 0.0 | 0.0 | 0.0 | 0.0 | 0.0 | 0.0 | 0.0 | 0.0 |
| Klk1b7-ps | 0.0 | 0.0 | 0.0 | 0.0 | 0.0 | 0.0 | 0.0 | 0.0 | 0.0 | 0.0 | 0.0 | 0.0 |
| Klk1b8 | 0.0 | 0.0 | 0.0 | 0.0 | 0.0 | 0.0 | 0.0 | 0.0 | 0.0 | 0.0 | 0.0 | 0.0 |
| Klk1b9 | 0.0 | 0.0 | 0.0 | 0.0 | 0.0 | 0.0 | 0.0 | 0.0 | 0.0 | 0.0 | 0.0 | 0.0 |
| Klk4 | 0.0 | 0.0 | 0.0 | 0.0 | 0.0 | 0.0 | 0.0 | 0.0 | 0.0 | 0.0 | 0.0 | 0.0 |
| Klk5 | 0.0 | 0.0 | 0.0 | 0.0 | 0.0 | 0.0 | 0.0 | 0.0 | 0.0 | 0.0 | 0.0 | 0.0 |
| Klra10 | 0.0 | 0.0 | 0.0 | 0.0 | 0.0 | 0.0 | 0.0 | 0.0 | 0.0 | 0.0 | 0.0 | 0.0 |
| Klra13-ps | 0.0 | 0.0 | 0.0 | 0.0 | 0.0 | 0.0 | 0.0 | 0.0 | 0.0 | 0.0 | 0.0 | 0.0 |
| Klra14 | 0.0 | 0.0 | 0.0 | 0.0 | 0.0 | 0.0 | 0.0 | 0.0 | 0.0 | 0.0 | 0.0 | 0.0 |
| Klra19 | 0.0 | 0.0 | 0.0 | 0.0 | 0.0 | 0.0 | 0.0 | 0.0 | 0.0 | 0.0 | 0.0 | 0.0 |
| Klra21 | 0.0 | 0.0 | 0.0 | 0.0 | 0.0 | 0.0 | 0.0 | 0.0 | 0.0 | 0.0 | 0.0 | 0.0 |
| Klra23 | 0.0 | 0.0 | 0.0 | 0.0 | 0.0 | 0.0 | 0.0 | 0.0 | 0.0 | 0.0 | 0.0 | 0.0 |
| Klra5 | 0.0 | 0.0 | 0.0 | 0.0 | 0.0 | 0.0 | 0.0 | 0.0 | 0.0 | 0.0 | 0.0 | 0.0 |
| Klra6 | 0.0 | 0.0 | 0.0 | 0.0 | 0.0 | 0.0 | 0.0 | 0.0 | 0.0 | 0.0 | 0.0 | 0.0 |
| Klra8 | 0.0 | 0.0 | 0.0 | 0.0 | 0.0 | 0.0 | 0.0 | 0.0 | 0.0 | 0.0 | 0.0 | 0.0 |
| Klra9 | 0.0 | 0.0 | 0.0 | 0.0 | 0.0 | 0.0 | 0.0 | 0.0 | 0.0 | 0.0 | 0.0 | 0.0 |
| Klrb1 | 0.0 | 0.0 | 0.0 | 0.0 | 0.0 | 0.0 | 0.0 | 0.0 | 0.0 | 0.0 | 0.0 | 0.0 |
| Klrb1b | 0.0 | 0.0 | 0.0 | 0.0 | 0.0 | 0.0 | 0.0 | 0.0 | 0.0 | 0.0 | 0.0 | 0.0 |
| Klrb1f | 0.0 | 0.0 | 0.0 | 0.0 | 0.0 | 0.0 | 0.0 | 0.0 | 0.0 | 0.0 | 0.0 | 0.0 |
| Klrc1 | 0.0 | 0.0 | 0.0 | 0.0 | 0.0 | 0.0 | 0.0 | 0.0 | 0.0 | 0.0 | 0.0 | 0.0 |
| Klrc2 | 0.0 | 0.0 | 0.0 | 0.0 | 0.0 | 0.0 | 0.0 | 0.0 | 0.0 | 0.0 | 0.0 | 0.0 |
| Klrc3 | 0.0 | 0.0 | 0.0 | 0.0 | 0.0 | 0.0 | 0.0 | 0.0 | 0.0 | 0.0 | 0.0 | 0.0 |
| Klrd1 | 0.0 | 0.0 | 0.0 | 0.0 | 0.0 | 0.0 | 0.0 | 0.0 | 0.0 | 0.0 | 0.0 | 0.0 |

Excluded Genes

| | | | | | | | | | | | | |
|---|---|---|---|---|---|---|---|---|---|---|---|---|
| Klrg1 | 0.0 | 0.0 | 0.0 | 0.0 | 0.0 | 0.0 | 0.0 | 0.0 | 0.0 | 0.0 | 0.0 | 0.0 |
| Klri1 | 0.0 | 0.0 | 0.0 | 0.0 | 0.0 | 0.0 | 0.0 | 0.0 | 0.0 | 0.0 | 0.0 | 0.0 |
| Kmo | 0.0 | 0.0 | 0.0 | 0.0 | 0.0 | 0.0 | 0.0 | 0.0 | 0.0 | 0.0 | 0.0 | 0.0 |
| Krt12 | 0.0 | 0.0 | 0.0 | 0.0 | 0.0 | 0.0 | 0.0 | 0.0 | 0.0 | 0.0 | 0.0 | 0.0 |
| Krt2 | 0.0 | 0.0 | 0.0 | 0.0 | 0.0 | 0.0 | 0.0 | 0.0 | 0.0 | 0.0 | 0.0 | 0.0 |
| Krt26 | 0.0 | 0.0 | 0.0 | 0.0 | 0.0 | 0.0 | 0.0 | 0.0 | 0.0 | 0.0 | 0.0 | 0.0 |
| Krt27 | 0.0 | 0.0 | 0.0 | 0.0 | 0.0 | 0.0 | 0.0 | 0.0 | 0.0 | 0.0 | 0.0 | 0.0 |
| Krt33b | 0.0 | 0.0 | 0.0 | 0.0 | 0.0 | 0.0 | 0.0 | 0.0 | 0.0 | 0.0 | 0.0 | 0.0 |
| Krt34 | 0.0 | 0.0 | 0.0 | 0.0 | 0.0 | 0.0 | 0.0 | 0.0 | 0.0 | 0.0 | 0.0 | 0.0 |
| Krt35 | 0.0 | 0.0 | 0.0 | 0.0 | 0.0 | 0.0 | 0.0 | 0.0 | 0.0 | 0.0 | 0.0 | 0.0 |
| Krt39 | 0.0 | 0.0 | 0.0 | 0.0 | 0.0 | 0.0 | 0.0 | 0.0 | 0.0 | 0.0 | 0.0 | 0.0 |
| Krt40 | 0.0 | 0.0 | 0.0 | 0.0 | 0.0 | 0.0 | 0.0 | 0.0 | 0.0 | 0.0 | 0.0 | 0.0 |
| Krt42 | 0.0 | 0.0 | 0.0 | 0.0 | 0.0 | 0.0 | 0.0 | 0.0 | 0.0 | 0.0 | 0.0 | 0.0 |
| Krt77 | 0.0 | 0.0 | 0.0 | 0.0 | 0.0 | 0.0 | 0.0 | 0.0 | 0.0 | 0.0 | 0.0 | 0.0 |
| Krt82 | 0.0 | 0.0 | 0.0 | 0.0 | 0.0 | 0.0 | 0.0 | 0.0 | 0.0 | 0.0 | 0.0 | 0.0 |
| Krt84 | 0.0 | 0.0 | 0.0 | 0.0 | 0.0 | 0.0 | 0.0 | 0.0 | 0.0 | 0.0 | 0.0 | 0.0 |
| Krtap10-10 | 0.0 | 0.0 | 0.0 | 0.0 | 0.0 | 0.0 | 0.0 | 0.0 | 0.0 | 0.0 | 0.0 | 0.0 |
| Krtap10-4 | 0.0 | 0.0 | 0.0 | 0.0 | 0.0 | 0.0 | 0.0 | 0.0 | 0.0 | 0.0 | 0.0 | 0.0 |
| Krtap12-1 | 0.0 | 0.0 | 0.0 | 0.0 | 0.0 | 0.0 | 0.0 | 0.0 | 0.0 | 0.0 | 0.0 | 0.0 |
| Krtap13 | 0.0 | 0.0 | 0.0 | 0.0 | 0.0 | 0.0 | 0.0 | 0.0 | 0.0 | 0.0 | 0.0 | 0.0 |
| Krtap1-3 | 0.0 | 0.0 | 0.0 | 0.0 | 0.0 | 0.0 | 0.0 | 0.0 | 0.0 | 0.0 | 0.0 | 0.0 |
| Krtap13-1 | 0.0 | 0.0 | 0.0 | 0.0 | 0.0 | 0.0 | 0.0 | 0.0 | 0.0 | 0.0 | 0.0 | 0.0 |
| Krtap14 | 0.0 | 0.0 | 0.0 | 0.0 | 0.0 | 0.0 | 0.0 | 0.0 | 0.0 | 0.0 | 0.0 | 0.0 |
| Krtap1-4 | 0.0 | 0.0 | 0.0 | 0.0 | 0.0 | 0.0 | 0.0 | 0.0 | 0.0 | 0.0 | 0.0 | 0.0 |
| Krtap15 | 0.0 | 0.0 | 0.0 | 0.0 | 0.0 | 0.0 | 0.0 | 0.0 | 0.0 | 0.0 | 0.0 | 0.0 |
| Krtap16-1 | 0.0 | 0.0 | 0.0 | 0.0 | 0.0 | 0.0 | 0.0 | 0.0 | 0.0 | 0.0 | 0.0 | 0.0 |
| Krtap16-3 | 0.0 | 0.0 | 0.0 | 0.0 | 0.0 | 0.0 | 0.0 | 0.0 | 0.0 | 0.0 | 0.0 | 0.0 |
| Krtap19-1 | 0.0 | 0.0 | 0.0 | 0.0 | 0.0 | 0.0 | 0.0 | 0.0 | 0.0 | 0.0 | 0.0 | 0.0 |
| Krtap19-3 | 0.0 | 0.0 | 0.0 | 0.0 | 0.0 | 0.0 | 0.0 | 0.0 | 0.0 | 0.0 | 0.0 | 0.0 |
| Krtap19-4 | 0.0 | 0.0 | 0.0 | 0.0 | 0.0 | 0.0 | 0.0 | 0.0 | 0.0 | 0.0 | 0.0 | 0.0 |
| Krtap19-5 | 0.0 | 0.0 | 0.0 | 0.0 | 0.0 | 0.0 | 0.0 | 0.0 | 0.0 | 0.0 | 0.0 | 0.0 |
| Krtap19-9b | 0.0 | 0.0 | 0.0 | 0.0 | 0.0 | 0.0 | 0.0 | 0.0 | 0.0 | 0.0 | 0.0 | 0.0 |
| Krtap20-2 | 0.0 | 0.0 | 0.0 | 0.0 | 0.0 | 0.0 | 0.0 | 0.0 | 0.0 | 0.0 | 0.0 | 0.0 |
| Krtap21-1 | 0.0 | 0.0 | 0.0 | 0.0 | 0.0 | 0.0 | 0.0 | 0.0 | 0.0 | 0.0 | 0.0 | 0.0 |
| Krtap22-2 | 0.0 | 0.0 | 0.0 | 0.0 | 0.0 | 0.0 | 0.0 | 0.0 | 0.0 | 0.0 | 0.0 | 0.0 |
| Krtap24-1 | 0.0 | 0.0 | 0.0 | 0.0 | 0.0 | 0.0 | 0.0 | 0.0 | 0.0 | 0.0 | 0.0 | 0.0 |
| Krtap26-1 | 0.0 | 0.0 | 0.0 | 0.0 | 0.0 | 0.0 | 0.0 | 0.0 | 0.0 | 0.0 | 0.0 | 0.0 |
| Krtap27-1 | 0.0 | 0.0 | 0.0 | 0.0 | 0.0 | 0.0 | 0.0 | 0.0 | 0.0 | 0.0 | 0.0 | 0.0 |
| Krtap3-1 | 0.0 | 0.0 | 0.0 | 0.0 | 0.0 | 0.0 | 0.0 | 0.0 | 0.0 | 0.0 | 0.0 | 0.0 |
| Krtap31-1 | 0.0 | 0.0 | 0.0 | 0.0 | 0.0 | 0.0 | 0.0 | 0.0 | 0.0 | 0.0 | 0.0 | 0.0 |
| Krtap31-2 | 0.0 | 0.0 | 0.0 | 0.0 | 0.0 | 0.0 | 0.0 | 0.0 | 0.0 | 0.0 | 0.0 | 0.0 |
| Krtap3-2 | 0.0 | 0.0 | 0.0 | 0.0 | 0.0 | 0.0 | 0.0 | 0.0 | 0.0 | 0.0 | 0.0 | 0.0 |
| Krtap3-3 | 0.0 | 0.0 | 0.0 | 0.0 | 0.0 | 0.0 | 0.0 | 0.0 | 0.0 | 0.0 | 0.0 | 0.0 |
| Krtap4-1 | 0.0 | 0.0 | 0.0 | 0.0 | 0.0 | 0.0 | 0.0 | 0.0 | 0.0 | 0.0 | 0.0 | 0.0 |
| Krtap4-13 | 0.0 | 0.0 | 0.0 | 0.0 | 0.0 | 0.0 | 0.0 | 0.0 | 0.0 | 0.0 | 0.0 | 0.0 |
| Krtap4-16 | 0.0 | 0.0 | 0.0 | 0.0 | 0.0 | 0.0 | 0.0 | 0.0 | 0.0 | 0.0 | 0.0 | 0.0 |
| Krtap4-2 | 0.0 | 0.0 | 0.0 | 0.0 | 0.0 | 0.0 | 0.0 | 0.0 | 0.0 | 0.0 | 0.0 | 0.0 |
| Krtap4-6 | 0.0 | 0.0 | 0.0 | 0.0 | 0.0 | 0.0 | 0.0 | 0.0 | 0.0 | 0.0 | 0.0 | 0.0 |
| Krtap4-7 | 0.0 | 0.0 | 0.0 | 0.0 | 0.0 | 0.0 | 0.0 | 0.0 | 0.0 | 0.0 | 0.0 | 0.0 |
| Krtap4-8 | 0.0 | 0.0 | 0.0 | 0.0 | 0.0 | 0.0 | 0.0 | 0.0 | 0.0 | 0.0 | 0.0 | 0.0 |
| Krtap4-9 | 0.0 | 0.0 | 0.0 | 0.0 | 0.0 | 0.0 | 0.0 | 0.0 | 0.0 | 0.0 | 0.0 | 0.0 |
| Krtap5-1 | 0.0 | 0.0 | 0.0 | 0.0 | 0.0 | 0.0 | 0.0 | 0.0 | 0.0 | 0.0 | 0.0 | 0.0 |
| Krtap5-2 | 0.0 | 0.0 | 0.0 | 0.0 | 0.0 | 0.0 | 0.0 | 0.0 | 0.0 | 0.0 | 0.0 | 0.0 |
| Krtap5-3 | 0.0 | 0.0 | 0.0 | 0.0 | 0.0 | 0.0 | 0.0 | 0.0 | 0.0 | 0.0 | 0.0 | 0.0 |
| Krtap5-4 | 0.0 | 0.0 | 0.0 | 0.0 | 0.0 | 0.0 | 0.0 | 0.0 | 0.0 | 0.0 | 0.0 | 0.0 |
| Krtap5-5 | 0.0 | 0.0 | 0.0 | 0.0 | 0.0 | 0.0 | 0.0 | 0.0 | 0.0 | 0.0 | 0.0 | 0.0 |
| Krtap6-1 | 0.0 | 0.0 | 0.0 | 0.0 | 0.0 | 0.0 | 0.0 | 0.0 | 0.0 | 0.0 | 0.0 | 0.0 |

Excluded Genes

| | | | | | | | | | | | |
|---|---|---|---|---|---|---|---|---|---|---|---|
| Krtap6-2 | 0.0 | 0.0 | 0.0 | 0.0 | 0.0 | 0.0 | 0.0 | 0.0 | 0.0 | 0.0 | 0.0 | 0.0 |
| Krtap6-5 | 0.0 | 0.0 | 0.0 | 0.0 | 0.0 | 0.0 | 0.0 | 0.0 | 0.0 | 0.0 | 0.0 | 0.0 |
| Krtap7-1 | 0.0 | 0.0 | 0.0 | 0.0 | 0.0 | 0.0 | 0.0 | 0.0 | 0.0 | 0.0 | 0.0 | 0.0 |
| Krtap8-1 | 0.0 | 0.0 | 0.0 | 0.0 | 0.0 | 0.0 | 0.0 | 0.0 | 0.0 | 0.0 | 0.0 | 0.0 |
| Krtap9-1 | 0.0 | 0.0 | 0.0 | 0.0 | 0.0 | 0.0 | 0.0 | 0.0 | 0.0 | 0.0 | 0.0 | 0.0 |
| Krtap9-3 | 0.0 | 0.0 | 0.0 | 0.0 | 0.0 | 0.0 | 0.0 | 0.0 | 0.0 | 0.0 | 0.0 | 0.0 |
| Krtdap | 0.0 | 0.0 | 0.0 | 0.0 | 0.0 | 0.0 | 0.0 | 0.0 | 0.0 | 0.0 | 0.0 | 0.0 |
| Lactbl1 | 0.0 | 0.0 | 0.0 | 0.0 | 0.0 | 0.0 | 0.0 | 0.0 | 0.0 | 0.0 | 0.0 | 0.0 |
| Lalba | 0.0 | 0.0 | 0.0 | 0.0 | 0.0 | 0.0 | 0.0 | 0.0 | 0.0 | 0.0 | 0.0 | 0.0 |
| Lamp5 | 0.0 | 0.0 | 0.0 | 0.0 | 0.0 | 0.0 | 0.0 | 0.0 | 0.0 | 0.0 | 0.0 | 0.0 |
| Lce1b | 0.0 | 0.0 | 0.0 | 0.0 | 0.0 | 0.0 | 0.0 | 0.0 | 0.0 | 0.0 | 0.0 | 0.0 |
| Lce1d | 0.0 | 0.0 | 0.0 | 0.0 | 0.0 | 0.0 | 0.0 | 0.0 | 0.0 | 0.0 | 0.0 | 0.0 |
| Lce1h | 0.0 | 0.0 | 0.0 | 0.0 | 0.0 | 0.0 | 0.0 | 0.0 | 0.0 | 0.0 | 0.0 | 0.0 |
| Lce1k | 0.0 | 0.0 | 0.0 | 0.0 | 0.0 | 0.0 | 0.0 | 0.0 | 0.0 | 0.0 | 0.0 | 0.0 |
| Lce1l | 0.0 | 0.0 | 0.0 | 0.0 | 0.0 | 0.0 | 0.0 | 0.0 | 0.0 | 0.0 | 0.0 | 0.0 |
| Lce3a | 0.0 | 0.0 | 0.0 | 0.0 | 0.0 | 0.0 | 0.0 | 0.0 | 0.0 | 0.0 | 0.0 | 0.0 |
| Lce3b | 0.0 | 0.0 | 0.0 | 0.0 | 0.0 | 0.0 | 0.0 | 0.0 | 0.0 | 0.0 | 0.0 | 0.0 |
| Lce3c | 0.0 | 0.0 | 0.0 | 0.0 | 0.0 | 0.0 | 0.0 | 0.0 | 0.0 | 0.0 | 0.0 | 0.0 |
| Lce3d | 0.0 | 0.0 | 0.0 | 0.0 | 0.0 | 0.0 | 0.0 | 0.0 | 0.0 | 0.0 | 0.0 | 0.0 |
| Lce3e | 0.0 | 0.0 | 0.0 | 0.0 | 0.0 | 0.0 | 0.0 | 0.0 | 0.0 | 0.0 | 0.0 | 0.0 |
| Lce3f | 0.0 | 0.0 | 0.0 | 0.0 | 0.0 | 0.0 | 0.0 | 0.0 | 0.0 | 0.0 | 0.0 | 0.0 |
| Lcn10 | 0.0 | 0.0 | 0.0 | 0.0 | 0.0 | 0.0 | 0.0 | 0.0 | 0.0 | 0.0 | 0.0 | 0.0 |
| Lcn11 | 0.0 | 0.0 | 0.0 | 0.0 | 0.0 | 0.0 | 0.0 | 0.0 | 0.0 | 0.0 | 0.0 | 0.0 |
| Lcn12 | 0.0 | 0.0 | 0.0 | 0.0 | 0.0 | 0.0 | 0.0 | 0.0 | 0.0 | 0.0 | 0.0 | 0.0 |
| Lcn3 | 0.0 | 0.0 | 0.0 | 0.0 | 0.0 | 0.0 | 0.0 | 0.0 | 0.0 | 0.0 | 0.0 | 0.0 |
| Lcn4 | 0.0 | 0.0 | 0.0 | 0.0 | 0.0 | 0.0 | 0.0 | 0.0 | 0.0 | 0.0 | 0.0 | 0.0 |
| Lcn5 | 0.0 | 0.0 | 0.0 | 0.0 | 0.0 | 0.0 | 0.0 | 0.0 | 0.0 | 0.0 | 0.0 | 0.0 |
| Lcn6 | 0.0 | 0.0 | 0.0 | 0.0 | 0.0 | 0.0 | 0.0 | 0.0 | 0.0 | 0.0 | 0.0 | 0.0 |
| Lcn9 | 0.0 | 0.0 | 0.0 | 0.0 | 0.0 | 0.0 | 0.0 | 0.0 | 0.0 | 0.0 | 0.0 | 0.0 |
| Ldoc1 | 0.0 | 0.0 | 0.0 | 0.0 | 0.0 | 0.0 | 0.0 | 0.0 | 0.0 | 0.0 | 0.0 | 0.0 |
| Lelp1 | 0.0 | 0.0 | 0.0 | 0.0 | 0.0 | 0.0 | 0.0 | 0.0 | 0.0 | 0.0 | 0.0 | 0.0 |
| Lgi1 | 0.0 | 0.0 | 0.0 | 0.0 | 0.0 | 0.0 | 0.0 | 0.0 | 0.0 | 0.0 | 0.0 | 0.0 |
| Lim2 | 0.0 | 0.0 | 0.0 | 0.0 | 0.0 | 0.0 | 0.0 | 0.0 | 0.0 | 0.0 | 0.0 | 0.0 |
| LOC100043315 | 0.0 | 0.0 | 0.0 | 0.0 | 0.0 | 0.0 | 0.0 | 0.0 | 0.0 | 0.0 | 0.0 | 0.0 |
| LOC666331 | 0.0 | 0.0 | 0.0 | 0.0 | 0.0 | 0.0 | 0.0 | 0.0 | 0.0 | 0.0 | 0.0 | 0.0 |
| Lrit2 | 0.0 | 0.0 | 0.0 | 0.0 | 0.0 | 0.0 | 0.0 | 0.0 | 0.0 | 0.0 | 0.0 | 0.0 |
| Lrrc72 | 0.0 | 0.0 | 0.0 | 0.0 | 0.0 | 0.0 | 0.0 | 0.0 | 0.0 | 0.0 | 0.0 | 0.0 |
| Lrriq4 | 0.0 | 0.0 | 0.0 | 0.0 | 0.0 | 0.0 | 0.0 | 0.0 | 0.0 | 0.0 | 0.0 | 0.0 |
| Lrrtm4 | 0.0 | 0.0 | 0.0 | 0.0 | 0.0 | 0.0 | 0.0 | 0.0 | 0.0 | 0.0 | 0.0 | 0.0 |
| Ly6g5c | 0.0 | 0.0 | 0.0 | 0.0 | 0.0 | 0.0 | 0.0 | 0.0 | 0.0 | 0.0 | 0.0 | 0.0 |
| Ly6g6e | 0.0 | 0.0 | 0.0 | 0.0 | 0.0 | 0.0 | 0.0 | 0.0 | 0.0 | 0.0 | 0.0 | 0.0 |
| Lyg1 | 0.0 | 0.0 | 0.0 | 0.0 | 0.0 | 0.0 | 0.0 | 0.0 | 0.0 | 0.0 | 0.0 | 0.0 |
| Lypd2 | 0.0 | 0.0 | 0.0 | 0.0 | 0.0 | 0.0 | 0.0 | 0.0 | 0.0 | 0.0 | 0.0 | 0.0 |
| Lypd5 | 0.0 | 0.0 | 0.0 | 0.0 | 0.0 | 0.0 | 0.0 | 0.0 | 0.0 | 0.0 | 0.0 | 0.0 |
| Lyzl1 | 0.0 | 0.0 | 0.0 | 0.0 | 0.0 | 0.0 | 0.0 | 0.0 | 0.0 | 0.0 | 0.0 | 0.0 |
| Lyzl6 | 0.0 | 0.0 | 0.0 | 0.0 | 0.0 | 0.0 | 0.0 | 0.0 | 0.0 | 0.0 | 0.0 | 0.0 |
| Magea1 | 0.0 | 0.0 | 0.0 | 0.0 | 0.0 | 0.0 | 0.0 | 0.0 | 0.0 | 0.0 | 0.0 | 0.0 |
| Magea10 | 0.0 | 0.0 | 0.0 | 0.0 | 0.0 | 0.0 | 0.0 | 0.0 | 0.0 | 0.0 | 0.0 | 0.0 |
| Magea2 | 0.0 | 0.0 | 0.0 | 0.0 | 0.0 | 0.0 | 0.0 | 0.0 | 0.0 | 0.0 | 0.0 | 0.0 |
| Magea3 | 0.0 | 0.0 | 0.0 | 0.0 | 0.0 | 0.0 | 0.0 | 0.0 | 0.0 | 0.0 | 0.0 | 0.0 |
| Magea4 | 0.0 | 0.0 | 0.0 | 0.0 | 0.0 | 0.0 | 0.0 | 0.0 | 0.0 | 0.0 | 0.0 | 0.0 |
| Magea6 | 0.0 | 0.0 | 0.0 | 0.0 | 0.0 | 0.0 | 0.0 | 0.0 | 0.0 | 0.0 | 0.0 | 0.0 |
| Mageb4 | 0.0 | 0.0 | 0.0 | 0.0 | 0.0 | 0.0 | 0.0 | 0.0 | 0.0 | 0.0 | 0.0 | 0.0 |
| Mageb5 | 0.0 | 0.0 | 0.0 | 0.0 | 0.0 | 0.0 | 0.0 | 0.0 | 0.0 | 0.0 | 0.0 | 0.0 |
| Mc3r | 0.0 | 0.0 | 0.0 | 0.0 | 0.0 | 0.0 | 0.0 | 0.0 | 0.0 | 0.0 | 0.0 | 0.0 |
| Mc4r | 0.0 | 0.0 | 0.0 | 0.0 | 0.0 | 0.0 | 0.0 | 0.0 | 0.0 | 0.0 | 0.0 | 0.0 |
| Mei1 | 0.0 | 0.0 | 0.0 | 0.0 | 0.0 | 0.0 | 0.0 | 0.0 | 0.0 | 0.0 | 0.0 | 0.0 |

| | | | | | | | | | | | | |
|---|---|---|---|---|---|---|---|---|---|---|---|---|
| Mgat4c | 0.0 | 0.0 | 0.0 | 0.0 | 0.0 | 0.0 | 0.0 | 0.0 | 0.0 | 0.0 | 0.0 | 0.0 |
| Mir100 | 0.0 | 0.0 | 0.0 | 0.0 | 0.0 | 0.0 | 0.0 | 0.0 | 0.0 | 0.0 | 0.0 | 0.0 |
| Mir101a | 0.0 | 0.0 | 0.0 | 0.0 | 0.0 | 0.0 | 0.0 | 0.0 | 0.0 | 0.0 | 0.0 | 0.0 |
| Mir101b | 0.0 | 0.0 | 0.0 | 0.0 | 0.0 | 0.0 | 0.0 | 0.0 | 0.0 | 0.0 | 0.0 | 0.0 |
| Mir101c | 0.0 | 0.0 | 0.0 | 0.0 | 0.0 | 0.0 | 0.0 | 0.0 | 0.0 | 0.0 | 0.0 | 0.0 |
| Mir103-1 | 0.0 | 0.0 | 0.0 | 0.0 | 0.0 | 0.0 | 0.0 | 0.0 | 0.0 | 0.0 | 0.0 | 0.0 |
| Mir103-2 | 0.0 | 0.0 | 0.0 | 0.0 | 0.0 | 0.0 | 0.0 | 0.0 | 0.0 | 0.0 | 0.0 | 0.0 |
| Mir105 | 0.0 | 0.0 | 0.0 | 0.0 | 0.0 | 0.0 | 0.0 | 0.0 | 0.0 | 0.0 | 0.0 | 0.0 |
| Mir106a | 0.0 | 0.0 | 0.0 | 0.0 | 0.0 | 0.0 | 0.0 | 0.0 | 0.0 | 0.0 | 0.0 | 0.0 |
| Mir106b | 0.0 | 0.0 | 0.0 | 0.0 | 0.0 | 0.0 | 0.0 | 0.0 | 0.0 | 0.0 | 0.0 | 0.0 |
| Mir107 | 0.0 | 0.0 | 0.0 | 0.0 | 0.0 | 0.0 | 0.0 | 0.0 | 0.0 | 0.0 | 0.0 | 0.0 |
| Mir10b | 0.0 | 0.0 | 0.0 | 0.0 | 0.0 | 0.0 | 0.0 | 0.0 | 0.0 | 0.0 | 0.0 | 0.0 |
| Mir1186b | 0.0 | 0.0 | 0.0 | 0.0 | 0.0 | 0.0 | 0.0 | 0.0 | 0.0 | 0.0 | 0.0 | 0.0 |
| Mir1187 | 0.0 | 0.0 | 0.0 | 0.0 | 0.0 | 0.0 | 0.0 | 0.0 | 0.0 | 0.0 | 0.0 | 0.0 |
| Mir1190 | 0.0 | 0.0 | 0.0 | 0.0 | 0.0 | 0.0 | 0.0 | 0.0 | 0.0 | 0.0 | 0.0 | 0.0 |
| Mir1191 | 0.0 | 0.0 | 0.0 | 0.0 | 0.0 | 0.0 | 0.0 | 0.0 | 0.0 | 0.0 | 0.0 | 0.0 |
| Mir1192 | 0.0 | 0.0 | 0.0 | 0.0 | 0.0 | 0.0 | 0.0 | 0.0 | 0.0 | 0.0 | 0.0 | 0.0 |
| Mir1195 | 0.0 | 0.0 | 0.0 | 0.0 | 0.0 | 0.0 | 0.0 | 0.0 | 0.0 | 0.0 | 0.0 | 0.0 |
| Mir1196 | 0.0 | 0.0 | 0.0 | 0.0 | 0.0 | 0.0 | 0.0 | 0.0 | 0.0 | 0.0 | 0.0 | 0.0 |
| Mir1198 | 0.0 | 0.0 | 0.0 | 0.0 | 0.0 | 0.0 | 0.0 | 0.0 | 0.0 | 0.0 | 0.0 | 0.0 |
| Mir1199 | 0.0 | 0.0 | 0.0 | 0.0 | 0.0 | 0.0 | 0.0 | 0.0 | 0.0 | 0.0 | 0.0 | 0.0 |
| Mir1224 | 0.0 | 0.0 | 0.0 | 0.0 | 0.0 | 0.0 | 0.0 | 0.0 | 0.0 | 0.0 | 0.0 | 0.0 |
| Mir122a | 0.0 | 0.0 | 0.0 | 0.0 | 0.0 | 0.0 | 0.0 | 0.0 | 0.0 | 0.0 | 0.0 | 0.0 |
| Mir1249 | 0.0 | 0.0 | 0.0 | 0.0 | 0.0 | 0.0 | 0.0 | 0.0 | 0.0 | 0.0 | 0.0 | 0.0 |
| Mir124a-1 | 0.0 | 0.0 | 0.0 | 0.0 | 0.0 | 0.0 | 0.0 | 0.0 | 0.0 | 0.0 | 0.0 | 0.0 |
| Mir124a-2 | 0.0 | 0.0 | 0.0 | 0.0 | 0.0 | 0.0 | 0.0 | 0.0 | 0.0 | 0.0 | 0.0 | 0.0 |
| Mir124a-3 | 0.0 | 0.0 | 0.0 | 0.0 | 0.0 | 0.0 | 0.0 | 0.0 | 0.0 | 0.0 | 0.0 | 0.0 |
| Mir1251 | 0.0 | 0.0 | 0.0 | 0.0 | 0.0 | 0.0 | 0.0 | 0.0 | 0.0 | 0.0 | 0.0 | 0.0 |
| Mir125a | 0.0 | 0.0 | 0.0 | 0.0 | 0.0 | 0.0 | 0.0 | 0.0 | 0.0 | 0.0 | 0.0 | 0.0 |
| Mir125b-1 | 0.0 | 0.0 | 0.0 | 0.0 | 0.0 | 0.0 | 0.0 | 0.0 | 0.0 | 0.0 | 0.0 | 0.0 |
| Mir125b-2 | 0.0 | 0.0 | 0.0 | 0.0 | 0.0 | 0.0 | 0.0 | 0.0 | 0.0 | 0.0 | 0.0 | 0.0 |
| Mir126 | 0.0 | 0.0 | 0.0 | 0.0 | 0.0 | 0.0 | 0.0 | 0.0 | 0.0 | 0.0 | 0.0 | 0.0 |
| Mir1264 | 0.0 | 0.0 | 0.0 | 0.0 | 0.0 | 0.0 | 0.0 | 0.0 | 0.0 | 0.0 | 0.0 | 0.0 |
| Mir127 | 0.0 | 0.0 | 0.0 | 0.0 | 0.0 | 0.0 | 0.0 | 0.0 | 0.0 | 0.0 | 0.0 | 0.0 |
| Mir128-1 | 0.0 | 0.0 | 0.0 | 0.0 | 0.0 | 0.0 | 0.0 | 0.0 | 0.0 | 0.0 | 0.0 | 0.0 |
| Mir128-2 | 0.0 | 0.0 | 0.0 | 0.0 | 0.0 | 0.0 | 0.0 | 0.0 | 0.0 | 0.0 | 0.0 | 0.0 |
| Mir129-1 | 0.0 | 0.0 | 0.0 | 0.0 | 0.0 | 0.0 | 0.0 | 0.0 | 0.0 | 0.0 | 0.0 | 0.0 |
| Mir129-2 | 0.0 | 0.0 | 0.0 | 0.0 | 0.0 | 0.0 | 0.0 | 0.0 | 0.0 | 0.0 | 0.0 | 0.0 |
| Mir1298 | 0.0 | 0.0 | 0.0 | 0.0 | 0.0 | 0.0 | 0.0 | 0.0 | 0.0 | 0.0 | 0.0 | 0.0 |
| Mir1306 | 0.0 | 0.0 | 0.0 | 0.0 | 0.0 | 0.0 | 0.0 | 0.0 | 0.0 | 0.0 | 0.0 | 0.0 |
| Mir130a | 0.0 | 0.0 | 0.0 | 0.0 | 0.0 | 0.0 | 0.0 | 0.0 | 0.0 | 0.0 | 0.0 | 0.0 |
| Mir130b | 0.0 | 0.0 | 0.0 | 0.0 | 0.0 | 0.0 | 0.0 | 0.0 | 0.0 | 0.0 | 0.0 | 0.0 |
| Mir132 | 0.0 | 0.0 | 0.0 | 0.0 | 0.0 | 0.0 | 0.0 | 0.0 | 0.0 | 0.0 | 0.0 | 0.0 |
| Mir133a-1 | 0.0 | 0.0 | 0.0 | 0.0 | 0.0 | 0.0 | 0.0 | 0.0 | 0.0 | 0.0 | 0.0 | 0.0 |
| Mir133a-2 | 0.0 | 0.0 | 0.0 | 0.0 | 0.0 | 0.0 | 0.0 | 0.0 | 0.0 | 0.0 | 0.0 | 0.0 |
| Mir133b | 0.0 | 0.0 | 0.0 | 0.0 | 0.0 | 0.0 | 0.0 | 0.0 | 0.0 | 0.0 | 0.0 | 0.0 |
| Mir134 | 0.0 | 0.0 | 0.0 | 0.0 | 0.0 | 0.0 | 0.0 | 0.0 | 0.0 | 0.0 | 0.0 | 0.0 |
| Mir135a-1 | 0.0 | 0.0 | 0.0 | 0.0 | 0.0 | 0.0 | 0.0 | 0.0 | 0.0 | 0.0 | 0.0 | 0.0 |
| Mir135a-2 | 0.0 | 0.0 | 0.0 | 0.0 | 0.0 | 0.0 | 0.0 | 0.0 | 0.0 | 0.0 | 0.0 | 0.0 |
| Mir135b | 0.0 | 0.0 | 0.0 | 0.0 | 0.0 | 0.0 | 0.0 | 0.0 | 0.0 | 0.0 | 0.0 | 0.0 |
| Mir136 | 0.0 | 0.0 | 0.0 | 0.0 | 0.0 | 0.0 | 0.0 | 0.0 | 0.0 | 0.0 | 0.0 | 0.0 |
| Mir137 | 0.0 | 0.0 | 0.0 | 0.0 | 0.0 | 0.0 | 0.0 | 0.0 | 0.0 | 0.0 | 0.0 | 0.0 |
| Mir138-1 | 0.0 | 0.0 | 0.0 | 0.0 | 0.0 | 0.0 | 0.0 | 0.0 | 0.0 | 0.0 | 0.0 | 0.0 |
| Mir138-2 | 0.0 | 0.0 | 0.0 | 0.0 | 0.0 | 0.0 | 0.0 | 0.0 | 0.0 | 0.0 | 0.0 | 0.0 |
| Mir139 | 0.0 | 0.0 | 0.0 | 0.0 | 0.0 | 0.0 | 0.0 | 0.0 | 0.0 | 0.0 | 0.0 | 0.0 |
| Mir140 | 0.0 | 0.0 | 0.0 | 0.0 | 0.0 | 0.0 | 0.0 | 0.0 | 0.0 | 0.0 | 0.0 | 0.0 |
| Mir141 | 0.0 | 0.0 | 0.0 | 0.0 | 0.0 | 0.0 | 0.0 | 0.0 | 0.0 | 0.0 | 0.0 | 0.0 |

| | | | | | | | | | | | | |
|---|---|---|---|---|---|---|---|---|---|---|---|---|
| Mir142 | 0.0 | 0.0 | 0.0 | 0.0 | 0.0 | 0.0 | 0.0 | 0.0 | 0.0 | 0.0 | 0.0 | 0.0 |
| Mir143 | 0.0 | 0.0 | 0.0 | 0.0 | 0.0 | 0.0 | 0.0 | 0.0 | 0.0 | 0.0 | 0.0 | 0.0 |
| Mir144 | 0.0 | 0.0 | 0.0 | 0.0 | 0.0 | 0.0 | 0.0 | 0.0 | 0.0 | 0.0 | 0.0 | 0.0 |
| Mir145 | 0.0 | 0.0 | 0.0 | 0.0 | 0.0 | 0.0 | 0.0 | 0.0 | 0.0 | 0.0 | 0.0 | 0.0 |
| Mir146 | 0.0 | 0.0 | 0.0 | 0.0 | 0.0 | 0.0 | 0.0 | 0.0 | 0.0 | 0.0 | 0.0 | 0.0 |
| Mir146b | 0.0 | 0.0 | 0.0 | 0.0 | 0.0 | 0.0 | 0.0 | 0.0 | 0.0 | 0.0 | 0.0 | 0.0 |
| Mir147 | 0.0 | 0.0 | 0.0 | 0.0 | 0.0 | 0.0 | 0.0 | 0.0 | 0.0 | 0.0 | 0.0 | 0.0 |
| Mir148a | 0.0 | 0.0 | 0.0 | 0.0 | 0.0 | 0.0 | 0.0 | 0.0 | 0.0 | 0.0 | 0.0 | 0.0 |
| Mir148b | 0.0 | 0.0 | 0.0 | 0.0 | 0.0 | 0.0 | 0.0 | 0.0 | 0.0 | 0.0 | 0.0 | 0.0 |
| Mir149 | 0.0 | 0.0 | 0.0 | 0.0 | 0.0 | 0.0 | 0.0 | 0.0 | 0.0 | 0.0 | 0.0 | 0.0 |
| Mir150 | 0.0 | 0.0 | 0.0 | 0.0 | 0.0 | 0.0 | 0.0 | 0.0 | 0.0 | 0.0 | 0.0 | 0.0 |
| Mir152 | 0.0 | 0.0 | 0.0 | 0.0 | 0.0 | 0.0 | 0.0 | 0.0 | 0.0 | 0.0 | 0.0 | 0.0 |
| Mir153 | 0.0 | 0.0 | 0.0 | 0.0 | 0.0 | 0.0 | 0.0 | 0.0 | 0.0 | 0.0 | 0.0 | 0.0 |
| Mir154 | 0.0 | 0.0 | 0.0 | 0.0 | 0.0 | 0.0 | 0.0 | 0.0 | 0.0 | 0.0 | 0.0 | 0.0 |
| Mir155 | 0.0 | 0.0 | 0.0 | 0.0 | 0.0 | 0.0 | 0.0 | 0.0 | 0.0 | 0.0 | 0.0 | 0.0 |
| Mir15a | 0.0 | 0.0 | 0.0 | 0.0 | 0.0 | 0.0 | 0.0 | 0.0 | 0.0 | 0.0 | 0.0 | 0.0 |
| Mir15b | 0.0 | 0.0 | 0.0 | 0.0 | 0.0 | 0.0 | 0.0 | 0.0 | 0.0 | 0.0 | 0.0 | 0.0 |
| Mir16-1 | 0.0 | 0.0 | 0.0 | 0.0 | 0.0 | 0.0 | 0.0 | 0.0 | 0.0 | 0.0 | 0.0 | 0.0 |
| Mir16-2 | 0.0 | 0.0 | 0.0 | 0.0 | 0.0 | 0.0 | 0.0 | 0.0 | 0.0 | 0.0 | 0.0 | 0.0 |
| Mir17 | 0.0 | 0.0 | 0.0 | 0.0 | 0.0 | 0.0 | 0.0 | 0.0 | 0.0 | 0.0 | 0.0 | 0.0 |
| Mir18 | 0.0 | 0.0 | 0.0 | 0.0 | 0.0 | 0.0 | 0.0 | 0.0 | 0.0 | 0.0 | 0.0 | 0.0 |
| Mir181a-1 | 0.0 | 0.0 | 0.0 | 0.0 | 0.0 | 0.0 | 0.0 | 0.0 | 0.0 | 0.0 | 0.0 | 0.0 |
| Mir181a-2 | 0.0 | 0.0 | 0.0 | 0.0 | 0.0 | 0.0 | 0.0 | 0.0 | 0.0 | 0.0 | 0.0 | 0.0 |
| Mir181b-1 | 0.0 | 0.0 | 0.0 | 0.0 | 0.0 | 0.0 | 0.0 | 0.0 | 0.0 | 0.0 | 0.0 | 0.0 |
| Mir181b-2 | 0.0 | 0.0 | 0.0 | 0.0 | 0.0 | 0.0 | 0.0 | 0.0 | 0.0 | 0.0 | 0.0 | 0.0 |
| Mir181c | 0.0 | 0.0 | 0.0 | 0.0 | 0.0 | 0.0 | 0.0 | 0.0 | 0.0 | 0.0 | 0.0 | 0.0 |
| Mir181d | 0.0 | 0.0 | 0.0 | 0.0 | 0.0 | 0.0 | 0.0 | 0.0 | 0.0 | 0.0 | 0.0 | 0.0 |
| Mir182 | 0.0 | 0.0 | 0.0 | 0.0 | 0.0 | 0.0 | 0.0 | 0.0 | 0.0 | 0.0 | 0.0 | 0.0 |
| Mir183 | 0.0 | 0.0 | 0.0 | 0.0 | 0.0 | 0.0 | 0.0 | 0.0 | 0.0 | 0.0 | 0.0 | 0.0 |
| Mir1839 | 0.0 | 0.0 | 0.0 | 0.0 | 0.0 | 0.0 | 0.0 | 0.0 | 0.0 | 0.0 | 0.0 | 0.0 |
| Mir184 | 0.0 | 0.0 | 0.0 | 0.0 | 0.0 | 0.0 | 0.0 | 0.0 | 0.0 | 0.0 | 0.0 | 0.0 |
| Mir1843 | 0.0 | 0.0 | 0.0 | 0.0 | 0.0 | 0.0 | 0.0 | 0.0 | 0.0 | 0.0 | 0.0 | 0.0 |
| Mir185 | 0.0 | 0.0 | 0.0 | 0.0 | 0.0 | 0.0 | 0.0 | 0.0 | 0.0 | 0.0 | 0.0 | 0.0 |
| Mir186 | 0.0 | 0.0 | 0.0 | 0.0 | 0.0 | 0.0 | 0.0 | 0.0 | 0.0 | 0.0 | 0.0 | 0.0 |
| Mir187 | 0.0 | 0.0 | 0.0 | 0.0 | 0.0 | 0.0 | 0.0 | 0.0 | 0.0 | 0.0 | 0.0 | 0.0 |
| Mir188 | 0.0 | 0.0 | 0.0 | 0.0 | 0.0 | 0.0 | 0.0 | 0.0 | 0.0 | 0.0 | 0.0 | 0.0 |
| Mir1893 | 0.0 | 0.0 | 0.0 | 0.0 | 0.0 | 0.0 | 0.0 | 0.0 | 0.0 | 0.0 | 0.0 | 0.0 |
| Mir1895 | 0.0 | 0.0 | 0.0 | 0.0 | 0.0 | 0.0 | 0.0 | 0.0 | 0.0 | 0.0 | 0.0 | 0.0 |
| Mir1897 | 0.0 | 0.0 | 0.0 | 0.0 | 0.0 | 0.0 | 0.0 | 0.0 | 0.0 | 0.0 | 0.0 | 0.0 |
| Mir1898 | 0.0 | 0.0 | 0.0 | 0.0 | 0.0 | 0.0 | 0.0 | 0.0 | 0.0 | 0.0 | 0.0 | 0.0 |
| Mir1899 | 0.0 | 0.0 | 0.0 | 0.0 | 0.0 | 0.0 | 0.0 | 0.0 | 0.0 | 0.0 | 0.0 | 0.0 |
| Mir18b | 0.0 | 0.0 | 0.0 | 0.0 | 0.0 | 0.0 | 0.0 | 0.0 | 0.0 | 0.0 | 0.0 | 0.0 |
| Mir190 | 0.0 | 0.0 | 0.0 | 0.0 | 0.0 | 0.0 | 0.0 | 0.0 | 0.0 | 0.0 | 0.0 | 0.0 |
| Mir1900 | 0.0 | 0.0 | 0.0 | 0.0 | 0.0 | 0.0 | 0.0 | 0.0 | 0.0 | 0.0 | 0.0 | 0.0 |
| Mir1901 | 0.0 | 0.0 | 0.0 | 0.0 | 0.0 | 0.0 | 0.0 | 0.0 | 0.0 | 0.0 | 0.0 | 0.0 |
| Mir1902 | 0.0 | 0.0 | 0.0 | 0.0 | 0.0 | 0.0 | 0.0 | 0.0 | 0.0 | 0.0 | 0.0 | 0.0 |
| Mir1904 | 0.0 | 0.0 | 0.0 | 0.0 | 0.0 | 0.0 | 0.0 | 0.0 | 0.0 | 0.0 | 0.0 | 0.0 |
| Mir1907 | 0.0 | 0.0 | 0.0 | 0.0 | 0.0 | 0.0 | 0.0 | 0.0 | 0.0 | 0.0 | 0.0 | 0.0 |
| Mir190b | 0.0 | 0.0 | 0.0 | 0.0 | 0.0 | 0.0 | 0.0 | 0.0 | 0.0 | 0.0 | 0.0 | 0.0 |
| Mir191 | 0.0 | 0.0 | 0.0 | 0.0 | 0.0 | 0.0 | 0.0 | 0.0 | 0.0 | 0.0 | 0.0 | 0.0 |
| Mir1912 | 0.0 | 0.0 | 0.0 | 0.0 | 0.0 | 0.0 | 0.0 | 0.0 | 0.0 | 0.0 | 0.0 | 0.0 |
| Mir192 | 0.0 | 0.0 | 0.0 | 0.0 | 0.0 | 0.0 | 0.0 | 0.0 | 0.0 | 0.0 | 0.0 | 0.0 |
| Mir1929 | 0.0 | 0.0 | 0.0 | 0.0 | 0.0 | 0.0 | 0.0 | 0.0 | 0.0 | 0.0 | 0.0 | 0.0 |
| Mir193 | 0.0 | 0.0 | 0.0 | 0.0 | 0.0 | 0.0 | 0.0 | 0.0 | 0.0 | 0.0 | 0.0 | 0.0 |
| Mir1930 | 0.0 | 0.0 | 0.0 | 0.0 | 0.0 | 0.0 | 0.0 | 0.0 | 0.0 | 0.0 | 0.0 | 0.0 |
| Mir1931 | 0.0 | 0.0 | 0.0 | 0.0 | 0.0 | 0.0 | 0.0 | 0.0 | 0.0 | 0.0 | 0.0 | 0.0 |
| Mir1932 | 0.0 | 0.0 | 0.0 | 0.0 | 0.0 | 0.0 | 0.0 | 0.0 | 0.0 | 0.0 | 0.0 | 0.0 |

Excluded Genes

| | | | | | | | | | | | |
|---|---|---|---|---|---|---|---|---|---|---|---|
| Mir1933 | 0.0 | 0.0 | 0.0 | 0.0 | 0.0 | 0.0 | 0.0 | 0.0 | 0.0 | 0.0 | 0.0 | 0.0 |
| Mir1934 | 0.0 | 0.0 | 0.0 | 0.0 | 0.0 | 0.0 | 0.0 | 0.0 | 0.0 | 0.0 | 0.0 | 0.0 |
| Mir1935 | 0.0 | 0.0 | 0.0 | 0.0 | 0.0 | 0.0 | 0.0 | 0.0 | 0.0 | 0.0 | 0.0 | 0.0 |
| Mir1936 | 0.0 | 0.0 | 0.0 | 0.0 | 0.0 | 0.0 | 0.0 | 0.0 | 0.0 | 0.0 | 0.0 | 0.0 |
| Mir193b | 0.0 | 0.0 | 0.0 | 0.0 | 0.0 | 0.0 | 0.0 | 0.0 | 0.0 | 0.0 | 0.0 | 0.0 |
| Mir1941 | 0.0 | 0.0 | 0.0 | 0.0 | 0.0 | 0.0 | 0.0 | 0.0 | 0.0 | 0.0 | 0.0 | 0.0 |
| Mir1942 | 0.0 | 0.0 | 0.0 | 0.0 | 0.0 | 0.0 | 0.0 | 0.0 | 0.0 | 0.0 | 0.0 | 0.0 |
| Mir194-2 | 0.0 | 0.0 | 0.0 | 0.0 | 0.0 | 0.0 | 0.0 | 0.0 | 0.0 | 0.0 | 0.0 | 0.0 |
| Mir1943 | 0.0 | 0.0 | 0.0 | 0.0 | 0.0 | 0.0 | 0.0 | 0.0 | 0.0 | 0.0 | 0.0 | 0.0 |
| Mir1945 | 0.0 | 0.0 | 0.0 | 0.0 | 0.0 | 0.0 | 0.0 | 0.0 | 0.0 | 0.0 | 0.0 | 0.0 |
| Mir1946b | 0.0 | 0.0 | 0.0 | 0.0 | 0.0 | 0.0 | 0.0 | 0.0 | 0.0 | 0.0 | 0.0 | 0.0 |
| Mir1947 | 0.0 | 0.0 | 0.0 | 0.0 | 0.0 | 0.0 | 0.0 | 0.0 | 0.0 | 0.0 | 0.0 | 0.0 |
| Mir1948 | 0.0 | 0.0 | 0.0 | 0.0 | 0.0 | 0.0 | 0.0 | 0.0 | 0.0 | 0.0 | 0.0 | 0.0 |
| Mir195 | 0.0 | 0.0 | 0.0 | 0.0 | 0.0 | 0.0 | 0.0 | 0.0 | 0.0 | 0.0 | 0.0 | 0.0 |
| Mir1950 | 0.0 | 0.0 | 0.0 | 0.0 | 0.0 | 0.0 | 0.0 | 0.0 | 0.0 | 0.0 | 0.0 | 0.0 |
| Mir1951 | 0.0 | 0.0 | 0.0 | 0.0 | 0.0 | 0.0 | 0.0 | 0.0 | 0.0 | 0.0 | 0.0 | 0.0 |
| Mir1952 | 0.0 | 0.0 | 0.0 | 0.0 | 0.0 | 0.0 | 0.0 | 0.0 | 0.0 | 0.0 | 0.0 | 0.0 |
| Mir1953 | 0.0 | 0.0 | 0.0 | 0.0 | 0.0 | 0.0 | 0.0 | 0.0 | 0.0 | 0.0 | 0.0 | 0.0 |
| Mir1954 | 0.0 | 0.0 | 0.0 | 0.0 | 0.0 | 0.0 | 0.0 | 0.0 | 0.0 | 0.0 | 0.0 | 0.0 |
| Mir1956 | 0.0 | 0.0 | 0.0 | 0.0 | 0.0 | 0.0 | 0.0 | 0.0 | 0.0 | 0.0 | 0.0 | 0.0 |
| Mir1958 | 0.0 | 0.0 | 0.0 | 0.0 | 0.0 | 0.0 | 0.0 | 0.0 | 0.0 | 0.0 | 0.0 | 0.0 |
| Mir1960 | 0.0 | 0.0 | 0.0 | 0.0 | 0.0 | 0.0 | 0.0 | 0.0 | 0.0 | 0.0 | 0.0 | 0.0 |
| Mir1961 | 0.0 | 0.0 | 0.0 | 0.0 | 0.0 | 0.0 | 0.0 | 0.0 | 0.0 | 0.0 | 0.0 | 0.0 |
| Mir1962 | 0.0 | 0.0 | 0.0 | 0.0 | 0.0 | 0.0 | 0.0 | 0.0 | 0.0 | 0.0 | 0.0 | 0.0 |
| Mir1963 | 0.0 | 0.0 | 0.0 | 0.0 | 0.0 | 0.0 | 0.0 | 0.0 | 0.0 | 0.0 | 0.0 | 0.0 |
| Mir1964 | 0.0 | 0.0 | 0.0 | 0.0 | 0.0 | 0.0 | 0.0 | 0.0 | 0.0 | 0.0 | 0.0 | 0.0 |
| Mir1965 | 0.0 | 0.0 | 0.0 | 0.0 | 0.0 | 0.0 | 0.0 | 0.0 | 0.0 | 0.0 | 0.0 | 0.0 |
| Mir1967 | 0.0 | 0.0 | 0.0 | 0.0 | 0.0 | 0.0 | 0.0 | 0.0 | 0.0 | 0.0 | 0.0 | 0.0 |
| Mir1968 | 0.0 | 0.0 | 0.0 | 0.0 | 0.0 | 0.0 | 0.0 | 0.0 | 0.0 | 0.0 | 0.0 | 0.0 |
| Mir1969 | 0.0 | 0.0 | 0.0 | 0.0 | 0.0 | 0.0 | 0.0 | 0.0 | 0.0 | 0.0 | 0.0 | 0.0 |
| Mir196a-1 | 0.0 | 0.0 | 0.0 | 0.0 | 0.0 | 0.0 | 0.0 | 0.0 | 0.0 | 0.0 | 0.0 | 0.0 |
| Mir196a-2 | 0.0 | 0.0 | 0.0 | 0.0 | 0.0 | 0.0 | 0.0 | 0.0 | 0.0 | 0.0 | 0.0 | 0.0 |
| Mir196b | 0.0 | 0.0 | 0.0 | 0.0 | 0.0 | 0.0 | 0.0 | 0.0 | 0.0 | 0.0 | 0.0 | 0.0 |
| Mir1970 | 0.0 | 0.0 | 0.0 | 0.0 | 0.0 | 0.0 | 0.0 | 0.0 | 0.0 | 0.0 | 0.0 | 0.0 |
| Mir1971 | 0.0 | 0.0 | 0.0 | 0.0 | 0.0 | 0.0 | 0.0 | 0.0 | 0.0 | 0.0 | 0.0 | 0.0 |
| Mir1981 | 0.0 | 0.0 | 0.0 | 0.0 | 0.0 | 0.0 | 0.0 | 0.0 | 0.0 | 0.0 | 0.0 | 0.0 |
| Mir1982 | 0.0 | 0.0 | 0.0 | 0.0 | 0.0 | 0.0 | 0.0 | 0.0 | 0.0 | 0.0 | 0.0 | 0.0 |
| Mir199a-1 | 0.0 | 0.0 | 0.0 | 0.0 | 0.0 | 0.0 | 0.0 | 0.0 | 0.0 | 0.0 | 0.0 | 0.0 |
| Mir199b | 0.0 | 0.0 | 0.0 | 0.0 | 0.0 | 0.0 | 0.0 | 0.0 | 0.0 | 0.0 | 0.0 | 0.0 |
| Mir19a | 0.0 | 0.0 | 0.0 | 0.0 | 0.0 | 0.0 | 0.0 | 0.0 | 0.0 | 0.0 | 0.0 | 0.0 |
| Mir19b-1 | 0.0 | 0.0 | 0.0 | 0.0 | 0.0 | 0.0 | 0.0 | 0.0 | 0.0 | 0.0 | 0.0 | 0.0 |
| Mir19b-2 | 0.0 | 0.0 | 0.0 | 0.0 | 0.0 | 0.0 | 0.0 | 0.0 | 0.0 | 0.0 | 0.0 | 0.0 |
| Mir1a-1 | 0.0 | 0.0 | 0.0 | 0.0 | 0.0 | 0.0 | 0.0 | 0.0 | 0.0 | 0.0 | 0.0 | 0.0 |
| Mir1a-2 | 0.0 | 0.0 | 0.0 | 0.0 | 0.0 | 0.0 | 0.0 | 0.0 | 0.0 | 0.0 | 0.0 | 0.0 |
| Mir1b | 0.0 | 0.0 | 0.0 | 0.0 | 0.0 | 0.0 | 0.0 | 0.0 | 0.0 | 0.0 | 0.0 | 0.0 |
| Mir200a | 0.0 | 0.0 | 0.0 | 0.0 | 0.0 | 0.0 | 0.0 | 0.0 | 0.0 | 0.0 | 0.0 | 0.0 |
| Mir200b | 0.0 | 0.0 | 0.0 | 0.0 | 0.0 | 0.0 | 0.0 | 0.0 | 0.0 | 0.0 | 0.0 | 0.0 |
| Mir200c | 0.0 | 0.0 | 0.0 | 0.0 | 0.0 | 0.0 | 0.0 | 0.0 | 0.0 | 0.0 | 0.0 | 0.0 |
| Mir201 | 0.0 | 0.0 | 0.0 | 0.0 | 0.0 | 0.0 | 0.0 | 0.0 | 0.0 | 0.0 | 0.0 | 0.0 |
| Mir202 | 0.0 | 0.0 | 0.0 | 0.0 | 0.0 | 0.0 | 0.0 | 0.0 | 0.0 | 0.0 | 0.0 | 0.0 |
| Mir203 | 0.0 | 0.0 | 0.0 | 0.0 | 0.0 | 0.0 | 0.0 | 0.0 | 0.0 | 0.0 | 0.0 | 0.0 |
| Mir204 | 0.0 | 0.0 | 0.0 | 0.0 | 0.0 | 0.0 | 0.0 | 0.0 | 0.0 | 0.0 | 0.0 | 0.0 |
| Mir205 | 0.0 | 0.0 | 0.0 | 0.0 | 0.0 | 0.0 | 0.0 | 0.0 | 0.0 | 0.0 | 0.0 | 0.0 |
| Mir206 | 0.0 | 0.0 | 0.0 | 0.0 | 0.0 | 0.0 | 0.0 | 0.0 | 0.0 | 0.0 | 0.0 | 0.0 |
| Mir208a | 0.0 | 0.0 | 0.0 | 0.0 | 0.0 | 0.0 | 0.0 | 0.0 | 0.0 | 0.0 | 0.0 | 0.0 |
| Mir208b | 0.0 | 0.0 | 0.0 | 0.0 | 0.0 | 0.0 | 0.0 | 0.0 | 0.0 | 0.0 | 0.0 | 0.0 |
| Mir20a | 0.0 | 0.0 | 0.0 | 0.0 | 0.0 | 0.0 | 0.0 | 0.0 | 0.0 | 0.0 | 0.0 | 0.0 |

Excluded Genes

| | | | | | | | | | | | | |
|---|---|---|---|---|---|---|---|---|---|---|---|---|
| Mir20b | 0.0 | 0.0 | 0.0 | 0.0 | 0.0 | 0.0 | 0.0 | 0.0 | 0.0 | 0.0 | 0.0 | 0.0 |
| Mir21 | 0.0 | 0.0 | 0.0 | 0.0 | 0.0 | 0.0 | 0.0 | 0.0 | 0.0 | 0.0 | 0.0 | 0.0 |
| Mir210 | 0.0 | 0.0 | 0.0 | 0.0 | 0.0 | 0.0 | 0.0 | 0.0 | 0.0 | 0.0 | 0.0 | 0.0 |
| Mir211 | 0.0 | 0.0 | 0.0 | 0.0 | 0.0 | 0.0 | 0.0 | 0.0 | 0.0 | 0.0 | 0.0 | 0.0 |
| Mir212 | 0.0 | 0.0 | 0.0 | 0.0 | 0.0 | 0.0 | 0.0 | 0.0 | 0.0 | 0.0 | 0.0 | 0.0 |
| Mir2136 | 0.0 | 0.0 | 0.0 | 0.0 | 0.0 | 0.0 | 0.0 | 0.0 | 0.0 | 0.0 | 0.0 | 0.0 |
| Mir215 | 0.0 | 0.0 | 0.0 | 0.0 | 0.0 | 0.0 | 0.0 | 0.0 | 0.0 | 0.0 | 0.0 | 0.0 |
| Mir216a | 0.0 | 0.0 | 0.0 | 0.0 | 0.0 | 0.0 | 0.0 | 0.0 | 0.0 | 0.0 | 0.0 | 0.0 |
| Mir216b | 0.0 | 0.0 | 0.0 | 0.0 | 0.0 | 0.0 | 0.0 | 0.0 | 0.0 | 0.0 | 0.0 | 0.0 |
| Mir217 | 0.0 | 0.0 | 0.0 | 0.0 | 0.0 | 0.0 | 0.0 | 0.0 | 0.0 | 0.0 | 0.0 | 0.0 |
| Mir218-1 | 0.0 | 0.0 | 0.0 | 0.0 | 0.0 | 0.0 | 0.0 | 0.0 | 0.0 | 0.0 | 0.0 | 0.0 |
| Mir218-2 | 0.0 | 0.0 | 0.0 | 0.0 | 0.0 | 0.0 | 0.0 | 0.0 | 0.0 | 0.0 | 0.0 | 0.0 |
| Mir219-2 | 0.0 | 0.0 | 0.0 | 0.0 | 0.0 | 0.0 | 0.0 | 0.0 | 0.0 | 0.0 | 0.0 | 0.0 |
| Mir221 | 0.0 | 0.0 | 0.0 | 0.0 | 0.0 | 0.0 | 0.0 | 0.0 | 0.0 | 0.0 | 0.0 | 0.0 |
| Mir222 | 0.0 | 0.0 | 0.0 | 0.0 | 0.0 | 0.0 | 0.0 | 0.0 | 0.0 | 0.0 | 0.0 | 0.0 |
| Mir223 | 0.0 | 0.0 | 0.0 | 0.0 | 0.0 | 0.0 | 0.0 | 0.0 | 0.0 | 0.0 | 0.0 | 0.0 |
| Mir23a | 0.0 | 0.0 | 0.0 | 0.0 | 0.0 | 0.0 | 0.0 | 0.0 | 0.0 | 0.0 | 0.0 | 0.0 |
| Mir24-2 | 0.0 | 0.0 | 0.0 | 0.0 | 0.0 | 0.0 | 0.0 | 0.0 | 0.0 | 0.0 | 0.0 | 0.0 |
| Mir25 | 0.0 | 0.0 | 0.0 | 0.0 | 0.0 | 0.0 | 0.0 | 0.0 | 0.0 | 0.0 | 0.0 | 0.0 |
| Mir26a-1 | 0.0 | 0.0 | 0.0 | 0.0 | 0.0 | 0.0 | 0.0 | 0.0 | 0.0 | 0.0 | 0.0 | 0.0 |
| Mir26a-2 | 0.0 | 0.0 | 0.0 | 0.0 | 0.0 | 0.0 | 0.0 | 0.0 | 0.0 | 0.0 | 0.0 | 0.0 |
| Mir26b | 0.0 | 0.0 | 0.0 | 0.0 | 0.0 | 0.0 | 0.0 | 0.0 | 0.0 | 0.0 | 0.0 | 0.0 |
| Mir27b | 0.0 | 0.0 | 0.0 | 0.0 | 0.0 | 0.0 | 0.0 | 0.0 | 0.0 | 0.0 | 0.0 | 0.0 |
| Mir28 | 0.0 | 0.0 | 0.0 | 0.0 | 0.0 | 0.0 | 0.0 | 0.0 | 0.0 | 0.0 | 0.0 | 0.0 |
| Mir28b | 0.0 | 0.0 | 0.0 | 0.0 | 0.0 | 0.0 | 0.0 | 0.0 | 0.0 | 0.0 | 0.0 | 0.0 |
| Mir28c | 0.0 | 0.0 | 0.0 | 0.0 | 0.0 | 0.0 | 0.0 | 0.0 | 0.0 | 0.0 | 0.0 | 0.0 |
| Mir290 | 0.0 | 0.0 | 0.0 | 0.0 | 0.0 | 0.0 | 0.0 | 0.0 | 0.0 | 0.0 | 0.0 | 0.0 |
| Mir291a | 0.0 | 0.0 | 0.0 | 0.0 | 0.0 | 0.0 | 0.0 | 0.0 | 0.0 | 0.0 | 0.0 | 0.0 |
| Mir291b | 0.0 | 0.0 | 0.0 | 0.0 | 0.0 | 0.0 | 0.0 | 0.0 | 0.0 | 0.0 | 0.0 | 0.0 |
| Mir292 | 0.0 | 0.0 | 0.0 | 0.0 | 0.0 | 0.0 | 0.0 | 0.0 | 0.0 | 0.0 | 0.0 | 0.0 |
| Mir293 | 0.0 | 0.0 | 0.0 | 0.0 | 0.0 | 0.0 | 0.0 | 0.0 | 0.0 | 0.0 | 0.0 | 0.0 |
| Mir294 | 0.0 | 0.0 | 0.0 | 0.0 | 0.0 | 0.0 | 0.0 | 0.0 | 0.0 | 0.0 | 0.0 | 0.0 |
| Mir295 | 0.0 | 0.0 | 0.0 | 0.0 | 0.0 | 0.0 | 0.0 | 0.0 | 0.0 | 0.0 | 0.0 | 0.0 |
| Mir296 | 0.0 | 0.0 | 0.0 | 0.0 | 0.0 | 0.0 | 0.0 | 0.0 | 0.0 | 0.0 | 0.0 | 0.0 |
| Mir297-1 | 0.0 | 0.0 | 0.0 | 0.0 | 0.0 | 0.0 | 0.0 | 0.0 | 0.0 | 0.0 | 0.0 | 0.0 |
| Mir297-2 | 0.0 | 0.0 | 0.0 | 0.0 | 0.0 | 0.0 | 0.0 | 0.0 | 0.0 | 0.0 | 0.0 | 0.0 |
| Mir297a-3 | 0.0 | 0.0 | 0.0 | 0.0 | 0.0 | 0.0 | 0.0 | 0.0 | 0.0 | 0.0 | 0.0 | 0.0 |
| Mir297a-4 | 0.0 | 0.0 | 0.0 | 0.0 | 0.0 | 0.0 | 0.0 | 0.0 | 0.0 | 0.0 | 0.0 | 0.0 |
| Mir297b | 0.0 | 0.0 | 0.0 | 0.0 | 0.0 | 0.0 | 0.0 | 0.0 | 0.0 | 0.0 | 0.0 | 0.0 |
| Mir297c | 0.0 | 0.0 | 0.0 | 0.0 | 0.0 | 0.0 | 0.0 | 0.0 | 0.0 | 0.0 | 0.0 | 0.0 |
| Mir298 | 0.0 | 0.0 | 0.0 | 0.0 | 0.0 | 0.0 | 0.0 | 0.0 | 0.0 | 0.0 | 0.0 | 0.0 |
| Mir299 | 0.0 | 0.0 | 0.0 | 0.0 | 0.0 | 0.0 | 0.0 | 0.0 | 0.0 | 0.0 | 0.0 | 0.0 |
| Mir29a | 0.0 | 0.0 | 0.0 | 0.0 | 0.0 | 0.0 | 0.0 | 0.0 | 0.0 | 0.0 | 0.0 | 0.0 |
| Mir29b-1 | 0.0 | 0.0 | 0.0 | 0.0 | 0.0 | 0.0 | 0.0 | 0.0 | 0.0 | 0.0 | 0.0 | 0.0 |
| Mir29b-2 | 0.0 | 0.0 | 0.0 | 0.0 | 0.0 | 0.0 | 0.0 | 0.0 | 0.0 | 0.0 | 0.0 | 0.0 |
| Mir29c | 0.0 | 0.0 | 0.0 | 0.0 | 0.0 | 0.0 | 0.0 | 0.0 | 0.0 | 0.0 | 0.0 | 0.0 |
| Mir300 | 0.0 | 0.0 | 0.0 | 0.0 | 0.0 | 0.0 | 0.0 | 0.0 | 0.0 | 0.0 | 0.0 | 0.0 |
| Mir301 | 0.0 | 0.0 | 0.0 | 0.0 | 0.0 | 0.0 | 0.0 | 0.0 | 0.0 | 0.0 | 0.0 | 0.0 |
| Mir301b | 0.0 | 0.0 | 0.0 | 0.0 | 0.0 | 0.0 | 0.0 | 0.0 | 0.0 | 0.0 | 0.0 | 0.0 |
| Mir302a | 0.0 | 0.0 | 0.0 | 0.0 | 0.0 | 0.0 | 0.0 | 0.0 | 0.0 | 0.0 | 0.0 | 0.0 |
| Mir302b | 0.0 | 0.0 | 0.0 | 0.0 | 0.0 | 0.0 | 0.0 | 0.0 | 0.0 | 0.0 | 0.0 | 0.0 |
| Mir302c | 0.0 | 0.0 | 0.0 | 0.0 | 0.0 | 0.0 | 0.0 | 0.0 | 0.0 | 0.0 | 0.0 | 0.0 |
| Mir302d | 0.0 | 0.0 | 0.0 | 0.0 | 0.0 | 0.0 | 0.0 | 0.0 | 0.0 | 0.0 | 0.0 | 0.0 |
| Mir3057 | 0.0 | 0.0 | 0.0 | 0.0 | 0.0 | 0.0 | 0.0 | 0.0 | 0.0 | 0.0 | 0.0 | 0.0 |
| Mir3058 | 0.0 | 0.0 | 0.0 | 0.0 | 0.0 | 0.0 | 0.0 | 0.0 | 0.0 | 0.0 | 0.0 | 0.0 |
| Mir3059 | 0.0 | 0.0 | 0.0 | 0.0 | 0.0 | 0.0 | 0.0 | 0.0 | 0.0 | 0.0 | 0.0 | 0.0 |
| Mir3060 | 0.0 | 0.0 | 0.0 | 0.0 | 0.0 | 0.0 | 0.0 | 0.0 | 0.0 | 0.0 | 0.0 | 0.0 |

Excluded Genes

| | | | | | | | | | | | |
|---|---|---|---|---|---|---|---|---|---|---|---|
| Mir3061 | 0.0 | 0.0 | 0.0 | 0.0 | 0.0 | 0.0 | 0.0 | 0.0 | 0.0 | 0.0 | 0.0 | 0.0 |
| Mir3062 | 0.0 | 0.0 | 0.0 | 0.0 | 0.0 | 0.0 | 0.0 | 0.0 | 0.0 | 0.0 | 0.0 | 0.0 |
| Mir3063 | 0.0 | 0.0 | 0.0 | 0.0 | 0.0 | 0.0 | 0.0 | 0.0 | 0.0 | 0.0 | 0.0 | 0.0 |
| Mir3065 | 0.0 | 0.0 | 0.0 | 0.0 | 0.0 | 0.0 | 0.0 | 0.0 | 0.0 | 0.0 | 0.0 | 0.0 |
| Mir3066 | 0.0 | 0.0 | 0.0 | 0.0 | 0.0 | 0.0 | 0.0 | 0.0 | 0.0 | 0.0 | 0.0 | 0.0 |
| Mir3067 | 0.0 | 0.0 | 0.0 | 0.0 | 0.0 | 0.0 | 0.0 | 0.0 | 0.0 | 0.0 | 0.0 | 0.0 |
| Mir3068 | 0.0 | 0.0 | 0.0 | 0.0 | 0.0 | 0.0 | 0.0 | 0.0 | 0.0 | 0.0 | 0.0 | 0.0 |
| Mir3071 | 0.0 | 0.0 | 0.0 | 0.0 | 0.0 | 0.0 | 0.0 | 0.0 | 0.0 | 0.0 | 0.0 | 0.0 |
| Mir3072 | 0.0 | 0.0 | 0.0 | 0.0 | 0.0 | 0.0 | 0.0 | 0.0 | 0.0 | 0.0 | 0.0 | 0.0 |
| Mir3073 | 0.0 | 0.0 | 0.0 | 0.0 | 0.0 | 0.0 | 0.0 | 0.0 | 0.0 | 0.0 | 0.0 | 0.0 |
| Mir3074-1 | 0.0 | 0.0 | 0.0 | 0.0 | 0.0 | 0.0 | 0.0 | 0.0 | 0.0 | 0.0 | 0.0 | 0.0 |
| Mir3074-2 | 0.0 | 0.0 | 0.0 | 0.0 | 0.0 | 0.0 | 0.0 | 0.0 | 0.0 | 0.0 | 0.0 | 0.0 |
| Mir3075 | 0.0 | 0.0 | 0.0 | 0.0 | 0.0 | 0.0 | 0.0 | 0.0 | 0.0 | 0.0 | 0.0 | 0.0 |
| Mir3076 | 0.0 | 0.0 | 0.0 | 0.0 | 0.0 | 0.0 | 0.0 | 0.0 | 0.0 | 0.0 | 0.0 | 0.0 |
| Mir3077 | 0.0 | 0.0 | 0.0 | 0.0 | 0.0 | 0.0 | 0.0 | 0.0 | 0.0 | 0.0 | 0.0 | 0.0 |
| Mir3078 | 0.0 | 0.0 | 0.0 | 0.0 | 0.0 | 0.0 | 0.0 | 0.0 | 0.0 | 0.0 | 0.0 | 0.0 |
| Mir3079 | 0.0 | 0.0 | 0.0 | 0.0 | 0.0 | 0.0 | 0.0 | 0.0 | 0.0 | 0.0 | 0.0 | 0.0 |
| Mir3081 | 0.0 | 0.0 | 0.0 | 0.0 | 0.0 | 0.0 | 0.0 | 0.0 | 0.0 | 0.0 | 0.0 | 0.0 |
| Mir3082 | 0.0 | 0.0 | 0.0 | 0.0 | 0.0 | 0.0 | 0.0 | 0.0 | 0.0 | 0.0 | 0.0 | 0.0 |
| Mir3083 | 0.0 | 0.0 | 0.0 | 0.0 | 0.0 | 0.0 | 0.0 | 0.0 | 0.0 | 0.0 | 0.0 | 0.0 |
| Mir3085 | 0.0 | 0.0 | 0.0 | 0.0 | 0.0 | 0.0 | 0.0 | 0.0 | 0.0 | 0.0 | 0.0 | 0.0 |
| Mir3086 | 0.0 | 0.0 | 0.0 | 0.0 | 0.0 | 0.0 | 0.0 | 0.0 | 0.0 | 0.0 | 0.0 | 0.0 |
| Mir3087 | 0.0 | 0.0 | 0.0 | 0.0 | 0.0 | 0.0 | 0.0 | 0.0 | 0.0 | 0.0 | 0.0 | 0.0 |
| Mir3088 | 0.0 | 0.0 | 0.0 | 0.0 | 0.0 | 0.0 | 0.0 | 0.0 | 0.0 | 0.0 | 0.0 | 0.0 |
| Mir3089 | 0.0 | 0.0 | 0.0 | 0.0 | 0.0 | 0.0 | 0.0 | 0.0 | 0.0 | 0.0 | 0.0 | 0.0 |
| Mir3091 | 0.0 | 0.0 | 0.0 | 0.0 | 0.0 | 0.0 | 0.0 | 0.0 | 0.0 | 0.0 | 0.0 | 0.0 |
| Mir3092 | 0.0 | 0.0 | 0.0 | 0.0 | 0.0 | 0.0 | 0.0 | 0.0 | 0.0 | 0.0 | 0.0 | 0.0 |
| Mir3093 | 0.0 | 0.0 | 0.0 | 0.0 | 0.0 | 0.0 | 0.0 | 0.0 | 0.0 | 0.0 | 0.0 | 0.0 |
| Mir3094 | 0.0 | 0.0 | 0.0 | 0.0 | 0.0 | 0.0 | 0.0 | 0.0 | 0.0 | 0.0 | 0.0 | 0.0 |
| Mir3095 | 0.0 | 0.0 | 0.0 | 0.0 | 0.0 | 0.0 | 0.0 | 0.0 | 0.0 | 0.0 | 0.0 | 0.0 |
| Mir3097 | 0.0 | 0.0 | 0.0 | 0.0 | 0.0 | 0.0 | 0.0 | 0.0 | 0.0 | 0.0 | 0.0 | 0.0 |
| Mir3098 | 0.0 | 0.0 | 0.0 | 0.0 | 0.0 | 0.0 | 0.0 | 0.0 | 0.0 | 0.0 | 0.0 | 0.0 |
| Mir3099 | 0.0 | 0.0 | 0.0 | 0.0 | 0.0 | 0.0 | 0.0 | 0.0 | 0.0 | 0.0 | 0.0 | 0.0 |
| Mir30a | 0.0 | 0.0 | 0.0 | 0.0 | 0.0 | 0.0 | 0.0 | 0.0 | 0.0 | 0.0 | 0.0 | 0.0 |
| Mir30b | 0.0 | 0.0 | 0.0 | 0.0 | 0.0 | 0.0 | 0.0 | 0.0 | 0.0 | 0.0 | 0.0 | 0.0 |
| Mir30c-1 | 0.0 | 0.0 | 0.0 | 0.0 | 0.0 | 0.0 | 0.0 | 0.0 | 0.0 | 0.0 | 0.0 | 0.0 |
| Mir30c-2 | 0.0 | 0.0 | 0.0 | 0.0 | 0.0 | 0.0 | 0.0 | 0.0 | 0.0 | 0.0 | 0.0 | 0.0 |
| Mir30d | 0.0 | 0.0 | 0.0 | 0.0 | 0.0 | 0.0 | 0.0 | 0.0 | 0.0 | 0.0 | 0.0 | 0.0 |
| Mir30e | 0.0 | 0.0 | 0.0 | 0.0 | 0.0 | 0.0 | 0.0 | 0.0 | 0.0 | 0.0 | 0.0 | 0.0 |
| Mir31 | 0.0 | 0.0 | 0.0 | 0.0 | 0.0 | 0.0 | 0.0 | 0.0 | 0.0 | 0.0 | 0.0 | 0.0 |
| Mir3100 | 0.0 | 0.0 | 0.0 | 0.0 | 0.0 | 0.0 | 0.0 | 0.0 | 0.0 | 0.0 | 0.0 | 0.0 |
| Mir3102 | 0.0 | 0.0 | 0.0 | 0.0 | 0.0 | 0.0 | 0.0 | 0.0 | 0.0 | 0.0 | 0.0 | 0.0 |
| Mir3103 | 0.0 | 0.0 | 0.0 | 0.0 | 0.0 | 0.0 | 0.0 | 0.0 | 0.0 | 0.0 | 0.0 | 0.0 |
| Mir3104 | 0.0 | 0.0 | 0.0 | 0.0 | 0.0 | 0.0 | 0.0 | 0.0 | 0.0 | 0.0 | 0.0 | 0.0 |
| Mir3106 | 0.0 | 0.0 | 0.0 | 0.0 | 0.0 | 0.0 | 0.0 | 0.0 | 0.0 | 0.0 | 0.0 | 0.0 |
| Mir3107 | 0.0 | 0.0 | 0.0 | 0.0 | 0.0 | 0.0 | 0.0 | 0.0 | 0.0 | 0.0 | 0.0 | 0.0 |
| Mir3108 | 0.0 | 0.0 | 0.0 | 0.0 | 0.0 | 0.0 | 0.0 | 0.0 | 0.0 | 0.0 | 0.0 | 0.0 |
| Mir3112 | 0.0 | 0.0 | 0.0 | 0.0 | 0.0 | 0.0 | 0.0 | 0.0 | 0.0 | 0.0 | 0.0 | 0.0 |
| Mir32 | 0.0 | 0.0 | 0.0 | 0.0 | 0.0 | 0.0 | 0.0 | 0.0 | 0.0 | 0.0 | 0.0 | 0.0 |
| Mir322 | 0.0 | 0.0 | 0.0 | 0.0 | 0.0 | 0.0 | 0.0 | 0.0 | 0.0 | 0.0 | 0.0 | 0.0 |
| Mir323 | 0.0 | 0.0 | 0.0 | 0.0 | 0.0 | 0.0 | 0.0 | 0.0 | 0.0 | 0.0 | 0.0 | 0.0 |
| Mir325 | 0.0 | 0.0 | 0.0 | 0.0 | 0.0 | 0.0 | 0.0 | 0.0 | 0.0 | 0.0 | 0.0 | 0.0 |
| Mir326 | 0.0 | 0.0 | 0.0 | 0.0 | 0.0 | 0.0 | 0.0 | 0.0 | 0.0 | 0.0 | 0.0 | 0.0 |
| Mir328 | 0.0 | 0.0 | 0.0 | 0.0 | 0.0 | 0.0 | 0.0 | 0.0 | 0.0 | 0.0 | 0.0 | 0.0 |
| Mir329 | 0.0 | 0.0 | 0.0 | 0.0 | 0.0 | 0.0 | 0.0 | 0.0 | 0.0 | 0.0 | 0.0 | 0.0 |
| Mir33 | 0.0 | 0.0 | 0.0 | 0.0 | 0.0 | 0.0 | 0.0 | 0.0 | 0.0 | 0.0 | 0.0 | 0.0 |
| Mir330 | 0.0 | 0.0 | 0.0 | 0.0 | 0.0 | 0.0 | 0.0 | 0.0 | 0.0 | 0.0 | 0.0 | 0.0 |

| | | | | | | | | | | | | |
|---|---|---|---|---|---|---|---|---|---|---|---|---|
| Mir335 | 0.0 | 0.0 | 0.0 | 0.0 | 0.0 | 0.0 | 0.0 | 0.0 | 0.0 | 0.0 | 0.0 | 0.0 |
| Mir337 | 0.0 | 0.0 | 0.0 | 0.0 | 0.0 | 0.0 | 0.0 | 0.0 | 0.0 | 0.0 | 0.0 | 0.0 |
| Mir338 | 0.0 | 0.0 | 0.0 | 0.0 | 0.0 | 0.0 | 0.0 | 0.0 | 0.0 | 0.0 | 0.0 | 0.0 |
| Mir339 | 0.0 | 0.0 | 0.0 | 0.0 | 0.0 | 0.0 | 0.0 | 0.0 | 0.0 | 0.0 | 0.0 | 0.0 |
| Mir340 | 0.0 | 0.0 | 0.0 | 0.0 | 0.0 | 0.0 | 0.0 | 0.0 | 0.0 | 0.0 | 0.0 | 0.0 |
| Mir341 | 0.0 | 0.0 | 0.0 | 0.0 | 0.0 | 0.0 | 0.0 | 0.0 | 0.0 | 0.0 | 0.0 | 0.0 |
| Mir344 | 0.0 | 0.0 | 0.0 | 0.0 | 0.0 | 0.0 | 0.0 | 0.0 | 0.0 | 0.0 | 0.0 | 0.0 |
| Mir344-2 | 0.0 | 0.0 | 0.0 | 0.0 | 0.0 | 0.0 | 0.0 | 0.0 | 0.0 | 0.0 | 0.0 | 0.0 |
| Mir344b | 0.0 | 0.0 | 0.0 | 0.0 | 0.0 | 0.0 | 0.0 | 0.0 | 0.0 | 0.0 | 0.0 | 0.0 |
| Mir344c | 0.0 | 0.0 | 0.0 | 0.0 | 0.0 | 0.0 | 0.0 | 0.0 | 0.0 | 0.0 | 0.0 | 0.0 |
| Mir344d-1 | 0.0 | 0.0 | 0.0 | 0.0 | 0.0 | 0.0 | 0.0 | 0.0 | 0.0 | 0.0 | 0.0 | 0.0 |
| Mir344d-2 | 0.0 | 0.0 | 0.0 | 0.0 | 0.0 | 0.0 | 0.0 | 0.0 | 0.0 | 0.0 | 0.0 | 0.0 |
| Mir344d-3 | 0.0 | 0.0 | 0.0 | 0.0 | 0.0 | 0.0 | 0.0 | 0.0 | 0.0 | 0.0 | 0.0 | 0.0 |
| Mir344e | 0.0 | 0.0 | 0.0 | 0.0 | 0.0 | 0.0 | 0.0 | 0.0 | 0.0 | 0.0 | 0.0 | 0.0 |
| Mir344f | 0.0 | 0.0 | 0.0 | 0.0 | 0.0 | 0.0 | 0.0 | 0.0 | 0.0 | 0.0 | 0.0 | 0.0 |
| Mir344g | 0.0 | 0.0 | 0.0 | 0.0 | 0.0 | 0.0 | 0.0 | 0.0 | 0.0 | 0.0 | 0.0 | 0.0 |
| Mir345 | 0.0 | 0.0 | 0.0 | 0.0 | 0.0 | 0.0 | 0.0 | 0.0 | 0.0 | 0.0 | 0.0 | 0.0 |
| Mir346 | 0.0 | 0.0 | 0.0 | 0.0 | 0.0 | 0.0 | 0.0 | 0.0 | 0.0 | 0.0 | 0.0 | 0.0 |
| Mir3470a | 0.0 | 0.0 | 0.0 | 0.0 | 0.0 | 0.0 | 0.0 | 0.0 | 0.0 | 0.0 | 0.0 | 0.0 |
| Mir3473c | 0.0 | 0.0 | 0.0 | 0.0 | 0.0 | 0.0 | 0.0 | 0.0 | 0.0 | 0.0 | 0.0 | 0.0 |
| Mir3473d | 0.0 | 0.0 | 0.0 | 0.0 | 0.0 | 0.0 | 0.0 | 0.0 | 0.0 | 0.0 | 0.0 | 0.0 |
| Mir3474 | 0.0 | 0.0 | 0.0 | 0.0 | 0.0 | 0.0 | 0.0 | 0.0 | 0.0 | 0.0 | 0.0 | 0.0 |
| Mir3475 | 0.0 | 0.0 | 0.0 | 0.0 | 0.0 | 0.0 | 0.0 | 0.0 | 0.0 | 0.0 | 0.0 | 0.0 |
| Mir34a | 0.0 | 0.0 | 0.0 | 0.0 | 0.0 | 0.0 | 0.0 | 0.0 | 0.0 | 0.0 | 0.0 | 0.0 |
| Mir34c | 0.0 | 0.0 | 0.0 | 0.0 | 0.0 | 0.0 | 0.0 | 0.0 | 0.0 | 0.0 | 0.0 | 0.0 |
| Mir350 | 0.0 | 0.0 | 0.0 | 0.0 | 0.0 | 0.0 | 0.0 | 0.0 | 0.0 | 0.0 | 0.0 | 0.0 |
| Mir351 | 0.0 | 0.0 | 0.0 | 0.0 | 0.0 | 0.0 | 0.0 | 0.0 | 0.0 | 0.0 | 0.0 | 0.0 |
| Mir3572 | 0.0 | 0.0 | 0.0 | 0.0 | 0.0 | 0.0 | 0.0 | 0.0 | 0.0 | 0.0 | 0.0 | 0.0 |
| Mir362 | 0.0 | 0.0 | 0.0 | 0.0 | 0.0 | 0.0 | 0.0 | 0.0 | 0.0 | 0.0 | 0.0 | 0.0 |
| Mir363 | 0.0 | 0.0 | 0.0 | 0.0 | 0.0 | 0.0 | 0.0 | 0.0 | 0.0 | 0.0 | 0.0 | 0.0 |
| Mir365-1 | 0.0 | 0.0 | 0.0 | 0.0 | 0.0 | 0.0 | 0.0 | 0.0 | 0.0 | 0.0 | 0.0 | 0.0 |
| Mir365-2 | 0.0 | 0.0 | 0.0 | 0.0 | 0.0 | 0.0 | 0.0 | 0.0 | 0.0 | 0.0 | 0.0 | 0.0 |
| Mir367 | 0.0 | 0.0 | 0.0 | 0.0 | 0.0 | 0.0 | 0.0 | 0.0 | 0.0 | 0.0 | 0.0 | 0.0 |
| Mir369 | 0.0 | 0.0 | 0.0 | 0.0 | 0.0 | 0.0 | 0.0 | 0.0 | 0.0 | 0.0 | 0.0 | 0.0 |
| Mir370 | 0.0 | 0.0 | 0.0 | 0.0 | 0.0 | 0.0 | 0.0 | 0.0 | 0.0 | 0.0 | 0.0 | 0.0 |
| Mir374 | 0.0 | 0.0 | 0.0 | 0.0 | 0.0 | 0.0 | 0.0 | 0.0 | 0.0 | 0.0 | 0.0 | 0.0 |
| Mir374c | 0.0 | 0.0 | 0.0 | 0.0 | 0.0 | 0.0 | 0.0 | 0.0 | 0.0 | 0.0 | 0.0 | 0.0 |
| Mir375 | 0.0 | 0.0 | 0.0 | 0.0 | 0.0 | 0.0 | 0.0 | 0.0 | 0.0 | 0.0 | 0.0 | 0.0 |
| Mir376a | 0.0 | 0.0 | 0.0 | 0.0 | 0.0 | 0.0 | 0.0 | 0.0 | 0.0 | 0.0 | 0.0 | 0.0 |
| Mir376b | 0.0 | 0.0 | 0.0 | 0.0 | 0.0 | 0.0 | 0.0 | 0.0 | 0.0 | 0.0 | 0.0 | 0.0 |
| Mir376c | 0.0 | 0.0 | 0.0 | 0.0 | 0.0 | 0.0 | 0.0 | 0.0 | 0.0 | 0.0 | 0.0 | 0.0 |
| Mir377 | 0.0 | 0.0 | 0.0 | 0.0 | 0.0 | 0.0 | 0.0 | 0.0 | 0.0 | 0.0 | 0.0 | 0.0 |
| Mir378 | 0.0 | 0.0 | 0.0 | 0.0 | 0.0 | 0.0 | 0.0 | 0.0 | 0.0 | 0.0 | 0.0 | 0.0 |
| Mir378b | 0.0 | 0.0 | 0.0 | 0.0 | 0.0 | 0.0 | 0.0 | 0.0 | 0.0 | 0.0 | 0.0 | 0.0 |
| Mir379 | 0.0 | 0.0 | 0.0 | 0.0 | 0.0 | 0.0 | 0.0 | 0.0 | 0.0 | 0.0 | 0.0 | 0.0 |
| Mir380 | 0.0 | 0.0 | 0.0 | 0.0 | 0.0 | 0.0 | 0.0 | 0.0 | 0.0 | 0.0 | 0.0 | 0.0 |
| Mir381 | 0.0 | 0.0 | 0.0 | 0.0 | 0.0 | 0.0 | 0.0 | 0.0 | 0.0 | 0.0 | 0.0 | 0.0 |
| Mir382 | 0.0 | 0.0 | 0.0 | 0.0 | 0.0 | 0.0 | 0.0 | 0.0 | 0.0 | 0.0 | 0.0 | 0.0 |
| Mir383 | 0.0 | 0.0 | 0.0 | 0.0 | 0.0 | 0.0 | 0.0 | 0.0 | 0.0 | 0.0 | 0.0 | 0.0 |
| Mir384 | 0.0 | 0.0 | 0.0 | 0.0 | 0.0 | 0.0 | 0.0 | 0.0 | 0.0 | 0.0 | 0.0 | 0.0 |
| Mir3960 | 0.0 | 0.0 | 0.0 | 0.0 | 0.0 | 0.0 | 0.0 | 0.0 | 0.0 | 0.0 | 0.0 | 0.0 |
| Mir3962 | 0.0 | 0.0 | 0.0 | 0.0 | 0.0 | 0.0 | 0.0 | 0.0 | 0.0 | 0.0 | 0.0 | 0.0 |
| Mir3963 | 0.0 | 0.0 | 0.0 | 0.0 | 0.0 | 0.0 | 0.0 | 0.0 | 0.0 | 0.0 | 0.0 | 0.0 |
| Mir3964 | 0.0 | 0.0 | 0.0 | 0.0 | 0.0 | 0.0 | 0.0 | 0.0 | 0.0 | 0.0 | 0.0 | 0.0 |
| Mir3965 | 0.0 | 0.0 | 0.0 | 0.0 | 0.0 | 0.0 | 0.0 | 0.0 | 0.0 | 0.0 | 0.0 | 0.0 |
| Mir3966 | 0.0 | 0.0 | 0.0 | 0.0 | 0.0 | 0.0 | 0.0 | 0.0 | 0.0 | 0.0 | 0.0 | 0.0 |
| Mir3967 | 0.0 | 0.0 | 0.0 | 0.0 | 0.0 | 0.0 | 0.0 | 0.0 | 0.0 | 0.0 | 0.0 | 0.0 |

Excluded Genes

| | | | | | | | | | | | | |
|---|---|---|---|---|---|---|---|---|---|---|---|---|
| Mir3968 | 0.0 | 0.0 | 0.0 | 0.0 | 0.0 | 0.0 | 0.0 | 0.0 | 0.0 | 0.0 | 0.0 | 0.0 |
| Mir3969 | 0.0 | 0.0 | 0.0 | 0.0 | 0.0 | 0.0 | 0.0 | 0.0 | 0.0 | 0.0 | 0.0 | 0.0 |
| Mir3970 | 0.0 | 0.0 | 0.0 | 0.0 | 0.0 | 0.0 | 0.0 | 0.0 | 0.0 | 0.0 | 0.0 | 0.0 |
| Mir3971 | 0.0 | 0.0 | 0.0 | 0.0 | 0.0 | 0.0 | 0.0 | 0.0 | 0.0 | 0.0 | 0.0 | 0.0 |
| Mir409 | 0.0 | 0.0 | 0.0 | 0.0 | 0.0 | 0.0 | 0.0 | 0.0 | 0.0 | 0.0 | 0.0 | 0.0 |
| Mir410 | 0.0 | 0.0 | 0.0 | 0.0 | 0.0 | 0.0 | 0.0 | 0.0 | 0.0 | 0.0 | 0.0 | 0.0 |
| Mir411 | 0.0 | 0.0 | 0.0 | 0.0 | 0.0 | 0.0 | 0.0 | 0.0 | 0.0 | 0.0 | 0.0 | 0.0 |
| Mir412 | 0.0 | 0.0 | 0.0 | 0.0 | 0.0 | 0.0 | 0.0 | 0.0 | 0.0 | 0.0 | 0.0 | 0.0 |
| Mir423 | 0.0 | 0.0 | 0.0 | 0.0 | 0.0 | 0.0 | 0.0 | 0.0 | 0.0 | 0.0 | 0.0 | 0.0 |
| Mir429 | 0.0 | 0.0 | 0.0 | 0.0 | 0.0 | 0.0 | 0.0 | 0.0 | 0.0 | 0.0 | 0.0 | 0.0 |
| Mir431 | 0.0 | 0.0 | 0.0 | 0.0 | 0.0 | 0.0 | 0.0 | 0.0 | 0.0 | 0.0 | 0.0 | 0.0 |
| Mir434 | 0.0 | 0.0 | 0.0 | 0.0 | 0.0 | 0.0 | 0.0 | 0.0 | 0.0 | 0.0 | 0.0 | 0.0 |
| Mir448 | 0.0 | 0.0 | 0.0 | 0.0 | 0.0 | 0.0 | 0.0 | 0.0 | 0.0 | 0.0 | 0.0 | 0.0 |
| Mir449a | 0.0 | 0.0 | 0.0 | 0.0 | 0.0 | 0.0 | 0.0 | 0.0 | 0.0 | 0.0 | 0.0 | 0.0 |
| Mir449b | 0.0 | 0.0 | 0.0 | 0.0 | 0.0 | 0.0 | 0.0 | 0.0 | 0.0 | 0.0 | 0.0 | 0.0 |
| Mir449c | 0.0 | 0.0 | 0.0 | 0.0 | 0.0 | 0.0 | 0.0 | 0.0 | 0.0 | 0.0 | 0.0 | 0.0 |
| Mir450-1 | 0.0 | 0.0 | 0.0 | 0.0 | 0.0 | 0.0 | 0.0 | 0.0 | 0.0 | 0.0 | 0.0 | 0.0 |
| Mir450-2 | 0.0 | 0.0 | 0.0 | 0.0 | 0.0 | 0.0 | 0.0 | 0.0 | 0.0 | 0.0 | 0.0 | 0.0 |
| Mir450b | 0.0 | 0.0 | 0.0 | 0.0 | 0.0 | 0.0 | 0.0 | 0.0 | 0.0 | 0.0 | 0.0 | 0.0 |
| Mir451 | 0.0 | 0.0 | 0.0 | 0.0 | 0.0 | 0.0 | 0.0 | 0.0 | 0.0 | 0.0 | 0.0 | 0.0 |
| Mir452 | 0.0 | 0.0 | 0.0 | 0.0 | 0.0 | 0.0 | 0.0 | 0.0 | 0.0 | 0.0 | 0.0 | 0.0 |
| Mir453 | 0.0 | 0.0 | 0.0 | 0.0 | 0.0 | 0.0 | 0.0 | 0.0 | 0.0 | 0.0 | 0.0 | 0.0 |
| Mir455 | 0.0 | 0.0 | 0.0 | 0.0 | 0.0 | 0.0 | 0.0 | 0.0 | 0.0 | 0.0 | 0.0 | 0.0 |
| Mir463 | 0.0 | 0.0 | 0.0 | 0.0 | 0.0 | 0.0 | 0.0 | 0.0 | 0.0 | 0.0 | 0.0 | 0.0 |
| Mir465 | 0.0 | 0.0 | 0.0 | 0.0 | 0.0 | 0.0 | 0.0 | 0.0 | 0.0 | 0.0 | 0.0 | 0.0 |
| Mir466 | 0.0 | 0.0 | 0.0 | 0.0 | 0.0 | 0.0 | 0.0 | 0.0 | 0.0 | 0.0 | 0.0 | 0.0 |
| Mir4660 | 0.0 | 0.0 | 0.0 | 0.0 | 0.0 | 0.0 | 0.0 | 0.0 | 0.0 | 0.0 | 0.0 | 0.0 |
| Mir466b-2 | 0.0 | 0.0 | 0.0 | 0.0 | 0.0 | 0.0 | 0.0 | 0.0 | 0.0 | 0.0 | 0.0 | 0.0 |
| Mir466d | 0.0 | 0.0 | 0.0 | 0.0 | 0.0 | 0.0 | 0.0 | 0.0 | 0.0 | 0.0 | 0.0 | 0.0 |
| Mir466f-1 | 0.0 | 0.0 | 0.0 | 0.0 | 0.0 | 0.0 | 0.0 | 0.0 | 0.0 | 0.0 | 0.0 | 0.0 |
| Mir466f-2 | 0.0 | 0.0 | 0.0 | 0.0 | 0.0 | 0.0 | 0.0 | 0.0 | 0.0 | 0.0 | 0.0 | 0.0 |
| Mir466f-3 | 0.0 | 0.0 | 0.0 | 0.0 | 0.0 | 0.0 | 0.0 | 0.0 | 0.0 | 0.0 | 0.0 | 0.0 |
| Mir466h | 0.0 | 0.0 | 0.0 | 0.0 | 0.0 | 0.0 | 0.0 | 0.0 | 0.0 | 0.0 | 0.0 | 0.0 |
| Mir466i | 0.0 | 0.0 | 0.0 | 0.0 | 0.0 | 0.0 | 0.0 | 0.0 | 0.0 | 0.0 | 0.0 | 0.0 |
| Mir466n | 0.0 | 0.0 | 0.0 | 0.0 | 0.0 | 0.0 | 0.0 | 0.0 | 0.0 | 0.0 | 0.0 | 0.0 |
| Mir467b | 0.0 | 0.0 | 0.0 | 0.0 | 0.0 | 0.0 | 0.0 | 0.0 | 0.0 | 0.0 | 0.0 | 0.0 |
| Mir467c | 0.0 | 0.0 | 0.0 | 0.0 | 0.0 | 0.0 | 0.0 | 0.0 | 0.0 | 0.0 | 0.0 | 0.0 |
| Mir467d | 0.0 | 0.0 | 0.0 | 0.0 | 0.0 | 0.0 | 0.0 | 0.0 | 0.0 | 0.0 | 0.0 | 0.0 |
| Mir467e | 0.0 | 0.0 | 0.0 | 0.0 | 0.0 | 0.0 | 0.0 | 0.0 | 0.0 | 0.0 | 0.0 | 0.0 |
| Mir467f | 0.0 | 0.0 | 0.0 | 0.0 | 0.0 | 0.0 | 0.0 | 0.0 | 0.0 | 0.0 | 0.0 | 0.0 |
| Mir468 | 0.0 | 0.0 | 0.0 | 0.0 | 0.0 | 0.0 | 0.0 | 0.0 | 0.0 | 0.0 | 0.0 | 0.0 |
| Mir470 | 0.0 | 0.0 | 0.0 | 0.0 | 0.0 | 0.0 | 0.0 | 0.0 | 0.0 | 0.0 | 0.0 | 0.0 |
| Mir471 | 0.0 | 0.0 | 0.0 | 0.0 | 0.0 | 0.0 | 0.0 | 0.0 | 0.0 | 0.0 | 0.0 | 0.0 |
| Mir483 | 0.0 | 0.0 | 0.0 | 0.0 | 0.0 | 0.0 | 0.0 | 0.0 | 0.0 | 0.0 | 0.0 | 0.0 |
| Mir484 | 0.0 | 0.0 | 0.0 | 0.0 | 0.0 | 0.0 | 0.0 | 0.0 | 0.0 | 0.0 | 0.0 | 0.0 |
| Mir485 | 0.0 | 0.0 | 0.0 | 0.0 | 0.0 | 0.0 | 0.0 | 0.0 | 0.0 | 0.0 | 0.0 | 0.0 |
| Mir486 | 0.0 | 0.0 | 0.0 | 0.0 | 0.0 | 0.0 | 0.0 | 0.0 | 0.0 | 0.0 | 0.0 | 0.0 |
| Mir487b | 0.0 | 0.0 | 0.0 | 0.0 | 0.0 | 0.0 | 0.0 | 0.0 | 0.0 | 0.0 | 0.0 | 0.0 |
| Mir488 | 0.0 | 0.0 | 0.0 | 0.0 | 0.0 | 0.0 | 0.0 | 0.0 | 0.0 | 0.0 | 0.0 | 0.0 |
| Mir489 | 0.0 | 0.0 | 0.0 | 0.0 | 0.0 | 0.0 | 0.0 | 0.0 | 0.0 | 0.0 | 0.0 | 0.0 |
| Mir490 | 0.0 | 0.0 | 0.0 | 0.0 | 0.0 | 0.0 | 0.0 | 0.0 | 0.0 | 0.0 | 0.0 | 0.0 |
| Mir491 | 0.0 | 0.0 | 0.0 | 0.0 | 0.0 | 0.0 | 0.0 | 0.0 | 0.0 | 0.0 | 0.0 | 0.0 |
| Mir493 | 0.0 | 0.0 | 0.0 | 0.0 | 0.0 | 0.0 | 0.0 | 0.0 | 0.0 | 0.0 | 0.0 | 0.0 |
| Mir494 | 0.0 | 0.0 | 0.0 | 0.0 | 0.0 | 0.0 | 0.0 | 0.0 | 0.0 | 0.0 | 0.0 | 0.0 |
| Mir495 | 0.0 | 0.0 | 0.0 | 0.0 | 0.0 | 0.0 | 0.0 | 0.0 | 0.0 | 0.0 | 0.0 | 0.0 |
| Mir496 | 0.0 | 0.0 | 0.0 | 0.0 | 0.0 | 0.0 | 0.0 | 0.0 | 0.0 | 0.0 | 0.0 | 0.0 |
| Mir497 | 0.0 | 0.0 | 0.0 | 0.0 | 0.0 | 0.0 | 0.0 | 0.0 | 0.0 | 0.0 | 0.0 | 0.0 |

Excluded Genes

| | | | | | | | | | | | |
|---|---|---|---|---|---|---|---|---|---|---|---|
| Mir499 | 0.0 | 0.0 | 0.0 | 0.0 | 0.0 | 0.0 | 0.0 | 0.0 | 0.0 | 0.0 | 0.0 | 0.0 |
| Mir500 | 0.0 | 0.0 | 0.0 | 0.0 | 0.0 | 0.0 | 0.0 | 0.0 | 0.0 | 0.0 | 0.0 | 0.0 |
| Mir501 | 0.0 | 0.0 | 0.0 | 0.0 | 0.0 | 0.0 | 0.0 | 0.0 | 0.0 | 0.0 | 0.0 | 0.0 |
| Mir503 | 0.0 | 0.0 | 0.0 | 0.0 | 0.0 | 0.0 | 0.0 | 0.0 | 0.0 | 0.0 | 0.0 | 0.0 |
| Mir504 | 0.0 | 0.0 | 0.0 | 0.0 | 0.0 | 0.0 | 0.0 | 0.0 | 0.0 | 0.0 | 0.0 | 0.0 |
| Mir505 | 0.0 | 0.0 | 0.0 | 0.0 | 0.0 | 0.0 | 0.0 | 0.0 | 0.0 | 0.0 | 0.0 | 0.0 |
| Mir509 | 0.0 | 0.0 | 0.0 | 0.0 | 0.0 | 0.0 | 0.0 | 0.0 | 0.0 | 0.0 | 0.0 | 0.0 |
| Mir5097 | 0.0 | 0.0 | 0.0 | 0.0 | 0.0 | 0.0 | 0.0 | 0.0 | 0.0 | 0.0 | 0.0 | 0.0 |
| Mir5098 | 0.0 | 0.0 | 0.0 | 0.0 | 0.0 | 0.0 | 0.0 | 0.0 | 0.0 | 0.0 | 0.0 | 0.0 |
| Mir5100 | 0.0 | 0.0 | 0.0 | 0.0 | 0.0 | 0.0 | 0.0 | 0.0 | 0.0 | 0.0 | 0.0 | 0.0 |
| Mir5101 | 0.0 | 0.0 | 0.0 | 0.0 | 0.0 | 0.0 | 0.0 | 0.0 | 0.0 | 0.0 | 0.0 | 0.0 |
| Mir5103 | 0.0 | 0.0 | 0.0 | 0.0 | 0.0 | 0.0 | 0.0 | 0.0 | 0.0 | 0.0 | 0.0 | 0.0 |
| Mir5104 | 0.0 | 0.0 | 0.0 | 0.0 | 0.0 | 0.0 | 0.0 | 0.0 | 0.0 | 0.0 | 0.0 | 0.0 |
| Mir5106 | 0.0 | 0.0 | 0.0 | 0.0 | 0.0 | 0.0 | 0.0 | 0.0 | 0.0 | 0.0 | 0.0 | 0.0 |
| Mir5107 | 0.0 | 0.0 | 0.0 | 0.0 | 0.0 | 0.0 | 0.0 | 0.0 | 0.0 | 0.0 | 0.0 | 0.0 |
| Mir5108 | 0.0 | 0.0 | 0.0 | 0.0 | 0.0 | 0.0 | 0.0 | 0.0 | 0.0 | 0.0 | 0.0 | 0.0 |
| Mir511 | 0.0 | 0.0 | 0.0 | 0.0 | 0.0 | 0.0 | 0.0 | 0.0 | 0.0 | 0.0 | 0.0 | 0.0 |
| Mir5112 | 0.0 | 0.0 | 0.0 | 0.0 | 0.0 | 0.0 | 0.0 | 0.0 | 0.0 | 0.0 | 0.0 | 0.0 |
| Mir5115 | 0.0 | 0.0 | 0.0 | 0.0 | 0.0 | 0.0 | 0.0 | 0.0 | 0.0 | 0.0 | 0.0 | 0.0 |
| Mir5116 | 0.0 | 0.0 | 0.0 | 0.0 | 0.0 | 0.0 | 0.0 | 0.0 | 0.0 | 0.0 | 0.0 | 0.0 |
| Mir5119 | 0.0 | 0.0 | 0.0 | 0.0 | 0.0 | 0.0 | 0.0 | 0.0 | 0.0 | 0.0 | 0.0 | 0.0 |
| Mir5120 | 0.0 | 0.0 | 0.0 | 0.0 | 0.0 | 0.0 | 0.0 | 0.0 | 0.0 | 0.0 | 0.0 | 0.0 |
| Mir5123 | 0.0 | 0.0 | 0.0 | 0.0 | 0.0 | 0.0 | 0.0 | 0.0 | 0.0 | 0.0 | 0.0 | 0.0 |
| Mir5124 | 0.0 | 0.0 | 0.0 | 0.0 | 0.0 | 0.0 | 0.0 | 0.0 | 0.0 | 0.0 | 0.0 | 0.0 |
| Mir5125 | 0.0 | 0.0 | 0.0 | 0.0 | 0.0 | 0.0 | 0.0 | 0.0 | 0.0 | 0.0 | 0.0 | 0.0 |
| Mir5126 | 0.0 | 0.0 | 0.0 | 0.0 | 0.0 | 0.0 | 0.0 | 0.0 | 0.0 | 0.0 | 0.0 | 0.0 |
| Mir5127 | 0.0 | 0.0 | 0.0 | 0.0 | 0.0 | 0.0 | 0.0 | 0.0 | 0.0 | 0.0 | 0.0 | 0.0 |
| Mir5128 | 0.0 | 0.0 | 0.0 | 0.0 | 0.0 | 0.0 | 0.0 | 0.0 | 0.0 | 0.0 | 0.0 | 0.0 |
| Mir5129 | 0.0 | 0.0 | 0.0 | 0.0 | 0.0 | 0.0 | 0.0 | 0.0 | 0.0 | 0.0 | 0.0 | 0.0 |
| Mir5132 | 0.0 | 0.0 | 0.0 | 0.0 | 0.0 | 0.0 | 0.0 | 0.0 | 0.0 | 0.0 | 0.0 | 0.0 |
| Mir5134 | 0.0 | 0.0 | 0.0 | 0.0 | 0.0 | 0.0 | 0.0 | 0.0 | 0.0 | 0.0 | 0.0 | 0.0 |
| Mir5135 | 0.0 | 0.0 | 0.0 | 0.0 | 0.0 | 0.0 | 0.0 | 0.0 | 0.0 | 0.0 | 0.0 | 0.0 |
| Mir532 | 0.0 | 0.0 | 0.0 | 0.0 | 0.0 | 0.0 | 0.0 | 0.0 | 0.0 | 0.0 | 0.0 | 0.0 |
| Mir539 | 0.0 | 0.0 | 0.0 | 0.0 | 0.0 | 0.0 | 0.0 | 0.0 | 0.0 | 0.0 | 0.0 | 0.0 |
| Mir540 | 0.0 | 0.0 | 0.0 | 0.0 | 0.0 | 0.0 | 0.0 | 0.0 | 0.0 | 0.0 | 0.0 | 0.0 |
| Mir541 | 0.0 | 0.0 | 0.0 | 0.0 | 0.0 | 0.0 | 0.0 | 0.0 | 0.0 | 0.0 | 0.0 | 0.0 |
| Mir542 | 0.0 | 0.0 | 0.0 | 0.0 | 0.0 | 0.0 | 0.0 | 0.0 | 0.0 | 0.0 | 0.0 | 0.0 |
| Mir543 | 0.0 | 0.0 | 0.0 | 0.0 | 0.0 | 0.0 | 0.0 | 0.0 | 0.0 | 0.0 | 0.0 | 0.0 |
| Mir547 | 0.0 | 0.0 | 0.0 | 0.0 | 0.0 | 0.0 | 0.0 | 0.0 | 0.0 | 0.0 | 0.0 | 0.0 |
| Mir551b | 0.0 | 0.0 | 0.0 | 0.0 | 0.0 | 0.0 | 0.0 | 0.0 | 0.0 | 0.0 | 0.0 | 0.0 |
| Mir568 | 0.0 | 0.0 | 0.0 | 0.0 | 0.0 | 0.0 | 0.0 | 0.0 | 0.0 | 0.0 | 0.0 | 0.0 |
| Mir574 | 0.0 | 0.0 | 0.0 | 0.0 | 0.0 | 0.0 | 0.0 | 0.0 | 0.0 | 0.0 | 0.0 | 0.0 |
| Mir582 | 0.0 | 0.0 | 0.0 | 0.0 | 0.0 | 0.0 | 0.0 | 0.0 | 0.0 | 0.0 | 0.0 | 0.0 |
| Mir592 | 0.0 | 0.0 | 0.0 | 0.0 | 0.0 | 0.0 | 0.0 | 0.0 | 0.0 | 0.0 | 0.0 | 0.0 |
| Mir598 | 0.0 | 0.0 | 0.0 | 0.0 | 0.0 | 0.0 | 0.0 | 0.0 | 0.0 | 0.0 | 0.0 | 0.0 |
| Mir599 | 0.0 | 0.0 | 0.0 | 0.0 | 0.0 | 0.0 | 0.0 | 0.0 | 0.0 | 0.0 | 0.0 | 0.0 |
| Mir615 | 0.0 | 0.0 | 0.0 | 0.0 | 0.0 | 0.0 | 0.0 | 0.0 | 0.0 | 0.0 | 0.0 | 0.0 |
| Mir653 | 0.0 | 0.0 | 0.0 | 0.0 | 0.0 | 0.0 | 0.0 | 0.0 | 0.0 | 0.0 | 0.0 | 0.0 |
| Mir654 | 0.0 | 0.0 | 0.0 | 0.0 | 0.0 | 0.0 | 0.0 | 0.0 | 0.0 | 0.0 | 0.0 | 0.0 |
| Mir664 | 0.0 | 0.0 | 0.0 | 0.0 | 0.0 | 0.0 | 0.0 | 0.0 | 0.0 | 0.0 | 0.0 | 0.0 |
| Mir665 | 0.0 | 0.0 | 0.0 | 0.0 | 0.0 | 0.0 | 0.0 | 0.0 | 0.0 | 0.0 | 0.0 | 0.0 |
| Mir666 | 0.0 | 0.0 | 0.0 | 0.0 | 0.0 | 0.0 | 0.0 | 0.0 | 0.0 | 0.0 | 0.0 | 0.0 |
| Mir668 | 0.0 | 0.0 | 0.0 | 0.0 | 0.0 | 0.0 | 0.0 | 0.0 | 0.0 | 0.0 | 0.0 | 0.0 |
| Mir669a-1 | 0.0 | 0.0 | 0.0 | 0.0 | 0.0 | 0.0 | 0.0 | 0.0 | 0.0 | 0.0 | 0.0 | 0.0 |
| Mir669a-2 | 0.0 | 0.0 | 0.0 | 0.0 | 0.0 | 0.0 | 0.0 | 0.0 | 0.0 | 0.0 | 0.0 | 0.0 |
| Mir669a-3 | 0.0 | 0.0 | 0.0 | 0.0 | 0.0 | 0.0 | 0.0 | 0.0 | 0.0 | 0.0 | 0.0 | 0.0 |
| Mir669a-9 | 0.0 | 0.0 | 0.0 | 0.0 | 0.0 | 0.0 | 0.0 | 0.0 | 0.0 | 0.0 | 0.0 | 0.0 |

Excluded Genes

| | | | | | | | | | | | | |
|---|---|---|---|---|---|---|---|---|---|---|---|---|
| Mir669b | 0.0 | 0.0 | 0.0 | 0.0 | 0.0 | 0.0 | 0.0 | 0.0 | 0.0 | 0.0 | 0.0 | 0.0 |
| Mir669c | 0.0 | 0.0 | 0.0 | 0.0 | 0.0 | 0.0 | 0.0 | 0.0 | 0.0 | 0.0 | 0.0 | 0.0 |
| Mir669e | 0.0 | 0.0 | 0.0 | 0.0 | 0.0 | 0.0 | 0.0 | 0.0 | 0.0 | 0.0 | 0.0 | 0.0 |
| Mir669g | 0.0 | 0.0 | 0.0 | 0.0 | 0.0 | 0.0 | 0.0 | 0.0 | 0.0 | 0.0 | 0.0 | 0.0 |
| Mir669h | 0.0 | 0.0 | 0.0 | 0.0 | 0.0 | 0.0 | 0.0 | 0.0 | 0.0 | 0.0 | 0.0 | 0.0 |
| Mir669i | 0.0 | 0.0 | 0.0 | 0.0 | 0.0 | 0.0 | 0.0 | 0.0 | 0.0 | 0.0 | 0.0 | 0.0 |
| Mir669j | 0.0 | 0.0 | 0.0 | 0.0 | 0.0 | 0.0 | 0.0 | 0.0 | 0.0 | 0.0 | 0.0 | 0.0 |
| Mir669k | 0.0 | 0.0 | 0.0 | 0.0 | 0.0 | 0.0 | 0.0 | 0.0 | 0.0 | 0.0 | 0.0 | 0.0 |
| Mir669m-1 | 0.0 | 0.0 | 0.0 | 0.0 | 0.0 | 0.0 | 0.0 | 0.0 | 0.0 | 0.0 | 0.0 | 0.0 |
| Mir669m-2 | 0.0 | 0.0 | 0.0 | 0.0 | 0.0 | 0.0 | 0.0 | 0.0 | 0.0 | 0.0 | 0.0 | 0.0 |
| Mir669p-1 | 0.0 | 0.0 | 0.0 | 0.0 | 0.0 | 0.0 | 0.0 | 0.0 | 0.0 | 0.0 | 0.0 | 0.0 |
| Mir670 | 0.0 | 0.0 | 0.0 | 0.0 | 0.0 | 0.0 | 0.0 | 0.0 | 0.0 | 0.0 | 0.0 | 0.0 |
| Mir672 | 0.0 | 0.0 | 0.0 | 0.0 | 0.0 | 0.0 | 0.0 | 0.0 | 0.0 | 0.0 | 0.0 | 0.0 |
| Mir673 | 0.0 | 0.0 | 0.0 | 0.0 | 0.0 | 0.0 | 0.0 | 0.0 | 0.0 | 0.0 | 0.0 | 0.0 |
| Mir674 | 0.0 | 0.0 | 0.0 | 0.0 | 0.0 | 0.0 | 0.0 | 0.0 | 0.0 | 0.0 | 0.0 | 0.0 |
| Mir675 | 0.0 | 0.0 | 0.0 | 0.0 | 0.0 | 0.0 | 0.0 | 0.0 | 0.0 | 0.0 | 0.0 | 0.0 |
| Mir676 | 0.0 | 0.0 | 0.0 | 0.0 | 0.0 | 0.0 | 0.0 | 0.0 | 0.0 | 0.0 | 0.0 | 0.0 |
| Mir679 | 0.0 | 0.0 | 0.0 | 0.0 | 0.0 | 0.0 | 0.0 | 0.0 | 0.0 | 0.0 | 0.0 | 0.0 |
| Mir680-2 | 0.0 | 0.0 | 0.0 | 0.0 | 0.0 | 0.0 | 0.0 | 0.0 | 0.0 | 0.0 | 0.0 | 0.0 |
| Mir680-3 | 0.0 | 0.0 | 0.0 | 0.0 | 0.0 | 0.0 | 0.0 | 0.0 | 0.0 | 0.0 | 0.0 | 0.0 |
| Mir681 | 0.0 | 0.0 | 0.0 | 0.0 | 0.0 | 0.0 | 0.0 | 0.0 | 0.0 | 0.0 | 0.0 | 0.0 |
| Mir687 | 0.0 | 0.0 | 0.0 | 0.0 | 0.0 | 0.0 | 0.0 | 0.0 | 0.0 | 0.0 | 0.0 | 0.0 |
| Mir688 | 0.0 | 0.0 | 0.0 | 0.0 | 0.0 | 0.0 | 0.0 | 0.0 | 0.0 | 0.0 | 0.0 | 0.0 |
| Mir691 | 0.0 | 0.0 | 0.0 | 0.0 | 0.0 | 0.0 | 0.0 | 0.0 | 0.0 | 0.0 | 0.0 | 0.0 |
| Mir693 | 0.0 | 0.0 | 0.0 | 0.0 | 0.0 | 0.0 | 0.0 | 0.0 | 0.0 | 0.0 | 0.0 | 0.0 |
| Mir694 | 0.0 | 0.0 | 0.0 | 0.0 | 0.0 | 0.0 | 0.0 | 0.0 | 0.0 | 0.0 | 0.0 | 0.0 |
| Mir695 | 0.0 | 0.0 | 0.0 | 0.0 | 0.0 | 0.0 | 0.0 | 0.0 | 0.0 | 0.0 | 0.0 | 0.0 |
| Mir697 | 0.0 | 0.0 | 0.0 | 0.0 | 0.0 | 0.0 | 0.0 | 0.0 | 0.0 | 0.0 | 0.0 | 0.0 |
| Mir698 | 0.0 | 0.0 | 0.0 | 0.0 | 0.0 | 0.0 | 0.0 | 0.0 | 0.0 | 0.0 | 0.0 | 0.0 |
| Mir700 | 0.0 | 0.0 | 0.0 | 0.0 | 0.0 | 0.0 | 0.0 | 0.0 | 0.0 | 0.0 | 0.0 | 0.0 |
| Mir701 | 0.0 | 0.0 | 0.0 | 0.0 | 0.0 | 0.0 | 0.0 | 0.0 | 0.0 | 0.0 | 0.0 | 0.0 |
| Mir702 | 0.0 | 0.0 | 0.0 | 0.0 | 0.0 | 0.0 | 0.0 | 0.0 | 0.0 | 0.0 | 0.0 | 0.0 |
| Mir704 | 0.0 | 0.0 | 0.0 | 0.0 | 0.0 | 0.0 | 0.0 | 0.0 | 0.0 | 0.0 | 0.0 | 0.0 |
| Mir706 | 0.0 | 0.0 | 0.0 | 0.0 | 0.0 | 0.0 | 0.0 | 0.0 | 0.0 | 0.0 | 0.0 | 0.0 |
| Mir707 | 0.0 | 0.0 | 0.0 | 0.0 | 0.0 | 0.0 | 0.0 | 0.0 | 0.0 | 0.0 | 0.0 | 0.0 |
| Mir708 | 0.0 | 0.0 | 0.0 | 0.0 | 0.0 | 0.0 | 0.0 | 0.0 | 0.0 | 0.0 | 0.0 | 0.0 |
| Mir709 | 0.0 | 0.0 | 0.0 | 0.0 | 0.0 | 0.0 | 0.0 | 0.0 | 0.0 | 0.0 | 0.0 | 0.0 |
| Mir710 | 0.0 | 0.0 | 0.0 | 0.0 | 0.0 | 0.0 | 0.0 | 0.0 | 0.0 | 0.0 | 0.0 | 0.0 |
| Mir711 | 0.0 | 0.0 | 0.0 | 0.0 | 0.0 | 0.0 | 0.0 | 0.0 | 0.0 | 0.0 | 0.0 | 0.0 |
| Mir713 | 0.0 | 0.0 | 0.0 | 0.0 | 0.0 | 0.0 | 0.0 | 0.0 | 0.0 | 0.0 | 0.0 | 0.0 |
| Mir717 | 0.0 | 0.0 | 0.0 | 0.0 | 0.0 | 0.0 | 0.0 | 0.0 | 0.0 | 0.0 | 0.0 | 0.0 |
| Mir718 | 0.0 | 0.0 | 0.0 | 0.0 | 0.0 | 0.0 | 0.0 | 0.0 | 0.0 | 0.0 | 0.0 | 0.0 |
| Mir7-2 | 0.0 | 0.0 | 0.0 | 0.0 | 0.0 | 0.0 | 0.0 | 0.0 | 0.0 | 0.0 | 0.0 | 0.0 |
| Mir721 | 0.0 | 0.0 | 0.0 | 0.0 | 0.0 | 0.0 | 0.0 | 0.0 | 0.0 | 0.0 | 0.0 | 0.0 |
| Mir741 | 0.0 | 0.0 | 0.0 | 0.0 | 0.0 | 0.0 | 0.0 | 0.0 | 0.0 | 0.0 | 0.0 | 0.0 |
| Mir742 | 0.0 | 0.0 | 0.0 | 0.0 | 0.0 | 0.0 | 0.0 | 0.0 | 0.0 | 0.0 | 0.0 | 0.0 |
| Mir743 | 0.0 | 0.0 | 0.0 | 0.0 | 0.0 | 0.0 | 0.0 | 0.0 | 0.0 | 0.0 | 0.0 | 0.0 |
| Mir743b | 0.0 | 0.0 | 0.0 | 0.0 | 0.0 | 0.0 | 0.0 | 0.0 | 0.0 | 0.0 | 0.0 | 0.0 |
| Mir744 | 0.0 | 0.0 | 0.0 | 0.0 | 0.0 | 0.0 | 0.0 | 0.0 | 0.0 | 0.0 | 0.0 | 0.0 |
| Mir758 | 0.0 | 0.0 | 0.0 | 0.0 | 0.0 | 0.0 | 0.0 | 0.0 | 0.0 | 0.0 | 0.0 | 0.0 |
| Mir759 | 0.0 | 0.0 | 0.0 | 0.0 | 0.0 | 0.0 | 0.0 | 0.0 | 0.0 | 0.0 | 0.0 | 0.0 |
| Mir761 | 0.0 | 0.0 | 0.0 | 0.0 | 0.0 | 0.0 | 0.0 | 0.0 | 0.0 | 0.0 | 0.0 | 0.0 |
| Mir764 | 0.0 | 0.0 | 0.0 | 0.0 | 0.0 | 0.0 | 0.0 | 0.0 | 0.0 | 0.0 | 0.0 | 0.0 |
| Mir767 | 0.0 | 0.0 | 0.0 | 0.0 | 0.0 | 0.0 | 0.0 | 0.0 | 0.0 | 0.0 | 0.0 | 0.0 |
| Mir770 | 0.0 | 0.0 | 0.0 | 0.0 | 0.0 | 0.0 | 0.0 | 0.0 | 0.0 | 0.0 | 0.0 | 0.0 |
| Mir7b | 0.0 | 0.0 | 0.0 | 0.0 | 0.0 | 0.0 | 0.0 | 0.0 | 0.0 | 0.0 | 0.0 | 0.0 |
| Mir802 | 0.0 | 0.0 | 0.0 | 0.0 | 0.0 | 0.0 | 0.0 | 0.0 | 0.0 | 0.0 | 0.0 | 0.0 |

Excluded Genes

| | | | | | | | | | | | |
|---|---|---|---|---|---|---|---|---|---|---|---|
| Mir804 | 0.0 | 0.0 | 0.0 | 0.0 | 0.0 | 0.0 | 0.0 | 0.0 | 0.0 | 0.0 | 0.0 |
| Mir871 | 0.0 | 0.0 | 0.0 | 0.0 | 0.0 | 0.0 | 0.0 | 0.0 | 0.0 | 0.0 | 0.0 |
| Mir872 | 0.0 | 0.0 | 0.0 | 0.0 | 0.0 | 0.0 | 0.0 | 0.0 | 0.0 | 0.0 | 0.0 |
| Mir873 | 0.0 | 0.0 | 0.0 | 0.0 | 0.0 | 0.0 | 0.0 | 0.0 | 0.0 | 0.0 | 0.0 |
| Mir874 | 0.0 | 0.0 | 0.0 | 0.0 | 0.0 | 0.0 | 0.0 | 0.0 | 0.0 | 0.0 | 0.0 |
| Mir875 | 0.0 | 0.0 | 0.0 | 0.0 | 0.0 | 0.0 | 0.0 | 0.0 | 0.0 | 0.0 | 0.0 |
| Mir876 | 0.0 | 0.0 | 0.0 | 0.0 | 0.0 | 0.0 | 0.0 | 0.0 | 0.0 | 0.0 | 0.0 |
| Mir878 | 0.0 | 0.0 | 0.0 | 0.0 | 0.0 | 0.0 | 0.0 | 0.0 | 0.0 | 0.0 | 0.0 |
| Mir879 | 0.0 | 0.0 | 0.0 | 0.0 | 0.0 | 0.0 | 0.0 | 0.0 | 0.0 | 0.0 | 0.0 |
| Mir880 | 0.0 | 0.0 | 0.0 | 0.0 | 0.0 | 0.0 | 0.0 | 0.0 | 0.0 | 0.0 | 0.0 |
| Mir881 | 0.0 | 0.0 | 0.0 | 0.0 | 0.0 | 0.0 | 0.0 | 0.0 | 0.0 | 0.0 | 0.0 |
| Mir882 | 0.0 | 0.0 | 0.0 | 0.0 | 0.0 | 0.0 | 0.0 | 0.0 | 0.0 | 0.0 | 0.0 |
| Mir883a | 0.0 | 0.0 | 0.0 | 0.0 | 0.0 | 0.0 | 0.0 | 0.0 | 0.0 | 0.0 | 0.0 |
| Mir883b | 0.0 | 0.0 | 0.0 | 0.0 | 0.0 | 0.0 | 0.0 | 0.0 | 0.0 | 0.0 | 0.0 |
| Mir9-1 | 0.0 | 0.0 | 0.0 | 0.0 | 0.0 | 0.0 | 0.0 | 0.0 | 0.0 | 0.0 | 0.0 |
| Mir9-2 | 0.0 | 0.0 | 0.0 | 0.0 | 0.0 | 0.0 | 0.0 | 0.0 | 0.0 | 0.0 | 0.0 |
| Mir92-1 | 0.0 | 0.0 | 0.0 | 0.0 | 0.0 | 0.0 | 0.0 | 0.0 | 0.0 | 0.0 | 0.0 |
| Mir92-2 | 0.0 | 0.0 | 0.0 | 0.0 | 0.0 | 0.0 | 0.0 | 0.0 | 0.0 | 0.0 | 0.0 |
| Mir92b | 0.0 | 0.0 | 0.0 | 0.0 | 0.0 | 0.0 | 0.0 | 0.0 | 0.0 | 0.0 | 0.0 |
| Mir93 | 0.0 | 0.0 | 0.0 | 0.0 | 0.0 | 0.0 | 0.0 | 0.0 | 0.0 | 0.0 | 0.0 |
| Mir9-3 | 0.0 | 0.0 | 0.0 | 0.0 | 0.0 | 0.0 | 0.0 | 0.0 | 0.0 | 0.0 | 0.0 |
| Mir96 | 0.0 | 0.0 | 0.0 | 0.0 | 0.0 | 0.0 | 0.0 | 0.0 | 0.0 | 0.0 | 0.0 |
| Mir98 | 0.0 | 0.0 | 0.0 | 0.0 | 0.0 | 0.0 | 0.0 | 0.0 | 0.0 | 0.0 | 0.0 |
| Mir99a | 0.0 | 0.0 | 0.0 | 0.0 | 0.0 | 0.0 | 0.0 | 0.0 | 0.0 | 0.0 | 0.0 |
| Mirlet7b | 0.0 | 0.0 | 0.0 | 0.0 | 0.0 | 0.0 | 0.0 | 0.0 | 0.0 | 0.0 | 0.0 |
| Mirlet7c-1 | 0.0 | 0.0 | 0.0 | 0.0 | 0.0 | 0.0 | 0.0 | 0.0 | 0.0 | 0.0 | 0.0 |
| Mirlet7c-2 | 0.0 | 0.0 | 0.0 | 0.0 | 0.0 | 0.0 | 0.0 | 0.0 | 0.0 | 0.0 | 0.0 |
| Mirlet7d | 0.0 | 0.0 | 0.0 | 0.0 | 0.0 | 0.0 | 0.0 | 0.0 | 0.0 | 0.0 | 0.0 |
| Mirlet7e | 0.0 | 0.0 | 0.0 | 0.0 | 0.0 | 0.0 | 0.0 | 0.0 | 0.0 | 0.0 | 0.0 |
| Mirlet7f-2 | 0.0 | 0.0 | 0.0 | 0.0 | 0.0 | 0.0 | 0.0 | 0.0 | 0.0 | 0.0 | 0.0 |
| Mirlet7g | 0.0 | 0.0 | 0.0 | 0.0 | 0.0 | 0.0 | 0.0 | 0.0 | 0.0 | 0.0 | 0.0 |
| Mmp20 | 0.0 | 0.0 | 0.0 | 0.0 | 0.0 | 0.0 | 0.0 | 0.0 | 0.0 | 0.0 | 0.0 |
| Mmp7 | 0.0 | 0.0 | 0.0 | 0.0 | 0.0 | 0.0 | 0.0 | 0.0 | 0.0 | 0.0 | 0.0 |
| Moxd2 | 0.0 | 0.0 | 0.0 | 0.0 | 0.0 | 0.0 | 0.0 | 0.0 | 0.0 | 0.0 | 0.0 |
| Mpl | 0.0 | 0.0 | 0.0 | 0.0 | 0.0 | 0.0 | 0.0 | 0.0 | 0.0 | 0.0 | 0.0 |
| Mrgpra2b | 0.0 | 0.0 | 0.0 | 0.0 | 0.0 | 0.0 | 0.0 | 0.0 | 0.0 | 0.0 | 0.0 |
| Mrgprb3 | 0.0 | 0.0 | 0.0 | 0.0 | 0.0 | 0.0 | 0.0 | 0.0 | 0.0 | 0.0 | 0.0 |
| Mrgprb4 | 0.0 | 0.0 | 0.0 | 0.0 | 0.0 | 0.0 | 0.0 | 0.0 | 0.0 | 0.0 | 0.0 |
| Mrgprb5 | 0.0 | 0.0 | 0.0 | 0.0 | 0.0 | 0.0 | 0.0 | 0.0 | 0.0 | 0.0 | 0.0 |
| Mrgprx1 | 0.0 | 0.0 | 0.0 | 0.0 | 0.0 | 0.0 | 0.0 | 0.0 | 0.0 | 0.0 | 0.0 |
| Mrgprx2 | 0.0 | 0.0 | 0.0 | 0.0 | 0.0 | 0.0 | 0.0 | 0.0 | 0.0 | 0.0 | 0.0 |
| Mroh2b | 0.0 | 0.0 | 0.0 | 0.0 | 0.0 | 0.0 | 0.0 | 0.0 | 0.0 | 0.0 | 0.0 |
| Ms4a18 | 0.0 | 0.0 | 0.0 | 0.0 | 0.0 | 0.0 | 0.0 | 0.0 | 0.0 | 0.0 | 0.0 |
| Ms4a8a | 0.0 | 0.0 | 0.0 | 0.0 | 0.0 | 0.0 | 0.0 | 0.0 | 0.0 | 0.0 | 0.0 |
| Msgn1 | 0.0 | 0.0 | 0.0 | 0.0 | 0.0 | 0.0 | 0.0 | 0.0 | 0.0 | 0.0 | 0.0 |
| Mstn | 0.0 | 0.0 | 0.0 | 0.0 | 0.0 | 0.0 | 0.0 | 0.0 | 0.0 | 0.0 | 0.0 |
| Mt4 | 0.0 | 0.0 | 0.0 | 0.0 | 0.0 | 0.0 | 0.0 | 0.0 | 0.0 | 0.0 | 0.0 |
| Mtnr1b | 0.0 | 0.0 | 0.0 | 0.0 | 0.0 | 0.0 | 0.0 | 0.0 | 0.0 | 0.0 | 0.0 |
| Mug2 | 0.0 | 0.0 | 0.0 | 0.0 | 0.0 | 0.0 | 0.0 | 0.0 | 0.0 | 0.0 | 0.0 |
| Mug-ps1 | 0.0 | 0.0 | 0.0 | 0.0 | 0.0 | 0.0 | 0.0 | 0.0 | 0.0 | 0.0 | 0.0 |
| Mup17 | 0.0 | 0.0 | 0.0 | 0.0 | 0.0 | 0.0 | 0.0 | 0.0 | 0.0 | 0.0 | 0.0 |
| Mup20 | 0.0 | 0.0 | 0.0 | 0.0 | 0.0 | 0.0 | 0.0 | 0.0 | 0.0 | 0.0 | 0.0 |
| Mup21 | 0.0 | 0.0 | 0.0 | 0.0 | 0.0 | 0.0 | 0.0 | 0.0 | 0.0 | 0.0 | 0.0 |
| Mup3 | 0.0 | 0.0 | 0.0 | 0.0 | 0.0 | 0.0 | 0.0 | 0.0 | 0.0 | 0.0 | 0.0 |
| Mup4 | 0.0 | 0.0 | 0.0 | 0.0 | 0.0 | 0.0 | 0.0 | 0.0 | 0.0 | 0.0 | 0.0 |
| Mup5 | 0.0 | 0.0 | 0.0 | 0.0 | 0.0 | 0.0 | 0.0 | 0.0 | 0.0 | 0.0 | 0.0 |
| Mup6 | 0.0 | 0.0 | 0.0 | 0.0 | 0.0 | 0.0 | 0.0 | 0.0 | 0.0 | 0.0 | 0.0 |

| | | | | | | | | | | | | |
|---|---|---|---|---|---|---|---|---|---|---|---|---|
| Mycs | 0.0 | 0.0 | 0.0 | 0.0 | 0.0 | 0.0 | 0.0 | 0.0 | 0.0 | 0.0 | 0.0 | 0.0 |
| Myf6 | 0.0 | 0.0 | 0.0 | 0.0 | 0.0 | 0.0 | 0.0 | 0.0 | 0.0 | 0.0 | 0.0 | 0.0 |
| Ndp | 0.0 | 0.0 | 0.0 | 0.0 | 0.0 | 0.0 | 0.0 | 0.0 | 0.0 | 0.0 | 0.0 | 0.0 |
| Neurog1 | 0.0 | 0.0 | 0.0 | 0.0 | 0.0 | 0.0 | 0.0 | 0.0 | 0.0 | 0.0 | 0.0 | 0.0 |
| Neurog3 | 0.0 | 0.0 | 0.0 | 0.0 | 0.0 | 0.0 | 0.0 | 0.0 | 0.0 | 0.0 | 0.0 | 0.0 |
| Nipsnap3a | 0.0 | 0.0 | 0.0 | 0.0 | 0.0 | 0.0 | 0.0 | 0.0 | 0.0 | 0.0 | 0.0 | 0.0 |
| Nkg7 | 0.0 | 0.0 | 0.0 | 0.0 | 0.0 | 0.0 | 0.0 | 0.0 | 0.0 | 0.0 | 0.0 | 0.0 |
| Nlrp4a | 0.0 | 0.0 | 0.0 | 0.0 | 0.0 | 0.0 | 0.0 | 0.0 | 0.0 | 0.0 | 0.0 | 0.0 |
| Nlrp4b | 0.0 | 0.0 | 0.0 | 0.0 | 0.0 | 0.0 | 0.0 | 0.0 | 0.0 | 0.0 | 0.0 | 0.0 |
| Nlrp9c | 0.0 | 0.0 | 0.0 | 0.0 | 0.0 | 0.0 | 0.0 | 0.0 | 0.0 | 0.0 | 0.0 | 0.0 |
| Nme9 | 0.0 | 0.0 | 0.0 | 0.0 | 0.0 | 0.0 | 0.0 | 0.0 | 0.0 | 0.0 | 0.0 | 0.0 |
| Noxa1 | 0.0 | 0.0 | 0.0 | 0.0 | 0.0 | 0.0 | 0.0 | 0.0 | 0.0 | 0.0 | 0.0 | 0.0 |
| Npbwr1 | 0.0 | 0.0 | 0.0 | 0.0 | 0.0 | 0.0 | 0.0 | 0.0 | 0.0 | 0.0 | 0.0 | 0.0 |
| Npffr1 | 0.0 | 0.0 | 0.0 | 0.0 | 0.0 | 0.0 | 0.0 | 0.0 | 0.0 | 0.0 | 0.0 | 0.0 |
| Nphs1as | 0.0 | 0.0 | 0.0 | 0.0 | 0.0 | 0.0 | 0.0 | 0.0 | 0.0 | 0.0 | 0.0 | 0.0 |
| Npvf | 0.0 | 0.0 | 0.0 | 0.0 | 0.0 | 0.0 | 0.0 | 0.0 | 0.0 | 0.0 | 0.0 | 0.0 |
| Npy2r | 0.0 | 0.0 | 0.0 | 0.0 | 0.0 | 0.0 | 0.0 | 0.0 | 0.0 | 0.0 | 0.0 | 0.0 |
| Nr2e3 | 0.0 | 0.0 | 0.0 | 0.0 | 0.0 | 0.0 | 0.0 | 0.0 | 0.0 | 0.0 | 0.0 | 0.0 |
| Obox1 | 0.0 | 0.0 | 0.0 | 0.0 | 0.0 | 0.0 | 0.0 | 0.0 | 0.0 | 0.0 | 0.0 | 0.0 |
| Obox2 | 0.0 | 0.0 | 0.0 | 0.0 | 0.0 | 0.0 | 0.0 | 0.0 | 0.0 | 0.0 | 0.0 | 0.0 |
| Obox5 | 0.0 | 0.0 | 0.0 | 0.0 | 0.0 | 0.0 | 0.0 | 0.0 | 0.0 | 0.0 | 0.0 | 0.0 |
| Obp1a | 0.0 | 0.0 | 0.0 | 0.0 | 0.0 | 0.0 | 0.0 | 0.0 | 0.0 | 0.0 | 0.0 | 0.0 |
| Obp2a | 0.0 | 0.0 | 0.0 | 0.0 | 0.0 | 0.0 | 0.0 | 0.0 | 0.0 | 0.0 | 0.0 | 0.0 |
| Obp2b | 0.0 | 0.0 | 0.0 | 0.0 | 0.0 | 0.0 | 0.0 | 0.0 | 0.0 | 0.0 | 0.0 | 0.0 |
| Olah | 0.0 | 0.0 | 0.0 | 0.0 | 0.0 | 0.0 | 0.0 | 0.0 | 0.0 | 0.0 | 0.0 | 0.0 |
| Olfr1 | 0.0 | 0.0 | 0.0 | 0.0 | 0.0 | 0.0 | 0.0 | 0.0 | 0.0 | 0.0 | 0.0 | 0.0 |
| Olfr100 | 0.0 | 0.0 | 0.0 | 0.0 | 0.0 | 0.0 | 0.0 | 0.0 | 0.0 | 0.0 | 0.0 | 0.0 |
| Olfr1000 | 0.0 | 0.0 | 0.0 | 0.0 | 0.0 | 0.0 | 0.0 | 0.0 | 0.0 | 0.0 | 0.0 | 0.0 |
| Olfr1008 | 0.0 | 0.0 | 0.0 | 0.0 | 0.0 | 0.0 | 0.0 | 0.0 | 0.0 | 0.0 | 0.0 | 0.0 |
| Olfr1009 | 0.0 | 0.0 | 0.0 | 0.0 | 0.0 | 0.0 | 0.0 | 0.0 | 0.0 | 0.0 | 0.0 | 0.0 |
| Olfr1010 | 0.0 | 0.0 | 0.0 | 0.0 | 0.0 | 0.0 | 0.0 | 0.0 | 0.0 | 0.0 | 0.0 | 0.0 |
| Olfr1012 | 0.0 | 0.0 | 0.0 | 0.0 | 0.0 | 0.0 | 0.0 | 0.0 | 0.0 | 0.0 | 0.0 | 0.0 |
| Olfr1013 | 0.0 | 0.0 | 0.0 | 0.0 | 0.0 | 0.0 | 0.0 | 0.0 | 0.0 | 0.0 | 0.0 | 0.0 |
| Olfr1014 | 0.0 | 0.0 | 0.0 | 0.0 | 0.0 | 0.0 | 0.0 | 0.0 | 0.0 | 0.0 | 0.0 | 0.0 |
| Olfr1015 | 0.0 | 0.0 | 0.0 | 0.0 | 0.0 | 0.0 | 0.0 | 0.0 | 0.0 | 0.0 | 0.0 | 0.0 |
| Olfr1018 | 0.0 | 0.0 | 0.0 | 0.0 | 0.0 | 0.0 | 0.0 | 0.0 | 0.0 | 0.0 | 0.0 | 0.0 |
| Olfr1019 | 0.0 | 0.0 | 0.0 | 0.0 | 0.0 | 0.0 | 0.0 | 0.0 | 0.0 | 0.0 | 0.0 | 0.0 |
| Olfr102 | 0.0 | 0.0 | 0.0 | 0.0 | 0.0 | 0.0 | 0.0 | 0.0 | 0.0 | 0.0 | 0.0 | 0.0 |
| Olfr1020 | 0.0 | 0.0 | 0.0 | 0.0 | 0.0 | 0.0 | 0.0 | 0.0 | 0.0 | 0.0 | 0.0 | 0.0 |
| Olfr1022 | 0.0 | 0.0 | 0.0 | 0.0 | 0.0 | 0.0 | 0.0 | 0.0 | 0.0 | 0.0 | 0.0 | 0.0 |
| Olfr1023 | 0.0 | 0.0 | 0.0 | 0.0 | 0.0 | 0.0 | 0.0 | 0.0 | 0.0 | 0.0 | 0.0 | 0.0 |
| Olfr1024 | 0.0 | 0.0 | 0.0 | 0.0 | 0.0 | 0.0 | 0.0 | 0.0 | 0.0 | 0.0 | 0.0 | 0.0 |
| Olfr1026 | 0.0 | 0.0 | 0.0 | 0.0 | 0.0 | 0.0 | 0.0 | 0.0 | 0.0 | 0.0 | 0.0 | 0.0 |
| Olfr1028 | 0.0 | 0.0 | 0.0 | 0.0 | 0.0 | 0.0 | 0.0 | 0.0 | 0.0 | 0.0 | 0.0 | 0.0 |
| Olfr1029 | 0.0 | 0.0 | 0.0 | 0.0 | 0.0 | 0.0 | 0.0 | 0.0 | 0.0 | 0.0 | 0.0 | 0.0 |
| Olfr1030 | 0.0 | 0.0 | 0.0 | 0.0 | 0.0 | 0.0 | 0.0 | 0.0 | 0.0 | 0.0 | 0.0 | 0.0 |
| Olfr1031 | 0.0 | 0.0 | 0.0 | 0.0 | 0.0 | 0.0 | 0.0 | 0.0 | 0.0 | 0.0 | 0.0 | 0.0 |
| Olfr1036 | 0.0 | 0.0 | 0.0 | 0.0 | 0.0 | 0.0 | 0.0 | 0.0 | 0.0 | 0.0 | 0.0 | 0.0 |
| Olfr1037 | 0.0 | 0.0 | 0.0 | 0.0 | 0.0 | 0.0 | 0.0 | 0.0 | 0.0 | 0.0 | 0.0 | 0.0 |
| Olfr1038-ps | 0.0 | 0.0 | 0.0 | 0.0 | 0.0 | 0.0 | 0.0 | 0.0 | 0.0 | 0.0 | 0.0 | 0.0 |
| Olfr1039 | 0.0 | 0.0 | 0.0 | 0.0 | 0.0 | 0.0 | 0.0 | 0.0 | 0.0 | 0.0 | 0.0 | 0.0 |
| Olfr1040 | 0.0 | 0.0 | 0.0 | 0.0 | 0.0 | 0.0 | 0.0 | 0.0 | 0.0 | 0.0 | 0.0 | 0.0 |
| Olfr1042 | 0.0 | 0.0 | 0.0 | 0.0 | 0.0 | 0.0 | 0.0 | 0.0 | 0.0 | 0.0 | 0.0 | 0.0 |
| Olfr1043 | 0.0 | 0.0 | 0.0 | 0.0 | 0.0 | 0.0 | 0.0 | 0.0 | 0.0 | 0.0 | 0.0 | 0.0 |
| Olfr1044 | 0.0 | 0.0 | 0.0 | 0.0 | 0.0 | 0.0 | 0.0 | 0.0 | 0.0 | 0.0 | 0.0 | 0.0 |
| Olfr1045 | 0.0 | 0.0 | 0.0 | 0.0 | 0.0 | 0.0 | 0.0 | 0.0 | 0.0 | 0.0 | 0.0 | 0.0 |
| Olfr1046 | 0.0 | 0.0 | 0.0 | 0.0 | 0.0 | 0.0 | 0.0 | 0.0 | 0.0 | 0.0 | 0.0 | 0.0 |

| | | | | | | | | | | | |
|---|---|---|---|---|---|---|---|---|---|---|---|
| Olfr1047 | 0.0 | 0.0 | 0.0 | 0.0 | 0.0 | 0.0 | 0.0 | 0.0 | 0.0 | 0.0 | 0.0 |
| Olfr1048 | 0.0 | 0.0 | 0.0 | 0.0 | 0.0 | 0.0 | 0.0 | 0.0 | 0.0 | 0.0 | 0.0 |
| Olfr1049 | 0.0 | 0.0 | 0.0 | 0.0 | 0.0 | 0.0 | 0.0 | 0.0 | 0.0 | 0.0 | 0.0 |
| Olfr1051 | 0.0 | 0.0 | 0.0 | 0.0 | 0.0 | 0.0 | 0.0 | 0.0 | 0.0 | 0.0 | 0.0 |
| Olfr1052 | 0.0 | 0.0 | 0.0 | 0.0 | 0.0 | 0.0 | 0.0 | 0.0 | 0.0 | 0.0 | 0.0 |
| Olfr1053 | 0.0 | 0.0 | 0.0 | 0.0 | 0.0 | 0.0 | 0.0 | 0.0 | 0.0 | 0.0 | 0.0 |
| Olfr1054 | 0.0 | 0.0 | 0.0 | 0.0 | 0.0 | 0.0 | 0.0 | 0.0 | 0.0 | 0.0 | 0.0 |
| Olfr1055 | 0.0 | 0.0 | 0.0 | 0.0 | 0.0 | 0.0 | 0.0 | 0.0 | 0.0 | 0.0 | 0.0 |
| Olfr1056 | 0.0 | 0.0 | 0.0 | 0.0 | 0.0 | 0.0 | 0.0 | 0.0 | 0.0 | 0.0 | 0.0 |
| Olfr1057 | 0.0 | 0.0 | 0.0 | 0.0 | 0.0 | 0.0 | 0.0 | 0.0 | 0.0 | 0.0 | 0.0 |
| Olfr1058 | 0.0 | 0.0 | 0.0 | 0.0 | 0.0 | 0.0 | 0.0 | 0.0 | 0.0 | 0.0 | 0.0 |
| Olfr1061 | 0.0 | 0.0 | 0.0 | 0.0 | 0.0 | 0.0 | 0.0 | 0.0 | 0.0 | 0.0 | 0.0 |
| Olfr1062 | 0.0 | 0.0 | 0.0 | 0.0 | 0.0 | 0.0 | 0.0 | 0.0 | 0.0 | 0.0 | 0.0 |
| Olfr1065 | 0.0 | 0.0 | 0.0 | 0.0 | 0.0 | 0.0 | 0.0 | 0.0 | 0.0 | 0.0 | 0.0 |
| Olfr1066 | 0.0 | 0.0 | 0.0 | 0.0 | 0.0 | 0.0 | 0.0 | 0.0 | 0.0 | 0.0 | 0.0 |
| Olfr107 | 0.0 | 0.0 | 0.0 | 0.0 | 0.0 | 0.0 | 0.0 | 0.0 | 0.0 | 0.0 | 0.0 |
| Olfr1076 | 0.0 | 0.0 | 0.0 | 0.0 | 0.0 | 0.0 | 0.0 | 0.0 | 0.0 | 0.0 | 0.0 |
| Olfr1077-ps1 | 0.0 | 0.0 | 0.0 | 0.0 | 0.0 | 0.0 | 0.0 | 0.0 | 0.0 | 0.0 | 0.0 |
| Olfr108 | 0.0 | 0.0 | 0.0 | 0.0 | 0.0 | 0.0 | 0.0 | 0.0 | 0.0 | 0.0 | 0.0 |
| Olfr1080 | 0.0 | 0.0 | 0.0 | 0.0 | 0.0 | 0.0 | 0.0 | 0.0 | 0.0 | 0.0 | 0.0 |
| Olfr1082 | 0.0 | 0.0 | 0.0 | 0.0 | 0.0 | 0.0 | 0.0 | 0.0 | 0.0 | 0.0 | 0.0 |
| Olfr1084 | 0.0 | 0.0 | 0.0 | 0.0 | 0.0 | 0.0 | 0.0 | 0.0 | 0.0 | 0.0 | 0.0 |
| Olfr1086 | 0.0 | 0.0 | 0.0 | 0.0 | 0.0 | 0.0 | 0.0 | 0.0 | 0.0 | 0.0 | 0.0 |
| Olfr1087 | 0.0 | 0.0 | 0.0 | 0.0 | 0.0 | 0.0 | 0.0 | 0.0 | 0.0 | 0.0 | 0.0 |
| Olfr1089 | 0.0 | 0.0 | 0.0 | 0.0 | 0.0 | 0.0 | 0.0 | 0.0 | 0.0 | 0.0 | 0.0 |
| Olfr109 | 0.0 | 0.0 | 0.0 | 0.0 | 0.0 | 0.0 | 0.0 | 0.0 | 0.0 | 0.0 | 0.0 |
| Olfr1090 | 0.0 | 0.0 | 0.0 | 0.0 | 0.0 | 0.0 | 0.0 | 0.0 | 0.0 | 0.0 | 0.0 |
| Olfr1093 | 0.0 | 0.0 | 0.0 | 0.0 | 0.0 | 0.0 | 0.0 | 0.0 | 0.0 | 0.0 | 0.0 |
| Olfr1094 | 0.0 | 0.0 | 0.0 | 0.0 | 0.0 | 0.0 | 0.0 | 0.0 | 0.0 | 0.0 | 0.0 |
| Olfr1095 | 0.0 | 0.0 | 0.0 | 0.0 | 0.0 | 0.0 | 0.0 | 0.0 | 0.0 | 0.0 | 0.0 |
| Olfr1097 | 0.0 | 0.0 | 0.0 | 0.0 | 0.0 | 0.0 | 0.0 | 0.0 | 0.0 | 0.0 | 0.0 |
| Olfr1098 | 0.0 | 0.0 | 0.0 | 0.0 | 0.0 | 0.0 | 0.0 | 0.0 | 0.0 | 0.0 | 0.0 |
| Olfr1099 | 0.0 | 0.0 | 0.0 | 0.0 | 0.0 | 0.0 | 0.0 | 0.0 | 0.0 | 0.0 | 0.0 |
| Olfr11 | 0.0 | 0.0 | 0.0 | 0.0 | 0.0 | 0.0 | 0.0 | 0.0 | 0.0 | 0.0 | 0.0 |
| Olfr1100 | 0.0 | 0.0 | 0.0 | 0.0 | 0.0 | 0.0 | 0.0 | 0.0 | 0.0 | 0.0 | 0.0 |
| Olfr1101 | 0.0 | 0.0 | 0.0 | 0.0 | 0.0 | 0.0 | 0.0 | 0.0 | 0.0 | 0.0 | 0.0 |
| Olfr1102 | 0.0 | 0.0 | 0.0 | 0.0 | 0.0 | 0.0 | 0.0 | 0.0 | 0.0 | 0.0 | 0.0 |
| Olfr1104 | 0.0 | 0.0 | 0.0 | 0.0 | 0.0 | 0.0 | 0.0 | 0.0 | 0.0 | 0.0 | 0.0 |
| Olfr1105 | 0.0 | 0.0 | 0.0 | 0.0 | 0.0 | 0.0 | 0.0 | 0.0 | 0.0 | 0.0 | 0.0 |
| Olfr1106 | 0.0 | 0.0 | 0.0 | 0.0 | 0.0 | 0.0 | 0.0 | 0.0 | 0.0 | 0.0 | 0.0 |
| Olfr1107 | 0.0 | 0.0 | 0.0 | 0.0 | 0.0 | 0.0 | 0.0 | 0.0 | 0.0 | 0.0 | 0.0 |
| Olfr1109 | 0.0 | 0.0 | 0.0 | 0.0 | 0.0 | 0.0 | 0.0 | 0.0 | 0.0 | 0.0 | 0.0 |
| Olfr1110 | 0.0 | 0.0 | 0.0 | 0.0 | 0.0 | 0.0 | 0.0 | 0.0 | 0.0 | 0.0 | 0.0 |
| Olfr1111 | 0.0 | 0.0 | 0.0 | 0.0 | 0.0 | 0.0 | 0.0 | 0.0 | 0.0 | 0.0 | 0.0 |
| Olfr1112 | 0.0 | 0.0 | 0.0 | 0.0 | 0.0 | 0.0 | 0.0 | 0.0 | 0.0 | 0.0 | 0.0 |
| Olfr1113 | 0.0 | 0.0 | 0.0 | 0.0 | 0.0 | 0.0 | 0.0 | 0.0 | 0.0 | 0.0 | 0.0 |
| Olfr1115 | 0.0 | 0.0 | 0.0 | 0.0 | 0.0 | 0.0 | 0.0 | 0.0 | 0.0 | 0.0 | 0.0 |
| Olfr1116-ps | 0.0 | 0.0 | 0.0 | 0.0 | 0.0 | 0.0 | 0.0 | 0.0 | 0.0 | 0.0 | 0.0 |
| Olfr1118 | 0.0 | 0.0 | 0.0 | 0.0 | 0.0 | 0.0 | 0.0 | 0.0 | 0.0 | 0.0 | 0.0 |
| Olfr1120 | 0.0 | 0.0 | 0.0 | 0.0 | 0.0 | 0.0 | 0.0 | 0.0 | 0.0 | 0.0 | 0.0 |
| Olfr1121 | 0.0 | 0.0 | 0.0 | 0.0 | 0.0 | 0.0 | 0.0 | 0.0 | 0.0 | 0.0 | 0.0 |
| Olfr1122 | 0.0 | 0.0 | 0.0 | 0.0 | 0.0 | 0.0 | 0.0 | 0.0 | 0.0 | 0.0 | 0.0 |
| Olfr1124 | 0.0 | 0.0 | 0.0 | 0.0 | 0.0 | 0.0 | 0.0 | 0.0 | 0.0 | 0.0 | 0.0 |
| Olfr1126 | 0.0 | 0.0 | 0.0 | 0.0 | 0.0 | 0.0 | 0.0 | 0.0 | 0.0 | 0.0 | 0.0 |
| Olfr1128 | 0.0 | 0.0 | 0.0 | 0.0 | 0.0 | 0.0 | 0.0 | 0.0 | 0.0 | 0.0 | 0.0 |
| Olfr1129 | 0.0 | 0.0 | 0.0 | 0.0 | 0.0 | 0.0 | 0.0 | 0.0 | 0.0 | 0.0 | 0.0 |
| Olfr113 | 0.0 | 0.0 | 0.0 | 0.0 | 0.0 | 0.0 | 0.0 | 0.0 | 0.0 | 0.0 | 0.0 |

Excluded Genes

| | | | | | | | | | | | |
|---|---|---|---|---|---|---|---|---|---|---|---|
| Olfr1130 | 0.0 | 0.0 | 0.0 | 0.0 | 0.0 | 0.0 | 0.0 | 0.0 | 0.0 | 0.0 | 0.0 |
| Olfr1131 | 0.0 | 0.0 | 0.0 | 0.0 | 0.0 | 0.0 | 0.0 | 0.0 | 0.0 | 0.0 | 0.0 |
| Olfr1134 | 0.0 | 0.0 | 0.0 | 0.0 | 0.0 | 0.0 | 0.0 | 0.0 | 0.0 | 0.0 | 0.0 |
| Olfr1135 | 0.0 | 0.0 | 0.0 | 0.0 | 0.0 | 0.0 | 0.0 | 0.0 | 0.0 | 0.0 | 0.0 |
| Olfr1136 | 0.0 | 0.0 | 0.0 | 0.0 | 0.0 | 0.0 | 0.0 | 0.0 | 0.0 | 0.0 | 0.0 |
| Olfr1137 | 0.0 | 0.0 | 0.0 | 0.0 | 0.0 | 0.0 | 0.0 | 0.0 | 0.0 | 0.0 | 0.0 |
| Olfr1138 | 0.0 | 0.0 | 0.0 | 0.0 | 0.0 | 0.0 | 0.0 | 0.0 | 0.0 | 0.0 | 0.0 |
| Olfr1140 | 0.0 | 0.0 | 0.0 | 0.0 | 0.0 | 0.0 | 0.0 | 0.0 | 0.0 | 0.0 | 0.0 |
| Olfr1141 | 0.0 | 0.0 | 0.0 | 0.0 | 0.0 | 0.0 | 0.0 | 0.0 | 0.0 | 0.0 | 0.0 |
| Olfr1143 | 0.0 | 0.0 | 0.0 | 0.0 | 0.0 | 0.0 | 0.0 | 0.0 | 0.0 | 0.0 | 0.0 |
| Olfr1145 | 0.0 | 0.0 | 0.0 | 0.0 | 0.0 | 0.0 | 0.0 | 0.0 | 0.0 | 0.0 | 0.0 |
| Olfr1148 | 0.0 | 0.0 | 0.0 | 0.0 | 0.0 | 0.0 | 0.0 | 0.0 | 0.0 | 0.0 | 0.0 |
| Olfr1151 | 0.0 | 0.0 | 0.0 | 0.0 | 0.0 | 0.0 | 0.0 | 0.0 | 0.0 | 0.0 | 0.0 |
| Olfr1152 | 0.0 | 0.0 | 0.0 | 0.0 | 0.0 | 0.0 | 0.0 | 0.0 | 0.0 | 0.0 | 0.0 |
| Olfr1153 | 0.0 | 0.0 | 0.0 | 0.0 | 0.0 | 0.0 | 0.0 | 0.0 | 0.0 | 0.0 | 0.0 |
| Olfr1154 | 0.0 | 0.0 | 0.0 | 0.0 | 0.0 | 0.0 | 0.0 | 0.0 | 0.0 | 0.0 | 0.0 |
| Olfr1155 | 0.0 | 0.0 | 0.0 | 0.0 | 0.0 | 0.0 | 0.0 | 0.0 | 0.0 | 0.0 | 0.0 |
| Olfr1156 | 0.0 | 0.0 | 0.0 | 0.0 | 0.0 | 0.0 | 0.0 | 0.0 | 0.0 | 0.0 | 0.0 |
| Olfr1157 | 0.0 | 0.0 | 0.0 | 0.0 | 0.0 | 0.0 | 0.0 | 0.0 | 0.0 | 0.0 | 0.0 |
| Olfr116 | 0.0 | 0.0 | 0.0 | 0.0 | 0.0 | 0.0 | 0.0 | 0.0 | 0.0 | 0.0 | 0.0 |
| Olfr1160 | 0.0 | 0.0 | 0.0 | 0.0 | 0.0 | 0.0 | 0.0 | 0.0 | 0.0 | 0.0 | 0.0 |
| Olfr1161 | 0.0 | 0.0 | 0.0 | 0.0 | 0.0 | 0.0 | 0.0 | 0.0 | 0.0 | 0.0 | 0.0 |
| Olfr1162 | 0.0 | 0.0 | 0.0 | 0.0 | 0.0 | 0.0 | 0.0 | 0.0 | 0.0 | 0.0 | 0.0 |
| Olfr1163 | 0.0 | 0.0 | 0.0 | 0.0 | 0.0 | 0.0 | 0.0 | 0.0 | 0.0 | 0.0 | 0.0 |
| Olfr1164 | 0.0 | 0.0 | 0.0 | 0.0 | 0.0 | 0.0 | 0.0 | 0.0 | 0.0 | 0.0 | 0.0 |
| Olfr1168 | 0.0 | 0.0 | 0.0 | 0.0 | 0.0 | 0.0 | 0.0 | 0.0 | 0.0 | 0.0 | 0.0 |
| Olfr117 | 0.0 | 0.0 | 0.0 | 0.0 | 0.0 | 0.0 | 0.0 | 0.0 | 0.0 | 0.0 | 0.0 |
| Olfr1170 | 0.0 | 0.0 | 0.0 | 0.0 | 0.0 | 0.0 | 0.0 | 0.0 | 0.0 | 0.0 | 0.0 |
| Olfr1173 | 0.0 | 0.0 | 0.0 | 0.0 | 0.0 | 0.0 | 0.0 | 0.0 | 0.0 | 0.0 | 0.0 |
| Olfr1176 | 0.0 | 0.0 | 0.0 | 0.0 | 0.0 | 0.0 | 0.0 | 0.0 | 0.0 | 0.0 | 0.0 |
| Olfr1178 | 0.0 | 0.0 | 0.0 | 0.0 | 0.0 | 0.0 | 0.0 | 0.0 | 0.0 | 0.0 | 0.0 |
| Olfr1179 | 0.0 | 0.0 | 0.0 | 0.0 | 0.0 | 0.0 | 0.0 | 0.0 | 0.0 | 0.0 | 0.0 |
| Olfr118 | 0.0 | 0.0 | 0.0 | 0.0 | 0.0 | 0.0 | 0.0 | 0.0 | 0.0 | 0.0 | 0.0 |
| Olfr1180 | 0.0 | 0.0 | 0.0 | 0.0 | 0.0 | 0.0 | 0.0 | 0.0 | 0.0 | 0.0 | 0.0 |
| Olfr1181 | 0.0 | 0.0 | 0.0 | 0.0 | 0.0 | 0.0 | 0.0 | 0.0 | 0.0 | 0.0 | 0.0 |
| Olfr1182 | 0.0 | 0.0 | 0.0 | 0.0 | 0.0 | 0.0 | 0.0 | 0.0 | 0.0 | 0.0 | 0.0 |
| Olfr1183 | 0.0 | 0.0 | 0.0 | 0.0 | 0.0 | 0.0 | 0.0 | 0.0 | 0.0 | 0.0 | 0.0 |
| Olfr1184 | 0.0 | 0.0 | 0.0 | 0.0 | 0.0 | 0.0 | 0.0 | 0.0 | 0.0 | 0.0 | 0.0 |
| Olfr1186 | 0.0 | 0.0 | 0.0 | 0.0 | 0.0 | 0.0 | 0.0 | 0.0 | 0.0 | 0.0 | 0.0 |
| Olfr1188 | 0.0 | 0.0 | 0.0 | 0.0 | 0.0 | 0.0 | 0.0 | 0.0 | 0.0 | 0.0 | 0.0 |
| Olfr119 | 0.0 | 0.0 | 0.0 | 0.0 | 0.0 | 0.0 | 0.0 | 0.0 | 0.0 | 0.0 | 0.0 |
| Olfr1193 | 0.0 | 0.0 | 0.0 | 0.0 | 0.0 | 0.0 | 0.0 | 0.0 | 0.0 | 0.0 | 0.0 |
| Olfr1195 | 0.0 | 0.0 | 0.0 | 0.0 | 0.0 | 0.0 | 0.0 | 0.0 | 0.0 | 0.0 | 0.0 |
| Olfr1196 | 0.0 | 0.0 | 0.0 | 0.0 | 0.0 | 0.0 | 0.0 | 0.0 | 0.0 | 0.0 | 0.0 |
| Olfr1197 | 0.0 | 0.0 | 0.0 | 0.0 | 0.0 | 0.0 | 0.0 | 0.0 | 0.0 | 0.0 | 0.0 |
| Olfr1198 | 0.0 | 0.0 | 0.0 | 0.0 | 0.0 | 0.0 | 0.0 | 0.0 | 0.0 | 0.0 | 0.0 |
| Olfr1199 | 0.0 | 0.0 | 0.0 | 0.0 | 0.0 | 0.0 | 0.0 | 0.0 | 0.0 | 0.0 | 0.0 |
| Olfr120 | 0.0 | 0.0 | 0.0 | 0.0 | 0.0 | 0.0 | 0.0 | 0.0 | 0.0 | 0.0 | 0.0 |
| Olfr1200 | 0.0 | 0.0 | 0.0 | 0.0 | 0.0 | 0.0 | 0.0 | 0.0 | 0.0 | 0.0 | 0.0 |
| Olfr1201 | 0.0 | 0.0 | 0.0 | 0.0 | 0.0 | 0.0 | 0.0 | 0.0 | 0.0 | 0.0 | 0.0 |
| Olfr1202 | 0.0 | 0.0 | 0.0 | 0.0 | 0.0 | 0.0 | 0.0 | 0.0 | 0.0 | 0.0 | 0.0 |
| Olfr1204 | 0.0 | 0.0 | 0.0 | 0.0 | 0.0 | 0.0 | 0.0 | 0.0 | 0.0 | 0.0 | 0.0 |
| Olfr1205 | 0.0 | 0.0 | 0.0 | 0.0 | 0.0 | 0.0 | 0.0 | 0.0 | 0.0 | 0.0 | 0.0 |
| Olfr1206 | 0.0 | 0.0 | 0.0 | 0.0 | 0.0 | 0.0 | 0.0 | 0.0 | 0.0 | 0.0 | 0.0 |
| Olfr1209 | 0.0 | 0.0 | 0.0 | 0.0 | 0.0 | 0.0 | 0.0 | 0.0 | 0.0 | 0.0 | 0.0 |
| Olfr121 | 0.0 | 0.0 | 0.0 | 0.0 | 0.0 | 0.0 | 0.0 | 0.0 | 0.0 | 0.0 | 0.0 |
| Olfr1211 | 0.0 | 0.0 | 0.0 | 0.0 | 0.0 | 0.0 | 0.0 | 0.0 | 0.0 | 0.0 | 0.0 |

Excluded Genes

| | | | | | | | | | | | |
|---|---|---|---|---|---|---|---|---|---|---|---|
| Olfr1212 | 0.0 | 0.0 | 0.0 | 0.0 | 0.0 | 0.0 | 0.0 | 0.0 | 0.0 | 0.0 | 0.0 | 0.0 |
| Olfr1213 | 0.0 | 0.0 | 0.0 | 0.0 | 0.0 | 0.0 | 0.0 | 0.0 | 0.0 | 0.0 | 0.0 | 0.0 |
| Olfr1214 | 0.0 | 0.0 | 0.0 | 0.0 | 0.0 | 0.0 | 0.0 | 0.0 | 0.0 | 0.0 | 0.0 | 0.0 |
| Olfr1215 | 0.0 | 0.0 | 0.0 | 0.0 | 0.0 | 0.0 | 0.0 | 0.0 | 0.0 | 0.0 | 0.0 | 0.0 |
| Olfr1216 | 0.0 | 0.0 | 0.0 | 0.0 | 0.0 | 0.0 | 0.0 | 0.0 | 0.0 | 0.0 | 0.0 | 0.0 |
| Olfr1217 | 0.0 | 0.0 | 0.0 | 0.0 | 0.0 | 0.0 | 0.0 | 0.0 | 0.0 | 0.0 | 0.0 | 0.0 |
| Olfr1218 | 0.0 | 0.0 | 0.0 | 0.0 | 0.0 | 0.0 | 0.0 | 0.0 | 0.0 | 0.0 | 0.0 | 0.0 |
| Olfr1219 | 0.0 | 0.0 | 0.0 | 0.0 | 0.0 | 0.0 | 0.0 | 0.0 | 0.0 | 0.0 | 0.0 | 0.0 |
| Olfr122 | 0.0 | 0.0 | 0.0 | 0.0 | 0.0 | 0.0 | 0.0 | 0.0 | 0.0 | 0.0 | 0.0 | 0.0 |
| Olfr1220 | 0.0 | 0.0 | 0.0 | 0.0 | 0.0 | 0.0 | 0.0 | 0.0 | 0.0 | 0.0 | 0.0 | 0.0 |
| Olfr1221 | 0.0 | 0.0 | 0.0 | 0.0 | 0.0 | 0.0 | 0.0 | 0.0 | 0.0 | 0.0 | 0.0 | 0.0 |
| Olfr1222 | 0.0 | 0.0 | 0.0 | 0.0 | 0.0 | 0.0 | 0.0 | 0.0 | 0.0 | 0.0 | 0.0 | 0.0 |
| Olfr1223 | 0.0 | 0.0 | 0.0 | 0.0 | 0.0 | 0.0 | 0.0 | 0.0 | 0.0 | 0.0 | 0.0 | 0.0 |
| Olfr1225 | 0.0 | 0.0 | 0.0 | 0.0 | 0.0 | 0.0 | 0.0 | 0.0 | 0.0 | 0.0 | 0.0 | 0.0 |
| Olfr1226 | 0.0 | 0.0 | 0.0 | 0.0 | 0.0 | 0.0 | 0.0 | 0.0 | 0.0 | 0.0 | 0.0 | 0.0 |
| Olfr1228 | 0.0 | 0.0 | 0.0 | 0.0 | 0.0 | 0.0 | 0.0 | 0.0 | 0.0 | 0.0 | 0.0 | 0.0 |
| Olfr1229 | 0.0 | 0.0 | 0.0 | 0.0 | 0.0 | 0.0 | 0.0 | 0.0 | 0.0 | 0.0 | 0.0 | 0.0 |
| Olfr123 | 0.0 | 0.0 | 0.0 | 0.0 | 0.0 | 0.0 | 0.0 | 0.0 | 0.0 | 0.0 | 0.0 | 0.0 |
| Olfr1230 | 0.0 | 0.0 | 0.0 | 0.0 | 0.0 | 0.0 | 0.0 | 0.0 | 0.0 | 0.0 | 0.0 | 0.0 |
| Olfr1232 | 0.0 | 0.0 | 0.0 | 0.0 | 0.0 | 0.0 | 0.0 | 0.0 | 0.0 | 0.0 | 0.0 | 0.0 |
| Olfr1234 | 0.0 | 0.0 | 0.0 | 0.0 | 0.0 | 0.0 | 0.0 | 0.0 | 0.0 | 0.0 | 0.0 | 0.0 |
| Olfr1238 | 0.0 | 0.0 | 0.0 | 0.0 | 0.0 | 0.0 | 0.0 | 0.0 | 0.0 | 0.0 | 0.0 | 0.0 |
| Olfr1239 | 0.0 | 0.0 | 0.0 | 0.0 | 0.0 | 0.0 | 0.0 | 0.0 | 0.0 | 0.0 | 0.0 | 0.0 |
| Olfr124 | 0.0 | 0.0 | 0.0 | 0.0 | 0.0 | 0.0 | 0.0 | 0.0 | 0.0 | 0.0 | 0.0 | 0.0 |
| Olfr1240 | 0.0 | 0.0 | 0.0 | 0.0 | 0.0 | 0.0 | 0.0 | 0.0 | 0.0 | 0.0 | 0.0 | 0.0 |
| Olfr1241 | 0.0 | 0.0 | 0.0 | 0.0 | 0.0 | 0.0 | 0.0 | 0.0 | 0.0 | 0.0 | 0.0 | 0.0 |
| Olfr1242 | 0.0 | 0.0 | 0.0 | 0.0 | 0.0 | 0.0 | 0.0 | 0.0 | 0.0 | 0.0 | 0.0 | 0.0 |
| Olfr1243 | 0.0 | 0.0 | 0.0 | 0.0 | 0.0 | 0.0 | 0.0 | 0.0 | 0.0 | 0.0 | 0.0 | 0.0 |
| Olfr1245 | 0.0 | 0.0 | 0.0 | 0.0 | 0.0 | 0.0 | 0.0 | 0.0 | 0.0 | 0.0 | 0.0 | 0.0 |
| Olfr1246 | 0.0 | 0.0 | 0.0 | 0.0 | 0.0 | 0.0 | 0.0 | 0.0 | 0.0 | 0.0 | 0.0 | 0.0 |
| Olfr1248 | 0.0 | 0.0 | 0.0 | 0.0 | 0.0 | 0.0 | 0.0 | 0.0 | 0.0 | 0.0 | 0.0 | 0.0 |
| Olfr1249 | 0.0 | 0.0 | 0.0 | 0.0 | 0.0 | 0.0 | 0.0 | 0.0 | 0.0 | 0.0 | 0.0 | 0.0 |
| Olfr125 | 0.0 | 0.0 | 0.0 | 0.0 | 0.0 | 0.0 | 0.0 | 0.0 | 0.0 | 0.0 | 0.0 | 0.0 |
| Olfr1250 | 0.0 | 0.0 | 0.0 | 0.0 | 0.0 | 0.0 | 0.0 | 0.0 | 0.0 | 0.0 | 0.0 | 0.0 |
| Olfr1251 | 0.0 | 0.0 | 0.0 | 0.0 | 0.0 | 0.0 | 0.0 | 0.0 | 0.0 | 0.0 | 0.0 | 0.0 |
| Olfr1252 | 0.0 | 0.0 | 0.0 | 0.0 | 0.0 | 0.0 | 0.0 | 0.0 | 0.0 | 0.0 | 0.0 | 0.0 |
| Olfr1253 | 0.0 | 0.0 | 0.0 | 0.0 | 0.0 | 0.0 | 0.0 | 0.0 | 0.0 | 0.0 | 0.0 | 0.0 |
| Olfr1254 | 0.0 | 0.0 | 0.0 | 0.0 | 0.0 | 0.0 | 0.0 | 0.0 | 0.0 | 0.0 | 0.0 | 0.0 |
| Olfr1255 | 0.0 | 0.0 | 0.0 | 0.0 | 0.0 | 0.0 | 0.0 | 0.0 | 0.0 | 0.0 | 0.0 | 0.0 |
| Olfr1256 | 0.0 | 0.0 | 0.0 | 0.0 | 0.0 | 0.0 | 0.0 | 0.0 | 0.0 | 0.0 | 0.0 | 0.0 |
| Olfr1257 | 0.0 | 0.0 | 0.0 | 0.0 | 0.0 | 0.0 | 0.0 | 0.0 | 0.0 | 0.0 | 0.0 | 0.0 |
| Olfr1258 | 0.0 | 0.0 | 0.0 | 0.0 | 0.0 | 0.0 | 0.0 | 0.0 | 0.0 | 0.0 | 0.0 | 0.0 |
| Olfr1259 | 0.0 | 0.0 | 0.0 | 0.0 | 0.0 | 0.0 | 0.0 | 0.0 | 0.0 | 0.0 | 0.0 | 0.0 |
| Olfr126 | 0.0 | 0.0 | 0.0 | 0.0 | 0.0 | 0.0 | 0.0 | 0.0 | 0.0 | 0.0 | 0.0 | 0.0 |
| Olfr1260 | 0.0 | 0.0 | 0.0 | 0.0 | 0.0 | 0.0 | 0.0 | 0.0 | 0.0 | 0.0 | 0.0 | 0.0 |
| Olfr1262 | 0.0 | 0.0 | 0.0 | 0.0 | 0.0 | 0.0 | 0.0 | 0.0 | 0.0 | 0.0 | 0.0 | 0.0 |
| Olfr1263 | 0.0 | 0.0 | 0.0 | 0.0 | 0.0 | 0.0 | 0.0 | 0.0 | 0.0 | 0.0 | 0.0 | 0.0 |
| Olfr1264 | 0.0 | 0.0 | 0.0 | 0.0 | 0.0 | 0.0 | 0.0 | 0.0 | 0.0 | 0.0 | 0.0 | 0.0 |
| Olfr1265 | 0.0 | 0.0 | 0.0 | 0.0 | 0.0 | 0.0 | 0.0 | 0.0 | 0.0 | 0.0 | 0.0 | 0.0 |
| Olfr1269 | 0.0 | 0.0 | 0.0 | 0.0 | 0.0 | 0.0 | 0.0 | 0.0 | 0.0 | 0.0 | 0.0 | 0.0 |
| Olfr1271 | 0.0 | 0.0 | 0.0 | 0.0 | 0.0 | 0.0 | 0.0 | 0.0 | 0.0 | 0.0 | 0.0 | 0.0 |
| Olfr1272 | 0.0 | 0.0 | 0.0 | 0.0 | 0.0 | 0.0 | 0.0 | 0.0 | 0.0 | 0.0 | 0.0 | 0.0 |
| Olfr1273-ps | 0.0 | 0.0 | 0.0 | 0.0 | 0.0 | 0.0 | 0.0 | 0.0 | 0.0 | 0.0 | 0.0 | 0.0 |
| Olfr1275 | 0.0 | 0.0 | 0.0 | 0.0 | 0.0 | 0.0 | 0.0 | 0.0 | 0.0 | 0.0 | 0.0 | 0.0 |
| Olfr1276 | 0.0 | 0.0 | 0.0 | 0.0 | 0.0 | 0.0 | 0.0 | 0.0 | 0.0 | 0.0 | 0.0 | 0.0 |
| Olfr1277 | 0.0 | 0.0 | 0.0 | 0.0 | 0.0 | 0.0 | 0.0 | 0.0 | 0.0 | 0.0 | 0.0 | 0.0 |
| Olfr1278 | 0.0 | 0.0 | 0.0 | 0.0 | 0.0 | 0.0 | 0.0 | 0.0 | 0.0 | 0.0 | 0.0 | 0.0 |

| | | | | | | | | | | | | |
|---|---|---|---|---|---|---|---|---|---|---|---|---|
| Olfr1279 | 0.0 | 0.0 | 0.0 | 0.0 | 0.0 | 0.0 | 0.0 | 0.0 | 0.0 | 0.0 | 0.0 | 0.0 |
| Olfr128 | 0.0 | 0.0 | 0.0 | 0.0 | 0.0 | 0.0 | 0.0 | 0.0 | 0.0 | 0.0 | 0.0 | 0.0 |
| Olfr1282 | 0.0 | 0.0 | 0.0 | 0.0 | 0.0 | 0.0 | 0.0 | 0.0 | 0.0 | 0.0 | 0.0 | 0.0 |
| Olfr1283 | 0.0 | 0.0 | 0.0 | 0.0 | 0.0 | 0.0 | 0.0 | 0.0 | 0.0 | 0.0 | 0.0 | 0.0 |
| Olfr1284 | 0.0 | 0.0 | 0.0 | 0.0 | 0.0 | 0.0 | 0.0 | 0.0 | 0.0 | 0.0 | 0.0 | 0.0 |
| Olfr1286 | 0.0 | 0.0 | 0.0 | 0.0 | 0.0 | 0.0 | 0.0 | 0.0 | 0.0 | 0.0 | 0.0 | 0.0 |
| Olfr1287 | 0.0 | 0.0 | 0.0 | 0.0 | 0.0 | 0.0 | 0.0 | 0.0 | 0.0 | 0.0 | 0.0 | 0.0 |
| Olfr1288 | 0.0 | 0.0 | 0.0 | 0.0 | 0.0 | 0.0 | 0.0 | 0.0 | 0.0 | 0.0 | 0.0 | 0.0 |
| Olfr1289 | 0.0 | 0.0 | 0.0 | 0.0 | 0.0 | 0.0 | 0.0 | 0.0 | 0.0 | 0.0 | 0.0 | 0.0 |
| Olfr1290 | 0.0 | 0.0 | 0.0 | 0.0 | 0.0 | 0.0 | 0.0 | 0.0 | 0.0 | 0.0 | 0.0 | 0.0 |
| Olfr1294 | 0.0 | 0.0 | 0.0 | 0.0 | 0.0 | 0.0 | 0.0 | 0.0 | 0.0 | 0.0 | 0.0 | 0.0 |
| Olfr1295 | 0.0 | 0.0 | 0.0 | 0.0 | 0.0 | 0.0 | 0.0 | 0.0 | 0.0 | 0.0 | 0.0 | 0.0 |
| Olfr1298 | 0.0 | 0.0 | 0.0 | 0.0 | 0.0 | 0.0 | 0.0 | 0.0 | 0.0 | 0.0 | 0.0 | 0.0 |
| Olfr13 | 0.0 | 0.0 | 0.0 | 0.0 | 0.0 | 0.0 | 0.0 | 0.0 | 0.0 | 0.0 | 0.0 | 0.0 |
| Olfr130 | 0.0 | 0.0 | 0.0 | 0.0 | 0.0 | 0.0 | 0.0 | 0.0 | 0.0 | 0.0 | 0.0 | 0.0 |
| Olfr1300-ps1 | 0.0 | 0.0 | 0.0 | 0.0 | 0.0 | 0.0 | 0.0 | 0.0 | 0.0 | 0.0 | 0.0 | 0.0 |
| Olfr1301 | 0.0 | 0.0 | 0.0 | 0.0 | 0.0 | 0.0 | 0.0 | 0.0 | 0.0 | 0.0 | 0.0 | 0.0 |
| Olfr1302 | 0.0 | 0.0 | 0.0 | 0.0 | 0.0 | 0.0 | 0.0 | 0.0 | 0.0 | 0.0 | 0.0 | 0.0 |
| Olfr1303 | 0.0 | 0.0 | 0.0 | 0.0 | 0.0 | 0.0 | 0.0 | 0.0 | 0.0 | 0.0 | 0.0 | 0.0 |
| Olfr1305 | 0.0 | 0.0 | 0.0 | 0.0 | 0.0 | 0.0 | 0.0 | 0.0 | 0.0 | 0.0 | 0.0 | 0.0 |
| Olfr1308 | 0.0 | 0.0 | 0.0 | 0.0 | 0.0 | 0.0 | 0.0 | 0.0 | 0.0 | 0.0 | 0.0 | 0.0 |
| Olfr131 | 0.0 | 0.0 | 0.0 | 0.0 | 0.0 | 0.0 | 0.0 | 0.0 | 0.0 | 0.0 | 0.0 | 0.0 |
| Olfr1311 | 0.0 | 0.0 | 0.0 | 0.0 | 0.0 | 0.0 | 0.0 | 0.0 | 0.0 | 0.0 | 0.0 | 0.0 |
| Olfr1312 | 0.0 | 0.0 | 0.0 | 0.0 | 0.0 | 0.0 | 0.0 | 0.0 | 0.0 | 0.0 | 0.0 | 0.0 |
| Olfr132 | 0.0 | 0.0 | 0.0 | 0.0 | 0.0 | 0.0 | 0.0 | 0.0 | 0.0 | 0.0 | 0.0 | 0.0 |
| Olfr1320 | 0.0 | 0.0 | 0.0 | 0.0 | 0.0 | 0.0 | 0.0 | 0.0 | 0.0 | 0.0 | 0.0 | 0.0 |
| Olfr1321 | 0.0 | 0.0 | 0.0 | 0.0 | 0.0 | 0.0 | 0.0 | 0.0 | 0.0 | 0.0 | 0.0 | 0.0 |
| Olfr1322 | 0.0 | 0.0 | 0.0 | 0.0 | 0.0 | 0.0 | 0.0 | 0.0 | 0.0 | 0.0 | 0.0 | 0.0 |
| Olfr1323 | 0.0 | 0.0 | 0.0 | 0.0 | 0.0 | 0.0 | 0.0 | 0.0 | 0.0 | 0.0 | 0.0 | 0.0 |
| Olfr1324 | 0.0 | 0.0 | 0.0 | 0.0 | 0.0 | 0.0 | 0.0 | 0.0 | 0.0 | 0.0 | 0.0 | 0.0 |
| Olfr1325 | 0.0 | 0.0 | 0.0 | 0.0 | 0.0 | 0.0 | 0.0 | 0.0 | 0.0 | 0.0 | 0.0 | 0.0 |
| Olfr1328 | 0.0 | 0.0 | 0.0 | 0.0 | 0.0 | 0.0 | 0.0 | 0.0 | 0.0 | 0.0 | 0.0 | 0.0 |
| Olfr1329 | 0.0 | 0.0 | 0.0 | 0.0 | 0.0 | 0.0 | 0.0 | 0.0 | 0.0 | 0.0 | 0.0 | 0.0 |
| Olfr133 | 0.0 | 0.0 | 0.0 | 0.0 | 0.0 | 0.0 | 0.0 | 0.0 | 0.0 | 0.0 | 0.0 | 0.0 |
| Olfr1330 | 0.0 | 0.0 | 0.0 | 0.0 | 0.0 | 0.0 | 0.0 | 0.0 | 0.0 | 0.0 | 0.0 | 0.0 |
| Olfr1331 | 0.0 | 0.0 | 0.0 | 0.0 | 0.0 | 0.0 | 0.0 | 0.0 | 0.0 | 0.0 | 0.0 | 0.0 |
| Olfr1333 | 0.0 | 0.0 | 0.0 | 0.0 | 0.0 | 0.0 | 0.0 | 0.0 | 0.0 | 0.0 | 0.0 | 0.0 |
| Olfr1335 | 0.0 | 0.0 | 0.0 | 0.0 | 0.0 | 0.0 | 0.0 | 0.0 | 0.0 | 0.0 | 0.0 | 0.0 |
| Olfr1336 | 0.0 | 0.0 | 0.0 | 0.0 | 0.0 | 0.0 | 0.0 | 0.0 | 0.0 | 0.0 | 0.0 | 0.0 |
| Olfr1337 | 0.0 | 0.0 | 0.0 | 0.0 | 0.0 | 0.0 | 0.0 | 0.0 | 0.0 | 0.0 | 0.0 | 0.0 |
| Olfr1339 | 0.0 | 0.0 | 0.0 | 0.0 | 0.0 | 0.0 | 0.0 | 0.0 | 0.0 | 0.0 | 0.0 | 0.0 |
| Olfr134 | 0.0 | 0.0 | 0.0 | 0.0 | 0.0 | 0.0 | 0.0 | 0.0 | 0.0 | 0.0 | 0.0 | 0.0 |
| Olfr1340 | 0.0 | 0.0 | 0.0 | 0.0 | 0.0 | 0.0 | 0.0 | 0.0 | 0.0 | 0.0 | 0.0 | 0.0 |
| Olfr1341 | 0.0 | 0.0 | 0.0 | 0.0 | 0.0 | 0.0 | 0.0 | 0.0 | 0.0 | 0.0 | 0.0 | 0.0 |
| Olfr1342 | 0.0 | 0.0 | 0.0 | 0.0 | 0.0 | 0.0 | 0.0 | 0.0 | 0.0 | 0.0 | 0.0 | 0.0 |
| Olfr1346 | 0.0 | 0.0 | 0.0 | 0.0 | 0.0 | 0.0 | 0.0 | 0.0 | 0.0 | 0.0 | 0.0 | 0.0 |
| Olfr1347 | 0.0 | 0.0 | 0.0 | 0.0 | 0.0 | 0.0 | 0.0 | 0.0 | 0.0 | 0.0 | 0.0 | 0.0 |
| Olfr1348 | 0.0 | 0.0 | 0.0 | 0.0 | 0.0 | 0.0 | 0.0 | 0.0 | 0.0 | 0.0 | 0.0 | 0.0 |
| Olfr1349 | 0.0 | 0.0 | 0.0 | 0.0 | 0.0 | 0.0 | 0.0 | 0.0 | 0.0 | 0.0 | 0.0 | 0.0 |
| Olfr135 | 0.0 | 0.0 | 0.0 | 0.0 | 0.0 | 0.0 | 0.0 | 0.0 | 0.0 | 0.0 | 0.0 | 0.0 |
| Olfr1350 | 0.0 | 0.0 | 0.0 | 0.0 | 0.0 | 0.0 | 0.0 | 0.0 | 0.0 | 0.0 | 0.0 | 0.0 |
| Olfr1351 | 0.0 | 0.0 | 0.0 | 0.0 | 0.0 | 0.0 | 0.0 | 0.0 | 0.0 | 0.0 | 0.0 | 0.0 |
| Olfr1354 | 0.0 | 0.0 | 0.0 | 0.0 | 0.0 | 0.0 | 0.0 | 0.0 | 0.0 | 0.0 | 0.0 | 0.0 |
| Olfr1357 | 0.0 | 0.0 | 0.0 | 0.0 | 0.0 | 0.0 | 0.0 | 0.0 | 0.0 | 0.0 | 0.0 | 0.0 |
| Olfr1359 | 0.0 | 0.0 | 0.0 | 0.0 | 0.0 | 0.0 | 0.0 | 0.0 | 0.0 | 0.0 | 0.0 | 0.0 |
| Olfr136 | 0.0 | 0.0 | 0.0 | 0.0 | 0.0 | 0.0 | 0.0 | 0.0 | 0.0 | 0.0 | 0.0 | 0.0 |
| Olfr1360 | 0.0 | 0.0 | 0.0 | 0.0 | 0.0 | 0.0 | 0.0 | 0.0 | 0.0 | 0.0 | 0.0 | 0.0 |

Excluded Genes

| | | | | | | | | | | | | |
|---|---|---|---|---|---|---|---|---|---|---|---|---|
| Olfr1361 | 0.0 | 0.0 | 0.0 | 0.0 | 0.0 | 0.0 | 0.0 | 0.0 | 0.0 | 0.0 | 0.0 | 0.0 |
| Olfr1362 | 0.0 | 0.0 | 0.0 | 0.0 | 0.0 | 0.0 | 0.0 | 0.0 | 0.0 | 0.0 | 0.0 | 0.0 |
| Olfr1367 | 0.0 | 0.0 | 0.0 | 0.0 | 0.0 | 0.0 | 0.0 | 0.0 | 0.0 | 0.0 | 0.0 | 0.0 |
| Olfr1368 | 0.0 | 0.0 | 0.0 | 0.0 | 0.0 | 0.0 | 0.0 | 0.0 | 0.0 | 0.0 | 0.0 | 0.0 |
| Olfr137 | 0.0 | 0.0 | 0.0 | 0.0 | 0.0 | 0.0 | 0.0 | 0.0 | 0.0 | 0.0 | 0.0 | 0.0 |
| Olfr1371 | 0.0 | 0.0 | 0.0 | 0.0 | 0.0 | 0.0 | 0.0 | 0.0 | 0.0 | 0.0 | 0.0 | 0.0 |
| Olfr1373 | 0.0 | 0.0 | 0.0 | 0.0 | 0.0 | 0.0 | 0.0 | 0.0 | 0.0 | 0.0 | 0.0 | 0.0 |
| Olfr1377 | 0.0 | 0.0 | 0.0 | 0.0 | 0.0 | 0.0 | 0.0 | 0.0 | 0.0 | 0.0 | 0.0 | 0.0 |
| Olfr138 | 0.0 | 0.0 | 0.0 | 0.0 | 0.0 | 0.0 | 0.0 | 0.0 | 0.0 | 0.0 | 0.0 | 0.0 |
| Olfr1380 | 0.0 | 0.0 | 0.0 | 0.0 | 0.0 | 0.0 | 0.0 | 0.0 | 0.0 | 0.0 | 0.0 | 0.0 |
| Olfr1381 | 0.0 | 0.0 | 0.0 | 0.0 | 0.0 | 0.0 | 0.0 | 0.0 | 0.0 | 0.0 | 0.0 | 0.0 |
| Olfr1382 | 0.0 | 0.0 | 0.0 | 0.0 | 0.0 | 0.0 | 0.0 | 0.0 | 0.0 | 0.0 | 0.0 | 0.0 |
| Olfr1384 | 0.0 | 0.0 | 0.0 | 0.0 | 0.0 | 0.0 | 0.0 | 0.0 | 0.0 | 0.0 | 0.0 | 0.0 |
| Olfr1385 | 0.0 | 0.0 | 0.0 | 0.0 | 0.0 | 0.0 | 0.0 | 0.0 | 0.0 | 0.0 | 0.0 | 0.0 |
| Olfr1386 | 0.0 | 0.0 | 0.0 | 0.0 | 0.0 | 0.0 | 0.0 | 0.0 | 0.0 | 0.0 | 0.0 | 0.0 |
| Olfr1387 | 0.0 | 0.0 | 0.0 | 0.0 | 0.0 | 0.0 | 0.0 | 0.0 | 0.0 | 0.0 | 0.0 | 0.0 |
| Olfr1388 | 0.0 | 0.0 | 0.0 | 0.0 | 0.0 | 0.0 | 0.0 | 0.0 | 0.0 | 0.0 | 0.0 | 0.0 |
| Olfr139 | 0.0 | 0.0 | 0.0 | 0.0 | 0.0 | 0.0 | 0.0 | 0.0 | 0.0 | 0.0 | 0.0 | 0.0 |
| Olfr1390 | 0.0 | 0.0 | 0.0 | 0.0 | 0.0 | 0.0 | 0.0 | 0.0 | 0.0 | 0.0 | 0.0 | 0.0 |
| Olfr1391 | 0.0 | 0.0 | 0.0 | 0.0 | 0.0 | 0.0 | 0.0 | 0.0 | 0.0 | 0.0 | 0.0 | 0.0 |
| Olfr1394 | 0.0 | 0.0 | 0.0 | 0.0 | 0.0 | 0.0 | 0.0 | 0.0 | 0.0 | 0.0 | 0.0 | 0.0 |
| Olfr1395 | 0.0 | 0.0 | 0.0 | 0.0 | 0.0 | 0.0 | 0.0 | 0.0 | 0.0 | 0.0 | 0.0 | 0.0 |
| Olfr140 | 0.0 | 0.0 | 0.0 | 0.0 | 0.0 | 0.0 | 0.0 | 0.0 | 0.0 | 0.0 | 0.0 | 0.0 |
| Olfr1402 | 0.0 | 0.0 | 0.0 | 0.0 | 0.0 | 0.0 | 0.0 | 0.0 | 0.0 | 0.0 | 0.0 | 0.0 |
| Olfr1404 | 0.0 | 0.0 | 0.0 | 0.0 | 0.0 | 0.0 | 0.0 | 0.0 | 0.0 | 0.0 | 0.0 | 0.0 |
| Olfr1406 | 0.0 | 0.0 | 0.0 | 0.0 | 0.0 | 0.0 | 0.0 | 0.0 | 0.0 | 0.0 | 0.0 | 0.0 |
| Olfr1408 | 0.0 | 0.0 | 0.0 | 0.0 | 0.0 | 0.0 | 0.0 | 0.0 | 0.0 | 0.0 | 0.0 | 0.0 |
| Olfr141 | 0.0 | 0.0 | 0.0 | 0.0 | 0.0 | 0.0 | 0.0 | 0.0 | 0.0 | 0.0 | 0.0 | 0.0 |
| Olfr1410 | 0.0 | 0.0 | 0.0 | 0.0 | 0.0 | 0.0 | 0.0 | 0.0 | 0.0 | 0.0 | 0.0 | 0.0 |
| Olfr1411 | 0.0 | 0.0 | 0.0 | 0.0 | 0.0 | 0.0 | 0.0 | 0.0 | 0.0 | 0.0 | 0.0 | 0.0 |
| Olfr1412 | 0.0 | 0.0 | 0.0 | 0.0 | 0.0 | 0.0 | 0.0 | 0.0 | 0.0 | 0.0 | 0.0 | 0.0 |
| Olfr1413 | 0.0 | 0.0 | 0.0 | 0.0 | 0.0 | 0.0 | 0.0 | 0.0 | 0.0 | 0.0 | 0.0 | 0.0 |
| Olfr1414 | 0.0 | 0.0 | 0.0 | 0.0 | 0.0 | 0.0 | 0.0 | 0.0 | 0.0 | 0.0 | 0.0 | 0.0 |
| Olfr1415 | 0.0 | 0.0 | 0.0 | 0.0 | 0.0 | 0.0 | 0.0 | 0.0 | 0.0 | 0.0 | 0.0 | 0.0 |
| Olfr1416 | 0.0 | 0.0 | 0.0 | 0.0 | 0.0 | 0.0 | 0.0 | 0.0 | 0.0 | 0.0 | 0.0 | 0.0 |
| Olfr1419 | 0.0 | 0.0 | 0.0 | 0.0 | 0.0 | 0.0 | 0.0 | 0.0 | 0.0 | 0.0 | 0.0 | 0.0 |
| Olfr1423 | 0.0 | 0.0 | 0.0 | 0.0 | 0.0 | 0.0 | 0.0 | 0.0 | 0.0 | 0.0 | 0.0 | 0.0 |
| Olfr1424 | 0.0 | 0.0 | 0.0 | 0.0 | 0.0 | 0.0 | 0.0 | 0.0 | 0.0 | 0.0 | 0.0 | 0.0 |
| Olfr1425 | 0.0 | 0.0 | 0.0 | 0.0 | 0.0 | 0.0 | 0.0 | 0.0 | 0.0 | 0.0 | 0.0 | 0.0 |
| Olfr1426 | 0.0 | 0.0 | 0.0 | 0.0 | 0.0 | 0.0 | 0.0 | 0.0 | 0.0 | 0.0 | 0.0 | 0.0 |
| Olfr1427 | 0.0 | 0.0 | 0.0 | 0.0 | 0.0 | 0.0 | 0.0 | 0.0 | 0.0 | 0.0 | 0.0 | 0.0 |
| Olfr1428 | 0.0 | 0.0 | 0.0 | 0.0 | 0.0 | 0.0 | 0.0 | 0.0 | 0.0 | 0.0 | 0.0 | 0.0 |
| Olfr143 | 0.0 | 0.0 | 0.0 | 0.0 | 0.0 | 0.0 | 0.0 | 0.0 | 0.0 | 0.0 | 0.0 | 0.0 |
| Olfr1433 | 0.0 | 0.0 | 0.0 | 0.0 | 0.0 | 0.0 | 0.0 | 0.0 | 0.0 | 0.0 | 0.0 | 0.0 |
| Olfr1434 | 0.0 | 0.0 | 0.0 | 0.0 | 0.0 | 0.0 | 0.0 | 0.0 | 0.0 | 0.0 | 0.0 | 0.0 |
| Olfr1436 | 0.0 | 0.0 | 0.0 | 0.0 | 0.0 | 0.0 | 0.0 | 0.0 | 0.0 | 0.0 | 0.0 | 0.0 |
| Olfr1437 | 0.0 | 0.0 | 0.0 | 0.0 | 0.0 | 0.0 | 0.0 | 0.0 | 0.0 | 0.0 | 0.0 | 0.0 |
| Olfr1440 | 0.0 | 0.0 | 0.0 | 0.0 | 0.0 | 0.0 | 0.0 | 0.0 | 0.0 | 0.0 | 0.0 | 0.0 |
| Olfr1441 | 0.0 | 0.0 | 0.0 | 0.0 | 0.0 | 0.0 | 0.0 | 0.0 | 0.0 | 0.0 | 0.0 | 0.0 |
| Olfr1442 | 0.0 | 0.0 | 0.0 | 0.0 | 0.0 | 0.0 | 0.0 | 0.0 | 0.0 | 0.0 | 0.0 | 0.0 |
| Olfr1443 | 0.0 | 0.0 | 0.0 | 0.0 | 0.0 | 0.0 | 0.0 | 0.0 | 0.0 | 0.0 | 0.0 | 0.0 |
| Olfr1445 | 0.0 | 0.0 | 0.0 | 0.0 | 0.0 | 0.0 | 0.0 | 0.0 | 0.0 | 0.0 | 0.0 | 0.0 |
| Olfr1446 | 0.0 | 0.0 | 0.0 | 0.0 | 0.0 | 0.0 | 0.0 | 0.0 | 0.0 | 0.0 | 0.0 | 0.0 |
| Olfr1447 | 0.0 | 0.0 | 0.0 | 0.0 | 0.0 | 0.0 | 0.0 | 0.0 | 0.0 | 0.0 | 0.0 | 0.0 |
| Olfr1448 | 0.0 | 0.0 | 0.0 | 0.0 | 0.0 | 0.0 | 0.0 | 0.0 | 0.0 | 0.0 | 0.0 | 0.0 |
| Olfr1449 | 0.0 | 0.0 | 0.0 | 0.0 | 0.0 | 0.0 | 0.0 | 0.0 | 0.0 | 0.0 | 0.0 | 0.0 |
| Olfr145 | 0.0 | 0.0 | 0.0 | 0.0 | 0.0 | 0.0 | 0.0 | 0.0 | 0.0 | 0.0 | 0.0 | 0.0 |

Excluded Genes

| | | | | | | | | | | | | |
|---|---|---|---|---|---|---|---|---|---|---|---|---|
| Olfr1450 | 0.0 | 0.0 | 0.0 | 0.0 | 0.0 | 0.0 | 0.0 | 0.0 | 0.0 | 0.0 | 0.0 | 0.0 |
| Olfr1451 | 0.0 | 0.0 | 0.0 | 0.0 | 0.0 | 0.0 | 0.0 | 0.0 | 0.0 | 0.0 | 0.0 | 0.0 |
| Olfr1453 | 0.0 | 0.0 | 0.0 | 0.0 | 0.0 | 0.0 | 0.0 | 0.0 | 0.0 | 0.0 | 0.0 | 0.0 |
| Olfr1454 | 0.0 | 0.0 | 0.0 | 0.0 | 0.0 | 0.0 | 0.0 | 0.0 | 0.0 | 0.0 | 0.0 | 0.0 |
| Olfr1457 | 0.0 | 0.0 | 0.0 | 0.0 | 0.0 | 0.0 | 0.0 | 0.0 | 0.0 | 0.0 | 0.0 | 0.0 |
| Olfr1459 | 0.0 | 0.0 | 0.0 | 0.0 | 0.0 | 0.0 | 0.0 | 0.0 | 0.0 | 0.0 | 0.0 | 0.0 |
| Olfr146 | 0.0 | 0.0 | 0.0 | 0.0 | 0.0 | 0.0 | 0.0 | 0.0 | 0.0 | 0.0 | 0.0 | 0.0 |
| Olfr1461 | 0.0 | 0.0 | 0.0 | 0.0 | 0.0 | 0.0 | 0.0 | 0.0 | 0.0 | 0.0 | 0.0 | 0.0 |
| Olfr1462 | 0.0 | 0.0 | 0.0 | 0.0 | 0.0 | 0.0 | 0.0 | 0.0 | 0.0 | 0.0 | 0.0 | 0.0 |
| Olfr1463 | 0.0 | 0.0 | 0.0 | 0.0 | 0.0 | 0.0 | 0.0 | 0.0 | 0.0 | 0.0 | 0.0 | 0.0 |
| Olfr1465 | 0.0 | 0.0 | 0.0 | 0.0 | 0.0 | 0.0 | 0.0 | 0.0 | 0.0 | 0.0 | 0.0 | 0.0 |
| Olfr1466 | 0.0 | 0.0 | 0.0 | 0.0 | 0.0 | 0.0 | 0.0 | 0.0 | 0.0 | 0.0 | 0.0 | 0.0 |
| Olfr1467 | 0.0 | 0.0 | 0.0 | 0.0 | 0.0 | 0.0 | 0.0 | 0.0 | 0.0 | 0.0 | 0.0 | 0.0 |
| Olfr1469 | 0.0 | 0.0 | 0.0 | 0.0 | 0.0 | 0.0 | 0.0 | 0.0 | 0.0 | 0.0 | 0.0 | 0.0 |
| Olfr147 | 0.0 | 0.0 | 0.0 | 0.0 | 0.0 | 0.0 | 0.0 | 0.0 | 0.0 | 0.0 | 0.0 | 0.0 |
| Olfr1471 | 0.0 | 0.0 | 0.0 | 0.0 | 0.0 | 0.0 | 0.0 | 0.0 | 0.0 | 0.0 | 0.0 | 0.0 |
| Olfr1472 | 0.0 | 0.0 | 0.0 | 0.0 | 0.0 | 0.0 | 0.0 | 0.0 | 0.0 | 0.0 | 0.0 | 0.0 |
| Olfr1474 | 0.0 | 0.0 | 0.0 | 0.0 | 0.0 | 0.0 | 0.0 | 0.0 | 0.0 | 0.0 | 0.0 | 0.0 |
| Olfr1475 | 0.0 | 0.0 | 0.0 | 0.0 | 0.0 | 0.0 | 0.0 | 0.0 | 0.0 | 0.0 | 0.0 | 0.0 |
| Olfr1477 | 0.0 | 0.0 | 0.0 | 0.0 | 0.0 | 0.0 | 0.0 | 0.0 | 0.0 | 0.0 | 0.0 | 0.0 |
| Olfr148 | 0.0 | 0.0 | 0.0 | 0.0 | 0.0 | 0.0 | 0.0 | 0.0 | 0.0 | 0.0 | 0.0 | 0.0 |
| Olfr1484 | 0.0 | 0.0 | 0.0 | 0.0 | 0.0 | 0.0 | 0.0 | 0.0 | 0.0 | 0.0 | 0.0 | 0.0 |
| Olfr1489 | 0.0 | 0.0 | 0.0 | 0.0 | 0.0 | 0.0 | 0.0 | 0.0 | 0.0 | 0.0 | 0.0 | 0.0 |
| Olfr149 | 0.0 | 0.0 | 0.0 | 0.0 | 0.0 | 0.0 | 0.0 | 0.0 | 0.0 | 0.0 | 0.0 | 0.0 |
| Olfr1490 | 0.0 | 0.0 | 0.0 | 0.0 | 0.0 | 0.0 | 0.0 | 0.0 | 0.0 | 0.0 | 0.0 | 0.0 |
| Olfr1491 | 0.0 | 0.0 | 0.0 | 0.0 | 0.0 | 0.0 | 0.0 | 0.0 | 0.0 | 0.0 | 0.0 | 0.0 |
| Olfr1494 | 0.0 | 0.0 | 0.0 | 0.0 | 0.0 | 0.0 | 0.0 | 0.0 | 0.0 | 0.0 | 0.0 | 0.0 |
| Olfr1495 | 0.0 | 0.0 | 0.0 | 0.0 | 0.0 | 0.0 | 0.0 | 0.0 | 0.0 | 0.0 | 0.0 | 0.0 |
| Olfr1496 | 0.0 | 0.0 | 0.0 | 0.0 | 0.0 | 0.0 | 0.0 | 0.0 | 0.0 | 0.0 | 0.0 | 0.0 |
| Olfr1497 | 0.0 | 0.0 | 0.0 | 0.0 | 0.0 | 0.0 | 0.0 | 0.0 | 0.0 | 0.0 | 0.0 | 0.0 |
| Olfr1499 | 0.0 | 0.0 | 0.0 | 0.0 | 0.0 | 0.0 | 0.0 | 0.0 | 0.0 | 0.0 | 0.0 | 0.0 |
| Olfr150 | 0.0 | 0.0 | 0.0 | 0.0 | 0.0 | 0.0 | 0.0 | 0.0 | 0.0 | 0.0 | 0.0 | 0.0 |
| Olfr1500 | 0.0 | 0.0 | 0.0 | 0.0 | 0.0 | 0.0 | 0.0 | 0.0 | 0.0 | 0.0 | 0.0 | 0.0 |
| Olfr1501 | 0.0 | 0.0 | 0.0 | 0.0 | 0.0 | 0.0 | 0.0 | 0.0 | 0.0 | 0.0 | 0.0 | 0.0 |
| Olfr1502 | 0.0 | 0.0 | 0.0 | 0.0 | 0.0 | 0.0 | 0.0 | 0.0 | 0.0 | 0.0 | 0.0 | 0.0 |
| Olfr1504 | 0.0 | 0.0 | 0.0 | 0.0 | 0.0 | 0.0 | 0.0 | 0.0 | 0.0 | 0.0 | 0.0 | 0.0 |
| Olfr1505 | 0.0 | 0.0 | 0.0 | 0.0 | 0.0 | 0.0 | 0.0 | 0.0 | 0.0 | 0.0 | 0.0 | 0.0 |
| Olfr1507 | 0.0 | 0.0 | 0.0 | 0.0 | 0.0 | 0.0 | 0.0 | 0.0 | 0.0 | 0.0 | 0.0 | 0.0 |
| Olfr1508 | 0.0 | 0.0 | 0.0 | 0.0 | 0.0 | 0.0 | 0.0 | 0.0 | 0.0 | 0.0 | 0.0 | 0.0 |
| Olfr1509 | 0.0 | 0.0 | 0.0 | 0.0 | 0.0 | 0.0 | 0.0 | 0.0 | 0.0 | 0.0 | 0.0 | 0.0 |
| Olfr151 | 0.0 | 0.0 | 0.0 | 0.0 | 0.0 | 0.0 | 0.0 | 0.0 | 0.0 | 0.0 | 0.0 | 0.0 |
| Olfr1510 | 0.0 | 0.0 | 0.0 | 0.0 | 0.0 | 0.0 | 0.0 | 0.0 | 0.0 | 0.0 | 0.0 | 0.0 |
| Olfr1513 | 0.0 | 0.0 | 0.0 | 0.0 | 0.0 | 0.0 | 0.0 | 0.0 | 0.0 | 0.0 | 0.0 | 0.0 |
| Olfr152 | 0.0 | 0.0 | 0.0 | 0.0 | 0.0 | 0.0 | 0.0 | 0.0 | 0.0 | 0.0 | 0.0 | 0.0 |
| Olfr153 | 0.0 | 0.0 | 0.0 | 0.0 | 0.0 | 0.0 | 0.0 | 0.0 | 0.0 | 0.0 | 0.0 | 0.0 |
| Olfr1537 | 0.0 | 0.0 | 0.0 | 0.0 | 0.0 | 0.0 | 0.0 | 0.0 | 0.0 | 0.0 | 0.0 | 0.0 |
| Olfr155 | 0.0 | 0.0 | 0.0 | 0.0 | 0.0 | 0.0 | 0.0 | 0.0 | 0.0 | 0.0 | 0.0 | 0.0 |
| Olfr160 | 0.0 | 0.0 | 0.0 | 0.0 | 0.0 | 0.0 | 0.0 | 0.0 | 0.0 | 0.0 | 0.0 | 0.0 |
| Olfr161 | 0.0 | 0.0 | 0.0 | 0.0 | 0.0 | 0.0 | 0.0 | 0.0 | 0.0 | 0.0 | 0.0 | 0.0 |
| Olfr164 | 0.0 | 0.0 | 0.0 | 0.0 | 0.0 | 0.0 | 0.0 | 0.0 | 0.0 | 0.0 | 0.0 | 0.0 |
| Olfr166 | 0.0 | 0.0 | 0.0 | 0.0 | 0.0 | 0.0 | 0.0 | 0.0 | 0.0 | 0.0 | 0.0 | 0.0 |
| Olfr167 | 0.0 | 0.0 | 0.0 | 0.0 | 0.0 | 0.0 | 0.0 | 0.0 | 0.0 | 0.0 | 0.0 | 0.0 |
| Olfr168 | 0.0 | 0.0 | 0.0 | 0.0 | 0.0 | 0.0 | 0.0 | 0.0 | 0.0 | 0.0 | 0.0 | 0.0 |
| Olfr169 | 0.0 | 0.0 | 0.0 | 0.0 | 0.0 | 0.0 | 0.0 | 0.0 | 0.0 | 0.0 | 0.0 | 0.0 |
| Olfr17 | 0.0 | 0.0 | 0.0 | 0.0 | 0.0 | 0.0 | 0.0 | 0.0 | 0.0 | 0.0 | 0.0 | 0.0 |
| Olfr170 | 0.0 | 0.0 | 0.0 | 0.0 | 0.0 | 0.0 | 0.0 | 0.0 | 0.0 | 0.0 | 0.0 | 0.0 |
| Olfr171 | 0.0 | 0.0 | 0.0 | 0.0 | 0.0 | 0.0 | 0.0 | 0.0 | 0.0 | 0.0 | 0.0 | 0.0 |

Excluded Genes

| | | | | | | | | | | | | |
|---|---|---|---|---|---|---|---|---|---|---|---|---|
| Olfr181 | 0.0 | 0.0 | 0.0 | 0.0 | 0.0 | 0.0 | 0.0 | 0.0 | 0.0 | 0.0 | 0.0 | 0.0 |
| Olfr183 | 0.0 | 0.0 | 0.0 | 0.0 | 0.0 | 0.0 | 0.0 | 0.0 | 0.0 | 0.0 | 0.0 | 0.0 |
| Olfr186 | 0.0 | 0.0 | 0.0 | 0.0 | 0.0 | 0.0 | 0.0 | 0.0 | 0.0 | 0.0 | 0.0 | 0.0 |
| Olfr187 | 0.0 | 0.0 | 0.0 | 0.0 | 0.0 | 0.0 | 0.0 | 0.0 | 0.0 | 0.0 | 0.0 | 0.0 |
| Olfr19 | 0.0 | 0.0 | 0.0 | 0.0 | 0.0 | 0.0 | 0.0 | 0.0 | 0.0 | 0.0 | 0.0 | 0.0 |
| Olfr190 | 0.0 | 0.0 | 0.0 | 0.0 | 0.0 | 0.0 | 0.0 | 0.0 | 0.0 | 0.0 | 0.0 | 0.0 |
| Olfr192 | 0.0 | 0.0 | 0.0 | 0.0 | 0.0 | 0.0 | 0.0 | 0.0 | 0.0 | 0.0 | 0.0 | 0.0 |
| Olfr194 | 0.0 | 0.0 | 0.0 | 0.0 | 0.0 | 0.0 | 0.0 | 0.0 | 0.0 | 0.0 | 0.0 | 0.0 |
| Olfr196 | 0.0 | 0.0 | 0.0 | 0.0 | 0.0 | 0.0 | 0.0 | 0.0 | 0.0 | 0.0 | 0.0 | 0.0 |
| Olfr197 | 0.0 | 0.0 | 0.0 | 0.0 | 0.0 | 0.0 | 0.0 | 0.0 | 0.0 | 0.0 | 0.0 | 0.0 |
| Olfr198 | 0.0 | 0.0 | 0.0 | 0.0 | 0.0 | 0.0 | 0.0 | 0.0 | 0.0 | 0.0 | 0.0 | 0.0 |
| Olfr199 | 0.0 | 0.0 | 0.0 | 0.0 | 0.0 | 0.0 | 0.0 | 0.0 | 0.0 | 0.0 | 0.0 | 0.0 |
| Olfr2 | 0.0 | 0.0 | 0.0 | 0.0 | 0.0 | 0.0 | 0.0 | 0.0 | 0.0 | 0.0 | 0.0 | 0.0 |
| Olfr20 | 0.0 | 0.0 | 0.0 | 0.0 | 0.0 | 0.0 | 0.0 | 0.0 | 0.0 | 0.0 | 0.0 | 0.0 |
| Olfr201 | 0.0 | 0.0 | 0.0 | 0.0 | 0.0 | 0.0 | 0.0 | 0.0 | 0.0 | 0.0 | 0.0 | 0.0 |
| Olfr202 | 0.0 | 0.0 | 0.0 | 0.0 | 0.0 | 0.0 | 0.0 | 0.0 | 0.0 | 0.0 | 0.0 | 0.0 |
| Olfr204 | 0.0 | 0.0 | 0.0 | 0.0 | 0.0 | 0.0 | 0.0 | 0.0 | 0.0 | 0.0 | 0.0 | 0.0 |
| Olfr205 | 0.0 | 0.0 | 0.0 | 0.0 | 0.0 | 0.0 | 0.0 | 0.0 | 0.0 | 0.0 | 0.0 | 0.0 |
| Olfr206 | 0.0 | 0.0 | 0.0 | 0.0 | 0.0 | 0.0 | 0.0 | 0.0 | 0.0 | 0.0 | 0.0 | 0.0 |
| Olfr209 | 0.0 | 0.0 | 0.0 | 0.0 | 0.0 | 0.0 | 0.0 | 0.0 | 0.0 | 0.0 | 0.0 | 0.0 |
| Olfr211 | 0.0 | 0.0 | 0.0 | 0.0 | 0.0 | 0.0 | 0.0 | 0.0 | 0.0 | 0.0 | 0.0 | 0.0 |
| Olfr213 | 0.0 | 0.0 | 0.0 | 0.0 | 0.0 | 0.0 | 0.0 | 0.0 | 0.0 | 0.0 | 0.0 | 0.0 |
| Olfr214 | 0.0 | 0.0 | 0.0 | 0.0 | 0.0 | 0.0 | 0.0 | 0.0 | 0.0 | 0.0 | 0.0 | 0.0 |
| Olfr215 | 0.0 | 0.0 | 0.0 | 0.0 | 0.0 | 0.0 | 0.0 | 0.0 | 0.0 | 0.0 | 0.0 | 0.0 |
| Olfr218 | 0.0 | 0.0 | 0.0 | 0.0 | 0.0 | 0.0 | 0.0 | 0.0 | 0.0 | 0.0 | 0.0 | 0.0 |
| Olfr220 | 0.0 | 0.0 | 0.0 | 0.0 | 0.0 | 0.0 | 0.0 | 0.0 | 0.0 | 0.0 | 0.0 | 0.0 |
| Olfr222 | 0.0 | 0.0 | 0.0 | 0.0 | 0.0 | 0.0 | 0.0 | 0.0 | 0.0 | 0.0 | 0.0 | 0.0 |
| Olfr224 | 0.0 | 0.0 | 0.0 | 0.0 | 0.0 | 0.0 | 0.0 | 0.0 | 0.0 | 0.0 | 0.0 | 0.0 |
| Olfr225 | 0.0 | 0.0 | 0.0 | 0.0 | 0.0 | 0.0 | 0.0 | 0.0 | 0.0 | 0.0 | 0.0 | 0.0 |
| Olfr228 | 0.0 | 0.0 | 0.0 | 0.0 | 0.0 | 0.0 | 0.0 | 0.0 | 0.0 | 0.0 | 0.0 | 0.0 |
| Olfr229 | 0.0 | 0.0 | 0.0 | 0.0 | 0.0 | 0.0 | 0.0 | 0.0 | 0.0 | 0.0 | 0.0 | 0.0 |
| Olfr23 | 0.0 | 0.0 | 0.0 | 0.0 | 0.0 | 0.0 | 0.0 | 0.0 | 0.0 | 0.0 | 0.0 | 0.0 |
| Olfr235 | 0.0 | 0.0 | 0.0 | 0.0 | 0.0 | 0.0 | 0.0 | 0.0 | 0.0 | 0.0 | 0.0 | 0.0 |
| Olfr237-ps1 | 0.0 | 0.0 | 0.0 | 0.0 | 0.0 | 0.0 | 0.0 | 0.0 | 0.0 | 0.0 | 0.0 | 0.0 |
| Olfr24 | 0.0 | 0.0 | 0.0 | 0.0 | 0.0 | 0.0 | 0.0 | 0.0 | 0.0 | 0.0 | 0.0 | 0.0 |
| Olfr242 | 0.0 | 0.0 | 0.0 | 0.0 | 0.0 | 0.0 | 0.0 | 0.0 | 0.0 | 0.0 | 0.0 | 0.0 |
| Olfr243 | 0.0 | 0.0 | 0.0 | 0.0 | 0.0 | 0.0 | 0.0 | 0.0 | 0.0 | 0.0 | 0.0 | 0.0 |
| Olfr248 | 0.0 | 0.0 | 0.0 | 0.0 | 0.0 | 0.0 | 0.0 | 0.0 | 0.0 | 0.0 | 0.0 | 0.0 |
| Olfr25 | 0.0 | 0.0 | 0.0 | 0.0 | 0.0 | 0.0 | 0.0 | 0.0 | 0.0 | 0.0 | 0.0 | 0.0 |
| Olfr259 | 0.0 | 0.0 | 0.0 | 0.0 | 0.0 | 0.0 | 0.0 | 0.0 | 0.0 | 0.0 | 0.0 | 0.0 |
| Olfr26 | 0.0 | 0.0 | 0.0 | 0.0 | 0.0 | 0.0 | 0.0 | 0.0 | 0.0 | 0.0 | 0.0 | 0.0 |
| Olfr262 | 0.0 | 0.0 | 0.0 | 0.0 | 0.0 | 0.0 | 0.0 | 0.0 | 0.0 | 0.0 | 0.0 | 0.0 |
| Olfr263 | 0.0 | 0.0 | 0.0 | 0.0 | 0.0 | 0.0 | 0.0 | 0.0 | 0.0 | 0.0 | 0.0 | 0.0 |
| Olfr266 | 0.0 | 0.0 | 0.0 | 0.0 | 0.0 | 0.0 | 0.0 | 0.0 | 0.0 | 0.0 | 0.0 | 0.0 |
| Olfr27 | 0.0 | 0.0 | 0.0 | 0.0 | 0.0 | 0.0 | 0.0 | 0.0 | 0.0 | 0.0 | 0.0 | 0.0 |
| Olfr270 | 0.0 | 0.0 | 0.0 | 0.0 | 0.0 | 0.0 | 0.0 | 0.0 | 0.0 | 0.0 | 0.0 | 0.0 |
| Olfr272 | 0.0 | 0.0 | 0.0 | 0.0 | 0.0 | 0.0 | 0.0 | 0.0 | 0.0 | 0.0 | 0.0 | 0.0 |
| Olfr273 | 0.0 | 0.0 | 0.0 | 0.0 | 0.0 | 0.0 | 0.0 | 0.0 | 0.0 | 0.0 | 0.0 | 0.0 |
| Olfr279 | 0.0 | 0.0 | 0.0 | 0.0 | 0.0 | 0.0 | 0.0 | 0.0 | 0.0 | 0.0 | 0.0 | 0.0 |
| Olfr281 | 0.0 | 0.0 | 0.0 | 0.0 | 0.0 | 0.0 | 0.0 | 0.0 | 0.0 | 0.0 | 0.0 | 0.0 |
| Olfr282 | 0.0 | 0.0 | 0.0 | 0.0 | 0.0 | 0.0 | 0.0 | 0.0 | 0.0 | 0.0 | 0.0 | 0.0 |
| Olfr284 | 0.0 | 0.0 | 0.0 | 0.0 | 0.0 | 0.0 | 0.0 | 0.0 | 0.0 | 0.0 | 0.0 | 0.0 |
| Olfr285 | 0.0 | 0.0 | 0.0 | 0.0 | 0.0 | 0.0 | 0.0 | 0.0 | 0.0 | 0.0 | 0.0 | 0.0 |
| Olfr293 | 0.0 | 0.0 | 0.0 | 0.0 | 0.0 | 0.0 | 0.0 | 0.0 | 0.0 | 0.0 | 0.0 | 0.0 |
| Olfr294 | 0.0 | 0.0 | 0.0 | 0.0 | 0.0 | 0.0 | 0.0 | 0.0 | 0.0 | 0.0 | 0.0 | 0.0 |
| Olfr295 | 0.0 | 0.0 | 0.0 | 0.0 | 0.0 | 0.0 | 0.0 | 0.0 | 0.0 | 0.0 | 0.0 | 0.0 |
| Olfr297 | 0.0 | 0.0 | 0.0 | 0.0 | 0.0 | 0.0 | 0.0 | 0.0 | 0.0 | 0.0 | 0.0 | 0.0 |

Excluded Genes

| | | | | | | | | | | | | |
|---|---|---|---|---|---|---|---|---|---|---|---|---|
| Olfr29-ps1 | 0.0 | 0.0 | 0.0 | 0.0 | 0.0 | 0.0 | 0.0 | 0.0 | 0.0 | 0.0 | 0.0 | 0.0 |
| Olfr3 | 0.0 | 0.0 | 0.0 | 0.0 | 0.0 | 0.0 | 0.0 | 0.0 | 0.0 | 0.0 | 0.0 | 0.0 |
| Olfr30 | 0.0 | 0.0 | 0.0 | 0.0 | 0.0 | 0.0 | 0.0 | 0.0 | 0.0 | 0.0 | 0.0 | 0.0 |
| Olfr301 | 0.0 | 0.0 | 0.0 | 0.0 | 0.0 | 0.0 | 0.0 | 0.0 | 0.0 | 0.0 | 0.0 | 0.0 |
| Olfr304 | 0.0 | 0.0 | 0.0 | 0.0 | 0.0 | 0.0 | 0.0 | 0.0 | 0.0 | 0.0 | 0.0 | 0.0 |
| Olfr305 | 0.0 | 0.0 | 0.0 | 0.0 | 0.0 | 0.0 | 0.0 | 0.0 | 0.0 | 0.0 | 0.0 | 0.0 |
| Olfr309 | 0.0 | 0.0 | 0.0 | 0.0 | 0.0 | 0.0 | 0.0 | 0.0 | 0.0 | 0.0 | 0.0 | 0.0 |
| Olfr310 | 0.0 | 0.0 | 0.0 | 0.0 | 0.0 | 0.0 | 0.0 | 0.0 | 0.0 | 0.0 | 0.0 | 0.0 |
| Olfr312 | 0.0 | 0.0 | 0.0 | 0.0 | 0.0 | 0.0 | 0.0 | 0.0 | 0.0 | 0.0 | 0.0 | 0.0 |
| Olfr313 | 0.0 | 0.0 | 0.0 | 0.0 | 0.0 | 0.0 | 0.0 | 0.0 | 0.0 | 0.0 | 0.0 | 0.0 |
| Olfr314 | 0.0 | 0.0 | 0.0 | 0.0 | 0.0 | 0.0 | 0.0 | 0.0 | 0.0 | 0.0 | 0.0 | 0.0 |
| Olfr315 | 0.0 | 0.0 | 0.0 | 0.0 | 0.0 | 0.0 | 0.0 | 0.0 | 0.0 | 0.0 | 0.0 | 0.0 |
| Olfr316 | 0.0 | 0.0 | 0.0 | 0.0 | 0.0 | 0.0 | 0.0 | 0.0 | 0.0 | 0.0 | 0.0 | 0.0 |
| Olfr317 | 0.0 | 0.0 | 0.0 | 0.0 | 0.0 | 0.0 | 0.0 | 0.0 | 0.0 | 0.0 | 0.0 | 0.0 |
| Olfr318 | 0.0 | 0.0 | 0.0 | 0.0 | 0.0 | 0.0 | 0.0 | 0.0 | 0.0 | 0.0 | 0.0 | 0.0 |
| Olfr319 | 0.0 | 0.0 | 0.0 | 0.0 | 0.0 | 0.0 | 0.0 | 0.0 | 0.0 | 0.0 | 0.0 | 0.0 |
| Olfr32 | 0.0 | 0.0 | 0.0 | 0.0 | 0.0 | 0.0 | 0.0 | 0.0 | 0.0 | 0.0 | 0.0 | 0.0 |
| Olfr320 | 0.0 | 0.0 | 0.0 | 0.0 | 0.0 | 0.0 | 0.0 | 0.0 | 0.0 | 0.0 | 0.0 | 0.0 |
| Olfr323 | 0.0 | 0.0 | 0.0 | 0.0 | 0.0 | 0.0 | 0.0 | 0.0 | 0.0 | 0.0 | 0.0 | 0.0 |
| Olfr324 | 0.0 | 0.0 | 0.0 | 0.0 | 0.0 | 0.0 | 0.0 | 0.0 | 0.0 | 0.0 | 0.0 | 0.0 |
| Olfr325 | 0.0 | 0.0 | 0.0 | 0.0 | 0.0 | 0.0 | 0.0 | 0.0 | 0.0 | 0.0 | 0.0 | 0.0 |
| Olfr328 | 0.0 | 0.0 | 0.0 | 0.0 | 0.0 | 0.0 | 0.0 | 0.0 | 0.0 | 0.0 | 0.0 | 0.0 |
| Olfr329-ps | 0.0 | 0.0 | 0.0 | 0.0 | 0.0 | 0.0 | 0.0 | 0.0 | 0.0 | 0.0 | 0.0 | 0.0 |
| Olfr33 | 0.0 | 0.0 | 0.0 | 0.0 | 0.0 | 0.0 | 0.0 | 0.0 | 0.0 | 0.0 | 0.0 | 0.0 |
| Olfr331 | 0.0 | 0.0 | 0.0 | 0.0 | 0.0 | 0.0 | 0.0 | 0.0 | 0.0 | 0.0 | 0.0 | 0.0 |
| Olfr332 | 0.0 | 0.0 | 0.0 | 0.0 | 0.0 | 0.0 | 0.0 | 0.0 | 0.0 | 0.0 | 0.0 | 0.0 |
| Olfr338 | 0.0 | 0.0 | 0.0 | 0.0 | 0.0 | 0.0 | 0.0 | 0.0 | 0.0 | 0.0 | 0.0 | 0.0 |
| Olfr339 | 0.0 | 0.0 | 0.0 | 0.0 | 0.0 | 0.0 | 0.0 | 0.0 | 0.0 | 0.0 | 0.0 | 0.0 |
| Olfr340 | 0.0 | 0.0 | 0.0 | 0.0 | 0.0 | 0.0 | 0.0 | 0.0 | 0.0 | 0.0 | 0.0 | 0.0 |
| Olfr341 | 0.0 | 0.0 | 0.0 | 0.0 | 0.0 | 0.0 | 0.0 | 0.0 | 0.0 | 0.0 | 0.0 | 0.0 |
| Olfr342 | 0.0 | 0.0 | 0.0 | 0.0 | 0.0 | 0.0 | 0.0 | 0.0 | 0.0 | 0.0 | 0.0 | 0.0 |
| Olfr344 | 0.0 | 0.0 | 0.0 | 0.0 | 0.0 | 0.0 | 0.0 | 0.0 | 0.0 | 0.0 | 0.0 | 0.0 |
| Olfr345 | 0.0 | 0.0 | 0.0 | 0.0 | 0.0 | 0.0 | 0.0 | 0.0 | 0.0 | 0.0 | 0.0 | 0.0 |
| Olfr346 | 0.0 | 0.0 | 0.0 | 0.0 | 0.0 | 0.0 | 0.0 | 0.0 | 0.0 | 0.0 | 0.0 | 0.0 |
| Olfr347 | 0.0 | 0.0 | 0.0 | 0.0 | 0.0 | 0.0 | 0.0 | 0.0 | 0.0 | 0.0 | 0.0 | 0.0 |
| Olfr348 | 0.0 | 0.0 | 0.0 | 0.0 | 0.0 | 0.0 | 0.0 | 0.0 | 0.0 | 0.0 | 0.0 | 0.0 |
| Olfr350 | 0.0 | 0.0 | 0.0 | 0.0 | 0.0 | 0.0 | 0.0 | 0.0 | 0.0 | 0.0 | 0.0 | 0.0 |
| Olfr351 | 0.0 | 0.0 | 0.0 | 0.0 | 0.0 | 0.0 | 0.0 | 0.0 | 0.0 | 0.0 | 0.0 | 0.0 |
| Olfr352 | 0.0 | 0.0 | 0.0 | 0.0 | 0.0 | 0.0 | 0.0 | 0.0 | 0.0 | 0.0 | 0.0 | 0.0 |
| Olfr353 | 0.0 | 0.0 | 0.0 | 0.0 | 0.0 | 0.0 | 0.0 | 0.0 | 0.0 | 0.0 | 0.0 | 0.0 |
| Olfr354 | 0.0 | 0.0 | 0.0 | 0.0 | 0.0 | 0.0 | 0.0 | 0.0 | 0.0 | 0.0 | 0.0 | 0.0 |
| Olfr355 | 0.0 | 0.0 | 0.0 | 0.0 | 0.0 | 0.0 | 0.0 | 0.0 | 0.0 | 0.0 | 0.0 | 0.0 |
| Olfr356 | 0.0 | 0.0 | 0.0 | 0.0 | 0.0 | 0.0 | 0.0 | 0.0 | 0.0 | 0.0 | 0.0 | 0.0 |
| Olfr357 | 0.0 | 0.0 | 0.0 | 0.0 | 0.0 | 0.0 | 0.0 | 0.0 | 0.0 | 0.0 | 0.0 | 0.0 |
| Olfr358 | 0.0 | 0.0 | 0.0 | 0.0 | 0.0 | 0.0 | 0.0 | 0.0 | 0.0 | 0.0 | 0.0 | 0.0 |
| Olfr360 | 0.0 | 0.0 | 0.0 | 0.0 | 0.0 | 0.0 | 0.0 | 0.0 | 0.0 | 0.0 | 0.0 | 0.0 |
| Olfr361 | 0.0 | 0.0 | 0.0 | 0.0 | 0.0 | 0.0 | 0.0 | 0.0 | 0.0 | 0.0 | 0.0 | 0.0 |
| Olfr362 | 0.0 | 0.0 | 0.0 | 0.0 | 0.0 | 0.0 | 0.0 | 0.0 | 0.0 | 0.0 | 0.0 | 0.0 |
| Olfr365 | 0.0 | 0.0 | 0.0 | 0.0 | 0.0 | 0.0 | 0.0 | 0.0 | 0.0 | 0.0 | 0.0 | 0.0 |
| Olfr367-ps | 0.0 | 0.0 | 0.0 | 0.0 | 0.0 | 0.0 | 0.0 | 0.0 | 0.0 | 0.0 | 0.0 | 0.0 |
| Olfr371 | 0.0 | 0.0 | 0.0 | 0.0 | 0.0 | 0.0 | 0.0 | 0.0 | 0.0 | 0.0 | 0.0 | 0.0 |
| Olfr376 | 0.0 | 0.0 | 0.0 | 0.0 | 0.0 | 0.0 | 0.0 | 0.0 | 0.0 | 0.0 | 0.0 | 0.0 |
| Olfr38 | 0.0 | 0.0 | 0.0 | 0.0 | 0.0 | 0.0 | 0.0 | 0.0 | 0.0 | 0.0 | 0.0 | 0.0 |
| Olfr380 | 0.0 | 0.0 | 0.0 | 0.0 | 0.0 | 0.0 | 0.0 | 0.0 | 0.0 | 0.0 | 0.0 | 0.0 |
| Olfr381 | 0.0 | 0.0 | 0.0 | 0.0 | 0.0 | 0.0 | 0.0 | 0.0 | 0.0 | 0.0 | 0.0 | 0.0 |
| Olfr382 | 0.0 | 0.0 | 0.0 | 0.0 | 0.0 | 0.0 | 0.0 | 0.0 | 0.0 | 0.0 | 0.0 | 0.0 |
| Olfr384 | 0.0 | 0.0 | 0.0 | 0.0 | 0.0 | 0.0 | 0.0 | 0.0 | 0.0 | 0.0 | 0.0 | 0.0 |

| | | | | | | | | | | | |
|---|---|---|---|---|---|---|---|---|---|---|---|
| Olfr385 | 0.0 | 0.0 | 0.0 | 0.0 | 0.0 | 0.0 | 0.0 | 0.0 | 0.0 | 0.0 | 0.0 | 0.0 |
| Olfr389 | 0.0 | 0.0 | 0.0 | 0.0 | 0.0 | 0.0 | 0.0 | 0.0 | 0.0 | 0.0 | 0.0 | 0.0 |
| Olfr390 | 0.0 | 0.0 | 0.0 | 0.0 | 0.0 | 0.0 | 0.0 | 0.0 | 0.0 | 0.0 | 0.0 | 0.0 |
| Olfr391-ps | 0.0 | 0.0 | 0.0 | 0.0 | 0.0 | 0.0 | 0.0 | 0.0 | 0.0 | 0.0 | 0.0 | 0.0 |
| Olfr392 | 0.0 | 0.0 | 0.0 | 0.0 | 0.0 | 0.0 | 0.0 | 0.0 | 0.0 | 0.0 | 0.0 | 0.0 |
| Olfr393 | 0.0 | 0.0 | 0.0 | 0.0 | 0.0 | 0.0 | 0.0 | 0.0 | 0.0 | 0.0 | 0.0 | 0.0 |
| Olfr394 | 0.0 | 0.0 | 0.0 | 0.0 | 0.0 | 0.0 | 0.0 | 0.0 | 0.0 | 0.0 | 0.0 | 0.0 |
| Olfr395 | 0.0 | 0.0 | 0.0 | 0.0 | 0.0 | 0.0 | 0.0 | 0.0 | 0.0 | 0.0 | 0.0 | 0.0 |
| Olfr397 | 0.0 | 0.0 | 0.0 | 0.0 | 0.0 | 0.0 | 0.0 | 0.0 | 0.0 | 0.0 | 0.0 | 0.0 |
| Olfr398 | 0.0 | 0.0 | 0.0 | 0.0 | 0.0 | 0.0 | 0.0 | 0.0 | 0.0 | 0.0 | 0.0 | 0.0 |
| Olfr399 | 0.0 | 0.0 | 0.0 | 0.0 | 0.0 | 0.0 | 0.0 | 0.0 | 0.0 | 0.0 | 0.0 | 0.0 |
| Olfr401 | 0.0 | 0.0 | 0.0 | 0.0 | 0.0 | 0.0 | 0.0 | 0.0 | 0.0 | 0.0 | 0.0 | 0.0 |
| Olfr402 | 0.0 | 0.0 | 0.0 | 0.0 | 0.0 | 0.0 | 0.0 | 0.0 | 0.0 | 0.0 | 0.0 | 0.0 |
| Olfr403 | 0.0 | 0.0 | 0.0 | 0.0 | 0.0 | 0.0 | 0.0 | 0.0 | 0.0 | 0.0 | 0.0 | 0.0 |
| Olfr406 | 0.0 | 0.0 | 0.0 | 0.0 | 0.0 | 0.0 | 0.0 | 0.0 | 0.0 | 0.0 | 0.0 | 0.0 |
| Olfr410 | 0.0 | 0.0 | 0.0 | 0.0 | 0.0 | 0.0 | 0.0 | 0.0 | 0.0 | 0.0 | 0.0 | 0.0 |
| Olfr412 | 0.0 | 0.0 | 0.0 | 0.0 | 0.0 | 0.0 | 0.0 | 0.0 | 0.0 | 0.0 | 0.0 | 0.0 |
| Olfr414 | 0.0 | 0.0 | 0.0 | 0.0 | 0.0 | 0.0 | 0.0 | 0.0 | 0.0 | 0.0 | 0.0 | 0.0 |
| Olfr417 | 0.0 | 0.0 | 0.0 | 0.0 | 0.0 | 0.0 | 0.0 | 0.0 | 0.0 | 0.0 | 0.0 | 0.0 |
| Olfr418-ps1 | 0.0 | 0.0 | 0.0 | 0.0 | 0.0 | 0.0 | 0.0 | 0.0 | 0.0 | 0.0 | 0.0 | 0.0 |
| Olfr426 | 0.0 | 0.0 | 0.0 | 0.0 | 0.0 | 0.0 | 0.0 | 0.0 | 0.0 | 0.0 | 0.0 | 0.0 |
| Olfr427 | 0.0 | 0.0 | 0.0 | 0.0 | 0.0 | 0.0 | 0.0 | 0.0 | 0.0 | 0.0 | 0.0 | 0.0 |
| Olfr429 | 0.0 | 0.0 | 0.0 | 0.0 | 0.0 | 0.0 | 0.0 | 0.0 | 0.0 | 0.0 | 0.0 | 0.0 |
| Olfr43 | 0.0 | 0.0 | 0.0 | 0.0 | 0.0 | 0.0 | 0.0 | 0.0 | 0.0 | 0.0 | 0.0 | 0.0 |
| Olfr430 | 0.0 | 0.0 | 0.0 | 0.0 | 0.0 | 0.0 | 0.0 | 0.0 | 0.0 | 0.0 | 0.0 | 0.0 |
| Olfr432 | 0.0 | 0.0 | 0.0 | 0.0 | 0.0 | 0.0 | 0.0 | 0.0 | 0.0 | 0.0 | 0.0 | 0.0 |
| Olfr433 | 0.0 | 0.0 | 0.0 | 0.0 | 0.0 | 0.0 | 0.0 | 0.0 | 0.0 | 0.0 | 0.0 | 0.0 |
| Olfr434 | 0.0 | 0.0 | 0.0 | 0.0 | 0.0 | 0.0 | 0.0 | 0.0 | 0.0 | 0.0 | 0.0 | 0.0 |
| Olfr437 | 0.0 | 0.0 | 0.0 | 0.0 | 0.0 | 0.0 | 0.0 | 0.0 | 0.0 | 0.0 | 0.0 | 0.0 |
| Olfr44 | 0.0 | 0.0 | 0.0 | 0.0 | 0.0 | 0.0 | 0.0 | 0.0 | 0.0 | 0.0 | 0.0 | 0.0 |
| Olfr441 | 0.0 | 0.0 | 0.0 | 0.0 | 0.0 | 0.0 | 0.0 | 0.0 | 0.0 | 0.0 | 0.0 | 0.0 |
| Olfr444 | 0.0 | 0.0 | 0.0 | 0.0 | 0.0 | 0.0 | 0.0 | 0.0 | 0.0 | 0.0 | 0.0 | 0.0 |
| Olfr447 | 0.0 | 0.0 | 0.0 | 0.0 | 0.0 | 0.0 | 0.0 | 0.0 | 0.0 | 0.0 | 0.0 | 0.0 |
| Olfr449 | 0.0 | 0.0 | 0.0 | 0.0 | 0.0 | 0.0 | 0.0 | 0.0 | 0.0 | 0.0 | 0.0 | 0.0 |
| Olfr45 | 0.0 | 0.0 | 0.0 | 0.0 | 0.0 | 0.0 | 0.0 | 0.0 | 0.0 | 0.0 | 0.0 | 0.0 |
| Olfr450 | 0.0 | 0.0 | 0.0 | 0.0 | 0.0 | 0.0 | 0.0 | 0.0 | 0.0 | 0.0 | 0.0 | 0.0 |
| Olfr452 | 0.0 | 0.0 | 0.0 | 0.0 | 0.0 | 0.0 | 0.0 | 0.0 | 0.0 | 0.0 | 0.0 | 0.0 |
| Olfr453 | 0.0 | 0.0 | 0.0 | 0.0 | 0.0 | 0.0 | 0.0 | 0.0 | 0.0 | 0.0 | 0.0 | 0.0 |
| Olfr459 | 0.0 | 0.0 | 0.0 | 0.0 | 0.0 | 0.0 | 0.0 | 0.0 | 0.0 | 0.0 | 0.0 | 0.0 |
| Olfr46 | 0.0 | 0.0 | 0.0 | 0.0 | 0.0 | 0.0 | 0.0 | 0.0 | 0.0 | 0.0 | 0.0 | 0.0 |
| Olfr460 | 0.0 | 0.0 | 0.0 | 0.0 | 0.0 | 0.0 | 0.0 | 0.0 | 0.0 | 0.0 | 0.0 | 0.0 |
| Olfr462 | 0.0 | 0.0 | 0.0 | 0.0 | 0.0 | 0.0 | 0.0 | 0.0 | 0.0 | 0.0 | 0.0 | 0.0 |
| Olfr463 | 0.0 | 0.0 | 0.0 | 0.0 | 0.0 | 0.0 | 0.0 | 0.0 | 0.0 | 0.0 | 0.0 | 0.0 |
| Olfr464 | 0.0 | 0.0 | 0.0 | 0.0 | 0.0 | 0.0 | 0.0 | 0.0 | 0.0 | 0.0 | 0.0 | 0.0 |
| Olfr467 | 0.0 | 0.0 | 0.0 | 0.0 | 0.0 | 0.0 | 0.0 | 0.0 | 0.0 | 0.0 | 0.0 | 0.0 |
| Olfr469 | 0.0 | 0.0 | 0.0 | 0.0 | 0.0 | 0.0 | 0.0 | 0.0 | 0.0 | 0.0 | 0.0 | 0.0 |
| Olfr47 | 0.0 | 0.0 | 0.0 | 0.0 | 0.0 | 0.0 | 0.0 | 0.0 | 0.0 | 0.0 | 0.0 | 0.0 |
| Olfr470 | 0.0 | 0.0 | 0.0 | 0.0 | 0.0 | 0.0 | 0.0 | 0.0 | 0.0 | 0.0 | 0.0 | 0.0 |
| Olfr472 | 0.0 | 0.0 | 0.0 | 0.0 | 0.0 | 0.0 | 0.0 | 0.0 | 0.0 | 0.0 | 0.0 | 0.0 |
| Olfr473 | 0.0 | 0.0 | 0.0 | 0.0 | 0.0 | 0.0 | 0.0 | 0.0 | 0.0 | 0.0 | 0.0 | 0.0 |
| Olfr474 | 0.0 | 0.0 | 0.0 | 0.0 | 0.0 | 0.0 | 0.0 | 0.0 | 0.0 | 0.0 | 0.0 | 0.0 |
| Olfr476 | 0.0 | 0.0 | 0.0 | 0.0 | 0.0 | 0.0 | 0.0 | 0.0 | 0.0 | 0.0 | 0.0 | 0.0 |
| Olfr477 | 0.0 | 0.0 | 0.0 | 0.0 | 0.0 | 0.0 | 0.0 | 0.0 | 0.0 | 0.0 | 0.0 | 0.0 |
| Olfr478 | 0.0 | 0.0 | 0.0 | 0.0 | 0.0 | 0.0 | 0.0 | 0.0 | 0.0 | 0.0 | 0.0 | 0.0 |
| Olfr479 | 0.0 | 0.0 | 0.0 | 0.0 | 0.0 | 0.0 | 0.0 | 0.0 | 0.0 | 0.0 | 0.0 | 0.0 |
| Olfr48 | 0.0 | 0.0 | 0.0 | 0.0 | 0.0 | 0.0 | 0.0 | 0.0 | 0.0 | 0.0 | 0.0 | 0.0 |
| Olfr480 | 0.0 | 0.0 | 0.0 | 0.0 | 0.0 | 0.0 | 0.0 | 0.0 | 0.0 | 0.0 | 0.0 | 0.0 |

Excluded Genes

| | | | | | | | | | | | |
|---|---|---|---|---|---|---|---|---|---|---|---|
| Olfr481 | 0.0 | 0.0 | 0.0 | 0.0 | 0.0 | 0.0 | 0.0 | 0.0 | 0.0 | 0.0 | 0.0 | 0.0 |
| Olfr482 | 0.0 | 0.0 | 0.0 | 0.0 | 0.0 | 0.0 | 0.0 | 0.0 | 0.0 | 0.0 | 0.0 | 0.0 |
| Olfr484 | 0.0 | 0.0 | 0.0 | 0.0 | 0.0 | 0.0 | 0.0 | 0.0 | 0.0 | 0.0 | 0.0 | 0.0 |
| Olfr485 | 0.0 | 0.0 | 0.0 | 0.0 | 0.0 | 0.0 | 0.0 | 0.0 | 0.0 | 0.0 | 0.0 | 0.0 |
| Olfr487 | 0.0 | 0.0 | 0.0 | 0.0 | 0.0 | 0.0 | 0.0 | 0.0 | 0.0 | 0.0 | 0.0 | 0.0 |
| Olfr488 | 0.0 | 0.0 | 0.0 | 0.0 | 0.0 | 0.0 | 0.0 | 0.0 | 0.0 | 0.0 | 0.0 | 0.0 |
| Olfr490 | 0.0 | 0.0 | 0.0 | 0.0 | 0.0 | 0.0 | 0.0 | 0.0 | 0.0 | 0.0 | 0.0 | 0.0 |
| Olfr491 | 0.0 | 0.0 | 0.0 | 0.0 | 0.0 | 0.0 | 0.0 | 0.0 | 0.0 | 0.0 | 0.0 | 0.0 |
| Olfr492 | 0.0 | 0.0 | 0.0 | 0.0 | 0.0 | 0.0 | 0.0 | 0.0 | 0.0 | 0.0 | 0.0 | 0.0 |
| Olfr494 | 0.0 | 0.0 | 0.0 | 0.0 | 0.0 | 0.0 | 0.0 | 0.0 | 0.0 | 0.0 | 0.0 | 0.0 |
| Olfr495 | 0.0 | 0.0 | 0.0 | 0.0 | 0.0 | 0.0 | 0.0 | 0.0 | 0.0 | 0.0 | 0.0 | 0.0 |
| Olfr497 | 0.0 | 0.0 | 0.0 | 0.0 | 0.0 | 0.0 | 0.0 | 0.0 | 0.0 | 0.0 | 0.0 | 0.0 |
| Olfr5 | 0.0 | 0.0 | 0.0 | 0.0 | 0.0 | 0.0 | 0.0 | 0.0 | 0.0 | 0.0 | 0.0 | 0.0 |
| Olfr50 | 0.0 | 0.0 | 0.0 | 0.0 | 0.0 | 0.0 | 0.0 | 0.0 | 0.0 | 0.0 | 0.0 | 0.0 |
| Olfr503 | 0.0 | 0.0 | 0.0 | 0.0 | 0.0 | 0.0 | 0.0 | 0.0 | 0.0 | 0.0 | 0.0 | 0.0 |
| Olfr504 | 0.0 | 0.0 | 0.0 | 0.0 | 0.0 | 0.0 | 0.0 | 0.0 | 0.0 | 0.0 | 0.0 | 0.0 |
| Olfr506 | 0.0 | 0.0 | 0.0 | 0.0 | 0.0 | 0.0 | 0.0 | 0.0 | 0.0 | 0.0 | 0.0 | 0.0 |
| Olfr508 | 0.0 | 0.0 | 0.0 | 0.0 | 0.0 | 0.0 | 0.0 | 0.0 | 0.0 | 0.0 | 0.0 | 0.0 |
| Olfr509 | 0.0 | 0.0 | 0.0 | 0.0 | 0.0 | 0.0 | 0.0 | 0.0 | 0.0 | 0.0 | 0.0 | 0.0 |
| Olfr51 | 0.0 | 0.0 | 0.0 | 0.0 | 0.0 | 0.0 | 0.0 | 0.0 | 0.0 | 0.0 | 0.0 | 0.0 |
| Olfr510 | 0.0 | 0.0 | 0.0 | 0.0 | 0.0 | 0.0 | 0.0 | 0.0 | 0.0 | 0.0 | 0.0 | 0.0 |
| Olfr512 | 0.0 | 0.0 | 0.0 | 0.0 | 0.0 | 0.0 | 0.0 | 0.0 | 0.0 | 0.0 | 0.0 | 0.0 |
| Olfr517 | 0.0 | 0.0 | 0.0 | 0.0 | 0.0 | 0.0 | 0.0 | 0.0 | 0.0 | 0.0 | 0.0 | 0.0 |
| Olfr518 | 0.0 | 0.0 | 0.0 | 0.0 | 0.0 | 0.0 | 0.0 | 0.0 | 0.0 | 0.0 | 0.0 | 0.0 |
| Olfr519 | 0.0 | 0.0 | 0.0 | 0.0 | 0.0 | 0.0 | 0.0 | 0.0 | 0.0 | 0.0 | 0.0 | 0.0 |
| Olfr52 | 0.0 | 0.0 | 0.0 | 0.0 | 0.0 | 0.0 | 0.0 | 0.0 | 0.0 | 0.0 | 0.0 | 0.0 |
| Olfr520 | 0.0 | 0.0 | 0.0 | 0.0 | 0.0 | 0.0 | 0.0 | 0.0 | 0.0 | 0.0 | 0.0 | 0.0 |
| Olfr521 | 0.0 | 0.0 | 0.0 | 0.0 | 0.0 | 0.0 | 0.0 | 0.0 | 0.0 | 0.0 | 0.0 | 0.0 |
| Olfr522 | 0.0 | 0.0 | 0.0 | 0.0 | 0.0 | 0.0 | 0.0 | 0.0 | 0.0 | 0.0 | 0.0 | 0.0 |
| Olfr523 | 0.0 | 0.0 | 0.0 | 0.0 | 0.0 | 0.0 | 0.0 | 0.0 | 0.0 | 0.0 | 0.0 | 0.0 |
| Olfr524 | 0.0 | 0.0 | 0.0 | 0.0 | 0.0 | 0.0 | 0.0 | 0.0 | 0.0 | 0.0 | 0.0 | 0.0 |
| Olfr525 | 0.0 | 0.0 | 0.0 | 0.0 | 0.0 | 0.0 | 0.0 | 0.0 | 0.0 | 0.0 | 0.0 | 0.0 |
| Olfr527 | 0.0 | 0.0 | 0.0 | 0.0 | 0.0 | 0.0 | 0.0 | 0.0 | 0.0 | 0.0 | 0.0 | 0.0 |
| Olfr53 | 0.0 | 0.0 | 0.0 | 0.0 | 0.0 | 0.0 | 0.0 | 0.0 | 0.0 | 0.0 | 0.0 | 0.0 |
| Olfr530 | 0.0 | 0.0 | 0.0 | 0.0 | 0.0 | 0.0 | 0.0 | 0.0 | 0.0 | 0.0 | 0.0 | 0.0 |
| Olfr531 | 0.0 | 0.0 | 0.0 | 0.0 | 0.0 | 0.0 | 0.0 | 0.0 | 0.0 | 0.0 | 0.0 | 0.0 |
| Olfr532 | 0.0 | 0.0 | 0.0 | 0.0 | 0.0 | 0.0 | 0.0 | 0.0 | 0.0 | 0.0 | 0.0 | 0.0 |
| Olfr533 | 0.0 | 0.0 | 0.0 | 0.0 | 0.0 | 0.0 | 0.0 | 0.0 | 0.0 | 0.0 | 0.0 | 0.0 |
| Olfr535 | 0.0 | 0.0 | 0.0 | 0.0 | 0.0 | 0.0 | 0.0 | 0.0 | 0.0 | 0.0 | 0.0 | 0.0 |
| Olfr538 | 0.0 | 0.0 | 0.0 | 0.0 | 0.0 | 0.0 | 0.0 | 0.0 | 0.0 | 0.0 | 0.0 | 0.0 |
| Olfr539 | 0.0 | 0.0 | 0.0 | 0.0 | 0.0 | 0.0 | 0.0 | 0.0 | 0.0 | 0.0 | 0.0 | 0.0 |
| Olfr54 | 0.0 | 0.0 | 0.0 | 0.0 | 0.0 | 0.0 | 0.0 | 0.0 | 0.0 | 0.0 | 0.0 | 0.0 |
| Olfr541 | 0.0 | 0.0 | 0.0 | 0.0 | 0.0 | 0.0 | 0.0 | 0.0 | 0.0 | 0.0 | 0.0 | 0.0 |
| Olfr551 | 0.0 | 0.0 | 0.0 | 0.0 | 0.0 | 0.0 | 0.0 | 0.0 | 0.0 | 0.0 | 0.0 | 0.0 |
| Olfr552 | 0.0 | 0.0 | 0.0 | 0.0 | 0.0 | 0.0 | 0.0 | 0.0 | 0.0 | 0.0 | 0.0 | 0.0 |
| Olfr554 | 0.0 | 0.0 | 0.0 | 0.0 | 0.0 | 0.0 | 0.0 | 0.0 | 0.0 | 0.0 | 0.0 | 0.0 |
| Olfr555 | 0.0 | 0.0 | 0.0 | 0.0 | 0.0 | 0.0 | 0.0 | 0.0 | 0.0 | 0.0 | 0.0 | 0.0 |
| Olfr556 | 0.0 | 0.0 | 0.0 | 0.0 | 0.0 | 0.0 | 0.0 | 0.0 | 0.0 | 0.0 | 0.0 | 0.0 |
| Olfr557 | 0.0 | 0.0 | 0.0 | 0.0 | 0.0 | 0.0 | 0.0 | 0.0 | 0.0 | 0.0 | 0.0 | 0.0 |
| Olfr560 | 0.0 | 0.0 | 0.0 | 0.0 | 0.0 | 0.0 | 0.0 | 0.0 | 0.0 | 0.0 | 0.0 | 0.0 |
| Olfr561 | 0.0 | 0.0 | 0.0 | 0.0 | 0.0 | 0.0 | 0.0 | 0.0 | 0.0 | 0.0 | 0.0 | 0.0 |
| Olfr564 | 0.0 | 0.0 | 0.0 | 0.0 | 0.0 | 0.0 | 0.0 | 0.0 | 0.0 | 0.0 | 0.0 | 0.0 |
| Olfr566 | 0.0 | 0.0 | 0.0 | 0.0 | 0.0 | 0.0 | 0.0 | 0.0 | 0.0 | 0.0 | 0.0 | 0.0 |
| Olfr568 | 0.0 | 0.0 | 0.0 | 0.0 | 0.0 | 0.0 | 0.0 | 0.0 | 0.0 | 0.0 | 0.0 | 0.0 |
| Olfr571 | 0.0 | 0.0 | 0.0 | 0.0 | 0.0 | 0.0 | 0.0 | 0.0 | 0.0 | 0.0 | 0.0 | 0.0 |
| Olfr572 | 0.0 | 0.0 | 0.0 | 0.0 | 0.0 | 0.0 | 0.0 | 0.0 | 0.0 | 0.0 | 0.0 | 0.0 |
| Olfr574 | 0.0 | 0.0 | 0.0 | 0.0 | 0.0 | 0.0 | 0.0 | 0.0 | 0.0 | 0.0 | 0.0 | 0.0 |

Excluded Genes

| | | | | | | | | | | | | |
|---|---|---|---|---|---|---|---|---|---|---|---|---|
| Olfr576 | 0.0 | 0.0 | 0.0 | 0.0 | 0.0 | 0.0 | 0.0 | 0.0 | 0.0 | 0.0 | 0.0 | 0.0 |
| Olfr577 | 0.0 | 0.0 | 0.0 | 0.0 | 0.0 | 0.0 | 0.0 | 0.0 | 0.0 | 0.0 | 0.0 | 0.0 |
| Olfr578 | 0.0 | 0.0 | 0.0 | 0.0 | 0.0 | 0.0 | 0.0 | 0.0 | 0.0 | 0.0 | 0.0 | 0.0 |
| Olfr58 | 0.0 | 0.0 | 0.0 | 0.0 | 0.0 | 0.0 | 0.0 | 0.0 | 0.0 | 0.0 | 0.0 | 0.0 |
| Olfr582 | 0.0 | 0.0 | 0.0 | 0.0 | 0.0 | 0.0 | 0.0 | 0.0 | 0.0 | 0.0 | 0.0 | 0.0 |
| Olfr583 | 0.0 | 0.0 | 0.0 | 0.0 | 0.0 | 0.0 | 0.0 | 0.0 | 0.0 | 0.0 | 0.0 | 0.0 |
| Olfr584 | 0.0 | 0.0 | 0.0 | 0.0 | 0.0 | 0.0 | 0.0 | 0.0 | 0.0 | 0.0 | 0.0 | 0.0 |
| Olfr585 | 0.0 | 0.0 | 0.0 | 0.0 | 0.0 | 0.0 | 0.0 | 0.0 | 0.0 | 0.0 | 0.0 | 0.0 |
| Olfr586 | 0.0 | 0.0 | 0.0 | 0.0 | 0.0 | 0.0 | 0.0 | 0.0 | 0.0 | 0.0 | 0.0 | 0.0 |
| Olfr589 | 0.0 | 0.0 | 0.0 | 0.0 | 0.0 | 0.0 | 0.0 | 0.0 | 0.0 | 0.0 | 0.0 | 0.0 |
| Olfr59 | 0.0 | 0.0 | 0.0 | 0.0 | 0.0 | 0.0 | 0.0 | 0.0 | 0.0 | 0.0 | 0.0 | 0.0 |
| Olfr591 | 0.0 | 0.0 | 0.0 | 0.0 | 0.0 | 0.0 | 0.0 | 0.0 | 0.0 | 0.0 | 0.0 | 0.0 |
| Olfr592 | 0.0 | 0.0 | 0.0 | 0.0 | 0.0 | 0.0 | 0.0 | 0.0 | 0.0 | 0.0 | 0.0 | 0.0 |
| Olfr593 | 0.0 | 0.0 | 0.0 | 0.0 | 0.0 | 0.0 | 0.0 | 0.0 | 0.0 | 0.0 | 0.0 | 0.0 |
| Olfr596 | 0.0 | 0.0 | 0.0 | 0.0 | 0.0 | 0.0 | 0.0 | 0.0 | 0.0 | 0.0 | 0.0 | 0.0 |
| Olfr597 | 0.0 | 0.0 | 0.0 | 0.0 | 0.0 | 0.0 | 0.0 | 0.0 | 0.0 | 0.0 | 0.0 | 0.0 |
| Olfr598 | 0.0 | 0.0 | 0.0 | 0.0 | 0.0 | 0.0 | 0.0 | 0.0 | 0.0 | 0.0 | 0.0 | 0.0 |
| Olfr599 | 0.0 | 0.0 | 0.0 | 0.0 | 0.0 | 0.0 | 0.0 | 0.0 | 0.0 | 0.0 | 0.0 | 0.0 |
| Olfr6 | 0.0 | 0.0 | 0.0 | 0.0 | 0.0 | 0.0 | 0.0 | 0.0 | 0.0 | 0.0 | 0.0 | 0.0 |
| Olfr60 | 0.0 | 0.0 | 0.0 | 0.0 | 0.0 | 0.0 | 0.0 | 0.0 | 0.0 | 0.0 | 0.0 | 0.0 |
| Olfr600 | 0.0 | 0.0 | 0.0 | 0.0 | 0.0 | 0.0 | 0.0 | 0.0 | 0.0 | 0.0 | 0.0 | 0.0 |
| Olfr601 | 0.0 | 0.0 | 0.0 | 0.0 | 0.0 | 0.0 | 0.0 | 0.0 | 0.0 | 0.0 | 0.0 | 0.0 |
| Olfr605 | 0.0 | 0.0 | 0.0 | 0.0 | 0.0 | 0.0 | 0.0 | 0.0 | 0.0 | 0.0 | 0.0 | 0.0 |
| Olfr608 | 0.0 | 0.0 | 0.0 | 0.0 | 0.0 | 0.0 | 0.0 | 0.0 | 0.0 | 0.0 | 0.0 | 0.0 |
| Olfr609 | 0.0 | 0.0 | 0.0 | 0.0 | 0.0 | 0.0 | 0.0 | 0.0 | 0.0 | 0.0 | 0.0 | 0.0 |
| Olfr61 | 0.0 | 0.0 | 0.0 | 0.0 | 0.0 | 0.0 | 0.0 | 0.0 | 0.0 | 0.0 | 0.0 | 0.0 |
| Olfr610 | 0.0 | 0.0 | 0.0 | 0.0 | 0.0 | 0.0 | 0.0 | 0.0 | 0.0 | 0.0 | 0.0 | 0.0 |
| Olfr611 | 0.0 | 0.0 | 0.0 | 0.0 | 0.0 | 0.0 | 0.0 | 0.0 | 0.0 | 0.0 | 0.0 | 0.0 |
| Olfr612 | 0.0 | 0.0 | 0.0 | 0.0 | 0.0 | 0.0 | 0.0 | 0.0 | 0.0 | 0.0 | 0.0 | 0.0 |
| Olfr616 | 0.0 | 0.0 | 0.0 | 0.0 | 0.0 | 0.0 | 0.0 | 0.0 | 0.0 | 0.0 | 0.0 | 0.0 |
| Olfr617 | 0.0 | 0.0 | 0.0 | 0.0 | 0.0 | 0.0 | 0.0 | 0.0 | 0.0 | 0.0 | 0.0 | 0.0 |
| Olfr618 | 0.0 | 0.0 | 0.0 | 0.0 | 0.0 | 0.0 | 0.0 | 0.0 | 0.0 | 0.0 | 0.0 | 0.0 |
| Olfr619 | 0.0 | 0.0 | 0.0 | 0.0 | 0.0 | 0.0 | 0.0 | 0.0 | 0.0 | 0.0 | 0.0 | 0.0 |
| Olfr62 | 0.0 | 0.0 | 0.0 | 0.0 | 0.0 | 0.0 | 0.0 | 0.0 | 0.0 | 0.0 | 0.0 | 0.0 |
| Olfr620 | 0.0 | 0.0 | 0.0 | 0.0 | 0.0 | 0.0 | 0.0 | 0.0 | 0.0 | 0.0 | 0.0 | 0.0 |
| Olfr622 | 0.0 | 0.0 | 0.0 | 0.0 | 0.0 | 0.0 | 0.0 | 0.0 | 0.0 | 0.0 | 0.0 | 0.0 |
| Olfr623 | 0.0 | 0.0 | 0.0 | 0.0 | 0.0 | 0.0 | 0.0 | 0.0 | 0.0 | 0.0 | 0.0 | 0.0 |
| Olfr624 | 0.0 | 0.0 | 0.0 | 0.0 | 0.0 | 0.0 | 0.0 | 0.0 | 0.0 | 0.0 | 0.0 | 0.0 |
| Olfr628 | 0.0 | 0.0 | 0.0 | 0.0 | 0.0 | 0.0 | 0.0 | 0.0 | 0.0 | 0.0 | 0.0 | 0.0 |
| Olfr629 | 0.0 | 0.0 | 0.0 | 0.0 | 0.0 | 0.0 | 0.0 | 0.0 | 0.0 | 0.0 | 0.0 | 0.0 |
| Olfr63 | 0.0 | 0.0 | 0.0 | 0.0 | 0.0 | 0.0 | 0.0 | 0.0 | 0.0 | 0.0 | 0.0 | 0.0 |
| Olfr630 | 0.0 | 0.0 | 0.0 | 0.0 | 0.0 | 0.0 | 0.0 | 0.0 | 0.0 | 0.0 | 0.0 | 0.0 |
| Olfr631 | 0.0 | 0.0 | 0.0 | 0.0 | 0.0 | 0.0 | 0.0 | 0.0 | 0.0 | 0.0 | 0.0 | 0.0 |
| Olfr632 | 0.0 | 0.0 | 0.0 | 0.0 | 0.0 | 0.0 | 0.0 | 0.0 | 0.0 | 0.0 | 0.0 | 0.0 |
| Olfr633 | 0.0 | 0.0 | 0.0 | 0.0 | 0.0 | 0.0 | 0.0 | 0.0 | 0.0 | 0.0 | 0.0 | 0.0 |
| Olfr638 | 0.0 | 0.0 | 0.0 | 0.0 | 0.0 | 0.0 | 0.0 | 0.0 | 0.0 | 0.0 | 0.0 | 0.0 |
| Olfr639 | 0.0 | 0.0 | 0.0 | 0.0 | 0.0 | 0.0 | 0.0 | 0.0 | 0.0 | 0.0 | 0.0 | 0.0 |
| Olfr64 | 0.0 | 0.0 | 0.0 | 0.0 | 0.0 | 0.0 | 0.0 | 0.0 | 0.0 | 0.0 | 0.0 | 0.0 |
| Olfr640 | 0.0 | 0.0 | 0.0 | 0.0 | 0.0 | 0.0 | 0.0 | 0.0 | 0.0 | 0.0 | 0.0 | 0.0 |
| Olfr641 | 0.0 | 0.0 | 0.0 | 0.0 | 0.0 | 0.0 | 0.0 | 0.0 | 0.0 | 0.0 | 0.0 | 0.0 |
| Olfr642 | 0.0 | 0.0 | 0.0 | 0.0 | 0.0 | 0.0 | 0.0 | 0.0 | 0.0 | 0.0 | 0.0 | 0.0 |
| Olfr644 | 0.0 | 0.0 | 0.0 | 0.0 | 0.0 | 0.0 | 0.0 | 0.0 | 0.0 | 0.0 | 0.0 | 0.0 |
| Olfr645 | 0.0 | 0.0 | 0.0 | 0.0 | 0.0 | 0.0 | 0.0 | 0.0 | 0.0 | 0.0 | 0.0 | 0.0 |
| Olfr646 | 0.0 | 0.0 | 0.0 | 0.0 | 0.0 | 0.0 | 0.0 | 0.0 | 0.0 | 0.0 | 0.0 | 0.0 |
| Olfr648 | 0.0 | 0.0 | 0.0 | 0.0 | 0.0 | 0.0 | 0.0 | 0.0 | 0.0 | 0.0 | 0.0 | 0.0 |
| Olfr649 | 0.0 | 0.0 | 0.0 | 0.0 | 0.0 | 0.0 | 0.0 | 0.0 | 0.0 | 0.0 | 0.0 | 0.0 |
| Olfr65 | 0.0 | 0.0 | 0.0 | 0.0 | 0.0 | 0.0 | 0.0 | 0.0 | 0.0 | 0.0 | 0.0 | 0.0 |

| | | | | | | | | | | | | |
|---|---|---|---|---|---|---|---|---|---|---|---|---|
| Olfr651 | 0.0 | 0.0 | 0.0 | 0.0 | 0.0 | 0.0 | 0.0 | 0.0 | 0.0 | 0.0 | 0.0 | 0.0 |
| Olfr652 | 0.0 | 0.0 | 0.0 | 0.0 | 0.0 | 0.0 | 0.0 | 0.0 | 0.0 | 0.0 | 0.0 | 0.0 |
| Olfr653 | 0.0 | 0.0 | 0.0 | 0.0 | 0.0 | 0.0 | 0.0 | 0.0 | 0.0 | 0.0 | 0.0 | 0.0 |
| Olfr654 | 0.0 | 0.0 | 0.0 | 0.0 | 0.0 | 0.0 | 0.0 | 0.0 | 0.0 | 0.0 | 0.0 | 0.0 |
| Olfr655 | 0.0 | 0.0 | 0.0 | 0.0 | 0.0 | 0.0 | 0.0 | 0.0 | 0.0 | 0.0 | 0.0 | 0.0 |
| Olfr656 | 0.0 | 0.0 | 0.0 | 0.0 | 0.0 | 0.0 | 0.0 | 0.0 | 0.0 | 0.0 | 0.0 | 0.0 |
| Olfr657 | 0.0 | 0.0 | 0.0 | 0.0 | 0.0 | 0.0 | 0.0 | 0.0 | 0.0 | 0.0 | 0.0 | 0.0 |
| Olfr659 | 0.0 | 0.0 | 0.0 | 0.0 | 0.0 | 0.0 | 0.0 | 0.0 | 0.0 | 0.0 | 0.0 | 0.0 |
| Olfr66 | 0.0 | 0.0 | 0.0 | 0.0 | 0.0 | 0.0 | 0.0 | 0.0 | 0.0 | 0.0 | 0.0 | 0.0 |
| Olfr661 | 0.0 | 0.0 | 0.0 | 0.0 | 0.0 | 0.0 | 0.0 | 0.0 | 0.0 | 0.0 | 0.0 | 0.0 |
| Olfr663 | 0.0 | 0.0 | 0.0 | 0.0 | 0.0 | 0.0 | 0.0 | 0.0 | 0.0 | 0.0 | 0.0 | 0.0 |
| Olfr665 | 0.0 | 0.0 | 0.0 | 0.0 | 0.0 | 0.0 | 0.0 | 0.0 | 0.0 | 0.0 | 0.0 | 0.0 |
| Olfr666 | 0.0 | 0.0 | 0.0 | 0.0 | 0.0 | 0.0 | 0.0 | 0.0 | 0.0 | 0.0 | 0.0 | 0.0 |
| Olfr667 | 0.0 | 0.0 | 0.0 | 0.0 | 0.0 | 0.0 | 0.0 | 0.0 | 0.0 | 0.0 | 0.0 | 0.0 |
| Olfr669 | 0.0 | 0.0 | 0.0 | 0.0 | 0.0 | 0.0 | 0.0 | 0.0 | 0.0 | 0.0 | 0.0 | 0.0 |
| Olfr67 | 0.0 | 0.0 | 0.0 | 0.0 | 0.0 | 0.0 | 0.0 | 0.0 | 0.0 | 0.0 | 0.0 | 0.0 |
| Olfr670 | 0.0 | 0.0 | 0.0 | 0.0 | 0.0 | 0.0 | 0.0 | 0.0 | 0.0 | 0.0 | 0.0 | 0.0 |
| Olfr671 | 0.0 | 0.0 | 0.0 | 0.0 | 0.0 | 0.0 | 0.0 | 0.0 | 0.0 | 0.0 | 0.0 | 0.0 |
| Olfr672 | 0.0 | 0.0 | 0.0 | 0.0 | 0.0 | 0.0 | 0.0 | 0.0 | 0.0 | 0.0 | 0.0 | 0.0 |
| Olfr675 | 0.0 | 0.0 | 0.0 | 0.0 | 0.0 | 0.0 | 0.0 | 0.0 | 0.0 | 0.0 | 0.0 | 0.0 |
| Olfr676 | 0.0 | 0.0 | 0.0 | 0.0 | 0.0 | 0.0 | 0.0 | 0.0 | 0.0 | 0.0 | 0.0 | 0.0 |
| Olfr677 | 0.0 | 0.0 | 0.0 | 0.0 | 0.0 | 0.0 | 0.0 | 0.0 | 0.0 | 0.0 | 0.0 | 0.0 |
| Olfr68 | 0.0 | 0.0 | 0.0 | 0.0 | 0.0 | 0.0 | 0.0 | 0.0 | 0.0 | 0.0 | 0.0 | 0.0 |
| Olfr681 | 0.0 | 0.0 | 0.0 | 0.0 | 0.0 | 0.0 | 0.0 | 0.0 | 0.0 | 0.0 | 0.0 | 0.0 |
| Olfr683 | 0.0 | 0.0 | 0.0 | 0.0 | 0.0 | 0.0 | 0.0 | 0.0 | 0.0 | 0.0 | 0.0 | 0.0 |
| Olfr684 | 0.0 | 0.0 | 0.0 | 0.0 | 0.0 | 0.0 | 0.0 | 0.0 | 0.0 | 0.0 | 0.0 | 0.0 |
| Olfr685 | 0.0 | 0.0 | 0.0 | 0.0 | 0.0 | 0.0 | 0.0 | 0.0 | 0.0 | 0.0 | 0.0 | 0.0 |
| Olfr686 | 0.0 | 0.0 | 0.0 | 0.0 | 0.0 | 0.0 | 0.0 | 0.0 | 0.0 | 0.0 | 0.0 | 0.0 |
| Olfr688 | 0.0 | 0.0 | 0.0 | 0.0 | 0.0 | 0.0 | 0.0 | 0.0 | 0.0 | 0.0 | 0.0 | 0.0 |
| Olfr69 | 0.0 | 0.0 | 0.0 | 0.0 | 0.0 | 0.0 | 0.0 | 0.0 | 0.0 | 0.0 | 0.0 | 0.0 |
| Olfr691 | 0.0 | 0.0 | 0.0 | 0.0 | 0.0 | 0.0 | 0.0 | 0.0 | 0.0 | 0.0 | 0.0 | 0.0 |
| Olfr693 | 0.0 | 0.0 | 0.0 | 0.0 | 0.0 | 0.0 | 0.0 | 0.0 | 0.0 | 0.0 | 0.0 | 0.0 |
| Olfr694 | 0.0 | 0.0 | 0.0 | 0.0 | 0.0 | 0.0 | 0.0 | 0.0 | 0.0 | 0.0 | 0.0 | 0.0 |
| Olfr695 | 0.0 | 0.0 | 0.0 | 0.0 | 0.0 | 0.0 | 0.0 | 0.0 | 0.0 | 0.0 | 0.0 | 0.0 |
| Olfr697 | 0.0 | 0.0 | 0.0 | 0.0 | 0.0 | 0.0 | 0.0 | 0.0 | 0.0 | 0.0 | 0.0 | 0.0 |
| Olfr698 | 0.0 | 0.0 | 0.0 | 0.0 | 0.0 | 0.0 | 0.0 | 0.0 | 0.0 | 0.0 | 0.0 | 0.0 |
| Olfr699 | 0.0 | 0.0 | 0.0 | 0.0 | 0.0 | 0.0 | 0.0 | 0.0 | 0.0 | 0.0 | 0.0 | 0.0 |
| Olfr700 | 0.0 | 0.0 | 0.0 | 0.0 | 0.0 | 0.0 | 0.0 | 0.0 | 0.0 | 0.0 | 0.0 | 0.0 |
| Olfr701 | 0.0 | 0.0 | 0.0 | 0.0 | 0.0 | 0.0 | 0.0 | 0.0 | 0.0 | 0.0 | 0.0 | 0.0 |
| Olfr703 | 0.0 | 0.0 | 0.0 | 0.0 | 0.0 | 0.0 | 0.0 | 0.0 | 0.0 | 0.0 | 0.0 | 0.0 |
| Olfr704 | 0.0 | 0.0 | 0.0 | 0.0 | 0.0 | 0.0 | 0.0 | 0.0 | 0.0 | 0.0 | 0.0 | 0.0 |
| Olfr705 | 0.0 | 0.0 | 0.0 | 0.0 | 0.0 | 0.0 | 0.0 | 0.0 | 0.0 | 0.0 | 0.0 | 0.0 |
| Olfr706 | 0.0 | 0.0 | 0.0 | 0.0 | 0.0 | 0.0 | 0.0 | 0.0 | 0.0 | 0.0 | 0.0 | 0.0 |
| Olfr71 | 0.0 | 0.0 | 0.0 | 0.0 | 0.0 | 0.0 | 0.0 | 0.0 | 0.0 | 0.0 | 0.0 | 0.0 |
| Olfr710 | 0.0 | 0.0 | 0.0 | 0.0 | 0.0 | 0.0 | 0.0 | 0.0 | 0.0 | 0.0 | 0.0 | 0.0 |
| Olfr711 | 0.0 | 0.0 | 0.0 | 0.0 | 0.0 | 0.0 | 0.0 | 0.0 | 0.0 | 0.0 | 0.0 | 0.0 |
| Olfr714 | 0.0 | 0.0 | 0.0 | 0.0 | 0.0 | 0.0 | 0.0 | 0.0 | 0.0 | 0.0 | 0.0 | 0.0 |
| Olfr715 | 0.0 | 0.0 | 0.0 | 0.0 | 0.0 | 0.0 | 0.0 | 0.0 | 0.0 | 0.0 | 0.0 | 0.0 |
| Olfr716 | 0.0 | 0.0 | 0.0 | 0.0 | 0.0 | 0.0 | 0.0 | 0.0 | 0.0 | 0.0 | 0.0 | 0.0 |
| Olfr720 | 0.0 | 0.0 | 0.0 | 0.0 | 0.0 | 0.0 | 0.0 | 0.0 | 0.0 | 0.0 | 0.0 | 0.0 |
| Olfr722 | 0.0 | 0.0 | 0.0 | 0.0 | 0.0 | 0.0 | 0.0 | 0.0 | 0.0 | 0.0 | 0.0 | 0.0 |
| Olfr723 | 0.0 | 0.0 | 0.0 | 0.0 | 0.0 | 0.0 | 0.0 | 0.0 | 0.0 | 0.0 | 0.0 | 0.0 |
| Olfr724 | 0.0 | 0.0 | 0.0 | 0.0 | 0.0 | 0.0 | 0.0 | 0.0 | 0.0 | 0.0 | 0.0 | 0.0 |
| Olfr725 | 0.0 | 0.0 | 0.0 | 0.0 | 0.0 | 0.0 | 0.0 | 0.0 | 0.0 | 0.0 | 0.0 | 0.0 |
| Olfr726 | 0.0 | 0.0 | 0.0 | 0.0 | 0.0 | 0.0 | 0.0 | 0.0 | 0.0 | 0.0 | 0.0 | 0.0 |
| Olfr727 | 0.0 | 0.0 | 0.0 | 0.0 | 0.0 | 0.0 | 0.0 | 0.0 | 0.0 | 0.0 | 0.0 | 0.0 |
| Olfr728 | 0.0 | 0.0 | 0.0 | 0.0 | 0.0 | 0.0 | 0.0 | 0.0 | 0.0 | 0.0 | 0.0 | 0.0 |

Excluded Genes

| | | | | | | | | | | | |
|---|---|---|---|---|---|---|---|---|---|---|---|
| Olfr729 | 0.0 | 0.0 | 0.0 | 0.0 | 0.0 | 0.0 | 0.0 | 0.0 | 0.0 | 0.0 | 0.0 |
| Olfr73 | 0.0 | 0.0 | 0.0 | 0.0 | 0.0 | 0.0 | 0.0 | 0.0 | 0.0 | 0.0 | 0.0 |
| Olfr730 | 0.0 | 0.0 | 0.0 | 0.0 | 0.0 | 0.0 | 0.0 | 0.0 | 0.0 | 0.0 | 0.0 |
| Olfr731 | 0.0 | 0.0 | 0.0 | 0.0 | 0.0 | 0.0 | 0.0 | 0.0 | 0.0 | 0.0 | 0.0 |
| Olfr732 | 0.0 | 0.0 | 0.0 | 0.0 | 0.0 | 0.0 | 0.0 | 0.0 | 0.0 | 0.0 | 0.0 |
| Olfr733 | 0.0 | 0.0 | 0.0 | 0.0 | 0.0 | 0.0 | 0.0 | 0.0 | 0.0 | 0.0 | 0.0 |
| Olfr734 | 0.0 | 0.0 | 0.0 | 0.0 | 0.0 | 0.0 | 0.0 | 0.0 | 0.0 | 0.0 | 0.0 |
| Olfr736 | 0.0 | 0.0 | 0.0 | 0.0 | 0.0 | 0.0 | 0.0 | 0.0 | 0.0 | 0.0 | 0.0 |
| Olfr738 | 0.0 | 0.0 | 0.0 | 0.0 | 0.0 | 0.0 | 0.0 | 0.0 | 0.0 | 0.0 | 0.0 |
| Olfr739 | 0.0 | 0.0 | 0.0 | 0.0 | 0.0 | 0.0 | 0.0 | 0.0 | 0.0 | 0.0 | 0.0 |
| Olfr740 | 0.0 | 0.0 | 0.0 | 0.0 | 0.0 | 0.0 | 0.0 | 0.0 | 0.0 | 0.0 | 0.0 |
| Olfr741 | 0.0 | 0.0 | 0.0 | 0.0 | 0.0 | 0.0 | 0.0 | 0.0 | 0.0 | 0.0 | 0.0 |
| Olfr742 | 0.0 | 0.0 | 0.0 | 0.0 | 0.0 | 0.0 | 0.0 | 0.0 | 0.0 | 0.0 | 0.0 |
| Olfr744 | 0.0 | 0.0 | 0.0 | 0.0 | 0.0 | 0.0 | 0.0 | 0.0 | 0.0 | 0.0 | 0.0 |
| Olfr745 | 0.0 | 0.0 | 0.0 | 0.0 | 0.0 | 0.0 | 0.0 | 0.0 | 0.0 | 0.0 | 0.0 |
| Olfr746 | 0.0 | 0.0 | 0.0 | 0.0 | 0.0 | 0.0 | 0.0 | 0.0 | 0.0 | 0.0 | 0.0 |
| Olfr747 | 0.0 | 0.0 | 0.0 | 0.0 | 0.0 | 0.0 | 0.0 | 0.0 | 0.0 | 0.0 | 0.0 |
| Olfr748 | 0.0 | 0.0 | 0.0 | 0.0 | 0.0 | 0.0 | 0.0 | 0.0 | 0.0 | 0.0 | 0.0 |
| Olfr749 | 0.0 | 0.0 | 0.0 | 0.0 | 0.0 | 0.0 | 0.0 | 0.0 | 0.0 | 0.0 | 0.0 |
| Olfr75-ps1 | 0.0 | 0.0 | 0.0 | 0.0 | 0.0 | 0.0 | 0.0 | 0.0 | 0.0 | 0.0 | 0.0 |
| Olfr76 | 0.0 | 0.0 | 0.0 | 0.0 | 0.0 | 0.0 | 0.0 | 0.0 | 0.0 | 0.0 | 0.0 |
| Olfr761 | 0.0 | 0.0 | 0.0 | 0.0 | 0.0 | 0.0 | 0.0 | 0.0 | 0.0 | 0.0 | 0.0 |
| Olfr765 | 0.0 | 0.0 | 0.0 | 0.0 | 0.0 | 0.0 | 0.0 | 0.0 | 0.0 | 0.0 | 0.0 |
| Olfr767 | 0.0 | 0.0 | 0.0 | 0.0 | 0.0 | 0.0 | 0.0 | 0.0 | 0.0 | 0.0 | 0.0 |
| Olfr768 | 0.0 | 0.0 | 0.0 | 0.0 | 0.0 | 0.0 | 0.0 | 0.0 | 0.0 | 0.0 | 0.0 |
| Olfr769 | 0.0 | 0.0 | 0.0 | 0.0 | 0.0 | 0.0 | 0.0 | 0.0 | 0.0 | 0.0 | 0.0 |
| Olfr77 | 0.0 | 0.0 | 0.0 | 0.0 | 0.0 | 0.0 | 0.0 | 0.0 | 0.0 | 0.0 | 0.0 |
| Olfr770 | 0.0 | 0.0 | 0.0 | 0.0 | 0.0 | 0.0 | 0.0 | 0.0 | 0.0 | 0.0 | 0.0 |
| Olfr771 | 0.0 | 0.0 | 0.0 | 0.0 | 0.0 | 0.0 | 0.0 | 0.0 | 0.0 | 0.0 | 0.0 |
| Olfr772 | 0.0 | 0.0 | 0.0 | 0.0 | 0.0 | 0.0 | 0.0 | 0.0 | 0.0 | 0.0 | 0.0 |
| Olfr773 | 0.0 | 0.0 | 0.0 | 0.0 | 0.0 | 0.0 | 0.0 | 0.0 | 0.0 | 0.0 | 0.0 |
| Olfr774 | 0.0 | 0.0 | 0.0 | 0.0 | 0.0 | 0.0 | 0.0 | 0.0 | 0.0 | 0.0 | 0.0 |
| Olfr775 | 0.0 | 0.0 | 0.0 | 0.0 | 0.0 | 0.0 | 0.0 | 0.0 | 0.0 | 0.0 | 0.0 |
| Olfr776 | 0.0 | 0.0 | 0.0 | 0.0 | 0.0 | 0.0 | 0.0 | 0.0 | 0.0 | 0.0 | 0.0 |
| Olfr777 | 0.0 | 0.0 | 0.0 | 0.0 | 0.0 | 0.0 | 0.0 | 0.0 | 0.0 | 0.0 | 0.0 |
| Olfr780 | 0.0 | 0.0 | 0.0 | 0.0 | 0.0 | 0.0 | 0.0 | 0.0 | 0.0 | 0.0 | 0.0 |
| Olfr781 | 0.0 | 0.0 | 0.0 | 0.0 | 0.0 | 0.0 | 0.0 | 0.0 | 0.0 | 0.0 | 0.0 |
| Olfr782 | 0.0 | 0.0 | 0.0 | 0.0 | 0.0 | 0.0 | 0.0 | 0.0 | 0.0 | 0.0 | 0.0 |
| Olfr784 | 0.0 | 0.0 | 0.0 | 0.0 | 0.0 | 0.0 | 0.0 | 0.0 | 0.0 | 0.0 | 0.0 |
| Olfr786 | 0.0 | 0.0 | 0.0 | 0.0 | 0.0 | 0.0 | 0.0 | 0.0 | 0.0 | 0.0 | 0.0 |
| Olfr787 | 0.0 | 0.0 | 0.0 | 0.0 | 0.0 | 0.0 | 0.0 | 0.0 | 0.0 | 0.0 | 0.0 |
| Olfr788 | 0.0 | 0.0 | 0.0 | 0.0 | 0.0 | 0.0 | 0.0 | 0.0 | 0.0 | 0.0 | 0.0 |
| Olfr791 | 0.0 | 0.0 | 0.0 | 0.0 | 0.0 | 0.0 | 0.0 | 0.0 | 0.0 | 0.0 | 0.0 |
| Olfr792 | 0.0 | 0.0 | 0.0 | 0.0 | 0.0 | 0.0 | 0.0 | 0.0 | 0.0 | 0.0 | 0.0 |
| Olfr794 | 0.0 | 0.0 | 0.0 | 0.0 | 0.0 | 0.0 | 0.0 | 0.0 | 0.0 | 0.0 | 0.0 |
| Olfr796 | 0.0 | 0.0 | 0.0 | 0.0 | 0.0 | 0.0 | 0.0 | 0.0 | 0.0 | 0.0 | 0.0 |
| Olfr798 | 0.0 | 0.0 | 0.0 | 0.0 | 0.0 | 0.0 | 0.0 | 0.0 | 0.0 | 0.0 | 0.0 |
| Olfr799 | 0.0 | 0.0 | 0.0 | 0.0 | 0.0 | 0.0 | 0.0 | 0.0 | 0.0 | 0.0 | 0.0 |
| Olfr800 | 0.0 | 0.0 | 0.0 | 0.0 | 0.0 | 0.0 | 0.0 | 0.0 | 0.0 | 0.0 | 0.0 |
| Olfr801 | 0.0 | 0.0 | 0.0 | 0.0 | 0.0 | 0.0 | 0.0 | 0.0 | 0.0 | 0.0 | 0.0 |
| Olfr802 | 0.0 | 0.0 | 0.0 | 0.0 | 0.0 | 0.0 | 0.0 | 0.0 | 0.0 | 0.0 | 0.0 |
| Olfr803 | 0.0 | 0.0 | 0.0 | 0.0 | 0.0 | 0.0 | 0.0 | 0.0 | 0.0 | 0.0 | 0.0 |
| Olfr804 | 0.0 | 0.0 | 0.0 | 0.0 | 0.0 | 0.0 | 0.0 | 0.0 | 0.0 | 0.0 | 0.0 |
| Olfr805 | 0.0 | 0.0 | 0.0 | 0.0 | 0.0 | 0.0 | 0.0 | 0.0 | 0.0 | 0.0 | 0.0 |
| Olfr806 | 0.0 | 0.0 | 0.0 | 0.0 | 0.0 | 0.0 | 0.0 | 0.0 | 0.0 | 0.0 | 0.0 |
| Olfr807 | 0.0 | 0.0 | 0.0 | 0.0 | 0.0 | 0.0 | 0.0 | 0.0 | 0.0 | 0.0 | 0.0 |
| Olfr808 | 0.0 | 0.0 | 0.0 | 0.0 | 0.0 | 0.0 | 0.0 | 0.0 | 0.0 | 0.0 | 0.0 |

| | | | | | | | | | | | | |
|---|---|---|---|---|---|---|---|---|---|---|---|---|
| Olfr809 | 0.0 | 0.0 | 0.0 | 0.0 | 0.0 | 0.0 | 0.0 | 0.0 | 0.0 | 0.0 | 0.0 | 0.0 |
| Olfr810 | 0.0 | 0.0 | 0.0 | 0.0 | 0.0 | 0.0 | 0.0 | 0.0 | 0.0 | 0.0 | 0.0 | 0.0 |
| Olfr812 | 0.0 | 0.0 | 0.0 | 0.0 | 0.0 | 0.0 | 0.0 | 0.0 | 0.0 | 0.0 | 0.0 | 0.0 |
| Olfr813 | 0.0 | 0.0 | 0.0 | 0.0 | 0.0 | 0.0 | 0.0 | 0.0 | 0.0 | 0.0 | 0.0 | 0.0 |
| Olfr814 | 0.0 | 0.0 | 0.0 | 0.0 | 0.0 | 0.0 | 0.0 | 0.0 | 0.0 | 0.0 | 0.0 | 0.0 |
| Olfr815 | 0.0 | 0.0 | 0.0 | 0.0 | 0.0 | 0.0 | 0.0 | 0.0 | 0.0 | 0.0 | 0.0 | 0.0 |
| Olfr816 | 0.0 | 0.0 | 0.0 | 0.0 | 0.0 | 0.0 | 0.0 | 0.0 | 0.0 | 0.0 | 0.0 | 0.0 |
| Olfr818 | 0.0 | 0.0 | 0.0 | 0.0 | 0.0 | 0.0 | 0.0 | 0.0 | 0.0 | 0.0 | 0.0 | 0.0 |
| Olfr819 | 0.0 | 0.0 | 0.0 | 0.0 | 0.0 | 0.0 | 0.0 | 0.0 | 0.0 | 0.0 | 0.0 | 0.0 |
| Olfr820 | 0.0 | 0.0 | 0.0 | 0.0 | 0.0 | 0.0 | 0.0 | 0.0 | 0.0 | 0.0 | 0.0 | 0.0 |
| Olfr821 | 0.0 | 0.0 | 0.0 | 0.0 | 0.0 | 0.0 | 0.0 | 0.0 | 0.0 | 0.0 | 0.0 | 0.0 |
| Olfr822 | 0.0 | 0.0 | 0.0 | 0.0 | 0.0 | 0.0 | 0.0 | 0.0 | 0.0 | 0.0 | 0.0 | 0.0 |
| Olfr823 | 0.0 | 0.0 | 0.0 | 0.0 | 0.0 | 0.0 | 0.0 | 0.0 | 0.0 | 0.0 | 0.0 | 0.0 |
| Olfr824 | 0.0 | 0.0 | 0.0 | 0.0 | 0.0 | 0.0 | 0.0 | 0.0 | 0.0 | 0.0 | 0.0 | 0.0 |
| Olfr825 | 0.0 | 0.0 | 0.0 | 0.0 | 0.0 | 0.0 | 0.0 | 0.0 | 0.0 | 0.0 | 0.0 | 0.0 |
| Olfr826 | 0.0 | 0.0 | 0.0 | 0.0 | 0.0 | 0.0 | 0.0 | 0.0 | 0.0 | 0.0 | 0.0 | 0.0 |
| Olfr828 | 0.0 | 0.0 | 0.0 | 0.0 | 0.0 | 0.0 | 0.0 | 0.0 | 0.0 | 0.0 | 0.0 | 0.0 |
| Olfr829 | 0.0 | 0.0 | 0.0 | 0.0 | 0.0 | 0.0 | 0.0 | 0.0 | 0.0 | 0.0 | 0.0 | 0.0 |
| Olfr830 | 0.0 | 0.0 | 0.0 | 0.0 | 0.0 | 0.0 | 0.0 | 0.0 | 0.0 | 0.0 | 0.0 | 0.0 |
| Olfr832 | 0.0 | 0.0 | 0.0 | 0.0 | 0.0 | 0.0 | 0.0 | 0.0 | 0.0 | 0.0 | 0.0 | 0.0 |
| Olfr834 | 0.0 | 0.0 | 0.0 | 0.0 | 0.0 | 0.0 | 0.0 | 0.0 | 0.0 | 0.0 | 0.0 | 0.0 |
| Olfr835 | 0.0 | 0.0 | 0.0 | 0.0 | 0.0 | 0.0 | 0.0 | 0.0 | 0.0 | 0.0 | 0.0 | 0.0 |
| Olfr836 | 0.0 | 0.0 | 0.0 | 0.0 | 0.0 | 0.0 | 0.0 | 0.0 | 0.0 | 0.0 | 0.0 | 0.0 |
| Olfr837 | 0.0 | 0.0 | 0.0 | 0.0 | 0.0 | 0.0 | 0.0 | 0.0 | 0.0 | 0.0 | 0.0 | 0.0 |
| Olfr843 | 0.0 | 0.0 | 0.0 | 0.0 | 0.0 | 0.0 | 0.0 | 0.0 | 0.0 | 0.0 | 0.0 | 0.0 |
| Olfr845 | 0.0 | 0.0 | 0.0 | 0.0 | 0.0 | 0.0 | 0.0 | 0.0 | 0.0 | 0.0 | 0.0 | 0.0 |
| Olfr846 | 0.0 | 0.0 | 0.0 | 0.0 | 0.0 | 0.0 | 0.0 | 0.0 | 0.0 | 0.0 | 0.0 | 0.0 |
| Olfr849 | 0.0 | 0.0 | 0.0 | 0.0 | 0.0 | 0.0 | 0.0 | 0.0 | 0.0 | 0.0 | 0.0 | 0.0 |
| Olfr850 | 0.0 | 0.0 | 0.0 | 0.0 | 0.0 | 0.0 | 0.0 | 0.0 | 0.0 | 0.0 | 0.0 | 0.0 |
| Olfr851 | 0.0 | 0.0 | 0.0 | 0.0 | 0.0 | 0.0 | 0.0 | 0.0 | 0.0 | 0.0 | 0.0 | 0.0 |
| Olfr853 | 0.0 | 0.0 | 0.0 | 0.0 | 0.0 | 0.0 | 0.0 | 0.0 | 0.0 | 0.0 | 0.0 | 0.0 |
| Olfr854 | 0.0 | 0.0 | 0.0 | 0.0 | 0.0 | 0.0 | 0.0 | 0.0 | 0.0 | 0.0 | 0.0 | 0.0 |
| Olfr855 | 0.0 | 0.0 | 0.0 | 0.0 | 0.0 | 0.0 | 0.0 | 0.0 | 0.0 | 0.0 | 0.0 | 0.0 |
| Olfr859 | 0.0 | 0.0 | 0.0 | 0.0 | 0.0 | 0.0 | 0.0 | 0.0 | 0.0 | 0.0 | 0.0 | 0.0 |
| Olfr860 | 0.0 | 0.0 | 0.0 | 0.0 | 0.0 | 0.0 | 0.0 | 0.0 | 0.0 | 0.0 | 0.0 | 0.0 |
| Olfr866 | 0.0 | 0.0 | 0.0 | 0.0 | 0.0 | 0.0 | 0.0 | 0.0 | 0.0 | 0.0 | 0.0 | 0.0 |
| Olfr867 | 0.0 | 0.0 | 0.0 | 0.0 | 0.0 | 0.0 | 0.0 | 0.0 | 0.0 | 0.0 | 0.0 | 0.0 |
| Olfr868 | 0.0 | 0.0 | 0.0 | 0.0 | 0.0 | 0.0 | 0.0 | 0.0 | 0.0 | 0.0 | 0.0 | 0.0 |
| Olfr869 | 0.0 | 0.0 | 0.0 | 0.0 | 0.0 | 0.0 | 0.0 | 0.0 | 0.0 | 0.0 | 0.0 | 0.0 |
| Olfr870 | 0.0 | 0.0 | 0.0 | 0.0 | 0.0 | 0.0 | 0.0 | 0.0 | 0.0 | 0.0 | 0.0 | 0.0 |
| Olfr871 | 0.0 | 0.0 | 0.0 | 0.0 | 0.0 | 0.0 | 0.0 | 0.0 | 0.0 | 0.0 | 0.0 | 0.0 |
| Olfr872 | 0.0 | 0.0 | 0.0 | 0.0 | 0.0 | 0.0 | 0.0 | 0.0 | 0.0 | 0.0 | 0.0 | 0.0 |
| Olfr873 | 0.0 | 0.0 | 0.0 | 0.0 | 0.0 | 0.0 | 0.0 | 0.0 | 0.0 | 0.0 | 0.0 | 0.0 |
| Olfr874 | 0.0 | 0.0 | 0.0 | 0.0 | 0.0 | 0.0 | 0.0 | 0.0 | 0.0 | 0.0 | 0.0 | 0.0 |
| Olfr875 | 0.0 | 0.0 | 0.0 | 0.0 | 0.0 | 0.0 | 0.0 | 0.0 | 0.0 | 0.0 | 0.0 | 0.0 |
| Olfr876 | 0.0 | 0.0 | 0.0 | 0.0 | 0.0 | 0.0 | 0.0 | 0.0 | 0.0 | 0.0 | 0.0 | 0.0 |
| Olfr878 | 0.0 | 0.0 | 0.0 | 0.0 | 0.0 | 0.0 | 0.0 | 0.0 | 0.0 | 0.0 | 0.0 | 0.0 |
| Olfr881 | 0.0 | 0.0 | 0.0 | 0.0 | 0.0 | 0.0 | 0.0 | 0.0 | 0.0 | 0.0 | 0.0 | 0.0 |
| Olfr883 | 0.0 | 0.0 | 0.0 | 0.0 | 0.0 | 0.0 | 0.0 | 0.0 | 0.0 | 0.0 | 0.0 | 0.0 |
| Olfr884 | 0.0 | 0.0 | 0.0 | 0.0 | 0.0 | 0.0 | 0.0 | 0.0 | 0.0 | 0.0 | 0.0 | 0.0 |
| Olfr885 | 0.0 | 0.0 | 0.0 | 0.0 | 0.0 | 0.0 | 0.0 | 0.0 | 0.0 | 0.0 | 0.0 | 0.0 |
| Olfr887 | 0.0 | 0.0 | 0.0 | 0.0 | 0.0 | 0.0 | 0.0 | 0.0 | 0.0 | 0.0 | 0.0 | 0.0 |
| Olfr888 | 0.0 | 0.0 | 0.0 | 0.0 | 0.0 | 0.0 | 0.0 | 0.0 | 0.0 | 0.0 | 0.0 | 0.0 |
| Olfr889 | 0.0 | 0.0 | 0.0 | 0.0 | 0.0 | 0.0 | 0.0 | 0.0 | 0.0 | 0.0 | 0.0 | 0.0 |
| Olfr890 | 0.0 | 0.0 | 0.0 | 0.0 | 0.0 | 0.0 | 0.0 | 0.0 | 0.0 | 0.0 | 0.0 | 0.0 |
| Olfr891 | 0.0 | 0.0 | 0.0 | 0.0 | 0.0 | 0.0 | 0.0 | 0.0 | 0.0 | 0.0 | 0.0 | 0.0 |
| Olfr893 | 0.0 | 0.0 | 0.0 | 0.0 | 0.0 | 0.0 | 0.0 | 0.0 | 0.0 | 0.0 | 0.0 | 0.0 |

Excluded Genes

| | | | | | | | | | | | | |
|---|---|---|---|---|---|---|---|---|---|---|---|---|
| Olfr894 | 0.0 | 0.0 | 0.0 | 0.0 | 0.0 | 0.0 | 0.0 | 0.0 | 0.0 | 0.0 | 0.0 | 0.0 |
| Olfr895 | 0.0 | 0.0 | 0.0 | 0.0 | 0.0 | 0.0 | 0.0 | 0.0 | 0.0 | 0.0 | 0.0 | 0.0 |
| Olfr898 | 0.0 | 0.0 | 0.0 | 0.0 | 0.0 | 0.0 | 0.0 | 0.0 | 0.0 | 0.0 | 0.0 | 0.0 |
| Olfr899 | 0.0 | 0.0 | 0.0 | 0.0 | 0.0 | 0.0 | 0.0 | 0.0 | 0.0 | 0.0 | 0.0 | 0.0 |
| Olfr900 | 0.0 | 0.0 | 0.0 | 0.0 | 0.0 | 0.0 | 0.0 | 0.0 | 0.0 | 0.0 | 0.0 | 0.0 |
| Olfr901 | 0.0 | 0.0 | 0.0 | 0.0 | 0.0 | 0.0 | 0.0 | 0.0 | 0.0 | 0.0 | 0.0 | 0.0 |
| Olfr902 | 0.0 | 0.0 | 0.0 | 0.0 | 0.0 | 0.0 | 0.0 | 0.0 | 0.0 | 0.0 | 0.0 | 0.0 |
| Olfr904 | 0.0 | 0.0 | 0.0 | 0.0 | 0.0 | 0.0 | 0.0 | 0.0 | 0.0 | 0.0 | 0.0 | 0.0 |
| Olfr905 | 0.0 | 0.0 | 0.0 | 0.0 | 0.0 | 0.0 | 0.0 | 0.0 | 0.0 | 0.0 | 0.0 | 0.0 |
| Olfr906 | 0.0 | 0.0 | 0.0 | 0.0 | 0.0 | 0.0 | 0.0 | 0.0 | 0.0 | 0.0 | 0.0 | 0.0 |
| Olfr907 | 0.0 | 0.0 | 0.0 | 0.0 | 0.0 | 0.0 | 0.0 | 0.0 | 0.0 | 0.0 | 0.0 | 0.0 |
| Olfr908 | 0.0 | 0.0 | 0.0 | 0.0 | 0.0 | 0.0 | 0.0 | 0.0 | 0.0 | 0.0 | 0.0 | 0.0 |
| Olfr910 | 0.0 | 0.0 | 0.0 | 0.0 | 0.0 | 0.0 | 0.0 | 0.0 | 0.0 | 0.0 | 0.0 | 0.0 |
| Olfr911-ps1 | 0.0 | 0.0 | 0.0 | 0.0 | 0.0 | 0.0 | 0.0 | 0.0 | 0.0 | 0.0 | 0.0 | 0.0 |
| Olfr912 | 0.0 | 0.0 | 0.0 | 0.0 | 0.0 | 0.0 | 0.0 | 0.0 | 0.0 | 0.0 | 0.0 | 0.0 |
| Olfr913 | 0.0 | 0.0 | 0.0 | 0.0 | 0.0 | 0.0 | 0.0 | 0.0 | 0.0 | 0.0 | 0.0 | 0.0 |
| Olfr914 | 0.0 | 0.0 | 0.0 | 0.0 | 0.0 | 0.0 | 0.0 | 0.0 | 0.0 | 0.0 | 0.0 | 0.0 |
| Olfr915 | 0.0 | 0.0 | 0.0 | 0.0 | 0.0 | 0.0 | 0.0 | 0.0 | 0.0 | 0.0 | 0.0 | 0.0 |
| Olfr916 | 0.0 | 0.0 | 0.0 | 0.0 | 0.0 | 0.0 | 0.0 | 0.0 | 0.0 | 0.0 | 0.0 | 0.0 |
| Olfr917 | 0.0 | 0.0 | 0.0 | 0.0 | 0.0 | 0.0 | 0.0 | 0.0 | 0.0 | 0.0 | 0.0 | 0.0 |
| Olfr918 | 0.0 | 0.0 | 0.0 | 0.0 | 0.0 | 0.0 | 0.0 | 0.0 | 0.0 | 0.0 | 0.0 | 0.0 |
| Olfr919 | 0.0 | 0.0 | 0.0 | 0.0 | 0.0 | 0.0 | 0.0 | 0.0 | 0.0 | 0.0 | 0.0 | 0.0 |
| Olfr92 | 0.0 | 0.0 | 0.0 | 0.0 | 0.0 | 0.0 | 0.0 | 0.0 | 0.0 | 0.0 | 0.0 | 0.0 |
| Olfr921 | 0.0 | 0.0 | 0.0 | 0.0 | 0.0 | 0.0 | 0.0 | 0.0 | 0.0 | 0.0 | 0.0 | 0.0 |
| Olfr924 | 0.0 | 0.0 | 0.0 | 0.0 | 0.0 | 0.0 | 0.0 | 0.0 | 0.0 | 0.0 | 0.0 | 0.0 |
| Olfr926 | 0.0 | 0.0 | 0.0 | 0.0 | 0.0 | 0.0 | 0.0 | 0.0 | 0.0 | 0.0 | 0.0 | 0.0 |
| Olfr930 | 0.0 | 0.0 | 0.0 | 0.0 | 0.0 | 0.0 | 0.0 | 0.0 | 0.0 | 0.0 | 0.0 | 0.0 |
| Olfr933 | 0.0 | 0.0 | 0.0 | 0.0 | 0.0 | 0.0 | 0.0 | 0.0 | 0.0 | 0.0 | 0.0 | 0.0 |
| Olfr934 | 0.0 | 0.0 | 0.0 | 0.0 | 0.0 | 0.0 | 0.0 | 0.0 | 0.0 | 0.0 | 0.0 | 0.0 |
| Olfr935 | 0.0 | 0.0 | 0.0 | 0.0 | 0.0 | 0.0 | 0.0 | 0.0 | 0.0 | 0.0 | 0.0 | 0.0 |
| Olfr936 | 0.0 | 0.0 | 0.0 | 0.0 | 0.0 | 0.0 | 0.0 | 0.0 | 0.0 | 0.0 | 0.0 | 0.0 |
| Olfr937 | 0.0 | 0.0 | 0.0 | 0.0 | 0.0 | 0.0 | 0.0 | 0.0 | 0.0 | 0.0 | 0.0 | 0.0 |
| Olfr938 | 0.0 | 0.0 | 0.0 | 0.0 | 0.0 | 0.0 | 0.0 | 0.0 | 0.0 | 0.0 | 0.0 | 0.0 |
| Olfr94 | 0.0 | 0.0 | 0.0 | 0.0 | 0.0 | 0.0 | 0.0 | 0.0 | 0.0 | 0.0 | 0.0 | 0.0 |
| Olfr944 | 0.0 | 0.0 | 0.0 | 0.0 | 0.0 | 0.0 | 0.0 | 0.0 | 0.0 | 0.0 | 0.0 | 0.0 |
| Olfr945 | 0.0 | 0.0 | 0.0 | 0.0 | 0.0 | 0.0 | 0.0 | 0.0 | 0.0 | 0.0 | 0.0 | 0.0 |
| Olfr947-ps1 | 0.0 | 0.0 | 0.0 | 0.0 | 0.0 | 0.0 | 0.0 | 0.0 | 0.0 | 0.0 | 0.0 | 0.0 |
| Olfr948 | 0.0 | 0.0 | 0.0 | 0.0 | 0.0 | 0.0 | 0.0 | 0.0 | 0.0 | 0.0 | 0.0 | 0.0 |
| Olfr95 | 0.0 | 0.0 | 0.0 | 0.0 | 0.0 | 0.0 | 0.0 | 0.0 | 0.0 | 0.0 | 0.0 | 0.0 |
| Olfr951 | 0.0 | 0.0 | 0.0 | 0.0 | 0.0 | 0.0 | 0.0 | 0.0 | 0.0 | 0.0 | 0.0 | 0.0 |
| Olfr952 | 0.0 | 0.0 | 0.0 | 0.0 | 0.0 | 0.0 | 0.0 | 0.0 | 0.0 | 0.0 | 0.0 | 0.0 |
| Olfr954 | 0.0 | 0.0 | 0.0 | 0.0 | 0.0 | 0.0 | 0.0 | 0.0 | 0.0 | 0.0 | 0.0 | 0.0 |
| Olfr955 | 0.0 | 0.0 | 0.0 | 0.0 | 0.0 | 0.0 | 0.0 | 0.0 | 0.0 | 0.0 | 0.0 | 0.0 |
| Olfr957 | 0.0 | 0.0 | 0.0 | 0.0 | 0.0 | 0.0 | 0.0 | 0.0 | 0.0 | 0.0 | 0.0 | 0.0 |
| Olfr958 | 0.0 | 0.0 | 0.0 | 0.0 | 0.0 | 0.0 | 0.0 | 0.0 | 0.0 | 0.0 | 0.0 | 0.0 |
| Olfr959 | 0.0 | 0.0 | 0.0 | 0.0 | 0.0 | 0.0 | 0.0 | 0.0 | 0.0 | 0.0 | 0.0 | 0.0 |
| Olfr96 | 0.0 | 0.0 | 0.0 | 0.0 | 0.0 | 0.0 | 0.0 | 0.0 | 0.0 | 0.0 | 0.0 | 0.0 |
| Olfr960 | 0.0 | 0.0 | 0.0 | 0.0 | 0.0 | 0.0 | 0.0 | 0.0 | 0.0 | 0.0 | 0.0 | 0.0 |
| Olfr961 | 0.0 | 0.0 | 0.0 | 0.0 | 0.0 | 0.0 | 0.0 | 0.0 | 0.0 | 0.0 | 0.0 | 0.0 |
| Olfr963 | 0.0 | 0.0 | 0.0 | 0.0 | 0.0 | 0.0 | 0.0 | 0.0 | 0.0 | 0.0 | 0.0 | 0.0 |
| Olfr965 | 0.0 | 0.0 | 0.0 | 0.0 | 0.0 | 0.0 | 0.0 | 0.0 | 0.0 | 0.0 | 0.0 | 0.0 |
| Olfr967 | 0.0 | 0.0 | 0.0 | 0.0 | 0.0 | 0.0 | 0.0 | 0.0 | 0.0 | 0.0 | 0.0 | 0.0 |
| Olfr968 | 0.0 | 0.0 | 0.0 | 0.0 | 0.0 | 0.0 | 0.0 | 0.0 | 0.0 | 0.0 | 0.0 | 0.0 |
| Olfr969 | 0.0 | 0.0 | 0.0 | 0.0 | 0.0 | 0.0 | 0.0 | 0.0 | 0.0 | 0.0 | 0.0 | 0.0 |
| Olfr970 | 0.0 | 0.0 | 0.0 | 0.0 | 0.0 | 0.0 | 0.0 | 0.0 | 0.0 | 0.0 | 0.0 | 0.0 |
| Olfr972 | 0.0 | 0.0 | 0.0 | 0.0 | 0.0 | 0.0 | 0.0 | 0.0 | 0.0 | 0.0 | 0.0 | 0.0 |
| Olfr974 | 0.0 | 0.0 | 0.0 | 0.0 | 0.0 | 0.0 | 0.0 | 0.0 | 0.0 | 0.0 | 0.0 | 0.0 |

Excluded Genes

| | | | | | | | | | | | |
|---|---|---|---|---|---|---|---|---|---|---|---|
| Olfr975 | 0.0 | 0.0 | 0.0 | 0.0 | 0.0 | 0.0 | 0.0 | 0.0 | 0.0 | 0.0 | 0.0 |
| Olfr976 | 0.0 | 0.0 | 0.0 | 0.0 | 0.0 | 0.0 | 0.0 | 0.0 | 0.0 | 0.0 | 0.0 |
| Olfr978 | 0.0 | 0.0 | 0.0 | 0.0 | 0.0 | 0.0 | 0.0 | 0.0 | 0.0 | 0.0 | 0.0 |
| Olfr979 | 0.0 | 0.0 | 0.0 | 0.0 | 0.0 | 0.0 | 0.0 | 0.0 | 0.0 | 0.0 | 0.0 |
| Olfr980 | 0.0 | 0.0 | 0.0 | 0.0 | 0.0 | 0.0 | 0.0 | 0.0 | 0.0 | 0.0 | 0.0 |
| Olfr981 | 0.0 | 0.0 | 0.0 | 0.0 | 0.0 | 0.0 | 0.0 | 0.0 | 0.0 | 0.0 | 0.0 |
| Olfr983 | 0.0 | 0.0 | 0.0 | 0.0 | 0.0 | 0.0 | 0.0 | 0.0 | 0.0 | 0.0 | 0.0 |
| Olfr984 | 0.0 | 0.0 | 0.0 | 0.0 | 0.0 | 0.0 | 0.0 | 0.0 | 0.0 | 0.0 | 0.0 |
| Olfr985 | 0.0 | 0.0 | 0.0 | 0.0 | 0.0 | 0.0 | 0.0 | 0.0 | 0.0 | 0.0 | 0.0 |
| Olfr986 | 0.0 | 0.0 | 0.0 | 0.0 | 0.0 | 0.0 | 0.0 | 0.0 | 0.0 | 0.0 | 0.0 |
| Olfr987 | 0.0 | 0.0 | 0.0 | 0.0 | 0.0 | 0.0 | 0.0 | 0.0 | 0.0 | 0.0 | 0.0 |
| Olfr992 | 0.0 | 0.0 | 0.0 | 0.0 | 0.0 | 0.0 | 0.0 | 0.0 | 0.0 | 0.0 | 0.0 |
| Olfr993 | 0.0 | 0.0 | 0.0 | 0.0 | 0.0 | 0.0 | 0.0 | 0.0 | 0.0 | 0.0 | 0.0 |
| Olfr995 | 0.0 | 0.0 | 0.0 | 0.0 | 0.0 | 0.0 | 0.0 | 0.0 | 0.0 | 0.0 | 0.0 |
| Olfr996 | 0.0 | 0.0 | 0.0 | 0.0 | 0.0 | 0.0 | 0.0 | 0.0 | 0.0 | 0.0 | 0.0 |
| Olfr998 | 0.0 | 0.0 | 0.0 | 0.0 | 0.0 | 0.0 | 0.0 | 0.0 | 0.0 | 0.0 | 0.0 |
| Olig1 | 0.0 | 0.0 | 0.0 | 0.0 | 0.0 | 0.0 | 0.0 | 0.0 | 0.0 | 0.0 | 0.0 |
| Omt2a | 0.0 | 0.0 | 0.0 | 0.0 | 0.0 | 0.0 | 0.0 | 0.0 | 0.0 | 0.0 | 0.0 |
| Omt2b | 0.0 | 0.0 | 0.0 | 0.0 | 0.0 | 0.0 | 0.0 | 0.0 | 0.0 | 0.0 | 0.0 |
| Ooep | 0.0 | 0.0 | 0.0 | 0.0 | 0.0 | 0.0 | 0.0 | 0.0 | 0.0 | 0.0 | 0.0 |
| Oog4 | 0.0 | 0.0 | 0.0 | 0.0 | 0.0 | 0.0 | 0.0 | 0.0 | 0.0 | 0.0 | 0.0 |
| Otol1 | 0.0 | 0.0 | 0.0 | 0.0 | 0.0 | 0.0 | 0.0 | 0.0 | 0.0 | 0.0 | 0.0 |
| Otop3 | 0.0 | 0.0 | 0.0 | 0.0 | 0.0 | 0.0 | 0.0 | 0.0 | 0.0 | 0.0 | 0.0 |
| Otor | 0.0 | 0.0 | 0.0 | 0.0 | 0.0 | 0.0 | 0.0 | 0.0 | 0.0 | 0.0 | 0.0 |
| Otp | 0.0 | 0.0 | 0.0 | 0.0 | 0.0 | 0.0 | 0.0 | 0.0 | 0.0 | 0.0 | 0.0 |
| Otx2 | 0.0 | 0.0 | 0.0 | 0.0 | 0.0 | 0.0 | 0.0 | 0.0 | 0.0 | 0.0 | 0.0 |
| Otx2os1 | 0.0 | 0.0 | 0.0 | 0.0 | 0.0 | 0.0 | 0.0 | 0.0 | 0.0 | 0.0 | 0.0 |
| Oxt | 0.0 | 0.0 | 0.0 | 0.0 | 0.0 | 0.0 | 0.0 | 0.0 | 0.0 | 0.0 | 0.0 |
| Pbsn | 0.0 | 0.0 | 0.0 | 0.0 | 0.0 | 0.0 | 0.0 | 0.0 | 0.0 | 0.0 | 0.0 |
| Pck1 | 0.0 | 0.0 | 0.0 | 0.0 | 0.0 | 0.0 | 0.0 | 0.0 | 0.0 | 0.0 | 0.0 |
| Pcp4 | 0.0 | 0.0 | 0.0 | 0.0 | 0.0 | 0.0 | 0.0 | 0.0 | 0.0 | 0.0 | 0.0 |
| Pdc | 0.0 | 0.0 | 0.0 | 0.0 | 0.0 | 0.0 | 0.0 | 0.0 | 0.0 | 0.0 | 0.0 |
| Pdha2 | 0.0 | 0.0 | 0.0 | 0.0 | 0.0 | 0.0 | 0.0 | 0.0 | 0.0 | 0.0 | 0.0 |
| Pdilt | 0.0 | 0.0 | 0.0 | 0.0 | 0.0 | 0.0 | 0.0 | 0.0 | 0.0 | 0.0 | 0.0 |
| Pdx1 | 0.0 | 0.0 | 0.0 | 0.0 | 0.0 | 0.0 | 0.0 | 0.0 | 0.0 | 0.0 | 0.0 |
| Pfn3 | 0.0 | 0.0 | 0.0 | 0.0 | 0.0 | 0.0 | 0.0 | 0.0 | 0.0 | 0.0 | 0.0 |
| Pgc | 0.0 | 0.0 | 0.0 | 0.0 | 0.0 | 0.0 | 0.0 | 0.0 | 0.0 | 0.0 | 0.0 |
| Pgk2 | 0.0 | 0.0 | 0.0 | 0.0 | 0.0 | 0.0 | 0.0 | 0.0 | 0.0 | 0.0 | 0.0 |
| Pgr15l | 0.0 | 0.0 | 0.0 | 0.0 | 0.0 | 0.0 | 0.0 | 0.0 | 0.0 | 0.0 | 0.0 |
| Phox2b | 0.0 | 0.0 | 0.0 | 0.0 | 0.0 | 0.0 | 0.0 | 0.0 | 0.0 | 0.0 | 0.0 |
| Pifo | 0.0 | 0.0 | 0.0 | 0.0 | 0.0 | 0.0 | 0.0 | 0.0 | 0.0 | 0.0 | 0.0 |
| Pih1d3 | 0.0 | 0.0 | 0.0 | 0.0 | 0.0 | 0.0 | 0.0 | 0.0 | 0.0 | 0.0 | 0.0 |
| Pinc | 0.0 | 0.0 | 0.0 | 0.0 | 0.0 | 0.0 | 0.0 | 0.0 | 0.0 | 0.0 | 0.0 |
| Pip | 0.0 | 0.0 | 0.0 | 0.0 | 0.0 | 0.0 | 0.0 | 0.0 | 0.0 | 0.0 | 0.0 |
| Pisd-ps3 | 0.0 | 0.0 | 0.0 | 0.0 | 0.0 | 0.0 | 0.0 | 0.0 | 0.0 | 0.0 | 0.0 |
| Pla2g2a | 0.0 | 0.0 | 0.0 | 0.0 | 0.0 | 0.0 | 0.0 | 0.0 | 0.0 | 0.0 | 0.0 |
| Pla2g2f | 0.0 | 0.0 | 0.0 | 0.0 | 0.0 | 0.0 | 0.0 | 0.0 | 0.0 | 0.0 | 0.0 |
| Pla2g4d | 0.0 | 0.0 | 0.0 | 0.0 | 0.0 | 0.0 | 0.0 | 0.0 | 0.0 | 0.0 | 0.0 |
| Plac1l | 0.0 | 0.0 | 0.0 | 0.0 | 0.0 | 0.0 | 0.0 | 0.0 | 0.0 | 0.0 | 0.0 |
| Pldi | 0.0 | 0.0 | 0.0 | 0.0 | 0.0 | 0.0 | 0.0 | 0.0 | 0.0 | 0.0 | 0.0 |
| Plg | 0.0 | 0.0 | 0.0 | 0.0 | 0.0 | 0.0 | 0.0 | 0.0 | 0.0 | 0.0 | 0.0 |
| Pnpla5 | 0.0 | 0.0 | 0.0 | 0.0 | 0.0 | 0.0 | 0.0 | 0.0 | 0.0 | 0.0 | 0.0 |
| Pom121l12 | 0.0 | 0.0 | 0.0 | 0.0 | 0.0 | 0.0 | 0.0 | 0.0 | 0.0 | 0.0 | 0.0 |
| Poteg | 0.0 | 0.0 | 0.0 | 0.0 | 0.0 | 0.0 | 0.0 | 0.0 | 0.0 | 0.0 | 0.0 |
| Pou1f1 | 0.0 | 0.0 | 0.0 | 0.0 | 0.0 | 0.0 | 0.0 | 0.0 | 0.0 | 0.0 | 0.0 |
| Pou4f2 | 0.0 | 0.0 | 0.0 | 0.0 | 0.0 | 0.0 | 0.0 | 0.0 | 0.0 | 0.0 | 0.0 |
| Ppp1r14d | 0.0 | 0.0 | 0.0 | 0.0 | 0.0 | 0.0 | 0.0 | 0.0 | 0.0 | 0.0 | 0.0 |

Excluded Genes

| | | | | | | | | | | | | |
|---|---|---|---|---|---|---|---|---|---|---|---|---|
| Ppp1r2-ps7 | 0.0 | 0.0 | 0.0 | 0.0 | 0.0 | 0.0 | 0.0 | 0.0 | 0.0 | 0.0 | 0.0 | 0.0 |
| Ppp1r2-ps9 | 0.0 | 0.0 | 0.0 | 0.0 | 0.0 | 0.0 | 0.0 | 0.0 | 0.0 | 0.0 | 0.0 | 0.0 |
| Ppp3r2 | 0.0 | 0.0 | 0.0 | 0.0 | 0.0 | 0.0 | 0.0 | 0.0 | 0.0 | 0.0 | 0.0 | 0.0 |
| Ppy | 0.0 | 0.0 | 0.0 | 0.0 | 0.0 | 0.0 | 0.0 | 0.0 | 0.0 | 0.0 | 0.0 | 0.0 |
| Prame | 0.0 | 0.0 | 0.0 | 0.0 | 0.0 | 0.0 | 0.0 | 0.0 | 0.0 | 0.0 | 0.0 | 0.0 |
| Pramef6 | 0.0 | 0.0 | 0.0 | 0.0 | 0.0 | 0.0 | 0.0 | 0.0 | 0.0 | 0.0 | 0.0 | 0.0 |
| Pramel1 | 0.0 | 0.0 | 0.0 | 0.0 | 0.0 | 0.0 | 0.0 | 0.0 | 0.0 | 0.0 | 0.0 | 0.0 |
| Pramel4 | 0.0 | 0.0 | 0.0 | 0.0 | 0.0 | 0.0 | 0.0 | 0.0 | 0.0 | 0.0 | 0.0 | 0.0 |
| Pramel5 | 0.0 | 0.0 | 0.0 | 0.0 | 0.0 | 0.0 | 0.0 | 0.0 | 0.0 | 0.0 | 0.0 | 0.0 |
| Pramel6 | 0.0 | 0.0 | 0.0 | 0.0 | 0.0 | 0.0 | 0.0 | 0.0 | 0.0 | 0.0 | 0.0 | 0.0 |
| Pramel7 | 0.0 | 0.0 | 0.0 | 0.0 | 0.0 | 0.0 | 0.0 | 0.0 | 0.0 | 0.0 | 0.0 | 0.0 |
| Prb1 | 0.0 | 0.0 | 0.0 | 0.0 | 0.0 | 0.0 | 0.0 | 0.0 | 0.0 | 0.0 | 0.0 | 0.0 |
| Prg3 | 0.0 | 0.0 | 0.0 | 0.0 | 0.0 | 0.0 | 0.0 | 0.0 | 0.0 | 0.0 | 0.0 | 0.0 |
| Prh1 | 0.0 | 0.0 | 0.0 | 0.0 | 0.0 | 0.0 | 0.0 | 0.0 | 0.0 | 0.0 | 0.0 | 0.0 |
| Prhoxnb | 0.0 | 0.0 | 0.0 | 0.0 | 0.0 | 0.0 | 0.0 | 0.0 | 0.0 | 0.0 | 0.0 | 0.0 |
| Prl3b1 | 0.0 | 0.0 | 0.0 | 0.0 | 0.0 | 0.0 | 0.0 | 0.0 | 0.0 | 0.0 | 0.0 | 0.0 |
| Prl3c1 | 0.0 | 0.0 | 0.0 | 0.0 | 0.0 | 0.0 | 0.0 | 0.0 | 0.0 | 0.0 | 0.0 | 0.0 |
| Prl5a1 | 0.0 | 0.0 | 0.0 | 0.0 | 0.0 | 0.0 | 0.0 | 0.0 | 0.0 | 0.0 | 0.0 | 0.0 |
| Prl7c1 | 0.0 | 0.0 | 0.0 | 0.0 | 0.0 | 0.0 | 0.0 | 0.0 | 0.0 | 0.0 | 0.0 | 0.0 |
| Prl8a1 | 0.0 | 0.0 | 0.0 | 0.0 | 0.0 | 0.0 | 0.0 | 0.0 | 0.0 | 0.0 | 0.0 | 0.0 |
| Prl8a6 | 0.0 | 0.0 | 0.0 | 0.0 | 0.0 | 0.0 | 0.0 | 0.0 | 0.0 | 0.0 | 0.0 | 0.0 |
| Prl8a8 | 0.0 | 0.0 | 0.0 | 0.0 | 0.0 | 0.0 | 0.0 | 0.0 | 0.0 | 0.0 | 0.0 | 0.0 |
| Prm3 | 0.0 | 0.0 | 0.0 | 0.0 | 0.0 | 0.0 | 0.0 | 0.0 | 0.0 | 0.0 | 0.0 | 0.0 |
| Prodh2 | 0.0 | 0.0 | 0.0 | 0.0 | 0.0 | 0.0 | 0.0 | 0.0 | 0.0 | 0.0 | 0.0 | 0.0 |
| Prok1 | 0.0 | 0.0 | 0.0 | 0.0 | 0.0 | 0.0 | 0.0 | 0.0 | 0.0 | 0.0 | 0.0 | 0.0 |
| Prol1 | 0.0 | 0.0 | 0.0 | 0.0 | 0.0 | 0.0 | 0.0 | 0.0 | 0.0 | 0.0 | 0.0 | 0.0 |
| Prop1 | 0.0 | 0.0 | 0.0 | 0.0 | 0.0 | 0.0 | 0.0 | 0.0 | 0.0 | 0.0 | 0.0 | 0.0 |
| Prpmp5 | 0.0 | 0.0 | 0.0 | 0.0 | 0.0 | 0.0 | 0.0 | 0.0 | 0.0 | 0.0 | 0.0 | 0.0 |
| Prr23a | 0.0 | 0.0 | 0.0 | 0.0 | 0.0 | 0.0 | 0.0 | 0.0 | 0.0 | 0.0 | 0.0 | 0.0 |
| Prss1 | 0.0 | 0.0 | 0.0 | 0.0 | 0.0 | 0.0 | 0.0 | 0.0 | 0.0 | 0.0 | 0.0 | 0.0 |
| Prss21 | 0.0 | 0.0 | 0.0 | 0.0 | 0.0 | 0.0 | 0.0 | 0.0 | 0.0 | 0.0 | 0.0 | 0.0 |
| Prss28 | 0.0 | 0.0 | 0.0 | 0.0 | 0.0 | 0.0 | 0.0 | 0.0 | 0.0 | 0.0 | 0.0 | 0.0 |
| Prss29 | 0.0 | 0.0 | 0.0 | 0.0 | 0.0 | 0.0 | 0.0 | 0.0 | 0.0 | 0.0 | 0.0 | 0.0 |
| Prss3 | 0.0 | 0.0 | 0.0 | 0.0 | 0.0 | 0.0 | 0.0 | 0.0 | 0.0 | 0.0 | 0.0 | 0.0 |
| Prss40 | 0.0 | 0.0 | 0.0 | 0.0 | 0.0 | 0.0 | 0.0 | 0.0 | 0.0 | 0.0 | 0.0 | 0.0 |
[truncated: 44,365 more chars]
