# Supplementary material for: Fibroblast polarization over the myocardial infarction time continuum shifts roles from inflammation to angiogenesis
Source: Basic Res Cardiol. 2019 Jan 11;114(2):6. doi: 10.1007/s00395-019-0715-4 (PMC6329742; doi:10.1007/s00395-019-0715-4)
Supplement: Supplementary file 2 — Supplementary material 2 (PDF 13 kb) [file 395_2019_715_MOESM2_ESM.pdf]

Online Table 2. Cell Surface Markers Across the MI Time Course (Top 25 ranked by fold-change and p value)

**No change across MI**	**Selective for MI Day 1**	**Selective for MI Day 3**	**Selective for MI Day 7**
App	*Abcc1*	*Clca1*	*Acvr1*
Axl	*Abcc4*	*Clca2*	*Acvr2a*
Bcap31	*Adrb2*	*Epha4*	*Cd200*
Bsg	*Ager*	*Ephb4*	*Cd44*
Bst2	*B3gnt3*	*Erbb2*	*Dlg3*
Caprin1	*Cav2*	*Fzd5*	*Efnb1*
Cav1	*Cd40*	*Gp1bb*	*Ephb2*
Cd151	*Eng*	*Ldlr*	*Ereg*
Cd63	*Gpr137b*	*Mfsd2a*	*Fgfr2*
Clec2d	*Gpr35*	*Notch2*	*Flrt2*
Degs1	*Hvcn1*	*Pcdh1*	*Flt1*
Fads2	*Lrrc32*	*Pcdh18*	*Gpc1*
Ghr	*Lrrc8d*	*Pcdh19*	*Gpc3*
Il6st	*Mmd*	*Pcdhgc3*	*Gpr39*
Lamp1	*Mrgpre*	*Pkd1*	*Itgb3*
Maea	*Nagpa*	*Ptprb*	*Kcnj15*
Osmr	*Procr*	*Sigmar1*	*Olr1*
Pdgfrb	*Psen2*	*Slc1a4*	*Pcdh7*
Pdpn	*Relt*	*Slc26a6*	*Plscr1*
Pgrmc1	*Rtn4r*	*Slc39a14*	*Ptprd*
Sdcbp	*Slc6a6*	*Tbxa2r*	*Ror1*
Slc25a3	*Slc7a11*	*Tm7sf2*	*Slc16a4*
Slc25a4	*Stom*	*Tmem150a*	*St3gal5*
Slc25a5	*Tfrc*	*Trabd2b*	*Stbd1*
Tspan4	*Tnfrsf21*	*Zyx*	*Traf5*
