# Supplementary material for: Fibroblast polarization over the myocardial infarction time continuum shifts roles from inflammation to angiogenesis
Source: Basic Res Cardiol. 2019 Jan 11;114(2):6. doi: 10.1007/s00395-019-0715-4 (PMC6329742; doi:10.1007/s00395-019-0715-4)
Supplement: Supplementary file 3 — Supplementary material 3 (PDF 30 kb) [file 395_2019_715_MOESM3_ESM.pdf]

Online Table 3. Top 500 Genes Ranked by Average FPKM.									
1-50	51-100	101-150	151-200	201-250	251-300	301-350	351-400	401-450	451-500
Mir2137	Rps24	Col4a1	Ubc	Lhfp	Csde1	C3	Mfge8	Prkar1a	Nfe2l1
Rmrp	Actg1	Rps27a	Atp5b	Anxa5	Snord89	Atox1	Hspd1	Eif3a	Gnb1
Snord49b	Snora74a	Rps14	Igfbp2	Cox6c	Ppib	Capns1	Ndufb4	Lbh	Csnk1a1
Rpph1	Snord34	Rps7	Ccng1	Tuba1c	Ndufa3	Prelid1	Psmd8	Tmem66	Ccnd1
Ftl1	Rpl17	Rpl8	Esd	Ptma	Uqcrb	Ddx5	Ccnd2	Atp6ap1	Ndufa6
Eef1a1	Snord15b	Rpl36	Rpl5	Rpl36al	Myeov2	Edf1	Krtcap2	Commd3	Vaultrc5
Snord49a	Rpl13a	Snord33	Gpx4	Dstn	Crip1	Slc25a3	Bag1	Cct6a	Igfbp4
Malat1	Rps15	Rpl27a	Ifitm2	Cyb5r3	Atp5e	Mif	Nupr1	Gpx1	Atp6v1e1
Sparc	Rpl7	Rpl6	S100a10	Snord55	Atpif1	Gdi2	Psmb3	Copa	Pabpc1
Snord83b	Ctgf	Gnas	Tceb2	Fbn1	Col1a1	Mrfap1	Atp5o	Myo1c	Cct8
S100a4	Rps17	Hspa5	Cox4i1	Shfm1	Pgam1	Mrps21	Copb2	Rcn1	Gars
Snord104	Calr	Rps6	Rnu12	Rpl15	Loxl2	Sptan1	Chchd2	Psma7	Psmc3
S100a6	Rps8	Col8a1	S100a11	Rps27l	Tbca	Cap1	Sumo2	Eif3h	Hnrnpf
Col3a1	Hsp90b1	Fau	Itgb5	Snord16a	P4ha1	Tpm3	2010107E04Rik	Lman1	Ctnna1
Rps12	Rpl9	Ccdc80	Rps3	Vcp	Mgp	Arf1	Serpinb9b	Degs1	Tcp1
Rplp1	Rps4x	Acta2	Rpl31	Timp2	Myl12b	Cox7c	Nars	Mnf1	Cct4
Rpl41	Rps26	Tmsb10	Ephx1	Anxa3	Uqcr11	Clic4	Clic1	Mfap5	Psma3
Snord32a	Rps28	Lamp1	Canx	Ctsz	Cstb	Nedd8	Dad1	Eif4g1	Eif3c
Snord110	Rpl37a	Rpl36a	Rpl27	Uqcrh	Atp6v0c	Bcap31	Psmb1	Gsto1	Ran
Hspa8	Prdx1	Ahnak	Rpl21	Ddb1	Wls	Ctnnb1	Scd2	Ywhae	Spp1
Rpl23a	Rps21	Rpl18	Cox6b1	Prnp	Arl1	Cct5	42615	Heg1	Atp5f1
Igfbp7	Eef2	Rpl3	Sod1	Gapdh	Hint1	Capn2	Calm1	Snora28	Hist1h1c
Snord22	Rnu73b	Snord100	Serpinb6a	Btf3	Id3	Atp6v0e	Serinc1	Tcf21	Rpl22
Vim	Rps10	Snord99	Slc25a4	Atp5l	Tmem59	Rhoc	Tubb4b	Tcf25	Anxa6
Anxa1	Rpl11	Cald1	Calm2	Pdia6	Txndc5	Rnase4	Tubb2a	Vdac1	Sh3bgrl
Scarna2	Rpl4	Cst3	Mt1	Ldha	Usmg5	Mt2	Tbx20	Aldh2	Ndufa2
Gm15772	Rps11	Sqstm1	Ost4	Ndufa4	Dynlrb1	Serpina3n	H3f3a	Ostc	2410006H16Rik
Rn4.5s	Rpl28	Rpl14	Hmox1	Swi5	Actn1	Ifi27l1	Prdx4	Adipor1	Gabarapl1
Rpl35	Rps19	Hspb1	Postn	Prss23	Ybx1	Tmem45a	Ptrf	Atp5c1	Cav1
Rpl39	Rps9	Pkm	Snora78	Atp5h	Nap1l1	Serf2	Slc44a2	Gadd45g	Rras2
Rps20	9930013L23Rik	Ankrd1	Bsg	Cd151	Vdac2	Cct2	Atp5g2	Sec61b	Rexo2
Gm9846	Rpl34-ps1	Snord17	Sptbn1	Fam129b	Ndufa7	Pebp1	Psmb4	Uqcr10	Shisa5
Serpine1	Rpl13	Rps23	Myl12a	Eef1b2	Gpx8	Rab7	Phldb2	Psmb7	Tns3
Uba52	Gm12191	Calu	Mcfd2	Asah1	Cox7a2l	Ghr	Sec61g	Spcs1	Cct3
Rpl38	Tpm4	Tagln	Cryab	Hnrnpk	Eif5a	42620	Srsf5	Ddost	Rcn2
Rpl10	Rpsa	Rps13	Prdx5	Pdpn	Pdia4	Cox5b	Sec13	Eif3e	Nucb1
Snord82	Gnb2l1	Rpl18a	App	Mgst1	Srp14	Psma2	Atf4	Rabac1	Park7
Rps2	Rps5	Gas6	Col4a2	Gm10094	Pgk1	Crip2	Mrpl33	Pls3	Ubxn1
Rpl32	Anxa2	Dcn	Pdia3	Cpe	Lmna	Atp5a1	Ndfip1	Fam114a1	Hsbp1
Rplp0	Rps16	Scarna17	Eif4a1	Sfrp1	Eno1	Npc2	1810022K09Rik	Cox7a2	Sulf1
Fbln2	Hsp90ab1	Ctsl	Ccl2	Il6st	Tma7	Itm2c	Aplp2	Copz2	Kcne4
Lgals1	Rpl23	Rplp2	Ccl7	Snord15a	Aes	Fhl2	Pomp	Gm12669	42624
Myl6	Rpl35a	Snord35a	Prdx2	42628	Gnai2	Arpc2	Wdr1	Ywhaq	Eif4b
Ppia	Cd81	Rpl10a	Cox8a	Atp5k	Arpc3	Psma4	Csrp1	Ctps	Mdh2
Rpl19	Rpl7a	Npm1	Naca	AF357425	Pfdn5	Tmed3	Ecm1	F3	Caprin1
Rpl12	Nme2	Itgb1	Serpinf1	Cd63	Clta	Ssr4	Hdlbp	Scarb2	Ppp1ca
Snora7a	Col1a2	Lyz2	Cox6a1	Cdc42	Rpl37	Eif3k	Scel	Psmc6	Ap2m1
Rps18	Rps25	Scarna10	Gsn	Atp5j2	Calm3	Axl	Ctsb	Psmd2	Vcl
Rps3a1	Rpl24	Rpl29	Col6a1	Pea15a	Tmem258	Map1lc3b	Atp6v1f	Snora44	Tmem50a
Rps29	Snord12	Cfl1	Gabarap	Serping1	Oaz1	Dpysl2	Hspa9	Gpi1	Atp5g3
