# Supplementary material for: Fibroblast polarization over the myocardial infarction time continuum shifts roles from inflammation to angiogenesis
Source: Basic Res Cardiol. 2019 Jan 11;114(2):6. doi: 10.1007/s00395-019-0715-4 (PMC6329742; doi:10.1007/s00395-019-0715-4)
Supplement: Supplementary file 4 — Supplementary material 4 (PDF 21 kb) [file 395_2019_715_MOESM4_ESM.pdf]

Online Table 6. Top 10 upregulated and downregulated GO Biological Processes for each MI day ranked by combined score.

Day 1			Day 3			Day 7		
**Pathway**	**Score**	**p value**	**Pathway**	**Score**	**p value**	**Pathway**	**Score**	**p value**
**Upregulated**								
Regulation of T-helper 17 cell lineage commitment	17.14	0.007	Regulation of alcohol biosynthetic process	73.18	7.00E-18	Regulation of monocyte chemotaxis	25.26	7.00E-06
Gland morphogenesis	16.04	0.007	Regulation of steroid biosynthetic process	61.69	3.00E-14	Ear morphogenesis	23.71	1.00E-05
Positive regulation of janus kinase activity	15.64	0.007	Cholesterol biosynthetic process	61.23	4.00E-16	Positive regulation of bone mineralization	21.69	6.00E-06
Positive regulation of memory T cell differentiation	15.5	0.007	Sterol biosynthetic process	57.46	5.00E-15	Regulation of endothelial cell chemotaxis	19.05	0.001
Chondroitin sulfate metabolic process	15.3	0.0003	Isoprenoid biosynthetic process	33.45	2.00E-06	Negative regulation of mononuclear cell migration	18.98	0.001
Negative regulation of interleukin-10 production	15.04	0.002	Cell migration involved in sprouting angiogenesis	23.73	4.00E-05	Positive regulation of collagen metabolic process	18.87	0.0001
Positive regulation of metanephros development	14.77	0.01	Blood vessel endothelial cell migration	21.86	9.00E-06	Bleb assembly	18.74	0.002
Positive regulation of macrophage chemotaxis	14.76	0.01	Bleb assembly	20.47	0.001	Phospholipid scrambling	17.65	0.003
Negative regulation of cholesterol storage	14.59	0.007	Cellular response to laminar fluid shear stress	20.05	0.0003	Positive regulation of wound healing	17.38	0.0002
Amino acid transmembrane transport	14.49	0.003	Extracellular matrix organization	19.48	7.00E-06	Protein localization to basolateral plasma membrane	17.35	0.002
**Downregulated**								
Secondary alcohol biosynthetic process	60.37	1.60E-13	NADH metabolic process	23.26	0.003	glutathione derivative metabolic process	28.95	3.00E-06
Cholesterol biosynthetic process	57.47	3.70E-15	Glutathione derivative metabolic process	16.95	0.0006	glutathione derivative biosynthetic process	21.8	3.00E-06
Sterol biosynthetic process	52.41	3.70E-14	Regulation of epithelial to mesenchymal transition involved in endocardial cushion formation	16.88	0.003	Xenobiotic catabolic process	20.49	8.00E-06

Regulation of steroid biosynthetic process	46.63	6.00E-11	Regulation of MyD88-independent toll-like receptor signaling	15.91	0.008	regulation of epithelial to mesenchymal transition involved in endocardial cushion formation	17.76	0.003
Acetyl CoA metabolic process	27.78	0.00002	Regulation of transcription from RNA pol II promoter involved in heart development	14.85	0.005	Positive regulation of apoptotic cell clearance	15.46	0.004
Choline catabolic process	20.99	0.0005	Ventricular trabecula myocardium morphogenesis	14.31	0.008	Response to iron ion	14.88	0.002
Positive regulation of osteoblast proliferation	20.91	0.0005	Xenobiotic catabolic process	14	0.0003	Positive regulation of interleukin-2 biosynthetic process	14.12	0.006
Cellular biogenic amine catabolic process	19.05	0.001	Membrane lipid metabolic process	13.99	0.002	Endocardial cushion development	14.03	0.001
Outflow tract septum morphogenesis	18.97	0.0002	Positive regulation of interleukin-2 biosynthetic process	13.33	0.0006	Glutathione metabolic process	12.82	0.0003
Regulation of Wnt signaling pathway, planar cell polarity pathway	18.69	0.0008	Glutathione derivative biosynthetic process	12.77	0.0006	Drug transmembrane transport	12.65	0.01
