# Supplementary material for: Fibroblast polarization over the myocardial infarction time continuum shifts roles from inflammation to angiogenesis
Source: Basic Res Cardiol. 2019 Jan 11;114(2):6. doi: 10.1007/s00395-019-0715-4 (PMC6329742; doi:10.1007/s00395-019-0715-4)
Supplement: Supplementary file 5 — Supplementary material 5 (PDF 457 kb) [file 395_2019_715_MOESM5_ESM.pdf]

Fibroblast polarization over the myocardial infarction time continuum shifts roles from inflammation to angiogenesis

Basic Research in Cardiology

Alan J. Mouton[1], Yonggang Ma[1], Osvaldo J. Rivera Gonzalez[1], Michael J. Daseke II[1], Elizabeth R. Flynn[1], Tom C. Freeman,[2] Michael R. Garrett[3], Kristine Y. DeLeon-Pennell[1,4], and *Merry L. Lindsey[1,4]

[1]Mississippi Center for Heart Research, Department of Physiology and Biophysics, University of Mississippi Medical Center, Jackson, MS, 39216, USA.

[2]The Roslin Institute, University of Edinburgh, Easter Bush, Midlothian EH25 9RG, UK.

[3]Department of Pharmacology and Toxicology, University of Mississippi Medical Center, Jackson, MS, 39216, USA.

[4]Research Service, G.V. (Sonny) Montgomery Veterans Affairs Medical Center, Jackson, MS, 39216, USA.

***Address for Correspondence:**

Merry L. Lindsey, Ph.D.

Department of Physiology and Biophysics

University of Mississippi Medical Center

2500 North State St., Jackson, MS 39216-4505, USA

(phone) +1601-815-1329 (fax) +1601-984-1817

email: mllindsey@umc.edu

Supplemental Material

Online Resource 1. Proof of Successful MI. (a) Day 7 survival rate was 52% (13/25). (b) Infarct size did not differ between days 1, 3, and 7. (c) LV wall thinning occurred to a similar degree at all MI times. (d) End-diastolic dimension was increased at MI day 7, while (e) end-systolic dimension was increased at day 1 and 3 and further increased at day 7. (f) Fractional shortening decreased similarly across all MI days. n=3 per group. *p<0.05 versus day 0, #p<0.05 versus MI day 1, $p<0.05 versus MI day 3

Online Resource 2. Proof of Cell Purity and Pro-fibrotic Protein Secretion after MI. (a) FPKM values for cell markers for cardiac fibroblasts (*Acta2, Col1a1, Col3a1, Postn, and Tcf21*), endothelial cells, lymphocytes, macrophages, myocytes, and neutrophils. (Bottom) Cardiac fibroblast markers graphed as fold change over day 0. (b) Secretion of collagen-related peptides by mass spectrometry. (c) Postn expression in the myocardium at different MI time points. *p<0.05 versus day 0, #p<0.05 versus MI day 1

Online Resource 3. RT-PCR Validation of RNA-Seq. For *Acta2, Col3a1, Cx3cl1, Mmp14*, and *Postn*, FPKM values are graphed in the left column, $2^{-\Delta Ct}$ values are graphed in the middle, and correlations between FPKM and $2^{-\Delta Ct}$ values are displayed on the right. n=3 per group. *p<0.05 versus day 0, #p<0.05 versus MI day 1.

Online Resource 4. (a) Pro-fibrotic Markers, (b) Cell Death/Apoptosis Markers, (c) Markers of Origin, and (D) MMPs. n=3 per group. *****p<0.05 versus day 0, #p<0.05 versus MI day 1, $p<0.05 versus MI day 3.

Online Resource 5. MI Angiogenic Gene Expression. (a) Pro-angiogenic genes upregulated after MI. (b) Anti-angiogenic genes upregulated after MI. (c) Pro-angiogenic genes downregulated after MI. n=3 per group. *p<0.05 versus day 0, #p<0.05 versus MI day 1, $p<0.05 versus MI day 3.

Online Resource 6. Expression of CCL5, CX3CL1, and VEGF in MI Cardiac Fibroblasts. CCL5 (a) and CX3CL1 (b) in the day 1 infarct region and expression of VEGF (c) in the day 3 infarct region did not localize to the infarct fibroblast. Expression of these proteins within fibroblasts (co-localization with PDGFRα+ cells) were not significantly different from day 0.

Online Resource 1

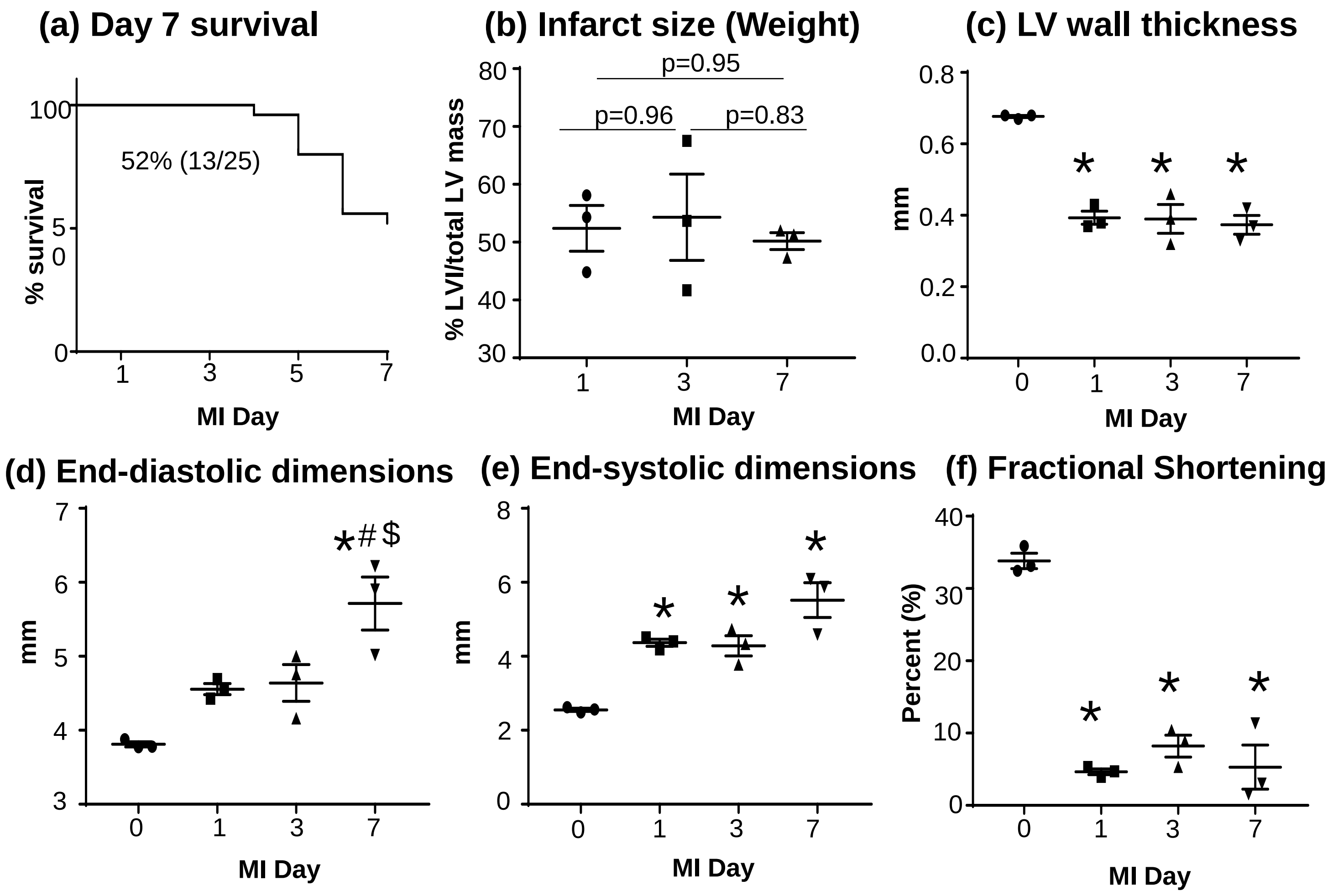

Online Resource 2

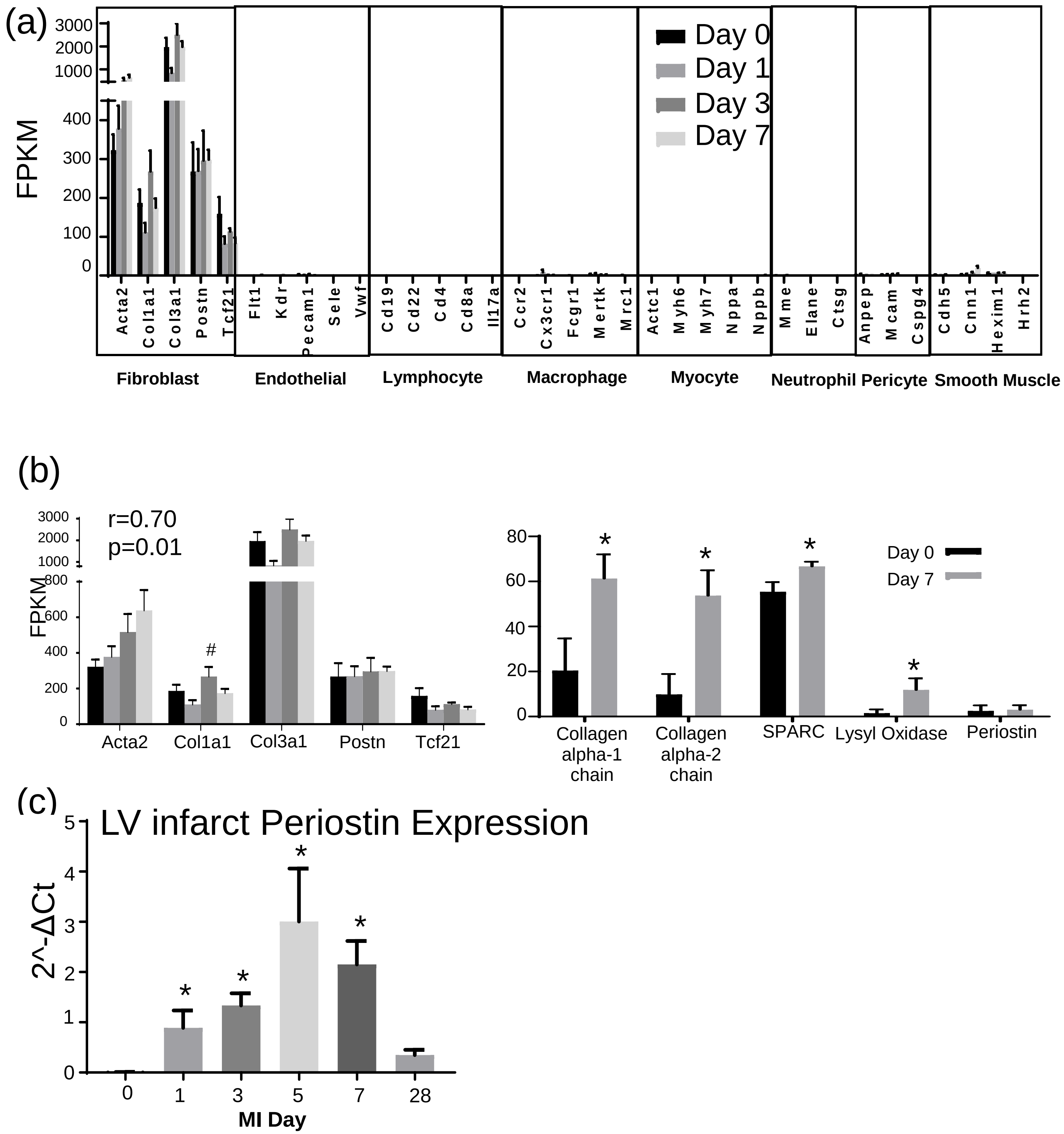

Online Resource 3

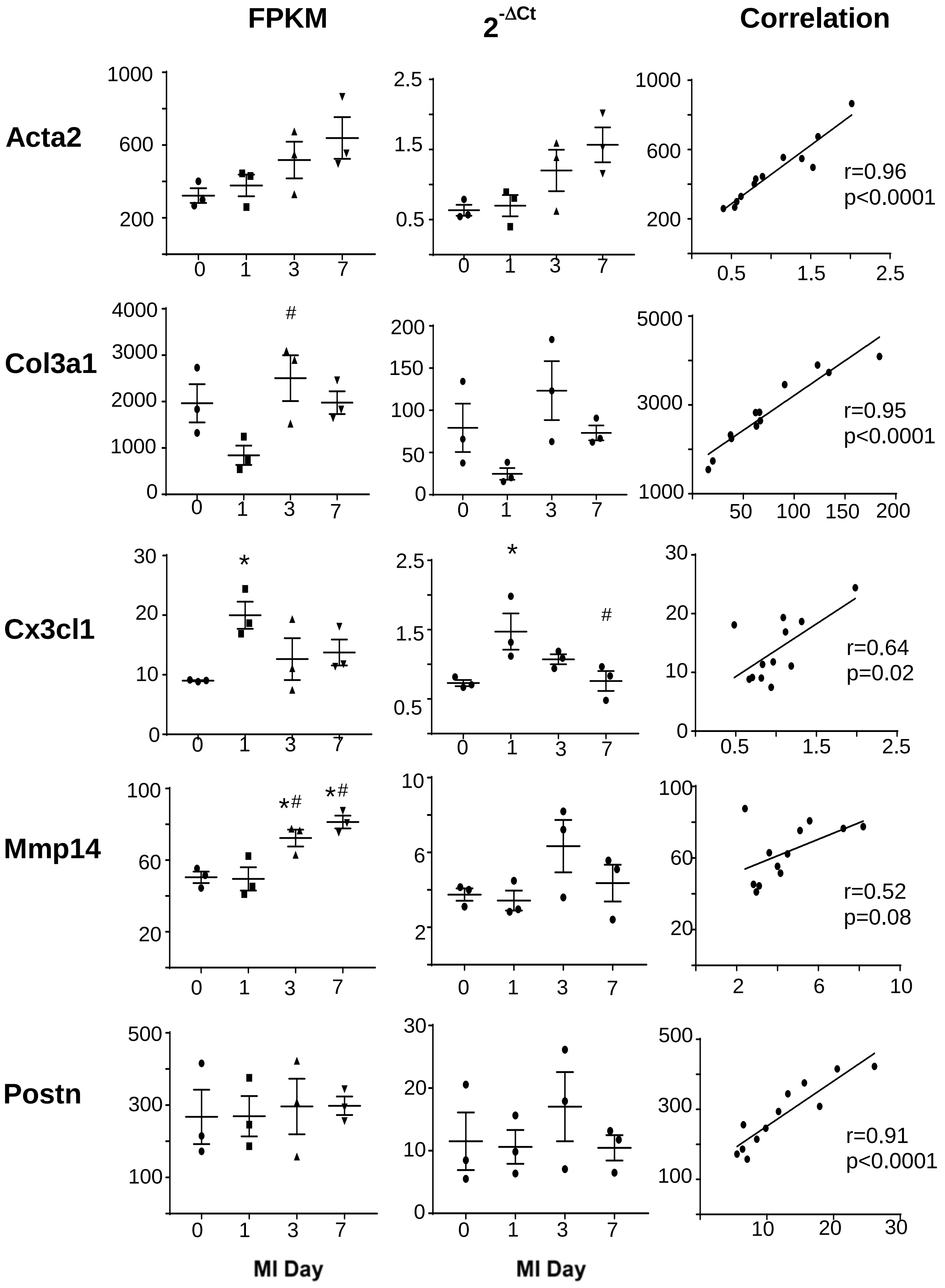

Online Resource 4

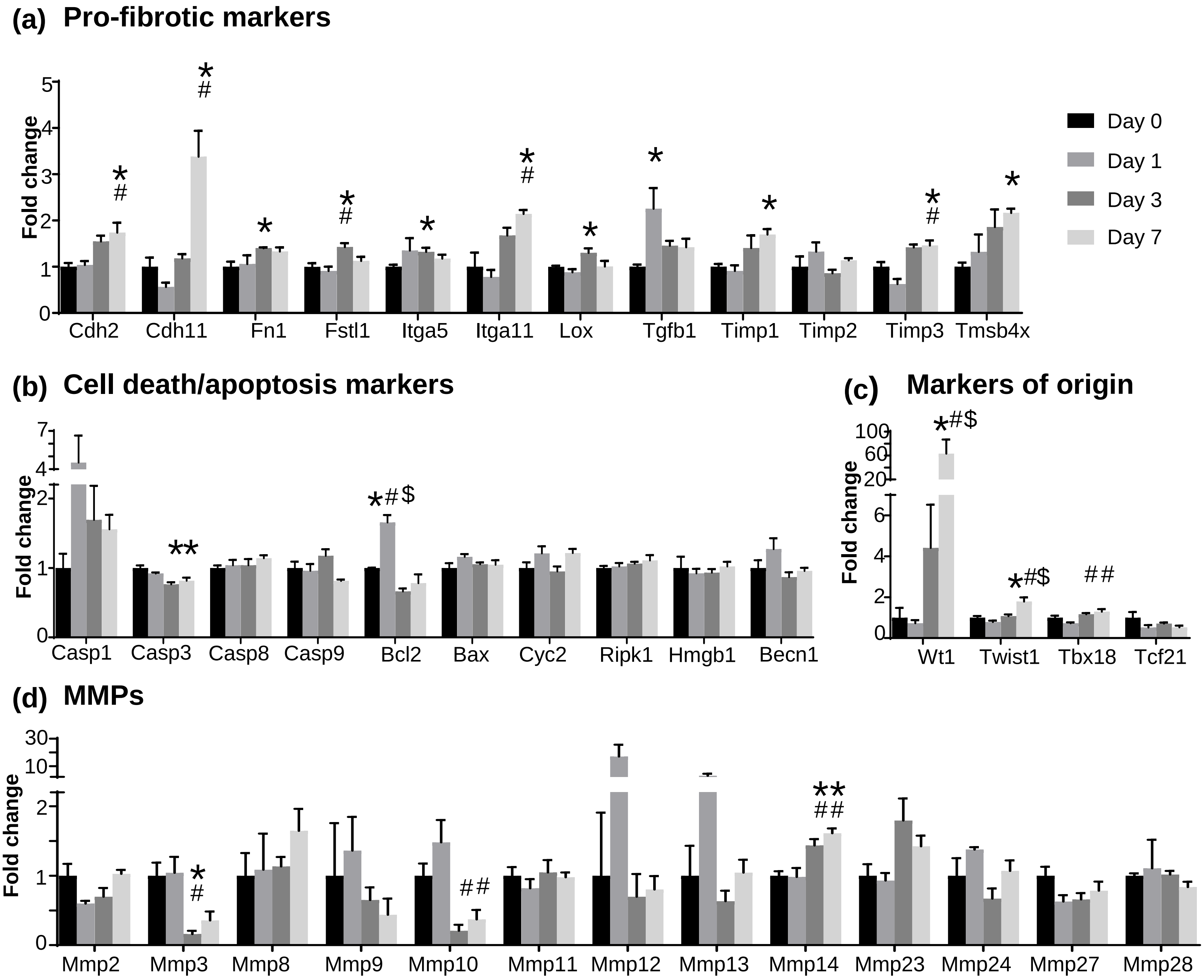

Online Resource 5

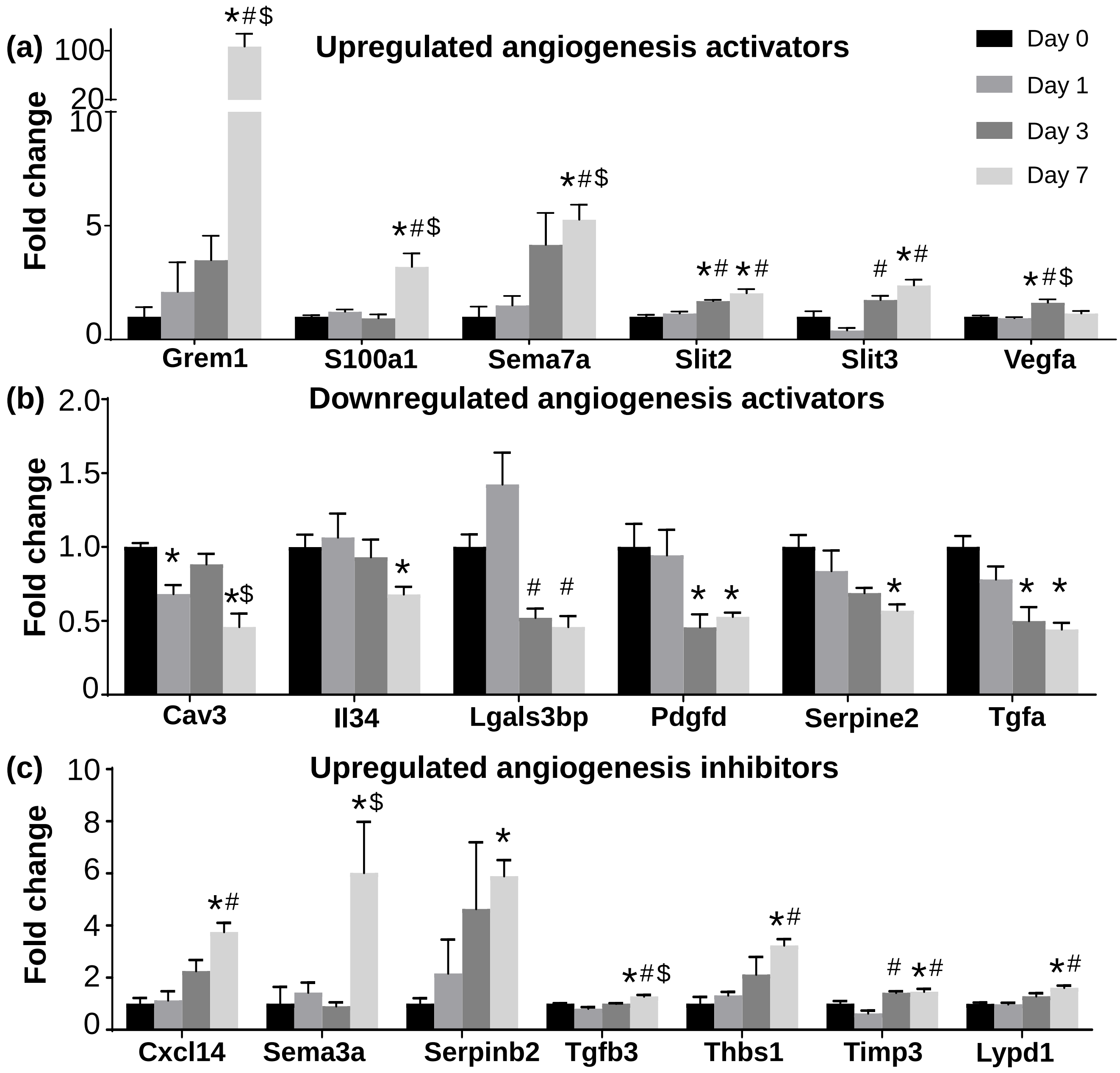

Online Resource 6

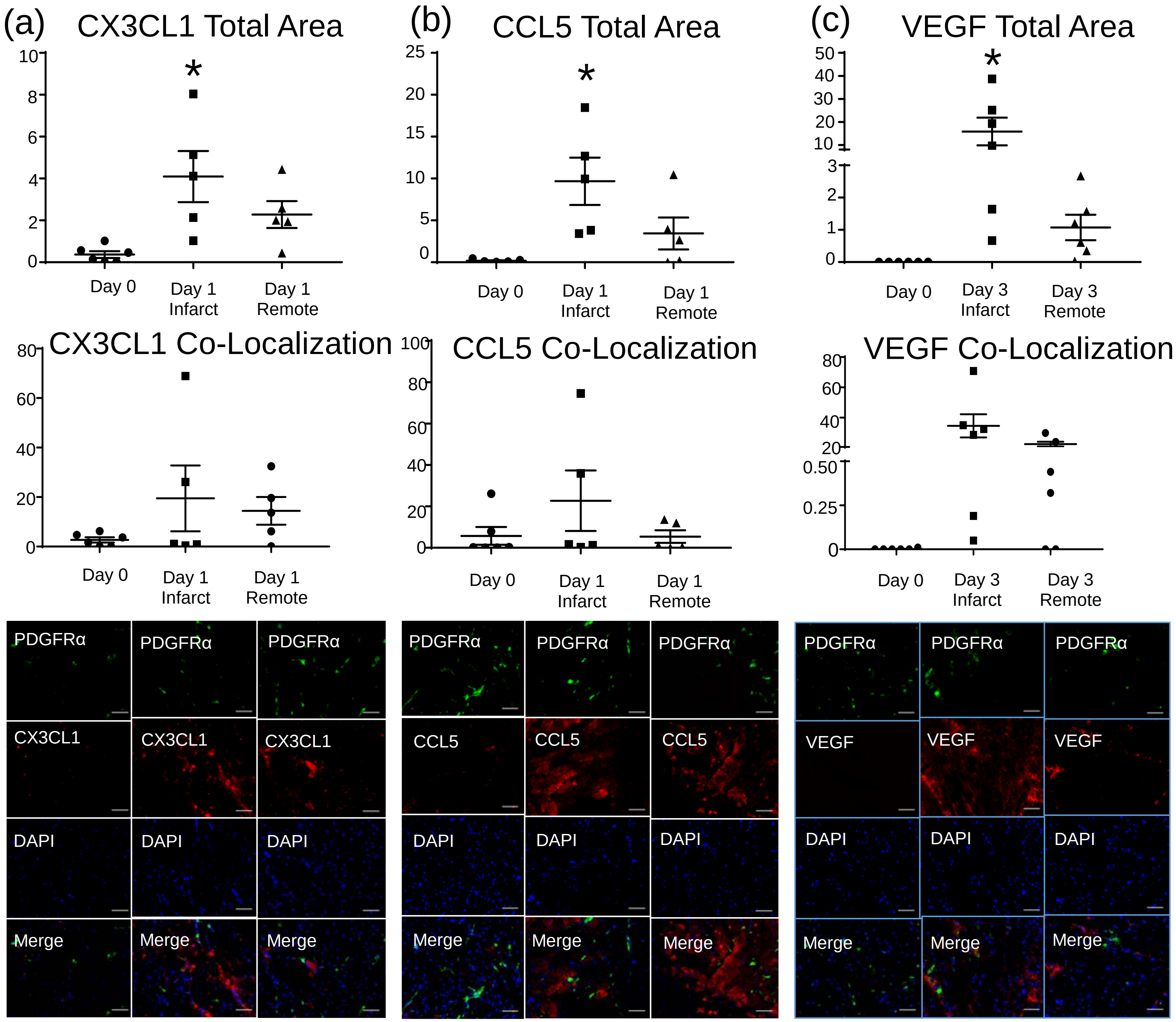

Online Tables 1, 2 and 3 are longer than one page and are included separately.

**Online Table 4.** Significantly enriched pathways in the day 0 phenotype and pan fibroblast marker phenotype.		
	**p value**	**Combined Score**
**Enriched pathways (Day 0 phenotype)**		
5-hydroxytryptamine degradation	0.010	3.51
Mannose metabolism	0.012	2.11
De novo pyrimidine ribonucleotides biosythesis	0.018	1.37
**Cell types corresponding to pan fibroblast markers**		
Mouse embryonic fibroblasts	0.005	8.92
Osteoblast day 14	0.005	8.37
Osteoblast day 21	0.045	4.32
**Pan cell surface markers**		
Met, Npr3, Pdgfra		
**Enriched pathways (pan fibroblast markers)**		
Integrin signaling	1.34E-8	32.29
Parkinson disease	1.02E-7	23.02
Cytoskeletal regulation by Rho	6.0E-5	12.15
CCKR signaling map ST	0.0009	9.28
Huntington disease	0.004	5.93
Inflammation mediated by chemokine and cytokine signaling	0.008	5.45
Glycolysis	0.0007	2.55

Online Table 5. Significantly upregulated and downregulated pathways by Panther ranked by p value. Differentially expressed genes for each pathway are listed.

Upregulated		Downregulated	
**Day 1**			
**pentose phosphate pathway**	*Taldo1, Tkt, Pgd*	**cholesterol biosynthesis**	*Fdft1, Fdps, Hmgcs1, Lss, Mvk, Mvd, Sqle*
**chemokine/cytokine-mediated inflammation**	*Arpc1b, Pak1, Ccl5, Cx3cl1, Cxcr6, Gng2, Gng5, Kras, Nfatc1*	**Transforming growth factor beta signaling**	*Bmp4, Bmp2, Nodal, Smad6, Smad7, Tgfb3*
**beta 2 adrenergic signaling**	*Adrb2, Prkar2b, Stx3*	**mannose metabolism**	*Gmds, Pmm1*
**CRF receptor signaling pathway**	*Gng2, Gng5, Stx3*		
**semaphorin-mediated axon guidance**	*Pak1, Plxna1*		
**Day 3**			
**cholesterol biosynthesis**	*Fdft1, Fdps, Hmgcr, Hmgcs1, Lss, Mvd, Mvk, Pmvk, Sqle*	**pyrimidine metabolism**	*Aldh6a1, Dpyd, Abat*
**integrin signaling**	*Actb, Actn4, Bcar1, Col11a1, Col16a1, Col27a1, Col2a1, Col5a1, Flna, Fn1, Itga5, Lama1, Pik3r3, Rhoa, Rhob, Vasp*	**5-hydroxytryptamine degradation**	*Aldh3a1, Maoa*
**cytoskeletal regulation by Rho GTPase**	*Actb, Arhgap1, Myh9, Pak4, Pfn1, Ssh1, Tubb3, Vasp*		
**cadherin signaling**	*Actb, Cdh2, Cdh10, Cdh24, Erbb2, Fzd5, Pcdh1, Pcdh18, Pcdh19, Pcdhga10, Pcdhgc3*		
**angiogenesis**	*Arhgap1, Fzd5, Kdr, Notch2, Pik3r3, Ptprb, Rhoa, Rhob, Vegfa*		
**Day 7**			
**cytoskeletal regulation by Rho GTPase**	*Actb, Actg2, Arhgap1, Arpc5, Enah, Myh9, Myh10, Myh11, Mylk, Stmn4*	**Gi and Gs alpha G protein signaling**	*Adcy2, Adcy9, Gng2, Grk1, Kcnj5, Prkar2b, Pygm, Rgs3, Rgs4, Rgs6, Rgs7*
**cadherin signaling**	*Actb, Actg2, Cdh2, Cdh3, Cdh10, Cdh11, Cdh24, Ctnnd1, Fzd3, Pcdh7, Pcdhga10, Wnt2, Wnt4*	**integrin signaling**	*Arhgap26, Col13a1, Col4a3, Col4a4, Col5a2, Elmo1, Itga2, Itga2b, Lama2, Lamb2, Lamb3, Pik3c2b*
**chemokine and cytokine-mediated inflammation**	*Ccr1, Camk2d, Arpc5, Actb, Actg2, Gnai1, Mylk, Gng5, Cxcr3, Grk6, Gnb3, Myh9, Myh10, Myh11*	**endothelin signaling**	*Adcy2, Adcy9, Ece1, Ednrb, Itpr2, Prkar2b, Pik3c2b*
**presenilin pathway**	*Actb2, Actg2, Cd44, Cdh3, Fzd3, Mmp14, Mmp17, Wnt2, Wnt4*		
**angiogenesis**	*Arhgap1, Efnb1, Ephb2, F2r, Fzd3, Mapapk3, Pdgfc, Pik3r3, Pla2g4c, Prkd1, Wnt2, Thbs1*		

Online Table 6 is larger than one page and is included separately.
